# The Tomato Hybrid Proline-rich Protein regulates the abscission zone competence to respond to ethylene signals

**DOI:** 10.1038/s41438-018-0033-2

**Published:** 2018-06-01

**Authors:** Srivignesh Sundaresan, Sonia Philosoph-Hadas, Chao Ma, Cai-Zhong Jiang, Joseph Riov, Raja Mugasimangalam, Betina Kochanek, Shoshana Salim, Michael S. Reid, Shimon Meir

**Affiliations:** 1 Department of Postharvest Science of Fresh Produce, Agricultural Research Organization (ARO), The Volcani Center, Rishon LeZiyon, Israel; 2 The Robert H. Smith Institute of Plant Sciences and Genetics in Agriculture, The Robert H. Smith Faculty of Agriculture, Food and Environment, The Hebrew University of Jerusalem, Rehovot, Israel; 3 Department of Plant Sciences, University of California, Davis, CA USA; 4 Crops Pathology & Genetic Research Unit, USDA-ARS, Davis, CA USA; 5 Department of Bioinformatics, QTLomics Technologies Pvt. Ltd, Bangalore, India; 6 Present Address: Department of Nano Science and Technology, Tamil Nadu Agricultural University, Coimbatore, India; 7 Present Address: Beijing Key Laboratory of Development and Quality Control of Ornamental Crops, Department of Ornamental Horticulture, China Agricultural University, Beijing, China

## Abstract

The Tomato Hybrid Proline-rich Protein (*THyPRP*) gene was specifically expressed in the tomato (*Solanum lycopersicum*) flower abscission zone (FAZ), and its stable antisense silencing under the control of an abscission zone (AZ)-specific promoter, *Tomato Abscission Polygalacturonase4*, significantly inhibited tomato pedicel abscission following flower removal. For understanding the THyPRP role in regulating pedicel abscission, a transcriptomic analysis of the FAZ of *THyPRP*-silenced plants was performed, using a newly developed AZ-specific tomato microarray chip. Decreased expression of *THyPRP* in the silenced plants was already observed before abscission induction, resulting in FAZ-specific altered gene expression of transcription factors, epigenetic modifiers, post-translational regulators, and transporters. Our data demonstrate that the effect of *THyPRP* silencing on pedicel abscission was not mediated by its effect on auxin balance, but by decreased ethylene biosynthesis and response. Additionally, *THyPRP* silencing revealed new players, which were demonstrated for the first time to be involved in regulating pedicel abscission processes. These include: gibberellin perception, Ca^2+^-Calmodulin signaling, Serpins and Small Ubiquitin-related Modifier proteins involved in post-translational modifications, Synthaxin and SNARE-like proteins, which participate in exocytosis, a process necessary for cell separation. These changes, occurring in the silenced plants early after flower removal, inhibited and/or delayed the acquisition of the competence of the FAZ cells to respond to ethylene signaling. Our results suggest that THyPRP acts as a master regulator of flower abscission in tomato, predominantly by playing a role in the regulation of the FAZ cell competence to respond to ethylene signals.

## Abscission: Uncovering a master regulatory gene

Investigations using tomato plant models provide insights into a key part of plants’ growth cycle. During healthy growth, plants deliberately drop unfertilized flowers and ripe fruit - a process called abscission. This process influences crop yields, yet the molecular mechanisms involved are not clear. Tomato plants provide a valuable model because they develop distinct abscission zones (AZ) at the stems (pedicels) attaching single flowers to the plant. Shimon Meir at the Agricultural Research Organization, The Volcani Center in Israel and co-workers uncovered the role of the tomato hybrid proline rich protein gene (*THyPRP*) in regulating the tomato flower AZ. Using a newly-developed microarray chip to monitor genes in the AZ, the team found that silencing *THyPRP* altered gene expression in the flower AZ, which in turn affected its competence to respond to ethylene signaling. They also identified several other proteins and signaling pathways involved in abscission.

## Introduction

Abscission is a natural process of plant development, in which subtended organs, leaves, flowers, fruits, and branches, separate from the parent plant^1,2^. The abscission process usually occurs in four phases (A–D)^3–6^. **A**, differentiation of undifferentiated cells to an anatomically discrete abscission zone (AZ); **B**, acquisition of the competence of the AZ cells to respond to abscission signals; **C**, activation of the AZ cells by the abscission signals (mainly ethylene), and synthesis of cell wall hydrolyzing enzymes, leading to organ separation (execution phase); **D**, trans-differentiation of the retained portion of the AZ to produce a protective defense layer. Much is already known about the anatomical events of phase A, and the physiology, biochemistry, and molecular basis of phase C^7–10^. The role of auxin depletion as a key event in the acquisition of the competence to respond to ethylene signaling in the AZ cells was recently reviewed^11^, and we are beginning to get an insight into the above events at the molecular level^12–18^.

Tomato (*Solanum lycopersicum*) is a convenient model system to study the abscission process, since tomato plants develop a distinct AZ in the midpoint of the flower pedicel, referred to as flower AZ (FAZ). In the first transcriptome microarray analysis of the tomato FAZ performed following abscission induction by auxin depletion, several genes were specifically expressed in the FAZ and not in the pedicel non-AZ (NAZ) region, including *KNOTTED1-LIKE HOMEOBOX PROTEIN1 (KD1)*, and *TOMATO PROLINE RICH PROTEIN* (*TPRP-F1*)^15^. A role of KD1 in tomato flower abscission was reported^19^, and the present study describes an attempt to elucidate the functional role of TPRP-F1 in abscission.

The *TPRP-F1* gene, first isolated from young tomato fruit, represents a single-copy gene in the tomato genome^20^. Sequence analysis of the deduced open reading frame of this gene revealed the existence of proline-rich repeated amino acid motifs^21^, but ignored the C-terminal domain that contains an eight-cysteine motif. Unlike this structure, the Hybrid Proline Rich Proteins (HyPRPs), which create a subgroup of structural cell wall proteins rich in proline^22^, are composed of a hydrophobic and two distinct domains: a proline-rich and a C-terminal domain. Regarding these findings, we refer to the tomato PRP-F1 protein in the present study as the Tomato HyPRP (THyPRP).

Protein domains binding proline-rich motifs are frequently involved in signaling events. The unique properties of proline provide a high discriminatory recognition without requiring high affinities, and therefore the structural features of proline-rich motif binding and specific recognition were investigated^23,24^. However, although HyPRPs are ubiquitous in plants, little is known about their roles other than the function as cell wall structural proteins^25–27^. Several reports indicated that subgroups of HyPRPs might have variable functions during specific developmental stages, and in response to biotic and abiotic stresses^28–30^. Ectopic expression of HyPRP in plants accelerated cell death, led to developmental abnormality with downregulation of reactive oxygen species-scavenging genes, and enhanced the susceptibility to pathogens by suppressing expression of defense-related genes^31^. Recent findings showed that another tomato *HyPRP1* gene (solyc12g009650) is a negative regulator of salt and oxidative stresses, and is probably involved in sulfite metabolism^32^.

The regulation of the FAZ and the tomato fruit to respond to ethylene involves a cross-talk between auxin and ethylene, as auxin depletion is a prerequisite for acquiring the competence for ripening or abscission induction by ethylene^11^. Thus, *THyPRP*, which was primarily expressed in immature green tomato fruit that are ethylene insensitive, was significantly downregulated upon transition to mature green fruit that ripen in response to ethylene^33^. Similarly, following flower removal, a significant decreased *THyPRP* expression in the tomato FAZ was obtained, which was not affected by the ethylene antagonist 1-methylcyclopropene (1-MCP)^15^. This indicates that the decreased *THyPRP* expression is a direct effect of auxin depletion.

The properties of THyPRP and its specific gene expression pattern in the tomato FAZ^15^ suggest, that ThyPRP has an important role in regulating the tissue competence to respond to ethylene signals. In the present report, we investigated the role of THyPRP in regulating tomato pedicel abscission induced by flower removal. For this purpose, we studied the effects of silencing the *THyPRP* gene under the control of the AZ-specific promoter, *Tomato Abscission Polygalacturonase4* (*TAPG4*), and performed a transcriptomic analysis of the FAZ in the wild type (WT) and the *THyPRP*-silenced plants.

## Materials and methods

### Plant material and abscission induction treatments

Tomato (*Solanum lycopersicum*, cv. New Yorker) seeds were obtained from the Tomato Genetics Resource Center, University of California, Davis, USA. The inflorescences of both the WT and the transgenic lines were harvested from 4-month-old greenhouse-grown plants between 08:00 and 10:00 a.m. Preparation of flower bunches, flower removal, experimental conditions, and pedicel abscission assays were performed as described before^15,34^. Pedicel abscission was evaluated by careful touching the distal side of the FAZ, and monitoring the abscised pedicels for calculating the percent of pedicel abscission. For each line, we used 10–12 plants, and the experiments were repeated independently three times with similar results.

### Vector construction and plant transformation

To generate *TAPG4::antisense THyPRP* transgenic plants, a 2379-bp fragment of the AZ-specific *TAPG4* promoter from tomato genomic DNA and a 227-bp fragment of the *THyPRP* gene from tomato cDNA were amplified, subcloned into the modified binary vector GSA1285 in an antisense orientation, and introduced into *Agrobacterium tumefaciens* (LBA4404) by electroporation^19^. Tomato was transformed by the tissue culture method as previously described^35^. Six independent transgenic lines were generated, and two representative transgenic lines (Lines 7 and 11) were selected for further analysis because they showed delayed pedicel abscission during 20 h after flower removal (Supplementary Figure S1). Line 5, which also showed a significantly delayed abscission, was not selected because it exhibited some additional morphological alterations (data not shown).

### Gene expression profiling using the Agilent platform

The plant samples of individual time points (0, 4, 8, 12, 16, and 20 h after flower removal) from FAZ and NAZ of the WT, and FAZ of *TAPG4::antisense THyPRP* line 7, were obtained from two different biological experiments displaced in time, and used for the gene expression and microarray studies. The RNA was isolated and processed as previously described^34^. Basically, the tissue samples (50 mg) were snap frozen and homogenized using a TOMY homogenizer and steel beads (TOMY Micro Smash MS-100, Tomy Medico Ltd., Tokyo, Japan). RNA was isolated by Qiagen RNeasy Plant mini kit (Qiagen, Hilden, Germany) according to the manufacturer’s protocol. In column DNase digestion was performed according to the protocol. The eluted RNA was stored at −70 °C until further processing. Total RNA purity was assessed using a NanoDrop® ND-1000 UV-Vis spectrophotometer (Nanodrop technologies, Rockland, DE, USA). Total RNA integrity was analyzed using RNA 6000 Nano Lab Chip on the 2100 Bioanalyzer (Agilent, Palo Alto, CA, USA) following the manufacturer’s protocol. A good quality RNA was defined based on the rRNA 28S/18S ratios and RNA integrity number >6.5.

### Microarray labeling, hybridization, and scanning

Samples for gene expression analysis were labeled using Agilent Quick-Amp labeling Kit one-color. Aliquots of 500 ng of each sample were incubated with a reverse transcription mixture at 40 °C and converted to double stranded cDNA primed by oligo (dT) with a T7 polymerase promoter. Synthesized double stranded cDNA was used as template for cRNA generation. The cRNA was generated by *in vitro* transcription, and the dye Cy3 CTP (Agilent) was incorporated during this step, both carried out at 40 °C. Labeled cRNA was cleaned up, and its quality was assessed using NanoDrop® ND-1000 UV-Vis spectrophotometer. The specific activity determination was based on the cRNA concentration and dye incorporation.

The labeled cRNA samples were hybridized onto an AZ-specific microarray chip, AMADID: 043310^34^ designed by Genotypic Technology Pvt. Ltd (Bangalore, India). Aliquots of 1650 ng of Cy3-labeled samples were fragmented and hybridized by using the Gene Expression Hybridization kit of Agilent. Hybridization was carried out in an Agilent SureHyb chamber at 65 °C for 16 h. The hybridized slides were washed using Agilent Gene Expression wash buffers, and scanned using the Agilent Scanner, Part Number G2600D. Data extraction from images was performed by using Feature Extraction software of Agilent V-11.5.

### Microarray data analysis

Feature extracted data were analyzed using Agilent GeneSpring GX Version 12 software. Normalization of the data was done in GeneSpring GX using the 75th Percentile shift. Significant genes that were upregulated and downregulated within the group of samples were identified. Statistical *t*-test *p*-values were calculated based on volcano plot using algorithm, which allows visualization of the relationship between fold-change and statistical significance (considering both the magnitude of change and variability). Differentially regulated genes were classified based on gene ontology functional category using GeneSpring GX Analysis software. Duplicate samples were analyzed for each time point. Duplicated probes within the array were averaged for each given transcript.

### Gene expression validation by quantitative PCR

Primers for qPCR were designed using the Gene Runner version 3.05 (Hastings Software Inc. Hastings, USA; http://www.generunner.net). The primers were designed to match the microarray probes, validated, and amplicon sizes were confirmed using 2% agarose gel (Supplementary Table S1). The same RNA samples were used for qPCR and microarray analysis, as previously described^34^. The relative expression levels of the genes were determined after normalization with *ACTIN* as the reference gene, using the comparative C_T_ method for calculating the value of 2^-∆∆C^T.

### Sequence deposition

The microarray data for the WT FAZ and NAZ (12 arrays each) samples were submitted under Gene Expression Omnibus database (NCBI-GEO) under the accession id GSE64221. The data for the *TAPG::antisense THyPRP* FAZ samples (12 arrays) were submitted under the NCBI-GEO accession id GSE64606. The data will be released for public access upon acceptance of the manuscript.

## Results and discussion

### Inhibition of abscission by *THyPRP* silencing

**Fig. 1:**
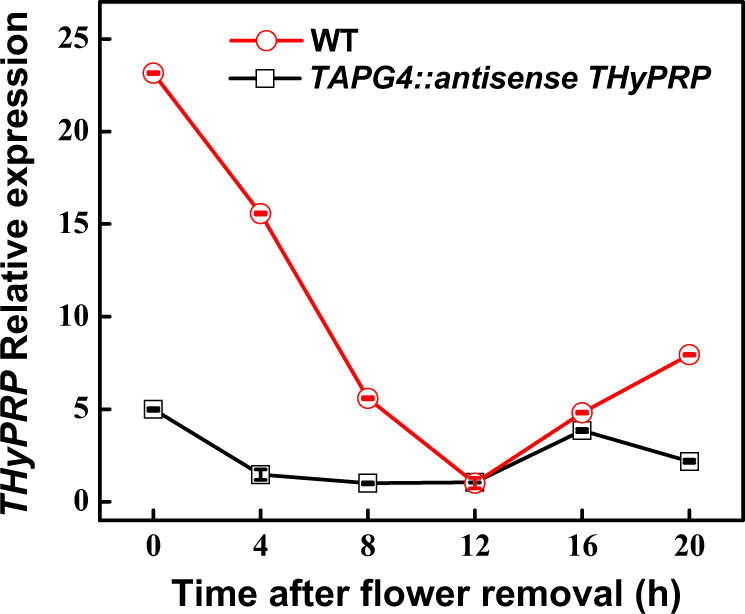
Effect of antisense silencing of *THyPRP* (ID—X57076/Solyc07g043000) on the kinetics of changes in qPCR expression of *THyPRP* in the FAZ at various time points after flower removal. qRT-PCR analysis of *THyPRP* expression in the FAZ of antisense transgenic line 11/generation T4 was compared to that in the WT at 0, 4, 8, 12, 16, and 20 h after flower removal. The relative quantification of the gene expression level in the qPCR assay was determined by the comparative C_T_ method 2^-∆∆CT^^95^, using *ACTIN* as the reference gene. The data represent the mean values (±SE) of duplicate experiments, each having three independent biological samples

We generated 12 transgenic lines in which the *THyPRP* antisense construct was driven by the AZ-specific promoter, *TAPG4*, that controls abscission^36^. For the abscission experiments, we focused on two lines, 7 and 11, of the *TAPG4::antisense THyPRP* transgenic plants, that showed significant delayed pedicel abscission phenotypes (Supplementary Figure S1). Line 11 was also used for transcriptome analysis. The *THyPRP* expression in the WT was downregulated after flower removal (Fig. 1), as previously reported^15^. The transcript abundance was significantly lower in the *THyPRP-*silenced lines than in the WT, and this was manifested already before removal of open flowers, suggesting that the *TAPG4* promoter was active in the FAZ of open flowers. The significantly reduced expression of *THyPRP* in the transgenic line 11 lasted up to 12 h after flower removal, during which the expression in the WT was gradually reduced as well (Fig. 1).

**Fig. 2:**
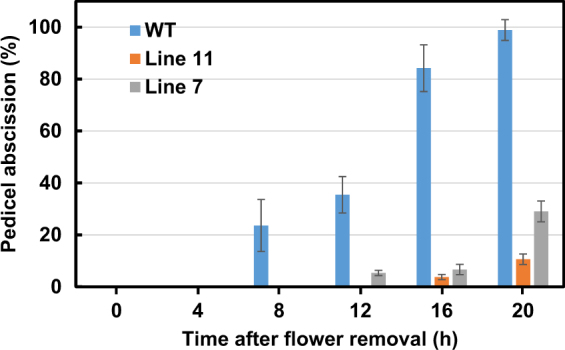
Effect of antisense silencing of *THyPRP* on the kinetics of flower pedicel abscission following flower removal. Flower explants were prepared and handled as previously described^15,34^. Wild type (WT) plants (cv. NY) and silenced lines 7 and 11/generation T4 were used. The percentage of accumulated pedicel abscission was monitored at 0, 4, 8, 12, 16, and 20 h after flower removal. The results are means of four replicates (*n* = 30 explants) ± SE

Stable silencing of the *THyPRP* gene under the control of an AZ-specific promoter (*TAPG4::antisense THyPRP*) significantly inhibited pedicel abscission during 20 h after flower removal (Fig. 2). This indicates that the efficient silencing of the *THyPRP* gene affected a significant regulatory function in the FAZ, which is important for organ abscission.

### Transcriptomic analysis of the FAZ of the *THyPRP*-silenced plants

The role of THyPRP in the regulation of abscission in the tomato flower model system was studied by performing a transcriptomic analysis, using an AZ-specific tomato microarray chip^34^. The results presented in Table 1 show that 157 genes were upregulated and 50 genes were downregulated at zero time in the transgenic plants (Supplementary Table S2). It is noteworthy that changes in the expression of many of the genes that occurred between 4 and 20 h after flower removal were detected at more than one time point (see below). This means that the total number of modified genes (log 2-based) was much lower than the sum of the modified genes listed in each time point in Table 1.

**Table 1:** Transcriptome responses of the FAZ tissues at various time points after flower removal in the transgenic (*TAPG4::antisense THyPRP*) line compared to the WT

Time after flower removal (h)	Number of differentially expressed transcripts in *TAPG::antisense THyPRP* FAZ vs. WT FAZ	
	Upregulated	Downregulated
0	157	50
4	101	192
8	162	573
12	141	296
16	504	544
20	450	193

The total numbers of differentially expressed transcripts (fold changes: downregulated ≤ −2; upregulated ≥ 2) at different time points during the abscission process are presented.

The microarray results were confirmed by two methods: (a) qPCR analyses for five selected genes (Supplementary Figure S2); (b) comparison of the abscission-related WT FAZ genes, such as genes associated with ethylene, cell wall degrading enzymes, and programmed cell death (PCD), to previous tomato FAZ microarray data^15^. The qPCR analyses successfully validated the microarray expression data of selected genes in all the samples from the WT and *THyPRP*-silenced lines (Supplementary Figure S2). We used the downregulation of cell wall modifying genes (Supplementary Figure S3), and of the *Ribonuclease T2* (*LX*) gene (Supplementary Figure S4), which were specifically upregulated in the WT FAZ following flower removal, to confirm the effect of *THyPRP* silencing in retarding pedicel abscission.

Enzymes associated with disassembly and modification of the cell wall include polyglacturonases (PGs), cellulases, endoglucanases, pectin methylesterases, pectate lyases, xyloglucan endotranglucosylase/hydrolases, and expansins^4,5,13,37,38^. The silencing of the *THyPRP* gene resulted in inhibition of 28 genes encoding these cell wall modifying enzymes that were specifically upregulated in the WT following flower removal (Supplementary Figure S3, Supplementary Tables S2-S7). The inhibited upregulation of the cell wall modifying genes was manifested by both lower expression levels and delayed upregulation after flower removal (Supplementary Figure S3), which highly fitted the abscission inhibited phenotype of the transgenic plants. The expression patterns of *TAPG1,2,4* in the WT FAZ (Supplementary Figure S2) were identical to those presented in a previous report^15^, confirming the results obtained from the AZ-specific tomato microarray chip analyses.

The *THyPRP* silencing also resulted in the inhibition of the induced expression of *LX*, which was specifically upregulated in the WT FAZ at 12–20 h after flower removal (Supplementary Figure S4, Supplementary Tables S5-S7). The *LX* gene, previously studied in tomato abscission^15,39^, was found in the group of 16 PCD-related genes that were specifically upregulated in the WT FAZ at a late stage (12–20 h) after flower removal^40^. Therefore, *LX* can serve as a late marker gene for the abscission execution phase in the tomato system.

It is noteworthy that the inhibitory effects of *THyPRP* silencing on the induced expression of cell wall-related and *LX* genes, resulted from the inhibitory effect of the silencing on pedicel abscission (Fig. 2). We assumed that the inhibition of pedicel abscission in the silenced plants was the result of the modified expression of regulatory genes before flower removal (zero time) and between 0 to 4 h after flower removal, when the competence of the FAZ cells to respond to ethylene is being acquired. Therefore, our transcriptomic analysis focused mainly on genes whose expression was changed at these time points. However, we cannot rule out the possibility that later regulatory events might occur also between 4 to 8 h after flower removal, coinciding with the execution of pedicel abscission, but it is difficult to distinguish at this stage between cause and effect of the abscission inhibition.

### *THyPRP* regulation of pedicel abscission is partially mediated by ethylene and gibberellin

**Fig. 3:**
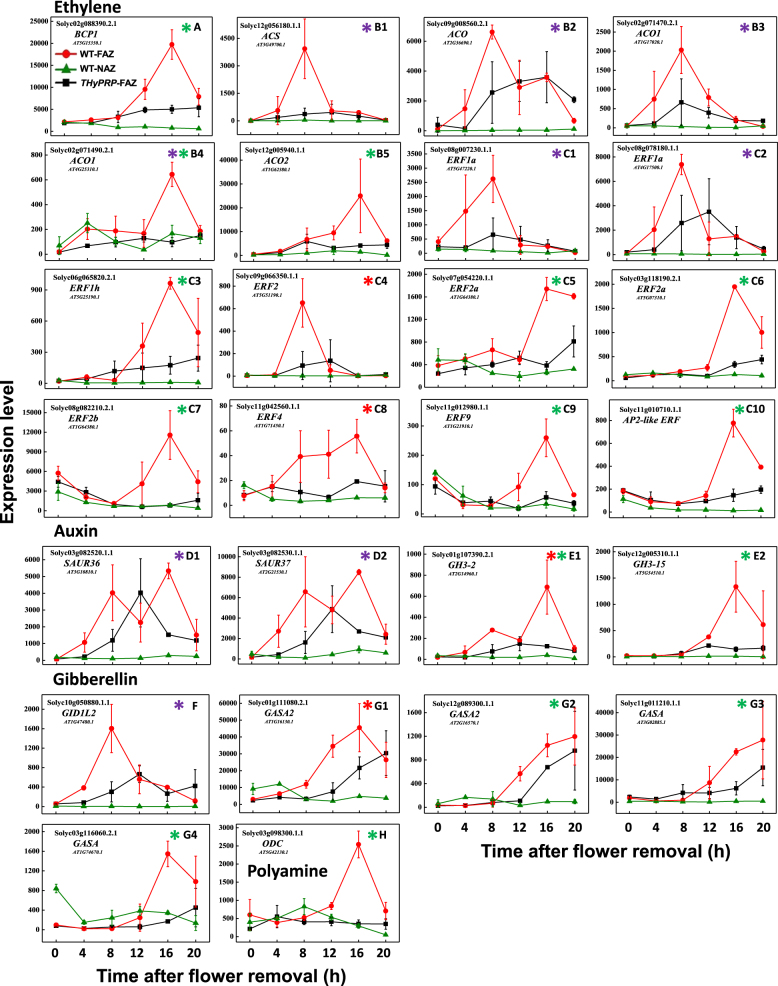
Effect of antisense silencing of *THyPRP* on the kinetics of changes in array-measured expression levels of genes related to the plant hormones. Ethylene (**a**–**c**), Auxin (**d**, **e**), and Gibberellin (**f**, **g**), and Polyamine (**h**), that were specifically upregulated in the WT FAZ at various time points after flower removal: early-4 h (blue*) or 8 h (red*), or late 12–20 h (green*).*TAPG4::antisense THyPRP*-silenced line 11/generation T4 was used. Expression levels were measured for *Blue copper-like protein1 (BCP1)* (**a**); *1-Aminocyclopropane-1-Carboxylate Synthase* (*ACS*) (**b**1); *1-Aminocyclo-propane-1-Carboxylate Oxidase* (*ACO*) genes (**b**2–**b**5); *Ethylene-Related Factor* (*ERF*) TF genes (**c**1–**c**10); *Small Auxin-Up RNA* (*SAUR*) genes (**d**1,** d**2); *Gretchen Hagen3* (*GH3*) which are *Indole-3-acetic acid-amido synthetase* genes (**e**1,** e**2); *GA receptor* (*GID1L2*) (**f**); *GA-regulated protein* (*GASA*) genes (**g**1–**g**4)**;** and *Ornithine Decarboxylase* (*ODC*) (**h**). Transcript identities are indicated by their gene ID and their Arabidopsis (At) gene number and/or their nucleotide accession number. The results are means of two independent biological replicates ± SD

Figure 3 presents the plant hormone-related genes associated with abscission that were specifically upregulated in the WT FAZ after flower removal and inhibited by *THyPRP* silencing. The majority of these genes are related to ethylene biosynthesis and response. Thus, *THyPRP* silencing inhibited the upregulation of a Cu transporter involved in ethylene signaling (Fig. 3a), 5 ethylene biosynthesis genes (Fig. 3b1–b5), and 10 ethylene response factors (*ERFs*) genes (Fig. 3c1–c10), which occurred in the WT FAZ after flower removal. It is noteworthy that two ethylene biosynthesis genes, *1-aminocyclopropane-1-carboxylate synthase* (*ACS*) and *1-amino-cyclopropane-1-carboxylate oxidase* (*ACO*), were upregulated early (4–8 h) in the WT FAZ after flower removal. On the other hand, only two *ERFs* genes were upregulated early at 4 h and two at 8 h, while the other six *ERFs* were upregulated at 12–20 h after flower removal (Fig. 3b1–c10, Supplementary Tables S3-S7). These findings suggest that *THyPRP* silencing can decrease ethylene biosynthesis and response. To the best of our knowledge, this is the first report that shows an effect of PRP on ethylene biosynthesis and ethylene response transcription factor (TF) genes (*ERFs*). Expression analysis during tomato fruit development indicated that the *THyPRP* gene might be downregulated by ethylene^33^. In soybean, *GmPRP* expression, upregulated in leaves infected with *Phytophthora sojae*, was affected by defense/stress signaling molecules, including ethylene, salicylic acid, abscisic acid, and jasmonic acid (JA)^41^. These results provide an indirect evidence which supports our data on the inhibition of ethylene biosynthesis and response by downregulation of *THyPRP* expression.

The role of auxin as a key regulatory factor of the AZ competence to respond to ethylene was extensively documented, and was recently reviewed^11^. In the present tomato system, auxin depletion is artificially induced by flower removal, resulting in downregulation of many auxin-related genes^11,15,19,42,43^. Similarly, 211 auxin-related genes were downregulated in the tomato FAZ and NAZ after flower removal^40^. However, unlike the ethylene-related genes, *THyPRP* silencing had no effect on the downregulation of auxin-related genes (data not shown). The downregulation of two early auxin-responsive genes, *Small Auxin Upregulated RNA* (*SAUR*) in the *THyPRP*-silenced plants at 4 and 16 h after flower removal (Fig. 3d1,d2), and the inhibition of the *Gretchen Hagen3* genes, which are related to indole-3-acetic acid (IAA) conjugation, at 16 h (Fig. 3e1,e2), do not seem to play a significant role in IAA depletion, as IAA levels are already very low at these time points^11^. Therefore, we rule out the possibility that the effect of *THyPRP* silencing on pedicel abscission is mediated by its effect on auxin balance, in contrary to the findings of tomato *KD1* silencing^19^. In the case of *KD1* silencing, the regulation of abscission by KD1 was associated with a changed abundance of auxin-related genes, and measurement of auxin content and activity showed that changes in *KD1* expression directly modulated the auxin concentration and response in the AZ.

Interestingly, we report here for the first time that five gibberellin (GA)-related genes, upregulated specifically in the WT FAZ, were inhibited in the *THyPRP*-silenced plants, including the gene encoding the GA receptor, *GIDIL2* (Fig. 3f) and the GA-responsive (*GASA*) genes (Fig. 3g1–g4, Supplementary Tables S3-S7). These results suggest that the inhibition of *GIDIL2* and *GASA* genes by *THyPRP* silencing might be involved in the inhibitory effect of *THyPRP* silencing on tomato pedicel abscission. To the best of our knowledge, there are no published reports on the effects of GA on tomato flower abscission. The GA promoting effects on bean and cotton leaf abscission were documented long ago, and were utilized for promoting the ethylene-induced abscission of cotton leaves^44–46^. GA spraying at bloom is widely used as a thinning treatment for grapevine, and the molecular pathways, which regulate the acquisition of flower abscission competence following GA application to seedless *Vitis vinifera*, were recently reported^47^. Additionally, the involvement of GA genes in rose petal abscission was recently demonstrated by a transcriptome profiling of the petal AZ^48^.

### Early (0–4 h) effects of *THyPRP* silencing on regulatory genes

*THyPRP* silencing in the FAZ at zero time altered the expression of some regulatory genes at this time point (Fig. 1). Our analysis focused on altered genes encoding functional proteins at five regulatory levels: transcriptional (homeobox and other TF genes); epigenetic; transport (peptide transporters and protein targeting, and exocytosis); post-translation related to protein degradation or phosphorylation/dephosphorylation; and signal transduction.

#### Transcriptional regulation

**Fig. 4:**
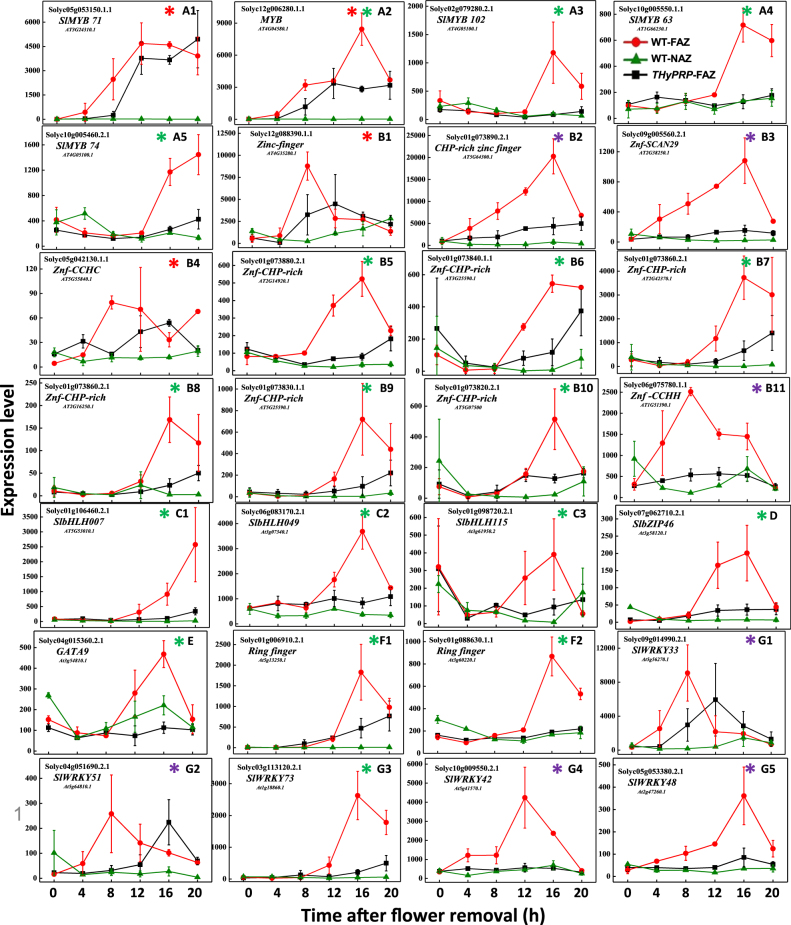
Effect of antisense silencing of *THyPRP* on the kinetics of changes in array-measured expression levels of genes related to transcription factors (TFs) that were specifically upregulated in the WT FAZ at various time points after flower removal: early—4 h (blue*) or 8 h (red*), or late 12–20 h (green*). *TAPG4::antisense THyPRP*-silenced line 11/generation T4 was used. Expression levels were measured for tomato *MYB* TF genes (**a**1–**a**5); *Zink finger (Znf)* TF genes (**b**1–**b**11); *Basic helix-loop-helix (bHLH)* TF genes (**c**1–**c**3); *Basic leucine zipper* (*bZIP*) (**d**); *GATA* TF (**e**); *Ring finger* TF genes (**f**1, **f**2); and *WRKY* TF genes (**g**1–**g**5). Transcript identities are indicated in the graphs by their gene ID and their Arabidopsis (At) gene number and/or their nucleotide accession number. The results are means of two independent biological replicates ± SD

Genes related to seven TF families were specifically upregulated in the WT FAZ at different time points after flower removal, including five *MYB* genes (Fig. 4a1–a5), 11 *Zink finger* (*Znf*) genes (Fig. 4b1–b11), three *basic helix-loop-helix* (*bHLH*) genes (Fig. 4c1–c3), one *basic leucine zipper* (*bZIP46*) gene (Fig. 4d), one *GATA9* gene (Fig. 4e), two *Ring finger* genes (Fig. 4f1–F2), and five *WRKY* genes (Fig. 4g1–g5). The specific upregulation in the WT FAZ of all these 28 TF genes was inhibited in the *THyPRP*-silenced plants (Supplementary Tables S3-S7).

Three Zinc Finger (Znf) TFs, *CHP-rich ZNF* (Solyc01g073890.2.1) (Fig. 4b2), *Znf-SCAN29* (Solyc09g005560.2.1) (Fig. 4b3), and *Znf–CCHH* (Solyc06g075780.1.1) (Fig. 4b11), were specifically upregulated in the WT FAZ at 4 h after flower removal, with a gradually increased expression up to 16 h, which was inhibited in the *THyPRP*-silenced plants. The involvement of *Znf* genes in abscission is well documented. In the soybean leaf abscission system, 20 out of 188 (11%) abscission-specific TF-induced genes were *Znfs*, and most of them were induced early (at 0 and 12 h) after ethylene treatment^40^. The Znf-ankyrin TF, which functions in a broad range of developmental processes and defense responses, was reported to play a role in the AZ establishment^49^. Five *Znf* genes were involved in calyx abscission of Korla fragrant pear^50^. Ten *Znf*s were upregulated early during induction of mature melon fruit abscission^51^. *AtDOF4.7*, a *ZNF BINDING PROTEIN2* (*ZFP2*) gene, was initially identified within a cluster of upregulated genes in Arabidopsis stamen AZ cells before abscission^8^. Transgenic plants with a constitutive ZFP2 activity also exhibited delayed abscission. ZFP2 was found to interact with AtDOF4.7, suggesting that they may function together in a transcriptional complex to modulate the expression of AZ PG and other enzymes during abscission^52^. Our results, as well as earlier reports demonstrating a regulatory role of different *Znf* genes in the early stages of abscission, imply that THyPRP partly regulates abscission in tomato by mediating *Znf* expression.

Four *WRKY* TF genes, *SlWRKY33* (*Solyc09g014990.2.1*) (Fig. 4g1), *SlWRKY51* (*Solyc04g051690.2.1*) (Fig. 4g2), *SlWRKY42* (*Solyc10g009550.2.1*) (Fig. 4g4), and *SlWRKY48* (*Solyc05g053380.2.1*) (Fig. 4g5), were specifically upregulated in the WT FAZ starting at 4 h after flower removal, and their upregulation was inhibited in the *THyPRP*-silenced plants. Similarly, eight *WRKY* TF genes were upregulated early during mature melon fruit abscission, implying that TFs of this family might be involved in triggering the transcriptional cascade during organ abscission^51^. In contrast, four *WRKY* genes were upregulated at the late stage of ethylene-induced soybean leaf abscission^53^. Recently, the *WRKY33* gene was found to be associated with leaf abscission in sugarcane^32^. WRKY33 proteins are evolutionarily conserved, having a critical role in broad stress responses, and two structural AtWRKY33 homologs were identified to function in tomato stress responses^54^. Additionally, the expression of the stress-induced ethylene biosynthesis genes, *ACS2*,*6*, was WRKY33-dependent^55^, while WRKY50,51 proteins mediated the signaling of the stress hormones salicylic acid and JA^56^. Accordingly, it is possible that the WRKY TFs are involved in tomato pedicel abscission by specifically regulating the induced expression of *ACS* and *ACO* genes in the FAZ (Fig. 3b1–b5).

**Fig. 5:**
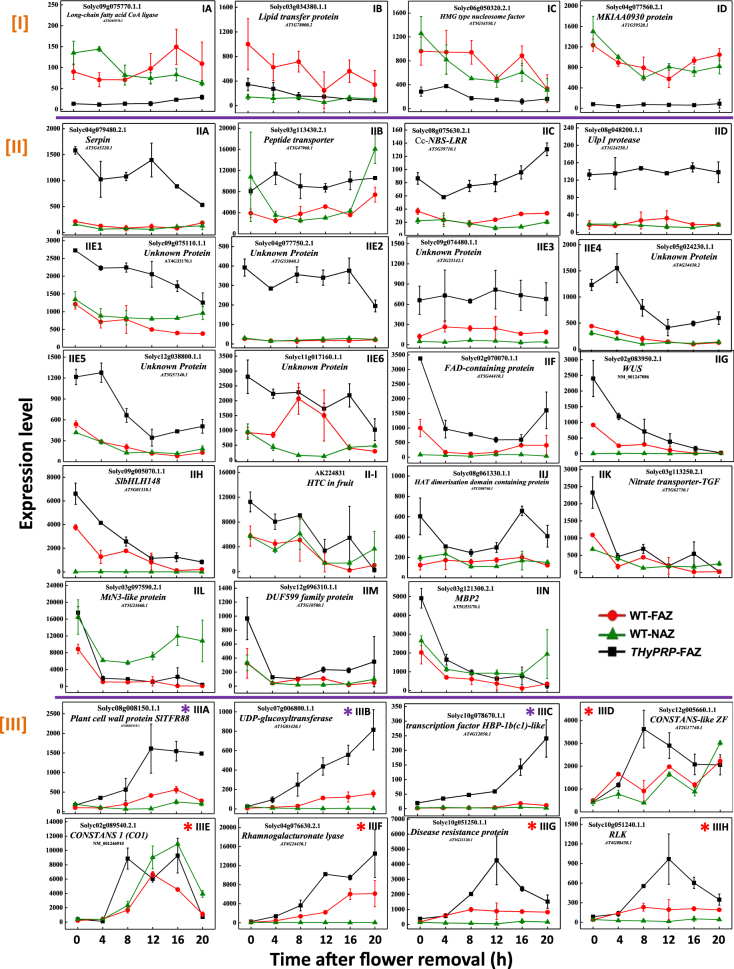
Line graphs showing the kinetics of changes in array-measured expression levels of genes that were specifically and continuously downregulated [I] or upregulated [II] at zero time and later on, or upregulated at 4 (blue*) or 8 (red*) h after flower removal [III] in the FAZ of *THyPRP-*silenced plants. *TAPG4::antisense THyPRP*-silenced line 11/generation T4 was used. Expression levels were measured for tomato *Long-chain fatty acyl-CoA synthetase* (**I**A); *Lipid transfer protein* (**I**B); *High Mobility Group* (*HMG*) *type nucleosome factor* (**I**C)**;** *MKIAA0930* (**I**D); *Serine protease inhibitor* (*Serpin*) (**II**A); *Peptide transporter* (**II**B); *Nucleotide Binding Site—Leucine-Rich Repeat* (*Cc-NBS-LRR*) (**II**C); *Ubiquitin-like protein1* (*Ulp1 protease*) (**II**D); *unknown proteins* (**II**E1–**E**6); *FAD-binding domain-containing protein* (**IIF**); *WUSCHEL-related homeobox-containing protein4 (WUS)* (**II**G); *SlbHLH transcription factor148* (**II**H); *HTC in fruit* (**II**–**I**); *HAT dimerization domain-containing protein* (**II**J); *Nitrate transporter-TGF* (**II**K); *MtN3-like protein* (**II**L); *DUF599 family protein* (**II**M); *Myrosinase-Binding protein2* (*MBP2*) (**II**N); *Plant cell wall protein SlTFR88* (**III**A); *Uridine 5′-diphospho (UDP)-glucuronosyltransferase* (**III**B); *Transcription factor HBP-1b(c1)-like* (**III**C); *CONSTANS-like ZF* (**III**D); *CONSTANS1* (*CO1*) TF (**III**E); *Rhamnogalacturonate endolyase* (**III**F); *Disease resistance protein* (**III**G); and defense-related *Receptor-Like protein Kinase* (*RLK*) (**III**H). Transcript identities are indicated in the graphs by their gene ID and their Arabidopsis (At) gene number and/or their nucleotide accession number. The results are means of two independent biological replicates ± SD

The downregulation of *THyPRP* expression in the FAZ of silenced plants already at zero time before flower removal (Fig. 1) resulted in altered expression of several genes in the FAZ at this time point. Four genes were downregulated at zero time in the FAZ of the transgenic plants and remained low during 20 h after flower removal (Fig. 5-I): *Long-chain Fatty acid CoA Ligase* (Fig. 5-IA); *Lipid Transfer Protein* (Fig. 5-IB); *High Mobility Group (HMG) Type Nucleosome Factor* (Fig. 5-IC), involved in chromatin remodeling that takes part in the regulation of gene transcription at the epigenetic level; and *MKIAA0930* *protein* (Fig. 5-ID) encoding a protein of an unknown function.

The expression of two TF genes, *WUSCHEL* (*WUS*) (Solyc02g083950.2.1) (Fig. 5-IIG) and *bHLH148* (Solyc09g005070.1.1) (Fig. 5-IIH), was specifically upregulated in the FAZ of *THyPRP-*silenced plants at zero time. Although the expression of these genes decreased in the FAZ after flower removal, it remained significantly higher in the silenced plants than in the WT during the first 8 h after flower removal. WUS is a homeodomain TF produced in the cells of the niche/organizing center of the shoot apical meristems. WUS specifies stem cell fate and also restricts its own level by activating a negative regulator, CLAVATA3, in adjacent cells of the central zone^57^. The tomato *WUS* homolog, *LeWUS*, was specifically expressed in the FAZ at anthesis, and was downregulated after flower removal^17,18,58^ and in the lines that do not differentiate AZs, JOINTLESS, and MACROCALYX-suppressed transformed plants^58^. This suggests that *LeWUS* expression in the FAZ might be involved in the regulation of the AZ development. Later studies suggested that *LeWUS* functions in the FAZ as a negative regulator of abscission^17,59^, which is in accordance with our results.

The *bHLH148* gene is predicted to encode a leucine-rich repeat receptor-like kinase (RLK) CLAVATA1 protein that is closely related to RLKs HAESA (HAE) and HAESALIKE2. It is well established that floral organ abscission in Arabidopsis is mediated by the small post-translational modified peptide ligand, INFLORESCENCE DEFICIENT IN ABSCISSION, which shares a similarity in key amino acids and post-translational modifications with CLAVATA3^60,61^. Our results, showing that *WUS* and *bHLH148* (*CLAVATA1*) were similarly upregulated in the FAZ following *THyPRP* silencing (Fig. 5-IIG,IIH), suggest a possible role of THyPRP in regulating pedicel abscission in tomato.

#### Epigenetic regulation

The gene encoding the HMG type nucleosome/chromatin assembly factor/HMG-box DNA-binding family protein (Solyc06g050320.2), involved in the regulation of gene transcription at the epigenetic level, was specifically downregulated in the *THyPRP*-silenced FAZ at zero time, and exhibited a low expression level during 20 h after flower removal (Fig. 5-IC). Unlike this, the expression of the *histone promoter-binding protein-1b(c1)-like TF* gene (Solyc10g078670.1.1) was gradually upregulated in the FAZ of the transgenic plants from zero time up to 20 h after flower removal (Fig. 5-IIIC, Supplementary Tables S3-S7). Since the expression of these two epigenetic genes in the FAZ of the *THyPRP*-silenced plants was altered very early, it is suggested that this modified expression may regulate a subsequent cascade of gene transcription, thereby resulting in the inhibition of pedicel abscission. The expression of HMG proteins is highly regulated and is affected by both developmental and environmental factors^62^.

#### Transport regulation—peptide transporters and protein targeting

**Fig. 6:**
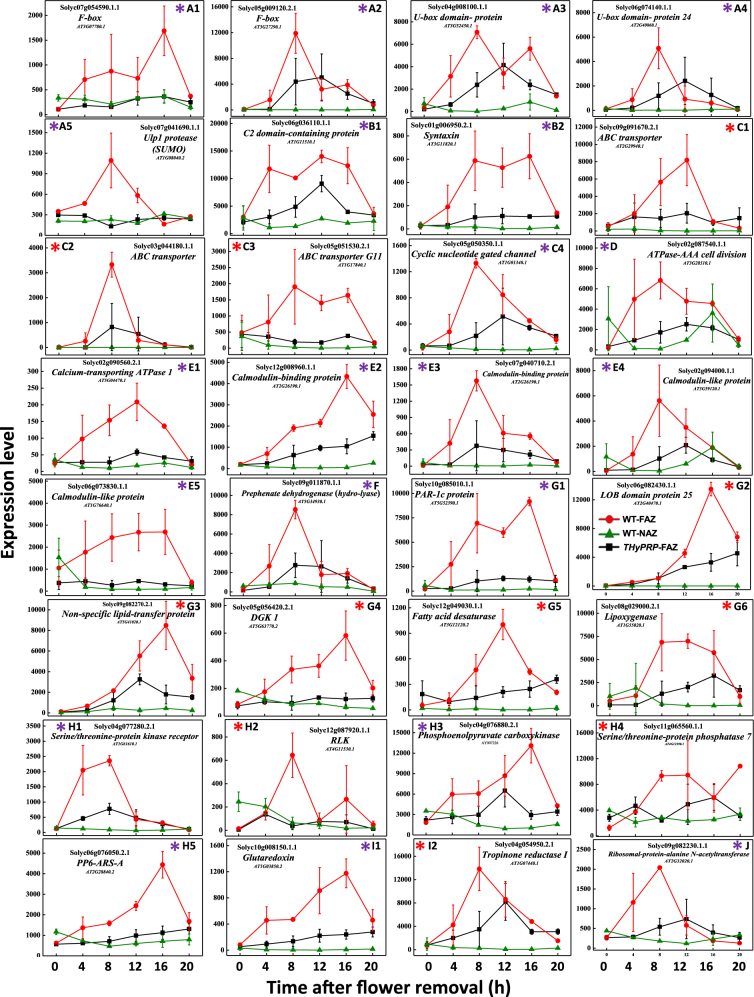
Effect of antisense silencing of *THyPRP* on the kinetics of changes in array-measured expression levels of genes that were specifically upregulated in the WT FAZ at various time points after flower removal: early—4 h (blue*) or 8 h (red*) or late 12–20 h (green*). *TAPG4::antisense THyPRP* silenced line 11/ generation T4 was used. Expression levels were measured for protein degradation–ubiquitin-related genes—*F-box* or *U-box* (**a**1–**a**5); genes related to transporters of macromolecules—*C2 domain-containing protein* (**b**1) and *Syntaxin* (**b**2); *ABC transporter* genes (**c**1–**c**3); *Cyclic nucleotide-gated ion channels* (**c**4); *ATPase-AAA cell division* (**d**); *Calcium-transporting ATPase1* (**e**1); *Calmodulin-binding protein* genes (**e**2,** e**3); *Calmodulin-like protein* genes (**e**4–**e**5); *Prephenate dehydrogenase hydrolyase* (**f**); Lateral organ boundaries (LOB) lipids and wax-related genes—*photoassimilate-responsive-1c* (*PAR-1c protein*) (**g**1); *LOB-domain protein25* (**g**2); *Non-specific lipid-transfer protein* (**g**3)*; Diacylglycerol kinase1* (*DGK1*) (**g**4); *Fatty acid desaturase* (**g**5); *Lipoxygenase* (**g**6); receptor kinase and protein phosphatase-related genes—S*erine/threonine-protein kinase receptor* (**h**1); *Receptor-Like protein Kinase* (*RLK*) (**h**2); *Phosphoenolpyruvate carboxykinase* (**h**3); *Serine/threonine-protein phosphatase7* (**h**4); *Serine/threonine-Protein Phosphatase6 regulatory Ankyrin Repeat Subunit A* (*PP6-ARS-A*) (**h**5); Redox regulation genes—*Glutaredoxin* (**i**1); *Tropinone reductase1* (**i**2); and *Ribosomal-protein-alanine N-acetyl-transferase* (**j**). Transcript identities are indicated in the graphs by their gene ID and their Arabidopsis (At) gene number, and/or their nucleotide accession number. The results are means of two biological independent replicates ± SD

A *peptide transporter* gene, that enables the directed movement of dipeptides into, out, within, or between cells, was specifically upregulated in the FAZ of the *THyPRP*-silenced plants at zero time, and its expression remained high during 20 h after flower removal (Fig. 5-IIB). An exocytosis-related gene, *Syntaxin*, upregulated between 4 and 16 h after flower removal in the WT FAZ, was inhibited in the *THyPRP*-silenced plants (Fig. 6b2). Syntaxins are a family of membrane integrated Q-SNARE proteins, which primarily mediate vesicle fusion with their target membrane-bound compartments participating in exocytosis^63^. It is well known that mobilization of the secretory pathway should enable the release of cell wall modifying enzymes to implement abscission^64,65^. Recently, the induction of several genes involved in vesicle trafficking, such as *SNARE-like* protein and *Syntaxin*, was demonstrated in the laminar AZ of abscising citrus leaves following a cycle of water stress/rehydration^66^. A sequential induction of genes encoding cell wall modifying enzymes, associated with the upregulation of genes involved in endocytosis and exocytosis during mature melon fruit abscission was reported^51^. Activation of vesicle trafficking involving small GTPases seems to be also required for cell wall modification during abscission of mature olive fruit^67^ and Arabidopsis floral organs^68–70^, as well as during self-pruning of spring shoots in sweet orange^71^. Our results suggest that THyPRP might regulate the secretion of cell wall modifying enzymes by exocytosis.

#### Other transporters

Two transporter genes, *nitrate transporter-TGF* (Fig. 5-IIK) and *Bidirectional sugar transporter SWEET12//MtN3-like protein* (Fig. 5-IIL), were upregulated in the FAZ of *THyPRP*-silenced plants at zero time, and their expression decreased to the levels found in the WT FAZ at 4 h after flower removal (Supplementary Table S3). Previous reports demonstrated the upregulation of *nitrate transporters* genes^15,51,67,72^ and *sugar transporters*, including the gene *Bidirectional sugar transporter SWEET12/MtN3-like*^15,67,73,74^, in various AZs during organ abscission induction and execution. Our results suggest that THyPRP might regulate the expression of these transporters in the tomato FAZ.

#### Post-translation regulation

The importance of post-translational regulation in plants is suggested by the observations that about 10% of the Arabidopsis genome is dedicated to protein ubiquitination and phosphorylation, two of the most frequent post-translational modifications^75,76^. The reversible conjugation of the Small Ubiquitin-related Modifier (SUMO) peptide to protein substrates (sumoylation) is arising as a major post-translational regulatory process in all eukaryotes, including plants^77–81^. Components of the SUMO conjugation and deconjugation systems are conserved in plants, including tomato^80^.

Our results indicate for the first time the possible involvement of SERPIN or SUMO proteins in regulating abscission. Thus, genes of a *serine protease inhibitor – SERPIN* (Solyc04g079480.2) (Fig. 5-IIA) and a *ubiquitin-like protein (ULP) SUMO protease1* (Solyc08g048200.1.1) (Fig. 5-IID) were specifically upregulated in the FAZ of the *THyPRP-*silenced plants at zero time, and their expression remained high during 20 h after flower removal. In contrast, the *Ulp1 protease* (*sumo*) gene was specifically upregulated in the WT FAZ at 4 h, peaked at 8 h, and remained high up to 12 h after flower removal, while in the *THyPRP*-silenced plants its expression remained at a low level, similar to that in the NAZ (Fig. 6a5, Supplementary Tables S3-S5). Each SERPIN with an inhibitory role is responsible for blocking the activity of one or more target proteins after binding, which induces an irreversible conformational change in the structure of the SERPIN, thereby disrupting its active site^82,83^.

Four protein ubiquitination-related genes (*F-box*, *U-box*) were specifically upregulated in the WT FAZ at 4 h, followed by a gradual increase in expression up to 8 or 16 h after flower removal (Fig. 6a1–a4). One of the *F-box* genes was completely inhibited in the transgenic plants (Fig. 6a1), while the expression of the other three was delayed, and was only partly inhibited in the *THyPRP*-silenced plants compared to the WT FAZ (Fig. 6a2–a4, Supplementary Tables S3-S7). Various genes related to selective ubiquitin-mediated protein degradation were specifically expressed in citrus leaf AZ after ethylene treatment^7^. Activity of the F-box protein HAWAIIAN SKIRT was linked to the control of petal abscission in Arabidopsis^84^, and a cysteine protease (*RbCP1*) gene was expressed in the petal AZ of rose flowers^85^. *RbCP1* expression increased during natural and ethylene-induced rose petal abscission, and this increase was inhibited by 1-MCP. A mutant of CORONATINE INSENSITIVE1, an F-box protein which serves as the JA co-receptor and previously defined as *Delayed Abscission4*, exhibited a delayed abscission phenotype in Arabidopsis^86^.

Protein phosphorylation and dephosphorylated are known for decades as major mechanisms for the transmission of stress signals^87,88^. Our results show that genes encoding several phosphorylation/dephosphorylation enzymes, such as Serine/threonine-protein kinase receptor (Solyc04g077280.2.1) (Fig. 6h1), Phosphoenolpyruvate carboxykinase (Solyc04g076880.2.1) (Fig. 6h3), and Serine/threonine-protein phosphatase6 regulatory ankyrin repeat subunitA (Solyc06g076050.2.1) (Fig. 6h5), were specifically upregulated in the WT FAZ starting at 4 h after flower removal, and this upregulation was inhibited in the *THyPRP*-silenced plants. The requirement of a MITOGEN-ACTIVATED PROTEIN kinase cascade and RLKs for abscission induction and execution was reported and extensively reviewed^13,89^. In a recent proteomic study, changes in proteins and phosphoproteins were examined in the tomato FAZ following ethylene and 1-MCP treatments^90^. A total of 450 phosphopeptides were detected (out of the 1429 quantified proteins), and the expression of 85 of them, corresponding to 73 phosphoproteins, was significantly modified by ethylene. These data demonstrate the unique features of the AZ phospho-proteomics, thereby suggesting the involvement of a complex network of kinase-substrate and phosphatase-substrate interactions in response to ethylene.

Another post-translational regulator gene that might be involved in abscission is *ATPase-AAA cell division*, which was upregulated between 4 and 16 h after flower removal in the WT FAZ, and its increased expression was inhibited in the *THyPRP*-silenced plants (Fig. 6d). The AAA modules were shown to behave as machines for folding/unfolding polypeptides, dissociation of protein–protein interactions, and production of unidirectional motion along tracks in which a usual feature is the assembly of its subunits to hexameric or heptameric rings^91^. Since the ATPase-AAA was inhibited in the silenced plants, it may indicate that the cell division and post-translational functions are involved in the abscission process.

#### Signal transduction regulation

Our results show that seven Ca-Calmodulin (Ca^2+^/CaM)-related genes with a similar expression pattern were specifically upregulated between 4 and 8 or at 16 h after flower removal in the WT FAZ, and were inhibited in the *THyPRP*-silenced plants: *Cyclic nucleotide-gated channel* (Fig. 6c4); *Calcium-transporting ATPase1* (Fig. 6e1); two *CaM-binding proteins* (Fig. 6e2,e3); two *CaM-like proteins* (Fig. 6e4,e5); and a *C2 domain-containing protein* (Fig. 6b1, Supplementary Tables S3-S7). C2 domain-containing proteins bind lipids and can regulate many cellular processes^92^. Other C2 domains were reported to act as modules for protein–protein interactions, or were suggested to play a critical role in protein localization^93^. These functions suggest that these proteins might be involved in the abscission process.

The Ca^2+^/CaM signal transduction plays a role in abscission processes. In citrus leaf abscission induced by a cycle of water stress/rehydration, a *CaM* gene was upregulated in the laminar AZ 1 h after rehydration^66^. During ethephon-induced litchi fruitlet abscission, 52 transcripts related to Ca^2+^ transport and perception displayed altered expression, among them, 19 and 33 genes were upregulated and downregulated, respectively^94^. Additionally, *CaM*, *CML*, and *Calcium-binding protein kinase* genes were upregulated in the AZ during mature olive fruit abscission^67^. These data suggest that Ca^2+^/CaM signaling plays an important role in the regulatory pathways of organ abscission. Our results, demonstrating an inhibition of the upregulation of a large number of Ca^2+^/CaM signaling-related genes in the *THyPRP*-silenced plants, suggest that THyPRP plays a significant role in regulating the Ca^2+^/CaM signal transduction in tomato pedicel abscission.

### Role of *THyPRP* in the regulation of pedicel abscission induced by flower removal

The aim of this study was to elucidate the possible role of THyPRP in regulating pedicel abscission induced by flower removal, by performing a transcriptome analysis of the FAZ in the *THyPRP*-silenced plants vs. the WT. In two previous transcriptome microarray analyses of the tomato FAZ vs. the NAZ, numerous genes were specifically expressed at zero time in the FAZ, including genes encoding TFs, hormone-related proteins, cell wall-modifying enzymes, lipid metabolism enzymes, and others^17,18^. *THyPRP* (*TPRP-F1*) was one of the specifically expressed genes at zero time in the tomato FAZ^15^, and we show here that its antisense silencing significantly inhibited tomato pedicel abscission induced by flower removal (Fig. 2).

**Fig. 7:**
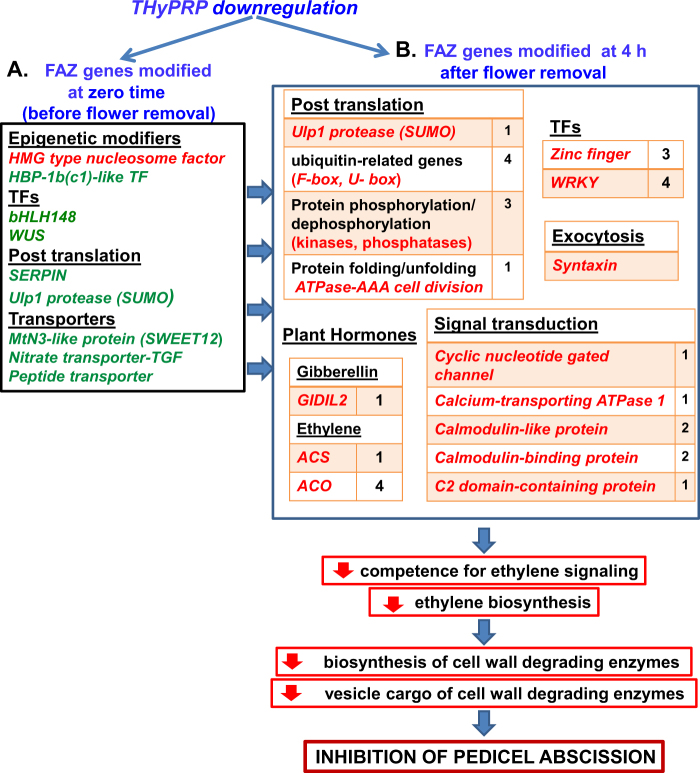
Summary of the genes modified in the FAZ of *TAPG4::antisense THyPRP* plants before (at zero time) and at 4 h after flower removal in response to *THyPRP* downregulation and abscission induction by flower removal, thereby leading to decrease of the postulated events resulting in the inhibition of pedicel abscission. Genes that were specifically upregulated or downregulated in the FAZ of *THyPRP-*silenced plants compared to the WT are marked in Green or Red, respectively. The numbers listed besides the gene names indicate the number of genes in the gene family that were affected in *THyPRP-*silenced plants

Figure 7 summarizes the postulated events leading to the inhibition of tomato pedicel abscission in the *THyPRP*-silenced plants. This sequence of events is based on regulatory genes whose expression was altered in the FAZ of the silenced plants at zero time and early (0–4 h) after flower removal, compared to their expression in the WT FAZ. The role of these regulatory genes, which are active at different regulatory levels in the abscission process, was described and discussed above.

The *TAPG4* promoter was found to be a very good candidate for controlling gene silencing in the FAZ, since *THyPRP* expression in the silenced plants already decreased at zero time. Consequently, alternation in the expression of regulatory genes, including epigenetic modifiers, TFs, post-translational regulators, and transporters, already occurred before flower removal (Fig. 7a). We detected genes that were specifically upregulated in the WT FAZ at 4 h after flower removal, and their expression continued to increase later on for different periods (Fig. 7b). These upregulated genes, including genes related to ethylene biosynthesis, GA perception, TFs, post-translational regulation, and exocytosis, were inhibited in the FAZ of the *THyPRP*-silenced plants. Downregulation of the ethylene biosynthesis genes (*ACS*, *ACO*) in the *THyPRP-*silenced plants (Fig. 3b1–b5) probably leads to reduced ethylene production in the FAZ of these plants at 4 h, and to downregulation of other genes listed in Fig. 7b, which delay and inhibit the acquisition of the competence of the FAZ cells to respond to ethylene signaling. This inhibition could have resulted from altered expression of regulatory genes at zero time (Fig. 7a), or from the low expression of *THyPRP* during the 4-h period after flower removal (Fig. 1). These two effects, resulting from the *THyPRP* silencing, lead in turn to downregulation of the genes involved in abscission execution (Supplementary Figures. S2, S3), finally resulting in the inhibition of the abscission phenotype (Fig. 2).

Our data suggest that the effect of *THyPRP* silencing on pedicel abscission was not mediated by its effect on auxin balance, but by decreased ethylene biosynthesis and response. Additionally, *THyPRP* silencing revealed new players, which were demonstrated for the first time to be involved in regulating pedicel abscission processes. These include: GA-perception; Ca^2+^/CaM signaling; SERPINS and SUMO proteins involved in post-translational modifications; Synthaxin and SNARE-like proteins, which participate in exocytosis, necessary for cell separation. Taken together, our results suggest that THyPRP is a master regulator of pedicel abscission in tomato, predominantly by playing a role in the regulation of the FAZ competence to respond to ethylene signals.

## Electronic supplementary material

Supplementary Information-Figs S1-S4-Table S1

Table S2

Table S3

Table S4

Table S5

Tabls S6

Table S7
