# Supplementary material for: The Tomato Hybrid Proline-rich Protein regulates the abscission zone competence to respond to ethylene signals
Source: Hortic Res. 2018 Jun 1;5:28. doi: 10.1038/s41438-018-0033-2 (PMC5981600; doi:10.1038/s41438-018-0033-2)
Supplement: Supplementary file 1 — Supplementary Information-Figs S1-S4-Table S1 [file 41438_2018_33_MOESM1_ESM.pdf]

Supplementary Material

The Tomato Hybrid Proline-Rich Protein regulates the abscission zone competence to respond to ethylene signals

By

Srivignesh Sundaresan, Sonia Philosoph-Hadas, Chao Ma, Cai-Zhong Jiang, Joseph Riov, Raja Mugasimangalam, Betina Kochanek, Shoshana Salim, Michael S. Reid and Shimon Meir

Figure S1

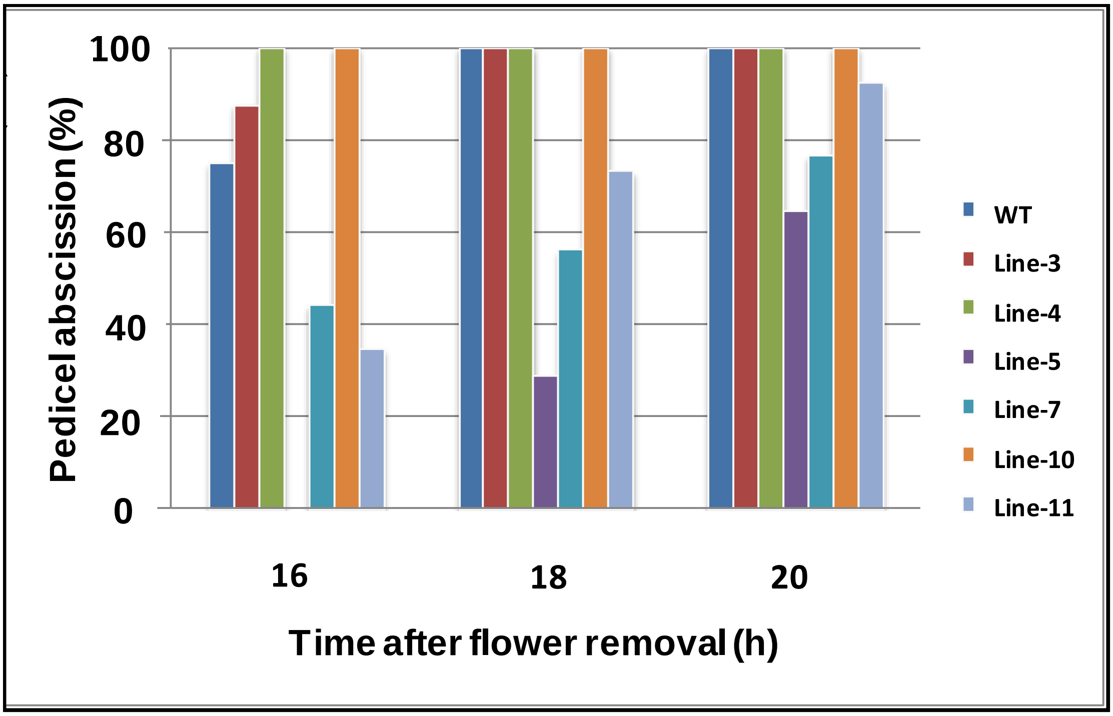

Figure S1. Screening of the kinetics of flower pedicel abscission following flower removal in several antisense silenced lines of the *THyPRP* gene. Flower explants were prepared and handled as previously described [15, 34]. Wild type (WT) plants (cv. NY) and six silenced lines generation T1 were examined. Each transgenic line contained 4-6 plants, and 3-5 flowers in the right stage were harvested from each plant for abscission assay. The percentage of accumulated pedicel abscission was monitored at 16, 18, and 20 h after flower removal. The results are means of all explants per line (n = 16-24 explants).

Figure S2

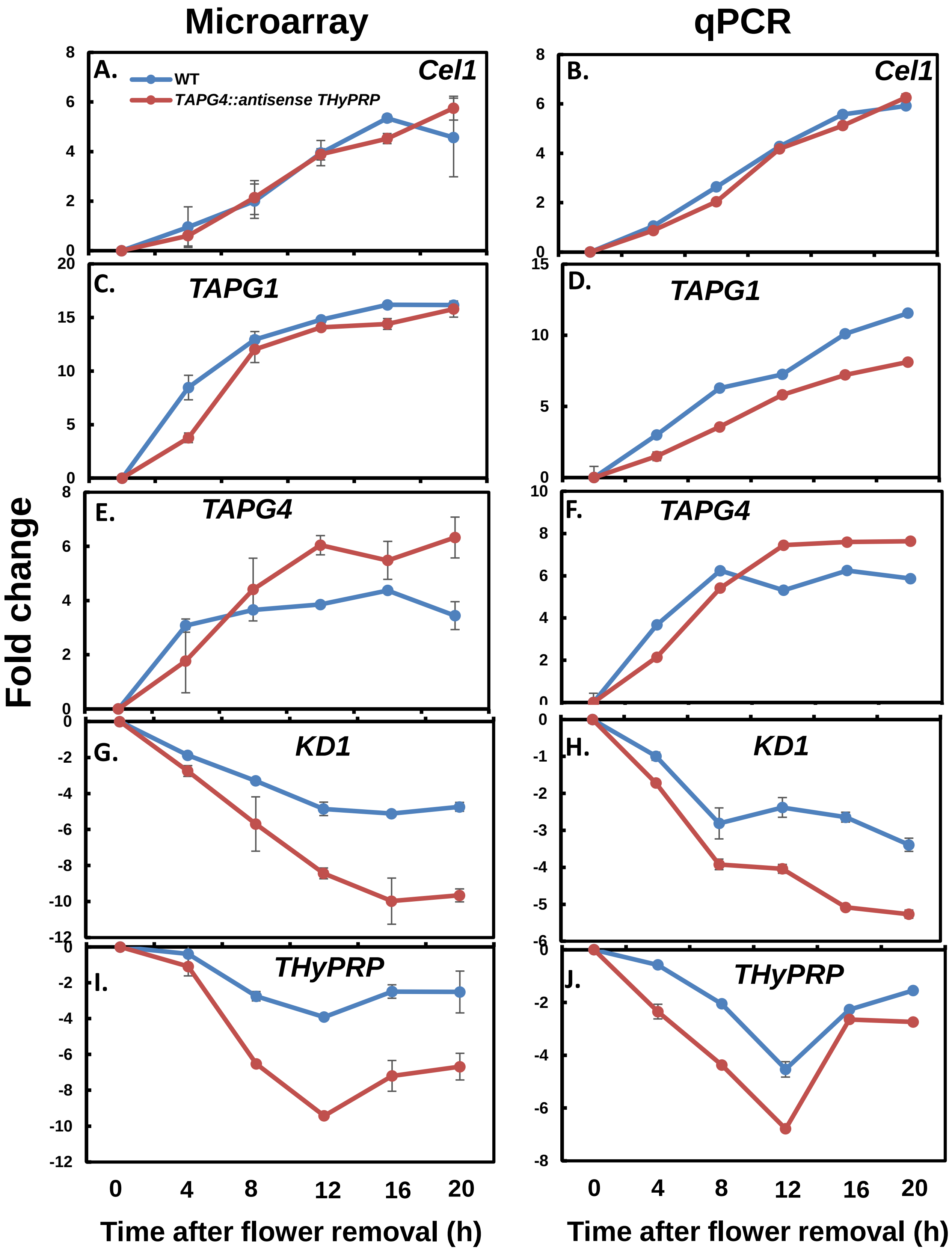

Microarray
qPCR
A.
WT
TAPG4::antisense THyPRP
Cel1
B.
Cel1
C.
TAPG1
D.
TAPG1
E.
TAPG4
F.
TAPG4
G.
KD1
H.
KD1
I.
THyPRP
J.
THyPRP
Fold change
0
4
8
12
16
20
Time after flower removal (h)
0
4
8
12
16
20
Time after flower removal (h)

Figure S2. Validation by qPCR (B, D, F, H, J) of the microarray results (A, C, E. G, I) of the kinetics of changes in expression patterns of selected genes in the FAZ of WT and antisense silenced *THyPRP* plants following abscission induction by flower removal. *TAPG4::antisense THyPRP*-silenced line 11/generation T4 was used. Expression levels were measured for tomato *Cellulase1* (*Cel1*) (ID - U13054/Solyc08g081620) (A, B); *Tomato Abscission Polygalacturonase1* (*TAPG1*) (ID - U23053/Solyc02g067630); (C, D); *TAPG4* (ID - U70481/Solyc12g096750) (E, F); *knotted1-like homeobox protein (KD1*) (ID - AF375969/Solyc06g072480) (G, H); and *THyPRP* (ID - X57076/Solyc07g043000) (I, J). The data indicate the fold change relative to time zero. The relative quantification of the gene expression level in the qPCR assay was determined by the comparative C_T method $2^{-\Delta\Delta C}T$ using *ACTIN* as a reference gene, and then calculating the fold change value. The ΔCT values for each gene were compared to the zero time ΔCT value for that gene to generate the $2^{-\Delta\Delta C}T$ values. The microarray and qPCR analyses were performed with different samples taken from independent biological replicates of two separate experiments. The results are means of two biological replicates ± SD.

Figure S3

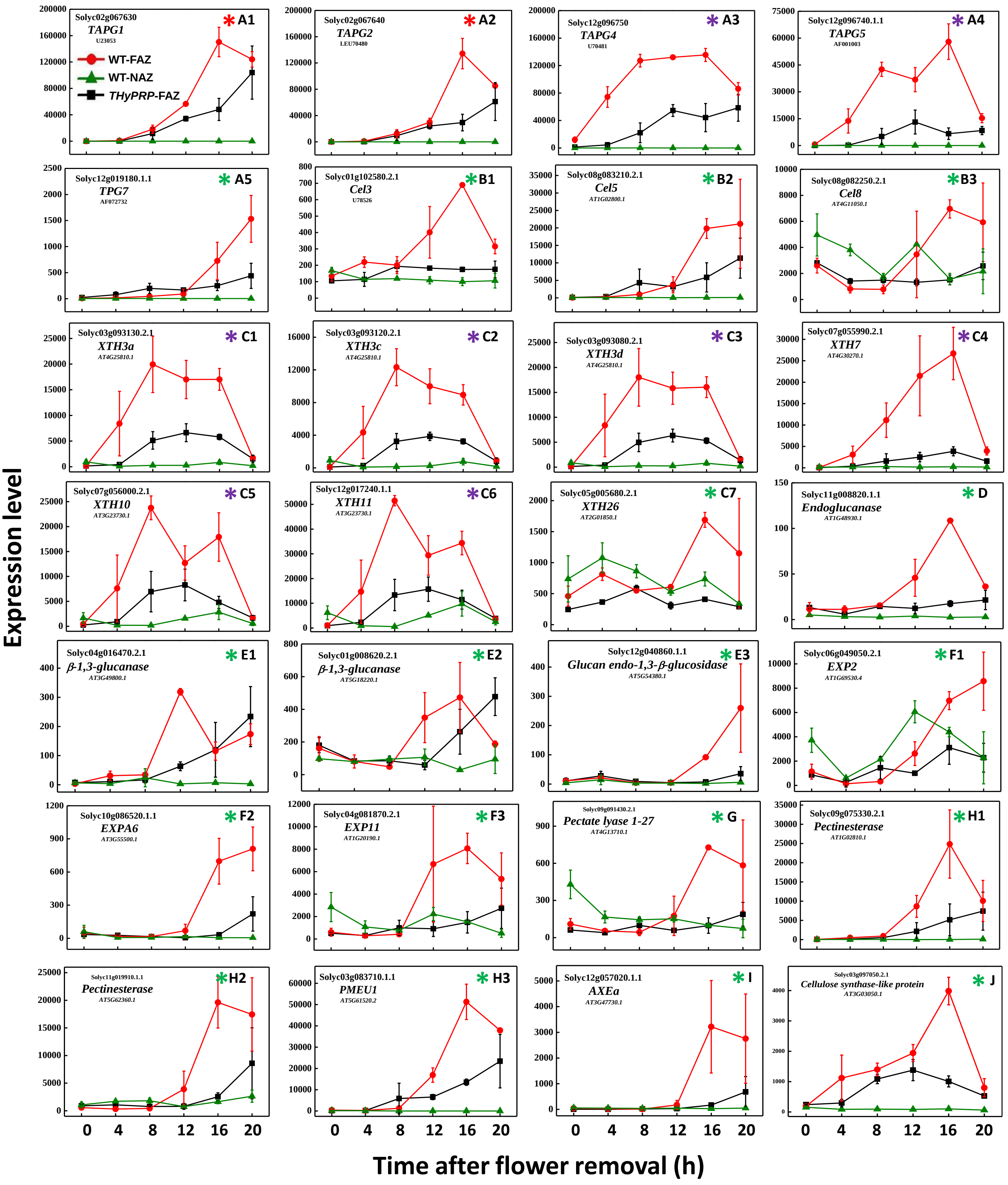

WT-FAZ
WT-NAZ
THyPRP-FAZ
Solyc02g067630 TAPG1 U23053 A1
Solyc02g067640 TAPG2 LEU70480 A2
Solyc12g096750 TAPG4 U70481 A3
Solyc12g096740.1.1 TAPG5 AF001003 A4
Solyc12g019180.1.1 TPG7 AF072732 A5
Solyc01g102580.2.1 Cel3 U78526 B1
Solyc08g083210.2.1 Cel5 AT1G02800.1 B2
Solyc08g082250.2.1 Cel8 AT4G11050.1 B3
Solyc03g093130.2.1 XTH3a AT4G25810.1 C1
Solyc03g093120.2.1 XTH3c AT4G25810.1 C2
Solyc03g093080.2.1 XTH3d AT4G25810.1 C3
Solyc07g055990.2.1 XTH7 AT4G30270.1 C4
Solyc07g056000.2.1 XTH10 AT3G23730.1 C5
Solyc12g017240.1.1 XTH11 AT3G23730.1 C6
Solyc05g005680.2.1 XTH26 AT2G01850.1 C7
Solyc11g008820.1.1 Endoglucanase AT1G48930.1 D
Solyc04g016470.2.1 β-1,3-glucanase AT3G49800.1 E1
Solyc01g008620.2.1 β-1,3-glucanase AT5G18220.1 E2
Solyc12g040860.1.1 Glucan endo-1,3-β-glucosidase AT5G54380.1 E3
Solyc06g049050.2.1 EXP2 AT1G69530.4 F1
Solyc10g086520.1.1 EXPA6 AT3G55500.1 F2
Solyc04g081870.2.1 EXP11 AT1G20190.1 F3
Solyc09g091430.2.1 Pectate lyase 1-27 AT4G13710.1 G
Solyc09g075330.2.1 Pectinesterase AT1G02810.1 H1
Solyc11g019910.1.1 Pectinesterase AT5G62360.1 H2
Solyc03g083710.1.1 PMEU1 AT5G61520.2 H3
Solyc12g057020.1.1 AXEa AT3G47730.1 I
Solyc03g097050.2.1 Cellulose synthase-like protein AT3G03050.1 J
Expression level
Time after flower removal (h)
0 4 8 12 16 20

<u>Figure S3.</u> Effect of antisense silencing of *THyPRP* on the kinetics of changes in array-measured expression levels of genes coding for **cell wall modifying enzymes** that **were** specifically upregulated in the WT FAZ at various time points after flower removal: early - 4 (*) or 8 (*) or late 12-20 (*) h. The *TAPG4::antisense THyPRP*-silenced line 11/generation T4 was used. Expression levels were measured for *Tomato abscission polygalacturonase* (*TAPG*) genes **(A1-A5)**; *Cellulase* (*Cel*) genes **(B1-B3)**; *Xyloglucan endotransglucosylase/hydrolase* (*XTH*) genes **(C1-C7)**; *Endoglucanase* **(D)**; *β-1,3-glucanase* genes **(E1-E3)**; *Expansin* (*EXP*) genes **(F1-F3)**; *Pectate lyase1-27* **(G)**; *Pectinesterase* genes **(H1-H2)**: *Pectin methyl esterase inhibitor U1* (*PMEU1*) **(H3)**; *Acetyl xylan esterase a* (*AXEa*) **(I)**; and *Cellulose synthase-like protein* **(J)**. Transcript identities are indicated in the graphs by their gene ID and their Arabidopsis (At) gene number, and/or their nucleotide accession number. The results are means of two biological replicates ± SD.

Figure S4

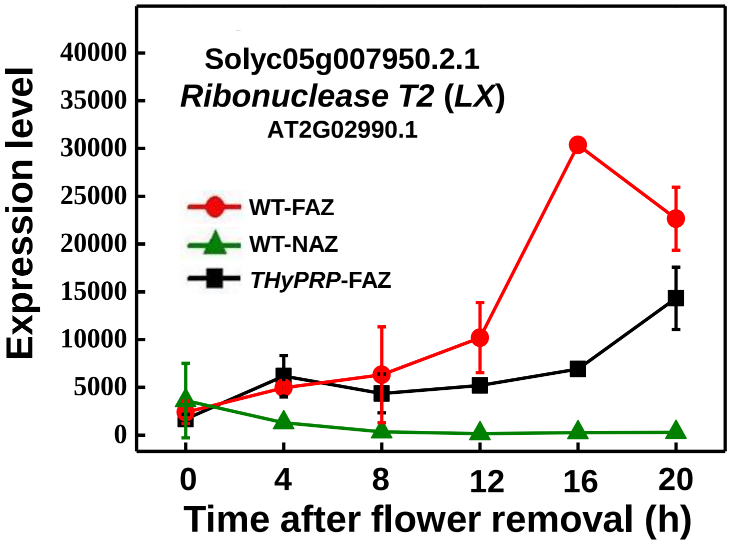

<u>Figure S4.</u> Effect of antisense silencing of *THyPRP* on the kinetics of changes in array-measured expression levels of *Ribonuclease T2* (*LX*) gene that was specifically upregulated in the WT FAZ at the late stages (12-20 h after flower removal) of abscission. The *TAPG4::antisense THyPRP*-silenced line 11/ generation T4 was used. The results are means of two biological replicates ± SD.

Table S1: List of primers and their sequences used in the qPCR assay.

No	Primer name	Gene bank ID	Solyc ID	Primer sequence (5' to 3')	Tm (°C)
1	Actin_F	AB199316	Solyc03g078400	GTGTTGGACTCTGGTGATGG	60
2	Actin_R			GTAGTCAAGAGCCACATAAGC	60
3	TAPG1_F	U23053	Solyc02g067630	GCAGTGAAACTTGATTGTAGC	56
4	TAPG1_R			CCATTCTTGTGATAGTATACAC	56
5	TAPG4_F	U70481	Solyc12g096750	GAACATCAGCTACAGAAATCG	60
6	TAPG4_R			ACCAGAAGCTCTTCCTCCAG	60
7	Cel1_F	U13054	Solyc08g081620	GGATTAATGCCAGAAAGTAGC	60
8	Cel1_R			GTAACTCCTCCTATTCCAGC	60
9	TPRP-F1_F	X57076	Solyc07g043000	GCACAACTATTAGACTCAAGC	60
10	TPRP-F1_R			TGCCTTCAACTATGACAATGC	60
11	KD1_F	AF375969	Solyc06g072480	CTCACTCACAATGGATCAACC	60
12	KD1_R			GGAGTGGAAGTAGAAGTAGG	60
