# Supplementary material for: The Tomato Hybrid Proline-rich Protein regulates the abscission zone competence to respond to ethylene signals
Source: Hortic Res. 2018 Jun 1;5:28. doi: 10.1038/s41438-018-0033-2 (PMC5981600; doi:10.1038/s41438-018-0033-2)
Supplement: Supplementary file 2 — Table S2 [file 41438_2018_33_MOESM2_ESM.pdf]

Table S2. Differentially regulated genes (up and down regulated) in the control compared with THyPRP silenced plants in FAZ at 0 h along with their expression values, fold changes, annotations, GO ids and terms.

Worksheet 2 & 3

Samples

Set1				
Line	Array	Details	Time	Analysis
Control – New Yorker	254331010025_1_1	0h_1_AZ	0h	Control
Control – New Yorker	254331010025_1_2	0h_2_AZ	0h	
TAPG + TPRP	254331010034_1_1	0h_37_AZ	0h	Treated
TAPG + TPRP	254331010034_1_2	0h_38_AZ	0h	

Note:

Fold change expression values are provided as logbase 2

Flagging Criteria:

Compromised: If the gProcessedSignal value is less than the background intensity

Detected: If the gProcessedSignal value is above background intensity

Column Header	Description
ProbeName	Unique probe identifier
GeneName	GeneSymbol
Fold	Fold change calculated is log base 2
Flag	Detected or Compromised
GeomeanFold	Average Fold change for the replicates calculated is log base 2
p-Value	t-test p- value
gProcessedSigna	Background substracted signal intensity

Cut off used to filter up and down regulated genes

Upregulated

For filtering upregulation we consider flag should be Detected in the treated sample and can be Compromised or Detected in the control sample and fold>=0.8 in the individual replicates and fold>=1 in the Geomean of treated samples.

Downregulated

For filtering downregulation we consider flag can be Detected or Compromised in the treated sample and should be Detected in the control sample and fold<=-0.8 in the individual replicates and fold<=-1 in the Geomean of treated samples.

Differentially Regulated Probes

Sample	Up	Down
0h_TPRP_ AZ vs. 0h_Control_AZ_Sense	1208	416
0h_TPRP_ AZ vs. 0h_Control_AZ_Antisense	862	216

Worksheet 4 & 5

This file contains the fold change for all the spots in the array

GO ID

Gene Ontology ID

GO ACCESSION

Gene Ontology Function name

p-value :

The probability of obtaining the specified GO accession number from a list of random entities. Less the p-value more significant is the GO accession number.

Significant pvalue<0.05

Count in Selection :

This refers to the number of genes in the selected entity (for example, from T-test) list which have that particular GO term.

%Count in Selection :

This refers to the percentage of genes in the input entity list which have that GO term.

Count in Total :

This refers to the number of genes in All Entities which have that GO term.

%Count in Total :

This refers to the percentage of genes in the All Entities list which have that GO term.

		0h_AZ_Control					0h_AZ_TAPG + TPRP vs. 0h_AZ_Control																															
Orientatio	ProbeNar	Fold 0h	Flag 0h	Fold 0h	Flag 0h	an 0h AZ	Fold 0h	Flag 0h	Fold 0h	Flag 0h	Geomean	P-value	ProbeNar	GeneNam	Systemat	Descripti	Sequence	Contig ID	ITAG ID	Parent Ge	ITAG Des	Ontology	Ontology	Contig ID	Target ID	Functiona	GO term	Transcrip	target ID	Symbols	TAIR Des	TAIR Chr	gProcess	gProcess	gProcess	gProcess	gProcess	gProcessedSignal 0h 38 AZ
Sense	A 96 P0(	0.025	Detected	-0.025	Detected	0.000	0.941	Detected	1.264	Detected	1.102	0.021	A 96 P0(	A 96 P0(	A 96 P0(	Unknown	TCACTTGCCAAGACATTAACACAACTCATGCTTATTTGAAATCAGGTACATTTTGCTACA																2636.56	2086.03	2196.38	1477.18	5184.47	6364.83
Sense	A 96 P01	-0.166	Detected	0.166	Detected	0.000	0.916	Detected	1.441	Detected	1.179	0.063	A 96 P01	wus	NM 0012	Solanum	GTTTGTAGTAAGTAAGTACTAATCTAATTTGGTATGTGCCAAGCTATTTGGACCTTATGG																902.021	930.293	700.468	707.556	1990.64	2810.61
Sense	A 96 P0(	-0.048	Detected	0.048	Detected	0.000	1.161	Detected	1.678	Detected	1.420	0.033	A 96 P0(	AK32256	AK32256	Solanum	GAACTTTCGTTTTTTCTGATTCAATTCCATGGTTTGAATGGATGGATATTGGTGGACATA																155.777	136.412	94.1587	173.392	375.292	527.288
Sense	A 96 P07	0.602	Detected	-0.602	Detected	0.000	2.014	Detected	1.643	Detected	1.829	0.101	A 96 P07	P69B	NM 0012	Solanum	GCCTAGTCAAAAATTGGCTATATAAGTGCATTAAGTACTCGGACATTTGAAAATGCTGTT																2332.77	829.649	622.174	3088.22	6471.18	4909.49
Sense	A 96 P07	-0.271	Detected	0.271	Detected	0.000	1.806	Detected	1.978	Detected	1.892	0.022	A 96 P07	LOC5439	AK24724	Solanum	GATGGCATGCTAATCACTATAATGTGGGCATCAAAGTTGTGTTATGTGTAACTACTTATT																1833.86	2186.84	3492.19	1405.56	8062.78	8912.85
Sense	A 96 P08	-1.077	Detected	1.077	Detected	0.000	0.806	Detected	1.500	Detected	1.153	0.416	A 96 P08	BW68867	BW68867	BW68867	ACTGGGCCGGAAAACGTCCTTATTAAGGTGTCGTTTATTTATGACATATTGCTAGTATTA																153.135	558.428	207.862	1506.18	588.592	934.671
Sense	A 96 P08	1.103	Detected	-1.103	Detected	0.000	1.592	Detected	1.157	Detected	1.375	0.346	A 96 P08	ODD	NM 0012	Solanum	AAATCTCTTGCAGCAAATTGGAGAGACTCACTTTACTCTGTCATGGCTCCTAATCCTGCT																77.4298	13.7509	24.4984	15.9711	113.3	82.2612
Sense	A 96 P08	-1.366	Detected	1.366	Detected	0.000	1.275	Detected	3.624	Detected	2.449	0.307	A 96 P08	TA36002	TA36002	Rep: Path	CCACGTACCATATGTTCATGGTATCGTGGCTTAGGTTCTTTACTTTTTCAAAATTATGAA																24.3014	132.179	35.072	232.937	157.936	789.477
Sense	A 96 P11	0.125	Comprom	-0.125	Comprom	0.000	1.402	Detected	1.459	Detected	1.431	0.008	A 96 P11	TA53569	TA53569	Unknown	CCTGTTAGGGCATAGCTTATCTCTATATATAGACCCTATGGGAAACCCTATTCTGTAATT																5.40343	3.72359	8.74962	3.0464	13.6518	13.9338
Sense	A 96 P11	-0.506	Detected	0.506	Detected	0.000	2.097	Detected	0.956	Detected	1.527	0.183	A 96 P11	AK32877	AK32877	Solanum	TCTTGTCATTTGCTGTTGTGGTATCTTGCAGCTGCTGGTTTACCTATGCCATGCTAGGAA																84.4235	139.55	134.506	92.7452	534.855	237.997
Sense	A 96 P12	1.446	Detected	-1.446	Comprom	0.000	1.797	Detected	1.126	Detected	1.462	0.429	A 96 P12	TA55074	TA55074	Rep: Sulf	CACATTCTTCACTGGGGCATTTCAAGCTGTTTTTGGACTATTCAGGTTGGGATTTCTAGT																21.2044	2.34056	20.6679	19.1242	28.1874	17.3799
Sense	A 96 P12	0.182	Detected	-0.182	Comprom	0.000	2.045	Detected	1.645	Detected	1.845	0.021	A 96 P12	NP92874	NP92874	GB	GCTGCCATCAGATGGAGAAAATTTCAAGCAGCAAAGACAGTGACCAATTGTCCAAATTGA																15.2443	9.70133	8.91089	16.1915	57.7907	42.989
Sense	A 96 P12	0.262	Detected	-0.262	Comprom	0.000	2.240	Detected	0.977	Detected	1.609	0.143	A 96 P12	NP92874	NP92874	GB	CTCACGAGTCTGCTCGGATAATATACATGAGGACATGATTGACTGTAGTATTAACAAGAA																14.1233	8.04108	10.206	23.6341	57.9718	23.7073
Sense	A 96 P13	-1.299	Detected	1.299	Detected	0.000	1.368	Detected	1.487	Detected	1.427	0.387	A 96 P13	LOC5440	BI931665	EST5515	GTTGTGTTAGTATTGGCCTATAGTAGCCATTTGACACATTAAATAAGTTTGTGACACATC																58.2207	288.864	199.307	97.3511	385.315	410.718
Sense	A 96 P13	-0.036	Detected	0.036	Detected	0.000	1.043	Detected	1.504	Detected	1.274	0.032	A 96 P13	AI487256	AI487256	Unknown	CTTAGCAATGTGTTAAACACATTAAAGTCTTCAACTTTCATGGCCGCTATGACTTTAGAT																1850.38	1593.33	1591.93	683.283	4073.08	5504.95
Sense	A 96 P13	0.388	Detected	-0.388	Comprom	0.000	1.041	Detected	1.179	Detected	1.110	0.106	A 96 P13	AI771618	AI771618	Unknown	TGCATTTGGGTGTATACTCATGGCACTTGTAGCAAAGTTGGATTACTTGGTATGGGTAAG																20.6785	9.89029	20.6314	24.7289	33.8809	36.5886
Sense	A 96 P13	-0.199	Comprom	0.199	Comprom	0.000	1.167	Detected	1.116	Detected	1.142	0.030	A 96 P13	AI776978	AI776978	Unknown	ACATGCCCATGTTCATTCCATTCCATGTCCTGTTTGTAAAATTTCCCTATTTTAGCTCCC																8.82688	9.52707	7.65367	12.2373	23.7173	22.4575
Sense	A 96 P13	-0.764	Detected	0.764	Detected	0.000	0.822	Detected	1.495	Detected	1.159	0.300	A 96 P13	AI779441	AI779441	Unknown	CTGTATCGGACAACGTCCTTACTAAAATGATGTATACTTATGACATGTTGCTAGTATTGA																7862.43	18577.3	7445.95	34805.3	24609.4	38509.7
Sense	A 96 P13	-0.673	Detected	0.673	Detected	0.000	1.828	Detected	2.248	Detected	2.038	0.102	A 96 P13	AI781976	AI781976	Unknown	ATATTGTGCACCTAAAATGCCACGTTCCAGATTATACTCAAGTAGTCTGGCGCAACTCAG																224.583	467.804	189.108	807.831	1325.08	1740.27
Sense	A 96 P14	-0.227	Detected	0.227	Detected	0.000	1.795	Detected	0.836	Detected	1.315	0.131	A 96 P14	AW03262	AW03262	Unknown	ATCAAATAAATCGCGAGCATGGAAGCCAGTTTTGTCTACCATTAGAGAATGGTCGGGTTT																33.6713	37.7639	20.3629	25.0759	142.458	71.9286
Sense	A 96 P16	0.598	Detected	-0.598	Comprom	0.000	2.142	Detected	1.975	Detected	2.058	0.076	A 96 P16	AW65032	AW65032	Unknown	ATGTTGGATCCGACTTCTAATTCAAGAGTCACTACTTCAAAGATCATCGATTCTTCATGG																14.3682	5.13763	12.3608	8.36146	43.6625	38.1631
Sense	A 96 P18	-0.373	Detected	0.373	Detected	0.000	0.911	Detected	1.326	Detected	1.119	0.120	A 96 P18	BG12772	BG12772	Unknown	TGGAGGTGATACTCAGTGCTTGGTTACTGGCTATTCTGATTCAGACATGCTGGAGATGTT																90.9081	124.972	366.603	206.738	230.852	302.059

Sense	GT Sens	-0.747	Comprom	0.747	Comprom	0.000	1.655	Detected	1.316	Detected	1.485	0.192	GT Sens
Sense	GT Sens	-0.539	Comprom	0.539	Detected	0.000	1.248	Detected	1.329	Detected	1.288	0.140	GT Sens
Sense	GT Sens	0.159	Detected	-0.159	Detected	0.000	1.293	Detected	0.904	Detected	1.098	0.049	GT Sens
Sense	GT Sens	1.636	Detected	-1.636	Comprom	0.000	0.934	Detected	2.560	Detected	1.747	0.440	GT Sens
Sense	GT Sens	0.000	Detected	0.000	Comprom	0.000	0.987	Detected	1.125	Detected	1.056	0.004	GT Sens
Sense	GT Sens	-0.890	Detected	0.890	Detected	0.000	1.353	Detected	1.861	Detected	1.607	0.225	GT Sens
Sense	GT Sens	1.744	Detected	-1.744	Detected	0.000	1.255	Detected	2.920	Detected	2.088	0.393	GT Sens
Sense	GT Sens	-0.129	Comprom	0.129	Comprom	0.000	1.085	Detected	1.240	Detected	1.162	0.016	GT Sens
Sense	GT Sens	0.975	Detected	-0.975	Detected	0.000	4.488	Detected	2.001	Detected	3.245	0.176	GT Sens
Sense	GT Sens	-0.149	Comprom	0.149	Comprom	0.000	1.721	Detected	1.722	Detected	1.721	0.007	GT Sens
Sense	GT Sens	-0.277	Comprom	0.277	Comprom	0.000	2.074	Detected	1.725	Detected	1.899	0.028	GT Sens
Sense	GT Sens	0.194	Detected	-0.194	Detected	0.000	1.108	Detected	1.312	Detected	1.210	0.031	GT Sens
Sense	GT Sens	-0.664	Comprom	0.664	Comprom	0.000	0.915	Detected	1.421	Detected	1.168	0.242	GT Sens
Sense	GT Sens	0.016	Comprom	-0.016	Comprom	0.000	1.331	Detected	1.069	Detected	1.200	0.012	GT Sens
Sense	GT Sens	-0.403	Detected	0.403	Detected	0.000	1.315	Detected	1.787	Detected	1.551	0.080	GT Sens
Sense	GT Sens	0.227	Detected	-0.227	Detected	0.000	0.812	Detected	1.368	Detected	1.090	0.093	GT Sens
Sense	GT Sens	1.478	Detected	-1.478	Comprom	0.000	1.108	Detected	2.361	Detected	1.735	0.393	GT Sens
Sense	GT Sens	0.057	Comprom	-0.057	Comprom	0.000	1.734	Detected	1.076	Detected	1.405	0.052	GT Sens
Sense	GT Sens	-0.006	Detected	0.006	Detected	0.000	1.389	Detected	1.422	Detected	1.405	0.000	GT Sens
Sense	GT Sens	0.442	Detected	-0.442	Detected	0.000	1.125	Detected	0.935	Detected	1.030	0.150	GT Sens
Sense	GT Sens	0.175	Detected	-0.175	Detected	0.000	1.143	Detected	1.262	Detected	1.202	0.023	GT Sens
Sense	GT Sens	0.124	Comprom	-0.124	Comprom	0.000	0.975	Detected	1.029	Detected	1.002	0.016	GT Sens
Sense	GT Sens	0.412	Detected	-0.412	Detected	0.000	1.399	Detected	1.362	Detected	1.381	0.079	GT Sens
Sense	GT Sens	0.482	Comprom	-0.482	Comprom	0.000	1.120	Detected	1.330	Detected	1.225	0.131	GT Sens
Sense	GT Sens	0.344	Comprom	-0.344	Comprom	0.000	1.484	Detected	1.047	Detected	1.265	0.090	GT Sens
Sense	GT Sens	0.080	Detected	-0.080	Detected	0.000	1.127	Detected	0.900	Detected	1.014	0.018	GT Sens
Sense	GT Sens	-0.409	Comprom	0.409	Comprom	0.000	0.925	Detected	1.296	Detected	1.110	0.132	GT Sens
Sense	GT Sens	0.168	Comprom	-0.168	Comprom	0.000	0.997	Detected	1.086	Detected	1.041	0.027	GT Sens
Sense	GT Sens	-0.824	Detected	0.824	Detected	0.000	1.332	Detected	1.734	Detected	1.533	0.213	GT Sens
Sense	GT Sens	-0.187	Comprom	0.187	Comprom	0.000	1.909	Detected	1.469	Detected	1.689	0.028	GT Sens
Sense	GT Sens	-0.302	Detected	0.302	Detected	0.000	0.816	Detected	1.591	Detected	1.203	0.134	GT Sens
Sense	GT Sens	-0.113	Detected	0.113	Detected	0.000	1.051	Detected	1.313	Detected	1.182	0.021	GT Sens
Sense	GT Sens	1.053	Detected	-1.053	Comprom	0.000	0.844	Detected	2.122	Detected	1.483	0.352	GT Sens
Sense	GT Sens	0.330	Comprom	-0.330	Comprom	0.000	2.128	Detected	2.761	Detected	2.445	0.033	GT Sens
Sense	GT Sens	-0.441	Comprom	0.441	Comprom	0.000	1.711	Detected	1.818	Detected	1.765	0.058	GT Sens
Sense	GT Sens	-0.253	Detected	0.253	Detected	0.000	0.851	Detected	1.187	Detected	1.019	0.078	GT Sens
Sense	GT Sens	1.334	Detected	-1.334	Detected	0.000	0.991	Detected	2.542	Detected	1.766	0.371	GT Sens
Sense	GT Sens	0.562	Detected	-0.562	Comprom	0.000	1.417	Detected	1.691	Detected	1.554	0.115	GT Sens
Sense	GT Sens	1.622	Detected	-1.622	Detected	0.000	0.876	Detected	2.567	Detected	1.721	0.446	GT Sens
Sense	GT Sens	0.617	Detected	-0.617	Detected	0.000	1.605	Detected	1.246	Detected	1.425	0.157	GT Sens
Sense	GT Sens	-0.131	Comprom	0.131	Comprom	0.000	2.609	Detected	1.366	Detected	1.988	0.089	GT Sens
Sense	GT Sens	-0.243	Comprom	0.243	Comprom	0.000	1.434	Detected	0.992	Detected	1.213	0.066	GT Sens
Sense	GT Sens	1.738	Detected	-1.738	Detected	0.000	0.966	Detected	2.576	Detected	1.771	0.453	GT Sens
Sense	GT Sens	0.572	Comprom	-0.572	Comprom	0.000	1.677	Detected	1.056	Detected	1.366	0.171	GT Sens
Sense	GT Sens	0.220	Comprom	-0.220	Comprom	0.000	1.544	Detected	1.243	Detected	1.393	0.035	GT Sens
Sense	GT Sens	0.380	Comprom	-0.380	Comprom	0.000	1.793	Detected	1.159	Detected	1.476	0.096	GT Sens
Sense	GT Sens	0.010	Detected	-0.010	Detected	0.000	1.321	Detected	1.362	Detected	1.342	0.000	GT Sens
Sense	GT Sens	1.443	Detected	-1.443	Comprom	0.000	0.801	Detected	2.458	Detected	1.630	0.431	GT Sens
Sense	GT Sens	-0.741	Comprom	0.741	Comprom	0.000	1.956	Detected	1.947	Detected	1.951	0.119	GT Sens
Sense	GT Sens	0.130	Comprom	-0.130	Comprom	0.000	1.697	Detected	1.610	Detected	1.654	0.007	GT Sens
Sense	GT Sens	-0.373	Comprom	0.373	Comprom	0.000	1.284	Detected	1.534	Detected	1.409	0.070	GT Sens
Sense	GT Sens	-0.762	Comprom	0.762	Comprom	0.000	1.335	Detected	1.806	Detected	1.571	0.188	GT Sens
Sense	GT Sens	-0.250	Comprom	0.250	Comprom	0.000	1.874	Detected	2.396	Detected	2.135	0.027	GT Sens
Sense	GT Sens	-0.490	Comprom	0.490	Comprom	0.000	1.201	Detected	1.197	Detected	1.199	0.134	GT Sens
Sense	GT Sens	1.266	Detected	-1.266	Detected	0.000	1.161	Detected	1.443	Detected	1.302	0.414	GT Sens
Sense	GT Sens	-0.408	Comprom	0.408	Comprom	0.000	2.237	Detected	1.705	Detected	1.971	0.056	GT Sens
Sense	GT Sens	-0.145	Detected	0.145	Detected	0.000	1.665	Detected	1.548	Detected	1.607	0.009	GT Sens
Sense	GT Sens	-0.256	Comprom	0.256	Comprom	0.000	2.216	Detected	1.968	Detected	2.092	0.018	GT Sens
Sense	GT Sens	0.023	Comprom	-0.023	Comprom	0.000	1.210	Detected	0.913	Detected	1.062	0.019	GT Sens
Sense	GT Sens	-0.478	Detected	0.478	Detected	0.000	1.201	Detected	1.739	Detected	1.470	0.115	GT Sens
Sense	GT Sens	-0.190	Comprom	0.190	Comprom	0.000	4.157	Detected	4.597	Detected	4.377	0.004	GT Sens
Sense	GT Sens	-0.093	Detected	0.093	Detected	0.000	1.050	Detected	1.200	Detected	1.125	0.011	GT Sens
Sense	GT Sens	-0.485	Comprom	0.485	Comprom	0.000	1.320	Detected	1.562	Detected	1.441	0.102	GT Sens
Sense	GT Sens	0.206	Comprom	-0.206	Comprom	0.000	0.952	Detected	1.543	Detected	1.247	0.074	GT Sens
Sense	GT Sens	-0.334	Comprom	0.334	Comprom	0.000	1.437	Detected	1.549	Detected	1.493	0.048	GT Sens
Sense	GT Sens	0.021	Detected	-0.021	Detected	0.000	1.245	Detected	1.307	Detected	1.276	0.001	GT Sens
Sense	GT Sens	0.132	Detected	-0.132	Detected	0.000	1.296	Detected	1.156	Detected	1.226	0.015	GT Sens
Sense	GT Sens	0.390	Detected	-0.390	Detected	0.000	2.126	Detected	0.892	Detected	1.509	0.175	GT Sens
Sense	GT Sens	-0.436	Detected	0.436	Detected	0.000	0.942	Detected	1.224	Detected	1.083	0.142	GT Sens
Sense	GT Sens	-0.173	Comprom	0.173	Comprom	0.000	1.112	Detected	1.394	Detected	1.253	0.030	GT Sens
Sense	GT Sens	-0.677	Comprom	0.677	Comprom	0.000	1.754	Detected	2.413	Detected	2.083	0.110	GT Sens
Sense	GT Sens	-0.455	Detected	0.455	Detected	0.000	0.996	Detected	1.241	Detected	1.119	0.141	GT Sens
Sense	GT Sens	0.019	Detected	-0.019	Detected	0.000	1.038	Detected	1.341	Detected	1.190	0.016	GT Sens
Sense	GT Sens	-0.081	Comprom	0.081	Comprom	0.000	1.672	Detected	1.986	Detected	1.829	0.009	GT Sens
Sense	GT Sens	-0.308	Comprom	0.308	Comprom	0.000	2.106	Detected	3.422	Detected	2.764	0.063	GT Sens
Sense	GT Sens	-0.051	Detected	0.051	Detected	0.000	1.201	Detected	1.348	Detected	1.275	0.005	GT Sens
Sense	GT Sens	-0.025	Comprom	0.025	Comprom	0.000	2.342	Detected	1.893	Detected	2.117	0.011	GT Sens
Sense	GT Sens	0.134	Comprom	-0.134	Comprom	0.000	2.892	Detected	2.829	Detected	2.860	0.002	GT Sens
Sense	GT Sens	-0.466	Comprom	0.466	Comprom	0.000	1.221	Detected	0.977	Detected	1.099	0.150	GT Sens
Sense	GT Sens	0.879	Detected	-0.879	Comprom	0.000	1.441	Detected	1.745	Detected	1.593	0.216	GT Sens
Sense	GT Sens	1.798	Detected	-1.798	Detected	0.000	1.208	Detected	2.818	Detected	2.013	0.414	GT Sens
Sense	GT Sens	0.133	Detected	-0.133	Detected	0.000	1.254	Detected	1.570	Detected	1.412	0.021	GT Sens
Sense	GT Sens	-0.031	Detected	0.031	Detected	0.000	1.209	Detected	1.090	Detected	1.150	0.003	GT Sens
Sense	GT Sens	0.177	Comprom	-0.177	Comprom	0.000	0.907	Detected	1.235	Detected	1.071	0.047	GT Sens
Sense	GT Sens	0.211	Detected	-0.211	Detected	0.000	1.356	Detected	1.065	Detected	1.211	0.042	GT Sens
Sense	GT Sens	-0.480	Comprom	0.480	Comprom	0.000	2.312	Detected	2.361	Detected	2.336	0.040	GT Sens
Sense	GT Sens	0.051	Detected	-0.051	Detected	0.000	1.021	Detected	1.009	Detected	1.015	0.003	GT Sens
Sense	GT Sens	0.205	Detected	-0.205	Detected	0.000	1.133	Detected	1.025	Detected	1.079	0.036	GT Sens
Sense	GT Sens	1.550	Detected	-1.550	Comprom	0.000	0.877	Detected	2.849	Detected	1.863	0.417	GT Sens
Sense	GT Sens	-0.499	Comprom	0.499	Comprom	0.000	1.599	Detected	1.998	Detected	1.799	0.079	GT Sens
Sense	GT Sens	-0.211	Comprom	0.211	Comprom	0.000	1.724	Detected	1.629	Detected	1.676	0.016	GT Sens
Sense	GT Sens	-0.267	Comprom	0.267	Comprom	0.000	1.905	Detected	3.596	Detected	2.750	0.090	GT Sens
Sense	GT Sens	-1.249	Detected	1.249	Detected	0.000	1.267	Detected	2.069	Detected	1.668	0.332	GT Sens
Sense	GT Sens	0.549	Comprom	-0.549	Comprom	0.000	0.839	Detected	1.352	Detected	1.095	0.213	GT Sens
Sense	GT Sens	0.237	Comprom	-0.237	Comprom	0.000	1.277	Detected	1.026	Detected	1.152	0.050	GT Sens
Sense	GT Sens	-0.030	Comprom	0.030	Comprom	0.000	1.176	Detected	0.825	Detected	1.000	0.030	GT Sens
Sense	GT Sens	-0.885	Comprom	0.885	Comprom	0.000	0.910	Detected	1.472	Detected	1.191	0.328	GT Sens
Sense	GT Sens	-0.403	Comprom	0.403	Comprom	0.000	1.613	Detected	3.482	Detected	2.548	0.129	GT Sens
Sense	GT Sens	-0.022	Comprom	0.022	Comprom	0.000	1.505	Detected	1.964	Detected	1.734	0.017	GT Sens
Sense	GT Sens	0.388	Comprom	-0.388	Comprom	0.000	1.158	Detected	1.414	Detected	1.286	0.088	GT Sens
Sense	GT Sens	1.759	Detected	-1.759	Comprom	0.000	1.024	Detected	2.541	Detected	1.783	0.450	GT Sens
Sense	GT Sens	0.136	Detected	-0.136	Detected	0.000	0.996	Detected	1.093	Detected	1.045	0.019	GT Sens
Sense	GT Sens	0.113	Comprom	-0.113	Comprom	0.000	1.146	Detected	0.997	Detected	1.072	0.016	GT Sens
Sense	GT Sens	0.138	Comprom	-0.138	Comprom	0.000	1.246	Detected	1.421	Detected	1.334	0.015	GT Sens
Sense	GT Sens	1.642	Detected	-1.642	Comprom	0.000	1.134	Detected	2.777	Detected	1.955	0.399	GT Sens
Sense	GT Sens	0.313	Detected	-0.313	Comprom	0.000	1.589	Detected	1.730	Detected	1.659	0.035	GT Sens
Sense	GT Sens	0.216	Detected	-0.216	Detected	0.000	1.116	Detected	2.188	Detected	1.652	0.104	GT Sens
Sense	GT Sens	0.418	Comprom	-0.418	Comprom	0.000	1.098	Detected	1.531	Detected	1.314	0.108	GT Sens
Sense	GT Sens	-0.159	Comprom	0.159	Comprom	0.000	1.869	Detected	2.133	Detected	2.001	0.010	GT Sens
Sense	GT Sens	-0.231	Comprom	0.231	Comprom	0.000	1.953	Detected	1.552	Detected	1.752	0.029	GT Sens
Sense	GT Sens	1.416	Detected	-1.416	Detected	0.000	0.918	Detected	2.485	Detected	1.702	0.403	GT Sens
Sense	GT Sens	-0.203	Detected	0.203	Detected	0.000	1.326	Detected	1.553	Detected	1.439	0.025	GT Sens
Sense	GT Sens	-0.053	Comprom	0.053	Comprom	0.000	1.588	Detected	1.457	Detected	1.523	0.003	GT Sens
Sense	GT Sens	0.141	Comprom	-0.141	Comprom	0.000	2.569	Detected	2.275	Detected	2.422	0.007	GT Sens
Sense	GT Sens	-0.548	Detected	0.548	Detected	0.000	1.051	Detected	2.195	Detected	1.623	0.177	GT Sens
Sense	GT Sens	-0.123	Comprom	0.123	Comprom	0.000	2.142	Detected	1.520	Detected	1.831	0.032	GT Sens
Sense	GT Sens	-0.201	Detected	0.201	Detected	0.000	2.143	Detected	2.189	Detected	2.166	0.009	GT Sens
Sense	GT Sens	0.981	Detected	-0.981	Comprom	0.000	1.066	Detected	1.715	Detected	1.391	0.311	GT Sens
Sense	GT Sens	0.391	Detected	-0.391	Comprom	0.000	2.030	Detected	0.854	Detected	1.442	0.178	GT Sens
Sense	GT Sens	-0.177	Comprom	0.177	Comprom	0.000	1.472	Detected	1.313	Detected	1.393	0.019	GT Sens
Sense	GT Sens	0.001	Detected	-0.001	Detected	0.000	2.036	Detected	1.996	Detected	2.016	0.000	GT Sens
Sense	GT Sens	-0.180	Detected	0.180	Detected	0.000	1.570	Detected	1.613	Detected	1.592	0.013	GT Sens
Sense	GT Sens	0.120	Detected	-0.120	Detected	0.000	1.158	Detected	1.192	Detected	1.175	0.010	GT Sens
Sense	GT Sens	-0.246	Comprom	0.246	Comprom	0.000	1.729	Detected	1.497	Detected	1.613	0.027	GT Sens
Sense	GT Sens	-0.171	Comprom	0.171	Comprom	0.000	2.577	Detected	2.820	Detected	2.698	0.006	GT Sens
Sense	GT Sens	-0.735	Comprom	0.735	Detected	0.000	1.283	Detected	1.643	Detected	1.463	0.193	GT Sens
Sense	GT Sens	0.253	Comprom	-0.253	Comprom	0.000	1.794	Detected	1.596	Detected	1.695	0.025	GT Sens
Sense	GT Sens	0.053	Comprom	-0.053	Comprom	0.000	2.376	Detected	2.165	Detected	2.271	0.003	GT Sens
Sense	GT Sens	-0.183	Comprom	0.183	Comprom	0.000	3.048	Detected	2.249	Detected	2.649	0.026	GT Sens
Sense	GT Sens	-0.916	Comprom	0.916	Comprom	0.000	1.454	Detected	4.685	Detected	3.069	0.240	GT Sens
Sense	GT Sens	-0.105	Comprom	0.105	Comprom	0.000	0.968	Detected	1.132	Detected	1.050	0.016	GT Sens
Sense	GT Sens	-0.022	Detected	0.022	Detected	0.000	1.307	Detected	1.234	Detected	1.271	0.001	GT Sens
Sense	GT Sens	-0.109	Detected	0.109	Detected	0.000	1.791	Detected	1.642	Detected	1.717	0.006	GT Sens
Sense	GT Sens	-0.191	Detected	0.191	Detected	0.000	2.104	Detected	2.811	Detected	2.457	0.026	GT Sens
Sense	GT Sens	0.012	Detected	-0.012	Detected	0.000	1.724	Detected	1.506	Detected	1.615	0.005	GT Sens
Sense	GT Sens	0.108	Comprom	-0.108	Comprom	0.000	1.388	Detected	0.814	Detected	1.101	0.070	GT Sens
Sense	GT Sens	0.404	Detected	-0.404	Comprom	0.000	1.547	Detected	0.944	Detected	1.245	0.132	GT Sens
Sense	GT Sens	-0.403	Comprom	0.403	Comprom	0.000	2.391	Detected	2.126	Detected	2.258	0.034	GT Sens
Sense	GT Sens	0.151	Detected	-0.151	Detected	0.000	1.588	Detected	1.770	Detected	1.679	0.011	GT Sens
Sense	GT Sens	-0.379	Detected	0.379	Detected	0.000	0.819	Detected	1.226	Detected	1.023	0.141	GT Sens
Sense	GT Sens	0.287	Detected	-0.287	Detected	0.000	1.259	Detected	0.831	Detected	1.045	0.100	GT Sens
Sense	GT Sens	-0.288	Detected	0.288	Detected	0.000	1.644	Detected	1.842	Detected	1.743	0.029	GT Sens
Sense	GT Sens	0.449	Comprom	-0.449	Comprom	0.000	1.229	Detected	1.082	Detected	1.155	0.126	GT Sens
Sense	GT Sens	-0.580	Comprom	0.580	Detected	0.000	3.047	Detected	1.208	Detected	2.127	0.190	GT Sens
Sense	GT Sens	-0.104	Detected	0.104	Detected	0.000	1.532	Detected	1.484	Detected	1.508	0.005	GT Sens
Sense	GT Sens	-0.740	Detected	0.740	Detected	0.000	1.411	Detected	1.205	Detected	1.308	0.222	GT Sens
Sense	GT Sens	-0.345	Comprom	0.345	Comprom	0.000	0.921	Detected	1.842	Detected	1.381	0.138	GT Sens
Sense	GT Sens	0.288	Detected	-0.288	Detected	0.000	1.375	Detected	1.181	Detected	1.278	0.052	GT Sens
Sense	GT Sens	0.148	Detected	-0.148	Detected	0.000	0.819	Detected	1.291	Detected	1.055	0.063	GT Sens
Sense	GT Sens	-0.142	Detected	0.142	Detected	0.000	1.031	Detected	1.054	Detected	1.043	0.018	GT Sens
Sense	GT Sens	-0.017	Detected	0.017	Detected	0.000	1.139	Detected	0.959	Detected	1.049	0.008	GT Sens
Sense	GT Sens	-0.505	Comprom	0.505	Comprom	0.000	1.714	Detected	1.662	Detected	1.688	0.079	GT Sens
Sense	GT Sens	-0.084	Detected	0.084	Detected	0.000	1.146	Detected	1.215	Detected	1.180	0.006	GT Sens
Sense	GT Sens	0.034	Detected	-0.034	Detected	0.000	1.034	Detected	0.967	Detected	1.001	0.002	GT Sens
Sense	GT Sens	0.272	Comprom	-0.272	Comprom	0.000	1.104	Detected	1.504	Detected	1.304	0.061	GT Sens
Sense	GT Sens	0.191	Comprom	-0.191	Comprom	0.000	1.087	Detected	1.691	Detected	1.389	0.060	GT Sens
Sense	GT Sens	-0.009	Comprom	0.009	Comprom	0.000	1.099	Detected	1.816	Detected	1.458	0.056	GT Sens
Sense	GT Sens	0.357	Comprom	-0.357	Comprom	0.000	1.791	Detected	1.643	Detected	1.717	0.042	GT Sens
Sense	GT Sens	-0.322	Comprom	0.322	Comprom	0.000	1.649	Detected	1.771	Detected	1.710	0.035	GT Sens
Sense	GT Sens	0.124	Comprom	-0.124	Comprom	0.000	2.320	Detected	2.963	Detected	2.641	0.017	GT Sens
Sense	GT Sens	0.081	Comprom	-0.081	Comprom	0.000	2.429	Detected	2.141	Detected	2.285	0.005	GT Sens
Sense	GT Sens	0.137	Comprom	-0.137	Comprom	0.000	2.707	Detected	1.604	Detected	2.155	0.063	GT Sens
Sense	GT Sens	0.209	Comprom	-0.209	Comprom	0.000	1.209	Detected	1.277	Detected	1.243	0.028	GT Sens
Sense	GT Sens	-0.125	Comprom	0.125	Comprom	0.000	1.710	Detected	1.898	Detected	1.804	0.007	GT Sens
Sense	GT Sens	-0.305	Comprom	0.305	Comprom	0.000	1.207	Detected	1.880	Detected	1.544	0.077	GT Sens
Sense	GT Sens	-0.076	Comprom	0.076	Comprom	0.000	1.717	Detected	1.026	Detected	1.371	0.061	GT Sens
Sense	GT Sens	-0.099	Comprom	0.099	Comprom	0.000	0.927	Detected	1.596	Detected	1.261	0.069	GT Sens
Sense	GT Sens	0.198	Comprom	-0.198	Comprom	0.000	1.582	Detected	1.140	Detected	1.361	0.045	GT Sens
Sense	GT Sens	-0.817	Detected	0.817	Detected	0.000	1.736	Detected	2.186	Detected	1.961	0.147	GT Sens
Sense	GT Sens	-0.620	Detected	0.620	Detected	0.000	1.544	Detected	2.107	Detected	1.826	0.115	GT Sens
Sense	GT Sens	0.242	Comprom	-0.242	Comprom	0.000	1.306	Detected	1.774	Detected	1.540	0.045	GT Sens
Sense	GT Sens	-0.171	Comprom	0.171	Comprom	0.000	0.850	Detected	1.256	Detected	1.053	0.058	GT Sens
Sense	GT Sens	-0.059	Comprom	0.059	Comprom	0.000	3.910	Detected	1.841	Detected	2.875	0.109	GT Sens
Sense	GT Sens	0.394	Detected	-0.394	Comprom	0.000	0.839	Detected	1.390	Detected	1.114	0.146	GT Sens
Sense	GT Sens	-0.004	Comprom	0.004	Comprom	0.000	1.155	Detected	1.461	Detected	1.308	0.013	GT Sens
Sense	GT Sens	-0.167	Comprom	0.167	Comprom	0.000	1.379	Detected	2.147	Detected	1.763	0.052	GT Sens
Sense	GT Sens	0.218	Comprom	-0.218	Comprom	0.000	1.258	Detected	1.488	Detected	1.373	0.031	GT Sens
Sense	GT Sens	0.129	Comprom	-0.129	Comprom	0.000	1.033	Detected	1.119	Detected	1.076	0.016	GT Sens
Sense	GT Sens	0.177	Comprom	-0.177	Comprom	0.000	1.079	Detected	1.515	Detected	1.297	0.044	GT Sens
Sense	GT Sens	-0.034	Comprom	0.034	Comprom	0.000	1.315	Detected	1.655	Detected	1.485	0.013	GT Sens
Sense	GT Sens	-0.865	Detected	0.865	Detected	0.000	1.183	Detected	1.858	Detected	1.521	0.243	GT Sens
Sense	GT Sens	-0.217	Comprom	0.217	Comprom	0.000	1.353	Detected	1.250	Detected	1.302	0.028	GT Sens
Sense	GT Sens	-0.295	Comprom	0.295	Comprom	0.000	1.125	Detected	1.370	Detected	1.248	0.060	GT Sens
Sense	GT Sens	-0.525	Comprom	0.525	Comprom	0.000	2.156	Detected	1.305	Detected	1.730	0.125	GT Sens
Sense	GT Sens	-0.365	Comprom	0.365	Comprom	0.000	1.272	Detected	1.892	Detected	1.582	0.081	GT Sens
Sense	GT Sens	0.145	Comprom	-0.145	Comprom	0.000	1.459	Detected	1.748	Detected	1.604	0.016	GT Sens
Sense	GT Sens	0.375	Detected	-0.375	Detected	0.000	0.952	Detected	1.091	Detected	1.022	0.116	GT Sens
Sense	GT Sens	-0.561	Comprom	0.561	Comprom	0.000	2.348	Detected	2.622	Detected	2.485	0.050	GT Sens
Sense	GT Sens	-0.465	Detected	0.465	Detected	0.000	1.917	Detected	2.101	Detected	2.009	0.051	GT Sens
Sense	GT Sens	-0.143	Detected	0.143	Detected	0.000	1.213	Detected	1.347	Detected	1.280	0.015	GT Sens
Sense	GT Sens	-0.165	Detected	0.165	Comprom	0.000	1.009	Detected	1.098	Detected	1.053	0.025	GT Sens
Sense	GT Sens	0.044	Comprom	-0.044	Comprom	0.000	0.992	Detected	1.347	Detected	1.169	0.024	GT Sens
Sense	GT Sens	-0.454	Comprom	0.454	Comprom	0.000	1.261	Detected	1.396	Detected	1.329	0.102	GT Sens
Sense	GT Sens	-0.325	Comprom	0.325	Comprom	0.000	1.886	Detected	1.431	Detected	1.659	0.053	GT Sens
Sense	GT Sens	-0.038	Comprom	0.038	Comprom	0.000	1.774	Detected	1.780	Detected	1.777	0.000	GT Sens
Sense	GT Sens	-0.614	Comprom	0.614	Comprom	0.000	1.431	Detected	1.659	Detected	1.545	0.132	GT Sens
Sense	GT Sens	0.602	Comprom	-0.602	Comprom	0.000	1.357	Detected	1.112	Detected	1.234	0.182	GT Sens
Sense	GT Sens	0.213	Detected	-0.213	Detected	0.000	1.044	Detected	1.288	Detected	1.166	0.042	GT Sens
Sense	GT Sens	0.124	Detected	-0.124	Detected	0.000	0.942	Detected	1.105	Detected	1.024	0.020	GT Sens
Sense	GT Sens	0.188	Detected	-0.188	Detected	0.000	1.474	Detected	1.316	Detected	1.395	0.021	GT Sens
Sense	GT Sens	0.135	Comprom	-0.135	Comprom	0.000	1.106	Detected	1.655	Detected	1.380	0.046	GT Sens
Sense	GT Sens	0.053	Comprom	-0.053	Comprom	0.000	0.993	Detected	1.205	Detected	1.099	0.011	GT Sens
Sense	GT Sens	0.300	Comprom	-0.300	Comprom	0.000	1.414	Detected	0.936	Detected	1.175	0.092	GT Sens
Sense	GT Sens	-0.206	Comprom	0.206	Comprom	0.000	1.783	Detected	1.784	Detected	1.783	0.013	GT Sens
Sense	GT Sens	0.193	Comprom	-0.193	Comprom	0.000	1.674	Detected	0.993	Detected	1.334	0.077	GT Sens
Sense	GT Sens	0.354	Detected	-0.354	Comprom	0.000	0.874	Detected	1.141	Detected	1.008	0.117	GT Sens
Sense	GT Sens	0.834	Detected	-0.834	Comprom	0.000	1.246	Detected	1.231	Detected	1.238	0.276	GT Sens
Sense	GT Sens	0.012	Comprom	-0.012	Comprom	0.000	1.660	Detected	1.563	Detected	1.611	0.001	GT Sens
Sense	GT Sens	0.602	Comprom	-0.602	Comprom	0.000	0.894	Detected	1.302	Detected	1.098	0.226	GT Sens
Sense	GT Sens	-0.055	Comprom	0.055	Comprom	0.000	1.482	Detected	1.715	Detected	1.599	0.006	GT Sens
Sense	GT Sens	0.204	Detected	-0.204	Comprom	0.000	1.089	Detected	1.326	Detected	1.207	0.036	GT Sens
Sense	GT Sens	0.501	Comprom	-0.501	Comprom	0.000	0.977	Detected	1.080	Detected	1.028	0.178	GT Sens
Sense	GT Sens	-0.601	Comprom	0.601	Comprom	0.000	1.068	Detected	0.980	Detected	1.024	0.231	GT Sens
Sense	GT Sens	0.306	Comprom	-0.306	Comprom	0.000	1.203	Detected	1.589	Detected	1.396	0.061	GT Sens
Sense	GT Sens	0.304	Comprom	-0.304	Comprom	0.000	1.116	Detected	1.714	Detected	1.415	0.080	GT Sens
Sense	GT Sens	-0.405	Comprom	0.405	Comprom	0.000	1.659	Detected	1.982	Detected	1.821	0.053	GT Sens
Sense	GT Sens	-0.419	Comprom	0.419	Comprom	0.000	1.357	Detected	1.560	Detected	1.459	0.077	GT Sens
Sense	GT Sens	-0.537	Comprom	0.537	Comprom	0.000	1.292	Detected	1.294	Detected	1.293	0.138	GT Sens
Sense	GT Sens	-0.391	Detected	0.391	Detected	0.000	1.834	Detected	2.563	Detected	2.198	0.054	GT Sens
Sense	GT Sens	-0.449	Detected	0.449	Detected	0.000	1.568	Detected	2.313	Detected	1.941	0.080	GT Sens
Sense	GT Sens	-0.450	Comprom	0.450	Detected	0.000	2.268	Detected	2.722	Detected	2.495	0.038	GT Sens
Sense	GT Sens	-0.518	Detected	0.518	Detected	0.000	1.370	Detected	2.004	Detected	1.687	0.109	GT Sens
Sense	GT Sens	-0.333	Comprom	0.333	Comprom	0.000	1.744	Detected	2.412	Detected	2.078	0.048	GT Sens
Sense	GT Sens	0.525	Detected	-0.525	Comprom	0.000	3.443	Detected	1.406	Detected	2.424	0.169	GT Sens
Sense	GT Sens	-0.074	Detected	0.074	Detected	0.000	1.575	Detected	1.580	Detected	1.578	0.002	GT Sens
Sense	GT Sens	-0.378	Comprom	0.378	Comprom	0.000	0.927	Detected	1.702	Detected	1.314	0.136	GT Sens
Sense	GT Sens	1.080	Detected	-1.080	Comprom	0.000	0.961	Detected	3.287	Detected	2.124	0.313	GT Sens
Sense	GT Sens	-0.187	Comprom	0.187	Comprom	0.000	1.430	Detected	1.801	Detected	1.616	0.025	GT Sens
Sense	GT Sens	0.046	Comprom	-0.046	Comprom	0.000	1.627	Detected	1.805	Detected	1.716	0.003	GT Sens
Sense	GT Sens	0.029	Comprom	-0.029	Comprom	0.000	1.188	Detected	1.519	Detected	1.354	0.015	GT Sens
Sense	GT Sens	-0.078	Comprom	0.078	Comprom	0.000	0.925	Detected	1.409	Detected	1.167	0.044	GT Sens
Sense	GT Sens	0.208	Comprom	-0.208	Comprom	0.000	1.448	Detected	1.939	Detected	1.694	0.034	GT Sens
Sense	GT Sens	0.112	Detected	-0.112	Comprom	0.000	1.066	Detected	1.301	Detected	1.184	0.018	GT Sens
Sense	GT Sens	0.016	Comprom	-0.016	Comprom	0.000	0.804	Detected	1.328	Detected	1.066	0.056	GT Sens
Sense	GT Sens	-0.328	Comprom	0.328	Comprom	0.000	0.900	Detected	1.204	Detected	1.052	0.100	GT Sens
Sense	GT Sens	-0.245	Comprom	0.245	Comprom	0.000	2.473	Detected	2.663	Detected	2.568	0.010	GT Sens
Sense	GT Sens	-0.275	Detected	0.275	Detected	0.000	0.975	Detected	1.342	Detected	1.158	0.073	GT Sens
Sense	GT Sens	-0.108	Detected	0.108	Detected	0.000	1.158	Detected	1.396	Detected	1.277	0.015	GT Sens
Sense	GT Sens	0.392	Comprom	-0.392	Comprom	0.000	1.219	Detected	1.429	Detected	1.324	0.083	GT Sens
Sense	GT Sens	0.313	Detected	-0.313	Comprom	0.000	1.482	Detected	1.578	Detected	1.530	0.040	GT Sens
Sense	GT Sens	-0.202	Comprom	0.202	Comprom	0.000	1.736	Detected	1.377	Detected	1.556	0.029	GT Sens
Sense	GT Sens	0.141	Comprom	-0.141	Comprom	0.000	1.594	Detected	1.787	Detected	1.690	0.010	GT Sens
Sense	GT Sens	0.168	Detected	-0.168	Detected	0.000	1.101	Detected	1.190	Detected	1.145	0.022	GT Sens
Sense	GT Sens	0.061	Comprom	-0.061	Comprom	0.000	1.450	Detected	1.187	Detected	1.319	0.012	GT Sens

Sense	GT Sens	-0.584	Comprom	0.584	Comprom	0.000	1.201	Detected	0.900	Detected	1.050	0.223	[illegible]
Sense	GT Sens	0.620	Comprom	-0.620	Comprom	0.000	1.075	Detected	1.164	Detected	1.120	0.213	[illegible]
Sense	GT Sens	0.056	Comprom	-0.056	Comprom	0.000	1.091	Detected	1.505	Detected	1.298	0.026	[illegible]
Sense	GT Sens	-0.197	Comprom	0.197	Comprom	0.000	1.612	Detected	1.965	Detected	1.789	0.021	[illegible]
Sense	GT Sens	-0.140	Detected	0.140	Detected	0.000	0.839	Detected	1.251	Detected	1.045	0.052	[illegible]
Sense	GT Sens	0.113	Comprom	-0.113	Comprom	0.000	1.884	Detected	1.914	Detected	1.899	0.004	[illegible]
Sense	GT Sens	0.034	Detected	-0.034	Detected	0.000	0.963	Detected	1.235	Detected	1.099	0.016	[illegible]
Sense	GT Sens	-0.191	Comprom	0.191	Comprom	0.000	2.199	Detected	2.223	Detected	2.211	0.007	[illegible]
Sense	GT Sens	-0.318	Comprom	0.318	Comprom	0.000	2.011	Detected	1.678	Detected	1.844	0.036	[illegible]
Sense	GT Sens	0.419	Detected	-0.419	Comprom	0.000	1.945	Detected	2.065	Detected	2.005	0.042	[illegible]
Sense	GT Sens	0.411	Comprom	-0.411	Comprom	0.000	1.345	Detected	1.621	Detected	1.483	0.076	[illegible]
Sense	GT Sens	0.337	Comprom	-0.337	Comprom	0.000	1.026	Detected	1.556	Detected	1.291	0.095	[illegible]
Sense	GT Sens	0.681	Detected	-0.681	Comprom	0.000	1.104	Detected	1.708	Detected	1.406	0.200	[illegible]
Sense	GT Sens	0.945	Detected	-0.945	Comprom	0.000	0.896	Detected	1.357	Detected	1.126	0.367	[illegible]
Sense	GT Sens	-0.584	Detected	0.584	Detected	0.000	1.415	Detected	1.946	Detected	1.680	0.120	[illegible]
Sense	GT Sens	-0.428	Detected	0.428	Detected	0.000	1.286	Detected	1.964	Detected	1.625	0.097	[illegible]
Sense	GT Sens	-0.448	Comprom	0.448	Comprom	0.000	1.503	Detected	1.697	Detected	1.600	0.073	[illegible]
Sense	GT Sens	0.225	Detected	-0.225	Detected	0.000	1.473	Detected	0.947	Detected	1.210	0.073	[illegible]
Sense	GT Sens	-0.366	Comprom	0.366	Comprom	0.000	2.741	Detected	1.502	Detected	2.122	0.098	[illegible]
Sense	GT Sens	-0.031	Comprom	0.031	Comprom	0.000	1.492	Detected	1.776	Detected	1.634	0.008	[illegible]
Sense	GT Sens	-0.219	Comprom	0.219	Comprom	0.000	1.240	Detected	1.267	Detected	1.254	0.029	[illegible]
Sense	GT Sens	-0.340	Comprom	0.340	Comprom	0.000	0.925	Detected	1.164	Detected	1.045	0.101	[illegible]
Sense	GT Sens	-0.528	Comprom	0.528	Comprom	0.000	0.846	Detected	1.170	Detected	1.008	0.210	[illegible]
Sense	GT Sens	0.363	Comprom	-0.363	Comprom	0.000	1.387	Detected	1.839	Detected	1.613	0.064	[illegible]
Sense	GT Sens	0.614	Comprom	-0.614	Comprom	0.000	1.765	Detected	1.616	Detected	1.691	0.112	[illegible]
Sense	GT Sens	0.328	Detected	-0.328	Comprom	0.000	1.310	Detected	1.653	Detected	1.481	0.057	[illegible]
Sense	GT Sens	0.040	Comprom	-0.040	Comprom	0.000	1.279	Detected	1.538	Detected	1.408	0.009	[illegible]
Sense	GT Sens	-0.558	Detected	0.558	Detected	0.000	1.020	Detected	1.074	Detected	1.047	0.202	[illegible]
Sense	GT Sens	-0.158	Detected	0.158	Detected	0.000	1.587	Detected	1.750	Detected	1.668	0.011	[illegible]
Sense	GT Sens	-0.093	Comprom	0.093	Comprom	0.000	1.036	Detected	1.413	Detected	1.224	0.028	[illegible]
Sense	GT Sens	0.037	Comprom	-0.037	Comprom	0.000	2.456	Detected	0.945	Detected	1.700	0.154	[illegible]
Sense	GT Sens	0.632	Detected	-0.632	Comprom	0.000	1.997	Detected	2.628	Detected	2.313	0.082	[illegible]
Sense	GT Sens	0.290	Detected	-0.290	Comprom	0.000	0.998	Detected	1.560	Detected	1.279	0.087	[illegible]
Sense	GT Sens	-0.883	Comprom	0.883	Comprom	0.000	0.984	Detected	1.589	Detected	1.287	0.302	[illegible]
Sense	GT Sens	-0.480	Comprom	0.480	Comprom	0.000	0.956	Detected	1.226	Detected	1.091	0.160	[illegible]
Sense	GT Sens	0.183	Comprom	-0.183	Comprom	0.000	1.575	Detected	1.394	Detected	1.484	0.018	[illegible]
Sense	GT Sens	-0.195	Comprom	0.195	Comprom	0.000	5.137	Detected	1.313	Detected	[illegible]	[illegible]	[illegible]
Sense	GT Sens	0.416	Detected	-0.416	Detected	0.000	1.154	Detected	0.985	Detected	[illegible]	[illegible]	[illegible]
Sense	GT Sens	0.182	Comprom	-0.182	Comprom	0.000	1.960	Detected	2.278	Detected	[illegible]	[illegible]	[illegible]
Sense	GT Sens	-0.492	Comprom	0.492	Comprom	0.000	1.012	Detected	1.265	Detected	[illegible]	[illegible]	[illegible]
Sense	GT Sens	-0.623	Detected	0.623	Detected	0.000	1.817	Detected	2.037	Detected	[illegible]	[illegible]	[illegible]
Sense	GT Sens	-0.458	Comprom	0.458	Detected	0.000	1.629	Detected	2.056	Detected	[illegible]	[illegible]	[illegible]
Sense	GT Sens	-0.540	Detected	0.540	Detected	0.000	1.072	Detected	1.581	Detected	[illegible]	[illegible]	[illegible]
Sense	GT Sens	-0.292	Detected	0.292	Detected	0.000	1.097	Detected	1.299	Detected	[illegible]	[illegible]	[illegible]
Sense	GT Sens	0.056	Detected	-0.056	Detected	0.000	1.253	Detected	1.105	Detected	[illegible]	[illegible]	[illegible]
Sense	GT Sens	0.608	Comprom	-0.608	Comprom	0.000	1.117	Detected	1.231	Detected	[illegible]	[illegible]	[illegible]
Sense	GT Sens	0.048	Comprom	-0.048	Comprom	0.000	1.215	Detected	1.568	Detected	[illegible]	[illegible]	[illegible]
Sense	GT Sens	0.496	Detected	-0.496	Comprom	0.000	1.408	Detected	1.022	Detected	[illegible]	[illegible]	[illegible]
Sense	GT Sens	-0.013	Comprom	0.013	Comprom	0.000	1.443	Detected	1.181	Detected	[illegible]	[illegible]	[illegible]
Sense	GT Sens	0.264	Detected	-0.264	Comprom	0.000	1.255	Detected	0.987	Detected	[illegible]	[illegible]	[illegible]
Sense	GT Sens	-0.116	Comprom	0.116	Comprom	0.000	1.202	Detected	0.965	Detected	[illegible]	[illegible]	[illegible]
Sense	GT Sens	-1.166	Comprom	1.166	Comprom	0.000	1.222	Detected	1.133	Detected	[illegible]	[illegible]	[illegible]
Sense	GT Sens	-0.489	Detected	0.489	Detected	0.000	1.357	Detected	1.922	Detected	[illegible]	[illegible]	[illegible]
Sense	GT Sens	-0.680	Detected	0.680	Detected	0.000	1.761	Detected	2.070	Detected	[illegible]	[illegible]	[illegible]
Sense	GT Sens	-0.060	Comprom	0.060	Comprom	0.000	1.548	Detected	0.827	Detected	[illegible]	[illegible]	[illegible]
Sense	GT Sens	-0.451	Comprom	0.451	Comprom	0.000	1.645	Detected	2.026	Detected	[illegible]	[illegible]	[illegible]
[illegible]	[illegible]	[illegible]	[illegible]	[illegible]	[illegible]	[illegible]	[illegible]	[illegible]	[illegible]	[illegible]	[illegible]	[illegible]	[illegible]
Sense	GT Sens	-0.445	Comprom	0.445	Comprom	0.000	2.027	Detected	1.810	Detected	[illegible]	[illegible]	[illegible]
Sense	GT Sens	0.922	Detected	-0.922	Comprom	0.000	0.991	Detected	1.059	Detected	[illegible]	[illegible]	[illegible]
Sense	GT Sens	0.586	Comprom	-0.586	Comprom	0.000	0.971	Detected	1.097	Detected	[illegible]	[illegible]	[illegible]
Sense	GT Sens	-0.197	Detected	0.197	Detected	0.000	1.029	Detected	0.993	Detected	[illegible]	[illegible]	[illegible]
Sense	GT Sens	0.275	Detected	-0.275	Comprom	0.000	1.508	Detected	1.043	Detected	[illegible]	[illegible]	[illegible]
Sense	GT Sens	-0.542	Comprom	0.542	Comprom	0.000	2.232	Detected	2.161	Detected	[illegible]	[illegible]	[illegible]
Sense	GT Sens	0.711	Detected	-0.711	Comprom	0.000	1.259	Detected	0.819	Detected	[illegible]	[illegible]	[illegible]
Sense	GT Sens	-0.083	Comprom	0.083	Comprom	0.000	0.979	Detected	1.552	Detected	[illegible]	[illegible]	[illegible]
Sense	GT Sens	-0.178	Detected	0.178	Detected	0.000	0.979	Detected	1.598	Detected	[illegible]	[illegible]	[illegible]
Sense	GT Sens	-0.090	Detected	0.090	Detected	0.000	1.102	Detected	1.039	Detected	[illegible]	[illegible]	[illegible]
Sense	GT Sens	0.288	Detected	-0.288	Detected	0.000	0.953	Detected	1.105	Detected	[illegible]	[illegible]	[illegible]
Sense	GT Sens	0.312	Comprom	-0.312	Comprom	0.000	2.001	Detected	1.649	Detected	[illegible]	[illegible]	[illegible]
Sense	GT Sens	-0.045	Comprom	0.045	Comprom	0.000	2.086	Detected	2.558	Detected	[illegible]	[illegible]	[illegible]
Sense	GT Sens	0.626	Detected	-0.626	Comprom	0.000	2.071	Detected	1.991	Detected	[illegible]	[illegible]	[illegible]
Sense	GT Sens	0.606	Detected	-0.606	Comprom	0.000	0.809	Detected	1.451	Detected	[illegible]	[illegible]	[illegible]
Sense	GT Sens	-0.486	Comprom	0.486	Comprom	0.000	1.090	Detected	1.328	Detected	[illegible]	[illegible]	[illegible]
Sense	GT Sens	0.030	Comprom	-0.030	Comprom	0.000	1.640	Detected	1.300	Detected	[illegible]	[illegible]	[illegible]
Sense	GT Sens	0.075	Comprom	-0.075	Comprom	0.000	0.829	Detected	1.959	Detected	[illegible]	[illegible]	[illegible]
Sense	GT Sens	-0.174	Comprom	0.174	Comprom	0.000	1.123	Detected	1.682	Detected	[illegible]	[illegible]	[illegible]
Sense	GT Sens	-0.184	Comprom	0.184	Comprom	0.000	1.375	Detected	2.077	Detected	[illegible]	[illegible]	[illegible]
Sense	GT Sens	-0.253	Comprom	0.253	Comprom	0.000	1.323	Detected	1.697	Detected	[illegible]	[illegible]	[illegible]
Sense	GT Sens	0.705	Detected	-0.705	Comprom	0.000	0.872	Detected	1.299	Detected	[illegible]	[illegible]	[illegible]
Sense	GT Sens	0.736	Comprom	-0.736	Comprom	0.000	1.305	Detected	1.164	Detected	[illegible]	[illegible]	[illegible]
Sense	GT Sens	-0.614	Comprom	0.614	Comprom	0.000	1.566	Detected	1.261	Detected	[illegible]	[illegible]	[illegible]
Sense	GT Sens	-0.135	Detected	0.135	Detected	0.000	1.372	Detected	1.434	Detected	[illegible]	[illegible]	[illegible]
Sense	GT Sens	0.289	Comprom	-0.289	Comprom	0.000	1.269	Detected	1.169	Detected	[illegible]	[illegible]	[illegible]
Sense	GT Sens	-0.164	Comprom	0.164	Comprom	0.000	1.864	Detected	2.509	Detected	[illegible]	[illegible]	[illegible]
Sense	GT Sens	0.160	Comprom	-0.160	Comprom	0.000	1.632	Detected	1.821	Detected	[illegible]	[illegible]	[illegible]
Sense	GT Sens	-0.444	Comprom	0.444	Comprom	0.000	1.573	Detected	2.005	Detected	[illegible]	[illegible]	[illegible]
Sense	GT Sens	-0.035	Detected	0.035	Detected	0.000	0.857	Detected	1.279	Detected	[illegible]	[illegible]	[illegible]
Sense	GT Sens	-0.071	Detected	0.071	Detected	0.000	1.476	Detected	1.471	Detected	[illegible]	[illegible]	[illegible]
Sense	GT Sens	0.426	Comprom	-0.426	Comprom	0.000	1.590	Detected	1.279	Detected	[illegible]	[illegible]	[illegible]
Sense	GT Sens	0.610	Detected	-0.610	Comprom	0.000	0.991	Detected	1.140	Detected	[illegible]	[illegible]	[illegible]
Sense	GT Sens	-0.175	Detected	0.175	Detected	0.000	0.926	Detected	1.139	Detected	[illegible]	[illegible]	[illegible]
Sense	GT Sens	-0.605	Comprom	0.605	Comprom	0.000	2.376	Detected	2.271	Detected	[illegible]	[illegible]	[illegible]
Sense	GT Sens	0.198	Comprom	-0.198	Comprom	0.000	2.620	Detected	2.896	Detected	[illegible]	[illegible]	[illegible]
Sense	GT Sens	-0.266	Comprom	0.266	Comprom	0.000	1.888	Detected	2.187	Detected	[illegible]	[illegible]	[illegible]
Sense	GT Sens	-0.835	Comprom	0.835	Comprom	0.000	1.006	Detected	1.283	Detected	[illegible]	[illegible]	[illegible]
Sense	GT Sens	0.202	Detected	-0.202	Detected	0.000	0.968	Detected	1.709	Detected	[illegible]	[illegible]	[illegible]
Sense	GT Sens	0.138	Detected	-0.138	Detected	0.000	0.910	Detected	1.698	Detected	[illegible]	[illegible]	[illegible]
Sense	GT Sens	0.440	Detected	-0.440	Comprom	0.000	0.987	Detected	1.345	Detected	[illegible]	[illegible]	[illegible]
Sense	GT Sens	-0.412	Comprom	0.412	Detected	0.000	1.508	Detected	1.861	Detected	[illegible]	[illegible]	[illegible]
Sense	GT Sens	-0.017	Comprom	0.017	Comprom	0.000	1.611	Detected	2.251	Detected	[illegible]	[illegible]	[illegible]
Sense	GT Sens	-0.067	Detected	0.067	Detected	0.000	1.397	Detected	1.039	Detected	[illegible]	[illegible]	[illegible]
Sense	GT Sens	-0.372	Comprom	0.372	Comprom	0.000	1.262	Detected	1.623	Detected	[illegible]	[illegible]	[illegible]
Sense	GT Sens	-0.078	Comprom	0.078	Comprom	0.000	1.748	Detected	1.918	Detected	[illegible]	[illegible]	[illegible]
Sense	GT Sens	-0.130	Detected	0.130	Detected	0.000	1.035	Detected	0.998	Detected	[illegible]	[illegible]	[illegible]
Sense	GT Sens	-0.536	Comprom	0.536	Comprom	0.000	1.331	Detected	1.123	Detected	[illegible]	[illegible]	[illegible]
Sense	GT Sens	-0.140	Comprom	0.140	Comprom	0.000	0.888	Detected	1.123	Detected	[illegible]	[illegible]	[illegible]
Sense	GT Sens	-0.267	Comprom	0.267	Comprom	0.000	1.592	Detected	2.010	Detected	[illegible]	[illegible]	[illegible]
Sense	GT Sens	0.033	Comprom	-0.033	Comprom	0.000	1.525	Detected	2.187	Detected	[illegible]	[illegible]	[illegible]
Sense	GT Sens	-0.648	Comprom	0.648	Comprom	0.000	1.560	Detected	1.641	Detected	[illegible]	[illegible]	[illegible]
Sense	GT Sens	-0.958	Comprom	0.958	Detected	0.000	1.023	Detected	1.255	Detected	[illegible]	[illegible]	[illegible]
[illegible]	[illegible]	[illegible]	[illegible]	[illegible]	[illegible]	[illegible]	[illegible]	[illegible]	[illegible]	[illegible]	[illegible]	[illegible]	[illegible]
Sense	GT Sens	-0.455	Detected	0.455	Detected	0.000	2.861	Detected	2.171	Detected	2.516	0.048	[illegible]
Sense	GT Sens	0.039	Detected	-0.039	Detected	0.000	3.159	Detected	1.696	Detected	2.428	0.080	[illegible]
Sense	GT Sens	-0.174	Comprom	0.174	Comprom	0.000	1.300	Detected	1.401	Detected	1.350	0.018	[illegible]
Sense	GT Sens	-0.094	Comprom	0.094	Comprom	0.000	1.383	Detected	1.619	Detected	1.501	0.010	[illegible]
Sense	GT Sens	0.483	Detected	-0.483	Comprom	0.000	1.216	Detected	1.143	Detected	1.180	0.135	[illegible]
Sense	GT Sens	-0.420	Comprom	0.420	Comprom	0.000	1.210	Detected	1.395	Detected	1.303	0.094	[illegible]
Sense	GT Sens	-0.350	Detected	0.350	Detected	0.000	0.825	Detected	1.280	Detected	1.053	0.128	[illegible]
Sense	GT Sens	-1.124	Comprom	1.124	Comprom	0.000	1.978	Detected	1.201	Detected	1.589	0.313	[illegible]
Sense	GT Sens	0.313	Comprom	-0.313	Comprom	0.000	1.013	Detected	1.376	Detected	1.195	0.081	[illegible]
Sense	GT Sens	0.346	Comprom	-0.346	Comprom	0.000	1.345	Detected	1.915	Detected	1.630	0.068	[illegible]
Sense	GT Sens	0.056	Detected	-0.056	Detected	0.000	0.962	Detected	1.286	Detected	1.124	0.022	[illegible]
Sense	GT Sens	-0.180	Detected	0.180	Detected	0.000	1.499	Detected	1.235	Detected	1.367	0.026	[illegible]
Sense	GT Sens	0.463	Comprom	-0.463	Comprom	0.000	1.098	Detected	1.325	Detected	1.211	0.126	[illegible]
Sense	GT Sens	-0.583	Comprom	0.583	Comprom	0.000	0.936	Detected	1.137	Detected	1.036	0.222	[illegible]
Sense	GT Sens	-0.059	Comprom	0.059	Comprom	0.000	1.435	Detected	1.361	Detected	1.398	0.002	[illegible]
Sense	GT Sens	0.115	Comprom	-0.115	Comprom	0.000	1.420	Detected	1.038	Detected	1.229	0.031	[illegible]
Sense	GT Sens	-1.035	Comprom	1.035	Detected	0.000	1.835	Detected	2.453	Detected	2.144	0.186	[illegible]
Sense	GT Sens	-0.200	Detected	0.200	Detected	0.000	1.189	Detected	0.929	Detected	1.059	0.047	[illegible]
Sense	GT Sens	0.599	Comprom	-0.599	Comprom	0.000	1.247	Detected	1.120	Detected	1.183	0.189	[illegible]
Sense	GT Sens	0.163	Comprom	-0.163	Comprom	0.000	1.009	Detected	1.498	Detected	1.253	0.051	[illegible]
Sense	GT Sens	0.091	Detected	-0.091	Comprom	0.000	1.139	Detected	0.998	Detected	1.069	0.011	[illegible]
Sense	GT Sens	0.166	Comprom	-0.166	Comprom	0.000	0.840	Detected	2.072	Detected	1.456	0.150	[illegible]
Sense	GT Sens	-0.045	Detected	0.045	Detected	0.000	1.135	Detected	1.020	Detected	1.077	0.005	[illegible]
Sense	GT Sens	0.119	Detected	-0.119	Detected	0.000	0.973	Detected	1.265	Detected	1.119	0.027	[illegible]
Sense	GT Sens	-0.263	Comprom	0.263	Comprom	0.000	1.307	Detected	1.360	Detected	1.333	0.037	[illegible]
Sense	GT Sens	0.097	Comprom	-0.097	Comprom	0.000	1.894	Detected	1.956	Detected	1.925	0.003	[illegible]
Sense	GT Sens	0.152	Comprom	-0.152	Comprom	0.000	1.428	Detected	1.556	Detected	1.492	0.012	[illegible]
Sense	GT Sens	0.361	Comprom	-0.361	Comprom	0.000	0.892	Detected	1.275	Detected	1.083	0.118	[illegible]
Sense	GT Sens	0.338	Comprom	-0.338	Comprom	0.000	1.148	Detected	1.246	Detected	1.197	0.073	[illegible]
Sense	GT Sens	-0.353	Comprom	0.353	Comprom	0.000	1.678	Detected	2.218	Detected	1.948	0.048	[illegible]
Sense	GT Sens	0.317	Comprom	-0.317	Comprom	0.000	1.145	Detected	1.120	Detected	1.132	0.070	[illegible]
Sense	GT Sens	0.392	Comprom	-0.392	Comprom	0.000	1.011	Detected	1.054	Detected	1.032	0.119	[illegible]
Sense	GT Sens	0.133	Comprom	-0.133	Comprom	0.000	1.115	Detected	0.976	Detected	1.045	0.020	[illegible]
Sense	GT Sens	-0.040	Comprom	0.040	Comprom	0.000	2.269	Detected	3.495	Detected	2.882	0.043	[illegible]
Sense	GT Sens	-0.179	Comprom	0.179	Comprom	0.000	1.313	Detected	1.483	Detected	1.398	0.019	[illegible]
Sense	GT Sens	-0.820	Comprom	0.820	Comprom	0.000	0.971	Detected	1.248	Detected	1.109	0.314	[illegible]
Sense	GT Sens	0.205	Detected	-0.205	Comprom	0.000	1.460	Detected	0.941	Detected	1.200	0.068	[illegible]
Sense	GT Sens	0.140	Comprom	-0.140	Comprom	0.000	1.175	Detected	1.389	Detected	1.282	0.018	[illegible]
Sense	GT Sens	0.273	Comprom	-0.273	Comprom	0.000	1.279	Detected	1.742	Detected	1.511	0.052	[illegible]
Sense	GT Sens	-0.442	Detected	0.442	Detected	0.000	1.982	Detected	2.171	Detected	2.077	0.044	[illegible]
Sense	GT Sens	0.920	Detected	-0.920	Comprom	0.000	1.172	Detected	1.225	Detected	1.199	0.323	[illegible]
Sense	GT Sens	0.349	Comprom	-0.349	Comprom	0.000	1.131	Detected	1.121	Detected	1.126	0.084	[illegible]
[illegible]	[illegible]	[illegible]	[illegible]	[illegible]	[illegible]	[illegible]	[illegible]	[illegible]	[illegible]	[illegible]	[illegible]	[illegible]	[illegible]

Sense	GT Sens	-0.548	Comprom	0.548	Comprom	0.000	1.401	Detected	1.782	Detected	1.592	0.111	GT Sense	[illegible]
Sense	GT Sens	-0.118	Comprom	0.118	Comprom	0.000	0.810	Detected	1.898	Detected	1.354	0.135	GT Sense	[illegible]
Sense	GT Sens	0.048	Comprom	-0.048	Comprom	0.000	1.744	Detected	1.775	Detected	1.760	0.001	GT Sense	[illegible]
Sense	GT Sens	-0.457	Detected	0.457	Detected	0.000	1.048	Detected	1.315	Detected	1.181	0.131	GT Sense	[illegible]
Sense	GT Sens	0.420	Detected	-0.420	Detected	0.000	0.988	Detected	1.107	Detected	1.047	0.133	GT Sense	[illegible]
Sense	GT Sens	0.628	Detected	-0.628	Comprom	0.000	1.783	Detected	1.878	Detected	1.831	0.101	GT Sense	[illegible]
Sense	GT Sens	-1.106	Comprom	1.106	Detected	0.000	0.930	Detected	1.134	Detected	1.032	0.451	GT Sense	[illegible]
Sense	GT Sens	-0.273	Detected	0.273	Detected	0.000	1.553	Detected	1.798	Detected	1.675	0.030	GT Sense	[illegible]
Sense	GT Sens	-0.708	Detected	0.708	Detected	0.000	1.847	Detected	2.094	Detected	1.970	0.111	GT Sense	[illegible]
Sense	GT Sens	0.084	Detected	-0.084	Detected	0.000	1.434	Detected	1.263	Detected	1.348	0.008	GT Sense	[illegible]
Sense	GT Sens	0.477	Comprom	-0.477	Comprom	0.000	1.063	Detected	1.448	Detected	1.256	0.135	GT Sense	[illegible]
Sense	GT Sens	-0.120	Comprom	0.120	Comprom	0.000	2.233	Detected	2.437	Detected	2.335	0.005	GT Sense	[illegible]
Sense	GT Sens	-0.337	Detected	0.337	Detected	0.000	0.834	Detected	1.178	Detected	1.006	0.117	GT Sense	[illegible]
Sense	GT Sens	-0.170	Comprom	0.170	Comprom	0.000	1.598	Detected	1.496	Detected	1.547	0.013	GT Sense	[illegible]
Sense	GT Sens	0.452	Comprom	-0.452	Comprom	0.000	1.142	Detected	0.963	Detected	1.052	0.150	GT Sense	[illegible]
Sense	GT Sens	0.056	Comprom	-0.056	Comprom	0.000	1.290	Detected	1.322	Detected	1.306	0.002	GT Sense	[illegible]
Sense	GT Sens	-0.302	Comprom	0.302	Comprom	0.000	0.973	Detected	1.143	Detected	1.058	0.078	GT Sense	[illegible]
Sense	GT Sens	-0.484	Detected	0.484	Detected	0.000	1.366	Detected	1.801	Detected	1.584	0.096	GT Sense	[illegible]
Sense	GT Sens	-0.240	Detected	0.240	Detected	0.000	1.106	Detected	1.501	Detected	1.304	0.052	GT Sense	[illegible]
Sense	GT Sens	-0.124	Comprom	0.124	Comprom	0.000	0.886	Detected	1.223	Detected	1.054	0.037	GT Sense	[illegible]
Sense	GT Sens	-0.006	Comprom	0.006	Comprom	0.000	2.524	Detected	2.195	Detected	2.360	0.005	GT Sense	[illegible]
Sense	GT Sens	0.118	Comprom	-0.118	Comprom	0.000	1.592	Detected	1.702	Detected	1.647	0.006	GT Sense	[illegible]
Sense	GT Sens	-0.368	Comprom	0.368	Detected	0.000	1.006	Detected	1.312	Detected	1.159	0.101	GT Sense	[illegible]
Sense	GT Sens	-0.166	Comprom	0.166	Comprom	0.000	2.044	Detected	2.608	Detected	2.326	0.019	GT Sense	[illegible]
Sense	GT Sens	-0.015	Comprom	0.015	Comprom	0.000	2.107	Detected	2.307	Detected	2.207	0.002	GT Sense	[illegible]
Sense	GT Sens	-0.208	Comprom	0.208	Comprom	0.000	0.972	Detected	1.847	Detected	1.409	0.101	GT Sense	[illegible]
Sense	GT Sens	-0.224	Comprom	0.224	Comprom	0.000	1.469	Detected	2.884	Detected	2.177	0.099	GT Sense	[illegible]
Sense	GT Sens	-0.272	Comprom	0.272	Comprom	0.000	1.176	Detected	1.382	Detected	1.279	0.048	GT Sense	[illegible]
Sense	GT Sens	0.150	Comprom	-0.150	Comprom	0.000	0.956	Detected	1.178	Detected	1.067	0.029	GT Sense	[illegible]
Sense	GT Sens	0.080	Comprom	-0.080	Comprom	0.000	1.292	Detected	1.594	Detected	1.443	0.014	GT Sense	[illegible]
Sense	GT Sens	-0.220	Detected	0.220	Detected	0.000	0.937	Detected	1.259	Detected	1.098	0.056	GT Sense	[illegible]
Sense	GT Sens	0.532	Detected	-0.532	Comprom	0.000	1.792	Detected	2.029	Detected	1.911	0.073	GT Sense	[illegible]
Sense	GT Sens	-0.148	Detected	0.148	Detected	0.000	0.931	Detected	1.071	Detected	1.001	0.026	GT Sense	[illegible]
Sense	GT Sens	-0.343	Detected	0.343	Detected	0.000	1.043	Detected	1.188	Detected	1.115	0.086	GT Sense	[illegible]
Sense	GT Sens	0.963	Detected	-0.963	Comprom	0.000	1.779	Detected	1.792	Detected	1.786	0.205	GT Sense	[illegible]
Sense	GT Sens	0.254	Detected	-0.254	Detected	0.000	1.258	Detected	1.303	Detected	1.281	0.037	GT Sense	[illegible]
Sense	GT Sens	-0.190	Detected	0.190	Detected	0.000	1.031	Detected	1.197	Detected	1.114	0.033	GT Sense	[illegible]
Sense	GT Sens	-0.232	Comprom	0.232	Comprom	0.000	1.513	Detected	1.827	Detected	1.670	0.027	GT Sense	[illegible]
Sense	GT Sens	-0.290	Detected	0.290	Detected	0.000	1.124	Detected	0.931	Detected	1.027	0.078	GT Sense	[illegible]
Sense	GT Sens	-0.604	Detected	0.604	Detected	0.000	0.808	Detected	1.379	Detected	1.094	0.243	GT Sense	[illegible]
Sense	GT Sens	-0.505	Detected	0.505	Detected	0.000	0.903	Detected	1.281	Detected	1.092	0.180	GT Sense	[illegible]
Sense	GT Sens	0.233	Detected	-0.233	Detected	0.000	1.575	Detected	1.707	Detected	1.641	0.021	GT Sense	[illegible]
Sense	GT Sens	0.159	Comprom	-0.159	Comprom	0.000	1.230	Detected	1.209	Detected	1.220	0.017	GT Sense	[illegible]
Sense	GT Sens	0.360	Comprom	-0.360	Comprom	0.000	1.533	Detected	1.515	Detected	1.524	0.052	GT Sense	[illegible]
Sense	GT Sens	0.098	Comprom	-0.098	Comprom	0.000	2.724	Detected	2.615	Detected	2.669	0.002	GT Sense	[illegible]
Sense	GT Sens	-0.128	Detected	0.128	Detected	0.000	1.302	Detected	0.990	Detected	1.146	0.030	GT Sense	[illegible]
Sense	GT Sens	0.475	Comprom	-0.475	Comprom	0.000	0.992	Detected	1.137	Detected	1.065	0.157	GT Sense	[illegible]
Sense	GT Sens	0.124	Comprom	-0.124	Comprom	0.000	1.347	Detected	1.840	Detected	1.593	0.029	GT Sense	[illegible]
Sense	GT Sens	-0.366	Detected	0.366	Detected	0.000	1.666	Detected	1.728	Detected	1.697	0.044	GT Sense	[illegible]
Sense	GT Sens	-0.799	Comprom	0.799	Comprom	0.000	0.965	Detected	1.441	Detected	1.203	0.286	GT Sense	[illegible]
Sense	GT Sens	0.109	Comprom	-0.109	Comprom	0.000	2.176	Detected	2.436	Detected	2.306	0.005	GT Sense	[illegible]
Sense	GT Sens	0.111	Comprom	-0.111	Comprom	0.000	1.436	Detected	1.870	Detected	1.653	0.021	GT Sense	[illegible]
Sense	GT Sens	-0.021	Comprom	0.021	Comprom	0.000	1.348	Detected	1.063	Detected	1.206	0.014	GT Sense	[illegible]
Sense	GT Sens	-0.309	Comprom	0.309	Comprom	0.000	1.753	Detected	1.568	Detected	1.660	0.036	GT Sense	[illegible]
Sense	GT Sens	-0.850	Detected	0.850	Detected	0.000	2.061	Detected	2.331	Detected	2.196	0.125	GT Sense	[illegible]
Sense	GT Sens	-0.298	Comprom	0.298	Comprom	0.000	2.347	Detected	2.693	Detected	2.520	0.018	GT Sense	[illegible]
Sense	GT Sens	0.590	Comprom	-0.590	Comprom	0.000	1.461	Detected	0.974	Detected	1.217	0.196	GT Sense	[illegible]
Sense	GT Sens	0.328	Comprom	-0.328	Comprom	0.000	3.067	Detected	3.195	Detected	3.131	0.011	GT Sense	[illegible]
Sense	GT Sens	-0.121	Comprom	0.121	Comprom	0.000	1.372	Detected	1.215	Detected	1.294	0.012	GT Sense	[illegible]
Sense	GT Sens	0.051	Comprom	-0.051	Comprom	0.000	1.178	Detected	3.080	Detected	2.129	0.155	GT Sense	[illegible]
Sense	GT Sens	-0.356	Comprom	0.356	Comprom	0.000	1.386	Detected	1.716	Detected	1.551	0.058	GT Sense	[illegible]
Sense	GT Sens	-0.167	Comprom	0.167	Comprom	0.000	1.466	Detected	1.532	Detected	1.499	0.013	GT Sense	[illegible]
Sense	GT Sens	0.001	Comprom	-0.001	Comprom	0.000	1.139	Detected	0.898	Detected	1.019	0.014	GT Sense	[illegible]
Sense	GT Sens	-0.019	Detected	0.019	Detected	0.000	1.009	Detected	1.224	Detected	1.117	0.009	GT Sense	[illegible]
Sense	GT Sens	0.865	Detected	-0.865	Comprom	0.000	1.004	Detected	2.107	Detected	1.556	0.269	GT Sense	[illegible]
Sense	GT Sens	-0.301	Comprom	0.301	Comprom	0.000	1.109	Detected	1.030	Detected	1.070	0.072	GT Sense	[illegible]
Sense	GT Sens	-0.070	Comprom	0.070	Comprom	0.000	1.032	Detected	1.344	Detected	1.188	0.020	GT Sense	[illegible]
Sense	GT Sens	-0.211	Comprom	0.211	Comprom	0.000	3.248	Detected	3.123	Detected	3.185	0.005	GT Sense	[illegible]
Sense	GT Sens	-0.484	Detected	0.484	Detected	0.000	1.207	Detected	1.849	Detected	1.528	0.119	GT Sense	[illegible]
Sense	GT Sens	-0.198	Detected	0.198	Detected	0.000	1.179	Detected	1.737	Detected	1.458	0.051	GT Sense	[illegible]
Sense	GT Sens	-0.089	Detected	0.089	Detected	0.000	0.962	Detected	1.202	Detected	1.082	0.019	GT Sense	[illegible]
Sense	GT Sens	0.331	Comprom	-0.331	Comprom	0.000	0.968	Detected	1.129	Detected	1.049	0.092	GT Sense	[illegible]
Sense	GT Sens	0.103	Comprom	-0.103	Comprom	0.000	1.197	Detected	1.097	Detected	1.147	0.010	GT Sense	[illegible]
Sense	GT Sens	-0.292	Detected	0.292	Detected	0.000	0.983	Detected	1.503	Detected	1.243	0.086	GT Sense	[illegible]
Sense	GT Sens	-0.145	Detected	0.145	Detected	0.000	1.118	Detected	1.190	Detected	1.154	0.016	GT Sense	[illegible]
Sense	GT Sens	-0.329	Comprom	0.329	Comprom	0.000	0.964	Detected	1.781	Detected	1.373	0.120	GT Sense	[illegible]
Sense	GT Sens	0.267	Detected	-0.267	Comprom	0.000	0.881	Detected	1.319	Detected	1.100	0.086	GT Sense	[illegible]
Sense	GT Sens	-0.090	Comprom	0.090	Comprom	0.000	1.580	Detected	1.491	Detected	1.536	0.004	GT Sense	[illegible]
Sense	GT Sens	0.311	Comprom	-0.311	Comprom	0.000	1.152	Detected	1.093	Detected	1.123	0.069	GT Sense	[illegible]
Sense	GT Sens	0.149	Comprom	-0.149	Comprom	0.000	1.280	Detected	1.442	Detected	1.361	0.015	GT Sense	[illegible]
Sense	GT Sens	-0.023	Detected	0.023	Detected	0.000	1.189	Detected	0.830	Detected	1.010	0.031	GT Sense	[illegible]
Sense	GT Sens	-0.216	Detected	0.216	Detected	0.000	1.074	Detected	1.316	Detected	1.195	0.040	GT Sense	[illegible]
Sense	GT Sens	0.042	Comprom	-0.042	Comprom	0.000	2.035	Detected	2.040	Detected	2.038	0.000	GT Sense	[illegible]
Sense	GT Sens	-0.256	Comprom	0.256	Comprom	0.000	0.889	Detected	1.415	Detected	1.152	0.088	GT Sense	[illegible]
Sense	GT Sens	-0.403	Detected	0.403	Detected	0.000	0.846	Detected	1.332	Detected	1.089	0.147	GT Sense	[illegible]
Sense	GT Sens	0.088	Comprom	-0.088	Comprom	0.000	1.589	Detected	1.781	Detected	1.685	0.006	GT Sense	[illegible]
Sense	GT Sens	-0.282	Comprom	0.282	Comprom	0.000	1.197	Detected	1.924	Detected	1.561	0.077	GT Sense	[illegible]
Sense	GT Sens	-0.232	Detected	0.232	Detected	0.000	1.442	Detected	1.841	Detected	1.642	0.033	GT Sense	[illegible]
Sense	GT Sens	-0.193	Detected	0.193	Detected	0.000	0.915	Detected	1.243	Detected	1.079	0.051	GT Sense	[illegible]
Sense	GT Sens	0.018	Comprom	-0.018	Comprom	0.000	1.157	Detected	1.151	Detected	1.154	0.000	GT Sense	[illegible]
Sense	GT Sens	-0.141	Comprom	0.141	Comprom	0.000	1.015	Detected	1.390	Detected	1.202	0.036	GT Sense	[illegible]
Sense	GT Sens	0.132	Comprom	-0.132	Comprom	0.000	1.030	Detected	1.326	Detected	1.178	0.027	GT Sense	[illegible]
Sense	GT Sens	0.523	Comprom	-0.523	Comprom	0.000	1.135	Detected	1.901	Detected	1.518	0.144	GT Sense	[illegible]
Sense	GT Sens	0.248	Detected	-0.248	Comprom	0.000	1.165	Detected	1.516	Detected	1.340	0.048	GT Sense	[illegible]
Sense	GT Sens	-0.388	Detected	0.388	Detected	0.000	1.258	Detected	1.524	Detected	1.391	0.077	GT Sense	[illegible]
Sense	GT Sens	0.002	Detected	-0.002	Detected	0.000	0.999	Detected	1.547	Detected	1.273	0.043	GT Sense	[illegible]
Sense	GT Sens	-0.112	Detected	0.112	Detected	0.000	0.884	Detected	1.311	Detected	1.097	0.045	GT Sense	[illegible]
Sense	GT Sens	0.190	Comprom	-0.190	Comprom	0.000	2.431	Detected	2.456	Detected	2.444	0.006	GT Sense	[illegible]
Sense	GT Sens	0.056	Detected	-0.056	Detected	0.000	1.151	Detected	1.154	Detected	1.153	0.002	GT Sense	[illegible]
Sense	GT Sens	-0.077	Detected	0.077	Comprom	0.000	1.029	Detected	1.237	Detected	1.133	0.013	GT Sense	[illegible]
Sense	GT Sens	-0.420	Comprom	0.420	Comprom	0.000	1.426	Detected	1.400	Detected	1.413	0.078	GT Sense	[illegible]
Sense	GT Sens	0.057	Detected	-0.057	Detected	0.000	0.972	Detected	1.207	Detected	1.089	0.014	GT Sense	[illegible]
Sense	GT Sens	-0.452	Detected	0.452	Detected	0.000	1.015	Detected	1.235	Detected	1.125	0.137	GT Sense	[illegible]
Sense	GT Sens	-0.123	Comprom	0.123	Comprom	0.000	1.912	Detected	2.322	Detected	2.117	0.013	GT Sense	[illegible]
Sense	GT Sens	-0.659	Comprom	0.659	Comprom	0.000	2.763	Detected	2.681	Detected	2.722	0.054	GT Sense	[illegible]
Sense	GT Sens	1.394	Detected	-1.394	Comprom	0.000	1.049	Detected	2.829	Detected	1.939	0.362	GT Sense	[illegible]
Sense	GT Sens	0.106	Detected	-0.106	Comprom	0.000	1.058	Detected	1.027	Detected	1.042	0.010	GT Sense	[illegible]
Sense	GT Sens	-0.164	Detected	0.164	Detected	0.000	1.372	Detected	0.931	Detected	1.151	0.053	GT Sense	[illegible]
Sense	GT Sens	-0.472	Detected	0.472	Detected	0.000	1.136	Detected	1.753	Detected	1.444	0.125	GT Sense	[illegible]
Sense	GT Sens	0.371	Detected	-0.371	Detected	0.000	1.552	Detected	1.045	Detected	1.299	0.102	GT Sense	[illegible]
Sense	GT Sens	-0.615	Comprom	0.615	Comprom	0.000	1.342	Detected	2.012	Detected	1.677	0.139	GT Sense	[illegible]
Sense	GT Sens	-0.021	Comprom	0.021	Comprom	0.000	1.652	Detected	1.502	Detected	1.577	0.002	GT Sense	[illegible]
Sense	GT Sens	0.208	Detected	-0.208	Comprom	0.000	1.980	Detected	1.958	Detected	1.969	0.011	GT Sense	[illegible]
Sense	GT Sens	-0.041	Detected	0.041	Detected	0.000	0.924	Detected	1.123	Detected	1.024	0.011	GT Sense	[illegible]
Sense	GT Sens	-0.674	Detected	0.674	Detected	0.000	1.806	Detected	1.918	Detected	1.862	0.111	GT Sense	[illegible]
Sense	GT Sens	-0.479	Detected	0.479	Detected	0.000	1.915	Detected	0.864	Detected	1.390	0.190	GT Sense	[illegible]
Sense	GT Sens	-0.326	Detected	0.326	Detected	0.000	2.315	Detected	1.750	Detected	2.033	0.042	GT Sense	[illegible]
Sense	GT Sens	1.497	Detected	-1.497	Detected	0.000	0.924	Detected	2.524	Detected	1.724	0.417	GT Sense	[illegible]
Sense	GT Sens	0.430	Detected	-0.430	Comprom	0.000	1.585	Detected	1.446	Detected	1.516	0.073	GT Sense	[illegible]
Sense	GT Sens	0.074	Detected	-0.074	Comprom	0.000	1.475	Detected	1.565	Detected	1.520	0.003	GT Sense	[illegible]
Sense	GT Sens	0.403	Detected	-0.403	Comprom	0.000	1.090	Detected	1.019	Detected	1.054	0.121	GT Sense	[illegible]
Sense	GT Sens	1.406	Detected	-1.406	Comprom	0.000	1.105	Detected	2.560	Detected	1.832	0.367	GT Sense	[illegible]
Sense	GT Sens	-0.460	Detected	0.460	Detected	0.000	0.802	Detected	1.260	Detected	1.031	0.183	GT Sense	[illegible]
Sense	GT Sens	-0.046	Comprom	0.046	Comprom	0.000	1.579	Detected	1.799	Detected	1.689	0.005	GT Sense	[illegible]
Sense	GT Sens	-0.309	Detected	0.309	Detected	0.000	1.476	Detected	1.847	Detected	1.661	0.044	GT Sense	[illegible]
Sense	GT Sens	-0.415	Comprom	0.415	Comprom	0.000	1.479	Detected	1.779	Detected	1.629	0.066	GT Sense	[illegible]
Sense	GT Sens	0.151	Comprom	-0.151	Comprom	0.000	1.087	Detected	1.450	Detected	1.268	0.033	GT Sense	[illegible]
Sense	GT Sens	-0.288	Comprom	0.288	Comprom	0.000	1.883	Detected	1.214	Detected	1.548	0.072	GT Sense	[illegible]
Sense	GT Sens	-0.767	Comprom	0.767	Comprom	0.000	1.097	Detected	0.935	Detected	1.016	0.318	GT Sense	[illegible]
Sense	GT Sens	0.003	Detected	-0.003	Detected	0.000	0.987	Detected	1.271	Detected	1.129	0.015	GT Sense	[illegible]
Sense	GT Sens	-0.641	Comprom	0.641	Comprom	0.000	1.040	Detected	0.973	Detected	1.006	0.258	GT Sense	[illegible]
Sense	GT Sens	0.011	Detected	-0.011	Detected	0.000	1.047	Detected	1.342	Detected	1.194	0.015	GT Sense	[illegible]
Sense	GT Sens	-0.845	Detected	0.845	Detected	0.000	1.197	Detected	1.629	Detected	1.413	0.247	GT Sense	[illegible]
Sense	GT Sens	0.962	Detected	-0.962	Detected	0.000	1.198	Detected	1.576	Detected	1.387	0.293	GT Sense	[illegible]
Sense	GT Sens	-0.265	Comprom	0.265	Comprom	0.000	1.841	Detected	2.115	Detected	1.978	0.022	GT Sense	[illegible]
Sense	GT Sens	0.308	Detected	-0.308	Comprom	0.000	1.174	Detected	1.378	Detected	1.276	0.059	GT Sense	[illegible]
Sense	GT Sens	0.185	Comprom	-0.185	Comprom	0.000	1.346	Detected	1.744	Detected	1.545	0.030	GT Sense	[illegible]
Sense	GT Sens	0.028	Detected	-0.028	Comprom	0.000	1.142	Detected	1.417	Detected	1.279	0.012	GT Sense	[illegible]
Sense	GT Sens	-0.376	Detected	0.376	Detected	0.000	1.128	Detected	1.310	Detected	1.219	0.088	GT Sense	[illegible]
Sense	GT Sens	0.114	Comprom	-0.114	Comprom	0.000	1.532	Detected	1.990	Detected	1.761	0.021	GT Sense	[illegible]
Sense	GT Sens	-0.113	Comprom	0.113	Comprom	0.000	1.396	Detected	1.291	Detected	1.344	0.008	GT Sense	[illegible]
Sense	GT Sens	-0.505	Comprom	0.505	Comprom	0.000	2.877	Detected	3.063	Detected	2.970	0.029	GT Sense	[illegible]
Sense	GT Sens	-0.019	Comprom	0.019	Comprom	0.000	1.469	Detected	2.512	Detected	1.991	0.062	GT Sense	[illegible]
Sense	GT Sens	1.610	Detected	-1.610	Detected	0.000	0.881	Detected	2.513	Detected	1.697	0.446	GT Sense	[illegible]
Sense	GT Sens	0.209	Detected	-0.209	Detected	0.000	1.208	Detected	0.895	Detected	1.052	0.056	GT Sense	[illegible]
Sense	GT Sens	-0.051	Detected	0.051	Detected	0.000	0.915	Detected	1.096	Detected	1.005	0.010	GT Sense	[illegible]
Sense	GT Sens	-0.423	Detected	0.423	Detected	0.000	1.447	Detected	1.598	Detected	1.522	0.071	GT Sense	[illegible]
Sense	GT Sens	0.383	Comprom	-0.383	Comprom	0.000	1.391	Detected	2.209	Detected	1.800	0.085	GT Sense	[illegible]
Sense	GT Sens	0.155	Comprom	-0.155	Comprom	0.000	1.605	Detected	1.651	Detected	1.628	0.009	GT Sense	[illegible]
Sense	GT Sens	-0.078	Comprom	0.078	Comprom	0.000	1.624	Detected	1.946	Detected	1.785	0.010	GT Sense	[illegible]
Sense	GT Sens	1.719	Detected	-1.719	Detected	0.000	1.039	Detected	2.888	Detected	1.963	0.420	GT Sense	[illegible]
Sense	GT Sens	-0.274	Detected	0.274	Detected	0.000	1.465	Detected	1.855	Detected	1.660	0.039	GT Sense	[illegible]
Sense	GT Sens	-0.466	Detected	0.466	Detected	0.000	1.413	Detected	1.422	Detected	1.417	0.093	GT Sense	[illegible]
Sense	GT Sens	-0.233	Detected	0.233	Detected	0.000	2.671	Detected	2.792	Detected	2.732	0.008	GT Sense	[illegible]
Sense	GT Sens	-0.623	Detected	0.623	Detected	0.000	1.750	Detected	2.273	Detected	2.011	0.097	GT Sense	[illegible]
Sense	GT Sens	-0.046	Detected	0.046	Detected	0.000	0.999	Detected	2.212	Detected	1.606	0.119	GT Sense	[illegible]
Sense	GT Sens	-0.028	Detected	0.028	Detected	0.000	0.930	Detected	1.130	Detected	1.030	0.010	GT Sense	[illegible]
Sense	GT Sens	-0.460	Detected	0.460	Detected	0.000	1.196	Detected	1.417	Detected	1.307	0.110	GT Sense	[illegible]
Sense	GT Sens	1.688	Detected	-1.688	Detected	0.000	1.058	Detected	2.728	Detected	1.893	0.421	GT Sense	[illegible]
Sense	GT Sens	0.072	Comprom	-0.072	Comprom	0.000	0.939	Detected	1.520	Detected	1.229	0.055	GT Sense	[illegible]
Sense	GT Sens	-0.817	Detected	0.817	Detected	0.000	1.113	Detected	1.633	Detected	1.373	0.250	GT Sense	[illegible]
Sense	GT Sens	-0.362	Detected	0.362	Detected	0.000	0.932	Detected	1.196	Detected	1.064	0.110	GT Sense	[illegible]
Sense	GT Sens	-0.351	Detected	0.351	Detected	0.000	1.401	Detected	1.844	Detected	1.622	0.060	GT Sense	[illegible]
Sense	GT Sens	-0.532	Detected	0.532	Detected	0.000	2.022	Detected	1.554	Detected	1.788	0.092	GT Sense	[illegible]
Sense	GT Sens	-0.758	Detected	0.758	Detected	0.000	2.001	Detected	2.132	Detected	2.067	0.113	GT Sense	[illegible]
Sense	GT Sens	0.164	Detected	-0.164	Detected	0.000	1.123	Detected	1.011	Detected	1.067	0.025	GT Sense	[illegible]
Sense	GT Sens	0.051	Detected	-0.051	Detected	0.000	1.028	Detected	1.438	Detected	1.233	0.028	GT Sense	[illegible]
Sense	GT Sens	-0.189	Comprom	0.189	Comprom	0.000	3.353	Detected	3.184	Detected	3.268	0.004	GT Sense	[illegible]
Sense	GT Sens	0.341	Comprom	-0.341	Comprom	0.000	1.532	Detected	1.078	Detected	1.305	0.086	GT Sense	[illegible]
Sense	GT Sens	-0.176	Detected	0.176	Detected	0.000	1.708	Detected	1.126	Detected	1.417	0.053	GT Sense	[illegible]
Sense	GT Sens	0.147	Detected	-0.147	Detected	0.000	1.019	Detected	1.170	Detected	1.094	0.022	GT Sense	[illegible]
Sense	GT Sens	0.302	Detected	-0.302	Comprom	0.000	1.789	Detected	1.837	Detected	1.813	0.027	GT Sense	[illegible]
Sense	GT Sens	1.547	Detected	-1.547	Detected	0.000	0.909	Detected	2.683	Detected	1.796	0.420	GT Sense	[illegible]
Sense	GT Sens	1.743	Detected	-1.743	Comprom	0.000	1.443	Detected	2.823	Detected	2.133	0.373	GT Sense	[illegible]
Sense	GT Sens	0.107	Comprom	-0.107	Comprom	0.000	1.488	Detected	2.281	Detected	1.884	0.044	GT Sense	[illegible]
Sense	GT Sens	0.109	Comprom	-0.109	Comprom	0.000	1.747	Detected	1.509	Detected	1.628	0.010	GT Sense	[illegible]
Sense	GT Sens	0.673	Comprom	-0.673	Comprom	0.000	1.027	Detected	1.170	Detected	1.098	0.246	GT Sense	[illegible]
Sense	GT Sens	0.808	Detected	-0.808	Comprom	0.000	1.600	Detected	1.022	Detected	1.311	0.266	GT Sense	[illegible]
Sense	GT Sens	1.270	Detected	-1.270	Comprom	0.000	1.229	Detected	2.929	Detected	2.079	0.307	GT Sense	[illegible]
Sense	GT Sens	-0.063	Detected	0.063	Comprom	0.000	1.096	Detected	1.687	Detected	1.392	0.044	GT Sense	[illegible]
Sense	GT Sens	-0.547	Detected	0.547	Detected	0.000	0.879	Detected	1.465	Detected	1.172	0.200	GT Sense	[illegible]
Sense	GT Sens	-0.202	Detected	0.202	Detected	0.000	1.187	Detected	1.511	Detected	1.349	0.035	GT Sense	[illegible]
Sense	GT Sens	-0.008	Comprom	0.008	Comprom	0.000	1.558	Detected	1.743	Detected	1.650	0.003	GT Sense	[illegible]
Sense	GT Sens	1.590	Detected	-1.590	Comprom	0.000	0.943	Detected	1.100	Detected	1.021	0.587	GT Sense	[illegible]
Sense	GT Sens	-0.498	Detected	0.498	Detected	0.000	1.509	Detected	1.809	Detected	1.659	0.086	GT Sense	[illegible]
Sense	GT Sens	0.246	Detected	-0.246	Detected	0.000	1.753	Detected	1.440	Detected	1.597	0.032	GT Sense	[illegible]
Sense	GT Sens	0.594	Comprom	-0.594	Comprom	0.000	1.127	Detected	1.689	Detected	1.408	0.165	GT Sense	[illegible]
Sense	GT Sens	0.592	Comprom	-0.592	Comprom	0.000	1.243	Detected	1.416	Detected	1.329	0.156	GT Sense	[illegible]
Sense	GT Sens	-0.358	Comprom	0.358	Comprom	0.000	2.697	Detected	3.123	Detected	2.910	0.020	GT Sense	[illegible]
Sense	GT Sens	0.962	Detected	-0.962	Comprom	0.000	1.117	Detected	0.892	Detected	1.004	0.409	GT Sense	[illegible]
Sense	GT Sens	-0.089	Detected	0.089	Detected	0.000	1.103	Detected	1.164	Detected	1.133	0.007	GT Sense	[illegible]
Sense	GT Sens	0.251	Detected	-0.251	Detected	0.000	1.055	Detected	1.156	Detected	1.105	0.050	GT Sense	[illegible]
Sense	GT Sens	0.809	Comprom	-0.809	Comprom	0.000	1.075	Detected	2.425	Detected	1.750	0.239	GT Sense	[illegible]
Sense	GT Sens	1.245	Detected	-1.245	Comprom	0.000	0.864	Detected	1.962	Detected	1.413	0.408	GT Sense	[illegible]
Sense	GT Sens	-0.396	Detected	0.396	Detected	0.000	1.030	Detected	1.352	Detected	1.191	0.108	GT Sense	[illegible]
Sense	GT Sens	0.033	Detected	-0.033	Detected	0.000	1.133	Detected	1.132	Detected	1.132	0.001	GT Sense	[illegible]
Sense	GT Sens	0.138	Detected	-0.138	Detected	0.000	1.058	Detected	1.256	Detected	1.157	0.021	GT Sense	[illegible]
Sense	GT Sens	-0.036	Comprom	0.036	Comprom	0.000	1.415	Detected	0.893	Detected	1.154	0.048	GT Sense	[illegible]
Sense	GT Sens	0.054	Comprom	-0.054	Comprom	0.000	1.627	Detected	1.751	Detected	1.689	0.002	GT Sense	[illegible]
Sense	GT Sens	0.151	Detected	-0.151	Detected	0.000	1.941	Detected	1.950	Detected	1.946	0.006	GT Sense	[illegible]
Sense	GT Sens	0.021	Comprom	-0.021	Comprom	0.000	0.968	Detected	2.173	Detected	1.571	0.121	GT Sense	[illegible]
Sense	GT Sens	-0.603	Comprom	0.603	Detected	0.000	0.884	Detected	1.784	Detected	1.334	0.218	GT Sense	[illegible]
Sense	GT Sens	0.125	Comprom	-0.125	Comprom	0.000	1.049	Detected	1.356	Detected	1.202	0.026	GT Sense	[illegible]
Sense	GT Sens	-0.189	Detected	0.189	Detected	0.000	1.057	Detected	1.168	Detected	1.112	0.030	GT Sense	[illegible]
Sense	GT Sens	-0.194	Comprom	0.194	Comprom	0.000	0.979	Detected	1.192	Detected	1.086	0.039	GT Sense	[illegible]
Sense	GT Sens	-0.104	Detected	0.104	Detected	0.000	0.969	Detected	1.077	Detected	1.023	0.013	GT Sense	[illegible]
Sense	GT Sens	-0.343	Detected	0.343	Detected	0.000	1.136	Detected	1.683	Detected	1.410	0.085	GT Sense	[illegible]
Sense	GT Sens	-1.273	Comprom	1.273	Detected	0.000	1.072	Detected	1.130	Detected	1.101	0.479	GT Sense	[illegible]
Sense	GT Sens	-0.950	Detected	0.950	Detected	0.000	0.890	Detected	1.537	Detected	1.213	0.350	GT Sense	[illegible]
Sense	GT Sens	-0.525	Comprom	0.525	Comprom	0.000	1.962	Detected	2.306	Detected	2.134	0.061	GT Sense	[illegible]
Sense	GT Sens	0.158	Comprom	-0.158	Comprom	0.000	1.588	Detected	0.950	Detected	1.269	0.071	GT Sense	[illegible]
Sense	GT Sens	0.225	Detected	-0.225	Detected	0.000	0.980	Detected	1.144	Detected	1.062	0.047	GT Sense	[illegible]
Sense	GT Sens	-0.539	Detected	0.539	Detected	0.000	0.866	Detected	1.445	Detected	1.156	0.200	GT Sense	[illegible]
Sense	GT Sens	0.164	Detected	-0.164	Detected	0.000	2.005	Detected	2.521	Detected	2.263	0.018	GT Sense	[illegible]
Sense	GT Sens	0.701	Comprom	-0.701	Comprom	0.000	2.053	Detected	1.540	Detected	1.797	0.138	GT Sense	[illegible]
Sense	GT Sens	-0.271	Comprom	0.271	Comprom	0.000	2.161	Detected	2.109	Detected	2.135	0.016	GT Sense	[illegible]
Sense	GT Sens	0.332	Detected	-0.332	Comprom	0.000	2.388	Detected	2.881	Detected	2.634	0.024	GT Sense	[illegible]
Sense	GT Sens	-0.579	Detected	0.579	Detected	0.000	1.092	Detected	1.629	Detected	1.361	0.167	GT Sense	[illegible]
Sense	GT Sens	0.562	Comprom	-0.562	Comprom	0.000	1.898	Detected	1.869	Detected	1.884	0.079	GT Sense	[illegible]
Sense	GT Sens	0.602	Comprom	-0.602	Comprom	0.000	0.968	Detected	1.247	Detected	1.107	0.215	GT Sense	[illegible]
Sense	GT Sens	-0.439	Detected	0.439	Detected	0.000	1.630	Detected	1.651	Detected	1.640	0.065	GT Sense	[illegible]
Sense	GT Sens	-0.640	Detected	0.640	Detected	0.000	1.280	Detected	1.896	Detected	1.588	0.155	GT Sense	[illegible]
Sense	GT Sens	-0.899	Comprom	0.899	Comprom	0.000	2.085	Detected	2.072	Detected	2.078	0.147	GT Sense	[illegible]
Sense	GT Sens	-0.522	Detected	0.522	Detected	0.000	1.108	Detected	1.685	Detected	1.396	0.144	GT Sense	[illegible]
Sense	GT Sens	-0.138	Comprom	0.138	Comprom	0.000	1.214	Detected	1.225	Detected	1.219	0.013	GT Sense	[illegible]
Sense	GT Sens	0.239	Comprom	-0.239	Comprom	0.000	2.882	Detected	1.221	Detected	2.052	0.141	GT Sense	[illegible]
Sense	GT Sens	-0.254	Detected	0.254	Detected	0.000	1.455	Detected	1.185	Detected	1.320	0.044	GT Sense	[illegible]
Sense	GT Sens	-0.069	Detected	0.069	Detected	0.000	1.079	Detected	1.380	Detected	1.230	0.018	GT Sense	[illegible]
Sense	GT Sens	-0.639	Detected	0.639	Detected	0.000	1.106	Detected	1.698	Detected	1.402	0.185	GT Sense	[illegible]
Sense	GT Sens	0.365	Comprom	-0.365	Comprom	0.000	1.956	Detected	2.110	Detected	2.033	0.032	GT Sense	[illegible]
Sense	GT Sens	0.247	Comprom	-0.247	Comprom	0.000	1.529	Detected	1.199	Detected	1.364	0.044	GT Sense	[illegible]
Sense	GT Sens	0.068	Detected	-0.068	Detected	0.000	1.163	Detected	1.919	Detected	1.541	0.057	GT Sense	[illegible]
Sense	GT Sens	0.270	Detected	-0.270	Detected	0.000	0.931	Detected	1.183	Detected	1.057	0.071	GT Sense	[illegible]
Sense	GT Sens	-0.439	Detected	0.439	Detected	0.000	1.288	Detected	2.057	Detected	1.673	0.103	GT Sense	[illegible]
Sense	GT Sens	-0.707	Detected	0.707	Detected	0.000	1.299	Detected	1.888	Detected	1.594	0.173	GT Sense	[illegible]
Sense	GT Sens	0.335	Detected	-0.335	Detected	0.000	1.477	Detected	0.900	Detected	1.188	0.115	GT Sense	[illegible]
Sense	GT Sens	-0.633	Detected	0.633	Detected	0.000	0.968	Detected	1.656	Detected	1.312	0.210	GT Sense	[illegible]
Sense	GT Sens	-0.519	Detected	0.519	Detected	0.000	1.094	Detected	1.721	Detected	1.407	0.146	GT Sense	[illegible]
Sense	GT Sens	-0.068	Detected	0.068	Detected	0.000	2.456	Detected	2.364	Detected	2.410	0.001	GT Sense	[illegible]
Sense	GT Sens	0.271	Detected	-0.271	Detected	0.000	1.864	Detected	1.052	Detected	1.458	0.096	GT Sense	[illegible]
Sense	GT Sens	-0.222	Comprom	0.222	Comprom	0.000	1.904	Detected	2.426	Detected	2.165	0.024	GT Sense	[illegible]
Sense	GT Sens	0.273	Detected	-0.273	Detected	0.000	1.490	Detected	1.110	Detected	1.300	0.060	GT Sense	[illegible]
Sense	GT Sens	0.438	Comprom	-0.438	Comprom	0.000	1.235	Detected	1.223	Detected	1.229	0.107	GT Sense	[illegible]
Sense	GT Sens	0.268	Comprom	-0.268	Comprom	0.000	1.357	Detected	1.534	Detected	1.446	0.036	GT Sense	[illegible]

Sense	GT Sens	-0.907	Comprom	0.907	Comprom	0.000	1.413	Detected	1.279	Detected	1.346	0.277	GT Sense	contig135	contig135	Mutator-li	TTGAAAT	contig135	Solyc10g	Solyc10g	Mutator-like transposase (AHF			contig135	Solyc10g	Mutator-like transpo		SL2.40ch	AT1G751	JAC1	JAC1 (J-l	chr1:281	2.20796	6.36349	3.44932	12.1695	11.4937	10.2837
Sense	GT Sens	-0.339	Detected	0.339	Detected	0.000	1.185	Detected	1.626	Detected	1.406	0.074	GT Sense	contig136	contig136	Unknown	TGCAAA/	contig136	Solyc11g	Solyc11g	Unknown Protein (AHRD V1)			contig136	Solyc11g	Unknown Protein (A		SL2.40ch	#N/A	#N/A	#N/A	#N/A	803.301	1052.42	1082.26	1082.61	2406.92	3208.1
Sense	GT Sens	0.030	Detected	-0.030	Detected	0.000	1.040	Detected	1.072	Detected	1.056	0.001	GT Sense	contig136	contig136	Histone H	GAAGAA(	contig136	Solyc10g	Solyc10g	Histone H	GO:0005(	GO:0005(	contig136	Solyc02g	Histone H	GO:0005!	SL2.40ch	AT5G228	H2B. HTB	HTB2: DI	chr5:765	186.589	146.668	195.085	195.946	391.738	392.931
Sense	GT Sens	0.049	Comprom	-0.049	Comprom	0.000	1.537	Detected	1.924	Detected	1.730	0.013	GT Sense	contig136	contig136	Unknown	TAAGTCT	contig136	Solyc04g	Solyc04g	Unknown Protein (AHRD V1)			contig136	Solyc06g	Unknown Protein (A		SL2.40ch	AT5G229	ATCHX3.	ATCHX3:	chr5:765	7.09258	5.42946	16.5823	16.1153	20.7384	26.6063
Sense	GT Sens	-0.586	Detected	0.586	Detected	0.000	0.865	Detected	1.494	Detected	1.179	0.218	GT Sense	contig136	contig136	Pol polyp	TAACCAT	contig136	Solyc11g	Solyc11g	Pol polyp	GO:0006:	GO:0006:	contig136	Solyc11g	Pol polyp	GO:0006:	SL2.40ch	AT5G101	ATMIPS3	MIPS3 (M	chr5:318	243.035	448.876	1163.1	663.109	692.433	1051.3
Sense	GT Sens	-0.036	Detected	0.036	Detected	0.000	1.410	Detected	1.628	Detected	1.519	0.006	GT Sense	contig138	contig138	Unknown	ATAGTGT	contig138	Solyc12g	Solyc12g	Unknown Protein (AHRD V1)			contig138	Solyc12g	Unknown Protein (A		SL2.40ch	AT2G36080.1		DNA-bind	chr2:151	114.407	98.5089	178.765	135.507	324.918	370.855
Sense	GT Sens	0.939	Detected	-0.939	Comprom	0.000	1.092	Detected	1.345	Detected	1.219	0.327	GT Sense	contig141	contig141	Hydrolase	GCAAAA(	contig141	Solyc01g	Solyc01g	Hydrolase alpha/beta fold fami			contig141	Solyc01g	Hydrolase alpha/bet		SL2.40ch	AT2G356	MOR1. GI	MOR1 (M	chr2:149	49.8425	11.1065	20.5738	25.0188	57.7539	67.5541
Sense	GT Sens	0.013	Detected	-0.013	Detected	0.000	1.893	Detected	1.559	Detected	1.726	0.009	GT Sense	contig141	contig141	Phenylala	ATGCGGT	contig141	Solyc09g	Solyc09g	Phenylala	GO:0004:	GO:0004:	contig141	Solyc09g	Phenylala	GO:0004:	SL2.40ch	AT2G370	pal1. ATP	pal1 (Phe	chr2:155	95.2238	76.6379	148.771	116.27	365.237	284.379
Sense	GT Sens	-0.540	Detected	0.540	Detected	0.000	1.347	Detected	1.661	Detected	1.504	0.116	GT Sense	contig144	contig144	Pol polyp	TGACTT/	contig144	Solyc10g	Solyc10g	Pol polyp	GO:0006:	GO:0006:	contig144	Solyc10g	Pol polyp	GO:0003(	SL2.40ch	AT1G27170.2		ATP bind	chr1:943	38.5089	66.7114	204.704	98.8004	148.453	181.121
Sense	GT Sens	0.669	Comprom	-0.669	Comprom	0.000	1.239	Detected	1.033	Detected	1.136	0.235	GT Sense	contig147	contig147	Mutator-li	AGAAGA/	contig147	Solyc05g	Solyc05g	Mutator-like transposase (AHF			contig147	Solyc07g	Mutator-li	GO:0008:	SL2.40ch	AT5G59450.1		scarecro	chr5:239	8.80117	2.85041	2.67435	5.14633	13.61	11.5832
Sense	GT Sens	-0.881	Comprom	0.881	Comprom	0.000	1.341	Detected	1.509	Detected	1.425	0.249	GT Sense	contig148	contig148	Pol polyp	CGTCAA/	contig148	Solyc11g	Solyc11g	Pol polyp	GO:0015(	GO:0015(	contig148	Solyc11g	Transpos	GO:0003(	SL2.40ch	AT5G12220.1		las1-like	chr5:395	3.17088	8.80551	12.7125	7.82424	15.4123	16.995
Sense	GT Sens	-0.281	Detected	0.281	Detected	0.000	0.939	Detected	1.284	Detected	1.112	0.078	GT Sense	contig148	contig148	Unknown	ATTGAG(	contig148	Solyc01g	Solyc01g	Unknown Protein (AHRD V1)%:			contig148	Solyc01g	Unknown Protein (A		SL2.40ch	AT4G21600.1				16.9012	20.4337	23.9693	28.4248	41.0216	51.1498
Sense	GT Sens	-0.652	Detected	0.652	Detected	0.000	0.809	Detected	1.431	Detected	1.120	0.261	GT Sense	contig148	contig148	Unknown	GACATA(	contig148	Solyc08g	Solyc08g	Unknown Protein (AHRD V1)			contig148	Solyc03g	&aposchromo&apo		SL2.40ch	AT3G51120.1		DNA bind	chr3:189	331.378	669.826	1744.51	1017.72	950.29	1435.46
Sense	GT Sens	0.261	Detected	-0.261	Detected	0.000	1.619	Detected	1.497	Detected	1.558	0.028	GT Sense	contig149	contig149	MuDR fan	GAAGTA/	contig149	Solyc08g	Solyc08g	MuDR family transposase con			contig149	Solyc10g	Mutator-li	GO:0008:	SL2.40ch	AT2G46360.1		unknowr	chr2:190	22.0915	12.5965	45.4512	45.4382	58.9955	53.229
Sense	GT Sens	-0.423	Detected	0.423	Detected	0.000	1.209	Detected	1.496	Detected	1.353	0.094	GT Sense	contig152	contig152	Unknown	GGACCT(	contig152	Solyc03g	Solyc03g	Unknown Protein (AHRD V1)			contig152	Solyc03g	Unknown Protein (A		SL2.40ch	AT1G06800.2				96.0777	141.554	248.152	208.299	310.472	371.821
Sense	GT Sens	-0.153	Detected	0.153	Detected	0.000	1.689	Detected	1.693	Detected	1.691	0.008	GT Sense	contig154	contig154	CBL-inter	CCTGCA/	contig154	Solyc06g	Solyc06g	CBL-inter	GO:0004(	GO:0004(	contig154	Solyc06g	CBL-inter	GO:0004(	SL2.40ch	AT5G583	CIPK10. F	SIP1 (SO	chr5:235	22.6296	22.9288	36.3822	26.0584	84.5703	83.2163
Sense	GT Sens	-0.254	Detected	0.254	Detected	0.000	1.085	Detected	1.515	Detected	1.300	0.060	GT Sense	contig154	contig154	Unknown	GATACC(	contig154	Solyc03g	Solyc03g	Unknown Protein (AHRD V1)			contig154	Solyc03g	Unknown Protein (A		SL2.40ch	AT1G502	ALATS. A	ALATS (/	chr1:185	122.196	142.427	416.084	340.216	322.135	426.148
Sense	GT Sens	-0.532	Detected	0.532	Detected	0.000	1.178	Detected	1.340	Detected	1.259	0.144	GT Sense	contig154	contig154	Reverse t	AACTCA/	contig154	Solyc05g	Solyc05g	Reverse transcriptase (Fragm			contig154	Solyc10g	Pol polyp	GO:0003(	SL2.40ch	AT5G50330.2		LOCATE	chr5:204	13.8224	23.6568	43.6065	31.6235	47.1056	51.727
Sense	GT Sens	0.436	Comprom	-0.436	Comprom	0.000	1.291	Detected	1.874	Detected	1.583	0.094	GT Sense	contig157	contig157	Receptor	CCATTC(	contig157	Solyc12g	Solyc12g	Receptor	GO:0005!	GO:0005!	contig157	Solyc12g	LRR recel	GO:0005!	SL2.40ch	AT1G26230.1		chaperor	chr1:907	6.14429	2.75117	1.97597	6.9346	11.587	17.0309
Sense	GT Sens	0.004	Detected	-0.004	Detected	0.000	1.280	Detected	1.589	Detected	1.435	0.011	GT Sense	contig158	contig158	Unknown	GTATCC/	contig158	Solyc02g	Solyc02g	Unknown Protein (AHRD V1)			contig158	Solyc09g	Unknown Protein (A		SL2.40ch	AT3G46640.2				21.2972	17.3469	48.8709	40.9834	53.7531	65.3721
[illegible]																																							

		0h_AZ_Control					0h_AZ_TAPG + TPRP vs. 0h_AZ_Control						
Orientatio	ProbeNar	Fold 0h	Flag 0h	Fold 0h	Flag 0h	an 0h AZ	Fold 0h	Flag 0h	Fold 0h	Flag 0h	Geomean	P-value	ProbeNar
Sense	A 96 P01	-0.022	Detected	0.022	Detected	0.000	-1.579	Detected	-1.530	Detected	-1.555	0.000	A 96 P01
Sense	A 96 P02	1.000	Detected	-1.000	Detected	0.000	-1.828	Detected	-1.317	Detected	-1.573	0.267	A 96 P02
Sense	A 96 P03	0.036	Detected	-0.036	Detected	0.000	-2.177	Comprom	-1.680	Comprom	-1.928	0.017	A 96 P03
Sense	A 96 P04	0.679	Detected	-0.679	Detected	0.000	-1.250	Detected	-0.965	Detected	-1.108	0.252	A 96 P04
Sense	A 96 P07	0.267	Detected	-0.267	Detected	0.000	-0.977	Detected	-1.366	Detected	-1.172	0.071	A 96 P07
Sense	A 96 P08	0.811	Detected	-0.811	Detected	0.000	-1.352	Detected	-1.727	Detected	-1.539	0.206	A 96 P08
Sense	A 96 P08	0.766	Detected	-0.766	Detected	0.000	-1.609	Detected	-1.044	Detected	-1.327	0.246	A 96 P08
Sense	A 96 P08	0.148	Detected	-0.148	Detected	0.000	-1.165	Detected	-1.684	Detected	-1.425	0.041	A 96 P08
Sense	A 96 P08	0.845	Detected	-0.845	Detected	0.000	-2.525	Detected	-1.638	Detected	-2.082	0.161	A 96 P08
Sense	A 96 P13	0.695	Detected	-0.695	Detected	0.000	-1.655	Detected	-1.652	Detected	-1.654	0.140	A 96 P13
Sense	A 96 P17	0.338	Detected	-0.338	Detected	0.000	-1.243	Detected	-1.036	Detected	-1.139	0.084	A 96 P17
Sense	A 96 P18	0.689	Detected	-0.689	Detected	0.000	-1.232	Detected	-0.853	Detected	-1.043	0.282	A 96 P18
Sense	A 96 P19	0.282	Detected	-0.282	Detected	0.000	-0.960	Detected	-1.354	Detected	-1.157	0.078	A 96 P19
Sense	A 96 P20	0.673	Detected	-0.673	Detected	0.000	-1.163	Detected	-1.451	Detected	-1.307	0.198	A 96 P20
Sense	A 96 P20	0.275	Detected	-0.275	Detected	0.000	-0.908	Detected	-1.196	Detected	-1.052	0.077	A 96 P20
Sense	A 96 P21	0.193	Detected	-0.193	Detected	0.000	-0.871	Detected	-1.710	Detected	-1.290	0.108	A 96 P21
Sense	A 96 P24	0.161	Detected	-0.161	Detected	0.000	-1.130	Detected	-1.303	Detected	-1.216	0.022	A 96 P24
Sense	A 96 P24	0.272	Detected	-0.272	Detected	0.000	-1.778	Detected	-1.024	Detected	-1.401	0.095	A 96 P24
Sense	A 96 P26	0.911	Detected	-0.911	Detected	0.000	-1.866	Detected	-1.670	Detected	-1.768	0.193	A 96 P26
Sense	GT Sens	0.051	Detected	-0.051	Detected	0.000	-1.478	Detected	-1.314	Detected	-1.396	0.005	GT Sense
Sense	GT Sens	0.017	Detected	-0.017	Detected	0.000	-1.275	Detected	-1.098	Detected	-1.187	0.006	GT Sense
Sense	GT Sens	0.425	Detected	-0.425	Detected	0.000	-1.151	Detected	-0.973	Detected	-1.062	0.134	GT Sense
Sense	GT Sens	0.627	Detected	-0.627	Detected	0.000	-1.890	Detected	-2.064	Detected	-1.977	0.089	GT Sense
Sense	GT Sens	-0.571	Detected	0.571	Detected	0.000	-1.369	Detected	-1.179	Detected	-1.274	0.159	GT Sense
Sense	GT Sens	0.759	Detected	-0.759	Detected	0.000	-1.816	Detected	-1.271	Detected	-1.543	0.196	GT Sense
Sense	GT Sens	0.256	Detected	-0.256	Detected	0.000	-1.675	Detected	-1.632	Detected	-1.653	0.023	GT Sense
Sense	GT Sens	0.829	Detected	-0.829	Detected	0.000	-1.445	Detected	-0.852	Detected	-1.149	0.322	GT Sense
Sense	GT Sens	-0.247	Detected	0.247	Detected	0.000	-0.828	Detected	-1.739	Comprom	-1.284	0.132	GT Sense
Sense	GT Sens	0.779	Detected	-0.779	Detected	0.000	-0.811	Detected	-1.503	Detected	-1.157	0.307	GT Sense
Sense	GT Sens	-0.503	Detected	0.503	Detected	0.000	-2.075	Detected	-1.814	Detected	-1.944	0.065	GT Sense
Sense	GT Sens	-0.963	Detected	0.963	Detected	0.000	-1.521	Detected	-1.148	Detected	-1.335	0.307	GT Sense
Sense	GT Sens	0.157	Detected	-0.157	Detected	0.000	-1.368	Detected	-0.974	Detected	-1.171	0.043	GT Sense
Sense	GT Sens	0.571	Detected	-0.571	Detected	0.000	-1.305	Detected	-1.004	Detected	-1.155	0.190	GT Sense
Sense	GT Sens	-0.842	Detected	0.842	Detected	0.000	-1.328	Detected	-1.344	Detected	-1.336	0.254	GT Sense
Sense	GT Sens	-0.307	Detected	0.307	Detected	0.000	-1.893	Detected	-1.047	Detected	-1.470	0.107	GT Sense
Sense	GT Sens	0.353	Detected	-0.353	Detected	0.000	-1.505	Detected	-1.113	Detected	-1.309	0.083	GT Sense
Sense	GT Sens	-0.152	Detected	0.152	Detected	0.000	-1.228	Detected	-1.188	Detected	-1.208	0.016	GT Sense
Sense	GT Sens	0.720	Detected	-0.720	Detected	0.000	-1.539	Detected	-0.982	Detected	-1.260	0.244	GT Sense
Sense	GT Sens	0.129	Detected	-0.129	Detected	0.000	-1.846	Detected	-1.285	Detected	-1.565	0.037	GT Sense
Sense	GT Sens	-0.220	Detected	0.220	Detected	0.000	-1.225	Comprom	-0.874	Detected	-1.050	0.065	GT Sense
Sense	GT Sens	-0.045	Detected	0.045	Detected	0.000	-4.082	Detected	-4.194	Detected	-4.138	0.000	GT Sense
Sense	GT Sens	0.336	Detected	-0.336	Detected	0.000	-0.918	Detected	-1.088	Detected	-1.003	0.102	GT Sense
Sense	GT Sens	-0.097	Detected	0.097	Detected	0.000	-1.077	Detected	-0.942	Detected	-1.009	0.013	GT Sense
Sense	GT Sens	0.637	Detected	-0.637	Detected	0.000	-1.323	Detected	-1.164	Detected	-1.244	0.192	GT Sense
Sense	GT Sens	0.658	Detected	-0.658	Detected	0.000	-1.196	Detected	-0.965	Detected	-1.080	0.247	GT Sense
Sense	GT Sens	0.101	Detected	-0.101	Detected	0.000	-3.219	Comprom	-1.375	Comprom	-2.297	0.132	GT Sense
Sense	GT Sens	0.055	Detected	-0.055	Detected	0.000	-1.304	Detected	-0.964	Detected	-1.134	0.024	GT Sense
Sense	GT Sens	-0.508	Detected	0.508	Detected	0.000	-1.332	Detected	-1.408	Detected	-1.370	0.115	GT Sense
Sense	GT Sens	-0.658	Detected	0.658	Detected	0.000	-3.318	Comprom	-1.585	Comprom	-2.451	0.153	GT Sense
Sense	GT Sens	0.697	Detected	-0.697	Detected	0.000	-1.483	Detected	-1.417	Detected	-1.450	0.173	GT Sense
Sense	GT Sens	0.744	Detected	-0.744	Detected	0.000	-1.312	Detected	-0.942	Detected	-1.127	0.279	GT Sense
Sense	GT Sens	0.721	Detected	-0.721	Detected	0.000	-1.659	Detected	-0.820	Detected	-1.240	0.276	GT Sense
Sense	GT Sens	-0.151	Detected	0.151	Detected	0.000	-1.266	Detected	-1.102	Detected	-1.184	0.021	GT Sense
Sense	GT Sens	0.478	Detected	-0.478	Detected	0.000	-1.020	Detected	-0.996	Detected	-1.008	0.169	GT Sense
Sense	GT Sens	-0.338	Detected	0.338	Detected	0.000	-2.342	Comprom	-2.505	Comprom	-2.423	0.020	GT Sense
Sense	GT Sens	-0.064	Detected	0.064	Detected	0.000	-1.152	Detected	-1.034	Detected	-1.093	0.006	GT Sense
Sense	GT Sens	-0.108	Detected	0.108	Detected	0.000	-0.834	Detected	-1.277	Comprom	-1.055	0.051	GT Sense
Sense	GT Sens	0.545	Detected	-0.545	Detected	0.000	-2.636	Comprom	-3.482	Comprom	-3.059	0.047	GT Sense
Sense	GT Sens	-0.342	Detected	0.342	Detected	0.000	-2.122	Comprom	-2.924	Comprom	-2.523	0.041	GT Sense
Sense	GT Sens	0.576	Detected	-0.576	Detected	0.000	-1.248	Detected	-1.743	Detected	-1.495	0.140	GT Sense
Sense	GT Sens	0.310	Detected	-0.310	Detected	0.000	-0.870	Detected	-1.479	Detected	-1.174	0.114	GT Sense
Sense	GT Sens	0.365	Detected	-0.365	Detected	0.000	-2.124	Detected	-3.708	Detected	-2.916	0.079	GT Sense
Sense	GT Sens	0.210	Detected	-0.210	Detected	0.000	-1.431	Detected	-1.362	Detected	-1.396	0.022	GT Sense
Sense	GT Sens	0.161	Detected	-0.161	Detected	0.000	-1.057	Detected	-1.049	Detected	-1.053	0.022	GT Sense
Sense	GT Sens	0.228	Detected	-0.228	Detected	0.000	-1.084	Detected	-1.184	Detected	-1.134	0.040	GT Sense
Sense	GT Sens	-1.119	Detected	1.119	Detected	0.000	-1.331	Detected	-1.312	Detected	-1.321	0.359	GT Sense
Sense	GT Sens	0.318	Detected	-0.318	Detected	0.000	-1.394	Detected	-2.948	Comprom	-2.171	0.123	GT Sense
Sense	GT Sens	0.928	Detected	-0.928	Detected	0.000	-1.176	Detected	-0.968	Detected	-1.072	0.370	GT Sense
Sense	GT Sens	0.345	Detected	-0.345	Detected	0.000	-0.869	Detected	-1.658	Detected	-1.264	0.138	GT Sense
Sense	GT Sens	-0.681	Detected	0.681	Detected	0.000	-2.049	Detected	-1.557	Detected	-1.803	0.131	GT Sense
Sense	GT Sens	-0.218	Detected	0.218	Detected	0.000	-2.212	Detected	-1.633	Detected	-1.922	0.034	GT Sense
Sense	GT Sens	0.032	Detected	-0.032	Detected	0.000	-1.276	Detected	-2.389	Detected	-1.832	0.081	GT Sense
Sense	GT Sens	0.761	Detected	-0.761	Detected	0.000	-1.520	Detected	-0.834	Detected	-1.177	0.294	GT Sense
Sense	GT Sens	0.552	Detected	-0.552	Detected	0.000	-1.739	Detected	-1.143	Detected	-1.441	0.148	GT Sense
Sense	GT Sens	-0.098	Detected	0.098	Detected	0.000	-0.836	Detected	-1.315	Detected	-1.075	0.053	GT Sense
Sense	GT Sens	-0.460	Detected	0.460	Detected	0.000	-1.216	Comprom	-1.954	Comprom	-1.585	0.115	GT Sense
Sense	GT Sens	0.511	Detected	-0.511	Detected	0.000	-2.408	Detected	-1.224	Detected	-1.816	0.146	GT Sense
Sense	GT Sens	0.224	Detected	-0.224	Detected	0.000	-1.212	Detected	-0.980	Detected	-1.096	0.049	GT Sense
Sense	GT Sens	0.003	Detected	-0.003	Detected	0.000	-1.134	Detected	-0.939	Detected	-1.036	0.009	GT Sense
Sense	GT Sens	0.056	Detected	-0.056	Detected	0.000	-1.418	Detected	-2.200	Detected	-1.809	0.044	GT Sense
Sense	GT Sens	0.649	Detected	-0.649	Detected	0.000	-1.483	Detected	-1.377	Detected	-1.430	0.159	GT Sense
Sense	GT Sens	0.468	Detected	-0.468	Detected	0.000	-1.227	Detected	-1.031	Detected	-1.129	0.142	GT Sense
Sense	GT Sens	-0.399	Detected	0.399	Detected	0.000	-1.076	Detected	-1.100	Detected	-1.088	0.112	GT Sense
Sense	GT Sens	-1.033	Detected	1.033	Detected	0.000	-1.106	Detected	-1.969	Detected	-1.537	0.303	GT Sense
Sense	GT Sens	0.588	Detected	-0.588	Detected	0.000	-1.075	Detected	-2.072	Detected	-1.573	0.178	GT Sense
Sense	GT Sens	-0.539	Detected	0.539	Detected	0.000	-1.179	Detected	-1.204	Detected	-1.192	0.158	GT Sense
Sense	GT Sens	0.361	Detected	-0.361	Detected	0.000	-1.417	Detected	-1.157	Detected	-1.287	0.078	GT Sense
Sense	GT Sens	0.852	Detected	-0.852	Detected	0.000	-1.148	Detected	-1.223	Detected	-1.186	0.299	GT Sense
Sense	GT Sens	1.058	Detected	-1.058	Detected	0.000	-1.808	Detected	-1.320	Detected	-1.564	0.286	GT Sense
Sense	GT Sens	0.521	Detected	-0.521	Detected	0.000	-2.072	Detected	-1.373	Detected	-1.722	0.111	GT Sense
Sense	GT Sens	0.382	Detected	-0.382	Detected	0.000	-1.099	Detected	-1.029	Detected	-1.064	0.109	GT Sense
Sense	GT Sens	0.018	Detected	-0.018	Detected	0.000	-1.001	Detected	-1.121	Detected	-1.061	0.004	GT Sense
Sense	GT Sens	0.018	Detected	-0.018	Detected	0.000	-1.178	Detected	-0.891	Detected	-1.035	0.019	GT Sense
Sense	GT Sens	-0.450	Detected	0.450	Detected	0.000	-0.948	Detected	-1.157	Detected	-1.053	0.150	GT Sense
Sense	GT Sens	-0.283	Detected	0.283	Detected	0.000	-2.056	Detected	-2.098	Detected	-2.077	0.018	GT Sense
Sense	GT Sens	0.519	Detected	-0.519	Detected	0.000	-1.396	Detected	-1.405	Detected	-1.401	0.114	GT Sense
Sense	GT Sens	0.360	Detected	-0.360	Detected	0.000	-1.380	Detected	-1.241	Detected	-1.311	0.070	GT Sense
Sense	GT Sens	0.171	Detected	-0.171	Detected	0.000	-1.374	Comprom	-1.940	Comprom	-1.657	0.038	GT Sense
Sense	GT Sens	-0.770	Detected	0.770	Detected	0.000	-1.221	Detected	-1.111	Detected	-1.166	0.270	GT Sense
Sense	GT Sens	0.486	Detected	-0.486	Detected	0.000	-1.596	Detected	-1.032	Detected	-1.314	0.144	GT Sense
Sense	GT Sens	-0.030	Detected	0.030	Detected	0.000	-1.016	Detected	-1.260	Detected	-1.138	0.012	GT Sense
Sense	GT Sens	0.591	Detected	-0.591	Detected	0.000	-0.908	Detected	-1.357	Detected	-1.133	0.215	GT Sense
Sense	GT Sens	-0.285	Detected	0.285	Detected	0.000	-3.373	Comprom	-2.647	Comprom	-3.010	0.023	GT Sense
Sense	GT Sens	0.261	Detected	-0.261	Detected	0.000	-0.912	Detected	-1.096	Detected	-1.004	0.068	GT Sense
Sense	GT Sens	-0.083	Detected	0.083	Detected	0.000	-1.410	Detected	-1.009	Detected	-1.210	0.031	GT Sense
Sense	GT Sens	0.308	Detected	-0.308	Detected	0.000	-1.283	Detected	-1.881	Comprom	-1.582	0.066	GT Sense
Sense	GT Sens	0.769	Detected	-0.769	Detected	0.000	-1.673	Detected	-0.960	Detected	-1.316	0.261	GT Sense
Sense	GT Sens	-0.727	Detected	0.727	Detected	0.000	-0.921	Detected	-1.511	Comprom	-1.216	0.261	GT Sense
Sense	GT Sens	0.102	Detected	-0.102	Detected	0.000	-1.118	Detected	-1.113	Detected	-1.115	0.008	GT Sense
Sense	GT Sens	-0.341	Detected	0.341	Detected	0.000	-3.669	Comprom	-3.563	Comprom	-3.616	0.009	GT Sense
Sense	GT Sens	-0.512	Detected	0.512	Detected	0.000	-1.349	Detected	-2.340	Detected	-1.844	0.122	GT Sense
Sense	GT Sens	0.686	Detected	-0.686	Detected	0.000	-1.361	Detected	-1.006	Detected	-1.184	0.237	GT Sense
Sense	GT Sens	0.010	Detected	-0.010	Detected	0.000	-1.322	Detected	-1.272	Detected	-1.297	0.000	GT Sense
Sense	GT Sens	0.483	Detected	-0.483	Detected	0.000	-1.616	Detected	-1.062	Detected	-1.339	0.138	GT Sense
Sense	GT Sens	0.326	Detected	-0.326	Detected	0.000	-1.943	Detected	-0.908	Detected	-1.426	0.145	GT Sense
Sense	GT Sens	1.104	Detected	-1.104	Detected	0.000	-1.599	Detected	-1.708	Detected	-1.653	0.273	GT Sense
Sense	GT Sens	-0.132	Detected	0.132	Detected	0.000	-1.384	Detected	-1.570	Detected	-1.477	0.012	GT Sense
Sense	GT Sens	-1.209	Detected	1.209	Detected	0.000	-2.284	Comprom	-1.323	Detected	-1.804	0.300	GT Sense
Sense	GT Sens	0.383	Detected	-0.383	Detected	0.000	-1.561	Detected	-1.074	Detected	-1.317	0.101	GT Sense
Sense	GT Sens	0.560	Detected	-0.560	Detected	0.000	-1.118	Detected	-0.988	Detected	-1.053	0.203	GT Sense
Sense	GT Sens	-0.302	Detected	0.302	Detected	0.000	-1.033	Detected	-1.130	Detected	-1.082	0.071	GT Sense
Sense	GT Sens	0.599	Detected	-0.599	Detected	0.000	-1.098	Detected	-0.945	Detected	-1.021	0.233	GT Sense
Sense	GT Sens	1.956	Detected	-1.956	Detected	0.000	-2.579	Detected	-3.168	Detected	-2.874	0.283	GT Sense
Sense	GT Sens	-0.003	Detected	0.003	Detected	0.000	-1.770	Detected	-1.690	Detected	-1.730	0.001	GT Sense
Sense	GT Sens	0.330	Detected	-0.330	Detected	0.000	-1.219	Detected	-1.299	Detected	-1.259	0.063	GT Sense
Sense	GT Sens	0.169	Detected	-0.169	Detected	0.000	-0.899	Detected	-1.962	Comprom	-1.431	0.124	GT Sense
Sense	GT Sens	0.120	Detected	-0.120	Detected	0.000	-1.195	Detected	-1.449	Detected	-1.322	0.017	GT Sense
Sense	GT Sens	0.512	Detected	-0.512	Detected	0.000	-1.676	Detected	-1.284	Detected	-1.480	0.114	GT Sense
Sense	GT Sens	0.741	Detected	-0.741	Detected	0.000	-1.099	Detected	-0.922	Detected	-1.010	0.309	GT Sense
Sense	GT Sens	2.415	Detected	-2.415	Detected	0.000	-1.445	Detected	-3.369	Detected	-2.407	0.452	GT Sense
Sense	GT Sens	0.485	Detected	-0.485	Detected	0.000	-0.989	Detected	-1.079	Detected	-1.034	0.168	GT Sense
Sense	GT Sens	0.643	Detected	-0.643	Detected	0.000	-1.282	Detected	-0.827	Detected	-1.055	0.262	GT Sense
Sense	GT Sens	-0.108	Detected	0.108	Detected	0.000	-0.969	Detected	-1.112	Detected	-1.040	0.015	GT Sense
Sense	GT Sens	0.169	Detected	-0.169	Detected	0.000	-3.407	Comprom	-2.252	Detected	-2.830	0.042	GT Sense
Sense	GT Sens	-0.257	Detected	0.257	Detected	0.000	-1.861	Comprom	-1.613	Comprom	-1.737	0.026	GT Sense
Sense	GT Sens	0.293	Detected	-0.293	Detected	0.000	-1.996	Detected	-1.372	Detected	-1.684	0.059	GT Sense
Sense	GT Sens	0.617	Detected	-0.617	Detected	0.000	-1.050	Detected	-1.255	Detected	-1.152	0.207	GT Sense
Sense	GT Sens	-0.585	Detected	0.585	Detected	0.000	-1.192	Detected	-0.877	Detected	-1.035	0.230	GT Sense
Sense	GT Sens	-0.134	Detected	0.134	Detected	0.000	-0.903	Detected	-1.122	Detected	-1.013	0.028	GT Sense
Sense	GT Sens	-0.331	Detected	0.331	Detected	0.000	-1.063	Detected	-1.041	Detected	-1.052	0.086	GT Sense
Sense	GT Sens	-0.390	Detected	0.390	Detected	0.000	-1.408	Comprom	-1.191	Comprom	-1.299	0.085	GT Sense
Sense	GT Sens	0.452	Detected	-0.452	Detected	0.000	-1.464	Detected	-1.964	Detected	-1.714	0.080	GT Sense
Sense	GT Sens	-0.242	Detected	0.242	Detected	0.000	-1.579	Comprom	-1.480	Comprom	-1.530	0.025	GT Sense
Sense	GT Sens	0.773	Detected	-0.773	Detected	0.000	-1.956	Detected	-1.365	Detected	-1.661	0.183	GT Sense
Sense	GT Sens	-0.274	Detected	0.274	Detected	0.000	-0.936	Detected	-1.461	Detected	-1.199	0.087	GT Sense
Sense	GT Sens	1.350	Detected	-1.350	Detected	0.000	-1.378	Detected	-1.427	Detected	-1.402	0.408	GT Sense
Sense	GT Sens	-0.122	Detected	0.122	Detected	0.000	-1.226	Detected	-0.958	Detected	-1.092	0.026	GT Sense
Sense	GT Sens	-0.539	Detected	0.539	Detected	0.000	-0.903	Detected	-1.121	Detected	-1.012	0.207	GT Sense
Sense	GT Sens	0.614	Detected	-0.614	Detected	0.000	-0.937	Detected	-1.412	Detected	-1.175	0.216	GT Sense
Sense	GT Sens	0.543	Detected	-0.543	Detected	0.000	-0.931	Detected	-1.504	Detected	-1.218	0.186	GT Sense
Sense	GT Sens	0.749	Detected	-0.749	Detected	0.000	-0.801	Detected	-2.591	Detected	-1.696	0.283	GT Sense
Sense	GT Sens	-0.201	Detected	0.201	Detected	0.000	-1.950	Comprom	-1.858	Comprom	-1.904	0.012	GT Sense
Sense	GT Sens	0.249	Detected	-0.249	Detected	0.000	-1.075	Detected	-0.996	Detected	-1.035	0.054	GT Sense
Sense	GT Sens	0.605	Detected	-0.605	Detected	0.000	-0.830	Detected	-1.893	Detected	-1.362	0.233	GT Sense
Sense	GT Sens	0.012	Detected	-0.012	Detected	0.000	-2.348	Detected	-1.884	Detected	-2.116	0.012	GT Sense
Sense	GT Sens	0.057	Detected	-0.057	Detected	0.000	-1.139	Detected	-1.438	Detected	-1.289	0.015	GT Sense
Sense	GT Sens	0.662	Detected	-0.662	Detected	0.000	-1.442	Detected	-0.982	Detected	-1.212	0.226	GT Sense
Sense	GT Sens	0.372	Detected	-0.372	Detected	0.000	-2.434	Detected	-1.466	Detected	-1.950	0.085	GT Sense
Sense	GT Sens	0.673	Detected	-0.673	Detected	0.000	-0.884	Detected	-1.824	Detected	-1.354	0.241	GT Sense
Sense	GT Sens	0.108	Detected	-0.108	Detected	0.000	-0.869	Detected	-1.592	Detected	-1.230	0.083	GT Sense
Sense	GT Sens	0.174	Detected	-0.174	Detected	0.000	-1.339	Detected	-1.150	Detected	-1.244	0.024	GT Sense
Sense	GT Sens	-0.002	Detected	0.002	Detected	0.000	-1.632	Detected	-1.619	Detected	-1.626	0.000	GT Sense
Sense	GT Sens	-0.357	Detected	0.357	Detected	0.000	-1.129	Detected	-1.013	Detected	-1.071	0.097	GT Sense
Sense	GT Sens	0.274	Detected	-0.274	Detected	0.000	-1.836	Comprom	-0.861	Detected	-1.348	0.137	GT Sense
Sense	GT Sens	-0.380	Detected	0.380	Detected	0.000	-1.221	Detected	-0.894	Detected	-1.058	0.125	GT Sense
Sense	GT Sens	1.380	Detected	-1.380	Detected	0.000	-1.627	Detected	-1.602	Detected	-1.615	0.362	GT Sense
Sense	GT Sens	0.080	Detected	-0.080	Detected	0.000	-0.909	Detected	-1.778	Detected	-1.343	0.093	GT Sense
Sense	GT Sens	-1.587	Detected	1.587	Detected	0.000	-2.405	Detected	-1.950	Detected	-2.178	0.307	GT Sense
Sense	GT Sens	0.044	Detected	-0.044	Detected	0.000	-1.345	Detected	-1.965	Detected	-1.655	0.034	GT Sense
Sense	GT Sens	0.739	Detected	-0.739	Detected	0.000	-1.901	Detected	-1.501	Detected	-1.701	0.156	GT Sense
Sense	GT Sens	0.122	Detected	-0.122	Detected	0.000	-1.329	Detected	-1.290	Detected	-1.309	0.009	GT Sense
Sense	GT Sens	0.598	Detected	-0.598	Detected	0.000	-1.314	Detected	-1.025	Detected	-1.169	0.198	GT Sense
Sense	GT Sens	0.917	Detected	-0.917	Detected	0.000	-1.739	Detected	-1.113	Detected	-1.426	0.279	GT Sense
Sense	GT Sens	0.339	Detected	-0.339	Detected	0.000	-1.978	Detected	-2.427	Comprom	-2.203	0.032	GT Sense
Sense	GT Sens	-0.539	Detected	0.539	Detected	0.000	-1.019	Detected	-1.296	Detected	-1.157	0.173	GT Sense
Sense	GT Sens	-1.183	Detected	1.183	Detected	0.000	-2.446	Detected	-1.254	Detected	-1.850	0.297	GT Sense
Sense	GT Sens	-0.081	Detected	0.081	Detected	0.000	-1.361	Detected	-1.266	Detected	-1.313	0.005	GT Sense
Sense	GT Sens	-0.087	Detected	0.087	Detected	0.000	-1.087	Detected	-1.036	Detected	-1.061	0.007	GT Sense
Sense	GT Sens	0.167	Detected	-0.167	Detected	0.000	-3.200	Comprom	-2.861	Comprom	-3.030	0.006	GT Sense
Sense	GT Sens	0.441	Detected	-0.441	Detected	0.000	-2.010	Detected	-2.251	Detected	-2.131	0.043	GT Sense
Sense	GT Sens	0.268	Detected	-0.268	Detected	0.000	-1.520	Detected	-1.057	Detected	-1.289	0.068	GT Sense
Sense	GT Sens	0.518	Detected	-0.518	Detected	0.000	-1.615	Detected	-1.323	Detected	-1.469	0.112	GT Sense
Sense	GT Sens	-0.474	Detected	0.474	Detected	0.000	-1.160	Detected	-1.422	Detected	-1.291	0.119	GT Sense
Sense	GT Sens	0.788	Detected	-0.788	Detected	0.000	-1.831	Detected	-1.495	Detected	-1.663	0.175	GT Sense
Sense	GT Sens	0.107	Detected	-0.107	Detected	0.000	-1.176	Detected	-1.229	Detected	-1.202	0.008	GT Sense
Sense	GT Sens	0.652	Detected	-0.652	Detected	0.000	-1.312	Detected	-0.875	Detected	-1.094	0.253	GT Sense
Sense	GT Sens	0.655	Detected	-0.655	Detected	0.000	-1.204	Detected	-0.853	Detected	-1.029	0.269	GT Sense
Sense	GT Sens	0.725	Detected	-0.725	Detected	0.000	-0.984	Detected	-1.381	Detected	-1.182	0.256	GT Sense
Sense	GT Sens	-2.078	Detected	2.078	Detected	0.000	-1.555	Detected	-1.857	Detected	-1.706	0.499	GT Sense
Sense	GT Sens	-0.311	Detected	0.311	Detected	0.000	-1.001	Detected	-1.545	Detected	-1.273	0.091	GT Sense
Sense	GT Sens	-0.299	Detected	0.299	Detected	0.000	-1.123	Detected	-1.017	Detected	-1.070	0.072	GT Sense
Sense	GT Sens	0.930	Detected	-0.930	Detected	0.000	-1.166	Detected	-1.026	Detected	-1.096	0.361	GT Sense
Sense	GT Sens	0.441	Detected	-0.441	Detected	0.000	-1.773	Detected	-1.976	Detected	-1.875	0.054	GT Sense
Sense	GT Sens	0.215	Detected	-0.215	Detected	0.000	-1.366	Detected	-0.988	Detected	-1.177	0.054	GT Sense
Sense	GT Sens	0.396	Detected	-0.396	Detected	0.000	-0.861	Detected	-1.151	Detected	-1.006	0.140	GT Sense
Sense	GT Sens	0.564	Detected	-0.564	Detected	0.000	-1.813	Detected	-0.969	Detected	-1.391	0.187	GT Sense
Sense	GT Sens	0.289	Detected	-0.289	Detected	0.000	-1.553	Detected	-1.161	Detected	-1.357	0.060	GT Sense
Sense	GT Sens	0.248	Detected	-0.248	Detected	0.000	-0.864	Detected	-2.055	Comprom	-1.459	0.152	GT Sense
Sense	GT Sens	0.097	Detected	-0.097	Detected	0.000	-1.336	Detected	-1.260	Detected	-1.298	0.006	GT Sense
Sense	GT Sens	0.875	Detected	-0.875	Detected	0.000	-1.999	Detected	-1.532	Detected	-1.766	0.191	GT Sense
Sense	GT Sens	-0.403	Detected	0.403	Detected	0.000	-0.948	Detected	-1.083	Detected	-1.016	0.131	GT Sense
Sense	GT Sens	0.583	Detected	-0.583	Detected	0.000	-1.598	Detected	-0.971	Detected	-1.285	0.192	GT Sense
Sense	GT Sens	0.681	Detected	-0.681	Detected	0.000	-1.497	Detected	-0.932	Detected	-1.214	0.241	GT Sense
Sense	GT Sens	-0.032	Detected	0.032	Detected	0.000	-1.192	Detected	-2.252	Comprom	-1.722	0.083	GT Sense
Sense	GT Sens	0.750	Detected	-0.750	Detected	0.000	-1.399	Detected	-1.107	Detected	-1.253	0.243	GT Sense
Sense	GT Sens	0.199	Detected	-0.199	Detected	0.000	-1.426	Detected	-0.809	Detected	-1.117	0.093	GT Sense
Sense	GT Sens	-0.184	Detected	0.184	Detected	0.000	-0.911	Comprom	-1.866	Comprom	-1.388	0.113	GT Sense
Sense	GT Sens	0.634	Detected	-0.634	Detected	0.000	-1.319	Detected	-1.158	Detected	-1.238	0.192	GT Sense
Sense	GT Sens	-0.824	Detected	0.824	Detected	0.000	-1.061	Detected	-1.360	Detected	-1.210	0.285	GT Sense
Sense	GT Sens	-0.644	Detected	0.644	Detected	0.000	-1.217	Detected	-1.088	Detected	-1.153	0.217	GT Sense
Sense	GT Sens	-0.612	Detected	0.612	Detected	0.000	-2.632	Comprom	-2.246	Comprom	-2.439	0.063	GT Sense
Sense	GT Sens	-0.779	Detected	0.779	Detected	0.000	-3.553	Comprom	-2.848	Comprom	-3.201	0.065	GT Sense
Sense	GT Sens	-0.066	Detected	0.066	Detected	0.000	-1.040	Detected	-1.102	Comprom	-1.071	0.005	GT Sense
Sense	GT Sens	-0.398	Detected	0.398	Detected	0.000	-1.205	Detected	-1.032	Detected	-1.119	0.111	GT Sense
Sense	GT Sens	-0.414	Detected	0.414	Detected	0.000	-0.974	Detected	-2.306	Comprom	-1.640	0.172	GT Sense
Sense	GT Sens	0.135	Detected	-0.135	Detected	0.000	-0.932	Detected	-1.108	Detected	-1.020	0.024	GT Sense
Sense	GT Sens	-0.186	Detected	0.186	Detected	0.000	-3.783	Detected	-4.068	Detected	-3.926	0.004	GT Sense
Sense	GT Sens	-0.436	Detected	0.436	Detected	0.000	-4.978	Comprom	-4.010	Comprom	-4.494	0.020	GT Sense
Sense	GT Sens	0.182	Detected	-0.182	Detected	0.000	-1.075	Detected	-1.186	Detected	-1.131	0.027	GT Sense
Sense	GT Sens	0.026	Detected	-0.026	Detected	0.000	-1.314	Detected	-1.315	Comprom	-1.315	0.000	GT Sense
Sense	GT Sens	0.776	Detected	-0.776	Detected	0.000	-2.060	Detected	-2.817	Comprom	-2.439	0.106	GT Sense
Sense	GT Sens	-0.317	Detected	0.317	Detected	0.000	-1.452	Detected	-1.779	Detected	-1.616	0.046	GT Sense
Sense	GT Sens	0.418	Detected	-0.418	Detected	0.000	-1.601	Detected	-1.911	Detected	-1.756	0.059	GT Sense
Sense	GT Sens	-0.911	Detected	0.911	Detected	0.000	-2.026	Comprom	-3.120	Comprom	-2.573	0.137	GT Sense
Sense	GT Sens	-1.062	Detected	1.062	Detected	0.000	-1.158	Detected	-1.219	Detected	-1.189	0.380	GT Sense
Sense	GT Sens	-0.258	Detected	0.258	Detected	0.000	-1.457	Comprom	-1.037	Comprom	-1.247	0.064	GT Sense
Sense	GT Sens	-0.605	Detected	0.605	Detected	0.000	-1.602	Comprom	-2.433	Comprom	-2.018	0.111	GT Sense
Sense	GT Sens	-0.309	Detected	0.309	Detected	0.000	-1.411	Detected	-0.989	Detected	-1.200	0.085	GT Sense
Sense	GT Sens	-1.805	Detected	1.805	Detected	0.000	-4.984	Comprom	-4.269	Comprom	-4.627	0.128	GT Sense
Sense	GT Sens	-0.475	Detected	0.475	Detected	0.000	-0.979	Comprom	-1.536	Comprom	-1.257	0.150	GT Sense
Sense	GT Sens	0.752	Detected	-0.752	Detected	0.000	-1.523	Detected	-0.932	Detected	-1.228	0.268	GT Sense
Sense	GT Sens	-0.754	Detected	0.754	Detected	0.000	-1.746	Detected	-1.285	Detected	-1.515	0.195	GT Sense
Sense	GT Sens	-0.079	Detected	0.079	Detected	0.000	-0.890	Detected	-1.144	Detected	-1.017	0.021	GT Sense
Sense	GT Sens	-0.162	Detected	0.162	Detected	0.000	-1.128	Detected	-1.495	Detected	-1.312	0.033	GT Sense
Sense	GT Sens	0.596	Detected	-0.596	Detected	0.000	-1.796	Detected	-1.571	Detected	-1.684	0.109	GT Sense
Sense	GT Sens	0.104	Detected	-0.104	Detected	0.000	-1.513	Comprom	-0.869	Detected	-1.191	0.072	GT Sense
Sense	GT Sens	-0.918	Detected	0.918	Detected	0.000	-1.624	Detected	-1.644	Detected	-1.634	0.217	GT Sense
Sense	GT Sens	0.239	Detected	-0.239	Detected	0.000	-2.073	Comprom	-1.796	Comprom	-1.934	0.020	GT Sense
Sense	GT Sens	0.070	Detected	-0.070	Detected	0.000	-1.328	Detected	-0.882	Detected	-1.105	0.042	GT Sense
Sense	GT Sens	-0.573	Detected	0.573	Detected	0.000	-1.309	Detected	-1.638	Comprom	-1.473	0.132	GT Sense
Sense	GT Sens	0.056	Detected	-0.056	Detected	0.000	-1.067	Detected	-3.438	Comprom	-2.252	0.198	GT Sense
Sense	GT Sens	0.113	Detected	-0.113	Detected	0.000	-1.640	Detected	-0.868	Detected	-1.254	0.089	GT Sense

Sense	GT Sens	-0.453	Detected	0.453	Detected	0.000	-1.006	Detected	-1.266	Comprom	-1.136	0.137	GT Sense	contig359	contig359	Unannota
Sense	GT Sens	-1.150	Detected	1.150	Detected	0.000	-1.544	Detected	-1.390	Detected	-1.467	0.331	GT Sense	contig360	contig360	Unannota
Sense	GT Sens	0.918	Detected	-0.918	Detected	0.000	-2.341	Comprom	-1.265	Detected	-1.803	0.232	GT Sense	contig365	contig365	Unannota
Sense	GT Sens	-0.256	Detected	0.256	Detected	0.000	-1.280	Detected	-1.080	Comprom	-1.180	0.050	GT Sense	contig365	contig365	Unannota
Sense	GT Sens	0.028	Detected	-0.028	Detected	0.000	-1.743	Comprom	-1.848	Comprom	-1.796	0.001	GT Sense	contig365	contig365	Unannota
Sense	GT Sens	0.361	Detected	-0.361	Detected	0.000	-2.150	Detected	-0.988	Detected	-1.569	0.149	GT Sense	contig367	contig367	Senescen
Sense	GT Sens	-0.034	Detected	0.034	Detected	0.000	-0.848	Detected	-1.354	Comprom	-1.101	0.050	GT Sense	contig368	contig368	Unannota
Sense	GT Sens	0.996	Detected	-0.996	Detected	0.000	-0.926	Detected	-1.656	Detected	-1.291	0.348	GT Sense	contig370	contig370	Unannota
Sense	GT Sens	0.380	Detected	-0.380	Detected	0.000	-1.130	Detected	-1.551	Detected	-1.340	0.091	GT Sense	contig372	contig372	Unannota
Sense	GT Sens	0.314	Detected	-0.314	Detected	0.000	-1.158	Detected	-0.960	Detected	-1.059	0.084	GT Sense	contig373	contig373	3-oxo-5-a
Sense	GT Sens	-0.018	Detected	0.018	Detected	0.000	-2.643	Comprom	-2.193	Comprom	-2.418	0.009	GT Sense	contig375	contig375	Unannota
Sense	GT Sens	0.106	Detected	-0.106	Detected	0.000	-3.339	Comprom	-2.258	Comprom	-2.798	0.037	GT Sense	contig375	contig375	Unannota
Sense	GT Sens	-0.026	Detected	0.026	Detected	0.000	-0.937	Detected	-1.238	Detected	-1.087	0.019	GT Sense	contig378	contig378	Unannota
Sense	GT Sens	-0.009	Detected	0.009	Detected	0.000	-3.285	Comprom	-4.135	Comprom	-3.710	0.013	GT Sense	contig379	contig379	Unannota
Sense	GT Sens	0.860	Detected	-0.860	Detected	0.000	-1.234	Detected	-0.985	Detected	-1.109	0.330	GT Sense	contig382	contig382	Chalcone
Sense	GT Sens	-1.774	Detected	1.774	Detected	0.000	-2.836	Comprom	-2.494	Comprom	-2.665	0.273	GT Sense	contig383	contig383	Unannota
Sense	GT Sens	-1.035	Detected	1.035	Detected	0.000	-2.418	Comprom	-3.264	Comprom	-2.841	0.126	GT Sense	contig385	contig385	Unannota
Sense	GT Sens	0.017	Detected	-0.017	Detected	0.000	-0.860	Detected	-1.325	Detected	-1.092	0.043	GT Sense	contig385	contig385	Unannota
Sense	GT Sens	-0.839	Detected	0.839	Detected	0.000	-0.995	Detected	-1.668	Comprom	-1.331	0.279	GT Sense	contig386	contig386	Unannota
Sense	GT Sens	-0.165	Detected	0.165	Detected	0.000	-0.956	Detected	-1.547	Comprom	-1.252	0.066	GT Sense	contig387	contig387	Unannota
Sense	GT Sens	-0.385	Detected	0.385	Detected	0.000	-2.043	Comprom	-1.339	Comprom	-1.691	0.083	GT Sense	contig389	contig389	Unannota
Sense	GT Sens	0.243	Detected	-0.243	Detected	0.000	-1.067	Detected	-1.348	Detected	-1.208	0.050	GT Sense	contig394	contig394	Unannota
Sense	GT Sens	-0.476	Detected	0.476	Detected	0.000	-0.958	Detected	-1.738	Comprom	-1.348	0.160	GT Sense	contig395	contig395	Unknown
Sense	GT Sens	-0.664	Detected	0.664	Detected	0.000	-1.588	Detected	-1.094	Detected	-1.341	0.199	GT Sense	contig397	contig397	Unannota
Sense	GT Sens	0.234	Detected	-0.234	Detected	0.000	-1.235	Detected	-1.043	Detected	-1.139	0.046	GT Sense	contig397	contig397	Unannota
Sense	GT Sens	0.272	Detected	-0.272	Detected	0.000	-1.492	Detected	-1.017	Detected	-1.255	0.074	GT Sense	contig397	contig397	Unannota
Sense	GT Sens	0.290	Detected	-0.290	Detected	0.000	-1.095	Detected	-1.355	Detected	-1.225	0.061	GT Sense	contig398	contig398	Glyoxalas
Sense	GT Sens	-1.095	Detected	1.095	Detected	0.000	-1.790	Detected	-2.067	Detected	-1.928	0.223	GT Sense	contig399	contig399	Unannota
Sense	GT Sens	0.444	Detected	-0.444	Detected	0.000	-7.209	Comprom	-6.945	Comprom	-7.077	0.004	GT Sense	contig399	contig399	Unannota
Sense	GT Sens	0.381	Detected	-0.381	Detected	0.000	-5.761	Comprom	-5.831	Comprom	-5.796	0.004	GT Sense	contig399	contig399	Unannota
Sense	GT Sens	-0.288	Detected	0.288	Detected	0.000	-1.141	Comprom	-0.905	Comprom	-1.023	0.081	GT Sense	contig400	contig400	Unannota
Sense	GT Sens	-0.538	Detected	0.538	Detected	0.000	-1.555	Detected	-1.020	Detected	-1.287	0.165	GT Sense	contig400	contig400	Unannota
Sense	GT Sens	-0.239	Detected	0.239	Detected	0.000	-2.056	Comprom	-0.906	Detected	-1.481	0.140	GT Sense	contig402	contig402	Unannota
Sense	GT Sens	0.467	Detected	-0.467	Detected	0.000	-0.941	Detected	-1.342	Detected	-1.142	0.154	GT Sense	contig403	contig403	Unannota
Sense	GT Sens	0.414	Detected	-0.414	Detected	0.000	-0.904	Detected	-1.681	Detected	-1.293	0.151	GT Sense	contig403	contig403	Unannota
Sense	GT Sens	-0.029	Detected	0.029	Detected	0.000	-0.884	Detected	-1.140	Comprom	-1.012	0.016	GT Sense	contig404	contig404	Unannota
Sense	GT Sens	-0.366	Detected	0.366	Detected	0.000	-1.144	Detected	-1.056	Detected	-1.100	0.096	GT Sense	contig404	contig404	Unannota
Sense	GT Sens	-0.331	Detected	0.331	Detected	0.000	-2.903	Comprom	-1.354	Comprom	-2.129	0.127	GT Sense	contig406	contig406	Unannota
Sense	GT Sens	-1.649	Detected	1.649	Detected	0.000	-1.239	Detected	-0.957	Detected	-1.098	0.575	GT Sense	contig407	contig407	Unannota
Sense	GT Sens	4.510	Detected	-4.510	Detected	0.000	-4.422	Detected	-4.452	Detected	-4.437	0.429	GT Sense	contig409	contig409	Unannota
Sense	GT Sens	-0.689	Detected	0.689	Detected	0.000	-2.448	Comprom	-0.848	Detected	-1.648	0.259	GT Sense	contig410	contig410	Unannota
Sense	GT Sens	0.341	Detected	-0.341	Detected	0.000	-0.941	Detected	-1.172	Detected	-1.057	0.099	GT Sense	contig413	contig413	MYB tran
Sense	GT Sens	0.428	Detected	-0.428	Detected	0.000	-1.311	Detected	-1.263	Detected	-1.287	0.095	GT Sense	contig414	contig414	Unannota
Sense	GT Sens	0.813	Detected	-0.813	Detected	0.000	-1.263	Detected	-0.843	Detected	-1.053	0.337	GT Sense	contig414	contig414	Unannota
Sense	GT Sens	0.360	Detected	-0.360	Detected	0.000	-1.199	Detected	-1.192	Detected	-1.195	0.080	GT Sense	contig414	contig414	Unannota
Sense	GT Sens	-0.301	Detected	0.301	Detected	0.000	-1.058	Detected	-1.283	Detected	-1.170	0.068	GT Sense	contig414	contig414	Unannota
Sense	GT Sens	-0.058	Detected	0.058	Detected	0.000	-1.199	Detected	-1.102	Detected	-1.150	0.004	GT Sense	contig415	contig415	Unannota
Sense	GT Sens	-0.572	Detected	0.572	Detected	0.000	-4.449	Comprom	-4.151	Comprom	-4.300	0.018	GT Sense	contig419	contig419	Unannota
Sense	GT Sens	-1.102	Detected	1.102	Detected	0.000	-1.202	Detected	-2.606	Comprom	-1.904	0.283	GT Sense	contig419	contig419	Unannota
Sense	GT Sens	0.224	Detected	-0.224	Detected	0.000	-1.616	Detected	-1.295	Detected	-1.455	0.034	GT Sense	contig421	contig421	Unannota
Sense	GT Sens	0.427	Detected	-0.427	Detected	0.000	-1.213	Detected	-1.298	Detected	-1.255	0.100	GT Sense	contig421	contig421	Unannota
Sense	GT Sens	-1.782	Detected	1.782	Detected	0.000	-1.791	Detected	-1.561	Detected	-1.676	0.447	GT Sense	contig422	contig422	Unannota
Sense	GT Sens	-0.764	Detected	0.764	Detected	0.000	-1.134	Detected	-1.228	Detected	-1.181	0.263	GT Sense	contig423	contig423	Unannota
Sense	GT Sens	-0.084	Detected	0.084	Detected	0.000	-0.853	Detected	-1.411	Comprom	-1.132	0.060	GT Sense	contig424	contig424	Unannota
Sense	GT Sens	0.021	Detected	-0.021	Detected	0.000	-0.800	Detected	-1.693	Detected	-1.247	0.108	GT Sense	contig425	contig425	Unknown
Sense	GT Sens	-0.414	Detected	0.414	Detected	0.000	-0.976	Detected	-1.337	Detected	-1.156	0.124	GT Sense	contig427	contig427	Auxin-res
Sense	GT Sens	-0.095	Detected	0.095	Detected	0.000	-0.974	Detected	-1.307	Detected	-1.140	0.027	GT Sense	contig428	contig428	Putative (
Sense	GT Sens	0.322	Detected	-0.322	Detected	0.000	-2.005	Comprom	-0.916	Detected	-1.460	0.147	GT Sense	contig431	contig431	Nicotia ta
Sense	GT Sens	-0.026	Detected	0.026	Detected	0.000	-1.058	Detected	-1.402	Detected	-1.230	0.019	GT Sense	contig437	contig437	Auxin res
Sense	GT Sens	-0.535	Detected	0.535	Detected	0.000	-0.872	Detected	-2.006	Comprom	-1.439	0.206	GT Sense	contig457	contig457	DNA bind
Sense	GT Sens	-0.149	Detected	0.149	Detected	0.000	-0.882	Detected	-1.406	Detected	-1.144	0.063	GT Sense	contig470	contig470	Cytochro
Sense	GT Sens	0.585	Detected	-0.585	Detected	0.000	-2.037	Detected	-1.205	Detected	-1.621	0.152	GT Sense	contig486	contig486	Acyltrans
Sense	GT Sens	-0.095	Detected	0.095	Detected	0.000	-1.330	Comprom	-1.806	Comprom	-1.568	0.026	GT Sense	contig531	contig531	UPF0497
Sense	GT Sens	-0.305	Detected	0.305	Detected	0.000	-0.826	Detected	-2.053	Comprom	-1.439	0.171	GT Sense	contig536	contig536	Unknown
Sense	GT Sens	0.322	Detected	-0.322	Detected	0.000	-1.328	Detected	-1.376	Detected	-1.352	0.052	GT Sense	contig547	contig547	Glycine-r
Sense	GT Sens	-0.106	Detected	0.106	Detected	0.000	-1.556	Detected	-0.870	Detected	-1.213	0.077	GT Sense	contig577	contig577	Cellulose
Sense	GT Sens	-0.071	Detected	0.071	Detected	0.000	-0.983	Detected	-1.184	Detected	-1.084	0.013	GT Sense	contig584	contig584	Peroxidas
Sense	GT Sens	0.752	Detected	-0.752	Detected	0.000	-1.118	Detected	-1.726	Detected	-1.422	0.222	GT Sense	contig587	contig587	Endogluc
Sense	GT Sens	-0.773	Detected	0.773	Detected	0.000	-2.217	Detected	-3.805	Comprom	-3.011	0.113	GT Sense	contig599	contig599	Nudix hyd
Sense	GT Sens	-1.924	Detected	1.924	Detected	0.000	-2.471	Detected	-2.733	Comprom	-2.602	0.310	GT Sense	contig602	contig602	Unknown
Sense	GT Sens	0.663	Detected	-0.663	Detected	0.000	-1.326	Detected	-0.867	Detected	-1.097	0.258	GT Sense	contig614	contig614	Dihydrofl
Sense	GT Sens	0.165	Detected	-0.165	Detected	0.000	-1.263	Detected	-1.353	Detected	-1.308	0.017	GT Sense	contig622	contig622	Leucine-r
Sense	GT Sens	-0.028	Detected	0.028	Detected	0.000	-1.290	Comprom	-1.154	Comprom	-1.222	0.004	GT Sense	contig623	contig623	Heat shoc
Sense	GT Sens	1.196	Detected	-1.196	Detected	0.000	-0.962	Detected	-1.914	Detected	-1.438	0.380	GT Sense	contig647	contig647	Aluminum
Sense	GT Sens	-0.144	Detected	0.144	Detected	0.000	-0.902	Detected	-1.199	Detected	-1.050	0.037	GT Sense	contig660	contig660	Pentatric
Sense	GT Sens	0.490	Detected	-0.490	Detected	0.000	-1.047	Detected	-1.235	Detected	-1.141	0.149	GT Sense	contig691	contig691	MuDR far
Sense	GT Sens	0.297	Detected	-0.297	Detected	0.000	-1.621	Detected	-0.899	Detected	-1.260	0.115	GT Sense	contig694	contig694	Cytochro
Sense	GT Sens	0.124	Detected	-0.124	Detected	0.000	-1.677	Detected	-2.485	Comprom	-2.081	0.039	GT Sense	contig699	contig699	Unknown
Sense	GT Sens	0.345	Detected	-0.345	Detected	0.000	-1.169	Detected	-1.090	Detected	-1.129	0.083	GT Sense	contig703	contig703	GDSL est
Sense	GT Sens	1.273	Detected	-1.273	Detected	0.000	-1.901	Detected	-0.970	Detected	-1.436	0.400	GT Sense	contig709	contig709	Cytochro
Sense	GT Sens	0.025	Detected	-0.025	Detected	0.000	-1.751	Comprom	-0.850	Detected	-1.301	0.102	GT Sense	contig726	contig726	Acyltrans
Sense	GT Sens	-0.337	Detected	0.337	Detected	0.000	-1.648	Comprom	-1.241	Comprom	-1.444	0.067	GT Sense	contig730	contig730	CBS dom
Sense	GT Sens	0.801	Detected	-0.801	Detected	0.000	-1.955	Detected	-1.144	Detected	-1.550	0.227	GT Sense	contig737	contig737	Receptor-
Sense	GT Sens	0.108	Detected	-0.108	Detected	0.000	-1.683	Detected	-2.221	Detected	-1.952	0.021	GT Sense	contig740	contig740	HMG type
Sense	GT Sens	-0.136	Detected	0.136	Detected	0.000	-1.466	Detected	-2.328	Detected	-1.897	0.052	GT Sense	contig751	contig751	Inositol 2-
Sense	GT Sens	0.631	Detected	-0.631	Detected	0.000	-0.962	Detected	-1.066	Detected	-1.014	0.250	GT Sense	contig769	contig769	Centrin 2
Sense	GT Sens	0.358	Detected	-0.358	Detected	0.000	-1.603	Detected	-1.091	Detected	-1.347	0.092	GT Sense	contig781	contig781	(+)-neome
Sense	GT Sens	0.537	Detected	-0.537	Detected	0.000	-1.314	Detected	-0.867	Detected	-1.091	0.201	GT Sense	contig790	contig790	Acetyl xy
Sense	GT Sens	0.724	Detected	-0.724	Detected	0.000	-0.893	Detected	-1.495	Detected	-1.194	0.267	GT Sense	contig804	contig804	Unknown
Sense	GT Sens	0.520	Detected	-0.520	Detected	0.000	-1.118	Detected	-1.059	Detected	-1.089	0.172	GT Sense	contig804	contig804	Progester
Sense	GT Sens	-0.358	Detected	0.358	Detected	0.000	-1.009	Detected	-2.494	Detected	-1.751	0.168	GT Sense	contig805	contig805	Dehydrat
Sense	GT Sens	0.221	Detected	-0.221	Detected	0.000	-1.159	Detected	-0.955	Detected	-1.057	0.049	GT Sense	contig807	contig807	Xylogluca
Sense	GT Sens	0.209	Detected	-0.209	Detected	0.000	-1.192	Detected	-0.864	Detected	-1.028	0.061	GT Sense	contig807	contig807	Cellulose
Sense	GT Sens	0.288	Detected	-0.288	Detected	0.000	-0.917	Detected	-1.255	Detected	-1.086	0.083	GT Sense	contig811	contig811	Dehydrin
Sense	GT Sens	-0.737	Detected	0.737	Detected	0.000	-1.287	Detected	-1.969	Detected	-1.628	0.183	GT Sense	contig811	contig811	Auxin res
Sense	GT Sens	-0.135	Detected	0.135	Detected	0.000	-1.172	Detected	-1.398	Detected	-1.285	0.018	GT Sense	contig815	contig815	Peptide tr
Sense	GT Sens	-0.709	Detected	0.709	Detected	0.000	-0.889	Detected	-1.872	Comprom	-1.380	0.251	GT Sense	contig820	contig820	Xylanase
Sense	GT Sens	0.498	Detected	-0.498	Detected	0.000	-0.909	Detected	-1.249	Detected	-1.079	0.177	GT Sense	contig869	contig869	Trehalose
Sense	GT Sens	0.124	Detected	-0.124	Detected	0.000	-1.118	Detected	-1.170	Detected	-1.144	0.012	GT Sense	contig873	contig873	Expansin
Sense	GT Sens	-0.025	Detected	0.025	Detected	0.000	-1.095	Detected	-1.023	Detected	-1.059	0.002	GT Sense	contig897	contig897	Unknown
Sense	GT Sens	0.305	Detected	-0.305	Detected	0.000	-1.419	Detected	-0.995	Detected	-1.207	0.083	GT Sense	contig912	contig912	3-phenylp
Sense	GT Sens	-0.021	Detected	0.021	Detected	0.000	-1.187	Detected	-1.624	Detected	-1.406	0.024	GT Sense	contig919	contig919	BZIP tran
Sense	GT Sens	1.594	Detected	-1.594	Detected	0.000	-1.745	Detected	-1.733	Detected	-1.739	0.389	GT Sense	contig945	contig945	Gibberelli
Sense	GT Sens	0.046	Detected	-0.046	Detected	0.000	-1.132	Detected	-0.914	Detected	-1.023	0.013	GT Sense	contig974	contig974	Short inte
Sense	GT Sens	-0.334	Detected	0.334	Detected	0.000	-0.904	Detected	-1.655	Comprom	-1.280	0.126	GT Sense	contig975	contig975	Unknown
Sense	GT Sens	0.941	Detected	-0.941	Detected	0.000	-1.957	Detected	-1.545	Detected	-1.751	0.211	GT Sense	contig986	contig986	Ent-kaure
Sense	GT Sens	-1.099	Detected	1.099	Detected	0.000	-1.194	Detected	-1.629	Detected	-1.411	0.335	GT Sense	contig104	contig104	Unknown
Sense	GT Sens	-1.083	Detected	1.083	Detected	0.000	-1.213	Detected	-1.869	Detected	-1.541	0.306	GT Sense	contig104	contig104	Unknown
Sense	GT Sens	0.231	Detected	-0.231	Detected	0.000	-1.659	Detected	-1.218	Detected	-1.438	0.046	GT Sense	contig103	contig103	Auxin-ind
Sense	GT Sens	0.541	Detected	-0.541	Detected	0.000	-1.144	Detected	-1.023	Detected	-1.084	0.185	GT Sense	contig107	contig107	Aminotra
Sense	GT Sens	0.827	Detected	-0.827	Detected	0.000	-1.272	Detected	-0.807	Detected	-1.040	0.350	GT Sense	contig120	contig120	Alpha-hyd
Sense	GT Sens	0.096	Detected	-0.096	Detected	0.000	-2.508	Detected	-1.796	Detected	-2.152	0.028	GT Sense	contig128	contig128	Long-cha
Sense	GT Sens	0.507	Detected	-0.507	Detected	0.000	-1.517	Detected	-1.452	Detected	-1.484	0.100	GT Sense	contig128	contig128	Auxin-res
Sense	GT Sens	0.285	Detected	-0.285	Detected	0.000	-1.632	Detected	-1.249	Detected	-1.440	0.052	GT Sense	contig136	contig136	Auxin-res
Sense	GT Sens	0.826	Detected	-0.826	Detected	0.000	-0.960	Detected	-1.590	Detected	-1.275	0.286	GT Sense	contig136	contig136	UDP-gluc
Sense	GT Sens	0.628	Detected	-0.628	Detected	0.000	-1.390	Detected	-1.901	Detected	-1.646	0.136	GT Sense	contig141	contig141	Major late
Sense	GT Sens	0.436	Detected	-0.436	Detected	0.000	-0.879	Detected	-1.400	Detected	-1.139	0.154	GT Sense	contig142	contig142	Plant vira
Sense	GT Sens	0.271	Detected	-0.271	Detected	0.000	-1.163	Detected	-1.762	Detected	-1.463	0.069	GT Sense	contig146	contig146	Zinc finge
Sense	GT Sens	0.338	Detected	-0.338	Detected	0.000	-1.647	Detected	-1.157	Detected	-1.402	0.078	GT Sense	contig148	contig148	Proteinas
Sense	GT Sens	-0.064	Detected	0.064	Detected	0.000	-0.977	Detected	-1.116	Detected	-1.047	0.008	GT Sense	contig157	contig157	Peroxidas
Sense	GT Sens	0.714	Detected	-0.714	Detected	0.000	-0.895	Detected	-1.535	Detected	-1.215	0.261	GT Sense	contig158	contig158	Zeatin O-
Sense	GT Sens	0.427	Detected	-0.427	Detected	0.000	-1.210	Detected	-0.919	Detected	-1.064	0.142	GT Sense	contig165	contig165	Unknown
Sense	GT Sens	0.180	Detected	-0.180	Detected	0.000	-1.272	Detected	-2.043	Detected	-1.658	0.060	GT Sense	contig167	contig167	FAD-bind
Sense	GT Sens	0.704	Detected	-0.704	Detected	0.000	-1.701	Detected	-1.408	Detected	-1.555	0.163	GT Sense	contig169	contig169	Sulfotran
Sense	GT Sens	0.741	Detected	-0.741	Detected	0.000	-1.979	Detected	-1.518	Detected	-1.748	0.153	GT Sense	contig174	contig174	Enoyl-Co
Sense	GT Sens	0.633	Detected	-0.633	Detected	0.000	-1.171	Detected	-1.127	Detected	-1.149	0.211	GT Sense	contig175	contig175	Pectinest
Sense	GT Sens	0.644	Detected	-0.644	Detected	0.000	-1.620	Detected	-1.348	Detected	-1.484	0.153	GT Sense	contig179	contig179	Ornithine
Sense	GT Sens	-0.271	Detected	0.271	Detected	0.000	-4.420	Detected	-4.773	Detected	-4.597	0.005	GT Sense	contig185	contig185	Polygalac
Sense	GT Sens	0.754	Detected	-0.754	Detected	0.000	-1.830	Detected	-1.337	Detected	-1.584	0.184	GT Sense	contig188	contig188	Sulfotran
Sense	GT Sens	-1.571	Detected	1.571	Detected	0.000	-1.937	Detected	-2.558	Detected	-2.247	0.296	GT Sense	contig190	contig190	Os12g06
Sense	GT Sens	0.718	Detected	-0.718	Detected	0.000	-1.317	Detected	-1.100	Detected	-1.209	0.238	GT Sense	contig194	contig194	Unknown
Sense	GT Sens	0.274	Detected	-0.274	Detected	0.000	-1.425	Detected	-1.076	Detected	-1.250	0.061	GT Sense	contig194	contig194	Auxin-res
Sense	GT Sens	0.392	Detected	-0.392	Detected	0.000	-1.697	Detected	-1.330	Detected	-1.513	0.073	GT Sense	contig195	contig195	Auxin-ind
Sense	GT Sens	1.167	Detected	-1.167	Detected	0.000	-1.844	Detected	-2.312	Detected	-2.078	0.223	GT Sense	contig198	contig198	Leucine-r
Sense	GT Sens	0.584	Detected	-0.584	Detected	0.000	-1.186	Detected	-1.222	Detected	-1.204	0.176	GT Sense	contig199	contig199	Auxin-res
Sense	GT Sens	-0.990	Detected	0.990	Detected	0.000	-1.024	Detected	-1.298	Detected	-1.161	0.365	GT Sense	contig215	contig215	Unknown
Sense	GT Sens	0.508	Detected	-0.508	Detected	0.000	-1.199	Detected	-0.850	Detected	-1.024	0.197	GT Sense	contig215	contig215	Hydroxyc
Sense	GT Sens	-0.956	Detected	0.956	Detected	0.000	-1.268	Detected	-0.893	Detected	-1.081	0.383	GT Sense	contig217	contig217	Serine/th
Sense	GT Sens	0.226	Detected	-0.226	Detected	0.000	-1.291	Detected	-0.960	Detected	-1.126	0.057	GT Sense	contig220	contig220	Myosin-li
Sense	GT Sens	0.240	Detected	-0.240	Detected	0.000	-1.355	Detected	-0.880	Detected	-1.118	0.080	GT Sense	contig226	contig226	Auxin-ind
Sense	GT Sens	0.234	Detected	-0.234	Detected	0.000	-1.263	Detected	-1.143	Detected	-1.203	0.038	GT Sense	contig226	contig226	Auxin-ind
Sense	GT Sens	0.782	Detected	-0.782	Detected	0.000	-1.177	Detected	-1.217	Detected	-1.197	0.266	GT Sense	contig228	contig228	UDP-gluc
Sense	GT Sens	0.135	Detected	-0.135	Detected	0.000	-1.180	Detected	-0.856	Detected	-1.018	0.040	GT Sense	contig231	contig231	Auxin-ind
Sense	GT Sens	0.492	Detected	-0.492	Detected	0.000	-1.237	Detected	-1.421	Detected	-1.329	0.118	GT Sense	contig239	contig239	Legumin
Sense	GT Sens	0.104	Detected	-0.104	Detected	0.000	-1.334	Detected	-1.100	Detected	-1.217	0.016	GT Sense	contig241	contig241	Auxin-ind
Sense	GT Sens	-0.338	Detected	0.338	Detected	0.000	-1.055	Detected	-1.063	Comprom	-1.059	0.088	GT Sense	contig243	contig243	Transpos
Sense	GT Sens	0.106	Detected	-0.106	Detected	0.000	-1.649	Detected	-1.080	Detected	-1.365	0.046	GT Sense	contig244	contig244	Auxin-ind
Sense	GT Sens	0.781	Detected	-0.781	Detected	0.000	-1.381	Detected	-1.250	Detected	-1.316	0.235	GT Sense	contig244	contig244	Cytochro
Sense	GT Sens	0.335	Detected	-0.335	Detected	0.000	-1.554	Detected	-1.056	Detected	-1.305	0.089	GT Sense	contig252	contig252	Auxin-res
Sense	GT Sens	0.126	Detected	-0.126	Detected	0.000	-1.267	Detected	-1.509	Detected	-1.388	0.016	GT Sense	contig257	contig257	Cytokinin
Sense	GT Sens	0.088	Detected	-0.088	Detected	0.000	-1.251	Detected	-1.395	Detected	-1.323	0.007	GT Sense	contig260	contig260	Cytochro
Sense	GT Sens	0.409	Detected	-0.409	Detected	0.000	-1.342	Detected	-0.980	Detected	-1.161	0.122	GT Sense	contig260	contig260	Auxin-ind
Sense	GT Sens	0.522	Detected	-0.522	Detected	0.000	-1.080	Detected	-1.285	Detected	-1.182	0.156	GT Sense	contig262	contig262	ATP synt
Sense	GT Sens	0.307	Detected	-0.307	Detected	0.000	-1.641	Detected	-1.308	Detected	-1.475	0.052	GT Sense	contig277	contig277	Auxin-res
Sense	GT Sens	0.408	Detected	-0.408	Detected	0.000	-1.622	Detected	-2.247	Detected	-1.934	0.064	GT Sense	contig278	contig278	MYB tran
Sense	GT Sens	-0.606	Detected	0.606	Detected	0.000	-1.112	Detected	-1.064	Detected	-1.088	0.215	GT Sense	contig285	contig285	Guanylat
Sense	GT Sens	0.130	Detected	-0.130	Detected	0.000	-1.403	Detected	-0.887	Detected	-1.145	0.058	GT Sense	contig291	contig291	Hydroxyc
Sense	GT Sens	0.553	Detected	-0.553	Detected	0.000	-1.403	Detected	-1.531	Detected	-1.467	0.119	GT Sense	contig292	contig292	Acetyl co
Sense	GT Sens	0.109	Detected	-0.109	Detected	0.000	-1.532	Detected	-1.148	Detected	-1.340	0.026	GT Sense	contig333	contig333	Transcrip
Sense	GT Sens	0.067	Detected	-0.067	Detected	0.000	-2.969	Detected	-2.891	Detected	-2.930	0.001	GT Sense	contig402	contig402	Long-cha
Sense	GT Sens	0.306	Detected	-0.306	Detected	0.000	-0.920	Detected	-1.306	Detected	-1.113	0.091	GT Sense	contig520	contig520	1-aminoc
Sense	GT Sens	0.934	Detected	-0.934	Detected	0.000	-1.712	Detected	-1.588	Detected	-1.650	0.220	GT Sense	contig568	contig568	Auxin-ind
Sense	GT Sens	0.194	Detected	-0.194	Detected	0.000	-1.144	Detected	-1.041	Detected	-1.093	0.032	GT Sense	contig570	contig570	Epoxide h
Sense	GT Sens	0.235	Detected	-0.235	Detected	0.000	-1.539	Detected	-0.993	Detected	-1.266	0.072	GT Sense	contig769	contig769	Proteinas
Sense	GT Sens	-0.171	Detected	0.171	Detected	0.000	-1.820	Detected	-2.672	Comprom	-2.246	0.039	GT Sense	contig816	contig816	Auxin-ind
Sense	GT Sens	0.306	Detected	-0.306	Detected	0.000	-2.195	Detected	-1.290	Detected	-1.742	0.086	GT Sense	contig834	contig834	Major late
Sense	GT Sens	0.010	Detected	-0.010	Detected	0.000	-1.089	Detected	-0.974	Detected	-1.031	0.003	GT Sense	contig843	contig843	Glutathio
Sense	GT Sens	0.304	Detected	-0.304	Detected	0.000	-1.564	Detected	-1.273	Detected	-1.419	0.052	GT Sense	contig849	contig849	Auxin-ind
Sense	GT Sens	0.742	Detected	-0.742	Detected	0.000	-1.527	Detected	-1.012	Detected	-1.269	0.247	GT Sense	contig862	contig862	Malate sy
Sense	GT Sens	0.757	Detected	-0.757	Detected	0.000	-1.421	Detected	-1.039	Detected	-1.230	0.256	GT Sense	contig888	contig888	Pectinest
Sense	GT Sens	0.789	Detected	-0.789	Detected	0.000	-1.124	Detected	-0.987	Detected	-1.056	0.314	GT Sense	contig958	contig958	Aromatic
Sense	GT Sens	-0.010	Detected	0.010	Detected	0.000	-0.906	Detected	-1.265	Comprom	-1.085	0.026	GT Sense	GmHAES	GmHAES	Cross hy
Sense	GT Sens	-0.487	Detected	0.487	Detected	0.000	-3.596	Detected	-3.122	Detected	-3.359	0.025	GT Sense	U70481	U70481	LEU7048
Sense	GT Sens	-0.478	Detected	0.478	Detected	0.000	-3.943	Detected	-3.489	Detected	-3.716	0.020	GT Sense	U70481	U70481	TAPG4: (

GO ID	GO ACCESSION	GO Term	p-value	Count in Selection	Count in Selecti	Count in Total	% Count in Total
4459	GO:0006259|GO:0055132	DNA metabolic process	0.00	41	11.357341	493	1.4237444
4476	GO:0006278	RNA-dependent DNA replication	0.00	27	7.479224	166	0.4793947
4460	GO:0006260|GO:0055133	DNA replication	0.00	27	7.479224	224	0.64689404
8493	GO:0015074	DNA integration	0.00	14	3.8781164	47	0.13573223
31895	GO:0090304	nucleic acid metabolic process	0.00	50	13.850415	2340	6.7577324
19473	GO:0043170|GO:0043283	macromolecule metabolic process	0.00	105	29.085873	6553	18.92454
20510	GO:0044260|GO:0034960	cellular macromolecule metabolic p	0.00	87	24.099724	5577	16.105928
4357	GO:0006139|GO:0055134	nucleobase, nucleoside, nucleotide	0.00	57	15.789474	3285	9.486816
7074	GO:0009815	1-aminocyclopropane-1-carboxylat	0.00	5	1.3850416	49	0.14150807
9722	GO:0016641	oxidoreductase activity, acting on t	0.00	5	1.3850416	69	0.19926646
17024	GO:0034645|GO:0034961	cellular macromolecule biosyntheti	0.00	36	9.9723	1999	5.7729516
6428	GO:0009059|GO:0043284	macromolecule biosynthetic proces	0.00	36	9.9723	2003	5.7845035
20488	GO:0044238	primary metabolic process	0.00	122	33.795013	9167	26.47356
17020	GO:0034641	cellular nitrogen compound metabo	0.00	57	15.789474	3678	10.621769
4906	GO:0006807	nitrogen compound metabolic proc	0.00	57	15.789474	3725	10.757502
9719	GO:0016638	oxidoreductase activity, acting on t	0.00	5	1.3850416	89	0.25702485
5791	GO:0008270	zinc ion binding	0.00	33	9.141274	1880	5.4292893
22415	GO:0046914	transition metal ion binding	0.01	41	11.357341	2599	7.5057034
31431	GO:0080054	low affinity nitrate transmembrane t	0.01	3	0.83102494	45	0.1299564
4641	GO:0006508	proteolysis	0.01	18	4.98615	965	2.7868426
21331	GO:0045735	nutrient reservoir activity	0.01	4	1.1080333	89	0.25702485
2986	GO:0004252	serine-type endopeptidase activity	0.02	5	1.3850416	144	0.41586044
2975	GO:0004175|GO:0016809	endopeptidase activity	0.02	8	2.2160666	320	0.9241344
5907	GO:0008413	8-oxo-7,8-dihydroguanosine tripho	0.02	1	0.27700832	2	0.00577584
11186	GO:0019177	dihydroneopterin triphosphate pyro	0.02	1	0.27700832	2	0.00577584
5636	GO:0008061	chitin binding	0.02	2	0.55401665	23	0.06642216
8526	GO:0015112	nitrate transmembrane transporter	0.03	3	0.83102494	61	0.1761631
8975	GO:0015706|GO:0006872	nitrate transport	0.03	3	0.83102494	61	0.1761631
9876	GO:0016837	carbon-oxygen lyase activity, actin	0.03	2	0.55401665	25	0.072197996
13054	GO:0030570	pectate lyase activity	0.03	2	0.55401665	25	0.072197996
22375	GO:0046872	metal ion binding	0.03	42	11.634349	2985	8.6204405
19470	GO:0043167	ion binding	0.03	42	11.634349	2997	8.655096
19472	GO:0043169	cation binding	0.03	42	11.634349	2997	8.655096
4990	GO:0006915|GO:0008632	apoptosis	0.03	6	1.6620499	227	0.6555578
3307	GO:0004675	transmembrane receptor protein se	0.04	8	2.2160666	367	1.0598665
5756	GO:0008219	cell death	0.04	6	1.6620499	239	0.69021285
8209	GO:0012501|GO:0016244	programmed cell death	0.04	6	1.6620499	239	0.69021285
9462	GO:0016265	death	0.04	6	1.6620499	239	0.69021285
2710	GO:0003860	3-hydroxyisobutyryl-CoA hydrolase	0.04	1	0.27700832	4	0.011551679
3717	GO:0005199	structural constituent of cell wall	0.04	2	0.55401665	32	0.09241343
11522	GO:0019538|GO:0006411	protein metabolic process	0.05	54	14.958448	4134	11.938661
12806	GO:0030247	polysaccharide binding	0.05	2	0.55401665	35	0.10107719
1169	GO:0001871	pattern binding	0.05	2	0.55401665	35	0.10107719
20499	GO:0044249	cellular biosynthetic process	0.06	36	9.9723	2635	7.6096687
2713	GO:0003863	3-methyl-2-oxobutanoate dehydrog	0.06	1	0.27700832	6	0.01732752
3073	GO:0004397	histidine ammonia-lyase activity	0.06	1	0.27700832	6	0.01732752
5810	GO:0008290	F-actin capping protein complex	0.06	1	0.27700832	6	0.01732752
14264	GO:0031849	olfactory receptor binding	0.06	1	0.27700832	6	0.01732752
5891	GO:0008395|GO:0008394	steroid hydroxylase activity	0.07	2	0.55401665	40	0.1155168
20487	GO:0044237	cellular metabolic process	0.07	98	27.146814	8185	23.637625
21149	GO:0045548	phenylalanine ammonia-lyase activ	0.07	1	0.27700832	7	0.020215439
8461	GO:0015020|GO:0003981	glucuronosyltransferase activity	0.07	2	0.55401665	42	0.121292636
13884	GO:0031461	cullin-RING ubiquitin ligase comple	0.07	2	0.55401665	42	0.121292636
5766	GO:0008236	serine-type peptidase activity	0.07	5	1.3850416	214	0.6180149
6427	GO:0009058	biosynthetic process	0.08	37	10.249308	2796	8.074624
10103	GO:0017171	serine hydrolase activity	0.08	5	1.3850416	220	0.63534236
4612	GO:0006468	protein phosphorylation	0.08	30	8.310249	2205	6.367863
11202	GO:0019199	transmembrane receptor protein ki	0.08	9	2.493075	504	1.4555116
7225	GO:0009978	allene oxide synthase activity	0.09	1	0.27700832	9	0.025991278
7506	GO:0010279	indole-3-acetic acid amido syntheta	0.09	1	0.27700832	9	0.025991278
19342	GO:0043027	caspase inhibitor activity	0.09	1	0.27700832	9	0.025991278
19343	GO:0043028	caspase regulator activity	0.09	1	0.27700832	9	0.025991278
19458	GO:0043154|GO:0001719	negative regulation of caspase acti	0.09	1	0.27700832	9	0.025991278
19579	GO:0043281|GO:0043026	regulation of caspase activity	0.09	1	0.27700832	9	0.025991278
3476	GO:0004872|GO:0019041	receptor activity	0.10	11	3.0470915	672	1.9406822
983	GO:0001664	G-protein-coupled receptor binding	0.10	1	0.27700832	10	0.0288792
3156	GO:0004497	monooxygenase activity	0.11	4	1.1080333	172	0.49672222
7690	GO:0010466	negative regulation of peptidase ac	0.11	3	0.83102494	110	0.31767118
12946	GO:0030414	peptidase inhibitor activity	0.11	3	0.83102494	110	0.31767118
25636	GO:0051346	negative regulation of hydrolase ac	0.11	3	0.83102494	110	0.31767118
6013	GO:0008553	hydrogen-exporting ATPase activit	0.11	1	0.27700832	11	0.03176712
5702	GO:0008146	sulfotransferase activity	0.11	2	0.55401665	54	0.15594767
9768	GO:0016706	oxidoreductase activity, acting on p	0.11	4	1.1080333	175	0.505386
26711	GO:0052547	regulation of peptidase activity	0.11	3	0.83102494	112	0.32344702
28100	GO:0061134	peptidase regulator activity	0.11	3	0.83102494	112	0.32344702
6946	GO:0009678	hydrogen-translocating pyrophosph	0.12	1	0.27700832	12	0.03465504
7526	GO:0010301|GO:0033710	xanthoxin dehydrogenase activity	0.12	1	0.27700832	12	0.03465504
3475	GO:0004871|GO:0005062	signal transducer activity	0.12	12	3.3240998	787	2.2727928
27063	GO:0060089	molecular transducer activity	0.12	12	3.3240998	787	2.2727928
24966	GO:0050660	flavin adenine dinucleotide binding	0.13	3	0.83102494	118	0.34077454
30700	GO:0072341	modified amino acid binding	0.13	2	0.55401665	59	0.17038727
7229	GO:0009987|GO:0008151	cellular process	0.13	121	33.518005	10601	30.614838
8149	GO:0010941	regulation of cell death	0.13	1	0.27700832	13	0.037542958
19298	GO:0042981	regulation of apoptosis	0.13	1	0.27700832	13	0.037542958
19381	GO:0043067|GO:0043070	regulation of programmed cell deat	0.13	1	0.27700832	13	0.037542958
6884	GO:0009607	response to biotic stimulus	0.14	3	0.83102494	122	0.3523262
8553	GO:0015140	malate transmembrane transporter	0.14	1	0.27700832	14	0.040430877
8859	GO:0015556	C4-dicarboxylate transmembrane t	0.14	1	0.27700832	14	0.040430877
9009	GO:0015740	C4-dicarboxylate transport	0.14	1	0.27700832	14	0.040430877
9012	GO:0015743	malate transport	0.14	1	0.27700832	14	0.040430877
9879	GO:0016841	ammonia-lyase activity	0.14	1	0.27700832	14	0.040430877
29787	GO:0071423	malate transmembrane transport	0.14	1	0.27700832	14	0.040430877
31408	GO:0080031	methyl salicylate esterase activity	0.14	1	0.27700832	14	0.040430877
3490	GO:0004888|GO:0004926	transmembrane receptor activity	0.15	9	2.493075	571	1.6490022
3306	GO:0004674|GO:0004695	protein serine/threonine kinase act	0.15	15	4.1551247	1062	3.0669708

3473	GO:0004867	serine-type endopeptidase inhibito	0.15	2	0.55401665	66	0.1906027
3742	GO:0005242	inward rectifier potassium channel	0.15	1	0.27700832	16	0.046206716
3782	GO:0005310|GO:0005312	dicarboxylic acid transmembrane tr	0.15	1	0.27700832	16	0.046206716
4928	GO:0006835|GO:0006841	dicarboxylic acid transport	0.15	1	0.27700832	16	0.046206716
9386	GO:0016157	sucrose synthase activity	0.15	1	0.27700832	16	0.046206716
9878	GO:0016840	carbon-nitrogen lyase activity	0.15	1	0.27700832	16	0.046206716
18955	GO:0042625	ATPase activity, coupled to transm	0.16	2	0.55401665	68	0.19637854
9874	GO:0016835	carbon-oxygen lyase activity	0.16	4	1.1080333	202	0.5833598
3801	GO:0005342	organic acid transmembrane transp	0.17	3	0.83102494	136	0.3927571
22444	GO:0046943	carboxylic acid transmembrane tra	0.17	3	0.83102494	136	0.3927571
4225	GO:0005985	sucrose metabolic process	0.17	1	0.27700832	18	0.051982556
12887	GO:0030332	cyclin binding	0.17	1	0.27700832	18	0.051982556
22917	GO:0047429	nucleoside-triphosphate diphospha	0.17	1	0.27700832	18	0.051982556
9832	GO:0016782	transferase activity, transferring su	0.18	2	0.55401665	74	0.21370606
2895	GO:0004089	carbonate dehydratase activity	0.18	1	0.27700832	19	0.054870475
9493	GO:0016310	phosphorylation	0.18	31	8.587257	2504	7.2313514
4609	GO:0006464	protein modification process	0.19	34	9.4182825	2781	8.031305
6201	GO:0008794	arsenate reductase (glutaredoxin)	0.19	1	0.27700832	20	0.0577584
9707	GO:0016624	oxidoreductase activity, acting on t	0.19	1	0.27700832	20	0.0577584
9784	GO:0016723	oxidoreductase activity, oxidizing n	0.19	1	0.27700832	20	0.0577584
13086	GO:0030611	arsenate reductase activity	0.19	1	0.27700832	20	0.0577584
13088	GO:0030613	oxidoreductase activity, acting on p	0.19	1	0.27700832	20	0.0577584
13089	GO:0030614	oxidoreductase activity, acting on p	0.19	1	0.27700832	20	0.0577584
225	GO:0000293	ferric-chelate reductase activity	0.19	1	0.27700832	20	0.0577584
3474	GO:0004869|GO:0004870	cysteine-type endopeptidase inhibi	0.20	1	0.27700832	21	0.060646318
5019	GO:0006952|GO:0002217	defense response	0.20	4	1.1080333	221	0.63823026
28378	GO:0070011	peptidase activity, acting on L-amin	0.20	8	2.2160666	537	1.550813
9682	GO:0016597	amino acid binding	0.20	2	0.55401665	80	0.2310336
9875	GO:0016836	hydro-lyase activity	0.20	2	0.55401665	80	0.2310336
19479	GO:0043176	amine binding	0.20	2	0.55401665	80	0.2310336
12660	GO:0030077	plasma membrane light-harvesting	0.21	1	0.27700832	22	0.06353424
13038	GO:0030551	cyclic nucleotide binding	0.21	1	0.27700832	22	0.06353424
19040	GO:0042716	plasma membrane-derived chroma	0.21	1	0.27700832	22	0.06353424
3827	GO:0005372	water transmembrane transporter a	0.21	2	0.55401665	81	0.23392151
4927	GO:0006833	water transport	0.21	2	0.55401665	81	0.23392151
8652	GO:0015250	water channel activity	0.21	2	0.55401665	81	0.23392151
18429	GO:0042044	fluid transport	0.21	2	0.55401665	81	0.23392151
7527	GO:0010302	2-oxoglutarate-dependent dioxyge	0.21	1	0.27700832	23	0.06642216
8517	GO:0015103	inorganic anion transmembrane tra	0.22	3	0.83102494	154	0.44473964
11931	GO:0020037	heme binding	0.22	8	2.2160666	550	1.5883559
3213	GO:0004568	chitinase activity	0.22	1	0.27700832	24	0.06931008
6248	GO:0008843	endochitinase activity	0.22	1	0.27700832	24	0.06931008
31407	GO:0080030	methyl indole-3-acetate esterase a	0.22	1	0.27700832	24	0.06931008
9114	GO:0015849	organic acid transport	0.22	3	0.83102494	156	0.45051548
22443	GO:0046942	carboxylic acid transport	0.22	3	0.83102494	156	0.45051548
3464	GO:0004857	enzyme inhibitor activity	0.23	3	0.83102494	157	0.4534034
22459	GO:0046961	proton-transporting ATPase activity	0.23	1	0.27700832	25	0.072197996
31493	GO:0080118	brassinosteroid sulfotransferase ac	0.23	1	0.27700832	25	0.072197996
119	GO:0000151	ubiquitin ligase complex	0.23	2	0.55401665	88	0.25413695
8496	GO:0015078	hydrogen ion transmembrane trans	0.23	3	0.83102494	160	0.4620672
3872	GO:0005506	iron ion binding	0.24	8	2.2160666	563	1.6258988
3210	GO:0004565	beta-galactosidase activity	0.24	1	0.27700832	26	0.075085916
3896	GO:0005544	calcium-dependent phospholipid bi	0.24	1	0.27700832	26	0.075085916
5763	GO:0008233	peptidase activity	0.24	8	2.2160666	565	1.6316746
2982	GO:0004197	cysteine-type endopeptidase activi	0.24	2	0.55401665	90	0.2599128
9869	GO:0016829	lyase activity	0.25	7	1.9390582	488	1.4093049
13144	GO:0030674	protein binding, bridging	0.25	1	0.27700832	28	0.080861755
13634	GO:0031177	phosphopantetheine binding	0.25	1	0.27700832	28	0.080861755
27064	GO:0060090	binding, bridging	0.25	1	0.27700832	28	0.080861755
3472	GO:0004866	endopeptidase inhibitor activity	0.26	2	0.55401665	95	0.27435237
8159	GO:0010951	negative regulation of endopeptida	0.26	2	0.55401665	95	0.27435237
26712	GO:0052548	regulation of endopeptidase activit	0.26	2	0.55401665	95	0.27435237
28101	GO:0061135	endopeptidase regulator activity	0.26	2	0.55401665	95	0.27435237
19684	GO:0043412	macromolecule modification	0.26	34	9.4182825	2900	8.374968
12805	GO:0030246	carbohydrate binding	0.27	4	1.1080333	251	0.7248679
2774	GO:0003950	NAD+ ADP-ribosyltransferase activ	0.27	1	0.27700832	30	0.086637594
6216	GO:0008810	cellulase activity	0.27	1	0.27700832	30	0.086637594
5705	GO:0008152	metabolic process	0.27	188	52.07756	17432	50.34222
13829	GO:0031406	carboxylic acid binding	0.27	2	0.55401665	98	0.28301615
19590	GO:0043295	glutathione binding	0.28	1	0.27700832	31	0.08952551
8967	GO:0015698	inorganic anion transport	0.28	3	0.83102494	176	0.5082739
3904	GO:0005576	extracellular region	0.28	3	0.83102494	178	0.5140497
13082	GO:0030599	pectinesterase activity	0.28	3	0.83102494	178	0.5140497
22409	GO:0046906	tetrapyrrole binding	0.28	8	2.2160666	596	1.7212002
3265	GO:0004630	phospholipase D activity	0.29	1	0.27700832	32	0.09241343
20517	GO:0044267	cellular protein metabolic process	0.29	37	10.249308	3225	9.313541
19253	GO:0042936	dipeptide transporter activity	0.30	2	0.55401665	105	0.3032316
19255	GO:0042938	dipeptide transport	0.30	2	0.55401665	105	0.3032316
3749	GO:0005249	voltage-gated potassium channel a	0.30	1	0.27700832	34	0.09818927
5195	GO:0007165|GO:0023033	signal transduction	0.30	12	3.3240998	961	2.775291
12602	GO:0023052|GO:0023046	signaling	0.30	12	3.3240998	961	2.775291
9480	GO:0016289	CoA hydrolase activity	0.31	1	0.27700832	35	0.10107719
21146	GO:0045544	gibberellin 20-oxidase activity	0.31	1	0.27700832	35	0.10107719
22049	GO:0046527	glucosyltransferase activity	0.31	4	1.1080333	270	0.77973837
19393	GO:0043086	negative regulation of catalytic acti	0.31	3	0.83102494	188	0.54292893
20342	GO:0044092	negative regulation of molecular fu	0.31	3	0.83102494	188	0.54292893
3804	GO:0005345	purine base transmembrane transp	0.31	1	0.27700832	36	0.10396511
4949	GO:0006863|GO:0015852	purine base transport	0.31	1	0.27700832	36	0.10396511
8739	GO:0015385|GO:0015502	sodium:hydrogen antiporter activity	0.31	1	0.27700832	36	0.10396511
9185	GO:0015925	galactosidase activity	0.31	1	0.27700832	36	0.10396511
3304	GO:0004672|GO:0050222	protein kinase activity	0.32	16	4.432133	1333	3.8495972
24968	GO:0050662	coenzyme binding	0.32	3	0.83102494	191	0.5515927
3318	GO:0004693|GO:0016537	cyclin-dependent protein kinase ac	0.32	1	0.27700832	37	0.10685303
3758	GO:0005267	potassium channel activity	0.32	1	0.27700832	37	0.10685303
8934	GO:0015662	ATPase activity, coupled to transm	0.32	1	0.27700832	37	0.10685303
29918	GO:0071554	cell wall organization or biogenesis	0.33	2	0.55401665	112	0.32344702
12538	GO:0022843	voltage-gated cation channel activi	0.33	1	0.27700832	38	0.10974095
25626	GO:0051336	regulation of hydrolase activity	0.34	3	0.83102494	196	0.5660323

8613	GO:0015205|GO:0015392	nucleobase transmembrane transp	0.34	1	0.27700832	40	0.1155168
9116	GO:0015851	nucleobase transport	0.34	1	0.27700832	40	0.1155168
8584	GO:0015171|GO:0015359	amino acid transmembrane transpo	0.35	2	0.55401665	118	0.34077454
2521	GO:0003333	amino acid transmembrane transpo	0.35	2	0.55401665	118	0.34077454
20627	GO:0044433	cytoplasmic vesicle part	0.35	1	0.27700832	41	0.118404716
30069	GO:0071705	nitrogen compound transport	0.35	3	0.83102494	202	0.5833598
12659	GO:0030076	light-harvesting complex	0.36	1	0.27700832	42	0.121292636
13833	GO:0031410	cytoplasmic vesicle	0.36	1	0.27700832	43	0.124180555
14393	GO:0031982	vesicle	0.36	1	0.27700832	43	0.124180555
4951	GO:0006865|GO:0006866	amino acid transport	0.37	2	0.55401665	123	0.35521415
9753	GO:0016682	oxidoreductase activity, acting on d	0.37	1	0.27700832	44	0.12706847
409	GO:0000502	proteasome complex	0.37	1	0.27700832	44	0.12706847
5692	GO:0008134	transcription factor binding	0.38	1	0.27700832	45	0.1299564
8666	GO:0015276	ligand-gated ion channel activity	0.38	1	0.27700832	45	0.1299564
11780	GO:0019829	cation-transporting ATPase activity	0.38	1	0.27700832	45	0.1299564
12530	GO:0022834	ligand-gated channel activity	0.38	1	0.27700832	45	0.1299564
18956	GO:0042626	ATPase activity, coupled to transm	0.38	3	0.83102494	211	0.6093511
3761	GO:0005275|GO:0005279	amine transmembrane transporter	0.38	2	0.55401665	126	0.3638779
9861	GO:0016820	hydrolase activity, acting on acid a	0.38	3	0.83102494	212	0.612239
11776	GO:0019825	oxygen binding	0.38	5	1.3850416	389	1.1234008
8495	GO:0015077	monovalent inorganic cation transn	0.39	3	0.83102494	214	0.6180149
9913	GO:0016881	acid-amino acid ligase activity	0.39	5	1.3850416	391	1.1291766
4188	GO:0005938	cell cortex	0.40	1	0.27700832	48	0.13862015
14591	GO:0032183	SUMO binding	0.40	1	0.27700832	48	0.13862015
20642	GO:0044448	cell cortex part	0.40	1	0.27700832	48	0.13862015
113	GO:0000145	exocyst	0.40	1	0.27700832	48	0.13862015
17617	GO:0035251	UDP-glucosyltransferase activity	0.40	3	0.83102494	218	0.62956655
3756	GO:0005261|GO:0015281	cation channel activity	0.40	1	0.27700832	49	0.14150807
3844	GO:0005451	monovalent cation:hydrogen antipo	0.40	1	0.27700832	49	0.14150807
31421	GO:0080044	quercetin 7-O-glucosyltransferase	0.40	1	0.27700832	49	0.14150807
12533	GO:0022838	substrate-specific channel activity	0.41	3	0.83102494	221	0.63823026
9845	GO:0016798	hydrolase activity, acting on glycos	0.41	5	1.3850416	401	1.1580559
5096	GO:0007047	cellular cell wall organization	0.41	1	0.27700832	50	0.14439599
6932	GO:0009664	plant-type cell wall organization	0.41	1	0.27700832	50	0.14439599
9783	GO:0016722	oxidoreductase activity, oxidizing m	0.41	1	0.27700832	50	0.14439599
20857	GO:0045229	external encapsulating structure or	0.41	1	0.27700832	50	0.14439599
30033	GO:0071669	plant-type cell wall organization or	0.41	1	0.27700832	50	0.14439599
30886	GO:0072530	purine-containing compound transn	0.41	1	0.27700832	50	0.14439599
9103	GO:0015837	amine transport	0.41	2	0.55401665	135	0.38986918
8908	GO:0015629	actin cytoskeleton	0.41	1	0.27700832	51	0.14728391
9975	GO:0016998	cell wall macromolecule catabolic p	0.41	1	0.27700832	51	0.14728391
4897	GO:0006793	phosphorus metabolic process	0.41	32	8.864266	2925	8.4471655
4900	GO:0006796	phosphate metabolic process	0.41	32	8.864266	2925	8.4471655
5704	GO:0008150|GO:0000004	biological_process	0.42	234	64.81995	22215	64.155136
8660	GO:0015267|GO:0015249	channel activity	0.42	3	0.83102494	226	0.6526699
12515	GO:0022803	passive transmembrane transporte	0.42	3	0.83102494	226	0.6526699
5097	GO:0007049	cell cycle	0.42	1	0.27700832	52	0.15017183
20287	GO:0044036	cell wall macromolecule metabolic	0.42	1	0.27700832	52	0.15017183
9812	GO:0016758	transferase activity, transferring he	0.42	6	1.6620499	501	1.4468478
8607	GO:0015197|GO:0015637	peptide transporter activity	0.43	2	0.55401665	139	0.40142086
8608	GO:0015198	oligopeptide transporter activity	0.43	2	0.55401665	139	0.40142086
9750	GO:0016679	oxidoreductase activity, acting on d	0.43	1	0.27700832	53	0.15305975
29246	GO:0070882	cellular cell wall organization or bio	0.43	1	0.27700832	53	0.15305975
3895	GO:0005543	phospholipid binding	0.43	2	0.55401665	140	0.40430877
5732	GO:0008194	UDP-glycosyltransferase activity	0.43	4	1.1080333	322	0.9299102
4944	GO:0006857	oligopeptide transport	0.43	2	0.55401665	141	0.4071967
9099	GO:0015833	peptide transport	0.43	2	0.55401665	141	0.4071967
9600	GO:0016491	oxidoreductase activity	0.44	19	5.263158	1724	4.9787736
3449	GO:0004842|GO:0004840	ubiquitin-protein ligase activity	0.44	4	1.1080333	326	0.94146186
4224	GO:0005984	disaccharide metabolic process	0.44	1	0.27700832	56	0.16172351
9369	GO:0016137	glycoside metabolic process	0.44	1	0.27700832	56	0.16172351
15576	GO:0033177	proton-transporting two-sector ATP	0.44	1	0.27700832	56	0.16172351
19758	GO:0043492	ATPase activity, coupled to movem	0.44	3	0.83102494	235	0.67866117
26959	GO:0055114	oxidation-reduction process	0.44	27	7.479224	2490	7.1909204
9767	GO:0016705	oxidoreductase activity, acting on p	0.45	4	1.1080333	329	0.95012563
9813	GO:0016759	cellulose synthase activity	0.45	1	0.27700832	57	0.16461143
4913	GO:0006814|GO:0006834	sodium ion transport	0.46	1	0.27700832	58	0.16749935
6665	GO:0009311	oligosaccharide metabolic process	0.46	1	0.27700832	58	0.16749935
8499	GO:0015081|GO:0022816	sodium ion transmembrane transpo	0.46	1	0.27700832	58	0.16749935
18086	GO:0035725	sodium ion transmembrane transpo	0.46	1	0.27700832	58	0.16749935
29800	GO:0071436	sodium ion export	0.46	1	0.27700832	58	0.16749935
29919	GO:0071555	cell wall organization	0.46	1	0.27700832	58	0.16749935
9654	GO:0016567	protein ubiquitination	0.46	4	1.1080333	332	0.9587894
14854	GO:0032446	protein modification by small protei	0.46	4	1.1080333	332	0.9587894
29011	GO:0070647	protein modification by small protei	0.46	4	1.1080333	332	0.9587894
2831	GO:0004022	alcohol dehydrogenase (NAD) acti	0.46	1	0.27700832	59	0.17038727
5652	GO:0008081|GO:0004434	phosphoric diester hydrolase activi	0.47	1	0.27700832	60	0.17327519
5981	GO:0008509	anion transmembrane transporter a	0.47	3	0.83102494	245	0.70754033
11747	GO:0019787|GO:0008639	small conjugating protein ligase ac	0.47	4	1.1080333	339	0.9790048
2818	GO:0004003	ATP-dependent DNA helicase activ	0.47	1	0.27700832	61	0.1761631
4917	GO:0006820|GO:0006822	anion transport	0.48	3	0.83102494	247	0.7133162
6424	GO:0009055|GO:0009053	electron carrier activity	0.48	2	0.55401665	154	0.44473964
9824	GO:0016773	phosphotransferase activity, alcoho	0.49	16	4.432133	1493	4.311664
9838	GO:0016790	thiolester hydrolase activity	0.49	1	0.27700832	65	0.18771479
2629	GO:0003678|GO:0003679	DNA helicase activity	0.50	1	0.27700832	67	0.19349062
5764	GO:0008234|GO:0004220	cysteine-type peptidase activity	0.51	2	0.55401665	163	0.47073093
8749	GO:0015399	primary active transmembrane tran	0.51	3	0.83102494	260	0.75085914
8753	GO:0015405	P-P-bond-hydrolysis-driven transm	0.51	3	0.83102494	260	0.75085914
31420	GO:0080043	quercetin 3-O-glucosyltransferase	0.52	1	0.27700832	69	0.19926646
9911	GO:0016879	ligase activity, forming carbon-nitro	0.52	5	1.3850416	456	1.3168914
9191	GO:0015931	nucleobase, nucleoside, nucleotide	0.52	1	0.27700832	70	0.20215438
9192	GO:0015932	nucleobase, nucleoside, nucleotide	0.52	1	0.27700832	70	0.20215438
25998	GO:0051716	cellular response to stimulus	0.52	12	3.3240998	1138	3.2864528
3744	GO:0005244	voltage-gated ion channel activity	0.53	1	0.27700832	72	0.20793022
12528	GO:0022832	voltage-gated channel activity	0.53	1	0.27700832	72	0.20793022
15280	GO:0032879	regulation of localization	0.53	1	0.27700832	72	0.20793022
17141	GO:0034762	regulation of transmembrane trans	0.53	1	0.27700832	72	0.20793022
17144	GO:0034765	regulation of ion transmembrane tr	0.53	1	0.27700832	72	0.20793022

19568	GO:0043269	regulation of ion transport	0.53	1	0.27700832	72	0.20793022
25348	GO:0051049	regulation of transport	0.53	1	0.27700832	72	0.20793022
12573	GO:0022891	substrate-specific transmembrane	0.53	13	3.601108	1241	3.5839086
3311	GO:0004683|GO:0004684	calmodulin-dependent protein kina	0.54	1	0.27700832	74	0.21370606
9817	GO:0016763	transferase activity, transferring pe	0.54	1	0.27700832	74	0.21370606
8810	GO:0015491	cation:cation antiporter activity	0.54	1	0.27700832	75	0.21659398
9488	GO:0016301	kinase activity	0.55	18	4.98615	1740	5.0249805
5663	GO:0008094|GO:0004011	DNA-dependent ATPase activity	0.55	1	0.27700832	76	0.2194819
8943	GO:0015672	monovalent inorganic cation transp	0.57	4	1.1080333	383	1.1060733
3787	GO:0005319	lipid transporter activity	0.58	1	0.27700832	83	0.23969735
12547	GO:0022857|GO:0005386	transmembrane transporter activity	0.58	15	4.1551247	1483	4.282785
3048	GO:0004364	glutathione transferase activity	0.59	1	0.27700832	84	0.24258527
4643	GO:0006511	ubiquitin-dependent protein catabo	0.59	1	0.27700832	84	0.24258527
4912	GO:0006813|GO:0015458	potassium ion transport	0.59	1	0.27700832	84	0.24258527
11864	GO:0019941	modification-dependent protein cat	0.59	1	0.27700832	84	0.24258527
19898	GO:0043632	modification-dependent macromole	0.59	1	0.27700832	84	0.24258527
20507	GO:0044257	cellular protein catabolic process	0.59	1	0.27700832	84	0.24258527
25887	GO:0051603	proteolysis involved in cellular prot	0.59	1	0.27700832	84	0.24258527
30168	GO:0071804	cellular potassium ion transport	0.59	1	0.27700832	84	0.24258527
30169	GO:0071805	potassium ion transmembrane tran	0.59	1	0.27700832	84	0.24258527
3716	GO:0005198	structural molecule activity	0.59	4	1.1080333	393	1.1349525
4916	GO:0006818	hydrogen transport	0.59	3	0.83102494	292	0.84327257
9246	GO:0015992	proton transport	0.59	3	0.83102494	292	0.84327257
25195	GO:0050896|GO:0051869	response to stimulus	0.59	19	5.263158	1881	5.432177
12734	GO:0030163	protein catabolic process	0.59	1	0.27700832	86	0.24836111
20515	GO:0044265|GO:0034962	cellular macromolecule catabolic p	0.59	1	0.27700832	86	0.24836111
20624	GO:0044430	cytoskeletal part	0.60	1	0.27700832	88	0.25413695
14590	GO:0032182	small conjugating protein binding	0.61	1	0.27700832	90	0.2599128
3644	GO:0005102	receptor binding	0.62	1	0.27700832	91	0.2628007
18633	GO:0042277	peptide binding	0.63	1	0.27700832	95	0.27435237
3200	GO:0004553|GO:0016800	hydrolase activity, hydrolyzing O-g	0.63	3	0.83102494	312	0.90103096
9811	GO:0016757|GO:0016932	transferase activity, transferring gly	0.64	7	1.9390582	729	2.1052935
4954	GO:0006869	lipid transport	0.64	2	0.55401665	208	0.6006873
8085	GO:0010876	lipid localization	0.64	2	0.55401665	208	0.6006873
16603	GO:0034220	ion transmembrane transport	0.64	8	2.2160666	833	2.4056373
8681	GO:0015299	solute:hydrogen antiporter activity	0.64	1	0.27700832	98	0.28301615
30066	GO:0071702	organic substance transport	0.65	5	1.3850416	529	1.5277096
16430	GO:0034046	poly(G) RNA binding	0.65	1	0.27700832	100	0.28879198
26931	GO:0055085	transmembrane transport	0.65	15	4.1551247	1548	4.4705
4408	GO:0006200	ATP catabolic process	0.66	6	1.6620499	641	1.8511566
9918	GO:0016887|GO:0004002	ATPase activity	0.66	6	1.6620499	641	1.8511566
21600	GO:0046034	ATP metabolic process	0.66	6	1.6620499	641	1.8511566
26845	GO:0052689	carboxylic ester hydrolase activity	0.66	5	1.3850416	538	1.5537009
23515	GO:0048037	cofactor binding	0.66	3	0.83102494	327	0.94434977
9579	GO:0016462	pyrophosphatase activity	0.67	9	2.493075	954	2.7550755
12574	GO:0022892	substrate-specific transporter activ	0.67	14	3.8781164	1463	4.2250266
9859	GO:0016818	hydrolase activity, acting on acid a	0.67	9	2.493075	956	2.7608514
2668	GO:0003779	actin binding	0.67	1	0.27700832	105	0.3032316
9858	GO:0016817	hydrolase activity, acting on acid a	0.67	9	2.493075	959	2.769515
6426	GO:0009057|GO:0043285	macromolecule catabolic process	0.67	1	0.27700832	106	0.3061195
12572	GO:0022890|GO:0015082	inorganic cation transmembrane tra	0.67	3	0.83102494	332	0.9587894
3942	GO:0005618	cell wall	0.67	1	0.27700832	107	0.30900744
12868	GO:0030312	external encapsulating structure	0.69	1	0.27700832	111	0.3205591
8680	GO:0015298	solute:cation antiporter activity	0.69	1	0.27700832	112	0.32344702
29081	GO:0070717	poly-purine tract binding	0.69	1	0.27700832	112	0.32344702
6796	GO:0009507	chloroplast	0.69	2	0.55401665	229	0.6613336
9933	GO:0016903	oxidoreductase activity, acting on t	0.69	1	0.27700832	113	0.32633495
4405	GO:0006195	purine nucleotide catabolic process	0.70	7	1.9390582	773	2.232362
6508	GO:0009143	nucleoside triphosphate catabolic p	0.70	7	1.9390582	773	2.232362
6509	GO:0009144	purine nucleoside triphosphate me	0.70	7	1.9390582	773	2.232362
6511	GO:0009146	purine nucleoside triphosphate cat	0.70	7	1.9390582	773	2.232362
6519	GO:0009154	purine ribonucleotide catabolic pro	0.70	7	1.9390582	773	2.232362
6530	GO:0009166	nucleotide catabolic process	0.70	7	1.9390582	773	2.232362
6567	GO:0009203	ribonucleoside triphosphate catabo	0.70	7	1.9390582	773	2.232362
6569	GO:0009205	purine ribonucleoside triphosphate	0.70	7	1.9390582	773	2.232362
6571	GO:0009207	purine ribonucleoside triphosphate	0.70	7	1.9390582	773	2.232362
6624	GO:0009261	ribonucleotide catabolic process	0.70	7	1.9390582	773	2.232362
17034	GO:0034655	nucleobase, nucleoside, nucleotide	0.70	7	1.9390582	773	2.232362
17035	GO:0034656	nucleobase, nucleoside and nucleo	0.70	7	1.9390582	773	2.232362
22209	GO:0046700	heterocycle catabolic process	0.70	7	1.9390582	773	2.232362
30879	GO:0072523	purine-containing compound catab	0.70	7	1.9390582	773	2.232362
10058	GO:0017111	nucleoside-triphosphatase activity	0.70	8	2.2160666	878	2.5355935
20520	GO:0044270	cellular nitrogen compound catabo	0.70	7	1.9390582	777	2.2439137
4215	GO:0005975	carbohydrate metabolic process	0.71	9	2.493075	990	2.8590407
20498	GO:0044248	cellular catabolic process	0.72	8	2.2160666	894	2.5818002
6563	GO:0009199	ribonucleoside triphosphate metab	0.72	7	1.9390582	790	2.2814567
6515	GO:0009150	purine ribonucleotide metabolic pro	0.72	7	1.9390582	791	2.2843447
9823	GO:0016772	transferase activity, transferring ph	0.72	18	4.98615	1932	5.579461
12532	GO:0022836	gated channel activity	0.72	1	0.27700832	122	0.3523262
3260	GO:0004620	phospholipase activity	0.73	1	0.27700832	123	0.35521415
6506	GO:0009141	nucleoside triphosphate metabolic	0.73	7	1.9390582	796	2.2987843
2660	GO:0003735|GO:0003736	structural constituent of ribosome	0.73	2	0.55401665	246	0.7104283
6824	GO:0009536	plastid	0.73	2	0.55401665	247	0.7133162
4121	GO:0005856	cytoskeleton	0.73	1	0.27700832	126	0.3638779
9583	GO:0016469	proton-transporting two-sector ATF	0.74	1	0.27700832	127	0.36676583
4373	GO:0006163	purine nucleotide metabolic proces	0.74	7	1.9390582	810	2.339215
6622	GO:0009259|GO:0009121	ribonucleotide metabolic process	0.74	7	1.9390582	812	2.344991
9906	GO:0016874	ligase activity	0.75	5	1.3850416	597	1.7240882
2758	GO:0003924	GTPase activity	0.75	1	0.27700832	132	0.3812054
4394	GO:0006184	GTP catabolic process	0.75	1	0.27700832	132	0.3812054
21605	GO:0046039	GTP metabolic process	0.75	1	0.27700832	132	0.3812054
5809	GO:0008289	lipid binding	0.75	2	0.55401665	258	0.74508333
30306	GO:0071944	cell periphery	0.75	2	0.55401665	259	0.74797124
3242	GO:0004601|GO:0016685	peroxidase activity	0.76	1	0.27700832	135	0.38986918
9754	GO:0016684	oxidoreductase activity, acting on p	0.76	1	0.27700832	135	0.38986918
8494	GO:0015075	ion transmembrane transporter act	0.76	7	1.9390582	827	2.3883097
30877	GO:0072521	purine-containing compound metab	0.76	7	1.9390582	827	2.3883097
19126	GO:0042803	protein homodimerization activity	0.77	2	0.55401665	265	0.7652988

5018	GO:0006950	response to stress	0.77	5	1.3850416	611	1.764519
6425	GO:0009056	catabolic process	0.77	9	2.493075	1051	3.0352037
3724	GO:0005216	ion channel activity	0.77	1	0.27700832	140	0.40430877
9266	GO:0016020	membrane	0.78	35	9.695291	3741	10.803708
19533	GO:0043234	protein complex	0.78	7	1.9390582	848	2.448956
4910	GO:0006811	ion transport	0.78	8	2.2160666	959	2.769515
20532	GO:0044282	small molecule catabolic process	0.79	7	1.9390582	854	2.4662836
12794	GO:0030234	enzyme regulator activity	0.79	3	0.83102494	403	1.1638317
8679	GO:0015297	antiporter activity	0.80	1	0.27700832	152	0.4389638
8682	GO:0015300	solute:solute antiporter activity	0.80	1	0.27700832	152	0.4389638
13656	GO:0031224	intrinsic to membrane	0.80	10	2.7700832	1193	3.4452884
3723	GO:0005215|GO:0005478	transporter activity	0.81	16	4.432133	1844	5.325324
3888	GO:0005529	sugar binding	0.81	1	0.27700832	158	0.45629135
18953	GO:0042623	ATPase activity, coupled	0.81	4	1.1080333	537	1.550813
5840	GO:0008324	cation transmembrane transporter	0.81	4	1.1080333	538	1.5537009
9422	GO:0016209	antioxidant activity	0.82	1	0.27700832	165	0.47650677
12516	GO:0022804	active transmembrane transporter a	0.83	6	1.6620499	787	2.2727928
4605	GO:0006457|GO:0007022	protein folding	0.84	1	0.27700832	173	0.49961013
4067	GO:0005783	endoplasmic reticulum	0.84	1	0.27700832	174	0.50249803
4109	GO:0005840|GO:0033279	ribosome	0.84	5	1.3850416	682	1.9695613
4867	GO:0006753	nucleoside phosphate metabolic pr	0.84	7	1.9390582	914	2.6395588
6484	GO:0009117	nucleotide metabolic process	0.84	7	1.9390582	914	2.6395588
11787	GO:0019842	vitamin binding	0.84	1	0.27700832	177	0.5111618
4572	GO:0006412|GO:0006416	translation	0.85	1	0.27700832	180	0.5198256
5610	GO:0008026	ATP-dependent helicase activity	0.85	1	0.27700832	183	0.52848935
28401	GO:0070035	purine NTP-dependent helicase ac	0.85	1	0.27700832	183	0.52848935
19125	GO:0042802	identical protein binding	0.85	5	1.3850416	697	2.01288
9486	GO:0016298	lipase activity	0.86	1	0.27700832	185	0.53426516
9818	GO:0016765|GO:0016766	transferase activity, transferring alk	0.86	1	0.27700832	185	0.53426516
9836	GO:0016787	hydrolase activity	0.86	33	9.141274	3723	10.751725
2657	GO:0003727|GO:0003728	single-stranded RNA binding	0.86	1	0.27700832	188	0.54292893
3068	GO:0004386	helicase activity	0.86	1	0.27700832	189	0.54581684
26932	GO:0055086	nucleobase, nucleoside and nucleo	0.86	7	1.9390582	944	2.7261963
2897	GO:0004091|GO:0004302	carboxylesterase activity	0.87	1	0.27700832	194	0.5602564
25095	GO:0050790	regulation of catalytic activity	0.88	3	0.83102494	480	1.3862015
28366	GO:0065009	regulation of molecular function	0.88	3	0.83102494	484	1.3977532
4909	GO:0006810|GO:0015457	transport	0.89	19	5.263158	2315	6.6855345
25529	GO:0051234	establishment of localization	0.89	19	5.263158	2315	6.6855345
13018	GO:0030529	ribonucleoprotein complex	0.89	5	1.3850416	741	2.1399486
25476	GO:0051179	localization	0.89	19	5.263158	2319	6.697086
21058	GO:0045454|GO:0030503	cell redox homeostasis	0.89	1	0.27700832	211	0.6093511
30204	GO:0071840	cellular component organization or	0.89	2	0.55401665	362	1.045427
22376	GO:0046873	metal ion transmembrane transport	0.89	1	0.27700832	213	0.6151269
4558	GO:0006396|GO:0006394	RNA processing	0.90	1	0.27700832	219	0.63245445
12608	GO:0030001	metal ion transport	0.90	2	0.55401665	374	1.080082
22479	GO:0046983	protein dimerization activity	0.91	3	0.83102494	517	1.4930545
13531	GO:0031072	heat shock protein binding	0.91	1	0.27700832	230	0.6642216
11695	GO:0019725	cellular homeostasis	0.91	1	0.27700832	234	0.67577326
15392	GO:0032991	macromolecular complex	0.92	12	3.3240998	1616	4.666878
4911	GO:0006812|GO:0006819	cation transport	0.92	4	1.1080333	662	1.9118029
3877	GO:0005516	calmodulin binding	0.92	1	0.27700832	236	0.6815491
11828	GO:0019899	enzyme binding	0.92	1	0.27700832	237	0.684437
18925	GO:0042592	homeostatic process	0.92	1	0.27700832	238	0.68732494
15436	GO:0033036	macromolecule localization	0.92	2	0.55401665	397	1.1465042
19527	GO:0043228	non-membrane-bounded organelle	0.92	6	1.6620499	923	2.66555
19531	GO:0043232	intracellular non-membrane-bound	0.92	6	1.6620499	923	2.66555
3885	GO:0005525	GTP binding	0.92	1	0.27700832	245	0.70754033
11032	GO:0019001	guanyl nucleotide binding	0.92	1	0.27700832	245	0.70754033
14967	GO:0032561	guanyl ribonucleotide binding	0.92	1	0.27700832	245	0.70754033
20619	GO:0044425	membrane part	0.92	11	3.0470915	1523	4.398302
20512	GO:0044262|GO:0006092	cellular carbohydrate metabolic pro	0.93	1	0.27700832	248	0.7162041
5661	GO:0008092	cytoskeletal protein binding	0.93	1	0.27700832	249	0.719092
9285	GO:0016043	cellular component organization	0.94	1	0.27700832	261	0.7537471
9699	GO:0016616	oxidoreductase activity, acting on t	0.94	1	0.27700832	266	0.7681867
22007	GO:0046483	heterocycle metabolic process	0.94	7	1.9390582	1094	3.1593843
9267	GO:0016021	integral to membrane	0.94	7	1.9390582	1103	3.1853757
3874	GO:0005509	calcium ion binding	0.95	1	0.27700832	279	0.8057296
18912	GO:0042578	phosphoric ester hydrolase activity	0.95	2	0.55401665	444	1.2822365
30205	GO:0071841	cellular component organization or	0.95	1	0.27700832	285	0.8230572
9697	GO:0016614	oxidoreductase activity, acting on C	0.95	1	0.27700832	293	0.84616053
28365	GO:0065008	regulation of biological quality	0.96	1	0.27700832	316	0.9125827
4849	GO:0006730|GO:0019753	one-carbon metabolic process	0.97	1	0.27700832	325	0.93857396
25098	GO:0050794|GO:0051244	regulation of cellular process	0.97	31	8.587257	3987	11.514136
20638	GO:0044444	cytoplasmic part	0.98	10	2.7700832	1644	4.7477403
25094	GO:0050789|GO:0050791	regulation of biological process	0.98	33	9.141274	4315	12.461374
9795	GO:0016740	transferase activity	0.98	28	7.7562327	3764	10.870131
9839	GO:0016791|GO:0016302	phosphatase activity	0.98	1	0.27700832	383	1.1060733
28364	GO:0065007	biological regulation	0.98	33	9.141274	4376	12.637537
4755	GO:0006629	lipid metabolic process	0.99	2	0.55401665	590	1.7038727
8673	GO:0015291|GO:0015290	secondary active transmembrane t	0.99	1	0.27700832	407	1.1753833
2674	GO:0003824	catalytic activity	0.99	93	25.761772	10750	31.045137
9494	GO:0016311	dephosphorylation	0.99	1	0.27700832	425	1.227366
9837	GO:0016788	hydrolase activity, acting on ester b	0.99	8	2.2160666	1534	4.430069
4530	GO:0006355|GO:0032583	regulation of transcription, DNA-de	0.99	16	4.432133	2591	7.4826
25544	GO:0051252	regulation of RNA metabolic proces	0.99	16	4.432133	2593	7.488376
4526	GO:0006351|GO:0006350	transcription, DNA-dependent	0.99	8	2.2160666	1575	4.548474
15176	GO:0032774	RNA biosynthetic process	0.99	8	2.2160666	1575	4.548474
7141	GO:0009889	regulation of biosynthetic process	0.99	16	4.432133	2605	7.523031
7772	GO:0010556	regulation of macromolecule biosyn	0.99	16	4.432133	2605	7.523031
13756	GO:0031326	regulation of cellular biosynthetic p	0.99	16	4.432133	2605	7.523031
32213	GO:2000112	regulation of cellular macromolecul	0.99	16	4.432133	2605	7.523031
4027	GO:0005737	cytoplasm	0.99	11	3.0470915	2001	5.7787275
7692	GO:0010468	regulation of gene expression	0.99	16	4.432133	2635	7.6096687
20640	GO:0044446	intracellular organelle part	1.00	2	0.55401665	710	2.0504231
27228	GO:0060255	regulation of macromolecule metab	1.00	16	4.432133	2660	7.6818666
20616	GO:0044422	organelle part	1.00	2	0.55401665	712	2.0561988
11221	GO:0019219	regulation of nucleobase, nucleosid	1.00	16	4.432133	2675	7.7251854
25468	GO:0051171	regulation of nitrogen compound m	1.00	16	4.432133	2675	7.7251854

131	GO:0000166	nucleotide binding	1.00	5	1.3850416	1213	3.5030468
3884	GO:0005524	ATP binding	1.00	1	0.27700832	524	1.51327
2628	GO:0003677	DNA binding	1.00	5	1.3850416	1224	3.534814
31465	GO:0080090	regulation of primary metabolic pro	1.00	16	4.432133	2710	7.826263
3876	GO:0005515|GO:0045308	protein binding	1.00	53	14.68144	6933	20.021948
13041	GO:0030554	adenyl nucleotide binding	1.00	1	0.27700832	550	1.5883559
14965	GO:0032559	adenyl ribonucleotide binding	1.00	1	0.27700832	550	1.5883559
11224	GO:0019222	regulation of metabolic process	1.00	19	5.263158	3115	8.995871
7691	GO:0010467	gene expression	1.00	10	2.7700832	1974	5.7007537
18000	GO:0035639	purine ribonucleoside triphosphate	1.00	2	0.55401665	769	2.2208104
9308	GO:0016070	RNA metabolic process	1.00	9	2.493075	1855	5.3570914
13753	GO:0031323	regulation of cellular metabolic pro	1.00	16	4.432133	2770	7.999538
14959	GO:0032553	ribonucleotide binding	1.00	2	0.55401665	795	2.2958963
14961	GO:0032555	purine ribonucleotide binding	1.00	2	0.55401665	795	2.2958963
10033	GO:0017076	purine nucleotide binding	1.00	2	0.55401665	797	2.3016722
2627	GO:0003676	nucleic acid binding	1.00	13	3.601108	2453	7.0840673
20531	GO:0044281	small molecule metabolic process	1.00	8	2.2160666	1788	5.1636004
3863	GO:0005488	binding	1.00	118	32.68698	13903	40.15075
3903	GO:0005575|GO:0008372	cellular_component	1.00	65	18.00554	8515	24.590637
3946	GO:0005623	cell	1.00	62	17.174515	8227	23.758917
20657	GO:0044464	cell part	1.00	62	17.174515	8227	23.758917
716	GO:0001071	nucleic acid binding transcription fa	1.00	5	1.3850416	1417	4.0921826
2643	GO:0003700|GO:0000130	sequence-specific DNA binding tra	1.00	5	1.3850416	1417	4.0921826
3955	GO:0005634	nucleus	1.00	5	1.3850416	1433	4.138389
19525	GO:0043226	organelle	1.00	15	4.1551247	3013	8.701303
19528	GO:0043229	intracellular organelle	1.00	15	4.1551247	3013	8.701303
2653	GO:0003723	RNA binding	1.00	1	0.27700832	797	2.3016722
19526	GO:0043227	membrane-bounded organelle	1.00	8	2.2160666	2142	6.185924
19530	GO:0043231	intracellular membrane-bounded o	1.00	8	2.2160666	2142	6.185924
20618	GO:0044424	intracellular part	1.00	20	5.5401664	3853	11.127155
3945	GO:0005622	intracellular	1.00	26	7.202216	4751	13.720507
2626	GO:0003674|GO:0005554	molecular_function	1.00	210	58.171745	24394	70.447914

GO ID	GO ACCESSION	GO Term	p-value	Count in Selection	% Count in Selection	Count in Total	% Count in Total
5704	GO:0008150|GO	biological_process	0.00	163	79.12621	22215	64.155136
5705	GO:0008152	metabolic process	0.00	135	65.53398	17432	50.34222
9869	GO:0016829	lyase activity	0.00	12	5.8252425	488	1.4093049
11776	GO:0019825	oxygen binding	0.00	10	4.854369	389	1.1234008
16391	GO:0034007	S-linalool synthase activity	0.00	2	0.9708738	4	0.011551679
9600	GO:0016491	oxidoreductase activity	0.00	22	10.679611	1724	4.9787736
26959	GO:0055114	oxidation-reduction process	0.00	28	13.592233	2490	7.1909204
9832	GO:0016782	transferase activity, transferring sulfur-containing gr	0.00	4	1.9417475	74	0.21370606
9877	GO:0016838	carbon-oxygen lyase activity, acting on phosphates	0.00	4	1.9417475	77	0.22236982
2864	GO:0004058|GO	aromatic-L-amino-acid decarboxylase activity	0.00	2	0.9708738	9	0.025991278
26845	GO:0052689	carboxylic ester hydrolase activity	0.00	10	4.854369	538	1.5537009
21148	GO:0045547	dehydrodolichyl diphosphate synthase activity	0.00	2	0.9708738	10	0.0288792
716	GO:0001071	nucleic acid binding transcription factor activity	0.00	18	8.7378645	1417	4.0921826
2643	GO:0003700|GO	sequence-specific DNA binding transcription factor	0.00	18	8.7378645	1417	4.0921826
9870	GO:0016830	carbon-carbon lyase activity	0.00	5	2.4271846	161	0.4649551
29787	GO:0071423	malate transmembrane transport	0.00	2	0.9708738	14	0.040430877
9012	GO:0015743	malate transport	0.00	2	0.9708738	14	0.040430877
8553	GO:0015140	malate transmembrane transporter activity	0.00	2	0.9708738	14	0.040430877
9009	GO:0015740	C4-dicarboxylate transport	0.00	2	0.9708738	14	0.040430877
8859	GO:0015556	C4-dicarboxylate transmembrane transporter activi	0.00	2	0.9708738	14	0.040430877
9767	GO:0016705	oxidoreductase activity, acting on paired donors, wi	0.00	7	3.3980582	329	0.95012563
3382	GO:0004768|GO	stearoyl-CoA 9-desaturase activity	0.00	2	0.9708738	16	0.046206716
4928	GO:0006835|GO	dicarboxylic acid transport	0.00	2	0.9708738	16	0.046206716
7521	GO:0010295	(+)-abscisic acid 8'-hydroxylase activity	0.00	2	0.9708738	16	0.046206716
3782	GO:0005310|GO	dicarboxylic acid transmembrane transporter activit	0.00	2	0.9708738	16	0.046206716
9427	GO:0016215	CoA desaturase activity	0.00	2	0.9708738	16	0.046206716
5702	GO:0008146	sulfotransferase activity	0.00	3	1.4563106	54	0.15594767
2674	GO:0003824	catalytic activity	0.00	82	39.805824	10750	31.045137
9871	GO:0016831	carboxy-lyase activity	0.01	4	1.9417475	115	0.3321108
8526	GO:0015112	nitrate transmembrane transporter activity	0.01	3	1.4563106	61	0.1761631
8975	GO:0015706|GO	nitrate transport	0.01	3	1.4563106	61	0.1761631
11931	GO:0020037	heme binding	0.01	9	4.3689322	550	1.5883559
3124	GO:0004462	lactoylglutathione lyase activity	0.01	2	0.9708738	20	0.0577584
15176	GO:0032774	RNA biosynthetic process	0.01	18	8.7378645	1575	4.548474
4526	GO:0006351|GO	transcription, DNA-dependent	0.01	18	8.7378645	1575	4.548474
3129	GO:0004467	long-chain fatty acid-CoA ligase activity	0.01	2	0.9708738	21	0.060646318
3872	GO:0005506	iron ion binding	0.01	9	4.3689322	563	1.6258988
9771	GO:0016709	oxidoreductase activity, acting on paired donors, wi	0.01	3	1.4563106	71	0.2050423
9779	GO:0016717	oxidoreductase activity, acting on paired donors, wi	0.01	2	0.9708738	24	0.06931008
31493	GO:0080118	brassinosteroid sulfotransferase activity	0.01	2	0.9708738	25	0.072197996
21145	GO:0045543	gibberellin 2-beta-dioxygenase activity	0.01	2	0.9708738	25	0.072197996
22409	GO:0046906	tetrapyrrole binding	0.01	9	4.3689322	596	1.7212002
9800	GO:0016746	transferase activity, transferring acyl groups	0.01	8	3.883495	498	1.438184
8919	GO:0015645	fatty acid ligase activity	0.01	2	0.9708738	27	0.077973835
3113	GO:0004451	isocitrate lyase activity	0.01	1	0.4854369	2	0.00577584
21946	GO:0046421	methylisocitrate lyase activity	0.01	1	0.4854369	2	0.00577584
22988	GO:0047501	(+)-neomenthol dehydrogenase activity	0.01	1	0.4854369	2	0.00577584
22991	GO:0047504	(-)-menthol dehydrogenase activity	0.01	1	0.4854369	2	0.00577584
8517	GO:0015103	inorganic anion transmembrane transporter activity	0.01	4	1.9417475	154	0.44473964
2870	GO:0004064	arylesterase activity	0.01	2	0.9708738	30	0.086637594
8683	GO:0015301|GO	anion:anion antiporter activity	0.01	2	0.9708738	30	0.086637594
5981	GO:0008509	anion transmembrane transporter activity	0.02	5	2.4271846	245	0.70754033
4917	GO:0006820|GO	anion transport	0.02	5	2.4271846	247	0.7133162
4319	GO:0006081	cellular aldehyde metabolic process	0.02	1	0.4854369	3	0.00866376
4330	GO:0006097	glyoxylate cycle	0.02	1	0.4854369	3	0.00866376
22011	GO:0046487	glyoxylate metabolic process	0.02	1	0.4854369	3	0.00866376
3134	GO:0004474	malate synthase activity	0.02	1	0.4854369	3	0.00866376
8159	GO:0010951	negative regulation of endopeptidase activity	0.02	3	1.4563106	95	0.27435237
26712	GO:0052548	regulation of endopeptidase activity	0.02	3	1.4563106	95	0.27435237
28101	GO:0061135	endopeptidase regulator activity	0.02	3	1.4563106	95	0.27435237
3472	GO:0004866	endopeptidase inhibitor activity	0.02	3	1.4563106	95	0.27435237
3156	GO:0004497	monooxygenase activity	0.02	4	1.9417475	172	0.49672222
9801	GO:0016747	transferase activity, transferring acyl groups other t	0.02	7	3.3980582	456	1.3168914
8967	GO:0015698	inorganic anion transport	0.02	4	1.9417475	176	0.5082739
5729	GO:0008191	metalloendopeptidase inhibitor activity	0.02	1	0.4854369	4	0.011551679
7157	GO:0009905	ent-copalyl diphosphate synthase activity	0.02	1	0.4854369	4	0.011551679
7792	GO:0010576	metalloenzyme regulator activity	0.02	1	0.4854369	4	0.011551679
9905	GO:0016872	intramolecular lyase activity	0.02	1	0.4854369	4	0.011551679
23942	GO:0048551	metalloenzyme inhibitor activity	0.02	1	0.4854369	4	0.011551679
24433	GO:0050113	inositol oxygenase activity	0.02	1	0.4854369	4	0.011551679
3661	GO:0005125	cytokine activity	0.02	1	0.4854369	4	0.011551679
3674	GO:0005138	interleukin-6 receptor binding	0.02	1	0.4854369	4	0.011551679
4320	GO:0006082	organic acid metabolic process	0.02	6	2.9126213	369	1.0656425
11716	GO:0019752	carboxylic acid metabolic process	0.02	6	2.9126213	369	1.0656425
19707	GO:0043436	oxoacid metabolic process	0.02	6	2.9126213	369	1.0656425
18541	GO:0042180	cellular ketone metabolic process	0.03	6	2.9126213	375	1.0829699
7690	GO:0010466	negative regulation of peptidase activity	0.03	3	1.4563106	110	0.31767118
12946	GO:0030414	peptidase inhibitor activity	0.03	3	1.4563106	110	0.31767118
25636	GO:0051346	negative regulation of hydrolase activity	0.03	3	1.4563106	110	0.31767118
9308	GO:0016070	RNA metabolic process	0.03	18	8.7378645	1855	5.3570914
2897	GO:0004091|GO	carboxylesterase activity	0.03	4	1.9417475	194	0.5602564
3229	GO:0004586	ornithine decarboxylase activity	0.03	1	0.4854369	5	0.0144396
26711	GO:0052547	regulation of peptidase activity	0.03	3	1.4563106	112	0.32344702
28100	GO:0061134	peptidase regulator activity	0.03	3	1.4563106	112	0.32344702
11224	GO:0019222	regulation of metabolic process	0.03	27	13.106796	3115	8.995871
9874	GO:0016835	carbon-oxygen lyase activity	0.03	4	1.9417475	202	0.5833598

26059	GO:0051777	ent-kaurenoate oxidase activity	**0.04**	1	0.4854369	6	0.01732752
4530	GO:0006355|GO	regulation of transcription, DNA-dependent	**0.04**	23	11.165049	2591	7.4826
25544	GO:0051252	regulation of RNA metabolic process	**0.04**	23	11.165049	2593	7.488376
6884	GO:0009607	response to biotic stimulus	**0.04**	3	1.4563106	122	0.3523262
3293	GO:0004659	prenyltransferase activity	**0.04**	2	0.9708738	51	0.14728391
7141	GO:0009889	regulation of biosynthetic process	**0.04**	23	11.165049	2605	7.523031
7772	GO:0010556	regulation of macromolecule biosynthetic process	**0.04**	23	11.165049	2605	7.523031
13756	GO:0031326	regulation of cellular biosynthetic process	**0.04**	23	11.165049	2605	7.523031
32213	GO:2000112	regulation of cellular macromolecule biosynthetic pr	**0.04**	23	11.165049	2605	7.523031
2626	GO:0003674|GO	molecular_function	**0.04**	157	76.21359	24394	70.447914
16115	GO:0033729	anthocyanidin reductase activity	**0.04**	1	0.4854369	7	0.020215439
7692	GO:0010468	regulation of gene expression	**0.04**	23	11.165049	2635	7.6096687
9883	GO:0016846	carbon-sulfur lyase activity	**0.04**	2	0.9708738	56	0.16172351
6427	GO:0009058	biosynthetic process	**0.04**	24	11.650485	2796	8.074624
7557	GO:0010333	terpene synthase activity	**0.05**	2	0.9708738	57	0.16461143
27228	GO:0060255	regulation of macromolecule metabolic process	**0.05**	23	11.165049	2660	7.6818666
8725	GO:0015367	oxoglutarate:malate antiporter activity	**0.05**	1	0.4854369	8	0.023103358
9011	GO:0015742	alpha-ketoglutarate transport	**0.05**	1	0.4854369	8	0.023103358
11196	GO:0019187	beta-1,4-mannosyltransferase activity	**0.05**	1	0.4854369	8	0.023103358
26035	GO:0051753	mannan synthase activity	**0.05**	1	0.4854369	8	0.023103358
9754	GO:0016684	oxidoreductase activity, acting on peroxide as acce	**0.05**	3	1.4563106	135	0.38986918
3242	GO:0004601|GO	peroxidase activity	**0.05**	3	1.4563106	135	0.38986918
11221	GO:0019219	regulation of nucleobase, nucleoside, nucleotide an	**0.05**	23	11.165049	2675	7.7251854
25468	GO:0051171	regulation of nitrogen compound metabolic process	**0.05**	23	11.165049	2675	7.7251854
7691	GO:0010467	gene expression	**0.05**	18	8.7378645	1974	5.7007537
7506	GO:0010279	indole-3-acetic acid amido synthetase activity	**0.05**	1	0.4854369	9	0.025991278
8661	GO:0015269	calcium-activated potassium channel activity	**0.05**	1	0.4854369	9	0.025991278
12534	GO:0022839	ion gated channel activity	**0.05**	1	0.4854369	9	0.025991278
2823	GO:0004014	adenosylmethionine decarboxylase activity	**0.05**	1	0.4854369	9	0.025991278
3734	GO:0005227	calcium activated cation channel activity	**0.05**	1	0.4854369	9	0.025991278
17024	GO:0034645|GO	cellular macromolecule biosynthetic process	**0.05**	18	8.7378645	1999	5.7729516
6428	GO:0009059|GO	macromolecule biosynthetic process	**0.05**	18	8.7378645	2003	5.7845035
31465	GO:0080090	regulation of primary metabolic process	**0.05**	23	11.165049	2710	7.826263
9710	GO:0016628	oxidoreductase activity, acting on the CH-CH group	0.06	2	0.9708738	65	0.18771479
4878	GO:0006772	thiamine metabolic process	0.06	1	0.4854369	10	0.0288792
6592	GO:0009228	thiamine biosynthetic process	0.06	1	0.4854369	10	0.0288792
23116	GO:0047631	ADP-ribose diphosphatase activity	0.06	1	0.4854369	10	0.0288792
29215	GO:0070851	growth factor receptor binding	0.06	1	0.4854369	10	0.0288792
3056	GO:0004372	glycine hydroxymethyltransferase activity	0.06	1	0.4854369	10	0.0288792
3403	GO:0004792	thiosulfate sulfurtransferase activity	0.06	1	0.4854369	10	0.0288792
3473	GO:0004867	serine-type endopeptidase inhibitor activity	0.06	2	0.9708738	66	0.1906027
9849	GO:0016803	ether hydrolase activity	0.06	1	0.4854369	11	0.03176712
2989	GO:0004301	epoxide hydrolase activity	0.06	1	0.4854369	11	0.03176712
4755	GO:0006629	lipid metabolic process	0.06	7	3.3980582	590	1.7038727
13753	GO:0031323	regulation of cellular metabolic process	0.07	23	11.165049	2770	7.999538
3464	GO:0004857	enzyme inhibitor activity	0.07	3	1.4563106	157	0.4534034
3888	GO:0005529	sugar binding	0.07	3	1.4563106	158	0.45629135
9764	GO:0016701	oxidoreductase activity, acting on single donors witl	0.07	2	0.9708738	72	0.20793022
7526	GO:0010301|GO	xanthoxin dehydrogenase activity	0.07	1	0.4854369	12	0.03465504
19047	GO:0042723	thiamine-containing compound metabolic process	0.07	1	0.4854369	12	0.03465504
19048	GO:0042724	thiamine-containing compound biosynthetic process	0.07	1	0.4854369	12	0.03465504
7551	GO:0010327	acetyl CoA:(Z)-3-hexen-1-ol acetyltransferase activ	0.07	1	0.4854369	13	0.037542958
2896	GO:0004090	carbonyl reductase (NADPH) activity	0.07	1	0.4854369	13	0.037542958
9422	GO:0016209	antioxidant activity	0.08	3	1.4563106	165	0.47650677
25094	GO:0050789|GO	regulation of biological process	0.08	33	16.019417	4315	12.461374
9909	GO:0016877	ligase activity, forming carbon-sulfur bonds	0.08	2	0.9708738	78	0.22525774
9847	GO:0016801	hydrolase activity, acting on ether bonds	0.08	1	0.4854369	14	0.040430877
9873	GO:0016833	oxo-acid-lyase activity	0.08	1	0.4854369	14	0.040430877
11157	GO:0019144	ADP-sugar diphosphatase activity	0.08	1	0.4854369	14	0.040430877
31408	GO:0080031	methyl salicylate esterase activity	0.08	1	0.4854369	14	0.040430877
3827	GO:0005372	water transmembrane transporter activity	0.08	2	0.9708738	81	0.23392151
4927	GO:0006833	water transport	0.08	2	0.9708738	81	0.23392151
8652	GO:0015250	water channel activity	0.08	2	0.9708738	81	0.23392151
18429	GO:0042044	fluid transport	0.08	2	0.9708738	81	0.23392151
9340	GO:0016108	tetraterpenoid metabolic process	0.09	1	0.4854369	15	0.043318797
9341	GO:0016109	tetraterpenoid biosynthetic process	0.09	1	0.4854369	15	0.043318797
9348	GO:0016116	carotenoid metabolic process	0.09	1	0.4854369	15	0.043318797
9349	GO:0016117	carotenoid biosynthetic process	0.09	1	0.4854369	15	0.043318797
14101	GO:0031683	G-protein beta/gamma-subunit complex binding	0.09	1	0.4854369	15	0.043318797
2868	GO:0004062	aryl sulfotransferase activity	0.09	1	0.4854369	15	0.043318797
22479	GO:0046983	protein dimerization activity	0.09	6	2.9126213	517	1.4930545
28364	GO:0065007	biological regulation	0.09	33	16.019417	4376	12.637537
5995	GO:0008524	glucose 6-phosphate:phosphate antiporter activity	0.09	1	0.4854369	16	0.046206716
8694	GO:0015315	organophosphate:inorganic phosphate antiporter ac	0.09	1	0.4854369	16	0.046206716
9797	GO:0016742	hydroxymethyl-, formyl- and related transferase act	0.09	1	0.4854369	16	0.046206716
31396	GO:0080019	fatty-acyl-CoA reductase (alcohol-forming) activity	0.09	1	0.4854369	16	0.046206716
219	GO:0000287	magnesium ion binding	0.09	1	0.4854369	16	0.046206716
9697	GO:0016614	oxidoreductase activity, acting on CH-OH group of	0.10	4	1.9417475	293	0.84616053
9486	GO:0016298	lipase activity	0.10	3	1.4563106	185	0.53426516
8662	GO:0015271	outward rectifier potassium channel activity	0.10	1	0.4854369	18	0.051982556
28368	GO:0070001	aspartic-type peptidase activity	0.10	1	0.4854369	18	0.051982556
2981	GO:0004190	aspartic-type endopeptidase activity	0.10	1	0.4854369	18	0.051982556
19393	GO:0043086	negative regulation of catalytic activity	0.10	3	1.4563106	188	0.54292893
20342	GO:0044092	negative regulation of molecular function	0.10	3	1.4563106	188	0.54292893
20499	GO:0044249	cellular biosynthetic process	0.10	21	10.194175	2635	7.6096687
24968	GO:0050662	coenzyme binding	0.11	3	1.4563106	191	0.5515927
25626	GO:0051336	regulation of hydrolase activity	0.11	3	1.4563106	196	0.5660323
9833	GO:0016783	sulfurtransferase activity	0.11	1	0.4854369	20	0.0577584
3628	GO:0005086	ARF guanyl-nucleotide exchange factor activity	0.11	1	0.4854369	20	0.0577584
24858	GO:0050551	myrcene synthase activity	0.12	1	0.4854369	22	0.06353424

9795	GO:0016740	transferase activity	0.13	28	13.592233	3764	10.870131
9709	GO:0016627	oxidoreductase activity, acting on the CH-CH group	0.13	2	0.9708738	104	0.30034366
22414	GO:0046912	transferase activity, transferring acyl groups, acyl g	0.13	1	0.4854369	23	0.06642216
3662	GO:0005126	cytokine receptor binding	0.13	1	0.4854369	23	0.06642216
19253	GO:0042936	dipeptide transporter activity	0.13	2	0.9708738	105	0.3032316
19255	GO:0042938	dipeptide transport	0.13	2	0.9708738	105	0.3032316
23515	GO:0048037	cofactor binding	0.13	4	1.9417475	327	0.94434977
4692	GO:0006563	L-serine metabolic process	0.13	1	0.4854369	24	0.06931008
25578	GO:0051287|GO	NAD binding	0.13	1	0.4854369	24	0.06931008
31407	GO:0080030	methyl indole-3-acetate esterase activity	0.13	1	0.4854369	24	0.06931008
31393	GO:0080016	(-)-E-beta-caryophyllene synthase activity	0.14	1	0.4854369	25	0.072197996
31394	GO:0080017	alpha-humulene synthase activity	0.14	1	0.4854369	25	0.072197996
17147	GO:0034768	(E)-beta-ocimene synthase activity	0.14	1	0.4854369	26	0.075085916
12533	GO:0022838	substrate-specific channel activity	0.15	3	1.4563106	221	0.63823026
25098	GO:0050794|GO	regulation of cellular process	0.15	29	14.07767	3987	11.514136
9346	GO:0016114	terpenoid biosynthetic process	0.15	1	0.4854369	27	0.077973835
9541	GO:0016413	O-acetyltransferase activity	0.15	1	0.4854369	27	0.077973835
9816	GO:0016762	xyloglucan:xyloglucosyl transferase activity	0.15	1	0.4854369	27	0.077973835
8660	GO:0015267|GO	channel activity	0.15	3	1.4563106	226	0.6526699
12515	GO:0022803	passive transmembrane transporter activity	0.15	3	1.4563106	226	0.6526699
3812	GO:0005355|GO	glucose transmembrane transporter activity	0.15	1	0.4854369	28	0.080861755
6438	GO:0009069	serine family amino acid metabolic process	0.15	1	0.4854369	28	0.080861755
9027	GO:0015758	glucose transport	0.15	1	0.4854369	28	0.080861755
24966	GO:0050660	flavin adenine dinucleotide binding	0.16	2	0.9708738	118	0.34077454
31895	GO:0090304	nucleic acid metabolic process	0.16	18	8.7378645	2340	6.7577324
4841	GO:0006721	terpenoid metabolic process	0.16	1	0.4854369	29	0.083749674
7558	GO:0010334	sesquiterpene synthase activity	0.16	1	0.4854369	29	0.083749674
23	GO:0000030	mannosyltransferase activity	0.16	1	0.4854369	30	0.086637594
3877	GO:0005516	calmodulin binding	0.17	3	1.4563106	236	0.6815491
7222	GO:0009975	cyclase activity	0.17	1	0.4854369	31	0.08952551
11202	GO:0019199	transmembrane receptor protein kinase activity	0.18	5	2.4271846	504	1.4555116
6072	GO:0008645	hexose transport	0.18	1	0.4854369	34	0.09818927
8562	GO:0015149	hexose transmembrane transporter activity	0.18	1	0.4854369	34	0.09818927
17794	GO:0035428	hexose transmembrane transport	0.18	1	0.4854369	34	0.09818927
3749	GO:0005249	voltage-gated potassium channel activity	0.18	1	0.4854369	34	0.09818927
6355	GO:0008970	phospholipase A1 activity	0.19	1	0.4854369	35	0.10107719
23199	GO:0047714	galactolipase activity	0.19	1	0.4854369	35	0.10107719
12805	GO:0030246	carbohydrate binding	0.19	3	1.4563106	251	0.7248679
4232	GO:0005992	trehalose biosynthetic process	0.19	1	0.4854369	36	0.10396511
9393	GO:0016165	lipoxygenase activity	0.19	1	0.4854369	36	0.10396511
3801	GO:0005342	organic acid transmembrane transporter activity	0.19	2	0.9708738	136	0.3927571
22444	GO:0046943	carboxylic acid transmembrane transporter activity	0.19	2	0.9708738	136	0.3927571
8980	GO:0015711	organic anion transport	0.20	1	0.4854369	37	0.10685303
3758	GO:0005267	potassium channel activity	0.20	1	0.4854369	37	0.10685303
4942	GO:0006855	drug transmembrane transport	0.20	2	0.9708738	139	0.40142086
8607	GO:0015197|GO	peptide transporter activity	0.20	2	0.9708738	139	0.40142086
8608	GO:0015198	oligopeptide transporter activity	0.20	2	0.9708738	139	0.40142086
8642	GO:0015238|GO	drug transmembrane transporter activity	0.20	2	0.9708738	139	0.40142086
9154	GO:0015893	drug transport	0.20	2	0.9708738	139	0.40142086
18832	GO:0042493|GO	response to drug	0.20	2	0.9708738	139	0.40142086
4231	GO:0005991	trehalose metabolic process	0.20	1	0.4854369	38	0.10974095
12538	GO:0022843	voltage-gated cation channel activity	0.20	1	0.4854369	38	0.10974095
9796	GO:0016741	transferase activity, transferring one-carbon groups	0.20	3	1.4563106	261	0.7537471
9837	GO:0016788	hydrolase activity, acting on ester bonds	0.20	12	5.8252425	1534	4.430069
4944	GO:0006857	oligopeptide transport	0.20	2	0.9708738	141	0.4071967
9099	GO:0015833	peptide transport	0.20	2	0.9708738	141	0.4071967
9699	GO:0016616	oxidoreductase activity, acting on the CH-OH group	0.21	3	1.4563106	266	0.7681867
5891	GO:0008395|GO	steroid hydroxylase activity	0.21	1	0.4854369	40	0.1155168
12794	GO:0030234	enzyme regulator activity	0.22	4	1.9417475	403	1.1638317
4874	GO:0006767	water-soluble vitamin metabolic process	0.22	1	0.4854369	42	0.121292636
5607	GO:0008022	protein C-terminus binding	0.22	1	0.4854369	42	0.121292636
6666	GO:0009312	oligosaccharide biosynthetic process	0.22	1	0.4854369	42	0.121292636
8461	GO:0015020|GO	glucuronosyltransferase activity	0.22	1	0.4854369	42	0.121292636
9370	GO:0016138	glycoside biosynthetic process	0.22	1	0.4854369	42	0.121292636
18713	GO:0042364	water-soluble vitamin biosynthetic process	0.22	1	0.4854369	42	0.121292636
21880	GO:0046351	disaccharide biosynthetic process	0.22	1	0.4854369	42	0.121292636
8679	GO:0015297	antiporter activity	0.23	2	0.9708738	152	0.4389638
8682	GO:0015300	solute:solute antiporter activity	0.23	2	0.9708738	152	0.4389638
16603	GO:0034220	ion transmembrane transport	0.23	7	3.3980582	833	2.4056373
4873	GO:0006766	vitamin metabolic process	0.23	1	0.4854369	44	0.12706847
6477	GO:0009110	vitamin biosynthetic process	0.23	1	0.4854369	44	0.12706847
20522	GO:0044272	sulfur compound biosynthetic process	0.23	1	0.4854369	44	0.12706847
6424	GO:0009055|GO	electron carrier activity	0.23	2	0.9708738	154	0.44473964
9114	GO:0015849	organic acid transport	0.24	2	0.9708738	156	0.45051548
22443	GO:0046942	carboxylic acid transport	0.24	2	0.9708738	156	0.45051548
4215	GO:0005975	carbohydrate metabolic process	0.24	8	3.883495	990	2.8590407
19831	GO:0043565	sequence-specific DNA binding	0.24	2	0.9708738	159	0.45917925
6750	GO:0009415	response to water	0.25	1	0.4854369	49	0.14150807
3756	GO:0005261|GO	cation channel activity	0.25	1	0.4854369	49	0.14150807
3490	GO:0004888|GO	transmembrane receptor activity	0.25	5	2.4271846	571	1.6490022
5096	GO:0007047	cellular cell wall organization	0.26	1	0.4854369	50	0.14439599
6932	GO:0009664	plant-type cell wall organization	0.26	1	0.4854369	50	0.14439599
20857	GO:0045229	external encapsulating structure organization	0.26	1	0.4854369	50	0.14439599
30033	GO:0071669	plant-type cell wall organization or biogenesis	0.26	1	0.4854369	50	0.14439599
7173	GO:0009922	fatty acid elongase activity	0.26	1	0.4854369	51	0.14728391
4915	GO:0006817	phosphate transport	0.27	1	0.4854369	52	0.15017183
8528	GO:0015114	phosphate transmembrane transporter activity	0.27	1	0.4854369	52	0.15017183
17801	GO:0035435	phosphate transmembrane transport	0.27	1	0.4854369	52	0.15017183
2809	GO:0003993	acid phosphatase activity	0.27	1	0.4854369	52	0.15017183
12547	GO:0022857|GO	transmembrane transporter activity	0.27	11	5.3398056	1483	4.282785
29246	GO:0070882	cellular cell wall organization or biogenesis	0.27	1	0.4854369	53	0.15305975
9768	GO:0016706	oxidoreductase activity, acting on paired donors, wi	0.28	2	0.9708738	175	0.505386

4894	GO:0006790	sulfur compound metabolic process	0.28	1	0.4854369	55	0.15883559
4224	GO:0005984	disaccharide metabolic process	0.28	1	0.4854369	56	0.16172351
9369	GO:0016137	glycoside metabolic process	0.28	1	0.4854369	56	0.16172351
13082	GO:0030599	pectinesterase activity	0.29	2	0.9708738	178	0.5140497
9813	GO:0016759	cellulose synthase activity	0.29	1	0.4854369	57	0.16461143
6166	GO:0008757	S-adenosylmethionine-dependent methyltransferas	0.29	2	0.9708738	180	0.5198256
5819	GO:0008299|GO	isoprenoid biosynthetic process	0.29	1	0.4854369	58	0.16749935
6665	GO:0009311	oligosaccharide metabolic process	0.29	1	0.4854369	58	0.16749935
8558	GO:0015145	monosaccharide transmembrane transporter activit	0.29	1	0.4854369	58	0.16749935
9018	GO:0015749	monosaccharide transport	0.29	1	0.4854369	58	0.16749935
29919	GO:0071555	cell wall organization	0.29	1	0.4854369	58	0.16749935
2831	GO:0004022	alcohol dehydrogenase (NAD) activity	0.30	1	0.4854369	59	0.17038727
4840	GO:0006720|GO	isoprenoid metabolic process	0.30	1	0.4854369	60	0.17327519
14811	GO:0032403	protein complex binding	0.30	1	0.4854369	60	0.17327519
9818	GO:0016765|GO	transferase activity, transferring alkyl or aryl (other t	0.30	2	0.9708738	185	0.53426516
6018	GO:0008559|GO	xenobiotic-transporting ATPase activity	0.31	1	0.4854369	62	0.17905103
19225	GO:0042908	xenobiotic transport	0.31	1	0.4854369	62	0.17905103
19227	GO:0042910	xenobiotic transporter activity	0.31	1	0.4854369	62	0.17905103
18582	GO:0042221	response to chemical stimulus	0.31	3	1.4563106	328	0.94723773
17016	GO:0034637	cellular carbohydrate biosynthetic process	0.31	1	0.4854369	63	0.18193895
26931	GO:0055085	transmembrane transport	0.32	11	5.3398056	1548	4.4705
9293	GO:0016051|GO	carbohydrate biosynthetic process	0.32	1	0.4854369	64	0.18482687
25095	GO:0050790	regulation of catalytic activity	0.32	4	1.9417475	480	1.3862015
12573	GO:0022891	substrate-specific transmembrane transporter activ	0.32	9	4.3689322	1241	3.5839086
28366	GO:0065009	regulation of molecular function	0.33	4	1.9417475	484	1.3977532
9765	GO:0016702	oxidoreductase activity, acting on single donors with	0.33	1	0.4854369	66	0.1906027
2999	GO:0004312	fatty acid synthase activity	0.33	1	0.4854369	68	0.19637854
21710	GO:0046148	pigment biosynthetic process	0.34	1	0.4854369	69	0.19926646
31420	GO:0080043	quercetin 3-O-glucosyltransferase activity	0.34	1	0.4854369	69	0.19926646
4910	GO:0006811	ion transport	0.35	7	3.3980582	959	2.769515
9812	GO:0016758	transferase activity, transferring hexosyl groups	0.35	4	1.9417475	501	1.4468478
12528	GO:0022832	voltage-gated channel activity	0.35	1	0.4854369	72	0.20793022
15280	GO:0032879	regulation of localization	0.35	1	0.4854369	72	0.20793022
17141	GO:0034762	regulation of transmembrane transport	0.35	1	0.4854369	72	0.20793022
17144	GO:0034765	regulation of ion transmembrane transport	0.35	1	0.4854369	72	0.20793022
19568	GO:0043269	regulation of ion transport	0.35	1	0.4854369	72	0.20793022
25348	GO:0051049	regulation of transport	0.35	1	0.4854369	72	0.20793022
3744	GO:0005244	voltage-gated ion channel activity	0.35	1	0.4854369	72	0.20793022
9703	GO:0016620	oxidoreductase activity, acting on the aldehyde or o	0.35	1	0.4854369	73	0.21081814
18786	GO:0042440	pigment metabolic process	0.35	1	0.4854369	73	0.21081814
25510	GO:0051213	dioxygenase activity	0.36	1	0.4854369	75	0.21659398
3627	GO:0005085|GO	guanyl-nucleotide exchange factor activity	0.36	1	0.4854369	75	0.21659398
8494	GO:0015075	ion transmembrane transporter activity	0.37	6	2.9126213	827	2.3883097
3476	GO:0004872|GO	receptor activity	0.37	5	2.4271846	672	1.9406822
12574	GO:0022892	substrate-specific transporter activity	0.37	10	4.854369	1463	4.2250266
15189	GO:0032787	monocarboxylic acid metabolic process	0.38	1	0.4854369	81	0.23392151
30066	GO:0071702	organic substance transport	0.39	4	1.9417475	529	1.5277096
4912	GO:0006813|GO	potassium ion transport	0.39	1	0.4854369	84	0.24258527
30168	GO:0071804	cellular potassium ion transport	0.39	1	0.4854369	84	0.24258527
30169	GO:0071805	potassium ion transmembrane transport	0.39	1	0.4854369	84	0.24258527
19125	GO:0042802	identical protein binding	0.40	5	2.4271846	697	2.01288
21331	GO:0045735	nutrient reservoir activity	0.41	1	0.4854369	89	0.25702485
3723	GO:0005215|GO	transporter activity	0.42	12	5.8252425	1844	5.325324
3644	GO:0005102	receptor binding	0.42	1	0.4854369	91	0.2628007
5712	GO:0008168|GO	methyltransferase activity	0.43	2	0.9708738	244	0.7046524
11712	GO:0019748	secondary metabolic process	0.43	1	0.4854369	95	0.27435237
20512	GO:0044262|GO	cellular carbohydrate metabolic process	0.43	2	0.9708738	248	0.7162041
2628	GO:0003677	DNA binding	0.44	8	3.883495	1224	3.534814
25195	GO:0050896|GO	response to stimulus	0.44	12	5.8252425	1881	5.432177
3306	GO:0004674|GO	protein serine/threonine kinase activity	0.45	7	3.3980582	1062	3.0669708
5874	GO:0008374	O-acyltransferase activity	0.46	1	0.4854369	102	0.29456782
19126	GO:0042803	protein homodimerization activity	0.47	2	0.9708738	265	0.7652988
22049	GO:0046527	glucosyltransferase activity	0.48	2	0.9708738	270	0.77973837
3626	GO:0005083	small GTPase regulator activity	0.48	1	0.4854369	111	0.3205591
5070	GO:0007017	microtubule-based process	0.49	1	0.4854369	112	0.32344702
5071	GO:0007018	microtubule-based movement	0.49	1	0.4854369	112	0.32344702
29918	GO:0071554	cell wall organization or biogenesis	0.49	1	0.4854369	112	0.32344702
9933	GO:0016903	oxidoreductase activity, acting on the aldehyde or o	0.49	1	0.4854369	113	0.32633495
4357	GO:0006139|GO	nucleobase, nucleoside, nucleotide and nucleic acid	0.49	20	9.708738	3285	9.486816
22478	GO:0046982	protein heterodimerization activity	0.49	1	0.4854369	114	0.32922286
3874	GO:0005509	calcium ion binding	0.50	2	0.9708738	279	0.8057296
9535	GO:0016407	acetyltransferase activity	0.50	1	0.4854369	115	0.3321108
20531	GO:0044281	small molecule metabolic process	0.50	11	5.3398056	1788	5.1636004
27063	GO:0060089	molecular transducer activity	0.50	5	2.4271846	787	2.2727928
3475	GO:0004871|GO	signal transducer activity	0.50	5	2.4271846	787	2.2727928
25416	GO:0051119	sugar transmembrane transporter activity	0.51	1	0.4854369	118	0.34077454
14667	GO:0032259	methylation	0.51	2	0.9708738	285	0.8230572
22375	GO:0046872	metal ion binding	0.51	18	8.7378645	2985	8.6204405
12532	GO:0022836	gated channel activity	0.52	1	0.4854369	122	0.3523262
19470	GO:0043167	ion binding	0.52	18	8.7378645	2997	8.655096
19472	GO:0043169	cation binding	0.52	18	8.7378645	2997	8.655096
3260	GO:0004620	phospholipase activity	0.52	1	0.4854369	123	0.35521415
17020	GO:0034641	cellular nitrogen compound metabolic process	0.52	22	10.679611	3678	10.621769
12741	GO:0030170	pyridoxal phosphate binding	0.53	1	0.4854369	126	0.3638779
28643	GO:0070279	vitamin B6 binding	0.53	1	0.4854369	126	0.3638779
4906	GO:0006807	nitrogen compound metabolic process	0.55	22	10.679611	3725	10.757502
3724	GO:0005216	ion channel activity	0.57	1	0.4854369	140	0.40430877
6046	GO:0008610	lipid biosynthetic process	0.57	1	0.4854369	141	0.4071967
13164	GO:0030695	GTPase regulator activity	0.57	1	0.4854369	142	0.4100846
8557	GO:0015144	carbohydrate transmembrane transporter activity	0.57	1	0.4854369	143	0.41297254
16602	GO:0034219	carbohydrate transmembrane transport	0.57	1	0.4854369	143	0.41297254
4849	GO:0006730|GO	one-carbon metabolic process	0.58	2	0.9708738	325	0.93857396

3873	GO:0005507	copper ion binding	0.58	1	0.4854369	146	0.42163628
6071	GO:0008643|GO	carbohydrate transport	0.58	1	0.4854369	147	0.42452422
5909	GO:0008415	acyltransferase activity	0.59	2	0.9708738	330	0.95301354
22415	GO:0046914	transition metal ion binding	0.59	15	7.2815533	2599	7.5057034
11426	GO:0019438	aromatic compound biosynthetic process	0.59	1	0.4854369	149	0.43030006
27562	GO:0060589	nucleoside-triphosphatase regulator activity	0.60	1	0.4854369	152	0.4389638
4909	GO:0006810|GO	transport	0.63	13	6.3106794	2315	6.6855345
25529	GO:0051234	establishment of localization	0.63	13	6.3106794	2315	6.6855345
25476	GO:0051179	localization	0.63	13	6.3106794	2319	6.697086
9811	GO:0016757|GO	transferase activity, transferring glycosyl groups	0.63	4	1.9417475	729	2.1052935
6899	GO:0009628	response to abiotic stimulus	0.64	1	0.4854369	170	0.49094638
3341	GO:0004722|GO	protein serine/threonine phosphatase activity	0.64	1	0.4854369	171	0.4938343
3307	GO:0004675	transmembrane receptor protein serine/threonine k	0.64	2	0.9708738	367	1.0598665
11787	GO:0019842	vitamin binding	0.65	1	0.4854369	177	0.5111618
9839	GO:0016791|GO	phosphatase activity	0.67	2	0.9708738	383	1.1060733
20505	GO:0044255	cellular lipid metabolic process	0.67	1	0.4854369	184	0.53137726
4844	GO:0006725	cellular aromatic compound metabolic process	0.67	1	0.4854369	185	0.53426516
5195	GO:0007165|GO	signal transduction	0.68	5	2.4271846	961	2.775291
12602	GO:0023052|GO	signaling	0.68	5	2.4271846	961	2.775291
3304	GO:0004672|GO	protein kinase activity	0.68	7	3.3980582	1333	3.8495972
9906	GO:0016874	ligase activity	0.69	3	1.4563106	597	1.7240882
10244	GO:0018130	heterocycle biosynthetic process	0.70	1	0.4854369	199	0.57469606
3340	GO:0004721	phosphoprotein phosphatase activity	0.70	1	0.4854369	199	0.57469606
8673	GO:0015291|GO	secondary active transmembrane transporter activi	0.70	2	0.9708738	407	1.1753833
4954	GO:0006869	lipid transport	0.71	1	0.4854369	208	0.6006873
8085	GO:0010876	lipid localization	0.71	1	0.4854369	208	0.6006873
18956	GO:0042626	ATPase activity, coupled to transmembrane moven	0.72	1	0.4854369	211	0.6093511
9861	GO:0016820	hydrolase activity, acting on acid anhydrides, cataly	0.72	1	0.4854369	212	0.612239
9494	GO:0016311	dephosphorylation	0.72	2	0.9708738	425	1.227366
17617	GO:0035251	UDP-glucosyltransferase activity	0.73	1	0.4854369	218	0.62956655
18912	GO:0042578	phosphoric ester hydrolase activity	0.74	2	0.9708738	444	1.2822365
19758	GO:0043492	ATPase activity, coupled to movement of substanc	0.76	1	0.4854369	235	0.67866117
3885	GO:0005525	GTP binding	0.77	1	0.4854369	245	0.70754033
11032	GO:0019001	guanyl nucleotide binding	0.77	1	0.4854369	245	0.70754033
14967	GO:0032561	guanyl ribonucleotide binding	0.77	1	0.4854369	245	0.70754033
9824	GO:0016773	phosphotransferase activity, alcohol group as acce	0.79	7	3.3980582	1493	4.311664
8749	GO:0015399	primary active transmembrane transporter activity	0.79	1	0.4854369	260	0.75085914
8753	GO:0015405	P-P-bond-hydrolysis-driven transmembrane transpo	0.79	1	0.4854369	260	0.75085914
9285	GO:0016043	cellular component organization	0.79	1	0.4854369	261	0.7537471
4649	GO:0006520|GO	cellular amino acid metabolic process	0.81	1	0.4854369	277	0.7999538
25998	GO:0051716	cellular response to stimulus	0.81	5	2.4271846	1138	3.2864528
30205	GO:0071841	cellular component organization or biogenesis at ce	0.82	1	0.4854369	285	0.8230572
20356	GO:0044106	cellular amine metabolic process	0.84	1	0.4854369	303	0.8750397
9889	GO:0016853	isomerase activity	0.84	1	0.4854369	306	0.8837035
6662	GO:0009308	amine metabolic process	0.85	1	0.4854369	314	0.9068068
12516	GO:0022804	active transmembrane transporter activity	0.85	3	1.4563106	787	2.2727928
2975	GO:0004175|GO	endopeptidase activity	0.85	1	0.4854369	320	0.9241344
9836	GO:0016787	hydrolase activity	0.85	18	8.7378645	3723	10.751725
5732	GO:0008194	UDP-glycosyltransferase activity	0.85	1	0.4854369	322	0.9299102
3955	GO:0005634	nucleus	0.86	6	2.9126213	1433	4.138389
20488	GO:0044238	primary metabolic process	0.87	48	23.30097	9167	26.47356
20533	GO:0044283	small molecule biosynthetic process	0.88	1	0.4854369	347	1.0021082
20487	GO:0044237	cellular metabolic process	0.88	42	20.38835	8185	23.637625
30204	GO:0071840	cellular component organization or biogenesis	0.89	1	0.4854369	362	1.045427
20521	GO:0044271	cellular nitrogen compound biosynthetic process	0.89	1	0.4854369	367	1.0598665
12608	GO:0030001	metal ion transport	0.89	1	0.4854369	374	1.080082
4408	GO:0006200	ATP catabolic process	0.90	2	0.9708738	641	1.8511566
9918	GO:0016887|GO	ATPase activity	0.90	2	0.9708738	641	1.8511566
21600	GO:0046034	ATP metabolic process	0.90	2	0.9708738	641	1.8511566
9488	GO:0016301	kinase activity	0.90	7	3.3980582	1740	5.0249805
8943	GO:0015672	monovalent inorganic cation transport	0.90	1	0.4854369	383	1.1060733
20510	GO:0044260|GO	cellular macromolecule metabolic process	0.90	27	13.106796	5577	16.105928
9913	GO:0016881	acid-amino acid ligase activity	0.90	1	0.4854369	391	1.1291766
15436	GO:0033036	macromolecule localization	0.91	1	0.4854369	397	1.1465042
9845	GO:0016798	hydrolase activity, acting on glycosyl bonds	0.91	1	0.4854369	401	1.1580559
4612	GO:0006468	protein phosphorylation	0.91	9	4.3689322	2205	6.367863
9579	GO:0016462	pyrophosphatase activity	0.93	3	1.4563106	954	2.7550755
9859	GO:0016818	hydrolase activity, acting on acid anhydrides, in pho	0.93	3	1.4563106	956	2.7608514
9858	GO:0016817	hydrolase activity, acting on acid anhydrides	0.93	3	1.4563106	959	2.769515
7229	GO:0009987|GO	cellular process	0.93	54	26.213593	10601	30.614838
4641	GO:0006508	proteolysis	0.93	3	1.4563106	965	2.7868426
9911	GO:0016879	ligase activity, forming carbon-nitrogen bonds	0.94	1	0.4854369	456	1.3168914
3876	GO:0005515|GO	protein binding	0.94	33	16.019417	6933	20.021948
9823	GO:0016772	transferase activity, transferring phosphorus-contai	0.95	7	3.3980582	1932	5.579461
4405	GO:0006195	purine nucleotide catabolic process	0.95	2	0.9708738	773	2.232362
6508	GO:0009143	nucleoside triphosphate catabolic process	0.95	2	0.9708738	773	2.232362
6509	GO:0009144	purine nucleoside triphosphate metabolic process	0.95	2	0.9708738	773	2.232362
6511	GO:0009146	purine nucleoside triphosphate catabolic process	0.95	2	0.9708738	773	2.232362
6519	GO:0009154	purine ribonucleotide catabolic process	0.95	2	0.9708738	773	2.232362
6530	GO:0009166	nucleotide catabolic process	0.95	2	0.9708738	773	2.232362
6567	GO:0009203	ribonucleoside triphosphate catabolic process	0.95	2	0.9708738	773	2.232362
6569	GO:0009205	purine ribonucleoside triphosphate metabolic proce	0.95	2	0.9708738	773	2.232362
6571	GO:0009207	purine ribonucleoside triphosphate catabolic proces	0.95	2	0.9708738	773	2.232362
6624	GO:0009261	ribonucleotide catabolic process	0.95	2	0.9708738	773	2.232362
17034	GO:0034655	nucleobase, nucleoside, nucleotide and nucleic acid	0.95	2	0.9708738	773	2.232362
17035	GO:0034656	nucleobase, nucleoside and nucleotide catabolic pr	0.95	2	0.9708738	773	2.232362
22209	GO:0046700	heterocycle catabolic process	0.95	2	0.9708738	773	2.232362
30879	GO:0072523	purine-containing compound catabolic process	0.95	2	0.9708738	773	2.232362
20520	GO:0044270	cellular nitrogen compound catabolic process	0.95	2	0.9708738	777	2.2439137
6563	GO:0009199	ribonucleoside triphosphate metabolic process	0.95	2	0.9708738	790	2.2814567
6515	GO:0009150	purine ribonucleotide metabolic process	0.95	2	0.9708738	791	2.2843447
6506	GO:0009141	nucleoside triphosphate metabolic process	0.95	2	0.9708738	796	2.2987843

4373	GO:0006163	purine nucleotide metabolic process	0.96	2	0.9708738	810	2.339215
6622	GO:0009259|GO	ribonucleotide metabolic process	0.96	2	0.9708738	812	2.344991
19473	GO:0043170|GO	macromolecule metabolic process	0.96	30	14.563107	6553	18.92454
30877	GO:0072521	purine-containing compound metabolic process	0.96	2	0.9708738	827	2.3883097
2627	GO:0003676	nucleic acid binding	0.96	9	4.3689322	2453	7.0840673
22007	GO:0046483	heterocycle metabolic process	0.96	3	1.4563106	1094	3.1593843
18953	GO:0042623	ATPase activity, coupled	0.96	1	0.4854369	537	1.550813
28378	GO:0070011	peptidase activity, acting on L-amino acid peptides	0.96	1	0.4854369	537	1.550813
5840	GO:0008324	cation transmembrane transporter activity	0.96	1	0.4854369	538	1.5537009
9267	GO:0016021	integral to membrane	0.96	3	1.4563106	1103	3.1853757
20532	GO:0044282	small molecule catabolic process	0.96	2	0.9708738	854	2.4662836
4897	GO:0006793	phosphorus metabolic process	0.97	11	5.3398056	2925	8.4471655
4900	GO:0006796	phosphate metabolic process	0.97	11	5.3398056	2925	8.4471655
9493	GO:0016310	phosphorylation	0.97	9	4.3689322	2504	7.2313514
5763	GO:0008233	peptidase activity	0.97	1	0.4854369	565	1.6316746
10058	GO:0017111	nucleoside-triphosphatase activity	0.97	2	0.9708738	878	2.5355935
5791	GO:0008270	zinc ion binding	0.97	6	2.9126213	1880	5.4292893
20498	GO:0044248	cellular catabolic process	0.97	2	0.9708738	894	2.5818002
3863	GO:0005488	binding	0.97	70	33.980583	13903	40.15075
4867	GO:0006753	nucleoside phosphate metabolic process	0.97	2	0.9708738	914	2.6395588
6484	GO:0009117	nucleotide metabolic process	0.97	2	0.9708738	914	2.6395588
13656	GO:0031224	intrinsic to membrane	0.97	3	1.4563106	1193	3.4452884
5018	GO:0006950	response to stress	0.97	1	0.4854369	611	1.764519
131	GO:0000166	nucleotide binding	0.98	3	1.4563106	1213	3.5030468
26932	GO:0055086	nucleobase, nucleoside and nucleotide metabolic p	0.98	2	0.9708738	944	2.7261963
4911	GO:0006812|GO	cation transport	0.98	1	0.4854369	662	1.9118029
4609	GO:0006464	protein modification process	0.99	9	4.3689322	2781	8.031305
6425	GO:0009056	catabolic process	0.99	2	0.9708738	1051	3.0352037
19526	GO:0043227	membrane-bounded organelle	0.99	6	2.9126213	2142	6.185924
19530	GO:0043231	intracellular membrane-bounded organelle	0.99	6	2.9126213	2142	6.185924
9266	GO:0016020	membrane	0.99	13	6.3106794	3741	10.803708
18000	GO:0035639	purine ribonucleoside triphosphate binding	0.99	1	0.4854369	769	2.2208104
19684	GO:0043412	macromolecule modification	0.99	9	4.3689322	2900	8.374968
14959	GO:0032553	ribonucleotide binding	0.99	1	0.4854369	795	2.2958963
14961	GO:0032555	purine ribonucleotide binding	0.99	1	0.4854369	795	2.2958963
10033	GO:0017076	purine nucleotide binding	0.99	1	0.4854369	797	2.3016722
2653	GO:0003723	RNA binding	0.99	1	0.4854369	797	2.3016722
20619	GO:0044425	membrane part	0.99	3	1.4563106	1523	4.398302
20517	GO:0044267	cellular protein metabolic process	1.00	9	4.3689322	3225	9.313541
11522	GO:0019538|GO	protein metabolic process	1.00	12	5.8252425	4134	11.938661
4027	GO:0005737	cytoplasm	1.00	3	1.4563106	2001	5.7787275
19525	GO:0043226	organelle	1.00	6	2.9126213	3013	8.701303
19528	GO:0043229	intracellular organelle	1.00	6	2.9126213	3013	8.701303
20618	GO:0044424	intracellular part	1.00	9	4.3689322	3853	11.127155
3945	GO:0005622	intracellular	1.00	11	5.3398056	4751	13.720507
3946	GO:0005623	cell	1.00	24	11.650485	8227	23.758917
20657	GO:0044464	cell part	1.00	24	11.650485	8227	23.758917
3903	GO:0005575|GO	cellular_component	1.00	24	11.650485	8515	24.590637
