# Supplementary material for: The Tomato Hybrid Proline-rich Protein regulates the abscission zone competence to respond to ethylene signals
Source: Hortic Res. 2018 Jun 1;5:28. doi: 10.1038/s41438-018-0033-2 (PMC5981600; doi:10.1038/s41438-018-0033-2)
Supplement: Supplementary file 3 — Table S3 [file 41438_2018_33_MOESM3_ESM.pdf]

Table S3. Differentially regulated genes (up and down regulated) in the control compared with THyPRP silenced plants in FAZ at 4 h along with their expression values, fold changes, annotations, GO ids and terms.

Worksheet 2 & 3

Set 2					
Line	Array	Details	Time	Treated vs. Con	Set
Control – New Yorker	254331010025_1_3	4h_3_AZ	4h	Control	Set 2
Control – New Yorker	254331010025_1_4	4h_4_AZ	4h		
TAPG + TPRP	254331010034_1_3	4h_39_AZ	4h	Treated	
TAPG + TPRP	254331010034_1_4	4h_40_AZ	4h		

Cut off used to filter up and down regulated genes

Upregulated

For filtering upregulation we consider flag should be Detected in the treated sample and can be Compromised or Detected in the control sample and fold>=0.8 in the individual replicates and fold>=1 in the Geomean of treated samples.

Downregulated

For filtering downregulation we consider flag can be Detected or Compromised in the treated sample and should be Detected in the control sample and fold<=-0.8 in the individual replicates and fold<=-1 in the Geomean of treated samples.

Differentially Regulated Probes

Sample	Up	Down
4h_TPRP_ AZ vs. 4h_Control_AZ_Sense	842	1064
4h_TPRP_ AZ vs. 4h_Control_AZ_Antisense	543	714

Note:

Fold change expression values are provided as logbase 2

Flagging Criteria:

Compromised: If the gProcessedSignal value is less than the background intensity

Detected: If the gProcessedSignal value is above background intensity

Column Header	Description
ProbeName	Unique probe identifier
GeneName	GeneSymbol
Fold	Fold change calculated is log base 2
Flag	Detected or Compromised
GeomeanFold	Average Fold change for the replicates calculated is log base 2
p-Value	t-test p- value
gProcessedSignal	Background substracted signal intensity

Worksheet 4 & 5

This file contains the fold change for all the spots in the array

GO ID

Gene Ontology ID

GO ACCESSION

Gene Ontology Function name

p-value :

The probability of obtaining the specified GO accession number from a list of random entities. Less the p-value more significant is the GO accession number.

Significant pvalue<0.05

Count in Selection :

This refers to the number of genes in the selected entity (for example, from T-test) list which have that particular GO term.

%Count in Selection :

This refers to the percentage of genes in the input entity list which have that GO term.

Count in Total :

This refers to the number of genes in All Entities which have that GO term.

%Count in Total :

This refers to the percentage of genes in the All Entities list which have that GO term.

		4h_AZ_Control					4h_AZ_TAPG + TPRP vs. 4h_AZ_Control						
ProbeNar	Orientatic	Fold 4h	Flag 4h	Fold 4h	Flag 4h	Geomean	Fold 4h	Flag 4h	Fold 4h	Flag 4h	Geomean	P-value	ProbeNar
A 96 P00	Sense	0.007	Detected	-0.007	Detected	0.000	1.020	Detected	1.179	Detected	1.099	0.005	A 96 P00
A 96 P00	Sense	-0.253	Detected	0.253	Detected	0.000	2.029	Detected	1.412	Detected	1.720	0.050	A 96 P00
A 96 P01	Sense	0.112	Detected	-0.112	Detected	0.000	1.590	Detected	1.088	Detected	1.339	0.040	A 96 P01
A 96 P01	Sense	-0.168	Detected	0.168	Detected	0.000	1.992	Detected	2.304	Detected	2.148	0.011	A 96 P01
A 96 P01	Sense	0.659	Comprom	-0.659	Comprom	0.000	1.169	Detected	2.148	Detected	1.658	0.181	A 96 P01
A 96 P01	Sense	-0.516	Detected	0.516	Detected	0.000	2.265	Detected	2.392	Detected	2.329	0.046	A 96 P01
A 96 P02	Sense	0.220	Detected	-0.220	Detected	0.000	1.155	Detected	1.114	Detected	1.134	0.036	A 96 P02
A 96 P03	Sense	0.152	Detected	-0.152	Detected	0.000	1.167	Detected	1.073	Detected	1.120	0.020	A 96 P03
A 96 P05	Sense	0.110	Detected	-0.110	Detected	0.000	1.194	Detected	0.944	Detected	1.069	0.023	A 96 P05
A 96 P06	Sense	0.164	Detected	-0.164	Detected	0.000	1.399	Detected	1.421	Detected	1.410	0.013	A 96 P06
A 96 P06	Sense	-0.554	Comprom	0.554	Comprom	0.000	1.303	Detected	1.866	Detected	1.584	0.125	A 96 P06
A 96 P07	Sense	0.177	Detected	-0.177	Detected	0.000	1.026	Detected	1.612	Detected	1.319	0.061	A 96 P07
A 96 P07	Sense	-0.349	Detected	0.349	Detected	0.000	1.500	Detected	1.882	Detected	1.691	0.051	A 96 P07
A 96 P07	Sense	0.220	Detected	-0.220	Detected	0.000	1.793	Detected	1.869	Detected	1.831	0.015	A 96 P07
A 96 P07	Sense	0.240	Detected	-0.240	Detected	0.000	1.069	Detected	1.980	Detected	1.524	0.098	A 96 P07
A 96 P08	Sense	0.066	Detected	-0.066	Detected	0.000	0.937	Detected	1.686	Detected	1.312	0.075	A 96 P08
A 96 P10	Sense	-0.016	Detected	0.016	Detected	0.000	2.289	Detected	1.846	Detected	2.068	0.011	A 96 P10
A 96 P10	Sense	-0.339	Comprom	0.339	Detected	0.000	2.273	Detected	2.252	Detected	2.263	0.022	A 96 P10
A 96 P11	Sense	-0.206	Detected	0.206	Detected	0.000	2.018	Detected	1.547	Detected	1.782	0.029	A 96 P11
A 96 P11	Sense	0.138	Comprom	-0.138	Comprom	0.000	1.714	Detected	2.624	Detected	2.169	0.045	A 96 P11
A 96 P11	Sense	0.443	Detected	-0.443	Detected	0.000	2.316	Detected	1.439	Detected	1.878	0.095	A 96 P11
A 96 P12	Sense	0.143	Detected	-0.143	Detected	0.000	1.914	Detected	2.083	Detected	1.998	0.007	A 96 P12
A 96 P12	Sense	-0.196	Comprom	0.196	Comprom	0.000	1.138	Detected	2.012	Detected	1.575	0.081	A 96 P12
A 96 P12	Sense	0.185	Detected	-0.185	Detected	0.000	2.048	Detected	1.534	Detected	1.791	0.030	A 96 P12
A 96 P12	Sense	-0.402	Comprom	0.402	Detected	0.000	1.687	Detected	2.065	Detected	1.876	0.052	A 96 P12
A 96 P13	Sense	0.214	Detected	-0.214	Detected	0.000	2.027	Detected	1.387	Detected	1.707	0.047	A 96 P13
A 96 P13	Sense	0.136	Detected	-0.136	Detected	0.000	1.269	Detected	1.605	Detected	1.437	0.022	A 96 P13
A 96 P13	Sense	-0.386	Detected	0.386	Detected	0.000	2.050	Detected	1.342	Detected	1.696	0.084	A 96 P13
A 96 P14	Sense	0.311	Detected	-0.311	Detected	0.000	1.354	Detected	1.349	Detected	1.351	0.049	A 96 P14
A 96 P15	Sense	0.404	Comprom	-0.404	Comprom	0.000	1.749	Detected	1.558	Detected	1.653	0.058	A 96 P15
A 96 P16	Sense	-0.733	Comprom	0.733	Detected	0.000	1.378	Detected	1.354	Detected	1.366	0.204	A 96 P16
A 96 P17	Sense	-0.226	Detected	0.226	Detected	0.000	1.765	Detected	1.073	Detected	1.419	0.075	A 96 P17
A 96 P18	Sense	0.078	Detected	-0.078	Detected	0.000	1.499	Detected	1.800	Detected	1.650	0.010	A 96 P18
A 96 P19	Sense	-0.046	Comprom	0.046	Comprom	0.000	1.537	Detected	1.610	Detected	1.573	0.001	A 96 P19
A 96 P19	Sense	-0.516	Detected	0.516	Detected	0.000	1.572	Detected	1.834	Detected	1.703	0.085	A 96 P19
A 96 P19	Sense	0.245	Detected	-0.245	Detected	0.000	1.191	Detected	1.237	Detected	1.214	0.039	A 96 P19
A 96 P19	Sense	0.311	Detected	-0.311	Detected	0.000	1.606	Detected	1.499	Detected	1.552	0.039	A 96 P19
A 96 P19	Sense	-0.336	Detected	0.336	Detected	0.000	1.884	Detected	1.206	Detected	1.545	0.084	A 96 P19
A 96 P19	Sense	0.158	Detected	-0.158	Detected	0.000	1.070	Detected	1.287	Detected	1.178	0.025	A 96 P19
A 96 P20	Sense	0.134	Detected	-0.134	Detected	0.000	1.365	Detected	1.347	Detected	1.356	0.010	A 96 P20
A 96 P20	Sense	0.294	Detected	-0.294	Detected	0.000	0.956	Detected	1.311	Detected	1.133	0.081	A 96 P20
A 96 P21	Sense	0.640	Detected	-0.640	Comprom	0.000	0.911	Detected	1.656	Detected	1.283	0.225	A 96 P21
A 96 P22	Sense	0.025	Detected	-0.025	Detected	0.000	0.995	Detected	1.660	Detected	1.328	0.058	A 96 P22
A 96 P22	Sense	-0.165	Detected	0.165	Detected	0.000	0.871	Detected	1.913	Detected	1.392	0.126	A 96 P22
A 96 P22	Sense	-0.166	Detected	0.166	Detected	0.000	0.814	Detected	1.572	Detected	1.193	0.102	A 96 P22
A 96 P22	Sense	0.074	Detected	-0.074	Detected	0.000	1.489	Detected	1.135	Detected	1.312	0.021	A 96 P22
A 96 P23	Sense	-0.607	Detected	0.607	Detected	0.000	1.485	Detected	1.845	Detected	1.665	0.119	A 96 P23
A 96 P24	Sense	0.131	Detected	-0.131	Detected	0.000	1.307	Detected	1.636	Detected	1.472	0.020	A 96 P24
A 96 P25	Sense	0.582	Comprom	-0.582	Comprom	0.000	1.581	Detected	1.129	Detected	1.355	0.162	A 96 P25
GT Sense	Sense	0.177	Detected	-0.177	Detected	0.000	1.285	Detected	0.859	Detected	1.072	0.061	GT Sens
GT Sense	Sense	0.052	Detected	-0.052	Detected	0.000	1.007	Detected	0.997	Detected	1.002	0.003	GT Sens
GT Sense	Sense	0.265	Detected	-0.265	Detected	0.000	0.964	Detected	1.080	Detected	1.022	0.064	GT Sens
GT Sense	Sense	0.173	Detected	-0.173	Detected	0.000	2.664	Detected	2.445	Detected	2.555	0.006	GT Sens
GT Sense	Sense	0.464	Detected	-0.464	Detected	0.000	1.094	Detected	1.461	Detected	1.277	0.125	GT Sens
GT Sense	Sense	0.638	Comprom	-0.638	Comprom	0.000	1.626	Detected	2.019	Detected	1.823	0.112	GT Sens
GT Sense	Sense	0.534	Detected	-0.534	Detected	0.000	1.249	Detected	1.200	Detected	1.225	0.149	GT Sens
GT Sense	Sense	-0.235	Detected	0.235	Detected	0.000	1.878	Detected	1.122	Detected	1.500	0.078	GT Sens
GT Sense	Sense	-0.219	Comprom	0.219	Comprom	0.000	2.515	Detected	2.604	Detected	2.559	0.008	GT Sens
GT Sense	Sense	0.096	Detected	-0.096	Detected	0.000	1.234	Detected	1.074	Detected	1.154	0.012	GT Sens
GT Sense	Sense	0.549	Detected	-0.549	Detected	0.000	1.287	Detected	1.124	Detected	1.205	0.162	GT Sens
GT Sense	Sense	0.212	Detected	-0.212	Detected	0.000	1.643	Detected	1.474	Detected	1.558	0.021	GT Sens
GT Sense	Sense	-0.030	Detected	0.030	Detected	0.000	1.545	Detected	1.544	Detected	1.544	0.000	GT Sens
GT Sense	Sense	0.363	Detected	-0.363	Detected	0.000	1.428	Detected	0.844	Detected	1.136	0.135	GT Sens
GT Sense	Sense	0.013	Detected	-0.013	Detected	0.000	2.590	Detected	1.077	Detected	1.833	0.136	GT Sens
GT Sense	Sense	0.249	Detected	-0.249	Detected	0.000	1.093	Detected	2.012	Detected	1.553	0.097	GT Sens
GT Sense	Sense	0.078	Comprom	-0.078	Comprom	0.000	2.087	Detected	2.177	Detected	2.132	0.002	GT Sens
GT Sense	Sense	-0.277	Detected	0.277	Detected	0.000	1.116	Detected	1.338	Detected	1.227	0.054	GT Sens
GT Sense	Sense	0.153	Detected	-0.153	Detected	0.000	1.507	Detected	1.310	Detected	1.408	0.016	GT Sens
GT Sense	Sense	-0.106	Detected	0.106	Detected	0.000	1.951	Detected	1.841	Detected	1.896	0.004	GT Sens
GT Sense	Sense	-0.288	Detected	0.288	Detected	0.000	1.089	Detected	1.181	Detected	1.135	0.060	GT Sens
GT Sense	Sense	-0.243	Detected	0.243	Detected	0.000	1.386	Detected	0.993	Detected	1.189	0.063	GT Sens
GT Sense	Sense	0.270	Detected	-0.270	Detected	0.000	1.032	Detected	1.019	Detected	1.026	0.063	GT Sens
GT Sense	Sense	0.280	Detected	-0.280	Detected	0.000	2.046	Detected	1.870	Detected	1.958	0.022	GT Sens
GT Sense	Sense	0.240	Detected	-0.240	Detected	0.000	1.557	Detected	1.619	Detected	1.588	0.022	GT Sens
GT Sense	Sense	0.431	Comprom	-0.431	Comprom	0.000	1.520	Detected	1.485	Detected	1.502	0.073	GT Sens
GT Sense	Sense	1.483	Detected	-1.483	Detected	0.000	1.484	Detected	1.286	Detected	1.385	0.450	GT Sens
GT Sense	Sense	-0.311	Detected	0.311	Detected	0.000	1.604	Detected	0.896	Detected	1.250	0.118	GT Sens
GT Sense	Sense	-0.242	Comprom	0.242	Comprom	0.000	1.249	Detected	1.583	Detected	1.416	0.041	GT Sens
GT Sense	Sense	0.867	Comprom	-0.867	Comprom	0.000	1.537	Detected	1.284	Detected	1.411	0.249	GT Sens
GT Sense	Sense	0.748	Detected	-0.748	Comprom	0.000	1.213	Detected	1.995	Detected	1.604	0.198	GT Sens
GT Sense	Sense	0.345	Detected	-0.345	Detected	0.000	1.520	Detected	1.498	Detected	1.509	0.049	GT Sens
GT Sense	Sense	0.020	Detected	-0.020	Detected	0.000	1.997	Detected	1.642	Detected	1.819	0.010	GT Sens
GT Sense	Sense	0.359	Detected	-0.359	Detected	0.000	0.860	Detected	1.317	Detected	1.089	0.125	GT Sens
GT Sense	Sense	0.527	Detected	-0.527	Detected	0.000	1.431	Detected	1.474	Detected	1.452	0.110	GT Sens
GT Sense	Sense	0.376	Detected	-0.376	Detected	0.000	1.440	Detected	1.624	Detected	1.532	0.058	GT Sens
GT Sense	Sense	0.243	Detected	-0.243	Detected	0.000	1.772	Detected	0.853	Detected	1.313	0.127	GT Sens
GT Sense	Sense	0.056	Detected	-0.056	Detected	0.000	1.229	Detected	1.328	Detected	1.279	0.003	GT Sens
GT Sense	Sense	0.419	Detected	-0.419	Detected	0.000	2.741	Detected	2.857	Detected	2.799	0.022	GT Sens
GT Sense	Sense	-0.317	Comprom	0.317	Comprom	0.000	0.836	Detected	1.501	Detected	1.168	0.126	GT Sens
GT Sense	Sense	-0.471	Comprom	0.471	Comprom	0.000	1.032	Detected	0.990	Detected	1.011	0.165	GT Sens
GT Sense	Sense	-0.351	Detected	0.351	Detected	0.000	1.176	Detected	1.034	Detected	1.105	0.091	GT Sens
GT Sense	Sense	0.520	Comprom	-0.520	Comprom	0.000	1.292	Detected	1.533	Detected	1.413	0.118	GT Sens
GT Sense	Sense	-0.091	Detected	0.091	Detected	0.000	1.288	Detected	1.251	Detected	1.269	0.005	GT Sens
GT Sense	Sense	1.353	Detected	-1.353	Comprom	0.000	2.664	Detected	1.089	Detected	1.877	0.353	GT Sens
GT Sense	Sense	0.241	Detected	-0.241	Detected	0.000	1.044	Detected	1.074	Detected	1.059	0.048	GT Sens
GT Sense	Sense	-0.150	Detected	0.150	Detected	0.000	1.320	Detected	0.920	Detected	1.120	0.046	GT Sens
GT Sense	Sense	0.003	Detected	-0.003	Detected	0.000	1.047	Detected	1.284	Detected	1.165	0.010	GT Sens
GT Sense	Sense	-0.843	Comprom	0.843	Comprom	0.000	3.765	Detected	1.845	Detected	2.805	0.159	GT Sens
GT Sense	Sense	0.737	Detected	-0.737	Detected	0.000	0.863	Detected	1.507	Detected	1.185	0.279	GT Sens
GT Sense	Sense	0.254	Detected	-0.254	Detected	0.000	0.855	Detected	1.241	Detected	1.048	0.082	GT Sens
GT Sense	Sense	0.062	Detected	-0.062	Detected	0.000	1.332	Detected	0.819	Detected	1.075	0.055	GT Sens
GT Sense	Sense	0.567	Comprom	-0.567	Comprom	0.000	0.998	Detected	1.764	Detected	1.381	0.181	GT Sens
GT Sense	Sense	0.136	Comprom	-0.136	Comprom	0.000	2.049	Detected	1.778	Detected	1.913	0.010	GT Sens
GT Sense	Sense	0.295	Detected	-0.295	Detected	0.000	1.431	Detected	1.313	Detected	1.372	0.045	GT Sens
GT Sense	Sense	-0.253	Detected	0.253	Detected	0.000	1.361	Detected	0.966	Detected	1.164	0.068	GT Sens
GT Sense	Sense	-0.124	Comprom	0.124	Comprom	0.000	1.065	Detected	1.056	Detected	1.060	0.013	GT Sens
GT Sense	Sense	0.276	Detected	-0.276	Detected	0.000	1.472	Detected	1.567	Detected	1.519	0.032	GT Sens
GT Sense	Sense	0.306	Detected	-0.306	Detected	0.000	1.025	Detected	1.028	Detected	1.027	0.078	GT Sens
GT Sense	Sense	0.007	Comprom	-0.007	Comprom	0.000	1.604	Detected	1.223	Detected	1.413	0.018	GT Sens
GT Sense	Sense	0.374	Comprom	-0.374	Comprom	0.000	1.207	Detected	1.712	Detected	1.460	0.084	GT Sens
GT Sense	Sense	-0.184	Comprom	0.184	Comprom	0.000	3.697	Detected	3.576	Detected	3.636	0.003	GT Sens
GT Sense	Sense	0.308	Detected	-0.308	Detected	0.000	1.279	Detected	1.263	Detected	1.271	0.054	GT Sens
GT Sense	Sense	0.162	Detected	-0.162	Detected	0.000	2.209	Detected	1.292	Detected	1.750	0.069	GT Sens
GT Sense	Sense	0.168	Detected	-0.168	Detected	0.000	1.196	Detected	1.329	Detected	1.262	0.020	GT Sens
GT Sense	Sense	-0.030	Comprom	0.030	Comprom	0.000	1.473	Detected	1.615	Detected	1.544	0.002	GT Sens
GT Sense	Sense	0.064	Detected	-0.064	Detected	0.000	3.306	Detected	2.892	Detected	3.099	0.005	GT Sens
GT Sense	Sense	-0.367	Comprom	0.367	Comprom	0.000	1.976	Detected	2.392	Detected	2.184	0.035	GT Sens
GT Sense	Sense	0.603	Detected	-0.603	Detected	0.000	0.920	Detected	1.434	Detected	1.177	0.215	GT Sens
GT Sense	Sense	0.056	Comprom	-0.056	Comprom	0.000	1.031	Detected	4.290	Detected	2.660	0.244	GT Sens
GT Sense	Sense	0.563	Detected	-0.563	Detected	0.000	0.983	Detected	1.131	Detected	1.057	0.204	GT Sens
GT Sense	Sense	1.022	Detected	-1.022	Comprom	0.000	1.099	Detected	1.542	Detected	1.321	0.334	GT Sens
GT Sense	Sense	0.094	Detected	-0.094	Detected	0.000	1.122	Detected	1.104	Detected	1.113	0.007	GT Sens
GT Sense	Sense	0.139	Detected	-0.139	Detected	0.000	1.081	Detected	1.303	Detected	1.192	0.022	GT Sens
GT Sense	Sense	-0.252	Comprom	0.252	Detected	0.000	1.555	Detected	1.445	Detected	1.500	0.028	GT Sens
GT Sense	Sense	-0.010	Detected	0.010	Detected	0.000	1.324	Detected	1.289	Detected	1.306	0.000	GT Sens
GT Sense	Sense	0.600	Detected	-0.600	Detected	0.000	1.725	Detected	1.447	Detected	1.586	0.123	GT Sens
GT Sense	Sense	0.182	Detected	-0.182	Detected	0.000	1.052	Detected	0.982	Detected	1.017	0.032	GT Sens
GT Sense	Sense	0.112	Detected	-0.112	Detected	0.000	0.934	Detected	1.138	Detected	1.036	0.021	GT Sens
GT Sense	Sense	0.743	Detected	-0.743	Detected	0.000	1.711	Detected	2.012	Detected	1.862	0.134	GT Sens
GT Sense	Sense	0.299	Detected	-0.299	Detected	0.000	1.921	Detected	1.813	Detected	1.867	0.025	GT Sens
GT Sense	Sense	0.288	Detected	-0.288	Detected	0.000	1.555	Detected	1.299	Detected	1.427	0.045	GT Sens
GT Sense	Sense	0.208	Detected	-0.208	Detected	0.000	1.338	Detected	1.971	Detected	1.655	0.049	GT Sens
GT Sense	Sense	0.205	Detected	-0.205	Detected	0.000	1.013	Detected	1.024	Detected	1.018	0.038	GT Sens
GT Sense	Sense	0.345	Detected	-0.345	Detected	0.000	1.297	Detected	1.233	Detected	1.265	0.068	GT Sens
GT Sense	Sense	-0.693	Detected	0.693	Detected	0.000	3.036	Detected	1.397	Detected	2.216	0.175	GT Sens
GT Sense	Sense	0.570	Detected	-0.570	Comprom	0.000	1.694	Detected	2.743	Detected	2.219	0.103	GT Sens
GT Sense	Sense	0.865	Comprom	-0.865	Comprom	0.000	1.259	Detected	1.887	Detected	1.573	0.230	GT Sens
GT Sense	Sense	0.243	Detected	-0.243	Detected	0.000	1.184	Detected	0.957	Detected	1.070	0.057	GT Sens
GT Sense	Sense	-0.049	Comprom	0.049	Comprom	0.000	1.693	Detected	1.641	Detected	1.667	0.001	GT Sens
GT Sense	Sense	-0.157	Detected	0.157	Detected	0.000	1.809	Detected	1.808	Detected	1.808	0.007	GT Sens
GT Sense	Sense	0.746	Detected	-0.746	Detected	0.000	1.696	Detected	1.676	Detected	1.686	0.152	GT Sens
GT Sense	Sense	-0.152	Detected	0.152	Detected	0.000	1.169	Detected	1.148	Detected	1.158	0.017	GT Sens
GT Sense	Sense	-0.696	Comprom	0.696	Detected	0.000	1.854	Detected	1.144	Detected	1.499	0.195	GT Sens
GT Sense	Sense	-0.370	Comprom	0.370	Comprom	0.000	1.203	Detected	1.190	Detected	1.196	0.084	GT Sens
GT Sense	Sense	0.639	Detected	-0.639	Detected	0.000	2.827	Detected	3.037	Detected	2.932	0.045	GT Sens
GT Sense	Sense	0.091	Detected	-0.091	Detected	0.000	1.141	Detected	2.011	Detected	1.576	0.071	GT Sens
GT Sense	Sense	-0.050	Detected	0.050	Detected	0.000	0.974	Detected	1.295	Detected	1.134	0.021	GT Sens
GT Sense	Sense	-0.355	Comprom	0.355	Comprom	0.000	1.199	Detected	1.485	Detected	1.342	0.073	GT Sens
GT Sense	Sense	0.268	Detected	-0.268	Detected	0.000	1.211	Detected	1.167	Detected	1.189	0.048	GT Sens
GT Sense	Sense	-0.107	Detected	0.107	Detected	0.000	1.232	Detected	0.978	Detected	1.105	0.022	GT Sens
GT Sense	Sense	-0.656	Comprom	0.656	Detected	0.000	1.628	Detected	1.778	Detected	1.703	0.123	GT Sens
GT Sense	Sense	0.169	Detected	-0.169	Detected	0.000	0.881	Detected	1.473	Detected	1.177	0.074	GT Sens
GT Sense	Sense	-0.037	Detected	0.037	Detected	0.000	2.173	Detected	1.937	Detected	2.055	0.004	GT Sens
GT Sense	Sense	-0.116	Comprom	0.116	Comprom	0.000	1.199	Detected	1.582	Detected	1.391	0.025	GT Sens
GT Sense	Sense	0.466	Detected	-0.466	Detected	0.000	1.473	Detected	2.120	Detected	1.796	0.087	GT Sens
GT Sense	Sense	-0.284	Comprom	0.284	Comprom	0.000	3.638	Detected	2.506	Detected	3.072	0.040	GT Sens
GT Sense	Sense	-0.465	Comprom	0.465	Detected	0.000	1.783	Detected	1.144	Detected	1.463	0.122	GT Sens
GT Sense	Sense	0.061	Detected	-0.061	Detected	0.000	0.911	Detected	1.141	Detected	1.026	0.016	GT Sens
GT Sense	Sense	0.336	Detected	-0.336	Detected	0.000	1.131	Detected	1.038	Detected	1.084	0.086	GT Sens
GT Sense	Sense	0.063	Detected	-0.063	Detected	0.000	1.063	Detected	1.050	Detected	1.057	0.004	GT Sens
GT Sense	Sense	-0.070	Detected	0.070	Detected	0.000	1.488	Detected	0.825	Detected	1.156	0.076	GT Sens
GT Sense	Sense	-1.260	Comprom	1.260	Detected	0.000	2.987	Detected	3.208	Detected	3.097	0.134	GT Sens
GT Sense	Sense	0.460	Detected	-0.460	Detected	0.000	1.320	Detected	1.311	Detected	1.316	0.104	GT Sens
GT Sense	Sense	0.071	Detected	-0.071	Detected	0.000	1.169	Detected	1.333	Detected	1.251	0.007	GT Sens
GT Sense	Sense	0.125	Detected	-0.125	Detected	0.000	1.536	Detected	1.609	Detected	1.573	0.007	GT Sens
GT Sense	Sense	0.234	Detected	-0.234	Detected	0.000	1.116	Detected	1.226	Detected	1.171	0.040	GT Sens
GT Sense	Sense	-0.351	Comprom	0.351	Comprom	0.000	1.101	Detected	1.251	Detected	1.176	0.082	GT Sens
GT Sense	Sense	0.088	Detected	-0.088	Detected	0.000	1.704	Detected	2.127	Detected	1.916	0.014	GT Sens
GT Sense	Sense	0.163	Detected	-0.163	Detected	0.000	1.135	Detected	0.877	Detected	1.006	0.040	GT Sens
GT Sense	Sense	0.473	Comprom	-0.473	Comprom	0.000	1.054	Detected	2.258	Detected	1.656	0.163	GT Sens
GT Sense	Sense	0.081	Detected	-0.081	Detected	0.000	0.989	Detected	1.572	Detected	1.280	0.052	GT Sens
GT Sense	Sense	0.310	Detected	-0.310	Detected	0.000	1.160	Detected	1.924	Detected	1.542	0.089	GT Sens
GT Sense	Sense	0.250	Comprom	-0.250	Comprom	0.000	1.446	Detected	1.940	Detected	1.693	0.041	GT Sens
GT Sense	Sense	0.413	Detected	-0.413	Detected	0.000	1.516	Detected	1.229	Detected	1.372	0.088	GT Sens
GT Sense	Sense	0.530	Detected	-0.530	Comprom	0.000	2.969	Detected	2.992	Detected	2.981	0.030	GT Sens
GT Sense	Sense	-0.502	Comprom	0.502	Comprom	0.000	2.216	Detected	0.956	Detected	1.586	0.188	GT Sens
GT Sense	Sense	-0.228	Detected	0.228	Detected	0.000	0.939	Detected	1.378	Detected	1.159	0.067	GT Sens
GT Sense	Sense	0.650	Detected	-0.650	Detected	0.000	1.213	Detected	1.043	Detected	1.128	0.228	GT Sens
GT Sense	Sense	0.600	Detected	-0.600	Detected	0.000	2.230	Detected	1.177	Detected	1.704	0.167	GT Sens
GT Sense	Sense	0.100	Detected	-0.100	Detected	0.000	2.748	Detected	2.217	Detected	2.483	0.013	GT Sens
GT Sense	Sense	0.574	Detected	-0.574	Detected	0.000	1.711	Detected	1.471	Detected	1.591	0.113	GT Sens
GT Sense	Sense	0.106	Detected	-0.106	Detected	0.000	1.453	Detected	1.506	Detected	1.479	0.005	GT Sens
GT Sense	Sense	-1.046	Comprom	1.046	Detected	0.000	2.035	Detected	1.106	Detected	1.570	0.304	GT Sens
GT Sense	Sense	0.223	Detected	-0.223	Detected	0.000	1.363	Detected	1.215	Detected	1.289	0.032	GT Sens
GT Sense	Sense	0.212	Detected	-0.212	Detected	0.000	1.023	Detected	1.008	Detected	1.016	0.041	GT Sens
GT Sense	Sense	0.227	Detected	-0.227	Detected	0.000	1.224	Detected	1.773	Detected	1.499	0.052	GT Sens
GT Sense	Sense	0.471	Detected	-0.471	Detected	0.000	1.097	Detected	1.314	Detected	1.206	0.130	GT Sens
GT Sense	Sense	-0.125	Comprom	0.125	Comprom	0.000	2.194	Detected	1.555	Detected	1.875	0.032	GT Sens
GT Sense	Sense	0.068	Detected	-0.068	Detected	0.000	1.426	Detected	1.656	Detected	1.541	0.007	GT Sens
GT Sense	Sense	0.584	Detected	-0.584	Detected	0.000	1.070	Detected	1.283	Detected	1.177	0.186	GT Sens
GT Sense	Sense	0.499	Detected	-0.499	Detected	0.000	1.805	Detected	1.300	Detected	1.552	0.109	GT Sens
GT Sense	Sense	-0.302	Detected	0.302	Detected	0.000	1.789	Detected	0.821	Detected	1.305	0.149	GT Sens
GT Sense	Sense	0.206	Detected	-0.206	Detected	0.000	1.553	Detected	0.885	Detected	1.219	0.090	GT Sens
GT Sense	Sense	0.114	Comprom	-0.114	Comprom	0.000	1.307	Detected	1.251	Detected	1.279	0.008	GT Sens
GT Sense	Sense	0.211	Detected	-0.211	Detected	0.000	0.986	Detected	1.086	Detected	1.036	0.041	GT Sens
GT Sense	Sense	-0.884	Detected	0.884	Detected	0.000	2.940	Detected	0.946	Detected	1.943	0.282	GT Sens
GT Sense	Sense	0.501	Detected	-0.501	Detected	0.000	1.250	Detected	2.207	Detected	1.728	0.130	GT Sens
GT Sense	Sense	0.167	Detected	-0.167	Detected	0.000	1.838	Detected	2.119	Detected	1.978	0.012	GT Sens
GT Sense	Sense	0.141	Detected	-0.141	Detected	0.000	1.244	Detected	0.822	Detected	1.033	0.055	GT Sens
GT Sense	Sense	0.300	Detected	-0.300	Detected	0.000	1.445	Detected	1.382	Detected	1.413	0.043	GT Sens
GT Sense	Sense	0.136	Detected	-0.136	Detected	0.000	1.370	Detected	3.195	Detected	2.282	0.132	GT Sens
GT Sense	Sense	-0.230	Comprom	0.230	Comprom	0.000	2.378	Detected	1.879	Detected	2.128	0.024	GT Sens
GT Sense	Sense	0.567	Detected	-0.567	Comprom	0.000	1.745	Detected	1.208	Detected	1.477	0.143	GT Sens
GT Sense	Sense	-0.484	Detected	0.484	Detected	0.000	4.506	Detected	2.888	Detected	3.697	0.059	GT Sens
GT Sense	Sense	0.173	Detected	-0.173	Detected	0.000	1.303	Detected	1.284	Detected	1.293	0.017	GT Sens
GT Sense	Sense	0.264	Detected	-0.264	Detected	0.000	2.182	Detected	2.616	Detected	2.399	0.020	GT Sens
GT Sense	Sense	0.059	Detected	-0.059	Detected	0.000	0.875	Detected	1.130	Detected	1.003	0.019	GT Sens
GT Sense	Sense	0.039	Detected	-0.039	Detected	0.000	0.946	Detected	1.249	Detected	1.098	0.020	GT Sens
GT Sense	Sense	0.421	Detected	-0.421	Detected	0.000	0.862	Detected	1.184	Detected	1.023	0.151	GT Sens
GT Sense	Sense	0.142	Detected	-0.142	Detected	0.000	1.681	Detected	1.338	Detected	1.510	0.021	GT Sens
GT Sense	Sense	0.311	Detected	-0.311	Detected	0.000	1.290	Detected	1.020	Detected	1.155	0.077	GT Sens
GT Sense	Sense	0.665	Detected	-0.665	Comprom	0.000	0.919	Detected	1.250	Detected	1.085	0.254	GT Sens
GT Sense	Sense	-0.600	Comprom	0.600	Comprom	0.000	1.544	Detected	2.059	Detected	1.802	0.110	GT Sens
GT Sense	Sense	0.468	Detected	-0.468	Comprom	0.000	1.145	Detected	1.236	Detected	1.191	0.127	GT Sens
GT Sense	Sense	0.768	Detected	-0.768	Detected	0.000	0.945	Detected	2.079	Detected	1.512	0.254	GT Sens
GT Sense	Sense	0.211	Detected	-0.211	Detected	0.000	1.054	Detected	1.335	Detected	1.195	0.042	GT Sens
GT Sense	Sense	0.567	Detected	-0.567	Detected	0.000	1.072	Detected	1.341	Detected	1.207	0.174	GT Sens
GT Sense	Sense	-0.040	Detected	0.040	Detected	0.000	1.886	Detected	2.155	Detected	2.021	0.005	GT Sens
GT Sense	Sense	0.525	Detected	-0.525	Detected	0.000	1.195	Detected	0.880	Detected	1.037	0.199	GT Sens
GT Sense	Sense	0.047	Comprom	-0.047	Comprom	0.000	2.548	Detected	4.383	Detected	3.466	0.064	GT Sens
GT Sense	Sense	0.114	Detected	-0.114	Detected	0.000	1.231	Detected	0.814	Detected	1.023	0.050	GT Sens
GT Sense	Sense	-0.479	Comprom	0.479	Comprom	0.000	1.975	Detected	1.979	Detected	1.977	0.054	GT Sens
GT Sense	Sense	0.278	Detected	-0.278	Detected	0.000	1.362	Detected	0.944	Detected	1.153	0.080	GT Sens
GT Sense	Sense	-0.039	Detected	0.039	Detected	0.000	1.123	Detected	0.966	Detected	1.045	0.007	GT Sens
GT Sense	Sense	0.579	Detected	-0.579	Comprom	0.000	2.497	Detected	1.267	Detected	1.882	0.156	GT Sens
GT Sense	Sense	-0.026	Detected	0.026	Detected	0.000	1.130	Detected	1.309	Detected	1.220	0.006	GT Sens
GT Sense	Sense	0.575	Detected	-0.575	Detected	0.000	2.386	Detected	1.375	Detected	1.881	0.133	GT Sens
GT Sense	Sense	-0.078	Comprom	0.078	Comprom	0.000	1.871	Detected	2.114	Detected	1.993	0.005	GT Sens
GT Sense	Sense	0.020	Detected	-0.020	Detected	0.000	1.337	Detected	1.076	Detected	1.207	0.012	GT Sens
GT Sense	Sense	-0.009	Comprom	0.009	Comprom	0.000	1.752	Detected	2.192	Detected	1.972	0.012	GT Sens
GT Sense	Sense	0.384	Detected	-0.384	Detected	0.000	1.223	Detected	1.135	Detected	1.179	0.093	GT Sens
GT Sense	Sense	0.279	Detected	-0.279	Detected	0.000	1.151	Detected	1.617	Detected	1.384	0.063	GT Sens
GT Sense	Sense	0.821	Detected	-0.821	Detected	0.000	1.203	Detected	1.707	Detected	1.455	0.232	GT Sens
GT Sense	Sense	1.067	Comprom	-1.067	Comprom	0.000	1.860	Detected	1.866	Detected	1.863	0.223	GT Sens
GT Sense	Sense	0.123	Detected	-0.123	Detected	0.000	0.829	Detected	1.256	Detected	1.042	0.051	GT Sens
GT Sense	Sense	0.097	Detected	-0.097	Detected	0.000	1.170	Detected	1.212	Detected	1.191	0.007	GT Sens
GT Sense	Sense	-1.571	Comprom	1.571	Detected	0.000	0.831	Detected	3.107	Detected	1.969	0.417	GT Sens
GT Sense	Sense	-0.646	Comprom	0.646	Comprom	0.000	0.873	Detected	1.315	Detected	1.094	0.250	GT Sens
GT Sense	Sense	0.772	Detected	-0.772	Detected	0.000	1.221	Detected	0.843	Detected	1.032	0.324	GT Sens
GT Sense	Sense	-0.873	Comprom	0.873	Detected	0.000	1.236	Detected	1.302	Detected	1.269	0.283	GT Sens
GT Sense	Sense	0.483	Detected	-0.483	Detected	0.000	1.445	Detected	1.502	Detected	1.474	0.093	GT Sens
GT Sense	Sense	0.043	Comprom	-0.043	Comprom	0.000	1.422	Detected	1.036	Detected	1.229	0.025	GT Sens

GT	Sense	Sense	-0.445	Comprom	0.445	Comprom	0.000	2.171	Detected	2.211	Detected	2.191	0.039	[illegible]
GT	Sense	Sense	-0.088	Detected	0.088	Detected	0.000	1.263	Detected	1.277	Detected	1.270	0.005	[illegible]
GT	Sense	Sense	0.119	Detected	-0.119	Detected	0.000	1.296	Detected	1.600	Detected	1.448	0.017	[illegible]
GT	Sense	Sense	-0.443	Detected	0.443	Detected	0.000	1.115	Detected	0.962	Detected	1.039	0.147	[illegible]
GT	Sense	Sense	0.111	Comprom	-0.111	Comprom	0.000	1.082	Detected	1.214	Detected	1.148	0.012	[illegible]
GT	Sense	Sense	0.502	Detected	-0.502	Detected	0.000	1.137	Detected	0.920	Detected	1.028	0.183	[illegible]
GT	Sense	Sense	0.343	Detected	-0.343	Detected	0.000	0.843	Detected	1.480	Detected	1.161	0.131	[illegible]
GT	Sense	Sense	0.858	Detected	-0.858	Comprom	0.000	1.600	Detected	1.110	Detected	1.355	0.268	[illegible]
GT	Sense	Sense	-0.690	Comprom	0.690	Comprom	0.000	1.963	Detected	2.505	Detected	2.234	0.095	[illegible]
GT	Sense	Sense	-0.064	Detected	0.064	Detected	0.000	1.061	Detected	1.011	Detected	1.036	0.004	[illegible]
GT	Sense	Sense	0.284	Detected	-0.284	Detected	0.000	0.852	Detected	1.351	Detected	1.101	0.100	[illegible]
GT	Sense	Sense	0.077	Comprom	-0.077	Comprom	0.000	1.608	Detected	1.811	Detected	1.710	0.006	[illegible]
GT	Sense	Sense	0.214	Detected	-0.214	Detected	0.000	1.232	Detected	1.013	Detected	1.123	0.043	[illegible]
GT	Sense	Sense	-0.162	Detected	0.162	Detected	0.000	1.218	Detected	1.286	Detected	1.252	0.017	[illegible]
GT	Sense	Sense	0.432	Detected	-0.432	Detected	0.000	1.055	Detected	1.111	Detected	1.083	0.129	[illegible]
GT	Sense	Sense	0.305	Detected	-0.305	Detected	0.000	1.967	Detected	1.022	Detected	1.495	0.117	[illegible]
GT	Sense	Sense	0.175	Detected	-0.175	Detected	0.000	1.070	Detected	1.086	Detected	1.078	0.025	[illegible]
GT	Sense	Sense	-0.030	Comprom	0.030	Comprom	0.000	1.435	Detected	1.264	Detected	1.350	0.005	[illegible]
GT	Sense	Sense	0.270	Detected	-0.270	Detected	0.000	1.060	Detected	1.206	Detected	1.133	0.056	[illegible]
GT	Sense	Sense	0.092	Detected	-0.092	Detected	0.000	1.152	Detected	1.027	Detected	1.089	0.010	[illegible]
GT	Sense	Sense	0.260	Comprom	-0.260	Comprom	0.000	1.921	Detected	1.492	Detected	1.706	0.037	[illegible]
GT	Sense	Sense	0.575	Detected	-0.575	Comprom	0.000	0.808	Detected	1.324	Detected	1.066	0.233	[illegible]
GT	Sense	Sense	0.061	Comprom	-0.061	Comprom	0.000	1.785	Detected	1.826	Detected	1.805	0.001	[illegible]
GT	Sense	Sense	0.191	Comprom	-0.191	Comprom	0.000	2.066	Detected	2.524	Detected	2.295	0.016	[illegible]
GT	Sense	Sense	-0.010	Detected	0.010	Detected	0.000	1.251	Detected	1.652	Detected	1.452	0.019	[illegible]
GT	Sense	Sense	-0.235	Comprom	0.235	Comprom	0.000	1.457	Detected	1.656	Detected	1.556	0.026	[illegible]
GT	Sense	Sense	0.597	Detected	-0.597	Detected	0.000	1.527	Detected	1.351	Detected	1.439	0.140	[illegible]
GT	Sense	Sense	0.296	Detected	-0.296	Detected	0.000	0.987	Detected	1.153	Detected	1.070	0.074	[illegible]
GT	Sense	Sense	-0.009	Detected	0.009	Detected	0.000	1.180	Detected	1.275	Detected	1.228	0.002	[illegible]
GT	Sense	Sense	0.411	Detected	-0.411	Detected	0.000	0.919	Detected	1.513	Detected	1.216	0.139	[illegible]
GT	Sense	Sense	0.252	Detected	-0.252	Detected	0.000	1.417	Detected	1.491	Detected	1.454	0.029	[illegible]
GT	Sense	Sense	-0.004	Detected	0.004	Detected	0.000	0.925	Detected	1.260	Detected	1.093	0.023	[illegible]
GT	Sense	Sense	0.078	Detected	-0.078	Detected	0.000	1.249	Detected	0.949	Detected	1.099	0.023	[illegible]
GT	Sense	Sense	0.050	Detected	-0.050	Detected	0.000	1.071	Detected	1.266	Detected	1.168	0.009	[illegible]
GT	Sense	Sense	0.512	Comprom	-0.512	Comprom	0.000	1.152	Detected	1.245	Detected	1.198	0.145	[illegible]
GT	Sense	Sense	0.378	Detected	-0.378	Detected	0.000	1.608	Detected	1.803	Detected	1.705	0.049	[illegible]
GT	Sense	Sense	0.008	Detected	-0.008	Detected	0.000	1.023	Detected	1.126	Detected	1.074	0.002	[illegible]
GT	Sense	Sense	-0.049	Detected	0.049	Detected	0.000	0.901	Detected	1.117	Detected	1.009	0.014	[illegible]
GT	Sense	Sense	0.092	Comprom	-0.092	Comprom	0.000	4.288	Detected	4.179	Detected	4.234	0.001	[illegible]
GT	Sense	Sense	0.385	Detected	-0.385	Detected	0.000	0.855	Detected	1.349	Detected	1.102	0.137	[illegible]
GT	Sense	Sense	0.172	Comprom	-0.172	Comprom	0.000	2.131	Detected	2.533	Detected	2.332	0.013	[illegible]
GT	Sense	Sense	1.296	Detected	-1.296	Comprom	0.000	1.094	Detected	1.107	Detected	1.101	0.485	[illegible]
GT	Sense	Sense	0.158	Detected	-0.158	Detected	0.000	1.843	Detected	1.217	Detected	1.530	0.049	[illegible]
GT	Sense	Sense	0.749	Detected	-0.749	Detected	0.000	1.628	Detected	1.497	Detected	1.562	0.173	[illegible]
GT	Sense	Sense	-0.445	Comprom	0.445	Comprom	0.000	1.254	Detected	1.716	Detected	1.485	0.097	[illegible]
GT	Sense	Sense	-0.484	Comprom	0.484	Comprom	0.000	1.478	Detected	1.732	Detected	1.605	0.085	[illegible]
GT	Sense	Sense	-0.026	Detected	0.026	Detected	0.000	1.097	Detected	0.998	Detected	1.048	0.003	[illegible]
GT	Sense	Sense	0.133	Detected	-0.133	Detected	0.000	0.892	Detected	1.386	Detected	1.139	0.056	[illegible]
GT	Sense	Sense	-0.052	Detected	0.052	Detected	0.000	1.328	Detected	1.071	Detected	1.200	0.013	[illegible]
GT	Sense	Sense	-0.105	Detected	0.105	Detected	0.000	1.588	Detected	1.159	Detected	1.373	0.029	[illegible]
GT	Sense	Sense	-0.019	Detected	0.019	Detected	0.000	1.418	Detected	1.017	Detected	1.218	0.026	[illegible]
GT	Sense	Sense	0.127	Comprom	-0.127	Comprom	0.000	1.265	Detected	1.538	Detected	1.402	0.017	[illegible]
GT	Sense	Sense	0.284	Comprom	-0.284	Comprom	0.000	1.641	Detected	2.221	Detected	1.931	0.042	[illegible]
GT	Sense	Sense	0.772	Detected	-0.772	Comprom	0.000	2.625	Detected	2.686	Detected	2.655	0.075	[illegible]
GT	Sense	Sense	0.085	Detected	-0.085	Detected	0.000	1.334	Detected	1.471	Detected	1.402	0.006	[illegible]
GT	Sense	Sense	0.177	Detected	-0.177	Detected	0.000	1.129	Detected	1.613	Detected	1.371	0.045	[illegible]
GT	Sense	Sense	-0.330	Detected	0.330	Detected	0.000	1.109	Detected	1.018	Detected	1.064	0.086	[illegible]
GT	Sense	Sense	0.072	Detected	-0.072	Detected	0.000	1.036	Detected	0.973	Detected	1.005	0.006	[illegible]
GT	Sense	Sense	-0.099	Detected	0.099	Detected	0.000	1.209	Detected	1.260	Detected	1.235	0.007	[illegible]
GT	Sense	Sense	0.598	Detected	-0.598	Detected	0.000	1.398	Detected	1.078	Detected	1.238	0.184	[illegible]
GT	Sense	Sense	0.481	Detected	-0.481	Detected	0.000	1.412	Detected	1.809	Detected	1.610	0.090	[illegible]
GT	Sense	Sense	-0.845	Comprom	0.845	Comprom	0.000	1.369	Detected	2.504	Detected	1.937	0.197	[illegible]
GT	Sense	Sense	-0.435	Comprom	0.435	Comprom	0.000	1.897	Detected	2.092	Detected	1.995	0.047	[illegible]
GT	Sense	Sense	-0.045	Detected	0.045	Detected	0.000	0.912	Detected	1.111	Detected	1.012	0.011	[illegible]
GT	Sense	Sense	0.540	Comprom	-0.540	Comprom	0.000	1.439	Detected	1.509	Detected	1.474	0.113	[illegible]
GT	Sense	Sense	0.438	Comprom	-0.438	Comprom	0.000	0.890	Detected	1.413	Detected	1.152	0.153	[illegible]
GT	Sense	Sense	0.537	Detected	-0.537	Detected	0.000	1.329	Detected	1.645	Detected	1.487	0.117	[illegible]
GT	Sense	Sense	-0.950	Detected	0.950	Detected	0.000	1.356	Detected	1.135	Detected	1.245	0.323	[illegible]
GT	Sense	Sense	-0.033	Detected	0.033	Detected	0.000	0.848	Detected	1.184	Detected	1.016	0.027	[illegible]
GT	Sense	Sense	-0.765	Detected	0.765	Detected	0.000	2.036	Detected	1.364	Detected	1.700	0.179	[illegible]
GT	Sense	Sense	0.413	Detected	-0.413	Detected	0.000	1.261	Detected	1.144	Detected	1.202	0.102	[illegible]
GT	Sense	Sense	-0.158	Detected	0.158	Detected	0.000	2.251	Detected	1.858	Detected	2.055	0.015	[illegible]
GT	Sense	Sense	0.017	Comprom	-0.017	Comprom	0.000	0.984	Detected	1.289	Detected	1.136	0.018	[illegible]
GT	Sense	Sense	-0.549	Comprom	0.549	Comprom	0.000	2.152	Detected	2.373	Detected	2.263	0.056	[illegible]
GT	Sense	Sense	-0.479	Detected	0.479	Detected	0.000	1.426	Detected	1.515	Detected	1.470	0.092	[illegible]
GT	Sense	Sense	0.335	Detected	-0.335	Detected	0.000	0.911	Detected	1.176	Detected	1.044	0.101	[illegible]
GT	Sense	Sense	-0.316	Detected	0.316	Detected	0.000	1.378	Detected	1.734	Detected	1.556	0.050	[illegible]
GT	Sense	Sense	0.620	Comprom	-0.620	Comprom	0.000	2.324	Detected	1.676	Detected	2.000	0.104	[illegible]
GT	Sense	Sense	0.285	Detected	-0.285	Detected	0.000	1.192	Detected	0.860	Detected	1.026	0.090	[illegible]
GT	Sense	Sense	-0.059	Detected	0.059	Detected	0.000	2.296	Detected	1.905	Detected	2.101	0.009	[illegible]
GT	Sense	Sense	0.059	Comprom	-0.059	Comprom	0.000	1.807	Detected	2.244	Detected	2.025	0.012	[illegible]
GT	Sense	Sense	0.254	Comprom	-0.254	Comprom	0.000	1.731	Detected	2.196	Detected	1.963	0.029	[illegible]
GT	Sense	Sense	0.216	Detected	-0.216	Detected	0.000	1.139	Detected	0.946	Detected	1.042	0.048	[illegible]
GT	Sense	Sense	-0.301	Comprom	0.301	Comprom	0.000	1.122	Detected	1.303	Detected	1.213	0.061	[illegible]
GT	Sense	Sense	0.274	Detected	-0.274	Detected	0.000	0.836	Detected	1.270	Detected	1.053	0.095	[illegible]
GT	Sense	Sense	-0.363	Comprom	0.363	Comprom	0.000	1.309	Detected	1.547	Detected	1.428	0.065	[illegible]
GT	Sense	Sense	0.168	Detected	-0.168	Detected	0.000	1.919	Detected	1.954	Detected	1.936	0.008	[illegible]
GT	Sense	Sense	-0.004	Comprom	0.004	Comprom	0.000	1.019	Detected	1.730	Detected	1.375	0.061	[illegible]
GT	Sense	Sense	-0.004	Comprom	0.004	Comprom	0.000	1.265	Detected	1.479	Detected	1.372	0.006	[illegible]
GT	Sense	Sense	0.139	Detected	-0.139	Detected	0.000	1.708	Detected	1.653	Detected	1.681	0.007	[illegible]
GT	Sense	Sense	0.248	Detected	-0.248	Detected	0.000	1.233	Detected	1.549	Detected	1.391	0.042	[illegible]
GT	Sense	Sense	0.076	Detected	-0.076	Detected	0.000	1.742	Detected	1.190	Detected	1.466	0.036	[illegible]
GT	Sense	Sense	-0.035	Detected	0.035	Detected	0.000	1.761	Detected	1.700	Detected	1.730	0.001	[illegible]
GT	Sense	Sense	0.043	Detected	-0.043	Detected	0.000	1.256	Detected	0.913	Detected	1.085	0.026	[illegible]
GT	Sense	Sense	0.221	Detected	-0.221	Detected	0.000	1.363	Detected	1.314	Detected	1.339	0.027	[illegible]
GT	Sense	Sense	0.314	Comprom	-0.314	Comprom	0.000	1.778	Detected	1.785	Detected	1.782	0.030	[illegible]
GT	Sense	Sense	0.102	Detected	-0.102	Detected	0.000	1.360	Detected	1.535	Detected	1.448	0.009	[illegible]
GT	Sense	Sense	0.085	Detected	-0.085	Detected	0.000	1.070	Detected	1.611	Detected	1.341	0.042	[illegible]
GT	Sense	Sense	0.321	Detected	-0.321	Detected	0.000	1.188	Detected	1.094	Detected	1.141	0.072	[illegible]
GT	Sense	Sense	0.010	Detected	-0.010	Detected	0.000	2.729	Detected	2.192	Detected	2.460	0.012	[illegible]
GT	Sense	Sense	0.208	Detected	-0.208	Detected	0.000	0.859	Detected	1.177	Detected	1.018	0.060	[illegible]
GT	Sense	Sense	0.198	Detected	-0.198	Detected	0.000	1.366	Detected	1.784	Detected	1.575	0.032	[illegible]
GT	Sense	Sense	-0.055	Detected	0.055	Detected	0.000	0.943	Detected	1.075	Detected	1.009	0.007	[illegible]
GT	Sense	Sense	-0.122	Detected	0.122	Detected	0.000	1.054	Detected	1.505	Detected	1.279	0.038	[illegible]
GT	Sense	Sense	-0.130	Comprom	0.130	Comprom	0.000	1.052	Detected	1.932	Detected	1.492	0.083	[illegible]
GT	Sense	Sense	1.240	Detected	-1.240	Comprom	0.000	0.901	Detected	1.268	Detected	1.085	0.478	[illegible]
GT	Sense	Sense	-0.263	Comprom	0.263	Comprom	0.000	2.043	Detected	2.143	Detected	2.093	0.016	[illegible]
GT	Sense	Sense	0.266	Comprom	-0.266	Comprom	0.000	1.687	Detected	1.954	Detected	1.821	0.026	[illegible]
GT	Sense	Sense	0.394	Comprom	-0.394	Comprom	0.000	1.237	Detected	1.239	Detected	1.238	0.088	[illegible]
GT	Sense	Sense	0.054	Detected	-0.054	Detected	0.000	1.522	Detected	1.631	Detected	1.577	0.002	[illegible]
GT	Sense	Sense	0.275	Detected	-0.275	Detected	0.000	1.363	Detected	1.748	Detected	1.556	0.044	[illegible]
GT	Sense	Sense	-0.071	Comprom	0.071	Comprom	0.000	1.389	Detected	1.927	Detected	1.658	0.027	[illegible]
GT	Sense	Sense	0.107	Detected	-0.107	Detected	0.000	1.738	Detected	1.066	Detected	1.402	0.058	[illegible]
GT	Sense	Sense	0.010	Comprom	-0.010	Comprom	0.000	0.964	Detected	1.097	Detected	1.030	0.004	[illegible]
GT	Sense	Sense	-0.099	Detected	0.099	Detected	0.000	0.996	Detected	1.109	Detected	1.053	0.012	[illegible]
GT	Sense	Sense	-0.118	Detected	0.118	Detected	0.000	1.716	Detected	1.618	Detected	1.667	0.006	[illegible]
GT	Sense	Sense	-0.481	Comprom	0.481	Comprom	0.000	1.296	Detected	1.246	Detected	1.271	0.119	[illegible]
GT	Sense	Sense	0.438	Detected	-0.438	Detected	0.000	1.396	Detected	1.802	Detected	1.599	0.080	[illegible]
GT	Sense	Sense	0.598	Comprom	-0.598	Comprom	0.000	1.030	Detected	2.093	Detected	1.562	0.190	[illegible]
GT	Sense	Sense	-0.017	Detected	0.017	Detected	0.000	1.294	Detected	1.551	Detected	1.422	0.008	[illegible]
GT	Sense	Sense	0.221	Detected	-0.221	Detected	0.000	1.583	Detected	1.155	Detected	1.369	0.047	[illegible]
GT	Sense	Sense	0.539	Detected	-0.539	Comprom	0.000	1.076	Detected	1.525	Detected	1.301	0.156	[illegible]
GT	Sense	Sense	0.152	Detected	-0.152	Detected	0.000	1.259	Detected	1.127	Detected	1.193	0.019	[illegible]
GT	Sense	Sense	-0.660	Comprom	0.660	Comprom	0.000	1.825	Detected	1.699	Detected	1.762	0.117	[illegible]
GT	Sense	Sense	-0.060	Detected	0.060	Detected	0.000	2.313	Detected	1.452	Detected	1.883	0.049	[illegible]
GT	Sense	Sense	0.232	Detected	-0.232	Detected	0.000	2.746	Detected	1.999	Detected	2.373	0.033	[illegible]
GT	Sense	Sense	0.144	Detected	-0.144	Detected	0.000	1.166	Detected	0.908	Detected	1.037	0.033	[illegible]
GT	Sense	Sense	0.167	Detected	-0.167	Detected	0.000	0.913	Detected	1.164	Detected	1.039	0.038	[illegible]
GT	Sense	Sense	0.290	Comprom	-0.290	Comprom	0.000	1.981	Detected	2.420	Detected	2.201	0.026	[illegible]
GT	Sense	Sense	0.162	Detected	-0.162	Detected	0.000	1.139	Detected	1.239	Detected	1.189	0.020	[illegible]
GT	Sense	Sense	0.149	Detected	-0.149	Detected	0.000	1.285	Detected	0.815	Detected	1.050	0.063	[illegible]
GT	Sense	Sense	0.468	Detected	-0.468	Detected	0.000	1.134	Detected	1.063	Detected	1.099	0.144	[illegible]
GT	Sense	Sense	-0.392	Comprom	0.392	Detected	0.000	0.933	Detected	1.352	Detected	1.142	0.124	[illegible]
GT	Sense	Sense	-0.023	Detected	0.023	Detected	0.000	1.165	Detected	0.982	Detected	1.073	0.008	[illegible]
GT	Sense	Sense	-1.105	Comprom	1.105	Detected	0.000	1.798	Detected	1.108	Detected	1.453	0.336	[illegible]
GT	Sense	Sense	0.279	Detected	-0.279	Detected	0.000	1.157	Detected	1.341	Detected	1.249	0.051	[illegible]
GT	Sense	Sense	0.677	Detected	-0.677	Comprom	0.000	1.250	Detected	1.662	Detected	1.456	0.176	[illegible]
GT	Sense	Sense	0.397	Detected	-0.397	Detected	0.000	1.040	Detected	1.353	Detected	1.196	0.107	[illegible]
GT	Sense	Sense	-0.831	Detected	0.831	Detected	0.000	1.106	Detected	0.971	Detected	1.038	0.339	[illegible]
GT	Sense	Sense	0.056	Detected	-0.056	Detected	0.000	1.035	Detected	1.132	Detected	1.084	0.005	[illegible]
GT	Sense	Sense	-0.312	Comprom	0.312	Comprom	0.000	1.374	Detected	1.270	Detected	1.322	0.053	[illegible]
GT	Sense	Sense	0.478	Comprom	-0.478	Comprom	0.000	1.175	Detected	1.728	Detected	1.452	0.119	[illegible]
GT	Sense	Sense	0.338	Detected	-0.338	Detected	0.000	1.681	Detected	1.131	Detected	1.406	0.084	[illegible]
GT	Sense	Sense	-0.016	Comprom	0.016	Comprom	0.000	2.724	Detected	2.733	Detected	2.728	0.000	[illegible]
GT	Sense	Sense	-0.648	Comprom	0.648	Detected	0.000	1.404	Detected	0.893	Detected	1.149	0.241	[illegible]
GT	Sense	Sense	0.091	Comprom	-0.091	Comprom	0.000	1.635	Detected	1.103	Detected	1.369	0.040	[illegible]
GT	Sense	Sense	0.453	Detected	-0.453	Detected	0.000	1.633	Detected	1.741	Detected	1.687	0.066	[illegible]
GT	Sense	Sense	0.157	Comprom	-0.157	Comprom	0.000	0.891	Detected	1.408	Detected	1.150	0.063	[illegible]
GT	Sense	Sense	0.183	Comprom	-0.183	Comprom	0.000	0.930	Detected	1.188	Detected	1.059	0.042	[illegible]
GT	Sense	Sense	-0.020	Comprom	0.020	Comprom	0.000	0.959	Detected	1.112	Detected	1.036	0.006	[illegible]
GT	Sense	Sense	-0.180	Detected	0.180	Detected	0.000	1.578	Detected	1.480	Detected	1.529	0.015	[illegible]
GT	Sense	Sense	0.503	Detected	-0.503	Detected	0.000	0.833	Detected	1.210	Detected	1.021	0.198	[illegible]
GT	Sense	Sense	-0.427	Comprom	0.427	Comprom	0.000	1.433	Detected	1.269	Detected	1.351	0.090	[illegible]
GT	Sense	Sense	-0.103	Comprom	0.103	Comprom	0.000	1.096	Detected	1.373	Detected	1.234	0.019	[illegible]
GT	Sense	Sense	-0.021	Comprom	0.021	Comprom	0.000	0.930	Detected	1.071	Detected	1.001	0.005	[illegible]
GT	Sense	Sense	0.003	Detected	-0.003	Detected	0.000	1.022	Detected	1.619	Detected	1.321	0.047	[illegible]
GT	Sense	Sense	-0.063	Comprom	0.063	Comprom	0.000	1.736	Detected	2.127	Detected	1.931	0.011	[illegible]
GT	Sense	Sense	0.212	Detected	-0.212	Detected	0.000	0.823	Detected	1.204	Detected	1.013	0.071	[illegible]
GT	Sense	Sense	-0.126	Detected	0.126	Detected	0.000	1.032	Detected	1.076	Detected	1.054	0.014	[illegible]
GT	Sense	Sense	0.089	Detected	-0.089	Detected	0.000	1.186	Detected	1.120	Detected	1.153	0.007	[illegible]
GT	Sense	Sense	-0.524	Comprom	0.524	Comprom	0.000	1.094	Detected	1.813	Detected	1.454	0.150	[illegible]
GT	Sense	Sense	0.342	Comprom	-0.342	Comprom	0.000	2.515	Detected	2.619	Detected	2.567	0.018	[illegible]
GT	Sense	Sense	-0.881	Comprom	0.881	Detected	0.000	1.129	Detected	0.911	Detected	1.020	0.369	[illegible]
GT	Sense	Sense	0.233	Comprom	-0.233	Comprom	0.000	1.173	Detected	1.815	Detected	1.494	0.064	[illegible]
GT	Sense	Sense	0.246	Detected	-0.246	Detected	0.000	1.225	Detected	1.524	Detected	1.374	0.041	[illegible]
GT	Sense	Sense	-0.260	Comprom	0.260	Comprom	0.000	1.165	Detected	1.611	Detected	1.388	0.056	[illegible]
GT	Sense	Sense	-0.049	Comprom	0.049	Comprom	0.000	1.779	Detected	2.991	Detected	2.385	0.059	[illegible]
GT	Sense	Sense	0.169	Detected	-0.169	Detected	0.000	1.522	Detected	0.920	Detected	1.221	0.071	[illegible]
GT	Sense	Sense	0.053	Detected	-0.053	Detected	0.000	1.215	Detected	1.160	Detected	1.188	0.002	[illegible]
GT	Sense	Sense	-0.248	Comprom	0.248	Comprom	0.000	0.908	Detected	1.202	Detected	1.055	0.067	[illegible]
GT	Sense	Sense	0.861	Comprom	-0.861	Comprom	0.000	1.573	Detected	2.971	Detected	2.272	0.177	[illegible]
GT	Sense	Sense	0.414	Detected	-0.414	Detected	0.000	0.843	Detected	1.309	Detected	1.076	0.152	[illegible]
GT	Sense	Sense	0.334	Detected	-0.334	Comprom	0.000	0.885	Detected	1.261	Detected	1.073	0.107	[illegible]
GT	Sense	Sense	-0.164	Comprom	0.164	Comprom	0.000	1.201	Detected	1.748	Detected	1.475	0.044	[illegible]
GT	Sense	Sense	0.875	Detected	-0.875	Comprom	0.000	1.285	Detected	1.208	Detected	1.246	0.291	[illegible]
GT	Sense	Sense	0.766	Comprom	-0.766	Comprom	0.000	1.635	Detected	1.574	Detected	1.605	0.172	[illegible]
GT	Sense	Sense	0.160	Detected	-0.160	Detected	0.000	1.848	Detected	2.353	Detected	2.100	0.020	[illegible]
GT	Sense	Sense	0.073	Detected	-0.073	Detected	0.000	0.867	Detected	1.504	Detected	1.186	0.068	[illegible]
GT	Sense	Sense	0.274	Comprom	-0.274	Comprom	0.000	1.340	Detected	0.885	Detected	1.113	0.089	[illegible]
GT	Sense	Sense	0.126	Detected	-0.126	Detected	0.000	1.162	Detected	1.387	Detected	1.275	0.017	[illegible]
GT	Sense	Sense	-0.209	Comprom	0.209	Comprom	0.000	1.558	Detected	1.734	Detected	1.646	0.018	[illegible]
GT	Sense	Sense	0.462	Comprom	-0.462	Comprom	0.000	1.246	Detected	1.750	Detected	1.498	0.104	[illegible]
GT	Sense	Sense	0.505	Comprom	-0.505	Comprom	0.000	0.836	Detected	1.608	Detected	1.222	0.194	[illegible]
GT	Sense	Sense	0.112	Detected	-0.112	Detected	0.000	0.991	Detected	1.173	Detected	1.082	0.017	[illegible]
GT	Sense	Sense	0.339	Comprom	-0.339	Comprom	0.000	0.807	Detected	1.411	Detected	1.109	0.135	[illegible]
GT	Sense	Sense	0.077	Comprom	-0.077	Comprom	0.000	1.039	Detected	1.343	Detected	1.191	0.020	[illegible]
GT	Sense	Sense	0.017	Comprom	-0.017	Comprom	0.000	1.104	Detected	1.743	Detected	1.423	0.047	[illegible]
GT	Sense	Sense	0.267	Comprom	-0.267	Comprom	0.000	0.855	Detected	1.490	Detected	1.173	0.106	[illegible]
GT	Sense	Sense	0.361	Detected	-0.361	Detected	0.000	1.151	Detected	1.169	Detected	1.160	0.085	[illegible]
GT	Sense	Sense	0.374	Comprom	-0.374	Comprom	0.000	1.215	Detected	1.677	Detected	1.446	0.081	[illegible]
GT	Sense	Sense	-0.500	Comprom	0.500	Comprom	0.000	1.499	Detected	1.321	Detected	1.410	0.109	[illegible]
GT	Sense	Sense	0.447	Detected	-0.447	Detected	0.000	1.512	Detected	1.066	Detected	1.289	0.123	[illegible]
GT	Sense	Sense	0.387	Detected	-0.387	Detected	0.000	1.169	Detected	1.114	Detected	1.141	0.099	[illegible]
GT	Sense	Sense	0.498	Detected	-0.498	Detected	0.000	0.827	Detected	1.528	Detected	1.177	0.193	[illegible]
GT	Sense	Sense	0.257	Detected	-0.257	Comprom	0.000	1.044	Detected	1.306	Detected	1.175	0.055	[illegible]
GT	Sense	Sense	0.393	Comprom	-0.393	Comprom	0.000	1.852	Detected	1.662	Detected	1.757	0.049	[illegible]
GT	Sense	Sense	-0.291	Comprom	0.291	Comprom	0.000	1.772	Detected	2.224	Detected	1.998	0.032	[illegible]
GT	Sense	Sense	0.273	Detected	-0.273	Detected	0.000	1.005	Detected	1.508	Detected	1.256	0.077	[illegible]
GT	Sense	Sense	-0.014	Detected	0.014	Detected	0.000	1.812	Detected	1.310	Detected	1.561	0.025	[illegible]
GT	Sense	Sense	0.247	Comprom	-0.247	Comprom	0.000	0.952	Detected	1.109	Detected	1.031	0.058	[illegible]
GT	Sense	Sense	-0.216	Comprom	0.216	Comprom	0.000	1.322	Detected	1.217	Detected	1.270	0.029	[illegible]
GT	Sense	Sense	0.666	Detected	-0.666	Comprom	0.000	0.894	Detected	1.360	Detected	1.127	0.251	[illegible]
GT	Sense	Sense	0.276	Detected	-0.276	Comprom	0.000	1.062	Detected	1.722	Detected	1.392	0.084	[illegible]
GT	Sense	Sense	-0.437	Detected	0.437	Detected	0.000	1.896	Detected	1.032	Detected	1.464	0.140	[illegible]
GT	Sense	Sense	0.179	Comprom	-0.179	Comprom	0.000	1.091	Detected	1.361	Detected	1.226	0.032	[illegible]
GT	Sense	Sense	0.312	Detected	-0.312	Detected	0.000	1.121	Detected	0.951	Detected	1.036	0.085	[illegible]
GT	Sense	Sense	0.342	Detected	-0.342	Detected	0.000	1.048	Detected	1.598	Detected	1.323	0.095	[illegible]
GT	Sense	Sense	0.235	Comprom	-0.235	Comprom	0.000	1.128	Detected	1.666	Detected	1.397	0.060	[illegible]
GT	Sense	Sense	0.004	Detected	-0.004	Detected	0.000	1.032	Detected	1.110	Detected	1.071	0.001	[illegible]
GT	Sense	Sense	0.234	Detected	-0.234	Detected	0.000	1.007	Detected	1.085	Detected	1.046	0.048	[illegible]
GT	Sense	Sense	0.216	Comprom	-0.216	Comprom	0.000	1.082	Detected	1.035	Detected	1.058	0.040	[illegible]
GT	Sense	Sense	0.117	Detected	-0.117	Detected	0.000	1.318	Detected	1.724	Detected	1.521	0.023	[illegible]
GT	Sense	Sense	-0.381	Comprom	0.381	Comprom	0.000	1.184	Detected	0.889	Detected	1.036	0.126	[illegible]
GT	Sense	Sense	0.211	Detected	-0.211	Detected	0.000	1.379	Detected	0.912	Detected	1.146	0.068	[illegible]
GT	Sense	Sense	0.294	Detected	-0.294	Detected	0.000	1.175	Detected	1.009	Detected	1.092	0.070	[illegible]
GT	Sense	Sense	0.340	Detected	-0.340	Detected	0.000	1.292	Detected	1.014	Detected	1.153	0.088	[illegible]
GT	Sense	Sense	0.446	Detected	-0.446	Detected	0.000	1.025	Detected	0.998	Detected	1.011	0.152	[illegible]
GT	Sense	Sense	0.395	Comprom	-0.395	Comprom	0.000	1.296	Detected	4.413	Detected	2.855	0.218	[illegible]
GT	Sense	Sense	-0.051	Detected	0.051	Detected	0.000	1.121	Detected	1.153	Detected	1.137	0.002	[illegible]
GT	Sense	Sense	0.162	Detected	-0.162	Detected	0.000	1.507	Detected	1.583	Detected	1.545	0.011	[illegible]
GT	Sense	Sense	-0.151	Detected	0.151	Detected	0.000	1.571	Detected	1.308	Detected	1.440	0.019	[illegible]
GT	Sense	Sense	-0.039	Detected	0.039	Detected	0.000	1.680	Detected	1.463	Detected	1.572	0.005	[illegible]
GT	Sense	Sense	0.419	Detected	-0.419	Detected	0.000	0.891	Detected	1.477	Detected	1.184	0.146	[illegible]
GT	Sense	Sense	0.043	Comprom	-0.043	Comprom	0.000	1.594	Detected	2.365	Detected	1.980	0.036	[illegible]
GT	Sense	Sense	0.161	Detected	-0.161	Detected	0.000	0.833	Detected	1.565	Detected	1.199	0.096	[illegible]
GT	Sense	Sense	-0.206	Comprom	0.206	Comprom	0.000	0.933	Detected	1.104	Detected	1.018	0.045	[illegible]
GT	Sense	Sense	-0.401	Comprom	0.401	Comprom	0.000	1.761	Detected	4.108	Detected	2.935	0.142	[illegible]
GT	Sense	Sense	0.065	Comprom	-0.065	Comprom	0.000	1.420	Detected	3.023	Detected	2.222	0.110	[illegible]
GT	Sense	Sense	-0.570	Comprom	0.570	Comprom	0.000	2.073	Detected	1.854	Detected	1.963	0.077	[illegible]
GT	Sense	Sense	-0.248	Detected	0.248	Detected	0.000	1.189	Detected	0.813	Detected	1.001	0.085	[illegible]
GT	Sense	Sense	0.119	Comprom	-0.119	Comprom	0.000	1.405	Detected	1.820	Detected	1.613	0.021	[illegible]
GT	Sense	Sense	0.340	Detected	-0.340	Detected	0.000	1.205	Detected	1.266	Detected	1.235	0.068	[illegible]
GT	Sense	Sense	0.297	Detected	-0.297	Detected	0.000	1.256	Detected	1.303	Detected	1.280	0.050	[illegible]
GT	Sense	Sense	0.992	Detected	-0.992	Comprom	0.000	1.061	Detected	1.185	Detected	1.123	0.376	[illegible]
GT	Sense	Sense	0.127	Comprom	-0.127	Comprom	0.000	1.640	Detected	1.224	Detected	1.432	0.028	[illegible]
GT	Sense	Sense	0.437	Comprom	-0.437	Comprom	0.000	0.983	Detected	1.276	Detected	1.129	0.134	[illegible]
GT	Sense	Sense	0.265	Detected	-0.265	Detected	0.000	1.026	Detected	1.233	Detected	1.130	0.058	[illegible]
GT	Sense	Sense	0.310	Detected	-0.310	Detected	0.000	1.152	Detected	1.367	Detected	1.260	0.062	[illegible]
GT	Sense	Sense	-0.303	Comprom	0.303	Comprom	0.000	2.197	Detected	2.441	Detected	2.319	0.019	[illegible]
GT	Sense	Sense	0.491	Comprom	-0.491	Comprom	0.000	1.132	Detected	1.330	Detected	1.231	0.133	[illegible]
GT	Sense	Sense	0.156	Comprom	-0.156	Comprom	0.000	1.781	Detected	1.748	Detected	1.764	0.008	[illegible]
GT	Sense	Sense	-0.383	Comprom	0.383	Comprom	0.000	1.344	Detected	1.897	Detected	1.620	0.076	[illegible]
GT	Sense	Sense	-0.026	Comprom	0.026	Comprom	0.000	1.083	Detected	1.578	Detected	1.331	0.033	[illegible]
GT	Sense	Sense	0.588	Detected	-0.588	Detected	0.000	1.367	Detected	1.459	Detected	1.413	0.139	[illegible]

[illegible]																																								
GT	Sens	Sense	0.445	Detected	-0.445	Detected	0.000	1.207	Detected	1.290	Detected	1.249	0.108	GT	Sens	contig499	contig499	Transcri	GTTCCA	contig499	Solyc12g	Solyc12g	Transcri	GO:00050	GO:00050	contig499	Solyc12g	Transcri	GO:00037	SL2.40ch	AT5G48560.1		basic he	chr5:196	207.967	110.898	374.016	202.17	390.534	376.005
GT	Sens	Sense	-0.053	Comprom	0.053	Comprom	0.000	1.353	Detected	1.068	Detected	1.210	0.015	GT	Sens	contig51	contig51	Unknown	CAATTA	contig51	Solyc08g	Solyc08g	Unknown Protein (AHRD V1)			contig51	Solyc08g	Unknown Protein (A		SL2.40ch	AT1G02670.1		DNA repa	chr1:576	5.53602	5.88928	13.9977	12.3555	16.24	12.1185
GT	Sens	Sense	0.741	Detected	-0.741	Detected	0.000	0.948	Detected	1.111	Detected	1.029	0.301	GT	Sens	contig510	contig510	ABC tran	GAGCAA	contig510	Solyc09g	Solyc09g	ABC tran	GO:00168	GO:00168	contig510	Solyc09g	ABC tran	GO:00085	SL2.40ch	AT2G37360.1		ABC tran	chr2:156	84.3591	29.8661	86.3774	78.7746	107.846	109.764
GT	Sens	Sense	0.239	Detected	-0.239	Detected	0.000	2.002	Detected	2.961	Detected	2.481	0.044	GT	Sens	contig514	contig514	Cationic	TAACGC	contig514	Solyc00g	Solyc00g	Cationic	GO:00055	GO:00055	contig514	Solyc02g	Cationic	GO:00055	SL2.40ch	AT1G188	WRKY61	WRKY61	chr1:650	15.8213	11.2228	13.5371	19.7521	59.4395	105.017
GT	Sens	Sense	0.581	Detected	-0.581	Detected	0.000	1.851	Detected	2.316	Detected	2.083	0.080	GT	Sens	contig516	contig516	Pectinest	GGGTTA	contig516	Solyc05g	Solyc05g	Pectinest	GO:00305	GO:00305	contig516	Solyc05g	Pectinest	GO:00050	SL2.40ch	AT2G26440.1		pectines	chr2:112	172.81	76.3726	336.079	722.391	461.424	579.324
GT	Sens	Sense	-0.081	Comprom	0.081	Comprom	0.000	1.295	Detected	1.163	Detected	1.229	0.007	GT	Sens	contig52	contig52	Unknown	CTCCTT	contig52	Solyc09g	Solyc09g	Unknown Protein (AHRD V1)			contig52	Solyc09g	Unknown Protein (A		SL2.40ch	AT5G24840.1		tRNA (gu	chr5:853	6.965	7.70713	10.8338	13.781	20.016	16.6106
GT	Sens	Sense	-0.094	Detected	0.094	Detected	0.000	1.829	Detected	1.255	Detected	1.542	0.036	GT	Sens	contig549	contig549	Unknown	AAGTGA	contig549	Solyc03g	Solyc03g	Unknown Protein (AHRD V1)%2			contig549	Solyc03g	Unknown Protein (A		SL2.40ch	AT4G33810.1		glycosyl	chr4:162	21.1543	23.829	25.8777	50.682	88.846	54.2703
GT	Sens	Sense	0.431	Detected	-0.431	Comprom	0.000	1.526	Detected	0.844	Detected	1.185	0.164	GT	Sens	contig557	contig557	Phospha	AAAGGA	contig557	Solyc07g	Solyc07g	Phospha	GO:00038	GO:00038	contig557	Solyc07g	Phospha	GO:00038	SL2.40ch	AT4G29530.1		2,3-diket	chr4:144	16.5493	8.99714	45.8241	41.0724	39.1449	22.1817
GT	Sens	Sense	0.076	Detected	-0.076	Detected	0.000	1.524	Detected	0.810	Detected	1.167	0.085	GT	Sens	contig557	contig557	Unknown	CGTTGG	contig557	Solyc03g	Solyc03g	Unknown Protein (AHRD V1)			contig557	Solyc03g	Unknown Protein (A		SL2.40ch	AT5G550	ATMYB12	MYB120	chr5:223	192.619	171.396	253.38	283.818	581.902	322.539
GT	Sens	Sense	0.593	Detected	-0.593	Detected	0.000	1.149	Detected	1.351	Detected	1.250	0.173	GT	Sens	contig566	contig566	Subtilisin	GAACGA	contig566	Solyc04g	Solyc04g	Subtilisin	GO:00042	GO:00042	contig566	Solyc04g	Subtilisin	GO:00042	SL2.40ch	AT5G517	ATSBT1.3	ATSBT1.	chr5:210	3257.45	1416.06	4325.08	3178.88	5306.13	5547.84
GT	Sens	Sense	0.103	Detected	-0.103	Detected	0.000	1.436	Detected	1.180	Detected	1.308	0.015	GT	Sens	contig599	contig599	Unknown	AAGAAA	contig599	Solyc02g	Solyc02g	Unknown Protein (AHRD V1)			contig599	Solyc02g	Unknown Protein (A		SL2.40ch	AT4G368	IRX14	IRX14 (ir	chr4:173	1801.04	1542.85	3382.62	2837.77	5024.74	3824.84
GT	Sens	Sense	0.566	Detected	-0.566	Detected	0.000	1.519	Detected	1.688	Detected	1.604	0.107	GT	Sens	contig610	contig610	Ferredox	ATTAAG	contig610	Solyc01g	Solyc01g	Ferredoxin-like (AHRD V1 ***- C			contig610	Solyc01g	Ferredoxin-like (AH		SL2.40ch	AT1G02180.1		ferredox	chr1:413	89.8095	40.5386	171.106	154.54	192.603	196.881
GT	Sens	Sense	-0.115	Detected	0.115	Comprom	0.000	1.385	Detected	1.636	Detected	1.510	0.012	GT	Sens	contig641	contig641	Cation/H(	ATTAGG	contig641	Solyc05g	Solyc05g	Cation/H(	GO:00153	GO:00153	contig641	Solyc05g	Cation/H(	GO:00153	SL2.40ch	AT4G237	ATCHX17	ATCHX17	chr4:123	5.24653	6.07987	9.11684	26.4845	16.4316	17.7663
GT	Sens	Sense	0.284	Detected	-0.284	Detected	0.000	1.703	Detected	2.208	Detected	1.956	0.036	GT	Sens	contig644	contig644	Os12g05	CCACAA	contig644	Solyc02g	Solyc02g	Os12g0581300 protein (Fragm			contig644	Solyc02g	Os12g0581300 prot		SL2.40ch	AT5G66740.1		unknown	chr5:266	28.5316	19.0142	71.3001	55.429	84.4734	108.951
GT	Sens	Sense	0.036	Detected	-0.036	Detected	0.000	1.857	Detected	1.911	Detected	1.884	0.001	GT	Sens	contig655	contig655	Leaf sene	AACTTA	contig655	Solyc02g	Solyc02g	Leaf senescence protein-like (			contig655	Solyc02g	Leaf senescence pr		SL2.40ch	AT3G28150.1		unknown	chr3:104	30.6354	28.794	59.0083	44.7404	119.852	113.099
GT	Sens	Sense	0.426	Detected	-0.426	Detected	0.000	2.537	Detected	1.987	Detected	2.262	0.047	GT	Sens	contig669	contig669	FAD-bind	CGAAGC	contig669	Solyc02g	Solyc02g	FAD-bind	GO:00090	GO:00090	contig669	Solyc02g	FAD-bind	GO:00090	SL2.40ch	AT5G44400.1		FAD-bind	chr5:178	125.938	69.0138	261.846	53.6744	602.696	374.099
GT	Sens	Sense	0.466	Comprom	-0.466	Comprom	0.000	2.125	Detected	1.049	Detected	1.587	0.155	GT	Sens	contig675	contig675	Centrome	ACGCTC	contig675	Solyc05g	Solyc05g	Centrome	GO:00081	GO:00081	contig675	Solyc05g	Centrome	GO:00081	SL2.40ch	AT2G39300.1		unknown	chr2:164	7.65736	3.96865	11.8893	14.7536	26.7723	11.5506
GT	Sens	Sense	0.059	Detected	-0.059	Detected	0.000	1.087	Detected	1.515	Detected	1.301	0.028	GT	Sens	contig679	contig679	Exostosi	GCTAAG	contig679	Solyc08g	Solyc08g	Exostosi	GO:00038	GO:00038	contig679	Solyc08g	Exostosi	GO:00038	SL2.40ch	AT5G25820.1		exostosi	chr5:899	17.4336	15.8857	22.0217	38.8912	39.3831	48.1471
GT	Sens	Sense	0.040	Detected	-0.040	Detected	0.000	1.039	Detected	1.223	Detected	1.131	0.008	GT	Sens	contig682	contig682	3-methyl-	GGAGAA	contig682	Solyc06g	Solyc06g	3-methyl-	GO:00081	GO:00081	contig682	Solyc06g	3-methyl-	GO:00038	SL2.40ch	AT5G09300.2		2-oxoiso	chr5:288	181.174	169.436	276.707	502.457	400.918	414.109
GT	Sens	Sense	-0.580	Comprom	0.580	Detected	0.000	1.607	Detected	1.521	Detected	1.564	0.115	GT	Sens	contig690	contig690	Peroxida	AACACA	contig690	Solyc01g	Solyc01g	Peroxida	GO:00055	GO:00055	contig690	Solyc01g	Peroxida	GO:00055	SL2.40ch	AT3G49960.1		peroxida	chr3:185	3.01212	6.6515	8.25218	2.15078	15.1822	13.0087
GT	Sens	Sense	0.565	Detected	-0.565	Detected	0.000	0.933	Detected	1.322	Detected	1.127	0.200	GT	Sens	contig702	contig702	Unknown	GAAAAC	contig702	Solyc02g	Solyc02g	Unknown Protein (AHRD V1)			contig702	Solyc02g	Unknown Protein (A		SL2.40ch	AT4G38340.1		RWP-RK	chr4:179	280.387	126.615	276.861	218.076	400.517	476.963
GT	Sens	Sense	1.288	Detected	-1.288	Comprom	0.000	2.014	Detected	3.607	Detected	2.811	0.205	GT	Sens	contig703	contig703	Beta-1 3-	ATTGAG	contig703	Solyc02g	Solyc02g	Beta-1 3-(	GO:00055	GO:00055	contig703	Solyc02g	Beta-1 3-(	GO:00055	SL2.40ch	AT4G29360.1		glycosyl	chr4:144	15.2156	2.52292	27.2871	8.43688	27.8753	76.4405
GT	Sens	Sense	-0.020	Detected	0.020	Detected	0.000	1.210	Detected	0.960	Detected	1.085	0.013	GT	Sens	contig706	contig706	Zinc finge	ATAATG	contig706	Solyc10g	Solyc10g	Zinc finge	GO:00050	GO:00050	contig706	Solyc10g	Zinc finge	GO:00050	SL2.40ch	AT5G066	GIS2	GIS2 (GL	chr5:204	21.7272	22.0896	36.4295	39.6486	56.4461	43.1579
GT	Sens	Sense	-0.298	Comprom	0.298	Detected	0.000	2.536	Detected	3.380	Detected	2.958	0.029	GT	Sens	contig711	contig711	Serpin (S	ATGGTA	contig711	Solyc04g	Solyc04g	Serpin (S	GO:00048	GO:00048	contig711	Solyc04g	Serpin (S	GO:00430	SL2.40ch	AT3G45220.1		serpin, p	chr3:165	98.5267	147.181	318.454	201.372	777.6	1268.87
GT	Sens	Sense	-0.686	Comprom	0.686	Detected	0.000	1.252	Detected	1.095	Detected	1.174	0.231	GT	Sens	contig724	contig724	Transcri	CCCTGT	contig724	Solyc11g	Solyc11g	Transcri	GO:00050	GO:00050	contig724	Solyc02g	60S ribos	GO:00037	SL2.40ch	AT3G52700.1		unknown	chr3:195	5.13693	13.1493	6.33652	6.7419	21.7992	17.7765
GT	Sens	Sense	-0.026	Comprom	0.026	Comprom	0.000	2.133	Detected	2.026	Detected	2.079	0.001	GT	Sens	contig735	contig735	Unknown	GACTTA	contig735	Solyc12g	Solyc12g	Unknown Protein (AHRD V1)			contig735	Solyc04g	Trehalos	GO:00081	SL2.40ch	#N/A	#N/A	#N/A	#N/A	4.4309	4.53989	3.48933	3.62614	21.9108	18.4925
GT	Sens	Sense	-0.064	Comprom	0.064	Comprom	0.000	1.245	Detected	1.384	Detected	1.315	0.005	GT	Sens	contig735	contig735	Zinc finge	TTGCTA	contig735	Solyc02g	Solyc02g	Zinc finge	GO:00050	GO:00050	contig735	Solyc02g	Zinc finge	GO:00050	SL2.40ch	AT4G38960.1		zinc fing	chr4:181	3.81165	4.1206	8.19584	17.1309	10.4605	10.4746
GT	Sens	Sense	-0.104	Detected	0.104	Detected	0.000	1.413	Detected	0.989	Detected	1.201	0.037	GT	Sens	contig738	contig738	High affin	AATCCT	contig738	Solyc04g	Solyc04g	High affin	GO:00153	GO:00153	contig738	Solyc04g	High affin	GO:00153	SL2.40ch	AT1G780	SULTR1:3	SULTR1:	chr1:293	78.3805	89.5039	172.192	200.232	248.481	168.331
GT	Sens	Sense	0.189	Detected	-0.189	Detected	0.000	1.408	Detected	0.897	Detected	1.152	0.068	GT	Sens	contig765	contig765	BES1/BZI	TAATGC	contig765	Solyc07g	Solyc07g	BES1/BZI	GO:00305	GO:00305	contig765	Solyc07g	BES1/BZI	GO:00305	SL2.40ch	AT1G78700.1		brassino	chr1:295	601.917	457.714	976.05	601.832	1551.12	989.33
GT	Sens	Sense	0.157	Detected	-0.157	Detected	0.000	0.859	Detected	1.209	Detected	1.034	0.048	GT	Sens	contig767	contig767	Os12g02	TCATTT	contig767	Solyc03g	Solyc03g	Os12g0236050 protein (Fragm			contig767	Solyc03g	Os12g0236050 prot		SL2.40ch	AT5G43230.1		unknown	chr5:173	77.9819	62.0493	117.569	92.4071	140.53	162.827
GT	Sens	Sense	0.558	Detected	-0.558	Detected	0.000	1.671	Detected	1.974	Detected	1.822	0.088	GT	Sens	contig781	contig781	Pectinest	CTCTGA	contig781	Solyc11g	Solyc11g	Pectinest	GO:00305	GO:00305	contig781	Solyc11g	Pectinest	GO:00305	SL2.40ch	AT5G62360.1		invertase	chr5:250	419.995	191.472	1002.81	1321.83	1005.78	1127.77
GT	Sens	Sense	0.572	Detected	-0.572	Detected	0.000	2.065	Detected	1.588	Detected	1.827	0.098	GT	Sens	contig790	contig790	Carboxyl	CCGATG	contig790	Solyc07g	Solyc07g	Carboxyl	GO:00055	GO:00055	contig790	Solyc07g	Carboxyl	GO:00055	SL2.40ch	AT4G23882.1				36.7006	16.4246	183.555	65.4045	114.449	74.7255
GT	Sens	Sense	-0.443	Comprom	0.443	Detected	0.000	2.029	Detected	1.610	Detected	1.819	0.066	GT	Sens	contig796	contig796	Unknown	TGAAGC	contig796	Solyc06g	Solyc06g	Unknown Protein (AHRD V1)			contig796	Solyc07g026990.1.1				AT5G24280.1		EXPRES	chr5:825	10.1853	18.6179	12.2864	17.0904	62.5954	42.5545
GT	Sens	Sense	-0.009	Detected	0.009	Detected	0.000	1.409	Detected	1.046	Detected	1.228	0.021	GT	Sens	contig805	contig805	Peroxida	CGAAGA	contig805	Solyc02g	Solyc02g	Peroxida	GO:00551	GO:00551	contig805	Solyc02g	Peroxida	GO:00040	SL2.40ch	AT1G05250.1		peroxida	chr1:152	255.085	255.261	700.407	249.055	754.616	533.58
GT	Sens	Sense	-0.301	Detected	0.301	Detected	0.000	2.399	Detected	1.330	Detected	1.865	0.093	GT	Sens	contig805	contig805	Gag-pol p	GAATTA	contig805	Solyc03g	Solyc03g	Gag-pol polyprotein (Fragmen			contig805	Solyc03g	Gag-pol polyprotein		SL2.40ch	AT1G76370.1				11.1882	16.7824	63.8551	67.0086	80.5158	34.8779
GT	Sens	Sense	0.349	Detected	-0.349	Detected	0.000	1.036	Detected	1.001	Detected	1.019	0.100	GT	Sens	contig806	contig806	Squamos	CCACTG	contig806	Solyc07g	Solyc07g	Squamos	GO:00050	GO:00050	contig806	Solyc07g	Squamos	GO:00037	SL2.40ch	AT5G50570.2		squamos	chr5:205	6245.53	3808.15	8088.5	6583.15	11134.4	9885.11
GT	Sens	Sense	0.381	Detected	-0.381	Detected	0.000	1.145	Detected	1.334	Detected	1.239	0.087	GT	Sens	contig808	contig808	Receptor	CAAGAG	contig808	Solyc07g	Solyc07g	Receptor	GO:00046	GO:00046	contig808	Solyc07g	Receptor	GO:00046	SL2.40ch	AT5G51350.1		leucine-r	chr5:208	775.423	452.197	1159.14	1107.93	1458.02	1510.91
GT	Sens	Sense	0.071	Comprom	-0.071	Comprom	0.000	1.321	Detected	1.072	Detected	1.197	0.014	GT	Sens	contig814	contig814	Unknown	CGTTAA	contig814	Solyc07g	Solyc07g	Unknown Protein (AHRD V1)			contig814	Solyc07g	Unknown Protein (A		SL2.40ch	AT1G137	AFP2	AFP2 (AE	chr1:471	7.15035	6.40593	8.04099	7.7533	18.8309	14.4088
GT	Sens	Sense	-0.567	Comprom	0.567	Detected	0.000	2.458	Detected	2.335	Detected	2.396	0.052	GT	Sens	contig815	contig815	Ring fing	CGTGAGA	contig815	Solyc04g	Solyc04g	Ring fing	GO:00082	GO:00082	contig815	Solyc04g	Protein p	GO:00055	SL2.40ch	AT2G18938.1				4.72313	10.2511	13.5931	9.55751	42.5723	35.548
GT	Sens	Sense	0.280	Detected	-0.280	Detected	0.000	0.867	Detected	1.189	Detected	1.028	0.086	GT	Sens	contig815	contig815	Phototro	GCCGTA	contig815	Solyc02g	Solyc02g	Phototro	GO:00094	GO:00094	contig815	Solyc02g	Phototro	GO:00055	SL2.40ch	AT5G67385.1		protein b	chr5:268	4222.35	2832.11	4008.63	5580.05	7024.93	7983.13
GT	Sens	Sense	0.292	Detected	-0.292	Detected	0.000	1.159	Detected	1.087	Detected	1.123	0.062	GT	Sens	contig817	contig817	Ras-relat	CTTCTG	contig817	Solyc01g	Solyc01g	Ras-relat	GO:00055	GO:00055	contig817	Solyc01g	Ras-relat	GO:00039	SL2.40ch	AT5G479	SMG1. AT	ATRABA	chr5:194	366.811	241.815	449.074	343.337	740.785	640.402
GT	Sens	Sense	-0.005	Comprom	0.005	Comprom	0.000	1.532	Detected	1.523	Detected	1.528	0.000	GT	Sens	contig821	contig821	Unknown	TAGGAA	contig821	Solyc01g	Solyc01g	Unknown Protein (AHRD V1)			contig821	Solyc01g	Unknown Protein (A		SL2.40ch	AT5G60390.3		elongatic	chr5:242	4.54041	4.52271	3.80681	10.7673	14.5925	13.1885
GT	Sens	Sense	0.045	Detected	-0.045	Detected	0.000	0.960	Detected	1.088	Detected	1.024	0.006	GT	Sens	contig835	contig835	Telomere	TTTCCA	contig835	Solyc11g	Solyc11g	Telomere	GO:00030	GO:00030	contig835	Solyc11g	Telomere	GO:00030	SL2.40ch	AT5G037	TRFL10	TRFL10	chr5:999	110.008	102.224	158.46	135.85	229.73	228.279
GT	Sens	Sense	-0.059	Detected	0.059	Detected	0.000	1.945	Detected	1.351	Detected	1.648	0.032	GT	Sens	contig835	contig835	Unknown	GAATAT	contig835	Solyc01g	Solyc01g	Unknown Protein (AHRD V1)			contig835	Solyc01g	Unknown Protein (A		SL2.40ch	AT3G09950.1		unknown	chr3:306	375.574	402.728	1038.19	1059.14	1667.18	1004.36
GT	Sens	Sense	0.684	Comprom	-0.684	Comprom	0.000	1.905	Detected	2.325	Detected	2.115	0.098	GT	Sens	contig850	contig850	Plant-spe	GTGGAG	contig850	Solyc10g	Solyc10g	Plant-specific domain TIGR01			contig850	Solyc10g	Plant-specific doma		SL2.40ch	AT5G02580.1		unknown	chr5:579	10.5654	4.04845	12.0712	19.1473	27.282	33.1838
GT	Sens	Sense	-0.284	Comprom	0.284	Comprom	0.000	2.252	Detected	2.837	Detected	2.544	0.025	GT	Sens	contig851	contig851	Unknown	CCAAAT	contig851	Solyc04g	Solyc04g	Unknown Protein (AHRD V1)			contig851	Solyc04g	Unknown Protein (A		SL2.40ch	AT2G433	ATGPX3	ATGPX3	chr2:180	3.45852	5.06894	20.5637	27.4244	22.2064	30.2794
GT	Sens	Sense	0.417	Detected	-0.417	Detected	0.000	0.867	Detected	1.232	Detected	1.050	0.148	GT	Sens	contig852	contig852	IQ-domai	GGCTTC	contig852	Solyc01g	Solyc01g	IQ-domai	GO:00055	GO:00055	contig852	Solyc01g	IQ-domai	GO:00055	SL2.40ch	AT5G620	IQD23	IQD23 (IC	chr5:249	4859.59	2693.84	5916.19	5019.64	7349.63	8606.2
GT	Sens	Sense	0.145	Detected	-0.145	Detected	0.000	1.359	Detected	1.122	Detected	1.241	0.022	GT	Sens	contig853	contig853	Zinc finge	GCATCT	contig853	Solyc03g	Solyc03g	Zinc finge	GO:00050	GO:00050	contig853	Solyc03g	Zinc finge	GO:00050	SL2.40ch	AT3G576	NTT	NTT (NO	chr3:213	102.138	82.5553	165.188	230.214	262.407	202.306
GT	Sens	Sense	0.182	Comprom	-0.182	Comprom	0.000	1.517	Detected	0.913	Detected	1.215	0.075	GT	Sens	contig866	contig866	B3 domai	ATGTGA	contig866	Solyc08g	Solyc08g	B3 domai	GO:00063	GO:00063	contig866	Solyc08g	B3 domai	GO:00063	SL2.40ch	AT4G31690.1		transcrip	chr4:153	8.17103	6.27625	12.4862	8.33453	22.8304	13.6508
GT	Sens	Sense	-0.327	Detected	0.327	Detected	0.000	2.499	Detected	2.059	Detected	2.279	0.029	GT	Sens	contig901	contig901	Hydrolas	TGTACA	contig901	Solyc08g	Solyc08g	Hydrolase alpha/beta fold fam			contig901	Solyc08g	Hydrolase alpha/be		SL2.40ch	AT4G24140.1		hydrolas	chr4:125	22.2237	34.5894	67.492	86.292	174.584	116.995
GT	Sens	Sense	0.185	Detected	-0.185	Detected	0.000	1.562	Detected	1.367	Detected	1.464	0.020	GT	Sens	contig908	contig908	Zinc ion b	AATATC	contig908	Solyc04g	Solyc04g	Zinc ion binding protein (AHRD			contig908	Solyc04g	Zinc ion binding pro		SL2.40ch	AT5G44005.1		unknown	chr5:177	343.39	262.588	381.332	362.784	987.263	784.218
GT	Sens	Sense	-0.348	Detected	0.348	Detected	0.000	0.997	Detected	1.345	Detected	1.171	0.095	GT	Sens	contig910	contig910	Unknown	ACTTTC	contig910	Solyc05g	Solyc05g	Unknown Protein (AHRD V1)			contig910	Solyc02g	Pectinace	GO:00040	SL2.40ch	AT1G26976.1				62.3345	99.8844	80.2902	95.8031	175.447	202.985
GT	Sens	Sense	-0.613	Comprom	0.613	Comprom	0.000	2.012	Detected	2.638	Detected	2.325	0.078	GT	Sens	contig911	contig911	EPIDERM	TAAGAA	contig911	Solyc02g	Solyc02g	EPIDERMAL PATTERNING FA(			contig911	Solyc02g	EPIDERMAL PATTE		SL2.40ch	AT5G47720.5		acetyl-Co	chr5:193	2.29733	5.31442	15.7665	2.21261	15.6944	22.0224
GT	Sens	Sense	0.323	Detected	-0.323	Detected	0.000	1.271	Detected	1.446	Detected	1.359	0.055	GT	Sens	contig914	contig914	Dirigent-l	TTCTGT	contig914	Solyc02g	Solyc02g	Dirigent-like protein 1 (AHRD V			contig914	Solyc02g	Dirigent-like protein		SL2.40ch	AT3G10810.1		zinc fing	chr3:338	1643.65	1039.03	3821.72	2156.03	3513.57	3604.73
GT	Sens	Sense	0.208	Detected	-0.208	Detected	0.000	1.368	Detected	1.157	Detected	1.263	0.032	GT	Sens	contig915	contig915	LINE-type	GGAGTT	contig915	Solyc11g	Solyc11g	LINE-type	GO:00030	GO:00030	contig915	Solyc03g046510.1.1				AT5G48510.1				37.2289	27.5823	79.0377	49.3473	92.1203	72.3458
GT	Sens	Sense	0.313	Comprom	-0.313	Comprom	0.000	2.978	Detected	3.116	Detected	3.047	0.011	GT	Sens	contig938	contig938	Unknown	GTAATG	contig938	Solyc07g	Solyc07g	Unknown Protein (AHRD V1)			contig938	Solyc07g	Unknown Protein (A		SL2.40ch	AT5G38600.1		proline-r	chr5:154	3.97617	2.54645	2.2089	4.09238	27.9302	27.9375
GT	Sens	Sense	-0.007	Detected	0.007	Detected	0.000	0.887	Detected	1.335	Detected	1.111	0.038	GT	Sens	contig951	contig951	Kelch rep	GCAGGG	contig951	Solyc03g	Solyc03g	Kelch repeat-containing F-box			contig951	Solyc03g	Kelch repeat-contai		SL2.40ch	AT1G15670.1		kelch rep	chr1:539	160.922	160.648	160.154	189.873	331.152	410.554
GT	Sens	Sense	-1.257	Comprom	1.257	Detected	0.000	1.523	Detected	1.612	Detected	1.567	0.339	GT	Sens	contig957	contig957	Unknown	TAAAAG	contig957	Solyc06g	Solyc06g	Unknown Protein (AHRD V1)			contig957	Solyc06g	Unknown Protein (A		SL2.40ch	AT2G174	ATMLO7	MLO7 (M	chr2:756	3.01043	17.0038	15.0288	35.0138	22.8927	22.1426
GT	Sens	Sense	-0.073	Detected	0.073	Detected	0.000	1.046	Detected	1.066	Detected	1.056	0.005	GT	Sens	contig975	contig975	B3 domai	GGAGGA	contig975	Solyc02g	Solyc02g	B3 domai	GO:00063	GO:00063	contig975	Solyc02g026080.1.1				AT3G44440.1				365.947	400.492	322.011	339.134	880.079	811.26
GT	Sens	Sense	0.381	Detected	-0.381	Detected	0.000	1.444	Detected	1.899	Detected	1.672	0.064	GT	Sens	contig978	contig978	Hydroxyc	GTGAGC	contig978	Solyc07g	Solyc07g	Hydroxyc	GO:00167	GO:00167	contig978	Solyc07g	Hydroxyc	GO:00167	SL2.40ch	AT1G03055.2		unknown	chr1:710	250.201	145.832	414.158	272.505	578.988	721.084
GT	Sens	Sense	-0.133	Comprom	0.133	Comprom	0.000	0.982	Detected	1.197	Detected	1.090	0.024	GT	Sens	contig982	contig982	Unknown	TTGACA	contig982	Solyc11g	Solyc11g	Unknown Protein (AHRD V1)			contig982	Solyc11g	Unknown Protein (A		SL2.40ch	AT3G467	PTAC16	PTAC16	chr3:172	4.96815	5.90783	6.13788	5.04685	11.9205	12.579
GT	Sens	Sense	0.397	Detected	-0.397	Detected	0.000	1.056	Detected	1.357	Detected	1.206	0.105	GT	Sens	contig987	contig987	Chromos	CTCACC	contig987	Solyc05g	Solyc05g	Chromosome 21 open readinc			contig987	Solyc05g	Chromosome 21 op		SL2.40ch	AT5G16420.1		pentatric	chr5:536	43.7792	24.9727	52.5528	48.4038	76.538	85.7311
GT	Sens	Sense	-0.040	Detected	0.040	Detected	0.000	0.903	Detected	1.110	Detected	1.007	0.012	GT	Sens	contig988	contig988	Unknown	TGCTGA	contig988	Solyc02g	Solyc02g	Unknown Protein (AHRD V1)			contig988	Solyc02g	Unknown Protein (A		SL2.40ch	AT5G37430.1		unknown	chr5:148	46.2796	48.3885	43.2723	50.1658	98.536	103.446
GT	Sens	Sense	0.370	Detected	-0.370	Detected	0.000	2.258	Detected	1.343	Detected	1.801	0.092	GT	Sens	contig991	contig991	Unknown	GTTGAA	contig991	Solyc01g	Solyc01g	Unknown Protein (AHRD V1)			contig991	Solyc01g	Unknown Protein (A		SL2.40ch	AT2G15020.1		unknown	chr2:649	40.2194	23.8221	81.0381	49.3976	164.95	79.4949
GT	Sens	Sense	0.019	Detected	-0.019	Detected	0.000	1.075	Detected	1.110	Detected	1.093	0.001	GT	Sens	contig999	contig999	Peptide n	TTGGAG	contig999	Solyc02g	Solyc02g	Peptide n	GO:00081	GO:00081	contig999	Solyc02g	Peptide n	GO:00081	SL2.40ch	AT4G35380.1		guanine	chr4:168	6428.86	6193.01	7027.78	7388.85	14808.3	13788.4
GT	Sens	Sense	-0.045	Detected	0.045	Detected	0.000	1.812	Detected	1.833	Detected	1.822	0.001	GT	Sens	HM00452	HM00452	ZmARF5	ACAATCATGGTCCATGTTGTGCGAAGTGTTGCCTGTGGTGGTTGTTGTGAAGAACTGAAT																709.512	746.932	878.453	897.309	2846.25	2626.33
GT	Sens	Sense	-0.081	Comprom	0.081	Comprom	0.000	0.982	Detected	1.200	Detected	1.091	0.015	GT	Sens	contig101	contig101	Protein F	AGATAA	contig101	Solyc02g	Solyc02g	Protein FAR1-RELATED SEOU			contig101	Solyc02g092170.1.1				AT5G189	FRS12	FRS12 (F	chr5:633	8.44597	9.34815	16.8131	14.1953	19.5473	20.6657
GT	Sens	Sense	-0.164	Comprom	0.164	Comprom	0.000	1.327	Detected	1.514	Detected	1.420	0.017	GT	Sens	contig103	contig103	Auxin-ind	GTGATT	contig103	Solyc11g	Solyc02g	Auxin-induced SAUR-like prote			contig103	Solyc11g	Auxin-induced SAU		SL2.40ch	AT1G80610.1		unknown	chr1:303	3.46299	4.29899	26.0123	25.2663	10.777	11.155
GT	Sens	Sense	0.407	Detected	-0.407	Detected	0.000	1.650	Detected	1.329	Detected	1.489	0.077	GT	Sens	contig104	contig104	Unknown	TACGAT	contig104	Solyc08g	Solyc08g	Unknown Protein (AHRD V1)			contig104	Solyc08g	Unknown Protein (A		SL2.40ch	AT5G14610.1		ATP binc	chr5:471	92.0866	51.7627	88.5143	80.5034	241.24	175.658
GT	Sens	Sense	-0.336	Detected	0.336	Detected	0.000	1.802	Detected	1.758	Detected	1.780	0.034	GT	Sens	contig104	contig104	MYB tran	ATGTCA	contig104	Solyc08g	Solyc08g	MYB tran	GO:0045449		contig104	Solyc04g	SMP-30/Gluconolac		SL2.40ch	AT5G44690.1		unknown	chr5:180	322.81	508.608	674.634	517.138	1573.27	1387.98
GT	Sens	Sense	0.358	Comprom	-0.358	Comprom	0.000	2.572	Detected	2.797	Detected	2.684	0.019	GT	Sens	contig106	contig106	Sulfotran	TTACAG	contig106	Solyc04g	Solyc04g	Sulfotran	GO:00081	GO:00081	contig106	Solyc04g	Sulfotran	GO:00081	SL2.40ch	AT1G59680.1				8.08574	4.86407	11.6536	12.2887	41.5299	44.1135
GT	Sens	Sense	0.346	Detected	-0.346	Comprom	0.000	1.621	Detected	1.419	Detected	1.520	0.052	GT	Sens	contig107	contig107	Glutared	AATGAG	contig107	Solyc04g	Solyc04g	Glutared	GO:00454	GO:00454	contig107	Solyc04g	Glutared	GO:00087	SL2.40ch	AT3G21460.1		electron	chr3:755	12.8199	7.84322	12.3109	28.9134	34.3617	27.1497
GT	Sens	Sense	0.287	Comprom	-0.287	Comprom	0.000	1.550	Detected	2.088	Detected	1.819	0.044	GT	Sens	contig108	contig108	Unknown	GAATCA	contig108	Solyc09g	Solyc09g	Unknown Protein (AHRD V1)			contig108	Solyc09g014920.1.1				ATCG011	YCF1.2	hypothet	chrC:123	10.9912	7.29671	27.0014	37.6676	29.2041	38.5442
GT	Sens	Sense	-0.161	Detected	0.161	Detected	0.000	1.466	Detected	1.382	Detected	1.424	0.013	GT	Sens	contig109	contig109	Nodulin-li	TTTTGT	contig109	Solyc01g	Solyc01g	Nodulin-li	GO:00160	GO:00160	contig109	Solyc01g	Nodulin-li	GO:00160	SL2.40ch	AT1G44800.1		nodulin	chr1:169	36.5691	45.1867	182.034	161.974	125.088	107.273
GT	Sens	Sense	0.477	Detected	-0.477	Detected	0.000	1.523	Detected	1.584	Detected	1.554	0.083	GT	Sens	contig111	contig111	Non-spec	GTGTTT	contig111	Solyc10g	Solyc10g	Non-spec	GO:00068	GO:00068	contig111	Solyc10g	Non-spec	GO:00068	SL2.40ch	AT3G52700.1		unknown	chr3:195	56.6436	28.9038	56.2866	55.1933	129.511	122.823
GT	Sens	Sense	-0.527	Detected	0.527	Detected	0.000	1.737	Detected	2.099	Detected	1.918	0.075	GT	Sens	contig113	contig113	Omega-6	ATTATA	contig113	Solyc12g	Solyc12g	Omega-6	GO:00423	GO:00423	contig113	Solyc12g	Omega-6	GO:00423	SL2.40ch	AT3G121	FAD2	FAD2 (FA	chr3:386	107.392	220.313	595.52	599.756	570.88	667.274
GT	Sens	Sense	0.694	Comprom	-0.694	Comprom	0.000	1.879	Detected	2.058	Detected	1.968	0.107	GT	Sens	contig114	contig114	Glutared	CCTTAA	contig114	Solyc04g	Solyc04g	Glutared	GO:00454	GO:00454	contig114	Solyc04g	Glutared	GO:00087	SL2.40ch	AT3G21460.1		electron	chr3:755	5.25239	1.98289	4.54701	15.2432	13.2165	13.6044
GT	Sens	Sense	-0.130	Detected	0.130	Detected	0.000	1.100	Detected	0.915	Detected	1.008	0.024	GT	Sens	contig119	contig119	Osmotin	GGTAGA	contig119	Solyc11g	Solyc11g	Osmotin 81 (Fragment) (AHRD			contig119	Solyc08g	Osmotin-l	GO:00055	SL2.40ch	AT4G26340.1		F-box far	chr4:133	135.491	160.35	132.024	96.1931	351.943	281.416
GT	Sens	Sense	0.183	Detected	-0.183	Detected	0.000	1.815	Detected	2.047	Detected	1.931	0.012	GT	Sens	contig120	contig120	Cytochro	GTGATT	contig120	Solyc04g	Solyc04g	Cytochro	GO:00198	GO:00198	contig120	Solyc04g	Cytochro	GO:00198	SL2.40ch	AT5G19640.1		proton-d	chr5:663	45.9233	35.2138	63.7243	52.8878	157.597	168.259
GT	Sens	Sense	-0.006	Detected	0.006	Detected	0.000	2.135	Detected	1.513	Detected	1.824	0.028	GT	Sens	contig125	contig125	F-box fan	TGGTGC	contig125	Solyc08g	Solyc08g	F-box family protein (AHRD V1			contig125	Solyc08g015890.1.1				AT3G47150.1		F-box far	chr3:173	53.4728	53.3009	578.514	817.952	261.125	154.296
GT	Sens	Sense	-0.439	Detected	0.439	Detected	0.000	3.025	Detected	2.672	Detected	2.848	0.027	GT	Sens	contig127	contig127	Major alle	TCTTAT	contig127	Solyc09g	Solyc09g	Major alle	GO:00090	GO:00090	contig127	Solyc09g	Major alle	GO:00090	SL2.40ch	AT5G434	ETFBETA	ETFBET	chr5:174	15.6819	28.4957	85.6927	97.3252	191.641	136.354
GT	Sens	Sense	-0.540	Comprom	0.540	Detected	0.000	1.267	Detected	1.237	Detected	1.252	0.147	GT	Sens	contig128	contig128	Tir-nbs-lr	TATTGG	contig128	Solyc00g	Solyc00g	Tir-nbs-lr	GO:00312	GO:00312	contig128	Solyc00g294230.1.1				AT2G271	AAO3	AAO3 (AI	chr2:116	23.4015	48.9335	40.9904	37.1916	90.6809	80.7281
GT	Sens	Sense	0.035	Detected	-0.035	Detected	0.000	2.366	Detected	2.520	Detected	2.443	0.001	GT	Sens	contig129	contig129	Unknown	GTGAAA	contig129	Solyc03g	Solyc03g	Unknown Protein (AHRD V1)			contig129	Solyc08g061470.1.1				AT3G45040.1		phospha	chr3:164	533.182	502.105	901.549	1030.2	2969.61	3005.6
GT	Sens	Sense	-0.130	Detected	0.130	Detected	0.000	1.784	Detected	0.826	Detected	1.305	0.119	GT	Sens	contig132	contig132	LRR rece	TGAATG	contig132	Solyc04g	Solyc04g	LRR rece	GO:00046	GO:00046	contig132	Solyc04g014200.1.1				AT4G208	FAH2. AT	FAH2 (FA	chr4:111	30.5924	36.1983	36.8698	94.1268	127.655	59.729
GT	Sens	Sense	0.100	Detected	-0.100	Detected	0.000	1.313	Detected	1.310	Detected	1.311	0.006	GT	Sens	contig136	contig136	Unknown	TGCAAA	contig136	Solyc11g	Solyc11g	Unknown Protein (AHRD V1)			contig136	Solyc11g	Unknown Protein (A		SL2.40ch	#N/A	#N/A	#N/A	#N/A	912.292	785.206	1787.68	1330.56	2342.26	2124.31
GT	Sens	Sense	-0.062	Detected	0.062	Detected	0.000	1.102	Detected	1.087	Detected	1.094	0.003	GT	Sens	contig138	contig138	Ulp1 prot	ACAGAA	contig138	Solyc11g	Solyc11g	Ulp1 protease family C-termina			contig138	Solyc11g	Ulp1 protease famil		SL2.40ch	AT4G02650.1		epsin N-t	chr4:115	23.2251	25.0182	46.7667	35.3489	57.6136	51.8454
GT	Sens	Sense	0.036	Detected	-0.036	Detected	0.000	1.382	Detected	1.153	Detected	1.267	0.009	GT	Sens	contig140	contig140	Methyl ja	GGCCCA	contig140	Solyc03g	Solyc03g	Methyl jasmonate esterase (AH			contig140	Solyc03g	Methyl ja	GO:00080	SL2.40ch	AT4G32350.1		unknown	chr4:156	37.6568	35.4359	49.2493	41.0349	106.032	82.2345
GT	Sens	Sense	0.086	Detected	-0.086	Detected	0.000	1.524	Detected	0.980	Detected	1.252	0.048	GT	Sens	contig140	contig140	Unknown	AATGAT	contig140	Solyc09g	Solyc09g	Unknown Protein (AHRD V1)			contig140	Solyc09g	Unknown Protein (A		SL2.40ch	AT2G25270.1		unknown	chr2:107	13.4433	11.7962	25.4122	33.4888	40.3299	25.1474
GT	Sens	Sense	0.191	Detected	-0.191	Detected	0.000	2.457	Detected	1.492	Detected	1.974	0.063	GT	Sens	contig141	contig141	Hydrolas	GCAAAA	contig141	Solyc01g	Solyc01g	Hydrolase alpha/beta fold fami			contig141	Solyc01g	Hydrolase alpha/be		SL2.40ch	AT2G356	MOR1. GI	MOR1 (M	chr2:149	33.7447	25.5967	60.3675	20.1329	179.734	83.6709
GT	Sens	Sense	0.224	Detected	-0.224	Detected	0.000	0.922	Detected	1.504	Detected	1.213	0.081	GT	Sens	contig141	contig141	Sulfotran	GAGGTG	contig141	Solyc05g	Solyc05g	Sulfotran	GO:00081	GO:00081	contig141	Solyc05g	Sulfotran	GO:00081	SL2.40ch	AT3G45070.1		sulfotran	chr3:164	2989.66	2166.03	4500.7	3543.17	5368.22	7307.62
GT	Sens	Sense	0.215	Detected	-0.215	Detected	0.000	1.205	Detected	1.080	Detected	1.142	0.036	GT	Sens	contig144	contig144	Naringen	GGTATT	contig144	Solyc04g	Solyc04g	Naringen	GO:00164	GO:00164	contig144	Solyc09g	1-aminoc	GO:00098	SL2.40ch	AT3G52140.1		tetratrico	chr3:193	112.223	82.38	186.644	176.477	246.855	205.732
GT	Sens	Sense	-0.895	Comprom	0.895	Comprom	0.000	1.798	Detected	1.956	Detected	1.877	0.172	GT	Sens	contig146	contig146	Cytochro	CAATAA	contig146	Solyc04g	Solyc04g	Cytochro	GO:00198	GO:00198	contig146	Solyc04g	Cytochro	GO:00198	SL2.40ch	AT3G148	MSL5	MSL5 (M	chr3:497	2.98259	10.1935	24.823	19.0526	21.356	21.6534
GT	Sens	Sense	-0.281	Detected	0.281	Detected	0.000	1.640	Detected	1.845	Detected	1.743	0.028	GT	Sens	contig149	contig149	MuDR far	GAAGTA	contig149	Solyc08g	Solyc08g	MuDR family transposase con			contig149	Solyc10g	Mutator-li	GO:00082	SL2.40ch	AT2G46360.1		unknown	chr2:190	11.5885	16.9046	36.1076	37.1017	48.5942	50.8999
GT	Sens	Sense	0.059	Comprom	-0.059	Comprom	0.000	1.853	Detected	1.326	Detected	1.589	0.028	GT	Sens	contig154	contig154	Mutator-li	GTTACA	contig154	Solyc11g	Solyc11g	Mutator-li	GO:00082	GO:00082	contig154	Solyc11g	Mutator-li	GO:00082	SL2.40ch	AT5G024	PLL2	PLL2: ca	chr5:513	5.31688	4.84519	13.3827	11.687	20.4177	12.8795
GT	Sens	Sense	0.193	Detected	-0.193	Detected	0.000	0.966	Detected	1.317	Detected	1.141	0.049	GT	Sens	contig154	contig154	Reverse t	AACTCA	contig154	Solyc05g	Solyc05g	Reverse transcriptase (Fragm			contig154	Solyc10g	Pol polyp	GO:00036	SL2.40ch	AT5G50330.2		LOCATE	chr5:204	38.377	29.0328	60.9038	63.9903	72.6004	84.1831
GT	Sens	Sense	0.065	Detected	-0.065	Detected	0.000	1.106	Detected	1.305	Detected	1.205	0.010	GT	Sens	contig156	contig156	Mutator-li	TAGGGC	contig156	Solyc10g	Solyc10g	Mutator-like transposase (AHF			contig156	Solyc10g	Mutator-like transpo		SL2.40ch	AT1G78950.1		beta-amy	chr1:296	45.3174	40.9356	100.062	116.907	103.257	107.71
GT	Sens	Sense	0.197	Detected	-0.197	Detected	0.000	1.163	Detected	1.560	Detected	1.361	0.040	GT	Sens	contig159	contig159	Unknown	TCTTAA	contig159	Solyc05g	Solyc05g	Unknown Protein (AHRD V1)			contig159	Solyc03g	Threonine endopep		SL2.40ch	AT5G10160.1		beta-hyd	chr5:318	89.1374	67.0648	99.9761	99.0509	192.797	230.78
GT	Sens	Sense	0.451	Comprom	-0.451	Comprom	0.000	2.193	Detected	2.290	Detected	2.242	0.039	GT	Sens	contig160	contig160	Glutared	CTTAAT	contig160	Solyc04g	Solyc04g	Glutared	GO:00454	GO:00454	contig160	Solyc04g	Glutared	GO:00087	SL2.40ch	AT1G03020.1		glutared	chr1:698	9.77127	5.17195	17.0592	28.274	36.206	35.2093
GT	Sens	Sense	-0.424	Detected	0.424	Detected	0.000	1.189	Detected	1.267	Detected	1.228	0.102	GT	Sens	contig160	contig160	Unknown	AAAGCA	contig160	Solyc08g	Solyc08g	Unknown Protein (AHRD V1)			contig160	Solyc11g	Unknown Protein (A		SL2.40ch	AT5G02350.1				19.1298	34.0259	24.9712	30.9597	64.8009	62.1615
GT	Sens	Sense	-0.014	Detected	0.014	Detected	0.000	1.482	Detected	1.599	Detected	1.541	0.002	GT	Sens	contig164	contig164	Transcrip	TCAGAT	contig164	Solyc06g	Solyc06g	Transcription factor homolog			contig164	Solyc07g	Nascent p	GO:00510	SL2.40ch	AT5G25500.1		unknown	chr5:888	250.093	252.054	266.57	294.967	781.203	769.967
GT	Sens	Sense	0.116	Comprom	-0.116	Comprom	0.000	0.923	Detected	1.164	Detected	1.043	0.025	GT	Sens	contig165	contig165	Unknown	GAGACC	contig165	Solyc05g	Solyc05g	Unknown Protein (AHRD V1)			contig165	Solyc05g026100.1.1				AT4G17210.1		myosin h	chr4:965	10.4952	8.83083	11.9358	39.6355	20.3225	21.8333
GT	Sens	Sense	0.196	Comprom	-0.196	Comprom	0.000	1.132	Detected	1.088	Detected	1.110	0.030	GT	Sens	contig171	contig171	B3 domai	AACTTG	contig171	Solyc00g	Solyc00g	B3 domai	GO:00063	GO:00063	contig171	Solyc00g	B3 domai	GO:00063	SL2.40ch	AT1G20350.1				7.81701	5.88905	10.2137	19.2255	16.5599	14.5998
GT	Sens	Sense	0.199	Comprom	-0.199	Comprom	0.000	2.477	Detected	3.047	Detected	2.762	0.015	GT	Sens	contig173	contig173	Glutared	TTTACG	contig173	Solyc04g	Solyc04g	Glutared	GO:00454	GO:00454	contig173	Solyc04g	Glutared	GO:00087	SL2.40ch	AT2G017	ATMAP70	ATMAP7	chr2:328	3.1534	2.36692	9.00349	11.6045	16.9392	22.8571
GT	Sens	Sense	0.047	Detected	-0.047	Detected	0.000	1.118	Detected	1.384	Detected	1.251	0.012	GT	Sens	contig176	contig176	S-phase k	AGACAT	contig176	Solyc10g	Solyc10g	S-phase k	GO:00065	GO:00065	contig176	Solyc10g	S-phase k	GO:00055	SL2.40ch	AT1G759	SKP1. AS	SKP1 (S	chr1:285	133.614	123.695	151.287	160.884	310.825	339.784
GT	Sens	Sense	-0.117	Comprom	0.117	Comprom	0.000	3.654	Detected	3.172	Detected	3.413	0.006	GT	Sens	contig176	contig176	Unknown	TTTGAT	contig176	Solyc08g	Solyc08g	Unknown Protein (AHRD V1)			contig176	Solyc08g015910.1.1				AT3G47150.1		F-box far	chr3:173	3.58819	4.17411	114.254	131.911	54.2515	35.3233
GT	Sens	Sense	0.262	Detected	-0.262	Detected	0.000	1.094	Detected	0.956	Detected	1.025	0.063	GT	Sens	contig181	contig181	Aldehyde	TGTGGC	contig181	Solyc11g	Solyc11g	Aldehyde	GO:00500	GO:00500	contig181	Solyc11g	Aldehyde	GO:00090	SL2.40ch	AT1G045	AAO4. AT	AAO4 (AI	chr1:125	133.111	91.4597	133.491	163.659	262.299	216.759
GT	Sens	Sense	-0.305	Detected	0.305	Detected	0.000	1.606	Detected	1.809	Detected	1.707	0.034	GT	Sens	contig184	contig184	MuDR far	TTTGAC	contig184	Solyc08g	Solyc08g	MuDR family transposase con			contig184	Solyc08g	MuDR family transp		SL2.40ch	AT5G132	SOL2. CR	CRN (CO	chr5:425	22.7677	34.3638	63.388	78.8484	94.8285	99.2074
GT	Sens	Sense	0.210	Detected	-0.210	Detected	0.000	2.884	Detected	2.681	Detected	2.783	0.007	GT	Sens	contig187	contig187	Unknown	TAAAAA	contig187	Solyc03g	Solyc03g	Unknown Protein (AHRD V1)			contig187	Solyc03g	Unknown Protein (A		SL2.40ch	ATMG005	MATR	hypothet	chrM:14	80.336	59.3786	171.539	102.489	567.769	448.498
GT	Sens	Sense	0.353	Detected	-0.353	Detected	0.000	2.243	Detected	1.564	Detected	1.904	0.060	GT	Sens	contig189	contig189	Unknown	TGAATC	contig189	Solyc01g	Solyc01g	Unknown Protein (AHRD V1)%2			contig189	Solyc01g	Unknown Protein (A		SL2.40ch	AT4G099	EMB1691	EMB169	chr4:624	18.2639	11.0676	29.3746	32.2855	74.9795	42.5495
GT	Sens	Sense	0.230	Detected	-0.230	Detected	0.000	1.329	Detected	1.563	Detected	1.446	0.030	GT	Sens	contig19	contig19	Unknown	ATAGGA	contig19	Solyc07g	Solyc07g	Unknown	GO:0045449		contig19	Solyc10g045250.1.1				AT3G02900.1		unknown	chr3:645	42.3159	30.418	32.0326	35.4125	100.377	107.327
GT	Sens	Sense	0.014	Detected	-0.014	Detected	0.000	0.897	Detected	1.124	Detected	1.011	0.012	GT	Sens	contig192	contig192	Unknown	GGTGGT	contig192	Solyc10g	Solyc10g	Unknown Protein (AHRD V1)			contig192	Solyc10g	Unknown Protein (A		SL2.40ch	AT4G030	BSL1	kelch rep	chr4:135	805.583	781.503	797.557	733.366	1646.01	1750.65
GT	Sens	Sense	0.425	Detected	-0.425	Detected	0.000	2.732	Detected	2.217	Detected	2.475	0.038	GT	Sens	contig195	contig195	FAD-bind	GAGCTT	contig195	Solyc10g	Solyc10g	FAD-bind	GO:00500	GO:00500	contig195	Solyc02g	FAD-bind	GO:00090	SL2.40ch	AT5G44410.1		FAD-bind	chr5:178	214.999	117.988	510.165	125.775	1178.85	749.721
GT	Sens	Sense	0.211	Detected	-0.211	Detected	0.000	0.941	Detected	1.379	Detected	1.160	0.062	GT	Sens	contig195	contig195	Unknown	TAAATT	contig195	Solyc12g	Solyc12g	Unknown	GO:00551	GO:00551	contig195	Solyc12g	Unknown	GO:00160	SL2.40ch	AT2G21420.1		zinc fing	chr2:916	360.082	265.769	507.982	522.567	661.609	814.583
GT	Sens	Sense	0.512	Detected	-0.512	Detected	0.000	0.869	Detected	1.399	Detected	1.134	0.188	GT	Sens	contig198	contig198	Lipase (F	TGTGAA	contig198	Solyc12g	Solyc12g	Lipase (F	GO:00066	GO:00066	contig198	Solyc12g	Lipase (F	GO:00066	SL2.40ch	AT2G31100.1		triacylgly	chr2:132	1857.43	903.223	1177.89	838.794	2634.91	3457.22
GT	Sens	Sense	0.388	Comprom	-0.388	Comprom	0.000	1.582	Detected	1.402	Detected	1.492	0.064	GT	Sens	contig198	contig198	Unknown	ATCAAG	contig198	Solyc03g	Solyc03g	Unknown Protein (AHRD V1)			contig198	Solyc03g	Unknown Protein (A		SL2.40ch	AT3G05470.1		formin h	chr3:157	13.5334	7.81211	23.3827	25.0029	34.2806	27.5143
GT	Sens	Sense	-0.577	Comprom	0.577	Comprom	0.000	3.660	Detected	2.130	Detected	2.895	0.094	GT	Sens	contig200	contig200	Leucine F	AAGACT	contig200	Solyc12g	Solyc12g	Leucine F	GO:00055	GO:00055	contig200	Solyc12g	LRR rece	GO:00046	SL2.40ch	AT5G41320.1		unknown	chr5:165	2.54319	5.59481	9.49302	14.7009	53.1086	16.7198
GT	Sens	Sense	-0.924	Comprom	0.924	Detected	0.000	1.484	Detected	1.389	Detected	1.437	0.261	GT	Sens	contig201	contig201	Unknown	ATTCAA	contig201	Solyc09g	Solyc09g	Unknown Protein (AHRD V1)			contig201	Solyc10g	RNA-bind	GO:00030	SL2.40ch	AT4G03930.1		enzyme	chr4:187	6.49974	23.1347	6.36725	2.97048	38.1981	32.5201
GT	Sens	Sense	0.209	Detected	-0.209	Detected	0.000	1.111	Detected	1.132	Detected	1.122	0.033	GT	Sens	contig203	contig203	Unknown	ATCGGA	contig203	Solyc06g	Solyc06g	Unknown Protein (AHRD V1)			contig203	Solyc06g	Unknown Protein (A		SL2.40ch	AT5G46520.1				14.2946	10.5811	10.2284	21.3717	29.584	27.2944
GT	Sens	Sense	0.102	Detected	-0.102	Detected	0.000	1.599	Detected	1.156	Detected	1.378	0.030	GT	Sens	contig205	contig205	Ovate pr	CATTAT	contig205	Solyc02g	Solyc02g	Ovate protein (AHRD V1 **-- O8			contig205	Solyc02g	Ovate protein (AHRI		SL2.40ch	AT5G16290.2		acetolac	chr5:533	1223.7	1050.93	1915.19	1975.17	3825.88	2558.61
GT	Sens	Sense	0.772	Detected	-0.772	Detected	0.000	1.738	Detected	1.801	Detected	1.769	0.149	GT	Sens	contig206	contig206	Glutared	GTTGAT	contig206	Solyc04g	Solyc04g	Glutared	GO:00454	GO:00454	contig206	Solyc04g	Glutared	GO:00454	SL2.40ch	AT2G32415.1		3'-5' exo	chr2:137	69.1426	23.4371	57.7139	49.9202	149.502	142.037

GT Sense	Sense	0.081	Detected	-0.081	Detected	0.000	2.894	Detected	1.743	Detected	2.319	0.058	GT Sens	contig209	contig209	1-aminoc	GACTAC	contig209	Solyc09g	Solyc09g	1-aminocyclopropane-1-carbo			contig209	Solyc09g	1-aminocyclopropa		SL2.40ch	AT3G49030.1		F-box far	chr3:181	34.0391	30.0735	183.571	123.801	264.935	108.41
GT Sense	Sense	1.048	Comprom	-1.048	Comprom	0.000	2.828	Detected	2.722	Detected	2.775	0.118	GT Sens	contig209	contig209	Glutared	ATTGAT	contig209	Solyc04g	Solyc04g	Glutared	GO:00454	GO:00454	contig209	Solyc04g	Glutared	GO:00087	SL2.40ch	AT2G017	ATMAP7	ATMAP7	chr2:328	8.30577	1.91978	12.2235	10.0674	31.5813	26.6707
GT Sense	Sense	-0.122	Detected	0.122	Detected	0.000	1.501	Detected	1.274	Detected	1.388	0.014	GT Sens	contig210	contig210	1-aminoc	CCCAAT	contig210	Solyc04g	Solyc04g	1-aminoc	GO:00164	GO:00164	contig210	Solyc04g	1-aminoc	GO:00098	SL2.40ch	AT2G30840.1		2-oxoglu	chr2:131	539.238	631.194	1091.4	1329.04	1838.52	1428.9
GT Sense	Sense	-0.019	Comprom	0.019	Comprom	0.000	1.082	Detected	1.210	Detected	1.146	0.003	GT Sens	contig211	contig211	Ulp1 prot	GAAAAA	contig211	Solyc06g	Solyc06g	Ulp1 protease family C-termina			contig211	Solyc06g065080.1.1				AT2G40070.2		FUNCTIO	chr2:167	5.6578	5.74154	5.99729	14.1296	13.4331	13.349
GT Sense	Sense	0.127	Detected	-0.127	Detected	0.000	1.231	Detected	0.956	Detected	1.093	0.028	GT Sens	contig213	contig213	Ulp1 prot	CATCAA	contig213	Solyc11g	Solyc11g	Ulp1 prot	GO:00065	GO:00065	contig213	Solyc12g	DUF599 family prote		SL2.40ch	AT5G60430.2		antiporte	chr5:243	28.293	23.4408	90.5009	64.0026	67.3251	50.5652
GT Sense	Sense	-0.046	Detected	0.046	Detected	0.000	1.482	Detected	1.639	Detected	1.561	0.003	GT Sens	contig213	contig213	Cullin 1-li	GATAAC	contig213	Solyc01g	Solyc01g	Cullin 1-li	GO:00303	GO:00303	contig213	Solyc01g	Cullin 1-li	GO:00303	SL2.40ch	AT4G025	ATCUL1.	ATCUL1	chr4:112	40.2935	42.4642	85.6762	50.8549	128.685	130.435
GT Sense	Sense	-0.142	Comprom	0.142	Comprom	0.000	1.146	Detected	1.945	Detected	1.545	0.068	GT Sens	contig213	contig213	Set prote	ACAACA	contig213	Solyc12g	Solyc12g	Set prote	GO:00056	GO:00056	contig213	Solyc12g	Set prote	GO:00056	SL2.40ch	AT5G37720.2		RNA and	chr5:149	4.60393	5.5435	2.40112	2.27162	12.4511	19.691
GT Sense	Sense	0.449	Comprom	-0.449	Comprom	0.000	1.847	Detected	2.097	Detected	1.972	0.052	GT Sens	contig214	contig214	Glutared	CCTTAA	contig214	Solyc04g	Solyc04g	Glutared	GO:00454	GO:00454	contig214	Solyc04g	Glutared	GO:00087	SL2.40ch	AT4G229	AGL19. G	AGL19 (A	chr4:120	8.30901	4.40785	12.4551	23.2877	24.2473	26.2117
GT Sense	Sense	-0.129	Comprom	0.129	Comprom	0.000	1.273	Detected	1.615	Detected	1.444	0.021	GT Sens	contig216	contig216	Unknown	TTCACT	contig216	Solyc05g	Solyc05g	Unknown Protein (AHRD V1)			contig216	Solyc05g	Unknown Protein (A		SL2.40ch	AT2G329	ATZW10	ATZW10	chr2:139	3.9391	4.65846	6.2711	10.3099	11.5268	13.2893
GT Sense	Sense	0.161	Detected	-0.161	Detected	0.000	1.035	Detected	1.457	Detected	1.246	0.043	GT Sens	contig219	contig219	UDP-gluc	GGATTC	contig219	Solyc04g	Solyc04g	UDP-gluc	GO:00081	GO:00081	contig219	Solyc04g	UDP-gluc	GO:00081	SL2.40ch	AT5G228	EMB1030	EMB1030	chr5:761	465.626	368.126	905.849	957.998	944.69	1150.73
GT Sense	Sense	0.137	Comprom	-0.137	Comprom	0.000	2.327	Detected	2.203	Detected	2.265	0.004	GT Sens	contig219	contig219	Nitrate tra	GGAGTA	contig219	Solyc06g	Solyc06g	Nitrate tra	GO:00800	GO:00800	contig219	Solyc06g	Nitrate tra	GO:00800	SL2.40ch	AT3G45680.1		proton-d	chr3:167	7.73783	6.32869	11.481	11.8467	39.1141	32.6119
GT Sense	Sense	0.133	Comprom	-0.133	Comprom	0.000	1.329	Detected	1.050	Detected	1.189	0.025	GT Sens	contig221	contig221	Unknown	GAAAAT	contig221	Solyc07g	Solyc07g	Unknown Protein (AHRD V1)			contig221	Solyc07g041670.1.1				AT1G69550.1				9.52522	7.83209	17.574	18.574	24.1726	18.1002
GT Sense	Sense	0.214	Detected	-0.214	Detected	0.000	0.817	Detected	1.292	Detected	1.054	0.081	GT Sens	contig221	contig221	Respons	CTGCTT	contig221	Solyc10g	Solyc10g	Respons	GO:00063	GO:00063	contig221	Solyc10g	Respons	GO:00063	SL2.40ch	AT3G570	ARR9. AT	ARR9 (RE	chr3:211	179.979	132.189	250.398	300.271	302.584	382.266
GT Sense	Sense	0.241	Detected	-0.241	Detected	0.000	1.515	Detected	1.490	Detected	1.502	0.025	GT Sens	contig223	contig223	Transcri	AGGAGA	contig223	Solyc03g	Solyc03g	Transcri	GO:0045449		contig223	Solyc11g	Cellular n	GO:00082	SL2.40ch	AT4G37250.1		leucine-r	chr4:175	93.879	66.4059	115.846	97.2266	251.326	224.467
GT Sense	Sense	0.355	Detected	-0.355	Detected	0.000	1.229	Detected	1.705	Detected	1.467	0.075	GT Sens	contig224	contig224	Unknown	AGAAGA	contig224	Solyc12g	Solyc12g	Unknown Protein (AHRD V1)			contig224	Solyc12g	Unknown Protein (A		SL2.40ch	AT5G509	CLPC. AT	CLPC1:	chr5:207	575.565	347.935	1287.69	1450.46	1167.9	1477.27
GT Sense	Sense	-0.174	Comprom	0.174	Comprom	0.000	1.624	Detected	2.110	Detected	1.867	0.025	GT Sens	contig225	contig225	Serine/thr	AATATT	contig225	Solyc09g	Solyc09g	Serine/threonine-protein phos			contig225	Solyc09g	Serine/threonine-pr		SL2.40ch	AT2G273	ATEPR1	ATEPR1:	chr2:117	2.70599	3.40422	10.8237	7.08074	10.4214	13.2681
GT Sense	Sense	0.193	Detected	-0.193	Detected	0.000	0.944	Detected	1.223	Detected	1.083	0.045	GT Sens	contig225	contig225	Sulfotran	TGGAGG	contig225	Solyc05g	Solyc05g	Sulfotran	GO:00081	GO:00081	contig225	Solyc05g	Sulfotran	GO:00801	SL2.40ch	AT5G49260.1		unknowr	chr5:199	39.2289	29.6586	15.9657	29.0126	73.1087	80.5866
GT Sense	Sense	-0.286	Detected	0.286	Detected	0.000	1.464	Detected	1.279	Detected	1.371	0.045	GT Sens	contig227	contig227	Transcri	GTTGCT	contig227	Solyc06g	Solyc06g	Transcri	GO:00056	GO:00056	contig227	Solyc06g	Transcri	GO:00056	SL2.40ch	AT4G118	AGL14	AGL14 (a	chr4:714	70.9851	104.28	137.019	128.729	264.308	211.401
GT Sense	Sense	0.178	Detected	-0.178	Detected	0.000	1.677	Detected	1.158	Detected	1.418	0.046	GT Sens	contig228	contig228	Defensin-	CCAAGC	contig228	Solyc07g	Solyc07g	Defensin-	GO:00069	GO:00069	contig228	Solyc07g	Defensin-	GO:00069	SL2.40ch	AT5G616	ATATH15	ATATH15	chr5:247	176.926	136.598	94.6953	237.616	553.734	351.332
GT Sense	Sense	-0.172	Detected	0.172	Detected	0.000	1.910	Detected	1.418	Detected	1.664	0.031	GT Sens	contig228	contig228	Unknown	GAGATC	contig228	Solyc09g	Solyc09g	Unknown Protein (AHRD V1)			contig228	Solyc07g	Unknown Protein (A		SL2.40ch	AT3G19520.2				36.107	45.2823	49.4117	37.3116	169.297	109.371
GT Sense	Sense	0.194	Detected	-0.194	Detected	0.000	2.343	Detected	1.498	Detected	1.921	0.054	GT Sens	contig230	contig230	1-aminoc	TCAGTT	contig230	Solyc09g	Solyc09g	1-aminoc	GO:00164	GO:00164	contig230	Solyc09g	1-aminoc	GO:00164	SL2.40ch	AT3G61400.1		2-oxoglu	chr3:227	71.7731	54.2361	389.706	332.22	352.545	178.464
GT Sense	Sense	0.178	Detected	-0.178	Detected	0.000	1.304	Detected	1.331	Detected	1.317	0.018	GT Sens	contig231	contig231	DNA-3-me	ATATGT	contig231	Solyc10g	Solyc10g	DNA-3-me	GO:00062	GO:00062	contig231	Solyc10g	DNA-3-me	GO:00062	SL2.40ch	AT1G43650.1		integral r	chr1:164	1025.47	792.507	836.376	1355.59	2478.83	2296.16
GT Sense	Sense	0.157	Detected	-0.157	Detected	0.000	1.411	Detected	1.773	Detected	1.592	0.022	GT Sens	contig231	contig231	Unknown	CCAAAG	contig231	Solyc11g	Solyc11g	Unknown Protein (AHRD V1)			contig231	Solyc07g	Hypersen	GO:00338	SL2.40ch	AT5G202	DIN10	DIN10 (D	chr5:683	568.556	452.24	459.115	366.867	1501.78	1754.9
GT Sense	Sense	-0.222	Detected	0.222	Detected	0.000	1.416	Detected	0.808	Detected	1.112	0.098	GT Sens	contig232	contig232	Cytochro	CTTTGT	contig232	Solyc07g	Solyc07g	Cytochro	GO:00198	GO:00198	contig232	Solyc07g	Cytochro	GO:00198	SL2.40ch	AT4G395	CYP96A1	CYP96A1	chr4:183	56.1051	75.457	81.8068	103.939	193.372	115.314
GT Sense	Sense	0.066	Detected	-0.066	Detected	0.000	1.248	Detected	1.348	Detected	1.298	0.004	GT Sens	contig233	contig233	Endonuc	CTTAGA	contig233	Solyc07g	Solyc07g	Endonuclease/exonuclease/pl			contig233	Solyc07g	Endonuclease/exon		SL2.40ch	AT5G618	GUT1	GUT1: ca	chr5:248	1760.4	1587.41	2462.64	2595.91	4423.4	4307.36
GT Sense	Sense	0.362	Detected	-0.362	Detected	0.000	1.322	Detected	1.047	Detected	1.184	0.092	GT Sens	contig234	contig234	N-hydrox	TTTGGG	contig234	Solyc01g	Solyc01g	N-hydrox	GO:00167	GO:00167	contig234	Solyc01g	N-hydrox	GO:00167	SL2.40ch	AT3G141	FLU	FLU (FLU	chr3:467	415.159	248.602	424.861	387.867	894.422	671.871
GT Sense	Sense	-0.288	Comprom	0.288	Comprom	0.000	2.253	Detected	2.403	Detected	2.328	0.016	GT Sens	contig235	contig235	Leucine-r	TGGCTT	contig235	Solyc12g	Solyc12g	Leucine-r	GO:00055	GO:00055	contig235	Solyc12g	LRR recei	GO:00046	SL2.40ch	AT1G055	SYTE. AT	NTMC2T	chr1:162	5.40123	7.96163	8.19156	14.9144	34.8129	35.1128
GT Sense	Sense	0.297	Detected	-0.297	Detected	0.000	1.718	Detected	2.017	Detected	1.867	0.030	GT Sens	contig236	contig236	Unknown	AGAAGA	contig236	Solyc01g	Solyc01g	Unknown Protein (AHRD V1)			contig236	Solyc03g	Iron-sulfu	GO:00081	SL2.40ch	AT1G01240.3				180.357	118.099	158.791	154.427	534.557	597.852
GT Sense	Sense	0.149	Detected	-0.149	Detected	0.000	1.103	Detected	1.054	Detected	1.079	0.019	GT Sens	contig238	contig238	Unknown	GCTGAT	contig238	Solyc07g	Solyc07g	Unknown Protein (AHRD V1)			contig238	Solyc07g032130.1.1				AT1G33560.1				20.7029	16.6453	9.04656	14.7186	44.4219	39.0318
GT Sense	Sense	0.287	Detected	-0.287	Detected	0.000	1.380	Detected	1.059	Detected	1.219	0.066	GT Sens	contig239	contig239	Chromod	GAAGTG	contig239	Solyc07g	Solyc07g	Chromod	GO:00082	GO:00082	contig239	Solyc09g	UDP-gluc	GO:00800	SL2.40ch	AT3G52700.1		unknowr	chr3:195	195.244	129.702	86.9961	67.6952	461.399	335.578
GT Sense	Sense	0.617	Comprom	-0.617	Comprom	0.000	1.132	Detected	1.436	Detected	1.284	0.181	GT Sens	contig239	contig239	Pol polyp	TATCAC	contig239	Solyc09g	Solyc09g	Pol polyprotein (AHRD V1 ***- (			contig239	Solyc09g	Pol polyprotein (AH		SL2.40ch	AT5G48470.1		unknowr	chr5:196	8.89405	3.73832	10.9636	17.7812	14.0755	15.7953
GT Sense	Sense	0.190	Detected	-0.190	Detected	0.000	1.067	Detected	1.071	Detected	1.069	0.030	GT Sens	contig240	contig240	Proteinas	TATTCA	contig240	Solyc03g	Solyc03g	Proteinas	GO:00048	GO:00048	contig240	Solyc03g	Proteinas	GO:00048	SL2.40ch	AT5G65920.1		U-box do	chr5:263	6031.52	4581.29	7048.32	5924.83	12262.8	11178.9
GT Sense	Sense	0.057	Detected	-0.057	Detected	0.000	2.308	Detected	2.079	Detected	2.194	0.003	GT Sens	contig246	contig246	Unknown	GACTTG	contig246	Solyc05g	Solyc05g	Unknown Protein (AHRD V1)			contig246	Solyc05g024230.1.1				AT4G344	CHB3. AT	CHB3: DI	chr4:164	331.601	303.019	256.539	275.934	1748.52	1355.97
GT Sense	Sense	0.380	Detected	-0.380	Detected	0.000	1.447	Detected	0.933	Detected	1.190	0.122	GT Sens	contig248	contig248	Gibberell	TCAATG	contig248	Solyc11g	Solyc11g	Gibberell	GO:00055	GO:00055	contig248	Solyc11g	Gibberell	GO:00055	SL2.40ch	AT3G028	GASA5	GASA5 ((	chr3:638	751.297	438.782	972.017	707.283	1743.43	1109.74
GT Sense	Sense	0.334	Detected	-0.334	Comprom	0.000	1.405	Detected	1.850	Detected	1.628	0.056	GT Sens	contig250	contig250	Unknown	GCTCTT	contig250	Solyc04g	Solyc04g	Unknown Protein (AHRD V1)			contig250	Solyc04g	Unknown Protein (A		SL2.40ch	AT3G03970.2		binding	chr3:102	14.0567	8.74892	12.3143	22.3869	32.703	40.4805
GT Sense	Sense	0.440	Detected	-0.440	Detected	0.000	1.134	Detected	1.898	Detected	1.516	0.121	GT Sens	contig251	contig251	Defensin	TGGCTA	contig251	Solyc07g	Solyc07g	Defensin	GO:00304	GO:00304	contig251	Solyc07g	Defensin	GO:00304	SL2.40ch	AT2G021	PDF2.1. L	PDF2.1:	chr2:538	1052.48	565.534	1159.2	2486.31	1885.32	2911.09
GT Sense	Sense	-0.043	Comprom	0.043	Comprom	0.000	1.199	Detected	1.524	Detected	1.362	0.015	GT Sens	contig251	contig251	MuDR fan	CGATAC	contig251	Solyc12g	Solyc12g	MuDR family transposase con			contig251	Solyc10g	Mutator-li	GO:00082	SL2.40ch	AT5G47250.1		disease	chr5:191	10.367	10.8822	20.6089	29.9337	27.1649	30.9367
GT Sense	Sense	-0.402	Comprom	0.402	Comprom	0.000	2.462	Detected	2.301	Detected	2.381	0.028	GT Sens	contig252	contig252	Cytochro	TGCCAT	contig252	Solyc07g	Solyc07g	Cytochro	GO:00200	GO:00200	contig252	Solyc07g	Cytochro	GO:00198	SL2.40ch	AT3G146	CYP72A7	CYP72A7	chr3:491	2.46916	4.26256	5.68072	2.31962	19.9066	16.1827
GT Sense	Sense	-0.033	Detected	0.033	Detected	0.000	0.861	Detected	1.221	Detected	1.041	0.030	GT Sens	contig252	contig252	Yippee-lil	CAGAAA	contig252	Solyc03g	Solyc03g	Yippee-lil	GO:00055	GO:00055	contig252	Solyc03g	Yippee-lik	GO:00055	SL2.40ch	AT2G40110.1		yippee fa	chr2:167	145.263	150.412	157.282	168.206	299.036	348.971
GT Sense	Sense	-0.320	Comprom	0.320	Comprom	0.000	1.117	Detected	1.299	Detected	1.208	0.068	GT Sens	contig253	contig253	Amino ac	TGGTAT	contig253	Solyc05g	Solyc05g	Amino acid transporter (AHRD			contig253	Solyc05g	Amino ac	GO:00151	SL2.40ch	AT5G14470.1				9.89088	15.2377	15.0393	8.51358	29.6631	30.5788
GT Sense	Sense	0.246	Detected	-0.246	Detected	0.000	2.086	Detected	2.105	Detected	2.095	0.014	GT Sens	contig253	contig253	Monooxy	CACAGG	contig253	Solyc06g	Solyc06g	Monooxy	GO:00044	GO:00044	contig253	Solyc03g	Proteinas	GO:00048	SL2.40ch	AT4G39610.1		unknowr	chr4:183	42.9359	30.168	46.222	37.8223	170.199	156.723
GT Sense	Sense	0.041	Detected	-0.041	Detected	0.000	1.371	Detected	1.234	Detected	1.303	0.004	GT Sens	contig261	contig261	Ribosoma	TTGAAG	contig261	Solyc11g	Solyc11g	Ribosoma	GO:00058	GO:00058	contig261	Solyc10g	30S ribos	GO:00058	SL2.40ch	AT5G02960.1		40S ribos	chr5:693	92.699	86.6252	104.204	106.067	258.216	213.378
GT Sense	Sense	-0.131	Comprom	0.131	Comprom	0.000	1.418	Detected	1.445	Detected	1.432	0.008	GT Sens	contig262	contig262	Mutator-li	GAAGAG	contig262	Solyc02g	Solyc02g	Mutator-li	GO:00082	GO:00082	contig262	Solyc08g	Mutator-li	GO:00082	SL2.40ch	AT4G29420.1		F-box far	chr4:144	5.71737	6.7738	11.2876	14.2191	18.5207	17.1545
GT Sense	Sense	0.757	Detected	-0.757	Comprom	0.000	1.014	Detected	1.400	Detected	1.207	0.262	GT Sens	contig264	contig264	Serine/thr	ATGACT	contig264	Solyc08g	Solyc08g	Serine/threonine-protein phos			contig264	Solyc10g	Serine/threonine-pr		SL2.40ch	AT2G329	ZFN2	ZFN2 (ZIN	chr2:139	11.7728	4.07696	16.9381	15.7729	15.5871	18.5063
GT Sense	Sense	0.686	Comprom	-0.686	Comprom	0.000	2.511	Detected	2.178	Detected	2.344	0.080	GT Sens	contig271	contig271	Unknown	GTTAAC	contig271	Solyc08g	Solyc08g	Unknown Protein (AHRD V1)			contig271	Solyc08g	F-box fan	GO:00048	SL2.40ch	AT1G72660.3		developr	chr1:273	6.96027	2.65923	83.1674	112.075	27.3125	19.7041
GT Sense	Sense	0.303	Detected	-0.303	Detected	0.000	1.168	Detected	1.250	Detected	1.209	0.059	GT Sens	contig272	contig272	Mutator-li	ATTATG	contig272	Solyc06g	Solyc06g	Mutator-like transposase (AHF			contig272	Solyc11g	Mutator-li	GO:00082	SL2.40ch	AT5G024	PLL2	PLL2: ca	chr5:513	22.845	14.833	40.8974	39.9979	46.0619	44.3261
GT Sense	Sense	0.021	Detected	-0.021	Detected	0.000	2.143	Detected	2.232	Detected	2.188	0.001	GT Sens	contig273	contig273	Dof zinc f	GTCTTT	contig273	Solyc00g	Solyc00g	Dof zinc finger protein DOF2.4			contig273	Solyc02g	GDSL est	GO:00040	SL2.40ch	AT4G03153.1		kinase in	chr4:139	42.5403	40.8778	62.848	57.7463	205.143	198.394
GT Sense	Sense	-0.383	Detected	0.383	Detected	0.000	1.215	Detected	1.253	Detected	1.234	0.085	GT Sens	contig278	contig278	Unknown	GGAAGG	contig278	Solyc10g	Solyc10g	Unknown Protein (AHRD V1)			contig278	Solyc10g	Unknown Protein (A		SL2.40ch	AT2G256	SPIK. AK	SPIK (Sh	chr2:108	13.854	23.295	35.1529	32.724	46.4554	43.3494
GT Sense	Sense	-0.173	Detected	0.173	Detected	0.000	1.460	Detected	1.770	Detected	1.615	0.020	GT Sens	contig278	contig278	Cellulose	CTTTTG	contig278	Solyc05g	Solyc05g	Cellulose	GO:00167	GO:00167	contig278	Solyc10g	Cytochro	GO:00198	SL2.40ch	AT2G43310.1				161.8	203.425	219.014	155.772	555.848	626.5
GT Sense	Sense	-0.310	Detected	0.310	Detected	0.000	1.040	Detected	1.227	Detected	1.133	0.073	GT Sens	contig279	contig279	Protein s	TTCGAA	contig279	Solyc07g	Solyc07g	Protein s	GO:00064	GO:00064	contig279	Solyc07g	Protein s	GO:00428	SL2.40ch	AT5G151	WRKY72.	WRKY72	chr5:490	24.0506	36.555	39.9861	49.7551	67.9076	70.2479
GT Sense	Sense	-0.415	Detected	0.415	Detected	0.000	1.102	Detected	0.931	Detected	1.016	0.138	GT Sens	contig280	contig280	Cytochro	TAGTCC	contig280	Solyc09g	Solyc09g	Cytochro	GO:00198	GO:00198	contig280	Solyc09g	Cytochro	GO:00198	SL2.40ch	AT3G262	CYP71B2	CYP71B2	chr3:963	33.8277	59.4291	70.0989	79.9007	107.198	86.5345
GT Sense	Sense	0.123	Detected	-0.123	Detected	0.000	2.069	Detected	2.344	Detected	2.207	0.007	GT Sens	contig282	contig282	F-box fan	GTTGAT	contig282	Solyc05g	Solyc05g	F-box family protein (AHRD V1			contig282	Solyc05g	F-box family protein		SL2.40ch	AT2G362	ABI5. GIA	ABI5 (AB	chr2:152	1684.57	1404.29	2575.45	2551.91	7187.82	7907.81
GT Sense	Sense	-0.346	Comprom	0.346	Comprom	0.000	2.257	Detected	1.949	Detected	2.103	0.031	GT Sens	contig282	contig282	Ulp1 prot	AAGTCG	contig282	Solyc12g	Solyc12g	Ulp1 protease family C-termina			contig282	Solyc12g	Ulp1 protease family		SL2.40ch	AT3G119	UBP13	UBP13 (U	chr3:376	2.21996	3.54645	2.66034	9.16855	14.9349	10.9676
GT Sense	Sense	-0.101	Comprom	0.101	Comprom	0.000	1.270	Detected	1.232	Detected	1.251	0.007	GT Sens	contig283	contig283	Mutator-li	TATTTAT	contig283	Solyc09g	Solyc09g	Mutator-li	GO:00082	GO:00082	contig283	Solyc09g	Mutator-li	GO:00082	SL2.40ch	AT1G604	ATVPS34	ATVPS34	chr1:222	4.97481	5.65501	5.88856	7.10309	14.2501	12.6165
GT Sense	Sense	-0.003	Detected	0.003	Detected	0.000	2.127	Detected	2.042	Detected	2.084	0.000	GT Sens	contig284	contig284	Unknown	GCTGAG	contig284	Solyc12g	Solyc12g	Unknown Protein (AHRD V1)			contig284	Solyc12g	Unknown Protein (A		SL2.40ch	AT5G571	ATPAP28	PAP28 (P	chr5:231	283.719	281.689	315.864	455.493	1375.08	1178.59
GT Sense	Sense	-0.665	Comprom	0.665	Detected	0.000	1.683	Detected	1.292	Detected	1.488	0.165	GT Sens	contig285	contig285	Mutator-li	TAATAG	contig285	Solyc10g	Solyc10g	Mutator-li	GO:00082	GO:00082	contig285	Solyc10g	Mutator-li	GO:00082	SL2.40ch	AT5G499	emb1441	emb1441	chr5:203	6.29471	15.6457	18.5978	25.6487	35.4872	24.6058
GT Sense	Sense	0.207	Detected	-0.207	Detected	0.000	1.397	Detected	1.630	Detected	1.514	0.024	GT Sens	contig285	contig285	Hcr2-p4.1	CAGAGT	contig285	Solyc01g	Solyc01g	Hcr2-p4.1 (AHRD V1 ***- O4G2\			contig285	Solyc01g	Hcr2-p4.1 (AHRD V1		SL2.40ch	AT4G22230.1				281.543	208.977	520.814	691.795	711.432	760.117
GT Sense	Sense	0.200	Detected	-0.200	Detected	0.000	1.196	Detected	0.835	Detected	1.015	0.064	GT Sens	contig287	contig287	MuDR fan	GAGTTT	contig287	Solyc06g	Solyc06g	MuDR family transposase con			contig287	Solyc12g	Unknown	GO:00160	SL2.40ch	AT5G46470.1		disease	chr5:188	3155.26	2365.43	3712.85	2807.22	6969.79	4934.42
GT Sense	Sense	-0.085	Detected	0.085	Detected	0.000	1.572	Detected	1.112	Detected	1.342	0.032	GT Sens	contig288	contig288	Unknown	AAAGAC	contig288	Solyc05g	Solyc05g	Unknown Protein (AHRD V1)			contig288	Solyc01g	Unknown Protein (A		SL2.40ch	AT3G09950.1		unknowr	chr3:306	1101.47	1224.87	2745.87	3019.99	3846.57	2542.67
GT Sense	Sense	-0.099	Detected	0.099	Detected	0.000	0.980	Detected	1.420	Detected	1.200	0.038	GT Sens	contig291	contig291	Mutator-li	GTCTTG	contig291	Solyc12g	Solyc12g	Mutator-like transposase (AHF			contig291	Solyc12g	Mutator-like transpo		SL2.40ch	AT4G138	AtRLP48	AtRLP48	chr4:802	33.4535	37.9131	54.1923	59.0378	78.2155	96.4671
GT Sense	Sense	-0.023	Detected	0.023	Detected	0.000	1.351	Detected	1.232	Detected	1.292	0.002	GT Sens	contig295	contig295	60S ribos	GGCTTC	contig295	Solyc09g	Solyc09g	60S ribos	GO:00037	GO:00037	contig295	Solyc09g	60S ribos	GO:00037	SL2.40ch	AT2G34480.1		60S ribos	chr2:145	60.5038	61.7331	57.347	69.3058	173.609	145.372
GT Sense	Sense	-0.010	Detected	0.010	Detected	0.000	1.151	Detected	1.150	Detected	1.151	0.000	GT Sens	contig296	contig296	Ulp1 prot	GAAAGG	contig296	Solyc05g	Solyc05g	Ulp1 protease family C-termina			contig296	Solyc05g	Ulp1 protease family		SL2.40ch	AT1G63430.1		leucine-r	chr1:235	133.205	133.533	255.553	207.519	329.86	299.684
GT Sense	Sense	0.181	Detected	-0.181	Comprom	0.000	1.079	Detected	1.015	Detected	1.047	0.029	GT Sens	contig299	contig299	Mutator-li	TCAACA	contig299	Solyc09g	Solyc09g	Mutator-like transposase-like (			contig299	Solyc09g	Mutator-like transpo		SL2.40ch	AT2G332	XID. ATXI	XID: mot	chr2:140	12.705	9.77842	25.5497	12.017	26.2167	22.805
GT Sense	Sense	0.117	Comprom	-0.117	Comprom	0.000	0.976	Detected	2.066	Detected	1.521	0.112	GT Sens	contig300	contig300	Pol polyp	TTTTAG	contig300	Solyc12g	Solyc12g	Pol polyp	GO:00062	GO:00062	contig300	Solyc08g	Pol polyp	GO:00082	SL2.40ch	AT5G40450.2		unknowr	chr5:161	6.35431	5.34302	6.89762	12.3845	12.7609	24.6962
GT Sense	Sense	-0.298	Detected	0.298	Detected	0.000	1.994	Detected	1.986	Detected	1.990	0.022	GT Sens	contig300	contig300	Cytochro	CGTTGA	contig300	Solyc12g	Solyc12g	Cytochro	GO:00551	GO:00551	contig300	Solyc12g	Cytochro	GO:00198	SL2.40ch	AT2G251	CYP82F1	CYP82F1	chr2:107	70.5752	105.397	200.596	107.492	382.602	345.934
GT Sense	Sense	-0.097	Detected	0.097	Detected	0.000	1.049	Detected	1.160	Detected	1.104	0.010	GT Sens	contig301	contig301	Unknown	AGTGAA	contig301	Solyc01g	Solyc01g	Unknown Protein (AHRD V1)			contig301	Solyc01g	Unknown Protein (A		SL2.40ch	AT5G28740.1		transcrip	chr5:107	153.517	173.615	249.829	264.535	376.089	369.336
GT Sense	Sense	-0.001	Comprom	0.001	Comprom	0.000	1.136	Detected	1.324	Detected	1.230	0.006	GT Sens	contig303	contig303	MuDR fan	TGAAAT	contig303	Solyc02g	Solyc02g	MuDR family transposase con			contig303	Solyc08g	MuDR family transp		SL2.40ch	AT5G62170.1		unknowr	chr5:249	9.68944	9.5883	16.9125	32.7594	23.59	24.4256
GT Sense	Sense	0.072	Detected	-0.072	Detected	0.000	1.345	Detected	0.824	Detected	1.084	0.057	GT Sens	contig303	contig303	Ovate prc	TTTTCC	contig303	Solyc02g	Solyc02g	Ovate protein (AHRD V1 ***- O8			contig303	Solyc02g	Ovate protein (AHRD		SL2.40ch	AT5G07820.1		FUNCTIO	chr5:249	496.231	444.164	649.244	633.181	1327.8	841.197
GT Sense	Sense	0.315	Detected	-0.315	Detected	0.000	0.835	Detected	1.370	Detected	1.102	0.117	GT Sens	contig306	contig306	Cytochro	GGAAAA	contig306	Solyc04g	Solyc04g	Cytochro	GO:00160	GO:00160	contig306	Solyc07g	Unknown Protein (A		SL2.40ch	AT5G537	MLO11. A	MLO11 (I	chr5:218	160.392	102.445	283.291	285.77	254.658	335.417
GT Sense	Sense	0.359	Detected	-0.359	Detected	0.000	1.306	Detected	1.776	Detected	1.541	0.070	GT Sens	contig306	contig306	Unknown	AAGAAT	contig306	Solyc07g	Solyc07g	Unknown Protein (AHRD V1)			contig306	Solyc07g	Unknown Protein (A		SL2.40ch	AT1G268	CUL3A. A	ATCUL3	chr1:929	606.62	364.523	1403.5	1320.79	1295.14	1630.73
GT Sense	Sense	-0.103	Detected	0.103	Detected	0.000	1.563	Detected	1.657	Detected	1.610	0.005	GT Sens	contig316	contig316	DNA-dire	AGCTCC	contig316	Solyc10g	Solyc10g	DNA-dire	GO:0006350		contig316	Solyc10g	DNA-dire	GO:00063	SL2.40ch	AT5G451	NRPC2	NRPC2:	chr5:182	40.1441	45.769	50.223	59.5724	141.088	136.834
GT Sense	Sense	-0.300	Comprom	0.300	Detected	0.000	1.289	Detected	1.332	Detected	1.311	0.049	GT Sens	contig324	contig324	MuDR fan	CCATTT	contig324	Solyc01g	Solyc01g	MuDR family transposase con			contig324	Solyc12g	Mutator-li	GO:00082	SL2.40ch	AT4G155	IAGLU	IAGLU (IN	chr4:887	9.92739	14.8837	18.9942	29.9607	33.0864	30.9884
GT Sense	Sense	0.020	Detected	-0.020	Detected	0.000	0.944	Detected	1.219	Detected	1.082	0.016	GT Sens	contig346	contig346	Proline-ri	TAGCCC	contig346	Solyc08g	Solyc08g	Proline-ri	GO:00082	GO:00082	contig346	Solyc08g	Proline-ri	GO:00082	SL2.40ch	AT4G12530.1		protease	chr4:742	134.341	129.231	134.045	289.279	282.258	310.622
GT Sense	Sense	0.203	Detected	-0.203	Detected	0.000	1.517	Detected	1.652	Detected	1.585	0.018	GT Sens	contig375	contig375	Leucine F	TTCTTA	contig375	Solyc12g	Solyc12g	Leucine F	GO:00055	GO:00055	contig375	Solyc12g	LRR recei	GO:00163	SL2.40ch	AT2G46915.1		unknowr	chr2:192	20.7355	15.4686	54.4879	27.8688	57.0873	56.9883
GT Sense	Sense	-0.018	Detected	0.018	Detected	0.000	0.936	Detected	1.189	Detected	1.062	0.014	GT Sens	contig381	contig381	Unannota	TTTTGCCTGGAGTGGAACAAAGCTTGAGATGACACACCAAATCCAATCT							contig381	Solyc11g	Cell divisi	GO:00064	SL2.40ch	AT3G487	CDKA:1.	CDC2 (CI	chr3:180	362.005	366.991	490.191	593.504	776.418	841.142
GT Sense	Sense	-0.051	Detected	0.051	Detected	0.000	1.711	Detected	1.628	Detected	1.670	0.002	GT Sens	contig386	contig386	Unknown	ATCGTT	contig386	Solyc06g	Solyc06g	Unknown Protein (AHRD V1)			contig386	Solyc06g009810.2.1				AT1G508	POLGAM	POLGAM	chr1:188	65.6683	69.7019	92.1901	113.743	246.627	211.775
GT Sense	Sense	0.241	Detected	-0.241	Detected	0.000	1.428	Detected	1.673	Detected	1.551	0.029	GT Sens	contig387	contig387	Terminal	AATTGT	contig387	Solyc01g	Solyc01g	Terminal ear1-like 2 protein (A			contig387	Solyc12g	Unknown Protein (A		SL2.40ch	AT1G53200.2				1218.48	861.99	1396.86	1398.55	3072.18	3307.7
GT Sense	Sense	0.195	Comprom	-0.195	Comprom	0.000	1.291	Detected	2.194	Detected	1.743	0.071	GT Sens	contig389	contig389	Receptor	TTTCGT	contig389	Solyc06g	Solyc06g	Receptor	GO:00046	GO:00046	contig389	Solyc06g	Receptor	GO:00428	SL2.40ch	AT5G071	ERL2	ERL2 (EF	chr5:222	6.36549	4.80167	13.1975	4.33864	15.0684	25.6127
GT Sense	Sense	0.506	Comprom	-0.506	Comprom	0.000	1.106	Detected	1.654	Detected	1.380	0.139	GT Sens	contig410	contig410	Unknown	TGGTTT	contig410	Solyc09g	Solyc09g	Unknown Protein (AHRD V1)			contig410	Solyc09g	Unknown Protein (A		SL2.40ch	AT5G259	AtRLP52	AtRLP52	chr5:903	10.6341	5.21045	7.2754	10.0884	17.8444	23.7134
GT Sense	Sense	0.009	Detected	-0.009	Detected	0.000	1.229	Detected	1.199	Detected	1.214	0.000	GT Sens	contig411	contig411	Unannota	AATAGATGTTGAATATTGATAGTTATTGAATTGGTTTTTATTAATGAGT							contig411	Solyc03g	Polyprote	GO:00065	SL2.40ch	AT3G24530.1		AAA-type	chr3:894	68.4156	66.8035	96.0982	92.9278	176.508	157.092
GT Sense	Sense	0.090	Detected	-0.090	Detected	0.000	1.053	Detected	1.519	Detected	1.286	0.036	GT Sens	contig413	contig413	Unannota	GAAGAACCGAAGTCAAGCAACTACTTCTCCAATGCCTTTCCTTTTCTTA							contig413	Solyc02g014100.1.1				AT4G24880.1		unknowr	chr4:128	36.8377	32.1523	67.6241	93.8636	79.5162	99.8441
GT Sense	Sense	0.115	Comprom	-0.115	Comprom	0.000	4.283	Detected	3.570	Detected	3.927	0.009	GT Sens	contig419	contig419	Unannota	CTCACCATTCAAATTTGTACATCATCGAATGGGAAATCTCTATCTTCTA							contig419	Solyc03g	Polyprote	GO:00065	SL2.40ch	AT5G64667.1				2.19591	1.85026	9.53782	6.57113	43.7108	24.236
GT Sense	Sense	-1.373	Detected	1.373	Detected	0.000	2.441	Detected	0.930	Detected	1.685	0.395	GT Sens	contig423	contig423	Fatty acid	CCAAGT	contig423	Solyc12g	Solyc12g	Fatty acid desaturase (AHRD V			contig423	Solyc12g100240.1.1				AT3G121	FAD2	FAD2 (FA	chr3:386	189.764	1258.01	642.236	668.381	2954.42	942.232
GT Sense	Sense	0.092	Detected	-0.092	Detected	0.000	1.048	Detected	1.051	Detected	1.050	0.008	GT Sens	contig425	contig425	Receptor	ATATCA	contig425	Solyc07g	Solyc07g	Receptor	GO:00046	GO:00046	contig425	Solyc07g	LRR recei	GO:00046	SL2.40ch	AT4G38470.1		protein k	chr4:179	28.4149	24.7401	35.3514	43.9237	61.0413	55.6386
GT Sense	Sense	-0.231	Detected	0.231	Detected	0.000	1.015	Detected	1.160	Detected	1.088	0.046	GT Sens	contig430	contig430	Cross hyl	ACCTCAATAGTTTTCGCGGAGAACCAGCTATTTCCCGGCTTGATTGGC							contig430	#N/A	#N/A	#N/A	#N/A	#N/A	#N/A	#N/A	#N/A	74.6817	101.762	74.8893	81.033	196.248	197.265
GT Sense	Sense	-0.752	Detected	0.752	Detected	0.000	1.376	Detected	1.096	Detected	1.236	0.248	GT Sens	contig434	contig434	Nicotia ta	ACTAGTACTCGTACTTGCCTTCACTCTAACTTTTGCATGGCCTTTATAC							contig434	#N/A	#N/A	#N/A	#N/A	#N/A	#N/A	#N/A	#N/A	396.575	1112.69	1165.18	1167.55	1920.04	1437.96
GT Sense	Sense	0.835	Comprom	-0.835	Comprom	0.000	2.660	Detected	2.787	Detected	2.724	0.083	GT Sens	contig462	contig462	Glutared	CAAACC	contig462	Solyc04g	Solyc04g	Glutared	GO:00454	GO:00454	contig462	Solyc04g	Glutared	GO:00454	SL2.40ch	ATMG009	NAD3	NADH de	chrM:26	6.41881	1.99411	12.1083	14.9454	25.1809	25.0035
GT Sense	Sense	0.402	Comprom	-0.402	Comprom	0.000	1.368	Detected	1.425	Detected	1.396	0.074	GT Sens	contig486	contig486	Unknown	GCGGAA	contig486	Solyc08g	Solyc08g	Unknown Protein (AHRD V1)			contig486	Solyc09g	Unknown Protein (A		SL2.40ch	AT5G422	VAR1. FT	VAR1 (VA	chr5:169	5.48332	3.10632	6.65895	2.40762	11.8601	11.2184
GT Sense	Sense	-0.215	Comprom	0.215	Comprom	0.000	1.776	Detected	1.295	Detected	1.536	0.042	GT Sens	contig499	contig499	Unknown	TCAAAT	contig499	Solyc12g	Solyc12g	Unknown Protein (AHRD V1)			contig499	Solyc12g	Unknown Protein (A		SL2.40ch	AT1G35330.1				4.51624	6.0189	10.6349	5.09654	19.8918	12.9505
GT Sense	Sense	0.425	Comprom	-0.425	Comprom	0.000	1.306	Detected	2.071	Detected	1.688	0.098	GT Sens	contig532	contig532	Mutator-li	TTTTATG	contig532	Solyc04g	Solyc04g	Mutator-li	GO:00082	GO:00082	contig532	Solyc07g	Mutator-li	GO:00082	SL2.40ch	AT4G29420.1		F-box far	chr4:144	7.8776	4.31887	15.9774	17.9955	16.0635	24.8069
GT Sense	Sense	0.205	Detected	-0.205	Detected	0.000	1.385	Detected	1.570	Detected	1.477	0.022	GT Sens	contig562	contig562	Mutator-li	CGTTGG	contig562	Solyc06g	Solyc06g	Mutator-li	GO:00082	GO:00082	contig562	Solyc12g	Mutator-li	GO:00082	SL2.40ch	AT5G059	ATROPGI	ROPGEF	chr5:178	1831.28	1362.69	1502.16	1156.68	4593.68	4749.16
GT Sense	Sense	0.081	Detected	-0.081	Detected	0.000	2.225	Detected	2.174	Detected	2.199	0.001	GT Sens	contig582	contig582	Helicase-	CAATGG	contig582	Solyc01g	Solyc01g	Helicase-like protein (AHRD V1			contig582	Solyc01g	Helicase-like proteir		SL2.40ch	AT3G20700.1				347.819	307.357	584.182	595.719	1702.42	1493.45
GT Sense	Sense	0.071	Comprom	-0.071	Comprom	0.000	2.617	Detected	2.415	Detected	2.516	0.002	GT Sens	contig606	contig606	Receptor	TTGTCA	contig606	Solyc09g	Solyc09g	Receptor	GO:00055	GO:00055	contig606	Solyc09g	Receptor	GO:00055	SL2.40ch	AT2G39360.1		protein k	chr2:164	2.18103	1.95327	2.03515	17.7265	14.0981	11.1458
GT Sense	Sense	0.842	Comprom	-0.842	Comprom	0.000	1.082	Detected	1.607	Detected	1.344	0.267	GT Sens	contig617	contig617	Mutator-li	AGTCCA	contig617	Solyc10g	Solyc10g	Mutator-li	GO:00082	GO:00082	contig617	Solyc12g	Mutator-li	GO:00082	SL2.40ch	AT3G227	SOL1	SOL1: tra	chr3:804	12.0779	3.71356	9.2689	14.6218	15.7862	20.6512
GT Sense	Sense	0.220	Detected	-0.220	Detected	0.000	1.019	Detected	1.150	Detected	1.084	0.042	GT Sens	contig623	contig623	Chromod	CTTGAT	contig623	Solyc10g	Solyc10g	Chromod	GO:00055	GO:00055	contig623	Solyc05g	Chromod	GO:00055	SL2.40ch	AT1G05120.1		SNF2 do	chr1:147	162.753	118.615	204.86	189.282	313.48	312.202
GT Sense	Sense	0.295	Detected	-0.295	Detected	0.000	0.919	Detected	1.403	Detected	1.161	0.093	GT Sens	contig702	contig702	Sulfotran	TGTTCT	contig702	Solyc09g	Solyc09g	Sulfotran	GO:00081	GO:00081	contig702	Solyc09g	Sulfotran	GO:00801	SL2.40ch	AT5G059	DHS	DHS (DE	chr5:177	93.1256	61.1361	99.0834	56.5809	158.946	202.015
GT Sense	Sense	-0.217	Detected	0.217	Detected	0.000	2.180	Detected	1.827	Detected	2.003	0.019	GT Sens	contig705	contig705	Unknown	ACAAAA	contig705	Solyc11g	Solyc11g	Unknown Protein (AHRD V1)			contig705	Solyc05g	Unknown Protein (A		SL2.40ch	AT3G52700.1		unknowr	chr3:195	206.332	275.715	239.366	564.981	1204.05	856.507
GT Sense	Sense	-0.173	Detected	0.173	Detected	0.000	0.991	Detected	1.256	Detected	1.124	0.036	GT Sens	contig742	contig742	Mutator-li	TGCTTT	contig742	Solyc04g	Solyc04g	Mutator-like transposase (AHF			contig742	Solyc12g	Mutator-like transpo		SL2.40ch	AT5G631	ATPAP29	ATPAP29	chr5:253	45.0232	56.5849	76.4398	105.515	111.763	122.079
GT Sense	Sense	-0.019	Detected	0.019	Detected	0.000	0.982	Detected	1.083	Detected	1.033	0.003	GT Sens	contig754	contig754	Mutator-li	CTTGCA	contig754	Solyc04g	Solyc04g	Mutator-like transposase (AHF			contig754	Solyc12g	Mutator-like transpo		SL2.40ch	AT5G631	ATPAP29	ATPAP29	chr5:253	49.324	50.0943	77.2538	90.9196	109.337	106.635
GT Sense	Sense	0.113	Detected	-0.113	Detected	0.000	1.231	Detected	1.304	Detected	1.268	0.009	GT Sens	contig787	contig787	Argonaut	CATCTT	contig787	Solyc06g	Solyc06g	Argonaut	GO:00036	GO:00036	contig787	Solyc06g	Argonaut	GO:00198	SL2.40ch	AT2G270	AGO4. OC	AGO4 (AI	chr2:115	1213.95	1026.43	1215.01	1208.42	2918.93	2790.67
GT Sense	Sense	0.160	Detected	-0.160	Detected	0.000	1.129	Detected	1.045	Detected	1.087	0.022	GT Sens	contig799	contig799	Hydroxyc	TTTCCA	contig799	Solyc07g	Solyc07g	Hydroxyc	GO:00167	GO:00167	contig799	Solyc07g	Hydroxyc	GO:00167	SL2.40ch	AT5G484	ATGLR1.	ATGLR1.	chr5:196	975.557	772.472	1343.25	452.169	2114.07	1813.66
GT Sense	Sense	0.155	Comprom	-0.155	Comprom	0.000	1.086	Detected	1.191	Detected	1.139	0.020	GT Sens	contig807	contig807	Leucine-r	TCAACA	contig807	Solyc06g	Solyc06g	Leucine-r	GO:00163	GO:00163	contig807	Solyc06g	LRR recei	GO:00163	SL2.40ch	AT2G368	ATGSL08	GSL8 (GI	chr2:154	12.1394	9.67565	12.0953	35.528	25.623	25.0545
GT Sense	Sense	0.342	Comprom	-0.342	Comprom	0.000	1.111	Detected	1.030	Detected	1.071	0.090	GT Sens	contig815	contig815	CONSTAI	TGGAAT	contig815	Solyc02g	Solyc02g	CONSTAI	GO:00037	GO:00037	contig815	Solyc02g	Tomato C	GO:00037	SL2.40ch	AT5G158	COL1. AT	COL1 (cc	chr5:517	9.94452	6.1158	13.1032	15.1903	18.764	16.1202
GT Sense	Sense	-0.212	Detected	0.212	Detected	0.000	0.990	Detected	1.033	Detected	1.012	0.042	GT Sens	contig844	contig844	B3 domai	GTGCTT	contig844	Solyc08g	Solyc08g	B3 domai	GO:00063	GO:00063	contig844	Solyc08g	B3 domai	GO:00063	SL2.40ch	AT3G189	VRN1. RE	VRN1 (RE	chr3:654	14.9404	19.8143	16.2927	13.3661	38.0518	35.6537
GT Sense	Sense	0.080	Detected	-0.080	Detected	0.000	1.288	Detected	1.403	Detected	1.346	0.005	GT Sens	contig890	contig890	Ribose-pl	AAAGCT	contig890	Solyc07g	Solyc07g	Ribose-pl	GO:00091	GO:00091	contig890	Solyc07g	Ribose-pl	GO:00428	SL2.40ch	AT2G42910.1		ribose-pl	chr2:178	290.535	257.065	502.535	440.978	743.453	731.491
GT Sense	Sense	0.352	Detected	-0.352	Detected	0.000	1.354	Detected	1.293	Detected	1.324	0.064	GT Sens	contig895	contig895	Polygalac	ATTAAG	contig895	Solyc00g	Solyc00g	Polygalac	GO:00059	GO:00059	contig895	Solyc01g	Polygalac	GO:00059	SL2.40ch	AT1G56710.1		glycosid	chr1:212	80.1879	48.6957	110.175	123.414	177.936	155.071
GT Sense	Sense	-0.260	Detected	0.260	Detected	0.000	0.990	Detected	1.062	Detected	1.026	0.060	GT Sens	contig895	contig895	Mutator-li	GTGAAG	contig895	Solyc10g	Solyc10g	Mutator-li	GO:00082	GO:00082	contig895	Solyc10g	Mutator-li	GO:00082	SL2.40ch	AT5G656	bHLH093	bHLH093	chr5:262	16.7185	23.6961	30.6606	42.6563	44.0432	42.0682
GT Sense	Sense	-0.003	Comprom	0.003	Comprom	0.000	1.334	Detected	1.132	Detected	1.233	0.007	GT Sens	contig904	contig904	Ulp1 prot	AAATCG	contig904	Solyc00g	Solyc00g	Ulp1 protease family C-termina			contig904	Solyc00g	Ulp1 protease family		SL2.40ch	AT4G255	MSH3. AT	MSH3 (A	chr4:130	7.80981	7.75677	15.5829	9.73921	21.8462	17.2741
GT Sense	Sense	-0.773	Comprom	0.773	Comprom	0.000	2.615	Detected	2.115	Detected	2.365	0.101	GT Sens	contig906	contig906	1-aminoc	GTCCAG	contig906	Solyc09g	Solyc09g	1-aminoc	GO:00164	GO:00164	contig906	Solyc09g	1-aminoc	GO:00098	SL2.40ch	AT1G06640.1		2-oxoglu	chr1:203	2.70077	7.79868	21.62	18.6497	31.3074	20.1297
GT Sense	Sense	-0.384	Comprom	0.384	Comprom	0.000	1.582	Detected	1.581	Detected	1.581	0.054	GT Sens	contig949	contig949	Unknown	GAAAAT	contig949	Solyc01g	Solyc01g	Unknown Protein (AHRD V1)			contig949	Solyc02g	Auxin-repressed pro		SL2.40ch	AT5G44578.1		unknowr	chr5:179	4.43671	7.47382	8.25519	7.56204	19.1959	17.4385
GT Sense	Sense	-0.039	Detected	0.039	Detected	0.000	0.973	Detected	1.299	Detected	1.136	0.021	GT Sens	contig956	contig956	Subtilisin	TACCAA	contig956	Solyc08g	Solyc08g	Subtilisin	GO:00042	GO:00042	contig956	Solyc08g	Subtilisin	GO:00042	SL2.40ch	AT5G673	ARA12	ARA12: s	chr5:268	646.196	674.143	1079.64	1014.48	1442.42	1644.76
GT Sense	Sense	0.051	Detected	-0.051	Detected	0.000	1.606	Detected	1.574	Detected	1.590	0.001	GT Sens	contig993	contig993	Unknown	TATTAGA	contig993	Solyc07g	Solyc07g	Unknown Protein (AHRD V1)			contig993	Solyc04g	30S ribos	GO:00055	SL2.40ch	AT3G460	RPS15AD	RPS15AD	chr3:169	1119.18	1031.09	1284.44	1379.85	3640.81	3238.88

		4h_AZ_Control					4h_AZ_TAPG + TPRP vs. 4h_AZ_Control					
ProbeNar	Orientatic	Fold 4h	Flag 4h	Fold 4h	Flag 4h	Geomean	Fold 4h	Flag 4h	Fold 4h	Flag 4h	Geomean	P-value
A 96 P0	Sense	-0.581	Detected	0.581	Detected	0.000	-0.959	Detected	-2.190	Detected	-1.574	0.204
A 96 P01	Sense	0.281	Detected	-0.281	Detected	0.000	-1.533	Detected	-1.543	Detected	-1.538	0.032
A 96 P01	Sense	0.052	Detected	-0.052	Detected	0.000	-0.837	Detected	-1.412	Detected	-1.125	0.061
A 96 P01	Sense	-0.931	Detected	0.931	Detected	0.000	-1.579	Detected	-1.679	Detected	-1.629	0.223
A 96 P02	Sense	0.293	Detected	-0.293	Detected	0.000	-2.174	Detected	-2.265	Detected	-2.219	0.017
A 96 P02	Sense	-0.484	Detected	0.484	Detected	0.000	-1.158	Detected	-1.513	Detected	-1.336	0.122
A 96 P02	Sense	-0.214	Detected	0.214	Detected	0.000	-0.986	Detected	-1.335	Detected	-1.160	0.052
A 96 P03	Sense	-0.503	Detected	0.503	Detected	0.000	-0.914	Detected	-1.091	Detected	-1.002	0.189
A 96 P03	Sense	-0.563	Detected	0.563	Detected	0.000	-1.058	Detected	-0.986	Detected	-1.022	0.212
A 96 P03	Sense	-0.494	Detected	0.494	Detected	0.000	-1.256	Detected	-0.957	Detected	-1.106	0.166
A 96 P03	Sense	-0.359	Detected	0.359	Detected	0.000	-1.361	Detected	-1.349	Detected	-1.355	0.064
A 96 P04	Sense	-0.162	Detected	0.162	Detected	0.000	-1.046	Detected	-1.117	Detected	-1.081	0.023
A 96 P04	Sense	-0.238	Detected	0.238	Detected	0.000	-1.056	Detected	-1.092	Detected	-1.074	0.046
A 96 P04	Sense	-0.278	Detected	0.278	Detected	0.000	-1.301	Detected	-1.346	Detected	-1.324	0.042
A 96 P04	Sense	0.102	Detected	-0.102	Detected	0.000	-1.315	Detected	-1.672	Detected	-1.493	0.018
A 96 P04	Sense	-0.178	Detected	0.178	Detected	0.000	-1.000	Detected	-1.094	Detected	-1.047	0.029
A 96 P04	Sense	0.431	Detected	-0.431	Detected	0.000	-1.247	Comprom	-1.644	Comprom	-1.445	0.093
A 96 P06	Sense	-0.307	Detected	0.307	Detected	0.000	-1.810	Detected	-1.347	Detected	-1.578	0.055
A 96 P06	Sense	-0.098	Detected	0.098	Detected	0.000	-1.628	Detected	-1.121	Detected	-1.374	0.037
A 96 P07	Sense	0.029	Detected	-0.029	Detected	0.000	-1.478	Detected	-1.325	Comprom	-1.401	0.003
A 96 P07	Sense	0.137	Detected	-0.137	Detected	0.000	-0.850	Detected	-1.388	Detected	-1.119	0.066
A 96 P07	Sense	-0.116	Detected	0.116	Detected	0.000	-0.866	Detected	-1.221	Detected	-1.043	0.039
A 96 P08	Sense	-0.393	Detected	0.393	Detected	0.000	-0.924	Detected	-1.590	Detected	-1.257	0.135
A 96 P08	Sense	0.157	Detected	-0.157	Detected	0.000	-3.141	Detected	-2.303	Detected	-2.722	0.026
A 96 P08	Sense	0.003	Detected	-0.003	Detected	0.000	-1.230	Detected	-0.969	Detected	-1.099	0.014
A 96 P08	Sense	0.338	Detected	-0.338	Detected	0.000	-1.684	Detected	-1.973	Detected	-1.828	0.038
A 96 P08	Sense	0.086	Detected	-0.086	Detected	0.000	-1.005	Detected	-1.009	Detected	-1.007	0.007
A 96 P08	Sense	0.396	Detected	-0.396	Detected	0.000	-1.348	Detected	-1.200	Detected	-1.274	0.087
A 96 P08	Sense	-0.720	Detected	0.720	Detected	0.000	-1.417	Detected	-1.160	Detected	-1.288	0.220
A 96 P09	Sense	-0.574	Detected	0.574	Detected	0.000	-2.053	Detected	-1.482	Detected	-1.768	0.110
A 96 P09	Sense	0.354	Detected	-0.354	Detected	0.000	-4.746	Comprom	-1.015	Detected	-2.880	0.269
A 96 P10	Sense	-0.120	Detected	0.120	Detected	0.000	-1.202	Detected	-1.550	Detected	-1.376	0.023
A 96 P10	Sense	-0.586	Detected	0.586	Detected	0.000	-0.838	Detected	-1.592	Comprom	-1.215	0.223
A 96 P10	Sense	-0.563	Detected	0.563	Detected	0.000	-1.590	Detected	-1.079	Detected	-1.335	0.164
A 96 P11	Sense	-0.312	Detected	0.312	Detected	0.000	-1.395	Detected	-1.481	Detected	-1.438	0.045
A 96 P11	Sense	0.177	Detected	-0.177	Detected	0.000	-1.301	Detected	-1.353	Detected	-1.327	0.018
A 96 P14	Sense	-0.603	Detected	0.603	Detected	0.000	-1.600	Detected	-1.236	Detected	-1.418	0.153
A 96 P14	Sense	-0.224	Detected	0.224	Detected	0.000	-1.006	Detected	-1.010	Detected	-1.008	0.046
A 96 P14	Sense	0.081	Detected	-0.081	Detected	0.000	-1.089	Detected	-1.250	Detected	-1.170	0.009
A 96 P15	Sense	-0.096	Detected	0.096	Detected	0.000	-0.942	Detected	-1.141	Detected	-1.041	0.017
A 96 P16	Sense	-0.535	Detected	0.535	Detected	0.000	-1.301	Detected	-1.002	Detected	-1.151	0.174
A 96 P16	Sense	-0.926	Detected	0.926	Detected	0.000	-1.111	Detected	-1.545	Detected	-1.328	0.298
A 96 P16	Sense	-0.249	Detected	0.249	Detected	0.000	-0.966	Detected	-1.229	Detected	-1.097	0.060
A 96 P17	Sense	-0.437	Detected	0.437	Detected	0.000	-0.981	Detected	-1.482	Detected	-1.231	0.134
A 96 P17	Sense	-0.437	Detected	0.437	Detected	0.000	-0.898	Detected	-1.338	Detected	-1.118	0.150
A 96 P17	Sense	-0.134	Detected	0.134	Detected	0.000	-0.964	Detected	-1.142	Detected	-1.053	0.023
A 96 P17	Sense	-1.080	Detected	1.080	Detected	0.000	-1.524	Detected	-2.012	Detected	-1.768	0.251
A 96 P17	Sense	-0.272	Detected	0.272	Detected	0.000	-0.899	Detected	-1.137	Detected	-1.018	0.075
A 96 P17	Sense	-0.731	Detected	0.731	Detected	0.000	-1.005	Detected	-1.763	Detected	-1.384	0.235
A 96 P18	Sense	0.201	Detected	-0.201	Detected	0.000	-1.111	Detected	-0.909	Detected	-1.010	0.046
A 96 P18	Sense	0.349	Detected	-0.349	Detected	0.000	-1.072	Detected	-1.097	Detected	-1.084	0.090
A 96 P18	Sense	-0.652	Detected	0.652	Detected	0.000	-0.817	Detected	-2.243	Detected	-1.530	0.254
A 96 P19	Sense	-0.927	Detected	0.927	Detected	0.000	-1.652	Detected	-1.713	Detected	-1.682	0.211
A 96 P20	Sense	-0.263	Detected	0.263	Detected	0.000	-1.191	Detected	-1.419	Detected	-1.305	0.045
A 96 P20	Sense	-0.580	Detected	0.580	Detected	0.000	-1.156	Detected	-0.945	Detected	-1.050	0.217
A 96 P20	Sense	-0.384	Detected	0.384	Detected	0.000	-1.203	Detected	-1.563	Detected	-1.383	0.083
A 96 P20	Sense	-0.483	Detected	0.483	Detected	0.000	-1.366	Detected	-0.913	Detected	-1.140	0.166
A 96 P20	Sense	-0.578	Detected	0.578	Detected	0.000	-0.965	Detected	-1.776	Detected	-1.370	0.192
A 96 P21	Sense	-0.291	Detected	0.291	Detected	0.000	-0.962	Detected	-1.063	Detected	-1.012	0.076
A 96 P21	Sense	-0.435	Detected	0.435	Detected	0.000	-1.718	Detected	-1.199	Detected	-1.459	0.102
A 96 P21	Sense	-0.249	Detected	0.249	Detected	0.000	-1.347	Detected	-2.115	Detected	-1.731	0.063
A 96 P22	Sense	0.375	Detected	-0.375	Detected	0.000	-1.304	Detected	-1.172	Detected	-1.238	0.083
A 96 P22	Sense	-0.286	Detected	0.286	Detected	0.000	-1.021	Detected	-1.457	Detected	-1.239	0.075
A 96 P23	Sense	-0.242	Detected	0.242	Detected	0.000	-1.065	Detected	-1.233	Detected	-1.149	0.046
A 96 P23	Sense	-0.395	Detected	0.395	Detected	0.000	-1.461	Detected	-1.576	Detected	-1.518	0.063
A 96 P23	Sense	-0.386	Detected	0.386	Detected	0.000	-1.025	Detected	-1.545	Detected	-1.285	0.110
A 96 P24	Sense	-0.630	Detected	0.630	Detected	0.000	-1.142	Detected	-1.345	Detected	-1.243	0.191
A 96 P24	Sense	-0.480	Detected	0.480	Detected	0.000	-1.380	Detected	-1.677	Detected	-1.529	0.093
A 96 P24	Sense	-0.736	Detected	0.736	Detected	0.000	-1.609	Detected	-1.246	Detected	-1.427	0.201
A 96 P25	Sense	-0.307	Detected	0.307	Detected	0.000	-2.269	Detected	-2.348	Detected	-2.309	0.017
A 96 P25	Sense	-0.072	Detected	0.072	Detected	0.000	-2.143	Detected	-4.164	Comprom	-3.153	0.090
A 96 P25	Sense	0.031	Detected	-0.031	Detected	0.000	-1.342	Comprom	-2.804	Comprom	-2.073	0.105
A 96 P26	Sense	0.277	Detected	-0.277	Detected	0.000	-1.385	Detected	-2.091	Detected	-1.738	0.061
A 96 P26	Sense	-0.311	Detected	0.311	Detected	0.000	-0.869	Detected	-1.243	Detected	-1.056	0.101
GT Sense	Sense	0.115	Detected	-0.115	Detected	0.000	-1.649	Comprom	-1.285	Comprom	-1.467	0.021
GT Sense	Sense	0.129	Detected	-0.129	Detected	0.000	-1.301	Comprom	-1.562	Comprom	-1.431	0.016
GT Sense	Sense	-0.702	Detected	0.702	Detected	0.000	-2.147	Detected	-1.785	Detected	-1.966	0.113
GT Sense	Sense	-1.719	Detected	1.719	Detected	0.000	-1.787	Detected	-2.755	Detected	-2.271	0.331
GT Sense	Sense	-0.676	Detected	0.676	Detected	0.000	-1.045	Detected	-1.162	Detected	-1.103	0.245
GT Sense	Sense	0.224	Detected	-0.224	Detected	0.000	-1.397	Detected	-2.071	Comprom	-1.734	0.050
GT Sense	Sense	0.204	Detected	-0.204	Detected	0.000	-1.194	Detected	-0.879	Detected	-1.036	0.057
GT Sense	Sense	-0.710	Detected	0.710	Detected	0.000	-1.972	Detected	-2.339	Detected	-2.155	0.099
GT Sense	Sense	-0.293	Detected	0.293	Detected	0.000	-1.152	Detected	-1.276	Detected	-1.214	0.056
GT Sense	Sense	0.362	Detected	-0.362	Detected	0.000	-1.698	Detected	-1.898	Detected	-1.798	0.041
GT Sense	Sense	-0.549	Detected	0.549	Detected	0.000	-2.657	Detected	-2.335	Detected	-2.496	0.049
GT Sense	Sense	0.217	Detected	-0.217	Detected	0.000	-2.125	Detected	-1.842	Detected	-1.983	0.017
GT Sense	Sense	-0.749	Detected	0.749	Detected	0.000	-0.996	Detected	-2.025	Detected	-1.511	0.238
GT Sense	Sense	0.173	Detected	-0.173	Detected	0.000	-1.114	Detected	-1.379	Detected	-1.247	0.029
GT Sense	Sense	-0.700	Detected	0.700	Detected	0.000	-2.319	Detected	-1.186	Detected	-1.753	0.191
GT Sense	Sense	-0.512	Detected	0.512	Detected	0.000	-1.232	Detected	-0.995	Detected	-1.113	0.168
GT Sense	Sense	-1.536	Detected	1.536	Detected	0.000	-1.173	Detected	-1.881	Detected	-1.527	0.435
GT Sense	Sense	-0.337	Detected	0.337	Detected	0.000	-1.485	Detected	-1.431	Detected	-1.458	0.050
GT Sense	Sense	-0.422	Detected	0.422	Detected	0.000	-1.119	Detected	-1.268	Detected	-1.193	0.108
GT Sense	Sense	0.201	Detected	-0.201	Detected	0.000	-1.980	Detected	-1.428	Detected	-1.704	0.038
GT Sense	Sense	-0.321	Detected	0.321	Detected	0.000	-1.293	Detected	-2.463	Detected	-1.878	0.106
GT Sense	Sense	-0.125	Detected	0.125	Detected	0.000	-1.201	Detected	-1.651	Detected	-1.426	0.031
GT Sense	Sense	-0.323	Detected	0.323	Detected	0.000	-1.872	Detected	-1.424	Detected	-1.648	0.053
GT Sense	Sense	-0.464	Detected	0.464	Detected	0.000	-1.163	Detected	-1.358	Detected	-1.261	0.117
GT Sense	Sense	-0.898	Detected	0.898	Detected	0.000	-1.618	Detected	-1.781	Detected	-1.699	0.200
GT Sense	Sense	-0.394	Detected	0.394	Detected	0.000	-1.619	Detected	-1.766	Detected	-1.692	0.052
GT Sense	Sense	0.613	Detected	-0.613	Detected	0.000	-2.924	Comprom	-1.557	Comprom	-2.240	0.135
GT Sense	Sense	-0.360	Detected	0.360	Detected	0.000	-1.024	Detected	-1.170	Detected	-1.097	0.096
GT Sense	Sense	0.125	Detected	-0.125	Detected	0.000	-1.337	Comprom	-1.271	Comprom	-1.304	0.010
GT Sense	Sense	-1.428	Detected	1.428	Detected	0.000	-2.580	Detected	-2.156	Detected	-2.368	0.243
GT Sense	Sense	-0.084	Detected	0.084	Detected	0.000	-1.133	Detected	-1.347	Detected	-1.240	0.012
GT Sense	Sense	-0.097	Detected	0.097	Detected	0.000	-1.838	Detected	-1.167	Detected	-1.502	0.050
GT Sense	Sense	0.581	Detected	-0.581	Detected	0.000	-1.833	Detected	-1.171	Detected	-1.502	0.154
GT Sense	Sense	0.019	Detected	-0.019	Detected	0.000	-1.424	Comprom	-2.371	Comprom	-1.897	0.057
GT Sense	Sense	0.344	Detected	-0.344	Detected	0.000	-1.286	Detected	-0.917	Detected	-1.101	0.106
GT Sense	Sense	0.157	Detected	-0.157	Detected	0.000	-0.957	Detected	-1.128	Detected	-1.043	0.028
GT Sense	Sense	0.453	Detected	-0.453	Detected	0.000	-1.390	Detected	-1.323	Comprom	-1.356	0.096
GT Sense	Sense	-0.899	Detected	0.899	Detected	0.000	-2.172	Detected	-2.800	Comprom	-2.486	0.121
GT Sense	Sense	-0.447	Detected	0.447	Detected	0.000	-1.463	Detected	-1.710	Detected	-1.586	0.076
GT Sense	Sense	1.128	Detected	-1.128	Detected	0.000	-0.916	Detected	-1.847	Comprom	-1.382	0.375
GT Sense	Sense	0.330	Detected	-0.330	Detected	0.000	-1.044	Detected	-1.256	Detected	-1.150	0.080
GT Sense	Sense	-0.663	Detected	0.663	Detected	0.000	-1.666	Detected	-2.117	Detected	-1.891	0.114
GT Sense	Sense	-0.645	Detected	0.645	Detected	0.000	-1.162	Detected	-1.145	Detected	-1.154	0.215
GT Sense	Sense	-1.161	Detected	1.161	Detected	0.000	-0.808	Detected	-2.862	Detected	-1.835	0.358
GT Sense	Sense	-0.136	Detected	0.136	Detected	0.000	-1.098	Detected	-1.378	Detected	-1.238	0.024
GT Sense	Sense	-0.271	Detected	0.271	Detected	0.000	-1.030	Detected	-2.201	Detected	-1.616	0.129
GT Sense	Sense	0.242	Detected	-0.242	Detected	0.000	-1.602	Detected	-2.399	Detected	-2.000	0.050
GT Sense	Sense	0.076	Detected	-0.076	Detected	0.000	-4.271	Detected	-4.760	Detected	-4.515	0.003
GT Sense	Sense	-1.339	Detected	1.339	Detected	0.000	-2.734	Detected	-4.671	Detected	-3.703	0.154
GT Sense	Sense	-0.410	Detected	0.410	Detected	0.000	-1.318	Detected	-1.049	Detected	-1.183	0.111
GT Sense	Sense	-0.362	Detected	0.362	Detected	0.000	-1.198	Detected	-1.109	Detected	-1.153	0.087
GT Sense	Sense	0.114	Detected	-0.114	Detected	0.000	-0.976	Detected	-1.380	Comprom	-1.178	0.037
GT Sense	Sense	-0.810	Detected	0.810	Detected	0.000	-1.439	Detected	-1.194	Detected	-1.317	0.249
GT Sense	Sense	-0.589	Detected	0.589	Detected	0.000	-1.532	Detected	-1.176	Detected	-1.354	0.159
GT Sense	Sense	0.144	Detected	-0.144	Detected	0.000	-1.185	Detected	-1.083	Detected	-1.134	0.018
GT Sense	Sense	-0.255	Detected	0.255	Detected	0.000	-0.857	Detected	-1.307	Detected	-1.082	0.086
GT Sense	Sense	-0.526	Detected	0.526	Detected	0.000	-2.048	Detected	-2.094	Detected	-2.071	0.059
GT Sense	Sense	-0.888	Detected	0.888	Detected	0.000	-1.767	Detected	-1.465	Detected	-1.616	0.215
GT Sense	Sense	-0.639	Detected	0.639	Detected	0.000	-2.711	Detected	-3.626	Comprom	-3.168	0.056
GT Sense	Sense	-1.309	Detected	1.309	Detected	0.000	-2.900	Detected	-2.980	Detected	-2.940	0.154
GT Sense	Sense	-0.501	Detected	0.501	Detected	0.000	-1.453	Detected	-1.732	Detected	-1.593	0.092
GT Sense	Sense	-0.694	Detected	0.694	Detected	0.000	-1.317	Detected	-1.265	Detected	-1.291	0.204
GT Sense	Sense	-0.405	Detected	0.405	Detected	0.000	-0.852	Detected	-1.665	Detected	-1.259	0.160
GT Sense	Sense	-1.241	Detected	1.241	Detected	0.000	-3.493	Comprom	-3.611	Comprom	-3.552	0.104
GT Sense	Sense	0.142	Detected	-0.142	Detected	0.000	-1.421	Detected	-2.379	Detected	-1.900	0.063
GT Sense	Sense	0.085	Detected	-0.085	Detected	0.000	-0.911	Detected	-1.353	Detected	-1.132	0.041
GT Sense	Sense	-0.270	Detected	0.270	Detected	0.000	-1.169	Detected	-1.010	Detected	-1.090	0.061
GT Sense	Sense	0.021	Detected	-0.021	Detected	0.000	-0.826	Comprom	-1.481	Comprom	-1.154	0.072
GT Sense	Sense	0.109	Detected	-0.109	Detected	0.000	-1.580	Detected	-1.217	Detected	-1.399	0.022
GT Sense	Sense	-0.979	Detected	0.979	Detected	0.000	-2.436	Comprom	-1.315	Detected	-1.876	0.238
GT Sense	Sense	0.124	Detected	-0.124	Detected	0.000	-1.396	Comprom	-1.736	Comprom	-1.566	0.018
GT Sense	Sense	-0.490	Detected	0.490	Detected	0.000	-1.067	Detected	-0.990	Detected	-1.028	0.171
GT Sense	Sense	-1.372	Detected	1.372	Detected	0.000	-3.786	Comprom	-2.236	Comprom	-3.011	0.196
GT Sense	Sense	0.132	Detected	-0.132	Detected	0.000	-1.526	Detected	-1.152	Detected	-1.339	0.028
GT Sense	Sense	0.321	Detected	-0.321	Detected	0.000	-0.853	Detected	-1.941	Comprom	-1.397	0.157
GT Sense	Sense	-0.288	Detected	0.288	Detected	0.000	-1.173	Detected	-1.600	Detected	-1.387	0.061
GT Sense	Sense	-1.133	Detected	1.133	Detected	0.000	-1.906	Detected	-0.803	Detected	-1.355	0.395
GT Sense	Sense	-0.634	Detected	0.634	Detected	0.000	-1.406	Detected	-1.545	Detected	-1.476	0.147
GT Sense	Sense	-0.337	Detected	0.337	Detected	0.000	-1.222	Detected	-0.912	Detected	-1.067	0.103
GT Sense	Sense	-0.575	Detected	0.575	Detected	0.000	-0.924	Detected	-1.401	Detected	-1.163	0.203
GT Sense	Sense	-0.774	Detected	0.774	Detected	0.000	-2.222	Detected	-1.697	Detected	-1.960	0.139
GT Sense	Sense	-0.722	Detected	0.722	Detected	0.000	-2.235	Comprom	-2.340	Comprom	-2.287	0.087
GT Sense	Sense	-1.048	Detected	1.048	Detected	0.000	-2.469	Detected	-4.266	Detected	-3.368	0.135
GT Sense	Sense	-0.781	Detected	0.781	Detected	0.000	-1.950	Detected	-1.620	Detected	-1.785	0.155
GT Sense	Sense	-0.934	Detected	0.934	Detected	0.000	-0.841	Detected	-1.639	Detected	-1.240	0.347
GT Sense	Sense	-1.094	Detected	1.094	Detected	0.000	-2.334	Comprom	-1.302	Detected	-1.818	0.272
GT Sense	Sense	-0.507	Detected	0.507	Detected	0.000	-1.678	Detected	-2.491	Detected	-2.085	0.085
GT Sense	Sense	-0.655	Detected	0.655	Detected	0.000	-1.775	Detected	-1.497	Detected	-1.636	0.135
GT Sense	Sense	0.100	Detected	-0.100	Detected	0.000	-1.736	Detected	-2.121	Detected	-1.928	0.012
GT Sense	Sense	-0.115	Detected	0.115	Detected	0.000	-1.066	Detected	-1.043	Detected	-1.054	0.012
GT Sense	Sense	-0.781	Detected	0.781	Detected	0.000	-1.378	Detected	-1.681	Detected	-1.530	0.195
GT Sense	Sense	0.035	Detected	-0.035	Detected	0.000	-1.113	Detected	-1.886	Detected	-1.500	0.061
GT Sense	Sense	-0.365	Detected	0.365	Detected	0.000	-2.803	Comprom	-1.748	Comprom	-2.275	0.071
GT Sense	Sense	-0.094	Detected	0.094	Detected	0.000	-1.450	Detected	-0.836	Detected	-1.143	0.071
GT Sense	Sense	-0.574	Detected	0.574	Detected	0.000	-1.208	Detected	-1.311	Detected	-1.260	0.161
GT Sense	Sense	-0.218	Detected	0.218	Detected	0.000	-1.068	Detected	-1.101	Detected	-1.085	0.038
GT Sense	Sense	-0.024	Detected	0.024	Detected	0.000	-1.586	Comprom	-2.331	Comprom	-1.958	0.034
GT Sense	Sense	0.063	Detected	-0.063	Detected	0.000	-1.287	Detected	-0.966	Detected	-1.127	0.023
GT Sense	Sense	-0.514	Detected	0.514	Detected	0.000	-1.123	Detected	-1.015	Detected	-1.069	0.174
GT Sense	Sense	0.209	Detected	-0.209	Detected	0.000	-1.114	Detected	-1.093	Detected	-1.103	0.034
GT Sense	Sense	-0.404	Detected	0.404	Detected	0.000	-1.645	Detected	-1.410	Detected	-1.527	0.068
GT Sense	Sense	0.019	Detected	-0.019	Detected	0.000	-1.981	Comprom	-1.773	Comprom	-1.877	0.003
GT Sense	Sense	-0.589	Detected	0.589	Detected	0.000	-1.250	Detected	-0.923	Detected	-1.087	0.217
GT Sense	Sense	1.140	Detected	-1.140	Detected	0.000	-1.207	Detected	-1.184	Detected	-1.196	0.404
GT Sense	Sense	-0.248	Detected	0.248	Detected	0.000	-0.949	Detected	-1.784	Detected	-1.367	0.107
GT Sense	Sense	-0.037	Detected	0.037	Detected	0.000	-1.235	Detected	-0.877	Detected	-1.056	0.029
GT Sense	Sense	-0.144	Detected	0.144	Detected	0.000	-1.070	Detected	-1.067	Detected	-1.069	0.018
GT Sense	Sense	0.001	Detected	-0.001	Detected	0.000	-1.395	Detected	-0.921	Detected	-1.158	0.039
GT Sense	Sense	0.155	Detected	-0.155	Detected	0.000	-1.519	Detected	-2.216	Detected	-1.868	0.039
GT Sense	Sense	0.376	Detected	-0.376	Detected	0.000	-1.489	Comprom	-1.107	Comprom	-1.298	0.091
GT Sense	Sense	-0.882	Detected	0.882	Detected	0.000	-1.893	Detected	-2.100	Detected	-1.997	0.153
GT Sense	Sense	-0.363	Detected	0.363	Detected	0.000	-1.567	Detected	-1.303	Detected	-1.435	0.065
GT Sense	Sense	-0.023	Detected	0.023	Detected	0.000	-2.188	Comprom	-0.902	Detected	-1.545	0.138
GT Sense	Sense	2.809	Detected	-2.809	Detected	0.000	-3.838	Detected	-1.103	Detected	-2.471	0.512
GT Sense	Sense	0.132	Detected	-0.132	Detected	0.000	-1.411	Detected	-1.562	Detected	-1.486	0.010
GT Sense	Sense	-0.745	Detected	0.745	Detected	0.000	-1.386	Detected	-1.573	Comprom	-1.479	0.188
GT Sense	Sense	-0.534	Detected	0.534	Detected	0.000	-1.290	Detected	-1.111	Detected	-1.201	0.157
GT Sense	Sense	-0.521	Detected	0.521	Detected	0.000	-1.965	Detected	-1.527	Detected	-1.746	0.091
GT Sense	Sense	-0.635	Detected	0.635	Detected	0.000	-1.061	Detected	-1.044	Detected	-1.053	0.239
GT Sense	Sense	-0.195	Detected	0.195	Detected	0.000	-1.408	Detected	-1.803	Detected	-1.605	0.029
GT Sense	Sense	0.244	Detected	-0.244	Detected	0.000	-1.231	Comprom	-1.234	Comprom	-1.233	0.037
GT Sense	Sense	0.513	Detected	-0.513	Detected	0.000	-1.275	Detected	-0.814	Detected	-1.044	0.204
GT Sense	Sense	-0.447	Detected	0.447	Detected	0.000	-1.415	Detected	-0.998	Detected	-1.206	0.134
GT Sense	Sense	-0.345	Detected	0.345	Detected	0.000	-1.218	Detected	-1.181	Detected	-1.200	0.074
GT Sense	Sense	-0.692	Detected	0.692	Detected	0.000	-2.079	Detected	-0.959	Detected	-1.519	0.230
GT Sense	Sense	-0.298	Detected	0.298	Detected	0.000	-1.159	Detected	-1.010	Detected	-1.084	0.072
GT Sense	Sense	-0.438	Detected	0.438	Detected	0.000	-1.055	Detected	-1.420	Detected	-1.237	0.121
GT Sense	Sense	-0.238	Detected	0.238	Detected	0.000	-0.856	Detected	-1.497	Detected	-1.177	0.098
GT Sense	Sense	-0.290	Detected	0.290	Detected	0.000	-1.016	Detected	-1.228	Detected	-1.122	0.068
GT Sense	Sense	-0.391	Detected	0.391	Detected	0.000	-1.793	Detected	-1.664	Detected	-1.728	0.049
GT Sense	Sense	-0.949	Detected	0.949	Detected	0.000	-2.203	Comprom	-3.117	Comprom	-2.660	0.127
GT Sense	Sense	0.158	Detected	-0.158	Detected	0.000	-1.238	Detected	-1.504	Detected	-1.371	0.022
GT Sense	Sense	-0.393	Detected	0.393	Detected	0.000	-0.971	Detected	-1.032	Detected	-1.001	0.126
GT Sense	Sense	-0.066	Detected	0.066	Detected	0.000	-1.517	Detected	-1.248	Detected	-1.383	0.012
GT Sense	Sense	0.083	Detected	-0.083	Detected	0.000	-4.019	Comprom	-4.130	Comprom	-4.074	0.001
GT Sense	Sense	-0.119	Detected	0.119	Detected	0.000	-1.941	Comprom	-1.397	Comprom	-1.669	0.030
GT Sense	Sense	-0.787	Detected	0.787	Detected	0.000	-1.809	Detected	-2.271	Detected	-2.040	0.131
GT Sense	Sense	-0.602	Detected	0.602	Detected	0.000	-1.676	Detected	-1.696	Detected	-1.686	0.107
GT Sense	Sense	-0.659	Detected	0.659	Detected	0.000	-1.107	Detected	-1.932	Comprom	-1.520	0.190
GT Sense	Sense	-1.320	Detected	1.320	Detected	0.000	-4.097	Comprom	-5.180	Comprom	-4.638	0.083
GT Sense	Sense	0.205	Detected	-0.205	Detected	0.000	-1.045	Comprom	-1.260	Comprom	-1.153	0.038
GT Sense	Sense	-0.214	Detected	0.214	Detected	0.000	-0.881	Detected	-1.218	Detected	-1.050	0.061
GT Sense	Sense	-0.855	Detected	0.855	Detected	0.000	-0.834	Detected	-1.629	Detected	-1.232	0.322
GT Sense	Sense	-0.658	Detected	0.658	Detected	0.000	-1.267	Detected	-1.068	Detected	-1.167	0.221
GT Sense	Sense	-1.038	Detected	1.038	Detected	0.000	-1.035	Detected	-1.911	Comprom	-1.473	0.321
GT Sense	Sense	0.111	Detected	-0.111	Detected	0.000	-1.308	Detected	-1.014	Detected	-1.161	0.024
GT Sense	Sense	-0.214	Detected	0.214	Detected	0.000	-0.844	Detected	-1.402	Detected	-1.123	0.086
GT Sense	Sense	-0.467	Detected	0.467	Detected	0.000	-0.883	Detected	-1.124	Detected	-1.003	0.173
GT Sense	Sense	-0.322	Detected	0.322	Detected	0.000	-1.360	Detected	-1.034	Detected	-1.197	0.080
GT Sense	Sense	-0.422	Detected	0.422	Detected	0.000	-0.904	Detected	-1.108	Detected	-1.006	0.146
GT Sense	Sense	-0.428	Detected	0.428	Detected	0.000	-1.798	Detected	-1.584	Detected	-1.691	0.062
GT Sense	Sense	-0.960	Detected	0.960	Detected	0.000	-3.041	Detected	-2.728	Detected	-2.885	0.097
GT Sense	Sense	-0.005	Detected	0.005	Detected	0.000	-0.885	Detected	-1.801	Detected	-1.343	0.099
GT Sense	Sense	-1.237	Detected	1.237	Detected	0.000	-0.922	Detected	-1.844	Detected	-1.383	0.405
GT Sense	Sense	-0.832	Detected	0.832	Detected	0.000	-1.798	Detected	-1.573	Detected	-1.685	0.183
GT Sense	Sense	0.416	Detected	-0.416	Detected	0.000	-1.512	Detected	-1.834	Detected	-1.673	0.064
GT Sense	Sense	-1.057	Detected	1.057	Detected	0.000	-2.498	Detected	-2.606	Detected	-2.552	0.137
GT Sense	Sense	-0.208	Detected	0.208	Detected	0.000	-1.037	Detected	-1.554	Detected	-1.296	0.060
GT Sense	Sense	-0.390	Detected	0.390	Detected	0.000	-1.692	Detected	-2.429	Detected	-2.060	0.062
GT Sense	Sense	-0.716	Detected	0.716	Detected	0.000	-2.973	Detected	-2.869	Detected	-2.921	0.055
GT Sense	Sense	0.276	Detected	-0.276	Detected	0.000	-1.880	Comprom	-1.251	Comprom	-1.565	0.065
GT Sense	Sense	0.122	Detected	-0.122	Detected	0.000	-1.120	Comprom	-1.419	Comprom	-1.269	0.022
GT Sense	Sense	-0.476	Detected	0.476	Detected	0.000	-0.990	Detected	-1.090	Detected	-1.040	0.162
GT Sense	Sense	-0.188	Detected	0.188	Detected	0.000	-1.133	Detected	-0.950	Detected	-1.042	0.038
GT Sense	Sense	0.102	Detected	-0.102	Detected	0.000	-1.900	Comprom	-1.452	Comprom	-1.676	0.021
GT Sense	Sense	-1.004	Detected	1.004	Detected	0.000	-2.117	Detected	-2.520	Detected	-2.318	0.152
GT Sense	Sense	-0.096	Detected	0.096	Detected	0.000	-2.046	Detected	-1.959	Detected	-2.003	0.003
GT Sense	Sense	-0.528	Detected	0.528	Detected	0.000	-0.892	Detected	-1.391	Detected	-1.142	0.190

GT	Sense	Sense	-0.427	Detected	0.427	Detected	0.000	-1.277	Detected	-1.958	Detected	-1.617	0.098	GT	Sens
GT	Sense	Sense	-0.323	Detected	0.323	Detected	0.000	-1.193	Detected	-1.193	Detected	-1.193	0.066	GT	Sens
GT	Sense	Sense	-0.094	Detected	0.094	Detected	0.000	-1.563	Detected	-1.721	Detected	-1.642	0.006	GT	Sens
GT	Sense	Sense	-0.442	Detected	0.442	Detected	0.000	-1.480	Detected	-1.012	Detected	-1.246	0.130	GT	Sens
GT	Sense	Sense	-0.087	Detected	0.087	Detected	0.000	-1.554	Detected	-0.976	Detected	-1.265	0.053	GT	Sens
GT	Sense	Sense	-0.255	Detected	0.255	Detected	0.000	-1.317	Comprom	-1.686	Comprom	-1.502	0.041	GT	Sens
GT	Sense	Sense	-1.101	Detected	1.101	Detected	0.000	-4.878	Comprom	-4.600	Comprom	-4.739	0.051	GT	Sens
GT	Sense	Sense	-1.631	Detected	1.631	Detected	0.000	-1.654	Detected	-1.538	Detected	-1.596	0.431	GT	Sens
GT	Sense	Sense	0.155	Detected	-0.155	Detected	0.000	-0.872	Detected	-1.997	Comprom	-1.435	0.133	GT	Sens
GT	Sense	Sense	0.232	Detected	-0.232	Detected	0.000	-0.965	Comprom	-1.460	Comprom	-1.213	0.070	GT	Sens
GT	Sense	Sense	-0.359	Detected	0.359	Detected	0.000	-1.145	Detected	-1.025	Detected	-1.085	0.096	GT	Sens
GT	Sense	Sense	-0.638	Detected	0.638	Detected	0.000	-0.954	Detected	-1.481	Detected	-1.218	0.220	GT	Sens
GT	Sense	Sense	0.048	Detected	-0.048	Detected	0.000	-0.823	Detected	-1.221	Comprom	-1.022	0.038	GT	Sens
GT	Sense	Sense	-0.261	Detected	0.261	Detected	0.000	-0.872	Detected	-1.336	Detected	-1.104	0.087	GT	Sens
GT	Sense	Sense	-1.146	Detected	1.146	Detected	0.000	-2.985	Detected	-3.450	Detected	-3.218	0.111	GT	Sens
GT	Sense	Sense	0.225	Detected	-0.225	Detected	0.000	-2.408	Detected	-1.869	Detected	-2.138	0.026	GT	Sens
GT	Sense	Sense	-0.019	Detected	0.019	Detected	0.000	-1.881	Detected	-2.167	Detected	-2.024	0.005	GT	Sens
GT	Sense	Sense	-0.462	Detected	0.462	Detected	0.000	-1.709	Detected	-1.310	Detected	-1.510	0.095	GT	Sens
GT	Sense	Sense	-1.712	Detected	1.712	Detected	0.000	-2.687	Detected	-3.379	Detected	-3.033	0.225	GT	Sens
GT	Sense	Sense	0.214	Detected	-0.214	Detected	0.000	-1.592	Detected	-1.187	Detected	-1.389	0.042	GT	Sens
GT	Sense	Sense	-0.513	Detected	0.513	Detected	0.000	-2.324	Comprom	-2.549	Comprom	-2.437	0.043	GT	Sens
GT	Sense	Sense	-0.496	Detected	0.496	Detected	0.000	-1.282	Detected	-1.329	Detected	-1.305	0.119	GT	Sens
GT	Sense	Sense	-0.067	Detected	0.067	Detected	0.000	-2.270	Detected	-2.118	Detected	-2.194	0.002	GT	Sens
GT	Sense	Sense	0.246	Detected	-0.246	Detected	0.000	-2.939	Comprom	-2.574	Comprom	-2.757	0.012	GT	Sens
GT	Sense	Sense	-1.009	Detected	1.009	Detected	0.000	-2.608	Detected	-3.832	Detected	-3.220	0.112	GT	Sens
GT	Sense	Sense	-1.217	Detected	1.217	Detected	0.000	-3.412	Detected	-3.102	Detected	-3.257	0.117	GT	Sens
GT	Sense	Sense	-0.789	Detected	0.789	Detected	0.000	-1.757	Detected	-1.936	Detected	-1.846	0.146	GT	Sens
GT	Sense	Sense	0.036	Detected	-0.036	Detected	0.000	-0.947	Detected	-2.308	Detected	-1.627	0.139	GT	Sens
GT	Sense	Sense	-0.404	Detected	0.404	Detected	0.000	-1.555	Detected	-1.440	Detected	-1.498	0.067	GT	Sens
GT	Sense	Sense	-1.094	Detected	1.094	Detected	0.000	-0.871	Detected	-2.208	Detected	-1.539	0.353	GT	Sens
GT	Sense	Sense	-0.760	Detected	0.760	Detected	0.000	-2.076	Comprom	-1.328	Detected	-1.702	0.182	GT	Sens
GT	Sense	Sense	-0.519	Detected	0.519	Detected	0.000	-2.084	Comprom	-1.284	Comprom	-1.684	0.124	GT	Sens
GT	Sense	Sense	-0.648	Detected	0.648	Detected	0.000	-1.542	Detected	-1.409	Detected	-1.476	0.152	GT	Sens
GT	Sense	Sense	0.353	Detected	-0.353	Detected	0.000	-2.461	Detected	-3.285	Detected	-2.873	0.034	GT	Sens
GT	Sense	Sense	-1.362	Detected	1.362	Detected	0.000	-3.054	Detected	-2.137	Detected	-2.596	0.213	GT	Sens
GT	Sense	Sense	-0.043	Detected	0.043	Detected	0.000	-1.303	Detected	-2.355	Comprom	-1.829	0.074	GT	Sens
GT	Sense	Sense	-0.173	Detected	0.173	Detected	0.000	-1.054	Detected	-1.139	Comprom	-1.097	0.025	GT	Sens
GT	Sense	Sense	-0.266	Detected	0.266	Detected	0.000	-1.146	Comprom	-1.717	Comprom	-1.431	0.067	GT	Sens
GT	Sense	Sense	-0.404	Detected	0.404	Detected	0.000	-1.253	Detected	-1.121	Detected	-1.187	0.101	GT	Sens
GT	Sense	Sense	-0.251	Detected	0.251	Detected	0.000	-0.848	Detected	-1.432	Detected	-1.140	0.098	GT	Sens
GT	Sense	Sense	-0.314	Detected	0.314	Detected	0.000	-0.992	Detected	-1.109	Detected	-1.051	0.082	GT	Sens
GT	Sense	Sense	-0.854	Detected	0.854	Detected	0.000	-1.317	Detected	-1.566	Detected	-1.441	0.237	GT	Sens
GT	Sense	Sense	0.029	Detected	-0.029	Detected	0.000	-1.197	Comprom	-1.823	Comprom	-1.510	0.041	GT	Sens
GT	Sense	Sense	-0.355	Detected	0.355	Detected	0.000	-3.034	Detected	-2.786	Detected	-2.910	0.016	GT	Sens
GT	Sense	Sense	-0.531	Detected	0.531	Detected	0.000	-1.054	Detected	-1.180	Detected	-1.117	0.172	GT	Sens
GT	Sense	Sense	-1.231	Detected	1.231	Detected	0.000	-1.589	Detected	-1.538	Detected	-1.564	0.332	GT	Sens
GT	Sense	Sense	-0.535	Detected	0.535	Detected	0.000	-1.450	Detected	-2.192	Detected	-1.821	0.107	GT	Sens
GT	Sense	Sense	-0.464	Detected	0.464	Detected	0.000	-1.335	Detected	-0.914	Detected	-1.125	0.158	GT	Sens
GT	Sense	Sense	-1.353	Detected	1.353	Detected	0.000	-0.951	Detected	-2.817	Detected	-1.884	0.370	GT	Sens
GT	Sense	Sense	0.175	Detected	-0.175	Detected	0.000	-1.674	Detected	-1.233	Detected	-1.454	0.036	GT	Sens
GT	Sense	Sense	0.176	Detected	-0.176	Detected	0.000	-1.662	Detected	-2.475	Comprom	-2.069	0.043	GT	Sens
GT	Sense	Sense	-1.219	Detected	1.219	Detected	0.000	-1.217	Detected	-1.653	Detected	-1.435	0.366	GT	Sens
GT	Sense	Sense	0.149	Detected	-0.149	Detected	0.000	-2.523	Detected	-1.958	Detected	-2.241	0.020	GT	Sens
GT	Sense	Sense	-1.032	Detected	1.032	Detected	0.000	-2.388	Detected	-2.973	Detected	-2.680	0.130	GT	Sens
GT	Sense	Sense	-0.195	Detected	0.195	Detected	0.000	-0.920	Detected	-1.093	Detected	-1.006	0.042	GT	Sens
GT	Sense	Sense	-0.061	Detected	0.061	Detected	0.000	-1.296	Comprom	-1.339	Comprom	-1.318	0.002	GT	Sens
GT	Sense	Sense	-0.268	Detected	0.268	Detected	0.000	-1.833	Detected	-1.592	Detected	-1.713	0.028	GT	Sens
GT	Sense	Sense	-0.374	Detected	0.374	Detected	0.000	-1.144	Detected	-1.046	Detected	-1.095	0.101	GT	Sens
GT	Sense	Sense	0.150	Detected	-0.150	Detected	0.000	-0.896	Detected	-1.266	Detected	-1.081	0.045	GT	Sens
GT	Sense	Sense	0.135	Detected	-0.135	Detected	0.000	-1.417	Detected	-2.079	Detected	-1.748	0.039	GT	Sens
GT	Sense	Sense	-0.668	Detected	0.668	Detected	0.000	-1.974	Detected	-2.157	Detected	-2.066	0.092	GT	Sens
GT	Sense	Sense	-0.783	Detected	0.783	Detected	0.000	-1.358	Detected	-1.396	Detected	-1.377	0.221	GT	Sens
GT	Sense	Sense	-0.130	Detected	0.130	Detected	0.000	-2.320	Detected	-2.309	Detected	-2.314	0.003	GT	Sens
GT	Sense	Sense	-0.206	Detected	0.206	Detected	0.000	-1.234	Detected	-0.933	Detected	-1.084	0.051	GT	Sens
GT	Sense	Sense	-0.664	Detected	0.664	Detected	0.000	-1.215	Detected	-1.233	Detected	-1.224	0.207	GT	Sens
GT	Sense	Sense	-0.603	Detected	0.603	Detected	0.000	-0.913	Detected	-1.573	Detected	-1.243	0.212	GT	Sens
GT	Sense	Sense	0.433	Detected	-0.433	Detected	0.000	-0.983	Detected	-1.123	Detected	-1.053	0.139	GT	Sens
GT	Sense	Sense	-0.260	Detected	0.260	Detected	0.000	-1.199	Comprom	-1.253	Comprom	-1.226	0.043	GT	Sens
GT	Sense	Sense	-0.672	Detected	0.672	Detected	0.000	-2.241	Detected	-2.011	Detected	-2.126	0.089	GT	Sens
GT	Sense	Sense	0.198	Detected	-0.198	Detected	0.000	-1.432	Detected	-1.645	Detected	-1.538	0.021	GT	Sens
GT	Sense	Sense	0.158	Detected	-0.158	Detected	0.000	-1.559	Detected	-2.265	Detected	-1.912	0.038	GT	Sens
GT	Sense	Sense	-0.454	Detected	0.454	Detected	0.000	-1.293	Detected	-0.973	Detected	-1.133	0.143	GT	Sens
GT	Sense	Sense	-0.180	Detected	0.180	Detected	0.000	-1.591	Comprom	-2.503	Comprom	-2.047	0.053	GT	Sens
GT	Sense	Sense	-0.403	Detected	0.403	Detected	0.000	-0.807	Detected	-1.751	Detected	-1.279	0.176	GT	Sens
GT	Sense	Sense	-1.865	Detected	1.865	Detected	0.000	-1.848	Detected	-1.756	Detected	-1.802	0.436	GT	Sens
GT	Sense	Sense	-0.386	Detected	0.386	Detected	0.000	-1.008	Detected	-1.713	Detected	-1.360	0.121	GT	Sens
GT	Sense	Sense	-0.284	Detected	0.284	Detected	0.000	-1.267	Detected	-1.135	Detected	-1.201	0.054	GT	Sens
GT	Sense	Sense	0.145	Detected	-0.145	Detected	0.000	-2.013	Detected	-0.989	Detected	-1.501	0.106	GT	Sens
GT	Sense	Sense	-0.004	Detected	0.004	Detected	0.000	-0.959	Detected	-1.665	Detected	-1.312	0.065	GT	Sens
GT	Sense	Sense	-0.477	Detected	0.477	Detected	0.000	-1.117	Detected	-1.131	Detected	-1.124	0.142	GT	Sens
GT	Sense	Sense	-0.592	Detected	0.592	Detected	0.000	-3.030	Detected	-2.483	Detected	-2.757	0.052	GT	Sens
GT	Sense	Sense	-0.270	Detected	0.270	Detected	0.000	-1.188	Detected	-1.136	Detected	-1.162	0.050	GT	Sens
GT	Sense	Sense	0.051	Detected	-0.051	Detected	0.000	-1.367	Comprom	-0.935	Comprom	-1.151	0.035	GT	Sens
GT	Sense	Sense	-0.619	Detected	0.619	Detected	0.000	-1.734	Detected	-1.468	Detected	-1.601	0.127	GT	Sens
GT	Sense	Sense	-0.584	Detected	0.584	Detected	0.000	-1.672	Detected	-1.569	Detected	-1.620	0.110	GT	Sens
GT	Sense	Sense	-0.241	Detected	0.241	Detected	0.000	-0.869	Detected	-1.266	Detected	-1.068	0.076	GT	Sens
GT	Sense	Sense	-0.004	Detected	0.004	Detected	0.000	-0.869	Detected	-1.171	Detected	-1.020	0.021	GT	Sens
GT	Sense	Sense	0.154	Detected	-0.154	Detected	0.000	-1.289	Detected	-0.802	Detected	-1.045	0.068	GT	Sens
GT	Sense	Sense	0.254	Detected	-0.254	Detected	0.000	-0.964	Detected	-1.434	Detected	-1.199	0.074	GT	Sens
GT	Sense	Sense	0.203	Detected	-0.203	Detected	0.000	-2.238	Detected	-3.366	Detected	-2.802	0.043	GT	Sens
GT	Sense	Sense	0.094	Detected	-0.094	Detected	0.000	-1.085	Detected	-1.329	Detected	-1.207	0.016	GT	Sens
GT	Sense	Sense	0.179	Detected	-0.179	Detected	0.000	-1.014	Detected	-1.249	Detected	-1.132	0.034	GT	Sens
GT	Sense	Sense	0.198	Detected	-0.198	Detected	0.000	-1.800	Comprom	-1.634	Comprom	-1.717	0.015	GT	Sens
GT	Sense	Sense	-0.385	Detected	0.385	Detected	0.000	-1.843	Comprom	-2.594	Comprom	-2.219	0.054	GT	Sens
GT	Sense	Sense	-0.602	Detected	0.602	Detected	0.000	-2.455	Detected	-2.228	Detected	-2.341	0.062	GT	Sens
GT	Sense	Sense	-1.358	Detected	1.358	Detected	0.000	-3.571	Detected	-3.221	Detected	-3.396	0.131	GT	Sens
GT	Sense	Sense	-0.950	Detected	0.950	Detected	0.000	-1.448	Detected	-1.330	Detected	-1.389	0.282	GT	Sens
GT	Sense	Sense	-0.654	Detected	0.654	Detected	0.000	-1.197	Detected	-1.207	Detected	-1.202	0.207	GT	Sens
GT	Sense	Sense	-0.325	Detected	0.325	Detected	0.000	-3.434	Comprom	-3.067	Comprom	-3.251	0.013	GT	Sens
GT	Sense	Sense	-0.246	Detected	0.246	Detected	0.000	-1.151	Detected	-1.143	Detected	-1.147	0.043	GT	Sens
GT	Sense	Sense	-0.727	Detected	0.727	Detected	0.000	-3.708	Detected	-3.316	Detected	-3.512	0.043	GT	Sens
GT	Sense	Sense	0.355	Detected	-0.355	Detected	0.000	-0.956	Detected	-1.629	Detected	-1.293	0.118	GT	Sens
GT	Sense	Sense	0.168	Detected	-0.168	Detected	0.000	-1.343	Detected	-1.049	Detected	-1.196	0.033	GT	Sens
GT	Sense	Sense	-0.896	Detected	0.896	Detected	0.000	-2.011	Comprom	-2.135	Comprom	-2.073	0.147	GT	Sens
GT	Sense	Sense	-1.821	Detected	1.821	Detected	0.000	-1.780	Detected	-4.624	Comprom	-3.202	0.300	GT	Sens
GT	Sense	Sense	-2.246	Detected	2.246	Detected	0.000	-1.880	Detected	-4.016	Comprom	-2.948	0.358	GT	Sens
GT	Sense	Sense	-0.308	Detected	0.308	Detected	0.000	-1.805	Detected	-1.712	Detected	-1.758	0.030	GT	Sens
GT	Sense	Sense	-0.101	Detected	0.101	Detected	0.000	-0.958	Detected	-1.686	Comprom	-1.322	0.073	GT	Sens
GT	Sense	Sense	-0.550	Detected	0.550	Detected	0.000	-0.918	Detected	-1.759	Comprom	-1.339	0.193	GT	Sens
GT	Sense	Sense	-0.243	Detected	0.243	Detected	0.000	-2.238	Detected	-2.471	Detected	-2.355	0.013	GT	Sens
GT	Sense	Sense	0.121	Detected	-0.121	Detected	0.000	-2.263	Comprom	-0.805	Detected	-1.534	0.174	GT	Sens
GT	Sense	Sense	-0.097	Detected	0.097	Detected	0.000	-1.589	Detected	-3.259	Comprom	-2.424	0.102	GT	Sens
GT	Sense	Sense	-0.432	Detected	0.432	Detected	0.000	-1.588	Detected	-1.432	Detected	-1.510	0.075	GT	Sens
GT	Sense	Sense	-0.407	Detected	0.407	Detected	0.000	-0.975	Detected	-1.242	Detected	-1.108	0.122	GT	Sens
GT	Sense	Sense	0.308	Detected	-0.308	Detected	0.000	-1.713	Detected	-1.661	Detected	-1.687	0.032	GT	Sens
GT	Sense	Sense	0.004	Detected	-0.004	Detected	0.000	-1.923	Detected	-1.282	Detected	-1.603	0.038	GT	Sens
GT	Sense	Sense	-0.943	Detected	0.943	Detected	0.000	-1.084	Detected	-1.376	Detected	-1.230	0.326	GT	Sens
GT	Sense	Sense	0.016	Detected	-0.016	Detected	0.000	-1.227	Comprom	-1.572	Comprom	-1.400	0.015	GT	Sens
GT	Sense	Sense	-0.010	Detected	0.010	Detected	0.000	-0.894	Detected	-2.368	Detected	-1.631	0.158	GT	Sens
GT	Sense	Sense	-0.818	Detected	0.818	Detected	0.000	-1.368	Detected	-1.646	Detected	-1.507	0.211	GT	Sens
GT	Sense	Sense	-0.570	Detected	0.570	Detected	0.000	-0.806	Detected	-1.415	Detected	-1.110	0.228	GT	Sens
GT	Sense	Sense	-0.099	Detected	0.099	Detected	0.000	-1.049	Detected	-1.474	Detected	-1.261	0.033	GT	Sens
GT	Sense	Sense	-1.356	Detected	1.356	Detected	0.000	-1.419	Detected	-1.920	Comprom	-1.670	0.350	GT	Sens
GT	Sense	Sense	-0.639	Detected	0.639	Detected	0.000	-2.015	Detected	-1.623	Detected	-1.819	0.113	GT	Sens
GT	Sense	Sense	0.192	Detected	-0.192	Detected	0.000	-1.078	Detected	-1.554	Comprom	-1.316	0.050	GT	Sens
GT	Sense	Sense	-0.143	Detected	0.143	Detected	0.000	-1.832	Comprom	-1.633	Comprom	-1.732	0.010	GT	Sens
GT	Sense	Sense	-0.646	Detected	0.646	Detected	0.000	-1.613	Detected	-1.645	Detected	-1.629	0.128	GT	Sens
GT	Sense	Sense	-0.759	Detected	0.759	Detected	0.000	-1.520	Detected	-2.241	Detected	-1.881	0.154	GT	Sens
GT	Sense	Sense	0.142	Detected	-0.142	Detected	0.000	-1.698	Comprom	-1.139	Detected	-1.419	0.046	GT	Sens
GT	Sense	Sense	0.135	Detected	-0.135	Detected	0.000	-1.383	Detected	-1.305	Detected	-1.344	0.011	GT	Sens
GT	Sense	Sense	-0.843	Detected	0.843	Detected	0.000	-0.831	Detected	-1.568	Detected	-1.199	0.322	GT	Sens
GT	Sense	Sense	-0.731	Detected	0.731	Detected	0.000	-1.057	Detected	-1.444	Detected	-1.251	0.240	GT	Sens
GT	Sense	Sense	-0.638	Detected	0.638	Detected	0.000	-1.224	Detected	-1.356	Detected	-1.290	0.182	GT	Sens
GT	Sense	Sense	-0.939	Detected	0.939	Detected	0.000	-1.554	Detected	-2.134	Detected	-1.844	0.202	GT	Sens
GT	Sense	Sense	-0.656	Detected	0.656	Detected	0.000	-1.327	Detected	-1.298	Detected	-1.312	0.183	GT	Sens
GT	Sense	Sense	-0.469	Detected	0.469	Detected	0.000	-1.218	Detected	-1.116	Detected	-1.167	0.132	GT	Sens
GT	Sense	Sense	0.105	Detected	-0.105	Detected	0.000	-1.932	Comprom	-1.841	Comprom	-1.887	0.004	GT	Sens
GT	Sense	Sense	-0.028	Detected	0.028	Detected	0.000	-1.647	Comprom	-1.103	Comprom	-1.375	0.037	GT	Sens
GT	Sense	Sense	-0.691	Detected	0.691	Detected	0.000	-1.903	Detected	-2.213	Detected	-2.058	0.101	GT	Sens
GT	Sense	Sense	0.032	Detected	-0.032	Detected	0.000	-1.254	Detected	-1.910	Detected	-1.582	0.041	GT	Sens
GT	Sense	Sense	0.094	Detected	-0.094	Detected	0.000	-1.432	Detected	-1.058	Detected	-1.245	0.027	GT	Sens
GT	Sense	Sense	-0.378	Detected	0.378	Detected	0.000	-1.037	Detected	-1.393	Detected	-1.215	0.100	GT	Sens
GT	Sense	Sense	1.531	Detected	-1.531	Detected	0.000	-3.714	Comprom	-2.799	Comprom	-3.256	0.178	GT	Sens
GT	Sense	Sense	-0.007	Detected	0.007	Detected	0.000	-1.443	Comprom	-1.983	Comprom	-1.713	0.024	GT	Sens
GT	Sense	Sense	-0.482	Detected	0.482	Detected	0.000	-1.570	Detected	-1.039	Detected	-1.304	0.141	GT	Sens
GT	Sense	Sense	0.215	Detected	-0.215	Detected	0.000	-1.038	Detected	-0.977	Detected	-1.007	0.043	GT	Sens
GT	Sense	Sense	-0.728	Detected	0.728	Detected	0.000	-1.368	Detected	-1.015	Detected	-1.192	0.253	GT	Sens
GT	Sense	Sense	-0.561	Detected	0.561	Detected	0.000	-1.956	Detected	-1.442	Detected	-1.699	0.110	GT	Sens
GT	Sense	Sense	-0.341	Detected	0.341	Detected	0.000	-0.981	Detected	-1.082	Detected	-1.032	0.096	GT	Sens
GT	Sense	Sense	-0.524	Detected	0.524	Detected	0.000	-1.154	Detected	-1.578	Detected	-1.366	0.137	GT	Sens
GT	Sense	Sense	0.520	Detected	-0.520	Detected	0.000	-1.407	Detected	-1.154	Detected	-1.281	0.139	GT	Sens
GT	Sense	Sense	-0.684	Detected	0.684	Detected	0.000	-1.690	Detected	-2.639	Detected	-2.164	0.121	GT	Sens
GT	Sense	Sense	0.012	Detected	-0.012	Detected	0.000	-1.036	Detected	-1.050	Detected	-1.043	0.000	GT	Sens
GT	Sense	Sense	0.373	Detected	-0.373	Detected	0.000	-1.961	Detected	-1.518	Detected	-1.740	0.057	GT	Sens
GT	Sense	Sense	0.392	Detected	-0.392	Detected	0.000	-1.017	Detected	-1.139	Detected	-1.078	0.113	GT	Sens
GT	Sense	Sense	-0.359	Detected	0.359	Detected	0.000	-0.997	Detected	-1.109	Detected	-1.053	0.101	GT	Sens
GT	Sense	Sense	-1.116	Detected	1.116	Detected	0.000	-1.824	Detected	-2.325	Detected	-2.074	0.211	GT	Sens
GT	Sense	Sense	-0.507	Detected	0.507	Detected	0.000	-1.460	Detected	-0.874	Detected	-1.167	0.184	GT	Sens
GT	Sense	Sense	-0.473	Detected	0.473	Detected	0.000	-1.065	Detected	-1.265	Detected	-1.165	0.138	GT	Sens
GT	Sense	Sense	0.234	Detected	-0.234	Detected	0.000	-1.179	Comprom	-2.262	Comprom	-1.721	0.100	GT	Sens
GT	Sense	Sense	-0.145	Detected	0.145	Detected	0.000	-1.360	Detected	-1.989	Detected	-1.675	0.040	GT	Sens
GT	Sense	Sense	0.095	Detected	-0.095	Detected	0.000	-1.328	Comprom	-0.968	Comprom	-1.148	0.030	GT	Sens
GT	Sense	Sense	-1.468	Detected	1.468	Detected	0.000	-1.725	Detected	-2.150	Comprom	-1.938	0.321	GT	Sens
GT	Sense	Sense	0.284	Detected	-0.284	Detected	0.000	-1.529	Detected	-1.029	Detected	-1.279	0.077	GT	Sens
GT	Sense	Sense	-0.685	Detected	0.685	Detected	0.000	-0.820	Detected	-1.313	Detected	-1.066	0.281	GT	Sens
GT	Sense	Sense	-0.805	Detected	0.805	Detected	0.000	-1.673	Detected	-1.426	Detected	-1.550	0.197	GT	Sens
GT	Sense	Sense	-0.633	Detected	0.633	Detected	0.000	-1.485	Detected	-1.742	Detected	-1.613	0.130	GT	Sens
GT	Sense	Sense	-0.575	Detected	0.575	Detected	0.000	-1.276	Detected	-1.233	Detected	-1.255	0.161	GT	Sens
GT	Sense	Sense	0.463	Detected	-0.463	Detected	0.000	-1.528	Detected	-0.885	Detected	-1.207	0.166	GT	Sens
GT	Sense	Sense	-0.499	Detected	0.499	Detected	0.000	-1.042	Detected	-1.383	Detected	-1.213	0.148	GT	Sens
GT	Sense	Sense	-0.109	Detected	0.109	Detected	0.000	-1.790	Detected	-1.395	Detected	-1.592	0.019	GT	Sens
GT	Sense	Sense	-0.345	Detected	0.345	Detected	0.000	-1.244	Detected	-0.830	Detected	-1.037	0.123	GT	Sens
GT	Sense	Sense	-0.752	Detected	0.752	Detected	0.000	-2.662	Detected	-3.770	Detected	-3.216	0.075	GT	Sens
GT	Sense	Sense	0.040	Detected	-0.040	Detected	0.000	-2.111	Comprom	-1.845	Comprom	-1.978	0.005	GT	Sens
GT	Sense	Sense	0.427	Detected	-0.427	Detected	0.000	-1.489	Detected	-1.726	Detected	-1.607	0.068	GT	Sens
GT	Sense	Sense	-0.922	Detected	0.922	Detected	0.000	-2.310	Detected	-1.725	Detected	-2.017	0.172	GT	Sens
GT	Sense	Sense	-0.258	Detected	0.258	Detected	0.000	-1.209	Detected	-0.991	Detected	-1.100	0.059	GT	Sens
GT	Sense	Sense	-0.468	Detected	0.468	Detected	0.000	-1.201	Detected	-1.594	Detected	-1.398	0.111	GT	Sens
GT	Sense	Sense	0.222	Detected	-0.222	Detected	0.000	-1.836	Detected	-1.995	Detected	-1.916	0.015	GT	Sens
GT	Sense	Sense	-1.284	Detected	1.284	Detected	0.000	-2.123	Detected	-1.438	Detected	-1.780	0.312	GT	Sens
GT	Sense	Sense	-0.957	Detected	0.957	Detected	0.000	-1.878	Detected	-2.053	Detected	-1.965	0.177	GT	Sens
GT	Sense	Sense	-0.416	Detected	0.416	Detected	0.000	-1.393	Detected	-0.924	Detected	-1.158	0.136	GT	Sens
GT	Sense	Sense	-0.492	Detected	0.492	Detected	0.000	-1.005	Detected	-1.201	Detected	-1.103	0.159	GT	Sens
GT	Sense	Sense	-0.350	Detected	0.350	Detected	0.000	-1.321	Detected	-1.429	Detected	-1.375	0.060	GT	Sens
GT	Sense	Sense	-0.139	Detected	0.139	Detected	0.000	-1.313	Comprom	-1.487	Comprom	-1.400	0.013	GT	Sens
GT	Sense	Sense	0.364	Detected	-0.364	Detected	0.000	-1.026	Detected	-1.085	Detected	-1.056	0.102	GT	Sens
GT	Sense	Sense	-1.115	Detected	1.115	Detected	0.000	-1.367	Detected	-2.840	Detected	-2.103	0.256	GT	Sens
GT	Sense	Sense	0.086	Detected	-0.086	Detected	0.000	-1.787	Detected	-1.621	Detected	-1.704	0.005	GT	Sens
GT	Sense	Sense	0.402	Detected	-0.402	Detected	0.000	-1.865	Detected	-1.694	Detected	-1.780	0.049	GT	Sens
GT	Sense	Sense	-0.577	Detected	0.577	Detected	0.000	-1.161	Detected	-1.485	Detected	-1.323	0.158	GT	Sens
GT	Sense	Sense	-0.705	Detected	0.705	Detected	0.000	-2.189	Detected	-1.725	Detected	-1.957	0.119	GT	Sens
GT	Sense	Sense	-1.896	Detected	1.896	Detected	0.000	-1.608	Detected	-2.676	Comprom	-2.142	0.390	GT	Sens
GT	Sense	Sense	0.376	Detected	-0.376	Detected	0.000	-2.346	Comprom	-2.298	Comprom	-2.322	0.025	GT	Sens
GT	Sense	Sense	-0.383	Detected	0.383	Detected	0.000	-1.944	Detected	-2.251	Detected	-2.098	0.037	GT	Sens
GT	Sense	Sense	0.222	Detected	-0.222	Detected	0.000	-2.220	Comprom	-1.367	Comprom	-1.793	0.065	GT	Sens
GT	Sense	Sense	-0.433	Detected	0.433	Detected	0.000	-3.397	Comprom	-1.930	Comprom	-2.663	0.089	GT	Sens
GT	Sense	Sense	-0.204	Detected	0.204	Detected	0.000	-1.245	Detected	-1.115	Detected	-1.180	0.031	GT	Sens
GT	Sense	Sense	-0.501	Detected	0.501	Detected	0.000	-1.587	Detected	-1.370	Detected	-1.478	0.102	GT	Sens
GT	Sense	Sense	0.116	Detected	-0.116	Detected	0.000	-1.180	Comprom	-1.323	Comprom	-1.251	0.012	GT	Sens
GT	Sense	Sense	-0.551	Detected	0.551	Detected	0.000	-0.941	Detected	-1.348	Detected	-1.144	0.191	GT	Sens
GT	Sense	Sense	-0.114	Detected	0.114	Detected	0.000	-1.662	Detected	-1.009	Detected	-1.335	0.061	GT	Sens
GT	Sense	Sense	-0.146	Detected	0.146	Detected	0.000	-0.907	Detected	-1.571	Comprom	-1.239	0.076	GT	Sens
GT	Sense	Sense	0.228	Detected	-0.228	Detected	0.000	-1.238	Detected	-0.884	Detected	-1.061	0.067	GT	Sens
GT	Sense	Sense	-0.063	Detected	0.063	Detected	0.000	-1.073	Detected	-1.245	Detected	-1.159	0.008	GT	Sens
GT	Sense	Sense	-2.060	Detected	2.060	Detected	0.000	-2.088	Detected	-3.071	Detected	-2.579	0.347	GT	Sens
GT	Sense	Sense	0.353	Detected	-0.353	Detected	0.000	-1.676	Detected	-1.727	Detected	-1.701	0.041	GT	Sens
GT	Sense	Sense	-1.368	Detected	1.368	Detected	0.000	-1.264	Detected	-1.826	Detected	-1.545	0.384	GT	Sens
GT	Sense	Sense	-0.480	Detected	0.480	Detected	0.000	-0.808	Detected	-2.439	Comprom	-1.623	0.228	GT	Sens
GT	Sense	Sense	-0.977	Detected	0.977	Detected	0.000	-1.082	Detected	-2.089	Detected	-1.585	0.286	GT	Sens
GT	Sense	Sense	-0.233	Detected	0.233	Detected	0.000	-1.641	Comprom	-1.397	Comprom	-1.519	0.029	GT	Sens
GT	Sense	Sense	-0.258	Detected	0.258	Detected	0.000	-0.886	Detected	-1.331	Detected	-1.108	0.083	GT	Sens
GT	Sense	Sense	-0.413	Detected	0.413	Detected	0.000	-0.931	Detected	-1.520	Detected	-1.226	0.137	GT	Sens
GT	Sense	Sense	0.069	Detected	-0.069	Detected	0.000	-0.939	Detected	-1.116	Detected	-1.027	0.012	GT	Sens
GT	Sense	Sense	-0.446	Detected	0.446	Detected	0.000	-1.538	Detected	-1.547	Detected	-1.542	0.074	GT	Sens
GT	Sense	Sense	-0.445	Detected	0.445	Detected	0.000	-1.171	Detected	-1.055	Detected	-1.113	0.131	GT	Sens
GT	Sense	Sense	0.277	Detected	-0.277	Detected	0.000	-1.440	Detected	-1.661	Detected	-1.550	0.035	GT	Sens
GT	Sense	Sense	-0.910	Detected	0.910	Detected	0.000	-1.809	Detected	-2.230	Detected	-2.020	0.163	GT	Sens
GT	Sense	Sense	-0.570	Detected	0.570	Detected	0.000	-0.948	Detected	-1.981	Detected	-1.465	0.197	GT	Sens
GT	Sense	Sense	-0.321	Detected	0.321	Detected	0.000	-1.238	Detected	-0.944	Detected	-1.091	0.091	GT	Sens
GT	Sense	Sense	0.209	Detected	-0.209	Detected	0.000	-1.690	Comprom	-2.742	Comprom	-2.216	0.060	GT	Sens
GT	Sense	Sense	0.275	Detected	-0.275	Detected	0.000	-1.131	Detected	-0.972	Detected	-1.051	0.067	GT	Sens
GT	Sense	Sense	-0.409	Detected	0.409	Detected	0.000	-1.023	Detected	-1.172	Detected	-1.098	0.118	GT	Sens
GT	Sense	Sense	-0.492	Detected	0.492	Detected	0.000	-1.940	Detected	-2.821	Detected	-2.380	0.069	GT	Sens
GT	Sense	Sense	-0.779	Detected	0.779	Detected	0.000	-1.215	Detected	-1.213	Detected	-1.214	0.259	GT	Sens
GT	Sense	Sense	0.450	Detected	-0.450	Detected	0.000	-1.119	Detected	-1.580	Detected	-1.349	0.116	GT	Sens
GT	Sense	Sense	0.095	Detected	-0.095	Detected	0.000	-1.297	Detected	-0.953	Detected	-1.125	0.029	GT	Sens
GT	Sense	Sense	-1.291	Detected	1.291	Detected	0.000	-3.269	Detected	-2.585	Detected	-2.927	0.160	GT	Sens
GT	Sense	Sense	0.100	Detected	-0.100	Detected	0.000	-1.074	Detected	-1.218	Detected	-1.146	0.011	GT	Sens
GT	Sense	Sense	0.258	Detected	-0.258	Detected	0.000	-0.976	Detected	-1.253	Detected	-1.115	0.062	GT	Sens
GT	Sense	Sense	-0.356	Detected	0.356	Detected	0.000	-1.430	Detected	-1.012	Detected	-1.221	0.098	GT	Sens
GT	Sense	Sense	-0.982	Detected	0.982	Detected	0.000	-1.387	Detected	-1.683	Detected	-1.535	0.262	GT	Sens
GT	Sense	Sense	-0.332	Detected	0.332	Detected	0.000	-1.178	Detected	-1.326	Detected	-1.252	0.067	GT	Sens
GT	Sense	Sense	-2.165	Detected	2.165	Detected	0.000	-4.559	Comprom	-5.473	Comprom	-5.016	0.152	GT	Sens
GT	Sense	Sense	-0.346	Detected	0.346	Detected	0.000	-1.191	Detected	-1.131	Detected	-1.161	0.079	GT	Sens
GT	Sense	Sense	0.304	Detected	-0.304	Detected	0.000	-1.842	Detected	-1.165	Detected	-1.504	0.081	GT	Sens
GT	Sense	Sense	-0.858	Detected	0.858	Detected	0.000	-0.855	Detected	-1.598	Comprom	-1.226	0.320	GT	Sens
GT	Sense	Sense	-0.394	Detected	0.394	Detected	0.000	-0.987	Detected	-1.743	Detected	-1.365	0.130	GT	Sens
GT	Sense	Sense	-0.988	Detected	0.988	Detected	0.000	-2.097	Detected	-2.158	Detected	-2.127	0.164	GT	Sens
GT	Sense	Sense	-1.375	Detected	1.375	Detected	0.000	-1.005	Detected	-3.250	Detected	-2.128	0.353	GT	Sens
GT	Sense	Sense	-0.524	Detected	0.524	Detected	0.000	-1.240	Detected	-1.651	Detected	-1.445	0.124	GT	Sens
GT	Sense	Sense	0.088	Detected	-0.088	Detected	0.000	-1.803	Detected	-1.638	Detected	-1.721	0.005	GT	Sens
GT	Sense	Sense	-0.305	Detected	0.305	Detected	0.000	-1.286	Detected	-1.371	Detected	-1.328	0.050	GT	Sens
GT	Sense	Sense	-0.269	Detected	0.269	Detected	0.000	-1.373	Detected	-1.539	Detected	-1.456	0.035	GT	Sens
GT	Sense	Sense	-0.100	Detected	0.100	Detected	0.000	-1.530	Detected	-1.131	Detected	-1.331	0.027	GT	Sens

GT	Sense	Sense	0.028	Detected	-0.028	Detected	0.000	-1.510	Detected	-1.688	Detected	-1.599	0.003	GT	Sens	contig303	contig303	Unknown	GGCAGT	contig303	Solvc06g	Solvc06g	Unknown Protein (AHRD V1)%			contig303	Solvc06g	Unknown Protein (A		SL2.40ch	AT5G61520.2		hexose t	chr5:247	163.218	155.201	110.401	148.621	62.2539	50.0126
GT	Sense	Sense	-0.999	Detected	0.999	Detected	0.000	-2.641	Detected	-2.096	Detected	-2.369	0.149	GT	Sens	contig304	contig304	WRKY-lik	TTCAAGT	contig304	Solvc09g	Solvc09g	WRKY-lik	GO:00037	GO:00037	contig304	Solvc09g	WRKY-lik	GO:0005	SL2.40ch	AT5G562	WRKY2. A	WRKY2:	chr5:227	1021.06	4030.16	475.286	916.667	362.07	480.297
GT	Sense	Sense	0.516	Detected	-0.516	Detected	0.000	-1.274	Detected	-0.878	Detected	-1.076	0.191	GT	Sens	contig305	contig305	Tropinon	TTCAGG	contig305	Solvc06g	Solvc06g	Tropinon	GO:00081	GO:00081	contig305	Solvc06g	Tropinon	GO:00081	SL2.40ch	AT2G29310.1		tropinone	chr2:125	5040.44	2435.78	1318.11	1529.85	1613.23	1929.94
GT	Sense	Sense	-0.164	Detected	0.164	Detected	0.000	-0.861	Detected	-1.280	Detected	-1.071	0.056	GT	Sens	contig305	contig305	F-box fam	TCCATT	contig305	Solvc03g	Solvc03g	F-box family protein (AHRD V1			contig305	Solvc03g	F-box family protein		SL2.40ch	AT5G10080.1		aspartyl	chr5:315	89.208	110.647	43.6457	50.0768	60.9148	41.4122
GT	Sense	Sense	0.045	Detected	-0.045	Detected	0.000	-1.114	Detected	-1.086	Detected	-1.100	0.002	GT	Sens	contig306	contig306	Protein B	GTAAGT	contig306	Solvc08g	Solvc08g	Protein B	GO:0042	GO:0042	contig306	Solvc08g	Protein B	GO:0042	SL2.40ch	AT1G318	NIP3:1. N	BRX (BRI	chr1:114	233.679	217.03	139.516	150.251	115.884	107.373
GT	Sense	Sense	0.258	Detected	-0.258	Detected	0.000	-1.846	Detected	-1.709	Detected	-1.777	0.022	GT	Sens	contig307	contig307	Undecapr	GCTTAC	contig307	Solvc08g	Solvc08g	Undecapr	GO:0045	GO:0045	contig307	Solvc08g	Undecapr	GO:0045	SL2.40ch	AT5G58770.1		dehydro	chr5:237	3249.84	2245.45	1351.15	3081.78	837.15	836.682
GT	Sense	Sense	-0.340	Detected	0.340	Detected	0.000	-0.957	Detected	-1.159	Detected	-1.058	0.097	GT	Sens	contig307	contig307	Alpha alp	TCTTCA	contig307	Solvc02g	Solvc02g	Alpha alp	GO:0003	GO:0003	contig307	Solvc02g	Alpha alp	GO:0003	SL2.40ch	AT1G064	ATTPS7.	ATTPS7:	chr1:195	942.558	1493.79	795.465	775.747	680.684	537.889
GT	Sense	Sense	-0.206	Detected	0.206	Detected	0.000	-1.935	Comprom	-3.287	Comprom	-2.611	0.066	GT	Sens	contig308	contig308	ATP-bind	GAAAAA	contig308	Solvc06g	Solvc06g	ATP-bind	GO:0017	GO:0017	contig308	Solvc03g	ATP-bind	GO:0016	SL2.40ch	AT3G477	ATATH1.	ATATH1:	chr3:175	19.493	25.6243	2.25831	8.57431	6.51161	2.31869
GT	Sense	Sense	-0.544	Detected	0.544	Detected	0.000	-1.242	Detected	-1.255	Detected	-1.249	0.149	GT	Sens	contig308	contig308	Bzip trans	ATGTGA	contig308	Solvc01g	Solvc01g	Bzip transcription factor-like (			contig308	Solvc01g	Bzip transcription fa		SL2.40ch	AT4G35240.1		unknown	chr4:167	168.719	354.771	150.739	172.849	115.217	103.767
GT	Sense	Sense	-0.506	Detected	0.506	Detected	0.000	-1.224	Detected	-1.356	Detected	-1.290	0.127	GT	Sens	contig309	contig309	Lysine-sr	AACAAG	contig309	Solvc08g	Solvc08g	Lysine-sr	GO:0005	GO:0005	contig309	Solvc08g	Lysine-sr	GO:0005	SL2.40ch	AT5G46910.1		transcrip	chr5:190	934.829	1863.39	628.238	806.582	629.47	522.168
GT	Sense	Sense	0.218	Detected	-0.218	Detected	0.000	-1.156	Detected	-1.726	Detected	-1.441	0.057	GT	Sens	contig309	contig309	Tumor-re	CAAATC	contig309	Solvc10g	Solvc10g	Tumor-related protein (AHRD V			contig309	Solvc10g	Tumor-related prote		SL2.40ch	AT5G523	CYP96A4	CYP96A4	chr5:212	718.714	525.374	402.823	745.498	307.013	188.101
GT	Sense	Sense	-0.496	Detected	0.496	Detected	0.000	-1.390	Detected	-1.364	Detected	-1.377	0.109	GT	Sens	contig310	contig310	Receptor	GTCGAG	contig310	Solvc03g	Solvc03g	Receptor-like kinase (AHRD V1			contig310	Solvc03g	Receptor-like kinase		SL2.40ch	AT3G28980.1		unknown	chr3:109	2760.09	5424.67	1943.82	2868.49	1644.03	1522.04
GT	Sense	Sense	-0.469	Detected	0.469	Detected	0.000	-0.919	Detected	-1.993	Detected	-1.456	0.178	GT	Sens	contig311	contig311	Cinnamyl	GGCATC	contig311	Solvc11g	Solvc11g	Cinnamyl	GO:0008	GO:0008	contig311	Solvc11g	Cinnamyl	GO:0045	SL2.40ch	AT4G393	ATCAD9.	CAD9 (CI	chr4:182	57.1851	108.282	19.9774	115.33	46.362	20.0181
GT	Sense	Sense	-0.685	Detected	0.685	Detected	0.000	-1.268	Detected	-1.319	Detected	-1.293	0.200	GT	Sens	contig311	contig311	Unknown	AGCTCA	contig311	Solvc03g	Solvc03g	Unknown Protein (AHRD V1)			contig311	Solvc03g	Unknown Protein (A		SL2.40ch	AT5G38990.1		protein k	chr5:156	164.76	420.896	108.066	185.983	121.801	106.876
GT	Sense	Sense	-1.311	Detected	1.311	Detected	0.000	-2.130	Detected	-2.475	Detected	-2.302	0.224	GT	Sens	contig311	contig311	Unknown	TTTATA	contig311	Solvc12g	Solvc12g	Unknown Protein (AHRD V1)%			contig311	Solvc12g	Unknown Protein (A		SL2.40ch	AT3G29075.1		glycine-r	chr3:110	149.754	911.13	60.1135	112.85	94.0013	67.2599
GT	Sense	Sense	-0.142	Detected	0.142	Detected	0.000	-1.134	Detected	-0.992	Detected	-1.063	0.022	GT	Sens	contig312	contig312	Unknown	GGAAAT	contig312	Solvc12g	Solvc12g	Unknown Protein (AHRD V1)			contig312	Solvc12g	Unknown Protein (A		SL2.40ch	AT3G15200.1		pentatric	chr3:511	23.6666	28.4806	29.7707	46.6305	13.1768	13.2135
GT	Sense	Sense	-0.825	Detected	0.825	Detected	0.000	-1.077	Detected	-1.183	Detected	-1.130	0.305	GT	Sens	contig312	contig312	Kunitz-ty	ATTTGT	contig312	Solvc03g	Solvc03g	Kunitz-ty	GO:0004	GO:0004	contig312	Solvc03g	Kunitz-ty	GO:0004	SL2.40ch	AT5G440	CESA4. IF	CESA4 (C	chr5:177	103.838	322.283	72.029	133.256	96.5522	81.6
GT	Sense	Sense	-0.099	Detected	0.099	Detected	0.000	-1.301	Comprom	-1.194	Comprom	-1.248	0.008	GT	Sens	contig313	contig313	Unannota	TATTAAAGCCTCAACCAGCCTAGATGAATTTGACTCTCTCATAATTGGC							contig313	Solvc08g042090.1.1				AT1G780	SULTR1::	SULTR1:	chr1:293	11.9145	13.5043	6.49667	6.52189	5.73307	5.61241
GT	Sense	Sense	-0.319	Detected	0.319	Detected	0.000	-1.330	Detected	-1.292	Detected	-1.311	0.055	GT	Sens	contig314	contig314	Unannota	TACATGCACTTACTAAACAATGAAAGCTGTAATCTGGAATATGTTTCTT							contig314	Solvc06g	Calcineur	GO:0005	SL2.40ch	AT4G29103.1				28.3284	43.5921	20.8136	20.8698	15.5628	14.526
GT	Sense	Sense	0.388	Detected	-0.388	Detected	0.000	-3.351	Comprom	-3.283	Comprom	-3.317	0.014	GT	Sens	contig314	contig314	Unannota	CTTGCACCAAAAACCCTAAAGAAAAAGGAACAAAAATGGTTTAGGTAGT							contig314	Solvc06g	Transmem	GO:0005	SL2.40ch	AT3G153	UBC25. P	UBC25 (U	chr3:518	32.3899	18.7004	2.6811	12.5953	2.68659	2.55979

GT	Sense	Sense	0.021	Detected	-0.021	Detected	0.000	-1.675	Comprom	-1.662	Comprom	-1.668	0.000	GT Sens
GT	Sense	Sense	0.024	Detected	-0.024	Detected	0.000	-1.407	Comprom	-1.085	Comprom	-1.246	0.017	GT Sens
GT	Sense	Sense	0.023	Detected	-0.023	Detected	0.000	-0.944	Comprom	-1.359	Comprom	-1.152	0.031	GT Sens
GT	Sense	Sense	0.226	Detected	-0.226	Detected	0.000	-0.905	Comprom	-1.648	Comprom	-1.276	0.099	GT Sens
GT	Sense	Sense	-0.475	Detected	0.475	Detected	0.000	-1.036	Detected	-1.055	Detected	-1.046	0.159	GT Sens
GT	Sense	Sense	0.086	Detected	-0.086	Detected	0.000	-1.970	Detected	-2.080	Detected	-2.025	0.003	GT Sens
GT	Sense	Sense	-0.641	Detected	0.641	Detected	0.000	-0.806	Detected	-1.327	Detected	-1.066	0.263	GT Sens
GT	Sense	Sense	-0.530	Detected	0.530	Detected	0.000	-1.130	Detected	-1.126	Detected	-1.128	0.167	GT Sens
GT	Sense	Sense	-0.300	Detected	0.300	Detected	0.000	-1.504	Comprom	-0.984	Comprom	-1.244	0.088	GT Sens
GT	Sense	Sense	-0.531	Detected	0.531	Detected	0.000	-1.219	Detected	-1.915	Detected	-1.567	0.132	GT Sens
GT	Sense	Sense	-0.510	Detected	0.510	Detected	0.000	-1.447	Detected	-1.376	Detected	-1.412	0.110	GT Sens
GT	Sense	Sense	0.084	Detected	-0.084	Detected	0.000	-1.085	Comprom	-0.982	Comprom	-1.034	0.009	GT Sens
GT	Sense	Sense	-0.402	Detected	0.402	Detected	0.000	-2.046	Detected	-1.688	Detected	-1.867	0.051	GT Sens
GT	Sense	Sense	-0.396	Detected	0.396	Detected	0.000	-1.997	Detected	-1.565	Detected	-1.781	0.059	GT Sens
GT	Sense	Sense	0.016	Detected	-0.016	Detected	0.000	-1.426	Comprom	-1.024	Comprom	-1.225	0.026	GT Sens
GT	Sense	Sense	0.327	Detected	-0.327	Detected	0.000	-1.007	Detected	-1.073	Detected	-1.040	0.087	GT Sens
GT	Sense	Sense	-0.383	Detected	0.383	Detected	0.000	-1.241	Detected	-1.528	Detected	-1.385	0.077	GT Sens
GT	Sense	Sense	-0.310	Detected	0.310	Detected	0.000	-0.973	Detected	-1.161	Detected	-1.067	0.081	GT Sens
GT	Sense	Sense	0.235	Detected	-0.235	Detected	0.000	-1.002	Detected	-1.797	Detected	-1.400	0.094	GT Sens
GT	Sense	Sense	0.261	Detected	-0.261	Detected	0.000	-0.887	Detected	-2.056	Detected	-1.471	0.148	GT Sens
GT	Sense	Sense	0.537	Detected	-0.537	Detected	0.000	-1.629	Detected	-0.947	Detected	-1.288	0.180	GT Sens
GT	Sense	Sense	-0.089	Detected	0.089	Detected	0.000	-1.991	Comprom	-2.134	Comprom	-2.062	0.003	GT Sens
GT	Sense	Sense	-0.678	Detected	0.678	Detected	0.000	-1.536	Detected	-0.866	Detected	-1.201	0.253	GT Sens
GT	Sense	Sense	-0.200	Detected	0.200	Detected	0.000	-3.400	Comprom	-3.311	Comprom	-3.356	0.004	GT Sens
GT	Sense	Sense	-0.141	Detected	0.141	Detected	0.000	-1.326	Detected	-1.249	Detected	-1.287	0.013	GT Sens
GT	Sense	Sense	-0.642	Detected	0.642	Detected	0.000	-0.905	Detected	-1.273	Detected	-1.089	0.244	GT Sens
GT	Sense	Sense	0.192	Detected	-0.192	Detected	0.000	-1.779	Detected	-0.932	Detected	-1.356	0.100	GT Sens
GT	Sense	Sense	-0.032	Detected	0.032	Detected	0.000	-0.997	Detected	-1.013	Detected	-1.005	0.001	GT Sens
GT	Sense	Sense	-1.090	Detected	1.090	Detected	0.000	-1.197	Detected	-1.431	Detected	-1.314	0.353	GT Sens
GT	Sense	Sense	0.895	Detected	-0.895	Detected	0.000	-1.508	Comprom	-1.048	Detected	-1.278	0.301	GT Sens
GT	Sense	Sense	-1.463	Detected	1.463	Detected	0.000	-0.902	Detected	-1.855	Detected	-1.378	0.465	GT Sens
GT	Sense	Sense	-0.368	Detected	0.368	Detected	0.000	-1.097	Detected	-1.518	Detected	-1.307	0.091	GT Sens
GT	Sense	Sense	-0.370	Detected	0.370	Detected	0.000	-1.335	Detected	-1.416	Detected	-1.376	0.066	GT Sens
GT	Sense	Sense	-0.151	Detected	0.151	Detected	0.000	-0.991	Comprom	-1.647	Comprom	-1.319	0.067	GT Sens
GT	Sense	Sense	0.296	Detected	-0.296	Detected	0.000	-2.035	Comprom	-1.957	Comprom	-1.996	0.022	GT Sens
GT	Sense	Sense	0.013	Detected	-0.013	Detected	0.000	-2.013	Comprom	-1.695	Comprom	-1.854	0.007	GT Sens
GT	Sense	Sense	-0.418	Detected	0.418	Detected	0.000	-1.377	Detected	-1.497	Detected	-1.437	0.076	GT Sens
GT	Sense	Sense	-0.268	Detected	0.268	Detected	0.000	-1.566	Detected	-1.763	Detected	-1.665	0.028	GT Sens
GT	Sense	Sense	-0.336	Detected	0.336	Detected	0.000	-0.928	Detected	-1.542	Comprom	-1.235	0.113	GT Sens
GT	Sense	Sense	1.253	Detected	-1.253	Detected	0.000	-1.181	Detected	-0.984	Detected	-1.083	0.480	GT Sens
GT	Sense	Sense	-0.124	Detected	0.124	Detected	0.000	-1.951	Comprom	-1.447	Comprom	-1.699	0.026	GT Sens
GT	Sense	Sense	-0.792	Detected	0.792	Detected	0.000	-0.849	Detected	-1.840	Detected	-1.345	0.287	GT Sens
GT	Sense	Sense	0.135	Detected	-0.135	Detected	0.000	-1.262	Comprom	-1.656	Comprom	-1.459	0.026	GT Sens
GT	Sense	Sense	-0.058	Detected	0.058	Detected	0.000	-1.251	Detected	-0.995	Detected	-1.123	0.015	GT Sens
GT	Sense	Sense	0.153	Detected	-0.153	Detected	0.000	-2.042	Comprom	-1.355	Comprom	-1.698	0.046	GT Sens
GT	Sense	Sense	-0.729	Detected	0.729	Detected	0.000	-1.767	Comprom	-2.272	Comprom	-2.019	0.120	GT Sens
GT	Sense	Sense	0.172	Detected	-0.172	Detected	0.000	-1.657	Detected	-1.084	Detected	-1.371	0.055	GT Sens
GT	Sense	Sense	0.086	Detected	-0.086	Detected	0.000	-0.980	Detected	-3.154	Comprom	-2.067	0.199	GT Sens
GT	Sense	Sense	0.248	Detected	-0.248	Detected	0.000	-1.060	Detected	-1.335	Detected	-1.197	0.052	GT Sens
GT	Sense	Sense	0.093	Detected	-0.093	Detected	0.000	-1.119	Detected	-1.529	Detected	-1.324	0.028	GT Sens
GT	Sense	Sense	0.052	Detected	-0.052	Detected	0.000	-1.252	Comprom	-1.949	Comprom	-1.601	0.045	GT Sens
GT	Sense	Sense	0.465	Detected	-0.465	Detected	0.000	-2.950	Comprom	-1.692	Comprom	-2.321	0.097	GT Sens
GT	Sense	Sense	-0.715	Detected	0.715	Detected	0.000	-0.846	Detected	-1.440	Detected	-1.143	0.278	GT Sens
GT	Sense	Sense	-0.004	Detected	0.004	Detected	0.000	-1.397	Comprom	-1.018	Comprom	-1.208	0.024	GT Sens
GT	Sense	Sense	0.273	Detected	-0.273	Detected	0.000	-2.095	Detected	-0.989	Detected	-1.542	0.130	GT Sens
GT	Sense	Sense	-0.214	Detected	0.214	Detected	0.000	-1.952	Comprom	-1.608	Comprom	-1.780	0.023	GT Sens
GT	Sense	Sense	0.024	Detected	-0.024	Detected	0.000	-1.703	Comprom	-1.890	Comprom	-1.796	0.003	GT Sens
GT	Sense	Sense	-0.452	Detected	0.452	Detected	0.000	-1.227	Detected	-1.176	Detected	-1.201	0.118	GT Sens
GT	Sense	Sense	-0.382	Detected	0.382	Detected	0.000	-0.947	Detected	-1.666	Comprom	-1.306	0.130	GT Sens
GT	Sense	Sense	0.076	Detected	-0.076	Detected	0.000	-1.495	Detected	-1.513	Detected	-1.504	0.003	GT Sens
GT	Sense	Sense	0.653	Detected	-0.653	Detected	0.000	-1.260	Detected	-1.100	Detected	-1.180	0.215	GT Sens
GT	Sense	Sense	0.013	Detected	-0.013	Detected	0.000	-1.098	Detected	-1.639	Comprom	-1.369	0.037	GT Sens
GT	Sense	Sense	-0.439	Detected	0.439	Detected	0.000	-0.986	Detected	-1.464	Detected	-1.225	0.134	GT Sens
GT	Sense	Sense	-0.192	Detected	0.192	Detected	0.000	-3.445	Comprom	-2.081	Comprom	-2.763	0.060	GT Sens
GT	Sense	Sense	-0.186	Detected	0.186	Detected	0.000	-0.838	Detected	-2.417	Detected	-1.627	0.169	GT Sens
GT	Sense	Sense	-0.294	Detected	0.294	Detected	0.000	-1.564	Detected	-1.001	Detected	-1.282	0.088	GT Sens
GT	Sense	Sense	-0.632	Detected	0.632	Detected	0.000	-1.550	Detected	-2.361	Detected	-1.956	0.121	GT Sens
GT	Sense	Sense	0.011	Detected	-0.011	Detected	0.000	-1.301	Detected	-1.150	Detected	-1.225	0.004	GT Sens
GT	Sense	Sense	-0.171	Detected	0.171	Detected	0.000	-0.913	Detected	-2.144	Detected	-1.529	0.055	GT Sens
GT	Sense	Sense	0.135	Detected	-0.135	Detected	0.000	-2.090	Detected	-1.168	Detected	-1.629	0.077	GT Sens
GT	Sense	Sense	0.024	Detected	-0.024	Detected	0.000	-0.809	Detected	-1.686	Comprom	-1.248	0.105	GT Sens
GT	Sense	Sense	-0.113	Detected	0.113	Detected	0.000	-0.955	Detected	-1.177	Detected	-1.066	0.021	GT Sens
GT	Sense	Sense	0.089	Detected	-0.089	Detected	0.000	-1.084	Detected	-1.495	Comprom	-1.289	0.029	GT Sens
GT	Sense	Sense	-0.333	Detected	0.333	Detected	0.000	-1.400	Detected	-1.408	Detected	-1.404	0.052	GT Sens
GT	Sense	Sense	-0.721	Detected	0.721	Detected	0.000	-2.783	Detected	-3.143	Detected	-2.963	0.058	GT Sens
GT	Sense	Sense	-0.534	Detected	0.534	Detected	0.000	-1.296	Detected	-1.760	Detected	-1.528	0.120	GT Sens
GT	Sense	Sense	-0.734	Detected	0.734	Detected	0.000	-1.267	Detected	-1.506	Detected	-1.386	0.203	GT Sens
GT	Sense	Sense	-0.338	Detected	0.338	Detected	0.000	-1.204	Detected	-1.524	Detected	-1.364	0.068	GT Sens
GT	Sense	Sense	0.381	Detected	-0.381	Detected	0.000	-1.114	Detected	-1.339	Comprom	-1.227	0.091	GT Sens
GT	Sense	Sense	-0.626	Detected	0.626	Detected	0.000	-1.470	Detected	-1.626	Detected	-1.548	0.133	GT Sens
GT	Sense	Sense	-0.341	Detected	0.341	Detected	0.000	-0.876	Detected	-1.206	Detected	-1.041	0.111	GT Sens
GT	Sense	Sense	-1.108	Detected	1.108	Detected	0.000	-2.099	Detected	-2.208	Detected	-2.153	0.192	GT Sens
GT	Sense	Sense	1.682	Detected	-1.682	Detected	0.000	-1.852	Detected	-1.660	Detected	-1.756	0.407	GT Sens
GT	Sense	Sense	-0.300	Detected	0.300	Detected	0.000	-1.251	Detected	-1.317	Detected	-1.284	0.051	GT Sens
GT	Sense	Sense	-0.304	Detected	0.304	Detected	0.000	-0.967	Detected	-1.325	Detected	-1.146	0.083	GT Sens
GT	Sense	Sense	-0.775	Detected	0.775	Detected	0.000	-2.360	Detected	-2.234	Detected	-2.297	0.098	GT Sens
GT	Sense	Sense	-0.475	Detected	0.475	Detected	0.000	-1.888	Detected	-1.580	Detected	-1.734	0.074	GT Sens
GT	Sense	Sense	0.405	Detected	-0.405	Detected	0.000	-1.908	Comprom	-2.463	Comprom	-2.186	0.047	GT Sens
GT	Sense	Sense	-0.293	Detected	0.293	Detected	0.000	-1.171	Detected	-0.962	Detected	-1.066	0.075	GT Sens
GT	Sense	Sense	-0.843	Detected	0.843	Detected	0.000	-2.465	Detected	-2.495	Detected	-2.480	0.099	GT Sens
GT	Sense	Sense	-0.638	Detected	0.638	Detected	0.000	-2.652	Detected	-2.585	Detected	-2.618	0.055	GT Sens
GT	Sense	Sense	-1.268	Detected	1.268	Detected	0.000	-1.517	Detected	-1.346	Detected	-1.432	0.377	GT Sens
GT	Sense	Sense	-0.169	Detected	0.169	Detected	0.000	-1.064	Detected	-1.999	Detected	-1.531	0.091	GT Sens
GT	Sense	Sense	0.371	Detected	-0.371	Detected	0.000	-1.606	Detected	-1.261	Detected	-1.433	0.073	GT Sens
GT	Sense	Sense	0.306	Detected	-0.306	Detected	0.000	-1.649	Detected	-1.645	Detected	-1.647	0.033	GT Sens
GT	Sense	Sense	-0.514	Detected	0.514	Detected	0.000	-0.962	Detected	-1.067	Detected	-1.014	0.189	GT Sens
GT	Sense	Sense	-0.010	Detected	0.010	Detected	0.000	-2.599	Detected	-2.864	Detected	-2.731	0.002	GT Sens
GT	Sense	Sense	-1.282	Detected	1.282	Detected	0.000	-2.155	Detected	-2.115	Detected	-2.135	0.238	GT Sens
GT	Sense	Sense	0.015	Detected	-0.015	Detected	0.000	-1.068	Detected	-1.855	Detected	-1.462	0.066	GT Sens
GT	Sense	Sense	-0.749	Detected	0.749	Detected	0.000	-1.123	Detected	-1.238	Detected	-1.181	0.257	GT Sens
GT	Sense	Sense	-0.509	Detected	0.509	Detected	0.000	-0.817	Detected	-1.226	Detected	-1.021	0.204	GT Sens
GT	Sense	Sense	-0.244	Detected	0.244	Detected	0.000	-2.214	Comprom	-1.326	Comprom	-1.770	0.073	GT Sens
GT	Sense	Sense	0.125	Detected	-0.125	Detected	0.000	-1.621	Detected	-1.234	Detected	-1.428	0.025	GT Sens
GT	Sense	Sense	-0.432	Detected	0.432	Detected	0.000	-1.343	Detected	-1.387	Detected	-1.365	0.088	GT Sens
GT	Sense	Sense	-0.431	Detected	0.431	Detected	0.000	-1.364	Detected	-1.812	Detected	-1.588	0.082	GT Sens
GT	Sense	Sense	0.116	Detected	-0.116	Detected	0.000	-0.922	Detected	-1.097	Detected	-1.009	0.020	GT Sens
GT	Sense	Sense	-0.744	Detected	0.744	Detected	0.000	-0.851	Detected	-1.156	Detected	-1.003	0.317	GT Sens
GT	Sense	Sense	-0.520	Detected	0.520	Detected	0.000	-2.376	Detected	-1.620	Detected	-1.998	0.090	GT Sens
GT	Sense	Sense	-0.248	Detected	0.248	Detected	0.000	-0.813	Detected	-1.249	Detected	-1.031	0.089	GT Sens
GT	Sense	Sense	-0.050	Detected	0.050	Detected	0.000	-2.083	Comprom	-1.048	Comprom	-1.565	0.095	GT Sens
GT	Sense	Sense	-0.341	Detected	0.341	Detected	0.000	-1.267	Detected	-1.478	Detected	-1.373	0.062	GT Sens
GT	Sense	Sense	-0.274	Detected	0.274	Detected	0.000	-1.087	Detected	-0.948	Detected	-1.018	0.069	GT Sens
GT	Sense	Sense	0.020	Detected	-0.020	Detected	0.000	-1.693	Detected	-1.412	Detected	-1.552	0.008	GT Sens
GT	Sense	Sense	-0.424	Detected	0.424	Detected	0.000	-0.810	Detected	-1.469	Detected	-1.140	0.168	GT Sens
GT	Sense	Sense	-0.230	Detected	0.230	Detected	0.000	-0.930	Detected	-1.489	Detected	-1.209	0.079	GT Sens
GT	Sense	Sense	-0.494	Detected	0.494	Detected	0.000	-1.837	Detected	-1.488	Detected	-1.662	0.087	GT Sens
GT	Sense	Sense	0.277	Detected	-0.277	Detected	0.000	-1.238	Detected	-0.920	Detected	-1.079	0.078	GT Sens
GT	Sense	Sense	-0.309	Detected	0.309	Detected	0.000	-1.754	Detected	-2.603	Detected	-2.178	0.054	GT Sens
GT	Sense	Sense	-0.278	Detected	0.278	Detected	0.000	-1.374	Detected	-1.198	Detected	-1.286	0.048	GT Sens
GT	Sense	Sense	-0.145	Detected	0.145	Detected	0.000	-1.500	Detected	-1.068	Detected	-1.284	0.039	GT Sens
GT	Sense	Sense	0.086	Detected	-0.086	Detected	0.000	-1.455	Detected	-1.178	Detected	-1.316	0.015	GT Sens
GT	Sense	Sense	-0.334	Detected	0.334	Detected	0.000	-1.133	Detected	-1.388	Detected	-1.261	0.072	GT Sens
GT	Sense	Sense	-0.089	Detected	0.089	Detected	0.000	-1.738	Detected	-1.103	Detected	-1.420	0.050	GT Sens
GT	Sense	Sense	0.145	Detected	-0.145	Detected	0.000	-2.229	Comprom	-1.293	Detected	-1.761	0.069	GT Sens
GT	Sense	Sense	-0.383	Detected	0.383	Detected	0.000	-0.959	Detected	-1.398	Detected	-1.178	0.116	GT Sens
GT	Sense	Sense	-0.854	Detected	0.854	Detected	0.000	-1.201	Detected	-1.549	Detected	-1.375	0.255	GT Sens
GT	Sense	Sense	-0.410	Detected	0.410	Detected	0.000	-0.945	Detected	-1.584	Detected	-1.264	0.136	GT Sens
GT	Sense	Sense	-0.615	Detected	0.615	Detected	0.000	-1.267	Detected	-1.526	Detected	-1.397	0.156	GT Sens
GT	Sense	Sense	-0.259	Detected	0.259	Detected	0.000	-1.182	Detected	-1.222	Detected	-1.202	0.044	GT Sens
GT	Sense	Sense	-0.588	Detected	0.588	Detected	0.000	-1.376	Detected	-1.427	Detected	-1.401	0.140	GT Sens
GT	Sense	Sense	0.027	Detected	-0.027	Detected	0.000	-1.641	Detected	-1.324	Detected	-1.482	0.012	GT Sens
GT	Sense	Sense	-0.200	Detected	0.200	Detected	0.000	-1.010	Detected	-1.052	Detected	-1.031	0.036	GT Sens
GT	Sense	Sense	-0.414	Detected	0.414	Detected	0.000	-1.409	Detected	-1.308	Detected	-1.359	0.083	GT Sens
GT	Sense	Sense	-0.485	Detected	0.485	Detected	0.000	-1.180	Detected	-2.456	Detected	-1.818	0.151	GT Sens
GT	Sense	Sense	0.318	Detected	-0.318	Detected	0.000	-1.160	Detected	-1.041	Detected	-1.100	0.076	GT Sens
GT	Sense	Sense	-0.290	Detected	0.290	Detected	0.000	-1.528	Detected	-2.073	Detected	-1.800	0.045	GT Sens
GT	Sense	Sense	-0.442	Detected	0.442	Detected	0.000	-1.067	Detected	-1.110	Detected	-1.089	0.133	GT Sens
GT	Sense	Sense	0.279	Detected	-0.279	Detected	0.000	-2.586	Comprom	-2.199	Comprom	-2.393	0.020	GT Sens
GT	Sense	Sense	0.492	Detected	-0.492	Detected	0.000	-1.215	Detected	-1.432	Detected	-1.324	0.119	GT Sens
GT	Sense	Sense	-0.047	Detected	0.047	Detected	0.000	-1.027	Comprom	-0.980	Comprom	-1.003	0.003	GT Sens
GT	Sense	Sense	-0.136	Detected	0.136	Detected	0.000	-1.219	Detected	-1.269	Detected	-1.244	0.012	GT Sens
GT	Sense	Sense	-0.223	Detected	0.223	Detected	0.000	-0.807	Detected	-1.202	Detected	-1.004	0.078	GT Sens
GT	Sense	Sense	-0.634	Detected	0.634	Detected	0.000	-2.668	Detected	-2.321	Detected	-2.495	0.063	GT Sens
GT	Sense	Sense	0.479	Detected	-0.479	Detected	0.000	-2.798	Comprom	-2.943	Comprom	-2.870	0.027	GT Sens
GT	Sense	Sense	-0.633	Detected	0.633	Detected	0.000	-1.074	Detected	-1.134	Detected	-1.104	0.224	GT Sens
GT	Sense	Sense	-0.462	Detected	0.462	Detected	0.000	-1.198	Detected	-1.066	Detected	-1.132	0.136	GT Sens
GT	Sense	Sense	-0.621	Detected	0.621	Detected	0.000	-1.181	Detected	-0.851	Detected	-1.016	0.255	GT Sens
GT	Sense	Sense	-0.242	Detected	0.242	Detected	0.000	-1.897	Detected	-1.987	Detected	-1.942	0.016	GT Sens
GT	Sense	Sense	-0.288	Detected	0.288	Detected	0.000	-0.820	Detected	-1.473	Detected	-1.147	0.119	GT Sens
GT	Sense	Sense	-0.473	Detected	0.473	Detected	0.000	-0.966	Detected	-1.807	Detected	-1.387	0.160	GT Sens
GT	Sense	Sense	-0.281	Detected	0.281	Detected	0.000	-1.437	Detected	-1.458	Detected	-1.447	0.036	GT Sens
GT	Sense	Sense	-0.586	Detected	0.586	Detected	0.000	-2.489	Detected	-2.022	Detected	-2.256	0.070	GT Sens
GT	Sense	Sense	-0.273	Detected	0.273	Detected	0.000	-1.190	Detected	-1.020	Detected	-1.105	0.061	GT Sens
GT	Sense	Sense	-0.064	Detected	0.064	Detected	0.000	-1.133	Detected	-1.353	Detected	-1.243	0.010	GT Sens
GT	Sense	Sense	-0.291	Detected	0.291	Detected	0.000	-1.019	Detected	-1.070	Detected	-1.044	0.070	GT Sens
GT	Sense	Sense	-0.813	Detected	0.813	Detected	0.000	-1.096	Detected	-1.607	Detected	-1.352	0.254	GT Sens
GT	Sense	Sense	0.082	Detected	-0.082	Detected	0.000	-0.927	Detected	-1.581	Detected	-1.254	0.065	GT Sens
GT	Sense	Sense	-0.595	Detected	0.595	Detected	0.000	-1.121	Detected	-1.280	Detected	-1.201	0.184	GT Sens
GT	Sense	Sense	-0.115	Detected	0.115	Detected	0.000	-2.116	Comprom	-1.770	Comprom	-1.943	0.011	GT Sens
GT	Sense	Sense	0.508	Detected	-0.508	Detected	0.000	-1.523	Detected	-1.039	Detected	-1.281	0.151	GT Sens
GT	Sense	Sense	-0.646	Detected	0.646	Detected	0.000	-2.088	Detected	-2.546	Detected	-2.317	0.077	GT Sens
GT	Sense	Sense	0.264	Detected	-0.264	Detected	0.000	-0.997	Detected	-1.167	Detected	-1.082	0.060	GT Sens
GT	Sense	Sense	-0.454	Detected	0.454	Detected	0.000	-1.086	Detected	-1.027	Detected	-1.056	0.146	GT Sens
GT	Sense	Sense	-0.370	Detected	0.370	Detected	0.000	-1.103	Detected	-1.032	Detected	-1.068	0.103	GT Sens
GT	Sense	Sense	-0.560	Detected	0.560	Detected	0.000	-1.212	Detected	-1.942	Comprom	-1.577	0.142	GT Sens
GT	Sense	Sense	0.061	Detected	-0.061	Detected	0.000	-2.457	Detected	-1.661	Detected	-2.059	0.036	GT Sens
GT	Sense	Sense	-0.506	Detected	0.506	Detected	0.000	-1.192	Detected	-1.337	Detected	-1.265	0.132	GT Sens
GT	Sense	Sense	-0.304	Detected	0.304	Detected	0.000	-0.855	Detected	-1.729	Detected	-1.292	0.136	GT Sens
GT	Sense	Sense	-0.347	Detected	0.347	Detected	0.000	-1.101	Detected	-1.032	Detected	-1.066	0.092	GT Sens
GT	Sense	Sense	-0.953	Detected	0.953	Detected	0.000	-1.189	Detected	-1.988	Detected	-1.589	0.264	GT Sens
GT	Sense	Sense	0.660	Detected	-0.660	Detected	0.000	-1.401	Comprom	-1.006	Comprom	-1.204	0.223	GT Sens
GT	Sense	Sense	0.149	Detected	-0.149	Detected	0.000	-1.725	Detected	-1.426	Detected	-1.575	0.018	GT Sens
GT	Sense	Sense	-0.126	Detected	0.126	Detected	0.000	-1.266	Detected	-1.581	Detected	-1.424	0.020	GT Sens
GT	Sense	Sense	-1.225	Detected	1.225	Detected	0.000	-2.116	Detected	-2.747	Detected	-2.431	0.195	GT Sens
GT	Sense	Sense	0.341	Detected	-0.341	Detected	0.000	-1.960	Detected	-1.763	Detected	-1.862	0.034	GT Sens
GT	Sense	Sense	-0.523	Detected	0.523	Detected	0.000	-1.156	Detected	-0.856	Detected	-1.006	0.206	GT Sens
GT	Sense	Sense	-0.087	Detected	0.087	Detected	0.000	-1.142	Detected	-0.949	Detected	-1.046	0.015	GT Sens
GT	Sense	Sense	0.203	Detected	-0.203	Detected	0.000	-1.584	Detected	-2.609	Detected	-2.096	0.063	GT Sens
GT	Sense	Sense	-0.396	Detected	0.396	Detected	0.000	-0.933	Detected	-1.225	Detected	-1.079	0.125	GT Sens
GT	Sense	Sense	-0.321	Detected	0.321	Detected	0.000	-0.935	Detected	-1.325	Detected	-1.130	0.095	GT Sens
GT	Sense	Sense	-0.019	Detected	0.019	Detected	0.000	-1.151	Detected	-1.124	Detected	-1.137	0.000	GT Sens
GT	Sense	Sense	-0.248	Detected	0.248	Detected	0.000	-1.271	Detected	-1.361	Detected	-1.316	0.035	GT Sens
GT	Sense	Sense	-0.291	Detected	0.291	Detected	0.000	-1.276	Detected	-1.511	Detected	-1.393	0.047	GT Sens
GT	Sense	Sense	-0.160	Detected	0.160	Detected	0.000	-1.190	Detected	-1.205	Detected	-1.198	0.017	GT Sens
GT	Sense	Sense	-0.050	Detected	0.050	Detected	0.000	-1.540	Detected	-1.506	Detected	-1.523	0.001	GT Sens
GT	Sense	Sense	-0.068	Detected	0.068	Detected	0.000	-1.020	Detected	-1.244	Detected	-1.132	0.013	GT Sens
GT	Sense	Sense	-0.810	Detected	0.810	Detected	0.000	-3.423	Detected	-3.633	Detected	-3.528	0.050	GT Sens
GT	Sense	Sense	0.160	Detected	-0.160	Detected	0.000	-1.040	Detected	-1.611	Comprom	-1.325	0.056	GT Sens
GT	Sense	Sense	-0.549	Detected	0.549	Detected	0.000	-0.868	Detected	-1.702	Detected	-1.285	0.203	GT Sens
GT	Sense	Sense	-1.124	Detected	1.124	Detected	0.000	-0.846	Detected	-2.898	Comprom	-1.872	0.344	GT Sens
GT	Sense	Sense	0.167	Detected	-0.167	Detected	0.000	-1.453	Detected	-0.960	Detected	-1.207	0.056	GT Sens
GT	Sense	Sense	-0.418	Detected	0.418	Detected	0.000	-0.944	Detected	-1.111	Detected	-1.027	0.138	GT Sens
GT	Sense	Sense	-0.434	Detected	0.434	Detected	0.000	-0.819	Detected	-1.538	Detected	-1.179	0.172	GT Sens
GT	Sense	Sense	-0.328	Detected	0.328	Detected	0.000	-1.007	Detected	-1.435	Detected	-1.221	0.089	GT Sens
GT	Sense	Sense	-0.347	Detected	0.347	Detected	0.000	-1.103	Detected	-1.655	Detected	-1.379	0.090	GT Sens
GT	Sense	Sense	-0.422	Detected	0.422	Detected	0.000	-1.703	Detected	-1.878	Detected	-1.790	0.053	GT Sens
GT	Sense	Sense	-0.002	Detected	0.002	Detected	0.000	-0.970	Comprom	-1.213	Comprom	-1.091	0.012	GT Sens
GT	Sense	Sense	-1.063	Detected	1.063	Detected	0.000	-1.496	Detected	-2.513	Detected	-2.004	0.231	GT Sens
GT	Sense	Sense	-0.933	Detected	0.933	Detected	0.000	-1.882	Detected	-0.971	Detected	-1.426	0.303	GT Sens
GT	Sense	Sense	-0.794	Detected	0.794	Detected	0.000	-3.925	Detected	-4.778	Detected	-4.352	0.040	GT Sens
GT	Sense	Sense	-0.034	Detected	0.034	Detected	0.000	-1.675	Detected	-1.826	Detected	-1.751	0.002	GT Sens
GT	Sense	Sense	-1.313	Detected	1.313	Detected	0.000	-2.936	Detected	-3.080	Detected	-3.008	0.149	GT Sens
GT	Sense	Sense	-0.755	Detected	0.755	Detected	0.000	-1.835	Detected	-1.877	Detected	-1.856	0.133	GT Sens
GT	Sense	Sense	0.100	Detected	-0.100	Detected	0.000	-2.323	Comprom	-3.175	Comprom	-2.749	0.024	GT Sens
GT	Sense	Sense	0.013	Detected	-0.013	Detected	0.000	-1.571	Detected	-1.726	Detected	-1.649	0.002	GT Sens
GT	Sense	Sense	-0.574	Detected	0.574	Detected	0.000	-1.242	Detected	-1.132	Detected	-1.187	0.176	GT Sens
GT	Sense	Sense	-0.032	Detected	0.032	Detected	0.000	-1.605	Comprom	-0.919	Comprom	-1.262	0.067	GT Sens
GT	Sense	Sense	-1.183	Detected	1.183	Detected	0.000	-1.937	Detected	-1.529	Detected	-1.733	0.286	GT Sens
GT	Sense	Sense	-0.523	Detected	0.523	Detected	0.000	-2.699	Detected	-3.718	Detected	-3.208	0.048	GT Sens
GT	Sense	Sense	0.096	Detected	-0.096	Detected	0.000	-0.804	Detected	-1.310	Detected	-1.057	0.060	GT Sens
GT	Sense	Sense	0.209	Detected	-0.209	Detected	0.000	-1.418	Detected	-1.320	Detected	-1.369	0.024	GT Sens
GT	Sense	Sense	-0.476	Detected	0.476	Detected	0.000	-0.885	Detected	-1.155	Detected	-1.020	0.175	GT Sens
GT	Sense	Sense	-0.528	Detected	0.528	Detected	0.000	-1.083	Detected	-1.020	Detected	-1.052	0.185	GT Sens
GT	Sense	Sense	-0.384	Detected	0.384	Detected	0.000	-1.266	Detected	-1.110	Detected	-1.188	0.094	GT Sens
GT	Sense	Sense	0.132	Detected	-0.132	Detected	0.000	-1.096	Detected	-0.908	Detected	-1.002	0.025	GT Sens
GT	Sense	Sense	-0.988	Detected	0.988	Detected	0.000	-1.214	Detected	-2.235	Detected	-1.725	0.261	GT Sens
GT	Sense	Sense	0.405	Detected	-0.405	Detected	0.000	-1.159	Detected	-2.535	Detected	-1.847	0.147	GT Sens
GT	Sense	Sense	-0.774	Detected	0.774	Detected	0.000	-1.591	Detected	-1.242	Detected	-1.417	0.216	GT Sens
GT	Sense	Sense	-0.038	Detected	0.038	Detected	0.000	-1.396	Detected	-2.937	Comprom	-2.167	0.107	GT Sens
GT	Sense	Sense	-0.321	Detected	0.321	Detected	0.000	-1.190	Detected	-1.425	Detected	-1.308	0.062	GT Sens
GT	Sense	Sense	0.154	Detected	-0.154	Detected	0.000	-1.253	Detected	-1.171	Detected	-1.212	0.017	GT Sens
GT	Sense	Sense	-0.800	Detected	0.800	Detected	0.000	-1.073	Detected	-1.219	Detected	-1.146	0.290	GT Sens
GT	Sense	Sense	-0.269	Detected	0.269	Detected	0.000	-1.008	Detected	-1.506	Detected	-1.257	0.075	GT Sens
GT	Sense	Sense	-0.440	Detected	0.440	Detected	0.000	-1.153	Detected	-0.905	Detected	-1.029	0.153	GT Sens
GT	Sense	Sense	-0.446	Detected	0.446	Detected	0.000	-1.538	Detected	-2.796	Detected	-2.167	0.107	GT Sens
GT	Sense	Sense	0.338	Detected	-0.338	Detected	0.000	-1.361	Detected	-1.834	Detected	-1.597	0.061	GT Sens
GT	Sense	Sense	0.051	Detected	-0.051	Detected	0.000	-1.702	Detected	-1.428	Detected	-1.565	0.009	GT Sens
GT	Sense	Sense	0.129	Detected	-0.129	Detected	0.000	-1.248	Detected	-2.002	Detected	-1.625	0.055	GT Sens
GT	Sense	Sense	-0.191	Detected	0.191	Detected	0.000	-3.073	Comprom	-2.751	Comprom	-2.912	0.007	GT Sens
GT	Sense	Sense	-0.841	Detected	0.841	Detected	0.000	-3.848	Detected	-3.901	Detected	-3.875	0.044	GT Sens
GT	Sense	Sense	-0.355	Detected	0.355	Detected	0.000	-1.145	Detected	-1.526	Detected	-1.336	0.080	GT Sens
GT	Sense	Sense	-0.512	Detected	0.512	Detected	0.000	-1.684	Detected	-2.028	Detected	-1.856	0.075	GT Sens
GT	Sense	Sense	-0.532	Detected	0.532	Detected	0.000	-5.495	Detected	-7.906	Detected	-6.701	0.037	GT Sens
GT	Sense	Sense	-0.472	Detected	0.472	Detected	0.000	-1.436	Detected	-0.980	Detected	-1.208	0.148	GT Sens
GT	Sense	Sense	0.218	Detected	-0.218	Detected	0.000	-1.415	Detected	-1.874	Detected	-1.644	0.035	GT Sens
GT	Sense	Sense	0.476	Detected	-0.476	Detected	0.000	-1.276	Comprom	-1.460	Comprom	-1.368	0.106	GT Sens
GT	Sense	Sense	-0.576	Detected	0.576	Detected	0.000	-3.251	Detected	-2.862	Detected	-3.056	0.037	GT Sens
GT	Sense	Sense	-1.085	Detected	1.085	Detected	0.000	-1.085	Detected	-1.965	Detected	-1.525	0.323	GT Sens
GT	Sense	Sense	-0.443	Detected	0.443	Detected	0.000	-1.402	Detected	-1.976	Detected	-1.689	0.085	GT Sens
GT	Sense	Sense	0.208	Detected	-0.208	Detected	0.000	-0.918	Detected	-1.236	Detected	-1.077	0.054	GT Sens
GT	Sense	Sense	-0.818	Detected	0.818	Detected	0.000	-3.694	Detected	-5.803	Detected	-4.749	0.071	GT Sens
GT	Sense	Sense	-0.498	Detected	0.498	Detected	0.000	-2.007	Comprom	-2.402	Comprom	-2.204	0.054	GT Sens
GT	Sense	Sense	-0.485	Detected	0.485	Detected	0.000	-1.525	Detected	-1.868	Detected	-1.697	0.081	GT Sens
GT	Sense	Sense	-0.111	Detected	0.111	Detected	0.000	-2.230	Detected	-3.085	Comprom	-2.657	0.027	GT Sens

GT Sense	Sense	-0.053	Detected	0.053	Detected	0.000	-0.826	Detected	-1.249	Detected	-1.037	0.041	GT Sens	contig205	contig205	Unknown	CTCCTC	contig205	Solyc00g	Solyc00g	Unknown Protein (AHRD V1)			contig205	Solyc00g075440.1.1				AT2G43150.1		proline-r	chr2:179	111.414	118.481	133.37	142.333	72.2015	48.9422
GT Sense	Sense	-0.230	Detected	0.230	Detected	0.000	-1.345	Detected	-1.707	Detected	-1.526	0.035	GT Sens	contig206	contig206	Leucine-r	GATGAC	contig206	Solyc12g	Solyc12g	Leucine-r	GO:0005	GO:0005	contig206	Solyc12g009690.1.1				AT1G25320.1		leucine-r	chr1:887	149.985	204.077	107.308	102.903	76.7156	54.2567
GT Sense	Sense	-0.417	Detected	0.417	Detected	0.000	-1.057	Detected	-1.141	Detected	-1.099	0.120	GT Sens	contig206	contig206	Serine/thr	TTATCC	contig206	Solyc04g	Solyc04g	Serine/thr	GO:0019	GO:0019	contig206	Solyc04g	Serine/thr	GO:0019	SL2.40ch	AT4G03230.1		ATP bind	chr4:141	45.9445	80.9989	54.261	55.291	32.663	27.9997
GT Sense	Sense	-0.038	Detected	0.038	Detected	0.000	-1.072	Comprom	-1.337	Comprom	-1.205	0.013	GT Sens	contig209	contig209	Unknown	GCCAAA	contig209	Solyc06g	Solyc06g	Unknown Protein (AHRD V1)			contig209	Solyc12g	Unknown	GO:0016	SL2.40ch	AT5G14370.1		LOCATE	chr5:463	13.6264	14.2027	11.1729	32.2002	7.36857	5.57611
GT Sense	Sense	-0.117	Detected	0.117	Detected	0.000	-1.131	Detected	-1.070	Detected	-1.101	0.012	GT Sens	contig210	contig210	Endochiti	TTCTTT	contig210	Solyc10g	Solyc10g	Endochiti	GO:0005	GO:0005	contig210	Solyc10g	Endochiti	GO:0008	SL2.40ch	AT3G125	ATHCHIB	ATHCHIB	chr3:396	34.4505	40.0334	18.1308	13.9064	18.8912	17.9031
GT Sense	Sense	0.252	Detected	-0.252	Detected	0.000	-1.297	Detected	-0.801	Detected	-1.049	0.097	GT Sens	contig210	contig210	Cortical c	TTTTTCA	contig210	Solyc08g	Solyc08g	Cortical c	GO:0031	GO:0031	contig210	Solyc08g	Cortical c	GO:0008	SL2.40ch	AT4G12490.1		protease	chr4:740	191.046	133.238	113.898	122.73	72.3344	92.6986
GT Sense	Sense	-0.539	Detected	0.539	Detected	0.000	-1.586	Detected	-1.933	Detected	-1.760	0.090	GT Sens	contig210	contig210	Os06g02	CACAGA	contig210	Solyc00g	Solyc00g	Os06g0220000 protein (Fragm			contig210	Solyc00g206460.1.1				AT4G089	EXO	EXO (EX	chr4:574	15972.1	33343	12126.6	13004.2	8561.72	6117.74
GT Sense	Sense	-0.384	Detected	0.384	Detected	0.000	-1.378	Detected	-1.636	Detected	-1.507	0.065	GT Sens	contig213	contig213	Unknown	TACTCA	contig213	Solyc12g	Solyc12g	Unknown Protein (AHRD V1)			contig213	Solyc12g	Unknown Protein (A		SL2.40ch	AT1G10390.2		nucleopo	chr1:340	761.64	1282.41	490.843	661.818	423.618	321.908
GT Sense	Sense	0.180	Detected	-0.180	Detected	0.000	-1.563	Detected	-1.074	Detected	-1.318	0.049	GT Sens	contig214	contig214	Proline-ri	CTTAGC	contig214	Solyc08g	Solyc08g	Proline-ri	GO:0008	GO:0008	contig214	Solyc08g078920.1.1				AT2G45180.1		protease	chr2:186	1683.45	1296.83	983.174	1306.24	557.108	710.596
GT Sense	Sense	0.186	Detected	-0.186	Detected	0.000	-1.783	Comprom	-1.138	Detected	-1.460	0.059	GT Sens	contig214	contig214	Unknown	ATGGGG	contig214	Solyc01g	Solyc01g	Unknown Protein (AHRD V1)			contig214	Solyc01g	Unknown Protein (A		SL2.40ch	AT4G351	PIP3. PIP	PIP3 (PL	chr4:167	30.77	23.5023	16.3103	25.7987	8.70149	12.375
GT Sense	Sense	-0.199	Detected	0.199	Detected	0.000	-0.996	Detected	-1.024	Detected	-1.010	0.037	GT Sens	contig214	contig214	Unknown	GCAGTT	contig214	Solyc03g	Solyc03g	Unknown Protein (AHRD V1)%			contig214	Solyc03g	Unknown Protein (A		SL2.40ch	AT3G16010.1		pentatric	chr3:543	27.4702	35.7953	23.6917	12.1478	17.5064	15.6072
GT Sense	Sense	0.086	Detected	-0.086	Detected	0.000	-1.239	Detected	-1.493	Detected	-1.366	0.012	GT Sens	contig214	contig214	Cortical c	TAGTTT	contig214	Solyc08g	Solyc08g	Cortical c	GO:0008	GO:0008	contig214	Solyc08g	Cortical c	GO:0008	SL2.40ch	AT2G45180.1		protease	chr2:186	871.932	764.666	256.103	443.645	385.169	293.644
GT Sense	Sense	0.016	Detected	-0.016	Detected	0.000	-2.148	Detected	-2.297	Detected	-2.222	0.001	GT Sens	contig214	contig214	Cytochro	CAAATG	contig214	Solyc09g	Solyc09g	Cytochro	GO:0019	GO:0019	contig214	Solyc09g	Cytochro	GO:0019	SL2.40ch	AT3G262	CYP71B2	CYP71B2	chr3:959	213.007	205.882	45.6097	50.4691	52.6285	43.1513
GT Sense	Sense	-0.217	Detected	0.217	Detected	0.000	-1.310	Detected	-1.734	Comprom	-1.522	0.038	GT Sens	contig215	contig215	Unknown	CCATCC	contig215	Solyc12g	Solyc12g	Unknown Protein (AHRD V1)%			contig215	Solyc08g	Homology to unkno		SL2.40ch	AT2G25800.1		INVOLVE	chr2:110	28.878	38.5751	20.4727	19.6068	14.9948	10.1552
GT Sense	Sense	-0.344	Detected	0.344	Detected	0.000	-1.188	Detected	-1.354	Detected	-1.271	0.070	GT Sens	contig215	contig215	Unknown	TACTCA	contig215	Solyc12g	Solyc12g	Unknown Protein (AHRD V1)			contig215	Solyc12g	Unknown Protein (A		SL2.40ch	AT4G013	ATMPK4.	ATMPK4	chr4:567	636.596	1014.29	420.145	576.217	392.825	318.179
GT Sense	Sense	-0.380	Detected	0.380	Detected	0.000	-0.977	Detected	-1.284	Detected	-1.131	0.110	GT Sens	contig215	contig215	UDP-gluc	TATTCT	contig215	Solyc02g	Solyc02g	UDP-gluc	GO:0003	GO:0003	contig215	Solyc02g	UDP-gluc	GO:0003	SL2.40ch	AT5G15490.1		UDP-gluc	chr5:502	7584.54	12703.7	4677.75	3602.43	5553.86	4081.07
GT Sense	Sense	-0.857	Detected	0.857	Detected	0.000	-4.179	Detected	-4.078	Detected	-4.129	0.041	GT Sens	contig215	contig215	Xylogluca	TCTTCT	contig215	Solyc03g	Solyc03g	Xylogluca	GO:0016	GO:0016	contig215	Solyc03g	Xylogluca	GO:0016	SL2.40ch	AT4G258	XTR6. XT	XTR6 (XY	chr4:131	3960.64	12848.5	1006.56	1242.49	438.556	427.575
GT Sense	Sense	-0.321	Detected	0.321	Detected	0.000	-0.973	Detected	-1.619	Detected	-1.296	0.104	GT Sens	contig217	contig217	Sesquiter	AGATTA	contig217	Solyc07g	Solyc07g	Sesquiterpene synthase (AHR			contig217	Solyc07g	Sesquiterpene synt		SL2.40ch	AT3G01160.1		unknown	chr3:546	44.2895	68.3061	37.6497	84.3883	31.2086	18.1324
GT Sense	Sense	-0.417	Detected	0.417	Detected	0.000	-1.392	Detected	-1.002	Detected	-1.197	0.121	GT Sens	contig217	contig217	Unknown	GCTGAA	contig217	Solyc12g	Solyc12g	Unknown Protein (AHRD V1)			contig217	Solyc07g	cDNA clone J013074		SL2.40ch	AT1G597	ARF1	ARF1 (AU	chr1:219	28.1428	49.5848	31.4875	43.7183	15.8518	18.8814
GT Sense	Sense	-0.871	Detected	0.871	Detected	0.000	-1.223	Detected	-1.791	Detected	-1.507	0.242	GT Sens	contig217	contig217	Cytochro	ATGGAC	contig217	Solyc03g	Solyc03g	Cytochro	GO:0019	GO:0019	contig217	Solyc03g	Cytochro	GO:0019	SL2.40ch	AT5G249	CYP71A1	CYP71A1	chr5:859	125.42	415.029	74.0699	91.2861	108.836	66.7421
GT Sense	Sense	-0.860	Detected	0.860	Detected	0.000	-4.177	Detected	-4.145	Detected	-4.161	0.040	GT Sens	contig218	contig218	Xylogluca	TCTTCT	contig218	Solyc03g	Solyc03g	Xylogluca	GO:0005	GO:0005	contig218	Solyc03g	Xylogluca	GO:0016	SL2.40ch	AT4G258	XTR6. XT	XTR6 (XY	chr4:131	3930	12806.5	981.101	1182.36	436.748	406.092
GT Sense	Sense	-0.606	Detected	0.606	Detected	0.000	-0.928	Detected	-1.189	Detected	-1.058	0.230	GT Sens	contig220	contig220	Genomic	GATGTT	contig220	Solyc07g	Solyc07g	Genomic	GO:0016	GO:0016	contig220	Solyc01g	Serine in	GO:0016	SL2.40ch	AT3G24460.1		TMS mem	chr3:888	139.802	320.338	149.003	165.853	123.863	93.99
GT Sense	Sense	-1.154	Detected	1.154	Detected	0.000	-1.992	Detected	-2.721	Comprom	-2.356	0.191	GT Sens	contig222	contig222	FAD-bind	GTCTAC	contig222	Solyc02g	Solyc02g	FAD-bind	GO:0050	GO:0050	contig222	Solyc02g070160.1.1				AT5G44400.1		FAD-bind	chr5:178	24.2843	118.844	10.3547	10.8443	15.0443	8.24922
GT Sense	Sense	-0.058	Detected	0.058	Detected	0.000	-0.974	Comprom	-1.361	Comprom	-1.168	0.029	GT Sens	contig227	contig227	Gibberelli	CGGAAG	contig227	Solyc06g	Solyc06g	Gibberelli	GO:0005	GO:0005	contig227	Solyc06g	Gibberelli	GO:0005	SL2.40ch	AT1G28281.1				13.3439	14.2948	12.931	7.58609	7.82885	5.44361
GT Sense	Sense	-0.308	Detected	0.308	Detected	0.000	-1.339	Detected	-1.628	Detected	-1.484	0.049	GT Sens	contig228	contig228	S-adenos	TCTGTG	contig228	Solyc10g	Solyc10g	S-adenos	GO:0006	GO:0006	contig228	Solyc10g	S-adenos	GO:0006	SL2.40ch	AT2G368	MAT3	MAT3 (m	chr2:154	3044.84	4615.42	1590.47	2140.96	1649.95	1227.71
GT Sense	Sense	-0.354	Detected	0.354	Detected	0.000	-2.257	Detected	-2.383	Detected	-2.320	0.023	GT Sens	contig228	contig228	Lysine ke	TGCTCA	contig228	Solyc05g	Solyc05g	Lysine ketoglutarate reductas			contig228	Solyc05g	Lysine ketoglutarate		SL2.40ch	AT1G13000.2		unknown	chr1:443	1588.29	2563.95	1059.99	1127.58	470.241	391.735
GT Sense	Sense	-0.106	Detected	0.106	Detected	0.000	-1.025	Detected	-1.003	Detected	-1.014	0.011	GT Sens	contig229	contig229	Receptor	AATAAAT	contig229	Solyc02g	Solyc02g	Receptor	GO:0006	GO:0006	contig229	Solyc02g	Receptor	GO:0006	SL2.40ch	AT4G23240.1				108.52	124.23	88.872	80.2723	63.5659	58.6622
GT Sense	Sense	0.159	Detected	-0.159	Detected	0.000	-1.345	Detected	-1.791	Detected	-1.568	0.029	GT Sens	contig234	contig234	Decarbox	ATTTCAT	contig234	Solyc08g	Solyc08g	Decarbox	GO:0019	GO:0019	contig234	Solyc08g	Decarbox	GO:0030	SL2.40ch	AT4G332	XI-I. ATXI	XI-I: moto	chr4:160	819.502	649.602	341.324	693.696	319.928	213.534
GT Sense	Sense	-0.374	Detected	0.374	Detected	0.000	-1.278	Detected	-1.028	Detected	-1.153	0.100	GT Sens	contig239	contig239	Transpor	TTTACAT	contig239	Solyc10g	Solyc10g	Transporter CorA family (AHR			contig239	Solyc08g	Zinc transport prote		SL2.40ch	AT5G62630.1				105.211	174.621	75.4606	76.5982	62.2446	67.3119
GT Sense	Sense	-0.206	Detected	0.206	Detected	0.000	-0.885	Detected	-1.361	Detected	-1.123	0.071	GT Sens	contig240	contig240	ATP synt	TAGTAA	contig240	Solyc11g	Solyc11g	ATP synt	GO:0045	GO:0045	contig240	Solyc11g	ATP synt	GO:0045	SL2.40ch	ATCG001	ATPA	Encodes	chrC:993	9888.28	13012.5	9255.88	7897.03	6843.01	4471.42
GT Sense	Sense	-0.746	Detected	0.746	Detected	0.000	-1.680	Detected	-1.410	Detected	-1.545	0.179	GT Sens	contig251	contig251	Unknown	CGACCC	contig251	Solyc03g	Solyc03g	Unknown Protein (AHRD V1)			contig251	Solyc03g	Unknown Protein (A		SL2.40ch	AT5G47490.1		unknown	chr5:192	36.3126	101.026	33.6911	46.2516	21.0469	23.0774
GT Sense	Sense	-0.607	Detected	0.607	Detected	0.000	-1.452	Detected	-1.165	Detected	-1.308	0.171	GT Sens	contig252	contig252	Histidine	CACTATC	contig252	Solyc05g	Solyc05g	Histidine	GO:0030	GO:0030	contig252	Solyc08g	Decarbox	GO:0030	SL2.40ch	AT5G28010.1				103.654	237.863	52.6454	89.4812	63.9148	70.9112
GT Sense	Sense	-0.753	Detected	0.753	Detected	0.000	-1.110	Detected	-1.090	Detected	-1.100	0.282	GT Sens	contig254	contig254	Phloem le	AATCACT	contig254	Solyc02g	Solyc02g	Phloem lectin (AHRD V1 --*- O8			contig254	Solyc02g	Phloem le	GO:0030	SL2.40ch	AT1G11270.3				300.707	844.796	150.482	363.068	260.135	239.713
GT Sense	Sense	-0.868	Detected	0.868	Detected	0.000	-1.340	Detected	-1.793	Detected	-1.566	0.223	GT Sens	contig255	contig255	Decarbox	AGAAAA	contig255	Solyc08g	Solyc08g	Decarbox	GO:0019	GO:0019	contig255	Solyc08g	Decarbox	GO:0030	SL2.40ch	AT1G437	emb1075	emb1075	chr1:164	67.1864	221.41	31.409	60.1099	53.6607	35.6433
GT Sense	Sense	-0.550	Detected	0.550	Detected	0.000	-0.976	Detected	-1.140	Detected	-1.058	0.197	GT Sens	contig256	contig256	Phosphat	AATGCA	contig256	Solyc10g	Solyc10g	Phosphatidylcholine:ceramide			contig256	Solyc10g	Phosphat	GO:0045	SL2.40ch	AT3G54020.1		phospha	chr3:200	1765.47	3741.78	1199.9	1710.67	1454.76	1180.74
GT Sense	Sense	-1.951	Detected	1.951	Detected	0.000	-2.824	Comprom	-4.542	Comprom	-3.683	0.226	GT Sens	contig257	contig257	Alpha-hu	CACTCAT	contig257	Solyc01g	Solyc01g	Alpha-hu	GO:0080	GO:0080	contig257	Solyc01g	Alpha-hu	GO:0080	SL2.40ch	AT5G60700.1		glycosylt	chr5:244	12.8568	190.082	5.29307	11.0859	7.77638	2.14862
GT Sense	Sense	-0.665	Detected	0.665	Detected	0.000	-1.711	Detected	-1.586	Detected	-1.648	0.132	GT Sens	contig257	contig257	Unknown	GAAAAG	contig257	Solyc10g	Solyc10g	Unknown Protein (AHRD V1)			contig257	Solyc03g	Unknown Protein (A		SL2.40ch	AT3G053	ALY2. AT	DNA bind	chr3:154	31.4297	78.0642	29.7675	37.3659	16.8551	16.7081
GT Sense	Sense	-0.252	Detected	0.252	Detected	0.000	-1.053	Detected	-1.535	Detected	-1.294	0.066	GT Sens	contig262	contig262	ATP synt	GTATAT	contig262	Solyc06g	Solyc06g	ATP synthase subunit alpha c			contig262	Solyc06g	ATP synthase subu		SL2.40ch	ATCG001	ATPA	Encodes	chrC:993	10670.9	14965.2	10182.3	7478.89	6781.7	4415.11
GT Sense	Sense	0.085	Detected	-0.085	Detected	0.000	-3.263	Comprom	-3.048	Comprom	-3.156	0.002	GT Sens	contig262	contig262	Cysteine-	TGCTGC	contig262	Solyc02g	Solyc02g	Cysteine-	GO:0016	GO:0016	contig262	Solyc02g	RLK. Rec	GO:0004	SL2.40ch	AT4G11890.2				17.7996	15.6437	4.39816	5.19898	1.93534	2.04245
GT Sense	Sense	-0.108	Detected	0.108	Detected	0.000	-1.753	Detected	-1.688	Detected	-1.720	0.004	GT Sens	contig264	contig264	Unknown	ATGAAA	contig264	Solyc01g	Solyc01g	Unknown Protein (AHRD V1)			contig264	Solyc01g	Unknown Protein (A		SL2.40ch	AT1G73870.1				1053.12	1208.94	271.164	747.95	372.874	354.534
GT Sense	Sense	-1.047	Detected	1.047	Detected	0.000	-2.173	Detected	-2.775	Detected	-2.474	0.151	GT Sens	contig268	contig268	Xylogluca	GCAGAT	contig268	Solyc12g	Solyc12g	Xylogluca	GO:0005	GO:0005	contig268	Solyc12g	Xylogluca	GO:0005	SL2.40ch	AT3G23730.1		xylogluc	chr3:855	5617.22	23711.7	3337.58	5427.65	2850.57	1707.43
GT Sense	Sense	-0.389	Detected	0.389	Detected	0.000	-1.156	Detected	-1.943	Comprom	-1.549	0.107	GT Sens	contig268	contig268	Adaptin N	AAAGTG	contig268	Solyc00g	Solyc00g	Adaptin N terminal region fami			contig268	Solyc11g	AP-2 com	GO:0030	SL2.40ch	AT1G595	CW7	CW7	chr1:218	26.0923	44.2314	21.2998	17.5272	16.9817	8.94366
GT Sense	Sense	-0.433	Detected	0.433	Detected	0.000	-1.482	Detected	-1.841	Detected	-1.661	0.071	GT Sens	contig268	contig268	Chitinase	AGCAAAT	contig268	Solyc07g	Solyc07g	Chitinase	GO:0008	GO:0008	contig268	Solyc07g	Chitinase	GO:0008	SL2.40ch	AT4G01290.2		unknown	chr4:538	13037.9	23489.1	10252.7	11310.5	6975.71	4946.78
GT Sense	Sense	-0.409	Detected	0.409	Detected	0.000	-0.955	Detected	-1.297	Detected	-1.126	0.126	GT Sens	contig270	contig270	Reductas	CACTGA	contig270	Solyc03g	Solyc03g	Reductas	GO:0045	GO:0045	contig270	Solyc12g	Reductas	GO:0032	SL2.40ch	AT1G59960.1		aldo/keto	chr1:220	13379.2	23307.5	9477.21	17809	10143.8	7274.9
GT Sense	Sense	-0.652	Detected	0.652	Detected	0.000	-0.825	Detected	-1.286	Detected	-1.055	0.267	GT Sens	contig272	contig272	Unknown	CTGTGA	contig272	Solyc02g	Solyc02g	Unknown Protein (AHRD V1)			contig272	Solyc02g	Unknown Protein (A		SL2.40ch	AT1G18170.1		immunop	chr1:625	105.321	257.198	108.512	136.96	103.454	68.3471
GT Sense	Sense	-0.763	Detected	0.763	Detected	0.000	-1.550	Detected	-1.011	Detected	-1.281	0.255	GT Sens	contig273	contig273	Histidine	GAGCAA	contig273	Solyc07g	Solyc07g	Histidine	GO:0030	GO:0030	contig273	Solyc08g	Decarbox	GO:0030	SL2.40ch	#N/A	#N/A	#N/A	#N/A	114.655	326.661	56.8354	90.269	73.6018	97.2199
GT Sense	Sense	1.575	Detected	-1.575	Detected	0.000	-1.497	Detected	-1.072	Detected	-1.285	0.504	GT Sens	contig274	contig274	Vicilin-lik	AAACAA	contig274	Solyc09g	Solyc09g	Vicilin-lik	GO:0045	GO:0045	contig274	Solyc09g	Vicilin-lik	GO:0045	SL2.40ch	AT5G60730.1		anion-tra	chr5:244	357.058	39.7587	598.398	121.516	47.0156	57.3637
GT Sense	Sense	-0.369	Detected	0.369	Detected	0.000	-1.114	Detected	-1.250	Detected	-1.182	0.088	GT Sens	contig275	contig275	Diacylglyc	TAGCTT	contig275	Solyc07g	Solyc07g	Diacylglyc	GO:0007	GO:0007	contig275	Solyc07g	Diacylglyc	GO:0004	SL2.40ch	AT2G209	DGK5. AT	diacylgly	chr2:898	3023.83	4983.13	2068.34	2104.82	1996.87	1652.48
GT Sense	Sense	-0.845	Detected	0.845	Detected	0.000	-2.904	Detected	-3.102	Detected	-3.003	0.072	GT Sens	contig277	contig277	FAD-bind	CGCGGT	contig277	Solyc02g	Solyc02g	FAD-bind	GO:0050	GO:0050	contig277	Solyc02g	FAD-bind	GO:0050	SL2.40ch	AT4G20860.1		FAD-bind	chr4:111	134.178	427.918	85.1323	95.6278	35.6436	28.2549
GT Sense	Sense	-0.291	Detected	0.291	Detected	0.000	-0.830	Detected	-1.196	Detected	-1.013	0.099	GT Sens	contig277	contig277	Fructose-	AATGAT	contig277	Solyc09g	Solyc09g	Fructose-	GO:0006	GO:0006	contig277	Solyc09g	Fructose-	GO:0005	SL2.40ch	AT3G52930.1		fructose-	chr3:196	7903.35	11699.9	4688.27	4553.74	6023.91	4249.52
GT Sense	Sense	-0.203	Detected	0.203	Detected	0.000	-1.248	Detected	-1.267	Detected	-1.258	0.025	GT Sens	contig278	contig278	UDP-gluc	CATGCA	contig278	Solyc01g	Solyc01g	UDP-gluc	GO:0010	GO:0010	contig278	Solyc05g	UDP-gluc	GO:0010	SL2.40ch	AT1G01390.1		UDP-gluc	chr1:148	10289.3	13487.5	7952.67	7058.04	5522.15	4955.61
GT Sense	Sense	0.267	Detected	-0.267	Detected	0.000	-1.151	Detected	-1.330	Detected	-1.241	0.048	GT Sens	contig284	contig284	Kunitz try	AATCAA	contig284	Solyc03g	Solyc03g	Kunitz try	GO:0004	GO:0004	contig284	Solyc03g	Kunitz try	GO:0004	SL2.40ch	AT5G173	HDG9	HDG9 (H	chr5:570	1171.51	799.886	307.219	549.664	485.554	389.729
GT Sense	Sense	0.256	Detected	-0.256	Detected	0.000	-2.168	Detected	-1.686	Detected	-1.927	0.032	GT Sens	contig285	contig285	CTP synt	TGAAAG	contig285	Solyc02g	Solyc02g	CTP synthase (AHRD V1 *-*- D7			contig285	Solyc02g	CTP synthase (AHR		SL2.40ch	AT1G30820.1		CTP synt	chr1:109	898.713	623.258	196.894	350.533	185.504	235.541
GT Sense	Sense	-0.348	Detected	0.348	Detected	0.000	-1.069	Detected	-1.734	Detected	-1.402	0.100	GT Sens	contig295	contig295	Chitinase	TAAGTG	contig295	Solyc10g	Solyc10g	Chitinase	GO:0008	GO:0008	contig295	Solyc10g017970.1.1				AT5G463	KCO2. AT	KCO2 (C	chr5:188	7397.31	11842.6	6526.98	7956.89	4968.92	2848.22
GT Sense	Sense	-0.365	Detected	0.365	Detected	0.000	-0.848	Detected	-1.362	Detected	-1.105	0.132	GT Sens	contig295	contig295	(-)-germa	GTTTAC	contig295	Solyc07g	Solyc07g	(-)-germa	GO:0016	GO:0016	contig295	Solyc07g	(-)-germa	GO:0016	SL2.40ch	AT5G05070.1		zinc ion	chr5:149	179.342	294.183	139.292	264.498	142.135	90.4569
GT Sense	Sense	-0.475	Detected	0.475	Detected	0.000	-1.312	Detected	-0.975	Detected	-1.143	0.151	GT Sens	contig299	contig299	Calmodul	CTCCTA	contig299	Solyc11g	Solyc11g	Calmodul	GO:0005	GO:0005	contig299	Solyc11g071750.1.1				AT5G423	CML39. C	CML37 (C	chr5:169	400.682	765.164	158.036	343.967	248.315	285.288
GT Sense	Sense	-0.498	Detected	0.498	Detected	0.000	-1.461	Detected	-2.094	Detected	-1.778	0.095	GT Sens	contig303	contig303	Os06g02	CTCACT	contig303	Solyc04g	Solyc04g	Os06g0220000 protein (Fragm			contig303	Solyc04g	Os06g0220000 prot		SL2.40ch	AT4G089	EXO	EXO (EX	chr4:574	352.154	694.797	203.502	242.649	200.046	117.29
GT Sense	Sense	0.182	Detected	-0.182	Detected	0.000	-1.255	Detected	-1.122	Detected	-1.189	0.026	GT Sens	contig305	contig305	Unknown	GAAAGC	contig305	Solyc04g	Solyc04g	Unknown	GO:0009	GO:0009	contig305	Solyc04g	Unknown	GO:0009	SL2.40ch	AT1G23720.1		proline-r	chr1:838	247.932	190.49	157.306	152.001	101.399	101.088
GT Sense	Sense	-0.790	Detected	0.790	Detected	0.000	-1.478	Detected	-2.114	Detected	-1.796	0.170	GT Sens	contig307	contig307	Cytochro	CTCCAC	contig307	Solyc03g	Solyc03g	Cytochro	GO:0019	GO:0019	contig307	Solyc03g	Cytochro	GO:0019	SL2.40ch	AT5G50540.1		unknown	chr5:205	75.0352	221.868	39.7083	47.6281	51.5831	30.1682
GT Sense	Sense	-0.864	Detected	0.864	Detected	0.000	-4.918	Detected	-5.509	Detected	-5.213	0.029	GT Sens	contig307	contig307	Polygalac	TGACAG	contig307	Solyc02g	Solyc02g	Polygalac	GO:0005	GO:0005	contig307	Solyc02g	Polygalac	GO:0005	SL2.40ch	AT2G43870.1		polygalac	chr2:181	320.966	1051.85	67.376	245.485	21.4108	12.9208
GT Sense	Sense	0.396	Detected	-0.396	Detected	0.000	-1.352	Comprom	-1.580	Comprom	-1.466	0.071	GT Sens	contig309	contig309	Phosphat	GCATAA	contig309	Solyc10g	Solyc10g	Phosphatidylglycerol/phospha			contig309	Solyc10g	Phosphatidylglycer		SL2.40ch	#N/A	#N/A	#N/A	#N/A	19.6944	11.2446	7.78286	3.85192	6.49319	5.03777
GT Sense	Sense	0.089	Detected	-0.089	Detected	0.000	-1.012	Detected	-0.989	Detected	-1.000	0.008	GT Sens	contig323	contig323	Legumin-	TCTCCC	contig323	Solyc01g	Solyc01g	Legumin-	GO:0008	GO:0008	contig323	Solyc01g	Legumin-	GO:0008	SL2.40ch	AT3G54600.1		DJ-1 fam	chr3:202	737.639	644.21	593.909	855.679	380.726	351.726
GT Sense	Sense	-0.431	Detected	0.431	Detected	0.000	-0.806	Detected	-1.763	Detected	-1.284	0.184	GT Sens	contig329	contig329	cDNA clo	ACATCA	contig329	Solyc02g	Solyc02g	cDNA clone J023049H21 full in			contig329	Solyc02g	BZIP tran	GO:0005	SL2.40ch	AT4G12700.1		unknown	chr4:748	116.641	209.542	115.245	31.4616	99.6108	46.6357
GT Sense	Sense	-0.256	Detected	0.256	Detected	0.000	-1.402	Detected	-0.844	Detected	-1.123	0.097	GT Sens	contig365	contig365	Unknown	AATACA	contig365	Solyc09g	Solyc09g	Unknown Protein (AHRD V1)			contig365	Solyc11g	Unknown Protein (A		SL2.40ch	AT4G09430.1		disease r	chr4:597	39.2943	55.3608	31.0119	32.0818	19.6494	26.3005
GT Sense	Sense	-1.062	Detected	1.062	Detected	0.000	-2.420	Detected	-3.308	Detected	-2.864	0.131	GT Sens	contig368	contig368	Xylogluca	AACGCT	contig368	Solyc07g	Solyc07g	Xylogluca	GO:0048	GO:0048	contig368	Solyc07g	Xylogluca	GO:0016	SL2.40ch	AT3G23730.1		xylogluc	chr3:855	2860.7	12326.5	1586.01	2636.21	1235.43	606.926
GT Sense	Sense	-0.043	Detected	0.043	Detected	0.000	-0.878	Detected	-1.125	Detected	-1.002	0.017	GT Sens	contig396	contig396	Alpha-hyd	CAGACA	contig396	Solyc07g	Solyc07g	Alpha-hyd	GO:0080	GO:0080	contig396	Solyc07g	Alpha-hyd	GO:0080	SL2.40ch	AT3G11130.1		clathrin h	chr3:348	119.634	125.597	68.6077	110.674	74.2925	56.8938
GT Sense	Sense	0.236	Detected	-0.236	Detected	0.000	-2.762	Detected	-2.654	Detected	-2.708	0.008	GT Sens	contig402	contig402	Long-cha	ATAGCA	contig402	Solyc09g	Solyc09g	Long-cha	GO:0008	GO:0008	contig402	Solyc09g	Long-cha	GO:0004	SL2.40ch	AT3G059	LACS6. A	LACS6 (lo	chr3:178	82.6864	58.9577	89.7559	87.2361	11.4664	11.2335
GT Sense	Sense	-0.233	Detected	0.233	Detected	0.000	-0.829	Detected	-1.259	Detected	-1.044	0.081	GT Sens	contig427	contig427	ECA1 gar	TAGTAGTAAGGTGGTGGAGGAGATGGTGAGGGTGGAGGAGGTGATTT							contig427	#N/A	#N/A	#N/A	#N/A	#N/A	#N/A	#N/A	#N/A	800.629	1093.71	806.589	597.592	586.492	395.759
GT Sense	Sense	0.172	Detected	-0.172	Detected	0.000	-1.294	Comprom	-1.952	Comprom	-1.623	0.048	GT Sens	contig446	contig446	Unknown	CCAAACT	contig446	Solyc00g	Solyc00g	Unknown Protein (AHRD V1)			contig446	Solyc00g	Unknown Protein (A		SL2.40ch	AT5G39980.1		pentatric	chr5:160	12.5278	9.76326	7.04392	3.60644	5.02178	2.89461
GT Sense	Sense	-0.263	Detected	0.263	Detected	0.000	-0.978	Detected	-1.708	Detected	-1.343	0.096	GT Sens	contig480	contig480	Sesquiter	CAGAGA	contig480	Solyc07g	Solyc07g	Sesquiterpene synthase (AHR			contig480	Solyc07g	Sesquiterpene synt		SL2.40ch	AT3G28770.1		unknown	chr3:107	60.0404	85.4958	51.0905	79.7817	40.5005	22.2068
GT Sense	Sense	-0.671	Detected	0.671	Detected	0.000	-1.858	Detected	-1.336	Detected	-1.597	0.157	GT Sens	contig489	contig489	Histidine	TAGGAA	contig489	Solyc07g	Solyc07g	Histidine	GO:0019	GO:0019	contig489	Solyc08g	Decarbox	GO:0030	SL2.40ch	AT5G25420.1		xanthine	chr5:883	564.881	1416.6	246.529	433.424	274.913	358.85
GT Sense	Sense	-0.154	Detected	0.154	Detected	0.000	-1.094	Detected	-1.667	Detected	-1.380	0.051	GT Sens	contig518	contig518	Xylogluca	GTGGAA	contig518	Solyc12g	Solyc12g	Xylogluca	GO:0005	GO:0005	contig518	Solyc12g	Xylogluca	GO:0005	SL2.40ch	AT3G23730.1		xylogluc	chr3:855	346.409	424.046	115.983	257.832	200.026	122.191
GT Sense	Sense	-0.227	Detected	0.227	Detected	0.000	-0.954	Detected	-1.088	Detected	-1.021	0.050	GT Sens	contig591	contig591	AP-2 com	CAAACT	contig591	Solyc11g	Solyc11g	AP-2 com	GO:0030	GO:0030	contig591	Solyc11g	AP-2 com	GO:0030	SL2.40ch	AT5G227	alpha-AD	alpha-AD	chr5:757	395.481	535.468	315.745	302.013	264.485	219.124
GT Sense	Sense	-0.595	Detected	0.595	Detected	0.000	-1.275	Detected	-0.995	Detected	-1.135	0.204	GT Sens	contig640	contig640	Unknown	TAAGCG	contig640	Solyc07g	Solyc07g	Unknown Protein (AHRD V1)			contig640	Solyc07g	Unknown Protein (A		SL2.40ch	AT5G174	IRX3. CES	IRX3 (IRR	chr5:573	38.7648	87.389	37.7656	42.9198	26.7868	29.5709
GT Sense	Sense	0.167	Detected	-0.167	Detected	0.000	-1.034	Comprom	-1.678	Comprom	-1.356	0.065	GT Sens	contig644	contig644	Unknown	TCTGCT	contig644	Solyc05g	Solyc05g	Unknown Protein (AHRD V1)			contig644	Solyc05g	Unknown Protein (A		SL2.40ch	AT4G170	CT-BMY.	CT-BMY	chr4:960	21.2528	16.662	5.90889	11.6767	10.2346	5.9526
GT Sense	Sense	0.163	Detected	-0.163	Detected	0.000	-1.722	Detected	-1.011	Detected	-1.367	0.073	GT Sens	contig657	contig657	Subtilisin	ACATTTT	contig657	Solyc08g	Solyc08g	Subtilisin	GO:0004	GO:0004	contig657	Solyc08g	Subtilisin	GO:0004	SL2.40ch	AT5G58830.1		identical	chr5:237	3188.17	2515.52	1625.88	2511.52	955.755	1423.14
GT Sense	Sense	-0.295	Detected	0.295	Detected	0.000	-2.279	Comprom	-2.214	Comprom	-2.247	0.017	GT Sens	contig683	contig683	Pyridine	GTGAAA	contig683	Solyc11g	Solyc11g	Pyridine	GO:0055	GO:0055	contig683	Solyc11g	Nitric oxide reducta		SL2.40ch	AT3G102	LYC	LYC (LYC	chr3:316	23.7694	35.3763	10.4377	17.8858	6.65217	6.32644
GT Sense	Sense	0.375	Detected	-0.375	Detected	0.000	-0.936	Detected	-1.953	Detected	-1.444	0.150	GT Sens	contig769	contig769	Proteinas	TACTCA	contig769	Solyc03g	Solyc03g	Proteinas	GO:0004	GO:0004	contig769	Solyc03g	Proteinas	GO:0004	SL2.40ch	AT4G15830.1		binding	chr4:899	15131.2	8899.52	6563.6	5830.48	6755.91	3034.29
GT Sense	Sense	-0.259	Detected	0.259	Detected	0.000	-2.356	Detected	-2.195	Detected	-2.275	0.014	GT Sens	contig816	contig816	Auxin-ind	TCTTCA	contig816	Solyc07g	Solyc07g	Auxin-induced protein-like (AH			contig816	Solyc07g	Auxin-induced prote		SL2.40ch	AT3G07730.1		unknown	chr3:246	49.1058	69.5534	8.43418	26.6495	12.7169	12.9258
GT Sense	Sense	-0.117	Detected	0.117	Detected	0.000	-0.925	Detected	-1.384	Detected	-1.154	0.046	GT Sens	contig816	contig816	Subtilisin	AGTGTC	contig816	Solyc01g	Solyc01g	Subtilisin	GO:0006	GO:0006	contig816	Solyc01g	Subtilisin	GO:0006	SL2.40ch	AT5G44530.1		subtilase	chr5:179	443.379	515.868	515.573	510.994	280.59	185.54
GT Sense	Sense	0.488	Detected	-0.488	Detected	0.000	-1.204	Detected	-0.819	Detected	-1.011	0.193	GT Sens	contig834	contig834	Major late	GTGATG	contig834	Solyc04g	Solyc04g	Major late	GO:0009	GO:0009	contig834	Solyc04g	Major late	GO:0009	SL2.40ch	AT3G427	AL3	AL3 (ALF	chr3:148	36.4324	18.3169	28.5438	44.906	12.492	14.8212
GT Sense	Sense	-0.188	Detected	0.188	Detected	0.000	-2.709	Detected	-2.410	Detected	-2.559	0.009	GT Sens	contig843	contig843	Glutathio	GGCGTA	contig843	Solyc01g	Solyc01g	Glutathione-S-transferase (AH			contig843	Solyc01g	Glutathione-S-trans		SL2.40ch	AT3G492	emb1796	emb1796	chr3:182	5037.75	6463.37	1129.53	1187.95	972.021	1087.34
GT Sense	Sense	-0.255	Detected	0.255	Detected	0.000	-1.529	Detected	-2.289	Detected	-1.909	0.053	GT Sens	contig862	contig862	Malate sy	ATGGCT	contig862	Solyc03g	Solyc03g	Malate sy	GO:0006	GO:0006	contig862	Solyc03g	Malate sy	GO:0004	SL2.40ch	AT5G038	MLS	MLS (MA	chr5:103	53.6849	75.5377	37.0418	40.1116	24.5818	13.1955
GT Sense	Sense	0.170	Detected	-0.170	Detected	0.000	-1.338	Detected	-1.585	Detected	-1.462	0.020	GT Sens	contig888	contig888	Pectinest	ATCATA	contig888	Solyc02g	Solyc02g	Pectinest	GO:0030	GO:0030	contig888	Solyc02g	Pectinest	GO:0005	SL2.40ch	AT1G02810.1		pectinest	chr1:618	2149.23	1678.45	1096.82	1891.27	836.824	640.789
GT Sense	Sense	0.018	Detected	-0.018	Detected	0.000	-0.920	Detected	-1.672	Detected	-1.296	0.075	GT Sens	contig958	contig958	Aromatic	ACCAAA	contig958	Solyc07g	Solyc07g	Aromatic	GO:0019	GO:0019	contig958	Solyc07g054860.1.1				AT2G20340.1		tyrosine	chr2:877	96.3453	92.8623	47.0697	72.5507	55.677	30.0459
GT Sense	Sense	-0.908	Detected	0.908	Detected	0.000	-5.202	Detected	-5.296	Detected	-5.249	0.029	GT Sens	U23053	U23053	LEU2305	CAAGAGTTAGGAGTCCAAAATGTGACAGTTAAGACTGTGACTTTCAGTGGAACAACAAAT																308.206	1073.3	61.8603	271.509	17.4008	14.8214
GT Sense	Sense	-0.805	Detected	0.805	Detected	0.000	-4.356	Detected	-4.992	Detected	-4.674	0.033	GT Sens	U23053	U23053	TAPG1: C	GCCAGAGGAAGAGTTTCTGGTTTACAAAAACCTACCAATTGCCTATTAAAAAGTTAGATA																423.297	1277.04	87.8408	304.214	39.9902	23.3835
GT Sense	Sense	-0.832	Detected	0.832	Detected	0.000	-4.368	Detected	-5.287	Detected	-4.828	0.037	GT Sens	U70480	U70480	LEU7048	GCTGGAAACAACAAGAGTTGGGAGTCCAAAATGTGACAGTTAAGACTGTGACTTTTAGTG																446.282	1398.8	87.6813	367.343	42.6094	20.485
GT Sense	Sense	-0.217	Detected	0.217	Detected	0.000	-3.585	Detected	-5.149	Detected	-4.367	0.033	GT Sens	U70481	U70481	LEU7048	CTGGAGGAAGAGCTTCTGGTTTTGAAGAACTTAGTAAATGCTTATAAATTATACAACAAA																63595.8	84911.7	55114.2	49292.9	6818.84	2097.41
GT Sense	Sense	-0.173	Detected	0.173	Detected	0.000	-4.195	Detected	-5.851	Detected	-5.023	0.027	GT Sens	U70481	U70481	TAPG4: C	AAGAGCAAGGAGTACAAAATGTGACAGTTAAGATGGTTAGCTTCACAAGTACTGAGAATG																46815.7	58856.6	34375.9	29312.8	3192.94	920.522

GO ID	GO ACCESSION	GO Term	p-value	Count in Selection	% Count in Selection	Count in Total	% Count in Total
6201	GO:0008794	arsenate reductase (glutared	0.00	6	1.8518518	20	0.0577584
13089	GO:0030614	oxidoreductase activity, actin	0.00	6	1.8518518	20	0.0577584
13088	GO:0030613	oxidoreductase activity, actin	0.00	6	1.8518518	20	0.0577584
13086	GO:0030611	arsenate reductase activity	0.00	6	1.8518518	20	0.0577584
28364	GO:0065007	biological regulation	0.00	72	22.222221	4376	12.637537
25094	GO:0050789|GO:0050791	regulation of biological proce	0.00	71	21.91358	4315	12.461374
25098	GO:0050794|GO:0051244	regulation of cellular process	0.00	64	19.753086	3987	11.514136
11224	GO:0019222	regulation of metabolic proce	0.00	48	14.814815	3115	8.995871
5243	GO:0007218	neuropeptide signaling pathw	0.00	2	0.61728394	5	0.0144396
21058	GO:0045454|GO:0030503|G	cell redox homeostasis	0.00	8	2.4691358	211	0.6093511
7074	GO:0009815	1-aminocyclopropane-1-carb	0.00	4	1.2345679	49	0.14150807
4530	GO:0006355|GO:0032583|G	regulation of transcription, DN	0.00	40	12.345679	2591	7.4826
25544	GO:0051252	regulation of RNA metabolic	0.00	40	12.345679	2593	7.488376
13756	GO:0031326	regulation of cellular biosynth	0.00	40	12.345679	2605	7.523031
7141	GO:0009889	regulation of biosynthetic pro	0.00	40	12.345679	2605	7.523031
32213	GO:2000112	regulation of cellular macrom	0.00	40	12.345679	2605	7.523031
7772	GO:0010556	regulation of macromolecule	0.00	40	12.345679	2605	7.523031
31493	GO:0080118	brassinosteroid sulfotransfera	0.00	3	0.9259259	25	0.072197996
5702	GO:0008146	sulfotransferase activity	0.00	4	1.2345679	54	0.15594767
11695	GO:0019725	cellular homeostasis	0.00	8	2.4691358	234	0.67577326
7692	GO:0010468	regulation of gene expression	0.00	40	12.345679	2635	7.6096687
18925	GO:0042592	homeostatic process	0.00	8	2.4691358	238	0.68732494
19393	GO:0043086	negative regulation of catalyt	0.00	7	2.1604939	188	0.54292893
20342	GO:0044092	negative regulation of molecu	0.00	7	2.1604939	188	0.54292893
27228	GO:0060255	regulation of macromolecule	0.00	40	12.345679	2660	7.6818666
25468	GO:0051171	regulation of nitrogen compo	0.00	40	12.345679	2675	7.7251854
11221	GO:0019219	regulation of nucleobase, nuc	0.00	40	12.345679	2675	7.7251854
31465	GO:0080090	regulation of primary metabo	0.00	40	12.345679	2710	7.826263
28365	GO:0065008	regulation of biological quality	0.00	9	2.7777777	316	0.9125827
19255	GO:0042938	dipeptide transport	0.00	5	1.5432099	105	0.3032316
19253	GO:0042936	dipeptide transporter activity	0.00	5	1.5432099	105	0.3032316
8517	GO:0015103	inorganic anion transmembra	0.00	6	1.8518518	154	0.44473964
3464	GO:0004857	enzyme inhibitor activity	0.00	6	1.8518518	157	0.4534034
12946	GO:0030414	peptidase inhibitor activity	0.00	5	1.5432099	110	0.31767118
7690	GO:0010466	negative regulation of peptida	0.00	5	1.5432099	110	0.31767118
25636	GO:0051346	negative regulation of hydrola	0.00	5	1.5432099	110	0.31767118
9722	GO:0016641	oxidoreductase activity, actin	0.00	4	1.2345679	69	0.19926646
28100	GO:0061134	peptidase regulator activity	0.00	5	1.5432099	112	0.32344702
26711	GO:0052547	regulation of peptidase activi	0.00	5	1.5432099	112	0.32344702
13753	GO:0031323	regulation of cellular metabol	0.00	40	12.345679	2770	7.999538
9832	GO:0016782	transferase activity, transferri	0.01	4	1.2345679	74	0.21370606
8967	GO:0015698	inorganic anion transport	0.01	6	1.8518518	176	0.5082739
13082	GO:0030599	pectinesterase activity	0.01	6	1.8518518	178	0.5140497
3307	GO:0004675	transmembrane receptor pro	0.01	9	2.7777777	367	1.0598665
31431	GO:0080054	low affinity nitrate transmemb	0.01	3	0.9259259	45	0.1299564
12608	GO:0030001	metal ion transport	0.01	9	2.7777777	374	1.080082
4910	GO:0006811	ion transport	0.01	17	5.2469134	959	2.769515
219	GO:0000287	magnesium ion binding	0.01	2	0.61728394	16	0.046206716
9719	GO:0016638	oxidoreductase activity, actin	0.01	4	1.2345679	89	0.25702485
8608	GO:0015198	oligopeptide transporter activ	0.01	5	1.5432099	139	0.40142086
8607	GO:0015197|GO:0015637	peptide transporter activity	0.01	5	1.5432099	139	0.40142086
9099	GO:0015833	peptide transport	0.01	5	1.5432099	141	0.4071967
4944	GO:0006857	oligopeptide transport	0.01	5	1.5432099	141	0.4071967
2986	GO:0004252	serine-type endopeptidase ac	0.01	5	1.5432099	144	0.41586044
5792	GO:0008271	secondary active sulfate tran	0.01	2	0.61728394	18	0.051982556
22917	GO:0047429	nucleoside-triphosphate diph	0.01	2	0.61728394	18	0.051982556
8736	GO:0015381	high affinity sulfate transmem	0.01	2	0.61728394	18	0.051982556
6023	GO:0008569	minus-end-directed microtub	0.02	1	0.30864197	2	0.00577584
8526	GO:0015112	nitrate transmembrane trans	0.02	3	0.9259259	61	0.1761631
8975	GO:0015706|GO:0006872	nitrate transport	0.02	3	0.9259259	61	0.1761631
5213	GO:0007186	G-protein coupled receptor p	0.02	3	0.9259259	62	0.17905103
8530	GO:0015116	sulfate transmembrane trans	0.02	2	0.61728394	26	0.075085916
8494	GO:0015075	ion transmembrane transport	0.02	14	4.3209877	827	2.3883097
16603	GO:0034220	ion transmembrane transport	0.03	14	4.3209877	833	2.4056373
5793	GO:0008272|GO:0006870	sulfate transport	0.03	2	0.61728394	28	0.080861755
5981	GO:0008509	anion transmembrane transp	0.03	6	1.8518518	245	0.70754033
4917	GO:0006820|GO:0006822	anion transport	0.03	6	1.8518518	247	0.7133162
26845	GO:0052689	carboxylic ester hydrolase ac	0.03	10	3.0864198	538	1.5537009
9600	GO:0016491	oxidoreductase activity	0.03	24	7.4074073	1724	4.9787736
4528	GO:0006353	transcription termination, DN	0.04	1	0.30864197	4	0.011551679
9351	GO:0016119	carotene metabolic process	0.04	1	0.30864197	4	0.011551679
9352	GO:0016120	carotene biosynthetic proces	0.04	1	0.30864197	4	0.011551679
9819	GO:0016767	geranylgeranyl-diphosphate g	0.04	1	0.30864197	4	0.011551679
18575	GO:0042214	terpene metabolic process	0.04	1	0.30864197	4	0.011551679
21791	GO:0046246	terpene biosynthetic process	0.04	1	0.30864197	4	0.011551679
22408	GO:0046905	phytoene synthase activity	0.04	1	0.30864197	4	0.011551679
5196	GO:0007166	cell surface receptor linked s	0.04	3	0.9259259	78	0.22525774
25626	GO:0051336	regulation of hydrolase activit	0.04	5	1.5432099	196	0.5660323
3490	GO:0004888|GO:0004926	transmembrane receptor act	0.04	10	3.0864198	571	1.6490022
8739	GO:0015385|GO:0015502	sodium:hydrogen antiporter a	0.04	2	0.61728394	36	0.10396511
4911	GO:0006812|GO:0006819|G	cation transport	0.05	11	3.3950617	662	1.9118029
11202	GO:0019199	transmembrane receptor pro	0.05	9	2.7777777	504	1.4555116
22376	GO:0046873	metal ion transmembrane tra	0.05	5	1.5432099	213	0.6151269
5766	GO:0008236	serine-type peptidase activity	0.05	5	1.5432099	214	0.6180149
3476	GO:0004872|GO:0019041	receptor activity	0.05	11	3.3950617	672	1.9406822
5810	GO:0008290	F-actin capping protein comp	0.05	1	0.30864197	6	0.01732752
8505	GO:0015089	high affinity copper ion transn	0.05	1	0.30864197	6	0.01732752
14264	GO:0031849	olfactory receptor binding	0.05	1	0.30864197	6	0.01732752
2713	GO:0003863	3-methyl-2-oxobutanoate deh	0.05	1	0.30864197	6	0.01732752
5704	GO:0008150|GO:0000004|G	biological_process	0.06	222	68.51852	22215	64.155136
10103	GO:0017171	serine hydrolase activity	0.06	5	1.5432099	220	0.63534236
4990	GO:0006915|GO:0008632	apoptosis	0.06	5	1.5432099	227	0.6555578
3021	GO:0004337	geranyltranstransferase activ	0.06	1	0.30864197	7	0.020215439
27063	GO:0060089	molecular transducer activity	0.07	12	3.7037036	787	2.2727928
3475	GO:0004871|GO:0005062|G	signal transducer activity	0.07	12	3.7037036	787	2.2727928
5195	GO:0007165|GO:0023033	signal transduction	0.07	14	4.3209877	961	2.775291
12602	GO:0023052|GO:0023046	signaling	0.07	14	4.3209877	961	2.775291
25998	GO:0051716	cellular response to stimulus	0.07	16	4.9382715	1138	3.2864528
7843	GO:0010628	positive regulation of gene ex	0.07	1	0.30864197	8	0.023103358
21461	GO:0045893|GO:0043193|G	positive regulation of transcri	0.07	1	0.30864197	8	0.023103358
3022	GO:0004338	glucan exo-1,3-beta-glucosid	0.07	1	0.30864197	8	0.023103358
3402	GO:0004791	thioredoxin-disulfide reductas	0.07	1	0.30864197	8	0.023103358
5756	GO:0008219	cell death	0.07	5	1.5432099	239	0.69021285
8209	GO:0012501|GO:0016244	programmed cell death	0.07	5	1.5432099	239	0.69021285
9462	GO:0016265	death	0.07	5	1.5432099	239	0.69021285
3844	GO:0005451	monovalent cation:hydrogen	0.08	2	0.61728394	49	0.14150807
25195	GO:0050896|GO:0051869	response to stimulus	0.08	24	7.4074073	1881	5.432177
19342	GO:0043027	caspase inhibitor activity	0.08	1	0.30864197	9	0.025991278
19343	GO:0043028	caspase regulator activity	0.08	1	0.30864197	9	0.025991278
19458	GO:0043154|GO:0001719	negative regulation of caspas	0.08	1	0.30864197	9	0.025991278
19579	GO:0043281|GO:0043026	regulation of caspase activity	0.08	1	0.30864197	9	0.025991278
2975	GO:0004175|GO:0016809	endopeptidase activity	0.08	6	1.8518518	320	0.9241344
25095	GO:0050790	regulation of catalytic activity	0.08	8	2.4691358	480	1.3862015
12794	GO:0030234	enzyme regulator activity	0.09	7	2.1604939	403	1.1638317
28366	GO:0065009	regulation of molecular functi	0.09	8	2.4691358	484	1.3977532

8673	GO:0015291|GO:0015290|G	secondary active transmemb	0.09	7	2.1604939	407	1.1753833
5678	GO:0008113|GO:0033742	peptide-methionine-(S)-S-oxi	0.09	1	0.30864197	10	0.0288792
9741	GO:0016668|GO:0016654	oxidoreductase activity, actin	0.09	1	0.30864197	10	0.0288792
983	GO:0001664	G-protein-coupled receptor b	0.09	1	0.30864197	10	0.0288792
12574	GO:0022892	substrate-specific transporter	0.10	19	5.8641977	1463	4.2250266
4909	GO:0006810|GO:0015457|G	transport	0.10	28	8.641975	2315	6.6855345
25529	GO:0051234	establishment of localization	0.10	28	8.641975	2315	6.6855345
24966	GO:0050660	flavin adenine dinucleotide bi	0.10	3	0.9259259	118	0.34077454
25476	GO:0051179	localization	0.10	28	8.641975	2319	6.697086
4913	GO:0006814|GO:0006834|G	sodium ion transport	0.10	2	0.61728394	58	0.16749935
8499	GO:0015081|GO:0022816	sodium ion transmembrane t	0.10	2	0.61728394	58	0.16749935
18086	GO:0035725	sodium ion transmembrane t	0.10	2	0.61728394	58	0.16749935
29800	GO:0071436	sodium ion export	0.10	2	0.61728394	58	0.16749935
4526	GO:0006351|GO:0006350|G	transcription, DNA-depender	0.10	20	6.1728396	1575	4.548474
15176	GO:0032774	RNA biosynthetic process	0.10	20	6.1728396	1575	4.548474
7526	GO:0010301|GO:0033710	xanthoxin dehydrogenase act	0.11	1	0.30864197	12	0.03465504
8748	GO:0015398	high affinity secondary active	0.11	1	0.30864197	12	0.03465504
21503	GO:0045935	positive regulation of nucleob	0.11	1	0.30864197	12	0.03465504
25470	GO:0051173	positive regulation of nitroger	0.11	1	0.30864197	12	0.03465504
25546	GO:0051254	positive regulation of RNA m	0.11	1	0.30864197	12	0.03465504
2843	GO:0004034	aldose 1-epimerase activity	0.11	1	0.30864197	12	0.03465504
8149	GO:0010941	regulation of cell death	0.12	1	0.30864197	13	0.037542958
19298	GO:0042981	regulation of apoptosis	0.12	1	0.30864197	13	0.037542958
19381	GO:0043067|GO:0043070	regulation of programmed ce	0.12	1	0.30864197	13	0.037542958
22416	GO:0046915	transition metal ion transmen	0.12	2	0.61728394	64	0.18482687
4641	GO:0006508	proteolysis	0.12	13	4.012346	965	2.7868426
12516	GO:0022804	active transmembrane transp	0.12	11	3.3950617	787	2.2727928
31408	GO:0080031	methyl salicylate esterase ac	0.12	1	0.30864197	14	0.040430877
12573	GO:0022891	substrate-specific transmemb	0.12	16	4.9382715	1241	3.5839086
3473	GO:0004867	serine-type endopeptidase in	0.13	2	0.61728394	66	0.1906027
7520	GO:0010294	abscisic acid glucosyltransfer	0.13	1	0.30864197	15	0.043318797
9340	GO:0016108	tetraterpenoid metabolic proc	0.13	1	0.30864197	15	0.043318797
9341	GO:0016109	tetraterpenoid biosynthetic pr	0.13	1	0.30864197	15	0.043318797
9348	GO:0016116	carotenoid metabolic process	0.13	1	0.30864197	15	0.043318797
9349	GO:0016117	carotenoid biosynthetic proce	0.13	1	0.30864197	15	0.043318797
26959	GO:0055114	oxidation-reduction process	0.13	29	8.950617	2490	7.1909204
4954	GO:0006869	lipid transport	0.13	4	1.2345679	208	0.6006873
8085	GO:0010876	lipid localization	0.13	4	1.2345679	208	0.6006873
32	GO:0000041	transition metal ion transport	0.13	2	0.61728394	68	0.19637854
5840	GO:0008324	cation transmembrane transp	0.13	8	2.4691358	538	1.5537009
3829	GO:0005381|GO:0005382|G	iron ion transmembrane trans	0.14	1	0.30864197	16	0.046206716
17134	GO:0034755	iron ion transmembrane trans	0.14	1	0.30864197	16	0.046206716
22412	GO:0046910	pectinesterase inhibitor activi	0.14	1	0.30864197	16	0.046206716
31396	GO:0080019	fatty-acyl-CoA reductase (alc	0.14	1	0.30864197	16	0.046206716
2667	GO:0003777	microtubule motor activity	0.14	1	0.30864197	16	0.046206716
3742	GO:0005242	inward rectifier potassium ch	0.14	1	0.30864197	16	0.046206716
9740	GO:0016667	oxidoreductase activity, actin	0.15	2	0.61728394	72	0.20793022
18738	GO:0042389	omega-3 fatty acid desaturas	0.15	1	0.30864197	17	0.049094636
31498	GO:0080123	jasmonate-amino synthetase	0.15	1	0.30864197	17	0.049094636
5019	GO:0006952|GO:0002217|G	defense response	0.15	4	1.2345679	221	0.63823026
7773	GO:0010557	positive regulation of macron	0.16	1	0.30864197	18	0.051982556
12887	GO:0030332	cyclin binding	0.16	1	0.30864197	18	0.051982556
8810	GO:0015491	cation:cation antiporter activi	0.16	2	0.61728394	75	0.21659398
12547	GO:0022857|GO:0005386|G	transmembrane transporter a	0.16	18	5.5555553	1483	4.282785
11776	GO:0019825	oxygen binding	0.16	6	1.8518518	389	1.1234008
5791	GO:0008270	zinc ion binding	0.17	22	6.7901235	1880	5.4292893
4921	GO:0006826|GO:0015681	iron ion transport	0.17	1	0.30864197	20	0.0577584
5991	GO:0008519	ammonium transmembrane t	0.17	1	0.30864197	20	0.0577584
8965	GO:0015696	ammonium transport	0.17	1	0.30864197	20	0.0577584
9707	GO:0016624	oxidoreductase activity, actin	0.17	1	0.30864197	20	0.0577584
30844	GO:0072488	ammonium transmembrane t	0.17	1	0.30864197	20	0.0577584
716	GO:0001071	nucleic acid binding transcrip	0.18	17	5.2469134	1417	4.0921826
2643	GO:0003700|GO:0000130	sequence-specific DNA bindi	0.18	17	5.2469134	1417	4.0921826
3474	GO:0004869|GO:0004870	cysteine-type endopeptidase	0.18	1	0.30864197	21	0.060646318
17024	GO:0034645|GO:0034961	cellular macromolecule biosy	0.18	23	7.0987654	1999	5.7729516
6428	GO:0009059|GO:0043284	macromolecule biosynthetic	0.18	23	7.0987654	2003	5.7845035
22415	GO:0046914	transition metal ion binding	0.19	29	8.950617	2599	7.5057034
3828	GO:0005375|GO:0005378|G	copper ion transmembrane tr	0.19	1	0.30864197	22	0.06353424
4920	GO:0006825	copper ion transport	0.19	1	0.30864197	22	0.06353424
7143	GO:0009891	positive regulation of biosynt	0.19	1	0.30864197	22	0.06353424
13038	GO:0030551	cyclic nucleotide binding	0.19	1	0.30864197	22	0.06353424
13758	GO:0031328	positive regulation of cellular	0.19	1	0.30864197	22	0.06353424
17800	GO:0035434	copper ion transmembrane tr	0.19	1	0.30864197	22	0.06353424
3955	GO:0005634	nucleus	0.19	17	5.2469134	1433	4.138389
7527	GO:0010302	2-oxoglutarate-dependent dio	0.19	1	0.30864197	23	0.06642216
12572	GO:0022890|GO:0015082	inorganic cation transmembra	0.20	5	1.5432099	332	0.9587894
31407	GO:0080030	methyl indole-3-acetate ester	0.20	1	0.30864197	24	0.06931008
26931	GO:0055085	transmembrane transport	0.20	18	5.5555553	1548	4.4705
4476	GO:0006278	RNA-dependent DNA replica	0.20	3	0.9259259	166	0.4793947
3723	GO:0005215|GO:0005478	transporter activity	0.21	21	6.4814816	1844	5.325324
31895	GO:0090304	nucleic acid metabolic proces	0.21	26	8.024692	2340	6.7577324
19125	GO:0042802	identical protein binding	0.21	9	2.7777777	697	2.01288
18747	GO:0042398	cellular modified amino acid	0.21	1	0.30864197	25	0.072197996
9308	GO:0016070	RNA metabolic process	0.21	21	6.4814816	1855	5.3570914
8516	GO:0015101	organic cation transmembrar	0.22	1	0.30864197	26	0.075085916
8964	GO:0015695	organic cation transport	0.22	1	0.30864197	26	0.075085916
9744	GO:0016671	oxidoreductase activity, actin	0.22	1	0.30864197	26	0.075085916
8159	GO:0010951	negative regulation of endope	0.22	2	0.61728394	95	0.27435237
26712	GO:0052548	regulation of endopeptidase a	0.22	2	0.61728394	95	0.27435237
28101	GO:0061135	endopeptidase regulator activ	0.22	2	0.61728394	95	0.27435237
3472	GO:0004866	endopeptidase inhibitor activi	0.22	2	0.61728394	95	0.27435237
9346	GO:0016114	terpenoid biosynthetic proces	0.22	1	0.30864197	27	0.077973835
3631	GO:0005089	Rho guanyl-nucleotide excha	0.22	1	0.30864197	27	0.077973835
6940	GO:0009672	auxin:hydrogen symporter ac	0.23	1	0.30864197	28	0.080861755
7145	GO:0009893	positive regulation of metabo	0.23	1	0.30864197	28	0.080861755
7819	GO:0010604	positive regulation of macron	0.23	1	0.30864197	28	0.080861755
13755	GO:0031325	positive regulation of cellular	0.23	1	0.30864197	28	0.080861755
8681	GO:0015299	solute:hydrogen antiporter ac	0.23	2	0.61728394	98	0.28301615
22375	GO:0046872	metal ion binding	0.23	32	9.876543	2985	8.6204405
19126	GO:0042803	protein homodimerization act	0.24	4	1.2345679	265	0.7652988
4704	GO:0006575	cellular modified amino acid	0.24	1	0.30864197	29	0.083749674
4841	GO:0006721	terpenoid metabolic process	0.24	1	0.30864197	29	0.083749674
19470	GO:0043167	ion binding	0.24	32	9.876543	2997	8.655096
19472	GO:0043169	cation binding	0.24	32	9.876543	2997	8.655096
5916	GO:0008422|GO:0016983	beta-glucosidase activity	0.25	1	0.30864197	30	0.086637594
6216	GO:0008810	cellulase activity	0.25	1	0.30864197	30	0.086637594
2666	GO:0003774	motor activity	0.25	1	0.30864197	30	0.086637594
2870	GO:0004064	arylesterase activity	0.25	1	0.30864197	30	0.086637594
3518	GO:0004930|GO:0001622|G	G-protein coupled receptor a	0.25	1	0.30864197	30	0.086637594
22049	GO:0046527	glucosyltransferase activity	0.25	4	1.2345679	270	0.77973837
11931	GO:0020037	heme binding	0.26	7	2.1604939	550	1.5883559
3785	GO:0005315|GO:0005317	inorganic phosphate transme	0.26	1	0.30864197	32	0.09241343
4481	GO:0006284	base-excision repair	0.26	1	0.30864197	32	0.09241343
12833	GO:0030275	LRR domain binding	0.26	1	0.30864197	32	0.09241343
23914	GO:0048522|GO:0051242	positive regulation of cellular	0.26	1	0.30864197	32	0.09241343
3717	GO:0005199	structural constituent of cell w	0.26	1	0.30864197	32	0.09241343
24968	GO:0050662	coenzyme binding	0.27	3	0.9259259	191	0.5515927
3749	GO:0005249	voltage-gated potassium cha	0.27	1	0.30864197	34	0.09818927
3872	GO:0005506	iron ion binding	0.28	7	2.1604939	563	1.6258988
6355	GO:0008970	phospholipase A1 activity	0.28	1	0.30864197	35	0.10107719
23199	GO:0047714	galactolipase activity	0.28	1	0.30864197	35	0.10107719
8680	GO:0015298	solute:cation antiporter activi	0.28	2	0.61728394	112	0.32344702

29918	GO:0071554	cell wall organization or bioge	0.28	2	0.61728394	112	0.32344702
9933	GO:0016903	oxidoreductase activity, actin	0.29	2	0.61728394	113	0.32633495
12626	GO:0030029	actin filament-based process	0.29	1	0.30864197	36	0.10396511
12633	GO:0030036	actin cytoskeleton organizatio	0.29	1	0.30864197	36	0.10396511
22478	GO:0046982	protein heterodimerization ac	0.29	2	0.61728394	114	0.32922286
3758	GO:0005267	potassium channel activity	0.29	1	0.30864197	37	0.10685303
5066	GO:0007010	cytoskeleton organization	0.30	1	0.30864197	38	0.10974095
12538	GO:0022843	voltage-gated cation channel	0.30	1	0.30864197	38	0.10974095
8584	GO:0015171|GO:0015359	amino acid transmembrane t	0.30	2	0.61728394	118	0.34077454
2521	GO:0003333	amino acid transmembrane t	0.30	2	0.61728394	118	0.34077454
7691	GO:0010467	gene expression	0.30	21	6.4814816	1974	5.7007537
23910	GO:0048518|GO:0043119	positive regulation of biologic	0.31	1	0.30864197	40	0.1155168
6884	GO:0009607	response to biotic stimulus	0.32	2	0.61728394	122	0.3523262
4951	GO:0006865|GO:0006866	amino acid transport	0.32	2	0.61728394	123	0.35521415
22409	GO:0046906	tetrapyrrole binding	0.33	7	2.1604939	596	1.7212002
8461	GO:0015020|GO:0003981	glucuronosyltransferase activ	0.33	1	0.30864197	42	0.121292636
13884	GO:0031461	cullin-RING ubiquitin ligase co	0.33	1	0.30864197	42	0.121292636
13656	GO:0031224	intrinsic to membrane	0.33	13	4.012346	1193	3.4452884
3761	GO:0005275|GO:0005279	amine transmembrane transp	0.33	2	0.61728394	126	0.3638779
17617	GO:0035251	UDP-glucosyltransferase act	0.33	3	0.9259259	218	0.62956655
9186	GO:0015926	glucosidase activity	0.34	1	0.30864197	44	0.12706847
8666	GO:0015276	ligand-gated ion channel acti	0.35	1	0.30864197	45	0.1299564
9893	GO:0016857	racemase and epimerase ac	0.35	1	0.30864197	45	0.1299564
12530	GO:0022834	ligand-gated channel activity	0.35	1	0.30864197	45	0.1299564
3630	GO:0005088	Ras guanyl-nucleotide excha	0.35	1	0.30864197	45	0.1299564
4460	GO:0006260|GO:0055133	DNA replication	0.35	3	0.9259259	224	0.64689404
22479	GO:0046983	protein dimerization activity	0.36	6	1.8518518	517	1.4930545
5732	GO:0008194	UDP-glycosyltransferase act	0.36	4	1.2345679	322	0.9299102
9103	GO:0015837	amine transport	0.36	2	0.61728394	135	0.38986918
14591	GO:0032183	SUMO binding	0.36	1	0.30864197	48	0.13862015
28930	GO:0070566	adenylyltransferase activity	0.36	1	0.30864197	48	0.13862015
3801	GO:0005342	organic acid transmembrane	0.36	2	0.61728394	136	0.3927571
22444	GO:0046943	carboxylic acid transmembra	0.36	2	0.61728394	136	0.3927571
3756	GO:0005261|GO:0015281|G	cation channel activity	0.37	1	0.30864197	49	0.14150807
6483	GO:0009116	nucleoside metabolic proces	0.37	1	0.30864197	49	0.14150807
31421	GO:0080044	quercetin 7-O-glucosyltransfe	0.37	1	0.30864197	49	0.14150807
30066	GO:0071702	organic substance transport	0.38	6	1.8518518	529	1.5277096
5096	GO:0007047	cellular cell wall organization	0.38	1	0.30864197	50	0.14439599
6932	GO:0009664	plant-type cell wall organizati	0.38	1	0.30864197	50	0.14439599
7166	GO:0009914	hormone transport	0.38	1	0.30864197	50	0.14439599
20857	GO:0045229	external encapsulating structu	0.38	1	0.30864197	50	0.14439599
27891	GO:0060918	auxin transport	0.38	1	0.30864197	50	0.14439599
30033	GO:0071669	plant-type cell wall organizati	0.38	1	0.30864197	50	0.14439599
31536	GO:0080161	auxin transmembrane transp	0.38	1	0.30864197	50	0.14439599
3877	GO:0005516	calmodulin binding	0.38	3	0.9259259	236	0.6815491
8908	GO:0015629	actin cytoskeleton	0.38	1	0.30864197	51	0.14728391
9975	GO:0016998	cell wall macromolecule cata	0.38	1	0.30864197	51	0.14728391
3293	GO:0004659	prenyltransferase activity	0.38	1	0.30864197	51	0.14728391
4915	GO:0006817	phosphate transport	0.39	1	0.30864197	52	0.15017183
8528	GO:0015114	phosphate transmembrane tr	0.39	1	0.30864197	52	0.15017183
17801	GO:0035435	phosphate transmembrane tr	0.39	1	0.30864197	52	0.15017183
20287	GO:0044036	cell wall macromolecule meta	0.39	1	0.30864197	52	0.15017183
28378	GO:0070011	peptidase activity, acting on l	0.39	6	1.8518518	537	1.550813
29246	GO:0070882	cellular cell wall organization	0.39	1	0.30864197	53	0.15305975
4478	GO:0006281	DNA repair	0.41	2	0.61728394	149	0.43030006
5037	GO:0006974|GO:0034984	response to DNA damage st	0.41	2	0.61728394	149	0.43030006
3306	GO:0004674|GO:0004695|G	protein serine/threonine kinas	0.41	11	3.3950617	1062	3.0669708
5715	GO:0008171	O-methyltransferase activity	0.42	1	0.30864197	57	0.16461143
9813	GO:0016759	cellulose synthase activity	0.42	1	0.30864197	57	0.16461143
8679	GO:0015297	antiporter activity	0.42	2	0.61728394	152	0.4389638
8682	GO:0015300	solute:solute antiporter activit	0.42	2	0.61728394	152	0.4389638
5819	GO:0008299|GO:0009241	isoprenoid biosynthetic proce	0.42	1	0.30864197	58	0.16749935
8028	GO:0010817	regulation of hormone levels	0.42	1	0.30864197	58	0.16749935
29919	GO:0071555	cell wall organization	0.42	1	0.30864197	58	0.16749935
6424	GO:0009055|GO:0009053|G	electron carrier activity	0.42	2	0.61728394	154	0.44473964
9801	GO:0016747	transferase activity, transferri	0.42	5	1.5432099	456	1.3168914
2831	GO:0004022	alcohol dehydrogenase (NAD	0.43	1	0.30864197	59	0.17038727
9114	GO:0015849	organic acid transport	0.43	2	0.61728394	156	0.45051548
22443	GO:0046942	carboxylic acid transport	0.43	2	0.61728394	156	0.45051548
4840	GO:0006720|GO:0016096	isoprenoid metabolic process	0.43	1	0.30864197	60	0.17327519
4357	GO:0006139|GO:0055134	nucleobase, nucleoside, nucl	0.43	32	9.876543	3285	9.486816
5763	GO:0008233	peptidase activity	0.43	6	1.8518518	565	1.6316746
5809	GO:0008289	lipid binding	0.43	3	0.9259259	258	0.74508333
2818	GO:0004003	ATP-dependent DNA helicas	0.44	1	0.30864197	61	0.1761631
6018	GO:0008559|GO:0005226|G	xenobiotic-transporting ATPa	0.44	1	0.30864197	62	0.17905103
19225	GO:0042908	xenobiotic transport	0.44	1	0.30864197	62	0.17905103
19227	GO:0042910	xenobiotic transporter activity	0.44	1	0.30864197	62	0.17905103
8496	GO:0015078	hydrogen ion transmembrane	0.44	2	0.61728394	160	0.4620672
9266	GO:0016020	membrane	0.46	36	11.111111	3741	10.803708
9267	GO:0016021	integral to membrane	0.46	11	3.3950617	1103	3.1853757
9422	GO:0016209	antioxidant activity	0.46	2	0.61728394	165	0.47650677
9765	GO:0016702	oxidoreductase activity, actin	0.46	1	0.30864197	66	0.1906027
9890	GO:0016854	racemase and epimerase ac	0.46	1	0.30864197	66	0.1906027
2629	GO:0003678|GO:0003679	DNA helicase activity	0.47	1	0.30864197	67	0.19349062
21710	GO:0046148	pigment biosynthetic process	0.48	1	0.30864197	69	0.19926646
31420	GO:0080043	quercetin 3-O-glucosyltransfe	0.48	1	0.30864197	69	0.19926646
15949	GO:0033554	cellular response to stress	0.48	2	0.61728394	173	0.49961013
8943	GO:0015672	monovalent inorganic cation	0.48	4	1.2345679	383	1.1060733
9768	GO:0016706	oxidoreductase activity, actin	0.49	2	0.61728394	175	0.505386
4459	GO:0006259|GO:0055132	DNA metabolic process	0.49	5	1.5432099	493	1.4237444
9764	GO:0016701	oxidoreductase activity, actin	0.49	1	0.30864197	72	0.20793022
12528	GO:0022832	voltage-gated channel activity	0.49	1	0.30864197	72	0.20793022
15280	GO:0032879	regulation of localization	0.49	1	0.30864197	72	0.20793022
17141	GO:0034762	regulation of transmembrane	0.49	1	0.30864197	72	0.20793022
17144	GO:0034765	regulation of ion transmembr	0.49	1	0.30864197	72	0.20793022
19568	GO:0043269	regulation of ion transport	0.49	1	0.30864197	72	0.20793022
25348	GO:0051049	regulation of transport	0.49	1	0.30864197	72	0.20793022
3744	GO:0005244	voltage-gated ion channel ac	0.49	1	0.30864197	72	0.20793022
9703	GO:0016620	oxidoreductase activity, actin	0.50	1	0.30864197	73	0.21081814
18786	GO:0042440	pigment metabolic process	0.50	1	0.30864197	73	0.21081814
9800	GO:0016746	transferase activity, transferri	0.50	5	1.5432099	498	1.438184
20499	GO:0044249	cellular biosynthetic process	0.50	25	7.716049	2635	7.6096687
3716	GO:0005198	structural molecule activity	0.50	4	1.2345679	393	1.1349525
25510	GO:0051213	dioxygenase activity	0.51	1	0.30864197	75	0.21659398
3627	GO:0005085|GO:0008433|G	guanyl-nucleotide exchange f	0.51	1	0.30864197	75	0.21659398
5018	GO:0006950	response to stress	0.51	6	1.8518518	611	1.764519
15436	GO:0033036	macromolecule localization	0.51	4	1.2345679	397	1.1465042
5663	GO:0008094|GO:0004011	DNA-dependent ATPase act	0.51	1	0.30864197	76	0.2194819
4916	GO:0006818	hydrogen transport	0.52	3	0.9259259	292	0.84327257
9246	GO:0015992	proton transport	0.52	3	0.9259259	292	0.84327257
9811	GO:0016757|GO:0016932	transferase activity, transferri	0.53	7	2.1604939	729	2.1052935
6668	GO:0009314	response to radiation	0.53	1	0.30864197	81	0.23392151
6751	GO:0009416	response to light stimulus	0.53	1	0.30864197	81	0.23392151
19473	GO:0043170|GO:0043283	macromolecule metabolic pro	0.54	61	18.82716	6553	18.92454
3787	GO:0005319	lipid transporter activity	0.54	1	0.30864197	83	0.23969735
6427	GO:0009058	biosynthetic process	0.54	26	8.024692	2796	8.074624
4643	GO:0006511	ubiquitin-dependent protein c	0.55	1	0.30864197	84	0.24258527
4912	GO:0006813|GO:0015458	potassium ion transport	0.55	1	0.30864197	84	0.24258527
11864	GO:0019941	modification-dependent prote	0.55	1	0.30864197	84	0.24258527
19898	GO:0043632	modification-dependent mac	0.55	1	0.30864197	84	0.24258527
20507	GO:0044257	cellular protein catabolic proc	0.55	1	0.30864197	84	0.24258527
25887	GO:0051603	proteolysis involved in cellula	0.55	1	0.30864197	84	0.24258527

30168	GO:0071804	cellular potassium ion transpo	0.55	1	0.30864197	84	0.24258527
30169	GO:0071805	potassium ion transmembran	0.55	1	0.30864197	84	0.24258527
12734	GO:0030163	protein catabolic process	0.55	1	0.30864197	86	0.24836111
20515	GO:0044265|GO:0034962	cellular macromolecule catab	0.55	1	0.30864197	86	0.24836111
20624	GO:0044430	cytoskeletal part	0.56	1	0.30864197	88	0.25413695
119	GO:0000151	ubiquitin ligase complex	0.56	1	0.30864197	88	0.25413695
30069	GO:0071705	nitrogen compound transport	0.57	2	0.61728394	202	0.5833598
21331	GO:0045735	nutrient reservoir activity	0.57	1	0.30864197	89	0.25702485
14590	GO:0032182	small conjugating protein bin	0.57	1	0.30864197	90	0.2599128
2982	GO:0004197	cysteine-type endopeptidase	0.57	1	0.30864197	90	0.2599128
3644	GO:0005102	receptor binding	0.58	1	0.30864197	91	0.2628007
18956	GO:0042626	ATPase activity, coupled to t	0.59	2	0.61728394	211	0.6093511
11712	GO:0019748	secondary metabolic process	0.59	1	0.30864197	95	0.27435237
9861	GO:0016820	hydrolase activity, acting on a	0.59	2	0.61728394	212	0.612239
23515	GO:0048037	cofactor binding	0.59	3	0.9259259	327	0.94434977
9767	GO:0016705	oxidoreductase activity, actin	0.60	3	0.9259259	329	0.95012563
8495	GO:0015077	monovalent inorganic cation	0.60	2	0.61728394	214	0.6180149
17020	GO:0034641	cellular nitrogen compound m	0.63	33	10.185185	3678	10.621769
2668	GO:0003779	actin binding	0.63	1	0.30864197	105	0.3032316
6426	GO:0009057|GO:0043285	macromolecule catabolic pro	0.63	1	0.30864197	106	0.3061195
11833	GO:0019904	protein domain specific bindi	0.64	1	0.30864197	109	0.31478328
19758	GO:0043492	ATPase activity, coupled to m	0.65	2	0.61728394	235	0.67866117
3626	GO:0005083	small GTPase regulator activ	0.65	1	0.30864197	111	0.3205591
5070	GO:0007017	microtubule-based process	0.65	1	0.30864197	112	0.32344702
5071	GO:0007018	microtubule-based movemen	0.65	1	0.30864197	112	0.32344702
9730	GO:0016651	oxidoreductase activity, actin	0.65	1	0.30864197	112	0.32344702
4906	GO:0006807	nitrogen compound metaboli	0.66	33	10.185185	3725	10.757502
30204	GO:0071840	cellular component organizat	0.66	3	0.9259259	362	1.045427
20619	GO:0044425	membrane part	0.67	13	4.012346	1523	4.398302
9579	GO:0016462	pyrophosphatase activity	0.67	8	2.4691358	954	2.7550755
2660	GO:0003735|GO:0003736|G	structural constituent of ribos	0.67	2	0.61728394	246	0.7104283
9859	GO:0016818	hydrolase activity, acting on a	0.67	8	2.4691358	956	2.7608514
9858	GO:0016817	hydrolase activity, acting on a	0.68	8	2.4691358	959	2.769515
4612	GO:0006468	protein phosphorylation	0.68	19	5.8641977	2205	6.367863
9795	GO:0016740	transferase activity	0.68	33	10.185185	3764	10.870131
12805	GO:0030246	carbohydrate binding	0.68	2	0.61728394	251	0.7248679
12532	GO:0022836	gated channel activity	0.68	1	0.30864197	122	0.3523262
3260	GO:0004620	phospholipase activity	0.69	1	0.30864197	123	0.35521415
9812	GO:0016758	transferase activity, transferri	0.69	4	1.2345679	501	1.4468478
4121	GO:0005856	cytoskeleton	0.69	1	0.30864197	126	0.3638779
8749	GO:0015399	primary active transmembran	0.70	2	0.61728394	260	0.75085914
8753	GO:0015405	P-P-bond-hydrolysis-driven tr	0.70	2	0.61728394	260	0.75085914
9285	GO:0016043	cellular component organizat	0.70	2	0.61728394	261	0.7537471
3304	GO:0004672|GO:0050222	protein kinase activity	0.71	11	3.3950617	1333	3.8495972
4215	GO:0005975	carbohydrate metabolic proc	0.71	8	2.4691358	990	2.8590407
4394	GO:0006184	GTP catabolic process	0.71	1	0.30864197	132	0.3812054
21605	GO:0046039	GTP metabolic process	0.71	1	0.30864197	132	0.3812054
2758	GO:0003924	GTPase activity	0.71	1	0.30864197	132	0.3812054
9754	GO:0016684	oxidoreductase activity, actin	0.72	1	0.30864197	135	0.38986918
3242	GO:0004601|GO:0016685|G	peroxidase activity	0.72	1	0.30864197	135	0.38986918
4942	GO:0006855	drug transmembrane transpo	0.73	1	0.30864197	139	0.40142086
8642	GO:0015238|GO:0015239|G	drug transmembrane transpo	0.73	1	0.30864197	139	0.40142086
9154	GO:0015893	drug transport	0.73	1	0.30864197	139	0.40142086
18832	GO:0042493|GO:0017035	response to drug	0.73	1	0.30864197	139	0.40142086
3724	GO:0005216	ion channel activity	0.73	1	0.30864197	140	0.40430877
6046	GO:0008610	lipid biosynthetic process	0.74	1	0.30864197	141	0.4071967
8676	GO:0015294	solute:cation symporter activ	0.74	1	0.30864197	141	0.4071967
13164	GO:0030695	GTPase regulator activity	0.74	1	0.30864197	142	0.4100846
30205	GO:0071841	cellular component organizat	0.75	2	0.61728394	285	0.8230572
9829	GO:0016779	nucleotidyltransferase activity	0.75	1	0.30864197	148	0.42741212
7229	GO:0009987|GO:0008151|G	cellular process	0.75	94	29.012346	10601	30.614838
9488	GO:0016301	kinase activity	0.76	14	4.3209877	1740	5.0249805
20510	GO:0044260|GO:0034960	cellular macromolecule meta	0.76	48	14.814815	5577	16.105928
27562	GO:0060589	nucleoside-triphosphatase re	0.76	1	0.30864197	152	0.4389638
3888	GO:0005529	sugar binding	0.77	1	0.30864197	158	0.45629135
19831	GO:0043565	sequence-specific DNA bindi	0.78	1	0.30864197	159	0.45917925
5764	GO:0008234|GO:0004220	cysteine-type peptidase activ	0.78	1	0.30864197	163	0.47073093
5055	GO:0006996	organelle organization	0.79	1	0.30864197	164	0.47361887
19526	GO:0043227	membrane-bounded organel	0.79	17	5.2469134	2142	6.185924
19530	GO:0043231	intracellular membrane-boun	0.79	17	5.2469134	2142	6.185924
3200	GO:0004553|GO:0016800	hydrolase activity, hydrolyzin	0.79	2	0.61728394	312	0.90103096
6077	GO:0008652	cellular amino acid biosynthe	0.80	1	0.30864197	169	0.48805845
6899	GO:0009628	response to abiotic stimulus	0.80	1	0.30864197	170	0.49094638
9493	GO:0016310	phosphorylation	0.80	20	6.1728396	2504	7.2313514
3156	GO:0004497	monooxygenase activity	0.80	1	0.30864197	172	0.49672222
4605	GO:0006457|GO:0007022|G	protein folding	0.80	1	0.30864197	173	0.49961013
8675	GO:0015293	symporter activity	0.80	1	0.30864197	173	0.49961013
4755	GO:0006629	lipid metabolic process	0.80	4	1.2345679	590	1.7038727
9823	GO:0016772	transferase activity, transferri	0.81	15	4.6296296	1932	5.579461
11522	GO:0019538|GO:0006411	protein metabolic process	0.81	34	10.493827	4134	11.938661
3904	GO:0005576	extracellular region	0.81	1	0.30864197	178	0.5140497
6663	GO:0009309	amine biosynthetic process	0.82	1	0.30864197	181	0.5227135
20488	GO:0044238	primary metabolic process	0.82	79	24.382715	9167	26.47356
5610	GO:0008026	ATP-dependent helicase acti	0.82	1	0.30864197	183	0.52848935
28401	GO:0070035	purine NTP-dependent helica	0.82	1	0.30864197	183	0.52848935
20505	GO:0044255	cellular lipid metabolic proces	0.82	1	0.30864197	184	0.53137726
9486	GO:0016298	lipase activity	0.83	1	0.30864197	185	0.53426516
9818	GO:0016765|GO:0016766	transferase activity, transferri	0.83	1	0.30864197	185	0.53426516
9824	GO:0016773	phosphotransferase activity,	0.83	11	3.3950617	1493	4.311664
3068	GO:0004386	helicase activity	0.83	1	0.30864197	189	0.54581684
10058	GO:0017111	nucleoside-triphosphatase ac	0.83	6	1.8518518	878	2.5355935
30206	GO:0071842	cellular component organizat	0.84	1	0.30864197	193	0.5573685
2897	GO:0004091|GO:0004302|G	carboxylesterase activity	0.84	1	0.30864197	194	0.5602564
20498	GO:0044248	cellular catabolic process	0.84	6	1.8518518	894	2.5818002
4405	GO:0006195	purine nucleotide catabolic pr	0.85	5	1.5432099	773	2.232362
6508	GO:0009143	nucleoside triphosphate cata	0.85	5	1.5432099	773	2.232362
6509	GO:0009144	purine nucleoside triphospha	0.85	5	1.5432099	773	2.232362
6511	GO:0009146	purine nucleoside triphospha	0.85	5	1.5432099	773	2.232362
6519	GO:0009154	purine ribonucleotide catabol	0.85	5	1.5432099	773	2.232362
6530	GO:0009166	nucleotide catabolic process	0.85	5	1.5432099	773	2.232362
6567	GO:0009203	ribonucleoside triphosphate c	0.85	5	1.5432099	773	2.232362
6569	GO:0009205	purine ribonucleoside triphos	0.85	5	1.5432099	773	2.232362
6571	GO:0009207	purine ribonucleoside triphos	0.85	5	1.5432099	773	2.232362
6624	GO:0009261	ribonucleotide catabolic proc	0.85	5	1.5432099	773	2.232362
17034	GO:0034655	nucleobase, nucleoside, nucl	0.85	5	1.5432099	773	2.232362
17035	GO:0034656	nucleobase, nucleoside and n	0.85	5	1.5432099	773	2.232362
22209	GO:0046700	heterocycle catabolic proces	0.85	5	1.5432099	773	2.232362
30879	GO:0072523	purine-containing compound	0.85	5	1.5432099	773	2.232362
4408	GO:0006200	ATP catabolic process	0.85	4	1.2345679	641	1.8511566
9918	GO:0016887|GO:0004002	ATPase activity	0.85	4	1.2345679	641	1.8511566
21600	GO:0046034	ATP metabolic process	0.85	4	1.2345679	641	1.8511566
9837	GO:0016788	hydrolase activity, acting on e	0.85	11	3.3950617	1534	4.430069
20520	GO:0044270	cellular nitrogen compound c	0.85	5	1.5432099	777	2.2439137
2626	GO:0003674|GO:0005554	molecular_function	0.86	220	67.90124	24394	70.447914
9295	GO:0016053	organic acid biosynthetic proc	0.86	1	0.30864197	211	0.6093511
21922	GO:0046394	carboxylic acid biosynthetic p	0.86	1	0.30864197	211	0.6093511
6563	GO:0009199	ribonucleoside triphosphate r	0.86	5	1.5432099	790	2.2814567
6425	GO:0009056	catabolic process	0.86	7	2.1604939	1051	3.0352037
6515	GO:0009150	purine ribonucleotide metabo	0.86	5	1.5432099	791	2.2843447
6506	GO:0009141	nucleoside triphosphate meta	0.87	5	1.5432099	796	2.2987843
4558	GO:0006396|GO:0006394	RNA processing	0.87	1	0.30864197	219	0.63245445
12533	GO:0022838	substrate-specific channel ac	0.88	1	0.30864197	221	0.63823026

4373	GO:0006163	purine nucleotide metabolic p	0.88	5	1.5432099	810	2.339215
26932	GO:0055086	nucleobase, nucleoside and	0.88	6	1.8518518	944	2.7261963
6622	GO:0009259|GO:0009121	ribonucleotide metabolic proc	0.88	5	1.5432099	812	2.344991
18953	GO:0042623	ATPase activity, coupled	0.88	3	0.9259259	537	1.550813
8660	GO:0015267|GO:0015249|G	channel activity	0.88	1	0.30864197	226	0.6526699
12515	GO:0022803	passive transmembrane trans	0.88	1	0.30864197	226	0.6526699
9913	GO:0016881	acid-amino acid ligase activit	0.88	2	0.61728394	391	1.1291766
4109	GO:0005840|GO:0033279	ribosome	0.88	4	1.2345679	682	1.9695613
30877	GO:0072521	purine-containing compound	0.89	5	1.5432099	827	2.3883097
2628	GO:0003677	DNA binding	0.89	8	2.4691358	1224	3.534814
9845	GO:0016798	hydrolase activity, acting on g	0.89	2	0.61728394	401	1.1580559
11828	GO:0019899	enzyme binding	0.89	1	0.30864197	237	0.684437
5712	GO:0008168|GO:0004480	methyltransferase activity	0.90	1	0.30864197	244	0.7046524
3885	GO:0005525	GTP binding	0.90	1	0.30864197	245	0.70754033
11032	GO:0019001	guanyl nucleotide binding	0.90	1	0.30864197	245	0.70754033
14967	GO:0032561	guanyl ribonucleotide binding	0.90	1	0.30864197	245	0.70754033
20532	GO:0044282	small molecule catabolic proc	0.90	5	1.5432099	854	2.4662836
5661	GO:0008092	cytoskeletal protein binding	0.90	1	0.30864197	249	0.719092
3876	GO:0005515|GO:0045308	protein binding	0.91	56	17.28395	6933	20.021948
9836	GO:0016787	hydrolase activity	0.91	28	8.641975	3723	10.751725
2674	GO:0003824	catalytic activity	0.91	90	27.777779	10750	31.045137
19525	GO:0043226	organelle	0.91	22	6.7901235	3013	8.701303
19528	GO:0043229	intracellular organelle	0.91	22	6.7901235	3013	8.701303
4609	GO:0006464	protein modification process	0.91	20	6.1728396	2781	8.031305
9796	GO:0016741	transferase activity, transferri	0.91	1	0.30864197	261	0.7537471
13018	GO:0030529	ribonucleoprotein complex	0.92	4	1.2345679	741	2.1399486
9699	GO:0016616	oxidoreductase activity, actin	0.92	1	0.30864197	266	0.7681867
5705	GO:0008152	metabolic process	0.92	151	46.60494	17432	50.34222
4897	GO:0006793	phosphorus metabolic proces	0.92	21	6.4814816	2925	8.4471655
4900	GO:0006796	phosphate metabolic process	0.92	21	6.4814816	2925	8.4471655
4649	GO:0006520|GO:0006519	cellular amino acid metabolic	0.93	1	0.30864197	277	0.7999538
3874	GO:0005509	calcium ion binding	0.93	1	0.30864197	279	0.8057296
9911	GO:0016879	ligase activity, forming carbo	0.93	2	0.61728394	456	1.3168914
4867	GO:0006753	nucleoside phosphate metab	0.93	5	1.5432099	914	2.6395588
6484	GO:0009117	nucleotide metabolic process	0.93	5	1.5432099	914	2.6395588
14667	GO:0032259	methylation	0.93	1	0.30864197	285	0.8230572
19527	GO:0043228	non-membrane-bounded org	0.93	5	1.5432099	923	2.66555
19531	GO:0043232	intracellular non-membrane-b	0.93	5	1.5432099	923	2.66555
3946	GO:0005623	cell	0.94	66	20.37037	8227	23.758917
20657	GO:0044464	cell part	0.94	66	20.37037	8227	23.758917
9697	GO:0016614	oxidoreductase activity, actin	0.94	1	0.30864197	293	0.84616053
20356	GO:0044106	cellular amine metabolic proc	0.94	1	0.30864197	303	0.8750397
19684	GO:0043412	macromolecule modification	0.94	20	6.1728396	2900	8.374968
9869	GO:0016829	lyase activity	0.94	2	0.61728394	488	1.4093049
9889	GO:0016853	isomerase activity	0.94	1	0.30864197	306	0.8837035
6662	GO:0009308	amine metabolic process	0.95	1	0.30864197	314	0.9068068
4849	GO:0006730|GO:0019753|G	one-carbon metabolic proces	0.95	1	0.30864197	325	0.93857396
3449	GO:0004842|GO:0004840|G	ubiquitin-protein ligase activit	0.95	1	0.30864197	326	0.94146186
18582	GO:0042221	response to chemical stimulu	0.95	1	0.30864197	328	0.94723773
9654	GO:0016567	protein ubiquitination	0.96	1	0.30864197	332	0.9587894
14854	GO:0032446	protein modification by small	0.96	1	0.30864197	332	0.9587894
29011	GO:0070647	protein modification by small	0.96	1	0.30864197	332	0.9587894
20517	GO:0044267	cellular protein metabolic pro	0.96	22	6.7901235	3225	9.313541
3903	GO:0005575|GO:0008372	cellular_component	0.96	67	20.679012	8515	24.590637
11747	GO:0019787|GO:0008639|G	small conjugating protein liga	0.96	1	0.30864197	339	0.9790048
20533	GO:0044283	small molecule biosynthetic p	0.96	1	0.30864197	347	1.0021082
20521	GO:0044271	cellular nitrogen compound b	0.97	1	0.30864197	367	1.0598665
4320	GO:0006082	organic acid metabolic proce	0.97	1	0.30864197	369	1.0656425
11716	GO:0019752	carboxylic acid metabolic pro	0.97	1	0.30864197	369	1.0656425
19707	GO:0043436	oxoacid metabolic process	0.97	1	0.30864197	369	1.0656425
18541	GO:0042180	cellular ketone metabolic pro	0.97	1	0.30864197	375	1.0829699
9839	GO:0016791|GO:0016302	phosphatase activity	0.97	1	0.30864197	383	1.1060733
9906	GO:0016874	ligase activity	0.98	2	0.61728394	597	1.7240882
22007	GO:0046483	heterocycle metabolic proces	0.98	5	1.5432099	1094	3.1593843
9494	GO:0016311	dephosphorylation	0.98	1	0.30864197	425	1.227366
18912	GO:0042578	phosphoric ester hydrolase a	0.99	1	0.30864197	444	1.2822365
20487	GO:0044237	cellular metabolic process	0.99	59	18.209877	8185	23.637625
20618	GO:0044424	intracellular part	0.99	23	7.0987654	3853	11.127155
3945	GO:0005622	intracellular	0.99	30	9.259259	4751	13.720507
20531	GO:0044281	small molecule metabolic pro	0.99	8	2.4691358	1788	5.1636004
19533	GO:0043234	protein complex	1.00	2	0.61728394	848	2.448956
3863	GO:0005488	binding	1.00	106	32.71605	13903	40.15075
15392	GO:0032991	macromolecular complex	1.00	6	1.8518518	1616	4.666878
2627	GO:0003676	nucleic acid binding	1.00	11	3.3950617	2453	7.0840673
20640	GO:0044446	intracellular organelle part	1.00	1	0.30864197	710	2.0504231
20616	GO:0044422	organelle part	1.00	1	0.30864197	712	2.0561988
131	GO:0000166	nucleotide binding	1.00	3	0.9259259	1213	3.5030468
18000	GO:0035639	purine ribonucleoside triphos	1.00	1	0.30864197	769	2.2208104
14959	GO:0032553	ribonucleotide binding	1.00	1	0.30864197	795	2.2958963
14961	GO:0032555	purine ribonucleotide binding	1.00	1	0.30864197	795	2.2958963
10033	GO:0017076	purine nucleotide binding	1.00	1	0.30864197	797	2.3016722
2653	GO:0003723	RNA binding	1.00	1	0.30864197	797	2.3016722
20638	GO:0044444	cytoplasmic part	1.00	4	1.2345679	1644	4.7477403
4027	GO:0005737	cytoplasm	1.00	4	1.2345679	2001	5.7787275

GO ID	GO ACCESSION	GO Term	p-value	Count in Selection	% Count in Selection	Count in Total	% Count in Total
12946	GO:0030414	peptidase inhibitor activity	0.00	12	2.6200874	110	0.31767118
7690	GO:0010466	negative regulation of pep	0.00	12	2.6200874	110	0.31767118
25636	GO:0051346	negative regulation of hyd	0.00	12	2.6200874	110	0.31767118
26711	GO:0052547	regulation of peptidase ac	0.00	12	2.6200874	112	0.32344702
28100	GO:0061134	peptidase regulator activit	0.00	12	2.6200874	112	0.32344702
26712	GO:0052548	regulation of endopeptidas	0.00	11	2.4017467	95	0.27435237
3472	GO:0004866	endopeptidase inhibitor ac	0.00	11	2.4017467	95	0.27435237
28101	GO:0061135	endopeptidase regulator a	0.00	11	2.4017467	95	0.27435237
8159	GO:0010951	negative regulation of end	0.00	11	2.4017467	95	0.27435237
3464	GO:0004857	enzyme inhibitor activity	0.00	13	2.838428	157	0.4534034
25626	GO:0051336	regulation of hydrolase ac	0.00	14	3.0567687	196	0.5660323
9869	GO:0016829	lyase activity	0.00	22	4.8034935	488	1.4093049
4215	GO:0005975	carbohydrate metabolic pr	0.00	33	7.2052402	990	2.8590407
19393	GO:0043086	negative regulation of cata	0.00	13	2.838428	188	0.54292893
20342	GO:0044092	negative regulation of mol	0.00	13	2.838428	188	0.54292893
4320	GO:0006082	organic acid metabolic pro	0.00	17	3.7117903	369	1.0656425
11716	GO:0019752	carboxylic acid metabolic	0.00	17	3.7117903	369	1.0656425
19707	GO:0043436	oxoacid metabolic proces	0.00	17	3.7117903	369	1.0656425
18541	GO:0042180	cellular ketone metabolic	0.00	17	3.7117903	375	1.0829699
9816	GO:0016762	xyloglucan:xyloglucosyl tr	0.00	5	1.091703	27	0.077973835
3473	GO:0004867	serine-type endopeptidase	0.00	7	1.5283843	66	0.1906027
5704	GO:0008150|GO:0000004	biological_process	0.00	334	72.925766	22215	64.155136
9845	GO:0016798	hydrolase activity, acting o	0.00	16	3.4934497	401	1.1580559
5705	GO:0008152	metabolic process	0.00	270	58.951965	17432	50.34222
26845	GO:0052689	carboxylic ester hydrolase	0.00	19	4.148472	538	1.5537009
2674	GO:0003824	catalytic activity	0.00	179	39.08297	10750	31.045137
9812	GO:0016758	transferase activity, transf	0.00	18	3.930131	501	1.4468478
2864	GO:0004058|GO:0016400	aromatic-L-amino-acid de	0.00	3	0.65502185	9	0.025991278
5636	GO:0008061	chitin binding	0.00	4	0.8733624	23	0.06642216
25095	GO:0050790	regulation of catalytic acti	0.00	17	3.7117903	480	1.3862015
28366	GO:0065009	regulation of molecular fu	0.00	17	3.7117903	484	1.3977532
9811	GO:0016757|GO:0016932	transferase activity, transf	0.00	22	4.8034935	729	2.1052935
3874	GO:0005509	calcium ion binding	0.00	12	2.6200874	279	0.8057296
9877	GO:0016838	carbon-oxygen lyase activ	0.00	6	1.3100437	77	0.22236982
13082	GO:0030599	pectinesterase activity	0.00	9	1.9650655	178	0.5140497
7557	GO:0010333	terpene synthase activity	0.00	5	1.091703	57	0.16461143
3291	GO:0004657	proline dehydrogenase ac	0.00	2	0.4366812	4	0.011551679
12794	GO:0030234	enzyme regulator activity	0.00	14	3.0567687	403	1.1638317
1169	GO:0001871	pattern binding	0.00	4	0.8733624	35	0.10107719
12806	GO:0030247	polysaccharide binding	0.00	4	0.8733624	35	0.10107719
9393	GO:0016165	lipoxygenase activity	0.00	4	0.8733624	36	0.10396511
4232	GO:0005992	trehalose biosynthetic pro	0.00	4	0.8733624	36	0.10396511
9870	GO:0016830	carbon-carbon lyase activ	0.00	8	1.7467248	161	0.4649551
17016	GO:0034637	cellular carbohydrate bios	0.00	5	1.091703	63	0.18193895
4231	GO:0005991	trehalose metabolic proce	0.00	4	0.8733624	38	0.10974095
9293	GO:0016051|GO:0006093	carbohydrate biosynthetic	0.00	5	1.091703	64	0.18482687
20512	GO:0044262|GO:0006092	cellular carbohydrate meta	0.00	10	2.183406	248	0.7162041
26959	GO:0055114	oxidation-reduction proces	0.00	50	10.91703	2490	7.1909204
3124	GO:0004462	lactoylglutathione lyase ac	0.00	3	0.65502185	20	0.0577584
21880	GO:0046351	disaccharide biosynthetic	0.00	4	0.8733624	42	0.121292636
6666	GO:0009312	oligosaccharide biosynthe	0.00	4	0.8733624	42	0.121292636
9370	GO:0016138	glycoside biosynthetic pro	0.00	4	0.8733624	42	0.121292636
3129	GO:0004467	long-chain fatty acid-CoA	0.00	3	0.65502185	21	0.060646318
9764	GO:0016701	oxidoreductase activity, ac	0.00	5	1.091703	72	0.20793022
9600	GO:0016491	oxidoreductase activity	0.00	37	8.078603	1724	4.9787736
25510	GO:0051213	dioxygenase activity	0.00	5	1.091703	75	0.21659398
31394	GO:0080017	alpha-humulene synthase	0.00	3	0.65502185	25	0.072197996
31393	GO:0080016	(-)-E-beta-caryophyllene s	0.00	3	0.65502185	25	0.072197996
9871	GO:0016831	carboxy-lyase activity	0.00	6	1.3100437	115	0.3321108
7168	GO:0009916	alternative oxidase activity	0.00	2	0.4366812	8	0.023103358
3415	GO:0004805	trehalose-phosphatase ac	0.00	3	0.65502185	26	0.075085916
8919	GO:0015645	fatty acid ligase activity	0.01	3	0.65502185	27	0.077973835
9883	GO:0016846	carbon-sulfur lyase activity	0.01	4	0.8733624	56	0.16172351
4224	GO:0005984	disaccharide metabolic pr	0.01	4	0.8733624	56	0.16172351
9369	GO:0016137	glycoside metabolic proce	0.01	4	0.8733624	56	0.16172351
7558	GO:0010334	sesquiterpene synthase a	0.01	3	0.65502185	29	0.083749674
12805	GO:0030246	carbohydrate binding	0.01	9	1.9650655	251	0.7248679
21331	GO:0045735	nutrient reservoir activity	0.01	5	1.091703	89	0.25702485
28643	GO:0070279	vitamin B6 binding	0.01	6	1.3100437	126	0.3638779
12741	GO:0030170	pyridoxal phosphate bindi	0.01	6	1.3100437	126	0.3638779
6665	GO:0009311	oligosaccharide metabolic	0.01	4	0.8733624	58	0.16749935
6464	GO:0009095	aromatic amino acid famil	0.01	2	0.4366812	10	0.0288792
21148	GO:0045547	dehydrodolichyl diphospha	0.01	2	0.4366812	10	0.0288792
7222	GO:0009975	cyclase activity	0.01	3	0.65502185	31	0.08952551
4250	GO:0006012	galactose metabolic proce	0.01	2	0.4366812	11	0.03176712
11787	GO:0019842	vitamin binding	0.01	7	1.5283843	177	0.5111618
11206	GO:0019203	carbohydrate phosphatase	0.01	3	0.65502185	35	0.10107719
11311	GO:0019318	hexose metabolic process	0.01	5	1.091703	101	0.2916799
9765	GO:0016702	oxidoreductase activity, ac	0.01	4	0.8733624	66	0.1906027
23515	GO:0048037	cofactor binding	0.01	10	2.183406	327	0.94434977
11224	GO:0019222	regulation of metabolic pro	0.02	55	12.008734	3115	8.995871
9874	GO:0016835	carbon-oxygen lyase activ	0.02	7	1.5283843	202	0.5833598
31396	GO:0080019	fatty-acyl-CoA reductase (	0.02	2	0.4366812	16	0.046206716
9909	GO:0016877	ligase activity, forming car	0.02	4	0.8733624	78	0.22525774
9753	GO:0016682	oxidoreductase activity, ac	0.02	3	0.65502185	44	0.12706847
4954	GO:0006869	lipid transport	0.02	7	1.5283843	208	0.6006873
8085	GO:0010876	lipid localization	0.02	7	1.5283843	208	0.6006873
9837	GO:0016788	hydrolase activity, acting o	0.02	30	6.550218	1534	4.430069
28368	GO:0070001	aspartic-type peptidase ac	0.02	2	0.4366812	18	0.051982556
2981	GO:0004190	aspartic-type endopeptida	0.02	2	0.4366812	18	0.051982556
4236	GO:0005996	monosaccharide metaboli	0.03	5	1.091703	125	0.36099
4699	GO:0006570	tyrosine metabolic proces	0.03	1	0.2183406	2	0.00577584
4700	GO:0006571	tyrosine biosynthetic proc	0.03	1	0.2183406	2	0.00577584
21946	GO:0046421	methylisocitrate lyase acti	0.03	1	0.2183406	2	0.00577584
192	GO:0000249	C-22 sterol desaturase ac	0.03	1	0.2183406	2	0.00577584
532	GO:0000823	inositol trisphosphate 6-ki	0.03	1	0.2183406	2	0.00577584
3113	GO:0004451	isocitrate lyase activity	0.03	1	0.2183406	2	0.00577584
9725	GO:0016645	oxidoreductase activity, ac	0.03	3	0.65502185	49	0.14150807
8726	GO:0015368	calcium:cation antiporter a	0.03	2	0.4366812	20	0.0577584
3293	GO:0004659	prenyltransferase activity	0.03	3	0.65502185	51	0.14728391
3904	GO:0005576	extracellular region	0.03	6	1.3100437	178	0.5140497
9750	GO:0016679	oxidoreductase activity, ac	0.03	3	0.65502185	53	0.15305975
8489	GO:0015066	alpha-amylase inhibitor ac	0.03	2	0.4366812	22	0.06353424
24858	GO:0050551	myrcene synthase activity	0.03	2	0.4366812	22	0.06353424
5702	GO:0008146	sulfotransferase activity	0.03	3	0.65502185	54	0.15594767
22414	GO:0046912	transferase activity, transf	0.04	2	0.4366812	23	0.06642216
4319	GO:0006081	cellular aldehyde metabol	0.04	1	0.2183406	3	0.00866376

4330	GO:0006097	glyoxylate cycle	0.04	1	0.2183406	3	0.00866376
13651	GO:0031219	levanase activity	0.04	1	0.2183406	3	0.00866376
22011	GO:0046487	glyoxylate metabolic proce	0.04	1	0.2183406	3	0.00866376
3134	GO:0004474	malate synthase activity	0.04	1	0.2183406	3	0.00866376
3252	GO:0004612	phosphoenolpyruvate carb	0.04	1	0.2183406	3	0.00866376
9779	GO:0016717	oxidoreductase activity, ac	0.04	2	0.4366812	24	0.06931008
25094	GO:0050789|GO:0050791	regulation of biological pro	0.04	70	15.283843	4315	12.461374
4304	GO:0006066	alcohol metabolic process	0.04	5	1.091703	143	0.41297254
31493	GO:0080118	brassinosteroid sulfotrans	0.04	2	0.4366812	25	0.072197996
2897	GO:0004091|GO:0004302	carboxylesterase activity	0.04	6	1.3100437	194	0.5602564
17147	GO:0034768	(E)-beta-ocimene synthas	0.05	2	0.4366812	26	0.075085916
4328	GO:0006094	gluconeogenesis	0.05	1	0.2183406	4	0.011551679
5928	GO:0008440	inositol trisphosphate 3-ki	0.05	1	0.2183406	4	0.011551679
7157	GO:0009905	ent-copalyl diphosphate s	0.05	1	0.2183406	4	0.011551679
9905	GO:0016872	intramolecular lyase activi	0.05	1	0.2183406	4	0.011551679
11312	GO:0019319	hexose biosynthetic proce	0.05	1	0.2183406	4	0.011551679
16391	GO:0034007	S-linalool synthase activity	0.05	1	0.2183406	4	0.011551679
22059	GO:0046538	2,3-bisphosphoglycerate-d	0.05	1	0.2183406	4	0.011551679
23251	GO:0047769	arogenate dehydratase ac	0.05	1	0.2183406	4	0.011551679
23923	GO:0048531	beta-1,3-galactosyltransfe	0.05	1	0.2183406	4	0.011551679
24433	GO:0050113	inositol oxygenase activity	0.05	1	0.2183406	4	0.011551679
26048	GO:0051766	inositol trisphosphate kina	0.05	1	0.2183406	4	0.011551679
2796	GO:0003978	UDP-glucose 4-epimerase	0.05	1	0.2183406	4	0.011551679
2829	GO:0004020	adenylylsulfate kinase act	0.05	1	0.2183406	4	0.011551679
2872	GO:0004066	asparagine synthase (glut	0.05	1	0.2183406	4	0.011551679
3093	GO:0004421	hydroxymethylglutaryl-CoA	0.05	1	0.2183406	4	0.011551679
28364	GO:0065007	biological regulation	0.05	70	15.283843	4376	12.637537
11829	GO:0019900	kinase binding	0.06	3	0.65502185	66	0.1906027
3888	GO:0005529	sugar binding	0.06	5	1.091703	158	0.45629135
6216	GO:0008810	cellulase activity	0.06	2	0.4366812	30	0.086637594
2870	GO:0004064	arylesterase activity	0.06	2	0.4366812	30	0.086637594
14422	GO:0032012	regulation of ARF protein	0.06	2	0.4366812	31	0.08952551
14720	GO:0032312	regulation of ARF GTPase	0.06	2	0.4366812	31	0.08952551
20908	GO:0045290	D-arabinose 1-dehydroge	0.06	1	0.2183406	5	0.0144396
23522	GO:0048046	apoplast	0.07	3	0.65502185	70	0.20215438
22049	GO:0046527	glucosyltransferase activit	0.07	7	1.5283843	270	0.77973837
24966	GO:0050660	flavin adenine dinucleotid	0.07	4	0.8733624	118	0.34077454
2985	GO:0004222	metalloendopeptidase act	0.07	2	0.4366812	34	0.09818927
9832	GO:0016782	transferase activity, transf	0.08	3	0.65502185	74	0.21370606
11776	GO:0019825	oxygen binding	0.08	9	1.9650655	389	1.1234008
2626	GO:0003674|GO:0005554	molecular_function	0.08	337	73.58079	24394	70.447914
20531	GO:0044281	small molecule metabolic	0.08	31	6.768559	1788	5.1636004
3845	GO:0005452	inorganic anion exchange	0.08	1	0.2183406	6	0.01732752
4657	GO:0006528	asparagine metabolic proc	0.08	1	0.2183406	6	0.01732752
4658	GO:0006529	asparagine biosynthetic p	0.08	1	0.2183406	6	0.01732752
5966	GO:0008486	diphosphoinositol-polypho	0.08	1	0.2183406	6	0.01732752
6317	GO:0008923	lysine decarboxylase activ	0.08	1	0.2183406	6	0.01732752
11899	GO:0019992	diacylglycerol binding	0.08	1	0.2183406	6	0.01732752
13471	GO:0031012	extracellular matrix	0.08	1	0.2183406	6	0.01732752
19761	GO:0043495	protein anchor	0.08	1	0.2183406	6	0.01732752
21727	GO:0046165	alcohol biosynthetic proce	0.08	1	0.2183406	6	0.01732752
21893	GO:0046364	monosaccharide biosynthe	0.08	1	0.2183406	6	0.01732752
23230	GO:0047746	chlorophyllase activity	0.08	1	0.2183406	6	0.01732752
135	GO:0000170	sphingosine hydroxylase a	0.08	1	0.2183406	6	0.01732752
3259	GO:0004619	phosphoglycerate mutase	0.08	1	0.2183406	6	0.01732752
21146	GO:0045544	gibberellin 20-oxidase act	0.08	2	0.4366812	35	0.10107719
9836	GO:0016787	hydrolase activity	0.08	59	12.882096	3723	10.751725
8501	GO:0015085	calcium ion transmembran	0.09	2	0.4366812	38	0.10974095
15189	GO:0032787	monocarboxylic acid meta	0.09	3	0.65502185	81	0.23392151
9486	GO:0016298	lipase activity	0.10	5	1.091703	185	0.53426516
4687	GO:0006558	L-phenylalanine metabolic	0.10	1	0.2183406	8	0.023103358
6378	GO:0008999	ribosomal-protein-alanine	0.10	1	0.2183406	8	0.023103358
6463	GO:0009094|GO:0019274	L-phenylalanine biosynthe	0.10	1	0.2183406	8	0.023103358
9680	GO:0016595	glutamate binding	0.10	1	0.2183406	8	0.023103358
18639	GO:0042284	sphingolipid delta-4 desat	0.10	1	0.2183406	8	0.023103358
29068	GO:0070704	sterol desaturase activity	0.10	1	0.2183406	8	0.023103358
3098	GO:0004427	inorganic diphosphatase a	0.10	1	0.2183406	8	0.023103358
4246	GO:0006006	glucose metabolic proces	0.11	3	0.65502185	87	0.25124902
5694	GO:0008137	NADH dehydrogenase (ub	0.11	2	0.4366812	43	0.124180555
119	GO:0000151	ubiquitin ligase complex	0.11	3	0.65502185	88	0.25413695
79	GO:0000103|GO:0019378	sulfate assimilation	0.11	1	0.2183406	9	0.025991278
3251	GO:0004611	phosphoenolpyruvate carb	0.11	1	0.2183406	9	0.025991278
4914	GO:0006816	calcium ion transport	0.11	2	0.4366812	44	0.12706847
28952	GO:0070588	calcium ion transmembran	0.11	2	0.4366812	44	0.12706847
2642	GO:0003697|GO:0003698	single-stranded DNA bind	0.11	2	0.4366812	44	0.12706847
11931	GO:0020037	heme binding	0.12	11	2.4017467	550	1.5883559
9893	GO:0016857	racemase and epimerase	0.12	2	0.4366812	45	0.1299564
14726	GO:0032318	regulation of Ras GTPase	0.12	2	0.4366812	45	0.1299564
22096	GO:0046578	regulation of Ras protein s	0.12	2	0.4366812	45	0.1299564
24456	GO:0050136	NADH dehydrogenase (qu	0.12	2	0.4366812	45	0.1299564
25355	GO:0051056	regulation of small GTPas	0.12	2	0.4366812	45	0.1299564
4329	GO:0006096|GO:0019641	glycolysis	0.12	2	0.4366812	46	0.13284431
2636	GO:0003690	double-stranded DNA bind	0.12	2	0.4366812	46	0.13284431
3842	GO:0005432	calcium:sodium antiporter	0.12	1	0.2183406	10	0.0288792
3849	GO:0005459	UDP-galactose transmem	0.12	1	0.2183406	10	0.0288792
5911	GO:0008417	fucosyltransferase activity	0.12	1	0.2183406	10	0.0288792
7175	GO:0009924	octadecanal decarbonylas	0.12	1	0.2183406	10	0.0288792
8727	GO:0015369	calcium:hydrogen antiport	0.12	1	0.2183406	10	0.0288792
9054	GO:0015785	UDP-galactose transport	0.12	1	0.2183406	10	0.0288792
30135	GO:0071771	aldehyde decarbonylase a	0.12	1	0.2183406	10	0.0288792
30693	GO:0072334	UDP-galactose transmem	0.12	1	0.2183406	10	0.0288792
2797	GO:0003979	UDP-glucose 6-dehydroge	0.12	1	0.2183406	10	0.0288792
6442	GO:0009073|GO:0016089	aromatic amino acid famil	0.13	2	0.4366812	47	0.13573223
21943	GO:0046417	chorismate metabolic proc	0.13	2	0.4366812	47	0.13573223
5809	GO:0008289	lipid binding	0.13	6	1.3100437	258	0.74508333
3872	GO:0005506	iron ion binding	0.13	11	2.4017467	563	1.6258988
716	GO:0001071	nucleic acid binding trans	0.13	24	5.240175	1417	4.0921826
2643	GO:0003700|GO:0000130	sequence-specific DNA bi	0.13	24	5.240175	1417	4.0921826
11152	GO:0019139|GO:0046420	cytokinin dehydrogenase	0.14	1	0.2183406	11	0.03176712
24696	GO:0050378	UDP-glucuronate 4-epime	0.14	1	0.2183406	11	0.03176712
3490	GO:0004888|GO:0004926	transmembrane receptor a	0.14	11	2.4017467	571	1.6490022
5096	GO:0007047	cellular cell wall organizat	0.14	2	0.4366812	50	0.14439599
6932	GO:0009664	plant-type cell wall organiz	0.14	2	0.4366812	50	0.14439599
19914	GO:0043648	dicarboxylic acid metaboli	0.14	2	0.4366812	50	0.14439599
20857	GO:0045229	external encapsulating str	0.14	2	0.4366812	50	0.14439599
30033	GO:0071669	plant-type cell wall organiz	0.14	2	0.4366812	50	0.14439599
7173	GO:0009922	fatty acid elongase activity	0.15	2	0.4366812	51	0.14728391
7213	GO:0009966|GO:0035466	regulation of signal transd	0.15	2	0.4366812	51	0.14728391
9733	GO:0016655	oxidoreductase activity, ac	0.15	2	0.4366812	51	0.14728391
12601	GO:0023051	regulation of signaling	0.15	2	0.4366812	51	0.14728391
7526	GO:0010301|GO:0033710	xanthoxin dehydrogenase	0.15	1	0.2183406	12	0.03465504
20615	GO:0044421	extracellular region part	0.15	1	0.2183406	12	0.03465504

25436	GO:0051139	metal ion:hydrogen antipo	0.15	1	0.2183406	12	0.03465504
9767	GO:0016705	oxidoreductase activity, ac	0.15	7	1.5283843	329	0.95012563
9295	GO:0016053	organic acid biosynthetic p	0.15	5	1.091703	211	0.6093511
21922	GO:0046394	carboxylic acid biosynthet	0.15	5	1.091703	211	0.6093511
9795	GO:0016740	transferase activity	0.16	57	12.445415	3764	10.870131
29246	GO:0070882	cellular cell wall organizat	0.16	2	0.4366812	53	0.15305975
2778	GO:0003954	NADH dehydrogenase act	0.16	2	0.4366812	53	0.15305975
22375	GO:0046872	metal ion binding	0.16	46	10.043668	2985	8.6204405
6436	GO:0009067	aspartate family amino ac	0.16	1	0.2183406	13	0.037542958
8578	GO:0015165	pyrimidine nucleotide suga	0.16	1	0.2183406	13	0.037542958
9050	GO:0015781	pyrimidine nucleotide-sug	0.16	1	0.2183406	13	0.037542958
4649	GO:0006520|GO:0006519	cellular amino acid metab	0.16	6	1.3100437	277	0.7999538
19470	GO:0043167	ion binding	0.16	46	10.043668	2997	8.655096
19472	GO:0043169	cation binding	0.16	46	10.043668	2997	8.655096
17617	GO:0035251	UDP-glucosyltransferase a	0.16	5	1.091703	218	0.62956655
30066	GO:0071702	organic substance transpo	0.17	10	2.183406	529	1.5277096
5767	GO:0008237	metallopeptidase activity	0.17	2	0.4366812	56	0.16172351
22409	GO:0046906	tetrapyrrole binding	0.17	11	2.4017467	596	1.7212002
4670	GO:0006541	glutamine metabolic proce	0.17	1	0.2183406	14	0.040430877
9399	GO:0016174	NAD(P)H oxidase activity	0.17	1	0.2183406	14	0.040430877
9873	GO:0016833	oxo-acid-lyase activity	0.17	1	0.2183406	14	0.040430877
24970	GO:0050664	oxidoreductase activity, ac	0.17	1	0.2183406	14	0.040430877
31408	GO:0080031	methyl salicylate esterase	0.17	1	0.2183406	14	0.040430877
270	GO:0000339	RNA cap binding	0.17	1	0.2183406	14	0.040430877
2998	GO:0004311	farnesyltranstransferase a	0.17	1	0.2183406	14	0.040430877
3204	GO:0004558|GO:0004562	alpha-glucosidase activity	0.17	1	0.2183406	14	0.040430877
6441	GO:0009072	aromatic amino acid famil	0.17	2	0.4366812	57	0.16461143
9813	GO:0016759	cellulose synthase activity	0.17	2	0.4366812	57	0.16461143
23973	GO:0048583	regulation of response to s	0.17	2	0.4366812	57	0.16461143
29919	GO:0071555	cell wall organization	0.18	2	0.4366812	58	0.16749935
7520	GO:0010294	abscisic acid glucosyltrans	0.18	1	0.2183406	15	0.043318797
8635	GO:0015229	L-ascorbic acid transporte	0.18	1	0.2183406	15	0.043318797
9144	GO:0015882	L-ascorbic acid transport	0.18	1	0.2183406	15	0.043318797
2868	GO:0004062	aryl sulfotransferase activ	0.18	1	0.2183406	15	0.043318797
3209	GO:0004564	beta-fructofuranosidase a	0.18	1	0.2183406	15	0.043318797
3476	GO:0004872|GO:0019041	receptor activity	0.18	12	2.6200874	672	1.9406822
2831	GO:0004022	alcohol dehydrogenase (N	0.18	2	0.4366812	59	0.17038727
9730	GO:0016651	oxidoreductase activity, ac	0.19	3	0.65502185	112	0.32344702
29918	GO:0071554	cell wall organization or bi	0.19	3	0.65502185	112	0.32344702
6077	GO:0008652	cellular amino acid biosyn	0.19	4	0.8733624	169	0.48805845
9427	GO:0016215	CoA desaturase activity	0.19	1	0.2183406	16	0.046206716
18757	GO:0042409	caffeoyl-CoA O-methyltran	0.19	1	0.2183406	16	0.046206716
219	GO:0000287	magnesium ion binding	0.19	1	0.2183406	16	0.046206716
3334	GO:0004713|GO:0004718	protein tyrosine kinase act	0.19	1	0.2183406	16	0.046206716
3382	GO:0004768|GO:0016214	stearoyl-CoA 9-desaturase	0.19	1	0.2183406	16	0.046206716
8526	GO:0015112	nitrate transmembrane tra	0.19	2	0.4366812	61	0.1761631
8975	GO:0015706|GO:0006872	nitrate transport	0.19	2	0.4366812	61	0.1761631
13753	GO:0031323	regulation of cellular meta	0.20	42	9.170305	2770	7.999538
5213	GO:0007186	G-protein coupled recepto	0.20	2	0.4366812	62	0.17905103
9768	GO:0016706	oxidoreductase activity, ac	0.20	4	0.8733624	175	0.505386
30887	GO:0072531	pyrimidine-containing com	0.20	1	0.2183406	17	0.049094636
31465	GO:0080090	regulation of primary meta	0.21	41	8.951965	2710	7.826263
16595	GO:0034212	peptide N-acetyltransferas	0.21	1	0.2183406	18	0.051982556
3239	GO:0004596	peptide alpha-N-acetyltran	0.21	1	0.2183406	18	0.051982556
20356	GO:0044106	cellular amine metabolic p	0.21	6	1.3100437	303	0.8750397
9890	GO:0016854	racemase and epimerase	0.22	2	0.4366812	66	0.1906027
6663	GO:0009309	amine biosynthetic proces	0.22	4	0.8733624	181	0.5227135
6884	GO:0009607	response to biotic stimulus	0.22	3	0.65502185	122	0.3523262
3799	GO:0005338|GO:0005339	nucleotide-sugar transmem	0.22	1	0.2183406	19	0.054870475
5230	GO:0007205	activation of protein kinas	0.22	1	0.2183406	19	0.054870475
6435	GO:0009066	aspartate family amino ac	0.22	1	0.2183406	19	0.054870475
9049	GO:0015780	nucleotide-sugar transpor	0.22	1	0.2183406	19	0.054870475
14556	GO:0032147	activation of protein kinas	0.22	1	0.2183406	19	0.054870475
5981	GO:0008509	anion transmembrane tran	0.23	5	1.091703	245	0.70754033
11202	GO:0019199	transmembrane receptor p	0.23	9	1.9650655	504	1.4555116
2999	GO:0004312	fatty acid synthase activity	0.23	2	0.4366812	68	0.19637854
4917	GO:0006820|GO:0006822	anion transport	0.23	5	1.091703	247	0.7133162
11221	GO:0019219	regulation of nucleobase,	0.23	40	8.733624	2675	7.7251854
25468	GO:0051171	regulation of nitrogen com	0.23	40	8.733624	2675	7.7251854
31420	GO:0080043	quercetin 3-O-glucosyltran	0.23	2	0.4366812	69	0.19926646
3200	GO:0004553|GO:0016800	hydrolase activity, hydroly	0.23	6	1.3100437	312	0.90103096
25477	GO:0051180	vitamin transport	0.23	1	0.2183406	20	0.0577584
25480	GO:0051183	vitamin transporter activity	0.23	1	0.2183406	20	0.0577584
2675	GO:0003825	alpha,alpha-trehalose-pho	0.23	1	0.2183406	20	0.0577584
3328	GO:0004707|GO:0008338	MAP kinase activity	0.23	1	0.2183406	20	0.0577584
9583	GO:0016469	proton-transporting two-se	0.24	3	0.65502185	127	0.36676583
15524	GO:0033124	regulation of GTP catabol	0.24	2	0.4366812	70	0.20215438
19394	GO:0043087	regulation of GTPase activ	0.24	2	0.4366812	70	0.20215438
6662	GO:0009308	amine metabolic process	0.24	6	1.3100437	314	0.9068068
25098	GO:0050794|GO:0051244	regulation of cellular proce	0.24	58	12.663755	3987	11.514136
5878	GO:0008378	galactosyltransferase activ	0.24	1	0.2183406	21	0.060646318
9902	GO:0016868|GO:0016777	intramolecular transferase	0.24	1	0.2183406	21	0.060646318
12999	GO:0030506	ankyrin binding	0.24	1	0.2183406	21	0.060646318
23518	GO:0048040	UDP-glucuronate decarbo	0.24	1	0.2183406	21	0.060646318
27063	GO:0060089	molecular transducer activ	0.25	13	2.838428	787	2.2727928
3475	GO:0004871|GO:0005062	signal transducer activity	0.25	13	2.838428	787	2.2727928
24968	GO:0050662	coenzyme binding	0.25	4	0.8733624	191	0.5515927
2975	GO:0004175|GO:0016809	endopeptidase activity	0.25	6	1.3100437	320	0.9241344
4247	GO:0006007	glucose catabolic process	0.25	2	0.4366812	73	0.21081814
9703	GO:0016620	oxidoreductase activity, ac	0.25	2	0.4366812	73	0.21081814
11313	GO:0019320	hexose catabolic process	0.25	2	0.4366812	73	0.21081814
20525	GO:0044275	cellular carbohydrate cata	0.25	2	0.4366812	73	0.21081814
21726	GO:0046164	alcohol catabolic process	0.25	2	0.4366812	73	0.21081814
21894	GO:0046365	monosaccharide catabolic	0.25	2	0.4366812	73	0.21081814
21152	GO:0045551	cinnamyl-alcohol dehydro	0.25	1	0.2183406	22	0.06353424
5732	GO:0008194	UDP-glycosyltransferase a	0.26	6	1.3100437	322	0.9299102
8810	GO:0015491	cation:cation antiporter ac	0.26	2	0.4366812	75	0.21659398
9420	GO:0016207	4-coumarate-CoA ligase a	0.26	1	0.2183406	23	0.06642216
16063	GO:0033674	positive regulation of kina	0.26	1	0.2183406	23	0.06642216
19815	GO:0043549	regulation of kinase activi	0.26	1	0.2183406	23	0.06642216
21437	GO:0045859	regulation of protein kinas	0.26	1	0.2183406	23	0.06642216
21438	GO:0045860	positive regulation of prote	0.26	1	0.2183406	23	0.06642216
22753	GO:0047262|GO:0050375	polygalacturonate 4-alpha	0.26	1	0.2183406	23	0.06642216
25628	GO:0051338	regulation of transferase a	0.26	1	0.2183406	23	0.06642216
25637	GO:0051347	positive regulation of trans	0.26	1	0.2183406	23	0.06642216
4526	GO:0006351|GO:0006350	transcription, DNA-depend	0.27	24	5.240175	1575	4.548474
15176	GO:0032774	RNA biosynthetic process	0.27	24	5.240175	1575	4.548474
6248	GO:0008843	endochitinase activity	0.27	1	0.2183406	24	0.06931008
13657	GO:0031225	anchored to membrane	0.27	1	0.2183406	24	0.06931008
13659	GO:0031227	intrinsic to endoplasmic re	0.27	1	0.2183406	24	0.06931008
31407	GO:0080030	methyl indole-3-acetate es	0.27	1	0.2183406	24	0.06931008
3213	GO:0004568	chitinase activity	0.27	1	0.2183406	24	0.06931008
27228	GO:0060255	regulation of macromolecu	0.27	39	8.515284	2660	7.6818866

15436	GO:0033036	macromolecule localizatio	0.27	7	1.5283843	397	1.1465042
19126	GO:0042803	protein homodimerization	0.27	5	1.091703	265	0.7652988
5196	GO:0007166	cell surface receptor linke	0.28	2	0.4366812	78	0.22525774
19832	GO:0043566	structure-specific DNA bin	0.28	2	0.4366812	78	0.22525774
30865	GO:0072509	divalent inorganic cation t	0.28	2	0.4366812	78	0.22525774
4530	GO:0006355|GO:0032583	regulation of transcription,	0.28	38	8.296944	2591	7.4826
9699	GO:0016616	oxidoreductase activity, ac	0.28	5	1.091703	266	0.7681867
25544	GO:0051252	regulation of RNA metabo	0.28	38	8.296944	2593	7.488376
13822	GO:0031399	regulation of protein modi	0.28	1	0.2183406	25	0.072197996
21145	GO:0045543	gibberellin 2-beta-dioxyge	0.28	1	0.2183406	25	0.072197996
1225	GO:0001932	regulation of protein phos	0.28	1	0.2183406	25	0.072197996
4358	GO:0006140	regulation of nucleotide m	0.29	2	0.4366812	80	0.2310336
7146	GO:0009894	regulation of catabolic pro	0.29	2	0.4366812	80	0.2310336
9682	GO:0016597	amino acid binding	0.29	2	0.4366812	80	0.2310336
13278	GO:0030811	regulation of nucleotide ca	0.29	2	0.4366812	80	0.2310336
13759	GO:0031329	regulation of cellular catab	0.29	2	0.4366812	80	0.2310336
15521	GO:0033121	regulation of purine nucle	0.29	2	0.4366812	80	0.2310336
19479	GO:0043176	amine binding	0.29	2	0.4366812	80	0.2310336
7141	GO:0009889	regulation of biosynthetic	0.29	38	8.296944	2605	7.523031
7772	GO:0010556	regulation of macromolecu	0.29	38	8.296944	2605	7.523031
13756	GO:0031326	regulation of cellular biosy	0.29	38	8.296944	2605	7.523031
32213	GO:2000112	regulation of cellular macr	0.29	38	8.296944	2605	7.523031
18674	GO:0042325	regulation of phosphorylat	0.29	2	0.4366812	81	0.23392151
4030	GO:0005741	mitochondrial outer memb	0.29	1	0.2183406	26	0.075085916
9884	GO:0016847|GO:0034100	1-aminocyclopropane-1-ca	0.29	1	0.2183406	26	0.075085916
10911	GO:0018871	1-aminocyclopropane-1-ca	0.29	1	0.2183406	26	0.075085916
18579	GO:0042218	1-aminocyclopropane-1-ca	0.29	1	0.2183406	26	0.075085916
30689	GO:0072330	monocarboxylic acid biosy	0.29	1	0.2183406	26	0.075085916
5195	GO:0007165|GO:0023033	signal transduction	0.29	15	3.275109	961	2.775291
12602	GO:0023052|GO:0023046	signaling	0.29	15	3.275109	961	2.775291
29202	GO:0070838	divalent metal ion transpo	0.30	2	0.4366812	82	0.23680943
6433	GO:0009064	glutamine family amino ac	0.30	1	0.2183406	27	0.077973835
3873	GO:0005507	copper ion binding	0.30	3	0.65502185	146	0.42163628
4327	GO:0006091	generation of precursor m	0.31	2	0.4366812	84	0.24258527
30867	GO:0072511	divalent inorganic cation t	0.31	2	0.4366812	84	0.24258527
11222	GO:0019220	regulation of phosphate m	0.31	2	0.4366812	85	0.24547319
25471	GO:0051174	regulation of phosphorus	0.31	2	0.4366812	85	0.24547319
13634	GO:0031177	phosphopantetheine bindi	0.31	1	0.2183406	28	0.080861755
139	GO:0000175	3'-5'-exoribonuclease acti	0.31	1	0.2183406	28	0.080861755
3284	GO:0004650	polygalacturonase activity	0.31	1	0.2183406	28	0.080861755
20533	GO:0044283	small molecule biosynthet	0.31	6	1.3100437	347	1.0021082
7692	GO:0010468	regulation of gene expres	0.31	38	8.296944	2635	7.6096687
11426	GO:0019438	aromatic compound biosy	0.32	3	0.65502185	149	0.43030006
4029	GO:0005740	mitochondrial envelope	0.32	3	0.65502185	151	0.4360759
8679	GO:0015297	antiporter activity	0.33	3	0.65502185	152	0.4389638
8682	GO:0015300	solute:solute antiporter ac	0.33	3	0.65502185	152	0.4389638
8683	GO:0015301|GO:0015380	anion:anion antiporter acti	0.33	1	0.2183406	30	0.086637594
2774	GO:0003950	NAD+ ADP-ribosyltransfer	0.33	1	0.2183406	30	0.086637594
3518	GO:0004930|GO:0001622	G-protein coupled recepto	0.33	1	0.2183406	30	0.086637594
6424	GO:0009055|GO:0009053	electron carrier activity	0.33	3	0.65502185	154	0.44473964
8517	GO:0015103	inorganic anion transmem	0.33	3	0.65502185	154	0.44473964
9294	GO:0016052|GO:0006095	carbohydrate catabolic pro	0.34	2	0.4366812	91	0.2628007
9697	GO:0016614	oxidoreductase activity, ac	0.35	5	1.091703	293	0.84616053
3785	GO:0005315|GO:0005317	inorganic phosphate trans	0.35	1	0.2183406	32	0.09241343
4757	GO:0006633|GO:0000037	fatty acid biosynthetic proc	0.35	1	0.2183406	32	0.09241343
9927	GO:0016896	exoribonuclease activity,	0.35	1	0.2183406	32	0.09241343
14381	GO:0031968	organelle outer membrane	0.35	1	0.2183406	32	0.09241343
21041	GO:0045431	flavonol synthase activity	0.35	1	0.2183406	32	0.09241343
25836	GO:0051552	flavone metabolic process	0.35	1	0.2183406	32	0.09241343
25837	GO:0051553	flavone biosynthetic proce	0.35	1	0.2183406	32	0.09241343
25838	GO:0051554	flavonol metabolic proces	0.35	1	0.2183406	32	0.09241343
25839	GO:0051555	flavonol biosynthetic proc	0.35	1	0.2183406	32	0.09241343
3190	GO:0004532	exoribonuclease activity	0.35	1	0.2183406	32	0.09241343
3265	GO:0004630	phospholipase D activity	0.35	1	0.2183406	32	0.09241343
20885	GO:0045261	proton-transporting ATP s	0.36	1	0.2183406	33	0.09530135
5827	GO:0008308|GO:0022844	voltage-gated anion chan	0.36	1	0.2183406	34	0.09818927
20321	GO:0044070	regulation of anion transp	0.36	1	0.2183406	34	0.09818927
13829	GO:0031406	carboxylic acid binding	0.37	2	0.4366812	98	0.28301615
18912	GO:0042578	phosphoric ester hydrolas	0.37	7	1.5283843	444	1.2822365
20623	GO:0044429	mitochondrial part	0.37	3	0.65502185	165	0.47650677
3877	GO:0005516	calmodulin binding	0.38	4	0.8733624	236	0.6815491
5902	GO:0008408	3'-5' exonuclease activity	0.38	1	0.2183406	36	0.10396511
9915	GO:0016884|GO:0016003	carbon-nitrogen ligase act	0.39	1	0.2183406	37	0.10685303
9839	GO:0016791|GO:0016302	phosphatase activity	0.40	6	1.3100437	383	1.1060733
9637	GO:0016538|GO:0003751	cyclin-dependent protein k	0.40	1	0.2183406	38	0.10974095
4067	GO:0005783	endoplasmic reticulum	0.41	3	0.65502185	174	0.50249803
13656	GO:0031224	intrinsic to membrane	0.41	17	3.7117903	1193	3.4452884
8967	GO:0015698	inorganic anion transport	0.41	3	0.65502185	176	0.5082739
5891	GO:0008395|GO:0008394	steroid hydroxylase activit	0.41	1	0.2183406	40	0.1155168
7072	GO:0009813	flavonoid biosynthetic pro	0.41	1	0.2183406	40	0.1155168
9843	GO:0016796	exonuclease activity, activ	0.41	1	0.2183406	40	0.1155168
11804	GO:0019867	outer membrane	0.41	1	0.2183406	40	0.1155168
3942	GO:0005618	cell wall	0.41	2	0.4366812	107	0.30900744
6166	GO:0008757	S-adenosylmethionine-de	0.43	3	0.65502185	180	0.5198256
3753	GO:0005253	anion channel activity	0.43	1	0.2183406	42	0.121292636
4756	GO:0006631	fatty acid metabolic proce	0.43	1	0.2183406	42	0.121292636
4849	GO:0006730|GO:0019753	one-carbon metabolic proc	0.43	5	1.091703	325	0.93857396
12868	GO:0030312	external encapsulating str	0.43	2	0.4366812	111	0.3205591
14676	GO:0032268	regulation of cellular prote	0.44	1	0.2183406	43	0.124180555
25538	GO:0051246	regulation of protein meta	0.44	1	0.2183406	43	0.124180555
8680	GO:0015298	solute:cation antiporter ac	0.44	2	0.4366812	112	0.32344702
9933	GO:0016903	oxidoreductase activity, ac	0.44	2	0.4366812	113	0.32633495
7071	GO:0009812	flavonoid metabolic proce	0.44	1	0.2183406	44	0.12706847
9186	GO:0015926	glucosidase activity	0.44	1	0.2183406	44	0.12706847
13730	GO:0031300	intrinsic to organelle mem	0.44	1	0.2183406	44	0.12706847
4844	GO:0006725	cellular aromatic compour	0.44	3	0.65502185	185	0.53426516
9818	GO:0016765|GO:0016766	transferase activity, transf	0.44	3	0.65502185	185	0.53426516
20499	GO:0044249	cellular biosynthetic proce	0.44	36	7.860262	2635	7.6096687
22478	GO:0046982	protein heterodimerization	0.45	2	0.4366812	114	0.32922286
20619	GO:0044425	membrane part	0.45	21	4.5851526	1523	4.398302
6427	GO:0009058	biosynthetic process	0.46	38	8.296944	2796	8.074624
4073	GO:0005789	endoplasmic reticulum me	0.46	1	0.2183406	46	0.13284431
18536	GO:0042175	nuclear outer membrane-e	0.46	1	0.2183406	46	0.13284431
20626	GO:0044432	endoplasmic reticulum pa	0.46	1	0.2183406	46	0.13284431
5807	GO:0008287	protein serine/threonine p	0.47	1	0.2183406	47	0.13573223
11830	GO:0019901	protein kinase binding	0.48	1	0.2183406	49	0.14150807
31421	GO:0080044	quercetin 7-O-glucosyltran	0.48	1	0.2183406	49	0.14150807
3260	GO:0004620	phospholipase activity	0.49	2	0.4366812	123	0.35521415
3185	GO:0004527|GO:0008857	exonuclease activity	0.49	1	0.2183406	50	0.14439599
3340	GO:0004721	phosphoprotein phosphata	0.49	3	0.65502185	199	0.57469606
9494	GO:0016311	dephosphorylation	0.49	6	1.3100437	425	1.227366
9267	GO:0016021	integral to membrane	0.49	15	3.275109	1103	3.1853757
9975	GO:0016998	cell wall macromolecule c	0.49	1	0.2183406	51	0.14728391

3099	GO:0004428	inositol or phosphatidylino	0.49	1	0.2183406	51	0.14728391
14380	GO:0031967	organelle envelope	0.50	3	0.65502185	201	0.5804719
4915	GO:0006817	phosphate transport	0.50	1	0.2183406	52	0.15017183
6965	GO:0009699	phenylpropanoid biosynth	0.50	1	0.2183406	52	0.15017183
8528	GO:0015114	phosphate transmembran	0.50	1	0.2183406	52	0.15017183
17801	GO:0035435	phosphate transmembran	0.50	1	0.2183406	52	0.15017183
20287	GO:0044036	cell wall macromolecule m	0.50	1	0.2183406	52	0.15017183
14387	GO:0031975	envelope	0.51	3	0.65502185	205	0.59202355
4894	GO:0006790	sulfur compound metaboli	0.52	1	0.2183406	55	0.15883559
9533	GO:0016405	CoA-ligase activity	0.52	1	0.2183406	55	0.15883559
4755	GO:0006629	lipid metabolic process	0.52	8	1.7467248	590	1.7038727
15577	GO:0033178	proton-transporting two-se	0.53	1	0.2183406	56	0.16172351
22479	GO:0046983	protein dimerization activi	0.53	7	1.5283843	517	1.4930545
5715	GO:0008171	O-methyltransferase activ	0.53	1	0.2183406	57	0.16461143
9910	GO:0016878	acid-thiol ligase activity	0.53	1	0.2183406	57	0.16461143
9906	GO:0016874	ligase activity	0.53	8	1.7467248	597	1.7240882
9754	GO:0016684	oxidoreductase activity, ac	0.53	2	0.4366812	135	0.38986918
3242	GO:0004601|GO:0016685	peroxidase activity	0.53	2	0.4366812	135	0.38986918
4913	GO:0006814|GO:0006834	sodium ion transport	0.54	1	0.2183406	58	0.16749935
5819	GO:0008299|GO:0009241	isoprenoid biosynthetic pr	0.54	1	0.2183406	58	0.16749935
8499	GO:0015081|GO:0022816	sodium ion transmembran	0.54	1	0.2183406	58	0.16749935
11820	GO:0019887	protein kinase regulator ac	0.54	1	0.2183406	58	0.16749935
18086	GO:0035725	sodium ion transmembran	0.54	1	0.2183406	58	0.16749935
29800	GO:0071436	sodium ion export	0.54	1	0.2183406	58	0.16749935
3323	GO:0004702	receptor signaling protein	0.54	1	0.2183406	58	0.16749935
25998	GO:0051716	cellular response to stimu	0.54	15	3.275109	1138	3.2864528
9900	GO:0016866	intramolecular transferase	0.54	1	0.2183406	59	0.17038727
30700	GO:0072341	modified amino acid bindi	0.54	1	0.2183406	59	0.17038727
4840	GO:0006720|GO:0016096	isoprenoid metabolic proc	0.55	1	0.2183406	60	0.17327519
5652	GO:0008081|GO:0004434	phosphoric diester hydrola	0.55	1	0.2183406	60	0.17327519
6964	GO:0009698	phenylpropanoid metaboli	0.55	1	0.2183406	60	0.17327519
4028	GO:0005739	mitochondrion	0.56	3	0.65502185	219	0.63245445
6046	GO:0008610	lipid biosynthetic process	0.56	2	0.4366812	141	0.4071967
11210	GO:0019207	kinase regulator activity	0.56	1	0.2183406	62	0.17905103
20883	GO:0045259|GO:0045255	proton-transporting ATP s	0.56	1	0.2183406	62	0.17905103
8557	GO:0015144	carbohydrate transmembr	0.57	2	0.4366812	143	0.41297254
16602	GO:0034219	carbohydrate transmembr	0.57	2	0.4366812	143	0.41297254
9308	GO:0016070	RNA metabolic process	0.57	24	5.240175	1855	5.3570914
19125	GO:0042802	identical protein binding	0.58	9	1.9650655	697	2.01288
9889	GO:0016853	isomerase activity	0.58	4	0.8733624	306	0.8837035
9710	GO:0016628	oxidoreductase activity, ac	0.58	1	0.2183406	65	0.18771479
6071	GO:0008643|GO:0006861	carbohydrate transport	0.58	2	0.4366812	147	0.42452422
5651	GO:0008080	N-acetyltransferase activit	0.59	1	0.2183406	67	0.19349062
21710	GO:0046148	pigment biosynthetic proc	0.60	1	0.2183406	69	0.19926646
3620	GO:0005057	receptor signaling protein	0.60	1	0.2183406	69	0.19926646
5726	GO:0008187	poly-pyrimidine tract bindi	0.61	1	0.2183406	70	0.20215438
5788	GO:0008266	poly(U) RNA binding	0.61	1	0.2183406	70	0.20215438
11828	GO:0019899	enzyme binding	0.61	3	0.65502185	237	0.684437
3744	GO:0005244	voltage-gated ion channel	0.62	1	0.2183406	72	0.20793022
12528	GO:0022832	voltage-gated channel act	0.62	1	0.2183406	72	0.20793022
15280	GO:0032879	regulation of localization	0.62	1	0.2183406	72	0.20793022
17141	GO:0034762	regulation of transmembra	0.62	1	0.2183406	72	0.20793022
17144	GO:0034765	regulation of ion transmen	0.62	1	0.2183406	72	0.20793022
19568	GO:0043269	regulation of ion transport	0.62	1	0.2183406	72	0.20793022
25348	GO:0051049	regulation of transport	0.62	1	0.2183406	72	0.20793022
18786	GO:0042440	pigment metabolic proces	0.62	1	0.2183406	73	0.21081814
19831	GO:0043565	sequence-specific DNA bi	0.62	2	0.4366812	159	0.45917925
4641	GO:0006508	proteolysis	0.63	12	2.6200874	965	2.7868426
8673	GO:0015291|GO:0015290	secondary active transme	0.63	5	1.091703	407	1.1753833
9817	GO:0016763	transferase activity, transf	0.63	1	0.2183406	74	0.21370606
5712	GO:0008168|GO:0004480	methyltransferase activity	0.63	3	0.65502185	244	0.7046524
9538	GO:0016410	N-acyltransferase activity	0.64	1	0.2183406	77	0.22236982
9422	GO:0016209	antioxidant activity	0.64	2	0.4366812	165	0.47650677
9800	GO:0016746	transferase activity, transf	0.65	6	1.3100437	498	1.438184
3306	GO:0004674|GO:0004695	protein serine/threonine ki	0.65	13	2.838428	1062	3.0669708
9875	GO:0016836	hydro-lyase activity	0.66	1	0.2183406	80	0.2310336
3827	GO:0005372	water transmembrane tran	0.66	1	0.2183406	81	0.23392151
4927	GO:0006833	water transport	0.66	1	0.2183406	81	0.23392151
8652	GO:0015250	water channel activity	0.66	1	0.2183406	81	0.23392151
18429	GO:0042044	fluid transport	0.66	1	0.2183406	81	0.23392151
2658	GO:0003729	mRNA binding	0.66	1	0.2183406	82	0.23680943
9796	GO:0016741	transferase activity, transf	0.67	3	0.65502185	261	0.7537471
12692	GO:0030117	membrane coat	0.67	1	0.2183406	84	0.24258527
23870	GO:0048475	coated membrane	0.67	1	0.2183406	84	0.24258527
3304	GO:0004672|GO:0050222	protein kinase activity	0.69	16	3.4934497	1333	3.8495972
9824	GO:0016773	phosphotransferase activi	0.69	18	3.930131	1493	4.311664
7691	GO:0010467	gene expression	0.69	24	5.240175	1974	5.7007537
2982	GO:0004197	cysteine-type endopeptida	0.70	1	0.2183406	90	0.2599128
20505	GO:0044255	cellular lipid metabolic pro	0.70	2	0.4366812	184	0.53137726
3644	GO:0005102	receptor binding	0.70	1	0.2183406	91	0.2628007
3808	GO:0005351|GO:0005403	sugar:hydrogen symporter	0.71	1	0.2183406	94	0.27146447
3835	GO:0005402	cation:sugar symporter ac	0.71	1	0.2183406	94	0.27146447
28378	GO:0070011	peptidase activity, acting o	0.72	6	1.3100437	537	1.550813
17024	GO:0034645|GO:0034961	cellular macromolecule bi	0.72	24	5.240175	1999	5.7729516
20521	GO:0044271	cellular nitrogen compoun	0.72	4	0.8733624	367	1.0598665
3307	GO:0004675	transmembrane receptor p	0.72	4	0.8733624	367	1.0598665
11712	GO:0019748	secondary metabolic proc	0.72	1	0.2183406	95	0.27435237
6428	GO:0009059|GO:0043284	macromolecule biosynthet	0.72	24	5.240175	2003	5.7845035
14667	GO:0032259	methylation	0.73	3	0.65502185	285	0.8230572
8677	GO:0015295	solute:hydrogen symporte	0.73	1	0.2183406	98	0.28301615
8681	GO:0015299	solute:hydrogen antiporter	0.73	1	0.2183406	98	0.28301615
12608	GO:0030001	metal ion transport	0.73	4	0.8733624	374	1.080082
2628	GO:0003677	DNA binding	0.75	14	3.0567687	1224	3.534814
9709	GO:0016627	oxidoreductase activity, ac	0.75	1	0.2183406	104	0.30034366
19392	GO:0043085	positive regulation of cata	0.75	1	0.2183406	104	0.30034366
20343	GO:0044093	positive regulation of mole	0.75	1	0.2183406	104	0.30034366
22415	GO:0046914	transition metal ion bindin	0.75	31	6.768559	2599	7.5057034
19253	GO:0042936	dipeptide transporter activ	0.75	1	0.2183406	105	0.3032316
19255	GO:0042938	dipeptide transport	0.75	1	0.2183406	105	0.3032316
3195	GO:0004540	ribonuclease activity	0.76	1	0.2183406	106	0.3061195
5763	GO:0008233	peptidase activity	0.76	6	1.3100437	565	1.6316746
9488	GO:0016301	kinase activity	0.77	20	4.366812	1740	5.0249805
22376	GO:0046873	metal ion transmembrane	0.77	2	0.4366812	213	0.6151269
8495	GO:0015077	monovalent inorganic cati	0.78	2	0.4366812	214	0.6180149
14379	GO:0031966	mitochondrial membrane	0.78	1	0.2183406	114	0.32922286
9535	GO:0016407	acetyltransferase activity	0.78	1	0.2183406	115	0.3321108
5019	GO:0006952|GO:0002217	defense response	0.79	2	0.4366812	221	0.63823026
12533	GO:0022838	substrate-specific channe	0.79	2	0.4366812	221	0.63823026
25416	GO:0051119	sugar transmembrane tran	0.79	1	0.2183406	118	0.34077454
20488	GO:0044238	primary metabolic process	0.79	114	24.89083	9167	26.47356
13547	GO:0031090	organelle membrane	0.80	2	0.4366812	224	0.64689404
8660	GO:0015267|GO:0015249	channel activity	0.80	2	0.4366812	226	0.6526699
12515	GO:0022803	passive transmembrane tr	0.80	2	0.4366812	226	0.6526699

12532	GO:0022836	gated channel activity	0.80	1	0.2183406	122	0.3523262
4990	GO:0006915|GO:0008632	apoptosis	0.80	2	0.4366812	227	0.6555578
3449	GO:0004842|GO:0004840	ubiquitin-protein ligase ac	0.81	3	0.65502185	326	0.94146186
5909	GO:0008415	acyltransferase activity	0.81	3	0.65502185	330	0.95301354
9654	GO:0016567	protein ubiquitination	0.82	3	0.65502185	332	0.9587894
14854	GO:0032446	protein modification by sm	0.82	3	0.65502185	332	0.9587894
29011	GO:0070647	protein modification by sm	0.82	3	0.65502185	332	0.9587894
25195	GO:0050896|GO:0051869	response to stimulus	0.82	21	4.5851526	1881	5.432177
4910	GO:0006811	ion transport	0.82	10	2.183406	959	2.769515
5756	GO:0008219	cell death	0.83	2	0.4366812	239	0.69021285
8209	GO:0012501|GO:0016244	programmed cell death	0.83	2	0.4366812	239	0.69021285
9462	GO:0016265	death	0.83	2	0.4366812	239	0.69021285
11747	GO:0019787|GO:0008639	small conjugating protein	0.83	3	0.65502185	339	0.9790048
4394	GO:0006184	GTP catabolic process	0.83	1	0.2183406	132	0.3812054
21605	GO:0046039	GTP metabolic process	0.83	1	0.2183406	132	0.3812054
2758	GO:0003924	GTPase activity	0.83	1	0.2183406	132	0.3812054
8607	GO:0015197|GO:0015637	peptide transporter activity	0.84	1	0.2183406	139	0.40142086
8608	GO:0015198	oligopeptide transporter ac	0.84	1	0.2183406	139	0.40142086
3724	GO:0005216	ion channel activity	0.85	1	0.2183406	140	0.40430877
3895	GO:0005543	phospholipid binding	0.85	1	0.2183406	140	0.40430877
4944	GO:0006857	oligopeptide transport	0.85	1	0.2183406	141	0.4071967
8676	GO:0015294	solute:cation symporter ac	0.85	1	0.2183406	141	0.4071967
9099	GO:0015833	peptide transport	0.85	1	0.2183406	141	0.4071967
4897	GO:0006793	phosphorus metabolic pro	0.85	33	7.2052402	2925	8.4471655
4900	GO:0006796	phosphate metabolic proc	0.85	33	7.2052402	2925	8.4471655
2986	GO:0004252	serine-type endopeptidase	0.85	1	0.2183406	144	0.41586044
9801	GO:0016747	transferase activity, transf	0.86	4	0.8733624	456	1.3168914
9911	GO:0016879	ligase activity, forming car	0.86	4	0.8733624	456	1.3168914
8494	GO:0015075	ion transmembrane transp	0.86	8	1.7467248	827	2.3883097
30306	GO:0071944	cell periphery	0.86	2	0.4366812	259	0.74797124
30204	GO:0071840	cellular component organi	0.86	3	0.65502185	362	1.045427
9285	GO:0016043	cellular component organi	0.86	2	0.4366812	261	0.7537471
16603	GO:0034220	ion transmembrane transp	0.86	8	1.7467248	833	2.4056373
4612	GO:0006468	protein phosphorylation	0.86	24	5.240175	2205	6.367863
19533	GO:0043234	protein complex	0.87	8	1.7467248	848	2.448956
9114	GO:0015849	organic acid transport	0.88	1	0.2183406	156	0.45051548
22443	GO:0046942	carboxylic acid transport	0.88	1	0.2183406	156	0.45051548
8496	GO:0015078	hydrogen ion transmembr	0.88	1	0.2183406	160	0.4620672
8943	GO:0015672	monovalent inorganic cati	0.88	3	0.65502185	383	1.1060733
5764	GO:0008234|GO:0004220	cysteine-type peptidase a	0.89	1	0.2183406	163	0.47073093
9493	GO:0016310	phosphorylation	0.89	27	5.8951964	2504	7.2313514
9913	GO:0016881	acid-amino acid ligase act	0.89	3	0.65502185	391	1.1291766
30205	GO:0071841	cellular component organi	0.89	2	0.4366812	285	0.8230572
9823	GO:0016772	transferase activity, transf	0.90	20	4.366812	1932	5.579461
3341	GO:0004722|GO:0000158	protein serine/threonine p	0.90	1	0.2183406	171	0.4938343
3156	GO:0004497	monooxygenase activity	0.90	1	0.2183406	172	0.49672222
4916	GO:0006818	hydrogen transport	0.90	2	0.4366812	292	0.84327257
9246	GO:0015992	proton transport	0.90	2	0.4366812	292	0.84327257
8675	GO:0015293	symporter activity	0.90	1	0.2183406	173	0.49961013
2657	GO:0003727|GO:0003728	single-stranded RNA bind	0.92	1	0.2183406	188	0.54292893
31895	GO:0090304	nucleic acid metabolic pro	0.92	24	5.240175	2340	6.7577324
30069	GO:0071705	nitrogen compound transp	0.93	1	0.2183406	202	0.5833598
12572	GO:0022890|GO:0015082	inorganic cation transmem	0.94	2	0.4366812	332	0.9587894
20487	GO:0044237	cellular metabolic process	0.94	95	20.742357	8185	23.637625
4911	GO:0006812|GO:0006819	cation transport	0.94	5	1.091703	662	1.9118029
21058	GO:0045454|GO:0030503	cell redox homeostasis	0.94	1	0.2183406	211	0.6093511
5766	GO:0008236	serine-type peptidase acti	0.94	1	0.2183406	214	0.6180149
5791	GO:0008270	zinc ion binding	0.94	18	3.930131	1880	5.4292893
10103	GO:0017171	serine hydrolase activity	0.95	1	0.2183406	220	0.63534236
13531	GO:0031072	heat shock protein binding	0.95	1	0.2183406	230	0.6642216
11695	GO:0019725	cellular homeostasis	0.96	1	0.2183406	234	0.67577326
18925	GO:0042592	homeostatic process	0.96	1	0.2183406	238	0.68732494
8211	GO:0012505	endomembrane system	0.96	1	0.2183406	242	0.6988766
4909	GO:0006810|GO:0015457	transport	0.96	22	4.8034935	2315	6.6855345
25529	GO:0051234	establishment of localizati	0.96	22	4.8034935	2315	6.6855345
25476	GO:0051179	localization	0.96	22	4.8034935	2319	6.697086
5661	GO:0008092	cytoskeletal protein bindin	0.96	1	0.2183406	249	0.719092
4609	GO:0006464	protein modification proce	0.97	27	5.8951964	2781	8.031305
12573	GO:0022891	substrate-specific transme	0.97	10	2.183406	1241	3.5839086
3955	GO:0005634	nucleus	0.97	12	2.6200874	1433	4.138389
9266	GO:0016020	membrane	0.97	38	8.296944	3741	10.803708
9579	GO:0016462	pyrophosphatase activity	0.97	7	1.5283843	954	2.7550755
3884	GO:0005524	ATP binding	0.97	3	0.65502185	524	1.51327
9859	GO:0016818	hydrolase activity, acting o	0.97	7	1.5283843	956	2.7608514
20532	GO:0044282	small molecule catabolic p	0.97	6	1.3100437	854	2.4662836
9858	GO:0016817	hydrolase activity, acting o	0.97	7	1.5283843	959	2.769515
5840	GO:0008324	cation transmembrane tra	0.97	3	0.65502185	538	1.5537009
13041	GO:0030554	adenyl nucleotide binding	0.98	3	0.65502185	550	1.5883559
14965	GO:0032559	adenyl ribonucleotide bind	0.98	3	0.65502185	550	1.5883559
12516	GO:0022804	active transmembrane tra	0.98	5	1.091703	787	2.2727928
19684	GO:0043412	macromolecule modificati	0.98	27	5.8951964	2900	8.374968
20640	GO:0044446	intracellular organelle part	0.99	4	0.8733624	710	2.0504231
26931	GO:0055085	transmembrane transport	0.99	12	2.6200874	1548	4.4705
28365	GO:0065008	regulation of biological qu	0.99	1	0.2183406	316	0.9125827
20616	GO:0044422	organelle part	0.99	4	0.8733624	712	2.0561988
6425	GO:0009056	catabolic process	0.99	7	1.5283843	1051	3.0352037
19526	GO:0043227	membrane-bounded organ	0.99	18	3.930131	2142	6.185924
19530	GO:0043231	intracellular membrane-bo	0.99	18	3.930131	2142	6.185924
5018	GO:0006950	response to stress	0.99	3	0.65502185	611	1.764519
12547	GO:0022857|GO:0005386	transmembrane transporte	0.99	11	2.4017467	1483	4.282785
10058	GO:0017111	nucleoside-triphosphatase	0.99	5	1.091703	878	2.5355935
4408	GO:0006200	ATP catabolic process	0.99	3	0.65502185	641	1.8511566
9918	GO:0016887|GO:0004002	ATPase activity	0.99	3	0.65502185	641	1.8511566
21600	GO:0046034	ATP metabolic process	0.99	3	0.65502185	641	1.8511566
4405	GO:0006195	purine nucleotide catabolic	0.99	4	0.8733624	773	2.232362
6508	GO:0009143	nucleoside triphosphate c	0.99	4	0.8733624	773	2.232362
6509	GO:0009144	purine nucleoside triphosp	0.99	4	0.8733624	773	2.232362
6511	GO:0009146	purine nucleoside triphosp	0.99	4	0.8733624	773	2.232362
6519	GO:0009154	purine ribonucleotide cata	0.99	4	0.8733624	773	2.232362
6530	GO:0009166	nucleotide catabolic proce	0.99	4	0.8733624	773	2.232362
6567	GO:0009203	ribonucleoside triphospha	0.99	4	0.8733624	773	2.232362
6569	GO:0009205	purine ribonucleoside trip	0.99	4	0.8733624	773	2.232362
6571	GO:0009207	purine ribonucleoside trip	0.99	4	0.8733624	773	2.232362
6624	GO:0009261	ribonucleotide catabolic p	0.99	4	0.8733624	773	2.232362
17034	GO:0034655	nucleobase, nucleoside, n	0.99	4	0.8733624	773	2.232362
17035	GO:0034656	nucleobase, nucleoside a	0.99	4	0.8733624	773	2.232362
22209	GO:0046700	heterocycle catabolic proc	0.99	4	0.8733624	773	2.232362
30879	GO:0072523	purine-containing compou	0.99	4	0.8733624	773	2.232362
17020	GO:0034641	cellular nitrogen compoun	0.99	34	7.4235806	3678	10.621769
20520	GO:0044270	cellular nitrogen compoun	0.99	4	0.8733624	777	2.2439137
11522	GO:0019538|GO:0006411	protein metabolic process	0.99	39	8.515284	4134	11.938661
7229	GO:0009987|GO:0008151	cellular process	0.99	117	25.54585	10601	30.614838
6563	GO:0009199	ribonucleoside triphospha	0.99	4	0.8733624	790	2.2814567

6515	GO:0009150	purine ribonucleotide meta	0.99	4	0.8733624	791	2.2843447
6506	GO:0009141	nucleoside triphosphate m	0.99	4	0.8733624	796	2.2987843
2653	GO:0003723	RNA binding	0.99	4	0.8733624	797	2.3016722
4906	GO:0006807	nitrogen compound metab	0.99	34	7.4235806	3725	10.757502
12574	GO:0022892	substrate-specific transpo	0.99	10	2.183406	1463	4.2250266
4373	GO:0006163	purine nucleotide metabol	0.99	4	0.8733624	810	2.339215
6622	GO:0009259|GO:0009121	ribonucleotide metabolic p	0.99	4	0.8733624	812	2.344991
30877	GO:0072521	purine-containing compou	1.00	4	0.8733624	827	2.3883097
4357	GO:0006139|GO:0055134	nucleobase, nucleoside, n	1.00	28	6.1135373	3285	9.486816
3177	GO:0004518	nuclease activity	1.00	1	0.2183406	427	1.2331418
2627	GO:0003676	nucleic acid binding	1.00	19	4.148472	2453	7.0840673
20517	GO:0044267	cellular protein metabolic	1.00	27	5.8951964	3225	9.313541
3876	GO:0005515|GO:0045308	protein binding	1.00	69	15.065502	6933	20.021948
20498	GO:0044248	cellular catabolic process	1.00	4	0.8733624	894	2.5818002
4027	GO:0005737	cytoplasm	1.00	14	3.0567687	2001	5.7787275
3863	GO:0005488	binding	1.00	155	33.842796	13903	40.15075
18000	GO:0035639	purine ribonucleoside triph	1.00	3	0.65502185	769	2.2208104
19473	GO:0043170|GO:0043283	macromolecule metabolic	1.00	64	13.973799	6553	18.92454
4867	GO:0006753	nucleoside phosphate me	1.00	4	0.8733624	914	2.6395588
6484	GO:0009117	nucleotide metabolic proc	1.00	4	0.8733624	914	2.6395588
15392	GO:0032991	macromolecular complex	1.00	10	2.183406	1616	4.666878
14959	GO:0032553	ribonucleotide binding	1.00	3	0.65502185	795	2.2958963
14961	GO:0032555	purine ribonucleotide bind	1.00	3	0.65502185	795	2.2958963
10033	GO:0017076	purine nucleotide binding	1.00	3	0.65502185	797	2.3016722
3723	GO:0005215|GO:0005478	transporter activity	1.00	12	2.6200874	1844	5.325324
26932	GO:0055086	nucleobase, nucleoside a	1.00	4	0.8733624	944	2.7261963
4109	GO:0005840|GO:0033279	ribosome	1.00	2	0.4366812	682	1.9695613
22007	GO:0046483	heterocycle metabolic pro	1.00	5	1.091703	1094	3.1593843
20510	GO:0044260|GO:0034960	cellular macromolecule m	1.00	51	11.135371	5577	16.105928
18953	GO:0042623	ATPase activity, coupled	1.00	1	0.2183406	537	1.550813
13018	GO:0030529	ribonucleoprotein complex	1.00	2	0.4366812	741	2.1399486
20638	GO:0044444	cytoplasmic part	1.00	9	1.9650655	1644	4.7477403
20618	GO:0044424	intracellular part	1.00	30	6.550218	3853	11.127155
19525	GO:0043226	organelle	1.00	20	4.366812	3013	8.701303
19528	GO:0043229	intracellular organelle	1.00	20	4.366812	3013	8.701303
131	GO:0000166	nucleotide binding	1.00	4	0.8733624	1213	3.5030468
19527	GO:0043228	non-membrane-bounded o	1.00	2	0.4366812	923	2.66555
19531	GO:0043232	intracellular non-membran	1.00	2	0.4366812	923	2.66555
3903	GO:0005575|GO:0008372	cellular_component	1.00	78	17.030567	8515	24.590637
3945	GO:0005622	intracellular	1.00	35	7.6419215	4751	13.720507
3946	GO:0005623	cell	1.00	72	15.720524	8227	23.758917
20657	GO:0044464	cell part	1.00	72	15.720524	8227	23.758917
