# Supplementary material for: The Tomato Hybrid Proline-rich Protein regulates the abscission zone competence to respond to ethylene signals
Source: Hortic Res. 2018 Jun 1;5:28. doi: 10.1038/s41438-018-0033-2 (PMC5981600; doi:10.1038/s41438-018-0033-2)
Supplement: Supplementary file 4 — Table S4 [file 41438_2018_33_MOESM4_ESM.pdf]

Table S4. Differentially regulated genes (up and down regulated) in the control compared with THyPRP silenced plants in FAZ at 8 h along with their expression values, fold changes, annotations, GO ids and terms.

Worksheet 2 & 3

Cut off used to filter up and down regulated genes

Upregulated

For filtering upregulation we consider flag should be Detected in the treated sample and can be Compromised or Detected in the control sample and fold>=0.8 in the individual replicates and fold>=1 in the Geomean of treated samples.

Downregulated

For filtering downregulation we consider flag can be Detected or Compromised in the treated sample and should be Detected in the control sample and fold<=-0.8 in the individual replicates and fold<=-1 in the Geomean of treated samples.

Set3					
Line	Array	Details	Time	Treated vs. Con	Set
Control – New Yorker	254331010026_1_1	8h_5_AZ	8h	Control	Set 3
Control – New Yorker	254331010026_1_2	8h_6_AZ	8h		
TAPG + TPRP	254331010035_1_1	8h_41_AZ	8h	Treated	
TAPG + TPRP	254331010035_1_2	8h_42_AZ	8h		

Note:

Fold change expression values are provided as logbase 2

Flagging Criteria:

Compromised: If the gProcessedSignal value is less than the background intensity

Detected: If the gProcessedSignal value is above background intensity

Column Header	Description
ProbeName	Unique probe identifier
GeneName	GeneSymbol
Fold	Fold change calculated is log base 2
Flag	Detected or Compromised
GeomeanFold	Average Fold change for the replicates calculated is log base 2
p-Value	t-test p- value
gProcessedSigna	Background substracted signal intensity

Differentially Regulated Probes

Sample	Up	Down
8h_TPRP_ AZ vs. 8h_Control_AZ_Sense	1314	2139
8h_TPRP_ AZ vs. 8h_Control_AZ_Antisense	424	1983

Worksheet 4 & 5

This file contains the fold change for all the spots in the array

GO ID

Gene Ontology ID

GO ACCESSION

Gene Ontology Function name

p-value :

The probability of obtaining the specified GO accession number from a list of random entities. Less the p-value more significant is the GO accession number.

Significant pvalue<0.05

Count in Selection :

This refers to the number of genes in the selected entity (for example, from T-test) list which have that particular GO term.

%Count in Selection :

This refers to the percentage of genes in the input entity list which have that GO term.

Count in Total :

This refers to the number of genes in All Entities which have that GO term.

%Count in Total :

This refers to the percentage of genes in the All Entities list which have that GO term.

		8h_AZ_Control					8h_AZ_TAPG + TPRP vs. 8h_AZ_Control					
ProbeNar	Orientatio	Fold_8h	Flag_8h	Fold_8h	Flag_8h	Geomean	Fold_8h	Flag_8h	Fold_8h	Flag_8h	Geomean	P-value
A 96 P0	Sense	-0.258	Detected	0.258	Detected	0.000	0.934	Detected	1.130	Detected	1.032	0.065
A 96 P0	Sense	-0.518	Detected	0.518	Detected	0.000	3.917	Detected	1.676	Detected	2.796	0.152
A 96 P0	Sense	0.030	Detected	-0.030	Detected	0.000	1.607	Detected	1.029	Detected	1.318	0.045
A 96 P0	Sense	-0.462	Detected	0.462	Detected	0.000	1.670	Detected	1.278	Detected	1.474	0.099
A 96 P0	Sense	0.204	Detected	-0.204	Detected	0.000	1.288	Detected	1.298	Detected	1.293	0.024
A 96 P0	Sense	-0.070	Detected	0.070	Detected	0.000	1.490	Detected	1.642	Detected	1.566	0.004
A 96 P01	Sense	-0.023	Detected	0.023	Detected	0.000	1.612	Detected	1.610	Detected	1.611	0.000
A 96 P01	Sense	0.106	Detected	-0.106	Detected	0.000	1.581	Detected	0.966	Detected	1.274	0.059
A 96 P01	Sense	-0.113	Detected	0.113	Detected	0.000	1.539	Detected	1.004	Detected	1.272	0.048
A 96 P02	Sense	-0.078	Detected	0.078	Detected	0.000	1.530	Detected	1.320	Detected	1.425	0.008
A 96 P02	Sense	0.117	Detected	-0.117	Detected	0.000	1.309	Detected	1.180	Detected	1.244	0.011
A 96 P02	Sense	0.020	Detected	-0.020	Detected	0.000	1.364	Detected	1.075	Detected	1.219	0.014
A 96 P02	Sense	0.420	Detected	-0.420	Detected	0.000	1.247	Detected	1.436	Detected	1.342	0.089
A 96 P02	Sense	-0.079	Detected	0.079	Detected	0.000	1.247	Detected	1.035	Detected	1.141	0.013
A 96 P02	Sense	-0.152	Detected	0.152	Detected	0.000	1.611	Detected	1.141	Detected	1.376	0.039
A 96 P02	Sense	-0.170	Comprom	0.170	Comprom	0.000	1.859	Detected	1.739	Detected	1.799	0.010
A 96 P02	Sense	0.190	Detected	-0.190	Detected	0.000	2.908	Detected	1.912	Detected	2.410	0.046
A 96 P02	Sense	-0.033	Detected	0.033	Detected	0.000	1.555	Detected	1.225	Detected	1.390	0.014
A 96 P02	Sense	-0.178	Comprom	0.178	Comprom	0.000	2.171	Detected	1.726	Detected	1.948	0.021
A 96 P02	Sense	0.017	Detected	-0.017	Detected	0.000	1.437	Detected	1.007	Detected	1.222	0.030
A 96 P02	Sense	-0.088	Detected	0.088	Detected	0.000	1.369	Detected	1.051	Detected	1.210	0.022
A 96 P02	Sense	-0.230	Detected	0.230	Detected	0.000	1.839	Detected	1.234	Detected	1.536	0.056
A 96 P02	Sense	0.256	Detected	-0.256	Detected	0.000	1.597	Detected	0.964	Detected	1.280	0.088
A 96 P03	Sense	0.167	Detected	-0.167	Detected	0.000	1.823	Detected	1.674	Detected	1.749	0.011
A 96 P03	Sense	-0.388	Detected	0.388	Detected	0.000	1.264	Detected	0.815	Detected	1.039	0.146
A 96 P03	Sense	0.137	Detected	-0.137	Detected	0.000	1.173	Detected	1.219	Detected	1.196	0.013
A 96 P03	Sense	0.169	Detected	-0.169	Detected	0.000	1.289	Detected	0.996	Detected	1.143	0.036
A 96 P03	Sense	-0.196	Detected	0.196	Detected	0.000	1.661	Detected	1.317	Detected	1.489	0.029
A 96 P03	Sense	0.100	Detected	-0.100	Detected	0.000	1.240	Detected	0.905	Detected	1.072	0.031
A 96 P03	Sense	-0.181	Detected	0.181	Detected	0.000	1.139	Detected	0.924	Detected	1.031	0.039
A 96 P03	Sense	0.050	Detected	-0.050	Detected	0.000	1.791	Detected	1.338	Detected	1.564	0.021
A 96 P03	Sense	0.334	Detected	-0.334	Comprom	0.000	1.791	Detected	1.237	Detected	1.514	0.073
A 96 P03	Sense	-0.147	Detected	0.147	Detected	0.000	1.974	Detected	1.607	Detected	1.791	0.017
A 96 P04	Sense	0.117	Detected	-0.117	Detected	0.000	1.289	Detected	1.020	Detected	1.154	0.023
A 96 P04	Sense	0.396	Detected	-0.396	Detected	0.000	1.234	Detected	0.947	Detected	1.091	0.122
A 96 P04	Sense	0.202	Detected	-0.202	Detected	0.000	1.121	Detected	1.036	Detected	1.079	0.035
A 96 P04	Sense	0.084	Detected	-0.084	Detected	0.000	1.120	Detected	0.932	Detected	1.026	0.015
A 96 P04	Sense	0.140	Detected	-0.140	Detected	0.000	1.222	Detected	1.152	Detected	1.187	0.014
A 96 P05	Sense	-0.461	Comprom	0.461	Detected	0.000	1.438	Detected	1.088	Detected	1.263	0.125
A 96 P05	Sense	0.113	Detected	-0.113	Detected	0.000	1.484	Detected	1.206	Detected	1.345	0.017
A 96 P05	Sense	0.130	Detected	-0.130	Detected	0.000	1.803	Detected	0.916	Detected	1.359	0.099
A 96 P05	Sense	-0.230	Detected	0.230	Detected	0.000	1.650	Detected	1.333	Detected	1.491	0.033
A 96 P05	Sense	0.054	Detected	-0.054	Detected	0.000	1.474	Detected	1.224	Detected	1.349	0.010
A 96 P06	Sense	0.500	Detected	-0.500	Detected	0.000	2.012	Detected	1.944	Detected	1.978	0.059
A 96 P06	Sense	-0.012	Detected	0.012	Detected	0.000	1.087	Detected	0.954	Detected	1.020	0.004
A 96 P06	Sense	-0.178	Detected	0.178	Detected	0.000	1.105	Detected	0.997	Detected	1.051	0.030
A 96 P06	Sense	0.083	Detected	-0.083	Detected	0.000	1.235	Detected	1.139	Detected	1.187	0.006
A 96 P06	Sense	-0.006	Detected	0.006	Detected	0.000	1.397	Detected	0.974	Detected	1.185	0.030
A 96 P06	Sense	0.427	Comprom	-0.427	Comprom	0.000	3.711	Detected	3.046	Detected	3.379	0.025
A 96 P06	Sense	0.589	Detected	-0.589	Comprom	0.000	1.827	Detected	1.585	Detected	1.706	0.105
A 96 P06	Sense	0.099	Detected	-0.099	Detected	0.000	1.437	Detected	0.968	Detected	1.202	0.042
A 96 P07	Sense	-0.344	Detected	0.344	Detected	0.000	1.688	Detected	1.020	Detected	1.354	0.106
A 96 P07	Sense	-0.217	Detected	0.217	Detected	0.000	1.966	Detected	0.923	Detected	1.444	0.125
A 96 P07	Sense	0.036	Detected	-0.036	Detected	0.000	1.872	Detected	1.693	Detected	1.783	0.003
A 96 P07	Sense	-1.056	Detected	1.056	Detected	0.000	2.523	Detected	1.322	Detected	1.922	0.254
A 96 P07	Sense	-0.022	Detected	0.022	Detected	0.000	1.893	Detected	0.916	Detected	1.404	0.103
A 96 P07	Sense	0.023	Detected	-0.023	Detected	0.000	1.480	Detected	1.335	Detected	1.408	0.003
A 96 P07	Sense	0.206	Detected	-0.206	Detected	0.000	1.510	Detected	1.177	Detected	1.343	0.037
A 96 P07	Sense	-0.166	Detected	0.166	Detected	0.000	2.904	Detected	2.774	Detected	2.839	0.004
A 96 P08	Sense	0.065	Detected	-0.065	Detected	0.000	2.059	Detected	1.105	Detected	1.582	0.081
A 96 P08	Sense	-0.191	Detected	0.191	Detected	0.000	1.539	Detected	1.190	Detected	1.364	0.034
A 96 P08	Sense	0.294	Detected	-0.294	Detected	0.000	1.449	Detected	1.300	Detected	1.375	0.045
A 96 P08	Sense	-0.386	Detected	0.386	Detected	0.000	1.976	Detected	1.621	Detected	1.798	0.052
A 96 P08	Sense	-0.342	Detected	0.342	Detected	0.000	1.852	Detected	0.868	Detected	1.360	0.151
A 96 P08	Sense	0.156	Detected	-0.156	Detected	0.000	1.622	Detected	1.352	Detected	1.487	0.019
A 96 P08	Sense	0.090	Detected	-0.090	Detected	0.000	1.535	Detected	1.276	Detected	1.405	0.012
A 96 P08	Sense	0.179	Detected	-0.179	Detected	0.000	1.602	Detected	1.350	Detected	1.476	0.021
A 96 P08	Sense	-0.107	Detected	0.107	Detected	0.000	1.246	Detected	1.327	Detected	1.286	0.008
A 96 P09	Sense	0.166	Detected	-0.166	Detected	0.000	1.067	Detected	0.935	Detected	1.001	0.030
A 96 P09	Sense	0.057	Detected	-0.057	Detected	0.000	1.642	Detected	1.628	Detected	1.635	0.001
A 96 P09	Sense	0.022	Detected	-0.022	Detected	0.000	2.235	Detected	1.776	Detected	2.005	0.013
A 96 P09	Sense	0.010	Detected	-0.010	Detected	0.000	1.448	Detected	0.965	Detected	1.207	0.038
A 96 P09	Sense	0.126	Detected	-0.126	Detected	0.000	2.216	Detected	2.151	Detected	2.183	0.004
A 96 P09	Sense	0.326	Detected	-0.326	Detected	0.000	2.542	Detected	2.651	Detected	2.597	0.016
A 96 P09	Sense	0.035	Detected	-0.035	Detected	0.000	1.175	Detected	1.198	Detected	1.187	0.001
A 96 P09	Sense	-0.048	Detected	0.048	Detected	0.000	1.043	Detected	0.966	Detected	1.004	0.004
A 96 P09	Sense	-0.192	Detected	0.192	Detected	0.000	1.152	Detected	1.028	Detected	1.090	0.033
A 96 P09	Sense	0.177	Detected	-0.177	Detected	0.000	1.148	Detected	0.992	Detected	1.070	0.031
A 96 P10	Sense	0.010	Detected	-0.010	Detected	0.000	1.350	Detected	0.931	Detected	1.141	0.032
A 96 P10	Sense	-0.023	Detected	0.023	Detected	0.000	1.266	Detected	1.079	Detected	1.173	0.007
A 96 P10	Sense	0.189	Detected	-0.189	Detected	0.000	1.765	Detected	1.090	Detected	1.428	0.066
A 96 P10	Sense	-0.217	Detected	0.217	Detected	0.000	1.164	Detected	1.322	Detected	1.243	0.033
A 96 P10	Sense	0.006	Detected	-0.006	Detected	0.000	1.713	Detected	1.629	Detected	1.671	0.001
A 96 P10	Sense	0.060	Detected	-0.060	Detected	0.000	1.451	Detected	2.608	Detected	2.030	0.073
A 96 P11	Sense	-0.255	Detected	0.255	Detected	0.000	1.103	Detected	0.912	Detected	1.008	0.066
A 96 P11	Sense	0.036	Detected	-0.036	Detected	0.000	1.058	Detected	1.054	Detected	1.056	0.001
A 96 P11	Sense	0.501	Detected	-0.501	Comprom	0.000	1.481	Detected	1.448	Detected	1.464	0.100
A 96 P11	Sense	0.121	Detected	-0.121	Detected	0.000	1.020	Detected	0.999	Detected	1.009	0.014
A 96 P11	Sense	0.099	Detected	-0.099	Detected	0.000	1.590	Detected	1.042	Detected	1.316	0.046
A 96 P11	Sense	-0.088	Detected	0.088	Detected	0.000	1.278	Detected	0.968	Detected	1.123	0.024
A 96 P11	Sense	0.119	Detected	-0.119	Detected	0.000	1.239	Detected	1.207	Detected	1.223	0.010
A 96 P11	Sense	-0.007	Detected	0.007	Detected	0.000	1.070	Detected	0.967	Detected	1.019	0.003
A 96 P12	Sense	0.004	Detected	-0.004	Detected	0.000	1.464	Detected	1.210	Detected	1.337	0.009
A 96 P12	Sense	0.065	Detected	-0.065	Detected	0.000	1.320	Detected	1.139	Detected	1.230	0.008
A 96 P12	Sense	-0.326	Comprom	0.326	Comprom	0.000	1.419	Detected	1.503	Detected	1.461	0.047
A 96 P13	Sense	0.376	Detected	-0.376	Detected	0.000	1.269	Detected	1.099	Detected	1.184	0.091
A 96 P13	Sense	0.285	Detected	-0.285	Detected	0.000	1.221	Detected	1.281	Detected	1.251	0.049
A 96 P13	Sense	0.524	Detected	-0.524	Detected	0.000	1.151	Detected	0.891	Detected	1.021	0.199
A 96 P13	Sense	-0.462	Detected	0.462	Detected	0.000	1.949	Detected	1.261	Detected	1.605	0.108
A 96 P13	Sense	0.133	Detected	-0.133	Detected	0.000	2.751	Detected	2.732	Detected	2.742	0.002
A 96 P13	Sense	-0.687	Comprom	0.687	Detected	0.000	4.724	Detected	2.397	Detected	3.561	0.119
A 96 P13	Sense	0.194	Detected	-0.194	Detected	0.000	1.118	Detected	1.120	Detected	1.119	0.029
A 96 P13	Sense	-0.082	Detected	0.082	Detected	0.000	1.404	Detected	0.824	Detected	1.114	0.066
A 96 P13	Sense	-0.223	Detected	0.223	Detected	0.000	1.054	Detected	1.215	Detected	1.134	0.041
A 96 P14	Sense	0.219	Detected	-0.219	Detected	0.000	2.144	Detected	2.167	Detected	2.156	0.010
A 96 P14	Sense	0.086	Detected	-0.086	Detected	0.000	1.724	Detected	0.944	Detected	1.334	0.079
A 96 P14	Sense	-0.037	Detected	0.037	Detected	0.000	1.500	Detected	0.804	Detected	1.152	0.081
A 96 P15	Sense	0.343	Detected	-0.343	Detected	0.000	2.237	Detected	2.314	Detected	2.275	0.022
A 96 P15	Sense	-0.548	Detected	0.548	Detected	0.000	1.675	Detected	1.673	Detected	1.674	0.092
A 96 P15	Sense	0.245	Detected	-0.245	Detected	0.000	1.707	Detected	1.407	Detected	1.557	0.032
A 96 P15	Sense	0.356	Detected	-0.356	Detected	0.000	1.515	Detected	2.124	Detected	1.820	0.060
A 96 P15	Sense	0.080	Detected	-0.080	Detected	0.000	1.213	Detected	0.953	Detected	1.083	0.019
A 96 P16	Sense	0.269	Detected	-0.269	Detected	0.000	1.270	Detected	1.019	Detected	1.144	0.061
A 96 P16	Sense	0.090	Detected	-0.090	Detected	0.000	1.049	Detected	0.964	Detected	1.007	0.010
A 96 P16	Sense	0.071	Detected	-0.071	Detected	0.000	1.594	Detected	0.929	Detected	1.262	0.065
A 96 P16	Sense	-0.199	Detected	0.199	Detected	0.000	1.686	Detected	1.314	Detected	1.500	0.031
A 96 P16	Sense	-0.125	Detected	0.125	Detected	0.000	1.246	Detected	1.278	Detected	1.262	0.010
A 96 P16	Sense	-0.066	Detected	0.066	Detected	0.000	1.368	Detected	0.961	Detected	1.164	0.032
A 96 P16	Sense	0.477	Detected	-0.477	Detected	0.000	1.269	Detected	1.074	Detected	1.172	0.138
A 96 P16	Sense	0.575	Detected	-0.575	Comprom	0.000	1.807	Detected	1.218	Detected	1.513	0.144
A 96 P17	Sense	0.730	Detected	-0.730	Detected	0.000	1.071	Detected	1.021	Detected	1.046	0.288
A 96 P17	Sense	-0.015	Comprom	0.015	Comprom	0.000	1.187	Detected	1.502	Detected	1.345	0.014
A 96 P17	Sense	-0.101	Detected	0.101	Detected	0.000	1.702	Detected	1.197	Detected	1.450	0.033
A 96 P17	Sense	-0.213	Detected	0.213	Detected	0.000	1.137	Detected	0.946	Detected	1.041	0.047
A 96 P17	Sense	-0.035	Detected	0.035	Detected	0.000	1.079	Detected	1.226	Detected	1.152	0.005
A 96 P17	Sense	0.067	Detected	-0.067	Detected	0.000	1.605	Detected	1.490	Detected	1.547	0.003
A 96 P17	Sense	-0.211	Detected	0.211	Detected	0.000	0.959	Detected	1.316	Detected	1.138	0.054
A 96 P17	Sense	-0.280	Detected	0.280	Detected	0.000	0.834	Detected	1.245	Detected	1.039	0.096
A 96 P17	Sense	0.153	Detected	-0.153	Detected	0.000	1.489	Detected	1.243	Detected	1.366	0.020
A 96 P17	Sense	-0.281	Detected	0.281	Detected	0.000	0.981	Detected	1.283	Detected	1.132	0.071
A 96 P17	Sense	-0.030	Detected	0.030	Detected	0.000	1.661	Detected	0.819	Detected	1.240	0.099
A 96 P17	Sense	-0.650	Detected	0.650	Detected	0.000	4.783	Detected	2.627	Detected	3.705	0.099
A 96 P17	Sense	0.110	Detected	-0.110	Detected	0.000	1.099	Detected	0.984	Detected	1.041	0.014
A 96 P17	Sense	0.134	Detected	-0.134	Detected	0.000	1.232	Detected	0.904	Detected	1.068	0.037
A 96 P17	Sense	-0.136	Detected	0.136	Detected	0.000	1.673	Detected	1.215	Detected	1.444	0.032
A 96 P18	Sense	-0.074	Detected	0.074	Comprom	0.000	1.382	Detected	0.805	Detected	1.094	0.067
A 96 P18	Sense	0.390	Detected	-0.390	Detected	0.000	1.437	Detected	1.097	Detected	1.267	0.097
A 96 P18	Sense	0.358	Detected	-0.358	Detected	0.000	1.173	Detected	0.934	Detected	1.053	0.108
A 96 P19	Sense	0.186	Detected	-0.186	Detected	0.000	1.966	Detected	1.084	Detected	1.525	0.086
A 96 P19	Sense	0.150	Detected	-0.150	Detected	0.000	1.409	Detected	1.597	Detected	1.503	0.014
A 96 P19	Sense	0.239	Detected	-0.239	Detected	0.000	1.196	Detected	0.856	Detected	1.026	0.073
A 96 P19	Sense	-0.090	Detected	0.090	Detected	0.000	1.479	Detected	1.032	Detected	1.256	0.035
A 96 P19	Sense	0.540	Detected	-0.540	Detected	0.000	1.290	Detected	1.012	Detected	1.151	0.175
A 96 P19	Sense	-0.673	Detected	0.673	Detected	0.000	4.472	Detected	2.051	Detected	3.262	0.143
A 96 P20	Sense	0.127	Detected	-0.127	Detected	0.000	0.901	Detected	1.208	Detected	1.055	0.034
A 96 P20	Sense	-0.086	Detected	0.086	Detected	0.000	1.465	Detected	1.340	Detected	1.403	0.006
A 96 P20	Sense	0.211	Detected	-0.211	Detected	0.000	1.519	Detected	1.390	Detected	1.454	0.022
A 96 P20	Sense	0.329	Detected	-0.329	Detected	0.000	1.803	Detected	1.832	Detected	1.818	0.031
A 96 P20	Sense	-0.002	Detected	0.002	Detected	0.000	1.619	Detected	1.484	Detected	1.552	0.002
A 96 P20	Sense	-0.053	Detected	0.053	Detected	0.000	1.617	Detected	1.041	Detected	1.329	0.045
A 96 P20	Sense	0.258	Detected	-0.258	Comprom	0.000	1.826	Detected	1.747	Detected	1.787	0.021
A 96 P20	Sense	0.412	Detected	-0.412	Comprom	0.000	1.982	Detected	1.680	Detected	1.831	0.053
A 96 P20	Sense	0.040	Detected	-0.040	Detected	0.000	1.719	Detected	1.136	Detected	1.427	0.040
A 96 P21	Sense	-0.106	Detected	0.106	Detected	0.000	1.416	Detected	1.294	Detected	1.355	0.008
A 96 P21	Sense	0.338	Detected	-0.338	Detected	0.000	2.453	Detected	2.435	Detected	2.444	0.019
A 96 P21	Sense	0.067	Detected	-0.067	Detected	0.000	1.405	Detected	0.918	Detected	1.161	0.044
A 96 P21	Sense	0.039	Detected	-0.039	Detected	0.000	1.311	Detected	1.163	Detected	1.237	0.005
A 96 P21	Sense	0.206	Detected	-0.206	Detected	0.000	1.772	Detected	1.394	Detected	1.583	0.030
A 96 P21	Sense	0.001	Detected	-0.001	Detected	0.000	1.860	Detected	1.725	Detected	1.793	0.001
A 96 P21	Sense	0.086	Detected	-0.086	Detected	0.000	1.421	Detected	1.374	Detected	1.397	0.004
A 96 P21	Sense	-0.242	Detected	0.242	Detected	0.000	1.234	Detected	1.471	Detected	1.352	0.038
A 96 P21	Sense	-0.172	Detected	0.172	Detected	0.000	1.380	Detected	1.393	Detected	1.387	0.015
A 96 P21	Sense	-0.166	Detected	0.166	Detected	0.000	1.525	Detected	1.083	Detected	1.304	0.042
A 96 P21	Sense	0.217	Detected	-0.217	Detected	0.000	1.425	Detected	1.272	Detected	1.348	0.028
A 96 P21	Sense	0.082	Detected	-0.082	Detected	0.000	2.611	Detected	2.581	Detected	2.596	0.001
A 96 P21	Sense	-0.056	Comprom	0.056	Comprom	0.000	3.430	Detected	3.218	Detected	3.324	0.001
A 96 P21	Sense	-0.101	Detected	0.101	Detected	0.000	1.317	Detected	0.920	Detected	1.118	0.037
A 96 P21	Sense	0.137	Detected	-0.137	Detected	0.000	2.070	Detected	2.024	Detected	2.047	0.005
A 96 P21	Sense	-0.249	Detected	0.249	Detected	0.000	0.999	Detected	1.358	Detected	1.179	0.062
A 96 P22	Sense	0.094	Detected	-0.094	Detected	0.000	1.534	Detected	1.052	Detected	1.293	0.038
A 96 P22	Sense	0.020	Detected	-0.020	Detected	0.000	1.417	Detected	1.072	Detected	1.244	0.019
A 96 P22	Sense	0.058	Detected	-0.058	Detected	0.000	2.086	Detected	2.026	Detected	2.056	0.001
A 96 P22	Sense	0.081	Detected	-0.081	Detected	0.000	1.004	Detected	1.065	Detected	1.035	0.007
A 96 P22	Sense	-0.080	Detected	0.080	Detected	0.000	1.009	Detected	1.150	Detected	1.080	0.010
A 96 P22	Sense	0.219	Detected	-0.219	Detected	0.000	1.497	Detected	1.482	Detected	1.490	0.021
A 96 P22	Sense	0.138	Detected	-0.138	Detected	0.000	1.854	Detected	1.558	Detected	1.706	0.014
A 96 P22	Sense	0.124	Detected	-0.124	Detected	0.000	1.418	Detected	1.081	Detected	1.249	0.027
A 96 P22	Sense	0.235	Comprom	-0.235	Comprom	0.000	1.866	Detected	1.808	Detected	1.837	0.016
A 96 P22	Sense	-0.030	Detected	0.030	Detected	0.000	1.750	Detected	1.519	Detected	1.634	0.005
A 96 P22	Sense	-0.471	Detected	0.471	Detected	0.000	1.517	Detected	1.372	Detected	1.445	0.094
A 96 P22	Sense	-0.039	Detected	0.039	Detected	0.000	1.454	Detected	1.025	Detected	1.240	0.030
A 96 P22	Sense	-0.188	Detected	0.188	Detected	0.000	1.291	Detected	1.213	Detected	1.252	0.023
A 96 P23	Sense	0.213	Detected	-0.213	Detected	0.000	1.382	Detected	0.909	Detected	1.145	0.069
A 96 P23	Sense	-0.128	Detected	0.128	Detected	0.000	1.099	Detected	0.969	Detected	1.034	0.019
A 96 P23	Sense	-0.043	Detected	0.043	Detected	0.000	1.198	Detected	0.916	Detected	1.057	0.019
A 96 P23	Sense	0.356	Detected	-0.356	Detected	0.000	1.128	Detected	0.876	Detected	1.002	0.118
A 96 P23	Sense	0.283	Detected	-0.283	Detected	0.000	2.025	Detected	1.905	Detected	1.965	0.021
A 96 P23	Sense	-0.008	Detected	0.008	Detected	0.000	1.153	Detected	1.074	Detected	1.113	0.001
A 96 P23	Sense	-0.256	Comprom	0.256	Comprom	0.000	1.934	Detected	1.689	Detected	1.812	0.024
A 96 P23	Sense	0.045	Detected	-0.045	Detected	0.000	1.240	Detected	0.960	Detected	1.100	0.017
A 96 P23	Sense	0.131	Detected	-0.131	Detected	0.000	1.606	Detected	1.583	Detected	1.594	0.007
A 96 P24	Sense	-0.194	Comprom	0.194	Comprom	0.000	1.986	Detected	1.293	Detected	1.639	0.054
A 96 P24	Sense	0.506	Detected	-0.506	Detected	0.000	1.253	Detected	0.940	Detected	1.097	0.174
A 96 P25	Sense	-0.101	Detected	0.101	Detected	0.000	1.497	Detected	0.832	Detected	1.165	0.079
A 96 P25	Sense	0.147	Detected	-0.147	Detected	0.000	1.572	Detected	1.351	Detected	1.461	0.015
A 96 P25	Sense	-0.065	Detected	0.065	Detected	0.000	1.299	Detected	0.849	Detected	1.074	0.044
A 96 P25	Sense	-0.270	Detected	0.270	Detected	0.000	1.699	Detected	1.119	Detected	1.409	0.071
A 96 P25	Sense	-0.030	Detected	0.030	Comprom	0.000	2.054	Detected	0.913	Detected	1.484	0.122
A 96 P25	Sense	-0.291	Detected	0.291	Detected	0.000	1.769	Detected	1.372	Detected	1.570	0.047
A 96 P26	Sense	-0.231	Detected	0.231	Detected	0.000	2.110	Detected	1.756	Detected	1.933	0.022
GT Sense	Sense	-0.646	Comprom	0.646	Detected	0.000	1.741	Detected	1.712	Detected	1.727	0.116
GT Sense	Sense	-0.339	Comprom	0.339	Comprom	0.000	1.496	Detected	1.774	Detected	1.635	0.047
GT Sense	Sense	0.075	Detected	-0.075	Detected	0.000	1.052	Detected	0.975	Detected	1.014	0.007
GT Sense	Sense	-0.646	Detected	0.646	Detected	0.000	0.973	Detected	1.144	Detected	1.058	0.246
GT Sense	Sense	-0.240	Detected	0.240	Detected	0.000	1.150	Detected	0.989	Detected	1.069	0.052
GT Sense	Sense	0.396	Detected	-0.396	Comprom	0.000	0.991	Detected	1.083	Detected	1.037	0.122
GT Sense	Sense	0.110	Detected	-0.110	Detected	0.000	1.104	Detected	1.266	Detected	1.185	0.013
GT Sense	Sense	-0.159	Detected	0.159	Detected	0.000	1.061	Detected	1.450	Detected	1.255	0.038
GT Sense	Sense	0.396	Comprom	-0.396	Comprom	0.000	1.273	Detected	1.089	Detected	1.181	0.101
GT Sense	Sense	0.199	Detected	-0.199	Detected	0.000	1.085	Detected	1.216	Detected	1.151	0.032
GT Sense	Sense	0.724	Comprom	-0.724	Comprom	0.000	1.517	Detected	1.456	Detected	1.486	0.177
GT Sense	Sense	0.431	Detected	-0.431	Comprom	0.000	0.926	Detected	1.512	Detected	1.219	0.144
GT Sense	Sense	-0.788	Detected	0.788	Detected	0.000	1.083	Detected	1.143	Detected	1.113	0.293
GT Sense	Sense	0.723	Comprom	-0.723	Comprom	0.000	0.991	Detected	1.345	Detected	1.168	0.257
GT Sense	Sense	0.503	Detected	-0.503	Detected	0.000	1.310	Detected	2.213	Detected	1.761	0.121
GT Sense	Sense	0.682	Detected	-0.682	Detected	0.000	1.062	Detected	1.055	Detected	1.059	0.261
GT Sense	Sense	0.075	Detected	-0.075	Detected	0.000	1.681	Detected	1.684	Detected	1.683	0.002
GT Sense	Sense	-0.119	Detected	0.119	Detected	0.000	1.039	Detected	1.431	Detected	1.235	0.033
GT Sense	Sense	0.033	Detected	-0.033	Detected	0.000	1.272	Detected	1.028	Detected	1.150	0.012
GT Sense	Sense	0.370	Detected	-0.370	Detected	0.000	1.104	Detected	1.090	Detected	1.097	0.097
GT Sense	Sense	0.483	Detected	-0.483	Detected	0.000	1.575	Detected	2.684	Detected	2.130	0.101
GT Sense	Sense	0.589	Detected	-0.589	Detected	0.000	1.169	Detected	0.847	Detected	1.008	0.241
GT Sense	Sense	0.060	Detected	-0.060	Detected	0.000	1.380	Detected	1.047	Detected	1.214	0.021
GT Sense	Sense	0.378	Detected	-0.378	Detected	0.000	1.139	Detected	1.489	Detected	1.314	0.088
GT Sense	Sense	0.242	Comprom	-0.242	Comprom	0.000	2.049	Detected	1.355	Detected	1.702	0.057
GT Sense	Sense	-0.069	Detected	0.069	Detected	0.000	1.169	Detected	0.861	Detected	1.015	0.026
GT Sense	Sense	0.281	Detected	-0.281	Detected	0.000	1.558	Detected	1.222	Detected	1.390	0.051
GT Sense	Sense	-0.589	Comprom	0.589	Comprom	0.000	2.553	Detected	1.236	Detected	1.895	0.165
GT Sense	Sense	0.513	Comprom	-0.513	Comprom	0.000	1.264	Detected	1.471	Detected	1.368	0.120
GT Sense	Sense	0.672	Detected	-0.672	Detected	0.000	1.328	Detected	0.920	Detected	1.124	0.251
GT Sense	Sense	-0.424	Detected	0.424	Detected	0.000	1.273	Detected	0.968	Detected	1.121	0.131
GT Sense	Sense	-0.070	Detected	0.070	Detected	0.000	1.150	Detected	1.033	Detected	1.091	0.007
GT Sense	Sense	0.236	Detected	-0.236	Detected	0.000	1.181	Detected	1.252	Detected	1.216	0.036
GT Sense	Sense	-0.166	Comprom	0.166	Comprom	0.000	2.382	Detected	1.698	Detected	2.040	0.033
GT Sense	Sense	0.142	Detected	-0.142	Detected	0.000	2.523	Detected	1.824	Detected	2.173	0.029
GT Sense	Sense	-0.634	Detected	0.634	Detected	0.000	1.054	Detected	1.221	Detected	1.137	0.217
GT Sense	Sense	0.095	Comprom	-0.095	Comprom	0.000	1.992	Detected	1.337	Detected	1.664	0.040
GT Sense	Sense	-0.032	Detected	0.032	Detected	0.000	1.552	Detected	1.208	Detected	1.380	0.016
GT Sense	Sense	0.322	Detected	-0.322	Detected	0.000	1.870	Detected	1.437	Detected	1.654	0.051
GT Sense	Sense	0.048	Detected	-0.048	Detected	0.000	1.130	Detected	0.971	Detected	1.051	0.008
GT Sense	Sense	0.545	Detected	-0.545	Detected	0.000	1.279	Detected	1.385	Detected	1.332	0.136
GT Sense	Sense	0.816	Detected	-0.816	Comprom	0.000	1.537	Detected	1.728	Detected	1.632	0.185

ProbeNar	GeneNam	Systemat	Descripti	Sequence	Contig_ID	ITAG_ID	Parent Ge	ITAG Des	Ontology	Ontology	Contig ID	Target ID	Functiona	GO_terms	Transcrip	target_ID	Symbols	TAIR Des	TAIR Chr	gProcess	gProcess	gProcess	gProcess	gProcess	gProcessedSignal_8h_42_AZ
A 96 P0	TA37220	TA37220	Rep: Chr	GGAAAGCATTATACCTCTTTGGGAGGATCCTTTTCAGAAGCCCAGTTATCTTTTTGCATT																45.2336	50.5685	76.6549	73.6232	96.5485	106.768
A 96 P0	Dea1	NM_0012	Solanum	CTTATGAGCAATGTTCAAGTGTGTGGTAACTTCTTGTTTCCTTTGTAGCTTGAAATAAGT																79.2574	127.058	64.7581	137.866	1602.47	327.081
A 96 P0	BI933609	BI933609	EST5534	AGGCGTGGGCTCTACTCCACAAATTCGGTGGACATGAACAATTCTTATCGAATGAGCTAA																308.173	231.049	540.909	526.934	859.347	555.647
A 96 P0	AK24756	AK24756	Solanum	TTTTCCAGGAATTCTCCATATATAACATCCACTATTCTCTGGGTGTACTGGGGAGTTTGC																18.6288	27.6147	43.0165	51.1505	76.2648	56.1033
A 96 P0	BI933865	BI933865	EST5537	TGGACAGTACCTCAATTCAGATGATGATTTCTTCAAAAACTCAGTTGTTAAGGGCTATTA																1074.67	632.989	1165.82	972	2129.11	2069.95
A 96 P0	AK31964	AK31964	Solanum	CTCAGTAACTTCATACACACTGGGGCTCTTCATGATTGCCAAAGCTCCATGGTATCTACT																1341.92	1155.46	2212.86	2609.46	3695.43	3965
A 96 P01	TA37845	TA37845	Rep: Chl	ATCTGGCTTTCCCACTTATTTTGATTGGTGGTCTTTTCCTGCTATCAAGGCGGTCTAACG																570.21	459.916	883.58	1107.46	1653.97	1594.56
A 96 P01	LOC1007	NM_0012	Solanum	CAAGGTGTGGTTATTCTGAACGTTTGTTGGTGTATTACTGATGAATTTAGTGGATGTAAT																623.08	420.24	1505.8	1015.6	1617.68	1020.18
A 96 P01	LOC5438	NM_0012	Solanum	AATAATGATAGAGTAAGGAGGATGTCAATTCATGATCCCACACCCACCTACTGTAGGTAG																515.507	471.584	713.478	529.012	1514.41	1009.11
A 96 P02	BI933568	BI933568	EST5534	TCTCACATTTGTTAGGGTATATTCAGGAAAGCTTAGTGCAGGATCGTATGTATTGAATGG																260.71	227.016	567.464	726.186	742.756	619.675
A 96 P02	BI933475	BI933475	EST5533	CAGGTATGGGATGTCACAAGAAGTCAGCTTTTCATGGAGATGAGAGAGCATGAGAAACGT																373.002	247.994	563.866	627.627	796.279	703.214
A 96 P02	AK32538	AK32538	Solanum	TTGGTACCTGCTGATCACATTAGCAGGAGTTACAATGACACATATTATGTGGATTCTCAA																27.5526	20.9537	45.913	43.0429	65.3506	51.641
A 96 P02	BI923464	BI923464	EST5433	ATCCTCTTGGTCCCGTTTCTCAGGCAGATGATGATCTTGGTTTTCTTGATAGTAATCATG																149.475	65.2555	234.372	248.18	247.813	272.705
A 96 P02	AK32364	AK32364	Solanum	CCTGATAATGATCCTGATAGTGATATTCCTCCTGATGACAAGAATTTGCTTAAAAATAGG																108.324	94.4508	201.972	229.17	253.681	211.464
A 96 P02	AK32130	AK32130	Solanum	CAACATAATCGCTGTTTCTAGTTGCAAGGGAGGGGTTGGTAAATCAACTGTAGCTGTCAA																142.145	137.105	269.588	324.128	450.655	314.238
A 96 P02	DB71789	DB71789	DB71789	AAGGACTGTGGTTGTTGCAACAAAGCTTGATACCAAAATACCTCAGTTTGCACGAGCTTC																5.25628	5.20062	16.1839	18.3827	20.0415	17.8029
A 96 P02	AK32154	AK32154	Solanum	AACTTCTTCAAGAAATCATTTCACGTGCACCAACTGCGCATGATAGCGCTTGCTCACGTT																36.8381	22.13	68.113	126.608	226.493	109.656
A 96 P02	BG12753	BG12753	EST4732	GAAAGAATGGAAAGTAATGGATGATTCTTGTCCTCATAGATATGCTCCACTTTCTGAAGG																221.894	181.501	354.759	459.839	623.013	478.592

GT	Sense	Sense	-0.435	Detected	0.435	Detected	0.000	2.316	Detected	0.901	Detected	1.608	0.192	GT	Sens	contig116	contig116	Solute ca	CAATGC	contig116	Solyc01g	Solyc01g	Solute ca	GO:0016	GO:0016	contig116	Solyc01g	Solute ca	GO:0016	SL2.40ch	AT3G19940.1		sugar tra	chr3:693	21.5983	30.8442	13.3753	65.8284	135.803	49.1646
GT	Sense	Sense	0.269	Detected	-0.269	Detected	0.000	1.626	Detected	1.499	Detected	1.562	0.030	GT	Sens	contig117	contig117	Late elon	TTTAAG	contig117	Solyc10g	Solyc10g	Late elongated hypocotyl and			contig117	Solyc10g	Late elon	GO:0005	SL2.40ch	AT2G468	CCA1	CCA1 (CI	chr2:192	11231.9	6048.89	15724.2	17988.4	26879.6	23772.4
GT	Sense	Sense	-0.105	Detected	0.105	Detected	0.000	1.705	Detected	1.524	Detected	1.614	0.007	GT	Sens	contig117	contig117	IBR finge	TATTTAC	contig117	Solyc06g	Solyc06g	IBR finge	GO:0008	GO:0008	contig117	Solyc06g	IBR finge	GO:0008	SL2.40ch	AT4G19670.2		zinc fing	chr4:106	56.4161	51.0359	175.595	102.765	184.866	157.448
GT	Sense	Sense	0.305	Detected	-0.305	Detected	0.000	1.532	Detected	1.153	Detected	1.343	0.065	GT	Sens	contig117	contig117	Sodium/c	TAATAGT	contig117	Solyc03g	Solyc03g	Sodium/c	GO:0005	GO:0005	contig117	Solyc03g	Sodium/c	GO:0005	SL2.40ch	AT5G27440.1		unknown	chr5:968	42.7521	21.9068	62.1344	58.9817	93.5506	69.4562
GT	Sense	Sense	-0.215	Comprom	0.215	Comprom	0.000	1.526	Detected	1.649	Detected	1.588	0.019	GT	Sens	contig118	contig118	Cell morp	TTTCTGT	contig118	Solyc02g	Solyc02g	Cell morphogenesis protein (A			contig118	Solyc02g	Cell morphogenesis		SL2.40ch	AT5G15680.1		binding	chr5:510	9.91064	10.4408	25.1871	24.9353	30.9677	32.5486
GT	Sense	Sense	0.428	Detected	-0.428	Comprom	0.000	1.634	Detected	0.988	Detected	1.311	0.135	GT	Sens	contig119	contig119	Unknown	GGCATG	contig119	Solyc06g	Solyc06g	Unknown Protein (AHRD V1)			contig119	Solyc06g	Unknown Protein (A		SL2.40ch	AT5G22760.1		PHD fing	chr5:757	12.9719	5.59955	11.3315	16.834	27.9535	17.2424
GT	Sense	Sense	0.052	Detected	-0.052	Detected	0.000	1.381	Detected	1.331	Detected	1.356	0.002	GT	Sens	contig120	contig120	Cytochro	CACATC	contig120	Solyc04g	Solyc04g	Cytochro	GO:0019	GO:0019	contig120	Solyc04g	Cytochro	GO:0019	SL2.40ch	AT5G453	CYP707A	CYP707A	chr5:183	86.551	62.9491	77.7134	170.695	203.132	189.453
GT	Sense	Sense	-0.149	Detected	0.149	Detected	0.000	1.496	Detected	1.589	Detected	1.542	0.010	GT	Sens	contig120	contig120	Unknown	GCTTGT	contig120	Solyc05g	Solyc05g	Unknown Protein (AHRD V1)			contig120	Solyc05g	Unknown Protein (A		SL2.40ch	AT2G313	emb1381	emb1381	chr2:133	33.6497	32.3468	69.985	67.7276	98.3402	101.248
GT	Sense	Sense	0.159	Detected	-0.159	Detected	0.000	1.241	Detected	1.039	Detected	1.140	0.026	GT	Sens	contig120	contig120	Unknown	AATTTG	contig120	Solyc05g	Solyc05g	Unknown Protein (AHRD V1)			contig120	Solyc05g	Unknown Protein (A		SL2.40ch	AT1G27270.1				62.6114	39.2598	84.903	143.529	123.837	103.976
GT	Sense	Sense	0.012	Detected	-0.012	Detected	0.000	1.502	Detected	1.254	Detected	1.378	0.008	GT	Sens	contig120	contig120	Magnesiu	GAGTGA	contig120	Solyc10g	Solyc10g	Magnesiu	GO:0048	GO:0048	contig120	Solyc10g	Magnesiu	GO:0003	SL2.40ch	AT3G569	CRD1, CH	CRD1 (C	chr3:210	7213.15	5546.92	12254.5	13258.4	18933	15388.5
GT	Sense	Sense	-0.038	Detected	0.038	Detected	0.000	1.018	Detected	1.001	Detected	1.010	0.001	GT	Sens	contig121	contig121	RNA ligas	TTTCTT	contig121	Solyc11g	Solyc11g	RNA ligase isoform 2 (Fragmen			contig121	Solyc11g	RNA ligas	GO:0004	SL2.40ch	AT1G079	ATRNL, R	RNL (RN	chr1:244	334.504	275.582	469.026	432.088	650.024	620.145
GT	Sense	Sense	0.577	Comprom	-0.577	Comprom	0.000	2.306	Detected	1.658	Detected	1.982	0.096	GT	Sens	contig122	contig122	Pentatric	GAGGTT	contig122	Solyc01g	Solyc01g	Pentatricopeptide repeat-conta			contig122	Solyc01g	Pentatricopeptide re		SL2.40ch	AT4G39530.1		pentatric	chr4:183	6.28273	2.20551	10.4506	10.2219	19.4499	11.9892
GT	Sense	Sense	-0.255	Comprom	0.255	Comprom	0.000	1.036	Detected	0.986	Detected	1.011	0.059	GT	Sens	contig122	contig122	Os01g09	AAGGAT	contig122	Solyc09g	Solyc09g	Os01g09	GO:0003	GO:0003	contig122	Solyc09g	Os01g09	GO:0003	SL2.40ch	AT3G07210.1		unknown	chr3:229	7.26201	8.08061	17.5211	13.3056	16.6003	15.4822
GT	Sense	Sense	0.036	Detected	-0.036	Detected	0.000	1.755	Detected	1.470	Detected	1.613	0.008	GT	Sens	contig122	contig122	Uncharac	CCTTGT	contig122	Solyc02g	Solyc02g	Uncharac	GO:0016	GO:0016	contig122	Solyc02g	Uncharac	GO:0016	SL2.40ch	AT1G11390.1		ABC1 far	chr1:383	102.808	76.5005	234.584	249.305	316.405	250.681
GT	Sense	Sense	0.243	Detected	-0.243	Detected	0.000	1.651	Detected	1.597	Detected	1.624	0.022	GT	Sens	contig123	contig123	Os01g049	TTGGCT	contig123	Solyc04g	Solyc04g	Os01g0498200 protein (Fragm			contig123	Solyc04g	Os01g0498200 prot		SL2.40ch	AT3G02860.2		zinc ion	chr3:625	1362.61	760.175	2377.31	2425.58	3378.8	3141.83
GT	Sense	Sense	0.225	Detected	-0.225	Detected	0.000	1.035	Detected	1.229	Detected	1.132	0.044	GT	Sens	contig123	contig123	Aldose-1-	TATGCA	contig123	Solyc02g	Solyc02g	Aldose-1-	GO:0005	GO:0005	contig123	Solyc02g	Aldose-1-	GO:0005	SL2.40ch	AT5G551	CNX5, SIR	CNX5 (C	chr5:223	96.2581	55.0537	90.3524	114.027	157.66	174.107
GT	Sense	Sense	0.886	Detected	-0.886	Detected	0.000	1.199	Detected	1.183	Detected	1.191	0.311	GT	Sens	contig123	contig123	Nodulin-li	CAATCG	contig123	Solyc09g	Solyc09g	Nodulin-li	GO:0016	GO:0016	contig123	Solyc09g	Nodulin-li	GO:0016	SL2.40ch	AT2G37460.1		nodulin M	chr2:157	787.732	180.409	563.512	621.852	914.78	873.582
GT	Sense	Sense	-0.459	Detected	0.459	Detected	0.000	1.389	Detected	0.819	Detected	1.104	0.178	GT	Sens	contig123	contig123	Mvb fami	GTTGCA	contig123	Solyc07g	Solyc07g	Mvb fami	GO:0003	GO:0003	contig123	Solyc07g	Mvb famil	GO:0005	SL2.40ch	AT1G566	PAP1, MY	PAP1 (PF	chr1:212	15.2452	22.5012	37.7494	22.6536	51.2646	33.337
GT	Sense	Sense	-0.694	Detected	0.694	Detected	0.000	1.340	Detected	0.852	Detected	1.096	0.275	GT	Sens	contig123	contig123	Histidine	ACTAGC	contig123	Solyc01g	Solyc01g	Histidine	GO:0009	GO:0009	contig123	Solyc01g	Histidine	GO:0009	SL2.40ch	AT3G215	AHP1	AHP1 (HI	chr3:757	187.099	382.655	796.675	740.796	715.737	492.957
GT	Sense	Sense	-0.488	Detected	0.488	Detected	0.000	1.203	Detected	0.924	Detected	1.064	0.171	GT	Sens	contig123	contig123	Transcrip	AATTGT	contig123	Solyc03g	Solyc03g	Transcrip	GO:0005	GO:0005	contig123	Solyc03g	Transcrip	GO:0005	SL2.40ch	AT4G365	BEE2	BEE2 (BI	chr4:172	18.2177	28.0095	49.0705	56.6681	54.9676	43.7304
GT	Sense	Sense	0.433	Detected	-0.433	Detected	0.000	1.850	Detected	1.880	Detected	1.865	0.050	GT	Sens	contig124	contig124	Unknown	CACGAA	contig124	Solyc05g	Solyc05g	Unknown Protein (AHRD V1)			contig124	Solyc05g	Unknown Protein (A		SL2.40ch	ATCG006	PSBB	encodes	chrC:723	1118.65	479.493	1733.95	2256.1	2789.71	2751.01
GT	Sense	Sense	0.175	Detected	-0.175	Detected	0.000	1.489	Detected	1.791	Detected	1.640	0.019	GT	Sens	contig125	contig125	Acidic ch	ATAGCA	contig125	Solyc01g	Solyc01g	Acidic ch	GO:0005	GO:0005	contig125	Solyc01g	Acidic ch	GO:0005	SL2.40ch	AT5G24090.1		acidic en	chr5:814	20.9435	12.8397	19.8932	12.6679	48.6483	57.89
GT	Sense	Sense	-0.059	Detected	0.059	Detected	0.000	1.210	Detected	0.908	Detected	1.059	0.023	GT	Sens	contig125	contig125	Cc-nbs-lr	ATTGCA	contig125	Solyc10g	Solyc10g	Cc-nbs-lr	GO:0006	GO:0006	contig125	Solyc10g	Cc-nbs-lr	GO:0005	SL2.40ch	AT5G11890.1		FUNCTIO	chr5:383	235.91	200.136	309.442	310.867	531.23	416.086
GT	Sense	Sense	0.371	Detected	-0.371	Detected	0.000	2.226	Detected	1.807	Detected	2.016	0.042	GT	Sens	contig125	contig125	CONSTAI	AAGATG	contig125	Solyc12g	Solyc12g	CONSTAI	GO:0005	GO:0005	contig125	Solyc12g	CONSTAI	GO:0005	SL2.40ch	AT2G17740.1				1244.67	582.052	1560.84	1383.32	4207.75	3038.69
GT	Sense	Sense	0.175	Detected	-0.175	Detected	0.000	1.224	Detected	1.302	Detected	1.263	0.020	GT	Sens	contig127	contig127	RING fing	ACAAGT	contig127	Solyc12g	Solyc12g	RING fing	GO:0008	GO:0008	contig127	Solyc12g	RING fing	GO:0008	SL2.40ch	AT4G30400.1		zinc fing	chr4:148	2685.37	1646.26	3881.36	5795.41	5190.09	5290.11
GT	Sense	Sense	-0.118	Detected	0.118	Detected	0.000	1.443	Detected	1.144	Detected	1.294	0.021	GT	Sens	contig127	contig127	Photosys	AGAGGC	contig127	Solyc06g	Solyc06g	Photosys	GO:0009	GO:0009	contig127	Solyc06g	Photosys	GO:0009	SL2.40ch	AT2G202	PSAE-2	PSAE-2 (	chr2:873	1724.07	1587.31	2682.42	2932.8	4754.28	3730.32
GT	Sense	Sense	-0.509	Detected	0.509	Detected	0.000	1.808	Detected	1.162	Detected	1.485	0.133	GT	Sens	contig128	contig128	Strictosid	AATTTG	contig128	Solyc07g	Solyc07g	Strictosid	GO:0004	GO:0004	contig128	Solyc07g	Strictosid	GO:0004	SL2.40ch	AT3G57030.1		strictosid	chr3:211	55.9205	88.5725	140.67	100.128	260.422	160.735
GT	Sense	Sense	0.708	Detected	-0.708	Comprom	0.000	1.285	Detected	1.350	Detected	1.318	0.204	GT	Sens	contig128	contig128	Targeting	TATCAA	contig128	Solyc07g	Solyc07g	Targeting	GO:0008	GO:0008	contig128	Solyc07g	Targeting	GO:0008	SL2.40ch	AT1G037	TPX2	targeting	chr1:947	13.0058	3.80826	22.278	17.5005	18.1302	18.3111
GT	Sense	Sense	-0.163	Detected	0.163	Detected	0.000	1.346	Detected	1.022	Detected	1.184	0.036	GT	Sens	contig129	contig129	Protein ki	AAACTT	contig129	Solyc10g	Solyc10g	Protein ki	GO:0006	GO:0006	contig129	Solyc10g	Protein ki	GO:0006	SL2.40ch	AT5G019	STN8	STN8: ki	chr5:359	504.584	494.314	842.025	994.199	1342.17	1035.1
GT	Sense	Sense	0.519	Detected	-0.519	Detected	0.000	1.271	Detected	1.406	Detected	1.339	0.125	GT	Sens	contig129	contig129	Unknown	GAAGAG	contig129	Solyc07g	Solyc07g	Unknown Protein (AHRD V1)			contig129	Solyc07g	Unknown Protein (A		SL2.40ch	AT5G56120.1		unknown	chr5:227	124.672	47.4456	255.194	156.368	196.209	207.963
GT	Sense	Sense	-0.348	Detected	0.348	Detected	0.000	1.225	Detected	1.263	Detected	1.244	0.070	GT	Sens	contig129	contig129	pH-respo	TTATAG	contig129	Solyc04g	Solyc04g	pH-response regulator protein			contig129	Solyc04g	pH-response regula		SL2.40ch	AT1G15130.1		hydroxyl	chr1:520	174.97	221.463	367.774	309.053	486.145	482.067
GT	Sense	Sense	-0.029	Detected	0.029	Detected	0.000	0.964	Detected	1.194	Detected	1.079	0.012	GT	Sens	contig129	contig129	Ubiquilin-	TCTCCT	contig129	Solyc02g	Solyc02g	Ubiquilin-	GO:0019	GO:0019	contig129	Solyc02g	Ubiquilin-	GO:0019	SL2.40ch	AT2G17200.1		ubiquitin	chr2:748	204.325	166.33	319.381	267.298	379.974	430.256
GT	Sense	Sense	0.224	Detected	-0.224	Detected	0.000	1.378	Detected	0.895	Detected	1.136	0.075	GT	Sens	contig130	contig130	Os01g061	ATCATG	contig130	Solyc08g	Solyc08g	Os01g0611000 protein (Fragm			contig130	Solyc08g	Os01g0611000 prot		SL2.40ch	AT4G32460.2		FUNCTIO	chr4:156	10249.2	5868.82	16216.5	13667.9	21302.1	14712.3
GT	Sense	Sense	-0.510	Detected	0.510	Detected	0.000	1.312	Detected	1.569	Detected	1.441	0.111	GT	Sens	contig130	contig130	COBRA-li	AACACT	contig130	Solyc03g	Solyc03g	COBRA-like protein (AHRD V1			contig130	Solyc03g	COBRA-like protein		SL2.40ch	AT5G609	COB	COB (CO	chr5:245	262.852	416.37	362.699	413.84	867.914	1001.81
GT	Sense	Sense	-0.360	Detected	0.360	Detected	0.000	1.147	Detected	1.326	Detected	1.236	0.080	GT	Sens	contig131	contig131	Nucleopo	ATTGAA	contig131	Solyc07g	Solyc07g	Nucleoporin Nup43 (AHRD V1			contig131	Solyc07g	Nucleoporin Nup43		SL2.40ch	AT4G30840.1		WD-40 re	chr4:150	333.021	429.036	638.758	553.082	884.727	966.832
GT	Sense	Sense	0.056	Detected	-0.056	Detected	0.000	1.230	Detected	1.153	Detected	1.192	0.003	GT	Sens	contig131	contig131	GATA tra	GACTCT	contig131	Solyc05g	Solyc05g	GATA tra	GO:0006	GO:0006	contig131	Solyc05g	GATA tra	GO:0005	SL2.40ch	AT3G548	BME3-ZF	zinc fing	chr3:202	572.068	414.006	780.662	747.276	1206.57	1104.18
GT	Sense	Sense	-0.001	Detected	0.001	Detected	0.000	1.360	Detected	0.830	Detected	1.095	0.054	GT	Sens	contig131	contig131	Acid phos	CAAGGT	contig131	Solyc03g	Solyc03g	Acid phos	GO:0016	GO:0016	contig131	Solyc03g	Acid phos	GO:0004	SL2.40ch	AT2G018	PAP7, AT	PAP7 (PU	chr2:391	40.956	32.0532	38.2699	36.6055	98.3239	65.7198
GT	Sense	Sense	0.318	Detected	-0.318	Comprom	0.000	1.645	Detected	0.991	Detected	1.318	0.102	GT	Sens	contig132	contig132	Mitochon	CAAGCT	contig132	Solyc10g	Solyc10g	Mitochon	GO:0005	GO:0005	contig132	Solyc10g	Mitochon	GO:0005	SL2.40ch	AT3G570	BIGYIN, F	BIGYIN:	chr3:211	13.5383	6.80814	21.5715	24.6332	31.7308	19.4694
GT	Sense	Sense	-0.039	Detected	0.039	Detected	0.000	1.151	Detected	1.114	Detected	1.133	0.001	GT	Sens	contig132	contig132	Fructokin	AGATCA	contig132	Solyc05g	Solyc05g	Fructokinase-like protein 2 (AH			contig132	Solyc05g	Fructokinase-like pr		SL2.40ch	AT1G69200.1		kinase	chr1:260	215.35	177.792	363.365	318.726	459.321	432.21
GT	Sense	Sense	-0.242	Detected	0.242	Detected	0.000	1.102	Detected	0.908	Detected	1.005	0.061	GT	Sens	contig132	contig132	LRR rece	GATTTA	contig132	Solyc01g	Solyc01g	LRR rece	GO:0005	GO:0005	contig132	Solyc01g	LRR rece	GO:0004	SL2.40ch	AT1G192	FMO1	FMO1 (FI	chr1:665	431.156	471.109	993.836	841.628	1022.36	862.87
GT	Sense	Sense	0.047	Detected	-0.047	Detected	0.000	1.392	Detected	0.921	Detected	1.157	0.041	GT	Sens	contig132	contig132	Thioredo	AATCCA	contig132	Solyc00g	Solyc00g	Thioredo	GO:0045	GO:0045	contig132	Solyc00g	Thioredo	GO:0016	SL2.40ch	AT4G296	ACHT2	thioredo	chr4:145	1626.18	1190.59	2351.78	3143.62	3860.63	2687.98
GT	Sense	Sense	0.044	Detected	-0.044	Detected	0.000	1.231	Detected	1.044	Detected	1.137	0.008	GT	Sens	contig133	contig133	ABC tran	TTGTTC	contig133	Solyc01g	Solyc01g	ABC tran	GO:0016	GO:0016	contig133	Solyc01g	ABC tran	GO:0008	SL2.40ch	AT3G52310.1		ABC tran	chr3:193	14123.8	10381.3	16710.2	19714.2	30030.6	25471.3
GT	Sense	Sense	0.226	Detected	-0.226	Detected	0.000	1.359	Detected	0.958	Detected	1.159	0.062	GT	Sens	contig134	contig134	Glucan e	ACACCA	contig134	Solyc12g	Solyc12g	Glucan endo-1 3-beta-glucosid			contig134	Solyc12g	Glucan endo-1 3-be		SL2.40ch	AT4G13600.1		EXPRES	chr4:791	43.4674	24.8483	63.7882	58.3464	89.1032	65.1403
GT	Sense	Sense	0.050	Detected	-0.050	Detected	0.000	1.917	Detected	1.878	Detected	1.897	0.001	GT	Sens	contig135	contig135	WD-40 re	TAAACC	contig135	Solyc11g	Solyc11g	WD-40 re	GO:0008	GO:0008	contig135	Solyc11g	WD-40 re	GO:0008	SL2.40ch	AT2G329	COP1, AT	COP1 (C	chr2:139	99.6107	72.6608	208.521	268.176	339.575	318.976
GT	Sense	Sense	-0.115	Comprom	0.115	Comprom	0.000	1.526	Detected	1.360	Detected	1.443	0.010	GT	Sens	contig135	contig135	Long-cha	GGGGAT	contig135	Solyc08g	Solyc08g	Long-cha	GO:0008	GO:0008	contig135	Solyc08g	Long-cha	GO:0004	SL2.40ch	AT4G11030.1		long-cha	chr4:673	4.32911	3.971	14.1125	14.9967	12.6174	10.8602

[illegible]

GT	Sense	Sense	-0.056	Detected	0.056	Detected	0.000	1.272	Detected	0.927	Detected	1.100	0.026	GT	Sens	[illegible]
GT	Sense	Sense	0.156	Detected	-0.156	Detected	0.000	1.568	Detected	1.000	Detected	1.284	0.058	GT	Sens	[illegible]
GT	Sense	Sense	0.276	Detected	-0.276	Detected	0.000	1.199	Detected	1.248	Detected	1.223	0.048	GT	Sens	[illegible]
GT	Sense	Sense	0.244	Detected	-0.244	Detected	0.000	1.121	Detected	1.087	Detected	1.104	0.046	GT	Sens	[illegible]
GT	Sense	Sense	0.131	Detected	-0.131	Detected	0.000	1.444	Detected	1.239	Detected	1.342	0.015	GT	Sens	[illegible]
GT	Sense	Sense	0.040	Detected	-0.040	Detected	0.000	1.579	Detected	1.092	Detected	1.336	0.032	GT	Sens	[illegible]
GT	Sense	Sense	0.011	Detected	-0.011	Detected	0.000	1.335	Detected	0.867	Detected	1.101	0.043	GT	Sens	[illegible]
GT	Sense	Sense	-1.921	Comprom	1.921	Detected	0.000	1.495	Detected	1.845	Detected	1.670	0.478	GT	Sens	[illegible]
GT	Sense	Sense	0.148	Detected	-0.148	Detected	0.000	1.303	Detected	0.858	Detected	1.080	0.056	GT	Sens	[illegible]
GT	Sense	Sense	0.010	Detected	-0.010	Detected	0.000	1.388	Detected	1.085	Detected	1.236	0.015	GT	Sens	[illegible]
GT	Sense	Sense	-0.245	Detected	0.245	Detected	0.000	1.067	Detected	1.149	Detected	1.108	0.047	GT	Sens	[illegible]
GT	Sense	Sense	-0.173	Comprom	0.173	Comprom	0.000	1.151	Detected	1.047	Detected	1.099	0.026	GT	Sens	[illegible]
GT	Sense	Sense	-0.390	Comprom	0.390	Comprom	0.000	4.203	Detected	2.520	Detected	3.362	0.068	GT	Sens	[illegible]
GT	Sense	Sense	0.111	Detected	-0.111	Detected	0.000	1.760	Detected	1.722	Detected	1.741	0.004	GT	Sens	[illegible]
GT	Sense	Sense	-0.058	Detected	0.058	Detected	0.000	1.289	Detected	1.068	Detected	1.178	0.011	GT	Sens	[illegible]
GT	Sense	Sense	-1.683	Detected	1.683	Detected	0.000	1.449	Detected	1.797	Detected	1.623	0.439	GT	Sens	[illegible]
GT	Sense	Sense	-0.054	Detected	0.054	Detected	0.000	1.657	Detected	1.348	Detected	1.502	0.012	GT	Sens	[illegible]
GT	Sense	Sense	-0.753	Comprom	0.753	Comprom	0.000	1.167	Detected	1.477	Detected	1.322	0.228	GT	Sens	[illegible]
GT	Sense	Sense	0.586	Detected	-0.586	Comprom	0.000	0.916	Detected	1.086	Detected	1.001	0.233	GT	Sens	[illegible]
GT	Sense	Sense	-0.087	Detected	0.087	Detected	0.000	2.431	Detected	1.059	Detected	1.745	0.128	GT	Sens	[illegible]
GT	Sense	Sense	-0.643	Comprom	0.643	Comprom	0.000	1.589	Detected	0.820	Detected	1.205	0.249	GT	Sens	[illegible]
GT	Sense	Sense	0.214	Detected	-0.214	Detected	0.000	1.182	Detected	0.848	Detected	1.015	0.065	GT	Sens	[illegible]
GT	Sense	Sense	-0.055	Detected	0.055	Detected	0.000	1.109	Detected	0.911	Detected	1.010	0.012	GT	Sens	[illegible]
GT	Sense	Sense	-0.236	Comprom	0.236	Comprom	0.000	2.582	Detected	1.730	Detected	2.156	0.047	GT	Sens	[illegible]
GT	Sense	Sense	-1.336	Comprom	1.336	Detected	0.000	3.451	Detected	1.871	Detected	2.661	0.229	GT	Sens	[illegible]
GT	Sense	Sense	-0.049	Detected	0.049	Detected	0.000	0.871	Detected	1.488	Detected	1.180	0.063	GT	Sens	[illegible]
GT	Sense	Sense	0.163	Detected	-0.163	Detected	0.000	1.158	Detected	1.049	Detected	1.103	0.023	GT	Sens	[illegible]
GT	Sense	Sense	0.383	Detected	-0.383	Detected	0.000	1.951	Detected	0.845	Detected	1.398	0.173	GT	Sens	[illegible]
GT	Sense	Sense	-0.427	Comprom	0.427	Comprom	0.000	1.860	Detected	1.979	Detected	1.920	0.047	GT	Sens	[illegible]
GT	Sense	Sense	0.382	Detected	-0.382	Detected	0.000	1.034	Detected	3.165	Detected	2.100	0.205	GT	Sens	[illegible]
GT	Sense	Sense	-0.340	Detected	0.340	Detected	0.000	1.585	Detected	1.102	Detected	1.343	0.084	GT	Sens	[illegible]
GT	Sense	Sense	0.339	Comprom	-0.339	Comprom	0.000	0.920	Detected	1.109	Detected	1.015	0.102	GT	Sens	[illegible]
GT	Sense	Sense	0.232	Detected	-0.232	Detected	0.000	0.988	Detected	1.097	Detected	1.042	0.049	GT	Sens	[illegible]
GT	Sense	Sense	0.023	Detected	-0.023	Detected	0.000	1.366	Detected	1.250	Detected	1.308	0.002	GT	Sens	[illegible]
GT	Sense	Sense	0.325	Detected	-0.325	Detected	0.000	1.945	Detected	1.788	Detected	1.867	0.031	GT	Sens	[illegible]
GT	Sense	Sense	0.279	Detected	-0.279	Detected	0.000	1.114	Detected	1.091	Detected	1.102	0.059	GT	Sens	[illegible]
GT	Sense	Sense	-0.193	Detected	0.193	Detected	0.000	2.270	Detected	2.094	Detected	2.182	0.009	GT	Sens	[illegible]
GT	Sense	Sense	-0.015	Detected	0.015	Detected	0.000	1.000	Detected	1.821	Detected	1.410	0.075	GT	Sens	[illegible]
GT	Sense	Sense	0.010	Detected	-0.010	Detected	0.000	1.109	Detected	1.091	Detected	1.100	0.000	GT	Sens	[illegible]
GT	Sense	Sense	0.079	Detected	-0.079	Detected	0.000	1.261	Detected	0.985	Detected	1.123	0.019	GT	Sens	[illegible]
GT	Sense	Sense	-0.066	Detected	0.066	Detected	0.000	1.409	Detected	0.868	Detected	1.138	0.055	GT	Sens	[illegible]
GT	Sense	Sense	0.124	Detected	-0.124	Detected	0.000	0.928	Detected	1.291	Detected	1.109	0.037	GT	Sens	[illegible]
GT	Sense	Sense	-0.374	Detected	0.374	Detected	0.000	2.113	Detected	1.288	Detected	1.700	0.093	GT	Sens	[illegible]
GT	Sense	Sense	0.163	Detected	-0.163	Detected	0.000	1.343	Detected	1.192	Detected	1.268	0.019	GT	Sens	[illegible]
GT	Sense	Sense	0.024	Detected	-0.024	Detected	0.000	0.959	Detected	1.092	Detected	1.026	0.005	GT	Sens	[illegible]
GT	Sense	Sense	-0.605	Detected	0.605	Detected	0.000	1.162	Detected	1.170	Detected	1.166	0.194	GT	Sens	[illegible]
GT	Sense	Sense	-0.364	Detected	0.364	Detected	0.000	1.347	Detected	1.149	Detected	1.248	0.080	GT	Sens	[illegible]
GT	Sense	Sense	0.276	Detected	-0.276	Detected	0.000	1.125	Detected	0.885	Detected	1.005	0.079	GT	Sens	[illegible]
GT	Sense	Sense	-0.496	Comprom	0.496	Comprom	0.000	0.961	Detected	1.117	Detected	1.039	0.174	GT	Sens	[illegible]
GT	Sense	Sense	-0.125	Comprom	0.125	Comprom	0.000	1.092	Detected	1.481	Detected	1.286	0.031	GT	Sens	[illegible]
GT	Sense	Sense	0.136	Detected	-0.136	Detected	0.000	0.964	Detected	1.121	Detected	1.042	0.022	GT	Sens	[illegible]
GT	Sense	Sense	0.344	Detected	-0.344	Comprom	0.000	1.077	Detected	1.291	Detected	1.184	0.081	GT	Sens	[illegible]
GT	Sense	Sense	-0.726	Comprom	0.726	Detected	0.000	2.518	Detected	1.509	Detected	2.014	0.151	GT	Sens	[illegible]
GT	Sense	Sense	0.268	Detected	-0.268	Detected	0.000	1.080	Detected	1.344	Detected	1.212	0.056	GT	Sens	[illegible]
GT	Sense	Sense	-0.394	Comprom	0.394	Comprom	0.000	2.303	Detected	2.333	Detected	2.318	0.028	GT	Sens	[illegible]
GT	Sense	Sense	-0.734	Detected	0.734	Detected	0.000	2.790	Detected	1.144	Detected	1.967	0.216	GT	Sens	[illegible]
GT	Sense	Sense	-0.125	Detected	0.125	Detected	0.000	1.444	Detected	1.122	Detected	1.283	0.024	GT	Sens	[illegible]
GT	Sense	Sense	-0.138	Detected	0.138	Detected	0.000	1.863	Detected	0.962	Detected	1.412	0.096	GT	Sens	[illegible]
GT	Sense	Sense	-0.095	Detected	0.095	Detected	0.000	2.050	Detected	1.327	Detected	1.689	0.046	GT	Sens	[illegible]
GT	Sense	Sense	-0.882	Detected	0.882	Detected	0.000	1.382	Detected	1.418	Detected	1.400	0.253	GT	Sens	[illegible]
GT	Sense	Sense	-0.712	Comprom	0.712	Detected	0.000	3.269	Detected	2.124	Detected	2.697	0.098	GT	Sens	[illegible]
GT	Sense	Sense	0.505	Comprom	-0.505	Comprom	0.000	1.577	Detected	1.782	Detected	1.680	0.083	GT	Sens	[illegible]
GT	Sense	Sense	-0.685	Comprom	0.685	Comprom	0.000	2.132	Detected	1.804	Detected	1.968	0.108	GT	Sens	[illegible]
GT	Sense	Sense	-0.099	Detected	0.099	Detected	0.000	1.983	Detected	2.354	Detected	2.169	0.009	GT	Sens	[illegible]
GT	Sense	Sense	-0.020	Detected	0.020	Detected	0.000	1.161	Detected	0.960	Detected	1.061	0.009	GT	Sens	[illegible]
GT	Sense	Sense	-0.724	Detected	0.724	Detected	0.000	1.514	Detected	1.483	Detected	1.499	0.174	GT	Sens	[illegible]
GT	Sense	Sense	0.521	Detected	-0.521	Comprom	0.000	1.335	Detected	1.194	Detected	1.265	0.138	GT	Sens	[illegible]
GT	Sense	Sense	-0.036	Detected	0.036	Detected	0.000	1.538	Detected	1.350	Detected	1.444	0.005	GT	Sens	[illegible]
GT	Sense	Sense	0.386	Detected	-0.386	Detected	0.000	1.709	Detected	1.209	Detected	1.459	0.087	GT	Sens	[illegible]
GT	Sense	Sense	-0.352	Detected	0.352	Detected	0.000	1.442	Detected	0.873	Detected	1.157	0.125	GT	Sens	[illegible]
GT	Sense	Sense	0.092	Detected	-0.092	Detected	0.000	1.571	Detected	1.303	Detected	1.437	0.013	GT	Sens	[illegible]
GT	Sense	Sense	-0.164	Detected	0.164	Detected	0.000	1.209	Detected	0.989	Detected	1.099	0.031	GT	Sens	[illegible]
GT	Sense	Sense	0.146	Detected	-0.146	Detected	0.000	1.005	Detected	1.313	Detected	1.159	0.032	GT	Sens	[illegible]
GT	Sense	Sense	0.211	Detected	-0.211	Detected	0.000	1.559	Detected	0.929	Detected	1.244	0.082	GT	Sens	[illegible]
GT	Sense	Sense	0.708	Detected	-0.708	Comprom	0.000	0.861	Detected	1.886	Detected	1.374	0.257	GT	Sens	[illegible]
GT	Sense	Sense	-0.261	Detected	0.261	Detected	0.000	1.634	Detected	0.899	Detected	1.267	0.107	GT	Sens	[illegible]
GT	Sense	Sense	-0.257	Detected	0.257	Detected	0.000	1.367	Detected	1.252	Detected	1.309	0.038	GT	Sens	[illegible]
GT	Sense	Sense	0.145	Detected	-0.145	Detected	0.000	0.964	Detected	1.048	Detected	1.006	0.022	GT	Sens	[illegible]
GT	Sense	Sense	-0.233	Comprom	0.233	Comprom	0.000	4.466	Detected	4.553	Detected	4.510	0.003	GT	Sens	[illegible]
GT	Sense	Sense	0.379	Detected	-0.379	Detected	0.000	1.211	Detected	1.540	Detected	1.375	0.080	GT	Sens	[illegible]
GT	Sense	Sense	-0.889	Detected	0.889	Detected	0.000	1.308	Detected	1.317	Detected	1.312	0.278	GT	Sens	[illegible]
GT	Sense	Sense	0.027	Detected	-0.027	Detected	0.000	1.682	Detected	1.546	Detected	1.614	0.002	GT	Sens	[illegible]
GT	Sense	Sense	0.034	Detected	-0.034	Detected	0.000	1.699	Detected	0.898	Detected	1.298	0.084	GT	Sens	[illegible]
GT	Sense	Sense	-0.202	Comprom	0.202	Comprom	0.000	2.239	Detected	1.864	Detected	2.051	0.018	GT	Sens	[illegible]
GT	Sense	Sense	0.204	Detected	-0.204	Detected	0.000	1.070	Detected	0.938	Detected	1.004	0.043	GT	Sens	[illegible]
GT	Sense	Sense	-0.092	Detected	0.092	Detected	0.000	0.851	Detected	1.460	Detected	1.156	0.068	GT	Sens	[illegible]
GT	Sense	Sense	0.498	Detected	-0.498	Detected	0.000	2.137	Detected	3.642	Detected	2.889	0.085	GT	Sens	[illegible]
GT	Sense	Sense	-0.167	Detected	0.167	Detected	0.000	1.296	Detected	0.953	Detected	1.124	0.042	GT	Sens	[illegible]
GT	Sense	Sense	0.341	Detected	-0.341	Detected	0.000	0.916	Detected	1.113	Detected	1.015	0.104	GT	Sens	[illegible]
GT	Sense	Sense	-0.090	Detected	0.090	Detected	0.000	1.217	Detected	0.923	Detected	1.070	0.025	GT	Sens	[illegible]
GT	Sense	Sense	0.135	Detected	-0.135	Detected	0.000	1.030	Detected	1.064	Detected	1.047	0.017	GT	Sens	[illegible]
GT	Sense	Sense	-0.676	Detected	0.676	Detected	0.000	1.603	Detected	1.592	Detected	1.598	0.142	GT	Sens	[illegible]
GT	Sense	Sense	-0.105	Detected	0.105	Detected	0.000	1.481	Detected	1.133	Detected	1.307	0.023	GT	Sens	[illegible]
GT	Sense	Sense	-0.343	Detected	0.343	Detected	0.000	2.603	Detected	1.373	Detected	1.988	0.106	GT	Sens	[illegible]
GT	Sense	Sense	-0.301	Detected	0.301	Detected	0.000	1.081	Detected	0.995	Detected	1.038	0.076	GT	Sens	[illegible]
GT	Sense	Sense	0.226	Detected	-0.226	Detected	0.000	2.262	Detected	1.238	Detected	1.750	0.089	GT	Sens	[illegible]
GT	Sense	Sense	-1.020	Comprom	1.020	Detected	0.000	3.245	Detected	1.040	Detected	2.143	0.290	GT	Sens	[illegible]
GT	Sense	Sense	0.042	Detected	-0.042	Detected	0.000	1.151	Detected	1.227	Detected	1.189	0.002	GT	Sens	[illegible]
GT	Sense	Sense	-1.204	Detected	1.204	Detected	0.000	0.933	Detected	1.081	Detected	1.007	0.492	GT	Sens	[illegible]
GT	Sense	Sense	-0.795	Detected	0.795	Detected	0.000	1.158	Detected	1.218	Detected	1.188	0.274	GT	Sens	[illegible]
GT	Sense	Sense	0.284	Comprom	-0.284	Comprom	0.000	1.251	Detected	1.049	Detected	1.150	0.062	GT	Sens	[illegible]
GT	Sense	Sense	-0.172	Detected	0.172	Detected	0.000	3.307	Detected	2.265	Detected	2.786	0.037	GT	Sens	[illegible]
GT	Sense	Sense	0.968	Detected	-0.968	Detected	0.000	1.094	Detected	1.663	Detected	1.379	0.305	GT	Sens	[illegible]
GT	Sense	Sense	0.573	Detected	-0.573	Comprom	0.000	1.157	Detected	0.855	Detected	1.006	0.232	GT	Sens	[illegible]
GT	Sense	Sense	-0.326	Detected	0.326	Detected	0.000	2.819	Detected	1.089	Detected	1.954	0.169	GT	Sens	[illegible]
GT	Sense	Sense	-0.068	Detected	0.068	Detected	0.000	1.410	Detected	1.244	Detected	1.327	0.006	GT	Sens	[illegible]
GT	Sense	Sense	0.017	Detected	-0.017	Detected	0.000	1.415	Detected	1.085	Detected	1.250	0.017	GT	Sens	[illegible]
GT	Sense	Sense	-0.007	Detected	0.007	Detected	0.000	1.394	Detected	1.394	Detected	1.394	0.000	GT	Sens	[illegible]
GT	Sense	Sense	0.551	Detected	-0.551	Detected	0.000	1.147	Detected	0.944	Detected	1.046	0.203	GT	Sens	[illegible]
GT	Sense	Sense	-0.024	Detected	0.024	Detected	0.000	1.334	Detected	1.028	Detected	1.181	0.017	GT	Sens	[illegible]
GT	Sense	Sense	0.449	Detected	-0.449	Detected	0.000	2.440	Detected	1.789	Detected	2.114	0.062	GT	Sens	[illegible]
GT	Sense	Sense	0.003	Detected	-0.003	Detected	0.000	2.199	Detected	0.805	Detected	1.502	0.164	GT	Sens	[illegible]
GT	Sense	Sense	-0.274	Detected	0.274	Detected	0.000	1.205	Detected	0.945	Detected	1.075	0.071	GT	Sens	[illegible]
GT	Sense	Sense	0.608	Comprom	-0.608	Comprom	0.000	0.955	Detected	1.161	Detected	1.058	0.228	GT	Sens	[illegible]
GT	Sense	Sense	-1.483	Detected	1.483	Detected	0.000	1.442	Detected	1.595	Detected	1.518	0.414	GT	Sens	[illegible]
GT	Sense	Sense	0.247	Comprom	-0.247	Comprom	0.000	1.603	Detected	1.359	Detected	1.481	0.033	GT	Sens	[illegible]
GT	Sense	Sense	0.343	Detected	-0.343	Detected	0.000	1.746	Detected	0.852	Detected	1.299	0.148	GT	Sens	[illegible]
GT	Sense	Sense	-0.205	Detected	0.205	Detected	0.000	1.404	Detected	1.359	Detected	1.381	0.021	GT	Sens	[illegible]
GT	Sense	Sense	-0.588	Detected	0.588	Detected	0.000	1.415	Detected	1.312	Detected	1.364	0.147	GT	Sens	[illegible]
GT	Sense	Sense	-0.400	Comprom	0.400	Comprom	0.000	3.779	Detected	2.082	Detected	2.930	0.089	GT	Sens	[illegible]
GT	Sense	Sense	0.106	Detected	-0.106	Detected	0.000	1.201	Detected	1.243	Detected	1.222	0.008	GT	Sens	[illegible]
GT	Sense	Sense	0.922	Detected	-0.922	Detected	0.000	1.507	Detected	1.331	Detected	1.419	0.265	GT	Sens	[illegible]
GT	Sense	Sense	0.329	Comprom	-0.329	Comprom	0.000	1.094	Detected	1.447	Detected	1.270	0.077	GT	Sens	[illegible]
GT	Sense	Sense	-0.094	Comprom	0.094	Comprom	0.000	2.949	Detected	1.204	Detected	2.076	0.142	GT	Sens	[illegible]
GT	Sense	Sense	0.353	Detected	-0.353	Detected	0.000	1.267	Detected	1.652	Detected	1.460	0.068	GT	Sens	[illegible]
GT	Sense	Sense	-0.331	Comprom	0.331	Comprom	0.000	1.443	Detected	3.426	Detected	2.435	0.145	GT	Sens	[illegible]
GT	Sense	Sense	0.102	Detected	-0.102	Detected	0.000	1.445	Detected	0.873	Detected	1.159	0.062	GT	Sens	[illegible]
GT	Sense	Sense	-0.068	Detected	0.068	Detected	0.000	1.090	Detected	1.008	Detected	1.049	0.006	GT	Sens	[illegible]
GT	Sense	Sense	0.162	Detected	-0.162	Detected	0.000	1.353	Detected	1.219	Detected	1.286	0.018	GT	Sens	[illegible]
GT	Sense	Sense	0.060	Comprom	-0.060	Comprom	0.000	2.033	Detected	6.346	Detected	4.189	0.192	GT	Sens	[illegible]
GT	Sense	Sense	0.308	Detected	-0.308	Detected	0.000	1.514	Detected	1.233	Detected	1.373	0.056	GT	Sens	[illegible]
GT	Sense	Sense	-0.251	Detected	0.251	Detected	0.000	1.337	Detected	2.564	Detected	1.950	0.099	GT	Sens	[illegible]
GT	Sense	Sense	-0.064	Detected	0.064	Detected	0.000	1.400	Detected	1.087	Detected	1.243	0.018	GT	Sens	[illegible]
GT	Sense	Sense	0.585	Comprom	-0.585	Comprom	0.000	1.719	Detected	1.412	Detected	1.566	0.122	GT	Sens	[illegible]
GT	Sense	Sense	-1.008	Detected	1.008	Detected	0.000	1.087	Detected	1.275	Detected	1.181	0.364	GT	Sens	[illegible]
GT	Sense	Sense	-0.188	Detected	0.188	Detected	0.000	1.416	Detected	1.719	Detected	1.568	0.023	GT	Sens	[illegible]
GT	Sense	Sense	-0.224	Comprom	0.224	Detected	0.000	3.082	Detected	1.728	Detected	2.405	0.078	GT	Sens	[illegible]
GT	Sense	Sense	0.360	Detected	-0.360	Comprom	0.000	1.205	Detected	1.007	Detected	1.106	0.098	GT	Sens	[illegible]
GT	Sense	Sense	0.344	Detected	-0.344	Detected	0.000	1.796	Detected	2.189	Detected	1.992	0.037	GT	Sens	[illegible]
GT	Sense	Sense	0.709	Detected	-0.709	Comprom	0.000	1.134	Detected	1.169	Detected	1.151	0.246	GT	Sens	[illegible]
GT	Sense	Sense	0.119	Comprom	-0.119	Comprom	0.000	0.873	Detected	1.412	Detected	1.143	0.060	GT	Sens	[illegible]
GT	Sense	Sense	0.705	Detected	-0.705	Comprom	0.000	1.593	Detected	1.467	Detected	1.530	0.163	GT	Sens	[illegible]
GT	Sense	Sense	-0.405	Comprom	0.405	Comprom	0.000	1.202	Detected	1.706	Detected	1.454	0.093	GT	Sens	[illegible]
GT	Sense	Sense	-0.726	Comprom	0.726	Comprom	0.000	2.943	Detected	1.168	Detected	2.056	0.215	GT	Sens	[illegible]
GT	Sense	Sense	0.006	Detected	-0.006	Detected	0.000	1.070	Detected	1.044	Detected	1.057	0.000	GT	Sens	[illegible]
GT	Sense	Sense	0.030	Detected	-0.030	Detected	0.000	1.707	Detected	0.880	Detected	1.293	0.089	GT	Sens	[illegible]
GT	Sense	Sense	0.600	Detected	-0.600	Detected	0.000	0.998	Detected	1.924	Detected	1.461	0.194	GT	Sens	[illegible]
GT	Sense	Sense	0.003	Detected	-0.003	Detected	0.000	1.038	Detected	1.125	Detected	1.082	0.002	GT	Sens	[illegible]
GT	Sense	Sense	0.379	Detected	-0.379	Detected	0.000	1.118	Detected	1.043	Detected	1.081	0.105	GT	Sens	[illegible]
GT	Sense	Sense	0.077	Detected	-0.077	Detected	0.000	1.222	Detected	0.944	Detected	1.083	0.021	GT	Sens	[illegible]
GT	Sense	Sense	-0.442	Detected	0.442	Detected	0.000	1.499	Detected	1.821	Detected	1.660	0.072	GT	Sens	[illegible]
GT	Sense	Sense	-0.415	Comprom	0.415	Comprom	0.000	1.090	Detected	1.346	Detected	1.218	0.107	GT	Sens	[illegible]
GT	Sense	Sense	-0.190	Detected	0.190	Detected	0.000	1.433	Detected	1.694	Detected	1.564	0.021	GT	Sens	[illegible]
GT	Sense	Sense	-0.148	Detected	0.148	Detected	0.000	1.220	Detected	0.857	Detected	1.039	0.047	GT	Sens	[illegible]
GT	Sense	Sense	-0.936	Comprom	0.936	Detected	0.000	2.653	Detected	0.905	Detected	1.779	0.299	GT	Sens	[illegible]
GT	Sense	Sense	-0.253	Detected	0.253	Detected	0.000	1.033	Detected	0.985	Detected	1.009	0.058	GT	Sens	[illegible]
GT	Sense	Sense	-0.354	Detected	0.354	Detected	0.000	1.125	Detected	1.261	Detected	1.193	0.080	GT	Sens	[illegible]
GT	Sense	Sense	-0.081	Detected	0.081	Detected	0.000	2.150	Detected	1.541	Detected	1.846	0.028	GT	Sens	[illegible]
GT	Sense	Sense	0.335	Detected	-0.335	Detected	0.000	1.449	Detected	1.362	Detected	1.406	0.053	GT	Sens	[illegible]
GT	Sense	Sense	0.098	Detected	-0.098	Detected	0.000	1.537	Detected	1.381	Detected	1.459	0.007	GT	Sens	[illegible]
GT	Sense	Sense	-0.199	Detected	0.199	Detected	0.000	1.341	Detected	0.949	Detected	1.145	0.055	GT	Sens	[illegible]
GT	Sense	Sense	0.061	Detected	-0.061	Detected	0.000	1.499	Detected	1.006	Detected	1.252	0.039	GT	Sens	[illegible]
GT	Sense	Sense	0.454	Detected	-0.454	Detected	0.000	2.690	Detected	2.404	Detected	2.547	0.033	GT	Sens	[illegible]
GT	Sense	Sense	-0.135	Detected	0.135	Detected	0.000	1.213	Detected	1.140	Detected	1.176	0.014	GT	Sens	[illegible]
GT	Sense	Sense	-0.173	Detected	0.173	Detected	0.000	1.018	Detected	1.131	Detected	1.074	0.027	GT	Sens	[illegible]
GT	Sense	Sense	0.503	Detected	-0.503	Detected	0.000	2.611	Detected	3.668	Detected	3.139	0.050	GT	Sens	[illegible]
GT	Sense	Sense	0.238	Detected	-0.238	Detected	0.000	0.888	Detected	1.143	Detected	1.015	0.064	GT	Sens	[illegible]
GT	Sense	Sense	-0.405	Detected	0.405	Detected	0.000	1.000	Detected	1.701	Detected	1.350	0.128	GT	Sens	[illegible]
GT	Sense	Sense	0.049	Detected	-0.049	Detected	0.000	0.995	Detected	1.073	Detected	1.034	0.004	GT	Sens	[illegible]
GT	Sense	Sense	0.341	Detected	-0.341	Detected	0.000	1.370	Detected	1.009	Detected	1.190	0.091	GT	Sens	[illegible]
GT	Sense	Sense	-0.026	Detected	0.026	Detected	0.000	1.293	Detected	0.864	Detected	1.078	0.038	GT	Sens	[illegible]
GT	Sense	Sense	-0.101	Comprom	0.101	Comprom	0.000	1.976	Detected	2.048	Detected	2.012	0.003	GT	Sens	[illegible]
GT	Sense	Sense	-0.285	Detected	0.285	Detected	0.000	0.894	Detected	1.344	Detected	1.119	0.091	GT	Sens	[illegible]
GT	Sense	Sense	0.258	Detected	-0.258	Comprom	0.000	0.970	Detected	1.138	Detected	1.054	0.060	GT	Sens	[illegible]
GT	Sense	Sense	0.082	Detected	-0.082	Detected	0.000	1.356	Detected	1.320	Detected	1.338	0.004	GT	Sens	[illegible]
GT	Sense	Sense	-0.020	Detected	0.020	Detected	0.000	1.102	Detected	1.189	Detected	1.146	0.002	GT	Sens	[illegible]
GT	Sense	Sense	0.519	Detected	-0.519	Detected	0.000	1.326	Detected	1.385	Detected	1.355	0.121	GT	Sens	[illegible]
GT	Sense	Sense	-0.496	Detected	0.496	Detected	0.000	1.531	Detected	1.142	Detected	1.337	0.129	GT	Sens	[illegible]
GT	Sense	Sense	0.178	Detected	-0.178	Detected	0.000	1.198	Detected	0.961	Detected	1.079	0.037	GT	Sens	[illegible]
GT	Sense	Sense	-0.435	Detected	0.435	Detected	0.000	1.007	Detected	1.336	Detected	1.172	0.128	GT	Sens	[illegible]
GT	Sense	Sense	0.164	Detected	-0.164	Detected	0.000	2.305	Detected	1.619	Detected	1.962	0.036	GT	Sens	[illegible]
GT	Sense	Sense	-0.329	Detected	0.329	Detected	0.000	2.105	Detected	1.022	Detected	1.564	0.132	GT	Sens	[illegible]
GT	Sense	Sense	0.164	Comprom	-0.164	Comprom	0.000	2.485	Detected	1.238	Detected	1.861	0.102	GT	Sens	[illegible]
GT	Sense	Sense	0.027	Detected	-0.027	Detected	0.000	1.214	Detected	0.988	Detected	1.101	0.011	GT	Sens	[illegible]
GT	Sense	Sense	0.174	Detected	-0.174	Detected	0.000	1.210	Detected	1.081	Detected	1.145	0.025	GT	Sens	[illegible]
GT	Sense	Sense	0.123	Detected	-0.123	Detected	0.000	1.248	Detected	1.118	Detected	1.183	0.014	GT	Sens	[illegible]
GT	Sense	Sense	0.092	Detected	-0.092	Detected	0.000	1.395	Detected	0.842	Detected	1.119	0.062	GT	Sens	[illegible]
GT	Sense	Sense	0.071	Detected	-0.071	Detected	0.000	2.826	Detected	2.261	Detected	2.544	0.013	GT	Sens	[illegible]
GT	Sense	Sense	0.041	Detected	-0.041	Detected	0.000	0.870	Detected	1.392	Detected	1.131	0.050	GT	Sens	[illegible]
GT	Sense	Sense	-0.328	Detected	0.328	Detected	0.000	2.214	Detected	0.914	Detected	1.564	0.165	GT	Sens	[illegible]
GT	Sense	Sense	0.120	Detected	-0.120	Detected	0.000	1.597	Detected	1.524	Detected	1.560	0.006	GT	Sens	[illegible]
GT	Sense	Sense	0.247	Detected	-0.247	Detected	0.000	1.056	Detected	1.358	Detected	1.207	0.053	GT	Sens	[illegible]
GT	Sense	Sense	-1.031	Detected	1.031	Detected	0.000	1.592	Detected	1.319	Detected	1.455	0.297	GT	Sens	[illegible]
GT	Sense	Sense	-0.341	Comprom	0.341	Comprom	0.000	1.227	Detected	1.808	Detected	1.518	0.077	GT	Sens	[illegible]
GT	Sense	Sense	-0.083	Comprom	0.083	Comprom	0.000	2.290	Detected	1.576	Detected	1.933	0.034	GT	Sens	[illegible]
GT	Sense	Sense	-0.117	Detected	0.117	Comprom	0.000	0.969	Detected	1.456	Detected	1.213	0.046	GT	Sens	[illegible]
GT	Sense	Sense	-0.110	Detected	0.110	Detected	0.000	1.643	Detected	1.162	Detected	1.403	0.034	GT	Sens	[illegible]
GT	Sense	Sense	0.549	Detected	-0.549	Comprom	0.000	3.369	Detected	3.212	Detected	3.290	0.027	GT	Sens	[illegible]
GT	Sense	Sense	-0.063	Detected	0.063	Detected	0.000	1.413	Detected	0.849	Detected	1.131	0.059	GT	Sens	[illegible]
GT	Sense	Sense	-0.482	Detected	0.482	Detected	0.000	1.256	Detected	1.399	Detected	1.327	0.112	GT	Sens	[illegible]
GT	Sense	Sense	0.099	Detected	-0.099	Detected	0.000	2.125	Detected	1.943	Detected	2.034	0.004	GT	Sens	[illegible]
GT	Sense	Sense	-0.207	Detected	0.207	Detected	0.000	1.791	Detected	0.876	Detected	1.333	0.117	GT	Sens	[illegible]
GT	Sense	Sense	0.376	Detected	-0.376	Detected	0.000	1.309	Detected	1.245	Detected	1.277	0.078	GT	Sens	[illegible]
GT	Sense	Sense	0.095	Detected	-0.095	Detected	0.000	1.192	Detected	1.156	Detected	1.174	0.007	GT	Sens	[illegible]
GT	Sense	Sense	-1.302	Comprom	1.302	Detected	0.000	2.790	Detected	1.520	Detected	2.155	0.275	GT	Sens	[illegible]
GT	Sense	Sense	-0.292	Detected	0.292	Detected	0.000	1.740	Detected	1.359	Detected	1.549	0.047	GT	Sens	[illegible]
GT	Sense	Sense	0.088	Detected	-0.088	Detected	0.000	1.419	Detected	0.946	Detected	1.183	0.043	GT	Sens	[illegible]
GT	Sense	Sense	0.963	Detected	-0.963	Comprom	0.000	1.352	Detected	1.083	Detected	1.217	0.337	GT	Sens	[illegible]
GT	Sense	Sense	-0.125	Detected	0.125	Detected	0.000	0.901	Detected	1.228	Detected	1.064	0.035	GT	Sens	[illegible]
GT	Sense	Sense	-0.033	Comprom	0.033	Comprom	0.000	2.570	Detected	2.385	Detected	2.478	0.002	GT	Sens	[illegible]
GT	Sense	Sense	0.190	Comprom	-0.190	Comprom	0.000	1.602	Detected	1.563	Detected	1.582	0.014	GT	Sens	[illegible]
GT	Sense	Sense	1.749	Detected	-1.749	Comprom	0.000	1.921	Detected	1.701	Detected	1.811	0.410	GT	Sens	[illegible]
GT	Sense	Sense	-0.365	Comprom	0.365	Detected	0.000	1.986	Detected	1.133	Detected	1.560	0.109	GT	Sens	[illegible]
GT	Sense	Sense	0.445	Detected	-0.445	Comprom	0.000	0.994	Detected	1.006	Detected	1.000	0.154	GT	Sens	[illegible]
GT	Sense	Sense	-0.729	Comprom	0.729	Comprom	0.000	2.657	Detected	2.842	Detected	2.750	0.065	GT	Sens	[illegible]
GT	Sense	Sense	-0.381	Comprom	0.381	Comprom	0.000	1.099	Detected	1.154	Detected	1.127	0.098	GT	Sens	[illegible]
GT	Sense	Sense	0.570	Detected	-0.570	Comprom	0.000	1.519	Detected	1.648	Detected	1.584	0.110	GT	Sens	[illegible]
GT	Sense	Sense	0.327	Comprom	-0.327	Comprom	0.000	1.623	Detected	1.405	Detected	1.514	0.048	GT	Sens	[illegible]
GT	Sense	Sense	0.183	Comprom	-0.183	Comprom	0.000	0.881	Detected	1.280	Detected	1.081	0.057	GT	Sens	[illegible]
GT	Sense	Sense	0.319	Comprom	-0.319	Comprom	0.000	3.758	Detected	1.973	Detected	2.865	0.094	GT	Sens	[illegible]
GT	Sense	Sense	0.418	Comprom	-0.418	Comprom	0.000	1.975	Detected	1.229	Detected	1.602	0.104	GT	Sens	[illegible]
GT	Sense	Sense	0.101	Detected	-0.101	Comprom	0.000	1.065	Detected	1.133	Detected	1.099	0.009	GT	Sens	[illegible]
GT	Sense	Sense	-0.098	Detected	0.098	Detected	0.000	1.084	Detected	1.144	Detected	1.114	0.008	GT	Sens	[illegible]
GT	Sense	Sense	0.069	Detected	-0.069	Detected	0.000	1.233	Detected	1.315	Detected	1.274	0.004	GT	Sens	[illegible]
GT	Sense	Sense	1.143	Detected	-1.143	Comprom	0.000	1.882	Detected	1.649	Detected	1.766	0.264	GT	Sens	[illegible]
GT	Sense	Sense	0.305	Detected	-0.305	Detected	0.000	1.373	Detected	1.371	Detected	1.372	0.046	GT	Sens	[illegible]
GT	Sense	Sense	-0.195	Detected	0.195	Detected	0.000	1.069	Detected	1.019	Detected	1.044	0.034	GT	Sens	[illegible]
GT	Sense	Sense	-0.786	Comprom	0.786	Comprom	0.000	1.441	Detected	1.336	Detected	1.388	0.220	GT	Sens	[illegible]
GT	Sense	Sense	-0.243	Comprom	0.243	Comprom	0.000	1.034	Detected	1.424	Detected	1.229	0.059	GT	Sens	[illegible]
GT	Sense	Sense	-0.809	Comprom	0.809	Comprom	0.000	3.646	Detected	2.503	Detected	3.074	0.090	GT	Sens	[illegible]
GT	Sense	Sense	-0.697	Comprom	0.697	Detected	0.000	1.043	Detected	1.316	Detected	1.179	0.239	GT	Sens	[illegible]
GT	Sense	Sense	-0.067	Comprom	0.067	Comprom	0.000	1.820	Detected	1.914	Detected	1.867	0.002	GT	Sens	[illegible]
GT	Sense	Sense	-0.085	Detected	0.085	Detected	0.000	1.238	Detected	0.903	Detected	1.070	0.029	GT	Sens	[illegible]
GT	Sense	Sense	1.549	Detected	-1.549	Comprom	0.000	1.608	Detected	1.293	Detected	1.450	0.450	GT	Sens	[illegible]
GT	Sense	Sense	-0.482	Comprom	0.482	Comprom	0.000	1.687	Detected	0.939	Detected	1.313	0.164	GT	Sens	[illegible]
GT	Sense	Sense	-0.025	Detected	0.025	Comprom	0.000	2.151	Detected	1.545	Detected	1.848	0.026	GT	Sens	[illegible]
GT	Sense	Sense	0.072	Detected	-0.072	Comprom	0.000	0.937	Detected	1.075	Detected	1.006	0.010	GT	Sens	[illegible]
GT	Sense	Sense	0.037	Detected	-0.037	Detected	0.000	1.117	Detected	0.977	Detected	1.047	0.006	GT	Sens	[illegible]
GT	Sense	Sense	-0.072	Detected	0.072	Comprom	0.000	1.405	Detected	1.391	Detected	1.398	0.003	GT	Sens	[illegible]
GT	Sense	Sense	0.849	Detected	-0.849	Comprom	0.000	2.229	Detected	1.991	Detected	2.110	0.133	GT	Sens	[illegible]
GT	Sense	Sense	-0.523	Comprom	0.523	Comprom	0.000	0.886	Detected	1.215	Detected	1.051	0.195	GT	Sens	[illegible]
GT	Sense	Sense	-0.168	Detected	0.168	Detected	0.000	0.956	Detected	1.475	Detected	1.216	0.059	GT	Sens	[illegible]
GT	Sense	Sense	0.717	Comprom	-0.717	Comprom	0.000	2.580	Detected	1.917	Detected	2.248	0.104	GT	Sens	[illegible]
GT	Sense	Sense	0.015	Detected	-0.015	Detected	0.000	1.317	Detected	0.928	Detected	1.123	0.029	GT	Sens	[illegible]

GT	Sense	Sense	-0.702	Comprom	0.702	Detected	0.000	0.918	Detected	1.485	Detected	1.202	0.253	GT	Sens	contig327	contig327	Unannota	[illegible]
GT	Sense	Sense	0.023	Detected	-0.023	Detected	0.000	1.502	Detected	1.362	Detected	1.432	0.003	GT	Sens	contig327	contig327	Unannota	[illegible]
GT	Sense	Sense	0.068	Detected	-0.068	Detected	0.000	1.410	Detected	1.050	Detected	1.230	0.024	GT	Sens	contig327	contig327	Unannota	[illegible]
GT	Sense	Sense	-0.732	Comprom	0.732	Comprom	0.000	0.972	Detected	1.289	Detected	1.130	0.270	GT	Sens	contig327	contig327	Unannota	[illegible]
GT	Sense	Sense	0.237	Comprom	-0.237	Comprom	0.000	1.315	Detected	0.862	Detected	1.089	0.080	GT	Sens	contig327	contig327	Unannota	[illegible]
GT	Sense	Sense	0.254	Comprom	-0.254	Comprom	0.000	1.262	Detected	1.164	Detected	1.213	0.043	GT	Sens	contig327	contig327	Unannota	[illegible]
GT	Sense	Sense	0.196	Comprom	-0.196	Comprom	0.000	2.204	Detected	1.619	Detected	1.911	0.032	GT	Sens	contig328	contig328	Unannota	[illegible]
GT	Sense	Sense	-0.542	Detected	0.542	Detected	0.000	1.078	Detected	1.146	Detected	1.112	0.177	GT	Sens	contig328	contig328	Unannota	[illegible]
GT	Sense	Sense	0.064	Detected	-0.064	Detected	0.000	1.577	Detected	1.140	Detected	1.358	0.027	GT	Sens	contig328	contig328	Unannota	[illegible]
GT	Sense	Sense	0.804	Detected	-0.804	Comprom	0.000	1.348	Detected	1.195	Detected	1.271	0.256	GT	Sens	contig329	contig329	Unannota	[illegible]
GT	Sense	Sense	-0.412	Detected	0.412	Detected	0.000	1.349	Detected	1.452	Detected	1.401	0.078	GT	Sens	contig329	contig329	Unannota	[illegible]
GT	Sense	Sense	-0.256	Detected	0.256	Detected	0.000	1.293	Detected	1.558	Detected	1.426	0.038	GT	Sens	contig329	contig329	Unannota	[illegible]
GT	Sense	Sense	0.769	Detected	-0.769	Detected	0.000	1.341	Detected	0.914	Detected	1.128	0.293	GT	Sens	contig331	contig331	Unannota	[illegible]
GT	Sense	Sense	-0.073	Comprom	0.073	Comprom	0.000	1.213	Detected	1.065	Detected	1.139	0.008	GT	Sens	contig331	contig331	Unannota	[illegible]
GT	Sense	Sense	0.735	Detected	-0.735	Comprom	0.000	0.953	Detected	2.900	Detected	1.926	0.255	GT	Sens	contig332	contig332	Unannota	[illegible]
GT	Sense	Sense	0.219	Detected	-0.219	Detected	0.000	1.471	Detected	0.987	Detected	1.229	0.064	GT	Sens	contig332	contig332	Peptide t	[illegible]
GT	Sense	Sense	-0.110	Comprom	0.110	Comprom	0.000	0.975	Detected	1.027	Detected	1.001	0.012	GT	Sens	contig332	contig332	Endonuc	[illegible]
GT	Sense	Sense	0.083	Detected	-0.083	Detected	0.000	1.230	Detected	0.859	Detected	1.044	0.036	GT	Sens	contig332	contig332	Unannota	[illegible]
GT	Sense	Sense	0.393	Comprom	-0.393	Comprom	0.000	1.797	Detected	1.418	Detected	1.607	0.067	GT	Sens	contig333	contig333	Unannota	[illegible]
GT	Sense	Sense	0.324	Detected	-0.324	Detected	0.000	1.964	Detected	1.810	Detected	1.887	0.030	GT	Sens	contig333	contig333	Unknown	[illegible]
GT	Sense	Sense	0.118	Detected	-0.118	Detected	0.000	1.307	Detected	0.908	Detected	1.107	0.041	GT	Sens	contig333	contig333	Unannota	[illegible]
GT	Sense	Sense	0.050	Detected	-0.050	Detected	0.000	1.444	Detected	1.007	Detected	1.226	0.032	GT	Sens	contig333	contig333	Unannota	[illegible]
GT	Sense	Sense	1.030	Detected	-1.030	Comprom	0.000	0.979	Detected	2.941	Detected	1.960	0.302	GT	Sens	contig333	contig333	Unannota	[illegible]
GT	Sense	Sense	-0.019	Comprom	0.019	Comprom	0.000	1.831	Detected	1.021	Detected	1.426	0.072	GT	Sens	contig334	contig334	Unannota	[illegible]
GT	Sense	Sense	-0.260	Comprom	0.260	Comprom	0.000	1.054	Detected	1.500	Detected	1.277	0.065	GT	Sens	contig335	contig335	Unannota	[illegible]
GT	Sense	Sense	-0.174	Detected	0.174	Detected	0.000	1.744	Detected	1.166	Detected	1.455	0.050	GT	Sens	contig335	contig335	Rhodane	[illegible]
GT	Sense	Sense	-0.171	Comprom	0.171	Comprom	0.000	2.471	Detected	2.288	Detected	2.380	0.007	GT	Sens	contig335	contig335	Unannota	[illegible]
GT	Sense	Sense	0.036	Detected	-0.036	Detected	0.000	1.589	Detected	1.482	Detected	1.535	0.002	GT	Sens	contig335	contig335	Unannota	[illegible]
GT	Sense	Sense	-0.242	Detected	0.242	Detected	0.000	1.233	Detected	1.139	Detected	1.186	0.040	GT	Sens	contig335	contig335	Unannota	[illegible]
GT	Sense	Sense	-0.712	Comprom	0.712	Comprom	0.000	1.157	Detected	1.195	Detected	1.176	0.240	GT	Sens	contig336	contig336	Unannota	[illegible]
GT	Sense	Sense	-0.144	Comprom	0.144	Comprom	0.000	1.425	Detected	1.072	Detected	1.249	0.032	GT	Sens	contig337	contig337	Unannota	[illegible]
GT	Sense	Sense	-0.582	Comprom	0.582	Comprom	0.000	1.467	Detected	1.899	Detected	1.683	0.113	GT	Sens	contig338	contig338	Unannota	[illegible]
GT	Sense	Sense	0.353	Comprom	-0.353	Comprom	0.000	1.796	Detected	1.776	Detected	1.786	0.037	GT	Sens	contig338	contig338	Unannota	[illegible]
GT	Sense	Sense	0.446	Detected	-0.446	Comprom	0.000	1.748	Detected	1.922	Detected	1.835	0.056	GT	Sens	contig338	contig338	Unannota	[illegible]
GT	Sense	Sense	-0.091	Detected	0.091	Detected	0.000	1.331	Detected	0.826	Detected	1.079	0.057	GT	Sens	contig338	contig338	Unannota	[illegible]
GT	Sense	Sense	-0.268	Detected	0.268	Detected	0.000	1.549	Detected	1.382	Detected	1.466	0.035	GT	Sens	contig338	contig338	Unannota	[illegible]
GT	Sense	Sense	-0.172	Detected	0.172	Detected	0.000	1.788	Detected	1.270	Detected	1.529	0.039	GT	Sens	contig338	contig338	Unannota	[illegible]
GT	Sense	Sense	-0.066	Detected	0.066	Detected	0.000	1.175	Detected	1.003	Detected	1.089	0.010	GT	Sens	contig339	contig339	Unannota	[illegible]
GT	Sense	Sense	-0.478	Comprom	0.478	Comprom	0.000	1.666	Detected	1.871	Detected	1.768	0.069	GT	Sens	contig339	contig339	NBS-codi	[illegible]
GT	Sense	Sense	0.110	Detected	-0.110	Detected	0.000	1.049	Detected	1.295	Detected	1.172	0.019	GT	Sens	contig339	contig339	Heat sho	[illegible]
GT	Sense	Sense	1.546	Detected	-1.546	Comprom	0.000	1.297	Detected	1.459	Detected	1.378	0.467	GT	Sens	contig340	contig340	Unannota	[illegible]
GT	Sense	Sense	-0.223	Comprom	0.223	Comprom	0.000	1.324	Detected	3.689	Detected	2.506	0.173	GT	Sens	contig341	contig341	Unannota	[illegible]
GT	Sense	Sense	0.081	Detected	-0.081	Detected	0.000	0.877	Detected	1.574	Detected	1.225	0.076	GT	Sens	contig341	contig341	Endogluc	[illegible]
GT	Sense	Sense	0.242	Detected	-0.242	Comprom	0.000	0.997	Detected	1.023	Detected	1.010	0.053	GT	Sens	contig341	contig341	Unannota	[illegible]
GT	Sense	Sense	0.445	Detected	-0.445	Comprom	0.000	0.812	Detected	1.712	Detected	1.262	0.184	GT	Sens	contig341	contig341	Unannota	[illegible]
GT	Sense	Sense	0.180	Detected	-0.180	Comprom	0.000	0.923	Detected	1.381	Detected	1.152	0.058	GT	Sens	contig342	contig342	Unannota	[illegible]
GT	Sense	Sense	-0.180	Detected	0.180	Detected	0.000	1.212	Detected	0.887	Detected	1.049	0.049	GT	Sens	contig342	contig342	Unannota	[illegible]
GT	Sense	Sense	-0.252	Detected	0.252	Detected	0.000	1.292	Detected	0.848	Detected	1.070	0.086	GT	Sens	contig342	contig342	Unannota	[illegible]
GT	Sense	Sense	0.000	Comprom	0.000	Comprom	0.000	3.522	Detected	2.466	Detected	2.994	0.030	GT	Sens	contig343	contig343	Unannota	[illegible]
GT	Sense	Sense	0.175	Comprom	-0.175	Comprom	0.000	3.313	Detected	2.575	Detected	2.944	0.019	GT	Sens	contig343	contig343	Unannota	[illegible]
GT	Sense	Sense	0.245	Detected	-0.245	Detected	0.000	1.029	Detected	1.094	Detected	1.061	0.050	GT	Sens	contig343	contig343	MutL DN	[illegible]
GT	Sense	Sense	0.982	Detected	-0.982	Comprom	0.000	1.175	Detected	1.126	Detected	1.150	0.362	GT	Sens	contig343	contig343	Unannota	[illegible]
GT	Sense	Sense	0.028	Comprom	-0.028	Comprom	0.000	2.305	Detected	1.465	Detected	1.885	0.046	GT	Sens	contig344	contig344	Unannota	[illegible]
GT	Sense	Sense	0.156	Detected	-0.156	Detected	0.000	1.121	Detected	1.517	Detected	1.319	0.035	GT	Sens	contig345	contig345	Unannota	[illegible]
GT	Sense	Sense	0.305	Comprom	-0.305	Comprom	0.000	1.927	Detected	1.807	Detected	1.867	0.027	GT	Sens	contig345	contig345	Unannota	[illegible]
GT	Sense	Sense	0.066	Detected	-0.066	Detected	0.000	1.399	Detected	1.054	Detected	1.226	0.022	GT	Sens	contig345	contig345	Unannota	[illegible]
GT	Sense	Sense	0.286	Detected	-0.286	Comprom	0.000	0.988	Detected	1.059	Detected	1.024	0.071	GT	Sens	contig345	contig345	Unannota	[illegible]
GT	Sense	Sense	-0.139	Detected	0.139	Detected	0.000	1.564	Detected	1.069	Detected	1.317	0.043	GT	Sens	contig345	contig345	Uncharac	[illegible]
GT	Sense	Sense	0.072	Comprom	-0.072	Comprom	0.000	2.047	Detected	1.765	Detected	1.906	0.007	GT	Sens	contig345	contig345	Unannota	[illegible]
GT	Sense	Sense	0.027	Comprom	-0.027	Comprom	0.000	1.225	Detected	1.448	Detected	1.337	0.007	GT	Sens	contig346	contig346	Unannota	[illegible]
GT	Sense	Sense	0.325	Detected	-0.325	Detected	0.000	1.711	Detected	1.429	Detected	1.570	0.047	GT	Sens	contig346	contig346	Unannota	[illegible]
GT	Sense	Sense	0.306	Detected	-0.306	Detected	0.000	1.322	Detected	1.107	Detected	1.215	0.065	GT	Sens	contig347	contig347	Unannota	[illegible]
GT	Sense	Sense	-0.197	Comprom	0.197	Comprom	0.000	1.526	Detected	1.167	Detected	1.346	0.037	GT	Sens	contig347	contig347	Unannota	[illegible]
GT	Sense	Sense	0.607	Comprom	-0.607	Comprom	0.000	1.189	Detected	1.695	Detected	1.442	0.160	GT	Sens	contig348	contig348	Unannota	[illegible]
GT	Sense	Sense	0.079	Comprom	-0.079	Comprom	0.000	1.170	Detected	1.143	Detected	1.156	0.005	GT	Sens	contig348	contig348	Unannota	[illegible]
GT	Sense	Sense	-0.148	Comprom	0.148	Comprom	0.000	1.414	Detected	1.192	Detected	1.303	0.020	GT	Sens	contig349	contig349	Unannota	[illegible]
GT	Sense	Sense	0.323	Detected	-0.323	Comprom	0.000	1.872	Detected	1.670	Detected	1.771	0.035	GT	Sens	contig350	contig350	Unknown	[illegible]
GT	Sense	Sense	0.959	Detected	-0.959	Comprom	0.000	2.088	Detected	1.456	Detected	1.772	0.221	GT	Sens	contig351	contig351	Unannota	[illegible]
GT	Sense	Sense	0.384	Detected	-0.384	Comprom	0.000	1.476	Detected	1.292	Detected	1.384	0.073	GT	Sens	contig352	contig352	Unannota	[illegible]
GT	Sense	Sense	0.005	Detected	-0.005	Detected	0.000	0.967	Detected	1.053	Detected	1.010	0.002	GT	Sens	contig352	contig352	Unannota	[illegible]
GT	Sense	Sense	-0.550	Comprom	0.550	Detected	0.000	0.907	Detected	1.476	Detected	1.191	0.194	GT	Sens	contig353	contig353	Unannota	[illegible]
GT	Sense	Sense	0.035	Detected	-0.035	Comprom	0.000	1.418	Detected	1.504	Detected	1.461	0.001	GT	Sens	contig353	contig353	Unannota	[illegible]
GT	Sense	Sense	0.275	Comprom	-0.275	Comprom	0.000	1.258	Detected	1.017	Detected	1.138	0.063	GT	Sens	contig353	contig353	Unannota	[illegible]
GT	Sense	Sense	0.136	Comprom	-0.136	Comprom	0.000	1.568	Detected	1.096	Detected	1.332	0.039	GT	Sens	contig354	contig354	Unannota	[illegible]
GT	Sense	Sense	0.683	Detected	-0.683	Comprom	0.000	0.848	Detected	1.280	Detected	1.064	0.276	GT	Sens	contig354	contig354	Unannota	[illegible]
GT	Sense	Sense	0.115	Detected	-0.115	Detected	0.000	1.087	Detected	0.923	Detected	1.005	0.019	GT	Sens	contig355	contig355	Unannota	[illegible]
GT	Sense	Sense	0.042	Comprom	-0.042	Comprom	0.000	1.020	Detected	1.087	Detected	1.053	0.003	GT	Sens	contig356	contig356	Low-temp	[illegible]
GT	Sense	Sense	-0.477	Comprom	0.477	Comprom	0.000	2.814	Detected	3.214	Detected	3.014	0.028	GT	Sens	contig356	contig356	Unannota	[illegible]
GT	Sense	Sense	-0.208	Comprom	0.208	Comprom	0.000	1.426	Detected	1.908	Detected	1.667	0.035	GT	Sens	contig356	contig356	Unannota	[illegible]
GT	Sense	Sense	0.768	Detected	-0.768	Comprom	0.000	0.839	Detected	1.167	Detected	1.003	0.330	GT	Sens	contig357	contig357	Unannota	[illegible]
GT	Sense	Sense	-0.168	Detected	0.168	Detected	0.000	1.402	Detected	0.993	Detected	1.198	0.045	GT	Sens	contig357	contig357	Unannota	[illegible]
GT	Sense	Sense	0.045	Detected	-0.045	Detected	0.000	2.157	Detected	1.886	Detected	2.022	0.005	GT	Sens	contig357	contig357	Unannota	[illegible]
GT	Sense	Sense	0.075	Detected	-0.075	Detected	0.000	2.162	Detected	1.804	Detected	1.983	0.009	GT	Sens	contig357	contig357	Unannota	[illegible]
GT	Sense	Sense	0.806	Comprom	-0.806	Comprom	0.000	1.478	Detected	3.030	Detected	2.254	0.182	GT	Sens	contig358	contig358	Unannota	[illegible]
GT	Sense	Sense	-0.211	Comprom	0.211	Comprom	0.000	2.329	Detected	2.116	Detected	2.223	0.011	GT	Sens	contig358	contig358	Unannota	[illegible]
GT	Sense	Sense	-0.049	Comprom	0.049	Comprom	0.000	1.851	Detected	2.035	Detected	1.943	0.003	GT	Sens	contig358	contig358	Unannota	[illegible]
GT	Sense	Sense	0.362	Detected	-0.362	Detected	0.000	0.882	Detected	1.372	Detected	1.127	0.123	GT	Sens	contig360	contig360	Unannota	[illegible]
GT	Sense	Sense	0.203	Detected	-0.203	Detected	0.000	0.995	Detected	1.504	Detected	1.250	0.062	GT	Sens	contig360	contig360	Unannota	[illegible]
GT	Sense	Sense	0.467	Detected	-0.467	Comprom	0.000	0.928	Detected	1.801	Detected	1.365	0.166	GT	Sens	contig361	contig361	Malate sv	[illegible]
GT	Sense	Sense	-0.618	Comprom	0.618	Comprom	0.000	1.129	Detected	1.056	Detected	1.092	0.219	GT	Sens	contig362	contig362	Unannota	[illegible]
GT	Sense	Sense	-0.140	Detected	0.140	Detected	0.000	1.248	Detected	1.092	Detected	1.170	0.018	GT	Sens	contig364	contig364	UDP-gluc	[illegible]
GT	Sense	Sense	-0.632	Comprom	0.632	Comprom	0.000	0.979	Detected	1.142	Detected	1.061	0.238	GT	Sens	contig364	contig364	Unannota	[illegible]
GT	Sense	Sense	0.002	Detected	-0.002	Detected	0.000	1.416	Detected	1.051	Detected	1.233	0.021	GT	Sens	contig365	contig365	Phytoene	[illegible]
GT	Sense	Sense	-0.121	Detected	0.121	Detected	0.000	1.251	Detected	0.984	Detected	1.118	0.025	GT	Sens	contig366	contig366	Unannota	[illegible]
GT	Sense	Sense	-0.389	Detected	0.389	Detected	0.000	1.114	Detected	0.915	Detected	1.015	0.127	GT	Sens	contig366	contig366	Unannota	[illegible]
GT	Sense	Sense	-0.155	Detected	0.155	Detected	0.000	1.029	Detected	1.062	Detected	1.046	0.021	GT	Sens	contig366	contig366	Unannota	[illegible]
GT	Sense	Sense	0.515	Detected	-0.515	Comprom	0.000	1.003	Detected	1.875	Detected	1.439	0.166	GT	Sens	contig366	contig366	Unannota	[illegible]
GT	Sense	Sense	0.004	Detected	-0.004	Detected	0.000	1.576	Detected	1.977	Detected	1.776	0.013	GT	Sens	contig366	contig366	Unannota	[illegible]
GT	Sense	Sense	-0.059	Detected	0.059	Detected	0.000	1.056	Detected	1.128	Detected	1.092	0.004	GT	Sens	contig367	contig367	Unannota	[illegible]
GT	Sense	Sense	-0.115	Detected	0.115	Detected	0.000	1.342	Detected	1.223	Detected	1.283	0.010	GT	Sens	contig367	contig367	Alpha ma	[illegible]
GT	Sense	Sense	-0.941	Detected	0.941	Detected	0.000	1.517	Detected	1.682	Detected	1.599	0.232	GT	Sens	contig369	contig369	HAT fami	[illegible]
GT	Sense	Sense	-0.022	Detected	0.022	Detected	0.000	1.462	Detected	1.526	Detected	1.494	0.001	GT	Sens	contig369	contig369	Unannota	[illegible]
GT	Sense	Sense	0.212	Detected	-0.212	Detected	0.000	1.403	Detected	1.381	Detected	1.392	0.022	GT	Sens	contig369	contig369	Unannota	[illegible]
GT	Sense	Sense	0.266	Detected	-0.266	Comprom	0.000	1.100	Detected	1.144	Detected	1.122	0.052	GT	Sens	contig369	contig369	Unannota	[illegible]
GT	Sense	Sense	-0.025	Detected	0.025	Detected	0.000	1.315	Detected	1.129	Detected	1.222	0.006	GT	Sens	contig371	contig371	Unannota	[illegible]
GT	Sense	Sense	-0.494	Comprom	0.494	Comprom	0.000	1.665	Detected	2.677	Detected	2.171	0.092	GT	Sens	contig371	contig371	Unannota	[illegible]
GT	Sense	Sense	-0.088	Comprom	0.088	Comprom	0.000	1.073	Detected	1.044	Detected	1.058	0.007	GT	Sens	contig371	contig371	Unannota	[illegible]
GT	Sense	Sense	0.233	Comprom	-0.233	Comprom	0.000	1.816	Detected	1.232	Detected	1.524	0.055	GT	Sens	contig371	contig371	Unannota	[illegible]
GT	Sense	Sense	0.363	Comprom	-0.363	Comprom	0.000	2.714	Detected	2.495	Detected	2.605	0.021	GT	Sens	contig372	contig372	Unannota	[illegible]
GT	Sense	Sense	0.991	Detected	-0.991	Comprom	0.000	2.644	Detected	2.959	Detected	2.802	0.108	GT	Sens	contig372	contig372	Unannota	[illegible]
GT	Sense	Sense	0.068	Comprom	-0.068	Comprom	0.000	2.045	Detected	2.533	Detected	2.289	0.012	GT	Sens	contig372	contig372	Unannota	[illegible]
GT	Sense	Sense	0.426	Detected	-0.426	Detected	0.000	1.541	Detected	1.533	Detected	1.537	0.069	GT	Sens	contig372	contig372	Unannota	[illegible]
GT	Sense	Sense	0.396	Detected	-0.396	Detected	0.000	1.196	Detected	1.130	Detected	1.163	0.100	GT	Sens	contig372	contig372	Unannota	[illegible]
GT	Sense	Sense	-0.086	Comprom	0.086	Comprom	0.000	1.026	Detected	1.713	Detected	1.369	0.061	GT	Sens	contig372	contig372	Unannota	[illegible]
GT	Sense	Sense	0.071	Detected	-0.071	Detected	0.000	1.140	Detected	1.255	Detected	1.197	0.006	GT	Sens	contig373	contig373	Unannota	[illegible]
GT	Sense	Sense	0.174	Detected	-0.174	Detected	0.000	1.260	Detected	1.308	Detected	1.284	0.018	GT	Sens	contig373	contig373	Unannota	[illegible]
GT	Sense	Sense	0.246	Detected	-0.246	Detected	0.000	1.765	Detected	1.407	Detected	1.586	0.035	GT	Sens	contig373	contig373	Unannota	[illegible]
GT	Sense	Sense	0.294	Detected	-0.294	Detected	0.000	1.727	Detected	1.336	Detected	1.532	0.049	GT	Sens	contig373	contig373	Unannota	[illegible]
GT	Sense	Sense	0.462	Comprom	-0.462	Comprom	0.000	1.244	Detected	0.957	Detected	1.101	0.151	GT	Sens	contig373	contig373	Unannota	[illegible]
GT	Sense	Sense	0.016	Comprom	-0.016	Comprom	0.000	3.144	Detected	1.308	Detected	2.226	0.136	GT	Sens	contig376	contig376	Unannota	[illegible]
GT	Sense	Sense	0.102	Detected	-0.102	Detected	0.000	1.035	Detected	1.022	Detected	1.028	0.010	GT	Sens	contig376	contig376	Unannota	[illegible]
GT	Sense	Sense	0.894	Detected	-0.894	Comprom	0.000	1.577	Detected	1.685	Detected	1.631	0.210	GT	Sens	contig378	contig378	Unannota	[illegible]
GT	Sense	Sense	-0.094	Detected	0.094	Detected	0.000	1.049	Detected	1.118	Detected	1.084	0.008	GT	Sens	contig379	contig379	Unannota	[illegible]
GT	Sense	Sense	0.083	Detected	-0.083	Detected	0.000	1.634	Detected	1.394	Detected	1.514	0.009	GT	Sens	contig379	contig379	Unannota	[illegible]
GT	Sense	Sense	0.096	Detected	-0.096	Detected	0.000	1.630	Detected	1.282	Detected	1.456	0.018	GT	Sens	contig379	contig379	Unannota	[illegible]
GT	Sense	Sense	0.707	Comprom	-0.707	Comprom	0.000	1.230	Detected	1.519	Detected	1.374	0.197	GT	Sens	contig379	contig379	Unannota	[illegible]
GT	Sense	Sense	0.481	Detected	-0.481	Comprom	0.000	1.321	Detected	0.959	Detected	1.140	0.157	GT	Sens	contig379	contig379	Unannota	[illegible]
GT	Sense	Sense	-0.288	Comprom	0.288	Comprom	0.000	1.073	Detected	1.151	Detected	1.112	0.062	GT	Sens	contig379	contig379	Unannota	[illegible]
GT	Sense	Sense	0.116	Comprom	-0.116	Comprom	0.000	2.344	Detected	2.190	Detected	2.267	0.004	GT	Sens	contig380	contig380	Unannota	[illegible]
GT	Sense	Sense	-0.911	Comprom	0.911	Comprom	0.000	1.251	Detected	1.524	Detected	1.387	0.271	GT	Sens	contig381	contig381	Unannota	[illegible]
GT	Sense	Sense	0.229	Detected	-0.229	Detected	0.000	1.292	Detected	1.171	Detected	1.231	0.035	GT	Sens	contig381	contig381	Unannota	[illegible]
GT	Sense	Sense	-0.527	Comprom	0.527	Comprom	0.000	1.019	Detected	1.042	Detected	1.030	0.190	GT	Sens	contig382	contig382	Unannota	[illegible]
GT	Sense	Sense	0.200	Detected	-0.200	Detected	0.000	1.179	Detected	1.068	Detected	1.124	0.032	GT	Sens	contig383	contig383	Aquapori	[illegible]
GT	Sense	Sense	-0.263	Detected	0.263	Detected	0.000	1.160	Detected	1.031	Detected	1.095	0.056	GT	Sens	contig386	contig386	Unannota	[illegible]
GT	Sense	Sense	-0.994	Comprom	0.994	Comprom	0.000	2.117	Detected	1.113	Detected	1.615	0.284	GT	Sens	contig386	contig386	Unannota	[illegible]
GT	Sense	Sense	0.527	Detected	-0.527	Comprom	0.000	1.308	Detected	1.712	Detected	1.510	0.116	GT	Sens	contig388	contig388	Unannota	[illegible]
GT	Sense	Sense	0.281	Detected	-0.281	Detected	0.000	1.374	Detected	1.305	Detected	1.340	0.042	GT	Sens	contig389	contig389	Unannota	[illegible]
GT	Sense	Sense	0.265	Detected	-0.265	Detected	0.000	1.154	Detected	0.942	Detected	1.048	0.067	GT	Sens	contig390	contig390	Unannota	[illegible]
GT	Sense	Sense	-0.197	Comprom	0.197	Comprom	0.000	1.035	Detected	1.160	Detected	1.098	0.034	GT	Sens	contig391	contig391	Unannota	[illegible]
GT	Sense	Sense	0.406	Comprom	-0.406	Comprom	0.000	1.081	Detected	1.289	Detected	1.185	0.106	GT	Sens	contig392	contig392	Unannota	[illegible]
GT	Sense	Sense	0.307	Detected	-0.307	Detected	0.000	1.161	Detected	1.122	Detected	1.141	0.065	GT	Sens	contig392	contig392	Unannota	[illegible]
GT	Sense	Sense	0.259	Detected	-0.259	Detected	0.000	1.457	Detected	1.404	Detected	1.430	0.032	GT	Sens	contig392	contig392	Unannota	[illegible]
GT	Sense	Sense	0.822	Detected	-0.822	Comprom	0.000	1.280	Detected	1.033	Detected	1.156	0.299	GT	Sens	contig392	contig392	Unannota	[illegible]
GT	Sense	Sense	0.052	Comprom	-0.052	Comprom	0.000	2.000	Detected	3.248	Detected	2.624	0.053	GT	Sens	contig392	contig392	F-box fan	[illegible]
GT	Sense	Sense	0.066	Detected	-0.066	Detected	0.000	1.416	Detected	1.446	Detected	1.431	0.002	GT	Sens	contig393	contig393	LRR rece	[illegible]
GT	Sense	Sense	0.046	Comprom	-0.046	Comprom	0.000	1.095	Detected	1.159	Detected	1.127	0.002	GT	Sens	contig393	contig393	Unannota	[illegible]
GT	Sense	Sense	-0.206	Comprom	0.206	Comprom	0.000	1.614	Detected	2.091	Detected	1.853	0.028	GT	Sens	contig393	contig393	Unannota	[illegible]
GT	Sense	Sense	-0.103	Detected	0.103	Detected	0.000	1.173	Detected	0.866	Detected	1.020	0.031	GT	Sens	contig393	contig393	Unannota	[illegible]
GT	Sense	Sense	0.120	Comprom	-0.120	Comprom	0.000	2.201	Detected	1.992	Detected	2.097	0.006	GT	Sens	contig393	contig393	Unknown	[illegible]
GT	Sense	Sense	0.082	Detected	-0.082	Detected	0.000	1.128	Detected	1.015	Detected	1.072	0.009	GT	Sens	contig394	contig394	Unannota	[illegible]
GT	Sense	Sense	0.699	Comprom	-0.699	Comprom	0.000	0.875	Detected	1.255	Detected	1.065	0.279	GT	Sens	contig395	contig395	Unannota	[illegible]
GT	Sense	Sense	0.385	Detected	-0.385	Detected	0.000	2.102	Detected	1.173	Detected	1.638	0.113	GT	Sens	contig395	contig395	Unannota	[illegible]
GT	Sense	Sense	0.103	Detected	-0.103	Detected	0.000	1.053	Detected	1.083	Detected	1.068	0.009	GT	Sens	contig396	contig396	Solute ca	[illegible]
GT	Sense	Sense	0.214	Detected	-0.214	Detected	0.000	1.450	Detected	0.814	Detected	1.132	0.098	GT	Sens	contig396	contig396	Unannota	[illegible]
GT	Sense	Sense	0.405	Comprom	-0.405	Comprom	0.000	4.364	Detected	3.345	Detected	3.855	0.027	GT	Sens	contig397	contig397	Unannota	[illegible]
GT	Sense	Sense	-0.183	Detected	0.183	Detected	0.000	1.130	Detected	0.873	Detected	1.001	0.047	GT	Sens	contig397	contig397	Unannota	[illegible]
GT	Sense	Sense	-0.071	Detected	0.071	Detected	0.000	1.757	Detected	1.535	Detected	1.646	0.006	GT	Sens	contig397	contig397	Unannota	[illegible]
GT	Sense	Sense	-0.443	Detected	0.443	Detected	0.000	0.967	Detected	1.243	Detected	1.105	0.140	GT	Sens	contig397	contig397	Unannota	[illegible]
GT	Sense	Sense	-1.035	Detected	1.035	Detected	0.000	1.365	Detected	1.498	Detected	1.432	0.302	GT	Sens	contig397	contig397	Unannota	[illegible]
GT	Sense	Sense	1.162	Detected	-1.162	Comprom	0.000	2.356	Detected	1.946	Detected	2.151	0.210	GT	Sens	contig397	contig397	Unannota	[illegible]
GT	Sense	Sense	-0.135	Detected	0.135	Detected	0.000	1.975	Detected	1.565	Detected	1.770	0.019	GT	Sens	contig397	contig397	Unannota	[illegible]
GT	Sense	Sense	0.095	Detected	-0.095	Detected	0.000	2.068	Detected	1.995	Detected	2.031	0.002	GT	Sens	contig397	contig397	Unannota	[illegible]
GT	Sense	Sense	0.875	Detected	-0.875	Comprom	0.000	4.846	Detected	4.668	Detected	4.757	0.033	GT	Sens	contig397	contig397	Unknown	[illegible]
GT	Sense	Sense	0.047	Detected	-0.047	Detected	0.000	1.060	Detected	1.015	Detected	1.038	0.003	GT	Sens	contig399	contig399	Unannota	[illegible]
GT	Sense	Sense	0.209	Detected	-0.209	Detected	0.000	1.762	Detected	1.524	Detected	1.643	0.021	GT	Sens	contig400	contig400	Unannota	[illegible]
GT	Sense	Sense	0.109	Detected	-0.109	Detected	0.000	1.804	Detected	1.620	Detected	1.712	0.007	GT	Sens	contig400	contig400	Unannota	[illegible]
GT	Sense	Sense	-0.279	Comprom	0.279	Comprom	0.000	1.568	Detected	2.049	Detected	1.809	0.039	GT	Sens	contig401	contig401	Unannota	[illegible]
GT	Sense	Sense	-1.623	Comprom	1.623	Detected	0.000	1.163	Detected	1.319	Detected	1.241	0.525	GT	Sens	contig401	contig401	Unknown	[illegible]
GT	Sense	Sense	-0.243	Detected	0.243	Detected	0.000	1.216	Detected	1.141	Detected	1.179	0.041	GT	Sens	contig401	contig401	Unannota	[illegible]
GT	Sense	Sense	-0.226	Detected	0.226	Detected	0.000	1.287	Detected	0.910	Detected	1.099	0.065	GT	Sens	contig401	contig401	Unannota	[illegible]
GT	Sense	Sense	-1.147	Comprom	1.147	Detected	0.000	1.995	Detected	0.974	Detected	1.484	0.359	GT	Sens	contig401	contig401	Unannota	[illegible]
GT	Sense	Sense	-1.295	Comprom	1.295	Detected	0.000	1.115	Detected	1.677	Detected	1.396	0.403	GT	Sens	contig401	contig401	Unannota	[illegible]
GT	Sense	Sense	-0.070	Comprom	0.070	Comprom	0.000	1.983	Detected	1.875	Detected	1.929	0.002	GT	Sens	contig401	contig401	Unannota	[illegible]
GT	Sense	Sense	0.836	Detected	-0.836	Comprom	0.000	1.771	Detected	1.642	Detected	1.707	0.179	GT	Sens	contig401	contig401	Unannota	[illegible]
GT	Sense	Sense	-0.151	Comprom	0.151	Comprom	0.000	1.040	Detected	1.331	Detected	1.186	0.030	GT	Sens	contig402	contig402	Unannota	[illegible]
GT	Sense	Sense	0.078	Comprom	-0.078	Comprom	0.000	2.069	Detected	2.036	Detected	2.053	0.002	GT	Sens	contig402	contig402	Unannota	[illegible]
GT	Sense	Sense	0.242	Detected	-0.242	Detected	0.000	1.775	Detected	1.516	Detected	1.646	0.027	GT	Sens	contig402	contig402	HAD supe	[illegible]
GT	Sense	Sense	0.290	Detected	-0.290	Detected	0.000	1.748	Detected	1.344	Detected	1.546	0.048	GT	Sens	contig402	contig402	Unannota	[illegible]
GT	Sense	Sense	0.567	Comprom	-0.567	Comprom	0.000	1.977	Detected	2.146	Detected	2.062	0.069	GT	Sens	contig402	contig402	Unannota	[illegible]
GT	Sense	Sense	0.319	Comprom	-0.319	Comprom	0.000	1.406	Detected	1.296	Detected	1.351	0.053	GT	Sens	contig403	contig403	Unannota	[illegible]
GT	Sense	Sense	0.136	Comprom	-0.136	Comprom	0.000	1.120	Detected	1.429	Detected	1.274	0.025	GT	Sens	contig403	contig403	Unannota	[illegible]
GT	Sense	Sense	-0.077	Detected	0.077	Detected	0.000	0.803	Detected	1.447	Detected	1.125	0.077	GT	Sens	contig403	contig403	Unannota	[illegible]
GT	Sense	Sense	0.091	Detected	-0.091	Detected	0.000	1.338	Detected	0.927	Detected	1.132	0.037	GT	Sens	contig403	contig403	Unannota	[illegible]
GT	Sense	Sense	-0.344	Comprom	0.344	Comprom	0.000	1.466	Detected	0.826	Detected	1.146	0.135	GT	Sens	contig404	contig404	Unannota	[illegible]
GT	Sense	Sense	0.221	Detected	-0.221	Detected	0.000	1.159	Detected	1.148	Detected	1.154	0.035	GT	Sens	contig405	contig405	Unannota	[illegible]
GT	Sense	Sense	-0.083	Comprom	0.083	Comprom	0.000	1.064	Detected	1.089	Detected	1.076	0.006	GT	Sens	contig405	contig405	Unannota	[illegible]
GT	Sense	Sense	0.485	Detected	-0.485	Comprom	0.000	1.108	Detected	1.285	Detected	1.197	0.136	GT	Sens	contig406	contig406	Unannota	[illegible]
GT	Sense	Sense	0.183	Detected	-0.183	Detected	0.000	1.092	Detected	1.130	Detected	1.111	0.026	GT	Sens	contig406	contig406	Unannota	[illegible]
GT	Sense	Sense	-0.285	Comprom	0.285	Comprom	0.000	1.315	Detected	1.313	Detected	1.314	0.044	GT	Sens	contig406	contig406	Unannota	[illegible]
GT	Sense	Sense	0.731	Detected	-0.731	Comprom	0.000	1.090	Detected	0.995	Detected	1.042	0.291	GT	Sens	contig407	contig407	Unannota	[illegible]
GT	Sense	Sense	0.387	Comprom	-0.387	Comprom	0.000	1.270	Detected	1.714	Detected	1.492	0.079	GT	Sens	contig408	contig408	Unannota	[illegible]
GT	Sense	Sense	0.660	Detected	-0.660	Detected	0.000	1.007	Detected	1.309	Detected	1.158	0.229	GT	Sens	contig409	contig409	Unannota	[illegible]
GT	Sense	Sense	0.605	Detected	-0.605	Detected	0.000	0.927	Detected	1.411	Detected	1.169	0.215	GT	Sens	contig409	contig409	Unannota	[illegible]
GT	Sense	Sense	0.257	Detected	-0.257	Detected	0.000	1.002	Detected	1.015	Detected	1.009	0.059	GT	Sens	contig410	contig410	Unannota	[illegible]
GT	Sense	Sense	0.116	Comprom	-0.116	Comprom	0.000	0.918	Detected	1.366	Detected	1.142	0.045	GT	Sens	contig410	contig410	Unannota	[illegible]
GT	Sense	Sense	0.036	Detected	-0.036	Detected	0.000	1.349	Detected	1.128	Detected	1.238	0.009	GT	Sens	contig410	contig410	Unannota	[illegible]
GT	Sense	Sense	-0.829	Comprom	0.829	Comprom	0.000	2.778	Detected	0.849	Detected	1.814	0.290	GT	Sens	contig411	contig411	Unannota	[illegible]
GT	Sense	Sense	0.215	Detected	-0.215	Detected	0.000	1.039	Detected	1.217	Detected	1.128	0.040	GT	Sens	contig411	contig411	Unannota	[illegible]
GT	Sense	Sense	-0.378	Detected	0.378	Detected	0.000	0.957	Detected	1.424	Detected	1.191	0.116	GT	Sens	contig411	contig411	LOB dom	[illegible]
GT	Sense	Sense	0.356	Detected	-0.356	Detected	0.000	1.477	Detected	1.645	Detected	1.561	0.051	GT	Sens	contig411	contig411	Unannota	[illegible]
GT	Sense	Sense	0.314	Detected	-0.314	Detected	0.000	1.281	Detected	1.308	Detected	1.294	0.054	GT	Sens	contig411	contig411	Unannota	[illegible]
GT	Sense	Sense	0.759	Detected	-0.759	Comprom	0.000	0.825	Detected	1.229	Detected	1.027	0.321	GT	Sens	contig411	contig411	Unannota	[illegible]
GT	Sense	Sense	-0.467	Detected	0.467	Detected	0.000	1.079	Detected	0.996	Detected	1.037	0.157	GT	Sens	contig412	contig412	Unannota	[illegible]
GT	Sense	Sense	-0.557	Comprom	0.557	Comprom	0.000	1.587	Detected	1.369	Detected	1.478	0.121	GT	Sens	contig412	contig412	Unannota	[illegible]
GT	Sense	Sense	0.390	Detected	-0.390	Comprom	0.000	1.855	Detected	1.540	Detected	1.698	0.056	GT	Sens	contig412	contig412	Unannota	[illegible]
GT	Sense	Sense	-0.122	Detected	0.122	Detected	0.000	1.052	Detected	1.082	Detected	1.067	0.013	GT	Sens	contig412	contig412	Short-cha	[illegible]
GT	Sense	Sense	-0.242	Comprom	0.242	Comprom	0.000	0.997	Detected	1.226	Detected	1.112	0.053	GT	Sens	contig413	contig413	Unannota	[illegible]
GT	Sense	Sense	0.385	Comprom	-0.385	Comprom	0.000	2.036	Detected	1.874	Detected	1.955	0.038	GT	Sens	contig413	contig413	Unannota	[illegible]
GT	Sense	Sense	-0.088	Detected	0.088	Detected	0.000	1.525	Detected	1.201	Detected	1.363	0.018	GT	Sens	contig414	contig414	Unannota	[illegible]
GT	Sense	Sense	-0.092	Detected	0.092	Detected	0.000	1.335	Detected	0.995	Detected	1.165	0.027	GT	Sens	contig414	contig414	Unannota	[illegible]
GT	Sense	Sense	-0.850	Comprom	0.850	Comprom	0.000	0.930	Detected	1.376	Detected	1.153	0.320	GT	Sens	contig414	contig414	Unannota	[illegible]
GT	Sense	Sense	0.113	Comprom	-0.113	Comprom	0.000	1.666	Detected	1.006	Detected	1.336	0.062	GT	Sens	contig415	contig415	Unannota	[illegible]
GT	Sense	Sense	-0.258	Detected	0.258	Detected	0.000	1.222	Detected	0.950	Detected	1.086	0.065	GT	Sens	contig415	contig415	Unannota	[illegible]
GT	Sense	Sense	0.110	Detected	-0.110	Detected	0.000	1.283	Detected	1.202	Detected	1.242	0.009	GT	Sens	contig416	contig416	Unannota	[illegible]
GT	Sense	Sense	-0.412	Comprom	0.412	Comprom	0.000	1.141	Detected	1.007	Detected	1.074	0.124	GT	Sens	contig416	contig416	Unannota	[illegible]
GT	Sense	Sense	-0.409	Comprom	0.409	Comprom	0.000	1.852	Detected	1.328	Detected	1.590	0.082	GT	Sens	contig416	contig416	Unannota	[illegible]
GT	Sense	Sense	-0.012	Comprom	0.012	Comprom	0.000	1.242	Detected	1.937	Detected	1.590	0.045	GT	Sens	contig417	contig417	Unannota	[illegible]
GT	Sense	Sense	-0.145	Comprom	0.145	Comprom	0.000	1.320	Detected	1.263	Detected	1.291	0.013	GT	Sens	contig418	contig418	Unannota	[illegible]
GT	Sense	Sense	-0.075	Detected	0.075	Detected	0.000	1.640	Detected	1.077	Detected	1.358	0.043	GT	Sens	contig418	contig418	Unannota	[illegible]
GT	Sense	Sense	0.271	Comprom	-0.271	Comprom	0.000	2.033	Detected	1.611	Detected	1.822	0.034	GT	Sens	contig418	contig418	Unannota	[illegible]
GT	Sense	Sense	-0.643	Comprom	0.643	Comprom	0.000	1.545	Detected	3.635	Detected	2.590	0.169	GT	Sens	contig419	contig419	Unannota	[illegible]
GT	Sense	Sense	-0.286	Comprom	0.286	Comprom	0.000	1.191	Detected	0.878	Detected	1.035	0.087	GT	Sens	contig419	contig419	Unannota	[illegible]
GT	Sense	Sense	0.129	Detected	-0.129	Detected	0.000	0.937	Detected	1.184	Detected	1.060	0.027	GT	Sens	contig419	contig419	Unannota	[illegible]
GT	Sense	Sense	-0.147	Detected	0.147	Detected	0.000	1.222	Detected	1.414	Detected	1.318	0.017	GT	Sens	contig419	contig419	Unannota	[illegible]
GT	Sense	Sense	-0.119	Detected	0.119	Detected	0.000	1.405	Detected	1.699	Detected	1.552	0.014	GT	Sens	contig419	contig419	Unannota	[illegible]
GT	Sense	Sense	-0.534	Comprom	0.534	Comprom	0.000	1.487	Detected	2.293	Detected	1.890	0.106	GT	Sens	contig420	contig420	Unannota	[illegible]
GT	Sense	Sense	0.165	Detected	-0.165	Comprom	0.000	1.212	Detected	1.356	Detected	1.284	0.019	GT	Sens	contig420	contig420	Unannota	[illegible]
GT	Sense	Sense	0.218	Comprom	-0.218	Comprom	0.000	1.280	Detected	0.914	Detected	1.097	0.061	GT	Sens	contig420	contig420	Unannota	[illegible]
GT	Sense	Sense	0.170	Detected	-0.170	Detected	0.000	1.155	Detected	1.728	Detected	1.442	0.049	GT	Sens	contig420	contig420	Unknown	[illegible]
GT	Sense	Sense	0.124	Detected	-0.124	Detected	0.000	1.058	Detected	0.979	Detected	1.018	0.016	GT	Sens	contig421	contig421	Unannota	[illegible]
GT	Sense	Sense	0.012	Detected	-0.012	Detected	0.000	1.069	Detected	1.252	Detected	1.160	0.006	GT	Sens	contig421	contig421	Unannota	[illegible]
GT	Sense	Sense	-0.035	Detected	0.035	Detected	0.000	1.002	Detected	1.361	Detected	1.182	0.023	GT	Sens	contig422	contig422	Unannota	[illegible]
GT	Sense	Sense	-0.111	Detected	0.111	Detected	0.000	0.885	Detected	1.121	Detected	1.003	0.025	GT	Sens	contig422	contig422	Unannota	[illegible]
GT	Sense	Sense	0.687	Detected	-0.687	Comprom	0.000	1.101	Detected	0.930	Detected	1.016	0.280	GT	Sens	contig422	contig422	Unannota	[illegible]
GT	Sense	Sense	0.206	Detected	-0.206	Detected	0.000	0.966	Detected	1.171	Detected	1.068	0.044	GT	Sens	contig422	contig422	Unannota	[illegible]
GT	Sense	Sense	0.788	Detected	-0.788	Comprom	0.000	1.605	Detected	0.862	Detected	1.233	0.292	GT	Sens	contig423	contig423	Unannota	[illegible]
GT	Sense	Sense	0.287	Comprom	-0.287	Comprom	0.000	1.213	Detected	1.348	Detected	1.280	0.049	GT	Sens	contig424	contig424	Unannota	[illegible]
GT	Sense	Sense	-0.626	Comprom	0.626	Comprom	0.000	1.761	Detected	1.327	Detected	1.544	0.145	GT	Sens	contig424	contig424	Unannota	[illegible]
GT	Sense	Sense	1.238	Detected	-1.238	Comprom	0.000	1.807	Detected	0.905	Detected	1.356	0.412	GT	Sens	contig425	contig425	Glutared	[illegible]
GT	Sense	Sense	-0.176	Comprom	0.176	Comprom	0.000	1.271	Detected	1.435	Detected	1.353	0.020	GT	Sens	contig425	contig425	Unannota	[illegible]
GT	Sense	Sense	0.121	Detected	-0.121	Detected	0.000	1.091	Detected	1.106	Detected	1.098	0.012	GT	Sens	contig426	contig426	Putative	[illegible]
GT	Sense	Sense	0.057	Detected	-0.057	Detected	0.000	1.300	Detected	0.910	Detected	1.105	0.032	GT	Sens	contig426	contig426	Putative	[illegible]
GT	Sense	Sense	0.353	Detected	-0.353	Comprom	0.000	0.840	Detected	1.306	Detected	1.073	0.126	GT	Sens	contig426	contig426	AT3G627	[illegible]
GT	Sense	Sense	0.205	Detected	-0.205	Detected	0.000	1.255	Detected	1.286	Detected	1.271	0.025	GT	Sens	contig427	contig427	Probable	[illegible]

GT	Sense	Sense	0.138	Detected	-0.138	Comprom	0.000	1.198	Detected	1.029	Detected	1.113	0.020	GT	Sens	contig427	contig427	FACT cor	TTGTTAAGACTGT		[illegible]
GT	Sense	Sense	-0.608	Detected	0.608	Detected	0.000	1.336	Detected	1.467	Detected	1.402	0.149	GT	Sens	contig427	contig427	Putative (	TCAGTACCGGGC		[illegible]
GT	Sense	Sense	0.279	Detected	-0.279	Detected	0.000	0.893	Detected	1.236	Detected	1.064	0.083	GT	Sens	contig428	contig428	O-Glycos	TGTTCAAGGACAC		[illegible]
GT	Sense	Sense	0.174	Detected	-0.174	Comprom	0.000	1.536	Detected	1.368	Detected	1.452	0.017	GT	Sens	contig428	contig428	Zinc finge	TTTCTGATTCATC		[illegible]
GT	Sense	Sense	0.108	Detected	-0.108	Detected	0.000	1.656	Detected	1.199	Detected	1.427	0.030	GT	Sens	contig429	contig429	Uncharac	TCTTGG	contig42	[illegible]
GT	Sense	Sense	-0.039	Detected	0.039	Detected	0.000	1.186	Detected	0.956	Detected	1.071	0.013	GT	Sens	contig430	contig430	Unknown	AGGAAAATGGCAC		[illegible]
GT	Sense	Sense	0.140	Detected	-0.140	Detected	0.000	1.490	Detected	1.217	Detected	1.353	0.020	GT	Sens	contig430	contig430	Unknown	TTGAACCGAATTC		[illegible]
GT	Sense	Sense	0.297	Comprom	-0.297	Comprom	0.000	1.253	Detected	1.047	Detected	1.150	0.067	GT	Sens	contig431	contig431	Nicotia ta	ACTCCTATCTTTA		[illegible]
GT	Sense	Sense	0.019	Detected	-0.019	Detected	0.000	1.321	Detected	1.067	Detected	1.194	0.011	GT	Sens	contig431	contig431	Nicotia ta	GCATAGAGGACAC		[illegible]
GT	Sense	Sense	0.484	Comprom	-0.484	Comprom	0.000	1.560	Detected	1.303	Detected	1.432	0.104	GT	Sens	contig432	contig432	Nicotia ta	CCTTAAAGATAAT		[illegible]
GT	Sense	Sense	0.186	Detected	-0.186	Detected	0.000	1.310	Detected	1.142	Detected	1.226	0.027	GT	Sens	contig433	contig433	Nicotia ta	CTAGTGTTTTGCT		[illegible]
GT	Sense	Sense	0.195	Detected	-0.195	Detected	0.000	1.423	Detected	0.870	Detected	1.147	0.077	GT	Sens	contig434	contig434	N.tabacu	GGTTCCTTGTTTT		[illegible]
GT	Sense	Sense	-1.069	Comprom	1.069	Detected	0.000	1.212	Detected	0.994	Detected	1.103	0.413	GT	Sens	contig434	contig434	Nicotia ta	GGTTTCTCCTAAA		[illegible]
GT	Sense	Sense	0.113	Detected	-0.113	Detected	0.000	1.114	Detected	1.043	Detected	1.079	0.012	GT	Sens	contig434	contig434	Nicotia ta	GGACCTGCAAAGA		[illegible]
GT	Sense	Sense	0.150	Detected	-0.150	Detected	0.000	2.890	Detected	2.396	Detected	2.643	0.012	GT	Sens	contig434	contig434	Chloroph	ATTAGG	contig43	[illegible]
GT	Sense	Sense	-0.457	Detected	0.457	Detected	0.000	1.189	Detected	0.986	Detected	1.088	0.146	GT	Sens	contig434	contig434	Nicotia ta	TCTCCGGAAACAC		[illegible]
GT	Sense	Sense	-0.252	Detected	0.252	Detected	0.000	1.293	Detected	1.053	Detected	1.173	0.052	GT	Sens	contig434	contig434	Mitochon	GTCTCT	contig43	[illegible]
GT	Sense	Sense	0.433	Detected	-0.433	Comprom	0.000	3.024	Detected	2.140	Detected	2.582	0.053	GT	Sens	contig435	contig435	Leucine-r	CTGGAA	contig43	[illegible]
GT	Sense	Sense	-0.131	Comprom	0.131	Comprom	0.000	2.590	Detected	2.549	Detected	2.569	0.003	GT	Sens	contig445	contig445	Unknown	CTCAGC	contig44	[illegible]
GT	Sense	Sense	-1.395	Comprom	1.395	Detected	0.000	1.731	Detected	2.296	Detected	2.013	0.293	GT	Sens	contig452	contig452	Reverse t	CGCCTC	contig45	[illegible]
GT	Sense	Sense	0.029	Detected	-0.029	Detected	0.000	1.086	Detected	1.174	Detected	1.130	0.002	GT	Sens	contig456	contig456	BHLH tra	TGATCC	contig45	[illegible]
GT	Sense	Sense	-0.172	Detected	0.172	Detected	0.000	1.748	Detected	1.744	Detected	1.746	0.010	GT	Sens	contig457	contig457	NHL repe	GCAATT	contig45	[illegible]
GT	Sense	Sense	-0.096	Detected	0.096	Detected	0.000	1.090	Detected	1.023	Detected	1.056	0.009	GT	Sens	contig459	contig459	Unknown	GGTGTT	contig45	[illegible]
GT	Sense	Sense	0.086	Detected	-0.086	Detected	0.000	1.222	Detected	1.139	Detected	1.180	0.006	GT	Sens	contig460	contig460	Formin 3	GACAGT	contig46	[illegible]
GT	Sense	Sense	-0.201	Comprom	0.201	Comprom	0.000	1.018	Detected	1.330	Detected	1.174	0.044	GT	Sens	contig462	contig462	Cation/H	GCATCT	contig46	[illegible]
GT	Sense	Sense	0.065	Comprom	-0.065	Comprom	0.000	3.614	Detected	3.700	Detected	3.657	0.000	GT	Sens	contig466	contig466	UPF0497	TTTGGT	contig46	[illegible]
GT	Sense	Sense	-0.604	Detected	0.604	Detected	0.000	2.269	Detected	0.977	Detected	1.623	0.208	GT	Sens	contig472	contig472	Blue copi	CTTGGT	contig47	[illegible]
GT	Sense	Sense	0.184	Detected	-0.184	Detected	0.000	1.229	Detected	0.896	Detected	1.062	0.051	GT	Sens	contig474	contig474	Nodulin M	CTCCAT	contig47	[illegible]
GT	Sense	Sense	0.335	Detected	-0.335	Comprom	0.000	1.104	Detected	0.914	Detected	1.009	0.101	GT	Sens	contig476	contig476	General t	GAAGAA	contig47	[illegible]
GT	Sense	Sense	0.150	Detected	-0.150	Detected	0.000	1.750	Detected	1.332	Detected	1.541	0.027	GT	Sens	contig477	contig477	Magnesiu	AAATCC	contig47	[illegible]
GT	Sense	Sense	-0.001	Detected	0.001	Detected	0.000	1.192	Detected	1.163	Detected	1.178	0.000	GT	Sens	contig491	contig491	Zinc finge	GGTTCC	contig49	[illegible]
GT	Sense	Sense	0.231	Detected	-0.231	Detected	0.000	1.076	Detected	1.204	Detected	1.140	0.041	GT	Sens	contig494	contig494	RNA-bind	CAGGAA	contig49	[illegible]
GT	Sense	Sense	-0.224	Detected	0.224	Detected	0.000	1.129	Detected	0.900	Detected	1.015	0.056	GT	Sens	contig495	contig495	Auxin-res	GAAAAA	contig49	[illegible]
GT	Sense	Sense	-0.083	Detected	0.083	Detected	0.000	1.064	Detected	0.954	Detected	1.009	0.010	GT	Sens	contig508	contig508	Heat stre	ATGGAT	contig50	[illegible]
GT	Sense	Sense	0.120	Detected	-0.120	Detected	0.000	1.073	Detected	1.045	Detected	1.059	0.013	GT	Sens	contig509	contig509	Ulp1 prot	AGAAAC	contig50	[illegible]
GT	Sense	Sense	0.470	Comprom	-0.470	Comprom	0.000	1.239	Detected	1.102	Detected	1.170	0.133	GT	Sens	contig51	contig51	Unknown	CAATTA	contig51	[illegible]
GT	Sense	Sense	0.030	Detected	-0.030	Detected	0.000	0.989	Detected	1.171	Detected	1.080	0.008	GT	Sens	contig512	contig512	Unknown	TGGATC	contig51	[illegible]
GT	Sense	Sense	0.145	Detected	-0.145	Detected	0.000	1.157	Detected	1.327	Detected	1.242	0.018	GT	Sens	contig515	contig515	Beta-1%2	TTGACA	contig51	[illegible]
GT	Sense	Sense	-1.014	Comprom	1.014	Detected	0.000	3.634	Detected	2.093	Detected	2.863	0.154	GT	Sens	contig515	contig515	Pectate l	AATCTG	contig51	[illegible]
GT	Sense	Sense	0.016	Detected	-0.016	Detected	0.000	2.270	Detected	2.139	Detected	2.205	0.001	GT	Sens	contig519	contig519	Ribulose-	GCTGCA	contig51	[illegible]
GT	Sense	Sense	-0.145	Detected	0.145	Detected	0.000	1.650	Detected	0.969	Detected	1.309	0.071	GT	Sens	contig523	contig523	Zinc tran	CGTGGA	contig52	[illegible]
GT	Sense	Sense	-0.281	Detected	0.281	Detected	0.000	1.665	Detected	0.846	Detected	1.255	0.127	GT	Sens	contig529	contig529	UDP-gluc	GATTTC	contig52	[illegible]
GT	Sense	Sense	0.013	Detected	-0.013	Detected	0.000	1.503	Detected	1.293	Detected	1.398	0.006	GT	Sens	contig532	contig532	Receptor	AAGAGA	contig53	[illegible]
GT	Sense	Sense	0.003	Detected	-0.003	Detected	0.000	1.413	Detected	1.315	Detected	1.364	0.001	GT	Sens	contig534	contig534	Nucleoso	GAAAGG	contig53	[illegible]
GT	Sense	Sense	0.041	Detected	-0.041	Detected	0.000	1.629	Detected	1.242	Detected	1.435	0.018	GT	Sens	contig537	contig537	Cc-nbs-lr	TTGCTC	contig53	[illegible]
GT	Sense	Sense	-0.085	Detected	0.085	Detected	0.000	1.087	Detected	0.932	Detected	1.010	0.013	GT	Sens	contig544	contig544	Dihydrofl	GGGTAT	contig54	[illegible]
GT	Sense	Sense	0.094	Detected	-0.094	Comprom	0.000	1.509	Detected	1.371	Detected	1.440	0.006	GT	Sens	contig548	contig548	Non-spec	TCTCTT	contig54	[illegible]
GT	Sense	Sense	-0.259	Detected	0.259	Detected	0.000	1.105	Detected	1.025	Detected	1.065	0.056	GT	Sens	contig559	contig559	Polyaden	TGGTTT	contig55	[illegible]
GT	Sense	Sense	0.639	Detected	-0.639	Comprom	0.000	1.645	Detected	0.855	Detected	1.250	0.238	GT	Sens	contig563	contig563	Unknown	ATCGTC	contig56	[illegible]
GT	Sense	Sense	0.169	Detected	-0.169	Detected	0.000	1.223	Detected	0.820	Detected	1.021	0.060	GT	Sens	contig565	contig565	Ferric red	CGACAA	contig56	[illegible]
GT	Sense	Sense	-0.155	Detected	0.155	Detected	0.000	1.548	Detected	1.117	Detected	1.332	0.037	GT	Sens	contig566	contig566	Subtilisin	GAACGA	contig56	[illegible]
GT	Sense	Sense	-0.128	Detected	0.128	Detected	0.000	1.096	Detected	0.921	Detected	1.008	0.023	GT	Sens	contig569	contig569	Os02g07	ATAAATT	contig56	[illegible]
GT	Sense	Sense	-0.107	Detected	0.107	Detected	0.000	1.482	Detected	1.196	Detected	1.339	0.017	GT	Sens	contig573	contig573	Vesicle-a	AGAATT	contig57	[illegible]
GT	Sense	Sense	0.162	Detected	-0.162	Detected	0.000	1.959	Detected	1.159	Detected	1.559	0.069	GT	Sens	contig576	contig576	NAC dom	CATGAA	contig57	[illegible]
GT	Sense	Sense	-0.088	Detected	0.088	Detected	0.000	0.950	Detected	1.083	Detected	1.016	0.012	GT	Sens	contig577	contig577	Cellulose	TGTGGA	contig57	[illegible]
GT	Sense	Sense	0.106	Detected	-0.106	Detected	0.000	0.820	Detected	1.189	Detected	1.005	0.042	GT	Sens	contig579	contig579	Phloem p	AAATCC	contig57	[illegible]
GT	Sense	Sense	-0.047	Detected	0.047	Detected	0.000	1.363	Detected	1.030	Detected	1.197	0.020	GT	Sens	contig584	contig584	Aldehyde	TCCGTT	contig58	[illegible]
GT	Sense	Sense	-0.097	Detected	0.097	Detected	0.000	1.658	Detected	1.140	Detected	1.399	0.037	GT	Sens	contig585	contig585	Oxygen-e	AGGCAT	contig58	[illegible]
GT	Sense	Sense	0.083	Detected	-0.083	Detected	0.000	1.588	Detected	1.530	Detected	1.559	0.003	GT	Sens	contig587	contig587	Acyl- (AH	CTACTC	contig58	[illegible]
GT	Sense	Sense	-0.210	Detected	0.210	Detected	0.000	1.490	Detected	1.025	Detected	1.257	0.057	GT	Sens	contig595	contig595	Glycosylt	CACCTG	contig59	[illegible]
GT	Sense	Sense	0.615	Detected	-0.615	Comprom	0.000	1.549	Detected	1.518	Detected	1.533	0.130	GT	Sens	contig597	contig597	Genomic	AGCAGT	contig59	[illegible]
GT	Sense	Sense	0.020	Detected	-0.020	Detected	0.000	0.956	Detected	1.069	Detected	1.012	0.003	GT	Sens	contig598	contig598	Nuclear p	ATAAAC	contig59	[illegible]
GT	Sense	Sense	-0.394	Detected	0.394	Detected	0.000	1.134	Detected	0.982	Detected	1.058	0.119	GT	Sens	contig610	contig610	Ferredox	ATTAAG	contig61	[illegible]
GT	Sense	Sense	0.616	Detected	-0.616	Comprom	0.000	1.493	Detected	1.143	Detected	1.318	0.176	GT	Sens	contig611	contig611	CWF19-lil	ACAGAA	contig61	[illegible]
GT	Sense	Sense	0.212	Detected	-0.212	Detected	0.000	1.186	Detected	1.668	Detected	1.427	0.047	GT	Sens	contig612	contig612	Expresse	TCATGA	contig61	[illegible]
GT	Sense	Sense	-0.266	Detected	0.266	Detected	0.000	1.978	Detected	2.243	Detected	2.110	0.019	GT	Sens	contig614	contig614	Expansin	CTAAAT	contig61	[illegible]
GT	Sense	Sense	0.065	Detected	-0.065	Detected	0.000	1.063	Detected	1.062	Detected	1.063	0.004	GT	Sens	contig615	contig615	Carboxyl	CCACAA	contig61	[illegible]
GT	Sense	Sense	-0.222	Detected	0.222	Detected	0.000	1.730	Detected	1.249	Detected	1.489	0.045	GT	Sens	contig625	contig625	FAD depe	ACTTGA	contig62	[illegible]
GT	Sense	Sense	0.180	Comprom	-0.180	Comprom	0.000	2.093	Detected	2.273	Detected	2.183	0.008	GT	Sens	contig628	contig628	F-box fan	AGATGG	contig62	[illegible]
GT	Sense	Sense	-1.036	Comprom	1.036	Detected	0.000	3.088	Detected	1.163	Detected	2.126	0.272	GT	Sens	contig628	contig628	Pollen all	GTTCGT	contig62	[illegible]
GT	Sense	Sense	-0.047	Comprom	0.047	Comprom	0.000	2.920	Detected	2.260	Detected	2.590	0.016	GT	Sens	contig630	contig630	Syntaxin	ATTTGA	contig63	[illegible]
GT	Sense	Sense	0.051	Detected	-0.051	Detected	0.000	1.642	Detected	0.909	Detected	1.275	0.075	GT	Sens	contig635	contig635	Cellulose	AGGAAG	contig63	[illegible]
GT	Sense	Sense	0.422	Detected	-0.422	Detected	0.000	1.061	Detected	1.259	Detected	1.160	0.116	GT	Sens	contig637	contig637	3-oxoacy	AACCCC	contig63	[illegible]
GT	Sense	Sense	-0.414	Detected	0.414	Detected	0.000	1.135	Detected	1.238	Detected	1.186	0.105	GT	Sens	contig638	contig638	Protein p	AAGGTG	contig63	[illegible]
GT	Sense	Sense	-0.558	Comprom	0.558	Comprom	0.000	2.480	Detected	1.447	Detected	1.964	0.123	GT	Sens	contig641	contig641	Cation/H	ATTAGG	contig64	[illegible]
GT	Sense	Sense	0.047	Detected	-0.047	Detected	0.000	1.226	Detected	0.993	Detected	1.110	0.013	GT	Sens	contig644	contig644	Os12g05	CCACAA	contig64	[illegible]
GT	Sense	Sense	0.035	Detected	-0.035	Detected	0.000	1.324	Detected	1.156	Detected	1.240	0.005	GT	Sens	contig646	contig646	NAD-depe	ATGTAT	contig64	[illegible]
GT	Sense	Sense	-0.120	Detected	0.120	Detected	0.000	1.409	Detected	1.502	Detected	1.456	0.008	GT	Sens	contig648	contig648	Rhamnog	ATCAGC	contig64	[illegible]
GT	Sense	Sense	-0.481	Detected	0.481	Detected	0.000	2.355	Detected	1.193	Detected	1.774	0.143	GT	Sens	contig655	contig655	GPI-anch	CGAAAA	contig65	[illegible]
GT	Sense	Sense	0.508	Detected	-0.508	Detected	0.000	0.817	Detected	1.592	Detected	1.204	0.200	GT	Sens	contig655	contig655	Leaf sene	AACTTA	contig65	[illegible]
GT	Sense	Sense	-0.051	Comprom	0.051	Comprom	0.000	1.113	Detected	1.505	Detected	1.309	0.023	GT	Sens	contig658	contig658	Rop-inter	GAAACA	contig65	[illegible]
GT	Sense	Sense	0.020	Detected	-0.020	Detected	0.000	2.012	Detected	1.773	Detected	1.893	0.004	GT	Sens	contig663	contig663	Unknown	CTCTAA	contig66	[illegible]
GT	Sense	Sense	-0.792	Detected	0.792	Detected	0.000	1.441	Detected	1.629	Detected	1.535	0.194	GT	Sens	contig663	contig663	Transcrip	CAGTTA	contig66	[illegible]
GT	Sense	Sense	0.203	Detected	-0.203	Detected	0.000	2.854	Detected	2.671	Detected	2.762	0.006	GT	Sens	contig669	contig669	FAD-bind	CGAAGC	contig66	[illegible]
GT	Sense	Sense	0.027	Detected	-0.027	Detected	0.000	1.116	Detected	0.965	Detected	1.041	0.006	GT	Sens	contig674	contig674	MYB tran	TCCTAG	contig67	[illegible]
GT	Sense	Sense	0.112	Detected	-0.112	Detected	0.000	2.306	Detected	2.351	Detected	2.329	0.002	GT	Sens	contig678	contig678	3-oxo-5-a	CAAACA	contig67	[illegible]
GT	Sense	Sense	0.054	Detected	-0.054	Detected	0.000	1.624	Detected	1.425	Detected	1.524	0.005	GT	Sens	contig680	contig680	Magnesiu	TTAAGA	contig68	[illegible]
GT	Sense	Sense	-0.413	Detected	0.413	Detected	0.000	1.627	Detected	1.020	Detected	1.323	0.123	GT	Sens	contig688	contig688	BHLH tra	GGAGTT	contig68	[illegible]
GT	Sense	Sense	-0.122	Detected	0.122	Detected	0.000	0.968	Detected	1.134	Detected	1.051	0.019	GT	Sens	contig706	contig706	Ribonucl	GTTGAA	contig70	[illegible]
GT	Sense	Sense	-0.032	Comprom	0.032	Comprom	0.000	1.868	Detected	1.377	Detected	1.623	0.022	GT	Sens	contig707	contig707	Crossove	GTTATT	contig70	[illegible]
GT	Sense	Sense	0.961	Detected	-0.961	Detected	0.000	2.003	Detected	1.324	Detected	1.664	0.244	GT	Sens	contig709	contig709	Cytochro	ATTGGC	contig70	[illegible]
GT	Sense	Sense	0.293	Detected	-0.293	Detected	0.000	0.840	Detected	1.305	Detected	1.072	0.103	GT	Sens	contig710	contig710	MazG nu	TATCTC	contig71	[illegible]
GT	Sense	Sense	0.536	Detected	-0.536	Detected	0.000	3.735	Detected	3.938	Detected	3.836	0.020	GT	Sens	contig711	contig711	Serpin (S	ATGGTA	contig71	[illegible]
GT	Sense	Sense	1.484	Detected	-1.484	Comprom	0.000	0.855	Detected	1.766	Detected	1.310	0.488	GT	Sens	contig711	contig711	Clathrin a	TTTTAGT	contig71	[illegible]
GT	Sense	Sense	-0.121	Detected	0.121	Detected	0.000	1.597	Detected	0.907	Detected	1.252	0.076	GT	Sens	contig712	contig712	Genomic	TGGCAG	contig71	[illegible]
GT	Sense	Sense	0.487	Detected	-0.487	Detected	0.000	1.688	Detected	1.321	Detected	1.504	0.102	GT	Sens	contig717	contig717	Non-spec	TTGCTC	contig71	[illegible]
GT	Sense	Sense	-0.086	Detected	0.086	Detected	0.000	1.139	Detected	0.905	Detected	1.022	0.020	GT	Sens	contig719	contig719	Granule-b	TAAAAA	contig71	[illegible]
GT	Sense	Sense	-0.592	Comprom	0.592	Comprom	0.000	1.811	Detected	2.689	Detected	2.250	0.093	GT	Sens	contig721	contig721	Unknown	AGTTCG	contig72	[illegible]
GT	Sense	Sense	-0.135	Detected	0.135	Detected	0.000	1.065	Detected	1.030	Detected	1.048	0.016	GT	Sens	contig725	contig725	POT fami	AATGTC	contig72	[illegible]
GT	Sense	Sense	-1.040	Detected	1.040	Detected	0.000	0.941	Detected	1.087	Detected	1.014	0.433	GT	Sens	contig730	contig730	Cation tra	GATTAC	contig73	[illegible]
GT	Sense	Sense	1.204	Detected	-1.204	Comprom	0.000	1.850	Detected	1.115	Detected	1.482	0.360	GT	Sens	contig733	contig733	Transme	ACCACC	contig73	[illegible]
GT	Sense	Sense	-0.143	Detected	0.143	Detected	0.000	1.835	Detected	1.320	Detected	1.577	0.033	GT	Sens	contig733	contig733	Membran	TATTCAT	contig73	[illegible]
GT	Sense	Sense	0.115	Detected	-0.115	Detected	0.000	2.220	Detected	1.984	Detected	2.102	0.006	GT	Sens	contig735	contig735	Zinc finge	TTGCTA	contig73	[illegible]
GT	Sense	Sense	0.152	Detected	-0.152	Detected	0.000	0.950	Detected	1.535	Detected	1.242	0.064	GT	Sens	contig737	contig737	Glycosylt	TCCATA	contig73	[illegible]
GT	Sense	Sense	0.113	Detected	-0.113	Detected	0.000	2.512	Detected	2.719	Detected	2.616	0.003	GT	Sens	contig737	contig737	Early ligh	TAGTGT	contig73	[illegible]
GT	Sense	Sense	-0.215	Detected	0.215	Detected	0.000	1.046	Detected	1.693	Detected	1.369	0.072	GT	Sens	contig740	contig740	Integral m	CTAGCA	contig74	[illegible]
GT	Sense	Sense	-0.246	Detected	0.246	Detected	0.000	1.927	Detected	1.202	Detected	1.564	0.070	GT	Sens	contig741	contig741	Alliinase	TTTGCC	contig74	[illegible]
GT	Sense	Sense	0.256	Detected	-0.256	Detected	0.000	1.271	Detected	1.462	Detected	1.367	0.038	GT	Sens	contig742	contig742	Acidic ch	CATCTG	contig74	[illegible]
GT	Sense	Sense	-0.890	Detected	0.890	Detected	0.000	3.280	Detected	1.537	Detected	2.408	0.193	GT	Sens	contig744	contig744	Polygalac	TTGTAG	contig74	[illegible]
GT	Sense	Sense	-0.158	Detected	0.158	Detected	0.000	1.511	Detected	0.906	Detected	1.208	0.071	GT	Sens	contig765	contig765	BES1/BZI	TAATGC	contig76	[illegible]
GT	Sense	Sense	0.053	Detected	-0.053	Detected	0.000	1.249	Detected	1.131	Detected	1.190	0.004	GT	Sens	contig766	contig766	Gibberell	GGAACC	contig76	[illegible]
GT	Sense	Sense	0.104	Detected	-0.104	Detected	0.000	1.167	Detected	0.994	Detected	1.081	0.015	GT	Sens	contig767	contig767	Fasciclin-	AAGTTT	contig76	[illegible]
GT	Sense	Sense	-0.362	Detected	0.362	Detected	0.000	0.973	Detected	1.445	Detected	1.209	0.108	GT	Sens	contig771	contig771	Glutathio	GCTATA	contig77	[illegible]
GT	Sense	Sense	0.060	Detected	-0.060	Detected	0.000	2.525	Detected	1.965	Detected	2.245	0.016	GT	Sens	contig775	contig775	Chloroph	TGCTTT	contig77	[illegible]
GT	Sense	Sense	-0.533	Detected	0.533	Detected	0.000	3.380	Detected	2.565	Detected	2.972	0.047	GT	Sens	contig790	contig790	Carboxyl	CCGATG	contig79	[illegible]
GT	Sense	Sense	0.261	Detected	-0.261	Detected	0.000	1.351	Detected	1.077	Detected	1.214	0.054	GT	Sens	contig792	contig792	Endo-1 4-	ATAGTC	contig79	[illegible]
GT	Sense	Sense	-0.427	Detected	0.427	Detected	0.000	1.028	Detected	1.610	Detected	1.319	0.125	GT	Sens	contig792	contig792	ABC tran	CTCTGT	contig79	[illegible]
GT	Sense	Sense	0.186	Detected	-0.186	Detected	0.000	2.222	Detected	1.622	Detected	1.922	0.032	GT	Sens	contig798	contig798	UBA/TS-N	ATACAT	contig79	[illegible]
GT	Sense	Sense	0.513	Detected	-0.513	Detected	0.000	1.052	Detected	1.324	Detected	1.188	0.154	GT	Sens	contig799	contig799	Cytochro	GTTCCT	contig79	[illegible]
GT	Sense	Sense	-0.313	Comprom	0.313	Comprom	0.000	1.227	Detected	1.047	Detected	1.137	0.073	GT	Sens	contig800	contig800	F-box fan	CCATCG	contig80	[illegible]
GT	Sense	Sense	0.165	Detected	-0.165	Detected	0.000	1.183	Detected	1.843	Detected	1.513	0.055	GT	Sens	contig805	contig805	Gag-pol p	GAATTA	contig80	[illegible]
GT	Sense	Sense	0.425	Detected	-0.425	Detected	0.000	1.643	Detected	1.813	Detected	1.728	0.057	GT	Sens	contig808	contig808	Xylogluca	GATCTT	contig80	[illegible]
GT	Sense	Sense	0.565	Detected	-0.565	Comprom	0.000	1.050	Detected	1.073	Detected	1.061	0.201	GT	Sens	contig811	contig811	Pentatric	ACCCAA	contig81	[illegible]
GT	Sense	Sense	-0.357	Comprom	0.357	Comprom	0.000	3.885	Detected	2.755	Detected	3.320	0.038	GT	Sens	contig813	contig813	Cysteine-	ACTTTC	contig81	[illegible]
GT	Sense	Sense	0.036	Detected	-0.036	Detected	0.000	1.134	Detected	0.933	Detected	1.034	0.010	GT	Sens	contig813	contig813	Protein s	CGTCAC	contig81	[illegible]
GT	Sense	Sense	-0.036	Detected	0.036	Detected	0.000	1.289	Detected	0.912	Detected	1.100	0.029	GT	Sens	contig814	contig814	Ethylene-	ATCTTC	contig81	[illegible]
GT	Sense	Sense	-0.243	Detected	0.243	Detected	0.000	1.289	Detected	1.560	Detected	1.424	0.036	GT	Sens	contig821	contig821	Genomic	GACGAA	contig82	[illegible]
GT	Sense	Sense	-0.166	Comprom	0.166	Comprom	0.000	0.925	Detected	1.227	Detected	1.076	0.041	GT	Sens	contig823	contig823	Acidic ch	TTAGCA	contig82	[illegible]
GT	Sense	Sense	-0.577	Comprom	0.577	Comprom	0.000	4.749	Detected	3.370	Detected	4.059	0.046	GT	Sens	contig824	contig824	Pectinest	GGAAGG	contig82	[illegible]
GT	Sense	Sense	-0.052	Detected	0.052	Detected	0.000	1.003	Detected	1.217	Detected	1.110	0.011	GT	Sens	contig825	contig825	Undecapr	TTACGC	contig82	[illegible]
GT	Sense	Sense	-0.419	Comprom	0.419	Comprom	0.000	2.918	Detected	1.712	Detected	2.315	0.088	GT	Sens	contig826	contig826	Glucan s	CTTCTC	contig82	[illegible]
GT	Sense	Sense	-0.015	Comprom	0.015	Comprom	0.000	1.119	Detected	1.344	Detected	1.232	0.008	GT	Sens	contig829	contig829	Resistanc	CTAAAA	contig82	[illegible]
GT	Sense	Sense	-0.096	Comprom	0.096	Comprom	0.000	2.235	Detected	2.982	Detected	2.608	0.021	GT	Sens	contig831	contig831	Nodulin f	ATCAAT	contig83	[illegible]
GT	Sense	Sense	0.224	Detected	-0.224	Detected	0.000	1.373	Detected	0.928	Detected	1.150	0.068	GT	Sens	contig835	contig835	Unknown	GAATAT	contig83	[illegible]
GT	Sense	Sense	0.186	Detected	-0.186	Detected	0.000	1.260	Detected	1.978	Detected	1.619	0.057	GT	Sens	contig836	contig836	Chaperon	AATCAT	contig83	[illegible]
GT	Sense	Sense	0.113	Detected	-0.113	Detected	0.000	1.257	Detected	1.191	Detected	1.224	0.009	GT	Sens	contig837	contig837	DNA helic	CTGTGA	contig83	[illegible]
GT	Sense	Sense	0.100	Detected	-0.100	Detected	0.000	1.665	Detected	1.272	Detected	1.469	0.022	GT	Sens	contig837	contig837	Unknown	TTCAAA	contig83	[illegible]
GT	Sense	Sense	0.689	Comprom	-0.689	Comprom	0.000	1.472	Detected	1.544	Detected	1.508	0.160	GT	Sens	contig839	contig839	Pentatric	TGAGGA	contig83	[illegible]
GT	Sense	Sense	-0.001	Detected	0.001	Detected	0.000	1.650	Detected	1.188	Detected	1.419	0.025	GT	Sens	contig841	contig841	Homolog	ATAAAC	contig84	[illegible]
GT	Sense	Sense	-0.191	Detected	0.191	Detected	0.000	1.283	Detected	1.128	Detected	1.205	0.028	GT	Sens	contig844	contig844	Unknown	AAGTTC	contig84	[illegible]
GT	Sense	Sense	0.180	Detected	-0.180	Detected	0.000	2.182	Detected	2.537	Detected	2.359	0.011	GT	Sens	contig850	contig850	Plant-spe	GTGGAG	contig85	[illegible]
GT	Sense	Sense	-0.203	Detected	0.203	Detected	0.000	1.270	Detected	1.203	Detected	1.237	0.027	GT	Sens	contig852	contig852	IQ-domai	GGCTTC	contig85	[illegible]
GT	Sense	Sense	0.110	Detected	-0.110	Detected	0.000	1.730	Detected	1.481	Detected	1.605	0.010	GT	Sens	contig867	contig867	COL dom	AAATCA	contig86	[illegible]
GT	Sense	Sense	-0.196	Detected	0.196	Detected	0.000	1.230	Detected	0.934	Detected	1.082	0.048	GT	Sens	contig868	contig868	Rhodane	TAAAGT	contig86	[illegible]
GT	Sense	Sense	-0.065	Detected	0.065	Detected	0.000	1.812	Detected	1.666	Detected	1.739	0.003	GT	Sens	contig868	contig868	Hydrolas	CTGAAA	contig86	[illegible]
GT	Sense	Sense	0.167	Detected	-0.167	Detected	0.000	1.881	Detected	1.546	Detected	1.714	0.018	GT	Sens	contig869	contig869	Chloroph	TTCTAT	contig86	[illegible]
GT	Sense	Sense	0.015	Detected	-0.015	Detected	0.000	1.005	Detected	1.084	Detected	1.044	0.002	GT	Sens	contig870	contig870	Unknown	GAAATC	contig87	[illegible]
GT	Sense	Sense	0.430	Detected	-0.430	Detected	0.000	1.751	Detected	1.705	Detected	1.728	0.057	GT	Sens	contig871	contig871	Plant-spe	AAGAGT	contig87	[illegible]
GT	Sense	Sense	-0.284	Detected	0.284	Detected	0.000	1.898	Detected	1.296	Detected	1.597	0.061	GT	Sens	contig879	contig879	Boron tra	GTCCAA	contig87	[illegible]
GT	Sense	Sense	0.324	Detected	-0.324	Detected	0.000	0.911	Detected	1.197	Detected	1.054	0.097	GT	Sens	contig893	contig893	Isoamyl a	CGGTAA	contig89	[illegible]
GT	Sense	Sense	0.109	Detected	-0.109	Detected	0.000	1.975	Detected	1.641	Detected	1.808	0.012	GT	Sens	contig901	contig901	Hydrolas	TGTACA	contig90	[illegible]
GT	Sense	Sense	0.169	Comprom	-0.169	Comprom	0.000	1.571	Detected	1.455	Detected	1.513	0.014	GT	Sens	contig902	contig902	Inositol-1	CAGTTC	contig90	[illegible]
GT	Sense	Sense	-0.099	Detected	0.099	Detected	0.000	1.046	Detected	1.117	Detected	1.081	0.009	GT	Sens	contig903	contig903	Dihydrox	AAAGAA	contig90	[illegible]
GT	Sense	Sense	0.282	Detected	-0.282	Detected	0.000	1.215	Detected	0.984	Detected	1.100	0.069	GT	Sens	contig917	contig917	MYB tran	TTATCG	contig91	[illegible]
GT	Sense	Sense	0.193	Detected	-0.193	Detected	0.000	1.241	Detected	1.131	Detected	1.186	0.028	GT	Sens	contig925	contig925	MATE eff	GATTAT	contig92	[illegible]
GT	Sense	Sense	-0.507	Detected	0.507	Detected	0.000	0.932	Detected	1.071	Detected	1.002	0.189	GT	Sens	contig930	contig930	Unknown	ATTGAT	contig93	[illegible]
GT	Sense	Sense	0.290	Comprom	-0.290	Comprom	0.000	1.463	Detected	1.712	Detected	1.588	0.037	GT	Sens	contig941	contig941	Unknown	CTTTCA	contig94	[illegible]
GT	Sense	Sense	-0.273	Detected	0.273	Detected	0.000	1.644	Detected	1.672	Detected	1.658	0.026	GT	Sens	contig941	contig941	GDSL est	AACATA	contig94	[illegible]
GT	Sense	Sense	0.733	Detected	-0.733	Detected	0.000	0.900	Detected	1.130	Detected	1.015	0.305	GT	Sens	contig945	contig945	Cytokinin	TGGAGG	contig94	[illegible]
GT	Sense	Sense	-0.020	Detected	0.020	Detected	0.000	1.714	Detected	1.381	Detected	1.547	0.012	GT	Sens	contig950	contig950	Thaumati	CTAGCA	contig95	[illegible]
GT	Sense	Sense	0.062	Detected	-0.062	Detected	0.000	1.276	Detected	1.015	Detected	1.146	0.016	GT	Sens	contig961	contig961	Expresse	AATGTC	contig96	[illegible]
GT	Sense	Sense	0.328	Detected	-0.328	Detected	0.000	1.248	Detected	1.190	Detected	1.219	0.066	GT	Sens	contig961	contig961	Non-spec	AAAGCG	contig96	[illegible]
GT	Sense	Sense	0.207	Detected	-0.207	Detected	0.000	0.955	Detected	1.057	Detected	1.006	0.042	GT	Sens	contig966	contig966	Dehydrat	ATGGAT	contig96	[illegible]
GT	Sense	Sense	-0.341	Detected	0.341	Detected	0.000	1.145	Detected	0.869	Detected	1.007	0.112	GT	Sens	contig967	contig967	Gibberell	TTTCAT	contig96	[illegible]
GT	Sense	Sense	-0.265	Detected	0.265	Detected	0.000	1.075	Detected	1.135	Detected	1.105	0.053	GT	Sens	contig968	contig968	Enhancer	GCCTAT	contig96	[illegible]
GT	Sense	Sense	0.402	Detected	-0.402	Detected	0.000	1.306	Detected	0.921	Detected	1.113	0.130	GT	Sens	contig969	contig969	Dihydrofl	CATTGA	contig96	[illegible]
GT	Sense	Sense	0.003	Detected	-0.003	Detected	0.000	1.382	Detected	1.054	Detected	1.218	0.018	GT	Sens	contig970	contig970	D-xylose-	ATACTC	contig97	[illegible]
GT	Sense	Sense	0.085	Detected	-0.085	Detected	0.000	1.145	Detected	1.116	Detected	1.131	0.006	GT	Sens	contig971	contig971	chaperon	GAATGC	contig97	[illegible]
GT	Sense	Sense	-1.346	Detected	1.346	Detected	0.000	1.774	Detected	1.930	Detected	1.852	0.303	GT	Sens	contig976	contig976	Lipase cl	CCATCA	contig97	[illegible]
GT	Sense	Sense	0.089	Comprom	-0.089	Comprom	0.000	1.111	Detected	2.693	Detected	1.902	0.139	GT	Sens	contig986	contig986	Unknown	TAGTAG	contig98	[illegible]
GT	Sense	Sense	0.106	Detected	-0.106	Detected	0.000	1.360	Detected	1.210	Detected	1.285	0.010	GT	Sens	contig986	contig986	Kinesin-li	AAATCA	contig98	[illegible]
GT	Sense	Sense	-1.014	Detected	1.014	Detected	0.000	1.929	Detected	1.806	Detected	1.868	0.207	GT	Sens	contig988	contig988	Ariadne-li	AAGATA	contig98	[illegible]
GT	Sense	Sense	0.306	Detected	-0.306	Detected	0.000	1.500	Detected	1.077	Detected	1.289	0.074	GT	Sens	contig989	contig989	Multidrug	TATCAG	contig98	[illegible]
GT	Sense	Sense	0.175	Detected	-0.175	Detected	0.000	1.876	Detected	1.161	Detected	1.518	0.062	GT	Sens	contig991	contig991	Unknown	GTTGAA	contig99	[illegible]
GT	Sense	Sense	0.295	Comprom	-0.295	Comprom	0.000	3.795	Detected	1.982	Detected	2.889	0.094	GT	Sens	LeIDA3 C	LeIDA3 C	Unknown	TAAAAAACACAAC		[illegible]
GT	Sense	Sense	0.078	Detected	-0.078	Detected	0.000	2.506	Detected	2.215	Detected	2.360	0.005	GT	Sens	NM 0012	NM 0012	Solanum	CAAAGAGAAGATC		[illegible]
GT	Sense	Sense	0.139	Detected	-0.139	Comprom	0.000	1.350	Detected	0.884	Detected	1.117	0.054	GT	Sens	contig100	contig100	Heat sho	CCCTGT	contig10	[illegible]
GT	Sense	Sense	0.015	Detected	-0.015	Detected	0.000	1.395	Detected	0.980	Detected	1.187	0.029	GT	Sens	contig100	contig100	Heat sho	AATGGA	contig10	[illegible]
GT	Sense	Sense	-0.639	Detected	0.639	Detected	0.000	1.185	Detected	1.135	Detected	1.160	0.211	GT	Sens	contig102	contig102	TBC1 do	AAGAAT	contig10	[illegible]
GT	Sense	Sense	-0.407	Detected	0.407	Detected	0.000	1.046	Detected	1.371	Detected	1.208	0.110	GT	Sens	contig103	contig103	Unknown	TATATCA	contig10	[illegible]
GT	Sense	Sense	-0.510	Detected	0.510	Detected	0.000	1.242	Detected	1.051	Detected	1.146	0.158	GT	Sens	contig106	contig106	AT hook	AATTCT	contig10	[illegible]
GT	Sense	Sense	-0.015	Detected	0.015	Detected	0.000	1.327	Detected	1.131	Detected	1.229	0.006	GT	Sens	contig100	contig100	Ammoniu	ATTGAC	contig10	[illegible]
GT	Sense	Sense	0.205	Detected	-0.205	Detected	0.000	1.335	Detected	0.979	Detected	1.157	0.051	GT	Sens	contig106	contig106	Chloroph	CCTCTT	contig10	[illegible]
GT	Sense	Sense	-0.250	Detected	0.250	Detected	0.000	1.219	Detected	1.009	Detected	1.114	0.054	GT	Sens	contig107	contig107	Transme	ATCGCC	contig10	[illegible]
GT	Sense	Sense	-0.023	Detected	0.023	Detected	0.000	2.147	Detected	1.433	Detected	1.790	0.038	GT	Sens	contig107	contig107	Unknown	AACATC	contig10	[illegible]
GT	Sense	Sense	-0.111	Comprom	0.111	Comprom	0.000	1.201	Detected	1.189	Detected	1.195	0.009	GT	Sens	contig108	contig108	DNA topo	GTATAT	contig10	[illegible]
GT	Sense	Sense	-0.204	Comprom	0.204	Comprom	0.000	2.391	Detected	2.719	Detected	2.555	0.010	GT	Sens	contig110	contig110	Cytochro	GTGATA	contig11	[illegible]
GT	Sense	Sense	-0.232	Detected	0.232	Detected	0.000	1.893	Detected	1.845	Detected	1.869	0.015	GT	Sens	contig110	contig110	Ycf2 (AH	TTTCCT	contig11	[illegible]
GT	Sense	Sense	0.061	Detected	-0.061	Detected	0.000	1.282	Detected	0.957	Detected	1.120	0.023	GT	Sens	contig111	contig111	Inositol-3	GCTGGT	contig11	[illegible]
GT	Sense	Sense	0.081	Detected	-0.081	Detected	0.000	2.045	Detected	1.590	Detected	1.818	0.017	GT	Sens	contig118	contig118	Chloroph	TCTGCC	contig11	[illegible]
GT	Sense	Sense	0.309	Detected	-0.309	Detected	0.000	2.181	Detected	1.629	Detected	1.905	0.044	GT	Sens	contig118	contig118	Chloroph	AAATTT	contig11	[illegible]
GT	Sense	Sense	-0.299	Detected	0.299	Detected	0.000	0.888	Detected	1.139	Detected	1.014	0.089	GT	Sens	contig119	contig119	Galactoki	GAAGGC	contig11	[illegible]
GT	Sense	Sense	0.058	Detected	-0.058	Detected	0.000	1.618	Detected	0.877	Detected	1.248	0.080	GT	Sens	contig123	contig123	1-AMINO	CCATTC	contig12	[illegible]
GT	Sense	Sense	-0.114	Detected	0.114	Detected	0.000	1.409	Detected	1.049	Detected	1.229	0.029	GT	Sens	contig123	contig123	Aquapori	AATTCT	contig12	[illegible]
GT	Sense	Sense	-0.289	Detected	0.289	Detected	0.000	1.328	Detected	1.087	Detected	1.208	0.061	GT	Sens	contig124	contig124	Aquapori	GCCATG	contig12	[illegible]
GT	Sense	Sense	0.243	Detected	-0.243	Detected	0.000	1.863	Detected	2.158	Detected	2.010	0.019	GT	Sens	contig125	contig125	F-box fan	TGGTGC	contig12	[illegible]
GT	Sense	Sense	-0.086	Detected	0.086	Detected	0.000	1.279	Detected	1.167	Detected	1.223	0.007	GT	Sens	contig125	contig125	UDP-gluc	AAAGTT	contig12	[illegible]
GT	Sense	Sense	-0.002	Detected	0.002	Detected	0.000	1.202	Detected	0.979	Detected	1.091	0.010	GT	Sens	contig127	contig127	Conserve	AGAATG	contig12	[illegible]
GT	Sense	Sense	-0.041	Detected	0.041	Detected	0.000	1.346	Detected	0.839	Detected	1.093	0.051	GT	Sens	contig127	contig127	Glucose-	TTGCCA	contig12	[illegible]
GT	Sense	Sense	0.157	Detected	-0.157	Detected	0.000	2.155	Detected	2.149	Detected	2.152	0.005	GT	Sens	contig127	contig127	Major alle	TCTTATT	contig12	[illegible]
GT	Sense	Sense	0.459	Detected	-0.459	Comprom	0.000	0.916	Detected	1.283	Detected	1.100	0.156	GT	Sens	contig131	contig131	Homeobo	AGTCTC	contig13	[illegible]
GT	Sense	Sense	0.247	Detected	-0.247	Detected	0.000	1.251	Detected	1.186	Detected	1.219	0.039	GT	Sens	contig132	contig132	LRR rece	TGAATG	contig13	[illegible]
GT	Sense	Sense	0.079	Detected	-0.079	Comprom	0.000	1.363	Detected	1.029	Detected	1.196	0.023	GT	Sens	contig132	contig132	Unknown	TGTAAT	contig13	[illegible]
GT	Sense	Sense	-0.153	Detected	0.153	Detected	0.000	1.082	Detected	0.939	Detected	1.010	0.027	GT	Sens	contig132	contig132	class I he	GAGCAA	contig13	[illegible]
GT	Sense	Sense	0.044	Detected	-0.044	Detected	0.000	1.100	Detected	0.959	Detected	1.030	0.006	GT	Sens	contig137	contig137	WD-40 re	TCCTCT	contig13	[illegible]
GT	Sense	Sense	0.065	Detected	-0.065	Detected	0.000	1.152	Detected	0.985	Detected	1.069	0.010	GT	Sens	contig140	contig140	Chloroph	TACCCT	contig14	[illegible]
GT	Sense	Sense	0.137	Detected	-0.137	Detected	0.000	1.531	Detected	1.279	Detected	1.405	0.017	GT	Sens	contig140	contig140	Chloroph	TTCTTC	contig14	[illegible]
GT	Sense	Sense	-0.038	Detected	0.038	Detected	0.000	1.103	Detected	0.965	Detected	1.034	0.006	GT	Sens	contig144	contig144	WD-40 re	AATGTT	contig14	[illegible]
GT	Sense	Sense	-0.131	Detected	0.131	Detected	0.000	1.733	Detected	1.447	Detected	1.590	0.014	GT	Sens	contig144	contig144	Naringeni	GGTATT	contig14	[illegible]
GT	Sense	Sense	0.343	Detected	-0.343	Detected	0.000	0.857	Detected	1.220	Detected	1.039	0.116	GT	Sens	contig147	contig147	Actin-bin	CTGAAA	contig14	[illegible]
GT	Sense	Sense	-0.776	Comprom	0.776	Detected	0.000	1.244	Detected	0.888	Detected	1.066	0.313	GT	Sens	contig150	contig150	CK25 (AH	TGAAAC	contig15	[illegible]
GT	Sense	Sense	0.196	Detected	-0.196	Detected	0.000	1.152	Detected	1.276	Detected	1.214	0.028	GT	Sens	contig150	contig150	Auxin res	CAATTA	contig15	[illegible]
GT	Sense	Sense	0.103	Detected	-0.103	Comprom	0.000	0.960	Detected	1.685	Detected	1.323	0.072	GT	Sens	contig152	contig152	Nucleopo	TTGCTT	contig15	[illegible]
GT	Sense	Sense	-0.139	Detected	0.139	Detected	0.000	1.474	Detected	0.892	Detected	1.183	0.067	GT	Sens	contig154	contig154	Glycerald	AATTGC	contig15	[illegible]
GT	Sense	Sense	0.141	Detected	-0.141	Detected	0.000	2.198	Detected	1.588	Detected	1.893	0.030	GT	Sens	contig157	contig157	Chloroph	TCTGCC	contig15	[illegible]
GT	Sense	Sense	-0.066	Detected	0.066	Detected	0.000	1.360	Detected	0.816	Detected	1.088	0.060	GT	Sens	contig157	contig157	Catalase	ATGCTC	contig15	[illegible]
GT	Sense	Sense	-0.597	Comprom	0.597	Comprom	0.000	2.914	Detected	1.758	Detected	2.336	0.107	GT	Sens	contig166	contig166	Pollen all	TGCATT	contig16	[illegible]
GT	Sense	Sense	-0.538	Detected	0.538	Detected	0.000	1.982	Detected	1.346	Detected	1.664	0.117	GT	Sens	contig166	contig166	Cytochro	GGCAAT	contig16	[illegible]
GT	Sense	Sense	0.571	Detected	-0.571	Detected	0.000	1.063	Detected	1.466	Detected	1.264	0.172	GT	Sens	contig166	contig166	Subtilisin	ACGAAA	contig16	[illegible]
GT	Sense	Sense	-0.102	Detected	0.102	Detected	0.000	1.024	Detected	1.420	Detected	1.222	0.032	GT	Sens	contig167	contig167	Gibberell	TCAGCT	contig16	[illegible]
GT	Sense	Sense	0.514	Comprom	-0.514	Comprom	0.000	6.351	Detected	8.321	Detected	7.336	0.022	GT	Sens	contig170	contig170	Gag poly	CTGCAT	contig17	[illegible]
GT	Sense	Sense	0.243	Detected	-0.243	Detected	0.000	1.283	Detected	0.922	Detected	1.103	0.068	GT	Sens	contig171	contig171	Chloroph	TCTTCT	contig17	[illegible]
GT	Sense	Sense	0.008	Detected	-0.008	Detected	0.000	1.400	Detected	0.898	Detected	1.149	0.045	GT	Sens	contig173	contig173	Oxygen-e	AAGACA	contig17	[illegible]
GT	Sense	Sense	-0.962	Detected	0.962	Detected	0.000	0.960	Detected	1.177	Detected	1.068	0.385	GT	Sens	contig173	contig173	Unknown	GAATTCA	contig17	[illegible]
GT	Sense	Sense	-0.093	Detected	0.093	Detected	0.000	1.200	Detected	1.124	Detected	1.162	0.007	GT	Sens	contig176	contig176	Kinase fa	AAAGAA	contig17	[illegible]
GT	Sense	Sense	1.036	Detected	-1.036	Comprom	0.000	2.595	Detected	3.213	Detected	2.904	0.115	GT	Sens	contig176	contig176	Unknown	TTTGATT	contig17	[illegible]
GT	Sense	Sense	0.145	Comprom	-0.145	Comprom	0.000	1.885	Detected	1.327	Detected	1.606	0.036	GT	Sens	contig177	contig177	Unknown	AACTTG	contig17	[illegible]
GT	Sense	Sense	-1.728	Comprom	1.728	Detected	0.000	1.887	Detected	1.790	Detected	1.838	0.399	GT	Sens	contig178	contig178	Equilibrat	TATGTC	contig17	[illegible]
GT	Sense	Sense	0.236	Detected	-0.236	Comprom	0.000	2.280	Detected	2.386	Detected	2.333	0.011	GT	Sens	contig179	contig179	Leucine F	TCTCAA	contig17	[illegible]
GT	Sense	Sense	-0.050	Detected	0.050	Comprom	0.000	1.221	Detected	1.171	Detected	1.196	0.002	GT	Sens	contig183	contig183	SAM (And	TTTATTA	contig18	[illegible]
GT	Sense	Sense	0.080	Detected	-0.080	Detected	0.000	0.986	Detected	1.162	Detected	1.074	0.012	GT	Sens	contig184	contig184	Menaquin	TGACCT	contig18	[illegible]
GT	Sense	Sense	-0.220	Detected	0.220	Detected	0.000	1.300	Detected	0.917	Detected	1.108	0.063	GT	Sens	contig185	contig185	Aquapori	TCATGT	contig18	[illegible]
GT	Sense	Sense	0.003	Detected	-0.003	Detected	0.000	1.289	Detected	0.984	Detected	1.137	0.018	GT	Sens	contig186	contig186	Cytochro	GGTGAA	contig18	[illegible]
GT	Sense	Sense	0.588	Comprom	-0.588	Comprom	0.000	1.392	Detected	1.902	Detected	1.647	0.124	GT	Sens	contig188	contig188	Subtilisin	ATGGTA	contig18	[illegible]
GT	Sense	Sense	0.067	Detected	-0.067	Detected	0.000	1.154	Detected	0.985	Detected	1.070	0.010	GT	Sens	contig190	contig190	Ankyrin r	ACAACA	contig19	[illegible]
GT	Sense	Sense	-0.055	Detected	0.055	Detected	0.000	1.167	Detected	1.316	Detected	1.241	0.006	GT	Sens	contig191	contig191	F-box fan	GTGCTG	contig19	[illegible]
GT	Sense	Sense	0.152	Detected	-0.152	Detected	0.000	2.814	Detected	2.717	Detected	2.766	0.003	GT	Sens	contig195	contig195	FAD-bind	GAGCTT	contig19	[illegible]
GT	Sense	Sense	0.229	Comprom	-0.229	Comprom	0.000	1.258	Detected	1.433	Detected	1.346	0.032	GT	Sens	contig198	contig198	Unknown	GTTGTC	contig19	[illegible]
GT	Sense	Sense	-0.497	Detected	0.497	Detected	0.000	1.222	Detected	0.972	Detected	1.097	0.166	GT	Sens	contig202	contig202	GH3 famil	TTACCA	contig20	[illegible]
GT	Sense	Sense	-1.634	Detected	1.634	Detected	0.000	1.807	Detected	1.997	Detected	1.902	0.365	GT	Sens	contig202	contig202	Ulp1 prot	AGACAT	contig20	[illegible]
GT	Sense	Sense	0.004	Detected	-0.004	Detected	0.000	1.705	Detected	1.609	Detected	1.657	0.001	GT	Sens	contig202	contig202	GDSL est	CATGTA	contig20	[illegible]
GT	Sense	Sense	-0.123	Detected	0.123	Detected	0.000	1.359	Detected	0.880	Detected	1.119	0.053	GT	Sens	contig205	contig205	Ovate pro	CATTAT	contig20	[illegible]
GT	Sense	Sense	-0.341	Comprom	0.341	Comprom	0.000	1.460	Detected	1.709	Detected	1.584	0.049	GT	Sens	contig205	contig205	Guanine	AGCTGC	contig20	[illegible]
GT	Sense	Sense	0.156	Detected	-0.156	Detected	0.000	1.149	Detected	1.179	Detected	1.164	0.018	GT	Sens	contig207	contig207	Cyclin B	GGTCCC	contig20	[illegible]

GT	Sense	Sense	-0.041	Detected	0.041	Detected	0.000	2.896	Detected	2.849	Detected	2.873	0.000	GT	Sens	contig209	contig209	1-aminoc	GACTAC	contig209	Solyc09g	Solyc09g	1-aminocyclopropane-1-carbo			contig209	Solyc09g	1-aminocyclopropa		SL2.40ch	AT3G49030.1		F-box fa	chr3:181	55.1769	45.6697	223.471	208.057	395.03	368.986
GT	Sense	Sense	-0.044	Detected	0.044	Detected	0.000	1.630	Detected	0.871	Detected	1.251	0.082	GT	Sens	contig209	contig209	Flavonol	TTGTGT	contig209	Solyc11g	Solyc11g	Flavonol	GO:0016	GO:0016	contig209	Solyc11g	1-AMINO	GO:0016	SL2.40ch	AT5G24800.1				892.594	741.851	1650.6	1981.94	2662.62	1518.44
GT	Sense	Sense	0.028	Detected	-0.028	Detected	0.000	1.019	Detected	1.192	Detected	1.106	0.007	GT	Sens	contig213	contig213	Tubulin b	AATGGC	contig213	Solyc10g	Solyc10g	Tubulin b	GO:0005	GO:0005	contig213	Solyc10g	Tubulin b	GO:0005	SL2.40ch	AT5G122	TUB6	TUB6 (B	chr5:396	2701.91	2032.84	3178.39	2617.96	5020.42	5464.95
GT	Sense	Sense	-0.020	Comprom	0.020	Comprom	0.000	1.742	Detected	1.399	Detected	1.570	0.012	GT	Sens	contig214	contig214	Phosphat	ATTACT	contig214	Solyc06g	Solyc06g	Phosphat	GO:0003	GO:0003	contig214	Solyc06g	Phosphat	GO:0003	SL2.40ch	AT1G17710.1		phospha	chr1:609	7.16407	5.75729	13.0262	10.7223	22.7094	17.2759
GT	Sense	Sense	-0.111	Detected	0.111	Detected	0.000	1.662	Detected	1.017	Detected	1.339	0.059	GT	Sens	contig215	contig215	Cytochro	GAATTA	contig215	Solyc03g	Solyc03g	Cytochro	GO:0020	GO:0020	contig215	Solyc03g	Cytochro	GO:0019	SL2.40ch	AT5G35715.1				49.5301	45.1724	85.8518	176.866	158.144	97.6838
GT	Sense	Sense	0.112	Detected	-0.112	Detected	0.000	0.927	Detected	1.336	Detected	1.132	0.040	GT	Sens	contig215	contig215	Pectinest	AATGGA	contig215	Solyc07g	Solyc07g	Pectinest	GO:0030	GO:0030	contig215	Solyc07g	Pectinest	GO:0005	SL2.40ch	AT1G115	ATPMEP	PMEPCR	chr1:388	258.456	172.947	273.312	212.939	424.83	544.701
GT	Sense	Sense	-0.244	Detected	0.244	Detected	0.000	1.021	Detected	1.128	Detected	1.075	0.050	GT	Sens	contig217	contig217	Flowerin	GTCGTC	contig217	Solyc01g	Solyc01g	Flowering promoting factor-lik			contig217	Solyc01g	Flowering promotin		SL2.40ch	AT2G42270.1		U5 small	chr2:176	211.175	231.398	259.226	177.926	474.12	493.116
GT	Sense	Sense	0.098	Detected	-0.098	Detected	0.000	1.540	Detected	1.183	Detected	1.362	0.022	GT	Sens	contig219	contig219	UDP-gluc	GGATTC	contig219	Solyc04g	Solyc04g	UDP-gluc	GO:0008	GO:0008	contig219	Solyc04g	UDP-gluc	GO:0008	SL2.40ch	AT5G228	EMB1030	EMB103	chr5:761	583.652	398.055	1131.15	1034.52	1481.23	1117.01
GT	Sense	Sense	-0.107	Detected	0.107	Detected	0.000	1.727	Detected	1.504	Detected	1.616	0.009	GT	Sens	contig221	contig221	Respons	CTGCTT	contig221	Solyc10g	Solyc10g	Respons	GO:0006	GO:0006	contig221	Solyc10g	Respons	GO:0006	SL2.40ch	AT3G570	ARR9. AT	ARR9 (R	chr3:211	258.787	234.735	654.522	796.328	862.578	713.426
GT	Sense	Sense	0.405	Comprom	-0.405	Comprom	0.000	1.103	Detected	1.417	Detected	1.260	0.101	GT	Sens	contig227	contig227	Steroid s	TTTCTG	contig227	Solyc05g	Solyc05g	Steroid s	GO:0008	GO:0008	contig227	Solyc05g	Steroid s	GO:0050	SL2.40ch	AT3G45070.1		sulfotran	chr3:164	6.60057	2.94243	8.58408	10.6647	10.0078	12.0044
GT	Sense	Sense	0.185	Detected	-0.185	Detected	0.000	2.322	Detected	1.718	Detected	2.020	0.029	GT	Sens	contig229	contig229	Chloroph	AAATCT	contig229	Solyc02g	Solyc02g	Chloroph	GO:0016	GO:0016	contig229	Solyc02g	Chloroph	GO:0016	SL2.40ch	AT1G299	CAB1. AB	CAB1 (C	chr1:104	451.796	273.218	856.765	934.338	1857.14	1179.04
GT	Sense	Sense	0.069	Detected	-0.069	Detected	0.000	2.299	Detected	1.494	Detected	1.896	0.043	GT	Sens	contig230	contig230	1-aminoc	TCAGTT	contig230	Solyc09g	Solyc09g	1-aminoc	GO:0016	GO:0016	contig230	Solyc09g	1-aminoc	GO:0016	SL2.40ch	AT3G61400.1		2-oxoglu	chr3:227	551.224	391.6	2180.98	3875.41	2416.47	1335.01
GT	Sense	Sense	-0.005	Detected	0.005	Detected	0.000	1.299	Detected	1.088	Detected	1.194	0.008	GT	Sens	contig234	contig234	Cytochro	AAGTAT	contig234	Solyc02g	Solyc02g	Cytochro	GO:0020	GO:0020	contig234	Solyc02g	Cytochro	GO:0019	SL2.40ch	AT5G249	CYP71A1	CYP71A1	chr5:859	3336.44	2624.39	4703.46	6128.01	7696.69	6419.79
GT	Sense	Sense	-0.194	Comprom	0.194	Comprom	0.000	1.444	Detected	1.110	Detected	1.277	0.038	GT	Sens	contig235	contig235	Leucine-r	TGGCTT	contig235	Solyc12g	Solyc12g	Leucine-r	GO:0005	GO:0005	contig235	Solyc12g	LRR rece	GO:0004	SL2.40ch	AT1G055	SYTE. AT	NTMC2T	chr1:162	5.98092	6.11371	8.52664	22.6346	17.385	13.314
GT	Sense	Sense	-0.451	Detected	0.451	Detected	0.000	1.359	Detected	1.516	Detected	1.438	0.088	GT	Sens	contig235	contig235	Subtilisin	CTACCA	contig235	Solyc08g	Solyc08g	Subtilisin	GO:0004	GO:0004	contig235	Solyc08g	Subtilisin	GO:0004	SL2.40ch	AT3G19540.1		unknown	chr3:678	145.309	212.299	203.758	181.616	476.223	512.626
GT	Sense	Sense	-0.017	Detected	0.017	Detected	0.000	1.712	Detected	1.396	Detected	1.554	0.010	GT	Sens	contig235	contig235	Inositol-3	GATTTC	contig235	Solyc05g	Solyc05g	Inositol-3	GO:0008	GO:0008	contig235	Solyc05g	Inositol-3	GO:0042	SL2.40ch	AT2G222	ATMIPS2	MIPS2 (M	chr2:945	2450.04	1961.37	3163.82	4632.16	7592.18	5886.84
GT	Sense	Sense	-0.235	Detected	0.235	Detected	0.000	1.325	Detected	1.243	Detected	1.284	0.033	GT	Sens	contig237	contig237	Dynein lic	CCAAGC	contig237	Solyc12g	Solyc12g	Dynein lic	GO:0042	GO:0042	contig237	Solyc12g	Dynein lic	GO:0042	SL2.40ch	AT1G522	ATROPGI	ROPGEF	chr1:194	152.737	165.298	480.244	256.76	420.706	383.631
GT	Sense	Sense	-0.347	Detected	0.347	Detected	0.000	1.540	Detected	1.025	Detected	1.282	0.097	GT	Sens	contig237	contig237	Leucine-r	TTCCAT	contig237	Solyc12g	Solyc12g	Leucine-rich repeat receptor-li			contig237	Solyc01g	Receptor	GO:0042	SL2.40ch	AT3G04190.1				28.1615	35.634	56.9255	74.9832	97.3777	65.7534
GT	Sense	Sense	-0.022	Detected	0.022	Detected	0.000	1.079	Detected	1.168	Detected	1.124	0.002	GT	Sens	contig238	contig238	Eukaryot	TGTGTG	contig238	Solyc01g	Solyc01g	Eukaryot	GO:0005	GO:0005	contig238	Solyc01g102570.2.1				AT3G561	EIF3C. AT	EIF3C (E	chr3:208	58.6604	47.2967	110.133	88.1128	117.631	120.752
GT	Sense	Sense	0.055	Detected	-0.055	Detected	0.000	1.172	Detected	1.183	Detected	1.177	0.002	GT	Sens	contig239	contig239	Unknown	AATATC	contig239	Solyc02g	Solyc02g	Unknown Protein (AHRD V1)			contig239	Solyc02g	Unknown Protein (A		SL2.40ch	AT4G14368.1		zinc ion	chr4:827	28.4554	20.6125	56.6905	44.4608	57.6533	56.0913
GT	Sense	Sense	0.016	Detected	-0.016	Detected	0.000	1.184	Detected	1.156	Detected	1.170	0.000	GT	Sens	contig245	contig245	F-box/LR	CTGAAG	contig245	Solyc07g	Solyc07g	F-box/LRR-repeat protein 13 (A			contig245	Solyc07g	F-box/LRR-repeat p		SL2.40ch	AT4G20720.1		dentin si	chr4:111	74.2441	56.7862	111.662	117.359	155.888	147.637
GT	Sense	Sense	0.097	Detected	-0.097	Detected	0.000	2.146	Detected	1.788	Detected	1.967	0.011	GT	Sens	contig246	contig246	Unknown	GACTTG	contig246	Solyc05g	Solyc05g	Unknown Protein (AHRD V1)			contig246	Solyc05g024230.1.1				AT4G344	CHB3. AT	CHB3: D	chr4:164	233.798	159.703	237.216	156.127	903.837	680.866
GT	Sense	Sense	-0.395	Detected	0.395	Detected	0.000	1.373	Detected	0.808	Detected	1.091	0.154	GT	Sens	contig248	contig248	Glucose-	TGCAGA	contig248	Solyc08g	Solyc08g	Glucose-methanol-choline oxi			contig248	Solyc08g	Glucose-methanol-c		SL2.40ch	AT5G285	BIP1	BIP1: AT	chr5:105	13.8979	18.7948	47.8755	37.217	44.2561	28.8784
GT	Sense	Sense	-0.072	Detected	0.072	Detected	0.000	1.095	Detected	1.072	Detected	1.083	0.004	GT	Sens	contig249	contig249	Auxin res	TTCACA	contig249	Solyc03g	Solyc03g	Auxin res	GO:0003	GO:0003	contig249	Solyc03g	Auxin res	GO:0003	SL2.40ch	AT5G370	ARF8	ARF8 (A	chr5:146	756.947	653.74	1164.94	1182.68	1588.19	1508.71
GT	Sense	Sense	0.127	Detected	-0.127	Detected	0.000	1.927	Detected	1.516	Detected	1.722	0.019	GT	Sens	contig249	contig249	Chloroph	AAATCA	contig249	Solyc02g	Solyc02g	Chloroph	GO:0005	GO:0005	contig249	Solyc02g	Chloroph	GO:0005	SL2.40ch	AT2G344	LHB1B2.	LHB1B2:	chr2:145	157.083	102.975	302.361	368.603	511.171	371.126
GT	Sense	Sense	-0.437	Detected	0.437	Detected	0.000	1.032	Detected	1.019	Detected	1.025	0.143	GT	Sens	contig250	contig250	Disease r	ATCTAT	contig250	Solyc11g	Solyc11g	Disease r	GO:0005	GO:0005	contig250	Solyc11g	Cc-nbs-lr	GO:0005	SL2.40ch	AT5G10500.1		kinase in	chr5:330	46.7947	66.9909	89.2993	72.8267	120.984	115.742
GT	Sense	Sense	-0.073	Detected	0.073	Detected	0.000	1.490	Detected	1.234	Detected	1.362	0.012	GT	Sens	contig253	contig253	Elongatio	AGTAGT	contig253	Solyc12g	Solyc12g	Elongatio	GO:0005	GO:0005	contig253	Solyc08g	Elongatio	GO:0003	SL2.40ch	AT1G627	ATSCO1.	SCO1 (S	chr1:232	1142.71	988.18	1887.49	2418.97	3155.09	2550.54
GT	Sense	Sense	-1.099	Comprom	1.099	Detected	0.000	1.447	Detected	1.092	Detected	1.269	0.372	GT	Sens	contig261	contig261	Hcr9-4E (	CCATAA	contig261	Solyc12g	Solyc12g	Hcr9-4E (AHRD V1 *-*- O50024			contig261	Solyc12g	LRR rece	GO:0016	SL2.40ch	AT3G09035.1		legume l	chr3:275	3.44402	12.3544	13.382	8.4148	18.7893	14.1901
GT	Sense	Sense	0.249	Detected	-0.249	Detected	0.000	2.510	Detected	2.717	Detected	2.614	0.010	GT	Sens	contig261	contig261	Early ligh	CTAGCT	contig261	Solyc09g	Solyc09g	Early light-induced protein 7 (A			contig261	Solyc09g	Early ligh	GO:0016	SL2.40ch	AT4G146	ELIP2	ELIP2 (E	chr4:841	2457.39	1360.53	4576.69	6712.15	11008.1	12270.6
GT	Sense	Sense	-0.946	Comprom	0.946	Detected	0.000	1.716	Detected	1.991	Detected	1.853	0.192	GT	Sens	contig263	contig263	Pentatric	TAGATA	contig263	Solyc11g	Solyc11g	Pentatricopeptide repeat-cont			contig263	Solyc11g	Pentatricopeptide r		SL2.40ch	AT3G49740.1		pentatric	chr3:184	4.91357	14.2532	24.8606	19.1928	29.0471	33.9431
GT	Sense	Sense	-0.021	Detected	0.021	Detected	0.000	1.034	Detected	1.039	Detected	1.037	0.000	GT	Sens	contig263	contig263	Unknown	GAAAGA	contig263	Solyc05g	Solyc05g	Unknown Protein (AHRD V1)			contig263	Solyc05g	Unknown Protein (A		SL2.40ch	AT4G22440.1				184.017	148.054	249.359	278.492	357.325	345.99
GT	Sense	Sense	0.078	Detected	-0.078	Detected	0.000	1.196	Detected	0.837	Detected	1.016	0.035	GT	Sens	contig267	contig267	F-box fan	GGCATA	contig267	Solyc04g	Solyc04g	F-box family protein (AHRD V1			contig267	Solyc04g	F-box family protein		SL2.40ch	AT3G05070.1		FUNCTIC	chr3:141	497.219	348.674	608.753	551.645	1008.09	758.866
GT	Sense	Sense	-0.684	Comprom	0.684	Comprom	0.000	3.562	Detected	2.886	Detected	3.224	0.052	GT	Sens	contig270	contig270	FAM65A	TTGGAA	contig270	Solyc09g	Solyc09g	FAM65A protein related (AHRD			contig270	Solyc09g	FAM65A protein rela		SL2.40ch	AT4G373	YDK1. GH	BRU6: in	chr4:175	3.40407	6.86931	4.97498	4.40682	60.3787	36.4655
GT	Sense	Sense	0.096	Detected	-0.096	Detected	0.000	1.028	Detected	1.005	Detected	1.016	0.009	GT	Sens	contig270	contig270	Hydroxyc	AGAACA	contig270	Solyc12g	Solyc12g	Hydroxyc	GO:0016	GO:0016	contig270	Solyc12g	Hydroxyc	GO:0016	SL2.40ch	AT4G15390.1		transfera	chr4:879	127.212	87.1001	180.678	158.898	226.82	215.497
GT	Sense	Sense	0.235	Comprom	-0.235	Comprom	0.000	1.793	Detected	2.344	Detected	2.069	0.029	GT	Sens	contig271	contig271	Unknown	GTTAACT	contig271	Solyc08g	Solyc08g	Unknown Protein (AHRD V1)			contig271	Solyc08g	F-box fan	GO:0004	SL2.40ch	AT1G72660.3		developr	chr1:273	8.27512	4.66894	82.6135	91.3305	22.7631	32.2111
GT	Sense	Sense	0.035	Detected	-0.035	Detected	0.000	0.854	Detected	1.434	Detected	1.144	0.059	GT	Sens	contig277	contig277	Cytochro	ATTCCG	contig277	Solyc03g	Solyc03g	Cytochro	GO:0020	GO:0020	contig277	Solyc03g	Cytochro	GO:0019	SL2.40ch	AT3G201	CYP705A	CYP705A	chr3:702	54.5038	40.6085	58.6259	57.9799	89.9008	129.705
GT	Sense	Sense	-0.116	Detected	0.116	Detected	0.000	1.425	Detected	1.183	Detected	1.304	0.016	GT	Sens	contig279	contig279	Oxygen-e	TTCCTT	contig279	Solyc07g	Solyc07g	Oxygen-e	GO:0019	GO:0019	contig279	Solyc02g	Oxygen-e	GO:0008	SL2.40ch	AT3G508	PSBO2. P	PSBO2 (	chr3:188	3122.32	2866.13	6214	6172.93	8488.67	6929.62
GT	Sense	Sense	-0.211	Detected	0.211	Detected	0.000	2.328	Detected	1.846	Detected	2.087	0.023	GT	Sens	contig280	contig280	Chloroph	TTAGAG	contig280	Solyc12g	Solyc12g	Chloroph	GO:0016	GO:0016	contig280	Solyc12g	Chloroph	GO:0016	SL2.40ch	AT5G542	LHCB3. L	LHCB3 (L	chr5:220	243.932	255.463	750.145	928.825	1325.11	915.782
GT	Sense	Sense	0.249	Detected	-0.249	Comprom	0.000	1.353	Detected	1.349	Detected	1.351	0.032	GT	Sens	contig282	contig282	Unknown	GATCTT	contig282	Solyc01g	Solyc01g	Unknown Protein (AHRD V1)			contig282	Solyc01g	Unknown Protein (A		SL2.40ch	AT4G336	AGD2	AGD2 (A	chr4:161	13.6794	7.56817	23.7928	16.1949	27.4702	26.4536
GT	Sense	Sense	0.045	Detected	-0.045	Detected	0.000	1.760	Detected	1.505	Detected	1.633	0.007	GT	Sens	contig284	contig284	Unknown	GCTGAG	contig284	Solyc12g	Solyc12g	Unknown Protein (AHRD V1)			contig284	Solyc12g	Unknown Protein (A		SL2.40ch	AT5G571	ATPAP28	PAP28 (P	chr5:231	239.404	175.809	305.805	294.947	734.35	594.189
GT	Sense	Sense	-1.830	Comprom	1.830	Detected	0.000	2.558	Detected	2.103	Detected	2.331	0.334	GT	Sens	contig287	contig287	Sulfotran	ATCTTT	contig287	Solyc11g	Solyc11g	Sulfotran	GO:0008	GO:0008	contig287	Solyc11g	Sulfotran	GO:0008	SL2.40ch	AT1G470	VFB1	VFB1 (V	chr1:172	10.692	105.672	144.883	248.258	209.231	147.331
GT	Sense	Sense	0.078	Detected	-0.078	Detected	0.000	1.545	Detected	1.025	Detected	1.285	0.042	GT	Sens	contig288	contig288	Unknown	AAAGAC	contig288	Solyc05g	Solyc05g	Unknown Protein (AHRD V1)			contig288	Solyc01g	Unknown Protein (A		SL2.40ch	AT3G09950.1		unknown	chr3:306	8384.35	5880.54	17823.8	15402.2	21659.8	14575.2
GT	Sense	Sense	0.520	Detected	-0.520	Detected	0.000	1.810	Detected	0.902	Detected	1.356	0.188	GT	Sens	contig292	contig292	Acetyl co	CTACTC	contig292	Solyc05g	Solyc05g	Acetyl co	GO:0010	GO:0010	contig292	Solyc05g	Acetyl co	GO:0010	SL2.40ch	AT5G17540.1		transfera	chr5:578	1886	716.679	1825.62	2071.01	4308.06	2217.21
GT	Sense	Sense	-0.055	Detected	0.055	Detected	0.000	1.179	Detected	1.360	Detected	1.270	0.007	GT	Sens	contig295	contig295	class II he	ATTTGA	contig295	Solyc08g	Solyc08g	class II he	GO:0051	GO:0051	contig295	Solyc08g	class II he	GO:0051	SL2.40ch	AT5G120	HSP17.6II	HSP17.6I	chr5:388	18.0198	15.1911	32.6136	22.801	39.604	43.3245
GT	Sense	Sense	0.280	Detected	-0.280	Detected	0.000	0.966	Detected	1.224	Detected	1.095	0.071	GT	Sens	contig298	contig298	Pectinace	AGTATT	contig298	Solyc03g	Solyc03g	Pectinacetylesterase (Fragme			contig298	Solyc03g	Pectinacetylesteras		SL2.40ch	AT5G23870.1		pectinac	chr5:804	85.862	45.5421	96.2992	133.579	129.055	149.06
GT	Sense	Sense	0.007	Detected	-0.007	Detected	0.000	2.434	Detected	2.151	Detected	2.293	0.004	GT	Sens	contig299	contig299	CONSTAN	GGAGTA	contig299	Solyc02g	Solyc02g	CONSTAN	GO:0003	GO:0003	contig299	Solyc02g	Tomato C	GO:0003	SL2.40ch	AT5G158	COL1. AT	COL1 (c	chr5:517	382.591	296.198	1061.23	1618.25	1922.68	1525.61
GT	Sense	Sense	0.294	Detected	-0.294	Comprom	0.000	1.321	Detected	0.898	Detected	1.109	0.092	GT	Sens	contig306	contig306	Ulp1 prot	CACTTT	contig306	Solyc07g	Solyc07g	Ulp1 prot	GO:0006	GO:0006	contig306	Solyc07g	Ulp1 prot	GO:0006	SL2.40ch	AT1G42470.1		patched	chr1:159	20.7593	10.7922	27.2508	35.3432	39.5096	28.456
GT	Sense	Sense	0.171	Detected	-0.171	Detected	0.000	1.509	Detected	1.227	Detected	1.368	0.025	GT	Sens	contig309	contig309	Chloroph	TTAATT	contig309	Solyc01g	Solyc01g	Chloroph	GO:0016	GO:0016	contig309	Solyc01g	Chloroph	GO:0016	SL2.40ch	AT1G158	LHCB6. C	LHCB6 (L	chr1:544	9055.38	5584.12	13318.8	16076.3	21394.2	16989.9
GT	Sense	Sense	-0.361	Detected	0.361	Detected	0.000	1.132	Detected	0.941	Detected	1.036	0.109	GT	Sens	contig311	contig311	GH3 famil	GCAGTG	contig311	Solyc10g	Solyc10g	GH3 famil	GO:0070	GO:0070	contig311	Solyc10g	GH3 famil	GO:0080	SL2.40ch	AT2G463	JAR1. FIN	JAR1 (JA	chr2:190	673.631	868.582	1296.89	1972.95	1771.98	1498.19
GT	Sense	Sense	-0.250	Detected	0.250	Detected	0.000	1.684	Detected	0.894	Detected	1.289	0.110	GT	Sens	contig312	contig312	Subtilisin	CAAGTG	contig312	Solyc08g	Solyc08g	Subtilisin	GO:0004	GO:0004	contig312	Solyc08g	Subtilisin	GO:0004	SL2.40ch	AT4G10510.1		subtilase	chr4:649	367.444	406.172	618.454	753.028	1312.09	732.597
GT	Sense	Sense	-0.330	Detected	0.330	Detected	0.000	1.355	Detected	1.204	Detected	1.280	0.063	GT	Sens	contig323	contig323	Indole-3-a	AATGGG	contig323	Solyc00g	Solyc00g	Indole-3-acetic acid-amido syn			contig323	Solyc10g	GH3 famil	GO:0080	SL2.40ch	AT5G38340.1		disease r	chr5:153	231.394	285.853	457.145	680.054	695.321	604.653
GT	Sense	Sense	0.093	Detected	-0.093	Detected	0.000	1.232	Detected	1.221	Detected	1.227	0.006	GT	Sens	contig340	contig340	Receptor	TATTAG	contig340	Solyc10g	Solyc10g	Receptor	GO:0005	GO:0005	contig340	Solyc10g	LRR rece	GO:0005	SL2.40ch	AT4G08450.1		disease r	chr4:536	275.426	189.159	311.053	215.034	566.491	543.071
GT	Sense	Sense	0.408	Detected	-0.408	Detected	0.000	0.814	Detected	1.247	Detected	1.031	0.155	GT	Sens	contig355	contig355	Endogluc	TTACAA	contig355	Solyc08g	Solyc08g	Endogluc	GO:0005	GO:0005	contig355	Solyc08g	Endogluc	GO:0030	SL2.40ch	AT1G643	AtGH9C2	AtGH9C2	chr1:239	60.8762	27.0283	70.5424	34.3085	75.3734	98.2443
GT	Sense	Sense	-0.094	Comprom	0.094	Comprom	0.000	2.787	Detected	2.871	Detected	2.829	0.001	GT	Sens	contig375	contig375	Leucine F	TTCTTA	contig375	Solyc12g	Solyc12g	Leucine F	GO:0005	GO:0005	contig375	Solyc12g	LRR rece	GO:0016	SL2.40ch	AT2G46915.1		unknown	chr2:192	9.21183	8.19957	12.5481	55.7702	63.3812	64.8771
GT	Sense	Sense	0.161	Detected	-0.161	Detected	0.000	1.387	Detected	1.133	Detected	1.260	0.025	GT	Sens	contig386	contig386	Unknown	ATCGTT	contig386	Solyc06g	Solyc06g	Unknown Protein (AHRD V1)			contig386	Solyc06g009810.2.1				AT1G508	POLGAM	POLGAM	chr1:188	62.6129	39.1668	75.5282	61.7393	136.873	110.821
GT	Sense	Sense	0.173	Detected	-0.173	Detected	0.000	0.991	Detected	1.382	Detected	1.186	0.045	GT	Sens	contig390	contig390	Digalacto	GATATT	contig390	Solyc10g	Solyc10g	Digalactosyldiacylglycerol syn			contig390	Solyc10g	Digalactosyldiacylg		SL2.40ch	AT3G116	DGD1	DGD1 (D	chr3:368	18.9888	11.6731	26.1108	22.2622	31.2792	39.5844
GT	Sense	Sense	0.076	Comprom	-0.076	Comprom	0.000	1.964	Detected	3.102	Detected	2.533	0.048	GT	Sens	contig416	contig416	Aldehyde	TCTTTC	contig416	Solyc11g	Solyc11g	Aldehyde	GO:0055	GO:0055	contig416	Solyc11g	Aldehyde	GO:0009	SL2.40ch	AT5G209	AAO1. AC	AAO1 (A	chr5:711	3.14601	2.21174	10.8062	18.0265	10.8754	23.1168
GT	Sense	Sense	2.123	Detected	-2.123	Comprom	0.000	1.644	Detected	2.759	Detected	2.201	0.421	GT	Sens	contig419	contig419	Unannota	CTCACCATTCAAATTTGTACATCATCGAATGGGAAATCTCTATCTTCTA							contig419	Solyc03g	Polyprote	GO:0006	SL2.40ch	AT5G64667.1				50.6456	2.08765	10.4512	6.49879	33.968	70.9997
GT	Sense	Sense	-0.224	Detected	0.224	Detected	0.000	1.994	Detected	1.480	Detected	1.737	0.036	GT	Sens	contig429	contig429	Nicotia ta	CAAGTAAGAATCCTTCTCAAATTGTTATTTGTGCATCTTGATCATCATG							contig429	#N/A	#N/A	#N/A	#N/A	#N/A	#N/A	#N/A	#N/A	48.0533	51.247	111.458	125.397	208.937	141.28
GT	Sense	Sense	0.002	Detected	-0.002	Detected	0.000	1.195	Detected	0.831	Detected	1.013	0.031	GT	Sens	contig441	contig441	Nodulin-li	CCGCTC	contig441	Solyc08g	Solyc08g	Nodulin-li	GO:0016	GO:0016	contig441	Solyc08g	Nodulin-li	GO:0016	SL2.40ch	AT5G45370.3		nodulin-r	chr5:183	406.507	316.669	627.683	739.121	868.006	651.354
GT	Sense	Sense	0.168	Detected	-0.168	Detected	0.000	1.954	Detected	1.858	Detected	1.906	0.008	GT	Sens	contig446	contig446	Auxin-ind	CATGAT	contig446	Solyc03g	Solyc03g	Auxin-ind	GO:0005	GO:0005	contig446	Solyc03g	Auxin-ind	GO:0005	SL2.40ch	AT2G21220.1		auxin-res	chr2:908	71.1491	44.0712	142.457	125.122	229.366	207.069
GT	Sense	Sense	-0.094	Detected	0.094	Detected	0.000	1.248	Detected	1.261	Detected	1.254	0.006	GT	Sens	contig456	contig456	ClpB cha	GAGTTA	contig456	Solyc06g	Solyc06g	ClpB cha	GO:0005	GO:0005	contig456	Solyc03g	ClpB cha	GO:0019	SL2.40ch	AT1G743	ATHSP10	ATHSP1	chr1:279	822.129	731.823	623.454	947.106	1946.75	1896.64
GT	Sense	Sense	-0.151	Detected	0.151	Detected	0.000	1.421	Detected	0.910	Detected	1.166	0.059	GT	Sens	contig461	contig461	Unknown	GGCTAC	contig461	Solyc00g	Solyc00g	Unknown Protein (AHRD V1)			contig461	Solyc00g	Unknown Protein (A		SL2.40ch	AT2G38160.1				13.1661	12.6922	16.9508	6.78287	36.583	24.7857
GT	Sense	Sense	-0.007	Detected	0.007	Detected	0.000	1.277	Detected	0.936	Detected	1.107	0.023	GT	Sens	contig476	contig476	Tryptoph	CACGGC	contig476	Solyc10g	Solyc10g	Tryptoph	GO:0008	GO:0008	contig476	Solyc10g	Tryptoph	GO:0004	SL2.40ch	AT3G12100.2		cation ef	chr3:385	2819.85	2226.02	3199.11	4304.07	6416.6	4891.32
GT	Sense	Sense	-0.204	Detected	0.204	Detected	0.000	1.850	Detected	1.732	Detected	1.791	0.014	GT	Sens	contig480	contig480	Unknown	AATAAT	contig480	Solyc06g	Solyc06g	Unknown Protein (AHRD V1)%:			contig480	Solyc12g	H-ATPase	GO:0016	SL2.40ch	AT2G13720.1				1399.45	1451.69	1685	1444.44	5430.01	4832
GT	Sense	Sense	-0.443	Comprom	0.443	Comprom	0.000	1.367	Detected	1.498	Detected	1.432	0.085	GT	Sens	contig482	contig482	Helicase-	TCTAAG	contig482	Solyc03g	Solyc03g	Helicase-like protein (AHRD V1			contig482	Solyc07g	Helicase-like protein		SL2.40ch	AT5G482	EMB1276	ACAT2 (A	chr5:195	3.18713	4.60089	8.9	6.77541	10.4408	11.0328
GT	Sense	Sense	0.014	Detected	-0.014	Detected	0.000	1.488	Detected	1.405	Detected	1.447	0.001	GT	Sens	contig483	contig483	Metacasp	CAGAAG	contig483	Solyc01g	Solyc01g	Metacasp	GO:0005	GO:0005	contig483	Solyc01g	Metacasp	GO:0006	SL2.40ch	AT5G502	OS. OLD5	OS (OUI	chr5:204	88.9679	68.1758	167.083	102.778	230.906	210.475
GT	Sense	Sense	0.067	Detected	-0.067	Detected	0.000	1.149	Detected	1.085	Detected	1.117	0.004	GT	Sens	contig502	contig502	F-box/LR	AATCAT	contig502	Solyc08g	Solyc08g	F-box/LRR-repeat protein At3g			contig502	Solyc08g	F-box/LRR-repeat p		SL2.40ch	AT4G20720.1		dentin si	chr4:111	153.984	109.75	454.819	395.825	304.733	281.381
GT	Sense	Sense	-0.029	Detected	0.029	Detected	0.000	1.148	Detected	1.121	Detected	1.135	0.001	GT	Sens	contig512	contig512	Topoison	TTGAGA	contig512	Solyc11g	Solyc11g	Topoisomerase 6 subunit B-lik			contig512	Solyc11g	Topoisomerase 6 s		SL2.40ch	AT3G294	ATEXO70	ATEXO7	chr3:112	34.719	28.2437	53.5356	51.8261	73.3539	69.5176
GT	Sense	Sense	-0.438	Detected	0.438	Detected	0.000	1.619	Detected	1.350	Detected	1.484	0.084	GT	Sens	contig531	contig531	PHD finge	GGATGA	contig531	Solyc01g	Solyc01g	PHD finger family protein (AHR			contig531	Solyc01g	PHD finger family p		SL2.40ch	AT3G52100.1		PHD fing	chr3:193	18.6965	26.8231	55.8783	42.6711	72.6904	58.2455
GT	Sense	Sense	0.141	Comprom	-0.141	Comprom	0.000	1.011	Detected	1.324	Detected	1.167	0.031	GT	Sens	contig535	contig535	Auxin res	GCTAGC	contig535	Solyc05g	Solyc05g	Auxin res	GO:0005	GO:0005	contig535	Solyc05g	Auxin res	GO:0005	SL2.40ch	AT3G19240.1		FUNCTIC	chr3:666	10.9936	7.06378	21.4678	21.2009	18.7695	22.5075
GT	Sense	Sense	0.346	Detected	-0.346	Detected	0.000	2.198	Detected	1.667	Detected	1.932	0.047	GT	Sens	contig536	contig536	Chloroph	AATTTCT	contig536	Solyc02g	Solyc02g	Chloroph	GO:0005	GO:0005	contig536	Solyc02g	Chloroph	GO:0005	SL2.40ch	AT1G299	CAB3. AB	CAB3 (C	chr1:104	347.626	168.146	621.439	626.834	1172.43	783.333
GT	Sense	Sense	-0.721	Comprom	0.721	Detected	0.000	1.477	Detected	0.933	Detected	1.205	0.258	GT	Sens	contig551	contig551	Cytochro	TCAGTT	contig551	Solyc10g	Solyc10g	Cytochro	GO:0020	GO:0020	contig551	Solyc10g	cytochro	GO:0019	SL2.40ch	AT5G51900.1				8.43982	17.9302	25.9475	20.4483	36.1887	23.9717
GT	Sense	Sense	0.121	Detected	-0.121	Detected	0.000	1.293	Detected	1.149	Detected	1.221	0.013	GT	Sens	contig60	contig60	WD-40 re	TCCCCT	contig60	Solyc12g	Solyc12g	WD-40 re	GO:0006	GO:0006	contig60	Solyc12g	WD-40 re	GO:0006	SL2.40ch	AT3G153	SPA3	SPA3 (S	chr3:516	1211.26	800.655	2119.71	2350.46	2550.04	2228.93
GT	Sense	Sense	0.000	Detected	0.000	Detected	0.000	1.825	Detected	1.530	Detected	1.678	0.008	GT	Sens	contig600	contig600	F-box fan	CTCAAG	contig600	Solyc07g	Solyc07g	F-box family protein (AHRD V1			contig600	Solyc07g	F-box family protein		SL2.40ch	AT4G20720.1		dentin si	chr4:111	62.9007	49.157	397.505	293.533	208.265	163.896
GT	Sense	Sense	-0.975	Comprom	0.975	Detected	0.000	1.015	Detected	2.354	Detected	1.685	0.290	GT	Sens	contig624	contig624	Unknown	CCAAAG	contig624	Solyc00g	Solyc00g	Unknown Protein (AHRD V1)			contig624	Solyc00g	Unknown Protein (A		SL2.40ch	AT1G31090.1		F-box fa	chr1:110	5.87173	17.7345	23.7024	13.9381	21.7971	53.2452
GT	Sense	Sense	0.669	Detected	-0.669	Comprom	0.000	2.784	Detected	2.048	Detected	2.416	0.087	GT	Sens	contig629	contig629	Malonyl	ACCATG	contig629	Solyc10g	Solyc10g	Malonyl CoA anthocyanin 5-O-			contig629	Solyc10g	Malonyl CoA anthoc		SL2.40ch	AT3G50840.1		phototro	chr3:188	14.3505	4.43432	26.8549	20.9333	58.0874	33.6663
GT	Sense	Sense	-0.049	Detected	0.049	Detected	0.000	1.138	Detected	1.004	Detected	1.071	0.006	GT	Sens	contig634	contig634	RNA bind	TGCTCT	contig634	Solyc09g	Solyc09g	RNA binding protein (AHRD V1			contig634	Solyc09g	RNA binding protein		SL2.40ch	AT5G01590.1		unknown	chr5:224	159.593	133.601	214.857	198.647	339.569	298.843
GT	Sense	Sense	0.091	Detected	-0.091	Detected	0.000	2.271	Detected	1.666	Detected	1.969	0.025	GT	Sens	contig660	contig660	Chloroph	AAACTC	contig660	Solyc02g	Solyc02g	Chloroph	GO:0005	GO:0005	contig660	Solyc02g070970.1.1				AT2G344	LHB1B2.	LHB1B2:	chr2:145	227.721	156.986	483.496	523.927	964.311	612.338
GT	Sense	Sense	0.242	Detected	-0.242	Detected	0.000	1.229	Detected	1.090	Detected	1.160	0.044	GT	Sens	contig666	contig666	Kinesin-li	AGATAT	contig666	Solyc12g	Solyc12g	Kinesin-like calmodulin bindin			contig666	Solyc12g	Kinesin-like calmod		SL2.40ch	AT1G11420.1		agenet d	chr1:384	170.083	95.1029	258.879	279.106	315.15	276.212
GT	Sense	Sense	-1.021	Comprom	1.021	Detected	0.000	1.133	Detected	2.576	Detected	1.854	0.276	GT	Sens	contig671	contig671	Cytochro	GTTCAA	contig671	Solyc10g	Solyc10g	Cytochro	GO:0020	GO:0020	contig671	Solyc10g	Cytochro	GO:0046	SL2.40ch	AT2G408	CYP98A3	CYP98A3	chr2:170	3.92301	12.6199	1.92968	1.81817	16.307	42.7961
GT	Sense	Sense	0.005	Detected	-0.005	Detected	0.000	1.549	Detected	1.060	Detected	1.305	0.033	GT	Sens	contig725	contig725	Salicylic	TCCTTG	contig725	Solyc09g	Solyc09g	Salicylic	GO:0008	GO:0008	contig725	Solyc09g	Salicylic	GO:0008	SL2.40ch	AT4G36470.1		S-adeno	chr4:172	202.465	157.099	242.325	403.955	551.672	379.455
GT	Sense	Sense	0.184	Comprom	-0.184	Comprom	0.000	2.347	Detected	2.855	Detected	2.601	0.014	GT	Sens	contig731	contig731	Leucine-r	ATTAGA	contig731	Solyc12g	Solyc12g	Leucine-r	GO:0005	GO:0005	contig731	Solyc12g	LRR rece	GO:0016	SL2.40ch	AT4G142	RHF1A	RHF1A (R	chr4:819	4.27342	2.58891	6.19118	6.19868	17.8838	24.5584
GT	Sense	Sense	0.554	Comprom	-0.554	Comprom	0.000	1.230	Detected	1.100	Detected	1.165	0.172	GT	Sens	contig790	contig790	Receptor	TTACAG	contig790	Solyc10g	Solyc10g	Receptor protein kinase-like (A			contig790	Solyc01g	Receptor	GO:0042	SL2.40ch	AT1G24090.1				8.95618	3.24943	14.8198	20.4175	13.3723	11.8031
GT	Sense	Sense	-0.078	Detected	0.078	Detected	0.000	2.352	Detected	0.972	Detected	1.662	0.139	GT	Sens	contig799	contig799	Hydroxyc	TTTCCA	contig799	Solyc07g	Solyc07g	Hydroxyc	GO:0016	GO:0016	contig799	Solyc07g	Hydroxyc	GO:0016	SL2.40ch	AT5G484	ATGLR1.	ATGLR1.	chr5:196	702.884	611.882	1995.59	979.212	3538.68	1312.22
GT	Sense	Sense	0.035	Detected	-0.035	Detected	0.000	2.674	Detected	2.363	Detected	2.518	0.004	GT	Sens	contig815	contig815	CONSTAN	TGGAAT	contig815	Solyc02g	Solyc02g	CONSTAN	GO:0003	GO:0003	contig815	Solyc02g	Tomato C	GO:0003	SL2.40ch	AT5G158	COL1. AT	COL1 (c	chr5:517	584.687	435.472	1711.06	2530.55	3402.97	2648.85
GT	Sense	Sense	0.005	Detected	-0.005	Detected	0.000	1.441	Detected	1.212	Detected	1.326	0.007	GT	Sens	contig844	contig844	B3 domai	GTGCTT	contig844	Solyc08g	Solyc08g	B3 domai	GO:0006	GO:0006	contig844	Solyc08g	B3 domai	GO:0006	SL2.40ch	AT3G189	VRN1. RE	VRN1 (R	chr3:654	14.7672	11.463	28.4262	22.1869	37.3215	30.759
GT	Sense	Sense	0.246	Detected	-0.246	Detected	0.000	1.139	Detected	1.035	Detected	1.087	0.050	GT	Sens	contig879	contig879	Protein ki	CCATTG	contig879	Solyc07g	Solyc07g	Protein kinase family protein (			contig879	Solyc01g	Receptor	GO:0006	SL2.40ch	AT1G09440.1		protein k	chr1:304	488.665	271.486	618.908	466.339	847.545	761.757
GT	Sense	Sense	-0.129	Detected	0.129	Detected	0.000	1.528	Detected	1.119	Detected	1.324	0.032	GT	Sens	contig886	contig886	Cytochro	TACCAA	contig886	Solyc03g	Solyc03g	Cytochro	GO:0019	GO:0019	contig886	Solyc03g	Cytochro	GO:0019	SL2.40ch	AT3G483	CYP71A2	CYP71A2	chr3:178	1467.73	1372.83	2288.69	4501.8	4326.66	3145.96
GT	Sense	Sense	-0.131	Comprom	0.131	Comprom	0.000	1.152	Detected	1.107	Detected	1.129	0.014	GT	Sens	contig889	contig889	GDSL est	CAAAAA	contig889	Solyc03g	Solyc03g	GDSL est	GO:0006	GO:0006	contig889	Solyc03g	GDSL est	GO:0004	SL2.40ch	AT3G16370.1		GDSL-m	chr3:555	7.31906	6.86023	28.5729	18.7628	16.6378	15.5759
GT	Sense	Sense	0.030	Detected	-0.030	Comprom	0.000	2.553	Detected	2.481	Detected	2.517	0.000	GT	Sens	contig906	contig906	1-aminoc	GTCCAG	contig906	Solyc09g	Solyc09g	1-aminoc	GO:0016	GO:0016	contig906	Solyc09g	1-aminoc	GO:0009	SL2.40ch	AT1G06640.1		2-oxoglu	chr1:203	11.4376	8.5736	32.0604	32.8921	61.4151	56.4026
GT	Sense	Sense	-0.225	Detected	0.225	Detected	0.000	1.284	Detected	0.809	Detected	1.047	0.085	GT	Sens	contig920	contig920	Photosys	TGTGTT	contig920	Solyc02g	Solyc02g	Photosys	GO:0009	GO:0009	contig920	Solyc02g	Photosys	GO:0009	SL2.40ch	AT1G313	PSAF	PSAF (p	chr1:112	147.814	157.794	311.273	280.456	393.13	272.965
GT	Sense	Sense	-0.530	Detected	0.530	Detected	0.000	2.176	Detected	1.014	Detected	1.597	0.180	GT	Sens	contig951	contig951	Subtilisin	TACCAA	contig951	Solyc08g	Solyc08g	Subtilisin	GO:0004	GO:0004	contig951	Solyc08g	Subtilisin	GO:0004	SL2.40ch	AT5G673	ARA12	ARA12:	chr5:268	289.645	472.154	523.128	671.51	1770.13	762.252
GT	Sense	Sense	0.133	Detected	-0.133	Detected	0.000	2.157	Detected	1.877	Detected	2.017	0.015	GT	Sens	contig955	contig955	Chloroph	AGTGAA	contig955	Solyc02g	Solyc02g	Chloroph	GO:0005	GO:0005	contig955	Solyc02g	Chloroph	GO:0005	SL2.40ch	AT2G344	LHB1B2.	LHB1B2:	chr2:145	173.365	112.697	358.878	397.379	811.217	561.411
GT	Sense	Sense	-0.471	Detected	0.471	Detected	0.000	1.483	Detected	1.560	Detected	1.522	0.084	GT	Sens	contig97	contig97	GPCR-typ	TCTGTT	contig97	Solyc07g	Solyc07g	GPCR-type G protein 1 (AHRD			contig97	Solyc07g	Golgi pH	GO:0051	SL2.40ch	AT4G276	GTG2	GTG2 (G	chr4:137	247.815	371.958	598.21	603.39	897.007	913.559
GT	Sense	Sense	-0.079	Detected	0.079	Detected	0.000	1.257	Detected	1.025	Detected	1.141	0.015	GT	Sens	EF66734	EF66734	SlARF8:	CACAACTAAATACGTGCTCCAACTCATCATCACTTCCAACTTACGGGAAAGATACATTCA																503.909	439.429	957.956	835.719	1188.56	977.096

		8h_AZ_Control					8h_AZ_TAPG + TPRP vs. 8h_AZ_Control					
ProbeNar	Orientatio	Fold 8h	Flag 8h	Fold 8h	Flag 8h	Geomean	Fold 8h	Flag 8h	Fold 8h	Flag 8h	Geomean	P-value
A 96 P0(	Sense	-0.359	Detected	0.359	Detected	0.000	-1.434	Detected	-0.911	Detected	-1.172	0.118
A 96 P01	Sense	-0.017	Detected	0.017	Detected	0.000	-2.344	Detected	-1.393	Detected	-1.869	0.059
A 96 P01	Sense	-0.610	Detected	0.610	Detected	0.000	-1.266	Detected	-1.801	Detected	-1.533	0.148
A 96 P01	Sense	-0.192	Detected	0.192	Detected	0.000	-1.137	Detected	-1.312	Detected	-1.225	0.028
A 96 P02	Sense	-0.265	Detected	0.265	Detected	0.000	-1.792	Detected	-1.421	Detected	-1.607	0.038
A 96 P03	Sense	-0.223	Detected	0.223	Detected	0.000	-1.081	Detected	-1.050	Comprom	-1.065	0.041
A 96 P04	Sense	-0.581	Detected	0.581	Detected	0.000	-1.493	Detected	-0.855	Detected	-1.174	0.219
A 96 P05	Sense	-0.335	Detected	0.335	Detected	0.000	-1.528	Detected	-0.986	Detected	-1.257	0.100
A 96 P06	Sense	0.609	Detected	-0.609	Detected	0.000	-1.878	Detected	-1.139	Detected	-1.508	0.169
A 96 P06	Sense	-0.368	Detected	0.368	Detected	0.000	-1.267	Comprom	-2.144	Comprom	-1.705	0.097
A 96 P08	Sense	0.086	Detected	-0.086	Detected	0.000	-2.130	Detected	-1.061	Detected	-1.596	0.099
A 96 P08	Sense	0.191	Detected	-0.191	Detected	0.000	-0.862	Detected	-1.640	Detected	-1.251	0.102
A 96 P08	Sense	0.098	Detected	-0.098	Detected	0.000	-1.130	Detected	-1.067	Detected	-1.098	0.009
A 96 P08	Sense	-0.510	Detected	0.510	Detected	0.000	-2.043	Detected	-2.270	Detected	-2.156	0.054
A 96 P08	Sense	-0.261	Detected	0.261	Detected	0.000	-1.590	Detected	-1.357	Detected	-1.473	0.036
A 96 P08	Sense	-1.127	Detected	1.127	Detected	0.000	-1.793	Detected	-1.490	Detected	-1.642	0.286
A 96 P08	Sense	0.132	Detected	-0.132	Detected	0.000	-1.136	Detected	-1.146	Detected	-1.141	0.013
A 96 P09	Sense	-0.182	Detected	0.182	Detected	0.000	-1.473	Detected	-0.918	Detected	-1.195	0.069
A 96 P09	Sense	-0.941	Detected	0.941	Detected	0.000	-1.565	Detected	-1.484	Detected	-1.524	0.247
A 96 P11	Sense	-0.179	Detected	0.179	Detected	0.000	-1.597	Detected	-1.792	Detected	-1.695	0.014
A 96 P11	Sense	0.167	Detected	-0.167	Detected	0.000	-2.182	Comprom	-1.936	Comprom	-2.059	0.010
A 96 P11	Sense	-0.268	Detected	0.268	Detected	0.000	-1.480	Detected	-1.372	Detected	-1.426	0.035
A 96 P11	Sense	-0.316	Detected	0.316	Detected	0.000	-0.967	Detected	-1.109	Detected	-1.038	0.085
A 96 P13	Sense	0.046	Detected	-0.046	Detected	0.000	-1.419	Detected	-1.432	Detected	-1.425	0.001
A 96 P14	Sense	0.291	Detected	-0.291	Detected	0.000	-2.176	Detected	-1.572	Detected	-1.874	0.047
A 96 P16	Sense	-0.157	Detected	0.157	Detected	0.000	-1.926	Detected	-1.772	Detected	-1.849	0.009
A 96 P18	Sense	-0.262	Detected	0.262	Detected	0.000	-1.064	Detected	-0.988	Detected	-1.026	0.061
A 96 P18	Sense	-0.111	Detected	0.111	Detected	0.000	-2.098	Detected	-2.105	Detected	-2.101	0.003
A 96 P19	Sense	-0.441	Detected	0.441	Detected	0.000	-2.013	Detected	-0.975	Detected	-1.494	0.160
A 96 P19	Sense	-0.259	Detected	0.259	Detected	0.000	-2.514	Detected	-2.035	Detected	-2.275	0.023
A 96 P20	Sense	0.147	Detected	-0.147	Detected	0.000	-2.357	Detected	-1.681	Detected	-2.019	0.032
A 96 P22	Sense	-0.699	Detected	0.699	Detected	0.000	-1.476	Detected	-2.571	Detected	-2.023	0.150
A 96 P22	Sense	-0.219	Detected	0.219	Detected	0.000	-1.557	Comprom	-1.980	Comprom	-1.769	0.028
A 96 P22	Sense	-0.940	Detected	0.940	Detected	0.000	-1.752	Detected	-1.891	Detected	-1.821	0.193
A 96 P23	Sense	-0.406	Detected	0.406	Detected	0.000	-0.955	Detected	-1.157	Detected	-1.056	0.128
A 96 P24	Sense	0.130	Detected	-0.130	Detected	0.000	-1.922	Detected	-1.011	Detected	-1.466	0.090
A 96 P24	Sense	-1.036	Detected	1.036	Detected	0.000	-1.317	Detected	-1.241	Detected	-1.279	0.343
A 96 P25	Sense	-1.654	Detected	1.654	Detected	0.000	-2.453	Detected	-1.268	Detected	-1.861	0.401
A 96 P25	Sense	-0.216	Detected	0.216	Detected	0.000	-1.248	Detected	-1.228	Detected	-1.238	0.029
A 96 P26	Sense	-0.344	Detected	0.344	Detected	0.000	-1.793	Detected	-0.930	Detected	-1.361	0.133
GT Sense	Sense	0.065	Detected	-0.065	Detected	0.000	-1.911	Detected	-1.691	Detected	-1.801	0.005
GT Sense	Sense	0.238	Detected	-0.238	Detected	0.000	-3.561	Comprom	-4.956	Comprom	-4.258	0.029
GT Sense	Sense	0.081	Detected	-0.081	Detected	0.000	-1.363	Detected	-1.284	Detected	-1.323	0.005
GT Sense	Sense	-0.699	Detected	0.699	Detected	0.000	-1.700	Comprom	-3.000	Comprom	-2.350	0.133
GT Sense	Sense	0.094	Detected	-0.094	Detected	0.000	-1.517	Detected	-0.978	Detected	-1.248	0.048
GT Sense	Sense	0.086	Detected	-0.086	Detected	0.000	-0.890	Detected	-1.167	Detected	-1.029	0.024
GT Sense	Sense	0.800	Detected	-0.800	Detected	0.000	-1.410	Detected	-1.626	Detected	-1.518	0.201
GT Sense	Sense	-0.124	Detected	0.124	Detected	0.000	-1.678	Detected	-0.864	Detected	-1.271	0.096
GT Sense	Sense	-0.923	Detected	0.923	Detected	0.000	-1.927	Detected	-0.972	Detected	-1.450	0.298
GT Sense	Sense	-0.272	Detected	0.272	Detected	0.000	-2.051	Detected	-1.877	Detected	-1.964	0.021
GT Sense	Sense	-0.141	Detected	0.141	Detected	0.000	-1.091	Detected	-1.165	Detected	-1.128	0.016
GT Sense	Sense	-0.008	Detected	0.008	Detected	0.000	-1.552	Detected	-1.950	Detected	-1.751	0.013
GT Sense	Sense	-0.834	Detected	0.834	Detected	0.000	-1.910	Detected	-1.027	Detected	-1.468	0.260
GT Sense	Sense	-0.199	Detected	0.199	Detected	0.000	-1.750	Detected	-1.087	Detected	-1.418	0.067
GT Sense	Sense	-0.092	Detected	0.092	Detected	0.000	-1.632	Detected	-1.571	Detected	-1.602	0.004
GT Sense	Sense	0.065	Detected	-0.065	Detected	0.000	-1.213	Detected	-1.229	Detected	-1.221	0.003
GT Sense	Sense	-0.103	Detected	0.103	Detected	0.000	-1.536	Detected	-1.402	Detected	-1.469	0.007
GT Sense	Sense	-1.103	Detected	1.103	Detected	0.000	-1.239	Detected	-1.222	Detected	-1.230	0.381
GT Sense	Sense	-0.375	Detected	0.375	Detected	0.000	-1.527	Detected	-1.530	Detected	-1.529	0.055
GT Sense	Sense	-0.411	Detected	0.411	Detected	0.000	-1.316	Detected	-0.805	Detected	-1.060	0.160
GT Sense	Sense	0.487	Detected	-0.487	Detected	0.000	-2.071	Detected	-2.037	Detected	-2.054	0.052
GT Sense	Sense	-0.460	Detected	0.460	Detected	0.000	-3.657	Detected	-2.522	Detected	-3.089	0.052
GT Sense	Sense	0.364	Detected	-0.364	Detected	0.000	-2.170	Detected	-2.470	Comprom	-2.320	0.028
GT Sense	Sense	-0.809	Detected	0.809	Detected	0.000	-2.661	Comprom	-1.601	Detected	-2.131	0.158
GT Sense	Sense	-0.229	Detected	0.229	Detected	0.000	-2.100	Detected	-0.939	Detected	-1.520	0.135
GT Sense	Sense	-0.550	Detected	0.550	Detected	0.000	-1.223	Detected	-1.072	Detected	-1.147	0.175
GT Sense	Sense	0.180	Detected	-0.180	Detected	0.000	-1.636	Detected	-1.349	Detected	-1.493	0.023
GT Sense	Sense	-0.330	Detected	0.330	Detected	0.000	-2.626	Detected	-2.290	Detected	-2.458	0.022
GT Sense	Sense	-0.064	Detected	0.064	Detected	0.000	-2.453	Detected	-1.258	Detected	-1.855	0.091
GT Sense	Sense	-0.520	Detected	0.520	Detected	0.000	-1.282	Detected	-1.043	Detected	-1.162	0.161
GT Sense	Sense	-0.281	Detected	0.281	Detected	0.000	-1.995	Comprom	-1.743	Comprom	-1.869	0.026
GT Sense	Sense	0.065	Detected	-0.065	Detected	0.000	-1.567	Detected	-1.231	Detected	-1.399	0.016
GT Sense	Sense	-0.148	Detected	0.148	Detected	0.000	-1.957	Detected	-1.534	Detected	-1.746	0.021
GT Sense	Sense	0.061	Detected	-0.061	Detected	0.000	-1.126	Detected	-1.617	Comprom	-1.372	0.032
GT Sense	Sense	-0.739	Detected	0.739	Detected	0.000	-3.106	Detected	-2.030	Detected	-2.568	0.107
GT Sense	Sense	-0.139	Detected	0.139	Detected	0.000	-2.208	Detected	-0.935	Detected	-1.571	0.137
GT Sense	Sense	0.066	Detected	-0.066	Detected	0.000	-1.094	Detected	-1.136	Detected	-1.115	0.004
GT Sense	Sense	-0.106	Detected	0.106	Detected	0.000	-1.188	Detected	-1.649	Detected	-1.419	0.031
GT Sense	Sense	0.205	Detected	-0.205	Detected	0.000	-1.636	Detected	-0.801	Detected	-1.218	0.120
GT Sense	Sense	-0.289	Detected	0.289	Detected	0.000	-2.253	Detected	-1.997	Detected	-2.125	0.021
GT Sense	Sense	-0.751	Detected	0.751	Detected	0.000	-4.532	Comprom	-2.754	Detected	-3.643	0.089
GT Sense	Sense	0.038	Detected	-0.038	Detected	0.000	-2.629	Comprom	-1.418	Detected	-2.023	0.079
GT Sense	Sense	0.133	Detected	-0.133	Detected	0.000	-1.260	Detected	-1.485	Detected	-1.373	0.016
GT Sense	Sense	-0.949	Detected	0.949	Detected	0.000	-3.023	Detected	-1.781	Detected	-2.402	0.168
GT Sense	Sense	-0.171	Detected	0.171	Detected	0.000	-1.421	Detected	-1.023	Detected	-1.222	0.043
GT Sense	Sense	-0.969	Detected	0.969	Detected	0.000	-1.443	Detected	-1.411	Detected	-1.427	0.279
GT Sense	Sense	-0.249	Detected	0.249	Detected	0.000	-3.666	Detected	-1.102	Detected	-2.384	0.209
GT Sense	Sense	0.347	Detected	-0.347	Detected	0.000	-1.258	Detected	-1.270	Detected	-1.264	0.068
GT Sense	Sense	-0.059	Detected	0.059	Detected	0.000	-1.282	Detected	-1.150	Detected	-1.216	0.005
GT Sense	Sense	0.188	Detected	-0.188	Detected	0.000	-1.177	Detected	-1.124	Detected	-1.150	0.026
GT Sense	Sense	-0.220	Detected	0.220	Detected	0.000	-2.678	Comprom	-1.335	Detected	-2.007	0.105
GT Sense	Sense	0.119	Detected	-0.119	Detected	0.000	-1.359	Detected	-1.510	Detected	-1.434	0.009
GT Sense	Sense	0.172	Detected	-0.172	Detected	0.000	-3.456	Comprom	-2.265	Comprom	-2.861	0.044
GT Sense	Sense	0.202	Detected	-0.202	Detected	0.000	-1.012	Detected	-1.826	Comprom	-1.419	0.089
GT Sense	Sense	0.050	Detected	-0.050	Detected	0.000	-3.671	Comprom	-2.245	Comprom	-2.958	0.054
GT Sense	Sense	0.071	Detected	-0.071	Detected	0.000	-1.072	Detected	-0.951	Detected	-1.012	0.008
GT Sense	Sense	-0.009	Detected	0.009	Detected	0.000	-1.362	Detected	-1.210	Detected	-1.286	0.003
GT Sense	Sense	0.011	Detected	-0.011	Detected	0.000	-3.229	Detected	-1.227	Detected	-2.228	0.156
GT Sense	Sense	0.107	Detected	-0.107	Detected	0.000	-1.952	Comprom	-3.086	Comprom	-2.519	0.049
GT Sense	Sense	-0.958	Detected	0.958	Detected	0.000	-3.851	Detected	-2.644	Detected	-3.247	0.103
GT Sense	Sense	-0.684	Detected	0.684	Detected	0.000	-2.837	Detected	-0.953	Detected	-1.895	0.245
GT Sense	Sense	0.042	Detected	-0.042	Detected	0.000	-1.816	Detected	-2.355	Comprom	-2.085	0.017
GT Sense	Sense	0.479	Detected	-0.479	Detected	0.000	-1.487	Detected	-1.635	Detected	-1.561	0.084
GT Sense	Sense	0.050	Detected	-0.050	Detected	0.000	-1.347	Detected	-1.708	Detected	-1.528	0.015
GT Sense	Sense	-0.413	Detected	0.413	Detected	0.000	-2.119	Detected	-1.601	Detected	-1.860	0.062
GT Sense	Sense	-0.577	Detected	0.577	Detected	0.000	-1.234	Detected	-0.849	Detected	-1.042	0.229
GT Sense	Sense	-0.028	Detected	0.028	Detected	0.000	-1.782	Comprom	-1.100	Comprom	-1.441	0.052
GT Sense	Sense	0.072	Detected	-0.072	Detected	0.000	-1.051	Comprom	-2.018	Comprom	-1.535	0.088
GT Sense	Sense	-0.199	Detected	0.199	Detected	0.000	-1.505	Detected	-1.155	Detected	-1.330	0.038
GT Sense	Sense	-0.054	Detected	0.054	Detected	0.000	-2.081	Detected	-1.973	Detected	-2.027	0.001
GT Sense	Sense	0.216	Detected	-0.216	Detected	0.000	-0.911	Detected	-1.484	Detected	-1.198	0.079
GT Sense	Sense	-0.088	Detected	0.088	Detected	0.000	-0.938	Detected	-1.237	Detected	-1.087	0.025
GT Sense	Sense	0.002	Detected	-0.002	Detected	0.000	-1.555	Comprom	-2.379	Comprom	-1.967	0.041
GT Sense	Sense	-0.172	Detected	0.172	Detected	0.000	-1.072	Comprom	-2.184	Comprom	-1.628	0.108
GT Sense	Sense	-0.333	Detected	0.333	Detected	0.000	-1.088	Comprom	-2.299	Comprom	-1.694	0.134
GT Sense	Sense	0.181	Detected	-0.181	Detected	0.000	-1.343	Detected	-1.336	Detected	-1.339	0.018
GT Sense	Sense	0.201	Detected	-0.201	Detected	0.000	-2.160	Comprom	-2.879	Comprom	-2.520	0.026
GT Sense	Sense	-0.044	Detected	0.044	Detected	0.000	-1.095	Detected	-1.314	Detected	-1.204	0.010
GT Sense	Sense	-0.629	Detected	0.629	Detected	0.000	-1.845	Detected	-1.738	Detected	-1.791	0.105
GT Sense	Sense	0.238	Detected	-0.238	Detected	0.000	-0.915	Detected	-1.415	Detected	-1.165	0.078
GT Sense	Sense	0.011	Detected	-0.011	Detected	0.000	-2.182	Detected	-2.286	Detected	-2.234	0.001
GT Sense	Sense	0.010	Detected	-0.010	Detected	0.000	-1.693	Detected	-1.250	Detected	-1.471	0.022
GT Sense	Sense	0.151	Detected	-0.151	Detected	0.000	-1.375	Detected	-0.959	Detected	-1.167	0.045
GT Sense	Sense	-0.197	Detected	0.197	Detected	0.000	-2.217	Detected	-1.215	Detected	-1.716	0.086
GT Sense	Sense	-0.214	Detected	0.214	Detected	0.000	-0.964	Detected	-1.619	Detected	-1.292	0.081
GT Sense	Sense	0.153	Detected	-0.153	Detected	0.000	-2.176	Detected	-1.367	Detected	-1.771	0.055
GT Sense	Sense	-0.410	Detected	0.410	Detected	0.000	-1.133	Detected	-1.156	Detected	-1.145	0.108
GT Sense	Sense	-0.092	Detected	0.092	Detected	0.000	-1.799	Detected	-1.863	Detected	-1.831	0.003
GT Sense	Sense	0.088	Detected	-0.088	Detected	0.000	-2.730	Detected	-2.179	Detected	-2.454	0.014
GT Sense	Sense	0.034	Detected	-0.034	Detected	0.000	-0.878	Detected	-1.326	Comprom	-1.102	0.040
GT Sense	Sense	0.144	Detected	-0.144	Detected	0.000	-1.835	Detected	-1.977	Detected	-1.906	0.007
GT Sense	Sense	0.177	Detected	-0.177	Detected	0.000	-1.870	Detected	-1.848	Detected	-1.859	0.009
GT Sense	Sense	-0.619	Detected	0.619	Detected	0.000	-3.718	Detected	-1.053	Detected	-2.385	0.246
GT Sense	Sense	0.372	Detected	-0.372	Detected	0.000	-1.266	Detected	-0.867	Detected	-1.066	0.127
GT Sense	Sense	-0.219	Detected	0.219	Detected	0.000	-4.387	Detected	-1.377	Detected	-2.882	0.199
GT Sense	Sense	-0.108	Detected	0.108	Detected	0.000	-1.987	Detected	-1.070	Detected	-1.529	0.083
GT Sense	Sense	-0.339	Detected	0.339	Detected	0.000	-1.080	Detected	-1.678	Detected	-1.379	0.093
GT Sense	Sense	-0.173	Detected	0.173	Detected	0.000	-1.671	Detected	-0.888	Detected	-1.279	0.096
GT Sense	Sense	0.258	Detected	-0.258	Detected	0.000	-4.880	Detected	-2.683	Detected	-3.782	0.079
GT Sense	Sense	-0.023	Detected	0.023	Detected	0.000	-1.978	Comprom	-2.267	Comprom	-2.123	0.005
GT Sense	Sense	-0.056	Detected	0.056	Detected	0.000	-2.060	Comprom	-1.836	Comprom	-1.948	0.004
GT Sense	Sense	0.097	Detected	-0.097	Detected	0.000	-3.258	Detected	-3.572	Detected	-3.415	0.003
GT Sense	Sense	-0.655	Detected	0.655	Detected	0.000	-4.348	Detected	-1.554	Detected	-2.951	0.196
GT Sense	Sense	-0.046	Detected	0.046	Detected	0.000	-1.099	Detected	-0.953	Detected	-1.026	0.007
GT Sense	Sense	-0.100	Detected	0.100	Detected	0.000	-1.622	Detected	-0.895	Detected	-1.258	0.079
GT Sense	Sense	-0.175	Detected	0.175	Detected	0.000	-2.294	Detected	-2.184	Detected	-2.239	0.007
GT Sense	Sense	-0.303	Detected	0.303	Detected	0.000	-1.436	Detected	-1.191	Detected	-1.313	0.057
GT Sense	Sense	0.310	Detected	-0.310	Detected	0.000	-1.420	Detected	-1.182	Detected	-1.301	0.060
GT Sense	Sense	0.165	Detected	-0.165	Detected	0.000	-2.012	Detected	-1.224	Detected	-1.618	0.063
GT Sense	Sense	0.055	Detected	-0.055	Detected	0.000	-1.882	Comprom	-1.366	Comprom	-1.624	0.025
GT Sense	Sense	-0.850	Detected	0.850	Detected	0.000	-5.804	Comprom	-2.437	Detected	-4.120	0.161
GT Sense	Sense	0.202	Detected	-0.202	Detected	0.000	-1.543	Detected	-1.503	Detected	-1.523	0.017
GT Sense	Sense	0.298	Detected	-0.298	Detected	0.000	-1.095	Detected	-1.196	Detected	-1.146	0.063
GT Sense	Sense	-0.102	Detected	0.102	Detected	0.000	-1.139	Detected	-1.191	Detected	-1.165	0.008
GT Sense	Sense	-0.129	Detected	0.129	Detected	0.000	-1.727	Detected	-1.077	Detected	-1.402	0.057
GT Sense	Sense	-0.209	Detected	0.209	Detected	0.000	-2.308	Comprom	-1.308	Comprom	-1.808	0.079
GT Sense	Sense	0.140	Detected	-0.140	Detected	0.000	-1.771	Comprom	-1.631	Comprom	-1.701	0.008
GT Sense	Sense	-0.553	Detected	0.553	Detected	0.000	-1.370	Detected	-0.841	Detected	-1.105	0.213
GT Sense	Sense	-0.480	Detected	0.480	Detected	0.000	-3.235	Detected	-2.573	Detected	-2.904	0.038
GT Sense	Sense	0.394	Detected	-0.394	Detected	0.000	-0.969	Detected	-1.033	Detected	-1.001	0.127
GT Sense	Sense	-0.144	Detected	0.144	Detected	0.000	-1.454	Detected	-0.910	Detected	-1.182	0.062
GT Sense	Sense	-0.141	Detected	0.141	Detected	0.000	-2.667	Detected	-1.268	Detected	-1.968	0.110
GT Sense	Sense	-0.422	Detected	0.422	Detected	0.000	-2.185	Detected	-1.253	Detected	-1.719	0.112
GT Sense	Sense	-0.393	Detected	0.393	Detected	0.000	-1.325	Detected	-1.367	Detected	-1.346	0.076
GT Sense	Sense	-0.170	Detected	0.170	Detected	0.000	-1.356	Detected	-2.087	Detected	-1.721	0.051
GT Sense	Sense	0.010	Detected	-0.010	Detected	0.000	-2.503	Detected	-0.867	Detected	-1.685	0.176
GT Sense	Sense	-0.158	Detected	0.158	Detected	0.000	-2.356	Comprom	-1.707	Comprom	-2.032	0.030
GT Sense	Sense	-0.650	Detected	0.650	Detected	0.000	-3.269	Detected	-2.067	Detected	-2.668	0.095
GT Sense	Sense	-0.920	Detected	0.920	Detected	0.000	-1.856	Detected	-1.625	Detected	-1.741	0.201
GT Sense	Sense	-0.581	Detected	0.581	Detected	0.000	-2.475	Detected	-1.996	Detected	-2.235	0.071
GT Sense	Sense	-0.440	Detected	0.440	Detected	0.000	-2.265	Detected	-0.883	Detected	-1.574	0.195
GT Sense	Sense	-0.313	Detected	0.313	Detected	0.000	-2.195	Detected	-1.092	Detected	-1.644	0.122
GT Sense	Sense	0.159	Detected	-0.159	Detected	0.000	-1.223	Detected	-1.148	Detected	-1.185	0.019
GT Sense	Sense	0.092	Detected	-0.092	Detected	0.000	-1.676	Detected	-0.993	Detected	-1.334	0.064
GT Sense	Sense	-0.143	Detected	0.143	Detected	0.000	-1.669	Detected	-1.778	Detected	-1.723	0.008
GT Sense	Sense	0.042	Detected	-0.042	Detected	0.000	-1.863	Detected	-1.142	Detected	-1.502	0.054
GT Sense	Sense	0.023	Detected	-0.023	Detected	0.000	-1.240	Detected	-1.833	Detected	-1.536	0.035
GT Sense	Sense	-0.524	Detected	0.524	Detected	0.000	-2.112	Detected	-1.495	Detected	-1.803	0.097
GT Sense	Sense	-0.176	Detected	0.176	Detected	0.000	-2.593	Detected	-2.852	Detected	-2.723	0.006
GT Sense	Sense	-0.058	Detected	0.058	Detected	0.000	-1.387	Detected	-1.518	Detected	-1.452	0.004
GT Sense	Sense	-0.233	Detected	0.233	Detected	0.000	-1.194	Detected	-1.203	Detected	-1.199	0.036
GT Sense	Sense	-0.099	Detected	0.099	Detected	0.000	-1.422	Detected	-0.841	Detected	-1.132	0.066
GT Sense	Sense	-0.309	Detected	0.309	Detected	0.000	-1.870	Detected	-1.116	Detected	-1.493	0.092
GT Sense	Sense	-0.124	Detected	0.124	Detected	0.000	-0.867	Detected	-1.340	Detected	-1.103	0.054
GT Sense	Sense	-0.624	Detected	0.624	Detected	0.000	-3.190	Comprom	-2.941	Comprom	-3.065	0.040
GT Sense	Sense	-0.206	Detected	0.206	Detected	0.000	-1.652	Detected	-1.660	Detected	-1.656	0.015
GT Sense	Sense	0.110	Detected	-0.110	Detected	0.000	-2.440	Detected	-1.704	Detected	-2.072	0.033
GT Sense	Sense	-0.452	Detected	0.452	Detected	0.000	-3.824	Detected	-1.998	Detected	-2.911	0.104
GT Sense	Sense	-0.169	Detected	0.169	Detected	0.000	-1.724	Detected	-1.675	Detected	-1.699	0.010
GT Sense	Sense	-0.182	Detected	0.182	Detected	0.000	-1.495	Detected	-0.912	Detected	-1.204	0.073
GT Sense	Sense	-0.013	Detected	0.013	Detected	0.000	-1.385	Detected	-1.132	Detected	-1.258	0.010
GT Sense	Sense	-0.500	Detected	0.500	Detected	0.000	-4.818	Comprom	-2.035	Detected	-3.426	0.146
GT Sense	Sense	-0.190	Detected	0.190	Detected	0.000	-1.687	Detected	-1.693	Detected	-1.690	0.012
GT Sense	Sense	-0.275	Detected	0.275	Detected	0.000	-2.436	Comprom	-1.826	Comprom	-2.131	0.035
GT Sense	Sense	-0.177	Detected	0.177	Detected	0.000	-2.365	Detected	-1.423	Detected	-1.894	0.064
GT Sense	Sense	-0.405	Detected	0.405	Detected	0.000	-2.523	Detected	-1.950	Detected	-2.237	0.046
GT Sense	Sense	0.378	Detected	-0.378	Detected	0.000	-4.180	Comprom	-1.558	Detected	-2.869	0.170
GT Sense	Sense	-0.515	Detected	0.515	Detected	0.000	-1.472	Detected	-0.890	Detected	-1.181	0.184
GT Sense	Sense	-1.062	Detected	1.062	Detected	0.000	-2.539	Comprom	-0.889	Detected	-1.714	0.331
GT Sense	Sense	-0.386	Detected	0.386	Detected	0.000	-1.644	Comprom	-2.206	Comprom	-1.925	0.056
GT Sense	Sense	0.167	Detected	-0.167	Detected	0.000	-2.135	Detected	-2.794	Comprom	-2.465	0.022
GT Sense	Sense	0.051	Detected	-0.051	Detected	0.000	-1.433	Detected	-0.811	Detected	-1.122	0.070
GT Sense	Sense	-0.276	Detected	0.276	Detected	0.000	-1.542	Detected	-1.269	Detected	-1.405	0.045
GT Sense	Sense	-0.656	Detected	0.656	Detected	0.000	-5.407	Detected	-2.715	Detected	-4.061	0.113
GT Sense	Sense	-0.615	Detected	0.615	Detected	0.000	-1.814	Detected	-1.543	Detected	-1.679	0.117
GT Sense	Sense	0.810	Detected	-0.810	Detected	0.000	-1.921	Comprom	-2.308	Comprom	-2.114	0.126
GT Sense	Sense	-0.505	Detected	0.505	Detected	0.000	-2.965	Comprom	-1.591	Detected	-2.278	0.116
GT Sense	Sense	-0.075	Detected	0.075	Detected	0.000	-1.339	Detected	-1.001	Detected	-1.170	0.024
GT Sense	Sense	0.320	Detected	-0.320	Detected	0.000	-1.760	Detected	-1.082	Detected	-1.421	0.093
GT Sense	Sense	-0.121	Detected	0.121	Detected	0.000	-0.917	Detected	-1.325	Detected	-1.121	0.042
GT Sense	Sense	0.083	Detected	-0.083	Detected	0.000	-1.183	Detected	-0.820	Detected	-1.002	0.037
GT Sense	Sense	-0.537	Detected	0.537	Detected	0.000	-2.330	Detected	-0.858	Detected	-1.594	0.222
GT Sense	Sense	0.366	Detected	-0.366	Detected	0.000	-1.253	Detected	-1.374	Detected	-1.313	0.071
GT Sense	Sense	-0.217	Detected	0.217	Detected	0.000	-2.616	Detected	-2.002	Detected	-2.309	0.026
GT Sense	Sense	-0.222	Detected	0.222	Detected	0.000	-1.237	Detected	-0.840	Detected	-1.039	0.073
GT Sense	Sense	-0.076	Detected	0.076	Detected	0.000	-1.294	Detected	-1.439	Detected	-1.366	0.006
GT Sense	Sense	0.034	Detected	-0.034	Detected	0.000	-1.872	Detected	-0.810	Detected	-1.341	0.128
GT Sense	Sense	0.277	Detected	-0.277	Detected	0.000	-1.234	Detected	-1.451	Detected	-1.343	0.046
GT Sense	Sense	-0.217	Detected	0.217	Detected	0.000	-1.461	Detected	-1.486	Detected	-1.474	0.021
GT Sense	Sense	0.064	Detected	-0.064	Detected	0.000	-1.540	Comprom	-1.275	Comprom	-1.408	0.011
GT Sense	Sense	-0.294	Detected	0.294	Detected	0.000	-2.875	Comprom	-1.690	Comprom	-2.282	0.075
GT Sense	Sense	-0.136	Detected	0.136	Detected	0.000	-1.154	Detected	-1.088	Detected	-1.121	0.015
GT Sense	Sense	-0.656	Detected	0.656	Detected	0.000	-3.124	Detected	-0.906	Detected	-2.015	0.258
GT Sense	Sense	-0.640	Detected	0.640	Detected	0.000	-1.375	Detected	-1.960	Comprom	-1.668	0.141
GT Sense	Sense	0.099	Detected	-0.099	Detected	0.000	-1.589	Detected	-1.530	Detected	-1.560	0.004
GT Sense	Sense	1.781	Detected	-1.781	Detected	0.000	-1.870	Detected	-1.577	Detected	-1.723	0.437
GT Sense	Sense	-1.107	Detected	1.107	Detected	0.000	-3.150	Detected	-1.624	Detected	-2.387	0.218
GT Sense	Sense	0.009	Detected	-0.009	Detected	0.000	-1.424	Detected	-1.777	Detected	-1.601	0.012
GT Sense	Sense	0.128	Detected	-0.128	Detected	0.000	-3.506	Detected	-5.422	Detected	-4.464	0.044
GT Sense	Sense	-0.157	Detected	0.157	Detected	0.000	-1.231	Detected	-1.165	Detected	-1.198	0.018
GT Sense	Sense	0.006	Detected	-0.006	Detected	0.000	-1.531	Detected	-1.374	Detected	-1.453	0.003
GT Sense	Sense	-0.159	Detected	0.159	Detected	0.000	-2.148	Detected	-2.321	Detected	-2.235	0.007
GT Sense	Sense	-0.019	Detected	0.019	Detected	0.000	-1.421	Comprom	-2.610	Comprom	-2.016	0.077
GT Sense	Sense	0.072	Detected	-0.072	Detected	0.000	-0.835	Detected	-1.547	Detected	-1.191	0.082
GT Sense	Sense	-0.339	Detected	0.339	Detected	0.000	-1.811	Detected	-1.026	Detected	-1.418	0.112
GT Sense	Sense	-0.230	Detected	0.230	Detected	0.000	-1.553	Comprom	-2.006	Comprom	-1.780	0.031
GT Sense	Sense	-0.153	Detected	0.153	Detected	0.000	-1.091	Detected	-1.182	Detected	-1.136	0.019
GT Sense	Sense	-0.381	Detected	0.381	Detected	0.000	-1.130	Detected	-1.403	Detected	-1.267	0.089
GT Sense	Sense	-0.003	Detected	0.003	Detected	0.000	-2.205	Comprom	-1.173	Comprom	-1.689	0.082
GT Sense	Sense	-0.497	Detected	0.497	Detected	0.000	-1.320	Detected	-0.995	Detected	-1.157	0.157
GT Sense	Sense	-0.204	Detected	0.204	Detected	0.000	-1.674	Detected	-0.952	Detected	-1.313	0.087
GT Sense	Sense	0.085	Detected	-0.085	Detected	0.000	-1.057	Detected	-0.954	Detected	-1.006	0.010

GT	Sense	Sense	-0.609	Detected	0.609	Detected	0.000	-2.257	Comprom	-2.495	Comprom	-2.376	0.062	GT Sens
GT	Sense	Sense	-0.032	Detected	0.032	Detected	0.000	-1.636	Detected	-0.880	Detected	-1.258	0.080	GT Sens
GT	Sense	Sense	-0.249	Detected	0.249	Detected	0.000	-2.035	Comprom	-1.398	Comprom	-1.716	0.051	GT Sens
GT	Sense	Sense	-0.228	Detected	0.228	Detected	0.000	-1.274	Detected	-1.401	Detected	-1.337	0.030	GT Sens
GT	Sense	Sense	0.353	Detected	-0.353	Detected	0.000	-2.321	Detected	-1.624	Detected	-1.973	0.058	GT Sens
GT	Sense	Sense	0.291	Detected	-0.291	Detected	0.000	-1.377	Detected	-0.873	Detected	-1.125	0.100	GT Sens
GT	Sense	Sense	-0.273	Detected	0.273	Detected	0.000	-1.982	Detected	-1.104	Detected	-1.543	0.096	GT Sens
GT	Sense	Sense	-0.104	Detected	0.104	Detected	0.000	-1.610	Detected	-1.139	Detected	-1.375	0.033	GT Sens
GT	Sense	Sense	-0.389	Detected	0.389	Detected	0.000	-3.165	Detected	-2.711	Detected	-2.938	0.023	GT Sens
GT	Sense	Sense	-0.193	Detected	0.193	Detected	0.000	-1.919	Detected	-1.924	Detected	-1.921	0.010	GT Sens
GT	Sense	Sense	-0.473	Detected	0.473	Detected	0.000	-2.058	Detected	-1.109	Detected	-1.584	0.142	GT Sens
GT	Sense	Sense	0.148	Detected	-0.148	Detected	0.000	-3.918	Detected	-3.292	Detected	-3.605	0.009	GT Sens
GT	Sense	Sense	-0.126	Detected	0.126	Detected	0.000	-1.397	Detected	-0.819	Detected	-1.108	0.072	GT Sens
GT	Sense	Sense	-0.748	Detected	0.748	Detected	0.000	-1.621	Detected	-0.897	Detected	-1.259	0.269	GT Sens
GT	Sense	Sense	-0.063	Detected	0.063	Detected	0.000	-2.369	Detected	-2.092	Detected	-2.231	0.005	GT Sens
GT	Sense	Sense	-0.294	Detected	0.294	Detected	0.000	-1.415	Detected	-0.873	Detected	-1.144	0.104	GT Sens
GT	Sense	Sense	0.260	Detected	-0.260	Detected	0.000	-1.509	Detected	-1.632	Detected	-1.571	0.028	GT Sens
GT	Sense	Sense	0.549	Detected	-0.549	Detected	0.000	-1.264	Detected	-1.761	Detected	-1.513	0.129	GT Sens
GT	Sense	Sense	-0.221	Detected	0.221	Detected	0.000	-2.201	Comprom	-0.814	Comprom	-1.508	0.174	GT Sens
GT	Sense	Sense	-0.377	Detected	0.377	Detected	0.000	-1.312	Detected	-1.542	Detected	-1.427	0.069	GT Sens
[illegible]														

GT	Sense	Sense	-0.309	Detected	0.309	Detected	0.000	-2.068	Detected	-1.660	Detected	-1.864	0.037	GT	Sens
GT	Sense	Sense	-0.081	Detected	0.081	Detected	0.000	-1.621	Detected	-1.692	Detected	-1.657	0.003	GT	Sens
GT	Sense	Sense	-0.058	Detected	0.058	Detected	0.000	-1.178	Detected	-1.139	Detected	-1.159	0.003	GT	Sens
GT	Sense	Sense	-0.205	Detected	0.205	Detected	0.000	-2.960	Comprom	-1.110	Detected	-2.035	0.165	GT	Sens
GT	Sense	Sense	-0.499	Detected	0.499	Detected	0.000	-1.190	Detected	-0.874	Detected	-1.032	0.188	GT	Sens
GT	Sense	Sense	-0.183	Detected	0.183	Detected	0.000	-2.336	Detected	-0.986	Detected	-1.661	0.141	GT	Sens
GT	Sense	Sense	-0.109	Detected	0.109	Detected	0.000	-1.866	Detected	-1.548	Detected	-1.707	0.012	GT	Sens
GT	Sense	Sense	-0.040	Detected	0.040	Detected	0.000	-2.754	Detected	-1.550	Detected	-2.152	0.070	GT	Sens
GT	Sense	Sense	0.279	Detected	-0.279	Detected	0.000	-3.255	Comprom	-1.283	Detected	-2.269	0.157	GT	Sens
GT	Sense	Sense	-0.299	Detected	0.299	Detected	0.000	-5.109	Detected	-1.191	Detected	-3.150	0.253	GT	Sens
GT	Sense	Sense	-0.095	Detected	0.095	Detected	0.000	-2.355	Detected	-2.423	Detected	-2.389	0.002	GT	Sens
GT	Sense	Sense	-0.412	Detected	0.412	Detected	0.000	-1.897	Comprom	-1.802	Comprom	-1.850	0.047	GT	Sens
GT	Sense	Sense	-0.133	Detected	0.133	Detected	0.000	-1.129	Detected	-0.927	Detected	-1.028	0.025	GT	Sens
GT	Sense	Sense	-0.834	Detected	0.834	Detected	0.000	-3.762	Detected	-2.394	Detected	-3.078	0.104	GT	Sens
GT	Sense	Sense	-0.459	Detected	0.459	Detected	0.000	-1.992	Detected	-1.515	Detected	-1.753	0.077	GT	Sens
GT	Sense	Sense	0.221	Detected	-0.221	Detected	0.000	-4.717	Comprom	-2.646	Comprom	-3.682	0.074	GT	Sens
GT	Sense	Sense	-0.508	Detected	0.508	Detected	0.000	-1.621	Detected	-0.801	Detected	-1.211	0.205	GT	Sens
GT	Sense	Sense	-0.433	Detected	0.433	Detected	0.000	-1.366	Detected	-0.947	Detected	-1.156	0.138	GT	Sens
GT	Sense	Sense	-0.118	Detected	0.118	Detected	0.000	-3.152	Detected	-1.401	Detected	-2.276	0.123	GT	Sens
GT	Sense	Sense	0.064	Detected	-0.064	Detected	0.000	-2.710	Detected	-1.867	Detected	-2.289	0.033	GT	Sens
GT	Sense	Sense	-0.874	Detected	0.874	Detected	0.000	-3.324	Comprom	-2.076	Detected	-2.700	0.128	GT	Sens
GT	Sense	Sense	-0.805	Detected	0.805	Detected	0.000	-6.258	Detected	-1.703	Detected	-3.981	0.241	GT	Sens
GT	Sense	Sense	-0.729	Detected	0.729	Detected	0.000	-5.090	Detected	-2.516	Detected	-3.803	0.124	GT	Sens
GT	Sense	Sense	0.275	Detected	-0.275	Detected	0.000	-1.004	Detected	-1.106	Detected	-1.055	0.064	GT	Sens
GT	Sense	Sense	0.258	Detected	-0.258	Detected	0.000	-1.031	Detected	-0.993	Detected	-1.012	0.060	GT	Sens
GT	Sense	Sense	-0.272	Detected	0.272	Detected	0.000	-1.335	Comprom	-2.147	Comprom	-1.741	0.071	GT	Sens
GT	Sense	Sense	0.080	Detected	-0.080	Detected	0.000	-1.991	Comprom	-1.908	Comprom	-1.949	0.002	GT	Sens
GT	Sense	Sense	0.057	Detected	-0.057	Detected	0.000	-1.445	Detected	-1.654	Detected	-1.549	0.006	GT	Sens
GT	Sense	Sense	-0.619	Detected	0.619	Detected	0.000	-3.811	Detected	-0.983	Detected	-2.397	0.261	GT	Sens
GT	Sense	Sense	-1.037	Detected	1.037	Detected	0.000	-1.317	Detected	-1.359	Detected	-1.338	0.326	GT	Sens
GT	Sense	Sense	-0.368	Detected	0.368	Detected	0.000	-2.795	Comprom	-1.063	Detected	-1.929	0.177	GT	Sens
GT	Sense	Sense	-0.387	Detected	0.387	Detected	0.000	-1.206	Detected	-0.983	Detected	-1.094	0.113	GT	Sens
GT	Sense	Sense	-0.614	Detected	0.614	Detected	0.000	-2.970	Comprom	-1.242	Detected	-2.106	0.185	GT	Sens
GT	Sense	Sense	0.734	Detected	-0.734	Detected	0.000	-1.998	Comprom	-1.918	Comprom	-1.958	0.117	GT	Sens
GT	Sense	Sense	0.290	Detected	-0.290	Detected	0.000	-2.736	Detected	-1.140	Detected	-1.938	0.150	GT	Sens
GT	Sense	Sense	0.549	Detected	-0.549	Detected	0.000	-4.750	Comprom	-2.163	Comprom	-3.456	0.133	GT	Sens
GT	Sense	Sense	-0.045	Detected	0.045	Detected	0.000	-2.331	Detected	-0.857	Detected	-1.594	0.164	GT	Sens
GT	Sense	Sense	0.028	Detected	-0.028	Detected	0.000	-1.619	Detected	-1.552	Detected	-1.585	0.001	GT	Sens
GT	Sense	Sense	1.280	Detected	-1.280	Detected	0.000	-1.745	Detected	-2.392	Detected	-2.068	0.258	GT	Sens
GT	Sense	Sense	-0.451	Detected	0.451	Detected	0.000	-2.404	Detected	-1.996	Detected	-2.200	0.047	GT	Sens
GT	Sense	Sense	0.213	Detected	-0.213	Detected	0.000	-3.856	Detected	-0.961	Detected	-2.408	0.242	GT	Sens
GT	Sense	Sense	-0.427	Detected	0.427	Detected	0.000	-1.246	Detected	-0.862	Detected	-1.054	0.153	GT	Sens
GT	Sense	Sense	-0.152	Detected	0.152	Detected	0.000	-1.031	Detected	-1.152	Detected	-1.092	0.022	GT	Sens
GT	Sense	Sense	-0.173	Detected	0.173	Detected	0.000	-1.970	Detected	-2.168	Detected	-2.069	0.009	GT	Sens
GT	Sense	Sense	-0.149	Detected	0.149	Detected	0.000	-1.684	Comprom	-0.805	Detected	-1.244	0.116	GT	Sens
GT	Sense	Sense	-0.219	Detected	0.219	Detected	0.000	-1.430	Detected	-0.962	Detected	-1.196	0.065	GT	Sens
GT	Sense	Sense	-0.642	Detected	0.642	Detected	0.000	-2.921	Comprom	-3.061	Comprom	-2.991	0.044	GT	Sens
GT	Sense	Sense	-0.736	Detected	0.736	Detected	0.000	-1.718	Detected	-1.977	Detected	-1.848	0.132	GT	Sens
GT	Sense	Sense	-0.157	Detected	0.157	Detected	0.000	-1.949	Detected	-1.784	Detected	-1.866	0.009	GT	Sens
GT	Sense	Sense	-0.075	Detected	0.075	Detected	0.000	-2.125	Comprom	-1.723	Comprom	-1.924	0.012	GT	Sens
GT	Sense	Sense	-0.720	Detected	0.720	Detected	0.000	-3.513	Detected	-1.942	Detected	-2.727	0.125	GT	Sens
GT	Sense	Sense	-0.672	Detected	0.672	Detected	0.000	-5.482	Detected	-1.160	Detected	-3.321	0.280	GT	Sens
GT	Sense	Sense	0.132	Detected	-0.132	Detected	0.000	-1.392	Comprom	-1.549	Comprom	-1.471	0.011	GT	Sens
GT	Sense	Sense	-0.314	Detected	0.314	Detected	0.000	-1.569	Detected	-0.802	Detected	-1.185	0.139	GT	Sens
GT	Sense	Sense	-0.203	Detected	0.203	Detected	0.000	-2.007	Detected	-2.236	Comprom	-2.121	0.012	GT	Sens
GT	Sense	Sense	-0.019	Detected	0.019	Detected	0.000	-1.594	Detected	-1.011	Detected	-1.302	0.047	GT	Sens
GT	Sense	Sense	-0.001	Detected	0.001	Detected	0.000	-2.518	Comprom	-2.188	Comprom	-2.353	0.005	GT	Sens
GT	Sense	Sense	-0.174	Detected	0.174	Detected	0.000	-1.332	Comprom	-2.070	Comprom	-1.701	0.053	GT	Sens
GT	Sense	Sense	-0.053	Detected	0.053	Detected	0.000	-1.049	Detected	-1.191	Detected	-1.120	0.006	GT	Sens
GT	Sense	Sense	0.053	Detected	-0.053	Detected	0.000	-1.154	Detected	-0.972	Detected	-1.063	0.010	GT	Sens
GT	Sense	Sense	-0.645	Detected	0.645	Detected	0.000	-4.486	Detected	-2.469	Detected	-3.478	0.101	GT	Sens
GT	Sense	Sense	0.315	Detected	-0.315	Detected	0.000	-2.481	Comprom	-1.310	Detected	-1.895	0.104	GT	Sens
GT	Sense	Sense	-0.107	Detected	0.107	Detected	0.000	-1.326	Detected	-1.132	Detected	-1.229	0.014	GT	Sens
GT	Sense	Sense	0.298	Detected	-0.298	Detected	0.000	-1.438	Detected	-0.941	Detected	-1.189	0.092	GT	Sens
GT	Sense	Sense	-1.289	Detected	1.289	Detected	0.000	-1.350	Detected	-1.363	Detected	-1.357	0.403	GT	Sens
GT	Sense	Sense	0.007	Detected	-0.007	Detected	0.000	-1.304	Detected	-1.099	Detected	-1.201	0.007	GT	Sens
GT	Sense	Sense	0.005	Detected	-0.005	Detected	0.000	-1.150	Comprom	-1.706	Comprom	-1.428	0.036	GT	Sens
GT	Sense	Sense	-0.082	Detected	0.082	Detected	0.000	-1.929	Detected	-1.704	Detected	-1.816	0.006	GT	Sens
GT	Sense	Sense	-0.339	Detected	0.339	Detected	0.000	-1.336	Detected	-1.501	Detected	-1.418	0.055	GT	Sens
GT	Sense	Sense	0.025	Detected	-0.025	Detected	0.000	-0.916	Detected	-1.125	Detected	-1.020	0.011	GT	Sens
GT	Sense	Sense	-0.451	Detected	0.451	Detected	0.000	-2.159	Detected	-1.436	Detected	-1.798	0.090	GT	Sens
GT	Sense	Sense	0.046	Detected	-0.046	Detected	0.000	-1.886	Detected	-1.981	Detected	-1.933	0.001	GT	Sens
GT	Sense	Sense	-0.109	Detected	0.109	Detected	0.000	-1.048	Detected	-1.055	Detected	-1.052	0.011	GT	Sens
GT	Sense	Sense	0.240	Detected	-0.240	Detected	0.000	-1.399	Detected	-1.138	Detected	-1.268	0.044	GT	Sens
GT	Sense	Sense	-0.076	Detected	0.076	Detected	0.000	-1.236	Detected	-1.683	Detected	-1.460	0.025	GT	Sens
GT	Sense	Sense	-0.226	Detected	0.226	Detected	0.000	-1.309	Detected	-0.901	Detected	-1.105	0.068	GT	Sens
GT	Sense	Sense	0.225	Detected	-0.225	Detected	0.000	-1.969	Detected	-2.339	Detected	-2.154	0.018	GT	Sens
GT	Sense	Sense	0.101	Detected	-0.101	Detected	0.000	-1.433	Detected	-1.397	Detected	-1.415	0.005	GT	Sens
GT	Sense	Sense	-0.175	Detected	0.175	Detected	0.000	-2.425	Detected	-2.121	Detected	-2.273	0.010	GT	Sens
GT	Sense	Sense	-0.133	Detected	0.133	Detected	0.000	-1.322	Detected	-1.186	Detected	-1.254	0.014	GT	Sens
GT	Sense	Sense	-0.139	Detected	0.139	Detected	0.000	-1.482	Detected	-0.983	Detected	-1.233	0.050	GT	Sens
GT	Sense	Sense	-0.255	Detected	0.255	Detected	0.000	-0.928	Detected	-1.233	Detected	-1.080	0.068	GT	Sens
GT	Sense	Sense	0.444	Detected	-0.444	Detected	0.000	-1.248	Detected	-1.083	Detected	-1.165	0.123	GT	Sens
GT	Sense	Sense	-0.061	Detected	0.061	Detected	0.000	-3.026	Comprom	-2.844	Comprom	-2.935	0.001	GT	Sens
GT	Sense	Sense	-1.661	Detected	1.661	Detected	0.000	-5.194	Comprom	-5.089	Comprom	-5.142	0.091	GT	Sens
GT	Sense	Sense	-0.488	Detected	0.488	Detected	0.000	-1.118	Detected	-1.333	Detected	-1.225	0.134	GT	Sens
GT	Sense	Sense	-0.384	Detected	0.384	Detected	0.000	-1.894	Detected	-1.171	Detected	-1.532	0.101	GT	Sens
GT	Sense	Sense	-0.163	Detected	0.163	Detected	0.000	-1.638	Detected	-0.931	Detected	-1.284	0.081	GT	Sens
GT	Sense	Sense	-0.118	Detected	0.118	Detected	0.000	-2.263	Detected	-1.008	Detected	-1.635	0.125	GT	Sens
GT	Sense	Sense	-0.064	Detected	0.064	Detected	0.000	-3.082	Comprom	-2.777	Comprom	-2.929	0.003	GT	Sens
GT	Sense	Sense	0.335	Detected	-0.335	Detected	0.000	-1.999	Comprom	-1.038	Detected	-1.519	0.122	GT	Sens
GT	Sense	Sense	0.117	Detected	-0.117	Detected	0.000	-1.332	Detected	-1.298	Detected	-1.315	0.008	GT	Sens
GT	Sense	Sense	-0.221	Detected	0.221	Detected	0.000	-3.770	Detected	-1.910	Detected	-2.840	0.097	GT	Sens
GT	Sense	Sense	0.161	Detected	-0.161	Detected	0.000	-1.591	Detected	-1.520	Detected	-1.556	0.011	GT	Sens
GT	Sense	Sense	-0.599	Detected	0.599	Detected	0.000	-1.156	Detected	-1.157	Detected	-1.156	0.193	GT	Sens
GT	Sense	Sense	-0.018	Detected	0.018	Detected	0.000	-1.173	Detected	-0.860	Detected	-1.017	0.023	GT	Sens
GT	Sense	Sense	-0.123	Detected	0.123	Detected	0.000	-1.424	Detected	-1.183	Detected	-1.303	0.017	GT	Sens
GT	Sense	Sense	-0.062	Detected	0.062	Detected	0.000	-2.997	Detected	-0.842	Detected	-1.919	0.217	GT	Sens
GT	Sense	Sense	0.359	Detected	-0.359	Detected	0.000	-1.180	Detected	-0.842	Detected	-1.011	0.126	GT	Sens
GT	Sense	Sense	-0.106	Detected	0.106	Detected	0.000	-1.145	Detected	-1.087	Detected	-1.116	0.009	GT	Sens
GT	Sense	Sense	0.013	Detected	-0.013	Detected	0.000	-2.197	Detected	-1.915	Detected	-2.056	0.005	GT	Sens
GT	Sense	Sense	-1.359	Detected	1.359	Detected	0.000	-6.236	Comprom	-2.263	Detected	-4.250	0.220	GT	Sens
GT	Sense	Sense	-0.774	Detected	0.774	Detected	0.000	-1.045	Detected	-0.969	Detected	-1.007	0.323	GT	Sens
GT	Sense	Sense	-0.214	Detected	0.214	Detected	0.000	-2.475	Detected	-1.493	Detected	-1.984	0.066	GT	Sens
GT	Sense	Sense	0.272	Detected	-0.272	Detected	0.000	-2.816	Comprom	-3.715	Comprom	-3.265	0.025	GT	Sens
GT	Sense	Sense	-0.027	Detected	0.027	Detected	0.000	-1.999	Comprom	-0.820	Detected	-1.410	0.139	GT	Sens
GT	Sense	Sense	0.570	Detected	-0.570	Detected	0.000	-3.798	Comprom	-3.576	Comprom	-3.687	0.024	GT	Sens
GT	Sense	Sense	-0.200	Detected	0.200	Detected	0.000	-1.730	Detected	-1.003	Detected	-1.366	0.081	GT	Sens
GT	Sense	Sense	0.052	Detected	-0.052	Detected	0.000	-1.433	Detected	-0.922	Detected	-1.178	0.046	GT	Sens
GT	Sense	Sense	0.266	Detected	-0.266	Detected	0.000	-1.948	Detected	-1.130	Detected	-1.539	0.088	GT	Sens
GT	Sense	Sense	-0.278	Detected	0.278	Detected	0.000	-1.795	Detected	-0.898	Detected	-1.347	0.125	GT	Sens
GT	Sense	Sense	-0.189	Detected	0.189	Detected	0.000	-1.973	Detected	-2.246	Detected	-2.109	0.012	GT	Sens
GT	Sense	Sense	-0.342	Detected	0.342	Detected	0.000	-3.651	Detected	-1.483	Detected	-2.567	0.152	GT	Sens
GT	Sense	Sense	0.147	Detected	-0.147	Detected	0.000	-0.840	Detected	-1.273	Detected	-1.056	0.056	GT	Sens
GT	Sense	Sense	0.079	Detected	-0.079	Detected	0.000	-1.527	Detected	-1.609	Detected	-1.568	0.003	GT	Sens
GT	Sense	Sense	-0.335	Detected	0.335	Detected	0.000	-2.428	Detected	-0.915	Detected	-1.671	0.181	GT	Sens
GT	Sense	Sense	-0.775	Detected	0.775	Detected	0.000	-6.896	Detected	-1.338	Detected	-4.117	0.290	GT	Sens
GT	Sense	Sense	-0.174	Detected	0.174	Detected	0.000	-2.444	Detected	-2.499	Detected	-2.472	0.005	GT	Sens
GT	Sense	Sense	0.152	Detected	-0.152	Detected	0.000	-2.390	Detected	-1.425	Detected	-1.907	0.064	GT	Sens
GT	Sense	Sense	-1.555	Detected	1.555	Detected	0.000	-3.603	Detected	-2.806	Detected	-3.204	0.184	GT	Sens
GT	Sense	Sense	0.077	Detected	-0.077	Detected	0.000	-1.315	Detected	-1.013	Detected	-1.164	0.021	GT	Sens
GT	Sense	Sense	-0.033	Detected	0.033	Detected	0.000	-3.020	Comprom	-2.870	Comprom	-2.945	0.001	GT	Sens
GT	Sense	Sense	-0.249	Detected	0.249	Detected	0.000	-2.056	Detected	-1.537	Detected	-1.797	0.038	GT	Sens
GT	Sense	Sense	-0.082	Detected	0.082	Detected	0.000	-1.942	Detected	-1.421	Detected	-1.681	0.025	GT	Sens
GT	Sense	Sense	-0.136	Detected	0.136	Detected	0.000	-1.291	Detected	-0.837	Detected	-1.064	0.057	GT	Sens
GT	Sense	Sense	0.146	Detected	-0.146	Detected	0.000	-1.573	Detected	-1.132	Detected	-1.353	0.036	GT	Sens
GT	Sense	Sense	-0.373	Detected	0.373	Detected	0.000	-2.140	Detected	-1.790	Detected	-1.965	0.041	GT	Sens
GT	Sense	Sense	-0.206	Detected	0.206	Detected	0.000	-2.463	Comprom	-1.572	Comprom	-2.018	0.054	GT	Sens
GT	Sense	Sense	-0.206	Detected	0.206	Detected	0.000	-2.702	Detected	-1.350	Detected	-2.026	0.103	GT	Sens
GT	Sense	Sense	-0.270	Detected	0.270	Detected	0.000	-1.435	Detected	-0.860	Detected	-1.148	0.101	GT	Sens
GT	Sense	Sense	-0.246	Detected	0.246	Detected	0.000	-1.786	Detected	-1.279	Detected	-1.532	0.049	GT	Sens
GT	Sense	Sense	-0.275	Detected	0.275	Detected	0.000	-1.320	Detected	-0.818	Detected	-1.069	0.103	GT	Sens
GT	Sense	Sense	0.029	Detected	-0.029	Detected	0.000	-1.092	Detected	-1.591	Comprom	-1.342	0.033	GT	Sens
GT	Sense	Sense	0.322	Detected	-0.322	Detected	0.000	-1.608	Detected	-1.394	Detected	-1.501	0.048	GT	Sens
GT	Sense	Sense	-0.137	Detected	0.137	Detected	0.000	-1.106	Detected	-1.032	Detected	-1.069	0.017	GT	Sens
GT	Sense	Sense	-0.076	Detected	0.076	Detected	0.000	-1.344	Detected	-0.889	Detected	-1.117	0.043	GT	Sens
GT	Sense	Sense	-0.467	Detected	0.467	Detected	0.000	-2.309	Detected	-1.211	Detected	-1.760	0.135	GT	Sens
GT	Sense	Sense	-0.158	Detected	0.158	Detected	0.000	-1.112	Detected	-1.124	Detected	-1.118	0.019	GT	Sens
GT	Sense	Sense	-0.422	Detected	0.422	Detected	0.000	-2.024	Detected	-1.526	Detected	-1.775	0.068	GT	Sens
GT	Sense	Sense	-0.134	Detected	0.134	Detected	0.000	-1.914	Detected	-2.164	Detected	-2.039	0.008	GT	Sens
GT	Sense	Sense	-0.398	Detected	0.398	Detected	0.000	-2.214	Detected	-1.282	Detected	-1.748	0.104	GT	Sens
GT	Sense	Sense	-0.293	Detected	0.293	Detected	0.000	-2.643	Comprom	-1.709	Comprom	-2.176	0.059	GT	Sens
GT	Sense	Sense	-0.164	Detected	0.164	Detected	0.000	-3.874	Comprom	-0.998	Detected	-2.436	0.234	GT	Sens
GT	Sense	Sense	-0.170	Detected	0.170	Detected	0.000	-0.940	Detected	-1.447	Detected	-1.193	0.060	GT	Sens
GT	Sense	Sense	0.240	Detected	-0.240	Detected	0.000	-1.612	Detected	-1.595	Detected	-1.604	0.022	GT	Sens
GT	Sense	Sense	-0.029	Detected	0.029	Detected	0.000	-2.628	Comprom	-1.525	Comprom	-2.076	0.064	GT	Sens
GT	Sense	Sense	-0.026	Detected	0.026	Detected	0.000	-1.605	Detected	-1.027	Detected	-1.316	0.045	GT	Sens
GT	Sense	Sense	-1.995	Detected	1.995	Detected	0.000	-1.694	Detected	-1.778	Detected	-1.736	0.476	GT	Sens
GT	Sense	Sense	0.391	Detected	-0.391	Detected	0.000	-1.138	Detected	-1.438	Detected	-1.288	0.091	GT	Sens
GT	Sense	Sense	-0.035	Detected	0.035	Detected	0.000	-1.837	Detected	-0.953	Detected	-1.395	0.088	GT	Sens
GT	Sense	Sense	-0.533	Detected	0.533	Detected	0.000	-1.448	Detected	-2.275	Detected	-1.862	0.110	GT	Sens
GT	Sense	Sense	0.056	Detected	-0.056	Detected	0.000	-0.946	Detected	-1.086	Detected	-1.016	0.008	GT	Sens
GT	Sense	Sense	-0.195	Detected	0.195	Detected	0.000	-2.461	Detected	-1.594	Detected	-2.027	0.051	GT	Sens
GT	Sense	Sense	-0.489	Detected	0.489	Detected	0.000	-3.045	Comprom	-2.107	Comprom	-2.576	0.063	GT	Sens
GT	Sense	Sense	-1.005	Detected	1.005	Detected	0.000	-2.484	Detected	-2.041	Detected	-2.263	0.159	GT	Sens
GT	Sense	Sense	-0.274	Detected	0.274	Detected	0.000	-1.558	Detected	-0.881	Detected	-1.219	0.107	GT	Sens
GT	Sense	Sense	-0.200	Detected	0.200	Detected	0.000	-0.928	Detected	-3.010	Comprom	-1.969	0.204	GT	Sens
GT	Sense	Sense	-0.004	Detected	0.004	Detected	0.000	-2.058	Detected	-1.089	Detected	-1.574	0.083	GT	Sens
GT	Sense	Sense	0.074	Detected	-0.074	Detected	0.000	-1.556	Detected	-0.825	Detected	-1.191	0.086	GT	Sens
GT	Sense	Sense	0.046	Detected	-0.046	Detected	0.000	-2.433	Comprom	-1.974	Comprom	-2.204	0.011	GT	Sens
GT	Sense	Sense	0.152	Detected	-0.152	Detected	0.000	-1.214	Detected	-1.830	Comprom	-1.522	0.047	GT	Sens
GT	Sense	Sense	-0.167	Detected	0.167	Detected	0.000	-1.917	Comprom	-1.390	Comprom	-1.653	0.034	GT	Sens
GT	Sense	Sense	-0.251	Detected	0.251	Detected	0.000	-1.339	Detected	-0.881	Detected	-1.110	0.082	GT	Sens
GT	Sense	Sense	-0.015	Detected	0.015	Detected	0.000	-1.766	Detected	-0.970	Detected	-1.368	0.075	GT	Sens
GT	Sense	Sense	-0.045	Detected	0.045	Detected	0.000	-1.588	Detected	-1.587	Detected	-1.588	0.001	GT	Sens
GT	Sense	Sense	-0.131	Detected	0.131	Detected	0.000	-1.063	Detected	-1.367	Detected	-1.215	0.026	GT	Sens
GT	Sense	Sense	-0.093	Detected	0.093	Detected	0.000	-1.331	Detected	-1.619	Detected	-1.475	0.013	GT	Sens
GT	Sense	Sense	-0.264	Detected	0.264	Detected	0.000	-1.647	Comprom	-1.312	Comprom	-1.479	0.042	GT	Sens
GT	Sense	Sense	-0.104	Detected	0.104	Detected	0.000	-0.961	Detected	-1.264	Detected	-1.113	0.026	GT	Sens
GT	Sense	Sense	-0.136	Detected	0.136	Detected	0.000	-0.876	Detected	-1.549	Detected	-1.212	0.079	GT	Sens
GT	Sense	Sense	-0.292	Detected	0.292	Detected	0.000	-1.220	Detected	-1.043	Detected	-1.132	0.066	GT	Sens
GT	Sense	Sense	-0.669	Detected	0.669	Detected	0.000	-3.627	Detected	-0.945	Detected	-2.286	0.267	GT	Sens
GT	Sense	Sense	0.107	Detected	-0.107	Detected	0.000	-1.208	Detected	-0.837	Detected	-1.022	0.041	GT	Sens
GT	Sense	Sense	-0.471	Detected	0.471	Detected	0.000	-1.197	Detected	-1.284	Detected	-1.241	0.120	GT	Sens
GT	Sense	Sense	-0.003	Detected	0.003	Detected	0.000	-1.983	Detected	-1.508	Detected	-1.746	0.018	GT	Sens
GT	Sense	Sense	0.186	Detected	-0.186	Detected	0.000	-1.473	Detected	-1.541	Detected	-1.507	0.015	GT	Sens
GT	Sense	Sense	0.013	Detected	-0.013	Detected	0.000	-2.895	Detected	-1.106	Detected	-2.001	0.155	GT	Sens
GT	Sense	Sense	-0.239	Detected	0.239	Detected	0.000	-1.439	Comprom	-2.303	Comprom	-1.871	0.063	GT	Sens
GT	Sense	Sense	-0.261	Detected	0.261	Detected	0.000	-2.732	Detected	-1.014	Detected	-1.873	0.172	GT	Sens
GT	Sense	Sense	-0.285	Detected	0.285	Detected	0.000	-2.010	Detected	-0.842	Detected	-1.426	0.160	GT	Sens
GT	Sense	Sense	-0.317	Detected	0.317	Detected	0.000	-2.189	Detected	-1.677	Detected	-1.933	0.042	GT	Sens
GT	Sense	Sense	0.443	Detected	-0.443	Detected	0.000	-1.046	Detected	-0.973	Detected	-1.009	0.151	GT	Sens
GT	Sense	Sense	-0.536	Detected	0.536	Detected	0.000	-3.610	Detected	-1.909	Detected	-2.759	0.111	GT	Sens
GT	Sense	Sense	-0.210	Detected	0.210	Detected	0.000	-3.590	Comprom	-1.941	Comprom	-2.766	0.083	GT	Sens
GT	Sense	Sense	-0.918	Detected	0.918	Detected	0.000	-2.097	Comprom	-3.171	Comprom	-2.634	0.132	GT	Sens
GT	Sense	Sense	0.089	Detected	-0.089	Detected	0.000	-1.350	Detected	-1.529	Detected	-1.440	0.008	GT	Sens
GT	Sense	Sense	-0.121	Detected	0.121	Detected	0.000	-1.143	Detected	-2.132	Comprom	-1.637	0.085	GT	Sens
GT	Sense	Sense	0.448	Detected	-0.448	Detected	0.000	-1.634	Detected	-1.671	Detected	-1.652	0.067	GT	Sens
GT	Sense	Sense	-0.144	Detected	0.144	Detected	0.000	-1.404	Detected	-1.834	Detected	-1.619	0.025	GT	Sens
GT	Sense	Sense	-0.009	Detected	0.009	Detected	0.000	-1.490	Detected	-1.465	Detected	-1.477	0.000	GT	Sens
GT	Sense	Sense	-0.690	Detected	0.690	Detected	0.000	-2.984	Detected	-1.298	Detected	-2.141	0.188	GT	Sens
GT	Sense	Sense	-0.312	Detected	0.312	Detected	0.000	-1.223	Detected	-0.876	Detected	-1.050	0.099	GT	Sens
GT	Sense	Sense	-0.141	Detected	0.141	Detected	0.000	-2.111	Detected	-2.362	Detected	-2.237	0.007	GT	Sens
GT	Sense	Sense	-0.403	Detected	0.403	Detected	0.000	-1.354	Detected	-1.198	Detected	-1.276	0.090	GT	Sens
GT	Sense	Sense	-0.122	Detected	0.122	Detected	0.000	-2.656	Detected	-1.302	Detected	-1.979	0.103	GT	Sens
GT	Sense	Sense	-0.111	Detected	0.111	Detected	0.000	-1.387	Detected	-1.642	Detected	-1.515	0.012	GT	Sens
GT	Sense	Sense	-0.079	Detected	0.079	Detected	0.000	-1.152	Detected	-1.123	Detected	-1.137	0.005	GT	Sens
GT	Sense	Sense	0.250	Detected	-0.250	Detected	0.000	-1.168	Detected	-1.205	Detected	-1.187	0.042	GT	Sens
GT	Sense	Sense	0.089	Detected	-0.089	Detected	0.000	-1.980	Detected	-2.063	Detected	-2.022	0.002	GT	Sens
GT	Sense	Sense	0.242	Detected	-0.242	Detected	0.000	-2.501	Detected	-2.332	Detected	-2.417	0.011	GT	Sens
GT	Sense	Sense	-0.165	Detected	0.165	Detected	0.000	-1.612	Comprom	-1.266	Comprom	-1.439	0.026	GT	Sens
GT	Sense	Sense	0.142	Detected	-0.142	Detected	0.000	-1.214	Detected	-1.108	Detected	-1.161	0.017	GT	Sens
GT	Sense	Sense	-0.715	Detected	0.715	Detected	0.000	-4.019	Detected	-1.658	Detected	-2.838	0.176	GT	Sens
GT	Sense	Sense	-0.289	Detected	0.289	Detected	0.000	-1.277	Detected	-1.041	Detected	-1.159	0.066	GT	Sens
GT	Sense	Sense	0.130	Detected	-0.130	Detected	0.000	-1.434	Detected	-0.857	Detected	-1.145	0.069	GT	Sens
GT	Sense	Sense	-0.164	Detected	0.164	Detected	0.000	-0.901	Detected	-1.694	Detected	-1.298	0.094	GT	Sens
GT	Sense	Sense	0.139	Detected	-0.139	Detected	0.000	-1.900	Comprom	-1.740	Comprom	-1.820	0.008	GT	Sens
GT	Sense	Sense	-0.452	Detected	0.452	Detected	0.000	-1.918	Detected	-1.370	Detected	-1.644	0.090	GT	Sens
GT	Sense	Sense	-0.139	Detected	0.139	Detected	0.000	-1.241	Detected	-0.842	Detected	-1.041	0.051	GT	Sens
GT	Sense	Sense	-0.475	Detected	0.475	Detected	0.000	-0.958	Detected	-1.206	Detected	-1.082	0.158	GT	Sens
GT	Sense	Sense	-0.350	Detected	0.350	Detected	0.000	-2.077	Detected	-1.195	Detected	-1.636	0.101	GT	Sens
GT	Sense	Sense	-0.044	Detected	0.044	Detected	0.000	-0.822	Detected	-1.391	Detected	-1.107	0.061	GT	Sens
GT	Sense	Sense	-0.331	Detected	0.331	Detected	0.000	-4.533	Detected	-1.179	Detected	-2.856	0.237	GT	Sens
GT	Sense	Sense	-0.097	Detected	0.097	Detected	0.000	-2.675	Detected	-1.416	Detected	-2.046	0.085	GT	Sens
GT	Sense	Sense	-0.090	Detected	0.090	Detected	0.000	-1.539	Detected	-1.080	Detected	-1.310	0.034	GT	Sens
GT	Sense	Sense	-0.030	Detected	0.030	Detected	0.000	-1.295	Detected	-1.243	Detected	-1.269	0.001	GT	Sens
GT	Sense	Sense	0.227	Detected	-0.227	Detected	0.000	-1.140	Detected	-0.988	Detected	-1.064	0.047	GT	Sens
GT	Sense	Sense	-0.139	Detected	0.139	Detected	0.000	-2.009	Detected	-0.968	Detected	-1.489	0.110	GT	Sens
GT	Sense	Sense	-0.498	Detected	0.498	Detected	0.000	-2.345	Detected	-1.052	Detected	-1.698	0.173	GT	Sens
GT	Sense	Sense	-0.367	Detected	0.367	Detected	0.000	-0.892	Detected	-1.515	Detected	-1.203	0.130	GT	Sens
GT	Sense	Sense	0.035	Detected	-0.035	Detected	0.000	-1.841	Comprom	-1.872	Comprom	-1.856	0.000	GT	Sens
GT	Sense	Sense	-0.274	Detected	0.274	Detected	0.000	-2.393	Detected	-1.014	Detected	-1.704	0.149	GT	Sens
GT	Sense	Sense	-0.063	Detected	0.063	Detected	0.000	-2.267	Detected	-1.247	Detected	-1.757	0.076	GT	Sens
GT	Sense	Sense	0.165	Detected	-0.165	Detected	0.000	-3.566	Detected	-1.379	Detected	-2.472	0.155	GT	Sens
GT	Sense	Sense	-0.036	Detected	0.036	Detected	0.000	-1.530	Detected	-0.948	Detected	-1.239	0.052	GT	Sens
GT	Sense	Sense	-0.193	Detected	0.193	Detected	0.000	-1.876	Comprom	-1.431	Comprom	-1.653	0.030	GT	Sens
GT	Sense	Sense	0.186	Detected	-0.186	Detected	0.000	-1.087	Detected	-0.928	Detected	-1.008	0.038	GT	Sens
GT	Sense	Sense	-0.137	Detected	0.137	Detected	0.000	-2.146	Detected	-2.222	Detected	-2.184	0.004	GT	Sens
GT	Sense	Sense	0.499	Detected	-0.499	Detected	0.000	-1.705	Detected	-1.272	Detected	-1.488	0.112	GT	Sens
GT	Sense	Sense	0.203	Detected	-0.203	Detected	0.000	-2.524	Detected	-1.050	Detected	-1.787	0.144	GT	Sens
GT	Sense	Sense	-0.144	Detected	0.144	Detected	0.000	-1.929	Detected	-1.886	Detected	-1.907	0.006	GT	Sens
GT	Sense	Sense	-0.013	Detected	0.013	Detected	0.000	-1.007	Detected	-1.613	Detected	-1.310	0.050	GT	Sens
GT	Sense	Sense	-0.674	Detected	0.674	Detected	0.000	-2.383	Detected	-1.770	Detected	-2.077	0.107	GT	Sens
GT	Sense	Sense	-0.244	Detected	0.244	Detected	0.000	-1.068	Detected	-1.929	Comprom	-1.499	0.094	GT	Sens
GT	Sense	Sense	-0.365	Detected	0.365	Detected	0.000	-1.261	Detected	-1.279	Detected	-1.270	0.074	GT	Sens
GT	Sense	Sense	-0.137	Detected	0.137	Detected	0.000	-2.299	Detected	-0.899	Detected	-1.599	0.154	GT	Sens
GT	Sense	Sense	-0.225	Detected	0.225	Detected	0.000	-1.081	Detected	-0.960	Detected	-1.020	0.049	GT	Sens
GT	Sense	Sense	0.168	Detected	-0.168	Detected	0.000	-1.753	Comprom	-1.380	Comprom	-1.567	0.025	GT	Sens
GT	Sense	Sense	-0.421	Detected	0.421	Detected	0.000	-1.473	Detected	-1.235	Detected	-1.354	0.090	GT	Sens
GT	Sense	Sense	-0.409	Detected	0.409	Detected	0.000	-1.914	Detected	-1.677	Detected	-1.796	0.052	GT	Sens
GT	Sense	Sense	-0.212	Detected	0.212	Detected	0.000	-2.238	Comprom	-2.027	Comprom	-2.132	0.012	GT	Sens
GT	Sense	Sense	-0.106	Detected	0.106	Detected	0.000	-3.704	Detected	-2.989	Detected	-3.347	0.012	GT	Sens
GT	Sense	Sense	-0.668	Detected	0.668	Detected	0.000	-1.851	Detected	-1.509	Detected	-1.680	0.135	GT	Sens
GT	Sense	Sense	-1.140	Detected	1.140	Detected	0.000	-2.883	Detected	-0.875	Detected	-1.879	0.342	GT	Sens

GT	Sense	Sense	-0.424	Detected	0.424	Detected	0.000	-2.375	Detected	-1.962	Detected	-2.169	0.044	GT Sens	contig309	contig309
GT	Sense	Sense	-0.036	Detected	0.036	Detected	0.000	-1.341	Detected	-1.365	Detected	-1.353	0.001	GT Sens	contig310	contig310
GT	Sense	Sense	-0.768	Detected	0.768	Detected	0.000	-1.273	Detected	-1.132	Detected	-1.202	0.259	GT Sens	contig310	contig310
GT	Sense	Sense	-0.306	Detected	0.306	Detected	0.000	-1.442	Detected	-1.221	Detected	-1.331	0.055	GT Sens	contig310	contig310
GT	Sense	Sense	-0.207	Detected	0.207	Detected	0.000	-2.545	Detected	-0.816	Detected	-1.680	0.199	GT Sens	contig310	contig310
GT	Sense	Sense	-0.001	Detected	0.001	Detected	0.000	-1.405	Detected	-2.801	Comprom	-2.103	0.095	GT Sens	contig311	contig311
GT	Sense	Sense	0.306	Detected	-0.306	Detected	0.000	-1.752	Detected	-1.267	Detected	-1.510	0.061	GT Sens	contig311	contig311
GT	Sense	Sense	-0.196	Detected	0.196	Detected	0.000	-1.111	Detected	-1.177	Detected	-1.144	0.029	GT Sens	contig311	contig311
GT	Sense	Sense	-0.260	Detected	0.260	Detected	0.000	-3.754	Detected	-1.459	Detected	-2.606	0.157	GT Sens	contig311	contig311
GT	Sense	Sense	-1.313	Detected	1.313	Detected	0.000	-5.287	Detected	-0.847	Detected	-3.067	0.356	GT Sens	contig312	contig312
GT	Sense	Sense	-0.094	Detected	0.094	Detected	0.000	-1.355	Detected	-0.940	Detected	-1.148	0.037	GT Sens	contig312	contig312
GT	Sense	Sense	-0.075	Detected	0.075	Detected	0.000	-1.726	Detected	-0.979	Detected	-1.352	0.071	GT Sens	contig312	contig312
GT	Sense	Sense	0.271	Detected	-0.271	Detected	0.000	-1.967	Detected	-1.470	Detected	-1.719	0.043	GT Sens	contig312	contig312
GT	Sense	Sense	-0.125	Detected	0.125	Detected	0.000	-1.518	Detected	-1.066	Detected	-1.292	0.038	GT Sens	contig313	contig313
GT	Sense	Sense	-0.104	Detected	0.104	Detected	0.000	-1.360	Detected	-0.958	Detected	-1.159	0.036	GT Sens	contig313	contig313
GT	Sense	Sense	-0.175	Detected	0.175	Detected	0.000	-1.292	Detected	-0.841	Detected	-1.067	0.065	GT Sens	contig313	contig313
GT	Sense	Sense	-0.207	Detected	0.207	Detected	0.000	-1.481	Detected	-1.476	Detected	-1.478	0.019	GT Sens	contig313	contig313
GT	Sense	Sense	-0.085	Detected	0.085	Detected	0.000	-1.283	Comprom	-1.903	Comprom	-1.593	0.038	GT Sens	contig313	contig313
GT	Sense	Sense	-0.012	Detected	0.012	Detected	0.000	-1.021	Detected	-1.285	Detected	-1.153	0.013	GT Sens	contig313	contig313
GT	Sense	Sense	-0.142	Detected	0.142	Detected	0.000	-1.274	Detected	-1.286	Detected	-1.280	0.012	GT Sens	contig313	contig313
GT	Sense	Sense	-0.320	Detected	0.320	Detected	0.000	-1.159	Comprom	-1.416	Comprom	-1.287	0.065	GT Sens	contig314	contig314
GT	Sense	Sense	0.101	Detected	-0.101	Detected	0.000	-2.777	Comprom	-1.809	Comprom	-2.293	0.044	GT Sens	contig314	contig314
GT	Sense	Sense	-0.057	Detected	0.057	Detected	0.000	-1.704	Detected	-0.856	Detected	-1.280	0.096	GT Sens	contig314	contig314
GT	Sense	Sense	-0.233	Detected	0.233	Detected	0.000	-0.844	Detected	-1.228	Detected	-1.036	0.076	GT Sens	contig314	contig314

[illegible]

GT	Sense	Sense	-0.057	Detected	0.057	Detected	0.000	-1.780	Comprom	-1.751	Comprom	-1.765	0.001	GT Sens	contig352	contig352	Unannota	AGCGAGGAATAA[illegible]
GT	Sense	Sense	-0.094	Detected	0.094	Detected	0.000	-1.995	Comprom	-0.818	Detected	-1.407	0.142	GT Sens	contig352	contig352	Unannota	GGAAGAGTACTAT[illegible]
GT	Sense	Sense	0.121	Detected	-0.121	Detected	0.000	-1.466	Comprom	-2.578	Comprom	-2.022	0.071	GT Sens	contig353	contig353	Unannota	GGCCAAAACTTCT[illegible]
GT	Sense	Sense	-0.062	Detected	0.062	Detected	0.000	-1.394	Detected	-1.337	Detected	-1.366	0.002	GT Sens	contig353	contig353	Unannota	TTGTTGAACATAC[illegible]
GT	Sense	Sense	-0.254	Detected	0.254	Detected	0.000	-1.385	Detected	-0.966	Detected	-1.175	0.070	GT Sens	contig353	contig353	Unannota	CTTCAATCCATTC[illegible]
GT	Sense	Sense	0.015	Detected	-0.015	Detected	0.000	-1.989	Detected	-1.689	Detected	-1.839	0.007	GT Sens	contig353	contig353	Unannota	CCACGAAATTGG[illegible]
GT	Sense	Sense	0.856	Detected	-0.856	Detected	0.000	-1.297	Detected	-1.357	Detected	-1.327	0.261	GT Sens	contig353	contig353	Unannota	TCGACAAATTCTT[illegible]
GT	Sense	Sense	0.133	Detected	-0.133	Detected	0.000	-1.718	Detected	-2.572	Detected	-2.145	0.041	GT Sens	contig353	contig353	Unannota	CCTTTTTTATTCC[illegible]
GT	Sense	Sense	0.103	Detected	-0.103	Detected	0.000	-1.765	Detected	-2.077	Detected	-1.921	0.009	GT Sens	contig353	contig353	Unannota	CTTCTAAAAGCAA[illegible]
GT	Sense	Sense	0.942	Detected	-0.942	Detected	0.000	-1.716	Detected	-3.224	Comprom	-2.470	0.177	GT Sens	contig353	contig353	Unannota	ATTTTACACCGTG[illegible]
GT	Sense	Sense	-0.295	Detected	0.295	Detected	0.000	-1.799	Detected	-0.943	Detected	-1.371	0.119	GT Sens	contig353	contig353	Unannota	TCAGACTCAAAGA[illegible]
GT	Sense	Sense	0.699	Detected	-0.699	Detected	0.000	-1.219	Detected	-1.231	Detected	-1.225	0.222	GT Sens	contig354	contig354	Unannota	TCTACTCACCCCA[illegible]
GT	Sense	Sense	-0.025	Detected	0.025	Detected	0.000	-1.548	Detected	-1.192	Detected	-1.370	0.017	GT Sens	contig354	contig354	Unannota	TCGTAAGTTGCTC[illegible]
GT	Sense	Sense	-0.113	Detected	0.113	Detected	0.000	-1.488	Detected	-1.759	Detected	-1.624	0.012	GT Sens	contig354	contig354	Unannota	TTTTCATTTTACA[illegible]
GT	Sense	Sense	-0.110	Detected	0.110	Detected	0.000	-1.542	Comprom	-1.082	Comprom	-1.312	0.036	GT Sens	contig354	contig354	Unannota	TCTCAGATAAAAG[illegible]
GT	Sense	Sense	-0.376	Detected	0.376	Detected	0.000	-2.920	Comprom	-2.243	Comprom	-2.581	0.036	GT Sens	contig354	contig354	Unannota	AAATTAATCGGAC[illegible]
GT	Sense	Sense	-0.064	Detected	0.064	Detected	0.000	-1.397	Comprom	-1.514	Comprom	-1.455	0.004	GT Sens	contig355	contig355	Unannota	AACAATTTTCATA[illegible]
GT	Sense	Sense	-0.032	Detected	0.032	Detected	0.000	-1.344	Comprom	-1.447	Comprom	-1.396	0.002	GT Sens	contig355	contig355	Unannota	TCTTTTTCGAGGC[illegible]
GT	Sense	Sense	0.424	Detected	-0.424	Detected	0.000	-1.453	Detected	-1.004	Detected	-1.228	0.125	GT Sens	contig355	contig355	Unannota	AAGAAGACATACA[illegible]
GT	Sense	Sense	-0.113	Detected	0.113	Detected	0.000	-1.818	Comprom	-2.230	Comprom	-2.024	0.013	GT Sens	contig355	contig355	Unannota	GTACAAACAACAA[illegible]
[illegible]																		

GT	Sense	Sense	-0.119	Detected	0.119	Detected	0.000	-5.538	Comprom	-5.146	Comprom	-5.342	0.002	GT	Sens	[illegible]
GT	Sense	Sense	0.170	Detected	-0.170	Detected	0.000	-1.271	Detected	-1.108	Detected	-1.189	0.024	GT	Sens	[illegible]
GT	Sense	Sense	0.346	Detected	-0.346	Detected	0.000	-1.307	Detected	-0.968	Detected	-1.138	0.098	GT	Sens	[illegible]
GT	Sense	Sense	0.379	Detected	-0.379	Detected	0.000	-1.388	Detected	-1.095	Detected	-1.242	0.093	GT	Sens	[illegible]
GT	Sense	Sense	-0.471	Detected	0.471	Detected	0.000	-1.209	Comprom	-1.204	Comprom	-1.206	0.124	GT	Sens	[illegible]
GT	Sense	Sense	0.110	Detected	-0.110	Detected	0.000	-1.311	Detected	-0.991	Detected	-1.151	0.027	GT	Sens	[illegible]
GT	Sense	Sense	-0.073	Detected	0.073	Detected	0.000	-1.822	Detected	-0.890	Detected	-1.356	0.103	GT	Sens	[illegible]
GT	Sense	Sense	0.111	Detected	-0.111	Detected	0.000	-2.039	Detected	-0.960	Detected	-1.500	0.113	GT	Sens	[illegible]
GT	Sense	Sense	0.090	Detected	-0.090	Detected	0.000	-2.199	Comprom	-1.971	Comprom	-2.085	0.005	GT	Sens	[illegible]
GT	Sense	Sense	0.034	Detected	-0.034	Detected	0.000	-2.019	Comprom	-1.863	Comprom	-1.941	0.002	GT	Sens	[illegible]
GT	Sense	Sense	-0.231	Detected	0.231	Detected	0.000	-1.770	Comprom	-1.322	Comprom	-1.546	0.041	GT	Sens	[illegible]
GT	Sense	Sense	0.123	Detected	-0.123	Detected	0.000	-1.728	Detected	-1.057	Detected	-1.393	0.060	GT	Sens	[illegible]
GT	Sense	Sense	-0.375	Detected	0.375	Detected	0.000	-1.022	Detected	-0.988	Detected	-1.005	0.116	GT	Sens	[illegible]
GT	Sense	Sense	-0.489	Detected	0.489	Detected	0.000	-3.838	Detected	-2.714	Detected	-3.276	0.048	GT	Sens	[illegible]
GT	Sense	Sense	-0.658	Detected	0.658	Detected	0.000	-3.276	Detected	-2.028	Detected	-2.652	0.100	GT	Sens	[illegible]
GT	Sense	Sense	-0.178	Detected	0.178	Detected	0.000	-1.467	Detected	-0.979	Detected	-1.223	0.056	GT	Sens	[illegible]
GT	Sense	Sense	-0.118	Detected	0.118	Detected	0.000	-1.478	Detected	-1.031	Detected	-1.254	0.038	GT	Sens	[illegible]
GT	Sense	Sense	-0.094	Detected	0.094	Detected	0.000	-1.174	Comprom	-0.950	Comprom	-1.062	0.018	GT	Sens	[illegible]
GT	Sense	Sense	-0.065	Detected	0.065	Detected	0.000	-1.676	Detected	-1.942	Detected	-1.809	0.007	GT	Sens	[illegible]
GT	Sense	Sense	-0.257	Detected	0.257	Detected	0.000	-1.536	Detected	-1.029	Detected	-1.283	0.071	GT	Sens	[illegible]
GT	Sense	Sense	-0.439	Detected	0.439	Detected	0.000	-1.368	Detected	-1.049	Detected	-1.208	0.122	GT	Sens	[illegible]
GT	Sense	Sense	0.055	Detected	-0.055	Detected	0.000	-2.191	Comprom	-1.121	Detected	-1.656	0.091	GT	Sens	[illegible]
GT	Sense	Sense	-0.668	Detected	0.668	Detected	0.000	-2.316	Detected	-1.409	Detected	-1.862	0.147	GT	Sens	[illegible]
GT	Sense	Sense	-0.609	Detected	0.609	Detected	0.000	-2.769	Detected	-1.258	Detected	-2.014	0.174	GT	Sens	[illegible]
GT	Sense	Sense	-0.149	Detected	0.149	Detected	0.000	-1.787	Detected	-1.659	Detected	-1.723	0.009	GT	Sens	[illegible]
GT	Sense	Sense	-0.607	Detected	0.607	Detected	0.000	-3.395	Comprom	-2.285	Comprom	-2.840	0.075	GT	Sens	[illegible]
GT	Sense	Sense	-0.568	Detected	0.568	Detected	0.000	-2.264	Comprom	-1.330	Comprom	-1.797	0.134	GT	Sens	[illegible]
GT	Sense	Sense	-0.076	Detected	0.076	Detected	0.000	-2.329	Comprom	-0.969	Comprom	-1.649	0.138	GT	Sens	[illegible]
GT	Sense	Sense	-3.349	Detected	3.349	Detected	0.000	-5.220	Comprom	-2.891	Detected	-4.056	0.371	GT	Sens	[illegible]
GT	Sense	Sense	-0.093	Detected	0.093	Detected	0.000	-1.166	Detected	-0.863	Detected	-1.015	0.029	GT	Sens	[illegible]
GT	Sense	Sense	0.322	Detected	-0.322	Detected	0.000	-1.097	Detected	-1.057	Detected	-1.077	0.079	GT	Sens	[illegible]
GT	Sense	Sense	-0.388	Detected	0.388	Detected	0.000	-1.278	Detected	-1.167	Detected	-1.222	0.089	GT	Sens	[illegible]
GT	Sense	Sense	-0.019	Detected	0.019	Detected	0.000	-2.034	Detected	-2.871	Comprom	-2.453	0.028	GT	Sens	[illegible]
GT	Sense	Sense	-0.309	Detected	0.309	Detected	0.000	-1.486	Comprom	-0.811	Detected	-1.149	0.129	GT	Sens	[illegible]
GT	Sense	Sense	-0.465	Detected	0.465	Detected	0.000	-1.333	Comprom	-1.983	Comprom	-1.658	0.100	GT	Sens	[illegible]
GT	Sense	Sense	0.148	Detected	-0.148	Detected	0.000	-1.711	Detected	-1.711	Detected	-1.711	0.007	GT	Sens	[illegible]
GT	Sense	Sense	-0.033	Detected	0.033	Detected	0.000	-1.699	Detected	-1.859	Detected	-1.779	0.002	GT	Sens	[illegible]
GT	Sense	Sense	-0.205	Detected	0.205	Detected	0.000	-1.963	Detected	-1.930	Detected	-1.947	0.011	GT	Sens	[illegible]
GT	Sense	Sense	-0.085	Detected	0.085	Detected	0.000	-4.078	Comprom	-3.866	Comprom	-3.972	0.001	GT	Sens	[illegible]
GT	Sense	Sense	0.912	Detected	-0.912	Detected	0.000	-2.414	Comprom	-1.413	Detected	-1.913	0.207	GT	Sens	[illegible]
GT	Sense	Sense	-0.499	Detected	0.499	Detected	0.000	-1.326	Comprom	-1.657	Comprom	-1.492	0.105	GT	Sens	[illegible]
GT	Sense	Sense	-0.041	Detected	0.041	Detected	0.000	-0.980	Detected	-1.061	Detected	-1.021	0.003	GT	Sens	[illegible]
GT	Sense	Sense	-0.728	Detected	0.728	Detected	0.000	-1.270	Detected	-0.866	Detected	-1.068	0.293	GT	Sens	[illegible]
GT	Sense	Sense	-0.028	Detected	0.028	Detected	0.000	-1.234	Detected	-1.212	Detected	-1.223	0.001	GT	Sens	[illegible]
GT	Sense	Sense	3.780	Detected	-3.780	Detected	0.000	-6.110	Comprom	-5.425	Comprom	-5.767	0.268	GT	Sens	[illegible]
GT	Sense	Sense	0.216	Detected	-0.216	Detected	0.000	-2.085	Comprom	-2.404	Comprom	-2.245	0.014	GT	Sens	[illegible]
GT	Sense	Sense	-0.340	Detected	0.340	Detected	0.000	-1.257	Comprom	-2.124	Comprom	-1.691	0.092	GT	Sens	[illegible]
GT	Sense	Sense	-0.097	Detected	0.097	Detected	0.000	-2.019	Comprom	-2.473	Comprom	-2.246	0.012	GT	Sens	[illegible]
GT	Sense	Sense	-0.125	Detected	0.125	Detected	0.000	-1.408	Comprom	-1.041	Comprom	-1.225	0.031	GT	Sens	[illegible]
GT	Sense	Sense	0.594	Detected	-0.594	Detected	0.000	-1.623	Detected	-1.050	Detected	-1.337	0.180	GT	Sens	[illegible]
GT	Sense	Sense	0.634	Detected	-0.634	Detected	0.000	-2.252	Detected	-1.471	Detected	-1.862	0.130	GT	Sens	[illegible]
GT	Sense	Sense	-0.104	Detected	0.104	Detected	0.000	-2.270	Detected	-1.944	Detected	-2.107	0.008	GT	Sens	[illegible]
GT	Sense	Sense	-0.087	Detected	0.087	Detected	0.000	-1.785	Comprom	-1.669	Comprom	-1.727	0.004	GT	Sens	[illegible]
GT	Sense	Sense	-0.205	Detected	0.205	Detected	0.000	-1.640	Detected	-0.981	Detected	-1.310	0.078	GT	Sens	[illegible]
GT	Sense	Sense	-0.150	Detected	0.150	Detected	0.000	-2.824	Detected	-2.716	Detected	-2.770	0.003	GT	Sens	[illegible]
GT	Sense	Sense	-0.135	Detected	0.135	Detected	0.000	-1.881	Detected	-1.546	Detected	-1.713	0.015	GT	Sens	[illegible]
GT	Sense	Sense	-0.152	Detected	0.152	Detected	0.000	-2.138	Detected	-1.891	Detected	-2.015	0.009	GT	Sens	[illegible]
GT	Sense	Sense	0.112	Detected	-0.112	Detected	0.000	-2.552	Detected	-2.569	Detected	-2.560	0.002	GT	Sens	[illegible]
GT	Sense	Sense	-0.092	Detected	0.092	Detected	0.000	-2.388	Detected	-2.355	Detected	-2.372	0.002	GT	Sens	[illegible]
GT	Sense	Sense	-0.082	Detected	0.082	Detected	0.000	-1.996	Detected	-1.991	Detected	-1.994	0.002	GT	Sens	[illegible]
GT	Sense	Sense	-0.118	Detected	0.118	Detected	0.000	-2.219	Detected	-1.956	Detected	-2.088	0.007	GT	Sens	[illegible]
GT	Sense	Sense	-0.137	Detected	0.137	Detected	0.000	-1.977	Detected	-1.870	Detected	-1.924	0.006	GT	Sens	[illegible]
GT	Sense	Sense	-0.020	Detected	0.020	Detected	0.000	-1.755	Detected	-1.384	Detected	-1.570	0.014	GT	Sens	[illegible]
GT	Sense	Sense	0.023	Detected	-0.023	Detected	0.000	-3.084	Comprom	-2.350	Comprom	-2.717	0.018	GT	Sens	[illegible]
GT	Sense	Sense	-0.151	Detected	0.151	Detected	0.000	-1.770	Detected	-1.732	Detected	-1.751	0.007	GT	Sens	[illegible]
GT	Sense	Sense	-0.228	Detected	0.228	Detected	0.000	-1.046	Detected	-1.231	Comprom	-1.139	0.044	GT	Sens	[illegible]
GT	Sense	Sense	-0.092	Detected	0.092	Detected	0.000	-1.739	Detected	-1.639	Detected	-1.689	0.004	GT	Sens	[illegible]
GT	Sense	Sense	-0.087	Detected	0.087	Detected	0.000	-1.065	Detected	-1.033	Detected	-1.049	0.007	GT	Sens	[illegible]
GT	Sense	Sense	-0.160	Detected	0.160	Detected	0.000	-2.354	Detected	-2.402	Detected	-2.378	0.005	GT	Sens	[illegible]
GT	Sense	Sense	-0.020	Detected	0.020	Detected	0.000	-0.855	Detected	-1.205	Detected	-1.030	0.028	GT	Sens	[illegible]
GT	Sense	Sense	0.051	Detected	-0.051	Detected	0.000	-1.073	Detected	-1.275	Detected	-1.174	0.009	GT	Sens	[illegible]
GT	Sense	Sense	-0.044	Detected	0.044	Detected	0.000	-1.281	Detected	-1.005	Detected	-1.143	0.016	GT	Sens	[illegible]
GT	Sense	Sense	0.052	Detected	-0.052	Detected	0.000	-1.750	Comprom	-1.423	Comprom	-1.587	0.012	GT	Sens	[illegible]
GT	Sense	Sense	-0.083	Detected	0.083	Detected	0.000	-2.257	Detected	-2.191	Detected	-2.224	0.002	GT	Sens	[illegible]
GT	Sense	Sense	0.060	Detected	-0.060	Detected	0.000	-2.491	Comprom	-1.595	Comprom	-2.043	0.046	GT	Sens	[illegible]
GT	Sense	Sense	0.059	Detected	-0.059	Detected	0.000	-1.955	Comprom	-1.062	Detected	-1.509	0.079	GT	Sens	[illegible]
GT	Sense	Sense	0.055	Detected	-0.055	Detected	0.000	-1.583	Comprom	-1.497	Comprom	-1.540	0.002	GT	Sens	[illegible]
GT	Sense	Sense	-0.163	Detected	0.163	Detected	0.000	-1.252	Comprom	-0.853	Detected	-1.053	0.055	GT	Sens	[illegible]
GT	Sense	Sense	-0.297	Detected	0.297	Detected	0.000	-2.083	Comprom	-2.121	Comprom	-2.102	0.019	GT	Sens	[illegible]
GT	Sense	Sense	-0.073	Detected	0.073	Detected	0.000	-2.015	Comprom	-2.767	Comprom	-2.391	0.025	GT	Sens	[illegible]
GT	Sense	Sense	-0.095	Detected	0.095	Detected	0.000	-1.303	Detected	-1.052	Detected	-1.177	0.017	GT	Sens	[illegible]
GT	Sense	Sense	0.018	Detected	-0.018	Detected	0.000	-1.299	Detected	-0.871	Detected	-1.085	0.037	GT	Sens	[illegible]
GT	Sense	Sense	-0.076	Detected	0.076	Detected	0.000	-1.208	Detected	-1.150	Detected	-1.179	0.005	GT	Sens	[illegible]
GT	Sense	Sense	-0.169	Detected	0.169	Detected	0.000	-1.777	Detected	-1.715	Detected	-1.746	0.010	GT	Sens	[illegible]
GT	Sense	Sense	-0.032	Detected	0.032	Detected	0.000	-0.958	Comprom	-1.937	Comprom	-1.447	0.098	GT	Sens	[illegible]
GT	Sense	Sense	-0.404	Detected	0.404	Detected	0.000	-1.265	Comprom	-1.145	Comprom	-1.205	0.098	GT	Sens	[illegible]
GT	Sense	Sense	-0.087	Detected	0.087	Detected	0.000	-2.211	Detected	-2.219	Detected	-2.215	0.002	GT	Sens	[illegible]
GT	Sense	Sense	-0.109	Detected	0.109	Detected	0.000	-2.431	Detected	-2.277	Detected	-2.354	0.003	GT	Sens	[illegible]
GT	Sense	Sense	-0.145	Detected	0.145	Detected	0.000	-1.129	Detected	-1.042	Detected	-1.086	0.019	GT	Sens	[illegible]
GT	Sense	Sense	-0.453	Detected	0.453	Detected	0.000	-1.169	Comprom	-1.083	Comprom	-1.126	0.132	GT	Sens	[illegible]
GT	Sense	Sense	-0.044	Detected	0.044	Detected	0.000	-2.070	Detected	-1.938	Detected	-2.004	0.002	GT	Sens	[illegible]
GT	Sense	Sense	-0.062	Detected	0.062	Detected	0.000	-1.145	Detected	-1.184	Detected	-1.164	0.003	GT	Sens	[illegible]
GT	Sense	Sense	-0.281	Detected	0.281	Detected	0.000	-1.337	Detected	-1.459	Detected	-1.398	0.040	GT	Sens	[illegible]
GT	Sense	Sense	-0.005	Detected	0.005	Detected	0.000	-2.143	Detected	-1.134	Detected	-1.639	0.083	GT	Sens	[illegible]
GT	Sense	Sense	0.010	Detected	-0.010	Detected	0.000	-2.511	Detected	-1.366	Detected	-1.938	0.077	GT	Sens	[illegible]
GT	Sense	Sense	-0.208	Detected	0.208	Detected	0.000	-2.574	Comprom	-1.982	Comprom	-2.278	0.024	GT	Sens	[illegible]
GT	Sense	Sense	0.237	Detected	-0.237	Detected	0.000	-1.426	Detected	-1.084	Detected	-1.255	0.050	GT	Sens	[illegible]
GT	Sense	Sense	-0.091	Detected	0.091	Detected	0.000	-0.926	Comprom	-1.176	Comprom	-1.051	0.021	GT	Sens	[illegible]
GT	Sense	Sense	0.112	Detected	-0.112	Detected	0.000	-1.137	Detected	-0.999	Detected	-1.068	0.015	GT	Sens	[illegible]
GT	Sense	Sense	-0.010	Detected	0.010	Detected	0.000	-2.536	Comprom	-0.810	Detected	-1.673	0.192	GT	Sens	[illegible]
GT	Sense	Sense	-0.411	Detected	0.411	Detected	0.000	-1.310	Comprom	-1.858	Comprom	-1.584	0.085	GT	Sens	[illegible]
GT	Sense	Sense	-0.203	Detected	0.203	Detected	0.000	-1.640	Detected	-1.570	Detected	-1.605	0.016	GT	Sens	[illegible]
GT	Sense	Sense	-0.094	Detected	0.094	Detected	0.000	-2.229	Detected	-2.226	Detected	-2.227	0.002	GT	Sens	[illegible]
GT	Sense	Sense	-0.101	Detected	0.101	Detected	0.000	-3.456	Comprom	-1.078	Comprom	-2.267	0.198	GT	Sens	[illegible]
GT	Sense	Sense	-0.441	Detected	0.441	Detected	0.000	-2.235	Comprom	-3.418	Comprom	-2.827	0.062	GT	Sens	[illegible]
GT	Sense	Sense	-0.555	Detected	0.555	Detected	0.000	-1.658	Detected	-0.847	Detected	-1.252	0.210	GT	Sens	[illegible]
GT	Sense	Sense	-0.347	Detected	0.347	Detected	0.000	-1.619	Detected	-1.168	Detected	-1.393	0.078	GT	Sens	[illegible]
GT	Sense	Sense	0.142	Detected	-0.142	Detected	0.000	-1.276	Detected	-1.121	Detected	-1.199	0.018	GT	Sens	[illegible]
GT	Sense	Sense	-0.201	Detected	0.201	Detected	0.000	-1.207	Detected	-0.978	Detected	-1.093	0.042	GT	Sens	[illegible]
GT	Sense	Sense	-0.142	Detected	0.142	Detected	0.000	-1.635	Detected	-1.622	Detected	-1.629	0.007	GT	Sens	[illegible]
GT	Sense	Sense	0.122	Detected	-0.122	Detected	0.000	-1.062	Detected	-1.163	Detected	-1.112	0.014	GT	Sens	[illegible]
GT	Sense	Sense	-0.197	Detected	0.197	Detected	0.000	-1.088	Detected	-1.021	Detected	-1.055	0.034	GT	Sens	[illegible]
GT	Sense	Sense	0.013	Detected	-0.013	Detected	0.000	-1.211	Detected	-1.293	Detected	-1.252	0.001	GT	Sens	[illegible]
GT	Sense	Sense	-0.130	Detected	0.130	Detected	0.000	-1.384	Detected	-1.286	Detected	-1.335	0.011	GT	Sens	[illegible]
GT	Sense	Sense	-1.454	Detected	1.454	Detected	0.000	-1.288	Detected	-1.271	Detected	-1.280	0.472	GT	Sens	[illegible]
GT	Sense	Sense	0.109	Detected	-0.109	Detected	0.000	-3.672	Detected	-3.336	Detected	-3.504	0.003	GT	Sens	[illegible]
GT	Sense	Sense	-1.083	Detected	1.083	Detected	0.000	-1.960	Comprom	-1.648	Comprom	-1.804	0.241	GT	Sens	[illegible]
GT	Sense	Sense	-0.013	Detected	0.013	Detected	0.000	-1.740	Detected	-2.570	Comprom	-2.155	0.035	GT	Sens	[illegible]
GT	Sense	Sense	-0.140	Detected	0.140	Detected	0.000	-1.276	Detected	-1.769	Detected	-1.522	0.033	GT	Sens	[illegible]
GT	Sense	Sense	0.043	Detected	-0.043	Detected	0.000	-1.141	Comprom	-1.307	Comprom	-1.224	0.006	GT	Sens	[illegible]
GT	Sense	Sense	-1.209	Detected	1.209	Detected	0.000	-1.316	Detected	-1.645	Detected	-1.480	0.349	GT	Sens	[illegible]
GT	Sense	Sense	0.166	Detected	-0.166	Detected	0.000	-1.838	Detected	-1.424	Detected	-1.631	0.025	GT	Sens	[illegible]
GT	Sense	Sense	-0.152	Detected	0.152	Detected	0.000	-0.990	Detected	-1.032	Detected	-1.011	0.022	GT	Sens	[illegible]
GT	Sense	Sense	-0.089	Detected	0.089	Detected	0.000	-3.118	Detected	-3.131	Detected	-3.124	0.001	GT	Sens	[illegible]
GT	Sense	Sense	-0.269	Detected	0.269	Detected	0.000	-1.634	Detected	-1.205	Detected	-1.419	0.054	GT	Sens	[illegible]
GT	Sense	Sense	0.028	Detected	-0.028	Detected	0.000	-3.667	Comprom	-3.193	Comprom	-3.430	0.005	GT	Sens	[illegible]
GT	Sense	Sense	-0.099	Detected	0.099	Detected	0.000	-2.468	Detected	-2.483	Detected	-2.476	0.002	GT	Sens	[illegible]
GT	Sense	Sense	-0.197	Detected	0.197	Detected	0.000	-1.725	Detected	-1.570	Detected	-1.648	0.016	GT	Sens	[illegible]
GT	Sense	Sense	-0.330	Detected	0.330	Detected	0.000	-1.336	Detected	-1.205	Detected	-1.270	0.064	GT	Sens	[illegible]
GT	Sense	Sense	0.185	Detected	-0.185	Detected	0.000	-1.769	Detected	-2.699	Comprom	-2.234	0.047	GT	Sens	[illegible]
GT	Sense	Sense	-0.088	Detected	0.088	Detected	0.000	-1.133	Detected	-1.137	Detected	-1.135	0.006	GT	Sens	[illegible]
GT	Sense	Sense	-0.139	Detected	0.139	Detected	0.000	-2.156	Detected	-2.326	Detected	-2.241	0.005	GT	Sens	[illegible]
GT	Sense	Sense	-0.053	Detected	0.053	Detected	0.000	-2.876	Detected	-3.176	Detected	-3.026	0.003	GT	Sens	[illegible]
GT	Sense	Sense	0.006	Detected	-0.006	Detected	0.000	-2.564	Comprom	-1.316	Comprom	-1.940	0.090	GT	Sens	[illegible]
GT	Sense	Sense	-0.067	Detected	0.067	Detected	0.000	-1.633	Detected	-1.912	Detected	-1.772	0.008	GT	Sens	[illegible]
GT	Sense	Sense	-0.006	Detected	0.006	Detected	0.000	-1.145	Detected	-1.223	Detected	-1.184	0.001	GT	Sens	[illegible]
GT	Sense	Sense	0.107	Detected	-0.107	Detected	0.000	-1.801	Comprom	-1.297	Comprom	-1.549	0.030	GT	Sens	[illegible]
GT	Sense	Sense	0.040	Detected	-0.040	Detected	0.000	-2.013	Detected	-1.859	Detected	-1.936	0.002	GT	Sens	[illegible]
GT	Sense	Sense	-0.003	Detected	0.003	Detected	0.000	-1.089	Comprom	-1.575	Comprom	-1.332	0.032	GT	Sens	[illegible]
GT	Sense	Sense	-0.057	Detected	0.057	Detected	0.000	-1.863	Comprom	-1.744	Comprom	-1.804	0.002	GT	Sens	[illegible]
GT	Sense	Sense	-0.137	Detected	0.137	Detected	0.000	-2.560	Detected	-2.049	Detected	-2.305	0.015	GT	Sens	[illegible]
GT	Sense	Sense	-0.353	Detected	0.353	Detected	0.000	-1.679	Comprom	-1.842	Comprom	-1.761	0.040	GT	Sens	[illegible]
GT	Sense	Sense	-0.223	Detected	0.223	Detected	0.000	-0.936	Detected	-1.169	Detected	-1.052	0.053	GT	Sens	[illegible]
GT	Sense	Sense	0.667	Detected	-0.667	Detected	0.000	-3.544	Comprom	-2.106	Comprom	-2.825	0.102	GT	Sens	[illegible]
GT	Sense	Sense	-0.067	Detected	0.067	Detected	0.000	-2.072	Detected	-1.874	Detected	-1.973	0.004	GT	Sens	[illegible]
GT	Sense	Sense	-0.064	Detected	0.064	Detected	0.000	-1.260	Detected	-1.291	Detected	-1.276	0.003	GT	Sens	[illegible]
GT	Sense	Sense	-0.146	Detected	0.146	Detected	0.000	-1.284	Detected	-1.027	Detected	-1.155	0.027	GT	Sens	[illegible]
GT	Sense	Sense	-0.469	Detected	0.469	Detected	0.000	-1.122	Comprom	-0.882	Comprom	-1.002	0.174	GT	Sens	[illegible]
GT	Sense	Sense	-0.413	Detected	0.413	Detected	0.000	-4.696	Comprom	-1.858	Detected	-3.277	0.157	GT	Sens	[illegible]
GT	Sense	Sense	0.025	Detected	-0.025	Detected	0.000	-1.066	Detected	-1.098	Detected	-1.082	0.001	GT	Sens	[illegible]
GT	Sense	Sense	-0.103	Detected	0.103	Detected	0.000	-1.262	Detected	-2.144	Comprom	-1.703	0.064	GT	Sens	[illegible]
GT	Sense	Sense	0.021	Detected	-0.021	Detected	0.000	-1.515	Detected	-1.612	Detected	-1.563	0.001	GT	Sens	[illegible]
GT	Sense	Sense	-0.057	Detected	0.057	Detected	0.000	-1.400	Detected	-1.261	Detected	-1.330	0.005	GT	Sens	[illegible]
GT	Sense	Sense	-0.175	Detected	0.175	Detected	0.000	-1.323	Detected	-0.904	Detected	-1.114	0.055	GT	Sens	[illegible]
GT	Sense	Sense	-0.497	Detected	0.497	Detected	0.000	-1.134	Detected	-1.459	Detected	-1.297	0.131	GT	Sens	[illegible]
GT	Sense	Sense	-0.150	Detected	0.150	Detected	0.000	-0.804	Detected	-1.250	Comprom	-1.027	0.062	GT	Sens	[illegible]
GT	Sense	Sense	-0.152	Detected	0.152	Detected	0.000	-2.208	Detected	-2.294	Detected	-2.251	0.005	GT	Sens	[illegible]
GT	Sense	Sense	-0.141	Detected	0.141	Detected	0.000	-2.273	Detected	-2.340	Detected	-2.306	0.004	GT	Sens	[illegible]
GT	Sense	Sense	0.084	Detected	-0.084	Detected	0.000	-2.258	Detected	-1.373	Detected	-1.815	0.056	GT	Sens	[illegible]
GT	Sense	Sense	-1.133	Detected	1.133	Detected	0.000	-6.492	Detected	-3.590	Detected	-5.041	0.111	GT	Sens	[illegible]
GT	Sense	Sense	-1.011	Detected	1.011	Detected	0.000	-6.097	Detected	-3.378	Detected	-4.738	0.108	GT	Sens	[illegible]
GT	Sense	Sense	0.451	Detected	-0.451	Detected	0.000	-2.003	Detected	-1.885	Detected	-1.944	0.051	GT	Sens	[illegible]
GT	Sense	Sense	-0.017	Detected	0.017	Detected	0.000	-0.944	Detected	-1.152	Detected	-1.048	0.010	GT	Sens	[illegible]
GT	Sense	Sense	-0.182	Detected	0.182	Detected	0.000	-1.358	Detected	-1.174	Detected	-1.266	0.025	GT	Sens	[illegible]
GT	Sense	Sense	-0.081	Detected	0.081	Detected	0.000	-1.624	Detected	-1.506	Detected	-1.565	0.004	GT	Sens	[illegible]
GT	Sense	Sense	0.292	Detected	-0.292	Detected	0.000	-1.191	Detected	-1.628	Detected	-1.409	0.061	GT	Sens	[illegible]
GT	Sense	Sense	-0.206	Detected	0.206	Detected	0.000	-1.199	Detected	-1.825	Comprom	-1.512	0.056	GT	Sens	[illegible]
GT	Sense	Sense	-0.709	Detected	0.709	Detected	0.000	-1.123	Detected	-1.847	Detected	-1.485	0.203	GT	Sens	[illegible]
GT	Sense	Sense	-0.398	Detected	0.398	Detected	0.000	-2.428	Detected	-1.128	Detected	-1.778	0.145	GT	Sens	[illegible]
GT	Sense	Sense	-0.030	Detected	0.030	Detected	0.000	-1.955	Detected	-1.163	Detected	-1.559	0.059	GT	Sens	[illegible]
GT	Sense	Sense	-1.405	Detected	1.405	Detected	0.000	-3.072	Comprom	-2.513	Comprom	-2.793	0.191	GT	Sens	[illegible]
GT	Sense	Sense	-0.204	Detected	0.204	Detected	0.000	-1.200	Detected	-0.943	Detected	-1.071	0.047	GT	Sens	[illegible]
GT	Sense	Sense	-0.238	Detected	0.238	Detected	0.000	-1.270	Detected	-1.281	Detected	-1.276	0.033	GT	Sens	[illegible]
GT	Sense	Sense	-0.134	Detected	0.134	Detected	0.000	-2.194	Detected	-2.353	Detected	-2.273	0.005	GT	Sens	[illegible]
GT	Sense	Sense	-0.155	Detected	0.155	Detected	0.000	-2.968	Comprom	-2.498	Comprom	-2.733	0.010	GT	Sens	[illegible]
GT	Sense	Sense	-0.084	Detected	0.084	Detected	0.000	-1.562	Detected	-1.718	Detected	-1.640	0.005	GT	Sens	[illegible]
GT	Sense	Sense	0.056	Detected	-0.056	Detected	0.000	-1.050	Detected	-1.153	Detected	-1.101	0.005	GT	Sens	[illegible]
GT	Sense	Sense	0.145	Detected	-0.145	Detected	0.000	-1.399	Detected	-1.208	Detected	-1.304	0.017	GT	Sens	[illegible]
GT	Sense	Sense	-0.055	Detected	0.055	Detected	0.000	-1.337	Detected	-1.144	Detected	-1.240	0.008	GT	Sens	[illegible]
GT	Sense	Sense	-0.216	Detected	0.216	Detected	0.000	-2.412	Comprom	-1.517	Comprom	-1.965	0.058	GT	Sens	[illegible]
GT	Sense	Sense	-0.234	Detected	0.234	Detected	0.000	-2.604	Comprom	-1.668	Comprom	-2.136	0.055	GT	Sens	[illegible]
GT	Sense	Sense	0.169	Detected	-0.169	Detected	0.000	-1.187	Comprom	-0.842	Comprom	-1.015	0.052	GT	Sens	[illegible]
GT	Sense	Sense	0.146	Detected	-0.146	Detected	0.000	-1.318	Detected	-0.930	Detected	-1.124	0.044	GT	Sens	[illegible]
GT	Sense	Sense	-0.085	Detected	0.085	Detected	0.000	-1.355	Comprom	-0.879	Comprom	-1.117	0.048	GT	Sens	[illegible]
GT	Sense	Sense	-0.023	Detected	0.023	Detected	0.000	-0.850	Detected	-1.281	Detected	-1.066	0.039	GT	Sens	[illegible]
GT	Sense	Sense	0.237	Detected	-0.237	Detected	0.000	-3.616	Comprom	-1.221	Detected	-2.419	0.186	GT	Sens	[illegible]
GT	Sense	Sense	0.343	Detected	-0.343	Detected	0.000	-4.047	Detected	-1.081	Detected	-2.564	0.234	GT	Sens	[illegible]
GT	Sense	Sense	0.089	Detected	-0.089	Detected	0.000	-1.254	Detected	-0.993	Detected	-1.124	0.019	GT	Sens	[illegible]
GT	Sense	Sense	0.027	Detected	-0.027	Detected	0.000	-1.328	Detected	-1.143	Detected	-1.235	0.006	GT	Sens	[illegible]
GT	Sense	Sense	0.201	Detected	-0.201	Detected	0.000	-0.972	Comprom	-1.483	Comprom	-1.228	0.063	GT	Sens	[illegible]
GT	Sense	Sense	-0.474	Detected	0.474	Detected	0.000	-0.993	Comprom	-1.897	Comprom	-1.445	0.158	GT	Sens	[illegible]
GT	Sense	Sense	-0.139	Detected	0.139	Detected	0.000	-2.238	Comprom	-2.477	Comprom	-2.358	0.006	GT	Sens	[illegible]
GT	Sense	Sense	-0.143	Detected	0.143	Detected	0.000	-2.236	Detected	-2.394	Detected	-2.315	0.005	GT	Sens	[illegible]
GT	Sense	Sense	-0.013	Detected	0.013	Detected	0.000	-2.337	Detected	-2.910	Comprom	-2.624	0.012	GT	Sens	[illegible]
GT	Sense	Sense	-0.093	Detected	0.093	Detected	0.000	-1.691	Detected	-1.664	Detected	-1.677	0.003	GT	Sens	[illegible]
GT	Sense	Sense	-0.250	Detected	0.250	Detected	0.000	-2.234	Detected	-0.899	Detected	-1.566	0.159	GT	Sens	[illegible]
GT	Sense	Sense	-0.142	Detected	0.142	Detected	0.000	-2.200	Detected	-2.082	Detected	-2.141	0.005	GT	Sens	[illegible]
GT	Sense	Sense	-0.028	Detected	0.028	Detected	0.000	-0.986	Detected	-1.020	Detected	-1.003	0.001	GT	Sens	[illegible]
GT	Sense	Sense	0.109	Detected	-0.109	Detected	0.000	-1.728	Comprom	-0.807	Detected	-1.267	0.116	GT	Sens	[illegible]
GT	Sense	Sense	-0.526	Detected	0.526	Detected	0.000	-0.824	Detected	-1.330	Comprom	-1.077	0.206	GT	Sens	[illegible]
GT	Sense	Sense	-0.062	Detected	0.062	Detected	0.000	-2.536	Detected	-2.680	Detected	-2.608	0.001	GT	Sens	[illegible]
GT	Sense	Sense	-0.048	Detected	0.048	Detected	0.000	-2.185	Detected	-2.213	Detected	-2.199	0.001	GT	Sens	[illegible]
GT	Sense	Sense	-0.066	Detected	0.066	Detected	0.000	-1.243	Detected	-0.992	Detected	-1.118	0.016	GT	Sens	[illegible]
GT	Sense	Sense	0.190	Detected	-0.190	Detected	0.000	-2.047	Detected	-2.010	Detected	-2.028	0.009	GT	Sens	[illegible]
GT	Sense	Sense	0.393	Detected	-0.393	Detected	0.000	-2.578	Detected	-2.419	Detected	-2.498	0.025	GT	Sens	[illegible]
GT	Sense	Sense	-0.023	Detected	0.023	Detected	0.000	-1.211	Detected	-0.910	Detected	-1.060	0.020	GT	Sens	[illegible]
GT	Sense	Sense	-0.227	Detected	0.227	Detected	0.000	-1.510	Detected	-0.960	Detected	-1.235	0.074	GT	Sens	[illegible]
GT	Sense	Sense	-0.395	Detected	0.395	Detected	0.000	-1.717	Comprom	-1.511	Comprom	-1.614	0.059	GT	Sens	[illegible]
GT	Sense	Sense	-0.309	Detected	0.309	Detected	0.000	-1.069	Detected	-1.004	Comprom	-1.036	0.079	GT	Sens	[illegible]
GT	Sense	Sense	-0.204	Detected	0.204	Detected	0.000	-1.031	Comprom	-1.069	Comprom	-1.050	0.036	GT	Sens	[illegible]
GT	Sense	Sense	0.369	Detected	-0.369	Detected	0.000	-1.534	Comprom	-0.846	Detected	-1.190	0.143	GT	Sens	[illegible]
GT	Sense	Sense	-0.218	Detected	0.218	Detected	0.000	-0.957	Detected	-1.335	Detected	-1.146	0.058	GT	Sens	[illegible]
GT	Sense	Sense	0.239	Detected	-0.239	Detected	0.000	-1.497	Detected	-1.396	Detected	-1.446	0.027	GT	Sens	[illegible]
GT	Sense	Sense	0.039	Detected	-0.039	Detected	0.000	-2.325	Comprom	-2.407	Comprom	-2.366	0.001	GT	Sens	[illegible]
GT	Sense	Sense	0.120	Detected	-0.120	Detected	0.000	-0.932	Detected	-1.072	Detected	-1.002	0.019	GT	Sens	[illegible]
GT	Sense	Sense	-0.078	Detected	0.078	Detected	0.000	-1.542	Detected	-1.247	Detected	-1.394	0.014	GT	Sens	[illegible]
GT	Sense	Sense	-0.415	Detected	0.415	Detected	0.000	-1.540	Detected	-1.089	Detected	-1.315	0.108	GT	Sens	[illegible]
GT	Sense	Sense	-0.042	Detected	0.042	Detected	0.000	-1.167	Detected	-1.225	Detected	-1.196	0.002	GT	Sens	[illegible]
GT	Sense	Sense	-0.163	Detected	0.163	Detected	0.000	-4.254	Comprom	-1.919	Detected	-3.087	0.120	GT	Sens	[illegible]
GT	Sense	Sense	-0.112	Detected	0.112	Detected	0.000	-0.966	Detected	-1.637	Detected	-1.301	0.067	GT	Sens	[illegible]
GT	Sense	Sense	-0.065	Detected	0.065	Detected	0.000	-0.828	Detected	-1.192	Detected	-1.010	0.035	GT	Sens	[illegible]
GT	Sense	Sense	0.070	Detected	-0.070	Detected	0.000	-1.715	Detected	-1.120	Detected	-1.418	0.044	GT	Sens	[illegible]
GT	Sense	Sense	-0.035	Detected	0.035	Detected	0.000	-1.165	Detected	-1.653	Detected	-1.409	0.029	GT	Sens	[illegible]
GT	Sense	Sense	-1.156	Detected	1.156	Detected	0.000	-4.750	Detected	-2.333	Detected	-3.542	0.168	GT	Sens	[illegible]
GT	Sense	Sense	0.651	Detected	-0.651	Detected	0.000	-2.324	Comprom	-2.501	Comprom	-2.413	0.067	GT	Sens	[illegible]
GT	Sense	Sense	-0.280	Detected	0.280	Detected	0.000	-1.159	Detected	-1.435	Comprom	-1.297	0.053	GT	Sens	[illegible]
GT	Sense	Sense	-0.314	Detected	0.314	Detected	0.000	-1.040	Detected	-1.029	Detected	-1.034	0.081	GT	Sens	[illegible]
GT	Sense	Sense	-0.104	Detected	0.104	Detected	0.000	-2.481	Detected	-1.445	Detected	-1.963	0.065	GT	Sens	[illegible]
GT	Sense	Sense	-0.122	Detected	0.122	Detected	0.000	-1.600	Detected	-1.718	Detected	-1.659	0.007	GT	Sens	[illegible]
GT	Sense	Sense	-0.664	Detected	0.664	Detected	0.000	-4.454	Detected	-2.457	Detected	-3.456	0.102	GT	Sens	[illegible]
GT	Sense	Sense	0.098	Detected	-0.098	Detected	0.000	-2.077	Comprom	-1.040	Comprom	-1.559	0.098	GT	Sens	[illegible]
GT	Sense	Sense	-0.027	Detected	0.027	Detected	0.000	-1.453	Detected	-0.831	Detected	-1.142	0.067	GT	Sens	[illegible]
GT	Sense	Sense	-0.370	Detected	0.370	Detected	0.000	-1.259	Detected	-1.021	Detected	-1.140	0.099	GT	Sens	[illegible]
GT	Sense	Sense	-0.468	Detected	0.468	Detected	0.000	-1.841	Detected	-1.408	Detected	-1.624	0.088	GT	Sens	[illegible]
GT	Sense	Sense	-0.295	Detected	0.295	Detected	0.000	-2.213	Detected	-1.687	Detected	-1.950	0.039	GT	Sens	[illegible]
GT	Sense	Sense	-0.724	Detected	0.724	Detected	0.000	-1.726	Detected	-0.874	Detected	-1.300	0.262	GT	Sens	[illegible]
GT	Sense	Sense	-0.279	Detected	0.279	Detected	0.000	-1.035	Detected	-1.394	Detected	-1.215	0.067	GT	Sens	[illegible]
GT	Sense	Sense	-0.018	Detected	0.018	Detected	0.000	-1.575	Detected	-1.121	Detected	-1.348	0.027	GT	Sens	[illegible]
GT	Sense	Sense	-0.245	Detected	0.245	Detected	0.000	-3.286	Comprom	-2.229	Comprom	-2.758	0.042	GT	Sens	[illegible]
GT	Sense	Sense	-0.214	Detected	0.214	Detected	0.000	-1.202	Detected	-1.429	Detected	-1.315	0.032	GT	Sens	[illegible]
GT	Sense	Sense	-0.188	Detected	0.188	Detected	0.000	-2.239	Detected	-1.236	Detected	-1.738	0.083	GT	Sens	[illegible]
GT	Sense	Sense	-0.274	Detected	0.274	Detected	0.000	-1.393	Comprom	-2.213	Comprom	-1.803	0.067	GT	Sens	[illegible]
GT	Sense	Sense	-0.088	Detected	0.088	Detected	0.000	-1.047	Detected	-1.191	Detected	-1.119	0.010	GT	Sens	[illegible]
GT	Sense	Sense	0.215	Detected	-0.215	Detected	0.000	-1.142	Detected	-1.364	Comprom	-1.253	0.035	GT	Sens	[illegible]

GT	Sense	Sense	-0.036	Detected	0.036	Detected	0.000	-1.783	Detected	-1.429	Detected	-1.606	0.012	GT	Sens	contig434	contig434	N.tabacu	AAATGATGTGATTATTTAATAACTTGGATTAATTAGAAGAATGATCATG							[illegible]
GT	Sense	Sense	-0.783	Detected	0.783	Detected	0.000	-1.593	Comprom	-2.611	Comprom	-2.102	0.153	GT	Sens	contig436	contig436	Unknown	CACTCAT	contig436	Solyc01g	Solyc01g	Unknown Protein (AHRD V1)			[illegible]
GT	Sense	Sense	-0.689	Detected	0.689	Detected	0.000	-1.609	Detected	-1.057	Detected	-1.333	0.214	GT	Sens	contig442	contig442	Adenylate	TATAGG	contig442	Solyc02g	Solyc02g	Adenylate	GO:0006	GO:0006	[illegible]
GT	Sense	Sense	-0.167	Detected	0.167	Detected	0.000	-1.230	Detected	-1.234	Detected	-1.232	0.018	GT	Sens	contig442	contig442	Unknown	CTTAGA	contig442	Solyc09g	Solyc09g	Unknown Protein (AHRD V1)			[illegible]
GT	Sense	Sense	-0.158	Detected	0.158	Detected	0.000	-1.440	Detected	-1.298	Detected	-1.369	0.016	GT	Sens	contig443	contig443	Polyprote	CTCACA	contig443	Solyc07g	Solyc07g	Polyprotein (AHRD V1 **-- O49			[illegible]
GT	Sense	Sense	0.009	Detected	-0.009	Detected	0.000	-2.169	Detected	-2.848	Comprom	-2.509	0.018	GT	Sens	contig443	contig443	Mutator-li	ATATGCT	contig443	Solyc06g	Solyc06g	Mutator-li	GO:0008	GO:0008	[illegible]
GT	Sense	Sense	-0.009	Detected	0.009	Detected	0.000	-0.871	Detected	-1.310	Comprom	-1.091	0.038	GT	Sens	contig443	contig443	BZIP dom	TGTGTT	contig443	Solyc06g	Solyc06g	BZIP dom	GO:0005	GO:0005	[illegible]
GT	Sense	Sense	-0.180	Detected	0.180	Detected	0.000	-1.974	Detected	-0.884	Detected	-1.429	0.131	GT	Sens	contig448	contig448	Receptor	TTAGAAT	contig448	Solyc03g	Solyc03g	Receptor	GO:0005	GO:0005	[illegible]
GT	Sense	Sense	-0.808	Detected	0.808	Detected	0.000	-1.493	Detected	-1.633	Detected	-1.563	0.194	GT	Sens	contig461	contig461	Aquapori	TAATGTA	contig461	Solyc03g	Solyc03g	Aquapori	GO:0016	GO:0016	[illegible]
GT	Sense	Sense	-0.171	Detected	0.171	Detected	0.000	-1.502	Detected	-0.829	Detected	-1.166	0.091	GT	Sens	contig463	contig463	NB-ARC d	GAAGTT	contig463	Solyc11g	Solyc11g	NB-ARC domain containing pr			[illegible]
GT	Sense	Sense	0.143	Detected	-0.143	Detected	0.000	-1.126	Detected	-1.141	Detected	-1.134	0.016	GT	Sens	contig466	contig466	DNA-dire	GGATAT	contig466	Solyc01g	Solyc01g	DNA-dire	GO:0006350		[illegible]
GT	Sense	Sense	-0.069	Detected	0.069	Detected	0.000	-2.223	Comprom	-2.349	Comprom	-2.286	0.002	GT	Sens	contig466	contig466	Unknown	TCTCATA	contig466	Solyc09g	Solyc09g	Unknown Protein (AHRD V1)			[illegible]
GT	Sense	Sense	-0.092	Detected	0.092	Detected	0.000	-1.882	Detected	-1.921	Detected	-1.901	0.002	GT	Sens	contig471	contig471	Reverse t	AGTTGAT	contig471	Solyc07g	Solyc07g	Reverse t	GO:0006	GO:0006	[illegible]
GT	Sense	Sense	-0.106	Detected	0.106	Detected	0.000	-1.041	Detected	-1.069	Detected	-1.055	0.010	GT	Sens	contig472	contig472	PG1 prot	TTCCTT	contig472	Solyc01g	Solyc01g	PG1 protein (Fragment) (AHRD			[illegible]
GT	Sense	Sense	-0.161	Detected	0.161	Detected	0.000	-1.452	Detected	-1.499	Detected	-1.475	0.012	GT	Sens	contig473	contig473	Pol polyp	AGAAGC	contig473	Solyc03g	Solyc03g	Pol polyp	GO:0006	GO:0006	[illegible]
GT	Sense	Sense	-0.759	Detected	0.759	Detected	0.000	-1.813	Detected	-0.877	Detected	-1.345	0.270	GT	Sens	contig481	contig481	Cytochro	GTACAC	contig481	Solyc10g	Solyc10g	Cytochro	GO:0019	GO:0019	[illegible]
GT	Sense	Sense	1.230	Detected	-1.230	Detected	0.000	-3.717	Detected	-3.024	Detected	-3.371	0.119	GT	Sens	contig482	contig482	Unknown	TCACCA	contig482	Solyc09g	Solyc09g	Unknown Protein (AHRD V1)			[illegible]
GT	Sense	Sense	0.065	Detected	-0.065	Detected	0.000	-1.844	Detected	-1.329	Detected	-1.587	0.027	GT	Sens	contig491	contig491	Myb-relat	CAAGTT	contig491	Solyc09g	Solyc09g	Myb-relat	GO:0003	GO:0003	[illegible]
GT	Sense	Sense	-0.227	Detected	0.227	Detected	0.000	-2.391	Detected	-0.853	Detected	-1.622	0.180	GT	Sens	contig496	contig496	Ring H2 fi	AATGAT	contig496	Solyc03g	Solyc03g	Ring H2 fi	GO:0008	GO:0008	[illegible]
GT	Sense	Sense	-0.221	Detected	0.221	Detected	0.000	-1.500	Comprom	-1.775	Comprom	-1.637	0.024	GT	Sens	contig501	contig501	Unknown	ATTGCT	contig501	Solyc08g	Solyc08g	Unknown Protein (AHRD V1)			[illegible]

[illegible]

GT	Sense	Sense	-0.132	Detected	0.132	Detected	0.000	-1.709	Detected	-1.844	Detected	-1.777	0.007	GT	Sens	contig129	contig129	Unknown	TTGCAG	[illegible]
GT	Sense	Sense	0.267	Detected	-0.267	Detected	0.000	-1.445	Detected	-1.705	Detected	-1.575	0.034	GT	Sens	contig129	contig129	Unknown	GTGAAA	[illegible]
GT	Sense	Sense	0.078	Detected	-0.078	Detected	0.000	-0.935	Detected	-1.686	Detected	-1.311	0.076	GT	Sens	contig130	contig130	Cysteine	GGAATCT	[illegible]
GT	Sense	Sense	-0.641	Detected	0.641	Detected	0.000	-2.429	Detected	-0.847	Detected	-1.638	0.249	GT	Sens	contig131	contig131	Glycosylt	GGCTCA	[illegible]
GT	Sense	Sense	-0.247	Detected	0.247	Detected	0.000	-1.760	Detected	-1.879	Detected	-1.819	0.019	GT	Sens	contig131	contig131	Unknown	AGGTGT	[illegible]
GT	Sense	Sense	-0.032	Detected	0.032	Detected	0.000	-2.300	Detected	-2.185	Detected	-2.242	0.001	GT	Sens	contig131	contig131	NADH del	CTGTTG	[illegible]
GT	Sense	Sense	-0.209	Detected	0.209	Detected	0.000	-1.182	Comprom	-1.659	Comprom	-1.421	0.046	GT	Sens	contig131	contig131	Gag-Pol p	CTCCGA	[illegible]
GT	Sense	Sense	-0.091	Detected	0.091	Detected	0.000	-2.519	Comprom	-1.729	Detected	-2.124	0.035	GT	Sens	contig132	contig132	F-box fam	TATAGTA	[illegible]
GT	Sense	Sense	-0.413	Detected	0.413	Detected	0.000	-1.473	Detected	-1.972	Comprom	-1.723	0.070	GT	Sens	contig133	contig133	Cytochro	GTACGAT	[illegible]
GT	Sense	Sense	-0.189	Detected	0.189	Detected	0.000	-1.621	Detected	-1.777	Detected	-1.699	0.014	GT	Sens	contig133	contig133	Unknown	TGTGAC	[illegible]
GT	Sense	Sense	-0.068	Detected	0.068	Detected	0.000	-2.507	Comprom	-2.114	Comprom	-2.310	0.008	GT	Sens	contig133	contig133	Transpos	ATCACG	[illegible]
GT	Sense	Sense	-0.079	Detected	0.079	Detected	0.000	-1.235	Detected	-1.635	Detected	-1.435	0.022	GT	Sens	contig134	contig134	Ycf2 (AHF	GATACTT	[illegible]
GT	Sense	Sense	-0.261	Detected	0.261	Detected	0.000	-2.195	Detected	-2.375	Detected	-2.285	0.014	GT	Sens	contig134	contig134	Polyprote	TACAGAT	[illegible]
GT	Sense	Sense	0.320	Detected	-0.320	Detected	0.000	-1.488	Comprom	-0.803	Detected	-1.145	0.134	GT	Sens	contig135	contig135	Ycf2 (AHF	TTACATA	[illegible]
GT	Sense	Sense	0.045	Detected	-0.045	Detected	0.000	-2.194	Comprom	-1.868	Comprom	-2.031	0.007	GT	Sens	contig135	contig135	Gag-Pol p	AGTCTT	[illegible]
GT	Sense	Sense	-0.492	Detected	0.492	Detected	0.000	-1.127	Detected	-0.971	Detected	-1.049	0.170	GT	Sens	contig136	contig136	UDP-gluc	AACCGTT	[illegible]
GT	Sense	Sense	-0.226	Detected	0.226	Detected	0.000	-1.808	Detected	-1.226	Detected	-1.517	0.054	GT	Sens	contig136	contig136	Unknown	TAAGTCT	[illegible]
GT	Sense	Sense	-0.018	Detected	0.018	Detected	0.000	-2.287	Comprom	-2.215	Comprom	-2.251	0.000	GT	Sens	contig136	contig136	Transpos	GGGTCC	[illegible]
GT	Sense	Sense	-0.155	Detected	0.155	Detected	0.000	-2.104	Detected	-2.143	Detected	-2.123	0.005	GT	Sens	contig136	contig136	Pol polyp	TAACCAT	[illegible]
GT	Sense	Sense	0.028	Detected	-0.028	Detected	0.000	-1.272	Detected	-1.037	Detected	-1.155	0.011	GT	Sens	contig138	contig138	Unknown	ATAGTGT	[illegible]
GT	Sense	Sense	-0.234	Detected	0.234	Detected	0.000	-4.246	Detected	-1.913	Detected	-3.079	0.123	GT	Sens	contig140	contig140	Cyclic nu	CAGATCA	[illegible]
GT	Sense	Sense	0.008	Detected	-0.008	Detected	0.000	-1.067	Detected	-1.051	Detected	-1.059	0.000	GT	Sens	contig140	contig140	Mutator-li	TTCCCGT	[illegible]
GT	Sense	Sense	-0.448	Detected	0.448	Detected	0.000	-1.616	Detected	-1.790	Detected	-1.703	0.065	GT	Sens	contig140	contig140	Unknown	TTTGGA	[illegible]
GT	Sense	Sense	0.244	Detected	-0.244	Detected	0.000	-1.324	Detected	-1.747	Detected	-1.535	0.042	GT	Sens	contig141	contig141	Phenylala	ATGCGG	[illegible]
GT	Sense	Sense	1.454	Detected	-1.454	Detected	0.000	-0.844	Detected	-2.477	Comprom	-1.661	0.424	GT	Sens	contig141	contig141	Major late	CCTCTC	[illegible]
GT	Sense	Sense	-0.246	Detected	0.246	Detected	0.000	-1.209	Comprom	-1.145	Comprom	-1.177	0.042	GT	Sens	contig141	contig141	Unknown	GGAAAAT	[illegible]
GT	Sense	Sense	-0.260	Detected	0.260	Detected	0.000	-2.609	Detected	-0.823	Detected	-1.716	0.206	GT	Sens	contig142	contig142	Laccase	CAACTAT	[illegible]
GT	Sense	Sense	-0.316	Detected	0.316	Detected	0.000	-1.417	Detected	-0.950	Detected	-1.184	0.095	GT	Sens	contig142	contig142	Pectinest	CTGATT	[illegible]
GT	Sense	Sense	-0.081	Detected	0.081	Detected	0.000	-1.501	Detected	-0.882	Detected	-1.191	0.065	GT	Sens	contig143	contig143	Caffeoyl-	TATGGCT	[illegible]
GT	Sense	Sense	-0.105	Detected	0.105	Detected	0.000	-2.660	Detected	-2.673	Detected	-2.666	0.002	GT	Sens	contig144	contig144	Pol polyp	TGACTTA	[illegible]
GT	Sense	Sense	0.093	Detected	-0.093	Detected	0.000	-1.214	Detected	-0.882	Detected	-1.048	0.032	GT	Sens	contig145	contig145	Unknown	ATAAGAA	[illegible]
GT	Sense	Sense	0.087	Detected	-0.087	Detected	0.000	-1.336	Detected	-1.087	Detected	-1.212	0.015	GT	Sens	contig146	contig146	Unknown	TGGAGT	[illegible]
GT	Sense	Sense	-0.191	Detected	0.191	Detected	0.000	-2.205	Detected	-2.340	Detected	-2.273	0.008	GT	Sens	contig146	contig146	Retroviru	TGCCGC	[illegible]
GT	Sense	Sense	-0.148	Detected	0.148	Detected	0.000	-1.606	Detected	-1.365	Detected	-1.485	0.016	GT	Sens	contig147	contig147	Ethylene-	TCATCGT	[illegible]
GT	Sense	Sense	-0.097	Detected	0.097	Detected	0.000	-1.671	Detected	-1.653	Comprom	-1.662	0.003	GT	Sens	contig147	contig147	Mutator-li	AGAAGA	[illegible]
GT	Sense	Sense	0.015	Detected	-0.015	Detected	0.000	-3.252	Detected	-2.242	Detected	-2.747	0.032	GT	Sens	contig148	contig148	Receptor	ATCTTCA	[illegible]
GT	Sense	Sense	-0.142	Detected	0.142	Detected	0.000	-3.516	Detected	-1.367	Detected	-2.442	0.153	GT	Sens	contig148	contig148	Decarbox	GTGCAA	[illegible]
GT	Sense	Sense	-0.216	Detected	0.216	Detected	0.000	-2.053	Detected	-1.115	Detected	-1.584	0.092	GT	Sens	contig148	contig148	Proteinas	TGGATTA	[illegible]
GT	Sense	Sense	-0.233	Detected	0.233	Detected	0.000	-2.172	Detected	-2.255	Detected	-2.214	0.011	GT	Sens	contig148	contig148	Retroviru	AAATTCT	[illegible]
GT	Sense	Sense	0.161	Detected	-0.161	Detected	0.000	-1.241	Detected	-1.178	Detected	-1.209	0.018	GT	Sens	contig148	contig148	Unknown	ATTGAG	[illegible]
GT	Sense	Sense	-0.160	Detected	0.160	Detected	0.000	-1.747	Detected	-1.950	Detected	-1.849	0.010	GT	Sens	contig148	contig148	Unknown	GACATA	[illegible]
GT	Sense	Sense	0.061	Detected	-0.061	Detected	0.000	-1.378	Detected	-1.116	Detected	-1.247	0.013	GT	Sens	contig149	contig149	Calcium-	ATCTCA	[illegible]
GT	Sense	Sense	0.205	Detected	-0.205	Detected	0.000	-1.107	Detected	-1.220	Detected	-1.163	0.032	GT	Sens	contig150	contig150	Serine/thi	CTGCAGT	[illegible]
GT	Sense	Sense	-0.386	Detected	0.386	Detected	0.000	-1.334	Detected	-0.931	Detected	-1.133	0.122	GT	Sens	contig150	contig150	MADS-bo	AAGAAAT	[illegible]
GT	Sense	Sense	-0.100	Detected	0.100	Detected	0.000	-1.217	Detected	-1.794	Detected	-1.506	0.039	GT	Sens	contig151	contig151	Photosys	GAGGATT	[illegible]
GT	Sense	Sense	-0.008	Detected	0.008	Detected	0.000	-1.163	Detected	-1.538	Detected	-1.350	0.019	GT	Sens	contig151	contig151	Unknown	AAATAAA	[illegible]
GT	Sense	Sense	-0.172	Detected	0.172	Detected	0.000	-1.579	Detected	-1.609	Detected	-1.594	0.012	GT	Sens	contig152	contig152	Unknown	GGACCT	[illegible]
GT	Sense	Sense	-0.104	Detected	0.104	Detected	0.000	-2.041	Comprom	-1.911	Comprom	-1.976	0.004	GT	Sens	contig152	contig152	Unknown	GTCCTC	[illegible]
GT	Sense	Sense	0.065	Detected	-0.065	Detected	0.000	-1.284	Comprom	-1.091	Comprom	-1.187	0.009	GT	Sens	contig153	contig153	Unknown	CTTTCAA	[illegible]
GT	Sense	Sense	-0.171	Detected	0.171	Detected	0.000	-0.899	Detected	-1.426	Detected	-1.163	0.066	GT	Sens	contig154	contig154	Ycf2 (AHF	AATGATT	[illegible]
GT	Sense	Sense	0.145	Detected	-0.145	Detected	0.000	-1.365	Detected	-1.889	Comprom	-1.627	0.032	GT	Sens	contig154	contig154	Mutator-li	GTTACA	[illegible]
GT	Sense	Sense	0.152	Detected	-0.152	Detected	0.000	-2.326	Detected	-1.414	Detected	-1.870	0.060	GT	Sens	contig154	contig154	CBL-inter	CCTGCA	[illegible]
GT	Sense	Sense	-0.103	Detected	0.103	Detected	0.000	-1.347	Detected	-1.472	Detected	-1.410	0.007	GT	Sens	contig154	contig154	Unknown	GATACC	[illegible]
GT	Sense	Sense	-0.177	Detected	0.177	Detected	0.000	-1.354	Detected	-1.115	Detected	-1.235	0.029	GT	Sens	contig154	contig154	Reverse t	AACTCAA	[illegible]
GT	Sense	Sense	-0.273	Detected	0.273	Detected	0.000	-2.153	Detected	-2.282	Detected	-2.218	0.016	GT	Sens	contig154	contig154	%26apos	TACAGAT	[illegible]
GT	Sense	Sense	0.034	Detected	-0.034	Detected	0.000	-1.920	Detected	-2.128	Detected	-2.024	0.003	GT	Sens	contig156	contig156	Gag-Pol p	GATACCA	[illegible]
GT	Sense	Sense	-0.107	Detected	0.107	Detected	0.000	-1.118	Detected	-2.379	Detected	-1.748	0.112	GT	Sens	contig156	contig156	Mutator-li	TAGGGCT	[illegible]
GT	Sense	Sense	0.114	Detected	-0.114	Detected	0.000	-1.483	Detected	-1.893	Detected	-1.688	0.019	GT	Sens	contig157	contig157	Ycf2 (AHF	GGATCTT	[illegible]
GT	Sense	Sense	-0.333	Detected	0.333	Detected	0.000	-1.535	Detected	-1.505	Detected	-1.520	0.045	GT	Sens	contig157	contig157	Unknown	TTATTG	[illegible]
GT	Sense	Sense	0.066	Detected	-0.066	Detected	0.000	-1.418	Detected	-1.011	Detected	-1.215	0.030	GT	Sens	contig158	contig158	Unknown	GTATCCA	[illegible]
GT	Sense	Sense	-0.098	Detected	0.098	Detected	0.000	-2.231	Detected	-1.201	Detected	-1.716	0.082	GT	Sens	contig158	contig158	Caffeoyl-	TTAAGGT	[illegible]
GT	Sense	Sense	-0.507	Detected	0.507	Detected	0.000	-1.564	Detected	-1.999	Detected	-1.782	0.084	GT	Sens	contig159	contig159	Pathogen	TGCAATT	[illegible]
GT	Sense	Sense	0.030	Detected	-0.030	Detected	0.000	-1.645	Detected	-2.735	Comprom	-2.190	0.057	GT	Sens	contig159	contig159	Mutator-li	TGGCATT	[illegible]
GT	Sense	Sense	-0.573	Detected	0.573	Detected	0.000	-2.054	Comprom	-1.543	Comprom	-1.798	0.103	GT	Sens	contig159	contig159	Serine/thi	TATAGGA	[illegible]
GT	Sense	Sense	-0.374	Detected	0.374	Detected	0.000	-1.538	Comprom	-1.212	Comprom	-1.375	0.078	GT	Sens	contig159	contig159	Pol polyp	TTGATAT	[illegible]
GT	Sense	Sense	-0.127	Detected	0.127	Detected	0.000	-0.934	Detected	-2.108	Comprom	-1.521	0.127	GT	Sens	contig160	contig160	Ycf1 (AHF	ACTCCA	[illegible]
GT	Sense	Sense	-0.099	Detected	0.099	Detected	0.000	-3.497	Comprom	-2.400	Comprom	-2.948	0.034	GT	Sens	contig160	contig160	Reverse t	GGGTGT	[illegible]
GT	Sense	Sense	0.147	Detected	-0.147	Detected	0.000	-1.826	Detected	-1.516	Detected	-1.671	0.016	GT	Sens	contig160	contig160	Unknown	AAAGCA	[illegible]
GT	Sense	Sense	-0.160	Detected	0.160	Detected	0.000	-1.990	Detected	-2.089	Detected	-2.039	0.007	GT	Sens	contig161	contig161	Reverse t	TAACTAT	[illegible]
GT	Sense	Sense	-0.463	Detected	0.463	Detected	0.000	-3.359	Detected	-2.332	Detected	-2.845	0.054	GT	Sens	contig161	contig161	Unknown	CCTGTG	[illegible]
GT	Sense	Sense	-0.042	Detected	0.042	Detected	0.000	-2.212	Detected	-2.129	Detected	-2.170	0.001	GT	Sens	contig162	contig162	Polynucl	CTGGGT	[illegible]
GT	Sense	Sense	-0.288	Detected	0.288	Detected	0.000	-1.348	Detected	-0.822	Detected	-1.085	0.109	GT	Sens	contig163	contig163	Unknown	TGTTCTT	[illegible]
GT	Sense	Sense	-0.041	Detected	0.041	Detected	0.000	-2.818	Comprom	-2.189	Comprom	-2.503	0.016	GT	Sens	contig163	contig163	Stearoyl-	AAGAAG	[illegible]
GT	Sense	Sense	-0.131	Detected	0.131	Detected	0.000	-1.860	Detected	-1.086	Detected	-1.473	0.069	GT	Sens	contig163	contig163	Unknown	ACTGAA	[illegible]
GT	Sense	Sense	-0.131	Detected	0.131	Detected	0.000	-1.158	Detected	-1.395	Detected	-1.277	0.019	GT	Sens	contig164	contig164	Ycf2 (AHF	AGGATCA	[illegible]
GT	Sense	Sense	-0.183	Detected	0.183	Detected	0.000	-1.147	Detected	-1.515	Detected	-1.331	0.036	GT	Sens	contig164	contig164	Ycf2 (AHF	CCTGAAT	[illegible]
GT	Sense	Sense	-0.064	Detected	0.064	Detected	0.000	-1.986	Detected	-1.785	Detected	-1.886	0.004	GT	Sens	contig164	contig164	%26apos	GCAGGT	[illegible]
GT	Sense	Sense	-0.190	Detected	0.190	Detected	0.000	-2.489	Detected	-2.146	Detected	-2.317	0.012	GT	Sens	contig165	contig165	Unknown	ACGACC	[illegible]
GT	Sense	Sense	-0.227	Detected	0.227	Detected	0.000	-1.490	Detected	-1.258	Detected	-1.374	0.033	GT	Sens	contig165	contig165	NAC dom	AACAAGT	[illegible]
GT	Sense	Sense	0.818	Detected	-0.818	Detected	0.000	-1.509	Detected	-1.462	Detected	-1.485	0.211	GT	Sens	contig165	contig165	Unknown	GAGACC	[illegible]
GT	Sense	Sense	-0.228	Detected	0.228	Detected	0.000	-2.303	Detected	-1.286	Detected	-1.795	0.085	GT	Sens	contig165	contig165	Unknown	GCATAA	[illegible]
GT	Sense	Sense	-0.050	Detected	0.050	Detected	0.000	-1.368	Detected	-2.000	Detected	-1.684	0.034	GT	Sens	contig166	contig166	NAD(P)H-	CCCTTT	[illegible]
GT	Sense	Sense	0.131	Detected	-0.131	Detected	0.000	-2.187	Detected	-1.475	Detected	-1.831	0.040	GT	Sens	contig166	contig166	Histidine	TAGCAC	[illegible]
GT	Sense	Sense	-0.418	Detected	0.418	Detected	0.000	-3.131	Detected	-1.516	Detected	-2.324	0.125	GT	Sens	contig167	contig167	FAD-bind	TGGGGT	[illegible]
GT	Sense	Sense	-0.169	Detected	0.169	Detected	0.000	-1.903	Detected	-2.068	Detected	-1.986	0.009	GT	Sens	contig168	contig168	Pol polyp	AGAAGA	[illegible]
GT	Sense	Sense	-0.481	Detected	0.481	Detected	0.000	-1.640	Detected	-1.105	Detected	-1.373	0.130	GT	Sens	contig169	contig169	Sulfotran	GAAGAG	[illegible]
GT	Sense	Sense	0.454	Detected	-0.454	Detected	0.000	-1.119	Detected	-1.534	Comprom	-1.327	0.117	GT	Sens	contig169	contig169	Ulp1 prot	AAATGAA	[illegible]
GT	Sense	Sense	-0.245	Detected	0.245	Detected	0.000	-2.207	Detected	-2.362	Detected	-2.285	0.012	GT	Sens	contig170	contig170	Pol polyp	TACAGAT	[illegible]
GT	Sense	Sense	-0.358	Detected	0.358	Detected	0.000	-1.886	Comprom	-0.867	Detected	-1.377	0.157	GT	Sens	contig170	contig170	Laccase	GCCTGC	[illegible]
GT	Sense	Sense	-0.132	Detected	0.132	Detected	0.000	-2.324	Detected	-2.394	Detected	-2.359	0.003	GT	Sens	contig170	contig170	Pol polyp	GTTAGC	[illegible]
GT	Sense	Sense	-0.139	Detected	0.139	Detected	0.000	-1.495	Detected	-1.663	Detected	-1.579	0.010	GT	Sens	contig170	contig170	Retrotran	TGGTGG	[illegible]
GT	Sense	Sense	-0.920	Detected	0.920	Detected	0.000	-0.952	Detected	-1.071	Detected	-1.012	0.387	GT	Sens	contig171	contig171	Acetylorn	TCAGGG	[illegible]
GT	Sense	Sense	-0.047	Detected	0.047	Detected	0.000	-2.105	Comprom	-3.234	Comprom	-2.669	0.042	GT	Sens	contig171	contig171	B3 domai	AACTTGT	[illegible]
GT	Sense	Sense	0.064	Detected	-0.064	Detected	0.000	-2.019	Comprom	-1.587	Comprom	-1.803	0.015	GT	Sens	contig172	contig172	Ulp1 prot	CATTACT	[illegible]
GT	Sense	Sense	-0.174	Detected	0.174	Detected	0.000	-1.194	Detected	-1.757	Detected	-1.475	0.047	GT	Sens	contig173	contig173	Unknown	ACAGCC	[illegible]
GT	Sense	Sense	-0.081	Detected	0.081	Detected	0.000	-2.538	Detected	-2.767	Detected	-2.652	0.003	GT	Sens	contig173	contig173	Pol polyp	TTCTGGT	[illegible]
GT	Sense	Sense	-0.382	Detected	0.382	Detected	0.000	-3.281	Comprom	-2.742	Comprom	-3.011	0.023	GT	Sens	contig174	contig174	Peroxida	ATAAAA	[illegible]
GT	Sense	Sense	-0.476	Detected	0.476	Detected	0.000	-1.935	Detected	-1.162	Detected	-1.549	0.128	GT	Sens	contig174	contig174	Enoyl-Co	TTGTCAT	[illegible]
GT	Sense	Sense	-0.032	Detected	0.032	Detected	0.000	-1.252	Detected	-1.086	Detected	-1.169	0.006	GT	Sens	contig175	contig175	Ulp1 prot	ACAACAT	[illegible]
GT	Sense	Sense	0.355	Detected	-0.355	Detected	0.000	-2.327	Comprom	-3.096	Comprom	-2.712	0.035	GT	Sens	contig175	contig175	Unknown	GCAACAT	[illegible]
GT	Sense	Sense	-0.157	Detected	0.157	Detected	0.000	-1.611	Detected	-1.825	Detected	-1.718	0.012	GT	Sens	contig175	contig175	Unknown	TGTGAC	[illegible]
GT	Sense	Sense	-0.229	Detected	0.229	Detected	0.000	-2.201	Detected	-2.341	Detected	-2.271	0.011	GT	Sens	contig176	contig176	%26apos	ACAGATT	[illegible]
GT	Sense	Sense	0.003	Detected	-0.003	Detected	0.000	-1.851	Comprom	-1.419	Detected	-1.635	0.017	GT	Sens	contig177	contig177	Reverse t	TTTGAT	[illegible]
GT	Sense	Sense	0.212	Detected	-0.212	Detected	0.000	-2.249	Comprom	-0.808	Detected	-1.528	0.179	GT	Sens	contig177	contig177	Unknown	GGTTTT	[illegible]
GT	Sense	Sense	-0.096	Detected	0.096	Detected	0.000	-2.403	Detected	-2.314	Detected	-2.359	0.002	GT	Sens	contig178	contig178	Retrotran	CCTTCA	[illegible]
GT	Sense	Sense	-0.152	Detected	0.152	Detected	0.000	-1.049	Detected	-1.083	Detected	-1.066	0.020	GT	Sens	contig179	contig179	Unknown	TGTCTAT	[illegible]
GT	Sense	Sense	-0.134	Detected	0.134	Detected	0.000	-1.898	Detected	-1.885	Detected	-1.892	0.005	GT	Sens	contig180	contig180	%26apos	TGTATA	[illegible]
GT	Sense	Sense	0.038	Detected	-0.038	Detected	0.000	-1.012	Detected	-1.243	Comprom	-1.128	0.011	GT	Sens	contig180	contig180	Unknown	AGAAAG	[illegible]
GT	Sense	Sense	0.089	Detected	-0.089	Detected	0.000	-2.094	Detected	-1.162	Detected	-1.628	0.075	GT	Sens	contig180	contig180	Caffeoyl-	AGATTAA	[illegible]
GT	Sense	Sense	-0.366	Detected	0.366	Detected	0.000	-2.330	Detected	-1.668	Detected	-1.999	0.056	GT	Sens	contig181	contig181	Xylogluca	TCTTCTT	[illegible]
GT	Sense	Sense	-0.161	Detected	0.161	Detected	0.000	-0.969	Detected	-2.000	Detected	-1.484	0.111	GT	Sens	contig184	contig184	SWIM zin	ATGGTT	[illegible]
GT	Sense	Sense	-0.083	Detected	0.083	Detected	0.000	-4.571	Detected	-2.397	Detected	-3.484	0.086	GT	Sens	contig185	contig185	Polygalac	AAAGAG	[illegible]
GT	Sense	Sense	-0.533	Detected	0.533	Detected	0.000	-1.010	Detected	-1.451	Detected	-1.230	0.166	GT	Sens	contig186	contig186	Aberrant	ATTTGC	[illegible]
GT	Sense	Sense	-0.048	Detected	0.048	Detected	0.000	-1.708	Detected	-1.442	Detected	-1.575	0.008	GT	Sens	contig186	contig186	Pol polyp	TGAGAGT	[illegible]
GT	Sense	Sense	-0.110	Detected	0.110	Detected	0.000	-1.102	Detected	-1.431	Detected	-1.266	0.024	GT	Sens	contig187	contig187	Ycf2 (AHF	TGTGGG	[illegible]
GT	Sense	Sense	-0.440	Detected	0.440	Detected	0.000	-1.787	Detected	-1.217	Detected	-1.502	0.103	GT	Sens	contig188	contig188	Sulfotran	GAAGAG	[illegible]
GT	Sense	Sense	0.006	Detected	-0.006	Detected	0.000	-2.245	Detected	-1.957	Detected	-2.101	0.005	GT	Sens	contig189	contig189	Unknown	TGAATC	[illegible]
GT	Sense	Sense	-0.252	Detected	0.252	Detected	0.000	-1.086	Detected	-1.206	Detected	-1.146	0.047	GT	Sens	contig189	contig189	Major late	GTGTGAT	[illegible]
GT	Sense	Sense	-0.582	Detected	0.582	Detected	0.000	-2.307	Detected	-2.326	Detected	-2.317	0.058	GT	Sens	contig189	contig189	Mutant re	CTCCAA	[illegible]
GT	Sense	Sense	0.218	Detected	-0.218	Detected	0.000	-2.811	Detected	-2.663	Detected	-2.737	0.007	GT	Sens	contig19	contig19	Unknown	ATAGGAT	[illegible]
GT	Sense	Sense	-0.177	Detected	0.177	Detected	0.000	-2.294	Detected	-2.212	Detected	-2.253	0.006	GT	Sens	contig190	contig190	Gag-Pol p	CATTTGA	[illegible]
GT	Sense	Sense	-0.085	Detected	0.085	Detected	0.000	-1.998	Detected	-1.661	Detected	-1.829	0.011	GT	Sens	contig191	contig191	Unknown	TCTTTT	[illegible]
GT	Sense	Sense	-0.108	Detected	0.108	Detected	0.000	-1.664	Detected	-1.655	Detected	-1.659	0.004	GT	Sens	contig192	contig192	Unknown	GGTGGT	[illegible]
GT	Sense	Sense	0.235	Detected	-0.235	Detected	0.000	-3.160	Comprom	-1.405	Comprom	-2.282	0.129	GT	Sens	contig193	contig193	Transpos	CAATGAT	[illegible]
GT	Sense	Sense	-0.509	Detected	0.509	Detected	0.000	-2.188	Detected	-1.647	Detected	-1.918	0.080	GT	Sens	contig194	contig194	Unknown	CAAATGA	[illegible]
GT	Sense	Sense	-0.116	Detected	0.116	Detected	0.000	-0.944	Detected	-1.172	Detected	-1.058	0.023	GT	Sens	contig194	contig194	Ycf2 (AHF	GAAGGCT	[illegible]
GT	Sense	Sense	-0.054	Detected	0.054	Detected	0.000	-1.424	Detected	-1.252	Detected	-1.338	0.006	GT	Sens	contig194	contig194	Gag-Pol p	TCATCTA	[illegible]
GT	Sense	Sense	0.026	Detected	-0.026	Detected	0.000	-1.078	Detected	-1.223	Comprom	-1.150	0.004	GT	Sens	contig195	contig195	Mutator-li	ACAAAAT	[illegible]
GT	Sense	Sense	-0.043	Detected	0.043	Detected	0.000	-2.757	Detected	-2.188	Detected	-2.472	0.013	GT	Sens	contig196	contig196	Unknown	AGGTACA	[illegible]
GT	Sense	Sense	-0.006	Detected	0.006	Detected	0.000	-1.298	Detected	-1.280	Detected	-1.289	0.000	GT	Sens	contig197	contig197	Acetyl-Co	GAACTA	[illegible]
GT	Sense	Sense	-0.004	Detected	0.004	Detected	0.000	-1.178	Detected	-1.074	Detected	-1.126	0.002	GT	Sens	contig198	contig198	Receptor	TTAAACT	[illegible]
GT	Sense	Sense	-0.221	Detected	0.221	Detected	0.000	-1.566	Detected	-1.587	Detected	-1.576	0.019	GT	Sens	contig199	contig199	Unknown	CCTGTG	[illegible]
GT	Sense	Sense	-1.103	Detected	1.103	Detected	0.000	-2.661	Detected	-1.850	Detected	-2.255	0.195	GT	Sens	contig200	contig200	Gibberell	TTTCGC	[illegible]
GT	Sense	Sense	0.084	Detected	-0.084	Detected	0.000	-1.075	Detected	-0.929	Detected	-1.002	0.012	GT	Sens	contig203	contig203	Unknown	TCTTGCT	[illegible]
GT	Sense	Sense	-0.196	Detected	0.196	Detected	0.000	-1.282	Detected	-1.554	Detected	-1.418	0.027	GT	Sens	contig203	contig203	Orf146 pr	TGAAGTT	[illegible]
GT	Sense	Sense	-0.031	Detected	0.031	Detected	0.000	-1.516	Detected	-1.386	Detected	-1.451	0.002	GT	Sens	contig203	contig203	Unknown	ATCGGAT	[illegible]
GT	Sense	Sense	-0.163	Detected	0.163	Detected	0.000	-1.796	Detected	-1.935	Detected	-1.865	0.009	GT	Sens	contig205	contig205	%26apos	GCCAAC	[illegible]
GT	Sense	Sense	-0.197	Detected	0.197	Detected	0.000	-1.805	Detected	-1.790	Detected	-1.797	0.012	GT	Sens	contig205	contig205	%26apos	AATTGT	[illegible]
GT	Sense	Sense	0.217	Detected	-0.217	Detected	0.000	-0.979	Detected	-1.532	Detected	-1.255	0.070	GT	Sens	contig208	contig208	Sulfotran	ACTCAAT	[illegible]
GT	Sense	Sense	0.005	Detected	-0.005	Detected	0.000	-2.214	Detected	-2.330	Detected	-2.272	0.001	GT	Sens	contig210	contig210	Polyprote	AGTGAA	[illegible]
GT	Sense	Sense	-0.166	Detected	0.166	Detected	0.000	-1.336	Detected	-1.350	Detected	-1.343	0.015	GT	Sens	contig210	contig210	Unknown	AAATGA	[illegible]
GT	Sense	Sense	-0.028	Detected	0.028	Detected	0.000	-1.293	Detected	-0.969	Detected	-1.131	0.020	GT	Sens	contig213	contig213	Gag-Pol p	TGATAG	[illegible]
GT	Sense	Sense	-0.279	Detected	0.279	Detected	0.000	-2.424	Detected	-1.542	Detected	-1.983	0.063	GT	Sens	contig213	contig213	Unknown	GAGTCTA	[illegible]
GT	Sense	Sense	-0.211	Detected	0.211	Detected	0.000	-0.968	Detected	-1.412	Detected	-1.190	0.060	GT	Sens	contig213	contig213	NADH del	CTAAAA	[illegible]
GT	Sense	Sense	0.217	Detected	-0.217	Detected	0.000	-1.297	Detected	-1.326	Detected	-1.311	0.026	GT	Sens	contig213	contig213	Ycf2 (AHF	GAAAGA	[illegible]
GT	Sense	Sense	-0.179	Detected	0.179	Detected	0.000	-1.107	Detected	-1.146	Detected	-1.126	0.025	GT	Sens	contig213	contig213	Unknown	TTGTCTA	[illegible]
GT	Sense	Sense	-0.299	Detected	0.299	Detected	0.000	-1.358	Detected	-0.827	Detected	-1.093	0.112	GT	Sens	contig214	contig214	Unknown	ATGGGG	[illegible]
GT	Sense	Sense	-0.462	Detected	0.462	Detected	0.000	-2.396	Detected	-1.656	Detected	-2.026	0.076	GT	Sens	contig215	contig215	Xylogluca	TCTTCTT	[illegible]
GT	Sense	Sense	0.315	Detected	-0.315	Detected	0.000	-3.112	Comprom	-2.929	Comprom	-3.021	0.012	GT	Sens	contig216	contig216	Unknown	TTCACT	[illegible]
GT	Sense	Sense	0.248	Detected	-0.248	Detected	0.000	-1.842	Detected	-1.845	Detected	-1.844	0.018	GT	Sens	contig216	contig216	Mutator-li	AGTATCA	[illegible]
GT	Sense	Sense	-0.107	Detected	0.107	Detected	0.000	-1.302	Detected	-1.015	Detected	-1.159	0.023	GT	Sens	contig216	contig216	Gag-Pol p	CTGGGA	[illegible]
GT	Sense	Sense	0.045	Detected	-0.045	Detected	0.000	-0.998	Detected	-1.005	Detected	-1.002	0.002	GT	Sens	contig216	contig216	Unknown	AAGAGT	[illegible]
GT	Sense	Sense	-0.042	Detected	0.042	Detected	0.000	-1.143	Detected	-1.845	Detected	-1.494	0.052	GT	Sens	contig216	contig216	NAD(P)H-	CTACTAT	[illegible]
GT	Sense	Sense	-0.004	Detected	0.004	Detected	0.000	-2.085	Detected	-1.003	Detected	-1.544	0.104	GT	Sens	contig217	contig217	Unknown	GCTGAAT	[illegible]
GT	Sense	Sense	-0.214	Detected	0.214	Detected	0.000	-1.288	Detected	-0.871	Detected	-1.080	0.069	GT	Sens	contig217	contig217	Pol polyp	ATAGCT	[illegible]
GT	Sense	Sense	-0.299	Detected	0.299	Detected	0.000	-2.801	Detected	-1.562	Detected	-2.181	0.087	GT	Sens	contig217	contig217	Cytochro	ATGGAC	[illegible]
GT	Sense	Sense	-0.511	Detected	0.511	Detected	0.000	-2.344	Detected	-1.512	Detected	-1.928	0.100	GT	Sens	contig218	contig218	Xylogluca	TCTTCTT	[illegible]
GT	Sense	Sense	-0.090	Detected	0.090	Detected	0.000	-1.987	Detected	-1.958	Detected	-1.972	0.002	GT	Sens	contig220	contig220	Pol polyp	TTGACA	[illegible]
GT	Sense	Sense	-0.447	Detected	0.447	Detected	0.000	-1.624	Detected	-1.942	Detected	-1.783	0.064	GT	Sens	contig221	contig221	Unknown	TCAGAG	[illegible]
GT	Sense	Sense	-0.152	Detected	0.152	Detected	0.000	-0.804	Detected	-1.433	Detected	-1.118	0.085	GT	Sens	contig221	contig221	NADH-qui	TTATTTG	[illegible]
GT	Sense	Sense	-0.322	Detected	0.322	Detected	0.000	-1.177	Detected	-1.220	Detected	-1.199	0.066	GT	Sens	contig222	contig222	Trachear	GAACCT	[illegible]
GT	Sense	Sense	0.016	Detected	-0.016	Detected	0.000	-1.212	Detected	-1.332	Detected	-1.272	0.002	GT	Sens	contig223	contig223	Unknown	GGGCAA	[illegible]
GT	Sense	Sense	0.082	Detected	-0.082	Detected	0.000	-1.455	Detected	-1.240	Detected	-1.348	0.010	GT	Sens	contig223	contig223	Transcrip	AGGAGA	[illegible]
GT	Sense	Sense	0.205	Detected	-0.205	Detected	0.000	-1.071	Detected	-2.077	Detected	-1.574	0.101	GT	Sens	contig223	contig223	Serine/thi	ATTCGG	[illegible]
GT	Sense	Sense	0.182	Detected	-0.182	Detected	0.000	-1.554	Detected	-1.373	Detected	-1.464	0.019	GT	Sens	contig223	contig223	Periaxin-l	AGGAAG	[illegible]
GT	Sense	Sense	-0.097	Detected	0.097	Detected	0.000	-2.027	Detected	-2.002	Detected	-2.014	0.002	GT	Sens	contig224	contig224	Unknown	CAGGCA	[illegible]
GT	Sense	Sense	0.192	Detected	-0.192	Detected	0.000	-0.821	Detected	-2.965	Comprom	-1.893	0.224	GT	Sens	contig225	contig225	Serine/thi	AATATTT	[illegible]
GT	Sense	Sense	-0.079	Detected	0.079	Detected	0.000	-2.112	Detected	-2.289	Detected	-2.200	0.003	GT	Sens	contig227	contig227	Pol polyp	ATGTGT	[illegible]
GT	Sense	Sense	0.193	Detected	-0.193	Detected	0.000	-1.515	Detected	-1.196	Detected	-1.356	0.032	GT	Sens	contig228	contig228	Unknown	GAGATC	[illegible]
GT	Sense	Sense	0.442	Detected	-0.442	Detected	0.000	-1.272	Detected	-1.370	Detected	-1.321	0.097	GT	Sens	contig228	contig228	Unknown	ATTGGA	[illegible]
GT	Sense	Sense	-0.125	Detected	0.125	Detected	0.000	-1.630	Comprom	-2.488	Comprom	-2.059	0.044	GT	Sens	contig229	contig229	Unknown	GATGCT	[illegible]
GT	Sense	Sense	-0.260	Detected	0.260	Detected	0.000	-1.617	Detected	-1.821	Detected	-1.719	0.025	GT	Sens	contig229	contig229	Receptor	AATAAAT	[illegible]
GT	Sense	Sense	-0.019	Detected	0.019	Detected	0.000	-1.875	Comprom	-0.953	Comprom	-1.414	0.092	GT	Sens	contig229	contig229	Mutator-li	GGTATT	[illegible]
GT	Sense	Sense	-0.119	Detected	0.119	Detected	0.000	-1.372	Comprom	-1.759	Comprom	-1.566	0.020	GT	Sens	contig229	contig229	Unknown	GGAAGA	[illegible]
GT	Sense	Sense	0.004	Detected	-0.004	Detected	0.000	-1.833	Comprom	-2.218	Comprom	-2.025	0.009	GT	Sens	contig229	contig229	Ulp1 prot	GACCAT	[illegible]
GT	Sense	Sense	-0.161	Detected	0.161	Detected	0.000	-0.876	Detected	-3.168	Comprom	-2.022	0.223	GT	Sens	contig230	contig230	Mutator-li	ATGGTT	[illegible]
GT	Sense	Sense	-0.497	Detected	0.497	Detected	0.000	-0.952	Detected	-1.304	Detected	-1.128	0.166	GT	Sens	contig230	contig230	Trachear	ATTCGGT	[illegible]
GT	Sense	Sense	-0.216	Detected	0.216	Detected	0.000	-2.572	Detected	-2.823	Detected	-2.698	0.008	GT	Sens	contig230	contig230	Unknown	GCTACG	[illegible]
GT	Sense	Sense	-0.153	Detected	0.153	Detected	0.000	-2.462	Detected	-1.040	Detected	-1.751	0.138	GT	Sens	contig230	contig230	CXE carb	GAATAA	[illegible]
GT	Sense	Sense	-0.248	Detected	0.248	Detected	0.000	-2.110	Detected	-2.285	Detected	-2.198	0.014	GT	Sens	contig231	contig231	Pro-Pol p	ATTCAG	[illegible]
GT	Sense	Sense	-0.024	Detected	0.024	Detected	0.000	-1.965	Comprom	-1.773	Comprom	-1.869	0.003	GT	Sens	contig231	contig231	Unknown	TATTGG	[illegible]
GT	Sense	Sense	-0.822	Detected	0.822	Detected	0.000	-2.943	Comprom	-3.315	Comprom	-3.129	0.065	GT	Sens	contig231	contig231	Mutator-li	AGTTAG	[illegible]
GT	Sense	Sense	-0.035	Detected	0.035	Detected	0.000	-1.188	Detected	-1.588	Detected	-1.388	0.021	GT	Sens	contig231	contig231	Unknown	CCAAAG	[illegible]
GT	Sense	Sense	-0.111	Detected	0.111	Detected	0.000	-4.747	Comprom	-1.772	Detected	-3.259	0.160	GT	Sens	contig233	contig233	Alpha-hu	CAGTTAT	[illegible]
GT	Sense	Sense	-0.419	Detected	0.419	Detected	0.000	-1.698	Detected	-1.174	Detected	-1.436	0.101	GT	Sens	contig234	contig234	Decarbox	ATTTCAT	[illegible]
GT	Sense	Sense	-0.165	Detected	0.165	Detected	0.000	-2.593	Detected	-2.749	Detected	-2.671	0.005	GT	Sens	contig235	contig235	Reverse t	ATGAGTT	[illegible]
GT	Sense	Sense	0.083	Detected	-0.083	Detected	0.000	-1.561	Detected	-1.512	Detected	-1.536	0.003	GT	Sens	contig236	contig236	Unknown	AGAAGA	[illegible]
GT	Sense	Sense	-0.258	Detected	0.258	Detected	0.000	-1.376	Detected	-0.848	Detected	-1.112	0.095	GT	Sens	contig237	contig237	Unknown	CTTGTCT	[illegible]
GT	Sense	Sense	-0.035	Detected	0.035	Detected	0.000	-1.264	Detected	-1.504	Detected	-1.384	0.008	GT	Sens	contig238	contig238	Ycf2 (AHF	TTATCA	[illegible]
GT	Sense	Sense	-0.193	Detected	0.193	Detected	0.000	-3.538	Comprom	-3.394	Comprom	-3.466	0.004	GT	Sens	contig238	contig238	Gag polyp	GGAAAC	[illegible]
GT	Sense	Sense	-0.238	Detected	0.238	Detected	0.000	-1.235	Detected	-1.423	Detected	-1.329	0.035	GT	Sens	contig238	contig238	Unknown	ACACGG	[illegible]
GT	Sense	Sense	-0.066	Detected	0.066	Detected	0.000	-1.216	Detected	-1.544	Detected	-1.380	0.016	GT	Sens	contig239	contig239	Mutator-li	ATAGGT	[illegible]
GT	Sense	Sense	0.073	Detected	-0.073	Detected	0.000	-1.307	Detected	-1.264	Detected	-1.286	0.003	GT	Sens	contig239	contig239	Chromod	GAAGTG	[illegible]
GT	Sense	Sense	-0.222	Detected	0.222	Detected	0.000	-1.209	Detected	-1.825	Detected	-1.517	0.057	GT	Sens	contig240	contig240	Reverse t	AGCTAA	[illegible]
GT	Sense	Sense	0.044	Detected	-0.044	Detected	0.000	-1.904	Detected	-2.449	Detected	-2.177	0.016	GT	Sens	contig240	contig240	Transpos	CAAGAT	[illegible]
GT	Sense	Sense	-0.239	Detected	0.239	Detected	0.000	-1.989	Detected	-2.053	Detected	-2.021	0.014	GT	Sens	contig241	contig241	Pol polyp	TCACAA	[illegible]
GT	Sense	Sense	-0.249	Detected	0.249	Detected	0.000	-2.829	Detected	-2.477	Detected	-2.653	0.013	GT	Sens	contig242	contig242	Unknown	TTGTTA	[illegible]
GT	Sense	Sense	-0.391	Detected	0.391	Detected	0.000	-1.551	Detected	-1.072	Detected	-1.311	0.104	GT	Sens	contig244	contig244	Cytochro	CAAGAA	[illegible]
GT	Sense	Sense	-0.233	Detected	0.233	Detected	0.000	-2.193	Detected	-1.812	Detected	-2.002	0.022	GT	Sens	contig245	contig245	Transpos	ACAGCGT	[illegible]
GT	Sense	Sense	-0.264	Detected	0.264	Detected	0.000	-2.011	Detected	-2.158	Detected	-2.084	0.017	GT	Sens	contig245	contig245	Gag-pol (	AGAAAA	[illegible]
GT	Sense	Sense	-0.006	Detected	0.006	Detected	0.000	-2.212	Detected	-1.430	Detected	-1.821	0.043	GT	Sens	contig245	contig245	Unknown	CTCAAG	[illegible]
GT	Sense	Sense	-0.490	Detected	0.490	Detected	0.000	-2.177	Comprom	-2.779	Comprom	-2.478	0.050	GT	Sens	contig247	contig247	Ulp1 prot	CTGAAAT	[illegible]
GT	Sense	Sense	-0.010	Detected	0.010	Detected	0.000	-1.447	Detected	-0.877	Detected	-1.162	0.055	GT	Sens	contig247	contig247	Mutator-li	TGGTGAT	[illegible]
GT	Sense	Sense	-0.151	Detected	0.151	Detected	0.000	-2.318	Detected	-2.336	Detected	-2.327	0.004	GT	Sens	contig248	contig248	Polyprote	CTTGTGT	[illegible]
GT	Sense	Sense	-0.093	Detected	0.093	Detected	0.000	-2.901	Detected	-3.786	Comprom	-3.344	0.018	GT	Sens	contig248	contig248	Pol polyp	CCATAT	[illegible]
GT	Sense	Sense	0.407	Detected	-0.407	Detected	0.000	-0.965	Detected	-2.074	Comprom	-1.520	0.158	GT	Sens	contig248	contig248	Unknown	GACAGAT	[illegible]
GT	Sense	Sense	-0.069	Detected	0.069	Detected	0.000	-1.991	Detected	-2.259	Comprom	-2.125	0.005	GT	Sens	contig249	contig249	Unknown	GGATATA	[illegible]
GT	Sense	Sense	-0.328	Detected	0.328	Detected	0.000	-1.287	Detected	-1.582	Detected	-1.435	0.058	GT	Sens	contig249	contig249	Os03g08	GGAAGG	[illegible]
GT	Sense	Sense	-0.145	Detected	0.145	Detected	0.000	-1.268	Detected	-1.491	Detected	-1.380	0.017	GT	Sens	contig250	contig250	NAD(P)H-	ATACAAT	[illegible]
GT	Sense	Sense	-0.114	Detected	0.114	Detected	0.000	-1.349	Detected	-0.970	Detected	-1.160	0.035	GT	Sens	contig250	contig250	Caffeoyl-	TAGCAG	[illegible]
GT	Sense	Sense	-0.176	Detected	0.176	Detected	0.000	-2.458	Detected	-2.218	Detected	-2.338	0.008	GT	Sens	contig251	contig251	Unknown	CGACCCT	[illegible]
GT	Sense	Sense	-0.232	Detected	0.232	Detected	0.000	-2.427	Detected	-2.656	Detected	-2.542	0.010	GT	Sens	contig251	contig251	Unknown	AGACAA	[illegible]
GT	Sense	Sense	0.171	Detected	-0.171	Detected	0.000	-1.972	Detected	-1.613	Detected	-1.792	0.019	GT	Sens	contig252	contig252	Histidine	CACTATC	[illegible]
GT	Sense	Sense	0.097	Detected	-0.097	Detected	0.000	-2.119	Detected	-1.507	Detected	-1.813	0.030	GT	Sens	contig253	contig253	6-phosph	GTGATAT	[illegible]
GT	Sense	Sense	-0.144	Detected	0.144	Detected	0.000	-2.143	Comprom	-1.778	Comprom	-1.961	0.014	GT	Sens	contig253	contig253	Laccase	TTGCCT	[illegible]
GT	Sense	Sense	-0.655	Detected	0.655	Detected	0.000	-1.595	Detected	-1.880	Detected	-1.738	0.122	GT	Sens	contig253	contig253	Unknown	AAGAAAT	[illegible]
GT	Sense	Sense	-0.004	Detected	0.004	Detected	0.000	-2.805	Detected	-1.452	Detected	-2.129	0.088	GT	Sens	contig255	contig255	Decarbox	AGAAAA	[illegible]
GT	Sense	Sense	-0.169	Detected	0.169	Detected	0.000	-1.666	Detected	-1.239	Detected	-1.453	0.033	GT	Sens	contig256	contig256	Cyclic nu	TTACCT	[illegible]
GT	Sense	Sense	-0.050	Detected	0.050	Detected	0.000	-1.622	Detected	-0.929	Detected	-1.276	0.068	GT	Sens	contig256	contig256	Homeobo	ACTCAAT	[illegible]
GT	Sense	Sense	0.038	Detected	-0.038	Detected	0.000	-1.521	Detected	-3.670	Comprom	-2.596	0.137	GT	Sens	contig256	contig256	Polyprote	CGGGAA	[illegible]
GT	Sense	Sense	0.148	Detected	-0.148	Detected	0.000	-0.953	Detected	-1.666	Detected	-1.309	0.077	GT	Sens	contig257	contig257	Unknown	ATGAGA	[illegible]
GT	Sense	Sense	-0.572	Detected	0.572	Detected	0.000	-4.946	Detected	-1.530	Detected	-3.238	0.214	GT	Sens	contig257	contig257	Alpha-hu	CACTCAT	[illegible]
GT	Sense	Sense	-0.119	Detected	0.119	Detected	0.000	-2.262	Detected	-1.792	Detected	-2.027	0.016	GT	Sens	contig257	contig257	Unknown	GAAAAG	[illegible]
GT	Sense	Sense	-0.114	Detected	0.114	Detected	0.000	-1.581	Detected	-1.261	Detected	-1.421	0.019	GT	Sens	contig258	contig258	Tyramine	GGTGAA	[illegible]
GT	Sense	Sense	-0.022	Detected	0.022	Detected	0.000	-2.110	Comprom	-2.397	Comprom	-2.253	0.004	GT	Sens	contig259	contig259	Mutator-li	TGTGGC	[illegible]
GT	Sense	Sense	0.020	Detected	-0.020	Detected	0.000	-1.303	Detected	-2.054	Detected	-1.679	0.047	GT	Sens	contig259	contig259	NAD(P)H-	ATCGAAT	[illegible]
GT	Sense	Sense	0.175	Detected	-0.175	Detected	0.000	-1.347	Detected	-1.663	Comprom	-1.505	0.024	GT	Sens	contig262	contig262	Mutator-li	GAAGAG	[illegible]
GT	Sense	Sense	-0.012	Detected	0.012	Detected	0.000	-1.643	Detected	-0.915	Detected	-1.279	0.072	GT	Sens	contig263	contig263	Mutator-li	GACATGT	[illegible]
GT	Sense	Sense	-0.725	Detected	0.725	Detected	0.000	-1.682	Detected	-1.290	Detected	-1.486	0.187	GT	Sens	contig264	contig264	Laccase	TTCGATA	[illegible]
GT	Sense	Sense	-0.171	Detected	0.171	Detected	0.000	-1.669	Detected	-1.597	Detected	-1.633	0.011	GT	Sens	contig267	contig267	Unknown	CTGAGG	[illegible]
GT	Sense	Sense	-0.187	Detected	0.187	Detected	0.000	-2.002	Detected	-2.052	Detected	-2.027	0.009	GT	Sens	contig267	contig267	Pol polyp	CCGCGA	[illegible]
GT	Sense	Sense	0.044	Detected	-0.044	Detected	0.000	-3.632	Detected	-1.363	Detected	-2.497	0.159	GT	Sens	contig267	contig267	Omega-6	GTTGTAT	[illegible]
GT	Sense	Sense	-0.320	Detected	0.320	Detected	0.000	-1.174	Detected	-0.933	Detected	-1.054	0.091	GT	Sens	contig267	contig267	L-lactate	AACAAGT	[illegible]
GT	Sense	Sense	-0.136	Detected	0.136	Detected	0.000	-2.618	Detected	-1.559	Detected	-2.089	0.062	GT	Sens	contig268	contig268	Xylogluca	GCAGAT	[illegible]
GT	Sense	Sense	-0.043	Detected	0.043	Detected	0.000	-2.462	Detected	-2.759	Detected	-2.610	0.004	GT	Sens	contig268	contig268	Pol polyp	CAGTACT	[illegible]
GT	Sense	Sense	-0.217	Detected	0.217	Detected	0.000	-1.588	Comprom	-1.428	Comprom	-1.508	0.023	GT	Sens	contig268	contig268	Ulp1 pept	CACAACA	[illegible]
GT	Sense	Sense	-0.368	Detected	0.368	Detected	0.000	-2.007	Comprom	-0.905	Detected	-1.456	0.159	GT	Sens	contig268	contig268	Thioester	ACAAACA	[illegible]
GT	Sense	Sense	0.026	Detected	-0.026	Detected	0.000	-1.879	Detected	-2.154	Detected	-2.017	0.005	GT	Sens	contig268	contig268	Unknown	GGTCTTT	[illegible]
GT	Sense	Sense	-0.147	Detected	0.147	Detected	0.000	-1.866	Detected	-1.974	Detected	-1.920	0.007	GT	Sens	contig27	contig27	Retrotran	AAATCT	[illegible]
GT	Sense	Sense	-0.204	Detected	0.204	Detected	0.000	-2.084	Detected	-1.708	Detected	-1.896	0.021	GT	Sens	contig271	contig271	Fibroblas	GCAGGC	[illegible]
GT	Sense	Sense	-0.052	Detected	0.052	Detected	0.000	-1.929	Detected	-1.213	Detected	-1.571	0.049	GT	Sens	contig272	contig272	Unknown	GCTTCA	[illegible]
GT	Sense	Sense	-0.487	Detected	0.487	Detected	0.000	-3.246	Detected	-2.114	Detected	-2.680	0.070	GT	Sens	contig272	contig272	Unknown	CTGTGAT	[illegible]
GT	Sense	Sense	-0.097	Detected	0.097	Detected	0.000	-2.667	Detected	-3.082	Detected	-2.875	0.006	GT	Sens	contig272	contig272	Pol polyp	CTATTCA	[illegible]
GT	Sense	Sense	0.308	Detected	-0.308	Detected	0.000	-2.069	Detected	-1.643	Detected	-1.856	0.038	GT	Sens	contig273	contig273	Histidine	GAGCAA	[illegible]

GT	Sense	Sense	-0.029	Detected	0.029	Detected	0.000	-1.813	Detected	-1.860	Detected	-1.836	0.000	GT	Sens	[illegible]
GT	Sense	Sense	-0.230	Detected	0.230	Detected	0.000	-1.863	Detected	-2.135	Detected	-1.999	0.017	GT	Sens	[illegible]
GT	Sense	Sense	-0.105	Detected	0.105	Detected	0.000	-1.329	Detected	-0.928	Detected	-1.128	0.038	GT	Sens	[illegible]
GT	Sense	Sense	-0.323	Detected	0.323	Detected	0.000	-1.667	Comprom	-1.126	Comprom	-1.397	0.080	GT	Sens	[illegible]
GT	Sense	Sense	0.383	Detected	-0.383	Detected	0.000	-1.223	Detected	-1.244	Detected	-1.233	0.085	GT	Sens	[illegible]
GT	Sense	Sense	-0.186	Detected	0.186	Detected	0.000	-1.482	Detected	-1.598	Detected	-1.540	0.016	GT	Sens	[illegible]
GT	Sense	Sense	-0.404	Detected	0.404	Detected	0.000	-1.243	Comprom	-2.952	Comprom	-2.098	0.157	GT	Sens	[illegible]
GT	Sense	Sense	-0.212	Detected	0.212	Detected	0.000	-2.578	Detected	-2.617	Detected	-2.598	0.007	GT	Sens	[illegible]
GT	Sense	Sense	-0.108	Detected	0.108	Detected	0.000	-1.501	Detected	-1.707	Detected	-1.604	0.009	GT	Sens	[illegible]
GT	Sense	Sense	-0.084	Detected	0.084	Detected	0.000	-1.735	Detected	-1.431	Detected	-1.583	0.012	GT	Sens	[illegible]
GT	Sense	Sense	0.234	Detected	-0.234	Detected	0.000	-1.006	Detected	-1.190	Detected	-1.098	0.049	GT	Sens	[illegible]
GT	Sense	Sense	0.202	Detected	-0.202	Detected	0.000	-2.083	Comprom	-2.159	Comprom	-2.121	0.009	GT	Sens	[illegible]
GT	Sense	Sense	-0.070	Detected	0.070	Detected	0.000	-1.035	Detected	-1.035	Detected	-1.035	0.005	GT	Sens	[illegible]
GT	Sense	Sense	-0.163	Detected	0.163	Detected	0.000	-0.969	Detected	-1.619	Detected	-1.294	0.071	GT	Sens	[illegible]
GT	Sense	Sense	-0.150	Detected	0.150	Detected	0.000	-1.698	Detected	-2.011	Comprom	-1.854	0.013	GT	Sens	[illegible]
GT	Sense	Sense	0.528	Detected	-0.528	Detected	0.000	-0.962	Detected	-1.117	Detected	-1.040	0.191	GT	Sens	[illegible]
GT	Sense	Sense	0.093	Detected	-0.093	Detected	0.000	-0.888	Detected	-1.797	Detected	-1.343	0.102	GT	Sens	[illegible]
GT	Sense	Sense	-0.110	Detected	0.110	Detected	0.000	-1.140	Detected	-0.956	Detected	-1.048	0.018	GT	Sens	[illegible]
GT	Sense	Sense	-0.152	Detected	0.152	Detected	0.000	-2.561	Detected	-2.717	Detected	-2.639	0.004	GT	Sens	[illegible]
GT	Sense	Sense	-0.225	Detected	0.225	Detected	0.000	-2.346	Detected	-2.503	Detected	-2.425	0.010	GT	Sens	[illegible]
GT	Sense	Sense	0.002	Detected	-0.002	Detected	0.000	-1.199	Detected	-1.445	Detected	-1.322	0.009	GT	Sens	[illegible]
GT	Sense	Sense	-0.452	Detected	0.452	Detected	0.000	-1.936	Detected	-2.043	Detected	-1.989	0.049	GT	Sens	[illegible]
GT	Sense	Sense	-0.499	Detected	0.499	Detected	0.000	-4.418	Detected	-1.456	Detected	-2.937	0.201	GT	Sens	[illegible]
GT	Sense	Sense	0.359	Detected	-0.359	Detected	0.000	-1.338	Detected	-1.215	Detected	-1.277	0.073	GT	Sens	[illegible]
GT	Sense	Sense	-0.130	Detected	0.130	Detected	0.000	-2.406	Detected	-2.602	Detected	-2.504	0.004	GT	Sens	[illegible]
GT	Sense	Sense	0.100	Detected	-0.100	Detected	0.000	-1.114	Detected	-1.174	Detected	-1.144	0.008	GT	Sens	[illegible]
GT	Sense	Sense	-0.359	Detected	0.359	Detected	0.000	-1.773	Detected	-1.903	Detected	-1.838	0.037	GT	Sens	[illegible]
GT	Sense	Sense	-0.094	Detected	0.094	Detected	0.000	-2.102	Detected	-2.124	Detected	-2.113	0.002	GT	Sens	[illegible]
GT	Sense	Sense	0.098	Detected	-0.098	Detected	0.000	-2.802	Detected	-1.813	Detected	-2.308	0.045	GT	Sens	[illegible]
GT	Sense	Sense	-1.068	Detected	1.068	Detected	0.000	-3.295	Detected	-1.577	Detected	-2.436	0.217	GT	Sens	[illegible]
GT	Sense	Sense	0.232	Detected	-0.232	Detected	0.000	-1.323	Detected	-1.227	Detected	-1.275	0.033	GT	Sens	[illegible]
GT	Sense	Sense	-0.158	Detected	0.158	Detected	0.000	-0.883	Detected	-1.761	Detected	-1.322	0.105	GT	Sens	[illegible]
GT	Sense	Sense	-0.207	Detected	0.207	Detected	0.000	-1.142	Detected	-1.531	Detected	-1.337	0.042	GT	Sens	[illegible]
GT	Sense	Sense	0.119	Detected	-0.119	Detected	0.000	-1.788	Detected	-0.802	Detected	-1.295	0.125	GT	Sens	[illegible]
GT	Sense	Sense	0.106	Detected	-0.106	Detected	0.000	-1.020	Detected	-1.049	Detected	-1.034	0.011	GT	Sens	[illegible]
GT	Sense	Sense	-0.211	Detected	0.211	Detected	0.000	-4.343	Comprom	-1.948	Comprom	-3.145	0.123	GT	Sens	[illegible]
GT	Sense	Sense	-0.219	Detected	0.219	Detected	0.000	-2.143	Detected	-2.308	Detected	-2.225	0.011	GT	Sens	[illegible]
GT	Sense	Sense	-0.052	Detected	0.052	Detected	0.000	-1.625	Detected	-2.371	Detected	-1.998	0.034	GT	Sens	[illegible]
GT	Sense	Sense	0.245	Detected	-0.245	Detected	0.000	-1.329	Detected	-1.381	Detected	-1.355	0.031	GT	Sens	[illegible]
GT	Sense	Sense	-0.199	Detected	0.199	Detected	0.000	-2.255	Detected	-2.527	Detected	-2.391	0.010	GT	Sens	[illegible]
GT	Sense	Sense	-0.264	Detected	0.264	Detected	0.000	-1.173	Comprom	-0.916	Comprom	-1.044	0.071	GT	Sens	[illegible]
GT	Sense	Sense	-0.250	Detected	0.250	Detected	0.000	-1.436	Detected	-0.977	Detected	-1.206	0.071	GT	Sens	[illegible]
GT	Sense	Sense	-0.170	Detected	0.170	Detected	0.000	-1.534	Detected	-1.846	Detected	-1.690	0.018	GT	Sens	[illegible]
GT	Sense	Sense	-0.106	Detected	0.106	Detected	0.000	-3.506	Comprom	-3.834	Comprom	-3.670	0.003	GT	Sens	[illegible]
GT	Sense	Sense	-0.097	Detected	0.097	Detected	0.000	-2.522	Detected	-2.443	Detected	-2.483	0.002	GT	Sens	[illegible]
GT	Sense	Sense	0.126	Detected	-0.126	Detected	0.000	-1.438	Detected	-1.286	Detected	-1.362	0.012	GT	Sens	[illegible]
GT	Sense	Sense	-0.103	Detected	0.103	Detected	0.000	-2.951	Detected	-2.899	Detected	-2.925	0.001	GT	Sens	[illegible]
GT	Sense	Sense	-0.227	Detected	0.227	Detected	0.000	-2.067	Detected	-1.705	Detected	-1.886	0.023	GT	Sens	[illegible]
GT	Sense	Sense	-0.054	Detected	0.054	Detected	0.000	-2.818	Comprom	-1.991	Detected	-2.404	0.029	GT	Sens	[illegible]
GT	Sense	Sense	0.060	Detected	-0.060	Detected	0.000	-1.426	Detected	-1.357	Detected	-1.391	0.002	GT	Sens	[illegible]
GT	Sense	Sense	-0.162	Detected	0.162	Detected	0.000	-1.746	Detected	-1.456	Detected	-1.601	0.018	GT	Sens	[illegible]
GT	Sense	Sense	-0.157	Detected	0.157	Detected	0.000	-2.387	Detected	-2.540	Detected	-2.464	0.005	GT	Sens	[illegible]
GT	Sense	Sense	-0.172	Detected	0.172	Detected	0.000	-1.977	Detected	-2.162	Detected	-2.069	0.009	GT	Sens	[illegible]
GT	Sense	Sense	-0.330	Detected	0.330	Detected	0.000	-0.943	Detected	-1.137	Detected	-1.040	0.094	GT	Sens	[illegible]
GT	Sense	Sense	-0.291	Detected	0.291	Detected	0.000	-3.544	Detected	-1.477	Detected	-2.510	0.144	GT	Sens	[illegible]
GT	Sense	Sense	-0.224	Detected	0.224	Detected	0.000	-0.976	Detected	-1.257	Detected	-1.116	0.052	GT	Sens	[illegible]
GT	Sense	Sense	0.048	Detected	-0.048	Detected	0.000	-1.283	Detected	-0.852	Detected	-1.067	0.040	GT	Sens	[illegible]
GT	Sense	Sense	0.156	Detected	-0.156	Detected	0.000	-1.880	Detected	-2.578	Comprom	-2.229	0.028	GT	Sens	[illegible]
GT	Sense	Sense	-0.297	Detected	0.297	Detected	0.000	-2.557	Detected	-0.846	Detected	-1.701	0.201	GT	Sens	[illegible]
GT	Sense	Sense	-0.048	Detected	0.048	Detected	0.000	-2.356	Detected	-2.500	Detected	-2.428	0.001	GT	Sens	[illegible]
GT	Sense	Sense	-0.180	Detected	0.180	Detected	0.000	-1.436	Detected	-1.371	Detected	-1.404	0.017	GT	Sens	[illegible]
GT	Sense	Sense	-0.142	Detected	0.142	Detected	0.000	-1.849	Detected	-1.878	Detected	-1.864	0.006	GT	Sens	[illegible]
GT	Sense	Sense	-0.152	Detected	0.152	Detected	0.000	-1.842	Detected	-1.873	Detected	-1.857	0.007	GT	Sens	[illegible]
GT	Sense	Sense	-0.343	Detected	0.343	Detected	0.000	-1.588	Detected	-0.870	Detected	-1.229	0.132	GT	Sens	[illegible]
GT	Sense	Sense	-0.377	Detected	0.377	Detected	0.000	-1.923	Comprom	-1.574	Comprom	-1.749	0.052	GT	Sens	[illegible]
GT	Sense	Sense	-0.417	Detected	0.417	Detected	0.000	-1.675	Comprom	-1.489	Comprom	-1.582	0.066	GT	Sens	[illegible]
GT	Sense	Sense	-0.170	Detected	0.170	Detected	0.000	-1.137	Detected	-1.152	Detected	-1.145	0.021	GT	Sens	[illegible]
GT	Sense	Sense	0.339	Detected	-0.339	Detected	0.000	-0.898	Detected	-1.244	Detected	-1.071	0.107	GT	Sens	[illegible]
GT	Sense	Sense	0.409	Detected	-0.409	Detected	0.000	-2.043	Comprom	-1.525	Comprom	-1.784	0.066	GT	Sens	[illegible]
GT	Sense	Sense	-0.532	Detected	0.532	Detected	0.000	-4.295	Comprom	-1.147	Detected	-2.721	0.243	GT	Sens	[illegible]
GT	Sense	Sense	-0.295	Detected	0.295	Detected	0.000	-0.862	Detected	-1.163	Detected	-1.013	0.093	GT	Sens	[illegible]
GT	Sense	Sense	-0.185	Detected	0.185	Detected	0.000	-1.265	Detected	-1.469	Detected	-1.367	0.023	GT	Sens	[illegible]
GT	Sense	Sense	-0.102	Detected	0.102	Detected	0.000	-1.136	Detected	-1.327	Detected	-1.232	0.013	GT	Sens	[illegible]
GT	Sense	Sense	-0.131	Detected	0.131	Detected	0.000	-1.697	Detected	-1.705	Detected	-1.701	0.006	GT	Sens	[illegible]
GT	Sense	Sense	-0.352	Detected	0.352	Detected	0.000	-1.325	Detected	-0.860	Detected	-1.093	0.122	GT	Sens	[illegible]
GT	Sense	Sense	-0.152	Detected	0.152	Detected	0.000	-2.596	Detected	-2.762	Detected	-2.679	0.004	GT	Sens	[illegible]
GT	Sense	Sense	-0.164	Detected	0.164	Detected	0.000	-1.777	Detected	-1.171	Detected	-1.474	0.051	GT	Sens	[illegible]
GT	Sense	Sense	-0.075	Detected	0.075	Detected	0.000	-2.614	Detected	-1.300	Detected	-1.957	0.098	GT	Sens	[illegible]
GT	Sense	Sense	-0.136	Detected	0.136	Detected	0.000	-2.347	Detected	-2.429	Detected	-2.388	0.004	GT	Sens	[illegible]
GT	Sense	Sense	-0.128	Detected	0.128	Detected	0.000	-2.128	Detected	-2.574	Detected	-2.351	0.012	GT	Sens	[illegible]
GT	Sense	Sense	0.098	Detected	-0.098	Detected	0.000	-1.617	Detected	-1.087	Detected	-1.352	0.041	GT	Sens	[illegible]
GT	Sense	Sense	-0.179	Detected	0.179	Detected	0.000	-1.362	Detected	-1.464	Detected	-1.413	0.017	GT	Sens	[illegible]
GT	Sense	Sense	-0.184	Detected	0.184	Detected	0.000	-2.474	Detected	-2.394	Detected	-2.434	0.006	GT	Sens	[illegible]
GT	Sense	Sense	-0.188	Detected	0.188	Detected	0.000	-1.302	Detected	-2.099	Detected	-1.701	0.061	GT	Sens	[illegible]
GT	Sense	Sense	0.173	Detected	-0.173	Detected	0.000	-1.263	Detected	-1.065	Detected	-1.164	0.028	GT	Sens	[illegible]
GT	Sense	Sense	-0.036	Detected	0.036	Detected	0.000	-1.552	Detected	-1.576	Detected	-1.564	0.001	GT	Sens	[illegible]
GT	Sense	Sense	-0.675	Detected	0.675	Detected	0.000	-2.344	Detected	-1.596	Detected	-1.970	0.125	GT	Sens	[illegible]
GT	Sense	Sense	-0.537	Detected	0.537	Detected	0.000	-1.518	Detected	-0.995	Detected	-1.256	0.170	GT	Sens	[illegible]
GT	Sense	Sense	-0.169	Detected	0.169	Detected	0.000	-2.480	Detected	-2.408	Detected	-2.444	0.005	GT	Sens	[illegible]
GT	Sense	Sense	-0.126	Detected	0.126	Detected	0.000	-2.746	Detected	-2.204	Detected	-2.475	0.014	GT	Sens	[illegible]
GT	Sense	Sense	-0.062	Detected	0.062	Detected	0.000	-1.160	Detected	-1.189	Detected	-1.174	0.003	GT	Sens	[illegible]
GT	Sense	Sense	-0.500	Detected	0.500	Detected	0.000	-1.383	Detected	-1.056	Detected	-1.219	0.146	GT	Sens	[illegible]
GT	Sense	Sense	0.079	Detected	-0.079	Detected	0.000	-1.102	Detected	-1.205	Detected	-1.154	0.007	GT	Sens	[illegible]
GT	Sense	Sense	0.125	Detected	-0.125	Detected	0.000	-1.305	Detected	-2.361	Comprom	-1.833	0.078	GT	Sens	[illegible]
GT	Sense	Sense	0.026	Detected	-0.026	Detected	0.000	-1.067	Detected	-1.101	Detected	-1.084	0.001	GT	Sens	[illegible]
GT	Sense	Sense	0.045	Detected	-0.045	Detected	0.000	-1.261	Detected	-0.938	Detected	-1.099	0.023	GT	Sens	[illegible]
GT	Sense	Sense	-0.114	Detected	0.114	Detected	0.000	-2.153	Detected	-1.841	Detected	-1.997	0.009	GT	Sens	[illegible]
GT	Sense	Sense	0.060	Detected	-0.060	Detected	0.000	-1.040	Detected	-1.283	Detected	-1.161	0.013	GT	Sens	[illegible]
GT	Sense	Sense	-0.031	Detected	0.031	Detected	0.000	-1.419	Comprom	-2.084	Comprom	-1.751	0.034	GT	Sens	[illegible]
GT	Sense	Sense	0.274	Detected	-0.274	Detected	0.000	-3.201	Comprom	-2.900	Comprom	-3.051	0.010	GT	Sens	[illegible]
GT	Sense	Sense	-0.074	Detected	0.074	Detected	0.000	-2.071	Detected	-1.921	Detected	-1.996	0.003	GT	Sens	[illegible]
GT	Sense	Sense	0.136	Detected	-0.136	Detected	0.000	-3.437	Comprom	-1.749	Detected	-2.593	0.094	GT	Sens	[illegible]
GT	Sense	Sense	-0.306	Detected	0.306	Detected	0.000	-1.157	Detected	-2.792	Detected	-1.974	0.152	GT	Sens	[illegible]
GT	Sense	Sense	-0.442	Detected	0.442	Detected	0.000	-1.218	Detected	-0.803	Detected	-1.011	0.175	GT	Sens	[illegible]
GT	Sense	Sense	0.474	Detected	-0.474	Detected	0.000	-0.989	Detected	-1.832	Comprom	-1.411	0.156	GT	Sens	[illegible]
GT	Sense	Sense	-0.700	Detected	0.700	Detected	0.000	-1.749	Detected	-1.012	Detected	-1.381	0.223	GT	Sens	[illegible]
GT	Sense	Sense	-0.015	Detected	0.015	Detected	0.000	-2.194	Detected	-1.623	Detected	-1.909	0.022	GT	Sens	[illegible]
GT	Sense	Sense	-0.287	Detected	0.287	Detected	0.000	-2.030	Comprom	-1.984	Comprom	-2.007	0.020	GT	Sens	[illegible]
GT	Sense	Sense	-0.015	Detected	0.015	Detected	0.000	-1.971	Detected	-2.095	Detected	-2.033	0.001	GT	Sens	[illegible]
GT	Sense	Sense	-0.072	Detected	0.072	Detected	0.000	-1.184	Detected	-1.568	Detected	-1.376	0.022	GT	Sens	[illegible]
GT	Sense	Sense	-0.062	Detected	0.062	Detected	0.000	-1.184	Detected	-1.863	Detected	-1.524	0.048	GT	Sens	[illegible]
GT	Sense	Sense	-0.101	Detected	0.101	Detected	0.000	-2.377	Detected	-2.604	Detected	-2.491	0.004	GT	Sens	[illegible]
GT	Sense	Sense	-0.207	Detected	0.207	Detected	0.000	-2.036	Detected	-0.832	Detected	-1.434	0.153	GT	Sens	[illegible]
GT	Sense	Sense	0.247	Detected	-0.247	Detected	0.000	-1.900	Detected	-1.365	Detected	-1.633	0.046	GT	Sens	[illegible]
GT	Sense	Sense	0.043	Detected	-0.043	Detected	0.000	-1.025	Detected	-1.908	Comprom	-1.466	0.080	GT	Sens	[illegible]
GT	Sense	Sense	-0.873	Detected	0.873	Detected	0.000	-4.096	Detected	-1.804	Detected	-2.950	0.177	GT	Sens	[illegible]
GT	Sense	Sense	-0.111	Detected	0.111	Detected	0.000	-2.157	Detected	-2.116	Detected	-2.136	0.003	GT	Sens	[illegible]
GT	Sense	Sense	0.024	Detected	-0.024	Detected	0.000	-2.381	Comprom	-1.457	Detected	-1.919	0.053	GT	Sens	[illegible]
GT	Sense	Sense	-0.178	Detected	0.178	Detected	0.000	-2.250	Detected	-2.512	Detected	-2.381	0.009	GT	Sens	[illegible]
GT	Sense	Sense	0.164	Detected	-0.164	Detected	0.000	-2.372	Comprom	-2.181	Comprom	-2.277	0.007	GT	Sens	[illegible]
GT	Sense	Sense	-0.200	Detected	0.200	Detected	0.000	-2.191	Detected	-2.362	Detected	-2.276	0.009	GT	Sens	[illegible]
GT	Sense	Sense	-0.195	Detected	0.195	Detected	0.000	-1.988	Detected	-2.019	Detected	-2.004	0.009	GT	Sens	[illegible]
GT	Sense	Sense	-0.239	Detected	0.239	Detected	0.000	-1.410	Detected	-1.791	Detected	-1.601	0.035	GT	Sens	[illegible]
GT	Sense	Sense	-0.009	Detected	0.009	Detected	0.000	-2.291	Comprom	-3.194	Comprom	-2.743	0.026	GT	Sens	[illegible]
GT	Sense	Sense	0.218	Detected	-0.218	Detected	0.000	-1.356	Detected	-1.395	Detected	-1.376	0.024	GT	Sens	[illegible]
GT	Sense	Sense	0.373	Detected	-0.373	Detected	0.000	-1.722	Detected	-2.006	Detected	-1.864	0.043	GT	Sens	[illegible]
GT	Sense	Sense	0.198	Detected	-0.198	Detected	0.000	-1.537	Detected	-1.740	Comprom	-1.638	0.018	GT	Sens	[illegible]
GT	Sense	Sense	-0.192	Detected	0.192	Detected	0.000	-1.779	Comprom	-1.170	Comprom	-1.474	0.055	GT	Sens	[illegible]
GT	Sense	Sense	0.072	Detected	-0.072	Detected	0.000	-2.110	Detected	-1.358	Detected	-1.734	0.045	GT	Sens	[illegible]
GT	Sense	Sense	-0.047	Detected	0.047	Detected	0.000	-1.803	Comprom	-2.148	Comprom	-1.975	0.008	GT	Sens	[illegible]
GT	Sense	Sense	0.261	Detected	-0.261	Detected	0.000	-2.401	Comprom	-1.741	Comprom	-2.071	0.039	GT	Sens	[illegible]
GT	Sense	Sense	-0.109	Detected	0.109	Detected	0.000	-1.118	Detected	-1.126	Detected	-1.122	0.009	GT	Sens	[illegible]
GT	Sense	Sense	0.211	Detected	-0.211	Detected	0.000	-1.313	Detected	-0.802	Detected	-1.058	0.086	GT	Sens	[illegible]
GT	Sense	Sense	-0.270	Detected	0.270	Detected	0.000	-1.206	Detected	-1.328	Detected	-1.267	0.044	GT	Sens	[illegible]
GT	Sense	Sense	-0.177	Detected	0.177	Detected	0.000	-1.461	Detected	-1.119	Detected	-1.290	0.034	GT	Sens	[illegible]
GT	Sense	Sense	-0.789	Detected	0.789	Detected	0.000	-3.183	Comprom	-2.033	Detected	-2.608	0.116	GT	Sens	[illegible]
GT	Sense	Sense	-0.123	Detected	0.123	Detected	0.000	-1.755	Comprom	-1.944	Comprom	-1.850	0.007	GT	Sens	[illegible]
GT	Sense	Sense	-0.227	Detected	0.227	Detected	0.000	-1.518	Detected	-1.212	Detected	-1.365	0.038	GT	Sens	[illegible]
GT	Sense	Sense	0.019	Detected	-0.019	Detected	0.000	-0.957	Detected	-1.553	Detected	-1.255	0.052	GT	Sens	[illegible]
GT	Sense	Sense	0.015	Detected	-0.015	Detected	0.000	-0.976	Detected	-1.060	Detected	-1.018	0.002	GT	Sens	[illegible]
GT	Sense	Sense	-0.094	Detected	0.094	Detected	0.000	-2.407	Detected	-1.734	Detected	-2.070	0.027	GT	Sens	[illegible]
GT	Sense	Sense	-0.120	Detected	0.120	Detected	0.000	-2.419	Detected	-2.420	Detected	-2.419	0.002	GT	Sens	[illegible]
GT	Sense	Sense	-0.610	Detected	0.610	Detected	0.000	-2.573	Detected	-0.836	Detected	-1.705	0.249	GT	Sens	[illegible]
GT	Sense	Sense	-0.173	Detected	0.173	Detected	0.000	-2.519	Detected	-2.691	Detected	-2.605	0.005	GT	Sens	[illegible]
GT	Sense	Sense	0.116	Detected	-0.116	Detected	0.000	-1.536	Detected	-2.155	Detected	-1.846	0.031	GT	Sens	[illegible]
GT	Sense	Sense	-0.153	Detected	0.153	Detected	0.000	-2.147	Detected	-2.244	Detected	-2.195	0.005	GT	Sens	[illegible]
GT	Sense	Sense	-0.196	Detected	0.196	Detected	0.000	-1.189	Detected	-2.046	Detected	-1.617	0.075	GT	Sens	[illegible]
GT	Sense	Sense	-0.194	Detected	0.194	Detected	0.000	-2.541	Detected	-1.075	Detected	-1.808	0.140	GT	Sens	[illegible]
GT	Sense	Sense	-0.083	Detected	0.083	Detected	0.000	-1.259	Detected	-1.265	Detected	-1.262	0.004	GT	Sens	[illegible]
GT	Sense	Sense	-1.683	Detected	1.683	Detected	0.000	-2.659	Detected	-0.802	Detected	-1.731	0.463	GT	Sens	[illegible]
GT	Sense	Sense	-0.165	Detected	0.165	Detected	0.000	-1.520	Detected	-1.421	Detected	-1.470	0.013	GT	Sens	[illegible]
GT	Sense	Sense	-0.162	Detected	0.162	Detected	0.000	-1.120	Detected	-2.056	Detected	-1.588	0.085	GT	Sens	[illegible]
GT	Sense	Sense	0.051	Detected	-0.051	Detected	0.000	-1.110	Detected	-1.009	Detected	-1.059	0.005	GT	Sens	[illegible]
GT	Sense	Sense	-0.304	Detected	0.304	Detected	0.000	-1.974	Detected	-1.024	Detected	-1.499	0.117	GT	Sens	[illegible]
GT	Sense	Sense	-0.097	Detected	0.097	Detected	0.000	-2.118	Detected	-2.283	Detected	-2.200	0.003	GT	Sens	[illegible]
GT	Sense	Sense	-0.154	Detected	0.154	Detected	0.000	-1.112	Detected	-1.136	Detected	-1.124	0.018	GT	Sens	[illegible]
GT	Sense	Sense	-0.174	Detected	0.174	Detected	0.000	-1.672	Detected	-1.602	Detected	-1.637	0.012	GT	Sens	[illegible]
GT	Sense	Sense	-0.188	Detected	0.188	Detected	0.000	-1.896	Detected	-1.961	Detected	-1.928	0.010	GT	Sens	[illegible]
GT	Sense	Sense	-0.958	Detected	0.958	Detected	0.000	-2.381	Comprom	-1.678	Detected	-2.029	0.185	GT	Sens	[illegible]
GT	Sense	Sense	0.081	Detected	-0.081	Detected	0.000	-1.055	Detected	-1.202	Detected	-1.129	0.009	GT	Sens	[illegible]
GT	Sense	Sense	-0.148	Detected	0.148	Detected	0.000	-1.495	Detected	-1.157	Detected	-1.326	0.028	GT	Sens	[illegible]
GT	Sense	Sense	-0.221	Detected	0.221	Detected	0.000	-2.881	Comprom	-2.746	Comprom	-2.813	0.007	GT	Sens	[illegible]
GT	Sense	Sense	-0.066	Detected	0.066	Detected	0.000	-2.695	Detected	-2.464	Detected	-2.579	0.003	GT	Sens	[illegible]
GT	Sense	Sense	-0.352	Detected	0.352	Detected	0.000	-1.822	Detected	-1.027	Detected	-1.424	0.115	GT	Sens	[illegible]
GT	Sense	Sense	0.156	Detected	-0.156	Detected	0.000	-1.742	Comprom	-3.245	Comprom	-2.493	0.083	GT	Sens	[illegible]
GT	Sense	Sense	-0.177	Detected	0.177	Detected	0.000	-0.978	Detected	-1.436	Detected	-1.207	0.053	GT	Sens	[illegible]
GT	Sense	Sense	-0.226	Detected	0.226	Detected	0.000	-1.050	Detected	-0.973	Detected	-1.012	0.048	GT	Sens	[illegible]
GT	Sense	Sense	0.014	Detected	-0.014	Detected	0.000	-1.475	Detected	-1.481	Detected	-1.478	0.000	GT	Sens	[illegible]
GT	Sense	Sense	-0.376	Detected	0.376	Detected	0.000	-1.954	Detected	-1.418	Detected	-1.686	0.067	GT	Sens	[illegible]
GT	Sense	Sense	0.064	Detected	-0.064	Detected	0.000	-1.636	Detected	-0.982	Detected	-1.309	0.059	GT	Sens	[illegible]
GT	Sense	Sense	0.009	Detected	-0.009	Detected	0.000	-0.868	Detected	-1.250	Detected	-1.059	0.031	GT	Sens	[illegible]
GT	Sense	Sense	-0.220	Detected	0.220	Detected	0.000	-1.227	Detected	-1.987	Detected	-1.607	0.067	GT	Sens	[illegible]
GT	Sense	Sense	-0.752	Detected	0.752	Detected	0.000	-2.199	Comprom	-2.090	Comprom	-2.145	0.105	GT	Sens	[illegible]
GT	Sense	Sense	-0.112	Detected	0.112	Detected	0.000	-1.842	Detected	-1.884	Detected	-1.863	0.004	GT	Sens	[illegible]
GT	Sense	Sense	-0.077	Detected	0.077	Detected	0.000	-2.004	Detected	-2.368	Detected	-2.186	0.008	GT	Sens	[illegible]
GT	Sense	Sense	0.846	Detected	-0.846	Detected	0.000	-2.289	Detected	-2.738	Detected	-2.513	0.103	GT	Sens	[illegible]
GT	Sense	Sense	0.268	Detected	-0.268	Detected	0.000	-3.620	Comprom	-2.743	Comprom	-3.181	0.025	GT	Sens	[illegible]
GT	Sense	Sense	-0.024	Detected	0.024	Detected	0.000	-1.932	Detected	-2.379	Detected	-2.155	0.011	GT	Sens	[illegible]
GT	Sense	Sense	-0.285	Detected	0.285	Detected	0.000	-2.006	Detected	-2.193	Detected	-2.099	0.020	GT	Sens	[illegible]
GT	Sense	Sense	-0.063	Detected	0.063	Detected	0.000	-2.843	Detected	-2.680	Detected	-2.762	0.001	GT	Sens	[illegible]
GT	Sense	Sense	-0.435	Detected	0.435	Detected	0.000	-1.172	Detected	-0.968	Detected	-1.070	0.139	GT	Sens	[illegible]
GT	Sense	Sense	-0.304	Detected	0.304	Detected	0.000	-3.196	Comprom	-1.468	Detected	-2.332	0.126	GT	Sens	[illegible]
GT	Sense	Sense	-0.193	Detected	0.193	Detected	0.000	-2.113	Detected	-2.208	Detected	-2.161	0.008	GT	Sens	[illegible]
GT	Sense	Sense	0.203	Detected	-0.203	Detected	0.000	-3.207	Detected	-2.879	Detected	-3.043	0.007	GT	Sens	[illegible]
GT	Sense	Sense	-0.585	Detected	0.585	Detected	0.000	-1.597	Detected	-1.153	Detected	-1.375	0.159	GT	Sens	[illegible]
GT	Sense	Sense	0.397	Detected	-0.397	Detected	0.000	-1.831	Comprom	-1.595	Comprom	-1.713	0.054	GT	Sens	[illegible]
GT	Sense	Sense	-0.553	Detected	0.553	Detected	0.000	-2.456	Detected	-0.885	Detected	-1.671	0.224	GT	Sens	[illegible]
GT	Sense	Sense	-0.104	Detected	0.104	Detected	0.000	-3.495	Detected	-2.005	Detected	-2.750	0.067	GT	Sens	[illegible]
GT	Sense	Sense	-0.046	Detected	0.046	Detected	0.000	-3.781	Detected	-2.142	Detected	-2.961	0.069	GT	Sens	[illegible]

GO ID	GO ACCESSION	GO Term	p-value	Count in Selection	% Count in Selection	Count in Total	% Count in Total
9396	GO:0016168	chlorophyll binding	0.00	12	2.169982	46	0.13284431
22409	GO:0046906	tetrapyrrole binding	0.00	33	5.96745	596	1.7212002
9266	GO:0016020	membrane	0.00	93	16.81736	3741	10.803708
3872	GO:0005506	iron ion binding	0.00	22	3.9783	563	1.6258988
11931	GO:0020037	heme binding	0.00	21	3.7974684	550	1.5883559
3827	GO:0005372	water transmembrane trans	0.00	7	1.2658228	81	0.23392151
8652	GO:0015250	water channel activity	0.00	7	1.2658228	81	0.23392151
4927	GO:0006833	water transport	0.00	7	1.2658228	81	0.23392151
18429	GO:0042044	fluid transport	0.00	7	1.2658228	81	0.23392151
2868	GO:0004062	aryl sulfotransferase activity	0.00	3	0.5424955	15	0.043318797
5702	GO:0008146	sulfotransferase activity	0.00	5	0.9041591	54	0.15594767
2986	GO:0004252	serine-type endopeptidase a	0.00	8	1.4466546	144	0.41586044
13082	GO:0030599	pectinesterase activity	0.00	9	1.6274865	178	0.5140497
9832	GO:0016782	transferase activity, transfe	0.01	5	0.9041591	74	0.21370606
6613	GO:0009250	glucan biosynthetic process	0.01	2	0.36166364	8	0.023103358
5766	GO:0008236	serine-type peptidase activi	0.01	9	1.6274865	214	0.6180149
10103	GO:0017171	serine hydrolase activity	0.01	9	1.6274865	220	0.63534236
4215	GO:0005975	carbohydrate metabolic pro	0.01	26	4.7016273	990	2.8590407
11776	GO:0019825	oxygen binding	0.01	13	2.3508136	389	1.1234008
6216	GO:0008810	cellulase activity	0.01	3	0.5424955	30	0.086637594
11827	GO:0019898|GO:0030396	extrinsic to membrane	0.01	3	0.5424955	30	0.086637594
3156	GO:0004497	monooxygenase activity	0.02	7	1.2658228	172	0.49672222
16081	GO:0033692	cellular polysaccharide bios	0.02	2	0.36166364	15	0.043318797
206	GO:0000271	polysaccharide biosynthetic	0.03	2	0.36166364	16	0.046206716
12533	GO:0022838	substrate-specific channel a	0.03	8	1.4466546	221	0.63823026
9771	GO:0016709	oxidoreductase activity, act	0.03	4	0.7233273	71	0.2050423
4909	GO:0006810|GO:0015457|	transport	0.03	49	8.86076	2315	6.6855345
25529	GO:0051234	establishment of localizatio	0.03	49	8.86076	2315	6.6855345
25476	GO:0051179	localization	0.03	49	8.86076	2319	6.697086
31498	GO:0080123	jasmonate-amino synthetas	0.03	2	0.36166364	17	0.049094636
8660	GO:0015267|GO:0015249|	channel activity	0.03	8	1.4466546	226	0.6526699
12515	GO:0022803	passive transmembrane tra	0.03	8	1.4466546	226	0.6526699
3013	GO:0004328|GO:0034566	formamidase activity	0.03	1	0.18083182	2	0.00577584
4429	GO:0006223	uracil salvage	0.03	1	0.18083182	2	0.00577584
4907	GO:0006808	regulation of nitrogen utiliza	0.03	1	0.18083182	2	0.00577584
6080	GO:0008655	pyrimidine-containing comp	0.03	1	0.18083182	2	0.00577584
6828	GO:0009540	zeaxanthin epoxidase [over	0.03	1	0.18083182	2	0.00577584
7504	GO:0010277|GO:0046407	chlorophyllide a oxygenase	0.03	1	0.18083182	2	0.00577584
7532	GO:0010307	acetylglutamate kinase regu	0.03	1	0.18083182	2	0.00577584
8612	GO:0015204|GO:0015287	urea transmembrane transp	0.03	1	0.18083182	2	0.00577584
9106	GO:0015840	urea transport	0.03	1	0.18083182	2	0.00577584
9766	GO:0016703	oxidoreductase activity, act	0.03	1	0.18083182	2	0.00577584
11797	GO:0019860	uracil metabolic process	0.03	1	0.18083182	2	0.00577584
14849	GO:0032441	pheophorbide a oxygenase	0.03	1	0.18083182	2	0.00577584
19204	GO:0042887	amide transmembrane tran	0.03	1	0.18083182	2	0.00577584
19407	GO:0043100	pyrimidine base salvage	0.03	1	0.18083182	2	0.00577584
23921	GO:0048529	magnesium-protoporphyrin	0.03	1	0.18083182	2	0.00577584
30280	GO:0071918	urea transmembrane transp	0.03	1	0.18083182	2	0.00577584
31514	GO:0080139	borate efflux transmembran	0.03	1	0.18083182	2	0.00577584
3723	GO:0005215|GO:0005478	transporter activity	0.03	40	7.233273	1844	5.325324
12547	GO:0022857|GO:0005386|	transmembrane transporter	0.04	33	5.96745	1483	4.282785
3877	GO:0005516	calmodulin binding	0.04	8	1.4466546	236	0.6815491
26931	GO:0055085	transmembrane transport	0.04	34	6.148282	1548	4.4705
9767	GO:0016705	oxidoreductase activity, act	0.04	10	1.8083183	329	0.95012563
9784	GO:0016723	oxidoreductase activity, oxi	0.04	2	0.36166364	20	0.0577584
225	GO:0000293	ferric-chelate reductase act	0.04	2	0.36166364	20	0.0577584
28930	GO:0070566	adenylyltransferase activity	0.04	3	0.5424955	48	0.13862015
7074	GO:0009815	1-aminocyclopropane-1-ca	0.04	3	0.5424955	49	0.14150807
31421	GO:0080044	quercetin 7-O-glucosyltrans	0.04	3	0.5424955	49	0.14150807
7166	GO:0009914	hormone transport	0.05	3	0.5424955	50	0.14439599
27891	GO:0060918	auxin transport	0.05	3	0.5424955	50	0.14439599
31536	GO:0080161	auxin transmembrane trans	0.05	3	0.5424955	50	0.14439599
12574	GO:0022892	substrate-specific transport	0.05	32	5.786618	1463	4.2250266
3134	GO:0004474	malate synthase activity	0.05	1	0.18083182	3	0.00866376
4319	GO:0006081	cellular aldehyde metabolic	0.05	1	0.18083182	3	0.00866376
4330	GO:0006097	glyoxylate cycle	0.05	1	0.18083182	3	0.00866376
21936	GO:0046409	p-coumarate 3-hydroxylase	0.05	1	0.18083182	3	0.00866376
22011	GO:0046487	glyoxylate metabolic proces	0.05	1	0.18083182	3	0.00866376
24613	GO:0050294	steroid sulfotransferase act	0.05	1	0.18083182	3	0.00866376
4954	GO:0006869	lipid transport	0.05	7	1.2658228	208	0.6006873
8085	GO:0010876	lipid localization	0.05	7	1.2658228	208	0.6006873
26845	GO:0052689	carboxylic ester hydrolase a	0.05	14	2.5316455	538	1.5537009
4311	GO:0006073	cellular glucan metabolic pr	0.06	2	0.36166364	24	0.06931008
20293	GO:0044042	glucan metabolic process	0.06	2	0.36166364	24	0.06931008
2969	GO:0004167|GO:0048059	dopachrome isomerase act	0.06	1	0.18083182	4	0.011551679
3057	GO:0004373	glycogen (starch) synthase	0.06	1	0.18083182	4	0.011551679
3320	GO:0004697|GO:0004701	protein kinase C activity	0.06	1	0.18083182	4	0.011551679
3452	GO:0004845	uracil phosphoribosyltransf	0.06	1	0.18083182	4	0.011551679
3834	GO:0005395	eye pigment precursor trans	0.06	1	0.18083182	4	0.011551679
4218	GO:0005978	glycogen biosynthetic proce	0.06	1	0.18083182	4	0.011551679
4413	GO:0006206	pyrimidine base metabolic	0.06	1	0.18083182	4	0.011551679
5942	GO:0008456	alpha-N-acetylgalactosamin	0.06	1	0.18083182	4	0.011551679
7538	GO:0010313	phytochrome binding	0.06	1	0.18083182	4	0.011551679
8637	GO:0015231	5-formyltetrahydrofolate tra	0.06	1	0.18083182	4	0.011551679
9147	GO:0015885	5-formyltetrahydrofolate tra	0.06	1	0.18083182	4	0.011551679
9351	GO:0016119	carotene metabolic process	0.06	1	0.18083182	4	0.011551679
9352	GO:0016120	carotene biosynthetic proce	0.06	1	0.18083182	4	0.011551679
9790	GO:0016730	oxidoreductase activity, act	0.06	1	0.18083182	4	0.011551679
9819	GO:0016767	geranylgeranyl-diphosphate	0.06	1	0.18083182	4	0.011551679
11857	GO:0019932	second-messenger-mediate	0.06	1	0.18083182	4	0.011551679
17811	GO:0035445	borate transmembrane tran	0.06	1	0.18083182	4	0.011551679
18575	GO:0042214	terpene metabolic process	0.06	1	0.18083182	4	0.011551679
18729	GO:0042380	hydroxymethylbutenyl pyro	0.06	1	0.18083182	4	0.011551679
21791	GO:0046246	terpene biosynthetic proces	0.06	1	0.18083182	4	0.011551679
22222	GO:0046713	borate transport	0.06	1	0.18083182	4	0.011551679
22224	GO:0046715	borate transmembrane tran	0.06	1	0.18083182	4	0.011551679
22366	GO:0046863|GO:0018236	ribulose-1,5-bisphosphate c	0.06	1	0.18083182	4	0.011551679
22408	GO:0046905	phytoene synthase activity	0.06	1	0.18083182	4	0.011551679
23493	GO:0048015	phosphatidylinositol-mediat	0.06	1	0.18083182	4	0.011551679
23495	GO:0048017	inositol lipid-mediated signa	0.06	1	0.18083182	4	0.011551679
25055	GO:0050750	low-density lipoprotein part	0.06	1	0.18083182	4	0.011551679
28689	GO:0070325	lipoprotein particle receptor	0.06	1	0.18083182	4	0.011551679
8673	GO:0015291|GO:0015290|	secondary active transmem	0.06	11	1.98915	407	1.1753833
8028	GO:0010817	regulation of hormone level	0.07	3	0.5424955	58	0.16749935
12573	GO:0022891	substrate-specific transmem	0.07	27	4.882459	1241	3.5839086
9541	GO:0016413	O-acetyltransferase activity	0.07	2	0.36166364	27	0.077973835
25379	GO:0051082	unfolded protein binding	0.07	4	0.7233273	98	0.28301615
2975	GO:0004175|GO:0016809	endopeptidase activity	0.07	9	1.6274865	320	0.9241344
6940	GO:0009672	auxin:hydrogen symporter a	0.07	2	0.36166364	28	0.080861755
8863	GO:0015562	efflux transmembrane trans	0.07	2	0.36166364	28	0.080861755
11210	GO:0019207	kinase regulator activity	0.08	3	0.5424955	62	0.17905103
2962	GO:0004160	dihydroxy-acid dehydratase	0.08	1	0.18083182	5	0.0144396
5989	GO:0008517	folic acid transporter activit	0.08	1	0.18083182	5	0.0144396
9146	GO:0015884	folic acid transport	0.08	1	0.18083182	5	0.0144396
2870	GO:0004064	arylesterase activity	0.08	2	0.36166364	30	0.086637594
20514	GO:0044264	cellular polysaccharide met	0.09	2	0.36166364	31	0.08952551
19253	GO:0042936	dipeptide transporter activit	0.09	4	0.7233273	105	0.3032316
19255	GO:0042938	dipeptide transport	0.09	4	0.7233273	105	0.3032316
3845	GO:0005452	inorganic anion exchanger	0.09	1	0.18083182	6	0.01732752
3940	GO:0005615	extracellular space	0.09	1	0.18083182	6	0.01732752
4118	GO:0005852	eukaryotic translation initiat	0.09	1	0.18083182	6	0.01732752
5559	GO:0007602	phototransduction	0.09	1	0.18083182	6	0.01732752
5775	GO:0008251	tRNA-specific adenosine de	0.09	1	0.18083182	6	0.01732752

6868	GO:0009585	red, far-red light phototrans	0.09	1	0.18083182	6	0.01732752	
7135	GO:0009883	red or far-red light photored	0.09	1	0.18083182	6	0.01732752	
7255	GO:0010017	red or far-red light signaling	0.09	1	0.18083182	6	0.01732752	
9248	GO:0015994	chlorophyll metabolic proce	0.09	1	0.18083182	6	0.01732752	
9249	GO:0015995	chlorophyll biosynthetic pro	0.09	1	0.18083182	6	0.01732752	
10045	GO:0017091	AU-rich element binding	0.09	1	0.18083182	6	0.01732752	
19203	GO:0042886	amide transport	0.09	1	0.18083182	6	0.01732752	
22188	GO:0046677	response to antibiotic	0.09	1	0.18083182	6	0.01732752	
29853	GO:0071489	cellular response to red or f	0.09	1	0.18083182	6	0.01732752	
5	GO:0000007	low-affinity zinc ion transmo	0.09	1	0.18083182	6	0.01732752	
19125	GO:0042802	identical protein binding	0.10	16	2.893309	697	2.01288	
2632	GO:0003682	chromatin binding	0.10	2	0.36166364	33	0.09530135	
9722	GO:0016641	oxidoreductase activity, act	0.10	3	0.5424955	69	0.19926646	
8517	GO:0015103	inorganic anion transmemb	0.10	5	0.9041591	154	0.44473964	
6004	GO:0008536	Ran GTPase binding	0.10	2	0.36166364	34	0.09818927	
3021	GO:0004337	geranyltranstransferase act	0.11	1	0.18083182	7	0.020215439	
19401	GO:0043094	cellular metabolic compour	0.11	1	0.18083182	7	0.020215439	
21107	GO:0045505	dynein intermediate chain b	0.11	1	0.18083182	7	0.020215439	
21146	GO:0045544	gibberellin 20-oxidase activ	0.11	2	0.36166364	35	0.10107719	
11202	GO:0019199	transmembrane receptor pr	0.11	12	2.169982	504	1.4555116	
8739	GO:0015385|GO:0015502	sodium:hydrogen antiporter	0.11	2	0.36166364	36	0.10396511	
9185	GO:0015925	galactosidase activity	0.11	2	0.36166364	36	0.10396511	
12626	GO:0030029	actin filament-based proces	0.11	2	0.36166364	36	0.10396511	
12633	GO:0030036	actin cytoskeleton organiza	0.11	2	0.36166364	36	0.10396511	
2816	GO:0004000	adenosine deaminase activ	0.12	1	0.18083182	8	0.023103358	
3803	GO:0005344|GO:0015033	oxygen transporter activity	0.12	1	0.18083182	8	0.023103358	
4961	GO:0006879	cellular iron ion homeostas	0.12	1	0.18083182	8	0.023103358	
7843	GO:0010628	positive regulation of gene	0.12	1	0.18083182	8	0.023103358	
8940	GO:0015669	gas transport	0.12	1	0.18083182	8	0.023103358	
8942	GO:0015671	oxygen transport	0.12	1	0.18083182	8	0.023103358	
11196	GO:0019187	beta-1,4-mannosyltransfera	0.12	1	0.18083182	8	0.023103358	
13262	GO:0030795|GO:0045546	jasmonate O-methyltransfe	0.12	1	0.18083182	8	0.023103358	
21461	GO:0045893|GO:0043193|	positive regulation of transc	0.12	1	0.18083182	8	0.023103358	
22702	GO:0047209	coniferyl-alcohol glucosyltra	0.12	1	0.18083182	8	0.023103358	
26035	GO:0051753	mannan synthase activity	0.12	1	0.18083182	8	0.023103358	
26918	GO:0055072	iron ion homeostasis	0.12	1	0.18083182	8	0.023103358	
5066	GO:0007010	cytoskeleton organization	0.12	2	0.36166364	38	0.10974095	
9637	GO:0016538|GO:0003751|	cyclin-dependent protein ki	0.12	2	0.36166364	38	0.10974095	
22479	GO:0046983	protein dimerization activity	0.13	12	2.169982	517	1.4930545	
3475	GO:0004871|GO:0005062|	signal transducer activity	0.13	17	3.074141	787	2.2727928	
27063	GO:0060089	molecular transducer activi	0.13	17	3.074141	787	2.2727928	
19126	GO:0042803	protein homodimerization a	0.13	7	1.2658228	265	0.7652988	
2837	GO:0004028	3-chloroallyl aldehyde dehy	0.13	1	0.18083182	9	0.025991278	
5735	GO:0008198	ferrous iron binding	0.13	1	0.18083182	9	0.025991278	
7225	GO:0009978	allene oxide synthase activi	0.13	1	0.18083182	9	0.025991278	
9769	GO:0016707	gibberellin 3-beta-dioxygen	0.13	1	0.18083182	9	0.025991278	
19342	GO:0043027	caspase inhibitor activity	0.13	1	0.18083182	9	0.025991278	
19343	GO:0043028	caspase regulator activity	0.13	1	0.18083182	9	0.025991278	
19458	GO:0043154|GO:0001719	negative regulation of casp	0.13	1	0.18083182	9	0.025991278	
19579	GO:0043281|GO:0043026	regulation of caspase activi	0.13	1	0.18083182	9	0.025991278	
21104	GO:0045502	dynein binding	0.13	1	0.18083182	9	0.025991278	
28365	GO:0065008	regulation of biological qua	0.14	8	1.4466546	316	0.9125827	
17617	GO:0035251	UDP-glucosyltransferase ac	0.14	6	1.084991	218	0.62956655	
18674	GO:0042325	regulation of phosphorylatio	0.14	3	0.5424955	81	0.23392151	
22049	GO:0046527	glucosyltransferase activity	0.14	7	1.2658228	270	0.77973837	
5019	GO:0006952|GO:0002217|	defense response	0.14	6	1.084991	221	0.63823026	
8675	GO:0015293	symporter activity	0.14	5	0.9041591	173	0.49961013	
3203	GO:0004557	alpha-galactosidase activity	0.15	1	0.18083182	10	0.0288792	
6275	GO:0008878	glucose-1-phosphate adeny	0.15	1	0.18083182	10	0.0288792	
6479	GO:0009112	nucleobase metabolic proce	0.15	1	0.18083182	10	0.0288792	
8651	GO:0015248	sterol transporter activity	0.15	1	0.18083182	10	0.0288792	
9178	GO:0015918	sterol transport	0.15	1	0.18083182	10	0.0288792	
10072	GO:0017127	cholesterol transporter activ	0.15	1	0.18083182	10	0.0288792	
12859	GO:0030301	cholesterol transport	0.15	1	0.18083182	10	0.0288792	
19443	GO:0043139	5'-3' DNA helicase activity	0.15	1	0.18083182	10	0.0288792	
19445	GO:0043141|GO:0008722	ATP-dependent 5'-3' DNA h	0.15	1	0.18083182	10	0.0288792	
26069	GO:0051787	misfolded protein binding	0.15	1	0.18083182	10	0.0288792	
29578	GO:0071214	cellular response to abiotic	0.15	1	0.18083182	10	0.0288792	
29842	GO:0071478	cellular response to radiatio	0.15	1	0.18083182	10	0.0288792	
29846	GO:0071482	cellular response to light st	0.15	1	0.18083182	10	0.0288792	
25095	GO:0050790	regulation of catalytic activi	0.15	11	1.98915	480	1.3862015	
25998	GO:0051716	cellular response to stimulu	0.15	23	4.159132	1138	3.2864528	
9768	GO:0016706	oxidoreductase activity, act	0.15	5	0.9041591	175	0.505386	
25195	GO:0050896|GO:0051869	response to stimulus	0.15	36	6.509946	1881	5.432177	
8967	GO:0015698	inorganic anion transport	0.15	5	0.9041591	176	0.5082739	
28378	GO:0070011	peptidase activity, acting or	0.15	12	2.169982	537	1.550813	
11222	GO:0019220	regulation of phosphate me	0.15	3	0.5424955	85	0.24547319	
25471	GO:0051174	regulation of phosphorus m	0.15	3	0.5424955	85	0.24547319	
28366	GO:0065009	regulation of molecular func	0.16	11	1.98915	484	1.3977532	
3904	GO:0005576	extracellular region	0.16	5	0.9041591	178	0.5140497	
31431	GO:0080054	low affinity nitrate transmer	0.16	2	0.36166364	45	0.1299564	
3416	GO:0004806	triglyceride lipase activity	0.16	1	0.18083182	11	0.03176712	
11152	GO:0019139|GO:0046420	cytokinin dehydrogenase ac	0.16	1	0.18083182	11	0.03176712	
9719	GO:0016638	oxidoreductase activity, act	0.17	3	0.5424955	89	0.25702485	
2843	GO:0004034	aldose 1-epimerase activity	0.18	1	0.18083182	12	0.03465504	
3049	GO:0004365	glyceraldehyde-3-phosphat	0.18	1	0.18083182	12	0.03465504	
3386	GO:0004773	steryl-sulfatase activity	0.18	1	0.18083182	12	0.03465504	
4217	GO:0005977	glycogen metabolic process	0.18	1	0.18083182	12	0.03465504	
4344	GO:0006112	energy reserve metabolic p	0.18	1	0.18083182	12	0.03465504	
5965	GO:0008484	sulfuric ester hydrolase acti	0.18	1	0.18083182	12	0.03465504	
6336	GO:0008943	glyceraldehyde-3-phosphat	0.18	1	0.18083182	12	0.03465504	
6826	GO:0009538	photosystem I reaction cen	0.18	1	0.18083182	12	0.03465504	
6864	GO:0009581	detection of external stimul	0.18	1	0.18083182	12	0.03465504	
6865	GO:0009582	detection of abiotic stimulus	0.18	1	0.18083182	12	0.03465504	
6866	GO:0009583	detection of light stimulus	0.18	1	0.18083182	12	0.03465504	
8748	GO:0015398	high affinity secondary activ	0.18	1	0.18083182	12	0.03465504	
9775	GO:0016713	oxidoreductase activity, act	0.18	1	0.18083182	12	0.03465504	
10743	GO:0018685|GO:0008393	alkane 1-monooxygenase a	0.18	1	0.18083182	12	0.03465504	
20615	GO:0044421	extracellular region part	0.18	1	0.18083182	12	0.03465504	
21503	GO:0045935	positive regulation of nucle	0.18	1	0.18083182	12	0.03465504	
25470	GO:0051173	positive regulation of nitrog	0.18	1	0.18083182	12	0.03465504	
25546	GO:0051254	positive regulation of RNA	0.18	1	0.18083182	12	0.03465504	
25890	GO:0051606	detection of stimulus	0.18	1	0.18083182	12	0.03465504	
2976	GO:0004176|GO:0004280	ATP-dependent peptidase a	0.18	2	0.36166364	48	0.13862015	
4942	GO:0006855	drug transmembrane transp	0.18	4	0.7233273	139	0.40142086	
8607	GO:0015197|GO:0015637	peptide transporter activity	0.18	4	0.7233273	139	0.40142086	
8608	GO:0015198	oligopeptide transporter act	0.18	4	0.7233273	139	0.40142086	
8642	GO:0015238|GO:0015239|	drug transmembrane transp	0.18	4	0.7233273	139	0.40142086	
9154	GO:0015893	drug transport	0.18	4	0.7233273	139	0.40142086	
18832	GO:0042493|GO:0017035	response to drug	0.18	4	0.7233273	139	0.40142086	
3844	GO:0005451	monovalent cation:hydroge	0.18	2	0.36166364	49	0.14150807	
8909	GO:0015630	microtubule cytoskeleton	0.18	2	0.36166364	49	0.14150807	
3002	GO:0004315	3-oxoacyl-[acyl-carrier-prot	0.19	1	0.18083182	13	0.037542958	
7551	GO:0010327	acetyl CoA:(Z)-3-hexen-1-o	0.19	1	0.18083182	13	0.037542958	
8149	GO:0010941	regulation of cell death	0.19	1	0.18083182	13	0.037542958	
13443	GO:0030984	kininogen binding	0.19	1	0.18083182	13	0.037542958	
19298	GO:0042981	regulation of apoptosis	0.19	1	0.18083182	13	0.037542958	
19381	GO:0043067|GO:0043070	regulation of programmed c	0.19	1	0.18083182	13	0.037542958	
4944	GO:0006857	oligopeptide transport	0.19	4	0.7233273	141	0.4071967	
8676	GO:0015294	solute:cation symporter act	0.19	4	0.7233273	141	0.4071967	
9099	GO:0015833	peptide transport	0.19	4	0.7233273	141	0.4071967	
4216	GO:0005976	polysaccharide metabolic p	0.19	2	0.36166364	50	0.14439599	
9783	GO:0016722	oxidoreductase activity, oxi	0.19	2	0.36166364	50	0.14439599	
9993	GO:0017016	Ras GTPase binding	0.19	2	0.36166364	50	0.14439599	
3476	GO:0004872|GO:0019041	receptor activity	0.19	14	2.5316455	672	1.9406822	
28364	GO:0065007	biological regulation	0.20	77	13.92405	4376	12.637537	
5763	GO:0008233	peptidase activity	0.20	12	2.169982	565	1.6316746	

12794	GO:0030234	enzyme regulator activity	0.20	9	1.6274865	403	1.1638317	
5981	GO:0008509	anion transmembrane trans	0.20	6	1.084991	245	0.70754033	
5195	GO:0007165|GO:0023033	signal transduction	0.20	19	3.4358046	961	2.775291	
12602	GO:0023052|GO:0023046	signaling	0.20	19	3.4358046	961	2.775291	
3629	GO:0005087	Ran guanyl-nucleotide exch	0.20	1	0.18083182	14	0.040430877	
11238	GO:0019239	deaminase activity	0.20	1	0.18083182	14	0.040430877	
4917	GO:0006820|GO:0006822	anion transport	0.20	6	1.084991	247	0.7133162	
25626	GO:0051336	regulation of hydrolase acti	0.21	5	0.9041591	196	0.5660323	
3490	GO:0004888|GO:0004926	transmembrane receptor ac	0.21	12	2.169982	571	1.6490022	
9600	GO:0016491	oxidoreductase activity	0.21	32	5.786618	1724	4.9787736	
6810	GO:0009522	photosystem I	0.21	1	0.18083182	15	0.043318797	
9340	GO:0016108	tetraterpenoid metabolic pr	0.21	1	0.18083182	15	0.043318797	
9341	GO:0016109	tetraterpenoid biosynthetic	0.21	1	0.18083182	15	0.043318797	
9348	GO:0016116	carotenoid metabolic proce	0.21	1	0.18083182	15	0.043318797	
9349	GO:0016117	carotenoid biosynthetic pro	0.21	1	0.18083182	15	0.043318797	
10079	GO:0017134	fibroblast growth factor bin	0.21	1	0.18083182	15	0.043318797	
4478	GO:0006281	DNA repair	0.22	4	0.7233273	149	0.43030006	
5037	GO:0006974|GO:0034984	response to DNA damage s	0.22	4	0.7233273	149	0.43030006	
13697	GO:0031267	small GTPase binding	0.23	2	0.36166364	56	0.16172351	
7133	GO:0009881	photoreceptor activity	0.23	1	0.18083182	16	0.046206716	
7178	GO:0009927	histidine phosphotransfer k	0.23	1	0.18083182	16	0.046206716	
7521	GO:0010295	(+)-abscisic acid 8'-hydroxy	0.23	1	0.18083182	16	0.046206716	
9189	GO:0015929	hexosaminidase activity	0.23	1	0.18083182	16	0.046206716	
9386	GO:0016157	sucrose synthase activity	0.23	1	0.18083182	16	0.046206716	
9898	GO:0016863	intramolecular oxidoreducta	0.23	1	0.18083182	16	0.046206716	
18757	GO:0042409	caffeoyl-CoA O-methyltrans	0.23	1	0.18083182	16	0.046206716	
30066	GO:0071702	organic substance transpor	0.23	11	1.98915	529	1.5277096	
3307	GO:0004675	transmembrane receptor pr	0.24	8	1.4466546	367	1.0598665	
4913	GO:0006814|GO:0006834|	sodium ion transport	0.24	2	0.36166364	58	0.16749935	
8499	GO:0015081|GO:0022816	sodium ion transmembrane	0.24	2	0.36166364	58	0.16749935	
11820	GO:0019887	protein kinase regulator act	0.24	2	0.36166364	58	0.16749935	
18086	GO:0035725	sodium ion transmembrane	0.24	2	0.36166364	58	0.16749935	
29800	GO:0071436	sodium ion export	0.24	2	0.36166364	58	0.16749935	
4135	GO:0005875	microtubule associated con	0.24	1	0.18083182	17	0.049094636	
4957	GO:0006875	cellular metal ion homeosta	0.24	1	0.18083182	17	0.049094636	
12610	GO:0030003	cellular cation homeostasis	0.24	1	0.18083182	17	0.049094636	
26912	GO:0055065	metal ion homeostasis	0.24	1	0.18083182	17	0.049094636	
26926	GO:0055080	cation homeostasis	0.24	1	0.18083182	17	0.049094636	
2831	GO:0004022	alcohol dehydrogenase (NA	0.24	2	0.36166364	59	0.17038727	
3942	GO:0005618	cell wall	0.24	3	0.5424955	107	0.30900744	
5704	GO:0008150|GO:0000004|	biological_process	0.25	363	65.64195	22215	64.155136	
25319	GO:0051020	GTPase binding	0.25	2	0.36166364	60	0.17327519	
2630	GO:0003680	AT DNA binding	0.25	1	0.18083182	18	0.051982556	
3963	GO:0005643|GO:0005644	nuclear pore	0.25	1	0.18083182	18	0.051982556	
4134	GO:0005874	microtubule	0.25	1	0.18083182	18	0.051982556	
4225	GO:0005985	sucrose metabolic process	0.25	1	0.18083182	18	0.051982556	
4252	GO:0006014	D-ribose metabolic process	0.25	1	0.18083182	18	0.051982556	
5792	GO:0008271	secondary active sulfate tra	0.25	1	0.18083182	18	0.051982556	
7773	GO:0010557	positive regulation of macro	0.25	1	0.18083182	18	0.051982556	
8662	GO:0015271	outward rectifier potassium	0.25	1	0.18083182	18	0.051982556	
8736	GO:0015381	high affinity sulfate transme	0.25	1	0.18083182	18	0.051982556	
12887	GO:0030332	cyclin binding	0.25	1	0.18083182	18	0.051982556	
22431	GO:0046930	pore complex	0.25	1	0.18083182	18	0.051982556	
22917	GO:0047429	nucleoside-triphosphate dip	0.25	1	0.18083182	18	0.051982556	
8494	GO:0015075	ion transmembrane transpo	0.25	16	2.893309	827	2.3883097	
8526	GO:0015112	nitrate transmembrane tran	0.25	2	0.36166364	61	0.1761631	
8975	GO:0015706|GO:0006872	nitrate transport	0.25	2	0.36166364	61	0.1761631	
25098	GO:0050794|GO:0051244	regulation of cellular proces	0.26	69	12.477396	3987	11.514136	
7690	GO:0010466	negative regulation of pepti	0.26	3	0.5424955	110	0.31767118	
12946	GO:0030414	peptidase inhibitor activity	0.26	3	0.5424955	110	0.31767118	
25636	GO:0051346	negative regulation of hydro	0.26	3	0.5424955	110	0.31767118	
5732	GO:0008194	UDP-glycosyltransferase ac	0.26	7	1.2658228	322	0.9299102	
16603	GO:0034220	ion transmembrane transpo	0.26	16	2.893309	833	2.4056373	
12868	GO:0030312	external encapsulating stru	0.26	3	0.5424955	111	0.3205591	
12985	GO:0030488	tRNA methylation	0.26	1	0.18083182	19	0.054870475	
26711	GO:0052547	regulation of peptidase acti	0.27	3	0.5424955	112	0.32344702	
28100	GO:0061134	peptidase regulator activity	0.27	3	0.5424955	112	0.32344702	
17016	GO:0034637	cellular carbohydrate biosy	0.27	2	0.36166364	63	0.18193895	
8943	GO:0015672	monovalent inorganic catio	0.27	8	1.4466546	383	1.1060733	
25094	GO:0050789|GO:0050791	regulation of biological proc	0.27	74	13.381556	4315	12.461374	
9293	GO:0016051|GO:0006093	carbohydrate biosynthetic p	0.27	2	0.36166364	64	0.18482687	
25820	GO:0051536	iron-sulfur cluster binding	0.27	2	0.36166364	64	0.18482687	
25824	GO:0051540	metal cluster binding	0.27	2	0.36166364	64	0.18482687	
4910	GO:0006811	ion transport	0.28	18	3.254973	959	2.769515	
3798	GO:0005337	nucleoside transmembrane	0.28	1	0.18083182	20	0.0577584	
3956	GO:0005635|GO:0005636	nuclear envelope	0.28	1	0.18083182	20	0.0577584	
5991	GO:0008519	ammonium transmembrane	0.28	1	0.18083182	20	0.0577584	
8965	GO:0015696	ammonium transport	0.28	1	0.18083182	20	0.0577584	
9122	GO:0015858	nucleoside transport	0.28	1	0.18083182	20	0.0577584	
9856	GO:0016814	hydrolase activity, acting or	0.28	1	0.18083182	20	0.0577584	
25477	GO:0051180	vitamin transport	0.28	1	0.18083182	20	0.0577584	
25480	GO:0051183	vitamin transporter activity	0.28	1	0.18083182	20	0.0577584	
30844	GO:0072488	ammonium transmembrane	0.28	1	0.18083182	20	0.0577584	
9811	GO:0016757|GO:0016932	transferase activity, transfe	0.28	14	2.5316455	729	2.1052935	
9812	GO:0016758	transferase activity, transfe	0.28	10	1.8083183	501	1.4468478	
3129	GO:0004467	long-chain fatty acid-CoA li	0.29	1	0.18083182	21	0.060646318	
3364	GO:0004745	retinol dehydrogenase activ	0.29	1	0.18083182	21	0.060646318	
3474	GO:0004869|GO:0004870	cysteine-type endopeptidas	0.29	1	0.18083182	21	0.060646318	
4955	GO:0006873	cellular ion homeostasis	0.29	1	0.18083182	21	0.060646318	
12999	GO:0030506	ankyrin binding	0.29	1	0.18083182	21	0.060646318	
25104	GO:0050801	ion homeostasis	0.29	1	0.18083182	21	0.060646318	
26928	GO:0055082	cellular chemical homeosta	0.29	1	0.18083182	21	0.060646318	
24966	GO:0050660	flavin adenine dinucleotide	0.29	3	0.5424955	118	0.34077454	
2662	GO:0003746|GO:0008182|	translation elongation facto	0.30	1	0.18083182	22	0.06353424	
3831	GO:0005385	zinc ion transmembrane tra	0.30	1	0.18083182	22	0.06353424	
4574	GO:0006414|GO:0006442|	translational elongation	0.30	1	0.18083182	22	0.06353424	
4882	GO:0006778	porphyrin metabolic proces	0.30	1	0.18083182	22	0.06353424	
4883	GO:0006779	porphyrin biosynthetic proc	0.30	1	0.18083182	22	0.06353424	
4924	GO:0006829	zinc ion transport	0.30	1	0.18083182	22	0.06353424	
7143	GO:0009891	positive regulation of biosyn	0.30	1	0.18083182	22	0.06353424	
13758	GO:0031328	positive regulation of cellula	0.30	1	0.18083182	22	0.06353424	
18524	GO:0042162	telomeric DNA binding	0.30	1	0.18083182	22	0.06353424	
21152	GO:0045551	cinnamyl-alcohol dehydrog	0.30	1	0.18083182	22	0.06353424	
29941	GO:0071577	zinc ion transmembrane tra	0.30	1	0.18083182	22	0.06353424	
15949	GO:0033554	cellular response to stress	0.30	4	0.7233273	173	0.49961013	
21710	GO:0046148	pigment biosynthetic proce	0.30	2	0.36166364	69	0.19926646	
15524	GO:0033124	regulation of GTP catabolic	0.31	2	0.36166364	70	0.20215438	
19394	GO:0043087	regulation of GTPase activi	0.31	2	0.36166364	70	0.20215438	
2633	GO:0003684	damaged DNA binding	0.31	1	0.18083182	23	0.06642216	
7527	GO:0010302	2-oxoglutarate-dependent d	0.31	1	0.18083182	23	0.06642216	
22414	GO:0046912	transferase activity, transfe	0.31	1	0.18083182	23	0.06642216	
9845	GO:0016798	hydrolase activity, acting or	0.31	8	1.4466546	401	1.1580559	
26959	GO:0055114	oxidation-reduction process	0.32	43	7.7757688	2490	7.1909204	
11695	GO:0019725	cellular homeostasis	0.32	5	0.9041591	234	0.67577326	
8559	GO:0015146	pentose transmembrane tra	0.32	1	0.18083182	24	0.06931008	
8561	GO:0015148	D-xylose transmembrane tr	0.32	1	0.18083182	24	0.06931008	
8829	GO:0015519	D-xylose:hydrogen symport	0.32	1	0.18083182	24	0.06931008	
9019	GO:0015750	pentose transport	0.32	1	0.18083182	24	0.06931008	
9022	GO:0015753	D-xylose transport	0.32	1	0.18083182	24	0.06931008	
4916	GO:0006818	hydrogen transport	0.32	6	1.084991	292	0.84327257	
9246	GO:0015992	proton transport	0.32	6	1.084991	292	0.84327257	
9703	GO:0016620	oxidoreductase activity, act	0.33	2	0.36166364	73	0.21081814	
18786	GO:0042440	pigment metabolic process	0.33	2	0.36166364	73	0.21081814	
3884	GO:0005524	ATP binding	0.33	10	1.8083183	524	1.51327	
11828	GO:0019899	enzyme binding	0.33	5	0.9041591	237	0.684437	
9817	GO:0016763	transferase activity, transfe	0.33	2	0.36166364	74	0.21370606	
973	GO:0001653	peptide receptor activity	0.33	1	0.18083182	25	0.072197996	

9876	GO:0016837	carbon-oxygen lyase activit	0.33	1	0.18083182	25	0.072197996
13054	GO:0030570	pectate lyase activity	0.33	1	0.18083182	25	0.072197996
24256	GO:0048878	chemical homeostasis	0.33	1	0.18083182	25	0.072197996
31493	GO:0080118	brassinosteroid sulfotransfe	0.33	1	0.18083182	25	0.072197996
18925	GO:0042592	homeostatic process	0.33	5	0.9041591	238	0.68732494
8810	GO:0015491	cation:cation antiporter acti	0.34	2	0.36166364	75	0.21659398
3210	GO:0004565	beta-galactosidase activity	0.34	1	0.18083182	26	0.075085916
5978	GO:0008506	sucrose:hydrogen symporte	0.34	1	0.18083182	26	0.075085916
5987	GO:0008515|GO:0019188	sucrose transmembrane tra	0.34	1	0.18083182	26	0.075085916
6907	GO:0009639	response to red or far red li	0.34	1	0.18083182	26	0.075085916
7000	GO:0009734	auxin mediated signaling pa	0.34	1	0.18083182	26	0.075085916
7540	GO:0010315	auxin efflux	0.34	1	0.18083182	26	0.075085916
7553	GO:0010329	auxin efflux transmembrane	0.34	1	0.18083182	26	0.075085916
8516	GO:0015101	organic cation transmembra	0.34	1	0.18083182	26	0.075085916
8530	GO:0015116	sulfate transmembrane tran	0.34	1	0.18083182	26	0.075085916
8567	GO:0015154	disaccharide transmembran	0.34	1	0.18083182	26	0.075085916
8964	GO:0015695	organic cation transport	0.34	1	0.18083182	26	0.075085916
9035	GO:0015766	disaccharide transport	0.34	1	0.18083182	26	0.075085916
9039	GO:0015770	sucrose transport	0.34	1	0.18083182	26	0.075085916
9744	GO:0016671	oxidoreductase activity, act	0.34	1	0.18083182	26	0.075085916
29729	GO:0071365	cellular response to auxin s	0.34	1	0.18083182	26	0.075085916
126	GO:0000160	two-component signal trans	0.34	1	0.18083182	26	0.075085916
18000	GO:0035639	purine ribonucleoside tripho	0.35	14	2.5316455	769	2.2208104
4562	GO:0006400|GO:0016549	tRNA modification	0.35	1	0.18083182	27	0.077973835
8919	GO:0015645	fatty acid ligase activity	0.35	1	0.18083182	27	0.077973835
9346	GO:0016114	terpenoid biosynthetic proc	0.35	1	0.18083182	27	0.077973835
25481	GO:0051184	cofactor transporter activity	0.35	1	0.18083182	27	0.077973835
19393	GO:0043086	negative regulation of catal	0.35	4	0.7233273	188	0.54292893
20342	GO:0044092	negative regulation of mole	0.35	4	0.7233273	188	0.54292893
5840	GO:0008324	cation transmembrane tran	0.36	10	1.8083183	538	1.5537009
2833	GO:0004024	alcohol dehydrogenase acti	0.36	1	0.18083182	28	0.080861755
5793	GO:0008272|GO:0006870	sulfate transport	0.36	1	0.18083182	28	0.080861755
6079	GO:0008654	phospholipid biosynthetic p	0.36	1	0.18083182	28	0.080861755
6809	GO:0009521|GO:0030090	photosystem	0.36	1	0.18083182	28	0.080861755
7145	GO:0009893	positive regulation of metab	0.36	1	0.18083182	28	0.080861755
7819	GO:0010604	positive regulation of macro	0.36	1	0.18083182	28	0.080861755
9238	GO:0015980	energy derivation by oxidati	0.36	1	0.18083182	28	0.080861755
13634	GO:0031177	phosphopantetheine binding	0.36	1	0.18083182	28	0.080861755
13755	GO:0031325	positive regulation of cellula	0.36	1	0.18083182	28	0.080861755
20512	GO:0044262|GO:0006092	cellular carbohydrate metab	0.36	5	0.9041591	248	0.7162041
4358	GO:0006140	regulation of nucleotide me	0.37	2	0.36166364	80	0.2310336
7146	GO:0009894	regulation of catabolic proc	0.37	2	0.36166364	80	0.2310336
9875	GO:0016836	hydro-lyase activity	0.37	2	0.36166364	80	0.2310336
13278	GO:0030811	regulation of nucleotide cat	0.37	2	0.36166364	80	0.2310336
13759	GO:0031329	regulation of cellular catabo	0.37	2	0.36166364	80	0.2310336
15521	GO:0033121	regulation of purine nucleot	0.37	2	0.36166364	80	0.2310336
4911	GO:0006812|GO:0006819|	cation transport	0.37	12	2.169982	662	1.9118029
4841	GO:0006721	terpenoid metabolic proces	0.37	1	0.18083182	29	0.083749674
4641	GO:0006508	proteolysis	0.37	17	3.074141	965	2.7868426
9869	GO:0016829	lyase activity	0.38	9	1.6274865	488	1.4093049
12516	GO:0022804	active transmembrane trans	0.38	14	2.5316455	787	2.2727928
3200	GO:0004553|GO:0016800	hydrolase activity, hydrolyz	0.38	6	1.084991	312	0.90103096
2774	GO:0003950	NAD+ ADP-ribosyltransfera	0.38	1	0.18083182	30	0.086637594
8683	GO:0015301|GO:0015380|	anion:anion antiporter activ	0.38	1	0.18083182	30	0.086637594
15414	GO:0033013	tetrapyrrole metabolic proce	0.38	1	0.18083182	30	0.086637594
15415	GO:0033014	tetrapyrrole biosynthetic pro	0.38	1	0.18083182	30	0.086637594
23	GO:0000030	mannosyltransferase activit	0.38	1	0.18083182	30	0.086637594
3787	GO:0005319	lipid transporter activity	0.38	2	0.36166364	83	0.23969735
16738	GO:0034357	photosynthetic membrane	0.38	2	0.36166364	83	0.23969735
13041	GO:0030554	adenyl nucleotide binding	0.38	10	1.8083183	550	1.5883559
14965	GO:0032559	adenyl ribonucleotide bindir	0.38	10	1.8083183	550	1.5883559
5018	GO:0006950	response to stress	0.39	11	1.98915	611	1.764519
4912	GO:0006813|GO:0015458	potassium ion transport	0.39	2	0.36166364	84	0.24258527
30168	GO:0071804	cellular potassium ion trans	0.39	2	0.36166364	84	0.24258527
30169	GO:0071805	potassium ion transmembra	0.39	2	0.36166364	84	0.24258527
12608	GO:0030001	metal ion transport	0.39	7	1.2658228	374	1.080082
14959	GO:0032553	ribonucleotide binding	0.39	14	2.5316455	795	2.2958963
14961	GO:0032555	purine ribonucleotide bindir	0.39	14	2.5316455	795	2.2958963
14422	GO:0032012	regulation of ARF protein si	0.39	1	0.18083182	31	0.08952551
14720	GO:0032312	regulation of ARF GTPase	0.39	1	0.18083182	31	0.08952551
25478	GO:0051181	cofactor transport	0.39	1	0.18083182	31	0.08952551
5624	GO:0008047	enzyme activator activity	0.39	2	0.36166364	85	0.24547319
10033	GO:0017076	purine nucleotide binding	0.40	14	2.5316455	797	2.3016722
3717	GO:0005199	structural constituent of cel	0.40	1	0.18083182	32	0.09241343
3785	GO:0005315|GO:0005317	inorganic phosphate transm	0.40	1	0.18083182	32	0.09241343
6942	GO:0009674	potassium:sodium symport	0.40	1	0.18083182	32	0.09241343
9041	GO:0015772	oligosaccharide transport	0.40	1	0.18083182	32	0.09241343
12522	GO:0022820	potassium ion symporter ac	0.40	1	0.18083182	32	0.09241343
23914	GO:0048522|GO:0051242	positive regulation of cellula	0.40	1	0.18083182	32	0.09241343
20624	GO:0044430	cytoskeletal part	0.41	2	0.36166364	88	0.25413695
119	GO:0000151	ubiquitin ligase complex	0.41	2	0.36166364	88	0.25413695
4493	GO:0006298|GO:0006300	mismatch repair	0.41	1	0.18083182	33	0.09530135
3749	GO:0005249	voltage-gated potassium ch	0.42	1	0.18083182	34	0.09818927
11831	GO:0019902	phosphatase binding	0.42	1	0.18083182	34	0.09818927
11832	GO:0019903	protein phosphatase bindin	0.42	1	0.18083182	34	0.09818927
9829	GO:0016779	nucleotidyltransferase activ	0.42	3	0.5424955	148	0.42741212
20630	GO:0044436	thylakoid part	0.42	2	0.36166364	90	0.2599128
18582	GO:0042221	response to chemical stimu	0.43	6	1.084991	328	0.94723773
16785	GO:0034404	nucleobase, nucleoside and	0.43	2	0.36166364	92	0.26568863
17033	GO:0034654	nucleobase, nucleoside, nu	0.43	2	0.36166364	92	0.26568863
21058	GO:0045454|GO:0030503|	cell redox homeostasis	0.44	4	0.7233273	211	0.6093511
8679	GO:0015297	antiporter activity	0.44	3	0.5424955	152	0.4389638
8682	GO:0015300	solute:solute antiporter acti	0.44	3	0.5424955	152	0.4389638
11314	GO:0019321	pentose metabolic process	0.44	1	0.18083182	36	0.10396511
22376	GO:0046873	metal ion transmembrane t	0.44	4	0.7233273	213	0.6151269
3808	GO:0005351|GO:0005403	sugar:hydrogen symporter	0.44	2	0.36166364	94	0.27146447
3835	GO:0005402	cation:sugar symporter acti	0.44	2	0.36166364	94	0.27146447
6863	GO:0009579	thylakoid	0.44	2	0.36166364	94	0.27146447
13656	GO:0031224	intrinsic to membrane	0.44	20	3.6166365	1193	3.4452884
15436	GO:0033036	macromolecule localization	0.45	7	1.2658228	397	1.1465042
3758	GO:0005267	potassium channel activity	0.45	1	0.18083182	37	0.10685303
3472	GO:0004866	endopeptidase inhibitor acti	0.45	2	0.36166364	95	0.27435237
8159	GO:0010951	negative regulation of endo	0.45	2	0.36166364	95	0.27435237
26712	GO:0052548	regulation of endopeptidase	0.45	2	0.36166364	95	0.27435237
28101	GO:0061135	endopeptidase regulator ac	0.45	2	0.36166364	95	0.27435237
12538	GO:0022843	voltage-gated cation chann	0.46	1	0.18083182	38	0.10974095
3464	GO:0004857	enzyme inhibitor activity	0.46	3	0.5424955	157	0.4534034
8677	GO:0015295	solute:hydrogen symporter	0.47	2	0.36166364	98	0.28301615
8681	GO:0015299	solute:hydrogen antiporter a	0.47	2	0.36166364	98	0.28301615
30883	GO:0072527	pyrimidine-containing comp	0.47	1	0.18083182	39	0.11262887
868	GO:0001510	RNA methylation	0.47	1	0.18083182	39	0.11262887
19831	GO:0043565	sequence-specific DNA bin	0.47	3	0.5424955	159	0.45917925
4755	GO:0006629	lipid metabolic process	0.47	10	1.8083183	590	1.7038727
6882	GO:0009605	response to external stimul	0.47	1	0.18083182	40	0.1155168
7021	GO:0009755	hormone-mediated signalin	0.47	1	0.18083182	40	0.1155168
15271	GO:0032870	cellular response to hormo	0.47	1	0.18083182	40	0.1155168
23910	GO:0048518|GO:0043119	positive regulation of biolog	0.47	1	0.18083182	40	0.1155168
16430	GO:0034046	poly(G) RNA binding	0.48	2	0.36166364	100	0.28879198
14667	GO:0032259	methylation	0.48	5	0.9041591	285	0.8230572
3638	GO:0005096	GTPase activator activity	0.48	1	0.18083182	41	0.118404716
19813	GO:0043547	positive regulation of GTPa	0.48	1	0.18083182	41	0.118404716
5874	GO:0008374	O-acyltransferase activity	0.49	2	0.36166364	102	0.29456782
9837	GO:0016788	hydrolase activity, acting or	0.49	25	4.520796	1534	4.430069
8461	GO:0015020|GO:0003981	glucuronosyltransferase ac	0.49	1	0.18083182	42	0.121292636
13884	GO:0031461	cullin-RING ubiquitin ligase	0.49	1	0.18083182	42	0.121292636
9267	GO:0016021	integral to membrane	0.50	18	3.254973	1103	3.1853757
19392	GO:0043085	positive regulation of cataly	0.50	2	0.36166364	104	0.30034366

20343	GO:0044093	positive regulation of molec	0.50	2	0.36166364	104	0.30034366	
13531	GO:0031072	heat shock protein binding	0.50	4	0.7233273	230	0.6642216	
11224	GO:0019222	regulation of metabolic proc	0.51	50	9.041592	3115	8.995871	
9753	GO:0016682	oxidoreductase activity, act	0.51	1	0.18083182	44	0.12706847	
29859	GO:0071495	cellular response to endoge	0.51	1	0.18083182	44	0.12706847	
3630	GO:0005088	Ras guanyl-nucleotide exch	0.52	1	0.18083182	45	0.1299564	
5692	GO:0008134	transcription factor binding	0.52	1	0.18083182	45	0.1299564	
9893	GO:0016857	racemase and epimerase a	0.52	1	0.18083182	45	0.1299564	
14726	GO:0032318	regulation of Ras GTPase a	0.52	1	0.18083182	45	0.1299564	
22096	GO:0046578	regulation of Ras protein si	0.52	1	0.18083182	45	0.1299564	
25355	GO:0051056	regulation of small GTPase	0.52	1	0.18083182	45	0.1299564	
2636	GO:0003690	double-stranded DNA bindi	0.52	1	0.18083182	46	0.13284431	
6999	GO:0009733	response to auxin stimulus	0.52	1	0.18083182	46	0.13284431	
9795	GO:0016740	transferase activity	0.53	60	10.84991	3764	10.870131	
8497	GO:0015079|GO:0022817	potassium ion transmembra	0.53	1	0.18083182	47	0.13573223	
8680	GO:0015298	solute:cation antiporter acti	0.54	2	0.36166364	112	0.32344702	
29081	GO:0070717	poly-purine tract binding	0.54	2	0.36166364	112	0.32344702	
13753	GO:0031323	regulation of cellular metab	0.54	44	7.9566	2770	7.999538	
9933	GO:0016903	oxidoreductase activity, act	0.54	2	0.36166364	113	0.32633495	
9800	GO:0016746	transferase activity, transfe	0.54	8	1.4466546	498	1.438184	
3756	GO:0005261|GO:0015281|	cation channel activity	0.55	1	0.18083182	49	0.14150807	
9725	GO:0016645	oxidoreductase activity, act	0.55	1	0.18083182	49	0.14150807	
11784	GO:0019838	growth factor binding	0.55	1	0.18083182	49	0.14150807	
6166	GO:0008757	S-adenosylmethionine-depe	0.55	3	0.5424955	180	0.5198256	
9535	GO:0016407	acetyltransferase activity	0.55	2	0.36166364	115	0.3321108	
3885	GO:0005525	GTP binding	0.55	4	0.7233273	245	0.70754033	
11032	GO:0019001	guanyl nucleotide binding	0.55	4	0.7233273	245	0.70754033	
14967	GO:0032561	guanyl ribonucleotide bindi	0.55	4	0.7233273	245	0.70754033	
29674	GO:0071310	cellular response to organic	0.55	1	0.18083182	50	0.14439599	
3903	GO:0005575|GO:0008372	cellular_component	0.56	135	24.412296	8515	24.590637	
20619	GO:0044425	membrane part	0.56	24	4.339964	1523	4.398302	
3293	GO:0004659	prenyltransferase activity	0.56	1	0.18083182	51	0.14728391	
7213	GO:0009966|GO:0035466	regulation of signal transdu	0.56	1	0.18083182	51	0.14728391	
12601	GO:0023051	regulation of signaling	0.56	1	0.18083182	51	0.14728391	
25416	GO:0051119	sugar transmembrane trans	0.56	2	0.36166364	118	0.34077454	
4915	GO:0006817	phosphate transport	0.57	1	0.18083182	52	0.15017183	
8528	GO:0015114	phosphate transmembrane	0.57	1	0.18083182	52	0.15017183	
17801	GO:0035435	phosphate transmembrane	0.57	1	0.18083182	52	0.15017183	
9818	GO:0016765|GO:0016766	transferase activity, transfe	0.57	3	0.5424955	185	0.53426516	
12805	GO:0030246	carbohydrate binding	0.57	4	0.7233273	251	0.7248679	
3946	GO:0005623	cell	0.57	130	23.508137	8227	23.758917	
20657	GO:0044464	cell part	0.57	130	23.508137	8227	23.758917	
9750	GO:0016679	oxidoreductase activity, act	0.57	1	0.18083182	53	0.15305975	
25635	GO:0051345	positive regulation of hydro	0.57	1	0.18083182	53	0.15305975	
2657	GO:0003727|GO:0003728	single-stranded RNA bindin	0.58	3	0.5424955	188	0.54292893	
17918	GO:0035556|GO:0007242|	intracellular signal transduc	0.58	1	0.18083182	54	0.15594767	
29251	GO:0070887	cellular response to chemic	0.58	1	0.18083182	54	0.15594767	
2628	GO:0003677	DNA binding	0.58	19	3.4358046	1224	3.534814	
4951	GO:0006865|GO:0006866	amino acid transport	0.59	2	0.36166364	123	0.35521415	
24968	GO:0050662	coenzyme binding	0.59	3	0.5424955	191	0.5515927	
5809	GO:0008289	lipid binding	0.59	4	0.7233273	258	0.74508333	
9801	GO:0016747	transferase activity, transfe	0.59	7	1.2658228	456	1.3168914	
4224	GO:0005984	disaccharide metabolic pro	0.59	1	0.18083182	56	0.16172351	
9369	GO:0016137	glycoside metabolic proces	0.59	1	0.18083182	56	0.16172351	
9883	GO:0016846	carbon-sulfur lyase activity	0.59	1	0.18083182	56	0.16172351	
4849	GO:0006730|GO:0019753|	one-carbon metabolic proce	0.59	5	0.9041591	325	0.93857396	
4121	GO:0005856	cytoskeleton	0.60	2	0.36166364	126	0.3638779	
5715	GO:0008171	O-methyltransferase activit	0.60	1	0.18083182	57	0.16461143	
9813	GO:0016759	cellulose synthase activity	0.60	1	0.18083182	57	0.16461143	
23973	GO:0048583	regulation of response to st	0.60	1	0.18083182	57	0.16461143	
3323	GO:0004702	receptor signaling protein s	0.61	1	0.18083182	58	0.16749935	
5819	GO:0008299|GO:0009241	isoprenoid biosynthetic pro	0.61	1	0.18083182	58	0.16749935	
6665	GO:0009311	oligosaccharide metabolic	0.61	1	0.18083182	58	0.16749935	
8558	GO:0015145	monosaccharide transmem	0.61	1	0.18083182	58	0.16749935	
9018	GO:0015749	monosaccharide transport	0.61	1	0.18083182	58	0.16749935	
3333	GO:0004712	protein serine/threonine/tyr	0.61	1	0.18083182	59	0.17038727	
18981	GO:0042651	thylakoid membrane	0.61	1	0.18083182	59	0.17038727	
30700	GO:0072341	modified amino acid bindin	0.61	1	0.18083182	59	0.17038727	
12572	GO:0022890|GO:0015082	inorganic cation transmem	0.61	5	0.9041591	332	0.9587894	
3340	GO:0004721	phosphoprotein phosphatas	0.62	3	0.5424955	199	0.57469606	
2980	GO:0004185	serine-type carboxypeptidas	0.62	1	0.18083182	60	0.17327519	
4840	GO:0006720|GO:0016096	isoprenoid metabolic proce	0.62	1	0.18083182	60	0.17327519	
28375	GO:0070008	serine-type exopeptidase a	0.62	1	0.18083182	60	0.17327519	
2818	GO:0004003	ATP-dependent DNA helica	0.63	1	0.18083182	61	0.1761631	
4768	GO:0006644	phospholipid metabolic pro	0.63	1	0.18083182	61	0.1761631	
9874	GO:0016835	carbon-oxygen lyase activit	0.63	3	0.5424955	202	0.5833598	
30069	GO:0071705	nitrogen compound transpo	0.63	3	0.5424955	202	0.5833598	
3876	GO:0005515|GO:0045308	protein binding	0.63	108	19.529837	6933	20.021948	
2978	GO:0004180	carboxypeptidase activity	0.63	1	0.18083182	62	0.17905103	
6018	GO:0008559|GO:0005226|	xenobiotic-transporting ATP	0.63	1	0.18083182	62	0.17905103	
19225	GO:0042908	xenobiotic transport	0.63	1	0.18083182	62	0.17905103	
19227	GO:0042910	xenobiotic transporter activ	0.63	1	0.18083182	62	0.17905103	
11221	GO:0019219	regulation of nucleobase, n	0.63	41	7.414105	2675	7.7251854	
25468	GO:0051171	regulation of nitrogen comp	0.63	41	7.414105	2675	7.7251854	
9852	GO:0016810	hydrolase activity, acting on	0.63	2	0.36166364	134	0.38698125	
9103	GO:0015837	amine transport	0.64	2	0.36166364	135	0.38986918	
3103	GO:0004437	inositol or phosphatidylinos	0.64	1	0.18083182	64	0.18482687	
22416	GO:0046915	transition metal ion transme	0.64	1	0.18083182	64	0.18482687	
11616	GO:0019637	organophosphate metabolic	0.65	1	0.18083182	65	0.18771479	
2626	GO:0003674|GO:0005554	molecular_function	0.65	386	69.80109	24394	70.447914	
9836	GO:0016787	hydrolase activity	0.65	57	10.307414	3723	10.751725	
3473	GO:0004867	serine-type endopeptidase	0.65	1	0.18083182	66	0.1906027	
4561	GO:0006399	tRNA metabolic process	0.65	1	0.18083182	66	0.1906027	
5615	GO:0008033	tRNA processing	0.65	1	0.18083182	66	0.1906027	
9890	GO:0016854	racemase and epimerase a	0.65	1	0.18083182	66	0.1906027	
11829	GO:0019900	kinase binding	0.65	1	0.18083182	66	0.1906027	
3895	GO:0005543	phospholipid binding	0.66	2	0.36166364	140	0.40430877	
2629	GO:0003678|GO:0003679	DNA helicase activity	0.66	1	0.18083182	67	0.19349062	
6046	GO:0008610	lipid biosynthetic process	0.66	2	0.36166364	141	0.4071967	
13164	GO:0030695	GTPase regulator activity	0.66	2	0.36166364	142	0.4100846	
31465	GO:0080090	regulation of primary metab	0.66	41	7.414105	2710	7.826263	
2999	GO:0004312	fatty acid synthase activity	0.67	1	0.18083182	68	0.19637854	
18742	GO:0042393	histone binding	0.67	1	0.18083182	68	0.19637854	
32	GO:0000041	transition metal ion transpo	0.67	1	0.18083182	68	0.19637854	
8495	GO:0015077	monovalent inorganic catio	0.67	3	0.5424955	214	0.6180149	
8557	GO:0015144	carbohydrate transmembra	0.67	2	0.36166364	143	0.41297254	
16602	GO:0034219	carbohydrate transmembra	0.67	2	0.36166364	143	0.41297254	
3620	GO:0005057	receptor signaling protein a	0.67	1	0.18083182	69	0.19926646	
4530	GO:0006355|GO:0032583|	regulation of transcription,	0.67	39	7.052441	2591	7.4826	
25544	GO:0051252	regulation of RNA metaboli	0.68	39	7.052441	2593	7.488376	
5726	GO:0008187	poly-pyrimidine tract bindin	0.68	1	0.18083182	70	0.20215438	
5788	GO:0008266	poly(U) RNA binding	0.68	1	0.18083182	70	0.20215438	
9191	GO:0015931	nucleobase, nucleoside, nu	0.68	1	0.18083182	70	0.20215438	
9192	GO:0015932	nucleobase, nucleoside, nu	0.68	1	0.18083182	70	0.20215438	
9853	GO:0016811	hydrolase activity, acting on	0.68	1	0.18083182	70	0.20215438	
23522	GO:0048046	apoplast	0.68	1	0.18083182	70	0.20215438	
3873	GO:0005507	copper ion binding	0.68	2	0.36166364	146	0.42163628	
6071	GO:0008643|GO:0006861|	carbohydrate transport	0.68	2	0.36166364	147	0.42452422	
3744	GO:0005244	voltage-gated ion channel a	0.69	1	0.18083182	72	0.20793022	
9740	GO:0016667	oxidoreductase activity, act	0.69	1	0.18083182	72	0.20793022	
9764	GO:0016701	oxidoreductase activity, act	0.69	1	0.18083182	72	0.20793022	
12528	GO:0022832	voltage-gated channel activ	0.69	1	0.18083182	72	0.20793022	
15280	GO:0032879	regulation of localization	0.69	1	0.18083182	72	0.20793022	
17141	GO:0034762	regulation of transmembrar	0.69	1	0.18083182	72	0.20793022	
17144	GO:0034765	regulation of ion transmem	0.69	1	0.18083182	72	0.20793022	
19568	GO:0043269	regulation of ion transport	0.69	1	0.18083182	72	0.20793022	
25348	GO:0051049	regulation of transport	0.69	1	0.18083182	72	0.20793022	

7141	GO:0009889	regulation of biosynthetic p	0.69	39	7.052441	2605	7.523031	
7772	GO:0010556	regulation of macromolecul	0.69	39	7.052441	2605	7.523031	
13756	GO:0031326	regulation of cellular biosyn	0.69	39	7.052441	2605	7.523031	
32213	GO:2000112	regulation of cellular macro	0.69	39	7.052441	2605	7.523031	
27562	GO:0060589	nucleoside-triphosphatase	0.70	2	0.36166364	152	0.4389638	
3627	GO:0005085|GO:0008433|	guanyl-nucleotide exchange	0.70	1	0.18083182	75	0.21659398	
4990	GO:0006915|GO:0008632	apoptosis	0.70	3	0.5424955	227	0.6555578	
5663	GO:0008094|GO:0004011	DNA-dependent ATPase ac	0.71	1	0.18083182	76	0.2194819	
6424	GO:0009055|GO:0009053|	electron carrier activity	0.71	2	0.36166364	154	0.44473964	
5604	GO:0008017	microtubule binding	0.71	1	0.18083182	77	0.22236982	
6529	GO:0009165	nucleotide biosynthetic proc	0.71	1	0.18083182	77	0.22236982	
7692	GO:0010468	regulation of gene expressio	0.71	39	7.052441	2635	7.6096687	
9114	GO:0015849	organic acid transport	0.71	2	0.36166364	156	0.45051548	
22443	GO:0046942	carboxylic acid transport	0.71	2	0.36166364	156	0.45051548	
9909	GO:0016877	ligase activity, forming carb	0.72	1	0.18083182	78	0.22525774	
19832	GO:0043566	structure-specific DNA bind	0.72	1	0.18083182	78	0.22525774	
30865	GO:0072509	divalent inorganic cation tra	0.72	1	0.18083182	78	0.22525774	
3888	GO:0005529	sugar binding	0.72	2	0.36166364	158	0.45629135	
9682	GO:0016597	amino acid binding	0.72	1	0.18083182	80	0.2310336	
19479	GO:0043176	amine binding	0.72	1	0.18083182	80	0.2310336	
8496	GO:0015078	hydrogen ion transmembra	0.73	2	0.36166364	160	0.4620672	
6668	GO:0009314	response to radiation	0.73	1	0.18083182	81	0.23392151	
6751	GO:0009416	response to light stimulus	0.73	1	0.18083182	81	0.23392151	
15189	GO:0032787	monocarboxylic acid metab	0.73	1	0.18083182	81	0.23392151	
6991	GO:0009725	response to hormone stimu	0.73	1	0.18083182	82	0.23680943	
29202	GO:0070838	divalent metal ion transport	0.73	1	0.18083182	82	0.23680943	
9839	GO:0016791|GO:0016302	phosphatase activity	0.73	5	0.9041591	383	1.1060733	
27228	GO:0060255	regulation of macromolecul	0.73	39	7.052441	2660	7.6818666	
5756	GO:0008219	cell death	0.74	3	0.5424955	239	0.69021285	
8209	GO:0012501|GO:0016244	programmed cell death	0.74	3	0.5424955	239	0.69021285	
9462	GO:0016265	death	0.74	3	0.5424955	239	0.69021285	
5055	GO:0006996	organelle organization	0.74	2	0.36166364	164	0.47361887	
3048	GO:0004364	glutathione transferase acti	0.74	1	0.18083182	84	0.24258527	
4327	GO:0006091	generation of precursor me	0.74	1	0.18083182	84	0.24258527	
30867	GO:0072511	divalent inorganic cation tra	0.74	1	0.18083182	84	0.24258527	
131	GO:0000166	nucleotide binding	0.74	17	3.074141	1213	3.5030468	
2643	GO:0003700|GO:0000130	sequence-specific DNA bin	0.75	20	3.6166365	1417	4.0921826	
716	GO:0001071	nucleic acid binding transcr	0.75	20	3.6166365	1417	4.0921826	
5712	GO:0008168|GO:0004480	methyltransferase activity	0.75	3	0.5424955	244	0.7046524	
5768	GO:0008238	exopeptidase activity	0.75	1	0.18083182	86	0.24836111	
6985	GO:0009719	response to endogenous st	0.75	1	0.18083182	86	0.24836111	
12734	GO:0030163	protein catabolic process	0.75	1	0.18083182	86	0.24836111	
3341	GO:0004722|GO:0000158|	protein serine/threonine pho	0.76	2	0.36166364	171	0.4938343	
21331	GO:0045735	nutrient reservoir activity	0.76	1	0.18083182	89	0.25702485	
2982	GO:0004197	cysteine-type endopeptidas	0.77	1	0.18083182	90	0.2599128	
4605	GO:0006457|GO:0007022|	protein folding	0.77	2	0.36166364	173	0.49961013	
3644	GO:0005102	receptor binding	0.77	1	0.18083182	91	0.2628007	
6784	GO:0009451|GO:0016547	RNA modification	0.77	1	0.18083182	91	0.2628007	
5693	GO:0008135	translation factor activity, n	0.77	1	0.18083182	92	0.26568863	
2674	GO:0003824	catalytic activity	0.77	164	29.65642	10750	31.045137	
11712	GO:0019748	secondary metabolic proce	0.78	1	0.18083182	95	0.27435237	
18633	GO:0042277	peptide binding	0.78	1	0.18083182	95	0.27435237	
30306	GO:0071944	cell periphery	0.78	3	0.5424955	259	0.74797124	
9796	GO:0016741	transferase activity, transfe	0.79	3	0.5424955	261	0.7537471	
9823	GO:0016772	transferase activity, transfe	0.79	27	4.882459	1932	5.579461	
5610	GO:0008026	ATP-dependent helicase ac	0.79	2	0.36166364	183	0.52848935	
28401	GO:0070035	purine NTP-dependent helic	0.79	2	0.36166364	183	0.52848935	
13829	GO:0031406	carboxylic acid binding	0.79	1	0.18083182	98	0.28301615	
20505	GO:0044255	cellular lipid metabolic proc	0.79	2	0.36166364	184	0.53137726	
9486	GO:0016298	lipase activity	0.80	2	0.36166364	185	0.53426516	
3999	GO:0005694	chromosome	0.80	1	0.18083182	99	0.28590405	
16851	GO:0034470	ncRNA processing	0.80	1	0.18083182	99	0.28590405	
17039	GO:0034660	ncRNA metabolic process	0.80	1	0.18083182	99	0.28590405	
9488	GO:0016301	kinase activity	0.80	24	4.339964	1740	5.0249805	
3304	GO:0004672|GO:0050222	protein kinase activity	0.80	18	3.254973	1333	3.8495972	
9699	GO:0016616	oxidoreductase activity, act	0.80	3	0.5424955	266	0.7681867	
7271	GO:0010033	response to organic substa	0.80	1	0.18083182	100	0.28879198	
4459	GO:0006259|GO:0055132	DNA metabolic process	0.80	6	1.084991	493	1.4237444	
5705	GO:0008152	metabolic process	0.80	269	48.64376	17432	50.34222	
3306	GO:0004674|GO:0004695|	protein serine/threonine kin	0.80	14	2.5316455	1062	3.0669708	
3068	GO:0004386	helicase activity	0.81	2	0.36166364	189	0.54581684	
9494	GO:0016311	dephosphorylation	0.81	5	0.9041591	425	1.227366	
30206	GO:0071842	cellular component organiz	0.82	2	0.36166364	193	0.5573685	
2897	GO:0004091|GO:0004302|	carboxylesterase activity	0.82	2	0.36166364	194	0.5602564	
6426	GO:0009057|GO:0043285	macromolecule catabolic pr	0.82	1	0.18083182	106	0.3061195	
2653	GO:0003723	RNA binding	0.82	10	1.8083183	797	2.3016722	
8910	GO:0015631	tubulin binding	0.82	1	0.18083182	107	0.30900744	
3178	GO:0004519	endonuclease activity	0.82	4	0.7233273	356	1.0280994	
3626	GO:0005083	small GTPase regulator act	0.83	1	0.18083182	111	0.3205591	
19686	GO:0043414	macromolecule methylation	0.84	1	0.18083182	113	0.32633495	
18912	GO:0042578	phosphoric ester hydrolase	0.84	5	0.9041591	444	1.2822365	
22478	GO:0046982	protein heterodimerization a	0.84	1	0.18083182	114	0.32922286	
4612	GO:0006468	protein phosphorylation	0.84	30	5.424955	2205	6.367863	
9697	GO:0016614	oxidoreductase activity, act	0.85	3	0.5424955	293	0.84616053	
2521	GO:0003333	amino acid transmembrane	0.85	1	0.18083182	118	0.34077454	
8584	GO:0015171|GO:0015359	amino acid transmembrane	0.85	1	0.18083182	118	0.34077454	
18956	GO:0042626	ATPase activity, coupled to	0.85	2	0.36166364	211	0.6093511	
9861	GO:0016820	hydrolase activity, acting on	0.85	2	0.36166364	212	0.612239	
9493	GO:0016310	phosphorylation	0.86	34	6.148282	2504	7.2313514	
18953	GO:0042623	ATPase activity, coupled	0.86	6	1.084991	537	1.550813	
6884	GO:0009607	response to biotic stimulus	0.86	1	0.18083182	122	0.3523262	
12532	GO:0022836	gated channel activity	0.86	1	0.18083182	122	0.3523262	
25485	GO:0051188	cofactor biosynthetic proce	0.86	1	0.18083182	123	0.35521415	
3863	GO:0005488	binding	0.86	210	37.97468	13903	40.15075	
4236	GO:0005996	monosaccharide metabolic	0.87	1	0.18083182	125	0.36099	
3761	GO:0005275|GO:0005279	amine transmembrane tran	0.87	1	0.18083182	126	0.3638779	
9913	GO:0016881	acid-amino acid ligase activ	0.87	4	0.7233273	391	1.1291766	
22415	GO:0046914	transition metal ion binding	0.87	35	6.329114	2599	7.5057034	
4526	GO:0006351|GO:0006350|	transcription, DNA-depende	0.88	20	3.6166365	1575	4.548474	
15176	GO:0032774	RNA biosynthetic process	0.88	20	3.6166365	1575	4.548474	
2758	GO:0003924	GTPase activity	0.88	1	0.18083182	132	0.3812054	
4394	GO:0006184	GTP catabolic process	0.88	1	0.18083182	132	0.3812054	
21605	GO:0046039	GTP metabolic process	0.88	1	0.18083182	132	0.3812054	
4408	GO:0006200	ATP catabolic process	0.89	7	1.2658228	641	1.8511566	
9918	GO:0016887|GO:0004002	ATPase activity	0.89	7	1.2658228	641	1.8511566	
21600	GO:0046034	ATP metabolic process	0.89	7	1.2658228	641	1.8511566	
3801	GO:0005342	organic acid transmembran	0.89	1	0.18083182	136	0.3927571	
22444	GO:0046943	carboxylic acid transmemb	0.89	1	0.18083182	136	0.3927571	
19758	GO:0043492	ATPase activity, coupled to	0.89	2	0.36166364	235	0.67866117	
3724	GO:0005216	ion channel activity	0.90	1	0.18083182	140	0.40430877	
23515	GO:0048037	cofactor binding	0.90	3	0.5424955	327	0.94434977	
5909	GO:0008415	acyltransferase activity	0.90	3	0.5424955	330	0.95301354	
4897	GO:0006793	phosphorus metabolic proc	0.90	39	7.052441	2925	8.4471655	
4900	GO:0006796	phosphate metabolic proce	0.90	39	7.052441	2925	8.4471655	
4304	GO:0006066	alcohol metabolic process	0.90	1	0.18083182	143	0.41297254	
9895	GO:0016860	intramolecular oxidoreducta	0.91	1	0.18083182	146	0.42163628	
6824	GO:0009536	plastid	0.91	2	0.36166364	247	0.7133162	
5661	GO:0008092	cytoskeletal protein binding	0.91	2	0.36166364	249	0.719092	
3177	GO:0004518	nuclease activity	0.91	4	0.7233273	427	1.2331418	
9824	GO:0016773	phosphotransferase activity	0.91	18	3.254973	1493	4.311664	
8749	GO:0015399	primary active transmembr	0.92	2	0.36166364	260	0.75085914	
8753	GO:0015405	P-P-bond-hydrolysis-driven	0.92	2	0.36166364	260	0.75085914	
9285	GO:0016043	cellular component organiz	0.92	2	0.36166364	261	0.7537471	
5764	GO:0008234|GO:0004220	cysteine-type peptidase act	0.93	1	0.18083182	163	0.47073093	
4405	GO:0006195	purine nucleotide catabolic	0.93	8	1.4466546	773	2.232362	
6508	GO:0009143	nucleoside triphosphate cat	0.93	8	1.4466546	773	2.232362	
6509	GO:0009144	purine nucleoside triphosph	0.93	8	1.4466546	773	2.232362	

6511	GO:0009146	purine nucleoside triphosph	0.93	8	1.4466546	773	2.232362	
6519	GO:0009154	purine ribonucleotide catab	0.93	8	1.4466546	773	2.232362	
6530	GO:0009166	nucleotide catabolic proces	0.93	8	1.4466546	773	2.232362	
6567	GO:0009203	ribonucleoside triphosphate	0.93	8	1.4466546	773	2.232362	
6569	GO:0009205	purine ribonucleoside tripho	0.93	8	1.4466546	773	2.232362	
6571	GO:0009207	purine ribonucleoside tripho	0.93	8	1.4466546	773	2.232362	
6624	GO:0009261	ribonucleotide catabolic pro	0.93	8	1.4466546	773	2.232362	
17034	GO:0034655	nucleobase, nucleoside, nu	0.93	8	1.4466546	773	2.232362	
17035	GO:0034656	nucleobase, nucleoside and	0.93	8	1.4466546	773	2.232362	
22209	GO:0046700	heterocycle catabolic proce	0.93	8	1.4466546	773	2.232362	
30879	GO:0072523	purine-containing compoun	0.93	8	1.4466546	773	2.232362	
25483	GO:0051186	cofactor metabolic process	0.93	1	0.18083182	164	0.47361887	
2627	GO:0003676	nucleic acid binding	0.93	31	5.605787	2453	7.0840673	
20520	GO:0044270	cellular nitrogen compound	0.93	8	1.4466546	777	2.2439137	
4476	GO:0006278	RNA-dependent DNA replic	0.93	1	0.18083182	166	0.4793947	
20521	GO:0044271	cellular nitrogen compound	0.93	3	0.5424955	367	1.0598665	
9911	GO:0016879	ligase activity, forming carb	0.93	4	0.7233273	456	1.3168914	
6899	GO:0009628	response to abiotic stimulus	0.94	1	0.18083182	170	0.49094638	
26932	GO:0055086	nucleobase, nucleoside and	0.94	10	1.8083183	944	2.7261963	
6563	GO:0009199	ribonucleoside triphosphate	0.94	8	1.4466546	790	2.2814567	
6515	GO:0009150	purine ribonucleotide metab	0.94	8	1.4466546	791	2.2843447	
3874	GO:0005509	calcium ion binding	0.94	2	0.36166364	279	0.8057296	
11522	GO:0019538|GO:0006411	protein metabolic process	0.94	55	9.94575	4134	11.938661	
6506	GO:0009141	nucleoside triphosphate me	0.94	8	1.4466546	796	2.2987843	
9579	GO:0016462	pyrophosphatase activity	0.94	10	1.8083183	954	2.7550755	
9859	GO:0016818	hydrolase activity, acting or	0.94	10	1.8083183	956	2.7608514	
10058	GO:0017111	nucleoside-triphosphatase a	0.94	9	1.6274865	878	2.5355935	
11787	GO:0019842	vitamin binding	0.94	1	0.18083182	177	0.5111618	
20622	GO:0044428	nuclear part	0.94	1	0.18083182	178	0.5140497	
30205	GO:0071841	cellular component organiz	0.94	2	0.36166364	285	0.8230572	
9858	GO:0016817	hydrolase activity, acting or	0.94	10	1.8083183	959	2.769515	
17024	GO:0034645|GO:0034961	cellular macromolecule bios	0.94	24	4.339964	1999	5.7729516	
4572	GO:0006412|GO:0006416|	translation	0.95	1	0.18083182	180	0.5198256	
6428	GO:0009059|GO:0043284	macromolecule biosynthetic	0.95	24	4.339964	2003	5.7845035	
4373	GO:0006163	purine nucleotide metabolic	0.95	8	1.4466546	810	2.339215	
6622	GO:0009259|GO:0009121	ribonucleotide metabolic pr	0.95	8	1.4466546	812	2.344991	
4844	GO:0006725	cellular aromatic compound	0.95	1	0.18083182	185	0.53426516	
30877	GO:0072521	purine-containing compoun	0.95	8	1.4466546	827	2.3883097	
4867	GO:0006753	nucleoside phosphate meta	0.96	9	1.6274865	914	2.6395588	
6484	GO:0009117	nucleotide metabolic proces	0.96	9	1.6274865	914	2.6395588	
9889	GO:0016853	isomerase activity	0.96	2	0.36166364	306	0.8837035	
10244	GO:0018130	heterocycle biosynthetic pro	0.96	1	0.18083182	199	0.57469606	
22375	GO:0046872	metal ion binding	0.96	37	6.690778	2985	8.6204405	
14380	GO:0031967	organelle envelope	0.96	1	0.18083182	201	0.5804719	
19470	GO:0043167	ion binding	0.96	37	6.690778	2997	8.655096	
19472	GO:0043169	cation binding	0.96	37	6.690778	2997	8.655096	
9906	GO:0016874	ligase activity	0.96	5	0.9041591	597	1.7240882	
14387	GO:0031975	envelope	0.96	1	0.18083182	205	0.59202355	
22007	GO:0046483	heterocycle metabolic proce	0.96	11	1.98915	1094	3.1593843	
20532	GO:0044282	small molecule catabolic pr	0.96	8	1.4466546	854	2.4662836	
9308	GO:0016070	RNA metabolic process	0.96	21	3.7974684	1855	5.3570914	
3449	GO:0004842|GO:0004840|	ubiquitin-protein ligase activ	0.97	2	0.36166364	326	0.94146186	
20488	GO:0044238	primary metabolic process	0.97	128	23.146473	9167	26.47356	
9654	GO:0016567	protein ubiquitination	0.97	2	0.36166364	332	0.9587894	
14854	GO:0032446	protein modification by sma	0.97	2	0.36166364	332	0.9587894	
29011	GO:0070647	protein modification by sma	0.97	2	0.36166364	332	0.9587894	
4558	GO:0006396|GO:0006394	RNA processing	0.97	1	0.18083182	219	0.63245445	
11747	GO:0019787|GO:0008639|	small conjugating protein lig	0.97	2	0.36166364	339	0.9790048	
31895	GO:0090304	nucleic acid metabolic proc	0.97	27	4.882459	2340	6.7577324	
4460	GO:0006260|GO:0055133	DNA replication	0.97	1	0.18083182	224	0.64689404	
7691	GO:0010467	gene expression	0.97	22	3.9783	1974	5.7007537	
20498	GO:0044248	cellular catabolic process	0.98	8	1.4466546	894	2.5818002	
6796	GO:0009507	chloroplast	0.98	1	0.18083182	229	0.6613336	
20533	GO:0044283	small molecule biosynthetic	0.98	2	0.36166364	347	1.0021082	
8211	GO:0012505	endomembrane system	0.98	1	0.18083182	242	0.6988766	
30204	GO:0071840	cellular component organiz	0.98	2	0.36166364	362	1.045427	
4609	GO:0006464	protein modification proces	0.98	32	5.786618	2781	8.031305	
19533	GO:0043234	protein complex	0.98	7	1.2658228	848	2.448956	
6427	GO:0009058	biosynthetic process	0.98	32	5.786618	2796	8.074624	
19684	GO:0043412	macromolecule modificatio	0.99	33	5.96745	2900	8.374968	
3716	GO:0005198	structural molecule activity	0.99	2	0.36166364	393	1.1349525	
6425	GO:0009056	catabolic process	0.99	9	1.6274865	1051	3.0352037	
20499	GO:0044249	cellular biosynthetic proces	0.99	29	5.244123	2635	7.6096687	
19473	GO:0043170|GO:0043283	macromolecule metabolic p	0.99	84	15.189874	6553	18.92454	
3955	GO:0005634	nucleus	0.99	13	2.3508136	1433	4.138389	
4357	GO:0006139|GO:0055134	nucleobase, nucleoside, nu	0.99	37	6.690778	3285	9.486816	
20517	GO:0044267	cellular protein metabolic p	0.99	36	6.509946	3225	9.313541	
20531	GO:0044281	small molecule metabolic p	0.99	17	3.074141	1788	5.1636004	
4320	GO:0006082	organic acid metabolic proc	1.00	1	0.18083182	369	1.0656425	
11716	GO:0019752	carboxylic acid metabolic p	1.00	1	0.18083182	369	1.0656425	
19707	GO:0043436	oxoacid metabolic process	1.00	1	0.18083182	369	1.0656425	
7229	GO:0009987|GO:0008151|	cellular process	1.00	140	25.316456	10601	30.614838	
18541	GO:0042180	cellular ketone metabolic pr	1.00	1	0.18083182	375	1.0829699	
20510	GO:0044260|GO:0034960	cellular macromolecule me	1.00	65	11.754068	5577	16.105928	
17020	GO:0034641	cellular nitrogen compound	1.00	38	6.871609	3678	10.621769	
20640	GO:0044446	intracellular organelle part	1.00	3	0.5424955	710	2.0504231	
20616	GO:0044422	organelle part	1.00	3	0.5424955	712	2.0561988	
4906	GO:0006807	nitrogen compound metabo	1.00	38	6.871609	3725	10.757502	
19527	GO:0043228	non-membrane-bounded or	1.00	4	0.7233273	923	2.66555	
19531	GO:0043232	intracellular non-membrane	1.00	4	0.7233273	923	2.66555	
20487	GO:0044237	cellular metabolic process	1.00	94	16.998192	8185	23.637625	
19526	GO:0043227	membrane-bounded organe	1.00	15	2.7124774	2142	6.185924	
19530	GO:0043231	intracellular membrane-bou	1.00	15	2.7124774	2142	6.185924	
4109	GO:0005840|GO:0033279	ribosome	1.00	1	0.18083182	682	1.9695613	
5791	GO:0008270	zinc ion binding	1.00	11	1.98915	1880	5.4292893	
15392	GO:0032991	macromolecular complex	1.00	8	1.4466546	1616	4.666878	
13018	GO:0030529	ribonucleoprotein complex	1.00	1	0.18083182	741	2.1399486	
19525	GO:0043226	organelle	1.00	19	3.4358046	3013	8.701303	
19528	GO:0043229	intracellular organelle	1.00	19	3.4358046	3013	8.701303	
3945	GO:0005622	intracellular	1.00	37	6.690778	4751	13.720507	
20618	GO:0044424	intracellular part	1.00	27	4.882459	3853	11.127155	
4027	GO:0005737	cytoplasm	1.00	7	1.2658228	2001	5.7787275	
20638	GO:0044444	cytoplasmic part	1.00	4	0.7233273	1644	4.7477403	

GO ID	GO ACCESSION	GO Term	p-value	Count in Selection	% Count in Selection	Count in Total	% Count in Total
4476	GO:0006278	RNA-dependent DNA re	0.00	38	5.322129	166	0.4793947
4459	GO:0006259|GO:00551	DNA metabolic process	0.00	59	8.263306	493	1.4237444
4460	GO:0006260|GO:00551	DNA replication	0.00	38	5.322129	224	0.64689404
8493	GO:0015074	DNA integration	0.00	21	2.9411764	47	0.13573223
31895	GO:0090304	nucleic acid metabolic p	0.00	94	13.165266	2340	6.7577324
31393	GO:0080016	(-)-E-beta-caryophyllene	0.00	9	1.2605042	25	0.072197996
31394	GO:0080017	alpha-humulene syntha	0.00	9	1.2605042	25	0.072197996
7558	GO:0010334	sesquiterpene synthase	0.00	9	1.2605042	29	0.083749674
7222	GO:0009975	cyclase activity	0.00	9	1.2605042	31	0.08952551
7557	GO:0010333	terpene synthase activit	0.00	11	1.5406163	57	0.16461143
9877	GO:0016838	carbon-oxygen lyase ac	0.00	12	1.6806723	77	0.22236982
4357	GO:0006139|GO:00551	nucleobase, nucleoside	0.00	111	15.546219	3285	9.486816
17024	GO:0034645|GO:00349	cellular macromolecule	0.00	73	10.22409	1999	5.7729516
6428	GO:0009059|GO:00432	macromolecule biosynth	0.00	73	10.22409	2003	5.7845035
17020	GO:0034641	cellular nitrogen compou	0.00	114	15.966387	3678	10.621769
4906	GO:0006807	nitrogen compound met	0.00	114	15.966387	3725	10.757502
6884	GO:0009607	response to biotic stimu	0.00	11	1.5406163	122	0.3523262
23522	GO:0048046	apoplast	0.00	8	1.1204482	70	0.20215438
5705	GO:0008152	metabolic process	0.00	408	57.142857	17432	50.34222
9869	GO:0016829	lyase activity	0.00	23	3.2212884	488	1.4093049
22375	GO:0046872	metal ion binding	0.00	89	12.464986	2985	8.6204405
9874	GO:0016835	carbon-oxygen lyase ac	0.00	13	1.8207283	202	0.5833598
19470	GO:0043167	ion binding	0.00	89	12.464986	2997	8.655096
19472	GO:0043169	cation binding	0.00	89	12.464986	2997	8.655096
20499	GO:0044249	cellular biosynthetic pro	0.00	80	11.204482	2635	7.6096687
3009	GO:0004322	ferroxidase activity	0.00	5	0.70028013	30	0.086637594
9785	GO:0016724	oxidoreductase activity,	0.00	5	0.70028013	30	0.086637594
6427	GO:0009058	biosynthetic process	0.00	83	11.62465	2796	8.074624
26959	GO:0055114	oxidation-reduction proc	0.00	75	10.504202	2490	7.1909204
2864	GO:0004058|GO:00164	aromatic-L-amino-acid d	0.00	3	0.42016807	9	0.025991278
3874	GO:0005509	calcium ion binding	0.00	15	2.1008403	279	0.8057296
3904	GO:0005576	extracellular region	0.00	11	1.5406163	178	0.5140497
3386	GO:0004773	steryl-sulfatase activity	0.00	3	0.42016807	12	0.03465504
5965	GO:0008484	sulfuric ester hydrolase	0.00	3	0.42016807	12	0.03465504
9816	GO:0016762	xyloglucan:xyloglucosyl	0.00	4	0.5602241	27	0.077973835
5704	GO:0008150|GO:00000	biological_process	0.00	494	69.187675	22215	64.155136
3473	GO:0004867	serine-type endopeptida	0.00	6	0.84033614	66	0.1906027
5791	GO:0008270	zinc ion binding	0.00	57	7.9831934	1880	5.4292893
14101	GO:0031683	G-protein beta/gamma-s	0.00	3	0.42016807	15	0.043318797
9783	GO:0016722	oxidoreductase activity,	0.00	5	0.70028013	50	0.14439599
18757	GO:0042409	caffeoyl-CoA O-methyltr	0.00	3	0.42016807	16	0.046206716
25837	GO:0051553	flavone biosynthetic pro	0.00	4	0.5602241	32	0.09241343
25838	GO:0051554	flavonol metabolic proce	0.00	4	0.5602241	32	0.09241343
25839	GO:0051555	flavonol biosynthetic pro	0.00	4	0.5602241	32	0.09241343
21041	GO:0045431	flavonol synthase activit	0.00	4	0.5602241	32	0.09241343
25836	GO:0051552	flavone metabolic proce	0.00	4	0.5602241	32	0.09241343
19707	GO:0043436	oxoacid metabolic proce	0.00	16	2.2408965	369	1.0656425
4320	GO:0006082	organic acid metabolic p	0.00	16	2.2408965	369	1.0656425
11716	GO:0019752	carboxylic acid metaboli	0.00	16	2.2408965	369	1.0656425
22415	GO:0046914	transition metal ion bind	0.00	73	10.22409	2599	7.5057034
5702	GO:0008146	sulfotransferase activity	0.01	5	0.70028013	54	0.15594767
18541	GO:0042180	cellular ketone metaboli	0.01	16	2.2408965	375	1.0829699
3888	GO:0005529	sugar binding	0.01	9	1.2605042	158	0.45629135
7072	GO:0009813	flavonoid biosynthetic p	0.01	4	0.5602241	40	0.1155168
13038	GO:0030551	cyclic nucleotide binding	0.01	3	0.42016807	22	0.06353424
3256	GO:0004616	phosphogluconate dehy	0.01	2	0.28011206	8	0.023103358
7071	GO:0009812	flavonoid metabolic proc	0.01	4	0.5602241	44	0.12706847
3472	GO:0004866	endopeptidase inhibitor	0.01	6	0.84033614	95	0.27435237
8159	GO:0010951	negative regulation of e	0.01	6	0.84033614	95	0.27435237
26712	GO:0052548	regulation of endopeptic	0.01	6	0.84033614	95	0.27435237
28101	GO:0061135	endopeptidase regulato	0.01	6	0.84033614	95	0.27435237
12805	GO:0030246	carbohydrate binding	0.02	11	1.5406163	251	0.7248679
6632	GO:0009269	response to desiccation	0.02	2	0.28011206	10	0.0288792
21148	GO:0045547	dehydrodolichyl diphosp	0.02	2	0.28011206	10	0.0288792
6750	GO:0009415	response to water	0.02	4	0.5602241	49	0.14150807
9832	GO:0016782	transferase activity, tran	0.02	5	0.70028013	74	0.21370606
9767	GO:0016705	oxidoreductase activity,	0.02	13	1.8207283	329	0.95012563
19473	GO:0043170|GO:00432	macromolecule metabol	0.02	157	21.988796	6553	18.92454
6965	GO:0009699	phenylpropanoid biosyn	0.02	4	0.5602241	52	0.15017183
7690	GO:0010466	negative regulation of p	0.03	6	0.84033614	110	0.31767118
12946	GO:0030414	peptidase inhibitor activ	0.03	6	0.84033614	110	0.31767118
25636	GO:0051346	negative regulation of h	0.03	6	0.84033614	110	0.31767118
26711	GO:0052547	regulation of peptidase	0.03	6	0.84033614	112	0.32344702
28100	GO:0061134	peptidase regulator acti	0.03	6	0.84033614	112	0.32344702
5715	GO:0008171	O-methyltransferase act	0.03	4	0.5602241	57	0.16461143
9871	GO:0016831	carboxy-lyase activity	0.03	6	0.84033614	115	0.3321108
11776	GO:0019825	oxygen binding	0.03	14	1.9607843	389	1.1234008
31408	GO:0080031	methyl salicylate esteras	0.03	2	0.28011206	14	0.040430877
6964	GO:0009698	phenylpropanoid metab	0.04	4	0.5602241	60	0.17327519
14811	GO:0032403	protein complex binding	0.04	4	0.5602241	60	0.17327519
9393	GO:0016165	lipoxygenase activity	0.04	3	0.42016807	36	0.10396511
6018	GO:0008559|GO:00052	xenobiotic-transporting	0.04	4	0.5602241	62	0.17905103
19225	GO:0042908	xenobiotic transport	0.04	4	0.5602241	62	0.17905103
19227	GO:0042910	xenobiotic transporter a	0.04	4	0.5602241	62	0.17905103
4699	GO:0006570	tyrosine metabolic proce	0.04	1	0.14005603	2	0.00577584
4700	GO:0006571	tyrosine biosynthetic pro	0.04	1	0.14005603	2	0.00577584
9419	GO:0016206	catechol O-methyltransf	0.04	1	0.14005603	2	0.00577584
3382	GO:0004768|GO:00162	stearoyl-CoA 9-desatura	0.04	2	0.28011206	16	0.046206716
9427	GO:0016215	CoA desaturase activity	0.04	2	0.28011206	16	0.046206716
31396	GO:0080019	fatty-acyl-CoA reductase	0.04	2	0.28011206	16	0.046206716
6749	GO:0009414	response to water depri	0.04	3	0.42016807	38	0.10974095
21710	GO:0046148	pigment biosynthetic pro	0.05	4	0.5602241	69	0.19926646
9812	GO:0016758	transferase activity, tran	0.06	16	2.2408965	501	1.4468478
13651	GO:0031219	levanase activity	0.06	1	0.14005603	3	0.00866376
9764	GO:0016701	oxidoreductase activity,	0.06	4	0.5602241	72	0.20793022
9753	GO:0016682	oxidoreductase activity,	0.06	3	0.42016807	44	0.12706847
18786	GO:0042440	pigment metabolic proce	0.06	4	0.5602241	73	0.21081814
8666	GO:0015276	ligand-gated ion channe	0.07	3	0.42016807	45	0.1299564
12530	GO:0022834	ligand-gated channel ac	0.07	3	0.42016807	45	0.1299564
23518	GO:0048040	UDP-glucuronate decar	0.07	2	0.28011206	21	0.060646318
3129	GO:0004467	long-chain fatty acid-Co	0.07	2	0.28011206	21	0.060646318

20510	GO:0044260|GO:00349	cellular macromolecule	0.07	130	18.207283	5577	16.105928
9768	GO:0016706	oxidoreductase activity,	0.07	7	0.98039216	175	0.505386
18956	GO:0042626	ATPase activity, couple	0.07	8	1.1204482	211	0.6093511
9861	GO:0016820	hydrolase activity, actin	0.07	8	1.1204482	212	0.612239
24858	GO:0050551	myrcene synthase activ	0.07	2	0.28011206	22	0.06353424
11787	GO:0019842	vitamin binding	0.07	7	0.98039216	177	0.5111618
23515	GO:0048037	cofactor binding	0.08	11	1.5406163	327	0.94434977
9811	GO:0016757|GO:00169	transferase activity, tran	0.08	21	2.9411764	729	2.1052935
3291	GO:0004657	proline dehydrogenase	0.08	1	0.14005603	4	0.011551679
3834	GO:0005395	eye pigment precursor t	0.08	1	0.14005603	4	0.011551679
4325	GO:0006089	lactate metabolic proces	0.08	1	0.14005603	4	0.011551679
4345	GO:0006113	fermentation	0.08	1	0.14005603	4	0.011551679
6422	GO:0009051	pentose-phosphate shu	0.08	1	0.14005603	4	0.011551679
8459	GO:0015018	galactosylgalactosylxylo	0.08	1	0.14005603	4	0.011551679
11632	GO:0019656	glucose catabolic proce	0.08	1	0.14005603	4	0.011551679
11635	GO:0019659	glucose catabolic proce	0.08	1	0.14005603	4	0.011551679
11638	GO:0019662	non-glycolytic fermentat	0.08	1	0.14005603	4	0.011551679
16391	GO:0034007	S-linalool synthase activ	0.08	1	0.14005603	4	0.011551679
22893	GO:0047405	pyrimidine-5'-nucleotide	0.08	1	0.14005603	4	0.011551679
24433	GO:0050113	inositol oxygenase activ	0.08	1	0.14005603	4	0.011551679
2753	GO:0003919	FMN adenylyltransferas	0.08	1	0.14005603	4	0.011551679
9779	GO:0016717	oxidoreductase activity,	0.09	2	0.28011206	24	0.06931008
31407	GO:0080030	methyl indole-3-acetate	0.09	2	0.28011206	24	0.06931008
3293	GO:0004659	prenyltransferase activit	0.09	3	0.42016807	51	0.14728391
9482	GO:0016291|GO:00087	acyl-CoA thioesterase a	0.09	2	0.28011206	25	0.072197996
31493	GO:0080118	brassinosteroid sulfotra	0.09	2	0.28011206	25	0.072197996
9750	GO:0016679	oxidoreductase activity,	0.10	3	0.42016807	53	0.15305975
5978	GO:0008506	sucrose:hydrogen symp	0.10	2	0.28011206	26	0.075085916
5987	GO:0008515|GO:00191	sucrose transmembrane	0.10	2	0.28011206	26	0.075085916
8567	GO:0015154	disaccharide transmem	0.10	2	0.28011206	26	0.075085916
9035	GO:0015766	disaccharide transport	0.10	2	0.28011206	26	0.075085916
9039	GO:0015770	sucrose transport	0.10	2	0.28011206	26	0.075085916
17147	GO:0034768	(E)-beta-ocimene synth	0.10	2	0.28011206	26	0.075085916
4641	GO:0006508	proteolysis	0.10	26	3.6414566	965	2.7868426
8919	GO:0015645	fatty acid ligase activity	0.11	2	0.28011206	27	0.077973835
3464	GO:0004857	enzyme inhibitor activity	0.11	6	0.84033614	157	0.4534034
119	GO:0000151	ubiquitin ligase complex	0.11	4	0.5602241	88	0.25413695
3260	GO:0004620	phospholipase activity	0.11	5	0.70028013	123	0.35521415
25626	GO:0051336	regulation of hydrolase	0.11	7	0.98039216	196	0.5660323
9813	GO:0016759	cellulose synthase activ	0.11	3	0.42016807	57	0.16461143
19758	GO:0043492	ATPase activity, couple	0.11	8	1.1204482	235	0.67866117
9870	GO:0016830	carbon-carbon lyase act	0.12	6	0.84033614	161	0.4649551
3376	GO:0004758	serine C-palmitoyltransf	0.12	1	0.14005603	6	0.01732752
3845	GO:0005452	inorganic anion exchang	0.12	1	0.14005603	6	0.01732752
4375	GO:0006165	nucleoside diphosphate	0.12	1	0.14005603	6	0.01732752
5966	GO:0008486	diphosphoinositol-polyp	0.12	1	0.14005603	6	0.01732752
6000	GO:0008531	riboflavin kinase activity	0.12	1	0.14005603	6	0.01732752
9572	GO:0016454	C-palmitoyltransferase a	0.12	1	0.14005603	6	0.01732752
9772	GO:0016710	trans-cinnamate 4-mon	0.12	1	0.14005603	6	0.01732752
11899	GO:0019992	diacylglycerol binding	0.12	1	0.14005603	6	0.01732752
19761	GO:0043495	protein anchor	0.12	1	0.14005603	6	0.01732752
3073	GO:0004397	histidine ammonia-lyase	0.12	1	0.14005603	6	0.01732752
3197	GO:0004550	nucleoside diphosphate	0.12	1	0.14005603	6	0.01732752
12741	GO:0030170	pyridoxal phosphate bin	0.12	5	0.70028013	126	0.3638779
28643	GO:0070279	vitamin B6 binding	0.12	5	0.70028013	126	0.3638779
2870	GO:0004064	arylesterase activity	0.13	2	0.28011206	30	0.086637594
28375	GO:0070008	serine-type exopeptidas	0.13	3	0.42016807	60	0.17327519
2980	GO:0004185	serine-type carboxypept	0.13	3	0.42016807	60	0.17327519
11712	GO:0019748	secondary metabolic pro	0.13	4	0.5602241	95	0.27435237
3255	GO:0004615|GO:00089	phosphomannomutase	0.14	1	0.14005603	7	0.020215439
16115	GO:0033729	anthocyanidin reductase	0.14	1	0.14005603	7	0.020215439
21149	GO:0045548	phenylalanine ammonia	0.14	1	0.14005603	7	0.020215439
2945	GO:0004143	diacylglycerol kinase ac	0.14	1	0.14005603	7	0.020215439
2978	GO:0004180	carboxypeptidase activit	0.14	3	0.42016807	62	0.17905103
6942	GO:0009674	potassium:sodium symp	0.14	2	0.28011206	32	0.09241343
9041	GO:0015772	oligosaccharide transpo	0.14	2	0.28011206	32	0.09241343
12522	GO:0022820	potassium ion symporte	0.14	2	0.28011206	32	0.09241343
9600	GO:0016491	oxidoreductase activity	0.15	42	5.882353	1724	4.9787736
3356	GO:0004737	pyruvate decarboxylase	0.15	1	0.14005603	8	0.023103358
3402	GO:0004791	thioredoxin-disulfide red	0.15	1	0.14005603	8	0.023103358
5953	GO:0008471	laccase activity	0.15	1	0.14005603	8	0.023103358
6378	GO:0008999	ribosomal-protein-alanin	0.15	1	0.14005603	8	0.023103358
6497	GO:0009132	nucleoside diphosphate	0.15	1	0.14005603	8	0.023103358
7003	GO:0009737	response to abscisic ac	0.15	1	0.14005603	8	0.023103358
7168	GO:0009916	alternative oxidase activ	0.15	1	0.14005603	8	0.023103358
9680	GO:0016595	glutamate binding	0.15	1	0.14005603	8	0.023103358
11196	GO:0019187	beta-1,4-mannosyltrans	0.15	1	0.14005603	8	0.023103358
18639	GO:0042284	sphingolipid delta-4 des	0.15	1	0.14005603	8	0.023103358
26035	GO:0051753	mannan synthase activi	0.15	1	0.14005603	8	0.023103358
2988	GO:0004300|GO:00165	enoyl-CoA hydratase ac	0.15	1	0.14005603	8	0.023103358
9765	GO:0016702	oxidoreductase activity,	0.16	3	0.42016807	66	0.1906027
11829	GO:0019900	kinase binding	0.16	3	0.42016807	66	0.1906027
4942	GO:0006855	drug transmembrane tra	0.16	5	0.70028013	139	0.40142086
8642	GO:0015238|GO:00152	drug transmembrane tra	0.16	5	0.70028013	139	0.40142086
9154	GO:0015893	drug transport	0.16	5	0.70028013	139	0.40142086
18832	GO:0042493|GO:00170	response to drug	0.16	5	0.70028013	139	0.40142086
9480	GO:0016289	CoA hydrolase activity	0.16	2	0.28011206	35	0.10107719
11931	GO:0020037	heme binding	0.17	15	2.1008403	550	1.5883559
8749	GO:0015399	primary active transmen	0.17	8	1.1204482	260	0.75085914
8753	GO:0015405	P-P-bond-hydrolysis-driv	0.17	8	1.1204482	260	0.75085914
4079	GO:0005795|GO:00169	Golgi stack	0.17	1	0.14005603	9	0.025991278
20488	GO:0044238	primary metabolic proce	0.18	200	28.011204	9167	26.47356
9486	GO:0016298	lipase activity	0.18	6	0.84033614	185	0.53426516
9818	GO:0016765|GO:00167	transferase activity, tran	0.18	6	0.84033614	185	0.53426516
5911	GO:0008417	fucosyltransferase activ	0.19	1	0.14005603	10	0.0288792
6464	GO:0009095	aromatic amino acid fam	0.19	1	0.14005603	10	0.0288792
8727	GO:0015369	calcium:hydrogen antipo	0.19	1	0.14005603	10	0.0288792
9741	GO:0016668|GO:00166	oxidoreductase activity,	0.19	1	0.14005603	10	0.0288792
23116	GO:0047631	ADP-ribose diphosphata	0.19	1	0.14005603	10	0.0288792
2797	GO:0003979	UDP-glucose 6-dehydro	0.19	1	0.14005603	10	0.0288792
4530	GO:0006355|GO:00325	regulation of transcriptio	0.19	60	8.403361	2591	7.4826
3872	GO:0005506	iron ion binding	0.19	15	2.1008403	563	1.6258988
25544	GO:0051252	regulation of RNA meta	0.19	60	8.403361	2593	7.488376
20487	GO:0044237	cellular metabolic proce	0.19	179	25.070028	8185	23.637625
19393	GO:0043086	negative regulation of c	0.19	6	0.84033614	188	0.54292893
20342	GO:0044092	negative regulation of m	0.19	6	0.84033614	188	0.54292893
11426	GO:0019438	aromatic compound bios	0.20	5	0.70028013	149	0.43030006

25510	GO:0051213	dioxygenase activity	0.20	3	0.42016807	75	0.21659398
7141	GO:0009889	regulation of biosyntheti	0.20	60	8.403361	2605	7.523031
7772	GO:0010556	regulation of macromole	0.20	60	8.403361	2605	7.523031
13756	GO:0031326	regulation of cellular bio	0.20	60	8.403361	2605	7.523031
32213	GO:2000112	regulation of cellular ma	0.20	60	8.403361	2605	7.523031
24968	GO:0050662	coenzyme binding	0.20	6	0.84033614	191	0.5515927
6288	GO:0008891	glycolate oxidase activit	0.20	1	0.14005603	11	0.03176712
11152	GO:0019139|GO:00464	cytokinin dehydrogenas	0.20	1	0.14005603	11	0.03176712
24696	GO:0050378	UDP-glucuronate 4-epim	0.20	1	0.14005603	11	0.03176712
2791	GO:0003973	(S)-2-hydroxy-acid oxida	0.20	1	0.14005603	11	0.03176712
9845	GO:0016798	hydrolase activity, acting	0.21	11	1.5406163	401	1.1580559
8461	GO:0015020|GO:00039	glucuronosyltransferase	0.21	2	0.28011206	42	0.121292636
3877	GO:0005516	calmodulin binding	0.22	7	0.98039216	236	0.6815491
9909	GO:0016877	ligase activity, forming c	0.22	3	0.42016807	78	0.22525774
25436	GO:0051139	metal ion:hydrogen anti	0.22	1	0.14005603	12	0.03465504
31380	GO:0080002	UDP-glucose:4-aminobe	0.22	1	0.14005603	12	0.03465504
11221	GO:0019219	regulation of nucleobase	0.22	61	8.543417	2675	7.7251854
25468	GO:0051171	regulation of nitrogen co	0.22	61	8.543417	2675	7.7251854
7692	GO:0010468	regulation of gene expre	0.23	60	8.403361	2635	7.6096687
9682	GO:0016597	amino acid binding	0.23	3	0.42016807	80	0.2310336
19479	GO:0043176	amine binding	0.23	3	0.42016807	80	0.2310336
13443	GO:0030984	kininogen binding	0.24	1	0.14005603	13	0.037542958
18582	GO:0042221	response to chemical st	0.24	9	1.2605042	328	0.94723773
12532	GO:0022836	gated channel activity	0.24	4	0.5602241	122	0.3523262
4912	GO:0006813|GO:00154	potassium ion transport	0.25	3	0.42016807	84	0.24258527
30168	GO:0071804	cellular potassium ion tr	0.25	3	0.42016807	84	0.24258527
30169	GO:0071805	potassium ion transmem	0.25	3	0.42016807	84	0.24258527
3048	GO:0004364	glutathione transferase	0.25	3	0.42016807	84	0.24258527
27228	GO:0060255	regulation of macromole	0.25	60	8.403361	2660	7.6818666
22409	GO:0046906	tetrapyrrole binding	0.25	15	2.1008403	596	1.7212002
8497	GO:0015079|GO:00228	potassium ion transmem	0.25	2	0.28011206	47	0.13573223
4670	GO:0006541	glutamine metabolic pro	0.25	1	0.14005603	14	0.040430877
6423	GO:0009052	pentose-phosphate shu	0.25	1	0.14005603	14	0.040430877
9399	GO:0016174	NAD(P)H oxidase activit	0.25	1	0.14005603	14	0.040430877
9879	GO:0016841	ammonia-lyase activity	0.25	1	0.14005603	14	0.040430877
11157	GO:0019144	ADP-sugar diphosphata	0.25	1	0.14005603	14	0.040430877
24970	GO:0050664	oxidoreductase activity,	0.25	1	0.14005603	14	0.040430877
2998	GO:0004311	farnesyltranstransferase	0.25	1	0.14005603	14	0.040430877
31465	GO:0080090	regulation of primary me	0.25	61	8.543417	2710	7.826263
9422	GO:0016209	antioxidant activity	0.25	5	0.70028013	165	0.47650677
5768	GO:0008238	exopeptidase activity	0.26	3	0.42016807	86	0.24836111
7074	GO:0009815	1-aminocyclopropane-1	0.27	2	0.28011206	49	0.14150807
8909	GO:0015630	microtubule cytoskeleto	0.27	2	0.28011206	49	0.14150807
9725	GO:0016645	oxidoreductase activity,	0.27	2	0.28011206	49	0.14150807
3209	GO:0004564	beta-fructofuranosidase	0.27	1	0.14005603	15	0.043318797
9536	GO:0016408	C-acyltransferase activit	0.27	1	0.14005603	15	0.043318797
2868	GO:0004062	aryl sulfotransferase act	0.27	1	0.14005603	15	0.043318797
21331	GO:0045735	nutrient reservoir activity	0.28	3	0.42016807	89	0.25702485
3334	GO:0004713|GO:00047	protein tyrosine kinase a	0.28	1	0.14005603	16	0.046206716
3742	GO:0005242	inward rectifier potassiu	0.28	1	0.14005603	16	0.046206716
7521	GO:0010295	(+)-abscisic acid 8'-hydr	0.28	1	0.14005603	16	0.046206716
9878	GO:0016840	carbon-nitrogen lyase a	0.28	1	0.14005603	16	0.046206716
13435	GO:0030976	thiamine pyrophosphate	0.28	1	0.14005603	16	0.046206716
4135	GO:0005875	microtubule associated	0.30	1	0.14005603	17	0.049094636
4446	GO:0006241	CTP biosynthetic proces	0.30	1	0.14005603	17	0.049094636
6507	GO:0009142	nucleoside triphosphate	0.30	1	0.14005603	17	0.049094636
6513	GO:0009148	pyrimidine nucleoside tr	0.30	1	0.14005603	17	0.049094636
6565	GO:0009201	ribonucleoside triphosph	0.30	1	0.14005603	17	0.049094636
6572	GO:0009208	pyrimidine ribonucleosid	0.30	1	0.14005603	17	0.049094636
6573	GO:0009209	pyrimidine ribonucleosid	0.30	1	0.14005603	17	0.049094636
9930	GO:0016899	oxidoreductase activity,	0.30	1	0.14005603	17	0.049094636
18738	GO:0042389	omega-3 fatty acid desa	0.30	1	0.14005603	17	0.049094636
21602	GO:0046036|GO:00062	CTP metabolic process	0.30	1	0.14005603	17	0.049094636
3242	GO:0004601|GO:00166	peroxidase activity	0.30	4	0.5602241	135	0.38986918
9754	GO:0016684	oxidoreductase activity,	0.30	4	0.5602241	135	0.38986918
12533	GO:0022838	substrate-specific chann	0.31	6	0.84033614	221	0.63823026
17918	GO:0035556|GO:00072	intracellular signal trans	0.31	2	0.28011206	54	0.15594767
3239	GO:0004596	peptide alpha-N-acetyltr	0.31	1	0.14005603	18	0.051982556
4134	GO:0005874	microtubule	0.31	1	0.14005603	18	0.051982556
5776	GO:0008252	nucleotidase activity	0.31	1	0.14005603	18	0.051982556
10899	GO:0018858	benzoate-CoA ligase ac	0.31	1	0.14005603	18	0.051982556
16595	GO:0034212	peptide N-acetyltransfer	0.31	1	0.14005603	18	0.051982556
13753	GO:0031323	regulation of cellular me	0.31	61	8.543417	2770	7.999538
26845	GO:0052689	carboxylic ester hydrola	0.32	13	1.8207283	538	1.5537009
8660	GO:0015267|GO:00152	channel activity	0.32	6	0.84033614	226	0.6526699
12515	GO:0022803	passive transmembrane	0.32	6	0.84033614	226	0.6526699
19887	GO:0043621	protein self-association	0.33	1	0.14005603	19	0.054870475
3724	GO:0005216	ion channel activity	0.33	4	0.5602241	140	0.40430877
13829	GO:0031406	carboxylic acid binding	0.33	3	0.42016807	98	0.28301615
4844	GO:0006725	cellular aromatic compo	0.33	5	0.70028013	185	0.53426516
3323	GO:0004702	receptor signaling prote	0.34	2	0.28011206	58	0.16749935
3328	GO:0004707|GO:00083	MAP kinase activity	0.34	1	0.14005603	20	0.0577584
6201	GO:0008794	arsenate reductase (glu	0.34	1	0.14005603	20	0.0577584
8726	GO:0015368	calcium:cation antiporte	0.34	1	0.14005603	20	0.0577584
13086	GO:0030611	arsenate reductase acti	0.34	1	0.14005603	20	0.0577584
13088	GO:0030613	oxidoreductase activity,	0.34	1	0.14005603	20	0.0577584
13089	GO:0030614	oxidoreductase activity,	0.34	1	0.14005603	20	0.0577584
30700	GO:0072341	modified amino acid bin	0.34	2	0.28011206	59	0.17038727
4526	GO:0006351|GO:00063	transcription, DNA-depe	0.35	35	4.901961	1575	4.548474
15176	GO:0032774	RNA biosynthetic proce	0.35	35	4.901961	1575	4.548474
4420	GO:0006213	pyrimidine nucleoside m	0.35	1	0.14005603	21	0.060646318
6582	GO:0009218	pyrimidine ribonucleotid	0.35	1	0.14005603	21	0.060646318
6584	GO:0009220	pyrimidine ribonucleotid	0.35	1	0.14005603	21	0.060646318
9902	GO:0016868|GO:00167	intramolecular transfera	0.35	1	0.14005603	21	0.060646318
21697	GO:0046131	pyrimidine ribonucleosid	0.35	1	0.14005603	21	0.060646318
8526	GO:0015112	nitrate transmembrane t	0.36	2	0.28011206	61	0.1761631
8975	GO:0015706|GO:00068	nitrate transport	0.36	2	0.28011206	61	0.1761631
12516	GO:0022804	active transmembrane t	0.36	18	2.5210085	787	2.2727928
8489	GO:0015066	alpha-amylase inhibitor	0.37	1	0.14005603	22	0.06353424
10032	GO:0017075	syntaxin-1 binding	0.37	1	0.14005603	22	0.06353424
11224	GO:0019222	regulation of metabolic p	0.38	67	9.383754	3115	8.995871
6512	GO:0009147	pyrimidine nucleoside tr	0.38	1	0.14005603	23	0.06642216
7052	GO:0009790|GO:00097	embryo development	0.38	1	0.14005603	23	0.06642216
9806	GO:0016752	sinapoyltransferase acti	0.38	1	0.14005603	23	0.06642216
9807	GO:0016753	O-sinapoyltransferase a	0.38	1	0.14005603	23	0.06642216
9808	GO:0016754	sinapoylglucose-malate	0.38	1	0.14005603	23	0.06642216
9710	GO:0016628	oxidoreductase activity,	0.39	2	0.28011206	65	0.18771479

9838	GO:0016790	thiolester hydrolase acti	0.39	2	0.28011206	65	0.18771479
6424	GO:0009055|GO:00090	electron carrier activity	0.39	4	0.5602241	154	0.44473964
3213	GO:0004568	chitinase activity	0.39	1	0.14005603	24	0.06931008
3262	GO:0004622|GO:00451	lysophospholipase activ	0.39	1	0.14005603	24	0.06931008
6248	GO:0008843	endochitinase activity	0.39	1	0.14005603	24	0.06931008
13657	GO:0031225	anchored to membrane	0.39	1	0.14005603	24	0.06931008
22440	GO:0046939	nucleotide phosphorylat	0.39	1	0.14005603	24	0.06931008
25578	GO:0051287|GO:00512	NAD binding	0.39	1	0.14005603	24	0.06931008
6824	GO:0009536	plastid	0.40	6	0.84033614	247	0.7133162
4331	GO:0006098	pentose-phosphate shu	0.41	1	0.14005603	25	0.072197996
4855	GO:0006739	NADP metabolic proces	0.41	1	0.14005603	25	0.072197996
4856	GO:0006740	NADPH regeneration	0.41	1	0.14005603	25	0.072197996
9846	GO:0016799	hydrolase activity, hydro	0.41	1	0.14005603	25	0.072197996
21145	GO:0045543	gibberellin 2-beta-dioxy	0.41	1	0.14005603	25	0.072197996
125	GO:0000159	protein phosphatase typ	0.41	1	0.14005603	25	0.072197996
3620	GO:0005057	receptor signaling prote	0.42	2	0.28011206	69	0.19926646
9722	GO:0016641	oxidoreductase activity,	0.42	2	0.28011206	69	0.19926646
3210	GO:0004565	beta-galactosidase activ	0.42	1	0.14005603	26	0.075085916
4030	GO:0005741	mitochondrial outer mer	0.42	1	0.14005603	26	0.075085916
9421	GO:0016208	AMP binding	0.42	1	0.14005603	26	0.075085916
20625	GO:0044431	Golgi apparatus part	0.42	1	0.14005603	26	0.075085916
3725	GO:0005217	intracellular ligand-gated	0.43	1	0.14005603	27	0.077973835
6433	GO:0009064	glutamine family amino	0.43	1	0.14005603	27	0.077973835
9771	GO:0016709	oxidoreductase activity,	0.43	2	0.28011206	71	0.2050423
3744	GO:0005244	voltage-gated ion chann	0.44	2	0.28011206	72	0.20793022
12528	GO:0022832	voltage-gated channel a	0.44	2	0.28011206	72	0.20793022
15280	GO:0032879	regulation of localization	0.44	2	0.28011206	72	0.20793022
17141	GO:0034762	regulation of transmemb	0.44	2	0.28011206	72	0.20793022
17144	GO:0034765	regulation of ion transm	0.44	2	0.28011206	72	0.20793022
19568	GO:0043269	regulation of ion transpo	0.44	2	0.28011206	72	0.20793022
25348	GO:0051049	regulation of transport	0.44	2	0.28011206	72	0.20793022
24966	GO:0050660	flavin adenine dinucleot	0.44	3	0.42016807	118	0.34077454
3284	GO:0004650	polygalacturonase activ	0.44	1	0.14005603	28	0.080861755
9238	GO:0015980	energy derivation by oxi	0.44	1	0.14005603	28	0.080861755
9827	GO:0016776	phosphotransferase act	0.44	1	0.14005603	28	0.080861755
13634	GO:0031177	phosphopantetheine bin	0.44	1	0.14005603	28	0.080861755
139	GO:0000175	3'-5'-exoribonuclease ac	0.44	1	0.14005603	28	0.080861755
2833	GO:0004024	alcohol dehydrogenase	0.44	1	0.14005603	28	0.080861755
25195	GO:0050896|GO:00518	response to stimulus	0.44	40	5.602241	1881	5.432177
9703	GO:0016620	oxidoreductase activity,	0.45	2	0.28011206	73	0.21081814
20531	GO:0044281	small molecule metaboli	0.45	38	5.322129	1788	5.1636004
5766	GO:0008236	serine-type peptidase a	0.45	5	0.70028013	214	0.6180149
4427	GO:0006221	pyrimidine nucleotide bi	0.45	1	0.14005603	29	0.083749674
11834	GO:0019905	syntaxin binding	0.45	1	0.14005603	29	0.083749674
20887	GO:0045263	proton-transporting ATP	0.45	1	0.14005603	29	0.083749674
3310	GO:0004679	AMP-activated protein k	0.46	1	0.14005603	30	0.086637594
8683	GO:0015301|GO:00153	anion:anion antiporter a	0.46	1	0.14005603	30	0.086637594
23	GO:0000030	mannosyltransferase ac	0.46	1	0.14005603	30	0.086637594
2774	GO:0003950	NAD+ ADP-ribosyltrans	0.46	1	0.14005603	30	0.086637594
6899	GO:0009628	response to abiotic stim	0.47	4	0.5602241	170	0.49094638
11202	GO:0019199	transmembrane recepto	0.47	11	1.5406163	504	1.4555116
716	GO:0001071	nucleic acid binding tran	0.47	30	4.2016807	1417	4.0921826
2643	GO:0003700|GO:00001	sequence-specific DNA	0.47	30	4.2016807	1417	4.0921826
4078	GO:0005794	Golgi apparatus	0.48	1	0.14005603	31	0.08952551
14422	GO:0032012	regulation of ARF protei	0.48	1	0.14005603	31	0.08952551
14720	GO:0032312	regulation of ARF GTPa	0.48	1	0.14005603	31	0.08952551
19590	GO:0043295	glutathione binding	0.48	1	0.14005603	31	0.08952551
22019	GO:0046496	nicotinamide nucleotide	0.48	1	0.14005603	31	0.08952551
30884	GO:0072528	pyrimidine-containing co	0.48	1	0.14005603	31	0.08952551
10103	GO:0017171	serine hydrolase activity	0.48	5	0.70028013	220	0.63534236
8675	GO:0015293	symporter activity	0.48	4	0.5602241	173	0.49961013
4121	GO:0005856	cytoskeleton	0.48	3	0.42016807	126	0.3638779
22049	GO:0046527	glucosyltransferase acti	0.48	6	0.84033614	270	0.77973837
3265	GO:0004630	phospholipase D activity	0.49	1	0.14005603	32	0.09241343
3717	GO:0005199	structural constituent of	0.49	1	0.14005603	32	0.09241343
3785	GO:0005315|GO:00053	inorganic phosphate tra	0.49	1	0.14005603	32	0.09241343
4510	GO:0006323	DNA packaging	0.49	1	0.14005603	32	0.09241343
4514	GO:0006334	nucleosome assembly	0.49	1	0.14005603	32	0.09241343
9537	GO:0016409	palmitoyltransferase act	0.49	1	0.14005603	32	0.09241343
9927	GO:0016896	exoribonuclease activity	0.49	1	0.14005603	32	0.09241343
13920	GO:0031497	chromatin assembly	0.49	1	0.14005603	32	0.09241343
14381	GO:0031968	organelle outer membra	0.49	1	0.14005603	32	0.09241343
17107	GO:0034728	nucleosome organizatio	0.49	1	0.14005603	32	0.09241343
28361	GO:0065004	protein-DNA complex as	0.49	1	0.14005603	32	0.09241343
29467	GO:0071103	DNA conformation chan	0.49	1	0.14005603	32	0.09241343
30188	GO:0071824	protein-DNA complex su	0.49	1	0.14005603	32	0.09241343
3190	GO:0004532	exoribonuclease activity	0.49	1	0.14005603	32	0.09241343
3827	GO:0005372	water transmembrane tr	0.50	2	0.28011206	81	0.23392151
4927	GO:0006833	water transport	0.50	2	0.28011206	81	0.23392151
8652	GO:0015250	water channel activity	0.50	2	0.28011206	81	0.23392151
15189	GO:0032787	monocarboxylic acid me	0.50	2	0.28011206	81	0.23392151
18429	GO:0042044	fluid transport	0.50	2	0.28011206	81	0.23392151
4849	GO:0006730|GO:00197	one-carbon metabolic p	0.51	7	0.98039216	325	0.93857396
3749	GO:0005249	voltage-gated potassiun	0.51	1	0.14005603	34	0.09818927
5270	GO:0007264	small GTPase mediated	0.51	1	0.14005603	34	0.09818927
5827	GO:0008308|GO:00228	voltage-gated anion cha	0.51	1	0.14005603	34	0.09818927
6486	GO:0009119	ribonucleoside metaboli	0.51	1	0.14005603	34	0.09818927
11208	GO:0019205	nucleobase, nucleoside	0.51	1	0.14005603	34	0.09818927
11831	GO:0019902	phosphatase binding	0.51	1	0.14005603	34	0.09818927
11832	GO:0019903	protein phosphatase bin	0.51	1	0.14005603	34	0.09818927
20321	GO:0044070	regulation of anion trans	0.51	1	0.14005603	34	0.09818927
6166	GO:0008757	S-adenosylmethionine-c	0.51	4	0.5602241	180	0.5198256
6796	GO:0009507	chloroplast	0.51	5	0.70028013	229	0.6613336
3490	GO:0004888|GO:00049	transmembrane recepto	0.51	12	1.6806723	571	1.6490022
4426	GO:0006220	pyrimidine nucleotide m	0.52	1	0.14005603	35	0.10107719
6355	GO:0008970	phospholipase A1 activi	0.52	1	0.14005603	35	0.10107719
21146	GO:0045544	gibberellin 20-oxidase a	0.52	1	0.14005603	35	0.10107719
23199	GO:0047714	galactolipase activity	0.52	1	0.14005603	35	0.10107719
4513	GO:0006333	chromatin assembly or c	0.53	1	0.14005603	36	0.10396511
5902	GO:0008408	3'-5' exonuclease activit	0.53	1	0.14005603	36	0.10396511
9185	GO:0015925	galactosidase activity	0.53	1	0.14005603	36	0.10396511
11314	GO:0019321	pentose metabolic proce	0.53	1	0.14005603	36	0.10396511
3758	GO:0005267	potassium channel activ	0.54	1	0.14005603	37	0.10685303
9193	GO:0015934	large ribosomal subunit	0.54	1	0.14005603	37	0.10685303
11355	GO:0019362	pyridine nucleotide meta	0.54	1	0.14005603	37	0.10685303
30880	GO:0072524	pyridine-containing com	0.54	1	0.14005603	37	0.10685303
20624	GO:0044430	cytoskeletal part	0.54	2	0.28011206	88	0.25413695

8501	GO:0015085	calcium ion transmembr	0.55	1	0.14005603	38	0.10974095
12538	GO:0022843	voltage-gated cation cha	0.55	1	0.14005603	38	0.10974095
117	GO:0000149	SNARE binding	0.55	1	0.14005603	38	0.10974095
2628	GO:0003677	DNA binding	0.55	25	3.5014005	1224	3.534814
9719	GO:0016638	oxidoreductase activity,	0.55	2	0.28011206	89	0.25702485
6623	GO:0009260	ribonucleotide biosynthe	0.56	1	0.14005603	39	0.11262887
30883	GO:0072527	pyrimidine-containing co	0.56	1	0.14005603	39	0.11262887
4408	GO:0006200	ATP catabolic process	0.56	13	1.8207283	641	1.8511566
9918	GO:0016887|GO:00040	ATPase activity	0.56	13	1.8207283	641	1.8511566
21600	GO:0046034	ATP metabolic process	0.56	13	1.8207283	641	1.8511566
9697	GO:0016614	oxidoreductase activity,	0.56	6	0.84033614	293	0.84616053
4058	GO:0005773	vacuole	0.57	1	0.14005603	40	0.1155168
5891	GO:0008395|GO:00083	steroid hydroxylase acti	0.57	1	0.14005603	40	0.1155168
9843	GO:0016796	exonuclease activity, ac	0.57	1	0.14005603	40	0.1155168
11804	GO:0019867	outer membrane	0.57	1	0.14005603	40	0.1155168
5712	GO:0008168|GO:00044	methyltransferase activi	0.57	5	0.70028013	244	0.7046524
2897	GO:0004091|GO:00043	carboxylesterase activity	0.57	4	0.5602241	194	0.5602564
5981	GO:0008509	anion transmembrane tr	0.57	5	0.70028013	245	0.70754033
4851	GO:0006733	oxidoreduction coenzym	0.57	1	0.14005603	41	0.118404716
25098	GO:0050794|GO:00512	regulation of cellular pro	0.57	81	11.344538	3987	11.514136
4917	GO:0006820|GO:00068	anion transport	0.58	5	0.70028013	247	0.7133162
3808	GO:0005351|GO:00054	sugar:hydrogen sympor	0.58	2	0.28011206	94	0.27146447
3835	GO:0005402	cation:sugar symporter	0.58	2	0.28011206	94	0.27146447
3753	GO:0005253	anion channel activity	0.58	1	0.14005603	42	0.121292636
4756	GO:0006631	fatty acid metabolic proc	0.58	1	0.14005603	42	0.121292636
9801	GO:0016747	transferase activity, tran	0.60	9	1.2605042	456	1.3168914
4914	GO:0006816	calcium ion transport	0.60	1	0.14005603	44	0.12706847
6822	GO:0009534	chloroplast thylakoid	0.60	1	0.14005603	44	0.12706847
6823	GO:0009535	chloroplast thylakoid me	0.60	1	0.14005603	44	0.12706847
14388	GO:0031976	plastid thylakoid	0.60	1	0.14005603	44	0.12706847
14395	GO:0031984	organelle subcompartm	0.60	1	0.14005603	44	0.12706847
26883	GO:0055035	plastid thylakoid membr	0.60	1	0.14005603	44	0.12706847
28952	GO:0070588	calcium ion transmembr	0.60	1	0.14005603	44	0.12706847
8677	GO:0015295	solute:hydrogen sympor	0.60	2	0.28011206	98	0.28301615
8681	GO:0015299	solute:hydrogen antipor	0.60	2	0.28011206	98	0.28301615
8673	GO:0015291|GO:00152	secondary active transm	0.60	8	1.1204482	407	1.1753833
25094	GO:0050789|GO:00507	regulation of biological p	0.61	87	12.184874	4315	12.461374
9893	GO:0016857	racemase and epimeras	0.61	1	0.14005603	45	0.1299564
14726	GO:0032318	regulation of Ras GTPa	0.61	1	0.14005603	45	0.1299564
17001	GO:0034622	cellular macromolecular	0.61	1	0.14005603	45	0.1299564
22096	GO:0046578	regulation of Ras protei	0.61	1	0.14005603	45	0.1299564
25355	GO:0051056	regulation of small GTP	0.61	1	0.14005603	45	0.1299564
28360	GO:0065003	macromolecular comple	0.61	1	0.14005603	45	0.1299564
8679	GO:0015297	antiporter activity	0.61	3	0.42016807	152	0.4389638
8682	GO:0015300	solute:solute antiporter	0.61	3	0.42016807	152	0.4389638
4073	GO:0005789	endoplasmic reticulum m	0.62	1	0.14005603	46	0.13284431
18536	GO:0042175	nuclear outer membrane	0.62	1	0.14005603	46	0.13284431
20626	GO:0044432	endoplasmic reticulum p	0.62	1	0.14005603	46	0.13284431
8517	GO:0015103	inorganic anion transme	0.62	3	0.42016807	154	0.44473964
5280	GO:0007275	multicellular organismal	0.62	2	0.28011206	101	0.2916799
5807	GO:0008287	protein serine/threonine	0.62	1	0.14005603	47	0.13573223
6442	GO:0009073|GO:00160	aromatic amino acid fan	0.62	1	0.14005603	47	0.13573223
21943	GO:0046417	chorismate metabolic pr	0.62	1	0.14005603	47	0.13573223
9796	GO:0016741	transferase activity, tran	0.63	5	0.70028013	261	0.7537471
14591	GO:0032183	SUMO binding	0.63	1	0.14005603	48	0.13862015
28930	GO:0070566	adenylyltransferase acti	0.63	1	0.14005603	48	0.13862015
9709	GO:0016627	oxidoreductase activity,	0.64	2	0.28011206	104	0.30034366
19831	GO:0043565	sequence-specific DNA	0.64	3	0.42016807	159	0.45917925
3756	GO:0005261|GO:00152	cation channel activity	0.64	1	0.14005603	49	0.14150807
6483	GO:0009116	nucleoside metabolic pr	0.64	1	0.14005603	49	0.14150807
11830	GO:0019901	protein kinase binding	0.64	1	0.14005603	49	0.14150807
31421	GO:0080044	quercetin 7-O-glucosyltr	0.64	1	0.14005603	49	0.14150807
19126	GO:0042803	protein homodimerizatio	0.64	5	0.70028013	265	0.7652988
14909	GO:0032501|GO:00508	multicellular organismal	0.64	2	0.28011206	105	0.3032316
2668	GO:0003779	actin binding	0.64	2	0.28011206	105	0.3032316
9795	GO:0016740	transferase activity	0.64	75	10.504202	3764	10.870131
8495	GO:0015077	monovalent inorganic ca	0.65	4	0.5602241	214	0.6180149
5096	GO:0007047	cellular cell wall organiz	0.65	1	0.14005603	50	0.14439599
6932	GO:0009664	plant-type cell wall orga	0.65	1	0.14005603	50	0.14439599
19914	GO:0043648	dicarboxylic acid metabo	0.65	1	0.14005603	50	0.14439599
20857	GO:0045229	external encapsulating s	0.65	1	0.14005603	50	0.14439599
30033	GO:0071669	plant-type cell wall orga	0.65	1	0.14005603	50	0.14439599
3185	GO:0004527|GO:00088	exonuclease activity	0.65	1	0.14005603	50	0.14439599
7213	GO:0009966|GO:00354	regulation of signal trans	0.65	1	0.14005603	51	0.14728391
9975	GO:0016998	cell wall macromolecule	0.65	1	0.14005603	51	0.14728391
12492	GO:0022607	cellular component asse	0.65	1	0.14005603	51	0.14728391
12601	GO:0023051	regulation of signaling	0.65	1	0.14005603	51	0.14728391
30208	GO:0071844	cellular component asse	0.65	1	0.14005603	51	0.14728391
3099	GO:0004428	inositol or phosphatidyli	0.65	1	0.14005603	51	0.14728391
28364	GO:0065007	biological regulation	0.66	87	12.184874	4376	12.637537
4915	GO:0006817	phosphate transport	0.66	1	0.14005603	52	0.15017183
8528	GO:0015114	phosphate transmembra	0.66	1	0.14005603	52	0.15017183
17801	GO:0035435	phosphate transmembra	0.66	1	0.14005603	52	0.15017183
20287	GO:0044036	cell wall macromolecule	0.66	1	0.14005603	52	0.15017183
6563	GO:0009199	ribonucleoside triphosph	0.66	15	2.1008403	790	2.2814567
29246	GO:0070882	cellular cell wall organiz	0.67	1	0.14005603	53	0.15305975
18953	GO:0042623	ATPase activity, couple	0.67	10	1.4005603	537	1.550813
6506	GO:0009141	nucleoside triphosphate	0.67	15	2.1008403	796	2.2987843
8680	GO:0015298	solute:cation antiporter	0.67	2	0.28011206	112	0.32344702
9730	GO:0016651	oxidoreductase activity,	0.67	2	0.28011206	112	0.32344702
29918	GO:0071554	cell wall organization or	0.67	2	0.28011206	112	0.32344702
8943	GO:0015672	monovalent inorganic ca	0.68	7	0.98039216	383	1.1060733
9933	GO:0016903	oxidoreductase activity,	0.68	2	0.28011206	113	0.32633495
9533	GO:0016405	CoA-ligase activity	0.68	1	0.14005603	55	0.15883559
15576	GO:0033177	proton-transporting two-	0.69	1	0.14005603	56	0.16172351
3156	GO:0004497	monooxygenase activity	0.69	3	0.42016807	172	0.49672222
6441	GO:0009072	aromatic amino acid fan	0.70	1	0.14005603	57	0.16461143
9910	GO:0016878	acid-thiol ligase activity	0.70	1	0.14005603	57	0.16461143
23973	GO:0048583	regulation of response t	0.70	1	0.14005603	57	0.16461143
9800	GO:0016746	transferase activity, tran	0.70	9	1.2605042	498	1.438184
5819	GO:0008299|GO:00092	isoprenoid biosynthetic	0.70	1	0.14005603	58	0.16749935
29919	GO:0071555	cell wall organization	0.70	1	0.14005603	58	0.16749935
14667	GO:0032259	methylation	0.70	5	0.70028013	285	0.8230572
8584	GO:0015171|GO:00153	amino acid transmembra	0.70	2	0.28011206	118	0.34077454
25416	GO:0051119	sugar transmembrane tr	0.70	2	0.28011206	118	0.34077454
2521	GO:0003333	amino acid transmembra	0.70	2	0.28011206	118	0.34077454
6622	GO:0009259|GO:00091	ribonucleotide metabolic	0.70	15	2.1008403	812	2.344991

8967	GO:0015698	inorganic anion transpo	0.71	3	0.42016807	176	0.5082739
9900	GO:0016866	intramolecular transfera	0.71	1	0.14005603	59	0.17038727
18981	GO:0042651	thylakoid membrane	0.71	1	0.14005603	59	0.17038727
2831	GO:0004022	alcohol dehydrogenase	0.71	1	0.14005603	59	0.17038727
13082	GO:0030599	pectinesterase activity	0.71	3	0.42016807	178	0.5140497
4840	GO:0006720|GO:00160	isoprenoid metabolic pro	0.71	1	0.14005603	60	0.17327519
5652	GO:0008081|GO:00044	phosphoric diester hydro	0.71	1	0.14005603	60	0.17327519
14910	GO:0032502	developmental process	0.72	2	0.28011206	121	0.3494383
2818	GO:0004003	ATP-dependent DNA he	0.72	1	0.14005603	61	0.1761631
11828	GO:0019899	enzyme binding	0.72	4	0.5602241	237	0.684437
4951	GO:0006865|GO:00068	amino acid transport	0.72	2	0.28011206	123	0.35521415
17000	GO:0034621	cellular macromolecular	0.73	1	0.14005603	62	0.17905103
20186	GO:0043933|GO:00346	macromolecular comple	0.73	1	0.14005603	62	0.17905103
20883	GO:0045259|GO:00452	proton-transporting ATP	0.73	1	0.14005603	62	0.17905103
4405	GO:0006195	purine nucleotide catab	0.73	14	1.9607843	773	2.232362
6508	GO:0009143	nucleoside triphosphate	0.73	14	1.9607843	773	2.232362
6509	GO:0009144	purine nucleoside tripho	0.73	14	1.9607843	773	2.232362
6511	GO:0009146	purine nucleoside tripho	0.73	14	1.9607843	773	2.232362
6519	GO:0009154	purine ribonucleotide ca	0.73	14	1.9607843	773	2.232362
6530	GO:0009166	nucleotide catabolic pro	0.73	14	1.9607843	773	2.232362
6567	GO:0009203	ribonucleoside triphosph	0.73	14	1.9607843	773	2.232362
6569	GO:0009205	purine ribonucleoside tr	0.73	14	1.9607843	773	2.232362
6571	GO:0009207	purine ribonucleoside tr	0.73	14	1.9607843	773	2.232362
6624	GO:0009261	ribonucleotide catabolic	0.73	14	1.9607843	773	2.232362
17034	GO:0034655	nucleobase, nucleoside	0.73	14	1.9607843	773	2.232362
17035	GO:0034656	nucleobase, nucleoside	0.73	14	1.9607843	773	2.232362
22209	GO:0046700	heterocycle catabolic pr	0.73	14	1.9607843	773	2.232362
30879	GO:0072523	purine-containing compo	0.73	14	1.9607843	773	2.232362
9308	GO:0016070	RNA metabolic process	0.73	35	4.901961	1855	5.3570914
20520	GO:0044270	cellular nitrogen compo	0.73	14	1.9607843	777	2.2439137
3476	GO:0004872|GO:00190	receptor activity	0.73	12	1.6806723	672	1.9406822
3761	GO:0005275|GO:00052	amine transmembrane t	0.74	2	0.28011206	126	0.3638779
25820	GO:0051536	iron-sulfur cluster bindin	0.74	1	0.14005603	64	0.18482687
25824	GO:0051540	metal cluster binding	0.74	1	0.14005603	64	0.18482687
22007	GO:0046483	heterocycle metabolic p	0.74	20	2.8011205	1094	3.1593843
25314	GO:0051015	actin filament binding	0.74	1	0.14005603	65	0.18771479
9890	GO:0016854	racemase and epimeras	0.75	1	0.14005603	66	0.1906027
3475	GO:0004871|GO:00050	signal transducer activit	0.75	14	1.9607843	787	2.2727928
27063	GO:0060089	molecular transducer ac	0.75	14	1.9607843	787	2.2727928
5651	GO:0008080	N-acetyltransferase acti	0.75	1	0.14005603	67	0.19349062
2629	GO:0003678|GO:00036	DNA helicase activity	0.75	1	0.14005603	67	0.19349062
6515	GO:0009150	purine ribonucleotide m	0.76	14	1.9607843	791	2.2843447
31420	GO:0080043	quercetin 3-O-glucosyltr	0.76	1	0.14005603	69	0.19926646
9579	GO:0016462	pyrophosphatase activit	0.76	17	2.3809524	954	2.7550755
9859	GO:0016818	hydrolase activity, acting	0.77	17	2.3809524	956	2.7608514
5726	GO:0008187	poly-pyrimidine tract bin	0.77	1	0.14005603	70	0.20215438
5788	GO:0008266	poly(U) RNA binding	0.77	1	0.14005603	70	0.20215438
15524	GO:0033124	regulation of GTP catab	0.77	1	0.14005603	70	0.20215438
19394	GO:0043087	regulation of GTPase ac	0.77	1	0.14005603	70	0.20215438
9103	GO:0015837	amine transport	0.77	2	0.28011206	135	0.38986918
3307	GO:0004675	transmembrane recepto	0.77	6	0.84033614	367	1.0598665
9858	GO:0016817	hydrolase activity, acting	0.77	17	2.3809524	959	2.769515
20532	GO:0044282	small molecule catabolic	0.77	15	2.1008403	854	2.4662836
3801	GO:0005342	organic acid transmemb	0.77	2	0.28011206	136	0.3927571
22444	GO:0046943	carboxylic acid transme	0.77	2	0.28011206	136	0.3927571
19125	GO:0042802	identical protein binding	0.78	12	1.6806723	697	2.01288
9740	GO:0016667	oxidoreductase activity,	0.78	1	0.14005603	72	0.20793022
5809	GO:0008289	lipid binding	0.78	4	0.5602241	258	0.74508333
4867	GO:0006753	nucleoside phosphate m	0.78	16	2.2408965	914	2.6395588
6484	GO:0009117	nucleotide metabolic pro	0.78	16	2.2408965	914	2.6395588
4247	GO:0006007	glucose catabolic proce	0.78	1	0.14005603	73	0.21081814
11313	GO:0019320	hexose catabolic proces	0.78	1	0.14005603	73	0.21081814
20525	GO:0044275	cellular carbohydrate ca	0.78	1	0.14005603	73	0.21081814
21726	GO:0046164	alcohol catabolic proces	0.78	1	0.14005603	73	0.21081814
21894	GO:0046365	monosaccharide catabo	0.78	1	0.14005603	73	0.21081814
8607	GO:0015197|GO:00156	peptide transporter activ	0.78	2	0.28011206	139	0.40142086
8608	GO:0015198	oligopeptide transporter	0.78	2	0.28011206	139	0.40142086
4373	GO:0006163	purine nucleotide metab	0.78	14	1.9607843	810	2.339215
3311	GO:0004683|GO:00046	calmodulin-dependent p	0.79	1	0.14005603	74	0.21370606
9817	GO:0016763	transferase activity, tran	0.79	1	0.14005603	74	0.21370606
20628	GO:0044434	chloroplast part	0.79	1	0.14005603	74	0.21370606
20629	GO:0044435	plastid part	0.79	1	0.14005603	74	0.21370606
3895	GO:0005543	phospholipid binding	0.79	2	0.28011206	140	0.40430877
4944	GO:0006857	oligopeptide transport	0.79	2	0.28011206	141	0.4071967
8676	GO:0015294	solute:cation symporter	0.79	2	0.28011206	141	0.4071967
9099	GO:0015833	peptide transport	0.79	2	0.28011206	141	0.4071967
8810	GO:0015491	cation:cation antiporter	0.79	1	0.14005603	75	0.21659398
5663	GO:0008094|GO:00040	DNA-dependent ATPas	0.80	1	0.14005603	76	0.2194819
5732	GO:0008194	UDP-glycosyltransferas	0.80	5	0.70028013	322	0.9299102
8557	GO:0015144	carbohydrate transmem	0.80	2	0.28011206	143	0.41297254
16602	GO:0034219	carbohydrate transmem	0.80	2	0.28011206	143	0.41297254
6529	GO:0009165	nucleotide biosynthetic	0.80	1	0.14005603	77	0.22236982
9538	GO:0016410	N-acyltransferase activit	0.80	1	0.14005603	77	0.22236982
2986	GO:0004252	serine-type endopeptida	0.80	2	0.28011206	144	0.41586044
9699	GO:0016616	oxidoreductase activity,	0.80	4	0.5602241	266	0.7681867
30865	GO:0072509	divalent inorganic cation	0.80	1	0.14005603	78	0.22525774
10058	GO:0017111	nucleoside-triphosphata	0.80	15	2.1008403	878	2.5355935
4954	GO:0006869	lipid transport	0.81	3	0.42016807	208	0.6006873
8085	GO:0010876	lipid localization	0.81	3	0.42016807	208	0.6006873
3873	GO:0005507	copper ion binding	0.81	2	0.28011206	146	0.42163628
8494	GO:0015075	ion transmembrane tran	0.81	14	1.9607843	827	2.3883097
30877	GO:0072521	purine-containing compo	0.81	14	1.9607843	827	2.3883097
6071	GO:0008643|GO:00068	carbohydrate transport	0.81	2	0.28011206	147	0.42452422
4215	GO:0005975	carbohydrate metabolic	0.81	17	2.3809524	990	2.8590407
4358	GO:0006140	regulation of nucleotide	0.81	1	0.14005603	80	0.2310336
7146	GO:0009894	regulation of catabolic p	0.81	1	0.14005603	80	0.2310336
9875	GO:0016836	hydro-lyase activity	0.81	1	0.14005603	80	0.2310336
13278	GO:0030811	regulation of nucleotide	0.81	1	0.14005603	80	0.2310336
13759	GO:0031329	regulation of cellular cat	0.81	1	0.14005603	80	0.2310336
15521	GO:0033121	regulation of purine nuc	0.81	1	0.14005603	80	0.2310336
16603	GO:0034220	ion transmembrane tran	0.82	14	1.9607843	833	2.4056373
22376	GO:0046873	metal ion transmembrar	0.82	3	0.42016807	213	0.6151269
6991	GO:0009725	response to hormone st	0.82	1	0.14005603	82	0.23680943
29202	GO:0070838	divalent metal ion transp	0.82	1	0.14005603	82	0.23680943
26932	GO:0055086	nucleobase, nucleoside	0.82	16	2.2408965	944	2.7261963
4029	GO:0005740	mitochondrial envelope	0.82	2	0.28011206	151	0.4360759
16738	GO:0034357	photosynthetic membra	0.82	1	0.14005603	83	0.23969735

4327	GO:0006091	generation of precursor	0.83	1	0.14005603	84	0.24258527
30867	GO:0072511	divalent inorganic cation	0.83	1	0.14005603	84	0.24258527
12547	GO:0022857|GO:00053	transmembrane transpo	0.83	26	3.6414566	1483	4.282785
17617	GO:0035251	UDP-glucosyltransferas	0.83	3	0.42016807	218	0.62956655
6985	GO:0009719	response to endogenou	0.83	1	0.14005603	86	0.24836111
9114	GO:0015849	organic acid transport	0.83	2	0.28011206	156	0.45051548
22443	GO:0046942	carboxylic acid transpor	0.83	2	0.28011206	156	0.45051548
4246	GO:0006006	glucose metabolic proce	0.84	1	0.14005603	87	0.25124902
7229	GO:0009987|GO:00081	cellular process	0.84	207	28.991596	10601	30.614838
26931	GO:0055085	transmembrane transpo	0.84	27	3.7815125	1548	4.4705
12794	GO:0030234	enzyme regulator activit	0.84	6	0.84033614	403	1.1638317
5195	GO:0007165|GO:00230	signal transduction	0.84	16	2.2408965	961	2.775291
12602	GO:0023052|GO:00230	signaling	0.84	16	2.2408965	961	2.775291
7691	GO:0010467	gene expression	0.84	35	4.901961	1974	5.7007537
8496	GO:0015078	hydrogen ion transmem	0.84	2	0.28011206	160	0.4620672
14590	GO:0032182	small conjugating protei	0.85	1	0.14005603	90	0.2599128
20630	GO:0044436	thylakoid part	0.85	1	0.14005603	90	0.2599128
2982	GO:0004197	cysteine-type endopepti	0.85	1	0.14005603	90	0.2599128
4990	GO:0006915|GO:00086	apoptosis	0.85	3	0.42016807	227	0.6555578
3644	GO:0005102	receptor binding	0.85	1	0.14005603	91	0.2628007
9294	GO:0016052|GO:00060	carbohydrate catabolic	0.85	1	0.14005603	91	0.2628007
5764	GO:0008234|GO:00042	cysteine-type peptidase	0.85	2	0.28011206	163	0.47073093
16785	GO:0034404	nucleobase, nucleoside	0.85	1	0.14005603	92	0.26568863
17033	GO:0034654	nucleobase, nucleoside	0.85	1	0.14005603	92	0.26568863
4916	GO:0006818	hydrogen transport	0.85	4	0.5602241	292	0.84327257
9246	GO:0015992	proton transport	0.85	4	0.5602241	292	0.84327257
20336	GO:0044085	cellular component biog	0.86	1	0.14005603	93	0.26857653
20623	GO:0044429	mitochondrial part	0.86	2	0.28011206	165	0.47650677
6863	GO:0009579	thylakoid	0.86	1	0.14005603	94	0.27146447
18633	GO:0042277	peptide binding	0.86	1	0.14005603	95	0.27435237
25095	GO:0050790	regulation of catalytic ac	0.87	7	0.98039216	480	1.3862015
5756	GO:0008219	cell death	0.87	3	0.42016807	239	0.69021285
8209	GO:0012501|GO:00162	programmed cell death	0.87	3	0.42016807	239	0.69021285
9462	GO:0016265	death	0.87	3	0.42016807	239	0.69021285
28366	GO:0065009	regulation of molecular	0.87	7	0.98039216	484	1.3977532
7271	GO:0010033	response to organic sub	0.88	1	0.14005603	100	0.28879198
4067	GO:0005783	endoplasmic reticulum	0.88	2	0.28011206	174	0.50249803
2674	GO:0003824	catalytic activity	0.88	208	29.131653	10750	31.045137
8211	GO:0012505	endomembrane system	0.88	3	0.42016807	242	0.6988766
11311	GO:0019318	hexose metabolic proce	0.88	1	0.14005603	101	0.2916799
5874	GO:0008374	O-acyltransferase activi	0.88	1	0.14005603	102	0.29456782
20498	GO:0044248	cellular catabolic proces	0.88	14	1.9607843	894	2.5818002
3306	GO:0004674|GO:00046	protein serine/threonine	0.89	17	2.3809524	1062	3.0669708
12608	GO:0030001	metal ion transport	0.89	5	0.70028013	374	1.080082
3200	GO:0004553|GO:00168	hydrolase activity, hydro	0.89	4	0.5602241	312	0.90103096
19253	GO:0042936	dipeptide transporter ac	0.89	1	0.14005603	105	0.3032316
19255	GO:0042938	dipeptide transport	0.89	1	0.14005603	105	0.3032316
3195	GO:0004540	ribonuclease activity	0.89	1	0.14005603	106	0.3061195
4910	GO:0006811	ion transport	0.89	15	2.1008403	959	2.769515
3942	GO:0005618	cell wall	0.89	1	0.14005603	107	0.30900744
20505	GO:0044255	cellular lipid metabolic p	0.90	2	0.28011206	184	0.53137726
12868	GO:0030312	external encapsulating s	0.90	1	0.14005603	111	0.3205591
4512	GO:0006325	chromatin organization	0.90	1	0.14005603	112	0.32344702
25568	GO:0051276|GO:00070	chromosome organizati	0.90	1	0.14005603	112	0.32344702
4850	GO:0006732|GO:00067	coenzyme metabolic pro	0.91	1	0.14005603	113	0.32633495
3449	GO:0004842|GO:00048	ubiquitin-protein ligase a	0.91	4	0.5602241	326	0.94146186
14379	GO:0031966	mitochondrial membran	0.91	1	0.14005603	114	0.32922286
22478	GO:0046982	protein heterodimerizati	0.91	1	0.14005603	114	0.32922286
9535	GO:0016407	acetyltransferase activit	0.91	1	0.14005603	115	0.3321108
22479	GO:0046983	protein dimerization acti	0.91	7	0.98039216	517	1.4930545
9654	GO:0016567	protein ubiquitination	0.91	4	0.5602241	332	0.9587894
12572	GO:0022890|GO:00150	inorganic cation transm	0.91	4	0.5602241	332	0.9587894
14854	GO:0032446	protein modification by s	0.91	4	0.5602241	332	0.9587894
29011	GO:0070647	protein modification by s	0.91	4	0.5602241	332	0.9587894
3955	GO:0005634	nucleus	0.91	23	3.2212884	1433	4.138389
4144	GO:0005886|GO:00059	plasma membrane	0.92	1	0.14005603	120	0.34655038
6425	GO:0009056	catabolic process	0.92	16	2.2408965	1051	3.0352037
11747	GO:0019787|GO:00086	small conjugating protei	0.92	4	0.5602241	339	0.9790048
14380	GO:0031967	organelle envelope	0.92	2	0.28011206	201	0.5804719
30066	GO:0071702	organic substance trans	0.92	7	0.98039216	529	1.5277096
4755	GO:0006629	lipid metabolic process	0.92	8	1.1204482	590	1.7038727
30069	GO:0071705	nitrogen compound tran	0.92	2	0.28011206	202	0.5833598
14387	GO:0031975	envelope	0.93	2	0.28011206	205	0.59202355
4236	GO:0005996	monosaccharide metabo	0.93	1	0.14005603	125	0.36099
4649	GO:0006520|GO:00065	cellular amino acid meta	0.93	3	0.42016807	277	0.7999538
634	GO:0000988	protein binding transcrip	0.93	1	0.14005603	126	0.3638779
635	GO:0000989	transcription factor bindi	0.93	1	0.14005603	126	0.3638779
2648	GO:0003712	transcription cofactor ac	0.93	1	0.14005603	126	0.3638779
28378	GO:0070011	peptidase activity, actin	0.93	7	0.98039216	537	1.550813
5840	GO:0008324	cation transmembrane t	0.93	7	0.98039216	538	1.5537009
9583	GO:0016469	proton-transporting two-	0.93	1	0.14005603	127	0.36676583
21058	GO:0045454|GO:00305	cell redox homeostasis	0.93	2	0.28011206	211	0.6093511
4394	GO:0006184	GTP catabolic process	0.94	1	0.14005603	132	0.3812054
21605	GO:0046039	GTP metabolic process	0.94	1	0.14005603	132	0.3812054
2758	GO:0003924	GTPase activity	0.94	1	0.14005603	132	0.3812054
9837	GO:0016788	hydrolase activity, actin	0.94	24	3.3613446	1534	4.430069
4028	GO:0005739	mitochondrion	0.94	2	0.28011206	219	0.63245445
13547	GO:0031090	organelle membrane	0.95	2	0.28011206	224	0.64689404
6046	GO:0008610	lipid biosynthetic proces	0.95	1	0.14005603	141	0.4071967
5763	GO:0008233	peptidase activity	0.95	7	0.98039216	565	1.6316746
4304	GO:0006066	alcohol metabolic proce	0.95	1	0.14005603	143	0.41297254
20356	GO:0044106	cellular amine metabolic	0.95	3	0.42016807	303	0.8750397
9488	GO:0016301	kinase activity	0.95	27	3.7815125	1740	5.0249805
9829	GO:0016779	nucleotidyltransferase a	0.95	1	0.14005603	148	0.42741212
11695	GO:0019725	cellular homeostasis	0.96	2	0.28011206	234	0.67577326
12573	GO:0022891	substrate-specific transı	0.96	18	2.5210085	1241	3.5839086
18925	GO:0042592	homeostatic process	0.96	2	0.28011206	238	0.68732494
6662	GO:0009308	amine metabolic proces	0.96	3	0.42016807	314	0.9068068
25998	GO:0051716	cellular response to stim	0.96	16	2.2408965	1138	3.2864528
9913	GO:0016881	acid-amino acid ligase a	0.96	4	0.5602241	391	1.1291766
2975	GO:0004175|GO:00168	endopeptidase activity	0.96	3	0.42016807	320	0.9241344
3716	GO:0005198	structural molecule activ	0.96	4	0.5602241	393	1.1349525
2660	GO:0003735|GO:00037	structural constituent of	0.96	2	0.28011206	246	0.7104283
9906	GO:0016874	ligase activity	0.96	7	0.98039216	597	1.7240882
4911	GO:0006812|GO:00068	cation transport	0.96	8	1.1204482	662	1.9118029
5661	GO:0008092	cytoskeletal protein bind	0.97	2	0.28011206	249	0.719092
3723	GO:0005215|GO:00054	transporter activity	0.97	28	3.9215686	1844	5.325324

3304	GO:0004672|GO:00502	protein kinase activity	0.97	19	2.6610644	1333	3.8495972
5055	GO:0006996	organelle organization	0.97	1	0.14005603	164	0.47361887
25483	GO:0051186	cofactor metabolic proce	0.97	1	0.14005603	164	0.47361887
5909	GO:0008415	acyltransferase activity	0.97	3	0.42016807	330	0.95301354
5018	GO:0006950	response to stress	0.97	7	0.98039216	611	1.764519
6077	GO:0008652	cellular amino acid biosy	0.97	1	0.14005603	169	0.48805845
19526	GO:0043227	membrane-bounded org	0.97	33	4.6218486	2142	6.185924
19530	GO:0043231	intracellular membrane-	0.97	33	4.6218486	2142	6.185924
30306	GO:0071944	cell periphery	0.97	2	0.28011206	259	0.74797124
9285	GO:0016043	cellular component orga	0.97	2	0.28011206	261	0.7537471
13656	GO:0031224	intrinsic to membrane	0.98	16	2.2408965	1193	3.4452884
6663	GO:0009309	amine biosynthetic proc	0.98	1	0.14005603	181	0.5227135
9824	GO:0016773	phosphotransferase act	0.98	21	2.9411764	1493	4.311664
5610	GO:0008026	ATP-dependent helicase	0.98	1	0.14005603	183	0.52848935
28401	GO:0070035	purine NTP-dependent	0.98	1	0.14005603	183	0.52848935
9836	GO:0016787	hydrolase activity	0.98	61	8.543417	3723	10.751725
2657	GO:0003727|GO:00037	single-stranded RNA bin	0.98	1	0.14005603	188	0.54292893
30204	GO:0071840	cellular component orga	0.98	3	0.42016807	362	1.045427
3068	GO:0004386	helicase activity	0.98	1	0.14005603	189	0.54581684
30205	GO:0071841	cellular component orga	0.98	2	0.28011206	285	0.8230572
30206	GO:0071842	cellular component orga	0.98	1	0.14005603	193	0.5573685
9267	GO:0016021	integral to membrane	0.98	14	1.9607843	1103	3.1853757
10244	GO:0018130	heterocycle biosynthetic	0.98	1	0.14005603	199	0.57469606
9911	GO:0016879	ligase activity, forming c	0.99	4	0.5602241	456	1.3168914
9889	GO:0016853	isomerase activity	0.99	2	0.28011206	306	0.8837035
9295	GO:0016053	organic acid biosyntheti	0.99	1	0.14005603	211	0.6093511
21922	GO:0046394	carboxylic acid biosynth	0.99	1	0.14005603	211	0.6093511
4612	GO:0006468	protein phosphorylation	0.99	32	4.481793	2205	6.367863
15436	GO:0033036	macromolecule localizat	0.99	3	0.42016807	397	1.1465042
9493	GO:0016310	phosphorylation	0.99	37	5.1820726	2504	7.2313514
9823	GO:0016772	transferase activity, tran	0.99	27	3.7815125	1932	5.579461
28365	GO:0065008	regulation of biological c	0.99	2	0.28011206	316	0.9125827
2627	GO:0003676	nucleic acid binding	0.99	36	5.042017	2453	7.0840673
12574	GO:0022892	substrate-specific transp	0.99	19	2.6610644	1463	4.2250266
5019	GO:0006952|GO:00022	defense response	0.99	1	0.14005603	221	0.63823026
20640	GO:0044446	intracellular organelle pa	0.99	7	0.98039216	710	2.0504231
20616	GO:0044422	organelle part	0.99	7	0.98039216	712	2.0561988
13531	GO:0031072	heat shock protein bindi	0.99	1	0.14005603	230	0.6642216
4909	GO:0006810|GO:00154	transport	0.99	33	4.6218486	2315	6.6855345
25529	GO:0051234	establishment of localiza	0.99	33	4.6218486	2315	6.6855345
25476	GO:0051179	localization	0.99	33	4.6218486	2319	6.697086
20533	GO:0044283	small molecule biosynth	0.99	2	0.28011206	347	1.0021082
20512	GO:0044262|GO:00060	cellular carbohydrate me	0.99	1	0.14005603	248	0.7162041
20521	GO:0044271	cellular nitrogen compou	1.00	2	0.28011206	367	1.0598665
19533	GO:0043234	protein complex	1.00	8	1.1204482	848	2.448956
20619	GO:0044425	membrane part	1.00	18	2.5210085	1523	4.398302
11522	GO:0019538|GO:00064	protein metabolic proces	1.00	62	8.683474	4134	11.938661
4109	GO:0005840|GO:00332	ribosome	1.00	5	0.70028013	682	1.9695613
19527	GO:0043228	non-membrane-bounde	1.00	8	1.1204482	923	2.66555
19531	GO:0043232	intracellular non-membr	1.00	8	1.1204482	923	2.66555
19525	GO:0043226	organelle	1.00	41	5.7422967	3013	8.701303
19528	GO:0043229	intracellular organelle	1.00	41	5.7422967	3013	8.701303
18912	GO:0042578	phosphoric ester hydrol	1.00	2	0.28011206	444	1.2822365
13041	GO:0030554	adenyl nucleotide bindir	1.00	3	0.42016807	550	1.5883559
14965	GO:0032559	adenyl ribonucleotide bi	1.00	3	0.42016807	550	1.5883559
4609	GO:0006464	protein modification pro	1.00	36	5.042017	2781	8.031305
13018	GO:0030529	ribonucleoprotein compl	1.00	5	0.70028013	741	2.1399486
4897	GO:0006793	phosphorus metabolic p	1.00	38	5.322129	2925	8.4471655
4900	GO:0006796	phosphate metabolic pr	1.00	38	5.322129	2925	8.4471655
9839	GO:0016791|GO:00163	phosphatase activity	1.00	1	0.14005603	383	1.1060733
3884	GO:0005524	ATP binding	1.00	2	0.28011206	524	1.51327
19684	GO:0043412	macromolecule modifica	1.00	36	5.042017	2900	8.374968
9494	GO:0016311	dephosphorylation	1.00	1	0.14005603	425	1.227366
3177	GO:0004518	nuclease activity	1.00	1	0.14005603	427	1.2331418
3863	GO:0005488	binding	1.00	240	33.613445	13903	40.15075
20618	GO:0044424	intracellular part	1.00	50	7.002801	3853	11.127155
20638	GO:0044444	cytoplasmic part	1.00	15	2.1008403	1644	4.7477403
131	GO:0000166	nucleotide binding	1.00	9	1.2605042	1213	3.5030468
9266	GO:0016020	membrane	1.00	46	6.442577	3741	10.803708
15392	GO:0032991	macromolecular comple	1.00	13	1.8207283	1616	4.666878
3945	GO:0005622	intracellular	1.00	62	8.683474	4751	13.720507
4027	GO:0005737	cytoplasm	1.00	18	2.5210085	2001	5.7787275
14959	GO:0032553	ribonucleotide binding	1.00	3	0.42016807	795	2.2958963
14961	GO:0032555	purine ribonucleotide bi	1.00	3	0.42016807	795	2.2958963
10033	GO:0017076	purine nucleotide bindin	1.00	3	0.42016807	797	2.3016722
20517	GO:0044267	cellular protein metaboli	1.00	36	5.042017	3225	9.313541
18000	GO:0035639	purine ribonucleoside tri	1.00	2	0.28011206	769	2.2208104
2626	GO:0003674|GO:00055	molecular_function	1.00	446	62.464985	24394	70.447914
3903	GO:0005575|GO:00083	cellular_component	1.00	119	16.666666	8515	24.590637
2653	GO:0003723	RNA binding	1.00	1	0.14005603	797	2.3016722
3876	GO:0005515|GO:00453	protein binding	1.00	87	12.184874	6933	20.021948
3946	GO:0005623	cell	1.00	108	15.12605	8227	23.758917
20657	GO:0044464	cell part	1.00	108	15.12605	8227	23.758917
