# Supplementary material for: The Tomato Hybrid Proline-rich Protein regulates the abscission zone competence to respond to ethylene signals
Source: Hortic Res. 2018 Jun 1;5:28. doi: 10.1038/s41438-018-0033-2 (PMC5981600; doi:10.1038/s41438-018-0033-2)
Supplement: Supplementary file 5 — Table S5 [file 41438_2018_33_MOESM5_ESM.pdf]

Table S5. Differentially regulated genes (up and down regulated) in the control compared with THyPRP silenced plants in FAZ at 12 h along with their expression values, fold changes, annotations, GO ids and terms.

Worksheet 2 & 3

Set4					
Line	Array	Details	Time	Treated vs. Con	Set
Control – New Yorker	254331010026_1_3	12h_7_AZ	12h	Control	Set 4
Control – New Yorker	254331010026_1_4	12h_8_AZ	12h		
TAPG + TPRP	254331010035_1_3	12h_43_AZ	12h	Treated	
TAPG + TPRP	254331010035_1_4	12h_44_AZ	12h		

Note:

Fold change expression values are provided as logbase 2

Flagging Criteria:

Compromised: If the gProcessedSignal value is less than the background intensity
Detected: If the gProcessedSignal value is above background intensity

Column Header	Description
ProbeName	Unique probe identifier
GeneName	GeneSymbol
Fold	Fold change calculated is log base 2
Flag	Detected or Compromised
GeomeanFold	Average Fold change for the replicates calculated is log base 2
p-Value	t-test p- value
gProcessedSigna	Background substracted signal intensity

Cut off used to filter up and down regulated genes

Upregulated

For filtering upregulation we consider flag should be Detected in the treated sample and can be Compromised or Detected in the control sample and fold>=0.8 in the individual replicates and fold>=1 in the Geomean of treated samples.

Downregulated

For filtering downregulation we consider flag can be Detected or Compromised in the treated sample and should be Detected in the control sample and fold<=-0.8 in the individual replicates and fold<=-1 in the Geomean of treated samples.

Differentially Regulated Probes

Sample	Up	Down
4h_TPRP_ AZ vs. 4h_Control_AZ_Sense	1415	1392
4h_TPRP_ AZ vs. 4h_Control_AZ_Antisense	536	872

Worksheet 4 & 5

This file contains the fold change for all the spots in the array

GO ID
Gene Ontology ID
GO ACCESSION
Gene Ontology Function name
p-value :
The probability of obtaining the specified GO accession number from a list of random entities. Less the p-value more significant is the GO accession number.
Significant pvalue<0.05
Count in Selection :
This refers to the number of genes in the selected entity (for example, from T-test) list which have that particular GO term.
%Count in Selection :
This refers to the percentage of genes in the input entity list which have that GO term.
Count in Total :
This refers to the number of genes in All Entities which have that GO term.
%Count in Total :
This refers to the percentage of genes in the All Entities list which have that GO term.

		12h_AZ_Control					12h_AZ_TAPG + TPRP vs. 12h_AZ_Control																																
ProbeNar	Orientatio	Fold_12h	Flag_12h	Fold_12h	Flag_12h	Geomean	Fold_12h	Flag_12h	Fold_12h	Flag_12h	Geomean	P-value	ProbeNar	GeneNam	Systemat	Descripti	Sequence	Contig_ID	ITAG_ID	Parent Ge	ITAG Des	Ontology	Ontology	Contig ID	Target ID	Functiona	GO_term	Transcrip	target_ID	Symbols	TAIR Des	TAIR Chr	gProcess	gProcess	gProcess	gProcess	gProcess	gProcessedSignal_12h_44_AZ
A_96_P0(	Sense	0.582	Detected	-0.582	Detected	0.000	0.959	Detected	1.109	Detected	1.034	0.220	A_96_P0(	AK24756(	AK24756(	Solanum	TTTTCCAGGAATTCTCCATATATAACATCCACTATTCTCTGGGTGTACTGGGGAGTTTGC																51.141	20.8124	38.8679	92.1459	60.5696	70.8007
A_96_P01	Sense	0.515	Detected	-0.515	Detected	0.000	1.430	Detected	1.692	Detected	1.561	0.099	A_96_P01	AK32373	AK32373	Solanum	TGGGAATCAAGCCAATTTTCATTACAGGGACGAAACAGAATTTCAAACAAGTAAGGATCG																13246.3	5915.71	11768.6	22613.3	22767.1	28773
A_96_P01	Sense	-0.074	Detected	0.074	Detected	0.000	0.958	Detected	1.371	Detected	1.165	0.034	A_96_P01	AW94475	AW94475	EST3368(	ATCGAGTTCGTCTTTTTGTTAACATTTTGGGTGATGTTGTACAAATTCCCAGAGTGACTT																10744	10860	8796.27	27764.4	20032.1	28114.4
A_96_P01	Sense	0.282	Detected	-0.282	Detected	0.000	1.399	Detected	2.101	Detected	1.750	0.060	A_96_P01	AK32807	AK32807	Solanum	AGCCAAAATTAAAATCATCGGTGATTGCTATTTTCATAGCGAGTGAAGAGAATGCATCTA																583.903	359.929	711.641	1916.85	1154.5	1977.93
A_96_P01	Sense	0.177	Detected	-0.177	Detected	0.000	1.101	Detected	1.779	Detected	1.440	0.064	A_96_P01	A_96_P01	A_96_P01	Unknown	TGATGGGATATTGGTACCTGTTTCCCAGTATCCACTATACTCAGCTTCATACAATATGGG																375.6	267.841	362.757	976.431	649.492	1095.16
A_96_P01	Sense	0.873	Detected	-0.873	Detected	0.000	1.144	Detected	1.277	Detected	1.210	0.301	A_96_P01	ARG2	NM_0012	Solanum	ATATCCTTCACCTTGATGCTCATCCTGACATTTATGATGCATTTGAAGGAAACAAATACT																28235.4	7679.46	12255.5	35896.8	31078.3	35885.8
A_96_P01	Sense	0.572	Detected	-0.572	Detected	0.000	0.932	Detected	1.399	Detected	1.166	0.200	A_96_P01	AK32589	AK32589	Solanum	AGGTTCGAGAATCGATAGTATCATCGTTGCTTTGCATTTGGCAGGAGAGTTTAGCTGAGG																17661.4	7281.04	9955.69	20888.6	20658	30090.4
A_96_P01	Sense	0.460	Detected	-0.460	Detected	0.000	1.542	Detected	2.015	Detected	1.778	0.075	A_96_P01	TA42576	TA42576	Rep: Wou	TGGGATGGGCCTCATTTTCAGATGACTATATGAAGAAGCTGTTGTTGCATGCCATGGAAA																412.797	198.913	491.526	1190.92	796.473	1164.92
A_96_P01	Sense	0.379	Detected	-0.379	Detected	0.000	1.376	Detected	1.338	Detected	1.357	0.070	A_96_P01	LOC5440	NM_0012	Solanum	GACTTATTCATTTCTTATGAGGAACGCGAAAATGTTCAAGTTAGACGCTTGGCTGCCTCG																25238.1	13612.4	29162.8	49546.8	45939.1	47128.6
A_96_P01	Sense	2.042	Detected	-2.042	Detected	0.000	2.647	Detected	2.240	Detected	2.444	0.356	A_96_P01	LOX1.1	NM_0012	Solanum	ACTTACAGGCAAAGGAATTCCAAACAGTGTGTCAATATAGAAGAGTTTTGAGTACACATG																17598	946.35	15499.4	44998.2	24410.1	19390.5
A_96_P01	Sense	0.806	Detected	-0.806	Detected	0.000	1.475	Detected	1.303	Detected	1.389	0.229	A_96_P01	LOC5438	NM_0012	Solanum	AATAATGATAGAGTAAGGAGGATGTCAATTCATGATCCCACACCCACCTACTGTAGGTAG																734.644	219.048	217.447	905.058	1065.02	995.435
A_96_P01	Sense	1.208	Detected	-1.208	Detected	0.000	2.935	Detected	1.360	Detected	2.148	0.275	A_96_P01	wus	NM_0012	Solanum	GTTTGTAGTAAGTAAGTACTAATCTAATTTGGTATGTGCCAAGCTATTTGGACCTTATGG																186.69	31.8873	78.9923	103.217	563.433	199.297
A_96_P01	Sense	0.563	Detected	-0.563	Detected	0.000	1.001	Detected	1.592	Detected	1.297	0.178	A_96_P01	TA37803	TA37803	Rep: Chr	GTGAATGTTTCGTCAGAGGCTCATCGATTTGCATACAGAGAAGGAATTCGGTTTGACAAA																1638.51	684.533	1013.76	3077.17	2024.2	3212.78
A_96_P02	Sense	0.653	Detected	-0.653	Comprom	0.000	0.882	Detected	1.526	Detected	1.204	0.240	A_96_P02	AK32538	AK32538	Solanum	TTGGTACCTGCTGATCACATTAGCAGGAGTTACAATGACACATATTATGTGGATTCTCAA																22.9236	8.45826	19.3284	34.4051	24.5019	40.3318
A_96_P02	Sense	0.257	Detected	-0.257	Detected	0.000	0.952	Detected	1.164	Detected	1.058	0.063	A_96_P02	BI924670	BI924670	EST5445	GAATCAGCAAAGCCAGTACCACTTGATCGCTTGAAAAGCAGATTGATCTTCCATGACGGT																996.988	636.599	432.227	1414.26	1471.61	1796.06
A_96_P02	Sense	0.444	Detected	-0.444	Detected	0.000	1.524	Detected	1.988	Detected	1.756	0.073	A_96_P02	AK32130	AK32130	Solanum	CAACATAATCGCTGTTTCTAGTTGCAAGGGAGGGGTTGGTAAATCAACTGTAGCTGTCAA																145.775	71.7937	119.931	379.482	280.821	408.327
A_96_P02	Sense	-0.418	Comprom	0.418	Comprom	0.000	1.049	Detected	1.401	Detected	1.225	0.114	A_96_P02	DB71789	DB71789	DB71789	AAGGACTGTGGTTGTTGCAACAAAGCTTGATACCAAAATACCTCAGTTTGCACGAGCTTC																4.30957	7.01807	4.67081	8.8903	10.8648	14.6099
A_96_P02	Sense	1.388	Detected	-1.388	Comprom	0.000	1.293	Detected	1.433	Detected	1.363	0.430	A_96_P02	BP89733	BP89733	BP89733	CTTTATGTACATTGATCAAGTGAAGAGTTCTCTAACTGATAGGATTGAGATTTCTGGTCA																16.3923	2.1816	14.2359	28.5483	13.9887	16.2466

[illegible]

GT	Sense	Sense	0.069	Comprom	-0.069	Comprom	0.000	0.897	Detected	1.271	Detected	1.084	0.032	GT	Sens
GT	Sense	Sense	1.393	Detected	-1.393	Detected	0.000	2.077	Detected	2.252	Detected	2.164	0.261	GT	Sens
GT	Sense	Sense	0.567	Detected	-0.567	Detected	0.000	1.400	Detected	1.144	Detected	1.272	0.160	GT	Sens
GT	Sense	Sense	0.348	Detected	-0.348	Detected	0.000	0.844	Detected	1.311	Detected	1.078	0.124	GT	Sens
GT	Sense	Sense	1.495	Detected	-1.495	Comprom	0.000	1.712	Detected	2.388	Detected	2.050	0.313	GT	Sens
GT	Sense	Sense	1.160	Detected	-1.160	Detected	0.000	1.879	Detected	1.762	Detected	1.821	0.258	GT	Sens
GT	Sense	Sense	0.373	Detected	-0.373	Detected	0.000	1.045	Detected	1.950	Detected	1.497	0.125	GT	Sens
GT	Sense	Sense	0.359	Detected	-0.359	Detected	0.000	1.441	Detected	1.437	Detected	1.439	0.057	GT	Sens
GT	Sense	Sense	0.495	Detected	-0.495	Detected	0.000	0.920	Detected	1.262	Detected	1.091	0.173	GT	Sens
GT	Sense	Sense	0.575	Detected	-0.575	Detected	0.000	1.318	Detected	1.097	Detected	1.207	0.175	GT	Sens
GT	Sense	Sense	0.201	Detected	-0.201	Detected	0.000	0.822	Detected	1.291	Detected	1.057	0.076	GT	Sens
GT	Sense	Sense	-0.149	Comprom	0.149	Comprom	0.000	2.167	Detected	2.284	Detected	2.226	0.005	GT	Sens
GT	Sense	Sense	0.132	Detected	-0.132	Detected	0.000	1.053	Detected	1.177	Detected	1.115	0.017	GT	Sens
GT	Sense	Sense	0.204	Detected	-0.204	Detected	0.000	1.191	Detected	1.262	Detected	1.227	0.027	GT	Sens
GT	Sense	Sense	0.259	Detected	-0.259	Detected	0.000	1.064	Detected	0.970	Detected	1.017	0.061	GT	Sens
GT	Sense	Sense	0.658	Detected	-0.658	Detected	0.000	1.463	Detected	1.821	Detected	1.642	0.138	GT	Sens
GT	Sense	Sense	0.056	Comprom	-0.056	Comprom	0.000	1.419	Detected	1.087	Detected	1.253	0.019	GT	Sens
GT	Sense	Sense	0.882	Detected	-0.882	Detected	0.000	1.083	Detected	1.353	Detected	1.218	0.305	GT	Sens
GT	Sense	Sense	0.159	Detected	-0.159	Detected	0.000	0.970	Detected	1.272	Detected	1.121	0.036	GT	Sens
GT	Sense	Sense	0.503	Detected	-0.503	Detected	0.000	1.379	Detected	1.410	Detected	1.394	0.109	GT	Sens
GT	Sense	Sense	1.139	Detected	-1.139	Detected	0.000	1.361	Detected	1.739	Detected	1.550	0.311	GT	Sens
GT	Sense	Sense	1.024	Detected	-1.024	Detected	0.000	0.884	Detected	1.239	Detected	1.062	0.414	GT	Sens
GT	Sense	Sense	0.646	Detected	-0.646	Detected	0.000	1.801	Detected	2.052	Detected	1.926	0.100	GT	Sens
GT	Sense	Sense	0.975	Detected	-0.975	Detected	0.000	1.659	Detected	1.044	Detected	1.351	0.317	GT	Sens
GT	Sense	Sense	-0.522	Comprom	0.522	Comprom	0.000	1.924	Detected	1.705	Detected	1.815	0.077	GT	Sens
GT	Sense	Sense	0.237	Detected	-0.237	Detected	0.000	0.842	Detected	1.179	Detected	1.010	0.074	GT	Sens
GT	Sense	Sense	0.885	Detected	-0.885	Detected	0.000	1.873	Detected	1.984	Detected	1.928	0.162	GT	Sens
GT	Sense	Sense	0.378	Detected	-0.378	Detected	0.000	0.802	Detected	1.263	Detected	1.033	0.145	GT	Sens
GT	Sense	Sense	0.357	Comprom	-0.357	Comprom	0.000	1.920	Detected	1.109	Detected	1.514	0.107	GT	Sens
GT	Sense	Sense	1.015	Detected	-1.015	Detected	0.000	2.219	Detected	1.777	Detected	1.998	0.194	GT	Sens
GT	Sense	Sense	1.191	Detected	-1.191	Comprom	0.000	1.725	Detected	1.575	Detected	1.650	0.301	GT	Sens
GT	Sense	Sense	0.548	Detected	-0.548	Detected	0.000	1.053	Detected	1.120	Detected	1.087	0.186	GT	Sens
GT	Sense	Sense	0.332	Comprom	-0.332	Comprom	0.000	4.117	Detected	4.020	Detected	4.069	0.007	GT	Sens
GT	Sense	Sense	0.245	Detected	-0.245	Detected	0.000	1.504	Detected	1.659	Detected	1.581	0.025	GT	Sens
GT	Sense	Sense	0.084	Detected	-0.084	Detected	0.000	1.835	Detected	1.285	Detected	1.560	0.032	GT	Sens
GT	Sense	Sense	-0.089	Detected	0.089	Detected	0.000	0.803	Detected	1.250	Detected	1.026	0.051	GT	Sens
GT	Sense	Sense	0.742	Detected	-0.742	Detected	0.000	1.490	Detected	0.976	Detected	1.233	0.257	GT	Sens
GT	Sense	Sense	0.433	Detected	-0.433	Detected	0.000	1.099	Detected	1.423	Detected	1.261	0.112	GT	Sens
GT	Sense	Sense	0.109	Detected	-0.109	Detected	0.000	1.127	Detected	1.320	Detected	1.223	0.014	GT	Sens
GT	Sense	Sense	-0.029	Detected	0.029	Detected	0.000	1.085	Detected	2.054	Detected	1.569	0.084	GT	Sens
GT	Sense	Sense	0.114	Comprom	-0.114	Comprom	0.000	1.544	Detected	1.706	Detected	1.625	0.007	GT	Sens
GT	Sense	Sense	0.380	Detected	-0.380	Detected	0.000	0.869	Detected	1.212	Detected	1.040	0.130	GT	Sens
GT	Sense	Sense	1.343	Detected	-1.343	Comprom	0.000	2.580	Detected	2.670	Detected	2.625	0.190	GT	Sens
GT	Sense	Sense	-0.628	Detected	0.628	Detected	0.000	2.217	Detected	2.180	Detected	2.198	0.073	GT	Sens
GT	Sense	Sense	0.343	Detected	-0.343	Detected	0.000	1.333	Detected	1.492	Detected	1.413	0.057	GT	Sens
GT	Sense	Sense	-0.925	Comprom	0.925	Comprom	0.000	1.792	Detected	2.465	Detected	2.129	0.163	GT	Sens
GT	Sense	Sense	0.021	Detected	-0.021	Detected	0.000	0.986	Detected	1.098	Detected	1.042	0.003	GT	Sens
GT	Sense	Sense	0.542	Detected	-0.542	Detected	0.000	1.131	Detected	1.212	Detected	1.172	0.164	GT	Sens
GT	Sense	Sense	0.777	Detected	-0.777	Detected	0.000	0.846	Detected	2.081	Detected	1.463	0.278	GT	Sens
GT	Sense	Sense	-0.044	Detected	0.044	Detected	0.000	1.061	Detected	0.946	Detected	1.004	0.005	GT	Sens
GT	Sense	Sense	0.457	Detected	-0.457	Detected	0.000	1.187	Detected	1.081	Detected	1.134	0.133	GT	Sens
GT	Sense	Sense	0.352	Detected	-0.352	Detected	0.000	1.498	Detected	1.598	Detected	1.548	0.049	GT	Sens
GT	Sense	Sense	0.329	Detected	-0.329	Detected	0.000	1.073	Detected	1.134	Detected	1.104	0.079	GT	Sens
GT	Sense	Sense	0.949	Detected	-0.949	Detected	0.000	2.021	Detected	0.809	Detected	1.415	0.336	GT	Sens
GT	Sense	Sense	0.180	Detected	-0.180	Detected	0.000	1.020	Detected	1.632	Detected	1.326	0.065	GT	Sens
GT	Sense	Sense	0.129	Detected	-0.129	Detected	0.000	0.956	Detected	1.085	Detected	1.021	0.019	GT	Sens
GT	Sense	Sense	0.556	Detected	-0.556	Detected	0.000	1.211	Detected	1.096	Detected	1.153	0.175	GT	Sens
GT	Sense	Sense	-0.583	Detected	0.583	Detected	0.000	1.598	Detected	1.304	Detected	1.451	0.137	GT	Sens
GT	Sense	Sense	0.302	Detected	-0.302	Detected	0.000	1.120	Detected	1.191	Detected	1.155	0.063	GT	Sens
GT	Sense	Sense	0.128	Detected	-0.128	Detected	0.000	0.831	Detected	1.581	Detected	1.206	0.093	GT	Sens
GT	Sense	Sense	0.479	Detected	-0.479	Detected	0.000	1.119	Detected	0.955	Detected	1.037	0.166	GT	Sens
GT	Sense	Sense	0.745	Detected	-0.745	Detected	0.000	1.222	Detected	1.785	Detected	1.503	0.200	GT	Sens
GT	Sense	Sense	0.853	Detected	-0.853	Detected	0.000	1.752	Detected	1.844	Detected	1.798	0.170	GT	Sens
GT	Sense	Sense	-0.113	Detected	0.113	Detected	0.000	0.884	Detected	1.275	Detected	1.080	0.041	GT	Sens
GT	Sense	Sense	0.221	Detected	-0.221	Detected	0.000	1.191	Detected	1.082	Detected	1.136	0.038	GT	Sens
GT	Sense	Sense	-0.048	Detected	0.048	Detected	0.000	0.914	Detected	1.863	Detected	1.389	0.100	GT	Sens
GT	Sense	Sense	-0.092	Detected	0.092	Detected	0.000	1.236	Detected	1.248	Detected	1.242	0.005	GT	Sens
GT	Sense	Sense	0.367	Detected	-0.367	Detected	0.000	1.317	Detected	1.429	Detected	1.373	0.066	GT	Sens
GT	Sense	Sense	-0.023	Detected	0.023	Detected	0.000	0.886	Detected	1.166	Detected	1.026	0.019	GT	Sens
GT	Sense	Sense	0.296	Comprom	-0.296	Comprom	0.000	3.048	Detected	2.120	Detected	2.584	0.042	GT	Sens
GT	Sense	Sense	0.155	Detected	-0.155	Detected	0.000	1.067	Detected	1.574	Detected	1.321	0.047	GT	Sens
GT	Sense	Sense	0.282	Detected	-0.282	Detected	0.000	1.415	Detected	1.636	Detected	1.526	0.037	GT	Sens
GT	Sense	Sense	0.310	Detected	-0.310	Detected	0.000	1.042	Detected	1.407	Detected	1.225	0.077	GT	Sens
GT	Sense	Sense	0.601	Detected	-0.601	Detected	0.000	1.289	Detected	1.446	Detected	1.368	0.153	GT	Sens
GT	Sense	Sense	0.178	Detected	-0.178	Detected	0.000	0.875	Detected	1.479	Detected	1.177	0.078	GT	Sens
GT	Sense	Sense	1.245	Detected	-1.245	Detected	0.000	1.759	Detected	3.923	Detected	2.841	0.227	GT	Sens
GT	Sense	Sense	0.150	Detected	-0.150	Detected	0.000	1.294	Detected	1.559	Detected	1.427	0.019	GT	Sens
GT	Sense	Sense	0.512	Detected	-0.512	Detected	0.000	1.014	Detected	1.128	Detected	1.071	0.173	GT	Sens
GT	Sense	Sense	0.303	Detected	-0.303	Detected	0.000	1.492	Detected	1.514	Detected	1.503	0.038	GT	Sens
GT	Sense	Sense	0.323	Detected	-0.323	Detected	0.000	1.077	Detected	1.207	Detected	1.142	0.074	GT	Sens
GT	Sense	Sense	0.621	Detected	-0.621	Detected	0.000	0.998	Detected	1.097	Detected	1.047	0.235	GT	Sens
GT	Sense	Sense	0.617	Detected	-0.617	Detected	0.000	1.045	Detected	1.231	Detected	1.138	0.210	GT	Sens
GT	Sense	Sense	0.222	Detected	-0.222	Detected	0.000	1.031	Detected	1.179	Detected	1.105	0.042	GT	Sens
GT	Sense	Sense	-0.298	Comprom	0.298	Comprom	0.000	1.220	Detected	1.906	Detected	1.563	0.075	GT	Sens
GT	Sense	Sense	0.015	Detected	-0.015	Detected	0.000	1.570	Detected	2.308	Detected	1.939	0.034	GT	Sens
GT	Sense	Sense	0.445	Detected	-0.445	Detected	0.000	1.122	Detected	1.257	Detected	1.189	0.118	GT	Sens
GT	Sense	Sense	0.954	Detected	-0.954	Comprom	0.000	2.124	Detected	1.198	Detected	1.661	0.258	GT	Sens
GT	Sense	Sense	-1.118	Comprom	1.118	Detected	0.000	1.135	Detected	1.216	Detected	1.175	0.404	GT	Sens
GT	Sense	Sense	0.399	Comprom	-0.399	Comprom	0.000	1.329	Detected	1.706	Detected	1.517	0.075	GT	Sens
GT	Sense	Sense	0.128	Detected	-0.128	Detected	0.000	0.819	Detected	1.188	Detected	1.004	0.046	GT	Sens
GT	Sense	Sense	0.216	Detected	-0.216	Detected	0.000	1.176	Detected	1.361	Detected	1.268	0.033	GT	Sens
GT	Sense	Sense	0.017	Detected	-0.017	Detected	0.000	1.692	Detected	1.843	Detected	1.767	0.002	GT	Sens
GT	Sense	Sense	0.145	Detected	-0.145	Detected	0.000	1.061	Detected	1.087	Detected	1.074	0.018	GT	Sens
GT	Sense	Sense	0.874	Detected	-0.874	Detected	0.000	1.602	Detected	1.720	Detected	1.661	0.198	GT	Sens
GT	Sense	Sense	0.232	Detected	-0.232	Detected	0.000	1.161	Detected	1.267	Detected	1.214	0.036	GT	Sens
GT	Sense	Sense	0.075	Detected	-0.075	Detected	0.000	1.326	Detected	1.930	Detected	1.628	0.035	GT	Sens
GT	Sense	Sense	0.640	Detected	-0.640	Detected	0.000	1.687	Detected	2.065	Detected	1.876	0.107	GT	Sens
GT	Sense	Sense	1.104	Detected	-1.104	Comprom	0.000	1.602	Detected	1.006	Detected	1.304	0.372	GT	Sens
GT	Sense	Sense	1.233	Detected	-1.233	Detected	0.000	1.902	Detected	2.509	Detected	2.206	0.225	GT	Sens
GT	Sense	Sense	0.664	Detected	-0.664	Detected	0.000	0.921	Detected	1.233	Detected	1.077	0.255	GT	Sens
GT	Sense	Sense	0.325	Detected	-0.325	Detected	0.000	1.170	Detected	1.451	Detected	1.311	0.066	GT	Sens
GT	Sense	Sense	0.521	Detected	-0.521	Detected	0.000	0.927	Detected	1.207	Detected	1.067	0.186	GT	Sens
GT	Sense	Sense	0.523	Detected	-0.523	Detected	0.000	0.907	Detected	1.134	Detected	1.021	0.197	GT	Sens
GT	Sense	Sense	0.202	Detected	-0.202	Detected	0.000	1.509	Detected	1.789	Detected	1.649	0.022	GT	Sens
GT	Sense	Sense	0.063	Detected	-0.063	Detected	0.000	1.082	Detected	0.930	Detected	1.006	0.010	GT	Sens
GT	Sense	Sense	0.045	Detected	-0.045	Detected	0.000	0.930	Detected	1.585	Detected	1.258	0.063	GT	Sens
GT	Sense	Sense	0.011	Detected	-0.011	Detected	0.000	1.101	Detected	1.159	Detected	1.130	0.001	GT	Sens
GT	Sense	Sense	0.803	Detected	-0.803	Detected	0.000	1.730	Detected	1.931	Detected	1.830	0.152	GT	Sens
GT	Sense	Sense	0.475	Detected	-0.475	Detected	0.000	0.859	Detected	1.341	Detected	1.100	0.175	GT	Sens
GT	Sense	Sense	0.414	Detected	-0.414	Detected	0.000	0.854	Detected	1.248	Detected	1.051	0.149	GT	Sens
GT	Sense	Sense	0.944	Detected	-0.944	Detected	0.000	1.639	Detected	1.371	Detected	1.505	0.255	GT	Sens
GT	Sense	Sense	0.386	Detected	-0.386	Detected	0.000	1.042	Detected	1.667	Detected	1.355	0.112	GT	Sens
GT	Sense	Sense	0.411	Detected	-0.411	Detected	0.000	1.302	Detected	1.762	Detected	1.532	0.083	GT	Sens
GT	Sense	Sense	0.687	Detected	-0.687	Detected	0.000	1.098	Detected	1.502	Detected	1.300	0.211	GT	Sens
GT	Sense	Sense	0.168	Detected	-0.168	Detected	0.000	2.420	Detected	2.766	Detected	2.593	0.009	GT	Sens
GT	Sense	Sense	0.385	Detected	-0.385	Detected	0.000	1.160	Detected	1.015	Detected	1.088	0.109	GT	Sens
GT	Sense	Sense	0.490	Detected	-0.490	Detected	0.000	0.884	Detected	1.253	Detected	1.068	0.178	GT	Sens
GT	Sense	Sense	0.561	Detected	-0.561	Comprom	0.000	1.101	Detected	0.900	Detected	1.001	0.221	GT	Sens
GT	Sense	Sense	0.435	Detected	-0.435	Detected	0.000	1.020	Detected	1.467	Detected	1.244	0.126	GT	Sens
GT	Sense	Sense	1.113	Detected	-1.113	Detected	0.000	0.838	Detected	1.277	Detected	1.057	0.450	GT	Sens
GT	Sense	Sense	0.581	Detected	-0.581	Detected	0.000	1.798	Detected	1.574	Detected	1.686	0.104	GT	Sens
GT	Sense	Sense	-0.158	Detected	0.158	Detected	0.000	1.083	Detected	1.388	Detected	1.236	0.030	GT	Sens
GT	Sense	Sense	0.601	Detected	-0.601	Detected	0.000	0.929	Detected	1.110	Detected	1.019	0.235	GT	Sens
GT	Sense	Sense	0.321	Detected	-0.321	Detected	0.000	1.341	Detected	1.383	Detected	1.362	0.051	GT	Sens
GT	Sense	Sense	-0.282	Comprom	0.282	Comprom	0.000	1.312	Detected	1.117	Detected	1.214	0.055	GT	Sens
GT	Sense	Sense	1.011	Detected	-1.011	Detected	0.000	2.445	Detected	1.212	Detected	1.829	0.263	GT	Sens
GT	Sense	Sense	0.667	Detected	-0.667	Detected	0.000	1.862	Detected	1.891	Detected	1.877	0.107	GT	Sens
GT	Sense	Sense	1.114	Detected	-1.114	Comprom	0.000	1.553	Detected	1.171	Detected	1.362	0.351	GT	Sens
GT	Sense	Sense	0.133	Detected	-0.133	Detected	0.000	2.932	Detected	0.820	Detected	1.876	0.220	GT	Sens
GT	Sense	Sense	0.134	Detected	-0.134	Detected	0.000	1.782	Detected	2.379	Detected	2.080	0.024	GT	Sens
GT	Sense	Sense	0.720	Detected	-0.720	Detected	0.000	1.180	Detected	2.080	Detected	1.630	0.195	GT	Sens
GT	Sense	Sense	1.025	Detected	-1.025	Detected	0.000	1.046	Detected	1.557	Detected	1.302	0.343	GT	Sens
GT	Sense	Sense	0.408	Detected	-0.408	Detected	0.000	0.908	Detected	1.216	Detected	1.062	0.135	GT	Sens
GT	Sense	Sense	0.264	Comprom	-0.264	Comprom	0.000	2.406	Detected	1.983	Detected	2.194	0.023	GT	Sens
GT	Sense	Sense	0.030	Detected	-0.030	Detected	0.000	1.201	Detected	0.840	Detected	1.020	0.031	GT	Sens
GT	Sense	Sense	1.536	Detected	-1.536	Comprom	0.000	2.321	Detected	4.408	Detected	3.365	0.212	GT	Sens
GT	Sense	Sense	0.234	Detected	-0.234	Detected	0.000	0.969	Detected	1.269	Detected	1.119	0.057	GT	Sens
GT	Sense	Sense	0.451	Detected	-0.451	Detected	0.000	0.944	Detected	1.872	Detected	1.408	0.162	GT	Sens
GT	Sense	Sense	0.278	Detected	-0.278	Detected	0.000	1.322	Detected	1.465	Detected	1.393	0.040	GT	Sens
GT	Sense	Sense	0.057	Detected	-0.057	Detected	0.000	1.267	Detected	1.336	Detected	1.301	0.003	GT	Sens
GT	Sense	Sense	0.905	Detected	-0.905	Detected	0.000	1.433	Detected	1.949	Detected	1.691	0.214	GT	Sens
GT	Sense	Sense	0.446	Detected	-0.446	Detected	0.000	0.886	Detected	1.269	Detected	1.077	0.157	GT	Sens
GT	Sense	Sense	0.918	Detected	-0.918	Detected	0.000	0.841	Detected	1.423	Detected	1.132	0.361	GT	Sens
GT	Sense	Sense	0.135	Detected	-0.135	Detected	0.000	1.118	Detected	1.029	Detected	1.074	0.017	GT	Sens
GT	Sense	Sense	0.130	Detected	-0.130	Detected	0.000	1.448	Detected	1.637	Detected	1.543	0.011	GT	Sens
GT	Sense	Sense	0.021	Comprom	-0.021	Comprom	0.000	1.065	Detected	1.507	Detected	1.286	0.029	GT	Sens
GT	Sense	Sense	0.476	Comprom	-0.476	Comprom	0.000	1.003	Detected	1.230	Detected	1.116	0.150	GT	Sens
GT	Sense	Sense	0.885	Detected	-0.885	Detected	0.000	1.026	Detected	2.145	Detected	1.586	0.269	GT	Sens
GT	Sense	Sense	0.102	Detected	-0.102	Detected	0.000	1.123	Detected	0.982	Detected	1.052	0.014	GT	Sens
GT	Sense	Sense	-0.787	Comprom	0.787	Detected	0.000	1.681	Detected	1.083	Detected	1.382	0.242	GT	Sens
GT	Sense	Sense	-0.946	Comprom	0.946	Comprom	0.000	1.573	Detected	1.328	Detected	1.451	0.268	GT	Sens
GT	Sense	Sense	0.319	Detected	-0.319	Detected	0.000	1.046	Detected	1.791	Detected	1.419	0.102	GT	Sens
GT	Sense	Sense	2.106	Detected	-2.106	Comprom	0.000	1.846	Detected	1.057	Detected	1.451	0.568	GT	Sens
GT	Sense	Sense	-0.054	Detected	0.054	Detected	0.000	1.359	Detected	1.254	Detected	1.306	0.003	GT	Sens
GT	Sense	Sense	0.175	Detected	-0.175	Detected	0.000	1.139	Detected	1.265	Detected	1.202	0.023	GT	Sens
GT	Sense	Sense	0.673	Detected	-0.673	Detected	0.000	1.252	Detected	1.190	Detected	1.221	0.211	GT	Sens
GT	Sense	Sense	0.372	Comprom	-0.372	Comprom	0.000	2.827	Detected	3.910	Detected	3.368	0.036	GT	Sens
GT	Sense	Sense	0.493	Detected	-0.493	Detected	0.000	1.119	Detected	1.121	Detected	1.120	0.151	GT	Sens
GT	Sense	Sense	-0.627	Comprom	0.627	Comprom	0.000	3.167	Detected	1.979	Detected	2.573	0.097	GT	Sens
GT	Sense	Sense	0.493	Comprom	-0.493	Comprom	0.000	1.309	Detected	1.289	Detected	1.299	0.119	GT	Sens
GT	Sense	Sense	0.825	Detected	-0.825	Detected	0.000	1.172	Detected	2.161	Detected	1.667	0.225	GT	Sens
GT	Sense	Sense	0.400	Detected	-0.400	Detected	0.000	0.961	Detected	1.183	Detected	1.072	0.123	GT	Sens
GT	Sense	Sense	1.366	Detected	-1.366	Comprom	0.000	1.108	Detected	1.811	Detected	1.460	0.410	GT	Sens
GT	Sense	Sense	0.204	Detected	-0.204	Comprom	0.000	1.902	Detected	1.193	Detected	1.548	0.063	GT	Sens
GT	Sense	Sense	1.028	Detected	-1.028	Detected	0.000	2.611	Detected	1.675	Detected	2.143	0.198	GT	Sens
GT	Sense	Sense	0.239	Detected	-0.239	Detected	0.000	1.482	Detected	1.448	Detected	1.465	0.026	GT	Sens
GT	Sense	Sense	-0.258	Comprom	0.258	Comprom	0.000	1.627	Detected	2.259	Detected	1.943	0.041	GT	Sens
GT	Sense	Sense	0.893	Detected	-0.893	Detected	0.000	1.734	Detected	2.267	Detected	2.000	0.165	GT	Sens
GT	Sense	Sense	0.556	Detected	-0.556	Detected	0.000	1.224	Detected	1.263	Detected	1.244	0.155	GT	Sens
GT	Sense	Sense	0.572	Detected	-0.572	Detected	0.000	1.095	Detected	1.054	Detected	1.075	0.201	GT	Sens
GT	Sense	Sense	0.213	Detected	-0.213	Detected	0.000	1.185	Detected	1.399	Detected	1.292	0.033	GT	Sens
GT	Sense	Sense	0.506	Detected	-0.506	Detected	0.000	1.251	Detected	2.346	Detected	1.798	0.137	GT	Sens
GT	Sense	Sense	0.245	Detected	-0.245	Detected	0.000	1.185	Detected	1.123	Detected	1.154	0.043	GT	Sens
GT	Sense	Sense	1.245	Detected	-1.245	Detected	0.000	1.474	Detected	3.407	Detected	2.440	0.262	GT	Sens
GT	Sense	Sense	0.535	Detected	-0.535	Detected	0.000	0.994	Detected	1.515	Detected	1.254	0.169	GT	Sens
GT	Sense	Sense	0.360	Detected	-0.360	Detected	0.000	0.979	Detected	1.406	Detected	1.192	0.104	GT	Sens
GT	Sense	Sense	0.340	Detected	-0.340	Detected	0.000	1.391	Detected	1.322	Detected	1.357	0.058	GT	Sens
GT	Sense	Sense	0.654	Detected	-0.654	Detected	0.000	1.076	Detected	0.956	Detected	1.016	0.262	GT	Sens
GT	Sense	Sense	1.269	Detected	-1.269	Comprom	0.000	3.744	Detected	2.908	Detected	3.326	0.130	GT	Sens
GT	Sense	Sense	0.283	Detected	-0.283	Detected	0.000	0.882	Detected	1.284	Detected	1.083	0.089	GT	Sens
GT	Sense	Sense	0.362	Detected	-0.362	Detected	0.000	0.871	Detected	1.537	Detected	1.204	0.134	GT	Sens
GT	Sense	Sense	0.777	Detected	-0.777	Detected	0.000	2.380	Detected	1.572	Detected	1.976	0.153	GT	Sens
GT	Sense	Sense	-0.070	Detected	0.070	Detected	0.000	1.073	Detected	1.438	Detected	1.255	0.023	GT	Sens
GT	Sense	Sense	0.191	Detected	-0.191	Detected	0.000	1.537	Detected	1.374	Detected	1.455	0.020	GT	Sens
GT	Sense	Sense	1.135	Detected	-1.135	Comprom	0.000	1.450	Detected	2.243	Detected	1.846	0.264	GT	Sens
GT	Sense	Sense	0.147	Detected	-0.147	Detected	0.000	0.847	Detected	1.279	Detected	1.063	0.055	GT	Sens
GT	Sense	Sense	0.294	Detected	-0.294	Detected	0.000	1.214	Detected	1.607	Detected	1.411	0.057	GT	Sens
GT	Sense	Sense	0.380	Comprom	-0.380	Comprom	0.000	1.851	Detected	2.001	Detected	1.926	0.038	GT	Sens
GT	Sense	Sense	0.425	Detected	-0.425	Detected	0.000	0.860	Detected	1.279	Detected	1.069	0.153	GT	Sens
GT	Sense	Sense	0.472	Detected	-0.472	Detected	0.000	1.077	Detected	1.179	Detected	1.128	0.141	GT	Sens
GT	Sense	Sense	0.301	Detected	-0.301	Detected	0.000	1.056	Detected	1.117	Detected	1.087	0.070	GT	Sens
GT	Sense	Sense	0.320	Detected	-0.320	Detected	0.000	0.834	Detected	1.598	Detected	1.216	0.135	GT	Sens
GT	Sense	Sense	0.578	Comprom	-0.578	Comprom	0.000	1.710	Detected	1.534	Detected	1.622	0.109	GT	Sens
GT	Sense	Sense	0.057	Detected	-0.057	Detected	0.000	1.117	Detected	1.404	Detected	1.260	0.015	GT	Sens
GT	Sense	Sense	0.357	Detected	-0.357	Detected	0.000	1.039	Detected	1.155	Detected	1.097	0.094	GT	Sens
GT	Sense	Sense	0.483	Detected	-0.483	Detected	0.000	0.895	Detected	1.145	Detected	1.020	0.177	GT	Sens
GT	Sense	Sense	1.172	Detected	-1.172	Comprom	0.000	1.223	Detected	1.211	Detected	1.217	0.408	GT	Sens
GT	Sense	Sense	0.338	Detected	-0.338	Detected	0.000	1.181	Detected	1.161	Detected	1.171	0.074	GT	Sens
GT	Sense	Sense	-0.367	Comprom	0.367	Comprom	0.000	1.430	Detected	1.398	Detected	1.414	0.061	GT	Sens
GT	Sense	Sense	-0.327	Detected	0.327	Detected	0.000	1.213	Detected	1.551	Detected	1.382	0.064	GT	Sens
GT	Sense	Sense	0.526	Detected	-0.526	Detected	0.000	1.067	Detected	1.282	Detected	1.174	0.160	GT	Sens
GT	Sense	Sense	0.274	Detected	-0.274	Detected	0.000	1.379	Detected	1.128	Detected	1.254	0.053	GT	Sens
GT	Sense	Sense	0.972	Detected	-0.972	Detected	0.000	1.357	Detected	2.147	Detected	1.752	0.237	GT	Sens
GT	Sense	Sense	0.704	Detected	-0.704	Comprom	0.000	1.200	Detected	1.080	Detected	1.140	0.248	GT	Sens
GT	Sense	Sense	0.500	Detected	-0.500	Detected	0.000	1.229	Detected	2.044	Detected	1.637	0.127	GT	Sens
GT	Sense	Sense	0.064	Detected	-0.064	Detected	0.000	0.948	Detected	1.103	Detected	1.026	0.009	GT	Sens
GT	Sense	Sense	0.225	Detected	-0.225	Detected	0.000	1.172	Detected	1.211	Detected	1.192	0.034	GT	Sens
GT	Sense	Sense	0.986	Detected	-0.986	Comprom	0.000	0.829	Detected	1.202	Detected	1.015	0.418	GT	Sens
GT	Sense	Sense	0.123	Detected	-0.123	Detected	0.000	1.703	Detected	1.203	Detected	1.453	0.035	GT	Sens
GT	Sense	Sense	0.561	Detected	-0.561	Detected	0.000	0.961	Detected	1.307	Detected	1.134	0.193	GT	Sens
GT	Sense	Sense	0.176	Comprom	-0.176	Comprom	0.000	1.472	Detected	1.015	Detected	1.244	0.050	GT	Sens
GT	Sense	Sense	0.822	Comprom	-0.822	Comprom	0.000	1.535	Detected	1.775	Detected	1.655	0.185	GT	Sens
GT	Sense	Sense	0.161	Detected	-0.161	Detected	0.000	1.031	Detected	1.463	Detected	1.247	0.044	GT	Sens
GT	Sense	Sense	1.157	Detected	-1.157	Comprom	0.000	0.868	Detected	3.092	Detected	1.980	0.343	GT	Sens
GT	Sense	Sense	0.773	Detected	-0.773	Comprom	0.000	1.594	Detected	1.126	Detected	1.360	0.234	GT	Sens
GT	Sense	Sense	0.491	Detected	-0.491	Detected	0.000	1.904	Detected	1.653	Detected	1.779	0.072	GT	Sens
GT	Sense	Sense	0.821	Detected	-0.821	Detected	0.000	1.651	Detected	2.743	Detected	2.197	0.156	GT	Sens
GT	Sense	Sense	1.479	Detected	-1.479	Detected	0.000	2.239	Detected	1.349	Detected	1.794	0.365	GT	Sens
GT	Sense	Sense	0.815	Comprom	-0.815	Comprom	0.000	1.540	Detected	1.440	Detected	1.490	0.209	GT	Sens
GT	Sense	Sense	0.951	Detected	-0.951	Comprom	0.000	1.142	Detected	1.277	Detected	1.209	0.332	GT	Sens
GT	Sense	Sense	0.834	Detected	-0.834	Detected	0.000	2.072	Detected	1.668	Detected	1.870	0.161	GT	Sens
GT	Sense	Sense	0.038	Detected	-0.038	Detected	0.000	1.328	Detected	1.598	Detected	1.463	0.009	GT	Sens
GT	Sense	Sense	0.706	Detected	-0.706	Detected	0.000	1.021	Detected	1.158	Detected	1.090	0.264	GT	Sens
GT	Sense	Sense	-0.352	Detected	0.352	Detected	0.000	1.108	Detected	1.007	Detected	1.058	0.097	GT	Sens
GT	Sense	Sense	0.786	Detected	-0.786	Detected	0.000	1.410	Detected	1.125	Detected	1.268	0.253	GT	Sens
GT	Sense	Sense	0.747	Detected	-0.747	Detected	0.000	1.010	Detected	2.141	Detected	1.575	0.234	GT	Sens
GT	Sense	Sense	1.634	Detected	-1.634	Detected	0.000	2.955	Detected	3.193	Detected	3.074	0.201	GT	Sens
GT	Sense	Sense	-0.209	Comprom	0.209	Detected	0.000	1.506	Detected	1.095	Detected	1.301	0.047	GT	Sens
GT	Sense	Sense	-0.018	Detected	0.018	Detected	0.000	1.750	Detected	1.149	Detected	1.450	0.041	GT	Sens
GT	Sense	Sense	-0.109	Detected	0.109	Detected	0.000	1.160	Detected	2.085	Detected	1.623	0.076	GT	Sens
GT	Sense	Sense	0.573	Detected	-0.573	Detected	0.000	0.873	Detected	1.169	Detected	1.021	0.227	GT	Sens
GT	Sense	Sense	0.495	Detected	-0.495	Detected	0.000	0.928	Detected	1.766	Detected	1.347	0.173	GT	Sens
GT	Sense	Sense	1.085	Detected	-1.085	Detected	0.000	2.802	Detected	1.330	Detected	2.066	0.256	GT	Sens
GT	Sense	Sense	0.424	Detected	-0.424	Detected	0.000	1.591	Detected	1.414	Detected	1.503	0.074	GT	Sens
GT	Sense	Sense	0.561	Detected	-0.561	Detected	0.000	1.160	Detected	0.855	Detected	1.007	0.225	GT	Sens
GT	Sense	Sense	0.608	Detected	-0.608	Detected	0.000	1.145	Detected	1.352	Detected	1.248	0.180	GT	Sens
GT	Sense	Sense	0.006	Detected	-0.006	Detected	0.000	1.165	Detected	0.891	Detected	1.028	0.017	GT	Sens
GT	Sense	Sense	-0.107	Detected	0.107	Detected	0.000	0.962	Detected	1.354	Detected	1.158	0.035	GT	Sens
GT	Sense	Sense	0.529	Comprom	-0.529	Comprom	0.000	1.596	Detected	1.099	Detected	1.348	0.147	GT	Sens
GT	Sense	Sense	0.031	Detected	-0.031	Detected	0.000	1.239	Detected	1.311	Detected	1.275	0.001	GT	Sens
GT	Sense	Sense	0.283	Detected	-0.283	Detected	0.000	1.203	Detected	0.868	Detected	1.036	0.088	GT	Sens
GT	Sense	Sense	-0.035	Detected	0.035	Detected	0.000	1.308	Detected	1.630	Detected	1.469	0.012	GT	Sens
GT	Sense	Sense	0.268	Detected	-0.268	Detected	0.000	0.964	Detected	1.258	Detected	1.111	0.068	GT	Sens
GT	Sense	Sense	0.353	Detected	-0.353	Detected	0.000	1.031	Detected	1.214	Detected	1.123	0.091	GT	Sens

GT	Sense	Sense	1.227	Detected	-1.227	Detected	0.000	2.187	Detected	1.931	Detected	2.059	0.237	GT	Sens
GT	Sense	Sense	0.527	Detected	-0.527	Detected	0.000	0.890	Detected	1.162	Detected	1.026	0.200	GT	Sens
GT	Sense	Sense	-0.331	Detected	0.331	Detected	0.000	1.250	Detected	1.638	Detected	1.444	0.064	GT	Sens
GT	Sense	Sense	-0.245	Comprom	0.245	Comprom	0.000	2.214	Detected	4.765	Detected	3.489	0.115	GT	Sens
GT	Sense	Sense	0.317	Detected	-0.317	Detected	0.000	0.868	Detected	1.263	Detected	1.066	0.104	GT	Sens
GT	Sense	Sense	0.467	Detected	-0.467	Detected	0.000	1.375	Detected	1.079	Detected	1.227	0.129	GT	Sens
GT	Sense	Sense	0.188	Detected	-0.188	Detected	0.000	1.021	Detected	1.412	Detected	1.216	0.046	GT	Sens
GT	Sense	Sense	0.221	Detected	-0.221	Detected	0.000	0.976	Detected	1.429	Detected	1.202	0.063	GT	Sens
GT	Sense	Sense	0.794	Detected	-0.794	Detected	0.000	1.774	Detected	1.693	Detected	1.733	0.161	GT	Sens
GT	Sense	Sense	0.438	Detected	-0.438	Detected	0.000	1.232	Detected	1.233	Detected	1.233	0.106	GT	Sens
GT	Sense	Sense	0.745	Detected	-0.745	Detected	0.000	1.764	Detected	2.280	Detected	2.022	0.124	GT	Sens
GT	Sense	Sense	-0.190	Comprom	0.190	Comprom	0.000	0.983	Detected	1.453	Detected	1.218	0.056	GT	Sens
GT	Sense	Sense	0.994	Detected	-0.994	Comprom	0.000	1.061	Detected	0.986	Detected	1.024	0.411	GT	Sens
GT	Sense	Sense	0.413	Detected	-0.413	Detected	0.000	0.898	Detected	1.462	Detected	1.180	0.142	GT	Sens
GT	Sense	Sense	0.330	Detected	-0.330	Detected	0.000	0.945	Detected	1.087	Detected	1.016	0.095	GT	Sens
GT	Sense	Sense	0.716	Detected	-0.716	Comprom	0.000	1.017	Detected	1.040	Detected	1.029	0.287	GT	Sens
GT	Sense	Sense	0.288	Detected	-0.288	Detected	0.000	0.950	Detected	1.407	Detected	1.179	0.085	GT	Sens
GT	Sense	Sense	0.656	Detected	-0.656	Detected	0.000	1.592	Detected	1.735	Detected	1.663	0.128	GT	Sens
GT	Sense	Sense	0.511	Detected	-0.511	Detected	0.000	1.214	Detected	1.257	Detected	1.236	0.137	GT	Sens
GT	Sense	Sense	-0.077	Comprom	0.077	Comprom	0.000	1.562	Detected	1.240	Detected	1.401	0.016	GT	Sens
GT	Sense	Sense	0.401	Detected	-0.401	Detected	0.000	1.102	Detected	1.698	Detected	1.400	0.107	GT	Sens
GT	Sense	Sense	0.168	Detected	-0.168	Detected	0.000	0.922	Detected	1.081	Detected	1.001	0.033	GT	Sens
GT	Sense	Sense	1.100	Detected	-1.100	Comprom	0.000	1.288	Detected	1.341	Detected	1.315	0.355	GT	Sens
GT	Sense	Sense	0.128	Detected	-0.128	Detected	0.000	1.012	Detected	1.105	Detected	1.059	0.016	GT	Sens
GT	Sense	Sense	0.252	Detected	-0.252	Detected	0.000	0.979	Detected	1.122	Detected	1.051	0.057	GT	Sens
GT	Sense	Sense	1.422	Detected	-1.422	Detected	0.000	2.983	Detected	2.246	Detected	2.614	0.217	GT	Sens
GT	Sense	Sense	0.176	Detected	-0.176	Detected	0.000	1.489	Detected	1.826	Detected	1.657	0.021	GT	Sens
GT	Sense	Sense	-0.601	Comprom	0.601	Detected	0.000	1.405	Detected	2.345	Detected	1.875	0.133	GT	Sens
GT	Sense	Sense	0.861	Detected	-0.861	Detected	0.000	1.797	Detected	0.840	Detected	1.319	0.313	GT	Sens
GT	Sense	Sense	0.471	Detected	-0.471	Detected	0.000	1.351	Detected	2.116	Detected	1.734	0.104	GT	Sens
GT	Sense	Sense	0.637	Detected	-0.637	Detected	0.000	1.434	Detected	1.292	Detected	1.363	0.167	GT	Sens
GT	Sense	Sense	0.417	Detected	-0.417	Detected	0.000	0.808	Detected	1.248	Detected	1.028	0.161	GT	Sens
GT	Sense	Sense	-0.254	Detected	0.254	Detected	0.000	1.725	Detected	1.643	Detected	1.684	0.023	GT	Sens
GT	Sense	Sense	-0.206	Detected	0.206	Detected	0.000	1.443	Detected	1.923	Detected	1.683	0.034	GT	Sens
GT	Sense	Sense	0.774	Detected	-0.774	Detected	0.000	1.674	Detected	1.663	Detected	1.669	0.164	GT	Sens
GT	Sense	Sense	0.747	Detected	-0.747	Detected	0.000	1.289	Detected	0.809	Detected	1.049	0.313	GT	Sens
GT	Sense	Sense	-0.404	Comprom	0.404	Comprom	0.000	1.452	Detected	1.154	Detected	1.303	0.094	GT	Sens
GT	Sense	Sense	0.270	Detected	-0.270	Detected	0.000	0.920	Detected	1.421	Detected	1.171	0.086	GT	Sens
GT	Sense	Sense	0.367	Detected	-0.367	Detected	0.000	0.970	Detected	1.303	Detected	1.136	0.106	GT	Sens
GT	Sense	Sense	0.264	Detected	-0.264	Detected	0.000	0.987	Detected	1.556	Detected	1.271	0.082	GT	Sens
GT	Sense	Sense	0.111	Detected	-0.111	Detected	0.000	0.992	Detected	1.171	Detected	1.082	0.017	GT	Sens
GT	Sense	Sense	-1.048	Comprom	1.048	Detected	0.000	0.872	Detected	1.506	Detected	1.189	0.391	GT	Sens
GT	Sense	Sense	0.275	Detected	-0.275	Detected	0.000	0.888	Detected	1.181	Detected	1.035	0.080	GT	Sens
GT	Sense	Sense	0.462	Detected	-0.462	Detected	0.000	0.861	Detected	1.535	Detected	1.198	0.171	GT	Sens
GT	Sense	Sense	0.269	Detected	-0.269	Detected	0.000	0.950	Detected	1.553	Detected	1.252	0.090	GT	Sens
GT	Sense	Sense	1.419	Detected	-1.419	Comprom	0.000	2.598	Detected	2.789	Detected	2.694	0.199	GT	Sens
GT	Sense	Sense	0.034	Detected	-0.034	Detected	0.000	0.824	Detected	1.436	Detected	1.130	0.067	GT	Sens
GT	Sense	Sense	0.670	Detected	-0.670	Comprom	0.000	0.801	Detected	1.570	Detected	1.186	0.265	GT	Sens
GT	Sense	Sense	0.125	Detected	-0.125	Detected	0.000	0.958	Detected	1.271	Detected	1.115	0.031	GT	Sens
GT	Sense	Sense	0.232	Detected	-0.232	Detected	0.000	0.990	Detected	1.020	Detected	1.005	0.050	GT	Sens
GT	Sense	Sense	0.742	Detected	-0.742	Detected	0.000	0.834	Detected	1.558	Detected	1.196	0.285	GT	Sens
GT	Sense	Sense	-0.519	Comprom	0.519	Detected	0.000	1.109	Detected	1.302	Detected	1.205	0.150	GT	Sens
GT	Sense	Sense	0.639	Detected	-0.639	Detected	0.000	0.893	Detected	1.167	Detected	1.030	0.255	GT	Sens
GT	Sense	Sense	-0.834	Comprom	0.834	Comprom	0.000	1.129	Detected	1.024	Detected	1.077	0.327	GT	Sens
GT	Sense	Sense	-0.124	Detected	0.124	Detected	0.000	1.001	Detected	1.206	Detected	1.103	0.021	GT	Sens
GT	Sense	Sense	0.269	Detected	-0.269	Detected	0.000	1.012	Detected	1.042	Detected	1.027	0.062	GT	Sens
GT	Sense	Sense	-0.331	Comprom	0.331	Comprom	0.000	1.449	Detected	1.291	Detected	1.370	0.056	GT	Sens
GT	Sense	Sense	-0.244	Detected	0.244	Detected	0.000	1.240	Detected	0.920	Detected	1.080	0.066	GT	Sens
GT	Sense	Sense	0.669	Detected	-0.669	Detected	0.000	1.257	Detected	1.151	Detected	1.204	0.215	GT	Sens
GT	Sense	Sense	0.238	Comprom	-0.238	Comprom	0.000	1.414	Detected	1.619	Detected	1.516	0.028	GT	Sens
GT	Sense	Sense	0.491	Detected	-0.491	Detected	0.000	0.846	Detected	1.172	Detected	1.009	0.190	GT	Sens
GT	Sense	Sense	1.998	Detected	-1.998	Detected	0.000	2.764	Detected	2.418	Detected	2.591	0.326	GT	Sens
GT	Sense	Sense	0.111	Detected	-0.111	Detected	0.000	0.981	Detected	1.615	Detected	1.298	0.061	GT	Sens
GT	Sense	Sense	0.389	Comprom	-0.389	Comprom	0.000	1.201	Detected	1.048	Detected	1.124	0.105	GT	Sens
GT	Sense	Sense	-0.071	Detected	0.071	Detected	0.000	1.536	Detected	1.456	Detected	1.496	0.003	GT	Sens
GT	Sense	Sense	0.081	Detected	-0.081	Detected	0.000	1.186	Detected	1.599	Detected	1.393	0.024	GT	Sens
GT	Sense	Sense	0.355	Detected	-0.355	Detected	0.000	1.237	Detected	1.464	Detected	1.351	0.068	GT	Sens
GT	Sense	Sense	0.814	Detected	-0.814	Detected	0.000	1.519	Detected	1.784	Detected	1.652	0.183	GT	Sens
GT	Sense	Sense	1.478	Detected	-1.478	Detected	0.000	1.929	Detected	2.016	Detected	1.973	0.314	GT	Sens
GT	Sense	Sense	0.147	Comprom	-0.147	Comprom	0.000	1.322	Detected	1.158	Detected	1.240	0.018	GT	Sens
GT	Sense	Sense	0.824	Detected	-0.824	Detected	0.000	1.312	Detected	1.350	Detected	1.331	0.248	GT	Sens
GT	Sense	Sense	0.922	Detected	-0.922	Comprom	0.000	1.990	Detected	2.041	Detected	2.016	0.160	GT	Sens
GT	Sense	Sense	0.542	Detected	-0.542	Detected	0.000	1.379	Detected	1.594	Detected	1.486	0.115	GT	Sens
GT	Sense	Sense	0.766	Detected	-0.766	Comprom	0.000	1.485	Detected	1.320	Detected	1.403	0.210	GT	Sens
GT	Sense	Sense	0.591	Detected	-0.591	Detected	0.000	1.008	Detected	1.050	Detected	1.029	0.224	GT	Sens
GT	Sense	Sense	-0.301	Detected	0.301	Detected	0.000	0.814	Detected	1.372	Detected	1.093	0.117	GT	Sens
GT	Sense	Sense	0.494	Detected	-0.494	Detected	0.000	1.155	Detected	1.807	Detected	1.481	0.129	GT	Sens
GT	Sense	Sense	0.221	Detected	-0.221	Detected	0.000	1.057	Detected	0.946	Detected	1.002	0.048	GT	Sens
GT	Sense	Sense	0.507	Detected	-0.507	Detected	0.000	1.074	Detected	0.965	Detected	1.020	0.183	GT	Sens
GT	Sense	Sense	0.279	Detected	-0.279	Detected	0.000	1.394	Detected	1.453	Detected	1.424	0.037	GT	Sens
GT	Sense	Sense	0.506	Detected	-0.506	Detected	0.000	1.413	Detected	1.596	Detected	1.505	0.100	GT	Sens
GT	Sense	Sense	0.258	Detected	-0.258	Detected	0.000	0.975	Detected	1.238	Detected	1.106	0.062	GT	Sens
GT	Sense	Sense	0.320	Detected	-0.320	Detected	0.000	0.906	Detected	1.199	Detected	1.053	0.096	GT	Sens
GT	Sense	Sense	-0.008	Detected	0.008	Detected	0.000	1.347	Detected	1.113	Detected	1.230	0.009	GT	Sens
GT	Sense	Sense	0.520	Detected	-0.520	Detected	0.000	1.092	Detected	1.011	Detected	1.052	0.182	GT	Sens
GT	Sense	Sense	0.525	Detected	-0.525	Detected	0.000	0.876	Detected	1.982	Detected	1.429	0.202	GT	Sens
GT	Sense	Sense	0.530	Detected	-0.530	Detected	0.000	1.293	Detected	1.531	Detected	1.412	0.122	GT	Sens
GT	Sense	Sense	0.532	Detected	-0.532	Detected	0.000	1.925	Detected	1.366	Detected	1.645	0.111	GT	Sens
GT	Sense	Sense	0.530	Detected	-0.530	Detected	0.000	1.148	Detected	1.108	Detected	1.128	0.167	GT	Sens
GT	Sense	Sense	0.309	Detected	-0.309	Detected	0.000	1.142	Detected	1.052	Detected	1.097	0.072	GT	Sens
GT	Sense	Sense	0.505	Detected	-0.505	Detected	0.000	1.217	Detected	1.747	Detected	1.482	0.122	GT	Sens
GT	Sense	Sense	-0.226	Detected	0.226	Detected	0.000	0.847	Detected	1.401	Detected	1.124	0.088	GT	Sens
GT	Sense	Sense	0.504	Detected	-0.504	Detected	0.000	1.439	Detected	1.033	Detected	1.236	0.151	GT	Sens
GT	Sense	Sense	0.488	Detected	-0.488	Detected	0.000	1.125	Detected	1.223	Detected	1.174	0.139	GT	Sens
GT	Sense	Sense	0.128	Detected	-0.128	Detected	0.000	1.097	Detected	1.945	Detected	1.521	0.075	GT	Sens
GT	Sense	Sense	-0.230	Comprom	0.230	Detected	0.000	1.404	Detected	0.834	Detected	1.119	0.093	GT	Sens
GT	Sense	Sense	0.757	Detected	-0.757	Detected	0.000	1.137	Detected	1.299	Detected	1.218	0.251	GT	Sens
GT	Sense	Sense	-0.029	Detected	0.029	Detected	0.000	2.697	Detected	2.997	Detected	2.847	0.003	GT	Sens
GT	Sense	Sense	0.405	Detected	-0.405	Detected	0.000	0.948	Detected	1.109	Detected	1.028	0.130	GT	Sens
GT	Sense	Sense	0.585	Detected	-0.585	Detected	0.000	0.901	Detected	1.276	Detected	1.089	0.218	GT	Sens
GT	Sense	Sense	0.275	Detected	-0.275	Detected	0.000	0.857	Detected	1.359	Detected	1.108	0.097	GT	Sens
GT	Sense	Sense	-0.063	Comprom	0.063	Comprom	0.000	1.799	Detected	1.316	Detected	1.557	0.025	GT	Sens
GT	Sense	Sense	0.618	Detected	-0.618	Detected	0.000	1.038	Detected	1.535	Detected	1.286	0.193	GT	Sens
GT	Sense	Sense	-0.022	Detected	0.022	Detected	0.000	1.242	Detected	1.528	Detected	1.385	0.011	GT	Sens
GT	Sense	Sense	0.642	Detected	-0.642	Comprom	0.000	1.432	Detected	1.904	Detected	1.668	0.135	GT	Sens
GT	Sense	Sense	0.415	Detected	-0.415	Comprom	0.000	1.647	Detected	1.693	Detected	1.670	0.057	GT	Sens
GT	Sense	Sense	0.380	Detected	-0.380	Detected	0.000	1.351	Detected	2.088	Detected	1.719	0.083	GT	Sens
GT	Sense	Sense	0.131	Detected	-0.131	Detected	0.000	1.500	Detected	1.175	Detected	1.337	0.024	GT	Sens
GT	Sense	Sense	-0.165	Detected	0.165	Detected	0.000	0.884	Detected	1.240	Detected	1.062	0.048	GT	Sens
GT	Sense	Sense	0.765	Detected	-0.765	Detected	0.000	1.042	Detected	1.540	Detected	1.291	0.250	GT	Sens
GT	Sense	Sense	0.223	Detected	-0.223	Detected	0.000	1.028	Detected	1.162	Detected	1.095	0.043	GT	Sens
GT	Sense	Sense	0.215	Detected	-0.215	Detected	0.000	1.287	Detected	1.064	Detected	1.176	0.040	GT	Sens
GT	Sense	Sense	-0.435	Comprom	0.435	Comprom	0.000	4.882	Detected	4.706	Detected	4.794	0.008	GT	Sens
GT	Sense	Sense	0.297	Detected	-0.297	Detected	0.000	1.208	Detected	0.993	Detected	1.100	0.073	GT	Sens
GT	Sense	Sense	0.338	Detected	-0.338	Comprom	0.000	1.083	Detected	1.215	Detected	1.149	0.079	GT	Sens
GT	Sense	Sense	0.680	Detected	-0.680	Detected	0.000	1.147	Detected	1.017	Detected	1.082	0.254	GT	Sens
GT	Sense	Sense	1.170	Detected	-1.170	Detected	0.000	1.190	Detected	2.390	Detected	1.790	0.307	GT	Sens
GT	Sense	Sense	0.968	Detected	-0.968	Detected	0.000	1.031	Detected	2.202	Detected	1.616	0.289	GT	Sens
GT	Sense	Sense	1.116	Detected	-1.116	Detected	0.000	1.945	Detected	1.568	Detected	1.757	0.261	GT	Sens
GT	Sense	Sense	0.567	Detected	-0.567	Detected	0.000	0.870	Detected	1.739	Detected	1.305	0.209	GT	Sens
GT	Sense	Sense	-0.142	Detected	0.142	Detected	0.000	1.241	Detected	1.490	Detected	1.366	0.019	GT	Sens
GT	Sense	Sense	-1.121	Comprom	1.121	Detected	0.000	1.140	Detected	1.412	Detected	1.276	0.376	GT	Sens
GT	Sense	Sense	1.107	Detected	-1.107	Comprom	0.000	1.086	Detected	1.499	Detected	1.292	0.370	GT	Sens
GT	Sense	Sense	0.425	Detected	-0.425	Comprom	0.000	1.732	Detected	3.061	Detected	2.397	0.093	GT	Sens
GT	Sense	Sense	0.349	Detected	-0.349	Detected	0.000	1.052	Detected	1.111	Detected	1.082	0.091	GT	Sens
GT	Sense	Sense	0.839	Detected	-0.839	Detected	0.000	1.859	Detected	1.891	Detected	1.875	0.155	GT	Sens
GT	Sense	Sense	0.524	Comprom	-0.524	Comprom	0.000	2.379	Detected	3.777	Detected	3.078	0.072	GT	Sens
GT	Sense	Sense	1.031	Detected	-1.031	Detected	0.000	1.104	Detected	1.236	Detected	1.170	0.375	GT	Sens
GT	Sense	Sense	1.014	Detected	-1.014	Detected	0.000	1.058	Detected	2.745	Detected	1.902	0.286	GT	Sens
GT	Sense	Sense	0.541	Detected	-0.541	Detected	0.000	1.541	Detected	1.356	Detected	1.449	0.119	GT	Sens
GT	Sense	Sense	0.896	Detected	-0.896	Detected	0.000	1.283	Detected	2.128	Detected	1.705	0.227	GT	Sens
GT	Sense	Sense	0.533	Detected	-0.533	Detected	0.000	0.901	Detected	1.244	Detected	1.073	0.195	GT	Sens
GT	Sense	Sense	0.255	Detected	-0.255	Detected	0.000	1.279	Detected	1.060	Detected	1.169	0.052	GT	Sens
GT	Sense	Sense	0.360	Detected	-0.360	Detected	0.000	1.393	Detected	1.111	Detected	1.252	0.083	GT	Sens
GT	Sense	Sense	-0.337	Comprom	0.337	Comprom	0.000	1.020	Detected	1.198	Detected	1.109	0.086	GT	Sens
GT	Sense	Sense	1.124	Detected	-1.124	Detected	0.000	1.148	Detected	1.103	Detected	1.125	0.422	GT	Sens
GT	Sense	Sense	0.620	Detected	-0.620	Detected	0.000	0.998	Detected	1.298	Detected	1.148	0.214	GT	Sens
GT	Sense	Sense	0.591	Detected	-0.591	Detected	0.000	1.491	Detected	1.461	Detected	1.476	0.130	GT	Sens
GT	Sense	Sense	0.102	Detected	-0.102	Detected	0.000	1.041	Detected	1.451	Detected	1.246	0.032	GT	Sens
GT	Sense	Sense	1.205	Detected	-1.205	Comprom	0.000	1.346	Detected	0.893	Detected	1.119	0.458	GT	Sens
GT	Sense	Sense	0.175	Detected	-0.175	Detected	0.000	1.237	Detected	2.157	Detected	1.697	0.075	GT	Sens
GT	Sense	Sense	0.611	Detected	-0.611	Detected	0.000	1.065	Detected	2.319	Detected	1.692	0.193	GT	Sens
GT	Sense	Sense	0.659	Detected	-0.659	Detected	0.000	1.319	Detected	1.601	Detected	1.460	0.163	GT	Sens
GT	Sense	Sense	0.430	Detected	-0.430	Detected	0.000	0.990	Detected	1.782	Detected	1.386	0.141	GT	Sens
GT	Sense	Sense	1.015	Detected	-1.015	Detected	0.000	0.959	Detected	1.886	Detected	1.423	0.330	GT	Sens
GT	Sense	Sense	1.052	Detected	-1.052	Detected	0.000	1.272	Detected	1.952	Detected	1.612	0.282	GT	Sens
GT	Sense	Sense	0.637	Detected	-0.637	Detected	0.000	0.894	Detected	1.529	Detected	1.212	0.231	GT	Sens
GT	Sense	Sense	0.495	Detected	-0.495	Detected	0.000	0.885	Detected	1.676	Detected	1.281	0.181	GT	Sens
GT	Sense	Sense	0.084	Detected	-0.084	Detected	0.000	1.008	Detected	1.088	Detected	1.048	0.008	GT	Sens
GT	Sense	Sense	-0.164	Detected	0.164	Detected	0.000	0.802	Detected	1.513	Detected	1.157	0.098	GT	Sens
GT	Sense	Sense	1.353	Detected	-1.353	Detected	0.000	0.986	Detected	2.674	Detected	1.830	0.370	GT	Sens
GT	Sense	Sense	0.108	Detected	-0.108	Detected	0.000	1.320	Detected	0.984	Detected	1.152	0.029	GT	Sens
GT	Sense	Sense	0.282	Detected	-0.282	Detected	0.000	0.851	Detected	1.372	Detected	1.112	0.102	GT	Sens
GT	Sense	Sense	-0.623	Comprom	0.623	Detected	0.000	0.869	Detected	1.311	Detected	1.090	0.241	GT	Sens
GT	Sense	Sense	0.314	Detected	-0.314	Detected	0.000	0.877	Detected	1.141	Detected	1.009	0.097	GT	Sens
GT	Sense	Sense	-0.734	Comprom	0.734	Detected	0.000	1.343	Detected	1.493	Detected	1.418	0.194	GT	Sens
GT	Sense	Sense	0.387	Detected	-0.387	Detected	0.000	1.228	Detected	0.872	Detected	1.050	0.133	GT	Sens
GT	Sense	Sense	0.168	Detected	-0.168	Detected	0.000	1.232	Detected	0.957	Detected	1.095	0.037	GT	Sens
GT	Sense	Sense	0.267	Detected	-0.267	Detected	0.000	1.044	Detected	1.471	Detected	1.258	0.067	GT	Sens
GT	Sense	Sense	0.574	Detected	-0.574	Detected	0.000	1.204	Detected	1.634	Detected	1.419	0.147	GT	Sens
GT	Sense	Sense	0.258	Detected	-0.258	Detected	0.000	2.416	Detected	1.105	Detected	1.761	0.130	GT	Sens
GT	Sense	Sense	0.422	Comprom	-0.422	Comprom	0.000	1.139	Detected	2.091	Detected	1.615	0.126	GT	Sens
GT	Sense	Sense	-0.410	Detected	0.410	Detected	0.000	1.962	Detected	2.946	Detected	2.454	0.062	GT	Sens
GT	Sense	Sense	1.355	Detected	-1.355	Comprom	0.000	1.084	Detected	1.211	Detected	1.148	0.486	GT	Sens
GT	Sense	Sense	0.465	Detected	-0.465	Detected	0.000	1.441	Detected	1.590	Detected	1.515	0.085	GT	Sens
GT	Sense	Sense	1.097	Detected	-1.097	Comprom	0.000	1.060	Detected	1.550	Detected	1.305	0.366	GT	Sens
GT	Sense	Sense	0.098	Detected	-0.098	Detected	0.000	1.178	Detected	1.215	Detected	1.196	0.007	GT	Sens
GT	Sense	Sense	1.466	Detected	-1.466	Comprom	0.000	1.851	Detected	3.423	Detected	2.637	0.254	GT	Sens
GT	Sense	Sense	0.795	Detected	-0.795	Comprom	0.000	1.377	Detected	0.945	Detected	1.161	0.294	GT	Sens
GT	Sense	Sense	1.649	Detected	-1.649	Comprom	0.000	0.930	Detected	1.139	Detected	1.035	0.595	GT	Sens
GT	Sense	Sense	0.548	Detected	-0.548	Detected	0.000	1.776	Detected	1.712	Detected	1.744	0.086	GT	Sens
GT	Sense	Sense	1.930	Detected	-1.930	Detected	0.000	3.068	Detected	2.614	Detected	2.841	0.281	GT	Sens
GT	Sense	Sense	-0.130	Detected	0.130	Detected	0.000	0.937	Detected	1.637	Detected	1.287	0.075	GT	Sens
GT	Sense	Sense	0.475	Detected	-0.475	Detected	0.000	0.807	Detected	1.198	Detected	1.003	0.190	GT	Sens
GT	Sense	Sense	0.473	Detected	-0.473	Detected	0.000	1.261	Detected	1.856	Detected	1.559	0.108	GT	Sens
GT	Sense	Sense	0.855	Detected	-0.855	Comprom	0.000	1.129	Detected	1.241	Detected	1.185	0.301	GT	Sens
GT	Sense	Sense	1.182	Detected	-1.182	Comprom	0.000	0.906	Detected	1.250	Detected	1.078	0.462	GT	Sens
GT	Sense	Sense	0.561	Detected	-0.561	Detected	0.000	1.162	Detected	1.705	Detected	1.434	0.148	GT	Sens
GT	Sense	Sense	1.552	Detected	-1.552	Comprom	0.000	2.384	Detected	2.238	Detected	2.311	0.275	GT	Sens
GT	Sense	Sense	-0.411	Comprom	0.411	Comprom	0.000	1.085	Detected	0.971	Detected	1.028	0.132	GT	Sens
GT	Sense	Sense	0.265	Detected	-0.265	Detected	0.000	1.591	Detected	1.322	Detected	1.457	0.039	GT	Sens
GT	Sense	Sense	0.049	Comprom	-0.049	Comprom	0.000	1.697	Detected	2.874	Detected	2.286	0.061	GT	Sens
GT	Sense	Sense	0.266	Detected	-0.266	Detected	0.000	1.191	Detected	1.148	Detected	1.170	0.048	GT	Sens
GT	Sense	Sense	1.112	Detected	-1.112	Detected	0.000	0.807	Detected	1.825	Detected	1.316	0.394	GT	Sens
GT	Sense	Sense	0.264	Detected	-0.264	Detected	0.000	1.101	Detected	0.962	Detected	1.032	0.064	GT	Sens
GT	Sense	Sense	-0.187	Comprom	0.187	Comprom	0.000	2.409	Detected	3.657	Detected	3.033	0.043	GT	Sens
GT	Sense	Sense	0.667	Detected	-0.667	Detected	0.000	0.898	Detected	1.135	Detected	1.016	0.272	GT	Sens
GT	Sense	Sense	0.406	Detected	-0.406	Detected	0.000	1.030	Detected	1.355	Detected	1.193	0.112	GT	Sens
GT	Sense	Sense	0.248	Detected	-0.248	Detected	0.000	1.008	Detected	1.563	Detected	1.286	0.075	GT	Sens
GT	Sense	Sense	0.345	Detected	-0.345	Detected	0.000	1.762	Detected	1.810	Detected	1.786	0.036	GT	Sens
GT	Sense	Sense	0.678	Detected	-0.678	Detected	0.000	1.218	Detected	1.054	Detected	1.136	0.238	GT	Sens
GT	Sense	Sense	0.222	Detected	-0.222	Detected	0.000	1.112	Detected	1.377	Detected	1.245	0.041	GT	Sens
GT	Sense	Sense	0.656	Detected	-0.656	Detected	0.000	0.827	Detected	1.195	Detected	1.011	0.276	GT	Sens
GT	Sense	Sense	1.434	Detected	-1.434	Comprom	0.000	2.579	Detected	1.009	Detected	1.794	0.387	GT	Sens
GT	Sense	Sense	0.833	Detected	-0.833	Detected	0.000	1.727	Detected	1.530	Detected	1.629	0.192	GT	Sens
GT	Sense	Sense	1.105	Detected	-1.105	Comprom	0.000	1.984	Detected	1.299	Detected	1.641	0.292	GT	Sens
GT	Sense	Sense	-1.274	Comprom	1.274	Detected	0.000	1.202	Detected	0.914	Detected	1.058	0.496	GT	Sens
GT	Sense	Sense	0.817	Detected	-0.817	Comprom	0.000	1.091	Detected	1.632	Detected	1.361	0.254	GT	Sens
GT	Sense	Sense	0.013	Detected	-0.013	Detected	0.000	1.012	Detected	1.057	Detected	1.034	0.001	GT	Sens
GT	Sense	Sense	-0.016	Detected	0.016	Detected	0.000	1.296	Detected	1.220	Detected	1.258	0.001	GT	Sens
GT	Sense	Sense	-0.778	Comprom	0.778	Comprom	0.000	1.483	Detected	1.334	Detected	1.408	0.213	GT	Sens
GT	Sense	Sense	1.065	Detected	-1.065	Detected	0.000	2.081	Detected	1.827	Detected	1.954	0.210	GT	Sens
GT	Sense	Sense	0.006	Detected	-0.006	Detected	0.000	0.940	Detected	1.131	Detected	1.036	0.008	GT	Sens
GT	Sense	Sense	0.385	Detected	-0.385	Detected	0.000	0.962	Detected	1.391	Detected	1.177	0.116	GT	Sens
GT	Sense	Sense	0.365	Detected	-0.365	Detected	0.000	0.848	Detected	1.318	Detected	1.083	0.130	GT	Sens
GT	Sense	Sense	0.703	Detected	-0.703	Detected	0.000	0.976	Detected	1.065	Detected	1.020	0.284	GT	Sens
GT	Sense	Sense	0.333	Detected	-0.333	Detected	0.000	1.664	Detected	1.560	Detected	1.612	0.041	GT	Sens
GT	Sense	Sense	1.605	Detected	-1.605	Comprom	0.000	1.896	Detected	1.575	Detected	1.736	0.394	GT	Sens
GT	Sense	Sense	0.350	Detected	-0.350	Detected	0.000	0.891	Detected	1.275	Detected	1.083	0.113	GT	Sens
GT	Sense	Sense	-0.011	Detected	0.011	Detected	0.000	1.189	Detected	1.764	Detected	1.476	0.036	GT	Sens
GT	Sense	Sense	0.386	Detected	-0.386	Detected	0.000	0.876	Detected	1.178	Detected	1.027	0.131	GT	Sens
GT	Sense	Sense	0.492	Detected	-0.492	Comprom	0.000	2.193	Detected	2.171	Detected	2.182	0.047	GT	Sens
GT	Sense	Sense	0.654	Detected	-0.654	Detected	0.000	0.952	Detected	1.800	Detected	1.376	0.219	GT	Sens
GT	Sense	Sense	0.252	Detected	-0.252	Detected	0.000	1.233	Detected	1.133	Detected	1.183	0.044	GT	Sens
GT	Sense	Sense	0.298	Detected	-0.298	Detected	0.000	1.191	Detected	0.915	Detected	1.053	0.085	GT	Sens
GT	Sense	Sense	0.131	Detected	-0.131	Detected	0.000	1.867	Detected	2.827	Detected	2.347	0.042	GT	Sens
GT	Sense	Sense	0.576	Detected	-0.576	Detected	0.000	0.836	Detected	1.577	Detected	1.206	0.220	GT	Sens
GT	Sense	Sense	0.537	Detected	-0.537	Comprom	0.000	1.463	Detected	1.267	Detected	1.365	0.130	GT	Sens
GT	Sense	Sense	1.457	Detected	-1.457	Detected	0.000	1.330	Detected	1.425	Detected	1.377	0.444	GT	Sens
GT	Sense	Sense	0.211	Detected	-0.211	Detected	0.000	0.940	Detected	1.445	Detected	1.192	0.068	GT	Sens
GT	Sense	Sense	0.138	Detected	-0.138	Detected	0.000	1.488	Detected	0.802	Detected	1.145	0.090	GT	Sens
GT	Sense	Sense	0.344	Detected	-0.344	Detected	0.000	1.832	Detected	2.027	Detected	1.929	0.033	GT	Sens
GT	Sense	Sense	0.389	Detected	-0.389	Detected	0.000	1.672	Detected	1.295	Detected	1.483	0.075	GT	Sens
GT	Sense	Sense	1.738	Detected	-1.738	Comprom	0.000	3.130	Detected	2.442	Detected	2.786	0.256	GT	Sens
GT	Sense	Sense	0.404	Detected	-0.404	Detected	0.000	1.101	Detected	1.481	Detected	1.291	0.102	GT	Sens
GT	Sense	Sense	-0.156	Detected	0.156	Detected	0.000	1.805	Detected	2.151	Detected	1.978	0.014	GT	Sens
GT	Sense	Sense	-0.037	Detected	0.037	Detected	0.000	2.249	Detected	2.236	Detected	2.242	0.000	GT	Sens
GT	Sense	Sense	0.658	Detected	-0.658	Detected	0.000	1.853	Detected	2.202	Detected	2.028	0.097	GT	Sens
GT	Sense	Sense	0.265	Detected	-0.265	Detected	0.000	1.319	Detected	0.970	Detected	1.144	0.069	GT	Sens
GT	Sense	Sense	1.269	Detected	-1.269	Detected	0.000	3.122	Detected	1.853	Detected	2.488	0.222	GT	Sens
GT	Sense	Sense	0.603	Detected	-0.603	Detected	0.000	1.015	Detected	1.959	Detected	1.487	0.192	GT	Sens
GT	Sense	Sense	0.246	Comprom	-0.246	Comprom	0.000	2.423	Detected	2.751	Detected	2.587	0.013	GT	Sens
GT	Sense	Sense	-0.246	Detected	0.246	Detected	0.000	0.830	Detected	1.289	Detected	1.059	0.088	GT	Sens
GT	Sense	Sense	0.221	Detected	-0.221	Detected	0.000	1.021	Detected	1.173	Detected	1.097	0.043	GT	Sens
GT	Sense	Sense	0.598	Detected	-0.598	Detected	0.000	1.421	Detected	1.180	Detected	1.300	0.166	GT	Sens
GT	Sense	Sense	1.348	Detected	-1.348	Detected	0.000	2.317	Detected	1.891	Detected	2.104	0.263	GT	Sens
GT	Sense	Sense	-1.111	Comprom	1.111	Detected	0.000	1.199	Detected	1.556	Detected	1.377	0.345	GT	Sens
GT	Sense	Sense	0.738	Detected	-0.738	Detected	0.000	0.912	Detected	1.495	Detected	1.203	0.269	GT	Sens
GT	Sense	Sense	0.433	Detected	-0.433	Detected	0.000	1.181	Detected	1.369	Detected	1.275	0.102	GT	Sens
GT	Sense	Sense	0.451	Comprom	-0.451	Comprom	0.000	2.231	Detected	1.007	Detected	1.619	0.167	GT	Sens
GT	Sense	Sense	0.390	Detected	-0.390	Detected	0.000	1.488	Detected	1.241	Detected	1.364	0.079	GT	Sens
GT	Sense	Sense	0.744	Detected	-0.744	Detected	0.000	1.218	Detected	1.685	Detected	1.451	0.204	GT	Sens
GT	Sense	Sense	0.224	Comprom	-0.224	Comprom	0.000	1.350	Detected	1.135	Detected	1.243	0.038	GT	Sens
GT	Sense	Sense	0.557	Detected	-0.557	Detected	0.000	1.000	Detected	1.372	Detected	1.186	0.181	GT	Sens

GT	Sense	Sense	0.851	Detected	-0.851	Detected	0.000	1.084	Detected	1.411	Detected	1.247	0.287	GT	Sens	contig301	contig301	Dihydrofl
GT	Sense	Sense	1.589	Detected	-1.589	Detected	0.000	1.053	Detected	2.820	Detected	1.937	0.398	GT	Sens	contig302	contig302	U-box do
GT	Sense	Sense	0.426	Comprom	-0.426	Comprom	0.000	1.243	Detected	1.101	Detected	1.172	0.113	GT	Sens	contig302	contig302	Condens
GT	Sense	Sense	0.973	Detected	-0.973	Detected	0.000	1.547	Detected	3.210	Detected	2.378	0.204	GT	Sens	contig302	contig302	Peroxida
GT	Sense	Sense	0.897	Detected	-0.897	Detected	0.000	1.294	Detected	2.005	Detected	1.649	0.230	GT	Sens	contig303	contig303	Ring H2 fi
GT	Sense	Sense	-0.374	Comprom	0.374	Comprom	0.000	1.788	Detected	1.644	Detected	1.716	0.046	GT	Sens	contig303	contig303	Short-cha
GT	Sense	Sense	0.190	Detected	-0.190	Detected	0.000	1.580	Detected	1.473	Detected	1.527	0.016	GT	Sens	contig303	contig303	Unknown
GT	Sense	Sense	0.068	Detected	-0.068	Detected	0.000	0.982	Detected	1.250	Detected	1.116	0.018	GT	Sens	contig304	contig304	Chloropla
GT	Sense	Sense	0.964	Detected	-0.964	Detected	0.000	0.838	Detected	2.382	Detected	1.610	0.322	GT	Sens	contig304	contig304	WRKY-lik
GT	Sense	Sense	-0.036	Detected	0.036	Detected	0.000	2.000	Detected	2.330	Detected	2.165	0.006	GT	Sens	contig305	contig305	At5g2428
GT	Sense	Sense	0.404	Comprom	-0.404	Comprom	0.000	2.678	Detected	2.225	Detected	2.451	0.034	GT	Sens	contig305	contig305	Ethylene-
GT	Sense	Sense	-0.742	Comprom	0.742	Detected	0.000	1.440	Detected	0.956	Detected	1.198	0.264	GT	Sens	contig305	contig305	Phospho
GT	Sense	Sense	0.414	Detected	-0.414	Detected	0.000	1.179	Detected	1.169	Detected	1.174	0.105	GT	Sens	contig306	contig306	Calmodul
GT	Sense	Sense	0.203	Detected	-0.203	Detected	0.000	1.028	Detected	0.993	Detected	1.011	0.038	GT	Sens	contig306	contig306	Leucine-r
GT	Sense	Sense	-0.533	Comprom	0.533	Detected	0.000	1.440	Detected	1.775	Detected	1.608	0.103	GT	Sens	contig307	contig307	Nucleopo
GT	Sense	Sense	0.312	Detected	-0.312	Detected	0.000	1.051	Detected	1.452	Detected	1.252	0.078	GT	Sens	contig307	contig307	Protein re
GT	Sense	Sense	0.412	Detected	-0.412	Detected	0.000	2.539	Detected	2.858	Detected	2.698	0.026	GT	Sens	contig309	contig309	Unknown
GT	Sense	Sense	0.496	Detected	-0.496	Detected	0.000	0.852	Detected	1.527	Detected	1.190	0.186	GT	Sens	contig309	contig309	Receptor
GT	Sense	Sense	0.190	Detected	-0.190	Detected	0.000	3.126	Detected	3.513	Detected	3.320	0.007	GT	Sens	contig309	contig309	Pentatric
GT	Sense	Sense	0.944	Detected	-0.944	Comprom	0.000	1.210	Detected	2.266	Detected	1.738	0.249	GT	Sens	contig310	contig310	Os01g06
GT	Sense	Sense	0.481	Detected	-0.481	Detected	0.000	0.819	Detected	1.258	Detected	1.038	0.189	GT	Sens	contig310	contig310	DsRNA-bi
GT	Sense	Sense	-0.060	Detected	0.060	Detected	0.000	1.631	Detected	1.889	Detected	1.760	0.007	GT	Sens	contig310	contig310	NBS-LRR
GT	Sense	Sense	0.483	Detected	-0.483	Detected	0.000	1.225	Detected	1.286	Detected	1.255	0.122	GT	Sens	contig311	contig311	Caffeoyl-
GT	Sense	Sense	0.128	Detected	-0.128	Detected	0.000	1.009	Detected	1.015	Detected	1.012	0.016	GT	Sens	contig311	contig311	Glucose-
GT	Sense	Sense	0.315	Comprom	-0.315	Comprom	0.000	1.223	Detected	1.948	Detected	1.586	0.081	GT	Sens	contig311	contig311	Protein-t
GT	Sense	Sense	1.788	Detected	-1.788	Comprom	0.000	2.307	Detected	2.007	Detected	2.157	0.352	GT	Sens	contig312	contig312	Laccase-
GT	Sense	Sense	0.403	Detected	-0.403	Detected	0.000	1.070	Detected	1.112	Detected	1.091	0.114	GT	Sens	contig312	contig312	Spermidi
GT	Sense	Sense	0.498	Detected	-0.498	Detected	0.000	0.868	Detected	1.498	Detected	1.183	0.182	GT	Sens	contig312	contig312	N utilizati
GT	Sense	Sense	0.014	Comprom	-0.014	Comprom	0.000	1.958	Detected	2.277	Detected	2.118	0.006	GT	Sens	contig314	contig314	Unannota
GT	Sense	Sense	0.398	Comprom	-0.398	Comprom	0.000	0.970	Detected	1.175	Detected	1.073	0.121	GT	Sens	contig314	contig314	Unannota
GT	Sense	Sense	1.139	Detected	-1.139	Detected	0.000	1.532	Detected	1.959	Detected	1.745	0.271	GT	Sens	contig316	contig316	Unannota
GT	Sense	Sense	1.263	Detected	-1.263	Detected	0.000	1.499	Detected	2.021	Detected	1.760	0.306	GT	Sens	contig316	contig316	Unannota
GT	Sense	Sense	1.137	Detected	-1.137	Comprom	0.000	2.885	Detected	1.132	Detected	2.009	0.297	GT	Sens	contig316	contig316	Unannota
GT	Sense	Sense	0.078	Detected	-0.078	Detected	0.000	1.110	Detected	1.143	Detected	1.127	0.005	GT	Sens	contig317	contig317	Unannota
GT	Sense	Sense	0.541	Comprom	-0.541	Comprom	0.000	1.250	Detected	1.319	Detected	1.284	0.141	GT	Sens	contig317	contig317	Unannota
GT	Sense	Sense	0.209	Detected	-0.209	Detected	0.000	1.383	Detected	0.974	Detected	1.179	0.056	GT	Sens	contig317	contig317	DUF599 f
GT	Sense	Sense	0.582	Detected	-0.582	Comprom	0.000	1.131	Detected	1.361	Detected	1.246	0.170	GT	Sens	contig318	contig318	Unannota
GT	Sense	Sense	0.466	Comprom	-0.466	Comprom	0.000	1.189	Detected	1.922	Detected	1.555	0.120	GT	Sens	contig318	contig318	Unannota
GT	Sense	Sense	-0.743	Comprom	0.743	Comprom	0.000	1.311	Detected	2.458	Detected	1.885	0.182	GT	Sens	contig318	contig318	Unannota
GT	Sense	Sense	0.113	Comprom	-0.113	Comprom	0.000	0.963	Detected	1.112	Detected	1.038	0.017	GT	Sens	contig318	contig318	Unannota
GT	Sense	Sense	0.375	Detected	-0.375	Detected	0.000	1.708	Detected	1.803	Detected	1.756	0.043	GT	Sens	contig319	contig319	Calcium-r
GT	Sense	Sense	-0.384	Detected	0.384	Detected	0.000	1.452	Detected	1.083	Detected	1.267	0.097	GT	Sens	contig319	contig319	Unannota
GT	Sense	Sense	0.582	Detected	-0.582	Comprom	0.000	0.870	Detected	1.528	Detected	1.199	0.215	GT	Sens	contig320	contig320	Glucose/s
GT	Sense	Sense	0.310	Detected	-0.310	Detected	0.000	1.105	Detected	2.737	Detected	1.921	0.159	GT	Sens	contig320	contig320	Glyoxal o
GT	Sense	Sense	0.986	Detected	-0.986	Comprom	0.000	1.157	Detected	1.330	Detected	1.244	0.336	GT	Sens	contig320	contig320	Unannota
GT	Sense	Sense	0.194	Detected	-0.194	Detected	0.000	0.979	Detected	1.315	Detected	1.147	0.047	GT	Sens	contig320	contig320	Unannota
GT	Sense	Sense	0.345	Detected	-0.345	Detected	0.000	0.949	Detected	1.323	Detected	1.136	0.102	GT	Sens	contig320	contig320	Unannota
GT	Sense	Sense	0.171	Detected	-0.171	Detected	0.000	1.316	Detected	1.176	Detected	1.246	0.021	GT	Sens	contig320	contig320	Unannota
GT	Sense	Sense	0.220	Comprom	-0.220	Comprom	0.000	1.134	Detected	1.165	Detected	1.150	0.035	GT	Sens	contig321	contig321	Unannota
GT	Sense	Sense	1.154	Detected	-1.154	Comprom	0.000	1.344	Detected	0.923	Detected	1.133	0.436	GT	Sens	contig321	contig321	Unannota
GT	Sense	Sense	0.878	Comprom	-0.878	Comprom	0.000	1.043	Detected	1.899	Detected	1.471	0.271	GT	Sens	contig322	contig322	Unannota
GT	Sense	Sense	0.509	Detected	-0.509	Comprom	0.000	1.197	Detected	1.330	Detected	1.263	0.133	GT	Sens	contig322	contig322	Unannota
GT	Sense	Sense	0.427	Detected	-0.427	Detected	0.000	1.122	Detected	0.915	Detected	1.018	0.146	GT	Sens	contig323	contig323	Unannota
GT	Sense	Sense	0.201	Comprom	-0.201	Comprom	0.000	2.352	Detected	2.929	Detected	2.641	0.017	GT	Sens	contig323	contig323	Unannota
GT	Sense	Sense	0.028	Detected	-0.028	Detected	0.000	0.997	Detected	1.031	Detected	1.014	0.001	GT	Sens	contig323	contig323	Unannota
GT	Sense	Sense	0.774	Detected	-0.774	Detected	0.000	0.908	Detected	1.204	Detected	1.056	0.312	GT	Sens	contig324	contig324	Unannota
GT	Sense	Sense	0.868	Detected	-0.868	Comprom	0.000	1.325	Detected	1.453	Detected	1.389	0.251	GT	Sens	contig324	contig324	Unannota
GT	Sense	Sense	0.649	Comprom	-0.649	Comprom	0.000	1.918	Detected	1.518	Detected	1.718	0.127	GT	Sens	contig325	contig325	Unannota
GT	Sense	Sense	0.692	Detected	-0.692	Detected	0.000	1.665	Detected	1.650	Detected	1.658	0.139	GT	Sens	contig325	contig325	Unannota
GT	Sense	Sense	0.722	Detected	-0.722	Detected	0.000	0.830	Detected	1.470	Detected	1.150	0.283	GT	Sens	contig325	contig325	Unannota
GT	Sense	Sense	0.668	Detected	-0.668	Detected	0.000	0.805	Detected	1.363	Detected	1.084	0.273	GT	Sens	contig325	contig325	Unannota
GT	Sense	Sense	0.365	Detected	-0.365	Detected	0.000	0.855	Detected	1.458	Detected	1.157	0.134	GT	Sens	contig325	contig325	Unannota
GT	Sense	Sense	0.333	Detected	-0.333	Detected	0.000	0.900	Detected	1.267	Detected	1.083	0.104	GT	Sens	contig325	contig325	Unannota
GT	Sense	Sense	0.424	Detected	-0.424	Detected	0.000	0.814	Detected	1.418	Detected	1.116	0.165	GT	Sens	contig326	contig326	Unannota
GT	Sense	Sense	-0.152	Comprom	0.152	Comprom	0.000	1.133	Detected	1.233	Detected	1.183	0.018	GT	Sens	contig326	contig326	Unannota
GT	Sense	Sense	1.190	Detected	-1.190	Comprom	0.000	1.149	Detected	2.501	Detected	1.825	0.314	GT	Sens	contig326	contig326	Unannota
GT	Sense	Sense	0.652	Comprom	-0.652	Comprom	0.000	1.359	Detected	1.258	Detected	1.309	0.183	GT	Sens	contig327	contig327	Unannota
GT	Sense	Sense	0.617	Comprom	-0.617	Comprom	0.000	1.205	Detected	1.666	Detected	1.435	0.161	GT	Sens	contig327	contig327	Unannota
GT	Sense	Sense	1.040	Detected	-1.040	Comprom	0.000	1.153	Detected	1.741	Detected	1.447	0.312	GT	Sens	contig327	contig327	Unannota
GT	Sense	Sense	0.359	Detected	-0.359	Detected	0.000	0.824	Detected	1.207	Detected	1.016	0.130	GT	Sens	contig327	contig327	Unannota
GT	Sense	Sense	0.439	Detected	-0.439	Detected	0.000	1.341	Detected	1.668	Detected	1.505	0.085	GT	Sens	contig327	contig327	Unannota
GT	Sense	Sense	0.452	Detected	-0.452	Detected	0.000	1.261	Detected	1.534	Detected	1.398	0.098	GT	Sens	contig327	contig327	Unannota
GT	Sense	Sense	0.090	Comprom	-0.090	Comprom	0.000	1.161	Detected	1.020	Detected	1.091	0.011	GT	Sens	contig328	contig328	Unannota
GT	Sense	Sense	0.508	Detected	-0.508	Detected	0.000	2.226	Detected	1.520	Detected	1.873	0.094	GT	Sens	contig329	contig329	Unannota
GT	Sense	Sense	0.817	Detected	-0.817	Detected	0.000	2.151	Detected	1.217	Detected	1.684	0.215	GT	Sens	contig329	contig329	Unannota
GT	Sense	Sense	-0.300	Comprom	0.300	Detected	0.000	1.162	Detected	0.853	Detected	1.008	0.096	GT	Sens	contig330	contig330	Unannota
GT	Sense	Sense	0.172	Detected	-0.172	Detected	0.000	1.149	Detected	1.082	Detected	1.116	0.024	GT	Sens	contig330	contig330	Unannota
GT	Sense	Sense	0.246	Comprom	-0.246	Comprom	0.000	1.164	Detected	0.856	Detected	1.010	0.074	GT	Sens	contig331	contig331	Unannota
GT	Sense	Sense	0.284	Comprom	-0.284	Comprom	0.000	1.182	Detected	0.916	Detected	1.049	0.079	GT	Sens	contig332	contig332	Unannota
GT	Sense	Sense	-0.001	Comprom	0.001	Comprom	0.000	3.010	Detected	2.499	Detected	2.754	0.008	GT	Sens	contig333	contig333	Unannota
GT	Sense	Sense	0.273	Detected	-0.273	Detected	0.000	0.914	Detected	1.462	Detected	1.188	0.092	GT	Sens	contig333	contig333	Unannota
GT	Sense	Sense	0.551	Detected	-0.551	Detected	0.000	1.065	Detected	2.276	Detected	1.670	0.178	GT	Sens	contig333	contig333	Unannota
GT	Sense	Sense	0.980	Detected	-0.980	Detected	0.000	1.298	Detected	1.211	Detected	1.255	0.329	GT	Sens	contig333	contig333	Unannota
GT	Sense	Sense	0.955	Detected	-0.955	Detected	0.000	1.212	Detected	1.416	Detected	1.314	0.304	GT	Sens	contig333	contig333	Unannota
GT	Sense	Sense	-0.221	Comprom	0.221	Comprom	0.000	1.134	Detected	1.804	Detected	1.469	0.067	GT	Sens	contig334	contig334	Unannota
GT	Sense	Sense	0.089	Comprom	-0.089	Comprom	0.000	1.365	Detected	1.010	Detected	1.187	0.027	GT	Sens	contig334	contig334	Unannota
GT	Sense	Sense	0.071	Comprom	-0.071	Comprom	0.000	1.514	Detected	1.242	Detected	1.378	0.012	GT	Sens	contig334	contig334	Unannota
GT	Sense	Sense	0.751	Detected	-0.751	Comprom	0.000	1.515	Detected	0.892	Detected	1.204	0.277	GT	Sens	contig334	contig334	Unannota
GT	Sense	Sense	-0.365	Detected	0.365	Detected	0.000	1.813	Detected	0.935	Detected	1.374	0.138	GT	Sens	contig334	contig334	Unannota
GT	Sense	Sense	1.040	Detected	-1.040	Comprom	0.000	1.068	Detected	0.942	Detected	1.005	0.437	GT	Sens	contig335	contig335	Unannota
GT	Sense	Sense	0.233	Detected	-0.233	Detected	0.000	1.381	Detected	1.319	Detected	1.350	0.029	GT	Sens	contig335	contig335	Rhodane
GT	Sense	Sense	0.742	Detected	-0.742	Detected	0.000	0.924	Detected	1.171	Detected	1.048	0.299	GT	Sens	contig335	contig335	Unannota
GT	Sense	Sense	0.475	Detected	-0.475	Comprom	0.000	1.397	Detected	1.049	Detected	1.223	0.137	GT	Sens	contig335	contig335	Unannota
GT	Sense	Sense	1.370	Detected	-1.370	Comprom	0.000	1.356	Detected	0.901	Detected	1.129	0.502	GT	Sens	contig336	contig336	Unannota
GT	Sense	Sense	0.394	Detected	-0.394	Detected	0.000	1.365	Detected	1.903	Detected	1.634	0.076	GT	Sens	contig337	contig337	Unannota
GT	Sense	Sense	0.252	Detected	-0.252	Comprom	0.000	0.805	Detected	1.865	Detected	1.335	0.151	GT	Sens	contig337	contig337	Unannota
GT	Sense	Sense	0.444	Detected	-0.444	Detected	0.000	0.932	Detected	1.094	Detected	1.013	0.154	GT	Sens	contig338	contig338	Unannota
GT	Sense	Sense	0.189	Detected	-0.189	Detected	0.000	1.187	Detected	1.824	Detected	1.506	0.055	GT	Sens	contig339	contig339	Unannota
GT	Sense	Sense	0.399	Detected	-0.399	Detected	0.000	1.027	Detected	1.033	Detected	1.030	0.123	GT	Sens	contig339	contig339	Unannota
GT	Sense	Sense	0.226	Detected	-0.226	Detected	0.000	1.028	Detected	1.166	Detected	1.097	0.043	GT	Sens	contig340	contig340	Unannota
GT	Sense	Sense	0.394	Detected	-0.394	Detected	0.000	1.163	Detected	1.842	Detected	1.502	0.102	GT	Sens	contig341	contig341	Unannota
GT	Sense	Sense	0.368	Detected	-0.368	Detected	0.000	1.089	Detected	1.887	Detected	1.488	0.111	GT	Sens	contig341	contig341	Unannota
GT	Sense	Sense	-0.038	Comprom	0.038	Comprom	0.000	1.815	Detected	1.157	Detected	1.486	0.046	GT	Sens	contig341	contig341	Unannota
GT	Sense	Sense	0.267	Comprom	-0.267	Comprom	0.000	1.343	Detected	1.112	Detected	1.228	0.052	GT	Sens	contig342	contig342	Unannota
GT	Sense	Sense	-0.463	Comprom	0.463	Comprom	0.000	1.393	Detected	1.457	Detected	1.425	0.092	GT	Sens	contig342	contig342	Unannota
GT	Sense	Sense	0.061	Comprom	-0.061	Comprom	0.000	1.103	Detected	1.278	Detected	1.190	0.008	GT	Sens	contig344	contig344	Unannota
GT	Sense	Sense	0.798	Detected	-0.798	Detected	0.000	1.345	Detected	0.994	Detected	1.170	0.289	GT	Sens	contig345	contig345	Unannota
GT	Sense	Sense	0.532	Detected	-0.532	Detected	0.000	1.366	Detected	0.850	Detected	1.108	0.202	GT	Sens	contig345	contig345	Unannota
GT	Sense	Sense	-0.205	Detected	0.205	Detected	0.000	0.919	Detected	1.140	Detected	1.029	0.047	GT	Sens	contig346	contig346	Unannota
GT	Sense	Sense	0.065	Detected	-0.065	Detected	0.000	1.113	Detected	1.222	Detected	1.168	0.005	GT	Sens	contig346	contig346	Sodium/h
GT	Sense	Sense	0.777	Detected	-0.777	Comprom	0.000	1.325	Detected	2.279	Detected	1.802	0.187	GT	Sens	contig347	contig347	Unannota
GT	Sense	Sense	0.643	Detected	-0.643	Detected	0.000	1.229	Detected	1.227	Detected	1.228	0.197	GT	Sens	contig348	contig348	Unannota
GT	Sense	Sense	0.250	Detected	-0.250	Detected	0.000	0.979	Detected	1.104	Detected	1.041	0.056	GT	Sens	contig349	contig349	Unannota
GT	Sense	Sense	0.368	Comprom	-0.368	Comprom	0.000	1.037	Detected	1.017	Detected	1.027	0.108	GT	Sens	contig349	contig349	Unannota
GT	Sense	Sense	0.510	Detected	-0.510	Comprom	0.000	1.114	Detected	1.400	Detected	1.257	0.141	GT	Sens	contig350	contig350	Unknown
GT	Sense	Sense	0.597	Detected	-0.597	Detected	0.000	0.943	Detected	1.148	Detected	1.046	0.226	GT	Sens	contig350	contig350	Unannota
GT	Sense	Sense	0.510	Detected	-0.510	Detected	0.000	1.106	Detected	1.346	Detected	1.226	0.144	GT	Sens	contig350	contig350	Unannota
GT	Sense	Sense	0.182	Detected	-0.182	Detected	0.000	1.062	Detected	1.039	Detected	1.051	0.029	GT	Sens	contig352	contig352	Genomic
GT	Sense	Sense	0.818	Detected	-0.818	Comprom	0.000	1.312	Detected	1.197	Detected	1.254	0.266	GT	Sens	contig353	contig353	Potassiu
GT	Sense	Sense	0.752	Detected	-0.752	Comprom	0.000	1.129	Detected	2.232	Detected	1.681	0.213	GT	Sens	contig353	contig353	Unannota
GT	Sense	Sense	0.711	Detected	-0.711	Detected	0.000	1.263	Detected	1.126	Detected	1.194	0.237	GT	Sens	contig353	contig353	Unannota
GT	Sense	Sense	0.524	Detected	-0.524	Detected	0.000	1.477	Detected	1.001	Detected	1.239	0.164	GT	Sens	contig354	contig354	RNA-depe
GT	Sense	Sense	-0.330	Comprom	0.330	Comprom	0.000	2.027	Detected	1.080	Detected	1.554	0.115	GT	Sens	contig354	contig354	Unannota
GT	Sense	Sense	1.307	Detected	-1.307	Comprom	0.000	1.234	Detected	1.695	Detected	1.464	0.385	GT	Sens	contig355	contig355	Unannota
GT	Sense	Sense	0.322	Detected	-0.322	Detected	0.000	1.055	Detected	1.312	Detected	1.183	0.076	GT	Sens	contig355	contig355	Unannota
GT	Sense	Sense	0.270	Detected	-0.270	Detected	0.000	1.065	Detected	1.546	Detected	1.306	0.069	GT	Sens	contig356	contig356	Unannota
GT	Sense	Sense	0.575	Detected	-0.575	Detected	0.000	1.090	Detected	1.602	Detected	1.346	0.166	GT	Sens	contig357	contig357	Unannota
GT	Sense	Sense	0.499	Detected	-0.499	Detected	0.000	0.841	Detected	1.666	Detected	1.253	0.192	GT	Sens	contig357	contig357	Unannota
GT	Sense	Sense	0.238	Detected	-0.238	Detected	0.000	1.287	Detected	0.881	Detected	1.084	0.074	GT	Sens	contig357	contig357	Unannota
GT	Sense	Sense	0.221	Detected	-0.221	Detected	0.000	1.315	Detected	0.934	Detected	1.125	0.061	GT	Sens	contig357	contig357	Unannota
GT	Sense	Sense	0.589	Detected	-0.589	Detected	0.000	1.192	Detected	1.189	Detected	1.190	0.181	GT	Sens	contig357	contig357	Transcrip
GT	Sense	Sense	0.195	Detected	-0.195	Detected	0.000	1.360	Detected	1.622	Detected	1.491	0.024	GT	Sens	contig358	contig358	Unannota
GT	Sense	Sense	0.473	Comprom	-0.473	Comprom	0.000	1.389	Detected	1.395	Detected	1.392	0.098	GT	Sens	contig358	contig358	Unannota
GT	Sense	Sense	0.179	Detected	-0.179	Detected	0.000	1.499	Detected	1.629	Detected	1.564	0.015	GT	Sens	contig358	contig358	chaperon
GT	Sense	Sense	0.077	Comprom	-0.077	Comprom	0.000	1.500	Detected	1.382	Detected	1.441	0.005	GT	Sens	contig359	contig359	Unannota
GT	Sense	Sense	0.670	Detected	-0.670	Detected	0.000	1.567	Detected	1.167	Detected	1.367	0.190	GT	Sens	contig359	contig359	Heat sho
GT	Sense	Sense	0.724	Detected	-0.724	Comprom	0.000	1.150	Detected	1.081	Detected	1.116	0.264	GT	Sens	contig360	contig360	Unannota
GT	Sense	Sense	0.634	Detected	-0.634	Detected	0.000	1.413	Detected	2.116	Detected	1.765	0.135	GT	Sens	contig360	contig360	Unannota
GT	Sense	Sense	0.577	Detected	-0.577	Detected	0.000	1.335	Detected	1.844	Detected	1.590	0.128	GT	Sens	contig360	contig360	Unannota
GT	Sense	Sense	0.103	Comprom	-0.103	Comprom	0.000	1.442	Detected	1.644	Detected	1.543	0.009	GT	Sens	contig360	contig360	Unannota
GT	Sense	Sense	0.164	Detected	-0.164	Detected	0.000	0.856	Detected	1.403	Detected	1.130	0.071	GT	Sens	contig360	contig360	Unannota
GT	Sense	Sense	-0.043	Comprom	0.043	Comprom	0.000	1.251	Detected	1.128	Detected	1.189	0.004	GT	Sens	contig360	contig360	Unannota
GT	Sense	Sense	0.260	Comprom	-0.260	Comprom	0.000	1.343	Detected	1.287	Detected	1.315	0.037	GT	Sens	contig361	contig361	Unannota
GT	Sense	Sense	1.008	Comprom	-1.008	Comprom	0.000	1.140	Detected	1.528	Detected	1.334	0.323	GT	Sens	contig361	contig361	Unannota
GT	Sense	Sense	-0.339	Detected	0.339	Detected	0.000	2.073	Detected	1.089	Detected	1.581	0.118	GT	Sens	contig362	contig362	Unannota
GT	Sense	Sense	0.638	Detected	-0.638	Detected	0.000	0.957	Detected	1.546	Detected	1.251	0.217	GT	Sens	contig362	contig362	Unknown
GT	Sense	Sense	0.449	Detected	-0.449	Detected	0.000	0.899	Detected	1.492	Detected	1.196	0.156	GT	Sens	contig364	contig364	Unannota
GT	Sense	Sense	0.390	Detected	-0.390	Detected	0.000	1.033	Detected	1.465	Detected	1.249	0.107	GT	Sens	contig364	contig364	Unannota
GT	Sense	Sense	-0.692	Comprom	0.692	Detected	0.000	1.277	Detected	1.412	Detected	1.344	0.193	GT	Sens	contig364	contig364	Unannota
GT	Sense	Sense	0.373	Detected	-0.373	Detected	0.000	1.091	Detected	1.364	Detected	1.228	0.091	GT	Sens	contig364	contig364	Genomic
GT	Sense	Sense	-0.030	Detected	0.030	Detected	0.000	0.850	Detected	1.266	Detected	1.058	0.037	GT	Sens	contig364	contig364	Unannota
GT	Sense	Sense	0.277	Detected	-0.277	Detected	0.000	1.019	Detected	1.638	Detected	1.328	0.085	GT	Sens	contig365	contig365	Unannota
GT	Sense	Sense	0.323	Detected	-0.323	Detected	0.000	1.106	Detected	1.436	Detected	1.271	0.073	GT	Sens	contig365	contig365	Unannota
GT	Sense	Sense	0.063	Comprom	-0.063	Comprom	0.000	1.536	Detected	0.932	Detected	1.234	0.057	GT	Sens	contig366	contig366	Unannota
GT	Sense	Sense	0.234	Detected	-0.234	Detected	0.000	1.380	Detected	0.827	Detected	1.104	0.093	GT	Sens	contig366	contig366	Unannota
GT	Sense	Sense	-0.601	Comprom	0.601	Detected	0.000	2.565	Detected	2.618	Detected	2.592	0.050	GT	Sens	contig366	contig366	Unannota
GT	Sense	Sense	-0.181	Detected	0.181	Detected	0.000	1.902	Detected	1.962	Detected	1.932	0.009	GT	Sens	contig366	contig366	Unannota
GT	Sense	Sense	0.428	Detected	-0.428	Detected	0.000	1.730	Detected	1.834	Detected	1.782	0.054	GT	Sens	contig366	contig366	Unannota
GT	Sense	Sense	0.595	Detected	-0.595	Detected	0.000	1.589	Detected	1.713	Detected	1.651	0.110	GT	Sens	contig366	contig366	Unannota
GT	Sense	Sense	0.673	Comprom	-0.673	Comprom	0.000	2.387	Detected	2.248	Detected	2.317	0.076	GT	Sens	contig367	contig367	Unannota
GT	Sense	Sense	0.356	Detected	-0.356	Detected	0.000	0.835	Detected	1.197	Detected	1.016	0.126	GT	Sens	contig368	contig368	Pentatric
GT	Sense	Sense	0.948	Comprom	-0.948	Comprom	0.000	1.845	Detected	2.405	Detected	2.125	0.165	GT	Sens	contig370	contig370	Unannota
GT	Sense	Sense	0.321	Detected	-0.321	Detected	0.000	1.276	Detected	0.975	Detected	1.126	0.087	GT	Sens	contig371	contig371	Unannota
GT	Sense	Sense	1.632	Detected	-1.632	Comprom	0.000	1.879	Detected	2.994	Detected	2.436	0.293	GT	Sens	contig372	contig372	Unannota
GT	Sense	Sense	0.191	Detected	-0.191	Detected	0.000	1.261	Detected	1.491	Detected	1.376	0.025	GT	Sens	contig372	contig372	Plant-spe
GT	Sense	Sense	-0.107	Comprom	0.107	Comprom	0.000	1.324	Detected	1.133	Detected	1.228	0.013	GT	Sens	contig373	contig373	Unannota
GT	Sense	Sense	-1.181	Comprom	1.181	Detected	0.000	1.303	Detected	1.525	Detected	1.414	0.356	GT	Sens	contig373	contig373	Unannota
GT	Sense	Sense	0.826	Detected	-0.826	Detected	0.000	1.142	Detected	1.518	Detected	1.330	0.257	GT	Sens	contig374	contig374	Unannota
GT	Sense	Sense	0.864	Detected	-0.864	Comprom	0.000	1.311	Detected	1.489	Detected	1.400	0.248	GT	Sens	contig374	contig374	Unannota
GT	Sense	Sense	0.594	Comprom	-0.594	Comprom	0.000	1.161	Detected	1.164	Detected	1.162	0.189	GT	Sens	contig374	contig374	Unannota
GT	Sense	Sense	0.485	Detected	-0.485	Detected	0.000	1.097	Detected	1.340	Detected	1.219	0.135	GT	Sens	contig374	contig374	Unannota
GT	Sense	Sense	0.798	Detected	-0.798	Detected	0.000	1.191	Detected	2.203	Detected	1.697	0.214	GT	Sens	contig375	contig375	Unannota
GT	Sense	Sense	0.759	Detected	-0.759	Detected	0.000	0.962	Detected	1.986	Detected	1.474	0.249	GT	Sens	contig375	contig375	Unannota
GT	Sense	Sense	0.513	Detected	-0.513	Detected	0.000	1.113	Detected	1.103	Detected	1.108	0.163	GT	Sens	contig376	contig376	Unannota
GT	Sense	Sense	0.827	Comprom	-0.827	Comprom	0.000	1.401	Detected	0.872	Detected	1.137	0.321	GT	Sens	contig377	contig377	Unannota
GT	Sense	Sense	1.047	Detected	-1.047	Comprom	0.000	1.392	Detected	1.080	Detected	1.236	0.363	GT	Sens	contig377	contig377	Unannota
GT	Sense	Sense	-0.109	Detected	0.109	Detected	0.000	1.045	Detected	1.541	Detected	1.293	0.041	GT	Sens	contig378	contig378	Unannota
GT	Sense	Sense	1.184	Detected	-1.184	Comprom	0.000	0.976	Detected	1.352	Detected	1.164	0.434	GT	Sens	contig378	contig378	Unknown
GT	Sense	Sense	0.078	Comprom	-0.078	Comprom	0.000	1.751	Detected	0.827	Detected	1.289	0.111	GT	Sens	contig378	contig378	Unannota
GT	Sense	Sense	0.735	Detected	-0.735	Detected	0.000	1.110	Detected	1.472	Detected	1.291	0.230	GT	Sens	contig379	contig379	Glyoxal o
GT	Sense	Sense	0.732	Detected	-0.732	Detected	0.000	1.030	Detected	0.979	Detected	1.005	0.304	GT	Sens	contig380	contig380	E3 ubiqui
GT	Sense	Sense	0.412	Comprom	-0.412	Comprom	0.000	2.382	Detected	1.951	Detected	2.166	0.043	GT	Sens	contig380	contig380	Unannota
GT	Sense	Sense	0.779	Comprom	-0.779	Comprom	0.000	1.577	Detected	5.206	Detected	3.391	0.228	GT	Sens	contig381	contig381	Unannota
GT	Sense	Sense	0.257	Detected	-0.257	Detected	0.000	1.221	Detected	0.886	Detected	1.054	0.075	GT	Sens	contig381	contig381	Unannota
GT	Sense	Sense	0.954	Detected	-0.954	Detected	0.000	1.149	Detected	2.033	Detected	1.591	0.269	GT	Sens	contig381	contig381	Unannota
GT	Sense	Sense	0.999	Detected	-0.999	Detected	0.000	1.165	Detected	2.179	Detected	1.672	0.274	GT	Sens	contig381	contig381	Unannota
GT	Sense	Sense	0.559	Detected	-0.559	Detected	0.000	1.591	Detected	2.622	Detected	2.106	0.109	GT	Sens	contig381	contig381	Unannota
GT	Sense	Sense	0.588	Detected	-0.588	Detected	0.000	1.644	Detected	2.596	Detected	2.120	0.107	GT	Sens	contig381	contig381	Unannota
GT	Sense	Sense	1.058	Detected	-1.058	Comprom	0.000	1.600	Detected	1.040	Detected	1.320	0.351	GT	Sens	contig383	contig383	Unannota
GT	Sense	Sense	0.391	Detected	-0.391	Detected	0.000	1.884	Detected	1.440	Detected	1.662	0.066	GT	Sens	contig384	contig384	Unannota
GT	Sense	Sense	-0.122	Comprom	0.122	Detected	0.000	1.553	Detected	1.162	Detected	1.357	0.028	GT	Sens	contig384	contig384	Unannota
GT	Sense	Sense	1.671	Detected	-1.671	Detected	0.000	1.416	Detected	1.188	Detected	1.302	0.518	GT	Sens	contig384	contig384	Unannota
GT	Sense	Sense	1.707	Detected	-1.707	Detected	0.000	1.584	Detected	1.411	Detected	1.497	0.473	GT	Sens	contig384	contig384	Unannota
GT	Sense	Sense	0.300	Detected	-0.300	Comprom	0.000	1.269	Detected	1.731	Detected	1.500	0.058	GT	Sens	contig384	contig384	Unannota
GT	Sense	Sense	0.408	Detected	-0.408	Detected	0.000	1.102	Detected	1.165	Detected	1.133	0.109	GT	Sens	contig387	contig387	Zinc finge
GT	Sense	Sense	-0.296	Comprom	0.296	Comprom	0.000	1.123	Detected	1.095	Detected	1.109	0.065	GT	Sens	contig388	contig388	Unannota
GT	Sense	Sense	0.329	Detected	-0.329	Detected	0.000	0.960	Detected	1.221	Detected	1.091	0.091	GT	Sens	contig389	contig389	Unannota
GT	Sense	Sense	0.193	Detected	-0.193	Detected	0.000	1.095	Detected	1.286	Detected	1.190	0.031	GT	Sens	contig389	contig389	Unannota
GT	Sense	Sense	0.011	Comprom	-0.011	Comprom	0.000	1.638	Detected	1.441	Detected	1.540	0.004	GT	Sens	contig389	contig389	Unannota
GT	Sense	Sense	0.528	Detected	-0.528	Detected	0.000	1.056	Detected	1.314	Detected	1.185	0.161	GT	Sens	contig389	contig389	Unannota
GT	Sense	Sense	0.501	Detected	-0.501	Detected	0.000	0.993	Detected	1.394	Detected	1.193	0.158	GT	Sens	contig389	contig389	Unannota
GT	Sense	Sense	-0.083	Comprom	0.083	Comprom	0.000	2.399	Detected	3.016	Detected	2.708	0.014	GT	Sens	contig389	contig389	Unannota
GT	Sense	Sense	0.591	Detected	-0.591	Detected	0.000	0.951	Detected	1.278	Detected	1.115	0.211	GT	Sens	contig389	contig389	Receptor
GT	Sense	Sense	0.494	Comprom	-0.494	Comprom	0.000	1.529	Detected	1.697	Detected	1.613	0.085	GT	Sens	contig389	contig389	Unannota
GT	Sense	Sense	0.518	Comprom	-0.518	Comprom	0.000	1.783	Detected	3.044	Detected	2.414	0.098	GT	Sens	contig390	contig390	Unannota
GT	Sense	Sense	0.898	Detected	-0.898	Detected	0.000	2.049	Detected	1.524	Detected	1.786	0.196	GT	Sens	contig390	contig390	2-oxoglut
GT	Sense	Sense	0.635	Detected	-0.635	Detected	0.000	1.266	Detected	1.018	Detected	1.142	0.220	GT	Sens	contig391	contig391	Unannota
GT	Sense	Sense	0.395	Detected	-0.395	Detected	0.000	1.099	Detected	1.685	Detected	1.392	0.105	GT	Sens	contig391	contig391	Exocyst c
GT	Sense	Sense	0.592	Comprom	-0.592	Comprom	0.000	1.631	Detected	1.676	Detected	1.654	0.108	GT	Sens	contig391	contig391	Unannota
GT	Sense	Sense	0.329	Detected	-0.329	Detected	0.000	0.891	Detected	1.229	Detected	1.060	0.103	GT	Sens	contig392	contig392	Unannota
GT	Sense	Sense	0.070	Comprom	-0.070	Comprom	0.000	1.812	Detected	0.881	Detected	1.347	0.104	GT	Sens	contig393	contig393	Unannota
GT	Sense	Sense	0.769	Detected	-0.769	Comprom	0.000	1.326	Detected	1.280	Detected	1.303	0.232	GT	Sens	contig394	contig394	Unannota
GT	Sense	Sense	-0.275	Detected	0.275	Detected	0.000	1.717	Detected	1.576	Detected	1.647	0.028	GT	Sens	contig394	contig394	Unannota
GT	Sense	Sense	-0.200	Detected	0.200	Detected	0.000	1.601	Detected	1.591	Detected	1.596	0.015	GT	Sens	contig394	contig394	Unannota
GT	Sense	Sense	0.032	Detected	-0.032	Detected	0.000	0.983	Detected	1.088	Detected	1.036	0.003	GT	Sens	contig395	contig395	Unannota
GT	Sense	Sense	0.270	Comprom	-0.270	Comprom	0.000	2.108	Detected	1.621	Detected	1.865	0.036	GT	Sens	contig395	contig395	Unannota
GT	Sense	Sense	-0.025	Comprom	0.025	Comprom	0.000	3.201	Detected	2.986	Detected	3.094	0.001	GT	Sens	contig395	contig395	Unannota
GT	Sense	Sense	-0.105	Detected	0.105	Detected	0.000	0.897	Detected	1.333	Detected	1.115	0.044	GT	Sens	contig396	contig396	Unannota
GT	Sense	Sense	-0.033	Detected	0.033	Detected	0.000	1.573	Detected	1.360	Detected	1.467	0.006	GT	Sens	contig396	contig396	Unannota
GT	Sense	Sense	0.290	Detected	-0.290	Detected	0.000	1.126	Detected	0.997	Detected	1.061	0.070	GT	Sens	contig396	contig396	Unannota
GT	Sense	Sense	0.193	Detected	-0.193	Detected	0.000	1.427	Detected	1.274	Detected	1.351	0.023	GT	Sens	contig396	contig396	Unannota
GT	Sense	Sense	0.028	Detected	-0.028	Detected	0.000	1.023	Detected	1.155	Detected	1.089	0.004	GT	Sens	contig397	contig397	Unannota
GT	Sense	Sense	0.182	Detected	-0.182	Detected	0.000	1.149	Detected	1.058	Detected	1.104	0.028	GT	Sens	contig397	contig397	Unannota
GT	Sense	Sense	0.720	Detected	-0.720	Comprom	0.000	1.409	Detected	1.310	Detected	1.360	0.200	GT	Sens	contig397	contig397	Unannota
GT	Sense	Sense	-0.947	Comprom	0.947	Detected	0.000	0.950	Detected	1.580	Detected	1.265	0.333	GT	Sens	contig397	contig397	Unannota
GT	Sense	Sense	0.042	Detected	-0.042	Detected	0.000	4.384	Detected	4.073	Detected	4.228	0.001	GT	Sens	contig397	contig397	Unknown
GT	Sense	Sense	0.543	Comprom	-0.543	Comprom	0.000	1.928	Detected	1.896	Detected	1.912	0.072	GT	Sens	contig397	contig397	Unannota
GT	Sense	Sense	0.894	Detected	-0.894	Detected	0.000	1.189	Detected	3.504	Detected	2.347	0.250	GT	Sens	contig397	contig397	Unannota
GT	Sense	Sense	2.007	Detected	-2.007	Comprom	0.000	2.609	Detected	4.520	Detected	3.565	0.250	GT	Sens	contig397	contig397	Unannota
GT	Sense	Sense	0.398	Detected	-0.398	Comprom	0.000	1.037	Detected	1.573	Detected	1.305	0.113	GT	Sens	contig398	contig398	Unannota
GT	Sense	Sense	0.391	Comprom	-0.391	Comprom	0.000	1.450	Detected	1.657	Detected	1.554	0.061	GT	Sens	contig398	contig398	Unannota
GT	Sense	Sense	0.596	Detected	-0.596	Detected	0.000	0.942	Detected	1.561	Detected	1.251	0.203	GT	Sens	contig399	contig399	Unannota
GT	Sense	Sense	0.594	Detected	-0.594	Detected	0.000	1.083	Detected	1.480	Detected	1.281	0.177	GT	Sens	contig399	contig399	Unannota
GT	Sense	Sense	0.529	Detected	-0.529	Detected	0.000	0.854	Detected	1.575	Detected	1.214	0.198	GT	Sens	contig400	contig400	Unannota
GT	Sense	Sense	0.502	Detected	-0.502	Detected	0.000	1.041	Detected	1.043	Detected	1.042	0.173	GT	Sens	contig400	contig400	Unannota
GT	Sense	Sense	0.279	Detected	-0.279	Detected	0.000	0.835	Detected	2.673	Detected	1.754	0.209	GT	Sens	contig400	contig400	Unknown
GT	Sense	Sense	0.667	Detected	-0.667	Detected	0.000	0.913	Detected	2.109	Detected	1.511	0.234	GT	Sens	contig400	contig400	Unannota
GT	Sense	Sense	0.853	Detected	-0.853	Comprom	0.000	1.199	Detected	1.635	Detected	1.417	0.249	GT	Sens	contig401	contig401	Unannota
GT	Sense	Sense	0.250	Detected	-0.250	Detected	0.000	1.032	Detected	1.657	Detected	1.345	0.078	GT	Sens	contig401	contig401	Unannota
GT	Sense	Sense	-0.318	Comprom	0.318	Comprom	0.000	1.563	Detected	1.896	Detected	1.729	0.040	GT	Sens	contig401	contig401	Unannota
GT	Sense	Sense	1.108	Detected	-1.108	Detected	0.000	1.521	Detected	1.906	Detected	1.714	0.267	GT	Sens	contig401	contig401	Unannota
GT	Sense	Sense	1.213	Detected	-1.213	Detected	0.000	1.826	Detected	1.914	Detected	1.870	0.263	GT	Sens	contig401	contig401	Unannota
GT	Sense	Sense	0.734	Detected	-0.734	Detected	0.000	1.816	Detected	1.502	Detected	1.659	0.158	GT	Sens	contig402	contig402	Unannota
GT	Sense	Sense	0.492	Detected	-0.492	Detected	0.000	1.816	Detected	1.121	Detected	1.468	0.135	GT	Sens	contig402	contig402	Unannota

GT	Sense	Sense	-0.864	Comprom	0.864	Comprom	0.000	1.250	Detected	1.587	Detected	1.419	0.248	GT	Sens	contig402	contig402	Unannota
GT	Sense	Sense	0.241	Comprom	-0.241	Comprom	0.000	0.816	Detected	1.344	Detected	1.080	0.094	GT	Sens	contig402	contig402	Unannota
GT	Sense	Sense	0.088	Comprom	-0.088	Comprom	0.000	1.235	Detected	1.490	Detected	1.363	0.013	GT	Sens	contig403	contig403	Unannota
GT	Sense	Sense	0.763	Detected	-0.763	Detected	0.000	1.252	Detected	1.586	Detected	1.419	0.211	GT	Sens	contig403	contig403	Unannota
GT	Sense	Sense	-0.015	Detected	0.015	Detected	0.000	1.337	Detected	1.116	Detected	1.227	0.008	GT	Sens	contig403	contig403	Unannota
GT	Sense	Sense	0.097	Detected	-0.097	Detected	0.000	0.884	Detected	1.245	Detected	1.064	0.035	GT	Sens	contig403	contig403	50S ribos
GT	Sense	Sense	0.659	Detected	-0.659	Detected	0.000	1.107	Detected	1.005	Detected	1.056	0.251	GT	Sens	contig405	contig405	Unannota
GT	Sense	Sense	-0.058	Comprom	0.058	Comprom	0.000	0.897	Detected	1.846	Detected	1.371	0.103	GT	Sens	contig405	contig405	Unannota
GT	Sense	Sense	0.038	Detected	-0.038	Detected	0.000	1.338	Detected	1.792	Detected	1.565	0.021	GT	Sens	contig405	contig405	Unannota
GT	Sense	Sense	0.023	Detected	-0.023	Detected	0.000	1.192	Detected	1.548	Detected	1.370	0.017	GT	Sens	contig405	contig405	Unannota
GT	Sense	Sense	0.442	Detected	-0.442	Detected	0.000	1.242	Detected	1.230	Detected	1.236	0.108	GT	Sens	contig406	contig406	Amino ac
GT	Sense	Sense	-0.118	Comprom	0.118	Comprom	0.000	1.549	Detected	1.499	Detected	1.524	0.006	GT	Sens	contig406	contig406	Unannota
GT	Sense	Sense	-0.049	Detected	0.049	Detected	0.000	0.949	Detected	1.252	Detected	1.101	0.020	GT	Sens	contig407	contig407	Unannota
GT	Sense	Sense	0.233	Detected	-0.233	Detected	0.000	1.321	Detected	1.518	Detected	1.420	0.030	GT	Sens	contig407	contig407	Unannota
GT	Sense	Sense	0.279	Detected	-0.279	Detected	0.000	1.126	Detected	1.026	Detected	1.076	0.063	GT	Sens	contig407	contig407	Unannota
GT	Sense	Sense	0.389	Detected	-0.389	Detected	0.000	1.644	Detected	1.529	Detected	1.587	0.056	GT	Sens	contig407	contig407	Unannota
GT	Sense	Sense	0.439	Detected	-0.439	Comprom	0.000	1.543	Detected	1.161	Detected	1.352	0.106	GT	Sens	contig408	contig408	Unannota
GT	Sense	Sense	0.438	Detected	-0.438	Detected	0.000	1.181	Detected	1.179	Detected	1.180	0.114	GT	Sens	contig408	contig408	Unannota
GT	Sense	Sense	0.307	Detected	-0.307	Detected	0.000	1.120	Detected	0.935	Detected	1.027	0.085	GT	Sens	contig410	contig410	Unannota
GT	Sense	Sense	0.523	Detected	-0.523	Comprom	0.000	0.879	Detected	1.262	Detected	1.070	0.195	GT	Sens	contig410	contig410	Unannota
GT	Sense	Sense	0.772	Detected	-0.772	Comprom	0.000	1.243	Detected	1.092	Detected	1.168	0.271	GT	Sens	contig410	contig410	Unannota
GT	Sense	Sense	0.592	Comprom	-0.592	Comprom	0.000	1.790	Detected	1.559	Detected	1.674	0.109	GT	Sens	contig411	contig411	Unannota
GT	Sense	Sense	2.134	Detected	-2.134	Detected	0.000	2.761	Detected	1.637	Detected	2.199	0.424	GT	Sens	contig411	contig411	Genomic
GT	Sense	Sense	1.047	Detected	-1.047	Comprom	0.000	1.252	Detected	0.818	Detected	1.035	0.435	GT	Sens	contig411	contig411	Unannota
GT	Sense	Sense	-0.040	Detected	0.040	Detected	0.000	1.089	Detected	1.048	Detected	1.069	0.002	GT	Sens	contig411	contig411	Unannota
GT	Sense	Sense	0.059	Detected	-0.059	Detected	0.000	1.407	Detected	1.150	Detected	1.279	0.012	GT	Sens	contig411	contig411	Unannota
GT	Sense	Sense	0.447	Comprom	-0.447	Comprom	0.000	1.925	Detected	2.791	Detected	2.358	0.063	GT	Sens	contig411	contig411	Unannota
GT	Sense	Sense	-0.269	Comprom	0.269	Comprom	0.000	1.405	Detected	1.584	Detected	1.495	0.034	GT	Sens	contig411	contig411	Unannota
GT	Sense	Sense	0.336	Detected	-0.336	Comprom	0.000	0.823	Detected	1.725	Detected	1.274	0.152	GT	Sens	contig411	contig411	Unannota
GT	Sense	Sense	0.378	Detected	-0.378	Detected	0.000	1.354	Detected	0.900	Detected	1.127	0.125	GT	Sens	contig411	contig411	LOB dom
GT	Sense	Sense	0.007	Detected	-0.007	Detected	0.000	1.917	Detected	2.202	Detected	2.060	0.005	GT	Sens	contig411	contig411	Unannota
GT	Sense	Sense	0.002	Detected	-0.002	Detected	0.000	1.554	Detected	1.752	Detected	1.653	0.004	GT	Sens	contig411	contig411	Unannota
GT	Sense	Sense	0.485	Detected	-0.485	Detected	0.000	1.201	Detected	1.135	Detected	1.168	0.138	GT	Sens	contig411	contig411	Unannota
GT	Sense	Sense	1.562	Detected	-1.562	Detected	0.000	1.258	Detected	1.191	Detected	1.224	0.515	GT	Sens	contig412	contig412	Lipase-lik
GT	Sense	Sense	-0.063	Detected	0.063	Detected	0.000	0.983	Detected	1.076	Detected	1.029	0.006	GT	Sens	contig412	contig412	Unannota
GT	Sense	Sense	0.224	Detected	-0.224	Detected	0.000	1.094	Detected	1.557	Detected	1.325	0.054	GT	Sens	contig413	contig413	Unannota
GT	Sense	Sense	-0.483	Comprom	0.483	Comprom	0.000	1.039	Detected	1.120	Detected	1.079	0.156	GT	Sens	contig413	contig413	Unannota
GT	Sense	Sense	0.604	Detected	-0.604	Detected	0.000	1.022	Detected	0.987	Detected	1.005	0.238	GT	Sens	contig414	contig414	Unannota
GT	Sense	Sense	0.716	Detected	-0.716	Detected	0.000	0.847	Detected	1.712	Detected	1.279	0.266	GT	Sens	contig414	contig414	Unannota
GT	Sense	Sense	0.218	Detected	-0.218	Detected	0.000	0.868	Detected	1.341	Detected	1.104	0.075	GT	Sens	contig415	contig415	Unannota
GT	Sense	Sense	0.214	Detected	-0.214	Detected	0.000	1.239	Detected	1.275	Detected	1.257	0.028	GT	Sens	contig415	contig415	Unannota
GT	Sense	Sense	-0.356	Comprom	0.356	Detected	0.000	1.225	Detected	1.034	Detected	1.130	0.092	GT	Sens	contig415	contig415	Unannota
GT	Sense	Sense	0.340	Comprom	-0.340	Comprom	0.000	1.506	Detected	1.522	Detected	1.514	0.047	GT	Sens	contig416	contig416	Unannota
GT	Sense	Sense	0.244	Comprom	-0.244	Comprom	0.000	1.566	Detected	1.880	Detected	1.723	0.027	GT	Sens	contig417	contig417	Unannota
GT	Sense	Sense	-1.219	Comprom	1.219	Detected	0.000	3.115	Detected	3.961	Detected	3.538	0.111	GT	Sens	contig418	contig418	Unannota
GT	Sense	Sense	-0.513	Comprom	0.513	Comprom	0.000	1.465	Detected	1.355	Detected	1.410	0.112	GT	Sens	contig420	contig420	Unannota
GT	Sense	Sense	1.511	Detected	-1.511	Detected	0.000	2.121	Detected	1.976	Detected	2.048	0.308	GT	Sens	contig421	contig421	Beta-gluc

GT	Sense	Sense	0.490	Detected	-0.490	Detected	0.000	0.810	Detected	1.804	Detected	1.307	0.202	GT	Sens	[illegible]
GT	Sense	Sense	0.348	Detected	-0.348	Detected	0.000	1.373	Detected	0.805	Detected	1.089	0.136	GT	Sens	[illegible]
GT	Sense	Sense	0.391	Detected	-0.391	Detected	0.000	1.014	Detected	1.210	Detected	1.112	0.110	GT	Sens	[illegible]
GT	Sense	Sense	0.530	Detected	-0.530	Detected	0.000	1.206	Detected	2.195	Detected	1.700	0.144	GT	Sens	[illegible]
GT	Sense	Sense	0.287	Detected	-0.287	Detected	0.000	0.933	Detected	2.028	Detected	1.480	0.139	GT	Sens	[illegible]
GT	Sense	Sense	-0.039	Detected	0.039	Detected	0.000	0.918	Detected	1.642	Detected	1.280	0.072	GT	Sens	[illegible]
GT	Sense	Sense	0.488	Detected	-0.488	Detected	0.000	0.848	Detected	2.084	Detected	1.466	0.204	GT	Sens	[illegible]
GT	Sense	Sense	0.563	Detected	-0.563	Detected	0.000	1.022	Detected	2.423	Detected	1.723	0.195	GT	Sens	[illegible]
GT	Sense	Sense	1.175	Detected	-1.175	Detected	0.000	1.348	Detected	1.018	Detected	1.183	0.424	GT	Sens	[illegible]
GT	Sense	Sense	1.149	Detected	-1.149	Detected	0.000	0.909	Detected	2.162	Detected	1.536	0.362	GT	Sens	[illegible]
GT	Sense	Sense	1.109	Detected	-1.109	Detected	0.000	2.006	Detected	1.364	Detected	1.685	0.282	GT	Sens	[illegible]
GT	Sense	Sense	-0.122	Detected	0.122	Detected	0.000	1.584	Detected	1.045	Detected	1.315	0.047	GT	Sens	[illegible]
GT	Sense	Sense	0.496	Detected	-0.496	Detected	0.000	1.096	Detected	1.146	Detected	1.121	0.152	GT	Sens	[illegible]
GT	Sense	Sense	0.322	Detected	-0.322	Detected	0.000	1.173	Detected	1.208	Detected	1.190	0.066	GT	Sens	[illegible]
GT	Sense	Sense	0.442	Detected	-0.442	Detected	0.000	0.803	Detected	1.804	Detected	1.304	0.190	GT	Sens	[illegible]
GT	Sense	Sense	0.671	Detected	-0.671	Detected	0.000	1.298	Detected	1.358	Detected	1.328	0.187	GT	Sens	[illegible]
GT	Sense	Sense	-0.455	Comprom	0.455	Comprom	0.000	2.279	Detected	2.963	Detected	2.621	0.044	GT	Sens	[illegible]
GT	Sense	Sense	0.473	Detected	-0.473	Detected	0.000	1.126	Detected	1.013	Detected	1.070	0.154	GT	Sens	[illegible]
GT	Sense	Sense	-0.198	Detected	0.198	Detected	0.000	1.482	Detected	1.691	Detected	1.586	0.019	GT	Sens	[illegible]
GT	Sense	Sense	0.036	Detected	-0.036	Detected	0.000	1.142	Detected	0.968	Detected	1.055	0.008	GT	Sens	[illegible]
GT	Sense	Sense	-0.058	Detected	0.058	Detected	0.000	1.297	Detected	1.041	Detected	1.169	0.014	GT	Sens	[illegible]
GT	Sense	Sense	0.377	Detected	-0.377	Detected	0.000	1.167	Detected	1.478	Detected	1.323	0.083	GT	Sens	[illegible]
GT	Sense	Sense	0.521	Detected	-0.521	Detected	0.000	1.075	Detected	1.547	Detected	1.311	0.149	GT	Sens	[illegible]
GT	Sense	Sense	0.278	Detected	-0.278	Detected	0.000	1.189	Detected	0.861	Detected	1.025	0.087	GT	Sens	[illegible]
GT	Sense	Sense	-1.201	Comprom	1.201	Detected	0.000	1.770	Detected	1.414	Detected	1.592	0.320	GT	Sens	[illegible]
GT	Sense	Sense	0.269	Comprom	-0.269	Comprom	0.000	1.121	Detected	1.221	Detected	1.171	0.050	GT	Sens	[illegible]
GT	Sense	Sense	0.232	Detected	-0.232	Detected	0.000	1.097	Detected	1.176	Detected	1.137	0.040	GT	Sens	[illegible]
GT	Sense	Sense	-0.789	Comprom	0.789	Comprom	0.000	1.356	Detected	1.005	Detected	1.180	0.282	GT	Sens	[illegible]
GT	Sense	Sense	0.694	Detected	-0.694	Detected	0.000	1.343	Detected	1.574	Detected	1.458	0.174	GT	Sens	[illegible]
GT	Sense	Sense	1.427	Detected	-1.427	Comprom	0.000	1.436	Detected	1.840	Detected	1.638	0.374	GT	Sens	[illegible]
GT	Sense	Sense	2.499	Detected	-2.499	Detected	0.000	2.049	Detected	4.326	Detected	3.188	0.366	GT	Sens	[illegible]
GT	Sense	Sense	0.115	Detected	-0.115	Detected	0.000	1.481	Detected	1.646	Detected	1.563	0.008	GT	Sens	[illegible]
GT	Sense	Sense	1.541	Detected	-1.541	Detected	0.000	2.183	Detected	1.601	Detected	1.892	0.351	GT	Sens	[illegible]
GT	Sense	Sense	1.388	Detected	-1.388	Detected	0.000	1.718	Detected	2.610	Detected	2.164	0.276	GT	Sens	[illegible]
GT	Sense	Sense	0.260	Detected	-0.260	Detected	0.000	1.260	Detected	1.275	Detected	1.268	0.040	GT	Sens	[illegible]
GT	Sense	Sense	0.958	Detected	-0.958	Detected	0.000	1.362	Detected	1.775	Detected	1.569	0.250	GT	Sens	[illegible]
GT	Sense	Sense	0.342	Detected	-0.342	Detected	0.000	0.885	Detected	1.553	Detected	1.219	0.125	GT	Sens	[illegible]
GT	Sense	Sense	-1.387	Comprom	1.387	Detected	0.000	1.929	Detected	1.664	Detected	1.797	0.326	GT	Sens	[illegible]
GT	Sense	Sense	0.218	Comprom	-0.218	Comprom	0.000	2.147	Detected	2.120	Detected	2.134	0.010	GT	Sens	[illegible]
GT	Sense	Sense	0.292	Detected	-0.292	Detected	0.000	1.245	Detected	0.998	Detected	1.121	0.071	GT	Sens	[illegible]
GT	Sense	Sense	0.630	Detected	-0.630	Detected	0.000	0.916	Detected	1.411	Detected	1.164	0.228	GT	Sens	[illegible]
GT	Sense	Sense	1.366	Detected	-1.366	Comprom	0.000	1.300	Detected	1.718	Detected	1.509	0.389	GT	Sens	[illegible]
GT	Sense	Sense	-0.039	Detected	0.039	Detected	0.000	0.851	Detected	1.194	Detected	1.023	0.028	GT	Sens	[illegible]
GT	Sense	Sense	0.489	Detected	-0.489	Detected	0.000	1.087	Detected	1.740	Detected	1.413	0.138	GT	Sens	[illegible]
GT	Sense	Sense	0.375	Detected	-0.375	Detected	0.000	1.333	Detected	1.789	Detected	1.561	0.071	GT	Sens	[illegible]
GT	Sense	Sense	0.745	Detected	-0.745	Detected	0.000	1.225	Detected	1.318	Detected	1.271	0.230	GT	Sens	[illegible]
GT	Sense	Sense	0.579	Detected	-0.579	Detected	0.000	0.866	Detected	2.034	Detected	1.450	0.220	GT	Sens	[illegible]
GT	Sense	Sense	0.297	Detected	-0.297	Detected	0.000	0.973	Detected	1.077	Detected	1.025	0.077	GT	Sens	[illegible]
GT	Sense	Sense	-0.397	Comprom	0.397	Comprom	0.000	8.353	Detected	7.400	Detected	7.876	0.006	GT	Sens	[illegible]
GT	Sense	Sense	0.250	Detected	-0.250	Detected	0.000	1.040	Detected	1.156	Detected	1.098	0.051	GT	Sens	[illegible]
GT	Sense	Sense	0.168	Detected	-0.168	Detected	0.000	2.981	Detected	2.474	Detected	2.728	0.012	GT	Sens	[illegible]
GT	Sense	Sense	0.337	Detected	-0.337	Detected	0.000	1.266	Detected	0.925	Detected	1.096	0.101	GT	Sens	[illegible]
GT	Sense	Sense	0.602	Detected	-0.602	Detected	0.000	0.818	Detected	1.289	Detected	1.053	0.245	GT	Sens	[illegible]
GT	Sense	Sense	0.595	Detected	-0.595	Detected	0.000	1.243	Detected	1.561	Detected	1.402	0.151	GT	Sens	[illegible]
GT	Sense	Sense	0.080	Detected	-0.080	Detected	0.000	1.863	Detected	2.025	Detected	1.944	0.003	GT	Sens	[illegible]
GT	Sense	Sense	0.428	Detected	-0.428	Detected	0.000	1.319	Detected	1.440	Detected	1.380	0.086	GT	Sens	[illegible]
GT	Sense	Sense	0.564	Detected	-0.564	Detected	0.000	1.056	Detected	1.659	Detected	1.357	0.168	GT	Sens	[illegible]
GT	Sense	Sense	0.576	Detected	-0.576	Detected	0.000	1.188	Detected	1.902	Detected	1.545	0.150	GT	Sens	[illegible]
GT	Sense	Sense	0.588	Detected	-0.588	Detected	0.000	1.480	Detected	1.552	Detected	1.516	0.124	GT	Sens	[illegible]
GT	Sense	Sense	0.266	Detected	-0.266	Detected	0.000	1.538	Detected	0.955	Detected	1.246	0.087	GT	Sens	[illegible]
GT	Sense	Sense	0.306	Detected	-0.306	Detected	0.000	1.427	Detected	1.659	Detected	1.543	0.042	GT	Sens	[illegible]
GT	Sense	Sense	0.607	Detected	-0.607	Detected	0.000	1.728	Detected	1.490	Detected	1.609	0.122	GT	Sens	[illegible]
GT	Sense	Sense	0.267	Detected	-0.267	Detected	0.000	1.566	Detected	1.382	Detected	1.474	0.035	GT	Sens	[illegible]
GT	Sense	Sense	-0.460	Detected	0.460	Detected	0.000	2.089	Detected	1.720	Detected	1.905	0.061	GT	Sens	[illegible]
GT	Sense	Sense	-0.559	Comprom	0.559	Detected	0.000	1.039	Detected	1.259	Detected	1.149	0.181	GT	Sens	[illegible]
GT	Sense	Sense	-0.163	Detected	0.163	Detected	0.000	0.972	Detected	1.759	Detected	1.365	0.085	GT	Sens	[illegible]
GT	Sense	Sense	0.801	Detected	-0.801	Detected	0.000	1.392	Detected	1.229	Detected	1.310	0.245	GT	Sens	[illegible]
GT	Sense	Sense	0.890	Comprom	-0.890	Comprom	0.000	1.536	Detected	1.783	Detected	1.659	0.206	GT	Sens	[illegible]
GT	Sense	Sense	0.429	Detected	-0.429	Detected	0.000	1.048	Detected	1.000	Detected	1.024	0.140	GT	Sens	[illegible]
GT	Sense	Sense	0.132	Detected	-0.132	Detected	0.000	3.461	Detected	3.014	Detected	3.238	0.006	GT	Sens	[illegible]
GT	Sense	Sense	0.292	Detected	-0.292	Detected	0.000	1.460	Detected	0.846	Detected	1.153	0.113	GT	Sens	[illegible]
GT	Sense	Sense	0.627	Detected	-0.627	Detected	0.000	1.068	Detected	1.996	Detected	1.532	0.189	GT	Sens	[illegible]
GT	Sense	Sense	0.463	Detected	-0.463	Detected	0.000	1.625	Detected	1.721	Detected	1.673	0.069	GT	Sens	[illegible]
GT	Sense	Sense	0.082	Detected	-0.082	Detected	0.000	1.089	Detected	1.537	Detected	1.313	0.031	GT	Sens	[illegible]
GT	Sense	Sense	0.179	Detected	-0.179	Detected	0.000	1.060	Detected	1.223	Detected	1.141	0.028	GT	Sens	[illegible]
GT	Sense	Sense	0.161	Detected	-0.161	Detected	0.000	1.458	Detected	1.638	Detected	1.548	0.014	GT	Sens	[illegible]
GT	Sense	Sense	0.656	Detected	-0.656	Comprom	0.000	1.666	Detected	1.348	Detected	1.507	0.155	GT	Sens	[illegible]
GT	Sense	Sense	0.098	Comprom	-0.098	Comprom	0.000	2.487	Detected	2.059	Detected	2.273	0.011	GT	Sens	[illegible]
GT	Sense	Sense	-0.058	Detected	0.058	Detected	0.000	0.869	Detected	1.646	Detected	1.257	0.085	GT	Sens	[illegible]
GT	Sense	Sense	-0.295	Detected	0.295	Detected	0.000	1.013	Detected	1.062	Detected	1.037	0.073	GT	Sens	[illegible]
GT	Sense	Sense	0.332	Detected	-0.332	Comprom	0.000	1.094	Detected	1.346	Detected	1.220	0.075	GT	Sens	[illegible]
GT	Sense	Sense	-0.359	Detected	0.359	Detected	0.000	1.912	Detected	1.853	Detected	1.882	0.035	GT	Sens	[illegible]
GT	Sense	Sense	-0.607	Comprom	0.607	Comprom	0.000	2.115	Detected	1.731	Detected	1.923	0.094	GT	Sens	[illegible]
GT	Sense	Sense	2.382	Detected	-2.382	Detected	0.000	3.032	Detected	4.642	Detected	3.837	0.267	GT	Sens	[illegible]
GT	Sense	Sense	0.900	Detected	-0.900	Detected	0.000	0.897	Detected	1.748	Detected	1.322	0.316	GT	Sens	[illegible]
GT	Sense	Sense	0.238	Detected	-0.238	Detected	0.000	0.971	Detected	1.376	Detected	1.173	0.064	GT	Sens	[illegible]
GT	Sense	Sense	-0.031	Detected	0.031	Detected	0.000	1.222	Detected	1.179	Detected	1.200	0.001	GT	Sens	[illegible]
GT	Sense	Sense	-0.086	Detected	0.086	Detected	0.000	1.165	Detected	1.221	Detected	1.193	0.006	GT	Sens	[illegible]
GT	Sense	Sense	0.132	Detected	-0.132	Detected	0.000	1.023	Detected	1.464	Detected	1.243	0.040	GT	Sens	[illegible]
GT	Sense	Sense	0.268	Detected	-0.268	Detected	0.000	0.965	Detected	1.202	Detected	1.083	0.066	GT	Sens	[illegible]
GT	Sense	Sense	1.025	Detected	-1.025	Detected	0.000	1.447	Detected	2.506	Detected	1.976	0.229	GT	Sens	[illegible]
GT	Sense	Sense	0.797	Detected	-0.797	Detected	0.000	1.087	Detected	0.944	Detected	1.015	0.332	GT	Sens	[illegible]
GT	Sense	Sense	0.071	Detected	-0.071	Detected	0.000	1.963	Detected	1.098	Detected	1.531	0.073	GT	Sens	[illegible]
GT	Sense	Sense	0.588	Detected	-0.588	Detected	0.000	0.810	Detected	1.265	Detected	1.038	0.242	GT	Sens	[illegible]
GT	Sense	Sense	0.358	Detected	-0.358	Detected	0.000	1.067	Detected	1.656	Detected	1.361	0.099	GT	Sens	[illegible]
GT	Sense	Sense	0.080	Detected	-0.080	Detected	0.000	1.106	Detected	1.194	Detected	1.150	0.006	GT	Sens	[illegible]
GT	Sense	Sense	-0.643	Detected	0.643	Detected	0.000	1.644	Detected	1.327	Detected	1.486	0.154	GT	Sens	[illegible]
GT	Sense	Sense	0.026	Detected	-0.026	Detected	0.000	1.929	Detected	2.893	Detected	2.411	0.038	GT	Sens	[illegible]
GT	Sense	Sense	0.923	Comprom	-0.923	Comprom	0.000	2.290	Detected	1.943	Detected	2.116	0.153	GT	Sens	[illegible]
GT	Sense	Sense	-0.355	Comprom	0.355	Comprom	0.000	2.213	Detected	2.744	Detected	2.478	0.031	GT	Sens	[illegible]
GT	Sense	Sense	0.667	Detected	-0.667	Detected	0.000	1.242	Detected	1.087	Detected	1.165	0.225	GT	Sens	[illegible]
GT	Sense	Sense	-0.558	Detected	0.558	Detected	0.000	1.195	Detected	1.235	Detected	1.215	0.161	GT	Sens	[illegible]
GT	Sense	Sense	0.501	Detected	-0.501	Detected	0.000	1.131	Detected	1.916	Detected	1.524	0.139	GT	Sens	[illegible]
GT	Sense	Sense	0.586	Detected	-0.586	Detected	0.000	1.416	Detected	1.502	Detected	1.459	0.131	GT	Sens	[illegible]
GT	Sense	Sense	0.370	Detected	-0.370	Detected	0.000	0.822	Detected	1.412	Detected	1.117	0.142	GT	Sens	[illegible]
GT	Sense	Sense	0.268	Comprom	-0.268	Comprom	0.000	1.328	Detected	1.635	Detected	1.482	0.041	GT	Sens	[illegible]
GT	Sense	Sense	0.503	Detected	-0.503	Detected	0.000	1.122	Detected	0.999	Detected	1.061	0.171	GT	Sens	[illegible]
GT	Sense	Sense	0.708	Detected	-0.708	Comprom	0.000	1.230	Detected	2.418	Detected	1.824	0.187	GT	Sens	[illegible]
GT	Sense	Sense	0.555	Detected	-0.555	Detected	0.000	1.027	Detected	1.348	Detected	1.188	0.176	GT	Sens	[illegible]
GT	Sense	Sense	0.002	Detected	-0.002	Detected	0.000	1.611	Detected	1.676	Detected	1.644	0.000	GT	Sens	[illegible]
GT	Sense	Sense	0.754	Detected	-0.754	Detected	0.000	0.947	Detected	2.169	Detected	1.558	0.250	GT	Sens	[illegible]
GT	Sense	Sense	0.301	Comprom	-0.301	Comprom	0.000	1.814	Detected	1.212	Detected	1.513	0.071	GT	Sens	[illegible]
GT	Sense	Sense	0.613	Detected	-0.613	Detected	0.000	1.471	Detected	1.060	Detected	1.266	0.189	GT	Sens	[illegible]
GT	Sense	Sense	0.599	Detected	-0.599	Detected	0.000	1.102	Detected	1.344	Detected	1.223	0.183	GT	Sens	[illegible]
GT	Sense	Sense	0.992	Detected	-0.992	Comprom	0.000	1.020	Detected	1.017	Detected	1.019	0.412	GT	Sens	[illegible]
GT	Sense	Sense	0.228	Detected	-0.228	Detected	0.000	1.361	Detected	1.678	Detected	1.519	0.032	GT	Sens	[illegible]
GT	Sense	Sense	0.384	Detected	-0.384	Detected	0.000	2.431	Detected	2.859	Detected	2.645	0.027	GT	Sens	[illegible]
GT	Sense	Sense	0.363	Detected	-0.363	Detected	0.000	1.042	Detected	1.375	Detected	1.208	0.094	GT	Sens	[illegible]
GT	Sense	Sense	0.036	Detected	-0.036	Detected	0.000	0.844	Detected	1.357	Detected	1.101	0.051	GT	Sens	[illegible]
GT	Sense	Sense	0.171	Detected	-0.171	Detected	0.000	1.271	Detected	1.349	Detected	1.310	0.018	GT	Sens	[illegible]
GT	Sense	Sense	-0.279	Detected	0.279	Detected	0.000	1.150	Detected	1.226	Detected	1.188	0.052	GT	Sens	[illegible]
GT	Sense	Sense	-0.015	Detected	0.015	Detected	0.000	1.362	Detected	1.208	Detected	1.285	0.004	GT	Sens	[illegible]
GT	Sense	Sense	0.236	Detected	-0.236	Detected	0.000	1.181	Detected	1.128	Detected	1.155	0.040	GT	Sens	[illegible]
GT	Sense	Sense	0.544	Detected	-0.544	Comprom	0.000	1.264	Detected	0.904	Detected	1.084	0.199	GT	Sens	[illegible]
GT	Sense	Sense	0.235	Comprom	-0.235	Comprom	0.000	1.124	Detected	2.408	Detected	1.766	0.123	GT	Sens	[illegible]
GT	Sense	Sense	-0.339	Comprom	0.339	Detected	0.000	0.865	Detected	1.172	Detected	1.019	0.112	GT	Sens	[illegible]
GT	Sense	Sense	0.072	Detected	-0.072	Detected	0.000	1.933	Detected	1.125	Detected	1.529	0.065	GT	Sens	[illegible]
GT	Sense	Sense	0.200	Detected	-0.200	Detected	0.000	1.357	Detected	1.172	Detected	1.265	0.029	GT	Sens	[illegible]
GT	Sense	Sense	0.193	Detected	-0.193	Detected	0.000	0.860	Detected	1.250	Detected	1.055	0.061	GT	Sens	[illegible]
GT	Sense	Sense	0.135	Detected	-0.135	Detected	0.000	1.266	Detected	1.226	Detected	1.246	0.012	GT	Sens	[illegible]
GT	Sense	Sense	0.270	Detected	-0.270	Detected	0.000	0.958	Detected	1.308	Detected	1.133	0.072	GT	Sens	[illegible]
GT	Sense	Sense	-0.977	Comprom	0.977	Detected	0.000	1.187	Detected	1.226	Detected	1.207	0.342	GT	Sens	[illegible]
GT	Sense	Sense	0.115	Detected	-0.115	Detected	0.000	1.063	Detected	1.504	Detected	1.284	0.036	GT	Sens	[illegible]
GT	Sense	Sense	0.295	Detected	-0.295	Detected	0.000	1.515	Detected	1.408	Detected	1.462	0.040	GT	Sens	[illegible]
GT	Sense	Sense	0.451	Detected	-0.451	Detected	0.000	1.142	Detected	1.323	Detected	1.233	0.116	GT	Sens	[illegible]
GT	Sense	Sense	0.186	Detected	-0.186	Detected	0.000	0.960	Detected	1.198	Detected	1.079	0.039	GT	Sens	[illegible]
GT	Sense	Sense	1.559	Detected	-1.559	Comprom	0.000	2.121	Detected	2.376	Detected	2.248	0.287	GT	Sens	[illegible]
GT	Sense	Sense	0.413	Detected	-0.413	Detected	0.000	1.619	Detected	1.964	Detected	1.792	0.057	GT	Sens	[illegible]
GT	Sense	Sense	0.649	Detected	-0.649	Detected	0.000	1.401	Detected	0.919	Detected	1.160	0.236	GT	Sens	[illegible]
GT	Sense	Sense	-0.074	Detected	0.074	Detected	0.000	1.288	Detected	1.212	Detected	1.250	0.004	GT	Sens	[illegible]
GT	Sense	Sense	0.089	Detected	-0.089	Detected	0.000	1.310	Detected	1.517	Detected	1.413	0.009	GT	Sens	[illegible]
GT	Sense	Sense	-0.411	Comprom	0.411	Detected	0.000	1.187	Detected	1.067	Detected	1.127	0.113	GT	Sens	[illegible]
GT	Sense	Sense	0.063	Detected	-0.063	Detected	0.000	1.033	Detected	1.290	Detected	1.161	0.015	GT	Sens	[illegible]
GT	Sense	Sense	0.909	Detected	-0.909	Detected	0.000	1.636	Detected	1.491	Detected	1.563	0.229	GT	Sens	[illegible]
GT	Sense	Sense	-0.838	Comprom	0.838	Detected	0.000	1.571	Detected	1.286	Detected	1.429	0.235	GT	Sens	[illegible]
GT	Sense	Sense	0.222	Comprom	-0.222	Comprom	0.000	3.657	Detected	5.464	Detected	4.560	0.039	GT	Sens	[illegible]
GT	Sense	Sense	1.275	Detected	-1.275	Comprom	0.000	0.888	Detected	2.301	Detected	1.595	0.388	GT	Sens	[illegible]
GT	Sense	Sense	0.350	Detected	-0.350	Detected	0.000	1.143	Detected	1.470	Detected	1.306	0.077	GT	Sens	[illegible]
GT	Sense	Sense	0.358	Detected	-0.358	Detected	0.000	0.885	Detected	1.502	Detected	1.193	0.128	GT	Sens	[illegible]
GT	Sense	Sense	0.850	Detected	-0.850	Detected	0.000	2.173	Detected	2.933	Detected	2.553	0.111	GT	Sens	[illegible]
GT	Sense	Sense	0.616	Detected	-0.616	Detected	0.000	2.045	Detected	2.765	Detected	2.405	0.078	GT	Sens	[illegible]
GT	Sense	Sense	-0.016	Comprom	0.016	Comprom	0.000	1.015	Detected	1.104	Detected	1.060	0.002	GT	Sens	[illegible]
GT	Sense	Sense	1.260	Detected	-1.260	Comprom	0.000	2.246	Detected	1.934	Detected	2.090	0.242	GT	Sens	[illegible]
GT	Sense	Sense	-1.163	Comprom	1.163	Detected	0.000	1.015	Detected	1.341	Detected	1.178	0.422	GT	Sens	[illegible]
GT	Sense	Sense	-0.040	Comprom	0.040	Comprom	0.000	3.602	Detected	2.893	Detected	3.248	0.012	GT	Sens	[illegible]
GT	Sense	Sense	-0.056	Comprom	0.056	Comprom	0.000	4.045	Detected	4.091	Detected	4.068	0.000	GT	Sens	[illegible]
GT	Sense	Sense	0.163	Detected	-0.163	Detected	0.000	0.936	Detected	1.381	Detected	1.159	0.052	GT	Sens	[illegible]
GT	Sense	Sense	0.094	Detected	-0.094	Detected	0.000	1.284	Detected	1.291	Detected	1.287	0.005	GT	Sens	[illegible]
GT	Sense	Sense	0.836	Detected	-0.836	Detected	0.000	1.677	Detected	2.094	Detected	1.886	0.160	GT	Sens	[illegible]
GT	Sense	Sense	0.201	Detected	-0.201	Detected	0.000	0.834	Detected	1.539	Detected	1.187	0.100	GT	Sens	[illegible]
GT	Sense	Sense	0.549	Detected	-0.549	Detected	0.000	0.942	Detected	1.874	Detected	1.408	0.190	GT	Sens	[illegible]
GT	Sense	Sense	0.205	Detected	-0.205	Detected	0.000	1.121	Detected	1.351	Detected	1.236	0.034	GT	Sens	[illegible]
GT	Sense	Sense	0.290	Detected	-0.290	Detected	0.000	1.022	Detected	1.120	Detected	1.071	0.068	GT	Sens	[illegible]
GT	Sense	Sense	1.034	Detected	-1.034	Detected	0.000	1.371	Detected	2.499	Detected	1.935	0.242	GT	Sens	[illegible]
GT	Sense	Sense	0.270	Detected	-0.270	Detected	0.000	0.853	Detected	1.544	Detected	1.199	0.112	GT	Sens	[illegible]
GT	Sense	Sense	0.922	Detected	-0.922	Comprom	0.000	0.864	Detected	1.299	Detected	1.082	0.372	GT	Sens	[illegible]
GT	Sense	Sense	0.203	Detected	-0.203	Detected	0.000	1.237	Detected	0.890	Detected	1.063	0.058	GT	Sens	[illegible]
GT	Sense	Sense	0.670	Detected	-0.670	Detected	0.000	1.078	Detected	1.505	Detected	1.291	0.208	GT	Sens	[illegible]
GT	Sense	Sense	0.722	Detected	-0.722	Detected	0.000	1.173	Detected	1.147	Detected	1.160	0.249	GT	Sens	[illegible]
GT	Sense	Sense	0.043	Detected	-0.043	Detected	0.000	1.251	Detected	1.253	Detected	1.252	0.001	GT	Sens	[illegible]
GT	Sense	Sense	0.237	Detected	-0.237	Detected	0.000	1.042	Detected	1.400	Detected	1.221	0.054	GT	Sens	[illegible]
GT	Sense	Sense	1.033	Comprom	-1.033	Comprom	0.000	1.609	Detected	1.825	Detected	1.717	0.240	GT	Sens	[illegible]
GT	Sense	Sense	0.336	Detected	-0.336	Detected	0.000	1.241	Detected	0.876	Detected	1.058	0.110	GT	Sens	[illegible]
GT	Sense	Sense	-0.092	Detected	0.092	Detected	0.000	1.235	Detected	1.407	Detected	1.321	0.009	GT	Sens	[illegible]
GT	Sense	Sense	0.340	Detected	-0.340	Detected	0.000	1.063	Detected	1.467	Detected	1.265	0.085	GT	Sens	[illegible]
GT	Sense	Sense	0.564	Detected	-0.564	Detected	0.000	0.823	Detected	1.184	Detected	1.003	0.232	GT	Sens	[illegible]
GT	Sense	Sense	0.439	Detected	-0.439	Detected	0.000	1.736	Detected	1.881	Detected	1.809	0.056	GT	Sens	[illegible]
GT	Sense	Sense	0.501	Comprom	-0.501	Comprom	0.000	1.679	Detected	3.281	Detected	2.480	0.120	GT	Sens	[illegible]
GT	Sense	Sense	0.301	Detected	-0.301	Detected	0.000	1.177	Detected	1.214	Detected	1.195	0.058	GT	Sens	[illegible]
GT	Sense	Sense	1.670	Detected	-1.670	Comprom	0.000	3.113	Detected	2.139	Detected	2.626	0.270	GT	Sens	[illegible]
GT	Sense	Sense	0.387	Detected	-0.387	Detected	0.000	1.063	Detected	1.194	Detected	1.128	0.103	GT	Sens	[illegible]
GT	Sense	Sense	0.484	Detected	-0.484	Detected	0.000	1.009	Detected	1.448	Detected	1.229	0.147	GT	Sens	[illegible]
GT	Sense	Sense	0.669	Detected	-0.669	Detected	0.000	1.021	Detected	0.994	Detected	1.008	0.271	GT	Sens	[illegible]
GT	Sense	Sense	1.437	Detected	-1.437	Detected	0.000	2.962	Detected	1.928	Detected	2.445	0.250	GT	Sens	[illegible]
GT	Sense	Sense	0.172	Detected	-0.172	Detected	0.000	0.992	Detected	1.441	Detected	1.216	0.050	GT	Sens	[illegible]
GT	Sense	Sense	1.018	Detected	-1.018	Comprom	0.000	1.822	Detected	1.475	Detected	1.648	0.252	GT	Sens	[illegible]
GT	Sense	Sense	0.330	Detected	-0.330	Detected	0.000	0.872	Detected	1.521	Detected	1.196	0.123	GT	Sens	[illegible]
GT	Sense	Sense	0.715	Detected	-0.715	Detected	0.000	2.225	Detected	2.830	Detected	2.527	0.083	GT	Sens	[illegible]
GT	Sense	Sense	0.434	Detected	-0.434	Detected	0.000	1.168	Detected	1.103	Detected	1.135	0.121	GT	Sens	[illegible]
GT	Sense	Sense	0.513	Detected	-0.513	Comprom	0.000	1.399	Detected	1.007	Detected	1.203	0.160	GT	Sens	[illegible]
GT	Sense	Sense	0.339	Detected	-0.339	Detected	0.000	0.924	Detected	1.108	Detected	1.016	0.101	GT	Sens	[illegible]
GT	Sense	Sense	-0.085	Comprom	0.085	Comprom	0.000	4.212	Detected	4.525	Detected	4.368	0.002	GT	Sens	[illegible]
GT	Sense	Sense	0.726	Detected	-0.726	Detected	0.000	1.001	Detected	1.042	Detected	1.022	0.295	GT	Sens	[illegible]
GT	Sense	Sense	-0.352	Detected	0.352	Detected	0.000	1.109	Detected	1.201	Detected	1.155	0.083	GT	Sens	[illegible]
GT	Sense	Sense	0.369	Detected	-0.369	Detected	0.000	1.073	Detected	1.075	Detected	1.074	0.101	GT	Sens	[illegible]
GT	Sense	Sense	0.603	Detected	-0.603	Detected	0.000	1.225	Detected	1.560	Detected	1.393	0.156	GT	Sens	[illegible]
GT	Sense	Sense	0.891	Detected	-0.891	Detected	0.000	1.015	Detected	1.006	Detected	1.011	0.374	GT	Sens	[illegible]

		12h_AZ_Control					12h_AZ_TAPG + TPRP vs. 12h_AZ_Control																															
ProbeNar	Orientatio	Fold 12h	Flag 12h	Fold 12h	Flag 12h	Geomean	Fold 12h	Flag 12h	Fold 12h	Flag 12h	Geomean	P-value	ProbeNar	GeneNam	Systemat	Descripti	Sequence	Contig ID	ITAG ID	Parent Ge	ITAG Des	Ontology	Ontology	Contig ID	Target ID	Functiona	GO terms	Transcrip	target ID	Symbols	TAIR Des	TAIR Chr	gProcess	gProcess	gProcess	gProcess	gProcess	gProcessedSignal 12h 44 AZ
A 96 P01	Sense	-0.556	Detected	0.556	Detected	0.000	-2.687	Detected	-1.778	Detected	-2.232	0.090	A 96 P01	LOC5439	NM 0012	Solanum	TTGTTGGTCCAGCAATGGAAAATATTTACAACGCGTTATCATCAGCAGGGTTGCAAAATC																103.308	203.701	140.132	163.853	21.5112	42.5567
A 96 P01	Sense	-0.297	Detected	0.297	Detected	0.000	-0.909	Detected	-1.714	Detected	-1.312	0.120	A 96 P01	XTH7	NM 0012	Solanum	ACCTTTGGTTCGATCCATCCGCTGATTTTCACACATACACCATTTTTTGGAACCATCATC																252.961	348.118	115.949	579.404	150.906	90.9803
A 96 P01	Sense	-0.081	Detected	0.081	Detected	0.000	-1.049	Detected	-1.106	Detected	-1.077	0.006	A 96 P01	PR1b1	NM 0012	Solanum	CAACTGGATCGGACAACGTCCTTACTAAAATGATGTATACTTATGACATGTTGCTAGTAT																136770	139438	148606	129326	63730.8	64550.9
A 96 P01	Sense	-0.118	Detected	0.118	Detected	0.000	-2.770	Detected	-3.610	Detected	-3.190	0.018	A 96 P01	cevi19	NM 0012	Solanum	GAGATTATGGTGTGTTAAATAAAGTGTTCCACAATGTTACGGATACACATGTTTTGCATC																608.975	654.114	559.27	29.6032	88.345	51.9942
A 96 P01	Sense	-0.549	Detected	0.549	Detected	0.000	-1.936	Detected	-1.649	Detected	-1.792	0.087	A 96 P01	AK31939	AK31939	Solanum	CCTTCTAAACATGAATGAATGAATACTAGTGTGCAATGTGTGTGTGATTGGAAGTTGTTT																3949.54	7702.63	2628.01	4620.54	1376.59	1768.88
A 96 P02	Sense	-0.114	Detected	0.114	Detected	0.000	-1.435	Detected	-1.645	Detected	-1.540	0.010	A 96 P02	AK32545	AK32545	Solanum	CACCATGTGAAAAAACCTTCTTTTCCAACACATGAAGTAGTTGAAATCAAAATCCCAACT																179.75	191.817	59.7818	114.572	65.5798	59.7103
A 96 P03	Sense	-0.027	Detected	0.027	Detected	0.000	-1.578	Comprom	-1.702	Comprom	-1.640	0.002	A 96 P03	TA49945	TA49945	Rep: Cyto	TAAGGAAGCGTCACGCTTTTTGAAAGGATTTGATGTAGCTGATATATTTCCATCACTAAA																27.3638	25.8981	5.73155	18.7081	8.51088	8.22868
A 96 P03	Sense	-0.341	Detected	0.341	Detected	0.000	-1.009	Detected	-2.908	Detected	-1.958	0.192	A 96 P03	BI931270	BI931270	EST5511	TCAGGGATGCATTTTCAGGATTTGATGCAGAAGTTGTTTGGCAAATACATGAAAAGAAGA																509.149	744.851	192.093	236.56	292.244	82.5251
A 96 P04	Sense	-0.196	Detected	0.196	Detected	0.000	-1.921	Detected	-2.168	Detected	-2.045	0.013	A 96 P04	AK32045	AK32045	Solanum	TGAAGCAGATGAAATTGATATTGTGATTCATAATCCAGCTGTCGAGAAGGACCCGGCTTG																278.899	333.659	317.383	293.013	76.9254	68.2736
A 96 P05	Sense	-0.107	Detected	0.107	Detected	0.000	-1.040	Detected	-0.985	Detected	-1.012	0.012	A 96 P05	BI922989	BI922989	EST5428	GAAGGAATATTCCGGGTTCAAATGTCAAAGCGGATTAGTAAAAGCCATTGTTGTATACGG																685.512	725.158	475.147	656.974	327.489	358.319
A 96 P06	Sense	-0.246	Detected	0.246	Detected	0.000	-0.957	Detected	-1.467	Comprom	-1.212	0.076	A 96 P06	DB68507	DB68507	DB68507	TTGCGGTTTTTTATATTCTACTAGCAATGTGTTTTGGTGTTGGGATGGATGATGAAGAAA																19.6472	25.1752	5.59147	32.9002	10.9408	8.09418
A 96 P07	Sense	-1.031	Detected	1.031	Detected	0.000	-3.149	Detected	-4.200	Detected	-3.674	0.086	A 96 P07	TA37448	TA37448	Rep: Non	TCGATATTGATCCTGCTTAAATCACGGATTGTGTCTGTTTCTCTCTTTAGCTTCTTTGTG																177.238	674.447	335.981	392.886	37.2244	18.9215
A 96 P08	Sense	-0.360	Detected	0.360	Detected	0.000	-1.357	Detected	-1.667	Detected	-1.512	0.061	A 96 P08	AW62139	AW62139	Unknown	ACACGAGAAATTTGAGCTTGAAGAACAAAAAATGAAACCAGTGCTCATTTCTGTGAAAGC																1201.63	1803.92	968.695	996.355	548.849	466.559
A 96 P08	Sense	0.091	Detected	-0.091	Detected	0.000	-1.508	Detected	-1.502	Detected	-1.505	0.004	A 96 P08	BW68867	BW68867	BW68867	ACTGGGCCGGAAAACGTCCTTATTAAGGTGTCGTTTATTTATGACATATTGCTAGTATTA																4419.79	3551.2	7768.13	4067.43	1329.94	1407.52
A 96 P08	Sense	-0.027	Detected	0.027	Detected	0.000	-4.300	Comprom	-2.222	Detected	-3.261	0.088	A 96 P08	AK24744	AK24744	Solanum	CTACCACCTACATGATATCAAGACTCGAAAGATCAAGTTTGATGATCTAGACTTATCAAA																62.2118	58.8646	75.1715	108.813	2.93412	13.0509
A 96 P08	Sense	0.308	Detected	-0.308	Detected	0.000	-1.641	Detected	-3.788	Detected	-2.714	0.136	A 96 P08	TA36002	TA36002	Rep: Path	CCACGTACCATATGTTCATGGTATCGTGGCTTAGGTTCTTTACTTTTTCAAAATTATGAA																4231.47	2516.61	5917.23	1849.67	999.249	237.638
A 96 P08	Sense	-0.094	Detected	0.094	Detected	0.000	-1.227	Detected	-1.177	Detected	-1.202	0.006	A 96 P08	PR1b1	NM 0012	Solanum	AACTCAAGAGCTGGTGATTGTAACTTGATTCATTCTGGTGCTGGGGAGAATCTTGCCAAG																97144.1	100840	139445	96569.3	40363.9	44033.3
A 96 P10	Sense	-0.396	Detected	0.396	Detected	0.000	-1.334	Detected	-1.065	Detected	-1.199	0.103	A 96 P10	TA46353	TA46353	Rep: Arab	GAGAATCACAACAACAACAATGATGCTTTCTCGGAGAATCACAACCCACAACATGATGGG																195.491	308.538	79.813	167.441	93.0471	118.1
[illegible]	[illegible]	[illegible]	[illegible]	[illegible]	[illegible]	[illegible]	[illegible]	[illegible]	[illegible]	[illegible]	[illegible]	[illegible]	[illegible]	[illegible]	[illegible]	[illegible]	[illegible]	[illegible]	[illegible]	[illegible]	[illegible]	[illegible]	[illegible]	[illegible]	[illegible]	[illegible]	[illegible]	[illegible]	[illegible]	[illegible]	[illegible]	[illegible]	[illegible]	[illegible]	[illegible]	[illegible]	[illegible]	[illegible]

GT	Sense	Sense	-0.050	Detected	0.050	Detected	0.000	-2.941	Comprom	-3.004	Comprom	-2.972	0.000	GT	Sens	contig168	contig168
GT	Sense	Sense	-0.150	Detected	0.150	Detected	0.000	-1.449	Detected	-3.808	Comprom	-2.629	0.158	GT	Sens	contig168	contig168
GT	Sense	Sense	-0.276	Detected	0.276	Detected	0.000	-0.806	Detected	-1.290	Detected	-1.048	0.104	GT	Sens	contig168	contig168
GT	Sense	Sense	-0.221	Detected	0.221	Detected	0.000	-2.724	Detected	-2.257	Detected	-2.491	0.016	GT	Sens	contig169	contig169
GT	Sense	Sense	-0.204	Detected	0.204	Detected	0.000	-1.092	Detected	-1.452	Detected	-1.272	0.043	GT	Sens	contig169	contig169
GT	Sense	Sense	-0.057	Detected	0.057	Detected	0.000	-0.950	Detected	-2.512	Detected	-1.731	0.158	GT	Sens	contig170	contig170
GT	Sense	Sense	-0.668	Detected	0.668	Detected	0.000	-1.548	Detected	-1.340	Detected	-1.444	0.166	GT	Sens	contig170	contig170
GT	Sense	Sense	-0.660	Detected	0.660	Detected	0.000	-1.316	Detected	-2.667	Detected	-1.991	0.170	GT	Sens	contig171	contig171
GT	Sense	Sense	0.097	Detected	-0.097	Detected	0.000	-1.868	Detected	-2.059	Detected	-1.964	0.005	GT	Sens	contig171	contig171
GT	Sense	Sense	-0.304	Detected	0.304	Detected	0.000	-1.437	Detected	-4.419	Comprom	-2.928	0.194	GT	Sens	contig172	contig172
GT	Sense	Sense	-0.043	Detected	0.043	Detected	0.000	-0.946	Detected	-1.286	Detected	-1.116	0.024	GT	Sens	contig172	contig172
GT	Sense	Sense	-1.075	Detected	1.075	Detected	0.000	-2.108	Detected	-4.720	Detected	-3.414	0.181	GT	Sens	contig172	contig172
GT	Sense	Sense	-0.023	Detected	0.023	Detected	0.000	-1.254	Detected	-2.279	Detected	-1.766	0.075	GT	Sens	contig173	contig173
GT	Sense	Sense	-0.446	Detected	0.446	Detected	0.000	-1.274	Detected	-1.747	Detected	-1.511	0.096	GT	Sens	contig173	contig173
GT	Sense	Sense	0.167	Detected	-0.167	Detected	0.000	-1.707	Detected	-0.961	Detected	-1.334	0.082	GT	Sens	contig174	contig174
GT	Sense	Sense	-0.267	Detected	0.267	Detected	0.000	-3.544	Detected	-2.479	Detected	-3.012	0.037	GT	Sens	contig174	contig174
GT	Sense	Sense	-0.459	Detected	0.459	Detected	0.000	-1.322	Detected	-2.667	Detected	-1.994	0.134	GT	Sens	contig174	contig174
GT	Sense	Sense	-0.098	Detected	0.098	Detected	0.000	-2.196	Detected	-0.875	Detected	-1.536	0.148	GT	Sens	contig175	contig175
GT	Sense	Sense	-0.271	Detected	0.271	Detected	0.000	-1.189	Detected	-1.553	Detected	-1.371	0.052	GT	Sens	contig175	contig175
GT	Sense	Sense	-0.270	Detected	0.270	Detected	0.000	-1.132	Detected	-3.631	Detected	-2.382	0.203	GT	Sens	contig176	contig176
GT	Sense	Sense	0.133	Detected	-0.133	Detected	0.000	-3.655	Comprom	-2.072	Comprom	-2.863	0.070	GT	Sens	contig177	contig177
GT	Sense	Sense	-0.986	Detected	0.986	Detected	0.000	-2.007	Detected	-2.458	Detected	-2.233	0.158	GT	Sens	contig178	contig178
GT	Sense	Sense	-0.355	Detected	0.355	Detected	0.000	-0.961	Detected	-1.681	Detected	-1.321	0.121	GT	Sens	contig178	contig178
GT	Sense	Sense	-0.415	Detected	0.415	Detected	0.000	-1.146	Detected	-1.022	Detected	-1.084	0.123	GT	Sens	contig178	contig178
GT	Sense	Sense	-0.294	Detected	0.294	Detected	0.000	-1.389	Detected	-1.037	Detected	-1.213	0.071	GT	Sens	contig178	contig178
GT	Sense	Sense	0.842	Detected	-0.842	Detected	0.000	-1.453	Detected	-1.423	Detected	-1.438	0.230	GT	Sens	contig178	contig178
GT	Sense	Sense	-0.215	Detected	0.215	Detected	0.000	-0.819	Detected	-1.354	Detected	-1.086	0.087	GT	Sens	contig178	contig178

GT	Sense	Sense	-0.160	Detected	0.160	Detected	0.000	-0.943	Detected	-1.183	Detected	-1.063	0.034	GT Sens	[illegible]
GT	Sense	Sense	-0.014	Detected	0.014	Detected	0.000	-3.126	Detected	-2.829	Detected	-2.978	0.003	GT Sens	[illegible]
GT	Sense	Sense	0.753	Detected	-0.753	Detected	0.000	-1.060	Detected	-1.175	Detected	-1.118	0.277	GT Sens	[illegible]
GT	Sense	Sense	-0.134	Detected	0.134	Detected	0.000	-1.147	Detected	-0.967	Detected	-1.057	0.023	GT Sens	[illegible]
GT	Sense	Sense	-0.155	Detected	0.155	Detected	0.000	-3.540	Comprom	-1.747	Detected	-2.643	0.101	GT Sens	[illegible]
GT	Sense	Sense	-0.292	Detected	0.292	Detected	0.000	-1.357	Detected	-0.966	Detected	-1.161	0.081	GT Sens	[illegible]
GT	Sense	Sense	-0.082	Detected	0.082	Detected	0.000	-1.208	Detected	-1.597	Detected	-1.402	0.022	GT Sens	[illegible]
GT	Sense	Sense	-0.086	Detected	0.086	Detected	0.000	-1.171	Comprom	-1.560	Comprom	-1.365	0.023	GT Sens	[illegible]
GT	Sense	Sense	-0.048	Detected	0.048	Detected	0.000	-1.098	Detected	-1.057	Detected	-1.077	0.002	GT Sens	[illegible]
GT	Sense	Sense	-0.516	Detected	0.516	Detected	0.000	-1.374	Detected	-1.174	Detected	-1.274	0.136	GT Sens	[illegible]
GT	Sense	Sense	0.948	Detected	-0.948	Detected	0.000	-2.438	Comprom	-2.846	Comprom	-2.642	0.112	GT Sens	[illegible]
GT	Sense	Sense	-0.088	Detected	0.088	Detected	0.000	-1.116	Detected	-1.393	Detected	-1.255	0.017	GT Sens	[illegible]
GT	Sense	Sense	-0.005	Detected	0.005	Detected	0.000	-2.170	Detected	-2.836	Detected	-2.503	0.017	GT Sens	[illegible]
GT	Sense	Sense	-0.395	Detected	0.395	Detected	0.000	-2.299	Detected	-2.697	Detected	-2.498	0.030	GT Sens	[illegible]
GT	Sense	Sense	0.128	Detected	-0.128	Detected	0.000	-2.078	Comprom	-1.139	Detected	-1.609	0.081	GT Sens	[illegible]
GT	Sense	Sense	0.045	Detected	-0.045	Detected	0.000	-1.274	Detected	-1.467	Detected	-1.371	0.006	GT Sens	[illegible]
GT	Sense	Sense	-0.013	Detected	0.013	Detected	0.000	-1.548	Detected	-1.126	Detected	-1.337	0.024	GT Sens	[illegible]
GT	Sense	Sense	-0.473	Detected	0.473	Detected	0.000	-1.668	Comprom	-2.539	Comprom	-2.104	0.082	GT Sens	[illegible]
GT	Sense	Sense	-0.466	Detected	0.466	Detected	0.000	-3.214	Comprom	-3.231	Comprom	-3.222	0.020	GT Sens	[illegible]
GT	Sense	Sense	-0.289	Detected	0.289	Detected	0.000	-1.101	Detected	-0.911	Detected	-1.006	0.080	GT Sens	[illegible]
GT	Sense	Sense	0.133	Detected	-0.133	Detected	0.000	-0.921	Detected	-1.368	Detected	-1.145	0.048	GT Sens	[illegible]
GT	Sense	Sense	-0.439	Detected	0.439	Detected	0.000	-1.744	Detected	-2.523	Detected	-2.133	0.068	GT Sens	[illegible]
GT	Sense	Sense	0.133	Detected	-0.133	Detected	0.000	-3.237	Detected	-3.044	Detected	-3.140	0.003	GT Sens	[illegible]
GT	Sense	Sense	-1.164	Detected	1.164	Detected	0.000	-1.053	Detected	-1.311	Detected	-1.182	0.419	GT Sens	[illegible]
GT	Sense	Sense	-0.827	Detected	0.827	Detected	0.000	-1.672	Detected	-3.366	Detected	-2.519	0.167	GT Sens	[illegible]
GT	Sense	Sense	0.024	Detected	-0.024	Detected	0.000	-2.711	Detected	-2.823	Detected	-2.767	0.000	GT Sens	[illegible]
GT	Sense	Sense	-0.765	Detected	0.765	Detected	0.000	-1.368	Detected	-1.649	Detected	-1.509	0.192	GT Sens	[illegible]
GT	Sense	Sense	-0.582	Detected	0.582	Detected	0.000	-1.436	Detected	-2.795	Detected	-2.116	0.142	GT Sens	[illegible]
GT	Sense	Sense	-0.242	Detected	0.242	Detected	0.000	-3.254	Comprom	-2.729	Comprom	-2.991	0.014	GT Sens	[illegible]
GT	Sense	Sense	-1.220	Detected	1.220	Detected	0.000	-0.841	Detected	-1.891	Detected	-1.366	0.412	GT Sens	[illegible]
GT	Sense	Sense	-0.027	Detected	0.027	Detected	0.000	-1.830	Comprom	-1.044	Detected	-1.437	0.068	GT Sens	[illegible]
GT	Sense	Sense	-0.470	Detected	0.470	Detected	0.000	-1.643	Detected	-1.014	Detected	-1.328	0.143	GT Sens	[illegible]
GT	Sense	Sense	-0.049	Detected	0.049	Detected	0.000	-1.669	Detected	-1.108	Detected	-1.388	0.040	GT Sens	[illegible]
GT	Sense	Sense	-0.815	Detected	0.815	Detected	0.000	-3.270	Comprom	-3.799	Comprom	-3.535	0.054	GT Sens	[illegible]
GT	Sense	Sense	-0.119	Detected	0.119	Detected	0.000	-1.702	Detected	-1.806	Detected	-1.754	0.005	GT Sens	[illegible]
GT	Sense	Sense	-0.056	Detected	0.056	Detected	0.000	-1.936	Detected	-1.711	Detected	-1.824	0.005	GT Sens	[illegible]
GT	Sense	Sense	-0.306	Detected	0.306	Detected	0.000	-1.044	Detected	-1.209	Detected	-1.126	0.071	GT Sens	[illegible]
GT	Sense	Sense	-0.485	Detected	0.485	Detected	0.000	-1.040	Detected	-1.155	Detected	-1.098	0.154	GT Sens	[illegible]
GT	Sense	Sense	0.226	Detected	-0.226	Detected	0.000	-2.555	Comprom	-1.033	Comprom	-1.794	0.152	GT Sens	[illegible]
GT	Sense	Sense	-0.346	Detected	0.346	Detected	0.000	-0.976	Detected	-2.091	Detected	-1.533	0.144	GT Sens	[illegible]
GT	Sense	Sense	-0.693	Detected	0.693	Detected	0.000	-1.126	Detected	-1.056	Detected	-1.091	0.256	GT Sens	[illegible]
GT	Sense	Sense	-0.415	Detected	0.415	Detected	0.000	-2.769	Comprom	-3.421	Comprom	-3.095	0.028	GT Sens	[illegible]
GT	Sense	Sense	0.101	Detected	-0.101	Detected	0.000	-1.291	Detected	-1.445	Detected	-1.368	0.009	GT Sens	[illegible]
GT	Sense	Sense	-0.643	Detected	0.643	Detected	0.000	-0.814	Detected	-1.799	Detected	-1.306	0.248	GT Sens	[illegible]
GT	Sense	Sense	-0.985	Detected	0.985	Detected	0.000	-1.126	Detected	-0.875	Detected	-1.000	0.420	GT Sens	[illegible]
GT	Sense	Sense	0.003	Detected	-0.003	Detected	0.000	-0.904	Comprom	-2.371	Comprom	-1.638	0.155	GT Sens	[illegible]
GT	Sense	Sense	-0.132	Detected	0.132	Detected	0.000	-1.123	Detected	-1.048	Detected	-1.085	0.016	GT Sens	[illegible]
GT	Sense	Sense	0.399	Detected	-0.399	Detected	0.000	-1.500	Detected	-1.194	Detected	-1.347	0.088	GT Sens	[illegible]
GT	Sense	Sense	-0.106	Detected	0.106	Detected	0.000	-1.599	Detected	-1.509	Detected	-1.554	0.005	GT Sens	[illegible]
GT	Sense	Sense	-0.137	Detected	0.137	Detected	0.000	-1.015	Detected	-1.238	Detected	-1.126	0.024	GT Sens	[illegible]
GT	Sense	Sense	-0.103	Detected	0.103	Detected	0.000	-1.703	Detected	-1.583	Detected	-1.643	0.005	GT Sens	[illegible]
GT	Sense	Sense	0.082	Detected	-0.082	Detected	0.000	-2.731	Comprom	-1.507	Comprom	-2.119	0.075	GT Sens	[illegible]
GT	Sense	Sense	-0.283	Detected	0.283	Detected	0.000	-1.179	Comprom	-0.842	Comprom	-1.011	0.092	GT Sens	[illegible]
GT	Sense	Sense	-0.482	Detected	0.482	Detected	0.000	-1.059	Comprom	-1.475	Comprom	-1.267	0.137	GT Sens	[illegible]
GT	Sense	Sense	0.376	Detected	-0.376	Detected	0.000	-1.149	Detected	-2.396	Comprom	-1.773	0.135	GT Sens	[illegible]
GT	Sense	Sense	-0.292	Detected	0.292	Detected	0.000	-0.919	Detected	-1.272	Detected	-1.096	0.085	GT Sens	[illegible]
GT	Sense	Sense	-0.200	Detected	0.200	Detected	0.000	-1.384	Detected	-1.007	Detected	-1.195	0.049	GT Sens	[illegible]
GT	Sense	Sense	0.026	Detected	-0.026	Detected	0.000	-0.811	Detected	-1.203	Detected	-1.007	0.036	GT Sens	[illegible]
GT	Sense	Sense	-0.538	Detected	0.538	Detected	0.000	-3.435	Detected	-2.992	Detected	-3.214	0.031	GT Sens	[illegible]
GT	Sense	Sense	-0.377	Detected	0.377	Detected	0.000	-0.953	Detected	-2.486	Detected	-1.720	0.182	GT Sens	[illegible]
GT	Sense	Sense	-0.373	Detected	0.373	Detected	0.000	-1.981	Detected	-2.929	Detected	-2.455	0.055	GT Sens	[illegible]
GT	Sense	Sense	-0.060	Detected	0.060	Detected	0.000	-2.159	Detected	-1.035	Detected	-1.597	0.106	GT Sens	[illegible]
GT	Sense	Sense	-0.495	Detected	0.495	Detected	0.000	-1.892	Detected	-1.830	Detected	-1.861	0.064	GT Sens	[illegible]
GT	Sense	Sense	-0.397	Detected	0.397	Detected	0.000	-1.063	Detected	-1.203	Detected	-1.133	0.107	GT Sens	[illegible]
GT	Sense	Sense	-0.532	Detected	0.532	Detected	0.000	-1.224	Detected	-1.706	Detected	-1.465	0.129	GT Sens	[illegible]
GT	Sense	Sense	-0.003	Detected	0.003	Detected	0.000	-1.629	Detected	-1.176	Detected	-1.402	0.025	GT Sens	[illegible]
GT	Sense	Sense	-0.450	Detected	0.450	Detected	0.000	-2.122	Detected	-3.193	Comprom	-2.658	0.063	GT Sens	[illegible]
GT	Sense	Sense	0.004	Detected	-0.004	Detected	0.000	-1.658	Detected	-1.198	Detected	-1.428	0.025	GT Sens	[illegible]
GT	Sense	Sense	-0.463	Detected	0.463	Detected	0.000	-1.589	Detected	-1.853	Detected	-1.721	0.070	GT Sens	[illegible]
GT	Sense	Sense	-0.380	Detected	0.380	Detected	0.000	-1.772	Detected	-1.562	Detected	-1.667	0.052	GT Sens	[illegible]
GT	Sense	Sense	0.084	Detected	-0.084	Detected	0.000	-2.187	Detected	-1.041	Detected	-1.614	0.108	GT Sens	[illegible]
GT	Sense	Sense	-0.402	Detected	0.402	Detected	0.000	-1.080	Detected	-1.196	Detected	-1.138	0.107	GT Sens	[illegible]
GT	Sense	Sense	-0.808	Detected	0.808	Detected	0.000	-1.068	Detected	-2.362	Detected	-1.715	0.239	GT Sens	[illegible]
GT	Sense	Sense	-0.049	Detected	0.049	Detected	0.000	-1.859	Detected	-1.587	Detected	-1.723	0.007	GT Sens	[illegible]
GT	Sense	Sense	0.663	Detected	-0.663	Detected	0.000	-2.652	Detected	-1.386	Detected	-2.019	0.159	GT Sens	[illegible]
GT	Sense	Sense	-0.646	Detected	0.646	Detected	0.000	-2.153	Detected	-1.798	Detected	-1.975	0.098	GT Sens	[illegible]
GT	Sense	Sense	0.093	Detected	-0.093	Detected	0.000	-1.098	Detected	-1.999	Detected	-1.548	0.078	GT Sens	[illegible]
GT	Sense	Sense	-0.205	Detected	0.205	Detected	0.000	-1.410	Detected	-1.282	Detected	-1.346	0.025	GT Sens	[illegible]
GT	Sense	Sense	-0.695	Detected	0.695	Detected	0.000	-0.940	Detected	-1.177	Detected	-1.058	0.272	GT Sens	[illegible]
GT	Sense	Sense	-0.329	Detected	0.329	Detected	0.000	-1.194	Detected	-1.046	Detected	-1.120	0.080	GT Sens	[illegible]
GT	Sense	Sense	-0.070	Detected	0.070	Detected	0.000	-2.609	Detected	-1.302	Detected	-1.956	0.097	GT Sens	[illegible]
GT	Sense	Sense	0.103	Detected	-0.103	Detected	0.000	-2.275	Comprom	-2.681	Comprom	-2.478	0.008	GT Sens	[illegible]
GT	Sense	Sense	-0.685	Detected	0.685	Detected	0.000	-2.280	Comprom	-2.602	Comprom	-2.441	0.074	GT Sens	[illegible]
GT	Sense	Sense	-0.306	Detected	0.306	Detected	0.000	-0.930	Detected	-1.247	Detected	-1.088	0.087	GT Sens	[illegible]
GT	Sense	Sense	-0.264	Detected	0.264	Detected	0.000	-1.191	Comprom	-1.187	Comprom	-1.189	0.046	GT Sens	[illegible]
GT	Sense	Sense	-0.303	Detected	0.303	Detected	0.000	-3.802	Comprom	-2.337	Detected	-3.069	0.061	GT Sens	[illegible]
GT	Sense	Sense	-1.384	Detected	1.384	Detected	0.000	-0.935	Detected	-5.581	Comprom	-3.258	0.351	GT Sens	[illegible]
GT	Sense	Sense	0.040	Detected	-0.040	Detected	0.000	-1.969	Comprom	-2.705	Comprom	-2.337	0.024	GT Sens	[illegible]
GT	Sense	Sense	-0.807	Detected	0.807	Detected	0.000	-0.908	Detected	-1.691	Detected	-1.299	0.284	GT Sens	[illegible]
GT	Sense	Sense	-0.377	Detected	0.377	Detected	0.000	-1.775	Detected	-1.428	Detected	-1.602	0.061	GT Sens	[illegible]
GT	Sense	Sense	0.021	Detected	-0.021	Detected	0.000	-1.702	Detected	-1.719	Detected	-1.710	0.000	GT Sens	[illegible]
GT	Sense	Sense	-0.392	Detected	0.392	Detected	0.000	-1.455	Comprom	-1.628	Comprom	-1.542	0.062	GT Sens	[illegible]
GT	Sense	Sense	-0.563	Detected	0.563	Detected	0.000	-1.336	Detected	-2.733	Detected	-2.034	0.151	GT Sens	[illegible]
GT	Sense	Sense	-0.895	Detected	0.895	Detected	0.000	-1.148	Detected	-1.374	Detected	-1.261	0.297	GT Sens	[illegible]
GT	Sense	Sense	-0.003	Detected	0.003	Detected	0.000	-1.328	Detected	-0.979	Detected	-1.153	0.022	GT Sens	[illegible]
GT	Sense	Sense	-0.674	Detected	0.674	Detected	0.000	-1.950	Comprom	-1.654	Comprom	-1.802	0.121	GT Sens	[illegible]
GT	Sense	Sense	-0.087	Detected	0.087	Detected	0.000	-1.604	Detected	-1.032	Detected	-1.318	0.048	GT Sens	[illegible]
GT	Sense	Sense	-0.153	Detected	0.153	Detected	0.000	-1.226	Detected	-1.023	Detected	-1.124	0.026	GT Sens	[illegible]
GT	Sense	Sense	-0.094	Detected	0.094	Detected	0.000	-1.061	Detected	-2.683	Comprom	-1.872	0.149	GT Sens	[illegible]
GT	Sense	Sense	-0.022	Detected	0.022	Detected	0.000	-1.564	Detected	-1.439	Detected	-1.502	0.002	GT Sens	[illegible]
GT	Sense	Sense	0.020	Detected	-0.020	Detected	0.000	-1.250	Detected	-1.410	Detected	-1.330	0.004	GT Sens	[illegible]
GT	Sense	Sense	-0.168	Detected	0.168	Detected	0.000	-1.090	Comprom	-1.105	Comprom	-1.098	0.023	GT Sens	[illegible]
GT	Sense	Sense	-0.018	Detected	0.018	Detected	0.000	-1.189	Detected	-1.127	Detected	-1.158	0.001	GT Sens	[illegible]
GT	Sense	Sense	-0.444	Detected	0.444	Detected	0.000	-2.609	Detected	-1.481	Detected	-2.045	0.104	GT Sens	[illegible]
GT	Sense	Sense	-0.112	Detected	0.112	Detected	0.000	-1.801	Comprom	-1.323	Comprom	-1.562	0.027	GT Sens	[illegible]
GT	Sense	Sense	-0.439	Detected	0.439	Detected	0.000	-0.849	Detected	-1.654	Detected	-1.252	0.170	GT Sens	[illegible]
GT	Sense	Sense	-0.352	Detected	0.352	Detected	0.000	-1.554	Detected	-1.588	Detected	-1.571	0.047	GT Sens	[illegible]
GT	Sense	Sense	-0.064	Detected	0.064	Detected	0.000	-1.801	Comprom	-0.963	Comprom	-1.382	0.083	GT Sens	[illegible]
GT	Sense	Sense	-0.555	Detected	0.555	Detected	0.000	-1.188	Detected	-2.150	Detected	-1.669	0.151	GT Sens	[illegible]
GT	Sense	Sense	-0.473	Detected	0.473	Detected	0.000	-1.516	Detected	-2.536	Detected	-2.026	0.100	GT Sens	[illegible]
GT	Sense	Sense	-0.281	Detected	0.281	Detected	0.000	-1.514	Detected	-2.303	Detected	-1.909	0.059	GT Sens	[illegible]
GT	Sense	Sense	0.470	Detected	-0.470	Detected	0.000	-1.953	Detected	-1.446	Detected	-1.700	0.086	GT Sens	[illegible]
GT	Sense	Sense	0.068	Detected	-0.068	Detected	0.000	-1.580	Detected	-0.860	Detected	-1.220	0.080	GT Sens	[illegible]
GT	Sense	Sense	0.089	Detected	-0.089	Detected	0.000	-1.968	Detected	-1.103	Detected	-1.535	0.074	GT Sens	[illegible]
GT	Sense	Sense	-0.814	Detected	0.814	Detected	0.000	-1.930	Comprom	-3.954	Comprom	-2.942	0.152	GT Sens	[illegible]
GT	Sense	Sense	-0.059	Detected	0.059	Detected	0.000	-1.008	Detected	-1.138	Detected	-1.073	0.007	GT Sens	[illegible]
GT	Sense	Sense	0.397	Detected	-0.397	Detected	0.000	-2.836	Detected	-2.677	Detected	-2.757	0.021	GT Sens	[illegible]
GT	Sense	Sense	-0.278	Detected	0.278	Detected	0.000	-1.584	Detected	-1.061	Detected	-1.322	0.074	GT Sens	[illegible]
GT	Sense	Sense	-0.299	Detected	0.299	Detected	0.000	-1.902	Detected	-1.291	Detected	-1.596	0.065	GT Sens	[illegible]
GT	Sense	Sense	-0.177	Detected	0.177	Detected	0.000	-1.644	Comprom	-1.267	Detected	-1.456	0.030	GT Sens	[illegible]
GT	Sense	Sense	-0.813	Detected	0.813	Detected	0.000	-1.034	Detected	-2.002	Detected	-1.518	0.250	GT Sens	[illegible]
GT	Sense	Sense	-0.332	Detected	0.332	Detected	0.000	-0.990	Detected	-1.689	Detected	-1.340	0.109	GT Sens	[illegible]
GT	Sense	Sense	-0.148	Detected	0.148	Detected	0.000	-1.877	Detected	-0.895	Detected	-1.386	0.114	GT Sens	[illegible]
GT	Sense	Sense	-0.270	Detected	0.270	Detected	0.000	-1.111	Detected	-1.898	Detected	-1.504	0.088	GT Sens	[illegible]
GT	Sense	Sense	0.064	Detected	-0.064	Detected	0.000	-1.065	Detected	-1.932	Detected	-1.499	0.076	GT Sens	[illegible]
GT	Sense	Sense	-0.527	Detected	0.527	Detected	0.000	-2.833	Detected	-1.994	Detected	-2.413	0.070	GT Sens	[illegible]
GT	Sense	Sense	-0.548	Detected	0.548	Detected	0.000	-1.467	Detected	-2.257	Detected	-1.862	0.110	GT Sens	[illegible]
GT	Sense	Sense	-0.430	Detected	0.430	Detected	0.000	-1.155	Detected	-2.065	Comprom	-1.610	0.124	GT Sens	[illegible]
GT	Sense	Sense	-0.155	Detected	0.155	Detected	0.000	-1.720	Detected	-0.952	Detected	-1.336	0.084	GT Sens	[illegible]
GT	Sense	Sense	-0.528	Detected	0.528	Detected	0.000	-1.809	Detected	-2.836	Detected	-2.322	0.088	GT Sens	[illegible]
GT	Sense	Sense	0.115	Detected	-0.115	Detected	0.000	-1.420	Detected	-1.414	Detected	-1.417	0.006	GT Sens	[illegible]
GT	Sense	Sense	-0.497	Detected	0.497	Detected	0.000	-1.825	Detected	-1.733	Detected	-1.779	0.070	GT Sens	[illegible]
GT	Sense	Sense	-0.412	Detected	0.412	Detected	0.000	-1.524	Detected	-1.535	Detected	-1.529	0.066	GT Sens	[illegible]
GT	Sense	Sense	-0.384	Detected	0.384	Detected	0.000	-0.964	Detected	-1.455	Detected	-1.210	0.117	GT Sens	[illegible]
GT	Sense	Sense	0.156	Detected	-0.156	Detected	0.000	-2.381	Detected	-0.891	Detected	-1.636	0.165	GT Sens	[illegible]
GT	Sense	Sense	-0.182	Detected	0.182	Detected	0.000	-1.239	Detected	-2.445	Detected	-1.842	0.100	GT Sens	[illegible]
GT	Sense	Sense	0.406	Detected	-0.406	Detected	0.000	-1.053	Detected	-1.063	Detected	-1.058	0.121	GT Sens	[illegible]
GT	Sense	Sense	-0.257	Detected	0.257	Detected	0.000	-1.325	Detected	-0.864	Detected	-1.095	0.086	GT Sens	[illegible]
GT	Sense	Sense	-0.098	Detected	0.098	Detected	0.000	-2.896	Detected	-2.343	Detected	-2.619	0.012	GT Sens	[illegible]
GT	Sense	Sense	-0.095	Detected	0.095	Detected	0.000	-1.177	Detected	-2.350	Detected	-1.763	0.097	GT Sens	[illegible]
GT	Sense	Sense	-0.011	Detected	0.011	Detected	0.000	-2.094	Detected	-1.078	Detected	-1.586	0.089	GT Sens	[illegible]
GT	Sense	Sense	-0.558	Detected	0.558	Detected	0.000	-1.274	Detected	-2.399	Detected	-1.837	0.146	GT Sens	[illegible]
GT	Sense	Sense	-0.251	Detected	0.251	Detected	0.000	-0.973	Detected	-1.056	Detected	-1.014	0.057	GT Sens	[illegible]
GT	Sense	Sense	-0.021	Detected	0.021	Detected	0.000	-1.760	Comprom	-1.723	Comprom	-1.741	0.000	GT Sens	[illegible]
GT	Sense	Sense	-0.037	Detected	0.037	Detected	0.000	-2.181	Detected	-1.435	Detected	-1.808	0.040	GT Sens	[illegible]
GT	Sense	Sense	-0.018	Detected	0.018	Detected	0.000	-3.228	Comprom	-3.201	Comprom	-3.214	0.000	GT Sens	[illegible]
GT	Sense	Sense	-0.291	Detected	0.291	Detected	0.000	-3.673	Detected	-1.820	Detected	-2.746	0.106	GT Sens	[illegible]
GT	Sense	Sense	-0.320	Detected	0.320	Detected	0.000	-1.693	Detected	-2.654	Detected	-2.173	0.064	GT Sens	[illegible]
GT	Sense	Sense	-0.295	Detected	0.295	Detected	0.000	-0.920	Detected	-1.367	Detected	-1.144	0.091	GT Sens	[illegible]
GT	Sense	Sense	-0.268	Detected	0.268	Detected	0.000	-1.422	Detected	-1.656	Detected	-1.539	0.034	GT Sens	[illegible]
GT	Sense	Sense	0.004	Detected	-0.004	Detected	0.000	-2.196	Comprom	-2.612	Comprom	-2.404	0.007	GT Sens	[illegible]
GT	Sense	Sense	-0.746	Detected	0.746	Detected	0.000	-0.901	Detected	-1.341	Detected	-1.121	0.286	GT Sens	[illegible]
GT	Sense	Sense	-0.885	Detected	0.885	Detected	0.000	-1.072	Detected	-1.554	Detected	-1.313	0.288	GT Sens	[illegible]
GT	Sense	Sense	-0.271	Detected	0.271	Detected	0.000	-1.118	Detected	-1.071	Detected	-1.095	0.057	GT Sens	[illegible]
GT	Sense	Sense	-0.004	Detected	0.004	Detected	0.000	-1.077	Comprom	-2.120	Comprom	-1.598	0.092	GT Sens	[illegible]
GT	Sense	Sense	-0.082	Detected	0.082	Detected	0.000	-1.458	Detected	-0.812	Detected	-1.135	0.077	GT Sens	[illegible]
GT	Sense	Sense	0.174	Detected	-0.174	Detected	0.000	-1.515	Comprom	-1.873	Comprom	-1.694	0.021	GT Sens	[illegible]
GT	Sense	Sense	-0.240	Detected	0.240	Detected	0.000	-0.980	Detected	-1.312	Comprom	-1.146	0.059	GT Sens	[illegible]
GT	Sense	Sense	-0.419	Detected	0.419	Detected	0.000	-1.096	Detected	-1.200	Detected	-1.148	0.113	GT Sens	[illegible]
GT	Sense	Sense	-0.223	Detected	0.223	Detected	0.000	-0.916	Detected	-1.509	Detected	-1.213	0.082	GT Sens	[illegible]
GT	Sense	Sense	-0.079	Detected	0.079	Detected	0.000	-1.669	Comprom	-1.627	Comprom	-1.648	0.002	GT Sens	[illegible]
GT	Sense	Sense	-0.417	Detected	0.417	Detected	0.000	-1.986	Comprom	-1.194	Comprom	-1.590	0.110	GT Sens	[illegible]
GT	Sense	Sense	0.189	Detected	-0.189	Detected	0.000	-1.003	Comprom	-1.633	Comprom	-1.318	0.070	GT Sens	[illegible]
GT	Sense	Sense	-0.144	Detected	0.144	Detected	0.000	-1.399	Comprom	-1.228	Comprom	-1.314	0.016	GT Sens	[illegible]
GT	Sense	Sense	-0.676	Detected	0.676	Detected	0.000	-1.143	Detected	-2.268	Comprom	-1.706	0.192	GT Sens	[illegible]
GT	Sense	Sense	-0.392	Detected	0.392	Detected	0.000	-1.753	Comprom	-1.604	Comprom	-1.678	0.052	GT Sens	[illegible]
GT	Sense	Sense	-0.182	Detected	0.182	Detected	0.000	-1.246	Detected	-1.189	Detected	-1.217	0.022	GT Sens	[illegible]
GT	Sense	Sense	-0.328	Detected	0.328	Detected	0.000	-2.807	Comprom	-1.682	Comprom	-2.244	0.075	GT Sens	[illegible]
GT	Sense	Sense	-0.377	Detected	0.377	Detected	0.000	-1.403	Detected	-1.571	Detected	-1.487	0.061	GT Sens	[illegible]
GT	Sense	Sense	-0.376	Detected	0.376	Detected	0.000	-1.206	Detected	-1.932	Detected	-1.569	0.095	GT Sens	[illegible]
GT	Sense	Sense	-0.416	Detected	0.416	Detected	0.000	-1.334	Detected	-2.059	Detected	-1.696	0.092	GT Sens	[illegible]
GT	Sense	Sense	-1.045	Detected	1.045	Detected	0.000	-1.533	Detected	-1.078	Detected	-1.306	0.346	GT Sens	[illegible]
GT	Sense	Sense	0.323	Detected	-0.323	Detected	0.000	-1.114	Detected	-1.004	Detected	-1.059	0.084	GT Sens	[illegible]
GT	Sense	Sense	0.109	Detected	-0.109	Detected	0.000	-1.717	Detected	-1.683	Detected	-1.700	0.004	GT Sens	[illegible]
GT	Sense	Sense	0.046	Detected	-0.046	Detected	0.000	-2.262	Comprom	-1.594	Comprom	-1.928	0.029	GT Sens	[illegible]
GT	Sense	Sense	-0.134	Detected	0.134	Detected	0.000	-1.339	Detected	-1.780	Detected	-1.560	0.026	GT Sens	[illegible]
GT	Sense	Sense	0.408	Detected	-0.408	Detected	0.000	-3.130	Comprom	-4.607	Comprom	-3.868	0.044	GT Sens	[illegible]
GT	Sense	Sense	0.124	Detected	-0.124	Detected	0.000	-2.710	Comprom	-2.729	Comprom	-2.719	0.002	GT Sens	[illegible]
GT	Sense	Sense	-0.230	Detected	0.230	Detected	0.000	-1.018	Detected	-1.198	Detected	-1.108	0.046	GT Sens	[illegible]
GT	Sense	Sense	-0.332	Detected	0.332	Detected	0.000	-1.263	Detected	-1.342	Detected	-1.302	0.060	GT Sens	[illegible]
GT	Sense	Sense	-0.127	Detected	0.127	Detected	0.000	-1.026	Detected	-1.215	Detected	-1.120	0.019	GT Sens	[illegible]
GT	Sense	Sense	0.096	Detected	-0.096	Detected	0.000	-0.869	Detected	-1.209	Detected	-1.039	0.034	GT Sens	[illegible]
GT	Sense	Sense	-0.045	Detected	0.045	Detected	0.000	-1.233	Comprom	-3.046	Comprom	-2.140	0.142	GT Sens	[illegible]
GT	Sense	Sense	-0.198	Detected	0.198	Detected	0.000	-1.401	Detected	-1.477	Detected	-1.439	0.019	GT Sens	[illegible]
GT	Sense	Sense	-0.073	Detected	0.073	Detected	0.000	-1.794	Detected	-0.832	Detected	-1.313	0.114	GT Sens	[illegible]
GT	Sense	Sense	-0.389	Detected	0.389	Detected	0.000	-1.275	Detected	-1.203	Detected	-1.239	0.087	GT Sens	[illegible]
GT	Sense	Sense	0.295	Detected	-0.295	Detected	0.000	-1.316	Detected	-1.053	Detected	-1.184	0.067	GT Sens	[illegible]
GT	Sense	Sense	-0.317	Detected	0.317	Detected	0.000	-1.494	Comprom	-1.827	Comprom	-1.660	0.043	GT Sens	[illegible]
GT	Sense	Sense	0.204	Detected	-0.204	Detected	0.000	-1.971	Comprom	-1.892	Comprom	-1.932	0.011	GT Sens	[illegible]
GT	Sense	Sense	-0.221	Detected	0.221	Detected	0.000	-1.299	Detected	-2.086	Detected	-1.693	0.064	GT Sens	[illegible]
GT	Sense	Sense	-0.078	Detected	0.078	Detected	0.000	-0.961	Detected	-1.728	Detected	-1.345	0.075	GT Sens	[illegible]
GT	Sense	Sense	0.015	Detected	-0.015	Detected	0.000	-1.049	Detected	-1.466	Detected	-1.257	0.027	GT Sens	[illegible]
GT	Sense	Sense	0.184	Detected	-0.184	Detected	0.000	-1.310	Detected	-1.007	Detected	-1.158	0.040	GT Sens	[illegible]
GT	Sense	Sense	-0.293	Detected	0.293	Detected	0.000	-1.145	Detected	-1.443	Detected	-1.294	0.059	GT Sens	[illegible]
GT	Sense	Sense	-0.212	Detected	0.212	Detected	0.000	-3.436	Comprom	-2.598	Comprom	-3.017	0.023	GT Sens	[illegible]
GT	Sense	Sense	-0.149	Detected	0.149	Detected	0.000	-1.258	Detected	-1.483	Detected	-1.371	0.018	GT Sens	[illegible]
GT	Sense	Sense	0.153	Detected	-0.153	Detected	0.000	-0.930	Detected	-2.021	Comprom	-1.476	0.121	GT Sens	[illegible]
GT	Sense	Sense	-0.022	Detected	0.022	Detected	0.000	-1.133	Detected	-1.583	Detected	-1.358	0.027	GT Sens	[illegible]
GT	Sense	Sense	-0.040	Detected	0.040	Detected	0.000	-1.246	Detected	-1.505	Detected	-1.375	0.010	GT Sens	[illegible]
GT	Sense	Sense	-0.683	Detected	0.683	Detected	0.000	-1.598	Comprom	-1.868	Comprom	-1.733	0.131	GT Sens	[illegible]
GT	Sense	Sense	-0.521	Detected	0.521	Detected	0.000	-1.370	Detected	-1.565	Detected	-1.468	0.109	GT Sens	[illegible]
GT	Sense	Sense	-0.030	Detected	0.030	Detected	0.000	-0.845	Detected	-2.194	Comprom	-1.519	0.153	GT Sens	[illegible]
GT	Sense	Sense	-0.283	Detected	0.283	Detected	0.000	-2.089	Detected	-2.409	Detected	-2.249	0.020	GT Sens	[illegible]
GT	Sense	Sense	-0.272	Detected	0.272	Detected	0.000	-1.855	Detected	-2.242	Detected	-2.048	0.026	GT Sens	[illegible]
GT	Sense	Sense	-0.162	Detected	0.162	Detected	0.000	-1.304	Comprom	-1.625	Comprom	-1.464	0.023	GT Sens	[illegible]
GT	Sense	Sense	1.305	Detected	-1.305	Detected	0.000	-2.611	Comprom	-1.435	Detected	-2.023	0.293	GT Sens	[illegible]
GT	Sense	Sense	-0.035	Detected	0.035	Detected	0.000	-1.185	Comprom	-1.471	Comprom	-1.328	0.012	GT Sens	[illegible]
GT	Sense	Sense	0.100	Detected	-0.100	Detected	0.000	-1.345	Comprom	-1.249	Comprom	-1.297	0.007	GT Sens	[illegible]
GT	Sense	Sense	0.073	Detected	-0.073	Detected	0.000	-1.294	Detected	-0.850	Detected	-1.072	0.044	GT Sens	[illegible]
GT	Sense	Sense	-0.838	Detected	0.838	Detected	0.000	-0.841	Detected	-1.850	Comprom	-1.345	0.303	GT Sens	[illegible]
GT	Sense	Sense	-0.312	Detected	0.312	Detected	0.000	-1.546	Detected	-1.046	Detected	-1.296	0.084	GT Sens	[illegible]
GT	Sense	Sense	0.006	Detected	-0.006	Detected	0.000	-0.931	Detected	-1.352	Detected	-1.141	0.032	GT Sens	[illegible]
GT	Sense	Sense	-0.156	Detected	0.156	Detected	0.000	-1.249	Detected	-1.594	Detected	-1.422	0.026	GT Sens	[illegible]
GT	Sense	Sense	0.951	Detected	-0.951	Detected	0.000	-0.884	Detected	-2.698	Detected	-1.791	0.306	GT Sens	[illegible]
GT	Sense	Sense	-0.532	Detected	0.532	Detected	0.000	-0.925	Detected	-2.889	Detected	-1.907	0.230	GT Sens	[illegible]
GT	Sense	Sense	-0.089	Detected	0.089	Detected	0.000	-0.948	Detected	-1.602	Detected	-1.275	0.064	GT Sens	[illegible]
GT	Sense	Sense	-0.202	Detected	0.202	Detected	0.000	-0.975	Detected	-1.782	Detected	-1.379	0.092	GT Sens	[illegible]
GT	Sense	Sense	-0.348	Detected	0.348	Detected	0.000	-2.196	Comprom	-1.707	Comprom	-1.951	0.044	GT Sens	[illegible]
GT	Sense	Sense	-0.071	Detected	0.071	Detected	0.000	-1.086	Comprom	-1.989	Comprom	-1.537	0.078	GT Sens	[illegible]
GT	Sense	Sense	-0.319	Detected	0.319	Detected	0.000	-0.967	Detected	-1.464	Detected	-1.216	0.095	GT Sens	[illegible]
GT	Sense	Sense	-0.379	Detected	0.379	Detected	0.000	-1.068	Detected	-1.444	Detected	-1.256	0.097	GT Sens	[illegible]
GT	Sense	Sense	-0.745	Detected	0.745	Detected	0.000	-0.963	Detected	-1.977	Detected	-1.470	0.244	GT Sens	[illegible]
GT	Sense	Sense	0.018	Detected	-0.018	Detected	0.000	-1.578	Detected	-0.982	Detected	-1.280	0.050	GT Sens	[illegible]
GT	Sense	Sense	-1.005	Detected	1.005	Detected	0.000	-0.992	Detected	-1.054	Detected	-1.023	0.416	GT Sens	[illegible]
GT	Sense	Sense	0.185	Detected	-0.185	Detected	0.000	-1.653	Comprom	-1.237	Comprom	-1.445	0.035	GT Sens	[illegible]
GT	Sense	Sense	-0.533	Detected	0.533	Detected	0.000	-1.230	Detected	-1.519	Detected	-1.374	0.131	GT Sens	[illegible]
GT	Sense	Sense	-0.128	Detected	0.128	Detected	0.000	-1.049	Detected	-1.485	Detected	-1.267	0.038	GT Sens	[illegible]
GT	Sense	Sense	-1.021	Detected	1.021	Detected	0.000	-1.089	Detected	-1.524	Detected	-1.306	0.337	GT Sens	[illegible]
GT	Sense	Sense	-1.031	Detected	1.031	Detected	0.000	-0.981	Detected	-1.545	Detected	-1.263	0.359	GT Sens	[illegible]
GT	Sense	Sense	-0.175	Detected	0.175	Detected	0.000	-3.195	Comprom	-1.775	Detected	-2.485	0.077	GT Sens	[illegible]
GT	Sense	Sense	-0.192	Detected	0.192	Detected	0.000	-2.137	Comprom	-0.845	Comprom	-1.491	0.157	GT Sens	[illegible]
GT	Sense	Sense	-0.023	Detected	0.023	Detected	0.000	-1.053	Detected	-1.078	Detected	-1.065	0.001	GT Sens	[illegible]
GT	Sense	Sense	-0.477	Detected	0.477	Detected	0.000	-3.061	Comprom	-2.101	Comprom	-2.581	0.062	GT Sens	[illegible]
GT	Sense	Sense	-0.223	Detected	0.223	Detected	0.000	-1.881	Comprom	-1.245	Detected	-1.563	0.056	GT Sens	[illegible]
GT	Sense	Sense	0.853	Detected	-0.853	Detected	0.000	-2.986	Comprom	-1.739	Detected	-2.362	0.155	GT Sens	[illegible]
GT	Sense	Sense	0.020	Detected	-0.020	Detected	0.000	-2.834	Detected	-2.484	Detected	-2.659	0.004	GT Sens	[illegible]
GT	Sense	Sense	0.404	Detected	-0.404	Detected	0.000	-0.833	Detected	-1.622	Comprom	-1.228	0.162	GT Sens	[illegible]
GT	Sense	Sense	-0.546	Detected	0.546	Detected	0.000	-2.152	Detected	-2.292	Detected	-2.222	0.056	GT Sens	[illegible]
GT	Sense	Sense	-0.149	Detected	0.149	Detected	0.000	-2.162	Comprom	-2.241	Comprom	-2.202	0.005	GT Sens	[illegible]
GT	Sense	Sense	-0.292	Detected	0.292	Detected	0.000	-0.997	Detected	-1.020	Detected	-1.009	0.075	GT Sens	[illegible]
GT	Sense	Sense	0.148	Detected	-0.148	Detected	0.000	-1.829	Comprom	-1.304	Comprom	-1.567	0.035	GT Sens	[illegible]
GT	Sense	Sense	-0.785	Detected	0.785	Detected	0.000	-2.184	Detected	-2.118	Detected	-2.151	0.111	GT Sens	[illegible]
GT	Sense	Sense	-1.242	Detected	1.242	Detected	0.000	-1.170	Detected	-1.397	Detected	-1.283	0.412	GT Sens	[illegible]
GT	Sense	Sense	-0.084	Detected	0.084	Detected	0.000	-1.866	Detected	-1.359	Detected	-1.613	0.026	GT Sens	[illegible]

GT	Sense	Sense	-0.040	Detected	0.040	Detected	0.000	-1.211	Comprom	-1.861	Comprom	-1.536	0.042	GT	Sens	contig345	contig345	Unannota	[illegible]
GT	Sense	Sense	0.612	Detected	-0.612	Detected	0.000	-2.808	Comprom	-2.180	Comprom	-2.494	0.068	GT	Sens	contig345	contig345	Unknown	[illegible]
GT	Sense	Sense	-0.048	Detected	0.048	Detected	0.000	-0.978	Comprom	-1.329	Comprom	-1.154	0.024	GT	Sens	contig347	contig347	Unannota	[illegible]
GT	Sense	Sense	-0.133	Detected	0.133	Detected	0.000	-1.217	Detected	-2.725	Detected	-1.971	0.124	GT	Sens	contig347	contig347	Unannota	[illegible]
GT	Sense	Sense	-0.261	Detected	0.261	Detected	0.000	-1.534	Detected	-3.194	Detected	-2.364	0.113	GT	Sens	contig347	contig347	Unannota	[illegible]
GT	Sense	Sense	-0.652	Detected	0.652	Detected	0.000	-2.333	Comprom	-2.661	Comprom	-2.497	0.065	GT	Sens	contig348	contig348	Unannota	[illegible]
GT	Sense	Sense	0.032	Detected	-0.032	Detected	0.000	-1.902	Comprom	-1.543	Comprom	-1.723	0.011	GT	Sens	contig349	contig349	Unannota	[illegible]
GT	Sense	Sense	-0.133	Detected	0.133	Detected	0.000	-0.980	Detected	-2.059	Comprom	-1.519	0.112	GT	Sens	contig349	contig349	class I he	[illegible]
GT	Sense	Sense	-0.078	Detected	0.078	Detected	0.000	-2.403	Comprom	-1.096	Comprom	-1.750	0.117	GT	Sens	contig350	contig350	Unannota	[illegible]
GT	Sense	Sense	-0.474	Detected	0.474	Detected	0.000	-0.928	Detected	-1.465	Detected	-1.196	0.159	GT	Sens	contig351	contig351	Unannota	[illegible]
GT	Sense	Sense	0.059	Detected	-0.059	Detected	0.000	-2.847	Comprom	-1.944	Comprom	-2.396	0.034	GT	Sens	contig352	contig352	Unannota	[illegible]
GT	Sense	Sense	-0.316	Detected	0.316	Detected	0.000	-1.055	Detected	-1.988	Detected	-1.521	0.114	GT	Sens	contig352	contig352	Unannota	[illegible]
GT	Sense	Sense	-0.274	Detected	0.274	Detected	0.000	-0.915	Detected	-1.927	Detected	-1.421	0.132	GT	Sens	contig352	contig352	Unannota	[illegible]
GT	Sense	Sense	0.330	Detected	-0.330	Detected	0.000	-0.974	Detected	-1.947	Comprom	-1.460	0.131	GT	Sens	contig352	contig352	Unannota	[illegible]
GT	Sense	Sense	-0.174	Detected	0.174	Detected	0.000	-0.976	Detected	-1.047	Detected	-1.011	0.029	GT	Sens	contig352	contig352	Unannota	[illegible]
GT	Sense	Sense	-0.501	Detected	0.501	Detected	0.000	-1.356	Detected	-1.280	Detected	-1.318	0.120	GT	Sens	contig353	contig353	Unannota	[illegible]
GT	Sense	Sense	-0.332	Detected	0.332	Detected	0.000	-1.081	Comprom	-1.166	Comprom	-1.124	0.078	GT	Sens	contig353	contig353	Unannota	[illegible]
GT	Sense	Sense	-0.007	Detected	0.007	Detected	0.000	-1.679	Comprom	-1.024	Detected	-1.352	0.054	GT	Sens	contig354	contig354	Unannota	[illegible]
GT	Sense	Sense	-0.042	Detected	0.042	Detected	0.000	-1.115	Comprom	-1.558	Comprom	-1.336	0.027	GT	Sens	contig355	contig355	Unannota	[illegible]
GT	Sense	Sense	0.908	Detected	-0.908	Detected	0.000	-1.868	Comprom	-2.447	Comprom	-2.157	0.152	GT	Sens	contig355	contig355	Unannota	[illegible]
GT	Sense	Sense	0.424	Detected	-0.424	Detected	0.000	-1.487	Detected	-1.214	Detected	-1.350	0.094	GT	Sens	contig355	contig355	Unannota	[illegible]
GT	Sense	Sense	-0.531	Detected	0.531	Detected	0.000	-2.931	Detected	-4.528	Detected	-3.730	0.060	GT	Sens	contig356	contig356	Unknown	[illegible]
GT	Sense	Sense	-0.286	Detected	0.286	Detected	0.000	-1.387	Comprom	-0.903	Comprom	-1.145	0.092	GT	Sens	contig356	contig356	Unannota	[illegible]
GT	Sense	Sense	-0.055	Detected	0.055	Detected	0.000	-0.998	Detected	-1.216	Detected	-1.107	0.012	GT	Sens	contig357	contig357	Os05g02	[illegible]
GT	Sense	Sense	-0.640	Detected	0.640	Detected	0.000	-0.843	Detected	-1.216	Detected	-1.029	0.263	GT	Sens	contig357	contig357	Unannota	[illegible]
GT	Sense	Sense	-0.243	Detected	0.243	Detected	0.000	-1.095	Comprom	-1.134	Comprom	-1.115	0.045	GT	Sens	contig357	contig357	Unknown	[illegible]
GT	Sense	Sense	-0.002	Detected	0.002	Detected	0.000	-0.802	Comprom	-1.617	Comprom	-1.209	0.097	GT	Sens	contig357	contig357	Unannota	[illegible]
GT	Sense	Sense	0.078	Detected	-0.078	Detected	0.000	-1.562	Comprom	-0.995	Comprom	-1.278	0.049	GT	Sens	contig357	contig357	Unannota	[illegible]
GT	Sense	Sense	-0.351	Detected	0.351	Detected	0.000	-1.382	Detected	-1.416	Detected	-1.399	0.058	GT	Sens	contig357	contig357	Unannota	[illegible]
GT	Sense	Sense	-0.425	Detected	0.425	Detected	0.000	-1.056	Detected	-1.437	Detected	-1.247	0.116	GT	Sens	contig357	contig357	Unannota	[illegible]
GT	Sense	Sense	0.103	Detected	-0.103	Detected	0.000	-1.258	Detected	-2.302	Comprom	-1.780	0.079	GT	Sens	contig358	contig358	Unannota	[illegible]
GT	Sense	Sense	-0.179	Detected	0.179	Detected	0.000	-1.859	Comprom	-2.038	Comprom	-1.948	0.010	GT	Sens	contig358	contig358	Unannota	[illegible]
GT	Sense	Sense	-0.064	Detected	0.064	Detected	0.000	-0.964	Comprom	-1.407	Comprom	-1.185	0.036	GT	Sens	contig358	contig358	Unannota	[illegible]
GT	Sense	Sense	0.499	Detected	-0.499	Detected	0.000	-1.097	Detected	-1.388	Detected	-1.242	0.140	GT	Sens	contig358	contig358	Unannota	[illegible]
GT	Sense	Sense	0.542	Detected	-0.542	Detected	0.000	-1.931	Comprom	-2.477	Comprom	-2.204	0.068	GT	Sens	contig358	contig358	Unannota	[illegible]
GT	Sense	Sense	-0.511	Detected	0.511	Detected	0.000	-1.431	Detected	-1.314	Detected	-1.373	0.116	GT	Sens	contig359	contig359	Unannota	[illegible]
GT	Sense	Sense	-0.348	Detected	0.348	Detected	0.000	-0.855	Detected	-1.249	Detected	-1.052	0.119	GT	Sens	contig359	contig359	Unannota	[illegible]
GT	Sense	Sense	-0.033	Detected	0.033	Detected	0.000	-1.274	Comprom	-2.611	Comprom	-1.943	0.101	GT	Sens	contig359	contig359	Unannota	[illegible]
GT	Sense	Sense	0.044	Detected	-0.044	Detected	0.000	-1.566	Comprom	-1.850	Comprom	-1.708	0.008	GT	Sens	contig360	contig360	Unannota	[illegible]
GT	Sense	Sense	-0.146	Detected	0.146	Detected	0.000	-2.905	Detected	-1.768	Detected	-2.337	0.058	GT	Sens	contig361	contig361	Calmodul	[illegible]
GT	Sense	Sense	-0.408	Detected	0.408	Detected	0.000	-1.252	Detected	-0.844	Detected	-1.048	0.149	GT	Sens	contig361	contig361	Unannota	[illegible]
GT	Sense	Sense	-0.528	Detected	0.528	Detected	0.000	-0.831	Detected	-1.170	Detected	-1.000	0.213	GT	Sens	contig361	contig361	Unannota	[illegible]
GT	Sense	Sense	-0.495	Detected	0.495	Detected	0.000	-1.905	Comprom	-2.552	Comprom	-2.229	0.064	GT	Sens	contig362	contig362	Unannota	[illegible]
GT	Sense	Sense	-0.516	Detected	0.516	Detected	0.000	-1.999	Detected	-2.081	Detected	-2.040	0.059	GT	Sens	contig362	contig362	Unannota	[illegible]
GT	Sense	Sense	0.246	Detected	-0.246	Detected	0.000	-1.163	Detected	-1.023	Detected	-1.093	0.051	GT	Sens	contig362	contig362	Unannota	[illegible]
GT	Sense	Sense	0.154	Detected	-0.154	Detected	0.000	-1.286	Detected	-0.963	Detected	-1.124	0.037	GT	Sens	contig362	contig362	Unannota	[illegible]
GT	Sense	Sense	-0.146	Detected	0.146	Detected	0.000	-1.393	Detected	-1.552	Detected	-1.472	0.012	GT	Sens	contig362	contig362	Unannota	[illegible]
GT	Sense	Sense	-0.096	Detected	0.096	Detected	0.000	-0.925	Detected	-1.140	Detected	-1.033	0.019	GT	Sens	contig362	contig362	Unannota	[illegible]
GT	Sense	Sense	-0.558	Detected	0.558	Detected	0.000	-1.038	Detected	-1.499	Comprom	-1.269	0.171	GT	Sens	contig363	contig363	Unannota	[illegible]
GT	Sense	Sense	-0.565	Detected	0.565	Detected	0.000	-2.249	Comprom	-2.873	Comprom	-2.561	0.058	GT	Sens	contig363	contig363	Ethylene-	[illegible]
GT	Sense	Sense	-0.246	Detected	0.246	Detected	0.000	-1.270	Detected	-0.943	Detected	-1.107	0.065	GT	Sens	contig363	contig363	Receptor	[illegible]
GT	Sense	Sense	-0.752	Detected	0.752	Detected	0.000	-1.879	Detected	-2.418	Detected	-2.149	0.115	GT	Sens	contig364	contig364	Unannota	[illegible]
GT	Sense	Sense	-0.702	Detected	0.702	Detected	0.000	-1.754	Detected	-2.585	Comprom	-2.169	0.117	GT	Sens	contig364	contig364	Unannota	[illegible]
GT	Sense	Sense	-0.266	Detected	0.266	Detected	0.000	-1.022	Detected	-1.104	Detected	-1.063	0.058	GT	Sens	contig364	contig364	Unannota	[illegible]
GT	Sense	Sense	-0.167	Detected	0.167	Detected	0.000	-1.307	Comprom	-1.532	Comprom	-1.419	0.019	GT	Sens	contig364	contig364	Unannota	[illegible]
GT	Sense	Sense	-0.181	Detected	0.181	Detected	0.000	-0.859	Detected	-1.460	Detected	-1.159	0.081	GT	Sens	contig365	contig365	Unannota	[illegible]
GT	Sense	Sense	0.256	Detected	-0.256	Detected	0.000	-0.841	Detected	-1.303	Detected	-1.072	0.090	GT	Sens	contig365	contig365	Unannota	[illegible]
GT	Sense	Sense	0.019	Detected	-0.019	Detected	0.000	-1.402	Detected	-1.492	Detected	-1.447	0.001	GT	Sens	contig365	contig365	Unannota	[illegible]
GT	Sense	Sense	1.414	Detected	-1.414	Detected	0.000	-2.663	Comprom	-3.973	Comprom	-3.318	0.167	GT	Sens	contig365	contig365	Unannota	[illegible]
GT	Sense	Sense	0.481	Detected	-0.481	Detected	0.000	-2.561	Detected	-3.386	Detected	-2.973	0.043	GT	Sens	contig365	contig365	Unannota	[illegible]
GT	Sense	Sense	0.654	Detected	-0.654	Detected	0.000	-1.911	Detected	-2.827	Detected	-2.369	0.097	GT	Sens	contig365	contig365	Unannota	[illegible]
GT	Sense	Sense	-0.371	Detected	0.371	Detected	0.000	-1.512	Comprom	-1.934	Comprom	-1.723	0.056	GT	Sens	contig365	contig365	Unannota	[illegible]
GT	Sense	Sense	-0.087	Detected	0.087	Detected	0.000	-1.241	Comprom	-2.130	Comprom	-1.685	0.065	GT	Sens	contig366	contig366	Unannota	[illegible]
GT	Sense	Sense	0.119	Detected	-0.119	Detected	0.000	-1.365	Comprom	-0.823	Detected	-1.094	0.066	GT	Sens	contig366	contig366	Unannota	[illegible]
GT	Sense	Sense	-0.220	Detected	0.220	Detected	0.000	-1.276	Detected	-1.775	Detected	-1.526	0.044	GT	Sens	contig366	contig366	Unannota	[illegible]
GT	Sense	Sense	-0.577	Detected	0.577	Detected	0.000	-1.151	Detected	-1.614	Detected	-1.382	0.156	GT	Sens	contig366	contig366	Unannota	[illegible]
GT	Sense	Sense	0.055	Detected	-0.055	Detected	0.000	-3.984	Comprom	-3.948	Comprom	-3.966	0.000	GT	Sens	contig366	contig366	Unannota	[illegible]
GT	Sense	Sense	-0.459	Detected	0.459	Detected	0.000	-1.475	Detected	-2.068	Detected	-1.772	0.083	GT	Sens	contig367	contig367	Unannota	[illegible]
GT	Sense	Sense	-0.389	Detected	0.389	Detected	0.000	-1.028	Detected	-1.739	Detected	-1.384	0.120	GT	Sens	contig367	contig367	Unannota	[illegible]
GT	Sense	Sense	-0.033	Detected	0.033	Detected	0.000	-1.302	Detected	-0.946	Detected	-1.124	0.025	GT	Sens	contig367	contig367	Chloropla	[illegible]
GT	Sense	Sense	-0.008	Detected	0.008	Detected	0.000	-2.083	Comprom	-2.323	Comprom	-2.203	0.003	GT	Sens	contig367	contig367	Unannota	[illegible]
GT	Sense	Sense	-0.064	Detected	0.064	Detected	0.000	-3.922	Comprom	-4.050	Comprom	-3.986	0.001	GT	Sens	contig367	contig367	Unannota	[illegible]
GT	Sense	Sense	0.195	Detected	-0.195	Detected	0.000	-0.840	Detected	-2.787	Comprom	-1.813	0.209	GT	Sens	contig367	contig367	Unannota	[illegible]
GT	Sense	Sense	0.258	Detected	-0.258	Detected	0.000	-1.013	Detected	-1.099	Comprom	-1.056	0.056	GT	Sens	contig367	contig367	Unannota	[illegible]
GT	Sense	Sense	-0.157	Detected	0.157	Detected	0.000	-1.111	Comprom	-0.976	Comprom	-1.043	0.026	GT	Sens	contig367	contig367	Unannota	[illegible]
GT	Sense	Sense	-0.171	Detected	0.171	Detected	0.000	-2.208	Comprom	-1.247	Comprom	-1.728	0.077	GT	Sens	contig367	contig367	Unannota	[illegible]
GT	Sense	Sense	-0.074	Detected	0.074	Detected	0.000	-1.012	Detected	-1.087	Detected	-1.049	0.006	GT	Sens	contig368	contig368	Unannota	[illegible]
GT	Sense	Sense	-0.235	Detected	0.235	Detected	0.000	-1.497	Comprom	-1.207	Comprom	-1.352	0.039	GT	Sens	contig369	contig369	Unannota	[illegible]
GT	Sense	Sense	-0.465	Detected	0.465	Detected	0.000	-0.815	Detected	-1.191	Comprom	-1.003	0.183	GT	Sens	contig369	contig369	Unannota	[illegible]
GT	Sense	Sense	0.025	Detected	-0.025	Detected	0.000	-1.496	Detected	-2.126	Detected	-1.811	0.029	GT	Sens	contig370	contig370	Unannota	[illegible]
GT	Sense	Sense	0.027	Detected	-0.027	Detected	0.000	-1.623	Detected	-1.884	Detected	-1.753	0.006	GT	Sens	contig370	contig370	Unannota	[illegible]
GT	Sense	Sense	-0.159	Detected	0.159	Detected	0.000	-1.354	Detected	-0.816	Detected	-1.085	0.074	GT	Sens	contig370	contig370	Unannota	[illegible]
GT	Sense	Sense	-1.161	Detected	1.161	Detected	0.000	-1.324	Detected	-1.456	Detected	-1.390	0.354	GT	Sens	contig370	contig370	Unannota	[illegible]
GT	Sense	Sense	-0.100	Detected	0.100	Detected	0.000	-0.814	Comprom	-1.256	Comprom	-1.035	0.051	GT	Sens	contig370	contig370	Unannota	[illegible]
GT	Sense	Sense	0.222	Detected	-0.222	Detected	0.000	-1.498	Comprom	-1.461	Comprom	-1.479	0.022	GT	Sens	contig371	contig371	26S prote	[illegible]
GT	Sense	Sense	-0.117	Detected	0.117	Detected	0.000	-1.315	Detected	-1.487	Detected	-1.401	0.011	GT	Sens	contig371	contig371	Unannota	[illegible]
GT	Sense	Sense	0.331	Detected	-0.331	Detected	0.000	-2.109	Detected	-1.045	Detected	-1.577	0.128	GT	Sens	contig371	contig371	Unannota	[illegible]
GT	Sense	Sense	0.174	Detected	-0.174	Detected	0.000	-1.976	Detected	-1.089	Detected	-1.533	0.085	GT	Sens	contig371	contig371	Unannota	[illegible]
GT	Sense	Sense	-0.228	Detected	0.228	Detected	0.000	-0.986	Detected	-2.181	Comprom	-1.584	0.132	GT	Sens	contig372	contig372	Unannota	[illegible]
GT	Sense	Sense	0.348	Detected	-0.348	Detected	0.000	-2.202	Detected	-2.292	Detected	-2.247	0.024	GT	Sens	contig372	contig372	Unannota	[illegible]
GT	Sense	Sense	0.084	Detected	-0.084	Detected	0.000	-1.024	Detected	-1.494	Detected	-1.259	0.037	GT	Sens	contig372	contig372	Unannota	[illegible]
GT	Sense	Sense	0.756	Detected	-0.756	Detected	0.000	-1.588	Detected	-2.261	Comprom	-1.925	0.146	GT	Sens	contig372	contig372	Unannota	[illegible]
GT	Sense	Sense	-0.111	Detected	0.111	Detected	0.000	-1.103	Comprom	-1.166	Comprom	-1.134	0.010	GT	Sens	contig372	contig372	Unannota	[illegible]
GT	Sense	Sense	-0.062	Detected	0.062	Detected	0.000	-1.241	Detected	-1.553	Detected	-1.397	0.014	GT	Sens	contig373	contig373	Unannota	[illegible]
GT	Sense	Sense	-0.220	Detected	0.220	Detected	0.000	-1.280	Detected	-1.543	Detected	-1.412	0.031	GT	Sens	contig373	contig373	Unannota	[illegible]
GT	Sense	Sense	-0.198	Detected	0.198	Detected	0.000	-1.471	Comprom	-1.447	Comprom	-1.459	0.018	GT	Sens	contig373	contig373	Unannota	[illegible]
GT	Sense	Sense	0.362	Detected	-0.362	Detected	0.000	-1.557	Comprom	-1.360	Comprom	-1.458	0.060	GT	Sens	contig374	contig374	Unannota	[illegible]
GT	Sense	Sense	-0.171	Detected	0.171	Detected	0.000	-2.412	Comprom	-1.127	Comprom	-1.770	0.117	GT	Sens	contig374	contig374	Unannota	[illegible]
GT	Sense	Sense	-0.329	Detected	0.329	Detected	0.000	-1.104	Comprom	-1.337	Comprom	-1.220	0.073	GT	Sens	contig375	contig375	Unannota	[illegible]
GT	Sense	Sense	-0.261	Detected	0.261	Detected	0.000	-1.184	Detected	-0.937	Detected	-1.060	0.067	GT	Sens	contig375	contig375	Unannota	[illegible]
GT	Sense	Sense	-0.275	Detected	0.275	Detected	0.000	-0.936	Detected	-1.618	Detected	-1.277	0.100	GT	Sens	contig375	contig375	Unannota	[illegible]
GT	Sense	Sense	-0.636	Detected	0.636	Detected	0.000	-1.219	Detected	-2.137	Detected	-1.678	0.166	GT	Sens	contig375	contig375	Unannota	[illegible]
GT	Sense	Sense	-0.161	Detected	0.161	Detected	0.000	-1.468	Comprom	-1.580	Comprom	-1.524	0.012	GT	Sens	contig375	contig375	Unannota	[illegible]
GT	Sense	Sense	-0.094	Detected	0.094	Detected	0.000	-1.690	Detected	-1.607	Detected	-1.648	0.004	GT	Sens	contig376	contig376	Unannota	[illegible]
GT	Sense	Sense	-0.440	Detected	0.440	Detected	0.000	-2.192	Detected	-2.955	Detected	-2.573	0.048	GT	Sens	contig376	contig376	Unannota	[illegible]
GT	Sense	Sense	0.145	Detected	-0.145	Detected	0.000	-1.349	Comprom	-1.174	Comprom	-1.262	0.018	GT	Sens	contig376	contig376	Unannota	[illegible]
GT	Sense	Sense	-0.181	Detected	0.181	Detected	0.000	-1.404	Detected	-1.061	Detected	-1.232	0.039	GT	Sens	contig377	contig377	Unannota	[illegible]
GT	Sense	Sense	-0.610	Detected	0.610	Detected	0.000	-1.978	Detected	-2.307	Detected	-2.143	0.077	GT	Sens	contig377	contig377	Ethylene-	[illegible]
GT	Sense	Sense	-0.192	Detected	0.192	Detected	0.000	-1.188	Detected	-1.355	Detected	-1.271	0.026	GT	Sens	contig377	contig377	Unannota	[illegible]
GT	Sense	Sense	-0.145	Detected	0.145	Detected	0.000	-2.432	Comprom	-2.296	Comprom	-2.364	0.005	GT	Sens	contig377	contig377	Gelsolin (	[illegible]
GT	Sense	Sense	-0.206	Detected	0.206	Detected	0.000	-1.233	Detected	-1.075	Detected	-1.154	0.035	GT	Sens	contig378	contig378	Unannota	[illegible]
GT	Sense	Sense	-0.260	Detected	0.260	Detected	0.000	-1.282	Detected	-1.119	Detected	-1.201	0.048	GT	Sens	contig378	contig378	Unannota	[illegible]
GT	Sense	Sense	-0.248	Detected	0.248	Detected	0.000	-1.031	Detected	-1.579	Detected	-1.305	0.072	GT	Sens	contig378	contig378	Unannota	[illegible]
GT	Sense	Sense	-0.476	Detected	0.476	Detected	0.000	-1.379	Detected	-1.108	Detected	-1.244	0.128	GT	Sens	contig379	contig379	Unannota	[illegible]
GT	Sense	Sense	-0.425	Detected	0.425	Detected	0.000	-1.500	Detected	-1.269	Detected	-1.384	0.088	GT	Sens	contig379	contig379	Unannota	[illegible]
GT	Sense	Sense	-0.243	Detected	0.243	Detected	0.000	-0.863	Detected	-1.354	Comprom	-1.109	0.085	GT	Sens	contig379	contig379	Unannota	[illegible]
GT	Sense	Sense	-0.253	Detected	0.253	Detected	0.000	-1.212	Comprom	-1.598	Comprom	-1.405	0.048	GT	Sens	contig381	contig381	Unannota	[illegible]
GT	Sense	Sense	-0.314	Detected	0.314	Detected	0.000	-1.297	Detected	-1.590	Detected	-1.444	0.053	GT	Sens	contig381	contig381	Unannota	[illegible]
GT	Sense	Sense	-0.062	Detected	0.062	Detected	0.000	-1.582	Comprom	-1.354	Comprom	-1.468	0.008	GT	Sens	contig381	contig381	Unannota	[illegible]
GT	Sense	Sense	-0.094	Detected	0.094	Detected	0.000	-0.984	Detected	-2.107	Detected	-1.546	0.113	GT	Sens	contig381	contig381	Unannota	[illegible]
GT	Sense	Sense	-0.027	Detected	0.027	Detected	0.000	-1.328	Detected	-0.827	Detected	-1.078	0.051	GT	Sens	contig382	contig382	Unannota	[illegible]
GT	Sense	Sense	0.108	Detected	-0.108	Detected	0.000	-1.265	Comprom	-1.281	Comprom	-1.273	0.007	GT	Sens	contig382	contig382	Unannota	[illegible]
GT	Sense	Sense	-0.257	Detected	0.257	Detected	0.000	-0.956	Detected	-1.136	Detected	-1.046	0.062	GT	Sens	contig382	contig382	Unannota	[illegible]
GT	Sense	Sense	0.088	Detected	-0.088	Detected	0.000	-1.348	Detected	-1.248	Detected	-1.298	0.006	GT	Sens	contig382	contig382	Unannota	[illegible]
GT	Sense	Sense	0.217	Detected	-0.217	Detected	0.000	-1.332	Comprom	-2.407	Comprom	-1.869	0.084	GT	Sens	contig383	contig383	Unannota	[illegible]
GT	Sense	Sense	-0.011	Detected	0.011	Detected	0.000	-2.786	Comprom	-3.549	Comprom	-3.168	0.014	GT	Sens	contig384	contig384	C4-dicarb	[illegible]
GT	Sense	Sense	-0.716	Detected	0.716	Detected	0.000	-1.658	Detected	-3.756	Detected	-2.707	0.167	GT	Sens	contig386	contig386	WRKY tra	[illegible]
GT	Sense	Sense	-1.074	Detected	1.074	Detected	0.000	-1.105	Detected	-1.869	Detected	-1.487	0.322	GT	Sens	contig386	contig386	Unannota	[illegible]
GT	Sense	Sense	-0.198	Detected	0.198	Detected	0.000	-0.986	Detected	-1.032	Detected	-1.009	0.037	GT	Sens	contig386	contig386	Unannota	[illegible]
GT	Sense	Sense	-0.083	Detected	0.083	Detected	0.000	-2.086	Comprom	-1.189	Detected	-1.638	0.070	GT	Sens	contig386	contig386	Unannota	[illegible]
GT	Sense	Sense	-0.839	Detected	0.839	Detected	0.000	-1.238	Detected	-2.360	Detected	-1.799	0.217	GT	Sens	contig387	contig387	Unannota	[illegible]
GT	Sense	Sense	-0.311	Detected	0.311	Detected	0.000	-0.882	Detected	-1.543	Detected	-1.213	0.116	GT	Sens	contig387	contig387	Unannota	[illegible]
GT	Sense	Sense	-0.374	Detected	0.374	Detected	0.000	-0.972	Detected	-1.651	Detected	-1.312	0.122	GT	Sens	contig387	contig387	Unannota	[illegible]
GT	Sense	Sense	0.037	Detected	-0.037	Detected	0.000	-1.279	Comprom	-1.370	Comprom	-1.325	0.002	GT	Sens	contig387	contig387	Unannota	[illegible]
GT	Sense	Sense	-0.536	Detected	0.536	Detected	0.000	-1.056	Detected	-1.747	Comprom	-1.402	0.159	GT	Sens	contig387	contig387	Unannota	[illegible]
GT	Sense	Sense	-0.289	Detected	0.289	Detected	0.000	-0.995	Comprom	-1.793	Comprom	-1.394	0.106	GT	Sens	contig388	contig388	Unannota	[illegible]
GT	Sense	Sense	0.088	Detected	-0.088	Detected	0.000	-1.283	Comprom	-1.151	Comprom	-1.217	0.008	GT	Sens	contig388	contig388	Unannota	[illegible]
GT	Sense	Sense	-1.169	Detected	1.169	Detected	0.000	-2.185	Detected	-3.373	Detected	-2.779	0.168	GT	Sens	contig389	contig389	Unannota	[illegible]
GT	Sense	Sense	-1.108	Detected	1.108	Detected	0.000	-2.028	Detected	-3.345	Detected	-2.687	0.172	GT	Sens	contig389	contig389	Unannota	[illegible]
GT	Sense	Sense	1.030	Detected	-1.030	Detected	0.000	-2.538	Comprom	-1.175	Comprom	-1.857	0.272	GT	Sens	contig391	contig391	Unannota	[illegible]
GT	Sense	Sense	0.270	Detected	-0.270	Detected	0.000	-1.241	Detected	-1.026	Detected	-1.133	0.060	GT	Sens	contig392	contig392	Unannota	[illegible]
GT	Sense	Sense	0.196	Detected	-0.196	Detected	0.000	-0.925	Detected	-1.287	Detected	-1.106	0.054	GT	Sens	contig392	contig392	Germin-li	[illegible]
GT	Sense	Sense	0.155	Detected	-0.155	Detected	0.000	-1.498	Comprom	-1.093	Comprom	-1.295	0.037	GT	Sens	contig393	contig393	Unannota	[illegible]
GT	Sense	Sense	-0.569	Detected	0.569	Detected	0.000	-1.251	Detected	-1.793	Detected	-1.522	0.137	GT	Sens	contig394	contig394	Unannota	[illegible]
GT	Sense	Sense	-0.486	Detected	0.486	Detected	0.000	-1.129	Detected	-2.519	Comprom	-1.824	0.164	GT	Sens	contig394	contig394	Unannota	[illegible]
GT	Sense	Sense	-0.252	Detected	0.252	Detected	0.000	-1.222	Detected	-0.921	Detected	-1.072	0.068	GT	Sens	contig394	contig394	Unannota	[illegible]
GT	Sense	Sense	-0.222	Detected	0.222	Detected	0.000	-1.345	Detected	-1.030	Detected	-1.188	0.049	GT	Sens	contig394	contig394	Unannota	[illegible]
GT	Sense	Sense	-0.174	Detected	0.174	Detected	0.000	-1.601	Comprom	-1.823	Comprom	-1.712	0.014	GT	Sens	contig394	contig394	Unannota	[illegible]
GT	Sense	Sense	-0.658	Detected	0.658	Detected	0.000	-1.092	Detected	-1.897	Detected	-1.495	0.192	GT	Sens	contig396	contig396	Unannota	[illegible]
GT	Sense	Sense	-0.566	Detected	0.566	Detected	0.000	-0.940	Detected	-1.958	Detected	-1.449	0.197	GT	Sens	contig396	contig396	Unannota	[illegible]
GT	Sense	Sense	-0.064	Detected	0.064	Detected	0.000	-1.038	Detected	-1.053	Detected	-1.045	0.004	GT	Sens	contig396	contig396	Unannota	[illegible]
GT	Sense	Sense	-0.352	Detected	0.352	Detected	0.000	-1.162	Comprom	-1.433	Comprom	-1.298	0.075	GT	Sens	contig396	contig396	Unannota	[illegible]
GT	Sense	Sense	-0.036	Detected	0.036	Detected	0.000	-1.888	Comprom	-0.832	Comprom	-1.360	0.124	GT	Sens	contig396	contig396	Unannota	[illegible]
GT	Sense	Sense	0.444	Detected	-0.444	Detected	0.000	-1.644	Comprom	-1.597	Comprom	-1.620	0.068	GT	Sens	contig397	contig397	Unannota	[illegible]
GT	Sense	Sense	-0.721	Detected	0.721	Detected	0.000	-1.562	Comprom	-2.088	Comprom	-1.825	0.140	GT	Sens	contig397	contig397	Unannota	[illegible]
GT	Sense	Sense	-0.759	Detected	0.759	Detected	0.000	-1.949	Detected	-3.287	Detected	-2.618	0.122	GT	Sens	contig398	contig398	Unannota	[illegible]
GT	Sense	Sense	-0.587	Detected	0.587	Detected	0.000	-1.597	Detected	-3.078	Detected	-2.337	0.132	GT	Sens	contig398	contig398	Unannota	[illegible]
GT	Sense	Sense	-0.268	Detected	0.268	Detected	0.000	-1.536	Detected	-1.224	Detected	-1.380	0.047	GT	Sens	contig398	contig398	Unannota	[illegible]
GT	Sense	Sense	-0.110	Detected	0.110	Detected	0.000	-1.412	Detected	-1.275	Detected	-1.344	0.009	GT	Sens	contig398	contig398	Unannota	[illegible]
GT	Sense	Sense	-0.094	Detected	0.094	Detected	0.000	-1.065	Detected	-1.254	Detected	-1.160	0.013	GT	Sens	contig399	contig399	Unannota	[illegible]
GT	Sense	Sense	0.535	Detected	-0.535	Detected	0.000	-1.033	Detected	-1.686	Comprom	-1.359	0.162	GT	Sens	contig399	contig399	Unannota	[illegible]
GT	Sense	Sense	-0.307	Detected	0.307	Detected	0.000	-0.898	Comprom	-1.890	Comprom	-1.394	0.139	GT	Sens	contig399	contig399	Unannota	[illegible]
GT	Sense	Sense	-0.330	Detected	0.330	Detected	0.000	-1.004	Detected	-2.188	Comprom	-1.596	0.143	GT	Sens	contig399	contig399	Unannota	[illegible]
GT	Sense	Sense	0.255	Detected	-0.255	Detected	0.000	-6.981	Comprom	-7.130	Comprom	-7.055	0.001	GT	Sens	contig399	contig399	Unannota	[illegible]
GT	Sense	Sense	0.297	Detected	-0.297	Detected	0.000	-6.187	Comprom	-6.081	Comprom	-6.134	0.002	GT	Sens	contig399	contig399	Unannota	[illegible]
GT	Sense	Sense	0.539	Detected	-0.539	Detected	0.000	-1.736	Detected	-1.467	Detected	-1.601	0.102	GT	Sens	contig400	contig400	Unannota	[illegible]
GT	Sense	Sense	-0.102	Detected	0.102	Detected	0.000	-1.335	Comprom	-1.116	Comprom	-1.226	0.015	GT	Sens	contig400	contig400	Unannota	[illegible]
GT	Sense	Sense	0.213	Detected	-0.213	Detected	0.000	-2.131	Detected	-2.587	Detected	-2.359	0.017	GT	Sens	contig400	contig400	Unannota	[illegible]
GT	Sense	Sense	0.189	Detected	-0.189	Detected	0.000	-1.343	Detected	-1.899	Detected	-1.621	0.040	GT	Sens	contig400	contig400	Unannota	[illegible]
GT	Sense	Sense	-0.026	Detected	0.026	Detected	0.000	-1.289	Comprom	-1.220	Comprom	-1.255	0.001	GT	Sens	contig400	contig400	Unannota	[illegible]
GT	Sense	Sense	-1.030	Detected	1.030	Detected	0.000	-1.659	Detected	-1.957	Detected	-1.808	0.224	GT	Sens	contig400	contig400	Unannota	[illegible]
GT	Sense	Sense	-1.089	Detected	1.089	Detected	0.000	-1.660	Detected	-2.206	Detected	-1.933	0.227	GT	Sens	contig400	contig400	Unannota	[illegible]
GT	Sense	Sense	-0.621	Detected	0.621	Detected	0.000	-1.356	Detected	-1.441	Detected	-1.398	0.154	GT	Sens	contig400	contig400	Unannota	[illegible]
GT	Sense	Sense	-0.175	Detected	0.175	Detected	0.000	-1.810	Comprom	-0.907	Comprom	-1.359	0.107	GT	Sens	contig401	contig401	Pentatric	[illegible]
GT	Sense	Sense	-0.127	Detected	0.127	Detected	0.000	-1.671	Detected	-1.594	Detected	-1.633	0.007	GT	Sens	contig401	contig401	Unannota	[illegible]
GT	Sense	Sense	-0.397	Detected	0.397	Detected	0.000	-0.913	Detected	-1.631	Detected	-1.272	0.141	GT	Sens	contig401	contig401	Unannota	[illegible]
GT	Sense	Sense	0.991	Detected	-0.991	Detected	0.000	-2.072	Detected	-1.406	Detected	-1.739	0.238	GT	Sens	contig401	contig401	Unannota	[illegible]
GT	Sense	Sense	-0.119	Detected	0.119	Detected	0.000	-2.037	Comprom	-2.193	Comprom	-2.115	0.004	GT	Sens	contig401	contig401	Unannota	[illegible]
GT	Sense	Sense	-0.375	Detected	0.375	Detected	0.000	-1.239	Detected	-1.619	Detected	-1.429	0.077	GT	Sens	contig401	contig401	NAC dom	[illegible]
GT	Sense	Sense	0.013	Detected	-0.013	Detected	0.000	-0.876	Comprom	-1.241	Comprom	-1.059	0.028	GT	Sens	contig402	contig402	Unannota	[illegible]
GT	Sense	Sense	-0.091	Detected	0.091	Detected	0.000	-0.944	Detected	-1.091	Detected	-1.018	0.013	GT	Sens	contig402	contig402	Unannota	[illegible]
GT	Sense	Sense	-0.430	Detected	0.430	Detected	0.000	-1.169	Detected	-1.407	Detected	-1.288	0.102	GT	Sens	contig403	contig403	Unannota	[illegible]
GT	Sense	Sense	-0.410	Detected	0.410	Detected	0.000	-1.340	Detected	-1.548	Detected	-1.444	0.076	GT	Sens	contig403	contig403	Receptor	[illegible]
GT	Sense	Sense	0.827	Detected	-0.827	Detected	0.000	-1.695	Detected	-1.791	Detected	-1.743	0.170	GT	Sens	contig403	contig403	Unannota	[illegible]
GT	Sense	Sense	-0.447	Detected	0.447	Detected	0.000	-1.064	Detected	-2.576	Detected	-1.820	0.174	GT	Sens	contig403	contig403	Unannota	[illegible]
GT	Sense	Sense	-0.352	Detected	0.352	Detected	0.000	-1.101	Comprom	-0.927	Comprom	-1.014	0.107	GT	Sens	contig403	contig403	Unannota	[illegible]
GT	Sense	Sense	-0.330	Detected	0.330	Detected	0.000	-0.913	Detected	-1.553	Detected	-1.233	0.116	GT	Sens	contig403	contig403	Unannota	[illegible]
GT	Sense	Sense	-0.217	Detected	0.217	Detected	0.000	-0.969	Comprom	-2.424	Comprom	-1.696	0.155	GT	Sens	contig403	contig403	Unannota	[illegible]
GT	Sense	Sense	-0.615	Detected	0.615	Detected	0.000	-1.872	Detected	-1.697	Detected	-1.784	0.103	GT	Sens	contig404	contig404	Unannota	[illegible]
GT	Sense	Sense	-0.443	Detected	0.443	Detected	0.000	-1.259	Comprom	-2.227	Comprom	-1.743	0.117	GT	Sens	contig404	contig404	Unannota	[illegible]
GT	Sense	Sense	-0.388	Detected	0.388	Detected	0.000	-1.659	Detected	-1.668	Detected	-1.664	0.050	GT	Sens	contig405	contig405	Unknown	[illegible]
GT	Sense	Sense	2.249	Detected	-2.249	Detected	0.000	-3.557	Comprom	-3.399	Comprom	-3.478	0.262	GT	Sens	contig405	contig405	Unannota	[illegible]
GT	Sense	Sense	2.713	Detected	-2.713	Detected	0.000	-3.412	Detected	-3.697	Detected	-3.554	0.321	GT	Sens	contig405	contig405	Unannota	[illegible]
GT	Sense	Sense	-0.076	Detected	0.076	Detected	0.000	-1.352	Detected	-0.950	Detected	-1.151	0.033	GT	Sens	contig405	contig405	Unannota	[illegible]
GT	Sense	Sense	-0.256	Detected	0.256	Detected	0.000	-1.740	Comprom	-1.276	Comprom	-1.508	0.049	GT	Sens	contig405	contig405	Unannota	[illegible]
GT	Sense	Sense	0.282	Detected	-0.282	Detected	0.000	-1.007	Detected	-1.343	Detected	-1.175	0.070	GT	Sens	contig405	contig405	Unannota	[illegible]
GT	Sense	Sense	0.336	Detected	-0.336	Detected	0.000	-2.081	Comprom	-1.729	Comprom	-1.905	0.037	GT	Sens	contig405	contig405	Unannota	[illegible]
GT	Sense	Sense	-0.281	Detected	0.281	Detected	0.000	-0.816	Detected	-1.295	Detected	-1.055	0.104	GT	Sens	contig406	contig406	Unannota	[illegible]
GT	Sense	Sense	-0.326	Detected	0.326	Detected	0.000	-1.065	Detected	-1.303	Detected	-1.184	0.076	GT	Sens	contig406	contig406	Unannota	[illegible]
GT	Sense	Sense	-0.292	Detected	0.292	Detected	0.000	-0.827	Detected	-1.332	Detected	-1.079	0.108	GT	Sens	contig407	contig407	Unannota	[illegible]
GT	Sense	Sense	-0.211	Detected	0.211	Detected	0.000	-1.370	Detected	-2.092	Comprom	-1.731	0.054	GT	Sens	contig407	contig407	Unannota	[illegible]
GT	Sense	Sense	-0.891	Detected	0.891	Detected	0.000	-1.885	Detected	-0.937	Detected	-1.411	0.297	GT	Sens	contig407	contig407	Unannota	[illegible]
GT	Sense	Sense	1.060	Detected	-1.060	Detected	0.000	-1.779	Comprom	-2.361	Comprom	-2.070	0.200	GT	Sens	contig407	contig407	Unannota	[illegible]
GT	Sense	Sense	-0.533	Detected	0.533	Detected	0.000	-1.496	Detected	-2.053	Detected	-1.774	0.098	GT	Sens	contig408	contig408	Ankyrin r	[illegible]
GT	Sense	Sense	-0.540	Detected	0.540	Detected	0.000	-2.317	Detected	-3.947	Detected	-3.132	0.085	GT	Sens	contig408	contig408	Glycosylt	[illegible]
GT	Sense	Sense	-0.504	Detected	0.504	Detected	0.000	-1.477	Comprom	-1.073	Comprom	-1.275	0.143	GT	Sens	contig408	contig408	SAR8.2 pr	[illegible]
GT	Sense	Sense	-0.491	Detected	0.491	Detected	0.000	-1.040	Detected	-1.042	Detected	-1.041	0.168	GT	Sens	contig409	contig409	Unannota	[illegible]
GT	Sense	Sense	0.522	Detected	-0.522	Detected	0.000	-1.670	Comprom	-1.378	Comprom	-1.524	0.107	GT	Sens	contig409	contig409	Unannota	[illegible]
GT	Sense	Sense	-0.026	Detected	0.026	Detected	0.000	-1.023	Detected	-1.595	Comprom	-1.309	0.045	GT	Sens	contig409	contig409	Unannota	[illegible]
GT	Sense	Sense	-0.134	Detected	0.134	Detected	0.000	-0.808	Detected	-1.290	Detected	-1.049	0.063	GT	Sens	contig410	contig410	Unannota	[illegible]
GT	Sense	Sense	0.866	Detected	-0.866	Detected	0.000	-2.133	Comprom	-1.527	Comprom	-1.830	0.184	GT	Sens	contig410	contig410	Unannota	[illegible]
GT	Sense	Sense	-0.069	Detected	0.069	Detected	0.000	-1.092	Comprom	-1.381	Comprom	-1.237	0.016	GT	Sens	contig410	contig410	Unannota	[illegible]
GT	Sense	Sense	0.555	Detected	-0.555	Detected	0.000	-2.886	Detected	-2.081	Detected	-2.484	0.068	GT	Sens	contig411	contig411	Unannota	[illegible]
GT	Sense	Sense	-0.510	Detected	0.510	Detected	0.000	-2.061	Detected	-2.804	Detected	-2.432	0.061	GT	Sens	contig411	contig411	CHP-rich	[illegible]
GT	Sense	Sense	-0.369	Detected	0.369	Detected	0.000	-1.715	Comprom	-1.284	Comprom	-1.499	0.073	GT	Sens	contig412	contig412	Unannota	[illegible]
GT	Sense	Sense	0.449	Detected	-0.449	Detected	0.000	-0.997	Detected	-1.772	Comprom	-1.384	0.145	GT	Sens	contig412	contig412	Unannota	[illegible]
GT	Sense	Sense	-1.135	Detected	1.135	Detected	0.000	-1.055	Detected	-2.333	Detected	-1.694	0.323	GT	Sens	contig412	contig412	Unannota	[illegible]
GT	Sense	Sense	-0.086	Detected	0.086	Detected	0.000	-0.931	Detected	-1.106	Detected	-1.019	0.014	GT	Sens	contig413	contig413	Unannota	[illegible]
GT	Sense	Sense	-0.911	Detected	0.911	Detected	0.000	-0.918	Detected	-2.252	Detected	-1.585	0.296	GT	Sens	contig413	contig413	Unannota	[illegible]
GT	Sense	Sense	0.005	Detected	-0.005	Detected	0.000	-1.884	Comprom	-2.022	Comprom	-1.953	0.001	GT	Sens	contig413	contig413	Unannota	[illegible]
GT	Sense	Sense	0.279	Detected	-0.279	Detected	0.000	-1.943	Comprom	-2.323	Comprom	-2.133	0.024	GT	Sens	contig414	contig414	Unannota	[illegible]
GT	Sense	Sense	0.130	Detected	-0.130	Detected	0.000	-0.999	Detected	-1.077	Detected	-1.038	0.017	GT	Sens	contig414	contig414	Unannota	[illegible]
GT	Sense	Sense	-0.066	Detected	0.066	Detected	0.000	-1.482	Detected	-1.086	Detected	-1.284	0.025	GT	Sens	contig414	contig414	Unannota	[illegible]
GT	Sense	Sense	-0.192	Detected	0.192	Detected	0.000	-1.106	Detected	-1.744	Comprom	-1.425	0.062	GT	Sens	contig415	contig415	Unannota	[illegible]
GT	Sense	Sense	0.473	Detected	-0.473	Detected	0.000	-1.291	Detected	-1.185	Detected	-1.238	0.122	GT	Sens	contig415	contig415	Unannota	[illegible]
GT	Sense	Sense	-0.353	Detected	0.353	Detected	0.000	-1.516	Detected	-1.658	Detected	-1.587	0.048	GT	Sens	contig415	contig415	Unannota	[illegible]
GT	Sense	Sense	-0.120	Detected	0.120	Detected	0.000	-1.989	Comprom	-2.231	Comprom	-2.110	0.006	GT	Sens	contig415	contig415	Unannota	[illegible]
GT	Sense	Sense	1.687	Detected	-1.687	Detected	0.000	-3.666	Comprom	-1.714	Detected	-2.690	0.302	GT	Sens	contig416	contig416	Unannota	[illegible]
GT	Sense	Sense	-0.242	Detected	0.242	Detected	0.000	-2.248	Detected	-3.842	Comprom	-3.045	0.067	GT	Sens	contig416	contig416	Kunitz try	[illegible]
GT	Sense	Sense	-0.259	Detected	0.259	Detected	0.000	-1.405	Detected	-1.413	Detected	-1.409	0.032	GT	Sens	contig416	contig416	Unannota	[illegible]
GT	Sense	Sense	-0.546	Detected	0.546	Detected	0.000	-1.384	Detected	-2.912	Detected	-2.148	0.149	GT	Sens	contig416	contig416	Unannota	[illegible]
GT	Sense	Sense	-0.674	Detected	0.674	Detected	0.000	-1.414	Detected	-3.705	Detected	-2.560	0.194	GT	Sens	contig416	contig416	Unannota	[illegible]
GT	Sense	Sense	0.206	Detected	-0.206	Detected	0.000	-3.049	Detected	-4.899	Detected	-3.974	0.052	GT	Sens	contig416	contig416	Unannota	[illegible]
GT	Sense	Sense	0.274	Detected	-0.274	Detected	0.000	-2.724	Detected	-4.905	Detected	-3.814	0.077	GT	Sens	contig416	contig416	Unannota	[illegible]
GT	Sense	Sense	-0.407	Detected	0.407	Detected	0.000	-1.593	Detected	-1.924	Detected	-1.759	0.057	GT	Sens	contig417	contig417	Unannota	[illegible]
GT	Sense	Sense	-0.080	Detected	0.080	Detected	0.000	-1.023	Comprom	-1.995	Comprom	-1.509	0.092	GT	Sens	contig417	contig417	Unannota	[illegible]
GT	Sense	Sense	0.239	Detected	-0.239	Detected	0.000	-0.925	Detected	-2.452	Comprom	-1.688	0.169	GT	Sens	contig417	contig417	Unannota	[illegible]
GT	Sense	Sense	-0.365	Detected	0.365	Detected	0.000	-1.022	Detected	-1.932	Detected	-1.477	0.127	GT	Sens	contig417	contig417	Unannota	[illegible]
GT	Sense	Sense	-0.304	Detected	0.304	Detected	0.000	-0.909	Detected	-1.279	Detected	-1.094	0.092	GT	Sens	contig418	contig418	Transpos	[illegible]
GT	Sense	Sense	0.059	Detected	-0.059	Detected	0.000	-1.220	Detected	-1.809	Detected	-1.515	0.037	GT	Sens	contig418	contig418	Unannota	[illegible]
GT	Sense	Sense	0.010	Detected	-0.010	Detected	0.000	-2.275	Comprom	-2.353	Comprom	-2.314	0.000	GT	Sens	contig418	contig418	Unannota	[illegible]
GT	Sense	Sense	-0.035	Detected	0.035	Detected	0.000	-1.054	Detected	-1.226	Detected	-1.140	0.007	GT	Sens	contig419	contig419	Unannota	[illegible]
GT	Sense	Sense	-0.045	Detected	0.045	Detected	0.000	-1.994	Detected	-1.813	Detected	-1.904	0.003	GT	Sens	contig419	contig419	Unannota	[illegible]
GT	Sense	Sense	-0.074	Detected	0.074	Detected	0.000	-2.196	Detected	-1.257	Detected	-1.727	0.068	GT	Sens	contig419	contig419	Unannota	[illegible]

GT	Sens	Sense	-0.048	Detected	0.048	Detected	0.000	-2.208	Detected	-1.370	Detected	-1.789	0.051	GT	Sens	[illegible]
GT	Sens	Sense	-0.111	Detected	0.111	Detected	0.000	-1.313	Comprom	-0.946	Comprom	-1.129	0.034	GT	Sens	[illegible]
GT	Sens	Sense	-0.019	Detected	0.019	Detected	0.000	-1.460	Detected	-1.620	Detected	-1.540	0.003	GT	Sens	[illegible]
GT	Sens	Sense	-0.118	Detected	0.118	Detected	0.000	-1.428	Comprom	-1.182	Comprom	-1.305	0.017	GT	Sens	[illegible]
GT	Sens	Sense	-0.729	Detected	0.729	Detected	0.000	-2.151	Detected	-5.740	Comprom	-3.945	0.179	GT	Sens	[illegible]
GT	Sens	Sense	-0.261	Detected	0.261	Detected	0.000	-1.657	Comprom	-1.923	Comprom	-1.790	0.026	GT	Sens	[illegible]
GT	Sens	Sense	-0.190	Detected	0.190	Detected	0.000	-0.856	Detected	-1.262	Detected	-1.059	0.063	GT	Sens	[illegible]
GT	Sens	Sense	0.788	Detected	-0.788	Detected	0.000	-1.764	Detected	-1.270	Detected	-1.517	0.208	GT	Sens	[illegible]
GT	Sens	Sense	0.114	Detected	-0.114	Detected	0.000	-0.940	Detected	-2.100	Detected	-1.520	0.124	GT	Sens	[illegible]
GT	Sens	Sense	0.088	Detected	-0.088	Detected	0.000	-1.126	Comprom	-1.082	Comprom	-1.104	0.007	GT	Sens	[illegible]
GT	Sens	Sense	-0.067	Detected	0.067	Detected	0.000	-1.183	Detected	-1.612	Detected	-1.398	0.025	GT	Sens	[illegible]
GT	Sens	Sense	-0.202	Detected	0.202	Detected	0.000	-0.988	Detected	-1.415	Detected	-1.201	0.055	GT	Sens	[illegible]
GT	Sens	Sense	-0.819	Detected	0.819	Detected	0.000	-1.008	Detected	-1.992	Detected	-1.500	0.257	GT	Sens	[illegible]
GT	Sens	Sense	-0.168	Detected	0.168	Detected	0.000	-0.850	Comprom	-1.219	Comprom	-1.035	0.054	GT	Sens	[illegible]
GT	Sens	Sense	-0.364	Detected	0.364	Detected	0.000	-1.586	Comprom	-1.058	Comprom	-1.322	0.099	GT	Sens	[illegible]
GT	Sens	Sense	-0.139	Detected	0.139	Detected	0.000	-1.063	Detected	-1.150	Detected	-1.106	0.017	GT	Sens	[illegible]
GT	Sens	Sense	-0.100	Detected	0.100	Detected	0.000	-1.097	Detected	-1.480	Detected	-1.289	0.027	GT	Sens	[illegible]
GT	Sens	Sense	0.834	Detected	-0.834	Detected	0.000	-1.445	Detected	-1.351	Detected	-1.398	0.236	GT	Sens	[illegible]
GT	Sens	Sense	-0.131	Detected	0.131	Detected	0.000	-1.437	Detected	-1.072	Detected	-1.254	0.031	GT	Sens	[illegible]
GT	Sens	Sense	0.041	Detected	-0.041	Detected	0.000	-1.198	Detected	-2.320	Detected	-1.759	0.089	GT	Sens	[illegible]
GT	Sens	Sense	-0.073	Detected	0.073	Detected	0.000	-1.038	Detected	-1.176	Detected	-1.107	0.008	GT	Sens	[illegible]
GT	Sens	Sense	-0.100	Detected	0.100	Detected	0.000	-0.878	Detected	-1.767	Detected	-1.322	0.101	GT	Sens	[illegible]
GT	Sens	Sense	0.064	Detected	-0.064	Detected	0.000	-1.164	Detected	-1.355	Detected	-1.260	0.008	GT	Sens	[illegible]
GT	Sens	Sense	0.174	Detected	-0.174	Detected	0.000	-1.552	Detected	-4.064	Detected	-2.808	0.157	GT	Sens	[illegible]
GT	Sens	Sense	-0.488	Detected	0.488	Detected	0.000	-0.897	Detected	-1.781	Detected	-1.339	0.179	GT	Sens	[illegible]
GT	Sens	Sense	-0.105	Detected	0.105	Detected	0.000	-1.296	Comprom	-1.253	Comprom	-1.275	0.007	GT	Sens	[illegible]
GT	Sens	Sense	-0.082	Detected	0.082	Detected	0.000	-1.748	Detected	-2.185	Detected	-1.966	0.014	GT	Sens	[illegible]
GT	Sens	Sense	-0.415	Detected	0.415	Detected	0.000	-1.079	Detected	-1.204	Detected	-1.141	0.113	GT	Sens	[illegible]
GT	Sens	Sense	-0.343	Detected	0.343	Detected	0.000	-1.833	Detected	-2.908	Detected	-2.370	0.065	GT	Sens	[illegible]
GT	Sens	Sense	-0.887	Detected	0.887	Detected	0.000	-0.809	Detected	-1.817	Detected	-1.313	0.327	GT	Sens	[illegible]
GT	Sens	Sense	-0.987	Detected	0.987	Detected	0.000	-1.360	Detected	-2.072	Detected	-1.716	0.244	GT	Sens	[illegible]
GT	Sens	Sense	0.259	Detected	-0.259	Detected	0.000	-3.112	Comprom	-2.068	Detected	-2.590	0.047	GT	Sens	[illegible]
GT	Sens	Sense	0.026	Detected	-0.026	Detected	0.000	-0.826	Comprom	-1.789	Comprom	-1.308	0.113	GT	Sens	[illegible]
GT	Sens	Sense	-0.509	Detected	0.509	Detected	0.000	-1.245	Detected	-1.108	Detected	-1.177	0.149	GT	Sens	[illegible]
GT	Sens	Sense	0.014	Detected	-0.014	Detected	0.000	-1.362	Detected	-1.290	Detected	-1.326	0.001	GT	Sens	[illegible]
GT	Sens	Sense	-0.296	Detected	0.296	Detected	0.000	-1.033	Detected	-1.275	Detected	-1.154	0.069	GT	Sens	[illegible]
GT	Sens	Sense	-0.348	Detected	0.348	Detected	0.000	-1.317	Detected	-1.290	Detected	-1.303	0.065	GT	Sens	[illegible]
GT	Sens	Sense	-0.028	Detected	0.028	Detected	0.000	-1.313	Detected	-0.826	Detected	-1.069	0.049	GT	Sens	[illegible]
GT	Sens	Sense	-0.365	Detected	0.365	Detected	0.000	-1.142	Detected	-1.071	Detected	-1.106	0.094	GT	Sens	[illegible]
GT	Sens	Sense	0.102	Detected	-0.102	Detected	0.000	-1.119	Detected	-1.122	Detected	-1.120	0.008	GT	Sens	[illegible]
GT	Sens	Sense	-0.119	Detected	0.119	Detected	0.000	-1.095	Comprom	-1.049	Comprom	-1.072	0.013	GT	Sens	[illegible]
GT	Sens	Sense	-0.304	Detected	0.304	Detected	0.000	-1.232	Detected	-1.649	Detected	-1.441	0.060	GT	Sens	[illegible]
GT	Sens	Sense	-0.375	Detected	0.375	Detected	0.000	-2.202	Detected	-1.879	Detected	-2.041	0.038	GT	Sens	[illegible]
GT	Sens	Sense	-0.301	Detected	0.301	Detected	0.000	-1.562	Detected	-1.992	Detected	-1.777	0.041	GT	Sens	[illegible]
GT	Sens	Sense	-0.398	Detected	0.398	Detected	0.000	-0.841	Detected	-1.279	Detected	-1.060	0.145	GT	Sens	[illegible]
GT	Sens	Sense	-0.501	Detected	0.501	Detected	0.000	-3.333	Detected	-2.684	Detected	-3.009	0.037	GT	Sens	[illegible]
GT	Sens	Sense	-0.483	Detected	0.483	Detected	0.000	-1.371	Detected	-1.569	Detected	-1.470	0.097	GT	Sens	[illegible]
GT	Sens	Sense	-0.470	Detected	0.470	Detected	0.000	-0.912	Detected	-2.771	Detected	-1.842	0.219	GT	Sens	[illegible]
GT	Sens	Sense	-0.470	Detected	0.470	Detected	0.000	-1.316	Detected	-0.972	Detected	-1.144	0.150	GT	Sens	[illegible]
GT	Sens	Sense	-0.239	Detected	0.239	Detected	0.000	-0.919	Detected	-1.517	Detected	-1.218	0.086	GT	Sens	[illegible]
GT	Sens	Sense	-0.461	Detected	0.461	Detected	0.000	-2.426	Detected	-3.341	Detected	-2.884	0.047	GT	Sens	[illegible]
GT	Sens	Sense	-0.172	Detected	0.172	Detected	0.000	-1.152	Comprom	-1.122	Comprom	-1.137	0.022	GT	Sens	[illegible]
GT	Sens	Sense	-0.139	Detected	0.139	Detected	0.000	-2.917	Comprom	-1.743	Comprom	-2.330	0.061	GT	Sens	[illegible]
GT	Sens	Sense	-0.110	Detected	0.110	Detected	0.000	-2.188	Detected	-1.678	Detected	-1.933	0.020	GT	Sens	[illegible]
GT	Sens	Sense	0.119	Detected	-0.119	Detected	0.000	-1.023	Comprom	-1.379	Comprom	-1.201	0.030	GT	Sens	[illegible]
GT	Sens	Sense	-0.182	Detected	0.182	Detected	0.000	-1.521	Detected	-1.466	Detected	-1.494	0.015	GT	Sens	[illegible]
GT	Sens	Sense	-0.002	Detected	0.002	Detected	0.000	-1.039	Detected	-1.176	Detected	-1.108	0.004	GT	Sens	[illegible]
GT	Sens	Sense	-0.321	Detected	0.321	Detected	0.000	-0.968	Comprom	-1.150	Comprom	-1.059	0.086	GT	Sens	[illegible]
GT	Sens	Sense	-0.276	Detected	0.276	Detected	0.000	-0.909	Detected	-1.596	Detected	-1.253	0.105	GT	Sens	[illegible]
GT	Sens	Sense	-0.286	Detected	0.286	Detected	0.000	-1.084	Detected	-1.553	Detected	-1.319	0.070	GT	Sens	[illegible]
GT	Sens	Sense	-0.353	Detected	0.353	Detected	0.000	-1.381	Detected	-2.202	Detected	-1.791	0.080	GT	Sens	[illegible]
GT	Sens	Sense	0.024	Detected	-0.024	Detected	0.000	-1.595	Detected	-1.309	Detected	-1.452	0.010	GT	Sens	[illegible]
GT	Sens	Sense	-0.726	Detected	0.726	Detected	0.000	-2.188	Detected	-3.462	Comprom	-2.825	0.100	GT	Sens	[illegible]
GT	Sens	Sense	-0.581	Detected	0.581	Detected	0.000	-1.686	Detected	-2.542	Detected	-2.114	0.099	GT	Sens	[illegible]
GT	Sens	Sense	-0.487	Detected	0.487	Detected	0.000	-1.837	Comprom	-2.603	Comprom	-2.220	0.070	GT	Sens	[illegible]
GT	Sens	Sense	-0.726	Detected	0.726	Detected	0.000	-0.961	Detected	-1.834	Detected	-1.398	0.241	GT	Sens	[illegible]
GT	Sens	Sense	-0.001	Detected	0.001	Detected	0.000	-1.464	Detected	-1.201	Detected	-1.333	0.010	GT	Sens	[illegible]
GT	Sens	Sense	0.266	Detected	-0.266	Detected	0.000	-2.948	Detected	-1.715	Detected	-2.332	0.074	GT	Sens	[illegible]
GT	Sens	Sense	-0.077	Detected	0.077	Detected	0.000	-1.285	Detected	-0.883	Detected	-1.084	0.037	GT	Sens	[illegible]
GT	Sens	Sense	0.018	Detected	-0.018	Detected	0.000	-1.308	Comprom	-2.979	Comprom	-2.144	0.124	GT	Sens	[illegible]
GT	Sens	Sense	-0.641	Detected	0.641	Detected	0.000	-1.396	Detected	-2.346	Comprom	-1.871	0.144	GT	Sens	[illegible]
GT	Sens	Sense	-0.302	Detected	0.302	Detected	0.000	-1.907	Detected	-2.165	Detected	-2.036	0.025	GT	Sens	[illegible]
GT	Sens	Sense	-0.410	Detected	0.410	Detected	0.000	-0.966	Detected	-1.163	Detected	-1.065	0.128	GT	Sens	[illegible]
GT	Sens	Sense	0.047	Detected	-0.047	Detected	0.000	-1.449	Detected	-1.252	Detected	-1.351	0.006	GT	Sens	[illegible]
GT	Sens	Sense	-0.286	Detected	0.286	Detected	0.000	-2.605	Detected	-1.243	Detected	-1.924	0.121	GT	Sens	[illegible]
GT	Sens	Sense	-0.504	Detected	0.504	Detected	0.000	-1.440	Detected	-1.286	Detected	-1.363	0.116	GT	Sens	[illegible]
GT	Sens	Sense	-0.987	Detected	0.987	Detected	0.000	-1.282	Detected	-1.407	Detected	-1.345	0.307	GT	Sens	[illegible]
GT	Sens	Sense	-0.855	Detected	0.855	Detected	0.000	-1.019	Detected	-1.083	Detected	-1.051	0.344	GT	Sens	[illegible]
GT	Sens	Sense	-0.872	Detected	0.872	Detected	0.000	-1.964	Detected	-3.887	Detected	-2.925	0.153	GT	Sens	[illegible]
GT	Sens	Sense	-0.080	Detected	0.080	Detected	0.000	-1.805	Detected	-0.847	Detected	-1.326	0.112	GT	Sens	[illegible]
GT	Sens	Sense	-0.223	Detected	0.223	Detected	0.000	-1.132	Detected	-1.079	Detected	-1.106	0.039	GT	Sens	[illegible]
GT	Sens	Sense	-0.211	Detected	0.211	Detected	0.000	-1.196	Detected	-1.045	Detected	-1.120	0.038	GT	Sens	[illegible]
GT	Sens	Sense	-0.017	Detected	0.017	Detected	0.000	-1.261	Detected	-0.969	Detected	-1.115	0.017	GT	Sens	[illegible]
GT	Sens	Sense	-0.247	Detected	0.247	Detected	0.000	-2.042	Detected	-3.320	Detected	-2.681	0.059	GT	Sens	[illegible]
GT	Sens	Sense	-0.395	Detected	0.395	Detected	0.000	-0.969	Detected	-1.181	Comprom	-1.075	0.119	GT	Sens	[illegible]
GT	Sens	Sense	-0.125	Detected	0.125	Detected	0.000	-1.656	Detected	-0.824	Detected	-1.240	0.104	GT	Sens	[illegible]
GT	Sens	Sense	0.043	Detected	-0.043	Detected	0.000	-2.574	Comprom	-1.437	Comprom	-2.006	0.072	GT	Sens	[illegible]
GT	Sens	Sense	-0.124	Detected	0.124	Detected	0.000	-1.670	Detected	-1.976	Detected	-1.823	0.011	GT	Sens	[illegible]
GT	Sens	Sense	-0.129	Detected	0.129	Detected	0.000	-1.065	Detected	-1.015	Detected	-1.040	0.016	GT	Sens	[illegible]
GT	Sens	Sense	-0.676	Detected	0.676	Detected	0.000	-1.268	Detected	-2.109	Detected	-1.689	0.168	GT	Sens	[illegible]
GT	Sens	Sense	-0.100	Detected	0.100	Detected	0.000	-1.037	Detected	-1.599	Detected	-1.318	0.048	GT	Sens	[illegible]
GT	Sens	Sense	-0.339	Detected	0.339	Detected	0.000	-0.987	Detected	-1.195	Detected	-1.091	0.091	GT	Sens	[illegible]
GT	Sens	Sense	-0.231	Detected	0.231	Detected	0.000	-1.060	Detected	-1.445	Detected	-1.252	0.053	GT	Sens	[illegible]
GT	Sens	Sense	0.090	Detected	-0.090	Detected	0.000	-1.125	Comprom	-1.474	Comprom	-1.299	0.022	GT	Sens	[illegible]
GT	Sens	Sense	-0.923	Detected	0.923	Detected	0.000	-1.158	Detected	-2.144	Detected	-1.651	0.255	GT	Sens	[illegible]
GT	Sens	Sense	-0.515	Detected	0.515	Detected	0.000	-0.993	Detected	-2.746	Comprom	-1.870	0.207	GT	Sens	[illegible]
GT	Sens	Sense	-0.268	Detected	0.268	Detected	0.000	-1.862	Comprom	-1.506	Comprom	-1.684	0.035	GT	Sens	[illegible]
GT	Sens	Sense	-0.620	Detected	0.620	Detected	0.000	-1.552	Detected	-1.451	Detected	-1.502	0.137	GT	Sens	[illegible]
GT	Sens	Sense	-0.357	Detected	0.357	Detected	0.000	-1.832	Comprom	-1.460	Comprom	-1.646	0.055	GT	Sens	[illegible]
GT	Sens	Sense	0.023	Detected	-0.023	Detected	0.000	-1.301	Detected	-1.568	Detected	-1.434	0.009	GT	Sens	[illegible]
GT	Sens	Sense	-1.299	Detected	1.299	Detected	0.000	-3.516	Comprom	-4.677	Comprom	-4.096	0.102	GT	Sens	[illegible]
GT	Sens	Sense	-0.766	Detected	0.766	Detected	0.000	-1.371	Detected	-3.327	Comprom	-2.349	0.199	GT	Sens	[illegible]
GT	Sens	Sense	-0.318	Detected	0.318	Detected	0.000	-1.527	Detected	-1.065	Detected	-1.296	0.081	GT	Sens	[illegible]
GT	Sens	Sense	0.007	Detected	-0.007	Detected	0.000	-1.313	Comprom	-1.076	Comprom	-1.194	0.010	GT	Sens	[illegible]
GT	Sens	Sense	-0.901	Detected	0.901	Detected	0.000	-1.228	Detected	-1.105	Detected	-1.167	0.326	GT	Sens	[illegible]
GT	Sens	Sense	-0.321	Detected	0.321	Detected	0.000	-1.643	Detected	-1.782	Detected	-1.713	0.035	GT	Sens	[illegible]
GT	Sens	Sense	-0.469	Detected	0.469	Detected	0.000	-1.118	Detected	-1.018	Detected	-1.068	0.152	GT	Sens	[illegible]
GT	Sens	Sense	-0.023	Detected	0.023	Detected	0.000	-0.944	Detected	-1.081	Detected	-1.013	0.005	GT	Sens	[illegible]
GT	Sens	Sense	0.108	Detected	-0.108	Detected	0.000	-2.477	Comprom	-0.912	Comprom	-1.695	0.165	GT	Sens	[illegible]
GT	Sens	Sense	-0.385	Detected	0.385	Detected	0.000	-1.642	Detected	-1.433	Detected	-1.537	0.061	GT	Sens	[illegible]
GT	Sens	Sense	-0.356	Detected	0.356	Detected	0.000	-1.261	Detected	-1.129	Detected	-1.195	0.081	GT	Sens	[illegible]
GT	Sens	Sense	-0.398	Detected	0.398	Detected	0.000	-0.890	Detected	-1.631	Detected	-1.261	0.146	GT	Sens	[illegible]
GT	Sens	Sense	-0.885	Detected	0.885	Detected	0.000	-1.119	Detected	-1.150	Detected	-1.134	0.328	GT	Sens	[illegible]
GT	Sens	Sense	0.773	Detected	-0.773	Detected	0.000	-1.600	Detected	-2.640	Comprom	-2.120	0.151	GT	Sens	[illegible]
GT	Sens	Sense	-0.162	Detected	0.162	Detected	0.000	-1.522	Detected	-2.662	Detected	-2.092	0.072	GT	Sens	[illegible]
GT	Sens	Sense	0.035	Detected	-0.035	Detected	0.000	-2.149	Comprom	-1.215	Comprom	-1.682	0.070	GT	Sens	[illegible]
GT	Sens	Sense	0.856	Detected	-0.856	Detected	0.000	-1.595	Detected	-1.476	Detected	-1.536	0.215	GT	Sens	[illegible]
GT	Sens	Sense	-0.132	Detected	0.132	Detected	0.000	-0.905	Detected	-1.181	Detected	-1.043	0.032	GT	Sens	[illegible]
GT	Sens	Sense	-0.412	Detected	0.412	Detected	0.000	-1.551	Detected	-1.980	Detected	-1.765	0.063	GT	Sens	[illegible]
GT	Sens	Sense	0.036	Detected	-0.036	Detected	0.000	-1.624	Detected	-1.161	Detected	-1.393	0.027	GT	Sens	[illegible]
GT	Sens	Sense	-0.040	Detected	0.040	Detected	0.000	-1.831	Detected	-1.628	Detected	-1.729	0.004	GT	Sens	[illegible]
GT	Sens	Sense	-0.072	Detected	0.072	Detected	0.000	-1.065	Comprom	-1.400	Comprom	-1.233	0.021	GT	Sens	[illegible]
GT	Sens	Sense	-0.255	Detected	0.255	Detected	0.000	-2.697	Detected	-2.535	Detected	-2.616	0.010	GT	Sens	[illegible]
GT	Sens	Sense	-0.039	Detected	0.039	Detected	0.000	-0.919	Detected	-1.985	Detected	-1.452	0.113	GT	Sens	[illegible]
GT	Sens	Sense	-0.256	Detected	0.256	Detected	0.000	-1.466	Detected	-1.692	Detected	-1.579	0.030	GT	Sens	[illegible]
GT	Sens	Sense	-0.674	Detected	0.674	Detected	0.000	-1.035	Detected	-0.969	Detected	-1.002	0.276	GT	Sens	[illegible]
GT	Sens	Sense	-0.582	Detected	0.582	Detected	0.000	-1.109	Detected	-1.657	Comprom	-1.383	0.164	GT	Sens	[illegible]
GT	Sens	Sense	0.071	Detected	-0.071	Detected	0.000	-1.037	Detected	-1.086	Detected	-1.061	0.005	GT	Sens	[illegible]
GT	Sens	Sense	-0.375	Detected	0.375	Detected	0.000	-2.153	Detected	-1.666	Detected	-1.909	0.051	GT	Sens	[illegible]
GT	Sens	Sense	-1.082	Detected	1.082	Detected	0.000	-0.916	Detected	-2.636	Detected	-1.776	0.327	GT	Sens	[illegible]
GT	Sens	Sense	-1.049	Detected	1.049	Detected	0.000	-1.360	Detected	-2.912	Detected	-2.136	0.243	GT	Sens	[illegible]
GT	Sens	Sense	-1.156	Detected	1.156	Detected	0.000	-2.786	Comprom	-3.159	Comprom	-2.973	0.126	GT	Sens	[illegible]
GT	Sens	Sense	0.060	Detected	-0.060	Detected	0.000	-1.599	Detected	-1.075	Detected	-1.337	0.038	GT	Sens	[illegible]
GT	Sens	Sense	-0.123	Detected	0.123	Detected	0.000	-1.203	Comprom	-2.191	Comprom	-1.697	0.079	GT	Sens	[illegible]
GT	Sens	Sense	0.402	Detected	-0.402	Detected	0.000	-2.071	Detected	-3.820	Detected	-2.946	0.092	GT	Sens	[illegible]
GT	Sens	Sense	0.412	Detected	-0.412	Detected	0.000	-1.947	Detected	-1.112	Detected	-1.529	0.121	GT	Sens	[illegible]
GT	Sens	Sense	-0.143	Detected	0.143	Detected	0.000	-1.443	Detected	-1.669	Detected	-1.556	0.013	GT	Sens	[illegible]
GT	Sens	Sense	-0.947	Detected	0.947	Detected	0.000	-1.726	Detected	-1.055	Detected	-1.391	0.300	GT	Sens	[illegible]
GT	Sens	Sense	-0.953	Detected	0.953	Detected	0.000	-1.941	Detected	-3.723	Detected	-2.832	0.162	GT	Sens	[illegible]
GT	Sens	Sense	0.027	Detected	-0.027	Detected	0.000	-3.092	Detected	-2.178	Detected	-2.635	0.029	GT	Sens	[illegible]
GT	Sens	Sense	-0.062	Detected	0.062	Detected	0.000	-1.626	Detected	-1.294	Detected	-1.460	0.014	GT	Sens	[illegible]
GT	Sens	Sense	-0.146	Detected	0.146	Detected	0.000	-1.194	Comprom	-2.586	Comprom	-1.890	0.117	GT	Sens	[illegible]
GT	Sens	Sense	-0.252	Detected	0.252	Detected	0.000	-1.787	Detected	-1.716	Detected	-1.752	0.020	GT	Sens	[illegible]
GT	Sens	Sense	0.009	Detected	-0.009	Detected	0.000	-0.977	Detected	-1.053	Detected	-1.015	0.001	GT	Sens	[illegible]
GT	Sens	Sense	-0.737	Detected	0.737	Detected	0.000	-1.437	Comprom	-1.388	Comprom	-1.412	0.195	GT	Sens	[illegible]
GT	Sens	Sense	-0.112	Detected	0.112	Detected	0.000	-1.125	Detected	-0.982	Detected	-1.053	0.015	GT	Sens	[illegible]
GT	Sens	Sense	-0.289	Detected	0.289	Detected	0.000	-0.859	Detected	-1.217	Detected	-1.038	0.093	GT	Sens	[illegible]
GT	Sens	Sense	-0.732	Detected	0.732	Detected	0.000	-0.809	Detected	-1.500	Detected	-1.155	0.290	GT	Sens	[illegible]
GT	Sens	Sense	-0.831	Detected	0.831	Detected	0.000	-0.974	Detected	-1.882	Detected	-1.428	0.270	GT	Sens	[illegible]
GT	Sens	Sense	-0.412	Detected	0.412	Detected	0.000	-1.612	Detected	-2.279	Detected	-1.945	0.067	GT	Sens	[illegible]
GT	Sens	Sense	-0.096	Detected	0.096	Detected	0.000	-1.335	Detected	-1.308	Detected	-1.322	0.005	GT	Sens	[illegible]
GT	Sens	Sense	-0.439	Detected	0.439	Detected	0.000	-0.897	Detected	-3.294	Comprom	-2.096	0.242	GT	Sens	[illegible]
GT	Sens	Sense	-0.191	Detected	0.191	Detected	0.000	-0.885	Detected	-1.490	Detected	-1.187	0.080	GT	Sens	[illegible]
GT	Sens	Sense	-0.492	Detected	0.492	Detected	0.000	-1.798	Detected	-1.000	Detected	-1.399	0.158	GT	Sens	[illegible]
GT	Sens	Sense	-0.570	Detected	0.570	Detected	0.000	-1.107	Detected	-1.075	Detected	-1.091	0.196	GT	Sens	[illegible]
GT	Sens	Sense	0.067	Detected	-0.067	Detected	0.000	-1.050	Detected	-1.177	Detected	-1.113	0.007	GT	Sens	[illegible]
GT	Sens	Sense	-0.463	Detected	0.463	Detected	0.000	-0.843	Detected	-2.491	Comprom	-1.667	0.220	GT	Sens	[illegible]
GT	Sens	Sense	0.175	Detected	-0.175	Detected	0.000	-1.301	Detected	-1.221	Detected	-1.261	0.020	GT	Sens	[illegible]
GT	Sens	Sense	0.113	Detected	-0.113	Detected	0.000	-1.220	Detected	-1.733	Detected	-1.476	0.034	GT	Sens	[illegible]
GT	Sens	Sense	-0.203	Detected	0.203	Detected	0.000	-1.507	Detected	-1.187	Detected	-1.347	0.035	GT	Sens	[illegible]
GT	Sens	Sense	-0.321	Detected	0.321	Detected	0.000	-1.305	Detected	-1.284	Detected	-1.295	0.056	GT	Sens	[illegible]
GT	Sens	Sense	-0.363	Detected	0.363	Detected	0.000	-1.224	Detected	-1.893	Comprom	-1.559	0.087	GT	Sens	[illegible]
GT	Sens	Sense	-0.337	Detected	0.337	Detected	0.000	-1.642	Detected	-1.795	Detected	-1.719	0.038	GT	Sens	[illegible]
GT	Sens	Sense	-0.537	Detected	0.537	Detected	0.000	-0.858	Detected	-1.598	Detected	-1.228	0.200	GT	Sens	[illegible]
GT	Sens	Sense	-0.011	Detected	0.011	Detected	0.000	-1.139	Detected	-1.295	Detected	-1.217	0.004	GT	Sens	[illegible]
GT	Sens	Sense	0.440	Detected	-0.440	Detected	0.000	-0.861	Detected	-1.361	Detected	-1.111	0.159	GT	Sens	[illegible]
GT	Sens	Sense	0.516	Detected	-0.516	Detected	0.000	-0.871	Detected	-1.570	Detected	-1.220	0.189	GT	Sens	[illegible]
GT	Sens	Sense	-0.182	Detected	0.182	Detected	0.000	-0.896	Detected	-1.381	Detected	-1.138	0.064	GT	Sens	[illegible]
GT	Sens	Sense	-0.417	Detected	0.417	Detected	0.000	-0.925	Detected	-1.260	Detected	-1.093	0.136	GT	Sens	[illegible]
GT	Sens	Sense	0.025	Detected	-0.025	Detected	0.000	-1.211	Detected	-1.161	Detected	-1.186	0.001	GT	Sens	[illegible]
GT	Sens	Sense	-0.229	Detected	0.229	Detected	0.000	-1.492	Detected	-1.463	Detected	-1.477	0.023	GT	Sens	[illegible]
GT	Sens	Sense	-0.030	Detected	0.030	Detected	0.000	-1.739	Detected	-0.870	Detected	-1.305	0.096	GT	Sens	[illegible]
GT	Sens	Sense	-0.031	Detected	0.031	Detected	0.000	-2.530	Detected	-2.113	Detected	-2.321	0.008	GT	Sens	[illegible]
GT	Sens	Sense	-0.359	Detected	0.359	Detected	0.000	-0.948	Detected	-1.935	Detected	-1.441	0.142	GT	Sens	[illegible]
GT	Sens	Sense	0.124	Detected	-0.124	Detected	0.000	-1.018	Detected	-1.323	Detected	-1.171	0.027	GT	Sens	[illegible]
GT	Sens	Sense	-0.214	Detected	0.214	Detected	0.000	-1.271	Detected	-1.113	Detected	-1.192	0.035	GT	Sens	[illegible]
GT	Sens	Sense	0.035	Detected	-0.035	Detected	0.000	-2.630	Comprom	-2.531	Comprom	-2.581	0.001	GT	Sens	[illegible]
GT	Sens	Sense	0.142	Detected	-0.142	Detected	0.000	-1.914	Detected	-2.063	Detected	-1.988	0.006	GT	Sens	[illegible]
GT	Sens	Sense	-0.494	Detected	0.494	Detected	0.000	-1.729	Detected	-2.824	Detected	-2.276	0.091	GT	Sens	[illegible]
GT	Sens	Sense	0.113	Detected	-0.113	Detected	0.000	-1.153	Detected	-1.503	Detected	-1.328	0.024	GT	Sens	[illegible]
GT	Sens	Sense	0.144	Detected	-0.144	Detected	0.000	-1.000	Detected	-1.175	Detected	-1.088	0.023	GT	Sens	[illegible]
GT	Sens	Sense	-0.260	Detected	0.260	Detected	0.000	-1.895	Detected	-2.181	Detected	-2.038	0.021	GT	Sens	[illegible]
GT	Sens	Sense	-0.408	Detected	0.408	Detected	0.000	-1.525	Detected	-2.201	Detected	-1.863	0.072	GT	Sens	[illegible]
GT	Sens	Sense	-0.723	Detected	0.723	Detected	0.000	-1.237	Detected	-1.905	Detected	-1.571	0.187	GT	Sens	[illegible]
GT	Sens	Sense	-0.279	Detected	0.279	Detected	0.000	-1.166	Detected	-1.178	Detected	-1.172	0.052	GT	Sens	[illegible]
GT	Sens	Sense	0.186	Detected	-0.186	Detected	0.000	-1.242	Detected	-1.039	Detected	-1.141	0.033	GT	Sens	[illegible]
GT	Sens	Sense	-0.473	Detected	0.473	Detected	0.000	-1.147	Detected	-2.148	Detected	-1.648	0.139	GT	Sens	[illegible]
GT	Sens	Sense	0.133	Detected	-0.133	Detected	0.000	-1.599	Detected	-1.936	Detected	-1.768	0.014	GT	Sens	[illegible]
GT	Sens	Sense	0.194	Detected	-0.194	Detected	0.000	-2.435	Comprom	-2.254	Comprom	-2.345	0.008	GT	Sens	[illegible]
GT	Sens	Sense	-0.438	Detected	0.438	Detected	0.000	-1.922	Detected	-1.347	Detected	-1.635	0.089	GT	Sens	[illegible]
GT	Sens	Sense	-0.292	Detected	0.292	Detected	0.000	-1.716	Detected	-1.476	Detected	-1.596	0.037	GT	Sens	[illegible]
GT	Sens	Sense	-0.549	Detected	0.549	Detected	0.000	-2.385	Detected	-1.592	Detected	-1.988	0.099	GT	Sens	[illegible]
GT	Sens	Sense	-0.433	Detected	0.433	Detected	0.000	-0.950	Detected	-1.880	Detected	-1.415	0.156	GT	Sens	[illegible]
GT	Sens	Sense	0.021	Detected	-0.021	Detected	0.000	-2.064	Detected	-2.169	Detected	-2.116	0.001	GT	Sens	[illegible]
GT	Sens	Sense	-0.318	Detected	0.318	Detected	0.000	-1.827	Comprom	-1.340	Detected	-1.584	0.058	GT	Sens	[illegible]
GT	Sens	Sense	-0.594	Detected	0.594	Detected	0.000	-1.445	Detected	-1.336	Detected	-1.390	0.145	GT	Sens	[illegible]
GT	Sens	Sense	-0.733	Detected	0.733	Detected	0.000	-1.043	Detected	-2.094	Detected	-1.568	0.224	GT	Sens	[illegible]
GT	Sens	Sense	-0.318	Detected	0.318	Detected	0.000	-2.045	Detected	-1.371	Detected	-1.708	0.066	GT	Sens	[illegible]
GT	Sens	Sense	0.053	Detected	-0.053	Detected	0.000	-1.887	Detected	-2.512	Detected	-2.200	0.020	GT	Sens	[illegible]
GT	Sens	Sense	-0.163	Detected	0.163	Detected	0.000	-0.947	Detected	-1.991	Detected	-1.469	0.115	GT	Sens	[illegible]
GT	Sens	Sense	-0.842	Detected	0.842	Detected	0.000	-0.854	Detected	-2.226	Detected	-1.540	0.292	GT	Sens	[illegible]
GT	Sens	Sense	-0.464	Detected	0.464	Detected	0.000	-1.857	Detected	-1.673	Detected	-1.765	0.065	GT	Sens	[illegible]
GT	Sens	Sense	-0.175	Detected	0.175	Detected	0.000	-1.056	Detected	-1.351	Detected	-1.204	0.034	GT	Sens	[illegible]
GT	Sens	Sense	-0.240	Detected	0.240	Detected	0.000	-1.129	Detected	-1.399	Detected	-1.264	0.044	GT	Sens	[illegible]
GT	Sens	Sense	-0.377	Detected	0.377	Detected	0.000	-1.688	Detected	-1.199	Detected	-1.443	0.085	GT	Sens	[illegible]
GT	Sens	Sense	-0.696	Detected	0.696	Detected	0.000	-0.870	Detected	-1.808	Detected	-1.339	0.252	GT	Sens	[illegible]
GT	Sens	Sense	0.427	Detected	-0.427	Detected	0.000	-1.201	Detected	-0.847	Detected	-1.024	0.157	GT	Sens	[illegible]
GT	Sens	Sense	0.125	Detected	-0.125	Detected	0.000	-1.557	Detected	-1.106	Detected	-1.332	0.035	GT	Sens	[illegible]
GT	Sens	Sense	0.230	Detected	-0.230	Detected	0.000	-1.388	Detected	-1.172	Detected	-1.280	0.037	GT	Sens	[illegible]
GT	Sens	Sense	0.042	Detected	-0.042	Detected	0.000	-1.557	Detected	-0.926	Detected	-1.241	0.060	GT	Sens	[illegible]
GT	Sens	Sense	0.143	Detected	-0.143	Detected	0.000	-1.415	Detected	-1.229	Detected	-1.322	0.016	GT	Sens	[illegible]
GT	Sens	Sense	0.375	Detected	-0.375	Detected	0.000	-2.368	Detected	-1.075	Detected	-1.722	0.148	GT	Sens	[illegible]
GT	Sens	Sense	-0.804	Detected	0.804	Detected	0.000	-1.616	Detected	-2.667	Detected	-2.142	0.155	GT	Sens	[illegible]
GT	Sens	Sense	-0.266	Detected	0.266	Detected	0.000	-0.952	Comprom	-3.230	Comprom	-2.091	0.216	GT	Sens	[illegible]
GT	Sens	Sense	-0.264	Detected	0.264	Detected	0.000	-4.021	Detected	-3.163	Detected	-3.592	0.019	GT	Sens	[illegible]
GT	Sens	Sense	-0.722	Detected	0.722	Detected	0.000	-1.050	Detected	-2.161	Comprom	-1.605	0.220	GT	Sens	[illegible]
GT	Sens	Sense	-0.402	Detected	0.402	Detected	0.000	-1.350	Detected	-2.080	Detected	-1.715	0.087	GT	Sens	[illegible]
GT	Sens	Sense	-0.322	Detected	0.322	Detected	0.000	-1.509	Detected	-2.268	Comprom	-1.889	0.063	GT	Sens	[illegible]
GT	Sens	Sense	0.221	Detected	-0.221	Detected	0.000	-1.315	Detected	-1.231	Detected	-1.273	0.030	GT	Sens	[illegible]
GT	Sens	Sense	-0.553	Detected	0.553	Detected	0.000	-1.037	Comprom	-1.505	Comprom	-1.271	0.168	GT	Sens	[illegible]
GT	Sens	Sense	0.097	Detected	-0.097	Detected	0.000	-1.169	Comprom	-1.321	Comprom	-1.245	0.010	GT	Sens	[illegible]
GT	Sens	Sense	-0.089	Detected	0.089	Detected	0.000	-1.584	Detected	-1.836	Detected	-1.710	0.008	GT	Sens	[illegible]
GT	Sens	Sense	-0.846	Detected	0.846	Detected	0.000	-3.186	Comprom	-4.496	Comprom	-3.841	0.070	GT	Sens	[illegible]
GT	Sens	Sense	-0.154	Detected	0.154	Detected	0.000	-1.399	Detected	-0.816	Detected	-1.108	0.078	GT	Sens	[illegible]
GT	Sens	Sense	-0.706	Detected	0.706	Detected	0.000	-1.833	Comprom	-3.421	Comprom	-2.627	0.132	GT	Sens	[illegible]
GT	Sens	Sense	-0.113	Detected	0.113	Detected	0.000	-1.477	Detected	-1.155	Detected	-1.316	0.022	GT	Sens	[illegible]
GT	Sens	Sense	0.047	Detected	-0.047	Detected	0.000	-1.306	Detected	-0.874	Detected	-1.090	0.039	GT	Sens	[illegible]
GT	Sens	Sense	-0.562	Detected	0.562	Detected	0.000	-0.893	Detected	-1.121	Detected	-1.007	0.221	GT	Sens	[illegible]
GT	Sens	Sense	-0.389	Detected	0.389	Detected	0.000	-1.004	Comprom	-1.600	Comprom	-1.302	0.117	GT	Sens	[illegible]
GT	Sens	Sense	0.411	Detected	-0.411	Detected	0.000	-2.548	Detected	-1.132	Detected	-1.840	0.154	GT	Sens	[illegible]
GT	Sens	Sense	0.594	Detected	-0.594	Detected	0.000	-0.815	Detected	-1.350	Detected	-1.083	0.238	GT	Sens	[illegible]
GT	Sens	Sense	-0.836	Detected	0.836	Detected	0.000	-0.852	Detected	-1.986	Detected	-1.419	0.295	GT	Sens	[illegible]
GT	Sens	Sense	-0.748	Detected	0.748	Detected	0.000	-2.196	Detected	-1.601	Detected	-1.898	0.142	GT	Sens	[illegible]
GT	Sens	Sense	-0.455	Detected	0.455	Detected	0.000	-1.379	Detected	-1.042	Detected	-1.210	0.130	GT	Sens	[illegible]
GT	Sens	Sense	0.169	Detected	-0.169	Detected	0.000	-1.238	Detected	-1.175	Detected	-1.206	0.020	GT	Sens	[illegible]
GT	Sens	Sense	-0.154	Detected	0.154	Detected	0.000	-2.150	Comprom	-1.708	Comprom	-1.929	0.019	GT	Sens	[illegible]
GT	Sens	Sense	0.482	Detected	-0.482	Detected	0.000	-1.215	Detected	-2.006	Detected	-1.610	0.123	GT	Sens	[illegible]
GT	Sens	Sense	0.207	Detected	-0.207	Detected	0.000	-2.271	Detected	-1.844	Detected	-2.057	0.020	GT	Sens	[illegible]
GT	Sens	Sense	0.372	Detected	-0.372	Detected	0.000	-2.251	Detected	-1.964	Detected	-2.108	0.034	GT	Sens	[illegible]
GT	Sens	Sense	0.010	Detected	-0.010	Detected	0.000	-3.755	Comprom	-3.024	Comprom	-3.389	0.011	GT	Sens	[illegible]
GT	Sens	Sense	-0.036	Detected	0.036	Detected	0.000	-1.246	Detected	-0.928	Detected	-1.087	0.022	GT	Sens	[illegible]
GT	Sens	Sense	0.035	Detected	-0.035	Detected	0.000	-1.488	Detected	-1.773	Detected	-1.630	0.008	GT	Sens	[illegible]
GT	Sens	Sense	0.247	Detected	-0.247	Detected	0.000	-1.661	Detected	-1.158	Detected	-1.409	0.057	GT	Sens	[illegible]
GT	Sens	Sense	-0.067	Detected	0.067	Detected	0.000	-1.571	Detected	-1.742	Detected	-1.657	0.004	GT	Sens	[illegible]

GT	Sense	Sense	0.592	Detected	-0.592	Detected	0.000	-4.373	Comprom	-3.271	Comprom	-3.822	0.042	GT Sens	[illegible]
GT	Sense	Sense	0.095	Detected	-0.095	Detected	0.000	-0.920	Detected	-1.283	Detected	-1.102	0.033	GT Sens	[illegible]
GT	Sense	Sense	-0.312	Detected	0.312	Detected	0.000	-1.867	Detected	-1.647	Detected	-1.757	0.034	GT Sens	[illegible]
GT	Sense	Sense	-0.253	Detected	0.253	Detected	0.000	-1.146	Detected	-2.341	Comprom	-1.743	0.115	GT Sens	[illegible]
GT	Sense	Sense	-0.335	Detected	0.335	Detected	0.000	-1.176	Comprom	-2.732	Comprom	-1.954	0.147	GT Sens	[illegible]
GT	Sense	Sense	0.153	Detected	-0.153	Detected	0.000	-1.160	Detected	-1.504	Detected	-1.332	0.029	GT Sens	[illegible]
GT	Sense	Sense	-0.548	Detected	0.548	Detected	0.000	-1.392	Detected	-2.145	Comprom	-1.769	0.117	GT Sens	[illegible]
GT	Sense	Sense	0.121	Detected	-0.121	Detected	0.000	-2.048	Detected	-1.041	Detected	-1.544	0.097	GT Sens	[illegible]
GT	Sense	Sense	-0.130	Detected	0.130	Detected	0.000	-1.191	Detected	-1.184	Detected	-1.187	0.012	GT Sens	[illegible]
GT	Sense	Sense	-0.110	Detected	0.110	Detected	0.000	-1.156	Detected	-0.928	Detected	-1.042	0.022	GT Sens	[illegible]
GT	Sense	Sense	0.127	Detected	-0.127	Detected	0.000	-1.462	Detected	-0.829	Detected	-1.146	0.078	GT Sens	[illegible]
GT	Sense	Sense	-0.322	Detected	0.322	Detected	0.000	-0.981	Detected	-1.209	Detected	-1.095	0.085	GT Sens	[illegible]
GT	Sense	Sense	-0.217	Detected	0.217	Detected	0.000	-1.671	Detected	-2.480	Detected	-2.076	0.046	GT Sens	[illegible]
GT	Sense	Sense	-0.072	Detected	0.072	Detected	0.000	-3.263	Detected	-2.401	Detected	-2.832	0.023	GT Sens	[illegible]
GT	Sense	Sense	-0.749	Detected	0.749	Detected	0.000	-1.408	Detected	-1.152	Detected	-1.280	0.234	GT Sens	[illegible]
GT	Sense	Sense	-0.231	Detected	0.231	Detected	0.000	-0.874	Detected	-1.262	Detected	-1.068	0.071	GT Sens	[illegible]
GT	Sense	Sense	0.125	Detected	-0.125	Detected	0.000	-1.222	Detected	-1.033	Detected	-1.127	0.019	GT Sens	[illegible]
GT	Sense	Sense	-0.210	Detected	0.210	Detected	0.000	-1.139	Detected	-1.049	Detected	-1.094	0.037	GT Sens	[illegible]
GT	Sense	Sense	-0.099	Detected	0.099	Detected	0.000	-1.442	Detected	-1.750	Detected	-1.596	0.013	GT Sens	[illegible]
GT	Sense	Sense	0.041	Detected	-0.041	Detected	0.000	-1.591	Detected	-1.143	Detected	-1.367	0.027	GT Sens	[illegible]
GT	Sense	Sense	0.090	Detected	-0.090	Detected	0.000	-2.228	Detected	-1.362	Detected	-1.795	0.056	GT Sens	[illegible]
GT	Sense	Sense	0.134	Detected	-0.134	Detected	0.000	-1.879	Detected	-1.521	Detected	-1.700	0.017	GT Sens	[illegible]
GT	Sense	Sense	-0.003	Detected	0.003	Detected	0.000	-0.996	Detected	-1.254	Detected	-1.125	0.013	GT Sens	[illegible]
GT	Sense	Sense	-0.465	Detected	0.465	Detected	0.000	-1.099	Detected	-1.402	Comprom	-1.251	0.125	GT Sens	[illegible]
GT	Sense	Sense	0.061	Detected	-0.061	Detected	0.000	-2.790	Detected	-1.674	Detected	-2.232	0.058	GT Sens	[illegible]
GT	Sense	Sense	-0.613	Detected	0.613	Detected	0.000	-0.915	Detected	-1.363	Detected	-1.139	0.223	GT Sens	[illegible]
GT	Sense	Sense	-0.258	Detected	0.258	Detected	0.000	-1.219	Detected	-2.095	Detected	-1.657	0.083	GT Sens	[illegible]
GT	Sense	Sense	-0.168	Detected	0.168	Detected	0.000	-1.042	Detected	-1.234	Detected	-1.138	0.028	GT Sens	[illegible]
GT	Sense	Sense	0.136	Detected	-0.136	Detected	0.000	-2.144	Comprom	-1.244	Detected	-1.694	0.069	GT Sens	[illegible]
GT	Sense	Sense	-0.042	Detected	0.042	Detected	0.000	-1.154	Detected	-1.755	Detected	-1.455	0.041	GT Sens	[illegible]
GT	Sense	Sense	-0.150	Detected	0.150	Detected	0.000	-1.114	Detected	-1.423	Detected	-1.269	0.028	GT Sens	[illegible]
GT	Sense	Sense	-0.048	Detected	0.048	Detected	0.000	-2.014	Comprom	-1.590	Comprom	-1.802	0.014	GT Sens	[illegible]
GT	Sense	Sense	-0.616	Detected	0.616	Detected	0.000	-2.197	Detected	-2.043	Detected	-2.120	0.076	GT Sens	[illegible]
GT	Sense	Sense	0.099	Detected	-0.099	Detected	0.000	-1.092	Detected	-0.927	Detected	-1.009	0.016	GT Sens	[illegible]
GT	Sense	Sense	0.031	Detected	-0.031	Detected	0.000	-1.379	Detected	-0.983	Detected	-1.181	0.028	GT Sens	[illegible]
GT	Sense	Sense	0.158	Detected	-0.158	Detected	0.000	-1.026	Detected	-1.644	Detected	-1.335	0.062	GT Sens	[illegible]
GT	Sense	Sense	0.143	Detected	-0.143	Detected	0.000	-1.045	Detected	-1.536	Detected	-1.290	0.045	GT Sens	[illegible]
GT	Sense	Sense	-0.429	Detected	0.429	Detected	0.000	-1.187	Detected	-0.900	Detected	-1.043	0.147	GT Sens	[illegible]
GT	Sense	Sense	-0.319	Detected	0.319	Detected	0.000	-1.244	Detected	-1.062	Detected	-1.153	0.074	GT Sens	[illegible]
GT	Sense	Sense	-0.290	Detected	0.290	Detected	0.000	-1.430	Comprom	-1.093	Detected	-1.262	0.064	GT Sens	[illegible]
GT	Sense	Sense	0.013	Detected	-0.013	Detected	0.000	-1.034	Detected	-1.011	Detected	-1.023	0.000	GT Sens	[illegible]
GT	Sense	Sense	0.203	Detected	-0.203	Detected	0.000	-1.187	Detected	-1.084	Detected	-1.136	0.032	GT Sens	[illegible]
GT	Sense	Sense	-0.097	Detected	0.097	Detected	0.000	-2.503	Comprom	-2.343	Detected	-2.423	0.003	GT Sens	[illegible]
GT	Sense	Sense	-0.128	Detected	0.128	Detected	0.000	-1.825	Detected	-1.355	Detected	-1.590	0.027	GT Sens	[illegible]
GT	Sense	Sense	-0.114	Detected	0.114	Detected	0.000	-1.642	Detected	-2.152	Detected	-1.897	0.021	GT Sens	[illegible]
GT	Sense	Sense	-0.057	Detected	0.057	Detected	0.000	-1.596	Comprom	-1.254	Detected	-1.425	0.016	GT Sens	[illegible]
GT	Sense	Sense	-0.726	Detected	0.726	Detected	0.000	-1.107	Detected	-2.713	Detected	-1.910	0.220	GT Sens	[illegible]
GT	Sense	Sense	-0.821	Detected	0.821	Detected	0.000	-0.892	Detected	-1.909	Detected	-1.401	0.284	GT Sens	[illegible]
GT	Sense	Sense	-0.345	Detected	0.345	Detected	0.000	-1.296	Comprom	-0.935	Detected	-1.116	0.103	GT Sens	[illegible]
GT	Sense	Sense	-0.273	Detected	0.273	Detected	0.000	-0.920	Comprom	-1.602	Comprom	-1.261	0.102	GT Sens	[illegible]
GT	Sense	Sense	-0.121	Detected	0.121	Detected	0.000	-1.227	Detected	-1.271	Detected	-1.249	0.010	GT Sens	[illegible]
GT	Sense	Sense	0.075	Detected	-0.075	Detected	0.000	-2.047	Detected	-0.866	Detected	-1.457	0.134	GT Sens	[illegible]
GT	Sense	Sense	-0.387	Detected	0.387	Detected	0.000	-1.363	Comprom	-2.069	Comprom	-1.716	0.082	GT Sens	[illegible]
GT	Sense	Sense	-0.761	Detected	0.761	Detected	0.000	-2.557	Detected	-1.564	Detected	-2.060	0.152	GT Sens	[illegible]
GT	Sense	Sense	0.206	Detected	-0.206	Detected	0.000	-1.785	Detected	-1.633	Detected	-1.709	0.016	GT Sens	[illegible]
GT	Sense	Sense	0.264	Detected	-0.264	Detected	0.000	-0.965	Detected	-1.082	Detected	-1.023	0.063	GT Sens	[illegible]
GT	Sense	Sense	0.089	Detected	-0.089	Detected	0.000	-2.575	Detected	-2.844	Detected	-2.710	0.004	GT Sens	[illegible]
GT	Sense	Sense	-0.936	Detected	0.936	Detected	0.000	-0.949	Detected	-1.877	Detected	-1.413	0.309	GT Sens	[illegible]
GT	Sense	Sense	-0.796	Detected	0.796	Detected	0.000	-0.864	Detected	-2.373	Detected	-1.618	0.278	GT Sens	[illegible]
GT	Sense	Sense	-0.909	Detected	0.909	Detected	0.000	-1.051	Detected	-1.156	Detected	-1.103	0.349	GT Sens	[illegible]
GT	Sense	Sense	-0.754	Detected	0.754	Detected	0.000	-2.818	Detected	-1.970	Detected	-2.394	0.109	GT Sens	[illegible]
GT	Sense	Sense	-0.825	Detected	0.825	Detected	0.000	-1.334	Detected	-3.332	Comprom	-2.333	0.213	GT Sens	[illegible]
GT	Sense	Sense	-0.107	Detected	0.107	Detected	0.000	-2.608	Comprom	-2.646	Comprom	-2.627	0.002	GT Sens	[illegible]
GT	Sense	Sense	0.014	Detected	-0.014	Detected	0.000	-1.412	Detected	-1.632	Detected	-1.522	0.005	GT Sens	[illegible]
GT	Sense	Sense	-0.834	Detected	0.834	Detected	0.000	-0.842	Detected	-2.445	Detected	-1.644	0.291	GT Sens	[illegible]
GT	Sense	Sense	-0.609	Detected	0.609	Detected	0.000	-1.415	Detected	-2.253	Detected	-1.834	0.131	GT Sens	[illegible]
GT	Sense	Sense	-0.171	Detected	0.171	Detected	0.000	-1.609	Detected	-2.333	Detected	-1.971	0.039	GT Sens	[illegible]
GT	Sense	Sense	-0.778	Detected	0.778	Detected	0.000	-1.048	Detected	-1.980	Comprom	-1.514	0.237	GT Sens	[illegible]
GT	Sense	Sense	0.093	Detected	-0.093	Detected	0.000	-1.080	Detected	-1.194	Detected	-1.137	0.009	GT Sens	[illegible]
GT	Sense	Sense	0.590	Detected	-0.590	Detected	0.000	-0.935	Detected	-2.126	Comprom	-1.531	0.209	GT Sens	[illegible]
GT	Sense	Sense	-0.533	Detected	0.533	Detected	0.000	-1.137	Detected	-1.450	Detected	-1.293	0.145	GT Sens	[illegible]
GT	Sense	Sense	-0.063	Detected	0.063	Detected	0.000	-1.142	Comprom	-2.014	Comprom	-1.578	0.070	GT Sens	[illegible]
GT	Sense	Sense	-0.111	Detected	0.111	Detected	0.000	-2.792	Detected	-2.860	Detected	-2.826	0.002	GT Sens	[illegible]
GT	Sense	Sense	-0.152	Detected	0.152	Detected	0.000	-2.565	Comprom	-3.512	Comprom	-3.039	0.026	GT Sens	[illegible]
GT	Sense	Sense	0.169	Detected	-0.169	Detected	0.000	-1.302	Comprom	-0.915	Comprom	-1.109	0.050	GT Sens	[illegible]
GT	Sense	Sense	0.324	Detected	-0.324	Detected	0.000	-1.702	Detected	-0.916	Detected	-1.309	0.124	GT Sens	[illegible]
GT	Sense	Sense	-0.647	Detected	0.647	Detected	0.000	-0.831	Detected	-2.360	Detected	-1.595	0.252	GT Sens	[illegible]
GT	Sense	Sense	0.267	Detected	-0.267	Detected	0.000	-1.620	Comprom	-2.385	Comprom	-2.002	0.050	GT Sens	[illegible]
GT	Sense	Sense	0.293	Detected	-0.293	Detected	0.000	-1.936	Comprom	-1.297	Detected	-1.616	0.065	GT Sens	[illegible]
GT	Sense	Sense	-0.176	Detected	0.176	Detected	0.000	-1.001	Detected	-1.262	Detected	-1.131	0.035	GT Sens	[illegible]
GT	Sense	Sense	-0.449	Detected	0.449	Detected	0.000	-2.339	Detected	-1.887	Detected	-2.113	0.052	GT Sens	[illegible]
GT	Sense	Sense	0.201	Detected	-0.201	Detected	0.000	-1.848	Detected	-1.576	Detected	-1.712	0.020	GT Sens	[illegible]
GT	Sense	Sense	-0.866	Detected	0.866	Detected	0.000	-2.406	Detected	-2.306	Detected	-2.356	0.113	GT Sens	[illegible]
GT	Sense	Sense	-0.695	Detected	0.695	Detected	0.000	-1.352	Detected	-2.590	Detected	-1.971	0.169	GT Sens	[illegible]
GT	Sense	Sense	-0.075	Detected	0.075	Detected	0.000	-1.247	Detected	-1.487	Detected	-1.367	0.010	GT Sens	[illegible]
GT	Sense	Sense	0.622	Detected	-0.622	Detected	0.000	-2.620	Detected	-4.031	Comprom	-3.325	0.072	GT Sens	[illegible]
GT	Sense	Sense	-0.374	Detected	0.374	Detected	0.000	-1.405	Comprom	-2.054	Comprom	-1.730	0.073	GT Sens	[illegible]
GT	Sense	Sense	0.077	Detected	-0.077	Detected	0.000	-1.432	Detected	-1.193	Detected	-1.312	0.012	GT Sens	[illegible]
GT	Sense	Sense	-0.680	Detected	0.680	Detected	0.000	-0.994	Detected	-2.346	Detected	-1.670	0.224	GT Sens	[illegible]
GT	Sense	Sense	-0.767	Detected	0.767	Detected	0.000	-0.835	Detected	-2.501	Detected	-1.668	0.279	GT Sens	[illegible]
GT	Sense	Sense	0.061	Detected	-0.061	Detected	0.000	-2.669	Detected	-1.203	Detected	-1.936	0.119	GT Sens	[illegible]
GT	Sense	Sense	-0.145	Detected	0.145	Detected	0.000	-1.371	Detected	-1.491	Detected	-1.431	0.012	GT Sens	[illegible]
GT	Sense	Sense	0.192	Detected	-0.192	Detected	0.000	-1.339	Detected	-1.421	Detected	-1.380	0.020	GT Sens	[illegible]
GT	Sense	Sense	-0.438	Detected	0.438	Detected	0.000	-2.148	Comprom	-3.918	Comprom	-3.033	0.092	GT Sens	[illegible]
GT	Sense	Sense	-0.046	Detected	0.046	Detected	0.000	-2.508	Detected	-1.782	Detected	-2.145	0.028	GT Sens	[illegible]
GT	Sense	Sense	-0.385	Detected	0.385	Detected	0.000	-0.925	Detected	-1.876	Detected	-1.400	0.149	GT Sens	[illegible]
GT	Sense	Sense	-0.360	Detected	0.360	Detected	0.000	-1.312	Detected	-1.043	Detected	-1.177	0.092	GT Sens	[illegible]
GT	Sense	Sense	-0.184	Detected	0.184	Detected	0.000	-1.410	Comprom	-1.805	Comprom	-1.607	0.027	GT Sens	[illegible]
GT	Sense	Sense	-0.131	Detected	0.131	Detected	0.000	-1.175	Detected	-1.769	Comprom	-1.472	0.045	GT Sens	[illegible]
GT	Sense	Sense	0.015	Detected	-0.015	Detected	0.000	-1.286	Detected	-0.887	Detected	-1.086	0.032	GT Sens	[illegible]
GT	Sense	Sense	-0.013	Detected	0.013	Detected	0.000	-1.358	Detected	-0.990	Detected	-1.174	0.024	GT Sens	[illegible]
GT	Sense	Sense	-0.331	Detected	0.331	Detected	0.000	-1.303	Detected	-2.096	Comprom	-1.699	0.081	GT Sens	[illegible]
GT	Sense	Sense	-0.471	Detected	0.471	Detected	0.000	-1.058	Detected	-1.908	Detected	-1.483	0.144	GT Sens	[illegible]
GT	Sense	Sense	-0.181	Detected	0.181	Detected	0.000	-2.143	Detected	-1.890	Detected	-2.017	0.012	GT Sens	[illegible]
GT	Sense	Sense	-0.558	Detected	0.558	Detected	0.000	-1.406	Detected	-2.657	Detected	-2.031	0.136	GT Sens	[illegible]
GT	Sense	Sense	-0.491	Detected	0.491	Detected	0.000	-2.422	Detected	-1.346	Detected	-1.884	0.123	GT Sens	[illegible]
GT	Sense	Sense	-0.883	Detected	0.883	Detected	0.000	-1.011	Detected	-2.083	Detected	-1.547	0.273	GT Sens	[illegible]
GT	Sense	Sense	-0.731	Detected	0.731	Detected	0.000	-0.995	Detected	-1.340	Detected	-1.167	0.260	GT Sens	[illegible]
GT	Sense	Sense	-0.381	Detected	0.381	Detected	0.000	-0.944	Detected	-1.185	Detected	-1.064	0.117	GT Sens	[illegible]
GT	Sense	Sense	-0.223	Detected	0.223	Detected	0.000	-1.867	Detected	-2.515	Detected	-2.191	0.031	GT Sens	[illegible]
GT	Sense	Sense	-0.416	Detected	0.416	Detected	0.000	-0.904	Detected	-1.211	Detected	-1.057	0.140	GT Sens	[illegible]
GT	Sense	Sense	-0.478	Detected	0.478	Detected	0.000	-1.052	Detected	-1.045	Detected	-1.049	0.159	GT Sens	[illegible]
GT	Sense	Sense	-0.059	Detected	0.059	Detected	0.000	-1.691	Detected	-1.187	Detected	-1.439	0.031	GT Sens	[illegible]
GT	Sense	Sense	-1.060	Detected	1.060	Detected	0.000	-0.889	Detected	-1.233	Detected	-1.061	0.428	GT Sens	[illegible]
GT	Sense	Sense	-0.734	Detected	0.734	Detected	0.000	-0.827	Detected	-2.294	Detected	-1.560	0.271	GT Sens	[illegible]
GT	Sense	Sense	-0.793	Detected	0.793	Detected	0.000	-0.912	Detected	-2.151	Detected	-1.531	0.267	GT Sens	[illegible]
GT	Sense	Sense	-0.694	Detected	0.694	Detected	0.000	-0.811	Detected	-2.443	Detected	-1.627	0.268	GT Sens	[illegible]
GT	Sense	Sense	-0.761	Detected	0.761	Detected	0.000	-1.075	Detected	-1.767	Detected	-1.421	0.231	GT Sens	[illegible]
GT	Sense	Sense	0.065	Detected	-0.065	Detected	0.000	-1.172	Detected	-0.869	Detected	-1.021	0.025	GT Sens	[illegible]
GT	Sense	Sense	-0.220	Detected	0.220	Detected	0.000	-0.829	Detected	-1.668	Detected	-1.248	0.119	GT Sens	[illegible]
GT	Sense	Sense	-0.380	Detected	0.380	Detected	0.000	-1.803	Detected	-2.348	Detected	-2.075	0.047	GT Sens	[illegible]
GT	Sense	Sense	-0.449	Detected	0.449	Detected	0.000	-3.159	Detected	-2.465	Detected	-2.812	0.038	GT Sens	[illegible]
GT	Sense	Sense	-0.305	Detected	0.305	Detected	0.000	-1.732	Detected	-2.196	Detected	-1.964	0.036	GT Sens	[illegible]
GT	Sense	Sense	-0.184	Detected	0.184	Detected	0.000	-0.907	Detected	-1.284	Detected	-1.095	0.053	GT Sens	[illegible]
GT	Sense	Sense	-0.295	Detected	0.295	Detected	0.000	-1.365	Comprom	-1.163	Comprom	-1.264	0.056	GT Sens	[illegible]
GT	Sense	Sense	-0.074	Detected	0.074	Detected	0.000	-1.018	Detected	-1.146	Detected	-1.082	0.008	GT Sens	[illegible]
GT	Sense	Sense	-0.466	Detected	0.466	Detected	0.000	-1.792	Detected	-1.854	Detected	-1.823	0.060	GT Sens	[illegible]
GT	Sense	Sense	-0.259	Detected	0.259	Detected	0.000	-1.279	Detected	-1.740	Detected	-1.509	0.049	GT Sens	[illegible]
GT	Sense	Sense	-0.214	Detected	0.214	Detected	0.000	-1.319	Detected	-1.524	Detected	-1.422	0.027	GT Sens	[illegible]
GT	Sense	Sense	-0.904	Detected	0.904	Detected	0.000	-1.202	Detected	-2.859	Comprom	-2.031	0.240	GT Sens	[illegible]
GT	Sense	Sense	-0.370	Detected	0.370	Detected	0.000	-2.110	Detected	-2.608	Detected	-2.359	0.034	GT Sens	[illegible]
GT	Sense	Sense	0.401	Detected	-0.401	Detected	0.000	-2.980	Detected	-2.897	Detected	-2.939	0.018	GT Sens	[illegible]
GT	Sense	Sense	-0.396	Detected	0.396	Detected	0.000	-1.994	Detected	-3.022	Detected	-2.508	0.061	GT Sens	[illegible]
GT	Sense	Sense	-0.161	Detected	0.161	Detected	0.000	-1.505	Comprom	-2.098	Comprom	-1.801	0.033	GT Sens	[illegible]
GT	Sense	Sense	-0.754	Detected	0.754	Detected	0.000	-0.991	Detected	-2.062	Detected	-1.526	0.241	GT Sens	[illegible]
GT	Sense	Sense	1.061	Detected	-1.061	Detected	0.000	-1.029	Detected	-2.189	Detected	-1.609	0.315	GT Sens	[illegible]
GT	Sense	Sense	0.261	Detected	-0.261	Detected	0.000	-1.791	Comprom	-1.864	Comprom	-1.827	0.020	GT Sens	[illegible]
GT	Sense	Sense	-0.269	Detected	0.269	Detected	0.000	-1.490	Detected	-1.186	Detected	-1.338	0.049	GT Sens	[illegible]
GT	Sense	Sense	0.096	Detected	-0.096	Detected	0.000	-1.201	Comprom	-1.072	Detected	-1.137	0.010	GT Sens	[illegible]
GT	Sense	Sense	-0.543	Detected	0.543	Detected	0.000	-0.880	Detected	-2.214	Detected	-1.547	0.214	GT Sens	[illegible]
GT	Sense	Sense	-0.733	Detected	0.733	Detected	0.000	-0.800	Detected	-2.129	Detected	-1.465	0.277	GT Sens	[illegible]
GT	Sense	Sense	0.189	Detected	-0.189	Detected	0.000	-1.693	Detected	-1.641	Detected	-1.667	0.013	GT Sens	[illegible]
GT	Sense	Sense	-0.432	Detected	0.432	Detected	0.000	-1.430	Detected	-1.395	Detected	-1.412	0.082	GT Sens	[illegible]
GT	Sense	Sense	-0.962	Detected	0.962	Detected	0.000	-3.975	Detected	-5.736	Comprom	-4.856	0.065	GT Sens	[illegible]
GT	Sense	Sense	0.297	Detected	-0.297	Detected	0.000	-1.506	Detected	-1.880	Detected	-1.693	0.040	GT Sens	[illegible]
GT	Sense	Sense	-0.394	Detected	0.394	Detected	0.000	-0.870	Detected	-1.236	Comprom	-1.053	0.136	GT Sens	[illegible]
GT	Sense	Sense	-0.901	Detected	0.901	Detected	0.000	-2.643	Comprom	-1.975	Detected	-2.309	0.138	GT Sens	[illegible]
GT	Sense	Sense	0.040	Detected	-0.040	Detected	0.000	-1.183	Detected	-1.765	Comprom	-1.474	0.038	GT Sens	[illegible]
GT	Sense	Sense	0.377	Detected	-0.377	Detected	0.000	-1.866	Detected	-3.104	Detected	-2.485	0.076	GT Sens	[illegible]
GT	Sense	Sense	-0.352	Detected	0.352	Detected	0.000	-2.075	Detected	-2.628	Detected	-2.352	0.034	GT Sens	[illegible]
GT	Sense	Sense	-0.320	Detected	0.320	Detected	0.000	-1.268	Detected	-1.625	Detected	-1.446	0.059	GT Sens	[illegible]
GT	Sense	Sense	-0.163	Detected	0.163	Detected	0.000	-2.546	Detected	-3.681	Detected	-3.114	0.034	GT Sens	[illegible]
GT	Sense	Sense	1.235	Detected	-1.235	Detected	0.000	-1.167	Detected	-1.970	Detected	-1.568	0.351	GT Sens	[illegible]
GT	Sense	Sense	-0.332	Detected	0.332	Detected	0.000	-1.391	Detected	-1.379	Detected	-1.385	0.053	GT Sens	[illegible]
GT	Sense	Sense	0.432	Detected	-0.432	Detected	0.000	-2.489	Detected	-1.842	Detected	-2.165	0.057	GT Sens	[illegible]
GT	Sense	Sense	-0.289	Detected	0.289	Detected	0.000	-2.278	Detected	-4.424	Comprom	-3.351	0.095	GT Sens	[illegible]
GT	Sense	Sense	0.205	Detected	-0.205	Detected	0.000	-3.526	Comprom	-3.195	Detected	-3.361	0.006	GT Sens	[illegible]
GT	Sense	Sense	-0.165	Detected	0.165	Detected	0.000	-1.201	Detected	-1.948	Detected	-1.575	0.061	GT Sens	[illegible]
GT	Sense	Sense	0.321	Detected	-0.321	Detected	0.000	-3.354	Detected	-3.596	Detected	-3.475	0.010	GT Sens	[illegible]
GT	Sense	Sense	-0.264	Detected	0.264	Detected	0.000	-1.023	Detected	-1.126	Detected	-1.074	0.057	GT Sens	[illegible]
GT	Sense	Sense	-0.732	Detected	0.732	Detected	0.000	-1.463	Detected	-2.049	Detected	-1.756	0.156	GT Sens	[illegible]
GT	Sense	Sense	0.063	Detected	-0.063	Detected	0.000	-1.356	Detected	-0.813	Detected	-1.085	0.060	GT Sens	[illegible]
GT	Sense	Sense	-0.050	Detected	0.050	Detected	0.000	-3.432	Comprom	-2.208	Detected	-2.820	0.044	GT Sens	[illegible]
GT	Sense	Sense	-0.687	Detected	0.687	Detected	0.000	-1.062	Detected	-1.375	Detected	-1.218	0.226	GT Sens	[illegible]
GT	Sense	Sense	0.006	Detected	-0.006	Detected	0.000	-2.704	Comprom	-3.470	Comprom	-3.087	0.015	GT Sens	[illegible]
GT	Sense	Sense	-0.456	Detected	0.456	Detected	0.000	-1.572	Detected	-1.649	Detected	-1.611	0.072	GT Sens	[illegible]
GT	Sense	Sense	-0.435	Detected	0.435	Detected	0.000	-2.243	Detected	-3.190	Detected	-2.717	0.052	GT Sens	[illegible]
GT	Sense	Sense	-0.326	Detected	0.326	Detected	0.000	-1.394	Detected	-1.422	Detected	-1.408	0.050	GT Sens	[illegible]
GT	Sense	Sense	0.120	Detected	-0.120	Detected	0.000	-0.962	Detected	-1.667	Detected	-1.315	0.072	GT Sens	[illegible]
GT	Sense	Sense	-0.095	Detected	0.095	Detected	0.000	-2.178	Detected	-1.585	Detected	-1.881	0.026	GT Sens	[illegible]
GT	Sense	Sense	-0.087	Detected	0.087	Detected	0.000	-1.638	Detected	-0.803	Detected	-1.221	0.104	GT Sens	[illegible]
GT	Sense	Sense	-0.678	Detected	0.678	Detected	0.000	-1.161	Comprom	-1.159	Comprom	-1.160	0.229	GT Sens	[illegible]
GT	Sense	Sense	-0.047	Detected	0.047	Detected	0.000	-1.381	Detected	-1.134	Detected	-1.257	0.011	GT Sens	[illegible]
GT	Sense	Sense	0.055	Detected	-0.055	Detected	0.000	-1.779	Detected	-1.274	Detected	-1.527	0.027	GT Sens	[illegible]

GO ID	GO ACCESSION	GO Term	p-value	Count in Selection	% Count in Selection	Count in Total	% Count in Total
8530	GO:0015116	sulfate transmembran	0.00	5	0.74294204	26	0.075085916
5793	GO:0008272|GO:0006	sulfate transport	0.00	5	0.74294204	28	0.080861755
8736	GO:0015381	high affinity sulfate tra	0.00	4	0.5943536	18	0.051982556
5792	GO:0008271	secondary active sulfa	0.00	4	0.5943536	18	0.051982556
8584	GO:0015171|GO:0015	amino acid transmemb	0.00	9	1.3372957	118	0.34077454
2521	GO:0003333	amino acid transmemb	0.00	9	1.3372957	118	0.34077454
4951	GO:0006865|GO:0006	amino acid transport	0.00	9	1.3372957	123	0.35521415
3761	GO:0005275|GO:0005	amine transmembrane	0.00	9	1.3372957	126	0.3638779
9806	GO:0016752	sinapoyltransferase ac	0.00	4	0.5943536	23	0.06642216
9807	GO:0016753	O-sinapoyltransferase	0.00	4	0.5943536	23	0.06642216
9808	GO:0016754	sinapoylglucose-malat	0.00	4	0.5943536	23	0.06642216
9114	GO:0015849	organic acid transport	0.00	10	1.4858841	156	0.45051548
22443	GO:0046942	carboxylic acid transp	0.00	10	1.4858841	156	0.45051548
9600	GO:0016491	oxidoreductase activity	0.00	52	7.7265973	1724	4.9787736
9103	GO:0015837	amine transport	0.00	9	1.3372957	135	0.38986918
3801	GO:0005342	organic acid transmen	0.00	9	1.3372957	136	0.3927571
22444	GO:0046943	carboxylic acid transm	0.00	9	1.3372957	136	0.3927571
5729	GO:0008191	metalloendopeptidase	0.00	2	0.2971768	4	0.011551679
23942	GO:0048551	metalloenzyme inhibito	0.00	2	0.2971768	4	0.011551679
7792	GO:0010576	metalloenzyme regula	0.00	2	0.2971768	4	0.011551679
8967	GO:0015698	inorganic anion transp	0.00	10	1.4858841	176	0.5082739
11827	GO:0019898|GO:0030	extrinsic to membrane	0.00	4	0.5943536	30	0.086637594
3884	GO:0005524	ATP binding	0.00	20	2.9717681	524	1.51327
21949	GO:0046424	ferulate 5-hydroxylase	0.00	2	0.2971768	5	0.0144396
7220	GO:0009973	adenylyl-sulfate reduc	0.01	2	0.2971768	6	0.01732752
7690	GO:0010466	negative regulation of	0.01	7	1.0401188	110	0.31767118
12946	GO:0030414	peptidase inhibitor act	0.01	7	1.0401188	110	0.31767118
25636	GO:0051346	negative regulation of	0.01	7	1.0401188	110	0.31767118
13041	GO:0030554	adenyl nucleotide bind	0.01	20	2.9717681	550	1.5883559
14965	GO:0032559	adenyl ribonucleotide	0.01	20	2.9717681	550	1.5883559
12547	GO:0022857|GO:0005	transmembrane transp	0.01	43	6.389302	1483	4.282785
26711	GO:0052547	regulation of peptidase	0.01	7	1.0401188	112	0.32344702
28100	GO:0061134	peptidase regulator ac	0.01	7	1.0401188	112	0.32344702
26931	GO:0055085	transmembrane transp	0.01	44	6.53789	1548	4.4705
5981	GO:0008509	anion transmembrane	0.01	11	1.6344725	245	0.70754033
5704	GO:0008150|GO:0000	biological_process	0.01	461	68.49926	22215	64.155136
4917	GO:0006820|GO:0006	anion transport	0.01	11	1.6344725	247	0.7133162
26959	GO:0055114	oxidation-reduction pro	0.01	65	9.658247	2490	7.1909204
11776	GO:0019825	oxygen binding	0.01	15	2.228826	389	1.1234008
3472	GO:0004866	endopeptidase inhibito	0.01	6	0.89153045	95	0.27435237
8159	GO:0010951	negative regulation of	0.01	6	0.89153045	95	0.27435237
26712	GO:0052548	regulation of endopept	0.01	6	0.89153045	95	0.27435237
28101	GO:0061135	endopeptidase regulat	0.01	6	0.89153045	95	0.27435237
8517	GO:0015103	inorganic anion transm	0.01	8	1.1887072	154	0.44473964
3723	GO:0005215|GO:0005	transporter activity	0.01	50	7.4294205	1844	5.325324
19393	GO:0043086	negative regulation of	0.01	9	1.3372957	188	0.54292893
20342	GO:0044092	negative regulation of	0.01	9	1.3372957	188	0.54292893
3464	GO:0004857	enzyme inhibitor activi	0.01	8	1.1887072	157	0.4534034
12516	GO:0022804	active transmembrane	0.01	25	3.7147102	787	2.2727928
25379	GO:0051082	unfolded protein bindir	0.01	6	0.89153045	98	0.28301615
12574	GO:0022892	substrate-specific tran	0.01	41	6.092125	1463	4.2250266
4878	GO:0006772	thiamine metabolic pro	0.02	2	0.2971768	10	0.0288792
6592	GO:0009228	thiamine biosynthetic p	0.02	2	0.2971768	10	0.0288792
8651	GO:0015248	sterol transporter activ	0.02	2	0.2971768	10	0.0288792
9178	GO:0015918	sterol transport	0.02	2	0.2971768	10	0.0288792
10072	GO:0017127	cholesterol transporter	0.02	2	0.2971768	10	0.0288792
12859	GO:0030301	cholesterol transport	0.02	2	0.2971768	10	0.0288792
30069	GO:0071705	nitrogen compound tra	0.02	9	1.3372957	202	0.5833598
3380	GO:0004766	spermidine synthase a	0.02	2	0.2971768	11	0.03176712
12573	GO:0022891	substrate-specific tran	0.02	35	5.2005944	1241	3.5839086
4261	GO:0006023	aminoglycan biosynthe	0.02	1	0.1485884	1	0.00288792
4262	GO:0006024	glycosaminoglycan bio	0.02	1	0.1485884	1	0.00288792
6615	GO:0009252|GO:0009	peptidoglycan biosynth	0.02	1	0.1485884	1	0.00288792
7606	GO:0010382	cellular cell wall macro	0.02	1	0.1485884	1	0.00288792
12766	GO:0030203	glycosaminoglycan me	0.02	1	0.1485884	1	0.00288792
20289	GO:0044038	cell wall macromolecu	0.02	1	0.1485884	1	0.00288792
28953	GO:0070589	cellular component ma	0.02	1	0.1485884	1	0.00288792
205	GO:0000270|GO:0009	peptidoglycan metabo	0.02	1	0.1485884	1	0.00288792
3827	GO:0005372	water transmembrane	0.02	5	0.74294204	81	0.23392151
4927	GO:0006833	water transport	0.02	5	0.74294204	81	0.23392151
8652	GO:0015250	water channel activity	0.02	5	0.74294204	81	0.23392151
18429	GO:0042044	fluid transport	0.02	5	0.74294204	81	0.23392151
6826	GO:0009538	photosystem I reaction	0.02	2	0.2971768	12	0.03465504
19047	GO:0042723	thiamine-containing co	0.02	2	0.2971768	12	0.03465504
19048	GO:0042724	thiamine-containing co	0.02	2	0.2971768	12	0.03465504
4894	GO:0006790	sulfur compound meta	0.02	4	0.5943536	55	0.15883559
6515	GO:0009150	purine ribonucleotide r	0.02	24	3.5661218	791	2.2843447
9933	GO:0016903	oxidoreductase activity	0.02	6	0.89153045	113	0.32633495
4493	GO:0006298|GO:0006	mismatch repair	0.03	3	0.44576523	33	0.09530135
24966	GO:0050660	flavin adenine dinucleo	0.03	6	0.89153045	118	0.34077454
4373	GO:0006163	purine nucleotide meta	0.03	24	3.5661218	810	2.339215
18000	GO:0035639	purine ribonucleoside	0.03	23	3.4175334	769	2.2208104
6622	GO:0009259|GO:0009	ribonucleotide metabo	0.03	24	3.5661218	812	2.344991
12533	GO:0022838	substrate-specific cha	0.03	9	1.3372957	221	0.63823026
9719	GO:0016638	oxidoreductase activity	0.03	5	0.74294204	89	0.25702485
4405	GO:0006195	purine nucleotide cata	0.03	23	3.4175334	773	2.232362
6508	GO:0009143	nucleoside triphospha	0.03	23	3.4175334	773	2.232362
6509	GO:0009144	purine nucleoside triph	0.03	23	3.4175334	773	2.232362
6511	GO:0009146	purine nucleoside triph	0.03	23	3.4175334	773	2.232362
6519	GO:0009154	purine ribonucleotide c	0.03	23	3.4175334	773	2.232362

6530	GO:0009166	nucleotide catabolic pr	0.03	23	3.4175334	773	2.232362
6567	GO:0009203	ribonucleoside triphos	0.03	23	3.4175334	773	2.232362
6569	GO:0009205	purine ribonucleoside	0.03	23	3.4175334	773	2.232362
6571	GO:0009207	purine ribonucleoside	0.03	23	3.4175334	773	2.232362
6624	GO:0009261	ribonucleotide catabol	0.03	23	3.4175334	773	2.232362
17034	GO:0034655	nucleobase, nucleosid	0.03	23	3.4175334	773	2.232362
17035	GO:0034656	nucleobase, nucleosid	0.03	23	3.4175334	773	2.232362
22209	GO:0046700	heterocycle catabolic	0.03	23	3.4175334	773	2.232362
30879	GO:0072523	purine-containing com	0.03	23	3.4175334	773	2.232362
20520	GO:0044270	cellular nitrogen comp	0.03	23	3.4175334	777	2.2439137
8660	GO:0015267|GO:0015	channel activity	0.03	9	1.3372957	226	0.6526699
12515	GO:0022803	passive transmembra	0.03	9	1.3372957	226	0.6526699
6810	GO:0009522	photosystem I	0.03	2	0.2971768	15	0.043318797
12650	GO:0030060	L-malate dehydrogena	0.03	2	0.2971768	15	0.043318797
24968	GO:0050662	coenzyme binding	0.03	8	1.1887072	191	0.5515927
30877	GO:0072521	purine-containing com	0.03	24	3.5661218	827	2.3883097
6563	GO:0009199	ribonucleoside triphos	0.04	23	3.4175334	790	2.2814567
3334	GO:0004713|GO:0004	protein tyrosine kinase	0.04	2	0.2971768	16	0.046206716
206	GO:0000271	polysaccharide biosyn	0.04	2	0.2971768	16	0.046206716
2878	GO:0004072	aspartate kinase activ	0.04	1	0.1485884	2	0.00577584
3013	GO:0004328|GO:0034	formamidase activity	0.04	1	0.1485884	2	0.00577584
3069	GO:0004392	heme oxygenase (dec	0.04	1	0.1485884	2	0.00577584
3335	GO:0004714	transmembrane recep	0.04	1	0.1485884	2	0.00577584
3337	GO:0004716	receptor signaling prot	0.04	1	0.1485884	2	0.00577584
3434	GO:0004825	methionine-tRNA ligas	0.04	1	0.1485884	2	0.00577584
3595	GO:0005017	platelet-derived growt	0.04	1	0.1485884	2	0.00577584
3664	GO:0005128	erythropoietin recepto	0.04	1	0.1485884	2	0.00577584
3709	GO:0005176	ErbB-2 class receptor	0.04	1	0.1485884	2	0.00577584
3751	GO:0005251	delayed rectifier potas	0.04	1	0.1485884	2	0.00577584
3899	GO:0005547	phosphatidylinositol-3,	0.04	1	0.1485884	2	0.00577584
4907	GO:0006808	regulation of nitrogen	0.04	1	0.1485884	2	0.00577584
5907	GO:0008413	8-oxo-7,8-dihydroguar	0.04	1	0.1485884	2	0.00577584
5923	GO:0008430	selenium binding	0.04	1	0.1485884	2	0.00577584
6023	GO:0008569	minus-end-directed m	0.04	1	0.1485884	2	0.00577584
7532	GO:0010307	acetylglutamate kinas	0.04	1	0.1485884	2	0.00577584
8513	GO:0015098	molybdate ion transm	0.04	1	0.1485884	2	0.00577584
8958	GO:0015689	molybdate ion transpo	0.04	1	0.1485884	2	0.00577584
9380	GO:0016151	nickel ion binding	0.04	1	0.1485884	2	0.00577584
11186	GO:0019177	dihydroneopterin triph	0.04	1	0.1485884	2	0.00577584
23486	GO:0048008	platelet-derived growt	0.04	1	0.1485884	2	0.00577584
24640	GO:0050321	tau-protein kinase acti	0.04	1	0.1485884	2	0.00577584
24919	GO:0050613|GO:000	delta14-sterol reducta	0.04	1	0.1485884	2	0.00577584
24987	GO:0050681	androgen receptor bin	0.04	1	0.1485884	2	0.00577584
190	GO:0000247	C-8 sterol isomerase	0.04	1	0.1485884	2	0.00577584
10058	GO:0017111	nucleoside-triphospha	0.04	25	3.7147102	878	2.5355935
14959	GO:0032553	ribonucleotide binding	0.04	23	3.4175334	795	2.2958963
14961	GO:0032555	purine ribonucleotide	0.04	23	3.4175334	795	2.2958963
3473	GO:0004867	serine-type endopeptid	0.04	4	0.5943536	66	0.1906027
6888	GO:0009611|GO:0002	response to wounding	0.04	4	0.5943536	66	0.1906027
6506	GO:0009141	nucleoside triphospha	0.04	23	3.4175334	796	2.2987843
10033	GO:0017076	purine nucleotide bind	0.04	23	3.4175334	797	2.3016722
9923	GO:0016892	endoribonuclease acti	0.04	2	0.2971768	17	0.049094636
9925	GO:0016894	endonuclease activity,	0.04	2	0.2971768	17	0.049094636
9930	GO:0016899	oxidoreductase activity	0.04	2	0.2971768	17	0.049094636
16281	GO:0033897	ribonuclease T2 activi	0.04	2	0.2971768	17	0.049094636
11828	GO:0019899	enzyme binding	0.04	9	1.3372957	237	0.684437
22409	GO:0046906	tetrapyrrole binding	0.05	18	2.6745913	596	1.7212002
4408	GO:0006200	ATP catabolic process	0.05	19	2.8231797	641	1.8511566
9918	GO:0016887|GO:0004	ATPase activity	0.05	19	2.8231797	641	1.8511566
21600	GO:0046034	ATP metabolic proces	0.05	19	2.8231797	641	1.8511566
20532	GO:0044282	small molecule catabo	0.05	24	3.5661218	854	2.4662836
5874	GO:0008374	O-acyltransferase acti	0.05	5	0.74294204	102	0.29456782
3744	GO:0005244	voltage-gated ion cha	0.05	4	0.5943536	72	0.20793022
12528	GO:0022832	voltage-gated channel	0.05	4	0.5943536	72	0.20793022
15280	GO:0032879	regulation of localizati	0.05	4	0.5943536	72	0.20793022
17141	GO:0034762	regulation of transmen	0.05	4	0.5943536	72	0.20793022
17144	GO:0034765	regulation of ion trans	0.05	4	0.5943536	72	0.20793022
19568	GO:0043269	regulation of ion trans	0.05	4	0.5943536	72	0.20793022
25348	GO:0051049	regulation of transport	0.05	4	0.5943536	72	0.20793022
2895	GO:0004089	carbonate dehydratas	0.05	2	0.2971768	19	0.054870475
20522	GO:0044272	sulfur compound biosy	0.05	3	0.44576523	44	0.12706847
19253	GO:0042936	dipeptide transporter a	0.05	5	0.74294204	105	0.3032316
19255	GO:0042938	dipeptide transport	0.05	5	0.74294204	105	0.3032316
9579	GO:0016462	pyrophosphatase activ	0.05	26	3.8632987	954	2.7550755
9859	GO:0016818	hydrolase activity, acti	0.06	26	3.8632987	956	2.7608514
9768	GO:0016706	oxidoreductase activity	0.06	7	1.0401188	175	0.505386
30066	GO:0071702	organic substance tra	0.06	16	2.3774145	529	1.5277096
4867	GO:0006753	nucleoside phosphate	0.06	25	3.7147102	914	2.6395588
6484	GO:0009117	nucleotide metabolic p	0.06	25	3.7147102	914	2.6395588
31431	GO:0080054	low affinity nitrate tran	0.06	3	0.44576523	45	0.1299564
3747	GO:0005247	voltage-gated chloride	0.06	2	0.2971768	20	0.0577584
3754	GO:0005254	chloride channel activi	0.06	2	0.2971768	20	0.0577584
6201	GO:0008794	arsenate reductase (g	0.06	2	0.2971768	20	0.0577584
9707	GO:0016624	oxidoreductase activity	0.06	2	0.2971768	20	0.0577584
13086	GO:0030611	arsenate reductase ac	0.06	2	0.2971768	20	0.0577584
13088	GO:0030613	oxidoreductase activity	0.06	2	0.2971768	20	0.0577584
13089	GO:0030614	oxidoreductase activity	0.06	2	0.2971768	20	0.0577584
6636	GO:0009273	peptidoglycan-based c	0.06	1	0.1485884	3	0.00866376
18884	GO:0042546	cell wall biogenesis	0.06	1	0.1485884	3	0.00866376
9858	GO:0016817	hydrolase activity, acti	0.06	26	3.8632987	959	2.769515
28364	GO:0065007	biological regulation	0.06	99	14.710253	4376	12.637537

9396	GO:0016168	chlorophyll binding	0.06	3	0.44576523	46	0.13284431
3129	GO:0004467	long-chain fatty acid-C	0.06	2	0.2971768	21	0.060646318
9698	GO:0016615	malate dehydrogenase	0.06	2	0.2971768	21	0.060646318
2986	GO:0004252	serine-type endopeptid	0.06	6	0.89153045	144	0.41586044
7074	GO:0009815	1-aminocyclopropane-	0.07	3	0.44576523	49	0.14150807
25094	GO:0050789|GO:0050	regulation of biological	0.07	97	14.413075	4315	12.461374
4755	GO:0006629	lipid metabolic process	0.07	17	2.526003	590	1.7038727
4478	GO:0006281	DNA repair	0.07	6	0.89153045	149	0.43030006
5037	GO:0006974|GO:0034	response to DNA dam	0.07	6	0.89153045	149	0.43030006
4918	GO:0006821	chloride transport	0.07	2	0.2971768	23	0.06642216
4216	GO:0005976	polysaccharide metab	0.07	3	0.44576523	50	0.14439599
7166	GO:0009914	hormone transport	0.07	3	0.44576523	50	0.14439599
27891	GO:0060918	auxin transport	0.07	3	0.44576523	50	0.14439599
31536	GO:0080161	auxin transmembrane	0.07	3	0.44576523	50	0.14439599
26932	GO:0055086	nucleobase, nucleosid	0.08	25	3.7147102	944	2.7261963
2789	GO:0003968	RNA-directed RNA po	0.08	1	0.1485884	4	0.011551679
3057	GO:0004373	glycogen (starch) synt	0.08	1	0.1485884	4	0.011551679
3084	GO:0004412	homoserine dehydrog	0.08	1	0.1485884	4	0.011551679
3336	GO:0004715	non-membrane spann	0.08	1	0.1485884	4	0.011551679
3557	GO:0004970	ionotropic glutamate re	0.08	1	0.1485884	4	0.011551679
4039	GO:0005751|GO:0005	mitochondrial respirato	0.08	1	0.1485884	4	0.011551679
5641	GO:0008066	glutamate receptor act	0.08	1	0.1485884	4	0.011551679
5942	GO:0008456	alpha-N-acetylgalacto	0.08	1	0.1485884	4	0.011551679
7170	GO:0009918	sterol delta7 reductase	0.08	1	0.1485884	4	0.011551679
7538	GO:0010313	phytochrome binding	0.08	1	0.1485884	4	0.011551679
8594	GO:0015184	L-cystine transmembra	0.08	1	0.1485884	4	0.011551679
8598	GO:0015188	L-isoleucine transmem	0.08	1	0.1485884	4	0.011551679
9072	GO:0015803	branched-chain alipha	0.08	1	0.1485884	4	0.011551679
9080	GO:0015811	L-cystine transport	0.08	1	0.1485884	4	0.011551679
9086	GO:0015818	isoleucine transport	0.08	1	0.1485884	4	0.011551679
9745	GO:0016672	oxidoreductase activity	0.08	1	0.1485884	4	0.011551679
10089	GO:0017151	DEAD/H-box RNA hel	0.08	1	0.1485884	4	0.011551679
15186	GO:0032784	regulation of transcript	0.08	1	0.1485884	4	0.011551679
15188	GO:0032786	positive regulation of t	0.08	1	0.1485884	4	0.011551679
15369	GO:0032968|GO:0090	positive regulation of t	0.08	1	0.1485884	4	0.011551679
16626	GO:0034243|GO:0090	regulation of transcript	0.08	1	0.1485884	4	0.011551679
17623	GO:0035257	nuclear hormone rece	0.08	1	0.1485884	4	0.011551679
17624	GO:0035258	steroid hormone recep	0.08	1	0.1485884	4	0.011551679
20810	GO:0045174	glutathione dehydroge	0.08	1	0.1485884	4	0.011551679
20901	GO:0045277|GO:0045	respiratory chain comp	0.08	1	0.1485884	4	0.011551679
23084	GO:0047598	7-dehydrocholesterol r	0.08	1	0.1485884	4	0.011551679
25055	GO:0050750	low-density lipoprotein	0.08	1	0.1485884	4	0.011551679
25711	GO:0051427	hormone receptor bind	0.08	1	0.1485884	4	0.011551679
28689	GO:0070325	lipoprotein particle rec	0.08	1	0.1485884	4	0.011551679
31507	GO:0080132	fatty acid alpha-hydrox	0.08	1	0.1485884	4	0.011551679
75	GO:0000099	sulfur amino acid trans	0.08	1	0.1485884	4	0.011551679
77	GO:0000101	sulfur amino acid trans	0.08	1	0.1485884	4	0.011551679
4311	GO:0006073	cellular glucan metabo	0.08	2	0.2971768	24	0.06931008
4692	GO:0006563	L-serine metabolic pro	0.08	2	0.2971768	24	0.06931008
20293	GO:0044042	glucan metabolic proc	0.08	2	0.2971768	24	0.06931008
2809	GO:0003993	acid phosphatase acti	0.08	3	0.44576523	52	0.15017183
6424	GO:0009055|GO:0009	electron carrier activity	0.08	6	0.89153045	154	0.44473964
5705	GO:0008152	metabolic process	0.08	357	53.046062	17432	50.34222
21145	GO:0045543	gibberellin 2-beta-diox	0.08	2	0.2971768	25	0.072197996
22007	GO:0046483	heterocycle metabolic	0.09	28	4.1604753	1094	3.1593843
25626	GO:0051336	regulation of hydrolase	0.09	7	1.0401188	196	0.5660323
11224	GO:0019222	regulation of metabolic	0.09	71	10.549777	3115	8.995871
28365	GO:0065008	regulation of biological	0.09	10	1.4858841	316	0.9125827
2962	GO:0004160	dihydroxy-acid dehydr	0.09	1	0.1485884	5	0.0144396
3392	GO:0004779	sulfate adenylyltransfe	0.09	1	0.1485884	5	0.0144396
3394	GO:0004781	sulfate adenylyltransfe	0.09	1	0.1485884	5	0.0144396
5243	GO:0007218	neuropeptide signaling	0.09	1	0.1485884	5	0.0144396
14047	GO:0031625	ubiquitin protein ligase	0.09	1	0.1485884	5	0.0144396
24603	GO:0050284	sinapate 1-glucosyltra	0.09	1	0.1485884	5	0.0144396
4909	GO:0006810|GO:0015	transport	0.09	54	8.023774	2315	6.6855345
25529	GO:0051234	establishment of locali	0.09	54	8.023774	2315	6.6855345
21331	GO:0045735	nutrient reservoir activ	0.10	4	0.5943536	89	0.25702485
8919	GO:0015645	fatty acid ligase activit	0.10	2	0.2971768	27	0.077973835
12794	GO:0030234	enzyme regulator activ	0.10	12	1.7830609	403	1.1638317
25476	GO:0051179	localization	0.10	54	8.023774	2319	6.697086
8673	GO:0015291|GO:0015	secondary active trans	0.10	12	1.7830609	407	1.1753833
6438	GO:0009069	serine family amino ac	0.10	2	0.2971768	28	0.080861755
6809	GO:0009521|GO:0030	photosystem	0.10	2	0.2971768	28	0.080861755
6940	GO:0009672	auxin:hydrogen sympo	0.10	2	0.2971768	28	0.080861755
8028	GO:0010817	regulation of hormone	0.10	3	0.44576523	58	0.16749935
9422	GO:0016209	antioxidant activity	0.10	6	0.89153045	165	0.47650677
20498	GO:0044248	cellular catabolic proc	0.11	23	3.4175334	894	2.5818002
2694	GO:0003844	1,4-alpha-glucan bran	0.11	1	0.1485884	6	0.01732752
2713	GO:0003863	3-methyl-2-oxobutano	0.11	1	0.1485884	6	0.01732752
3029	GO:0004345	glucose-6-phosphate	0.11	1	0.1485884	6	0.01732752
3058	GO:0004375	glycine dehydrogenas	0.11	1	0.1485884	6	0.01732752
3065	GO:0004382	guanosine-diphosphat	0.11	1	0.1485884	6	0.01732752
3777	GO:0005302|GO:0015	L-tyrosine transmemb	0.11	1	0.1485884	6	0.01732752
3845	GO:0005452	inorganic anion excha	0.11	1	0.1485884	6	0.01732752
4532	GO:0006357|GO:0006	regulation of transcript	0.11	1	0.1485884	6	0.01732752
4953	GO:0006868|GO:0015	glutamine transport	0.11	1	0.1485884	6	0.01732752
8586	GO:0015173	aromatic amino acid tr	0.11	1	0.1485884	6	0.01732752
8596	GO:0015186	L-glutamine transmem	0.11	1	0.1485884	6	0.01732752
9070	GO:0015801	aromatic amino acid tr	0.11	1	0.1485884	6	0.01732752
9096	GO:0015828	tyrosine transport	0.11	1	0.1485884	6	0.01732752
9723	GO:0016642	oxidoreductase activity	0.11	1	0.1485884	6	0.01732752
9825	GO:0016774	phosphotransferase a	0.11	1	0.1485884	6	0.01732752
11169	GO:0019156	isoamylase activity	0.11	1	0.1485884	6	0.01732752
13832	GO:0031409	pigment binding	0.11	1	0.1485884	6	0.01732752
22095	GO:0046577	long-chain-alcohol oxi	0.11	1	0.1485884	6	0.01732752

23230	GO:0047746	chlorophyllase activity	0.11	1	0.1485884	6	0.01732752
30707	GO:0072348	sulfur compound trans	0.11	1	0.1485884	6	0.01732752
191	GO:0000248	C-5 sterol desaturase	0.11	1	0.1485884	6	0.01732752
9266	GO:0016020	membrane	0.11	83	12.332838	3741	10.803708
4215	GO:0005975	carbohydrate metaboli	0.11	25	3.7147102	990	2.8590407
3886	GO:0005527	macrolide binding	0.11	2	0.2971768	30	0.086637594
3887	GO:0005528	FK506 binding	0.11	2	0.2971768	30	0.086637594
8526	GO:0015112	nitrate transmembrane	0.12	3	0.44576523	61	0.1761631
8975	GO:0015706|GO:0006	nitrate transport	0.12	3	0.44576523	61	0.1761631
21058	GO:0045454|GO:0030	cell redox homeostasis	0.12	7	1.0401188	211	0.6093511
11931	GO:0020037	heme binding	0.12	15	2.228826	550	1.5883559
20514	GO:0044264	cellular polysaccharide	0.12	2	0.2971768	31	0.08952551
15949	GO:0033554	cellular response to st	0.12	6	0.89153045	173	0.49961013
3242	GO:0004601|GO:0016	peroxidase activity	0.12	5	0.74294204	135	0.38986918
9754	GO:0016684	oxidoreductase activity	0.12	5	0.74294204	135	0.38986918
5766	GO:0008236	serine-type peptidase	0.13	7	1.0401188	214	0.6180149
3717	GO:0005199	structural constituent c	0.13	2	0.2971768	32	0.09241343
21041	GO:0045431	flavonol synthase activ	0.13	2	0.2971768	32	0.09241343
25836	GO:0051552	flavone metabolic proc	0.13	2	0.2971768	32	0.09241343
25837	GO:0051553	flavone biosynthetic pr	0.13	2	0.2971768	32	0.09241343
25838	GO:0051554	flavonol metabolic pro	0.13	2	0.2971768	32	0.09241343
25839	GO:0051555	flavonol biosynthetic p	0.13	2	0.2971768	32	0.09241343
9293	GO:0016051|GO:0006	carbohydrate biosynth	0.13	3	0.44576523	64	0.18482687
3357	GO:0004738	pyruvate dehydrogena	0.13	1	0.1485884	7	0.020215439
3358	GO:0004739	pyruvate dehydrogena	0.13	1	0.1485884	7	0.020215439
3367	GO:0004748|GO:0016	ribonucleoside-diphos	0.13	1	0.1485884	7	0.020215439
4260	GO:0006022	aminoglycan metabolic	0.13	1	0.1485884	7	0.020215439
9786	GO:0016725	oxidoreductase activity	0.13	1	0.1485884	7	0.020215439
9789	GO:0016728	oxidoreductase activity	0.13	1	0.1485884	7	0.020215439
21107	GO:0045505	dynein intermediate ch	0.13	1	0.1485884	7	0.020215439
2632	GO:0003682	chromatin binding	0.13	2	0.2971768	33	0.09530135
4942	GO:0006855	drug transmembrane t	0.14	5	0.74294204	139	0.40142086
8607	GO:0015197|GO:0015	peptide transporter act	0.14	5	0.74294204	139	0.40142086
8608	GO:0015198	oligopeptide transporte	0.14	5	0.74294204	139	0.40142086
8642	GO:0015238|GO:0015	drug transmembrane t	0.14	5	0.74294204	139	0.40142086
9154	GO:0015893	drug transport	0.14	5	0.74294204	139	0.40142086
18832	GO:0042493|GO:0017	response to drug	0.14	5	0.74294204	139	0.40142086
25098	GO:0050794|GO:0051	regulation of cellular p	0.14	87	12.927192	3987	11.514136
3872	GO:0005506	iron ion binding	0.14	15	2.228826	563	1.6258988
10103	GO:0017171	serine hydrolase activ	0.14	7	1.0401188	220	0.63534236
4944	GO:0006857	oligopeptide transport	0.14	5	0.74294204	141	0.4071967
9099	GO:0015833	peptide transport	0.14	5	0.74294204	141	0.4071967
3749	GO:0005249	voltage-gated potassiu	0.14	2	0.2971768	34	0.09818927
5700	GO:0008144	drug binding	0.14	2	0.2971768	34	0.09818927
5827	GO:0008308|GO:0022	voltage-gated anion ch	0.14	2	0.2971768	34	0.09818927
20321	GO:0044070	regulation of anion tran	0.14	2	0.2971768	34	0.09818927
2932	GO:0004130	cytochrome-c peroxida	0.15	1	0.1485884	8	0.023103358
2935	GO:0004133	glycogen debranching	0.15	1	0.1485884	8	0.023103358
3280	GO:0004645	phosphorylase activity	0.15	1	0.1485884	8	0.023103358
3356	GO:0004737	pyruvate decarboxylas	0.15	1	0.1485884	8	0.023103358
3803	GO:0005344|GO:0015	oxygen transporter act	0.15	1	0.1485884	8	0.023103358
4035	GO:0005746	mitochondrial respirato	0.15	1	0.1485884	8	0.023103358
4486	GO:0006289|GO:0045	nucleotide-excision re	0.15	1	0.1485884	8	0.023103358
4664	GO:0006535	cysteine biosynthetic p	0.15	1	0.1485884	8	0.023103358
5724	GO:0008184	glycogen phosphorylas	0.15	1	0.1485884	8	0.023103358
6613	GO:0009250	glucan biosynthetic pro	0.15	1	0.1485884	8	0.023103358
8940	GO:0015669	gas transport	0.15	1	0.1485884	8	0.023103358
8942	GO:0015671	oxygen transport	0.15	1	0.1485884	8	0.023103358
10057	GO:0017110	nucleoside-diphosphat	0.15	1	0.1485884	8	0.023103358
12818	GO:0030259	lipid glycosylation	0.15	1	0.1485884	8	0.023103358
22702	GO:0047209	coniferyl-alcohol gluco	0.15	1	0.1485884	8	0.023103358
28833	GO:0070469	respiratory chain	0.15	1	0.1485884	8	0.023103358
29068	GO:0070704	sterol desaturase activ	0.15	1	0.1485884	8	0.023103358
30696	GO:0072337	modified amino acid tr	0.15	1	0.1485884	8	0.023103358
30708	GO:0072349	modified amino acid tr	0.15	1	0.1485884	8	0.023103358
4530	GO:0006355|GO:0032	regulation of transcript	0.15	58	8.618128	2591	7.4826
25095	GO:0050790	regulation of catalytic a	0.15	13	1.9316493	480	1.3862015
25544	GO:0051252	regulation of RNA met	0.15	58	8.618128	2593	7.488376
9722	GO:0016641	oxidoreductase activity	0.15	3	0.44576523	69	0.19926646
28366	GO:0065009	regulation of molecula	0.15	13	1.9316493	484	1.3977532
9185	GO:0015925	galactosidase activity	0.15	2	0.2971768	36	0.10396511
12626	GO:0030029	actin filament-based p	0.15	2	0.2971768	36	0.10396511
12633	GO:0030036	actin cytoskeleton orga	0.15	2	0.2971768	36	0.10396511
7141	GO:0009889	regulation of biosynthe	0.16	58	8.618128	2605	7.523031
7772	GO:0010556	regulation of macromo	0.16	58	8.618128	2605	7.523031
13756	GO:0031326	regulation of cellular b	0.16	58	8.618128	2605	7.523031
32213	GO:2000112	regulation of cellular m	0.16	58	8.618128	2605	7.523031
3758	GO:0005267	potassium channel act	0.16	2	0.2971768	37	0.10685303
2655	GO:0003725	double-stranded RNA	0.16	1	0.1485884	9	0.025991278
2837	GO:0004028	3-chloroallyl aldehyde	0.16	1	0.1485884	9	0.025991278
19342	GO:0043027	caspase inhibitor activ	0.16	1	0.1485884	9	0.025991278
19343	GO:0043028	caspase regulator acti	0.16	1	0.1485884	9	0.025991278
19458	GO:0043154|GO:0001	negative regulation of	0.16	1	0.1485884	9	0.025991278
19579	GO:0043281|GO:0043	regulation of caspase	0.16	1	0.1485884	9	0.025991278
21104	GO:0045502	dynein binding	0.16	1	0.1485884	9	0.025991278
79	GO:0000103|GO:0019	sulfate assimilation	0.16	1	0.1485884	9	0.025991278
9740	GO:0016667	oxidoreductase activity	0.16	3	0.44576523	72	0.20793022
18953	GO:0042623	ATPase activity, coupl	0.17	14	2.0802376	537	1.550813
5066	GO:0007010	cytoskeleton organizat	0.17	2	0.2971768	38	0.10974095
8588	GO:0015175	neutral amino acid tran	0.17	2	0.2971768	38	0.10974095
9073	GO:0015804	neutral amino acid tran	0.17	2	0.2971768	38	0.10974095
12538	GO:0022843	voltage-gated cation c	0.17	2	0.2971768	38	0.10974095
9703	GO:0016620	oxidoreductase activity	0.17	3	0.44576523	73	0.21081814
11695	GO:0019725	cellular homeostasis	0.17	7	1.0401188	234	0.67577326
7692	GO:0010468	regulation of gene exp	0.18	58	8.618128	2635	7.6096687

2897	GO:0004091|GO:0004	carboxylesterase activ	0.18	6	0.89153045	194	0.5602564
3056	GO:0004372	glycine hydroxymethyl	0.18	1	0.1485884	10	0.0288792
3203	GO:0004557	alpha-galactosidase a	0.18	1	0.1485884	10	0.0288792
3362	GO:0004743	pyruvate kinase activit	0.18	1	0.1485884	10	0.0288792
4663	GO:0006534	cysteine metabolic pro	0.18	1	0.1485884	10	0.0288792
8470	GO:0015037	peptide disulfide oxido	0.18	1	0.1485884	10	0.0288792
8471	GO:0015038	glutathione disulfide o	0.18	1	0.1485884	10	0.0288792
9659	GO:0016572	histone phosphorylatio	0.18	1	0.1485884	10	0.0288792
10220	GO:0018105	peptidyl-serine phosph	0.18	1	0.1485884	10	0.0288792
10320	GO:0018209	peptidyl-serine modific	0.18	1	0.1485884	10	0.0288792
11205	GO:0019202	amino acid kinase acti	0.18	1	0.1485884	10	0.0288792
11337	GO:0019344	cysteine biosynthetic p	0.18	1	0.1485884	10	0.0288792
13472	GO:0031013	troponin I binding	0.18	1	0.1485884	10	0.0288792
17539	GO:0035173	histone kinase activity	0.18	1	0.1485884	10	0.0288792
17540	GO:0035174	histone serine kinase a	0.18	1	0.1485884	10	0.0288792
17541	GO:0035175|GO:0044	histone kinase activity	0.18	1	0.1485884	10	0.0288792
17770	GO:0035404	histone-serine phosph	0.18	1	0.1485884	10	0.0288792
20239	GO:0043987|GO:0043	histone H3-S10 phosp	0.18	1	0.1485884	10	0.0288792
23419	GO:0047938	glucose-6-phosphate 1	0.18	1	0.1485884	10	0.0288792
26022	GO:0051740	ethylene binding	0.18	1	0.1485884	10	0.0288792
28451	GO:0070085	glycosylation	0.18	1	0.1485884	10	0.0288792
30687	GO:0072328	alkene binding	0.18	1	0.1485884	10	0.0288792
3627	GO:0005085|GO:0008	guanyl-nucleotide excl	0.18	3	0.44576523	75	0.21659398
9801	GO:0016747	transferase activity, tra	0.18	12	1.7830609	456	1.3168914
7072	GO:0009813	flavonoid biosynthetic	0.18	2	0.2971768	40	0.1155168
18925	GO:0042592	homeostatic process	0.18	7	1.0401188	238	0.68732494
5663	GO:0008094|GO:0004	DNA-dependent ATPa	0.18	3	0.44576523	76	0.2194819
8494	GO:0015075	ion transmembrane tra	0.19	20	2.9717681	827	2.3883097
23515	GO:0048037	cofactor binding	0.19	9	1.3372957	327	0.94434977
9767	GO:0016705	oxidoreductase activity	0.19	9	1.3372957	329	0.95012563
5196	GO:0007166	cell surface receptor li	0.19	3	0.44576523	78	0.22525774
2791	GO:0003973	(S)-2-hydroxy-acid oxi	0.19	1	0.1485884	11	0.03176712
2989	GO:0004301	epoxide hydrolase act	0.19	1	0.1485884	11	0.03176712
3202	GO:0004556	alpha-amylase activity	0.19	1	0.1485884	11	0.03176712
6288	GO:0008891	glycolate oxidase activ	0.19	1	0.1485884	11	0.03176712
9849	GO:0016803	ether hydrolase activit	0.19	1	0.1485884	11	0.03176712
11152	GO:0019139|GO:0046	cytokinin dehydrogena	0.19	1	0.1485884	11	0.03176712
3753	GO:0005253	anion channel activity	0.20	2	0.2971768	42	0.121292636
4874	GO:0006767	water-soluble vitamin	0.20	2	0.2971768	42	0.121292636
8461	GO:0015020|GO:0003	glucuronosyltransferas	0.20	2	0.2971768	42	0.121292636
18713	GO:0042364	water-soluble vitamin	0.20	2	0.2971768	42	0.121292636
27228	GO:0060255	regulation of macromo	0.20	58	8.618128	2660	7.6818666
16603	GO:0034220	ion transmembrane tra	0.20	20	2.9717681	833	2.4056373
131	GO:0000166	nucleotide binding	0.20	28	4.1604753	1213	3.5030468
8589	GO:0015179	L-amino acid transmer	0.20	2	0.2971768	43	0.124180555
9076	GO:0015807	L-amino acid transport	0.20	2	0.2971768	43	0.124180555
9875	GO:0016836	hydro-lyase activity	0.20	3	0.44576523	80	0.2310336
11221	GO:0019219	regulation of nucleoba	0.21	58	8.618128	2675	7.7251854
25468	GO:0051171	regulation of nitrogen	0.21	58	8.618128	2675	7.7251854
2838	GO:0004029	aldehyde dehydrogena	0.21	1	0.1485884	12	0.03465504
3037	GO:0004353	glutamate dehydrogen	0.21	1	0.1485884	12	0.03465504
3049	GO:0004365	glyceraldehyde-3-phos	0.21	1	0.1485884	12	0.03465504
3246	GO:0004605	phosphatidate cytidyly	0.21	1	0.1485884	12	0.03465504
4217	GO:0005977	glycogen metabolic pr	0.21	1	0.1485884	12	0.03465504
4344	GO:0006112	energy reserve metab	0.21	1	0.1485884	12	0.03465504
6336	GO:0008943	glyceraldehyde-3-phos	0.21	1	0.1485884	12	0.03465504
6439	GO:0009070	serine family amino ac	0.21	1	0.1485884	12	0.03465504
21503	GO:0045935	positive regulation of n	0.21	1	0.1485884	12	0.03465504
25470	GO:0051173	positive regulation of n	0.21	1	0.1485884	12	0.03465504
25546	GO:0051254	positive regulation of R	0.21	1	0.1485884	12	0.03465504
37	GO:0000049	tRNA binding	0.21	1	0.1485884	12	0.03465504
4873	GO:0006766	vitamin metabolic proc	0.21	2	0.2971768	44	0.12706847
6477	GO:0009110	vitamin biosynthetic pr	0.21	2	0.2971768	44	0.12706847
7071	GO:0009812	flavonoid metabolic pr	0.21	2	0.2971768	44	0.12706847
12532	GO:0022836	gated channel activity	0.21	4	0.5943536	122	0.3523262
3630	GO:0005088	Ras guanyl-nucleotide	0.22	2	0.2971768	45	0.1299564
3787	GO:0005319	lipid transporter activit	0.22	3	0.44576523	83	0.23969735
4912	GO:0006813|GO:0015	potassium ion transpo	0.22	3	0.44576523	84	0.24258527
30168	GO:0071804	cellular potassium ion	0.22	3	0.44576523	84	0.24258527
30169	GO:0071805	potassium ion transme	0.22	3	0.44576523	84	0.24258527
4357	GO:0006139|GO:0055	nucleobase, nucleosid	0.22	70	10.401189	3285	9.486816
2896	GO:0004090	carbonyl reductase (N	0.23	1	0.1485884	13	0.037542958
7551	GO:0010327	acetyl CoA:(Z)-3-hexe	0.23	1	0.1485884	13	0.037542958
8149	GO:0010941	regulation of cell death	0.23	1	0.1485884	13	0.037542958
13443	GO:0030984	kininogen binding	0.23	1	0.1485884	13	0.037542958
19298	GO:0042981	regulation of apoptosis	0.23	1	0.1485884	13	0.037542958
19381	GO:0043067|GO:0043	regulation of programm	0.23	1	0.1485884	13	0.037542958
6425	GO:0009056	catabolic process	0.24	24	3.5661218	1051	3.0352037
31465	GO:0080090	regulation of primary m	0.24	58	8.618128	2710	7.826263
2976	GO:0004176|GO:0004	ATP-dependent peptid	0.24	2	0.2971768	48	0.13862015
2977	GO:0004177	aminopeptidase activit	0.24	1	0.1485884	14	0.040430877
3204	GO:0004558|GO:0004	alpha-glucosidase acti	0.24	1	0.1485884	14	0.040430877
3629	GO:0005087	Ran guanyl-nucleotide	0.24	1	0.1485884	14	0.040430877
5197	GO:0007167	enzyme linked recepto	0.24	1	0.1485884	14	0.040430877
5199	GO:0007169	transmembrane recep	0.24	1	0.1485884	14	0.040430877
7413	GO:0010181	FMN binding	0.24	1	0.1485884	14	0.040430877
9720	GO:0016639	oxidoreductase activity	0.24	1	0.1485884	14	0.040430877
9847	GO:0016801	hydrolase activity, acti	0.24	1	0.1485884	14	0.040430877
28373	GO:0070006	metalloaminopeptidas	0.24	1	0.1485884	14	0.040430877
3756	GO:0005261|GO:0015	cation channel activity	0.25	2	0.2971768	49	0.14150807
9725	GO:0016645	oxidoreductase activity	0.25	2	0.2971768	49	0.14150807
4605	GO:0006457|GO:0007	protein folding	0.25	5	0.74294204	173	0.49961013
8675	GO:0015293	symporter activity	0.25	5	0.74294204	173	0.49961013
13753	GO:0031323	regulation of cellular m	0.25	59	8.766716	2770	7.999538
8635	GO:0015229	L-ascorbic acid transp	0.26	1	0.1485884	15	0.043318797

9144	GO:0015882	L-ascorbic acid transp	0.26	1	0.1485884	15	0.043318797
16081	GO:0033692	cellular polysaccharide	0.26	1	0.1485884	15	0.043318797
2758	GO:0003924	GTPase activity	0.26	4	0.5943536	132	0.3812054
4394	GO:0006184	GTP catabolic process	0.26	4	0.5943536	132	0.3812054
21605	GO:0046039	GTP metabolic process	0.26	4	0.5943536	132	0.3812054
4906	GO:0006807	nitrogen compound m	0.26	78	11.589896	3725	10.757502
19126	GO:0042803	protein homodimerizat	0.26	7	1.0401188	265	0.7652988
30207	GO:0071843	cellular component bio	0.26	2	0.2971768	51	0.14728391
9800	GO:0016746	transferase activity, tra	0.26	12	1.7830609	498	1.438184
6965	GO:0009699	phenylpropanoid biosy	0.27	2	0.2971768	52	0.15017183
2667	GO:0003777	microtubule motor acti	0.27	1	0.1485884	16	0.046206716
3742	GO:0005242	inward rectifier potass	0.27	1	0.1485884	16	0.046206716
7178	GO:0009927	histidine phosphotrans	0.27	1	0.1485884	16	0.046206716
9189	GO:0015929	hexosaminidase activi	0.27	1	0.1485884	16	0.046206716
9727	GO:0016647	oxidoreductase activity	0.27	1	0.1485884	16	0.046206716
9797	GO:0016742	hydroxymethyl-, formy	0.27	1	0.1485884	16	0.046206716
9898	GO:0016863	intramolecular oxidore	0.27	1	0.1485884	16	0.046206716
13435	GO:0030976	thiamine pyrophospha	0.27	1	0.1485884	16	0.046206716
18757	GO:0042409	caffeoyl-CoA O-methy	0.27	1	0.1485884	16	0.046206716
22110	GO:0046592	polyamine oxidase act	0.27	1	0.1485884	16	0.046206716
72	GO:0000096	sulfur amino acid meta	0.27	1	0.1485884	16	0.046206716
73	GO:0000097	sulfur amino acid bios	0.27	1	0.1485884	16	0.046206716
219	GO:0000287	magnesium ion bindin	0.27	1	0.1485884	16	0.046206716
2643	GO:0003700|GO:000	sequence-specific DN	0.27	31	4.6062407	1417	4.0921826
716	GO:0001071	nucleic acid binding tra	0.27	31	4.6062407	1417	4.0921826
8585	GO:0015172	acidic amino acid tran	0.28	1	0.1485884	17	0.049094636
9069	GO:0015800	acidic amino acid tran	0.28	1	0.1485884	17	0.049094636
2674	GO:0003824	catalytic activity	0.29	216	32.095097	10750	31.045137
3724	GO:0005216	ion channel activity	0.29	4	0.5943536	140	0.40430877
8676	GO:0015294	solute:cation symporte	0.29	4	0.5943536	141	0.4071967
5767	GO:0008237	metallopeptidase activ	0.30	2	0.2971768	56	0.16172351
5765	GO:0008235	metalloexopeptidase a	0.30	1	0.1485884	18	0.051982556
6491	GO:0009126	purine nucleoside mon	0.30	1	0.1485884	18	0.051982556
6492	GO:0009127	purine nucleoside mon	0.30	1	0.1485884	18	0.051982556
6517	GO:0009152	purine ribonucleotide b	0.30	1	0.1485884	18	0.051982556
6531	GO:0009167	purine ribonucleoside	0.30	1	0.1485884	18	0.051982556
6532	GO:0009168	purine ribonucleoside	0.30	1	0.1485884	18	0.051982556
8469	GO:0015036	disulfide oxidoreducta	0.30	1	0.1485884	18	0.051982556
8662	GO:0015271	outward rectifier potas	0.30	1	0.1485884	18	0.051982556
22917	GO:0047429	nucleoside-triphospha	0.30	1	0.1485884	18	0.051982556
28931	GO:0070567	cytidylyltransferase ac	0.30	1	0.1485884	18	0.051982556
17020	GO:0034641	cellular nitrogen comp	0.30	76	11.292719	3678	10.621769
5763	GO:0008233	peptidase activity	0.31	13	1.9316493	565	1.6316746
20531	GO:0044281	small molecule metab	0.31	38	5.6463594	1788	5.1636004
3290	GO:0004656	procollagen-proline 4-	0.31	1	0.1485884	19	0.054870475
11752	GO:0019798	procollagen-proline dio	0.31	1	0.1485884	19	0.054870475
13966	GO:0031543	peptidyl-proline dioxyg	0.31	1	0.1485884	19	0.054870475
13968	GO:0031545	peptidyl-proline 4-diox	0.31	1	0.1485884	19	0.054870475
4910	GO:0006811	ion transport	0.32	21	3.1203566	959	2.769515
2626	GO:0003674|GO:0005	molecular_function	0.32	480	71.32243	24394	70.447914
2675	GO:0003825	alpha,alpha-trehalose-	0.32	1	0.1485884	20	0.0577584
3628	GO:0005086	ARF guanyl-nucleotide	0.32	1	0.1485884	20	0.0577584
9706	GO:0016623	oxidoreductase activity	0.32	1	0.1485884	20	0.0577584
9784	GO:0016723	oxidoreductase activity	0.32	1	0.1485884	20	0.0577584
24482	GO:0050162	oxalate oxidase activit	0.32	1	0.1485884	20	0.0577584
25477	GO:0051180	vitamin transport	0.32	1	0.1485884	20	0.0577584
25480	GO:0051183	vitamin transporter act	0.32	1	0.1485884	20	0.0577584
122	GO:0000154|GO:001	rRNA modification	0.32	1	0.1485884	20	0.0577584
225	GO:0000293	ferric-chelate reducta	0.32	1	0.1485884	20	0.0577584
6964	GO:0009698	phenylpropanoid meta	0.33	2	0.2971768	60	0.17327519
8943	GO:0015672	monovalent inorganic	0.33	9	1.3372957	383	1.1060733
11426	GO:0019438	aromatic compound bi	0.33	4	0.5943536	149	0.43030006
2818	GO:0004003	ATP-dependent DNA	0.33	2	0.2971768	61	0.1761631
3474	GO:0004869|GO:0004	cysteine-type endope	0.34	1	0.1485884	21	0.060646318
3195	GO:0004540	ribonuclease activity	0.34	3	0.44576523	106	0.3061195
5213	GO:0007186	G-protein coupled rec	0.34	2	0.2971768	62	0.17905103
9697	GO:0016614	oxidoreductase activity	0.34	7	1.0401188	293	0.84616053
17016	GO:0034637	cellular carbohydrate	0.35	2	0.2971768	63	0.18193895
6521	GO:0009156	ribonucleoside monop	0.35	1	0.1485884	22	0.06353424
6525	GO:0009161	ribonucleoside monop	0.35	1	0.1485884	22	0.06353424
7143	GO:0009891	positive regulation of	0.35	1	0.1485884	22	0.06353424
8489	GO:0015066	alpha-amylase inhibito	0.35	1	0.1485884	22	0.06353424
8774	GO:0015431	glutathione S-conjugat	0.35	1	0.1485884	22	0.06353424
13031	GO:0030544	Hsp70 protein binding	0.35	1	0.1485884	22	0.06353424
13758	GO:0031328	positive regulation of	0.35	1	0.1485884	22	0.06353424
24858	GO:0050551	myrcene synthase act	0.35	1	0.1485884	22	0.06353424
30359	GO:0071997	glutathione S-conjugat	0.35	1	0.1485884	22	0.06353424
28378	GO:0070011	peptidase activity, acti	0.35	12	1.7830609	537	1.550813
9710	GO:0016628	oxidoreductase activity	0.36	2	0.2971768	65	0.18771479
2633	GO:0003684	damaged DNA binding	0.36	1	0.1485884	23	0.06642216
3662	GO:0005126	cytokine receptor bind	0.36	1	0.1485884	23	0.06642216
7527	GO:0010302	2-oxoglutarate-depend	0.36	1	0.1485884	23	0.06642216
3626	GO:0005083	small GTPase regulat	0.37	3	0.44576523	111	0.3205591
3180	GO:0004521	endoribonuclease acti	0.37	2	0.2971768	66	0.1906027
9765	GO:0016702	oxidoreductase activity	0.37	2	0.2971768	66	0.1906027
11829	GO:0019900	kinase binding	0.37	2	0.2971768	66	0.1906027
2629	GO:0003678|GO:0003	DNA helicase activity	0.38	2	0.2971768	67	0.19349062
8559	GO:0015146	pentose transmembra	0.38	1	0.1485884	24	0.06931008
8561	GO:0015148	D-xylose transmembra	0.38	1	0.1485884	24	0.06931008
8829	GO:0015519	D-xylose:hydrogen sy	0.38	1	0.1485884	24	0.06931008
9019	GO:0015750	pentose transport	0.38	1	0.1485884	24	0.06931008
9022	GO:0015753	D-xylose transport	0.38	1	0.1485884	24	0.06931008
9779	GO:0016717	oxidoreductase activity	0.38	1	0.1485884	24	0.06931008
20488	GO:0044238	primary metabolic pro	0.38	182	27.04309	9167	26.47356
9836	GO:0016787	hydrolase activity	0.39	75	11.144131	3723	10.751725

3620	GO:0005057	receptor signaling prot	0.39	2	0.2971768	69	0.19926646
21710	GO:0046148	pigment biosynthetic p	0.39	2	0.2971768	69	0.19926646
2649	GO:0003713	transcription coactivat	0.40	2	0.2971768	70	0.20215438
5726	GO:0008187	poly-pyrimidine tract b	0.40	2	0.2971768	70	0.20215438
5788	GO:0008266	poly(U) RNA binding	0.40	2	0.2971768	70	0.20215438
3210	GO:0004565	beta-galactosidase ac	0.40	1	0.1485884	26	0.075085916
3415	GO:0004805	trehalose-phosphatase	0.40	1	0.1485884	26	0.075085916
7000	GO:0009734	auxin mediated signali	0.40	1	0.1485884	26	0.075085916
7540	GO:0010315	auxin efflux	0.40	1	0.1485884	26	0.075085916
7553	GO:0010329	auxin efflux transmem	0.40	1	0.1485884	26	0.075085916
17147	GO:0034768	(E)-beta-ocimene synt	0.40	1	0.1485884	26	0.075085916
29729	GO:0071365	cellular response to au	0.40	1	0.1485884	26	0.075085916
126	GO:0000160	two-component signal	0.40	1	0.1485884	26	0.075085916
9764	GO:0016701	oxidoreductase activity	0.41	2	0.2971768	72	0.20793022
5018	GO:0006950	response to stress	0.41	13	1.9316493	611	1.764519
3631	GO:0005089	Rho guanyl-nucleotide	0.41	1	0.1485884	27	0.077973835
9389	GO:0016160	amylase activity	0.41	1	0.1485884	27	0.077973835
9541	GO:0016413	O-acetyltransferase ac	0.41	1	0.1485884	27	0.077973835
18786	GO:0042440	pigment metabolic pro	0.42	2	0.2971768	73	0.21081814
17617	GO:0035251	UDP-glucosyltransfera	0.42	5	0.74294204	218	0.62956655
3284	GO:0004650	polygalacturonase act	0.42	1	0.1485884	28	0.080861755
4374	GO:0006164	purine nucleotide bios	0.42	1	0.1485884	28	0.080861755
6079	GO:0008654	phospholipid biosynthe	0.42	1	0.1485884	28	0.080861755
7145	GO:0009893	positive regulation of r	0.42	1	0.1485884	28	0.080861755
7819	GO:0010604	positive regulation of r	0.42	1	0.1485884	28	0.080861755
8863	GO:0015562	efflux transmembrane	0.42	1	0.1485884	28	0.080861755
9238	GO:0015980	energy derivation by o	0.42	1	0.1485884	28	0.080861755
13144	GO:0030674	protein binding, bridgin	0.42	1	0.1485884	28	0.080861755
13755	GO:0031325	positive regulation of c	0.42	1	0.1485884	28	0.080861755
27064	GO:0060090	binding, bridging	0.42	1	0.1485884	28	0.080861755
139	GO:0000175	3'-5'-exoribonuclease	0.42	1	0.1485884	28	0.080861755
4526	GO:0006351|GO:0006	transcription, DNA-dep	0.42	32	4.754829	1575	4.548474
15176	GO:0032774	RNA biosynthetic proc	0.42	32	4.754829	1575	4.548474
6884	GO:0009607	response to biotic stim	0.42	3	0.44576523	122	0.3523262
25195	GO:0050896|GO:0051	response to stimulus	0.43	38	5.6463594	1881	5.432177
22049	GO:0046527	glucosyltransferase ac	0.43	6	0.89153045	270	0.77973837
3260	GO:0004620	phospholipase activity	0.43	3	0.44576523	123	0.35521415
2975	GO:0004175|GO:0016	endopeptidase activity	0.43	7	1.0401188	320	0.9241344
25510	GO:0051213	dioxygenase activity	0.43	2	0.2971768	75	0.21659398
3490	GO:0004888|GO:0004	transmembrane recep	0.43	12	1.7830609	571	1.6490022
4968	GO:0006886	intracellular protein tra	0.43	1	0.1485884	29	0.083749674
16992	GO:0034613|GO:0016	cellular protein localiza	0.43	1	0.1485884	29	0.083749674
29091	GO:0070727	cellular macromolecule	0.43	1	0.1485884	29	0.083749674
5732	GO:0008194	UDP-glycosyltransfera	0.44	7	1.0401188	322	0.9299102
6529	GO:0009165	nucleotide biosynthetic	0.44	2	0.2971768	77	0.22236982
2666	GO:0003774	motor activity	0.45	1	0.1485884	30	0.086637594
3518	GO:0004930|GO:0001	G-protein coupled rece	0.45	1	0.1485884	30	0.086637594
6216	GO:0008810	cellulase activity	0.45	1	0.1485884	30	0.086637594
6488	GO:0009123	nucleoside monophos	0.45	1	0.1485884	30	0.086637594
6489	GO:0009124	nucleoside monophos	0.45	1	0.1485884	30	0.086637594
8683	GO:0015301|GO:0015	anion:anion antiporter	0.45	1	0.1485884	30	0.086637594
5681	GO:0008116	prostaglandin-I syntha	0.45	2	0.2971768	78	0.22525774
9909	GO:0016877	ligase activity, forming	0.45	2	0.2971768	78	0.22525774
25998	GO:0051716	cellular response to st	0.45	23	3.4175334	1138	3.2864528
19590	GO:0043295	glutathione binding	0.46	1	0.1485884	31	0.08952551
3190	GO:0004532	exoribonuclease activi	0.47	1	0.1485884	32	0.09241343
3265	GO:0004630	phospholipase D activ	0.47	1	0.1485884	32	0.09241343
6942	GO:0009674	potassium:sodium syn	0.47	1	0.1485884	32	0.09241343
9927	GO:0016896	exoribonuclease activi	0.47	1	0.1485884	32	0.09241343
12522	GO:0022820	potassium ion sympor	0.47	1	0.1485884	32	0.09241343
12833	GO:0030275	LRR domain binding	0.47	1	0.1485884	32	0.09241343
23914	GO:0048522|GO:0051	positive regulation of c	0.47	1	0.1485884	32	0.09241343
30878	GO:0072522	purine-containing com	0.48	1	0.1485884	33	0.09530135
16738	GO:0034357	photosynthetic membr	0.48	2	0.2971768	83	0.23969735
4844	GO:0006725	cellular aromatic comp	0.49	4	0.5943536	185	0.53426516
9486	GO:0016298	lipase activity	0.49	4	0.5943536	185	0.53426516
9818	GO:0016765|GO:0016	transferase activity, tra	0.49	4	0.5943536	185	0.53426516
2985	GO:0004222	metalloendopeptidase	0.49	1	0.1485884	34	0.09818927
6004	GO:0008536	Ran GTPase binding	0.49	1	0.1485884	34	0.09818927
4327	GO:0006091	generation of precurso	0.49	2	0.2971768	84	0.24258527
19125	GO:0042802	identical protein bindin	0.49	14	2.0802376	697	2.01288
4538	GO:0006364|GO:0006	rRNA processing	0.50	1	0.1485884	35	0.10107719
6355	GO:0008970	phospholipase A1 acti	0.50	1	0.1485884	35	0.10107719
9310	GO:0016072	rRNA metabolic proce	0.50	1	0.1485884	35	0.10107719
11206	GO:0019203	carbohydrate phospha	0.50	1	0.1485884	35	0.10107719
12817	GO:0030258	lipid modification	0.50	1	0.1485884	35	0.10107719
21146	GO:0045544	gibberellin 20-oxidase	0.50	1	0.1485884	35	0.10107719
23199	GO:0047714	galactolipase activity	0.50	1	0.1485884	35	0.10107719
2657	GO:0003727|GO:0003	single-stranded RNA b	0.50	4	0.5943536	188	0.54292893
5768	GO:0008238	exopeptidase activity	0.50	2	0.2971768	86	0.24836111
4916	GO:0006818	hydrogen transport	0.50	6	0.89153045	292	0.84327257
9246	GO:0015992	proton transport	0.50	6	0.89153045	292	0.84327257
4232	GO:0005992	trehalose biosynthetic	0.51	1	0.1485884	36	0.10396511
5902	GO:0008408	3'-5' exonuclease activ	0.51	1	0.1485884	36	0.10396511
6794	GO:0009505	plant-type cell wall	0.51	1	0.1485884	36	0.10396511
8739	GO:0015385|GO:0015	sodium:hydrogen antip	0.51	1	0.1485884	36	0.10396511
9393	GO:0016165	lipoxygenase activity	0.51	1	0.1485884	36	0.10396511
20649	GO:0044455	mitochondrial membra	0.51	1	0.1485884	36	0.10396511
4641	GO:0006508	proteolysis	0.51	19	2.8231797	965	2.7868426
3318	GO:0004693|GO:0016	cyclin-dependent prote	0.52	1	0.1485884	37	0.10685303
11202	GO:0019199	transmembrane recep	0.52	10	1.4858841	504	1.4555116
13164	GO:0030695	GTPase regulator acti	0.52	3	0.44576523	142	0.4100846
20630	GO:0044436	thylakoid part	0.52	2	0.2971768	90	0.2599128
4231	GO:0005991	trehalose metabolic pr	0.53	1	0.1485884	38	0.10974095
9308	GO:0016070	RNA metabolic proces	0.53	36	5.3491826	1855	5.3570914

3644	GO:0005102	receptor binding	0.53	2	0.2971768	91	0.2628007
6784	GO:0009451|GO:0016	RNA modification	0.53	2	0.2971768	91	0.2628007
6623	GO:0009260	ribonucleotide biosynth	0.54	1	0.1485884	39	0.11262887
16785	GO:0034404	nucleobase, nucleosid	0.54	2	0.2971768	92	0.26568863
17033	GO:0034654	nucleobase, nucleosid	0.54	2	0.2971768	92	0.26568863
12805	GO:0030246	carbohydrate binding	0.54	5	0.74294204	251	0.7248679
3178	GO:0004519	endonuclease activity	0.54	7	1.0401188	356	1.0280994
9895	GO:0016860	intramolecular oxidore	0.54	3	0.44576523	146	0.42163628
20336	GO:0044085	cellular component bio	0.54	2	0.2971768	93	0.26857653
5891	GO:0008395|GO:0008	steroid hydroxylase ac	0.54	1	0.1485884	40	0.1155168
7021	GO:0009755	hormone-mediated sig	0.54	1	0.1485884	40	0.1155168
9843	GO:0016796	exonuclease activity, a	0.54	1	0.1485884	40	0.1155168
15271	GO:0032870	cellular response to ho	0.54	1	0.1485884	40	0.1155168
16446	GO:0034062	RNA polymerase activ	0.54	1	0.1485884	40	0.1155168
23910	GO:0048518|GO:0043	positive regulation of b	0.54	1	0.1485884	40	0.1155168
3808	GO:0005351|GO:0005	sugar:hydrogen sympo	0.55	2	0.2971768	94	0.27146447
3835	GO:0005402	cation:sugar symporte	0.55	2	0.2971768	94	0.27146447
6863	GO:0009579	thylakoid	0.55	2	0.2971768	94	0.27146447
22479	GO:0046983	protein dimerization ac	0.55	10	1.4858841	517	1.4930545
9829	GO:0016779	nucleotidyltransferase	0.55	3	0.44576523	148	0.42741212
22415	GO:0046914	transition metal ion bin	0.55	50	7.4294205	2599	7.5057034
31895	GO:0090304	nucleic acid metabolic	0.55	45	6.6864786	2340	6.7577324
11712	GO:0019748	secondary metabolic p	0.55	2	0.2971768	95	0.27435237
9874	GO:0016835	carbon-oxygen lyase a	0.55	4	0.5943536	202	0.5833598
6666	GO:0009312	oligosaccharide biosyn	0.56	1	0.1485884	42	0.121292636
9370	GO:0016138	glycoside biosynthetic	0.56	1	0.1485884	42	0.121292636
21880	GO:0046351	disaccharide biosynthe	0.56	1	0.1485884	42	0.121292636
3200	GO:0004553|GO:0016	hydrolase activity, hyd	0.57	6	0.89153045	312	0.90103096
8679	GO:0015297	antiporter activity	0.57	3	0.44576523	152	0.4389638
8682	GO:0015300	solute:solute antiporte	0.57	3	0.44576523	152	0.4389638
27562	GO:0060589	nucleoside-triphospha	0.57	3	0.44576523	152	0.4389638
8677	GO:0015295	solute:hydrogen symp	0.57	2	0.2971768	98	0.28301615
8681	GO:0015299	solute:hydrogen antipo	0.57	2	0.2971768	98	0.28301615
20619	GO:0044425	membrane part	0.57	29	4.309064	1523	4.398302
3307	GO:0004675	transmembrane recep	0.57	7	1.0401188	367	1.0598665
16851	GO:0034470	ncRNA processing	0.58	2	0.2971768	99	0.28590405
17039	GO:0034660	ncRNA metabolic proc	0.58	2	0.2971768	99	0.28590405
4954	GO:0006869	lipid transport	0.58	4	0.5943536	208	0.6006873
8085	GO:0010876	lipid localization	0.58	4	0.5943536	208	0.6006873
5797	GO:0008276	protein methyltransfera	0.58	1	0.1485884	44	0.12706847
9186	GO:0015926	glucosidase activity	0.58	1	0.1485884	44	0.12706847
9753	GO:0016682	oxidoreductase activity	0.58	1	0.1485884	44	0.12706847
29859	GO:0071495	cellular response to en	0.58	1	0.1485884	44	0.12706847
16430	GO:0034046	poly(G) RNA binding	0.58	2	0.2971768	100	0.28879198
7229	GO:0009987|GO:0008	cellular process	0.58	204	30.312037	10601	30.614838
8666	GO:0015276	ligand-gated ion chann	0.59	1	0.1485884	45	0.1299564
9893	GO:0016857	racemase and epimera	0.59	1	0.1485884	45	0.1299564
12530	GO:0022834	ligand-gated channel a	0.59	1	0.1485884	45	0.1299564
18956	GO:0042626	ATPase activity, coupl	0.59	4	0.5943536	211	0.6093511
3177	GO:0004518	nuclease activity	0.59	8	1.1887072	427	1.2331418
9699	GO:0016616	oxidoreductase activity	0.59	5	0.74294204	266	0.7681867
9861	GO:0016820	hydrolase activity, acti	0.59	4	0.5943536	212	0.612239
4329	GO:0006096|GO:0019	glycolysis	0.59	1	0.1485884	46	0.13284431
6999	GO:0009733	response to auxin stim	0.59	1	0.1485884	46	0.13284431
12498	GO:0022613	ribonucleoprotein com	0.59	1	0.1485884	46	0.13284431
18614	GO:0042254|GO:0007	ribosome biogenesis	0.59	1	0.1485884	46	0.13284431
26845	GO:0052689	carboxylic ester hydro	0.60	10	1.4858841	538	1.5537009
3421	GO:0004812|GO:0017	aminoacyl-tRNA ligase	0.60	1	0.1485884	47	0.13573223
8497	GO:0015079|GO:0022	potassium ion transme	0.60	1	0.1485884	47	0.13573223
9907	GO:0016875	ligase activity, forming	0.60	1	0.1485884	47	0.13573223
9908	GO:0016876	ligase activity, forming	0.60	1	0.1485884	47	0.13573223
9709	GO:0016627	oxidoreductase activity	0.60	2	0.2971768	104	0.30034366
4849	GO:0006730|GO:0019	one-carbon metabolic	0.61	6	0.89153045	325	0.93857396
9869	GO:0016829	lyase activity	0.61	9	1.3372957	488	1.4093049
4188	GO:0005938	cell cortex	0.61	1	0.1485884	48	0.13862015
20642	GO:0044448	cell cortex part	0.61	1	0.1485884	48	0.13862015
28930	GO:0070566	adenylyltransferase ac	0.61	1	0.1485884	48	0.13862015
113	GO:0000145	exocyst	0.61	1	0.1485884	48	0.13862015
18582	GO:0042221	response to chemical	0.62	6	0.89153045	328	0.94723773
3844	GO:0005451	monovalent cation:hyd	0.62	1	0.1485884	49	0.14150807
31421	GO:0080044	quercetin 7-O-glucosy	0.62	1	0.1485884	49	0.14150807
5055	GO:0006996	organelle organization	0.62	3	0.44576523	164	0.47361887
5909	GO:0008415	acyltransferase activity	0.62	6	0.89153045	330	0.95301354
4459	GO:0006259|GO:0055	DNA metabolic proces	0.62	9	1.3372957	493	1.4237444
3185	GO:0004527|GO:0008	exonuclease activity	0.63	1	0.1485884	50	0.14439599
4622	GO:0006479	protein methylation	0.63	1	0.1485884	50	0.14439599
5750	GO:0008213	protein alkylation	0.63	1	0.1485884	50	0.14439599
9783	GO:0016722	oxidoreductase activity	0.63	1	0.1485884	50	0.14439599
9993	GO:0017016	Ras GTPase binding	0.63	1	0.1485884	50	0.14439599
29674	GO:0071310	cellular response to or	0.63	1	0.1485884	50	0.14439599
13656	GO:0031224	intrinsic to membrane	0.63	22	3.268945	1193	3.4452884
22375	GO:0046872	metal ion binding	0.63	56	8.3209505	2985	8.6204405
7173	GO:0009922	fatty acid elongase act	0.63	1	0.1485884	51	0.14728391
22410	GO:0046907	intracellular transport	0.63	1	0.1485884	51	0.14728391
25925	GO:0051641	cellular localization	0.63	1	0.1485884	51	0.14728391
25933	GO:0051649	establishment of locali	0.63	1	0.1485884	51	0.14728391
5097	GO:0007049	cell cycle	0.64	1	0.1485884	52	0.15017183
20287	GO:0044036	cell wall macromolecu	0.64	1	0.1485884	52	0.15017183
9812	GO:0016758	transferase activity, tra	0.64	9	1.3372957	501	1.4468478
19470	GO:0043167	ion binding	0.64	56	8.3209505	2997	8.655096
19472	GO:0043169	cation binding	0.64	56	8.3209505	2997	8.655096
5070	GO:0007017	microtubule-based pro	0.64	2	0.2971768	112	0.32344702
5071	GO:0007018	microtubule-based mo	0.64	2	0.2971768	112	0.32344702
8680	GO:0015298	solute:cation antiporte	0.64	2	0.2971768	112	0.32344702
9730	GO:0016651	oxidoreductase activity	0.64	2	0.2971768	112	0.32344702

29081	GO:0070717	poly-purine tract bindir	0.64	2	0.2971768	112	0.32344702
17024	GO:0034645|GO:0034	cellular macromolecule	0.65	37	5.4977713	1999	5.7729516
9750	GO:0016679	oxidoreductase activity	0.65	1	0.1485884	53	0.15305975
29246	GO:0070882	cellular cell wall organ	0.65	1	0.1485884	53	0.15305975
6428	GO:0009059|GO:0043	macromolecule biosyn	0.65	37	5.4977713	2003	5.7845035
30205	GO:0071841	cellular component org	0.65	5	0.74294204	285	0.8230572
3156	GO:0004497	monooxygenase activ	0.65	3	0.44576523	172	0.49672222
29251	GO:0070887	cellular response to ch	0.65	1	0.1485884	54	0.15594767
9267	GO:0016021	integral to membrane	0.66	20	2.9717681	1103	3.1853757
3476	GO:0004872|GO:0019	receptor activity	0.66	12	1.7830609	672	1.9406822
4224	GO:0005984	disaccharide metabolic	0.67	1	0.1485884	56	0.16172351
9369	GO:0016137	glycoside metabolic pr	0.67	1	0.1485884	56	0.16172351
9883	GO:0016846	carbon-sulfur lyase ac	0.67	1	0.1485884	56	0.16172351
13697	GO:0031267	small GTPase binding	0.67	1	0.1485884	56	0.16172351
3955	GO:0005634	nucleus	0.67	26	3.8632987	1433	4.138389
20533	GO:0044283	small molecule biosyn	0.67	6	0.89153045	347	1.0021082
3475	GO:0004871|GO:0005	signal transducer activ	0.67	14	2.0802376	787	2.2727928
27063	GO:0060089	molecular transducer a	0.67	14	2.0802376	787	2.2727928
25416	GO:0051119	sugar transmembrane	0.67	2	0.2971768	118	0.34077454
19758	GO:0043492	ATPase activity, coupl	0.67	4	0.5943536	235	0.67866117
5715	GO:0008171	O-methyltransferase a	0.67	1	0.1485884	57	0.16461143
7557	GO:0010333	terpene synthase activ	0.67	1	0.1485884	57	0.16461143
9813	GO:0016759	cellulose synthase act	0.67	1	0.1485884	57	0.16461143
3877	GO:0005516	calmodulin binding	0.68	4	0.5943536	236	0.6815491
2628	GO:0003677	DNA binding	0.68	22	3.268945	1224	3.534814
9824	GO:0016773	phosphotransferase ac	0.68	27	4.011887	1493	4.311664
3304	GO:0004672|GO:0050	protein kinase activity	0.68	24	3.5661218	1333	3.8495972
3323	GO:0004702	receptor signaling prot	0.68	1	0.1485884	58	0.16749935
4913	GO:0006814|GO:0006	sodium ion transport	0.68	1	0.1485884	58	0.16749935
6665	GO:0009311	oligosaccharide metab	0.68	1	0.1485884	58	0.16749935
8499	GO:0015081|GO:0022	sodium ion transmemb	0.68	1	0.1485884	58	0.16749935
8558	GO:0015145	monosaccharide trans	0.68	1	0.1485884	58	0.16749935
9018	GO:0015749	monosaccharide trans	0.68	1	0.1485884	58	0.16749935
18086	GO:0035725	sodium ion transmemb	0.68	1	0.1485884	58	0.16749935
29800	GO:0071436	sodium ion export	0.68	1	0.1485884	58	0.16749935
30700	GO:0072341	modified amino acid b	0.69	1	0.1485884	59	0.17038727
5195	GO:0007165|GO:0023	signal transduction	0.69	17	2.526003	961	2.775291
12602	GO:0023052|GO:0023	signaling	0.69	17	2.526003	961	2.775291
9823	GO:0016772	transferase activity, tra	0.69	35	5.2005944	1932	5.579461
2980	GO:0004185	serine-type carboxype	0.69	1	0.1485884	60	0.17327519
5652	GO:0008081|GO:0004	phosphoric diester hyd	0.69	1	0.1485884	60	0.17327519
14811	GO:0032403	protein complex bindir	0.69	1	0.1485884	60	0.17327519
25319	GO:0051020	GTPase binding	0.69	1	0.1485884	60	0.17327519
28375	GO:0070008	serine-type exopeptida	0.69	1	0.1485884	60	0.17327519
5610	GO:0008026	ATP-dependent helica	0.69	3	0.44576523	183	0.52848935
28401	GO:0070035	purine NTP-dependen	0.69	3	0.44576523	183	0.52848935
4768	GO:0006644	phospholipid metaboli	0.70	1	0.1485884	61	0.1761631
2978	GO:0004180	carboxypeptidase activ	0.70	1	0.1485884	62	0.17905103
6018	GO:0008559|GO:0005	xenobiotic-transporting	0.70	1	0.1485884	62	0.17905103
11210	GO:0019207	kinase regulator activi	0.70	1	0.1485884	62	0.17905103
17457	GO:0035091	phosphatidylinositol bi	0.70	1	0.1485884	62	0.17905103
19225	GO:0042908	xenobiotic transport	0.70	1	0.1485884	62	0.17905103
19227	GO:0042910	xenobiotic transporter	0.70	1	0.1485884	62	0.17905103
2648	GO:0003712	transcription cofactor a	0.71	2	0.2971768	126	0.3638779
634	GO:0000988	protein binding transcr	0.71	2	0.2971768	126	0.3638779
635	GO:0000989	transcription factor bin	0.71	2	0.2971768	126	0.3638779
20512	GO:0044262|GO:0006	cellular carbohydrate r	0.71	4	0.5943536	248	0.7162041
3068	GO:0004386	helicase activity	0.71	3	0.44576523	189	0.54581684
25820	GO:0051536	iron-sulfur cluster bind	0.72	1	0.1485884	64	0.18482687
25824	GO:0051540	metal cluster binding	0.72	1	0.1485884	64	0.18482687
20521	GO:0044271	cellular nitrogen comp	0.72	6	0.89153045	367	1.0598665
5840	GO:0008324	cation transmembrane	0.72	9	1.3372957	538	1.5537009
11616	GO:0019637	organophosphate met	0.72	1	0.1485884	65	0.18771479
4561	GO:0006399	tRNA metabolic proce	0.73	1	0.1485884	66	0.1906027
5615	GO:0008033	tRNA processing	0.73	1	0.1485884	66	0.1906027
9890	GO:0016854	racemase and epimera	0.73	1	0.1485884	66	0.1906027
30206	GO:0071842	cellular component org	0.73	3	0.44576523	193	0.5573685
9837	GO:0016788	hydrolase activity, acti	0.73	27	4.011887	1534	4.430069
6662	GO:0009308	amine metabolic proce	0.73	5	0.74294204	314	0.9068068
2999	GO:0004312	fatty acid synthase act	0.74	1	0.1485884	68	0.19637854
7691	GO:0010467	gene expression	0.74	35	5.2005944	1974	5.7007537
5809	GO:0008289	lipid binding	0.74	4	0.5943536	258	0.74508333
31420	GO:0080043	quercetin 3-O-glucosy	0.74	1	0.1485884	69	0.19926646
10244	GO:0018130	heterocycle biosynthet	0.75	3	0.44576523	199	0.57469606
8749	GO:0015399	primary active transme	0.75	4	0.5943536	260	0.75085914
8753	GO:0015405	P-P-bond-hydrolysis-d	0.75	4	0.5943536	260	0.75085914
9853	GO:0016811	hydrolase activity, acti	0.75	1	0.1485884	70	0.20215438
9796	GO:0016741	transferase activity, tra	0.75	4	0.5943536	261	0.7537471
20499	GO:0044249	cellular biosynthetic pr	0.75	47	6.9836555	2635	7.6096687
6427	GO:0009058	biosynthetic process	0.75	50	7.4294205	2796	8.074624
4612	GO:0006468	protein phosphorylatio	0.75	39	5.794948	2205	6.367863
3306	GO:0004674|GO:0004	protein serine/threonin	0.76	18	2.6745913	1062	3.0669708
9839	GO:0016791|GO:0016	phosphatase activity	0.76	6	0.89153045	383	1.1060733
3895	GO:0005543	phospholipid binding	0.76	2	0.2971768	140	0.40430877
9811	GO:0016757|GO:0016	transferase activity, tra	0.76	12	1.7830609	729	2.1052935
4247	GO:0006007	glucose catabolic proc	0.76	1	0.1485884	73	0.21081814
11313	GO:0019320	hexose catabolic proc	0.76	1	0.1485884	73	0.21081814
20525	GO:0044275	cellular carbohydrate c	0.76	1	0.1485884	73	0.21081814
21726	GO:0046164	alcohol catabolic proce	0.76	1	0.1485884	73	0.21081814
21894	GO:0046365	monosaccharide catab	0.76	1	0.1485884	73	0.21081814
18912	GO:0042578	phosphoric ester hydro	0.76	7	1.0401188	444	1.2822365
6046	GO:0008610	lipid biosynthetic proce	0.76	2	0.2971768	141	0.4071967
8557	GO:0015144	carbohydrate transme	0.77	2	0.2971768	143	0.41297254
16602	GO:0034219	carbohydrate transme	0.77	2	0.2971768	143	0.41297254
8810	GO:0015491	cation:cation antiporte	0.77	1	0.1485884	75	0.21659398

9488	GO:0016301	kinase activity	0.78	30	4.457652	1740	5.0249805
3873	GO:0005507	copper ion binding	0.78	2	0.2971768	146	0.42163628
9493	GO:0016310	phosphorylation	0.78	44	6.53789	2504	7.2313514
9877	GO:0016838	carbon-oxygen lyase a	0.78	1	0.1485884	77	0.22236982
6071	GO:0008643|GO:0006	carbohydrate transpor	0.78	2	0.2971768	147	0.42452422
2819	GO:0004004	ATP-dependent RNA	0.78	1	0.1485884	78	0.22525774
5725	GO:0008186|GO:0004	RNA-dependent ATPa	0.78	1	0.1485884	78	0.22525774
8495	GO:0015077	monovalent inorganic	0.79	3	0.44576523	214	0.6180149
4649	GO:0006520|GO:0006	cellular amino acid me	0.79	4	0.5943536	277	0.7999538
2654	GO:0003724	RNA helicase activity	0.79	1	0.1485884	80	0.2310336
9655	GO:0016568	chromatin modification	0.79	1	0.1485884	80	0.2310336
9656	GO:0016569	covalent chromatin mo	0.79	1	0.1485884	80	0.2310336
9657	GO:0016570	histone modification	0.79	1	0.1485884	80	0.2310336
9682	GO:0016597	amino acid binding	0.79	1	0.1485884	80	0.2310336
19479	GO:0043176	amine binding	0.79	1	0.1485884	80	0.2310336
9845	GO:0016798	hydrolase activity, acti	0.79	6	0.89153045	401	1.1580559
9795	GO:0016740	transferase activity	0.80	67	9.955423	3764	10.870131
18674	GO:0042325	regulation of phosphor	0.80	1	0.1485884	81	0.23392151
6991	GO:0009725	response to hormone	0.80	1	0.1485884	82	0.23680943
4558	GO:0006396|GO:0006	RNA processing	0.80	3	0.44576523	219	0.63245445
3048	GO:0004364	glutathione transferase	0.81	1	0.1485884	84	0.24258527
5624	GO:0008047	enzyme activator activ	0.81	1	0.1485884	85	0.24547319
11222	GO:0019220	regulation of phosphat	0.81	1	0.1485884	85	0.24547319
25471	GO:0051174	regulation of phosphor	0.81	1	0.1485884	85	0.24547319
4460	GO:0006260|GO:0055	DNA replication	0.81	3	0.44576523	224	0.64689404
3888	GO:0005529	sugar binding	0.81	2	0.2971768	158	0.45629135
6985	GO:0009719	response to endogeno	0.82	1	0.1485884	86	0.24836111
4246	GO:0006006	glucose metabolic pro	0.82	1	0.1485884	87	0.25124902
4990	GO:0006915|GO:0008	apoptosis	0.82	3	0.44576523	227	0.6555578
8496	GO:0015078	hydrogen ion transmem	0.82	2	0.2971768	160	0.4620672
4032	GO:0005743	mitochondrial inner me	0.82	1	0.1485884	88	0.25413695
119	GO:0000151	ubiquitin ligase comple	0.82	1	0.1485884	88	0.25413695
6796	GO:0009507	chloroplast	0.82	3	0.44576523	229	0.6613336
10304	GO:0018193	peptidyl-amino acid m	0.83	1	0.1485884	89	0.25702485
4911	GO:0006812|GO:0006	cation transport	0.83	10	1.4858841	662	1.9118029
9294	GO:0016052|GO:0006	carbohydrate catabolic	0.83	1	0.1485884	91	0.2628007
30204	GO:0071840	cellular component org	0.83	5	0.74294204	362	1.045427
4476	GO:0006278	RNA-dependent DNA	0.84	2	0.2971768	166	0.4793947
9494	GO:0016311	dephosphorylation	0.84	6	0.89153045	425	1.227366
11803	GO:0019866	organelle inner membr	0.84	1	0.1485884	94	0.27146447
20356	GO:0044106	cellular amine metabo	0.84	4	0.5943536	303	0.8750397
6077	GO:0008652	cellular amino acid bio	0.84	2	0.2971768	169	0.48805845
18633	GO:0042277	peptide binding	0.85	1	0.1485884	95	0.27435237
5756	GO:0008219	cell death	0.85	3	0.44576523	239	0.69021285
8209	GO:0012501|GO:0016	programmed cell deat	0.85	3	0.44576523	239	0.69021285
9462	GO:0016265	death	0.85	3	0.44576523	239	0.69021285
3341	GO:0004722|GO:0000	protein serine/threonin	0.85	2	0.2971768	171	0.4938343
9889	GO:0016853	isomerase activity	0.85	4	0.5943536	306	0.8837035
4897	GO:0006793	phosphorus metabolic	0.85	50	7.4294205	2925	8.4471655
4900	GO:0006796	phosphate metabolic p	0.85	50	7.4294205	2925	8.4471655
5791	GO:0008270	zinc ion binding	0.85	31	4.6062407	1880	5.4292893
2653	GO:0003723	RNA binding	0.85	12	1.7830609	797	2.3016722
12608	GO:0030001	metal ion transport	0.85	5	0.74294204	374	1.080082
13829	GO:0031406	carboxylic acid binding	0.85	1	0.1485884	98	0.28301615
4067	GO:0005783	endoplasmic reticulum	0.85	2	0.2971768	174	0.50249803
5712	GO:0008168|GO:0004	methyltransferase acti	0.86	3	0.44576523	244	0.7046524
3885	GO:0005525	GTP binding	0.86	3	0.44576523	245	0.70754033
11032	GO:0019001	guanyl nucleotide bind	0.86	3	0.44576523	245	0.70754033
14967	GO:0032561	guanyl ribonucleotide	0.86	3	0.44576523	245	0.70754033
7271	GO:0010033	response to organic su	0.86	1	0.1485884	100	0.28879198
11787	GO:0019842	vitamin binding	0.86	2	0.2971768	177	0.5111618
6824	GO:0009536	plastid	0.86	3	0.44576523	247	0.7133162
11311	GO:0019318	hexose metabolic proc	0.86	1	0.1485884	101	0.2916799
2627	GO:0003676	nucleic acid binding	0.86	41	6.092125	2453	7.0840673
13082	GO:0030599	pectinesterase activity	0.86	2	0.2971768	178	0.5140497
6166	GO:0008757	S-adenosylmethionine	0.87	2	0.2971768	180	0.5198256
6663	GO:0009309	amine biosynthetic pro	0.87	2	0.2971768	181	0.5227135
19392	GO:0043085	positive regulation of c	0.87	1	0.1485884	104	0.30034366
20343	GO:0044093	positive regulation of m	0.87	1	0.1485884	104	0.30034366
2668	GO:0003779	actin binding	0.87	1	0.1485884	105	0.3032316
20505	GO:0044255	cellular lipid metabolic	0.88	2	0.2971768	184	0.53137726
20487	GO:0044237	cellular metabolic proc	0.88	147	21.842497	8185	23.637625
3942	GO:0005618	cell wall	0.88	1	0.1485884	107	0.30900744
19473	GO:0043170|GO:0043	macromolecule metab	0.88	116	17.236256	6553	18.92454
11833	GO:0019904	protein domain specifi	0.88	1	0.1485884	109	0.31478328
9285	GO:0016043	cellular component org	0.88	3	0.44576523	261	0.7537471
15436	GO:0033036	macromolecule localiz	0.89	5	0.74294204	397	1.1465042
12868	GO:0030312	external encapsulating	0.89	1	0.1485884	111	0.3205591
4512	GO:0006325	chromatin organization	0.89	1	0.1485884	112	0.32344702
25568	GO:0051276|GO:0007	chromosome organiza	0.89	1	0.1485884	112	0.32344702
29918	GO:0071554	cell wall organization c	0.89	1	0.1485884	112	0.32344702
19686	GO:0043414	macromolecule methy	0.89	1	0.1485884	113	0.32633495
14379	GO:0031966	mitochondrial membra	0.89	1	0.1485884	114	0.32922286
22478	GO:0046982	protein heterodimeriza	0.89	1	0.1485884	114	0.32922286
9535	GO:0016407	acetyltransferase activ	0.90	1	0.1485884	115	0.3321108
9871	GO:0016831	carboxy-lyase activity	0.90	1	0.1485884	115	0.3321108
20510	GO:0044260|GO:0034	cellular macromolecule	0.90	97	14.413075	5577	16.105928
3340	GO:0004721	phosphoprotein phosp	0.90	2	0.2971768	199	0.57469606
4236	GO:0005996	monosaccharide meta	0.91	1	0.1485884	125	0.36099
12741	GO:0030170	pyridoxal phosphate b	0.92	1	0.1485884	126	0.3638779
28643	GO:0070279	vitamin B6 binding	0.92	1	0.1485884	126	0.3638779
14667	GO:0032259	methylation	0.92	3	0.44576523	285	0.8230572
9295	GO:0016053	organic acid biosynthe	0.92	2	0.2971768	211	0.6093511
21922	GO:0046394	carboxylic acid biosyn	0.92	2	0.2971768	211	0.6093511
22376	GO:0046873	metal ion transmembr	0.92	2	0.2971768	213	0.6151269

9852	GO:0016810	hydrolase activity, acti	0.93	1	0.1485884	134	0.38698125
4320	GO:0006082	organic acid metabolic	0.93	4	0.5943536	369	1.0656425
11716	GO:0019752	carboxylic acid metab	0.93	4	0.5943536	369	1.0656425
19707	GO:0043436	oxoacid metabolic pro	0.93	4	0.5943536	369	1.0656425
5019	GO:0006952|GO:0002	defense response	0.93	2	0.2971768	221	0.63823026
18541	GO:0042180	cellular ketone metabo	0.93	4	0.5943536	375	1.0829699
13531	GO:0031072	heat shock protein bin	0.94	2	0.2971768	230	0.6642216
4304	GO:0006066	alcohol metabolic proc	0.94	1	0.1485884	143	0.41297254
11522	GO:0019538|GO:0006	protein metabolic proc	0.94	68	10.104012	4134	11.938661
4029	GO:0005740	mitochondrial envelop	0.95	1	0.1485884	151	0.4360759
19526	GO:0043227	membrane-bounded o	0.95	32	4.754829	2142	6.185924
19530	GO:0043231	intracellular membrane	0.95	32	4.754829	2142	6.185924
3449	GO:0004842|GO:0004	ubiquitin-protein ligase	0.95	3	0.44576523	326	0.94146186
4609	GO:0006464	protein modification pr	0.95	43	6.389302	2781	8.031305
5661	GO:0008092	cytoskeletal protein bin	0.96	2	0.2971768	249	0.719092
19684	GO:0043412	macromolecule modifi	0.96	45	6.6864786	2900	8.374968
19831	GO:0043565	sequence-specific DN	0.96	1	0.1485884	159	0.45917925
9654	GO:0016567	protein ubiquitination	0.96	3	0.44576523	332	0.9587894
12572	GO:0022890|GO:0015	inorganic cation transm	0.96	3	0.44576523	332	0.9587894
14854	GO:0032446	protein modification by	0.96	3	0.44576523	332	0.9587894
29011	GO:0070647	protein modification by	0.96	3	0.44576523	332	0.9587894
9870	GO:0016830	carbon-carbon lyase a	0.96	1	0.1485884	161	0.4649551
5764	GO:0008234|GO:0004	cysteine-type peptidas	0.96	1	0.1485884	163	0.47073093
20623	GO:0044429	mitochondrial part	0.96	1	0.1485884	165	0.47650677
11747	GO:0019787|GO:0008	small conjugating prot	0.96	3	0.44576523	339	0.9790048
30306	GO:0071944	cell periphery	0.96	2	0.2971768	259	0.74797124
3946	GO:0005623	cell	0.97	140	20.802378	8227	23.758917
20657	GO:0044464	cell part	0.97	140	20.802378	8227	23.758917
3874	GO:0005509	calcium ion binding	0.97	2	0.2971768	279	0.8057296
8466	GO:0015031|GO:0015	protein transport	0.97	1	0.1485884	185	0.53426516
20820	GO:0045184	establishment of prote	0.97	1	0.1485884	185	0.53426516
20517	GO:0044267	cellular protein metabo	0.97	49	7.2808323	3225	9.313541
3863	GO:0005488	binding	0.98	246	36.55275	13903	40.15075
5669	GO:0008104	protein localization	0.98	1	0.1485884	189	0.54581684
9906	GO:0016874	ligase activity	0.98	6	0.89153045	597	1.7240882
14380	GO:0031967	organelle envelope	0.98	1	0.1485884	201	0.5804719
14387	GO:0031975	envelope	0.98	1	0.1485884	205	0.59202355
9913	GO:0016881	acid-amino acid ligase	0.98	3	0.44576523	391	1.1291766
4028	GO:0005739	mitochondrion	0.99	1	0.1485884	219	0.63245445
13547	GO:0031090	organelle membrane	0.99	1	0.1485884	224	0.64689404
3903	GO:0005575|GO:0008	cellular_component	0.99	140	20.802378	8515	24.590637
9911	GO:0016879	ligase activity, forming	0.99	3	0.44576523	456	1.3168914
3716	GO:0005198	structural molecule ac	1.00	2	0.2971768	393	1.1349525
3876	GO:0005515|GO:0045	protein binding	1.00	108	16.047548	6933	20.021948
19533	GO:0043234	protein complex	1.00	5	0.74294204	848	2.448956
19525	GO:0043226	organelle	1.00	34	5.052006	3013	8.701303
19528	GO:0043229	intracellular organelle	1.00	34	5.052006	3013	8.701303
20618	GO:0044424	intracellular part	1.00	47	6.9836555	3853	11.127155
4027	GO:0005737	cytoplasm	1.00	18	2.6745913	2001	5.7787275
3945	GO:0005622	intracellular	1.00	59	8.766716	4751	13.720507
4109	GO:0005840|GO:0033	ribosome	1.00	2	0.2971768	682	1.9695613
13018	GO:0030529	ribonucleoprotein com	1.00	2	0.2971768	741	2.1399486
20640	GO:0044446	intracellular organelle	1.00	1	0.1485884	710	2.0504231
20616	GO:0044422	organelle part	1.00	1	0.1485884	712	2.0561988
19527	GO:0043228	non-membrane-bound	1.00	2	0.2971768	923	2.66555
19531	GO:0043232	intracellular non-meml	1.00	2	0.2971768	923	2.66555
20638	GO:0044444	cytoplasmic part	1.00	9	1.3372957	1644	4.7477403
15392	GO:0032991	macromolecular comp	1.00	7	1.0401188	1616	4.666878

GO ID	GO ACCESSION	GO Term	p-value	Count in Selection	% Count in Selection	Count in Total	% Count in Total
5704	GO:0008150|GO:0000004|G	biological_process	0.00	449	72.41936	22215	64.155136
28101	GO:0061135	endopeptidase regulator acti	0.00	10	1.6129032	95	0.27435237
8159	GO:0010951	negative regulation of endope	0.00	10	1.6129032	95	0.27435237
3472	GO:0004866	endopeptidase inhibitor activi	0.00	10	1.6129032	95	0.27435237
26712	GO:0052548	regulation of endopeptidase a	0.00	10	1.6129032	95	0.27435237
3473	GO:0004867	serine-type endopeptidase in	0.00	8	1.2903225	66	0.1906027
25636	GO:0051346	negative regulation of hydrola	0.00	10	1.6129032	110	0.31767118
12946	GO:0030414	peptidase inhibitor activity	0.00	10	1.6129032	110	0.31767118
7690	GO:0010466	negative regulation of peptida	0.00	10	1.6129032	110	0.31767118
26711	GO:0052547	regulation of peptidase activi	0.00	10	1.6129032	112	0.32344702
28100	GO:0061134	peptidase regulator activity	0.00	10	1.6129032	112	0.32344702
3874	GO:0005509	calcium ion binding	0.00	16	2.580645	279	0.8057296
25626	GO:0051336	regulation of hydrolase activi	0.00	12	1.9354838	196	0.5660323
3167	GO:0004508	steroid 17-alpha-monooxyge	0.00	2	0.32258064	2	0.00577584
3464	GO:0004857	enzyme inhibitor activity	0.00	10	1.6129032	157	0.4534034
3048	GO:0004364	glutathione transferase activi	0.00	7	1.1290323	84	0.24258527
4476	GO:0006278	RNA-dependent DNA replica	0.00	10	1.6129032	166	0.4793947
3877	GO:0005516	calmodulin binding	0.00	12	1.9354838	236	0.6815491
8919	GO:0015645	fatty acid ligase activity	0.00	4	0.6451613	27	0.077973835
9816	GO:0016762	xyloglucan:xyloglucosyl trans	0.00	4	0.6451613	27	0.077973835
9869	GO:0016829	lyase activity	0.00	19	3.064516	488	1.4093049
6932	GO:0009664	plant-type cell wall organizati	0.00	5	0.8064516	50	0.14439599
5096	GO:0007047	cellular cell wall organization	0.00	5	0.8064516	50	0.14439599
20857	GO:0045229	external encapsulating structu	0.00	5	0.8064516	50	0.14439599
30033	GO:0071669	plant-type cell wall organizati	0.00	5	0.8064516	50	0.14439599
12805	GO:0030246	carbohydrate binding	0.00	12	1.9354838	251	0.7248679
19393	GO:0043086	negative regulation of catalyt	0.00	10	1.6129032	188	0.54292893
20342	GO:0044092	negative regulation of molecu	0.00	10	1.6129032	188	0.54292893
14101	GO:0031683	G-protein beta/gamma-subun	0.00	3	0.48387095	15	0.043318797
4460	GO:0006260|GO:0055133	DNA replication	0.00	11	1.7741935	224	0.64689404
29246	GO:0070882	cellular cell wall organization	0.00	5	0.8064516	53	0.15305975
9422	GO:0016209	antioxidant activity	0.00	9	1.451613	165	0.47650677
3242	GO:0004601|GO:0016685|G	peroxidase activity	0.00	8	1.2903225	135	0.38986918
9754	GO:0016684	oxidoreductase activity, actin	0.00	8	1.2903225	135	0.38986918
11224	GO:0019222	regulation of metabolic proce	0.00	76	12.258064	3115	8.995871
4459	GO:0006259|GO:0055132	DNA metabolic process	0.00	18	2.903226	493	1.4237444
29919	GO:0071555	cell wall organization	0.00	5	0.8064516	58	0.16749935
9393	GO:0016165	lipoxygenase activity	0.00	4	0.6451613	36	0.10396511
29918	GO:0071554	cell wall organization or bioge	0.00	7	1.1290323	112	0.32344702
9096	GO:0015828	tyrosine transport	0.00	2	0.32258064	6	0.01732752
8586	GO:0015173	aromatic amino acid transme	0.00	2	0.32258064	6	0.01732752
9070	GO:0015801	aromatic amino acid transpo	0.00	2	0.32258064	6	0.01732752
3777	GO:0005302|GO:0015508	L-tyrosine transmembrane tr	0.00	2	0.32258064	6	0.01732752
3904	GO:0005576	extracellular region	0.00	9	1.451613	178	0.5140497
21331	GO:0045735	nutrient reservoir activity	0.01	6	0.9677419	89	0.25702485
5705	GO:0008152	metabolic process	0.01	344	55.48387	17432	50.34222
3129	GO:0004467	long-chain fatty acid-CoA liga	0.01	3	0.48387095	21	0.060646318
9818	GO:0016765|GO:0016766	transferase activity, transferr	0.01	9	1.451613	185	0.53426516
6884	GO:0009607	response to biotic stimulus	0.01	7	1.1290323	122	0.3523262
8489	GO:0015066	alpha-amylase inhibitor activi	0.01	3	0.48387095	22	0.06353424
5636	GO:0008061	chitin binding	0.01	3	0.48387095	23	0.06642216
3888	GO:0005529	sugar binding	0.01	8	1.2903225	158	0.45629135
4530	GO:0006355|GO:0032583|G	regulation of transcription, DN	0.01	63	10.16129	2591	7.4826
25544	GO:0051252	regulation of RNA metabolic	0.01	63	10.16129	2593	7.488376
6248	GO:0008843	endochitinase activity	0.01	3	0.48387095	24	0.06931008
3213	GO:0004568	chitinase activity	0.01	3	0.48387095	24	0.06931008
7141	GO:0009889	regulation of biosynthetic pro	0.01	63	10.16129	2605	7.523031
13756	GO:0031326	regulation of cellular biosynth	0.01	63	10.16129	2605	7.523031
32213	GO:2000112	regulation of cellular macrom	0.01	63	10.16129	2605	7.523031
7772	GO:0010556	regulation of macromolecule	0.01	63	10.16129	2605	7.523031
9764	GO:0016701	oxidoreductase activity, actin	0.01	5	0.8064516	72	0.20793022
8493	GO:0015074	DNA integration	0.01	4	0.6451613	47	0.13573223
2864	GO:0004058|GO:0016400	aromatic-L-amino-acid decar	0.01	2	0.32258064	9	0.025991278
25094	GO:0050789|GO:0050791	regulation of biological proce	0.01	97	15.645162	4315	12.461374
13753	GO:0031323	regulation of cellular metabol	0.01	66	10.645162	2770	7.999538
11221	GO:0019219	regulation of nucleobase, nuc	0.01	64	10.32258	2675	7.7251854
25468	GO:0051171	regulation of nitrogen compo	0.01	64	10.32258	2675	7.7251854
28364	GO:0065007	biological regulation	0.01	98	15.806452	4376	12.637537
7692	GO:0010468	regulation of gene expressior	0.01	63	10.16129	2635	7.6096687
21148	GO:0045547	dehydrodolichyl diphosphate	0.01	2	0.32258064	10	0.0288792
23116	GO:0047631	ADP-ribose diphosphatase a	0.01	2	0.32258064	10	0.0288792
9909	GO:0016877	ligase activity, forming carbo	0.01	5	0.8064516	78	0.22525774
9845	GO:0016798	hydrolase activity, acting on g	0.01	14	2.2580645	401	1.1580559
27228	GO:0060255	regulation of macromolecule	0.01	63	10.16129	2660	7.6818666
31465	GO:0080090	regulation of primary metabo	0.01	64	10.32258	2710	7.826263
13082	GO:0030599	pectinesterase activity	0.02	8	1.2903225	178	0.5140497
5702	GO:0008146	sulfotransferase activity	0.02	4	0.6451613	54	0.15594767
9871	GO:0016831	carboxy-lyase activity	0.02	6	0.9677419	115	0.3321108
26845	GO:0052689	carboxylic ester hydrolase ac	0.02	17	2.7419355	538	1.5537009
6424	GO:0009055|GO:0009053|G	electron carrier activity	0.02	7	1.1290323	154	0.44473964
25098	GO:0050794|GO:0051244	regulation of cellular process	0.02	88	14.193548	3987	11.514136
12806	GO:0030247	polysaccharide binding	0.02	3	0.48387095	35	0.10107719
1169	GO:0001871	pattern binding	0.02	3	0.48387095	35	0.10107719
11157	GO:0019144	ADP-sugar diphosphatase ac	0.03	2	0.32258064	14	0.040430877
9870	GO:0016830	carbon-carbon lyase activity	0.03	7	1.1290323	161	0.4649551
9765	GO:0016702	oxidoreductase activity, actin	0.03	4	0.6451613	66	0.1906027
3382	GO:0004768|GO:0016214|G	stearoyl-CoA 9-desaturase a	0.03	2	0.32258064	16	0.046206716
9427	GO:0016215	CoA desaturase activity	0.03	2	0.32258064	16	0.046206716
26959	GO:0055114	oxidation-reduction process	0.03	57	9.193548	2490	7.1909204
5952	GO:0008470	isovaleryl-CoA dehydrogenas	0.04	1	0.16129032	2	0.00577584
6593	GO:0009229	thiamine diphosphate biosyn	0.04	1	0.16129032	2	0.00577584
9558	GO:0016433	rRNA (adenine) methyltransf	0.04	1	0.16129032	2	0.00577584
9595	GO:0016485	protein processing	0.04	1	0.16129032	2	0.00577584
9638	GO:0016539	intein-mediated protein splici	0.04	1	0.16129032	2	0.00577584
9686	GO:0016602	CCAAT-binding factor compl	0.04	1	0.16129032	2	0.00577584
12948	GO:0030417	nicotianamine metabolic proc	0.04	1	0.16129032	2	0.00577584
12949	GO:0030418	nicotianamine biosynthetic pr	0.04	1	0.16129032	2	0.00577584
13372	GO:0030908	protein splicing	0.04	1	0.16129032	2	0.00577584
18706	GO:0042357	thiamine diphosphate metabo	0.04	1	0.16129032	2	0.00577584
22759	GO:0047268	galactinol-raffinose galactosy	0.04	1	0.16129032	2	0.00577584
25888	GO:0051604	protein maturation	0.04	1	0.16129032	2	0.00577584
30709	GO:0072350	tricarboxylic acid metabolic p	0.04	1	0.16129032	2	0.00577584
30710	GO:0072351	tricarboxylic acid biosynthetic	0.04	1	0.16129032	2	0.00577584
143	GO:0000179|GO:0043790	rRNA (adenine-N6,N6-)-dime	0.04	1	0.16129032	2	0.00577584
3148	GO:0004489|GO:0008702	methylenetetrahydrofolate re	0.04	1	0.16129032	2	0.00577584
18738	GO:0042389	omega-3 fatty acid desaturas	0.04	2	0.32258064	17	0.049094636
23522	GO:0048046	apoplast	0.04	4	0.6451613	70	0.20215438
10899	GO:0018858	benzoate-CoA ligase activity	0.04	2	0.32258064	18	0.051982556
2674	GO:0003824	catalytic activity	0.04	213	34.35484	10750	31.045137
9832	GO:0016782	transferase activity, transferr	0.04	4	0.6451613	74	0.21370606
18750	GO:0042401	cellular biogenic amine biosy	0.04	2	0.32258064	19	0.054870475
25510	GO:0051213	dioxygenase activity	0.05	4	0.6451613	75	0.21659398
3124	GO:0004462	lactoylglutathione lyase activi	0.05	2	0.32258064	20	0.0577584
9877	GO:0016838	carbon-oxygen lyase activity,	0.05	4	0.6451613	77	0.22236982

9486	GO:0016298	lipase activity	**0.05**	7	1.1290323	185	0.53426516
3853	GO:0005463	UDP-N-acetylgalactosamine	**0.05**	1	0.16129032	3	0.00866376
9058	GO:0015789	UDP-N-acetylgalactosamine	**0.05**	1	0.16129032	3	0.00866376
13651	GO:0031219	levanase activity	**0.05**	1	0.16129032	3	0.00866376
21936	GO:0046409	p-coumarate 3-hydroxylase a	**0.05**	1	0.16129032	3	0.00866376
9600	GO:0016491	oxidoreductase activity	0.06	40	6.451613	1724	4.9787736
24858	GO:0050551	myrcene synthase activity	0.06	2	0.32258064	22	0.06353424
9783	GO:0016722	oxidoreductase activity, oxidi	0.06	3	0.48387095	50	0.14439599
12794	GO:0030234	enzyme regulator activity	0.06	12	1.9354838	403	1.1638317
9779	GO:0016717	oxidoreductase activity, actin	0.07	2	0.32258064	24	0.06931008
13657	GO:0031225	anchored to membrane	0.07	2	0.32258064	24	0.06931008
25578	GO:0051287|GO:0051288	NAD binding	0.07	2	0.32258064	24	0.06931008
3399	GO:0004788	thiamine diphosphokinase ac	0.07	1	0.16129032	4	0.011551679
3834	GO:0005395	eye pigment precursor transp	0.07	1	0.16129032	4	0.011551679
5826	GO:0008307	structural constituent of musc	0.07	1	0.16129032	4	0.011551679
5896	GO:0008401	retinoic acid 4-hydroxylase ac	0.07	1	0.16129032	4	0.011551679
5934	GO:0008446	GDP-mannose 4,6-dehydrata	0.07	1	0.16129032	4	0.011551679
6293	GO:0008898	homocysteine S-methyltransf	0.07	1	0.16129032	4	0.011551679
7511	GO:0010285|GO:0043742	L,L-diaminopimelate aminotr	0.07	1	0.16129032	4	0.011551679
8130	GO:0010921	regulation of phosphatase ac	0.07	1	0.16129032	4	0.011551679
8598	GO:0015188	L-isoleucine transmembrane	0.07	1	0.16129032	4	0.011551679
8877	GO:0015578|GO:0015589	mannose transmembrane tra	0.07	1	0.16129032	4	0.011551679
9030	GO:0015761	mannose transport	0.07	1	0.16129032	4	0.011551679
9072	GO:0015803	branched-chain aliphatic ami	0.07	1	0.16129032	4	0.011551679
9086	GO:0015818	isoleucine transport	0.07	1	0.16129032	4	0.011551679
16391	GO:0034007	S-linalool synthase activity	0.07	1	0.16129032	4	0.011551679
17669	GO:0035303	regulation of dephosphorylati	0.07	1	0.16129032	4	0.011551679
19932	GO:0043666	regulation of phosphoprotein	0.07	1	0.16129032	4	0.011551679
24433	GO:0050113	inositol oxygenase activity	0.07	1	0.16129032	4	0.011551679
2689	GO:0003839	gamma-glutamylcyclotransfe	0.07	1	0.16129032	4	0.011551679
2753	GO:0003919	FMN adenylyltransferase act	0.07	1	0.16129032	4	0.011551679
3291	GO:0004657	proline dehydrogenase activi	0.07	1	0.16129032	4	0.011551679
9482	GO:0016291|GO:0008778|G	acyl-CoA thioesterase activity	0.07	2	0.32258064	25	0.072197996
17147	GO:0034768	(E)-beta-ocimene synthase a	0.08	2	0.32258064	26	0.075085916
7557	GO:0010333	terpene synthase activity	0.08	3	0.48387095	57	0.16461143
18956	GO:0042626	ATPase activity, coupled to t	0.09	7	1.1290323	211	0.6093511
22765	GO:0047274	galactinol-sucrose galactosyl	0.09	1	0.16129032	5	0.0144396
3035	GO:0004351	glutamate decarboxylase act	0.09	1	0.16129032	5	0.0144396
9861	GO:0016820	hydrolase activity, acting on a	0.09	7	1.1290323	212	0.612239
30700	GO:0072341	modified amino acid binding	0.09	3	0.48387095	59	0.17038727
14811	GO:0032403	protein complex binding	0.09	3	0.48387095	60	0.17327519
25095	GO:0050790	regulation of catalytic activity	0.09	13	2.096774	480	1.3862015
28366	GO:0065009	regulation of molecular functi	0.10	13	2.096774	484	1.3977532
6216	GO:0008810	cellulase activity	0.10	2	0.32258064	30	0.086637594
9785	GO:0016724	oxidoreductase activity, oxidi	0.10	2	0.32258064	30	0.086637594
2870	GO:0004064	arylesterase activity	0.10	2	0.32258064	30	0.086637594
3009	GO:0004322	ferroxidase activity	0.10	2	0.32258064	30	0.086637594
4953	GO:0006868|GO:0015815	glutamine transport	0.10	1	0.16129032	6	0.01732752
6000	GO:0008531	riboflavin kinase activity	0.10	1	0.16129032	6	0.01732752
6317	GO:0008923	lysine decarboxylase activity	0.10	1	0.16129032	6	0.01732752
8505	GO:0015089	high affinity copper ion transr	0.10	1	0.16129032	6	0.01732752
8596	GO:0015186	L-glutamine transmembrane	0.10	1	0.16129032	6	0.01732752
11899	GO:0019992	diacylglycerol binding	0.10	1	0.16129032	6	0.01732752
19761	GO:0043495	protein anchor	0.10	1	0.16129032	6	0.01732752
31395	GO:0080018	anthocyanin 5-O-glucosyltrar	0.10	1	0.16129032	6	0.01732752
5	GO:0000007	low-affinity zinc ion transmen	0.10	1	0.16129032	6	0.01732752
2713	GO:0003863	3-methyl-2-oxobutanoate del	0.10	1	0.16129032	6	0.01732752
2811	GO:0003995|GO:0019109	acyl-CoA dehydrogenase act	0.10	1	0.16129032	6	0.01732752
19590	GO:0043295	glutathione binding	0.11	2	0.32258064	31	0.08952551
3717	GO:0005199	structural constituent of cell w	0.11	2	0.32258064	32	0.09241343
6942	GO:0009674	potassium:sodium symporter	0.11	2	0.32258064	32	0.09241343
12522	GO:0022820	potassium ion symporter acti	0.11	2	0.32258064	32	0.09241343
4705	GO:0006576	cellular biogenic amine meta	0.12	2	0.32258064	33	0.09530135
9812	GO:0016758	transferase activity, transferr	0.12	13	2.096774	501	1.4468478
3443	GO:0004834	tryptophan synthase activity	0.12	1	0.16129032	7	0.020215439
21776	GO:0046219	indolalkylamine biosynthetic	0.12	1	0.16129032	7	0.020215439
128	GO:0000162|GO:0009096	tryptophan biosynthetic proce	0.12	1	0.16129032	7	0.020215439
2945	GO:0004143	diacylglycerol kinase activity	0.12	1	0.16129032	7	0.020215439
3255	GO:0004615|GO:0008971	phosphomannomutase activi	0.12	1	0.16129032	7	0.020215439
11931	GO:0020037	heme binding	0.12	14	2.2580645	550	1.5883559
3873	GO:0005507	copper ion binding	0.12	5	0.8064516	146	0.42163628
22375	GO:0046872	metal ion binding	0.12	62	10	2985	8.6204405
4320	GO:0006082	organic acid metabolic proce	0.13	10	1.6129032	369	1.0656425
11716	GO:0019752	carboxylic acid metabolic pro	0.13	10	1.6129032	369	1.0656425
19707	GO:0043436	oxoacid metabolic process	0.13	10	1.6129032	369	1.0656425
9480	GO:0016289	CoA hydrolase activity	0.13	2	0.32258064	35	0.10107719
24968	GO:0050662	coenzyme binding	0.13	6	0.9677419	191	0.5515927
19470	GO:0043167	ion binding	0.13	62	10	2997	8.655096
19472	GO:0043169	cation binding	0.13	62	10	2997	8.655096
19758	GO:0043492	ATPase activity, coupled to n	0.13	7	1.1290323	235	0.67866117
3402	GO:0004791	thioredoxin-disulfide reductas	0.13	1	0.16129032	8	0.023103358
6378	GO:0008999	ribosomal-protein-alanine N-	0.13	1	0.16129032	8	0.023103358
4	GO:0000006	high affinity zinc uptake trans	0.13	1	0.16129032	8	0.023103358
3872	GO:0005506	iron ion binding	0.14	14	2.2580645	563	1.6258988
18541	GO:0042180	cellular ketone metabolic pro	0.14	10	1.6129032	375	1.0829699
9767	GO:0016705	oxidoreductase activity, actin	0.14	9	1.451613	329	0.95012563
3306	GO:0004674|GO:0004695|G	protein serine/threonine kinas	0.15	24	3.8709676	1062	3.0669708
3694	GO:0005160	transforming growth factor be	0.15	1	0.16129032	9	0.025991278
4697	GO:0006568	tryptophan metabolic process	0.15	1	0.16129032	9	0.025991278
4715	GO:0006586	indolalkylamine metabolic pro	0.15	1	0.16129032	9	0.025991278
7506	GO:0010279	indole-3-acetic acid amido sy	0.15	1	0.16129032	9	0.025991278
13224	GO:0030755	quercetin 3-O-methyltransfer	0.15	1	0.16129032	9	0.025991278
19254	GO:0042937	tripeptide transporter activity	0.15	1	0.16129032	9	0.025991278
19342	GO:0043027	caspase inhibitor activity	0.15	1	0.16129032	9	0.025991278
19343	GO:0043028	caspase regulator activity	0.15	1	0.16129032	9	0.025991278
19458	GO:0043154|GO:0001719	negative regulation of caspas	0.15	1	0.16129032	9	0.025991278
19579	GO:0043281|GO:0043026	regulation of caspase activity	0.15	1	0.16129032	9	0.025991278
9874	GO:0016835	carbon-oxygen lyase activity	0.16	6	0.9677419	202	0.5833598
5891	GO:0008395|GO:0008394	steroid hydroxylase activity	0.16	2	0.32258064	40	0.1155168
24966	GO:0050660	flavin adenine dinucleotide bi	0.16	4	0.6451613	118	0.34077454
5716	GO:0008172	S-methyltransferase activity	0.17	1	0.16129032	10	0.0288792
7175	GO:0009924	octadecanal decarbonylase a	0.17	1	0.16129032	10	0.0288792
9741	GO:0016668|GO:0016654	oxidoreductase activity, actin	0.17	1	0.16129032	10	0.0288792
9828	GO:0016778	diphosphotransferase activity	0.17	1	0.16129032	10	0.0288792
15183	GO:0032781	positive regulation of ATPase	0.17	1	0.16129032	10	0.0288792
19728	GO:0043462	regulation of ATPase activity	0.17	1	0.16129032	10	0.0288792
27563	GO:0060590	ATPase regulator activity	0.17	1	0.16129032	10	0.0288792
30135	GO:0071771	aldehyde decarbonylase acti	0.17	1	0.16129032	10	0.0288792
989	GO:0001671	ATPase activator activity	0.17	1	0.16129032	10	0.0288792
2797	GO:0003979	UDP-glucose 6-dehydrogena	0.17	1	0.16129032	10	0.0288792
4954	GO:0006869	lipid transport	0.17	6	0.9677419	208	0.6006873
8085	GO:0010876	lipid localization	0.17	6	0.9677419	208	0.6006873
9682	GO:0016597	amino acid binding	0.17	3	0.48387095	80	0.2310336
19479	GO:0043176	amine binding	0.17	3	0.48387095	80	0.2310336
13884	GO:0031461	cullin-RING ubiquitin ligase c	0.17	2	0.32258064	42	0.121292636
9795	GO:0016740	transferase activity	0.18	75	12.096774	3764	10.870131
3260	GO:0004620	phospholipase activity	0.18	4	0.6451613	123	0.35521415
8589	GO:0015179	L-amino acid transmembrane	0.18	2	0.32258064	43	0.124180555
9076	GO:0015807	L-amino acid transport	0.18	2	0.32258064	43	0.124180555
16183	GO:0033799	myricetin 3'-O-methyltransfer	0.18	1	0.16129032	11	0.03176712
18781	GO:0042435	indole-containing compound	0.18	1	0.16129032	11	0.03176712

19256	GO:0042939	tripeptide transport	0.18	1	0.16129032	11	0.03176712
22376	GO:0046873	metal ion transmembrane tra	0.18	6	0.9677419	213	0.6151269
22409	GO:0046906	tetrapyrrole binding	0.19	14	2.2580645	596	1.7212002
8749	GO:0015399	primary active transmembrar	0.19	7	1.1290323	260	0.75085914
8753	GO:0015405	P-P-bond-hydrolysis-driven tr	0.19	7	1.1290323	260	0.75085914
4912	GO:0006813|GO:0015458	potassium ion transport	0.19	3	0.48387095	84	0.24258527
30168	GO:0071804	cellular potassium ion transpo	0.19	3	0.48387095	84	0.24258527
30169	GO:0071805	potassium ion transmembrar	0.19	3	0.48387095	84	0.24258527
3386	GO:0004773	steryl-sulfatase activity	0.19	1	0.16129032	12	0.03465504
4506	GO:0006313|GO:0006317|G	transposition, DNA-mediated	0.19	1	0.16129032	12	0.03465504
5965	GO:0008484	sulfuric ester hydrolase activi	0.19	1	0.16129032	12	0.03465504
9775	GO:0016713	oxidoreductase activity, actin	0.19	1	0.16129032	12	0.03465504
10743	GO:0018685|GO:0008393	alkane 1-monooxygenase ac	0.19	1	0.16129032	12	0.03465504
14604	GO:0032196	transposition	0.19	1	0.16129032	12	0.03465504
19047	GO:0042723	thiamine-containing compour	0.19	1	0.16129032	12	0.03465504
19048	GO:0042724	thiamine-containing compour	0.19	1	0.16129032	12	0.03465504
31380	GO:0080002	UDP-glucose:4-aminobenzoa	0.19	1	0.16129032	12	0.03465504
3156	GO:0004497	monooxygenase activity	0.20	5	0.8064516	172	0.49672222
3200	GO:0004553|GO:0016800	hydrolase activity, hydrolyzing	0.20	8	1.2903225	312	0.90103096
30204	GO:0071840	cellular component organizat	0.20	9	1.451613	362	1.045427
8497	GO:0015079|GO:0022817	potassium ion transmembrar	0.21	2	0.32258064	47	0.13573223
119	GO:0000151	ubiquitin ligase complex	0.21	3	0.48387095	88	0.25413695
7551	GO:0010327	acetyl CoA:(Z)-3-hexen-1-ol	0.21	1	0.16129032	13	0.037542958
8149	GO:0010941	regulation of cell death	0.21	1	0.16129032	13	0.037542958
8578	GO:0015165	pyrimidine nucleotide sugar t	0.21	1	0.16129032	13	0.037542958
9050	GO:0015781	pyrimidine nucleotide-sugar t	0.21	1	0.16129032	13	0.037542958
18777	GO:0042430|GO:0042434	indole-containing compound	0.21	1	0.16129032	13	0.037542958
19298	GO:0042981	regulation of apoptosis	0.21	1	0.16129032	13	0.037542958
19381	GO:0043067|GO:0043070	regulation of programmed ce	0.21	1	0.16129032	13	0.037542958
28930	GO:0070566	adenylyltransferase activity	0.21	2	0.32258064	48	0.13862015
9725	GO:0016645	oxidoreductase activity, actin	0.22	2	0.32258064	49	0.14150807
6075	GO:0008649	rRNA methyltransferase activ	0.22	1	0.16129032	14	0.040430877
31408	GO:0080031	methyl salicylate esterase ac	0.22	1	0.16129032	14	0.040430877
12608	GO:0030001	metal ion transport	0.23	9	1.451613	374	1.080082
9975	GO:0016998	cell wall macromolecule cata	0.23	2	0.32258064	51	0.14728391
3293	GO:0004659	prenyltransferase activity	0.23	2	0.32258064	51	0.14728391
23515	GO:0048037	cofactor binding	0.23	8	1.2903225	327	0.94434977
7520	GO:0010294	abscisic acid glucosyltransfer	0.24	1	0.16129032	15	0.043318797
11733	GO:0019773	proteasome core complex, a	0.24	1	0.16129032	15	0.043318797
2868	GO:0004062	aryl sulfotransferase activity	0.24	1	0.16129032	15	0.043318797
3209	GO:0004564	beta-fructofuranosidase activ	0.24	1	0.16129032	15	0.043318797
20287	GO:0044036	cell wall macromolecule meta	0.24	2	0.32258064	52	0.15017183
30066	GO:0071702	organic substance transport	0.24	12	1.9354838	529	1.5277096
3742	GO:0005242	inward rectifier potassium ch	0.25	1	0.16129032	16	0.046206716
13624	GO:0031167	rRNA methylation	0.25	1	0.16129032	16	0.046206716
31396	GO:0080019	fatty-acyl-CoA reductase (alc	0.25	1	0.16129032	16	0.046206716
30205	GO:0071841	cellular component organizat	0.25	7	1.1290323	285	0.8230572
13829	GO:0031406	carboxylic acid binding	0.26	3	0.48387095	98	0.28301615
2986	GO:0004252	serine-type endopeptidase ac	0.26	4	0.6451613	144	0.41586044
9533	GO:0016405	CoA-ligase activity	0.26	2	0.32258064	55	0.15883559
11776	GO:0019825	oxygen binding	0.26	9	1.451613	389	1.1234008
30887	GO:0072531	pyrimidine-containing compo	0.26	1	0.16129032	17	0.049094636
31498	GO:0080123	jasmonate-amino synthetase	0.26	1	0.16129032	17	0.049094636
9883	GO:0016846	carbon-sulfur lyase activity	0.27	2	0.32258064	56	0.16172351
9910	GO:0016878	acid-thiol ligase activity	0.27	2	0.32258064	57	0.16461143
9906	GO:0016874	ligase activity	0.28	13	2.096774	597	1.7240882
3832	GO:0005388	calcium-transporting ATPase	0.28	1	0.16129032	18	0.051982556
5776	GO:0008252	nucleotidase activity	0.28	1	0.16129032	18	0.051982556
9726	GO:0016646	oxidoreductase activity, actin	0.28	1	0.16129032	18	0.051982556
12887	GO:0030332	cyclin binding	0.28	1	0.16129032	18	0.051982556
16595	GO:0034212	peptide N-acetyltransferase a	0.28	1	0.16129032	18	0.051982556
28368	GO:0070001	aspartic-type peptidase activ	0.28	1	0.16129032	18	0.051982556
2981	GO:0004190	aspartic-type endopeptidase	0.28	1	0.16129032	18	0.051982556
3239	GO:0004596	peptide alpha-N-acetyltransfe	0.28	1	0.16129032	18	0.051982556
8558	GO:0015145	monosaccharide transmemb	0.28	2	0.32258064	58	0.16749935
9018	GO:0015749	monosaccharide transport	0.28	2	0.32258064	58	0.16749935
9800	GO:0016746	transferase activity, transferr	0.28	11	1.7741935	498	1.438184
3304	GO:0004672|GO:0050222	protein kinase activity	0.28	27	4.354839	1333	3.8495972
19253	GO:0042936	dipeptide transporter activity	0.29	3	0.48387095	105	0.3032316
19255	GO:0042938	dipeptide transport	0.29	3	0.48387095	105	0.3032316
3799	GO:0005338|GO:0005339	nucleotide-sugar transmemb	0.29	1	0.16129032	19	0.054870475
9049	GO:0015780	nucleotide-sugar transport	0.29	1	0.16129032	19	0.054870475
9809	GO:0016755	transferase activity, transferr	0.29	1	0.16129032	19	0.054870475
11202	GO:0019199	transmembrane receptor pro	0.29	11	1.7741935	504	1.4555116
8526	GO:0015112	nitrate transmembrane trans	0.30	2	0.32258064	61	0.1761631
8975	GO:0015706|GO:0006872	nitrate transport	0.30	2	0.32258064	61	0.1761631
9801	GO:0016747	transferase activity, transferr	0.30	10	1.6129032	456	1.3168914
3798	GO:0005337	nucleoside transmembrane t	0.30	1	0.16129032	20	0.0577584
6201	GO:0008794	arsenate reductase (glutared	0.30	1	0.16129032	20	0.0577584
9122	GO:0015858	nucleoside transport	0.30	1	0.16129032	20	0.0577584
9707	GO:0016624	oxidoreductase activity, actin	0.30	1	0.16129032	20	0.0577584
9784	GO:0016723	oxidoreductase activity, oxidi	0.30	1	0.16129032	20	0.0577584
13086	GO:0030611	arsenate reductase activity	0.30	1	0.16129032	20	0.0577584
13088	GO:0030613	oxidoreductase activity, actin	0.30	1	0.16129032	20	0.0577584
13089	GO:0030614	oxidoreductase activity, actin	0.30	1	0.16129032	20	0.0577584
122	GO:0000154|GO:0016548	rRNA modification	0.30	1	0.16129032	20	0.0577584
225	GO:0000293	ferric-chelate reductase activ	0.30	1	0.16129032	20	0.0577584
6018	GO:0008559|GO:0005226|G	xenobiotic-transporting ATPa	0.31	2	0.32258064	62	0.17905103
19225	GO:0042908	xenobiotic transport	0.31	2	0.32258064	62	0.17905103
19227	GO:0042910	xenobiotic transporter activity	0.31	2	0.32258064	62	0.17905103
3474	GO:0004869|GO:0004870	cysteine-type endopeptidase	0.32	1	0.16129032	21	0.060646318
5878	GO:0008378	galactosyltransferase activity	0.32	1	0.16129032	21	0.060646318
9902	GO:0016868|GO:0016777	intramolecular transferase ac	0.32	1	0.16129032	21	0.060646318
23518	GO:0048040	UDP-glucuronate decarboxyl	0.32	1	0.16129032	21	0.060646318
22416	GO:0046915	transition metal ion transmen	0.32	2	0.32258064	64	0.18482687
9838	GO:0016790	thiolester hydrolase activity	0.32	2	0.32258064	65	0.18771479
9285	GO:0016043	cellular component organizat	0.33	6	0.9677419	261	0.7537471
3490	GO:0004888|GO:0004926	transmembrane receptor act	0.33	12	1.9354838	571	1.6490022
3828	GO:0005375|GO:0005378|G	copper ion transmembrane tr	0.33	1	0.16129032	22	0.06353424
3831	GO:0005385	zinc ion transmembrane tran	0.33	1	0.16129032	22	0.06353424
4920	GO:0006825	copper ion transport	0.33	1	0.16129032	22	0.06353424
4924	GO:0006829	zinc ion transport	0.33	1	0.16129032	22	0.06353424
10032	GO:0017075	syntaxin-1 binding	0.33	1	0.16129032	22	0.06353424
13038	GO:0030551	cyclic nucleotide binding	0.33	1	0.16129032	22	0.06353424
17800	GO:0035434	copper ion transmembrane tr	0.33	1	0.16129032	22	0.06353424
29941	GO:0071577	zinc ion transmembrane tran	0.33	1	0.16129032	22	0.06353424
9811	GO:0016757|GO:0016932	transferase activity, transferr	0.33	15	2.419355	729	2.1052935
11829	GO:0019900	kinase binding	0.33	2	0.32258064	66	0.1906027
9837	GO:0016788	hydrolase activity, acting on e	0.34	30	4.83871	1534	4.430069
3662	GO:0005126	cytokine receptor binding	0.34	1	0.16129032	23	0.06642216
22753	GO:0047262|GO:0050375	polygalacturonate 4-alpha-ga	0.34	1	0.16129032	23	0.06642216
2633	GO:0003684	damaged DNA binding	0.34	1	0.16129032	23	0.06642216
32	GO:0000041	transition metal ion transport	0.34	2	0.32258064	68	0.19637854
9836	GO:0016787	hydrolase activity	0.35	70	11.290322	3723	10.751725
8559	GO:0015146	pentose transmembrane tran	0.35	1	0.16129032	24	0.06931008
8561	GO:0015148	D-xylose transmembrane tra	0.35	1	0.16129032	24	0.06931008
8829	GO:0015519	D-xylose:hydrogen symporte	0.35	1	0.16129032	24	0.06931008
9019	GO:0015750	pentose transport	0.35	1	0.16129032	24	0.06931008
9022	GO:0015753	D-xylose transport	0.35	1	0.16129032	24	0.06931008
31407	GO:0080030	methyl indole-3-acetate ester	0.35	1	0.16129032	24	0.06931008
3262	GO:0004622|GO:0045126	lysophospholipase activity	0.35	1	0.16129032	24	0.06931008
17617	GO:0035251	UDP-glucosyltransferase act	0.35	5	0.8064516	218	0.62956655

9824	GO:0016773	phosphotransferase activity,	0.35	29	4.677419	1493	4.311664
8584	GO:0015171|GO:0015359	amino acid transmembrane t	0.35	3	0.48387095	118	0.34077454
2521	GO:0003333	amino acid transmembrane t	0.35	3	0.48387095	118	0.34077454
22049	GO:0046527	glucosyltransferase activity	0.35	6	0.9677419	270	0.77973837
10103	GO:0017171	serine hydrolase activity	0.36	5	0.8064516	220	0.63534236
4503	GO:0006310	DNA recombination	0.36	1	0.16129032	25	0.072197996
21145	GO:0045543	gibberellin 2-beta-dioxygenas	0.36	1	0.16129032	25	0.072197996
31393	GO:0080016	(-)-E-beta-caryophyllene synt	0.36	1	0.16129032	25	0.072197996
31394	GO:0080017	alpha-humulene synthase ac	0.36	1	0.16129032	25	0.072197996
31493	GO:0080118	brassinosteroid sulfotransfera	0.36	1	0.16129032	25	0.072197996
8675	GO:0015293	symporter activity	0.37	4	0.6451613	173	0.49961013
9421	GO:0016208	AMP binding	0.37	1	0.16129032	26	0.075085916
9897	GO:0016862	intramolecular oxidoreductas	0.37	1	0.16129032	26	0.075085916
9899	GO:0016864	intramolecular oxidoreductas	0.37	1	0.16129032	26	0.075085916
25135	GO:0050832|GO:0042831	defense response to fungus	0.37	1	0.16129032	26	0.075085916
2665	GO:0003756	protein disulfide isomerase a	0.37	1	0.16129032	26	0.075085916
3210	GO:0004565	beta-galactosidase activity	0.37	1	0.16129032	26	0.075085916
4951	GO:0006865|GO:0006866	amino acid transport	0.38	3	0.48387095	123	0.35521415
12572	GO:0022890|GO:0015082	inorganic cation transmembra	0.38	7	1.1290323	332	0.9587894
9541	GO:0016413	O-acetyltransferase activity	0.39	1	0.16129032	27	0.077973835
3761	GO:0005275|GO:0005279	amine transmembrane transp	0.39	3	0.48387095	126	0.3638779
3332	GO:0004711	ribosomal protein S6 kinase	0.40	1	0.16129032	28	0.080861755
6940	GO:0009672	auxin:hydrogen symporter ac	0.40	1	0.16129032	28	0.080861755
13634	GO:0031177	phosphopantetheine binding	0.40	1	0.16129032	28	0.080861755
3284	GO:0004650	polygalacturonase activity	0.40	1	0.16129032	28	0.080861755
9913	GO:0016881	acid-amino acid ligase activit	0.40	8	1.2903225	391	1.1291766
6166	GO:0008757	S-adenosylmethionine-deper	0.40	4	0.6451613	180	0.5198256
7558	GO:0010334	sesquiterpene synthase activ	0.41	1	0.16129032	29	0.083749674
11834	GO:0019905	syntaxin binding	0.41	1	0.16129032	29	0.083749674
20887	GO:0045263	proton-transporting ATP synt	0.41	1	0.16129032	29	0.083749674
30865	GO:0072509	divalent inorganic cation tran	0.41	2	0.32258064	78	0.22525774
3975	GO:0005667	transcription factor complex	0.42	1	0.16129032	30	0.086637594
2774	GO:0003950	NAD+ ADP-ribosyltransferas	0.42	1	0.16129032	30	0.086637594
9875	GO:0016836	hydro-lyase activity	0.42	2	0.32258064	80	0.2310336
4612	GO:0006468	protein phosphorylation	0.42	41	6.612903	2205	6.367863
3476	GO:0004872|GO:0019041	receptor activity	0.43	13	2.096774	672	1.9406822
7222	GO:0009975	cyclase activity	0.43	1	0.16129032	31	0.08952551
29202	GO:0070838	divalent metal ion transport	0.43	2	0.32258064	82	0.23680943
9103	GO:0015837	amine transport	0.44	3	0.48387095	135	0.38986918
3787	GO:0005319	lipid transporter activity	0.44	2	0.32258064	83	0.23969735
4108	GO:0005839|GO:0000503	proteasome core complex	0.44	1	0.16129032	32	0.09241343
4481	GO:0006284	base-excision repair	0.44	1	0.16129032	32	0.09241343
4510	GO:0006323	DNA packaging	0.44	1	0.16129032	32	0.09241343
4514	GO:0006334	nucleosome assembly	0.44	1	0.16129032	32	0.09241343
12833	GO:0030275	LRR domain binding	0.44	1	0.16129032	32	0.09241343
13920	GO:0031497	chromatin assembly	0.44	1	0.16129032	32	0.09241343
17107	GO:0034728	nucleosome organization	0.44	1	0.16129032	32	0.09241343
28361	GO:0065004	protein-DNA complex assem	0.44	1	0.16129032	32	0.09241343
29467	GO:0071103	DNA conformation change	0.44	1	0.16129032	32	0.09241343
30188	GO:0071824	protein-DNA complex subuni	0.44	1	0.16129032	32	0.09241343
3265	GO:0004630	phospholipase D activity	0.44	1	0.16129032	32	0.09241343
3475	GO:0004871|GO:0005062|G	signal transducer activity	0.44	15	2.419355	787	2.2727928
12516	GO:0022804	active transmembrane transp	0.44	15	2.419355	787	2.2727928
27063	GO:0060089	molecular transducer activity	0.44	15	2.419355	787	2.2727928
3801	GO:0005342	organic acid transmembrane	0.44	3	0.48387095	136	0.3927571
22444	GO:0046943	carboxylic acid transmembra	0.44	3	0.48387095	136	0.3927571
30867	GO:0072511	divalent inorganic cation tran	0.45	2	0.32258064	84	0.24258527
4493	GO:0006298|GO:0006300	mismatch repair	0.45	1	0.16129032	33	0.09530135
11222	GO:0019220	regulation of phosphate meta	0.45	2	0.32258064	85	0.24547319
25471	GO:0051174	regulation of phosphorus me	0.45	2	0.32258064	85	0.24547319
31895	GO:0090304	nucleic acid metabolic proce	0.45	43	6.935484	2340	6.7577324
4942	GO:0006855	drug transmembrane transpo	0.45	3	0.48387095	139	0.40142086
8607	GO:0015197|GO:0015637	peptide transporter activity	0.45	3	0.48387095	139	0.40142086
8608	GO:0015198	oligopeptide transporter activ	0.45	3	0.48387095	139	0.40142086
8642	GO:0015238|GO:0015239|G	drug transmembrane transpo	0.45	3	0.48387095	139	0.40142086
9154	GO:0015893	drug transport	0.45	3	0.48387095	139	0.40142086
18832	GO:0042493|GO:0017035	response to drug	0.45	3	0.48387095	139	0.40142086
2897	GO:0004091|GO:0004302|G	carboxylesterase activity	0.46	4	0.6451613	194	0.5602564
3749	GO:0005249	voltage-gated potassium cha	0.46	1	0.16129032	34	0.09818927
5717	GO:0008173	RNA methyltransferase activ	0.46	1	0.16129032	34	0.09818927
6072	GO:0008645	hexose transport	0.46	1	0.16129032	34	0.09818927
8562	GO:0015149	hexose transmembrane trans	0.46	1	0.16129032	34	0.09818927
11831	GO:0019902	phosphatase binding	0.46	1	0.16129032	34	0.09818927
11832	GO:0019903	protein phosphatase binding	0.46	1	0.16129032	34	0.09818927
17794	GO:0035428	hexose transmembrane trans	0.46	1	0.16129032	34	0.09818927
4944	GO:0006857	oligopeptide transport	0.46	3	0.48387095	141	0.4071967
9099	GO:0015833	peptide transport	0.46	3	0.48387095	141	0.4071967
4538	GO:0006364|GO:0006365	rRNA processing	0.47	1	0.16129032	35	0.10107719
6355	GO:0008970	phospholipase A1 activity	0.47	1	0.16129032	35	0.10107719
9310	GO:0016072	rRNA metabolic process	0.47	1	0.16129032	35	0.10107719
23199	GO:0047714	galactolipase activity	0.47	1	0.16129032	35	0.10107719
8557	GO:0015144	carbohydrate transmembran	0.47	3	0.48387095	143	0.41297254
16602	GO:0034219	carbohydrate transmembran	0.47	3	0.48387095	143	0.41297254
4513	GO:0006333	chromatin assembly or disas	0.48	1	0.16129032	36	0.10396511
9185	GO:0015925	galactosidase activity	0.48	1	0.16129032	36	0.10396511
3758	GO:0005267	potassium channel activity	0.49	1	0.16129032	37	0.10685303
8934	GO:0015662	ATPase activity, coupled to t	0.49	1	0.16129032	37	0.10685303
30069	GO:0071705	nitrogen compound transport	0.49	4	0.6451613	202	0.5833598
6071	GO:0008643|GO:0006861|G	carbohydrate transport	0.49	3	0.48387095	147	0.42452422
5840	GO:0008324	cation transmembrane transp	0.50	10	1.6129032	538	1.5537009
6893	GO:0009620|GO:0009621	response to fungus	0.50	1	0.16129032	38	0.10974095
8501	GO:0015085	calcium ion transmembrane	0.50	1	0.16129032	38	0.10974095
8588	GO:0015175	neutral amino acid transmem	0.50	1	0.16129032	38	0.10974095
9073	GO:0015804	neutral amino acid transport	0.50	1	0.16129032	38	0.10974095
9637	GO:0016538|GO:0003751|G	cyclin-dependent protein kina	0.50	1	0.16129032	38	0.10974095
12538	GO:0022843	voltage-gated cation channel	0.50	1	0.16129032	38	0.10974095
117	GO:0000149	SNARE binding	0.50	1	0.16129032	38	0.10974095
20336	GO:0044085	cellular component biogenes	0.50	2	0.32258064	93	0.26857653
868	GO:0001510	RNA methylation	0.51	1	0.16129032	39	0.11262887
18633	GO:0042277	peptide binding	0.51	2	0.32258064	95	0.27435237
5732	GO:0008194	UDP-glycosyltransferase act	0.52	6	0.9677419	322	0.9299102
2628	GO:0003677	DNA binding	0.52	22	3.548387	1224	3.534814
4911	GO:0006812|GO:0006819|G	cation transport	0.52	12	1.9354838	662	1.9118029
3449	GO:0004842|GO:0004840|G	ubiquitin-protein ligase activit	0.53	6	0.9677419	326	0.94146186
8943	GO:0015672	monovalent inorganic cation	0.53	7	1.1290323	383	1.1060733
9114	GO:0015849	organic acid transport	0.53	3	0.48387095	156	0.45051548
22443	GO:0046942	carboxylic acid transport	0.53	3	0.48387095	156	0.45051548
4874	GO:0006767	water-soluble vitamin metabo	0.53	1	0.16129032	42	0.121292636
18713	GO:0042364	water-soluble vitamin biosynt	0.53	1	0.16129032	42	0.121292636
5766	GO:0008236	serine-type peptidase activity	0.54	4	0.6451613	214	0.6180149
8495	GO:0015077	monovalent inorganic cation	0.54	4	0.6451613	214	0.6180149
9654	GO:0016567	protein ubiquitination	0.55	6	0.9677419	332	0.9587894
14854	GO:0032446	protein modification by small	0.55	6	0.9677419	332	0.9587894
29011	GO:0070647	protein modification by small	0.55	6	0.9677419	332	0.9587894
4873	GO:0006766	vitamin metabolic process	0.55	1	0.16129032	44	0.12706847
4914	GO:0006816	calcium ion transport	0.55	1	0.16129032	44	0.12706847
6477	GO:0009110	vitamin biosynthetic process	0.55	1	0.16129032	44	0.12706847
20522	GO:0044272	sulfur compound biosyntheti	0.55	1	0.16129032	44	0.12706847
28952	GO:0070588	calcium ion transmembrane	0.55	1	0.16129032	44	0.12706847
409	GO:0000502	proteasome complex	0.55	1	0.16129032	44	0.12706847
5195	GO:0007165|GO:0023033	signal transduction	0.55	17	2.7419355	961	2.775291
12602	GO:0023052|GO:0023046	signaling	0.55	17	2.7419355	961	2.775291

8666	GO:0015276	ligand-gated ion channel acti	0.56	1	0.16129032	45	0.1299564
11780	GO:0019829	cation-transporting ATPase a	0.56	1	0.16129032	45	0.1299564
12530	GO:0022834	ligand-gated channel activity	0.56	1	0.16129032	45	0.1299564
17001	GO:0034622	cellular macromolecular com	0.56	1	0.16129032	45	0.1299564
28360	GO:0065003	macromolecular complex ass	0.56	1	0.16129032	45	0.1299564
31431	GO:0080054	low affinity nitrate transmemb	0.56	1	0.16129032	45	0.1299564
9709	GO:0016627	oxidoreductase activity, actin	0.56	2	0.32258064	104	0.30034366
4641	GO:0006508	proteolysis	0.56	17	2.7419355	965	2.7868426
2668	GO:0003779	actin binding	0.56	2	0.32258064	105	0.3032316
12498	GO:0022613	ribonucleoprotein complex bi	0.56	1	0.16129032	46	0.13284431
18614	GO:0042254|GO:0007046	ribosome biogenesis	0.56	1	0.16129032	46	0.13284431
11747	GO:0019787|GO:0008639|G	small conjugating protein liga	0.57	6	0.9677419	339	0.9790048
9911	GO:0016879	ligase activity, forming carbo	0.57	8	1.2903225	456	1.3168914
5714	GO:0008170	N-methyltransferase activity	0.57	1	0.16129032	47	0.13573223
6442	GO:0009073|GO:0016089	aromatic amino acid family b	0.57	1	0.16129032	47	0.13573223
21943	GO:0046417	chorismate metabolic proces	0.57	1	0.16129032	47	0.13573223
17024	GO:0034645|GO:0034961	cellular macromolecule biosy	0.58	35	5.645161	1999	5.7729516
14591	GO:0032183	SUMO binding	0.58	1	0.16129032	48	0.13862015
6428	GO:0009059|GO:0043284	macromolecule biosynthetic	0.58	35	5.645161	2003	5.7845035
3756	GO:0005261|GO:0015281|G	cation channel activity	0.59	1	0.16129032	49	0.14150807
7074	GO:0009815	1-aminocyclopropane-1-carb	0.59	1	0.16129032	49	0.14150807
11830	GO:0019901	protein kinase binding	0.59	1	0.16129032	49	0.14150807
31421	GO:0080044	quercetin 7-O-glucosyltransfe	0.59	1	0.16129032	49	0.14150807
7166	GO:0009914	hormone transport	0.60	1	0.16129032	50	0.14439599
19914	GO:0043648	dicarboxylic acid metabolic p	0.60	1	0.16129032	50	0.14439599
27891	GO:0060918	auxin transport	0.60	1	0.16129032	50	0.14439599
31536	GO:0080161	auxin transmembrane transp	0.60	1	0.16129032	50	0.14439599
7173	GO:0009922	fatty acid elongase activity	0.60	1	0.16129032	51	0.14728391
12492	GO:0022607	cellular component assembly	0.60	1	0.16129032	51	0.14728391
30207	GO:0071843	cellular component biogenes	0.60	1	0.16129032	51	0.14728391
30208	GO:0071844	cellular component assembly	0.60	1	0.16129032	51	0.14728391
9933	GO:0016903	oxidoreductase activity, actin	0.60	2	0.32258064	113	0.32633495
4215	GO:0005975	carbohydrate metabolic proc	0.60	17	2.7419355	990	2.8590407
22478	GO:0046982	protein heterodimerization ac	0.61	2	0.32258064	114	0.32922286
9535	GO:0016407	acetyltransferase activity	0.61	2	0.32258064	115	0.3321108
25635	GO:0051345	positive regulation of hydrola	0.62	1	0.16129032	53	0.15305975
11787	GO:0019842	vitamin binding	0.62	3	0.48387095	177	0.5111618
17918	GO:0035556|GO:0007242|G	intracellular signal transductio	0.62	1	0.16129032	54	0.15594767
25416	GO:0051119	sugar transmembrane transp	0.63	2	0.32258064	118	0.34077454
4894	GO:0006790	sulfur compound metabolic p	0.63	1	0.16129032	55	0.15883559
9493	GO:0016310	phosphorylation	0.64	43	6.935484	2504	7.2313514
15576	GO:0033177	proton-transporting two-secto	0.64	1	0.16129032	56	0.16172351
5712	GO:0008168|GO:0004480	methyltransferase activity	0.64	4	0.6451613	244	0.7046524
5715	GO:0008171	O-methyltransferase activity	0.64	1	0.16129032	57	0.16461143
5964	GO:0008483	transaminase activity	0.64	1	0.16129032	57	0.16461143
6441	GO:0009072	aromatic amino acid family m	0.64	1	0.16129032	57	0.16461143
9813	GO:0016759	cellulose synthase activity	0.64	1	0.16129032	57	0.16461143
9821	GO:0016769	transferase activity, transferr	0.64	1	0.16129032	57	0.16461143
25195	GO:0050896|GO:0051869	response to stimulus	0.64	32	5.16129	1881	5.432177
4913	GO:0006814|GO:0006834|G	sodium ion transport	0.65	1	0.16129032	58	0.16749935
8028	GO:0010817	regulation of hormone levels	0.65	1	0.16129032	58	0.16749935
8499	GO:0015081|GO:0022816	sodium ion transmembrane t	0.65	1	0.16129032	58	0.16749935
11820	GO:0019887	protein kinase regulator activ	0.65	1	0.16129032	58	0.16749935
18086	GO:0035725	sodium ion transmembrane t	0.65	1	0.16129032	58	0.16749935
25989	GO:0051707|GO:0009613|G	response to other organism	0.65	1	0.16129032	58	0.16749935
29800	GO:0071436	sodium ion export	0.65	1	0.16129032	58	0.16749935
3323	GO:0004702	receptor signaling protein ser	0.65	1	0.16129032	58	0.16749935
13656	GO:0031224	intrinsic to membrane	0.65	20	3.2258065	1193	3.4452884
25998	GO:0051716	cellular response to stimulus	0.65	19	3.064516	1138	3.2864528
9900	GO:0016866	intramolecular transferase ac	0.66	1	0.16129032	59	0.17038727
2831	GO:0004022	alcohol dehydrogenase (NAD	0.66	1	0.16129032	59	0.17038727
5652	GO:0008081|GO:0004434|G	phosphoric diester hydrolase	0.66	1	0.16129032	60	0.17327519
12741	GO:0030170	pyridoxal phosphate binding	0.66	2	0.32258064	126	0.3638779
28643	GO:0070279	vitamin B6 binding	0.66	2	0.32258064	126	0.3638779
2818	GO:0004003	ATP-dependent DNA helicas	0.67	1	0.16129032	61	0.1761631
22415	GO:0046914	transition metal ion binding	0.67	44	7.096774	2599	7.5057034
11210	GO:0019207	kinase regulator activity	0.67	1	0.16129032	62	0.17905103
17000	GO:0034621	cellular macromolecular com	0.67	1	0.16129032	62	0.17905103
17457	GO:0035091	phosphatidylinositol binding	0.67	1	0.16129032	62	0.17905103
20186	GO:0043933|GO:0034600	macromolecular complex sub	0.67	1	0.16129032	62	0.17905103
20883	GO:0045259|GO:0045255	proton-transporting ATP synt	0.67	1	0.16129032	62	0.17905103
25987	GO:0051704|GO:0051706	multi-organism process	0.67	1	0.16129032	62	0.17905103
2975	GO:0004175|GO:0016809	endopeptidase activity	0.68	5	0.8064516	320	0.9241344
5809	GO:0008289	lipid binding	0.68	4	0.6451613	258	0.74508333
9488	GO:0016301	kinase activity	0.68	29	4.677419	1740	5.0249805
4609	GO:0006464	protein modification process	0.68	47	7.580645	2781	8.031305
25820	GO:0051536	iron-sulfur cluster binding	0.69	1	0.16129032	64	0.18482687
25824	GO:0051540	metal cluster binding	0.69	1	0.16129032	64	0.18482687
9796	GO:0016741	transferase activity, transferr	0.69	4	0.6451613	261	0.7537471
9710	GO:0016628	oxidoreductase activity, actin	0.69	1	0.16129032	65	0.18771479
25314	GO:0051015	actin filament binding	0.69	1	0.16129032	65	0.18771479
10244	GO:0018130	heterocycle biosynthetic proc	0.69	3	0.48387095	199	0.57469606
5651	GO:0008080	N-acetyltransferase activity	0.70	1	0.16129032	67	0.19349062
2629	GO:0003678|GO:0003679	DNA helicase activity	0.70	1	0.16129032	67	0.19349062
18955	GO:0042625	ATPase activity, coupled to t	0.71	1	0.16129032	68	0.19637854
2999	GO:0004312	fatty acid synthase activity	0.71	1	0.16129032	68	0.19637854
3620	GO:0005057	receptor signaling protein act	0.71	1	0.16129032	69	0.19926646
9722	GO:0016641	oxidoreductase activity, actin	0.71	1	0.16129032	69	0.19926646
31420	GO:0080043	quercetin 3-O-glucosyltransfe	0.71	1	0.16129032	69	0.19926646
4408	GO:0006200	ATP catabolic process	0.71	10	1.6129032	641	1.8511566
9918	GO:0016887|GO:0004002	ATPase activity	0.71	10	1.6129032	641	1.8511566
21600	GO:0046034	ATP metabolic process	0.71	10	1.6129032	641	1.8511566
716	GO:0001071	nucleic acid binding transcrip	0.71	23	3.7096775	1417	4.0921826
2643	GO:0003700|GO:0000130	sequence-specific DNA bindi	0.71	23	3.7096775	1417	4.0921826
15436	GO:0033036	macromolecule localization	0.72	6	0.9677419	397	1.1465042
3895	GO:0005543	phospholipid binding	0.72	2	0.32258064	140	0.40430877
9191	GO:0015931	nucleobase, nucleoside, nucl	0.72	1	0.16129032	70	0.20215438
9192	GO:0015932	nucleobase, nucleoside, nucl	0.72	1	0.16129032	70	0.20215438
8676	GO:0015294	solute:cation symporter activ	0.72	2	0.32258064	141	0.4071967
12547	GO:0022857|GO:0005386|G	transmembrane transporter a	0.72	24	3.8709676	1483	4.282785
3744	GO:0005244	voltage-gated ion channel ac	0.73	1	0.16129032	72	0.20793022
6475	GO:0009108	coenzyme biosynthetic proce	0.73	1	0.16129032	72	0.20793022
9740	GO:0016667	oxidoreductase activity, actin	0.73	1	0.16129032	72	0.20793022
12528	GO:0022832	voltage-gated channel activit	0.73	1	0.16129032	72	0.20793022
15280	GO:0032879	regulation of localization	0.73	1	0.16129032	72	0.20793022
17141	GO:0034762	regulation of transmembrane	0.73	1	0.16129032	72	0.20793022
17144	GO:0034765	regulation of ion transmembr	0.73	1	0.16129032	72	0.20793022
19568	GO:0043269	regulation of ion transport	0.73	1	0.16129032	72	0.20793022
25348	GO:0051049	regulation of transport	0.73	1	0.16129032	72	0.20793022
21058	GO:0045454|GO:0030503|G	cell redox homeostasis	0.73	3	0.48387095	211	0.6093511
9703	GO:0016620	oxidoreductase activity, actin	0.73	1	0.16129032	73	0.21081814
19684	GO:0043412	macromolecule modification	0.74	48	7.7419353	2900	8.374968
9817	GO:0016763	transferase activity, transferr	0.74	1	0.16129032	74	0.21370606
3965	GO:0005654	nucleoplasm	0.74	1	0.16129032	75	0.21659398
20645	GO:0044451	nucleoplasm part	0.74	1	0.16129032	75	0.21659398
9829	GO:0016779	nucleotidyltransferase activity	0.75	2	0.32258064	148	0.42741212
5663	GO:0008094|GO:0004011	DNA-dependent ATPase act	0.75	1	0.16129032	76	0.2194819
18953	GO:0042623	ATPase activity, coupled	0.75	8	1.2903225	537	1.550813
4478	GO:0006281	DNA repair	0.75	2	0.32258064	149	0.43030006
5037	GO:0006974|GO:0034984	response to DNA damage st	0.75	2	0.32258064	149	0.43030006
11426	GO:0019438	aromatic compound biosynth	0.75	2	0.32258064	149	0.43030006
9538	GO:0016410	N-acyltransferase activity	0.75	1	0.16129032	77	0.22236982

14667	GO:0032259	methylation	0.75	4	0.6451613	285	0.8230572
9823	GO:0016772	transferase activity, transferr	0.76	31	5	1932	5.579461
4358	GO:0006140	regulation of nucleotide meta	0.76	1	0.16129032	80	0.2310336
7146	GO:0009894	regulation of catabolic proces	0.76	1	0.16129032	80	0.2310336
13278	GO:0030811	regulation of nucleotide catab	0.76	1	0.16129032	80	0.2310336
13759	GO:0031329	regulation of cellular cataboli	0.76	1	0.16129032	80	0.2310336
15521	GO:0033121	regulation of purine nucleotid	0.76	1	0.16129032	80	0.2310336
8517	GO:0015103	inorganic anion transmembra	0.77	2	0.32258064	154	0.44473964
4357	GO:0006139|GO:0055134	nucleobase, nucleoside, nucl	0.77	54	8.709678	3285	9.486816
18674	GO:0042325	regulation of phosphorylation	0.77	1	0.16129032	81	0.23392151
4990	GO:0006915|GO:0008632	apoptosis	0.77	3	0.48387095	227	0.6555578
19831	GO:0043565	sequence-specific DNA bindi	0.78	2	0.32258064	159	0.45917925
5624	GO:0008047	enzyme activator activity	0.79	1	0.16129032	85	0.24547319
3307	GO:0004675	transmembrane receptor pro	0.79	5	0.8064516	367	1.0598665
19473	GO:0043170|GO:0043283	macromolecule metabolic pr	0.79	110	17.741936	6553	18.92454
3723	GO:0005215|GO:0005478	transporter activity	0.79	29	4.677419	1844	5.325324
11695	GO:0019725	cellular homeostasis	0.79	3	0.48387095	234	0.67577326
26931	GO:0055085	transmembrane transport	0.79	24	3.8709676	1548	4.4705
11828	GO:0019899	enzyme binding	0.80	3	0.48387095	237	0.684437
9719	GO:0016638	oxidoreductase activity, actin	0.80	1	0.16129032	89	0.25702485
18925	GO:0042592	homeostatic process	0.80	3	0.48387095	238	0.68732494
4897	GO:0006793	phosphorus metabolic proces	0.80	47	7.580645	2925	8.4471655
4900	GO:0006796	phosphate metabolic process	0.80	47	7.580645	2925	8.4471655
5756	GO:0008219	cell death	0.80	3	0.48387095	239	0.69021285
8209	GO:0012501|GO:0016244	programmed cell death	0.80	3	0.48387095	239	0.69021285
9462	GO:0016265	death	0.80	3	0.48387095	239	0.69021285
14590	GO:0032182	small conjugating protein bin	0.80	1	0.16129032	90	0.2599128
3644	GO:0005102	receptor binding	0.81	1	0.16129032	91	0.2628007
6784	GO:0009451|GO:0016547	RNA modification	0.81	1	0.16129032	91	0.2628007
8494	GO:0015075	ion transmembrane transport	0.81	12	1.9354838	827	2.3883097
9579	GO:0016462	pyrophosphatase activity	0.81	14	2.2580645	954	2.7550755
9859	GO:0016818	hydrolase activity, acting on a	0.81	14	2.2580645	956	2.7608514
16603	GO:0034220	ion transmembrane transpor	0.82	12	1.9354838	833	2.4056373
4910	GO:0006811	ion transport	0.82	14	2.2580645	959	2.769515
9858	GO:0016817	hydrolase activity, acting on a	0.82	14	2.2580645	959	2.769515
3808	GO:0005351|GO:0005403	sugar:hydrogen symporter ac	0.82	1	0.16129032	94	0.27146447
3835	GO:0005402	cation:sugar symporter activi	0.82	1	0.16129032	94	0.27146447
15949	GO:0033554	cellular response to stress	0.82	2	0.32258064	173	0.49961013
4526	GO:0006351|GO:0006350|G	transcription, DNA-dependen	0.82	24	3.8709676	1575	4.548474
15176	GO:0032774	RNA biosynthetic process	0.82	24	3.8709676	1575	4.548474
28365	GO:0065008	regulation of biological quality	0.82	4	0.6451613	316	0.9125827
4067	GO:0005783	endoplasmic reticulum	0.82	2	0.32258064	174	0.50249803
8967	GO:0015698	inorganic anion transport	0.83	2	0.32258064	176	0.5082739
8677	GO:0015295	solute:hydrogen symporter a	0.83	1	0.16129032	98	0.28301615
8681	GO:0015299	solute:hydrogen antiporter ac	0.83	1	0.16129032	98	0.28301615
25379	GO:0051082	unfolded protein binding	0.83	1	0.16129032	98	0.28301615
4909	GO:0006810|GO:0015457|G	transport	0.83	36	5.806452	2315	6.6855345
25529	GO:0051234	establishment of localization	0.83	36	5.806452	2315	6.6855345
16851	GO:0034470	ncRNA processing	0.83	1	0.16129032	99	0.28590405
17039	GO:0034660	ncRNA metabolic process	0.83	1	0.16129032	99	0.28590405
3716	GO:0005198	structural molecule activity	0.83	5	0.8064516	393	1.1349525
25476	GO:0051179	localization	0.84	36	5.806452	2319	6.697086
4849	GO:0006730|GO:0019753|G	one-carbon metabolic proces	0.84	4	0.6451613	325	0.93857396
16430	GO:0034046	poly(G) RNA binding	0.84	1	0.16129032	100	0.28879198
6663	GO:0009309	amine biosynthetic process	0.84	2	0.32258064	181	0.5227135
5874	GO:0008374	O-acyltransferase activity	0.84	1	0.16129032	102	0.29456782
4844	GO:0006725	cellular aromatic compound	0.85	2	0.32258064	185	0.53426516
19392	GO:0043085	positive regulation of catalyti	0.85	1	0.16129032	104	0.30034366
20343	GO:0044093	positive regulation of molecu	0.85	1	0.16129032	104	0.30034366
12573	GO:0022891	substrate-specific transmem	0.85	18	2.903226	1241	3.5839086
8673	GO:0015291|GO:0015290|G	secondary active transmemb	0.86	5	0.8064516	407	1.1753833
3942	GO:0005618	cell wall	0.86	1	0.16129032	107	0.30900744
11833	GO:0019904	protein domain specific bindi	0.86	1	0.16129032	109	0.31478328
10058	GO:0017111	nucleoside-triphosphatase ac	0.86	12	1.9354838	878	2.5355935
17020	GO:0034641	cellular nitrogen compound m	0.86	58	9.354838	3678	10.621769
12868	GO:0030312	external encapsulating struct	0.87	1	0.16129032	111	0.3205591
4512	GO:0006325	chromatin organization	0.87	1	0.16129032	112	0.32344702
5070	GO:0007017	microtubule-based process	0.87	1	0.16129032	112	0.32344702
5071	GO:0007018	microtubule-based movemer	0.87	1	0.16129032	112	0.32344702
8680	GO:0015298	solute:cation antiporter activi	0.87	1	0.16129032	112	0.32344702
9730	GO:0016651	oxidoreductase activity, actin	0.87	1	0.16129032	112	0.32344702
25568	GO:0051276|GO:0007001|G	chromosome organization	0.87	1	0.16129032	112	0.32344702
29081	GO:0070717	poly-purine tract binding	0.87	1	0.16129032	112	0.32344702
4850	GO:0006732|GO:0006752	coenzyme metabolic process	0.87	1	0.16129032	113	0.32633495
19686	GO:0043414	macromolecule methylation	0.87	1	0.16129032	113	0.32633495
20510	GO:0044260|GO:0034960	cellular macromolecule meta	0.87	90	14.5161295	5577	16.105928
12574	GO:0022892	substrate-specific transporte	0.88	21	3.387097	1463	4.2250266
11522	GO:0019538|GO:0006411	protein metabolic process	0.88	65	10.4838705	4134	11.938661
4906	GO:0006807	nitrogen compound metaboli	0.89	58	9.354838	3725	10.757502
4405	GO:0006195	purine nucleotide catabolic p	0.89	10	1.6129032	773	2.232362
6508	GO:0009143	nucleoside triphosphate cata	0.89	10	1.6129032	773	2.232362
6509	GO:0009144	purine nucleoside triphospha	0.89	10	1.6129032	773	2.232362
6511	GO:0009146	purine nucleoside triphospha	0.89	10	1.6129032	773	2.232362
6519	GO:0009154	purine ribonucleotide catabol	0.89	10	1.6129032	773	2.232362
6530	GO:0009166	nucleotide catabolic process	0.89	10	1.6129032	773	2.232362
6567	GO:0009203	ribonucleoside triphosphate c	0.89	10	1.6129032	773	2.232362
6569	GO:0009205	purine ribonucleoside triphos	0.89	10	1.6129032	773	2.232362
6571	GO:0009207	purine ribonucleoside triphos	0.89	10	1.6129032	773	2.232362
6624	GO:0009261	ribonucleotide catabolic proc	0.89	10	1.6129032	773	2.232362
17034	GO:0034655	nucleobase, nucleoside, nucl	0.89	10	1.6129032	773	2.232362
17035	GO:0034656	nucleobase, nucleoside and	0.89	10	1.6129032	773	2.232362
22209	GO:0046700	heterocycle catabolic proces	0.89	10	1.6129032	773	2.232362
30879	GO:0072523	purine-containing compound	0.89	10	1.6129032	773	2.232362
12532	GO:0022836	gated channel activity	0.89	1	0.16129032	122	0.3523262
9267	GO:0016021	integral to membrane	0.89	15	2.419355	1103	3.1853757
20520	GO:0044270	cellular nitrogen compound c	0.89	10	1.6129032	777	2.2439137
14392	GO:0031981	nuclear lumen	0.89	1	0.16129032	123	0.35521415
25485	GO:0051188	cofactor biosynthetic process	0.89	1	0.16129032	123	0.35521415
9295	GO:0016053	organic acid biosynthetic pro	0.89	2	0.32258064	211	0.6093511
21922	GO:0046394	carboxylic acid biosynthetic p	0.89	2	0.32258064	211	0.6093511
4916	GO:0006818	hydrogen transport	0.90	3	0.48387095	292	0.84327257
9246	GO:0015992	proton transport	0.90	3	0.48387095	292	0.84327257
9583	GO:0016469	proton-transporting two-secto	0.90	1	0.16129032	127	0.36676583
6563	GO:0009199	ribonucleoside triphosphate m	0.90	10	1.6129032	790	2.2814567
6515	GO:0009150	purine ribonucleotide metabo	0.90	10	1.6129032	791	2.2843447
22479	GO:0046983	protein dimerization activity	0.90	6	0.9677419	517	1.4930545
6506	GO:0009141	nucleoside triphosphate meta	0.91	10	1.6129032	796	2.2987843
20356	GO:0044106	cellular amine metabolic proc	0.91	3	0.48387095	303	0.8750397
20619	GO:0044425	membrane part	0.91	21	3.387097	1523	4.398302
19532	GO:0043233	organelle lumen	0.92	1	0.16129032	137	0.39564502
28380	GO:0070013	intracellular organelle lumen	0.92	1	0.16129032	137	0.39564502
4373	GO:0006163	purine nucleotide metabolic p	0.92	10	1.6129032	810	2.339215
6622	GO:0009259|GO:0009121	ribonucleotide metabolic proc	0.92	10	1.6129032	812	2.344991
13531	GO:0031072	heat shock protein binding	0.92	2	0.32258064	230	0.6642216
20531	GO:0044281	small molecule metabolic pro	0.92	25	4.032258	1788	5.1636004
28378	GO:0070011	peptidase activity, acting on l	0.92	6	0.9677419	537	1.550813
3724	GO:0005216	ion channel activity	0.92	1	0.16129032	140	0.40430877
6662	GO:0009308	amine metabolic process	0.92	3	0.48387095	314	0.9068068
30877	GO:0072521	purine-containing compound	0.93	10	1.6129032	827	2.3883097
9895	GO:0016860	intramolecular oxidoreductas	0.93	1	0.16129032	146	0.42163628
5791	GO:0008270	zinc ion binding	0.93	26	4.193548	1880	5.4292893
20499	GO:0044249	cellular biosynthetic process	0.93	38	6.129032	2635	7.6096687

18582	GO:0042221	response to chemical stimulu	0.93	3	0.48387095	328	0.94723773
5981	GO:0008509	anion transmembrane transp	0.94	2	0.32258064	245	0.70754033
8679	GO:0015297	antiporter activity	0.94	1	0.16129032	152	0.4389638
8682	GO:0015300	solute:solute antiporter activit	0.94	1	0.16129032	152	0.4389638
27562	GO:0060589	nucleoside-triphosphatase re	0.94	1	0.16129032	152	0.4389638
2660	GO:0003735|GO:0003736|G	structural constituent of ribos	0.94	2	0.32258064	246	0.7104283
5909	GO:0008415	acyltransferase activity	0.94	3	0.48387095	330	0.95301354
4917	GO:0006820|GO:0006822	anion transport	0.94	2	0.32258064	247	0.7133162
14386	GO:0031974	membrane-enclosed lumen	0.94	1	0.16129032	154	0.44473964
5661	GO:0008092	cytoskeletal protein binding	0.94	2	0.32258064	249	0.719092
5763	GO:0008233	peptidase activity	0.94	6	0.9677419	565	1.6316746
20532	GO:0044282	small molecule catabolic proc	0.94	10	1.6129032	854	2.4662836
8496	GO:0015078	hydrogen ion transmembrane	0.94	1	0.16129032	160	0.4620672
20517	GO:0044267	cellular protein metabolic pro	0.94	47	7.580645	3225	9.313541
6427	GO:0009058	biosynthetic process	0.95	40	6.451613	2796	8.074624
9308	GO:0016070	RNA metabolic process	0.95	25	4.032258	1855	5.3570914
9494	GO:0016311	dephosphorylation	0.95	4	0.6451613	425	1.227366
5764	GO:0008234|GO:0004220	cysteine-type peptidase activ	0.95	1	0.16129032	163	0.47073093
5055	GO:0006996	organelle organization	0.95	1	0.16129032	164	0.47361887
25483	GO:0051186	cofactor metabolic process	0.95	1	0.16129032	164	0.47361887
20533	GO:0044283	small molecule biosynthetic p	0.95	3	0.48387095	347	1.0021082
19126	GO:0042803	protein homodimerization act	0.95	2	0.32258064	265	0.7652988
9699	GO:0016616	oxidoreductase activity, actin	0.95	2	0.32258064	266	0.7681867
6077	GO:0008652	cellular amino acid biosynthe	0.95	1	0.16129032	169	0.48805845
4755	GO:0006629	lipid metabolic process	0.95	6	0.9677419	590	1.7038727
22007	GO:0046483	heterocycle metabolic proces	0.96	13	2.096774	1094	3.1593843
3955	GO:0005634	nucleus	0.96	18	2.903226	1433	4.138389
9768	GO:0016706	oxidoreductase activity, actin	0.96	1	0.16129032	175	0.505386
18912	GO:0042578	phosphoric ester hydrolase a	0.96	4	0.6451613	444	1.2822365
4649	GO:0006520|GO:0006519	cellular amino acid metabolic	0.96	2	0.32258064	277	0.7999538
20498	GO:0044248	cellular catabolic process	0.96	10	1.6129032	894	2.5818002
20622	GO:0044428	nuclear part	0.96	1	0.16129032	178	0.5140497
20521	GO:0044271	cellular nitrogen compound b	0.96	3	0.48387095	367	1.0598665
4109	GO:0005840|GO:0033279	ribosome	0.96	7	1.1290323	682	1.9695613
7691	GO:0010467	gene expression	0.96	26	4.193548	1974	5.7007537
5018	GO:0006950	response to stress	0.96	6	0.9677419	611	1.764519
5610	GO:0008026	ATP-dependent helicase acti	0.96	1	0.16129032	183	0.52848935
28401	GO:0070035	purine NTP-dependent helica	0.96	1	0.16129032	183	0.52848935
6425	GO:0009056	catabolic process	0.97	12	1.9354838	1051	3.0352037
4867	GO:0006753	nucleoside phosphate metab	0.97	10	1.6129032	914	2.6395588
6484	GO:0009117	nucleotide metabolic process	0.97	10	1.6129032	914	2.6395588
2657	GO:0003727|GO:0003728	single-stranded RNA binding	0.97	1	0.16129032	188	0.54292893
3068	GO:0004386	helicase activity	0.97	1	0.16129032	189	0.54581684
9697	GO:0016614	oxidoreductase activity, actin	0.97	2	0.32258064	293	0.84616053
9839	GO:0016791|GO:0016302	phosphatase activity	0.97	3	0.48387095	383	1.1060733
2626	GO:0003674|GO:0005554	molecular_function	0.97	416	67.09677	24394	70.447914
30206	GO:0071842	cellular component organizat	0.97	1	0.16129032	193	0.5573685
20488	GO:0044238	primary metabolic process	0.97	144	23.225807	9167	26.47356
3340	GO:0004721	phosphoprotein phosphatase	0.97	1	0.16129032	199	0.57469606
9889	GO:0016853	isomerase activity	0.97	2	0.32258064	306	0.8837035
26932	GO:0055086	nucleobase, nucleoside and	0.98	10	1.6129032	944	2.7261963
13018	GO:0030529	ribonucleoprotein complex	0.98	7	1.1290323	741	2.1399486
4558	GO:0006396|GO:0006394	RNA processing	0.98	1	0.16129032	219	0.63245445
5019	GO:0006952|GO:0002217|G	defense response	0.98	1	0.16129032	221	0.63823026
12533	GO:0022838	substrate-specific channel ac	0.98	1	0.16129032	221	0.63823026
8660	GO:0015267|GO:0015249|G	channel activity	0.98	1	0.16129032	226	0.6526699
12515	GO:0022803	passive transmembrane tran	0.98	1	0.16129032	226	0.6526699
8211	GO:0012505	endomembrane system	0.99	1	0.16129032	242	0.6988766
3178	GO:0004519	endonuclease activity	0.99	2	0.32258064	356	1.0280994
20487	GO:0044237	cellular metabolic process	0.99	123	19.838709	8185	23.637625
30306	GO:0071944	cell periphery	0.99	1	0.16129032	259	0.74797124
2627	GO:0003676	nucleic acid binding	0.99	30	4.83871	2453	7.0840673
3177	GO:0004518	nuclease activity	1.00	2	0.32258064	427	1.2331418
13041	GO:0030554	adenyl nucleotide binding	1.00	3	0.48387095	550	1.5883559
14965	GO:0032559	adenyl ribonucleotide binding	1.00	3	0.48387095	550	1.5883559
19527	GO:0043228	non-membrane-bounded org	1.00	7	1.1290323	923	2.66555
19531	GO:0043232	intracellular non-membrane-b	1.00	7	1.1290323	923	2.66555
19533	GO:0043234	protein complex	1.00	6	0.9677419	848	2.448956
3876	GO:0005515|GO:0045308	protein binding	1.00	97	15.645162	6933	20.021948
7229	GO:0009987|GO:0008151|G	cellular process	1.00	157	25.32258	10601	30.614838
3863	GO:0005488	binding	1.00	212	34.19355	13903	40.15075
3884	GO:0005524	ATP binding	1.00	2	0.32258064	524	1.51327
19125	GO:0042802	identical protein binding	1.00	3	0.48387095	697	2.01288
19526	GO:0043227	membrane-bounded organel	1.00	20	3.2258065	2142	6.185924
19530	GO:0043231	intracellular membrane-boun	1.00	20	3.2258065	2142	6.185924
15392	GO:0032991	macromolecular complex	1.00	13	2.096774	1616	4.666878
9266	GO:0016020	membrane	1.00	42	6.774194	3741	10.803708
131	GO:0000166	nucleotide binding	1.00	8	1.2903225	1213	3.5030468
14959	GO:0032553	ribonucleotide binding	1.00	3	0.48387095	795	2.2958963
14961	GO:0032555	purine ribonucleotide binding	1.00	3	0.48387095	795	2.2958963
10033	GO:0017076	purine nucleotide binding	1.00	3	0.48387095	797	2.3016722
18000	GO:0035639	purine ribonucleoside triphos	1.00	2	0.32258064	769	2.2208104
19525	GO:0043226	organelle	1.00	27	4.354839	3013	8.701303
19528	GO:0043229	intracellular organelle	1.00	27	4.354839	3013	8.701303
2653	GO:0003723	RNA binding	1.00	2	0.32258064	797	2.3016722
20640	GO:0044446	intracellular organelle part	1.00	1	0.16129032	710	2.0504231
20616	GO:0044422	organelle part	1.00	1	0.16129032	712	2.0561988
3945	GO:0005622	intracellular	1.00	49	7.903226	4751	13.720507
4027	GO:0005737	cytoplasm	1.00	13	2.096774	2001	5.7787275
20638	GO:0044444	cytoplasmic part	1.00	9	1.451613	1644	4.7477403
20618	GO:0044424	intracellular part	1.00	36	5.806452	3853	11.127155
3903	GO:0005575|GO:0008372	cellular_component	1.00	101	16.290323	8515	24.590637
3946	GO:0005623	cell	1.00	92	14.83871	8227	23.758917
20657	GO:0044464	cell part	1.00	92	14.83871	8227	23.758917
