# Supplementary material for: The Tomato Hybrid Proline-rich Protein regulates the abscission zone competence to respond to ethylene signals
Source: Hortic Res. 2018 Jun 1;5:28. doi: 10.1038/s41438-018-0033-2 (PMC5981600; doi:10.1038/s41438-018-0033-2)
Supplement: Supplementary file 6 — Tabls S6 [file 41438_2018_33_MOESM6_ESM.pdf]

Table S6. Differentially regulated genes (up and down regulated) in the control compared with THyPRP silenced plants in FAZ at 16 h along with their expression values, fold changes, annotations, GO ids and terms.

Worksheet 2 & 3

Cut off used to filter up and down regulated genes

Upregulated

For filtering upregulation we consider flag should be Detected in the treated sample and can be Compromised or Detected in the control sample and fold>=0.8 in the individual replicates and fold>=1 in the Geomean of treated samples.

Downregulated

For filtering downregulation we consider flag can be Detected or Compromised in the treated sample and should be Detected in the control sample and fold<=-0.8 in the individual replicates and fold<=-1 in the Geomean of treated samples.

Set5					
Line	Array	Details	Time	Treated vs. Con	Set
Control – New Yorker	254331010027_1_1	16h_9_AZ	16h	Control	Set 5
Control – New Yorker	254331010027_1_2	16h_10_AZ	16h		
TAPG + TPRP	254331010036_1_1	16h_45_AZ	16h	Treated	
TAPG + TPRP	254331010036_1_2	16h_46_AZ	16h		

Note:

Fold change expression values are provided as logbase 2

Flagging Criteria:

Compromised: If the gProcessedSignal value is less than the background intensity

Detected: If the gProcessedSignal value is above background intensity

Column Header	Description
ProbeName	Unique probe identifier
GeneName	GeneSymbol
Fold	Fold change calculated is log base 2
Flag	Detected or Compromised
GeomeanFold	Average Fold change for the replicates calculated is log base 2
p-Value	t-test p- value
gProcessedSigna	Background substracted signal intensity

Differentially Regulated Probes

Sample	Up	Down
16h_TPRP_ AZ vs. 16h_Control_AZ_Sense	2408	2491
16h_TPRP_ AZ vs. 16h_Control_AZ_Antisense	1483	895

Worksheet 4 & 5

This file contains the fold change for all the spots in the array

GO ID

Gene Ontology ID

GO ACCESSION

Gene Ontology Function name

p-value :

The probability of obtaining the specified GO accession number from a list of random entities. Less the p-value more significant is the GO accession number.

Significant pvalue<0.05

Count in Selection :

This refers to the number of genes in the selected entity (for example, from T-test) list which have that particular GO term.

%Count in Selection :

This refers to the percentage of genes in the input entity list which have that GO term.

Count in Total :

This refers to the number of genes in All Entities which have that GO term.

%Count in Total :

This refers to the percentage of genes in the All Entities list which have that GO term.

		16h_AZ_Control					16h_AZ_TAPG + TPRP vs. 16h_AZ_Control																													
ProbeNar	Orientatic	Fold 16h	Flag 16h	Fold 16h	Flag 16h	Geomean	Fold 16h	Flag 16h	Fold 16h	Flag 16h	Geomean	P-value	ProbeNar	GeneNam	Systemat	Descripti	Sequence	Contig ID	ITAG ID	Parent Ge	ITAG Des	Ontology	Ontology	Contig ID	Target ID	Functiona	GO term	Transcrip	target ID	Symbols	TAIR Des	TAIR Chr	gProcess	gProcess	gProcess	gProcess	gProcess	gProcessedSignal 16h 46 AZ
A 96 P0(	Sense	0.462	Detected	-0.462	Detected	0.000	2.830	Detected	1.913	Detected	2.372	0.068	A 96 P0(	A 96 P0(	A 96 P0(	Unknown	TCACTTGCCAAGACATTAACACAACTCATGCTTATTTGAAATCAGGTACATTTTGCTACA																1876.44	930.273	1701.76	955.311	10016.5	5291.61
A 96 P0(	Sense	0.070	Detected	-0.070	Detected	0.000	1.421	Detected	0.920	Detected	1.171	0.046	A 96 P0(	AK32048	AK32048	Solanum	TGATGGGGGCAAAAGAGTACACGAAATTATGCGATGAAGAGGAAATTCATTGAAACTTGA																1232.31	1051.67	4861.47	1823.61	3250.9	2290.68
A 96 P01	Sense	0.509	Detected	-0.509	Detected	0.000	1.753	Detected	0.950	Detected	1.352	0.172	A 96 P01	AK32373	AK32373	Solanum	TGGGAATCAAGCCAATTTTCATTACAGGGACGAAACAGAATTTCAAACAAGTAAGGATCG																13830.7	6429.8	12932.2	8810.26	33893.7	19374.7
A 96 P01	Sense	0.994	Detected	-0.994	Detected	0.000	4.888	Detected	3.934	Detected	4.411	0.057	A 96 P01	AW94475	AW94475	EST3368	ATCGAGTTCGTCTTTTTGTTAACATTTTGGGTGATGTTGTACAAATTCCCAGAGTGACTT																2523.05	598.356	2602.05	1105.06	38796	19973.3
A 96 P01	Sense	0.133	Detected	-0.133	Detected	0.000	3.916	Detected	3.084	Detected	3.500	0.015	A 96 P01	BI933034	BI933034	EST5529	CGAGACTTATGCCTTTGGCTTTAGCAAAGTAAACAACCATCTTGATGCATAGTTAATTAT																1496.63	1170.69	2784.44	1537.92	21313.4	11937.5
A 96 P01	Sense	0.171	Detected	-0.171	Detected	0.000	1.990	Detected	2.294	Detected	2.142	0.011	A 96 P01	AW73822	AW73822	EST3396	AGCAGGCTATGTGGCGTCATGGTTTCAGATTAAGGAAGTTGAAGGTAATAATTCCACTGA																692.481	513.998	1089.75	222.226	2527.63	3110.58
A 96 P01	Sense	0.379	Detected	-0.379	Detected	0.000	2.247	Detected	1.247	Detected	1.747	0.108	A 96 P01	LOC5440	NM 0012	Solanum	GACTTATTCATTTCTTATGAGGAACGCGAAAATGTTCAAGTTAGACGCTTGGCTGCCTCG																13751.9	7654.68	23710.2	11685	51935	25899.6
A 96 P01	Sense	-0.089	Detected	0.089	Detected	0.000	2.318	Detected	1.667	Detected	1.993	0.028	A 96 P01	LOX1.1	NM 0012	Solanum	ACTTACAGGCAAAGGAATTCCAAACAGTGTGTCAATATAGAAGAGTTTTGAGTACACATG																2412.08	2569.31	2592.72	2151.81	13240.6	8406.01
A 96 P01	Sense	-0.197	Detected	0.197	Detected	0.000	1.931	Detected	1.885	Detected	1.908	0.011	A 96 P01	LOC5438	NM 0012	Solanum	AATAATGATAGAGTAAGGAGGATGTCAATTCATGATCCCACACCCACCTACTGTAGGTAG																179.517	221.986	249.297	262.65	811.61	783.78
A 96 P01	Sense	-0.167	Detected	0.167	Detected	0.000	1.043	Detected	1.014	Detected	1.028	0.026	A 96 P01	PPCK2	NM 0012	Solanum	CAAGTTCTCAGGCATCCATGGATGACTAGCAACGGAGAAATCAGAACAGTTGCTCCATTT																663.137	786.322	995.893	860.128	1586.46	1550.23
A 96 P01	Sense	0.762	Detected	-0.762	Comprom	0.000	4.293	Detected	3.368	Detected	3.830	0.050	A 96 P01	wus	NM 0012	Solanum	GTTTGTAGTAAGTAAGTACTAATCTAATTTGGTATGTGCCAAGCTATTTGGACCTTATGG																18.018	5.89878	143.013	35.8825	215.504	113.17
A 96 P01	Sense	-0.180	Detected	0.180	Detected	0.000	1.915	Detected	1.441	Detected	1.678	0.030	A 96 P01	TA37803	TA37803	Rep: Chr	GTGAATGTTTCGTCAGAGGCTCATCGATTTGCATACAGAGAAGGAATTCGGTTTGACAAA																835.087	1007.9	1539.63	1104.63	3688.16	2648.76
A 96 P03	Sense	0.486	Detected	-0.486	Detected	0.000	1.858	Detected	1.088	Detected	1.473	0.141	A 96 P03	Td	M61914	L.esculer	CTGAGATGGATGAGTTCAAAAATCAAGCTGATAAGCTTGGTTATCCATATGAACTTGATA																33276.7	15967.8	23198.6	15219.3	89078.3	52110.3
A 96 P03	Sense	-0.006	Detected	0.006	Detected	0.000	1.275	Detected	0.917	Detected	1.096	0.026	A 96 P03	AK32134	AK32134	Solanum	TAGCTTCAAACCCCTGGCAGAAGCTTCTGATGAAGATTTAATTCAAGTTGTCACTACAAA																155.497	147.529	271.917	163.442	390.822	304.119
A 96 P05	Sense	-0.056	Detected	0.056	Detected	0.000	2.737	Detected	1.903	Detected	2.320	0.031	A 96 P05	GO37623	GO37623	ctsd3j1 T	ATTGCGTTTGTAAGTCTGGTTGAAAAATCCTGGATAGCAAAGACTCCTTTTCACCTACCA																25.5763	25.9917	131.375	42.9089	183.353	102.503
A 96 P05	Sense	-0.030	Detected	0.030	Detected	0.000	1.494	Detected	0.891	Detected	1.193	0.059	A 96 P05	LOC7783	NM 0012	Solanum	CAAAAACTTCGACGAACTATACCTTTATTGTTATTATGTTGCTGGTACGGTTGGGTTGAT																1854.84	1820.26	2370.97	1835.5	5520.2	3623.34
A 96 P05	Sense	0.077	Detected	-0.077	Detected	0.000	0.993	Detected	1.173	Detected	1.083	0.012	A 96 P05	AI483528	AI483528	EST2493	TATATTTGTTCGTAGTTTACTAGCTAGTCTGAGTCGTATAGGGTCCTAAAGGGAGAAATA																4834.56	4090.49	6132.36	7274.19	9441.55	10659.5
A 96 P05	Sense	0.681	Detected	-0.681	Detected	0.000	4.678	Detected	2.944	Detected	3.811	0.074	A 96 P05	LOC1001	Z75519	L.esculer	CAGGATGATTCGTTTGTATCATTATTGTCGTATTTGTGAACCTGCTCATGTTAATGTTGT																71.2742	26.0841	2877.43	1577.54	1177.16	352.75
A 96 P05	Sense	0.386	Detected	-0.386	Detected	0.000	2.099	Detected	1.306	Detected	1.702	0.091	A 96 P05	AK32258	AK32258	Solanum	[illegible]																[illegible]	[illegible]	[illegible]	[illegible]	[illegible]	[illegible]
A 96 P05	Sense	0.152	Detected	-0.152	Detected	0.000	1.712	Detected	0.805	Detected	1.258	0.119	A 96 P05	DB69307	DB69307	DB69307	[illegible]																[illegible]	[illegible]	[illegible]	[illegible]	[illegible]	[illegible]
A 96 P06	Sense	-0.372	Detected	0.372	Detected	0.000	1.365	Detected	1.029	Detected	1.197	0.100	A 96 P06	AK32477	AK32477	Solanum	[illegible]																[illegible]	[illegible]	[illegible]	[illegible]	[illegible]	[illegible]
A 96 P06	Sense	-0.008	Detected	0.008	Detected	0.000	4.087	Detected	2.170	Detected	3.129	0.082	A 96 P06	DB69309	DB69309	DB69309	[illegible]																[illegible]	[illegible]	[illegible]	[illegible]	[illegible]	[illegible]
A 96 P06	Sense	0.486	Detected	-0.486	Detected	0.000	1.317	Detected	1.919	Detected	1.618	0.106	A 96 P06	AK31923	AK31923	Solanum	[illegible]																[illegible]	[illegible]	[illegible]	[illegible]	[illegible]	[illegible]
A 96 P06	Sense	-0.381	Detected	0.381	Detected	0.000	5.397	Detected	3.110	Detected	4.253	0.072	A 96 P06	AK22483	AK22483	Solanum	[illegible]																[illegible]	[illegible]	[illegible]	[illegible]	[illegible]	[illegible]
A 96 P07	Sense	-0.289	Detected	0.289	Detected	0.000	1.744	Detected	1.166	Detected	1.455	0.070	A 96 P07	DB71124	DB71124	DB71124	[illegible]																[illegible]	[illegible]	[illegible]	[illegible]	[illegible]	[illegible]
A 96 P07	Sense	0.020	Detected	-0.020	Detected	0.000	1.213	Detected	1.568	Detected	1.390	0.016	A 96 P07	DB70200	DB70200	DB70200	[illegible]																[illegible]	[illegible]	[illegible]	[illegible]	[illegible]	[illegible]
A 96 P07	Sense	0.868	Detected	-0.868	Detected	0.000	2.284	Detected	1.397	Detected	1.841	0.200	A 96 P07	LOC5439	AK24724	Solanum	[illegible]																[illegible]	[illegible]	[illegible]	[illegible]	[illegible]	[illegible]
A 96 P07	Sense	1.091	Detected	-1.091	Detected	0.000	3.876	Detected	2.490	Detected	3.183	0.133	A 96 P07	LOC5440	NM 0012	Solanum	[illegible]																[illegible]	[illegible]	[illegible]	[illegible]	[illegible]	[illegible]
A 96 P07	Sense	-0.497	Detected	0.497	Detected	0.000	1.337	Detected	3.236	Detected	2.287	0.166	A 96 P07	ADH2	NM 0012	Solanum	[illegible]																[illegible]	[illegible]	[illegible]	[illegible]	[illegible]	[illegible]
A 96 P07	Sense	0.547	Detected	-0.547	Detected	0.000	2.227	Detected	0.943	Detected	1.585	0.201	A 96 P07	LOC5442	NM 0012	Solanum	[illegible]																[illegible]	[illegible]	[illegible]	[illegible]	[illegible]	[illegible]
A 96 P07	Sense	-0.247	Detected	0.247	Detected	0.000	1.291	Detected	1.633	Detected	1.462	0.040	A 96 P07	TA36339	TA36339	Unknown	[illegible]																[illegible]	[illegible]	[illegible]	[illegible]	[illegible]	[illegible]
A 96 P08	Sense	-0.146	Detected	0.146	Detected	0.000	1.388	Detected	1.147	Detected	1.267	0.022	A 96 P08	EG55367	EG55367	to00628 T	[illegible]																[illegible]	[illegible]	[illegible]	[illegible]	[illegible]	[illegible]
A 96 P08	Sense	-0.146	Comprom	0.146	Comprom	0.000	2.994	Detected	2.525	Detected	2.759	0.010	A 96 P08	BG12915	BG12915	EST4748	[illegible]																[illegible]	[illegible]	[illegible]	[illegible]	[illegible]	[illegible]
A 96 P08	Sense	0.207	Detected	-0.207	Detected	0.000	1.477	Detected	0.843	Detected	1.160	0.092	A 96 P08	TA35649	TA35649	Unknown	[illegible]																[illegible]	[illegible]	[illegible]	[illegible]	[illegible]	[illegible]
[illegible]																																						

GT	Sense	Sense	-0.648	Comprom	0.648	Detected	0.000	1.571	Detected	1.207	Detected	1.389	0.175	[illegible]
GT	Sense	Sense	-0.217	Detected	0.217	Detected	0.000	1.169	Detected	1.048	Detected	1.108	0.039	[illegible]
GT	Sense	Sense	0.026	Detected	-0.026	Detected	0.000	0.997	Detected	1.011	Detected	1.004	0.001	[illegible]
GT	Sense	Sense	-0.685	Comprom	0.685	Comprom	0.000	1.499	Detected	1.492	Detected	1.495	0.161	[illegible]
GT	Sense	Sense	-0.225	Detected	0.225	Detected	0.000	1.349	Detected	1.082	Detected	1.216	0.043	[illegible]
GT	Sense	Sense	-0.216	Detected	0.216	Detected	0.000	1.539	Detected	1.233	Detected	1.386	0.035	[illegible]
GT	Sense	Sense	-0.305	Detected	0.305	Detected	0.000	1.396	Detected	0.866	Detected	1.131	0.108	[illegible]
GT	Sense	Sense	-0.074	Detected	0.074	Detected	0.000	1.295	Detected	0.942	Detected	1.119	0.028	[illegible]
GT	Sense	Sense	-0.316	Detected	0.316	Detected	0.000	2.074	Detected	1.215	Detected	1.644	0.091	[illegible]
GT	Sense	Sense	-0.349	Detected	0.349	Detected	0.000	3.298	Detected	2.245	Detected	2.772	0.048	[illegible]
GT	Sense	Sense	0.348	Detected	-0.348	Detected	0.000	3.009	Detected	2.115	Detected	2.562	0.046	[illegible]
GT	Sense	Sense	0.025	Comprom	-0.025	Comprom	0.000	1.070	Detected	1.180	Detected	1.125	0.003	[illegible]
GT	Sense	Sense	0.707	Comprom	-0.707	Comprom	0.000	3.152	Detected	1.788	Detected	2.470	0.129	[illegible]
GT	Sense	Sense	0.539	Comprom	-0.539	Comprom	0.000	1.607	Detected	1.980	Detected	1.793	0.088	[illegible]
GT	Sense	Sense	0.240	Detected	-0.240	Detected	0.000	1.463	Detected	1.509	Detected	1.486	0.025	[illegible]
GT	Sense	Sense	-0.851	Comprom	0.851	Detected	0.000	1.847	Detected	2.189	Detected	2.018	0.146	[illegible]
GT	Sense	Sense	-0.045	Detected	0.045	Detected	0.000	1.118	Detected	0.971	Detected	1.045	0.007	[illegible]
GT	Sense	Sense	0.283	Comprom	-0.283	Comprom	0.000	1.713	Detected	1.562	Detected	1.637	0.031	[illegible]
GT	Sense	Sense	-0.221	Comprom	0.221	Comprom	0.000	2.641	Detected	2.218	Detected	2.430	0.015	[illegible]
GT	Sense	Sense	-0.580	Comprom	0.580	Detected	0.000	1.272	Detected	1.032	Detected	1.152	0.191	[illegible]
GT	Sense	Sense	-0.860	Comprom	0.860	Comprom	0.000	5.541	Detected	5.337	Detected	5.439	0.024	[illegible]
GT	Sense	Sense	0.186	Detected	-0.186	Detected	0.000	1.470	Detected	1.287	Detected	1.378	0.022	[illegible]
GT	Sense	Sense	0.777	Comprom	-0.777	Comprom	0.000	1.674	Detected	1.555	Detected	1.614	0.174	[illegible]
GT	Sense	Sense	0.135	Detected	-0.135	Detected	0.000	2.002	Detected	1.737	Detected	1.870	0.010	[illegible]
GT	Sense	Sense	0.314	Detected	-0.314	Detected	0.000	2.776	Detected	1.852	Detected	2.314	0.054	[illegible]
GT	Sense	Sense	0.584	Detected	-0.584	Comprom	0.000	1.758	Detected	1.916	Detected	1.837	0.089	[illegible]
GT	Sense	Sense	-0.135	Comprom	0.135	Comprom	0.000	1.690	Detected	1.177	Detected	1.434	0.038	[illegible]
GT	Sense	Sense	-0.318	Comprom	0.318	Comprom	0.000	1.247	Detected	1.003	Detected	1.125	0.081	[illegible]
GT	Sense	Sense	-0.358	Detected	0.358	Detected	0.000	2.094	Detected	1.665	Detected	1.879	0.046	[illegible]
GT	Sense	Sense	-0.032	Detected	0.032	Detected	0.000	1.600	Detected	1.032	Detected	1.316	0.044	[illegible]
GT	Sense	Sense	0.282	Detected	-0.282	Detected	0.000	2.325	Detected	1.367	Detected	1.846	0.080	[illegible]
GT	Sense	Sense	-0.208	Detected	0.208	Detected	0.000	1.665	Detected	1.360	Detected	1.513	0.028	[illegible]
GT	Sense	Sense	0.072	Detected	-0.072	Detected	0.000	0.894	Detected	1.215	Detected	1.055	0.027	[illegible]
GT	Sense	Sense	-0.200	Detected	0.200	Detected	0.000	1.427	Detected	1.123	Detected	1.275	0.037	[illegible]
GT	Sense	Sense	-0.224	Detected	0.224	Detected	0.000	1.356	Detected	0.914	Detected	1.135	0.069	[illegible]
GT	Sense	Sense	0.184	Comprom	-0.184	Comprom	0.000	1.920	Detected	3.320	Detected	2.620	0.069	[illegible]
GT	Sense	Sense	-0.056	Detected	0.056	Detected	0.000	3.676	Detected	2.934	Detected	3.305	0.013	[illegible]
GT	Sense	Sense	-0.562	Comprom	0.562	Detected	0.000	1.139	Detected	1.033	Detected	1.086	0.194	[illegible]
GT	Sense	Sense	0.067	Detected	-0.067	Detected	0.000	1.195	Detected	1.316	Detected	1.255	0.005	[illegible]
GT	Sense	Sense	-0.249	Detected	0.249	Detected	0.000	1.143	Detected	1.051	Detected	1.097	0.049	[illegible]
GT	Sense	Sense	0.028	Detected	-0.028	Detected	0.000	3.827	Detected	1.922	Detected	2.874	0.095	[illegible]
GT	Sense	Sense	0.190	Detected	-0.190	Detected	0.000	1.262	Detected	0.923	Detected	1.092	0.050	[illegible]
GT	Sense	Sense	1.031	Detected	-1.031	Comprom	0.000	2.218	Detected	1.720	Detected	1.969	0.205	[illegible]
GT	Sense	Sense	0.060	Detected	-0.060	Detected	0.000	1.785	Detected	1.255	Detected	1.520	0.031	[illegible]
GT	Sense	Sense	0.173	Detected	-0.173	Detected	0.000	1.692	Detected	1.303	Detected	1.497	0.029	[illegible]
GT	Sense	Sense	-0.200	Detected	0.200	Detected	0.000	1.144	Detected	1.058	Detected	1.101	0.033	[illegible]
GT	Sense	Sense	-0.717	Comprom	0.717	Detected	0.000	1.614	Detected	1.271	Detected	1.442	0.189	[illegible]
GT	Sense	Sense	-0.397	Detected	0.397	Detected	0.000	1.541	Detected	1.515	Detected	1.528	0.061	[illegible]
GT	Sense	Sense	-0.103	Comprom	0.103	Comprom	0.000	1.567	Detected	1.964	Detected	1.765	0.016	[illegible]
GT	Sense	Sense	0.050	Detected	-0.050	Detected	0.000	1.687	Detected	1.049	Detected	1.368	0.051	[illegible]
GT	Sense	Sense	0.057	Detected	-0.057	Detected	0.000	1.855	Detected	1.136	Detected	1.496	0.055	[illegible]
GT	Sense	Sense	-0.192	Detected	0.192	Detected	0.000	1.217	Detected	0.959	Detected	1.088	0.042	[illegible]
GT	Sense	Sense	-0.309	Comprom	0.309	Comprom	0.000	2.952	Detected	3.534	Detected	3.243	0.017	[illegible]
GT	Sense	Sense	-0.125	Comprom	0.125	Comprom	0.000	1.651	Detected	1.271	Detected	1.461	0.023	[illegible]
GT	Sense	Sense	-0.564	Detected	0.564	Detected	0.000	1.476	Detected	1.125	Detected	1.301	0.159	[illegible]
GT	Sense	Sense	0.004	Comprom	-0.004	Comprom	0.000	5.012	Detected	5.238	Detected	5.125	0.000	[illegible]
GT	Sense	Sense	0.189	Detected	-0.189	Detected	0.000	2.956	Detected	1.989	Detected	2.472	0.041	[illegible]
GT	Sense	Sense	-0.677	Detected	0.677	Detected	0.000	2.475	Detected	2.067	Detected	2.271	0.085	[illegible]
GT	Sense	Sense	-0.243	Comprom	0.243	Comprom	0.000	2.372	Detected	2.099	Detected	2.236	0.015	[illegible]
GT	Sense	Sense	0.096	Detected	-0.096	Detected	0.000	1.230	Detected	0.929	Detected	1.079	0.026	[illegible]
GT	Sense	Sense	-1.253	Comprom	1.253	Detected	0.000	2.639	Detected	1.243	Detected	1.941	0.309	[illegible]
GT	Sense	Sense	0.160	Detected	-0.160	Comprom	0.000	1.331	Detected	1.236	Detected	1.284	0.016	[illegible]
GT	Sense	Sense	0.044	Detected	-0.044	Detected	0.000	0.984	Detected	1.157	Detected	1.071	0.008	[illegible]
GT	Sense	Sense	0.031	Detected	-0.031	Detected	0.000	2.216	Detected	1.732	Detected	1.974	0.015	[illegible]
GT	Sense	Sense	-0.481	Detected	0.481	Detected	0.000	3.094	Detected	2.292	Detected	2.693	0.050	[illegible]
GT	Sense	Sense	-0.561	Comprom	0.561	Comprom	0.000	1.583	Detected	2.179	Detected	1.881	0.098	[illegible]
GT	Sense	Sense	-0.498	Detected	0.498	Detected	0.000	1.499	Detected	1.153	Detected	1.326	0.128	[illegible]
GT	Sense	Sense	-0.334	Detected	0.334	Detected	0.000	1.177	Detected	0.966	Detected	1.071	0.092	[illegible]
GT	Sense	Sense	0.467	Detected	-0.467	Comprom	0.000	1.034	Detected	1.708	Detected	1.371	0.140	[illegible]
GT	Sense	Sense	0.006	Detected	-0.006	Detected	0.000	4.178	Detected	3.089	Detected	3.634	0.022	[illegible]
GT	Sense	Sense	0.537	Comprom	-0.537	Comprom	0.000	2.818	Detected	1.659	Detected	2.239	0.105	[illegible]
GT	Sense	Sense	-0.153	Detected	0.153	Detected	0.000	3.195	Detected	1.685	Detected	2.440	0.087	[illegible]
GT	Sense	Sense	-0.022	Detected	0.022	Detected	0.000	2.169	Detected	2.097	Detected	2.133	0.000	[illegible]
GT	Sense	Sense	-0.124	Detected	0.124	Detected	0.000	2.134	Detected	1.459	Detected	1.796	0.038	[illegible]
GT	Sense	Sense	-0.340	Detected	0.340	Detected	0.000	1.983	Detected	1.629	Detected	1.806	0.042	[illegible]
GT	Sense	Sense	0.192	Detected	-0.192	Detected	0.000	0.926	Detected	1.267	Detected	1.096	0.051	[illegible]
GT	Sense	Sense	-0.029	Detected	0.029	Detected	0.000	1.246	Detected	0.881	Detected	1.063	0.029	[illegible]
GT	Sense	Sense	0.171	Detected	-0.171	Detected	0.000	1.810	Detected	1.108	Detected	1.459	0.065	[illegible]
GT	Sense	Sense	-0.185	Detected	0.185	Detected	0.000	1.404	Detected	1.076	Detected	1.240	0.038	[illegible]
GT	Sense	Sense	-0.333	Detected	0.333	Detected	0.000	2.169	Detected	1.763	Detected	1.966	0.037	[illegible]
GT	Sense	Sense	0.025	Detected	-0.025	Detected	0.000	2.675	Detected	3.083	Detected	2.879	0.005	[illegible]
GT	Sense	Sense	0.126	Detected	-0.126	Detected	0.000	1.555	Detected	0.916	Detected	1.235	0.069	[illegible]
GT	Sense	Sense	-0.080	Detected	0.080	Detected	0.000	1.651	Detected	0.948	Detected	1.300	0.069	[illegible]
GT	Sense	Sense	-0.940	Comprom	0.940	Comprom	0.000	1.652	Detected	1.495	Detected	1.573	0.237	[illegible]
GT	Sense	Sense	-0.048	Detected	0.048	Detected	0.000	1.175	Detected	1.022	Detected	1.098	0.007	[illegible]
GT	Sense	Sense	0.081	Detected	-0.081	Detected	0.000	1.138	Detected	1.106	Detected	1.122	0.005	[illegible]
GT	Sense	Sense	-0.066	Detected	0.066	Detected	0.000	1.146	Detected	1.173	Detected	1.159	0.003	[illegible]
GT	Sense	Sense	0.099	Detected	-0.099	Detected	0.000	2.063	Detected	1.310	Detected	1.687	0.049	[illegible]
GT	Sense	Sense	0.255	Comprom	-0.255	Comprom	0.000	1.726	Detected	1.685	Detected	1.705	0.022	[illegible]
GT	Sense	Sense	0.230	Detected	-0.230	Detected	0.000	1.518	Detected	1.013	Detected	1.265	0.066	[illegible]
GT	Sense	Sense	0.245	Comprom	-0.245	Comprom	0.000	2.890	Detected	2.359	Detected	2.624	0.018	[illegible]
GT	Sense	Sense	-0.259	Detected	0.259	Detected	0.000	1.210	Detected	0.843	Detected	1.027	0.084	[illegible]
GT	Sense	Sense	0.163	Detected	-0.163	Detected	0.000	2.896	Detected	3.043	Detected	2.970	0.004	[illegible]
GT	Sense	Sense	-0.008	Detected	0.008	Detected	0.000	1.248	Detected	1.279	Detected	1.263	0.000	[illegible]
GT	Sense	Sense	-0.636	Comprom	0.636	Detected	0.000	1.129	Detected	1.603	Detected	1.366	0.182	[illegible]
GT	Sense	Sense	0.157	Detected	-0.157	Detected	0.000	1.704	Detected	1.424	Detected	1.564	0.018	[illegible]
GT	Sense	Sense	-0.120	Detected	0.120	Detected	0.000	1.082	Detected	0.963	Detected	1.023	0.017	[illegible]
GT	Sense	Sense	-0.017	Detected	0.017	Detected	0.000	1.860	Detected	0.969	Detected	1.415	0.087	[illegible]
GT	Sense	Sense	0.259	Detected	-0.259	Detected	0.000	1.704	Detected	0.900	Detected	1.302	0.113	[illegible]
GT	Sense	Sense	-0.131	Detected	0.131	Detected	0.000	1.039	Detected	1.031	Detected	1.035	0.016	[illegible]
GT	Sense	Sense	-0.098	Detected	0.098	Detected	0.000	1.010	Detected	1.030	Detected	1.020	0.009	[illegible]
GT	Sense	Sense	0.662	Detected	-0.662	Detected	0.000	2.756	Detected	1.421	Detected	2.088	0.156	[illegible]
GT	Sense	Sense	0.060	Detected	-0.060	Detected	0.000	1.448	Detected	0.994	Detected	1.221	0.035	[illegible]
GT	Sense	Sense	-0.060	Detected	0.060	Detected	0.000	1.095	Detected	0.950	Detected	1.022	0.008	[illegible]
GT	Sense	Sense	0.195	Detected	-0.195	Detected	0.000	1.272	Detected	0.805	Detected	1.039	0.076	[illegible]
GT	Sense	Sense	0.550	Detected	-0.550	Detected	0.000	0.919	Detected	2.380	Detected	1.650	0.213	[illegible]
GT	Sense	Sense	-0.432	Comprom	0.432	Comprom	0.000	1.925	Detected	1.623	Detected	1.774	0.060	[illegible]
GT	Sense	Sense	-0.002	Detected	0.002	Detected	0.000	1.179	Detected	0.946	Detected	1.063	0.012	[illegible]
GT	Sense	Sense	-0.143	Detected	0.143	Detected	0.000	2.437	Detected	1.620	Detected	2.029	0.043	[illegible]
GT	Sense	Sense	-0.252	Detected	0.252	Detected	0.000	1.179	Detected	1.189	Detected	1.184	0.042	[illegible]
GT	Sense	Sense	-0.324	Detected	0.324	Detected	0.000	1.655	Detected	1.051	Detected	1.353	0.093	[illegible]
GT	Sense	Sense	-0.240	Detected	0.240	Detected	0.000	0.933	Detected	1.305	Detected	1.119	0.066	[illegible]
GT	Sense	Sense	0.329	Detected	-0.329	Detected	0.000	3.377	Detected	2.171	Detected	2.774	0.056	[illegible]
GT	Sense	Sense	-0.462	Detected	0.462	Detected	0.000	1.011	Detected	1.028	Detected	1.019	0.158	[illegible]
GT	Sense	Sense	-0.148	Detected	0.148	Detected	0.000	1.808	Detected	1.174	Detected	1.491	0.051	[illegible]
GT	Sense	Sense	-0.317	Comprom	0.317	Comprom	0.000	1.989	Detected	1.742	Detected	1.865	0.032	[illegible]
GT	Sense	Sense	0.095	Comprom	-0.095	Comprom	0.000	1.631	Detected	0.949	Detected	1.290	0.068	[illegible]
GT	Sense	Sense	-0.190	Detected	0.190	Detected	0.000	1.290	Detected	1.002	Detected	1.146	0.041	[illegible]
GT	Sense	Sense	0.090	Detected	-0.090	Detected	0.000	2.646	Detected	1.793	Detected	2.219	0.036	[illegible]
GT	Sense	Sense	0.104	Detected	-0.104	Detected	0.000	1.986	Detected	1.390	Detected	1.688	0.033	[illegible]
GT	Sense	Sense	0.134	Detected	-0.134	Detected	0.000	1.664	Detected	1.571	Detected	1.617	0.008	[illegible]
GT	Sense	Sense	0.015	Detected	-0.015	Detected	0.000	1.346	Detected	1.252	Detected	1.299	0.001	[illegible]
GT	Sense	Sense	-0.211	Detected	0.211	Detected	0.000	1.405	Detected	1.170	Detected	1.287	0.033	[illegible]
GT	Sense	Sense	-0.146	Detected	0.146	Detected	0.000	2.347	Detected	1.795	Detected	2.071	0.022	[illegible]
GT	Sense	Sense	-0.150	Detected	0.150	Detected	0.000	1.606	Detected	1.013	Detected	1.309	0.059	[illegible]
GT	Sense	Sense	-0.188	Detected	0.188	Detected	0.000	1.086	Detected	1.148	Detected	1.117	0.028	[illegible]
GT	Sense	Sense	-0.043	Detected	0.043	Detected	0.000	2.912	Detected	2.016	Detected	2.464	0.032	[illegible]
GT	Sense	Sense	0.183	Detected	-0.183	Detected	0.000	3.131	Detected	1.936	Detected	2.533	0.056	[illegible]
GT	Sense	Sense	-0.276	Detected	0.276	Detected	0.000	2.259	Detected	1.720	Detected	1.990	0.036	[illegible]
GT	Sense	Sense	0.050	Detected	-0.050	Comprom	0.000	2.873	Detected	2.025	Detected	2.449	0.029	[illegible]
GT	Sense	Sense	0.206	Comprom	-0.206	Comprom	0.000	1.521	Detected	2.520	Detected	2.021	0.065	[illegible]
GT	Sense	Sense	-0.104	Detected	0.104	Detected	0.000	3.642	Detected	2.039	Detected	2.841	0.072	[illegible]
GT	Sense	Sense	0.865	Detected	-0.865	Detected	0.000	3.366	Detected	2.079	Detected	2.722	0.128	[illegible]
GT	Sense	Sense	0.111	Detected	-0.111	Detected	0.000	1.634	Detected	1.375	Detected	1.505	0.013	[illegible]
GT	Sense	Sense	-0.064	Detected	0.064	Detected	0.000	1.632	Detected	0.997	Detected	1.315	0.056	[illegible]
GT	Sense	Sense	0.267	Detected	-0.267	Detected	0.000	1.235	Detected	0.817	Detected	1.026	0.094	[illegible]
GT	Sense	Sense	-0.072	Detected	0.072	Detected	0.000	2.922	Detected	1.496	Detected	2.209	0.091	[illegible]
GT	Sense	Sense	0.361	Detected	-0.361	Detected	0.000	1.673	Detected	0.811	Detected	1.242	0.158	[illegible]
GT	Sense	Sense	0.553	Detected	-0.553	Detected	0.000	5.371	Detected	3.006	Detected	4.188	0.085	[illegible]
GT	Sense	Sense	-0.475	Detected	0.475	Detected	0.000	1.541	Detected	1.362	Detected	1.451	0.095	[illegible]
GT	Sense	Sense	-0.274	Detected	0.274	Detected	0.000	1.353	Detected	0.842	Detected	1.098	0.099	[illegible]
GT	Sense	Sense	-0.165	Detected	0.165	Detected	0.000	1.443	Detected	1.338	Detected	1.390	0.015	[illegible]
GT	Sense	Sense	-0.447	Detected	0.447	Detected	0.000	1.093	Detected	1.060	Detected	1.077	0.138	[illegible]
GT	Sense	Sense	0.112	Detected	-0.112	Detected	0.000	1.200	Detected	1.538	Detected	1.369	0.021	[illegible]
GT	Sense	Sense	-0.154	Detected	0.154	Detected	0.000	2.012	Detected	1.814	Detected	1.913	0.009	[illegible]
GT	Sense	Sense	-0.359	Comprom	0.359	Comprom	0.000	2.136	Detected	2.125	Detected	2.131	0.027	[illegible]
GT	Sense	Sense	-0.264	Comprom	0.264	Detected	0.000	1.574	Detected	1.071	Detected	1.322	0.068	[illegible]
GT	Sense	Sense	-0.305	Detected	0.305	Detected	0.000	1.579	Detected	1.147	Detected	1.363	0.067	[illegible]
GT	Sense	Sense	-0.596	Comprom	0.596	Comprom	0.000	1.546	Detected	1.188	Detected	1.367	0.159	[illegible]
GT	Sense	Sense	0.031	Detected	-0.031	Detected	0.000	1.159	Detected	1.592	Detected	1.375	0.024	[illegible]
GT	Sense	Sense	0.020	Comprom	-0.020	Comprom	0.000	2.836	Detected	1.722	Detected	2.279	0.055	[illegible]
GT	Sense	Sense	-0.101	Detected	0.101	Detected	0.000	1.277	Detected	1.058	Detected	1.168	0.016	[illegible]
GT	Sense	Sense	-0.211	Comprom	0.211	Comprom	0.000	2.344	Detected	1.675	Detected	2.010	0.037	[illegible]
GT	Sense	Sense	-0.247	Detected	0.247	Detected	0.000	1.294	Detected	1.297	Detected	1.295	0.035	[illegible]
GT	Sense	Sense	-0.207	Detected	0.207	Detected	0.000	1.547	Detected	1.197	Detected	1.372	0.037	[illegible]
GT	Sense	Sense	0.164	Comprom	-0.164	Comprom	0.000	1.540	Detected	4.138	Detected	2.839	0.162	[illegible]
GT	Sense	Sense	0.394	Detected	-0.394	Detected	0.000	2.205	Detected	1.715	Detected	1.960	0.052	[illegible]
GT	Sense	Sense	-0.339	Detected	0.339	Detected	0.000	2.568	Detected	2.055	Detected	2.311	0.032	[illegible]
GT	Sense	Sense	-0.117	Comprom	0.117	Comprom	0.000	2.459	Detected	2.195	Detected	2.327	0.006	[illegible]
GT	Sense	Sense	-0.109	Comprom	0.109	Comprom	0.000	1.348	Detected	0.915	Detected	1.132	0.043	[illegible]
GT	Sense	Sense	0.198	Detected	-0.198	Detected	0.000	1.184	Detected	1.401	Detected	1.293	0.029	[illegible]
GT	Sense	Sense	-0.626	Detected	0.626	Detected	0.000	1.006	Detected	2.363	Detected	1.685	0.210	[illegible]
GT	Sense	Sense	0.105	Comprom	-0.105	Comprom	0.000	1.473	Detected	2.436	Detected	1.955	0.058	[illegible]
GT	Sense	Sense	-0.286	Comprom	0.286	Comprom	0.000	1.583	Detected	1.826	Detected	1.704	0.032	[illegible]
GT	Sense	Sense	0.332	Detected	-0.332	Detected	0.000	1.752	Detected	1.142	Detected	1.447	0.085	[illegible]
GT	Sense	Sense	0.208	Comprom	-0.208	Comprom	0.000	2.584	Detected	2.009	Detected	2.296	0.023	[illegible]
GT	Sense	Sense	-0.202	Detected	0.202	Detected	0.000	1.261	Detected	0.880	Detected	1.070	0.061	[illegible]
GT	Sense	Sense	-0.204	Detected	0.204	Detected	0.000	1.355	Detected	1.029	Detected	1.192	0.045	[illegible]
GT	Sense	Sense	0.237	Detected	-0.237	Detected	0.000	1.310	Detected	0.894	Detected	1.102	0.073	[illegible]
GT	Sense	Sense	-0.433	Detected	0.433	Detected	0.000	2.837	Detected	1.532	Detected	2.185	0.108	[illegible]
GT	Sense	Sense	-0.009	Detected	0.009	Detected	0.000	1.129	Detected	1.163	Detected	1.146	0.000	[illegible]
GT	Sense	Sense	-0.759	Comprom	0.759	Detected	0.000	1.882	Detected	1.019	Detected	1.450	0.239	[illegible]
GT	Sense	Sense	0.007	Comprom	-0.007	Comprom	0.000	2.474	Detected	2.598	Detected	2.536	0.001	[illegible]
GT	Sense	Sense	-0.755	Detected	0.755	Detected	0.000	1.423	Detected	0.853	Detected	1.138	0.294	[illegible]
GT	Sense	Sense	-0.071	Detected	0.071	Detected	0.000	1.167	Detected	1.009	Detected	1.088	0.009	[illegible]
GT	Sense	Sense	0.143	Detected	-0.143	Comprom	0.000	1.933	Detected	1.334	Detected	1.633	0.039	[illegible]
GT	Sense	Sense	-0.184	Detected	0.184	Detected	0.000	1.002	Detected	1.195	Detected	1.098	0.034	[illegible]
GT	Sense	Sense	0.187	Detected	-0.187	Detected	0.000	1.013	Detected	1.078	Detected	1.045	0.031	[illegible]
GT	Sense	Sense	-0.132	Detected	0.132	Detected	0.000	1.621	Detected	1.052	Detected	1.336	0.051	[illegible]
GT	Sense	Sense	-0.068	Detected	0.068	Detected	0.000	1.414	Detected	2.218	Detected	1.816	0.047	[illegible]
GT	Sense	Sense	-0.609	Comprom	0.609	Comprom	0.000	1.453	Detected	1.596	Detected	1.524	0.131	[illegible]
GT	Sense	Sense	-0.010	Detected	0.010	Detected	0.000	1.945	Detected	0.856	Detected	1.400	0.124	[illegible]
GT	Sense	Sense	0.296	Detected	-0.296	Detected	0.000	1.312	Detected	1.190	Detected	1.251	0.054	[illegible]
GT	Sense	Sense	0.505	Detected	-0.505	Comprom	0.000	1.622	Detected	1.607	Detected	1.614	0.086	[illegible]
GT	Sense	Sense	-0.243	Detected	0.243	Detected	0.000	1.576	Detected	1.420	Detected	1.498	0.028	[illegible]
GT	Sense	Sense	-0.123	Detected	0.123	Detected	0.000	1.163	Detected	0.863	Detected	1.013	0.035	[illegible]
GT	Sense	Sense	-0.303	Comprom	0.303	Comprom	0.000	2.223	Detected	2.090	Detected	2.156	0.020	[illegible]
GT	Sense	Sense	-0.214	Detected	0.214	Detected	0.000	1.979	Detected	1.559	Detected	1.769	0.028	[illegible]
GT	Sense	Sense	-0.504	Comprom	0.504	Comprom	0.000	2.501	Detected	2.091	Detected	2.296	0.052	[illegible]
GT	Sense	Sense	0.444	Detected	-0.444	Detected	0.000	1.482	Detected	1.756	Detected	1.619	0.073	[illegible]
GT	Sense	Sense	0.431	Detected	-0.431	Detected	0.000	1.784	Detected	1.083	Detected	1.434	0.123	[illegible]
GT	Sense	Sense	-0.277	Detected	0.277	Detected	0.000	1.325	Detected	0.897	Detected	1.111	0.087	[illegible]
GT	Sense	Sense	-0.142	Detected	0.142	Detected	0.000	1.134	Detected	0.911	Detected	1.022	0.030	[illegible]
GT	Sense	Sense	-0.210	Detected	0.210	Detected	0.000	2.874	Detected	2.416	Detected	2.645	0.014	[illegible]
GT	Sense	Sense	-0.528	Detected	0.528	Detected	0.000	1.997	Detected	1.824	Detected	1.911	0.070	[illegible]
GT	Sense	Sense	-0.666	Comprom	0.666	Detected	0.000	2.095	Detected	0.846	Detected	1.471	0.249	[illegible]
GT	Sense	Sense	0.186	Detected	-0.186	Detected	0.000	1.895	Detected	1.065	Detected	1.480	0.083	[illegible]
GT	Sense	Sense	0.102	Detected	-0.102	Detected	0.000	2.688	Detected	1.007	Detected	1.848	0.161	[illegible]
GT	Sense	Sense	-0.040	Detected	0.040	Detected	0.000	2.567	Detected	1.395	Detected	1.981	0.078	[illegible]
GT	Sense	Sense	-0.305	Detected	0.305	Detected	0.000	1.187	Detected	0.834	Detected	1.011	0.103	[illegible]
GT	Sense	Sense	0.417	Comprom	-0.417	Comprom	0.000	2.039	Detected	1.924	Detected	1.981	0.042	[illegible]
GT	Sense	Sense	-0.105	Detected	0.105	Detected	0.000	1.146	Detected	0.918	Detected	1.032	0.022	[illegible]
GT	Sense	Sense	0.192	Detected	-0.192	Detected	0.000	1.471	Detected	1.151	Detected	1.311	0.035	[illegible]
GT	Sense	Sense	-0.208	Comprom	0.208	Comprom	0.000	3.373	Detected	2.403	Detected	2.888	0.032	[illegible]
GT	Sense	Sense	-0.117	Detected	0.117	Detected	0.000	1.165	Detected	0.836	Detected	1.000	0.038	[illegible]
GT	Sense	Sense	0.078	Detected	-0.078	Detected	0.000	1.460	Detected	1.009	Detected	1.235	0.035	[illegible]
GT	Sense	Sense	-0.207	Detected	0.207	Detected	0.000	1.499	Detected	0.927	Detected	1.213	0.075	[illegible]
GT	Sense	Sense	0.595	Detected	-0.595	Detected	0.000	2.189	Detected	0.905	Detected	1.547	0.219	[illegible]
GT	Sense	Sense	0.001	Detected	-0.001	Detected	0.000	1.508	Detected	0.905	Detected	1.207	0.057	[illegible]
GT	Sense	Sense	0.035	Detected	-0.035	Detected	0.000	1.735	Detected	1.587	Detected	1.661	0.002	[illegible]
GT	Sense	Sense	-0.638	Comprom	0.638	Comprom	0.000	0.843	Detected	1.215	Detected	1.029	0.261	[illegible]
GT	Sense	Sense	-0.206	Comprom	0.206	Comprom	0.000	1.934	Detected	1.864	Detected	1.899	0.012	[illegible]
GT	Sense	Sense	-0.116	Detected	0.116	Detected	0.000	2.601	Detected	1.134	Detected	1.868	0.128	[illegible]
GT	Sense	Sense	0.100	Detected	-0.100	Detected	0.000	2.418	Detected	2.129	Detected	2.273	0.006	[illegible]
GT	Sense	Sense	0.228	Detected	-0.228	Detected	0.000	0.830	Detected	1.283	Detected	1.057	0.081	[illegible]
GT	Sense	Sense	0.193	Detected	-0.193	Detected	0.000	1.400	Detected	1.477	Detected	1.438	0.018	[illegible]
GT	Sense	Sense	-0.489	Detected	0.489	Detected	0.000	1.968	Detected	1.523	Detected	1.746	0.083	[illegible]
GT	Sense	Sense	-0.079	Detected	0.079	Detected	0.000	2.399	Detected	1.161	Detected	1.780	0.104	[illegible]
GT	Sense	Sense	0.086	Detected	-0.086	Detected	0.000	1.941	Detected	1.482	Detected	1.711	0.020	[illegible]
GT	Sense	Sense	0.327	Detected	-0.327	Detected	0.000	1.733	Detected	0.878	Detected	1.305	0.136	[illegible]
GT	Sense	Sense	-0.059	Detected	0.059	Detected	0.000	1.815	Detected	1.555	Detected	1.685	0.007	[illegible]
GT	Sense	Sense	-0.122	Detected	0.122	Detected	0.000	1.219	Detected	0.827	Detected	1.023	0.048	[illegible]
GT	Sense	Sense	0.234	Detected	-0.234	Detected	0.000	1.320	Detected	1.022	Detected	1.171	0.052	[illegible]
GT	Sense	Sense	0.177	Detected	-0.177	Detected	0.000	1.270	Detected	1.024	Detected	1.147	0.034	[illegible]
GT	Sense	Sense	-0.134	Detected	0.134	Detected	0.000	1.421	Detected	1.048	Detected	1.234	0.033	[illegible]
GT	Sense	Sense	-0.048	Detected	0.048	Detected	0.000	1.146	Detected	0.958	Detected	1.052	0.010	[illegible]
GT	Sense	Sense	-0.449	Comprom	0.449	Detected	0.000	2.593	Detected	2.021	Detected	2.307	0.049	[illegible]
GT	Sense	Sense	0.189	Detected	-0.189	Detected	0.000	1.306	Detected	0.902	Detected	1.104	0.057	[illegible]
GT	Sense	Sense	0.274	Detected	-0.274	Detected	0.000	1.642	Detected	1.984	Detected	1.813	0.030	[illegible]
GT	Sense	Sense	-0.134	Detected	0.134	Detected	0.000	1.814	Detected	1.701	Detected	1.758	0.007	[illegible]
GT	Sense	Sense	0.129	Detected	-0.129	Detected	0.000	1.263	Detected	0.830	Detected	1.046	0.053	[illegible]
GT	Sense	Sense	-0.663	Detected	0.663	Detected	0.000	3.890	Detected	2.744	Detected	3.317	0.063	[illegible]
GT	Sense	Sense	0.086	Detected	-0.086	Detected	0.000	1.229	Detected	1.526	Detected	1.377	0.015	[illegible]
GT	Sense	Sense	0.007	Detected	-0.007	Detected	0.000	1.376	Detected	1.109	Detected	1.242	0.011	[illegible]
GT	Sense	Sense	-0.147	Comprom	0.147	Comprom	0.000	1.961	Detected	2.797	Detected	2.379	0.033	[illegible]
GT	Sense	Sense	-0.400	Detected	0.400	Detected	0.000	1.569	Detected	1.532	Detected	1.550	0.061	[illegible]
GT	Sense	Sense	-0.251	Detected	0.251	Detected	0.000	1.630	Detected	1.122	Detected	1.376	0.061	[illegible]
GT	Sense	Sense	-0.150	Comprom	0.150	Comprom	0.000	1.800	Detected	1.348	Detected	1.574	0.028	[illegible]
GT	Sense	Sense	-0.120	Detected	0.120	Detected	0.000	1.531	Detected	1.199	Detected	1.365	0.022	[illegible]
GT	Sense	Sense	-0.226	Detected	0.226	Detected	0.000	1.771	Detected	2.210	Detected	1.990	0.024	[illegible]
GT	Sense	Sense	0.135	Detected	-0.135	Detected	0.000	1.400	Detected	0.880	Detected	1.140	0.060	[illegible]
GT	Sense	Sense	-1.032	Comprom	1.032	Detected	0.000	5.856	Detected	4.446	Detected	5.151	0.054	[illegible]
GT	Sense	Sense	-0.110	Detected	0.110	Detected	0.000	1.189	Detected	1.582	Detected	1.385	0.025	[illegible]
GT	Sense	Sense	0.141	Detected	-0.141	Detected	0.000	1.567	Detected	0.812	Detected	1.189	0.098	[illegible]

GT	Sense	Sense	0.369	Detected	-0.369	Comprom	0.000	2.519	Detected	2.311	Detected	2.415	0.024	GT Sens	contig182	contig182	Unknown
GT	Sense	Sense	-0.693	Comprom	0.693	Comprom	0.000	1.982	Detected	2.101	Detected	2.041	0.099	GT Sens	contig182	contig182	Auxin Effl
GT	Sense	Sense	-0.163	Detected	0.163	Detected	0.000	1.153	Detected	0.891	Detected	1.022	0.039	GT Sens	contig182	contig182	Unknown
GT	Sense	Sense	-0.136	Detected	0.136	Detected	0.000	1.230	Detected	1.121	Detected	1.176	0.015	GT Sens	contig182	contig182	Charac
GT	Sense	Sense	0.619	Detected	-0.619	Detected	0.000	1.466	Detected	1.161	Detected	1.314	0.175	GT Sens	contig182	contig182	Cytochro
GT	Sense	Sense	-0.133	Detected	0.133	Detected	0.000	1.294	Detected	0.881	Detected	1.087	0.047	GT Sens	contig182	contig182	Catalase
GT	Sense	Sense	-0.248	Detected	0.248	Detected	0.000	2.037	Detected	1.516	Detected	1.776	0.039	GT Sens	contig183	contig183	Auxin-rep
GT	Sense	Sense	-0.262	Detected	0.262	Detected	0.000	1.719	Detected	1.312	Detected	1.516	0.045	GT Sens	contig183	contig183	Photosys
GT	Sense	Sense	-0.062	Detected	0.062	Detected	0.000	1.600	Detected	1.198	Detected	1.399	0.022	GT Sens	contig183	contig183	Unknown
GT	Sense	Sense	-0.165	Detected	0.165	Detected	0.000	1.366	Detected	1.308	Detected	1.337	0.015	GT Sens	contig183	contig183	Lipase (A
GT	Sense	Sense	-0.108	Detected	0.108	Detected	0.000	2.139	Detected	0.956	Detected	1.547	0.124	GT Sens	contig184	contig184	LRR recei
GT	Sense	Sense	0.429	Comprom	-0.429	Comprom	0.000	2.888	Detected	2.618	Detected	2.753	0.026	GT Sens	contig184	contig184	Abc trans
GT	Sense	Sense	0.090	Comprom	-0.090	Comprom	0.000	2.907	Detected	2.038	Detected	2.472	0.031	GT Sens	contig184	contig184	Aspartic
GT	Sense	Sense	-0.020	Comprom	0.020	Comprom	0.000	4.435	Detected	3.770	Detected	4.103	0.007	GT Sens	contig184	contig184	Leucine-r
GT	Sense	Sense	-0.186	Detected	0.186	Detected	0.000	1.623	Detected	1.129	Detected	1.376	0.047	GT Sens	contig185	contig185	Serine/thr
GT	Sense	Sense	-0.081	Comprom	0.081	Comprom	0.000	1.201	Detected	1.476	Detected	1.339	0.014	GT Sens	contig185	contig185	Gag-Pol
GT	Sense	Sense	0.258	Detected	-0.258	Detected	0.000	1.802	Detected	1.448	Detected	1.625	0.035	GT Sens	contig185	contig185	Glycosyl
GT	Sense	Sense	0.029	Detected	-0.029	Detected	0.000	1.175	Detected	1.342	Detected	1.258	0.005	GT Sens	contig185	contig185	Unknown
GT	Sense	Sense	-0.182	Detected	0.182	Detected	0.000	1.113	Detected	1.166	Detected	1.139	0.025	GT Sens	contig185	contig185	Nbs-lrr re
GT	Sense	Sense	-0.457	Detected	0.457	Detected	0.000	1.523	Detected	1.366	Detected	1.444	0.089	GT Sens	contig185	contig185	Amino ac
GT	Sense	Sense	1.126	Detected	-1.126	Comprom	0.000	3.278	Detected	2.540	Detected	2.909	0.133	GT Sens	contig185	contig185	GDSL est
GT	Sense	Sense	-0.659	Comprom	0.659	Detected	0.000	1.093	Detected	1.283	Detected	1.188	0.216	GT Sens	contig185	contig185	Serine/thr
GT	Sense	Sense	-0.294	Detected	0.294	Detected	0.000	2.211	Detected	1.371	Detected	1.791	0.073	GT Sens	contig186	contig186	Light-dep
GT	Sense	Sense	-0.345	Detected	0.345	Detected	0.000	1.372	Detected	1.210	Detected	1.291	0.068	GT Sens	contig186	contig186	Gibberell
[illegible]																	

GT	Sense	Sense	0.204	Detected	-0.204	Detected	0.000	1.152	Detected	0.988	Detected	1.070	0.040	GT Sens [illegible]
GT	Sense	Sense	0.259	Comprom	-0.259	Comprom	0.000	2.499	Detected	2.220	Detected	2.360	0.015	GT Sens [illegible]
GT	Sense	Sense	0.193	Detected	-0.193	Detected	0.000	1.359	Detected	0.815	Detected	1.087	0.082	GT Sens [illegible]
GT	Sense	Sense	-0.400	Detected	0.400	Detected	0.000	1.120	Detected	0.896	Detected	1.008	0.136	GT Sens [illegible]
GT	Sense	Sense	-0.150	Detected	0.150	Detected	0.000	1.193	Detected	0.833	Detected	1.013	0.049	GT Sens [illegible]
GT	Sense	Sense	-0.157	Comprom	0.157	Comprom	0.000	1.636	Detected	1.689	Detected	1.663	0.009	GT Sens [illegible]
GT	Sense	Sense	-0.114	Detected	0.114	Detected	0.000	1.330	Detected	0.804	Detected	1.067	0.065	GT Sens [illegible]
GT	Sense	Sense	-0.011	Detected	0.011	Detected	0.000	1.619	Detected	1.188	Detected	1.403	0.023	GT Sens [illegible]
GT	Sense	Sense	-0.189	Comprom	0.189	Comprom	0.000	1.242	Detected	0.867	Detected	1.055	0.058	GT Sens [illegible]
GT	Sense	Sense	-0.181	Detected	0.181	Detected	0.000	1.514	Detected	0.847	Detected	1.180	0.090	GT Sens [illegible]
GT	Sense	Sense	0.866	Detected	-0.866	Comprom	0.000	2.199	Detected	1.001	Detected	1.600	0.268	GT Sens [illegible]
GT	Sense	Sense	-0.202	Detected	0.202	Detected	0.000	2.175	Detected	1.385	Detected	1.780	0.057	GT Sens [illegible]
GT	Sense	Sense	-0.141	Detected	0.141	Detected	0.000	2.219	Detected	1.048	Detected	1.633	0.113	GT Sens [illegible]
GT	Sense	Sense	-0.765	Comprom	0.765	Comprom	0.000	2.436	Detected	1.985	Detected	2.210	0.109	GT Sens [illegible]
GT	Sense	Sense	-0.151	Detected	0.151	Detected	0.000	1.619	Detected	1.651	Detected	1.635	0.009	GT Sens [illegible]
GT	Sense	Sense	0.854	Detected	-0.854	Detected	0.000	3.673	Detected	2.520	Detected	3.096	0.095	GT Sens [illegible]
GT	Sense	Sense	-0.111	Comprom	0.111	Comprom	0.000	1.304	Detected	1.418	Detected	1.361	0.008	GT Sens [illegible]
GT	Sense	Sense	-0.239	Detected	0.239	Detected	0.000	2.386	Detected	1.294	Detected	1.840	0.091	GT Sens [illegible]
GT	Sense	Sense	-0.107	Comprom	0.107	Comprom	0.000	1.377	Detected	1.411	Detected	1.394	0.006	GT Sens [illegible]
GT	Sense	Sense	-0.160	Detected	0.160	Detected	0.000	1.034	Detected	1.228	Detected	1.131	0.026	GT Sens [illegible]
[illegible]														

GT	Sense	Sense	-0.212	Detected	0.212	Detected	0.000	1.413	Detected	1.075	Detected	1.244	0.044	GT Sens	contig289
GT	Sense	Sense	-0.745	Comprom	0.745	Comprom	0.000	3.288	Detected	2.983	Detected	3.136	0.054	GT Sens	contig290
GT	Sense	Sense	0.338	Detected	-0.338	Detected	0.000	2.087	Detected	1.956	Detected	2.022	0.028	GT Sens	contig290
GT	Sense	Sense	0.741	Detected	-0.741	Comprom	0.000	2.371	Detected	2.365	Detected	2.368	0.086	GT Sens	contig290
GT	Sense	Sense	0.476	Detected	-0.476	Comprom	0.000	1.479	Detected	2.296	Detected	1.888	0.095	GT Sens	contig290
GT	Sense	Sense	0.927	Detected	-0.927	Detected	0.000	0.812	Detected	1.625	Detected	1.218	0.352	GT Sens	contig290
GT	Sense	Sense	-0.110	Detected	0.110	Detected	0.000	1.400	Detected	0.963	Detected	1.181	0.040	GT Sens	contig290
GT	Sense	Sense	0.238	Detected	-0.238	Detected	0.000	1.908	Detected	2.221	Detected	2.065	0.019	GT Sens	contig290
GT	Sense	Sense	-0.127	Detected	0.127	Detected	0.000	1.328	Detected	0.882	Detected	1.105	0.050	GT Sens	contig290
GT	Sense	Sense	-0.089	Detected	0.089	Detected	0.000	1.633	Detected	1.126	Detected	1.380	0.036	GT Sens	contig290
GT	Sense	Sense	0.387	Detected	-0.387	Detected	0.000	1.464	Detected	1.070	Detected	1.267	0.100	GT Sens	contig291
GT	Sense	Sense	-0.152	Detected	0.152	Detected	0.000	1.181	Detected	0.832	Detected	1.007	0.049	GT Sens	contig291
GT	Sense	Sense	-0.404	Detected	0.404	Detected	0.000	2.891	Detected	2.235	Detected	2.563	0.039	GT Sens	contig291
GT	Sense	Sense	0.066	Detected	-0.066	Detected	0.000	1.313	Detected	1.428	Detected	1.370	0.004	GT Sens	contig292
GT	Sense	Sense	-0.336	Detected	0.336	Detected	0.000	1.754	Detected	0.992	Detected	1.373	0.114	GT Sens	contig292
GT	Sense	Sense	-0.180	Detected	0.180	Detected	0.000	1.480	Detected	0.847	Detected	1.163	0.086	GT Sens	contig292
GT	Sense	Sense	0.055	Comprom	-0.055	Comprom	0.000	0.961	Detected	1.362	Detected	1.162	0.030	GT Sens	contig292
GT	Sense	Sense	-0.412	Detected	0.412	Detected	0.000	2.556	Detected	1.518	Detected	2.037	0.092	GT Sens	contig292
GT	Sense	Sense	-0.109	Detected	0.109	Detected	0.000	1.247	Detected	1.091	Detected	1.169	0.013	GT Sens	contig292
GT	Sense	Sense	-0.223	Detected	0.223	Detected	0.000	1.157	Detected	1.104	Detected	1.130	0.037	GT Sens	contig292

GT	Sense	Sense	-0.245	Detected	0.245	Detected	0.000	2.019	Detected	1.520	Detected	1.769	0.037	GT Sens	contig335	contig335	Rhodane
GT	Sense	Sense	0.029	Comprom	-0.029	Comprom	0.000	1.746	Detected	2.066	Detected	1.906	0.007	GT Sens	contig335	contig335	Unannota
GT	Sense	Sense	-0.355	Comprom	0.355	Comprom	0.000	1.517	Detected	2.094	Detected	1.805	0.059	GT Sens	contig335	contig335	Unannota
GT	Sense	Sense	0.255	Detected	-0.255	Comprom	0.000	2.063	Detected	1.310	Detected	1.687	0.066	GT Sens	contig335	contig335	Unannota
GT	Sense	Sense	0.001	Detected	-0.001	Detected	0.000	1.255	Detected	1.441	Detected	1.348	0.005	GT Sens	contig335	contig335	Unannota
GT	Sense	Sense	-0.786	Comprom	0.786	Comprom	0.000	1.350	Detected	1.580	Detected	1.465	0.206	GT Sens	contig335	contig335	Unannota
GT	Sense	Sense	-0.174	Comprom	0.174	Detected	0.000	1.072	Detected	1.084	Detected	1.078	0.025	GT Sens	contig335	contig335	Unannota
GT	Sense	Sense	0.066	Detected	-0.066	Detected	0.000	1.345	Detected	1.021	Detected	1.183	0.021	GT Sens	contig335	contig335	Unannota
GT	Sense	Sense	0.374	Comprom	-0.374	Comprom	0.000	1.753	Detected	1.413	Detected	1.583	0.061	GT Sens	contig335	contig335	Unknown
GT	Sense	Sense	-0.012	Detected	0.012	Detected	0.000	1.263	Detected	0.921	Detected	1.092	0.024	GT Sens	contig335	contig335	Unannota
GT	Sense	Sense	-0.414	Comprom	0.414	Detected	0.000	0.926	Detected	1.089	Detected	1.007	0.140	GT Sens	contig335	contig335	Unannota
GT	Sense	Sense	0.383	Detected	-0.383	Detected	0.000	1.224	Detected	1.445	Detected	1.335	0.079	GT Sens	contig336	contig336	Unannota
GT	Sense	Sense	-0.198	Comprom	0.198	Comprom	0.000	0.830	Detected	1.212	Detected	1.021	0.065	GT Sens	contig336	contig336	Unannota
GT	Sense	Sense	-0.203	Detected	0.203	Detected	0.000	0.939	Detected	1.249	Detected	1.094	0.051	GT Sens	contig336	contig336	Unannota
GT	Sense	Sense	0.291	Detected	-0.291	Detected	0.000	1.033	Detected	1.188	Detected	1.111	0.066	GT Sens	contig336	contig336	Unannota
GT	Sense	Sense	-0.178	Detected	0.178	Detected	0.000	1.508	Detected	1.411	Detected	1.459	0.016	GT Sens	contig337	contig337	Unannota
GT	Sense	Sense	-0.254	Comprom	0.254	Comprom	0.000	1.456	Detected	1.407	Detected	1.431	0.030	GT Sens	contig337	contig337	Unannota
GT	Sense	Sense	0.126	Detected	-0.126	Detected	0.000	1.206	Detected	1.133	Detected	1.170	0.012	GT Sens	contig337	contig337	Unannota
GT	Sense	Sense	-0.095	Comprom	0.095	Comprom	0.000	0.937	Detected	1.162	Detected	1.049	0.019	GT Sens	contig338	contig338	Unannota
GT	Sense	Sense	-0.034	Comprom	0.034	Comprom	0.000	1.433	Detected	1.432	Detected	1.432	0.001	GT Sens	contig338	contig338	Unannota
GT	Sense	Sense	-0.492	Comprom	0.492	Comprom	0.000	3.186	Detected	4.816	Detected	4.001	0.052	GT Sens	contig339	contig339	Unannota
GT	Sense	Sense	-0.634	Comprom	0.634	Comprom	0.000	2.080	Detected	2.177	Detected	2.128	0.079	GT Sens	contig339	contig339	Unannota
GT	Sense	Sense	-0.008	Detected	0.008	Detected	0.000	1.294	Detected	1.227	Detected	1.260	0.001	GT Sens	contig339	contig339	Unannota
GT	Sense	Sense	0.535	Comprom	-0.535	Comprom	0.000	0.851	Detected	1.859	Detected	1.355	0.207	GT Sens	contig339	contig339	Unannota
GT	Sense	Sense	-0.044	Comprom	0.044	Comprom	0.000	1.385	Detected	1.353	Detected	1.369	0.001	GT Sens	contig339	contig339	Unannota
GT	Sense	Sense	-0.111	Detected	0.111	Detected	0.000	1.233	Detected	1.160	Detected	1.197	0.009	GT Sens	contig340	contig340	Unannota
GT	Sense	Sense	-0.015	Comprom	0.015	Comprom	0.000	1.239	Detected	1.212	Detected	1.226	0.000	GT Sens	contig340	contig340	Unannota
GT	Sense	Sense	0.146	Detected	-0.146	Detected	0.000	1.157	Detected	1.039	Detected	1.098	0.020	GT Sens	contig340	contig340	Unannota

[illegible]

GT	Sense	Sense	0.759	Comprom	-0.759	Comprom	0.000	1.480	Detected	2.255	Detected	1.867	0.160	GT Sens
GT	Sense	Sense	0.403	Detected	-0.403	Detected	0.000	1.534	Detected	0.915	Detected	1.224	0.138	GT Sens
GT	Sense	Sense	-0.523	Comprom	0.523	Comprom	0.000	2.036	Detected	1.828	Detected	1.932	0.068	GT Sens
GT	Sense	Sense	-0.108	Detected	0.108	Detected	0.000	1.360	Detected	1.120	Detected	1.240	0.017	GT Sens
GT	Sense	Sense	0.016	Detected	-0.016	Detected	0.000	1.308	Detected	1.034	Detected	1.171	0.014	GT Sens
GT	Sense	Sense	-0.252	Comprom	0.252	Comprom	0.000	1.043	Detected	1.415	Detected	1.229	0.059	GT Sens
GT	Sense	Sense	0.203	Detected	-0.203	Detected	0.000	1.253	Detected	1.014	Detected	1.134	0.040	GT Sens
GT	Sense	Sense	0.115	Detected	-0.115	Detected	0.000	1.503	Detected	1.215	Detected	1.359	0.018	GT Sens
GT	Sense	Sense	-0.137	Detected	0.137	Detected	0.000	1.406	Detected	1.550	Detected	1.478	0.011	GT Sens
GT	Sense	Sense	0.098	Detected	-0.098	Detected	0.000	1.256	Detected	1.181	Detected	1.218	0.007	GT Sens
GT	Sense	Sense	-0.003	Detected	0.003	Detected	0.000	1.748	Detected	1.492	Detected	1.620	0.006	GT Sens
GT	Sense	Sense	0.851	Detected	-0.851	Comprom	0.000	1.649	Detected	2.592	Detected	2.121	0.161	GT Sens
GT	Sense	Sense	-0.069	Comprom	0.069	Comprom	0.000	2.667	Detected	3.618	Detected	3.143	0.023	GT Sens
GT	Sense	Sense	0.169	Detected	-0.169	Detected	0.000	2.468	Detected	2.001	Detected	2.234	0.016	GT Sens
GT	Sense	Sense	0.107	Detected	-0.107	Detected	0.000	2.493	Detected	1.993	Detected	2.243	0.014	GT Sens
GT	Sense	Sense	-0.071	Comprom	0.071	Comprom	0.000	1.606	Detected	1.168	Detected	1.387	0.026	GT Sens
GT	Sense	Sense	-0.042	Comprom	0.042	Comprom	0.000	3.251	Detected	2.912	Detected	3.081	0.003	GT Sens
GT	Sense	Sense	0.247	Comprom	-0.247	Comprom	0.000	2.256	Detected	2.182	Detected	2.219	0.012	GT Sens
GT	Sense	Sense	0.091	Detected	-0.091	Detected	0.000	1.872	Detected	1.710	Detected	1.791	0.005	GT Sens
GT	Sense	Sense	0.098	Detected	-0.098	Detected	0.000	1.217	Detected	0.930	Detected	1.074	0.025	GT Sens
GT	Sense	Sense	0.082	Detected	-0.082	Detected	0.000	1.045	Detected	1.104	Detected	1.074	0.007	GT Sens
GT	Sense	Sense	0.158	Detected	-0.158	Detected	0.000	1.053	Detected	1.071	Detected	1.062	0.022	GT Sens
GT	Sense	Sense	0.165	Detected	-0.165	Detected	0.000	2.394	Detected	1.268	Detected	1.831	0.089	GT Sens
GT	Sense	Sense	-0.748	Comprom	0.748	Comprom	0.000	1.413	Detected	1.889	Detected	1.651	0.170	GT Sens
GT	Sense	Sense	-0.267	Comprom	0.267	Detected	0.000	1.906	Detected	2.386	Detected	2.146	0.027	GT Sens
GT	Sense	Sense	0.195	Comprom	-0.195	Comprom	0.000	2.816	Detected	2.588	Detected	2.702	0.007	GT Sens
GT	Sense	Sense	0.137	Detected	-0.137	Detected	0.000	1.283	Detected	0.959	Detected	1.121	0.034	GT Sens
GT	Sense	Sense	-0.304	Detected	0.304	Detected	0.000	3.197	Detected	2.181	Detected	2.689	0.045	GT Sens
GT	Sense	Sense	-0.627	Detected	0.627	Detected	0.000	2.487	Detected	1.597	Detected	2.042	0.117	GT Sens
GT	Sense	Sense	-0.026	Detected	0.026	Detected	0.000	0.927	Detected	1.142	Detected	1.035	0.011	GT Sens
GT	Sense	Sense	-0.180	Detected	0.180	Detected	0.000	1.019	Detected	0.989	Detected	1.004	0.031	GT Sens
GT	Sense	Sense	-0.113	Detected	0.113	Detected	0.000	1.136	Detected	0.963	Detected	1.050	0.018	GT Sens
GT	Sense	Sense	-0.422	Comprom	0.422	Detected	0.000	2.443	Detected	1.505	Detected	1.974	0.089	GT Sens
GT	Sense	Sense	0.300	Comprom	-0.300	Comprom	0.000	1.138	Detected	1.504	Detected	1.321	0.064	GT Sens
GT	Sense	Sense	-0.136	Comprom	0.136	Comprom	0.000	0.902	Detected	1.549	Detected	1.225	0.073	GT Sens
GT	Sense	Sense	0.325	Detected	-0.325	Comprom	0.000	2.121	Detected	1.113	Detected	1.617	0.114	GT Sens
GT	Sense	Sense	-0.325	Detected	0.325	Detected	0.000	1.380	Detected	1.250	Detected	1.315	0.058	GT Sens
GT	Sense	Sense	-0.157	Detected	0.157	Detected	0.000	2.254	Detected	1.771	Detected	2.013	0.020	GT Sens
GT	Sense	Sense	-0.115	Detected	0.115	Detected	0.000	2.210	Detected	1.737	Detected	1.974	0.017	GT Sens
GT	Sense	Sense	0.311	Comprom	-0.311	Comprom	0.000	1.309	Detected	1.576	Detected	1.442	0.051	GT Sens
GT	Sense	Sense	0.738	Detected	-0.738	Comprom	0.000	1.638	Detected	1.302	Detected	1.470	0.192	GT Sens
GT	Sense	Sense	0.329	Detected	-0.329	Detected	0.000	1.891	Detected	1.662	Detected	1.777	0.036	GT Sens
GT	Sense	Sense	-0.694	Comprom	0.694	Comprom	0.000	0.991	Detected	1.679	Detected	1.335	0.227	GT Sens
GT	Sense	Sense	-0.993	Comprom	0.993	Detected	0.000	1.440	Detected	1.058	Detected	1.249	0.342	GT Sens
GT	Sense	Sense	-0.033	Detected	0.033	Detected	0.000	1.117	Detected	0.929	Detected	1.023	0.009	GT Sens
GT	Sense	Sense	-0.363	Detected	0.363	Detected	0.000	1.921	Detected	1.792	Detected	1.856	0.037	GT Sens
GT	Sense	Sense	-0.267	Detected	0.267	Detected	0.000	1.945	Detected	1.960	Detected	1.952	0.018	GT Sens
GT	Sense	Sense	0.005	Detected	-0.005	Detected	0.000	0.832	Detected	1.671	Detected	1.252	0.096	GT Sens
GT	Sense	Sense	-0.013	Comprom	0.013	Comprom	0.000	1.364	Detected	1.258	Detected	1.311	0.002	GT Sens
GT	Sense	Sense	-0.004	Detected	0.004	Detected	0.000	1.108	Detected	1.066	Detected	1.087	0.000	GT Sens
GT	Sense	Sense	0.053	Comprom	-0.053	Comprom	0.000	1.828	Detected	0.830	Detected	1.329	0.118	GT Sens
GT	Sense	Sense	-0.254	Comprom	0.254	Comprom	0.000	2.022	Detected	1.016	Detected	1.519	0.114	GT Sens
GT	Sense	Sense	0.421	Detected	-0.421	Detected	0.000	0.993	Detected	1.026	Detected	1.010	0.139	GT Sens
GT	Sense	Sense	-0.039	Detected	0.039	Detected	0.000	1.353	Detected	1.072	Detected	1.212	0.014	GT Sens
GT	Sense	Sense	-0.152	Detected	0.152	Detected	0.000	1.397	Detected	0.961	Detected	1.179	0.047	GT Sens
GT	Sense	Sense	-0.075	Comprom	0.075	Comprom	0.000	1.888	Detected	1.903	Detected	1.896	0.002	GT Sens
GT	Sense	Sense	0.137	Comprom	-0.137	Comprom	0.000	1.790	Detected	1.641	Detected	1.715	0.008	GT Sens
GT	Sense	Sense	-0.205	Detected	0.205	Detected	0.000	0.828	Detected	1.441	Detected	1.135	0.091	GT Sens
GT	Sense	Sense	0.717	Comprom	-0.717	Comprom	0.000	3.335	Detected	3.212	Detected	3.273	0.045	GT Sens
GT	Sense	Sense	0.350	Detected	-0.350	Detected	0.000	1.174	Detected	1.043	Detected	1.109	0.089	GT Sens
GT	Sense	Sense	0.258	Detected	-0.258	Detected	0.000	1.080	Detected	1.000	Detected	1.040	0.058	GT Sens
GT	Sense	Sense	-0.264	Detected	0.264	Detected	0.000	2.131	Detected	2.665	Detected	2.398	0.024	GT Sens
GT	Sense	Sense	-0.292	Detected	0.292	Detected	0.000	1.258	Detected	1.866	Detected	1.562	0.066	GT Sens
GT	Sense	Sense	-0.128	Detected	0.128	Detected	0.000	1.149	Detected	1.453	Detected	1.301	0.023	GT Sens
GT	Sense	Sense	-0.086	Detected	0.086	Detected	0.000	0.994	Detected	1.266	Detected	1.130	0.020	GT Sens
GT	Sense	Sense	-1.030	Comprom	1.030	Detected	0.000	2.612	Detected	2.019	Detected	2.315	0.163	GT Sens
GT	Sense	Sense	-1.013	Comprom	1.013	Detected	0.000	2.224	Detected	1.965	Detected	2.094	0.177	GT Sens
GT	Sense	Sense	-0.125	Comprom	0.125	Comprom	0.000	2.187	Detected	1.046	Detected	1.616	0.110	GT Sens
GT	Sense	Sense	0.262	Detected	-0.262	Detected	0.000	1.327	Detected	1.117	Detected	1.222	0.049	GT Sens
GT	Sense	Sense	0.025	Detected	-0.025	Detected	0.000	1.062	Detected	1.033	Detected	1.048	0.001	GT Sens
GT	Sense	Sense	0.059	Comprom	-0.059	Comprom	0.000	1.482	Detected	1.358	Detected	1.420	0.004	GT Sens
GT	Sense	Sense	-0.430	Detected	0.430	Detected	0.000	1.263	Detected	1.124	Detected	1.194	0.111	GT Sens
GT	Sense	Sense	0.587	Comprom	-0.587	Comprom	0.000	1.410	Detected	1.288	Detected	1.349	0.149	GT Sens
GT	Sense	Sense	-0.387	Comprom	0.387	Comprom	0.000	1.620	Detected	1.649	Detected	1.634	0.052	GT Sens
GT	Sense	Sense	0.147	Detected	-0.147	Detected	0.000	1.335	Detected	1.330	Detected	1.333	0.012	GT Sens
GT	Sense	Sense	-0.274	Detected	0.274	Detected	0.000	1.224	Detected	1.051	Detected	1.138	0.058	GT Sens
GT	Sense	Sense	-0.019	Detected	0.019	Detected	0.000	1.253	Detected	1.325	Detected	1.289	0.001	GT Sens
GT	Sense	Sense	1.174	Detected	-1.174	Comprom	0.000	2.323	Detected	1.857	Detected	2.090	0.223	GT Sens
GT	Sense	Sense	-0.192	Comprom	0.192	Comprom	0.000	2.852	Detected	2.175	Detected	2.514	0.023	GT Sens
GT	Sense	Sense	-0.732	Comprom	0.732	Detected	0.000	1.135	Detected	1.152	Detected	1.144	0.259	GT Sens
GT	Sense	Sense	0.582	Detected	-0.582	Comprom	0.000	0.826	Detected	1.421	Detected	1.124	0.228	GT Sens
GT	Sense	Sense	-0.214	Detected	0.214	Detected	0.000	1.913	Detected	1.484	Detected	1.699	0.030	GT Sens
GT	Sense	Sense	-0.102	Detected	0.102	Detected	0.000	1.140	Detected	1.119	Detected	1.130	0.008	GT Sens
GT	Sense	Sense	-0.323	Detected	0.323	Detected	0.000	1.133	Detected	1.201	Detected	1.167	0.069	GT Sens
GT	Sense	Sense	-0.139	Detected	0.139	Detected	0.000	2.063	Detected	0.868	Detected	1.465	0.139	GT Sens
GT	Sense	Sense	-0.300	Detected	0.300	Detected	0.000	1.282	Detected	1.309	Detected	1.295	0.050	GT Sens
GT	Sense	Sense	-0.267	Comprom	0.267	Comprom	0.000	1.898	Detected	1.081	Detected	1.489	0.092	GT Sens
GT	Sense	Sense	-0.590	Comprom	0.590	Comprom	0.000	2.166	Detected	1.667	Detected	1.916	0.096	GT Sens
GT	Sense	Sense	0.088	Detected	-0.088	Detected	0.000	4.553	Detected	4.206	Detected	4.380	0.002	GT Sens
GT	Sense	Sense	-0.380	Comprom	0.380	Comprom	0.000	1.902	Detected	1.673	Detected	1.787	0.046	GT Sens
GT	Sense	Sense	1.400	Detected	-1.400	Comprom	0.000	4.171	Detected	1.348	Detected	2.759	0.300	GT Sens
GT	Sense	Sense	-0.435	Comprom	0.435	Comprom	0.000	1.225	Detected	1.433	Detected	1.329	0.097	GT Sens
GT	Sense	Sense	-0.260	Detected	0.260	Detected	0.000	1.661	Detected	1.069	Detected	1.365	0.074	GT Sens
GT	Sense	Sense	-0.804	Comprom	0.804	Detected	0.000	1.487	Detected	1.041	Detected	1.264	0.269	GT Sens
GT	Sense	Sense	-0.170	Detected	0.170	Detected	0.000	0.817	Detected	1.231	Detected	1.024	0.062	GT Sens
GT	Sense	Sense	-0.031	Comprom	0.031	Comprom	0.000	1.208	Detected	0.906	Detected	1.057	0.021	GT Sens
GT	Sense	Sense	-0.508	Comprom	0.508	Comprom	0.000	1.582	Detected	1.312	Detected	1.447	0.110	GT Sens
GT	Sense	Sense	-1.019	Comprom	1.019	Detected	0.000	1.206	Detected	0.907	Detected	1.056	0.413	GT Sens
GT	Sense	Sense	0.201	Detected	-0.201	Detected	0.000	1.667	Detected	0.819	Detected	1.243	0.118	GT Sens
GT	Sense	Sense	-0.030	Detected	0.030	Detected	0.000	1.894	Detected	1.248	Detected	1.571	0.040	GT Sens
GT	Sense	Sense	-0.164	Detected	0.164	Detected	0.000	1.302	Detected	1.310	Detected	1.306	0.015	GT Sens
GT	Sense	Sense	0.089	Detected	-0.089	Detected	0.000	1.107	Detected	0.924	Detected	1.015	0.015	GT Sens
GT	Sense	Sense	-0.047	Comprom	0.047	Comprom	0.000	1.959	Detected	2.022	Detected	1.991	0.001	GT Sens
GT	Sense	Sense	-0.092	Comprom	0.092	Comprom	0.000	2.198	Detected	1.229	Detected	1.713	0.074	GT Sens
GT	Sense	Sense	0.274	Detected	-0.274	Detected	0.000	1.277	Detected	1.489	Detected	1.383	0.042	GT Sens
GT	Sense	Sense	-0.108	Detected	0.108	Detected	0.000	1.303	Detected	1.233	Detected	1.268	0.008	GT Sens
GT	Sense	Sense	-0.037	Detected	0.037	Detected	0.000	1.155	Detected	1.095	Detected	1.125	0.002	GT Sens
GT	Sense	Sense	-0.058	Comprom	0.058	Comprom	0.000	2.010	Detected	2.124	Detected	2.067	0.002	GT Sens
GT	Sense	Sense	-0.159	Detected	0.159	Detected	0.000	1.009	Detected	1.063	Detected	1.036	0.023	GT Sens
GT	Sense	Sense	0.548	Detected	-0.548	Comprom	0.000	2.417	Detected	1.733	Detected	2.075	0.085	GT Sens
GT	Sense	Sense	0.027	Comprom	-0.027	Comprom	0.000	1.668	Detected	1.475	Detected	1.572	0.004	GT Sens
GT	Sense	Sense	0.216	Detected	-0.216	Detected	0.000	1.251	Detected	0.986	Detected	1.119	0.048	GT Sens
GT	Sense	Sense	-0.154	Comprom	0.154	Comprom	0.000	1.318	Detected	1.364	Detected	1.341	0.013	GT Sens
GT	Sense	Sense	-0.250	Detected	0.250	Detected	0.000	1.228	Detected	0.950	Detected	1.089	0.063	GT Sens
GT	Sense	Sense	-0.579	Comprom	0.579	Comprom	0.000	1.631	Detected	1.236	Detected	1.434	0.144	GT Sens
GT	Sense	Sense	-0.003	Comprom	0.003	Comprom	0.000	1.315	Detected	1.376	Detected	1.346	0.001	GT Sens
GT	Sense	Sense	-0.432	Comprom	0.432	Comprom	0.000	1.078	Detected	1.880	Detected	1.479	0.129	GT Sens
GT	Sense	Sense	-0.285	Detected	0.285	Detected	0.000	2.236	Detected	1.594	Detected	1.915	0.047	GT Sens
GT	Sense	Sense	-0.076	Detected	0.076	Detected	0.000	1.905	Detected	1.269	Detected	1.587	0.040	GT Sens
GT	Sense	Sense	0.251	Detected	-0.251	Detected	0.000	1.349	Detected	1.509	Detected	1.429	0.032	GT Sens
GT	Sense	Sense	-0.900	Comprom	0.900	Detected	0.000	1.706	Detected	0.924	Detected	1.315	0.312	GT Sens
GT	Sense	Sense	-0.385	Detected	0.385	Detected	0.000	1.603	Detected	1.363	Detected	1.483	0.067	GT Sens
GT	Sense	Sense	0.044	Detected	-0.044	Detected	0.000	0.968	Detected	1.074	Detected	1.021	0.005	GT Sens
GT	Sense	Sense	0.203	Detected	-0.203	Detected	0.000	1.279	Detected	1.054	Detected	1.166	0.037	GT Sens
GT	Sense	Sense	-0.099	Detected	0.099	Detected	0.000	1.423	Detected	1.160	Detected	1.291	0.016	GT Sens
GT	Sense	Sense	-0.171	Detected	0.171	Detected	0.000	1.568	Detected	1.290	Detected	1.429	0.023	GT Sens
GT	Sense	Sense	-0.171	Detected	0.171	Detected	0.000	1.641	Detected	1.343	Detected	1.492	0.022	GT Sens
GT	Sense	Sense	-0.146	Comprom	0.146	Comprom	0.000	3.001	Detected	1.421	Detected	2.211	0.110	GT Sens
GT	Sense	Sense	0.210	Detected	-0.210	Comprom	0.000	0.876	Detected	1.196	Detected	1.036	0.059	GT Sens
GT	Sense	Sense	-0.128	Comprom	0.128	Comprom	0.000	2.568	Detected	2.460	Detected	2.514	0.003	GT Sens
GT	Sense	Sense	-0.315	Comprom	0.315	Comprom	0.000	1.229	Detected	1.073	Detected	1.151	0.071	GT Sens
GT	Sense	Sense	0.126	Detected	-0.126	Detected	0.000	1.087	Detected	1.188	Detected	1.138	0.014	GT Sens
GT	Sense	Sense	-0.089	Detected	0.089	Detected	0.000	1.689	Detected	1.604	Detected	1.647	0.004	GT Sens
GT	Sense	Sense	0.013	Detected	-0.013	Detected	0.000	1.865	Detected	1.466	Detected	1.666	0.014	GT Sens
GT	Sense	Sense	0.279	Comprom	-0.279	Comprom	0.000	1.988	Detected	2.086	Detected	2.037	0.019	GT Sens
GT	Sense	Sense	0.083	Detected	-0.083	Detected	0.000	1.421	Detected	1.287	Detected	1.354	0.006	GT Sens
GT	Sense	Sense	-0.682	Comprom	0.682	Comprom	0.000	0.924	Detected	2.012	Detected	1.468	0.234	GT Sens
GT	Sense	Sense	-0.217	Comprom	0.217	Comprom	0.000	2.232	Detected	2.284	Detected	2.258	0.009	GT Sens
GT	Sense	Sense	0.155	Comprom	-0.155	Comprom	0.000	4.414	Detected	1.205	Detected	2.809	0.223	GT Sens
GT	Sense	Sense	-0.019	Detected	0.019	Detected	0.000	1.281	Detected	0.879	Detected	1.080	0.033	GT Sens
GT	Sense	Sense	-0.040	Detected	0.040	Detected	0.000	1.103	Detected	1.278	Detected	1.190	0.006	GT Sens
GT	Sense	Sense	0.207	Comprom	-0.207	Comprom	0.000	1.549	Detected	1.701	Detected	1.625	0.018	GT Sens
GT	Sense	Sense	0.484	Comprom	-0.484	Comprom	0.000	1.126	Detected	0.959	Detected	1.043	0.168	GT Sens
GT	Sense	Sense	-0.507	Comprom	0.507	Detected	0.000	0.897	Detected	1.309	Detected	1.103	0.181	GT Sens
GT	Sense	Sense	-0.682	Comprom	0.682	Comprom	0.000	1.784	Detected	1.812	Detected	1.798	0.119	GT Sens
GT	Sense	Sense	-0.338	Detected	0.338	Detected	0.000	1.599	Detected	0.831	Detected	1.215	0.141	GT Sens
GT	Sense	Sense	0.029	Detected	-0.029	Detected	0.000	1.995	Detected	0.954	Detected	1.475	0.105	GT Sens
GT	Sense	Sense	0.128	Detected	-0.128	Detected	0.000	1.450	Detected	0.964	Detected	1.207	0.048	GT Sens
GT	Sense	Sense	-0.237	Comprom	0.237	Comprom	0.000	1.387	Detected	1.582	Detected	1.484	0.029	GT Sens
GT	Sense	Sense	0.227	Detected	-0.227	Detected	0.000	1.051	Detected	1.615	Detected	1.333	0.067	GT Sens
GT	Sense	Sense	0.236	Detected	-0.236	Detected	0.000	0.867	Detected	1.416	Detected	1.142	0.088	GT Sens
GT	Sense	Sense	0.196	Detected	-0.196	Detected	0.000	0.900	Detected	1.147	Detected	1.023	0.048	GT Sens
GT	Sense	Sense	0.122	Comprom	-0.122	Comprom	0.000	2.320	Detected	2.028	Detected	2.174	0.008	GT Sens
GT	Sense	Sense	-0.323	Comprom	0.323	Detected	0.000	1.407	Detected	1.262	Detected	1.334	0.056	GT Sens
GT	Sense	Sense	0.004	Detected	-0.004	Detected	0.000	0.927	Detected	1.106	Detected	1.017	0.008	GT Sens
GT	Sense	Sense	0.090	Detected	-0.090	Comprom	0.000	0.946	Detected	1.421	Detected	1.183	0.043	GT Sens
GT	Sense	Sense	0.929	Detected	-0.929	Comprom	0.000	0.827	Detected	1.226	Detected	1.026	0.393	GT Sens
GT	Sense	Sense	-0.041	Comprom	0.041	Comprom	0.000	1.220	Detected	1.351	Detected	1.286	0.004	GT Sens
GT	Sense	Sense	-0.203	Comprom	0.203	Comprom	0.000	1.698	Detected	1.842	Detected	1.770	0.015	GT Sens
GT	Sense	Sense	-0.049	Comprom	0.049	Comprom	0.000	1.179	Detected	1.458	Detected	1.319	0.012	GT Sens
GT	Sense	Sense	0.160	Detected	-0.160	Detected	0.000	0.865	Detected	1.300	Detected	1.083	0.057	GT Sens
GT	Sense	Sense	0.262	Comprom	-0.262	Comprom	0.000	1.599	Detected	1.304	Detected	1.451	0.040	GT Sens
GT	Sense	Sense	-1.164	Comprom	1.164	Detected	0.000	1.138	Detected	1.519	Detected	1.328	0.377	GT Sens
GT	Sense	Sense	0.085	Detected	-0.085	Detected	0.000	1.520	Detected	1.168	Detected	1.344	0.020	GT Sens
GT	Sense	Sense	0.183	Comprom	-0.183	Comprom	0.000	1.721	Detected	1.208	Detected	1.464	0.043	GT Sens
GT	Sense	Sense	0.826	Detected	-0.826	Comprom	0.000	1.231	Detected	1.386	Detected	1.309	0.256	GT Sens
GT	Sense	Sense	0.105	Detected	-0.105	Detected	0.000	0.827	Detected	1.377	Detected	1.102	0.065	GT Sens
GT	Sense	Sense	-0.207	Detected	0.207	Detected	0.000	1.749	Detected	0.981	Detected	1.365	0.089	GT Sens
GT	Sense	Sense	0.277	Detected	-0.277	Detected	0.000	0.859	Detected	1.378	Detected	1.118	0.098	GT Sens
GT	Sense	Sense	0.042	Detected	-0.042	Detected	0.000	1.183	Detected	0.923	Detected	1.053	0.016	GT Sens
GT	Sense	Sense	-0.038	Detected	0.038	Detected	0.000	1.537	Detected	1.151	Detected	1.344	0.021	GT Sens
GT	Sense	Sense	-0.433	Comprom	0.433	Comprom	0.000	2.181	Detected	1.703	Detected	1.942	0.059	GT Sens
GT	Sense	Sense	-0.121	Detected	0.121	Detected	0.000	1.447	Detected	1.105	Detected	1.276	0.026	GT Sens
GT	Sense	Sense	-0.670	Comprom	0.670	Comprom	0.000	1.532	Detected	2.793	Detected	2.162	0.143	GT Sens
GT	Sense	Sense	0.226	Detected	-0.226	Detected	0.000	1.384	Detected	1.737	Detected	1.560	0.032	GT Sens
GT	Sense	Sense	-0.868	Detected	0.868	Detected	0.000	1.341	Detected	1.059	Detected	1.200	0.306	GT Sens
GT	Sense	Sense	-0.207	Detected	0.207	Detected	0.000	0.897	Detected	1.120	Detected	1.008	0.050	GT Sens
GT	Sense	Sense	-0.101	Comprom	0.101	Comprom	0.000	1.615	Detected	1.583	Detected	1.599	0.004	GT Sens
GT	Sense	Sense	-0.087	Comprom	0.087	Comprom	0.000	1.197	Detected	0.880	Detected	1.039	0.029	GT Sens
GT	Sense	Sense	0.351	Detected	-0.351	Comprom	0.000	1.053	Detected	1.417	Detected	1.235	0.089	GT Sens
GT	Sense	Sense	-0.747	Comprom	0.747	Comprom	0.000	1.122	Detected	1.109	Detected	1.115	0.274	GT Sens
GT	Sense	Sense	0.624	Detected	-0.624	Comprom	0.000	1.951	Detected	2.091	Detected	2.021	0.085	GT Sens
GT	Sense	Sense	0.221	Detected	-0.221	Detected	0.000	1.403	Detected	1.253	Detected	1.328	0.029	GT Sens
GT	Sense	Sense	0.104	Detected	-0.104	Detected	0.000	1.333	Detected	1.282	Detected	1.308	0.007	GT Sens
GT	Sense	Sense	0.074	Comprom	-0.074	Comprom	0.000	1.299	Detected	1.028	Detected	1.164	0.017	GT Sens
GT	Sense	Sense	0.520	Detected	-0.520	Detected	0.000	1.616	Detected	1.508	Detected	1.562	0.096	GT Sens
GT	Sense	Sense	0.247	Detected	-0.247	Comprom	0.000	1.303	Detected	1.242	Detected	1.272	0.036	GT Sens
GT	Sense	Sense	0.293	Detected	-0.293	Detected	0.000	1.207	Detected	0.884	Detected	1.046	0.089	GT Sens
GT	Sense	Sense	-0.868	Comprom	0.868	Comprom	0.000	1.577	Detected	1.411	Detected	1.494	0.229	GT Sens
GT	Sense	Sense	-0.269	Comprom	0.269	Comprom	0.000	1.187	Detected	1.180	Detected	1.183	0.048	GT Sens
GT	Sense	Sense	0.407	Detected	-0.407	Comprom	0.000	2.219	Detected	2.023	Detected	2.121	0.037	GT Sens
GT	Sense	Sense	-0.026	Detected	0.026	Detected	0.000	1.341	Detected	1.139	Detected	1.240	0.007	GT Sens
GT	Sense	Sense	-0.467	Detected	0.467	Detected	0.000	3.225	Detected	2.308	Detected	2.767	0.052	GT Sens
GT	Sense	Sense	0.262	Detected	-0.262	Detected	0.000	1.970	Detected	1.279	Detected	1.625	0.064	GT Sens
GT	Sense	Sense	-0.200	Detected	0.200	Detected	0.000	0.877	Detected	1.167	Detected	1.022	0.054	GT Sens
GT	Sense	Sense	-0.319	Detected	0.319	Detected	0.000	1.485	Detected	1.163	Detected	1.324	0.066	GT Sens
GT	Sense	Sense	-0.203	Detected	0.203	Detected	0.000	1.429	Detected	1.037	Detected	1.233	0.049	GT Sens
GT	Sense	Sense	-0.142	Detected	0.142	Detected	0.000	1.348	Detected	0.883	Detected	1.116	0.055	GT Sens
GT	Sense	Sense	-0.065	Detected	0.065	Detected	0.000	2.091	Detected	1.916	Detected	2.003	0.003	GT Sens
GT	Sense	Sense	0.013	Detected	-0.013	Detected	0.000	1.855	Detected	1.683	Detected	1.769	0.002	GT Sens
GT	Sense	Sense	0.248	Detected	-0.248	Detected	0.000	1.705	Detected	1.050	Detected	1.378	0.079	GT Sens
GT	Sense	Sense	0.244	Comprom	-0.244	Comprom	0.000	4.927	Detected	2.856	Detected	3.892	0.067	GT Sens
GT	Sense	Sense	-0.022	Detected	0.022	Detected	0.000	1.378	Detected	1.409	Detected	1.394	0.000	GT Sens
GT	Sense	Sense	-0.134	Comprom	0.134	Comprom	0.000	1.205	Detected	1.585	Detected	1.395	0.027	GT Sens
GT	Sense	Sense	0.394	Detected	-0.394	Comprom	0.000	0.959	Detected	1.142	Detected	1.051	0.122	GT Sens
GT	Sense	Sense	-0.055	Detected	0.055	Detected	0.000	1.293	Detected	1.302	Detected	1.297	0.002	GT Sens
GT	Sense	Sense	-0.320	Comprom	0.320	Comprom	0.000	1.272	Detected	1.480	Detected	1.376	0.055	GT Sens
GT	Sense	Sense	-0.743	Comprom	0.743	Comprom	0.000	1.582	Detected	1.411	Detected	1.496	0.184	GT Sens
GT	Sense	Sense	0.763	Comprom	-0.763	Comprom	0.000	2.427	Detected	1.657	Detected	2.042	0.139	GT Sens
GT	Sense	Sense	-0.233	Comprom	0.233	Comprom	0.000	1.895	Detected	1.307	Detected	1.601	0.051	GT Sens
GT	Sense	Sense	0.830	Detected	-0.830	Comprom	0.000	1.219	Detected	1.554	Detected	1.386	0.243	GT Sens
GT	Sense	Sense	-0.513	Comprom	0.513	Comprom	0.000	0.940	Detected	1.619	Detected	1.279	0.173	GT Sens
GT	Sense	Sense	0.558	Detected	-0.558	Comprom	0.000	2.360	Detected	2.760	Detected	2.560	0.050	GT Sens
GT	Sense	Sense	0.092	Detected	-0.092	Detected	0.000	1.656	Detected	2.042	Detected	1.849	0.013	GT Sens
GT	Sense	Sense	0.583	Detected	-0.583	Detected	0.000	2.344	Detected	1.123	Detected	1.733	0.176	GT Sens
GT	Sense	Sense	-0.087	Detected	0.087	Detected	0.000	1.215	Detected	0.995	Detected	1.105	0.016	GT Sens
GT	Sense	Sense	-0.282	Detected	0.282	Detected	0.000	0.928	Detected	1.142	Detected	1.035	0.076	GT Sens
GT	Sense	Sense	-0.182	Detected	0.182	Detected	0.000	1.038	Detected	1.245	Detected	1.142	0.032	GT Sens
GT	Sense	Sense	0.022	Detected	-0.022	Detected	0.000	1.265	Detected	1.348	Detected	1.307	0.001	GT Sens
GT	Sense	Sense	-0.275	Comprom	0.275	Comprom	0.000	2.059	Detected	2.067	Detected	2.063	0.017	GT Sens
GT	Sense	Sense	-0.126	Detected	0.126	Detected	0.000	1.033	Detected	0.999	Detected	1.016	0.015	GT Sens
GT	Sense	Sense	-0.302	Detected	0.302	Detected	0.000	1.700	Detected	1.507	Detected	1.603	0.037	GT Sens
GT	Sense	Sense	0.186	Comprom	-0.186	Comprom	0.000	2.252	Detected	2.773	Detected	2.512	0.016	GT Sens
GT	Sense	Sense	0.153	Detected	-0.153	Detected	0.000	1.568	Detected	1.066	Detected	1.317	0.046	GT Sens
GT	Sense	Sense	0.147	Detected	-0.147	Detected	0.000	1.217	Detected	0.907	Detected	1.062	0.038	GT Sens
GT	Sense	Sense	0.064	Detected	-0.064	Detected	0.000	1.116	Detected	0.953	Detected	1.034	0.010	GT Sens
GT	Sense	Sense	0.072	Detected	-0.072	Detected	0.000	1.698	Detected	1.324	Detected	1.511	0.017	GT Sens
GT	Sense	Sense	0.225	Detected	-0.225	Comprom	0.000	0.937	Detected	1.271	Detected	1.104	0.059	GT Sens
GT	Sense	Sense	0.219	Detected	-0.219	Comprom	0.000	1.284	Detected	1.476	Detected	1.380	0.029	GT Sens
GT	Sense	Sense	-0.445	Comprom	0.445	Comprom	0.000	1.687	Detected	2.076	Detected	1.882	0.061	GT Sens
GT	Sense	Sense	-0.660	Comprom	0.660	Comprom	0.000	1.282	Detected	0.853	Detected	1.068	0.264	GT Sens
GT	Sense	Sense	-0.358	Comprom	0.358	Comprom	0.000	1.054	Detected	1.761	Detected	1.407	0.108	GT Sens
GT	Sense	Sense	0.066	Detected	-0.066	Detected	0.000	1.317	Detected	1.163	Detected	1.240	0.007	GT Sens
GT	Sense	Sense	-0.732	Comprom	0.732	Comprom	0.000	2.024	Detected	2.184	Detected	2.104	0.104	GT Sens
GT	Sense	Sense	0.174	Comprom	-0.174	Comprom	0.000	1.904	Detected	7.106	Detected	4.505	0.226	GT Sens
GT	Sense	Sense	-0.027	Detected	0.027	Detected	0.000	1.102	Detected	0.911	Detected	1.007	0.010	GT Sens
GT	Sense	Sense	0.891	Detected	-0.891	Comprom	0.000	2.227	Detected	2.476	Detected	2.351	0.121	GT Sens
GT	Sense	Sense	0.369	Comprom	-0.369	Comprom	0.000	1.079	Detected	1.168	Detected	1.123	0.094	GT Sens
GT	Sense	Sense	0.227	Detected	-0.227	Detected	0.000	1.988	Detected	1.755	Detected	1.871	0.018	GT Sens
GT	Sense	Sense	1.002	Detected	-1.002	Comprom	0.000	1.491	Detected	2.104	Detected	1.798	0.228	GT Sens
GT	Sense	Sense	0.034	Comprom	-0.034	Comprom	0.000	1.125	Detected	1.429	Detected	1.277	0.015	GT Sens
GT	Sense	Sense	-0.044	Detected	0.044	Detected	0.000	1.394	Detected	0.901	Detected	1.148	0.044	GT Sens
GT	Sense	Sense	-0.432	Comprom	0.432	Comprom	0.000	1.090	Detected	1.037	Detected	1.064	0.133	GT Sens
GT	Sense	Sense	-0.694	Detected	0.694	Detected	0.000	2.188	Detected	1.970	Detected	2.079	0.098	GT Sens

GT	Sense	Sense	-0.131	Detected	0.131	Detected	0.000	1.810	Detected	1.472	Detected	1.641	0.017	GT	Sens	contig419	contig419	Unannota	GAAGTTTTATTCT
GT	Sense	Sense	-0.169	Detected	0.169	Detected	0.000	1.438	Detected	1.206	Detected	1.322	0.023	GT	Sens	contig419	contig419	Unannota	GTCATTAATTGAT
GT	Sense	Sense	-0.879	Comprom	0.879	Detected	0.000	1.799	Detected	1.775	Detected	1.787	0.179	GT	Sens	contig420	contig420	Unannota	AAATAGCCTGAGG
GT	Sense	Sense	0.077	Detected	-0.077	Detected	0.000	1.049	Detected	1.077	Detected	1.063	0.005	GT	Sens	contig420	contig420	Unannota	ATTGTAGTCGTCG
GT	Sense	Sense	-0.143	Detected	0.143	Detected	0.000	1.481	Detected	1.309	Detected	1.395	0.014	GT	Sens	contig420	contig420	Unannota	GAGTTCACGTGTC
GT	Sense	Sense	-0.437	Comprom	0.437	Comprom	0.000	1.281	Detected	1.968	Detected	1.625	0.100	GT	Sens	contig420	contig420	Unannota	TTCTAGGCTTGAG
GT	Sense	Sense	0.013	Detected	-0.013	Detected	0.000	1.153	Detected	0.942	Detected	1.048	0.010	GT	Sens	contig420	contig420	Unannota	GAGCTAGCTTTTG
GT	Sense	Sense	-0.159	Detected	0.159	Detected	0.000	1.568	Detected	1.116	Detected	1.342	0.040	GT	Sens	contig421	contig421	Histidine	TACCCTT contig42
GT	Sense	Sense	-0.568	Comprom	0.568	Comprom	0.000	0.906	Detected	1.161	Detected	1.034	0.218	GT	Sens	contig421	contig421	Unannota	ATGGAAGAATCAG
GT	Sense	Sense	0.082	Detected	-0.082	Detected	0.000	1.042	Detected	1.079	Detected	1.061	0.006	GT	Sens	contig421	contig421	Unannota	GGACTCCAACATA
GT	Sense	Sense	-0.190	Detected	0.190	Detected	0.000	1.240	Detected	0.890	Detected	1.065	0.054	GT	Sens	contig421	contig421	Unannota	CGATCGATAAAAA
GT	Sense	Sense	-0.844	Comprom	0.844	Comprom	0.000	1.683	Detected	1.492	Detected	1.588	0.202	GT	Sens	contig421	contig421	Unannota	TAAGCATAAGGTA
GT	Sense	Sense	0.132	Detected	-0.132	Detected	0.000	1.637	Detected	1.633	Detected	1.635	0.006	GT	Sens	contig421	contig421	Unannota	TATTTTAATATGT
GT	Sense	Sense	-0.403	Detected	0.403	Detected	0.000	2.893	Detected	3.167	Detected	3.030	0.019	GT	Sens	contig421	contig421	Beta-gluc	AGGGAAT contig42
GT	Sense	Sense	0.122	Detected	-0.122	Detected	0.000	1.197	Detected	1.020	Detected	1.108	0.018	GT	Sens	contig421	contig421	Unannota	CCTATGTTCATCG
GT	Sense	Sense	-0.466	Comprom	0.466	Comprom	0.000	0.904	Detected	1.243	Detected	1.073	0.163	GT	Sens	contig422	contig422	Unannota	GGAATTACCCGAA
GT	Sense	Sense	-0.131	Detected	0.131	Detected	0.000	1.626	Detected	1.081	Detected	1.353	0.047	GT	Sens	contig422	contig422	Unannota	TATTCTCCGTCAT
GT	Sense	Sense	-0.208	Detected	0.208	Detected	0.000	1.691	Detected	1.138	Detected	1.415	0.055	GT	Sens	contig422	contig422	Unannota	TACCTTTTACTCT
GT	Sense	Sense	-0.579	Comprom	0.579	Comprom	0.000	1.307	Detected	1.767	Detected	1.537	0.132	GT	Sens	contig422	contig422	Unannota	ATCCCAAGGAGTC
GT	Sense	Sense	0.745	Detected	-0.745	Comprom	0.000	1.374	Detected	1.796	Detected	1.585	0.177	GT	Sens	contig422	contig422	Unannota	CCAACATTGAGTT
GT	Sense	Sense	0.171	Detected	-0.171	Detected	0.000	1.349	Detected	1.128	Detected	1.239	0.026	GT	Sens	contig422	contig422	Unannota	TAGAACTCTTCTC
GT	Sense	Sense	-0.121	Comprom	0.121	Comprom	0.000	8.256	Detected	4.397	Detected	6.327	0.082	GT	Sens	contig423	contig423	Unannota	GGAAATTGGTTTG
GT	Sense	Sense	-0.214	Detected	0.214	Detected	0.000	2.184	Detected	2.756	Detected	2.470	0.020	GT	Sens	contig423	contig423	Unannota	TTGCCTTTCGAGA

[illegible]

GT	Sense	Sense	-0.548	Detected	0.548	Detected	0.000	2.212	Detected	1.970	Detected	2.091	0.065	GT	Sens	contig815	contig815	Phototro	GCCGTA	contig81
GT	Sense	Sense	0.425	Detected	-0.425	Comprom	0.000	1.301	Detected	0.893	Detected	1.097	0.145	GT	Sens	contig823	contig823	Calmodul	ACATTG	contig82
GT	Sense	Sense	0.026	Detected	-0.026	Detected	0.000	1.318	Detected	1.170	Detected	1.244	0.004	GT	Sens	contig827	contig827	Unknown	CAGTTT	contig82
GT	Sense	Sense	0.089	Comprom	-0.089	Comprom	0.000	1.818	Detected	1.729	Detected	1.774	0.003	GT	Sens	contig829	contig829	Ankyrin r	GTTCAT	contig82
GT	Sense	Sense	-1.273	Comprom	1.273	Detected	0.000	3.754	Detected	3.392	Detected	3.573	0.109	GT	Sens	contig835	contig835	Telomere	TTTCCA	contig83
GT	Sense	Sense	-0.329	Detected	0.329	Detected	0.000	1.949	Detected	1.881	Detected	1.915	0.029	GT	Sens	contig835	contig835	Unknown	GAATAT	contig83
GT	Sense	Sense	-0.133	Detected	0.133	Detected	0.000	1.540	Detected	1.136	Detected	1.338	0.031	GT	Sens	contig839	contig839	Strictosid	GATGAT	contig83
GT	Sense	Sense	-0.053	Detected	0.053	Detected	0.000	1.582	Detected	0.963	Detected	1.272	0.056	GT	Sens	contig843	contig843	Pentatric	GGGTCT	contig84
GT	Sense	Sense	-0.131	Detected	0.131	Detected	0.000	1.660	Detected	1.198	Detected	1.429	0.033	GT	Sens	contig844	contig844	Ribonucl	GTCGAT	contig84
GT	Sense	Sense	-0.218	Comprom	0.218	Comprom	0.000	1.172	Detected	1.257	Detected	1.215	0.032	GT	Sens	contig849	contig849	Sigma fac	CCAAAA	contig84
GT	Sense	Sense	0.527	Detected	-0.527	Comprom	0.000	1.131	Detected	1.334	Detected	1.232	0.149	GT	Sens	contig851	contig851	Unknown	CCAAAT	contig85
GT	Sense	Sense	-0.191	Detected	0.191	Detected	0.000	1.956	Detected	1.413	Detected	1.685	0.037	GT	Sens	contig851	contig851	LRR rece	ACGTAT	contig85
GT	Sense	Sense	-0.138	Detected	0.138	Detected	0.000	1.780	Detected	1.442	Detected	1.611	0.018	GT	Sens	contig853	contig853	Zinc finge	GCATCT	contig85
GT	Sense	Sense	-0.140	Comprom	0.140	Comprom	0.000	2.548	Detected	1.806	Detected	2.177	0.032	GT	Sens	contig856	contig856	BURP do	AGTCCA	contig85
GT	Sense	Sense	-0.142	Comprom	0.142	Comprom	0.000	1.139	Detected	1.279	Detected	1.209	0.017	GT	Sens	contig857	contig857	Unknown	GAACCA	contig85
GT	Sense	Sense	0.028	Detected	-0.028	Detected	0.000	1.395	Detected	1.021	Detected	1.208	0.024	GT	Sens	contig857	contig857	Ammoniu	TTGGAA	contig85
GT	Sense	Sense	0.102	Detected	-0.102	Detected	0.000	1.075	Detected	0.929	Detected	1.002	0.015	GT	Sens	contig862	contig862	Cysteine-	TTAGTT	contig86
GT	Sense	Sense	-0.144	Detected	0.144	Detected	0.000	1.636	Detected	1.385	Detected	1.510	0.016	GT	Sens	contig863	contig863	Ring H2 fi	GGATAT	contig86
GT	Sense	Sense	-0.220	Detected	0.220	Detected	0.000	2.115	Detected	1.357	Detected	1.736	0.058	GT	Sens	contig869	contig869	Dof zinc f	CCAAAG	contig86
GT	Sense	Sense	-0.210	Detected	0.210	Detected	0.000	1.445	Detected	1.161	Detected	1.303	0.036	GT	Sens	contig869	contig869	Chloroph	TTCTAT	contig86
GT	Sense	Sense	-0.215	Detected	0.215	Detected	0.000	1.550	Detected	1.369	Detected	1.459	0.025	GT	Sens	contig870	contig870	Unknown	GAAATC	contig87
GT	Sense	Sense	-0.204	Comprom	0.204	Comprom	0.000	1.208	Detected	1.070	Detected	1.139	0.034	GT	Sens	contig872	contig872	Glucose-	GAAGCG	contig87
GT	Sense	Sense	-0.689	Comprom	0.689	Detected	0.000	0.980	Detected	1.185	Detected	1.082	0.260	GT	Sens	contig877	contig877	S-locus r	GACTCA	contig87
GT	Sense	Sense	0.291	Detected	-0.291	Detected	0.000	1.733	Detected	2.271	Detected	2.002	0.037	GT	Sens	contig878	contig878	1-aminoc	GCCATT	contig87
GT	Sense	Sense	-0.072	Detected	0.072	Detected	0.000	1.158	Detected	0.853	Detected	1.005	0.027	GT	Sens	contig881	contig881	Cathepsi	TTAAAA	contig88
GT	Sense	Sense	0.396	Comprom	-0.396	Comprom	0.000	1.693	Detected	1.238	Detected	1.466	0.085	GT	Sens	contig884	contig884	Unknown	GAAGCA	contig88
GT	Sense	Sense	-0.275	Detected	0.275	Detected	0.000	0.978	Detected	1.076	Detected	1.027	0.067	GT	Sens	contig887	contig887	Chitinase	AGCAAG	contig88
[illegible]																				

GT	Sense	Sense	-0.105	Detected	0.105	Detected	0.000	1.767	Detected	1.053	Detected	1.410	0.063	GT	Sens	contig223	contig223	RING-fing	[illegible]	contig223	Solvc12g	Solvc12g	RING-fing	GO:00082	GO:00082	contig223	Solvc03g	Cellulose	GO:00167	SL2.40ch	AT3G518	HMGB1. I	HMGB1 (	chr3:192	123.277	134.199	288.526	189.704	466.866	283.699
GT	Sense	Sense	-0.156	Detected	0.156	Detected	0.000	1.101	Detected	1.093	Detected	1.097	0.020	GT	Sens	contig224	contig224	Ulp1 prot	[illegible]	contig224	Solvc01g	Solvc01g	Ulp1 protease family C-termina			contig224	Solvc07g	Lysine ketoglutarate		SL2.40ch	AT5G39870.1		unknown	chr5:159	214.884	250.99	352.356	270.15	531.226	526.598
GT	Sense	Sense	0.779	Detected	-0.779	Detected	0.000	1.836	Detected	1.626	Detected	1.760	0.156	GT	Sens	contig224	contig224	Unknown	[illegible]	contig224	Solvc12g	Solvc12g	Unknown Protein (AHRD V1)			contig224	Solvc12g	Unknown Protein (A		SL2.40ch	AT5G509	CLPC. AT	CLPC1:	chr5:207	216.572	600.284	1485.44	590.694	1429.04	1183.41
GT	Sense	Sense	-0.012	Detected	0.012	Detected	0.000	1.692	Detected	1.240	Detected	1.466	0.023	GT	Sens	contig225	contig225	Subtilisin	[illegible]	contig225	Solvc08g	Solvc08g	Subtilisin	GO:00042	GO:00042	contig225	Solvc08g	Subtilisin	GO:00042	SL2.40ch	AT3G52700.1		unknown	chr3:195	51.7874	49.5488	112.461	45.3844	174.49	127.232
GT	Sense	Sense	-0.342	Comprom	0.342	Comprom	0.000	4.813	Detected	4.768	Detected	4.791	0.009	GT	Sens	contig226	contig226	Ectonucl	[illegible]	contig226	Solvc12g	Solvc12g	Ectonucl	GO:00167	GO:00167	contig226	Solvc11g	Somethin	GO:00056	SL2.40ch	AT5G65520.1				1.9957	3.01712	35.7349	19.7019	78.8317	53.8843
GT	Sense	Sense	0.588	Detected	-0.588	Detected	0.000	3.020	Detected	2.395	Detected	2.708	0.044	GT	Sens	contig228	contig228	Defensin	[illegible]	contig228	Solvc07g	Solvc07g	Defensin-	GO:00069	GO:00069	contig228	Solvc07g	Defensin-	GO:00069	SL2.40ch	AT5G616	ATATH15	ATATH1	chr5:247	68.6934	28.6181	36.8124	38.9189	674.895	365.949
GT	Sense	Sense	0.304	Detected	-0.304	Detected	0.000	1.577	Detected	1.858	Detected	1.718	0.036	GT	Sens	contig228	contig228	Unknown	[illegible]	contig228	Solvc09g	Solvc09g	Unknown Protein (AHRD V1)			contig228	Solvc07g	Unknown Protein (A		SL2.40ch	AT3G19520.2				53.2742	32.9014	47.1089	47.2239	133.228	161.414
GT	Sense	Sense	-0.164	Comprom	0.164	Comprom	0.000	2.174	Detected	1.124	Detected	1.649	0.096	GT	Sens	contig229	contig229	Ulp1 prot	[illegible]	contig229	Solvc04g	Solvc04g	Ulp1 protease family C-termina			contig229	Solvc10g	Ulp1 protease family		SL2.40ch	AT4G38090.2		unknown	chr4:178	6.20577	7.32988	25.9611	24.9784	32.4483	15.6298
GT	Sense	Sense	0.033	Detected	-0.033	Detected	0.000	2.193	Detected	2.059	Detected	2.126	0.002	GT	Sens	contig230	contig230	1-aminoc	[illegible]	contig230	Solvc09g	Solvc09g	1-aminoc	GO:00164	GO:00164	contig230	Solvc09g	1-aminoc	GO:00164	SL2.40ch	AT3G61400.1		2-oxoglu	chr3:227	568.761	511.551	4841.14	2301.45	2638.76	2346.98
GT	Sense	Sense	-0.278	Comprom	0.278	Comprom	0.000	1.285	Detected	1.783	Detected	1.534	0.054	GT	Sens	contig231	contig231	Unknown	[illegible]	contig231	Solvc11g	Solvc11g	Unknown Protein (AHRD V1)			contig231	Solvc01g	Unknown Protein (A		SL2.40ch	AT4G12590.1		unknown	chr4:745	4.27633	5.91343	10.3747	11.2392	13.0707	18.3901
GT	Sense	Sense	0.420	Detected	-0.420	Detected	0.000	2.583	Detected	1.681	Detected	2.132	0.042	GT	Sens	contig231	contig231	Hypersen	[illegible]	contig231	Solvc11g	Solvc11g	Unknown Protein (AHRD V1)			contig231	Solvc07g	Hypersen	GO:00338	SL2.40ch	AT5G202	DIN10	DIN10 (D	chr5:683	240.769	126.639	202.053	116.26	2124.07	1077.78
GT	Sense	Sense	0.514	Comprom	-0.514	Comprom	0.000	1.613	Detected	2.398	Detected	2.006	0.090	GT	Sens	contig232	contig232	Unknown	[illegible]	contig232	Solvc12g	Solvc12g	Unknown Protein (AHRD V1)			contig232	Solvc04g	Unknown Protein (A		SL2.40ch	AT4G19570.1		DNAJ he	chr4:106	4.80753	2.21819	5.73532	4.27969	10.6535	18.1814
GT	Sense	Sense	-0.240	Detected	0.240	Detected	0.000	1.260	Detected	1.448	Detected	1.354	0.034	GT	Sens	contig232	contig232	Cytochro	[illegible]	contig232	Solvc07g	Solvc07g	Cytochro	GO:00198	GO:00198	contig232	Solvc07g	Cytochro	GO:00198	SL2.40ch	AT4G395	CYP96A1	CYP96A1	chr4:183	542.422	711.739	1225.02	1170.8	1586.78	1798.13
GT	Sense	Sense	-0.481	Comprom	0.481	Detected	0.000	1.949	Detected	1.478	Detected	1.713	0.085	GT	Sens	contig233	contig233	Unknown	[illegible]	contig233	Solvc07g	Solvc07g	Unknown Protein (AHRD V1)			contig233	Solvc07g	Unknown Protein (A		SL2.40ch	AT2G479	SCC3. AT	SCC3 (SI	chr2:196	3.03462	5.56496	7.98976	4.26984	16.9147	12.1704
GT	Sense	Sense	0.018	Detected	-0.018	Detected	0.000	1.939	Detected	1.887	Detected	1.913	0.000	GT	Sens	contig233	contig233	Endonuc	[illegible]	contig233	Solvc07g	Solvc07g	Endonuclease/exonuclease/pl			contig233	Solvc07g	Endonuclease/exon		SL2.40ch	AT5G618	GUT1	GUT1: ca	chr5:248	963.235	883.587	2562.9	1791.2	3773.15	3613.99
GT	Sense	Sense	0.334	Detected	-0.334	Detected	0.000	1.026	Detected	1.163	Detected	1.095	0.066	GT	Sens	contig234	contig234	Hydroxy	[illegible]	contig234	Solvc05g	Solvc05g	Hydroxyc	GO:00167	GO:00167	contig234	Solvc05g	Hydroxyc	GO:00167	SL2.40ch	AT2G39980.1		transfera	chr2:166	814.586	482.59	401.984	836.507	4641.63	1492.22
GT	Sense	Sense	0.093	Detected	-0.093	Detected	0.000	1.385	Detected	1.011	Detected	1.198	0.029	GT	Sens	contig236	contig236	Acetyl es	[illegible]	contig236	Solvc01g	Solvc01g	Acetyl es	GO:00040	GO:00040	contig236	Solvc01g	Acetyl es	GO:00040	SL2.40ch	AT1G19190.1		hydrolas	chr1:662	1044.25	864.135	2165.46	1487.38	2646.49	2035.49
GT	Sense	Sense	0.160	Detected	-0.160	Detected	0.000	1.280	Detected	1.803	Detected	1.542	0.023	GT	Sens	contig236	contig236	Unknown	[illegible]	contig236	Solvc01g	Solvc01g	Unknown Protein (AHRD V1)			contig236	Solvc03g	Iron-sulfu	GO:00081	SL2.40ch	AT1G01240.3				208.862	157.326	254.84	241.749	711.637	643.142
GT	Sense	Sense	0.801	Comprom	-0.801	Comprom	0.000	1.547	Detected	1.792	Detected	1.669	0.176	GT	Sens	contig237	contig237	Non-LTR	ATTCAA	contig237	Solvc06g	Solvc06g	Non-LTR retroelement reverse			contig237	Solvc02g	Nitric oxid	GO:00055	SL2.40ch	AT1G61620.1		phospho	chr1:227	7.2918	2.26012	7.07775	3.71569	12.6469	14.9474
GT	Sense	Sense	-0.176	Detected	0.176	Detected	0.000	2.459	Detected	1.359	Detected	1.909	0.081	GT	Sens	contig237	contig237	Subtilisin	TTTTTTA	contig237	Solvc08g	Solvc08g	Subtilisin	GO:00042	GO:00042	contig237	Solvc08g	Subtilisin	GO:00042	SL2.40ch	AT1G019	ATSBT1.1	SBTI1.1:	chr1:310	26.8134	32.1967	142.683	35.3451	172.226	80.143
GT	Sense	Sense	-0.114	Detected	0.114	Detected	0.000	1.574	Detected	1.170	Detected	1.372	0.028	GT	Sens	contig238	contig238	Receptor	TGGCTT	contig238	Solvc04g	Solvc04g	Receptor	GO:00046	GO:00046	contig238	Solvc04g	Receptor	GO:00046	SL2.40ch	AT2G43700.1		lectin pro	chr2:181	143.604	158.327	280.853	227.647	478.753	360.623
GT	Sense	Sense	1.150	Detected	-1.150	Detected	0.000	5.399	Detected	4.308	Detected	4.854	0.062	GT	Sens	contig238	contig238	Chymotry	CCATAG	contig238	Solvc09g	Solvc09g	Chymotry	GO:00096	GO:00096	contig238	Solvc09g	Chymotry	GO:00096	SL2.40ch	AT5G28350.2		FUNCTIO	chr5:103	1076.6	205.752	1114.01	574.524	21174.6	9914.83
GT	Sense	Sense	-0.427	Comprom	0.427	Comprom	0.000	1.495	Detected	0.853	Detected	1.174	0.159	GT	Sens	contig238	contig238	Solute ca	TGAAGA	contig238	Solvc04g	Solvc04g	Solute ca	GO:00080	GO:00080	contig238	Solvc04g	Solute ca	GO:00080	SL2.40ch	AT5G46040.1		proton-d	chr5:186	5.82832	9.91928	14.7945	10.2122	22.85	14.5987
GT	Sense	Sense	-0.016	Detected	0.016	Detected	0.000	1.793	Detected	1.310	Detected	1.552	0.024	GT	Sens	contig239	contig239	Mutator-li	ATAGGT	contig239	Solvc08g	Solvc08g	Mutator-like transposase (AHR			contig239	Solvc08g	Mutator-like transpo		SL2.40ch	AT5G30490.2		FUNCTIO	chr5:116	11.6522	11.2032	21.8634	36.8207	42.2362	30.1119
GT	Sense	Sense	0.294	Detected	-0.294	Detected	0.000	1.246	Detected	1.278	Detected	1.262	0.050	GT	Sens	contig239	contig239	Chromod	GAAGTG	contig239	Solvc07g	Solvc07g	Chromod	GO:00082	GO:00082	contig239	Solvc09g	UDP-gluc	GO:00080	SL2.40ch	AT3G52700.1		unknown	chr3:195	695.702	435.66	338.83	369.221	1392.4	1419.71
GT	Sense	Sense	0.359	Detected	-0.359	Detected	0.000	2.096	Detected	1.274	Detected	1.685	0.091	GT	Sens	contig240	contig240	Proteinas	TATTCA	contig240	Solvc03g	Solvc03g	Proteinas	GO:00048	GO:00048	contig240	Solvc03g	Proteinas	GO:00048	SL2.40ch	AT5G65920.1		U-box do	chr5:263	8248.06	4720.08	8113.61	4213.49	28454.8	16046.3
GT	Sense	Sense	-0.188	Detected	0.188	Detected	0.000	2.184	Detected	2.378	Detected	2.281	0.009	GT	Sens	contig246	contig246	Unknown	GACTTG	contig246	Solvc05g	Solvc05g	Unknown Protein (AHRD V1)			contig246	Solvc05g024230.1.1				AT4G344	CHB3. AT	CHB3: DI	chr4:164	86.0548	105.097	69.3597	64.115	460.725	525.446
GT	Sense	Sense	0.176	Detected	-0.176	Detected	0.000	1.251	Detected	1.768	Detected	1.510	0.040	GT	Sens	contig246	contig246	Unknown	CGAGAA	contig246	Solvc12g	Solvc12g	Unknown Protein (AHRD V1)			contig246	Solvc12g	Unknown Protein (A		SL2.40ch	AT2G173	HMG2. HM	HMG2 (3-	chr2:755	40.9615	30.2057	64.3856	54.9848	89.3046	127.382
GT	Sense	Sense	-0.192	Detected	0.192	Detected	0.000	1.741	Detected	1.997	Detected	1.869	0.015	GT	Sens	contig247	contig247	Unknown	CTGTAT	contig247	Solvc00g	Solvc00g	Unknown Protein (AHRD V1)			contig247	Solvc04g	Subtilisin	GO:00042	SL2.40ch	AT1G64300.2		protein k	chr1:238	9267.84	11377.6	96831.9	55010.9	36591.4	43580.5
GT	Sense	Sense	-0.512	Comprom	0.512	Comprom	0.000	2.193	Detected	1.076	Detected	1.634	0.164	GT	Sens	contig248	contig248	Glucose-I	TGCAGA	contig248	Solvc08g	Solvc08g	Glucose-methanol-choline oxi			contig248	Solvc08g	Glucose-methanol-c		SL2.40ch	AT5G285	BIP1	BIP1: AT	chr5:105	4.47838	8.57167	8.27817	12.9064	30.2162	13.8845
GT	Sense	Sense	0.023	Detected	-0.023	Detected	0.000	1.428	Detected	1.155	Detected	1.291	0.011	GT	Sens	contig248	contig248	Unknown	TCCTGC	contig248	Solvc10g	Solvc10g	Unknown Protein (AHRD V1)			contig248	Solvc12g	Kinesin-like calmod		SL2.40ch	AT1G03590.1		catalytic/	chr1:894	70.5667	64.2778	103.643	103.579	193.258	159.403
GT	Sense	Sense	0.721	Detected	-0.721	Detected	0.000	0.929	Detected	1.660	Detected	1.295	0.251	GT	Sens	contig248	contig248	Pol polyp	CCATAT	contig248	Solvc08g	Solvc08g	Pol polyp	GO:00082	GO:00082	contig248	Solvc08g	Pol polyp	GO:00082	SL2.40ch	AT5G28210.1		mRNA ca	chr5:101	35.56	12.3137	27.6953	55.9737	42.4791	70.322
GT	Sense	Sense	0.175	Detected	-0.175	Detected	0.000	1.533	Detected	2.823	Detected	2.178	0.083	GT	Sens	contig249	contig249	Alcohol d	GAATTG	contig249	Solvc09g	Solvc09g	Alcohol dehydrogenase (AHRD			contig249	Solvc09g	Alcohol dehydrogen		SL2.40ch	AT1G01760.1		RNA bind	chr1:276	17.4188	12.853	28.9439	29.1475	46.1819	112.562
GT	Sense	Sense	-0.123	Detected	0.123	Detected	0.000	1.168	Detected	1.281	Detected	1.225	0.012	GT	Sens	contig249	contig249	Chloroph	AAATCA	contig249	Solvc02g	Solvc02g	Chloroph	GO:00055	GO:00055	contig249	Solvc02g	Chloroph	GO:00055	SL2.40ch	AT2G344	LHB1B2.	LHB1B2:	chr2:145	290.441	323.97	612.073	379.645	735.22	792.437
GT	Sense	Sense	0.008	Detected	-0.008	Detected	0.000	0.999	Detected	1.584	Detected	1.291	0.048	GT	Sens	contig250	contig250	Unknown	GCTCTT	contig250	Solvc04g	Solvc04g	Unknown Protein (AHRD V1)			contig250	Solvc04g	Unknown Protein (A		SL2.40ch	AT3G03970.2		binding	chr3:102	18.1768	16.9075	15.4243	26.3012	37.3629	55.8623
GT	Sense	Sense	0.039	Detected	-0.039	Detected	0.000	1.117	Detected	1.240	Detected	1.178	0.004	GT	Sens	contig250	contig250	Serine/th	GACGTA	contig250	Solvc06g	Solvc06g	Serine/threonine-protein phos			contig250	Solvc06g050390.1.1				AT5G40380.1		protein k	chr5:161	15.6086	13.9068	67.0543	76.9075	34.0661	36.9951
GT	Sense	Sense	-0.122	Detected	0.122	Detected	0.000	1.559	Detected	1.932	Detected	1.746	0.016	GT	Sens	contig251	contig251	Unknown	GAGTTG	contig251	Solvc01g	Solvc01g	Unknown Protein (AHRD V1)			contig251	Solvc01g	Unknown Protein (A		SL2.40ch	AT1G11500.1				11669.5	13009.3	116032	66977.3	38717.6	49987.7
GT	Sense	Sense	0.273	Detected	-0.273	Comprom	0.000	1.019	Detected	0.982	Detected	1.001	0.067	GT	Sens	contig251	contig251	MuDR far	CGATAC	contig251	Solvc12g	Solvc12g	MuDR family transposase con			contig251	Solvc10g	Mutator-li	GO:00082	SL2.40ch	AT5G47250.1		disease r	chr5:191	15.776	10.1661	18.1862	16.7508	27.3767	26.5908
GT	Sense	Sense	0.007	Detected	-0.007	Detected	0.000	1.525	Detected	1.383	Detected	1.454	0.002	GT	Sens	contig252	contig252	Yippee-lik	CAGAAA	contig252	Solvc03g	Solvc03g	Yippee-lik	GO:00055	GO:00055	contig252	Solvc03g	Yippee-lik	GO:00055	SL2.40ch	AT2G40110.1		yippee fa	chr2:167	163.275	152.134	264.812	221.791	483.72	437.15
GT	Sense	Sense	-0.728	Comprom	0.728	Comprom	0.000	1.599	Detected	1.141	Detected	1.370	0.215	GT	Sens	contig253	contig253	Amino ac	TGGTAT	contig253	Solvc05g	Solvc05g	Amino acid transporter (AHRD			contig253	Solvc05g	Amino ac	GO:00151	SL2.40ch	AT5G14470.1				4.19197	10.8213	4.61434	6.70937	21.7578	15.7875
GT	Sense	Sense	1.174	Detected	-1.174	Comprom	0.000	2.737	Detected	1.455	Detected	2.096	0.258	GT	Sens	contig253	contig253	Unknown	AAGAAA	contig253	Solvc05g	Solvc05g	Unknown Protein (AHRD V1)			contig253	Solvc05g	Unknown Protein (A		SL2.40ch	AT2G237	BLH4. SA	BLH4 (BE	chr2:101	26.8278	4.9604	18.1434	31.0204	81.9973	33.6251
GT	Sense	Sense	0.474	Detected	-0.474	Comprom	0.000	0.917	Detected	2.800	Detected	1.859	0.220	GT	Sens	contig253	contig253	Carboxyl	CCTATG	contig253	Solvc07g	Solvc07g	Carboxyl-	GO:00055	GO:00055	contig253	Solvc07g	Carboxyl-	GO:00055	SL2.40ch	AT2G472	LACS1	long-cha	chr2:193	14.6092	7.12247	143.9	43.6541	20.5443	75.5532
GT	Sense	Sense	0.507	Detected	-0.507	Detected	0.000	3.974	Detected	2.959	Detected	3.467	0.040	GT	Sens	contig253	contig253	Monooxy	CACAGG	contig253	Solvc06g	Solvc06g	Monooxy	GO:00044	GO:00044	contig253	Solvc03g	Proteinas	GO:00048	SL2.40ch	AT4G39610.1		unknown	chr4:183	39.7514	18.524	39.5176	14.5476	454.784	224.345
GT	Sense	Sense	-0.172	Detected	0.172	Detected	0.000	1.150	Detected	0.947	Detected	1.048	0.035	GT	Sens	contig255	contig255	Unknown	AAGGGT	contig255	Solvc08g	Solvc08g	Unknown Protein (AHRD V1)			contig255	Solvc08g	Unknown Protein (A		SL2.40ch	AT4G26190.1		FUNCTIO	chr4:132	49.2172	58.8109	53.176	49.6081	127.304	110.25
GT	Sense	Sense	-0.126	Detected	0.126	Detected	0.000	1.932	Detected	0.982	Detected	1.457	0.097	GT	Sens	contig258	contig258	Glutared	ATAGAG	contig258	Solvc01g	Solvc01g	Glutared	GO:00454	GO:00454	contig258	Solvc01g	Glutared	GO:00454	SL2.40ch	AT3G020	ROXY1	ROXY1:	chr3:332	57.9671	64.9554	95.0603	83.6427	249.77	128.857
GT	Sense	Sense	-0.227	Comprom	0.227	Comprom	0.000	1.286	Detected	1.131	Detected	1.209	0.037	GT	Sens	contig259	contig259	Mutator-li	TGTGGC	contig259	Solvc10g	Solvc10g	Mutator-li	GO:00082	GO:00082	contig259	Solvc10g	Mutator-li	GO:00082	SL2.40ch	AT5G20690.1		ATP bind	chr5:700	6.29077	8.10693	7.98593	14.3422	18.5749	16.6295
GT	Sense	Sense	0.052	Detected	-0.052	Detected	0.000	1.114	Detected	1.275	Detected	1.195	0.006	GT	Sens	contig261	contig261	Ribosom	TTGAAG	contig261	Solvc11g	Solvc11g	Ribosom	GO:00058	GO:00058	contig261	Solvc10g	30S ribos	GO:00058	SL2.40ch	AT5G02960.1		40S ribos	chr5:693	64.9076	56.8198	59.3294	55.6292	140.194	156.25
GT	Sense	Sense	0.032	Comprom	-0.032	Comprom	0.000	0.884	Detected	1.198	Detected	1.041	0.023	GT	Sens	contig261	contig261	Hcr9-4E (	CCATAA	contig261	Solvc12g	Solvc12g	Hcr9-4E (AHRD V1 *-*- O50024			contig261	Solvc12g	LRR rece	GO:00163	SL2.40ch	AT3G09035.1		legume l	chr3:275	8.30461	7.47626	10.6642	6.98017	15.5082	19.2167
GT	Sense	Sense	0.213	Detected	-0.213	Comprom	0.000	3.117	Detected	2.138	Detected	2.627	0.039	GT	Sens	contig262	contig262	Acid pho	CACTTA	contig262	Solvc06g	Solvc06g	Acid pho	GO:00039	GO:00039	contig262	Solvc06g	Acid pho	GO:00039	SL2.40ch	AT5G247	VSP2. AT	VSP2 (VE	chr5:850	15.8577	11.1042	17.835	21.2728	122.742	62.0954
GT	Sense	Sense	0.363	Comprom	-0.363	Comprom	0.000	0.879	Detected	1.552	Detected	1.216	0.134	GT	Sens	contig262	contig262	Mutator-li	GAAGAG	contig262	Solvc02g	Solvc02g	Mutator-li	GO:00082	GO:00082	contig262	Solvc08g	Mutator-li	GO:00082	SL2.40ch	AT4G29420.1		F-box far	chr4:144	12.7156	7.22909	20.5764	19.2131	18.8015	29.8982
GT	Sense	Sense	-0.798	Comprom	0.798	Detected	0.000	1.762	Detected	1.185	Detected	1.473	0.225	GT	Sens	contig266	contig266	Very simi	GCTCAA	contig266	Solvc03g	Solvc03g	Very similar to retrotransposo			contig266	Solvc03g	Unknown Protein (A		SL2.40ch	AT3G089	ATERDJ3	ATERDJ3	chr3:273	5.91525	16.8215	25.2758	16.6708	36.0896	24.1055
GT	Sense	Sense	-0.053	Detected	0.053	Detected	0.000	1.267	Detected	1.013	Detected	1.140	0.014	GT	Sens	contig267	contig267	F-box fan	GGCATA	contig267	Solvc04g	Solvc04g	F-box family protein (AHRD V1			contig267	Solvc04g	F-box family protein		SL2.40ch	AT3G05070.1		FUNCTIO	chr3:141	264.062	267.291	405.655	261.8	681.738	569.956
GT	Sense	Sense	0.007	Comprom	-0.007	Comprom	0.000	1.741	Detected	2.323	Detected	2.032	0.020	GT	Sens	contig268	contig268	Ulp1 pept	CACAAC	contig268	Solvc08g	Solvc08g	Ulp1 pept	GO:00065	GO:00065	contig268	Solvc08g	Ulp1 pept	GO:00065	SL2.40ch	AT3G26140.1		glycosyl	chr3:955	3.04092	2.83556	9.18784	22.6039	10.47	15.6243
GT	Sense	Sense	0.101	Detected	-0.101	Comprom	0.000	1.320	Detected	1.283	Detected	1.301	0.006	GT	Sens	contig268	contig268	Thioester	ACAAAC	contig268	Solvc03g	Solvc03g	Thioester	GO:00055	GO:00055	contig268	Solvc03g	Thioester	GO:00055	SL2.40ch	AT2G47250.1				11.18	9.14879	13.7465	13.0235	26.9267	26.1572
GT	Sense	Sense	-0.082	Detected	0.082	Detected	0.000	1.977	Detected	1.408	Detected	1.693	0.029	GT	Sens	contig273	contig273	Dehydroc	ACCAAT	contig273	Solvc03g	Solvc03g	Dehydroc	GO:00469	GO:00469	contig273	Solvc03g	Dehydroc	GO:00469	SL2.40ch	AT4G23430.2		short-cha	chr4:122	1259.78	1328.36	2392.4	1590.04	5430.79	3649.99
GT	Sense	Sense	-0.096	Comprom	0.096	Comprom	0.000	2.848	Detected	2.877	Detected	2.862	0.001	GT	Sens	contig273	contig273	Unknown	AGAGGA	contig273	Solvc11g	Solvc11g	Unknown Protein (AHRD V1)			contig273	Solvc11g	Unknown Protein (A		SL2.40ch	AT4G17210.1		myosin h	chr4:965	2.11476	2.27186	12.8581	9.51695	16.8247	17.1148
GT	Sense	Sense	-1.215	Comprom	1.215	Comprom	0.000	3.833	Detected	2.561	Detected	3.197	0.145	GT	Sens	contig273	contig273	Salicylic	TTTGAA	contig273	Solvc09g	Solvc09g	Salicylic	GO:00081	GO:00081	contig273	Solvc09g	Salicylic	GO:00081	SL2.40ch	AT3G448	FAMT	FAMT (fa	chr3:163	2.09482	10.6178	14.4595	6.05584	71.6594	29.585
GT	Sense	Sense	0.114	Detected	-0.114	Detected	0.000	1.861	Detected	1.679	Detected	1.770	0.007	GT	Sens	contig273	contig273	Dof zinc	GTCTTT	contig273	Solvc00g	Solvc00g	Dof zinc finger protein DOF2.4			contig273	Solvc02g	GDSL est	GO:00040	SL2.40ch	AT4G03153.1		kinase in	chr4:139	29.5741	23.7674	39.7857	29.6883	102.733	90.2506
GT	Sense	Sense	0.138	Detected	-0.138	Detected	0.000	1.329	Detected	0.967	Detected	1.148	0.037	GT	Sens	contig275	contig275	Os01g01	AATTAT	contig275	Solvc07g	Solvc07g	Os01g0101200 protein (Fragm			contig275	Solvc07g	Os01g0101200 prot		SL2.40ch	AT5G202	PI	PI (PISTI	chr5:682	43.901	34.1335	24.9951	20.9534	103.697	80.4562
GT	Sense	Sense	-0.127	Detected	0.127	Detected	0.000	1.827	Detected	1.533	Detected	1.680	0.013	GT	Sens	contig276	contig276	Unknown	ACTACA	contig276	Solvc08g	Solvc08g	Unknown Protein (AHRD V1)			contig276	Solvc08g	Unknown Protein (A		SL2.40ch	AT3G45850.1		kinesin m	chr3:168	58.0966	65.1504	102.378	90.2958	232.766	189.316
GT	Sense	Sense	-0.001	Detected	0.001	Detected	0.000	1.128	Detected	0.952	Detected	1.040	0.007	GT	Sens	contig276	contig276	Unknown	CCTTCA	contig276	Solvc00g	Solvc00g	Unknown Protein (AHRD V1)			contig276	Solvc00g	Unknown Protein (A		SL2.40ch	AT4G25800.2		calmodu	chr4:131	47.7243	44.9492	56.1333	56.7159	107.947	95.2748
GT	Sense	Sense	0.427	Detected	-0.427	Detected	0.000	1.101	Detected	1.472	Detected	1.286	0.110	GT	Sens	contig278	contig278	Unknown	GGAAGG	contig278	Solvc10g	Solvc10g	Unknown Protein (AHRD V1)			contig278	Solvc10g	Unknown Protein (A		SL2.40ch	AT2G256	SPIK. AK	SPIK (Sh	chr2:108	34.3024	17.8573	29.4304	43.4658	56.6076	72.9808
GT	Sense	Sense	0.078	Detected	-0.078	Detected	0.000	3.578	Detected	3.568	Detected	3.573	0.000	GT	Sens	contig278	contig278	Cellulose	CTTTTG	contig278	Solvc05g	Solvc05g	Cellulose	GO:00167	GO:00167	contig278	Solvc10g	Cytochro	GO:00198	SL2.40ch	AT2G43310.1				53.4696	45.1659	338.345	177.877	625.917	619.508
GT	Sense	Sense	0.654	Detected	-0.654	Detected	0.000	2.941	Detected	1.943	Detected	2.442	0.097	GT	Sens	contig278	contig278	Proteinas	ATCCAT	contig278	Solvc09g	Solvc09g	Proteinas	GO:00096	GO:00096	contig278	Solvc09g	Proteinas	GO:00096	SL2.40ch	AT3G426	CHR38. C	CHR38 (C	chr3:147	1778.24	675.329	961.061	289.734	8975.18	4480.24
GT	Sense	Sense	0.101	Detected	-0.101	Detected	0.000	1.479	Detected	1.095	Detected	1.287	0.027	GT	Sens	contig279	contig279	Pentatric	GTTCAG	contig279	Solvc11g	Solvc11g	Pentatricopeptide repeat-conta			contig279	Solvc11g	Pentatricopeptide re		SL2.40ch	AT5G64510.1		unknown	chr5:257	83.729	68.4556	298.578	126.572	224.982	172.001
GT	Sense	Sense	0.000	Detected	0.000	Detected	0.000	1.518	Detected	0.836	Detected	1.177	0.075	GT	Sens	contig279	contig279	Unknown	ACTGCG	contig279	Solvc05g	Solvc05g	Unknown Protein (AHRD V1)			contig279	Solvc06g	Protein-P	GO:00081	SL2.40ch	AT2G438	UGT74F1	UDP-gluc	chr2:181	299.589	281.734	531.312	122.773	887.177	551.447
GT	Sense	Sense	-0.808	Comprom	0.808	Comprom	0.000	2.355	Detected	2.033	Detected	2.194	0.117	GT	Sens	contig279	contig279	Serine/th	TGATAG	contig279	Solvc05g	Solvc05g	Serine/threonine-protein phos			contig279	Solvc09g	Serine/threonine-pr		SL2.40ch	AT4G046	APR1. AP	APR1 (AF	chr4:232	2.02742	5.84465	9.1853	5.04575	18.7763	14.9742
GT	Sense	Sense	-0.211	Detected	0.211	Detected	0.000	1.573	Detected	1.290	Detected	1.431	0.030	GT	Sens	contig280	contig280	Chloroph	TTAGAG	contig280	Solvc12g	Solvc12g	Chloroph	GO:00160	GO:00160	contig280	Solvc12g	Chloroph	GO:00160	SL2.40ch	AT5G542	LHCB3. L	LHCB3 (L	chr5:220	527.478	665.372	1150.05	652.641	1879.1	1539.78
GT	Sense	Sense	-0.004	Detected	0.004	Detected	0.000	1.216	Detected	1.092	Detected	1.154	0.003	GT	Sens	contig280	contig280	Mutator-li	GTGCAG	contig280	Solvc12g	Solvc12g	Mutator-li	GO:00082	GO:00082	contig280	Solvc12g	Mutator-li	GO:00082	SL2.40ch	AT2G37080.1		myosin h	chr2:155	47.708	45.1606	53.6422	64.2059	114.947	105.221
GT	Sense	Sense	-0.017	Detected	0.017	Detected	0.000	1.143	Detected	1.153	Detected	1.148	0.000	GT	Sens	contig280	contig280	Cytochro	TAGTCC	contig280	Solvc09g	Solvc09g	Cytochro	GO:00198	GO:00198	contig280	Solvc09g	Cytochro	GO:00198	SL2.40ch	AT3G262	CYP71B2	CYP71B2	chr3:963	271.313	261.234	417.923	453.646	626.759	629.346
GT	Sense	Sense	-0.203	Detected	0.203	Detected	0.000	2.430	Detected	1.351	Detected	1.890	0.082	GT	Sens	contig280	contig280	Pol polyp	ATTGCG	contig280	Solvc08g	Solvc08g	Pol polyp	GO:00150	GO:00150	contig280	Solvc11g	Pol polyp	GO:00030	SL2.40ch	AT3G58090.1		FUNCTIO	chr3:215	125.999	157.125	522.215	193.961	808.393	381.499
GT	Sense	Sense	0.413	Comprom	-0.413	Comprom	0.000	2.260	Detected	2.031	Detected	2.145	0.038	GT	Sens	contig281	contig281	Serine/th	GGATGG	contig281	Solvc08g	Solvc08g	Serine/threonine-protein phos			contig281	Solvc05g	Serine/threonine-pr		SL2.40ch	AT4G046	APR1. AP	APR1 (AF	chr4:232	4.14204	2.19894	8.09663	13.3297	15.4122	13.1117
GT	Sense	Sense	0.085	Detected	-0.085	Comprom	0.000	1.670	Detected	1.238	Detected	1.454	0.024	GT	Sens	contig281	contig281	Ulp1 prot	TTCTGT	contig281	Solvc02g	Solvc02g	Ulp1 protease family C-termina			contig281	Solvc04g045500.1.1				AT4G13340.1		leucine-r	chr4:775	11.2098	9.37825	15.6153	20.8617	34.796	25.7204
GT	Sense	Sense	0.043	Detected	-0.043	Detected	0.000	3.232	Detected	3.003	Detected	3.118	0.002	GT	Sens	contig282	contig282	F-box fan	GTTGAT	contig282	Solvc05g	Solvc05g	F-box family protein (AHRD V1			contig282	Solvc05g	F-box family protein		SL2.40ch	AT2G362	ABI5. GIA	ABI5 (AB	chr2:152	1559.92	1382.07	4694.94	3375.88	14714.9	12513.3
GT	Sense	Sense	0.449	Comprom	-0.449	Comprom	0.000	1.531	Detected	1.437	Detected	1.484	0.082	GT	Sens	contig282	contig282	Ulp1 prot	AAGTCG	contig282	Solvc12g	Solvc12g	Ulp1 protease family C-termina			contig282	Solvc12g	Ulp1 protease family		SL2.40ch	AT3G119	UBP13	UBP13 (U	chr3:376	7.77711	3.92436	11.0176	20.7383	17.0253	15.9009
GT	Sense	Sense	-0.057	Comprom	0.057	Comprom	0.000	1.644	Detected	0.888	Detected	1.266	0.080	GT	Sens	contig283	contig283	Mutator-li	TATTTAT	contig283	Solvc09g	Solvc09g	Mutator-li	GO:00082	GO:00082	contig283	Solvc09g	Mutator-li	GO:00082	SL2.40ch	AT1G604	ATVPS34	ATVPS34	chr1:222	9.41623	9.59247	20.531	19.6313	31.6767	18.6965
GT	Sense	Sense	-0.023	Detected	0.023	Detected	0.000	2.349	Detected	2.289	Detected	2.319	0.000	GT	Sens	contig284	contig284	Unknown	GCTGAG	contig284	Solvc12g	Solvc12g	Unknown Protein (AHRD V1)			contig284	Solvc12g	Unknown Protein (A		SL2.40ch	AT5G571	ATPAP28	PAP28 (F	chr5:231	82.9116	80.5936	77.7959	64.7884	444.131	424.893
GT	Sense	Sense	0.009	Detected	-0.009	Detected	0.000	1.680	Detected	1.942	Detected	1.811	0.005	GT	Sens	contig284	contig284	Kunitz try	AATCAA	contig284	Solvc03g	Solvc03g	Kunitz try	GO:00048	GO:00048	contig284	Solvc03g	Kunitz try	GO:00048	SL2.40ch	AT5G173	HDG9	HDG9 (H	chr5:570	280.191	260.353	424.695	146.595	922.875	1103.13
GT	Sense	Sense	0.083	Detected	-0.083	Detected	0.000	1.262	Detected	0.974	Detected	1.118	0.021	GT	Sens	contig285	contig285	Sieve ele	CTCATC	contig285	Solvc04g	Solvc04g	Sieve element-occluding prote			contig285	Solvc04g	Sieve element-occlu		SL2.40ch	AT1G31130.1		unknown	chr1:111	527.027	442.304	607.277	565.767	1234.68	1008.1
GT	Sense	Sense	0.094	Detected	-0.094	Comprom	0.000	1.373	Detected	1.537	Detected	1.455	0.007	GT	Sens	contig285	contig285	Mutator-li	TAATAG	contig285	Solvc10g	Solvc10g	Mutator-li	GO:00082	GO:00082	contig285	Solvc10g	Mutator-li	GO:00082	SL2.40ch	AT5G499	emb1441	emb1441	chr5:203	12.6389	10.4354	17.1739	29.556	31.7287	35.4422
GT	Sense	Sense	0.255	Detected	-0.255	Detected	0.000	1.203	Detected	1.015	Detected	1.109	0.055	GT	Sens	contig285	contig285	Hcr2-p4.1	CAGAGT	contig285	Solvc01g	Solvc01g	Hcr2-p4.1 (AHRD V1 ***- O4G2V			contig285	Solvc01g	Hcr2-p4.1 (AHRD V1		SL2.40ch	AT4G22230.1				123.157	81.3775	201.774	184.418	245.817	215.073
GT	Sense	Sense	0.076	Comprom	-0.076	Comprom	0.000	1.923	Detected	1.239	Detected	1.581	0.046	GT	Sens	contig287	contig287	Salicylic	GCGAAC	contig287	Solvc09g	Solvc09g	Salicylic	GO:00081	GO:00081	contig287	Solvc09g	Salicylic	GO:00081	SL2.40ch	AT3G448	FAMT	FAMT (fa	chr3:163	6.30812	5.34475	7.79732	3.73994	23.4735	14.5686
GT	Sense	Sense	-0.126	Detected	0.126	Detected	0.000	1.130	Detected	0.941	Detected	1.036	0.022	GT	Sens	contig287	contig287	Malate de	AGATCT	contig287	Solvc11g	Solvc11g	Malate de	GO:00300	GO:00300	contig287	Solvc11g	Malate de	GO:00300	SL2.40ch	AT5G58330.1		malate d	chr5:235	4341.24	4861.13	8013.81	5320.8	10722.2	9377.99
GT	Sense	Sense	-0.006	Detected	0.006	Detected	0.000	1.582	Detected	1.151	Detected	1.367	0.024	GT	Sens	contig287	contig287	Genomic	TTAGCT	contig287	Solvc05g	Solvc05g	Genomic	GO:00055	GO:00055	contig287	Solvc05g	Genomic	GO:00055	SL2.40ch	AT5G501	ATFRO8.	FRO8 (FE	chr5:204	29.9987	28.4813	51.3996	37.8095	93.3499	69.024
GT	Sense	Sense	1.153	Detected	-1.153	Detected	0.000	5.615	Detected	4.560	Detected	5.088	0.057	GT	Sens	contig288	contig288	Chymotry	CCATAT	contig288	Solvc09g	Solvc09g	Chymotry	GO:00096	GO:00096	contig288	Solvc09g	Chymotry	GO:00096	SL2.40ch	AT4G123	CYP706A	CYP706A	chr4:731	547.311	104.147	637.572	310.892	12481	5986.21
GT	Sense	Sense	-0.391	Detected	0.391	Detected	0.000	1.643	Detected	1.595	Detected	1.619	0.054	GT	Sens	contig288	contig288	Unknown	AAAGAC	contig288	Solvc05g	Solvc05g	Unknown Protein (AHRD V1)			contig288	Solvc01g	Unknown Protein (A		SL2.40ch	AT3G09950.1		unknown	chr3:306	3249.67	5260.36	14519.1	9475.51	13773.2	13283.4
GT	Sense	Sense	-0.136	Detected	0.136	Detected	0.000	1.158	Detected	1.075	Detected	1.116	0.016	GT	Sens	contig288	contig288	Mutator-li	TCATGT	contig288	Solvc10g	Solvc10g	Mutator-like transposase (AHR			contig288	Solvc10g	Mutator-like transpo		SL2.40ch	AT5G44730.1		haloacid	chr5:180	48.443	55.0014	58.0042	75.0711	122.794	115.593
GT	Sense	Sense	-0.004	Detected	0.004	Detected	0.000	1.690	Detected	1.377	Detected	1.534	0.010	GT	Sens	contig289	contig289	Unknown	ACCTCG	contig289	Solvc05g	Solvc05g	Unknown Protein (AHRD V1)			contig289	Solvc11g	Ulp1 protease family		SL2.40ch	AT5G28740.1		transcrip	chr5:107	2591.35	2452.21	4382.07	5011.04	8674.92	6961.78
GT	Sense	Sense	0.171	Comprom	-0.171	Comprom	0.000	2.009	Detected	2.264	Detected	2.137	0.010	GT	Sens	contig290	contig290	Ulp1 prot	TGAGGA	contig290	Solvc06g	Solvc06g	Ulp1 prot	GO:00065	GO:00065	contig290	Solvc11g	Ulp1 protease family		SL2.40ch	AT1G03060.1		WD-40 re	chr1:712	3.74198	2.77613	5.39312	14.0585	13.8385	16.46
GT	Sense	Sense	-0.056	Detected	0.056	Detected	0.000	1.159	Detected	0.878	Detected	1.019	0.021	GT	Sens	contig290	contig290	Purine pe	GGTTGA	contig290	Solvc02g	Solvc02g	Purine pe	GO:00053	GO:00053	contig290	Solvc02g	Purine pe	GO:00053	SL2.40ch	AT4G25730.1		FtsJ-like	chr4:131	595.856	605.619	1206.78	1067.38	1430.82	1173.61
GT	Sense	Sense	0.009	Comprom	-0.009	Comprom	0.000	1.208	Detected	1.026	Detected	1.117	0.007	GT	Sens	contig290	contig290	Unknown	GAAGTT	contig290	Solvc08g	Solvc08g	Unknown	GO:00082	GO:00082	contig290	Solvc09g	GRF zinc	GO:00082	SL2.40ch	AT5G59600.1		pentatric	chr5:240	9.78684	9.09272	16.2266	14.8513	23.2369	20.4182
GT	Sense	Sense	-0.273	Detected	0.273	Detected	0.000	1.351	Detected	0.969	Detected	1.160	0.073	GT	Sens	contig291	contig291	Unknown	GTAACT	contig291	Solvc05g	Solvc05g	Unknown Protein (AHRD V1)			contig291	Solvc05g	Unknown Protein (A		SL2.40ch	AT1G19230.1		respirato	chr1:664	303.756	417.015	313.862	708.484	968.366	740.614
GT	Sense	Sense	0.027	Detected	-0.027	Detected	0.000	1.641	Detected	1.477	Detected	1.559	0.003	GT	Sens	contig291	contig291	Ulp1 prot	CGAACT	contig291	Solvc09g	Solvc09g	Ulp1 prot	GO:00065	GO:00065	contig291	Solvc11g	Ulp1 protease family		SL2.40ch	AT5G28740.1		transcrip	chr5:107	2468.46	2237.19	4485.57	4832.68	7813.1	6955.57
GT	Sense	Sense	0.107	Detected	-0.107	Comprom	0.000	1.467	Detected	0.880	Detected	1.173	0.064	GT	Sens	contig291	contig291	Oxidored	TTGGAA	contig291	Solvc03g	Solvc03g	Oxidored	GO:00055	GO:00055	contig291	Solvc03g	Oxidored	GO:00055	SL2.40ch	AT5G16990.1		NADP-de	chr5:558	13.3659	10.8356	6.35726	2.70491	35.4878	23.5405
GT	Sense	Sense	0.422	Detected	-0.422	Detected	0.000	1.811	Detected	1.768	Detected	1.789	0.051	GT	Sens	contig294	contig294	Carbonyl	TAAAAA	contig294	Solvc01g	Solvc01g	Carbonyl	GO:00040	GO:00040	contig294	Solvc01g	Carbonyl	GO:00040	SL2.40ch	AT3G61220.1		short-cha	chr3:226	37.2454	19.5288	90.2433	20.5093	100.909	97.6345
GT	Sense	Sense	-0.218	Detected	0.218	Detected	0.000	1.194	Detected	1.160	Detected	1.177	0.033	GT	Sens	contig295	contig295	Replicatio	TATGAG	contig295	Solvc01g	Solvc01g	Replicatio	GO:00036	GO:00036	contig295	Solvc01g	Replicatio	GO:00055	SL2.40ch	AT5G277	EMB2775	EMB2775	chr5:982	81.5948	103.796	144.855	87.0237	224.468	218.682
GT	Sense	Sense	-0.161	Comprom	0.161	Comprom	0.000	2.866	Detected	1.226	Detected	2.046	0.134	GT	Sens	contig295	contig295	class II h	ATTTGA	contig295	Solvc08g	Solvc08g	class II he	GO:00510	GO:00510	contig295	Solvc08g	class II he	GO:00510	SL2.40ch	AT5G120	HSP17.6II	HSP17.6I	chr5:388	9.55267	11.2342	11.9926	13.1148	80.5204	25.7718
GT	Sense	Sense	-0.070	Detected	0.070	Detected	0.000	1.109	Detected	1.284	Detected	1.197	0.009	GT	Sens	contig295	contig295	60S ribos	GGCTTC	contig295	Solvc09g	Solvc09g	60S ribos	GO:00037	GO:00037	contig295	Solvc09g	60S ribos	GO:00037	SL2.40ch	AT2G34480.1		60S ribos	chr2:145	38.9914	40.447	39.3658	37.4732	91.3351	102.828
GT	Sense	Sense	-0.557	Comprom	0.557	Comprom	0.000	1.095	Detected	1.278	Detected	1.187	0.170	GT	Sens	contig297	contig297	Ulp1 prot	AATATG	contig297	Solvc09g	Solvc09g	Ulp1 protease family C-termina			contig297	Solvc09g	Ulp1 protease family		SL2.40ch	AT5G47160.1				3.24925	6.61822	4.99075	10.638	10.5667	11.9582
GT	Sense	Sense	0.404	Comprom	-0.404	Comprom	0.000	1.450	Detected	0.934	Detected	1.192	0.131	GT	Sens	contig297	contig297	Unknown	AAGCAT	contig297	Solvc03g	Solvc03g	Unknown Protein (AHRD V1)			contig297	Solvc06g	Unknown Protein (A		SL2.40ch	AT2G021	TOM3	TOM3: p	chr2:560	10.5439	5.66364	8.96982	12.5305	22.5115	15.7017
GT	Sense	Sense	-0.696	Comprom	0.696	Detected	0.000	1.815	Detected	1.444	Detected	1.629	0.152	GT	Sens	contig298	contig298	Ulp1 prot	CTTCCT	contig298	Solvc04g	Solvc04g	Ulp1 protease family C-termina			contig298	Solvc12g	Ulp1 prot	GO:00065	SL2.40ch	AT5G612	AtGUS1	AtGUS1 (	chr5:246	7.25297	17.9229	31.277	25.7011	42.7823	32.9726
GT	Sense	Sense	-0.456	Comprom	0.456	Comprom	0.000	1.194	Detected	2.297	Detected	1.746	0.135	GT	Sens	contig299	contig299	Thaumati	ATCGTA	contig299	Solvc12g	Solvc12g	Thaumatin-like protein (AHRD			contig299	Solvc12g	Thaumatin-like prot		SL2.40ch	AT4G116	ATOSM34	ATOSM3	chr4:702	3.69386	6.54332	13.6725	14.1302	11.9943	25.6879
GT	Sense	Sense	1.377	Detected	-1.377	Comprom	0.000	1.460	Detected	2.571	Detected	2.015	0.308	GT	Sens	contig299	contig299	Unknown	TCGGTG	contig299	Solvc03g	Solvc03g	Unknown Protein (AHRD V1)			contig299	Solvc09g	Ethylene-	GO:00030	SL2.40ch	AT2G30280.1		unknown	chr2:129	16.4314	2.29164	3.51861	11.6789	17.9984	38.76
GT	Sense	Sense	-0.108	Detected	0.108	Detected	0.000	1.589	Detected	1.024	Detected	1.306	0.050	GT	Sens	contig299	contig299	CONSTAI	GGAGTA	contig299	Solvc02g	Solvc02g	CONSTAI	GO:00037	GO:00037	contig299	Solvc02g	Tomato C	GO:00037	SL2.40ch	AT5G158	COL1. AT	COL1 (co	chr5:517	719.405	786.237	1522.11	839.291	2412.49	1625.91
GT	Sense	Sense	0.042	Comprom	-0.042	Comprom	0.000	1.452	Detected	2.086	Detected	1.769	0.031	GT	Sens	contig300	contig300	Pol polyp	TTTTAG	contig300	Solvc12g	Solvc12g	Pol polyp	GO:00062	GO:00062	contig300	Solvc08g	Pol polyp	GO:00082	SL2.40ch	AT5G40450.2		unknown	chr5:161	7.63799	6.78167	16.0777	19.9826	21.0024	32.4914
GT	Sense	Sense	-0.351	Detected	0.351	Detected	0.000	1.154	Detected	0.922	Detected	1.038	0.107	GT	Sens	contig301	contig301	Unknown	AGTGAA	contig301	Solvc01g	Solvc01g	Unknown Protein (AHRD V1)			contig301	Solvc01g	Unknown Protein (A		SL2.40ch	AT5G28740.1		transcrip	chr5:107	147.696	226.206	179.67	216.869	433.656	368.144
GT	Sense	Sense	-0.070	Detected	0.070	Detected	0.000	1.089	Detected	1.054	Detected	1.072	0.005	GT	Sens	contig302	contig302	Ras-relat	TCGAGA	contig302	Solvc03g	Solvc03g	Ras-relat	GO:00055	GO:00055	contig302	Solvc03g006270.2.1				AT5G457	AtRABA1	AtRABA1	chr5:185	91.7348	95.1236	120.939	76.9521	211.939	206.265
GT	Sense	Sense	0.002	Comprom	-0.002	Comprom	0.000	0.802	Detected	1.232	Detected	1.017	0.042	GT	Sens	contig302	contig302	Unknown	ACACAG	contig302	Solvc03g	Solvc03g	Unknown Protein (AHRD V1)			contig302	Solvc03g	Unknown Protein (A		SL2.40ch	AT4G17210.1		myosin h	chr4:965	7.34843	6.89943	15.7222	18.7811	13.2425	17.7825
GT	Sense	Sense	0.221	Detected	-0.221	Detected	0.000	0.938	Detected	1.579	Detected	1.258	0.084	GT	Sens	contig302	contig302	Pol polyp	CAGTAT	contig302	Solvc01g	Solvc01g	Pol polyprotein (AHRD V1 *-*- F			contig302	Solvc11g	Pol polyp	GO:00065	SL2.40ch	AT3G27025.1				77.0916	53.377	96.5492	144.535	131.067	203.735
GT	Sense	Sense	-0.111	Detected	0.111	Detected	0.000	1.935	Detected	1.712	Detected	1.823	0.007	GT	Sens	contig303	contig303	Ovate pro	TTTTCC	contig303	Solvc02g	Solvc02g	Ovate protein (AHRD V1 ***- O8			contig303	Solvc02g	Ovate protein (AHRD		SL2.40ch	AT5G07820.1		FUNCTIO	chr5:249	123.451	135.548	126.478	136.898	527.385	450.526
GT	Sense	Sense	0.062	Detected	-0.062	Detected	0.000	1.415	Detected	1.088	Detected	1.251	0.019	GT	Sens	contig304	contig304	Cellulose	CTCAAC	contig304	Solvc08g	Solvc08g	Cellulose	GO:00167	GO:00167	contig304	Solvc08g	Cellulose	GO:00167	SL2.40ch	AT4G240	ATCSLG1	ATCSLG	chr4:124	1174.85	1014.76	3168.23	1406.94	3104.48	2467.71
GT	Sense	Sense	-0.450	Comprom	0.450	Comprom	0.000	2.163	Detected	1.855	Detected	2.009	0.052	GT	Sens	contig304	contig304	Unknown	TCCAAT	contig304	Solvc01g	Solvc01g	Unknown Protein (AHRD V1)			contig304	Solvc11g	Ulp1 protease family		SL2.40ch	AT4G098	RPS18C	RPS18C	chr4:617	2.91058	5.11152	4.38765	5.28329	18.4195	14.8309
GT	Sense	Sense	-0.712	Detected	0.712	Detected	0.000	1.810	Detected	1.477	Detected	1.644	0.153	GT	Sens	contig306	contig306	Cytochro	GGAAAA	contig306	Solvc04g	Solvc04g	Cytochro	GO:00160	GO:00160	contig306	Solvc07g	Unknown Protein (A		SL2.40ch	AT5G537	MLO11. A	MLO11 (I	chr5:218	55.2941	139.515	262.893	126.368	328.429	260.005
GT	Sense	Sense	0.777	Detected	-0.777	Comprom	0.000	2.033	Detected	1.474	Detected	1.754	0.168	GT	Sens	contig306	contig306	Major late	GATTAA	contig306	Solvc04g	Solvc04g	Major late	GO:00096	GO:00096	contig306	Solvc04g	Major late	GO:00096	SL2.40ch	AT5G643	AGP1. AT	AGP1 (AI	chr5:257	25.2773	8.09892	9.8497	6.04235	62.4524	42.2556
GT	Sense	Sense	-0.543	Comprom	0.543	Detected	0.000	2.044	Detected	1.475	Detected	1.760	0.103	GT	Sens	contig306	contig306	Ulp1 prot	CACTTT	contig306	Solvc07g	Solvc07g	Ulp1 prot	GO:00065	GO:00065	contig306	Solvc07g	Ulp1 prot	GO:00065	SL2.40ch	AT1G42470.1		patched	chr1:159	6.52386	13.0393	9.71815	12.4566	40.5726	27.2539
GT	Sense	Sense	-0.794	Detected	0.794	Detected	0.000	2.005	Detected	1.804	Detected	1.904	0.140	GT	Sens	contig306	contig306	Unknown	AAGAAT	contig306	Solvc07g	Solvc07g	Unknown Protein (AHRD V1)			contig306	Solvc07g	Unknown Protein (A		SL2.40ch	AT1G268	CUL3A. A	ATCUL3	chr1:929	201.637	570.551	1394.05	594.338	1451.89	1259.07
GT	Sense	Sense	-0.060	Detected	0.060	Detected	0.000	0.915	Detected	1.126	Detected	1.020	0.014	GT	Sens	contig308	contig308	Transpor	ACTGCA	contig308	Solvc05g	Solvc05g	Transporter-like protein (AHRD			contig308	Solvc05g	Transporter-like pro		SL2.40ch	AT5G10190.1		transpor	chr5:319	31.9467	32.6621	38.2509	29.9815	64.9339	74.9365
GT	Sense	Sense	-0.174	Detected	0.174	Detected	0.000	1.525	Detected	1.328	Detected	1.426	0.019	GT	Sens	contig309	contig309	Chloroph	TTAATT	contig309	Solvc01g	Solvc01g	Chloroph	GO:00161	GO:00161	contig309	Solvc01g	Chloroph	GO:00161	SL2.40ch	AT1G158	LHCB6. C	LHCB6 (L	chr1:544	7372.36	8835.08	19553.3	13326.1	24764.9	21535.7
GT	Sense	Sense	-0.059	Detected	0.059	Detected	0.000	1.363	Detected	1.244	Detected	1.304	0.004	GT	Sens	contig310	contig310	Yippee zi	TTGCCC	contig310	Solvc03g	Solvc03g	Yippee zi	GO:00055	GO:00055	contig310	Solvc03g	Yippee zi	GO:00055	SL2.40ch	AT2G40110.2		yippee fa	chr2:167	185.161	189.157	254.09	217.984	513.369	471.262
GT	Sense	Sense	0.026	Comprom	-0.026	Comprom	0.000	1.685	Detected	0.937	Detected	1.311	0.073	GT	Sens	contig310	contig310	Cytochro	ATATAT	contig310	Solvc11g	Solvc11g	Cytochro	GO:00200	GO:00200	contig310	Solvc11g	Cytochro	GO:00198	SL2.40ch	AT5G251	CYP71B1	CYP71B1	chr5:866	5.96577	5.41718	8.32797	8.66113	19.4862	11.5707
GT	Sense	Sense	-0.221	Detected	0.221	Detected	0.000	2.282	Detected	1.614	Detected	1.948	0.040	GT	Sens	contig316	contig316	DNA-direc	AGCTCC	contig316	Solvc10g	Solvc10g	DNA-direc	GO:0006350		contig316	Solvc10g	DNA-direc	GO:00063	SL2.40ch	AT5G451	NRPC2	NRPC2:	chr5:182	12.7677	16.3275	34.5036	21.388	74.8699	47.0017
GT	Sense	Sense	0.492	Detected	-0.492	Comprom	0.000	1.687	Detected	1.626	Detected	1.656	0.078	GT	Sens	contig324	contig324	MuDR far	CCATTT	contig324	Solvc01g	Solvc01g	MuDR family transposase con			contig324	Solvc12g	Mutator-li	GO:00082	SL2.40ch	AT4G155	IAGLU	IAGLU (IN	chr4:887	12.3364	5.86962	17.856	28.4036	29.2225	27.9182
GT	Sense	Sense	-0.199	Detected	0.199	Detected	0.000	1.585	Detected	1.296	Detected	1.441	0.028	GT	Sens	contig340	contig340	Receptor	TATTAG	contig340	Solvc10g	Solvc10g	Receptor	GO:00055	GO:00055	contig340	Solvc10g	LRR rece	GO:00055	SL2.40ch	AT4G08450.1		disease r	chr4:536	186.84	231.712	226.579	212.452	665.655	543.373
GT	Sense	Sense	-0.141	Detected	0.141	Detected	0.000	1.442	Detected	1.315	Detected	1.379	0.012	GT	Sens	contig342	contig342	Disease r	TTCAAG	contig342	Solvc10g	Solvc10g	Disease resistance protein (AH			contig342	Solvc10g	Lrr. resistance prot		SL2.40ch	AT3G231	AtRLP38	AtRLP38	chr3:822	799.403	914.941	1324.22	1245.75	2478.45	2262.45
GT	Sense	Sense	0.004	Comprom	-0.004	Comprom	0.000	2.159	Detected	1.654	Detected	1.906	0.017	GT	Sens	contig358	contig358	Cytochro	CCAACT	contig358	Solvc04g	Solvc04g	Cytochro	GO:00200	GO:00200	contig358	Solvc07g	Cytochro	GO:00198	SL2.40ch	AT3G16370.1		GDSL-m	chr3:555	9.10251	8.51997	9.78822	12.0647	41.9297	29.4612
GT	Sense	Sense	-0.255	Detected	0.255	Detected	0.000	1.279	Detected	1.064	Detected	1.171	0.051	GT	Sens	contig365	contig365	Unannota	ATGTTGTTTCAATCATGAAGTCGCTTGTGATTATCACGTTATCCATCGT							contig365	Solvc11g	Unknown Protein (A		SL2.40ch	AT3G47570.1		leucine-r	chr3:175	167.771	224.694	232.19	247.621	502.476	431.495
GT	Sense	Sense	0.076	Detected	-0.076	Detected	0.000	1.629	Detected	1.331	Detected	1.480	0.013	GT	Sens	contig370	contig370	Legumin	AACCCA	contig370	Solvc11g	Solvc11g	Legumin	GO:00457	GO:00457	contig370	Solvc11g	Legumin	GO:00457	SL2.40ch	AT1G07750.1		cupin far	chr1:240	20.6726	17.5061	18.9431	16.8918	62.7529	50.8811
GT	Sense	Sense	-0.071	Comprom	0.071	Comprom	0.000	1.074	Detected	1.070	Detected	1.072	0.004	GT	Sens	contig376	contig376	Pathogen	GAAAGA	contig376	Solvc02g	Solvc02g	Pathogenesis-related protein-l			contig376	Solvc04g	Pathogenesis-relate		SL2.40ch	AT3G52140.1		tetratrico	chr3:193	9.80705	10.1826	11.3398	12.0284	22.4392	22.3
GT	Sense	Sense	-0.338	Detected	0.338	Detected	0.000	1.907	Detected	1.888	Detected	1.898	0.030	GT	Sens	contig386	contig386	Unknown	ATCGTT	contig386	Solvc06g	Solvc06g	Unknown Protein (AHRD V1)			contig386	Solvc06g009810.2.1				AT1G508	POLGAM	POLGAM	chr1:188	20.7035	31.1407	28.2527	39.0429	101.571	99.9274
GT	Sense	Sense	0.153	Detected	-0.153	Detected	0.000	1.752	Detected	1.650	Detected	1.701	0.009	GT	Sens	contig387	contig387	Terminal	AATTGT	contig387	Solvc01g	Solvc01g	Terminal ear1-like 2 protein (A			contig387	Solvc12g	Unknown Protein (A		SL2.40ch	AT1G53200.2				1087.18	827.853	2223.13	1339.94	3406.52	3164.99
GT	Sense	Sense	-0.757	Comprom	0.757	Comprom	0.000	2.245	Detected	4.225	Detected	3.235	0.122	GT	Sens	contig405	contig405	Unannota	CCATAAGCGGCAGCATGGTGTTATGCTGTAATTTTACTATCTCAAGAAA							contig405	Solvc06g	Beta-fruc	GO:00059	SL2.40ch	AT3G45800.1				2.00863	5.39916	99.5982	124.134	16.6451	65.4662
GT	Sense	Sense	0.161	Detected	-0.161	Detected	0.000	1.307	Detected	1.360	Detected	1.334	0.015	GT	Sens	contig405	contig405	Unannota	TAATCTCCTTTGCAAAATTTATGTCTCAACACAGAGAACCCCTTTTCTC							contig405	Solvc07g	LRR rece	GO:00046	SL2.40ch	AT5G27240.1		DNAJ he	chr5:959	18.8879	14.2262	22.7558	45.4016	43.2467	44.7416
GT	Sense	Sense	0.228	Detected	-0.228	Detected	0.000	1.056	Detected	0.995	Detected	1.026	0.047	GT	Sens	contig409	contig409	Unannota	AACTTAGAATCATTTCATGCATTTAACTTGGCTTTAAAAAATATTTCAAA							contig409	Solvc11g	Unknown Protein (A		SL2.40ch	AT5G463	FLS2	FLS2 (FL	chr5:187	41.1916	28.2366	40.4797	54.0983	75.6109	72.2883
GT	Sense	Sense	0.882	Comprom	-0.882	Comprom	0.000	1.093	Detected	1.962	Detected	1.528	0.261	GT	Sens	contig410	contig410	Unknown	TGGTTT	contig410	Solvc09g	Solvc09g	Unknown Protein (AHRD V1)			contig410	Solvc09g	Unknown Protein (A		SL2.40ch	AT5G259	AtRLP52	AtRLP52	chr5:903	7.70283	2.13394	6.18184	6.9929	9.2221	16.7978
GT	Sense	Sense	-0.893	Comprom	0.893	Comprom	0.000	1.592	Detected	2.017	Detected	1.804	0.188	GT	Sens	contig410	contig410	Unannota	AGAGAAACAATTGGATTGCTTCTAGGATTGTCGTTTTGTTACTTAGGTC							contig410	Solvc03g	Unknown Protein (A		SL2.40ch	AT1G019	EDA10	EDA10 (e	chr1:330	1.98924	6.45452	2.64089	5.39169	11.5173	15.4167
GT	Sense	Sense	0.390	Detected	-0.390	Detected	0.000	0.859	Detected	1.328	Detected	1.093	0.138	GT	Sens	contig413	contig413	Unannota	GAAGAACCGAAGTCAAGCAACTACTTCTCCAATGCCTTTCCTTTTCTTA							contig413	Solvc02g014100.1.1				AT4G24880.1		unknown	chr4:128	71.7861	39.3387	82.1639	169.37	102.757	141.878
GT	Sense	Sense	-0.123	Detected	0.123	Detected	0.000	1.135	Detected	0.916	Detected	1.026	0.025	GT	Sens	contig415	contig415	Unannota	CGCTGATAAACAGTAATTATCCGCTAAAGTACGATGCTAATAGTGACTA							contig415	Solvc12g	Ulp1 protease family		SL2.40ch	AT5G24880.1		INVOLVE	chr5:855	103.408	115.457	334.5	347.665	255.896	219.22
GT	Sense	Sense	-0.111	Comprom	0.111	Comprom	0.000	3.915	Detected	4.078	Detected	3.997	0.001	GT	Sens	contig419	contig419	Unannota	CTCACCATTCAAATTTGTACATCATCGAATGGGAAATCTCTATCTTCTA							contig419	Solvc03g	Polyprote	GO:00065	SL2.40ch	AT5G64667.1				2.024	2.2202	9.93461	8.2817	34.1105	38.074
GT	Sense	Sense	-0.647	Comprom	0.647	Detected	0.000	1.616	Detected	1.217	Detected	1.417	0.172	GT	Sens	contig420	contig420	Unannota	AGCAGTAAGCAACTAAGCACTGCTTAGTGCTTACAGCCTTACACCAAAA							contig420	Solvc02g	HAT family dimerisa		SL2.40ch	AT1G21060.1		unknown	chr1:737	5.37662	12.4088	12.8054	11.3895	26.7009	20.1907
GT	Sense	Sense	0.476	Detected	-0.476	Comprom	0.000	0.815	Detected	1.639	Detected	1.227	0.191	GT	Sens	contig421	contig421	Unannota	GCTCCTGTTGTGAAGTTAATCTCTATTTGAATTCTGTTGAGAATTGTTG							contig421	Solvc06g	Peroxiso	GO:00055	SL2.40ch	AT2G22400.1		NOL1/NO	chr2:950	18.6469	9.06951	25.5993	41.9516	24.3929	43.0629
GT	Sense	Sense	-0.394	Comprom	0.394	Comprom	0.000	3.027	Detected	2.301	Detected	2.664	0.038	GT	Sens	contig425	contig425	Receptor	ATATCA	contig425	Solvc07g	Solvc07g	Receptor	GO:00046	GO:00046	contig425	Solvc07g	LRR rece	GO:00046	SL2.40ch	AT4G38470.1		protein k	chr4:179	3.68545	5.99121	13.8252	14.7562	40.8568	24.6134
GT	Sense	Sense	-0.088	Detected	0.088	Detected	0.000	1.699	Detected	0.904	Detected	1.301	0.085	GT	Sens	contig428	contig428	AT1G689	ATCAAGTTCTCAGACGGCTAACAAGGGGACTGAACAAAGTAGTAAACCT							contig428	#N/A	#N/A	#N/A	#N/A	#N/A	#N/A	#N/A	#N/A	1320.54	1404.21	855.978	349.85	4714.46	2709.3
GT	Sense	Sense	0.191	Comprom	-0.191	Comprom	0.000	1.229	Detected	1.834	Detected	1.531	0.050	GT	Sens	contig430	contig430	Cross hyl	TCATTTCCTTGTCCGAAAGGTCTGGGTAATCTTGTTAAACTCTGTCGTC							contig430	#N/A	#N/A	#N/A	#N/A	#N/A	#N/A	#N/A	#N/A	6.02729	4.3536	7.14157	50.3533	12.8059	19.4136
GT	Sense	Sense	-0.234	Detected	0.234	Detected	0.000	1.033	Detected	1.132	Detected	1.083	0.045	GT	Sens	contig430	contig430	Cross hyl	ACCTCAATAGTTTTCGCGGAGAACCAGCTATTTCCCGGCTTGATTGGC							contig430	#N/A	#N/A	#N/A	#N/A	#N/A	#N/A	#N/A	#N/A	49.0611	63.8413	114.073	109.65	122.09	130.442
GT	Sense	Sense	0.020	Detected	-0.020	Detected	0.000	2.293	Detected	1.614	Detected	1.954	0.029	GT	Sens	contig432	contig432	Nicotia ta	GGAGGTATAGCTGTTGTTTGATGATGTGCTAAGTTGAATGTCATTATG							contig432	#N/A	#N/A	#N/A	#N/A	#N/A	#N/A	#N/A	#N/A	73.449	67.2346	61.6201	52.826	367.357	228.67
GT	Sense	Sense	-0.191	Detected	0.191	Detected	0.000	2.103	Detected	1.209	Detected	1.656	0.077	GT	Sens	contig435	contig435	Auxin-ind	CAAGAC	contig435	Solvc10g	Solvc10g	Auxin-ind	GO:00055	GO:00055	contig435	Solvc10g	Auxin-ind	GO:00055	SL2.40ch	AT5G18060.1		auxin-res	chr5:597	14.5975	17.9091	24.0365	28.9794	74.0913	39.7348
GT	Sense	Sense	-0.092	Comprom	0.092	Comprom	0.000	3.833	Detected	2.963	Detected	3.398	0.017	GT	Sens	contig438	contig438	Unknown	TTCTTT	contig438	Solvc04g	Solvc04g	Unknown Protein (AHRD V1)			contig438	Solvc04g	Unknown Protein (A		SL2.40ch	AT2G070	CAS1	CAS1 (cy	chr2:292	2.14936	2.29883	6.17688	4.74569	33.791	18.4291
GT	Sense	Sense	-0.222	Detected	0.222	Detected	0.000	2.283	Detected	0.841	Detected	1.562	0.174	GT	Sens	contig447	contig447	Ribonucl	TCCCAA	contig447	Solvc11g	Solvc11g	Ribonucl	GO:00063	GO:00063	contig447	Solvc11g	Ribonucl	GO:00063	SL2.40ch	AT3G033	DCL2. AT	DCL2 (DI	chr3:768	307.057	393.208	379.154	297.419	1803.56	661.924
GT	Sense	Sense	-0.010	Detected	0.010	Detected	0.000	1.599	Detected	1.190	Detected	1.394	0.021	GT	Sens	contig447	contig447	Inositol-3	GTGGAA	contig447	Solvc04g	Solvc04g	Inositol-3	GO:00055	GO:00055	contig447	Solvc04g	Inositol-3	GO:00055	SL2.40ch	AT2G222	ATMIPS2	MIPS2 (M	chr2:945	504.98	481.966	368.924	231.541	1593.36	1196.93
GT	Sense	Sense	0.017	Detected	-0.017	Detected	0.000	1.440	Detected	1.243	Detected	1.341	0.006	GT	Sens	contig447	contig447	Mutator-li	GAGCAG	contig447	Solvc10g	Solvc10g	Mutator-li	GO:00082	GO:00082	contig447	Solvc10g	Mutator-li	GO:00082	SL2.40ch	AT4G18580.2		unknown	chr4:102	42.3541	38.9314	41.9408	42.0346	117.449	102.154
GT	Sense	Sense	-0.183	Detected	0.183	Detected	0.000	1.670	Detected	1.123	Detected	1.397	0.051	GT	Sens	contig456	contig456	ClpB cha	GAGTTA	contig456	Solvc06g	Solvc06g	ClpB cha	GO:00055	GO:00055	contig456	Solvc03g	ClpB cha	GO:00195	SL2.40ch	AT1G743	ATHSP10	ATHSP1	chr1:279	806.572	978.473	668.845	637.077	3015.13	2057.74
GT	Sense	Sense	0.019	Detected	-0.019	Detected	0.000	1.176	Detected	1.203	Detected	1.189	0.000	GT	Sens	contig471	contig471	Light-dep	TTATCT	contig471	Solvc07g	Solvc07g	Light-dependent short hypoco			contig471	Solvc07g	Light-dependent sh		SL2.40ch	AT2G426	LSH10	LSH10 (L	chr2:177	61.7518	56.5967	24.2022	62.986	142.395	144.733
GT	Sense	Sense	-0.019	Comprom	0.019	Comprom	0.000	2.927	Detected	2.454	Detected	2.690	0.008	GT	Sens	contig482	contig482	Helicase-	TCTAAG	contig482	Solvc03g	Solvc03g	Helicase-like protein (AHRD V1			contig482	Solvc07g	Helicase-like protei		SL2.40ch	AT5G482	EMB1276	ACAT2 (A	chr5:195	2.21588	2.14055	7.09877	3.15031	17.6613	12.6855
GT	Sense	Sense	0.211	Comprom	-0.211	Comprom	0.000	1.681	Detected	1.289	Detected	1.485	0.036	GT	Sens	contig499	contig499	Unknown	TCAAAT	contig499	Solvc12g	Solvc12g	Unknown Protein (AHRD V1)			contig499	Solvc12g	Unknown Protein (A		SL2.40ch	AT1G35330.1				8.50019	5.96799	12.0948	18.172	24.352	18.507
GT	Sense	Sense	-0.324	Comprom	0.324	Comprom	0.000	3.801	Detected	2.171	Detected	2.986	0.077	GT	Sens	contig508	contig508	MYB tran	GTTTTC	contig508	Solvc05g	Solvc05g	MYB tran	GO:0045449		contig508	Solvc05g	MYB tran	GO:00030	SL2.40ch	AT5G153	ATMYB1	ATMYB1	chr5:497	4.06364	5.98966	18.8412	10.9081	73.3214	23.6151
GT	Sense	Sense	0.681	Comprom	-0.681	Comprom	0.000	2.297	Detected	1.712	Detected	2.005	0.114	GT	Sens	contig521	contig521	Disease r	AGCAGA	contig521	Solvc07g	Solvc07g	Disease r	GO:00069	GO:00069	contig521	Solvc07g	Nbs-lrr. re	GO:00069	SL2.40ch	AT3G51080.1		zinc fing	chr3:189	8.93431	3.27155	12.9891	15.3645	28.3321	18.8321
GT	Sense	Sense	-0.007	Detected	0.007	Detected	0.000	1.006	Detected	1.036	Detected	1.021	0.000	GT	Sens	contig536	contig536	Chloroph	AATTTCT	contig536	Solvc02g	Solvc02g	Chloroph	GO:00055	GO:00055	contig536	Solvc02g	Chloroph	GO:00055	SL2.40ch	AT1G299	CAB3. AB	CAB3 (Cl	chr1:104	695.444	660.661	1297.91	794.141	1451.35	1477.96
GT	Sense	Sense	-0.202	Detected	0.202	Detected	0.000	2.275	Detected	0.819	Detected	1.547	0.177	GT	Sens	contig538	contig538	Ribonucl	ATTCTT	contig538	Solvc11g	Solvc11g	Ribonucl	GO:00063	GO:00063	contig538	Solvc11g	Ribonucl	GO:00063	SL2.40ch	AT3G033	DCL2	DCL2 (DI	chr3:768	313.896	390.748	441.429	304.521	1807.29	657.017
GT	Sense	Sense	0.653	Comprom	-0.653	Comprom	0.000	1.280	Detected	1.086	Detected	1.183	0.215	GT	Sens	contig555	contig555	Transpos	AACAAT	contig555	Solvc10g	Solvc10g	Transposase (AHRD V1 *--- O6			contig555	Solvc08g	Transposase (AHRD		SL2.40ch	AT1G75900.1		family II e	chr1:284	9.6124	3.65941	15.0168	18.497	15.3563	13.3907
GT	Sense	Sense	-0.100	Detected	0.100	Detected	0.000	1.015	Detected	1.132	Detected	1.074	0.011	GT	Sens	contig557	contig557	Chloroph	CCAGAA	contig557	Solvc03g	Solvc03g	Chloroph	GO:00160	GO:00160	contig557	Solvc03g	Chloroph	GO:00160	SL2.40ch	AT5G542	LHCB3. L	LHCB3 (L	chr5:220	2746.58	2966.99	5731.36	3829.43	6153.41	6653.21
GT	Sense	Sense	0.455	Detected	-0.455	Detected	0.000	1.520	Detected	1.444	Detected	1.482	0.083	GT	Sens	contig562	contig562	Mutator-li	CGTTGG	contig562	Solvc06g	Solvc06g	Mutator-li	GO:00082	GO:00082	contig562	Solvc12g	Mutator-li	GO:00082	SL2.40ch	AT5G059	ATROPGI	ROPGEF	chr5:178	3392.28	1698.05	2122.61	1493.84	7339.32	6944.5
GT	Sense	Sense	0.498	Detected	-0.498	Detected	0.000	3.821	Detected	3.344	Detected	3.582	0.023	GT	Sens	contig582	contig582	Helicase-	CAATGG	contig582	Solvc01g	Solvc01g	Helicase-like protein (AHRD V1			contig582	Solvc01g	Helicase-like protei		SL2.40ch	AT3G20700.1				278.287	131.288	851.666	538.713	2880.57	2063.45
GT	Sense	Sense	1.324	Detected	-1.324	Comprom	0.000	1.679	Detected	1.901	Detected	1.790	0.310	GT	Sens	contig585	contig585	Unknown	CTCGTT	contig585	Solvc06g	Solvc06g	Unknown Protein (AHRD V1)			contig585	Solvc06g	Unknown Protein (A		SL2.40ch	AT5G09890.2		protein k	chr5:308	17.9923	2.70049	23.9269	22.5961	23.8021	27.674
GT	Sense	Sense	0.062	Detected	-0.062	Detected	0.000	2.000	Detected	0.954	Detected	1.477	0.107	GT	Sens	contig589	contig589	Subtilisin	TACCTC	contig589	Solvc10g	Solvc10g	Subtilisin	GO:00065	GO:00065	contig589	Solvc10g	Subtilisin	GO:00065	SL2.40ch	AT1G308	ARPC2A.	DIS2 (DIS	chr1:109	1163.41	1004.03	875.883	841.188	4611.64	2226.64
GT	Sense	Sense	0.314	Detected	-0.314	Detected	0.000	2.631	Detected	1.887	Detected	2.259	0.043	GT	Sens	contig607	contig607	Fructose-	GGTAGT	contig607	Solvc04g	Solvc04g	Fructose-	GO:00059	GO:00059	contig607	Solvc04g	Fructose-	GO:00421	SL2.40ch	AT1G43670.1		fructose-	chr1:164	584.989	356.298	2338.93	926.059	3014.99	1795.64
GT	Sense	Sense	0.094	Comprom	-0.094	Comprom	0.000	1.447	Detected	2.079	Detected	1.763	0.033	GT	Sens	contig617	contig617	Mutator-li	AGTCCA	contig617	Solvc10g	Solvc10g	Mutator-li	GO:00082	GO:00082	contig617	Solvc12g	Mutator-li	GO:00082	SL2.40ch	AT3G227	SOL1	SOL1: tra	chr3:804	8.03224	6.63784	14.2194	35.9673	21.2304	32.8023
GT	Sense	Sense	0.057	Detected	-0.057	Detected	0.000	2.232	Detected	1.658	Detected	1.945	0.022	GT	Sens	contig639	contig639	Susceptil	CCCAAA	contig639	Solvc01g	Solvc01g	Susceptil	GO:00055	GO:00055	contig639	Solvc01g	Susceptil	GO:00055	SL2.40ch	AT5G49600.1		unknown	chr5:201	398.474	346.582	962.898	703.768	1861.11	1247.03
GT	Sense	Sense	0.072	Detected	-0.072	Detected	0.000	1.663	Detected	1.408	Detected	1.536	0.009	GT	Sens	contig642	contig642	Ulp1 prot	AGAAGG	contig642	Solvc06g	Solvc06g	Ulp1 protease family C-termina			contig642	Solvc09g	Ulp1 protease family		SL2.40ch	AT5G651	ACX2. AT	ACX2 (AC	chr5:260	3457.34	2944.17	6012.87	6185.7	10773.4	9003.8
GT	Sense	Sense	0.036	Detected	-0.036	Detected	0.000	1.820	Detected	1.558	Detected	1.689	0.006	GT	Sens	contig645	contig645	Ulp1 prot	CCTCAA	contig645	Solvc08g	Solvc08g	Ulp1 prot	GO:00065	GO:00065	contig645	Solvc11g	Ulp1 protease family		SL2.40ch	AT5G38360.1		catalytic	chr5:153	2059.58	1843.42	3521.89	3982.13	7334.43	6101.78
GT	Sense	Sense	0.087	Detected	-0.087	Detected	0.000	1.119	Detected	0.956	Detected	1.038	0.013	GT	Sens	contig648	contig648	NBS-cont	TGAATA	contig648	Solvc05g	Solvc05g	NBS-containing resistance-like			contig648	Solvc05g	NBS-containing resi		SL2.40ch	AT4G171	FPS2	FPS2 (FA	chr4:964	30.0584	25.0628	45.2563	46.0953	63.5748	56.6079
GT	Sense	Sense	-0.071	Detected	0.071	Detected	0.000	2.353	Detected	2.011	Detected	2.182	0.007	GT	Sens	contig668	contig668	1-aminoc	TGCCAC	contig668	Solvc09g	Solvc09g	1-aminoc	GO:00164	GO:00164	contig668	Solvc09g	1-aminoc	GO:00164	SL2.40ch	AT1G06620.1		2-oxoglu	chr1:202	21.6959	22.5237	104.782	54.9482	120.445	94.7536
GT	Sense	Sense	0.051	Comprom	-0.051	Comprom	0.000	1.522	Detected	0.914	Detected	1.218	0.059	GT	Sens	contig682	contig682	Unknown	AGAGGA	contig682	Solvc00g	Solvc00g	Unknown Protein (AHRD V1)			contig682	Solvc00g	Unknown Protein (A		SL2.40ch	AT5G102	ANNAT7.	ANNAT7	chr5:320	7.36619	6.45936	9.8481	16.7017	21.121	13.8148
GT	Sense	Sense	-0.116	Detected	0.116	Detected	0.000	1.214	Detected	0.941	Detected	1.077	0.027	GT	Sens	contig689	contig689	Protease	TACTGC	contig689	Solvc10g	Solvc10g	Protease inhibitor/seed storag			contig689	Solvc10g	Protease inhibitor/s		SL2.40ch	AT5G35370.1		ATP bind	chr5:135	194.615	214.985	412.028	164.187	506.009	417.422
GT	Sense	Sense	0.759	Detected	-0.759	Detected	0.000	2.292	Detected	1.501	Detected	1.896	0.157	GT	Sens	contig690	contig690	Proteinas	CAGGAT	contig690	Solvc09g	Solvc09g	Proteinas	GO:00096	GO:00096	contig690	Solvc09g	Proteinas	GO:00096	SL2.40ch	AT5G58830.1		identical	chr5:237	23803.1	7824.62	22561	9776.76	71253.7	41073.1
GT	Sense	Sense	0.128	Detected	-0.128	Detected	0.000	1.647	Detected	1.048	Detected	1.347	0.054	GT	Sens	contig702	contig702	Sulfotran	TGTTCT	contig702	Solvc09g	Solvc09g	Sulfotran	GO:00081	GO:00081	contig702	Solvc09g	Sulfotran	GO:00801	SL2.40ch	AT5G059	DHS	DHS (DE	chr5:177	209.735	165.369	250.316	118.646	621.945	409.371
GT	Sense	Sense	-0.171	Detected	0.171	Detected	0.000	2.027	Detected	2.123	Detected	2.075	0.007	GT	Sens	contig715	contig715	Wound in	GCACAA	contig715	Solvc07g	Solvc07g	Wound induced protein (AHRD			contig715	Solvc07g	Wound induced pro		SL2.40ch	AT4G10270.1		wound-r	chr4:637	730.135	870.373	2634.03	1649.6	3465.66	3690.72
GT	Sense	Sense	0.804	Detected	-0.804	Detected	0.000	3.280	Detected	2.514	Detected	2.897	0.083	GT	Sens	contig717	contig717	Proteinas	GTGATC	contig717	Solvc09g	Solvc09g	Proteinas	GO:00096	GO:00096	contig717	Solvc09g	Proteinas	GO:00096	SL2.40ch	AT5G40340.1		PWWP d	chr5:161	10296	3179.95	13594.8	5899.66	59264.3	34741.8
GT	Sense	Sense	0.138	Detected	-0.138	Detected	0.000	1.467	Detected	1.073	Detected	1.270	0.034	GT	Sens	contig736	contig736	Unknown	CGGATG	contig736	Solvc04g	Solvc04g	Unknown Protein (AHRD V1)%:			contig736	Solvc04g	Unknown Protein (A		SL2.40ch	AT1G59990.1		DEAD/DE	chr1:220	155.291	120.706	239.917	159.247	403.534	306.323
GT	Sense	Sense	-0.288	Comprom	0.288	Comprom	0.000	2.120	Detected	2.135	Detected	2.127	0.018	GT	Sens	contig739	contig739	Unknown	ATGTTG	contig739	Solvc06g	Solvc06g	Unknown Protein (AHRD V1)%:			contig739	Solvc06g	Unknown Protein (A		SL2.40ch	AT4G27520.1		plastocy	chr4:137	2.38483	3.34711	6.42263	6.59303	13.0925	13.1979
GT	Sense	Sense	0.635	Detected	-0.635	Detected	0.000	2.136	Detected	1.482	Detected	1.809	0.127	GT	Sens	contig754	contig754	Proteinas	AGAGTT	contig754	Solvc09g	Solvc09g	Proteinas	GO:00096	GO:00096	contig754	Solvc09g	Proteinas	GO:00096	SL2.40ch	AT3G08947.1		binding /	chr3:272	38552.7	15039.2	42181.7	19266.9	112832	71534.9
GT	Sense	Sense	-0.227	Comprom	0.227	Comprom	0.000	1.207	Detected	1.032	Detected	1.120	0.044	GT	Sens	contig758	contig758	DOG1 alp	CACATG	contig758	Solvc02g	Solvc02g	DOG1 alpha splice variant (Fra			contig758	Solvc02g	DOG1 alpha splice v		SL2.40ch	AT3G44050.1		kinesin m	chr3:158	8.61208	11.1021	21.3267	11.8574	24.0687	21.2624
GT	Sense	Sense	-0.177	Detected	0.177	Detected	0.000	1.399	Detected	1.317	Detected	1.358	0.017	GT	Sens	contig763	contig763	Develop	TTATGG	contig763	Solvc12g	Solvc12g	Developmentally regulated GT			contig763	Solvc12g	Developmentally re		SL2.40ch	AT1G72660.3		develop	chr1:273	14.5486	17.5025	12.3194	17.9807	44.8871	42.2776
GT	Sense	Sense	-0.600	Comprom	0.600	Comprom	0.000	1.605	Detected	1.905	Detected	1.755	0.105	GT	Sens	contig770	contig770	Unknown	TGGAAT	contig770	Solvc05g	Solvc05g	Unknown Protein (AHRD V1)			contig770	Solvc05g	Unknown Protein (A		SL2.40ch	AT5G20360.1		octicosa	chr5:688	2.20839	4.77478	4.32486	4.66871	10.5341	12.9295
GT	Sense	Sense	-0.208	Detected	0.208	Detected	0.000	1.454	Detected	1.024	Detected	1.239	0.054	GT	Sens	contig782	contig782	Serine ca	GATGAT	contig782	Solvc10g	Solvc10g	Serine ca	GO:00065	GO:00065	contig782	Solvc10g	Serine ca	GO:00167	SL2.40ch	AT5G559	emb2731	emb2731	chr5:226	141.052	177.202	284.987	176.269	461.807	341.873
GT	Sense	Sense	-0.089	Detected	0.089	Detected	0.000	1.612	Detected	1.381	Detected	1.496	0.009	GT	Sens	contig787	contig787	Argonaut	CATCTT	contig787	Solvc06g	Solvc06g	Argonaut	GO:00036	GO:00036	contig787	Solvc06g	Argonaut	GO:00198	SL2.40ch	AT2G270	AGO4. O	AGO4 (A	chr2:115	430.642	458.596	553.488	387.018	1448.3	1230.48
GT	Sense	Sense	1.957	Detected	-1.957	Comprom	0.000	4.392	Detected	2.400	Detected	3.396	0.262	GT	Sens	contig799	contig799	Hydroxy	TTTCCA	contig799	Solvc07g	Solvc07g	Hydroxyc	GO:00167	GO:00167	contig799	Solvc07g	Hydroxyc	GO:00167	SL2.40ch	AT5G484	ATGLR1.	ATGLR1.	chr5:196	174.111	10.8617	114.901	13.1251	973.943	244.084
GT	Sense	Sense	0.348	Comprom	-0.348	Comprom	0.000	2.939	Detected	1.846	Detected	2.392	0.066	GT	Sens	contig803	contig803	Glutared	GTGAAG	contig803	Solvc01g	Solvc01g	Glutared	GO:00454	GO:00454	contig803	Solvc01g	Glutared	GO:00454	SL2.40ch	AT1G610	CPSF73-I	CPSF73-	chr1:224	7.77491	4.51322	13.8007	29.7124	48.4324	22.641
GT	Sense	Sense	-0.070	Detected	0.070	Detected	0.000	1.224	Detected	1.088	Detected	1.156	0.007	GT	Sens	contig804	contig804	Purine pe	GCACTT	contig804	Solvc12g	Solvc12g	Purine pe	GO:00063	GO:00063	contig804	Solvc12g	Purine pe	GO:00160	SL2.40ch	AT5G132	SOL2. CR	CRN (CO	chr5:425	2240.35	2321.77	6887.45	5323.95	5680.82	5156.28
GT	Sense	Sense	0.394	Detected	-0.394	Comprom	0.000	1.753	Detected	0.948	Detected	1.351	0.139	GT	Sens	contig813	contig813	Unknown	CGTTCT	contig813	Solvc03g	Solvc03g	Unknown Protein (AHRD V1)			contig813	Solvc03g063180.1.1				AT4G267	MAP65-2	MAP65-2	chr4:134	11.5923	6.31654	31.2554	19.3469	30.7594	17.5527
GT	Sense	Sense	-0.143	Detected	0.143	Detected	0.000	1.481	Detected	0.950	Detected	1.216	0.056	GT	Sens	contig815	contig815	CONSTAI	TGGAAT	contig815	Solvc02g	Solvc02g	CONSTAI	GO:00037	GO:00037	contig815	Solvc02g	Tomato C	GO:00037	SL2.40ch	AT5G158	COL1. AT	COL1 (co	chr5:517	1242.5	1425.87	2553.48	1317.51	3962.94	2733.83
GT	Sense	Sense	-0.086	Comprom	0.086	Comprom	0.000	1.178	Detected	1.128	Detected	1.153	0.006	GT	Sens	contig817	contig817	Ulp1 prot	AGCGCA	contig817	Solvc04g	Solvc04g	Ulp1 protease family C-termina			contig817	Solvc10g	Ulp1 protease family		SL2.40ch	AT4G14746.1				6.96646	7.38041	8.35593	7.86333	17.305	16.6642
GT	Sense	Sense	-0.572	Comprom	0.572	Detected	0.000	0.933	Detected	1.086	Detected	1.010	0.223	GT	Sens	contig828	contig828	2-oxoglut	TGAAGA	contig828	Solvc11g	Solvc11g	2-oxoglut	GO:00103	IGO:0016	contig828	Solvc11g	2-oxoglut	GO:00103	SL2.40ch	AT3G13610.1		oxidored	chr3:444	8.03771	16.7238	31.4372	11.5279	23.6086	26.1654
GT	Sense	Sense	-0.853	Detected	0.853	Detected	0.000	1.650	Detected	1.845	Detected	1.748	0.179	GT	Sens	contig837	contig837	MYB tran	TAAAAT	contig837	Solvc12g	Solvc12g	MYB transcription factor MYB			contig837	Solvc12g	MYB transcription f		SL2.40ch	AT1G566	PAP1. MY	PAP1 (PF	chr1:212	12.7257	39.0875	73.2438	39.6828	74.6423	85.186
GT	Sense	Sense	-0.007	Detected	0.007	Detected	0.000	1.679	Detected	1.388	Detected	1.533	0.009	GT	Sens	contig841	contig841	Unknown	GGAATT	contig841	Solvc10g	Solvc10g	Unknown Protein (AHRD V1)			contig841	Solvc10g	Unknown Protein (A		SL2.40ch	AT5G38720.1		unknown	chr5:155	1534.63	1458.79	2307.85	2799.83	5108.66	4162.05
GT	Sense	Sense	-0.324	Detected	0.324	Detected	0.000	1.645	Detected	1.397	Detected	1.521	0.048	GT	Sens	contig852	contig852	Wound in	ATCAGA	contig852	Solvc07g	Solvc07g	Wound induced protein (AHRD			contig852	Solvc07g	Wound induced pro		SL2.40ch	AT5G24010.1		protein k	chr5:811	87.0352	128.311	343.095	163.625	352.528	295.855
GT	Sense	Sense	-0.154	Detected	0.154	Detected	0.000	3.003	Detected	2.911	Detected	2.957	0.003	GT	Sens	contig860	contig860	Gibberell	TACTGT	contig860	Solvc04g	Solvc04g	Gibberellin-regulated family pr			contig860	Solvc04g	Gibberellin-regulate		SL2.40ch	AT1G10588.2		Encodes	chr1:350	21.1917	24.6723	137.749	67.2174	195.487	182.863
GT	Sense	Sense	-0.298	Comprom	0.298	Comprom	0.000	1.569	Detected	1.029	Detected	1.299	0.084	GT	Sens	contig875	contig875	Mutator-li	GAAAAA	contig875	Solvc10g	Solvc10g	Mutator-li	GO:00082	GO:00082	contig875	Solvc10g	Unknown Protein (A		SL2.40ch	AT5G60810.2				7.98645	11.3539	11.9063	5.96649	30.1349	20.6591
GT	Sense	Sense	-0.231	Detected	0.231	Detected	0.000	1.385	Detected	1.252	Detected	1.319	0.032	GT	Sens	contig886	contig886	Cytochro	TACCAA	contig886	Solvc03g	Solvc03g	Cytochro	GO:00198	GO:00198	contig886	Solvc03g	Cytochro	GO:00198	SL2.40ch	AT3G483	CYP71A2	CYP71A2	chr3:178	2596.24	3365.66	10176.6	4667.25	8234.9	7486.6
GT	Sense	Sense	-0.247	Detected	0.247	Detected	0.000	1.325	Detected	1.427	Detected	1.376	0.032	GT	Sens	contig890	contig890	Ribose-pl	AAAGCT	contig890	Solvc07g	Solvc07g	Ribose-pl	GO:00091	GO:00091	contig890	Solvc07g	Ribose-pl	GO:00428	SL2.40ch	AT2G42910.1		ribose-pl	chr2:178	131.06	173.633	218.975	198.983	403.065	431.181
GT	Sense	Sense	0.513	Detected	-0.513	Comprom	0.000	1.368	Detected	1.170	Detected	1.269	0.136	GT	Sens	contig895	contig895	Mutator-li	GTGAAG	contig895	Solvc10g	Solvc10g	Mutator-li	GO:00082	GO:00082	contig895	Solvc10g	Mutator-li	GO:00082	SL2.40ch	AT5G656	bHLH093	bHLH093	chr5:262	25.3282	11.7043	37.2776	26.5576	47.3821	41.1842
GT	Sense	Sense	0.657	Detected	-0.657	Detected	0.000	4.970	Detected	4.382	Detected	4.676	0.023	GT	Sens	contig905	contig905	Proteinas	GCAGAG	contig905	Solvc09g	Solvc09g	Proteinas	GO:00096	GO:00096	contig905	Solvc09g	Proteinas	GO:00096	SL2.40ch	AT3G30387.1		Encodes	chr3:119	49.0598	18.5603	40.3429	11.5482	1008.29	668.837

		16h_AZ_Control					16h_AZ_TAPG + TPRP vs. 16h_AZ_Control					
ProbeNar	Orientatic	Fold 16h	Flag 16h	Fold 16h	Flag 16h	Geomean	Fold 16h	Flag 16h	Fold 16h	Flag 16h	Geomean	P-value
A 96 P0	Sense	0.296	Detected	-0.296	Detected	0.000	-2.898	Detected	-1.658	Detected	-2.278	0.080
A 96 P0	Sense	0.083	Detected	-0.083	Detected	0.000	-1.956	Detected	-1.690	Detected	-1.823	0.007
A 96 P01	Sense	0.003	Detected	-0.003	Detected	0.000	-3.446	Comprom	-1.396	Comprom	-2.421	0.142
A 96 P01	Sense	0.036	Detected	-0.036	Detected	0.000	-1.352	Detected	-0.884	Detected	-1.118	0.042
A 96 P01	Sense	0.434	Detected	-0.434	Detected	0.000	-1.704	Detected	-1.146	Detected	-1.425	0.110
A 96 P02	Sense	-0.107	Detected	0.107	Detected	0.000	-1.371	Detected	-0.865	Detected	-1.118	0.055
A 96 P02	Sense	-0.025	Detected	0.025	Detected	0.000	-1.081	Detected	-1.067	Detected	-1.074	0.001
A 96 P02	Sense	0.222	Detected	-0.222	Detected	0.000	-2.055	Detected	-1.275	Detected	-1.665	0.066
A 96 P02	Sense	-0.104	Detected	0.104	Detected	0.000	-1.130	Detected	-1.193	Detected	-1.161	0.009
A 96 P02	Sense	0.123	Detected	-0.123	Detected	0.000	-2.197	Detected	-1.819	Detected	-2.008	0.012
A 96 P04	Sense	-0.011	Detected	0.011	Detected	0.000	-1.160	Detected	-1.237	Detected	-1.198	0.001
A 96 P04	Sense	0.039	Detected	-0.039	Detected	0.000	-1.661	Detected	-1.780	Detected	-1.721	0.002
A 96 P04	Sense	0.143	Detected	-0.143	Detected	0.000	-1.421	Detected	-1.073	Detected	-1.247	0.031
A 96 P04	Sense	0.477	Detected	-0.477	Detected	0.000	-3.118	Detected	-3.869	Detected	-3.493	0.029
A 96 P05	Sense	-0.140	Detected	0.140	Detected	0.000	-0.990	Detected	-1.146	Detected	-1.068	0.022
A 96 P05	Sense	0.140	Detected	-0.140	Detected	0.000	-1.171	Detected	-0.932	Detected	-1.052	0.029
A 96 P05	Sense	-0.279	Detected	0.279	Detected	0.000	-1.496	Comprom	-1.438	Comprom	-1.467	0.035
A 96 P05	Sense	0.033	Detected	-0.033	Detected	0.000	-1.263	Detected	-1.080	Detected	-1.172	0.007
A 96 P05	Sense	0.241	Detected	-0.241	Detected	0.000	-1.614	Detected	-1.434	Detected	-1.524	0.027
A 96 P05	Sense	0.053	Detected	-0.053	Detected	0.000	-1.460	Detected	-1.131	Detected	-1.296	0.017
A 96 P06	Sense	-0.025	Detected	0.025	Detected	0.000	-1.269	Detected	-1.377	Detected	-1.323	0.002
A 96 P06	Sense	0.014	Detected	-0.014	Detected	0.000	-1.186	Detected	-1.127	Detected	-1.156	0.001
A 96 P06	Sense	0.194	Detected	-0.194	Detected	0.000	-1.845	Comprom	-0.962	Detected	-1.404	0.101
A 96 P06	Sense	-0.025	Detected	0.025	Detected	0.000	-1.476	Detected	-1.852	Detected	-1.664	0.013
A 96 P06	Sense	0.089	Detected	-0.089	Detected	0.000	-1.392	Detected	-1.454	Detected	-1.423	0.004
A 96 P06	Sense	0.113	Detected	-0.113	Detected	0.000	-1.686	Detected	-1.671	Detected	-1.678	0.005
A 96 P06	Sense	0.167	Detected	-0.167	Detected	0.000	-1.221	Detected	-1.348	Detected	-1.284	0.019
A 96 P06	Sense	0.053	Detected	-0.053	Detected	0.000	-2.723	Detected	-2.670	Detected	-2.696	0.000
A 96 P06	Sense	-0.062	Detected	0.062	Detected	0.000	-1.272	Detected	-1.017	Detected	-1.144	0.015
A 96 P06	Sense	0.405	Detected	-0.405	Detected	0.000	-2.520	Comprom	-1.590	Detected	-2.055	0.079
A 96 P06	Sense	-0.037	Detected	0.037	Detected	0.000	-1.180	Detected	-1.089	Detected	-1.134	0.003
A 96 P07	Sense	0.053	Detected	-0.053	Detected	0.000	-1.619	Detected	-1.423	Detected	-1.521	0.005
A 96 P07	Sense	0.030	Detected	-0.030	Detected	0.000	-1.940	Detected	-1.712	Detected	-1.826	0.004
A 96 P07	Sense	0.330	Detected	-0.330	Detected	0.000	-1.169	Detected	-2.282	Detected	-1.726	0.117
A 96 P07	Sense	0.089	Detected	-0.089	Detected	0.000	-1.284	Detected	-1.335	Detected	-1.309	0.005
A 96 P07	Sense	-0.062	Detected	0.062	Detected	0.000	-1.202	Detected	-1.349	Detected	-1.276	0.006
A 96 P07	Sense	-0.025	Detected	0.025	Detected	0.000	-1.476	Detected	-1.530	Detected	-1.503	0.001
A 96 P07	Sense	0.037	Detected	-0.037	Detected	0.000	-0.993	Detected	-1.203	Detected	-1.098	0.010
A 96 P08	Sense	0.386	Detected	-0.386	Detected	0.000	-0.937	Detected	-2.317	Detected	-1.627	0.176
A 96 P08	Sense	0.075	Detected	-0.075	Detected	0.000	-1.161	Detected	-1.181	Detected	-1.171	0.004
A 96 P08	Sense	0.410	Detected	-0.410	Detected	0.000	-2.703	Detected	-1.878	Detected	-2.290	0.059
A 96 P08	Sense	0.129	Detected	-0.129	Detected	0.000	-1.095	Detected	-1.586	Detected	-1.340	0.040
A 96 P08	Sense	-0.073	Detected	0.073	Detected	0.000	-1.365	Detected	-1.066	Detected	-1.215	0.018
A 96 P08	Sense	0.211	Detected	-0.211	Detected	0.000	-0.962	Detected	-1.222	Detected	-1.092	0.048
A 96 P08	Sense	0.397	Detected	-0.397	Detected	0.000	-1.103	Detected	-2.368	Detected	-1.735	0.146
A 96 P09	Sense	0.184	Detected	-0.184	Detected	0.000	-1.071	Detected	-1.016	Detected	-1.044	0.030
A 96 P09	Sense	0.162	Detected	-0.162	Detected	0.000	-1.892	Detected	-1.632	Detected	-1.762	0.014
A 96 P09	Sense	0.333	Detected	-0.333	Detected	0.000	-1.906	Detected	-1.250	Detected	-1.578	0.078
A 96 P1	Sense	0.317	Detected	-0.317	Detected	0.000	-2.899	Detected	-2.063	Detected	-2.481	0.042
A 96 P1	Sense	0.468	Detected	-0.468	Detected	0.000	-1.724	Detected	-1.678	Comprom	-1.701	0.068
A 96 P1	Sense	0.462	Detected	-0.462	Detected	0.000	-1.374	Detected	-1.219	Detected	-1.296	0.110
A 96 P1	Sense	0.080	Detected	-0.080	Detected	0.000	-1.264	Detected	-1.296	Detected	-1.280	0.004
A 96 P1	Sense	-0.148	Detected	0.148	Detected	0.000	-0.926	Detected	-1.107	Detected	-1.016	0.028
A 96 P1	Sense	-0.036	Detected	0.036	Detected	0.000	-1.515	Detected	-1.013	Detected	-1.264	0.038
A 96 P11	Sense	-0.380	Detected	0.380	Detected	0.000	-1.679	Detected	-1.194	Detected	-1.437	0.086
A 96 P11	Sense	0.001	Detected	-0.001	Detected	0.000	-3.631	Detected	-1.036	Detected	-2.334	0.214
A 96 P12	Sense	0.185	Detected	-0.185	Detected	0.000	-1.875	Detected	-1.965	Detected	-1.920	0.010
A 96 P13	Sense	0.152	Detected	-0.152	Detected	0.000	-1.238	Detected	-1.282	Detected	-1.260	0.015
A 96 P13	Sense	0.347	Detected	-0.347	Detected	0.000	-1.004	Detected	-1.018	Detected	-1.011	0.100
A 96 P13	Sense	-0.548	Detected	0.548	Detected	0.000	-3.045	Comprom	-1.672	Comprom	-2.359	0.115
A 96 P13	Sense	0.072	Detected	-0.072	Detected	0.000	-1.199	Detected	-0.821	Detected	-1.010	0.038
A 96 P13	Sense	-0.153	Detected	0.153	Detected	0.000	-2.288	Detected	-1.048	Detected	-1.668	0.121
A 96 P13	Sense	0.312	Detected	-0.312	Detected	0.000	-2.690	Detected	-1.585	Detected	-2.137	0.078
A 96 P13	Sense	0.164	Detected	-0.164	Detected	0.000	-1.303	Detected	-1.954	Detected	-1.628	0.047
A 96 P14	Sense	0.334	Detected	-0.334	Detected	0.000	-1.901	Detected	-2.043	Detected	-1.972	0.029
A 96 P14	Sense	0.136	Detected	-0.136	Detected	0.000	-1.396	Detected	-1.537	Detected	-1.466	0.011
A 96 P14	Sense	0.385	Detected	-0.385	Detected	0.000	-1.787	Detected	-1.508	Detected	-1.648	0.056
A 96 P15	Sense	0.166	Detected	-0.166	Detected	0.000	-1.213	Detected	-0.982	Detected	-1.098	0.032
A 96 P15	Sense	-0.028	Detected	0.028	Detected	0.000	-1.307	Detected	-1.491	Detected	-1.399	0.005
A 96 P15	Sense	0.050	Detected	-0.050	Detected	0.000	-1.696	Detected	-1.492	Detected	-1.594	0.005
A 96 P15	Sense	0.160	Detected	-0.160	Detected	0.000	-1.027	Detected	-1.468	Detected	-1.248	0.045
A 96 P16	Sense	0.246	Detected	-0.246	Detected	0.000	-1.877	Detected	-2.041	Detected	-1.959	0.017
A 96 P16	Sense	0.125	Detected	-0.125	Detected	0.000	-1.270	Detected	-1.799	Detected	-1.535	0.034
A 96 P16	Sense	0.075	Detected	-0.075	Detected	0.000	-1.548	Detected	-1.399	Detected	-1.474	0.005
A 96 P16	Sense	-0.044	Detected	0.044	Detected	0.000	-1.036	Detected	-1.061	Detected	-1.048	0.002
A 96 P16	Sense	0.271	Detected	-0.271	Detected	0.000	-0.967	Detected	-2.080	Detected	-1.524	0.133
A 96 P16	Sense	0.023	Detected	-0.023	Detected	0.000	-1.405	Detected	-1.316	Detected	-1.361	0.001
A 96 P16	Sense	0.160	Detected	-0.160	Detected	0.000	-2.090	Detected	-1.797	Detected	-1.944	0.012
A 96 P17	Sense	0.188	Detected	-0.188	Detected	0.000	-3.048	Detected	-1.823	Detected	-2.436	0.063
A 96 P17	Sense	0.171	Detected	-0.171	Detected	0.000	-1.957	Detected	-1.688	Detected	-1.822	0.014
A 96 P17	Sense	-0.130	Detected	0.130	Detected	0.000	-1.447	Detected	-0.928	Detected	-1.187	0.055
A 96 P17	Sense	-0.156	Detected	0.156	Detected	0.000	-1.013	Detected	-0.991	Detected	-1.002	0.023
A 96 P18	Sense	0.153	Detected	-0.153	Detected	0.000	-1.594	Detected	-1.226	Detected	-1.410	0.028
A 96 P18	Sense	0.115	Detected	-0.115	Detected	0.000	-2.020	Detected	-1.724	Detected	-1.872	0.010
A 96 P19	Sense	0.176	Detected	-0.176	Detected	0.000	-2.847	Detected	-1.773	Detected	-2.310	0.055
A 96 P19	Sense	0.138	Detected	-0.138	Detected	0.000	-3.901	Detected	-3.000	Detected	-3.451	0.018
A 96 P19	Sense	0.476	Detected	-0.476	Detected	0.000	-1.708	Detected	-1.145	Detected	-1.426	0.123
A 96 P2	Sense	-0.030	Detected	0.030	Detected	0.000	-1.293	Detected	-1.023	Detected	-1.158	0.014
A 96 P2	Sense	-0.043	Detected	0.043	Detected	0.000	-1.503	Detected	-1.269	Detected	-1.386	0.008
A 96 P2	Sense	-0.113	Detected	0.113	Detected	0.000	-1.296	Detected	-1.369	Detected	-1.332	0.008
A 96 P2	Sense	0.288	Detected	-0.288	Detected	0.000	-2.400	Detected	-1.828	Detected	-2.114	0.035
A 96 P2	Sense	0.077	Detected	-0.077	Detected	0.000	-1.547	Detected	-1.271	Detected	-1.409	0.012
A 96 P2	Sense	0.349	Detected	-0.349	Detected	0.000	-1.493	Detected	-2.640	Comprom	-2.067	0.091
A 96 P2	Sense	-0.234	Detected	0.234	Detected	0.000	-1.163	Detected	-1.251	Detected	-1.207	0.037
A 96 P2	Sense	0.352	Detected	-0.352	Detected	0.000	-1.367	Detected	-2.307	Detected	-1.837	0.089
A 96 P21	Sense	-0.145	Detected	0.145	Detected	0.000	-1.070	Detected	-0.988	Detected	-1.029	0.021
A 96 P21	Sense	0.141	Detected	-0.141	Detected	0.000	-1.849	Detected	-3.812	Detected	-2.831	0.104
A 96 P21	Sense	0.079	Detected	-0.079	Detected	0.000	-1.220	Detected	-1.700	Detected	-1.460	0.029
A 96 P22	Sense	0.058	Detected	-0.058	Detected	0.000	-0.978	Detected	-1.177	Detected	-1.077	0.011
A 96 P22	Sense	0.020	Detected	-0.020	Detected	0.000	-1.794	Detected	-1.913	Detected	-1.854	0.001
A 96 P22	Sense	0.050	Detected	-0.050	Detected	0.000	-1.623	Detected	-1.312	Detected	-1.467	0.012
A 96 P23	Sense	0.211	Detected	-0.211	Detected	0.000	-1.577	Detected	-1.566	Detected	-1.571	0.018
A 96 P23	Sense	-0.109	Detected	0.109	Detected	0.000	-1.046	Detected	-1.255	Detected	-1.150	0.017
A 96 P23	Sense	0.204	Detected	-0.204	Detected	0.000	-1.181	Detected	-1.237	Detected	-1.209	0.028
A 96 P23	Sense	-0.026	Detected	0.026	Detected	0.000	-0.849	Detected	-1.281	Detected	-1.065	0.039
A 96 P23	Sense	0.063	Detected	-0.063	Detected	0.000	-0.854	Detected	-1.242	Detected	-1.048	0.036
A 96 P23	Sense	0.172	Detected	-0.172	Detected	0.000	-1.475	Detected	-1.362	Detected	-1.418	0.016
A 96 P24	Sense	0.101	Detected	-0.101	Detected	0.000	-1.104	Detected	-1.017	Detected	-1.061	0.011
A 96 P24	Sense	0.135	Detected	-0.135	Detected	0.000	-1.342	Detected	-0.910	Detected	-1.126	0.048
A 96 P24	Sense	0.173	Detected	-0.173	Detected	0.000	-0.958	Detected	-1.226	Detected	-1.092	0.038
A 96 P24	Sense	0.187	Detected	-0.187	Detected	0.000	-2.256	Detected	-1.722	Detected	-1.989	0.026
A 96 P24	Sense	-0.063	Detected	0.063	Detected	0.000	-0.844	Detected	-1.647	Detected	-1.246	0.092
A 96 P24	Sense	0.138	Detected	-0.138	Detected	0.000	-1.498	Detected	-1.456	Detected	-1.477	0.009
A 96 P25	Sense	-0.233	Detected	0.233	Detected	0.000	-2.463	Comprom	-2.635	Comprom	-2.549	0.009
A 96 P25	Sense	0.017	Detected	-0.017	Detected	0.000	-1.427	Detected	-1.401	Detected	-1.414	0.000
A 96 P25	Sense	-0.672	Detected	0.672	Detected	0.000	-1.131	Detected	-1.414	Detected	-1.273	0.205
A 96 P25	Sense	0.265	Detected	-0.265	Detected	0.000	-3.057	Detected	-1.935	Detected	-2.496	0.057
A 96 P25	Sense	-0.065	Detected	0.065	Detected	0.000	-1.032	Detected	-1.127	Detected	-1.080	0.005
A 96 P26	Sense	-0.196	Detected	0.196	Detected	0.000	-1.143	Detected	-1.689	Detected	-1.416	0.052
GT Sens	Sense	0.160	Detected	-0.160	Detected	0.000	-2.721	Detected	-2.220	Detected	-2.471	0.014
GT Sens	Sense	0.266	Detected	-0.266	Detected	0.000	-2.366	Detected	-1.152	Detected	-1.759	0.117
GT Sens	Sense	0.133	Detected	-0.133	Detected	0.000	-1.407	Detected	-2.113	Detected	-1.760	0.043
GT Sens	Sense	0.015	Detected	-0.015	Detected	0.000	-1.750	Detected	-1.349	Detected	-1.550	0.016
GT Sens	Sense	0.018	Detected	-0.018	Detected	0.000	-1.398	Detected	-1.540	Detected	-1.469	0.002
GT Sens	Sense	-0.079	Detected	0.079	Detected	0.000	-1.216	Detected	-1.135	Detected	-1.175	0.006
GT Sens	Sense	-0.106	Detected	0.106	Detected	0.000	-1.719	Detected	-1.310	Detected	-1.514	0.022
GT Sens	Sense	0.442	Detected	-0.442	Detected	0.000	-4.516	Detected	-3.775	Detected	-4.145	0.019
GT Sens	Sense	0.285	Detected	-0.285	Detected	0.000	-3.763	Detected	-1.828	Detected	-2.796	0.109
GT Sens	Sense	0.320	Detected	-0.320	Detected	0.000	-2.108	Detected	-1.664	Detected	-1.886	0.040
GT Sens	Sense	0.166	Detected	-0.166	Detected	0.000	-1.304	Comprom	-0.871	Detected	-1.088	0.058
GT Sens	Sense	-0.013	Detected	0.013	Detected	0.000	-1.392	Detected	-1.163	Detected	-1.278	0.008
GT Sens	Sense	0.116	Detected	-0.116	Detected	0.000	-1.656	Detected	-1.411	Detected	-1.534	0.012
GT Sens	Sense	-0.100	Detected	0.100	Detected	0.000	-1.070	Detected	-1.024	Detected	-1.047	0.009
GT Sens	Sense	0.329	Detected	-0.329	Detected	0.000	-1.265	Detected	-1.655	Comprom	-1.460	0.062
GT Sens	Sense	0.047	Detected	-0.047	Detected	0.000	-1.213	Detected	-0.967	Detected	-1.090	0.014
GT Sens	Sense	0.091	Detected	-0.091	Detected	0.000	-1.253	Detected	-0.986	Detected	-1.120	0.020
GT Sens	Sense	0.670	Detected	-0.670	Detected	0.000	-3.116	Detected	-3.070	Detected	-3.093	0.044
GT Sens	Sense	0.135	Detected	-0.135	Detected	0.000	-1.868	Detected	-1.916	Detected	-1.892	0.005
GT Sens	Sense	-0.066	Detected	0.066	Detected	0.000	-1.938	Detected	-1.979	Detected	-1.958	0.001
GT Sens	Sense	0.302	Detected	-0.302	Detected	0.000	-3.269	Detected	-3.275	Detected	-3.272	0.008
GT Sens	Sense	-0.080	Detected	0.080	Detected	0.000	-1.443	Detected	-1.127	Detected	-1.285	0.018
GT Sens	Sense	0.246	Detected	-0.246	Detected	0.000	-3.433	Detected	-2.472	Detected	-2.952	0.032
GT Sens	Sense	0.077	Detected	-0.077	Detected	0.000	-1.116	Detected	-0.888	Detected	-1.002	0.018
GT Sens	Sense	-0.403	Detected	0.403	Detected	0.000	-2.493	Comprom	-0.936	Detected	-1.715	0.190
GT Sens	Sense	-0.206	Detected	0.206	Detected	0.000	-1.106	Detected	-1.158	Detected	-1.132	0.032
GT Sens	Sense	-0.075	Detected	0.075	Detected	0.000	-1.175	Detected	-1.387	Detected	-1.281	0.010
GT Sens	Sense	0.483	Detected	-0.483	Detected	0.000	-0.854	Detected	-1.351	Detected	-1.102	0.180
GT Sens	Sense	0.324	Detected	-0.324	Detected	0.000	-1.062	Detected	-1.537	Detected	-1.299	0.084
GT Sens	Sense	0.131	Detected	-0.131	Detected	0.000	-1.625	Detected	-1.371	Detected	-1.498	0.015
GT Sens	Sense	0.119	Detected	-0.119	Detected	0.000	-1.545	Detected	-1.362	Detected	-1.453	0.011
GT Sens	Sense	0.187	Detected	-0.187	Detected	0.000	-1.549	Detected	-0.925	Detected	-1.237	0.077
GT Sens	Sense	0.045	Detected	-0.045	Detected	0.000	-1.205	Detected	-1.215	Detected	-1.210	0.001
GT Sens	Sense	0.041	Detected	-0.041	Detected	0.000	-1.676	Detected	-1.349	Detected	-1.513	0.012
GT Sens	Sense	0.177	Detected	-0.177	Detected	0.000	-2.545	Detected	-1.908	Detected	-2.226	0.026
GT Sens	Sense	0.257	Detected	-0.257	Detected	0.000	-1.402	Detected	-1.472	Detected	-1.437	0.031
GT Sens	Sense	0.082	Detected	-0.082	Detected	0.000	-1.226	Detected	-1.188	Detected	-1.207	0.005
GT Sens	Sense	0.064	Detected	-0.064	Detected	0.000	-1.159	Detected	-1.236	Detected	-1.198	0.004
GT Sens	Sense	0.290	Detected	-0.290	Detected	0.000	-2.023	Detected	-1.480	Detected	-1.752	0.048
GT Sens	Sense	0.190	Detected	-0.190	Detected	0.000	-1.211	Detected	-1.034	Detected	-1.122	0.033
GT Sens	Sense	0.127	Detected	-0.127	Detected	0.000	-1.248	Detected	-1.351	Detected	-1.300	0.011
GT Sens	Sense	0.115	Detected	-0.115	Detected	0.000	-2.926	Detected	-2.152	Detected	-2.539	0.024
GT Sens	Sense	0.100	Detected	-0.100	Detected	0.000	-1.621	Detected	-1.302	Detected	-1.462	0.016
GT Sens	Sense	0.309	Detected	-0.309	Detected	0.000	-1.188	Detected	-1.124	Detected	-1.156	0.065
GT Sens	Sense	0.265	Detected	-0.265	Detected	0.000	-1.007	Detected	-1.393	Detected	-1.200	0.067
GT Sens	Sense	0.167	Detected	-0.167	Detected	0.000	-1.948	Detected	-1.291	Detected	-1.620	0.048
GT Sens	Sense	0.313	Detected	-0.313	Detected	0.000	-2.646	Detected	-2.446	Detected	-2.546	0.016
GT Sens	Sense	0.253	Detected	-0.253	Detected	0.000	-2.136	Detected	-1.354	Detected	-1.745	0.064
GT Sens	Sense	0.136	Detected	-0.136	Detected	0.000	-2.611	Detected	-2.026	Detected	-2.318	0.019
GT Sens	Sense	-0.310	Detected	0.310	Detected	0.000	-1.844	Detected	-0.935	Detected	-1.389	0.128
GT Sens	Sense	0.087	Detected	-0.087	Detected	0.000	-2.061	Detected	-2.259	Detected	-2.160	0.004
GT Sens	Sense	0.087	Detected	-0.087	Detected	0.000	-1.346	Detected	-0.848	Detected	-1.097	0.053
GT Sens	Sense	0.236	Detected	-0.236	Detected	0.000	-2.786	Detected	-1.715	Detected	-2.251	0.062
GT Sens	Sense	0.482	Detected	-0.482	Detected	0.000	-1.984	Detected	-1.195	Detected	-1.589	0.125
GT Sens	Sense	-0.059	Detected	0.059	Detected	0.000	-2.133	Detected	-1.001	Detected	-1.567	0.110
GT Sens	Sense	0.215	Detected	-0.215	Detected	0.000	-1.632	Detected	-0.954	Detected	-1.293	0.085
GT Sens	Sense	0.055	Detected	-0.055	Detected	0.000	-1.567	Detected	-1.356	Detected	-1.461	0.007
GT Sens	Sense	-0.095	Detected	0.095	Detected	0.000	-1.011	Detected	-1.073	Detected	-1.042	0.009
GT Sens	Sense	0.078	Detected	-0.078	Detected	0.000	-2.164	Detected	-1.944	Detected	-2.054	0.004
GT Sens	Sense	-0.358	Detected	0.358	Detected	0.000	-1.477	Detected	-1.747	Detected	-1.612	0.052
GT Sens	Sense	0.155	Detected	-0.155	Detected	0.000	-1.642	Detected	-1.550	Detected	-1.596	0.010
GT Sens	Sense	0.031	Detected	-0.031	Detected	0.000	-1.119	Detected	-1.194	Detected	-1.157	0.002
GT Sens	Sense	0.164	Detected	-0.164	Detected	0.000	-2.073	Detected	-1.876	Detected	-1.974	0.009
GT Sens	Sense	0.079	Detected	-0.079	Detected	0.000	-2.297	Detected	-1.431	Detected	-1.864	0.051
GT Sens	Sense	-0.273	Detected	0.273	Detected	0.000	-1.555	Detected	-0.931	Detected	-1.243	0.096
GT Sens	Sense	0.101	Detected	-0.101	Detected	0.000	-1.060	Detected	-1.175	Detected	-1.118	0.011
GT Sens	Sense	0.169	Detected	-0.169	Detected	0.000	-1.321	Detected	-1.123	Detected	-1.222	0.025
GT Sens	Sense	-0.157	Detected	0.157	Detected	0.000	-0.903	Detected	-1.435	Detected	-1.169	0.063
GT Sens	Sense	0.097	Detected	-0.097	Detected	0.000	-1.249	Detected	-0.854	Detected	-1.051	0.041
GT Sens	Sense	1.808	Detected	-1.808	Detected	0.000	-1.590	Detected	-1.561	Detected	-1.576	0.475
GT Sens	Sense	0.242	Detected	-0.242	Detected	0.000	-2.171	Detected	-1.462	Detected	-1.817	0.052
GT Sens	Sense	0.129	Detected	-0.129	Detected	0.000	-1.727	Detected	-1.058	Detected	-1.393	0.060
GT Sens	Sense	0.062	Detected	-0.062	Detected	0.000	-1.292	Detected	-0.998	Detected	-1.145	0.019
GT Sens	Sense	0.212	Detected	-0.212	Detected	0.000	-2.973	Comprom	-1.079	Detected	-2.026	0.172
GT Sens	Sense	0.278	Detected	-0.278	Detected	0.000	-2.542	Detected	-2.052	Detected	-2.297	0.025
GT Sens	Sense	0.115	Detected	-0.115	Detected	0.000	-1.078	Detected	-1.266	Detected	-1.172	0.016
GT Sens	Sense	-0.083	Detected	0.083	Detected	0.000	-1.412	Detected	-1.780	Detected	-1.596	0.016
GT Sens	Sense	0.331	Detected	-0.331	Detected	0.000	-2.124	Comprom	-1.069	Detected	-1.596	0.125
GT Sens	Sense	0.241	Detected	-0.241	Detected	0.000	-2.325	Detected	-1.801	Detected	-2.063	0.028
GT Sens	Sense	0.110	Detected	-0.110	Detected	0.000	-2.292	Detected	-2.199	Detected	-2.245	0.003
GT Sens	Sense	0.037	Detected	-0.037	Detected	0.000	-1.332	Detected	-1.391	Detected	-1.362	0.001
GT Sens	Sense	-0.029	Detected	0.029	Detected	0.000	-1.020	Detected	-1.311	Detected	-1.166	0.016
GT Sens	Sense	-0.033	Detected	0.033	Detected	0.000	-2.121	Detected	-1.490	Detected	-1.805	0.029
GT Sens	Sense	0.213	Detected	-0.213	Detected	0.000	-2.136	Detected	-1.649	Detected	-1.892	0.028
GT Sens	Sense	-0.110	Detected	0.110	Detected	0.000	-2.410	Comprom	-1.891	Comprom	-2.151	0.017
GT Sens	Sense	0.099	Detected	-0.099	Detected	0.000	-1.226	Detected	-1.033	Detected	-1.130	0.015
GT Sens	Sense	0.514	Detected	-0.514	Detected	0.000	-1.307	Detected	-2.990	Detected	-2.148	0.161
GT Sens	Sense	0.151	Detected	-0.151	Detected	0.000	-0.985	Detected	-1.245	Detected	-1.115	0.031
GT Sens	Sense	0.040	Detected	-0.040	Detected	0.000	-1.198	Detected	-0.929	Detected	-1.064	0.017
GT Sens	Sense	0.265	Detected	-0.265	Detected	0.000	-2.181	Detected	-1.708	Detected	-1.944	0.032
GT Sens	Sense	-0.196	Detected	0.196	Detected	0.000	-1.745	Detected	-1.687	Detected	-1.716	0.013
GT Sens	Sense	0.107	Detected	-0.107	Detected	0.000	-1.281	Detected	-1.223	Detected	-1.252	0.008
GT Sens	Sense	0.022	Detected	-0.022	Detected	0.000	-4.356	Comprom	-4.128	Comprom	-4.242	0.001
GT Sens	Sense	0.040	Detected	-0.040	Detected	0.000	-1.416	Detected	-2.388	Comprom	-1.902	0.060
GT Sens	Sense	0.223	Detected	-0.223	Detected	0.000	-1.383	Detected	-1.066	Detected	-1.224	0.046
GT Sens	Sense	0.144	Detected	-0.144	Detected	0.000	-1.316	Detected	-1.892	Detected	-1.604	0.038
GT Sens	Sense	-0.087	Detected	0.087	Detected	0.000	-1.348	Detected	-1.044	Detected	-1.196	0.021
GT Sens	Sense	0.182	Detected	-0.182	Detected	0.000	-3.647	Detected	-1.414	Detected	-2.531	0.155
GT Sens	Sense	0.170	Detected	-0.170	Detected	0.000	-2.407	Detected	-1.811	Detected	-2.109	0.025
GT Sens	Sense	0.052	Detected	-0.052	Detected	0.000	-2.414	Comprom	-2.503	Comprom	-2.458	0.001
GT Sens	Sense	0.101	Detected	-0.101	Detected	0.000	-1.266	Detected	-1.058	Detected	-1.162	0.015
GT Sens	Sense	0.185	Detected	-0.185	Detected	0.000	-2.732	Detected	-1.983	Detected	-2.358	0.030
GT Sens	Sense	0.048	Detected	-0.048	Detected	0.000	-1.080	Detected	-1.118	Detected	-1.099	0.002
GT Sens	Sense	0.166	Detected	-0.166	Detected	0.000	-1.525	Detected	-1.154	Detected	-1.339	0.033
GT Sens	Sense	0.126	Detected	-0.126	Detected	0.000	-1.459	Detected	-1.158	Detected	-1.309	0.022
GT Sens	Sense	-0.125	Detected	0.125	Detected	0.000	-3.804	Comprom	-1.027	Detected	-2.415	0.225
GT Sens	Sense	-0.190	Detected	0.190	Detected	0.000	-1.172	Detected	-2.064	Detected	-1.618	0.079
GT Sens	Sense	0.095	Detected	-0.095	Detected	0.000	-1.369	Detected	-1.377	Detected	-1.373	0.005
GT Sens	Sense	0.389	Detected	-0.389	Detected	0.000	-1.191	Detected	-0.892	Detected	-1.041	0.130
GT Sens	Sense	0.129	Detected	-0.129	Detected	0.000	-2.251	Detected	-1.898	Detected	-2.075	0.011
GT Sens	Sense	0.307	Detected	-0.307	Detected	0.000	-1.674	Detected	-1.939	Comprom	-1.807	0.033
GT Sens	Sense	-0.356	Detected	0.356	Detected	0.000	-1.610	Comprom	-2.054	Comprom	-1.832	0.049
GT Sens	Sense	0.630	Detected	-0.630	Detected	0.000	-3.395	Detected	-4.663	Detected	-4.029	0.046
GT Sens	Sense	0.128	Detected	-0.128	Detected	0.000	-1.847	Detected	-1.761	Detected	-1.804	0.006
GT Sens	Sense	0.542	Detected	-0.542	Detected	0.000	-2.872	Detected	-2.481	Detected	-2.677	0.043
GT Sens	Sense	0.305	Detected	-0.305	Detected	0.000	-3.033	Comprom	-2.849	Comprom	-2.941	0.012
GT Sens	Sense	0.088	Detected	-0.088	Detected	0.000	-2.162	Detected	-1.253	Detected	-1.707	0.066
GT Sens	Sense	0.315	Detected	-0.315	Detected	0.000	-2.998	Detected	-2.368	Detected	-2.683	0.026
GT Sens	Sense	0.473	Detected	-0.473	Detected	0.000	-2.569	Detected	-1.292	Detected	-1.930	0.136
GT Sens	Sense	0.174	Detected	-0.174	Detected	0.000	-1.947	Detected	-2.020	Detected	-1.984	0.008
GT Sens	Sense	-0.076	Detected	0.076	Detected	0.000	-1.188	Detected	-0.930	Detected	-1.059	0.019
GT Sens	Sense	0.176	Detected	-0.176	Detected	0.000	-2.039	Detected	-2.501	Detected	-2.270	0.016
GT Sens	Sense	0.056	Detected	-0.056	Detected	0.000	-1.640	Detected	-1.856	Detected	-1.748	0.005

GT	Sense	Sense	-0.163	Detected	0.163	Detected	0.000	-1.157	Detected	-1.748	Detected	-1.452	0.050	GT	Sens
GT	Sense	Sense	-0.059	Detected	0.059	Detected	0.000	-1.167	Detected	-1.211	Detected	-1.189	0.003	GT	Sens
GT	Sense	Sense	0.196	Detected	-0.196	Detected	0.000	-1.624	Detected	-2.290	Comprom	-1.957	0.037	GT	Sens
GT	Sense	Sense	0.171	Detected	-0.171	Detected	0.000	-1.260	Detected	-0.878	Detected	-1.069	0.053	GT	Sens
GT	Sense	Sense	0.002	Detected	-0.002	Detected	0.000	-1.315	Detected	-1.201	Detected	-1.258	0.002	GT	Sens
GT	Sense	Sense	-0.061	Detected	0.061	Detected	0.000	-1.073	Detected	-1.502	Detected	-1.288	0.029	GT	Sens
GT	Sense	Sense	-0.146	Detected	0.146	Detected	0.000	-2.575	Comprom	-2.427	Comprom	-2.501	0.004	GT	Sens
GT	Sense	Sense	0.238	Detected	-0.238	Detected	0.000	-2.995	Detected	-1.693	Detected	-2.344	0.077	GT	Sens
GT	Sense	Sense	0.149	Detected	-0.149	Detected	0.000	-1.638	Detected	-1.463	Detected	-1.550	0.012	GT	Sens
GT	Sense	Sense	0.117	Detected	-0.117	Detected	0.000	-2.882	Detected	-2.205	Detected	-2.543	0.019	GT	Sens
GT	Sense	Sense	0.033	Detected	-0.033	Detected	0.000	-2.027	Detected	-1.925	Detected	-1.976	0.001	GT	Sens
GT	Sense	Sense	0.107	Detected	-0.107	Detected	0.000	-1.321	Detected	-0.917	Detected	-1.119	0.039	GT	Sens
GT	Sense	Sense	0.258	Detected	-0.258	Detected	0.000	-2.503	Detected	-3.104	Detected	-2.803	0.019	GT	Sens
GT	Sense	Sense	0.104	Detected	-0.104	Detected	0.000	-1.377	Detected	-1.206	Detected	-1.292	0.011	GT	Sens
GT	Sense	Sense	0.499	Detected	-0.499	Detected	0.000	-1.319	Detected	-1.206	Detected	-1.262	0.129	GT	Sens
GT	Sense	Sense	0.480	Detected	-0.480	Detected	0.000	-3.538	Comprom	-3.522	Comprom	-3.530	0.018	GT	Sens
GT	Sense	Sense	-0.281	Detected	0.281	Detected	0.000	-2.606	Comprom	-1.662	Comprom	-2.134	0.060	GT	Sens
GT	Sense	Sense	0.234	Detected	-0.234	Detected	0.000	-1.360	Detected	-1.370	Detected	-1.365	0.028	GT	Sens
GT	Sense	Sense	0.033	Detected	-0.033	Detected	0.000	-4.032	Detected	-4.005	Detected	-4.019	0.000	GT	Sens
GT	Sense	Sense	0.104	Detected	-0.104	Detected	0.000	-2.378	Detected	-1.789	Detected	-2.084	0.022	GT	Sens
GT	Sense	Sense	0.233	Detected	-0.233	Detected	0.000	-1.381	Detected	-1.398	Detected	-1.390	0.027	GT	Sens
GT	Sense	Sense	0.025	Detected	-0.025	Detected	0.000	-1.294	Detected	-1.064	Detected	-1.179	0.010	GT	Sens
GT	Sense	Sense	0.592	Detected	-0.592	Detected	0.000	-1.221	Detected	-1.005	Detected	-1.113	0.205	GT	Sens
GT	Sense	Sense	0.120	Detected	-0.120	Detected	0.000	-1.827	Detected	-1.076	Detected	-1.452	0.066	GT	Sens
GT	Sense	Sense	0.303	Detected	-0.303	Detected	0.000	-1.771	Detected	-1.505	Detected	-1.638	0.038	GT	Sens
GT	Sense	Sense	-0.075	Detected	0.075	Detected	0.000	-0.830	Detected	-1.517	Detected	-1.173	0.079	GT	Sens
GT	Sense	Sense	0.196	Detected	-0.196	Detected	0.000	-2.231	Detected	-2.037	Detected	-2.134	0.010	GT	Sens
GT	Sense	Sense	0.493	Detected	-0.493	Detected	0.000	-1.775	Comprom	-2.091	Comprom	-1.933	0.065	GT	Sens
GT	Sense	Sense	-0.060	Detected	0.060	Detected	0.000	-1.000	Detected	-1.529	Detected	-1.265	0.043	GT	Sens
GT	Sense	Sense	0.051	Detected	-0.051	Detected	0.000	-1.018	Detected	-1.419	Detected	-1.218	0.028	GT	Sens
GT	Sense	Sense	-0.039	Detected	0.039	Detected	0.000	-1.483	Detected	-1.360	Detected	-1.422	0.003	GT	Sens
GT	Sense	Sense	0.014	Detected	-0.014	Detected	0.000	-1.321	Detected	-3.052	Comprom	-2.187	0.127	GT	Sens
GT	Sense	Sense	-0.036	Detected	0.036	Detected	0.000	-2.926	Detected	-2.541	Detected	-2.733	0.005	GT	Sens
GT	Sense	Sense	0.292	Detected	-0.292	Detected	0.000	-3.447	Comprom	-2.066	Detected	-2.757	0.067	GT	Sens
GT	Sense	Sense	0.153	Detected	-0.153	Detected	0.000	-1.318	Detected	-2.406	Comprom	-1.862	0.081	GT	Sens
GT	Sense	Sense	0.078	Detected	-0.078	Detected	0.000	-1.673	Detected	-1.501	Detected	-1.587	0.005	GT	Sens
GT	Sense	Sense	0.035	Detected	-0.035	Detected	0.000	-2.473	Comprom	-0.966	Detected	-1.720	0.150	GT	Sens
GT	Sense	Sense	0.328	Detected	-0.328	Detected	0.000	-1.602	Detected	-1.495	Detected	-1.548	0.043	GT	Sens
GT	Sense	Sense	0.173	Detected	-0.173	Detected	0.000	-2.507	Detected	-2.254	Detected	-2.380	0.008	GT	Sens
GT	Sense	Sense	0.159	Detected	-0.159	Detected	0.000	-1.616	Detected	-1.045	Detected	-1.330	0.055	GT	Sens
GT	Sense	Sense	0.087	Detected	-0.087	Detected	0.000	-1.138	Detected	-0.886	Detected	-1.012	0.022	GT	Sens
GT	Sense	Sense	-0.382	Detected	0.382	Detected	0.000	-2.622	Comprom	-1.119	Detected	-1.871	0.157	GT	Sens
GT	Sense	Sense	0.089	Detected	-0.089	Detected	0.000	-1.113	Detected	-1.088	Detected	-1.100	0.007	GT	Sens
GT	Sense	Sense	0.031	Detected	-0.031	Detected	0.000	-1.281	Detected	-1.292	Detected	-1.287	0.001	GT	Sens
GT	Sense	Sense	-0.043	Detected	0.043	Detected	0.000	-1.486	Detected	-1.384	Detected	-1.435	0.002	GT	Sens
GT	Sense	Sense	-0.093	Detected	0.093	Detected	0.000	-1.188	Detected	-1.010	Detected	-1.099	0.014	GT	Sens
GT	Sense	Sense	-0.103	Detected	0.103	Detected	0.000	-0.989	Detected	-1.214	Detected	-1.101	0.019	GT	Sens
GT	Sense	Sense	0.250	Detected	-0.250	Detected	0.000	-2.081	Detected	-1.377	Detected	-1.729	0.057	GT	Sens
GT	Sense	Sense	0.608	Detected	-0.608	Detected	0.000	-3.765	Comprom	-5.150	Comprom	-4.458	0.040	GT	Sens
GT	Sense	Sense	-0.148	Detected	0.148	Detected	0.000	-0.972	Detected	-1.224	Detected	-1.098	0.030	GT	Sens
GT	Sense	Sense	0.170	Detected	-0.170	Detected	0.000	-2.634	Detected	-1.601	Detected	-2.118	0.060	GT	Sens
GT	Sense	Sense	0.525	Detected	-0.525	Detected	0.000	-1.800	Comprom	-0.832	Detected	-1.316	0.207	GT	Sens
GT	Sense	Sense	0.254	Detected	-0.254	Detected	0.000	-2.808	Detected	-1.051	Detected	-1.929	0.169	GT	Sens
GT	Sense	Sense	0.022	Detected	-0.022	Detected	0.000	-1.178	Detected	-1.083	Detected	-1.130	0.002	GT	Sens
GT	Sense	Sense	0.118	Detected	-0.118	Detected	0.000	-1.685	Detected	-1.718	Detected	-1.701	0.005	GT	Sens
GT	Sense	Sense	0.021	Detected	-0.021	Detected	0.000	-1.821	Detected	-1.802	Detected	-1.811	0.000	GT	Sens
GT	Sense	Sense	-0.256	Detected	0.256	Detected	0.000	-1.967	Detected	-1.593	Detected	-1.780	0.030	GT	Sens
GT	Sense	Sense	0.452	Detected	-0.452	Detected	0.000	-2.762	Detected	-2.163	Detected	-2.463	0.045	GT	Sens
GT	Sense	Sense	0.491	Detected	-0.491	Detected	0.000	-4.016	Detected	-2.008	Detected	-3.012	0.115	GT	Sens
GT	Sense	Sense	0.300	Detected	-0.300	Detected	0.000	-3.948	Detected	-2.693	Detected	-3.320	0.041	GT	Sens
GT	Sense	Sense	0.158	Detected	-0.158	Detected	0.000	-1.283	Detected	-0.825	Detected	-1.054	0.063	GT	Sens
GT	Sense	Sense	0.546	Detected	-0.546	Detected	0.000	-1.108	Detected	-1.273	Detected	-1.190	0.164	GT	Sens
GT	Sense	Sense	0.063	Detected	-0.063	Detected	0.000	-2.236	Detected	-1.435	Detected	-1.836	0.046	GT	Sens
GT	Sense	Sense	-0.102	Detected	0.102	Detected	0.000	-1.241	Detected	-3.229	Detected	-2.235	0.155	GT	Sens
GT	Sense	Sense	0.264	Detected	-0.264	Detected	0.000	-3.154	Detected	-1.566	Detected	-2.360	0.106	GT	Sens
GT	Sense	Sense	-0.167	Detected	0.167	Detected	0.000	-2.201	Detected	-1.772	Detected	-1.986	0.018	GT	Sens
GT	Sense	Sense	0.457	Detected	-0.457	Detected	0.000	-1.998	Detected	-1.061	Detected	-1.530	0.145	GT	Sens
GT	Sense	Sense	0.186	Detected	-0.186	Detected	0.000	-3.295	Detected	-1.700	Detected	-2.498	0.093	GT	Sens
GT	Sense	Sense	0.131	Detected	-0.131	Detected	0.000	-1.913	Detected	-1.137	Detected	-1.525	0.065	GT	Sens
GT	Sense	Sense	0.943	Detected	-0.943	Detected	0.000	-2.512	Comprom	-2.886	Comprom	-2.699	0.107	GT	Sens
GT	Sense	Sense	0.166	Detected	-0.166	Detected	0.000	-1.297	Detected	-1.043	Detected	-1.170	0.030	GT	Sens
GT	Sense	Sense	0.053	Detected	-0.053	Detected	0.000	-1.584	Detected	-1.531	Detected	-1.557	0.001	GT	Sens
GT	Sense	Sense	-0.010	Detected	0.010	Detected	0.000	-1.380	Detected	-1.022	Detected	-1.201	0.022	GT	Sens
GT	Sense	Sense	0.582	Detected	-0.582	Detected	0.000	-3.249	Detected	-2.196	Detected	-2.723	0.074	GT	Sens
GT	Sense	Sense	0.583	Detected	-0.583	Detected	0.000	-0.943	Detected	-1.524	Detected	-1.234	0.199	GT	Sens
GT	Sense	Sense	0.022	Detected	-0.022	Detected	0.000	-1.224	Detected	-0.863	Detected	-1.043	0.029	GT	Sens
GT	Sense	Sense	0.159	Detected	-0.159	Detected	0.000	-1.442	Detected	-1.377	Detected	-1.409	0.013	GT	Sens
GT	Sense	Sense	0.058	Detected	-0.058	Detected	0.000	-1.471	Detected	-0.957	Detected	-1.214	0.044	GT	Sens
GT	Sense	Sense	0.018	Detected	-0.018	Detected	0.000	-2.464	Detected	-1.367	Detected	-1.916	0.073	GT	Sens
GT	Sense	Sense	0.041	Detected	-0.041	Detected	0.000	-2.758	Detected	-1.219	Detected	-1.989	0.123	GT	Sens
GT	Sense	Sense	0.184	Detected	-0.184	Detected	0.000	-1.086	Detected	-0.924	Detected	-1.005	0.038	GT	Sens
GT	Sense	Sense	0.037	Detected	-0.037	Detected	0.000	-1.685	Detected	-1.381	Detected	-1.533	0.010	GT	Sens
GT	Sense	Sense	0.256	Detected	-0.256	Detected	0.000	-1.163	Detected	-1.149	Detected	-1.156	0.046	GT	Sens
GT	Sense	Sense	0.121	Detected	-0.121	Detected	0.000	-2.122	Detected	-1.895	Detected	-2.009	0.007	GT	Sens
GT	Sense	Sense	0.316	Detected	-0.316	Detected	0.000	-3.368	Detected	-2.129	Detected	-2.748	0.058	GT	Sens
GT	Sense	Sense	0.312	Detected	-0.312	Detected	0.000	-2.106	Detected	-0.968	Detected	-1.537	0.141	GT	Sens
GT	Sense	Sense	0.055	Detected	-0.055	Detected	0.000	-1.218	Detected	-1.014	Detected	-1.116	0.011	GT	Sens
GT	Sense	Sense	0.116	Detected	-0.116	Detected	0.000	-1.868	Detected	-0.816	Detected	-1.342	0.130	GT	Sens
GT	Sense	Sense	0.238	Detected	-0.238	Detected	0.000	-1.323	Detected	-0.910	Detected	-1.117	0.071	GT	Sens
GT	Sense	Sense	-0.032	Detected	0.032	Detected	0.000	-1.803	Detected	-1.415	Detected	-1.609	0.015	GT	Sens
GT	Sense	Sense	-0.129	Detected	0.129	Detected	0.000	-2.768	Detected	-1.618	Detected	-2.193	0.065	GT	Sens
GT	Sense	Sense	0.004	Detected	-0.004	Detected	0.000	-1.805	Detected	-1.285	Detected	-1.545	0.027	GT	Sens
GT	Sense	Sense	0.522	Detected	-0.522	Detected	0.000	-4.992	Detected	-4.504	Detected	-4.748	0.014	GT	Sens
GT	Sense	Sense	0.527	Detected	-0.527	Detected	0.000	-4.453	Detected	-1.828	Detected	-3.141	0.157	GT	Sens
GT	Sense	Sense	0.315	Detected	-0.315	Detected	0.000	-1.453	Detected	-1.535	Detected	-1.494	0.042	GT	Sens
GT	Sense	Sense	0.150	Detected	-0.150	Detected	0.000	-1.966	Detected	-2.050	Detected	-2.008	0.006	GT	Sens
GT	Sense	Sense	0.113	Detected	-0.113	Detected	0.000	-2.177	Detected	-2.109	Detected	-2.143	0.003	GT	Sens
GT	Sense	Sense	0.105	Detected	-0.105	Detected	0.000	-1.523	Detected	-1.443	Detected	-1.483	0.006	GT	Sens
GT	Sense	Sense	0.098	Detected	-0.098	Detected	0.000	-1.284	Detected	-1.425	Detected	-1.354	0.008	GT	Sens
GT	Sense	Sense	0.071	Detected	-0.071	Detected	0.000	-1.451	Detected	-0.851	Detected	-1.151	0.065	GT	Sens
GT	Sense	Sense	0.108	Detected	-0.108	Detected	0.000	-3.182	Detected	-1.684	Detected	-2.433	0.085	GT	Sens
GT	Sense	Sense	-0.241	Detected	0.241	Detected	0.000	-2.212	Detected	-2.716	Comprom	-2.464	0.019	GT	Sens
GT	Sense	Sense	0.042	Detected	-0.042	Detected	0.000	-1.235	Detected	-0.813	Detected	-1.024	0.041	GT	Sens
GT	Sense	Sense	0.208	Detected	-0.208	Detected	0.000	-1.065	Detected	-1.141	Detected	-1.103	0.035	GT	Sens
GT	Sense	Sense	-0.382	Detected	0.382	Detected	0.000	-0.981	Detected	-1.503	Detected	-1.242	0.115	GT	Sens
GT	Sense	Sense	0.069	Detected	-0.069	Detected	0.000	-1.003	Detected	-1.168	Detected	-1.085	0.010	GT	Sens
GT	Sense	Sense	-1.023	Detected	1.023	Detected	0.000	-1.698	Detected	-2.499	Comprom	-2.098	0.196	GT	Sens
GT	Sense	Sense	0.090	Detected	-0.090	Detected	0.000	-1.155	Detected	-1.016	Detected	-1.085	0.011	GT	Sens
GT	Sense	Sense	-0.142	Detected	0.142	Detected	0.000	-1.035	Detected	-1.295	Detected	-1.165	0.026	GT	Sens
GT	Sense	Sense	0.054	Detected	-0.054	Detected	0.000	-1.875	Detected	-2.509	Comprom	-2.192	0.021	GT	Sens
GT	Sense	Sense	0.183	Detected	-0.183	Detected	0.000	-1.278	Detected	-1.940	Detected	-1.609	0.051	GT	Sens
GT	Sense	Sense	0.154	Detected	-0.154	Detected	0.000	-1.420	Detected	-1.065	Detected	-1.242	0.034	GT	Sens
GT	Sense	Sense	0.208	Detected	-0.208	Detected	0.000	-3.397	Detected	-2.039	Detected	-2.718	0.062	GT	Sens
GT	Sense	Sense	0.053	Detected	-0.053	Detected	0.000	-1.146	Detected	-0.984	Detected	-1.065	0.008	GT	Sens
GT	Sense	Sense	0.102	Detected	-0.102	Detected	0.000	-1.704	Detected	-1.409	Detected	-1.557	0.013	GT	Sens
GT	Sense	Sense	-0.064	Detected	0.064	Detected	0.000	-1.630	Detected	-1.486	Detected	-1.558	0.004	GT	Sens
GT	Sense	Sense	0.190	Detected	-0.190	Detected	0.000	-2.238	Detected	-2.191	Detected	-2.214	0.007	GT	Sens
GT	Sense	Sense	-0.062	Detected	0.062	Detected	0.000	-1.211	Detected	-1.052	Detected	-1.131	0.008	GT	Sens
GT	Sense	Sense	0.342	Detected	-0.342	Detected	0.000	-1.031	Detected	-1.282	Detected	-1.156	0.087	GT	Sens
GT	Sense	Sense	-0.019	Detected	0.019	Detected	0.000	-1.675	Detected	-0.878	Detected	-1.276	0.085	GT	Sens
GT	Sense	Sense	0.076	Detected	-0.076	Detected	0.000	-1.193	Detected	-1.261	Detected	-1.227	0.005	GT	Sens
GT	Sense	Sense	0.124	Detected	-0.124	Detected	0.000	-1.534	Detected	-1.332	Detected	-1.433	0.012	GT	Sens
GT	Sense	Sense	0.021	Detected	-0.021	Detected	0.000	-1.008	Detected	-1.632	Comprom	-1.320	0.052	GT	Sens
GT	Sense	Sense	0.398	Detected	-0.398	Detected	0.000	-0.966	Detected	-1.469	Detected	-1.218	0.123	GT	Sens
GT	Sense	Sense	-0.041	Detected	0.041	Detected	0.000	-3.494	Detected	-1.483	Detected	-2.488	0.132	GT	Sens
GT	Sense	Sense	-0.003	Detected	0.003	Detected	0.000	-1.118	Detected	-1.797	Comprom	-1.458	0.050	GT	Sens
GT	Sense	Sense	0.022	Detected	-0.022	Detected	0.000	-2.074	Detected	-1.503	Detected	-1.788	0.025	GT	Sens
GT	Sense	Sense	0.057	Detected	-0.057	Detected	0.000	-1.849	Detected	-1.574	Detected	-1.711	0.007	GT	Sens
GT	Sense	Sense	0.106	Detected	-0.106	Detected	0.000	-1.233	Detected	-3.473	Detected	-2.353	0.171	GT	Sens
GT	Sense	Sense	0.322	Detected	-0.322	Detected	0.000	-1.931	Detected	-1.691	Detected	-1.811	0.034	GT	Sens
GT	Sense	Sense	0.013	Detected	-0.013	Detected	0.000	-1.280	Detected	-1.011	Detected	-1.146	0.014	GT	Sens
GT	Sense	Sense	0.207	Detected	-0.207	Detected	0.000	-1.207	Detected	-1.313	Detected	-1.260	0.027	GT	Sens
GT	Sense	Sense	0.639	Detected	-0.639	Detected	0.000	-2.419	Detected	-1.777	Detected	-2.098	0.099	GT	Sens
GT	Sense	Sense	0.140	Detected	-0.140	Detected	0.000	-1.091	Detected	-0.949	Detected	-1.020	0.023	GT	Sens
GT	Sense	Sense	0.128	Detected	-0.128	Detected	0.000	-1.604	Detected	-0.953	Detected	-1.278	0.067	GT	Sens
GT	Sense	Sense	-0.052	Detected	0.052	Detected	0.000	-1.354	Detected	-1.161	Detected	-1.257	0.007	GT	Sens
GT	Sense	Sense	0.215	Detected	-0.215	Detected	0.000	-0.901	Detected	-1.570	Detected	-1.236	0.090	GT	Sens
GT	Sense	Sense	-0.010	Detected	0.010	Detected	0.000	-1.393	Detected	-1.186	Detected	-1.290	0.006	GT	Sens
GT	Sense	Sense	0.282	Detected	-0.282	Detected	0.000	-1.886	Detected	-0.951	Detected	-1.418	0.122	GT	Sens
GT	Sense	Sense	0.303	Detected	-0.303	Detected	0.000	-2.173	Detected	-1.291	Detected	-1.732	0.084	GT	Sens
GT	Sense	Sense	-0.028	Detected	0.028	Detected	0.000	-1.096	Detected	-0.933	Detected	-1.014	0.007	GT	Sens
GT	Sense	Sense	0.120	Detected	-0.120	Detected	0.000	-1.855	Comprom	-0.963	Comprom	-1.409	0.093	GT	Sens
GT	Sense	Sense	0.289	Detected	-0.289	Detected	0.000	-4.021	Comprom	-1.459	Detected	-2.740	0.172	GT	Sens
GT	Sense	Sense	-0.016	Detected	0.016	Detected	0.000	-2.436	Detected	-1.841	Detected	-2.138	0.019	GT	Sens
GT	Sense	Sense	-0.643	Detected	0.643	Detected	0.000	-1.014	Detected	-1.589	Detected	-1.302	0.206	GT	Sens
GT	Sense	Sense	0.137	Detected	-0.137	Detected	0.000	-1.562	Detected	-1.160	Detected	-1.361	0.031	GT	Sens
GT	Sense	Sense	0.152	Detected	-0.152	Detected	0.000	-2.181	Detected	-1.612	Detected	-1.896	0.028	GT	Sens
GT	Sense	Sense	0.183	Detected	-0.183	Detected	0.000	-1.539	Detected	-1.626	Detected	-1.583	0.014	GT	Sens
GT	Sense	Sense	0.128	Detected	-0.128	Detected	0.000	-3.178	Detected	-3.363	Detected	-3.270	0.002	GT	Sens
GT	Sense	Sense	-0.075	Detected	0.075	Detected	0.000	-1.105	Detected	-1.104	Detected	-1.104	0.005	GT	Sens
GT	Sense	Sense	0.203	Detected	-0.203	Detected	0.000	-1.326	Detected	-1.024	Detected	-1.175	0.043	GT	Sens
GT	Sense	Sense	0.172	Detected	-0.172	Detected	0.000	-2.141	Comprom	-1.360	Detected	-1.751	0.055	GT	Sens
GT	Sense	Sense	-0.029	Detected	0.029	Detected	0.000	-1.064	Detected	-0.967	Detected	-1.016	0.003	GT	Sens
GT	Sense	Sense	-0.073	Detected	0.073	Detected	0.000	-1.373	Detected	-1.384	Detected	-1.378	0.003	GT	Sens
GT	Sense	Sense	0.070	Detected	-0.070	Detected	0.000	-1.016	Detected	-0.989	Detected	-1.003	0.005	GT	Sens
GT	Sense	Sense	0.114	Detected	-0.114	Detected	0.000	-1.585	Detected	-1.129	Detected	-1.357	0.034	GT	Sens
GT	Sense	Sense	0.058	Detected	-0.058	Detected	0.000	-1.106	Detected	-1.028	Detected	-1.067	0.004	GT	Sens
GT	Sense	Sense	-0.178	Detected	0.178	Detected	0.000	-2.789	Comprom	-2.047	Comprom	-2.418	0.028	GT	Sens
GT	Sense	Sense	-0.465	Detected	0.465	Detected	0.000	-1.491	Comprom	-2.306	Comprom	-1.899	0.092	GT	Sens
GT	Sense	Sense	0.024	Detected	-0.024	Detected	0.000	-1.616	Detected	-1.235	Detected	-1.425	0.018	GT	Sens
GT	Sense	Sense	1.851	Detected	-1.851	Detected	0.000	-1.691	Detected	-1.809	Detected	-1.750	0.444	GT	Sens
GT	Sense	Sense	0.426	Detected	-0.426	Detected	0.000	-2.894	Detected	-2.704	Detected	-2.799	0.023	GT	Sens
GT	Sense	Sense	0.055	Detected	-0.055	Detected	0.000	-1.198	Detected	-1.216	Detected	-1.207	0.002	GT	Sens
GT	Sense	Sense	0.291	Detected	-0.291	Detected	0.000	-3.975	Detected	-3.850	Detected	-3.913	0.006	GT	Sens
GT	Sense	Sense	0.366	Detected	-0.366	Detected	0.000	-1.244	Detected	-1.332	Detected	-1.288	0.073	GT	Sens
GT	Sense	Sense	0.042	Detected	-0.042	Detected	0.000	-1.407	Detected	-0.999	Detected	-1.203	0.029	GT	Sens
GT	Sense	Sense	-0.095	Detected	0.095	Detected	0.000	-1.668	Detected	-0.860	Detected	-1.264	0.093	GT	Sens
GT	Sense	Sense	0.181	Detected	-0.181	Detected	0.000	-1.849	Detected	-1.285	Detected	-1.567	0.043	GT	Sens
GT	Sense	Sense	-0.129	Detected	0.129	Detected	0.000	-1.269	Detected	-1.096	Detected	-1.182	0.017	GT	Sens
GT	Sense	Sense	0.255	Detected	-0.255	Detected	0.000	-2.215	Detected	-1.574	Detected	-1.895	0.044	GT	Sens
GT	Sense	Sense	0.078	Detected	-0.078	Detected	0.000	-1.954	Detected	-1.235	Detected	-1.594	0.049	GT	Sens
GT	Sense	Sense	0.197	Detected	-0.197	Detected	0.000	-1.454	Detected	-1.090	Detected	-1.272	0.042	GT	Sens
GT	Sense	Sense	0.560	Detected	-0.560	Detected	0.000	-1.569	Detected	-1.351	Detected	-1.460	0.125	GT	Sens
GT	Sense	Sense	0.016	Detected	-0.016	Detected	0.000	-1.132	Detected	-1.207	Detected	-1.169	0.001	GT	Sens
GT	Sense	Sense	-0.138	Detected	0.138	Detected	0.000	-1.641	Detected	-1.010	Detected	-1.325	0.061	GT	Sens
GT	Sense	Sense	0.344	Detected	-0.344	Detected	0.000	-1.832	Detected	-1.389	Detected	-1.611	0.059	GT	Sens
GT	Sense	Sense	0.120	Detected	-0.120	Detected	0.000	-1.900	Detected	-1.179	Detected	-1.539	0.056	GT	Sens
GT	Sense	Sense	0.127	Detected	-0.127	Detected	0.000	-1.155	Detected	-1.064	Detected	-1.110	0.015	GT	Sens
GT	Sense	Sense	0.696	Detected	-0.696	Detected	0.000	-1.898	Detected	-1.310	Detected	-1.604	0.168	GT	Sens
GT	Sense	Sense	0.409	Detected	-0.409	Detected	0.000	-2.829	Detected	-3.664	Detected	-3.247	0.031	GT	Sens
GT	Sense	Sense	-0.047	Detected	0.047	Detected	0.000	-1.285	Detected	-0.945	Detected	-1.115	0.024	GT	Sens
GT	Sense	Sense	-0.083	Detected	0.083	Detected	0.000	-1.310	Detected	-0.945	Detected	-1.127	0.030	GT	Sens
GT	Sense	Sense	0.025	Detected	-0.025	Detected	0.000	-0.807	Detected	-1.581	Detected	-1.194	0.091	GT	Sens
GT	Sense	Sense	0.070	Detected	-0.070	Detected	0.000	-1.738	Detected	-1.362	Detected	-1.550	0.016	GT	Sens
GT	Sense	Sense	0.004	Detected	-0.004	Detected	0.000	-2.322	Detected	-1.867	Detected	-2.095	0.012	GT	Sens
GT	Sense	Sense	0.259	Detected	-0.259	Detected	0.000	-2.940	Comprom	-1.267	Comprom	-2.104	0.138	GT	Sens
GT	Sense	Sense	0.132	Detected	-0.132	Detected	0.000	-1.563	Detected	-0.874	Detected	-1.219	0.081	GT	Sens
GT	Sense	Sense	-0.021	Detected	0.021	Detected	0.000	-1.584	Detected	-1.321	Detected	-1.453	0.008	GT	Sens
GT	Sense	Sense	0.196	Detected	-0.196	Detected	0.000	-1.923	Detected	-1.838	Detected	-1.880	0.011	GT	Sens
GT	Sense	Sense	0.142	Detected	-0.142	Detected	0.000	-1.779	Detected	-1.070	Detected	-1.424	0.065	GT	Sens
GT	Sense	Sense	-0.119	Detected	0.119	Detected	0.000	-3.024	Detected	-2.950	Detected	-2.987	0.002	GT	Sens
GT	Sense	Sense	0.144	Detected	-0.144	Detected	0.000	-1.281	Detected	-2.132	Comprom	-1.706	0.063	GT	Sens
GT	Sense	Sense	0.046	Detected	-0.046	Detected	0.000	-1.439	Detected	-0.982	Detected	-1.210	0.035	GT	Sens
GT	Sense	Sense	0.121	Detected	-0.121	Detected	0.000	-2.727	Comprom	-1.990	Comprom	-2.359	0.026	GT	Sens
GT	Sense	Sense	0.277	Detected	-0.277	Detected	0.000	-3.532	Comprom	-3.580	Comprom	-3.556	0.006	GT	Sens
GT	Sense	Sense	-0.045	Detected	0.045	Detected	0.000	-1.042	Detected	-1.577	Detected	-1.309	0.040	GT	Sens
GT	Sense	Sense	0.046	Detected	-0.046	Detected	0.000	-1.562	Detected	-1.300	Detected	-1.431	0.009	GT	Sens
GT	Sense	Sense	-0.027	Detected	0.027	Detected	0.000	-1.070	Detected	-1.363	Detected	-1.217	0.015	GT	Sens
GT	Sense	Sense	0.088	Detected	-0.088	Detected	0.000	-1.164	Detected	-1.373	Detected	-1.269	0.011	GT	Sens
GT	Sense	Sense	0.399	Detected	-0.399	Detected	0.000	-1.152	Detected	-1.629	Detected	-1.391	0.096	GT	Sens
GT	Sense	Sense	-0.040	Detected	0.040	Detected	0.000	-1.764	Detected	-1.576	Detected	-1.670	0.004	GT	Sens
GT	Sense	Sense	0.354	Detected	-0.354	Detected	0.000	-1.923	Detected	-2.149	Detected	-2.036	0.032	GT	Sens
GT	Sense	Sense	0.914	Detected	-0.914	Detected	0.000	-2.598	Comprom	-3.484	Comprom	-3.041	0.096	GT	Sens
GT	Sense	Sense	0.339	Detected	-0.339	Detected	0.000	-0.984	Detected	-1.069	Detected	-1.026	0.095	GT	Sens
GT	Sense	Sense	0.249	Detected	-0.249	Detected	0.000	-1.316	Detected	-2.389	Detected	-1.852	0.089	GT	Sens
GT	Sense	Sense	0.068	Detected	-0.068	Detected	0.000	-1.185	Detected	-0.871	Detected	-1.028	0.027	GT	Sens
GT	Sense	Sense	0.161	Detected	-0.161	Detected	0.000	-1.114	Detected	-1.054	Detected	-1.084	0.022	GT	Sens
GT	Sense	Sense	0.122	Detected	-0.122	Detected	0.000	-0.995	Detected	-1.025	Detected	-1.010	0.015	GT	Sens
GT	Sense	Sense	0.061	Detected	-0.061	Detected	0.000	-1.059	Detected	-0.968	Detected	-1.013	0.006	GT	Sens
GT	Sense	Sense	0.099	Detected	-0.099	Detected	0.000	-1.461	Detected	-1.697	Detected	-1.579	0.009	GT	Sens
GT	Sense	Sense	0.050	Detected	-0.050	Detected	0.000	-1.096	Detected	-1.144	Detected	-1.120	0.002	GT	Sens
GT	Sense	Sense	0.087	Detected	-0.087	Detected	0.000	-1.673	Detected	-1.032	Detected	-1.352	0.055	GT	Sens
GT	Sense	Sense	0.264	Detected	-0.264	Detected	0.000	-1.136	Detected	-1.028	Detected	-1.082	0.057	GT	Sens
GT	Sense	Sense	-0.048	Detected	0.048	Detected	0.000	-1.202	Detected	-0.961	Detected	-1.081	0.014	GT	Sens
GT	Sense	Sense	0.164	Detected	-0.164	Detected	0.000	-0.856	Detected	-1.374	Detected	-1.115	0.068	GT	Sens
GT	Sense	Sense	0.106	Detected	-0.106	Detected	0.000	-2.044	Detected	-1.751	Detected	-1.898	0.009	GT	Sens
GT	Sense	Sense	0.307	Detected	-0.307	Detected	0.000	-1.116	Detected	-2.168	Detected	-1.642	0.114	GT	Sens
GT	Sense	Sense	-0.017	Detected	0.017	Detected	0.000	-2.194	Detected	-1.832	Detected	-2.013	0.008	GT	Sens
GT	Sense	Sense	-0.070	Detected	0.070	Detected	0.000	-2.639	Comprom	-2.991	Comprom	-2.815	0.005	GT	Sens
GT	Sense	Sense	0.034	Detected	-0.034	Detected	0.000	-0.952	Detected	-1.364	Detected	-1.158	0.031	GT	Sens
GT	Sense	Sense	0.238	Detected	-0.238	Detected	0.000	-1.221	Comprom	-1.385	Comprom	-1.303	0.035	GT	Sens
GT	Sense	Sense	0.229	Detected	-0.229	Detected	0.000	-1.643	Detected	-1.063	Detected	-1.353	0.067	GT	Sens
GT	Sense	Sense	0.062	Detected	-0.062	Detected	0.000	-2.072	Detected	-1.580	Detected	-1.826	0.019	GT	Sens
GT	Sense	Sense	0.170	Detected	-0.170	Detected	0.000	-1.544	Detected	-1.717	Detected	-1.631	0.013	GT	Sens
GT	Sense	Sense	0.241	Detected	-0.241	Detected	0.000	-1.408	Detected	-0.948	Detected	-1.178	0.072	GT	Sens
GT	Sense	Sense	0.067	Detected	-0.067	Detected	0.000	-1.998	Detected	-1.204	Detected	-1.601	0.058	GT	Sens
GT	Sense	Sense	0.015	Detected	-0.015	Detected	0.000	-1.214	Detected	-0.801	Detected	-1.008	0.040	GT	Sens
GT	Sense	Sense	0.105	Detected	-0.105	Detected	0.000	-2.250	Detected	-1.155	Detected	-1.703	0.093	GT	Sens
GT	Sense	Sense	0.314	Detected	-0.314	Detected	0.000	-1.813	Comprom	-1.358	Detected	-1.586	0.055	GT	Sens
GT	Sense	Sense	0.277	Detected	-0.277	Detected	0.000	-1.026	Detected	-1.362	Detected	-1.194	0.066	GT	Sens
GT	Sense	Sense	0.330	Detected	-0.330	Detected	0.000	-2.701	Detected	-1.920	Detected	-2.311	0.046	GT	Sens
GT	Sense	Sense	0.150	Detected	-0.150	Detected	0.000	-1.623	Detected	-0.992	Detected	-1.308	0.065	GT	Sens
GT	Sense	Sense	0.298	Detected	-0.298	Detected	0.000	-1.897	Detected	-1.067	Detected	-1.482	0.101	GT	Sens
GT	Sense	Sense	0.196	Detected	-0.196	Detected	0.000	-2.625	Detected	-1.000	Detected	-1.813	0.162	GT	Sens
GT	Sense	Sense	0.201	Detected	-0.201	Detected	0.000	-1.343	Detected	-1.486	Detected	-1.414	0.022	GT	Sens
GT	Sense	Sense	0.315	Detected	-0.315	Detected	0.000	-2.384	Detected	-2.389	Detected	-2.387	0.017	GT	Sens
GT	Sense	Sense	0.189	Detected	-0.189	Detected	0.000	-1.669	Detected	-1.692	Detected	-1.681	0.012	GT	Sens
GT	Sense	Sense	0.326	Detected	-0.326	Detected	0.000	-1.718	Detected	-1.424	Detected	-1.571	0.048	GT	Sens
GT	Sense	Sense	-0.342	Detected	0.342	Detected	0.000	-2.037	Comprom	-2.399	Comprom	-2.218	0.029	GT	Sens
GT	Sense	Sense	0.192	Detected	-0.192	Detected	0.000	-1.569	Detected	-0.846	Detected	-1.208	0.098	GT	Sens
GT	Sense	Sense	0.076	Detected	-0.076	Detected	0.000	-1.583	Detected	-1.032	Detected	-1.308	0.045	GT	Sens
GT	Sense	Sense	-0.034	Detected	0.034	Detected	0.000	-1.142	Detected	-1.123	Detected	-1.132	0.001	GT	Sens
GT	Sense	Sense	0.340	Detected	-0.340	Detected	0.000	-2.351	Detected	-2.025	Detected	-2.188	0.028	GT	Sens
GT	Sense	Sense	0.183	Detected	-0.183	Detected	0.000	-2.538	Detected	-1.728	Detected	-2.133	0.041	GT	Sens

GT	Sense	Sense	0.101	Detected	-0.101	Detected	0.000	-1.123	Detected	-0.978	Detected	-1.051	0.014	GT Sens	contig167	contig167	Inner mer	TTTTCA
GT	Sense	Sense	0.095	Detected	-0.095	Detected	0.000	-2.836	Detected	-2.619	Detected	-2.728	0.003	GT Sens	contig167	contig167	Receptor	ACCATG
GT	Sense	Sense	0.279	Detected	-0.279	Detected	0.000	-1.877	Detected	-1.726	Detected	-1.802	0.025	GT Sens	contig167	contig167	Protein s	GTGGAC
GT	Sense	Sense	0.061	Detected	-0.061	Detected	0.000	-1.373	Detected	-1.132	Detected	-1.252	0.011	GT Sens	contig168	contig168	DUF803 d	TTGCCA
GT	Sense	Sense	0.012	Detected	-0.012	Detected	0.000	-0.886	Detected	-1.649	Detected	-1.267	0.080	GT Sens	contig168	contig168	Fatty acid	TTGCAG
GT	Sense	Sense	-0.013	Detected	0.013	Detected	0.000	-1.219	Detected	-0.851	Detected	-1.035	0.030	GT Sens	contig168	contig168	LysM rec	AGAATT
GT	Sense	Sense	0.378	Detected	-0.378	Detected	0.000	-1.269	Detected	-2.256	Comprom	-1.763	0.105	GT Sens	contig168	contig168	Cold sho	TCAGAA
GT	Sense	Sense	0.049	Detected	-0.049	Detected	0.000	-1.787	Detected	-1.395	Detected	-1.591	0.016	GT Sens	contig168	contig168	ADP-ribo	TAGTAA
GT	Sense	Sense	0.124	Detected	-0.124	Detected	0.000	-2.740	Detected	-1.997	Detected	-2.368	0.026	GT Sens	contig168	contig168	Lipase-lik	GAGGCT
GT	Sense	Sense	0.040	Detected	-0.040	Detected	0.000	-3.581	Detected	-2.040	Detected	-2.810	0.068	GT Sens	contig168	contig168	Hydrolas	TTATCAT
GT	Sense	Sense	-0.043	Detected	0.043	Detected	0.000	-1.843	Detected	-0.959	Detected	-1.401	0.088	GT Sens	contig168	contig168	Receptor	TCAAAT
GT	Sense	Sense	-0.010	Detected	0.010	Detected	0.000	-1.496	Detected	-1.502	Detected	-1.499	0.000	GT Sens	contig169	contig169	Hydrolas	GATTTT
GT	Sense	Sense	0.144	Detected	-0.144	Detected	0.000	-1.155	Detected	-0.968	Detected	-1.062	0.025	GT Sens	contig169	contig169	Adaptin e	ATCTGA
GT	Sense	Sense	-0.073	Detected	0.073	Detected	0.000	-1.276	Detected	-0.853	Detected	-1.065	0.042	GT Sens	contig169	contig169	Unknown	GCCTCG
GT	Sense	Sense	-0.120	Detected	0.120	Detected	0.000	-1.630	Comprom	-1.865	Comprom	-1.748	0.009	GT Sens	contig169	contig169	Dof zinc f	CAACAA
GT	Sense	Sense	0.109	Detected	-0.109	Detected	0.000	-0.988	Detected	-1.030	Detected	-1.009	0.012	GT Sens	contig169	contig169	Homeobo	TCATGA
GT	Sense	Sense	0.171	Detected	-0.171	Detected	0.000	-1.283	Detected	-1.009	Detected	-1.146	0.035	GT Sens	contig169	contig169	Dehydrat	TCGTAT
GT	Sense	Sense	0.013	Detected	-0.013	Detected	0.000	-1.205	Detected	-1.040	Detected	-1.122	0.005	GT Sens	contig169	contig169	Phospho	TGAAGA
GT	Sense	Sense	-0.083	Detected	0.083	Detected	0.000	-1.276	Detected	-0.815	Detected	-1.045	0.051	GT Sens	contig169	contig169	Unknown	GGAGAA
GT	Sense	Sense	0.126	Detected	-0.126	Detected	0.000	-1.037	Detected	-1.288	Detected	-1.163	0.023	GT Sens	contig170	contig170	Os12g05	TAAGTG

[illegible]

GT	Sense	Sense	0.276	Detected	-0.276	Detected	0.000	-1.408	Detected	-1.182	Detected	-1.295	0.049	GT	Sens	contig21(	contig21(	Carboxyl-	GTAGTG	contig21(	Solyc03g	Solyc03g	Carboxyl-terminal proteinase (			contig21(	[illegible]	[illegible]
GT	Sense	Sense	0.401	Detected	-0.401	Detected	0.000	-2.475	Detected	-3.232	Detected	-2.854	0.035	GT	Sens	contig21(	contig21(	Calmodul	CAACTG	contig21(	Solyc01g	Solyc01g	Calmodul	GO:0005	GO:0005	contig21(	[illegible]	[illegible]
GT	Sense	Sense	-0.018	Detected	0.018	Detected	0.000	-1.005	Detected	-1.022	Detected	-1.014	0.000	GT	Sens	contig211	contig211	AT-hook	ATGGTC	contig211	Solyc08g	Solyc08g	AT-hook	GO:0003	GO:0003	contig211	[illegible]	[illegible]
GT	Sense	Sense	-0.387	Detected	0.387	Detected	0.000	-0.817	Detected	-1.484	Detected	-1.151	0.153	GT	Sens	contig211	contig211	LRR rece	CCGAAA	contig211	Solyc09g	Solyc09g	LRR rece	GO:0005	GO:0005	contig211	[illegible]	[illegible]
GT	Sense	Sense	0.168	Detected	-0.168	Detected	0.000	-2.228	Detected	-1.414	Detected	-1.821	0.054	GT	Sens	contig211	contig211	Serine hy	CTCTTG	contig211	Solyc12g	Solyc12g	Serine hy	GO:0004	GO:0004	contig211	[illegible]	[illegible]
GT	Sense	Sense	0.590	Detected	-0.590	Detected	0.000	-0.822	Detected	-1.260	Detected	-1.041	0.240	GT	Sens	contig211	contig211	BCL-2 bir	GGGGTG	contig211	Solyc02g	Solyc02g	BCL-2 binding anthanogene-1			contig211	[illegible]	[illegible]
GT	Sense	Sense	0.512	Detected	-0.512	Detected	0.000	-1.892	Detected	-1.358	Detected	-1.625	0.106	GT	Sens	contig211	contig211	Phloem le	AGTATT	contig211	Solyc00g	Solyc00g	Phloem lectin (AHRD V1 ***- O8			contig211	[illegible]	[illegible]
GT	Sense	Sense	0.148	Detected	-0.148	Detected	0.000	-1.019	Detected	-1.073	Detected	-1.046	0.020	GT	Sens	contig211	contig211	Pentatric	GTTGAT	contig211	Solyc11g	Solyc11g	Pentatric	GO:0004	GO:0004	contig211	[illegible]	[illegible]
GT	Sense	Sense	-0.020	Detected	0.020	Detected	0.000	-1.255	Detected	-1.076	Detected	-1.166	0.006	GT	Sens	contig212	contig212	E3 ubiqui	ATGGGG	contig212	Solyc03g	Solyc03g	E3 ubiqui	GO:0005	GO:0005	contig212	[illegible]	[illegible]
GT	Sense	Sense	0.341	Detected	-0.341	Detected	0.000	-2.629	Detected	-1.559	Detected	-2.094	0.081	GT	Sens	contig212	contig212	Ring fing	CGGATG	contig212	Solyc01g	Solyc01g	Ring fing	GO:0008	GO:0008	contig212	[illegible]	[illegible]
GT	Sense	Sense	0.284	Detected	-0.284	Detected	0.000	-3.488	Detected	-2.703	Detected	-3.095	0.024	GT	Sens	contig212	contig212	Dehydrat	CAGAAG	contig212	Solyc08g	Solyc08g	Dehydration-responsive family			contig212	[illegible]	[illegible]
GT	Sense	Sense	0.334	Detected	-0.334	Detected	0.000	-2.164	Detected	-1.183	Detected	-1.673	0.106	GT	Sens	contig212	contig212	Os07g01	TTGGTT	contig212	Solyc12g	Solyc12g	Os07g0175100 protein (Fragm			contig212	[illegible]	[illegible]
GT	Sense	Sense	0.027	Detected	-0.027	Detected	0.000	-3.093	Detected	-2.778	Detected	-2.936	0.003	GT	Sens	contig212	contig212	U-box do	AACTCA	contig212	Solyc05g	Solyc05g	U-box do	GO:0005	GO:0005	contig212	[illegible]	[illegible]
GT	Sense	Sense	0.224	Detected	-0.224	Detected	0.000	-1.702	Detected	-1.333	Detected	-1.518	0.035	GT	Sens	contig212	contig212	FAD-bind	GGTATA	contig212	Solyc02g	Solyc02g	FAD-bind	GO:0009	GO:0009	contig212	[illegible]	[illegible]
GT	Sense	Sense	-0.115	Detected	0.115	Detected	0.000	-1.793	Detected	-1.062	Detected	-1.427	0.065	GT	Sens	contig212	contig212	Unknown	TGTAGG	contig212	Solyc02g	Solyc02g	Unknown Protein (AHRD V1)			contig212	[illegible]	[illegible]
GT	Sense	Sense	0.374	Detected	-0.374	Detected	0.000	-1.415	Detected	-1.330	Detected	-1.372	0.068	GT	Sens	contig213	contig213	Leaf sene	TGGCCC	contig213	Solyc02g	Solyc02g	Leaf senescence protein-like (			contig213	[illegible]	[illegible]
GT	Sense	Sense	0.032	Detected	-0.032	Detected	0.000	-2.776	Detected	-2.599	Detected	-2.687	0.001	GT	Sens	contig213	contig213	Dynamin-	TCTGAT	contig213	Solyc06g	Solyc06g	Dynamin-related protein 1E (A			contig213	[illegible]	[illegible]
GT	Sense	Sense	-0.032	Detected	0.032	Detected	0.000	-2.639	Detected	-2.417	Detected	-2.528	0.002	GT	Sens	contig213	contig213	Serine/thr	TTTGGG	contig213	Solyc01g	Solyc01g	Serine/thr	GO:0005	GO:0005	contig213	[illegible]	[illegible]
GT	Sense	Sense	0.070	Detected	-0.070	Detected	0.000	-2.708	Comprom	-2.820	Comprom	-2.764	0.001	GT	Sens	contig213	contig213	Harpin-in	AATTTT	contig213	Solyc05g	Solyc05g	Harpin-induced protein-relatec			contig213	[illegible]	[illegible]
GT	Sense	Sense	0.164	Detected	-0.164	Detected	0.000	-1.129	Detected	-1.210	Detected	-1.170	0.020	GT	Sens	contig213	contig213	Potassiu	CTGAAC	contig213	Solyc04g	Solyc04g	Potassiu	GO:0009	GO:0009	contig213	[illegible]	[illegible]
[illegible]																												

GT	Sense	Sense	-0.080	Detected	0.080	Detected	0.000	-1.447	Detected	-1.149	Detected	-1.298	0.017	GT	Sens	contig254	contig254	Glycosyl	AAGGAT	contig254	Solyc02g
GT	Sense	Sense	0.061	Detected	-0.061	Detected	0.000	-1.423	Detected	-1.112	Detected	-1.268	0.017	GT	Sens	contig255	contig255	Os03g01	CAAGAA	contig255	Solyc12g
GT	Sense	Sense	0.062	Detected	-0.062	Detected	0.000	-1.481	Detected	-1.466	Detected	-1.474	0.002	GT	Sens	contig255	contig255	Aquapori	GGGCCT	contig255	Solyc05g
GT	Sense	Sense	0.072	Detected	-0.072	Detected	0.000	-3.886	Comprom	-3.159	Detected	-3.523	0.011	GT	Sens	contig255	contig255	Transcrip	AGAGCA	contig255	Solyc04g
GT	Sense	Sense	0.118	Detected	-0.118	Detected	0.000	-1.352	Detected	-1.480	Detected	-1.416	0.009	GT	Sens	contig255	contig255	Uncharac	GTACCA	contig255	Solyc05g
GT	Sense	Sense	-0.078	Detected	0.078	Detected	0.000	-2.460	Detected	-1.678	Detected	-2.069	0.035	GT	Sens	contig255	contig255	Late emb	ATTGAT	contig255	Solyc01g
GT	Sense	Sense	-0.055	Detected	0.055	Detected	0.000	-1.327	Detected	-1.068	Detected	-1.198	0.014	GT	Sens	contig255	contig255	Xylanase	ATGTGT	contig255	Solyc01g
GT	Sense	Sense	0.240	Detected	-0.240	Detected	0.000	-1.547	Detected	-1.651	Detected	-1.599	0.023	GT	Sens	contig256	contig256	NAC dom	GGCTTT	contig256	Solyc02g
GT	Sense	Sense	0.083	Detected	-0.083	Detected	0.000	-1.298	Detected	-0.859	Detected	-1.079	0.044	GT	Sens	contig256	contig256	Phospho	CCCACA	contig256	Solyc06g
GT	Sense	Sense	-0.045	Detected	0.045	Detected	0.000	-1.408	Detected	-1.024	Detected	-1.216	0.025	GT	Sens	contig256	contig256	Glutathio	ACAAAA	contig256	Solyc07g
GT	Sense	Sense	0.082	Detected	-0.082	Detected	0.000	-1.668	Detected	-1.649	Detected	-1.658	0.002	GT	Sens	contig256	contig256	SRC2 hor	ATATCC	contig256	Solyc01g
GT	Sense	Sense	0.142	Detected	-0.142	Detected	0.000	-3.267	Comprom	-2.903	Comprom	-3.085	0.006	GT	Sens	contig256	contig256	Serine/th	GCCTAG	contig256	Solyc07g
GT	Sense	Sense	0.205	Detected	-0.205	Detected	0.000	-0.922	Detected	-1.671	Detected	-1.296	0.094	GT	Sens	contig256	contig256	AP2-like	AACAAT	contig256	Solyc05g
GT	Sense	Sense	0.214	Detected	-0.214	Detected	0.000	-1.657	Detected	-1.129	Detected	-1.393	0.055	GT	Sens	contig256	contig256	Mitotic ch	GCATCC	contig256	Solyc05g
GT	Sense	Sense	0.074	Detected	-0.074	Detected	0.000	-1.348	Detected	-1.491	Detected	-1.419	0.005	GT	Sens	contig256	contig256	UDP-gluc	ATCCTT	contig256	Solyc12g
GT	Sense	Sense	0.199	Detected	-0.199	Detected	0.000	-2.539	Detected	-1.852	Detected	-2.196	0.031	GT	Sens	contig257	contig257	ABC tran	CCGCGA	contig257	Solyc07g
GT	Sense	Sense	0.072	Detected	-0.072	Detected	0.000	-2.269	Detected	-1.896	Detected	-2.082	0.009	GT	Sens	contig257	contig257	Cellulose	CAGGAC	contig257	Solyc03g
GT	Sense	Sense	0.077	Detected	-0.077	Detected	0.000	-2.726	Detected	-2.043	Detected	-2.385	0.021	GT	Sens	contig257	contig257	Receptor	ACACTT	contig257	Solyc12g
GT	Sense	Sense	-0.195	Detected	0.195	Detected	0.000	-1.105	Detected	-1.129	Detected	-1.117	0.029	GT	Sens	contig257	contig257	Ring H2 fi	CAATAT	contig257	Solyc01g
GT	Sense	Sense	-0.001	Detected	0.001	Detected	0.000	-1.809	Detected	-3.572	Comprom	-2.690	0.093	GT	Sens	contig258	contig258	GRAS fan	CGGATG	contig258	Solyc02g
GT	Sense	Sense	-0.001	Detected	0.001	Detected	0.000	-1.951	Detected	-1.600	Detected	-1.775	0.010	GT	Sens	contig258	contig258	LRR rece	CAGTTG	contig258	Solyc02g
GT	Sense	Sense	0.101	Detected	-0.101	Detected	0.000	-1.283	Detected	-1.019	Detected	-1.151	0.020	GT	Sens	contig258	contig258	GDSL est	AACGGA	contig258	Solyc02g
GT	Sense	Sense	0.009	Detected	-0.009	Detected	0.000	-1.183	Detected	-1.168	Detected	-1.176	0.000	GT	Sens	contig259	contig259	Unknown	ATAAAA	contig259	Solyc10g
GT	Sense	Sense	0.336	Detected	-0.336	Detected	0.000	-2.572	Detected	-2.597	Detected	-2.585	0.016	GT	Sens	contig259	contig259	Omega-3	CAGGGG	contig259	Solyc06g
GT	Sense	Sense	0.032	Detected	-0.032	Detected	0.000	-1.336	Detected	-1.553	Detected	-1.444	0.006	GT	Sens	contig259	contig259	Kinesin h	CTTGTT	contig259	Solyc04g
GT	Sense	Sense	0.075	Detected	-0.075	Detected	0.000	-1.656	Detected	-1.573	Detected	-1.614	0.003	GT	Sens	contig259	contig259	MtN21 no	AATAACT	contig259	Solyc01g
GT	Sense	Sense	0.338	Detected	-0.338	Detected	0.000	-2.758	Detected	-3.703	Comprom	-3.230	0.031	GT	Sens	contig259	contig259	Dof zinc f	GAGGAG	contig259	Solyc04g
GT	Sense	Sense	0.059	Detected	-0.059	Detected	0.000	-2.287	Comprom	-1.954	Comprom	-2.121	0.007	GT	Sens	contig259	contig259	ATP-bind	GAACTA	contig259	Solyc06g
GT	Sense	Sense	0.306	Detected	-0.306	Detected	0.000	-1.315	Detected	-1.627	Detected	-1.471	0.050	GT	Sens	contig260	contig260	F-box fan	TGAGAG	contig260	Solyc08g
GT	Sense	Sense	0.578	Detected	-0.578	Detected	0.000	-1.344	Detected	-2.157	Comprom	-1.750	0.132	GT	Sens	contig260	contig260	Calmodul	AGACCT	contig260	Solyc10g
GT	Sense	Sense	0.118	Detected	-0.118	Detected	0.000	-0.942	Detected	-1.248	Detected	-1.095	0.030	GT	Sens	contig260	contig260	Glycerol-	AAGGGG	contig260	Solyc12g
GT	Sense	Sense	0.353	Detected	-0.353	Detected	0.000	-3.159	Detected	-3.204	Detected	-3.181	0.012	GT	Sens	contig260	contig260	Calmodul	TCGGAT	contig260	Solyc06g
GT	Sense	Sense	-0.152	Detected	0.152	Detected	0.000	-0.910	Detected	-1.109	Detected	-1.009	0.031	GT	Sens	contig260	contig260	Atcamba	TGTTTT	contig260	Solyc10g
GT	Sense	Sense	-0.377	Detected	0.377	Detected	0.000	-1.446	Detected	-1.199	Detected	-1.322	0.079	GT	Sens	contig260	contig260	UDP-gluc	TTTGCA	contig260	Solyc10g
GT	Sense	Sense	0.261	Detected	-0.261	Detected	0.000	-0.973	Detected	-1.118	Detected	-1.045	0.061	GT	Sens	contig260	contig260	F-box pro	AACAGT	contig260	Solyc01g
GT	Sense	Sense	0.042	Detected	-0.042	Detected	0.000	-1.203	Detected	-1.297	Detected	-1.250	0.003	GT	Sens	contig260	contig260	Integrin-li	TTATCT	contig260	Solyc01g
GT	Sense	Sense	0.131	Detected	-0.131	Detected	0.000	-2.257	Detected	-1.880	Detected	-2.068	0.012	GT	Sens	contig260	contig260	Blue cop	TGTTAT	contig260	Solyc02g
GT	Sense	Sense	-0.072	Detected	0.072	Detected	0.000	-1.396	Detected	-1.079	Detected	-1.238	0.019	GT	Sens	contig260	contig260	cDNA clo	GTTGAT	contig260	Solyc02g
GT	Sense	Sense	0.173	Detected	-0.173	Detected	0.000	-1.656	Detected	-0.911	Detected	-1.284	0.089	GT	Sens	contig261	contig261	Patatin-lik	GGCAAA	contig261	Solyc09g
GT	Sense	Sense	0.194	Detected	-0.194	Detected	0.000	-1.287	Detected	-1.358	Detected	-1.322	0.022	GT	Sens	contig261	contig261	Membran	TGTGTG	contig261	Solyc06g
GT	Sense	Sense	0.033	Detected	-0.033	Detected	0.000	-2.438	Comprom	-1.098	Comprom	-1.768	0.119	GT	Sens	contig261	contig261	Unknown	TCGTAG	contig261	Solyc09g
GT	Sense	Sense	0.566	Detected	-0.566	Detected	0.000	-3.810	Detected	-3.212	Detected	-3.511	0.032	GT	Sens	contig261	contig261	cDNA clo	CAGTTC	contig261	Solyc02g
GT	Sense	Sense	0.033	Detected	-0.033	Detected	0.000	-2.289	Detected	-2.470	Detected	-2.380	0.002	GT	Sens	contig261	contig261	Acyl-CoA	CCTCAG	contig261	Solyc12g
GT	Sense	Sense	-0.404	Detected	0.404	Detected	0.000	-1.886	Detected	-0.958	Detected	-1.422	0.147	GT	Sens	contig262	contig262	Endogluc	GAGGTC	contig262	Solyc11g
GT	Sense	Sense	0.000	Detected	0.000	Detected	0.000	-2.328	Detected	-1.463	Detected	-1.895	0.048	GT	Sens	contig262	contig262	Expansin	AATTGG	contig262	Solyc02g
GT	Sense	Sense	0.243	Detected	-0.243	Detected	0.000	-1.061	Detected	-1.093	Detected	-1.077	0.048	GT	Sens	contig262	contig262	Ribosom	TACAGA	contig262	Solyc01g
GT	Sense	Sense	-0.128	Detected	0.128	Detected	0.000	-1.040	Detected	-0.969	Detected	-1.004	0.017	GT	Sens	contig262	contig262	TPR dom	AAGCTG	contig262	Solyc08g
GT	Sense	Sense	0.150	Detected	-0.150	Detected	0.000	-1.478	Detected	-2.049	Detected	-1.764	0.032	GT	Sens	contig262	contig262	Unknown	TGCACA	contig262	Solyc03g
GT	Sense	Sense	-0.022	Detected	0.022	Detected	0.000	-3.876	Detected	-2.454	Detected	-3.165	0.047	GT	Sens	contig263	contig263	Pectate ly	AATTGC	contig263	Solyc09g
GT	Sense	Sense	0.101	Detected	-0.101	Detected	0.000	-1.324	Detected	-0.895	Detected	-1.109	0.043	GT	Sens	contig263	contig263	2-phosph	CGCTTC	contig263	Solyc11g
GT	Sense	Sense	-0.013	Detected	0.013	Detected	0.000	-1.297	Detected	-1.004	Detected	-1.150	0.016	GT	Sens	contig263	contig263	Low affin	GTGGTT	contig263	Solyc07g
GT	Sense	Sense	0.240	Detected	-0.240	Detected	0.000	-2.958	Detected	-1.863	Detected	-2.410	0.056	GT	Sens	contig263	contig263	Non-spec	CTCAAC	contig263	Solyc09g
GT	Sense	Sense	0.212	Detected	-0.212	Detected	0.000	-1.363	Detected	-1.178	Detected	-1.270	0.032	GT	Sens	contig263	contig263	Diacylgly	CATGCT	contig263	Solyc12g
GT	Sense	Sense	0.267	Detected	-0.267	Detected	0.000	-4.258	Detected	-3.102	Detected	-3.680	0.029	GT	Sens	contig263	contig263	Unknown	GCTACA	contig263	Solyc11g
GT	Sense	Sense	0.077	Detected	-0.077	Detected	0.000	-1.485	Detected	-1.317	Detected	-1.401	0.007	GT	Sens	contig263	contig263	Transcrip	AGTTCC	contig263	Solyc05g
GT	Sense	Sense	-0.103	Detected	0.103	Detected	0.000	-1.919	Detected	-1.667	Detected	-1.793	0.008	GT	Sens	contig263	contig263	Purple ac	ATAGAA	contig263	Solyc01g
GT	Sense	Sense	-0.492	Detected	0.492	Detected	0.000	-2.858	Detected	-2.884	Detected	-2.871	0.028	GT	Sens	contig264	contig264	Genomic	TCTACT	contig264	Solyc05g
GT	Sense	Sense	-0.073	Detected	0.073	Detected	0.000	-1.354	Detected	-1.618	Detected	-1.486	0.010	GT	Sens	contig264	contig264	Acid phos	CATTTT	contig264	Solyc03g
GT	Sense	Sense	0.292	Detected	-0.292	Detected	0.000	-3.304	Detected	-1.874	Detected	-2.589	0.079	GT	Sens	contig264	contig264	UDP-D-gl	ATGAGC	contig264	Solyc06g
GT	Sense	Sense	0.143	Detected	-0.143	Detected	0.000	-1.324	Detected	-1.074	Detected	-1.199	0.024	GT	Sens	contig264	contig264	Catalase	AACATT	contig264	Solyc04g
GT	Sense	Sense	0.010	Detected	-0.010	Detected	0.000	-2.838	Detected	-1.784	Detected	-2.311	0.048	GT	Sens	contig264	contig264	Genomic	TTATTG	contig264	Solyc03g
GT	Sense	Sense	0.395	Detected	-0.395	Detected	0.000	-1.352	Detected	-1.055	Detected	-1.203	0.104	GT	Sens	contig265	contig265	LRR rece	CTTGAG	contig265	Solyc07g
GT	Sense	Sense	-0.084	Detected	0.084	Detected	0.000	-0.991	Detected	-1.188	Detected	-1.089	0.014	GT	Sens	contig265	contig265	Verticilliu	CAGCAG	contig265	Solyc09g
GT	Sense	Sense	0.197	Detected	-0.197	Detected	0.000	-2.914	Detected	-1.647	Detected	-2.281	0.075	GT	Sens	contig265	contig265	Chloropla	TTGACG	contig265	Solyc05g
GT	Sense	Sense	0.072	Detected	-0.072	Detected	0.000	-1.293	Detected	-1.052	Detected	-1.173	0.014	GT	Sens	contig265	contig265	Expresse	AATTTG	contig265	Solyc01g
GT	Sense	Sense	0.170	Detected	-0.170	Detected	0.000	-4.958	Comprom	-2.905	Detected	-3.931	0.063	GT	Sens	contig265	contig265	Agenet d	GAATGG	contig265	Solyc06g
GT	Sense	Sense	0.036	Detected	-0.036	Detected	0.000	-1.166	Detected	-0.938	Detected	-1.052	0.013	GT	Sens	contig265	contig265	Os11g01	AGAAAA	contig265	Solyc04g
GT	Sense	Sense	0.338	Detected	-0.338	Detected	0.000	-2.771	Detected	-2.322	Detected	-2.547	0.024	GT	Sens	contig265	contig265	Pollen-sp	AGGAAG	contig265	Solyc03g
GT	Sense	Sense	0.290	Detected	-0.290	Detected	0.000	-1.682	Detected	-1.461	Detected	-1.572	0.037	GT	Sens	contig266	contig266	Arf-GAP	TATTGC	contig266	Solyc03g
GT	Sense	Sense	0.205	Detected	-0.205	Detected	0.000	-1.667	Detected	-1.293	Detected	-1.480	0.033	GT	Sens	contig266	contig266	Mannan e	AATTGT	contig266	Solyc10g
GT	Sense	Sense	0.015	Detected	-0.015	Detected	0.000	-1.629	Detected	-1.456	Detected	-1.543	0.003	GT	Sens	contig267	contig267	Receptor	TGTGTT	contig267	Solyc03g
GT	Sense	Sense	0.092	Detected	-0.092	Detected	0.000	-3.122	Detected	-1.504	Detected	-2.313	0.105	GT	Sens	contig267	contig267	CHP-rich	GTCCAA	contig267	Solyc02g
GT	Sense	Sense	0.167	Detected	-0.167	Detected	0.000	-3.138	Comprom	-2.964	Comprom	-3.051	0.004	GT	Sens	contig267	contig267	Xylanase	AATGGA	contig267	Solyc01g
GT	Sense	Sense	0.351	Detected	-0.351	Detected	0.000	-1.546	Detected	-1.551	Detected	-1.549	0.048	GT	Sens	contig268	contig268	Ethylene-	GAGTTA	contig268	Solyc12g
GT	Sense	Sense	-0.043	Detected	0.043	Detected	0.000	-2.103	Comprom	-1.658	Comprom	-1.880	0.014	GT	Sens	contig268	contig268	Os11g04	TAAGTT	contig268	Solyc03g
GT	Sense	Sense	0.066	Detected	-0.066	Detected	0.000	-1.391	Detected	-1.090	Detected	-1.241	0.017	GT	Sens	contig268	contig268	Ectonucl	TTTCAT	contig268	Solyc05g
GT	Sense	Sense	0.083	Detected	-0.083	Detected	0.000	-1.291	Detected	-1.267	Detected	-1.279	0.004	GT	Sens	contig268	contig268	Serine/th	TAAGGT	contig268	Solyc05g
GT	Sense	Sense	-0.023	Detected	0.023	Detected	0.000	-1.848	Detected	-2.324	Detected	-2.086	0.013	GT	Sens	contig268	contig268	Expansin	AAATCA	contig268	Solyc07g
GT	Sense	Sense	0.096	Detected	-0.096	Detected	0.000	-1.594	Detected	-0.815	Detected	-1.205	0.095	GT	Sens	contig268	contig268	Receptor	ATGTCT	contig268	Solyc02g
GT	Sense	Sense	-0.057	Detected	0.057	Detected	0.000	-3.047	Detected	-2.805	Detected	-2.926	0.002	GT	Sens	contig268	contig268	Unknown	TGTTGT	contig268	Solyc08g
GT	Sense	Sense	-0.001	Detected	0.001	Detected	0.000	-1.211	Detected	-0.818	Detected	-1.014	0.036	GT	Sens	contig268	contig268	Fructose-	ACCTTA	contig268	Solyc10g
GT	Sense	Sense	0.089	Detected	-0.089	Detected	0.000	-1.483	Detected	-1.282	Detected	-1.383	0.009	GT	Sens	contig268	contig268	MIP02663	CTCATT	contig268	Solyc08g
GT	Sense	Sense	0.148	Detected	-0.148	Detected	0.000	-1.649	Detected	-2.182	Detected	-1.915	0.024	GT	Sens	contig269	contig269	Stachyos	GCATAC	contig269	Solyc01g
GT	Sense	Sense	-0.319	Detected	0.319	Detected	0.000	-2.578	Comprom	-3.154	Comprom	-2.866	0.022	GT	Sens	contig269	contig269	2-oxoglut	CGGAGC	contig269	Solyc11g
GT	Sense	Sense	0.274	Detected	-0.274	Detected	0.000	-1.848	Detected	-1.542	Detected	-1.695	0.033	GT	Sens	contig269	contig269	WUSCHEL	TCCATA	contig269	Solyc04g
GT	Sense	Sense	-0.115	Detected	0.115	Detected	0.000	-1.596	Detected	-1.560	Detected	-1.578	0.005	GT	Sens	contig269	contig269	Cell divis	TCTCAG	contig269	Solyc03g
GT	Sense	Sense	0.176	Detected	-0.176	Detected	0.000	-2.027	Detected	-2.064	Detected	-2.045	0.007	GT	Sens	contig269	contig269	Ring H2 fi	GACACA	contig269	Solyc07g
GT	Sense	Sense	0.015	Detected	-0.015	Detected	0.000	-1.206	Detected	-0.886	Detected	-1.046	0.023	GT	Sens	contig269	contig269	Integral m	TTTAAG	contig269	Solyc12g
GT	Sense	Sense	0.221	Detected	-0.221	Detected	0.000	-1.103	Detected	-1.087	Detected	-1.095	0.038	GT	Sens	contig269	contig269	AP-2 com	CAATTC	contig269	Solyc06g
GT	Sense	Sense	0.214	Detected	-0.214	Detected	0.000	-1.722	Detected	-1.169	Detected	-1.445	0.054	GT	Sens	contig269	contig269	Glutamat	TTTTAA	contig269	Solyc10g
GT	Sense	Sense	-0.145	Detected	0.145	Detected	0.000	-1.012	Detected	-1.234	Detected	-1.123	0.025	GT	Sens	contig270	contig270	Unknown	ATGCTT	contig270	Solyc02g
GT	Sense	Sense	0.012	Detected	-0.012	Detected	0.000	-1.424	Detected	-0.986	Detected	-1.205	0.032	GT	Sens	contig270	contig270	Pectinest	TTAATA	contig270	Solyc06g
GT	Sense	Sense	0.388	Detected	-0.388	Detected	0.000	-2.179	Detected	-1.776	Detected	-1.978	0.046	GT	Sens	contig270	contig270	Short inte	GAGGAG	contig270	Solyc00g
GT	Sense	Sense	-0.231	Detected	0.231	Detected	0.000	-0.912	Detected	-2.362	Detected	-1.637	0.164	GT	Sens	contig271	contig271	Pre-mRN	GGGAAA	contig271	Solyc10g
GT	Sense	Sense	-0.125	Detected	0.125	Detected	0.000	-1.843	Comprom	-1.237	Comprom	-1.540	0.042	GT	Sens	contig271	contig271	Unknown	ATTAAT	contig271	Solyc09g
GT	Sense	Sense	0.102	Detected	-0.102	Detected	0.000	-1.629	Detected	-1.025	Detected	-1.327	0.053	GT	Sens	contig271	contig271	Hydroxyc	AATGCA	contig271	Solyc01g
GT	Sense	Sense	-0.110	Detected	0.110	Detected	0.000	-1.205	Detected	-1.161	Detected	-1.183	0.009	GT	Sens	contig271	contig271	Subtilisin	CACATT	contig271	Solyc12g
GT	Sense	Sense	0.109	Detected	-0.109	Detected	0.000	-1.091	Detected	-0.928	Detected	-1.010	0.018	GT	Sens	contig272	contig272	Kinesin li	AAAGTG	contig272	Solyc12g
GT	Sense	Sense	-0.119	Detected	0.119	Detected	0.000	-1.356	Detected	-1.654	Detected	-1.505	0.016	GT	Sens	contig272	contig272	HAD-sup	TTGAGG	contig272	Solyc07g
GT	Sense	Sense	0.140	Detected	-0.140	Detected	0.000	-1.618	Detected	-1.774	Detected	-1.696	0.009	GT	Sens	contig272	contig272	Zinc finge	CAACAA	contig272	Solyc05g
GT	Sense	Sense	0.150	Detected	-0.150	Detected	0.000	-1.595	Detected	-1.061	Detected	-1.328	0.049	GT	Sens	contig272	contig272	Mannan e	TGTAAT	contig272	Solyc04g
GT	Sense	Sense	0.204	Detected	-0.204	Detected	0.000	-2.664	Detected	-2.246	Detected	-2.455	0.014	GT	Sens	contig272	contig272	Lipoxyge	CACGAC	contig272	Solyc09g
GT	Sense	Sense	0.007	Detected	-0.007	Detected	0.000	-1.958	Detected	-1.770	Detected	-1.864	0.003	GT	Sens	contig272	contig272	Unknown	GTGCTA	contig272	Solyc12g
GT	Sense	Sense	0.202	Detected	-0.202	Detected	0.000	-1.123	Detected	-1.448	Comprom	-1.285	0.038	GT	Sens	contig272	contig272	Glycogen	GATGAA	contig272	Solyc02g
GT	Sense	Sense	0.175	Detected	-0.175	Detected	0.000	-2.774	Detected	-1.592	Detected	-2.183	0.071	GT	Sens	contig273	contig273	Unknown	CACTCG	contig273	Solyc01g
GT	Sense	Sense	0.354	Detected	-0.354	Detected	0.000	-3.365	Detected	-1.114	Detected	-2.240	0.198	GT	Sens	contig273	contig273	Predicted	GTCAAG	contig273	Solyc05g
GT	Sense	Sense	-0.060	Detected	0.060	Detected	0.000	-1.349	Detected	-1.083	Detected	-1.216	0.014	GT	Sens	contig273	contig273	Serine/th	TGTGTA	contig273	Solyc03g
GT	Sense	Sense	0.014	Detected	-0.014	Detected	0.000	-1.405	Detected	-0.985	Detected	-1.195	0.030	GT	Sens	contig273	contig273	Unknown	AGGCTT	contig273	Solyc11g
GT	Sense	Sense	0.040	Detected	-0.040	Detected	0.000	-1.241	Detected	-0.990	Detected	-1.115	0.014	GT	Sens	contig273	contig273	Calmodul	ATTGAA	contig273	Solyc06g
GT	Sense	Sense	0.057	Detected	-0.057	Detected	0.000	-1.343	Detected	-1.481	Detected	-1.412	0.004	GT	Sens	contig274	contig274	Acyltrans	TTGATG	contig274	Solyc05g
GT	Sense	Sense	0.165	Detected	-0.165	Detected	0.000	-1.748	Detected	-1.524	Detected	-1.636	0.014	GT	Sens	contig274	contig274	Calmodul	CAAAAA	contig274	Solyc02g
GT	Sense	Sense	-0.071	Detected	0.071	Detected	0.000	-0.812	Comprom	-1.431	Comprom	-1.121	0.072	GT	Sens	contig274	contig274	Kinase in	GCAGAT	contig274	Solyc10g
GT	Sense	Sense	0.208	Detected	-0.208	Detected	0.000	-1.451	Detected	-1.271	Detected	-1.361	0.027	GT	Sens	contig274	contig274	Plant syn	GGCTAT	contig274	Solyc09g
GT	Sense	Sense	0.141	Detected	-0.141	Detected	0.000	-1.761	Detected	-1.559	Detected	-1.660	0.011	GT	Sens	contig274	contig274	Myb-like	CTTCAC	contig274	Solyc12g
GT	Sense	Sense	0.016	Detected	-0.016	Detected	0.000	-2.305	Detected	-3.378	Detected	-2.842	0.034	GT	Sens	contig274	contig274	FAD-bind	ATTCTT	contig274	Solyc06g
GT	Sense	Sense	0.131	Detected	-0.131	Detected	0.000	-1.500	Detected	-1.304	Detected	-1.402	0.013	GT	Sens	contig275	contig275	Unknown	TCTGTG	contig275	Solyc02g
GT	Sense	Sense	0.111	Detected	-0.111	Detected	0.000	-3.256	Detected	-2.467	Detected	-2.862	0.020	GT	Sens	contig275	contig275	1-aminoc	AAGGAA	contig275	Solyc02g
GT	Sense	Sense	-0.151	Detected	0.151	Detected	0.000	-1.576	Detected	-1.434	Detected	-1.505	0.012	GT	Sens	contig275	contig275	Unknown	TTCAAG	contig275	Solyc06g
GT	Sense	Sense	0.069	Detected	-0.069	Detected	0.000	-1.309	Detected	-0.956	Detected	-1.133	0.027	GT	Sens	contig275	contig275	V-type pr	AGATTT	contig275	Solyc11g
GT	Sense	Sense	-0.056	Detected	0.056	Detected	0.000	-1.377	Comprom	-1.714	Comprom	-1.546	0.013	GT	Sens	contig275	contig275	Coiled-co	AGAATC	contig275	Solyc01g
GT	Sense	Sense	0.115	Detected	-0.115	Detected	0.000	-1.274	Detected	-0.866	Detected	-1.070	0.045	GT	Sens	contig276	contig276	CXE carb	AGCAGA	contig276	Solyc02g
GT	Sense	Sense	-0.301	Detected	0.301	Detected	0.000	-2.397	Detected	-1.490	Detected	-1.944	0.070	GT	Sens	contig276	contig276	Unknown	AGAATA	contig276	Solyc05g
GT	Sense	Sense	0.012	Detected	-0.012	Detected	0.000	-1.355	Detected	-1.290	Detected	-1.322	0.001	GT	Sens	contig276	contig276	Receptor	ATTGAA	contig276	Solyc03g
GT	Sense	Sense	0.121	Detected	-0.121	Detected	0.000	-2.736	Detected	-2.174	Detected	-2.455	0.015	GT	Sens	contig276	contig276	Aquapori	AAATAT	contig276	Solyc06g
GT	Sense	Sense	0.144	Detected	-0.144	Detected	0.000	-1.631	Detected	-1.364	Detected	-1.497	0.017	GT	Sens	contig276	contig276	Cytochro	TTTTGG	contig276	Solyc11g
GT	Sense	Sense	0.232	Detected	-0.232	Detected	0.000	-1.663	Detected	-0.907	Detected	-1.285	0.101	GT	Sens	contig276	contig276	Fatty acyl	GCATTC	contig276	Solyc11g
GT	Sense	Sense	-0.002	Detected	0.002	Detected	0.000	-1.287	Detected	-0.940	Detected	-1.114	0.023	GT	Sens	contig276	contig276	Patatin-lik	AAGAAG	contig276	Solyc02g
GT	Sense	Sense	0.171	Detected	-0.171	Detected	0.000	-1.411	Detected	-0.968	Detected	-1.190	0.051	GT	Sens	contig276	contig276	WRKY tra	CGAATT	contig276	Solyc01g
GT	Sense	Sense	0.009	Detected	-0.009	Detected	0.000	-1.471	Detected	-0.953	Detected	-1.212	0.043	GT	Sens	contig277	contig277	Glycosylt	CAAGGC	contig277	Solyc08g
GT	Sense	Sense	0.165	Detected	-0.165	Detected	0.000	-1.671	Detected	-1.462	Detected	-1.567	0.015	GT	Sens	contig277	contig277	Receptor	ACACCA	contig277	Solyc02g
GT	Sense	Sense	-0.127	Detected	0.127	Detected	0.000	-1.958	Detected	-2.611	Comprom	-2.285	0.023	GT	Sens	contig277	contig277	Unknown	GGGCAT	contig277	Solyc08g
GT	Sense	Sense	0.190	Detected	-0.190	Detected	0.000	-1.876	Detected	-1.675	Detected	-1.776	0.014	GT	Sens	contig277	contig277	ATP bind	AATACA	contig277	Solyc04g
GT	Sense	Sense	0.408	Detected	-0.408	Detected	0.000	-1.829	Detected	-1.262	Detected	-1.545	0.090	GT	Sens	contig278	contig278	Xylogluca	AATAAA	contig278	Solyc12g
GT	Sense	Sense	0.123	Detected	-0.123	Detected	0.000	-1.327	Detected	-1.176	Detected	-1.251	0.013	GT	Sens	contig278	contig278	Avr9/Cf-9	CGCGGA	contig278	Solyc08g
GT	Sense	Sense	0.092	Detected	-0.092	Detected	0.000	-0.973	Detected	-1.061	Detected	-1.017	0.010	GT	Sens	contig278	contig278	Cellulose	CTGACT	contig278	Solyc02g
GT	Sense	Sense	0.054	Detected	-0.054	Detected	0.000	-1.777	Detected	-1.163	Detected	-1.470	0.042	GT	Sens	contig278	contig278	Late emb	CCCTCT	contig278	Solyc01g
GT	Sense	Sense	0.166	Detected	-0.166	Detected	0.000	-2.603	Detected	-1.410	Detected	-2.007	0.083	GT	Sens	contig279	contig279	Unknown	GGAGGA	contig279	Solyc04g
GT	Sense	Sense	0.148	Detected	-0.148	Detected	0.000	-2.022	Detected	-1.970	Detected	-1.996	0.006	GT	Sens	contig279	contig279	Arabinog	CTGCAC	contig279	Solyc02g
GT	Sense	Sense	0.435	Detected	-0.435	Detected	0.000	-2.540	Detected	-1.514	Detected	-2.027	0.095	GT	Sens	contig279	contig279	BHLH tra	TTTCAG	contig279	Solyc08g
GT	Sense	Sense	0.065	Detected	-0.065	Detected	0.000	-1.235	Detected	-1.744	Detected	-1.490	0.030	GT	Sens	contig279	contig279	Hydrolase	AACAGA	contig279	Solyc03g
GT	Sense	Sense	0.353	Detected	-0.353	Detected	0.000	-3.972	Comprom	-1.462	Detected	-2.717	0.172	GT	Sens	contig280	contig280	Calmodul	CTTGTC	contig280	Solyc03g
GT	Sense	Sense	-0.013	Detected	0.013	Detected	0.000	-3.393	Detected	-1.377	Detected	-2.385	0.142	GT	Sens	contig280	contig280	Protein T	CTTCTA	contig280	Solyc08g
GT	Sense	Sense	0.085	Detected	-0.085	Detected	0.000	-1.628	Detected	-1.963	Detected	-1.796	0.011	GT	Sens	contig280	contig280	Unknown	TTGTTT	contig280	Solyc05g
GT	Sense	Sense	0.063	Detected	-0.063	Detected	0.000	-1.197	Detected	-0.903	Detected	-1.050	0.022	GT	Sens	contig280	contig280	Organic a	GTAGCT	contig280	Solyc05g
GT	Sense	Sense	0.307	Detected	-0.307	Detected	0.000	-1.598	Detected	-2.209	Detected	-1.903	0.048	GT	Sens	contig280	contig280	Unknown	TACGGC	contig280	Solyc08g
GT	Sense	Sense	0.104	Detected	-0.104	Detected	0.000	-1.435	Detected	-1.154	Detected	-1.295	0.018	GT	Sens	contig281	contig281	Universal	TGTATT	contig281	Solyc09g
GT	Sense	Sense	-0.033	Detected	0.033	Detected	0.000	-1.012	Detected	-0.994	Detected	-1.003	0.001	GT	Sens	contig281	contig281	Oxidored	ATCAAG	contig281	Solyc02g
GT	Sense	Sense	0.230	Detected	-0.230	Detected	0.000	-1.914	Detected	-1.339	Detected	-1.626	0.048	GT	Sens	contig282	contig282	Ribonucl	TGGGAA	contig282	Solyc05g
GT	Sense	Sense	0.603	Detected	-0.603	Detected	0.000	-3.187	Detected	-1.314	Detected	-2.250	0.181	GT	Sens	contig282	contig282	FAD-bind	TTCAGA	contig282	Solyc02g
GT	Sense	Sense	0.180	Detected	-0.180	Detected	0.000	-1.187	Detected	-0.988	Detected	-1.088	0.034	GT	Sens	contig282	contig282	Heat sho	CTATGA	contig282	Solyc01g
GT	Sense	Sense	0.097	Detected	-0.097	Detected	0.000	-2.689	Detected	-1.559	Detected	-2.124	0.066	GT	Sens	contig282	contig282	FIP1 (AHF	TTTTTG	contig282	Solyc11g
GT	Sense	Sense	0.008	Detected	-0.008	Detected	0.000	-1.093	Detected	-1.076	Detected	-1.085	0.000	GT	Sens	contig283	contig283	Glucosylt	CATAGG	contig283	Solyc01g
GT	Sense	Sense	-0.357	Detected	0.357	Detected	0.000	-1.695	Detected	-1.490	Detected	-1.593	0.050	GT	Sens	contig283	contig283	Cytochro	TTTCAC	contig283	Solyc01g
GT	Sense	Sense	0.279	Detected	-0.279	Detected	0.000	-1.577	Detected	-1.293	Detected	-1.435	0.044	GT	Sens	contig283	contig283	Myb fami	GTTCAG	contig283	Solyc02g
GT	Sense	Sense	0.275	Detected	-0.275	Detected	0.000	-1.292	Detected	-1.081	Detected	-1.186	0.057	GT	Sens	contig283	contig283	von Wille	ATTACA	contig283	Solyc02g
GT	Sense	Sense	0.001	Detected	-0.001	Detected	0.000	-1.290	Detected	-0.946	Detected	-1.118	0.023	GT	Sens	contig284	contig284	Os02g02	ATAACC	contig284	Solyc06g
GT	Sense	Sense	0.281	Detected	-0.281	Detected	0.000	-1.574	Detected	-1.902	Detected	-1.738	0.033	GT	Sens	contig284	contig284	Sulfate tr	ATAACA	contig284	Solyc12g
GT	Sense	Sense	0.348	Detected	-0.348	Detected	0.000	-1.063	Detected	-1.634	Detected	-1.348	0.096	GT	Sens	contig284	contig284	Auxin-ind	TAGTCC	contig284	Solyc03g
GT	Sense	Sense	0.228	Detected	-0.228	Detected	0.000	-2.596	Detected	-2.756	Detected	-2.676	0.008	GT	Sens	contig284	contig284	Pathogen	CATTAT	contig284	Solyc08g
GT	Sense	Sense	0.091	Detected	-0.091	Detected	0.000	-1.207	Detected	-1.364	Detected	-1.285	0.009	GT	Sens	contig284	contig284	TIR-NBS-	CAGATG	contig284	Solyc01g
GT	Sense	Sense	-0.099	Detected	0.099	Detected	0.000	-1.383	Detected	-2.104	Detected	-1.743	0.043	GT	Sens	contig284	contig284	Unknown	TTGCTT	contig284	Solyc07g
GT	Sense	Sense	0.306	Detected	-0.306	Detected	0.000	-1.837	Detected	-1.003	Detected	-1.420	0.111	GT	Sens	contig284	contig284	LRR rece	GTGTGA	contig284	Solyc08g
GT	Sense	Sense	0.183	Detected	-0.183	Detected	0.000	-2.741	Detected	-3.603	Detected	-3.172	0.021	GT	Sens	contig284	contig284	Germin-li	TTGAGC	contig284	Solyc10g
GT	Sense	Sense	0.084	Detected	-0.084	Detected	0.000	-2.146	Detected	-1.936	Detected	-2.041	0.004	GT	Sens	contig284	contig284	Cellulose	TGATTG	contig284	Solyc04g
GT	Sense	Sense	0.112	Detected	-0.112	Detected	0.000	-0.835	Detected	-1.214	Detected	-1.024	0.043	GT	Sens	contig284	contig284	Alpha-1 6	TGTCGA	contig284	Solyc07g
GT	Sense	Sense	0.019	Detected	-0.019	Detected	0.000	-1.755	Detected	-1.367	Detected	-1.561	0.015	GT	Sens	contig284	contig284	Beta-gala	GTCGAA	contig284	Solyc03g
GT	Sense	Sense	-0.056	Detected	0.056	Detected	0.000	-1.284	Detected	-1.211	Detected	-1.247	0.003	GT	Sens	contig284	contig284	E3 ubiqui	ATGCTA	contig284	Solyc06g
GT	Sense	Sense	-0.480	Detected	0.480	Detected	0.000	-0.833	Detected	-1.388	Detected	-1.110	0.183	GT	Sens	contig284	contig284	Beta-gala	TTCCAT	contig284	Solyc11g
GT	Sense	Sense	-0.020	Detected	0.020	Detected	0.000	-1.102	Detected	-1.052	Detected	-1.077	0.001	GT	Sens	contig284	contig284	Polyaden	GTAACT	contig284	Solyc12g
GT	Sense	Sense	0.139	Detected	-0.139	Detected	0.000	-1.267	Detected	-1.135	Detected	-1.201	0.016	GT	Sens	contig285	contig285	Myosin X	ATGTTA	contig285	Solyc09g
GT	Sense	Sense	-0.164	Detected	0.164	Detected	0.000	-1.986	Detected	-1.415	Detected	-1.701	0.035	GT	Sens	contig285	contig285	Gty37 pro	AATGGC	contig285	Solyc07g
GT	Sense	Sense	-0.079	Detected	0.079	Detected	0.000	-2.080	Detected	-1.628	Detected	-1.854	0.016	GT	Sens	contig285	contig285	Respirato	CATTTC	contig285	Solyc08g
GT	Sense	Sense	0.560	Detected	-0.560	Detected	0.000	-4.525	Detected	-3.986	Detected	-4.255	0.021	GT	Sens	contig286	contig286	Acetyl xyl	AGATGC	contig286	Solyc12g
GT	Sense	Sense	0.174	Detected	-0.174	Detected	0.000	-2.316	Detected	-1.385	Detected	-1.851	0.065	GT	Sens	contig286	contig286	Receptor	ATAGAT	contig286	Solyc07g
GT	Sense	Sense	0.186	Detected	-0.186	Detected	0.000	-1.078	Detected	-1.188	Detected	-1.133	0.028	GT	Sens	contig286	contig286	Aspartic	TTTCCA	contig286	Solyc07g
GT	Sense	Sense	0.148	Detected	-0.148	Detected	0.000	-3.053	Comprom	-1.058	Detected	-2.056	0.178	GT	Sens	contig286	contig286	CBS dom	AGTAGA	contig286	Solyc03g
GT	Sense	Sense	0.078	Detected	-0.078	Detected	0.000	-2.312	Comprom	-3.116	Comprom	-2.714	0.022	GT	Sens	contig286	contig286	Unknown	GCTTAT	contig286	Solyc06g
GT	Sense	Sense	0.141	Detected	-0.141	Detected	0.000	-1.539	Detected	-0.968	Detected	-1.254	0.059	GT	Sens	contig287	contig287	Cellulose	TTTTCT	contig287	Solyc11g
GT	Sense	Sense	-0.261	Detected	0.261	Detected	0.000	-0.981	Detected	-1.308	Detected	-1.145	0.065	GT	Sens	contig287	contig287	Mitogen-a	AGTATC	contig287	Solyc06g
GT	Sense	Sense	0.039	Detected	-0.039	Detected	0.000	-1.292	Detected	-1.118	Detected	-1.205	0.006	GT	Sens	contig288	contig288	CBS dom	ACGAAA	contig288	Solyc01g
GT	Sense	Sense	0.268	Detected	-0.268	Detected	0.000	-1.240	Detected	-1.050	Detected	-1.145	0.056	GT	Sens	contig288	contig288	Pyrophos	TTGATT	contig288	Solyc12g
GT	Sense	Sense	-0.182	Detected	0.182	Detected	0.000	-0.919	Detected	-1.533	Comprom	-1.226	0.075	GT	Sens	contig288	contig288	Transket	AAACGT	contig288	Solyc01g
GT	Sense	Sense	0.192	Detected	-0.192	Detected	0.000	-1.285	Detected	-1.212	Detected	-1.249	0.024	GT	Sens	contig288	contig288	Unknown	TCGATG	contig288	Solyc09g
GT	Sense	Sense	0.214	Detected	-0.214	Detected	0.000	-0.823	Detected	-1.726	Comprom	-1.275	0.125	GT	Sens	contig288	contig288	LRR rece	TTGTCT	contig288	Solyc02g
GT	Sense	Sense	-0.067	Detected	0.067	Detected	0.000	-1.645	Detected	-0.997	Detected	-1.321	0.057	GT	Sens	contig289	contig289	Phosphat	ATGGAA	contig289	Solyc10g
GT	Sense	Sense	-0.114	Detected	0.114	Detected	0.000	-1.122	Detected	-1.306	Detected	-1.214	0.014	GT	Sens	contig289	contig289	Endo-1 4-	GGTGAA	contig289	Solyc01g
GT	Sense	Sense	0.180	Detected	-0.180	Detected	0.000	-2.084	Detected	-1.522	Detected	-1.803	0.033	GT	Sens	contig289	contig289	Gibberell	AATGCT	contig289	Solyc01g
GT	Sense	Sense	-0.127	Detected	0.127	Detected	0.000	-1.145	Detected	-1.088	Detected	-1.117	0.013	GT	Sens	contig289	contig289	Legume l	GAAAGG	contig289	Solyc10g
GT	Sense	Sense	0.174	Detected	-0.174	Detected	0.000	-2.838	Detected	-3.360	Comprom	-3.099	0.010	GT	Sens	contig289	contig289	Disease r	TCACGA	contig289	Solyc10g
GT	Sense	Sense	0.209	Detected	-0.209	Detected	0.000	-1.738	Detected	-1.336	Detected	-1.537	0.034	GT	Sens	contig289	contig289	Leucine r	GGGTTT	contig289	Solyc03g
GT	Sense	Sense	0.084	Detected	-0.084	Detected	0.000	-2.369	Detected	-1.300	Detected	-1.835	0.077	GT	Sens	contig290	contig290	Polygalac	AGGTTG	contig290	Solyc03g
GT	Sense	Sense	-0.135	Detected	0.135	Detected	0.000	-1.554	Detected	-1.309	Detected	-1.432	0.016	GT	Sens	contig290	contig290	Unknown	TCTCTG	contig290	Solyc01g
GT	Sense	Sense	0.024	Detected	-0.024	Detected	0.000	-1.154	Detected	-1.474	Detected	-1.314	0.015	GT	Sens	contig290	contig290	Lipid pho	GCATGA	contig290	Solyc05g
GT	Sense	Sense	0.084	Detected	-0.084	Detected	0.000	-1.490	Detected	-1.061	Detected	-1.275	0.031	GT	Sens	contig290	contig290	Sodium/c	TCTTTG	contig290	Solyc01g
GT	Sense	Sense	0.039	Detected	-0.039	Detected	0.000	-2.628	Comprom	-0.904	Detected	-1.766	0.177	GT	Sens	contig290	contig290	N-acetyltr	GATTTA	contig290	Solyc01g
GT	Sense	Sense	0.405	Detected	-0.405	Detected	0.000	-3.290	Detected	-2.586	Detected	-2.938	0.032	GT	Sens	contig290	contig290	Unknown	GAGGTT	contig290	Solyc02g
GT	Sense	Sense	0.284	Detected	-0.284	Detected	0.000	-1.743	Detected	-2.305	Detected	-2.024	0.037	GT	Sens	contig291	contig291	Serine/th	CCAAGA	contig291	Solyc02g
GT	Sense	Sense	0.006	Detected	-0.006	Detected	0.000	-1.371	Detected	-1.200	Detected	-1.286	0.004	GT	Sens	contig291	contig291	Aldehyde	TGTCCA	contig291	Solyc12g
GT	Sense	Sense	0.347	Detected	-0.347	Detected	0.000	-3.824	Detected	-3.224	Detected	-3.524	0.017	GT	Sens	contig291	contig291	Trehalose	ATGGGA	contig291	Solyc06g
GT	Sense	Sense	-0.218	Detected	0.218	Detected	0.000	-1.908	Detected	-1.846	Detected	-1.877	0.014	GT	Sens	contig292	contig292	Calmodul	TCTACG	contig292	Solyc04g
GT	Sense	Sense	0.059	Detected	-0.059	Detected	0.000	-2.352	Detected	-1.935	Detected	-2.143	0.010	GT	Sens	contig292	contig292	Receptor	AAGCAA	contig292	Solyc09g
GT	Sense	Sense	0.266	Detected	-0.266	Detected	0.000	-1.401	Detected	-1.231	Detected	-1.316	0.042	GT	Sens	contig292	contig292	Alpha-1 6	AAGGGA	contig292	Solyc03g
GT	Sense	Sense	0.088	Detected	-0.088	Detected	0.000	-2.523	Detected	-1.826	Detected	-2.174	0.026	GT	Sens	contig292	contig292	Calmodul	AACAAG	contig292	Solyc12g
GT	Sense	Sense	-0.010	Detected	0.010	Detected	0.000	-1.124	Detected	-1.293	Detected	-1.209	0.005	GT	Sens	contig292	contig292	Protein L	AGGCAG	contig292	Solyc08g
GT	Sense	Sense	0.109	Detected	-0.109	Detected	0.000	-0.959	Detected	-1.079	Detected	-1.019	0.014	GT	Sens	contig293	contig293	Eukaryoti	TGGAGC	contig293	Solyc05g
GT	Sense	Sense	0.080	Detected	-0.080	Detected	0.000	-1.996	Detected	-1.475	Detected	-1.735	0.024	GT	Sens	contig293	contig293	Vesicular	TCTAAT	contig293	Solyc09g
GT	Sense	Sense	0.167	Detected	-0.167	Detected	0.000	-1.393	Detected	-0.807	Detected	-1.100	0.082	GT	Sens	contig293	contig293	Alpha-1 4	CAAAAC	contig293	Solyc03g
GT	Sense	Sense	0.187	Detected	-0.187	Detected	0.000	-0.813	Detected	-1.350	Detected	-1.082	0.081	GT	Sens	contig293	contig293	Unknown	TGATGC	contig293	Solyc12g
GT	Sense	Sense	0.145	Detected	-0.145	Detected	0.000	-1.560	Detected	-0.803	Detected	-1.181	0.100	GT	Sens	contig293	contig293	Dehydrat	CATACT	contig293	Solyc03g
GT	Sense	Sense	0.101	Detected	-0.101	Detected	0.000	-1.072	Detected	-1.145	Detected	-1.109	0.009	GT	Sens	contig293	contig293	Enoyl-Co	GAGCTG	contig293	Solyc06g
GT	Sense	Sense	0.280	Detected	-0.280	Detected	0.000	-2.891	Detected	-2.639	Detected	-2.765	0.012	GT	Sens	contig294	contig294	Fasciclin-	TAAAAC	contig294	Solyc07g
GT	Sense	Sense	0.025	Detected	-0.025	Detected	0.000	-1.126	Detected	-2.957	Detected	-2.042	0.156	GT	Sens	contig294	contig294	Cold sho	GGGCTA	contig294	Solyc01g
GT	Sense	Sense	0.055	Detected	-0.055	Detected	0.000	-1.081	Detected	-0.951	Detected	-1.016	0.007	GT	Sens	contig294	contig294	Unknown	GAATCG	contig294	Solyc10g
GT	Sense	Sense	0.428	Detected	-0.428	Detected	0.000	-2.467	Detected	-2.622	Comprom	-2.545	0.028	GT	Sens	contig294	contig294	Inositol o	ACAACA	contig294	Solyc11g
GT	Sense	Sense	0.441	Detected	-0.441	Detected	0.000	-3.813	Detected	-3.747	Detected	-3.780	0.013	GT	Sens	contig294	contig294	MYB tran	CTACGC	contig294	Solyc02g
GT	Sense	Sense	0.152	Detected	-0.152	Detected	0.000	-1.096	Detected	-0.961	Detected	-1.028	0.025	GT	Sens	contig295	contig295	Group II i	TAACAG	contig295	Solyc07g
GT	Sense	Sense	0.158	Detected	-0.158	Detected	0.000	-2.438	Detected	-1.875	Detected	-2.156	0.022	GT	Sens	contig295	contig295	Receptor	AACAAA	contig295	Solyc06g
GT	Sense	Sense	0.272	Detected	-0.272	Detected	0.000	-2.200	Detected	-2.027	Detected	-2.113	0.018	GT	Sens	contig295	contig295	Voltage-g	CATTTG	contig295	Solyc01g
GT	Sense	Sense	0.199	Detected	-0.199	Detected	0.000	-2.538	Detected	-1.459	Detected	-1.999	0.074	GT	Sens	contig296	contig296	UTP-gluc	TTTTGG	contig296	Solyc05g
GT	Sense	Sense	0.146	Detected	-0.146	Detected	0.000	-1.754	Detected	-1.527	Detected	-1.640	0.012	GT	Sens	contig296	contig296	Transcrip	TGTTCT	contig296	Solyc09g
GT	Sense	Sense	0.149	Detected	-0.149	Detected	0.000	-2.712	Detected	-2.089	Detected	-2.401	0.020	GT	Sens	contig296	contig296	Glutaredo	GAAAAT	contig296	Solyc10g
GT	Sense	Sense	0.094	Detected	-0.094	Detected	0.000	-1.647	Detected	-0.969	Detected	-1.308	0.065	GT	Sens	contig296	contig296	Ethylene-	TGATAT	contig296	Solyc12g
GT	Sense	Sense	0.077	Detected	-0.077	Detected	0.000	-1.782	Detected	-1.590	Detected	-1.686	0.005	GT	Sens	contig296	contig296	Serine/th	TTACAG	contig296	Solyc06g
GT	Sense	Sense	0.083	Detected	-0.083	Detected	0.000	-1.180	Detected	-0.829	Detected	-1.005	0.035	GT	Sens	contig296	contig296	DNAJ cha	TGAAGG	contig296	Solyc01g
GT	Sense	Sense	0.185	Detected	-0.185	Detected	0.000	-3.220	Detected	-1.946	Detected	-2.583	0.060	GT	Sens	contig296	contig296	Glutamat	CGGCTA	contig296	Solyc04g
GT	Sense	Sense	0.268	Detected	-0.268	Detected	0.000	-1.572	Comprom	-1.233	Comprom	-1.402	0.047	GT	Sens	contig296	contig296	ATP-depe	AATGCC	contig296	Solyc11g
GT	Sense	Sense	-0.244	Detected	0.244	Detected	0.000	-2.493	Detected	-0.896	Detected	-1.695	0.180	GT	Sens	contig296	contig296	LOB dom	GTGAAC	contig296	Solyc06g
GT	Sense	Sense	0.441	Detected	-0.441	Detected	0.000	-2.309	Detected	-1.668	Detected	-1.989	0.068	GT	Sens	contig297	contig297	Galactosy	AGAGAT	contig297	Solyc07g
GT	Sense	Sense	0.064	Detected	-0.064	Detected	0.000	-1.731	Detected	-1.664	Detected	-1.697	0.002	GT	Sens	contig297	contig297	Hydrolase	GTCGAG	contig297	Solyc06g
GT	Sense	Sense	-0.215	Detected	0.215	Detected	0.000	-1.234	Detected	-1.144	Detected	-1.189	0.033	GT	Sens	contig297	contig297	Ras-relat	AAATAC	contig297	Solyc06g
GT	Sense	Sense	0.031	Detected	-0.031	Detected	0.000	-1.229	Detected	-0.991	Detected	-1.110	0.012	GT	Sens	contig297	contig297	Calmodul	CTTTCT	contig297	Solyc02g
GT	Sense	Sense	0.157	Detected	-0.157	Detected	0.000	-1.698	Detected	-1.612	Detected	-1.655	0.010	GT	Sens	contig297	contig297	Serine/th	ATTGAG	contig297	Solyc05g
GT	Sense	Sense	0.055	Detected	-0.055	Detected	0.000	-1.568	Detected	-2.176	Comprom	-1.872	0.026	GT	Sens	contig297	contig297	Small nuc	CTAAAG	contig297	Solyc12g
GT	Sense	Sense	0.046	Detected	-0.046	Detected	0.000	-1.615	Detected	-1.368	Detected	-1.491	0.008	GT	Sens	contig297	contig297	AT-hook	AATTAG	contig297	Solyc04g
GT	Sense	Sense	-0.184	Detected	0.184	Detected	0.000	-1.963	Detected	-1.718	Detected	-1.841	0.014	GT	Sens	contig298	contig298	Expansin	TTTTGG	contig298	Solyc06g
GT	Sense	Sense	-0.132	Detected	0.132	Detected	0.000	-2.143	Detected	-1.081	Detected	-1.612	0.098	GT	Sens	contig299	contig299	Octicosa	GTTCAA	contig299	Solyc03g
GT	Sense	Sense	0.270	Detected	-0.270	Detected	0.000	-1.956	Detected	-1.784	Detected	-1.870	0.022	GT	Sens	contig299	contig299	MAP-like	TGTAAC	contig299	Solyc02g
GT	Sense	Sense	0.177	Detected	-0.177	Detected	0.000	-1.276	Detected	-1.103	Detected	-1.190	0.026	GT	Sens	contig299	contig299	RING-H2	GAAAAT	contig299	Solyc01g
GT	Sense	Sense	0.051	Detected	-0.051	Detected	0.000	-1.353	Detected	-1.003	Detected	-1.178	0.023	GT	Sens	contig300	contig300	Endoplas	AAGGAT	contig300	Solyc12g
GT	Sense	Sense	0.304	Detected	-0.304	Detected	0.000	-0.967	Detected	-2.454	Detected	-1.710	0.167	GT	Sens	contig300	contig300	Gibberell	TGTTTA	contig300	Solyc04g
GT	Sense	Sense	0.239	Detected	-0.239	Detected	0.000	-2.791	Detected	-1.388	Detected	-2.089	0.106	GT	Sens	contig300	contig300	Xylanase	TATCAA	contig300	Solyc03g
GT	Sense	Sense	0.031	Detected	-0.031	Detected	0.000	-1.863	Detected	-1.008	Detected	-1.436	0.079	GT	Sens	contig300	contig300	Unknown	CTCATG	contig300	Solyc10g
GT	Sense	Sense	0.065	Detected	-0.065	Detected	0.000	-1.284	Detected	-0.932	Detected	-1.108	0.028	GT	Sens	contig300	contig300	Os03g08	AGTATA	contig300	Solyc10g
GT	Sense	Sense	-0.175	Detected	0.175	Detected	0.000	-3.279	Detected	-3.047	Detected	-3.163	0.004	GT	Sens	contig300	contig300	ST63-2 (A	GTTTCT	contig300	Solyc11g

GT	Sense	Sense	0.572	Detected	-0.572	Detected	0.000	-2.136	Comprom	-1.882	Comprom	-2.009	0.076	GT	Sens	contig300	contig300	Retinol de	[illegible]
GT	Sense	Sense	-0.381	Detected	0.381	Detected	0.000	-2.095	Detected	-1.973	Detected	-2.034	0.034	GT	Sens	contig300	contig300	Formin 3	[illegible]
GT	Sense	Sense	-0.142	Detected	0.142	Detected	0.000	-1.014	Detected	-1.097	Detected	-1.055	0.019	GT	Sens	contig301	contig301	F-box fan	[illegible]
GT	Sense	Sense	0.497	Detected	-0.497	Detected	0.000	-2.042	Detected	-1.062	Detected	-1.552	0.156	GT	Sens	contig301	contig301	ABC tran	[illegible]
GT	Sense	Sense	0.946	Detected	-0.946	Detected	0.000	-4.307	Comprom	-3.261	Detected	-3.784	0.073	GT	Sens	contig301	contig301	Expansin	[illegible]
GT	Sense	Sense	-0.030	Detected	0.030	Detected	0.000	-1.742	Detected	-1.374	Detected	-1.558	0.014	GT	Sens	contig301	contig301	Isoamyl a	[illegible]
GT	Sense	Sense	0.037	Detected	-0.037	Detected	0.000	-1.749	Detected	-1.289	Detected	-1.519	0.023	GT	Sens	contig301	contig301	WD-repea	[illegible]
GT	Sense	Sense	-0.043	Detected	0.043	Detected	0.000	-1.243	Detected	-1.333	Detected	-1.288	0.002	GT	Sens	contig301	contig301	Armadillo	[illegible]
GT	Sense	Sense	0.137	Detected	-0.137	Detected	0.000	-2.760	Comprom	-1.588	Detected	-2.174	0.069	GT	Sens	contig301	contig301	Phospho	[illegible]
GT	Sense	Sense	0.005	Detected	-0.005	Detected	0.000	-1.207	Detected	-1.038	Detected	-1.123	0.006	GT	Sens	contig302	contig302	Transduc	[illegible]
GT	Sense	Sense	-0.035	Detected	0.035	Detected	0.000	-3.218	Comprom	-2.659	Comprom	-2.939	0.009	GT	Sens	contig302	contig302	F1L3.5 (A	[illegible]
GT	Sense	Sense	0.347	Detected	-0.347	Detected	0.000	-1.570	Detected	-1.440	Detected	-1.505	0.051	GT	Sens	contig302	contig302	Plant-spe	[illegible]
GT	Sense	Sense	0.065	Detected	-0.065	Detected	0.000	-1.332	Detected	-0.928	Detected	-1.130	0.034	GT	Sens	contig302	contig302	DsRNA-bi	[illegible]
GT	Sense	Sense	0.444	Detected	-0.444	Detected	0.000	-1.951	Detected	-1.106	Detected	-1.529	0.130	GT	Sens	contig302	contig302	Unknown	[illegible]
GT	Sense	Sense	2.264	Detected	-2.264	Detected	0.000	-3.019	Comprom	-3.110	Comprom	-3.065	0.309	GT	Sens	contig303	contig303	Unknown	[illegible]
GT	Sense	Sense	-0.150	Detected	0.150	Detected	0.000	-1.170	Comprom	-1.102	Comprom	-1.136	0.018	GT	Sens	contig303	contig303	Phospho	[illegible]
GT	Sense	Sense	0.081	Detected	-0.081	Detected	0.000	-1.123	Detected	-1.370	Detected	-1.247	0.014	GT	Sens	contig303	contig303	ATP bind	[illegible]
GT	Sense	Sense	0.200	Detected	-0.200	Detected	0.000	-1.221	Detected	-1.093	Detected	-1.157	0.031	GT	Sens	contig303	contig303	UPF0503	[illegible]
GT	Sense	Sense	0.369	Detected	-0.369	Detected	0.000	-1.471	Detected	-1.088	Detected	-1.280	0.092	GT	Sens	contig303	contig303	Genomic	[illegible]
GT	Sense	Sense	0.045	Detected	-0.045	Detected	0.000	-1.628	Detected	-2.134	Detected	-1.881	0.018	GT	Sens	contig303	contig303	Unknown	[illegible]
GT	Sense	Sense	0.061	Detected	-0.061	Detected	0.000	-4.577	Comprom	-3.965	Comprom	-4.271	0.005	GT	Sens	contig303	contig303	Patatin-lik	[illegible]
GT	Sense	Sense	0.172	Detected	-0.172	Detected	0.000	-1.071	Detected	-0.990	Detected	-1.031	0.028	GT	Sens	contig304	contig304	TBC1 do	[illegible]
GT	Sense	Sense	-0.104	Detected	0.104	Detected	0.000	-3.020	Comprom	-1.650	Comprom	-2.335	0.078	GT	Sens	contig304	contig304	WRKY tra	[illegible]
GT	Sense	Sense	0.055	Detected	-0.055	Detected	0.000	-2.494	Detected	-1.401	Detected	-1.947	0.071	GT	Sens	contig304	contig304	WRKY tra	[illegible]
GT	Sense	Sense	0.062	Detected	-0.062	Detected	0.000	-1.294	Detected	-1.000	Detected	-1.147	0.019	GT	Sens	contig304	contig304	Phospho	[illegible]
GT	Sense	Sense	-0.268	Detected	0.268	Detected	0.000	-1.504	Detected	-1.629	Detected	-1.566	0.030	GT	Sens	contig304	contig304	Unknown	[illegible]
GT	Sense	Sense	0.191	Detected	-0.191	Detected	0.000	-1.627	Detected	-1.096	Detected	-1.362	0.053	GT	Sens	contig304	contig304	Jumonji	[illegible]
GT	Sense	Sense	-0.030	Detected	0.030	Detected	0.000	-2.427	Detected	-1.944	Detected	-2.185	0.012	GT	Sens	contig305	contig305	ATP-bind	[illegible]
GT	Sense	Sense	0.192	Detected	-0.192	Detected	0.000	-0.856	Detected	-2.500	Detected	-1.678	0.185	GT	Sens	contig305	contig305	Cathepsi	[illegible]
GT	Sense	Sense	0.346	Detected	-0.346	Detected	0.000	-3.470	Detected	-2.711	Detected	-3.091	0.027	GT	Sens	contig305	contig305	Tropinon	[illegible]
GT	Sense	Sense	0.042	Detected	-0.042	Detected	0.000	-1.659	Detected	-1.019	Detected	-1.339	0.053	GT	Sens	contig305	contig305	Multidrug	[illegible]
GT	Sense	Sense	0.183	Detected	-0.183	Detected	0.000	-2.457	Detected	-2.271	Detected	-2.364	0.007	GT	Sens	contig305	contig305	Guanine	[illegible]
GT	Sense	Sense	0.091	Detected	-0.091	Detected	0.000	-1.391	Detected	-1.202	Detected	-1.297	0.010	GT	Sens	contig305	contig305	CXE carb	[illegible]
GT	Sense	Sense	0.385	Detected	-0.385	Detected	0.000	-2.501	Detected	-3.599	Detected	-3.050	0.045	GT	Sens	contig305	contig305	Unknown	[illegible]
GT	Sense	Sense	-0.004	Detected	0.004	Detected	0.000	-1.422	Detected	-1.061	Detected	-1.241	0.021	GT	Sens	contig305	contig305	F-box fan	[illegible]
GT	Sense	Sense	0.012	Detected	-0.012	Detected	0.000	-3.230	Detected	-2.172	Detected	-2.701	0.036	GT	Sens	contig306	contig306	Unknown	[illegible]
GT	Sense	Sense	0.214	Detected	-0.214	Detected	0.000	-1.720	Detected	-1.512	Detected	-1.616	0.021	GT	Sens	contig306	contig306	Purple ac	[illegible]
GT	Sense	Sense	-0.128	Detected	0.128	Detected	0.000	-1.094	Detected	-0.924	Detected	-1.009	0.022	GT	Sens	contig306	contig306	Unknown	[illegible]
GT	Sense	Sense	-0.141	Detected	0.141	Detected	0.000	-1.099	Detected	-1.477	Detected	-1.288	0.032	GT	Sens	contig306	contig306	Nitrate tra	[illegible]
GT	Sense	Sense	0.138	Detected	-0.138	Detected	0.000	-0.860	Detected	-1.168	Detected	-1.014	0.039	GT	Sens	contig306	contig306	Palmitoyl	[illegible]
GT	Sense	Sense	0.043	Detected	-0.043	Detected	0.000	-1.348	Detected	-1.421	Detected	-1.385	0.002	GT	Sens	contig306	contig306	Hedgeho	[illegible]
GT	Sense	Sense	0.332	Detected	-0.332	Detected	0.000	-2.497	Detected	-1.704	Detected	-2.100	0.056	GT	Sens	contig306	contig306	Receptor	[illegible]
GT	Sense	Sense	0.062	Detected	-0.062	Detected	0.000	-1.572	Detected	-1.320	Detected	-1.446	0.009	GT	Sens	contig307	contig307	Receptor	[illegible]
GT	Sense	Sense	-0.120	Detected	0.120	Detected	0.000	-1.551	Detected	-1.345	Detected	-1.448	0.012	GT	Sens	contig308	contig308	Cys2/His	[illegible]
GT	Sense	Sense	0.153	Detected	-0.153	Detected	0.000	-2.465	Detected	-1.735	Detected	-2.100	0.034	GT	Sens	contig308	contig308	ATP-bind	[illegible]
GT	Sense	Sense	0.014	Detected	-0.014	Detected	0.000	-2.536	Detected	-2.376	Detected	-2.456	0.001	GT	Sens	contig308	contig308	Protein p	[illegible]
GT	Sense	Sense	0.376	Detected	-0.376	Detected	0.000	-1.357	Detected	-1.299	Detected	-1.328	0.072	GT	Sens	contig308	contig308	CHP-rich	[illegible]
GT	Sense	Sense	0.129	Detected	-0.129	Detected	0.000	-0.960	Detected	-1.042	Detected	-1.001	0.018	GT	Sens	contig309	contig309	Bifunctio	[illegible]
GT	Sense	Sense	0.124	Detected	-0.124	Detected	0.000	-1.884	Detected	-1.832	Detected	-1.858	0.005	GT	Sens	contig309	contig309	U-box do	[illegible]
GT	Sense	Sense	0.320	Detected	-0.320	Detected	0.000	-2.034	Detected	-2.046	Detected	-2.040	0.024	GT	Sens	contig309	contig309	IST1 hom	[illegible]
GT	Sense	Sense	0.084	Detected	-0.084	Detected	0.000	-2.191	Detected	-1.602	Detected	-1.896	0.025	GT	Sens	contig309	contig309	Receptor	[illegible]
GT	Sense	Sense	0.409	Detected	-0.409	Detected	0.000	-2.448	Comprom	-1.096	Detected	-1.772	0.154	GT	Sens	contig309	contig309	Os08g01	[illegible]
GT	Sense	Sense	-0.050	Detected	0.050	Detected	0.000	-1.895	Detected	-1.257	Detected	-1.576	0.040	GT	Sens	contig310	contig310	Receptor	[illegible]
GT	Sense	Sense	0.135	Detected	-0.135	Detected	0.000	-1.835	Detected	-1.570	Detected	-1.702	0.012	GT	Sens	contig310	contig310	Dynein li	[illegible]
GT	Sense	Sense	0.072	Detected	-0.072	Detected	0.000	-0.968	Detected	-1.070	Detected	-1.019	0.007	GT	Sens	contig310	contig310	ATP bind	[illegible]
GT	Sense	Sense	0.102	Detected	-0.102	Detected	0.000	-2.854	Detected	-1.251	Detected	-2.053	0.126	GT	Sens	contig310	contig310	Endogluc	[illegible]
GT	Sense	Sense	-0.049	Detected	0.049	Detected	0.000	-1.209	Detected	-0.818	Detected	-1.014	0.037	GT	Sens	contig310	contig310	Histone H	[illegible]
GT	Sense	Sense	0.095	Detected	-0.095	Detected	0.000	-1.760	Detected	-1.075	Detected	-1.417	0.058	GT	Sens	contig310	contig310	Aspartic	[illegible]
GT	Sense	Sense	-0.029	Detected	0.029	Detected	0.000	-1.684	Detected	-1.326	Detected	-1.505	0.014	GT	Sens	contig310	contig310	Neutral in	[illegible]
GT	Sense	Sense	0.041	Detected	-0.041	Detected	0.000	-1.319	Comprom	-2.472	Comprom	-1.896	0.082	GT	Sens	contig311	contig311	CHP-rich	[illegible]
GT	Sense	Sense	-0.083	Detected	0.083	Detected	0.000	-2.918	Detected	-4.052	Comprom	-3.485	0.026	GT	Sens	contig311	contig311	Cinnamyl	[illegible]
GT	Sense	Sense	0.222	Detected	-0.222	Detected	0.000	-1.433	Detected	-1.998	Detected	-1.716	0.041	GT	Sens	contig311	contig311	GDSL est	[illegible]
GT	Sense	Sense	-0.378	Detected	0.378	Detected	0.000	-0.857	Detected	-1.955	Detected	-1.406	0.169	GT	Sens	contig311	contig311	Unknown	[illegible]
GT	Sense	Sense	0.164	Detected	-0.164	Detected	0.000	-3.248	Detected	-2.646	Detected	-2.947	0.013	GT	Sens	contig312	contig312	Laccase	[illegible]
GT	Sense	Sense	0.343	Detected	-0.343	Detected	0.000	-1.387	Detected	-1.022	Detected	-1.205	0.090	GT	Sens	contig312	contig312	Ceramide	[illegible]
GT	Sense	Sense	0.255	Detected	-0.255	Detected	0.000	-1.335	Detected	-1.315	Detected	-1.325	0.035	GT	Sens	contig312	contig312	Leucine-r	[illegible]
GT	Sense	Sense	-0.095	Detected	0.095	Detected	0.000	-2.763	Detected	-0.971	Detected	-1.867	0.174	GT	Sens	contig312	contig312	Kunitz-ty	[illegible]
GT	Sense	Sense	-0.171	Detected	0.171	Detected	0.000	-1.024	Detected	-1.062	Detected	-1.043	0.026	GT	Sens	contig312	contig312	Hydroxyp	[illegible]
GT	Sense	Sense	0.164	Detected	-0.164	Detected	0.000	-1.177	Detected	-1.080	Detected	-1.129	0.022	GT	Sens	contig312	contig312	Unknown	[illegible]
GT	Sense	Sense	0.059	Detected	-0.059	Detected	0.000	-1.363	Detected	-2.078	Detected	-1.720	0.042	GT	Sens	contig312	contig312	Unknown	[illegible]
GT	Sense	Sense	0.187	Detected	-0.187	Detected	0.000	-1.882	Detected	-1.449	Detected	-1.665	0.028	GT	Sens	contig312	contig312	Harpin-in	[illegible]
GT	Sense	Sense	0.097	Detected	-0.097	Detected	0.000	-1.504	Detected	-1.454	Detected	-1.479	0.005	GT	Sens	contig312	contig312	Thioester	[illegible]
GT	Sense	Sense	0.071	Detected	-0.071	Detected	0.000	-2.226	Detected	-1.179	Detected	-1.703	0.084	GT	Sens	contig313	contig313	RING fing	[illegible]
GT	Sense	Sense	-0.022	Detected	0.022	Detected	0.000	-1.758	Detected	-0.963	Detected	-1.361	0.076	GT	Sens	contig313	contig313	Unannota	[illegible]
GT	Sense	Sense	-0.081	Detected	0.081	Detected	0.000	-1.072	Detected	-1.014	Detected	-1.043	0.007	GT	Sens	contig313	contig313	Unannota	[illegible]
GT	Sense	Sense	0.131	Detected	-0.131	Detected	0.000	-1.001	Detected	-1.029	Detected	-1.015	0.016	GT	Sens	contig313	contig313	Unannota	[illegible]
GT	Sense	Sense	0.116	Detected	-0.115	Detected	0.000	-2.450	Detected	-1.548	Detected	-1.999	0.050	GT	Sens	contig313	contig313	Unannota	[illegible]
GT	Sense	Sense	0.216	Detected	-0.216	Detected	0.000	-2.329	Detected	-1.477	Detected	-1.903	0.058	GT	Sens	contig313	contig313	Unannota	[illegible]
GT	Sense	Sense	0.177	Detected	-0.177	Detected	0.000	-2.129	Detected	-1.923	Detected	-2.026	0.010	GT	Sens	contig314	contig314	Unannota	[illegible]
GT	Sense	Sense	0.037	Detected	-0.037	Detected	0.000	-1.855	Detected	-1.767	Detected	-1.811	0.001	GT	Sens	contig314	contig314	Unannota	[illegible]
GT	Sense	Sense	0.133	Detected	-0.133	Detected	0.000	-1.229	Comprom	-1.453	Comprom	-1.341	0.016	GT	Sens	contig314	contig314	Unannota	[illegible]
GT	Sense	Sense	0.020	Detected	-0.020	Detected	0.000	-1.038	Detected	-1.036	Detected	-1.037	0.000	GT	Sens	contig315	contig315	Unannota	[illegible]
GT	Sense	Sense	0.043	Detected	-0.043	Detected	0.000	-1.595	Detected	-1.277	Detected	-1.436	0.013	GT	Sens	contig315	contig315	Unannota	[illegible]
GT	Sense	Sense	0.095	Detected	-0.095	Detected	0.000	-1.354	Detected	-1.157	Detected	-1.255	0.012	GT	Sens	contig315	contig315	Unannota	[illegible]
GT	Sense	Sense	-1.218	Detected	1.218	Detected	0.000	-1.175	Detected	-2.076	Comprom	-1.626	0.337	GT	Sens	contig316	contig316	Unannota	[illegible]
GT	Sense	Sense	0.066	Detected	-0.066	Detected	0.000	-1.368	Detected	-1.183	Detected	-1.275	0.008	GT	Sens	contig316	contig316	Unannota	[illegible]
GT	Sense	Sense	0.035	Detected	-0.035	Detected	0.000	-1.534	Detected	-1.314	Detected	-1.424	0.007	GT	Sens	contig316	contig316	Unannota	[illegible]
GT	Sense	Sense	-0.047	Detected	0.047	Detected	0.000	-1.075	Detected	-2.090	Detected	-1.582	0.090	GT	Sens	contig317	contig317	Pectate ly	[illegible]
GT	Sense	Sense	0.653	Detected	-0.653	Detected	0.000	-2.106	Comprom	-1.323	Comprom	-1.715	0.153	GT	Sens	contig317	contig317	Unannota	[illegible]
GT	Sense	Sense	0.023	Detected	-0.023	Detected	0.000	-2.110	Comprom	-1.703	Comprom	-1.906	0.011	GT	Sens	contig317	contig317	Unannota	[illegible]
GT	Sense	Sense	-0.079	Detected	0.079	Detected	0.000	-1.526	Detected	-1.254	Detected	-1.390	0.013	GT	Sens	contig317	contig317	Unannota	[illegible]
GT	Sense	Sense	0.114	Detected	-0.114	Detected	0.000	-2.001	Detected	-1.604	Detected	-1.802	0.016	GT	Sens	contig318	contig318	Unannota	[illegible]
GT	Sense	Sense	0.313	Detected	-0.313	Detected	0.000	-2.019	Detected	-1.147	Detected	-1.583	0.098	GT	Sens	contig318	contig318	Unannota	[illegible]
GT	Sense	Sense	0.513	Detected	-0.513	Detected	0.000	-0.894	Detected	-1.794	Detected	-1.344	0.188	GT	Sens	contig318	contig318	Unannota	[illegible]
GT	Sense	Sense	-0.021	Detected	0.021	Detected	0.000	-0.849	Detected	-1.155	Detected	-1.002	0.023	GT	Sens	contig318	contig318	Unannota	[illegible]
GT	Sense	Sense	0.078	Detected	-0.078	Detected	0.000	-2.349	Detected	-1.913	Detected	-2.131	0.012	GT	Sens	contig318	contig318	UDP-D-gl	[illegible]
GT	Sense	Sense	-0.019	Detected	0.019	Detected	0.000	-1.194	Detected	-1.011	Detected	-1.102	0.007	GT	Sens	contig319	contig319	Unannota	[illegible]
GT	Sense	Sense	-0.045	Detected	0.045	Detected	0.000	-1.187	Detected	-1.062	Detected	-1.125	0.005	GT	Sens	contig319	contig319	Unannota	[illegible]
GT	Sense	Sense	-0.054	Detected	0.054	Detected	0.000	-2.662	Comprom	-2.497	Comprom	-2.579	0.001	GT	Sens	contig319	contig319	Unannota	[illegible]
GT	Sense	Sense	-0.040	Detected	0.040	Detected	0.000	-4.479	Comprom	-2.572	Detected	-3.526	0.066	GT	Sens	contig319	contig319	Unannota	[illegible]
GT	Sense	Sense	-0.203	Detected	0.203	Detected	0.000	-3.248	Comprom	-1.994	Comprom	-2.621	0.058	GT	Sens	contig319	contig319	Unannota	[illegible]
GT	Sense	Sense	-0.043	Detected	0.043	Detected	0.000	-1.400	Detected	-1.161	Detected	-1.280	0.010	GT	Sens	contig319	contig319	Unannota	[illegible]
GT	Sense	Sense	0.125	Detected	-0.125	Detected	0.000	-2.047	Comprom	-0.992	Comprom	-1.519	0.107	GT	Sens	contig319	contig319	Unannota	[illegible]
GT	Sense	Sense	0.336	Detected	-0.336	Detected	0.000	-1.107	Detected	-1.623	Detected	-1.365	0.084	GT	Sens	contig320	contig320	Unannota	[illegible]
GT	Sense	Sense	0.183	Detected	-0.183	Detected	0.000	-1.288	Comprom	-1.402	Comprom	-1.345	0.020	GT	Sens	contig320	contig320	Unannota	[illegible]
GT	Sense	Sense	0.699	Detected	-0.699	Detected	0.000	-2.046	Comprom	-2.045	Comprom	-2.045	0.100	GT	Sens	contig320	contig320	Unannota	[illegible]
GT	Sense	Sense	1.134	Detected	-1.134	Detected	0.000	-2.803	Detected	-2.616	Detected	-2.710	0.140	GT	Sens	contig320	contig320	Glyoxal o	[illegible]
GT	Sense	Sense	0.260	Detected	-0.260	Detected	0.000	-1.147	Detected	-2.104	Comprom	-1.626	0.096	GT	Sens	contig320	contig320	Unannota	[illegible]
GT	Sense	Sense	-0.160	Detected	0.160	Detected	0.000	-1.182	Detected	-1.304	Detected	-1.243	0.019	GT	Sens	contig321	contig321	Unannota	[illegible]
GT	Sense	Sense	-0.082	Detected	0.082	Detected	0.000	-0.836	Detected	-2.459	Comprom	-1.648	0.181	GT	Sens	contig321	contig321	Unannota	[illegible]
GT	Sense	Sense	0.106	Detected	-0.106	Detected	0.000	-1.023	Detected	-1.083	Detected	-1.053	0.011	GT	Sens	contig322	contig322	CONSTAI	[illegible]
GT	Sense	Sense	-0.072	Detected	0.072	Detected	0.000	-1.204	Detected	-1.068	Detected	-1.136	0.008	GT	Sens	contig322	contig322	Myb-like	[illegible]
GT	Sense	Sense	-0.362	Detected	0.362	Detected	0.000	-4.154	Comprom	-3.849	Comprom	-4.002	0.009	GT	Sens	contig322	contig322	Unannota	[illegible]
GT	Sense	Sense	-0.380	Detected	0.380	Detected	0.000	-3.498	Comprom	-3.267	Comprom	-3.383	0.014	GT	Sens	contig322	contig322	Unannota	[illegible]
GT	Sense	Sense	0.717	Detected	-0.717	Detected	0.000	-2.060	Comprom	-1.500	Comprom	-1.780	0.147	GT	Sens	contig322	contig322	Unannota	[illegible]
GT	Sense	Sense	-0.027	Detected	0.027	Detected	0.000	-1.810	Detected	-1.268	Detected	-1.539	0.030	GT	Sens	contig322	contig322	Predicted	[illegible]
GT	Sense	Sense	-0.121	Detected	0.121	Detected	0.000	-1.021	Detected	-1.849	Comprom	-1.435	0.080	GT	Sens	contig322	contig322	Unannota	[illegible]
GT	Sense	Sense	0.162	Detected	-0.162	Detected	0.000	-1.664	Detected	-1.304	Detected	-1.484	0.026	GT	Sens	contig322	contig322	Unannota	[illegible]
GT	Sense	Sense	-0.116	Detected	0.116	Detected	0.000	-0.980	Detected	-1.021	Detected	-1.001	0.014	GT	Sens	contig322	contig322	Unannota	[illegible]
GT	Sense	Sense	0.364	Detected	-0.364	Detected	0.000	-1.290	Comprom	-1.927	Comprom	-1.608	0.080	GT	Sens	contig323	contig323	Unannota	[illegible]
GT	Sense	Sense	0.161	Detected	-0.161	Detected	0.000	-1.008	Comprom	-2.894	Comprom	-1.951	0.178	GT	Sens	contig323	contig323	Unannota	[illegible]
GT	Sense	Sense	-0.008	Detected	0.008	Detected	0.000	-2.565	Comprom	-2.414	Comprom	-2.489	0.001	GT	Sens	contig323	contig323	Unannota	[illegible]
GT	Sense	Sense	0.314	Detected	-0.314	Detected	0.000	-1.547	Detected	-1.447	Detected	-1.497	0.042	GT	Sens	contig323	contig323	Unannota	[illegible]
GT	Sense	Sense	-0.022	Detected	0.022	Detected	0.000	-0.894	Detected	-1.223	Detected	-1.058	0.024	GT	Sens	contig324	contig324	Unannota	[illegible]
GT	Sense	Sense	-0.016	Detected	0.016	Detected	0.000	-1.015	Detected	-1.429	Detected	-1.222	0.028	GT	Sens	contig324	contig324	Unannota	[illegible]
GT	Sense	Sense	0.104	Detected	-0.104	Detected	0.000	-1.114	Detected	-0.898	Detected	-1.006	0.021	GT	Sens	contig324	contig324	Unannota	[illegible]
GT	Sense	Sense	0.167	Detected	-0.167	Detected	0.000	-2.686	Detected	-1.971	Detected	-2.328	0.028	GT	Sens	contig325	contig325	MFS fami	[illegible]
GT	Sense	Sense	0.004	Detected	-0.004	Detected	0.000	-1.805	Detected	-1.682	Detected	-1.744	0.001	GT	Sens	contig325	contig325	Unannota	[illegible]
GT	Sense	Sense	0.098	Detected	-0.098	Detected	0.000	-1.714	Detected	-1.186	Detected	-1.450	0.036	GT	Sens	contig325	contig325	Unannota	[illegible]
GT	Sense	Sense	0.595	Detected	-0.595	Detected	0.000	-0.841	Detected	-1.680	Comprom	-1.261	0.225	GT	Sens	contig325	contig325	Unannota	[illegible]
GT	Sense	Sense	0.480	Detected	-0.480	Detected	0.000	-2.571	Comprom	-2.040	Detected	-2.305	0.052	GT	Sens	contig325	contig325	Unannota	[illegible]
GT	Sense	Sense	0.211	Detected	-0.211	Detected	0.000	-3.297	Detected	-2.583	Detected	-2.940	0.019	GT	Sens	contig325	contig325	Unannota	[illegible]
GT	Sense	Sense	0.179	Detected	-0.179	Detected	0.000	-1.725	Detected	-1.297	Detected	-1.511	0.032	GT	Sens	contig325	contig325	Unannota	[illegible]
GT	Sense	Sense	0.154	Detected	-0.154	Detected	0.000	-1.566	Detected	-1.263	Detected	-1.415	0.022	GT	Sens	contig325	contig325	Unannota	[illegible]
GT	Sense	Sense	0.163	Detected	-0.163	Detected	0.000	-1.727	Detected	-1.018	Detected	-1.373	0.072	GT	Sens	contig325	contig325	Unannota	[illegible]
GT	Sense	Sense	0.181	Detected	-0.181	Detected	0.000	-2.911	Comprom	-0.864	Comprom	-1.887	0.211	GT	Sens	contig325	contig325	Unannota	[illegible]
GT	Sense	Sense	-0.026	Detected	0.026	Detected	0.000	-1.403	Detected	-1.228	Detected	-1.315	0.005	GT	Sens	contig325	contig325	F-box fan	[illegible]
GT	Sense	Sense	0.306	Detected	-0.306	Detected	0.000	-2.031	Detected	-2.257	Detected	-2.144	0.022	GT	Sens	contig325	contig325	Unannota	[illegible]
GT	Sense	Sense	0.388	Detected	-0.388	Detected	0.000	-1.950	Detected	-2.041	Detected	-1.996	0.036	GT	Sens	contig325	contig325	Unannota	[illegible]
GT	Sense	Sense	0.004	Detected	-0.004	Detected	0.000	-0.909	Detected	-1.219	Detected	-1.064	0.021	GT	Sens	contig326	contig326	Unannota	[illegible]
GT	Sense	Sense	0.194	Detected	-0.194	Detected	0.000	-1.229	Detected	-1.183	Detected	-1.206	0.025	GT	Sens	contig326	contig326	Unannota	[illegible]
GT	Sense	Sense	0.037	Detected	-0.037	Detected	0.000	-1.255	Detected	-0.807	Detected	-1.031	0.045	GT	Sens	contig326	contig326	Aspartic	[illegible]
GT	Sense	Sense	-0.097	Detected	0.097	Detected	0.000	-1.144	Detected	-1.104	Detected	-1.124	0.008	GT	Sens	contig326	contig326	Unannota	[illegible]
GT	Sense	Sense	-0.029	Detected	0.029	Detected	0.000	-2.670	Comprom	-2.914	Comprom	-2.792	0.002	GT	Sens	contig326	contig326	Unannota	[illegible]
GT	Sense	Sense	0.079	Detected	-0.079	Detected	0.000	-1.239	Detected	-1.318	Detected	-1.279	0.005	GT	Sens	contig327	contig327	Unannota	[illegible]
GT	Sense	Sense	0.004	Detected	-0.004	Detected	0.000	-2.160	Detected	-1.460	Detected	-1.810	0.035	GT	Sens	contig327	contig327	Unannota	[illegible]
GT	Sense	Sense	-0.048	Detected	0.048	Detected	0.000	-1.011	Detected	-1.192	Detected	-1.102	0.009	GT	Sens	contig327	contig327	Unannota	[illegible]
GT	Sense	Sense	0.014	Detected	-0.014	Detected	0.000	-1.301	Detected	-1.045	Detected	-1.173	0.012	GT	Sens	contig327	contig327	Unannota	[illegible]
GT	Sense	Sense	0.967	Detected	-0.967	Detected	0.000	-1.848	Comprom	-1.116	Detected	-1.482	0.288	GT	Sens	contig327	contig327	Unannota	[illegible]
GT	Sense	Sense	0.121	Detected	-0.121	Detected	0.000	-1.295	Detected	-1.016	Detected	-1.156	0.025	GT	Sens	contig328	contig328	Unannota	[illegible]
GT	Sense	Sense	0.167	Detected	-0.167	Detected	0.000	-1.537	Detected	-1.083	Detected	-1.310	0.043	GT	Sens	contig328	contig328	Unannota	[illegible]
GT	Sense	Sense	0.636	Detected	-0.636	Detected	0.000	-1.066	Detected	-1.363	Detected	-1.215	0.204	GT	Sens	contig328	contig328	High mob	[illegible]
GT	Sense	Sense	0.043	Detected	-0.043	Detected	0.000	-1.582	Detected	-0.947	Detected	-1.264	0.059	GT	Sens	contig328	contig328	Unannota	[illegible]
GT	Sense	Sense	0.004	Detected	-0.004	Detected	0.000	-1.519	Detected	-1.002	Detected	-1.261	0.040	GT	Sens	contig328	contig328	Unannota	[illegible]
GT	Sense	Sense	-0.091	Detected	0.091	Detected	0.000	-1.109	Detected	-1.717	Comprom	-1.413	0.047	GT	Sens	contig328	contig328	Unannota	[illegible]
GT	Sense	Sense	0.120	Detected	-0.120	Detected	0.000	-1.159	Detected	-0.982	Detected	-1.071	0.019	GT	Sens	contig329	contig329	Unannota	[illegible]
GT	Sense	Sense	0.218	Detected	-0.218	Detected	0.000	-1.395	Comprom	-0.956	Comprom	-1.176	0.063	GT	Sens	contig329	contig329	Unannota	[illegible]
GT	Sense	Sense	0.197	Detected	-0.197	Detected	0.000	-1.235	Detected	-1.019	Detected	-1.127	0.037	GT	Sens	contig329	contig329	Unannota	[illegible]
GT	Sense	Sense	0.151	Detected	-0.151	Detected	0.000	-1.389	Detected	-1.100	Detected	-1.244	0.027	GT	Sens	contig329	contig329	Unannota	[illegible]
GT	Sense	Sense	0.124	Detected	-0.124	Detected	0.000	-1.117	Detected	-0.915	Detected	-1.016	0.024	GT	Sens	contig329	contig329	Rhomboi	[illegible]
GT	Sense	Sense	-0.430	Detected	0.430	Detected	0.000	-1.222	Comprom	-1.130	Comprom	-1.176	0.113	GT	Sens	contig329	contig329	Unannota	[illegible]
GT	Sense	Sense	0.151	Detected	-0.151	Detected	0.000	-2.072	Detected	-1.491	Detected	-1.782	0.032	GT	Sens	contig330	contig330	Calcineur	[illegible]
GT	Sense	Sense	-0.090	Detected	0.090	Detected	0.000	-1.430	Comprom	-1.012	Comprom	-1.221	0.033	GT	Sens	contig330	contig330	Unannota	[illegible]
GT	Sense	Sense	0.156	Detected	-0.156	Detected	0.000	-1.832	Detected	-1.626	Detected	-1.729	0.011	GT	Sens	contig331	contig331	Transcrip	[illegible]
GT	Sense	Sense	0.059	Detected	-0.059	Detected	0.000	-0.888	Detected	-1.359	Detected	-1.124	0.044	GT	Sens	contig332	contig332	Unannota	[illegible]
GT	Sense	Sense	0.047	Detected	-0.047	Detected	0.000	-1.196	Detected	-1.250	Detected	-1.223	0.002	GT	Sens	contig332	contig332	Ring zinc	[illegible]
GT	Sense	Sense	0.038	Detected	-0.038	Detected	0.000	-0.995	Detected	-1.052	Detected	-1.023	0.002	GT	Sens	contig332	contig332	Unannota	[illegible]
GT	Sense	Sense	0.357	Detected	-0.357	Detected	0.000	-1.994	Detected	-0.993	Detected	-1.493	0.136	GT	Sens	contig332	contig332	Respons	[illegible]
GT	Sense	Sense	0.142	Detected	-0.142	Detected	0.000	-1.311	Detected	-0.895	Detected	-1.103	0.048	GT	Sens	contig333	contig333	Unannota	[illegible]
GT	Sense	Sense	0.162	Detected	-0.162	Detected	0.000	-1.598	Detected	-1.184	Detected	-1.391	0.034	GT	Sens	contig334	contig334	LIM doma	[illegible]
GT	Sense	Sense	0.168	Detected	-0.168	Detected	0.000	-1.325	Detected	-1.345	Detected	-1.335	0.016	GT	Sens	contig334	contig334	Unannota	[illegible]
GT	Sense	Sense	0.136	Detected	-0.136	Detected	0.000	-1.455	Detected	-1.128	Detected	-1.292	0.026	GT	Sens	contig334	contig334	Unannota	[illegible]
GT	Sense	Sense	0.070	Detected	-0.070	Detected	0.000	-1.589	Detected	-1.737	Detected	-1.663	0.004	GT	Sens	contig334	contig334	cDNA clo	[illegible]
GT	Sense	Sense	-0.693	Detected	0.693	Detected	0.000	-0.981	Detected	-1.167	Comprom	-1.074	0.264	GT	Sens	contig334	contig334	Helitron h	[illegible]
GT	Sense	Sense	0.125	Detected	-0.125	Detected	0.000	-0.986	Detected	-1.046	Detected	-1.016	0.016	GT	Sens	contig335	contig335	Unannota	[illegible]
GT	Sense	Sense	0.142	Detected	-0.142	Detected	0.000	-1.275	Comprom	-1.193	Comprom	-1.234	0.014	GT	Sens	contig335	contig335	Unannota	[illegible]
GT	Sense	Sense	-0.003	Detected	0.003	Detected	0.000	-2.078	Detected	-1.523	Detected	-1.800	0.023	GT	Sens	contig335	contig335	Cysteine-	[illegible]
GT	Sense	Sense	0.126	Detected	-0.126	Detected	0.000	-2.101	Detected	-1.323	Detected	-1.712	0.053	GT	Sens	contig336	contig336	Unannota	[illegible]
GT	Sense	Sense	0.210	Detected	-0.210	Detected	0.000	-2.298	Detected	-1.507	Detected	-1.902	0.051	GT	Sens	contig336	contig336	Unannota	[illegible]
GT	Sense	Sense	0.154	Detected	-0.154	Detected	0.000	-0.976	Detected	-1.065	Detected	-1.021	0.024	GT	Sens	contig336	contig336	Unannota	[illegible]
GT	Sense	Sense	0.028	Detected	-0.028	Detected	0.000	-1.050	Detected	-1.080	Detected	-1.065	0.001	GT	Sens	contig336	contig336	Unannota	[illegible]
GT	Sense	Sense	0.399	Detected	-0.399	Detected	0.000	-3.427	Detected	-3.092	Detected	-3.260	0.017	GT	Sens	contig336	contig336	Unannota	[illegible]
GT	Sense	Sense	-0.004	Detected	0.004	Detected	0.000	-1.800	Detected	-0.905	Detected	-1.353	0.094	GT	Sens	contig336	contig336	Unannota	[illegible]
GT	Sense	Sense	0.365	Detected	-0.365	Detected	0.000	-1.687	Comprom	-1.491	Comprom	-1.589	0.052	GT	Sens	contig337	contig337	Unannota	[illegible]
GT	Sense	Sense	0.069	Detected	-0.069	Detected	0.000	-1.946	Detected	-1.628	Detected	-1.787	0.009	GT	Sens	contig337	contig337	Unknown	[illegible]
GT	Sense	Sense	0.251	Detected	-0.251	Detected	0.000	-0.994	Detected	-1.600	Detected	-1.297	0.081	GT	Sens	contig338	contig338	Aspartic	[illegible]
GT	Sense	Sense	0.621	Detected	-0.621	Detected	0.000	-2.377	Detected	-1.770	Detected	-2.074	0.095	GT	Sens	contig338	contig338	Calmodul	[illegible]
GT	Sense	Sense	0.081	Detected	-0.081	Detected	0.000	-1.750	Detected	-1.167	Detected	-1.459	0.041	GT	Sens	contig338	contig338	Unannota	[illegible]
GT	Sense	Sense	0.172	Detected	-0.172	Detected	0.000	-1.813	Detected	-2.242	Detected	-2.027	0.018	GT	Sens	contig338	contig338	Unannota	[illegible]
GT	Sense	Sense	0.145	Detected	-0.145	Detected	0.000	-1.624	Detected	-1.399	Detected	-1.511	0.014	GT	Sens	contig338	contig338	Unannota	[illegible]
GT	Sense	Sense	0.164	Detected	-0.164	Detected	0.000	-4.224	Detected	-2.768	Detected	-3.496	0.043	GT	Sens	contig338	contig338	Unannota	[illegible]
GT	Sense	Sense	0.196	Detected	-0.196	Detected	0.000	-4.378	Detected	-2.910	Detected	-3.644	0.041	GT	Sens	contig338	contig338	Unannota	[illegible]
GT	Sense	Sense	-0.051	Detected	0.051	Detected	0.000	-1.238	Detected	-0.971	Detected	-1.105	0.016	GT	Sens	contig339	contig339	Unannota	[illegible]
GT	Sense	Sense	0.174	Detected	-0.174	Detected	0.000	-1.890	Detected	-1.571	Detected	-1.730	0.018	GT	Sens	contig339	contig339	Unannota	[illegible]
GT	Sense	Sense	0.156	Detected	-0.156	Detected	0.000	-1.873	Detected	-1.570	Detected	-1.721	0.016	GT	Sens	contig339	contig339	Unannota	[illegible]
GT	Sense	Sense	0.150	Detected	-0.150	Detected	0.000	-1.132	Detected	-1.615	Detected	-1.373	0.040	GT	Sens	contig339	contig339	Unannota	[illegible]
GT	Sense	Sense	0.068	Detected	-0.068	Detected	0.000	-1.135	Detected	-2.303	Detected	-1.719	0.100	GT	Sens	contig339	contig339	Coenzym	[illegible]
GT	Sense	Sense	0.005	Detected	-0.005	Detected	0.000	-1.243	Detected	-1.345	Detected	-1.294	0.002	GT	Sens	contig339	contig339	Unannota	[illegible]
GT	Sense	Sense	0.222	Detected	-0.222	Detected	0.000	-1.959	Comprom	-1.403	Comprom	-1.681	0.042	GT	Sens	contig340	contig340	Unannota	[illegible]
GT	Sense	Sense	0.313	Detected	-0.313	Detected	0.000	-1.973	Detected	-1.490	Detected	-1.731	0.048	GT	Sens	contig340	contig340	Unannota	[illegible]
GT	Sense	Sense	0.294	Detected	-0.294	Detected	0.000	-1.786	Detected	-0.940	Detected	-1.363	0.118	GT	Sens	contig340	contig340	Unannota	[illegible]
GT	Sense	Sense	0.055	Detected	-0.055	Detected	0.000	-2.402	Detected	-0.957	Detected	-1.679	0.146	GT	Sens	contig340	contig340	Unannota	[illegible]
GT	Sense	Sense	-1.541	Detected	1.541	Detected	0.000	-3.348	Comprom	-4.039	Comprom	-3.693	0.144	GT	Sens	contig341	contig341	Unannota	[illegible]
GT	Sense	Sense	-0.103	Detected	0.103	Detected	0.000	-2.028	Detected	-1.313	Detected	-1.671	0.046	GT	Sens	contig341	contig341	Endogluc	[illegible]
GT	Sense	Sense	0.086	Detected	-0.086	Detected	0.000	-1.337	Detected	-1.171	Detected	-1.254	0.009	GT	Sens	contig341	contig341	Unannota	[illegible]
GT	Sense	Sense	0.040	Detected	-0.040	Detected	0.000	-1.578	Detected	-1.337	Detected	-1.457	0.008	GT	Sens	contig341	contig341	Unannota	[illegible]
GT	Sense	Sense	0.428	Detected	-0.428	Detected	0.000	-3.683	Detected	-2.037	Detected	-2.860	0.091	GT	Sens	contig341	contig341	LRR rece	[illegible]
GT	Sense	Sense	0.280	Detected	-0.280	Detected	0.000	-1.042	Detected	-1.116	Detected	-1.079	0.062	GT	Sens	contig341	contig341	Unannota	[illegible]
GT	Sense	Sense	-0.914	Detected	0.914	Detected	0.000	-2.571	Comprom	-2.797	Comprom	-2.684	0.100	GT	Sens	contig341	contig341	Unannota	[illegible]
GT	Sense	Sense	-1.529	Detected	1.529	Detected	0.000	-4.643	Comprom	-4.213	Comprom	-4.428	0.103	GT	Sens	contig342	contig342	Unannota	[illegible]
GT	Sense	Sense	-0.096	Detected	0.096	Detected	0.000	-1.003	Detected	-1.022	Detected	-1.012	0.009	GT	Sens	contig342	contig342	Unannota	[illegible]
GT	Sense	Sense	-0.219	Detected	0.219	Detected	0.000	-1.196	Detected	-1.013	Detected	-1.104	0.043	GT	Sens	contig342	contig342	Unannota	[illegible]
GT	Sense	Sense	0.078	Detected	-0.078	Detected	0.000	-1.244	Detected	-1.256	Detected	-1.250	0.004	GT	Sens	contig343	contig343	Unannota	[illegible]
GT	Sense	Sense	-0.051	Detected	0.051	Detected	0.000	-1.553	Detected	-1.318	Detected	-1.435	0.008	GT	Sens	contig343	contig343	Unannota	[illegible]
GT	Sense	Sense	0.051	Detected	-0.051	Detected	0.000	-1.277	Detected	-0.907	Detected	-1.092	0.030	GT	Sens	contig343	contig343	Unannota	[illegible]
GT	Sense	Sense	0.048	Detected	-0.048	Detected	0.000	-1.418	Detected	-0.832	Detected	-1.125	0.063	GT	Sens	contig343	contig343	Unannota	[illegible]
GT	Sense	Sense	0.240	Detected	-0.240	Detected	0.000	-1.127	Detected	-1.257	Detected	-1.192	0.041	GT	Sens	contig343	contig343	3-ketoacy	[illegible]
GT	Sense	Sense	0.166	Detected	-0.166	Detected	0.000	-1.824	Detected	-1.178	Detected	-1.501	0.054	GT	Sens	contig343	contig343	Unannota	[illegible]
GT	Sense	Sense	0.320	Detected	-0.320	Detected	0.000	-1.191	Detected	-0.903	Detected	-1.047	0.096	GT	Sens	contig344	contig344	Unannota	[illegible]
GT	Sense	Sense	-0.031	Detected	0.031	Detected	0.000	-1.295	Detected	-1.031	Detected	-1.163	0.013	GT	Sens	contig344	contig344	Unannota	[illegible]
GT	Sense	Sense	0.061	Detected	-0.061	Detected	0.000	-2.028	Detected	-1.471	Detected	-1.750	0.026	GT	Sens	contig344	contig344	Unannota	[illegible]
GT	Sense	Sense	0.111	Detected	-0.111	Detected	0.000	-1.965	Detected	-1.310	Detected	-1.637	0.042	GT	Sens	contig344	contig344	Unannota	[illegible]
GT	Sense	Sense	0.510	Detected	-0.510	Detected	0.000	-2.886	Detected	-2.615	Detected	-2.751	0.035	GT	Sens	contig345	contig345	Unknown	[illegible]
GT	Sense	Sense	0.368	Detected	-0.368	Detected	0.000	-1.534	Detected	-2.640	Comprom	-2.087	0.088	GT	Sens	contig345	contig345	Unannota	[illegible]
GT	Sense	Sense	0.412	Detected	-0.412	Detected	0.000	-2.310	Detected	-2.534	Detected	-2.422	0.030	GT	Sens	contig345	contig345	Unannota	[illegible]
GT	Sense	Sense	0.354	Detected	-0.354	Detected	0.000	-2.242	Comprom	-1.944	Comprom	-2.093	0.032	GT	Sens	contig346	contig346	Unannota	[illegible]
GT	Sense	Sense	-0.091	Detected	0.091	Detected	0.000	-2.010	Comprom	-1.087	Comprom	-1.549	0.081	GT	Sens	contig346	contig346	Unannota	[illegible]
GT	Sense	Sense	0.236	Detected	-0.236	Detected	0.000	-0.989	Detected	-1.989	Detected	-1.489	0.115	GT	Sens	contig347	contig347	Unannota	[illegible]
GT	Sense	Sense	0.268	Detected	-0.268	Detected	0.000	-1.671	Detected	-1.086	Detected	-1.379	0.074	GT	Sens	contig347	contig347	Unannota	[illegible]
GT	Sense	Sense	0.281	Detected	-0.281	Detected	0.000	-1.477	Detected	-0.848	Detected	-1.162	0.110	GT	Sens	contig347	contig347	Unannota	[illegible]
GT	Sense	Sense	-0.436	Detected	0.436	Detected	0.000	-2.130	Comprom	-1.264	Comprom	-1.697	0.110	GT	Sens	contig347	contig347	Unannota	[illegible]
GT	Sense	Sense	0.167	Detected	-0.167	Detected	0.000	-1.212	Detected	-1.016	Detected	-1.114	0.029	GT	Sens	contig348	contig348	Unannota	[illegible]
GT	Sense	Sense	-0.602	Detected	0.602	Detected	0.000	-0.977	Detected	-1.617	Detected	-1.297	0.197	GT	Sens	contig348	contig348	Unannota	[illegible]
GT	Sense	Sense	0.360	Detected	-0.360	Detected	0.000	-1.200	Detected	-1.297	Detected	-1.248	0.075	GT	Sens	contig348	contig348	Unannota	[illegible]
GT	Sense	Sense	0.346	Detected	-0.346	Detected	0.000	-1.302	Detected	-1.177	Detected	-1.240	0.072	GT	Sens	contig348	contig348	Unannota	[illegible]
GT	Sense	Sense	0.183	Detected	-0.183	Detected	0.000	-1.622	Detected	-1.218	Detected	-1.420	0.035	GT	Sens	contig348	contig348	Susceptil	[illegible]
GT	Sense	Sense	0.105	Detected	-0.105	Detected	0.000	-1.290	Detected	-1.258	Detected	-1.274	0.007	GT	Sens	contig349	contig349	Unannota	[illegible]
GT	Sense	Sense	-0.100	Detected	0.100	Detected	0.000	-1.206	Detected	-1.049	Detected	-1.128	0.013	GT	Sens	contig349	contig349	Unannota	[illegible]
GT	Sense	Sense	0.131	Detected	-0.131	Detected	0.000	-1.288	Detected	-0.821	Detected	-1.054	0.059	GT	Sens	contig349	contig349	Unannota	[illegible]
GT	Sense	Sense	-0.134	Detected	0.134	Detected	0.000	-1.232	Detected	-1.431	Comprom	-1.332	0.015	GT	Sens	contig350	contig350	Unannota	[illegible]
GT	Sense	Sense	-0.024	Detected	0.024	Detected	0.000	-1.843	Detected	-2.220	Detected	-2.031	0.009	GT	Sens	contig350	contig350	Unannota	[illegible]
GT	Sense	Sense	0.223	Detected	-0.223	Detected	0.000	-1.791	Detected	-2.094	Detected	-1.943	0.019	GT	Sens	contig350	contig350	Unannota	[illegible]
GT	Sense	Sense	-0.012	Detected	0.012	Detected	0.000	-1.923	Comprom	-1.298	Detected	-1.611	0.036	GT	Sens	contig350	contig350	Unannota	[illegible]
GT	Sense	Sense	0.110	Detected	-0.110	Detected	0.000	-1.743	Detected	-1.351	Detected	-1.547	0.020	GT	Sens	contig350	contig350	Unknown	[illegible]

GT	Sense	Sense	-0.060	Detected	0.060	Detected	0.000	-0.960	Detected	-1.183	Detected	-1.072	0.014	GT	Sens	contig350	contig350	Unannota	GGACTGATTAGTGCTTTTTTGTTATTATACCCTGACTGATTGTGCTCAA							[illegible]
GT	Sense	Sense	-0.023	Detected	0.023	Detected	0.000	-0.871	Detected	-1.285	Detected	-1.078	0.035	GT	Sens	contig350	contig350	Unannota	TTACTAGTGAAGTTAGGGTCTTATGTAGCAAGAGTCTTAATCAAATGAG							[illegible]
GT	Sense	Sense	-0.890	Detected	0.890	Detected	0.000	-1.939	Comprom	-2.110	Comprom	-2.025	0.152	GT	Sens	contig351	contig351	Unannota	GAATTGAATCTTATGCTATGTGAGAAATGTAATGTGTTCCTCTCGAATT							[illegible]
GT	Sense	Sense	-0.062	Detected	0.062	Detected	0.000	-1.418	Detected	-1.037	Detected	-1.228	0.026	GT	Sens	contig351	contig351	Unannota	GCAAAAGATAATTATGATCCATACAATCCCCTTCCAATGTTTTTTCATG							[illegible]
GT	Sense	Sense	-0.088	Detected	0.088	Detected	0.000	-1.323	Detected	-1.097	Detected	-1.210	0.014	GT	Sens	contig351	contig351	Unannota	TCAGTCTAGACCTTTGAGTTGCTAACTTGCATCATTTCTGATACTTCAA							[illegible]
GT	Sense	Sense	0.077	Detected	-0.077	Detected	0.000	-1.074	Detected	-0.927	Detected	-1.001	0.011	GT	Sens	contig351	contig351	Unannota	CATCCCCATGTAATTCCACGAGTCTCTTCTGGAAAAGGTAGAGTGTAT							[illegible]
GT	Sense	Sense	0.102	Detected	-0.102	Detected	0.000	-1.206	Detected	-0.839	Detected	-1.023	0.040	GT	Sens	contig351	contig351	Unannota	CTAAGCTTTGCTTTCCTGATGTTGTATCCTGATTTTTGACATCTTGTCC							[illegible]
GT	Sense	Sense	0.037	Detected	-0.037	Detected	0.000	-1.130	Detected	-0.971	Detected	-1.050	0.007	GT	Sens	contig351	contig351	Unannota	ATCGATTCTTGGGTTTTGAGCTTTGTGGGGGTGTTTATCTTGGTTCAT							[illegible]
GT	Sense	Sense	-0.078	Detected	0.078	Detected	0.000	-2.266	Comprom	-2.199	Comprom	-2.232	0.001	GT	Sens	contig352	contig352	Unannota	CTTCTTGCAATTAGGATGGAAGAATTAGGAGGTCGGAGGTTATTGCAG							[illegible]
GT	Sense	Sense	0.498	Detected	-0.498	Detected	0.000	-1.940	Detected	-1.663	Detected	-1.802	0.073	GT	Sens	contig352	contig352	Unannota	GTACACCATTCGAGCTGAACAGAAGTCTAAACACACCGACAACAAAAAA							[illegible]
GT	Sense	Sense	0.048	Detected	-0.048	Detected	0.000	-2.219	Detected	-1.953	Detected	-2.086	0.005	GT	Sens	contig353	contig353	Serine ca	ATATATA	contig353	Solyc03g	Solyc03g	Serine ca	GO:00041	GO:00041	[illegible]
GT	Sense	Sense	0.238	Detected	-0.238	Detected	0.000	-1.110	Comprom	-1.269	Comprom	-1.190	0.042	GT	Sens	contig353	contig353	Unannota	CAAAAAAGAGTGATAGTCAACATTACAAGCAGAGCCATCTAATTACTAT							[illegible]
GT	Sense	Sense	-0.157	Detected	0.157	Detected	0.000	-1.442	Comprom	-0.855	Comprom	-1.149	0.075	GT	Sens	contig353	contig353	Unannota	CTACAAAATCCAAGATGCGATTACCAAAAAGGAGCATCTCATCTAGTGC							[illegible]
GT	Sense	Sense	0.081	Detected	-0.081	Detected	0.000	-1.469	Detected	-1.057	Detected	-1.263	0.029	GT	Sens	contig353	contig353	Unannota	CTTATGGATTATTCAGTGTGAATTCCATTTCACCTTTAAATTATCATGT							[illegible]
GT	Sense	Sense	0.031	Detected	-0.031	Detected	0.000	-1.371	Detected	-1.019	Detected	-1.195	0.022	GT	Sens	contig353	contig353	Unannota	CAACATGGAGTATGCTTGTTCATCTCTTATCTTAAATCAGTAATTGTGA							[illegible]
GT	Sense	Sense	-0.048	Detected	0.048	Detected	0.000	-1.284	Detected	-1.407	Detected	-1.345	0.003	GT	Sens	contig353	contig353	Unannota	ACAGGTTAGATTCCGTTAGCACCATTTCTTGAAATTGCTTATTCTTTAT							[illegible]
GT	Sense	Sense	-0.117	Detected	0.117	Detected	0.000	-1.065	Detected	-1.350	Detected	-1.208	0.023	GT	Sens	contig353	contig353	Unannota	GCCCCCGAAGAGAAGAGATATCTAAAAAAGTTAAAACCTTCATTGCTAC							[illegible]
GT	Sense	Sense	0.640	Detected	-0.640	Detected	0.000	-1.884	Detected	-0.924	Detected	-1.404	0.222	GT	Sens	contig354	contig354	Unannota	TCTCAAGTCCAAACTTGTTGCTCAGGTGAACATTAAGTTTCACATTGGT							[illegible]
GT	Sense	Sense	0.056	Detected	-0.056	Detected	0.000	-1.230	Comprom	-1.712	Comprom	-1.471	0.027	GT	Sens	contig354	contig354	Unannota	GGCTGATGCAATGATTTAGGTTGTTATTTTTGTGGAGATTGGAGGAGC							[illegible]
GT	Sense	Sense	0.151	Detected	-0.151	Detected	0.000	-1.187	Detected	-0.837	Detected	-1.012	0.048	GT	Sens	contig354	contig354	Unannota	GTCTGGCAGTGAACGTAATTTGAGAATTTAAGATGGGTACACAATCCTA							[illegible]
GT	Sense	Sense	0.151	Detected	-0.151	Detected	0.000	-1.233	Detected	-0.881	Detected	-1.057	0.045	GT	Sens	contig355	contig355	Unannota	ATACTGTGTAGCTAGGATTCATATTCGGGGGTTTCGAAATCGAGACATA							[illegible]
GT	Sense	Sense	0.202	Detected	-0.202	Detected	0.000	-1.262	Detected	-0.860	Detected	-1.061	0.065	GT	Sens	contig355	contig355	Unannota	GGATGAAATAGTCTCGTCATTTCATTGTCTACGGTGAAACATAAAAGTT							[illegible]
GT	Sense	Sense	-0.112	Detected	0.112	Detected	0.000	-1.821	Detected	-0.935	Detected	-1.378	0.095	GT	Sens	contig355	contig355	Unannota	TTAAATTTACATGTGCACTTAAGGGTGTCAAACGGGGGCCGATTGGGC							[illegible]
GT	Sense	Sense	0.195	Detected	-0.195	Detected	0.000	-1.025	Detected	-0.977	Detected	-1.001	0.037	GT	Sens	contig355	contig355	Unannota	GGGTGTGTGGGTATTAGCAATTACAGTGATAAATTATTGAAATTGACAA							[illegible]
GT	Sense	Sense	-0.210	Detected	0.210	Detected	0.000	-1.145	Detected	-2.006	Detected	-1.576	0.081	GT	Sens	contig356	contig356	Unknown	TTTGGG	contig356	Solyc08g	Solyc08g	Unknown Protein (AHRD V1)%:			[illegible]
GT	Sense	Sense	0.548	Detected	-0.548	Detected	0.000	-1.884	Comprom	-1.062	Comprom	-1.473	0.164	GT	Sens	contig357	contig357	Unannota	CTACAGAATTTGTCCAGTAGTTATAACCTGTGATCTGAAGAGGTTATTA							[illegible]
GT	Sense	Sense	0.054	Detected	-0.054	Detected	0.000	-1.101	Detected	-1.225	Detected	-1.163	0.005	GT	Sens	contig357	contig357	Unannota	TTGCTTCTGGTAGGTTAACTTAGTCACATATACTAGCTTTTGCAGCTAT							[illegible]
GT	Sense	Sense	0.108	Detected	-0.108	Detected	0.000	-1.454	Detected	-1.315	Detected	-1.384	0.009	GT	Sens	contig357	contig357	Unannota	GGACGACTACAGTATACATGTTTCCTTCAATATTTTTCTGCTTATTCTT							[illegible]
GT	Sense	Sense	0.039	Detected	-0.039	Detected	0.000	-1.005	Detected	-1.180	Detected	-1.093	0.008	GT	Sens	contig358	contig358	Unannota	TACAAGAAGTACAAGGATCAAGGTTTGTCCAATTCTCCCGAACCAACTG							[illegible]
GT	Sense	Sense	-0.024	Detected	0.024	Detected	0.000	-1.533	Detected	-1.016	Detected	-1.275	0.039	GT	Sens	contig358	contig358	Unannota	GGCGTTCGCCCTGACATATTTCTGCAAAGAGAACTTAAATAAAATGGAT							[illegible]
GT	Sense	Sense	0.113	Detected	-0.113	Detected	0.000	-1.598	Detected	-1.288	Detected	-1.443	0.017	GT	Sens	contig358	contig358	Unannota	ATGGTGAGAACTTCAGCCAACTGGGTGGAACTAGGAAGTTGTTTTCTT							[illegible]
GT	Sense	Sense	-0.411	Detected	0.411	Detected	0.000	-0.863	Detected	-1.268	Comprom	-1.066	0.146	GT	Sens	contig358	contig358	Unannota	GGGACCAAAATCCAAAATTCACTAACTCTAACCCAGAATAGTTCCAACT							[illegible]
GT	Sense	Sense	0.143	Detected	-0.143	Detected	0.000	-1.304	Detected	-0.886	Detected	-1.095	0.050	GT	Sens	contig358	contig358	Unannota	AACGAGTATGGCATTTTGTGAGGTAGTTTTGATTTCCACATTTAGTTTT							[illegible]
GT	Sense	Sense	0.098	Detected	-0.098	Detected	0.000	-1.467	Detected	-1.037	Detected	-1.252	0.034	GT	Sens	contig358	contig358	Unannota	GCGACTTCCTTTGTTAGAGCCCTTTAGTTACTGGCTTTGCTTTATTACA							[illegible]
GT	Sense	Sense	0.230	Detected	-0.230	Detected	0.000	-1.154	Comprom	-0.888	Comprom	-1.021	0.062	GT	Sens	contig358	contig358	Unannota	ATCCTAAAATGCTTATAATTTCAAGATGAGTCGCTAGCACCGTCCCATG							[illegible]
GT	Sense	Sense	0.047	Detected	-0.047	Detected	0.000	-1.286	Comprom	-1.976	Comprom	-1.631	0.043	GT	Sens	contig359	contig359	Unannota	TTTGTAGTTGAGCTTACAGCAAGAACCAATAAAGAAGCCAAATCTCCTT							[illegible]
GT	Sense	Sense	0.217	Detected	-0.217	Detected	0.000	-1.501	Detected	-1.074	Detected	-1.288	0.052	GT	Sens	contig359	contig359	Unannota	GATAGTAGCTGCAAAATGTGCGGCGCTGCTTAAACTTGCTATTGATTTA							[illegible]
GT	Sense	Sense	0.427	Detected	-0.427	Detected	0.000	-1.928	Detected	-1.244	Detected	-1.586	0.101	GT	Sens	contig359	contig359	Unannota	GGGTCTGACAATTTTGATGATTTCGTTCAATATCAGTTGAAGAATTGGA							[illegible]
GT	Sense	Sense	-0.455	Detected	0.455	Detected	0.000	-1.676	Comprom	-1.952	Comprom	-1.814	0.062	GT	Sens	contig359	contig359	Unannota	TGCATCAGCTGGTGTTTATATGTGCTAGGAGGCGGAAGAGGATTTAGA							[illegible]
GT	Sense	Sense	0.004	Detected	-0.004	Detected	0.000	-1.876	Comprom	-1.219	Comprom	-1.547	0.042	GT	Sens	contig359	contig359	Unannota	AAATACATTGACTGGCAGGGACAACGACAGGTCTTTCGTGATGATTAT							[illegible]
GT	Sense	Sense	0.268	Detected	-0.268	Detected	0.000	-1.290	Comprom	-2.386	Comprom	-1.838	0.095	GT	Sens	contig360	contig360	Unannota	CATGTATGTGGTCGCTGAATATTTATCTTCAGTTCCTATTAATCTGTCA							[illegible]
GT	Sense	Sense	0.122	Detected	-0.122	Detected	0.000	-1.319	Detected	-1.292	Detected	-1.306	0.009	GT	Sens	contig360	contig360	Unannota	CTAGTTAATCGGTGACATAACTGCTGAACTAAGGATGATGATGTTTGTT							[illegible]
GT	Sense	Sense	0.110	Detected	-0.110	Detected	0.000	-1.595	Detected	-1.377	Detected	-1.486	0.011	GT	Sens	contig360	contig360	Unannota	AAGCTAGTGGTTTAGAAGTCTGGATGGAATAGCATACTCTTCTGACATT							[illegible]
GT	Sense	Sense	0.208	Detected	-0.208	Detected	0.000	-1.224	Detected	-0.838	Detected	-1.031	0.068	GT	Sens	contig360	contig360	Unannota	GAATTGTCCTGATAGCCTCAACTAAAACCAAACATGTTCCCTTGTCCAA							[illegible]
GT	Sense	Sense	-0.008	Detected	0.008	Detected	0.000	-1.030	Detected	-1.408	Detected	-1.219	0.023	GT	Sens	contig360	contig360	Unannota	CCTGCTATAATGTTCTTGCTTTGACCGGGGAAAAGTATCATTACTTGTT							[illegible]
GT	Sense	Sense	0.121	Detected	-0.121	Detected	0.000	-1.399	Detected	-1.547	Detected	-1.473	0.009	GT	Sens	contig360	contig360	Unannota	GATATAATTGGGTTAAAACTGTCTGTATGAGAAGTCAGTGTTACAGATT							[illegible]
GT	Sense	Sense	0.094	Detected	-0.094	Detected	0.000	-1.366	Detected	-1.484	Detected	-1.425	0.006	GT	Sens	contig360	contig360	Unannota	TTTCTCAATTGTTGCAAGTGGAAGTGGTGATATATCCTGGTTGTTGACA							[illegible]
GT	Sense	Sense	0.363	Detected	-0.363	Detected	0.000	-2.053	Detected	-1.540	Detected	-1.796	0.056	GT	Sens	contig361	contig361	Malate sy	AGCTAT	contig361	Solyc03g	Solyc03g	Malate sy	GO:0006	GO:0006	[illegible]
GT	Sense	Sense	0.352	Detected	-0.352	Detected	0.000	-1.078	Detected	-1.699	Comprom	-1.389	0.098	GT	Sens	contig361	contig361	Unannota	GAGAATATATTTGGTAACTCCAGATAAACATGTCATATATAAACAATAG							[illegible]
GT	Sense	Sense	0.220	Detected	-0.220	Detected	0.000	-1.683	Detected	-1.294	Detected	-1.489	0.037	GT	Sens	contig361	contig361	Unannota	GGCTTCAGTCCATCTTGTACTGATTCTTGGAGTGCATTATTGTTTATAT							[illegible]
GT	Sense	Sense	0.188	Detected	-0.188	Detected	0.000	-1.535	Detected	-1.049	Detected	-1.292	0.052	GT	Sens	contig361	contig361	Unannota	CCCACAAAGAGAGACAATAACAAGCTCAAACAACGAGAATCTTTATAGT							[illegible]
GT	Sense	Sense	0.736	Detected	-0.736	Detected	0.000	-1.754	Detected	-1.855	Detected	-1.805	0.134	GT	Sens	contig362	contig362	Unannota	TAACATTATACTGATAGTATGATAGTATAAACCATAACTCTTTATACTAT							[illegible]
GT	Sense	Sense	0.689	Detected	-0.689	Detected	0.000	-2.423	Detected	-2.045	Detected	-2.234	0.089	GT	Sens	contig362	contig362	Unannota	TTGAGTCAGAATAACATGACTTTTTGTTGGTTGAGCCCTAATGTGTTGC							[illegible]
GT	Sense	Sense	-0.036	Detected	0.036	Detected	0.000	-1.234	Detected	-1.096	Detected	-1.165	0.004	GT	Sens	contig362	contig362	Unannota	TAACTAGGTAACTTCACACATTCGAAGTACTCATGAACTTAGCTTTACG							[illegible]
GT	Sense	Sense	0.010	Detected	-0.010	Detected	0.000	-1.362	Detected	-1.158	Detected	-1.260	0.007	GT	Sens	contig362	contig362	Unannota	CCGGAGATTGTGAATTCCTCGGCTATTTATGTTAACATATCATGTATAT							[illegible]
GT	Sense	Sense	0.078	Detected	-0.078	Detected	0.000	-1.070	Detected	-1.177	Detected	-1.123	0.007	GT	Sens	contig362	contig362	Unannota	CAGATGAAACATGGTTTGTTTGTCTGAAATAGATAACTGAAGCCAGTTG							[illegible]
GT	Sense	Sense	0.310	Detected	-0.310	Detected	0.000	-1.458	Detected	-1.334	Detected	-1.396	0.048	GT	Sens	contig362	contig362	Unannota	TGAAGGAATTGCATTTGACTCTTACCAGAATTCTACTTTTAAGTTTAGG							[illegible]
GT	Sense	Sense	-0.011	Detected	0.011	Detected	0.000	-1.251	Detected	-0.985	Detected	-1.118	0.014	GT	Sens	contig363	contig363	Unannota	TTGTTATAAGCATCCTTTTCTTCCTCCCACTCAAGAGCACAGGATTCCC							[illegible]
GT	Sense	Sense	0.206	Detected	-0.206	Detected	0.000	-1.683	Detected	-1.528	Detected	-1.606	0.018	GT	Sens	contig363	contig363	Ethylene-	ATTAAT	contig363	Solyc11g	Solyc11g	Ethylene-	GO:00063	GO:00063	[illegible]
GT	Sense	Sense	0.220	Detected	-0.220	Detected	0.000	-2.142	Detected	-1.873	Detected	-2.007	0.016	GT	Sens	contig364	contig364	Unannota	AGACACGACAACAATTTTAAAGAGTCCAAGCACAAAAAAAGTAGAGTGG							[illegible]
GT	Sense	Sense	0.247	Detected	-0.247	Detected	0.000	-2.147	Detected	-1.780	Detected	-1.964	0.024	GT	Sens	contig364	contig364	Unannota	GAAATAAAAATGTATGGTTAGTTACTTTATTAACCTATTTAAGTAGGGA							[illegible]
GT	Sense	Sense	-1.863	Detected	1.863	Detected	0.000	-1.822	Detected	-1.067	Detected	-1.444	0.527	GT	Sens	contig364	contig364	Unannota	CCTGAATATAGCATAAACAACAACAACATACATGGTCTAGTCCTACAAA							[illegible]
GT	Sense	Sense	0.276	Detected	-0.276	Detected	0.000	-1.398	Comprom	-1.497	Comprom	-1.447	0.036	GT	Sens	contig364	contig364	Unannota	GCAAGAGATTTTAACACAAGACGAGGCCGTTTCATCAGAATTTACTACA							[illegible]
GT	Sense	Sense	-0.114	Detected	0.114	Detected	0.000	-1.906	Comprom	-1.483	Comprom	-1.694	0.020	GT	Sens	contig364	contig364	Unannota	TTTGGCGTAACTAGTAAAATTGTTGCCATGTGACTTGATGGTCATGGT							[illegible]
GT	Sense	Sense	-0.043	Detected	0.043	Detected	0.000	-1.124	Detected	-1.132	Detected	-1.128	0.001	GT	Sens	contig365	contig365	Unannota	CTCTTATCTTGTTCGCCAGTACTCTTATATGTGTCTGGTATAGCAGATA							[illegible]
GT	Sense	Sense	0.152	Detected	-0.152	Detected	0.000	-1.435	Detected	-1.705	Detected	-1.570	0.016	GT	Sens	contig365	contig365	Unannota	GGATGATTCAAGTTGTCGTCATTGTTGTGATCTTTCTACTTCTTGTCTA							[illegible]
GT	Sense	Sense	0.219	Detected	-0.219	Detected	0.000	-2.752	Detected	-1.798	Detected	-2.275	0.049	GT	Sens	contig365	contig365	Prephena	CTATTCA	contig365	Solyc06g	Solyc06g	Prephena	GO:00065	GO:00065	[illegible]
GT	Sense	Sense	0.166	Detected	-0.166	Detected	0.000	-1.426	Detected	-1.151	Detected	-1.288	0.027	GT	Sens	contig366	contig366	Alpha-1 6	TCCTAAT	contig366	Solyc01g	Solyc01g	Alpha-1 6	GO:00160	GO:00160	[illegible]
GT	Sense	Sense	0.042	Detected	-0.042	Detected	0.000	-1.967	Detected	-1.016	Detected	-1.491	0.089	GT	Sens	contig367	contig367	Unannota	AGTCGAAATCGAATCTCAGGTGTTGGTTCGGATTCTGAATCGATCGTC							[illegible]
GT	Sense	Sense	-0.074	Detected	0.074	Detected	0.000	-2.169	Detected	-1.183	Detected	-1.676	0.078	GT	Sens	contig367	contig367	Unannota	TTTGAAAGTTGGGTATCAGGTTGCGCGTCAAGGTCATATTTCGATTTGA							[illegible]
GT	Sense	Sense	0.123	Detected	-0.123	Detected	0.000	-1.197	Detected	-1.167	Detected	-1.182	0.011	GT	Sens	contig367	contig367	Unannota	GCACCAAATCTATGAAGATACATCGAAGGAACGATTCTTCAAACACGAA							[illegible]
GT	Sense	Sense	0.038	Detected	-0.038	Detected	0.000	-1.303	Detected	-1.459	Detected	-1.381	0.004	GT	Sens	contig367	contig367	Unannota	GACCGTCAAGAGGAAAGATTCCACAAGCAAGAAACCTCATCAACATTAT							[illegible]
GT	Sense	Sense	-0.013	Detected	0.013	Detected	0.000	-1.318	Detected	-1.490	Detected	-1.404	0.004	GT	Sens	contig370	contig370	Peptide tr	AAAGCT	contig370	Solyc09g	Solyc09g	Peptide tr	GO:00429	GO:00429	[illegible]
GT	Sense	Sense	-0.094	Detected	0.094	Detected	0.000	-1.364	Comprom	-1.198	Comprom	-1.281	0.009	GT	Sens	contig370	contig370	Unannota	CGGTAGTTACTTCAGATCTCTGACCTCATTTCACTATAGATGCACAAAC							[illegible]
GT	Sense	Sense	0.208	Detected	-0.208	Detected	0.000	-1.163	Detected	-1.040	Detected	-1.102	0.037	GT	Sens	contig371	contig371	Unannota	GTCTCCTTCCCTTAGAAGCCAATGGCCGATTTTTGGTATTAGAGGTGC							[illegible]
GT	Sense	Sense	0.097	Detected	-0.097	Detected	0.000	-1.754	Comprom	-1.691	Comprom	-1.722	0.003	GT	Sens	contig371	contig371	Unannota	ATGTTGACGTGCTGCTCAATGGAACGGTGTCAGTTGACACCACTTGAA							[illegible]
GT	Sense	Sense	0.412	Detected	-0.412	Detected	0.000	-1.619	Detected	-1.363	Detected	-1.491	0.075	GT	Sens	contig371	contig371	Unannota	TATTGATCAGTCTACCCGATTTGAACTGGGGAAAAAAGGATTTGCACAA							[illegible]
GT	Sense	Sense	0.526	Detected	-0.526	Detected	0.000	-3.893	Comprom	-3.799	Comprom	-3.846	0.018	GT	Sens	contig371	contig371	Unannota	GGTATCATTGCATCTTTGAGACTTCACACTCGTATTGATGCAATAAAAA							[illegible]
GT	Sense	Sense	0.719	Detected	-0.719	Detected	0.000	-3.041	Comprom	-3.621	Comprom	-3.331	0.050	GT	Sens	contig371	contig371	Unannota	CTGACTGATAAGATAGTAGCAAGTGCTAAGAAAAATGCTATCAAAAGTT							[illegible]
GT	Sense	Sense	0.371	Detected	-0.371	Detected	0.000	-1.803	Detected	-1.148	Detected	-1.475	0.096	GT	Sens	contig372	contig372	Unannota	TGAAAATGGGAAGTCTGAGTTGGTTTAGAAATTTGCTTTCAGCTGTTG							[illegible]
GT	Sense	Sense	0.162	Detected	-0.162	Detected	0.000	-1.326	Detected	-0.898	Detected	-1.112	0.054	GT	Sens	contig372	contig372	Unannota	GTTGCAAGTTATGTGCTGCACGAGTTCTATCATACTATTATGTAATCTT							[illegible]
GT	Sense	Sense	-0.188	Detected	0.188	Detected	0.000	-1.015	Detected	-1.013	Detected	-1.014	0.033	GT	Sens	contig372	contig372	Unannota	TTGGAGGATTCATGTTTTAAACTACCGTTTGACTCTAGTCTTCCATATT							[illegible]
GT	Sense	Sense	0.110	Detected	-0.110	Detected	0.000	-1.558	Detected	-2.397	Detected	-1.977	0.045	GT	Sens	contig372	contig372	Unannota	TGTGTGACAGTTTGAAGTACAACCACAACATACCATGTGTAACAATGTG							[illegible]
GT	Sense	Sense	0.167	Detected	-0.167	Detected	0.000	-0.927	Detected	-1.254	Detected	-1.091	0.043	GT	Sens	contig373	contig373	Dynamin	AAGTAT	contig373	Solyc10g	Solyc10g	Dynamin	GO:00055	GO:00055	[illegible]
GT	Sense	Sense	0.358	Detected	-0.358	Detected	0.000	-2.064	Detected	-1.017	Detected	-1.541	0.136	GT	Sens	contig373	contig373	Unannota	GAAAAATAGTTAGATTTGCAATAGAGATGGAGACGTGAAGGTATGCTG							[illegible]
GT	Sense	Sense	0.228	Detected	-0.228	Detected	0.000	-2.315	Detected	-1.962	Detected	-2.139	0.018	GT	Sens	contig373	contig373	Glycerol-	AGAGAA	contig373	Solyc04g	Solyc04g	Glycerol-	GO:00512	GO:00512	[illegible]
GT	Sense	Sense	0.061	Detected	-0.061	Detected	0.000	-1.770	Comprom	-1.093	Comprom	-1.432	0.053	GT	Sens	contig374	contig374	Unannota	GCTAATTAAGCCATCTGCTTTGTCCTTCCCATGCTCTTATGAGATGGTA							[illegible]
GT	Sense	Sense	0.121	Detected	-0.121	Detected	0.000	-1.423	Detected	-1.123	Detected	-1.273	0.022	GT	Sens	contig375	contig375	Unannota	ACTCCTCAAGAACGCAGTAGATAACTTAATCTACGATGTAGTTGCATGT							[illegible]
GT	Sense	Sense	-0.064	Detected	0.064	Detected	0.000	-1.643	Detected	-1.710	Detected	-1.676	0.002	GT	Sens	contig375	contig375	AP2-like e	TTAATT	contig375	Solyc11g	Solyc11g	AP2-like e	GO:00036	GO:00036	[illegible]
GT	Sense	Sense	0.010	Detected	-0.010	Detected	0.000	-0.988	Comprom	-1.396	Comprom	-1.192	0.028	GT	Sens	contig375	contig375	Solute ca	ATGATAT	contig375	Solyc11g	Solyc11g	Solute ca	GO:00160	GO:00160	[illegible]
GT	Sense	Sense	-0.122	Detected	0.122	Detected	0.000	-1.584	Detected	-1.440	Detected	-1.512	0.009	GT	Sens	contig375	contig375	NBS-LRR	GCTGCA	contig375	Solyc07g	Solyc07g	NBS-LRR class disease resista			[illegible]
GT	Sense	Sense	0.226	Detected	-0.226	Detected	0.000	-1.111	Detected	-1.131	Detected	-1.121	0.038	GT	Sens	contig375	contig375	Unannota	TTGTTGTCCCTGCTACCCGACAATTTTGGGCAATCGATCTCTCAAAGAT							[illegible]
GT	Sense	Sense	-0.017	Detected	0.017	Detected	0.000	-1.030	Detected	-1.320	Detected	-1.175	0.015	GT	Sens	contig375	contig375	Unannota	CTCACCATTCACGCTTATCTATTGCAATTCTCATAAATTTTGCAAGTTTG							[illegible]
GT	Sense	Sense	0.106	Detected	-0.106	Detected	0.000	-0.918	Detected	-1.282	Detected	-1.100	0.035	GT	Sens	contig375	contig375	Unannota	GAGGATATACCACTTCAAAATCACAAAGTTGAGGGTAAATCGAATCGTC							[illegible]
GT	Sense	Sense	0.163	Detected	-0.163	Detected	0.000	-1.269	Detected	-0.823	Detected	-1.046	0.063	GT	Sens	contig376	contig376	Unannota	GATTGATATTGATCAAACTTCTGGACCATTTTGTTCTTGATCTTTTCAC							[illegible]
GT	Sense	Sense	0.140	Detected	-0.140	Detected	0.000	-1.514	Detected	-1.515	Detected	-1.514	0.008	GT	Sens	contig376	contig376	Unannota	GGGTCTGGGGTTAATGGTGTGTATGCTAATGTCATCAACAATAATAACA							[illegible]
GT	Sense	Sense	0.721	Detected	-0.721	Detected	0.000	-2.049	Comprom	-2.233	Comprom	-2.141	0.099	GT	Sens	contig376	contig376	Unannota	GAAGAGTTTTTGGTGTATCAAAAGCATCTACTATATCCATCCCAGGTAA							[illegible]
GT	Sense	Sense	0.260	Detected	-0.260	Detected	0.000	-2.079	Detected	-1.469	Detected	-1.774	0.047	GT	Sens	contig377	contig377	Unannota	TGTCTTGGAATTTTAGCAACATCAACTCACTTCAATTAGGTTGGTTATT							[illegible]
GT	Sense	Sense	0.263	Detected	-0.263	Detected	0.000	-2.709	Detected	-1.935	Detected	-2.322	0.038	GT	Sens	contig377	contig377	Unannota	CCCACATTAGTTACCAAATGTACCTGTGGCTGTGAATCATAGTTAAAAT							[illegible]
GT	Sense	Sense	0.227	Detected	-0.227	Detected	0.000	-1.267	Detected	-1.008	Detected	-1.137	0.049	GT	Sens	contig377	contig377	Unannota	CGGTACCAAGGAGTTCATTGTCTACTGTACATACAAAAGAATCAATGTT							[illegible]
GT	Sense	Sense	0.028	Detected	-0.028	Detected	0.000	-1.397	Detected	-0.984	Detected	-1.191	0.029	GT	Sens	contig377	contig377	Porin/vol	AACAAG	contig377	Solyc03g	Solyc03g	Porin/vol	GO:00055	GO:00055	[illegible]
GT	Sense	Sense	0.211	Detected	-0.211	Detected	0.000	-2.672	Detected	-1.952	Detected	-2.312	0.031	GT	Sens	contig377	contig377	Ethylene-	CTTTGAT	contig377	Solyc11g	Solyc11g	Ethylene-	GO:00063	GO:00063	[illegible]
GT	Sense	Sense	0.110	Detected	-0.110	Detected	0.000	-1.341	Detected	-1.242	Detected	-1.291	0.009	GT	Sens	contig377	contig377	Unannota	TAACTATCCAGTTAAAGGCACATTCGTGGGATGCTGATGGAAACGACTC							[illegible]
GT	Sense	Sense	0.110	Detected	-0.110	Detected	0.000	-1.837	Detected	-1.005	Detected	-1.421	0.081	GT	Sens	contig378	contig378	Unannota	GGTGACGGGTCATATGTTAATTAGCATTATGGAAGCTTATTAACAATCG							[illegible]
GT	Sense	Sense	-0.031	Detected	0.031	Detected	0.000	-1.479	Detected	-0.919	Detected	-1.199	0.051	GT	Sens	contig378	contig378	Unannota	GCCACTAGCCCTAGGGGATTTTTCAGTTATAAAGTAGAAGTACATTAAA							[illegible]
GT	Sense	Sense	0.117	Detected	-0.117	Detected	0.000	-1.838	Detected	-1.407	Detected	-1.622	0.022	GT	Sens	contig378	contig378	Unannota	TTGAATGTGGAATTTGAGATAGGGCTCCTGTTTGACCTTTATTTACTAC							[illegible]
GT	Sense	Sense	0.085	Detected	-0.085	Detected	0.000	-1.642	Detected	-1.201	Detected	-1.422	0.027	GT	Sens	contig378	contig378	Adenylyl	GGTTCG	contig378	Solyc01g	Solyc01g	Adenylyl	GO:00037	GO:00037	[illegible]
GT	Sense	Sense	-0.027	Detected	0.027	Detected	0.000	-1.280	Detected	-1.617	Detected	-1.448	0.014	GT	Sens	contig379	contig379	Unannota	CCACTGGGTATATGTTGTTGTAGTTGCTTTGTTTAATGATCTTTGTTTT							[illegible]
GT	Sense	Sense	0.022	Detected	-0.022	Detected	0.000	-1.152	Detected	-1.411	Detected	-1.282	0.010	GT	Sens	contig379	contig379	Unannota	AACTTGAAATATGCCGGCGAAAGTCAAAAGGGTGCATATATTTTTATGT							[illegible]
GT	Sense	Sense	0.208	Detected	-0.208	Detected	0.000	-1.933	Detected	-0.914	Detected	-1.423	0.123	GT	Sens	contig379	contig379	Unannota	ATAGCCTTAAATGACAGTCCTGACTCTGCTACTAGCCAATAGCTAACGC							[illegible]
GT	Sense	Sense	0.132	Detected	-0.132	Detected	0.000	-1.477	Detected	-1.069	Detected	-1.273	0.035	GT	Sens	contig379	contig379	Unannota	GAAGTGTGAATGCTTCTATTTGCTGCTTGGAGGGTAAAACTTGTTTCTC							[illegible]
GT	Sense	Sense	-0.008	Detected	0.008	Detected	0.000	-1.390	Comprom	-1.703	Comprom	-1.547	0.010	GT	Sens	contig379	contig379	Unannota	CTTCCACGTAAAGTACAACTCTGTTTGTATTGTTTATGCTTTCTGACTT							[illegible]
GT	Sense	Sense	-0.025	Detected	0.025	Detected	0.000	-1.254	Comprom	-0.987	Comprom	-1.120	0.014	GT	Sens	contig379	contig379	Unannota	CGCGCCTTTAATTGTTGTTTGAAGTGCTAGTCTTACTTTCTTAATTTGG							[illegible]
GT	Sense	Sense	-0.221	Detected	0.221	Detected	0.000	-1.173	Comprom	-0.946	Comprom	-1.060	0.051	GT	Sens	contig379	contig379	Unannota	TAAGGAGGTTTATGTTCACACTCACGGCTTAAGGCCACCAGGCCCAAA							[illegible]
GT	Sense	Sense	0.066	Detected	-0.066	Detected	0.000	-1.036	Detected	-1.031	Detected	-1.034	0.004	GT	Sens	contig380	contig380	Unannota	AGTCTGAGGGAAGTCCCATGTAATGAATTCTTGTTTCATGCATGTTCTC							[illegible]
GT	Sense	Sense	0.074	Detected	-0.074	Detected	0.000	-1.116	Detected	-1.302	Detected	-1.209	0.010	GT	Sens	contig380	contig380	Unannota	ATCAAAATCACAATCAAATATTCATGGAAGAGTGCAACTATATATTCGCA							[illegible]
GT	Sense	Sense	0.304	Detected	-0.304	Detected	0.000	-1.410	Detected	-1.111	Detected	-1.260	0.065	GT	Sens	contig380	contig380	Unannota	ACGAACTCATCGTTGAAATTCTGAGAAAACTTCCTGCTAAGTCTCTAAT							[illegible]
GT	Sense	Sense	-0.062	Detected	0.062	Detected	0.000	-1.234	Detected	-0.903	Detected	-1.068	0.026	GT	Sens	contig380	contig380	Phosphat	ATCAAG	contig380	Solyc05g	Solyc05g	Phosphat	GO:00038	GO:00038	[illegible]
GT	Sense	Sense	0.057	Detected	-0.057	Detected	0.000	-2.233	Comprom	-1.540	Comprom	-1.887	0.033	GT	Sens	contig381	contig381	Unannota	GTTTTATAGACTGCAGGAAAATTTGATTCAGCAATTACTGGGACACACT							[illegible]
GT	Sense	Sense	0.202	Detected	-0.202	Detected	0.000	-1.859	Comprom	-0.913	Comprom	-1.386	0.115	GT	Sens	contig381	contig381	Unannota	CACTAATGGGGAGGTTGTTCTTTGAATTCTTGCAATAAAAAGATAATGA							[illegible]
GT	Sense	Sense	0.093	Detected	-0.093	Detected	0.000	-1.143	Detected	-0.949	Detected	-1.046	0.016	GT	Sens	contig381	contig381	Unannota	CTCTTTCTCGAACCTTACATAAACGGGATGTTTTATGTACTGAACTGGA							[illegible]
GT	Sense	Sense	0.158	Detected	-0.158	Detected	0.000	-1.308	Detected	-1.018	Detected	-1.163	0.032	GT	Sens	contig381	contig381	Unannota	GGAGGAGATGGGTTGAAGGATATATGGTTTTATTTTTAGTTATTTGCAT							[illegible]
GT	Sense	Sense	0.015	Detected	-0.015	Detected	0.000	-1.440	Detected	-1.615	Detected	-1.527	0.003	GT	Sens	contig381	contig381	E3 ubiqui	ATTTTCA	contig381	Solyc08g	Solyc08g	E3 ubiqui	GO:00055	GO:00055	[illegible]
GT	Sense	Sense	-0.023	Detected	0.023	Detected	0.000	-1.290	Detected	-0.962	Detected	-1.126	0.021	GT	Sens	contig382	contig382	Early nod	AAGGGT	contig382	Solyc02g	Solyc02g	Early nod	GO:00090	GO:00090	[illegible]
GT	Sense	Sense	0.231	Detected	-0.231	Detected	0.000	-0.974	Comprom	-1.866	Comprom	-1.420	0.106	GT	Sens	contig382	contig382	Unannota	GGCGAGAAAAAAACCTGGGAGGTAAGTTTCGGCTACTGAATGGGTTTG							[illegible]
GT	Sense	Sense	0.107	Detected	-0.107	Detected	0.000	-1.039	Detected	-1.152	Detected	-1.096	0.012	GT	Sens	contig382	contig382	Unannota	TTTAGGATTCCATGGTTATGGGGAAAAGATTGATTTTGCAAAGGCTGA							[illegible]
GT	Sense	Sense	0.002	Detected	-0.002	Detected	0.000	-1.175	Detected	-1.086	Detected	-1.131	0.002	GT	Sens	contig382	contig382	Unannota	TGTAGTTTTGCAACAGCAGGGGAAGGTTTTCGGTTGGGTTTTCGTTGA							[illegible]
GT	Sense	Sense	0.199	Detected	-0.199	Detected	0.000	-0.867	Detected	-1.172	Detected	-1.019	0.056	GT	Sens	contig383	contig383	Unannota	CCCATAGTACTTGTCTCCTTGTTTCTTGAAAATCCCACAAAAAATTGGG							[illegible]
GT	Sense	Sense	-0.039	Detected	0.039	Detected	0.000	-1.727	Detected	-1.062	Detected	-1.395	0.053	GT	Sens	contig383	contig383	Unannota	GCTCCCTTTGGTTCACACATAATTCCGGCTCTGTTTAATCTCCTCACTG							[illegible]
GT	Sense	Sense	-0.226	Detected	0.226	Detected	0.000	-1.535	Detected	-1.753	Comprom	-1.644	0.022	GT	Sens	contig383	contig383	Unannota	CCTGTCACTTTCTGCGAAAAGACTTTGCTAACAATGGAACAGACTAATT							[illegible]
GT	Sense	Sense	0.140	Detected	-0.140	Detected	0.000	-1.802	Detected	-1.883	Detected	-1.842	0.006	GT	Sens	contig383	contig383	Unknown	TTGTCT	contig383	Solyc07g	Solyc07g	Unknown Protein (AHRD V1)%:			[illegible]
GT	Sense	Sense	0.158	Detected	-0.158	Detected	0.000	-1.189	Detected	-1.418	Detected	-1.303	0.022	GT	Sens	contig383	contig383	Unannota	ACAAGAAGAAGGTTCTGTTAATTCGACGGTGAAGCGGAATTTGAGCTTC							[illegible]
GT	Sense	Sense	0.133	Detected	-0.133	Detected	0.000	-1.199	Detected	-1.474	Detected	-1.337	0.020	GT	Sens	contig383	contig383	Unannota	GAGAAACTTGAGTTTCAACGTGCCGATTCAACCACCGAGATCAATTTCC							[illegible]
GT	Sense	Sense	0.151	Detected	-0.151	Detected	0.000	-1.450	Detected	-1.180	Detected	-1.315	0.023	GT	Sens	contig383	contig383	Aquapori	CTTGTCA	contig383	Solyc03g	Solyc03g	Aquapori	GO:00160	GO:00160	[illegible]
GT	Sense	Sense	-0.003	Detected	0.003	Detected	0.000	-1.247	Detected	-0.991	Detected	-1.119	0.013	GT	Sens	contig384	contig384	Unannota	ATTGAAAAGTGTTGAACAACACAAAGTTCCCTTGGTCTGATTTGTAGAA							[illegible]
GT	Sense	Sense	0.150	Detected	-0.150	Detected	0.000	-0.913	Detected	-1.425	Comprom	-1.169	0.059	GT	Sens	contig384	contig384	Unannota	CCTCTTTCGATTATCAATGGAAACGTTCAACTTGAGCTAAATCAGGTTT							[illegible]
GT	Sense	Sense	0.155	Detected	-0.155	Detected	0.000	-1.160	Detected	-0.881	Detected	-1.020	0.039	GT	Sens	contig384	contig384	Unannota	CTTCCTCTTCTGATTAGAGCATCAACAATCAATTAAAACTACTATCCAC							[illegible]
GT	Sense	Sense	0.235	Detected	-0.235	Detected	0.000	-0.890	Detected	-1.554	Detected	-1.222	0.095	GT	Sens	contig384	contig384	Unannota	GGACGAAATTGGGGTTGTATTGTGTTATTTAATGATGGGTATTGAGTAA							[illegible]
GT	Sense	Sense	0.114	Detected	-0.114	Detected	0.000	-1.087	Detected	-1.361	Detected	-1.224	0.021	GT	Sens	contig384	contig384	Unannota	TGGTTAGTGTTGCTGAATATAGATTACACAAAATGGAAAGGGATGGAAC							[illegible]
GT	Sense	Sense	0.576	Detected	-0.576	Detected	0.000	-2.008	Detected	-2.504	Comprom	-2.256	0.069	GT	Sens	contig384	contig384	C4-dicarb	ACTGCT	contig384	Solyc07g	Solyc07g	C4-dicarb	GO:00160	GO:00160	[illegible]
GT	Sense	Sense	0.171	Detected	-0.171	Detected	0.000	-1.638	Detected	-1.396	Detected	-1.517	0.019	GT	Sens	contig386	contig386	Unannota	CAGACCTTACACAATCTGCACTGTTTTCCTTTTTACTTGTCATGCTTTC							[illegible]
GT	Sense	Sense	0.256	Detected	-0.256	Detected	0.000	-4.186	Detected	-3.319	Detected	-3.753	0.018	GT	Sens	contig386	contig386	WRKY tra	TGGCTG	contig386	Solyc03g	Solyc03g	WRKY tra	GO:0045449		[illegible]
GT	Sense	Sense	-0.309	Detected	0.309	Detected	0.000	-1.469	Comprom	-1.043	Comprom	-1.256	0.079	GT	Sens	contig386	contig386	Unannota	CCCATTTTGAATCAGTACTTGGTAGAGAAGACCATTTAAACGAAAAGAA							[illegible]
GT	Sense	Sense	1.136	Detected	-1.136	Detected	0.000	-1.872	Detected	-2.272	Comprom	-2.072	0.214	GT	Sens	contig387	contig387	Unannota	GACTTGATGATAGCAAGGATGCATGCAACAGCTGCATGATGCATTTGTA							[illegible]
GT	Sense	Sense	0.236	Detected	-0.236	Detected	0.000	-1.619	Detected	-0.858	Detected	-1.239	0.109	GT	Sens	contig387	contig387	Unannota	GGATTTTCACACTGAAAGCAAGTGTGTTATTGATTTCTGATGTTTCATT							[illegible]
GT	Sense	Sense	0.282	Detected	-0.282	Detected	0.000	-1.732	Detected	-0.986	Detected	-1.359	0.101	GT	Sens	contig387	contig387	Unannota	GATGGTCTGAACTGGTAAAATCTGTTTGTTTATCAACTTTGGTATTAGT							[illegible]
GT	Sense	Sense	0.613	Detected	-0.613	Detected	0.000	-4.103	Comprom	-1.420	Comprom	-2.762	0.202	GT	Sens	contig387	contig387	Unannota	ATAGCGAATGTGTTTGTTCTCTATTGGCTCATCTGCCTATTTTCCTTTC							[illegible]
GT	Sense	Sense	0.083	Detected	-0.083	Detected	0.000	-1.181	Detected	-0.900	Detected	-1.040	0.024	GT	Sens	contig387	contig387	Unannota	CAGCATATAGAACTGGCAGCTAGCAACCCCAAAAGGATTTTAAAGAGAA							[illegible]
GT	Sense	Sense	0.028	Detected	-0.028	Detected	0.000	-1.421	Detected	-1.021	Detected	-1.221	0.026	GT	Sens	contig387	contig387	Unannota	ACTAGTAAGATGGTTAACAGGACGACGAAGAGGAAAATTAGGATTCCGC							[illegible]
GT	Sense	Sense	-0.015	Detected	0.015	Detected	0.000	-1.395	Detected	-1.035	Detected	-1.215	0.021	GT	Sens	contig387	contig387	Unannota	TTTCGTTTTTTGTCTTTGTGGTGAGTGCACTCCTTGACTGACATGGTG							[illegible]
GT	Sense	Sense	-0.035	Detected	0.035	Detected	0.000	-1.903	Comprom	-1.632	Comprom	-1.768	0.006	GT	Sens	contig388	contig388	Unannota	TGTGATCCTTCTTTTGTTCAATGTTAATGCCAGACGCTTTGTGCACCAA							[illegible]
GT	Sense	Sense	-0.064	Detected	0.064	Detected	0.000	-1.231	Comprom	-0.958	Comprom	-1.095	0.018	GT	Sens	contig388	contig388	Unannota	TTCTTGCTGAAGATAATGGCACCCCCATTTTGTGGTTTAGCTTAAAGTT							[illegible]
GT	Sense	Sense	0.021	Detected	-0.021	Detected	0.000	-1.027	Detected	-1.150	Detected	-1.088	0.004	GT	Sens	contig388	contig388	Unannota	AAGGGTTGAGACTCCGAAGAAAAGCAATGAAATGGTTAAGCCTAGTCTC							[illegible]
GT	Sense	Sense	0.000	Detected	0.000	Detected	0.000	-0.913	Detected	-1.331	Detected	-1.122	0.033	GT	Sens	contig388	contig388	Unannota	AGATATACTGGTGGCACCAGTGAGTTACAAAGTCAGAGCAGCGGTGAT							[illegible]
GT	Sense	Sense	0.121	Detected	-0.121	Detected	0.000	-1.280	Detected	-1.304	Detected	-1.292	0.009	GT	Sens	contig389	contig389	Os01g09	GTACACT	contig389	Solyc06g	Solyc06g	Os01g09	GO:00038	GO:00038	[illegible]
GT	Sense	Sense	0.053	Detected	-0.053	Detected	0.000	-1.901	Detected	-1.442	Detected	-1.671	0.019	GT	Sens	contig389	contig389	Unannota	GGAAGATTCATCTGATACATCAGAAGAAGATATTCCTGATAACTGTTCT							[illegible]
GT	Sense	Sense	0.162	Detected	-0.162	Detected	0.000	-1.527	Detected	-1.094	Detected	-1.310	0.040	GT	Sens	contig389	contig389	Unannota	TACTTGGTGTTGTTACTTGTTGAGTTAATTACTTCTCTAGAATCTTAAG							[illegible]
GT	Sense	Sense	-0.119	Detected	0.119	Detected	0.000	-1.099	Comprom	-1.287	Comprom	-1.193	0.016	GT	Sens	contig389	contig389	Unannota	TCAGGAAATTACTCGATTGACGCCTACTGCTGCAATTTCCAGACTCATT							[illegible]
GT	Sense	Sense	-0.320	Detected	0.320	Detected	0.000	-1.656	Detected	-0.897	Detected	-1.277	0.124	GT	Sens	contig389	contig389	Unannota	TGGAGGAGTATCTGGCACTTTCTTAAATTACATCATCACTTCGCCTTAC							[illegible]
GT	Sense	Sense	0.294	Detected	-0.294	Detected	0.000	-3.483	Detected	-3.431	Detected	-3.457	0.007	GT	Sens	contig389	contig389	Unannota	ATCTATCTTATGACTAGTACTATTAAGCAGTGATGAATGTCTTATGTGT							[illegible]
GT	Sense	Sense	0.211	Detected	-0.211	Detected	0.000	-3.639	Detected	-3.650	Detected	-3.645	0.003	GT	Sens	contig389	contig389	Unannota	GATGAATGGACATATTTGAGCTCTATTTACTCCCAAGTATATGTATCAA							[illegible]
GT	Sense	Sense	-0.138	Detected	0.138	Detected	0.000	-1.166	Comprom	-1.006	Comprom	-1.086	0.021	GT	Sens	contig390	contig390	Unannota	AACGTATGCAAAACATTAGAAAACGTGCTACTAAATTCCTACAGCACAT							[illegible]
GT	Sense	Sense	-0.431	Detected	0.431	Detected	0.000	-1.510	Detected	-1.252	Detected	-1.381	0.092	GT	Sens	contig390	contig390	Unannota	GACGATCAAAGTTAACCCGCAATGAGCTTACTCTCACTCGTAGCCCCTC							[illegible]
GT	Sense	Sense	0.260	Detected	-0.260	Detected	0.000	-2.629	Detected	-1.656	Detected	-2.142	0.060	GT	Sens	contig391	contig391	Unannota	CCCCAAAAAAGATAATGTGTCCTCCTCTACAAATATTCTTTCGGTCTTT							[illegible]
GT	Sense	Sense	0.333	Detected	-0.333	Detected	0.000	-2.815	Detected	-1.651	Detected	-2.233	0.080	GT	Sens	contig391	contig391	Unannota	TTGAGCTCTTTGTTCGTCATTTGGAGTTCTAGTTATTTTTTCCCCCTTT							[illegible]
GT	Sense	Sense	-0.037	Detected	0.037	Detected	0.000	-1.392	Detected	-1.110	Detected	-1.251	0.013	GT	Sens	contig391	contig391	Unannota	GGTTAGAGTCTAATTAGTCTCAAAAGTTTCTGCCCTTTGGAAATAAAGG							[illegible]
GT	Sense	Sense	-0.035	Detected	0.035	Detected	0.000	-1.284	Comprom	-0.911	Comprom	-1.097	0.029	GT	Sens	contig391	contig391	Unannota	ACATTCTGAACTCACTAGTGGTAGATCTTGGAAATGCCTCTTCCTAAAG							[illegible]
GT	Sense	Sense	0.019	Detected	-0.019	Detected	0.000	-1.519	Detected	-1.088	Detected	-1.304	0.027	GT	Sens	contig392	contig392	Unannota	GCCTTTTAAGGTGTTTATCGACTTTGTTGATTGTTAGGTCACAATCTTA							[illegible]
GT	Sense	Sense	0.011	Detected	-0.011	Detected	0.000	-1.502	Detected	-1.021	Detected	-1.262	0.034	GT	Sens	contig392	contig392	Unannota	GTGATCAATTGATGTTGACTTCTTCACATTTCACCAAATTCCCAAGTTC							[illegible]
GT	Sense	Sense	0.070	Detected	-0.070	Detected	0.000	-1.159	Comprom	-1.977	Comprom	-1.568	0.063	GT	Sens	contig393	contig393	Unannota	ATAGTCATTAAGGAGGATAGGGGTTGAAGAAAATCTTGAGGCTGGTGT							[illegible]
GT	Sense	Sense	0.057	Detected	-0.057	Detected	0.000	-1.416	Detected	-1.386	Detected	-1.401	0.002	GT	Sens	contig393	contig393	Arginine	ATGGAA	contig393	Solyc01g	Solyc01g	Arginine	GO:00082	GO:00082	[illegible]
GT	Sense	Sense	-0.388	Detected	0.388	Detected	0.000	-1.712	Comprom	-2.205	Comprom	-1.959	0.051	GT	Sens	contig393	contig393	Unknown	ATATTTC	contig393	Solyc10g	Solyc10g	Unknown Protein (AHRD V1)			[illegible]
GT	Sense	Sense	0.024	Detected	-0.024	Detected	0.000	-1.080	Detected	-1.579	Detected	-1.330	0.034	GT	Sens	contig394	contig394	Unannota	ATAATGCGCACACACACGTTGTGGGACCCTACACCTACTTGAAGCATCA							[illegible]
GT	Sense	Sense	0.121	Detected	-0.121	Detected	0.000	-2.730	Detected	-2.129	Detected	-2.430	0.017	GT	Sens	contig394	contig394	Unannota	TATTACCTATATAACTGTGTATCCAACTCAAGCTGTGCGTAAACTGTAA							[illegible]
GT	Sense	Sense	0.101	Detected	-0.101	Detected	0.000	-2.437	Detected	-1.862	Detected	-2.149	0.019	GT	Sens	contig394	contig394	Unannota	GTGGATCTAAGATCAGAGTATATTGAAGCTCAAATAGAAACATCTGATT							[illegible]
GT	Sense	Sense	0.470	Detected	-0.470	Detected	0.000	-2.515	Comprom	-1.624	Comprom	-2.070	0.086	GT	Sens	contig394	contig394	Unannota	TATAATGCGTTGAAGCTTCCAGCACATTAATCGACCAAACCAGCAATCT							[illegible]
GT	Sense	Sense	0.211	Detected	-0.211	Detected	0.000	-1.362	Detected	-0.936	Detected	-1.149	0.062	GT	Sens	contig394	contig394	Unannota	GTTCGTCGAAAATTATTCAGAATTGAGATTGAGGCATAGTTTTTATGGT							[illegible]
GT	Sense	Sense	0.175	Detected	-0.175	Detected	0.000	-1.327	Detected	-1.570	Detected	-1.448	0.021	GT	Sens	contig394	contig394	Unannota	CCAAAAGGCGCGACCAAGACCTAGACAAGCACATAAGTTTGAGGACCT							[illegible]
GT	Sense	Sense	0.195	Detected	-0.195	Detected	0.000	-1.302	Detected	-1.510	Detected	-1.406	0.024	GT	Sens	contig394	contig394	Unannota	ACAAAGAGTTCCCATAGTACTTGAGATATAGTACACCACAAAGGGTATG							[illegible]
GT	Sense	Sense	0.504	Detected	-0.504	Detected	0.000	-1.293	Detected	-2.351	Detected	-1.822	0.130	GT	Sens	contig394	contig394	Unannota	CTGACTTCGTATTAGACTAAAATATGCCACAACAACTTATTGTGAACCT							[illegible]
GT	Sense	Sense	0.558	Detected	-0.558	Detected	0.000	-1.353	Detected	-2.470	Detected	-1.911	0.137	GT	Sens	contig394	contig394	Unannota	CAGTATTTGCAAGTGACGAAAAATTGGGATGCACATATGAAAAGTTAGC							[illegible]
GT	Sense	Sense	0.477	Detected	-0.477	Detected	0.000	-1.060	Detected	-2.588	Comprom	-1.824	0.180	GT	Sens	contig394	contig394	Unannota	GGCAACATGGGAGAGATCATTAGCCCTCCATTCTAACAAGCACATGCCA							[illegible]
GT	Sense	Sense	0.316	Detected	-0.316	Detected	0.000	-1.659	Detected	-0.923	Detected	-1.291	0.117	GT	Sens	contig395	contig395	Unannota	CAACCCCATGGTGTAAAAAATGTATGACATGGATATGCCTAGCTCGAAT							[illegible]
GT	Sense	Sense	0.106	Detected	-0.106	Detected	0.000	-1.163	Detected	-1.111	Detected	-1.137	0.009	GT	Sens	contig395	contig395	Unannota	GTTATTCCTAGCAGTGAGAAGGCCGAGATTTCCCGTTCTTACTCGTATT							[illegible]
GT	Sense	Sense	0.126	Detected	-0.126	Detected	0.000	-1.130	Detected	-1.095	Detected	-1.112	0.013	GT	Sens	contig396	contig396	CBS dom	ATATTCT	contig396	Solyc02g	Solyc02g	CBS domain containing protei			[illegible]
GT	Sense	Sense	0.237	Detected	-0.237	Detected	0.000	-3.883	Comprom	-1.717	Comprom	-2.800	0.127	GT	Sens	contig396	contig396	Unannota	CCCTACTAATTTCCTTATAGCCCACATACCCCTACGAATAATATGATGT							[illegible]
GT	Sense	Sense	0.140	Detected	-0.140	Detected	0.000	-1.492	Comprom	-2.477	Comprom	-1.984	0.061	GT	Sens	contig396	contig396	Unannota	GAAAATAACGTCCTAACGTTCCATTTGACAGGCCACATTCCCAACTCAA							[illegible]
GT	Sense	Sense	0.224	Detected	-0.224	Detected	0.000	-1.103	Detected	-1.205	Detected	-1.154	0.037	GT	Sens	contig396	contig396	Unannota	CTTGTTGGTTTGCCAAGAAAATAAAAACAGAATCTTTGCCCATCTGGTA							[illegible]
GT	Sense	Sense	0.325	Detected	-0.325	Detected	0.000	-1.282	Detected	-1.513	Detected	-1.398	0.056	GT	Sens	contig396	contig396	Unannota	CGGATGCACAGATTAAAGCATCTCTCAGTTAATCCCTTTTTTGTTTATC							[illegible]
GT	Sense	Sense	0.141	Detected	-0.141	Detected	0.000	-0.979	Detected	-1.659	Comprom	-1.319	0.070	GT	Sens	contig396	contig396	Unannota	ATTGGGGAATTTGAGTCCGACCCAGATGACATGAGGATTGGGAGGAGT							[illegible]
GT	Sense	Sense	0.333	Detected	-0.333	Detected	0.000	-1.591	Detected	-1.296	Detected	-1.443	0.058	GT	Sens	contig396	contig396	Unknown	TACGTTA	contig396	Solyc04g	Solyc04g	Unknown Protein (AHRD V1)			[illegible]
GT	Sense	Sense	0.077	Detected	-0.077	Detected	0.000	-1.159	Detected	-1.184	Detected	-1.172	0.004	GT	Sens	contig396	contig396	Unannota	TTGTTTATGTGTGTGTTGTCCCTATACTCCATTTAATAAGAGTCCTACG							[illegible]
GT	Sense	Sense	0.096	Detected	-0.096	Detected	0.000	-0.835	Detected	-2.023	Comprom	-1.429	0.141	GT	Sens	contig397	contig397	Unannota	GGTTGGGTTTTTATGTTGCTAGTAGAACTTATCAGTCAAGTGAACTGAT							[illegible]
GT	Sense	Sense	0.139	Detected	-0.139	Detected	0.000	-1.654	Comprom	-1.299	Comprom	-1.477	0.023	GT	Sens	contig397	contig397	Unannota	TAGTAGAGTTTGATTTGATTTGAAGTTCTGGTAGATTAGTTGGAAAGAC							[illegible]
GT	Sense	Sense	0.156	Detected	-0.156	Detected	0.000	-1.380	Detected	-0.930	Detected	-1.155	0.052	GT	Sens	contig397	contig397	TIM-barre	TATGTAC	contig397	Solyc02g	Solyc02g	TIM-barre	GO:00059	GO:00059	[illegible]
GT	Sense	Sense	-0.121	Detected	0.121	Detected	0.000	-1.882	Detected	-2.195	Detected	-2.038	0.009	GT	Sens	contig397	contig397	Unannota	AACAGCAACTTCCATCCAACATTTTGCTTCCCTTTTAATCGCCATTTCT							[illegible]
GT	Sense	Sense	0.125	Detected	-0.125	Detected	0.000	-2.238	Detected	-2.841	Detected	-2.539	0.016	GT	Sens	contig397	contig397	Unannota	CTACTCGTGAAAATTACACGAAATAGAGTGTTCAGTACTACTCTGCTCT							[illegible]
GT	Sense	Sense	0.224	Detected	-0.224	Detected	0.000	-1.877	Detected	-1.399	Detected	-1.638	0.038	GT	Sens	contig398	contig398	Unannota	TGTATAAAGATGTAGGAGGTTATAGTAAAGAAGTAGATGACATATAGTT							[illegible]
GT	Sense	Sense	0.114	Detected	-0.114	Detected	0.000	-2.055	Detected	-1.421	Detected	-1.738	0.036	GT	Sens	contig398	contig398	Unannota	TAGTGTTAAGAGATGAAAATGGAGTCAGGCTAAGGCAGCTTTAGTGTTA							[illegible]
GT	Sense	Sense	0.271	Detected	-0.271	Detected	0.000	-1.402	Detected	-1.898	Detected	-1.650	0.046	GT	Sens	contig398	contig398	Cytokinin	CTCATTA	contig398	Solyc04g	Solyc04g	Cytokinin	GO:00191	GO:00191	[illegible]
GT	Sense	Sense	0.228	Detected	-0.228	Detected	0.000	-1.593	Detected	-1.042	Detected	-1.318	0.066	GT	Sens	contig398	contig398	Unannota	GAATCTGTCTCATTCTCATTGTAATTAGCATCATTGGCCTTTTCATTTC							[illegible]
GT	Sense	Sense	0.238	Detected	-0.238	Detected	0.000	-1.477	Detected	-0.998	Detected	-1.238	0.067	GT	Sens	contig398	contig398	Unannota	CAATGCCTCTTTTAACTTTAAGTAAGATGGAAGAGATATGCCATTGTTT							[illegible]
GT	Sense	Sense	-0.290	Detected	0.290	Detected	0.000	-2.626	Comprom	-0.858	Comprom	-1.742	0.202	GT	Sens	contig399	contig399	Unannota	TCAGTGTTTGTAAATATCCAAGGATGTGTGCATTAATAAGGCTTACACC							[illegible]
GT	Sense	Sense	0.311	Detected	-0.311	Detected	0.000	-6.221	Comprom	-5.365	Comprom	-5.793	0.008	GT	Sens	contig399	contig399	Unannota	CAAAAACACATTTGTTCTAGTGGGAATTGCTTGTGGCAATTTAGATGGA							[illegible]
GT	Sense	Sense	0.322	Detected	-0.322	Detected	0.000	-4.787	Comprom	-5.035	Comprom	-4.911	0.005	GT	Sens	contig399	contig399	Unannota	GGCAATTAAAAAGGCTACATAATACAATTCAAAGAGGTAGGTTTCTCAT							[illegible]
GT	Sense	Sense	0.052	Detected	-0.052	Detected	0.000	-1.192	Detected	-0.943	Detected	-1.067	0.016	GT	Sens	contig4	contig4	Post-GPI	AACTTCT	contig4	Solyc11g	Solyc11g	Post-GPI attachment to protein			[illegible]
GT	Sense	Sense	-0.210	Detected	0.210	Detected	0.000	-1.474	Detected	-1.218	Detected	-1.346	0.032	GT	Sens	contig400	contig400	Ethylene-	AATTTTC	contig400	Solyc03g	Solyc03g	Ethylene-	GO:00165	GO:00165	[illegible]
GT	Sense	Sense	0.312	Detected	-0.312	Detected	0.000	-1.688	Detected	-0.899	Detected	-1.293	0.124	GT	Sens	contig400	contig400	Unannota	GTTTTCATTTGGGTAATAGACTGTTCTGGAAGATTTACTTGAAGTTGAG							[illegible]
GT	Sense	Sense	0.175	Detected	-0.175	Detected	0.000	-1.271	Detected	-1.119	Detected	-1.195	0.025	GT	Sens	contig400	contig400	Unannota	GGTTGTACTGAAGCTTTTCTGTGGAGTCAAGGATGCAGCCTCAAAGT							[illegible]
GT	Sense	Sense	0.267	Detected	-0.267	Detected	0.000	-1.807	Detected	-0.999	Detected	-1.403	0.101	GT	Sens	contig400	contig400	Unannota	GTGTATAGTCATACATGTGTTTCTTTTGCCATGTTTATACTGATTTTAG							[illegible]
GT	Sense	Sense	0.151	Detected	-0.151	Detected	0.000	-1.935	Detected	-1.147	Detected	-1.541	0.067	GT	Sens	contig400	contig400	Unannota	CATCAATCTACTTTTAGTGTCATGAGGAGATGTCTATTATAGGTCATAC							[illegible]
GT	Sense	Sense	0.271	Detected	-0.271	Detected	0.000	-1.658	Detected	-1.569	Detected	-1.613	0.028	GT	Sens	contig400	contig400	Unannota	TGGGCTAGTTTTATCCAAAAGTGAACAGCCCAAAGCCTAAAAAATAAGA							[illegible]
GT	Sense	Sense	0.253	Detected	-0.253	Detected	0.000	-3.155	Detected	-2.991	Detected	-3.073	0.007	GT	Sens	contig400	contig400	Unannota	CATGTTCAATTTATCCACCTATTGTAAAGATATAGTTGACCCCATGGCT							[illegible]
GT	Sense	Sense	0.379	Detected	-0.379	Detected	0.000	-3.254	Detected	-3.083	Detected	-3.169	0.015	GT	Sens	contig400	contig400	Unannota	GGTTGAGAAATTCAACGTATACCAAGTGGTTAGAGAAAAGGGACAATAC							[illegible]
GT	Sense	Sense	-0.042	Detected	0.042	Detected	0.000	-1.535	Comprom	-1.905	Comprom	-1.720	0.012	GT	Sens	contig401	contig401	Unannota	GAAGGACAAAATCAAATTTCTAGCTCCGCTCTTAATAGTAGATTTGACA							[illegible]
GT	Sense	Sense	0.113	Detected	-0.113	Detected	0.000	-2.303	Detected	-2.634	Detected	-2.468	0.007	GT	Sens	contig401	contig401	Unannota	TAAACCCTGGACACCAGAGTTCAAATTCCAGCAGAGCCATCTATTCCTC							[illegible]
GT	Sense	Sense	0.175	Detected	-0.175	Detected	0.000	-2.271	Detected	-2.793	Detected	-2.532	0.015	GT	Sens	contig401	contig401	Unannota	AAGAGAAAAGTTGTAACTAGACTGCATCCAACAGTGGCCAAGTGGTCAA							[illegible]
GT	Sense	Sense	-0.087	Detected	0.087	Detected	0.000	-1.206	Detected	-1.345	Detected	-1.275	0.008	GT	Sens	contig401	contig401	Unannota	CTTCTAAACTCAAAACTGGTTTGAAATGCCCATTTTGAGCTGGTTCAGG							[illegible]
GT	Sense	Sense	-0.094	Detected	0.094	Detected	0.000	-1.628	Detected	-1.342	Detected	-1.485	0.013	GT	Sens	contig401	contig401	Unannota	CGCTTATCTAGATTTCACTAATTGTGAGCTTCTGTTATTTTGGTCATTT							[illegible]
GT	Sense	Sense	0.045	Detected	-0.045	Detected	0.000	-2.145	Comprom	-1.716	Comprom	-1.931	0.013	GT	Sens	contig401	contig401	Unannota	TCTTGAAGGCCATATTTTGGATAGGAATTATATGGGTCCGAGTAGAGCA							[illegible]
GT	Sense	Sense	0.113	Detected	-0.113	Detected	0.000	-2.752	Comprom	-1.573	Comprom	-2.163	0.069	GT	Sens	contig401	contig401	Unannota	GGCTTGAGGCTTAGCTATTGAGTTGATTAAATATAGTTCTTACTATTGT							[illegible]
GT	Sense	Sense	-0.045	Detected	0.045	Detected	0.000	-1.273	Detected	-1.590	Detected	-1.431	0.013	GT	Sens	contig401	contig401	NAC dom	TGAACT	contig401	Solyc10g	Solyc10g	NAC dom	GO:0045449		[illegible]
GT	Sense	Sense	0.045	Detected	-0.045	Detected	0.000	-1.397	Detected	-0.906	Comprom	-1.151	0.044	GT	Sens	contig402	contig402	Unannota	TTGAGACTGAAGCATAGTTGTTGTTTTTGGTTCTCCTGGAATCACAGTC							[illegible]
GT	Sense	Sense	0.074	Detected	-0.074	Detected	0.000	-1.182	Detected	-1.172	Detected	-1.177	0.004	GT	Sens	contig402	contig402	Unannota	GCATGGAAATCTAAACCTCTCCAGGTAGAACCAAGGTATAGCAGAAGTC							[illegible]
GT	Sense	Sense	0.154	Detected	-0.154	Detected	0.000	-1.186	Detected	-1.056	Detected	-1.121	0.021	GT	Sens	contig402	contig402	Unannota	ATGCTTTGCTCGGGACCAGGTTTGAAGATGGGATTGAATTTTCTACTAA							[illegible]
GT	Sense	Sense	-0.129	Detected	0.129	Detected	0.000	-1.340	Detected	-0.936	Detected	-1.138	0.042	GT	Sens	contig402	contig402	Unannota	GGGGGTTCTAATTGGTATGCTCTTTCAAATGCCAATACTAACTTTTAAT							[illegible]
GT	Sense	Sense	0.051	Detected	-0.051	Detected	0.000	-1.642	Detected	-3.300	Comprom	-2.471	0.097	GT	Sens	contig402	contig402	Unannota	CTGATGTCTAGTCTAGTTGTCTCGCTATCCATGGTAAAAGTATATTAAA							[illegible]
GT	Sense	Sense	0.355	Detected	-0.355	Detected	0.000	-0.918	Detected	-1.266	Comprom	-1.092	0.110	GT	Sens	contig402	contig402	Unannota	TTAATGTGCACTTTAGCTTCGTCATCAAGGAACAAAGGCATACTTATCC							[illegible]
GT	Sense	Sense	0.046	Detected	-0.046	Detected	0.000	-3.230	Comprom	-2.571	Comprom	-2.901	0.013	GT	Sens	contig402	contig402	Unannota	CTAGTAAGTTCACTAAATCTGTGATGCCTGCATCATTAGCATTATGGCG							[illegible]
GT	Sense	Sense	0.073	Detected	-0.073	Detected	0.000	-1.326	Detected	-1.718	Comprom	-1.522	0.018	GT	Sens	contig402	contig402	Unannota	TCAAAACGACAAATTCCTGAAAACCTTTTTTCTTGCTGGCCACAATTC							[illegible]
GT	Sense	Sense	0.180	Detected	-0.180	Detected	0.000	-1.643	Detected	-1.421	Comprom	-1.532	0.019	GT	Sens	contig403	contig403	Unannota	GCCTTGTAAAAATTAGAGCATATATACGTCCTTCAATCTAACGGAAGAC							[illegible]
GT	Sense	Sense	0.251	Detected	-0.251	Detected	0.000	-1.415	Detected	-1.637	Detected	-1.526	0.031	GT	Sens	contig403	contig403	Unannota	GTGACACAATCGTCTTATCCGGCAATGTGATTTTTAATTAATGTCGAAT							[illegible]
GT	Sense	Sense	-0.243	Detected	0.243	Detected	0.000	-2.081	Comprom	-1.626	Comprom	-1.853	0.031	GT	Sens	contig403	contig403	Unannota	TTCTGATCTCCTTGATTTTCCTTTCTTGCGATGAGAGTGTGTCGGGAAC							[illegible]
GT	Sense	Sense	-0.113	Detected	0.113	Detected	0.000	-1.428	Comprom	-1.021	Comprom	-1.225	0.034	GT	Sens	contig404	contig404	Unannota	GTATCTTAACTAGGATGACTTGATGGGTGTACCAGTATAGGTAAGGATA							[illegible]
GT	Sense	Sense	-0.150	Detected	0.150	Detected	0.000	-1.131	Comprom	-0.905	Comprom	-1.018	0.032	GT	Sens	contig404	contig404	Unannota	CATAGATGTTAGGATTCATGGACATTCAGATGAGTAGAGTTCATTTAGT							[illegible]
GT	Sense	Sense	-0.129	Detected	0.129	Detected	0.000	-1.203	Comprom	-0.841	Comprom	-1.022	0.044	GT	Sens	contig404	contig404	Unannota	CTCTTCTTAAGCACAAACTTAAAAACAAACTAGGGTTCCATTGCATCCC							[illegible]
GT	Sense	Sense	0.353	Detected	-0.353	Detected	0.000	-1.335	Detected	-1.100	Detected	-1.218	0.082	GT	Sens	contig405	contig405	Unannota	CATTGTTGTGTTCCGCATTTGTTGCCTTTATTACCGCAAATATTATTTC							[illegible]
GT	Sense	Sense	0.198	Detected	-0.198	Detected	0.000	-2.133	Comprom	-1.061	Comprom	-1.597	0.108	GT	Sens	contig406	contig406	Unannota	TTACACGTGTCCCGCCCTACTCAAGTCCTGATATCTCACTTTCGCATAC							[illegible]
GT	Sense	Sense	0.105	Detected	-0.105	Detected	0.000	-1.415	Detected	-1.075	Detected	-1.245	0.025	GT	Sens	contig406	contig406	Hydrolas	AAGAAGT	contig406	Solyc10g	Solyc10g	Hydrolas	GO:00167	GO:00167	[illegible]
GT	Sense	Sense	0.155	Detected	-0.155	Detected	0.000	-1.127	Detected	-1.107	Detected	-1.117	0.019	GT	Sens	contig406	contig406	Unannota	ACTCAATGAAATAGTCGAGGTATGTGGCTTGAACAGCAGAGGTATGTGG							[illegible]
GT	Sense	Sense	0.011	Detected	-0.011	Detected	0.000	-1.671	Comprom	-1.878	Comprom	-1.775	0.003	GT	Sens	contig406	contig406	Unannota	GGACGTACTTTTGTACCTATTCAACCCAAGTATGAAAGGCAATATATTC							[illegible]
GT	Sense	Sense	0.146	Detected	-0.146	Detected	0.000	-1.822	Detected	-1.216	Detected	-1.519	0.046	GT	Sens	contig406	contig406	Glycerol-	TCATCGA	contig406	Solyc02g	Solyc02g	Glycerol-	GO:00093	GO:00093	[illegible]
GT	Sense	Sense	0.129	Detected	-0.129	Detected	0.000	-1.245	Detected	-0.803	Detected	-1.024	0.057	GT	Sens	contig406	contig406	Unannota	GCCGTTTGAAGTTATTTTTGTTTCTGTGCTACTCATAAGAGTTGCTTGA							[illegible]
GT	Sense	Sense	0.172	Detected	-0.172	Detected	0.000	-1.251	Detected	-0.963	Detected	-1.107	0.039	GT	Sens	contig406	contig406	Unannota	ATCGAAAACAACCTCTCTACTACTATTTTCCTCAAGGCTTAGACTGCAC							[illegible]

[illegible]	[illegible]	[illegible]	[illegible]	[illegible]	[illegible]	[illegible]	[illegible]	[illegible]	[illegible]	[illegible]	[illegible]	[illegible]	[illegible]	[illegible]	[illegible]	[illegible]
GT	Sense	Sense	-0.030	Detected	0.030	Detected	0.000	-1.249	Detected	-1.003	Detected	-1.126	0.012	GT	Sens	[illegible]
GT	Sense	Sense	0.048	Detected	-0.048	Detected	0.000	-0.916	Detected	-1.405	Comprom	-1.161	0.043	GT	Sens	[illegible]
GT	Sense	Sense	0.494	Detected	-0.494	Detected	0.000	-2.473	Comprom	-1.308	Detected	-1.890	0.132	GT	Sens	[illegible]
GT	Sense	Sense	0.346	Detected	-0.346	Detected	0.000	-2.455	Detected	-1.778	Detected	-2.117	0.049	GT	Sens	[illegible]
GT	Sense	Sense	0.352	Detected	-0.352	Detected	0.000	-1.417	Detected	-0.840	Detected	-1.129	0.131	GT	Sens	[illegible]
GT	Sense	Sense	0.156	Detected	-0.156	Detected	0.000	-1.145	Detected	-0.913	Detected	-1.029	0.034	GT	Sens	[illegible]
GT	Sense	Sense	0.039	Detected	-0.039	Detected	0.000	-1.604	Detected	-1.441	Detected	-1.523	0.004	GT	Sens	[illegible]
GT	Sense	Sense	0.097	Detected	-0.097	Detected	0.000	-1.687	Detected	-1.171	Detected	-1.429	0.035	GT	Sens	[illegible]
GT	Sense	Sense	-0.034	Detected	0.034	Detected	0.000	-1.619	Detected	-0.832	Detected	-1.225	0.090	GT	Sens	[illegible]
GT	Sense	Sense	0.165	Detected	-0.165	Detected	0.000	-1.000	Detected	-1.066	Detected	-1.033	0.026	GT	Sens	[illegible]
GT	Sense	Sense	0.084	Detected	-0.084	Detected	0.000	-1.238	Detected	-1.322	Detected	-1.280	0.005	GT	Sens	[illegible]
GT	Sense	Sense	0.119	Detected	-0.119	Detected	0.000	-0.882	Detected	-1.607	Comprom	-1.244	0.082	GT	Sens	[illegible]
GT	Sense	Sense	0.015	Detected	-0.015	Detected	0.000	-2.327	Detected	-1.118	Detected	-1.723	0.104	GT	Sens	[illegible]
GT	Sense	Sense	-0.856	Detected	0.856	Detected	0.000	-1.492	Detected	-0.921	Detected	-1.207	0.313	GT	Sens	[illegible]
GT	Sense	Sense	0.118	Detected	-0.118	Detected	0.000	-1.078	Detected	-1.039	Detected	-1.059	0.013	GT	Sens	[illegible]
GT	Sense	Sense	0.025	Detected	-0.025	Detected	0.000	-1.973	Detected	-1.240	Detected	-1.607	0.048	GT	Sens	[illegible]
GT	Sense	Sense	0.510	Detected	-0.510	Detected	0.000	-1.738	Comprom	-1.008	Detected	-1.373	0.160	GT	Sens	[illegible]
GT	Sense	Sense	0.053	Detected	-0.053	Detected	0.000	-1.390	Detected	-1.157	Detected	-1.273	0.010	GT	Sens	[illegible]
GT	Sense	Sense	0.046	Detected	-0.046	Detected	0.000	-1.497	Detected	-1.424	Detected	-1.460	0.002	GT	Sens	[illegible]
GT	Sense	Sense	0.208	Detected	-0.208	Detected	0.000	-2.281	Detected	-1.823	Detected	-2.052	0.022	GT	Sens	[illegible]
GT	Sense	Sense	0.121	Detected	-0.121	Detected	0.000	-1.471	Detected	-1.393	Detected	-1.432	0.008	GT	Sens	[illegible]
GT	Sense	Sense	-0.513	Detected	0.513	Detected	0.000	-2.688	Comprom	-1.932	Comprom	-2.310	0.068	GT	Sens	[illegible]
GT	Sense	Sense	0.602	Detected	-0.602	Detected	0.000	-2.070	Detected	-2.172	Detected	-2.121	0.072	GT	Sens	[illegible]
GT	Sense	Sense	0.494	Detected	-0.494	Detected	0.000	-2.097	Detected	-0.986	Detected	-1.542	0.174	GT	Sens	[illegible]
GT	Sense	Sense	0.007	Detected	-0.007	Detected	0.000	-1.713	Comprom	-1.328	Comprom	-1.521	0.016	GT	Sens	[illegible]
GT	Sense	Sense	0.060	Detected	-0.060	Detected	0.000	-1.380	Detected	-1.317	Detected	-1.349	0.002	GT	Sens	[illegible]
GT	Sense	Sense	-0.138	Detected	0.138	Detected	0.000	-2.229	Detected	-1.573	Detected	-1.901	0.033	GT	Sens	[illegible]
GT	Sense	Sense	-0.072	Detected	0.072	Detected	0.000	-1.147	Detected	-1.356	Detected	-1.252	0.010	GT	Sens	[illegible]
GT	Sense	Sense	0.177	Detected	-0.177	Detected	0.000	-1.474	Detected	-1.225	Detected	-1.349	0.025	GT	Sens	[illegible]
GT	Sense	Sense	0.300	Detected	-0.300	Detected	0.000	-2.249	Detected	-2.541	Detected	-2.395	0.019	GT	Sens	[illegible]
GT	Sense	Sense	-0.047	Detected	0.047	Detected	0.000	-2.834	Detected	-1.907	Detected	-2.370	0.037	GT	Sens	[illegible]
GT	Sense	Sense	0.110	Detected	-0.110	Detected	0.000	-1.257	Comprom	-1.531	Comprom	-1.394	0.016	GT	Sens	[illegible]
GT	Sense	Sense	0.374	Detected	-0.374	Detected	0.000	-3.761	Detected	-1.780	Detected	-2.770	0.120	GT	Sens	[illegible]
GT	Sense	Sense	0.062	Detected	-0.062	Detected	0.000	-1.343	Detected	-0.994	Detected	-1.168	0.024	GT	Sens	[illegible]
GT	Sense	Sense	0.051	Detected	-0.051	Detected	0.000	-1.571	Detected	-1.325	Detected	-1.448	0.008	GT	Sens	[illegible]
GT	Sense	Sense	0.305	Detected	-0.305	Detected	0.000	-1.234	Detected	-1.644	Detected	-1.439	0.059	GT	Sens	[illegible]
GT	Sense	Sense	0.106	Detected	-0.106	Detected	0.000	-1.175	Detected	-1.174	Detected	-1.175	0.008	GT	Sens	[illegible]
GT	Sense	Sense	-0.083	Detected	0.083	Detected	0.000	-1.357	Detected	-1.252	Detected	-1.304	0.006	GT	Sens	[illegible]
GT	Sense	Sense	0.363	Detected	-0.363	Detected	0.000	-1.671	Detected	-1.229	Detected	-1.450	0.076	GT	Sens	[illegible]
GT	Sense	Sense	0.053	Detected	-0.053	Detected	0.000	-1.568	Detected	-1.212	Detected	-1.390	0.017	GT	Sens	[illegible]
GT	Sense	Sense	0.091	Detected	-0.091	Detected	0.000	-3.180	Comprom	-2.506	Comprom	-2.843	0.015	GT	Sens	[illegible]
GT	Sense	Sense	0.008	Detected	-0.008	Detected	0.000	-1.476	Detected	-0.822	Detected	-1.149	0.072	GT	Sens	[illegible]
GT	Sense	Sense	0.091	Detected	-0.091	Detected	0.000	-3.176	Detected	-2.206	Detected	-2.691	0.032	GT	Sens	[illegible]
GT	Sense	Sense	0.117	Detected	-0.117	Detected	0.000	-1.162	Detected	-1.035	Detected	-1.099	0.014	GT	Sens	[illegible]
GT	Sense	Sense	-0.022	Detected	0.022	Detected	0.000	-0.875	Detected	-1.412	Detected	-1.144	0.051	GT	Sens	[illegible]
GT	Sense	Sense	0.108	Detected	-0.108	Detected	0.000	-1.350	Detected	-1.444	Detected	-1.397	0.007	GT	Sens	[illegible]
GT	Sense	Sense	0.262	Detected	-0.262	Detected	0.000	-1.383	Detected	-1.019	Detected	-1.201	0.064	GT	Sens	[illegible]
GT	Sense	Sense	0.126	Detected	-0.126	Detected	0.000	-1.348	Detected	-0.893	Detected	-1.120	0.050	GT	Sens	[illegible]
GT	Sense	Sense	0.298	Detected	-0.298	Detected	0.000	-2.081	Detected	-1.553	Detected	-1.817	0.045	GT	Sens	[illegible]
GT	Sense	Sense	0.068	Detected	-0.068	Detected	0.000	-1.379	Detected	-1.015	Detected	-1.197	0.025	GT	Sens	[illegible]
GT	Sense	Sense	0.080	Detected	-0.080	Detected	0.000	-1.924	Detected	-1.279	Detected	-1.602	0.040	GT	Sens	[illegible]
GT	Sense	Sense	0.272	Detected	-0.272	Detected	0.000	-1.521	Detected	-0.921	Detected	-1.221	0.095	GT	Sens	[illegible]
GT	Sense	Sense	0.120	Detected	-0.120	Detected	0.000	-1.226	Detected	-1.101	Detected	-1.163	0.013	GT	Sens	[illegible]
GT	Sense	Sense	0.249	Detected	-0.249	Detected	0.000	-1.027	Detected	-1.014	Detected	-1.020	0.055	GT	Sens	[illegible]
GT	Sense	Sense	0.155	Detected	-0.155	Detected	0.000	-1.738	Detected	-1.668	Detected	-1.703	0.009	GT	Sens	[illegible]
GT	Sense	Sense	-0.316	Detected	0.316	Detected	0.000	-2.889	Detected	-0.910	Detected	-1.899	0.209	GT	Sens	[illegible]
GT	Sense	Sense	-0.017	Detected	0.017	Detected	0.000	-1.653	Detected	-1.421	Detected	-1.537	0.006	GT	Sens	[illegible]
GT	Sense	Sense	0.277	Detected	-0.277	Detected	0.000	-2.688	Detected	-1.486	Detected	-2.087	0.087	GT	Sens	[illegible]
GT	Sense	Sense	0.172	Detected	-0.172	Detected	0.000	-1.269	Detected	-0.834	Detected	-1.052	0.063	GT	Sens	[illegible]
GT	Sense	Sense	0.180	Detected	-0.180	Detected	0.000	-2.031	Detected	-1.868	Detected	-1.949	0.010	GT	Sens	[illegible]
GT	Sense	Sense	0.209	Detected	-0.209	Detected	0.000	-1.355	Detected	-1.211	Detected	-1.283	0.028	GT	Sens	[illegible]
GT	Sense	Sense	0.003	Detected	-0.003	Detected	0.000	-1.712	Detected	-1.752	Detected	-1.732	0.000	GT	Sens	[illegible]
GT	Sense	Sense	0.149	Detected	-0.149	Detected	0.000	-1.570	Detected	-1.674	Detected	-1.622	0.009	GT	Sens	[illegible]
GT	Sense	Sense	0.132	Detected	-0.132	Detected	0.000	-1.202	Detected	-0.828	Detected	-1.015	0.047	GT	Sens	[illegible]
GT	Sense	Sense	0.449	Detected	-0.449	Detected	0.000	-0.977	Detected	-1.495	Comprom	-1.236	0.140	GT	Sens	[illegible]
GT	Sense	Sense	0.140	Detected	-0.140	Detected	0.000	-1.172	Detected	-0.849	Detected	-1.011	0.042	GT	Sens	[illegible]
GT	Sense	Sense	-0.175	Detected	0.175	Detected	0.000	-2.682	Comprom	-1.702	Detected	-2.192	0.052	GT	Sens	[illegible]
GT	Sense	Sense	0.058	Detected	-0.058	Detected	0.000	-2.169	Detected	-0.949	Detected	-1.559	0.126	GT	Sens	[illegible]
GT	Sense	Sense	0.106	Detected	-0.106	Detected	0.000	-1.538	Detected	-1.587	Detected	-1.563	0.005	GT	Sens	[illegible]
GT	Sense	Sense	0.133	Detected	-0.133	Detected	0.000	-1.522	Detected	-1.139	Detected	-1.330	0.029	GT	Sens	[illegible]
GT	Sense	Sense	-0.034	Detected	0.034	Detected	0.000	-1.393	Detected	-0.948	Detected	-1.171	0.035	GT	Sens	[illegible]
GT	Sense	Sense	0.164	Detected	-0.164	Detected	0.000	-1.486	Detected	-1.443	Detected	-1.465	0.013	GT	Sens	[illegible]
GT	Sense	Sense	0.379	Detected	-0.379	Detected	0.000	-2.143	Detected	-1.650	Detected	-1.897	0.052	GT	Sens	[illegible]
GT	Sense	Sense	0.758	Detected	-0.758	Detected	0.000	-1.813	Detected	-1.612	Detected	-1.713	0.154	GT	Sens	[illegible]
GT	Sense	Sense	0.101	Detected	-0.101	Detected	0.000	-1.420	Detected	-0.865	Detected	-1.142	0.061	GT	Sens	[illegible]
GT	Sense	Sense	-0.053	Detected	0.053	Detected	0.000	-0.980	Detected	-1.177	Detected	-1.078	0.011	GT	Sens	[illegible]
GT	Sense	Sense	0.083	Detected	-0.083	Detected	0.000	-1.405	Detected	-0.989	Detected	-1.197	0.033	GT	Sens	[illegible]
GT	Sense	Sense	-0.102	Detected	0.102	Detected	0.000	-1.586	Detected	-2.419	Detected	-2.003	0.043	GT	Sens	[illegible]
GT	Sense	Sense	0.046	Detected	-0.046	Detected	0.000	-0.956	Detected	-1.778	Detected	-1.367	0.081	GT	Sens	[illegible]
GT	Sense	Sense	0.113	Detected	-0.113	Detected	0.000	-2.377	Detected	-1.957	Detected	-2.167	0.012	GT	Sens	[illegible]
GT	Sense	Sense	0.185	Detected	-0.185	Detected	0.000	-0.998	Detected	-1.127	Detected	-1.062	0.032	GT	Sens	[illegible]
GT	Sense	Sense	0.291	Detected	-0.291	Detected	0.000	-2.835	Detected	-1.673	Detected	-2.254	0.074	GT	Sens	[illegible]
GT	Sense	Sense	0.122	Detected	-0.122	Detected	0.000	-2.130	Detected	-1.865	Detected	-1.997	0.008	GT	Sens	[illegible]
GT	Sense	Sense	0.478	Detected	-0.478	Detected	0.000	-2.036	Detected	-1.442	Detected	-1.739	0.091	GT	Sens	[illegible]
GT	Sense	Sense	-0.029	Detected	0.029	Detected	0.000	-2.020	Detected	-1.518	Detected	-1.769	0.020	GT	Sens	[illegible]
GT	Sense	Sense	-0.029	Detected	0.029	Detected	0.000	-1.058	Detected	-2.012	Comprom	-1.535	0.085	GT	Sens	[illegible]
GT	Sense	Sense	0.184	Detected	-0.184	Detected	0.000	-1.161	Detected	-0.840	Detected	-1.000	0.055	GT	Sens	[illegible]
GT	Sense	Sense	0.070	Detected	-0.070	Detected	0.000	-1.079	Detected	-2.378	Comprom	-1.729	0.118	GT	Sens	[illegible]
GT	Sense	Sense	0.192	Detected	-0.192	Detected	0.000	-1.178	Detected	-1.299	Detected	-1.239	0.025	GT	Sens	[illegible]
GT	Sense	Sense	0.160	Detected	-0.160	Detected	0.000	-1.154	Detected	-0.928	Detected	-1.041	0.033	GT	Sens	[illegible]
GT	Sense	Sense	0.411	Detected	-0.411	Detected	0.000	-1.467	Detected	-1.375	Detected	-1.421	0.075	GT	Sens	[illegible]
GT	Sense	Sense	0.445	Detected	-0.445	Detected	0.000	-4.390	Detected	-2.169	Detected	-3.280	0.111	GT	Sens	[illegible]
GT	Sense	Sense	0.314	Detected	-0.314	Detected	0.000	-1.399	Detected	-1.773	Detected	-1.586	0.049	GT	Sens	[illegible]
GT	Sense	Sense	0.425	Detected	-0.425	Detected	0.000	-2.045	Detected	-1.022	Detected	-1.533	0.148	GT	Sens	[illegible]
GT	Sense	Sense	0.175	Detected	-0.175	Detected	0.000	-1.825	Detected	-2.038	Detected	-1.932	0.011	GT	Sens	[illegible]
GT	Sense	Sense	-0.005	Detected	0.005	Detected	0.000	-1.112	Detected	-1.004	Detected	-1.058	0.003	GT	Sens	[illegible]
GT	Sense	Sense	-0.070	Detected	0.070	Detected	0.000	-1.550	Detected	-1.037	Detected	-1.293	0.040	GT	Sens	[illegible]
GT	Sense	Sense	0.121	Detected	-0.121	Detected	0.000	-1.124	Detected	-0.970	Detected	-1.047	0.018	GT	Sens	[illegible]
GT	Sense	Sense	0.174	Detected	-0.174	Detected	0.000	-3.287	Detected	-2.027	Detected	-2.657	0.055	GT	Sens	[illegible]
GT	Sense	Sense	0.095	Detected	-0.095	Detected	0.000	-2.872	Detected	-1.657	Detected	-2.265	0.066	GT	Sens	[illegible]
GT	Sense	Sense	0.231	Detected	-0.231	Detected	0.000	-2.926	Comprom	-3.529	Comprom	-3.228	0.014	GT	Sens	[illegible]
GT	Sense	Sense	0.100	Detected	-0.100	Detected	0.000	-1.166	Detected	-1.025	Detected	-1.096	0.012	GT	Sens	[illegible]
GT	Sense	Sense	0.162	Detected	-0.162	Detected	0.000	-2.275	Detected	-2.253	Detected	-2.264	0.005	GT	Sens	[illegible]
GT	Sense	Sense	-0.058	Detected	0.058	Detected	0.000	-2.437	Detected	-2.058	Detected	-2.248	0.008	GT	Sens	[illegible]
GT	Sense	Sense	0.066	Detected	-0.066	Detected	0.000	-1.744	Detected	-1.257	Detected	-1.501	0.027	GT	Sens	[illegible]
GT	Sense	Sense	0.307	Detected	-0.307	Detected	0.000	-2.090	Detected	-1.952	Detected	-2.021	0.023	GT	Sens	[illegible]
GT	Sense	Sense	0.059	Detected	-0.059	Detected	0.000	-1.264	Detected	-2.245	Detected	-1.754	0.071	GT	Sens	[illegible]
GT	Sense	Sense	0.194	Detected	-0.194	Detected	0.000	-1.547	Detected	-1.534	Detected	-1.540	0.016	GT	Sens	[illegible]
GT	Sense	Sense	0.094	Detected	-0.094	Detected	0.000	-1.361	Detected	-1.530	Detected	-1.445	0.008	GT	Sens	[illegible]
GT	Sense	Sense	0.124	Detected	-0.124	Detected	0.000	-0.872	Detected	-1.421	Detected	-1.146	0.063	GT	Sens	[illegible]
GT	Sense	Sense	0.031	Detected	-0.031	Detected	0.000	-1.621	Detected	-1.687	Detected	-1.654	0.001	GT	Sens	[illegible]
GT	Sense	Sense	0.052	Detected	-0.052	Detected	0.000	-1.051	Detected	-1.252	Detected	-1.151	0.009	GT	Sens	[illegible]
GT	Sense	Sense	-0.026	Detected	0.026	Detected	0.000	-1.107	Detected	-2.472	Detected	-1.789	0.120	GT	Sens	[illegible]
GT	Sense	Sense	0.449	Detected	-0.449	Detected	0.000	-2.523	Comprom	-2.441	Comprom	-2.482	0.031	GT	Sens	[illegible]
GT	Sense	Sense	-0.055	Detected	0.055	Detected	0.000	-1.267	Detected	-1.151	Detected	-1.209	0.004	GT	Sens	[illegible]
GT	Sense	Sense	-0.209	Detected	0.209	Detected	0.000	-1.070	Detected	-1.906	Detected	-1.488	0.086	GT	Sens	[illegible]
GT	Sense	Sense	0.236	Detected	-0.236	Detected	0.000	-1.739	Detected	-0.987	Detected	-1.363	0.092	GT	Sens	[illegible]
GT	Sense	Sense	0.163	Detected	-0.163	Detected	0.000	-1.769	Detected	-1.010	Detected	-1.389	0.078	GT	Sens	[illegible]
GT	Sense	Sense	-0.049	Detected	0.049	Detected	0.000	-1.158	Detected	-0.931	Detected	-1.044	0.014	GT	Sens	[illegible]
GT	Sense	Sense	-0.066	Detected	0.066	Detected	0.000	-0.952	Detected	-1.219	Detected	-1.085	0.018	GT	Sens	[illegible]
GT	Sense	Sense	0.222	Detected	-0.222	Detected	0.000	-1.315	Detected	-0.969	Detected	-1.142	0.056	GT	Sens	[illegible]
GT	Sense	Sense	0.142	Detected	-0.142	Detected	0.000	-1.594	Detected	-1.439	Detected	-1.517	0.011	GT	Sens	[illegible]
GT	Sense	Sense	0.061	Detected	-0.061	Detected	0.000	-2.033	Detected	-1.268	Detected	-1.651	0.051	GT	Sens	[illegible]
GT	Sense	Sense	0.099	Detected	-0.099	Detected	0.000	-1.231	Detected	-0.861	Detected	-1.046	0.038	GT	Sens	[illegible]
GT	Sense	Sense	0.119	Detected	-0.119	Detected	0.000	-2.313	Detected	-1.578	Detected	-1.945	0.037	GT	Sens	[illegible]
GT	Sense	Sense	-0.013	Detected	0.013	Detected	0.000	-1.710	Detected	-1.803	Detected	-1.757	0.001	GT	Sens	[illegible]
GT	Sense	Sense	0.149	Detected	-0.149	Detected	0.000	-1.225	Detected	-1.003	Detected	-1.114	0.027	GT	Sens	[illegible]
GT	Sense	Sense	0.110	Detected	-0.110	Detected	0.000	-1.379	Detected	-0.938	Detected	-1.158	0.042	GT	Sens	[illegible]
GT	Sense	Sense	0.105	Detected	-0.105	Detected	0.000	-3.226	Detected	-2.135	Detected	-2.681	0.040	GT	Sens	[illegible]
GT	Sense	Sense	0.065	Detected	-0.065	Detected	0.000	-1.634	Detected	-1.249	Detected	-1.442	0.019	GT	Sens	[illegible]
GT	Sense	Sense	-0.319	Detected	0.319	Detected	0.000	-1.320	Detected	-2.050	Detected	-1.685	0.074	GT	Sens	[illegible]
GT	Sense	Sense	0.321	Detected	-0.321	Detected	0.000	-3.025	Detected	-3.062	Detected	-3.044	0.011	GT	Sens	[illegible]
GT	Sense	Sense	0.310	Detected	-0.310	Detected	0.000	-1.039	Detected	-1.124	Detected	-1.081	0.074	GT	Sens	[illegible]
GT	Sense	Sense	0.168	Detected	-0.168	Detected	0.000	-1.404	Detected	-2.865	Detected	-2.135	0.104	GT	Sens	[illegible]
GT	Sense	Sense	0.133	Detected	-0.133	Detected	0.000	-0.924	Detected	-1.441	Detected	-1.182	0.056	GT	Sens	[illegible]
GT	Sense	Sense	0.086	Detected	-0.086	Detected	0.000	-1.529	Detected	-1.390	Detected	-1.459	0.006	GT	Sens	[illegible]
GT	Sense	Sense	0.295	Detected	-0.295	Detected	0.000	-1.863	Detected	-1.455	Detected	-1.659	0.044	GT	Sens	[illegible]
GT	Sense	Sense	0.130	Detected	-0.130	Detected	0.000	-1.013	Detected	-1.006	Detected	-1.009	0.016	GT	Sens	[illegible]
GT	Sense	Sense	-0.079	Detected	0.079	Detected	0.000	-1.190	Detected	-0.900	Detected	-1.045	0.024	GT	Sens	[illegible]
GT	Sense	Sense	0.289	Detected	-0.289	Detected	0.000	-2.290	Detected	-1.973	Detected	-2.132	0.023	GT	Sens	[illegible]
GT	Sense	Sense	0.020	Detected	-0.020	Detected	0.000	-5.055	Comprom	-2.309	Detected	-3.682	0.115	GT	Sens	[illegible]
GT	Sense	Sense	0.163	Detected	-0.163	Detected	0.000	-1.018	Detected	-1.248	Detected	-1.133	0.030	GT	Sens	[illegible]
GT	Sense	Sense	-0.043	Detected	0.043	Detected	0.000	-2.004	Detected	-0.931	Detected	-1.468	0.112	GT	Sens	[illegible]
GT	Sense	Sense	0.031	Detected	-0.031	Detected	0.000	-1.228	Detected	-0.927	Detected	-1.077	0.020	GT	Sens	[illegible]
GT	Sense	Sense	0.426	Detected	-0.426	Detected	0.000	-2.313	Detected	-1.798	Detected	-2.056	0.054	GT	Sens	[illegible]
GT	Sense	Sense	-0.011	Detected	0.011	Detected	0.000	-1.303	Detected	-1.122	Detected	-1.212	0.006	GT	Sens	[illegible]
GT	Sense	Sense	0.253	Detected	-0.253	Detected	0.000	-2.391	Detected	-2.249	Detected	-2.320	0.013	GT	Sens	[illegible]
GT	Sense	Sense	0.396	Detected	-0.396	Detected	0.000	-3.609	Comprom	-2.892	Comprom	-3.251	0.026	GT	Sens	[illegible]
GT	Sense	Sense	0.278	Detected	-0.278	Detected	0.000	-2.776	Detected	-2.458	Detected	-2.617	0.015	GT	Sens	[illegible]
GT	Sense	Sense	0.111	Detected	-0.111	Detected	0.000	-1.497	Detected	-0.980	Detected	-1.239	0.048	GT	Sens	[illegible]
GT	Sense	Sense	0.117	Detected	-0.117	Detected	0.000	-1.313	Detected	-1.048	Detected	-1.181	0.022	GT	Sens	[illegible]
GT	Sense	Sense	0.266	Detected	-0.266	Detected	0.000	-3.177	Detected	-2.510	Detected	-2.843	0.022	GT	Sens	[illegible]
GT	Sense	Sense	0.062	Detected	-0.062	Detected	0.000	-1.065	Detected	-1.170	Detected	-1.118	0.005	GT	Sens	[illegible]
GT	Sense	Sense	0.143	Detected	-0.143	Detected	0.000	-2.199	Detected	-1.751	Detected	-1.975	0.018	GT	Sens	[illegible]
GT	Sense	Sense	0.160	Detected	-0.160	Detected	0.000	-1.469	Detected	-1.223	Detected	-1.346	0.022	GT	Sens	[illegible]
GT	Sense	Sense	-0.004	Detected	0.004	Detected	0.000	-1.023	Detected	-1.031	Detected	-1.027	0.000	GT	Sens	[illegible]
GT	Sense	Sense	-0.686	Detected	0.686	Detected	0.000	-2.439	Comprom	-1.869	Comprom	-2.154	0.101	GT	Sens	[illegible]
GT	Sense	Sense	0.099	Detected	-0.099	Detected	0.000	-1.359	Detected	-0.920	Detected	-1.140	0.042	GT	Sens	[illegible]
GT	Sense	Sense	0.179	Detected	-0.179	Detected	0.000	-1.750	Detected	-1.187	Detected	-1.469	0.048	GT	Sens	[illegible]
GT	Sense	Sense	0.061	Detected	-0.061	Detected	0.000	-1.237	Detected	-1.273	Detected	-1.255	0.003	GT	Sens	[illegible]
GT	Sense	Sense	0.308	Detected	-0.308	Detected	0.000	-1.796	Detected	-0.824	Detected	-1.310	0.151	GT	Sens	[illegible]
GT	Sense	Sense	0.154	Detected	-0.154	Detected	0.000	-0.968	Detected	-2.613	Detected	-1.790	0.166	GT	Sens	[illegible]
GT	Sense	Sense	0.127	Detected	-0.127	Detected	0.000	-1.184	Detected	-1.193	Detected	-1.189	0.011	GT	Sens	[illegible]
GT	Sense	Sense	0.316	Detected	-0.316	Detected	0.000	-2.229	Detected	-1.343	Detected	-1.786	0.082	GT	Sens	[illegible]
GT	Sense	Sense	0.064	Detected	-0.064	Detected	0.000	-0.983	Detected	-1.585	Detected	-1.284	0.053	GT	Sens	[illegible]
GT	Sense	Sense	0.014	Detected	-0.014	Detected	0.000	-1.192	Detected	-0.941	Detected	-1.066	0.014	GT	Sens	[illegible]
GT	Sense	Sense	-0.144	Detected	0.144	Detected	0.000	-1.552	Detected	-1.978	Detected	-1.765	0.021	GT	Sens	[illegible]
GT	Sense	Sense	0.268	Detected	-0.268	Detected	0.000	-1.154	Detected	-1.066	Detected	-1.110	0.055	GT	Sens	[illegible]
GT	Sense	Sense	0.031	Detected	-0.031	Detected	0.000	-1.370	Detected	-1.659	Detected	-1.515	0.009	GT	Sens	[illegible]
GT	Sense	Sense	0.227	Detected	-0.227	Detected	0.000	-1.085	Detected	-1.374	Detected	-1.230	0.045	GT	Sens	[illegible]
GT	Sense	Sense	0.339	Detected	-0.339	Detected	0.000	-3.362	Comprom	-1.433	Comprom	-2.398	0.144	GT	Sens	[illegible]
[illegible]	[illegible]	[illegible]	[illegible]	[illegible]	[illegible]	[illegible]	[illegible]	[illegible]	[illegible]	[illegible]	[illegible]	[illegible]	[illegible]	[illegible]	[illegible]	[illegible]

GT	Sense	Sense	0.183	Detected	-0.183	Detected	0.000	-1.151	Detected	-1.056	Detected	-1.103	0.028	GT	Sens	[illegible]
GT	Sense	Sense	0.205	Detected	-0.205	Detected	0.000	-1.885	Detected	-1.796	Detected	-1.840	0.013	GT	Sens	[illegible]
GT	Sense	Sense	0.455	Detected	-0.455	Detected	0.000	-1.588	Detected	-1.542	Detected	-1.565	0.075	GT	Sens	[illegible]
GT	Sense	Sense	0.133	Detected	-0.133	Detected	0.000	-1.450	Detected	-0.816	Detected	-1.133	0.081	GT	Sens	[illegible]
GT	Sense	Sense	0.415	Detected	-0.415	Detected	0.000	-3.877	Detected	-3.003	Detected	-3.440	0.029	GT	Sens	[illegible]
GT	Sense	Sense	0.169	Detected	-0.169	Detected	0.000	-1.762	Detected	-1.090	Detected	-1.426	0.063	GT	Sens	[illegible]
GT	Sense	Sense	-0.007	Detected	0.007	Detected	0.000	-2.556	Detected	-1.644	Detected	-2.100	0.044	GT	Sens	[illegible]
GT	Sense	Sense	-0.093	Detected	0.093	Detected	0.000	-2.037	Detected	-1.849	Detected	-1.943	0.005	GT	Sens	[illegible]
GT	Sense	Sense	-0.203	Detected	0.203	Detected	0.000	-0.966	Comprom	-1.297	Comprom	-1.131	0.050	GT	Sens	[illegible]
GT	Sense	Sense	0.094	Detected	-0.094	Detected	0.000	-1.366	Detected	-1.147	Detected	-1.256	0.013	GT	Sens	[illegible]
GT	Sense	Sense	0.011	Detected	-0.011	Detected	0.000	-1.375	Detected	-0.839	Detected	-1.107	0.054	GT	Sens	[illegible]
GT	Sense	Sense	-0.050	Detected	0.050	Detected	0.000	-1.302	Detected	-0.889	Detected	-1.095	0.036	GT	Sens	[illegible]
GT	Sense	Sense	0.129	Detected	-0.129	Detected	0.000	-2.812	Comprom	-3.232	Comprom	-3.022	0.007	GT	Sens	[illegible]
GT	Sense	Sense	0.072	Detected	-0.072	Detected	0.000	-1.201	Detected	-1.118	Detected	-1.159	0.005	GT	Sens	[illegible]
GT	Sense	Sense	-0.100	Detected	0.100	Detected	0.000	-1.710	Detected	-1.015	Detected	-1.362	0.064	GT	Sens	[illegible]
GT	Sense	Sense	0.064	Detected	-0.064	Detected	0.000	-1.214	Detected	-0.960	Detected	-1.087	0.017	GT	Sens	[illegible]
GT	Sense	Sense	0.011	Detected	-0.011	Detected	0.000	-2.053	Detected	-2.614	Detected	-2.334	0.014	GT	Sens	[illegible]
GT	Sense	Sense	0.106	Detected	-0.106	Detected	0.000	-0.862	Detected	-2.328	Detected	-1.595	0.164	GT	Sens	[illegible]
GT	Sense	Sense	0.160	Detected	-0.160	Detected	0.000	-4.565	Comprom	-4.691	Comprom	-4.628	0.001	GT	Sens	[illegible]
GT	Sense	Sense	0.047	Detected	-0.047	Detected	0.000	-2.508	Detected	-1.949	Detected	-2.228	0.016	GT	Sens	[illegible]
GT	Sense	Sense	0.063	Detected	-0.063	Detected	0.000	-0.963	Detected	-1.286	Detected	-1.124	0.023	GT	Sens	[illegible]
GT	Sense	Sense	0.135	Detected	-0.135	Detected	0.000	-1.240	Detected	-1.012	Detected	-1.126	0.024	GT	Sens	[illegible]
GT	Sense	Sense	0.636	Detected	-0.636	Detected	0.000	-1.315	Detected	-0.839	Detected	-1.077	0.253	GT	Sens	[illegible]
GT	Sense	Sense	0.137	Detected	-0.137	Detected	0.000	-1.159	Detected	-1.040	Detected	-1.099	0.018	GT	Sens	[illegible]
GT	Sense	Sense	0.096	Detected	-0.096	Detected	0.000	-1.572	Comprom	-2.570	Comprom	-2.071	0.055	GT	Sens	[illegible]
GT	Sense	Sense	0.070	Detected	-0.070	Detected	0.000	-1.102	Comprom	-1.849	Comprom	-1.476	0.060	GT	Sens	[illegible]
GT	Sense	Sense	-0.043	Detected	0.043	Detected	0.000	-1.882	Detected	-1.020	Detected	-1.451	0.079	GT	Sens	[illegible]
GT	Sense	Sense	0.053	Detected	-0.053	Detected	0.000	-1.391	Detected	-2.602	Detected	-1.996	0.081	GT	Sens	[illegible]
GT	Sense	Sense	0.074	Detected	-0.074	Detected	0.000	-1.414	Detected	-1.031	Detected	-1.222	0.027	GT	Sens	[illegible]
GT	Sense	Sense	0.181	Detected	-0.181	Detected	0.000	-1.730	Detected	-1.324	Detected	-1.527	0.030	GT	Sens	[illegible]
GT	Sense	Sense	-0.002	Detected	0.002	Detected	0.000	-1.606	Detected	-1.609	Detected	-1.607	0.000	GT	Sens	[illegible]
GT	Sense	Sense	0.282	Detected	-0.282	Detected	0.000	-1.080	Detected	-0.933	Detected	-1.006	0.075	GT	Sens	[illegible]
GT	Sense	Sense	-0.191	Detected	0.191	Detected	0.000	-1.233	Detected	-0.802	Detected	-1.017	0.071	GT	Sens	[illegible]
GT	Sense	Sense	0.726	Detected	-0.726	Detected	0.000	-1.862	Detected	-2.516	Detected	-2.189	0.111	GT	Sens	[illegible]
GT	Sense	Sense	-0.210	Detected	0.210	Detected	0.000	-1.120	Detected	-1.511	Detected	-1.315	0.044	GT	Sens	[illegible]
GT	Sense	Sense	0.215	Detected	-0.215	Detected	0.000	-1.725	Detected	-1.334	Detected	-1.529	0.034	GT	Sens	[illegible]
GT	Sense	Sense	-0.298	Detected	0.298	Detected	0.000	-1.139	Detected	-1.647	Detected	-1.393	0.071	GT	Sens	[illegible]
GT	Sense	Sense	0.508	Detected	-0.508	Detected	0.000	-1.546	Comprom	-3.049	Comprom	-2.297	0.127	GT	Sens	[illegible]
GT	Sense	Sense	-0.187	Detected	0.187	Detected	0.000	-1.145	Detected	-0.999	Detected	-1.072	0.033	GT	Sens	[illegible]
GT	Sense	Sense	0.029	Detected	-0.029	Detected	0.000	-1.509	Detected	-1.228	Detected	-1.368	0.011	GT	Sens	[illegible]
GT	Sense	Sense	0.327	Detected	-0.327	Detected	0.000	-2.118	Detected	-1.398	Detected	-1.758	0.069	GT	Sens	[illegible]
GT	Sense	Sense	0.199	Detected	-0.199	Detected	0.000	-1.684	Detected	-1.426	Detected	-1.555	0.023	GT	Sens	[illegible]
GT	Sense	Sense	-0.128	Detected	0.128	Detected	0.000	-1.403	Detected	-1.291	Detected	-1.347	0.011	GT	Sens	[illegible]
GT	Sense	Sense	0.178	Detected	-0.178	Detected	0.000	-1.417	Detected	-1.466	Detected	-1.441	0.015	GT	Sens	[illegible]
GT	Sense	Sense	0.152	Detected	-0.152	Detected	0.000	-1.289	Detected	-1.078	Detected	-1.183	0.024	GT	Sens	[illegible]
GT	Sense	Sense	0.311	Detected	-0.311	Detected	0.000	-2.232	Detected	-1.579	Detected	-1.906	0.052	GT	Sens	[illegible]
GT	Sense	Sense	-0.060	Detected	0.060	Detected	0.000	-1.239	Detected	-1.496	Detected	-1.367	0.011	GT	Sens	[illegible]
GT	Sense	Sense	0.174	Detected	-0.174	Detected	0.000	-1.282	Detected	-1.416	Detected	-1.349	0.019	GT	Sens	[illegible]
GT	Sense	Sense	-0.070	Detected	0.070	Detected	0.000	-0.884	Detected	-2.954	Comprom	-1.919	0.206	GT	Sens	[illegible]
GT	Sense	Sense	0.632	Detected	-0.632	Detected	0.000	-2.646	Detected	-2.417	Detected	-2.531	0.059	GT	Sens	[illegible]
GT	Sense	Sense	0.212	Detected	-0.212	Detected	0.000	-2.082	Detected	-1.062	Detected	-1.572	0.105	GT	Sens	[illegible]
GT	Sense	Sense	0.016	Detected	-0.016	Detected	0.000	-1.800	Detected	-0.978	Detected	-1.389	0.078	GT	Sens	[illegible]
GT	Sense	Sense	0.064	Detected	-0.064	Detected	0.000	-1.192	Detected	-0.911	Detected	-1.051	0.021	GT	Sens	[illegible]
GT	Sense	Sense	0.145	Detected	-0.145	Detected	0.000	-2.381	Detected	-1.464	Detected	-1.923	0.057	GT	Sens	[illegible]
GT	Sense	Sense	0.266	Detected	-0.266	Detected	0.000	-1.280	Detected	-1.264	Detected	-1.272	0.041	GT	Sens	[illegible]
GT	Sense	Sense	-0.024	Detected	0.024	Detected	0.000	-1.641	Detected	-1.433	Detected	-1.537	0.005	GT	Sens	[illegible]
GT	Sense	Sense	0.065	Detected	-0.065	Detected	0.000	-2.759	Comprom	-3.033	Comprom	-2.896	0.003	GT	Sens	[illegible]
GT	Sense	Sense	0.733	Detected	-0.733	Detected	0.000	-2.308	Detected	-2.815	Detected	-2.561	0.081	GT	Sens	[illegible]
GT	Sense	Sense	-0.063	Detected	0.063	Detected	0.000	-2.176	Comprom	-2.963	Comprom	-2.569	0.023	GT	Sens	[illegible]
GT	Sense	Sense	0.009	Detected	-0.009	Detected	0.000	-1.497	Detected	-1.257	Detected	-1.377	0.008	GT	Sens	[illegible]
GT	Sense	Sense	-0.034	Detected	0.034	Detected	0.000	-0.967	Detected	-1.151	Detected	-1.059	0.008	GT	Sens	[illegible]
GT	Sense	Sense	-0.076	Detected	0.076	Detected	0.000	-1.134	Detected	-0.955	Detected	-1.044	0.012	GT	Sens	[illegible]
GT	Sense	Sense	0.154	Detected	-0.154	Detected	0.000	-2.477	Detected	-2.030	Detected	-2.254	0.014	GT	Sens	[illegible]
GT	Sense	Sense	0.296	Detected	-0.296	Detected	0.000	-1.518	Detected	-1.669	Detected	-1.593	0.035	GT	Sens	[illegible]
GT	Sense	Sense	0.203	Detected	-0.203	Detected	0.000	-1.325	Comprom	-1.695	Comprom	-1.510	0.031	GT	Sens	[illegible]
GT	Sense	Sense	0.089	Detected	-0.089	Detected	0.000	-2.559	Detected	-1.346	Detected	-1.952	0.086	GT	Sens	[illegible]
GT	Sense	Sense	0.526	Detected	-0.526	Detected	0.000	-0.971	Detected	-1.607	Detected	-1.289	0.171	GT	Sens	[illegible]
GT	Sense	Sense	0.036	Detected	-0.036	Detected	0.000	-1.397	Detected	-0.880	Detected	-1.139	0.049	GT	Sens	[illegible]
GT	Sense	Sense	0.027	Detected	-0.027	Detected	0.000	-1.576	Detected	-1.164	Detected	-1.370	0.022	GT	Sens	[illegible]
GT	Sense	Sense	0.045	Detected	-0.045	Detected	0.000	-1.134	Detected	-1.396	Detected	-1.265	0.012	GT	Sens	[illegible]
GT	Sense	Sense	0.060	Detected	-0.060	Detected	0.000	-1.297	Detected	-1.427	Detected	-1.362	0.004	GT	Sens	[illegible]
GT	Sense	Sense	0.039	Detected	-0.039	Detected	0.000	-1.028	Detected	-1.047	Detected	-1.037	0.001	GT	Sens	[illegible]
GT	Sense	Sense	0.075	Detected	-0.075	Detected	0.000	-1.107	Detected	-1.036	Detected	-1.072	0.006	GT	Sens	[illegible]
GT	Sense	Sense	0.146	Detected	-0.146	Detected	0.000	-3.415	Detected	-2.608	Detected	-3.012	0.020	GT	Sens	[illegible]
GT	Sense	Sense	0.291	Detected	-0.291	Detected	0.000	-1.932	Detected	-1.633	Detected	-1.783	0.032	GT	Sens	[illegible]
GT	Sense	Sense	0.137	Detected	-0.137	Detected	0.000	-1.493	Detected	-0.869	Detected	-1.181	0.074	GT	Sens	[illegible]
GT	Sense	Sense	0.198	Detected	-0.198	Detected	0.000	-1.738	Detected	-1.402	Detected	-1.570	0.026	GT	Sens	[illegible]
GT	Sense	Sense	0.399	Detected	-0.399	Detected	0.000	-1.507	Detected	-1.454	Detected	-1.481	0.066	GT	Sens	[illegible]
GT	Sense	Sense	0.229	Detected	-0.229	Detected	0.000	-4.357	Detected	-3.456	Detected	-3.906	0.016	GT	Sens	[illegible]
GT	Sense	Sense	0.082	Detected	-0.082	Detected	0.000	-1.495	Detected	-1.279	Detected	-1.387	0.009	GT	Sens	[illegible]
GT	Sense	Sense	-0.013	Detected	0.013	Detected	0.000	-2.574	Detected	-1.816	Detected	-2.195	0.029	GT	Sens	[illegible]
GT	Sense	Sense	0.078	Detected	-0.078	Detected	0.000	-1.235	Detected	-1.260	Detected	-1.248	0.004	GT	Sens	[illegible]
GT	Sense	Sense	0.033	Detected	-0.033	Detected	0.000	-3.119	Comprom	-3.484	Comprom	-3.301	0.003	GT	Sens	[illegible]
GT	Sense	Sense	0.113	Detected	-0.113	Detected	0.000	-1.269	Detected	-1.085	Detected	-1.177	0.015	GT	Sens	[illegible]
GT	Sense	Sense	0.254	Detected	-0.254	Detected	0.000	-1.132	Detected	-1.172	Detected	-1.152	0.045	GT	Sens	[illegible]
GT	Sense	Sense	-0.078	Detected	0.078	Detected	0.000	-1.781	Detected	-1.982	Detected	-1.882	0.005	GT	Sens	[illegible]
GT	Sense	Sense	0.120	Detected	-0.120	Detected	0.000	-2.889	Detected	-2.027	Detected	-2.458	0.032	GT	Sens	[illegible]
GT	Sense	Sense	0.005	Detected	-0.005	Detected	0.000	-2.348	Detected	-2.255	Detected	-2.302	0.000	GT	Sens	[illegible]
GT	Sense	Sense	0.072	Detected	-0.072	Detected	0.000	-1.950	Detected	-1.333	Detected	-1.641	0.035	GT	Sens	[illegible]
GT	Sense	Sense	-0.038	Detected	0.038	Detected	0.000	-1.337	Detected	-1.197	Detected	-1.267	0.004	GT	Sens	[illegible]
GT	Sense	Sense	0.444	Detected	-0.444	Detected	0.000	-2.715	Detected	-3.076	Detected	-2.896	0.026	GT	Sens	[illegible]
GT	Sense	Sense	0.002	Detected	-0.002	Detected	0.000	-1.285	Detected	-1.022	Detected	-1.154	0.013	GT	Sens	[illegible]
GT	Sense	Sense	-0.237	Detected	0.237	Detected	0.000	-1.151	Detected	-1.231	Detected	-1.191	0.038	GT	Sens	[illegible]
GT	Sense	Sense	-0.659	Detected	0.659	Detected	0.000	-1.981	Comprom	-2.374	Comprom	-2.177	0.087	GT	Sens	[illegible]
GT	Sense	Sense	0.158	Detected	-0.158	Detected	0.000	-1.070	Detected	-1.067	Detected	-1.069	0.021	GT	Sens	[illegible]
GT	Sense	Sense	0.134	Detected	-0.134	Detected	0.000	-1.992	Detected	-1.967	Detected	-1.980	0.005	GT	Sens	[illegible]
GT	Sense	Sense	0.200	Detected	-0.200	Detected	0.000	-3.336	Detected	-2.754	Detected	-3.045	0.013	GT	Sens	[illegible]
GT	Sense	Sense	0.514	Detected	-0.514	Detected	0.000	-2.326	Comprom	-1.389	Comprom	-1.857	0.116	GT	Sens	[illegible]
GT	Sense	Sense	0.361	Detected	-0.361	Detected	0.000	-2.821	Comprom	-1.688	Comprom	-2.255	0.078	GT	Sens	[illegible]
GT	Sense	Sense	0.189	Detected	-0.189	Detected	0.000	-3.451	Detected	-1.344	Detected	-2.397	0.155	GT	Sens	[illegible]
GT	Sense	Sense	0.158	Detected	-0.158	Detected	0.000	-1.011	Detected	-1.235	Detected	-1.123	0.028	GT	Sens	[illegible]
GT	Sense	Sense	0.225	Detected	-0.225	Detected	0.000	-3.833	Detected	-3.824	Detected	-3.829	0.003	GT	Sens	[illegible]
GT	Sense	Sense	0.029	Detected	-0.029	Detected	0.000	-1.210	Detected	-0.851	Detected	-1.030	0.030	GT	Sens	[illegible]
GT	Sense	Sense	-0.017	Detected	0.017	Detected	0.000	-0.975	Detected	-1.055	Detected	-1.015	0.002	GT	Sens	[illegible]
GT	Sense	Sense	0.128	Detected	-0.128	Detected	0.000	-3.403	Detected	-1.982	Detected	-2.692	0.065	GT	Sens	[illegible]
GT	Sense	Sense	0.231	Detected	-0.231	Detected	0.000	-2.044	Detected	-1.359	Detected	-1.701	0.054	GT	Sens	[illegible]
GT	Sense	Sense	0.355	Detected	-0.355	Detected	0.000	-1.619	Detected	-1.769	Detected	-1.694	0.043	GT	Sens	[illegible]
GT	Sense	Sense	0.421	Detected	-0.421	Detected	0.000	-2.066	Detected	-1.660	Detected	-1.863	0.057	GT	Sens	[illegible]
GT	Sense	Sense	0.029	Detected	-0.029	Detected	0.000	-2.084	Detected	-2.192	Detected	-2.138	0.001	GT	Sens	[illegible]
GT	Sense	Sense	0.112	Detected	-0.112	Detected	0.000	-2.286	Comprom	-2.681	Comprom	-2.483	0.008	GT	Sens	[illegible]
GT	Sense	Sense	0.109	Detected	-0.109	Detected	0.000	-1.202	Detected	-0.895	Detected	-1.049	0.031	GT	Sens	[illegible]
GT	Sense	Sense	0.015	Detected	-0.015	Detected	0.000	-1.292	Detected	-1.091	Detected	-1.191	0.007	GT	Sens	[illegible]
GT	Sense	Sense	-0.023	Detected	0.023	Detected	0.000	-2.480	Detected	-2.267	Detected	-2.373	0.002	GT	Sens	[illegible]
GT	Sense	Sense	0.074	Detected	-0.074	Detected	0.000	-1.781	Detected	-1.487	Detected	-1.634	0.010	GT	Sens	[illegible]
GT	Sense	Sense	-0.104	Detected	0.104	Detected	0.000	-0.924	Detected	-1.079	Detected	-1.001	0.016	GT	Sens	[illegible]
GT	Sense	Sense	0.007	Detected	-0.007	Detected	0.000	-1.819	Detected	-1.371	Detected	-1.595	0.019	GT	Sens	[illegible]
GT	Sense	Sense	0.067	Detected	-0.067	Detected	0.000	-1.347	Detected	-1.441	Detected	-1.394	0.003	GT	Sens	[illegible]
GT	Sense	Sense	-0.093	Detected	0.093	Detected	0.000	-1.226	Detected	-0.998	Detected	-1.112	0.017	GT	Sens	[illegible]
GT	Sense	Sense	0.232	Detected	-0.232	Detected	0.000	-1.549	Detected	-1.394	Detected	-1.471	0.026	GT	Sens	[illegible]
GT	Sense	Sense	0.014	Detected	-0.014	Detected	0.000	-1.140	Detected	-1.289	Detected	-1.215	0.004	GT	Sens	[illegible]
GT	Sense	Sense	0.363	Detected	-0.363	Detected	0.000	-1.655	Detected	-1.417	Detected	-1.536	0.057	GT	Sens	[illegible]
GT	Sense	Sense	0.147	Detected	-0.147	Detected	0.000	-2.207	Comprom	-1.989	Comprom	-2.098	0.008	GT	Sens	[illegible]
GT	Sense	Sense	0.321	Detected	-0.321	Detected	0.000	-2.109	Detected	-0.993	Detected	-1.551	0.138	GT	Sens	[illegible]
GT	Sense	Sense	0.225	Detected	-0.225	Detected	0.000	-2.105	Detected	-1.543	Detected	-1.824	0.037	GT	Sens	[illegible]
GT	Sense	Sense	0.053	Detected	-0.053	Detected	0.000	-1.091	Detected	-1.406	Detected	-1.249	0.017	GT	Sens	[illegible]
GT	Sense	Sense	0.254	Detected	-0.254	Detected	0.000	-3.174	Detected	-1.539	Detected	-2.356	0.111	GT	Sens	[illegible]
GT	Sense	Sense	0.023	Detected	-0.023	Detected	0.000	-1.001	Detected	-1.142	Detected	-1.072	0.005	GT	Sens	[illegible]
GT	Sense	Sense	0.191	Detected	-0.191	Detected	0.000	-2.771	Detected	-2.438	Detected	-2.604	0.009	GT	Sens	[illegible]
GT	Sense	Sense	0.198	Detected	-0.198	Detected	0.000	-2.011	Detected	-1.431	Detected	-1.721	0.039	GT	Sens	[illegible]
GT	Sense	Sense	0.089	Detected	-0.089	Detected	0.000	-2.255	Detected	-1.695	Detected	-1.975	0.021	GT	Sens	[illegible]
GT	Sense	Sense	0.294	Detected	-0.294	Detected	0.000	-2.690	Detected	-3.237	Detected	-2.964	0.018	GT	Sens	[illegible]
GT	Sense	Sense	0.050	Detected	-0.050	Detected	0.000	-1.308	Detected	-1.084	Detected	-1.196	0.010	GT	Sens	[illegible]
GT	Sense	Sense	0.058	Detected	-0.058	Detected	0.000	-1.300	Detected	-1.257	Detected	-1.279	0.002	GT	Sens	[illegible]
GT	Sense	Sense	-0.049	Detected	0.049	Detected	0.000	-1.176	Detected	-1.801	Detected	-1.488	0.042	GT	Sens	[illegible]
GT	Sense	Sense	0.394	Detected	-0.394	Detected	0.000	-1.572	Detected	-1.545	Detected	-1.558	0.058	GT	Sens	[illegible]
GT	Sense	Sense	0.151	Detected	-0.151	Detected	0.000	-2.305	Detected	-1.900	Detected	-2.102	0.014	GT	Sens	[illegible]
GT	Sense	Sense	0.135	Detected	-0.135	Detected	0.000	-1.792	Detected	-1.613	Detected	-1.703	0.009	GT	Sens	[illegible]
GT	Sense	Sense	0.258	Detected	-0.258	Detected	0.000	-2.371	Detected	-1.560	Detected	-1.965	0.055	GT	Sens	[illegible]
GT	Sense	Sense	0.126	Detected	-0.126	Detected	0.000	-1.457	Detected	-1.554	Detected	-1.505	0.008	GT	Sens	[illegible]
GT	Sense	Sense	0.477	Detected	-0.477	Detected	0.000	-4.781	Comprom	-4.693	Comprom	-4.737	0.010	GT	Sens	[illegible]
GT	Sense	Sense	0.096	Detected	-0.096	Detected	0.000	-2.037	Detected	-1.256	Detected	-1.647	0.055	GT	Sens	[illegible]
GT	Sense	Sense	0.209	Detected	-0.209	Detected	0.000	-1.851	Detected	-1.538	Detected	-1.694	0.023	GT	Sens	[illegible]
GT	Sense	Sense	0.133	Detected	-0.133	Detected	0.000	-1.740	Detected	-1.224	Detected	-1.482	0.036	GT	Sens	[illegible]
GT	Sense	Sense	0.249	Detected	-0.249	Detected	0.000	-2.852	Comprom	-1.551	Detected	-2.201	0.087	GT	Sens	[illegible]
GT	Sense	Sense	-0.092	Detected	0.092	Detected	0.000	-1.162	Detected	-1.056	Detected	-1.109	0.009	GT	Sens	[illegible]
GT	Sense	Sense	0.046	Detected	-0.046	Detected	0.000	-1.923	Detected	-1.870	Detected	-1.897	0.001	GT	Sens	[illegible]
GT	Sense	Sense	0.087	Detected	-0.087	Detected	0.000	-1.149	Detected	-1.087	Detected	-1.118	0.007	GT	Sens	[illegible]
GT	Sense	Sense	0.076	Detected	-0.076	Detected	0.000	-1.960	Detected	-1.340	Detected	-1.650	0.035	GT	Sens	[illegible]
GT	Sense	Sense	-0.071	Detected	0.071	Detected	0.000	-1.071	Detected	-1.060	Detected	-1.065	0.004	GT	Sens	[illegible]
GT	Sense	Sense	0.263	Detected	-0.263	Detected	0.000	-2.139	Detected	-2.232	Detected	-2.186	0.015	GT	Sens	[illegible]
GT	Sense	Sense	0.358	Detected	-0.358	Detected	0.000	-2.568	Detected	-1.409	Detected	-1.988	0.100	GT	Sens	[illegible]
GT	Sense	Sense	-0.011	Detected	0.011	Detected	0.000	-1.569	Detected	-1.616	Detected	-1.593	0.000	GT	Sens	[illegible]
GT	Sense	Sense	0.400	Detected	-0.400	Detected	0.000	-3.768	Detected	-3.319	Detected	-3.543	0.016	GT	Sens	[illegible]
GT	Sense	Sense	0.047	Detected	-0.047	Detected	0.000	-1.187	Detected	-0.863	Detected	-1.025	0.026	GT	Sens	[illegible]
GT	Sense	Sense	0.021	Detected	-0.021	Detected	0.000	-1.214	Detected	-1.002	Detected	-1.108	0.009	GT	Sens	[illegible]
GT	Sense	Sense	0.155	Detected	-0.155	Detected	0.000	-1.051	Detected	-0.969	Detected	-1.010	0.024	GT	Sens	[illegible]
GT	Sense	Sense	0.029	Detected	-0.029	Detected	0.000	-1.596	Detected	-1.673	Detected	-1.634	0.001	GT	Sens	[illegible]
GT	Sense	Sense	-0.072	Detected	0.072	Detected	0.000	-1.047	Detected	-0.962	Detected	-1.004	0.007	GT	Sens	[illegible]
GT	Sense	Sense	0.145	Detected	-0.145	Detected	0.000	-1.110	Detected	-0.939	Detected	-1.025	0.026	GT	Sens	[illegible]
GT	Sense	Sense	0.109	Detected	-0.109	Detected	0.000	-2.308	Detected	-1.705	Detected	-2.006	0.025	GT	Sens	[illegible]
GT	Sense	Sense	-0.224	Detected	0.224	Detected	0.000	-1.659	Detected	-2.098	Detected	-1.879	0.027	GT	Sens	[illegible]
GT	Sense	Sense	0.153	Detected	-0.153	Detected	0.000	-1.778	Detected	-1.099	Detected	-1.438	0.061	GT	Sens	[illegible]
GT	Sense	Sense	-0.038	Detected	0.038	Detected	0.000	-1.108	Detected	-1.084	Detected	-1.096	0.001	GT	Sens	[illegible]
GT	Sense	Sense	0.261	Detected	-0.261	Detected	0.000	-2.278	Detected	-2.037	Detected	-2.158	0.017	GT	Sens	[illegible]
GT	Sense	Sense	0.148	Detected	-0.148	Detected	0.000	-2.002	Detected	-1.544	Detected	-1.773	0.023	GT	Sens	[illegible]
GT	Sense	Sense	0.326	Detected	-0.326	Detected	0.000	-1.776	Detected	-2.622	Detected	-2.199	0.054	GT	Sens	[illegible]
GT	Sense	Sense	0.037	Detected	-0.037	Detected	0.000	-1.880	Detected	-1.853	Detected	-1.866	0.000	GT	Sens	[illegible]
GT	Sense	Sense	0.251	Detected	-0.251	Detected	0.000	-1.661	Detected	-1.130	Detected	-1.396	0.062	GT	Sens	[illegible]
GT	Sense	Sense	0.016	Detected	-0.016	Detected	0.000	-3.178	Detected	-2.124	Detected	-2.651	0.037	GT	Sens	[illegible]
GT	Sense	Sense	0.316	Detected	-0.316	Detected	0.000	-2.576	Comprom	-1.885	Comprom	-2.231	0.041	GT	Sens	[illegible]
GT	Sense	Sense	0.228	Detected	-0.228	Detected	0.000	-1.074	Detected	-1.038	Detected	-1.056	0.044	GT	Sens	[illegible]
GT	Sense	Sense	0.305	Detected	-0.305	Detected	0.000	-1.833	Detected	-1.594	Detected	-1.713	0.035	GT	Sens	[illegible]
GT	Sense	Sense	-0.052	Detected	0.052	Detected	0.000	-1.094	Detected	-1.436	Detected	-1.265	0.019	GT	Sens	[illegible]
GT	Sense	Sense	0.035	Detected	-0.035	Detected	0.000	-1.699	Detected	-1.214	Detected	-1.457	0.027	GT	Sens	[illegible]
GT	Sense	Sense	0.144	Detected	-0.144	Detected	0.000	-1.518	Detected	-1.134	Detected	-1.326	0.031	GT	Sens	[illegible]
GT	Sense	Sense	0.264	Detected	-0.264	Detected	0.000	-1.176	Detected	-0.846	Detected	-1.011	0.083	GT	Sens	[illegible]
GT	Sense	Sense	-0.159	Detected	0.159	Detected	0.000	-1.243	Detected	-1.328	Detected	-1.285	0.016	GT	Sens	[illegible]
GT	Sense	Sense	0.013	Detected	-0.013	Detected	0.000	-1.345	Detected	-1.339	Detected	-1.342	0.000	GT	Sens	[illegible]
GT	Sense	Sense	0.310	Detected	-0.310	Detected	0.000	-2.317	Detected	-2.133	Detected	-2.225	0.020	GT	Sens	[illegible]
GT	Sense	Sense	0.026	Detected	-0.026	Detected	0.000	-1.166	Detected	-0.922	Detected	-1.044	0.014	GT	Sens	[illegible]
GT	Sense	Sense	0.511	Detected	-0.511	Detected	0.000	-2.053	Detected	-2.106	Detected	-2.080	0.056	GT	Sens	[illegible]
GT	Sense	Sense	0.112	Detected	-0.112	Detected	0.000	-1.368	Detected	-1.253	Detected	-1.310	0.009	GT	Sens	[illegible]
GT	Sense	Sense	0.117	Detected	-0.117	Detected	0.000	-1.344	Detected	-0.987	Detected	-1.166	0.032	GT	Sens	[illegible]
GT	Sense	Sense	0.070	Detected	-0.070	Detected	0.000	-1.180	Detected	-0.837	Detected	-1.009	0.032	GT	Sens	[illegible]
GT	Sense	Sense	0.093	Detected	-0.093	Detected	0.000	-1.542	Detected	-2.400	Detected	-1.971	0.046	GT	Sens	[illegible]
GT	Sense	Sense	0.056	Detected	-0.056	Detected	0.000	-3.083	Detected	-2.281	Detected	-2.682	0.022	GT	Sens	[illegible]
GT	Sense	Sense	-0.034	Detected	0.034	Detected	0.000	-1.157	Detected	-0.965	Detected	-1.061	0.009	GT	Sens	[illegible]
GT	Sense	Sense	0.249	Detected	-0.249	Detected	0.000	-2.125	Detected	-1.492	Detected	-1.809	0.046	GT	Sens	[illegible]
GT	Sense	Sense	0.089	Detected	-0.089	Detected	0.000	-1.760	Detected	-1.743	Detected	-1.752	0.003	GT	Sens	[illegible]
GT	Sense	Sense	0.041	Detected	-0.041	Detected	0.000	-2.184	Detected	-1.545	Detected	-1.865	0.029	GT	Sens	[illegible]
GT	Sense	Sense	0.067	Detected	-0.067	Detected	0.000	-1.144	Detected	-1.294	Detected	-1.219	0.007	GT	Sens	[illegible]
GT	Sense	Sense	0.123	Detected	-0.123	Detected	0.000	-1.963	Detected	-1.469	Detected	-1.716	0.025	GT	Sens	[illegible]
GT	Sense	Sense	0.046	Detected	-0.046	Detected	0.000	-1.021	Detected	-1.037	Detected	-1.029	0.002	GT	Sens	[illegible]
GT	Sense	Sense	-0.053	Detected	0.053	Detected	0.000	-1.500	Detected	-1.097	Detected	-1.298	0.025	GT	Sens	[illegible]
GT	Sense	Sense	0.149	Detected	-0.149	Detected	0.000	-1.826	Detected	-1.393	Detected	-1.609	0.026	GT	Sens	[illegible]
GT	Sense	Sense	0.311	Detected	-0.311	Detected	0.000	-2.558	Detected	-1.428	Detected	-1.993	0.091	GT	Sens	[illegible]
GT	Sense	Sense	0.034	Detected	-0.034	Detected	0.000	-2.038	Detected	-1.218	Detected	-1.628	0.058	GT	Sens	[illegible]
GT	Sense	Sense	0.164	Detected	-0.164	Detected	0.000	-2.141	Detected	-1.964	Detected	-2.052	0.008	GT	Sens	[illegible]
GT	Sense	Sense	0.009	Detected	-0.009	Detected	0.000	-2.516	Detected	-2.052	Detected	-2.284	0.010	GT	Sens	[illegible]
GT	Sense	Sense	-0.026	Detected	0.026	Detected	0.000	-2.101	Detected	-1.781	Detected	-1.941	0.007	GT	Sens	[illegible]
GT	Sense	Sense	-0.045	Detected	0.045	Detected	0.000	-1.112	Detected	-1.113	Detected	-1.113	0.002	GT	Sens	[illegible]
GT	Sense	Sense	-0.051	Detected	0.051	Detected	0.000	-1.477	Comprom	-1.623	Comprom	-1.550	0.003	GT	Sens	[illegible]
GT	Sense	Sense	-0.053	Detected	0.053	Detected	0.000	-1.809	Detected	-1.977	Detected	-1.893	0.003	GT	Sens	[illegible]
GT	Sense	Sense	0.219	Detected	-0.219	Detected	0.000	-1.622	Detected	-1.692	Detected	-1.657	0.018	GT	Sens	[illegible]
GT	Sense	Sense	0.317	Detected	-0.317	Detected	0.000	-0.819	Detected	-1.435	Detected	-1.127	0.125	GT	Sens	[illegible]
GT	Sense	Sense	0.058	Detected	-0.058	Detected	0.000	-0.810	Detected	-1.412	Detected	-1.111	0.068	GT	Sens	[illegible]
GT	Sense	Sense	0.378	Detected	-0.378	Detected	0.000	-3.020	Detected	-2.099	Detected	-2.560	0.050	GT	Sens	[illegible]
GT	Sense	Sense	-0.098	Detected	0.098	Detected	0.000	-1.988	Comprom	-1.113	Detected	-1.551	0.074	GT	Sens	[illegible]
GT	Sense	Sense	0.161	Detected	-0.161	Detected	0.000	-1.061	Detected	-1.006	Detected	-1.034	0.024	GT	Sens	[illegible]
GT	Sense	Sense	-0.067	Detected	0.067	Detected	0.000	-1.343	Detected	-1.220	Detected	-1.281	0.005	GT	Sens	[illegible]
GT	Sense	Sense	0.236	Detected	-0.236	Detected	0.000	-1.175	Detected	-1.262	Detected	-1.218	0.037	GT	Sens	[illegible]
GT	Sense	Sense	0.113	Detected	-0.113	Detected	0.000	-1.917	Detected	-1.710	Detected	-1.813	0.007	GT	Sens	[illegible]
GT	Sense	Sense	0.412	Detected	-0.412	Detected	0.000	-2.097	Detected	-2.299	Detected	-2.198	0.035	GT	Sens	[illegible]
GT	Sense	Sense	0.051	Detected	-0.051	Detected	0.000	-1.827	Detected	-1.365	Detected	-1.596	0.021	GT	Sens	[illegible]
GT	Sense	Sense	0.319	Detected	-0.319	Detected	0.000	-1.390	Detected	-1.439	Detected	-1.414	0.048	GT	Sens	[illegible]
GT	Sense	Sense	-0.315	Detected	0.315	Detected	0.000	-2.901	Detected	-1.331	Detected	-2.116	0.129	GT	Sens	[illegible]
GT	Sense	Sense	-0.085	Detected	0.085	Detected	0.000	-1.466	Detected	-1.098	Detected	-1.282	0.024	GT	Sens	[illegible]
GT	Sense	Sense	0.010	Detected	-0.010	Detected	0.000	-2.234	Detected	-1.089	Detected	-1.662	0.101	GT	Sens	[illegible]
GT	Sense	Sense	-0.105	Detected	0.105	Detected	0.000	-1.374	Detected	-1.259	Detected	-1.317	0.008	GT	Sens	[illegible]
GT	Sense	Sense	0.293	Detected	-0.293	Detected	0.000	-1.768	Detected	-1.368	Detected	-1.568	0.048	GT	Sens	[illegible]
GT	Sense	Sense	0.049	Detected	-0.049	Detected	0.000	-2.237	Detected	-1.642	Detected	-1.940	0.023	GT	Sens	[illegible]
GT	Sense	Sense	0.221	Detected	-0.221	Detected	0.000	-1.155	Detected	-1.360	Comprom	-1.257	0.036	GT	Sens	[illegible]
GT	Sense	Sense	-0.201	Detected	0.201	Detected	0.000	-1.684	Detected	-0.941	Detected	-1.312	0.090	GT	Sens	[illegible]
GT	Sense	Sense	-0.024	Detected	0.024	Detected	0.000	-1.076	Detected	-0.987	Detected	-1.032	0.002	GT	Sens	[illegible]
GT	Sense	Sense	0.384	Detected	-0.384	Detected	0.000	-1.769	Detected	-1.298	Detected	-1.534	0.077	GT	Sens	[illegible]
GT	Sense	Sense	0.098	Detected	-0.098	Detected	0.000	-1.801	Detected	-1.214	Detected	-1.508	0.040	GT	Sens	[illegible]
GT	Sense	Sense	0.134	Detected	-0.134	Detected	0.000	-1.273	Detected	-1.145	Detected	-1.209	0.015	GT	Sens	[illegible]
GT	Sense	Sense	0.730	Detected	-0.730	Detected	0.000	-1.260	Detected	-1.741	Detected	-1.500	0.190	GT	Sens	[illegible]
GT	Sense	Sense	-0.012	Detected	0.012	Detected	0.000	-1.363	Detected	-1.044	Detected	-1.203	0.017	GT	Sens	[illegible]
GT	Sense	Sense	-0.145	Detected	0.145	Detected	0.000	-1.824	Detected	-2.550	Detected	-2.187	0.030	GT	Sens	[illegible]
GT	Sense	Sense	0.409	Detected	-0.409	Detected	0.000	-2.755	Detected	-2.533	Detected	-2.644	0.025	GT	Sens	[illegible]
GT	Sense	Sense	0.006	Detected	-0.006	Detected	0.000	-1.699	Detected	-1.022	Detected	-1.361	0.057	GT	Sens	[illegible]
GT	Sense	Sense	0.028	Detected	-0.028	Detected	0.000	-0.850	Detected	-1.799	Detected	-1.324	0.108	GT	Sens	[illegible]
GT	Sense	Sense	0.149	Detected	-0.149	Detected	0.000	-1.008	Detected	-1.615	Detected	-1.312	0.060	GT	Sens	[illegible]
GT	Sense	Sense	0.055	Detected	-0.055	Detected	0.000	-1.187	Detected	-1.095	Detected	-1.141	0.004	GT	Sens	[illegible]
GT	Sense	Sense	0.079	Detected	-0.079	Detected	0.000	-1.432	Detected	-1.228	Detected	-1.330	0.009	GT	Sens	[illegible]
GT	Sense	Sense	0.419	Detected	-0.419	Detected	0.000	-2.931	Detected	-3.069	Detected	-3.000	0.019	GT	Sens	[illegible]
GT	Sense	Sense	0.118	Detected	-0.118	Detected	0.000	-1.870	Detected	-1.632	Detected	-1.751	0.009	GT	Sens	[illegible]
GT	Sense	Sense	0.098	Detected	-0.098	Detected	0.000	-1.734	Detected	-1.575	Detected	-1.654	0.006	GT	Sens	[illegible]
GT	Sense	Sense	0.153	Detected	-0.153	Detected	0.000	-2.496	Detected	-1.990	Detected	-2.243	0.017	GT	Sens	[illegible]
GT	Sense	Sense	0.280	Detected	-0.280	Detected	0.000	-2.516	Detected	-2.022	Detected	-2.269	0.026	GT	Sens	[illegible]
GT	Sense	Sense	0.146	Detected	-0.146	Detected	0.000	-2.421	Detected	-0.950	Detected	-1.686	0.154	GT	Sens	[illegible]
GT	Sense	Sense	-0.122	Detected	0.122	Detected	0.000	-1.186	Detected	-1.282	Detected	-1.234	0.011	GT	Sens	[illegible]
GT	Sense	Sense	0.246	Detected	-0.246	Detected	0.000	-1.142	Detected	-1.430	Detected	-1.286	0.046	GT	Sens	[illegible]

GT	Sense	Sense	0.130	Detected	-0.130	Detected	0.000	-1.232	Detected	-0.897	Detected	-1.065	0.037	GT Sens	contig105	contig105	Adenosyl	TTAATGA	contig105	Solyc09g	Solyc09g	Adenosyl	GO:0004	GO:0004	contig105	Solyc09g	Adenosyl	GO:0004	SL2.40ch	AT4G139	HOG1. EM	MEE58 (M	chr4:805	8016.59	6298.25	2358.64	2417.52	3225.95	4056.32
GT	Sense	Sense	-0.072	Detected	0.072	Detected	0.000	-1.330	Detected	-1.060	Detected	-1.195	0.016	GT Sens	contig106	contig106	N-acetyltr	AAGTCTC	contig106	Solyc08g	Solyc08g	N-acetyltr	GO:0008	GO:0008	contig106	Solyc08g	N-acetyltr	GO:0008	SL2.40ch	AT4G37920.1		unknown	chr4:178	12899.8	13409.4	5524.32	5788.84	5579.07	6707.63
GT	Sense	Sense	0.250	Detected	-0.250	Detected	0.000	-3.909	Comprom	-3.902	Comprom	-3.905	0.004	GT Sens	contig109	contig109	Acyltrans	GGACAC	contig109	Solyc11g	Solyc11g	Acyltrans	GO:0016	GO:0016	contig109	Solyc11g	Acyltrans	GO:0016	SL2.40ch	AT5G241	SOP1	SOP1: sc	chr5:817	99.9373	66.4582	16.2616	19.1308	5.78549	5.79686
GT	Sense	Sense	-0.054	Detected	0.054	Detected	0.000	-3.929	Detected	-2.901	Detected	-3.415	0.022	GT Sens	contig109	contig109	HIN1-like	ATCCTAC	contig109	Solyc10g	Solyc10g	HIN1-like protein (Fragment) (A			contig109	Solyc10g	HIN1-like protein (Fr		SL2.40ch	AT5G63900.1		PHD fing	chr5:255	1640.01	1663.89	228.763	350.425	115.689	235.163
GT	Sense	Sense	-0.502	Detected	0.502	Detected	0.000	-0.954	Detected	-2.001	Comprom	-1.478	0.179	GT Sens	contig109	contig109	UDP-gluc	TTTGAAC	contig109	Solyc10g	Solyc10g	UDP-gluc	GO:0008	GO:0008	contig109	Solyc10g	UDP-gluc	GO:0008	SL2.40ch	AT2G36970.1		UDP-gluc	chr2:155	23.4798	44.3276	20.8631	17.3158	17.7616	8.56656
GT	Sense	Sense	-0.005	Detected	0.005	Detected	0.000	-1.653	Detected	-1.145	Detected	-1.399	0.031	GT Sens	contig110	contig110	Ycf2 (AHF	TTTCCTC	contig110	Solyc02g	Solyc02g	Ycf2 (AHF	GO:0009	GO:0009	contig110	Solyc09g	RING fing	GO:0006	SL2.40ch	AT3G218	ATXR2. S	ATXR2 (H	chr3:768	2442.68	2315.95	1075.3	822.965	806.331	1143.41
GT	Sense	Sense	-0.149	Detected	0.149	Detected	0.000	-2.129	Detected	-1.471	Detected	-1.800	0.038	GT Sens	contig110	contig110	Chitinase	TTTTTTC	contig110	Solyc10g	Solyc10g	Chitinase	GO:0008	GO:0008	contig110	Solyc10g	Chitinase	GO:0005	SL2.40ch	AT5G08300.1		succinyl-	chr5:266	2024	2342.13	1205.77	1003.73	530.97	835.219
GT	Sense	Sense	0.348	Detected	-0.348	Detected	0.000	-2.439	Detected	-2.565	Detected	-2.502	0.019	GT Sens	contig110	contig110	Auxin-res	ATTAACC	contig110	Solyc01g	Solyc01g	Auxin-responsive GH3 produc			contig110	Solyc01g	Auxin-responsive G		SL2.40ch	AT2G149	GH3.1	GH3.1	chr2:645	869.011	504.964	77.9446	169.987	130.293	118.981
GT	Sense	Sense	0.138	Detected	-0.138	Detected	0.000	-1.556	Detected	-1.084	Detected	-1.320	0.040	GT Sens	contig111	contig111	Plant syn	CCAGTTC	contig111	Solyc12g	Solyc12g	Plant synaptotagmin (AHRD V1			contig111	Solyc12g	Synaptotagmin-7 (A		SL2.40ch	AT1G055	SYTE. AT	NTMC2T	chr1:162	378.133	293.964	100.03	125.62	120.937	167.241
GT	Sense	Sense	0.411	Detected	-0.411	Detected	0.000	-1.361	Detected	-0.988	Detected	-1.175	0.121	GT Sens	contig112	contig112	Cc-nbs-lr	GAGAAG	contig112	Solyc11g	Solyc11g	Cc-nbs-lrr resistance protein (			contig112	Solyc05g014620.1.1				AT1G086	ZIGA4	ZIGA4 (A	chr1:276	22881.9	12174.2	7345.05	8106.73	6927.47	8946.83
GT	Sense	Sense	-0.080	Detected	0.080	Detected	0.000	-1.420	Detected	-1.274	Detected	-1.347	0.006	GT Sens	contig112	contig112	ATP-bind	TTGGAG	contig112	Solyc03g	Solyc03g	ATP-bind	GO:0016	GO:0016	contig112	Solyc03g	ATP-bind	GO:0042	SL2.40ch	AT3G477	ATATH6.	ATATH6:	chr3:176	1971.49	2071.56	704.097	986.065	805.207	888.188
GT	Sense	Sense	0.023	Detected	-0.023	Detected	0.000	-2.152	Detected	-1.637	Detected	-1.894	0.018	GT Sens	contig112	contig112	Unknown	ACTGGT	contig112	Solyc05g	Solyc05g	Unknown Protein (AHRD V1)			contig112	Solyc11g	Unknown Protein (A		SL2.40ch	AT3G27870.1		haloacid	chr3:103	88.2875	80.508	48.4368	34.9382	20.2353	28.8189
GT	Sense	Sense	0.046	Detected	-0.046	Detected	0.000	-1.317	Detected	-1.021	Detected	-1.169	0.017	GT Sens	contig113	contig113	Squalene	ACACTGT	contig113	Solyc10g	Solyc10g	Squalene	GO:0016	GO:0016	contig113	Solyc01g	Squalene	GO:0005	SL2.40ch	AT4G346	SOS1. ER	SOS1 (SC	chr4:165	1199.31	1058.87	405.906	406.456	482.398	590.237
GT	Sense	Sense	-0.094	Detected	0.094	Detected	0.000	-1.153	Detected	-1.040	Detected	-1.096	0.010	GT Sens	contig114	contig114	Receptor	ATTCACT	contig114	Solyc10g	Solyc10g	Receptor-	GO:0006	GO:0006	contig114	Solyc10g	Receptor-	GO:0006	SL2.40ch	AT1G075	APK1A. A	APK1A: k	chr1:233	4607.81	4940.99	1984.26	2106.26	2288.9	2467.82
GT	Sense	Sense	0.017	Detected	-0.017	Detected	0.000	-1.215	Detected	-1.618	Detected	-1.416	0.020	GT Sens	contig116	contig116	cDNA clo	ATATTTA	contig116	Solyc11g	Solyc11g	cDNA clo	GO:0016	GO:0016	contig116	Solyc05g	NPH3 domain conta		SL2.40ch	AT5G638	PP7. ATP	PP7 (SEF	chr5:255	1119.75	1028.68	281.946	429.577	492.973	371.791
GT	Sense	Sense	0.003	Detected	-0.003	Detected	0.000	-1.650	Detected	-1.492	Detected	-1.571	0.003	GT Sens	contig116	contig116	Phi-1 pro	TCTTGCA	contig116	Solyc04g	Solyc04g	Phi-1 protein (Fragment) (AHRI			contig116	Solyc04g	Phi-1 protein (Fragn		SL2.40ch	AT4G089	EXO	EXO (EX	chr4:574	2128.59	1994.39	504.567	855.075	700.289	778.74
GT	Sense	Sense	-0.037	Detected	0.037	Detected	0.000	-1.212	Detected	-1.005	Detected	-1.108	0.010	GT Sens	contig118	contig118	Unknown	CAAGAAC	contig118	Solyc10g	Solyc10g	Unknown Protein (AHRD V1)			contig118	Solyc10g	Unknown Protein (A		SL2.40ch	AT5G62350.1				801.589	794.18	340.868	481.268	367.343	422.71
GT	Sense	Sense	0.364	Detected	-0.364	Detected	0.000	-1.256	Detected	-1.102	Detected	-1.179	0.087	GT Sens	contig118	contig118	U-box do	TTTGAGT	contig118	Solyc01g	Solyc01g	U-box do	GO:0005	GO:0005	contig118	Solyc01g	U-box do	GO:0005	SL2.40ch	AT4G02430.2		pre-mRN	chr4:106	932.842	530.236	179.352	154.927	314	348.293
GT	Sense	Sense	0.210	Detected	-0.210	Detected	0.000	-1.807	Detected	-1.000	Detected	-1.403	0.091	GT Sens	contig119	contig119	Galactoki	GAAGGC	contig119	Solyc09g	Solyc09g	Galactoki	GO:0005	GO:0005	contig119	Solyc09g	Galactoki	GO:0005	SL2.40ch	AT3G10700.1		GHMP ki	chr3:334	1436.14	1010.01	300.33	427.199	367.075	640.215
GT	Sense	Sense	0.086	Detected	-0.086	Detected	0.000	-1.732	Detected	-1.640	Detected	-1.686	0.003	GT Sens	contig121	contig121	Unknown	AGTTGCT	contig121	Solyc02g	Solyc02g	Unknown Protein (AHRD V1)			contig121	Solyc02g	Unknown Protein (A		SL2.40ch	AT5G424	ARC6	ARC6 (AC	chr5:169	325.626	271.82	67.3772	109.442	95.5105	101.525

GT	Sense	Sense	0.211	Detected	-0.211	Detected	0.000	-0.839	Detected	-1.304	Detected	-1.072	0.076	GT	Sens	contig552	contig552	Pseudo-r	GCAGCT	contig552	Solvc03g	Solvc03g	Pseudo-r	GO:0006	GO:0006	contig552	Solvc03g	Pseudo-r	GO:0006	SL2.40ch	AT5G028	PRR7, AP	PRR7 (PS	chr5:638	206.789	145.241	58.0192	63.3428	103.278	74.6347
GT	Sense	Sense	-0.334	Detected	0.334	Detected	0.000	-1.627	Detected	-1.086	Detected	-1.356	0.087	GT	Sens	contig570	contig570	Epoxide h	GTATTG	contig570	Solvc01g	Solvc01g	Epoxide h	GO:0003	GO:0003	contig570	Solvc01g	Epoxide h	GO:0004	SL2.40ch	AT4G15960.1		catalytic/	chr4:904	9341.08	13962.4	6676.26	10229.9	3943.9	5720.11
GT	Sense	Sense	0.072	Detected	-0.072	Detected	0.000	-1.280	Detected	-1.092	Detected	-1.186	0.010	GT	Sens	contig591	contig591	AP-2 com	CAAACT	contig591	Solvc11g	Solvc11g	AP-2 com	GO:0030	GO:0030	contig591	Solvc11g	AP-2 com	GO:0030	SL2.40ch	AT5G227	alpha-AD	alpha-AD	chr5:757	1054.43	897.516	248.597	334.808	427.223	485.104
GT	Sense	Sense	0.254	Detected	-0.254	Detected	0.000	-2.672	Detected	-1.136	Detected	-1.904	0.143	GT	Sens	contig592	contig592	UDP-gluc	ACAAGA	contig592	Solvc04g	Solvc04g	UDP-gluc	GO:0008	GO:0008	contig592	Solvc04g	UDP-gluc	GO:0015	SL2.40ch	AT3G531	UGT73C7	UGT73C	chr3:197	235.585	155.9	31.0156	49.9844	32.0593	92.7328
GT	Sense	Sense	0.599	Detected	-0.599	Detected	0.000	-3.894	Detected	-4.041	Detected	-3.967	0.022	GT	Sens	contig600	contig600	Acyltrans	CCAAGT	contig600	Solvc11g	Solvc11g	Acyltrans	GO:0016	GO:0016	contig600	Solvc11g	Acyltrans	GO:0016	SL2.40ch	AT4G37950.1		lyase	chr4:178	1634.61	670.145	293.818	829.989	75.0738	67.6202
GT	Sense	Sense	0.070	Detected	-0.070	Detected	0.000	-3.092	Detected	-1.383	Detected	-2.238	0.121	GT	Sens	contig606	contig606	Receptor	TTGTCA	contig606	Solvc09g	Solvc09g	Receptor	GO:0005	GO:0005	contig606	Solvc09g	Receptor	GO:0005	SL2.40ch	AT2G39360.1		protein k	chr2:164	173.644	148.253	43.9537	32.3027	20.0631	65.4022
GT	Sense	Sense	0.081	Detected	-0.081	Detected	0.000	-2.468	Detected	-1.456	Detected	-1.962	0.062	GT	Sens	contig612	contig612	Cysteine	AAGTAC	contig612	Solvc11g	Solvc11g	Cysteine	GO:0008	GO:0008	contig612	Solvc11g	Cysteine	GO:0008	SL2.40ch	AT4G30260.1		integral r	chr4:148	258.021	217.078	53.5008	75.3693	45.6111	91.7332
GT	Sense	Sense	0.063	Detected	-0.063	Detected	0.000	-1.379	Detected	-1.127	Detected	-1.253	0.012	GT	Sens	contig623	contig623	Glycosylt	TACACT	contig623	Solvc09g	Solvc09g	Glycosylt	GO:0016	GO:0016	contig623	Solvc06g	Glycosylt	GO:0047	SL2.40ch	AT3G251	GAUT8, C	QUA1 (QU	chr3:915	8079.94	6965.03	2803.26	3571.92	3076.23	3651.32
GT	Sense	Sense	0.056	Detected	-0.056	Detected	0.000	-1.429	Detected	-1.291	Detected	-1.360	0.004	GT	Sens	contig625	contig625	Auxin-ind	GGCATT	contig625	Solvc01g	Solvc01g	Auxin-ind	GO:0005	GO:0005	contig625	Solvc01g	Auxin-ind	GO:0005	SL2.40ch	AT4G34760.1		auxin-res	chr4:165	530.004	461.615	151.996	328.891	195.87	214.953
GT	Sense	Sense	-0.030	Detected	0.030	Detected	0.000	-1.165	Detected	-0.871	Detected	-1.018	0.021	GT	Sens	contig640	contig640	UDP-gluc	AGATGT	contig640	Solvc02g	Solvc02g	UDP-gluc	GO:0006	GO:0006	contig640	Solvc02g	UDP-gluc	GO:0048	SL2.40ch	AT5G444	DUR	DUR (DEI	chr5:179	5123.41	5026.15	2205.22	2707.64	2413.23	2949.46
GT	Sense	Sense	0.029	Detected	-0.029	Detected	0.000	-1.221	Detected	-1.092	Detected	-1.156	0.004	GT	Sens	contig644	contig644	Unknown	GTAGAT	contig644	Solvc00g	Solvc00g	Unknown Protein (AHRD V1)			contig644	Solvc00g	Unknown Protein (A		SL2.40ch	AT3G559	CCR3, AT	CCR3 (AF	chr3:207	259.188	234.162	77.9808	98.4186	112.678	122.914
GT	Sense	Sense	0.170	Detected	-0.170	Detected	0.000	-1.085	Detected	-0.949	Detected	-1.017	0.031	GT	Sens	contig666	contig666	CBL-inter	GTCTTG	contig666	Solvc06g	Solvc06g	CBL-inter	GO:0004	GO:0004	contig666	Solvc06g050290.1.1				AT5G583	CIPK10, F	SIP1 (SO	chr5:235	5064.67	3764.51	1302.82	2431.02	2195.35	2405.55
GT	Sense	Sense	0.196	Detected	-0.196	Detected	0.000	-3.427	Comprom	-1.877	Detected	-2.652	0.080	GT	Sens	contig671	contig671	Cytochro	GTTCAA	contig671	Solvc10g	Solvc10g	Cytochro	GO:0020	GO:0020	contig671	Solvc10g	Cytochro	GO:0046	SL2.40ch	AT2G408	CYP98A3	CYP98A3	chr2:170	87.2294	62.5529	14.2201	12.7902	7.32582	21.3841
GT	Sense	Sense	0.381	Detected	-0.381	Detected	0.000	-3.149	Detected	-2.572	Detected	-2.861	0.027	GT	Sens	contig683	contig683	Pyridine r	GTGAAA	contig683	Solvc11g	Solvc11g	Pyridine r	GO:0055	GO:0055	contig683	Solvc11g	Nitric oxide reducta		SL2.40ch	AT3G102	LYC	LYC (LYC	chr3:316	348.06	193.192	36.3001	69.6139	31.1827	46.3492
GT	Sense	Sense	-0.002	Detected	0.002	Detected	0.000	-1.837	Detected	-1.468	Detected	-1.653	0.012	GT	Sens	contig690	contig690	Fatty acid	AAAGCA	contig690	Solvc10g	Solvc10g	Fatty acid	GO:0004	GO:0004	contig690	Solvc10g	Fatty acid	GO:0004	SL2.40ch	AT3G61580.1		delta-8 s	chr3:227	10452	9861.19	3446.19	3668.14	3029.32	3900.74
GT	Sense	Sense	0.206	Detected	-0.206	Detected	0.000	-1.302	Detected	-1.329	Detected	-1.316	0.024	GT	Sens	contig698	contig698	ABC trans	TGCTAG	contig698	Solvc06g	Solvc06g	ABC trans	GO:0017	GO:0017	contig698	Solvc03g	ABC trans	GO:0008	SL2.40ch	AT1G178	WBC11, A	WBC11 (W	chr1:614	185.033	130.892	55.8269	35.7373	67.293	65.8728
GT	Sense	Sense	-0.004	Detected	0.004	Detected	0.000	-1.071	Detected	-1.138	Detected	-1.105	0.001	GT	Sens	contig714	contig714	GTPase a	AATGAA	contig714	Solvc06g	Solvc06g	GTPase a	GO:0005	GO:0005	contig714	Solvc06g	GTPase a	GO:0005	SL2.40ch	AT3G49350.1		RAB GTP	chr3:182	720.374	682.031	202.102	268.772	355.863	338.549
GT	Sense	Sense	0.711	Detected	-0.711	Detected	0.000	-0.864	Detected	-1.502	Detected	-1.183	0.268	GT	Sens	contig725	contig725	Salicylic a	TCCTTG	contig725	Solvc09g	Solvc09g	Salicylic a	GO:0008	GO:0008	contig725	Solvc09g	Salicylic a	GO:0008	SL2.40ch	AT4G36470.1		S-adenos	chr4:172	165.567	58.1529	69.3762	3.2412	57.4706	36.8342
GT	Sense	Sense	0.159	Detected	-0.159	Detected	0.000	-2.531	Detected	-1.758	Detected	-2.144	0.036	GT	Sens	contig729	contig729	Beta-fruc	GAGAAT	contig729	Solvc09g	Solvc09g	Beta-fruc	GO:0005	GO:0005	contig729	Solvc10g	Beta-fruc	GO:0005	SL2.40ch	AT3G52700.1		unknown	chr3:195	62562.5	47232.7	12406.3	18487.2	10028.4	17092.4
GT	Sense	Sense	0.136	Detected	-0.136	Detected	0.000	-3.635	Detected	-2.992	Detected	-3.313	0.011	GT	Sens	contig732	contig732	Globulin-	AATCAA	contig732	Solvc09g	Solvc09g	Globulin-	GO:0045	GO:0045	contig732	Solvc09g	Vicilin-like	GO:0045	SL2.40ch	AT2G43560.1		immunor	chr2:180	174.683	136.165	23.8679	48.1812	13.2405	20.6153
GT	Sense	Sense	0.048	Detected	-0.048	Detected	0.000	-3.146	Detected	-2.447	Detected	-2.797	0.016	GT	Sens	contig749	contig749	Abscisic	ACCGTT	contig749	Solvc04g	Solvc04g	Abscisic	GO:0006	GO:0006	contig749	Solvc04g	Abscisic	GO:0006	SL2.40ch	AT1G76940.1		RNA reco	chr1:289	321.173	282.9	43.5141	43.544	36.3009	58.7916
GT	Sense	Sense	-0.135	Detected	0.135	Detected	0.000	-2.777	Detected	-1.970	Detected	-2.374	0.031	GT	Sens	contig756	contig756	Peroxidas	TTTTCA	contig756	Solvc02g	Solvc02g	Peroxidas	GO:0005	GO:0005	contig756	Solvc02g	Peroxidas	GO:0004	SL2.40ch	AT1G14540.1		anionic p	chr1:497	30472.3	34550.8	7303.67	5629.95	5048.85	8803.31
GT	Sense	Sense	0.635	Detected	-0.635	Detected	0.000	-4.162	Comprom	-2.086	Comprom	-3.124	0.124	GT	Sens	contig761	contig761	Cytochro	GAATGC	contig761	Solvc02g	Solvc02g	Cytochro	GO:0019	GO:0019	contig761	Solvc02g	Cytochro	GO:0008	SL2.40ch	AT5G384	CYP735A	CYP735A	chr5:153	50.5369	19.7139	6.01537	7.63431	1.87981	7.90172
GT	Sense	Sense	-0.310	Detected	0.310	Detected	0.000	-1.514	Detected	-1.600	Detected	-1.557	0.038	GT	Sens	contig769	contig769	Proteinas	TACTCA	contig769	Solvc03g	Solvc03g	Proteinas	GO:0004	GO:0004	contig769	Solvc03g	Proteinas	GO:0004	SL2.40ch	AT4G15830.1		binding	chr4:899	7399.16	10696.1	4832.43	7458.89	3321.66	3119.72
GT	Sense	Sense	0.667	Detected	-0.667	Detected	0.000	-0.914	Detected	-2.509	Detected	-1.712	0.241	GT	Sens	contig776	contig776	Unknown	TATGAT	contig776	Solvc12g	Solvc12g	Unknown Protein (AHRD V1)			contig776	Solvc12g	Unknown Protein (A		SL2.40ch	AT5G14020.1		INVOLVE	chr5:452	610.72	227.945	43.6162	26.0922	211.131	69.6704
GT	Sense	Sense	0.115	Detected	-0.115	Detected	0.000	-1.403	Detected	-1.092	Detected	-1.247	0.023	GT	Sens	contig797	contig797	Ras-like G	CAGCCA	contig797	Solvc02g	Solvc02g	Ras-like G	GO:0003	GO:0003	contig797	Solvc02g	Ras-like G	GO:0003	SL2.40ch	AT1G200	ARAC4, R	ROP2 (RH	chr1:696	6430.65	5162.32	1765.55	2742.21	2324.08	2874.46
GT	Sense	Sense	0.078	Detected	-0.078	Detected	0.000	-3.017	Detected	-2.335	Detected	-2.676	0.017	GT	Sens	contig810	contig810	Abscisic	CACCGT	contig810	Solvc04g	Solvc04g	Abscisic	GO:0006	GO:0006	contig810	Solvc04g071590.1.1				AT3G44590.1		60S acid	chr3:161	323.356	273.004	32.8925	56.4069	39.1472	62.5922
GT	Sense	Sense	0.067	Detected	-0.067	Detected	0.000	-3.049	Detected	-1.591	Detected	-2.320	0.087	GT	Sens	contig816	contig816	Auxin-ind	TCTTCA	contig816	Solvc07g	Solvc07g	Auxin-induced protein-like (AH			contig816	Solvc07g	Auxin-induced prote		SL2.40ch	AT3G07730.1		unknown	chr3:246	109.761	94.1702	24.2466	45.9353	13.0952	35.8714
GT	Sense	Sense	0.171	Detected	-0.171	Detected	0.000	-1.680	Detected	-1.336	Detected	-1.508	0.025	GT	Sens	contig837	contig837	Ras-relate	ATTCCG	contig837	Solvc07g	Solvc07g	Ras-relate	GO:0005	GO:0005	contig837	Solvc07g	Ras-relate	GO:0015	SL2.40ch	AT3G150	AtRABA1	AtRABA1	chr3:506	463.068	343.542	102.831	120.669	132.735	167.997
GT	Sense	Sense	-0.107	Detected	0.107	Detected	0.000	-3.528	Detected	-3.976	Detected	-3.752	0.004	GT	Sens	contig843	contig843	Glutathio	GGCGTA	contig843	Solvc01g	Solvc01g	Glutathione-S-transferase (AH			contig843	Solvc01g	Glutathione-S-trans		SL2.40ch	AT3G492	emb1796	emb1796	chr3:182	8061.34	8794.29	1176.48	809.895	778.404	569.078
GT	Sense	Sense	0.692	Detected	-0.692	Detected	0.000	-0.857	Detected	-2.063	Detected	-1.460	0.252	GT	Sens	contig854	contig854	Unknown	GATCAA	contig854	Solvc12g	Solvc12g	Unknown Protein (AHRD V1)			contig854	Solvc12g	Unknown Protein (A		SL2.40ch	AT3G236	PAKRP1L	KINESIN-	chr3:851	256.914	92.6733	20.8997	19.6888	90.824	39.2536
GT	Sense	Sense	-0.160	Detected	0.160	Detected	0.000	-0.951	Detected	-1.683	Detected	-1.317	0.081	GT	Sens	contig856	contig856	Cytochro	TCAAAA	contig856	Solvc03g	Solvc03g	Cytochro	GO:0020	GO:0020	contig856	Solvc03g	Cytochro	GO:0004	SL2.40ch	AT3G483	CYP71A2	CYP71A2	chr3:178	679.757	798.194	265.454	183.481	406.437	243.833
GT	Sense	Sense	0.026	Detected	-0.026	Detected	0.000	-1.364	Detected	-1.561	Detected	-1.462	0.005	GT	Sens	contig86	contig86	Calcium-d	TTTACG	contig86	Solvc04g	Solvc04g	Calcium-d	GO:0006	GO:0006	contig86	Solvc04g	Calcium-d	GO:0004	SL2.40ch	AT1G086	PPCK1	PPCK1 (P	chr1:275	1872.02	1699.27	657.508	718.556	739.132	642.616
GT	Sense	Sense	0.046	Detected	-0.046	Detected	0.000	-1.344	Detected	-0.871	Detected	-1.107	0.044	GT	Sens	contig870	contig870	Alpha-1 4	AAACTT	contig870	Solvc05g	Solvc05g	Alpha-1 4	GO:0016	GO:0016	contig870	Solvc05g	Alpha-1 4	GO:0016	SL2.40ch	AT3G022	RGP1, AT	RGP1 (RE	chr3:415	9109.1	8039.21	2896.27	4176.27	3595.28	4974.79
GT	Sense	Sense	0.034	Detected	-0.034	Detected	0.000	-1.344	Detected	-0.945	Detected	-1.144	0.030	GT	Sens	contig879	contig879	Pyrophos	AAAGAA	contig879	Solvc07g	Solvc07g	Pyrophos	GO:0016	GO:0016	contig879	Solvc07g	Pyrophos	GO:0009	SL2.40ch	AT1G156	AVP1, AT	AVP1: AT	chr1:539	3636.7	3262.19	1156.73	1263.76	1447.15	1902.05
GT	Sense	Sense	0.135	Detected	-0.135	Detected	0.000	-0.979	Detected	-1.187	Detected	-1.083	0.024	GT	Sens	contig879	contig879	Protein ki	CCATTG	contig879	Solvc07g	Solvc07g	Protein kinase family protein (A			contig879	Solvc01g	Receptor-	GO:0006	SL2.40ch	AT1G09440.1		protein k	chr1:304	938.671	732.983	226.175	229.817	448.703	387.335
GT	Sense	Sense	0.228	Detected	-0.228	Detected	0.000	-1.724	Comprom	-1.467	Comprom	-1.596	0.026	GT	Sens	contig909	contig909	Inosine-u	GCATAC	contig909	Solvc01g	Solvc01g	Inosine-u	GO:0016	GO:0016	contig909	Solvc01g	Inosine-u	GO:0016	SL2.40ch	AT5G18890.1		inosine-u	chr5:630	17.3706	11.9176	2.12974	16.7163	4.6435	5.53241
GT	Sense	Sense	-0.022	Detected	0.022	Detected	0.000	-1.938	Detected	-1.038	Detected	-1.488	0.081	GT	Sens	contig911	contig911	Pectate ly	ATTTTG	contig911	Solvc06g	Solvc06g	Pectate ly	GO:0016	GO:0016	contig911	Solvc06g071840.1.1				AT4G24780.1		pectate ly	chr4:127	3975.09	3854.04	1289.82	1240.72	1089.58	2026.16
GT	Sense	Sense	0.200	Detected	-0.200	Detected	0.000	-2.011	Detected	-1.287	Detected	-1.649	0.057	GT	Sens	contig931	contig931	Auxin-ind	ATACCT	contig931	Solvc01g	Solvc01g	Auxin-ind	GO:0005	GO:0005	contig931	Solvc01g	Auxin-ind	GO:0005	SL2.40ch	AT4G38840.1		auxin-res	chr4:181	6374.94	4547.93	1272.42	2582.89	1424.8	2345.66
GT	Sense	Sense	0.005	Detected	-0.005	Detected	0.000	-1.351	Detected	-1.611	Detected	-1.481	0.008	GT	Sens	contig938	contig938	Laccase	GTTATT	contig938	Solvc05g	Solvc05g	Laccase	GO:0048	GO:0048	contig938	Solvc07g	Laccase	GO:0005	SL2.40ch	AT5G589	LAC16	LAC16 (la	chr5:237	1120.02	1047.22	621.951	495.869	452.631	377.038
GT	Sense	Sense	-0.032	Detected	0.032	Detected	0.000	-2.908	Comprom	-2.910	Comprom	-2.909	0.000	GT	Sens	contig964	contig964	NifS-like	TAGAGA	contig964	Solvc11g	Solvc11g	NifS-like	GO:0008	GO:0008	contig964	Solvc11g005830.1.1				AT3G48670.2		XH/XS do	chr3:180	38.4059	37.7695	5.5804	9.30441	5.41116	5.38815
GT	Sense	Sense	0.146	Detected	-0.146	Detected	0.000	-1.121	Detected	-1.021	Detected	-1.071	0.020	GT	Sens	contig978	contig978	KH domai	ATTAGA	contig978	Solvc10g	Solvc10g	KH domai	GO:0003	GO:0003	contig978	Solvc10g	KH domai	GO:0003	SL2.40ch	AT3G08620.1		KH doma	chr3:261	3022.76	2321.91	1199.32	1015.18	1299.02	1388.15
GT	Sense	Sense	0.177	Detected	-0.177	Detected	0.000	-1.478	Detected	-1.429	Detected	-1.453	0.015	GT	Sens	contig979	contig979	Potassiur	CTCTCT	contig979	Solvc10g	Solvc10g	Potassiur	GO:0016	GO:0016	contig979	Solvc06g	Potassiur	GO:0009	SL2.40ch	AT1G703	KUP6	KUP6: po	chr1:264	86.1045	63.3532	30.4747	24.1633	28.2787	29.1607
GT	Sense	Sense	0.113	Detected	-0.113	Detected	0.000	-1.259	Detected	-1.046	Detected	-1.152	0.018	GT	Sens	contig979	contig979	Cytochro	ACTATA	contig979	Solvc05g	Solvc05g	Cytochro	GO:0016	GO:0016	contig979	Solvc05g	Cytochro	GO:0016	SL2.40ch	ATMG001	COX2	cytochro	chrM:40	1811.3	1456.64	736.182	668.851	723.776	836.458
GT	Sense	Sense	-0.739	Detected	0.739	Detected	0.000	-1.324	Detected	-0.892	Detected	-1.108	0.287	GT	Sens	contig995	contig995	Pectinest	ATTATA	contig995	Solvc07g	Solvc07g	Pectinest	GO:0030	GO:0030	contig995	Solvc07g	Pectinest	GO:0005	SL2.40ch	AT5G64640.1		pectinest	chr5:258	27.1557	71.1974	13.8221	19.0628	18.7267	25.2002
GT	Sense	Sense	-0.061	Detected	0.061	Detected	0.000	-1.572	Detected	-1.188	Detected	-1.380	0.021	GT	Sens	NM_0012	NM_0012	Solanum	CAGAACAGCCCATTATTATTCCAGCAGCCCAACAAAACAATTTGTTCACCAATAATAACA																2120.41	2170.06	1291.25	1107.29	769.399	1001.32
GT	Sense	Sense	0.105	Detected	-0.105	Detected	0.000	-1.279	Detected	-1.566	Detected	-1.423	0.015	GT	Sens	U13054	U13054	Cel1: Cro	GCCCAAACTTTACTAGCTAAGGAATACCTTAATGGAAAGAGCAATTTGGAAAAATTCAAG																16960.8	13795.4	5517.97	7993.57	6721.07	5491.68
GT	Sense	Sense	0.168	Detected	-0.168	Detected	0.000	-2.614	Detected	-1.849	Detected	-2.232	0.033	GT	Sens	U23053	U23053	LEU2305	CAAGAGTTAGGAGTCCAAAATGTGACAGTTAAGACTGTGACTTTCAGTGGAACAACAAAT																140820	105036	21734.1	40192.3	21180.3	35900.5
GT	Sense	Sense	0.108	Detected	-0.108	Detected	0.000	-2.135	Detected	-1.407	Detected	-1.771	0.043	GT	Sens	U23053	U23053	TAPG1: C	GCCAGAGGAAGAGTTTCTGGTTTACAAAAACCTACCAATTGCCTATTAAAAAGTTAGATA																166114	134545	44248.4	75243.7	36285.6	59928.1
GT	Sense	Sense	0.133	Detected	-0.133	Detected	0.000	-2.804	Detected	-1.893	Detected	-2.349	0.038	GT	Sens	U70480	U70480	LEU7048	GCTGGAAACAACAAGAGTTGGGAGTCCAAAATGTGACAGTTAAGACTGTGACTTTTAGTG																150729	118004	23161.7	42916.5	20359.5	38170.2
GT	Sense	Sense	0.028	Detected	-0.028	Detected	0.000	-2.283	Detected	-1.292	Detected	-1.788	0.069	GT	Sens	U70481	U70481	LEU7048	CTGGAGGAAGAGCTTCTGGTTTTGAAGAACTTAGTAAATGCTTATAAATTATACAACAAA																142211	128708	96985.6	113955	29637.8	58733.5
GT	Sense	Sense	0.051	Detected	-0.051	Detected	0.000	-3.100	Detected	-2.111	Detected	-2.605	0.035	GT	Sens	U70481	U70481	TAPG4: C	AAGAGCAAGGAGTACAAAATGTGACAGTTAAGATGGTTAGCTTCACAAGTACTGAGAATG																132592	116321	57908.7	75525.2	15449.5	30574.9
GT	Sense	Sense	-0.363	Detected	0.363	Detected	0.000	-1.944	Detected	-1.094	Detected	-1.519	0.113	GT	Sens	Y11268	Y11268	L.esculen	ATGAACGTACTGATTATAGTCATTCGGAGCCAGCTACTTACATTAATGCTGCTATTGTTG																1723.61	2680.96	818	515.736	595.677	1070.88

GO ID	GO ACCESSION	GO Term	p-value	Count in Selection	% Count in Selection	Count in Total	% Count in Total
6888	GO:0009611|GO:0002	response to wounding	0.00	14	1.4752371	66	0.1906027
9600	GO:0016491	oxidoreductase activity	0.00	81	8.5353	1724	4.9787736
3472	GO:0004866	endopeptidase inhibitor	0.00	13	1.369863	95	0.27435237
28101	GO:0061135	endopeptidase regulato	0.00	13	1.369863	95	0.27435237
8159	GO:0010951	negative regulation of e	0.00	13	1.369863	95	0.27435237
26712	GO:0052548	regulation of endopepti	0.00	13	1.369863	95	0.27435237
7690	GO:0010466	negative regulation of p	0.00	13	1.369863	110	0.31767118
12946	GO:0030414	peptidase inhibitor activ	0.00	13	1.369863	110	0.31767118
25636	GO:0051346	negative regulation of h	0.00	13	1.369863	110	0.31767118
28100	GO:0061134	peptidase regulator acti	0.00	13	1.369863	112	0.32344702
26711	GO:0052547	regulation of peptidase	0.00	13	1.369863	112	0.32344702
3473	GO:0004867	serine-type endopeptida	0.00	9	0.9483667	66	0.1906027
3464	GO:0004857	enzyme inhibitor activity	0.00	14	1.4752371	157	0.4534034
22409	GO:0046906	tetrapyrrole binding	0.00	33	3.4773445	596	1.7212002
19255	GO:0042938	dipeptide transport	0.00	11	1.1591148	105	0.3032316
19253	GO:0042936	dipeptide transporter ac	0.00	11	1.1591148	105	0.3032316
26959	GO:0055114	oxidation-reduction pro	0.00	98	10.326659	2490	7.1909204
4641	GO:0006508	proteolysis	0.00	46	4.8472075	965	2.7868426
9396	GO:0016168	chlorophyll binding	0.00	7	0.73761857	46	0.13284431
2896	GO:0004090	carbonyl reductase (NA	0.00	4	0.4214963	13	0.037542958
19393	GO:0043086	negative regulation of c	0.00	14	1.4752371	188	0.54292893
20342	GO:0044092	negative regulation of m	0.00	14	1.4752371	188	0.54292893
9813	GO:0016759	cellulose synthase activ	0.00	7	0.73761857	57	0.16461143
9768	GO:0016706	oxidoreductase activity,	0.00	13	1.369863	175	0.505386
8607	GO:0015197|GO:0015	peptide transporter acti	0.00	11	1.1591148	139	0.40142086
8608	GO:0015198	oligopeptide transporter	0.00	11	1.1591148	139	0.40142086
4944	GO:0006857	oligopeptide transport	0.00	11	1.1591148	141	0.4071967
9099	GO:0015833	peptide transport	0.00	11	1.1591148	141	0.4071967
9784	GO:0016723	oxidoreductase activity,	0.00	4	0.4214963	20	0.0577584
225	GO:0000293	ferric-chelate reductase	0.00	4	0.4214963	20	0.0577584
9266	GO:0016020	membrane	0.00	131	13.804005	3741	10.803708
28364	GO:0065007	biological regulation	0.00	150	15.806111	4376	12.637537
15570	GO:0033170	protein-DNA loading AT	0.00	2	0.21074815	3	0.00866376
2635	GO:0003689	DNA clamp loader activ	0.00	2	0.21074815	3	0.00866376
8489	GO:0015066	alpha-amylase inhibitor	0.00	4	0.4214963	22	0.06353424
3307	GO:0004675	transmembrane recepto	0.00	20	2.1074815	367	1.0598665
25626	GO:0051336	regulation of hydrolase	0.00	13	1.369863	196	0.5660323
9808	GO:0016754	sinapoylglucose-malate	0.00	4	0.4214963	23	0.06642216
9807	GO:0016753	O-sinapoyltransferase a	0.00	4	0.4214963	23	0.06642216
9806	GO:0016752	sinapoyltransferase act	0.00	4	0.4214963	23	0.06642216
5702	GO:0008146	sulfotransferase activity	0.00	6	0.63224447	54	0.15594767
6826	GO:0009538	photosystem I reaction	0.00	3	0.31612223	12	0.03465504
25094	GO:0050789|GO:0050	regulation of biological	0.00	146	15.384615	4315	12.461374
5729	GO:0008191	metalloendopeptidase i	0.00	2	0.21074815	4	0.011551679
23942	GO:0048551	metalloenzyme inhibitor	0.00	2	0.21074815	4	0.011551679
7792	GO:0010576	metalloenzyme regulato	0.00	2	0.21074815	4	0.011551679
9718	GO:0016636	oxidoreductase activity,	0.00	2	0.21074815	4	0.011551679
11931	GO:0020037	heme binding	0.01	26	2.739726	550	1.5883559
6809	GO:0009521|GO:0030	photosystem	0.01	4	0.4214963	28	0.080861755
6940	GO:0009672	auxin:hydrogen sympor	0.01	4	0.4214963	28	0.080861755
18502	GO:0042132	fructose 1,6-bisphospha	0.01	2	0.21074815	5	0.0144396
6810	GO:0009522	photosystem I	0.01	3	0.31612223	15	0.043318797
2868	GO:0004062	aryl sulfotransferase ac	0.01	3	0.31612223	15	0.043318797
3872	GO:0005506	iron ion binding	0.01	26	2.739726	563	1.6258988
31431	GO:0080054	low affinity nitrate trans	0.01	5	0.52687037	45	0.1299564
22049	GO:0046527	glucosyltransferase acti	0.01	15	1.5806112	270	0.77973837
3065	GO:0004382	guanosine-diphosphata	0.01	2	0.21074815	6	0.01732752
21331	GO:0045735	nutrient reservoir activit	0.01	7	0.73761857	89	0.25702485
11776	GO:0019825	oxygen binding	0.01	19	2.0021074	389	1.1234008
9783	GO:0016722	oxidoreductase activity,	0.01	5	0.52687037	50	0.14439599
20630	GO:0044436	thylakoid part	0.01	7	0.73761857	90	0.2599128
5792	GO:0008271	secondary active sulfat	0.01	3	0.31612223	18	0.051982556
8736	GO:0015381	high affinity sulfate tran	0.01	3	0.31612223	18	0.051982556
25098	GO:0050794|GO:0051	regulation of cellular pr	0.01	132	13.909378	3987	11.514136
9933	GO:0016903	oxidoreductase activity,	0.01	8	0.8429926	113	0.32633495
6863	GO:0009579	thylakoid	0.01	7	0.73761857	94	0.27146447
9832	GO:0016782	transferase activity, tra	0.02	6	0.63224447	74	0.21370606
6201	GO:0008794	arsenate reductase (glu	0.02	3	0.31612223	20	0.0577584
13086	GO:0030611	arsenate reductase acti	0.02	3	0.31612223	20	0.0577584
13088	GO:0030613	oxidoreductase activity,	0.02	3	0.31612223	20	0.0577584
13089	GO:0030614	oxidoreductase activity,	0.02	3	0.31612223	20	0.0577584
10057	GO:0017110	nucleoside-diphosphata	0.02	2	0.21074815	8	0.023103358
11202	GO:0019199	transmembrane recepto	0.02	22	2.3182297	504	1.4555116
8967	GO:0015698	inorganic anion transpo	0.02	10	1.0537407	176	0.5082739
9769	GO:0016707	gibberellin 3-beta-dioxy	0.02	2	0.21074815	9	0.025991278
24627	GO:0050308	sugar-phosphatase acti	0.02	2	0.21074815	9	0.025991278
8526	GO:0015112	nitrate transmembrane	0.03	5	0.52687037	61	0.1761631
8975	GO:0015706|GO:0006	nitrate transport	0.03	5	0.52687037	61	0.1761631
8517	GO:0015103	inorganic anion transme	0.03	9	0.9483667	154	0.44473964
16738	GO:0034357	photosynthetic membra	0.03	6	0.63224447	83	0.23969735
28365	GO:0065008	regulation of biological	0.03	15	1.5806112	316	0.9125827
8651	GO:0015248	sterol transporter activit	0.03	2	0.21074815	10	0.0288792
9178	GO:0015918	sterol transport	0.03	2	0.21074815	10	0.0288792
10072	GO:0017127	cholesterol transporter	0.03	2	0.21074815	10	0.0288792
12859	GO:0030301	cholesterol transport	0.03	2	0.21074815	10	0.0288792
26022	GO:0051740	ethylene binding	0.03	2	0.21074815	10	0.0288792
30687	GO:0072328	alkene binding	0.03	2	0.21074815	10	0.0288792
21145	GO:0045543	gibberellin 2-beta-dioxy	0.03	3	0.31612223	25	0.072197996
21058	GO:0045454|GO:0030	cell redox homeostasis	0.03	11	1.1591148	211	0.6093511
5704	GO:0008150|GO:0000	biological_process	0.03	636	67.017914	22215	64.155136
8673	GO:0015291|GO:0015	secondary active transr	0.03	18	1.8967334	407	1.1753833
8530	GO:0015116	sulfate transmembrane	0.03	3	0.31612223	26	0.075085916
9765	GO:0016702	oxidoreductase activity,	0.03	5	0.52687037	66	0.1906027
12574	GO:0022892	substrate-specific trans	0.03	52	5.479452	1463	4.2250266
4329	GO:0006096|GO:0019	glycolysis	0.04	4	0.4214963	46	0.13284431
5981	GO:0008509	anion transmembrane t	0.04	12	1.2644889	245	0.70754033
9767	GO:0016705	oxidoreductase activity,	0.04	15	1.5806112	329	0.95012563

22415	GO:0046914	transition metal ion binc	0.04	86	9.062171	2599	7.5057034
5793	GO:0008272|GO:00068	sulfate transport	0.04	3	0.31612223	28	0.080861755
4917	GO:0006820|GO:00068	anion transport	0.04	12	1.2644889	247	0.7133162
3386	GO:0004773	steryl-sulfatase activity	0.04	2	0.21074815	12	0.03465504
3397	GO:0004784|GO:00047	superoxide dismutase a	0.04	2	0.21074815	12	0.03465504
5965	GO:0008484	sulfuric ester hydrolase	0.04	2	0.21074815	12	0.03465504
9704	GO:0016621	cinnamoyl-CoA reducta	0.04	2	0.21074815	12	0.03465504
9782	GO:0016721	oxidoreductase activity,	0.04	2	0.21074815	12	0.03465504
3490	GO:0004888|GO:00049	transmembrane recepto	0.04	23	2.4236038	571	1.6490022
7074	GO:0009815	1-aminocyclopropane-1	0.04	4	0.4214963	49	0.14150807
2986	GO:0004252	serine-type endopeptida	0.05	8	0.8429926	144	0.41586044
9764	GO:0016701	oxidoreductase activity,	0.05	5	0.52687037	72	0.20793022
7166	GO:0009914	hormone transport	0.05	4	0.4214963	50	0.14439599
27891	GO:0060918	auxin transport	0.05	4	0.4214963	50	0.14439599
31536	GO:0080161	auxin transmembrane t	0.05	4	0.4214963	50	0.14439599
2870	GO:0004064	arylesterase activity	0.05	3	0.31612223	30	0.086637594
3013	GO:0004328|GO:00345	formamidase activity	0.05	1	0.105374075	2	0.00577584
3152	GO:0004493	methylmalonyl-CoA epi	0.05	1	0.105374075	2	0.00577584
3604	GO:0005031|GO:00050	tumor necrosis factor re	0.05	1	0.105374075	2	0.00577584
3606	GO:0005035	death receptor activity	0.05	1	0.105374075	2	0.00577584
5907	GO:0008413	8-oxo-7,8-dihydroguano	0.05	1	0.105374075	2	0.00577584
6790	GO:0009496	plastoquinol-plastocyan	0.05	1	0.105374075	2	0.00577584
6828	GO:0009540	zeaxanthin epoxidase [	0.05	1	0.105374075	2	0.00577584
8612	GO:0015204|GO:00152	urea transmembrane tra	0.05	1	0.105374075	2	0.00577584
9106	GO:0015840	urea transport	0.05	1	0.105374075	2	0.00577584
11186	GO:0019177	dihydroneopterin tripho	0.05	1	0.105374075	2	0.00577584
11223	GO:0019221	cytokine-mediated signa	0.05	1	0.105374075	2	0.00577584
11870	GO:0019955|GO:00199	cytokine binding	0.05	1	0.105374075	2	0.00577584
14849	GO:0032441	pheophorbide a oxygen	0.05	1	0.105374075	2	0.00577584
15608	GO:0033209	tumor necrosis factor-m	0.05	1	0.105374075	2	0.00577584
16481	GO:0034097	response to cytokine sti	0.05	1	0.105374075	2	0.00577584
16991	GO:0034612	response to tumor necr	0.05	1	0.105374075	2	0.00577584
18651	GO:0042300	beta-amyrin synthase a	0.05	1	0.105374075	2	0.00577584
19204	GO:0042887	amide transmembrane	0.05	1	0.105374075	2	0.00577584
19424	GO:0043120	tumor necrosis factor bi	0.05	1	0.105374075	2	0.00577584
21947	GO:0046422	violaxanthin de-epoxida	0.05	1	0.105374075	2	0.00577584
22490	GO:0046995	oxidoreductase activity,	0.05	1	0.105374075	2	0.00577584
24561	GO:0050242	pyruvate, phosphate dik	0.05	1	0.105374075	2	0.00577584
24767	GO:0050454	coenzyme F420 hydrog	0.05	1	0.105374075	2	0.00577584
24925	GO:0050619	phytochromobilin:ferred	0.05	1	0.105374075	2	0.00577584
24931	GO:0050625|GO:00185	2-hydroxy-1,4-benzoqu	0.05	1	0.105374075	2	0.00577584
26025	GO:0051743	red chlorophyll catabolit	0.05	1	0.105374075	2	0.00577584
29709	GO:0071345	cellular response to cyt	0.05	1	0.105374075	2	0.00577584
29720	GO:0071356	cellular response to tum	0.05	1	0.105374075	2	0.00577584
30280	GO:0071918	urea transmembrane tra	0.05	1	0.105374075	2	0.00577584
2748	GO:0003913	DNA photolyase activity	0.05	1	0.105374075	2	0.00577584
11224	GO:0019222	regulation of metabolic	0.05	100	10.537408	3115	8.995871
7413	GO:0010181	FMN binding	0.05	2	0.21074815	14	0.040430877
25510	GO:0051213	dioxygenase activity	0.06	5	0.52687037	75	0.21659398
9750	GO:0016679	oxidoreductase activity,	0.06	4	0.4214963	53	0.15305975
11695	GO:0019725	cellular homeostasis	0.06	11	1.1591148	234	0.67577326
25195	GO:0050896|GO:00518	response to stimulus	0.06	63	6.638567	1881	5.432177
4530	GO:0006355|GO:00325	regulation of transcriptio	0.06	84	8.851422	2591	7.4826
12650	GO:0030060	L-malate dehydrogenas	0.06	2	0.21074815	15	0.043318797
25544	GO:0051252	regulation of RNA meta	0.06	84	8.851422	2593	7.488376
18925	GO:0042592	homeostatic process	0.06	11	1.1591148	238	0.68732494
7692	GO:0010468	regulation of gene expr	0.07	85	8.956797	2635	7.6096687
12516	GO:0022804	active transmembrane t	0.07	29	3.0558484	787	2.2727928
7141	GO:0009889	regulation of biosynthet	0.07	84	8.851422	2605	7.523031
7772	GO:0010556	regulation of macromole	0.07	84	8.851422	2605	7.523031
13756	GO:0031326	regulation of cellular bio	0.07	84	8.851422	2605	7.523031
32213	GO:2000112	regulation of cellular ma	0.07	84	8.851422	2605	7.523031
3742	GO:0005242	inward rectifier potassiu	0.07	2	0.21074815	16	0.046206716
3829	GO:0005381|GO:00053	iron ion transmembrane	0.07	2	0.21074815	16	0.046206716
7178	GO:0009927	histidine phosphotransf	0.07	2	0.21074815	16	0.046206716
17134	GO:0034755	iron ion transmembrane	0.07	2	0.21074815	16	0.046206716
11206	GO:0019203	carbohydrate phosphat	0.07	3	0.31612223	35	0.10107719
22375	GO:0046872	metal ion binding	0.07	95	10.010537	2985	8.6204405
5766	GO:0008236	serine-type peptidase a	0.07	10	1.0537407	214	0.6180149
8028	GO:0010817	regulation of hormone l	0.07	4	0.4214963	58	0.16749935
8494	GO:0015075	ion transmembrane tran	0.07	30	3.1612225	827	2.3883097
9393	GO:0016165	lipoxygenase activity	0.08	3	0.31612223	36	0.10396511
19470	GO:0043167	ion binding	0.08	95	10.010537	2997	8.655096
19472	GO:0043169	cation binding	0.08	95	10.010537	2997	8.655096
4910	GO:0006811	ion transport	0.08	34	3.5827186	959	2.769515
27228	GO:0060255	regulation of macromole	0.08	85	8.956797	2660	7.6818666
5018	GO:0006950	response to stress	0.08	23	2.4236038	611	1.764519
3405	GO:0004794	L-threonine ammonia-ly	0.08	1	0.105374075	3	0.00866376
16603	GO:0034220	ion transmembrane tran	0.08	30	3.1612225	833	2.4056373
4327	GO:0006091	generation of precursor	0.08	5	0.52687037	84	0.24258527
10103	GO:0017171	serine hydrolase activity	0.08	10	1.0537407	220	0.63534236
2981	GO:0004190	aspartic-type endopepti	0.09	2	0.21074815	18	0.051982556
6450	GO:0009081	branched chain family a	0.09	2	0.21074815	18	0.051982556
10899	GO:0018858	benzoate-CoA ligase ac	0.09	2	0.21074815	18	0.051982556
22917	GO:0047429	nucleoside-triphosphate	0.09	2	0.21074815	18	0.051982556
28368	GO:0070001	aspartic-type peptidase	0.09	2	0.21074815	18	0.051982556
9267	GO:0016021	integral to membrane	0.09	38	4.004215	1103	3.1853757
9812	GO:0016758	transferase activity, tran	0.10	19	2.0021074	501	1.4468478
3475	GO:0004871|GO:00050	signal transducer activit	0.10	28	2.9504743	787	2.2727928
27063	GO:0060089	molecular transducer ac	0.10	28	2.9504743	787	2.2727928
4921	GO:0006826|GO:00156	iron ion transport	0.10	2	0.21074815	20	0.0577584
9706	GO:0016623	oxidoreductase activity,	0.10	2	0.21074815	20	0.0577584
9707	GO:0016624	oxidoreductase activity,	0.10	2	0.21074815	20	0.0577584
24482	GO:0050162	oxalate oxidase activity	0.10	2	0.21074815	20	0.0577584
2900	GO:0004096|GO:00169	catalase activity	0.11	1	0.105374075	4	0.011551679
2953	GO:0004151	dihydroorotase activity	0.11	1	0.105374075	4	0.011551679
3033	GO:0004349	glutamate 5-kinase acti	0.11	1	0.105374075	4	0.011551679
3057	GO:0004373	glycogen (starch) synth	0.11	1	0.105374075	4	0.011551679
3101	GO:0004435	phosphatidylinositol pho	0.11	1	0.105374075	4	0.011551679
4039	GO:0005751|GO:00057	mitochondrial respirator	0.11	1	0.105374075	4	0.011551679
5942	GO:0008456	alpha-N-acetylgalactosa	0.11	1	0.105374075	4	0.011551679

6350	GO:0008965	phosphoenolpyruvate-p	0.11	1	0.105374075	4	0.011551679
7170	GO:0009918	sterol delta7 reductase	0.11	1	0.105374075	4	0.011551679
7406	GO:0010174	nucleoside transmembr	0.11	1	0.105374075	4	0.011551679
7534	GO:0010309	acireductone dioxygena	0.11	1	0.105374075	4	0.011551679
8459	GO:0015018	galactosylgalactosylxylo	0.11	1	0.105374075	4	0.011551679
8594	GO:0015184	L-cystine transmembran	0.11	1	0.105374075	4	0.011551679
8885	GO:0015603	iron chelate transmemb	0.11	1	0.105374075	4	0.011551679
8902	GO:0015623|GO:0015	iron-chelate-transportin	0.11	1	0.105374075	4	0.011551679
8957	GO:0015688	iron chelate transport	0.11	1	0.105374075	4	0.011551679
9080	GO:0015811	L-cystine transport	0.11	1	0.105374075	4	0.011551679
9351	GO:0016119	carotene metabolic proc	0.11	1	0.105374075	4	0.011551679
9352	GO:0016120	carotene biosynthetic p	0.11	1	0.105374075	4	0.011551679
9760	GO:0016695	oxidoreductase activity,	0.11	1	0.105374075	4	0.011551679
9790	GO:0016730	oxidoreductase activity,	0.11	1	0.105374075	4	0.011551679
9810	GO:0016756|GO:0042	glutathione gamma-glut	0.11	1	0.105374075	4	0.011551679
9819	GO:0016767	geranylgeranyl-diphosp	0.11	1	0.105374075	4	0.011551679
10039	GO:0017084	delta1-pyrroline-5-carbo	0.11	1	0.105374075	4	0.011551679
11549	GO:0019566	arabinose metabolic pro	0.11	1	0.105374075	4	0.011551679
16639	GO:0034256	chlorophyll(ide) b reduc	0.11	1	0.105374075	4	0.011551679
18575	GO:0042214	terpene metabolic proce	0.11	1	0.105374075	4	0.011551679
20901	GO:0045277|GO:0045	respiratory chain compl	0.11	1	0.105374075	4	0.011551679
21791	GO:0046246	terpene biosynthetic pro	0.11	1	0.105374075	4	0.011551679
21902	GO:0046373	L-arabinose metabolic p	0.11	1	0.105374075	4	0.011551679
22408	GO:0046905	phytoene synthase activ	0.11	1	0.105374075	4	0.011551679
23084	GO:0047598	7-dehydrocholesterol re	0.11	1	0.105374075	4	0.011551679
24646	GO:0050327	testosterone 17-beta-de	0.11	1	0.105374075	4	0.011551679
31507	GO:0080132	fatty acid alpha-hydroxy	0.11	1	0.105374075	4	0.011551679
75	GO:0000099	sulfur amino acid transm	0.11	1	0.105374075	4	0.011551679
77	GO:0000101	sulfur amino acid transp	0.11	1	0.105374075	4	0.011551679
11221	GO:0019219	regulation of nucleobas	0.11	84	8.851422	2675	7.7251854
25468	GO:0051171	regulation of nitrogen co	0.11	84	8.851422	2675	7.7251854
8461	GO:0015020|GO:0003	glucuronosyltransferase	0.11	3	0.31612223	42	0.121292636
9698	GO:0016615	malate dehydrogenase	0.11	2	0.21074815	21	0.060646318
3476	GO:0004872|GO:0019	receptor activity	0.12	24	2.5289779	672	1.9406822
9699	GO:0016616	oxidoreductase activity,	0.12	11	1.1591148	266	0.7681867
9753	GO:0016682	oxidoreductase activity,	0.12	3	0.31612223	44	0.12706847
9722	GO:0016641	oxidoreductase activity,	0.12	4	0.4214963	69	0.19926646
8679	GO:0015297	antiporter activity	0.13	7	0.73761857	152	0.4389638
8682	GO:0015300	solute:solute antiporter	0.13	7	0.73761857	152	0.4389638
12573	GO:0022891	substrate-specific trans	0.13	41	4.3203373	1241	3.5839086
4678	GO:0006549	isoleucine metabolic pro	0.13	1	0.105374075	5	0.0144396
6465	GO:0009097	isoleucine biosynthetic	0.13	1	0.105374075	5	0.0144396
21949	GO:0046424	ferulate 5-hydroxylase a	0.13	1	0.105374075	5	0.0144396
31465	GO:0080090	regulation of primary me	0.13	84	8.851422	2710	7.826263
7527	GO:0010302	2-oxoglutarate-depende	0.13	2	0.21074815	23	0.06642216
3744	GO:0005244	voltage-gated ion chann	0.14	4	0.4214963	72	0.20793022
12528	GO:0022832	voltage-gated channel a	0.14	4	0.4214963	72	0.20793022
15280	GO:0032879	regulation of localizatio	0.14	4	0.4214963	72	0.20793022
17141	GO:0034762	regulation of transmem	0.14	4	0.4214963	72	0.20793022
17144	GO:0034765	regulation of ion transm	0.14	4	0.4214963	72	0.20793022
19568	GO:0043269	regulation of ion transp	0.14	4	0.4214963	72	0.20793022
25348	GO:0051049	regulation of transport	0.14	4	0.4214963	72	0.20793022
8493	GO:0015074	DNA integration	0.14	3	0.31612223	47	0.13573223
4247	GO:0006007	glucose catabolic proce	0.14	4	0.4214963	73	0.21081814
9703	GO:0016620	oxidoreductase activity,	0.14	4	0.4214963	73	0.21081814
11313	GO:0019320	hexose catabolic proces	0.14	4	0.4214963	73	0.21081814
20525	GO:0044275	cellular carbohydrate ca	0.14	4	0.4214963	73	0.21081814
21726	GO:0046164	alcohol catabolic proces	0.14	4	0.4214963	73	0.21081814
21894	GO:0046365	monosaccharide catabo	0.14	4	0.4214963	73	0.21081814
2976	GO:0004176|GO:0004	ATP-dependent peptida	0.14	3	0.31612223	48	0.13862015
12794	GO:0030234	enzyme regulator activi	0.14	15	1.5806112	403	1.1638317
13656	GO:0031224	intrinsic to membrane	0.15	39	4.109589	1193	3.4452884
13753	GO:0031323	regulation of cellular me	0.15	85	8.956797	2770	7.999538
973	GO:0001653	peptide receptor activity	0.15	2	0.21074815	25	0.072197996
12608	GO:0030001	metal ion transport	0.15	14	1.4752371	374	1.080082
8810	GO:0015491	cation:cation antiporter	0.15	4	0.4214963	75	0.21659398
3183	GO:0004525	ribonuclease III activity	0.15	1	0.105374075	6	0.01732752
3413	GO:0004802	transketolase activity	0.15	1	0.105374075	6	0.01732752
3845	GO:0005452	inorganic anion exchan	0.15	1	0.105374075	6	0.01732752
5810	GO:0008290	F-actin capping protein	0.15	1	0.105374075	6	0.01732752
7250	GO:0010012	steroid 22-alpha hydrox	0.15	1	0.105374075	6	0.01732752
9825	GO:0016774	phosphotransferase act	0.15	1	0.105374075	6	0.01732752
9831	GO:0016781	phosphotransferase act	0.15	1	0.105374075	6	0.01732752
12826	GO:0030267	glyoxylate reductase (N	0.15	1	0.105374075	6	0.01732752
13832	GO:0031409	pigment binding	0.15	1	0.105374075	6	0.01732752
13982	GO:0031559	oxidosqualene cyclase	0.15	1	0.105374075	6	0.01732752
14264	GO:0031849	olfactory receptor bindi	0.15	1	0.105374075	6	0.01732752
19203	GO:0042886	amide transport	0.15	1	0.105374075	6	0.01732752
23230	GO:0047746	chlorophyllase activity	0.15	1	0.105374075	6	0.01732752
30707	GO:0072348	sulfur compound transp	0.15	1	0.105374075	6	0.01732752
2713	GO:0003863	3-methyl-2-oxobutanoa	0.15	1	0.105374075	6	0.01732752
2846	GO:0004038	allantoinase activity	0.15	1	0.105374075	6	0.01732752
9709	GO:0016627	oxidoreductase activity,	0.16	5	0.52687037	104	0.30034366
9744	GO:0016671	oxidoreductase activity,	0.16	2	0.21074815	26	0.075085916
126	GO:0000160	two-component signal t	0.16	2	0.21074815	26	0.075085916
8919	GO:0015645	fatty acid ligase activity	0.17	2	0.21074815	27	0.077973835
2975	GO:0004175|GO:0016	endopeptidase activity	0.17	12	1.2644889	320	0.9241344
4909	GO:0006810|GO:0015	transport	0.18	71	7.4815598	2315	6.6855345
25529	GO:0051234	establishment of localiz	0.18	71	7.4815598	2315	6.6855345
5732	GO:0008194	UDP-glycosyltransferas	0.18	12	1.2644889	322	0.9299102
3021	GO:0004337	geranyltranstransferase	0.18	1	0.105374075	7	0.020215439
3255	GO:0004615|GO:0008	phosphomannomutase	0.18	1	0.105374075	7	0.020215439
3357	GO:0004738	pyruvate dehydrogenas	0.18	1	0.105374075	7	0.020215439
3358	GO:0004739	pyruvate dehydrogenas	0.18	1	0.105374075	7	0.020215439
6801	GO:0009512	cytochrome b6f comple	0.18	1	0.105374075	7	0.020215439
16115	GO:0033729	anthocyanidin reductas	0.18	1	0.105374075	7	0.020215439
2833	GO:0004024	alcohol dehydrogenase	0.18	2	0.21074815	28	0.080861755
25476	GO:0051179	localization	0.18	71	7.4815598	2319	6.697086
6668	GO:0009314	response to radiation	0.18	4	0.4214963	81	0.23392151
6751	GO:0009416	response to light stimul	0.18	4	0.4214963	81	0.23392151
9697	GO:0016614	oxidoreductase activity,	0.18	11	1.1591148	293	0.84616053
3787	GO:0005319	lipid transporter activity	0.19	4	0.4214963	83	0.23969735
8680	GO:0015298	solute:cation antiporter	0.19	5	0.52687037	112	0.32344702

9730	GO:0016651	oxidoreductase activity,	0.19	5	0.52687037	112	0.32344702
9883	GO:0016846	carbon-sulfur lyase acti	0.20	3	0.31612223	56	0.16172351
8683	GO:0015301|GO:0015	anion:anion antiporter a	0.20	2	0.21074815	30	0.086637594
3306	GO:0004674|GO:0004	protein serine/threonine	0.20	34	3.5827186	1062	3.0669708
3048	GO:0004364	glutathione transferase	0.20	4	0.4214963	84	0.24258527
3254	GO:0004614	phosphoglucomutase a	0.20	1	0.105374075	8	0.023103358
3402	GO:0004791	thioredoxin-disulfide re	0.20	1	0.105374075	8	0.023103358
4035	GO:0005746	mitochondrial respirator	0.20	1	0.105374075	8	0.023103358
5953	GO:0008471	laccase activity	0.20	1	0.105374075	8	0.023103358
6613	GO:0009250	glucan biosynthetic pro	0.20	1	0.105374075	8	0.023103358
18639	GO:0042284	sphingolipid delta-4 des	0.20	1	0.105374075	8	0.023103358
22076	GO:0046556	alpha-N-arabinofuranos	0.20	1	0.105374075	8	0.023103358
28833	GO:0070469	respiratory chain	0.20	1	0.105374075	8	0.023103358
30696	GO:0072337	modified amino acid tra	0.20	1	0.105374075	8	0.023103358
30708	GO:0072349	modified amino acid tra	0.20	1	0.105374075	8	0.023103358
5195	GO:0007165|GO:0023	signal transduction	0.20	31	3.2665963	961	2.775291
12602	GO:0023052|GO:0023	signaling	0.20	31	3.2665963	961	2.775291
4911	GO:0006812|GO:0006	cation transport	0.21	22	2.3182297	662	1.9118029
9811	GO:0016757|GO:0016	transferase activity, tran	0.21	24	2.5289779	729	2.1052935
19952	GO:0043687	post-translational protei	0.21	4	0.4214963	86	0.24836111
4913	GO:0006814|GO:0006	sodium ion transport	0.21	3	0.31612223	58	0.16749935
8499	GO:0015081|GO:0022	sodium ion transmembr	0.21	3	0.31612223	58	0.16749935
18086	GO:0035725	sodium ion transmembr	0.21	3	0.31612223	58	0.16749935
29800	GO:0071436	sodium ion export	0.21	3	0.31612223	58	0.16749935
4954	GO:0006869	lipid transport	0.21	8	0.8429926	208	0.6006873
8085	GO:0010876	lipid localization	0.21	8	0.8429926	208	0.6006873
4246	GO:0006006	glucose metabolic proc	0.22	4	0.4214963	87	0.25124902
4481	GO:0006284	base-excision repair	0.22	2	0.21074815	32	0.09241343
21041	GO:0045431	flavonol synthase activi	0.22	2	0.21074815	32	0.09241343
25836	GO:0051552	flavone metabolic proce	0.22	2	0.21074815	32	0.09241343
25837	GO:0051553	flavone biosynthetic pro	0.22	2	0.21074815	32	0.09241343
25838	GO:0051554	flavonol metabolic proc	0.22	2	0.21074815	32	0.09241343
25839	GO:0051555	flavonol biosynthetic pr	0.22	2	0.21074815	32	0.09241343
2831	GO:0004022	alcohol dehydrogenase	0.22	3	0.31612223	59	0.17038727
4322	GO:0006084	acetyl-CoA metabolic p	0.22	1	0.105374075	9	0.025991278
4332	GO:0006099	tricarboxylic acid cycle	0.22	1	0.105374075	9	0.025991278
6429	GO:0009060	aerobic respiration	0.22	1	0.105374075	9	0.025991278
6476	GO:0009109	coenzyme catabolic pro	0.22	1	0.105374075	9	0.025991278
6670	GO:0009317	acetyl-CoA carboxylase	0.22	1	0.105374075	9	0.025991278
7225	GO:0009978	allene oxide synthase a	0.22	1	0.105374075	9	0.025991278
19254	GO:0042937	tripeptide transporter ac	0.22	1	0.105374075	9	0.025991278
19342	GO:0043027	caspase inhibitor activit	0.22	1	0.105374075	9	0.025991278
19343	GO:0043028	caspase regulator activ	0.22	1	0.105374075	9	0.025991278
19458	GO:0043154|GO:0001	negative regulation of c	0.22	1	0.105374075	9	0.025991278
19579	GO:0043281|GO:0043	regulation of caspase a	0.22	1	0.105374075	9	0.025991278
21885	GO:0046356	acetyl-CoA catabolic pr	0.22	1	0.105374075	9	0.025991278
25484	GO:0051187	cofactor catabolic proce	0.22	1	0.105374075	9	0.025991278
2837	GO:0004028	3-chloroallyl aldehyde d	0.22	1	0.105374075	9	0.025991278
24966	GO:0050660	flavin adenine dinucleot	0.22	5	0.52687037	118	0.34077454
5840	GO:0008324	cation transmembrane	0.23	18	1.8967334	538	1.5537009
9719	GO:0016638	oxidoreductase activity,	0.23	4	0.4214963	89	0.25702485
3723	GO:0005215|GO:0005	transporter activity	0.23	56	5.9009485	1844	5.325324
22376	GO:0046873	metal ion transmembra	0.23	8	0.8429926	213	0.6151269
3749	GO:0005249	voltage-gated potassiu	0.24	2	0.21074815	34	0.09818927
5827	GO:0008308|GO:0022	voltage-gated anion cha	0.24	2	0.21074815	34	0.09818927
20321	GO:0044070	regulation of anion tran	0.24	2	0.21074815	34	0.09818927
9294	GO:0016052|GO:0006	carbohydrate catabolic	0.24	4	0.4214963	91	0.2628007
3040	GO:0004356	glutamate-ammonia liga	0.24	1	0.105374075	10	0.0288792
3203	GO:0004557	alpha-galactosidase act	0.24	1	0.105374075	10	0.0288792
3264	GO:0004629|GO:0042	phospholipase C activit	0.24	1	0.105374075	10	0.0288792
3842	GO:0005432	calcium:sodium antipor	0.24	1	0.105374075	10	0.0288792
5678	GO:0008113|GO:0033	peptide-methionine-(S)-	0.24	1	0.105374075	10	0.0288792
9424	GO:0016211	ammonia ligase activity	0.24	1	0.105374075	10	0.0288792
9741	GO:0016668|GO:0016	oxidoreductase activity,	0.24	1	0.105374075	10	0.0288792
9826	GO:0016775	phosphotransferase act	0.24	1	0.105374075	10	0.0288792
9912	GO:0016880	acid-ammonia (or amide	0.24	1	0.105374075	10	0.0288792
11205	GO:0019202	amino acid kinase activ	0.24	1	0.105374075	10	0.0288792
12918	GO:0030374	ligand-dependent nucle	0.24	1	0.105374075	10	0.0288792
31707	GO:0090116	C-5 methylation of cyto	0.24	1	0.105374075	10	0.0288792
983	GO:0001664	G-protein-coupled rece	0.24	1	0.105374075	10	0.0288792
2736	GO:0003886|GO:0008	DNA (cytosine-5-)-meth	0.24	1	0.105374075	10	0.0288792
4215	GO:0005975	carbohydrate metabolic	0.25	31	3.2665963	990	2.8590407
17016	GO:0034637	cellular carbohydrate bi	0.25	3	0.31612223	63	0.18193895
21146	GO:0045544	gibberellin 20-oxidase a	0.25	2	0.21074815	35	0.10107719
17617	GO:0035251	UDP-glucosyltransferas	0.25	8	0.8429926	218	0.62956655
26931	GO:0055085	transmembrane transpo	0.25	47	4.952582	1548	4.4705
9293	GO:0016051|GO:0006	carbohydrate biosynthe	0.26	3	0.31612223	64	0.18482687
4236	GO:0005996	monosaccharide metab	0.26	5	0.52687037	125	0.36099
4232	GO:0005992	trehalose biosynthetic p	0.26	2	0.21074815	36	0.10396511
8739	GO:0015385|GO:0015	sodium:hydrogen antipo	0.26	2	0.21074815	36	0.10396511
9185	GO:0015925	galactosidase activity	0.26	2	0.21074815	36	0.10396511
3380	GO:0004766	spermidine synthase ac	0.26	1	0.105374075	11	0.03176712
3416	GO:0004806	triglyceride lipase activi	0.26	1	0.105374075	11	0.03176712
11152	GO:0019139|GO:0046	cytokinin dehydrogenas	0.26	1	0.105374075	11	0.03176712
13809	GO:0031386	protein tag	0.26	1	0.105374075	11	0.03176712
19256	GO:0042939	tripeptide transport	0.26	1	0.105374075	11	0.03176712
9710	GO:0016628	oxidoreductase activity,	0.26	3	0.31612223	65	0.18771479
3761	GO:0005275|GO:0005	amine transmembrane	0.26	5	0.52687037	126	0.3638779
25998	GO:0051716	cellular response to stir	0.26	35	3.6880927	1138	3.2864528
3318	GO:0004693|GO:0016	cyclin-dependent protei	0.27	2	0.21074815	37	0.10685303
3758	GO:0005267	potassium channel activ	0.27	2	0.21074815	37	0.10685303
24968	GO:0050662	coenzyme binding	0.27	7	0.73761857	191	0.5515927
5791	GO:0008270	zinc ion binding	0.28	56	5.9009485	1880	5.4292893
4231	GO:0005991	trehalose metabolic pro	0.28	2	0.21074815	38	0.10974095
8501	GO:0015085	calcium ion transmemb	0.28	2	0.21074815	38	0.10974095
12538	GO:0022843	voltage-gated cation ch	0.28	2	0.21074815	38	0.10974095
6383	GO:0009008	DNA-methyltransferase	0.28	1	0.105374075	12	0.03465504
6451	GO:0009082	branched chain family a	0.28	1	0.105374075	12	0.03465504
7526	GO:0010301|GO:0033	xanthoxin dehydrogena	0.28	1	0.105374075	12	0.03465504
8748	GO:0015398	high affinity secondary	0.28	1	0.105374075	12	0.03465504
9235	GO:0015976|GO:0015	carbon utilization	0.28	1	0.105374075	12	0.03465504
14848	GO:0032440	2-alkenal reductase act	0.28	1	0.105374075	12	0.03465504
16148	GO:0033764	steroid dehydrogenase	0.28	1	0.105374075	12	0.03465504

20943	GO:0045333	cellular respiration	0.28	1	0.105374075	12	0.03465504
4990	GO:0006915|GO:00086	apoptosis	0.29	8	0.8429926	227	0.6555578
6796	GO:0009507	chloroplast	0.29	8	0.8429926	229	0.6613336
21710	GO:0046148	pigment biosynthetic pr	0.29	3	0.31612223	69	0.19926646
31420	GO:0080043	quercetin 3-O-glucosylt	0.29	3	0.31612223	69	0.19926646
9422	GO:0016209	antioxidant activity	0.30	6	0.63224447	165	0.47650677
7072	GO:0009813	flavonoid biosynthetic p	0.30	2	0.21074815	40	0.1155168
11311	GO:0019318	hexose metabolic proce	0.30	4	0.4214963	101	0.2916799
6811	GO:0009523	photosystem II	0.30	1	0.105374075	13	0.037542958
8149	GO:0010941	regulation of cell death	0.30	1	0.105374075	13	0.037542958
19043	GO:0042719	mitochondrial intermem	0.30	1	0.105374075	13	0.037542958
19298	GO:0042981	regulation of apoptosis	0.30	1	0.105374075	13	0.037542958
19381	GO:0043067|GO:00430	regulation of programm	0.30	1	0.105374075	13	0.037542958
5874	GO:0008374	O-acyltransferase activi	0.31	4	0.4214963	102	0.29456782
28378	GO:0070011	peptidase activity, actin	0.31	17	1.7913593	537	1.550813
4408	GO:0006200	ATP catabolic process	0.31	20	2.1074815	641	1.8511566
9918	GO:0016887|GO:0004C	ATPase activity	0.31	20	2.1074815	641	1.8511566
21600	GO:0046034	ATP metabolic process	0.31	20	2.1074815	641	1.8511566
9103	GO:0015837	amine transport	0.31	5	0.52687037	135	0.38986918
12547	GO:0022857|GO:00053	transmembrane transpo	0.31	44	4.6364594	1483	4.282785
9740	GO:0016667	oxidoreductase activity,	0.32	3	0.31612223	72	0.20793022
30069	GO:0071705	nitrogen compound tran	0.32	7	0.73761857	202	0.5833598
20532	GO:0044282	small molecule catabol	0.32	26	2.739726	854	2.4662836
3753	GO:0005253	anion channel activity	0.32	2	0.21074815	42	0.121292636
6666	GO:0009312	oligosaccharide biosynt	0.32	2	0.21074815	42	0.121292636
9370	GO:0016138	glycoside biosynthetic p	0.32	2	0.21074815	42	0.121292636
13884	GO:0031461	cullin-RING ubiquitin lig	0.32	2	0.21074815	42	0.121292636
21880	GO:0046351	disaccharide biosynthet	0.32	2	0.21074815	42	0.121292636
7844	GO:0010629	negative regulation of g	0.32	1	0.105374075	14	0.040430877
9399	GO:0016174	NAD(P)H oxidase activ	0.32	1	0.105374075	14	0.040430877
9433	GO:0016229	steroid dehydrogenase	0.32	1	0.105374075	14	0.040430877
9854	GO:0016812	hydrolase activity, actin	0.32	1	0.105374075	14	0.040430877
9879	GO:0016841	ammonia-lyase activity	0.32	1	0.105374075	14	0.040430877
15178	GO:0032776	DNA methylation on cyt	0.32	1	0.105374075	14	0.040430877
21460	GO:0045892|GO:00164	negative regulation of tr	0.32	1	0.105374075	14	0.040430877
21502	GO:0045934	negative regulation of n	0.32	1	0.105374075	14	0.040430877
24970	GO:0050664	oxidoreductase activity,	0.32	1	0.105374075	14	0.040430877
25469	GO:0051172	negative regulation of n	0.32	1	0.105374075	14	0.040430877
25545	GO:0051253	negative regulation of R	0.32	1	0.105374075	14	0.040430877
6899	GO:0009628	response to abiotic stim	0.32	6	0.63224447	170	0.49094638
18786	GO:0042440	pigment metabolic proc	0.32	3	0.31612223	73	0.21081814
5756	GO:0008219	cell death	0.33	8	0.8429926	239	0.69021285
8209	GO:0012501|GO:00162	programmed cell death	0.33	8	0.8429926	239	0.69021285
9462	GO:0016265	death	0.33	8	0.8429926	239	0.69021285
8675	GO:0015293	symporter activity	0.34	6	0.63224447	173	0.49961013
25095	GO:0050790	regulation of catalytic ac	0.34	15	1.5806112	480	1.3862015
4914	GO:0006816	calcium ion transport	0.34	2	0.21074815	44	0.12706847
7071	GO:0009812	flavonoid metabolic pro	0.34	2	0.21074815	44	0.12706847
28952	GO:0070588	calcium ion transmemb	0.34	2	0.21074815	44	0.12706847
7520	GO:0010294	abscisic acid glucosyltra	0.34	1	0.105374075	15	0.043318797
9340	GO:0016108	tetraterpenoid metaboli	0.34	1	0.105374075	15	0.043318797
9341	GO:0016109	tetraterpenoid biosynthe	0.34	1	0.105374075	15	0.043318797
9348	GO:0016116	carotenoid metabolic pr	0.34	1	0.105374075	15	0.043318797
9349	GO:0016117	carotenoid biosynthetic	0.34	1	0.105374075	15	0.043318797
10079	GO:0017134	fibroblast growth factor	0.34	1	0.105374075	15	0.043318797
16081	GO:0033692	cellular polysaccharide	0.34	1	0.105374075	15	0.043318797
8676	GO:0015294	solute:cation symporter	0.34	5	0.52687037	141	0.4071967
5663	GO:0008094|GO:0004C	DNA-dependent ATPas	0.35	3	0.31612223	76	0.2194819
28366	GO:0065009	regulation of molecular	0.35	15	1.5806112	484	1.3977532
8666	GO:0015276	ligand-gated ion channe	0.35	2	0.21074815	45	0.1299564
12530	GO:0022834	ligand-gated channel ac	0.35	2	0.21074815	45	0.1299564
4304	GO:0006066	alcohol metabolic proce	0.36	5	0.52687037	143	0.41297254
9795	GO:0016740	transferase activity	0.36	107	11.275026	3764	10.870131
5995	GO:0008524	glucose 6-phosphate:ph	0.36	1	0.105374075	16	0.046206716
7142	GO:0009890	negative regulation of b	0.36	1	0.105374075	16	0.046206716
7774	GO:0010558	negative regulation of n	0.36	1	0.105374075	16	0.046206716
7820	GO:0010605	negative regulation of n	0.36	1	0.105374075	16	0.046206716
8694	GO:0015315	organophosphate:inorg	0.36	1	0.105374075	16	0.046206716
9189	GO:0015929	hexosaminidase activity	0.36	1	0.105374075	16	0.046206716
9390	GO:0016161	beta-amylase activity	0.36	1	0.105374075	16	0.046206716
9727	GO:0016647	oxidoreductase activity,	0.36	1	0.105374075	16	0.046206716
9878	GO:0016840	carbon-nitrogen lyase a	0.36	1	0.105374075	16	0.046206716
13757	GO:0031327	negative regulation of c	0.36	1	0.105374075	16	0.046206716
18757	GO:0042409	caffeoyl-CoA O-methylt	0.36	1	0.105374075	16	0.046206716
22110	GO:0046592	polyamine oxidase activ	0.36	1	0.105374075	16	0.046206716
22412	GO:0046910	pectinesterase inhibitor	0.36	1	0.105374075	16	0.046206716
32214	GO:2000113	negative regulation of c	0.36	1	0.105374075	16	0.046206716
206	GO:0000271	polysaccharide biosynth	0.36	1	0.105374075	16	0.046206716
219	GO:0000287	magnesium ion binding	0.36	1	0.105374075	16	0.046206716
8943	GO:0015672	monovalent inorganic c	0.36	12	1.2644889	383	1.1060733
9909	GO:0016877	ligase activity, forming c	0.36	3	0.31612223	78	0.22525774
6425	GO:0009056	catabolic process	0.36	31	3.2665963	1051	3.0352037
6824	GO:0009536	plastid	0.37	8	0.8429926	247	0.7133162
20512	GO:0044262|GO:00060	cellular carbohydrate m	0.37	8	0.8429926	248	0.7162041
4044	GO:0005758|GO:00319	mitochondrial intermem	0.38	1	0.105374075	17	0.049094636
9923	GO:0016892	endoribonuclease activ	0.38	1	0.105374075	17	0.049094636
9925	GO:0016894	endonuclease activity, a	0.38	1	0.105374075	17	0.049094636
14383	GO:0031970	organelle envelope lum	0.38	1	0.105374075	17	0.049094636
16281	GO:0033897	ribonuclease T2 activity	0.38	1	0.105374075	17	0.049094636
20619	GO:0044425	membrane part	0.38	44	4.6364594	1523	4.398302
22478	GO:0046982	protein heterodimerizat	0.38	4	0.4214963	114	0.32922286
5763	GO:0008233	peptidase activity	0.38	17	1.7913593	565	1.6316746
3756	GO:0005261|GO:00152	cation channel activity	0.39	2	0.21074815	49	0.14150807
3844	GO:0005451	monovalent cation:hydr	0.39	2	0.21074815	49	0.14150807
9725	GO:0016645	oxidoreductase activity,	0.39	2	0.21074815	49	0.14150807
31421	GO:0080044	quercetin 7-O-glucosylt	0.39	2	0.21074815	49	0.14150807
3832	GO:0005388	calcium-transporting AT	0.39	1	0.105374075	18	0.051982556
4134	GO:0005874	microtubule	0.39	1	0.105374075	18	0.051982556
6416	GO:0009044	xylan 1,4-beta-xylosidas	0.39	1	0.105374075	18	0.051982556
12887	GO:0030332	cyclin binding	0.39	1	0.105374075	18	0.051982556
9818	GO:0016765|GO:00167	transferase activity, tran	0.40	6	0.63224447	185	0.53426516
4912	GO:0006813|GO:00154	potassium ion transport	0.41	3	0.31612223	84	0.24258527
30168	GO:0071804	cellular potassium ion tr	0.41	3	0.31612223	84	0.24258527

30169	GO:0071805	potassium ion transmer	0.41	3	0.31612223	84	0.24258527
8584	GO:0015171|GO:00153	amino acid transmembr	0.41	4	0.4214963	118	0.34077454
2521	GO:0003333	amino acid transmembr	0.41	4	0.4214963	118	0.34077454
7173	GO:0009922	fatty acid elongase activ	0.41	2	0.21074815	51	0.14728391
9733	GO:0016655	oxidoreductase activity,	0.41	2	0.21074815	51	0.14728391
9975	GO:0016998	cell wall macromolecule	0.41	2	0.21074815	51	0.14728391
9809	GO:0016755	transferase activity, trar	0.41	1	0.105374075	19	0.054870475
9891	GO:0016855	racemase and epimera	0.41	1	0.105374075	19	0.054870475
2895	GO:0004089	carbonate dehydratase	0.41	1	0.105374075	19	0.054870475
5097	GO:0007049	cell cycle	0.42	2	0.21074815	52	0.15017183
6965	GO:0009699	phenylpropanoid biosyn	0.42	2	0.21074815	52	0.15017183
20287	GO:0044036	cell wall macromolecule	0.42	2	0.21074815	52	0.15017183
3124	GO:0004462	lactoylglutathione lyase	0.43	1	0.105374075	20	0.0577584
3747	GO:0005247	voltage-gated chloride c	0.43	1	0.105374075	20	0.0577584
3754	GO:0005254	chloride channel activity	0.43	1	0.105374075	20	0.0577584
3798	GO:0005337	nucleoside transmembr	0.43	1	0.105374075	20	0.0577584
5991	GO:0008519	ammonium transmembr	0.43	1	0.105374075	20	0.0577584
8726	GO:0015368	calcium:cation antiporte	0.43	1	0.105374075	20	0.0577584
8965	GO:0015696	ammonium transport	0.43	1	0.105374075	20	0.0577584
9122	GO:0015858	nucleoside transport	0.43	1	0.105374075	20	0.0577584
9799	GO:0016744|GO:00167	transferase activity, trar	0.43	1	0.105374075	20	0.0577584
30844	GO:0072488	ammonium transmemb	0.43	1	0.105374075	20	0.0577584
2675	GO:0003825	alpha,alpha-trehalose-p	0.43	1	0.105374075	20	0.0577584
12532	GO:0022836	gated channel activity	0.43	4	0.4214963	122	0.3523262
119	GO:0000151	ubiquitin ligase complex	0.43	3	0.31612223	88	0.25413695
4951	GO:0006865|GO:00068	amino acid transport	0.44	4	0.4214963	123	0.35521415
2897	GO:0004091|GO:00043	carboxylesterase activit	0.44	6	0.63224447	194	0.5602564
3129	GO:0004467	long-chain fatty acid-Co	0.44	1	0.105374075	21	0.060646318
3474	GO:0004869|GO:00048	cysteine-type endopept	0.44	1	0.105374075	21	0.060646318
9902	GO:0016868|GO:00167	intramolecular transfera	0.44	1	0.105374075	21	0.060646318
12999	GO:0030506	ankyrin binding	0.44	1	0.105374075	21	0.060646318
23518	GO:0048040	UDP-glucuronate decar	0.44	1	0.105374075	21	0.060646318
716	GO:0001071	nucleic acid binding trar	0.45	40	4.214963	1417	4.0921826
2643	GO:0003700|GO:00001	sequence-specific DNA	0.45	40	4.214963	1417	4.0921826
9533	GO:0016405	CoA-ligase activity	0.45	2	0.21074815	55	0.15883559
4224	GO:0005984	disaccharide metabolic	0.46	2	0.21074815	56	0.16172351
9369	GO:0016137	glycoside metabolic pro	0.46	2	0.21074815	56	0.16172351
10032	GO:0017075	syntaxin-1 binding	0.46	1	0.105374075	22	0.06353424
12660	GO:0030077	plasma membrane light	0.46	1	0.105374075	22	0.06353424
13038	GO:0030551	cyclic nucleotide binding	0.46	1	0.105374075	22	0.06353424
19040	GO:0042716	plasma membrane-deri	0.46	1	0.105374075	22	0.06353424
19325	GO:0043008	ATP-dependent protein	0.46	1	0.105374075	22	0.06353424
24858	GO:0050551	myrcene synthase activ	0.46	1	0.105374075	22	0.06353424
4405	GO:0006195	purine nucleotide catab	0.46	22	2.3182297	773	2.232362
6508	GO:0009143	nucleoside triphosphate	0.46	22	2.3182297	773	2.232362
6509	GO:0009144	purine nucleoside tripho	0.46	22	2.3182297	773	2.232362
6511	GO:0009146	purine nucleoside tripho	0.46	22	2.3182297	773	2.232362
6519	GO:0009154	purine ribonucleotide ca	0.46	22	2.3182297	773	2.232362
6530	GO:0009166	nucleotide catabolic pro	0.46	22	2.3182297	773	2.232362
6567	GO:0009203	ribonucleoside triphosp	0.46	22	2.3182297	773	2.232362
6569	GO:0009205	purine ribonucleoside tr	0.46	22	2.3182297	773	2.232362
6571	GO:0009207	purine ribonucleoside tr	0.46	22	2.3182297	773	2.232362
6624	GO:0009261	ribonucleotide catabolic	0.46	22	2.3182297	773	2.232362
17034	GO:0034655	nucleobase, nucleoside	0.46	22	2.3182297	773	2.232362
17035	GO:0034656	nucleobase, nucleoside	0.46	22	2.3182297	773	2.232362
22209	GO:0046700	heterocycle catabolic pr	0.46	22	2.3182297	773	2.232362
30879	GO:0072523	purine-containing comp	0.46	22	2.3182297	773	2.232362
5715	GO:0008171	O-methyltransferase ac	0.47	2	0.21074815	57	0.16461143
9910	GO:0016878	acid-thiol ligase activity	0.47	2	0.21074815	57	0.16461143
20520	GO:0044270	cellular nitrogen compo	0.47	22	2.3182297	777	2.2439137
4918	GO:0006821	chloride transport	0.47	1	0.105374075	23	0.06642216
22753	GO:0047262|GO:00503	polygalacturonate 4-alp	0.47	1	0.105374075	23	0.06642216
2633	GO:0003684	damaged DNA binding	0.47	1	0.105374075	23	0.06642216
6665	GO:0009311	oligosaccharide metabo	0.47	2	0.21074815	58	0.16749935
9801	GO:0016747	transferase activity, trar	0.48	13	1.369863	456	1.3168914
30066	GO:0071702	organic substance trans	0.48	15	1.5806112	529	1.5277096
9900	GO:0016866	intramolecular transfera	0.48	2	0.21074815	59	0.17038727
18981	GO:0042651	thylakoid membrane	0.48	2	0.21074815	59	0.17038727
11712	GO:0019748	secondary metabolic pr	0.48	3	0.31612223	95	0.27435237
18633	GO:0042277	peptide binding	0.48	3	0.31612223	95	0.27435237
4311	GO:0006073	cellular glucan metaboli	0.49	1	0.105374075	24	0.06931008
7144	GO:0009892	negative regulation of n	0.49	1	0.105374075	24	0.06931008
13754	GO:0031324	negative regulation of c	0.49	1	0.105374075	24	0.06931008
20293	GO:0044042	glucan metabolic proce	0.49	1	0.105374075	24	0.06931008
20498	GO:0044248	cellular catabolic proces	0.49	25	2.634352	894	2.5818002
3304	GO:0004672|GO:00502	protein kinase activity	0.49	37	3.898841	1333	3.8495972
2980	GO:0004185	serine-type carboxypep	0.49	2	0.21074815	60	0.17327519
6964	GO:0009698	phenylpropanoid metab	0.49	2	0.21074815	60	0.17327519
28375	GO:0070008	serine-type exopeptidas	0.49	2	0.21074815	60	0.17327519
9800	GO:0016746	transferase activity, trar	0.50	14	1.4752371	498	1.438184
6563	GO:0009199	ribonucleoside triphosp	0.50	22	2.3182297	790	2.2814567
9482	GO:0016291|GO:00087	acyl-CoA thioesterase a	0.50	1	0.105374075	25	0.072197996
31493	GO:0080118	brassinosteroid sulfotra	0.50	1	0.105374075	25	0.072197996
6515	GO:0009150	purine ribonucleotide m	0.50	22	2.3182297	791	2.2843447
5712	GO:0008168|GO:00044	methyltransferase activ	0.50	7	0.73761857	244	0.7046524
8681	GO:0015299	solute:hydrogen antipor	0.51	3	0.31612223	98	0.28301615
25379	GO:0051082	unfolded protein binding	0.51	3	0.31612223	98	0.28301615
2978	GO:0004180	carboxypeptidase activi	0.51	2	0.21074815	62	0.17905103
3801	GO:0005342	organic acid transmemb	0.51	4	0.4214963	136	0.3927571
22444	GO:0046943	carboxylic acid transme	0.51	4	0.4214963	136	0.3927571
6506	GO:0009141	nucleoside triphosphate	0.51	22	2.3182297	796	2.2987843
3210	GO:0004565	beta-galactosidase activ	0.51	1	0.105374075	26	0.075085916
3415	GO:0004805	trehalose-phosphatase	0.51	1	0.105374075	26	0.075085916
4030	GO:0005741	mitochondrial outer mer	0.51	1	0.105374075	26	0.075085916
8516	GO:0015101	organic cation transmer	0.51	1	0.105374075	26	0.075085916
8964	GO:0015695	organic cation transport	0.51	1	0.105374075	26	0.075085916
9421	GO:0016208	AMP binding	0.51	1	0.105374075	26	0.075085916
9884	GO:0016847|GO:00341	1-aminocyclopropane-1	0.51	1	0.105374075	26	0.075085916
10911	GO:0018871	1-aminocyclopropane-1	0.51	1	0.105374075	26	0.075085916
17147	GO:0034768	(E)-beta-ocimene synth	0.51	1	0.105374075	26	0.075085916
18579	GO:0042218	1-aminocyclopropane-1	0.51	1	0.105374075	26	0.075085916
30689	GO:0072330	monocarboxylic acid bio	0.51	1	0.105374075	26	0.075085916
14667	GO:0032259	methylation	0.52	8	0.8429926	285	0.8230572

22416	GO:0046915	transition metal ion tran	0.53	2	0.21074815	64	0.18482687
25820	GO:0051536	iron-sulfur cluster bindir	0.53	2	0.21074815	64	0.18482687
25824	GO:0051540	metal cluster binding	0.53	2	0.21074815	64	0.18482687
9346	GO:0016114	terpenoid biosynthetic p	0.53	1	0.105374075	27	0.077973835
9389	GO:0016160	amylase activity	0.53	1	0.105374075	27	0.077973835
4849	GO:0006730|GO:0019	one-carbon metabolic p	0.53	9	0.9483667	325	0.93857396
10058	GO:0017111	nucleoside-triphosphata	0.53	24	2.5289779	878	2.5355935
25314	GO:0051015	actin filament binding	0.54	2	0.21074815	65	0.18771479
3724	GO:0005216	ion channel activity	0.54	4	0.4214963	140	0.40430877
9579	GO:0016462	pyrophosphatase activi	0.54	26	2.739726	954	2.7550755
3284	GO:0004650	polygalacturonase activ	0.54	1	0.105374075	28	0.080861755
3812	GO:0005355|GO:0015	glucose transmembrane	0.54	1	0.105374075	28	0.080861755
6079	GO:0008654	phospholipid biosynthet	0.54	1	0.105374075	28	0.080861755
9027	GO:0015758	glucose transport	0.54	1	0.105374075	28	0.080861755
9238	GO:0015980	energy derivation by ox	0.54	1	0.105374075	28	0.080861755
23911	GO:0048519|GO:0043	negative regulation of b	0.54	1	0.105374075	28	0.080861755
23915	GO:0048523|GO:0051	negative regulation of c	0.54	1	0.105374075	28	0.080861755
139	GO:0000175	3'-5'-exoribonuclease a	0.54	1	0.105374075	28	0.080861755
23515	GO:0048037	cofactor binding	0.54	9	0.9483667	327	0.94434977
3180	GO:0004521	endoribonuclease activ	0.54	2	0.21074815	66	0.1906027
9890	GO:0016854	racemase and epimera	0.54	2	0.21074815	66	0.1906027
9859	GO:0016818	hydrolase activity, actin	0.54	26	2.739726	956	2.7608514
4373	GO:0006163	purine nucleotide metab	0.55	22	2.3182297	810	2.339215
4916	GO:0006818	hydrogen transport	0.55	8	0.8429926	292	0.84327257
9246	GO:0015992	proton transport	0.55	8	0.8429926	292	0.84327257
6166	GO:0008757	S-adenosylmethionine-	0.55	5	0.52687037	180	0.5198256
9858	GO:0016817	hydrolase activity, actin	0.55	26	2.739726	959	2.769515
2668	GO:0003779	actin binding	0.55	3	0.31612223	105	0.3032316
6622	GO:0009259|GO:0009	ribonucleotide metaboli	0.55	22	2.3182297	812	2.344991
4755	GO:0006629	lipid metabolic process	0.55	16	1.6859852	590	1.7038727
4841	GO:0006721	terpenoid metabolic pro	0.55	1	0.105374075	29	0.083749674
11834	GO:0019905	syntaxin binding	0.55	1	0.105374075	29	0.083749674
3195	GO:0004540	ribonuclease activity	0.56	3	0.31612223	106	0.3061195
2999	GO:0004312	fatty acid synthase activ	0.56	2	0.21074815	68	0.19637854
32	GO:0000041	transition metal ion tran	0.56	2	0.21074815	68	0.19637854
12572	GO:0022890|GO:0015	inorganic cation transm	0.56	9	0.9483667	332	0.9587894
3009	GO:0004322	ferroxidase activity	0.57	1	0.105374075	30	0.086637594
3310	GO:0004679	AMP-activated protein k	0.57	1	0.105374075	30	0.086637594
3886	GO:0005527	macrolide binding	0.57	1	0.105374075	30	0.086637594
3887	GO:0005528	FK506 binding	0.57	1	0.105374075	30	0.086637594
4498	GO:0006305	DNA alkylation	0.57	1	0.105374075	30	0.086637594
4499	GO:0006306	DNA methylation	0.57	1	0.105374075	30	0.086637594
6216	GO:0008810	cellulase activity	0.57	1	0.105374075	30	0.086637594
9785	GO:0016724	oxidoreductase activity,	0.57	1	0.105374075	30	0.086637594
12533	GO:0022838	substrate-specific chan	0.57	6	0.63224447	221	0.63823026
3620	GO:0005057	receptor signaling prote	0.57	2	0.21074815	69	0.19926646
11522	GO:0019538|GO:0006	protein metabolic proce	0.57	112	11.801897	4134	11.938661
3873	GO:0005507	copper ion binding	0.57	4	0.4214963	146	0.42163628
9486	GO:0016298	lipase activity	0.57	5	0.52687037	185	0.53426516
9191	GO:0015931	nucleobase, nucleoside	0.58	2	0.21074815	70	0.20215438
9192	GO:0015932	nucleobase, nucleoside	0.58	2	0.21074815	70	0.20215438
9796	GO:0016741	transferase activity, tran	0.58	7	0.73761857	261	0.7537471
19590	GO:0043295	glutathione binding	0.58	1	0.105374075	31	0.08952551
20514	GO:0044264	cellular polysaccharide	0.58	1	0.105374075	31	0.08952551
4478	GO:0006281	DNA repair	0.59	4	0.4214963	149	0.43030006
5037	GO:0006974|GO:0034	response to DNA dama	0.59	4	0.4214963	149	0.43030006
30877	GO:0072521	purine-containing comp	0.59	22	2.3182297	827	2.3883097
8660	GO:0015267|GO:0015	channel activity	0.59	6	0.63224447	226	0.6526699
12515	GO:0022803	passive transmembrane	0.59	6	0.63224447	226	0.6526699
3190	GO:0004532	exoribonuclease activity	0.59	1	0.105374075	32	0.09241343
3717	GO:0005199	structural constituent of	0.59	1	0.105374075	32	0.09241343
4614	GO:0006470	protein dephosphorylati	0.59	1	0.105374075	32	0.09241343
6942	GO:0009674	potassium:sodium symp	0.59	1	0.105374075	32	0.09241343
9537	GO:0016409	palmitoyltransferase act	0.59	1	0.105374075	32	0.09241343
9927	GO:0016896	exoribonuclease activity	0.59	1	0.105374075	32	0.09241343
12522	GO:0022820	potassium ion symporte	0.59	1	0.105374075	32	0.09241343
12833	GO:0030275	LRR domain binding	0.59	1	0.105374075	32	0.09241343
14381	GO:0031968	organelle outer membra	0.59	1	0.105374075	32	0.09241343
4459	GO:0006259|GO:0055	DNA metabolic process	0.60	13	1.369863	493	1.4237444
4526	GO:0006351|GO:0006	transcription, DNA-depe	0.60	42	4.425711	1575	4.548474
15176	GO:0032774	RNA biosynthetic proce	0.60	42	4.425711	1575	4.548474
4493	GO:0006298|GO:0006	mismatch repair	0.60	1	0.105374075	33	0.09530135
2632	GO:0003682	chromatin binding	0.60	1	0.105374075	33	0.09530135
3955	GO:0005634	nucleus	0.61	38	4.004215	1433	4.138389
4497	GO:0006304	DNA modification	0.61	1	0.105374075	34	0.09818927
5270	GO:0007264	small GTPase mediated	0.61	1	0.105374075	34	0.09818927
5700	GO:0008144	drug binding	0.61	1	0.105374075	34	0.09818927
6004	GO:0008536	Ran GTPase binding	0.61	1	0.105374075	34	0.09818927
6072	GO:0008645	hexose transport	0.61	1	0.105374075	34	0.09818927
8562	GO:0015149	hexose transmembrane	0.61	1	0.105374075	34	0.09818927
17794	GO:0035428	hexose transmembrane	0.61	1	0.105374075	34	0.09818927
18382	GO:0040029	regulation of gene expr	0.61	1	0.105374075	34	0.09818927
6424	GO:0009055|GO:0009	electron carrier activity	0.61	4	0.4214963	154	0.44473964
2674	GO:0003824	catalytic activity	0.61	291	30.663857	10750	31.045137
9488	GO:0016301	kinase activity	0.62	46	4.8472075	1740	5.0249805
4538	GO:0006364|GO:0006	rRNA processing	0.62	1	0.105374075	35	0.10107719
9310	GO:0016072	rRNA metabolic proces	0.62	1	0.105374075	35	0.10107719
9480	GO:0016289	CoA hydrolase activity	0.62	1	0.105374075	35	0.10107719
9922	GO:0016891	endoribonuclease activ	0.62	1	0.105374075	35	0.10107719
9114	GO:0015849	organic acid transport	0.62	4	0.4214963	156	0.45051548
22443	GO:0046942	carboxylic acid transpor	0.62	4	0.4214963	156	0.45051548
5604	GO:0008017	microtubule binding	0.63	2	0.21074815	77	0.22236982
3877	GO:0005516	calmodulin binding	0.63	6	0.63224447	236	0.6815491
3888	GO:0005529	sugar binding	0.63	4	0.4214963	158	0.45629135
3804	GO:0005345	purine base transmemb	0.63	1	0.105374075	36	0.10396511
4949	GO:0006863|GO:0015	purine base transport	0.63	1	0.105374075	36	0.10396511
5902	GO:0008408	3'-5' exonuclease activi	0.63	1	0.105374075	36	0.10396511
11314	GO:0019321	pentose metabolic proc	0.63	1	0.105374075	36	0.10396511
20649	GO:0044455	mitochondrial membran	0.63	1	0.105374075	36	0.10396511
30865	GO:0072509	divalent inorganic cation	0.63	2	0.21074815	78	0.22525774
5705	GO:0008152	metabolic process	0.64	473	49.841938	17432	50.34222
19831	GO:0043565	sequence-specific DNA	0.64	4	0.4214963	159	0.45917925
8934	GO:0015662	ATPase activity, couple	0.64	1	0.105374075	37	0.10685303

3874	GO:0005509	calcium ion binding	0.65	7	0.73761857	279	0.8057296
9875	GO:0016836	hydro-lyase activity	0.65	2	0.21074815	80	0.2310336
9637	GO:0016538|GO:00037	cyclin-dependent protei	0.65	1	0.105374075	38	0.10974095
117	GO:0000149	SNARE binding	0.65	1	0.105374075	38	0.10974095
3827	GO:0005372	water transmembrane t	0.65	2	0.21074815	81	0.23392151
4927	GO:0006833	water transport	0.65	2	0.21074815	81	0.23392151
8652	GO:0015250	water channel activity	0.65	2	0.21074815	81	0.23392151
18429	GO:0042044	fluid transport	0.65	2	0.21074815	81	0.23392151
3260	GO:0004620	phospholipase activity	0.66	3	0.31612223	123	0.35521415
29202	GO:0070838	divalent metal ion trans	0.66	2	0.21074815	82	0.23680943
19473	GO:0043170|GO:00432	macromolecule metabo	0.66	175	18.440464	6553	18.92454
20488	GO:0044238	primary metabolic proce	0.66	246	25.922024	9167	26.47356
4476	GO:0006278	RNA-dependent DNA r	0.67	4	0.4214963	166	0.4793947
5891	GO:0008395|GO:00083	steroid hydroxylase acti	0.67	1	0.105374075	40	0.1155168
8613	GO:0015205|GO:00153	nucleobase transmemb	0.67	1	0.105374075	40	0.1155168
9116	GO:0015851	nucleobase transport	0.67	1	0.105374075	40	0.1155168
9843	GO:0016796	exonuclease activity, ac	0.67	1	0.105374075	40	0.1155168
11804	GO:0019867	outer membrane	0.67	1	0.105374075	40	0.1155168
4643	GO:0006511	ubiquitin-dependent pro	0.67	2	0.21074815	84	0.24258527
11864	GO:0019941	modification-dependent	0.67	2	0.21074815	84	0.24258527
19898	GO:0043632	modification-dependent	0.67	2	0.21074815	84	0.24258527
20507	GO:0044257	cellular protein catabolic	0.67	2	0.21074815	84	0.24258527
25887	GO:0051603	proteolysis involved in c	0.67	2	0.21074815	84	0.24258527
30867	GO:0072511	divalent inorganic cation	0.67	2	0.21074815	84	0.24258527
26932	GO:0055086	nucleobase, nucleoside	0.68	24	2.5289779	944	2.7261963
20627	GO:0044433	cytoplasmic vesicle par	0.68	1	0.105374075	41	0.118404716
5661	GO:0008092	cytoskeletal protein bind	0.68	6	0.63224447	249	0.719092
5768	GO:0008238	exopeptidase activity	0.69	2	0.21074815	86	0.24836111
12734	GO:0030163	protein catabolic proces	0.69	2	0.21074815	86	0.24836111
20515	GO:0044265|GO:00349	cellular macromolecule	0.69	2	0.21074815	86	0.24836111
12805	GO:0030246	carbohydrate binding	0.69	6	0.63224447	251	0.7248679
3718	GO:0005200	structural constituent of	0.69	1	0.105374075	42	0.121292636
5033	GO:0006970	response to osmotic str	0.69	1	0.105374075	42	0.121292636
12659	GO:0030076	light-harvesting comple	0.69	1	0.105374075	42	0.121292636
18956	GO:0042626	ATPase activity, couple	0.69	5	0.52687037	211	0.6093511
4867	GO:0006753	nucleoside phosphate r	0.69	23	2.4236038	914	2.6395588
6484	GO:0009117	nucleotide metabolic pr	0.69	23	2.4236038	914	2.6395588
9861	GO:0016820	hydrolase activity, actin	0.69	5	0.52687037	212	0.612239
3156	GO:0004497	monooxygenase activity	0.70	4	0.4214963	172	0.49672222
5694	GO:0008137	NADH dehydrogenase	0.70	1	0.105374075	43	0.124180555
8589	GO:0015179	L-amino acid transmem	0.70	1	0.105374075	43	0.124180555
9076	GO:0015807	L-amino acid transport	0.70	1	0.105374075	43	0.124180555
13833	GO:0031410	cytoplasmic vesicle	0.70	1	0.105374075	43	0.124180555
14393	GO:0031982	vesicle	0.70	1	0.105374075	43	0.124180555
20624	GO:0044430	cytoskeletal part	0.70	2	0.21074815	88	0.25413695
8495	GO:0015077	monovalent inorganic c	0.70	5	0.52687037	214	0.6180149
4605	GO:0006457|GO:00070	protein folding	0.70	4	0.4214963	173	0.49961013
15949	GO:0033554	cellular response to stre	0.70	4	0.4214963	173	0.49961013
9824	GO:0016773	phosphotransferase act	0.70	38	4.004215	1493	4.311664
2982	GO:0004197	cysteine-type endopept	0.71	2	0.21074815	90	0.2599128
18953	GO:0042623	ATPase activity, couple	0.71	13	1.369863	537	1.550813
8787	GO:0015450	P-P-bond-hydrolysis-dri	0.71	1	0.105374075	45	0.1299564
9924	GO:0016893	endonuclease activity, a	0.71	1	0.105374075	45	0.1299564
11780	GO:0019829	cation-transporting ATP	0.71	1	0.105374075	45	0.1299564
24456	GO:0050136	NADH dehydrogenase	0.71	1	0.105374075	45	0.1299564
26845	GO:0052689	carboxylic ester hydrola	0.72	13	1.369863	538	1.5537009
3242	GO:0004601|GO:00166	peroxidase activity	0.72	3	0.31612223	135	0.38986918
9754	GO:0016684	oxidoreductase activity,	0.72	3	0.31612223	135	0.38986918
8749	GO:0015399	primary active transmer	0.72	6	0.63224447	260	0.75085914
8753	GO:0015405	P-P-bond-hydrolysis-dri	0.72	6	0.63224447	260	0.75085914
4558	GO:0006396|GO:00063	RNA processing	0.72	5	0.52687037	219	0.63245445
12498	GO:0022613	ribonucleoprotein comp	0.72	1	0.105374075	46	0.13284431
18614	GO:0042254|GO:00070	ribosome biogenesis	0.72	1	0.105374075	46	0.13284431
31895	GO:0090304	nucleic acid metabolic p	0.72	60	6.322445	2340	6.7577324
5714	GO:0008170	N-methyltransferase ac	0.73	1	0.105374075	47	0.13573223
8497	GO:0015079|GO:00228	potassium ion transmer	0.73	1	0.105374075	47	0.13573223
9308	GO:0016070	RNA metabolic process	0.73	47	4.952582	1855	5.3570914
9823	GO:0016772	transferase activity, trar	0.73	49	5.1633296	1932	5.579461
14591	GO:0032183	SUMO binding	0.74	1	0.105374075	48	0.13862015
4460	GO:0006260|GO:00551	DNA replication	0.74	5	0.52687037	224	0.64689404
6483	GO:0009116	nucleoside metabolic pr	0.74	1	0.105374075	49	0.14150807
6750	GO:0009415	response to water	0.74	1	0.105374075	49	0.14150807
8909	GO:0015630	microtubule cytoskeleto	0.74	1	0.105374075	49	0.14150807
11784	GO:0019838	growth factor binding	0.74	1	0.105374075	49	0.14150807
3185	GO:0004527|GO:00088	exonuclease activity	0.75	1	0.105374075	50	0.14439599
4216	GO:0005976	polysaccharide metabo	0.75	1	0.105374075	50	0.14439599
9993	GO:0017016	Ras GTPase binding	0.75	1	0.105374075	50	0.14439599
29674	GO:0071310	cellular response to org	0.75	1	0.105374075	50	0.14439599
30886	GO:0072530	purine-containing comp	0.75	1	0.105374075	50	0.14439599
3293	GO:0004659	prenyltransferase activi	0.76	1	0.105374075	51	0.14728391
8908	GO:0015629	actin cytoskeleton	0.76	1	0.105374075	51	0.14728391
30207	GO:0071843	cellular component biog	0.76	1	0.105374075	51	0.14728391
2628	GO:0003677	DNA binding	0.76	30	3.1612225	1224	3.534814
15436	GO:0033036	macromolecule localiza	0.76	9	0.9483667	397	1.1465042
4915	GO:0006817	phosphate transport	0.76	1	0.105374075	52	0.15017183
8528	GO:0015114	phosphate transmembr	0.76	1	0.105374075	52	0.15017183
17801	GO:0035435	phosphate transmembr	0.76	1	0.105374075	52	0.15017183
2809	GO:0003993	acid phosphatase activi	0.76	1	0.105374075	52	0.15017183
2778	GO:0003954	NADH dehydrogenase	0.77	1	0.105374075	53	0.15305975
19758	GO:0043492	ATPase activity, couple	0.77	5	0.52687037	235	0.67866117
17918	GO:0035556|GO:00072	intracellular signal trans	0.78	1	0.105374075	54	0.15594767
29251	GO:0070887	cellular response to che	0.78	1	0.105374075	54	0.15594767
11828	GO:0019899	enzyme binding	0.78	5	0.52687037	237	0.684437
4029	GO:0005740	mitochondrial envelope	0.79	3	0.31612223	151	0.4360759
13697	GO:0031267	small GTPase binding	0.79	1	0.105374075	56	0.16172351
6426	GO:0009057|GO:00432	macromolecule catabol	0.79	2	0.21074815	106	0.3061195
7557	GO:0010333	terpene synthase activi	0.80	1	0.105374075	57	0.16461143
8910	GO:0015631	tubulin binding	0.80	2	0.21074815	107	0.30900744
4357	GO:0006139|GO:00551	nucleobase, nucleoside	0.80	83	8.746049	3285	9.486816
3323	GO:0004702	receptor signaling prote	0.80	1	0.105374075	58	0.16749935
5819	GO:0008299|GO:00092	isoprenoid biosynthetic	0.80	1	0.105374075	58	0.16749935
8558	GO:0015145	monosaccharide transn	0.80	1	0.105374075	58	0.16749935
9018	GO:0015749	monosaccharide transp	0.80	1	0.105374075	58	0.16749935

11820	GO:0019887	protein kinase regulator	0.80	1	0.105374075	58	0.16749935
3946	GO:0005623	cell	0.80	215	22.655426	8227	23.758917
20657	GO:0044464	cell part	0.80	215	22.655426	8227	23.758917
5909	GO:0008415	acyltransferase activity	0.80	7	0.73761857	330	0.95301354
11833	GO:0019904	protein domain specific	0.80	2	0.21074815	109	0.31478328
30700	GO:0072341	modified amino acid bin	0.81	1	0.105374075	59	0.17038727
4840	GO:0006720|GO:0016C	isoprenoid metabolic pr	0.81	1	0.105374075	60	0.17327519
5652	GO:0008081|GO:0004	phosphoric diester hydr	0.81	1	0.105374075	60	0.17327519
14811	GO:0032403	protein complex binding	0.81	1	0.105374075	60	0.17327519
25319	GO:0051020	GTPase binding	0.81	1	0.105374075	60	0.17327519
29918	GO:0071554	cell wall organization or	0.82	2	0.21074815	112	0.32344702
4768	GO:0006644	phospholipid metabolic	0.82	1	0.105374075	61	0.1761631
2818	GO:0004003	ATP-dependent DNA h	0.82	1	0.105374075	61	0.1761631
8496	GO:0015078	hydrogen ion transmem	0.82	3	0.31612223	160	0.4620672
6018	GO:0008559|GO:0005	xenobiotic-transporting	0.82	1	0.105374075	62	0.17905103
11210	GO:0019207	kinase regulator activity	0.82	1	0.105374075	62	0.17905103
19225	GO:0042908	xenobiotic transport	0.82	1	0.105374075	62	0.17905103
19227	GO:0042910	xenobiotic transporter a	0.82	1	0.105374075	62	0.17905103
14379	GO:0031966	mitochondrial membran	0.82	2	0.21074815	114	0.32922286
3103	GO:0004437	inositol or phosphatidyli	0.83	1	0.105374075	64	0.18482687
20623	GO:0044429	mitochondrial part	0.83	3	0.31612223	165	0.47650677
9838	GO:0016790	thiolester hydrolase act	0.84	1	0.105374075	65	0.18771479
11616	GO:0019637	organophosphate meta	0.84	1	0.105374075	65	0.18771479
19526	GO:0043227	membrane-bounded org	0.84	52	5.479452	2142	6.185924
19530	GO:0043231	intracellular membrane-	0.84	52	5.479452	2142	6.185924
25416	GO:0051119	sugar transmembrane t	0.84	2	0.21074815	118	0.34077454
5809	GO:0008289	lipid binding	0.84	5	0.52687037	258	0.74508333
2629	GO:0003678|GO:0003	DNA helicase activity	0.84	1	0.105374075	67	0.19349062
17024	GO:0034645|GO:0034	cellular macromolecule	0.85	48	5.0579557	1999	5.7729516
18955	GO:0042625	ATPase activity, couple	0.85	1	0.105374075	68	0.19637854
6884	GO:0009607	response to biotic stimu	0.85	2	0.21074815	122	0.3523262
6428	GO:0009059|GO:0043	macromolecule biosynt	0.85	48	5.0579557	2003	5.7845035
5726	GO:0008187	poly-pyrimidine tract bin	0.86	1	0.105374075	70	0.20215438
5788	GO:0008266	poly(U) RNA binding	0.86	1	0.105374075	70	0.20215438
5838	GO:0008320	protein transmembrane	0.86	1	0.105374075	70	0.20215438
9853	GO:0016811	hydrolase activity, actin	0.86	1	0.105374075	70	0.20215438
12568	GO:0022884	macromolecule transme	0.86	1	0.105374075	70	0.20215438
23522	GO:0048046	apoplast	0.86	1	0.105374075	70	0.20215438
2649	GO:0003713	transcription coactivato	0.86	1	0.105374075	70	0.20215438
4067	GO:0005783	endoplasmic reticulum	0.86	3	0.31612223	174	0.50249803
3200	GO:0004553|GO:0016	hydrolase activity, hydro	0.86	6	0.63224447	312	0.90103096
7691	GO:0010467	gene expression	0.86	47	4.952582	1974	5.7007537
9845	GO:0016798	hydrolase activity, actin	0.86	8	0.8429926	401	1.1580559
9771	GO:0016709	oxidoreductase activity,	0.86	1	0.105374075	71	0.2050423
9869	GO:0016829	lyase activity	0.86	10	1.0537407	488	1.4093049
4121	GO:0005856	cytoskeleton	0.86	2	0.21074815	126	0.3638779
20531	GO:0044281	small molecule metabol	0.87	42	4.425711	1788	5.1636004
3311	GO:0004683|GO:0004	calmodulin-dependent p	0.87	1	0.105374075	74	0.21370606
4394	GO:0006184	GTP catabolic process	0.88	2	0.21074815	132	0.3812054
21605	GO:0046039	GTP metabolic process	0.88	2	0.21074815	132	0.3812054
2758	GO:0003924	GTPase activity	0.88	2	0.21074815	132	0.3812054
6529	GO:0009165	nucleotide biosynthetic	0.88	1	0.105374075	77	0.22236982
9877	GO:0016838	carbon-oxygen lyase ac	0.88	1	0.105374075	77	0.22236982
9852	GO:0016810	hydrolase activity, actin	0.89	2	0.21074815	134	0.38698125
5196	GO:0007166	cell surface receptor lin	0.89	1	0.105374075	78	0.22525774
3903	GO:0005575|GO:0008	cellular_component	0.89	218	22.971548	8515	24.590637
22007	GO:0046483	heterocycle metabolic p	0.89	24	2.5289779	1094	3.1593843
9682	GO:0016597	amino acid binding	0.89	1	0.105374075	80	0.2310336
19479	GO:0043176	amine binding	0.89	1	0.105374075	80	0.2310336
15189	GO:0032787	monocarboxylic acid me	0.89	1	0.105374075	81	0.23392151
18674	GO:0042325	regulation of phosphory	0.89	1	0.105374075	81	0.23392151
4942	GO:0006855	drug transmembrane tra	0.90	2	0.21074815	139	0.40142086
8642	GO:0015238|GO:0015	drug transmembrane tra	0.90	2	0.21074815	139	0.40142086
9154	GO:0015893	drug transport	0.90	2	0.21074815	139	0.40142086
18832	GO:0042493|GO:0017	response to drug	0.90	2	0.21074815	139	0.40142086
2658	GO:0003729	mRNA binding	0.90	1	0.105374075	82	0.23680943
3895	GO:0005543	phospholipid binding	0.90	2	0.21074815	140	0.40430877
6046	GO:0008610	lipid biosynthetic proces	0.90	2	0.21074815	141	0.4071967
22479	GO:0046983	protein dimerization act	0.90	10	1.0537407	517	1.4930545
8557	GO:0015144	carbohydrate transmem	0.91	2	0.21074815	143	0.41297254
16602	GO:0034219	carbohydrate transmem	0.91	2	0.21074815	143	0.41297254
11222	GO:0019220	regulation of phosphate	0.91	1	0.105374075	85	0.24547319
25471	GO:0051174	regulation of phosphoru	0.91	1	0.105374075	85	0.24547319
4032	GO:0005743	mitochondrial inner mer	0.91	1	0.105374075	88	0.25413695
6071	GO:0008643|GO:0006	carbohydrate transport	0.91	2	0.21074815	147	0.42452422
14380	GO:0031967	organelle envelope	0.92	3	0.31612223	201	0.5804719
9874	GO:0016835	carbon-oxygen lyase ac	0.92	3	0.31612223	202	0.5833598
11426	GO:0019438	aromatic compound bio	0.92	2	0.21074815	149	0.43030006
14590	GO:0032182	small conjugating prote	0.92	1	0.105374075	90	0.2599128
3644	GO:0005102	receptor binding	0.92	1	0.105374075	91	0.2628007
14387	GO:0031975	envelope	0.92	3	0.31612223	205	0.59202355
16785	GO:0034404	nucleobase, nucleoside	0.92	1	0.105374075	92	0.26568863
17033	GO:0034654	nucleobase, nucleoside	0.92	1	0.105374075	92	0.26568863
20336	GO:0044085	cellular component biog	0.92	1	0.105374075	93	0.26857653
3808	GO:0005351|GO:0005	sugar:hydrogen sympor	0.93	1	0.105374075	94	0.27146447
3835	GO:0005402	cation:sugar symporter	0.93	1	0.105374075	94	0.27146447
11803	GO:0019866	organelle inner membra	0.93	1	0.105374075	94	0.27146447
3178	GO:0004519	endonuclease activity	0.93	6	0.63224447	356	1.0280994
4906	GO:0006807	nitrogen compound met	0.93	89	9.378293	3725	10.757502
4612	GO:0006468	protein phosphorylation	0.93	50	5.268704	2205	6.367863
19126	GO:0042803	protein homodimerizatio	0.93	4	0.4214963	265	0.7652988
8677	GO:0015295	solute:hydrogen sympo	0.93	1	0.105374075	98	0.28301615
13829	GO:0031406	carboxylic acid binding	0.93	1	0.105374075	98	0.28301615
16851	GO:0034470	ncRNA processing	0.94	1	0.105374075	99	0.28590405
17039	GO:0034660	ncRNA metabolic proce	0.94	1	0.105374075	99	0.28590405
13041	GO:0030554	adenyl nucleotide bindi	0.94	10	1.0537407	550	1.5883559
14965	GO:0032559	adenyl ribonucleotide b	0.94	10	1.0537407	550	1.5883559
9870	GO:0016830	carbon-carbon lyase ac	0.94	2	0.21074815	161	0.4649551
7271	GO:0010033	response to organic sul	0.94	1	0.105374075	100	0.28879198
16430	GO:0034046	poly(G) RNA binding	0.94	1	0.105374075	100	0.28879198
17020	GO:0034641	cellular nitrogen compo	0.94	87	9.167544	3678	10.621769
5764	GO:0008234|GO:0004	cysteine-type peptidase	0.94	2	0.21074815	163	0.47073093
4028	GO:0005739	mitochondrion	0.94	3	0.31612223	219	0.63245445

19125	GO:0042802	identical protein binding	0.95	13	1.369863	697	2.01288
4649	GO:0006520|GO:00065	cellular amino acid meta	0.95	4	0.4214963	277	0.7999538
9494	GO:0016311	dephosphorylation	0.95	7	0.73761857	425	1.227366
6077	GO:0008652	cellular amino acid bios	0.95	2	0.21074815	169	0.48805845
3177	GO:0004518	nuclease activity	0.95	7	0.73761857	427	1.2331418
3884	GO:0005524	ATP binding	0.95	9	0.9483667	524	1.51327
9839	GO:0016791|GO:00163	phosphatase activity	0.95	6	0.63224447	383	1.1060733
13531	GO:0031072	heat shock protein bind	0.95	3	0.31612223	230	0.6642216
9493	GO:0016310	phosphorylation	0.96	56	5.9009485	2504	7.2313514
29081	GO:0070717	poly-purine tract binding	0.96	1	0.105374075	112	0.32344702
4850	GO:0006732|GO:00067	coenzyme metabolic pr	0.96	1	0.105374075	113	0.32633495
19686	GO:0043414	macromolecule methyla	0.96	1	0.105374075	113	0.32633495
3904	GO:0005576	extracellular region	0.96	2	0.21074815	178	0.5140497
13082	GO:0030599	pectinesterase activity	0.96	2	0.21074815	178	0.5140497
9837	GO:0016788	hydrolase activity, actin	0.96	32	3.3719704	1534	4.430069
6020	GO:0008565|GO:00154	protein transporter activ	0.96	1	0.105374075	115	0.3321108
9871	GO:0016831	carboxy-lyase activity	0.96	1	0.105374075	115	0.3321108
6663	GO:0009309	amine biosynthetic proc	0.96	2	0.21074815	181	0.5227135
18912	GO:0042578	phosphoric ester hydrol	0.96	7	0.73761857	444	1.2822365
20505	GO:0044255	cellular lipid metabolic p	0.96	2	0.21074815	184	0.53137726
4844	GO:0006725	cellular aromatic compo	0.96	2	0.21074815	185	0.53426516
3885	GO:0005525	GTP binding	0.97	3	0.31612223	245	0.70754033
11032	GO:0019001	guanyl nucleotide bindin	0.97	3	0.31612223	245	0.70754033
14967	GO:0032561	guanyl ribonucleotide b	0.97	3	0.31612223	245	0.70754033
2657	GO:0003727|GO:00037	single-stranded RNA bi	0.97	2	0.21074815	188	0.54292893
20356	GO:0044106	cellular amine metaboli	0.97	4	0.4214963	303	0.8750397
9889	GO:0016853	isomerase activity	0.97	4	0.4214963	306	0.8837035
12741	GO:0030170	pyridoxal phosphate bin	0.97	1	0.105374075	126	0.3638779
28643	GO:0070279	vitamin B6 binding	0.97	1	0.105374075	126	0.3638779
634	GO:0000988	protein binding transcrip	0.97	1	0.105374075	126	0.3638779
635	GO:0000989	transcription factor bind	0.97	1	0.105374075	126	0.3638779
2648	GO:0003712	transcription cofactor ac	0.97	1	0.105374075	126	0.3638779
6662	GO:0009308	amine metabolic proces	0.97	4	0.4214963	314	0.9068068
19533	GO:0043234	protein complex	0.97	15	1.5806112	848	2.448956
20499	GO:0044249	cellular biosynthetic pro	0.98	57	6.0063224	2635	7.6096687
9836	GO:0016787	hydrolase activity	0.98	84	8.851422	3723	10.751725
3449	GO:0004842|GO:00048	ubiquitin-protein ligase	0.98	4	0.4214963	326	0.94146186
18582	GO:0042221	response to chemical st	0.98	4	0.4214963	328	0.94723773
9295	GO:0016053	organic acid biosyntheti	0.98	2	0.21074815	211	0.6093511
21922	GO:0046394	carboxylic acid biosynth	0.98	2	0.21074815	211	0.6093511
9654	GO:0016567	protein ubiquitination	0.98	4	0.4214963	332	0.9587894
14854	GO:0032446	protein modification by	0.98	4	0.4214963	332	0.9587894
29011	GO:0070647	protein modification by	0.98	4	0.4214963	332	0.9587894
3716	GO:0005198	structural molecule activ	0.98	5	0.52687037	393	1.1349525
11747	GO:0019787|GO:00086	small conjugating prote	0.98	4	0.4214963	339	0.9790048
4897	GO:0006793	phosphorus metabolic p	0.98	63	6.638567	2925	8.4471655
4900	GO:0006796	phosphate metabolic pr	0.98	63	6.638567	2925	8.4471655
5019	GO:0006952|GO:00022	defense response	0.98	2	0.21074815	221	0.63823026
14959	GO:0032553	ribonucleotide binding	0.99	13	1.369863	795	2.2958963
14961	GO:0032555	purine ribonucleotide bi	0.99	13	1.369863	795	2.2958963
10033	GO:0017076	purine nucleotide bindin	0.99	13	1.369863	797	2.3016722
13547	GO:0031090	organelle membrane	0.99	2	0.21074815	224	0.64689404
14386	GO:0031974	membrane-enclosed lu	0.99	1	0.105374075	154	0.44473964
4609	GO:0006464	protein modification pro	0.99	59	6.2170706	2781	8.031305
6427	GO:0009058	biosynthetic process	0.99	59	6.2170706	2796	8.074624
18000	GO:0035639	purine ribonucleoside tr	0.99	12	1.2644889	769	2.2208104
25483	GO:0051186	cofactor metabolic proc	0.99	1	0.105374075	164	0.47361887
4320	GO:0006082	organic acid metabolic	0.99	4	0.4214963	369	1.0656425
11716	GO:0019752	carboxylic acid metabol	0.99	4	0.4214963	369	1.0656425
19707	GO:0043436	oxoacid metabolic proc	0.99	4	0.4214963	369	1.0656425
2660	GO:0003735|GO:00037	structural constituent of	0.99	2	0.21074815	246	0.7104283
131	GO:0000166	nucleotide binding	0.99	21	2.2128556	1213	3.5030468
18541	GO:0042180	cellular ketone metabol	0.99	4	0.4214963	375	1.0829699
11787	GO:0019842	vitamin binding	0.99	1	0.105374075	177	0.5111618
9906	GO:0016874	ligase activity	0.99	8	0.8429926	597	1.7240882
19684	GO:0043412	macromolecule modifica	0.99	60	6.322445	2900	8.374968
20510	GO:0044260|GO:00349	cellular macromolecule	0.99	126	13.277134	5577	16.105928
5610	GO:0008026	ATP-dependent helicas	0.99	1	0.105374075	183	0.52848935
28401	GO:0070035	purine NTP-dependent	0.99	1	0.105374075	183	0.52848935
8466	GO:0015031|GO:00158	protein transport	0.99	1	0.105374075	185	0.53426516
20820	GO:0045184	establishment of protein	0.99	1	0.105374075	185	0.53426516
9913	GO:0016881	acid-amino acid ligase a	0.99	4	0.4214963	391	1.1291766
3068	GO:0004386	helicase activity	0.99	1	0.105374075	189	0.54581684
5669	GO:0008104	protein localization	0.99	1	0.105374075	189	0.54581684
9911	GO:0016879	ligase activity, forming c	1.00	5	0.52687037	456	1.3168914
3340	GO:0004721	phosphoprotein phosph	1.00	1	0.105374075	199	0.57469606
20533	GO:0044283	small molecule biosynth	1.00	3	0.31612223	347	1.0021082
2626	GO:0003674|GO:00055	molecular_function	1.00	630	66.385666	24394	70.447914
30204	GO:0071840	cellular component orga	1.00	3	0.31612223	362	1.045427
19525	GO:0043226	organelle	1.00	60	6.322445	3013	8.701303
19528	GO:0043229	intracellular organelle	1.00	60	6.322445	3013	8.701303
20517	GO:0044267	cellular protein metabol	1.00	65	6.849315	3225	9.313541
20521	GO:0044271	cellular nitrogen compo	1.00	3	0.31612223	367	1.0598665
7229	GO:0009987|GO:00081	cellular process	1.00	250	26.34352	10601	30.614838
20487	GO:0044237	cellular metabolic proce	1.00	185	19.494204	8185	23.637625
30205	GO:0071841	cellular component orga	1.00	1	0.105374075	285	0.8230572
20618	GO:0044424	intracellular part	1.00	75	7.9030557	3853	11.127155
3876	GO:0005515|GO:00453	protein binding	1.00	147	15.489989	6933	20.021948
20640	GO:0044446	intracellular organelle p	1.00	6	0.63224447	710	2.0504231
20616	GO:0044422	organelle part	1.00	6	0.63224447	712	2.0561988
3945	GO:0005622	intracellular	1.00	92	9.694415	4751	13.720507
2627	GO:0003676	nucleic acid binding	1.00	39	4.109589	2453	7.0840673
4109	GO:0005840|GO:00332	ribosome	1.00	5	0.52687037	682	1.9695613
20638	GO:0044444	cytoplasmic part	1.00	21	2.2128556	1644	4.7477403
13018	GO:0030529	ribonucleoprotein comp	1.00	5	0.52687037	741	2.1399486
15392	GO:0032991	macromolecular comple	1.00	20	2.1074815	1616	4.666878
19527	GO:0043228	non-membrane-bounde	1.00	7	0.73761857	923	2.66555
19531	GO:0043232	intracellular non-membr	1.00	7	0.73761857	923	2.66555
4027	GO:0005737	cytoplasm	1.00	25	2.634352	2001	5.7787275
3863	GO:0005488	binding	1.00	308	32.455215	13903	40.15075
2653	GO:0003723	RNA binding	1.00	3	0.31612223	797	2.3016722

GO ID	GO ACCESSION	GO Term	p-value	Count in Selection	% Count in Selection	Count in Total	% Count in Total
5704	GO:0008150|GO:000	biological_process	0.00	922	71.806854	22215	64.155136
2674	GO:0003824	catalytic activity	0.00	474	36.91589	10750	31.045137
4612	GO:0006468	protein phosphorylat	0.00	123	9.579439	2205	6.367863
9493	GO:0016310	phosphorylation	0.00	136	10.5919	2504	7.2313514
4897	GO:0006793	phosphorus metabol	0.00	154	11.99377	2925	8.4471655
4900	GO:0006796	phosphate metabolic	0.00	154	11.99377	2925	8.4471655
9795	GO:0016740	transferase activity	0.00	189	14.719626	3764	10.870131
5705	GO:0008152	metabolic process	0.00	721	56.15265	17432	50.34222
9488	GO:0016301	kinase activity	0.00	99	7.7102804	1740	5.0249805
9824	GO:0016773	phosphotransferase	0.00	86	6.697819	1493	4.311664
3304	GO:0004672|GO:00	protein kinase activit	0.00	78	6.074766	1333	3.8495972
4215	GO:0005975	carbohydrate metab	0.00	61	4.7507787	990	2.8590407
3874	GO:0005509	calcium ion binding	0.00	24	1.8691589	279	0.8057296
3306	GO:0004674|GO:000	protein serine/threon	0.00	63	4.906542	1062	3.0669708
3490	GO:0004888|GO:000	transmembrane rece	0.00	39	3.037383	571	1.6490022
9823	GO:0016772	transferase activity, t	0.00	102	7.9439254	1932	5.579461
24968	GO:0050662	coenzyme binding	0.00	18	1.4018692	191	0.5515927
3904	GO:0005576	extracellular region	0.00	17	1.3239875	178	0.5140497
3476	GO:0004872|GO:00	receptor activity	0.00	43	3.3489096	672	1.9406822
24966	GO:0050660	flavin adenine dinucl	0.00	13	1.0124611	118	0.34077454
9845	GO:0016798	hydrolase activity, ac	0.00	29	2.258567	401	1.1580559
9486	GO:0016298	lipase activity	0.00	17	1.3239875	185	0.53426516
11202	GO:0019199	transmembrane rece	0.00	34	2.647975	504	1.4555116
4609	GO:0006464	protein modification	0.00	135	10.514019	2781	8.031305
9813	GO:0016759	cellulose synthase a	0.00	8	0.62305295	57	0.16461143
3167	GO:0004508	steroid 17-alpha-mo	0.00	2	0.15576324	2	0.00577584
9847	GO:0016801	hydrolase activity, ac	0.00	4	0.31152648	14	0.040430877
25098	GO:0050794|GO:00	regulation of cellular	0.00	182	14.174455	3987	11.514136
3475	GO:0004871|GO:000	signal transducer act	0.00	46	3.5825546	787	2.2727928
27063	GO:0060089	molecular transduce	0.00	46	3.5825546	787	2.2727928
3260	GO:0004620	phospholipase activi	0.00	12	0.93457943	123	0.35521415
5981	GO:0008509	anion transmembran	0.00	19	1.4797508	245	0.70754033
8980	GO:0015711	organic anion transp	0.00	6	0.46728972	37	0.10685303
4917	GO:0006820|GO:000	anion transport	0.00	19	1.4797508	247	0.7133162
25094	GO:0050789|GO:00	regulation of biologic	0.00	194	15.109035	4315	12.461374
8159	GO:0010951	negative regulation o	0.00	10	0.7788162	95	0.27435237
3472	GO:0004866	endopeptidase inhibi	0.00	10	0.7788162	95	0.27435237
26712	GO:0052548	regulation of endope	0.00	10	0.7788162	95	0.27435237
28101	GO:0061135	endopeptidase regul	0.00	10	0.7788162	95	0.27435237
9816	GO:0016762	xyloglucan:xyloglucо	0.00	5	0.3894081	27	0.077973835
3473	GO:0004867	serine-type endopep	0.00	8	0.62305295	66	0.1906027
5891	GO:0008395|GO:000	steroid hydroxylase a	0.00	6	0.46728972	40	0.1155168
3723	GO:0005215|GO:000	transporter activity	0.00	91	7.0872273	1844	5.325324
19684	GO:0043412	macromolecule mod	0.00	135	10.514019	2900	8.374968
5986	GO:0008514	organic anion transm	0.00	5	0.3894081	29	0.083749674
9848	GO:0016802	trialkylsulfonium hyd	0.00	2	0.15576324	3	0.00866376
2822	GO:0004013	adenosylhomocystei	0.00	2	0.15576324	3	0.00866376
28364	GO:0065007	biological regulation	0.00	194	15.109035	4376	12.637537
3242	GO:0004601|GO:00	peroxidase activity	0.00	12	0.93457943	135	0.38986918
9754	GO:0016684	oxidoreductase activ	0.00	12	0.93457943	135	0.38986918
12602	GO:0023052|GO:00	signaling	0.00	52	4.0498443	961	2.775291
5195	GO:0007165|GO:00	signal transduction	0.00	52	4.0498443	961	2.775291
23116	GO:0047631	ADP-ribose diphosph	0.01	3	0.23364486	10	0.0288792
2797	GO:0003979	UDP-glucose 6-dehy	0.01	3	0.23364486	10	0.0288792
3307	GO:0004675	transmembrane rece	0.01	24	1.8691589	367	1.0598665
23515	GO:0048037	cofactor binding	0.01	22	1.7133956	327	0.94434977
26931	GO:0055085	transmembrane trans	0.01	77	5.996885	1548	4.4705
21331	GO:0045735	nutrient reservoir act	0.01	9	0.7009346	89	0.25702485
3464	GO:0004857	enzyme inhibitor acti	0.01	13	1.0124611	157	0.4534034
13082	GO:0030599	pectinesterase activi	0.01	14	1.0903426	178	0.5140497
3877	GO:0005516	calmodulin binding	0.01	17	1.3239875	236	0.6815491
7690	GO:0010466	negative regulation o	0.01	10	0.7788162	110	0.31767118
12946	GO:0030414	peptidase inhibitor a	0.01	10	0.7788162	110	0.31767118
25636	GO:0051346	negative regulation o	0.01	10	0.7788162	110	0.31767118
6293	GO:0008898	homocysteine S-met	0.01	2	0.15576324	4	0.011551679
16227	GO:0033843	xyloglucan 6-xylosylt	0.01	2	0.15576324	4	0.011551679
6018	GO:0008559|GO:000	xenobiotic-transporti	0.01	7	0.5451713	62	0.17905103
19225	GO:0042908	xenobiotic transport	0.01	7	0.5451713	62	0.17905103
19227	GO:0042910	xenobiotic transporte	0.01	7	0.5451713	62	0.17905103
12547	GO:0022857|GO:000	transmembrane trans	0.01	73	5.685358	1483	4.282785
26711	GO:0052547	regulation of peptida	0.01	10	0.7788162	112	0.32344702
28100	GO:0061134	peptidase regulator a	0.01	10	0.7788162	112	0.32344702
12806	GO:0030247	polysaccharide bindi	0.01	5	0.3894081	35	0.10107719
1169	GO:0001871	pattern binding	0.01	5	0.3894081	35	0.10107719
5636	GO:0008061	chitin binding	0.01	4	0.31152648	23	0.06642216
26845	GO:0052689	carboxylic ester hydr	0.01	31	2.4143302	538	1.5537009
9814	GO:0016760	cellulose synthase (U	0.01	3	0.23364486	13	0.037542958
3213	GO:0004568	chitinase activity	0.01	4	0.31152648	24	0.06931008
6248	GO:0008843	endochitinase activit	0.01	4	0.31152648	24	0.06931008
25578	GO:0051287|GO:00	NAD binding	0.01	4	0.31152648	24	0.06931008
3229	GO:0004586	ornithine decarboxyla	0.01	2	0.15576324	5	0.0144396
9812	GO:0016758	transferase activity, t	0.01	29	2.258567	501	1.4468478
2999	GO:0004312	fatty acid synthase a	0.01	7	0.5451713	68	0.19637854
11157	GO:0019144	ADP-sugar diphosph	0.01	3	0.23364486	14	0.040430877

4909	GO:0006810|GO:00[illegible]	transport	0.01	106	8.255452	2315	6.6855345
25529	GO:0051234	establishment of loca	0.01	106	8.255452	2315	6.6855345
4942	GO:0006855	drug transmembrane	0.01	11	0.8566978	139	0.40142086
8642	GO:0015238|GO:00[illegible]	drug transmembrane	0.01	11	0.8566978	139	0.40142086
9154	GO:0015893	drug transport	0.01	11	0.8566978	139	0.40142086
18832	GO:0042493|GO:00[illegible]	response to drug	0.01	11	0.8566978	139	0.40142086
25626	GO:0051336	regulation of hydrola	0.01	14	1.0903426	196	0.5660323
9811	GO:0016757|GO:00[illegible]	transferase activity, t	0.01	39	3.037383	729	2.1052935
25476	GO:0051179	localization	0.02	106	8.255452	2319	6.697086
119	GO:0000151	ubiquitin ligase comp	0.02	8	0.62305295	88	0.25413695
3209	GO:0004564	beta-fructofuranosida	0.02	3	0.23364486	15	0.043318797
3305	GO:0004673|GO:00[illegible]	protein histidine kina	0.02	2	0.15576324	6	0.01732752
4118	GO:0005852	eukaryotic translation	0.02	2	0.15576324	6	0.01732752
11899	GO:0019992	diacylglycerol binding	0.02	2	0.15576324	6	0.01732752
18640	GO:0042285	xylosyltransferase ac	0.02	2	0.15576324	6	0.01732752
3382	GO:0004768|GO:00[illegible]	stearoyl-CoA 9-desa	0.02	3	0.23364486	16	0.046206716
9427	GO:0016215	CoA desaturase activ	0.02	3	0.23364486	16	0.046206716
9422	GO:0016209	antioxidant activity	0.02	12	0.93457943	165	0.47650677
18738	GO:0042389	omega-3 fatty acid d	0.02	3	0.23364486	17	0.049094636
19393	GO:0043086	negative regulation o	0.02	13	1.0124611	188	0.54292893
20342	GO:0044092	negative regulation o	0.02	13	1.0124611	188	0.54292893
6216	GO:0008810	cellulase activity	0.02	4	0.31152648	30	0.086637594
3200	GO:0004553|GO:00[illegible]	hydrolase activity, hy	0.02	19	1.4797508	312	0.90103096
12805	GO:0030246	carbohydrate binding	0.03	16	1.2461059	251	0.7248679
6416	GO:0009044	xylan 1,4-beta-xylosi	0.03	3	0.23364486	18	0.051982556
9871	GO:0016831	carboxy-lyase activit	0.03	9	0.7009346	115	0.3321108
25998	GO:0051716	cellular response to	0.03	55	4.283489	1138	3.2864528
6424	GO:0009055|GO:00[illegible]	electron carrier activ	0.03	11	0.8566978	154	0.44473964
3265	GO:0004630	phospholipase D act	0.03	4	0.31152648	32	0.09241343
3785	GO:0005315|GO:00[illegible]	inorganic phosphate	0.03	4	0.31152648	32	0.09241343
19758	GO:0043492	ATPase activity, cou	0.03	15	1.1682243	235	0.67866117
3827	GO:0005372	water transmembran	0.03	7	0.5451713	81	0.23392151
4927	GO:0006833	water transport	0.03	7	0.5451713	81	0.23392151
8652	GO:0015250	water channel activit	0.03	7	0.5451713	81	0.23392151
18429	GO:0042044	fluid transport	0.03	7	0.5451713	81	0.23392151
9800	GO:0016746	transferase activity, t	0.03	27	2.1028037	498	1.438184
3051	GO:0004367	glycerol-3-phosphate	0.03	2	0.15576324	8	0.023103358
5698	GO:0008142	oxysterol binding	0.03	2	0.15576324	8	0.023103358
6684	GO:0009331	glycerol-3-phosphate	0.03	2	0.15576324	8	0.023103358
15335	GO:0032934|GO:00[illegible]	sterol binding	0.03	2	0.15576324	8	0.023103358
18639	GO:0042284	sphingolipid delta-4	0.03	2	0.15576324	8	0.023103358
4755	GO:0006629	lipid metabolic proce	0.03	31	2.4143302	590	1.7038727
6750	GO:0009415	response to water	0.03	5	0.3894081	49	0.14150807
11221	GO:0019219	regulation of nucleob	0.03	117	9.112149	2675	7.7251854
25468	GO:0051171	regulation of nitrogen	0.03	117	9.112149	2675	7.7251854
12533	GO:0022838	substrate-specific ch	0.04	14	1.0903426	221	0.63823026
21046	GO:0045437	uridine nucleosidase	0.04	1	0.07788162	1	0.00288792
24582	GO:0050263	ribosylpyrimidine nuc	0.04	1	0.07788162	1	0.00288792
31465	GO:0080090	regulation of primary	0.04	118	9.190031	2710	7.826263
9836	GO:0016787	hydrolase activity	0.04	158	12.305296	3723	10.751725
23199	GO:0047714	galactolipase activity	0.04	4	0.31152648	35	0.10107719
7213	GO:0009966|GO:00[illegible]	regulation of signal t	0.04	5	0.3894081	51	0.14728391
12601	GO:0023051	regulation of signalin	0.04	5	0.3894081	51	0.14728391
4530	GO:0006355|GO:00[illegible]	regulation of transcri	0.04	113	8.800623	2591	7.4826
25544	GO:0051252	regulation of RNA me	0.04	113	8.800623	2593	7.488376
23518	GO:0048040	UDP-glucuronate de	0.04	3	0.23364486	21	0.060646318
13753	GO:0031323	regulation of cellular	0.04	120	9.345795	2770	7.999538
3734	GO:0005227	calcium activated ca	0.04	2	0.15576324	9	0.025991278
8661	GO:0015269	calcium-activated po	0.04	2	0.15576324	9	0.025991278
12534	GO:0022839	ion gated channel ac	0.04	2	0.15576324	9	0.025991278
19254	GO:0042937	tripeptide transporter	0.04	2	0.15576324	9	0.025991278
20517	GO:0044267	cellular protein meta	0.04	138	10.7476635	3225	9.313541
4915	GO:0006817	phosphate transport	0.04	5	0.3894081	52	0.15017183
8528	GO:0015114	phosphate transmem	0.04	5	0.3894081	52	0.15017183
17801	GO:0035435	phosphate transmem	0.04	5	0.3894081	52	0.15017183
8660	GO:0015267|GO:00[illegible]	channel activity	0.04	14	1.0903426	226	0.6526699
12515	GO:0022803	passive transmembra	0.04	14	1.0903426	226	0.6526699
23522	GO:0048046	apoplast	0.05	6	0.46728972	70	0.20215438
7141	GO:0009889	regulation of biosynt	0.05	113	8.800623	2605	7.523031
7772	GO:0010556	regulation of macrom	0.05	113	8.800623	2605	7.523031
13756	GO:0031326	regulation of cellular	0.05	113	8.800623	2605	7.523031
32213	GO:2000112	regulation of cellular	0.05	113	8.800623	2605	7.523031
2626	GO:0003674|GO:00[illegible]	molecular_function	0.05	932	72.58567	24394	70.447914
8489	GO:0015066	alpha-amylase inhibi	0.05	3	0.23364486	22	0.06353424
11224	GO:0019222	regulation of metabo	0.05	133	10.358255	3115	8.995871
5716	GO:0008172	S-methyltransferase	0.05	2	0.15576324	10	0.0288792
6632	GO:0009269	response to desiccat	0.05	2	0.15576324	10	0.0288792
9826	GO:0016775	phosphotransferase	0.05	2	0.15576324	10	0.0288792
18956	GO:0042626	ATPase activity, cou	0.05	13	1.0124611	211	0.6093511
12644	GO:0030054	cell junction	0.05	3	0.23364486	23	0.06642216
22414	GO:0046912	transferase activity, t	0.05	3	0.23364486	23	0.06642216
9861	GO:0016820	hydrolase activity, ac	0.05	13	1.0124611	212	0.612239
8494	GO:0015075	ion transmembrane t	0.05	40	3.115265	827	2.3883097
9801	GO:0016747	transferase activity, t	0.06	24	1.8691589	456	1.3168914
9779	GO:0016717	oxidoreductase activ	0.06	3	0.23364486	24	0.06931008
27228	GO:0060255	regulation of macrom	0.06	114	8.878505	2660	7.6818666

2897	GO:0004091|GO:000	carboxylesterase act	0.06	12	0.93457943	194	0.5602564
7692	GO:0010468	regulation of gene ex	0.06	113	8.800623	2635	7.6096687
12574	GO:0022892	substrate-specific tra	0.06	66	5.140187	1463	4.2250266
16603	GO:0034220	ion transmembrane t	0.06	40	3.115265	833	2.4056373
23973	GO:0048583	regulation of respons	0.06	5	0.3894081	57	0.16461143
2989	GO:0004301	epoxide hydrolase ac	0.06	2	0.15576324	11	0.03176712
4250	GO:0006012	galactose metabolic	0.06	2	0.15576324	11	0.03176712
9849	GO:0016803	ether hydrolase activ	0.06	2	0.15576324	11	0.03176712
19256	GO:0042939	tripeptide transport	0.06	2	0.15576324	11	0.03176712
8517	GO:0015103	inorganic anion trans	0.06	10	0.7788162	154	0.44473964
8749	GO:0015399	primary active transm	0.06	15	1.1682243	260	0.75085914
8753	GO:0015405	P-P-bond-hydrolysis	0.06	15	1.1682243	260	0.75085914
22478	GO:0046982	protein heterodimeriz	0.06	8	0.62305295	114	0.32922286
8558	GO:0015145	monosaccharide tran	0.06	5	0.3894081	58	0.16749935
9018	GO:0015749	monosaccharide tran	0.06	5	0.3894081	58	0.16749935
8967	GO:0015698	inorganic anion trans	0.06	11	0.8566978	176	0.5082739
2831	GO:0004022	alcohol dehydrogena	0.07	5	0.3894081	59	0.17038727
9909	GO:0016877	ligase activity, formin	0.07	6	0.46728972	78	0.22525774
7000	GO:0009734	auxin mediated signa	0.07	3	0.23364486	26	0.075085916
7540	GO:0010315	auxin efflux	0.07	3	0.23364486	26	0.075085916
7553	GO:0010329	auxin efflux transmen	0.07	3	0.23364486	26	0.075085916
29729	GO:0071365	cellular response to a	0.07	3	0.23364486	26	0.075085916
3864	GO:0005496	steroid binding	0.07	2	0.15576324	12	0.03465504
6946	GO:0009678	hydrogen-translocati	0.07	2	0.15576324	12	0.03465504
7526	GO:0010301|GO:003	xanthoxin dehydroge	0.07	2	0.15576324	12	0.03465504
9699	GO:0016616	oxidoreductase activ	0.07	15	1.1682243	266	0.7681867
2804	GO:0003988	acetyl-CoA C-acyltra	0.07	1	0.07788162	2	0.00577584
2951	GO:0004149	dihydrolipoyllysine-re	0.07	1	0.07788162	2	0.00577584
2990	GO:0004303	estradiol 17-beta-de	0.07	1	0.07788162	2	0.00577584
3148	GO:0004489|GO:000	methylenetetrahydro	0.07	1	0.07788162	2	0.00577584
4699	GO:0006570	tyrosine metabolic pr	0.07	1	0.07788162	2	0.00577584
4700	GO:0006571	tyrosine biosynthetic	0.07	1	0.07788162	2	0.00577584
5941	GO:0008455	alpha-1,6-mannosylg	0.07	1	0.07788162	2	0.00577584
6593	GO:0009229	thiamine diphosphate	0.07	1	0.07788162	2	0.00577584
9190	GO:0015930	glutamate synthase a	0.07	1	0.07788162	2	0.00577584
9282	GO:0016040	glutamate synthase (	0.07	1	0.07788162	2	0.00577584
9419	GO:0016206	catechol O-methyltra	0.07	1	0.07788162	2	0.00577584
9686	GO:0016602	CCAAT-binding facto	0.07	1	0.07788162	2	0.00577584
9802	GO:0016748	succinyltransferase a	0.07	1	0.07788162	2	0.00577584
9805	GO:0016751	S-succinyltransferase	0.07	1	0.07788162	2	0.00577584
15586	GO:0033187	inositol hexakisphos	0.07	1	0.07788162	2	0.00577584
18706	GO:0042357	thiamine diphosphate	0.07	1	0.07788162	2	0.00577584
20817	GO:0045181	glutamate synthase a	0.07	1	0.07788162	2	0.00577584
22759	GO:0047268	galactinol-raffinose g	0.07	1	0.07788162	2	0.00577584
168	GO:0000221	vacuolar proton-trans	0.07	1	0.07788162	2	0.00577584
535	GO:0000826	inositol pyrophospha	0.07	1	0.07788162	2	0.00577584
537	GO:0000828	inositol hexakisphos	0.07	1	0.07788162	2	0.00577584
2699	GO:0003849	3-deoxy-7-phosphoh	0.07	1	0.07788162	2	0.00577584
9837	GO:0016788	hydrolase activity, ac	0.07	68	5.29595	1534	4.430069
8526	GO:0015112	nitrate transmembra	0.08	5	0.3894081	61	0.1761631
8975	GO:0015706|GO:000	nitrate transport	0.08	5	0.3894081	61	0.1761631
12573	GO:0022891	substrate-specific tra	0.08	56	4.3613706	1241	3.5839086
8919	GO:0015645	fatty acid ligase activ	0.08	3	0.23364486	27	0.077973835
9541	GO:0016413	O-acetyltransferase a	0.08	3	0.23364486	27	0.077973835
22049	GO:0046527	glucosyltransferase a	0.08	15	1.1682243	270	0.77973837
3002	GO:0004315	3-oxoacyl-[acyl-carri	0.08	2	0.15576324	13	0.037542958
7551	GO:0010327	acetyl CoA:(Z)-3-hex	0.08	2	0.15576324	13	0.037542958
8863	GO:0015562	efflux transmembran	0.08	3	0.23364486	28	0.080861755
14726	GO:0032318	regulation of Ras GT	0.08	4	0.31152648	45	0.1299564
22096	GO:0046578	regulation of Ras pro	0.08	4	0.31152648	45	0.1299564
25355	GO:0051056	regulation of small G	0.08	4	0.31152648	45	0.1299564
11931	GO:0020037	heme binding	0.09	27	2.1028037	550	1.5883559
12692	GO:0030117	membrane coat	0.09	6	0.46728972	84	0.24258527
23870	GO:0048475	coated membrane	0.09	6	0.46728972	84	0.24258527
3641	GO:0005099	Ras GTPase activato	0.09	2	0.15576324	14	0.040430877
9720	GO:0016639	oxidoreductase activ	0.09	2	0.15576324	14	0.040430877
14728	GO:0032320	positive regulation of	0.09	2	0.15576324	14	0.040430877
19253	GO:0042936	dipeptide transporter	0.10	7	0.5451713	105	0.3032316
19255	GO:0042938	dipeptide transport	0.10	7	0.5451713	105	0.3032316
3009	GO:0004322	ferroxidase activity	0.10	3	0.23364486	30	0.086637594
8683	GO:0015301|GO:001	anion:anion antiporte	0.10	3	0.23364486	30	0.086637594
9785	GO:0016724	oxidoreductase activ	0.10	3	0.23364486	30	0.086637594
5809	GO:0008289	lipid binding	0.10	14	1.0903426	258	0.74508333
3341	GO:0004722|GO:000	protein serine/threon	0.10	10	0.7788162	171	0.4938343
18912	GO:0042578	phosphoric ester hyd	0.10	22	1.7133956	444	1.2822365
25516	GO:0051219	phosphoprotein bind	0.10	2	0.15576324	15	0.043318797
18582	GO:0042221	response to chemica	0.11	17	1.3239875	328	0.94723773
3872	GO:0005506	iron ion binding	0.11	27	2.1028037	563	1.6258988
19590	GO:0043295	glutathione binding	0.11	3	0.23364486	31	0.08952551
9600	GO:0016491	oxidoreductase activ	0.11	74	5.76324	1724	4.9787736
3134	GO:0004474	malate synthase acti	0.11	1	0.07788162	3	0.00866376
3252	GO:0004612	phosphoenolpyruvate	0.11	1	0.07788162	3	0.00866376
4319	GO:0006081	cellular aldehyde me	0.11	1	0.07788162	3	0.00866376
4330	GO:0006097	glyoxylate cycle	0.11	1	0.07788162	3	0.00866376
4376	GO:0006166	purine ribonucleoside	0.11	1	0.07788162	3	0.00866376
9478	GO:0016286	small conductance c	0.11	1	0.07788162	3	0.00866376
13651	GO:0031219	levanase activity	0.11	1	0.07788162	3	0.00866376
19408	GO:0043101	purine-containing co	0.11	1	0.07788162	3	0.00866376
19477	GO:0043174	nucleoside salvage	0.11	1	0.07788162	3	0.00866376
21936	GO:0046409	p-coumarate 3-hydro	0.11	1	0.07788162	3	0.00866376
22011	GO:0046487	glyoxylate metabolic	0.11	1	0.07788162	3	0.00866376

11522	GO:0019538|GO:00(	protein metabolic pro	0.11	168	13.084112	4134	11.938661
9267	GO:0016021	integral to membrane	0.11	49	3.8161993	1103	3.1853757
5096	GO:0007047	cellular cell wall orga	0.11	4	0.31152648	50	0.14439599
6932	GO:0009664	plant-type cell wall o	0.11	4	0.31152648	50	0.14439599
20857	GO:0045229	external encapsulatin	0.11	4	0.31152648	50	0.14439599
30033	GO:0071669	plant-type cell wall o	0.11	4	0.31152648	50	0.14439599
20488	GO:0044238	primary metabolic pr	0.12	359	27.959501	9167	26.47356
12516	GO:0022804	active transmembran	0.12	36	2.8037384	787	2.2727928
4910	GO:0006811	ion transport	0.12	43	3.3489096	959	2.769515
3072	GO:0004396	hexokinase activity	0.12	2	0.15576324	16	0.046206716
3334	GO:0004713|GO:00(	protein tyrosine kinas	0.12	2	0.15576324	16	0.046206716
15688	GO:0033293	monocarboxylic acid	0.12	2	0.15576324	16	0.046206716
22412	GO:0046910	pectinesterase inhibi	0.12	2	0.15576324	16	0.046206716
2982	GO:0004197	cysteine-type endope	0.12	6	0.46728972	90	0.2599128
7173	GO:0009922	fatty acid elongase a	0.12	4	0.31152648	51	0.14728391
3340	GO:0004721	phosphoprotein phos	0.12	11	0.8566978	199	0.57469606
2809	GO:0003993	acid phosphatase ac	0.13	4	0.31152648	52	0.15017183
9923	GO:0016892	endoribonuclease ac	0.13	2	0.15576324	17	0.049094636
9925	GO:0016894	endonuclease activit	0.13	2	0.15576324	17	0.049094636
16281	GO:0033897	ribonuclease T2 activ	0.13	2	0.15576324	17	0.049094636
6072	GO:0008645	hexose transport	0.13	3	0.23364486	34	0.09818927
8562	GO:0015149	hexose transmembra	0.13	3	0.23364486	34	0.09818927
17794	GO:0035428	hexose transmembra	0.13	3	0.23364486	34	0.09818927
9697	GO:0016614	oxidoreductase activ	0.13	15	1.1682243	293	0.84616053
29246	GO:0070882	cellular cell wall orga	0.13	4	0.31152648	53	0.15305975
6355	GO:0008970	phospholipase A1 ac	0.14	3	0.23364486	35	0.10107719
5702	GO:0008146	sulfotransferase activ	0.14	4	0.31152648	54	0.15594767
2872	GO:0004066	asparagine synthase	0.14	1	0.07788162	4	0.011551679
2900	GO:0004096|GO:00	catalase activity	0.14	1	0.07788162	4	0.011551679
2967	GO:0004165|GO:00(	dodecenoyl-CoA deh	0.14	1	0.07788162	4	0.011551679
3093	GO:0004421	hydroxymethylglutary	0.14	1	0.07788162	4	0.011551679
3119	GO:0004457	lactate dehydrogena	0.14	1	0.07788162	4	0.011551679
3121	GO:0004459	L-lactate dehydroger	0.14	1	0.07788162	4	0.011551679
3291	GO:0004657	proline dehydrogena	0.14	1	0.07788162	4	0.011551679
3399	GO:0004788	thiamine diphosphok	0.14	1	0.07788162	4	0.011551679
3406	GO:0004795	threonine synthase a	0.14	1	0.07788162	4	0.011551679
3456	GO:0004849	uridine kinase activit	0.14	1	0.07788162	4	0.011551679
3639	GO:0005097	Rab GTPase activato	0.14	1	0.07788162	4	0.011551679
3661	GO:0005125	cytokine activity	0.14	1	0.07788162	4	0.011551679
3674	GO:0005138	interleukin-6 recepto	0.14	1	0.07788162	4	0.011551679
4328	GO:0006094	gluconeogenesis	0.14	1	0.07788162	4	0.011551679
4428	GO:0006222	UMP biosynthetic pro	0.14	1	0.07788162	4	0.011551679
5934	GO:0008446	GDP-mannose 4,6-d	0.14	1	0.07788162	4	0.011551679
6199	GO:0008792	arginine decarboxyla	0.14	1	0.07788162	4	0.011551679
6494	GO:0009129	pyrimidine nucleosid	0.14	1	0.07788162	4	0.011551679
6495	GO:0009130	pyrimidine nucleosid	0.14	1	0.07788162	4	0.011551679
6537	GO:0009173	pyrimidine ribonucle	0.14	1	0.07788162	4	0.011551679
6538	GO:0009174	pyrimidine ribonucle	0.14	1	0.07788162	4	0.011551679
7083	GO:0009824	AMP dimethylallyltra	0.14	1	0.07788162	4	0.011551679
7511	GO:0010285|GO:004	L,L-diaminopimelate	0.14	1	0.07788162	4	0.011551679
8637	GO:0015231	5-formyltetrahydrofol	0.14	1	0.07788162	4	0.011551679
8877	GO:0015578|GO:00	mannose transmemb	0.14	1	0.07788162	4	0.011551679
9030	GO:0015761	mannose transport	0.14	1	0.07788162	4	0.011551679
9147	GO:0015885	5-formyltetrahydrofol	0.14	1	0.07788162	4	0.011551679
9485	GO:0016297|GO:00	acyl-[acyl-carrier-pro	0.14	1	0.07788162	4	0.011551679
11312	GO:0019319	hexose biosynthetic	0.14	1	0.07788162	4	0.011551679
14721	GO:0032313	regulation of Rab GT	0.14	1	0.07788162	4	0.011551679
14891	GO:0032483	regulation of Rab pro	0.14	1	0.07788162	4	0.011551679
15252	GO:0032851	positive regulation of	0.14	1	0.07788162	4	0.011551679
21615	GO:0046049	UMP metabolic proc	0.14	1	0.07788162	4	0.011551679
22893	GO:0047405	pyrimidine-5'-nucleot	0.14	1	0.07788162	4	0.011551679
24433	GO:0050113	inositol oxygenase a	0.14	1	0.07788162	4	0.011551679
24692	GO:0050373	UDP-arabinose 4-ep	0.14	1	0.07788162	4	0.011551679
2753	GO:0003919	FMN adenylyltransfe	0.14	1	0.07788162	4	0.011551679
2799	GO:0003983	UTP:glucose-1-phos	0.14	1	0.07788162	4	0.011551679
5776	GO:0008252	nucleotidase activity	0.14	2	0.15576324	18	0.051982556
8213	GO:0012507|GO:00	ER to Golgi transpor	0.14	2	0.15576324	18	0.051982556
10899	GO:0018858	benzoate-CoA ligase	0.14	2	0.15576324	18	0.051982556
12702	GO:0030127	COPII vesicle coat	0.14	2	0.15576324	18	0.051982556
12708	GO:0030133	transport vesicle	0.14	2	0.15576324	18	0.051982556
12709	GO:0030134|GO:00	ER to Golgi transpor	0.14	2	0.15576324	18	0.051982556
13129	GO:0030658	transport vesicle me	0.14	2	0.15576324	18	0.051982556
9869	GO:0016829	lyase activity	0.14	23	1.7912773	488	1.4093049
9870	GO:0016830	carbon-carbon lyase	0.15	9	0.7009346	161	0.4649551
8607	GO:0015197|GO:00	peptide transporter a	0.15	8	0.62305295	139	0.40142086
8608	GO:0015198	oligopeptide transpo	0.15	8	0.62305295	139	0.40142086
4232	GO:0005992	trehalose biosyntheti	0.15	3	0.23364486	36	0.10396511
25416	GO:0051119	sugar transmembran	0.15	7	0.5451713	118	0.34077454
4944	GO:0006857	oligopeptide transpo	0.15	8	0.62305295	141	0.4071967
9099	GO:0015833	peptide transport	0.15	8	0.62305295	141	0.4071967
3290	GO:0004656	procollagen-proline 4	0.16	2	0.15576324	19	0.054870475
8212	GO:0012506	vesicle membrane	0.16	2	0.15576324	19	0.054870475
11752	GO:0019798	procollagen-proline d	0.16	2	0.15576324	19	0.054870475
12695	GO:0030120	vesicle coat	0.16	2	0.15576324	19	0.054870475
12710	GO:0030135|GO:00(	coated vesicle	0.16	2	0.15576324	19	0.054870475
13130	GO:0030659	cytoplasmic vesicle	0.16	2	0.15576324	19	0.054870475
13133	GO:0030662	coated vesicle meml	0.16	2	0.15576324	19	0.054870475
13966	GO:0031543	peptidyl-proline diox	0.16	2	0.15576324	19	0.054870475
13968	GO:0031545	peptidyl-proline 4-di	0.16	2	0.15576324	19	0.054870475
13829	GO:0031406	carboxylic acid bindi	0.16	6	0.46728972	98	0.28301615
3758	GO:0005267	potassium channel a	0.16	3	0.23364486	37	0.10685303

8557	GO:0015144	carbohydrate transm	0.16	8	0.62305295	143	0.41297254
16602	GO:0034219	carbohydrate transm	0.16	8	0.62305295	143	0.41297254
9767	GO:0016705	oxidoreductase activ	0.17	16	1.2461059	329	0.95012563
4231	GO:0005991	trehalose metabolic	0.17	3	0.23364486	38	0.10974095
8501	GO:0015085	calcium ion transmer	0.17	3	0.23364486	38	0.10974095
22409	GO:0046906	tetrapyrrole binding	0.17	27	2.1028037	596	1.7212002
29919	GO:0071555	cell wall organization	0.17	4	0.31152648	58	0.16749935
12532	GO:0022836	gated channel activit	0.17	7	0.5451713	122	0.3523262
3328	GO:0004707|GO:00(	MAP kinase activity	0.17	2	0.15576324	20	0.0577584
3798	GO:0005337	nucleoside transmem	0.17	2	0.15576324	20	0.0577584
8726	GO:0015368	calcium:cation antipo	0.17	2	0.15576324	20	0.0577584
9122	GO:0015858	nucleoside transport	0.17	2	0.15576324	20	0.0577584
25477	GO:0051180	vitamin transport	0.17	2	0.15576324	20	0.0577584
25480	GO:0051183	vitamin transporter a	0.17	2	0.15576324	20	0.0577584
3035	GO:0004351	glutamate decarboxy	0.17	1	0.07788162	5	0.0144396
3314	GO:0004689|GO:00(	phosphorylase kinas	0.17	1	0.07788162	5	0.0144396
5989	GO:0008517	folic acid transporter	0.17	1	0.07788162	5	0.0144396
9146	GO:0015884	folic acid transport	0.17	1	0.07788162	5	0.0144396
22765	GO:0047274	galactinol-sucrose ga	0.17	1	0.07788162	5	0.0144396
31506	GO:0080131	hydroxyjasmonate su	0.17	1	0.07788162	5	0.0144396
6071	GO:0008643|GO:00(	carbohydrate transpo	0.18	8	0.62305295	147	0.42452422
8673	GO:0015291|GO:00	secondary active tra	0.18	19	1.4797508	407	1.1753833
3129	GO:0004467	long-chain fatty acid-	0.18	2	0.15576324	21	0.060646318
9268	GO:0016023	cytoplasmic membra	0.18	2	0.15576324	21	0.060646318
14399	GO:0031988	membrane-bounded	0.18	2	0.15576324	21	0.060646318
9839	GO:0016791|GO:00	phosphatase activity	0.18	18	1.4018692	383	1.1060733
5652	GO:0008081|GO:00(	phosphoric diester h	0.18	4	0.31152648	60	0.17327519
7021	GO:0009755	hormone-mediated s	0.18	3	0.23364486	40	0.1155168
15271	GO:0032870	cellular response to	0.18	3	0.23364486	40	0.1155168
3156	GO:0004497	monooxygenase acti	0.19	9	0.7009346	172	0.49672222
24858	GO:0050551	myrcene synthase ac	0.20	2	0.15576324	22	0.06353424
25195	GO:0050896|GO:00	response to stimulus	0.20	77	5.996885	1881	5.432177
17457	GO:0035091	phosphatidylinositol	0.20	4	0.31152648	62	0.17905103
20619	GO:0044425	membrane part	0.20	63	4.906542	1523	4.398302
4912	GO:0006813|GO:00	potassium ion transp	0.20	5	0.3894081	84	0.24258527
30168	GO:0071804	cellular potassium io	0.20	5	0.3894081	84	0.24258527
30169	GO:0071805	potassium ion transn	0.20	5	0.3894081	84	0.24258527
2906	GO:0004103	choline kinase activit	0.20	1	0.07788162	6	0.01732752
3131	GO:0004470	malic enzyme activity	0.20	1	0.07788162	6	0.01732752
3133	GO:0004473	malate dehydrogena	0.20	1	0.07788162	6	0.01732752
3208	GO:0004563	beta-N-acetylhexosa	0.20	1	0.07788162	6	0.01732752
3269	GO:0004634	phosphopyruvate hy	0.20	1	0.07788162	6	0.01732752
3342	GO:0004723|GO:00(	calcium-dependent p	0.20	1	0.07788162	6	0.01732752
3413	GO:0004802	transketolase activity	0.20	1	0.07788162	6	0.01732752
3467	GO:0004860	protein kinase inhibit	0.20	1	0.07788162	6	0.01732752
3642	GO:0005100	Rho GTPase activat	0.20	1	0.07788162	6	0.01732752
3845	GO:0005452	inorganic anion exch	0.20	1	0.07788162	6	0.01732752
4059	GO:0005774	vacuolar membrane	0.20	1	0.07788162	6	0.01732752
4264	GO:0006026	aminoglycan catabol	0.20	1	0.07788162	6	0.01732752
4268	GO:0006030	chitin metabolic proc	0.20	1	0.07788162	6	0.01732752
4270	GO:0006032	chitin catabolic proce	0.20	1	0.07788162	6	0.01732752
4657	GO:0006528	asparagine metaboli	0.20	1	0.07788162	6	0.01732752
4658	GO:0006529	asparagine biosynthe	0.20	1	0.07788162	6	0.01732752
4953	GO:0006868|GO:00	glutamine transport	0.20	1	0.07788162	6	0.01732752
5713	GO:0008169	C-methyltransferase	0.20	1	0.07788162	6	0.01732752
6000	GO:0008531	riboflavin kinase acti	0.20	1	0.07788162	6	0.01732752
6344	GO:0008959	phosphate acetyltran	0.20	1	0.07788162	6	0.01732752
7651	GO:0010427	abscisic acid binding	0.20	1	0.07788162	6	0.01732752
8505	GO:0015089	high affinity copper io	0.20	1	0.07788162	6	0.01732752
8596	GO:0015186	L-glutamine transme	0.20	1	0.07788162	6	0.01732752
9435	GO:0016231	beta-N-acetylglucosa	0.20	1	0.07788162	6	0.01732752
9584	GO:0016471	vacuolar proton-trans	0.20	1	0.07788162	6	0.01732752
14727	GO:0032319	regulation of Rho GT	0.20	1	0.07788162	6	0.01732752
14729	GO:0032321	positive regulation of	0.20	1	0.07788162	6	0.01732752
17394	GO:0035023	regulation of Rho pro	0.20	1	0.07788162	6	0.01732752
19761	GO:0043495	protein anchor	0.20	1	0.07788162	6	0.01732752
20631	GO:0044437	vacuolar part	0.20	1	0.07788162	6	0.01732752
21727	GO:0046165	alcohol biosynthetic	0.20	1	0.07788162	6	0.01732752
21893	GO:0046364	monosaccharide bios	0.20	1	0.07788162	6	0.01732752
26030	GO:0051748	UTP-monosaccharid	0.20	1	0.07788162	6	0.01732752
5	GO:0000007	low-affinity zinc ion t	0.20	1	0.07788162	6	0.01732752
135	GO:0000170	sphingosine hydroxy	0.20	1	0.07788162	6	0.01732752
2688	GO:0003838	sterol 24-C-methyltra	0.20	1	0.07788162	6	0.01732752
2713	GO:0003863	3-methyl-2-oxobutan	0.20	1	0.07788162	6	0.01732752
3718	GO:0005200	structural constituent	0.20	3	0.23364486	42	0.121292636
6666	GO:0009312	oligosaccharide bios	0.20	3	0.23364486	42	0.121292636
8461	GO:0015020|GO:00(	glucuronosyltransfera	0.20	3	0.23364486	42	0.121292636
9370	GO:0016138	glycoside biosynthet	0.20	3	0.23364486	42	0.121292636
21880	GO:0046351	disaccharide biosynt	0.20	3	0.23364486	42	0.121292636
13656	GO:0031224	intrinsic to membran	0.20	50	3.894081	1193	3.4452884
8679	GO:0015297	antiporter activity	0.20	8	0.62305295	152	0.4389638
8682	GO:0015300	solute:solute antipor	0.20	8	0.62305295	152	0.4389638
17016	GO:0034637	cellular carbohydrate	0.21	4	0.31152648	63	0.18193895
3662	GO:0005126	cytokine receptor bin	0.21	2	0.15576324	23	0.06642216
22753	GO:0047262|GO:00	polygalacturonate 4-	0.21	2	0.15576324	23	0.06642216
9293	GO:0016051|GO:00(	carbohydrate biosyn	0.21	4	0.31152648	64	0.18482687
4914	GO:0006816	calcium ion transport	0.22	3	0.23364486	44	0.12706847
28952	GO:0070588	calcium ion transmer	0.22	3	0.23364486	44	0.12706847
29859	GO:0071495	cellular response to	0.22	3	0.23364486	44	0.12706847
2821	GO:0004012|GO:00(	phospholipid-translo	0.22	2	0.15576324	24	0.06931008
8559	GO:0015146	pentose transmembr	0.22	2	0.15576324	24	0.06931008

8561	GO:0015148	D-xylose transmemb	0.22	2	0.15576324	24	0.06931008
8829	GO:0015519	D-xylose:hydrogen s	0.22	2	0.15576324	24	0.06931008
9019	GO:0015750	pentose transport	0.22	2	0.15576324	24	0.06931008
9022	GO:0015753	D-xylose transport	0.22	2	0.15576324	24	0.06931008
2945	GO:0004143	diacylglycerol kinase	0.23	1	0.07788162	7	0.020215439
4260	GO:0006022	aminoglycan metabo	0.23	1	0.07788162	7	0.020215439
6527	GO:0009163	nucleoside biosynthe	0.23	1	0.07788162	7	0.020215439
18796	GO:0042451	purine nucleoside bio	0.23	1	0.07788162	7	0.020215439
18800	GO:0042455	ribonucleoside biosy	0.23	1	0.07788162	7	0.020215439
19401	GO:0043094	cellular metabolic co	0.23	1	0.07788162	7	0.020215439
21695	GO:0046129	purine ribonucleoside	0.23	1	0.07788162	7	0.020215439
8666	GO:0015276	ligand-gated ion cha	0.23	3	0.23364486	45	0.1299564
12530	GO:0022834	ligand-gated channe	0.23	3	0.23364486	45	0.1299564
31431	GO:0080054	low affinity nitrate tra	0.23	3	0.23364486	45	0.1299564
3888	GO:0005529	sugar binding	0.23	8	0.62305295	158	0.45629135
5070	GO:0007017	microtubule-based p	0.24	6	0.46728972	112	0.32344702
5071	GO:0007018	microtubule-based m	0.24	6	0.46728972	112	0.32344702
9846	GO:0016799	hydrolase activity, hy	0.24	2	0.15576324	25	0.072197996
31493	GO:0080118	brassinosteroid sulfo	0.24	2	0.15576324	25	0.072197996
973	GO:0001653	peptide receptor acti	0.24	2	0.15576324	25	0.072197996
6999	GO:0009733	response to auxin sti	0.24	3	0.23364486	46	0.13284431
20652	GO:0044459	plasma membrane p	0.24	3	0.23364486	46	0.13284431
5909	GO:0008415	acyltransferase activ	0.25	15	1.1682243	330	0.95301354
3415	GO:0004805	trehalose-phosphata	0.25	2	0.15576324	26	0.075085916
17147	GO:0034768	(E)-beta-ocimene sy	0.25	2	0.15576324	26	0.075085916
4773	GO:0006650|GO:000	glycerophospholipid	0.25	3	0.23364486	47	0.13573223
22010	GO:0046486	glycerolipid metaboli	0.25	3	0.23364486	47	0.13573223
31420	GO:0080043	quercetin 3-O-glucos	0.25	4	0.31152648	69	0.19926646
3098	GO:0004427	inorganic diphosphat	0.26	1	0.07788162	8	0.023103358
4778	GO:0006656	phosphatidylcholine	0.26	1	0.07788162	8	0.023103358
4779	GO:0006657	CDP-choline pathwa	0.26	1	0.07788162	8	0.023103358
5753	GO:0008216	spermidine metaboli	0.26	1	0.07788162	8	0.023103358
5815	GO:0008295	spermidine biosynthe	0.26	1	0.07788162	8	0.023103358
6262	GO:0008865	fructokinase activity	0.26	1	0.07788162	8	0.023103358
7779	GO:0010563	negative regulation o	0.26	1	0.07788162	8	0.023103358
8725	GO:0015367	oxoglutarate:malate	0.26	1	0.07788162	8	0.023103358
9011	GO:0015742	alpha-ketoglutarate t	0.26	1	0.07788162	8	0.023103358
11209	GO:0019206	nucleoside kinase ac	0.26	1	0.07788162	8	0.023103358
11213	GO:0019210	kinase inhibitor activ	0.26	1	0.07788162	8	0.023103358
12736	GO:0030165	PDZ domain binding	0.26	1	0.07788162	8	0.023103358
13262	GO:0030795|GO:004	jasmonate O-methylt	0.26	1	0.07788162	8	0.023103358
18675	GO:0042326	negative regulation o	0.26	1	0.07788162	8	0.023103358
18785	GO:0042439	ethanolamine-contai	0.26	1	0.07788162	8	0.023103358
21504	GO:0045936	negative regulation o	0.26	1	0.07788162	8	0.023103358
21995	GO:0046470	phosphatidylcholine	0.26	1	0.07788162	8	0.023103358
22076	GO:0046556	alpha-N-arabinofurar	0.26	1	0.07788162	8	0.023103358
22702	GO:0047209	coniferyl-alcohol gluc	0.26	1	0.07788162	8	0.023103358
23264	GO:0047782	coniferin beta-glucos	0.26	1	0.07788162	8	0.023103358
28933	GO:0070569	uridylyltransferase ac	0.26	1	0.07788162	8	0.023103358
4	GO:0000006	high affinity zinc upta	0.26	1	0.07788162	8	0.023103358
11	GO:0000015	phosphopyruvate hyd	0.26	1	0.07788162	8	0.023103358
47	GO:0000062	fatty-acyl-CoA bindin	0.26	1	0.07788162	8	0.023103358
2728	GO:0003878|GO:004	ATP citrate synthase	0.26	1	0.07788162	8	0.023103358
15524	GO:0033124	regulation of GTP ca	0.26	4	0.31152648	70	0.20215438
19394	GO:0043087	regulation of GTPase	0.26	4	0.31152648	70	0.20215438
4188	GO:0005938	cell cortex	0.26	3	0.23364486	48	0.13862015
20642	GO:0044448	cell cortex part	0.26	3	0.23364486	48	0.13862015
113	GO:0000145	exocyst	0.26	3	0.23364486	48	0.13862015
3724	GO:0005216	ion channel activity	0.26	7	0.5451713	140	0.40430877
3895	GO:0005543	phospholipid binding	0.26	7	0.5451713	140	0.40430877
3725	GO:0005217	intracellular ligand-ga	0.26	2	0.15576324	27	0.077973835
3808	GO:0005351|GO:000	sugar:hydrogen sym	0.27	5	0.3894081	94	0.27146447
3835	GO:0005402	cation:sugar symport	0.27	5	0.3894081	94	0.27146447
3756	GO:0005261|GO:001	cation channel activi	0.27	3	0.23364486	49	0.14150807
9725	GO:0016645	oxidoreductase activ	0.27	3	0.23364486	49	0.14150807
18633	GO:0042277	peptide binding	0.28	5	0.3894081	95	0.27435237
3744	GO:0005244	voltage-gated ion cha	0.28	4	0.31152648	72	0.20793022
12528	GO:0022832	voltage-gated chann	0.28	4	0.31152648	72	0.20793022
15280	GO:0032879	regulation of localiza	0.28	4	0.31152648	72	0.20793022
17141	GO:0034762	regulation of transme	0.28	4	0.31152648	72	0.20793022
17144	GO:0034765	regulation of ion tran	0.28	4	0.31152648	72	0.20793022
19568	GO:0043269	regulation of ion tran	0.28	4	0.31152648	72	0.20793022
25348	GO:0051049	regulation of transpo	0.28	4	0.31152648	72	0.20793022
3332	GO:0004711	ribosomal protein S6	0.28	2	0.15576324	28	0.080861755
3812	GO:0005355|GO:001	glucose transmembr	0.28	2	0.15576324	28	0.080861755
9027	GO:0015758	glucose transport	0.28	2	0.15576324	28	0.080861755
716	GO:0001071	nucleic acid binding	0.28	57	4.4392524	1417	4.0921826
2643	GO:0003700|GO:000	sequence-specific D	0.28	57	4.4392524	1417	4.0921826
4304	GO:0006066	alcohol metabolic pro	0.28	7	0.5451713	143	0.41297254
7166	GO:0009914	hormone transport	0.28	3	0.23364486	50	0.14439599
9783	GO:0016722	oxidoreductase activ	0.28	3	0.23364486	50	0.14439599
27891	GO:0060918	auxin transport	0.28	3	0.23364486	50	0.14439599
29674	GO:0071310	cellular response to	0.28	3	0.23364486	50	0.14439599
31536	GO:0080161	auxin transmembran	0.28	3	0.23364486	50	0.14439599
2801	GO:0003985	acetyl-CoA C-acetylt	0.29	1	0.07788162	9	0.025991278
3251	GO:0004611	phosphoenolpyruvate	0.29	1	0.07788162	9	0.025991278
3625	GO:0005080	protein kinase C bind	0.29	1	0.07788162	9	0.025991278
3694	GO:0005160	transforming growth	0.29	1	0.07788162	9	0.025991278
4322	GO:0006084	acetyl-CoA metaboli	0.29	1	0.07788162	9	0.025991278
4332	GO:0006099	tricarboxylic acid cyc	0.29	1	0.07788162	9	0.025991278
6429	GO:0009060	aerobic respiration	0.29	1	0.07788162	9	0.025991278

6476	GO:0009109	coenzyme catabolic	0.29	1	0.07788162	9	0.025991278
7506	GO:0010279	indole-3-acetic acid a	0.29	1	0.07788162	9	0.025991278
9571	GO:0016453	C-acetyltransferase a	0.29	1	0.07788162	9	0.025991278
11785	GO:0019840	isoprenoid binding	0.29	1	0.07788162	9	0.025991278
13224	GO:0030755	quercetin 3-O-methy	0.29	1	0.07788162	9	0.025991278
15579	GO:0033180	proton-transporting V	0.29	1	0.07788162	9	0.025991278
19342	GO:0043027	caspase inhibitor act	0.29	1	0.07788162	9	0.025991278
19343	GO:0043028	caspase regulator ac	0.29	1	0.07788162	9	0.025991278
19458	GO:0043154|GO:000	negative regulation c	0.29	1	0.07788162	9	0.025991278
19579	GO:0043281|GO:004	regulation of caspase	0.29	1	0.07788162	9	0.025991278
21885	GO:0046356	acetyl-CoA catabolic	0.29	1	0.07788162	9	0.025991278
25484	GO:0051187	cofactor catabolic pr	0.29	1	0.07788162	9	0.025991278
17617	GO:0035251	UDP-glucosyltransfe	0.29	10	0.7788162	218	0.62956655
4427	GO:0006221	pyrimidine nucleotide	0.29	2	0.15576324	29	0.083749674
3311	GO:0004683|GO:000	calmodulin-depende	0.29	4	0.31152648	74	0.21370606
9832	GO:0016782	transferase activity, t	0.29	4	0.31152648	74	0.21370606
8677	GO:0015295	solute:hydrogen sym	0.30	5	0.3894081	98	0.28301615
8810	GO:0015491	cation:cation antiport	0.30	4	0.31152648	75	0.21659398
2666	GO:0003774	motor activity	0.31	2	0.15576324	30	0.086637594
5732	GO:0008194	UDP-glycosyltransfe	0.31	14	1.0903426	322	0.9299102
3024	GO:0004340	glucokinase activity	0.31	1	0.07788162	10	0.0288792
3056	GO:0004372	glycine hydroxymeth	0.31	1	0.07788162	10	0.0288792
3813	GO:0005356|GO:000	hydrogen:glucose sy	0.31	1	0.07788162	10	0.0288792
3815	GO:0005358	high-affinity hydroge	0.31	1	0.07788162	10	0.0288792
3842	GO:0005432	calcium:sodium antip	0.31	1	0.07788162	10	0.0288792
3849	GO:0005459	UDP-galactose trans	0.31	1	0.07788162	10	0.0288792
3871	GO:0005504	fatty acid binding	0.31	1	0.07788162	10	0.0288792
4309	GO:0006071	glycerol metabolic pr	0.31	1	0.07788162	10	0.0288792
4724	GO:0006596	polyamine biosynthe	0.31	1	0.07788162	10	0.0288792
5678	GO:0008113|GO:003	peptide-methionine-(	0.31	1	0.07788162	10	0.0288792
6464	GO:0009095	aromatic amino acid	0.31	1	0.07788162	10	0.0288792
6947	GO:0009679	hexose:hydrogen syr	0.31	1	0.07788162	10	0.0288792
8727	GO:0015369	calcium:hydrogen an	0.31	1	0.07788162	10	0.0288792
9054	GO:0015785	UDP-galactose trans	0.31	1	0.07788162	10	0.0288792
9828	GO:0016778	diphosphotransferase	0.31	1	0.07788162	10	0.0288792
11391	GO:0019400	alditol metabolic pro	0.31	1	0.07788162	10	0.0288792
11715	GO:0019751	polyol metabolic pro	0.31	1	0.07788162	10	0.0288792
13472	GO:0031013	troponin I binding	0.31	1	0.07788162	10	0.0288792
17563	GO:0035197	siRNA binding	0.31	1	0.07788162	10	0.0288792
25453	GO:0051156|GO:000	glucose 6-phosphate	0.31	1	0.07788162	10	0.0288792
26069	GO:0051787	misfolded protein bin	0.31	1	0.07788162	10	0.0288792
26975	GO:0060001	minus-end directed r	0.31	1	0.07788162	10	0.0288792
29215	GO:0070851	growth factor recepto	0.31	1	0.07788162	10	0.0288792
30693	GO:0072334	UDP-galactose trans	0.31	1	0.07788162	10	0.0288792
31441	GO:0080065	4-alpha-methyl-delta	0.31	1	0.07788162	10	0.0288792
114	GO:0000146	microfilament motor	0.31	1	0.07788162	10	0.0288792
196	GO:0000254	C-4 methylsterol oxid	0.31	1	0.07788162	10	0.0288792
9494	GO:0016311	dephosphorylation	0.32	18	1.4018692	425	1.227366
26959	GO:0055114	oxidation-reduction p	0.32	97	7.5545173	2490	7.1909204
9877	GO:0016838	carbon-oxygen lyase	0.32	4	0.31152648	77	0.22236982
14422	GO:0032012	regulation of ARF pro	0.32	2	0.15576324	31	0.08952551
14720	GO:0032312	regulation of ARF GT	0.32	2	0.15576324	31	0.08952551
30884	GO:0072528	pyrimidine-containing	0.32	2	0.15576324	31	0.08952551
30066	GO:0071702	organic substance tra	0.32	22	1.7133956	529	1.5277096
29251	GO:0070887	cellular response to c	0.32	3	0.23364486	54	0.15594767
5874	GO:0008374	O-acyltransferase ac	0.33	5	0.3894081	102	0.29456782
12794	GO:0030234	enzyme regulator act	0.33	17	1.3239875	403	1.1638317
30865	GO:0072509	divalent inorganic ca	0.33	4	0.31152648	78	0.22525774
25095	GO:0050790	regulation of catalytic	0.33	20	1.5576324	480	1.3862015
3717	GO:0005199	structural constituent	0.33	2	0.15576324	32	0.09241343
9533	GO:0016405	CoA-ligase activity	0.33	3	0.23364486	55	0.15883559
5733	GO:0008195|GO:000	phosphatidate phosp	0.34	1	0.07788162	11	0.03176712
11152	GO:0019139|GO:004	cytokinin dehydroger	0.34	1	0.07788162	11	0.03176712
16183	GO:0033799	myricetin 3'-O-methy	0.34	1	0.07788162	11	0.03176712
24696	GO:0050378	UDP-glucuronate 4-e	0.34	1	0.07788162	11	0.03176712
2669	GO:0003785	actin monomer bindi	0.34	1	0.07788162	11	0.03176712
28366	GO:0065009	regulation of molecu	0.34	20	1.5576324	484	1.3977532
4224	GO:0005984	disaccharide metabo	0.34	3	0.23364486	56	0.16172351
9369	GO:0016137	glycoside metabolic	0.34	3	0.23364486	56	0.16172351
4358	GO:0006140	regulation of nucleoti	0.35	4	0.31152648	80	0.2310336
7146	GO:0009894	regulation of catabol	0.35	4	0.31152648	80	0.2310336
9682	GO:0016597	amino acid binding	0.35	4	0.31152648	80	0.2310336
13278	GO:0030811	regulation of nucleoti	0.35	4	0.31152648	80	0.2310336
13759	GO:0031329	regulation of cellular	0.35	4	0.31152648	80	0.2310336
15521	GO:0033121	regulation of purine r	0.35	4	0.31152648	80	0.2310336
19479	GO:0043176	amine binding	0.35	4	0.31152648	80	0.2310336
4705	GO:0006576	cellular biogenic ami	0.35	2	0.15576324	33	0.09530135
9576	GO:0016459	myosin complex	0.35	2	0.15576324	33	0.09530135
8943	GO:0015672	monovalent inorganic	0.35	16	1.2461059	383	1.1060733
6166	GO:0008757	S-adenosylmethionin	0.35	8	0.62305295	180	0.5198256
7557	GO:0010333	terpene synthase act	0.35	3	0.23364486	57	0.16461143
9910	GO:0016878	acid-thiol ligase activ	0.35	3	0.23364486	57	0.16461143
9266	GO:0016020	membrane	0.36	143	11.137072	3741	10.803708
3749	GO:0005249	voltage-gated potass	0.36	2	0.15576324	34	0.09818927
5827	GO:0008308|GO:002	voltage-gated anion	0.36	2	0.15576324	34	0.09818927
6486	GO:0009119	ribonucleoside metal	0.36	2	0.15576324	34	0.09818927
11203	GO:0019200	carbohydrate kinase	0.36	2	0.15576324	34	0.09818927
20321	GO:0044070	regulation of anion tr	0.36	2	0.15576324	34	0.09818927
22341	GO:0046835	carbohydrate phosph	0.36	2	0.15576324	34	0.09818927
29202	GO:0070838	divalent metal ion tra	0.36	4	0.31152648	82	0.23680943
3037	GO:0004353	glutamate dehydroge	0.36	1	0.07788162	12	0.03465504

3386	GO:0004773	steryl-sulfatase activ	0.36	1	0.07788162	12	0.03465504
4506	GO:0006313|GO:000	transposition, DNA-m	0.36	1	0.07788162	12	0.03465504
4786	GO:0006664	glycolipid metabolic	0.36	1	0.07788162	12	0.03465504
5965	GO:0008484	sulfuric ester hydrola	0.36	1	0.07788162	12	0.03465504
6024	GO:0008574	plus-end-directed mi	0.36	1	0.07788162	12	0.03465504
6610	GO:0009247	glycolipid biosynthet	0.36	1	0.07788162	12	0.03465504
14604	GO:0032196	transposition	0.36	1	0.07788162	12	0.03465504
14674	GO:0032266	phosphatidylinositol-	0.36	1	0.07788162	12	0.03465504
16148	GO:0033764	steroid dehydrogena	0.36	1	0.07788162	12	0.03465504
19047	GO:0042723	thiamine-containing	0.36	1	0.07788162	12	0.03465504
19048	GO:0042724	thiamine-containing	0.36	1	0.07788162	12	0.03465504
20943	GO:0045333	cellular respiration	0.36	1	0.07788162	12	0.03465504
21992	GO:0046467	membrane lipid biosy	0.36	1	0.07788162	12	0.03465504
25436	GO:0051139	metal ion:hydrogen a	0.36	1	0.07788162	12	0.03465504
31380	GO:0080002	UDP-glucose:4-amin	0.36	1	0.07788162	12	0.03465504
29	GO:0000036	acyl carrier activity	0.36	1	0.07788162	12	0.03465504
3323	GO:0004702	receptor signaling pr	0.36	3	0.23364486	58	0.16749935
6665	GO:0009311	oligosaccharide meta	0.36	3	0.23364486	58	0.16749935
8028	GO:0010817	regulation of hormon	0.36	3	0.23364486	58	0.16749935
4954	GO:0006869	lipid transport	0.37	9	0.7009346	208	0.6006873
8085	GO:0010876	lipid localization	0.37	9	0.7009346	208	0.6006873
4426	GO:0006220	pyrimidine nucleotide	0.37	2	0.15576324	35	0.10107719
11206	GO:0019203	carbohydrate phosph	0.37	2	0.15576324	35	0.10107719
22012	GO:0046488|GO:003	phosphatidylinositol	0.37	2	0.15576324	35	0.10107719
30700	GO:0072341	modified amino acid	0.37	3	0.23364486	59	0.17038727
3048	GO:0004364	glutathione transfera	0.38	4	0.31152648	84	0.24258527
30867	GO:0072511	divalent inorganic ca	0.38	4	0.31152648	84	0.24258527
9393	GO:0016165	lipoxygenase activity	0.39	2	0.15576324	36	0.10396511
12626	GO:0030029	actin filament-based	0.39	2	0.15576324	36	0.10396511
12633	GO:0030036	actin cytoskeleton or	0.39	2	0.15576324	36	0.10396511
6436	GO:0009067	aspartate family ami	0.39	1	0.07788162	13	0.037542958
8149	GO:0010941	regulation of cell dea	0.39	1	0.07788162	13	0.037542958
8578	GO:0015165	pyrimidine nucleotide	0.39	1	0.07788162	13	0.037542958
9050	GO:0015781	pyrimidine nucleotide	0.39	1	0.07788162	13	0.037542958
18634	GO:0042278	purine nucleoside me	0.39	1	0.07788162	13	0.037542958
19298	GO:0042981	regulation of apoptos	0.39	1	0.07788162	13	0.037542958
19381	GO:0043067|GO:004	regulation of progran	0.39	1	0.07788162	13	0.037542958
20923	GO:0045309	protein phosphorylat	0.39	1	0.07788162	13	0.037542958
21694	GO:0046128	purine ribonucleoside	0.39	1	0.07788162	13	0.037542958
25118	GO:0050815	phosphoserine bindi	0.39	1	0.07788162	13	0.037542958
4768	GO:0006644	phospholipid metabo	0.40	3	0.23364486	61	0.1761631
8680	GO:0015298	solute:cation antiport	0.40	5	0.3894081	112	0.32344702
29918	GO:0071554	cell wall organization	0.40	5	0.3894081	112	0.32344702
4670	GO:0006541	glutamine metabolic	0.41	1	0.07788162	14	0.040430877
4723	GO:0006595	polyamine metabolic	0.41	1	0.07788162	14	0.040430877
5875	GO:0008375	acetylglucosaminyltr	0.41	1	0.07788162	14	0.040430877
8553	GO:0015140	malate transmembra	0.41	1	0.07788162	14	0.040430877
8859	GO:0015556	C4-dicarboxylate tra	0.41	1	0.07788162	14	0.040430877
9009	GO:0015740	C4-dicarboxylate tra	0.41	1	0.07788162	14	0.040430877
9012	GO:0015743	malate transport	0.41	1	0.07788162	14	0.040430877
9399	GO:0016174	NAD(P)H oxidase ac	0.41	1	0.07788162	14	0.040430877
9433	GO:0016229	steroid dehydrogena	0.41	1	0.07788162	14	0.040430877
9711	GO:0016629	12-oxophytodienoate	0.41	1	0.07788162	14	0.040430877
20671	GO:0045017	glycerolipid biosynth	0.41	1	0.07788162	14	0.040430877
20791	GO:0045153	electron transporter,	0.41	1	0.07788162	14	0.040430877
21998	GO:0046474	glycerophospholipid	0.41	1	0.07788162	14	0.040430877
24970	GO:0050664	oxidoreductase activ	0.41	1	0.07788162	14	0.040430877
25040	GO:0050734	hydroxycinnamoyltra	0.41	1	0.07788162	14	0.040430877
29787	GO:0071423	malate transmembra	0.41	1	0.07788162	14	0.040430877
270	GO:0000339	RNA cap binding	0.41	1	0.07788162	14	0.040430877
20624	GO:0044430	cytoskeletal part	0.41	4	0.31152648	88	0.25413695
19533	GO:0043234	protein complex	0.41	33	2.5700934	848	2.448956
5066	GO:0007010	cytoskeleton organiz	0.41	2	0.15576324	38	0.10974095
6749	GO:0009414	response to water de	0.41	2	0.15576324	38	0.10974095
8588	GO:0015175	neutral amino acid tr	0.41	2	0.15576324	38	0.10974095
9073	GO:0015804	neutral amino acid tr	0.41	2	0.15576324	38	0.10974095
12538	GO:0022843	voltage-gated cation	0.41	2	0.15576324	38	0.10974095
9535	GO:0016407	acetyltransferase act	0.42	5	0.3894081	115	0.3321108
8676	GO:0015294	solute:cation sympor	0.42	6	0.46728972	141	0.4071967
30883	GO:0072527	pyrimidine-containin	0.43	2	0.15576324	39	0.11262887
2868	GO:0004062	aryl sulfotransferase	0.43	1	0.07788162	15	0.043318797
7520	GO:0010294	abscisic acid glucosy	0.43	1	0.07788162	15	0.043318797
8635	GO:0015229	L-ascorbic acid trans	0.43	1	0.07788162	15	0.043318797
8740	GO:0015386	potassium:hydrogen	0.43	1	0.07788162	15	0.043318797
9144	GO:0015882	L-ascorbic acid trans	0.43	1	0.07788162	15	0.043318797
9536	GO:0016408	C-acyltransferase ac	0.43	1	0.07788162	15	0.043318797
12523	GO:0022821	potassium ion antipo	0.43	1	0.07788162	15	0.043318797
11616	GO:0019637	organophosphate me	0.44	3	0.23364486	65	0.18771479
20512	GO:0044262|GO:000	cellular carbohydrate	0.44	10	0.7788162	248	0.7162041
11829	GO:0019900	kinase binding	0.44	3	0.23364486	66	0.1906027
3638	GO:0005096	GTPase activator ac	0.45	2	0.15576324	41	0.118404716
19813	GO:0043547	positive regulation of	0.45	2	0.15576324	41	0.118404716
20627	GO:0044433	cytoplasmic vesicle	0.45	2	0.15576324	41	0.118404716
3742	GO:0005242	inward rectifier potas	0.45	1	0.07788162	16	0.046206716
3782	GO:0005310|GO:000	dicarboxylic acid tran	0.45	1	0.07788162	16	0.046206716
4767	GO:0006643	membrane lipid meta	0.45	1	0.07788162	16	0.046206716
4928	GO:0006835|GO:000	dicarboxylic acid tran	0.45	1	0.07788162	16	0.046206716
5691	GO:0008131|GO:000	primary amine oxida	0.45	1	0.07788162	16	0.046206716
5995	GO:0008524	glucose 6-phosphate	0.45	1	0.07788162	16	0.046206716
7521	GO:0010295	(+)-abscisic acid 8'-h	0.45	1	0.07788162	16	0.046206716
8694	GO:0015315	organophosphate:ino	0.45	1	0.07788162	16	0.046206716

9189	GO:0015929	hexosaminidase acti	0.45	1	0.07788162	16	0.046206716
9797	GO:0016742	hydroxymethyl-, form	0.45	1	0.07788162	16	0.046206716
9898	GO:0016863	intramolecular oxidor	0.45	1	0.07788162	16	0.046206716
18757	GO:0042409	caffeoyl-CoA O-meth	0.45	1	0.07788162	16	0.046206716
18899	GO:0042562	hormone binding	0.45	1	0.07788162	16	0.046206716
31396	GO:0080019	fatty-acyl-CoA reduc	0.45	1	0.07788162	16	0.046206716
2667	GO:0003777	microtubule motor ac	0.45	1	0.07788162	16	0.046206716
22479	GO:0046983	protein dimerization a	0.46	20	1.5576324	517	1.4930545
3873	GO:0005507	copper ion binding	0.46	6	0.46728972	146	0.42163628
8675	GO:0015293	symporter activity	0.46	7	0.5451713	173	0.49961013
7229	GO:0009987|GO:000	cellular process	0.46	395	30.76324	10601	30.614838
3753	GO:0005253	anion channel activit	0.46	2	0.15576324	42	0.121292636
5607	GO:0008022	protein C-terminus b	0.46	2	0.15576324	42	0.121292636
3620	GO:0005057	receptor signaling pr	0.47	3	0.23364486	69	0.19926646
4135	GO:0005875	microtubule associat	0.47	1	0.07788162	17	0.049094636
8585	GO:0015172	acidic amino acid tra	0.47	1	0.07788162	17	0.049094636
9069	GO:0015800	acidic amino acid tra	0.47	1	0.07788162	17	0.049094636
30887	GO:0072531	pyrimidine-containing	0.47	1	0.07788162	17	0.049094636
3900	GO:0005548|GO:000	phospholipid transpo	0.48	2	0.15576324	43	0.124180555
9174	GO:0015914	phospholipid transpo	0.48	2	0.15576324	43	0.124180555
13833	GO:0031410	cytoplasmic vesicle	0.48	2	0.15576324	43	0.124180555
14393	GO:0031982	vesicle	0.48	2	0.15576324	43	0.124180555
9579	GO:0016462	pyrophosphatase act	0.48	36	2.8037384	954	2.7550755
9191	GO:0015931	nucleobase, nucleos	0.48	3	0.23364486	70	0.20215438
9192	GO:0015932	nucleobase, nucleos	0.48	3	0.23364486	70	0.20215438
9859	GO:0016818	hydrolase activity, ac	0.49	36	2.8037384	956	2.7608514
9753	GO:0016682	oxidoreductase activ	0.49	2	0.15576324	44	0.12706847
2642	GO:0003697|GO:000	single-stranded DNA	0.49	2	0.15576324	44	0.12706847
3832	GO:0005388	calcium-transporting	0.49	1	0.07788162	18	0.051982556
4134	GO:0005874	microtubule	0.49	1	0.07788162	18	0.051982556
4252	GO:0006014	D-ribose metabolic p	0.49	1	0.07788162	18	0.051982556
8662	GO:0015271	outward rectifier pota	0.49	1	0.07788162	18	0.051982556
9726	GO:0016646	oxidoreductase activ	0.49	1	0.07788162	18	0.051982556
12887	GO:0030332	cyclin binding	0.49	1	0.07788162	18	0.051982556
207	GO:0000272	polysaccharide catab	0.49	1	0.07788162	18	0.051982556
2737	GO:0003887|GO:000	DNA-directed DNA p	0.49	1	0.07788162	18	0.051982556
9858	GO:0016817	hydrolase activity, ac	0.49	36	2.8037384	959	2.769515
8681	GO:0015299	solute:hydrogen anti	0.49	4	0.31152648	98	0.28301615
4236	GO:0005996	monosaccharide met	0.50	5	0.3894081	125	0.36099
9893	GO:0016857	racemase and epime	0.50	2	0.15576324	45	0.1299564
11780	GO:0019829	cation-transporting A	0.50	2	0.15576324	45	0.1299564
9764	GO:0016701	oxidoreductase activ	0.50	3	0.23364486	72	0.20793022
3799	GO:0005338|GO:000	nucleotide-sugar tran	0.51	1	0.07788162	19	0.054870475
5230	GO:0007205	activation of protein	0.51	1	0.07788162	19	0.054870475
6435	GO:0009066	aspartate family ami	0.51	1	0.07788162	19	0.054870475
9049	GO:0015780	nucleotide-sugar tran	0.51	1	0.07788162	19	0.054870475
9490	GO:0016307	phosphatidylinositol	0.51	1	0.07788162	19	0.054870475
9491	GO:0016308|GO:000	1-phosphatidylinosito	0.51	1	0.07788162	19	0.054870475
14556	GO:0032147	activation of protein	0.51	1	0.07788162	19	0.054870475
18750	GO:0042401	cellular biogenic ami	0.51	1	0.07788162	19	0.054870475
2636	GO:0003690	double-stranded DN/	0.51	2	0.15576324	46	0.13284431
11311	GO:0019318	hexose metabolic pro	0.52	4	0.31152648	101	0.2916799
9817	GO:0016763	transferase activity, t	0.52	3	0.23364486	74	0.21370606
4916	GO:0006818	hydrogen transport	0.52	11	0.8566978	292	0.84327257
9246	GO:0015992	proton transport	0.52	11	0.8566978	292	0.84327257
12608	GO:0030001	metal ion transport	0.52	14	1.0903426	374	1.080082
6442	GO:0009073|GO:001	aromatic amino acid	0.52	2	0.15576324	47	0.13573223
8497	GO:0015079|GO:002	potassium ion transm	0.52	2	0.15576324	47	0.13573223
21943	GO:0046417	chorismate metaboli	0.52	2	0.15576324	47	0.13573223
25510	GO:0051213	dioxygenase activity	0.53	3	0.23364486	75	0.21659398
3747	GO:0005247	voltage-gated chlorid	0.53	1	0.07788162	20	0.0577584
3754	GO:0005254	chloride channel acti	0.53	1	0.07788162	20	0.0577584
6201	GO:0008794	arsenate reductase (	0.53	1	0.07788162	20	0.0577584
9707	GO:0016624	oxidoreductase activ	0.53	1	0.07788162	20	0.0577584
9799	GO:0016744|GO:001	transferase activity, t	0.53	1	0.07788162	20	0.0577584
13086	GO:0030611	arsenate reductase a	0.53	1	0.07788162	20	0.0577584
13088	GO:0030613	oxidoreductase activ	0.53	1	0.07788162	20	0.0577584
13089	GO:0030614	oxidoreductase activ	0.53	1	0.07788162	20	0.0577584
15575	GO:0033176	proton-transporting V	0.53	1	0.07788162	20	0.0577584
16445	GO:0034061	DNA polymerase act	0.53	1	0.07788162	20	0.0577584
22376	GO:0046873	metal ion transmemb	0.54	8	0.62305295	213	0.6151269
3844	GO:0005451	monovalent cation:hy	0.55	2	0.15576324	49	0.14150807
6483	GO:0009116	nucleoside metabolic	0.55	2	0.15576324	49	0.14150807
8909	GO:0015630	microtubule cytoskel	0.55	2	0.15576324	49	0.14150807
31421	GO:0080044	quercetin 7-O-glucos	0.55	2	0.15576324	49	0.14150807
3364	GO:0004745	retinol dehydrogenas	0.55	1	0.07788162	21	0.060646318
3474	GO:0004869|GO:000	cysteine-type endope	0.55	1	0.07788162	21	0.060646318
4420	GO:0006213	pyrimidine nucleosid	0.55	1	0.07788162	21	0.060646318
5878	GO:0008378	galactosyltransferase	0.55	1	0.07788162	21	0.060646318
6582	GO:0009218	pyrimidine ribonucle	0.55	1	0.07788162	21	0.060646318
6584	GO:0009220	pyrimidine ribonucle	0.55	1	0.07788162	21	0.060646318
9698	GO:0016615	malate dehydrogena	0.55	1	0.07788162	21	0.060646318
21697	GO:0046131	pyrimidine ribonucle	0.55	1	0.07788162	21	0.060646318
2668	GO:0003779	actin binding	0.55	4	0.31152648	105	0.3032316
3449	GO:0004842|GO:000	ubiquitin-protein liga	0.55	12	0.93457943	326	0.94146186
19914	GO:0043648	dicarboxylic acid me	0.56	2	0.15576324	50	0.14439599
3828	GO:0005375|GO:000	copper ion transmem	0.56	1	0.07788162	22	0.06353424
3831	GO:0005385	zinc ion transmembra	0.56	1	0.07788162	22	0.06353424
4920	GO:0006825	copper ion transport	0.56	1	0.07788162	22	0.06353424
4924	GO:0006829	zinc ion transport	0.56	1	0.07788162	22	0.06353424
6521	GO:0009156	ribonucleoside mono	0.56	1	0.07788162	22	0.06353424

6525	GO:0009161	ribonucleoside mono	0.56	1	0.07788162	22	0.06353424
9545	GO:0016417	S-acyltransferase ac	0.56	1	0.07788162	22	0.06353424
17800	GO:0035434	copper ion transmem	0.56	1	0.07788162	22	0.06353424
21152	GO:0045551	cinnamyl-alcohol deh	0.56	1	0.07788162	22	0.06353424
29941	GO:0071577	zinc ion transmembra	0.56	1	0.07788162	22	0.06353424
2691	GO:0003841|GO:000	1-acylglycerol-3-pho	0.56	1	0.07788162	22	0.06353424
5764	GO:0008234|GO:000	cysteine-type peptida	0.56	6	0.46728972	163	0.47073093
3099	GO:0004428	inositol or phosphatic	0.57	2	0.15576324	51	0.14728391
8908	GO:0015629	actin cytoskeleton	0.57	2	0.15576324	51	0.14728391
9654	GO:0016567	protein ubiquitination	0.58	12	0.93457943	332	0.9587894
14854	GO:0032446	protein modification b	0.58	12	0.93457943	332	0.9587894
29011	GO:0070647	protein modification b	0.58	12	0.93457943	332	0.9587894
11833	GO:0019904	protein domain spec	0.58	4	0.31152648	109	0.31478328
4918	GO:0006821	chloride transport	0.58	1	0.07788162	23	0.06642216
9420	GO:0016207	4-coumarate-CoA lig	0.58	1	0.07788162	23	0.06642216
9806	GO:0016752	sinapoyltransferase a	0.58	1	0.07788162	23	0.06642216
9807	GO:0016753	O-sinapoyltransferas	0.58	1	0.07788162	23	0.06642216
9808	GO:0016754	sinapoylglucose-mal	0.58	1	0.07788162	23	0.06642216
16063	GO:0033674	positive regulation of	0.58	1	0.07788162	23	0.06642216
19815	GO:0043549	regulation of kinase a	0.58	1	0.07788162	23	0.06642216
21437	GO:0045859	regulation of protein	0.58	1	0.07788162	23	0.06642216
21438	GO:0045860	positive regulation of	0.58	1	0.07788162	23	0.06642216
25628	GO:0051338	regulation of transfer	0.58	1	0.07788162	23	0.06642216
25637	GO:0051347	positive regulation of	0.58	1	0.07788162	23	0.06642216
2633	GO:0003684	damaged DNA bindi	0.58	1	0.07788162	23	0.06642216
15189	GO:0032787	monocarboxylic acid	0.58	3	0.23364486	81	0.23392151
18674	GO:0042325	regulation of phosph	0.58	3	0.23364486	81	0.23392151
11776	GO:0019825	oxygen binding	0.58	14	1.0903426	389	1.1234008
9750	GO:0016679	oxidoreductase activ	0.59	2	0.15576324	53	0.15305975
25635	GO:0051345	positive regulation of	0.59	2	0.15576324	53	0.15305975
2661	GO:0003743|GO:000	translation initiation f	0.59	2	0.15576324	53	0.15305975
6991	GO:0009725	response to hormone	0.59	3	0.23364486	82	0.23680943
4526	GO:0006351|GO:000	transcription, DNA-d	0.60	57	4.4392524	1575	4.548474
15176	GO:0032774	RNA biosynthetic pro	0.60	57	4.4392524	1575	4.548474
3262	GO:0004622|GO:004	lysophospholipase a	0.60	1	0.07788162	24	0.06931008
4692	GO:0006563	L-serine metabolic p	0.60	1	0.07788162	24	0.06931008
7144	GO:0009892	negative regulation o	0.60	1	0.07788162	24	0.06931008
12694	GO:0030119	AP-type membrane c	0.60	1	0.07788162	24	0.06931008
12706	GO:0030131	clathrin adaptor com	0.60	1	0.07788162	24	0.06931008
13754	GO:0031324	negative regulation o	0.60	1	0.07788162	24	0.06931008
15390	GO:0032989	cellular component m	0.60	1	0.07788162	24	0.06931008
551	GO:0000902|GO:000	cell morphogenesis	0.60	1	0.07788162	24	0.06931008
11747	GO:0019787|GO:000	small conjugating pro	0.61	12	0.93457943	339	0.9790048
4503	GO:0006310	DNA recombination	0.61	1	0.07788162	25	0.072197996
9482	GO:0016291|GO:000	acyl-CoA thioesteras	0.61	1	0.07788162	25	0.072197996
13822	GO:0031399	regulation of protein	0.61	1	0.07788162	25	0.072197996
21145	GO:0045543	gibberellin 2-beta-dio	0.61	1	0.07788162	25	0.072197996
22357	GO:0046854|GO:004	phosphatidylinositol	0.61	1	0.07788162	25	0.072197996
22459	GO:0046961	proton-transporting A	0.61	1	0.07788162	25	0.072197996
31393	GO:0080016	(-)-E-beta-caryophyll	0.61	1	0.07788162	25	0.072197996
31394	GO:0080017	alpha-humulene synt	0.61	1	0.07788162	25	0.072197996
125	GO:0000159	protein phosphatase	0.61	1	0.07788162	25	0.072197996
1225	GO:0001932	regulation of protein	0.61	1	0.07788162	25	0.072197996
11222	GO:0019220	regulation of phospha	0.61	3	0.23364486	85	0.24547319
25471	GO:0051174	regulation of phosph	0.61	3	0.23364486	85	0.24547319
5840	GO:0008324	cation transmembran	0.62	19	1.4797508	538	1.5537009
6985	GO:0009719	response to endogen	0.62	3	0.23364486	86	0.24836111
3896	GO:0005544	calcium-dependent p	0.63	1	0.07788162	26	0.075085916
4030	GO:0005741	mitochondrial outer m	0.63	1	0.07788162	26	0.075085916
5978	GO:0008506	sucrose:hydrogen sy	0.63	1	0.07788162	26	0.075085916
5987	GO:0008515|GO:001	sucrose transmembr	0.63	1	0.07788162	26	0.075085916
8567	GO:0015154	disaccharide transm	0.63	1	0.07788162	26	0.075085916
9035	GO:0015766	disaccharide transpo	0.63	1	0.07788162	26	0.075085916
9039	GO:0015770	sucrose transport	0.63	1	0.07788162	26	0.075085916
9421	GO:0016208	AMP binding	0.63	1	0.07788162	26	0.075085916
9539	GO:0016411	acylglycerol O-acyltr	0.63	1	0.07788162	26	0.075085916
9744	GO:0016671	oxidoreductase activ	0.63	1	0.07788162	26	0.075085916
9884	GO:0016847|GO:003	1-aminocyclopropane	0.63	1	0.07788162	26	0.075085916
9897	GO:0016862	intramolecular oxido	0.63	1	0.07788162	26	0.075085916
9899	GO:0016864	intramolecular oxido	0.63	1	0.07788162	26	0.075085916
10911	GO:0018871	1-aminocyclopropane	0.63	1	0.07788162	26	0.075085916
18579	GO:0042218	1-aminocyclopropane	0.63	1	0.07788162	26	0.075085916
30689	GO:0072330	monocarboxylic acid	0.63	1	0.07788162	26	0.075085916
2665	GO:0003756	protein disulfide ison	0.63	1	0.07788162	26	0.075085916
5715	GO:0008171	O-methyltransferase	0.63	2	0.15576324	57	0.16461143
6441	GO:0009072	aromatic amino acid	0.63	2	0.15576324	57	0.16461143
9768	GO:0016706	oxidoreductase activ	0.63	6	0.46728972	175	0.505386
9796	GO:0016741	transferase activity, t	0.63	9	0.7009346	261	0.7537471
4913	GO:0006814|GO:000	sodium ion transport	0.64	2	0.15576324	58	0.16749935
8499	GO:0015081|GO:002	sodium ion transmem	0.64	2	0.15576324	58	0.16749935
11820	GO:0019887	protein kinase regula	0.64	2	0.15576324	58	0.16749935
18086	GO:0035725	sodium ion transmem	0.64	2	0.15576324	58	0.16749935
29800	GO:0071436	sodium ion export	0.64	2	0.15576324	58	0.16749935
3329	GO:0004708	MAP kinase kinase a	0.64	1	0.07788162	27	0.077973835
3631	GO:0005089	Rho guanyl-nucleotid	0.64	1	0.07788162	27	0.077973835
6433	GO:0009064	glutamine family ami	0.64	1	0.07788162	27	0.077973835
22340	GO:0046834	lipid phosphorylation	0.64	1	0.07788162	27	0.077973835
25481	GO:0051184	cofactor transporter a	0.64	1	0.07788162	27	0.077973835
1029	GO:0001727	lipid kinase activity	0.64	1	0.07788162	27	0.077973835
9719	GO:0016638	oxidoreductase activ	0.65	3	0.23364486	89	0.25702485
19126	GO:0042803	protein homodimeriz	0.65	9	0.7009346	265	0.7652988

2833	GO:0004024	alcohol dehydrogena	0.65	1	0.07788162	28	0.080861755
3284	GO:0004650	polygalacturonase ac	0.65	1	0.07788162	28	0.080861755
6079	GO:0008654	phospholipid biosynt	0.65	1	0.07788162	28	0.080861755
6438	GO:0009069	serine family amino a	0.65	1	0.07788162	28	0.080861755
6940	GO:0009672	auxin:hydrogen symp	0.65	1	0.07788162	28	0.080861755
9238	GO:0015980	energy derivation by	0.65	1	0.07788162	28	0.080861755
9827	GO:0016776	phosphotransferase	0.65	1	0.07788162	28	0.080861755
13144	GO:0030674	protein binding, bridg	0.65	1	0.07788162	28	0.080861755
23911	GO:0048519|GO:004	negative regulation o	0.65	1	0.07788162	28	0.080861755
23915	GO:0048523|GO:005	negative regulation o	0.65	1	0.07788162	28	0.080861755
27064	GO:0060090	binding, bridging	0.65	1	0.07788162	28	0.080861755
20487	GO:0044237	cellular metabolic pro	0.65	298	23.208723	8185	23.637625
20510	GO:0044260|GO:003	cellular macromolecu	0.66	202	15.732087	5577	16.105928
2980	GO:0004185	serine-type carboxyp	0.66	2	0.15576324	60	0.17327519
28375	GO:0070008	serine-type exopeptid	0.66	2	0.15576324	60	0.17327519
3644	GO:0005102	receptor binding	0.66	3	0.23364486	91	0.2628007
4849	GO:0006730|GO:001	one-carbon metaboli	0.66	11	0.8566978	325	0.93857396
7558	GO:0010334	sesquiterpene syntha	0.67	1	0.07788162	29	0.083749674
9409	GO:0016192|GO:000	vesicle-mediated tra	0.67	1	0.07788162	29	0.083749674
16785	GO:0034404	nucleobase, nucleos	0.67	3	0.23364486	92	0.26568863
17033	GO:0034654	nucleobase, nucleos	0.67	3	0.23364486	92	0.26568863
2978	GO:0004180	carboxypeptidase ac	0.67	2	0.15576324	62	0.17905103
5213	GO:0007186	G-protein coupled re	0.67	2	0.15576324	62	0.17905103
11210	GO:0019207	kinase regulator acti	0.67	2	0.15576324	62	0.17905103
3310	GO:0004679	AMP-activated prote	0.68	1	0.07788162	30	0.086637594
3518	GO:0004930|GO:000	G-protein coupled re	0.68	1	0.07788162	30	0.086637594
3975	GO:0005667	transcription factor c	0.68	1	0.07788162	30	0.086637594
5916	GO:0008422|GO:001	beta-glucosidase act	0.68	1	0.07788162	30	0.086637594
6488	GO:0009123	nucleoside monopho	0.68	1	0.07788162	30	0.086637594
6489	GO:0009124	nucleoside monopho	0.68	1	0.07788162	30	0.086637594
24247	GO:0048869	cellular development	0.68	1	0.07788162	30	0.086637594
2774	GO:0003950	NAD+ ADP-ribosyltra	0.68	1	0.07788162	30	0.086637594
8495	GO:0015077	monovalent inorgani	0.68	7	0.5451713	214	0.6180149
9818	GO:0016765|GO:001	transferase activity, t	0.69	6	0.46728972	185	0.53426516
5712	GO:0008168|GO:000	methyltransferase ac	0.69	8	0.62305295	244	0.7046524
7222	GO:0009975	cyclase activity	0.69	1	0.07788162	31	0.08952551
25478	GO:0051181	cofactor transport	0.69	1	0.07788162	31	0.08952551
9114	GO:0015849	organic acid transpo	0.69	5	0.3894081	156	0.45051548
22443	GO:0046942	carboxylic acid trans	0.69	5	0.3894081	156	0.45051548
4121	GO:0005856	cytoskeleton	0.69	4	0.31152648	126	0.3638779
12741	GO:0030170	pyridoxal phosphate	0.69	4	0.31152648	126	0.3638779
28643	GO:0070279	vitamin B6 binding	0.69	4	0.31152648	126	0.3638779
22416	GO:0046915	transition metal ion t	0.69	2	0.15576324	64	0.18482687
9913	GO:0016881	acid-amino acid ligas	0.69	13	1.0124611	391	1.1291766
9583	GO:0016469	proton-transporting t	0.70	4	0.31152648	127	0.36676583
3861	GO:0005484|GO:000	SNAP receptor activ	0.70	2	0.15576324	65	0.18771479
9838	GO:0016790	thiolester hydrolase a	0.70	2	0.15576324	65	0.18771479
25314	GO:0051015	actin filament binding	0.70	2	0.15576324	65	0.18771479
4757	GO:0006633|GO:000	fatty acid biosyntheti	0.70	1	0.07788162	32	0.09241343
6942	GO:0009674	potassium:sodium sy	0.70	1	0.07788162	32	0.09241343
9041	GO:0015772	oligosaccharide trans	0.70	1	0.07788162	32	0.09241343
12522	GO:0022820	potassium ion sympo	0.70	1	0.07788162	32	0.09241343
12833	GO:0030275	LRR domain binding	0.70	1	0.07788162	32	0.09241343
14381	GO:0031968	organelle outer mem	0.70	1	0.07788162	32	0.09241343
21041	GO:0045431	flavonol synthase ac	0.70	1	0.07788162	32	0.09241343
25836	GO:0051552	flavone metabolic pro	0.70	1	0.07788162	32	0.09241343
25837	GO:0051553	flavone biosynthetic	0.70	1	0.07788162	32	0.09241343
25838	GO:0051554	flavonol metabolic pr	0.70	1	0.07788162	32	0.09241343
25839	GO:0051555	flavonol biosynthetic	0.70	1	0.07788162	32	0.09241343
10058	GO:0017111	nucleoside-triphosph	0.70	30	2.3364487	878	2.5355935
3180	GO:0004521	endoribonuclease ac	0.71	2	0.15576324	66	0.1906027
9765	GO:0016702	oxidoreductase activ	0.71	2	0.15576324	66	0.1906027
9890	GO:0016854	racemase and epime	0.71	2	0.15576324	66	0.1906027
9906	GO:0016874	ligase activity	0.71	20	1.5576324	597	1.7240882
10103	GO:0017171	serine hydrolase acti	0.71	7	0.5451713	220	0.63534236
8496	GO:0015078	hydrogen ion transm	0.71	5	0.3894081	160	0.4620672
4493	GO:0006298|GO:000	mismatch repair	0.71	1	0.07788162	33	0.09530135
30878	GO:0072522	purine-containing co	0.71	1	0.07788162	33	0.09530135
2632	GO:0003682	chromatin binding	0.71	1	0.07788162	33	0.09530135
4320	GO:0006082	organic acid metabo	0.72	12	0.93457943	369	1.0656425
11716	GO:0019752	carboxylic acid meta	0.72	12	0.93457943	369	1.0656425
19707	GO:0043436	oxoacid metabolic pr	0.72	12	0.93457943	369	1.0656425
7271	GO:0010033	response to organic	0.72	3	0.23364486	100	0.28879198
18955	GO:0042625	ATPase activity, cou	0.72	2	0.15576324	68	0.19637854
32	GO:0000041	transition metal ion t	0.72	2	0.15576324	68	0.19637854
6004	GO:0008536	Ran GTPase binding	0.72	1	0.07788162	34	0.09818927
11208	GO:0019205	nucleobase, nucleos	0.72	1	0.07788162	34	0.09818927
11831	GO:0019902	phosphatase binding	0.72	1	0.07788162	34	0.09818927
11832	GO:0019903	protein phosphatase	0.72	1	0.07788162	34	0.09818927
4394	GO:0006184	GTP catabolic proce	0.73	4	0.31152648	132	0.3812054
21605	GO:0046039	GTP metabolic proce	0.73	4	0.31152648	132	0.3812054
2758	GO:0003924	GTPase activity	0.73	4	0.31152648	132	0.3812054
19473	GO:0043170|GO:004	macromolecule meta	0.73	235	18.302181	6553	18.92454
4911	GO:0006812|GO:000	cation transport	0.73	22	1.7133956	662	1.9118029
9480	GO:0016289	CoA hydrolase activi	0.73	1	0.07788162	35	0.10107719
12817	GO:0030258	lipid modification	0.73	1	0.07788162	35	0.10107719
18541	GO:0042180	cellular ketone metal	0.74	12	0.93457943	375	1.0829699
3804	GO:0005345	purine base transme	0.74	1	0.07788162	36	0.10396511
4949	GO:0006863|GO:001	purine base transpor	0.74	1	0.07788162	36	0.10396511
8739	GO:0015385|GO:001	sodium:hydrogen ant	0.74	1	0.07788162	36	0.10396511
11314	GO:0019321	pentose metabolic pr	0.74	1	0.07788162	36	0.10396511

9771	GO:0016709	oxidoreductase activ	0.75	2	0.15576324	71	0.2050423
19392	GO:0043085	positive regulation of	0.75	3	0.23364486	104	0.30034366
20343	GO:0044093	positive regulation of	0.75	3	0.23364486	104	0.30034366
4408	GO:0006200	ATP catabolic proce	0.75	21	1.635514	641	1.8511566
9918	GO:0016887|GO:000	ATPase activity	0.75	21	1.635514	641	1.8511566
21600	GO:0046034	ATP metabolic proce	0.75	21	1.635514	641	1.8511566
2975	GO:0004175|GO:001	endopeptidase activi	0.75	10	0.7788162	320	0.9241344
8934	GO:0015662	ATPase activity, cou	0.75	1	0.07788162	37	0.10685303
9915	GO:0016884|GO:001	carbon-nitrogen ligas	0.75	1	0.07788162	37	0.10685303
6899	GO:0009628	response to abiotic s	0.76	5	0.3894081	170	0.49094638
9637	GO:0016538|GO:000	cyclin-dependent pro	0.76	1	0.07788162	38	0.10974095
12834	GO:0030276	clathrin binding	0.76	1	0.07788162	38	0.10974095
9874	GO:0016835	carbon-oxygen lyase	0.76	6	0.46728972	202	0.5833598
22375	GO:0046872	metal ion binding	0.76	104	8.099689	2985	8.6204405
6623	GO:0009260	ribonucleotide biosyn	0.77	1	0.07788162	39	0.11262887
6046	GO:0008610	lipid biosynthetic pro	0.77	4	0.31152648	141	0.4071967
4573	GO:0006413|GO:000	translational initiation	0.78	2	0.15576324	76	0.2194819
19470	GO:0043167	ion binding	0.78	104	8.099689	2997	8.655096
19472	GO:0043169	cation binding	0.78	104	8.099689	2997	8.655096
11828	GO:0019899	enzyme binding	0.78	7	0.5451713	237	0.684437
4058	GO:0005773	vacuole	0.78	1	0.07788162	40	0.1155168
7072	GO:0009813	flavonoid biosyntheti	0.78	1	0.07788162	40	0.1155168
8613	GO:0015205|GO:001	nucleobase transmen	0.78	1	0.07788162	40	0.1155168
9116	GO:0015851	nucleobase transpor	0.78	1	0.07788162	40	0.1155168
11804	GO:0019867	outer membrane	0.78	1	0.07788162	40	0.1155168
18953	GO:0042623	ATPase activity, cou	0.78	17	1.3239875	537	1.550813
5604	GO:0008017	microtubule binding	0.78	2	0.15576324	77	0.22236982
6529	GO:0009165	nucleotide biosynthe	0.78	2	0.15576324	77	0.22236982
3626	GO:0005083	small GTPase regula	0.78	3	0.23364486	111	0.3205591
2986	GO:0004252	serine-type endopep	0.79	4	0.31152648	144	0.41586044
4405	GO:0006195	purine nucleotide cat	0.79	25	1.9470406	773	2.232362
6508	GO:0009143	nucleoside triphosph	0.79	25	1.9470406	773	2.232362
6509	GO:0009144	purine nucleoside trip	0.79	25	1.9470406	773	2.232362
6511	GO:0009146	purine nucleoside trip	0.79	25	1.9470406	773	2.232362
6519	GO:0009154	purine ribonucleotide	0.79	25	1.9470406	773	2.232362
6530	GO:0009166	nucleotide catabolic	0.79	25	1.9470406	773	2.232362
6567	GO:0009203	ribonucleoside tripho	0.79	25	1.9470406	773	2.232362
6569	GO:0009205	purine ribonucleoside	0.79	25	1.9470406	773	2.232362
6571	GO:0009207	purine ribonucleoside	0.79	25	1.9470406	773	2.232362
6624	GO:0009261	ribonucleotide catab	0.79	25	1.9470406	773	2.232362
17034	GO:0034655	nucleobase, nucleos	0.79	25	1.9470406	773	2.232362
17035	GO:0034656	nucleobase, nucleos	0.79	25	1.9470406	773	2.232362
22209	GO:0046700	heterocycle catabolic	0.79	25	1.9470406	773	2.232362
30879	GO:0072523	purine-containing co	0.79	25	1.9470406	773	2.232362
12572	GO:0022890|GO:001	inorganic cation tran	0.79	10	0.7788162	332	0.9587894
5196	GO:0007166	cell surface receptor	0.79	2	0.15576324	78	0.22525774
19832	GO:0043566	structure-specific DN	0.79	2	0.15576324	78	0.22525774
20520	GO:0044270	cellular nitrogen com	0.79	25	1.9470406	777	2.2439137
4756	GO:0006631	fatty acid metabolic	0.80	1	0.07788162	42	0.121292636
4874	GO:0006767	water-soluble vitami	0.80	1	0.07788162	42	0.121292636
13884	GO:0031461	cullin-RING ubiquitin	0.80	1	0.07788162	42	0.121292636
18713	GO:0042364	water-soluble vitami	0.80	1	0.07788162	42	0.121292636
9911	GO:0016879	ligase activity, formi	0.80	14	1.0903426	456	1.3168914
9875	GO:0016836	hydro-lyase activity	0.80	2	0.15576324	80	0.2310336
5694	GO:0008137	NADH dehydrogenas	0.80	1	0.07788162	43	0.124180555
8589	GO:0015179	L-amino acid transm	0.80	1	0.07788162	43	0.124180555
9076	GO:0015807	L-amino acid transpo	0.80	1	0.07788162	43	0.124180555
14676	GO:0032268	regulation of cellular	0.80	1	0.07788162	43	0.124180555
25538	GO:0051246	regulation of protein	0.80	1	0.07788162	43	0.124180555
6663	GO:0009309	amine biosynthetic p	0.81	5	0.3894081	181	0.5227135
6622	GO:0009259|GO:000	ribonucleotide metab	0.81	26	2.0249221	812	2.344991
11426	GO:0019438	aromatic compound	0.81	4	0.31152648	149	0.43030006
5766	GO:0008236	serine-type peptidas	0.81	6	0.46728972	214	0.6180149
4873	GO:0006766	vitamin metabolic pro	0.81	1	0.07788162	44	0.12706847
6477	GO:0009110	vitamin biosynthetic	0.81	1	0.07788162	44	0.12706847
7071	GO:0009812	flavonoid metabolic	0.81	1	0.07788162	44	0.12706847
9186	GO:0015926	glucosidase activity	0.81	1	0.07788162	44	0.12706847
20522	GO:0044272	sulfur compound bios	0.81	1	0.07788162	44	0.12706847
20505	GO:0044255	cellular lipid metabol	0.82	5	0.3894081	184	0.53137726
2628	GO:0003677	DNA binding	0.82	40	3.115265	1224	3.534814
3630	GO:0005088	Ras guanyl-nucleotid	0.82	1	0.07788162	45	0.1299564
12693	GO:0030118|GO:001	clathrin coat	0.82	1	0.07788162	45	0.1299564
24456	GO:0050136	NADH dehydrogenas	0.82	1	0.07788162	45	0.1299564
3787	GO:0005319	lipid transporter activ	0.82	2	0.15576324	83	0.23969735
6563	GO:0009199	ribonucleoside tripho	0.82	25	1.9470406	790	2.2814567
5661	GO:0008092	cytoskeletal protein b	0.82	7	0.5451713	249	0.719092
6515	GO:0009150	purine ribonucleotide	0.82	25	1.9470406	791	2.2843447
6662	GO:0009308	amine metabolic pro	0.83	9	0.7009346	314	0.9068068
4144	GO:0005886|GO:000	plasma membrane	0.83	3	0.23364486	120	0.34655038
5624	GO:0008047	enzyme activator act	0.83	2	0.15576324	85	0.24547319
6506	GO:0009141	nucleoside triphosph	0.83	25	1.9470406	796	2.2987843
5807	GO:0008287	protein serine/threon	0.83	1	0.07788162	47	0.13573223
20533	GO:0044283	small molecule biosy	0.83	10	0.7788162	347	1.0021082
30877	GO:0072521	purine-containing co	0.83	26	2.0249221	827	2.3883097
14667	GO:0032259	methylation	0.83	8	0.62305295	285	0.8230572
5768	GO:0008238	exopeptidase activity	0.83	2	0.15576324	86	0.24836111
28930	GO:0070566	adenylyltransferase a	0.84	1	0.07788162	48	0.13862015
4246	GO:0006006	glucose metabolic pr	0.84	2	0.15576324	87	0.25124902
4951	GO:0006865|GO:000	amino acid transport	0.84	3	0.23364486	123	0.35521415
20531	GO:0044281	small molecule meta	0.84	59	4.5950155	1788	5.1636004
11830	GO:0019901	protein kinase bindin	0.84	1	0.07788162	49	0.14150807

19831	GO:0043565	sequence-specific D	0.84	4	0.31152648	159	0.45917925
30306	GO:0071944	cell periphery	0.85	7	0.5451713	259	0.74797124
4216	GO:0005976	polysaccharide meta	0.85	1	0.07788162	50	0.14439599
6921	GO:0009653	anatomical structure	0.85	1	0.07788162	50	0.14439599
9993	GO:0017016	Ras GTPase binding	0.85	1	0.07788162	50	0.14439599
30886	GO:0072530	purine-containing co	0.85	1	0.07788162	50	0.14439599
3876	GO:0005515|GO:00	protein binding	0.85	243	18.925234	6933	20.021948
4373	GO:0006163	purine nucleotide me	0.85	25	1.9470406	810	2.339215
9285	GO:0016043	cellular component o	0.85	7	0.5451713	261	0.7537471
9733	GO:0016655	oxidoreductase activ	0.85	1	0.07788162	51	0.14728391
9975	GO:0016998	cell wall macromolec	0.85	1	0.07788162	51	0.14728391
22410	GO:0046907	intracellular transpor	0.85	1	0.07788162	51	0.14728391
25925	GO:0051641	cellular localization	0.85	1	0.07788162	51	0.14728391
25933	GO:0051649	establishment of loca	0.85	1	0.07788162	51	0.14728391
13531	GO:0031072	heat shock protein bi	0.86	6	0.46728972	230	0.6642216
5693	GO:0008135	translation factor act	0.86	2	0.15576324	92	0.26568863
5097	GO:0007049	cell cycle	0.86	1	0.07788162	52	0.15017183
6965	GO:0009699	phenylpropanoid bios	0.86	1	0.07788162	52	0.15017183
20287	GO:0044036	cell wall macromolec	0.86	1	0.07788162	52	0.15017183
2778	GO:0003954	NADH dehydrogenas	0.87	1	0.07788162	53	0.15305975
17918	GO:0035556|GO:00	intracellular signal tr	0.87	1	0.07788162	54	0.15594767
30069	GO:0071705	nitrogen compound t	0.87	5	0.3894081	202	0.5833598
15436	GO:0033036	macromolecule local	0.87	11	0.8566978	397	1.1465042
4894	GO:0006790	sulfur compound me	0.88	1	0.07788162	55	0.15883559
6077	GO:0008652	cellular amino acid b	0.88	4	0.31152648	169	0.48805845
20356	GO:0044106	cellular amine metab	0.88	8	0.62305295	303	0.8750397
9883	GO:0016846	carbon-sulfur lyase a	0.88	1	0.07788162	56	0.16172351
13697	GO:0031267	small GTPase bindin	0.88	1	0.07788162	56	0.16172351
15576	GO:0033177	proton-transporting t	0.88	1	0.07788162	56	0.16172351
15577	GO:0033178	proton-transporting t	0.88	1	0.07788162	56	0.16172351
9308	GO:0016070	RNA metabolic proc	0.88	60	4.6728973	1855	5.3570914
9103	GO:0015837	amine transport	0.88	3	0.23364486	135	0.38986918
25379	GO:0051082	unfolded protein bind	0.88	2	0.15576324	98	0.28301615
3801	GO:0005342	organic acid transme	0.88	3	0.23364486	136	0.3927571
22444	GO:0046943	carboxylic acid trans	0.88	3	0.23364486	136	0.3927571
5964	GO:0008483	transaminase activity	0.88	1	0.07788162	57	0.16461143
9821	GO:0016769	transferase activity, t	0.88	1	0.07788162	57	0.16461143
3333	GO:0004712	protein serine/threon	0.89	1	0.07788162	59	0.17038727
9900	GO:0016866	intramolecular transf	0.89	1	0.07788162	59	0.17038727
9295	GO:0016053	organic acid biosynth	0.90	5	0.3894081	211	0.6093511
21058	GO:0045454|GO:00	cell redox homeostas	0.90	5	0.3894081	211	0.6093511
21922	GO:0046394	carboxylic acid biosy	0.90	5	0.3894081	211	0.6093511
6964	GO:0009698	phenylpropanoid me	0.90	1	0.07788162	60	0.17327519
25319	GO:0051020	GTPase binding	0.90	1	0.07788162	60	0.17327519
11787	GO:0019842	vitamin binding	0.90	4	0.31152648	177	0.5111618
4641	GO:0006508	proteolysis	0.90	29	2.258567	965	2.7868426
13164	GO:0030695	GTPase regulator ac	0.90	3	0.23364486	142	0.4100846
28365	GO:0065008	regulation of biologic	0.90	8	0.62305295	316	0.9125827
20639	GO:0044445	cytosolic part	0.90	1	0.07788162	62	0.17905103
3195	GO:0004540	ribonuclease activity	0.91	2	0.15576324	106	0.3061195
26932	GO:0055086	nucleobase, nucleos	0.91	28	2.1806853	944	2.7261963
4867	GO:0006753	nucleoside phosphat	0.91	27	2.1028037	914	2.6395588
6484	GO:0009117	nucleotide metabolic	0.91	27	2.1028037	914	2.6395588
20532	GO:0044282	small molecule catab	0.91	25	1.9470406	854	2.4662836
8910	GO:0015631	tubulin binding	0.91	2	0.15576324	107	0.30900744
9710	GO:0016628	oxidoreductase activ	0.91	1	0.07788162	65	0.18771479
4844	GO:0006725	cellular aromatic co	0.92	4	0.31152648	185	0.53426516
9829	GO:0016779	nucleotidyltransferas	0.92	3	0.23364486	148	0.42741212
4478	GO:0006281	DNA repair	0.92	3	0.23364486	149	0.43030006
5037	GO:0006974|GO:00	response to DNA da	0.92	3	0.23364486	149	0.43030006
4561	GO:0006399	tRNA metabolic proc	0.92	1	0.07788162	66	0.1906027
5615	GO:0008033	tRNA processing	0.92	1	0.07788162	66	0.1906027
20498	GO:0044248	cellular catabolic pro	0.92	26	2.0249221	894	2.5818002
9730	GO:0016651	oxidoreductase activ	0.92	2	0.15576324	112	0.32344702
27562	GO:0060589	nucleoside-triphosph	0.92	3	0.23364486	152	0.4389638
4850	GO:0006732|GO:00	coenzyme metabolic	0.93	2	0.15576324	113	0.32633495
9933	GO:0016903	oxidoreductase activ	0.93	2	0.15576324	113	0.32633495
9722	GO:0016641	oxidoreductase activ	0.93	1	0.07788162	69	0.19926646
21710	GO:0046148	pigment biosynthetic	0.93	1	0.07788162	69	0.19926646
5726	GO:0008187	poly-pyrimidine tract	0.93	1	0.07788162	70	0.20215438
5788	GO:0008266	poly(U) RNA binding	0.93	1	0.07788162	70	0.20215438
2649	GO:0003713	transcription coactiva	0.93	1	0.07788162	70	0.20215438
20521	GO:0044271	cellular nitrogen com	0.93	9	0.7009346	367	1.0598665
6020	GO:0008565|GO:00	protein transporter a	0.93	2	0.15576324	115	0.3321108
6475	GO:0009108	coenzyme biosynthe	0.93	1	0.07788162	72	0.20793022
9740	GO:0016667	oxidoreductase activ	0.93	1	0.07788162	72	0.20793022
8584	GO:0015171|GO:00	amino acid transmen	0.94	2	0.15576324	118	0.34077454
2521	GO:0003333	amino acid transmen	0.94	2	0.15576324	118	0.34077454
9703	GO:0016620	oxidoreductase activ	0.94	1	0.07788162	73	0.21081814
18786	GO:0042440	pigment metabolic p	0.94	1	0.07788162	73	0.21081814
19125	GO:0042802	identical protein bind	0.94	19	1.4797508	697	2.01288
11695	GO:0019725	cellular homeostasis	0.94	5	0.3894081	234	0.67577326
10244	GO:0018130	heterocycle biosynth	0.94	4	0.31152648	199	0.57469606
3627	GO:0005085|GO:00	guanyl-nucleotide ex	0.94	1	0.07788162	75	0.21659398
3965	GO:0005654	nucleoplasm	0.94	1	0.07788162	75	0.21659398
20645	GO:0044451	nucleoplasm part	0.94	1	0.07788162	75	0.21659398
18925	GO:0042592	homeostatic process	0.94	5	0.3894081	238	0.68732494
7691	GO:0010467	gene expression	0.94	61	4.7507787	1974	5.7007537
4649	GO:0006520|GO:00	cellular amino acid m	0.95	6	0.46728972	277	0.7999538
2819	GO:0004004	ATP-dependent RNA	0.95	1	0.07788162	78	0.22525774
4100	GO:0005829	cytosol	0.95	1	0.07788162	78	0.22525774

5725	GO:0008186|GO:000	RNA-dependent ATF	0.95	1	0.07788162	78	0.22525774
3761	GO:0005275|GO:000	amine transmembrar	0.95	2	0.15576324	126	0.3638779
634	GO:0000988	protein binding trans	0.95	2	0.15576324	126	0.3638779
635	GO:0000989	transcription factor b	0.95	2	0.15576324	126	0.3638779
2648	GO:0003712	transcription cofactor	0.95	2	0.15576324	126	0.3638779
2654	GO:0003724	RNA helicase activity	0.95	1	0.07788162	80	0.2310336
22007	GO:0046483	heterocycle metaboli	0.95	31	2.4143302	1094	3.1593843
2658	GO:0003729	mRNA binding	0.96	1	0.07788162	82	0.23680943
30205	GO:0071841	cellular component o	0.96	6	0.46728972	285	0.8230572
15949	GO:0033554	cellular response to	0.96	3	0.23364486	173	0.49961013
4327	GO:0006091	generation of precurs	0.96	1	0.07788162	84	0.24258527
4067	GO:0005783	endoplasmic reticulu	0.96	3	0.23364486	174	0.50249803
30204	GO:0071840	cellular component o	0.96	8	0.62305295	362	1.045427
24235	GO:0048856	anatomical structure	0.97	1	0.07788162	90	0.2599128
6428	GO:0009059|GO:004	macromolecule biosy	0.97	60	4.6728973	2003	5.7845035
9294	GO:0016052|GO:000	carbohydrate catabo	0.97	1	0.07788162	91	0.2628007
13547	GO:0031090	organelle membrane	0.97	4	0.31152648	224	0.64689404
13041	GO:0030554	adenyl nucleotide bir	0.97	13	1.0124611	550	1.5883559
14965	GO:0032559	adenyl ribonucleotide	0.97	13	1.0124611	550	1.5883559
11712	GO:0019748	secondary metabolic	0.97	1	0.07788162	95	0.27435237
9895	GO:0016860	intramolecular oxidor	0.97	2	0.15576324	146	0.42163628
17024	GO:0034645|GO:00	cellular macromolecu	0.97	59	4.5950155	1999	5.7729516
3884	GO:0005524	ATP binding	0.97	12	0.93457943	524	1.51327
6425	GO:0009056	catabolic process	0.98	28	2.1806853	1051	3.0352037
16851	GO:0034470	ncRNA processing	0.98	1	0.07788162	99	0.28590405
17039	GO:0034660	ncRNA metabolic pro	0.98	1	0.07788162	99	0.28590405
16430	GO:0034046	poly(G) RNA binding	0.98	1	0.07788162	100	0.28879198
5763	GO:0008233	peptidase activity	0.98	13	1.0124611	565	1.6316746
28378	GO:0070011	peptidase activity, ac	0.98	12	0.93457943	537	1.550813
9709	GO:0016627	oxidoreductase activ	0.98	1	0.07788162	104	0.30034366
3885	GO:0005525	GTP binding	0.98	4	0.31152648	245	0.70754033
11032	GO:0019001	guanyl nucleotide bir	0.98	4	0.31152648	245	0.70754033
14967	GO:0032561	guanyl ribonucleotide	0.98	4	0.31152648	245	0.70754033
6426	GO:0009057|GO:004	macromolecule catab	0.98	1	0.07788162	106	0.3061195
3942	GO:0005618	cell wall	0.98	1	0.07788162	107	0.30900744
12868	GO:0030312	external encapsulatir	0.99	1	0.07788162	111	0.3205591
5055	GO:0006996	organelle organizatio	0.99	2	0.15576324	164	0.47361887
25483	GO:0051186	cofactor metabolic p	0.99	2	0.15576324	164	0.47361887
29081	GO:0070717	poly-purine tract bind	0.99	1	0.07788162	112	0.32344702
14379	GO:0031966	mitochondrial memb	0.99	1	0.07788162	114	0.32922286
9889	GO:0016853	isomerase activity	0.99	5	0.3894081	306	0.8837035
5019	GO:0006952|GO:000	defense response	0.99	3	0.23364486	221	0.63823026
14910	GO:0032502	developmental proce	0.99	1	0.07788162	121	0.3494383
6884	GO:0009607	response to biotic sti	0.99	1	0.07788162	122	0.3523262
14392	GO:0031981	nuclear lumen	0.99	1	0.07788162	123	0.35521415
25485	GO:0051188	cofactor biosynthetic	0.99	1	0.07788162	123	0.35521415
131	GO:0000166	nucleotide binding	0.99	31	2.4143302	1213	3.5030468
4572	GO:0006412|GO:000	translation	0.99	2	0.15576324	180	0.5198256
8466	GO:0015031|GO:00	protein transport	0.99	2	0.15576324	185	0.53426516
20820	GO:0045184	establishment of prot	0.99	2	0.15576324	185	0.53426516
3863	GO:0005488	binding	0.99	474	36.91589	13903	40.15075
20499	GO:0044249	cellular biosynthetic	0.99	76	5.919003	2635	7.6096687
2657	GO:0003727|GO:000	single-stranded RNA	0.99	2	0.15576324	188	0.54292893
5669	GO:0008104	protein localization	0.99	2	0.15576324	189	0.54581684
30206	GO:0071842	cellular component o	0.99	2	0.15576324	193	0.5573685
19532	GO:0043233	organelle lumen	0.99	1	0.07788162	137	0.39564502
28380	GO:0070013	intracellular organelle	0.99	1	0.07788162	137	0.39564502
22415	GO:0046914	transition metal ion b	0.99	74	5.76324	2599	7.5057034
6427	GO:0009058	biosynthetic process	1.00	80	6.23053	2796	8.074624
5018	GO:0006950	response to stress	1.00	12	0.93457943	611	1.764519
14959	GO:0032553	ribonucleotide bindin	1.00	17	1.3239875	795	2.2958963
14961	GO:0032555	purine ribonucleotide	1.00	17	1.3239875	795	2.2958963
3955	GO:0005634	nucleus	1.00	36	2.8037384	1433	4.138389
10033	GO:0017076	purine nucleotide bir	1.00	17	1.3239875	797	2.3016722
18000	GO:0035639	purine ribonucleoside	1.00	16	1.2461059	769	2.2208104
4029	GO:0005740	mitochondrial envelo	1.00	1	0.07788162	151	0.4360759
3716	GO:0005198	structural molecule a	1.00	6	0.46728972	393	1.1349525
31895	GO:0090304	nucleic acid metabol	1.00	64	4.9844236	2340	6.7577324
14386	GO:0031974	membrane-enclosed	1.00	1	0.07788162	154	0.44473964
4558	GO:0006396|GO:000	RNA processing	1.00	2	0.15576324	219	0.63245445
20623	GO:0044429	mitochondrial part	1.00	1	0.07788162	165	0.47650677
4990	GO:0006915|GO:000	apoptosis	1.00	2	0.15576324	227	0.6555578
4357	GO:0006139|GO:005	nucleobase, nucleos	1.00	93	7.2429905	3285	9.486816
4605	GO:0006457|GO:000	protein folding	1.00	1	0.07788162	173	0.49961013
5756	GO:0008219	cell death	1.00	2	0.15576324	239	0.69021285
8209	GO:0012501|GO:00	programmed cell dea	1.00	2	0.15576324	239	0.69021285
9462	GO:0016265	death	1.00	2	0.15576324	239	0.69021285
20622	GO:0044428	nuclear part	1.00	1	0.07788162	178	0.5140497
8211	GO:0012505	endomembrane syste	1.00	2	0.15576324	242	0.6988766
5610	GO:0008026	ATP-dependent helic	1.00	1	0.07788162	183	0.52848935
28401	GO:0070035	purine NTP-depende	1.00	1	0.07788162	183	0.52848935
3068	GO:0004386	helicase activity	1.00	1	0.07788162	189	0.54581684
3178	GO:0004519	endonuclease activit	1.00	4	0.31152648	356	1.0280994
14380	GO:0031967	organelle envelope	1.00	1	0.07788162	201	0.5804719
14387	GO:0031975	envelope	1.00	1	0.07788162	205	0.59202355
17020	GO:0034641	cellular nitrogen com	1.00	102	7.9439254	3678	10.621769
2653	GO:0003723	RNA binding	1.00	14	1.0903426	797	2.3016722
4906	GO:0006807	nitrogen compound r	1.00	103	8.021807	3725	10.757502
2627	GO:0003676	nucleic acid binding	1.00	62	4.8286605	2453	7.0840673
4028	GO:0005739	mitochondrion	1.00	1	0.07788162	219	0.63245445
6796	GO:0009507	chloroplast	1.00	1	0.07788162	229	0.6613336

5791	GO:0008270	zinc ion binding	1.00	43	3.3489096	1880	5.4292893
6824	GO:0009536	plastid	1.00	1	0.07788162	247	0.7133162
3177	GO:0004518	nuclease activity	1.00	4	0.31152648	427	1.2331418
3903	GO:0005575|GO:000	cellular_component	1.00	260	20.249222	8515	24.590637
15392	GO:0032991	macromolecular com	1.00	34	2.647975	1616	4.666878
20640	GO:0044446	intracellular organell	1.00	9	0.7009346	710	2.0504231
20616	GO:0044422	organelle part	1.00	9	0.7009346	712	2.0561988
4459	GO:0006259|GO:005	DNA metabolic proce	1.00	4	0.31152648	493	1.4237444
3946	GO:0005623	cell	1.00	242	18.847351	8227	23.758917
20657	GO:0044464	cell part	1.00	242	18.847351	8227	23.758917
19526	GO:0043227	membrane-bounded	1.00	44	3.4267912	2142	6.185924
19530	GO:0043231	intracellular membra	1.00	44	3.4267912	2142	6.185924
3945	GO:0005622	intracellular	1.00	106	8.255452	4751	13.720507
4027	GO:0005737	cytoplasm	1.00	29	2.258567	2001	5.7787275
4109	GO:0005840|GO:003	ribosome	1.00	1	0.07788162	682	1.9695613
13018	GO:0030529	ribonucleoprotein co	1.00	1	0.07788162	741	2.1399486
19525	GO:0043226	organelle	1.00	49	3.8161993	3013	8.701303
19527	GO:0043228	non-membrane-bour	1.00	5	0.3894081	923	2.66555
19528	GO:0043229	intracellular organell	1.00	49	3.8161993	3013	8.701303
19531	GO:0043232	intracellular non-mer	1.00	5	0.3894081	923	2.66555
20618	GO:0044424	intracellular part	1.00	78	6.074766	3853	11.127155
20638	GO:0044444	cytoplasmic part	1.00	21	1.635514	1644	4.7477403
