# Supplementary material for: The Tomato Hybrid Proline-rich Protein regulates the abscission zone competence to respond to ethylene signals
Source: Hortic Res. 2018 Jun 1;5:28. doi: 10.1038/s41438-018-0033-2 (PMC5981600; doi:10.1038/s41438-018-0033-2)
Supplement: Supplementary file 7 — Table S7 [file 41438_2018_33_MOESM7_ESM.pdf]

Table S7. Differentially regulated genes (up and down regulated) in the control compared with THyPRP silenced plants in FAZ at 20 h along with their expression values, fold changes, annotations, GO ids and terms.

Worksheet 2 & 3

Set6					
Line	Array	Details	Time	Treated vs. Con	Set
Control – New Yorker	254331010027_1_3	20h_11_AZ	20h	Control	Set 6
Control – New Yorker	254331010027_1_4	20h_12_AZ	20h		
TAPG + TPRP	254331010036_1_3	20h_47_AZ	20h	Treated	
TAPG + TPRP	254331010036_1_4	20h_48_AZ	20h		

Note:

Fold change expression values are provided as logbase 2

Flagging Criteria:

Compromised: If the gProcessedSignal value is less than the background intensity

Detected: If the gProcessedSignal value is above background intensity

Column Header	Description
ProbeName	Unique probe identifier
GeneName	GeneSymbol
Fold	Fold change calculated is log base 2
Flag	Detected or Compromised
GeomeanFold	Average Fold change for the replicates calculated is log base 2
p-Value	t-test p- value
gProcessedSigna	Background substracted signal intensity

Cut off used to filter up and down regulated genes

Upregulated

For filtering upregulation we consider flag should be Detected in the treated sample and can be Compromised or Detected in the control sample and fold>=0.8 in the individual replicates and fold>=1 in the Geomean of treated samples.

Downregulated

For filtering downregulation we consider flag can be Detected or Compromised in the treated sample and should be Detected in the control sample and fold<=-0.8 in the individual replicates and fold<=-1 in the Geomean of treated samples.

Differentially Regulated Probes

Sample	Up	Down
20h_TPRP_ AZ vs. 20h_Control_AZ_Sense	1663	1160
20h_TPRP_ AZ vs. 20h_Control_AZ_Antisense	1029	807

Worksheet 4 & 5

This file contains the fold change for all the spots in the array

GO ID

Gene Ontology ID

GO ACCESSION

Gene Ontology Function name

p-value :

The probability of obtaining the specified GO accession number from a list of random entities. Less the p-value more significant is the GO accession number.

Significant pvalue<0.05

Count in Selection :

This refers to the number of genes in the selected entity (for example, from T-test) list which have that particular GO term.

%Count in Selection :

This refers to the percentage of genes in the input entity list which have that GO term.

Count in Total :

This refers to the number of genes in All Entities which have that GO term.

%Count in Total :

This refers to the percentage of genes in the All Entities list which have that GO term.

		20h_AZ_Control					20h_AZ_TAPG + TPRP vs. 20h_AZ_Control																														
ProbeNar	Orientatic	Fold 20h	Flag 20h	Fold 20h	Flag 20h	Geomean	Fold 20h	Flag 20h	Fold 20h	Flag 20h	Geomean	P-value	ProbeNar	GeneNam	Systemat	Descripti	Sequence	Contig ID	ITAG ID	Parent Ge	ITAG Des	Ontology	Ontology	Contig ID	Target ID	Functiona	GO terms	Transcrip	target ID	Symbols	TAIR Des	TAIR Chr	gProcess	gProcess	gProcess	gProcess	gProcess	gProcessedSignal 20h 48 AZ
A 96 P0(	Sense	0.305	Detected	-0.305	Detected	0.000	1.159	Detected	1.751	Detected	1.455	0.076	A 96 P0(	Ls	NM 0012	Solanum	CGCGTGGAGTGAAAACCCTAAATAACCAGATTTTCTAATGAAGTTGTAGTAGTAGAAATT																33.5284	26.1563	53.0063	38.8545	74.4734	94.2477
A 96 P01	Sense	-0.615	Detected	0.615	Detected	0.000	1.856	Detected	2.337	Detected	2.097	0.087	A 96 P01	AW94475	AW94475	EST3368	ATCGAGTTCGTCTTTTTGTTAACATTTTGGGTGATGTTGTACAAATTCCCAGAGTGACTT																583.877	1630.91	1875.08	618.03	3979.07	4663.16
A 96 P01	Sense	-0.262	Detected	0.262	Detected	0.000	1.181	Detected	1.499	Detected	1.340	0.049	A 96 P01	BI933034	BI933034	EST5529	CGAGACTTATGCCTTTGGCTTTAGCAAAGTAAACAACCATCTTGATGCATAGTTAATTAT																847.895	1451.07	2813.03	1139.95	2832.5	2964.92
A 96 P01	Sense	-0.487	Detected	0.487	Detected	0.000	2.952	Detected	1.262	Detected	2.107	0.163	A 96 P01	LOC5438	NM 0012	Solanum	AATAATGATAGAGTAAGGAGGATGTCAATTCATGATCCCACACCCACCTACTGTAGGTAG																77.8097	181.972	297.864	238.629	1037.05	269.908
A 96 P01	Sense	0.249	Comprom	-0.249	Comprom	0.000	3.566	Detected	3.208	Detected	3.387	0.008	A 96 P01	cevi19	NM 0012	Solanum	GAGATTATGGTGTGTTAAATAAAGTGTTCCACAATGTTACGGATACACATGTTTTGCATC																9.10174	7.67665	100.436	42.6946	111.52	73.024
A 96 P04	Sense	0.205	Detected	-0.205	Detected	0.000	1.372	Detected	0.843	Detected	1.108	0.080	A 96 P04	BI925751	BI925751	EST5456	CCCGTATACATTATCGGGCGACGATATACACTTGTTTTTAAAAAATGATACAGGGTATAA																278.702	249.802	626.253	584.776	769.352	447.631
A 96 P05	Sense	0.053	Detected	-0.053	Detected	0.000	2.165	Detected	1.518	Detected	1.841	0.030	A 96 P05	BM53511	BM53511	EST5881	CAAAGCTTACTCATGAAGCTGCAACTAACTTGGTCCTGTCGAAATCTTTTGTTTCAAAGA																2385.64	2637.09	7236.5	8177.48	12671.2	6791.29
A 96 P05	Sense	0.549	Detected	-0.549	Detected	0.000	1.830	Detected	1.577	Detected	1.704	0.094	A 96 P05	AI483528	AI483528	EST2493	TATATTTGTTCGTAGTTTACTAGCTAGTCTGAGTCGTATAGGGTCCTAAAGGGAGAAATA																2148.08	1194.1	4600.82	3141.76	6415.69	4518.2
A 96 P05	Sense	-0.284	Detected	0.284	Detected	0.000	1.608	Detected	1.338	Detected	1.473	0.043	A 96 P05	FS203485	FS203485	FS203485	AATCCTATTTCCGCAATCTCCGCTCGCGGAAATAACTAATTTCGGTATTATTATTATTTG																417.316	736.77	994.655	1055.14	1904.49	1325.58
A 96 P07	Sense	0.214	Detected	-0.214	Detected	0.000	1.236	Detected	1.679	Detected	1.457	0.042	A 96 P07	DB70200	DB70200	DB70200	GGAGCGCTTTATCTCTAATGCACCTACTCGATGTAAATTTGTGAATCTTAATAGTAAGTG																245.797	217.452	223.614	378.567	613.336	699.715
A 96 P07	Sense	0.365	Detected	-0.365	Detected	0.000	1.707	Detected	1.778	Detected	1.742	0.042	A 96 P07	P69B	NM 0012	Solanum	GCCTAGTCAAAAATTGGCTATATAAGTGCATTAAGTACTCGGACATTTGAAAATGCTGTT																486.301	348.956	1057.14	1411.27	1514.59	1335.99
A 96 P07	Sense	-0.903	Detected	0.903	Detected	0.000	1.011	Detected	1.422	Detected	1.217	0.319	A 96 P07	LOC5440	NM 0012	Solanum	CTTTGTCCATTTTGCCGTGGTGATGGCTTCTGTGCAAAAGTGGGTGTAATTAATCAAGAT																4180.2	17394.4	14493.2	5672.07	19364.1	21611.5
A 96 P07	Sense	-0.606	Detected	0.606	Detected	0.000	4.154	Detected	1.461	Detected	2.807	0.198	A 96 P07	ADH2	NM 0012	Solanum	GCTTAGATGTTGTGCTTTGCCATATCTCTTTCGATTCTTGTAAAAAATGCAAATTCTCTC																62.4152	172.107	249.151	232.143	2078.85	269.714
A 96 P07	Sense	0.403	Detected	-0.403	Detected	0.000	1.406	Detected	1.444	Detected	1.425	0.072	A 96 P07	TA36339	TA36339	Unknown	CATGCTGTGCCTCTGGTTGTAACTGATAATATTATTAAGACTGATGTTGGAATTTGTGGT																17092.9	11646.6	24902.9	22790.1	42105.4	36306.6
A 96 P08	Sense	-0.057	Detected	0.057	Detected	0.000	1.080	Detected	1.838	Detected	1.459	0.063	A 96 P08	LOC7783	AK32079	Solanum	TATGTTTAGCATTCGGCGGTGTAGCCAATGTTGATAAAGGACAAGCTGTGAAGGATGCTG																55.2333	71.1616	183.734	124.91	149.252	211.968
A 96 P08	Sense	-0.010	Detected	0.010	Detected	0.000	1.098	Detected	1.008	Detected	1.053	0.002	A 96 P08	P69B	NM 0012	Solanum	TGATACACCATTCGAGGACTATGTACCTTATTTATGTGGTTTGAAGTACACAGATCAACA																232.561	280.658	442.953	405.768	616.223	485.922
A 96 P08	Sense	0.437	Detected	-0.437	Detected	0.000	1.288	Detected	1.361	Detected	1.324	0.094	A 96 P08	TA36340	TA36340	Rep: Hist	AAATAAGCAAAGCAGGGGAAGTTATAGCTGAATGCGGAGAAGGTTGCATTTGCTTTGTGG																11935.7	7749.87	14702	12342.8	26454.6	23350.6
A 96 P08	Sense	0.176	Detected	-0.176	Detected	0.000	1.162	Detected	1.554	Detected	1.358	0.036	A 96 P08	TA36375	TA36375	Unknown	GCTCTGGACAGAGAGCTTCATTAAGATTAAAACTACTACAACGACATAAATTGGACTTAA																26.8401	25.0308	46.94	36.9084	65.313	71.9757
[illegible]	[illegible]	[illegible]	[illegible]	[illegible]	[illegible]	[illegible]	[illegible]	[illegible]	[illegible]	[illegible]	[illegible]	[illegible]	[illegible]	[illegible]	[illegible]	[illegible]	[illegible]	[illegible]	[illegible]	[illegible]	[illegible]	[illegible]	[illegible]	[illegible]	[illegible]	[illegible]	[illegible]	[illegible]	[illegible]	[illegible]	[illegible]	[illegible]	[illegible]	[illegible]	[illegible]	[illegible]	[illegible]	[illegible]

GT	Sense	Sense	-0.172	Comprom	0.172	Comprom	0.000	0.838	Detected	1.841	Detected	1.340	0.127	GT	Sens	contig168	contig168	Cytokinin	CCCAGC	contig168	Solyc11g	Solyc11g	Cytokinin riboside 5%26apos%	[illegible]
GT	Sense	Sense	0.031	Comprom	-0.031	Comprom	0.000	1.401	Detected	1.605	Detected	1.503	0.005	GT	Sens	contig168	contig168	Unknown	CATTAG	contig168	Solyc01g	Solyc01g	Unknown Protein (AHRD V1)%:	[illegible]
GT	Sense	Sense	-0.467	Comprom	0.467	Comprom	0.000	1.926	Detected	2.635	Detected	2.281	0.060	GT	Sens	contig168	contig168	Multidrug	GAACAC	contig168	Solyc06g	Solyc06g	Multidrug GO:0015 GO:0015	[illegible]
GT	Sense	Sense	-0.274	Detected	0.274	Detected	0.000	3.031	Detected	2.637	Detected	2.834	0.014	GT	Sens	contig169	contig169	40S ribos	TGATGT	contig169	Solyc05g	Solyc05g	40S ribos GO:0003 GO:0003	[illegible]
GT	Sense	Sense	-0.254	Detected	0.254	Detected	0.000	1.297	Detected	1.878	Detected	1.588	0.054	GT	Sens	contig169	contig169	Unknown	TCTTTT	contig169	Solyc03g	Solyc03g	Unknown Protein (AHRD V1)	[illegible]
GT	Sense	Sense	0.170	Detected	-0.170	Detected	0.000	1.129	Detected	1.049	Detected	1.089	0.025	GT	Sens	contig170	contig170	Prolyl 4-h	CAGCAG	contig170	Solyc02g	Solyc02g	Prolyl 4-h GO:0016 GO:0016	[illegible]
GT	Sense	Sense	0.187	Detected	-0.187	Detected	0.000	1.084	Detected	1.479	Detected	1.282	0.042	GT	Sens	contig171	contig171	Protein L	GAGCAT	contig171	Solyc03g	Solyc03g	Protein LSM14 homolog A-B (A	[illegible]
GT	Sense	Sense	0.731	Comprom	-0.731	Comprom	0.000	0.878	Detected	1.710	Detected	1.294	0.264	GT	Sens	contig171	contig171	Heavy me	ATTTGT	contig171	Solyc07g	Solyc07g	Heavy me GO:0030 GO:0030	[illegible]
GT	Sense	Sense	0.434	Detected	-0.434	Detected	0.000	1.372	Detected	1.877	Detected	1.624	0.084	GT	Sens	contig171	contig171	Myb-relat	TTGGAC	contig171	Solyc12g	Solyc12g	Myb-relat GO:0045449	[illegible]
GT	Sense	Sense	-0.730	Detected	0.730	Detected	0.000	1.282	Detected	0.921	Detected	1.101	0.280	GT	Sens	contig172	contig172	Transme	AGTAAG	contig172	Solyc07g	Solyc07g	Transme GO:0016 GO:0016	[illegible]
GT	Sense	Sense	0.336	Comprom	-0.336	Comprom	0.000	2.328	Detected	2.452	Detected	2.390	0.020	GT	Sens	contig172	contig172	GTP bind	AATTTT	contig172	Solyc08g	Solyc08g	GTP binding protein (AHRD V1	[illegible]
GT	Sense	Sense	0.315	Detected	-0.315	Detected	0.000	1.260	Detected	1.415	Detected	1.337	0.054	GT	Sens	contig173	contig173	Zinc finge	ATAGAT	contig173	Solyc09g	Solyc09g	Zinc finge GO:0005 GO:0005	[illegible]
GT	Sense	Sense	0.287	Comprom	-0.287	Comprom	0.000	1.695	Detected	1.491	Detected	1.593	0.035	GT	Sens	contig173	contig173	Transpos	GTCAGA	contig173	Solyc02g	Solyc02g	Transposase (Fragment) (AHR	[illegible]
GT	Sense	Sense	-0.116	Detected	0.116	Detected	0.000	1.586	Detected	1.462	Detected	1.524	0.007	GT	Sens	contig173	contig173	Unknown	AATTTC	contig173	Solyc00g	Solyc00g	Unknown Protein (AHRD V1)	[illegible]
GT	Sense	Sense	0.197	Detected	-0.197	Detected	0.000	0.998	Detected	1.173	Detected	1.085	0.037	GT	Sens	contig174	contig174	LINE-type	TTGTTT	contig174	Solyc04g	Solyc04g	LINE-type retrotransposon LIb	[illegible]
GT	Sense	Sense	-0.388	Detected	0.388	Detected	0.000	4.327	Detected	2.158	Detected	3.242	0.106	GT	Sens	contig174	contig174	Ferredox	CTCGTG	contig174	Solyc11g	Solyc11g	Ferredox GO:0051 GO:0051	[illegible]
GT	Sense	Sense	-0.064	Detected	0.064	Detected	0.000	1.006	Detected	1.226	Detected	1.116	0.013	GT	Sens	contig174	contig174	Unknown	TTTAAT	contig174	Solyc02g	Solyc02g	Unknown Protein (AHRD V1)	[illegible]
GT	Sense	Sense	-0.227	Detected	0.227	Detected	0.000	2.334	Detected	1.945	Detected	2.140	0.019	GT	Sens	contig175	contig175	ATP-bind	GTGATT	contig175	Solyc11g	Solyc11g	ATP-bind GO:0016 GO:0016	[illegible]
GT	Sense	Sense	0.799	Detected	-0.799	Detected	0.000	1.425	Detected	1.240	Detected	1.332	0.239	GT	Sens	contig175	contig175	Pollen all	GGCAGA	contig175	Solyc06g	Solyc06g	Pollen allergen (AHRD V1 *-* C	[illegible]
GT	Sense	Sense	0.279	Detected	-0.279	Detected	0.000	0.993	Detected	1.380	Detected	1.187	0.073	GT	Sens	contig175	contig175	Unknown	TCTGGT	contig175	Solyc04g	Solyc04g	Unknown Protein (AHRD V1)	[illegible]
GT	Sense	Sense	0.081	Detected	-0.081	Detected	0.000	1.464	Detected	1.380	Detected	1.422	0.004	GT	Sens	contig175	contig175	Magnesiu	TGGTAT	contig175	Solyc03g	Solyc03g	Magnesiu GO:0005 GO:0005	[illegible]
GT	Sense	Sense	0.128	Detected	-0.128	Detected	0.000	1.169	Detected	1.791	Detected	1.480	0.048	GT	Sens	contig176	contig176	Genomic	TGAACA	contig176	Solyc10g	Solyc10g	Genomic DNA chromosome 5	[illegible]
GT	Sense	Sense	0.232	Detected	-0.232	Detected	0.000	1.241	Detected	1.003	Detected	1.122	0.050	GT	Sens	contig177	contig177	Abscisic	AATTAC	contig177	Solyc03g	Solyc03g	Abscisic GO:0004 GO:0004	[illegible]
GT	Sense	Sense	0.104	Detected	-0.104	Detected	0.000	1.601	Detected	1.090	Detected	1.345	0.040	GT	Sens	contig177	contig177	L-tyrosin	GGTATT	contig177	Solyc12g	Solyc12g	L-tyrosine transporter (AHRD V	[illegible]
GT	Sense	Sense	-0.192	Detected	0.192	Detected	0.000	1.093	Detected	0.941	Detected	1.017	0.039	GT	Sens	contig178	contig178	Unknown	CAATTG	contig178	Solyc01g	Solyc01g	Unknown Protein (AHRD V1)	[illegible]
GT	Sense	Sense	0.527	Detected	-0.527	Detected	0.000	1.273	Detected	1.884	Detected	1.579	0.122	GT	Sens	contig178	contig178	Glutathio	ATACAT	contig178	Solyc10g	Solyc10g	Glutathio GO:0004 GO:0004	[illegible]
GT	Sense	Sense	-0.581	Detected	0.581	Detected	0.000	4.337	Detected	1.005	Detected	2.671	0.269	GT	Sens	contig179	contig179	Cytochro	TTCGAC	contig179	Solyc06g	Solyc06g	Cytochro GO:0019 GO:0019	[illegible]
[illegible]																								

GT	Sense	Sense	0.415	Comprom	-0.415	Comprom	0.000	1.869	Detected	3.376	Detected	2.622	0.093	GT	Sens	contig240	contig240	Collagen	CTAGTG
GT	Sense	Sense	-0.316	Comprom	0.316	Comprom	0.000	2.434	Detected	2.679	Detected	2.556	0.017	GT	Sens	contig241	contig241	Unknown	AGCTCA
GT	Sense	Sense	-0.078	Comprom	0.078	Comprom	0.000	5.379	Detected	2.761	Detected	4.070	0.090	GT	Sens	contig241	contig241	F-box fan	AATCGA
GT	Sense	Sense	-0.105	Detected	0.105	Detected	0.000	1.159	Detected	1.087	Detected	1.123	0.010	GT	Sens	contig241	contig241	Unknown	CAGAAA
GT	Sense	Sense	0.089	Detected	-0.089	Detected	0.000	1.289	Detected	1.857	Detected	1.573	0.034	GT	Sens	contig241	contig241	cDNA clo	TCTGAT
GT	Sense	Sense	0.220	Detected	-0.220	Detected	0.000	0.855	Detected	1.409	Detected	1.132	0.085	GT	Sens	contig242	contig242	Sensitivit	CCTTAA
GT	Sense	Sense	-0.015	Detected	0.015	Detected	0.000	1.303	Detected	1.263	Detected	1.283	0.000	GT	Sens	contig242	contig242	YABBY-li	TCTACA
GT	Sense	Sense	-0.283	Detected	0.283	Detected	0.000	1.094	Detected	1.710	Detected	1.402	0.079	GT	Sens	contig242	contig242	Glucan e	GTGATT
GT	Sense	Sense	-0.065	Comprom	0.065	Comprom	0.000	0.901	Detected	2.247	Detected	1.574	0.145	GT	Sens	contig242	contig242	Unknown	CCCGGA
GT	Sense	Sense	-0.173	Comprom	0.173	Comprom	0.000	1.625	Detected	3.761	Detected	2.693	0.131	GT	Sens	contig242	contig242	Unknown	AGGCAA
GT	Sense	Sense	0.061	Detected	-0.061	Detected	0.000	1.228	Detected	1.913	Detected	1.570	0.046	GT	Sens	contig243	contig243	Thioredo	CCACAA
GT	Sense	Sense	0.430	Detected	-0.430	Detected	0.000	1.545	Detected	1.338	Detected	1.441	0.083	GT	Sens	contig243	contig243	Nuclear t	TTGATG
GT	Sense	Sense	0.018	Comprom	-0.018	Comprom	0.000	1.401	Detected	2.552	Detected	1.977	0.075	GT	Sens	contig243	contig243	Non-LTR	AATCCA
GT	Sense	Sense	-0.238	Comprom	0.238	Comprom	0.000	1.146	Detected	1.646	Detected	1.396	0.056	GT	Sens	contig244	contig244	DNA repa	ATGAGG
GT	Sense	Sense	0.536	Detected	-0.536	Detected	0.000	1.517	Detected	1.200	Detected	1.358	0.136	GT	Sens	contig245	contig245	Basic hel	TTTCTC
GT	Sense	Sense	0.238	Comprom	-0.238	Comprom	0.000	2.197	Detected	2.878	Detected	2.538	0.026	GT	Sens	contig245	contig245	Unknown	AGGATT
GT	Sense	Sense	0.324	Comprom	-0.324	Comprom	0.000	1.859	Detected	1.528	Detected	1.694	0.043	GT	Sens	contig246	contig246	PVR3-like	TATGCA
GT	Sense	Sense	0.748	Detected	-0.748	Detected	0.000	1.098	Detected	0.932	Detected	1.015	0.310	GT	Sens	contig246	contig246	Glutamat	ATAACA
GT	Sense	Sense	0.049	Comprom	-0.049	Comprom	0.000	1.245	Detected	2.143	Detected	1.694	0.064	GT	Sens	contig246	contig246	TNFR/CD	GCCAAG
GT	Sense	Sense	-0.030	Comprom	0.030	Comprom	0.000	1.974	Detected	2.558	Detected	2.266	0.016	GT	Sens	contig247	contig247	Cytochro	CAAGCA
GT	Sense	Sense	0.971	Comprom	-0.971	Comprom	0.000	1.381	Detected	2.607	Detected	1.994	0.225	GT	Sens	contig247	contig247	F-box/LR	ATTGTA
GT	Sense	Sense	0.343	Detected	-0.343	Detected	0.000	2.168	Detected	1.430	Detected	1.799	0.070	GT	Sens	contig247	contig247	Unknown	GAAGCT
GT	Sense	Sense	0.472	Detected	-0.472	Detected	0.000	1.432	Detected	1.894	Detected	1.663	0.087	GT	Sens	contig248	contig248	Unknown	CATGGG
GT	Sense	Sense	-0.357	Comprom	0.357	Detected	0.000	0.869	Detected	1.470	Detected	1.169	0.129	GT	Sens	contig248	contig248	Mutator-li	TCGAAA
GT	Sense	Sense	-0.316	Comprom	0.316	Comprom	0.000	0.849	Detected	2.027	Detected	1.438	0.165	GT	Sens	contig248	contig248	Pentatric	AAGACC
GT	Sense	Sense	-0.387	Comprom	0.387	Comprom	0.000	1.483	Detected	2.166	Detected	1.825	0.071	GT	Sens	contig248	contig248	Unknown	ATTCTT

[illegible]

GT	Sense	Sense	-0.076	Comprom	0.076	Comprom	0.000	1.128	Detected	2.293	Detected	1.710	0.100	GT	Sens	contig317	contig317	Unannota	GAACAATGAGGG
GT	Sense	Sense	-0.338	Comprom	0.338	Comprom	0.000	1.481	Detected	2.230	Detected	1.856	0.067	GT	Sens	contig317	contig317	Unannota	AAGTGAGTGAGA
GT	Sense	Sense	0.491	Comprom	-0.491	Comprom	0.000	2.606	Detected	2.755	Detected	2.681	0.033	GT	Sens	contig317	contig317	Unannota	CACCCCAAGCTG
GT	Sense	Sense	0.725	Detected	-0.725	Detected	0.000	3.538	Detected	1.125	Detected	2.331	0.239	GT	Sens	contig317	contig317	DUF599 f	TTCTTGT contig31
GT	Sense	Sense	0.185	Detected	-0.185	Detected	0.000	1.381	Detected	1.508	Detected	1.444	0.018	GT	Sens	contig318	contig318	Unannota	GGTATTGAAAAA
GT	Sense	Sense	-1.453	Comprom	1.453	Detected	0.000	1.728	Detected	1.882	Detected	1.805	0.341	GT	Sens	contig318	contig318	Unannota	GGAATGCAACAT
GT	Sense	Sense	-0.790	Comprom	0.790	Detected	0.000	0.927	Detected	1.146	Detected	1.036	0.323	GT	Sens	contig318	contig318	Unannota	ACAAAACCAGCA
GT	Sense	Sense	0.217	Comprom	-0.217	Comprom	0.000	2.028	Detected	3.077	Detected	2.552	0.046	GT	Sens	contig318	contig318	Unannota	TTACGGAAGAGT
GT	Sense	Sense	0.971	Detected	-0.971	Detected	0.000	1.172	Detected	1.215	Detected	1.194	0.344	GT	Sens	contig318	contig318	Unannota	AGATCATAACTG
GT	Sense	Sense	-0.744	Comprom	0.744	Detected	0.000	1.811	Detected	2.090	Detected	1.950	0.123	GT	Sens	contig319	contig319	Unannota	CTATATTTTTAG
GT	Sense	Sense	0.056	Detected	-0.056	Detected	0.000	0.922	Detected	1.569	Detected	1.246	0.063	GT	Sens	contig319	contig319	Unannota	CCACCAACTTCA
GT	Sense	Sense	0.485	Detected	-0.485	Detected	0.000	1.054	Detected	1.032	Detected	1.043	0.165	GT	Sens	contig319	contig319	Unannota	AGGAGAGGGAG
GT	Sense	Sense	0.642	Detected	-0.642	Detected	0.000	1.379	Detected	1.109	Detected	1.244	0.198	GT	Sens	contig319	contig319	Unannota	AGCCTGATAAAG
GT	Sense	Sense	0.199	Comprom	-0.199	Comprom	0.000	1.172	Detected	2.311	Detected	1.741	0.102	GT	Sens	contig320	contig320	Unannota	GGGAGGCAGAAT
GT	Sense	Sense	0.940	Detected	-0.940	Comprom	0.000	0.977	Detected	1.110	Detected	1.043	0.383	GT	Sens	contig320	contig320	Unannota	ATTCAATAATTGA
GT	Sense	Sense	0.200	Detected	-0.200	Detected	0.000	1.406	Detected	1.658	Detected	1.532	0.023	GT	Sens	contig320	contig320	Unannota	CCAAGGCTTCAC
GT	Sense	Sense	0.947	Detected	-0.947	Detected	0.000	1.000	Detected	1.303	Detected	1.151	0.353	GT	Sens	contig320	contig320	Unannota	CAGAAATCTGTC
GT	Sense	Sense	-0.585	Comprom	0.585	Comprom	0.000	1.824	Detected	1.277	Detected	1.550	0.138	GT	Sens	contig320	contig320	Unannota	TCTGTAGGAAGT
GT	Sense	Sense	-0.147	Detected	0.147	Detected	0.000	1.884	Detected	1.316	Detected	1.600	0.038	GT	Sens	contig320	contig320	Unannota	AAACGTGTCACC
GT	Sense	Sense	0.160	Detected	-0.160	Detected	0.000	1.063	Detected	1.199	Detected	1.131	0.023	GT	Sens	contig320	contig320	Unannota	GGTAGAGAGATT
GT	Sense	Sense	0.013	Detected	-0.013	Detected	0.000	1.108	Detected	0.944	Detected	1.026	0.006	GT	Sens	contig321	contig321	Unannota	GGAGAGCTGACC
GT	Sense	Sense	-0.291	Detected	0.291	Detected	0.000	1.706	Detected	1.834	Detected	1.770	0.027	GT	Sens	contig321	contig321	Unannota	GGTATAAAGATG
GT	Sense	Sense	-0.074	Detected	0.074	Detected	0.000	1.375	Detected	0.990	Detected	1.182	0.029	GT	Sens	contig321	contig321	Unannota	CATCGCACAAGT
GT	Sense	Sense	-0.144	Detected	0.144	Detected	0.000	1.504	Detected	1.434	Detected	1.469	0.010	GT	Sens	contig321	contig321	Unannota	TACAGTGTCCTT
GT	Sense	Sense	-0.596	Comprom	0.596	Comprom	0.000	0.850	Detected	1.618	Detected	1.234	0.224	GT	Sens	contig322	contig322	Unannota	AGAAAGAAGAGA
GT	Sense	Sense	0.088	Comprom	-0.088	Comprom	0.000	0.935	Detected	1.100	Detected	1.018	0.014	GT	Sens	contig323	contig323	Ycf2 (AHF	AAATAAC contig32

[illegible]

GT	Sense	Sense	-0.457	Comprom	0.457	Detected	0.000	1.681	Detected	1.826	Detected	1.754	0.063	GT Sens	[illegible]
GT	Sense	Sense	-0.444	Detected	0.444	Detected	0.000	1.187	Detected	1.419	Detected	1.303	0.105	GT Sens	[illegible]
GT	Sense	Sense	0.105	Detected	-0.105	Detected	0.000	0.946	Detected	1.151	Detected	1.048	0.019	GT Sens	[illegible]
GT	Sense	Sense	1.085	Detected	-1.085	Comprom	0.000	1.100	Detected	1.445	Detected	1.272	0.367	GT Sens	[illegible]
GT	Sense	Sense	0.311	Comprom	-0.311	Comprom	0.000	0.808	Detected	1.723	Detected	1.265	0.149	GT Sens	[illegible]
GT	Sense	Sense	-0.065	Detected	0.065	Detected	0.000	1.726	Detected	2.051	Detected	1.889	0.009	GT Sens	[illegible]
GT	Sense	Sense	0.063	Detected	-0.063	Detected	0.000	0.942	Detected	1.207	Detected	1.074	0.018	GT Sens	[illegible]
GT	Sense	Sense	0.292	Detected	-0.292	Comprom	0.000	1.462	Detected	2.285	Detected	1.873	0.065	GT Sens	[illegible]
GT	Sense	Sense	0.232	Comprom	-0.232	Comprom	0.000	1.422	Detected	1.725	Detected	1.574	0.030	GT Sens	[illegible]
GT	Sense	Sense	-0.215	Comprom	0.215	Comprom	0.000	2.591	Detected	2.596	Detected	2.594	0.007	GT Sens	[illegible]
GT	Sense	Sense	0.556	Detected	-0.556	Detected	0.000	1.663	Detected	2.417	Detected	2.040	0.093	GT Sens	[illegible]
GT	Sense	Sense	0.332	Detected	-0.332	Detected	0.000	1.086	Detected	1.099	Detected	1.093	0.081	GT Sens	[illegible]
GT	Sense	Sense	0.446	Comprom	-0.446	Comprom	0.000	1.162	Detected	1.830	Detected	1.496	0.115	GT Sens	[illegible]
GT	Sense	Sense	0.362	Comprom	-0.362	Comprom	0.000	2.093	Detected	1.583	Detected	1.838	0.053	GT Sens	[illegible]
GT	Sense	Sense	0.338	Comprom	-0.338	Comprom	0.000	2.517	Detected	1.392	Detected	1.954	0.097	GT Sens	[illegible]
GT	Sense	Sense	-0.536	Detected	0.536	Detected	0.000	1.441	Detected	1.009	Detected	1.225	0.168	GT Sens	[illegible]
GT	Sense	Sense	0.029	Detected	-0.029	Detected	0.000	1.925	Detected	1.939	Detected	1.932	0.000	GT Sens	[illegible]
GT	Sense	Sense	0.387	Comprom	-0.387	Comprom	0.000	1.258	Detected	1.523	Detected	1.390	0.077	GT Sens	[illegible]
GT	Sense	Sense	-0.204	Detected	0.204	Detected	0.000	0.925	Detected	1.524	Detected	1.225	0.077	GT Sens	[illegible]
GT	Sense	Sense	-0.140	Comprom	0.140	Comprom	0.000	0.940	Detected	1.838	Detected	1.389	0.098	GT Sens	[illegible]
GT	Sense	Sense	0.118	Detected	-0.118	Detected	0.000	2.448	Detected	3.503	Detected	2.976	0.031	GT Sens	[illegible]
GT	Sense	Sense	0.165	Comprom	-0.165	Comprom	0.000	0.986	Detected	1.408	Detected	1.197	0.047	GT Sens	[illegible]
GT	Sense	Sense	0.274	Detected	-0.274	Detected	0.000	2.521	Detected	1.232	Detected	1.877	0.116	GT Sens	[illegible]
GT	Sense	Sense	0.193	Detected	-0.193	Detected	0.000	2.938	Detected	1.436	Detected	2.187	0.106	GT Sens	[illegible]
GT	Sense	Sense	0.097	Detected	-0.097	Detected	0.000	2.279	Detected	0.894	Detected	1.586	0.151	GT Sens	[illegible]
GT	Sense	Sense	-0.062	Comprom	0.062	Comprom	0.000	1.370	Detected	2.868	Detected	2.119	0.106	GT Sens	[illegible]
GT	Sense	Sense	-0.056	Comprom	0.056	Comprom	0.000	2.498	Detected	2.151	Detected	2.324	0.006	GT Sens	[illegible]
GT	Sense	Sense	0.285	Detected	-0.285	Detected	0.000	1.254	Detected	1.044	Detected	1.149	0.063	GT Sens	[illegible]
GT	Sense	Sense	0.545	Comprom	-0.545	Comprom	0.000	1.379	Detected	2.040	Detected	1.710	0.115	GT Sens	[illegible]
GT	Sense	Sense	0.389	Detected	-0.389	Detected	0.000	1.322	Detected	1.673	Detected	1.498	0.072	GT Sens	[illegible]
GT	Sense	Sense	-0.055	Detected	0.055	Detected	0.000	1.278	Detected	1.446	Detected	1.362	0.005	GT Sens	[illegible]
GT	Sense	Sense	-0.436	Comprom	0.436	Comprom	0.000	1.707	Detected	2.351	Detected	2.029	0.064	GT Sens	[illegible]
GT	Sense	Sense	-0.180	Comprom	0.180	Comprom	0.000	1.622	Detected	3.770	Detected	2.696	0.132	GT Sens	[illegible]
GT	Sense	Sense	0.555	Comprom	-0.555	Comprom	0.000	1.349	Detected	2.393	Detected	1.871	0.133	GT Sens	[illegible]
GT	Sense	Sense	-0.188	Detected	0.188	Detected	0.000	1.591	Detected	1.003	Detected	1.297	0.065	GT Sens	[illegible]
GT	Sense	Sense	0.063	Detected	-0.063	Detected	0.000	1.207	Detected	1.387	Detected	1.297	0.007	GT Sens	[illegible]
GT	Sense	Sense	0.160	Detected	-0.160	Detected	0.000	2.236	Detected	2.416	Detected	2.326	0.006	GT Sens	[illegible]
GT	Sense	Sense	0.133	Detected	-0.133	Detected	0.000	2.201	Detected	0.945	Detected	1.573	0.134	GT Sens	[illegible]
GT	Sense	Sense	-0.024	Comprom	0.024	Comprom	0.000	1.051	Detected	3.279	Detected	2.165	0.191	GT Sens	[illegible]
GT	Sense	Sense	0.404	Detected	-0.404	Detected	0.000	1.159	Detected	0.843	Detected	1.001	0.148	GT Sens	[illegible]
GT	Sense	Sense	0.225	Comprom	-0.225	Comprom	0.000	1.355	Detected	1.961	Detected	1.658	0.048	GT Sens	[illegible]
GT	Sense	Sense	-0.544	Detected	0.544	Detected	0.000	1.242	Detected	1.296	Detected	1.269	0.145	GT Sens	[illegible]
GT	Sense	Sense	0.163	Comprom	-0.163	Comprom	0.000	2.169	Detected	2.829	Detected	2.499	0.021	GT Sens	[illegible]
GT	Sense	Sense	-0.728	Comprom	0.728	Detected	0.000	0.860	Detected	1.378	Detected	1.119	0.285	GT Sens	[illegible]
GT	Sense	Sense	-0.043	Detected	0.043	Detected	0.000	0.893	Detected	1.190	Detected	1.041	0.021	GT Sens	[illegible]
GT	Sense	Sense	-0.019	Detected	0.019	Detected	0.000	1.223	Detected	1.029	Detected	1.126	0.008	GT Sens	[illegible]
GT	Sense	Sense	-0.211	Comprom	0.211	Comprom	0.000	0.991	Detected	2.088	Detected	1.540	0.120	GT Sens	[illegible]
GT	Sense	Sense	0.240	Comprom	-0.240	Comprom	0.000	1.600	Detected	2.952	Detected	2.276	0.087	GT Sens	[illegible]
GT	Sense	Sense	-0.363	Detected	0.363	Detected	0.000	1.376	Detected	1.191	Detected	1.284	0.076	GT Sens	[illegible]
GT	Sense	Sense	0.558	Comprom	-0.558	Comprom	0.000	1.206	Detected	2.528	Detected	1.867	0.163	GT Sens	[illegible]
GT	Sense	Sense	0.268	Detected	-0.268	Detected	0.000	1.463	Detected	1.022	Detected	1.243	0.070	GT Sens	[illegible]
GT	Sense	Sense	0.117	Detected	-0.117	Detected	0.000	2.598	Detected	2.045	Detected	2.321	0.016	GT Sens	[illegible]
GT	Sense	Sense	-0.429	Comprom	0.429	Detected	0.000	1.080	Detected	1.403	Detected	1.242	0.113	GT Sens	[illegible]
GT	Sense	Sense	0.271	Detected	-0.271	Detected	0.000	1.640	Detected	0.930	Detected	1.285	0.102	GT Sens	[illegible]
GT	Sense	Sense	-0.121	Detected	0.121	Detected	0.000	1.254	Detected	0.888	Detected	1.071	0.039	GT Sens	[illegible]
GT	Sense	Sense	-0.193	Comprom	0.193	Detected	0.000	1.319	Detected	1.042	Detected	1.181	0.038	GT Sens	[illegible]
GT	Sense	Sense	0.312	Detected	-0.312	Detected	0.000	1.781	Detected	1.416	Detected	1.599	0.047	GT Sens	[illegible]
GT	Sense	Sense	0.277	Detected	-0.277	Detected	0.000	1.756	Detected	1.484	Detected	1.620	0.034	GT Sens	[illegible]
GT	Sense	Sense	0.334	Detected	-0.334	Detected	0.000	1.314	Detected	1.666	Detected	1.490	0.059	GT Sens	[illegible]
GT	Sense	Sense	-1.093	Comprom	1.093	Detected	0.000	1.253	Detected	1.277	Detected	1.265	0.367	GT Sens	[illegible]
GT	Sense	Sense	-0.570	Comprom	0.570	Comprom	0.000	2.149	Detected	1.967	Detected	2.058	0.070	GT Sens	[illegible]
GT	Sense	Sense	-0.483	Comprom	0.483	Comprom	0.000	3.752	Detected	5.593	Detected	4.672	0.046	GT Sens	[illegible]
GT	Sense	Sense	0.838	Detected	-0.838	Detected	0.000	2.263	Detected	1.560	Detected	1.911	0.170	GT Sens	[illegible]
GT	Sense	Sense	0.911	Detected	-0.911	Detected	0.000	2.124	Detected	1.281	Detected	1.702	0.232	GT Sens	[illegible]
GT	Sense	Sense	-0.302	Comprom	0.302	Comprom	0.000	1.118	Detected	1.938	Detected	1.528	0.095	GT Sens	[illegible]
GT	Sense	Sense	0.382	Detected	-0.382	Detected	0.000	0.887	Detected	1.280	Detected	1.083	0.128	GT Sens	[illegible]
GT	Sense	Sense	0.309	Detected	-0.309	Detected	0.000	1.260	Detected	1.565	Detected	1.412	0.055	GT Sens	[illegible]
GT	Sense	Sense	0.722	Comprom	-0.722	Comprom	0.000	3.691	Detected	3.778	Detected	3.735	0.036	GT Sens	[illegible]
GT	Sense	Sense	0.136	Comprom	-0.136	Comprom	0.000	0.816	Detected	1.485	Detected	1.150	0.086	GT Sens	[illegible]
GT	Sense	Sense	0.006	Detected	-0.006	Detected	0.000	1.376	Detected	1.801	Detected	1.589	0.017	GT Sens	[illegible]
GT	Sense	Sense	0.452	Comprom	-0.452	Comprom	0.000	3.046	Detected	1.742	Detected	2.394	0.095	GT Sens	[illegible]
GT	Sense	Sense	-0.447	Detected	0.447	Detected	0.000	1.377	Detected	0.808	Detected	1.092	0.176	GT Sens	[illegible]
GT	Sense	Sense	-0.507	Detected	0.507	Detected	0.000	1.628	Detected	1.131	Detected	1.379	0.134	GT Sens	[illegible]
GT	Sense	Sense	-0.107	Detected	0.107	Detected	0.000	1.341	Detected	0.882	Detected	1.111	0.048	GT Sens	[illegible]
GT	Sense	Sense	-0.147	Detected	0.147	Detected	0.000	1.256	Detected	0.951	Detected	1.104	0.035	GT Sens	[illegible]
GT	Sense	Sense	0.268	Comprom	-0.268	Comprom	0.000	2.834	Detected	0.998	Detected	1.916	0.183	GT Sens	[illegible]
GT	Sense	Sense	-0.051	Detected	0.051	Detected	0.000	1.391	Detected	0.990	Detected	1.191	0.029	GT Sens	[illegible]
GT	Sense	Sense	-0.400	Comprom	0.400	Comprom	0.000	1.551	Detected	3.625	Detected	2.588	0.145	GT Sens	[illegible]
GT	Sense	Sense	-0.162	Comprom	0.162	Comprom	0.000	1.358	Detected	2.004	Detected	1.681	0.043	GT Sens	[illegible]
GT	Sense	Sense	-0.142	Comprom	0.142	Comprom	0.000	1.484	Detected	2.001	Detected	1.742	0.027	GT Sens	[illegible]
GT	Sense	Sense	0.941	Comprom	-0.941	Comprom	0.000	1.417	Detected	2.447	Detected	1.932	0.213	GT Sens	[illegible]
GT	Sense	Sense	-0.344	Comprom	0.344	Detected	0.000	3.621	Detected	1.092	Detected	2.357	0.214	GT Sens	[illegible]
GT	Sense	Sense	0.418	Detected	-0.418	Detected	0.000	1.118	Detected	1.507	Detected	1.313	0.104	GT Sens	[illegible]
GT	Sense	Sense	0.348	Detected	-0.348	Detected	0.000	1.207	Detected	1.463	Detected	1.335	0.069	GT Sens	[illegible]
GT	Sense	Sense	-0.024	Detected	0.024	Detected	0.000	1.623	Detected	0.920	Detected	1.271	0.069	GT Sens	[illegible]
GT	Sense	Sense	0.161	Comprom	-0.161	Comprom	0.000	4.056	Detected	2.664	Detected	3.360	0.042	GT Sens	[illegible]
GT	Sense	Sense	0.661	Detected	-0.661	Comprom	0.000	1.241	Detected	1.272	Detected	1.257	0.198	GT Sens	[illegible]
GT	Sense	Sense	-0.214	Comprom	0.214	Comprom	0.000	1.313	Detected	2.433	Detected	1.873	0.089	GT Sens	[illegible]
GT	Sense	Sense	0.187	Detected	-0.187	Detected	0.000	0.851	Detected	1.161	Detected	1.006	0.054	GT Sens	[illegible]
GT	Sense	Sense	0.109	Comprom	-0.109	Comprom	0.000	1.303	Detected	1.532	Detected	1.417	0.012	GT Sens	[illegible]
GT	Sense	Sense	0.139	Comprom	-0.139	Comprom	0.000	2.060	Detected	2.216	Detected	2.138	0.005	GT Sens	[illegible]
GT	Sense	Sense	0.201	Comprom	-0.201	Comprom	0.000	4.828	Detected	2.369	Detected	3.599	0.102	GT Sens	[illegible]
GT	Sense	Sense	-0.358	Detected	0.358	Detected	0.000	3.220	Detected	3.155	Detected	3.188	0.012	GT Sens	[illegible]
GT	Sense	Sense	-0.082	Comprom	0.082	Detected	0.000	1.348	Detected	1.634	Detected	1.491	0.012	GT Sens	[illegible]
GT	Sense	Sense	-0.259	Comprom	0.259	Comprom	0.000	1.129	Detected	1.780	Detected	1.454	0.073	GT Sens	[illegible]
GT	Sense	Sense	-0.065	Comprom	0.065	Comprom	0.000	1.586	Detected	2.405	Detected	1.995	0.041	GT Sens	[illegible]
GT	Sense	Sense	-0.689	Comprom	0.689	Detected	0.000	3.162	Detected	3.981	Detected	3.572	0.047	GT Sens	[illegible]
GT	Sense	Sense	0.446	Detected	-0.446	Comprom	0.000	1.537	Detected	1.474	Detected	1.505	0.078	GT Sens	[illegible]
GT	Sense	Sense	0.933	Detected	-0.933	Comprom	0.000	1.273	Detected	3.254	Detected	2.263	0.238	GT Sens	[illegible]
GT	Sense	Sense	0.255	Comprom	-0.255	Comprom	0.000	1.579	Detected	3.359	Detected	2.469	0.117	GT Sens	[illegible]
GT	Sense	Sense	0.252	Detected	-0.252	Detected	0.000	1.620	Detected	2.051	Detected	1.836	0.031	GT Sens	[illegible]
GT	Sense	Sense	0.027	Detected	-0.027	Detected	0.000	1.129	Detected	1.069	Detected	1.099	0.001	GT Sens	[illegible]
GT	Sense	Sense	-0.596	Comprom	0.596	Comprom	0.000	3.035	Detected	3.103	Detected	3.069	0.036	GT Sens	[illegible]
GT	Sense	Sense	-0.146	Comprom	0.146	Comprom	0.000	3.169	Detected	1.897	Detected	2.533	0.060	GT Sens	[illegible]
GT	Sense	Sense	-0.435	Comprom	0.435	Comprom	0.000	1.333	Detected	2.048	Detected	1.690	0.095	GT Sens	[illegible]
GT	Sense	Sense	0.307	Detected	-0.307	Comprom	0.000	2.027	Detected	0.910	Detected	1.468	0.148	GT Sens	[illegible]
GT	Sense	Sense	-0.159	Comprom	0.159	Comprom	0.000	1.948	Detected	2.518	Detected	2.233	0.021	GT Sens	[illegible]
GT	Sense	Sense	-0.659	Comprom	0.659	Detected	0.000	2.372	Detected	3.814	Detected	3.093	0.087	GT Sens	[illegible]
GT	Sense	Sense	1.000	Comprom	-1.000	Comprom	0.000	1.271	Detected	1.451	Detected	1.361	0.308	GT Sens	[illegible]
GT	Sense	Sense	0.399	Comprom	-0.399	Comprom	0.000	1.653	Detected	3.042	Detected	2.347	0.099	GT Sens	[illegible]
GT	Sense	Sense	-0.845	Comprom	0.845	Detected	0.000	1.119	Detected	2.157	Detected	1.638	0.240	GT Sens	[illegible]
GT	Sense	Sense	0.010	Detected	-0.010	Detected	0.000	1.591	Detected	1.079	Detected	1.335	0.035	GT Sens	[illegible]
GT	Sense	Sense	0.294	Comprom	-0.294	Comprom	0.000	1.032	Detected	1.574	Detected	1.303	0.083	GT Sens	[illegible]
GT	Sense	Sense	-0.108	Detected	0.108	Detected	0.000	1.064	Detected	0.973	Detected	1.019	0.013	GT Sens	[illegible]
GT	Sense	Sense	0.274	Detected	-0.274	Detected	0.000	0.932	Detected	1.135	Detected	1.034	0.071	GT Sens	[illegible]
GT	Sense	Sense	0.048	Detected	-0.048	Detected	0.000	1.280	Detected	0.958	Detected	1.119	0.022	GT Sens	[illegible]
GT	Sense	Sense	0.908	Detected	-0.908	Comprom	0.000	0.955	Detected	1.161	Detected	1.058	0.366	GT Sens	[illegible]
GT	Sense	Sense	-0.268	Comprom	0.268	Comprom	0.000	1.072	Detected	2.203	Detected	1.637	0.120	GT Sens	[illegible]
GT	Sense	Sense	-0.203	Detected	0.203	Detected	0.000	1.799	Detected	0.867	Detected	1.333	0.120	GT Sens	[illegible]
GT	Sense	Sense	0.808	Detected	-0.808	Detected	0.000	1.497	Detected	0.828	Detected	1.162	0.315	GT Sens	[illegible]
GT	Sense	Sense	0.827	Detected	-0.827	Detected	0.000	1.241	Detected	0.819	Detected	1.030	0.351	GT Sens	[illegible]
GT	Sense	Sense	-0.022	Comprom	0.022	Comprom	0.000	1.933	Detected	3.059	Detected	2.496	0.047	GT Sens	[illegible]
GT	Sense	Sense	0.258	Detected	-0.258	Detected	0.000	1.282	Detected	1.039	Detected	1.160	0.056	GT Sens	[illegible]
GT	Sense	Sense	0.325	Comprom	-0.325	Comprom	0.000	1.154	Detected	1.750	Detected	1.452	0.081	GT Sens	[illegible]
GT	Sense	Sense	0.100	Detected	-0.100	Detected	0.000	0.878	Detected	1.174	Detected	1.026	0.029	GT Sens	[illegible]
GT	Sense	Sense	0.137	Detected	-0.137	Detected	0.000	1.387	Detected	1.259	Detected	1.323	0.013	GT Sens	[illegible]
GT	Sense	Sense	0.250	Detected	-0.250	Detected	0.000	1.271	Detected	0.980	Detected	1.126	0.060	GT Sens	[illegible]
GT	Sense	Sense	0.040	Comprom	-0.040	Comprom	0.000	1.610	Detected	1.974	Detected	1.792	0.011	GT Sens	[illegible]
GT	Sense	Sense	0.086	Comprom	-0.086	Comprom	0.000	1.557	Detected	1.912	Detected	1.734	0.013	GT Sens	[illegible]
GT	Sense	Sense	-0.328	Comprom	0.328	Comprom	0.000	1.353	Detected	3.201	Detected	2.277	0.146	GT Sens	[illegible]
GT	Sense	Sense	-0.138	Detected	0.138	Detected	0.000	1.780	Detected	2.334	Detected	2.057	0.022	GT Sens	[illegible]
GT	Sense	Sense	0.135	Comprom	-0.135	Comprom	0.000	0.989	Detected	1.241	Detected	1.115	0.026	GT Sens	[illegible]
GT	Sense	Sense	1.103	Detected	-1.103	Comprom	0.000	0.947	Detected	1.718	Detected	1.332	0.372	GT Sens	[illegible]
GT	Sense	Sense	0.006	Detected	-0.006	Detected	0.000	1.086	Detected	0.944	Detected	1.015	0.005	GT Sens	[illegible]
GT	Sense	Sense	-0.074	Comprom	0.074	Comprom	0.000	5.673	Detected	3.015	Detected	4.344	0.082	GT Sens	[illegible]
GT	Sense	Sense	0.155	Comprom	-0.155	Comprom	0.000	3.505	Detected	3.488	Detected	3.497	0.002	GT Sens	[illegible]
GT	Sense	Sense	0.223	Detected	-0.223	Detected	0.000	0.991	Detected	1.257	Detected	1.124	0.049	GT Sens	[illegible]
GT	Sense	Sense	-0.365	Detected	0.365	Detected	0.000	0.959	Detected	1.075	Detected	1.017	0.110	GT Sens	[illegible]
GT	Sense	Sense	-0.241	Detected	0.241	Detected	0.000	1.129	Detected	1.287	Detected	1.208	0.041	GT Sens	[illegible]
GT	Sense	Sense	0.450	Comprom	-0.450	Comprom	0.000	1.950	Detected	1.939	Detected	1.945	0.050	GT Sens	[illegible]
GT	Sense	Sense	1.072	Detected	-1.072	Comprom	0.000	1.974	Detected	1.456	Detected	1.715	0.260	GT Sens	[illegible]
GT	Sense	Sense	-0.456	Comprom	0.456	Comprom	0.000	1.827	Detected	2.470	Detected	2.149	0.061	GT Sens	[illegible]
GT	Sense	Sense	0.317	Detected	-0.317	Detected	0.000	1.424	Detected	1.195	Detected	1.310	0.060	GT Sens	[illegible]
GT	Sense	Sense	-0.452	Comprom	0.452	Detected	0.000	1.166	Detected	1.309	Detected	1.238	0.114	GT Sens	[illegible]
GT	Sense	Sense	-0.742	Comprom	0.742	Detected	0.000	1.498	Detected	1.212	Detected	1.355	0.215	GT Sens	[illegible]
GT	Sense	Sense	0.219	Detected	-0.219	Detected	0.000	0.987	Detected	1.298	Detected	1.142	0.051	GT Sens	[illegible]
GT	Sense	Sense	-0.338	Comprom	0.338	Comprom	0.000	1.154	Detected	1.506	Detected	1.330	0.073	GT Sens	[illegible]
GT	Sense	Sense	0.972	Comprom	-0.972	Comprom	0.000	1.019	Detected	2.195	Detected	1.607	0.293	GT Sens	[illegible]
GT	Sense	Sense	-0.113	Detected	0.113	Detected	0.000	0.977	Detected	1.037	Detected	1.007	0.013	GT Sens	[illegible]
GT	Sense	Sense	-0.242	Detected	0.242	Detected	0.000	1.086	Detected	1.622	Detected	1.354	0.064	GT Sens	[illegible]
GT	Sense	Sense	-0.288	Comprom	0.288	Comprom	0.000	1.123	Detected	1.437	Detected	1.280	0.060	GT Sens	[illegible]
GT	Sense	Sense	-0.338	Comprom	0.338	Comprom	0.000	1.289	Detected	1.715	Detected	1.502	0.064	GT Sens	[illegible]
GT	Sense	Sense	0.123	Detected	-0.123	Detected	0.000	1.618	Detected	1.132	Detected	1.375	0.037	GT Sens	[illegible]
GT	Sense	Sense	0.042	Detected	-0.042	Detected	0.000	1.174	Detected	0.947	Detected	1.061	0.013	GT Sens	[illegible]
GT	Sense	Sense	0.026	Comprom	-0.026	Comprom	0.000	4.405	Detected	2.628	Detected	3.517	0.058	GT Sens	[illegible]
GT	Sense	Sense	0.716	Detected	-0.716	Comprom	0.000	1.287	Detected	1.318	Detected	1.302	0.211	GT Sens	[illegible]
GT	Sense	Sense	0.222	Detected	-0.222	Detected	0.000	0.903	Detected	1.460	Detected	1.181	0.080	GT Sens	[illegible]
GT	Sense	Sense	0.554	Detected	-0.554	Detected	0.000	0.950	Detected	1.651	Detected	1.300	0.186	GT Sens	[illegible]
GT	Sense	Sense	0.449	Detected	-0.449	Comprom	0.000	2.757	Detected	2.163	Detected	2.460	0.045	GT Sens	[illegible]
GT	Sense	Sense	0.235	Detected	-0.235	Detected	0.000	1.600	Detected	1.923	Detected	1.762	0.025	GT Sens	[illegible]
GT	Sense	Sense	-0.685	Comprom	0.685	Detected	0.000	1.023	Detected	1.543	Detected	1.283	0.222	GT Sens	[illegible]
GT	Sense	Sense	1.251	Detected	-1.251	Comprom	0.000	0.871	Detected	1.498	Detected	1.185	0.455	GT Sens	[illegible]
GT	Sense	Sense	-0.446	Comprom	0.446	Comprom	0.000	2.460	Detected	2.015	Detected	2.238	0.046	GT Sens	[illegible]
GT	Sense	Sense	0.284	Detected	-0.284	Detected	0.000	1.300	Detected	1.359	Detected	1.330	0.043	GT Sens	[illegible]
GT	Sense	Sense	0.088	Detected	-0.088	Detected	0.000	1.749	Detected	1.555	Detected	1.652	0.006	GT Sens	[illegible]
GT	Sense	Sense	0.099	Detected	-0.099	Detected	0.000	1.562	Detected	1.313	Detected	1.438	0.012	GT Sens	[illegible]
GT	Sense	Sense	0.664	Detected	-0.664	Detected	0.000	2.452	Detected	0.994	Detected	1.723	0.223	GT Sens	[illegible]
GT	Sense	Sense	-0.279	Comprom	0.279	Comprom	0.000	1.679	Detected	2.996	Detected	2.337	0.082	GT Sens	[illegible]
GT	Sense	Sense	0.215	Comprom	-0.215	Comprom	0.000	3.459	Detected	3.604	Detected	3.531	0.004	GT Sens	[illegible]
GT	Sense	Sense	-0.041	Comprom	0.041	Comprom	0.000	1.093	Detected	2.521	Detected	1.807	0.127	GT Sens	[illegible]
GT	Sense	Sense	0.547	Comprom	-0.547	Comprom	0.000	1.086	Detected	1.576	Detected	1.331	0.157	GT Sens	[illegible]
GT	Sense	Sense	0.349	Detected	-0.349	Detected	0.000	1.158	Detected	1.278	Detected	1.218	0.075	GT Sens	[illegible]
GT	Sense	Sense	0.135	Comprom	-0.135	Comprom	0.000	1.384	Detected	1.483	Detected	1.433	0.010	GT Sens	[illegible]
GT	Sense	Sense	0.690	Detected	-0.690	Comprom	0.000	2.378	Detected	1.511	Detected	1.944	0.140	GT Sens	[illegible]
GT	Sense	Sense	-0.183	Detected	0.183	Detected	0.000	1.303	Detected	0.917	Detected	1.110	0.053	GT Sens	[illegible]
GT	Sense	Sense	0.397	Detected	-0.397	Detected	0.000	1.443	Detected	1.011	Detected	1.227	0.113	GT Sens	[illegible]
GT	Sense	Sense	0.754	Detected	-0.754	Detected	0.000	1.233	Detected	1.021	Detected	1.127	0.277	GT Sens	[illegible]
GT	Sense	Sense	0.443	Comprom	-0.443	Comprom	0.000	1.414	Detected	1.668	Detected	1.541	0.079	GT Sens	[illegible]
GT	Sense	Sense	-0.316	Comprom	0.316	Detected	0.000	1.209	Detected	1.761	Detected	1.485	0.071	GT Sens	[illegible]
GT	Sense	Sense	-0.110	Comprom	0.110	Comprom	0.000	0.833	Detected	1.950	Detected	1.392	0.134	GT Sens	[illegible]
GT	Sense	Sense	0.168	Detected	-0.168	Detected	0.000	1.571	Detected	1.265	Detected	1.418	0.025	GT Sens	[illegible]
GT	Sense	Sense	0.276	Comprom	-0.276	Comprom	0.000	1.866	Detected	2.967	Detected	2.416	0.059	GT Sens	[illegible]
GT	Sense	Sense	0.558	Comprom	-0.558	Comprom	0.000	1.186	Detected	2.142	Detected	1.664	0.152	GT Sens	[illegible]
GT	Sense	Sense	-0.235	Detected	0.235	Detected	0.000	1.435	Detected	0.984	Detected	1.210	0.066	GT Sens	[illegible]
GT	Sense	Sense	0.198	Detected	-0.198	Detected	0.000	1.287	Detected	1.363	Detected	1.325	0.022	GT Sens	[illegible]
GT	Sense	Sense	0.726	Comprom	-0.726	Comprom	0.000	1.618	Detected	2.973	Detected	2.296	0.147	GT Sens	[illegible]
GT	Sense	Sense	-0.514	Comprom	0.514	Detected	0.000	1.119	Detected	1.165	Detected	1.142	0.157	GT Sens	[illegible]
GT	Sense	Sense	-0.099	Comprom	0.099	Comprom	0.000	0.992	Detected	1.138	Detected	1.065	0.013	GT Sens	[illegible]
GT	Sense	Sense	-0.101	Detected	0.101	Detected	0.000	2.608	Detected	1.099	Detected	1.854	0.135	GT Sens	[illegible]
GT	Sense	Sense	0.203	Comprom	-0.203	Comprom	0.000	1.339	Detected	1.220	Detected	1.280	0.026	GT Sens	[illegible]
GT	Sense	Sense	0.086	Detected	-0.086	Detected	0.000	1.266	Detected	1.258	Detected	1.262	0.005	GT Sens	[illegible]
GT	Sense	Sense	-0.233	Comprom	0.233	Comprom	0.000	1.415	Detected	2.342	Detected	1.879	0.069	GT Sens	[illegible]
GT	Sense	Sense	0.155	Comprom	-0.155	Comprom	0.000	1.508	Detected	1.492	Detected	1.500	0.011	GT Sens	[illegible]
GT	Sense	Sense	0.340	Detected	-0.340	Detected	0.000	1.382	Detected	1.108	Detected	1.245	0.077	GT Sens	[illegible]
GT	Sense	Sense	0.069	Comprom	-0.069	Comprom	0.000	1.357	Detected	1.183	Detected	1.270	0.008	GT Sens	[illegible]
GT	Sense	Sense	0.153	Comprom	-0.153	Comprom	0.000	2.529	Detected	2.888	Detected	2.709	0.007	GT Sens	[illegible]
GT	Sense	Sense	0.172	Comprom	-0.172	Comprom	0.000	3.161	Detected	2.575	Detected	2.868	0.014	GT Sens	[illegible]
GT	Sense	Sense	0.318	Comprom	-0.318	Comprom	0.000	2.955	Detected	2.810	Detected	2.883	0.013	GT Sens	[illegible]
GT	Sense	Sense	0.430	Detected	-0.430	Detected	0.000	1.182	Detected	1.754	Detected	1.468	0.105	GT Sens	[illegible]
GT	Sense	Sense	0.563	Comprom	-0.563	Comprom	0.000	1.194	Detected	1.988	Detected	1.591	0.147	GT Sens	[illegible]
GT	Sense	Sense	0.197	Comprom	-0.197	Comprom	0.000	1.413	Detected	1.778	Detected	1.596	0.027	GT Sens	[illegible]
GT	Sense	Sense	-0.058	Comprom	0.058	Comprom	0.000	1.373	Detected	3.517	Detected	2.445	0.150	GT Sens	[illegible]
GT	Sense	Sense	-0.008	Comprom	0.008	Comprom	0.000	6.720	Detected	2.341	Detected	4.531	0.174	GT Sens	[illegible]
GT	Sense	Sense	0.217	Detected	-0.217	Detected	0.000	0.887	Detected	1.245	Detected	1.066	0.063	GT Sens	[illegible]
GT	Sense	Sense	-0.369	Comprom	0.369	Comprom	0.000	1.002	Detected	1.156	Detected	1.079	0.103	GT Sens	[illegible]
GT	Sense	Sense	0.010	Comprom	-0.010	Comprom	0.000	2.020	Detected	2.346	Detected	2.183	0.006	GT Sens	[illegible]
GT	Sense	Sense	-0.250	Comprom	0.250	Comprom	0.000	1.889	Detected	2.716	Detected	2.302	0.041	GT Sens	[illegible]
GT	Sense	Sense	0.608	Detected	-0.608	Comprom	0.000	1.285	Detected	0.887	Detected	1.086	0.232	GT Sens	[illegible]
GT	Sense	Sense	0.293	Detected	-0.293	Detected	0.000	1.613	Detected	1.971	Detected	1.792	0.035	GT Sens	[illegible]
GT	Sense	Sense	-0.657	Comprom	0.657	Comprom	0.000	1.423	Detected	6.120	Detected	3.772	0.262	GT Sens	[illegible]
GT	Sense	Sense	-0.889	Comprom	0.889	Comprom	0.000	1.026	Detected	2.860	Detected	1.943	0.268	GT Sens	[illegible]
GT	Sense	Sense	0.093	Detected	-0.093	Detected	0.000	1.476	Detected	1.668	Detected	1.572	0.007	GT Sens	[illegible]
GT	Sense	Sense	-0.033	Detected	0.033	Detected	0.000	1.035	Detected	1.307	Detected	1.171	0.014	GT Sens	[illegible]
GT	Sense	Sense	0.605	Comprom	-0.605	Comprom	0.000	1.527	Detected	2.184	Detected	1.855	0.115	GT Sens	[illegible]
GT	Sense	Sense	-0.539	Comprom	0.539	Detected	0.000	1.147	Detected	2.203	Detected	1.675	0.157	GT Sens	[illegible]
GT	Sense	Sense	0.147	Detected	-0.147	Detected	0.000	0.908	Detected	1.367	Detected	1.138	0.053	GT Sens	[illegible]
GT	Sense	Sense	0.035	Detected	-0.035	Detected	0.000	1.093	Detected	1.461	Detected	1.277	0.021	GT Sens	[illegible]
GT	Sense	Sense	1.234	Detected	-1.234	Comprom	0.000	1.554	Detected	1.101	Detected	1.328	0.401	GT Sens	[illegible]
GT	Sense	Sense	-0.090	Comprom	0.090	Comprom	0.000	1.017	Detected	2.182	Detected	1.600	0.113	GT Sens	[illegible]
GT	Sense	Sense	-0.048	Detected	0.048	Detected	0.000	1.428	Detected	1.012	Detected	1.220	0.029	GT Sens	[illegible]
GT	Sense	Sense	0.236	Detected	-0.236	Detected	0.000	1.750	Detected	1.105	Detected	1.427	0.070	GT Sens	[illegible]
GT	Sense	Sense	0.101	Detected	-0.101	Detected	0.000	1.065	Detected	1.280	Detected	1.173	0.015	GT Sens	[illegible]
GT	Sense	Sense	-0.091	Detected	0.091	Detected	0.000	1.326	Detected	2.379	Detected	1.853	0.074	GT Sens	[illegible]
GT	Sense	Sense	-0.772	Comprom	0.772	Comprom	0.000	1.298	Detected	2.149	Detected	1.724	0.190	GT Sens	[illegible]
GT	Sense	Sense	0.161	Comprom	-0.161	Comprom	0.000	2.953	Detected	2.623	Detected	2.788	0.007	GT Sens	[illegible]
GT	Sense	Sense	0.406	Comprom	-0.406	Comprom	0.000	1.628	Detected	2.405	Detected	2.017	0.070	GT Sens	[illegible]
GT	Sense	Sense	-0.251	Comprom	0.251	Comprom	0.000	1.057	Detected	2.594	Detected	1.826	0.153	GT Sens	[illegible]
GT	Sense	Sense	0.062	Detected	-0.062	Detected	0.000	1.175	Detected	1.102	Detected	1.139	0.004	GT Sens	[illegible]
GT	Sense	Sense	0.869	Comprom	-0.869	Comprom	0.000	1.089	Detected	1.738	Detected	1.413	0.267	GT Sens	[illegible]
GT	Sense	Sense	-0.219	Comprom	0.219	Comprom	0.000	1.023	Detected	2.046	Detected	1.535	0.110	GT Sens	[illegible]
GT	Sense	Sense	0.358	Comprom	-0.358	Comprom	0.000	1.097	Detected	1.831	Detected	1.464	0.104	GT Sens	[illegible]
GT	Sense	Sense	-0.889	Detected	0.889	Detected	0.000	7.882	Detected	5.755	Detected	6.819	0.039	GT Sens	[illegible]
GT	Sense	Sense	-0.090	Comprom	0.090	Comprom	0.000	5.735	Detected	3.718	Detected	4.726	0.043	GT Sens	[illegible]
GT	Sense	Sense	0.165	Comprom	-0.165	Comprom	0.000	3.046	Detected	3.051	Detected	3.048	0.003	GT Sens	[illegible]
GT	Sense	Sense	0.580	Comprom	-0.580	Comprom	0.000	1.586	Detected	1.627	Detected	1.607	0.110	GT Sens	[illegible]
GT	Sense	Sense	0.162	Comprom	-0.162	Comprom	0.000	2.460	Detected	2.788	Detected	2.624	0.008	GT Sens	[illegible]
GT	Sense	Sense	0.057	Comprom	-0.057	Detected	0.000	1.059	Detected	1.787	Detected	1.423	0.061	GT Sens	[illegible]
GT	Sense	Sense	0.016	Detected	-0.016	Detected	0.000	1.138	Detected	1.178	Detected	1.158	0.000	GT Sens	[illegible]
GT	Sense	Sense	0.122	Detected	-0.122	Detected	0.000	1.035	Detected	1.198	Detected	1.116	0.017	GT Sens	[illegible]
GT	Sense	Sense	0.269	Comprom	-0.269	Comprom	0.000	1.086	Detected	3.215	Detected	2.150	0.189	GT Sens	[illegible]
GT	Sense	Sense	0.903	Detected	-0.903	Comprom	0.000	1.078	Detected	1.196	Detected	1.137	0.336	GT Sens	[illegible]
GT	Sense	Sense	0.123	Comprom	-0.123	Comprom	0.000	1.208	Detected	2.376	Detected	1.792	0.095	GT Sens	[illegible]
GT	Sense	Sense	-0.156	Detected	0.156	Detected	0.000	0.930	Detected	1.589	Detected	1.259	0.074	GT Sens	[illegible]
GT	Sense	Sense	0.775	Comprom	-0.775	Comprom	0.000	2.452	Detected	2.866	Detected	2.659	0.080	GT Sens	[illegible]

GT	Sense	Sense	-0.003	Comprom	0.003	Comprom	0.000	0.926	Detected	1.384	Detected	1.155	0.037	GT	Sens	[illegible]
GT	Sense	Sense	0.739	Detected	-0.739	Comprom	0.000	1.679	Detected	1.649	Detected	1.664	0.153	GT	Sens	[illegible]
GT	Sense	Sense	0.123	Detected	-0.123	Detected	0.000	1.225	Detected	1.643	Detected	1.434	0.027	GT	Sens	[illegible]
GT	Sense	Sense	0.048	Comprom	-0.048	Comprom	0.000	0.872	Detected	2.403	Detected	1.638	0.166	GT	Sens	[illegible]
GT	Sense	Sense	0.589	Comprom	-0.589	Comprom	0.000	1.017	Detected	1.711	Detected	1.364	0.184	GT	Sens	[illegible]
GT	Sense	Sense	-0.371	Comprom	0.371	Comprom	0.000	1.647	Detected	1.631	Detected	1.639	0.048	GT	Sens	[illegible]
GT	Sense	Sense	0.165	Detected	-0.165	Detected	0.000	1.476	Detected	1.728	Detected	1.602	0.016	GT	Sens	[illegible]
GT	Sense	Sense	-0.008	Detected	0.008	Detected	0.000	1.516	Detected	0.871	Detected	1.193	0.066	GT	Sens	[illegible]
GT	Sense	Sense	0.376	Detected	-0.376	Detected	0.000	1.025	Detected	1.706	Detected	1.365	0.115	GT	Sens	[illegible]
GT	Sense	Sense	0.455	Detected	-0.455	Detected	0.000	1.194	Detected	0.957	Detected	1.076	0.149	GT	Sens	[illegible]
GT	Sense	Sense	0.509	Detected	-0.509	Detected	0.000	1.241	Detected	1.372	Detected	1.306	0.126	GT	Sens	[illegible]
GT	Sense	Sense	0.735	Detected	-0.735	Detected	0.000	1.592	Detected	1.699	Detected	1.646	0.155	GT	Sens	[illegible]
GT	Sense	Sense	0.127	Detected	-0.127	Detected	0.000	1.065	Detected	1.071	Detected	1.068	0.014	GT	Sens	[illegible]
GT	Sense	Sense	0.709	Detected	-0.709	Detected	0.000	0.992	Detected	1.497	Detected	1.245	0.240	GT	Sens	[illegible]
GT	Sense	Sense	-0.111	Comprom	0.111	Comprom	0.000	2.289	Detected	2.079	Detected	2.184	0.005	GT	Sens	[illegible]
GT	Sense	Sense	0.135	Detected	-0.135	Detected	0.000	1.091	Detected	1.154	Detected	1.122	0.015	GT	Sens	[illegible]
GT	Sense	Sense	-0.023	Comprom	0.023	Comprom	0.000	1.068	Detected	1.822	Detected	1.445	0.062	GT	Sens	[illegible]
GT	Sense	Sense	0.003	Detected	-0.003	Detected	0.000	2.358	Detected	1.120	Detected	1.739	0.107	GT	Sens	[illegible]
GT	Sense	Sense	0.443	Detected	-0.443	Detected	0.000	1.076	Detected	1.142	Detected	1.109	0.130	GT	Sens	[illegible]
GT	Sense	Sense	0.059	Detected	-0.059	Detected	0.000	1.506	Detected	1.746	Detected	1.626	0.007	GT	Sens	[illegible]
GT	Sense	Sense	0.135	Comprom	-0.135	Comprom	0.000	1.399	Detected	2.442	Detected	1.920	0.070	GT	Sens	[illegible]
GT	Sense	Sense	0.192	Comprom	-0.192	Comprom	0.000	2.430	Detected	2.823	Detected	2.627	0.011	GT	Sens	[illegible]
GT	Sense	Sense	-0.220	Detected	0.220	Detected	0.000	1.873	Detected	1.928	Detected	1.901	0.013	GT	Sens	[illegible]
GT	Sense	Sense	-0.302	Comprom	0.302	Comprom	0.000	1.774	Detected	1.676	Detected	1.725	0.030	GT	Sens	[illegible]
GT	Sense	Sense	-0.948	Comprom	0.948	Detected	0.000	2.076	Detected	1.436	Detected	1.756	0.221	GT	Sens	[illegible]
GT	Sense	Sense	-0.057	Detected	0.057	Detected	0.000	1.577	Detected	2.096	Detected	1.836	0.020	GT	Sens	[illegible]
GT	Sense	Sense	0.053	Detected	-0.053	Detected	0.000	0.898	Detected	2.698	Detected	1.798	0.184	GT	Sens	[illegible]
GT	Sense	Sense	0.277	Detected	-0.277	Detected	0.000	2.253	Detected	2.590	Detected	2.422	0.017	GT	Sens	[illegible]
GT	Sense	Sense	-0.815	Detected	0.815	Detected	0.000	0.947	Detected	1.396	Detected	1.171	0.300	GT	Sens	[illegible]
GT	Sense	Sense	0.107	Detected	-0.107	Detected	0.000	1.397	Detected	0.826	Detected	1.112	0.068	GT	Sens	[illegible]
GT	Sense	Sense	0.018	Detected	-0.018	Detected	0.000	0.893	Detected	1.398	Detected	1.145	0.046	GT	Sens	[illegible]
GT	Sense	Sense	-1.122	Comprom	1.122	Detected	0.000	2.157	Detected	1.132	Detected	1.644	0.314	GT	Sens	[illegible]
GT	Sense	Sense	0.790	Detected	-0.790	Detected	0.000	1.856	Detected	2.797	Detected	2.327	0.127	GT	Sens	[illegible]
GT	Sense	Sense	-1.621	Comprom	1.621	Detected	0.000	0.935	Detected	1.867	Detected	1.401	0.494	GT	Sens	[illegible]
GT	Sense	Sense	-0.227	Comprom	0.227	Comprom	0.000	0.932	Detected	2.274	Detected	1.603	0.152	GT	Sens	[illegible]
GT	Sense	Sense	-0.688	Comprom	0.688	Comprom	0.000	4.255	Detected	2.370	Detected	3.313	0.105	GT	Sens	[illegible]
GT	Sense	Sense	0.382	Detected	-0.382	Detected	0.000	1.807	Detected	2.433	Detected	2.120	0.050	GT	Sens	[illegible]
GT	Sense	Sense	0.103	Detected	-0.103	Detected	0.000	1.193	Detected	1.754	Detected	1.474	0.039	GT	Sens	[illegible]
GT	Sense	Sense	-0.083	Comprom	0.083	Detected	0.000	1.803	Detected	2.083	Detected	1.943	0.007	GT	Sens	[illegible]
GT	Sense	Sense	-0.007	Detected	0.007	Detected	0.000	1.177	Detected	1.330	Detected	1.253	0.004	GT	Sens	[illegible]
GT	Sense	Sense	0.167	Detected	-0.167	Detected	0.000	1.366	Detected	1.186	Detected	1.276	0.021	GT	Sens	[illegible]
GT	Sense	Sense	-0.348	Detected	0.348	Detected	0.000	0.851	Detected	1.430	Detected	1.141	0.128	GT	Sens	[illegible]
GT	Sense	Sense	-0.210	Comprom	0.210	Comprom	0.000	0.934	Detected	1.250	Detected	1.092	0.053	GT	Sens	[illegible]
GT	Sense	Sense	0.383	Detected	-0.383	Detected	0.000	1.073	Detected	1.114	Detected	1.093	0.104	GT	Sens	[illegible]
GT	Sense	Sense	0.609	Detected	-0.609	Detected	0.000	2.137	Detected	0.826	Detected	1.481	0.240	GT	Sens	[illegible]
GT	Sense	Sense	0.200	Comprom	-0.200	Comprom	0.000	1.924	Detected	2.371	Detected	2.148	0.019	GT	Sens	[illegible]
GT	Sense	Sense	0.217	Comprom	-0.217	Comprom	0.000	1.433	Detected	2.091	Detected	1.762	0.047	GT	Sens	[illegible]
GT	Sense	Sense	0.095	Comprom	-0.095	Comprom	0.000	3.515	Detected	2.347	Detected	2.931	0.038	GT	Sens	[illegible]
GT	Sense	Sense	-1.042	Comprom	1.042	Detected	0.000	1.090	Detected	2.087	Detected	1.589	0.303	GT	Sens	[illegible]
GT	Sense	Sense	-0.204	Detected	0.204	Detected	0.000	1.990	Detected	1.147	Detected	1.568	0.079	GT	Sens	[illegible]
GT	Sense	Sense	-0.215	Comprom	0.215	Comprom	0.000	1.387	Detected	3.463	Detected	2.425	0.149	GT	Sens	[illegible]
GT	Sense	Sense	0.048	Detected	-0.048	Detected	0.000	0.987	Detected	1.462	Detected	1.225	0.037	GT	Sens	[illegible]
GT	Sense	Sense	0.246	Detected	-0.246	Detected	0.000	1.469	Detected	1.719	Detected	1.594	0.029	GT	Sens	[illegible]
GT	Sense	Sense	0.127	Comprom	-0.127	Comprom	0.000	1.581	Detected	1.834	Detected	1.708	0.011	GT	Sens	[illegible]
GT	Sense	Sense	0.423	Detected	-0.423	Detected	0.000	1.297	Detected	1.768	Detected	1.532	0.087	GT	Sens	[illegible]
GT	Sense	Sense	0.191	Detected	-0.191	Detected	0.000	2.312	Detected	0.857	Detected	1.585	0.170	GT	Sens	[illegible]
GT	Sense	Sense	0.004	Detected	-0.004	Detected	0.000	1.716	Detected	2.322	Detected	2.019	0.022	GT	Sens	[illegible]
GT	Sense	Sense	-0.323	Detected	0.323	Detected	0.000	1.343	Detected	1.404	Detected	1.374	0.052	GT	Sens	[illegible]
GT	Sense	Sense	-0.824	Detected	0.824	Detected	0.000	2.147	Detected	2.700	Detected	2.423	0.108	GT	Sens	[illegible]
GT	Sense	Sense	0.092	Detected	-0.092	Detected	0.000	1.413	Detected	1.679	Detected	1.546	0.011	GT	Sens	[illegible]
GT	Sense	Sense	0.343	Detected	-0.343	Detected	0.000	2.178	Detected	3.169	Detected	2.674	0.047	GT	Sens	[illegible]
GT	Sense	Sense	1.536	Detected	-1.536	Detected	0.000	0.877	Detected	1.225	Detected	1.051	0.567	GT	Sens	[illegible]
GT	Sense	Sense	0.124	Comprom	-0.124	Comprom	0.000	1.664	Detected	2.132	Detected	1.898	0.019	GT	Sens	[illegible]
GT	Sense	Sense	-0.034	Comprom	0.034	Detected	0.000	1.183	Detected	1.393	Detected	1.288	0.007	GT	Sens	[illegible]
GT	Sense	Sense	-0.581	Detected	0.581	Detected	0.000	1.621	Detected	2.097	Detected	1.859	0.098	GT	Sens	[illegible]
GT	Sense	Sense	0.250	Comprom	-0.250	Comprom	0.000	0.973	Detected	1.664	Detected	1.318	0.091	GT	Sens	[illegible]
GT	Sense	Sense	0.387	Comprom	-0.387	Comprom	0.000	1.307	Detected	1.657	Detected	1.482	0.073	GT	Sens	[illegible]
GT	Sense	Sense	0.696	Comprom	-0.696	Comprom	0.000	1.257	Detected	1.527	Detected	1.392	0.189	GT	Sens	[illegible]
GT	Sense	Sense	0.351	Detected	-0.351	Detected	0.000	1.224	Detected	1.127	Detected	1.176	0.080	GT	Sens	[illegible]
GT	Sense	Sense	0.004	Detected	-0.004	Detected	0.000	0.840	Detected	1.241	Detected	1.041	0.035	GT	Sens	[illegible]
GT	Sense	Sense	0.176	Comprom	-0.176	Comprom	0.000	5.263	Detected	2.854	Detected	4.059	0.079	GT	Sens	[illegible]
GT	Sense	Sense	0.232	Detected	-0.232	Detected	0.000	0.873	Detected	1.386	Detected	1.129	0.082	GT	Sens	[illegible]
GT	Sense	Sense	-0.192	Detected	0.192	Detected	0.000	1.145	Detected	1.012	Detected	1.079	0.034	GT	Sens	[illegible]
GT	Sense	Sense	-0.614	Comprom	0.614	Comprom	0.000	1.151	Detected	3.752	Detected	2.452	0.230	GT	Sens	[illegible]
GT	Sense	Sense	0.291	Comprom	-0.291	Comprom	0.000	0.983	Detected	1.394	Detected	1.189	0.079	GT	Sens	[illegible]
GT	Sense	Sense	0.218	Comprom	-0.218	Comprom	0.000	1.412	Detected	2.173	Detected	1.792	0.055	GT	Sens	[illegible]
GT	Sense	Sense	0.036	Detected	-0.036	Detected	0.000	1.593	Detected	0.963	Detected	1.278	0.056	GT	Sens	[illegible]
GT	Sense	Sense	-0.231	Comprom	0.231	Comprom	0.000	2.545	Detected	3.121	Detected	2.833	0.017	GT	Sens	[illegible]
GT	Sense	Sense	-0.335	Comprom	0.335	Comprom	0.000	0.927	Detected	1.835	Detected	1.381	0.134	GT	Sens	[illegible]
GT	Sense	Sense	0.221	Detected	-0.221	Detected	0.000	1.357	Detected	1.468	Detected	1.413	0.025	GT	Sens	[illegible]
GT	Sense	Sense	-0.667	Comprom	0.667	Detected	0.000	1.140	Detected	1.667	Detected	1.403	0.190	GT	Sens	[illegible]
GT	Sense	Sense	-0.316	Comprom	0.316	Comprom	0.000	3.836	Detected	2.224	Detected	3.030	0.073	GT	Sens	[illegible]
GT	Sense	Sense	-0.102	Detected	0.102	Detected	0.000	3.069	Detected	2.182	Detected	2.625	0.029	GT	Sens	[illegible]
GT	Sense	Sense	-0.751	Detected	0.751	Detected	0.000	2.209	Detected	1.628	Detected	1.919	0.140	GT	Sens	[illegible]
GT	Sense	Sense	0.153	Detected	-0.153	Detected	0.000	1.067	Detected	1.801	Detected	1.434	0.069	GT	Sens	[illegible]
GT	Sense	Sense	0.276	Detected	-0.276	Detected	0.000	1.077	Detected	1.032	Detected	1.054	0.062	GT	Sens	[illegible]
GT	Sense	Sense	-0.076	Comprom	0.076	Comprom	0.000	0.973	Detected	2.307	Detected	1.640	0.135	GT	Sens	[illegible]
GT	Sense	Sense	0.130	Detected	-0.130	Detected	0.000	1.816	Detected	2.606	Detected	2.211	0.034	GT	Sens	[illegible]
GT	Sense	Sense	0.455	Detected	-0.455	Detected	0.000	1.156	Detected	1.254	Detected	1.205	0.119	GT	Sens	[illegible]
GT	Sense	Sense	-0.175	Detected	0.175	Detected	0.000	3.715	Detected	1.370	Detected	2.543	0.165	GT	Sens	[illegible]
GT	Sense	Sense	0.212	Comprom	-0.212	Comprom	0.000	2.198	Detected	2.157	Detected	2.177	0.009	GT	Sens	[illegible]
GT	Sense	Sense	-0.915	Comprom	0.915	Detected	0.000	1.489	Detected	1.761	Detected	1.625	0.221	GT	Sens	[illegible]
GT	Sense	Sense	-0.404	Comprom	0.404	Comprom	0.000	1.376	Detected	3.164	Detected	2.270	0.147	GT	Sens	[illegible]
GT	Sense	Sense	0.015	Detected	-0.015	Detected	0.000	1.405	Detected	1.549	Detected	1.477	0.002	GT	Sens	[illegible]
GT	Sense	Sense	-0.579	Detected	0.579	Detected	0.000	1.747	Detected	1.041	Detected	1.394	0.176	GT	Sens	[illegible]
GT	Sense	Sense	-0.058	Detected	0.058	Detected	0.000	1.185	Detected	1.656	Detected	1.421	0.028	GT	Sens	[illegible]
GT	Sense	Sense	0.581	Comprom	-0.581	Comprom	0.000	0.958	Detected	1.438	Detected	1.198	0.197	GT	Sens	[illegible]
GT	Sense	Sense	-0.327	Detected	0.327	Detected	0.000	1.310	Detected	1.302	Detected	1.306	0.057	GT	Sens	[illegible]
GT	Sense	Sense	0.316	Detected	-0.316	Detected	0.000	2.015	Detected	0.844	Detected	1.429	0.165	GT	Sens	[illegible]
GT	Sense	Sense	-0.611	Detected	0.611	Detected	0.000	2.087	Detected	1.232	Detected	1.659	0.156	GT	Sens	[illegible]
GT	Sense	Sense	0.449	Detected	-0.449	Detected	0.000	1.161	Detected	1.663	Detected	1.412	0.111	GT	Sens	[illegible]
GT	Sense	Sense	0.139	Detected	-0.139	Detected	0.000	1.383	Detected	1.415	Detected	1.399	0.010	GT	Sens	[illegible]
GT	Sense	Sense	0.110	Detected	-0.110	Detected	0.000	1.721	Detected	1.262	Detected	1.491	0.028	GT	Sens	[illegible]
GT	Sense	Sense	-0.395	Comprom	0.395	Comprom	0.000	0.920	Detected	2.358	Detected	1.639	0.184	GT	Sens	[illegible]
GT	Sense	Sense	-0.114	Comprom	0.114	Detected	0.000	1.465	Detected	0.918	Detected	1.192	0.057	GT	Sens	[illegible]
GT	Sense	Sense	-0.085	Detected	0.085	Detected	0.000	1.270	Detected	1.604	Detected	1.437	0.017	GT	Sens	[illegible]
GT	Sense	Sense	0.119	Detected	-0.119	Detected	0.000	1.670	Detected	1.443	Detected	1.556	0.011	GT	Sens	[illegible]
GT	Sense	Sense	-0.305	Comprom	0.305	Detected	0.000	2.984	Detected	0.867	Detected	1.926	0.222	GT	Sens	[illegible]
GT	Sense	Sense	0.308	Detected	-0.308	Detected	0.000	1.149	Detected	1.411	Detected	1.280	0.062	GT	Sens	[illegible]
GT	Sense	Sense	-0.209	Comprom	0.209	Comprom	0.000	2.406	Detected	1.865	Detected	2.136	0.025	GT	Sens	[illegible]
GT	Sense	Sense	1.611	Detected	-1.611	Detected	0.000	1.395	Detected	1.359	Detected	1.377	0.483	GT	Sens	[illegible]
GT	Sense	Sense	0.211	Detected	-0.211	Detected	0.000	3.762	Detected	1.131	Detected	2.447	0.208	GT	Sens	[illegible]
GT	Sense	Sense	-0.392	Detected	0.392	Detected	0.000	1.138	Detected	1.040	Detected	1.089	0.110	GT	Sens	[illegible]
GT	Sense	Sense	-0.064	Detected	0.064	Detected	0.000	1.397	Detected	0.869	Detected	1.133	0.053	GT	Sens	[illegible]
GT	Sense	Sense	0.200	Detected	-0.200	Detected	0.000	2.665	Detected	1.313	Detected	1.989	0.106	GT	Sens	[illegible]
GT	Sense	Sense	0.780	Detected	-0.780	Comprom	0.000	2.658	Detected	1.106	Detected	1.882	0.229	GT	Sens	[illegible]
GT	Sense	Sense	0.350	Comprom	-0.350	Comprom	0.000	1.112	Detected	1.610	Detected	1.361	0.087	GT	Sens	[illegible]
GT	Sense	Sense	-0.131	Detected	0.131	Detected	0.000	2.092	Detected	2.784	Detected	2.438	0.022	GT	Sens	[illegible]
GT	Sense	Sense	0.048	Detected	-0.048	Detected	0.000	1.881	Detected	2.187	Detected	2.034	0.006	GT	Sens	[illegible]
GT	Sense	Sense	0.270	Detected	-0.270	Detected	0.000	3.812	Detected	3.263	Detected	3.538	0.012	GT	Sens	[illegible]
GT	Sense	Sense	-0.205	Comprom	0.205	Comprom	0.000	2.025	Detected	2.437	Detected	2.231	0.017	GT	Sens	[illegible]
GT	Sense	Sense	0.402	Detected	-0.402	Detected	0.000	1.032	Detected	1.235	Detected	1.134	0.112	GT	Sens	[illegible]
GT	Sense	Sense	-0.288	Detected	0.288	Detected	0.000	1.855	Detected	1.899	Detected	1.877	0.023	GT	Sens	[illegible]
GT	Sense	Sense	-0.152	Detected	0.152	Detected	0.000	2.298	Detected	1.735	Detected	2.016	0.024	GT	Sens	[illegible]
GT	Sense	Sense	0.644	Comprom	-0.644	Comprom	0.000	0.821	Detected	1.588	Detected	1.205	0.249	GT	Sens	[illegible]
GT	Sense	Sense	-0.112	Detected	0.112	Detected	0.000	2.279	Detected	1.566	Detected	1.922	0.036	GT	Sens	[illegible]
GT	Sense	Sense	-0.459	Detected	0.459	Detected	0.000	1.706	Detected	1.764	Detected	1.735	0.064	GT	Sens	[illegible]
GT	Sense	Sense	-0.118	Comprom	0.118	Comprom	0.000	1.701	Detected	2.655	Detected	2.178	0.047	GT	Sens	[illegible]
GT	Sense	Sense	-0.171	Comprom	0.171	Comprom	0.000	1.574	Detected	3.054	Detected	2.314	0.093	GT	Sens	[illegible]
GT	Sense	Sense	-0.180	Comprom	0.180	Comprom	0.000	2.822	Detected	2.641	Detected	2.732	0.005	GT	Sens	[illegible]
GT	Sense	Sense	0.522	Detected	-0.522	Detected	0.000	0.845	Detected	1.204	Detected	1.024	0.205	GT	Sens	[illegible]
GT	Sense	Sense	-0.005	Detected	0.005	Detected	0.000	1.960	Detected	1.709	Detected	1.835	0.005	GT	Sens	[illegible]
GT	Sense	Sense	0.254	Comprom	-0.254	Comprom	0.000	1.431	Detected	2.177	Detected	1.804	0.057	GT	Sens	[illegible]
GT	Sense	Sense	0.014	Detected	-0.014	Detected	0.000	1.256	Detected	1.145	Detected	1.201	0.002	GT	Sens	[illegible]
GT	Sense	Sense	0.228	Detected	-0.228	Detected	0.000	2.415	Detected	2.022	Detected	2.218	0.018	GT	Sens	[illegible]
GT	Sense	Sense	-0.541	Comprom	0.541	Comprom	0.000	1.355	Detected	2.213	Detected	1.784	0.123	GT	Sens	[illegible]
GT	Sense	Sense	1.315	Detected	-1.315	Comprom	0.000	1.550	Detected	2.573	Detected	2.062	0.281	GT	Sens	[illegible]
GT	Sense	Sense	-0.324	Detected	0.324	Detected	0.000	1.954	Detected	1.271	Detected	1.612	0.076	GT	Sens	[illegible]
GT	Sense	Sense	-0.931	Comprom	0.931	Detected	0.000	1.075	Detected	1.152	Detected	1.113	0.355	GT	Sens	[illegible]
GT	Sense	Sense	1.335	Detected	-1.335	Detected	0.000	1.501	Detected	2.020	Detected	1.761	0.325	GT	Sens	[illegible]
GT	Sense	Sense	-0.075	Detected	0.075	Detected	0.000	0.986	Detected	1.081	Detected	1.034	0.007	GT	Sens	[illegible]
GT	Sense	Sense	0.221	Detected	-0.221	Detected	0.000	2.565	Detected	1.822	Detected	2.194	0.037	GT	Sens	[illegible]
GT	Sense	Sense	-0.919	Comprom	0.919	Detected	0.000	1.968	Detected	1.626	Detected	1.797	0.195	GT	Sens	[illegible]
GT	Sense	Sense	0.354	Detected	-0.354	Detected	0.000	2.123	Detected	1.392	Detected	1.758	0.075	GT	Sens	[illegible]
GT	Sense	Sense	0.198	Detected	-0.198	Detected	0.000	1.539	Detected	1.600	Detected	1.570	0.016	GT	Sens	[illegible]
GT	Sense	Sense	0.261	Detected	-0.261	Detected	0.000	2.337	Detected	2.972	Detected	2.655	0.023	GT	Sens	[illegible]
GT	Sense	Sense	0.198	Comprom	-0.198	Comprom	0.000	4.051	Detected	2.976	Detected	3.513	0.026	GT	Sens	[illegible]
GT	Sense	Sense	-0.049	Comprom	0.049	Comprom	0.000	1.430	Detected	2.718	Detected	2.074	0.085	GT	Sens	[illegible]
GT	Sense	Sense	-0.195	Comprom	0.195	Comprom	0.000	2.724	Detected	2.046	Detected	2.385	0.026	GT	Sens	[illegible]
GT	Sense	Sense	0.186	Detected	-0.186	Detected	0.000	1.728	Detected	1.195	Detected	1.461	0.046	GT	Sens	[illegible]
GT	Sense	Sense	0.330	Detected	-0.330	Detected	0.000	2.287	Detected	2.522	Detected	2.405	0.021	GT	Sens	[illegible]
GT	Sense	Sense	0.025	Comprom	-0.025	Comprom	0.000	1.474	Detected	2.796	Detected	2.135	0.084	GT	Sens	[illegible]
GT	Sense	Sense	-0.275	Comprom	0.275	Comprom	0.000	1.318	Detected	2.360	Detected	1.839	0.089	GT	Sens	[illegible]
GT	Sense	Sense	0.227	Detected	-0.227	Detected	0.000	1.230	Detected	1.699	Detected	1.465	0.046	GT	Sens	[illegible]
GT	Sense	Sense	-0.491	Comprom	0.491	Detected	0.000	0.803	Detected	3.053	Detected	1.928	0.257	GT	Sens	[illegible]
GT	Sense	Sense	-0.273	Comprom	0.273	Detected	0.000	1.481	Detected	1.432	Detected	1.456	0.034	GT	Sens	[illegible]
GT	Sense	Sense	-0.977	Comprom	0.977	Detected	0.000	0.966	Detected	1.671	Detected	1.318	0.332	GT	Sens	[illegible]
GT	Sense	Sense	0.765	Detected	-0.765	Comprom	0.000	0.913	Detected	1.128	Detected	1.020	0.318	GT	Sens	[illegible]
GT	Sense	Sense	0.212	Comprom	-0.212	Comprom	0.000	3.019	Detected	2.970	Detected	2.994	0.005	GT	Sens	[illegible]
GT	Sense	Sense	0.004	Detected	-0.004	Detected	0.000	0.809	Detected	1.968	Detected	1.389	0.139	GT	Sens	[illegible]
GT	Sense	Sense	-0.042	Detected	0.042	Detected	0.000	1.166	Detected	1.111	Detected	1.139	0.002	GT	Sens	[illegible]
GT	Sense	Sense	0.490	Comprom	-0.490	Comprom	0.000	1.876	Detected	2.254	Detected	2.065	0.059	GT	Sens	[illegible]
GT	Sense	Sense	-0.220	Comprom	0.220	Detected	0.000	0.867	Detected	1.875	Detected	1.371	0.130	GT	Sens	[illegible]
GT	Sense	Sense	0.470	Comprom	-0.470	Comprom	0.000	2.561	Detected	1.815	Detected	2.188	0.068	GT	Sens	[illegible]
GT	Sense	Sense	0.198	Detected	-0.198	Detected	0.000	1.372	Detected	1.015	Detected	1.193	0.046	GT	Sens	[illegible]
GT	Sense	Sense	0.026	Comprom	-0.026	Comprom	0.000	1.050	Detected	1.322	Detected	1.186	0.013	GT	Sens	[illegible]
GT	Sense	Sense	0.113	Comprom	-0.113	Comprom	0.000	2.152	Detected	2.124	Detected	2.138	0.003	GT	Sens	[illegible]
GT	Sense	Sense	0.172	Detected	-0.172	Detected	0.000	1.097	Detected	1.514	Detected	1.306	0.040	GT	Sens	[illegible]
GT	Sense	Sense	1.611	Detected	-1.611	Comprom	0.000	1.372	Detected	1.226	Detected	1.299	0.505	GT	Sens	[illegible]
GT	Sense	Sense	0.120	Detected	-0.120	Detected	0.000	1.201	Detected	0.983	Detected	1.092	0.021	GT	Sens	[illegible]
GT	Sense	Sense	0.323	Detected	-0.323	Detected	0.000	1.314	Detected	0.890	Detected	1.102	0.104	GT	Sens	[illegible]
GT	Sense	Sense	0.065	Detected	-0.065	Detected	0.000	1.043	Detected	1.291	Detected	1.167	0.014	GT	Sens	[illegible]
GT	Sense	Sense	0.295	Detected	-0.295	Detected	0.000	0.940	Detected	1.243	Detected	1.091	0.081	GT	Sens	[illegible]
GT	Sense	Sense	0.252	Comprom	-0.252	Comprom	0.000	1.092	Detected	2.544	Detected	1.818	0.142	GT	Sens	[illegible]
GT	Sense	Sense	-0.355	Detected	0.355	Detected	0.000	2.354	Detected	1.829	Detected	2.091	0.042	GT	Sens	[illegible]
GT	Sense	Sense	-0.648	Comprom	0.648	Detected	0.000	1.001	Detected	1.605	Detected	1.303	0.210	GT	Sens	[illegible]
GT	Sense	Sense	0.230	Detected	-0.230	Detected	0.000	1.449	Detected	1.443	Detected	1.446	0.024	GT	Sens	[illegible]
GT	Sense	Sense	0.553	Comprom	-0.553	Comprom	0.000	3.383	Detected	3.725	Detected	3.554	0.026	GT	Sens	[illegible]
GT	Sense	Sense	-0.567	Detected	0.567	Detected	0.000	2.534	Detected	2.316	Detected	2.425	0.052	GT	Sens	[illegible]
GT	Sense	Sense	-0.304	Detected	0.304	Detected	0.000	1.295	Detected	1.086	Detected	1.191	0.066	GT	Sens	[illegible]
GT	Sense	Sense	0.153	Detected	-0.153	Detected	0.000	1.227	Detected	1.137	Detected	1.182	0.018	GT	Sens	[illegible]
GT	Sense	Sense	0.146	Detected	-0.146	Detected	0.000	1.113	Detected	1.008	Detected	1.061	0.021	GT	Sens	[illegible]
GT	Sense	Sense	0.580	Detected	-0.580	Detected	0.000	3.390	Detected	0.934	Detected	2.162	0.252	GT	Sens	[illegible]
GT	Sense	Sense	0.282	Detected	-0.282	Detected	0.000	1.308	Detected	1.558	Detected	1.433	0.043	GT	Sens	[illegible]
GT	Sense	Sense	0.268	Detected	-0.268	Detected	0.000	2.233	Detected	2.514	Detected	2.374	0.016	GT	Sens	[illegible]
GT	Sense	Sense	-0.302	Comprom	0.302	Comprom	0.000	1.419	Detected	2.028	Detected	1.723	0.057	GT	Sens	[illegible]
GT	Sense	Sense	-1.294	Comprom	1.294	Detected	0.000	1.146	Detected	0.860	Detected	1.003	0.522	GT	Sens	[illegible]
GT	Sense	Sense	0.726	Detected	-0.726	Detected	0.000	1.150	Detected	0.939	Detected	1.044	0.291	GT	Sens	[illegible]
GT	Sense	Sense	-0.101	Detected	0.101	Detected	0.000	2.107	Detected	0.952	Detected	1.529	0.121	GT	Sens	[illegible]
GT	Sense	Sense	-0.082	Detected	0.082	Detected	0.000	1.759	Detected	0.884	Detected	1.322	0.097	GT	Sens	[illegible]
GT	Sense	Sense	-0.159	Detected	0.159	Detected	0.000	0.834	Detected	1.304	Detected	1.069	0.064	GT	Sens	[illegible]
GT	Sense	Sense	-0.093	Comprom	0.093	Detected	0.000	0.903	Detected	2.258	Detected	1.581	0.147	GT	Sens	[illegible]
GT	Sense	Sense	0.141	Comprom	-0.141	Comprom	0.000	1.338	Detected	1.525	Detected	1.431	0.014	GT	Sens	[illegible]
GT	Sense	Sense	-0.247	Detected	0.247	Detected	0.000	0.962	Detected	2.602	Detected	1.782	0.173	GT	Sens	[illegible]
GT	Sense	Sense	0.294	Detected	-0.294	Detected	0.000	1.360	Detected	0.928	Detected	1.144	0.088	GT	Sens	[illegible]
GT	Sense	Sense	0.811	Detected	-0.811	Detected	0.000	3.022	Detected	3.367	Detected	3.195	0.061	GT	Sens	[illegible]
GT	Sense	Sense	0.758	Detected	-0.758	Detected	0.000	1.305	Detected	0.802	Detected	1.053	0.318	GT	Sens	[illegible]
GT	Sense	Sense	-0.168	Comprom	0.168	Comprom	0.000	2.038	Detected	2.340	Detected	2.189	0.010	GT	Sens	[illegible]
GT	Sense	Sense	-0.007	Detected	0.007	Detected	0.000	2.029	Detected	1.117	Detected	1.573	0.075	GT	Sens	[illegible]
GT	Sense	Sense	0.412	Detected	-0.412	Detected	0.000	1.841	Detected	1.307	Detected	1.574	0.085	GT	Sens	[illegible]
GT	Sense	Sense	-0.076	Detected	0.076	Detected	0.000	1.146	Detected	0.909	Detected	1.027	0.018	GT	Sens	[illegible]
GT	Sense	Sense	0.042	Detected	-0.042	Detected	0.000	1.601	Detected	2.231	Detected	1.916	0.026	GT	Sens	[illegible]
GT	Sense	Sense	-0.204	Comprom	0.204	Comprom	0.000	4.796	Detected	2.296	Detected	3.546	0.107	GT	Sens	[illegible]
GT	Sense	Sense	0.126	Comprom	-0.126	Comprom	0.000	1.686	Detected	1.637	Detected	1.662	0.006	GT	Sens	[illegible]
GT	Sense	Sense	-0.182	Comprom	0.182	Comprom	0.000	1.546	Detected	1.473	Detected	1.509	0.015	GT	Sens	[illegible]
GT	Sense	Sense	-0.219	Comprom	0.219	Detected	0.000	1.435	Detected	1.461	Detected	1.448	0.022	GT	Sens	[illegible]
GT	Sense	Sense	0.081	Detected	-0.081	Detected	0.000	1.248	Detected	1.172	Detected	1.210	0.005	GT	Sens	[illegible]
GT	Sense	Sense	-0.068	Detected	0.068	Detected	0.000	0.921	Detected	1.465	Detected	1.193	0.051	GT	Sens	[illegible]
GT	Sense	Sense	-0.027	Detected	0.027	Detected	0.000	3.135	Detected	3.261	Detected	3.198	0.000	GT	Sens	[illegible]
GT	Sense	Sense	-0.176	Comprom	0.176	Comprom	0.000	1.870	Detected	2.800	Detected	2.335	0.042	GT	Sens	[illegible]
GT	Sense	Sense	0.150	Detected	-0.150	Detected	0.000	1.173	Detected	1.462	Detected	1.317	0.024	GT	Sens	[illegible]
GT	Sense	Sense	-0.083	Detected	0.083	Detected	0.000	1.394	Detected	1.430	Detected	1.412	0.004	GT	Sens	[illegible]
GT	Sense	Sense	-0.438	Comprom	0.438	Comprom	0.000	2.739	Detected	2.158	Detected	2.449	0.043	GT	Sens	[illegible]
GT	Sense	Sense	-0.002	Detected	0.002	Detected	0.000	3.119	Detected	2.382	Detected	2.751	0.017	GT	Sens	[illegible]
GT	Sense	Sense	-0.743	Comprom	0.743	Comprom	0.000	1.841	Detected	3.387	Detected	2.614	0.135	GT	Sens	[illegible]
GT	Sense	Sense	-0.536	Comprom	0.536	Comprom	0.000	1.355	Detected	1.598	Detected	1.477	0.115	GT	Sens	[illegible]
GT	Sense	Sense	0.228	Detected	-0.228	Detected	0.000	1.317	Detected	1.686	Detected	1.502	0.036	GT	Sens	[illegible]
GT	Sense	Sense	-0.065	Detected	0.065	Detected	0.000	1.136	Detected	2.683	Detected	1.909	0.133	GT	Sens	[illegible]
GT	Sense	Sense	-0.008	Detected	0.008	Detected	0.000	1.949	Detected	1.450	Detected	1.700	0.021	GT	Sens	[illegible]
GT	Sense	Sense	0.148	Detected	-0.148	Detected	0.000	1.302	Detected	1.664	Detected	1.483	0.024	GT	Sens	[illegible]
GT	Sense	Sense	0.267	Comprom	-0.267	Comprom	0.000	1.954	Detected	1.438	Detected	1.696	0.045	GT	Sens	[illegible]
GT	Sense	Sense	1.199	Detected	-1.199	Detected	0.000	2.275	Detected	2.230	Detected	2.253	0.201	GT	Sens	[illegible]
GT	Sense	Sense	0.249	Detected	-0.249	Detected	0.000	1.243	Detected	1.798	Detected	1.520	0.055	GT	Sens	[illegible]
GT	Sense	Sense	-0.571	Comprom	0.571	Comprom	0.000	1.875	Detected	1.883	Detected	1.879	0.081	GT	Sens	[illegible]
GT	Sense	Sense	0.000	Detected	0.000	Detected	0.000	1.315	Detected	1.037	Detected	1.176	0.014	GT	Sens	[illegible]
GT	Sense	Sense	0.546	Detected	-0.546	Detected	0.000	1.636	Detected	1.480	Detected	1.558	0.106	GT	Sens	[illegible]
GT	Sense	Sense	0.355	Detected	-0.355	Detected	0.000	0.952	Detected	1.099	Detected	1.025	0.106	GT	Sens	[illegible]
GT	Sense	Sense	-0.119	Detected	0.119	Detected	0.000	1.226	Detected	1.005	Detected	1.115	0.021	GT	Sens	[illegible]
GT	Sense	Sense	0.428	Detected	-0.428	Detected	0.000	1.595	Detected	1.635	Detected	1.615	0.064	GT	Sens	[illegible]
GT	Sense	Sense	0.072	Detected	-0.072	Detected	0.000	1.293	Detected	1.363	Detected	1.328	0.004	GT	Sens	[illegible]
GT	Sense	Sense	0.016	Detected	-0.016	Detected	0.000	1.036	Detected	1.376	Detected	1.206	0.019	GT	Sens	[illegible]
GT	Sense	Sense	-0.042	Detected	0.042	Detected	0.000	0.892	Detected	1.180	Detected	1.036	0.020	GT	Sens	[illegible]
GT	Sense	Sense	0.097	Detected	-0.097	Detected	0.000	1.622	Detected	1.579	Detected	1.600	0.004	GT	Sens	[illegible]
GT	Sense	Sense	0.283	Detected	-0.283	Detected	0.000	2.524	Detected	2.093	Detected	2.308	0.023	GT	Sens	[illegible]
GT	Sense	Sense	0.517	Detected	-0.517	Detected	0.000	1.437	Detected	1.032	Detected	1.235	0.156	GT	Sens	[illegible]
GT	Sense	Sense	0.415	Comprom	-0.415	Comprom	0.000	1.555	Detected	2.277	Detected	1.916	0.073	GT	Sens	[illegible]
GT	Sense	Sense	0.453	Detected	-0.453	Detected	0.000	1.016	Detected	1.116	Detected	1.066	0.144	GT	Sens	[illegible]
GT	Sense	Sense	0.303	Detected	-0.303	Detected	0.000	1.528	Detected	2.161	Detected	1.844	0.052	GT	Sens	[illegible]
GT	Sense	Sense	0.021	Detected	-0.021	Detected	0.000	1.518	Detected	1.914	Detected	1.716	0.013	GT	Sens	[illegible]
GT	Sense	Sense	0.124	Detected	-0.124	Detected	0.000	1.070	Detected	0.951	Detected	1.010	0.018	GT	Sens	[illegible]
GT	Sense	Sense	0.499	Comprom	-0.499	Comprom	0.000	1.368	Detected	2.016	Detected	1.692	0.105	GT	Sens	[illegible]
GT	Sense	Sense	-0.212	Detected	0.212	Detected	0.000	1.984	Detected	1.578	Detected	1.781	0.026	GT	Sens	[illegible]
GT	Sense	Sense	0.210	Detected	-0.210	Detected	0.000	1.296	Detected	1.222	Detected	1.259	0.028	GT	Sens	[illegible]
GT	Sense	Sense	-0.965	Comprom	0.965	Comprom	0.000	0.802	Detected	2.814	Detected	1.808	0.324	GT	Sens	[illegible]

GT	Sense	Sense	0.289	Comprom	-0.289	Comprom	0.000	1.135	Detected	0.994	Detected	1.064	0.070	GT	Sens	contig169	contig169	Ulp1 prot	AAATGA	[illegible]
GT	Sense	Sense	-0.873	Comprom	0.873	Detected	0.000	7.272	Detected	7.857	Detected	7.564	0.014	GT	Sens	contig170	contig170	Gag poly	CTGCAT	[illegible]
GT	Sense	Sense	0.202	Detected	-0.202	Detected	0.000	1.379	Detected	1.539	Detected	1.459	0.022	GT	Sens	contig171	contig171	Ulp1 prot	TGCAAC	[illegible]
GT	Sense	Sense	-0.166	Detected	0.166	Detected	0.000	1.336	Detected	0.822	Detected	1.079	0.072	GT	Sens	contig171	contig171	Ulp1 prot	CTCATA	[illegible]
GT	Sense	Sense	0.415	Detected	-0.415	Detected	0.000	2.117	Detected	1.405	Detected	1.761	0.084	GT	Sens	contig172	contig172	Wound in	CCAAGA	[illegible]
GT	Sense	Sense	-0.597	Comprom	0.597	Comprom	0.000	2.859	Detected	2.529	Detected	2.694	0.049	GT	Sens	contig172	contig172	Receptor	CTGAGG	[illegible]
GT	Sense	Sense	0.302	Detected	-0.302	Detected	0.000	1.455	Detected	1.310	Detected	1.382	0.047	GT	Sens	contig174	contig174	Plant syn	ATCGGT	[illegible]
GT	Sense	Sense	0.014	Detected	-0.014	Detected	0.000	1.419	Detected	1.471	Detected	1.445	0.000	GT	Sens	contig176	contig176	S-phase	AGACAT	[illegible]
GT	Sense	Sense	-0.048	Detected	0.048	Detected	0.000	0.979	Detected	1.126	Detected	1.053	0.007	GT	Sens	contig180	contig180	Caffeoyl-	AGATTA	[illegible]
GT	Sense	Sense	0.130	Detected	-0.130	Detected	0.000	0.828	Detected	1.458	Detected	1.143	0.079	GT	Sens	contig181	contig181	Aldehyde	TGTGGC	[illegible]
GT	Sense	Sense	0.291	Detected	-0.291	Detected	0.000	1.353	Detected	1.292	Detected	1.323	0.046	GT	Sens	contig182	contig182	Ubiquitin	TCTACC	[illegible]
GT	Sense	Sense	0.483	Detected	-0.483	Detected	0.000	1.246	Detected	1.033	Detected	1.139	0.148	GT	Sens	contig182	contig182	Unknown	TTATGT	[illegible]
GT	Sense	Sense	0.446	Detected	-0.446	Detected	0.000	1.742	Detected	2.265	Detected	2.003	0.061	GT	Sens	contig183	contig183	Photosys	CCTAGC	[illegible]
GT	Sense	Sense	0.189	Comprom	-0.189	Comprom	0.000	2.027	Detected	4.489	Detected	3.258	0.120	GT	Sens	contig185	contig185	Ethylene-	GTACAT	[illegible]
GT	Sense	Sense	0.134	Detected	-0.134	Detected	0.000	1.356	Detected	1.701	Detected	1.529	0.020	GT	Sens	contig185	contig185	Unknown	GGAGTT	[illegible]
GT	Sense	Sense	-0.080	Detected	0.080	Detected	0.000	1.639	Detected	1.187	Detected	1.413	0.028	GT	Sens	contig186	contig186	Ulp1 prot	GAGAAA	[illegible]
GT	Sense	Sense	-0.285	Detected	0.285	Detected	0.000	1.907	Detected	2.223	Detected	2.065	0.024	GT	Sens	contig187	contig187	Unknown	TAAAAA	[illegible]
GT	Sense	Sense	-0.949	Comprom	0.949	Detected	0.000	3.593	Detected	3.102	Detected	3.348	0.076	GT	Sens	contig19	contig19	Unknown	ATAGGA	[illegible]
GT	Sense	Sense	0.218	Detected	-0.218	Detected	0.000	0.983	Detected	1.607	Detected	1.295	0.077	GT	Sens	contig190	contig190	PIF-like tr	CTAGGG	[illegible]
GT	Sense	Sense	0.418	Detected	-0.418	Detected	0.000	0.920	Detected	1.779	Detected	1.349	0.153	GT	Sens	contig190	contig190	Rhamnos	CAAAGT	[illegible]
GT	Sense	Sense	-0.188	Detected	0.188	Detected	0.000	1.768	Detected	3.112	Detected	2.440	0.073	GT	Sens	contig194	contig194	Auxin-res	ATCAAT	[illegible]
GT	Sense	Sense	-0.219	Detected	0.219	Detected	0.000	1.431	Detected	2.865	Detected	2.148	0.103	GT	Sens	contig195	contig195	Auxin-ind	TGCTGT	[illegible]
GT	Sense	Sense	-0.864	Detected	0.864	Detected	0.000	2.474	Detected	1.907	Detected	2.191	0.138	GT	Sens	contig195	contig195	FAD-bind	GAGCTT	[illegible]
GT	Sense	Sense	-0.013	Detected	0.013	Detected	0.000	0.848	Detected	1.271	Detected	1.059	0.038	GT	Sens	contig195	contig195	Unknown	TAAATT	[illegible]
GT	Sense	Sense	-0.487	Comprom	0.487	Detected	0.000	1.250	Detected	1.029	Detected	1.140	0.150	GT	Sens	contig196	contig196	Cytochro	GTTGAG	[illegible]
GT	Sense	Sense	-0.656	Comprom	0.656	Comprom	0.000	1.665	Detected	2.426	Detected	2.045	0.114	GT	Sens	contig196	contig196	Peroxida	ATTCTA	[illegible]
GT	Sense	Sense	-0.315	Comprom	0.315	Comprom	0.000	1.848	Detected	3.039	Detected	2.443	0.068	GT	Sens	contig200	contig200	Leucine	AAGACT	[illegible]
GT	Sense	Sense	0.486	Detected	-0.486	Detected	0.000	1.091	Detected	2.241	Detected	1.666	0.157	GT	Sens	contig202	contig202	RING fing	TCGGTT	[illegible]
GT	Sense	Sense	-0.077	Detected	0.077	Detected	0.000	1.024	Detected	1.237	Detected	1.131	0.013	GT	Sens	contig203	contig203	Unknown	AAATTA	[illegible]
GT	Sense	Sense	0.205	Detected	-0.205	Detected	0.000	0.969	Detected	1.502	Detected	1.235	0.067	GT	Sens	contig203	contig203	Unknown	AATAGA	[illegible]
GT	Sense	Sense	0.553	Comprom	-0.553	Comprom	0.000	4.211	Detected	1.655	Detected	2.933	0.170	GT	Sens	contig204	contig204	Transcrip	TGTTTC	[illegible]
GT	Sense	Sense	-0.509	Detected	0.509	Detected	0.000	2.739	Detected	1.136	Detected	1.938	0.178	GT	Sens	contig205	contig205	Ovate pro	CATTAT	[illegible]
GT	Sense	Sense	0.057	Detected	-0.057	Detected	0.000	1.549	Detected	1.294	Detected	1.421	0.010	GT	Sens	contig207	contig207	Unknown	TGTCAT	[illegible]
GT	Sense	Sense	0.249	Detected	-0.249	Detected	0.000	1.094	Detected	1.766	Detected	1.430	0.076	GT	Sens	contig208	contig208	Unknown	TGTTGC	[illegible]
GT	Sense	Sense	-0.460	Detected	0.460	Detected	0.000	2.611	Detected	1.841	Detected	2.226	0.066	GT	Sens	contig209	contig209	1-aminoc	GACTAC	[illegible]
GT	Sense	Sense	-0.489	Comprom	0.489	Detected	0.000	1.131	Detected	2.824	Detected	1.978	0.181	GT	Sens	contig209	contig209	Exocyst	CAGCAG	[illegible]
GT	Sense	Sense	0.035	Detected	-0.035	Detected	0.000	2.153	Detected	1.259	Detected	1.706	0.063	GT	Sens	contig210	contig210	Wound in	TAGAAG	[illegible]
GT	Sense	Sense	-0.146	Detected	0.146	Detected	0.000	1.050	Detected	1.966	Detected	1.508	0.088	GT	Sens	contig210	contig210	1-aminoc	CCCAAT	[illegible]
GT	Sense	Sense	0.077	Detected	-0.077	Detected	0.000	1.292	Detected	0.911	Detected	1.102	0.033	GT	Sens	contig212	contig212	NBS resis	TCCTTA	[illegible]
GT	Sense	Sense	0.033	Detected	-0.033	Detected	0.000	1.307	Detected	1.585	Detected	1.446	0.010	GT	Sens	contig212	contig212	Unknown	GGAGTT	[illegible]
GT	Sense	Sense	0.187	Detected	-0.187	Detected	0.000	1.411	Detected	1.954	Detected	1.682	0.036	GT	Sens	contig213	contig213	Cullin 1-li	GATAAC	[illegible]
GT	Sense	Sense	0.372	Detected	-0.372	Detected	0.000	1.983	Detected	2.417	Detected	2.200	0.036	GT	Sens	contig214	contig214	Cortical c	TAGTTT	[illegible]
GT	Sense	Sense	0.049	Detected	-0.049	Detected	0.000	0.936	Detected	1.160	Detected	1.048	0.013	GT	Sens	contig215	contig215	Unknown	CAATGC	[illegible]
GT	Sense	Sense	-0.173	Detected	0.173	Detected	0.000	1.197	Detected	0.807	Detected	1.002	0.062	GT	Sens	contig216	contig216	Pentatric	TAATCT	[illegible]
GT	Sense	Sense	-0.064	Comprom	0.064	Comprom	0.000	1.324	Detected	7.083	Detected	4.203	0.282	GT	Sens	contig217	contig217	Unknown	AGTAAG	[illegible]
GT	Sense	Sense	-0.341	Comprom	0.341	Comprom	0.000	1.997	Detected	2.230	Detected	2.114	0.028	GT	Sens	contig219	contig219	Nitrate tra	GGAGTA	[illegible]
GT	Sense	Sense	0.195	Detected	-0.195	Detected	0.000	0.913	Detected	1.242	Detected	1.077	0.052	GT	Sens	contig220	contig220	Short-cha	GGTTAA	[illegible]
GT	Sense	Sense	0.325	Detected	-0.325	Detected	0.000	1.144	Detected	1.103	Detected	1.124	0.075	GT	Sens	contig221	contig221	Unknown	GAAAAT	[illegible]
GT	Sense	Sense	-0.184	Detected	0.184	Detected	0.000	1.186	Detected	0.965	Detected	1.076	0.038	GT	Sens	contig221	contig221	Unknown	TACATA	[illegible]
GT	Sense	Sense	-0.102	Detected	0.102	Detected	0.000	1.027	Detected	1.363	Detected	1.195	0.026	GT	Sens	contig222	contig222	Subtilisin	TAGTTT	[illegible]
GT	Sense	Sense	0.036	Detected	-0.036	Detected	0.000	0.916	Detected	1.684	Detected	1.300	0.078	GT	Sens	contig222	contig222	Sister-chr	CTCATT	[illegible]
GT	Sense	Sense	-0.388	Comprom	0.388	Comprom	0.000	2.320	Detected	2.933	Detected	2.626	0.034	GT	Sens	contig223	contig223	Unknown	GATGAG	[illegible]
GT	Sense	Sense	0.552	Detected	-0.552	Detected	0.000	1.951	Detected	2.422	Detected	2.186	0.068	GT	Sens	contig223	contig223	Transcrip	AGGAGA	[illegible]
GT	Sense	Sense	0.246	Detected	-0.246	Comprom	0.000	0.917	Detected	1.091	Detected	1.004	0.062	GT	Sens	contig223	contig223	Periaxin-l	AGGAAG	[illegible]
GT	Sense	Sense	0.213	Detected	-0.213	Detected	0.000	1.094	Detected	1.009	Detected	1.052	0.040	GT	Sens	contig225	contig225	Subtilisin	AAGTTG	[illegible]
GT	Sense	Sense	0.029	Detected	-0.029	Detected	0.000	1.081	Detected	2.301	Detected	1.691	0.109	GT	Sens	contig225	contig225	Pathogen	TGAAGA	[illegible]
GT	Sense	Sense	-0.825	Comprom	0.825	Comprom	0.000	1.977	Detected	2.396	Detected	2.186	0.124	GT	Sens	contig225	contig225	Sulfotran	TGGAGG	[illegible]
GT	Sense	Sense	-0.166	Comprom	0.166	Comprom	0.000	0.800	Detected	1.985	Detected	1.393	0.152	GT	Sens	contig226	contig226	Structura	TTCATT	[illegible]
GT	Sense	Sense	-0.132	Detected	0.132	Detected	0.000	0.931	Detected	1.135	Detected	1.033	0.025	GT	Sens	contig227	contig227	Unknown	TATGAA	[illegible]
GT	Sense	Sense	0.155	Detected	-0.155	Detected	0.000	1.008	Detected	1.014	Detected	1.011	0.023	GT	Sens	contig227	contig227	Transcrip	GTTGCT	[illegible]
GT	Sense	Sense	-0.180	Detected	0.180	Detected	0.000	2.146	Detected	2.064	Detected	2.105	0.008	GT	Sens	contig228	contig228	Unknown	GAGATC	[illegible]
GT	Sense	Sense	0.324	Detected	-0.324	Detected	0.000	1.129	Detected	1.212	Detected	1.170	0.070	GT	Sens	contig228	contig228	Lysine ke	GCCTTT	[illegible]
GT	Sense	Sense	0.137	Detected	-0.137	Detected	0.000	1.060	Detected	1.730	Detected	1.395	0.061	GT	Sens	contig229	contig229	Receptor	AATAAAT	[illegible]
GT	Sense	Sense	0.744	Comprom	-0.744	Comprom	0.000	2.845	Detected	2.591	Detected	2.718	0.069	GT	Sens	contig231	contig231	Omega-6	GCATGT	[illegible]
GT	Sense	Sense	0.250	Detected	-0.250	Comprom	0.000	0.990	Detected	1.041	Detected	1.015	0.056	GT	Sens	contig231	contig231	Solute ca	GGGATT	[illegible]
GT	Sense	Sense	0.112	Detected	-0.112	Detected	0.000	1.407	Detected	1.243	Detected	1.325	0.011	GT	Sens	contig231	contig231	Unknown	CCAAAG	[illegible]
GT	Sense	Sense	0.487	Detected	-0.487	Detected	0.000	0.867	Detected	1.381	Detected	1.124	0.178	GT	Sens	contig232	contig232	Cytochro	CTTTGT	[illegible]
GT	Sense	Sense	-0.087	Detected	0.087	Detected	0.000	1.810	Detected	1.655	Detected	1.733	0.004	GT	Sens	contig233	contig233	Endonuc	CTTAGA	[illegible]
GT	Sense	Sense	0.411	Detected	-0.411	Comprom	0.000	1.152	Detected	1.457	Detected	1.304	0.097	GT	Sens	contig234	contig234	Alpha-hy	GCATTG	[illegible]
GT	Sense	Sense	-0.824	Detected	0.824	Detected	0.000	5.259	Detected	1.121	Detected	3.190	0.288	GT	Sens	contig234	contig234	Hydroxy	AATTCA	[illegible]
GT	Sense	Sense	-0.510	Detected	0.510	Detected	0.000	1.851	Detected	2.715	Detected	2.283	0.076	GT	Sens	contig235	contig235	Subtilisin	CTACCA	[illegible]
GT	Sense	Sense	0.110	Detected	-0.110	Detected	0.000	1.961	Detected	2.175	Detected	2.068	0.005	GT	Sens	contig236	contig236	Unknown	AGAAGA	[illegible]
GT	Sense	Sense	0.000	Comprom	0.000	Comprom	0.000	1.850	Detected	1.786	Detected	1.818	0.000	GT	Sens	contig238	contig238	Unknown	AAACAG	[illegible]
GT	Sense	Sense	-0.591	Detected	0.591	Detected	0.000	2.099	Detected	2.672	Detected	2.385	0.068	GT	Sens	contig238	contig238	Chymotry	CCATAG	[illegible]
GT	Sense	Sense	-0.640	Comprom	0.640	Comprom	0.000	1.704	Detected	2.311	Detected	2.008	0.105	GT	Sens	contig238	contig238	Solute ca	TGAAGA	[illegible]
GT	Sense	Sense	0.045	Detected	-0.045	Detected	0.000	1.813	Detected	2.874	Detected	2.343	0.048	GT	Sens	contig239	contig239	Chromod	GAAGTG	[illegible]
GT	Sense	Sense	0.126	Detected	-0.126	Detected	0.000	1.877	Detected	2.378	Detected	2.128	0.017	GT	Sens	contig240	contig240	Calcium-l	GTTGAT	[illegible]
GT	Sense	Sense	1.064	Detected	-1.064	Detected	0.000	2.934	Detected	1.371	Detected	2.153	0.245	GT	Sens	contig240	contig240	EIN3-bind	CACAAG	[illegible]
GT	Sense	Sense	0.217	Detected	-0.217	Detected	0.000	0.981	Detected	1.105	Detected	1.043	0.044	GT	Sens	contig243	contig243	Unknown	GCGTGG	[illegible]
GT	Sense	Sense	-0.017	Detected	0.017	Detected	0.000	1.863	Detected	2.528	Detected	2.196	0.022	GT	Sens	contig246	contig246	Unknown	GACTTG	[illegible]
GT	Sense	Sense	-0.578	Detected	0.578	Detected	0.000	1.664	Detected	2.795	Detected	2.229	0.110	GT	Sens	contig247	contig247	Subtilisin	ACATTT	[illegible]
GT	Sense	Sense	0.478	Detected	-0.478	Detected	0.000	1.289	Detected	2.601	Detected	1.945	0.139	GT	Sens	contig247	contig247	Unknown	CTGTAT	[illegible]
GT	Sense	Sense	0.557	Comprom	-0.557	Comprom	0.000	5.115	Detected	2.729	Detected	3.922	0.097	GT	Sens	contig248	contig248	Unknown	GACAGA	[illegible]
GT	Sense	Sense	-0.005	Detected	0.005	Detected	0.000	3.316	Detected	0.963	Detected	2.139	0.211	GT	Sens	contig249	contig249	Alcohol d	GAATTG	[illegible]
GT	Sense	Sense	0.207	Detected	-0.207	Detected	0.000	1.372	Detected	1.965	Detected	1.668	0.044	GT	Sens	contig249	contig249	Os03g08	GGAAGG	[illegible]
GT	Sense	Sense	0.435	Detected	-0.435	Detected	0.000	1.091	Detected	2.482	Detected	1.787	0.161	GT	Sens	contig251	contig251	Unknown	GAGTTG	[illegible]
GT	Sense	Sense	0.335	Detected	-0.335	Detected	0.000	1.807	Detected	1.997	Detected	1.902	0.032	GT	Sens	contig252	contig252	Yippee-lik	CAGAAA	[illegible]
GT	Sense	Sense	0.418	Comprom	-0.418	Comprom	0.000	1.102	Detected	1.827	Detected	1.464	0.118	GT	Sens	contig253	contig253	Amino ac	TGGTAT	[illegible]
GT	Sense	Sense	-0.065	Detected	0.065	Detected	0.000	2.011	Detected	1.282	Detected	1.646	0.047	GT	Sens	contig253	contig253	Cytochro	AAATGA	[illegible]
GT	Sense	Sense	-0.077	Comprom	0.077	Comprom	0.000	1.364	Detected	1.912	Detected	1.638	0.029	GT	Sens	contig255	contig255	2-isoprop	TGAGTA	[illegible]
GT	Sense	Sense	-0.797	Comprom	0.797	Comprom	0.000	5.526	Detected	2.133	Detected	3.830	0.178	GT	Sens	contig256	contig256	Unknown	GCCATT	[illegible]
GT	Sense	Sense	0.270	Comprom	-0.270	Comprom	0.000	0.861	Detected	1.181	Detected	1.021	0.083	GT	Sens	contig256	contig256	Mutator-li	TATGGT	[illegible]
GT	Sense	Sense	0.440	Detected	-0.440	Detected	0.000	1.259	Detected	1.438	Detected	1.349	0.095	GT	Sens	contig256	contig256	Homeobo	ACTCAA	[illegible]
GT	Sense	Sense	0.437	Comprom	-0.437	Comprom	0.000	1.252	Detected	1.871	Detected	1.562	0.100	GT	Sens	contig256	contig256	Sister-chr	AATCAT	[illegible]
GT	Sense	Sense	-0.232	Detected	0.232	Detected	0.000	1.800	Detected	1.692	Detected	1.746	0.018	GT	Sens	contig261	contig261	Ribosoma	TTGAAG	[illegible]
GT	Sense	Sense	-0.961	Comprom	0.961	Detected	0.000	2.138	Detected	2.040	Detected	2.089	0.162	GT	Sens	contig262	contig262	Acid pho	CACTTA	[illegible]
GT	Sense	Sense	0.088	Detected	-0.088	Detected	0.000	0.887	Detected	1.164	Detected	1.026	0.025	GT	Sens	contig264	contig264	Receptor	AGCACT	[illegible]
GT	Sense	Sense	0.062	Comprom	-0.062	Comprom	0.000	1.529	Detected	2.020	Detected	1.774	0.020	GT	Sens	contig264	contig264	Phenylala	GGTTTT	[illegible]
GT	Sense	Sense	0.141	Detected	-0.141	Detected	0.000	1.305	Detected	1.329	Detected	1.317	0.011	GT	Sens	contig266	contig266	Pyridine	TTGATA	[illegible]
GT	Sense	Sense	0.318	Detected	-0.318	Detected	0.000	1.086	Detected	1.251	Detected	1.169	0.071	GT	Sens	contig267	contig267	Mutator-li	ATGGGG	[illegible]
GT	Sense	Sense	-0.146	Comprom	0.146	Detected	0.000	0.822	Detected	1.646	Detected	1.234	0.106	GT	Sens	contig268	contig268	Thioester	ACAAAC	[illegible]
GT	Sense	Sense	0.451	Detected	-0.451	Detected	0.000	1.074	Detected	0.996	Detected	1.035	0.150	GT	Sens	contig269	contig269	Aminotra	CCAGAA	[illegible]
GT	Sense	Sense	-0.151	Detected	0.151	Detected	0.000	1.198	Detected	1.131	Detected	1.164	0.017	GT	Sens	contig270	contig270	Cytochro	TTAGCA	[illegible]
GT	Sense	Sense	0.380	Detected	-0.380	Detected	0.000	1.118	Detected	1.512	Detected	1.315	0.092	GT	Sens	contig272	contig272	Unknown	ACCAGC	[illegible]
GT	Sense	Sense	-0.241	Detected	0.241	Detected	0.000	1.565	Detected	1.742	Detected	1.653	0.023	GT	Sens	contig273	contig273	Dof zinc f	GTCTTT	[illegible]
GT	Sense	Sense	0.226	Detected	-0.226	Detected	0.000	1.127	Detected	1.665	Detected	1.396	0.058	GT	Sens	contig274	contig274	F-box fam	TTTTTT	[illegible]
GT	Sense	Sense	0.482	Detected	-0.482	Detected	0.000	1.965	Detected	2.308	Detected	2.137	0.053	GT	Sens	contig274	contig274	Hydroxyc	TCTGAG	[illegible]
GT	Sense	Sense	1.289	Detected	-1.289	Comprom	0.000	0.908	Detected	1.690	Detected	1.299	0.437	GT	Sens	contig277	contig277	Cytochro	ATTCCG	[illegible]
GT	Sense	Sense	-0.287	Detected	0.287	Detected	0.000	1.237	Detected	2.787	Detected	2.012	0.135	GT	Sens	contig277	contig277	Auxin-res	TTCTTG	[illegible]
GT	Sense	Sense	0.194	Detected	-0.194	Detected	0.000	3.949	Detected	3.207	Detected	3.578	0.013	GT	Sens	contig278	contig278	Cellulose	CTTTTG	[illegible]
GT	Sense	Sense	0.009	Detected	-0.009	Detected	0.000	0.850	Detected	1.560	Detected	1.205	0.077	GT	Sens	contig280	contig280	Cytochro	TAGTCC	[illegible]
GT	Sense	Sense	0.794	Comprom	-0.794	Comprom	0.000	2.332	Detected	5.429	Detected	3.880	0.156	GT	Sens	contig280	contig280	Ethylene	GGGAAC	[illegible]
GT	Sense	Sense	-0.056	Detected	0.056	Detected	0.000	2.571	Detected	2.691	Detected	2.631	0.001	GT	Sens	contig282	contig282	F-box fam	GTTGAT	[illegible]
GT	Sense	Sense	0.363	Comprom	-0.363	Comprom	0.000	1.613	Detected	1.139	Detected	1.376	0.087	GT	Sens	contig283	contig283	Mutator-li	TATTTAT	[illegible]
GT	Sense	Sense	0.045	Detected	-0.045	Detected	0.000	1.576	Detected	2.229	Detected	1.902	0.029	GT	Sens	contig284	contig284	Unknown	GCTGAG	[illegible]
GT	Sense	Sense	0.513	Comprom	-0.513	Comprom	0.000	1.099	Detected	2.349	Detected	1.724	0.167	GT	Sens	contig284	contig284	Transpor	CATGGT	[illegible]
GT	Sense	Sense	0.071	Detected	-0.071	Detected	0.000	1.702	Detected	1.967	Detected	1.835	0.007	GT	Sens	contig285	contig285	CTP synt	TGAAAG	[illegible]
GT	Sense	Sense	-0.116	Detected	0.116	Detected	0.000	1.581	Detected	1.635	Detected	1.608	0.005	GT	Sens	contig285	contig285	Hcr2-p4.1	CAGAGT	[illegible]
GT	Sense	Sense	-0.566	Comprom	0.566	Comprom	0.000	1.337	Detected	2.090	Detected	1.713	0.128	GT	Sens	contig286	contig286	Unknown	AATCTT	[illegible]
GT	Sense	Sense	-0.171	Detected	0.171	Detected	0.000	1.518	Detected	1.195	Detected	1.356	0.029	GT	Sens	contig287	contig287	MuDR fam	GAGTTT	[illegible]
GT	Sense	Sense	-0.534	Detected	0.534	Detected	0.000	2.360	Detected	3.020	Detected	2.690	0.050	GT	Sens	contig288	contig288	Chymotry	CCATAT	[illegible]
GT	Sense	Sense	0.261	Detected	-0.261	Detected	0.000	1.254	Detected	2.147	Detected	1.701	0.081	GT	Sens	contig290	contig290	Cytochro	TGACAC	[illegible]
GT	Sense	Sense	-0.081	Detected	0.081	Detected	0.000	3.120	Detected	1.870	Detected	2.495	0.058	GT	Sens	contig291	contig291	Unknown	GTAACT	[illegible]
GT	Sense	Sense	0.015	Detected	-0.015	Detected	0.000	1.045	Detected	1.583	Detected	1.314	0.040	GT	Sens	contig291	contig291	Solute ca	GCTGCA	[illegible]
GT	Sense	Sense	0.255	Comprom	-0.255	Comprom	0.000	1.784	Detected	2.851	Detected	2.318	0.059	GT	Sens	contig291	contig291	Pentatric	GAAAAA	[illegible]
GT	Sense	Sense	-0.022	Detected	0.022	Detected	0.000	1.174	Detected	1.277	Detected	1.226	0.002	GT	Sens	contig292	contig292	Storekee	AGGTTG	[illegible]
GT	Sense	Sense	-0.045	Detected	0.045	Detected	0.000	1.387	Detected	0.838	Detected	1.113	0.057	GT	Sens	contig292	contig292	UDP-gluc	AGAGAT	[illegible]
GT	Sense	Sense	-0.300	Detected	0.300	Detected	0.000	2.058	Detected	1.720	Detected	1.889	0.032	GT	Sens	contig295	contig295	60S ribos	GGCTTC	[illegible]
GT	Sense	Sense	1.123	Detected	-1.123	Detected	0.000	1.961	Detected	1.998	Detected	1.979	0.220	GT	Sens	contig296	contig296	Ulp1 prot	GAAAGG	[illegible]
GT	Sense	Sense	0.444	Detected	-0.444	Detected	0.000	1.600	Detected	1.431	Detected	1.516	0.079	GT	Sens	contig299	contig299	Mutator-li	GCAAGA	[illegible]
GT	Sense	Sense	-0.431	Comprom	0.431	Comprom	0.000	2.237	Detected	2.343	Detected	2.290	0.034	GT	Sens	contig299	contig299	Unknown	TCGGTG	[illegible]
GT	Sense	Sense	-0.170	Comprom	0.170	Comprom	0.000	1.722	Detected	2.187	Detected	1.954	0.021	GT	Sens	contig306	contig306	Ulp1 prot	CACTTT	[illegible]
GT	Sense	Sense	-0.263	Comprom	0.263	Detected	0.000	1.468	Detected	0.842	Detected	1.155	0.106	GT	Sens	contig307	contig307	Cytochro	CTCCAC	[illegible]
GT	Sense	Sense	-0.099	Detected	0.099	Detected	0.000	1.648	Detected	2.070	Detected	1.859	0.015	GT	Sens	contig308	contig308	Transpor	ACTGCA	[illegible]
GT	Sense	Sense	0.491	Detected	-0.491	Detected	0.000	1.721	Detected	1.879	Detected	1.800	0.069	GT	Sens	contig310	contig310	Yippee zi	TTGCCC	[illegible]
GT	Sense	Sense	0.229	Detected	-0.229	Detected	0.000	0.984	Detected	1.136	Detected	1.060	0.048	GT	Sens	contig311	contig311	Photosys	AAGAAA	[illegible]
GT	Sense	Sense	-0.006	Detected	0.006	Detected	0.000	2.151	Detected	1.623	Detected	1.887	0.019	GT	Sens	contig312	contig312	CER1 (AH	TGAGAA	[illegible]
GT	Sense	Sense	-0.480	Comprom	0.480	Detected	0.000	2.055	Detected	1.043	Detected	1.549	0.157	GT	Sens	contig316	contig316	DNA-dire	AGCTCC	[illegible]
GT	Sense	Sense	-0.224	Detected	0.224	Detected	0.000	1.352	Detected	1.658	Detected	1.505	0.031	GT	Sens	contig329	contig329	cDNA clo	ACATCA	[illegible]
GT	Sense	Sense	0.006	Detected	-0.006	Detected	0.000	1.438	Detected	1.778	Detected	1.608	0.011	GT	Sens	contig337	contig337	Unknown	GGGATG	[illegible]
GT	Sense	Sense	0.616	Detected	-0.616	Comprom	0.000	0.962	Detected	1.671	Detected	1.316	0.205	GT	Sens	contig344	contig344	Serine/thr	ATCAAC	[illegible]
GT	Sense	Sense	-0.097	Comprom	0.097	Detected	0.000	2.037	Detected	2.239	Detected	2.138	0.004	GT	Sens	contig345	contig345	Unknown	TGGAAA	[illegible]
GT	Sense	Sense	0.369	Comprom	-0.369	Comprom	0.000	1.399	Detected	1.382	Detected	1.390	0.064	GT	Sens	contig358	contig358	Cytochro	CCAACT	[illegible]
GT	Sense	Sense	-0.085	Comprom	0.085	Detected	0.000	2.023	Detected	2.071	Detected	2.047	0.002	GT	Sens	contig370	contig370	Legumin	AACCCA	[illegible]
GT	Sense	Sense	0.379	Comprom	-0.379	Comprom	0.000	1.681	Detected	2.324	Detected	2.002	0.056	GT	Sens	contig376	contig376	Pathogen	GAAAGA	[illegible]
GT	Sense	Sense	0.222	Detected	-0.222	Detected	0.000	1.011	Detected	1.818	Detected	1.415	0.091	GT	Sens	contig386	contig386	Unknown	ATCGTT	[illegible]
GT	Sense	Sense	0.243	Detected	-0.243	Detected	0.000	2.190	Detected	2.630	Detected	2.410	0.018	GT	Sens	contig387	contig387	Terminal	AATTGT	[illegible]
GT	Sense	Sense	-0.371	Comprom	0.371	Detected	0.000	2.043	Detected	3.118	Detected	2.581	0.058	GT	Sens	contig405	contig405	Unannota	CCATAAGCGGCA	[illegible]
GT	Sense	Sense	0.137	Detected	-0.137	Detected	0.000	1.376	Detected	1.281	Detected	1.329	0.012	GT	Sens	contig411	contig411	Unannota	AATAGATGTTGAA	[illegible]
GT	Sense	Sense	-0.565	Comprom	0.565	Comprom	0.000	1.930	Detected	2.950	Detected	2.440	0.085	GT	Sens	contig418	contig418	Mutator-li	GCTGAT	[illegible]
GT	Sense	Sense	0.176	Comprom	-0.176	Comprom	0.000	4.407	Detected	3.408	Detected	3.908	0.018	GT	Sens	contig419	contig419	Unannota	CTCACCATTCAAA	[illegible]
GT	Sense	Sense	-0.193	Comprom	0.193	Detected	0.000	1.084	Detected	1.023	Detected	1.054	0.033	GT	Sens	contig461	contig461	Unknown	GGCTAC	[illegible]
GT	Sense	Sense	0.651	Detected	-0.651	Detected	0.000	0.992	Detected	1.082	Detected	1.037	0.253	GT	Sens	contig462	contig462	Glutaredo	CAAACC	[illegible]
GT	Sense	Sense	-0.594	Comprom	0.594	Detected	0.000	1.086	Detected	1.820	Detected	1.453	0.173	GT	Sens	contig489	contig489	Unknown	AGTGAT	[illegible]
GT	Sense	Sense	1.126	Detected	-1.126	Detected	0.000	2.786	Detected	1.199	Detected	1.993	0.285	GT	Sens	contig497	contig497	EIN3-bind	GCTGCT	[illegible]
GT	Sense	Sense	0.802	Comprom	-0.802	Comprom	0.000	1.731	Detected	2.095	Detected	1.913	0.146	GT	Sens	contig499	contig499	Unknown	TCAAAT	[illegible]
GT	Sense	Sense	-0.189	Comprom	0.189	Comprom	0.000	2.048	Detected	2.489	Detected	2.269	0.016	GT	Sens	contig500	contig500	Cytochro	GCAGGA	[illegible]
GT	Sense	Sense	0.392	Comprom	-0.392	Comprom	0.000	1.191	Detected	1.332	Detected	1.262	0.087	GT	Sens	contig516	contig516	Phenylco	GATGGC	[illegible]
GT	Sense	Sense	-0.139	Comprom	0.139	Detected	0.000	1.722	Detected	1.454	Detected	1.588	0.014	GT	Sens	contig518	contig518	Glutathio	AGGAAT	[illegible]
GT	Sense	Sense	0.535	Comprom	-0.535	Comprom	0.000	1.079	Detected	1.441	Detected	1.260	0.156	GT	Sens	contig521	contig521	Disease r	AGCAGA	[illegible]
GT	Sense	Sense	0.186	Detected	-0.186	Detected	0.000	1.387	Detected	0.822	Detected	1.104	0.082	GT	Sens	contig577	contig577	Myb-relat	CTCCAA	[illegible]
GT	Sense	Sense	-0.229	Detected	0.229	Detected	0.000	3.177	Detected	2.508	Detected	2.842	0.020	GT	Sens	contig582	contig582	Helicase-	CAATGG	[illegible]
GT	Sense	Sense	0.853	Detected	-0.853	Detected	0.000	1.460	Detected	1.399	Detected	1.429	0.236	GT	Sens	contig584	contig584	Epoxide	GTATTG	[illegible]
GT	Sense	Sense	0.173	Comprom	-0.173	Comprom	0.000	2.396	Detected	3.332	Detected	2.864	0.029	GT	Sens	contig584	contig584	Nonribos	GTTATT	[illegible]
GT	Sense	Sense	-0.209	Comprom	0.209	Comprom	0.000	3.551	Detected	3.069	Detected	3.310	0.009	GT	Sens	contig608	contig608	Aldo/keto	TGGTAT	[illegible]
GT	Sense	Sense	0.019	Detected	-0.019	Detected	0.000	1.239	Detected	1.310	Detected	1.275	0.001	GT	Sens	contig614	contig614	Unknown	GACTCC	[illegible]
GT	Sense	Sense	-0.015	Detected	0.015	Detected	0.000	1.373	Detected	1.546	Detected	1.459	0.004	GT	Sens	contig618	contig618	Cyclin-de	ACTTAT	[illegible]
GT	Sense	Sense	-0.161	Detected	0.161	Detected	0.000	1.350	Detected	0.828	Detected	1.089	0.071	GT	Sens	contig642	contig642	Ulp1 prot	AGAAGG	[illegible]
GT	Sense	Sense	-0.173	Detected	0.173	Detected	0.000	1.385	Detected	0.877	Detected	1.131	0.066	GT	Sens	contig645	contig645	Ulp1 prot	CCTCAA	[illegible]
GT	Sense	Sense	-0.667	Detected	0.667	Detected	0.000	1.153	Detected	1.296	Detected	1.225	0.209	GT	Sens	contig690	contig690	Proteinas	CAGGAT	[illegible]
GT	Sense	Sense	0.244	Comprom	-0.244	Comprom	0.000	0.826	Detected	1.805	Detected	1.315	0.138	GT	Sens	contig696	contig696	Peroxida	TTCAGA	[illegible]
GT	Sense	Sense	-0.134	Detected	0.134	Detected	0.000	0.808	Detected	2.275	Detected	1.541	0.175	GT	Sens	contig705	contig705	Unknown	ACAAAA	[illegible]
GT	Sense	Sense	-0.148	Comprom	0.148	Comprom	0.000	1.793	Detected	1.893	Detected	1.843	0.007	GT	Sens	contig712	contig712	Unknown	AACTAA	[illegible]
GT	Sense	Sense	-0.294	Detected	0.294	Detected	0.000	3.010	Detected	1.792	Detected	2.401	0.071	GT	Sens	contig715	contig715	Wound in	GCACAA	[illegible]
GT	Sense	Sense	-0.350	Detected	0.350	Detected	0.000	1.214	Detected	0.807	Detected	1.011	0.130	GT	Sens	contig736	contig736	Unknown	CGGATG	[illegible]
GT	Sense	Sense	-0.009	Comprom	0.009	Comprom	0.000	2.027	Detected	3.571	Detected	2.799	0.068	GT	Sens	contig739	contig739	Unknown	ATGTTG	[illegible]
GT	Sense	Sense	1.118	Detected	-1.118	Comprom	0.000	1.024	Detected	1.434	Detected	1.229	0.393	GT	Sens	contig752	contig752	Phosphat	AGAGTA	[illegible]
GT	Sense	Sense	-0.640	Detected	0.640	Detected	0.000	0.934	Detected	1.169	Detected	1.052	0.247	GT	Sens	contig754	contig754	Proteinas	AGAGTT	[illegible]
GT	Sense	Sense	-0.072	Detected	0.072	Detected	0.000	1.176	Detected	1.288	Detected	1.232	0.005	GT	Sens	contig763	contig763	Developm	TTATGG	[illegible]
GT	Sense	Sense	0.802	Detected	-0.802	Detected	0.000	0.850	Detected	1.289	Detected	1.069	0.327	GT	Sens	contig776	contig776	Cytochro	AAACTC	[illegible]
GT	Sense	Sense	-0.633	Comprom	0.633	Detected	0.000	1.015	Detected	1.843	Detected	1.429	0.199	GT	Sens	contig777	contig777	Unknown	GTAAAT	[illegible]
GT	Sense	Sense	0.335	Detected	-0.335	Detected	0.000	1.689	Detected	1.017	Detected	1.353	0.104	GT	Sens	contig782	contig782	Ankyrin r	GCAAGT	[illegible]
GT	Sense	Sense	0.967	Detected	-0.967	Comprom	0.000	1.963	Detected	1.425	Detected	1.694	0.233	GT	Sens	contig802	contig802	Glutathio	GATGAG	[illegible]
GT	Sense	Sense	0.150	Comprom	-0.150	Comprom	0.000	1.669	Detected	2.085	Detected	1.877	0.018	GT	Sens	contig817	contig817	Ulp1 prot	AGCGCA	[illegible]
GT	Sense	Sense	0.909	Detected	-0.909	Comprom	0.000	1.924	Detected	1.623	Detected	1.774	0.194	GT	Sens	contig827	contig827	Hydrolase	CAACTT	[illegible]
GT	Sense	Sense	0.242	Detected	-0.242	Detected	0.000	0.939	Detected	1.212	Detected	1.076	0.061	GT	Sens	contig828	contig828	Yippee zi	CTGAAA	[illegible]
GT	Sense	Sense	-0.338	Detected	0.338	Detected	0.000	0.905	Detected	2.444	Detected	1.675	0.185	GT	Sens	contig849	contig849	Auxin-ind	TGCTGT	[illegible]
GT	Sense	Sense	1.015	Comprom	-1.015	Comprom	0.000	1.362	Detected	2.814	Detected	2.088	0.236	GT	Sens	contig875	contig875	Mutator-li	GAAAAT	[illegible]
GT	Sense	Sense	0.098	Detected	-0.098	Detected	0.000	2.050	Detected	1.842	Detected	1.946	0.005	GT	Sens	contig890	contig890	Ribose-pl	AAAGCT	[illegible]
GT	Sense	Sense	0.540	Detected	-0.540	Comprom	0.000	1.143	Detected	1.411	Detected	1.277	0.149	GT	Sens	contig895	contig895	Mutator-li	GTGAAG	[illegible]
GT	Sense	Sense	1.067	Detected	-1.067	Detected	0.000	1.841	Detected	1.722	Detected	1.782	0.237	GT	Sens	contig900	contig900	Ulp1 prot	ATGCAG	[illegible]
GT	Sense	Sense	0.113	Comprom	-0.113	Detected	0.000	0.873	Detected	1.315	Detected	1.094	0.048	GT	Sens	contig904	contig904	Ulp1 prot	AAATCG	[illegible]
GT	Sense	Sense	-0.019	Detected	0.019	Detected	0.000	2.470	Detected	1.913	Detected	2.192	0.016	GT	Sens	contig906	contig906	1-aminoc	GTCCAG	[illegible]
GT	Sense	Sense	-0.610	Comprom	0.610	Comprom	0.000	1.998	Detected	2.551	Detected	2.274	0.077	GT	Sens	contig909	contig909	Inosine-u	GCATAC	[illegible]
GT	Sense	Sense	-0.414	Detected	0.414	Detected	0.000	1.284	Detected	0.807	Detected	1.045	0.160	GT	Sens	contig934	contig934	Fatty acid	TGTAGA	[illegible]
GT	Sense	Sense	-0.397	Comprom	0.397	Comprom	0.000	2.457	Detected	3.407	Detected	2.932	0.042	GT	Sens	contig949	contig949	Unknown	GAAAAT	[illegible]
GT	Sense	Sense	0.044	Comprom	-0.044	Comprom	0.000	1.320	Detected	1.870	Detected	1.595	0.029	GT	Sens	contig950	contig950	Unknown	AAACAA	[illegible]
GT	Sense	Sense	0.099	Detected	-0.099	Detected	0.000	1.432	Detected	1.343	Detected	1.387	0.006	GT	Sens	contig956	contig956	Subtilisin	TACCAA	[illegible]
GT	Sense	Sense	-0.054	Detected	0.054	Detected	0.000	1.815	Detected	1.890	Detected	1.852	0.001	GT	Sens	contig993	contig993	Glutathio	CACGCA	[illegible]
GT	Sense	Sense	-0.237	Detected	0.237	Detected	0.000	2.517	Detected	2.034	Detected	2.276	0.021	GT	Sens	contig993	contig993	Unknown	TATTAGA	[illegible]
GT	Sense	Sense	0.733	Detected	-0.733	Comprom	0.000	1.477	Detected	1.256	Detected	1.366	0.206	GT	Sens	contig998	contig998	E3 ubiqui	GGATTC	[illegible]

		20h_AZ_Control					20h_AZ_TAPG + TPRP vs. 20h_AZ_Control					
ProbeNar	Orientatio	Fold 20h	Flag 20h	Fold 20h	Flag 20h	Geomean	Fold 20h	Flag 20h	Fold 20h	Flag 20h	Geomean	P-value
A 96 P0	Sense	-1.335	Detected	1.335	Detected	0.000	-1.452	Detected	-2.550	Detected	-2.001	0.300
A 96 P0	Sense	-0.058	Detected	0.058	Detected	0.000	-0.885	Detected	-1.516	Detected	-1.200	0.064
A 96 P0	Sense	0.122	Detected	-0.122	Detected	0.000	-1.284	Detected	-1.567	Detected	-1.425	0.017
A 96 P0	Sense	0.607	Detected	-0.607	Detected	0.000	-1.136	Detected	-1.044	Detected	-1.090	0.215
A 96 P01	Sense	-0.428	Detected	0.428	Detected	0.000	-1.208	Detected	-1.518	Detected	-1.363	0.096
A 96 P02	Sense	0.163	Detected	-0.163	Detected	0.000	-1.220	Detected	-1.437	Detected	-1.329	0.021
A 96 P02	Sense	0.011	Detected	-0.011	Detected	0.000	-1.177	Detected	-1.470	Detected	-1.323	0.012
A 96 P02	Sense	0.173	Detected	-0.173	Detected	0.000	-1.124	Detected	-1.004	Detected	-1.064	0.028
A 96 P02	Sense	-0.104	Detected	0.104	Detected	0.000	-0.864	Detected	-1.243	Detected	-1.054	0.040
A 96 P02	Sense	0.581	Detected	-0.581	Detected	0.000	-1.219	Detected	-2.926	Comprom	-2.072	0.183
A 96 P02	Sense	-0.099	Detected	0.099	Detected	0.000	-0.983	Detected	-1.232	Detected	-1.107	0.020
A 96 P02	Sense	-0.041	Detected	0.041	Detected	0.000	-1.459	Detected	-1.069	Detected	-1.264	0.024
A 96 P03	Sense	0.086	Detected	-0.086	Detected	0.000	-1.366	Detected	-1.534	Detected	-1.450	0.007
A 96 P03	Sense	0.199	Detected	-0.199	Detected	0.000	-1.600	Comprom	-1.692	Comprom	-1.646	0.015
A 96 P03	Sense	-0.084	Detected	0.084	Detected	0.000	-1.027	Detected	-1.199	Detected	-1.113	0.011
A 96 P03	Sense	-1.152	Detected	1.152	Detected	0.000	-4.323	Comprom	-2.245	Comprom	-3.284	0.169
A 96 P03	Sense	0.242	Detected	-0.242	Detected	0.000	-1.646	Detected	-2.029	Detected	-1.838	0.027
A 96 P03	Sense	0.074	Detected	-0.074	Detected	0.000	-0.959	Detected	-2.018	Detected	-1.489	0.108
A 96 P03	Sense	0.041	Detected	-0.041	Detected	0.000	-1.260	Detected	-1.075	Detected	-1.168	0.007
A 96 P04	Sense	-0.114	Detected	0.114	Detected	0.000	-1.367	Detected	-1.030	Detected	-1.198	0.028
A 96 P04	Sense	-0.070	Detected	0.070	Detected	0.000	-1.524	Detected	-0.828	Detected	-1.176	0.080
A 96 P04	Sense	-0.094	Detected	0.094	Detected	0.000	-3.021	Detected	-0.900	Detected	-1.961	0.207
A 96 P05	Sense	-0.269	Detected	0.269	Detected	0.000	-1.896	Detected	-1.401	Detected	-1.648	0.046
A 96 P05	Sense	-0.941	Detected	0.941	Detected	0.000	-2.872	Detected	-2.112	Detected	-2.492	0.134
A 96 P05	Sense	0.050	Detected	-0.050	Detected	0.000	-1.158	Detected	-1.503	Detected	-1.331	0.018
A 96 P05	Sense	-0.271	Detected	0.271	Detected	0.000	-1.193	Comprom	-2.434	Comprom	-1.814	0.116
A 96 P06	Sense	0.242	Detected	-0.242	Detected	0.000	-1.229	Detected	-0.894	Detected	-1.062	0.069
A 96 P06	Sense	0.064	Detected	-0.064	Detected	0.000	-3.332	Detected	-2.045	Detected	-2.689	0.053
A 96 P06	Sense	-0.060	Detected	0.060	Detected	0.000	-1.095	Detected	-0.974	Detected	-1.034	0.007
A 96 P06	Sense	0.374	Detected	-0.374	Detected	0.000	-1.187	Detected	-5.298	Detected	-3.243	0.261
A 96 P06	Sense	-0.066	Detected	0.066	Detected	0.000	-0.873	Detected	-1.246	Detected	-1.060	0.033
A 96 P07	Sense	-0.351	Detected	0.351	Detected	0.000	-1.106	Detected	-2.257	Comprom	-1.681	0.130
A 96 P07	Sense	0.016	Detected	-0.016	Detected	0.000	-1.310	Detected	-1.215	Detected	-1.263	0.002
A 96 P07	Sense	-0.054	Detected	0.054	Detected	0.000	-1.484	Detected	-1.132	Comprom	-1.308	0.019
A 96 P07	Sense	-0.624	Detected	0.624	Detected	0.000	-1.929	Detected	-1.032	Detected	-1.481	0.194
A 96 P07	Sense	-0.460	Detected	0.460	Detected	0.000	-1.081	Detected	-1.519	Detected	-1.300	0.125
A 96 P07	Sense	0.020	Detected	-0.020	Detected	0.000	-1.059	Detected	-1.030	Detected	-1.044	0.001
A 96 P07	Sense	0.369	Detected	-0.369	Detected	0.000	-1.217	Detected	-1.651	Detected	-1.434	0.079
A 96 P07	Sense	-0.057	Detected	0.057	Detected	0.000	-1.172	Detected	-2.326	Comprom	-1.749	0.095
A 96 P08	Sense	-0.110	Detected	0.110	Detected	0.000	-1.199	Detected	-1.685	Detected	-1.442	0.032
A 96 P08	Sense	0.216	Detected	-0.216	Detected	0.000	-0.887	Detected	-1.166	Detected	-1.026	0.058
A 96 P08	Sense	0.125	Detected	-0.125	Detected	0.000	-1.650	Detected	-1.877	Detected	-1.763	0.009
A 96 P08	Sense	0.184	Detected	-0.184	Detected	0.000	-1.632	Detected	-0.883	Detected	-1.258	0.095
A 96 P08	Sense	0.439	Detected	-0.439	Detected	0.000	-1.913	Detected	-1.383	Detected	-1.648	0.085
A 96 P08	Sense	0.422	Detected	-0.422	Detected	0.000	-0.879	Detected	-4.026	Detected	-2.452	0.271
A 96 P08	Sense	0.237	Detected	-0.237	Detected	0.000	-0.969	Detected	-1.133	Detected	-1.051	0.052
A 96 P08	Sense	0.235	Detected	-0.235	Detected	0.000	-0.985	Detected	-1.037	Detected	-1.011	0.051
A 96 P08	Sense	-0.580	Detected	0.580	Detected	0.000	-1.951	Detected	-0.808	Detected	-1.379	0.232
A 96 P09	Sense	-0.963	Detected	0.963	Detected	0.000	-1.257	Detected	-1.780	Detected	-1.518	0.268
A 96 P09	Sense	-0.224	Detected	0.224	Detected	0.000	-1.604	Detected	-1.739	Detected	-1.671	0.019
A 96 P09	Sense	0.263	Detected	-0.263	Detected	0.000	-0.898	Detected	-1.382	Detected	-1.140	0.086
A 96 P09	Sense	-0.033	Detected	0.033	Detected	0.000	-1.345	Detected	-0.810	Detected	-1.078	0.057
A 96 P10	Sense	0.076	Detected	-0.076	Detected	0.000	-1.808	Detected	-0.943	Detected	-1.376	0.089
A 96 P10	Sense	-0.184	Detected	0.184	Detected	0.000	-1.383	Detected	-1.526	Detected	-1.454	0.018
A 96 P10	Sense	-0.194	Detected	0.194	Detected	0.000	-2.099	Detected	-1.419	Detected	-1.759	0.046
A 96 P10	Sense	0.292	Detected	-0.292	Detected	0.000	-1.451	Detected	-1.957	Detected	-1.704	0.048
A 96 P10	Sense	-0.043	Detected	0.043	Detected	0.000	-1.250	Detected	-1.206	Detected	-1.228	0.002
A 96 P11	Sense	-0.278	Detected	0.278	Detected	0.000	-1.608	Detected	-1.532	Detected	-1.570	0.031
A 96 P12	Sense	-0.355	Detected	0.355	Detected	0.000	-2.511	Comprom	-1.567	Comprom	-2.039	0.075
A 96 P12	Sense	-0.404	Detected	0.404	Detected	0.000	-1.397	Detected	-1.623	Detected	-1.510	0.069
A 96 P12	Sense	0.090	Detected	-0.090	Detected	0.000	-1.206	Detected	-0.986	Detected	-1.096	0.016
A 96 P12	Sense	-0.064	Detected	0.064	Detected	0.000	-1.511	Detected	-2.648	Detected	-2.079	0.068
A 96 P12	Sense	0.139	Detected	-0.139	Detected	0.000	-1.129	Detected	-1.086	Detected	-1.108	0.016
A 96 P12	Sense	0.521	Detected	-0.521	Detected	0.000	-1.217	Detected	-1.700	Detected	-1.459	0.126
A 96 P12	Sense	-0.583	Detected	0.583	Detected	0.000	-1.363	Detected	-1.196	Detected	-1.280	0.162
A 96 P13	Sense	-0.025	Detected	0.025	Detected	0.000	-1.136	Detected	-1.125	Detected	-1.131	0.001
A 96 P13	Sense	0.556	Detected	-0.556	Detected	0.000	-1.340	Detected	-1.833	Detected	-1.587	0.121
A 96 P13	Sense	0.050	Detected	-0.050	Detected	0.000	-1.257	Detected	-0.955	Detected	-1.106	0.020
A 96 P13	Sense	-0.796	Detected	0.796	Detected	0.000	-3.368	Comprom	-2.304	Comprom	-2.836	0.098
A 96 P13	Sense	-0.228	Detected	0.228	Detected	0.000	-0.875	Detected	-1.539	Detected	-1.207	0.096
A 96 P13	Sense	0.260	Detected	-0.260	Detected	0.000	-1.361	Detected	-2.415	Detected	-1.888	0.085
A 96 P13	Sense	-1.374	Detected	1.374	Detected	0.000	-1.386	Detected	-2.588	Detected	-1.987	0.316
A 96 P13	Sense	-0.156	Detected	0.156	Detected	0.000	-0.866	Detected	-1.558	Detected	-1.212	0.086
A 96 P13	Sense	-0.277	Detected	0.277	Detected	0.000	-1.130	Detected	-1.973	Detected	-1.552	0.091
A 96 P14	Sense	0.516	Detected	-0.516	Detected	0.000	-1.062	Detected	-4.480	Detected	-2.771	0.261
A 96 P14	Sense	-0.199	Detected	0.199	Detected	0.000	-1.132	Detected	-2.009	Detected	-1.570	0.083
A 96 P15	Sense	-0.178	Detected	0.178	Detected	0.000	-1.404	Detected	-1.693	Comprom	-1.549	0.021
A 96 P16	Sense	0.214	Detected	-0.214	Detected	0.000	-1.291	Detected	-1.129	Detected	-1.210	0.034
A 96 P16	Sense	-0.663	Detected	0.663	Detected	0.000	-2.271	Detected	-1.072	Detected	-1.671	0.202
A 96 P16	Sense	0.466	Detected	-0.466	Detected	0.000	-0.876	Detected	-1.356	Detected	-1.116	0.167
A 96 P16	Sense	-0.097	Detected	0.097	Detected	0.000	-0.900	Detected	-1.475	Detected	-1.187	0.060
A 96 P16	Sense	-0.011	Detected	0.011	Detected	0.000	-1.156	Detected	-1.137	Detected	-1.146	0.000
A 96 P17	Sense	0.572	Detected	-0.572	Detected	0.000	-1.256	Detected	-1.804	Detected	-1.530	0.137
A 96 P17	Sense	0.187	Detected	-0.187	Detected	0.000	-1.693	Detected	-1.915	Detected	-1.804	0.014
A 96 P17	Sense	-0.154	Detected	0.154	Detected	0.000	-1.264	Detected	-1.645	Detected	-1.455	0.027
A 96 P17	Sense	-0.170	Detected	0.170	Detected	0.000	-1.022	Detected	-1.508	Detected	-1.265	0.051
A 96 P17	Sense	-0.003	Detected	0.003	Detected	0.000	-1.268	Detected	-1.158	Detected	-1.213	0.002
A 96 P17	Sense	-1.326	Detected	1.326	Detected	0.000	-1.763	Detected	-2.875	Detected	-2.319	0.248
A 96 P17	Sense	0.190	Detected	-0.190	Detected	0.000	-1.230	Detected	-0.868	Detected	-1.049	0.057
A 96 P17	Sense	0.513	Detected	-0.513	Detected	0.000	-0.831	Detected	-3.226	Detected	-2.029	0.260
A 96 P17	Sense	-0.105	Detected	0.105	Detected	0.000	-1.018	Detected	-1.708	Detected	-1.363	0.063
A 96 P17	Sense	-0.231	Detected	0.231	Detected	0.000	-1.769	Detected	-1.896	Comprom	-1.832	0.017
A 96 P18	Sense	-0.075	Detected	0.075	Detected	0.000	-1.068	Detected	-1.826	Comprom	-1.447	0.064
A 96 P18	Sense	0.514	Detected	-0.514	Detected	0.000	-1.059	Detected	-1.011	Detected	-1.035	0.182
A 96 P18	Sense	0.169	Detected	-0.169	Detected	0.000	-1.876	Detected	-2.129	Detected	-2.002	0.011
A 96 P18	Sense	-0.184	Detected	0.184	Detected	0.000	-0.975	Detected	-1.451	Detected	-1.213	0.056
A 96 P18	Sense	0.579	Detected	-0.579	Detected	0.000	-0.925	Detected	-1.089	Detected	-1.007	0.227
A 96 P19	Sense	-1.333	Detected	1.333	Detected	0.000	-1.657	Detected	-2.680	Detected	-2.168	0.268
A 96 P19	Sense	-0.520	Detected	0.520	Detected	0.000	-4.222	Detected	-2.194	Detected	-3.208	0.106
A 96 P20	Sense	-0.185	Detected	0.185	Detected	0.000	-0.852	Detected	-1.540	Detected	-1.196	0.092
A 96 P20	Sense	-0.669	Detected	0.669	Detected	0.000	-1.332	Detected	-1.307	Detected	-1.320	0.188
A 96 P20	Sense	-0.066	Detected	0.066	Detected	0.000	-1.664	Detected	-2.368	Comprom	-2.016	0.030
A 96 P20	Sense	0.465	Detected	-0.465	Detected	0.000	-1.094	Detected	-1.654	Detected	-1.374	0.127
A 96 P20	Sense	-0.267	Detected	0.267	Detected	0.000	-1.416	Detected	-2.261	Detected	-1.839	0.067
A 96 P20	Sense	0.154	Detected	-0.154	Detected	0.000	-1.532	Detected	-1.389	Comprom	-1.461	0.013
A 96 P21	Sense	0.020	Detected	-0.020	Detected	0.000	-1.744	Detected	-2.479	Detected	-2.112	0.029
A 96 P21	Sense	0.107	Detected	-0.107	Detected	0.000	-2.534	Comprom	-2.182	Comprom	-2.358	0.008
A 96 P21	Sense	0.260	Detected	-0.260	Detected	0.000	-1.289	Detected	-1.897	Detected	-1.593	0.058
A 96 P21	Sense	0.428	Detected	-0.428	Detected	0.000	-1.241	Detected	-0.802	Detected	-1.021	0.168
A 96 P21	Sense	0.150	Detected	-0.150	Detected	0.000	-1.684	Detected	-1.816	Detected	-1.750	0.009
A 96 P21	Sense	-0.113	Detected	0.113	Detected	0.000	-2.692	Detected	-1.412	Detected	-2.052	0.087
A 96 P22	Sense	0.035	Detected	-0.035	Detected	0.000	-0.956	Detected	-1.482	Detected	-1.219	0.044
A 96 P22	Sense	0.218	Detected	-0.218	Detected	0.000	-1.020	Detected	-0.989	Detected	-1.004	0.044
A 96 P22	Sense	-0.245	Detected	0.245	Detected	0.000	-1.049	Detected	-1.091	Detected	-1.070	0.049
A 96 P22	Sense	-0.490	Detected	0.490	Detected	0.000	-1.308	Comprom	-1.861	Comprom	-1.585	0.106
A 96 P22	Sense	-0.202	Detected	0.202	Detected	0.000	-1.227	Detected	-1.715	Detected	-1.471	0.043
A 96 P22	Sense	-0.076	Detected	0.076	Detected	0.000	-1.929	Detected	-2.056	Detected	-1.993	0.002
A 96 P23	Sense	0.145	Detected	-0.145	Detected	0.000	-1.021	Detected	-1.062	Detected	-1.041	0.019
A 96 P23	Sense	0.457	Detected	-0.457	Detected	0.000	-1.352	Detected	-1.885	Comprom	-1.619	0.092
A 96 P23	Sense	0.185	Detected	-0.185	Detected	0.000	-0.992	Detected	-1.234	Detected	-1.113	0.037
A 96 P23	Sense	0.583	Detected	-0.583	Detected	0.000	-1.058	Detected	-1.102	Detected	-1.080	0.205
A 96 P24	Sense	-1.099	Detected	1.099	Detected	0.000	-2.478	Detected	-3.096	Comprom	-2.787	0.135
A 96 P25	Sense	0.058	Detected	-0.058	Detected	0.000	-1.880	Detected	-1.212	Detected	-1.546	0.045
A 96 P25	Sense	-0.058	Detected	0.058	Detected	0.000	-1.720	Detected	-1.617	Detected	-1.669	0.002
A 96 P26	Sense	0.690	Detected	-0.690	Detected	0.000	-0.906	Detected	-1.170	Detected	-1.038	0.278
A 96 P26	Sense	-0.007	Detected	0.007	Detected	0.000	-1.414	Detected	-1.332	Detected	-1.373	0.001
A 96 P26	Sense	-1.030	Detected	1.030	Detected	0.000	-2.689	Detected	-2.646	Detected	-2.667	0.122
GT Sense	Sense	0.658	Detected	-0.658	Detected	0.000	-1.175	Detected	-1.739	Detected	-1.457	0.179
GT Sense	Sense	0.388	Detected	-0.388	Detected	0.000	-1.181	Detected	-1.081	Detected	-1.131	0.102
GT Sense	Sense	0.262	Detected	-0.262	Detected	0.000	-1.223	Detected	-1.521	Detected	-1.372	0.045
GT Sense	Sense	-0.427	Detected	0.427	Detected	0.000	-1.392	Detected	-0.871	Detected	-1.132	0.152
GT Sense	Sense	0.131	Detected	-0.131	Detected	0.000	-0.895	Detected	-1.370	Detected	-1.132	0.053
GT Sense	Sense	0.237	Detected	-0.237	Detected	0.000	-1.553	Detected	-1.596	Detected	-1.575	0.022
GT Sense	Sense	0.502	Detected	-0.502	Detected	0.000	-3.099	Detected	-2.335	Detected	-2.717	0.050
GT Sense	Sense	-0.045	Detected	0.045	Detected	0.000	-1.642	Detected	-1.788	Detected	-1.715	0.002
GT Sense	Sense	0.044	Detected	-0.044	Detected	0.000	-1.601	Detected	-2.181	Detected	-1.891	0.023
GT Sense	Sense	0.399	Detected	-0.399	Detected	0.000	-1.099	Detected	-1.558	Comprom	-1.329	0.102
GT Sense	Sense	-0.213	Detected	0.213	Detected	0.000	-1.999	Detected	-1.483	Detected	-1.741	0.035
GT Sense	Sense	0.100	Detected	-0.100	Detected	0.000	-1.732	Detected	-0.874	Detected	-1.303	0.098
GT Sense	Sense	-0.128	Detected	0.128	Detected	0.000	-1.483	Detected	-1.102	Detected	-1.293	0.030
GT Sense	Sense	2.536	Detected	-2.536	Detected	0.000	-1.438	Detected	-1.292	Detected	-1.365	0.644
GT Sense	Sense	-0.707	Detected	0.707	Detected	0.000	-1.200	Detected	-2.144	Detected	-1.672	0.188
GT Sense	Sense	0.261	Detected	-0.261	Detected	0.000	-0.826	Detected	-1.219	Detected	-1.023	0.089
GT Sense	Sense	0.165	Detected	-0.165	Detected	0.000	-1.456	Detected	-1.588	Detected	-1.522	0.013
GT Sense	Sense	-0.152	Detected	0.152	Detected	0.000	-0.940	Detected	-1.195	Detected	-1.067	0.033
GT Sense	Sense	-0.206	Detected	0.206	Detected	0.000	-0.934	Detected	-1.468	Comprom	-1.201	0.070
GT Sense	Sense	-0.178	Detected	0.178	Detected	0.000	-2.652	Detected	-1.217	Detected	-1.934	0.120
GT Sense	Sense	-0.893	Detected	0.893	Detected	0.000	-3.561	Detected	-2.031	Detected	-2.796	0.141
GT Sense	Sense	0.018	Detected	-0.018	Detected	0.000	-1.216	Comprom	-0.934	Comprom	-1.075	0.017
GT Sense	Sense	0.119	Detected	-0.119	Detected	0.000	-1.610	Detected	-1.820	Detected	-1.715	0.008
GT Sense	Sense	0.072	Detected	-0.072	Detected	0.000	-1.112	Detected	-1.107	Detected	-1.109	0.004
GT Sense	Sense	-0.070	Detected	0.070	Detected	0.000	-2.045	Comprom	-2.422	Comprom	-2.233	0.008
GT Sense	Sense	-1.395	Detected	1.395	Detected	0.000	-3.526	Detected	-1.462	Detected	-2.494	0.287
GT Sense	Sense	-0.247	Detected	0.247	Detected	0.000	-0.817	Detected	-1.270	Detected	-1.043	0.089
GT Sense	Sense	-1.371	Detected	1.371	Detected	0.000	-1.773	Detected	-2.308	Comprom	-2.040	0.282
GT Sense	Sense	0.073	Detected	-0.073	Detected	0.000	-0.984	Detected	-1.699	Detected	-1.341	0.067
GT Sense	Sense	-0.185	Detected	0.185	Detected	0.000	-1.312	Detected	-0.923	Comprom	-1.118	0.053
GT Sense	Sense	0.077	Detected	-0.077	Detected	0.000	-0.840	Detected	-1.181	Detected	-1.010	0.033
GT Sense	Sense	0.207	Detected	-0.207	Detected	0.000	-0.890	Detected	-1.894	Comprom	-1.392	0.124
GT Sense	Sense	0.138	Detected	-0.138	Detected	0.000	-1.358	Detected	-1.220	Detected	-1.289	0.014
GT Sense	Sense	0.068	Detected	-0.068	Detected	0.000	-1.323	Detected	-1.467	Detected	-1.395	0.005
GT Sense	Sense	0.140	Detected	-0.140	Detected	0.000	-1.447	Detected	-1.141	Detected	-1.294	0.025
GT Sense	Sense	-0.046	Detected	0.046	Detected	0.000	-2.367	Detected	-2.501	Detected	-2.434	0.001
GT Sense	Sense	-0.562	Detected	0.562	Detected	0.000	-1.606	Detected	-1.307	Detected	-1.456	0.129
GT Sense	Sense	-0.075	Detected	0.075	Detected	0.000	-1.229	Detected	-0.956	Detected	-1.092	0.020
GT Sense	Sense	-1.023	Detected	1.023	Detected	0.000	-2.546	Detected	-2.852	Detected	-2.699	0.121
GT Sense	Sense	0.516	Detected	-0.516	Detected	0.000	-1.367	Detected	-1.818	Detected	-1.593	0.106
GT Sense	Sense	0.231	Detected	-0.231	Detected	0.000	-1.462	Detected	-1.245	Detected	-1.353	0.034
GT Sense	Sense	-0.270	Detected	0.270	Detected	0.000	-3.070	Comprom	-1.142	Comprom	-2.106	0.170
GT Sense	Sense	-0.590	Detected	0.590	Detected	0.000	-1.724	Detected	-2.522	Detected	-2.123	0.096
GT Sense	Sense	-0.020	Detected	0.020	Detected	0.000	-1.474	Detected	-1.412	Detected	-1.443	0.001
GT Sense	Sense	-0.278	Detected	0.278	Detected	0.000	-2.673	Detected	-1.048	Detected	-1.860	0.163
GT Sense	Sense	-0.286	Detected	0.286	Detected	0.000	-1.417	Comprom	-1.337	Comprom	-1.377	0.041
GT Sense	Sense	0.073	Detected	-0.073	Detected	0.000	-1.782	Detected	-1.625	Detected	-1.704	0.004
GT Sense	Sense	1.112	Detected	-1.112	Detected	0.000	-1.359	Detected	-1.196	Detected	-1.278	0.370
GT Sense	Sense	-0.250	Detected	0.250	Detected	0.000	-1.775	Detected	-0.835	Detected	-1.305	0.134
GT Sense	Sense	-0.749	Detected	0.749	Detected	0.000	-1.141	Detected	-1.065	Detected	-1.103	0.279
GT Sense	Sense	0.322	Detected	-0.322	Detected	0.000	-1.998	Comprom	-1.085	Comprom	-1.541	0.110
GT Sense	Sense	0.449	Detected	-0.449	Detected	0.000	-2.344	Comprom	-0.983	Comprom	-1.663	0.178
GT Sense	Sense	0.260	Detected	-0.260	Detected	0.000	-1.413	Comprom	-1.864	Comprom	-1.639	0.041
GT Sense	Sense	0.009	Detected	-0.009	Detected	0.000	-2.975	Detected	-5.011	Detected	-3.993	0.059
GT Sense	Sense	0.086	Detected	-0.086	Detected	0.000	-2.063	Detected	-1.920	Detected	-1.992	0.003
GT Sense	Sense	-0.621	Detected	0.621	Detected	0.000	-1.043	Detected	-1.230	Detected	-1.137	0.212
GT Sense	Sense	-0.037	Detected	0.037	Detected	0.000	-0.964	Detected	-1.897	Comprom	-1.431	0.092
GT Sense	Sense	-0.073	Detected	0.073	Detected	0.000	-1.350	Detected	-1.045	Detected	-1.197	0.019
GT Sense	Sense	-1.044	Detected	1.044	Detected	0.000	-1.631	Detected	-1.120	Detected	-1.375	0.329
GT Sense	Sense	-0.180	Detected	0.180	Detected	0.000	-1.144	Detected	-1.050	Detected	-1.097	0.028
GT Sense	Sense	-0.022	Detected	0.022	Detected	0.000	-1.068	Detected	-1.661	Detected	-1.364	0.044
GT Sense	Sense	0.144	Detected	-0.144	Detected	0.000	-1.579	Comprom	-0.862	Comprom	-1.220	0.087
GT Sense	Sense	-0.163	Detected	0.163	Detected	0.000	-3.603	Comprom	-1.895	Comprom	-2.749	0.087
GT Sense	Sense	-0.329	Detected	0.329	Detected	0.000	-1.313	Detected	-1.318	Detected	-1.315	0.057
GT Sense	Sense	-1.039	Detected	1.039	Detected	0.000	-0.874	Detected	-1.198	Detected	-1.036	0.428
GT Sense	Sense	-0.132	Detected	0.132	Detected	0.000	-1.237	Detected	-1.529	Detected	-1.383	0.020
GT Sense	Sense	-0.322	Detected	0.322	Detected	0.000	-1.206	Detected	-1.126	Detected	-1.166	0.070
GT Sense	Sense	0.105	Detected	-0.105	Detected	0.000	-2.036	Detected	-0.929	Detected	-1.482	0.119
GT Sense	Sense	-0.622	Detected	0.622	Detected	0.000	-2.048	Detected	-1.120	Detected	-1.584	0.178
GT Sense	Sense	-0.142	Detected	0.142	Detected	0.000	-1.628	Detected	-1.325	Detected	-1.476	0.019
GT Sense	Sense	0.037	Detected	-0.037	Detected	0.000	-1.219	Detected	-2.580	Detected	-1.899	0.108
GT Sense	Sense	-0.083	Detected	0.083	Detected	0.000	-2.176	Detected	-1.456	Detected	-1.816	0.039
GT Sense	Sense	0.103	Detected	-0.103	Detected	0.000	-1.776	Detected	-1.875	Detected	-1.826	0.004
GT Sense	Sense	-0.011	Detected	0.011	Detected	0.000	-3.370	Detected	-1.456	Detected	-2.413	0.128
GT Sense	Sense	-0.337	Detected	0.337	Detected	0.000	-1.106	Detected	-0.986	Detected	-1.046	0.093
GT Sense	Sense	0.065	Detected	-0.065	Detected	0.000	-2.171	Detected	-1.828	Detected	-1.999	0.008
GT Sense	Sense	0.001	Detected	-0.001	Detected	0.000	-1.016	Detected	-1.412	Detected	-1.214	0.026
GT Sense	Sense	-0.021	Detected	0.021	Detected	0.000	-1.312	Detected	-1.722	Detected	-1.517	0.018
GT Sense	Sense	0.087	Detected	-0.087	Detected	0.000	-1.656	Detected	-1.696	Detected	-1.676	0.003
GT Sense	Sense	-0.388	Detected	0.388	Detected	0.000	-1.792	Detected	-1.881	Detected	-1.836	0.042
GT Sense	Sense	0.035	Detected	-0.035	Detected	0.000	-1.356	Detected	-0.809	Detected	-1.083	0.059
GT Sense	Sense	0.413	Detected	-0.413	Detected	0.000	-1.669	Comprom	-0.985	Comprom	-1.327	0.132
GT Sense	Sense	0.167	Detected	-0.167	Detected	0.000	-1.618	Detected	-1.759	Detected	-1.688	0.011
GT Sense	Sense	0.094	Detected	-0.094	Detected	0.000	-1.736	Detected	-2.034	Detected	-1.885	0.009
GT Sense	Sense	0.262	Detected	-0.262	Detected	0.000	-2.076	Comprom	-1.825	Comprom	-1.950	0.022
GT Sense	Sense	-0.203	Detected	0.203	Detected	0.000	-1.137	Detected	-0.956	Comprom	-1.046	0.042
GT Sense	Sense	-0.100	Detected	0.100	Detected	0.000	-1.375	Detected	-2.636	Detected	-2.006	0.088
GT Sense	Sense	-0.194	Detected	0.194	Detected	0.000	-1.245	Detected	-1.753	Detected	-1.499	0.042
GT Sense	Sense	0.109	Detected	-0.109	Detected	0.000	-1.947	Detected	-2.053	Detected	-2.000	0.004
GT Sense	Sense	0.096	Detected	-0.096	Detected	0.000	-1.287	Detected	-1.739	Detected	-1.513	0.025
GT Sense	Sense	-0.126	Detected	0.126	Detected	0.000	-2.816	Detected	-1.323	Detected	-2.069	0.112
GT Sense	Sense	-0.163	Detected	0.163	Detected	0.000	-2.005	Detected	-0.854	Detected	-1.430	0.139
GT Sense	Sense	0.065	Detected	-0.065	Detected	0.000	-1.431	Detected	-1.110	Detected	-1.271	0.018
GT Sense	Sense	0.072	Detected	-0.072	Detected	0.000	-2.789	Detected	-1.825	Detected	-2.307	0.042
GT Sense	Sense	0.069	Detected	-0.069	Detected	0.000	-1.106	Detected	-1.350	Detected	-1.228	0.013
GT Sense	Sense	-0.156	Detected	0.156	Detected	0.000	-1.161	Detected	-0.845	Detected	-1.003	0.046
GT Sense	Sense	-0.429	Detected	0.429	Detected	0.000	-1.152	Detected	-1.375	Detected	-1.263	0.104
GT Sense	Sense	0.037	Detected	-0.037	Detected	0.000	-0.856	Detected	-1.279	Detected	-1.067	0.038
GT Sense	Sense	-0.057	Detected	0.057	Detected	0.000	-1.520	Detected	-1.938	Detected	-1.729	0.015
GT Sense	Sense	-0.027	Detected	0.027	Detected	0.000	-1.096	Detected	-1.095	Comprom	-1.096	0.001
GT Sense	Sense	0.022	Detected	-0.022	Detected	0.000	-1.484	Detected	-1.001	Detected	-1.243	0.036
GT Sense	Sense	0.050	Detected	-0.050	Detected	0.000	-1.316	Detected	-1.624	Detected	-1.470	0.012
GT Sense	Sense	0.059	Detected	-0.059	Detected	0.000	-2.045	Detected	-2.345	Detected	-2.195	0.005
GT Sense	Sense	-0.307	Detected	0.307	Detected	0.000	-2.810	Detected	-1.092	Detected	-1.951	0.166
GT Sense	Sense	-0.002	Detected	0.002	Detected	0.000	-1.255	Detected	-1.388	Detected	-1.322	0.003
GT Sense	Sense	0.017	Detected	-0.017	Detected	0.000	-1.106	Detected	-1.015	Detected	-1.060	0.002
GT Sense	Sense	-0.199	Detected	0.199	Detected	0.000	-0.983	Detected	-1.152	Detected	-1.067	0.039
GT Sense	Sense	-0.601	Detected	0.601	Detected	0.000	-2.120	Detected	-1.481	Detected	-1.800	0.118
GT Sense	Sense	0.253	Detected	-0.253	Detected	0.000	-1.182	Detected	-0.914	Comprom	-1.048	0.067
GT Sense	Sense	0.800	Detected	-0.800	Detected	0.000	-2.275	Comprom	-1.465	Detected	-1.870	0.172
GT Sense	Sense	0.157	Detected	-0.157	Detected	0.000	-1.204	Detected	-0.941	Detected	-1.073	0.035
GT Sense	Sense	-0.522	Detected	0.522	Detected	0.000	-2.792	Detected	-0.935	Detected	-1.864	0.222
GT Sense	Sense	0.836	Detected	-0.836	Detected	0.000	-4.285	Comprom	-1.543	Detected	-2.914	0.211
GT Sense	Sense	0.172	Detected	-0.172	Detected	0.000	-1.968	Detected	-1.946	Detected	-1.957	0.008
GT Sense	Sense	0.225	Detected	-0.225	Detected	0.000	-1.228	Detected	-1.152	Detected	-1.190	0.035

GT	Sense	Sense	-0.217	Detected	0.217	Detected	0.000	-1.215	Detected	-0.894	Detected	-1.055	0.060	GT	Sens	contig15	contig15	Cell divisi	CCGGCC
GT	Sense	Sense	0.448	Detected	-0.448	Detected	0.000	-0.852	Detected	-1.818	Comprom	-1.335	0.180	GT	Sens	contig15	contig15	Unknown	ACATGT
GT	Sense	Sense	0.048	Detected	-0.048	Detected	0.000	-2.124	Detected	-0.984	Detected	-1.554	0.113	GT	Sens	contig16	contig16	NAC-dom	TTAGAG
GT	Sense	Sense	-0.006	Detected	0.006	Detected	0.000	-1.029	Detected	-1.138	Detected	-1.083	0.003	GT	Sens	contig161	contig161	Phytosul	CACCAG
GT	Sense	Sense	0.243	Detected	-0.243	Detected	0.000	-1.311	Detected	-1.656	Detected	-1.483	0.038	GT	Sens	contig162	contig162	Unknown	CACCAT
GT	Sense	Sense	0.423	Detected	-0.423	Detected	0.000	-2.672	Comprom	-1.329	Comprom	-2.001	0.128	GT	Sens	contig163	contig163	Hexokina	GGTGTC
GT	Sense	Sense	-0.320	Detected	0.320	Detected	0.000	-1.296	Detected	-0.858	Detected	-1.077	0.109	GT	Sens	contig164	contig164	Gun4-like	ATTTGG
GT	Sense	Sense	0.097	Detected	-0.097	Detected	0.000	-2.087	Detected	-2.027	Detected	-2.057	0.002	GT	Sens	contig164	contig164	Unknown	TTATTA
GT	Sense	Sense	-0.090	Detected	0.090	Detected	0.000	-1.023	Comprom	-1.699	Comprom	-1.361	0.060	GT	Sens	contig164	contig164	Glutared	AGTACC
GT	Sense	Sense	0.163	Detected	-0.163	Detected	0.000	-1.157	Detected	-1.259	Detected	-1.208	0.019	GT	Sens	contig166	contig166	Mutator-li	GTTGTC
GT	Sense	Sense	-0.555	Detected	0.555	Detected	0.000	-2.287	Detected	-1.242	Detected	-1.764	0.147	GT	Sens	contig167	contig167	Receptor	ACCATG
GT	Sense	Sense	-0.030	Detected	0.030	Detected	0.000	-2.086	Comprom	-0.876	Comprom	-1.481	0.134	GT	Sens	contig167	contig167	Subtilisin	TACAGT
GT	Sense	Sense	-0.093	Detected	0.093	Detected	0.000	-1.850	Detected	-0.896	Detected	-1.373	0.106	GT	Sens	contig168	contig168	Fatty acid	TTGCAG
GT	Sense	Sense	0.014	Detected	-0.014	Detected	0.000	-1.226	Detected	-1.509	Detected	-1.367	0.011	GT	Sens	contig168	contig168	T24D18.4	AAGGGC
GT	Sense	Sense	-1.323	Detected	1.323	Detected	0.000	-3.505	Comprom	-2.210	Comprom	-2.858	0.192	GT	Sens	contig17	contig17	Universal	AATGTG
GT	Sense	Sense	-0.009	Detected	0.009	Detected	0.000	-2.789	Detected	-1.966	Detected	-2.378	0.029	GT	Sens	contig171	contig171	MYB tran	AGCAGC
GT	Sense	Sense	-0.212	Detected	0.212	Detected	0.000	-1.113	Detected	-1.020	Detected	-1.066	0.039	GT	Sens	contig172	contig172	Gamma-g	AGATGT
GT	Sense	Sense	0.031	Detected	-0.031	Detected	0.000	-1.240	Detected	-0.926	Detected	-1.083	0.021	GT	Sens	contig172	contig172	F-box/kel	TTATGA
GT	Sense	Sense	-0.291	Detected	0.291	Detected	0.000	-1.604	Detected	-1.072	Detected	-1.338	0.077	GT	Sens	contig173	contig173	UPF0503	TCACCT
GT	Sense	Sense	0.148	Detected	-0.148	Detected	0.000	-1.014	Detected	-1.098	Detected	-1.056	0.021	GT	Sens	contig173	contig173	WRKY tra	GATCAG
GT	Sense	Sense	-0.032	Detected	0.032	Detected	0.000	-1.199	Detected	-1.913	Detected	-1.556	0.049	GT	Sens	contig173	contig173	Hydrolas	AACTGA
GT	Sense	Sense	-0.613	Detected	0.613	Detected	0.000	-1.255	Detected	-1.999	Detected	-1.627	0.151	GT	Sens	contig173	contig173	Peroxidas	ATGCCT
GT	Sense	Sense	-0.789	Detected	0.789	Detected	0.000	-1.230	Detected	-1.029	Detected	-1.129	0.291	GT	Sens	contig175	contig175	Pectinest	TATCGT
GT	Sense	Sense	-0.548	Detected	0.548	Detected	0.000	-2.668	Detected	-0.912	Detected	-1.790	0.226	GT	Sens	contig176	contig176	Xylanase	GACAAT
GT	Sense	Sense	0.277	Detected	-0.277	Detected	0.000	-1.245	Detected	-1.278	Detected	-1.261	0.045	GT	Sens	contig176	contig176	Zinc finge	ATGAGT
GT	Sense	Sense	-0.504	Detected	0.504	Detected	0.000	-3.835	Detected	-2.090	Detected	-2.963	0.099	GT	Sens	contig176	contig176	Glucan e	GCGATG
GT	Sense	Sense	-0.466	Detected	0.466	Detected	0.000	-0.852	Detected	-1.820	Detected	-1.336	0.185	GT	Sens	contig176	contig176	Metal ion	TTTGGA

[illegible]

GT	Sense	Sense	0.154	Detected	-0.154	Detected	0.000	-0.937	Detected	-2.771	Detected	-1.854	0.184	GT	Sens	contig293
GT	Sense	Sense	0.506	Detected	-0.506	Detected	0.000	-1.165	Detected	-1.003	Detected	-1.084	0.169	GT	Sens	contig293
GT	Sense	Sense	0.305	Detected	-0.305	Detected	0.000	-1.378	Comprom	-0.861	Comprom	-1.120	0.107	GT	Sens	contig293
GT	Sense	Sense	0.336	Detected	-0.336	Detected	0.000	-1.019	Detected	-1.209	Detected	-1.114	0.086	GT	Sens	contig294
GT	Sense	Sense	-0.286	Detected	0.286	Detected	0.000	-2.938	Detected	-1.389	Detected	-2.164	0.120	GT	Sens	contig294
GT	Sense	Sense	0.088	Detected	-0.088	Detected	0.000	-1.064	Detected	-1.274	Detected	-1.169	0.013	GT	Sens	contig296
GT	Sense	Sense	-0.022	Detected	0.022	Detected	0.000	-1.762	Detected	-1.489	Detected	-1.625	0.007	GT	Sens	contig296
GT	Sense	Sense	0.184	Detected	-0.184	Detected	0.000	-1.584	Comprom	-2.319	Comprom	-1.952	0.042	GT	Sens	contig297
GT	Sense	Sense	-0.026	Detected	0.026	Detected	0.000	-2.227	Comprom	-1.321	Comprom	-1.774	0.060	GT	Sens	contig297
GT	Sense	Sense	0.150	Detected	-0.150	Detected	0.000	-0.904	Detected	-1.809	Detected	-1.357	0.104	GT	Sens	contig300
GT	Sense	Sense	0.148	Detected	-0.148	Detected	0.000	-0.821	Detected	-1.591	Comprom	-1.206	0.100	GT	Sens	contig300
GT	Sense	Sense	-0.130	Detected	0.130	Detected	0.000	-2.486	Detected	-1.610	Detected	-2.048	0.046	GT	Sens	contig300
GT	Sense	Sense	-0.859	Detected	0.859	Detected	0.000	-1.216	Detected	-1.195	Detected	-1.206	0.295	GT	Sens	contig300
GT	Sense	Sense	0.187	Detected	-0.187	Detected	0.000	-1.099	Detected	-1.270	Detected	-1.185	0.029	GT	Sens	contig300
GT	Sense	Sense	0.115	Detected	-0.115	Detected	0.000	-1.355	Detected	-0.886	Detected	-1.121	0.050	GT	Sens	contig301
GT	Sense	Sense	-0.444	Detected	0.444	Detected	0.000	-2.891	Detected	-1.485	Detected	-2.188	0.119	GT	Sens	contig301
GT	Sense	Sense	-0.208	Detected	0.208	Detected	0.000	-1.375	Detected	-1.676	Detected	-1.526	0.027	GT	Sens	contig301
GT	Sense	Sense	0.409	Detected	-0.409	Detected	0.000	-2.854	Detected	-1.106	Detected	-1.980	0.177	GT	Sens	contig303
GT	Sense	Sense	0.084	Detected	-0.084	Detected	0.000	-1.672	Detected	-1.459	Detected	-1.565	0.007	GT	Sens	contig304
GT	Sense	Sense	-0.351	Detected	0.351	Detected	0.000	-1.767	Detected	-1.290	Detected	-1.529	0.069	GT	Sens	contig304
GT	Sense	Sense	-0.047	Detected	0.047	Detected	0.000	-1.131	Detected	-1.064	Detected	-1.098	0.003	GT	Sens	contig304
GT	Sense	Sense	-0.017	Detected	0.017	Detected	0.000	-1.440	Detected	-2.142	Detected	-1.791	0.036	GT	Sens	contig305
GT	Sense	Sense	-1.296	Detected	1.296	Detected	0.000	-2.299	Detected	-2.122	Comprom	-2.210	0.231	GT	Sens	contig305
GT	Sense	Sense	0.011	Detected	-0.011	Detected	0.000	-1.328	Detected	-1.561	Detected	-1.445	0.006	GT	Sens	contig305
GT	Sense	Sense	-0.278	Detected	0.278	Detected	0.000	-1.354	Detected	-0.911	Detected	-1.132	0.086	GT	Sens	contig306
GT	Sense	Sense	-0.344	Detected	0.344	Detected	0.000	-2.275	Detected	-1.022	Detected	-1.649	0.147	GT	Sens	contig306
GT	Sense	Sense	-0.246	Detected	0.246	Detected	0.000	-1.964	Detected	-2.192	Comprom	-2.078	0.017	GT	Sens	contig307
GT	Sense	Sense	0.581	Detected	-0.581	Detected	0.000	-1.185	Detected	-1.291	Detected	-1.238	0.168	GT	Sens	contig307
GT	Sense	Sense	-0.651	Detected	0.651	Detected	0.000	-1.409	Detected	-1.108	Detected	-1.258	0.201	GT	Sens	contig307
GT	Sense	Sense	0.328	Detected	-0.328	Detected	0.000	-1.309	Detected	-1.619	Comprom	-1.464	0.056	GT	Sens	contig307
GT	Sense	Sense	-0.149	Detected	0.149	Detected	0.000	-1.134	Detected	-1.223	Detected	-1.179	0.017	GT	Sens	contig307
GT	Sense	Sense	-0.225	Detected	0.225	Detected	0.000	-1.424	Detected	-0.821	Detected	-1.122	0.097	GT	Sens	contig307
GT	Sense	Sense	0.623	Detected	-0.623	Detected	0.000	-1.594	Detected	-0.994	Detected	-1.294	0.202	GT	Sens	contig308
GT	Sense	Sense	0.029	Detected	-0.029	Detected	0.000	-3.386	Detected	-1.920	Detected	-2.653	0.069	GT	Sens	contig308
GT	Sense	Sense	0.343	Detected	-0.343	Detected	0.000	-2.027	Comprom	-1.751	Comprom	-1.889	0.036	GT	Sens	contig309
GT	Sense	Sense	1.041	Detected	-1.041	Detected	0.000	-1.925	Detected	-1.519	Detected	-1.722	0.246	GT	Sens	contig309
GT	Sense	Sense	0.326	Detected	-0.326	Detected	0.000	-1.059	Detected	-1.084	Detected	-1.072	0.082	GT	Sens	contig309
GT	Sense	Sense	-0.134	Detected	0.134	Detected	0.000	-0.956	Detected	-2.268	Comprom	-1.612	0.138	GT	Sens	contig310
GT	Sense	Sense	0.074	Detected	-0.074	Detected	0.000	-1.533	Detected	-1.431	Detected	-1.482	0.004	GT	Sens	contig311
GT	Sense	Sense	-0.274	Detected	0.274	Detected	0.000	-0.995	Detected	-1.528	Detected	-1.261	0.081	GT	Sens	contig311
GT	Sense	Sense	0.935	Detected	-0.935	Detected	0.000	-1.991	Detected	-1.240	Detected	-1.615	0.250	GT	Sens	contig312
GT	Sense	Sense	0.323	Detected	-0.323	Detected	0.000	-1.501	Detected	-0.877	Detected	-1.189	0.118	GT	Sens	contig313
GT	Sense	Sense	-0.018	Detected	0.018	Detected	0.000	-2.147	Detected	-2.326	Detected	-2.236	0.002	GT	Sens	contig313
GT	Sense	Sense	0.142	Detected	-0.142	Detected	0.000	-1.036	Detected	-1.194	Detected	-1.115	0.020	GT	Sens	contig314
GT	Sense	Sense	-0.059	Detected	0.059	Detected	0.000	-1.522	Comprom	-1.970	Comprom	-1.746	0.017	GT	Sens	contig315
GT	Sense	Sense	0.098	Detected	-0.098	Detected	0.000	-1.718	Detected	-2.212	Detected	-1.965	0.018	GT	Sens	contig316
GT	Sense	Sense	0.058	Detected	-0.058	Detected	0.000	-1.953	Detected	-2.244	Detected	-2.098	0.006	GT	Sens	contig316
GT	Sense	Sense	-0.197	Detected	0.197	Detected	0.000	-0.849	Detected	-2.299	Comprom	-1.574	0.171	GT	Sens	contig316
GT	Sense	Sense	0.087	Detected	-0.087	Detected	0.000	-1.446	Detected	-1.666	Detected	-1.556	0.008	GT	Sens	contig317
GT	Sense	Sense	-0.903	Detected	0.903	Detected	0.000	-1.508	Detected	-2.384	Comprom	-1.946	0.192	GT	Sens	contig317
GT	Sense	Sense	-0.054	Detected	0.054	Detected	0.000	-1.544	Comprom	-1.961	Comprom	-1.753	0.015	GT	Sens	contig318
GT	Sense	Sense	0.125	Detected	-0.125	Detected	0.000	-1.380	Detected	-1.844	Detected	-1.612	0.026	GT	Sens	contig319
GT	Sense	Sense	0.163	Detected	-0.163	Detected	0.000	-1.695	Detected	-1.976	Detected	-1.836	0.013	GT	Sens	contig319
GT	Sense	Sense	0.125	Detected	-0.125	Detected	0.000	-1.098	Detected	-1.398	Detected	-1.248	0.024	GT	Sens	contig319
GT	Sense	Sense	0.506	Detected	-0.506	Detected	0.000	-1.034	Detected	-1.522	Comprom	-1.278	0.151	GT	Sens	contig319
GT	Sense	Sense	0.000	Detected	0.000	Detected	0.000	-1.207	Comprom	-1.229	Comprom	-1.218	0.000	GT	Sens	contig320
GT	Sense	Sense	-0.220	Detected	0.220	Detected	0.000	-1.608	Detected	-1.761	Detected	-1.685	0.019	GT	Sens	contig321
GT	Sense	Sense	0.669	Detected	-0.669	Detected	0.000	-1.081	Detected	-1.301	Detected	-1.191	0.221	GT	Sens	contig321
GT	Sense	Sense	-0.207	Detected	0.207	Detected	0.000	-1.138	Detected	-1.466	Detected	-1.302	0.039	GT	Sens	contig321
GT	Sense	Sense	0.034	Detected	-0.034	Detected	0.000	-5.745	Comprom	-5.470	Comprom	-5.608	0.001	GT	Sens	contig322
GT	Sense	Sense	-0.037	Detected	0.037	Detected	0.000	-4.373	Comprom	-3.588	Comprom	-3.980	0.010	GT	Sens	contig322
GT	Sense	Sense	0.253	Detected	-0.253	Detected	0.000	-1.261	Comprom	-1.600	Comprom	-1.431	0.042	GT	Sens	contig322
GT	Sense	Sense	-0.020	Detected	0.020	Detected	0.000	-0.882	Detected	-1.692	Comprom	-1.287	0.086	GT	Sens	contig322
GT	Sense	Sense	-0.364	Detected	0.364	Detected	0.000	-1.607	Detected	-1.048	Detected	-1.327	0.101	GT	Sens	contig322
GT	Sense	Sense	-1.360	Detected	1.360	Detected	0.000	-3.965	Comprom	-2.711	Comprom	-3.338	0.156	GT	Sens	contig323
GT	Sense	Sense	0.012	Detected	-0.012	Detected	0.000	-1.074	Detected	-0.999	Detected	-1.036	0.001	GT	Sens	contig323
GT	Sense	Sense	-0.089	Detected	0.089	Detected	0.000	-0.940	Detected	-1.077	Detected	-1.008	0.012	GT	Sens	contig323
GT	Sense	Sense	-0.113	Detected	0.113	Detected	0.000	-0.947	Detected	-1.297	Detected	-1.122	0.033	GT	Sens	contig324
GT	Sense	Sense	-0.510	Detected	0.510	Detected	0.000	-1.230	Detected	-2.262	Comprom	-1.746	0.138	GT	Sens	contig325
GT	Sense	Sense	-0.054	Detected	0.054	Detected	0.000	-1.736	Detected	-1.184	Detected	-1.460	0.035	GT	Sens	contig325
GT	Sense	Sense	-0.172	Detected	0.172	Detected	0.000	-1.631	Detected	-1.151	Detected	-1.391	0.042	GT	Sens	contig325
GT	Sense	Sense	-0.053	Detected	0.053	Detected	0.000	-1.110	Detected	-1.327	Detected	-1.219	0.010	GT	Sens	contig326
GT	Sense	Sense	0.007	Detected	-0.007	Detected	0.000	-1.349	Detected	-1.153	Detected	-1.251	0.006	GT	Sens	contig326
GT	Sense	Sense	-0.015	Detected	0.015	Detected	0.000	-1.785	Detected	-1.543	Detected	-1.664	0.005	GT	Sens	contig327
GT	Sense	Sense	-0.437	Detected	0.437	Detected	0.000	-4.569	Comprom	-3.850	Comprom	-4.209	0.018	GT	Sens	contig327
GT	Sense	Sense	0.555	Detected	-0.555	Detected	0.000	-0.963	Detected	-1.359	Detected	-1.161	0.188	GT	Sens	contig327
GT	Sense	Sense	-0.602	Detected	0.602	Detected	0.000	-1.112	Detected	-2.218	Detected	-1.665	0.179	GT	Sens	contig328
GT	Sense	Sense	0.262	Detected	-0.262	Detected	0.000	-1.542	Detected	-1.658	Detected	-1.600	0.027	GT	Sens	contig329
GT	Sense	Sense	0.232	Detected	-0.232	Detected	0.000	-1.832	Detected	-1.861	Detected	-1.846	0.015	GT	Sens	contig329
GT	Sense	Sense	0.095	Detected	-0.095	Detected	0.000	-1.501	Detected	-2.147	Detected	-1.824	0.032	GT	Sens	contig332
GT	Sense	Sense	0.110	Detected	-0.110	Detected	0.000	-1.858	Detected	-2.574	Detected	-2.216	0.027	GT	Sens	contig332
GT	Sense	Sense	-0.001	Detected	0.001	Detected	0.000	-1.469	Detected	-1.787	Detected	-1.628	0.009	GT	Sens	contig332
GT	Sense	Sense	-0.045	Detected	0.045	Detected	0.000	-1.422	Detected	-1.781	Detected	-1.601	0.013	GT	Sens	contig332
GT	Sense	Sense	0.176	Detected	-0.176	Detected	0.000	-1.069	Detected	-1.871	Detected	-1.470	0.078	GT	Sens	contig332
GT	Sense	Sense	0.100	Detected	-0.100	Detected	0.000	-1.254	Detected	-1.527	Detected	-1.391	0.014	GT	Sens	contig332
GT	Sense	Sense	0.078	Detected	-0.078	Detected	0.000	-0.880	Detected	-1.251	Detected	-1.066	0.034	GT	Sens	contig332
GT	Sense	Sense	-0.158	Detected	0.158	Detected	0.000	-1.509	Detected	-1.518	Detected	-1.513	0.011	GT	Sens	contig333
GT	Sense	Sense	-0.156	Detected	0.156	Detected	0.000	-1.298	Detected	-0.920	Detected	-1.109	0.046	GT	Sens	contig333
GT	Sense	Sense	-0.084	Detected	0.084	Detected	0.000	-1.834	Detected	-1.179	Detected	-1.507	0.047	GT	Sens	contig333
GT	Sense	Sense	-0.097	Detected	0.097	Detected	0.000	-1.771	Detected	-1.865	Detected	-1.818	0.004	GT	Sens	contig333
GT	Sense	Sense	0.056	Detected	-0.056	Detected	0.000	-2.124	Comprom	-2.505	Comprom	-2.315	0.007	GT	Sens	contig333
GT	Sense	Sense	0.447	Detected	-0.447	Detected	0.000	-1.112	Detected	-1.121	Detected	-1.117	0.130	GT	Sens	contig333
GT	Sense	Sense	0.397	Detected	-0.397	Detected	0.000	-1.387	Comprom	-1.164	Comprom	-1.275	0.091	GT	Sens	contig334
GT	Sense	Sense	0.142	Detected	-0.142	Detected	0.000	-1.342	Detected	-1.292	Detected	-1.317	0.012	GT	Sens	contig334
GT	Sense	Sense	0.181	Detected	-0.181	Detected	0.000	-1.398	Detected	-1.071	Detected	-1.235	0.037	GT	Sens	contig334
GT	Sense	Sense	0.191	Detected	-0.191	Detected	0.000	-1.410	Comprom	-1.930	Comprom	-1.670	0.035	GT	Sens	contig334
GT	Sense	Sense	0.146	Detected	-0.146	Detected	0.000	-1.124	Detected	-1.447	Detected	-1.285	0.028	GT	Sens	contig335
GT	Sense	Sense	1.764	Detected	-1.764	Detected	0.000	-2.032	Detected	-1.058	Detected	-1.545	0.487	GT	Sens	contig335
GT	Sense	Sense	0.098	Detected	-0.098	Detected	0.000	-1.501	Detected	-1.843	Detected	-1.672	0.014	GT	Sens	contig336
GT	Sense	Sense	0.026	Detected	-0.026	Detected	0.000	-1.494	Detected	-2.200	Detected	-1.847	0.035	GT	Sens	contig336
GT	Sense	Sense	0.043	Detected	-0.043	Detected	0.000	-1.528	Detected	-2.030	Detected	-1.779	0.020	GT	Sens	contig336
GT	Sense	Sense	0.079	Detected	-0.079	Detected	0.000	-1.888	Detected	-2.239	Detected	-2.064	0.009	GT	Sens	contig338
GT	Sense	Sense	0.023	Detected	-0.023	Detected	0.000	-1.907	Detected	-2.118	Detected	-2.013	0.003	GT	Sens	contig338
GT	Sense	Sense	-0.289	Detected	0.289	Detected	0.000	-1.039	Comprom	-2.237	Comprom	-1.638	0.133	GT	Sens	contig338
GT	Sense	Sense	0.106	Detected	-0.106	Detected	0.000	-1.948	Comprom	-3.114	Comprom	-2.531	0.051	GT	Sens	contig338
GT	Sense	Sense	0.194	Detected	-0.194	Detected	0.000	-3.596	Comprom	-2.217	Comprom	-2.907	0.056	GT	Sens	contig338
GT	Sense	Sense	-0.352	Detected	0.352	Detected	0.000	-1.197	Detected	-0.881	Detected	-1.039	0.115	GT	Sens	contig339
GT	Sense	Sense	0.010	Detected	-0.010	Detected	0.000	-1.073	Comprom	-1.333	Comprom	-1.203	0.011	GT	Sens	contig340
GT	Sense	Sense	-0.573	Detected	0.573	Detected	0.000	-2.149	Detected	-0.910	Detected	-1.530	0.212	GT	Sens	contig341
GT	Sense	Sense	-1.107	Detected	1.107	Detected	0.000	-2.205	Detected	-2.291	Detected	-2.248	0.180	GT	Sens	contig341
GT	Sense	Sense	0.205	Detected	-0.205	Detected	0.000	-1.408	Detected	-1.875	Detected	-1.641	0.034	GT	Sens	contig341
GT	Sense	Sense	0.192	Detected	-0.192	Detected	0.000	-1.691	Detected	-1.313	Detected	-1.502	0.031	GT	Sens	contig341
GT	Sense	Sense	-0.280	Detected	0.280	Detected	0.000	-0.937	Detected	-1.211	Detected	-1.074	0.075	GT	Sens	contig341
GT	Sense	Sense	-0.087	Detected	0.087	Detected	0.000	-0.865	Comprom	-2.225	Comprom	-1.545	0.153	GT	Sens	contig342
GT	Sense	Sense	-0.036	Detected	0.036	Detected	0.000	-1.806	Detected	-1.356	Detected	-1.581	0.020	GT	Sens	contig344
GT	Sense	Sense	-0.018	Detected	0.018	Detected	0.000	-1.359	Detected	-1.413	Detected	-1.386	0.001	GT	Sens	contig344
GT	Sense	Sense	-0.280	Detected	0.280	Detected	0.000	-1.047	Detected	-1.357	Detected	-1.202	0.064	GT	Sens	contig345
GT	Sense	Sense	1.620	Detected	-1.620	Detected	0.000	-0.932	Detected	-1.976	Comprom	-1.454	0.483	GT	Sens	contig345
GT	Sense	Sense	0.151	Detected	-0.151	Detected	0.000	-1.070	Detected	-1.833	Comprom	-1.451	0.072	GT	Sens	contig345
GT	Sense	Sense	0.071	Detected	-0.071	Detected	0.000	-0.906	Detected	-1.411	Comprom	-1.159	0.048	GT	Sens	contig345
GT	Sense	Sense	0.126	Detected	-0.126	Detected	0.000	-1.272	Comprom	-0.810	Comprom	-1.041	0.058	GT	Sens	contig346
GT	Sense	Sense	0.110	Detected	-0.110	Detected	0.000	-1.910	Detected	-2.057	Detected	-1.984	0.004	GT	Sens	contig346
GT	Sense	Sense	0.180	Detected	-0.180	Detected	0.000	-1.564	Detected	-1.890	Detected	-1.727	0.019	GT	Sens	contig346
GT	Sense	Sense	0.085	Detected	-0.085	Detected	0.000	-1.969	Detected	-2.016	Detected	-1.993	0.002	GT	Sens	contig347
GT	Sense	Sense	0.058	Detected	-0.058	Detected	0.000	-1.825	Detected	-2.052	Detected	-1.938	0.004	GT	Sens	contig347
GT	Sense	Sense	0.303	Detected	-0.303	Detected	0.000	-1.038	Detected	-1.556	Comprom	-1.297	0.083	GT	Sens	contig347
GT	Sense	Sense	0.153	Detected	-0.153	Detected	0.000	-1.311	Comprom	-1.935	Comprom	-1.623	0.043	GT	Sens	contig347
GT	Sense	Sense	0.096	Detected	-0.096	Detected	0.000	-1.481	Comprom	-1.123	Comprom	-1.302	0.023	GT	Sens	contig348
GT	Sense	Sense	0.042	Detected	-0.042	Detected	0.000	-2.403	Detected	-2.230	Detected	-2.316	0.002	GT	Sens	contig348
GT	Sense	Sense	0.053	Detected	-0.053	Detected	0.000	-2.053	Detected	-2.027	Detected	-2.040	0.001	GT	Sens	contig348
GT	Sense	Sense	0.127	Detected	-0.127	Detected	0.000	-1.089	Detected	-0.913	Detected	-1.001	0.023	GT	Sens	contig349
GT	Sense	Sense	-0.206	Detected	0.206	Detected	0.000	-1.067	Detected	-1.057	Detected	-1.062	0.036	GT	Sens	contig350
GT	Sense	Sense	0.131	Detected	-0.131	Detected	0.000	-1.660	Comprom	-1.241	Comprom	-1.451	0.028	GT	Sens	contig351
GT	Sense	Sense	0.049	Detected	-0.049	Detected	0.000	-0.958	Detected	-1.348	Detected	-1.153	0.029	GT	Sens	contig351
GT	Sense	Sense	0.166	Detected	-0.166	Detected	0.000	-1.299	Detected	-1.594	Detected	-1.446	0.023	GT	Sens	contig351
GT	Sense	Sense	-0.073	Detected	0.073	Detected	0.000	-2.292	Comprom	-1.676	Comprom	-1.984	0.025	GT	Sens	contig351
GT	Sense	Sense	0.072	Detected	-0.072	Detected	0.000	-1.574	Comprom	-0.889	Comprom	-1.231	0.072	GT	Sens	contig352
GT	Sense	Sense	0.033	Detected	-0.033	Detected	0.000	-1.494	Comprom	-1.060	Comprom	-1.277	0.028	GT	Sens	contig353
GT	Sense	Sense	0.074	Detected	-0.074	Detected	0.000	-1.648	Detected	-2.530	Comprom	-2.089	0.043	GT	Sens	contig353
GT	Sense	Sense	-0.091	Detected	0.091	Detected	0.000	-1.386	Comprom	-1.757	Comprom	-1.572	0.017	GT	Sens	contig354
GT	Sense	Sense	0.063	Detected	-0.063	Detected	0.000	-1.452	Comprom	-2.417	Comprom	-1.935	0.058	GT	Sens	contig356
GT	Sense	Sense	0.191	Detected	-0.191	Detected	0.000	-0.988	Comprom	-2.001	Comprom	-1.495	0.110	GT	Sens	contig356
GT	Sense	Sense	-0.420	Detected	0.420	Detected	0.000	-1.368	Detected	-0.987	Comprom	-1.177	0.125	GT	Sens	contig356
GT	Sense	Sense	0.246	Detected	-0.246	Detected	0.000	-1.856	Detected	-1.006	Detected	-1.431	0.100	GT	Sens	contig357
GT	Sense	Sense	0.184	Detected	-0.184	Detected	0.000	-2.270	Detected	-1.037	Detected	-1.654	0.124	GT	Sens	contig357
GT	Sense	Sense	1.446	Detected	-1.446	Detected	0.000	-1.252	Detected	-1.060	Detected	-1.156	0.509	GT	Sens	contig358
GT	Sense	Sense	0.380	Detected	-0.380	Detected	0.000	-1.115	Detected	-1.971	Comprom	-1.543	0.114	GT	Sens	contig360
GT	Sense	Sense	-0.332	Detected	0.332	Detected	0.000	-1.017	Detected	-1.239	Comprom	-1.128	0.084	GT	Sens	contig360
GT	Sense	Sense	0.165	Detected	-0.165	Detected	0.000	-1.011	Detected	-1.927	Comprom	-1.469	0.094	GT	Sens	contig360
GT	Sense	Sense	-0.039	Detected	0.039	Detected	0.000	-1.560	Detected	-1.916	Detected	-1.738	0.011	GT	Sens	contig361
GT	Sense	Sense	-0.050	Detected	0.050	Detected	0.000	-1.531	Comprom	-2.574	Comprom	-2.053	0.059	GT	Sens	contig361
GT	Sense	Sense	0.133	Detected	-0.133	Detected	0.000	-1.272	Detected	-0.922	Detected	-1.097	0.038	GT	Sens	contig362
GT	Sense	Sense	0.238	Detected	-0.238	Detected	0.000	-1.534	Detected	-1.703	Detected	-1.618	0.023	GT	Sens	contig362
GT	Sense	Sense	0.143	Detected	-0.143	Detected	0.000	-1.175	Detected	-1.347	Detected	-1.261	0.017	GT	Sens	contig362
GT	Sense	Sense	0.855	Detected	-0.855	Detected	0.000	-1.834	Comprom	-1.389	Comprom	-1.611	0.210	GT	Sens	contig364
GT	Sense	Sense	-0.240	Detected	0.240	Detected	0.000	-1.159	Comprom	-1.150	Comprom	-1.155	0.041	GT	Sens	contig365
GT	Sense	Sense	0.347	Detected	-0.347	Detected	0.000	-1.632	Detected	-1.807	Detected	-1.720	0.041	GT	Sens	contig368
GT	Sense	Sense	0.270	Detected	-0.270	Detected	0.000	-1.253	Comprom	-1.713	Comprom	-1.483	0.053	GT	Sens	contig369
GT	Sense	Sense	0.161	Detected	-0.161	Detected	0.000	-1.874	Detected	-2.300	Detected	-2.087	0.016	GT	Sens	contig370
GT	Sense	Sense	0.021	Detected	-0.021	Detected	0.000	-1.612	Detected	-2.318	Detected	-1.965	0.031	GT	Sens	contig370
GT	Sense	Sense	0.030	Detected	-0.030	Detected	0.000	-0.899	Detected	-1.147	Detected	-1.023	0.015	GT	Sens	contig370
GT	Sense	Sense	0.124	Detected	-0.124	Detected	0.000	-2.342	Detected	-2.322	Detected	-2.332	0.003	GT	Sens	contig370
GT	Sense	Sense	0.205	Detected	-0.205	Detected	0.000	-1.944	Detected	-2.438	Comprom	-2.191	0.021	GT	Sens	contig370
GT	Sense	Sense	0.161	Detected	-0.161	Detected	0.000	-1.755	Detected	-2.068	Detected	-1.911	0.014	GT	Sens	contig371
GT	Sense	Sense	0.089	Detected	-0.089	Detected	0.000	-1.709	Detected	-1.259	Detected	-1.484	0.026	GT	Sens	contig371
GT	Sense	Sense	-0.196	Detected	0.196	Detected	0.000	-1.408	Detected	-1.249	Detected	-1.328	0.024	GT	Sens	contig372
GT	Sense	Sense	-0.253	Detected	0.253	Detected	0.000	-1.401	Detected	-1.255	Detected	-1.328	0.037	GT	Sens	contig372
GT	Sense	Sense	-0.029	Detected	0.029	Detected	0.000	-1.702	Detected	-2.505	Detected	-2.104	0.035	GT	Sens	contig372
GT	Sense	Sense	0.005	Detected	-0.005	Detected	0.000	-1.505	Detected	-2.689	Detected	-2.097	0.071	GT	Sens	contig372
GT	Sense	Sense	0.345	Detected	-0.345	Detected	0.000	-1.082	Detected	-1.316	Comprom	-1.199	0.081	GT	Sens	contig372
GT	Sense	Sense	0.105	Detected	-0.105	Detected	0.000	-1.229	Detected	-1.438	Detected	-1.334	0.012	GT	Sens	contig372
GT	Sense	Sense	1.528	Detected	-1.528	Detected	0.000	-1.410	Detected	-2.979	Comprom	-2.194	0.330	GT	Sens	contig373
GT	Sense	Sense	0.139	Detected	-0.139	Detected	0.000	-1.063	Detected	-1.110	Detected	-1.086	0.016	GT	Sens	contig373
GT	Sense	Sense	-0.016	Detected	0.016	Detected	0.000	-1.577	Detected	-1.264	Detected	-1.421	0.012	GT	Sens	contig373
GT	Sense	Sense	0.116	Detected	-0.116	Detected	0.000	-1.556	Detected	-1.605	Detected	-1.581	0.006	GT	Sens	contig373
GT	Sense	Sense	0.319	Detected	-0.319	Detected	0.000	-1.878	Comprom	-2.698	Comprom	-2.288	0.048	GT	Sens	contig373
GT	Sense	Sense	0.106	Detected	-0.106	Detected	0.000	-0.866	Detected	-1.549	Detected	-1.208	0.078	GT	Sens	contig374
GT	Sense	Sense	-0.064	Detected	0.064	Detected	0.000	-1.856	Comprom	-0.936	Comprom	-1.396	0.095	GT	Sens	contig374
GT	Sense	Sense	0.823	Detected	-0.823	Detected	0.000	-2.628	Comprom	-1.253	Comprom	-1.941	0.212	GT	Sens	contig375
GT	Sense	Sense	0.384	Detected	-0.384	Detected	0.000	-1.445	Detected	-1.683	Comprom	-1.564	0.060	GT	Sens	contig375
GT	Sense	Sense	-0.032	Detected	0.032	Detected	0.000	-2.697	Comprom	-1.465	Comprom	-2.081	0.078	GT	Sens	contig376
GT	Sense	Sense	-0.360	Detected	0.360	Detected	0.000	-0.823	Detected	-1.284	Comprom	-1.054	0.133	GT	Sens	contig377
GT	Sense	Sense	-1.016	Detected	1.016	Detected	0.000	-1.202	Detected	-1.175	Detected	-1.188	0.363	GT	Sens	contig378
GT	Sense	Sense	-0.247	Detected	0.247	Detected	0.000	-1.271	Detected	-0.958	Detected	-1.115	0.063	GT	Sens	contig378
GT	Sense	Sense	-0.098	Detected	0.098	Detected	0.000	-1.822	Detected	-1.899	Comprom	-1.860	0.003	GT	Sens	contig378
GT	Sense	Sense	0.130	Detected	-0.130	Detected	0.000	-1.283	Detected	-1.570	Detected	-1.427	0.018	GT	Sens	contig379
GT	Sense	Sense	0.166	Detected	-0.166	Detected	0.000	-1.212	Detected	-0.909	Detected	-1.060	0.042	GT	Sens	contig379
GT	Sense	Sense	0.167	Detected	-0.167	Detected	0.000	-0.928	Detected	-1.216	Detected	-1.072	0.040	GT	Sens	contig381
GT	Sense	Sense	0.203	Detected	-0.203	Detected	0.000	-0.950	Detected	-1.521	Detected	-1.235	0.072	GT	Sens	contig381
GT	Sense	Sense	0.355	Detected	-0.355	Detected	0.000	-1.167	Detected	-1.047	Detected	-1.107	0.091	GT	Sens	contig382
GT	Sense	Sense	-0.304	Detected	0.304	Detected	0.000	-1.717	Detected	-1.401	Detected	-1.559	0.045	GT	Sens	contig383
GT	Sense	Sense	0.072	Detected	-0.072	Detected	0.000	-1.385	Detected	-1.685	Detected	-1.535	0.012	GT	Sens	contig385
GT	Sense	Sense	0.083	Detected	-0.083	Detected	0.000	-1.586	Detected	-1.905	Detected	-1.745	0.010	GT	Sens	contig385
GT	Sense	Sense	-0.392	Detected	0.392	Detected	0.000	-1.419	Detected	-1.013	Comprom	-1.216	0.110	GT	Sens	contig386
GT	Sense	Sense	-0.095	Detected	0.095	Detected	0.000	-2.601	Detected	-1.308	Detected	-1.955	0.096	GT	Sens	contig386
GT	Sense	Sense	0.088	Detected	-0.088	Detected	0.000	-0.802	Detected	-1.281	Comprom	-1.042	0.055	GT	Sens	contig386
GT	Sense	Sense	0.014	Detected	-0.014	Detected	0.000	-2.172	Detected	-1.863	Detected	-2.018	0.006	GT	Sens	contig387
GT	Sense	Sense	0.073	Detected	-0.073	Detected	0.000	-2.543	Detected	-2.345	Detected	-2.444	0.003	GT	Sens	contig387
GT	Sense	Sense	0.090	Detected	-0.090	Detected	0.000	-1.483	Detected	-1.885	Detected	-1.684	0.017	GT	Sens	contig387
GT	Sense	Sense	0.119	Detected	-0.119	Detected	0.000	-0.990	Detected	-1.066	Comprom	-1.028	0.015	GT	Sens	contig388
GT	Sense	Sense	0.193	Detected	-0.193	Detected	0.000	-0.882	Detected	-1.731	Comprom	-1.307	0.107	GT	Sens	contig389
GT	Sense	Sense	-0.380	Detected	0.380	Detected	0.000	-1.831	Detected	-1.362	Detected	-1.597	0.070	GT	Sens	contig394
GT	Sense	Sense	-0.099	Detected	0.099	Detected	0.000	-1.184	Detected	-1.228	Detected	-1.206	0.007	GT	Sens	contig394
GT	Sense	Sense	-0.616	Detected	0.616	Detected	0.000	-1.673	Detected	-2.354	Comprom	-2.013	0.103	GT	Sens	contig394
GT	Sense	Sense	0.118	Detected	-0.118	Detected	0.000	-1.755	Detected	-1.511	Detected	-1.633	0.011	GT	Sens	contig396
GT	Sense	Sense	0.094	Detected	-0.094	Detected	0.000	-2.319	Detected	-2.320	Detected	-2.319	0.002	GT	Sens	contig396
GT	Sense	Sense	-0.066	Detected	0.066	Detected	0.000	-1.701	Detected	-1.600	Detected	-1.651	0.003	GT	Sens	contig397
GT	Sense	Sense	-0.052	Detected	0.052	Detected	0.000	-3.202	Detected	-6.097	Comprom	-4.650	0.085	GT	Sens	contig399
GT	Sense	Sense	-0.113	Detected	0.113	Detected	0.000	-4.976	Comprom	-4.575	Comprom	-4.775	0.002	GT	Sens	contig399
GT	Sense	Sense	-0.181	Detected	0.181	Detected	0.000	-1.533	Comprom	-1.048	Comprom	-1.291	0.051	GT	Sens	contig400
GT	Sense	Sense	-0.030	Detected	0.030	Detected	0.000	-1.086	Detected	-1.305	Comprom	-1.195	0.009	GT	Sens	contig400
GT	Sense	Sense	-0.426	Detected	0.426	Detected	0.000	-3.623	Detected	-2.689	Detected	-3.156	0.038	GT	Sens	contig400
GT	Sense	Sense	-0.387	Detected	0.387	Detected	0.000	-3.421	Detected	-2.434	Detected	-2.927	0.043	GT	Sens	contig400
GT	Sense	Sense	0.146	Detected	-0.146	Detected	0.000	-1.840	Comprom	-0.972	Comprom	-1.406	0.092	GT	Sens	contig401
GT	Sense	Sense	0.040	Detected	-0.040	Detected	0.000	-0.969	Detected	-1.322	Detected	-1.146	0.024	GT	Sens	contig401
GT	Sense	Sense	-0.024	Detected	0.024	Detected	0.000	-1.141	Detected	-1.092	Detected	-1.117	0.001	GT	Sens	contig401
GT	Sense	Sense	-0.014	Detected	0.014	Detected	0.000	-1.540	Comprom	-0.809	Comprom	-1.175	0.085	GT	Sens	contig401
GT	Sense	Sense	0.793	Detected	-0.793	Detected	0.000	-2.159	Comprom	-2.865	Comprom	-2.512	0.102	GT	Sens	contig402
GT	Sense	Sense	0.173	Detected	-0.173	Detected	0.000	-1.446	Comprom	-2.352	Comprom	-1.899	0.059	GT	Sens	contig402
GT	Sense	Sense	-0.170	Detected	0.170	Detected	0.000	-1.862	Detected	-2.976	Comprom	-2.419	0.053	GT	Sens	contig402
GT	Sense	Sense	-0.159	Detected	0.159	Detected	0.000	-1.235	Detected	-1.206	Detected	-1.221	0.017	GT	Sens	contig403
GT	Sense	Sense	0.026	Detected	-0.026	Detected	0.000	-1.053	Detected	-1.045	Detected	-1.049	0.001	GT	Sens	contig403
GT	Sense	Sense	0.265	Detected	-0.265	Detected	0.000	-1.218	Detected	-1.795	Comprom	-1.506	0.061	GT	Sens	contig403
GT	Sense	Sense	-1.399	Detected	1.399	Detected	0.000	-1.268	Detected	-2.123	Detected	-1.696	0.366	GT	Sens	contig404
GT	Sense	Sense	0.090	Detected	-0.090	Detected	0.000	-1.946	Detected	-2.230	Detected	-2.088	0.006	GT	Sens	contig405
GT	Sense	Sense	0.094	Detected	-0.094	Detected	0.000	-1.096	Detected	-1.579	Detected	-1.338	0.035	GT	Sens	contig405
GT	Sense	Sense	0.099	Detected	-0.099	Detected	0.000	-1.389	Detected	-1.860	Detected	-1.624	0.024	GT	Sens	contig405
GT	Sense	Sense	0.086	Detected	-0.086	Detected	0.000	-1.437	Detected	-0.998	Detected	-1.217	0.035	GT	Sens	contig405
GT	Sense	Sense	0.218	Detected	-0.218	Detected	0.000	-1.636	Detected	-1.761	Detected	-1.699	0.017	GT	Sens	contig405
GT	Sense	Sense	0.098	Detected	-0.098	Detected	0.000	-1.521	Detected	-2.490	Comprom	-2.006	0.056	GT	Sens	contig405
GT	Sense	Sense	0.085	Detected	-0.085	Detected	0.000	-1.621	Detected	-1.745	Detected	-1.683	0.004	GT	Sens	contig405
GT	Sense	Sense	0.119	Detected	-0.119	Detected	0.000	-1.814	Detected	-1.831	Detected	-1.822	0.004	GT	Sens	contig405
GT	Sense	Sense	0.084	Detected	-0.084	Detected	0.000	-0.969	Detected	-1.157	Detected	-1.063	0.014	GT	Sens	contig405
GT	Sense	Sense	0.278	Detected	-0.278	Detected	0.000	-1.143	Comprom	-2.490	Comprom	-1.816	0.130	GT	Sens	contig405
GT	Sense	Sense	0.105	Detected	-0.105	Detected	0.000	-1.899	Detected	-1.923	Detected	-1.911	0.003	GT	Sens	contig405
GT	Sense	Sense	0.068	Detected	-0.068	Detected	0.000	-1.717	Detected	-1.582	Detected	-1.650	0.003	GT	Sens	contig406
GT	Sense	Sense	0.112	Detected	-0.112	Detected	0.000	-1.266	Detected	-1.100	Detected	-1.183	0.014	GT	Sens	contig406
GT	Sense	Sense	-1.066	Detected	1.066	Detected	0.000	-1.547	Detected	-2.084	Comprom	-1.815	0.240	GT	Sens	contig406
GT	Sense	Sense	0.029	Detected	-0.029	Detected	0.000	-1.835	Detected	-1.794	Detected	-1.814	0.000	GT	Sens	contig407
GT	Sense	Sense	0.073	Detected	-0.073	Detected	0.000	-1.820	Detected	-1.552	Detected	-1.686	0.008	GT	Sens	contig407
GT	Sense	Sense	0.068	Detected	-0.068	Detected	0.000	-1.063	Detected	-1.272	Detected	-1.167	0.011	GT	Sens	contig407
GT	Sense	Sense	0.168	Detected	-0.168	Detected	0.000	-1.476	Detected	-1.347	Detected	-1.412	0.016	GT	Sens	contig409
GT	Sense	Sense	0.172	Detected	-0.172	Detected	0.000	-1.584	Detected	-1.445	Detected	-1.514	0.015	GT	Sens	contig409
GT	Sense	Sense	-0.112	Detected	0.112	Detected	0.000	-1.109	Detected	-1.579	Detected	-1.344	0.036	GT	Sens	contig410

GT	Sense	Sense	0.092	Detected	-0.092	Detected	0.000	-1.713	Detected	-1.807	Detected	-1.760	0.003	[illegible]
GT	Sense	Sense	0.103	Detected	-0.103	Detected	0.000	-1.242	Comprom	-0.922	Comprom	-1.082	0.030	[illegible]
GT	Sense	Sense	0.724	Detected	-0.724	Detected	0.000	-2.684	Comprom	-1.965	Comprom	-2.325	0.103	[illegible]
GT	Sense	Sense	0.050	Detected	-0.050	Detected	0.000	-1.041	Detected	-1.068	Detected	-1.055	0.002	[illegible]
GT	Sense	Sense	0.090	Detected	-0.090	Detected	0.000	-1.528	Detected	-1.504	Detected	-1.516	0.004	[illegible]
GT	Sense	Sense	0.080	Detected	-0.080	Detected	0.000	-0.930	Detected	-1.102	Detected	-1.016	0.013	[illegible]
GT	Sense	Sense	-0.076	Detected	0.076	Detected	0.000	-1.534	Detected	-1.820	Detected	-1.677	0.009	[illegible]
GT	Sense	Sense	-0.030	Detected	0.030	Detected	0.000	-1.414	Detected	-1.366	Detected	-1.390	0.001	[illegible]
GT	Sense	Sense	-0.026	Detected	0.026	Detected	0.000	-1.187	Detected	-1.022	Detected	-1.104	0.006	[illegible]
GT	Sense	Sense	-0.054	Detected	0.054	Detected	0.000	-1.628	Detected	-1.604	Comprom	-1.616	0.001	[illegible]
GT	Sense	Sense	-0.799	Detected	0.799	Detected	0.000	-3.157	Detected	-3.062	Detected	-3.110	0.060	[illegible]
GT	Sense	Sense	0.218	Detected	-0.218	Detected	0.000	-1.090	Detected	-1.837	Detected	-1.463	0.077	[illegible]
GT	Sense	Sense	0.121	Detected	-0.121	Detected	0.000	-0.901	Detected	-1.146	Comprom	-1.023	0.027	[illegible]
GT	Sense	Sense	0.111	Detected	-0.111	Detected	0.000	-2.708	Comprom	-1.595	Comprom	-2.152	0.063	[illegible]
GT	Sense	Sense	0.148	Detected	-0.148	Detected	0.000	-1.135	Detected	-1.149	Detected	-1.142	0.016	[illegible]
GT	Sense	Sense	0.493	Detected	-0.493	Detected	0.000	-2.884	Comprom	-1.552	Comprom	-2.218	0.116	[illegible]
GT	Sense	Sense	0.113	Detected	-0.113	Detected	0.000	-2.154	Detected	-2.421	Detected	-2.288	0.006	[illegible]
GT	Sense	Sense	0.088	Detected	-0.088	Detected	0.000	-1.292	Detected	-1.295	Detected	-1.293	0.005	[illegible]
GT	Sense	Sense	0.202	Detected	-0.202	Detected	0.000	-1.080	Detected	-0.925	Detected	-1.002	0.044	[illegible]
GT	Sense	Sense	0.056	Detected	-0.056	Detected	0.000	-0.953	Detected	-1.589	Detected	-1.271	0.059	[illegible]
GT	Sense	Sense	0.194	Detected	-0.194	Detected	0.000	-0.943	Detected	-1.076	Detected	-1.009	0.039	[illegible]
GT	Sense	Sense	0.340	Detected	-0.340	Detected	0.000	-1.477	Detected	-0.841	Detected	-1.159	0.130	[illegible]
GT	Sense	Sense	-0.249	Detected	0.249	Detected	0.000	-1.285	Comprom	-2.465	Comprom	-1.875	0.100	[illegible]
GT	Sense	Sense	0.180	Detected	-0.180	Detected	0.000	-1.990	Detected	-1.941	Detected	-1.966	0.008	[illegible]
GT	Sense	Sense	0.223	Detected	-0.223	Detected	0.000	-1.853	Detected	-1.775	Detected	-1.814	0.015	[illegible]
GT	Sense	Sense	-0.781	Detected	0.781	Detected	0.000	-1.652	Detected	-1.007	Detected	-1.329	0.256	[illegible]
GT	Sense	Sense	-0.501	Detected	0.501	Detected	0.000	-1.136	Detected	-0.889	Detected	-1.012	0.189	[illegible]
GT	Sense	Sense	-1.387	Detected	1.387	Detected	0.000	-2.121	Detected	-2.009	Comprom	-2.065	0.275	[illegible]
GT	Sense	Sense	0.057	Detected	-0.057	Detected	0.000	-1.025	Detected	-1.386	Detected	-1.205	0.024	[illegible]
GT	Sense	Sense	0.361	Detected	-0.361	Detected	0.000	-1.085	Detected	-1.527	Detected	-1.306	0.091	[illegible]
GT	Sense	Sense	0.123	Detected	-0.123	Detected	0.000	-0.916	Detected	-1.366	Detected	-1.141	0.047	[illegible]
GT	Sense	Sense	0.697	Detected	-0.697	Detected	0.000	-1.134	Detected	-2.053	Comprom	-1.594	0.196	[illegible]
GT	Sense	Sense	0.075	Detected	-0.075	Detected	0.000	-0.891	Detected	-1.483	Detected	-1.187	0.060	[illegible]
GT	Sense	Sense	0.183	Detected	-0.183	Detected	0.000	-1.398	Comprom	-1.425	Comprom	-1.412	0.016	[illegible]
GT	Sense	Sense	-0.124	Detected	0.124	Detected	0.000	-2.423	Detected	-1.014	Detected	-1.719	0.138	[illegible]
GT	Sense	Sense	0.080	Detected	-0.080	Detected	0.000	-1.971	Detected	-1.597	Detected	-1.784	0.013	[illegible]
GT	Sense	Sense	0.349	Detected	-0.349	Detected	0.000	-1.264	Detected	-1.414	Comprom	-1.339	0.064	[illegible]
GT	Sense	Sense	0.386	Detected	-0.386	Detected	0.000	-1.413	Detected	-1.583	Comprom	-1.498	0.063	[illegible]
GT	Sense	Sense	0.102	Detected	-0.102	Detected	0.000	-2.067	Detected	-1.521	Detected	-1.794	0.025	[illegible]
GT	Sense	Sense	-0.049	Detected	0.049	Detected	0.000	-1.632	Detected	-1.240	Detected	-1.436	0.019	[illegible]
GT	Sense	Sense	0.007	Detected	-0.007	Detected	0.000	-1.231	Detected	-1.265	Detected	-1.248	0.000	[illegible]
GT	Sense	Sense	0.192	Detected	-0.192	Detected	0.000	-1.034	Comprom	-1.179	Comprom	-1.107	0.033	[illegible]
GT	Sense	Sense	0.369	Detected	-0.369	Detected	0.000	-2.866	Detected	-0.994	Detected	-1.930	0.195	[illegible]
GT	Sense	Sense	-0.003	Detected	0.003	Detected	0.000	-0.940	Detected	-1.125	Detected	-1.033	0.008	[illegible]
GT	Sense	Sense	-0.017	Detected	0.017	Detected	0.000	-0.983	Comprom	-1.568	Comprom	-1.275	0.049	[illegible]
GT	Sense	Sense	0.166	Detected	-0.166	Detected	0.000	-1.472	Detected	-1.927	Detected	-1.699	0.026	[illegible]
GT	Sense	Sense	-0.119	Detected	0.119	Detected	0.000	-1.985	Comprom	-0.894	Comprom	-1.440	0.123	[illegible]
GT	Sense	Sense	-0.007	Detected	0.007	Detected	0.000	-1.354	Detected	-1.574	Detected	-1.464	0.006	[illegible]
GT	Sense	Sense	0.326	Detected	-0.326	Detected	0.000	-1.596	Detected	-1.354	Detected	-1.475	0.051	[illegible]
GT	Sense	Sense	0.033	Detected	-0.033	Detected	0.000	-0.905	Detected	-1.545	Detected	-1.225	0.063	[illegible]
GT	Sense	Sense	0.241	Detected	-0.241	Detected	0.000	-0.926	Detected	-2.131	Comprom	-1.528	0.143	[illegible]
GT	Sense	Sense	0.050	Detected	-0.050	Detected	0.000	-1.377	Detected	-0.920	Detected	-1.148	0.039	[illegible]
GT	Sense	Sense	0.349	Detected	-0.349	Detected	0.000	-1.044	Detected	-0.970	Detected	-1.007	0.103	[illegible]
GT	Sense	Sense	-0.122	Detected	0.122	Detected	0.000	-1.182	Detected	-0.839	Detected	-1.010	0.041	[illegible]
GT	Sense	Sense	-0.024	Detected	0.024	Detected	0.000	-1.066	Detected	-1.175	Detected	-1.120	0.003	[illegible]
GT	Sense	Sense	-0.323	Detected	0.323	Detected	0.000	-2.414	Detected	-4.140	Comprom	-3.277	0.071	[illegible]
GT	Sense	Sense	0.155	Detected	-0.155	Detected	0.000	-1.101	Detected	-1.464	Detected	-1.282	0.033	[illegible]
GT	Sense	Sense	0.111	Detected	-0.111	Detected	0.000	-1.532	Detected	-1.743	Detected	-1.637	0.009	[illegible]
GT	Sense	Sense	0.007	Detected	-0.007	Detected	0.000	-1.444	Detected	-1.372	Comprom	-1.408	0.001	[illegible]
GT	Sense	Sense	-0.051	Detected	0.051	Detected	0.000	-1.533	Comprom	-1.132	Comprom	-1.333	0.023	[illegible]
GT	Sense	Sense	-0.121	Detected	0.121	Detected	0.000	-0.950	Detected	-1.077	Detected	-1.014	0.018	[illegible]
GT	Sense	Sense	0.736	Detected	-0.736	Detected	0.000	-1.461	Detected	-1.322	Detected	-1.391	0.201	[illegible]
GT	Sense	Sense	-0.180	Detected	0.180	Detected	0.000	-1.227	Detected	-1.387	Detected	-1.307	0.022	[illegible]
GT	Sense	Sense	0.085	Detected	-0.085	Detected	0.000	-1.944	Detected	-2.091	Detected	-2.018	0.003	[illegible]
GT	Sense	Sense	-0.846	Detected	0.846	Detected	0.000	-2.006	Comprom	-2.422	Comprom	-2.214	0.126	[illegible]
GT	Sense	Sense	0.194	Detected	-0.194	Detected	0.000	-1.284	Detected	-1.338	Detected	-1.311	0.022	[illegible]
GT	Sense	Sense	0.075	Detected	-0.075	Detected	0.000	-1.860	Detected	-1.469	Detected	-1.664	0.015	[illegible]
GT	Sense	Sense	-0.185	Detected	0.185	Detected	0.000	-0.965	Detected	-1.278	Detected	-1.122	0.044	[illegible]
GT	Sense	Sense	0.253	Detected	-0.253	Detected	0.000	-2.212	Detected	-2.630	Detected	-2.421	0.018	[illegible]
GT	Sense	Sense	0.033	Detected	-0.033	Detected	0.000	-1.351	Detected	-3.440	Comprom	-2.395	0.149	[illegible]
GT	Sense	Sense	-0.612	Detected	0.612	Detected	0.000	-2.054	Detected	-1.106	Detected	-1.580	0.178	[illegible]
GT	Sense	Sense	0.062	Detected	-0.062	Detected	0.000	-1.233	Detected	-2.810	Comprom	-2.022	0.125	[illegible]
GT	Sense	Sense	0.092	Detected	-0.092	Detected	0.000	-1.233	Detected	-1.154	Detected	-1.194	0.007	[illegible]
GT	Sense	Sense	0.225	Detected	-0.225	Detected	0.000	-1.559	Detected	-1.087	Detected	-1.323	0.056	[illegible]
GT	Sense	Sense	0.071	Detected	-0.071	Detected	0.000	-1.287	Detected	-1.162	Detected	-1.225	0.006	[illegible]
GT	Sense	Sense	-0.447	Detected	0.447	Detected	0.000	-1.895	Detected	-2.235	Detected	-2.065	0.050	[illegible]
GT	Sense	Sense	-0.074	Detected	0.074	Detected	0.000	-0.870	Detected	-1.513	Detected	-1.191	0.069	[illegible]
GT	Sense	Sense	0.337	Detected	-0.337	Detected	0.000	-1.184	Comprom	-1.526	Comprom	-1.355	0.070	[illegible]
GT	Sense	Sense	0.122	Detected	-0.122	Detected	0.000	-1.319	Detected	-1.412	Detected	-1.366	0.009	[illegible]
GT	Sense	Sense	0.074	Detected	-0.074	Detected	0.000	-0.967	Detected	-1.668	Detected	-1.317	0.067	[illegible]
GT	Sense	Sense	-0.606	Detected	0.606	Detected	0.000	-1.819	Detected	-0.970	Detected	-1.395	0.200	[illegible]
GT	Sense	Sense	-0.256	Detected	0.256	Detected	0.000	-1.120	Detected	-1.074	Detected	-1.097	0.051	[illegible]
GT	Sense	Sense	0.068	Detected	-0.068	Detected	0.000	-1.487	Detected	-2.216	Detected	-1.852	0.038	[illegible]
GT	Sense	Sense	-0.310	Detected	0.310	Detected	0.000	-2.189	Detected	-1.528	Detected	-1.859	0.055	[illegible]
GT	Sense	Sense	0.013	Detected	-0.013	Detected	0.000	-1.364	Detected	-1.013	Detected	-1.189	0.021	[illegible]
GT	Sense	Sense	0.097	Detected	-0.097	Detected	0.000	-2.123	Detected	-2.248	Detected	-2.185	0.003	[illegible]
GT	Sense	Sense	0.018	Detected	-0.018	Detected	0.000	-1.171	Detected	-1.604	Detected	-1.387	0.024	[illegible]
GT	Sense	Sense	0.580	Detected	-0.580	Detected	0.000	-0.975	Detected	-2.072	Comprom	-1.524	0.196	[illegible]
GT	Sense	Sense	0.600	Detected	-0.600	Detected	0.000	-1.706	Detected	-1.263	Detected	-1.484	0.146	[illegible]
GT	Sense	Sense	0.202	Detected	-0.202	Detected	0.000	-1.024	Detected	-1.482	Detected	-1.253	0.055	[illegible]
GT	Sense	Sense	0.106	Detected	-0.106	Detected	0.000	-1.398	Detected	-1.433	Detected	-1.416	0.006	[illegible]
GT	Sense	Sense	-0.163	Detected	0.163	Detected	0.000	-2.845	Detected	-1.296	Detected	-2.071	0.120	[illegible]
GT	Sense	Sense	0.117	Detected	-0.117	Detected	0.000	-1.065	Detected	-1.025	Detected	-1.045	0.013	[illegible]
GT	Sense	Sense	-0.219	Detected	0.219	Detected	0.000	-1.570	Detected	-1.242	Detected	-1.406	0.036	[illegible]
GT	Sense	Sense	0.068	Detected	-0.068	Detected	0.000	-1.229	Detected	-1.183	Detected	-1.206	0.004	[illegible]
GT	Sense	Sense	-0.171	Detected	0.171	Detected	0.000	-2.565	Detected	-0.984	Detected	-1.774	0.160	[illegible]
GT	Sense	Sense	0.148	Detected	-0.148	Detected	0.000	-1.221	Detected	-1.335	Detected	-1.278	0.015	[illegible]
GT	Sense	Sense	-0.181	Detected	0.181	Detected	0.000	-1.576	Detected	-0.871	Detected	-1.224	0.091	[illegible]
GT	Sense	Sense	-0.031	Detected	0.031	Detected	0.000	-1.216	Detected	-0.818	Detected	-1.017	0.037	[illegible]
GT	Sense	Sense	-0.118	Detected	0.118	Detected	0.000	-2.730	Detected	-1.295	Detected	-2.013	0.109	[illegible]
GT	Sense	Sense	-0.174	Detected	0.174	Detected	0.000	-2.634	Detected	-1.579	Detected	-2.106	0.063	[illegible]
GT	Sense	Sense	-0.383	Detected	0.383	Detected	0.000	-0.934	Detected	-3.258	Detected	-2.096	0.229	[illegible]
GT	Sense	Sense	0.003	Detected	-0.003	Detected	0.000	-1.866	Detected	-1.895	Detected	-1.881	0.000	[illegible]
GT	Sense	Sense	-0.034	Detected	0.034	Detected	0.000	-1.974	Detected	-1.542	Detected	-1.758	0.015	[illegible]
GT	Sense	Sense	-1.159	Detected	1.159	Detected	0.000	-2.790	Detected	-1.608	Detected	-2.199	0.233	[illegible]
GT	Sense	Sense	-0.231	Detected	0.231	Detected	0.000	-3.434	Comprom	-1.935	Comprom	-2.685	0.076	[illegible]
GT	Sense	Sense	0.117	Detected	-0.117	Detected	0.000	-1.554	Detected	-1.345	Detected	-1.450	0.011	[illegible]
GT	Sense	Sense	0.134	Detected	-0.134	Detected	0.000	-1.561	Detected	-1.999	Detected	-1.780	0.020	[illegible]
GT	Sense	Sense	-0.241	Detected	0.241	Detected	0.000	-1.664	Detected	-2.090	Detected	-1.877	0.028	[illegible]
GT	Sense	Sense	0.494	Detected	-0.494	Detected	0.000	-3.000	Detected	-2.441	Detected	-2.721	0.041	[illegible]
GT	Sense	Sense	-0.243	Detected	0.243	Detected	0.000	-1.170	Detected	-1.416	Detected	-1.293	0.042	[illegible]
GT	Sense	Sense	-0.080	Detected	0.080	Detected	0.000	-2.064	Comprom	-1.688	Comprom	-1.876	0.012	[illegible]
GT	Sense	Sense	0.525	Detected	-0.525	Detected	0.000	-1.214	Detected	-2.179	Comprom	-1.697	0.140	[illegible]
GT	Sense	Sense	-0.099	Detected	0.099	Detected	0.000	-1.824	Comprom	-1.166	Comprom	-1.495	0.049	[illegible]
GT	Sense	Sense	0.303	Detected	-0.303	Detected	0.000	-1.032	Detected	-3.309	Comprom	-2.170	0.207	[illegible]
GT	Sense	Sense	0.116	Detected	-0.116	Detected	0.000	-1.009	Detected	-1.019	Detected	-1.014	0.013	[illegible]
GT	Sense	Sense	0.113	Detected	-0.113	Detected	0.000	-2.340	Detected	-2.508	Detected	-2.424	0.003	[illegible]
GT	Sense	Sense	0.743	Detected	-0.743	Detected	0.000	-1.738	Detected	-1.036	Detected	-1.387	0.233	[illegible]
GT	Sense	Sense	0.180	Detected	-0.180	Detected	0.000	-2.100	Detected	-2.007	Detected	-2.054	0.008	[illegible]
GT	Sense	Sense	0.057	Detected	-0.057	Detected	0.000	-2.393	Detected	-1.900	Detected	-2.146	0.014	[illegible]
GT	Sense	Sense	0.293	Detected	-0.293	Detected	0.000	-1.238	Detected	-1.293	Comprom	-1.265	0.050	[illegible]
GT	Sense	Sense	0.089	Detected	-0.089	Detected	0.000	-1.868	Detected	-2.120	Detected	-1.994	0.006	[illegible]
GT	Sense	Sense	-0.259	Detected	0.259	Detected	0.000	-1.058	Detected	-1.773	Detected	-1.416	0.085	[illegible]
GT	Sense	Sense	0.121	Detected	-0.121	Detected	0.000	-1.175	Detected	-1.015	Detected	-1.095	0.017	[illegible]
GT	Sense	Sense	-0.094	Detected	0.094	Detected	0.000	-1.090	Detected	-1.179	Detected	-1.135	0.008	[illegible]
GT	Sense	Sense	0.061	Detected	-0.061	Detected	0.000	-1.057	Detected	-1.300	Detected	-1.178	0.013	[illegible]
GT	Sense	Sense	-0.509	Detected	0.509	Detected	0.000	-0.962	Detected	-1.139	Detected	-1.051	0.179	[illegible]
GT	Sense	Sense	0.195	Detected	-0.195	Detected	0.000	-1.298	Detected	-1.475	Detected	-1.386	0.023	[illegible]
GT	Sense	Sense	-0.212	Detected	0.212	Detected	0.000	-1.741	Detected	-1.964	Detected	-1.852	0.016	[illegible]
GT	Sense	Sense	-0.097	Detected	0.097	Detected	0.000	-3.194	Detected	-0.976	Detected	-2.085	0.202	[illegible]
GT	Sense	Sense	-0.755	Detected	0.755	Detected	0.000	-2.039	Detected	-1.546	Detected	-1.792	0.153	[illegible]
GT	Sense	Sense	-0.003	Detected	0.003	Detected	0.000	-4.272	Comprom	-3.089	Comprom	-3.680	0.025	[illegible]
GT	Sense	Sense	0.182	Detected	-0.182	Detected	0.000	-1.480	Comprom	-1.324	Comprom	-1.402	0.019	[illegible]
GT	Sense	Sense	0.217	Detected	-0.217	Detected	0.000	-1.170	Detected	-2.198	Comprom	-1.684	0.094	[illegible]
GT	Sense	Sense	0.413	Detected	-0.413	Detected	0.000	-1.220	Detected	-1.220	Detected	-1.220	0.098	[illegible]
GT	Sense	Sense	-0.003	Detected	0.003	Detected	0.000	-1.233	Detected	-0.984	Detected	-1.108	0.012	[illegible]
GT	Sense	Sense	0.151	Detected	-0.151	Detected	0.000	-2.011	Detected	-1.486	Detected	-1.749	0.029	[illegible]
GT	Sense	Sense	0.005	Detected	-0.005	Detected	0.000	-2.335	Detected	-1.598	Detected	-1.966	0.033	[illegible]
GT	Sense	Sense	0.054	Detected	-0.054	Detected	0.000	-4.341	Comprom	-2.215	Detected	-3.278	0.091	[illegible]
GT	Sense	Sense	0.015	Detected	-0.015	Detected	0.000	-1.304	Detected	-1.166	Detected	-1.235	0.003	[illegible]
GT	Sense	Sense	0.120	Detected	-0.120	Detected	0.000	-1.012	Detected	-1.525	Comprom	-1.269	0.046	[illegible]
GT	Sense	Sense	0.065	Detected	-0.065	Detected	0.000	-0.942	Detected	-1.339	Comprom	-1.141	0.032	[illegible]
GT	Sense	Sense	0.101	Detected	-0.101	Detected	0.000	-1.861	Detected	-1.776	Detected	-1.819	0.004	[illegible]
GT	Sense	Sense	0.236	Detected	-0.236	Detected	0.000	-1.782	Detected	-1.653	Detected	-1.717	0.020	[illegible]
GT	Sense	Sense	0.030	Detected	-0.030	Detected	0.000	-2.157	Comprom	-2.660	Comprom	-2.408	0.011	[illegible]
GT	Sense	Sense	-0.006	Detected	0.006	Detected	0.000	-1.948	Detected	-2.103	Detected	-2.026	0.001	[illegible]
GT	Sense	Sense	-0.244	Detected	0.244	Detected	0.000	-2.334	Detected	-1.245	Detected	-1.789	0.096	[illegible]
GT	Sense	Sense	-0.162	Detected	0.162	Detected	0.000	-1.785	Comprom	-1.100	Comprom	-1.443	0.063	[illegible]
GT	Sense	Sense	-0.202	Detected	0.202	Detected	0.000	-2.054	Detected	-1.088	Detected	-1.571	0.095	[illegible]
GT	Sense	Sense	0.047	Detected	-0.047	Detected	0.000	-2.049	Detected	-2.143	Detected	-2.096	0.001	[illegible]
GT	Sense	Sense	0.153	Detected	-0.153	Detected	0.000	-1.340	Detected	-1.319	Detected	-1.329	0.013	[illegible]
GT	Sense	Sense	-0.078	Detected	0.078	Detected	0.000	-1.339	Detected	-1.020	Detected	-1.180	0.022	[illegible]
GT	Sense	Sense	0.012	Detected	-0.012	Detected	0.000	-1.882	Detected	-2.370	Detected	-2.126	0.013	[illegible]
GT	Sense	Sense	-0.066	Detected	0.066	Detected	0.000	-1.325	Detected	-0.878	Detected	-1.102	0.042	[illegible]
GT	Sense	Sense	0.133	Detected	-0.133	Detected	0.000	-1.314	Detected	-1.339	Detected	-1.326	0.010	[illegible]
GT	Sense	Sense	0.048	Detected	-0.048	Detected	0.000	-1.242	Detected	-1.734	Detected	-1.488	0.027	[illegible]
GT	Sense	Sense	-0.218	Detected	0.218	Detected	0.000	-1.408	Detected	-1.859	Detected	-1.634	0.035	[illegible]
GT	Sense	Sense	1.207	Detected	-1.207	Detected	0.000	-2.604	Detected	-0.987	Detected	-1.795	0.342	[illegible]
GT	Sense	Sense	0.429	Detected	-0.429	Detected	0.000	-1.374	Detected	-1.657	Detected	-1.516	0.079	[illegible]
GT	Sense	Sense	-0.004	Detected	0.004	Detected	0.000	-1.781	Detected	-2.895	Detected	-2.338	0.052	[illegible]
GT	Sense	Sense	0.127	Detected	-0.127	Detected	0.000	-0.949	Detected	-1.122	Detected	-1.035	0.021	[illegible]
GT	Sense	Sense	-0.276	Detected	0.276	Detected	0.000	-1.636	Detected	-1.326	Detected	-1.481	0.043	[illegible]
GT	Sense	Sense	0.181	Detected	-0.181	Detected	0.000	-1.322	Detected	-0.917	Detected	-1.120	0.054	[illegible]
GT	Sense	Sense	0.148	Detected	-0.148	Detected	0.000	-2.124	Detected	-2.186	Detected	-2.155	0.005	[illegible]
GT	Sense	Sense	-0.046	Detected	0.046	Detected	0.000	-1.625	Detected	-2.032	Detected	-1.828	0.013	[illegible]
GT	Sense	Sense	0.116	Detected	-0.116	Detected	0.000	-1.292	Detected	-1.269	Detected	-1.281	0.008	[illegible]
GT	Sense	Sense	0.048	Detected	-0.048	Detected	0.000	-2.079	Detected	-1.780	Detected	-1.929	0.007	[illegible]
GT	Sense	Sense	-0.077	Detected	0.077	Detected	0.000	-2.109	Comprom	-1.210	Detected	-1.660	0.068	[illegible]
GT	Sense	Sense	-0.085	Detected	0.085	Detected	0.000	-1.073	Detected	-1.330	Detected	-1.201	0.016	[illegible]
GT	Sense	Sense	0.068	Detected	-0.068	Detected	0.000	-1.814	Detected	-1.741	Detected	-1.777	0.002	[illegible]
GT	Sense	Sense	0.079	Detected	-0.079	Detected	0.000	-2.486	Detected	-2.038	Detected	-2.262	0.011	[illegible]
GT	Sense	Sense	0.439	Detected	-0.439	Detected	0.000	-5.219	Comprom	-3.190	Detected	-4.204	0.063	[illegible]
GT	Sense	Sense	0.002	Detected	-0.002	Detected	0.000	-2.329	Detected	-2.425	Detected	-2.377	0.000	[illegible]
GT	Sense	Sense	0.121	Detected	-0.121	Detected	0.000	-2.019	Detected	-1.964	Detected	-1.991	0.004	[illegible]
GT	Sense	Sense	0.076	Detected	-0.076	Detected	0.000	-2.091	Detected	-2.230	Detected	-2.161	0.002	[illegible]
GT	Sense	Sense	-0.421	Detected	0.421	Detected	0.000	-0.819	Detected	-1.212	Detected	-1.016	0.160	[illegible]
GT	Sense	Sense	0.082	Detected	-0.082	Detected	0.000	-0.960	Detected	-1.091	Detected	-1.025	0.010	[illegible]
GT	Sense	Sense	0.112	Detected	-0.112	Detected	0.000	-1.874	Detected	-1.827	Detected	-1.850	0.004	[illegible]
GT	Sense	Sense	-0.030	Detected	0.030	Detected	0.000	-1.435	Detected	-1.870	Detected	-1.653	0.017	[illegible]
GT	Sense	Sense	0.005	Detected	-0.005	Detected	0.000	-2.009	Detected	-2.174	Detected	-2.092	0.002	[illegible]
GT	Sense	Sense	0.097	Detected	-0.097	Detected	0.000	-1.529	Detected	-1.471	Detected	-1.500	0.005	[illegible]
GT	Sense	Sense	0.056	Detected	-0.056	Detected	0.000	-1.305	Detected	-1.223	Detected	-1.264	0.003	[illegible]
GT	Sense	Sense	0.133	Detected	-0.133	Detected	0.000	-1.386	Detected	-1.716	Detected	-1.551	0.018	[illegible]
GT	Sense	Sense	-0.032	Detected	0.032	Detected	0.000	-2.003	Detected	-2.182	Detected	-2.092	0.002	[illegible]
GT	Sense	Sense	-0.079	Detected	0.079	Detected	0.000	-1.627	Detected	-1.139	Detected	-1.383	0.033	[illegible]
GT	Sense	Sense	-0.074	Detected	0.074	Detected	0.000	-1.408	Detected	-1.413	Detected	-1.410	0.003	[illegible]
GT	Sense	Sense	0.054	Detected	-0.054	Detected	0.000	-2.066	Detected	-1.846	Detected	-1.956	0.004	[illegible]
GT	Sense	Sense	-0.411	Detected	0.411	Detected	0.000	-1.305	Detected	-1.320	Detected	-1.312	0.086	[illegible]
GT	Sense	Sense	0.034	Detected	-0.034	Detected	0.000	-1.064	Detected	-1.397	Detected	-1.230	0.018	[illegible]
GT	Sense	Sense	1.801	Detected	-1.801	Detected	0.000	-1.911	Detected	-1.888	Detected	-1.900	0.402	[illegible]
GT	Sense	Sense	-0.194	Detected	0.194	Detected	0.000	-2.168	Detected	-1.583	Detected	-1.875	0.033	[illegible]
GT	Sense	Sense	0.085	Detected	-0.085	Detected	0.000	-1.672	Detected	-1.290	Detected	-1.481	0.019	[illegible]
GT	Sense	Sense	0.104	Detected	-0.104	Detected	0.000	-2.033	Detected	-1.854	Detected	-1.944	0.005	[illegible]
GT	Sense	Sense	0.138	Detected	-0.138	Detected	0.000	-1.429	Detected	-1.314	Detected	-1.371	0.012	[illegible]
GT	Sense	Sense	-0.158	Detected	0.158	Detected	0.000	-0.969	Comprom	-1.478	Comprom	-1.224	0.055	[illegible]
GT	Sense	Sense	0.126	Detected	-0.126	Detected	0.000	-1.584	Detected	-1.364	Detected	-1.474	0.013	[illegible]
GT	Sense	Sense	-0.063	Detected	0.063	Detected	0.000	-1.824	Detected	-1.530	Comprom	-1.677	0.009	[illegible]
GT	Sense	Sense	-0.244	Detected	0.244	Detected	0.000	-1.040	Detected	-1.013	Detected	-1.027	0.052	[illegible]
GT	Sense	Sense	0.819	Detected	-0.819	Detected	0.000	-4.336	Detected	-4.085	Detected	-4.211	0.037	[illegible]
GT	Sense	Sense	0.053	Detected	-0.053	Detected	0.000	-1.786	Detected	-1.636	Detected	-1.711	0.003	[illegible]
GT	Sense	Sense	0.041	Detected	-0.041	Detected	0.000	-2.167	Detected	-1.759	Detected	-1.963	0.011	[illegible]
GT	Sense	Sense	0.038	Detected	-0.038	Detected	0.000	-1.965	Detected	-1.266	Detected	-1.616	0.044	[illegible]
GT	Sense	Sense	0.691	Detected	-0.691	Detected	0.000	-1.521	Detected	-1.177	Detected	-1.349	0.199	[illegible]
GT	Sense	Sense	0.082	Detected	-0.082	Detected	0.000	-1.891	Detected	-1.621	Detected	-1.756	0.008	[illegible]
GT	Sense	Sense	-0.762	Detected	0.762	Detected	0.000	-2.163	Detected	-0.814	Detected	-1.489	0.281	[illegible]
GT	Sense	Sense	-0.083	Detected	0.083	Detected	0.000	-1.058	Detected	-1.259	Detected	-1.158	0.012	[illegible]
GT	Sense	Sense	0.003	Detected	-0.003	Detected	0.000	-2.174	Detected	-1.183	Detected	-1.679	0.077	[illegible]
GT	Sense	Sense	-0.092	Detected	0.092	Detected	0.000	-2.894	Detected	-1.648	Detected	-2.271	0.069	[illegible]
GT	Sense	Sense	0.321	Detected	-0.321	Detected	0.000	-0.933	Comprom	-2.402	Comprom	-1.668	0.173	[illegible]
GT	Sense	Sense	0.173	Detected	-0.173	Detected	0.000	-1.610	Detected	-1.507	Detected	-1.558	0.013	[illegible]
GT	Sense	Sense	0.064	Detected	-0.064	Detected	0.000	-2.011	Detected	-1.674	Detected	-1.842	0.009	[illegible]
GT	Sense	Sense	0.431	Detected	-0.431	Detected	0.000	-1.006	Detected	-1.034	Comprom	-1.020	0.142	[illegible]
GT	Sense	Sense	0.207	Detected	-0.207	Detected	0.000	-2.140	Detected	-2.078	Comprom	-2.109	0.010	[illegible]
GT	Sense	Sense	0.090	Detected	-0.090	Detected	0.000	-2.055	Detected	-1.754	Detected	-1.905	0.008	[illegible]
GT	Sense	Sense	0.076	Detected	-0.076	Detected	0.000	-1.832	Detected	-1.771	Detected	-1.801	0.002	[illegible]
GT	Sense	Sense	-0.027	Detected	0.027	Detected	0.000	-1.649	Detected	-1.776	Detected	-1.712	0.002	[illegible]
GT	Sense	Sense	-0.044	Detected	0.044	Detected	0.000	-1.217	Detected	-1.336	Detected	-1.276	0.003	[illegible]
GT	Sense	Sense	0.070	Detected	-0.070	Detected	0.000	-1.672	Detected	-2.043	Detected	-1.858	0.011	[illegible]
GT	Sense	Sense	0.022	Detected	-0.022	Detected	0.000	-2.278	Detected	-1.853	Detected	-2.065	0.011	[illegible]
GT	Sense	Sense	0.038	Detected	-0.038	Detected	0.000	-1.314	Detected	-1.752	Detected	-1.533	0.020	[illegible]
GT	Sense	Sense	-0.032	Detected	0.032	Detected	0.000	-1.183	Detected	-1.619	Detected	-1.401	0.024	[illegible]
GT	Sense	Sense	-0.157	Detected	0.157	Detected	0.000	-1.887	Detected	-1.507	Detected	-1.697	0.021	[illegible]
GT	Sense	Sense	-0.812	Detected	0.812	Detected	0.000	-3.961	Comprom	-3.184	Comprom	-3.572	0.058	[illegible]
GT	Sense	Sense	-0.306	Detected	0.306	Detected	0.000	-1.511	Detected	-1.622	Detected	-1.567	0.037	[illegible]
GT	Sense	Sense	-0.010	Detected	0.010	Detected	0.000	-0.854	Detected	-1.388	Detected	-1.121	0.052	[illegible]
GT	Sense	Sense	0.238	Detected	-0.238	Detected	0.000	-1.366	Detected	-1.325	Detected	-1.345	0.030	[illegible]
GT	Sense	Sense	0.117	Detected	-0.117	Detected	0.000	-1.832	Detected	-1.604	Detected	-1.718	0.009	[illegible]
GT	Sense	Sense	-0.054	Detected	0.054	Detected	0.000	-1.202	Detected	-1.144	Detected	-1.173	0.003	[illegible]
GT	Sense	Sense	-0.049	Detected	0.049	Detected	0.000	-1.061	Detected	-1.191	Detected	-1.126	0.005	[illegible]
GT	Sense	Sense	-0.004	Detected	0.004	Detected	0.000	-0.994	Detected	-1.128	Detected	-1.061	0.004	[illegible]
GT	Sense	Sense	-0.198	Detected	0.198	Detected	0.000	-1.075	Detected	-1.161	Detected	-1.118	0.031	[illegible]
GT	Sense	Sense	0.078	Detected	-0.078	Detected	0.000	-2.303	Detected	-2.330	Detected	-2.316	0.001	[illegible]
GT	Sense	Sense	0.530	Detected	-0.530	Detected	0.000	-1.134	Detected	-1.317	Detected	-1.226	0.150	[illegible]
GT	Sense	Sense	0.011	Detected	-0.011	Detected	0.000	-2.212	Detected	-1.752	Detected	-1.982	0.013	[illegible]
GT	Sense	Sense	0.080	Detected	-0.080	Detected	0.000	-1.895	Detected	-1.547	Detected	-1.721	0.012	[illegible]
GT	Sense	Sense	0.056	Detected	-0.056	Detected	0.000	-1.496	Detected	-1.501	Detected	-1.498	0.001	[illegible]
GT	Sense	Sense	-0.044	Detected	0.044	Detected	0.000	-1.257	Detected	-1.176	Detected	-1.216	0.002	[illegible]
GT	Sense	Sense	0.032	Detected	-0.032	Detected	0.000	-1.836	Detected	-1.452	Detected	-1.644	0.014	[illegible]
GT	Sense	Sense	0.479	Detected	-0.479	Detected	0.000	-1.111	Detected	-0.951	Detected	-1.031	0.168	[illegible]
GT	Sense	Sense	0.127	Detected	-0.127	Detected	0.000	-1.620	Detected	-1.519	Detected	-1.570	0.007	[illegible]
GT	Sense	Sense	0.051	Detected	-0.051	Detected	0.000	-2.169	Detected	-1.766	Detected	-1.967	0.011	[illegible]
GT	Sense	Sense	0.090	Detected	-0.090	Detected	0.000	-1.874	Detected	-1.852	Detected	-1.863	0.002	[illegible]
GT	Sense	Sense	0.198	Detected	-0.198	Detected	0.000	-1.268	Detected	-1.244	Detected	-1.256	0.024	[illegible]
GT	Sense	Sense	-0.231	Detected	0.231	Detected	0.000	-2.626	Detected	-1.612	Detected	-2.119	0.063	[illegible]

GT	Sense	Sense	-0.072	Detected	0.072	Detected	0.000	-1.420	Detected	-2.095	Detected	-1.757	0.037	GT	Sens	contig182	contig182	DNA-dire	TGAATC
GT	Sense	Sense	0.117	Detected	-0.117	Detected	0.000	-1.243	Detected	-1.010	Detected	-1.126	0.021	GT	Sens	contig184	contig184	Unknown	GATGGG
GT	Sense	Sense	0.080	Detected	-0.080	Detected	0.000	-1.623	Detected	-1.113	Detected	-1.368	0.036	GT	Sens	contig186	contig186	Pol polyp	TGAGAG
GT	Sense	Sense	0.018	Detected	-0.018	Detected	0.000	-1.484	Detected	-1.661	Detected	-1.572	0.003	GT	Sens	contig187	contig187	Ycf2 (AHF	TGTGGG
GT	Sense	Sense	0.555	Detected	-0.555	Detected	0.000	-0.957	Detected	-1.076	Detected	-1.016	0.210	GT	Sens	contig188	contig188	Sulfotran	GAAGAG
GT	Sense	Sense	-0.092	Detected	0.092	Detected	0.000	-1.519	Detected	-1.142	Detected	-1.331	0.024	GT	Sens	contig189	contig189	Unknown	TGAATC
GT	Sense	Sense	-0.180	Detected	0.180	Detected	0.000	-1.676	Detected	-0.937	Detected	-1.306	0.086	GT	Sens	contig190	contig190	Unknown	CATGGG
GT	Sense	Sense	0.106	Detected	-0.106	Detected	0.000	-1.738	Detected	-1.552	Detected	-1.645	0.007	GT	Sens	contig190	contig190	Gag-Pol r	CATTTGA
GT	Sense	Sense	-0.472	Detected	0.472	Detected	0.000	-2.015	Detected	-2.018	Detected	-2.016	0.051	GT	Sens	contig193	contig193	Alcohol d	GGTAAT
GT	Sense	Sense	-0.510	Detected	0.510	Detected	0.000	-1.783	Detected	-0.908	Detected	-1.346	0.183	GT	Sens	contig193	contig193	Purple ac	ATGTGG
GT	Sense	Sense	0.530	Detected	-0.530	Detected	0.000	-1.064	Detected	-1.102	Detected	-1.083	0.178	GT	Sens	contig194	contig194	UDP-gluc	AAACAT
GT	Sense	Sense	0.021	Detected	-0.021	Detected	0.000	-1.262	Detected	-1.414	Detected	-1.338	0.003	GT	Sens	contig194	contig194	Gag-Pol r	TCATCT
GT	Sense	Sense	0.162	Detected	-0.162	Detected	0.000	-1.199	Detected	-1.562	Detected	-1.381	0.030	GT	Sens	contig195	contig195	Ycf1 (AHF	AAGCGA
GT	Sense	Sense	0.080	Detected	-0.080	Detected	0.000	-2.014	Comprom	-1.829	Comprom	-1.921	0.004	GT	Sens	contig195	contig195	Mutator-li	ACAAAA
GT	Sense	Sense	0.099	Detected	-0.099	Detected	0.000	-1.680	Detected	-1.624	Detected	-1.652	0.004	GT	Sens	contig199	contig199	Unknown	CCTGTG
GT	Sense	Sense	0.019	Detected	-0.019	Detected	0.000	-1.013	Detected	-1.350	Detected	-1.182	0.020	GT	Sens	contig202	contig202	Ycf2 (AHF	GCAATT
GT	Sense	Sense	-0.061	Detected	0.061	Detected	0.000	-1.188	Detected	-1.474	Detected	-1.331	0.013	GT	Sens	contig203	contig203	Orf146 pr	TGAAGT
GT	Sense	Sense	0.154	Detected	-0.154	Detected	0.000	-1.944	Detected	-1.879	Detected	-1.912	0.007	GT	Sens	contig205	contig205	%26apos	GCCAAC
GT	Sense	Sense	0.078	Detected	-0.078	Detected	0.000	-1.954	Detected	-1.857	Detected	-1.905	0.002	GT	Sens	contig205	contig205	%26apos	AATTGT
GT	Sense	Sense	-0.947	Detected	0.947	Detected	0.000	-2.430	Detected	-1.120	Detected	-1.775	0.263	GT	Sens	contig206	contig206	Peroxida	GAAACC
GT	Sense	Sense	0.117	Detected	-0.117	Detected	0.000	-0.925	Detected	-1.078	Detected	-1.001	0.019	GT	Sens	contig207	contig207	Sieve ele	GCATAC
GT	Sense	Sense	1.162	Detected	-1.162	Detected	0.000	-0.907	Detected	-1.164	Detected	-1.036	0.469	GT	Sens	contig210	contig210	Endochiti	TTCTTT
GT	Sense	Sense	0.041	Detected	-0.041	Detected	0.000	-1.230	Detected	-1.071	Detected	-1.151	0.006	GT	Sens	contig210	contig210	Polyprote	AGTGAA
GT	Sense	Sense	0.131	Detected	-0.131	Detected	0.000	-1.538	Detected	-1.245	Detected	-1.392	0.019	GT	Sens	contig210	contig210	Unknown	AAATGA
GT	Sense	Sense	0.050	Detected	-0.050	Detected	0.000	-1.436	Detected	-0.900	Detected	-1.168	0.050	GT	Sens	contig213	contig213	Gag-Pol r	TGATAG
GT	Sense	Sense	-0.623	Detected	0.623	Detected	0.000	-0.863	Detected	-1.770	Detected	-1.317	0.230	GT	Sens	contig214	contig214	Proline-ri	CTTAGC
GT	Sense	Sense	0.019	Detected	-0.019	Detected	0.000	-2.862	Comprom	-3.163	Comprom	-3.013	0.003	GT	Sens	contig214	contig214	Unknown	GCAGTT
GT	Sense	Sense	-1.097	Detected	1.097	Detected	0.000	-3.087	Detected	-2.896	Detected	-2.992	0.113	GT	Sens	contig215	contig215	Hydroxyc	ACAACG
GT	Sense	Sense	0.184	Detected	-0.184	Detected	0.000	-1.737	Detected	-1.415	Detected	-1.576	0.023	GT	Sens	contig216	contig216	Mutator-li	AGTATC
GT	Sense	Sense	-0.106	Detected	0.106	Detected	0.000	-1.500	Detected	-1.518	Detected	-1.509	0.005	GT	Sens	contig216	contig216	NAD(P)H-	CTACTA
GT	Sense	Sense	0.132	Detected	-0.132	Detected	0.000	-1.148	Detected	-1.462	Detected	-1.305	0.024	GT	Sens	contig217	contig217	Pol polyp	ATAGCT
GT	Sense	Sense	-0.525	Detected	0.525	Detected	0.000	-1.632	Detected	-0.930	Detected	-1.281	0.180	GT	Sens	contig218	contig218	Aldose-1-	AAGTCA
GT	Sense	Sense	0.102	Detected	-0.102	Detected	0.000	-1.914	Detected	-2.061	Detected	-1.988	0.004	GT	Sens	contig220	contig220	Pol polyp	TTGACA
GT	Sense	Sense	0.056	Detected	-0.056	Detected	0.000	-1.259	Detected	-1.556	Detected	-1.408	0.012	GT	Sens	contig221	contig221	NADH-qui	TTATTT
GT	Sense	Sense	1.112	Detected	-1.112	Detected	0.000	-1.706	Detected	-1.140	Detected	-1.423	0.341	GT	Sens	contig222	contig222	FAD-bind	GTCTAC
GT	Sense	Sense	0.071	Detected	-0.071	Detected	0.000	-1.756	Detected	-2.141	Detected	-1.949	0.011	GT	Sens	contig223	contig223	Unknown	GGGCAA
GT	Sense	Sense	0.102	Detected	-0.102	Detected	0.000	-1.420	Detected	-1.664	Detected	-1.542	0.010	GT	Sens	contig224	contig224	Unknown	CAGGCA
GT	Sense	Sense	-0.052	Detected	0.052	Detected	0.000	-0.941	Detected	-1.129	Detected	-1.035	0.011	GT	Sens	contig226	contig226	NADH-qui	AGAATT
GT	Sense	Sense	0.133	Detected	-0.133	Detected	0.000	-1.527	Detected	-1.244	Detected	-1.386	0.019	GT	Sens	contig227	contig227	Pol polyp	ATGTGT
GT	Sense	Sense	0.119	Detected	-0.119	Detected	0.000	-1.060	Detected	-1.034	Detected	-1.047	0.013	GT	Sens	contig228	contig228	UDP-gluc	ATAGTG
GT	Sense	Sense	0.042	Detected	-0.042	Detected	0.000	-2.215	Detected	-1.710	Detected	-1.962	0.017	GT	Sens	contig230	contig230	Unknown	GCTACG
GT	Sense	Sense	0.023	Detected	-0.023	Detected	0.000	-2.351	Detected	-1.981	Detected	-2.166	0.007	GT	Sens	contig231	contig231	Pro-Pol p	ATTCAG
GT	Sense	Sense	0.136	Detected	-0.136	Detected	0.000	-1.523	Detected	-1.479	Detected	-1.501	0.008	GT	Sens	contig232	contig232	Unknown	CACGCA
GT	Sense	Sense	0.141	Detected	-0.141	Detected	0.000	-1.581	Detected	-1.367	Detected	-1.474	0.014	GT	Sens	contig235	contig235	Reverse t	ATGAGT
GT	Sense	Sense	0.129	Detected	-0.129	Detected	0.000	-1.329	Detected	-0.971	Detected	-1.150	0.035	GT	Sens	contig238	contig238	Unknown	ACACGG
GT	Sense	Sense	-0.841	Detected	0.841	Detected	0.000	-1.224	Detected	-1.102	Detected	-1.163	0.302	GT	Sens	contig239	contig239	Histone-ly	CTGAGG
GT	Sense	Sense	-0.211	Detected	0.211	Detected	0.000	-0.915	Detected	-1.203	Detected	-1.059	0.054	GT	Sens	contig240	contig240	ATP synt	TAGTAA
GT	Sense	Sense	0.056	Detected	-0.056	Detected	0.000	-2.060	Detected	-2.256	Detected	-2.158	0.003	GT	Sens	contig240	contig240	Reverse t	AGCTAA
GT	Sense	Sense	0.113	Detected	-0.113	Detected	0.000	-1.500	Detected	-2.031	Detected	-1.765	0.026	GT	Sens	contig240	contig240	Transpos	CAAGAT
GT	Sense	Sense	0.079	Detected	-0.079	Detected	0.000	-1.202	Detected	-1.533	Detected	-1.368	0.017	GT	Sens	contig240	contig240	Receptor-	GCTATG
GT	Sense	Sense	-0.852	Detected	0.852	Detected	0.000	-2.545	Detected	-1.084	Detected	-1.815	0.247	GT	Sens	contig241	contig241	Peroxida	AAGAAT
GT	Sense	Sense	0.068	Detected	-0.068	Detected	0.000	-2.249	Detected	-2.304	Detected	-2.276	0.001	GT	Sens	contig241	contig241	Pol polyp	TCACAA
GT	Sense	Sense	0.140	Detected	-0.140	Detected	0.000	-1.688	Detected	-3.151	Comprom	-2.419	0.083	GT	Sens	contig245	contig245	Transpos	ACAGCG
GT	Sense	Sense	-0.052	Detected	0.052	Detected	0.000	-1.896	Detected	-1.480	Detected	-1.688	0.016	GT	Sens	contig245	contig245	Gag-pol (	AGAAAA
GT	Sense	Sense	-0.028	Detected	0.028	Detected	0.000	-1.067	Detected	-1.616	Detected	-1.341	0.040	GT	Sens	contig247	contig247	DNA-dire	CACAAT
GT	Sense	Sense	0.104	Detected	-0.104	Detected	0.000	-1.844	Detected	-1.447	Detected	-1.645	0.018	GT	Sens	contig248	contig248	Polyprote	CTTGTG
GT	Sense	Sense	-0.074	Detected	0.074	Detected	0.000	-1.747	Detected	-1.191	Detected	-1.469	0.036	GT	Sens	contig248	contig248	Aldose-1-	TCCTTA
GT	Sense	Sense	-0.445	Detected	0.445	Detected	0.000	-2.597	Detected	-1.238	Detected	-1.917	0.142	GT	Sens	contig251	contig251	Cyclopro	ATTTCA
GT	Sense	Sense	0.092	Detected	-0.092	Detected	0.000	-2.490	Detected	-2.059	Detected	-2.274	0.010	GT	Sens	contig251	contig251	Unknown	AGACAA
GT	Sense	Sense	-0.035	Detected	0.035	Detected	0.000	-1.391	Detected	-1.736	Detected	-1.563	0.012	GT	Sens	contig253	contig253	Elongatio	AGTAGT
GT	Sense	Sense	0.700	Detected	-0.700	Detected	0.000	-3.186	Comprom	-1.821	Comprom	-2.504	0.125	GT	Sens	contig257	contig257	Alpha-gal	TTTCAT
GT	Sense	Sense	0.005	Detected	-0.005	Detected	0.000	-1.286	Detected	-1.000	Detected	-1.143	0.015	GT	Sens	contig259	contig259	NAD(P)H-	ATCGAA
GT	Sense	Sense	-0.007	Detected	0.007	Detected	0.000	-1.142	Detected	-1.457	Detected	-1.300	0.014	GT	Sens	contig260	contig260	Unknown	CTTAGT
GT	Sense	Sense	-0.010	Detected	0.010	Detected	0.000	-1.073	Detected	-1.088	Detected	-1.080	0.000	GT	Sens	contig260	contig260	Peptide tr	ATTGGC
GT	Sense	Sense	-0.008	Detected	0.008	Detected	0.000	-1.138	Detected	-0.967	Detected	-1.052	0.007	GT	Sens	contig261	contig261	Unknown	ACTCCA
GT	Sense	Sense	0.141	Detected	-0.141	Detected	0.000	-1.950	Detected	-1.920	Detected	-1.935	0.005	GT	Sens	contig261	contig261	Early ligh	CTAGCT
GT	Sense	Sense	-0.273	Detected	0.273	Detected	0.000	-1.051	Detected	-1.436	Detected	-1.244	0.065	GT	Sens	contig262	contig262	ATP synt	GTATAT
GT	Sense	Sense	0.100	Detected	-0.100	Detected	0.000	-1.129	Detected	-1.265	Detected	-1.197	0.010	GT	Sens	contig264	contig264	Unknown	TGGCTG
GT	Sense	Sense	0.122	Detected	-0.122	Detected	0.000	-1.731	Detected	-0.840	Detected	-1.286	0.108	GT	Sens	contig266	contig266	Unknown	ATGGAA
GT	Sense	Sense	0.073	Detected	-0.073	Detected	0.000	-2.094	Detected	-1.912	Detected	-2.003	0.003	GT	Sens	contig267	contig267	Pol polyp	CCGCGA
GT	Sense	Sense	-0.283	Detected	0.283	Detected	0.000	-2.233	Detected	-0.966	Detected	-1.600	0.148	GT	Sens	contig268	contig268	Hydroxyc	TTCAGC
GT	Sense	Sense	0.092	Detected	-0.092	Detected	0.000	-1.299	Detected	-0.905	Detected	-1.102	0.037	GT	Sens	contig268	contig268	Pol polyp	CAGTAC
GT	Sense	Sense	0.012	Detected	-0.012	Detected	0.000	-1.620	Detected	-1.299	Detected	-1.459	0.012	GT	Sens	contig27	contig27	Retrotran	AAATCT
GT	Sense	Sense	-0.037	Detected	0.037	Detected	0.000	-1.180	Detected	-1.252	Detected	-1.216	0.002	GT	Sens	contig270	contig270	Tir-nbs-lr	TCACTT
GT	Sense	Sense	-0.751	Detected	0.751	Detected	0.000	-0.917	Detected	-1.663	Detected	-1.290	0.264	GT	Sens	contig270	contig270	Purple ac	TATAAG
GT	Sense	Sense	-0.058	Detected	0.058	Detected	0.000	-1.213	Detected	-0.995	Detected	-1.104	0.012	GT	Sens	contig271	contig271	Zinc finge	TGCTAA
GT	Sense	Sense	1.872	Detected	-1.872	Detected	0.000	-2.160	Detected	-1.657	Detected	-1.908	0.419	GT	Sens	contig272	contig272	Acyl-CoA	ATCTTG
GT	Sense	Sense	0.039	Detected	-0.039	Detected	0.000	-1.831	Detected	-1.229	Detected	-1.530	0.037	GT	Sens	contig272	contig272	Pol polyp	CTATTC
GT	Sense	Sense	0.005	Detected	-0.005	Detected	0.000	-0.971	Detected	-1.382	Detected	-1.176	0.029	GT	Sens	contig273	contig273	Ycf2 (AHF	GATCTC
GT	Sense	Sense	0.085	Detected	-0.085	Detected	0.000	-1.397	Detected	-1.468	Detected	-1.433	0.004	GT	Sens	contig274	contig274	Gag-Pol r	GTCTTG
GT	Sense	Sense	0.116	Detected	-0.116	Detected	0.000	-2.247	Detected	-1.704	Detected	-1.976	0.022	GT	Sens	contig276	contig276	Unknown	ACAAGA
GT	Sense	Sense	-0.197	Detected	0.197	Detected	0.000	-3.452	Detected	-1.216	Detected	-2.334	0.176	GT	Sens	contig278	contig278	MYB tran	TTGGAA
GT	Sense	Sense	0.083	Detected	-0.083	Detected	0.000	-1.841	Detected	-1.388	Detected	-1.615	0.022	GT	Sens	contig281	contig281	Pol polyp	ACATTG
GT	Sense	Sense	0.011	Detected	-0.011	Detected	0.000	-1.353	Detected	-1.791	Detected	-1.572	0.019	GT	Sens	contig281	contig281	NADH del	TTTGTT
GT	Sense	Sense	-0.095	Detected	0.095	Detected	0.000	-0.940	Detected	-1.064	Detected	-1.002	0.013	GT	Sens	contig282	contig282	Cytochro	TTTTTG
GT	Sense	Sense	-0.780	Detected	0.780	Detected	0.000	-2.126	Detected	-0.801	Detected	-1.463	0.289	GT	Sens	contig284	contig284	Cytochro	ATTAAG
GT	Sense	Sense	0.117	Detected	-0.117	Detected	0.000	-1.814	Detected	-1.383	Detected	-1.598	0.023	GT	Sens	contig284	contig284	Polyprote	CTACTG
GT	Sense	Sense	0.038	Detected	-0.038	Detected	0.000	-2.119	Detected	-1.735	Detected	-1.927	0.010	GT	Sens	contig284	contig284	Polyprote	TTTTGT
GT	Sense	Sense	0.194	Detected	-0.194	Detected	0.000	-1.120	Detected	-1.413	Detected	-1.266	0.035	GT	Sens	contig286	contig286	Mutator-li	GTGCGT
GT	Sense	Sense	0.061	Detected	-0.061	Detected	0.000	-2.124	Detected	-1.692	Detected	-1.908	0.014	GT	Sens	contig287	contig287	Retrotran	GCATGG
GT	Sense	Sense	0.066	Detected	-0.066	Detected	0.000	-1.184	Detected	-1.120	Detected	-1.152	0.004	GT	Sens	contig287	contig287	Unknown	AGATTC
GT	Sense	Sense	0.133	Detected	-0.133	Detected	0.000	-1.483	Detected	-1.340	Detected	-1.412	0.011	GT	Sens	contig288	contig288	LINE-type	CAGCCG
GT	Sense	Sense	0.082	Detected	-0.082	Detected	0.000	-1.894	Detected	-1.676	Detected	-1.785	0.006	GT	Sens	contig289	contig289	Pol polyp	GGATGT
GT	Sense	Sense	-0.022	Detected	0.022	Detected	0.000	-0.946	Detected	-1.072	Detected	-1.009	0.004	GT	Sens	contig291	contig291	Photosys	TGGGCC
GT	Sense	Sense	-0.222	Detected	0.222	Detected	0.000	-1.252	Detected	-0.917	Detected	-1.084	0.060	GT	Sens	contig292	contig292	Acetyl co	CTACTC
GT	Sense	Sense	0.162	Detected	-0.162	Detected	0.000	-1.924	Detected	-1.610	Detected	-1.767	0.016	GT	Sens	contig294	contig294	Unknown	AAACCT
GT	Sense	Sense	0.046	Detected	-0.046	Detected	0.000	-1.651	Detected	-1.300	Detected	-1.475	0.015	GT	Sens	contig294	contig294	Gag-Pol r	ACGTAC
GT	Sense	Sense	-0.290	Detected	0.290	Detected	0.000	-1.182	Detected	-1.101	Detected	-1.141	0.060	GT	Sens	contig295	contig295	Serine ca	AGCTCC
GT	Sense	Sense	0.087	Detected	-0.087	Detected	0.000	-2.127	Detected	-1.758	Detected	-1.942	0.011	GT	Sens	contig296	contig296	Pro-Pol p	ACTTTA
GT	Sense	Sense	0.001	Detected	-0.001	Detected	0.000	-1.165	Detected	-1.378	Detected	-1.271	0.007	GT	Sens	contig296	contig296	Ycf2 (AHF	ATCAAA
GT	Sense	Sense	-0.216	Detected	0.216	Detected	0.000	-0.972	Detected	-1.191	Detected	-1.082	0.047	GT	Sens	contig299	contig299	CONSTAI	GGAGTA
GT	Sense	Sense	0.139	Detected	-0.139	Detected	0.000	-1.289	Detected	-1.340	Comprom	-1.314	0.011	GT	Sens	contig303	contig303	Transpos	ACTCTT
GT	Sense	Sense	-0.091	Detected	0.091	Detected	0.000	-1.716	Detected	-1.333	Detected	-1.525	0.019	GT	Sens	contig305	contig305	Reverse t	AGCATG
GT	Sense	Sense	0.094	Detected	-0.094	Detected	0.000	-1.856	Detected	-1.458	Detected	-1.657	0.017	GT	Sens	contig306	contig306	Pol polyp	TCTCCT
GT	Sense	Sense	0.025	Detected	-0.025	Detected	0.000	-1.541	Detected	-1.077	Detected	-1.309	0.030	GT	Sens	contig306	contig306	Pol polyp	AGTTTA
GT	Sense	Sense	0.044	Detected	-0.044	Detected	0.000	-1.247	Detected	-0.865	Detected	-1.056	0.033	GT	Sens	contig308	contig308	Unknown	AATGCA
GT	Sense	Sense	0.189	Detected	-0.189	Detected	0.000	-1.216	Detected	-2.016	Comprom	-1.616	0.067	GT	Sens	contig308	contig308	Gag-Pol r	AAGACA
GT	Sense	Sense	0.005	Detected	-0.005	Detected	0.000	-1.839	Detected	-1.422	Detected	-1.631	0.016	GT	Sens	contig310	contig310	Polyprote	TTGATC
GT	Sense	Sense	0.125	Detected	-0.125	Detected	0.000	-1.178	Detected	-1.264	Detected	-1.221	0.012	GT	Sens	contig311	contig311	Unknown	ACAGAC
GT	Sense	Sense	0.079	Detected	-0.079	Detected	0.000	-1.633	Detected	-1.472	Detected	-1.552	0.005	GT	Sens	contig314	contig314	Pol polyp	ATCTGA
GT	Sense	Sense	-0.244	Detected	0.244	Detected	0.000	-1.176	Detected	-1.088	Detected	-1.132	0.045	GT	Sens	contig323	contig323	Indole-3-a	AATGGG
GT	Sense	Sense	-0.630	Detected	0.630	Detected	0.000	-1.860	Detected	-1.299	Detected	-1.580	0.149	GT	Sens	contig323	contig323	Beta-gala	TTAATG
GT	Sense	Sense	-0.098	Detected	0.098	Detected	0.000	-1.033	Detected	-1.225	Detected	-1.129	0.014	GT	Sens	contig329	contig329	Ycf1 (Fra	CTGGAA
GT	Sense	Sense	0.184	Detected	-0.184	Detected	0.000	-1.471	Detected	-1.568	Detected	-1.519	0.015	GT	Sens	contig330	contig330	Unknown	CCATTG
GT	Sense	Sense	0.051	Detected	-0.051	Detected	0.000	-2.423	Detected	-2.597	Detected	-2.510	0.002	GT	Sens	contig333	contig333	Transcrip	GGAAGA
GT	Sense	Sense	0.158	Detected	-0.158	Detected	0.000	-1.467	Detected	-1.144	Detected	-1.306	0.029	GT	Sens	contig348	contig348	Unknown	CGTCGG
GT	Sense	Sense	0.017	Detected	-0.017	Detected	0.000	-1.337	Detected	-0.987	Detected	-1.162	0.022	GT	Sens	contig361	contig361	Ycf2 (AHF	GGTTAT
GT	Sense	Sense	0.146	Detected	-0.146	Detected	0.000	-1.459	Detected	-1.654	Detected	-1.557	0.012	GT	Sens	contig365	contig365	Unknown	GCATGG
GT	Sense	Sense	0.061	Detected	-0.061	Detected	0.000	-2.034	Detected	-1.600	Detected	-1.817	0.015	GT	Sens	contig366	contig366	Pol polyp	ATGCTT
GT	Sense	Sense	0.064	Detected	-0.064	Detected	0.000	-2.307	Detected	-1.816	Detected	-2.061	0.015	GT	Sens	contig371	contig371	Pol polyp	TCGCTA
GT	Sense	Sense	0.096	Detected	-0.096	Detected	0.000	-1.546	Detected	-1.478	Detected	-1.512	0.005	GT	Sens	contig374	contig374	Unknown	AACCTC
GT	Sense	Sense	0.118	Detected	-0.118	Detected	0.000	-1.601	Detected	-1.278	Detected	-1.439	0.019	GT	Sens	contig381	contig381	Unknown	GGGGCT
GT	Sense	Sense	0.141	Detected	-0.141	Detected	0.000	-1.550	Detected	-1.191	Detected	-1.370	0.027	GT	Sens	contig383	contig383	Gag-pol r	GATCTT
GT	Sense	Sense	-0.289	Detected	0.289	Detected	0.000	-2.466	Detected	-2.341	Detected	-2.403	0.015	GT	Sens	contig383	contig383	Pol polyp	CAACCA
GT	Sense	Sense	0.174	Detected	-0.174	Detected	0.000	-1.637	Detected	-0.926	Detected	-1.282	0.084	GT	Sens	contig387	contig387	Lipoxyge	TTGGTT
GT	Sense	Sense	-0.376	Detected	0.376	Detected	0.000	-2.185	Detected	-1.581	Detected	-1.883	0.060	GT	Sens	contig402	contig402	Long-cha	ATAGCA
GT	Sense	Sense	0.132	Detected	-0.132	Detected	0.000	-1.157	Detected	-1.399	Detected	-1.278	0.019	GT	Sens	contig405	contig405	Unannota	TAATCTCCTTTGC
GT	Sense	Sense	0.070	Detected	-0.070	Detected	0.000	-0.958	Detected	-1.414	Detected	-1.186	0.038	GT	Sens	contig409	contig409	Ycf2 (AHF	TGCAGA
GT	Sense	Sense	0.181	Detected	-0.181	Detected	0.000	-1.244	Detected	-2.071	Detected	-1.657	0.067	GT	Sens	contig413	contig413	Unannota	GAAGAACCGAAGT
GT	Sense	Sense	0.079	Detected	-0.079	Detected	0.000	-2.056	Detected	-1.808	Detected	-1.932	0.006	GT	Sens	contig422	contig422	Unannota	ATAGGGATCTGG
GT	Sense	Sense	0.219	Detected	-0.219	Detected	0.000	-1.633	Detected	-1.045	Detected	-1.339	0.067	GT	Sens	contig426	contig426	Lipoxyge	TTGCAA
GT	Sense	Sense	-2.003	Detected	2.003	Detected	0.000	-1.920	Detected	-1.152	Detected	-1.536	0.530	GT	Sens	contig427	contig427	ORMDL d	ACAGTGATATGTG
GT	Sense	Sense	-0.311	Detected	0.311	Detected	0.000	-1.659	Detected	-1.190	Detected	-1.424	0.067	GT	Sens	contig427	contig427	Uncharac	ATTAGGCGTCGA
GT	Sense	Sense	-0.296	Detected	0.296	Detected	0.000	-1.226	Detected	-0.818	Detected	-1.022	0.105	GT	Sens	contig429	contig429	Probable	GAGAGGTTGAGT
GT	Sense	Sense	-0.543	Detected	0.543	Detected	0.000	-1.424	Detected	-1.291	Detected	-1.358	0.131	GT	Sens	contig429	contig429	Nicotia ta	CAAGTAAGAATCC
GT	Sense	Sense	-0.507	Detected	0.507	Detected	0.000	-1.657	Detected	-1.038	Detected	-1.348	0.151	GT	Sens	contig430	contig430	Nicotia ta	AGAGGCCATACC
GT	Sense	Sense	-0.373	Detected	0.373	Detected	0.000	-1.043	Detected	-1.265	Detected	-1.154	0.097	GT	Sens	contig446	contig446	Auxin-ind	CATGAT
GT	Sense	Sense	0.062	Detected	-0.062	Detected	0.000	-1.628	Detected	-1.799	Detected	-1.713	0.004	GT	Sens	contig454	contig454	Gag-Pol r	AAGGTT
GT	Sense	Sense	-0.040	Detected	0.040	Detected	0.000	-1.449	Detected	-1.544	Detected	-1.496	0.002	GT	Sens	contig456	contig456	Ycf2 (AHF	GGGGAT
GT	Sense	Sense	-0.078	Detected	0.078	Detected	0.000	-1.555	Detected	-1.485	Detected	-1.520	0.003	GT	Sens	contig457	contig457	NAD(P)H-	ACTATA
GT	Sense	Sense	0.118	Detected	-0.118	Detected	0.000	-1.481	Detected	-1.060	Detected	-1.271	0.034	GT	Sens	contig460	contig460	Reverse t	TATACT
GT	Sense	Sense	0.545	Detected	-0.545	Detected	0.000	-1.166	Detected	-1.220	Detected	-1.193	0.160	GT	Sens	contig484	contig484	UDP-gluc	AATAGG
GT	Sense	Sense	0.071	Detected	-0.071	Detected	0.000	-1.298	Detected	-0.950	Detected	-1.124	0.027	GT	Sens	contig504	contig504	Pol polyp	TTGTGA
GT	Sense	Sense	-0.018	Detected	0.018	Detected	0.000	-1.021	Detected	-1.244	Detected	-1.132	0.010	GT	Sens	contig512	contig512	Topoisom	TTGAGA
GT	Sense	Sense	0.157	Detected	-0.157	Detected	0.000	-1.298	Detected	-0.966	Detected	-1.132	0.038	GT	Sens	contig526	contig526	Reverse t	AGAATG
GT	Sense	Sense	0.053	Detected	-0.053	Detected	0.000	-1.928	Detected	-1.698	Detected	-1.813	0.005	GT	Sens	contig545	contig545	Retrotran	ATATTG
GT	Sense	Sense	0.113	Detected	-0.113	Detected	0.000	-1.745	Detected	-1.651	Detected	-1.698	0.005	GT	Sens	contig545	contig545	Polyprote	ACACTT
GT	Sense	Sense	0.015	Detected	-0.015	Detected	0.000	-2.261	Detected	-1.982	Detected	-2.121	0.004	GT	Sens	contig547	contig547	Unknown	GGCGGG
GT	Sense	Sense	0.183	Detected	-0.183	Detected	0.000	-1.578	Comprom	-0.849	Comprom	-1.214	0.097	GT	Sens	contig555	contig555	Transpos	AACAAT
GT	Sense	Sense	0.670	Detected	-0.670	Detected	0.000	-3.837	Detected	-3.155	Detected	-3.496	0.043	GT	Sens	contig600	contig600	Acyltrans	CCAAGT
GT	Sense	Sense	-0.068	Detected	0.068	Detected	0.000	-1.865	Comprom	-1.182	Comprom	-1.524	0.048	GT	Sens	contig605	contig605	Retroviru	GTTTGA
GT	Sense	Sense	0.043	Detected	-0.043	Detected	0.000	-1.800	Detected	-1.626	Detected	-1.713	0.003	GT	Sens	contig606	contig606	Unknown	ACAGAC
GT	Sense	Sense	-0.129	Detected	0.129	Detected	0.000	-0.835	Detected	-2.540	Comprom	-1.688	0.189	GT	Sens	contig617	contig617	Mutator-li	AGTCCA
GT	Sense	Sense	0.114	Detected	-0.114	Detected	0.000	-0.950	Detected	-1.120	Detected	-1.035	0.018	GT	Sens	contig640	contig640	Unknown	TAAGCG
GT	Sense	Sense	0.070	Detected	-0.070	Detected	0.000	-1.649	Comprom	-1.384	Comprom	-1.517	0.010	GT	Sens	contig644	contig644	Unknown	TCTGCT
GT	Sense	Sense	0.405	Detected	-0.405	Detected	0.000	-1.627	Detected	-2.082	Detected	-1.855	0.057	GT	Sens	contig689	contig689	Protease	TACTGC
GT	Sense	Sense	-0.439	Detected	0.439	Detected	0.000	-1.372	Detected	-1.505	Detected	-1.438	0.083	GT	Sens	contig691	contig691	Serine ca	AGACAG
GT	Sense	Sense	-0.024	Detected	0.024	Detected	0.000	-1.309	Detected	-1.063	Detected	-1.186	0.011	GT	Sens	contig694	contig694	NAD(P)H-	TTCCTC
GT	Sense	Sense	0.094	Detected	-0.094	Detected	0.000	-1.348	Detected	-0.828	Detected	-1.088	0.059	GT	Sens	contig698	contig698	ABC tran	TGCTAG
GT	Sense	Sense	-0.200	Detected	0.200	Detected	0.000	-0.963	Detected	-1.054	Detected	-1.009	0.039	GT	Sens	contig702	contig702	Sulfotran	TGTTCT
GT	Sense	Sense	-0.115	Detected	0.115	Detected	0.000	-2.038	Detected	-3.187	Detected	-2.613	0.047	GT	Sens	contig725	contig725	Salicylic	TCCTTG
GT	Sense	Sense	0.048	Detected	-0.048	Detected	0.000	-1.736	Detected	-1.475	Detected	-1.605	0.007	GT	Sens	contig729	contig729	Pol polyp	CCTCCG
GT	Sense	Sense	0.026	Detected	-0.026	Detected	0.000	-2.460	Detected	-1.768	Detected	-2.114	0.026	GT	Sens	contig730	contig730	Retrotran	TAGTAC
GT	Sense	Sense	-0.077	Detected	0.077	Detected	0.000	-0.922	Detected	-1.338	Detected	-1.130	0.036	GT	Sens	contig737	contig737	NADH-qui	TGCTTA
GT	Sense	Sense	0.004	Detected	-0.004	Detected	0.000	-1.536	Detected	-1.544	Detected	-1.540	0.000	GT	Sens	contig739	contig739	Retrotran	CTTTGT
GT	Sense	Sense	-0.015	Detected	0.015	Detected	0.000	-1.728	Detected	-2.073	Detected	-1.900	0.008	GT	Sens	contig744	contig744	Ycf1 (Fra	ATCAAT
GT	Sense	Sense	-0.197	Detected	0.197	Detected	0.000	-2.233	Detected	-0.911	Detected	-1.572	0.150	GT	Sens	contig756	contig756	Peroxida	TTTTCA
GT	Sense	Sense	0.094	Detected	-0.094	Detected	0.000	-1.468	Detected	-1.440	Detected	-1.454	0.004	GT	Sens	contig768	contig768	Unknown	GAAGAG
GT	Sense	Sense	0.114	Detected	-0.114	Detected	0.000	-1.949	Detected	-1.646	Detected	-1.798	0.011	GT	Sens	contig791	contig791	Polyprote	GAATGG
GT	Sense	Sense	0.107	Detected	-0.107	Detected	0.000	-1.664	Detected	-1.628	Detected	-1.646	0.004	GT	Sens	contig812	contig812	Unknown	TGCAGG
GT	Sense	Sense	-0.231	Detected	0.231	Detected	0.000	-1.148	Detected	-1.634	Detected	-1.391	0.054	GT	Sens	contig815	contig815	CONSTAI	TGGAAT
GT	Sense	Sense	0.148	Detected	-0.148	Detected	0.000	-1.872	Detected	-1.786	Detected	-1.829	0.007	GT	Sens	contig816	contig816	Unknown	TTCAGA
GT	Sense	Sense	0.131	Detected	-0.131	Detected	0.000	-3.129	Detected	-3.185	Detected	-3.157	0.002	GT	Sens	contig843	contig843	Glutathio	GGCGTA
GT	Sense	Sense	0.081	Detected	-0.081	Detected	0.000	-1.584	Detected	-1.555	Detected	-1.569	0.003	GT	Sens	contig875	contig875	Unknown	TGCCGT
GT	Sense	Sense	0.418	Detected	-0.418	Detected	0.000	-1.568	Detected	-1.834	Detected	-1.701	0.060	GT	Sens	contig883	contig883	Protease	AATGCG
GT	Sense	Sense	0.193	Detected	-0.193	Detected	0.000	-1.143	Detected	-1.218	Detected	-1.181	0.027	GT	Sens	contig883	contig883	Unknown	AGCAGA
GT	Sense	Sense	-0.076	Detected	0.076	Detected	0.000	-1.295	Detected	-1.280	Detected	-1.287	0.003	GT	Sens	contig903	contig903	Ribulose	AGATGA
GT	Sense	Sense	0.046	Detected	-0.046	Detected	0.000	-1.521	Detected	-1.722	Detected	-1.622	0.005	GT	Sens	contig907	contig907	NADH-qui	TACACG
GT	Sense	Sense	0.313	Detected	-0.313	Detected	0.000	-1.118	Detected	-1.424	Detected	-1.271	0.067	GT	Sens	contig921	contig921	Reverse t	GATCGC
GT	Sense	Sense	0.054	Detected	-0.054	Detected	0.000	-1.994	Detected	-1.958	Detected	-1.976	0.001	GT	Sens	contig922	contig922	Retrotran	CGGAGT
GT	Sense	Sense	-0.041	Detected	0.041	Detected	0.000	-1.656	Detected	-0.891	Detected	-1.274	0.080	GT	Sens	contig927	contig927	Unknown	AGGATT
GT	Sense	Sense	-0.016	Detected	0.016	Detected	0.000	-1.639	Detected	-0.936	Detected	-1.288	0.067	GT	Sens	contig930	contig930	Mutator-li	ATAGTG
GT	Sense	Sense	0.086	Detected	-0.086	Detected	0.000	-2.295	Detected	-1.796	Detected	-2.045	0.016	GT	Sens	contig931	contig931	Pol polyp	ACATCT
GT	Sense	Sense	0.041	Detected	-0.041	Detected	0.000	-1.487	Detected	-1.287	Detected	-1.387	0.006	GT	Sens	contig931	contig931	Pol polyp	GTGACT
GT	Sense	Sense	-0.238	Detected	0.238	Detected	0.000	-2.366	Detected	-1.039	Detected	-1.702	0.137	GT	Sens	contig938	contig938	Laccase	GTTATT
GT	Sense	Sense	0.066	Detected	-0.066	Detected	0.000	-1.917	Detected	-1.510	Detected	-1.713	0.015	GT	Sens	contig943	contig943	Polyprote	TTCTTG

GO ID	GO ACCESSION	GO Term	p-value	Count in Selection	% Count in Selection	Count in Total	% Count in Total
11776	GO:0019825	oxygen binding	0.00	22	3.7225041	389	1.1234008
3048	GO:0004364	glutathione transferase activi	0.00	9	1.5228426	84	0.24258527
25094	GO:0050789|GO:0050791	regulation of biological proce	0.00	108	18.27411	4315	12.461374
11931	GO:0020037	heme binding	0.00	24	4.0609136	550	1.5883559
3872	GO:0005506	iron ion binding	0.00	24	4.0609136	563	1.6258988
28364	GO:0065007	biological regulation	0.00	108	18.27411	4376	12.637537
25098	GO:0050794|GO:0051244	regulation of cellular process	0.00	100	16.920473	3987	11.514136
11224	GO:0019222	regulation of metabolic proce	0.00	81	13.705584	3115	8.995871
4530	GO:0006355|GO:0032583|G	regulation of transcription, DI	0.00	70	11.844332	2591	7.4826
25544	GO:0051252	regulation of RNA metabolic	0.00	70	11.844332	2593	7.488376
22409	GO:0046906	tetrapyrrole binding	0.00	24	4.0609136	596	1.7212002
32213	GO:2000112	regulation of cellular macrom	0.00	70	11.844332	2605	7.523031
13756	GO:0031326	regulation of cellular biosynth	0.00	70	11.844332	2605	7.523031
7141	GO:0009889	regulation of biosynthetic pro	0.00	70	11.844332	2605	7.523031
7772	GO:0010556	regulation of macromolecule	0.00	70	11.844332	2605	7.523031
7692	GO:0010468	regulation of gene expressio	0.00	70	11.844332	2635	7.6096687
19590	GO:0043295	glutathione binding	0.00	5	0.8460237	31	0.08952551
7074	GO:0009815	1-aminocyclopropane-1-carb	0.00	6	1.0152284	49	0.14150807
27228	GO:0060255	regulation of macromolecule	0.00	70	11.844332	2660	7.6818666
13753	GO:0031323	regulation of cellular metabo	0.00	72	12.182741	2770	7.999538
25468	GO:0051171	regulation of nitrogen compo	0.00	70	11.844332	2675	7.7251854
11221	GO:0019219	regulation of nucleobase, nu	0.00	70	11.844332	2675	7.7251854
31465	GO:0080090	regulation of primary metabo	0.00	70	11.844332	2710	7.826263
19479	GO:0043176	amine binding	0.00	7	1.1844332	80	0.2310336
9682	GO:0016597	amino acid binding	0.00	7	1.1844332	80	0.2310336
30700	GO:0072341	modified amino acid binding	0.00	6	1.0152284	59	0.17038727
2986	GO:0004252	serine-type endopeptidase a	0.00	9	1.5228426	144	0.41586044
6888	GO:0009611|GO:0002245	response to wounding	0.00	6	1.0152284	66	0.1906027
9722	GO:0016641	oxidoreductase activity, actin	0.00	6	1.0152284	69	0.19926646
5766	GO:0008236	serine-type peptidase activity	0.00	11	1.8612521	214	0.6180149
4641	GO:0006508	proteolysis	0.00	30	5.0761423	965	2.7868426
13829	GO:0031406	carboxylic acid binding	0.00	7	1.1844332	98	0.28301615
10103	GO:0017171	serine hydrolase activity	0.00	11	1.8612521	220	0.63534236
9768	GO:0016706	oxidoreductase activity, actin	0.00	9	1.5228426	175	0.505386
9719	GO:0016638	oxidoreductase activity, actin	0.00	6	1.0152284	89	0.25702485
9767	GO:0016705	oxidoreductase activity, actin	0.00	13	2.1996615	329	0.95012563
9818	GO:0016765|GO:0016766	transferase activity, transferr	0.00	9	1.5228426	185	0.53426516
5704	GO:0008150|GO:0000004|G	biological_process	0.01	409	69.204735	22215	64.155136
20342	GO:0044092	negative regulation of molecu	0.01	9	1.5228426	188	0.54292893
19393	GO:0043086	negative regulation of catalyt	0.01	9	1.5228426	188	0.54292893
22375	GO:0046872	metal ion binding	0.01	69	11.675127	2985	8.6204405
19470	GO:0043167	ion binding	0.01	69	11.675127	2997	8.655096
19472	GO:0043169	cation binding	0.01	69	11.675127	2997	8.655096
716	GO:0001071	nucleic acid binding transcrip	0.01	37	6.2605753	1417	4.0921826
2643	GO:0003700|GO:0000130	sequence-specific DNA bind	0.01	37	6.2605753	1417	4.0921826
3356	GO:0004737	pyruvate decarboxylase activ	0.01	2	0.33840948	8	0.023103358
9600	GO:0016491	oxidoreductase activity	0.01	43	7.2758036	1724	4.9787736
5097	GO:0007049	cell cycle	0.01	4	0.67681897	52	0.15017183
5678	GO:0008113|GO:0033742	peptide-methionine-(S)-S-ox	0.01	2	0.33840948	10	0.0288792
4526	GO:0006351|GO:0006350|G	transcription, DNA-depender	0.01	39	6.5989847	1575	4.548474
15176	GO:0032774	RNA biosynthetic process	0.01	39	6.5989847	1575	4.548474
2870	GO:0004064	arylesterase activity	0.01	3	0.5076142	30	0.086637594
13809	GO:0031386	protein tag	0.01	2	0.33840948	11	0.03176712
26959	GO:0055114	oxidation-reduction process	0.01	57	9.6446705	2490	7.1909204
5715	GO:0008171	O-methyltransferase activity	0.02	4	0.67681897	57	0.16461143
12516	GO:0022804	active transmembrane transp	0.02	22	3.7225041	787	2.2727928
3464	GO:0004857	enzyme inhibitor activity	0.02	7	1.1844332	157	0.4534034
18633	GO:0042277	peptide binding	0.02	5	0.8460237	95	0.27435237
3318	GO:0004693|GO:0016537	cyclin-dependent protein kina	0.03	3	0.5076142	37	0.10685303
12805	GO:0030246	carbohydrate binding	0.03	9	1.5228426	251	0.7248679
3801	GO:0005342	organic acid transmembrane	0.03	6	1.0152284	136	0.3927571
22444	GO:0046943	carboxylic acid transmembra	0.03	6	1.0152284	136	0.3927571
13435	GO:0030976	thiamine pyrophosphate bind	0.03	2	0.33840948	16	0.046206716
18757	GO:0042409	caffeoyl-CoA O-methyltransf	0.03	2	0.33840948	16	0.046206716
22412	GO:0046910	pectinesterase inhibitor activi	0.03	2	0.33840948	16	0.046206716
22415	GO:0046914	transition metal ion binding	0.03	57	9.6446705	2599	7.5057034
18738	GO:0042389	omega-3 fatty acid desatura:	0.03	2	0.33840948	17	0.049094636
3719	GO:0005201	extracellular matrix structural	0.03	1	0.16920474	2	0.00577584
5907	GO:0008413	8-oxo-7,8-dihydroguanosine	0.03	1	0.16920474	2	0.00577584
7409	GO:0010177	2-(2'-methylthio)ethylmalate	0.03	1	0.16920474	2	0.00577584
7470	GO:0010241	ent-kaurene oxidation to kau	0.03	1	0.16920474	2	0.00577584
8509	GO:0015093	ferrous iron transmembrane	0.03	1	0.16920474	2	0.00577584
8612	GO:0015204|GO:0015287	urea transmembrane transpo	0.03	1	0.16920474	2	0.00577584
8953	GO:0015684	ferrous iron transport	0.03	1	0.16920474	2	0.00577584
9106	GO:0015840	urea transport	0.03	1	0.16920474	2	0.00577584
11186	GO:0019177	dihydroneopterin triphosphat	0.03	1	0.16920474	2	0.00577584
15726	GO:0033331	ent-kaurene metabolic proce	0.03	1	0.16920474	2	0.00577584
19204	GO:0042887	amide transmembrane trans	0.03	1	0.16920474	2	0.00577584
30280	GO:0071918	urea transmembrane transpo	0.03	1	0.16920474	2	0.00577584
10899	GO:0018858	benzoate-CoA ligase activity	0.04	2	0.33840948	18	0.051982556
22917	GO:0047429	nucleoside-triphosphate diph	0.04	2	0.33840948	18	0.051982556
31431	GO:0080054	low affinity nitrate transmem	0.04	3	0.5076142	45	0.1299564
2975	GO:0004175|GO:0016809	endopeptidase activity	0.05	10	1.6920474	320	0.9241344
19758	GO:0043492	ATPase activity, coupled to r	0.05	8	1.3536379	235	0.67866117
5921	GO:0008428	ribonuclease inhibitor activity	0.05	1	0.16920474	3	0.00866376
11784	GO:0019838	growth factor binding	0.05	3	0.5076142	49	0.14150807
31421	GO:0080044	quercetin 7-O-glucosyltransfe	0.05	3	0.5076142	49	0.14150807
3874	GO:0005509	calcium ion binding	0.05	9	1.5228426	279	0.8057296
9114	GO:0015849	organic acid transport	0.05	6	1.0152284	156	0.45051548
22443	GO:0046942	carboxylic acid transport	0.05	6	1.0152284	156	0.45051548
8584	GO:0015171|GO:0015359	amino acid transmembrane t	0.05	5	0.8460237	118	0.34077454
2521	GO:0003333	amino acid transmembrane t	0.05	5	0.8460237	118	0.34077454
8489	GO:0015066	alpha-amylase inhibitor activi	0.05	2	0.33840948	22	0.06353424
12608	GO:0030001	metal ion transport	0.06	11	1.8612521	374	1.080082
9806	GO:0016752	sinapoyltransferase activity	0.06	2	0.33840948	23	0.06642216
9807	GO:0016753	O-sinapoyltransferase activity	0.06	2	0.33840948	23	0.06642216
9808	GO:0016754	sinapoylglucose-malate O-si	0.06	2	0.33840948	23	0.06642216
4951	GO:0006865|GO:0006866	amino acid transport	0.06	5	0.8460237	123	0.35521415
19473	GO:0043170|GO:0043283	macromolecule metabolic pr	0.06	127	21.489002	6553	18.92454
8559	GO:0015146	pentose transmembrane trar	0.06	2	0.33840948	24	0.06931008
8561	GO:0015148	D-xylose transmembrane tra	0.06	2	0.33840948	24	0.06931008
8829	GO:0015519	D-xylose:hydrogen symporte	0.06	2	0.33840948	24	0.06931008
9019	GO:0015750	pentose transport	0.06	2	0.33840948	24	0.06931008
9022	GO:0015753	D-xylose transport	0.06	2	0.33840948	24	0.06931008
5702	GO:0008146	sulfotransferase activity	0.06	3	0.5076142	54	0.15594767
3761	GO:0005275|GO:0005279	amine transmembrane trans	0.07	5	0.8460237	126	0.3638779
11522	GO:0019538|GO:0006411	protein metabolic process	0.07	83	14.043993	4134	11.938661
4039	GO:0005751|GO:0005752	mitochondrial respiratory cha	0.07	1	0.16920474	4	0.011551679
7511	GO:0010285|GO:0043742	L,L-diaminopimelate aminotr	0.07	1	0.16920474	4	0.011551679
8594	GO:0015184	L-cystine transmembrane tra	0.07	1	0.16920474	4	0.011551679
9080	GO:0015811	L-cystine transport	0.07	1	0.16920474	4	0.011551679
20901	GO:0045277|GO:0045287	respiratory chain complex IV	0.07	1	0.16920474	4	0.011551679
21300	GO:0045703	ketoreductase activity	0.07	1	0.16920474	4	0.011551679
31418	GO:0080041	ADP-ribose pyrophosphohyd	0.07	1	0.16920474	4	0.011551679
31507	GO:0080132	fatty acid alpha-hydroxylase	0.07	1	0.16920474	4	0.011551679

75	GO:0000099	sulfur amino acid transmemb	0.07	1	0.16920474	4	0.011551679
77	GO:0000101	sulfur amino acid transport	0.07	1	0.16920474	4	0.011551679
21145	GO:0045543	gibberellin 2-beta-dioxygenas	0.07	2	0.33840948	25	0.072197996
18956	GO:0042626	ATPase activity, coupled to t	0.07	7	1.1844332	211	0.6093511
17024	GO:0034645|GO:0034961	cellular macromolecule biosy	0.07	43	7.2758036	1999	5.7729516
30066	GO:0071702	organic substance transport	0.07	14	2.3688664	529	1.5277096
9744	GO:0016671	oxidoreductase activity, actin	0.07	2	0.33840948	26	0.075085916
9861	GO:0016820	hydrolase activity, acting on a	0.07	7	1.1844332	212	0.612239
6428	GO:0009059|GO:0043284	macromolecule biosynthetic	0.07	43	7.2758036	2003	5.7845035
9308	GO:0016070	RNA metabolic process	0.08	40	6.7681894	1855	5.3570914
8749	GO:0015399	primary active transmembrar	0.08	8	1.3536379	260	0.75085914
8753	GO:0015405	P-P-bond-hydrolysis-driven t	0.08	8	1.3536379	260	0.75085914
17617	GO:0035251	UDP-glucosyltransferase act	0.08	7	1.1844332	218	0.62956655
9103	GO:0015837	amine transport	0.08	5	0.8460237	135	0.38986918
14850	GO:0032442	phenylcoumaran benzylic eth	0.08	1	0.16920474	5	0.0144396
21949	GO:0046424	ferulate 5-hydroxylase activit	0.08	1	0.16920474	5	0.0144396
11787	GO:0019842	vitamin binding	0.08	6	1.0152284	177	0.5111618
8526	GO:0015112	nitrate transmembrane trans	0.09	3	0.5076142	61	0.1761631
8975	GO:0015706|GO:0006872	nitrate transport	0.09	3	0.5076142	61	0.1761631
16430	GO:0034046	poly(G) RNA binding	0.09	4	0.67681897	100	0.28879198
3603	GO:0005030	neurotrophin receptor activity	0.09	2	0.33840948	30	0.086637594
6216	GO:0008810	cellulase activity	0.09	2	0.33840948	30	0.086637594
19425	GO:0043121	neurotrophin binding	0.09	2	0.33840948	30	0.086637594
22049	GO:0046527	glucosyltransferase activity	0.09	8	1.3536379	270	0.77973837
25820	GO:0051536	iron-sulfur cluster binding	0.10	3	0.5076142	64	0.18482687
25824	GO:0051540	metal cluster binding	0.10	3	0.5076142	64	0.18482687
3197	GO:0004550	nucleoside diphosphate kinas	0.10	1	0.16920474	6	0.01732752
3413	GO:0004802	transketolase activity	0.10	1	0.16920474	6	0.01732752
4375	GO:0006165	nucleoside diphosphate phos	0.10	1	0.16920474	6	0.01732752
7250	GO:0010012	steroid 22-alpha hydroxylase	0.10	1	0.16920474	6	0.01732752
9333	GO:0016101	diterpenoid metabolic proces	0.10	1	0.16920474	6	0.01732752
9772	GO:0016710	trans-cinnamate 4-monooxyg	0.10	1	0.16920474	6	0.01732752
19203	GO:0042886	amide transport	0.10	1	0.16920474	6	0.01732752
30707	GO:0072348	sulfur compound transport	0.10	1	0.16920474	6	0.01732752
2702	GO:0003852	2-isopropylmalate synthase a	0.10	1	0.16920474	6	0.01732752
2713	GO:0003863	3-methyl-2-oxobutanoate del	0.10	1	0.16920474	6	0.01732752
3073	GO:0004397	histidine ammonia-lyase activ	0.10	1	0.16920474	6	0.01732752
5732	GO:0008194	UDP-glycosyltransferase act	0.10	9	1.5228426	322	0.9299102
21041	GO:0045431	flavonol synthase activity	0.10	2	0.33840948	32	0.09241343
25836	GO:0051552	flavone metabolic process	0.10	2	0.33840948	32	0.09241343
25837	GO:0051553	flavone biosynthetic process	0.10	2	0.33840948	32	0.09241343
25838	GO:0051554	flavonol metabolic process	0.10	2	0.33840948	32	0.09241343
25839	GO:0051555	flavonol biosynthetic process	0.10	2	0.33840948	32	0.09241343
3473	GO:0004867	serine-type endopeptidase in	0.10	3	0.5076142	66	0.1906027
23515	GO:0048037	cofactor binding	0.11	9	1.5228426	327	0.94434977
24968	GO:0050662	coenzyme binding	0.11	6	1.0152284	191	0.5515927
7691	GO:0010467	gene expression	0.11	41	6.937394	1974	5.7007537
5114	GO:0007067	mitosis	0.11	1	0.16920474	7	0.020215439
16115	GO:0033729	anthocyanidin reductase acti	0.11	1	0.16920474	7	0.020215439
21149	GO:0045548	phenylalanine ammonia-lyas	0.11	1	0.16920474	7	0.020215439
23710	GO:0048285	organelle fission	0.11	1	0.16920474	7	0.020215439
64	GO:0000087	M phase of mitotic cell cycle	0.11	1	0.16920474	7	0.020215439
213	GO:0000279	M phase	0.11	1	0.16920474	7	0.020215439
214	GO:0000280	nuclear division	0.11	1	0.16920474	7	0.020215439
31420	GO:0080043	quercetin 3-O-glucosyltransfe	0.11	3	0.5076142	69	0.19926646
7690	GO:0010466	negative regulation of peptida	0.12	4	0.67681897	110	0.31767118
12946	GO:0030414	peptidase inhibitor activity	0.12	4	0.67681897	110	0.31767118
25636	GO:0051346	negative regulation of hydrola	0.12	4	0.67681897	110	0.31767118
8679	GO:0015297	antiporter activity	0.12	5	0.8460237	152	0.4389638
8682	GO:0015300	solute:solute antiporter activi	0.12	5	0.8460237	152	0.4389638
21146	GO:0045544	gibberellin 20-oxidase activity	0.12	2	0.33840948	35	0.10107719
9740	GO:0016667	oxidoreductase activity, actin	0.13	3	0.5076142	72	0.20793022
26711	GO:0052547	regulation of peptidase activi	0.13	4	0.67681897	112	0.32344702
28100	GO:0061134	peptidase regulator activity	0.13	4	0.67681897	112	0.32344702
29081	GO:0070717	poly-purine tract binding	0.13	4	0.67681897	112	0.32344702
8739	GO:0015385|GO:0015502	sodium:hydrogen antiporter a	0.13	2	0.33840948	36	0.10396511
3402	GO:0004791	thioredoxin-disulfide reductas	0.13	1	0.16920474	8	0.023103358
3803	GO:0005344|GO:0015033	oxygen transporter activity	0.13	1	0.16920474	8	0.023103358
4035	GO:0005746	mitochondrial respiratory cha	0.13	1	0.16920474	8	0.023103358
6497	GO:0009132	nucleoside diphosphate meta	0.13	1	0.16920474	8	0.023103358
7003	GO:0009737	response to abscisic acid stir	0.13	1	0.16920474	8	0.023103358
7843	GO:0010628	positive regulation of gene ex	0.13	1	0.16920474	8	0.023103358
8725	GO:0015367	oxoglutarate:malate antiporte	0.13	1	0.16920474	8	0.023103358
8940	GO:0015669	gas transport	0.13	1	0.16920474	8	0.023103358
8942	GO:0015671	oxygen transport	0.13	1	0.16920474	8	0.023103358
9011	GO:0015742	alpha-ketoglutarate transport	0.13	1	0.16920474	8	0.023103358
9680	GO:0016595	glutamate binding	0.13	1	0.16920474	8	0.023103358
21461	GO:0045893|GO:0043193|G	positive regulation of transcri	0.13	1	0.16920474	8	0.023103358
22702	GO:0047209	coniferyl-alcohol glucosyltran	0.13	1	0.16920474	8	0.023103358
28833	GO:0070469	respiratory chain	0.13	1	0.16920474	8	0.023103358
30696	GO:0072337	modified amino acid transpo	0.13	1	0.16920474	8	0.023103358
30708	GO:0072349	modified amino acid transme	0.13	1	0.16920474	8	0.023103358
3022	GO:0004338	glucan exo-1,3-beta-glucosid	0.13	1	0.16920474	8	0.023103358
9832	GO:0016782	transferase activity, transferr	0.13	3	0.5076142	74	0.21370606
30069	GO:0071705	nitrogen compound transport	0.13	6	1.0152284	202	0.5833598
26845	GO:0052689	carboxylic ester hydrolase ac	0.13	13	2.1996615	538	1.5537009
9637	GO:0016538|GO:0003751|G	cyclin-dependent protein kina	0.14	2	0.33840948	38	0.10974095
24966	GO:0050660	flavin adenine dinucleotide bi	0.14	4	0.67681897	118	0.34077454
7225	GO:0009978	allene oxide synthase activity	0.14	1	0.16920474	9	0.025991278
13224	GO:0030755	quercetin 3-O-methyltransfer	0.14	1	0.16920474	9	0.025991278
19342	GO:0043027	caspase inhibitor activity	0.14	1	0.16920474	9	0.025991278
19343	GO:0043028	caspase regulator activity	0.14	1	0.16920474	9	0.025991278
19458	GO:0043154|GO:0001719	negative regulation of caspas	0.14	1	0.16920474	9	0.025991278
19579	GO:0043281|GO:0043026	regulation of caspase activity	0.14	1	0.16920474	9	0.025991278
5891	GO:0008395|GO:0008394	steroid hydroxylase activity	0.15	2	0.33840948	40	0.1155168
7072	GO:0009813	flavonoid biosynthetic proces	0.15	2	0.33840948	40	0.1155168
21058	GO:0045454|GO:0030503|G	cell redox homeostasis	0.15	6	1.0152284	211	0.6093511
3856	GO:0005471|GO:0005349	ATP:ADP antiporter activity	0.16	1	0.16920474	10	0.0288792
4878	GO:0006772	thiamine metabolic process	0.16	1	0.16920474	10	0.0288792
6592	GO:0009228	thiamine biosynthetic proces	0.16	1	0.16920474	10	0.0288792
7175	GO:0009924	octadecanal decarbonylase a	0.16	1	0.16920474	10	0.0288792
9741	GO:0016668|GO:0016654	oxidoreductase activity, actin	0.16	1	0.16920474	10	0.0288792
26022	GO:0051740	ethylene binding	0.16	1	0.16920474	10	0.0288792
30135	GO:0071771	aldehyde decarbonylase acti	0.16	1	0.16920474	10	0.0288792
30687	GO:0072328	alkene binding	0.16	1	0.16920474	10	0.0288792
22376	GO:0046873	metal ion transmembrane tra	0.16	6	1.0152284	213	0.6151269
3718	GO:0005200	structural constituent of cytos	0.16	2	0.33840948	42	0.121292636
4911	GO:0006812|GO:0006819|G	cation transport	0.16	15	2.5380712	662	1.9118029
9849	GO:0016803	ether hydrolase activity	0.17	1	0.16920474	11	0.03176712
12469	GO:0022402	cell cycle process	0.17	1	0.16920474	11	0.03176712
12470	GO:0022403	cell cycle phase	0.17	1	0.16920474	11	0.03176712
16183	GO:0033799	myricetin 3'-O-methyltransfer	0.17	1	0.16920474	11	0.03176712
212	GO:0000278	mitotic cell cycle	0.17	1	0.16920474	11	0.03176712
2989	GO:0004301	epoxide hydrolase activity	0.17	1	0.16920474	11	0.03176712
7071	GO:0009812	flavonoid metabolic process	0.17	2	0.33840948	44	0.12706847
3476	GO:0004872|GO:0019041	receptor activity	0.18	15	2.5380712	672	1.9406822
8666	GO:0015276	ligand-gated ion channel acti	0.18	2	0.33840948	45	0.1299564
12530	GO:0022834	ligand-gated channel activity	0.18	2	0.33840948	45	0.1299564
4786	GO:0006664	glycolipid metabolic process	0.19	1	0.16920474	12	0.03465504
6610	GO:0009247	glycolipid biosynthetic proces	0.19	1	0.16920474	12	0.03465504
7526	GO:0010301|GO:0033710	xanthoxin dehydrogenase ac	0.19	1	0.16920474	12	0.03465504
9704	GO:0016621	cinnamoyl-CoA reductase ac	0.19	1	0.16920474	12	0.03465504

14848	GO:0032440	2-alkenal reductase activity	0.19	1	0.16920474	12	0.03465504
19047	GO:0042723	thiamine-containing compour	0.19	1	0.16920474	12	0.03465504
19048	GO:0042724	thiamine-containing compour	0.19	1	0.16920474	12	0.03465504
21503	GO:0045935	positive regulation of nucleob	0.19	1	0.16920474	12	0.03465504
21992	GO:0046467	membrane lipid biosynthetic	0.19	1	0.16920474	12	0.03465504
25470	GO:0051173	positive regulation of nitroger	0.19	1	0.16920474	12	0.03465504
25546	GO:0051254	positive regulation of RNA m	0.19	1	0.16920474	12	0.03465504
6427	GO:0009058	biosynthetic process	0.19	54	9.137055	2796	8.074624
4032	GO:0005743	mitochondrial inner membrar	0.19	3	0.5076142	88	0.25413695
3475	GO:0004871|GO:0005062|G	signal transducer activity	0.19	17	2.8764806	787	2.2727928
27063	GO:0060089	molecular transducer activity	0.19	17	2.8764806	787	2.2727928
12547	GO:0022857|GO:0005386|G	transmembrane transporter a	0.19	30	5.0761423	1483	4.282785
4990	GO:0006915|GO:0008632	apoptosis	0.19	6	1.0152284	227	0.6555578
21331	GO:0045735	nutrient reservoir activity	0.19	3	0.5076142	89	0.25702485
3449	GO:0004842|GO:0004840|G	ubiquitin-protein ligase activit	0.20	8	1.3536379	326	0.94146186
7551	GO:0010327	acetyl CoA:(Z)-3-hexen-1-ol	0.20	1	0.16920474	13	0.037542958
8149	GO:0010941	regulation of cell death	0.20	1	0.16920474	13	0.037542958
13443	GO:0030984	kininogen binding	0.20	1	0.16920474	13	0.037542958
19298	GO:0042981	regulation of apoptosis	0.20	1	0.16920474	13	0.037542958
19381	GO:0043067|GO:0043070	regulation of programmed ce	0.20	1	0.16920474	13	0.037542958
25095	GO:0050790	regulation of catalytic activity	0.20	11	1.8612521	480	1.3862015
4909	GO:0006810|GO:0015457|G	transport	0.20	45	7.614213	2315	6.6855345
25529	GO:0051234	establishment of localization	0.20	45	7.614213	2315	6.6855345
3844	GO:0005451	monovalent cation:hydrogen	0.20	2	0.33840948	49	0.14150807
6483	GO:0009116	nucleoside metabolic proces	0.20	2	0.33840948	49	0.14150807
25476	GO:0051179	localization	0.20	45	7.614213	2319	6.697086
28366	GO:0065009	regulation of molecular funct	0.21	11	1.8612521	484	1.3977532
9654	GO:0016567	protein ubiquitination	0.21	8	1.3536379	332	0.9587894
14854	GO:0032446	protein modification by small	0.21	8	1.3536379	332	0.9587894
29011	GO:0070647	protein modification by small	0.21	8	1.3536379	332	0.9587894
4408	GO:0006200	ATP catabolic process	0.21	14	2.3688664	641	1.8511566
9918	GO:0016887|GO:0004002	ATPase activity	0.21	14	2.3688664	641	1.8511566
21600	GO:0046034	ATP metabolic process	0.21	14	2.3688664	641	1.8511566
4910	GO:0006811	ion transport	0.21	20	3.3840947	959	2.769515
28378	GO:0070011	peptidase activity, acting on	0.21	12	2.0304568	537	1.550813
11695	GO:0019725	cellular homeostasis	0.21	6	1.0152284	234	0.67577326
5195	GO:0007165|GO:0023033	signal transduction	0.21	20	3.3840947	961	2.775291
12602	GO:0023052|GO:0023046	signaling	0.21	20	3.3840947	961	2.775291
7660	GO:0010436	carotenoid dioxygenase activ	0.21	1	0.16920474	14	0.040430877
8553	GO:0015140	malate transmembrane trans	0.21	1	0.16920474	14	0.040430877
8859	GO:0015556	C4-dicarboxylate transmemb	0.21	1	0.16920474	14	0.040430877
9009	GO:0015740	C4-dicarboxylate transport	0.21	1	0.16920474	14	0.040430877
9012	GO:0015743	malate transport	0.21	1	0.16920474	14	0.040430877
9399	GO:0016174	NAD(P)H oxidase activity	0.21	1	0.16920474	14	0.040430877
9847	GO:0016801	hydrolase activity, acting on	0.21	1	0.16920474	14	0.040430877
9879	GO:0016841	ammonia-lyase activity	0.21	1	0.16920474	14	0.040430877
11157	GO:0019144	ADP-sugar diphosphatase ac	0.21	1	0.16920474	14	0.040430877
17616	GO:0035250	UDP-galactosyltransferase a	0.21	1	0.16920474	14	0.040430877
21150	GO:0045549	9-cis-epoxycarotenoid dioxyc	0.21	1	0.16920474	14	0.040430877
24970	GO:0050664	oxidoreductase activity, actin	0.21	1	0.16920474	14	0.040430877
29787	GO:0071423	malate transmembrane trans	0.21	1	0.16920474	14	0.040430877
31408	GO:0080031	methyl salicylate esterase ac	0.21	1	0.16920474	14	0.040430877
3808	GO:0005351|GO:0005403	sugar:hydrogen symporter ac	0.22	3	0.5076142	94	0.27146447
3835	GO:0005402	cation:sugar symporter activi	0.22	3	0.5076142	94	0.27146447
11803	GO:0019866	organelle inner membrane	0.22	3	0.5076142	94	0.27146447
2657	GO:0003727|GO:0003728	single-stranded RNA binding	0.22	5	0.8460237	188	0.54292893
3472	GO:0004866	endopeptidase inhibitor activ	0.22	3	0.5076142	95	0.27435237
8159	GO:0010951	negative regulation of endop	0.22	3	0.5076142	95	0.27435237
11712	GO:0019748	secondary metabolic process	0.22	3	0.5076142	95	0.27435237
26712	GO:0052548	regulation of endopeptidase	0.22	3	0.5076142	95	0.27435237
28101	GO:0061135	endopeptidase regulator acti	0.22	3	0.5076142	95	0.27435237
6965	GO:0009699	phenylpropanoid biosynthetic	0.22	2	0.33840948	52	0.15017183
2809	GO:0003993	acid phosphatase activity	0.22	2	0.33840948	52	0.15017183
18925	GO:0042592	homeostatic process	0.22	6	1.0152284	238	0.68732494
11747	GO:0019787|GO:0008639|G	small conjugating protein liga	0.22	8	1.3536379	339	0.9790048
5756	GO:0008219	cell death	0.23	6	1.0152284	239	0.69021285
8209	GO:0012501|GO:0016244	programmed cell death	0.23	6	1.0152284	239	0.69021285
9462	GO:0016265	death	0.23	6	1.0152284	239	0.69021285
7520	GO:0010294	abscisic acid glucosyltransfe	0.23	1	0.16920474	15	0.043318797
10079	GO:0017134	fibroblast growth factor bindi	0.23	1	0.16920474	15	0.043318797
14101	GO:0031683	G-protein beta/gamma-subu	0.23	1	0.16920474	15	0.043318797
2868	GO:0004062	aryl sulfotransferase activity	0.23	1	0.16920474	15	0.043318797
8677	GO:0015295	solute:hydrogen symporter a	0.23	3	0.5076142	98	0.28301615
8681	GO:0015299	solute:hydrogen antiporter ac	0.23	3	0.5076142	98	0.28301615
20499	GO:0044249	cellular biosynthetic process	0.24	50	8.460237	2635	7.6096687
2897	GO:0004091|GO:0004302|G	carboxylesterase activity	0.24	5	0.8460237	194	0.5602564
3382	GO:0004768|GO:0016214|G	stearoyl-CoA 9-desaturase a	0.24	1	0.16920474	16	0.046206716
3742	GO:0005242	inward rectifier potassium ch	0.24	1	0.16920474	16	0.046206716
3782	GO:0005310|GO:0005312|G	dicarboxylic acid transmemb	0.24	1	0.16920474	16	0.046206716
3829	GO:0005381|GO:0005382|G	iron ion transmembrane trans	0.24	1	0.16920474	16	0.046206716
4767	GO:0006643	membrane lipid metabolic pr	0.24	1	0.16920474	16	0.046206716
4928	GO:0006835|GO:0006841	dicarboxylic acid transport	0.24	1	0.16920474	16	0.046206716
7178	GO:0009927	histidine phosphotransfer kin	0.24	1	0.16920474	16	0.046206716
9386	GO:0016157	sucrose synthase activity	0.24	1	0.16920474	16	0.046206716
9427	GO:0016215	CoA desaturase activity	0.24	1	0.16920474	16	0.046206716
9878	GO:0016840	carbon-nitrogen lyase activity	0.24	1	0.16920474	16	0.046206716
17134	GO:0034755	iron ion transmembrane trans	0.24	1	0.16920474	16	0.046206716
31396	GO:0080019	fatty-acyl-CoA reductase (alc	0.24	1	0.16920474	16	0.046206716
9533	GO:0016405	CoA-ligase activity	0.24	2	0.33840948	55	0.15883559
5874	GO:0008374	O-acyltransferase activity	0.25	3	0.5076142	102	0.29456782
12794	GO:0030234	enzyme regulator activity	0.25	9	1.5228426	403	1.1638317
4446	GO:0006241	CTP biosynthetic process	0.25	1	0.16920474	17	0.049094636
6507	GO:0009142	nucleoside triphosphate bios	0.25	1	0.16920474	17	0.049094636
6513	GO:0009148	pyrimidine nucleoside triphos	0.25	1	0.16920474	17	0.049094636
6565	GO:0009201	ribonucleoside triphosphate t	0.25	1	0.16920474	17	0.049094636
6572	GO:0009208	pyrimidine ribonucleoside trip	0.25	1	0.16920474	17	0.049094636
6573	GO:0009209	pyrimidine ribonucleoside trip	0.25	1	0.16920474	17	0.049094636
8585	GO:0015172	acidic amino acid transmemb	0.25	1	0.16920474	17	0.049094636
9069	GO:0015800	acidic amino acid transport	0.25	1	0.16920474	17	0.049094636
21602	GO:0046036|GO:0006243	CTP metabolic process	0.25	1	0.16920474	17	0.049094636
9910	GO:0016878	acid-thiol ligase activity	0.25	2	0.33840948	57	0.16461143
4913	GO:0006814|GO:0006834|G	sodium ion transport	0.26	2	0.33840948	58	0.16749935
8499	GO:0015081|GO:0022816	sodium ion transmembrane t	0.26	2	0.33840948	58	0.16749935
8558	GO:0015145	monosaccharide transmemb	0.26	2	0.33840948	58	0.16749935
9018	GO:0015749	monosaccharide transport	0.26	2	0.33840948	58	0.16749935
11820	GO:0019887	protein kinase regulator activ	0.26	2	0.33840948	58	0.16749935
18086	GO:0035725	sodium ion transmembrane t	0.26	2	0.33840948	58	0.16749935
29800	GO:0071436	sodium ion export	0.26	2	0.33840948	58	0.16749935
5763	GO:0008233	peptidase activity	0.26	12	2.0304568	565	1.6316746
4357	GO:0006139|GO:0055134	nucleobase, nucleoside, nucl	0.26	61	10.321489	3285	9.486816
8673	GO:0015291|GO:0015290|G	secondary active transmemb	0.26	9	1.5228426	407	1.1753833
26931	GO:0055085	transmembrane transport	0.26	30	5.0761423	1548	4.4705
3832	GO:0005388	calcium-transporting ATPase	0.27	1	0.16920474	18	0.051982556
4134	GO:0005874	microtubule	0.27	1	0.16920474	18	0.051982556
4225	GO:0005985	sucrose metabolic process	0.27	1	0.16920474	18	0.051982556
7773	GO:0010557	positive regulation of macron	0.27	1	0.16920474	18	0.051982556
12887	GO:0030332	cyclin binding	0.27	1	0.16920474	18	0.051982556
19253	GO:0042936	dipeptide transporter activity	0.27	3	0.5076142	105	0.3032316
19255	GO:0042938	dipeptide transport	0.27	3	0.5076142	105	0.3032316
3333	GO:0004712	protein serine/threonine/tyros	0.27	2	0.33840948	59	0.17038727
31895	GO:0090304	nucleic acid metabolic proce	0.27	44	7.4450083	2340	6.7577324
4840	GO:0006720|GO:0016096	isoprenoid metabolic process	0.27	2	0.33840948	60	0.17327519

6964	GO:0009698	phenylpropanoid metabolic p	0.27	2	0.33840948	60	0.17327519
14811	GO:0032403	protein complex binding	0.27	2	0.33840948	60	0.17327519
28375	GO:0070008	serine-type exopeptidase act	0.27	2	0.33840948	60	0.17327519
2980	GO:0004185	serine-type carboxypeptidase	0.27	2	0.33840948	60	0.17327519
3290	GO:0004656	procollagen-proline 4-dioxyge	0.28	1	0.16920474	19	0.054870475
11752	GO:0019798	procollagen-proline dioxygen	0.28	1	0.16920474	19	0.054870475
13966	GO:0031543	peptidyl-proline dioxygenase	0.28	1	0.16920474	19	0.054870475
13968	GO:0031545	peptidyl-proline 4-dioxygenas	0.28	1	0.16920474	19	0.054870475
5705	GO:0008152	metabolic process	0.28	305	51.607445	17432	50.34222
4954	GO:0006869	lipid transport	0.28	5	0.8460237	208	0.6006873
8085	GO:0010876	lipid localization	0.28	5	0.8460237	208	0.6006873
20488	GO:0044238	primary metabolic process	0.28	163	27.580372	9167	26.47356
3888	GO:0005529	sugar binding	0.28	4	0.67681897	158	0.45629135
6018	GO:0008559|GO:0005226|G	xenobiotic-transporting ATPa	0.29	2	0.33840948	62	0.17905103
11210	GO:0019207	kinase regulator activity	0.29	2	0.33840948	62	0.17905103
19225	GO:0042908	xenobiotic transport	0.29	2	0.33840948	62	0.17905103
19227	GO:0042910	xenobiotic transporter activity	0.29	2	0.33840948	62	0.17905103
2978	GO:0004180	carboxypeptidase activity	0.29	2	0.33840948	62	0.17905103
4921	GO:0006826|GO:0015681	iron ion transport	0.29	1	0.16920474	20	0.0577584
9707	GO:0016624	oxidoreductase activity, actin	0.29	1	0.16920474	20	0.0577584
9799	GO:0016744|GO:0016745	transferase activity, transferr	0.29	1	0.16920474	20	0.0577584
8680	GO:0015298	solute:cation antiporter activi	0.30	3	0.5076142	112	0.32344702
9730	GO:0016651	oxidoreductase activity, actin	0.30	3	0.5076142	112	0.32344702
3364	GO:0004745	retinol dehydrogenase activit	0.30	1	0.16920474	21	0.060646318
3474	GO:0004869|GO:0004870	cysteine-type endopeptidase	0.30	1	0.16920474	21	0.060646318
4420	GO:0006213	pyrimidine nucleoside metab	0.30	1	0.16920474	21	0.060646318
5878	GO:0008378	galactosyltransferase activity	0.30	1	0.16920474	21	0.060646318
6582	GO:0009218	pyrimidine ribonucleotide me	0.30	1	0.16920474	21	0.060646318
6584	GO:0009220	pyrimidine ribonucleotide bio	0.30	1	0.16920474	21	0.060646318
21697	GO:0046131	pyrimidine ribonucleoside me	0.30	1	0.16920474	21	0.060646318
9933	GO:0016903	oxidoreductase activity, actin	0.30	3	0.5076142	113	0.32633495
9710	GO:0016628	oxidoreductase activity, actin	0.30	2	0.33840948	65	0.18771479
14379	GO:0031966	mitochondrial membrane	0.31	3	0.5076142	114	0.32922286
9422	GO:0016209	antioxidant activity	0.31	4	0.67681897	165	0.47650677
5840	GO:0008324	cation transmembrane trans	0.31	11	1.8612521	538	1.5537009
7143	GO:0009891	positive regulation of biosynt	0.32	1	0.16920474	22	0.06353424
8774	GO:0015431	glutathione S-conjugate-expo	0.32	1	0.16920474	22	0.06353424
10032	GO:0017075	syntaxin-1 binding	0.32	1	0.16920474	22	0.06353424
12660	GO:0030077	plasma membrane light-harv	0.32	1	0.16920474	22	0.06353424
13038	GO:0030551	cyclic nucleotide binding	0.32	1	0.16920474	22	0.06353424
13758	GO:0031328	positive regulation of cellular	0.32	1	0.16920474	22	0.06353424
18438	GO:0042054	histone methyltransferase ac	0.32	1	0.16920474	22	0.06353424
19040	GO:0042716	plasma membrane-derived c	0.32	1	0.16920474	22	0.06353424
19325	GO:0043008	ATP-dependent protein bindi	0.32	1	0.16920474	22	0.06353424
30359	GO:0071997	glutathione S-conjugate-tran	0.32	1	0.16920474	22	0.06353424
4609	GO:0006464	protein modification process	0.32	51	8.629441	2781	8.031305
2674	GO:0003824	catalytic activity	0.32	189	31.979696	10750	31.045137
3986	GO:0005681	spliceosomal complex	0.33	1	0.16920474	23	0.06642216
5636	GO:0008061	chitin binding	0.33	1	0.16920474	23	0.06642216
6512	GO:0009147	pyrimidine nucleoside triphos	0.33	1	0.16920474	23	0.06642216
7527	GO:0010302	2-oxoglutarate-dependent di	0.33	1	0.16920474	23	0.06642216
22414	GO:0046912	transferase activity, transferr	0.33	1	0.16920474	23	0.06642216
25416	GO:0051119	sugar transmembrane transp	0.33	3	0.5076142	118	0.34077454
9795	GO:0016740	transferase activity	0.33	68	11.505922	3764	10.870131
3620	GO:0005057	receptor signaling protein act	0.33	2	0.33840948	69	0.19926646
21710	GO:0046148	pigment biosynthetic process	0.33	2	0.33840948	69	0.19926646
8943	GO:0015672	monovalent inorganic cation	0.33	8	1.3536379	383	1.1060733
3156	GO:0004497	monooxygenase activity	0.34	4	0.67681897	172	0.49672222
3213	GO:0004568	chitinase activity	0.34	1	0.16920474	24	0.06931008
6248	GO:0008843	endochitinase activity	0.34	1	0.16920474	24	0.06931008
9779	GO:0016717	oxidoreductase activity, actin	0.34	1	0.16920474	24	0.06931008
22440	GO:0046939	nucleotide phosphorylation	0.34	1	0.16920474	24	0.06931008
31407	GO:0080030	methyl indole-3-acetate este	0.34	1	0.16920474	24	0.06931008
2821	GO:0004012|GO:0008557	phospholipid-translocating AT	0.34	1	0.16920474	24	0.06931008
8494	GO:0015075	ion transmembrane transpor	0.34	16	2.7072759	827	2.3883097
8675	GO:0015293	symporter activity	0.34	4	0.67681897	173	0.49961013
3723	GO:0005215|GO:0005478	transporter activity	0.35	34	5.752961	1844	5.325324
9482	GO:0016291|GO:0008778|G	acyl-CoA thioesterase activit	0.35	1	0.16920474	25	0.072197996
31493	GO:0080118	brassinosteroid sulfotransfera	0.35	1	0.16920474	25	0.072197996
16603	GO:0034220	ion transmembrane transpor	0.35	16	2.7072759	833	2.4056373
9913	GO:0016881	acid-amino acid ligase activit	0.35	8	1.3536379	391	1.1291766
9812	GO:0016758	transferase activity, transferr	0.35	10	1.6920474	501	1.4468478
9703	GO:0016620	oxidoreductase activity, actin	0.35	2	0.33840948	73	0.21081814
18786	GO:0042440	pigment metabolic process	0.35	2	0.33840948	73	0.21081814
11202	GO:0019199	transmembrane receptor pro	0.36	10	1.6920474	504	1.4555116
9194	GO:0015935	small ribosomal subunit	0.36	1	0.16920474	26	0.075085916
9421	GO:0016208	AMP binding	0.36	1	0.16920474	26	0.075085916
9658	GO:0016571	histone methylation	0.36	1	0.16920474	26	0.075085916
126	GO:0000160	two-component signal transd	0.36	1	0.16920474	26	0.075085916
13082	GO:0030599	pectinesterase activity	0.36	4	0.67681897	178	0.5140497
12741	GO:0030170	pyridoxal phosphate binding	0.36	3	0.5076142	126	0.3638779
28643	GO:0070279	vitamin B6 binding	0.36	3	0.5076142	126	0.3638779
8810	GO:0015491	cation:cation antiporter activi	0.37	2	0.33840948	75	0.21659398
4612	GO:0006468	protein phosphorylation	0.37	40	6.7681894	2205	6.367863
3329	GO:0004708	MAP kinase kinase activity	0.37	1	0.16920474	27	0.077973835
3725	GO:0005217	intracellular ligand-gated ion	0.37	1	0.16920474	27	0.077973835
8919	GO:0015645	fatty acid ligase activity	0.37	1	0.16920474	27	0.077973835
9541	GO:0016413	O-acetyltransferase activity	0.37	1	0.16920474	27	0.077973835
12573	GO:0022891	substrate-specific transmem	0.37	23	3.8917089	1241	3.5839086
6563	GO:0009199	ribonucleoside triphosphate r	0.37	15	2.5380712	790	2.2814567
3877	GO:0005516	calmodulin binding	0.38	5	0.8460237	236	0.6815491
6529	GO:0009165	nucleotide biosynthetic proce	0.38	2	0.33840948	77	0.22236982
4916	GO:0006818	hydrogen transport	0.38	6	1.0152284	292	0.84327257
9246	GO:0015992	proton transport	0.38	6	1.0152284	292	0.84327257
3284	GO:0004650	polygalacturonase activity	0.38	1	0.16920474	28	0.080861755
4374	GO:0006164	purine nucleotide biosyntheti	0.38	1	0.16920474	28	0.080861755
7145	GO:0009893	positive regulation of metabo	0.38	1	0.16920474	28	0.080861755
7819	GO:0010604	positive regulation of macron	0.38	1	0.16920474	28	0.080861755
9827	GO:0016776	phosphotransferase activity,	0.38	1	0.16920474	28	0.080861755
13634	GO:0031177	phosphopantetheine binding	0.38	1	0.16920474	28	0.080861755
13755	GO:0031325	positive regulation of cellular	0.38	1	0.16920474	28	0.080861755
6506	GO:0009141	nucleoside triphosphate meta	0.38	15	2.5380712	796	2.2987843
9909	GO:0016877	ligase activity, forming carbo	0.39	2	0.33840948	78	0.22525774
30865	GO:0072509	divalent inorganic cation tran	0.39	2	0.33840948	78	0.22525774
25998	GO:0051716	cellular response to stimulus	0.39	21	3.5532994	1138	3.2864528
25195	GO:0050896|GO:0051869	response to stimulus	0.39	34	5.752961	1881	5.432177
20510	GO:0044260|GO:0034960	cellular macromolecule meta	0.39	98	16.582064	5577	16.105928
4427	GO:0006221	pyrimidine nucleotide biosynt	0.39	1	0.16920474	29	0.083749674
4841	GO:0006721	terpenoid metabolic process	0.39	1	0.16920474	29	0.083749674
11834	GO:0019905	syntaxin binding	0.39	1	0.16920474	29	0.083749674
3955	GO:0005634	nucleus	0.40	26	4.399323	1433	4.138389
18674	GO:0042325	regulation of phosphorylation	0.40	2	0.33840948	81	0.23392151
5916	GO:0008422|GO:0016983	beta-glucosidase activity	0.40	1	0.16920474	30	0.086637594
6814	GO:0009526	plastid envelope	0.40	1	0.16920474	30	0.086637594
7188	GO:0009941	chloroplast envelope	0.40	1	0.16920474	30	0.086637594
8683	GO:0015301|GO:0015380|G	anion:anion antiporter activity	0.40	1	0.16920474	30	0.086637594
5712	GO:0008168|GO:0004480	methyltransferase activity	0.40	5	0.8460237	244	0.7046524
3242	GO:0004601|GO:0016685|G	peroxidase activity	0.41	3	0.5076142	135	0.38986918
9754	GO:0016684	oxidoreductase activity, actin	0.41	3	0.5076142	135	0.38986918
29202	GO:0070838	divalent metal ion transport	0.41	2	0.33840948	82	0.23680943
4373	GO:0006163	purine nucleotide metabolic p	0.41	15	2.5380712	810	2.339215
30884	GO:0072528	pyrimidine-containing compo	0.41	1	0.16920474	31	0.08952551

6622	GO:0009259|GO:0009121	ribonucleotide metabolic pro	0.41	15	2.5380712	812	2.344991
3787	GO:0005319	lipid transporter activity	0.42	2	0.33840948	83	0.23969735
4912	GO:0006813|GO:0015458	potassium ion transport	0.42	2	0.33840948	84	0.24258527
30168	GO:0071804	cellular potassium ion transp	0.42	2	0.33840948	84	0.24258527
30169	GO:0071805	potassium ion transmembrar	0.42	2	0.33840948	84	0.24258527
30867	GO:0072511	divalent inorganic cation tran	0.42	2	0.33840948	84	0.24258527
3304	GO:0004672|GO:0050222	protein kinase activity	0.42	24	4.0609136	1333	3.8495972
3265	GO:0004630	phospholipase D activity	0.42	1	0.16920474	32	0.09241343
4614	GO:0006470	protein dephosphorylation	0.42	1	0.16920474	32	0.09241343
6942	GO:0009674	potassium:sodium symporter	0.42	1	0.16920474	32	0.09241343
12522	GO:0022820	potassium ion symporter act	0.42	1	0.16920474	32	0.09241343
23914	GO:0048522|GO:0051242	positive regulation of cellular	0.42	1	0.16920474	32	0.09241343
4942	GO:0006855	drug transmembrane transpo	0.42	3	0.5076142	139	0.40142086
8607	GO:0015197|GO:0015637	peptide transporter activity	0.42	3	0.5076142	139	0.40142086
8608	GO:0015198	oligopeptide transporter activ	0.42	3	0.5076142	139	0.40142086
8642	GO:0015238|GO:0015239|G	drug transmembrane transpo	0.42	3	0.5076142	139	0.40142086
9154	GO:0015893	drug transport	0.42	3	0.5076142	139	0.40142086
18832	GO:0042493|GO:0017035	response to drug	0.42	3	0.5076142	139	0.40142086
11222	GO:0019220	regulation of phosphate meta	0.43	2	0.33840948	85	0.24547319
25471	GO:0051174	regulation of phosphorus me	0.43	2	0.33840948	85	0.24547319
25626	GO:0051336	regulation of hydrolase activi	0.43	4	0.67681897	196	0.5660323
19684	GO:0043412	macromolecule modification	0.43	51	8.629441	2900	8.374968
5768	GO:0008238	exopeptidase activity	0.43	2	0.33840948	86	0.24836111
4944	GO:0006857	oligopeptide transport	0.43	3	0.5076142	141	0.4071967
8676	GO:0015294	solute:cation symporter activ	0.43	3	0.5076142	141	0.4071967
9099	GO:0015833	peptide transport	0.43	3	0.5076142	141	0.4071967
4493	GO:0006298|GO:0006300	mismatch repair	0.43	1	0.16920474	33	0.09530135
30878	GO:0072522	purine-containing compound	0.43	1	0.16920474	33	0.09530135
18953	GO:0042623	ATPase activity, coupled	0.43	10	1.6920474	537	1.550813
30877	GO:0072521	purine-containing compound	0.44	15	2.5380712	827	2.3883097
8557	GO:0015144	carbohydrate transmembran	0.44	3	0.5076142	143	0.41297254
16602	GO:0034219	carbohydrate transmembran	0.44	3	0.5076142	143	0.41297254
3749	GO:0005249	voltage-gated potassium cha	0.44	1	0.16920474	34	0.09818927
5270	GO:0007264	small GTPase mediated sign	0.44	1	0.16920474	34	0.09818927
6486	GO:0009119	ribonucleoside metabolic pro	0.44	1	0.16920474	34	0.09818927
11208	GO:0019205	nucleobase, nucleoside, nucl	0.44	1	0.16920474	34	0.09818927
11831	GO:0019902	phosphatase binding	0.44	1	0.16920474	34	0.09818927
11832	GO:0019903	protein phosphatase binding	0.44	1	0.16920474	34	0.09818927
12574	GO:0022892	substrate-specific transporte	0.44	26	4.399323	1463	4.2250266
26932	GO:0055086	nucleobase, nucleoside and	0.45	17	2.8764806	944	2.7261963
4405	GO:0006195	purine nucleotide catabolic p	0.45	14	2.3688664	773	2.232362
6508	GO:0009143	nucleoside triphosphate cata	0.45	14	2.3688664	773	2.232362
6509	GO:0009144	purine nucleoside triphospha	0.45	14	2.3688664	773	2.232362
6511	GO:0009146	purine nucleoside triphospha	0.45	14	2.3688664	773	2.232362
6519	GO:0009154	purine ribonucleotide catabol	0.45	14	2.3688664	773	2.232362
6530	GO:0009166	nucleotide catabolic process	0.45	14	2.3688664	773	2.232362
6567	GO:0009203	ribonucleoside triphosphate c	0.45	14	2.3688664	773	2.232362
6569	GO:0009205	purine ribonucleoside triphos	0.45	14	2.3688664	773	2.232362
6571	GO:0009207	purine ribonucleoside triphos	0.45	14	2.3688664	773	2.232362
6624	GO:0009261	ribonucleotide catabolic proc	0.45	14	2.3688664	773	2.232362
17034	GO:0034655	nucleobase, nucleoside, nucl	0.45	14	2.3688664	773	2.232362
17035	GO:0034656	nucleobase, nucleoside and	0.45	14	2.3688664	773	2.232362
22209	GO:0046700	heterocycle catabolic proces	0.45	14	2.3688664	773	2.232362
30879	GO:0072523	purine-containing compound	0.45	14	2.3688664	773	2.232362
14380	GO:0031967	organelle envelope	0.45	4	0.67681897	201	0.5804719
4426	GO:0006220	pyrimidine nucleotide metabo	0.45	1	0.16920474	35	0.10107719
9480	GO:0016289	CoA hydrolase activity	0.45	1	0.16920474	35	0.10107719
12806	GO:0030247	polysaccharide binding	0.45	1	0.16920474	35	0.10107719
1169	GO:0001871	pattern binding	0.45	1	0.16920474	35	0.10107719
28365	GO:0065008	regulation of biological quality	0.45	6	1.0152284	316	0.9125827
20520	GO:0044270	cellular nitrogen compound c	0.46	14	2.3688664	777	2.2439137
6071	GO:0008643|GO:0006861|G	carbohydrate transport	0.46	3	0.5076142	147	0.42452422
9796	GO:0016741	transferase activity, transferr	0.46	5	0.8460237	261	0.7537471
6794	GO:0009505	plant-type cell wall	0.46	1	0.16920474	36	0.10396511
12626	GO:0030029	actin filament-based process	0.46	1	0.16920474	36	0.10396511
12633	GO:0030036	actin cytoskeleton organizati	0.46	1	0.16920474	36	0.10396511
20649	GO:0044455	mitochondrial membrane par	0.46	1	0.16920474	36	0.10396511
9579	GO:0016462	pyrophosphatase activity	0.46	17	2.8764806	954	2.7550755
14387	GO:0031975	envelope	0.46	4	0.67681897	205	0.59202355
9859	GO:0016818	hydrolase activity, acting on a	0.47	17	2.8764806	956	2.7608514
16785	GO:0034404	nucleobase, nucleoside and	0.47	2	0.33840948	92	0.26568863
17033	GO:0034654	nucleobase, nucleoside, nucl	0.47	2	0.33840948	92	0.26568863
11426	GO:0019438	aromatic compound biosynth	0.47	3	0.5076142	149	0.43030006
3758	GO:0005267	potassium channel activity	0.47	1	0.16920474	37	0.10685303
8934	GO:0015662	ATPase activity, coupled to t	0.47	1	0.16920474	37	0.10685303
8980	GO:0015711	organic anion transport	0.47	1	0.16920474	37	0.10685303
9858	GO:0016817	hydrolase activity, acting on a	0.47	17	2.8764806	959	2.769515
5018	GO:0006950	response to stress	0.47	11	1.8612521	611	1.764519
9811	GO:0016757|GO:0016932	transferase activity, transferr	0.48	13	2.1996615	729	2.1052935
4029	GO:0005740	mitochondrial envelope	0.48	3	0.5076142	151	0.4360759
5066	GO:0007010	cytoskeleton organization	0.48	1	0.16920474	38	0.10974095
6749	GO:0009414	response to water deprivatio	0.48	1	0.16920474	38	0.10974095
8501	GO:0015085	calcium ion transmembrane	0.48	1	0.16920474	38	0.10974095
8588	GO:0015175	neutral amino acid transmem	0.48	1	0.16920474	38	0.10974095
9073	GO:0015804	neutral amino acid transport	0.48	1	0.16920474	38	0.10974095
12538	GO:0022843	voltage-gated cation channel	0.48	1	0.16920474	38	0.10974095
117	GO:0000149	SNARE binding	0.48	1	0.16920474	38	0.10974095
6515	GO:0009150	purine ribonucleotide metabo	0.48	14	2.3688664	791	2.2843447
6623	GO:0009260	ribonucleotide biosynthetic p	0.49	1	0.16920474	39	0.11262887
30883	GO:0072527	pyrimidine-containing compo	0.49	1	0.16920474	39	0.11262887
6424	GO:0009055|GO:0009053|G	electron carrier activity	0.49	3	0.5076142	154	0.44473964
8517	GO:0015103	inorganic anion transmembra	0.49	3	0.5076142	154	0.44473964
4867	GO:0006753	nucleoside phosphate metab	0.49	16	2.7072759	914	2.6395588
6484	GO:0009117	nucleotide metabolic process	0.49	16	2.7072759	914	2.6395588
8495	GO:0015077	monovalent inorganic cation	0.50	4	0.67681897	214	0.6180149
23910	GO:0048518|GO:0043119	positive regulation of biologic	0.50	1	0.16920474	40	0.1155168
12572	GO:0022890|GO:0015082	inorganic cation transmembr	0.50	6	1.0152284	332	0.9587894
22007	GO:0046483	heterocycle metabolic proces	0.50	19	3.21489	1094	3.1593843
20627	GO:0044433	cytoplasmic vesicle part	0.51	1	0.16920474	41	0.118404716
3716	GO:0005198	structural molecule activity	0.51	7	1.1844332	393	1.1349525
17020	GO:0034641	cellular nitrogen compound m	0.51	63	10.659899	3678	10.621769
3490	GO:0004888|GO:0004926	transmembrane receptor act	0.51	10	1.6920474	571	1.6490022
4874	GO:0006767	water-soluble vitamin metabo	0.51	1	0.16920474	42	0.121292636
8461	GO:0015020|GO:0003981	glucuronosyltransferase activ	0.51	1	0.16920474	42	0.121292636
12659	GO:0030076	light-harvesting complex	0.51	1	0.16920474	42	0.121292636
13884	GO:0031461	cullin-RING ubiquitin ligase c	0.51	1	0.16920474	42	0.121292636
18713	GO:0042364	water-soluble vitamin biosynt	0.51	1	0.16920474	42	0.121292636
8496	GO:0015078	hydrogen ion transmembran	0.52	3	0.5076142	160	0.4620672
9911	GO:0016879	ligase activity, forming carbo	0.52	8	1.3536379	456	1.3168914
9870	GO:0016830	carbon-carbon lyase activity	0.52	3	0.5076142	161	0.4649551
3900	GO:0005548|GO:0008497	phospholipid transporter activ	0.52	1	0.16920474	43	0.124180555
8589	GO:0015179	L-amino acid transmembran	0.52	1	0.16920474	43	0.124180555
9076	GO:0015807	L-amino acid transport	0.52	1	0.16920474	43	0.124180555
9174	GO:0015914	phospholipid transport	0.52	1	0.16920474	43	0.124180555
13833	GO:0031410	cytoplasmic vesicle	0.52	1	0.16920474	43	0.124180555
14393	GO:0031982	vesicle	0.52	1	0.16920474	43	0.124180555
4873	GO:0006766	vitamin metabolic process	0.53	1	0.16920474	44	0.12706847
4914	GO:0006816	calcium ion transport	0.53	1	0.16920474	44	0.12706847
5797	GO:0008276	protein methyltransferase ac	0.53	1	0.16920474	44	0.12706847
6477	GO:0009110	vitamin biosynthetic process	0.53	1	0.16920474	44	0.12706847
9186	GO:0015926	glucosidase activity	0.53	1	0.16920474	44	0.12706847
9753	GO:0016682	oxidoreductase activity, actin	0.53	1	0.16920474	44	0.12706847

20522	GO:0044272	sulfur compound biosynthetic	0.53	1	0.16920474	44	0.12706847
28952	GO:0070588	calcium ion transmembrane	0.53	1	0.16920474	44	0.12706847
9709	GO:0016627	oxidoreductase activity, actin	0.53	2	0.33840948	104	0.30034366
5055	GO:0006996	organelle organization	0.53	3	0.5076142	164	0.47361887
20623	GO:0044429	mitochondrial part	0.54	3	0.5076142	165	0.47650677
14667	GO:0032259	methylation	0.54	5	0.8460237	285	0.8230572
5692	GO:0008134	transcription factor binding	0.54	1	0.16920474	45	0.1299564
8787	GO:0015450	P-P-bond-hydrolysis-driven p	0.54	1	0.16920474	45	0.1299564
11780	GO:0019829	cation-transporting ATPase a	0.54	1	0.16920474	45	0.1299564
4476	GO:0006278	RNA-dependent DNA replica	0.54	3	0.5076142	166	0.4793947
3942	GO:0005618	cell wall	0.55	2	0.33840948	107	0.30900744
4906	GO:0006807	nitrogen compound metaboli	0.55	63	10.659899	3725	10.757502
8497	GO:0015079|GO:0022817	potassium ion transmembran	0.55	1	0.16920474	47	0.13573223
4188	GO:0005938	cell cortex	0.56	1	0.16920474	48	0.13862015
14591	GO:0032183	SUMO binding	0.56	1	0.16920474	48	0.13862015
20642	GO:0044448	cell cortex part	0.56	1	0.16920474	48	0.13862015
113	GO:0000145	exocyst	0.56	1	0.16920474	48	0.13862015
20498	GO:0044248	cellular catabolic process	0.56	15	2.5380712	894	2.5818002
12868	GO:0030312	external encapsulating struct	0.57	2	0.33840948	111	0.3205591
9906	GO:0016874	ligase activity	0.57	10	1.6920474	597	1.7240882
3756	GO:0005261|GO:0015281|G	cation channel activity	0.57	1	0.16920474	49	0.14150807
6750	GO:0009415	response to water	0.57	1	0.16920474	49	0.14150807
8909	GO:0015630	microtubule cytoskeleton	0.57	1	0.16920474	49	0.14150807
9493	GO:0016310	phosphorylation	0.57	42	7.106599	2504	7.2313514
4622	GO:0006479	protein methylation	0.58	1	0.16920474	50	0.14439599
5750	GO:0008213	protein alkylation	0.58	1	0.16920474	50	0.14439599
20517	GO:0044267	cellular protein metabolic pro	0.58	54	9.137055	3225	9.313541
8967	GO:0015698	inorganic anion transport	0.58	3	0.5076142	176	0.5082739
9871	GO:0016831	carboxy-lyase activity	0.59	2	0.33840948	115	0.3321108
6166	GO:0008757	S-adenosylmethionine-deper	0.60	3	0.5076142	180	0.5198256
20532	GO:0044282	small molecule catabolic pro	0.60	14	2.3688664	854	2.4662836
9750	GO:0016679	oxidoreductase activity, actin	0.60	1	0.16920474	53	0.15305975
9836	GO:0016787	hydrolase activity	0.60	62	10.490694	3723	10.751725
5981	GO:0008509	anion transmembrane transp	0.60	4	0.67681897	245	0.70754033
17918	GO:0035556|GO:0007242|G	intracellular signal transducti	0.61	1	0.16920474	54	0.15594767
4917	GO:0006820|GO:0006822	anion transport	0.61	4	0.67681897	247	0.7133162
20505	GO:0044255	cellular lipid metabolic proces	0.61	3	0.5076142	184	0.53137726
4894	GO:0006790	sulfur compound metabolic p	0.61	1	0.16920474	55	0.15883559
4844	GO:0006725	cellular aromatic compound	0.61	3	0.5076142	185	0.53426516
3200	GO:0004553|GO:0016800	hydrolase activity, hydrolyzin	0.62	5	0.8460237	312	0.90103096
12532	GO:0022836	gated channel activity	0.62	2	0.33840948	122	0.3523262
4224	GO:0005984	disaccharide metabolic proce	0.62	1	0.16920474	56	0.16172351
9369	GO:0016137	glycoside metabolic process	0.62	1	0.16920474	56	0.16172351
7229	GO:0009987|GO:0008151|G	cellular process	0.62	178	30.118443	10601	30.614838
5964	GO:0008483	transaminase activity	0.63	1	0.16920474	57	0.16461143
9813	GO:0016759	cellulose synthase activity	0.63	1	0.16920474	57	0.16461143
9821	GO:0016769	transferase activity, transferr	0.63	1	0.16920474	57	0.16461143
3323	GO:0004702	receptor signaling protein se	0.63	1	0.16920474	58	0.16749935
5819	GO:0008299|GO:0009241	isoprenoid biosynthetic proce	0.63	1	0.16920474	58	0.16749935
6665	GO:0009311	oligosaccharide metabolic pr	0.63	1	0.16920474	58	0.16749935
2831	GO:0004022	alcohol dehydrogenase (NAD	0.64	1	0.16920474	59	0.17038727
2626	GO:0003674|GO:0005554	molecular_function	0.64	413	69.88155	24394	70.447914
10058	GO:0017111	nucleoside-triphosphatase ac	0.64	14	2.3688664	878	2.5355935
3306	GO:0004674|GO:0004695|G	protein serine/threonine kinas	0.64	17	2.8764806	1062	3.0669708
30206	GO:0071842	cellular component organizat	0.64	3	0.5076142	193	0.5573685
5652	GO:0008081|GO:0004434|G	phosphoric diester hydrolase	0.64	1	0.16920474	60	0.17327519
9824	GO:0016773	phosphotransferase activity,	0.65	24	4.0609136	1493	4.311664
2818	GO:0004003	ATP-dependent DNA helicas	0.65	1	0.16920474	61	0.1761631
4849	GO:0006730|GO:0019753|G	one-carbon metabolic proces	0.65	5	0.8460237	325	0.93857396
9488	GO:0016301	kinase activity	0.65	28	4.737733	1740	5.0249805
22479	GO:0046983	protein dimerization activity	0.66	8	1.3536379	517	1.4930545
3340	GO:0004721	phosphoprotein phosphatase	0.66	3	0.5076142	199	0.57469606
10244	GO:0018130	heterocycle biosynthetic proc	0.66	3	0.5076142	199	0.57469606
19126	GO:0042803	protein homodimerization ac	0.66	4	0.67681897	265	0.7652988
22416	GO:0046915	transition metal ion transmen	0.67	1	0.16920474	64	0.18482687
15436	GO:0033036	macromolecule localization	0.67	6	1.0152284	397	1.1465042
9838	GO:0016790	thiolester hydrolase activity	0.67	1	0.16920474	65	0.18771479
5791	GO:0008270	zinc ion binding	0.67	30	5.0761423	1880	5.4292893
9765	GO:0016702	oxidoreductase activity, actin	0.68	1	0.16920474	66	0.1906027
2629	GO:0003678|GO:0003679	DNA helicase activity	0.68	1	0.16920474	67	0.19349062
4897	GO:0006793	phosphorus metabolic proce	0.69	47	7.952623	2925	8.4471655
4900	GO:0006796	phosphate metabolic process	0.69	47	7.952623	2925	8.4471655
18955	GO:0042625	ATPase activity, coupled to t	0.69	1	0.16920474	68	0.19637854
32	GO:0000041	transition metal ion transport	0.69	1	0.16920474	68	0.19637854
3724	GO:0005216	ion channel activity	0.69	2	0.33840948	140	0.40430877
6046	GO:0008610	lipid biosynthetic process	0.70	2	0.33840948	141	0.4071967
5726	GO:0008187	poly-pyrimidine tract binding	0.70	1	0.16920474	70	0.20215438
5788	GO:0008266	poly(U) RNA binding	0.70	1	0.16920474	70	0.20215438
5838	GO:0008320	protein transmembrane trans	0.70	1	0.16920474	70	0.20215438
12568	GO:0022884	macromolecule transmembra	0.70	1	0.16920474	70	0.20215438
9771	GO:0016709	oxidoreductase activity, actin	0.71	1	0.16920474	71	0.2050423
3744	GO:0005244	voltage-gated ion channel ac	0.71	1	0.16920474	72	0.20793022
9764	GO:0016701	oxidoreductase activity, actin	0.71	1	0.16920474	72	0.20793022
12528	GO:0022832	voltage-gated channel activit	0.71	1	0.16920474	72	0.20793022
15280	GO:0032879	regulation of localization	0.71	1	0.16920474	72	0.20793022
17141	GO:0034762	regulation of transmembrane	0.71	1	0.16920474	72	0.20793022
17144	GO:0034765	regulation of ion transmembr	0.71	1	0.16920474	72	0.20793022
19568	GO:0043269	regulation of ion transport	0.71	1	0.16920474	72	0.20793022
25348	GO:0051049	regulation of transport	0.71	1	0.16920474	72	0.20793022
3311	GO:0004683|GO:0004684|G	calmodulin-dependent protei	0.72	1	0.16920474	74	0.21370606
20628	GO:0044434	chloroplast part	0.72	1	0.16920474	74	0.21370606
20629	GO:0044435	plastid part	0.72	1	0.16920474	74	0.21370606
4028	GO:0005739	mitochondrion	0.72	3	0.5076142	219	0.63245445
25510	GO:0051213	dioxygenase activity	0.73	1	0.16920474	75	0.21659398
5019	GO:0006952|GO:0002217|G	defense response	0.73	3	0.5076142	221	0.63823026
5663	GO:0008094|GO:0004011	DNA-dependent ATPase act	0.73	1	0.16920474	76	0.2194819
5604	GO:0008017	microtubule binding	0.73	1	0.16920474	77	0.22236982
13656	GO:0031224	intrinsic to membrane	0.74	18	3.0456853	1193	3.4452884
4460	GO:0006260|GO:0055133	DNA replication	0.74	3	0.5076142	224	0.64689404
13547	GO:0031090	organelle membrane	0.74	3	0.5076142	224	0.64689404
9697	GO:0016614	oxidoreductase activity, actin	0.74	4	0.67681897	293	0.84616053
5681	GO:0008116	prostaglandin-I synthase acti	0.74	1	0.16920474	78	0.22525774
9800	GO:0016746	transferase activity, transferr	0.75	7	1.1844332	498	1.438184
9655	GO:0016568	chromatin modification	0.75	1	0.16920474	80	0.2310336
9656	GO:0016569	covalent chromatin modificat	0.75	1	0.16920474	80	0.2310336
9657	GO:0016570	histone modification	0.75	1	0.16920474	80	0.2310336
9875	GO:0016836	hydro-lyase activity	0.75	1	0.16920474	80	0.2310336
3307	GO:0004675	transmembrane receptor pro	0.75	5	0.8460237	367	1.0598665
19526	GO:0043227	membrane-bounded organel	0.75	33	5.5837564	2142	6.185924
19530	GO:0043231	intracellular membrane-boun	0.75	33	5.5837564	2142	6.185924
6991	GO:0009725	response to hormone stimulu	0.76	1	0.16920474	82	0.23680943
20487	GO:0044237	cellular metabolic process	0.76	133	22.50423	8185	23.637625
4643	GO:0006511	ubiquitin-dependent protein c	0.76	1	0.16920474	84	0.24258527
11864	GO:0019941	modification-dependent prote	0.76	1	0.16920474	84	0.24258527
19898	GO:0043632	modification-dependent mac	0.76	1	0.16920474	84	0.24258527
20507	GO:0044257	cellular protein catabolic proc	0.76	1	0.16920474	84	0.24258527
25887	GO:0051603	proteolysis involved in cellula	0.76	1	0.16920474	84	0.24258527
9837	GO:0016788	hydrolase activity, acting on e	0.77	23	3.8917089	1534	4.430069
2628	GO:0003677	DNA binding	0.77	18	3.0456853	1224	3.534814
6985	GO:0009719	response to endogenous stin	0.77	1	0.16920474	86	0.24836111
12734	GO:0030163	protein catabolic process	0.77	1	0.16920474	86	0.24836111
19952	GO:0043687	post-translational protein mo	0.77	1	0.16920474	86	0.24836111

20515	GO:0044265|GO:0034962	cellular macromolecule catal	0.77	1	0.16920474	86	0.24836111
9267	GO:0016021	integral to membrane	0.78	16	2.7072759	1103	3.1853757
20624	GO:0044430	cytoskeletal part	0.78	1	0.16920474	88	0.25413695
119	GO:0000151	ubiquitin ligase complex	0.78	1	0.16920474	88	0.25413695
14590	GO:0032182	small conjugating protein bin	0.79	1	0.16920474	90	0.2599128
2982	GO:0004197	cysteine-type endopeptidase	0.79	1	0.16920474	90	0.2599128
3644	GO:0005102	receptor binding	0.79	1	0.16920474	91	0.2628007
3341	GO:0004722|GO:0000158|G	protein serine/threonine phos	0.79	2	0.33840948	171	0.4938343
9801	GO:0016747	transferase activity, transferr	0.79	6	1.0152284	456	1.3168914
2660	GO:0003735|GO:0003736|G	structural constituent of ribos	0.79	3	0.5076142	246	0.7104283
6425	GO:0009056	catabolic process	0.79	15	2.5380712	1051	3.0352037
4067	GO:0005783	endoplasmic reticulum	0.80	2	0.33840948	174	0.50249803
18582	GO:0042221	response to chemical stimulu	0.81	4	0.67681897	328	0.94723773
9845	GO:0016798	hydrolase activity, acting on	0.82	5	0.8460237	401	1.1580559
5809	GO:0008289	lipid binding	0.82	3	0.5076142	258	0.74508333
30306	GO:0071944	cell periphery	0.82	3	0.5076142	259	0.74797124
7271	GO:0010033	response to organic substan	0.82	1	0.16920474	100	0.28879198
5280	GO:0007275	multicellular organismal deve	0.82	1	0.16920474	101	0.2916799
9285	GO:0016043	cellular component organizat	0.82	3	0.5076142	261	0.7537471
9486	GO:0016298	lipase activity	0.83	2	0.33840948	185	0.53426516
9699	GO:0016616	oxidoreductase activity, actin	0.83	3	0.5076142	266	0.7681867
14909	GO:0032501|GO:0050874	multicellular organismal proc	0.84	1	0.16920474	105	0.3032316
9823	GO:0016772	transferase activity, transferr	0.84	28	4.737733	1932	5.579461
6426	GO:0009057|GO:0043285	macromolecule catabolic pro	0.84	1	0.16920474	106	0.3061195
9869	GO:0016829	lyase activity	0.84	6	1.0152284	488	1.4093049
8910	GO:0015631	tubulin binding	0.84	1	0.16920474	107	0.30900744
9494	GO:0016311	dephosphorylation	0.85	5	0.8460237	425	1.227366
4512	GO:0006325	chromatin organization	0.86	1	0.16920474	112	0.32344702
5070	GO:0007017	microtubule-based process	0.86	1	0.16920474	112	0.32344702
5071	GO:0007018	microtubule-based movemen	0.86	1	0.16920474	112	0.32344702
25568	GO:0051276|GO:0007001|G	chromosome organization	0.86	1	0.16920474	112	0.32344702
19686	GO:0043414	macromolecule methylation	0.86	1	0.16920474	113	0.32633495
22478	GO:0046982	protein heterodimerization ac	0.86	1	0.16920474	114	0.32922286
6020	GO:0008565|GO:0015463	protein transporter activity	0.86	1	0.16920474	115	0.3321108
9535	GO:0016407	acetyltransferase activity	0.86	1	0.16920474	115	0.3321108
30205	GO:0071841	cellular component organizat	0.87	3	0.5076142	285	0.8230572
14910	GO:0032502	developmental process	0.88	1	0.16920474	121	0.3494383
18912	GO:0042578	phosphoric ester hydrolase a	0.88	5	0.8460237	444	1.2822365
4755	GO:0006629	lipid metabolic process	0.88	7	1.1844332	590	1.7038727
3260	GO:0004620	phospholipase activity	0.88	1	0.16920474	123	0.35521415
4121	GO:0005856	cytoskeleton	0.89	1	0.16920474	126	0.3638779
13018	GO:0030529	ribonucleoprotein complex	0.89	9	1.5228426	741	2.1399486
12533	GO:0022838	substrate-specific channel ac	0.89	2	0.33840948	221	0.63823026
9839	GO:0016791|GO:0016302	phosphatase activity	0.89	4	0.67681897	383	1.1060733
4109	GO:0005840|GO:0033279	ribosome	0.90	8	1.3536379	682	1.9695613
8660	GO:0015267|GO:0015249|G	channel activity	0.90	2	0.33840948	226	0.6526699
12515	GO:0022803	passive transmembrane tran	0.90	2	0.33840948	226	0.6526699
6796	GO:0009507	chloroplast	0.90	2	0.33840948	229	0.6613336
20531	GO:0044281	small molecule metabolic pro	0.91	24	4.0609136	1788	5.1636004
19125	GO:0042802	identical protein binding	0.91	8	1.3536379	697	2.01288
3873	GO:0005507	copper ion binding	0.92	1	0.16920474	146	0.42163628
9895	GO:0016860	intramolecular oxidoreductas	0.92	1	0.16920474	146	0.42163628
5909	GO:0008415	acyltransferase activity	0.92	3	0.5076142	330	0.95301354
4478	GO:0006281	DNA repair	0.92	1	0.16920474	149	0.43030006
5037	GO:0006974|GO:0034984	response to DNA damage st	0.92	1	0.16920474	149	0.43030006
6824	GO:0009536	plastid	0.93	2	0.33840948	247	0.7133162
19525	GO:0043226	organelle	0.93	42	7.106599	3013	8.701303
19528	GO:0043229	intracellular organelle	0.93	42	7.106599	3013	8.701303
19831	GO:0043565	sequence-specific DNA bind	0.94	1	0.16920474	159	0.45917925
20533	GO:0044283	small molecule biosynthetic	0.94	3	0.5076142	347	1.0021082
5764	GO:0008234|GO:0004220	cysteine-type peptidase activ	0.94	1	0.16920474	163	0.47073093
20619	GO:0044425	membrane part	0.94	19	3.21489	1523	4.398302
3178	GO:0004519	endonuclease activity	0.94	3	0.5076142	356	1.0280994
6899	GO:0009628	response to abiotic stimulus	0.95	1	0.16920474	170	0.49094638
30204	GO:0071840	cellular component organizat	0.95	3	0.5076142	362	1.045427
4605	GO:0006457|GO:0007022|G	protein folding	0.95	1	0.16920474	173	0.49961013
15949	GO:0033554	cellular response to stress	0.95	1	0.16920474	173	0.49961013
20521	GO:0044271	cellular nitrogen compound b	0.95	3	0.5076142	367	1.0598665
4215	GO:0005975	carbohydrate metabolic proc	0.95	11	1.8612521	990	2.8590407
3904	GO:0005576	extracellular region	0.95	1	0.16920474	178	0.5140497
20622	GO:0044428	nuclear part	0.95	1	0.16920474	178	0.5140497
4572	GO:0006412|GO:0006416|G	translation	0.96	1	0.16920474	180	0.5198256
5610	GO:0008026	ATP-dependent helicase act	0.96	1	0.16920474	183	0.52848935
28401	GO:0070035	purine NTP-dependent helica	0.96	1	0.16920474	183	0.52848935
8466	GO:0015031|GO:0015831	protein transport	0.96	1	0.16920474	185	0.53426516
20820	GO:0045184	establishment of protein loca	0.96	1	0.16920474	185	0.53426516
20640	GO:0044446	intracellular organelle part	0.96	7	1.1844332	710	2.0504231
20616	GO:0044422	organelle part	0.96	7	1.1844332	712	2.0561988
5669	GO:0008104	protein localization	0.96	1	0.16920474	189	0.54581684
3068	GO:0004386	helicase activity	0.96	1	0.16920474	189	0.54581684
9874	GO:0016835	carbon-oxygen lyase activity	0.97	1	0.16920474	202	0.5833598
4459	GO:0006259|GO:0055132	DNA metabolic process	0.97	4	0.67681897	493	1.4237444
3863	GO:0005488	binding	0.97	215	36.379017	13903	40.15075
9266	GO:0016020	membrane	0.98	50	8.460237	3741	10.803708
19527	GO:0043228	non-membrane-bounded org	0.98	9	1.5228426	923	2.66555
19531	GO:0043232	intracellular non-membrane-	0.98	9	1.5228426	923	2.66555
4558	GO:0006396|GO:0006394	RNA processing	0.98	1	0.16920474	219	0.63245445
3177	GO:0004518	nuclease activity	0.98	3	0.5076142	427	1.2331418
13531	GO:0031072	heat shock protein binding	0.98	1	0.16920474	230	0.6642216
2627	GO:0003676	nucleic acid binding	0.98	30	5.0761423	2453	7.0840673
11828	GO:0019899	enzyme binding	0.98	1	0.16920474	237	0.684437
2653	GO:0003723	RNA binding	0.98	7	1.1844332	797	2.3016722
13041	GO:0030554	adenyl nucleotide binding	0.99	4	0.67681897	550	1.5883559
14965	GO:0032559	adenyl ribonucleotide binding	0.99	4	0.67681897	550	1.5883559
20512	GO:0044262|GO:0006092	cellular carbohydrate metabo	0.99	1	0.16920474	248	0.7162041
5661	GO:0008092	cytoskeletal protein binding	0.99	1	0.16920474	249	0.719092
4649	GO:0006520|GO:0006519	cellular amino acid metabolic	0.99	1	0.16920474	277	0.7999538
3884	GO:0005524	ATP binding	0.99	3	0.5076142	524	1.51327
20356	GO:0044106	cellular amine metabolic proc	0.99	1	0.16920474	303	0.8750397
9889	GO:0016853	isomerase activity	0.99	1	0.16920474	306	0.8837035
6662	GO:0009308	amine metabolic process	1.00	1	0.16920474	314	0.9068068
20638	GO:0044444	cytoplasmic part	1.00	16	2.7072759	1644	4.7477403
19533	GO:0043234	protein complex	1.00	6	1.0152284	848	2.448956
15392	GO:0032991	macromolecular complex	1.00	15	2.5380712	1616	4.666878
4320	GO:0006082	organic acid metabolic proce	1.00	1	0.16920474	369	1.0656425
11716	GO:0019752	carboxylic acid metabolic pro	1.00	1	0.16920474	369	1.0656425
19707	GO:0043436	oxoacid metabolic process	1.00	1	0.16920474	369	1.0656425
20618	GO:0044424	intracellular part	1.00	45	7.614213	3853	11.127155
18541	GO:0042180	cellular ketone metabolic pro	1.00	1	0.16920474	375	1.0829699
3945	GO:0005622	intracellular	1.00	56	9.475466	4751	13.720507
14959	GO:0032553	ribonucleotide binding	1.00	4	0.67681897	795	2.2958963
14961	GO:0032555	purine ribonucleotide binding	1.00	4	0.67681897	795	2.2958963
10033	GO:0017076	purine nucleotide binding	1.00	4	0.67681897	797	2.3016722
3876	GO:0005515|GO:0045308	protein binding	1.00	88	14.890017	6933	20.021948
4027	GO:0005737	cytoplasm	1.00	17	2.8764806	2001	5.7787275
18000	GO:0035639	purine ribonucleoside triphos	1.00	3	0.5076142	769	2.2208104
3946	GO:0005623	cell	1.00	105	17.766497	8227	23.758917
20657	GO:0044464	cell part	1.00	105	17.766497	8227	23.758917
3903	GO:0005575|GO:0008372	cellular_component	1.00	108	18.27411	8515	24.590637
131	GO:0000166	nucleotide binding	1.00	6	1.0152284	1213	3.5030468

GO ID	GO ACCESSION	GO Term	p-value	Count in Selection	% Count in Selection	Count in Total	% Count in Total
6427	GO:0009058	biosynthetic process	0.00	42	15.730337	2796	8.074624
17024	GO:0034645|GO:0034961	cellular macromolecule bi	0.00	31	11.610487	1999	5.7729516
6428	GO:0009059|GO:0043284	macromolecule biosynthe	0.00	31	11.610487	2003	5.7845035
20499	GO:0044249	cellular biosynthetic proce	0.00	37	13.857677	2635	7.6096687
4476	GO:0006278	RNA-dependent DNA repl	0.00	7	2.621723	166	0.4793947
4109	GO:0005840|GO:0033279	ribosome	0.00	14	5.243446	682	1.9695613
6613	GO:0009250	glucan biosynthetic proce	0.00	2	0.7490637	8	0.023103358
4460	GO:0006260|GO:0055133	DNA replication	0.00	7	2.621723	224	0.64689404
13018	GO:0030529	ribonucleoprotein complex	0.00	14	5.243446	741	2.1399486
2837	GO:0004028	3-chloroallyl aldehyde deh	0.00	2	0.7490637	9	0.025991278
31895	GO:0090304	nucleic acid metabolic pro	0.00	31	11.610487	2340	6.7577324
6275	GO:0008878	glucose-1-phosphate ade	0.00	2	0.7490637	10	0.0288792
26959	GO:0055114	oxidation-reduction proce	0.00	32	11.985019	2490	7.1909204
5624	GO:0008047	enzyme activator activity	0.00	4	1.4981273	85	0.24547319
4459	GO:0006259|GO:0055132	DNA metabolic process	0.01	10	3.7453184	493	1.4237444
19527	GO:0043228	non-membrane-bounded	0.01	15	5.6179776	923	2.66555
19531	GO:0043232	intracellular non-membran	0.01	15	5.6179776	923	2.66555
8493	GO:0015074	DNA integration	0.01	3	1.1235955	47	0.13573223
16081	GO:0033692	cellular polysaccharide bi	0.01	2	0.7490637	15	0.043318797
206	GO:0000271	polysaccharide biosynthe	0.01	2	0.7490637	16	0.046206716
25635	GO:0051345	positive regulation of hydr	0.01	3	1.1235955	53	0.15305975
19392	GO:0043085	positive regulation of cata	0.01	4	1.4981273	104	0.30034366
20343	GO:0044093	positive regulation of mole	0.01	4	1.4981273	104	0.30034366
2662	GO:0003746|GO:0008182	translation elongation fact	0.01	2	0.7490637	22	0.06353424
4574	GO:0006414|GO:0006442	translational elongation	0.01	2	0.7490637	22	0.06353424
4357	GO:0006139|GO:0055134	nucleobase, nucleoside, r	0.01	37	13.857677	3285	9.486816
17016	GO:0034637	cellular carbohydrate bios	0.01	3	1.1235955	63	0.18193895
9293	GO:0016051|GO:0006093	carbohydrate biosynthetic	0.01	3	1.1235955	64	0.18482687
9767	GO:0016705	oxidoreductase activity, a	0.01	7	2.621723	329	0.95012563
4311	GO:0006073	cellular glucan metabolic	0.01	2	0.7490637	24	0.06931008
20293	GO:0044042	glucan metabolic process	0.01	2	0.7490637	24	0.06931008
3309	GO:0004677	DNA-dependent protein k	0.02	1	0.37453184	2	0.00577584
4600	GO:0006448	regulation of translational	0.02	1	0.37453184	2	0.00577584
4602	GO:0006450|GO:0000029	regulation of translational	0.02	1	0.37453184	2	0.00577584
14849	GO:0032441	pheophorbide a oxygenas	0.02	1	0.37453184	2	0.00577584
24925	GO:0050619	phytochromobilin:ferredox	0.02	1	0.37453184	2	0.00577584
26020	GO:0051738	xanthophyll binding	0.02	1	0.37453184	2	0.00577584
5809	GO:0008289	lipid binding	0.02	6	2.247191	258	0.74508333
17020	GO:0034641	cellular nitrogen compoun	0.02	40	14.981274	3678	10.621769
3896	GO:0005544	calcium-dependent phosp	0.02	2	0.7490637	26	0.075085916
9194	GO:0015935	small ribosomal subunit	0.02	2	0.7490637	26	0.075085916
9771	GO:0016709	oxidoreductase activity, a	0.02	3	1.1235955	71	0.2050423
9703	GO:0016620	oxidoreductase activity, a	0.02	3	1.1235955	73	0.21081814
4906	GO:0006807	nitrogen compound metab	0.02	40	14.981274	3725	10.757502
20487	GO:0044237	cellular metabolic process	0.02	78	29.213484	8185	23.637625
4526	GO:0006351|GO:0006350	transcription, DNA-depen	0.02	20	7.490637	1575	4.548474
15176	GO:0032774	RNA biosynthetic process	0.02	20	7.490637	1575	4.548474
20510	GO:0044260|GO:0034960	cellular macromolecule m	0.02	56	20.973783	5577	16.105928
11827	GO:0019898|GO:0030396	extrinsic to membrane	0.02	2	0.7490637	30	0.086637594
3895	GO:0005543	phospholipid binding	0.02	4	1.4981273	140	0.40430877
20514	GO:0044264	cellular polysaccharide m	0.02	2	0.7490637	31	0.08952551
4358	GO:0006140	regulation of nucleotide m	0.02	3	1.1235955	80	0.2310336
7146	GO:0009894	regulation of catabolic pro	0.02	3	1.1235955	80	0.2310336
13278	GO:0030811	regulation of nucleotide ca	0.02	3	1.1235955	80	0.2310336
13759	GO:0031329	regulation of cellular catal	0.02	3	1.1235955	80	0.2310336
15521	GO:0033121	regulation of purine nucle	0.02	3	1.1235955	80	0.2310336
2632	GO:0003682	chromatin binding	0.03	2	0.7490637	33	0.09530135
2985	GO:0004222	metalloendopeptidase act	0.03	2	0.7490637	34	0.09818927
2805	GO:0003989	acetyl-CoA carboxylase a	0.03	1	0.37453184	4	0.011551679
3033	GO:0004349	glutamate 5-kinase activit	0.03	1	0.37453184	4	0.011551679
3057	GO:0004373	glycogen (starch) synthas	0.03	1	0.37453184	4	0.011551679
3100	GO:0004430	1-phosphatidylinositol 4-k	0.03	1	0.37453184	4	0.011551679
3466	GO:0004859	phospholipase inhibitor ac	0.03	1	0.37453184	4	0.011551679
3640	GO:0005098	Ran GTPase activator act	0.03	1	0.37453184	4	0.011551679
4218	GO:0005978	glycogen biosynthetic pro	0.03	1	0.37453184	4	0.011551679
4859	GO:0006743	ubiquinone metabolic proc	0.03	1	0.37453184	4	0.011551679
4860	GO:0006744	ubiquinone biosynthetic p	0.03	1	0.37453184	4	0.011551679
5729	GO:0008191	metalloendopeptidase inh	0.03	1	0.37453184	4	0.011551679
7792	GO:0010576	metalloenzyme regulator a	0.03	1	0.37453184	4	0.011551679
8911	GO:0015633	zinc transporting ATPase	0.03	1	0.37453184	4	0.011551679
9718	GO:0016636	oxidoreductase activity, a	0.03	1	0.37453184	4	0.011551679
9790	GO:0016730	oxidoreductase activity, a	0.03	1	0.37453184	4	0.011551679
10039	GO:0017084	delta1-pyrroline-5-carboxy	0.03	1	0.37453184	4	0.011551679
11781	GO:0019834	phospholipase A2 inhibito	0.03	1	0.37453184	4	0.011551679
11857	GO:0019932	second-messenger-media	0.03	1	0.37453184	4	0.011551679
14425	GO:0032015	regulation of Ran protein s	0.03	1	0.37453184	4	0.011551679
14724	GO:0032316	regulation of Ran GTPase	0.03	1	0.37453184	4	0.011551679
15254	GO:0032853	positive regulation of Ran	0.03	1	0.37453184	4	0.011551679
20918	GO:0045300	acyl-[acyl-carrier-protein]	0.03	1	0.37453184	4	0.011551679
23493	GO:0048015	phosphatidylinositol-medi	0.03	1	0.37453184	4	0.011551679
23495	GO:0048017	inositol lipid-mediated sig	0.03	1	0.37453184	4	0.011551679
23942	GO:0048551	metalloenzyme inhibitor a	0.03	1	0.37453184	4	0.011551679
26947	GO:0055102	lipase inhibitor activity	0.03	1	0.37453184	4	0.011551679
7691	GO:0010467	gene expression	0.03	23	8.614232	1974	5.7007537
13791	GO:0031361	integral to thylakoid memb	0.04	1	0.37453184	5	0.0144396
3638	GO:0005096	GTPase activator activity	0.04	2	0.7490637	41	0.118404716
19813	GO:0043547	positive regulation of GTF	0.04	2	0.7490637	41	0.118404716
5607	GO:0008022	protein C-terminus binding	0.04	2	0.7490637	42	0.121292636
3029	GO:0004345	glucose-6-phosphate deh	0.05	1	0.37453184	6	0.01732752
3208	GO:0004563	beta-N-acetylhexosaminid	0.05	1	0.37453184	6	0.01732752
3342	GO:0004723|GO:0008598	calcium-dependent protei	0.05	1	0.37453184	6	0.01732752
5810	GO:0008290	F-actin capping protein co	0.05	1	0.37453184	6	0.01732752
7220	GO:0009973	adenylyl-sulfate reductase	0.05	1	0.37453184	6	0.01732752
9248	GO:0015994	chlorophyll metabolic proc	0.05	1	0.37453184	6	0.01732752
9249	GO:0015995	chlorophyll biosynthetic pr	0.05	1	0.37453184	6	0.01732752
9435	GO:0016231	beta-N-acetylglucosamini	0.05	1	0.37453184	6	0.01732752
9825	GO:0016774	phosphotransferase activi	0.05	1	0.37453184	6	0.01732752
13832	GO:0031409	pigment binding	0.05	1	0.37453184	6	0.01732752
14264	GO:0031849	olfactory receptor binding	0.05	1	0.37453184	6	0.01732752
18724	GO:0042375	quinone cofactor metabol	0.05	1	0.37453184	6	0.01732752
21036	GO:0045426	quinone cofactor biosynth	0.05	1	0.37453184	6	0.01732752
9600	GO:0016491	oxidoreductase activity	0.05	20	7.490637	1724	4.9787736
14726	GO:0032318	regulation of Ras GTPase	0.05	2	0.7490637	45	0.1299564
22096	GO:0046578	regulation of Ras protein s	0.05	2	0.7490637	45	0.1299564
25355	GO:0051056	regulation of small GTPas	0.05	2	0.7490637	45	0.1299564
19253	GO:0042936	dipeptide transporter activ	0.05	3	1.1235955	105	0.3032316
19255	GO:0042938	dipeptide transport	0.05	3	1.1235955	105	0.3032316

3904	GO:0005576	extracellular region	**0.05**	4	1.4981273	178	0.5140497
9308	GO:0016070	RNA metabolic process	**0.05**	21	7.8651686	1855	5.3570914
20638	GO:0044444	cytoplasmic part	**0.05**	19	7.116105	1644	4.7477403
28930	GO:0070566	adenylyltransferase activi	**0.05**	2	0.7490637	48	0.13862015
4216	GO:0005976	polysaccharide metabolic	0.06	2	0.7490637	50	0.14439599
5096	GO:0007047	cellular cell wall organizat	0.06	2	0.7490637	50	0.14439599
6932	GO:0009664	plant-type cell wall organiz	0.06	2	0.7490637	50	0.14439599
9783	GO:0016722	oxidoreductase activity, o	0.06	2	0.7490637	50	0.14439599
20857	GO:0045229	external encapsulating str	0.06	2	0.7490637	50	0.14439599
30033	GO:0071669	plant-type cell wall organiz	0.06	2	0.7490637	50	0.14439599
9933	GO:0016903	oxidoreductase activity, a	0.06	3	1.1235955	113	0.32633495
18912	GO:0042578	phosphoric ester hydrolas	0.06	7	2.621723	444	1.2822365
7213	GO:0009966|GO:003546	regulation of signal transd	0.06	2	0.7490637	51	0.14728391
12601	GO:0023051	regulation of signaling	0.06	2	0.7490637	51	0.14728391
3051	GO:0004367	glycerol-3-phosphate deh	0.06	1	0.37453184	8	0.023103358
3257	GO:0004617	phosphoglycerate dehydr	0.06	1	0.37453184	8	0.023103358
5635	GO:0008060	ARF GTPase activator ac	0.06	1	0.37453184	8	0.023103358
6684	GO:0009331	glycerol-3-phosphate deh	0.06	1	0.37453184	8	0.023103358
9549	GO:0016421	CoA carboxylase activity	0.06	1	0.37453184	8	0.023103358
9916	GO:0016885	ligase activity, forming ca	0.06	1	0.37453184	8	0.023103358
29246	GO:0070882	cellular cell wall organizat	0.06	2	0.7490637	53	0.15305975
25626	GO:0051336	regulation of hydrolase ac	0.07	4	1.4981273	196	0.5660323
3158	GO:0004499|GO:004707	flavin-containing monooxy	0.07	1	0.37453184	9	0.025991278
6670	GO:0009317	acetyl-CoA carboxylase c	0.07	1	0.37453184	9	0.025991278
11785	GO:0019840	isoprenoid binding	0.07	1	0.37453184	9	0.025991278
19254	GO:0042937	tripeptide transporter activ	0.07	1	0.37453184	9	0.025991278
3340	GO:0004721	phosphoprotein phosphat	0.07	4	1.4981273	199	0.57469606
5767	GO:0008237	metallopeptidase activity	0.07	2	0.7490637	56	0.16172351
23973	GO:0048583	regulation of response to	0.07	2	0.7490637	57	0.16461143
29919	GO:0071555	cell wall organization	0.07	2	0.7490637	58	0.16749935
11205	GO:0019202	amino acid kinase activity	0.07	1	0.37453184	10	0.0288792
15183	GO:0032781	positive regulation of ATP	0.07	1	0.37453184	10	0.0288792
19728	GO:0043462	regulation of ATPase acti	0.07	1	0.37453184	10	0.0288792
21148	GO:0045547	dehydrodolichyl diphosph	0.07	1	0.37453184	10	0.0288792
27563	GO:0060590	ATPase regulator activity	0.07	1	0.37453184	10	0.0288792
31441	GO:0080065	4-alpha-methyl-delta7-ste	0.07	1	0.37453184	10	0.0288792
196	GO:0000254	C-4 methylsterol oxidase	0.07	1	0.37453184	10	0.0288792
983	GO:0001664	G-protein-coupled recepto	0.07	1	0.37453184	10	0.0288792
989	GO:0001671	ATPase activator activity	0.07	1	0.37453184	10	0.0288792
9839	GO:0016791|GO:001630	phosphatase activity	0.08	6	2.247191	383	1.1060733
25095	GO:0050790	regulation of catalytic acti	0.08	7	2.621723	480	1.3862015
12579	GO:0022900	electron transport chain	0.08	1	0.37453184	11	0.03176712
19256	GO:0042939	tripeptide transport	0.08	1	0.37453184	11	0.03176712
19473	GO:0043170|GO:004328	macromolecule metabolic	0.08	60	22.47191	6553	18.92454
28366	GO:0065009	regulation of molecular fu	0.08	7	2.621723	484	1.3977532
6018	GO:0008559|GO:000522	xenobiotic-transporting AT	0.08	2	0.7490637	62	0.17905103
19225	GO:0042908	xenobiotic transport	0.08	2	0.7490637	62	0.17905103
19227	GO:0042910	xenobiotic transporter act	0.08	2	0.7490637	62	0.17905103
25820	GO:0051536	iron-sulfur cluster binding	0.09	2	0.7490637	64	0.18482687
25824	GO:0051540	metal cluster binding	0.09	2	0.7490637	64	0.18482687
3386	GO:0004773	steryl-sulfatase activity	0.09	1	0.37453184	12	0.03465504
4217	GO:0005977	glycogen metabolic proce	0.09	1	0.37453184	12	0.03465504
4344	GO:0006112	energy reserve metabolic	0.09	1	0.37453184	12	0.03465504
5965	GO:0008484	sulfuric ester hydrolase ac	0.09	1	0.37453184	12	0.03465504
8607	GO:0015197|GO:001563	peptide transporter activit	0.09	3	1.1235955	139	0.40142086
8608	GO:0015198	oligopeptide transporter a	0.09	3	1.1235955	139	0.40142086
12794	GO:0030234	enzyme regulator activity	0.09	6	2.247191	403	1.1638317
2628	GO:0003677	DNA binding	0.09	14	5.243446	1224	3.534814
7551	GO:0010327	acetyl CoA:(Z)-3-hexen-1-	0.10	1	0.37453184	13	0.037542958
4944	GO:0006857	oligopeptide transport	0.10	3	1.1235955	141	0.4071967
9099	GO:0015833	peptide transport	0.10	3	1.1235955	141	0.4071967
21710	GO:0046148	pigment biosynthetic proc	0.10	2	0.7490637	69	0.19926646
15524	GO:0033124	regulation of GTP catabol	0.10	2	0.7490637	70	0.20215438
19394	GO:0043087	regulation of GTPase acti	0.10	2	0.7490637	70	0.20215438
3641	GO:0005099	Ras GTPase activator act	0.10	1	0.37453184	14	0.040430877
14728	GO:0032320	positive regulation of Ras	0.10	1	0.37453184	14	0.040430877
25040	GO:0050734	hydroxycinnamoyltransfer	0.10	1	0.37453184	14	0.040430877
9740	GO:0016667	oxidoreductase activity, a	0.11	2	0.7490637	72	0.20793022
5705	GO:0008152	metabolic process	0.11	145	54.307117	17432	50.34222
18786	GO:0042440	pigment metabolic proces	0.11	2	0.7490637	73	0.21081814
9494	GO:0016311	dephosphorylation	0.11	6	2.247191	425	1.227366
27562	GO:0060589	nucleoside-triphosphatase	0.11	3	1.1235955	152	0.4389638
3382	GO:0004768|GO:001621	stearoyl-CoA 9-desaturas	0.12	1	0.37453184	16	0.046206716
7521	GO:0010295	(+)-abscisic acid 8'-hydrox	0.12	1	0.37453184	16	0.046206716
9189	GO:0015929	hexosaminidase activity	0.12	1	0.37453184	16	0.046206716
9427	GO:0016215	CoA desaturase activity	0.12	1	0.37453184	16	0.046206716
19338	GO:0043022|GO:003037	ribosome binding	0.12	1	0.37453184	16	0.046206716
15392	GO:0032991	macromolecular complex	0.12	17	6.367041	1616	4.666878
3885	GO:0005525	GTP binding	0.12	4	1.4981273	245	0.70754033
11032	GO:0019001	guanyl nucleotide binding	0.12	4	1.4981273	245	0.70754033
14967	GO:0032561	guanyl ribonucleotide bind	0.12	4	1.4981273	245	0.70754033
9923	GO:0016892	endoribonuclease activity	0.12	1	0.37453184	17	0.049094636
9925	GO:0016894	endonuclease activity, ac	0.12	1	0.37453184	17	0.049094636
16281	GO:0033897	ribonuclease T2 activity	0.12	1	0.37453184	17	0.049094636
15189	GO:0032787	monocarboxylic acid meta	0.13	2	0.7490637	81	0.23392151
2659	GO:0003730	mRNA 3'-UTR binding	0.13	1	0.37453184	18	0.051982556
4576	GO:0006417|GO:000644	regulation of translation	0.13	1	0.37453184	18	0.051982556
5776	GO:0008252	nucleotidase activity	0.13	1	0.37453184	18	0.051982556
8469	GO:0015036	disulfide oxidoreductase a	0.13	1	0.37453184	18	0.051982556
2658	GO:0003729	mRNA binding	0.13	2	0.7490637	82	0.23680943
3290	GO:0004656	procollagen-proline 4-diox	0.14	1	0.37453184	19	0.054870475
11752	GO:0019798	procollagen-proline dioxy	0.14	1	0.37453184	19	0.054870475
13966	GO:0031543	peptidyl-proline dioxygena	0.14	1	0.37453184	19	0.054870475
13968	GO:0031545	peptidyl-proline 4-dioxyge	0.14	1	0.37453184	19	0.054870475
2627	GO:0003676	nucleic acid binding	0.14	24	8.988764	2453	7.0840673
4327	GO:0006091	generation of precursor m	0.14	2	0.7490637	84	0.24258527
18000	GO:0035639	purine ribonucleoside tripl	0.14	9	3.3707864	769	2.2208104
4027	GO:0005737	cytoplasm	0.14	20	7.490637	2001	5.7787275
9784	GO:0016723	oxidoreductase activity, o	0.14	1	0.37453184	20	0.0577584
225	GO:0000293	ferric-chelate reductase a	0.14	1	0.37453184	20	0.0577584
3341	GO:0004722|GO:000015	protein serine/threonine p	0.15	3	1.1235955	171	0.4938343
3156	GO:0004497	monooxygenase activity	0.15	3	1.1235955	172	0.49672222
4605	GO:0006457|GO:000702	protein folding	0.15	3	1.1235955	173	0.49961013
9768	GO:0016706	oxidoreductase activity, a	0.15	3	1.1235955	175	0.505386
3831	GO:0005385	zinc ion transmembrane t	0.16	1	0.37453184	22	0.06353424
4882	GO:0006778	porphyrin metabolic proce	0.16	1	0.37453184	22	0.06353424
4883	GO:0006779	porphyrin biosynthetic pro	0.16	1	0.37453184	22	0.06353424
4924	GO:0006829	zinc ion transport	0.16	1	0.37453184	22	0.06353424
7823	GO:0010608	posttranscriptional regulat	0.16	1	0.37453184	22	0.06353424
29941	GO:0071577	zinc ion transmembrane t	0.16	1	0.37453184	22	0.06353424
5693	GO:0008135	translation factor activity,	0.16	2	0.7490637	92	0.26568863
14959	GO:0032553	ribonucleotide binding	0.16	9	3.3707864	795	2.2958963

14961	GO:0032555	purine ribonucleotide bind	0.16	9	3.3707864	795	2.2958963
10033	GO:0017076	purine nucleotide binding	0.17	9	3.3707864	797	2.3016722
3262	GO:0004622|GO:0045126	lysophospholipase activity	0.17	1	0.37453184	24	0.06931008
8559	GO:0015146	pentose transmembrane t	0.17	1	0.37453184	24	0.06931008
8561	GO:0015148	D-xylose transmembrane	0.17	1	0.37453184	24	0.06931008
8829	GO:0015519	D-xylose:hydrogen sympo	0.17	1	0.37453184	24	0.06931008
9019	GO:0015750	pentose transport	0.17	1	0.37453184	24	0.06931008
9022	GO:0015753	D-xylose transport	0.17	1	0.37453184	24	0.06931008
9779	GO:0016717	oxidoreductase activity, a	0.17	1	0.37453184	24	0.06931008
13657	GO:0031225	anchored to membrane	0.17	1	0.37453184	24	0.06931008
25578	GO:0051287|GO:0051288	NAD binding	0.17	1	0.37453184	24	0.06931008
22357	GO:0046854|GO:0046837	phosphatidylinositol phos	0.18	1	0.37453184	25	0.072197996
19393	GO:0043086	negative regulation of cat	0.18	3	1.1235955	188	0.54292893
20342	GO:0044092	negative regulation of mo	0.18	3	1.1235955	188	0.54292893
3415	GO:0004805	trehalose-phosphatase ac	0.18	1	0.37453184	26	0.075085916
9884	GO:0016847|GO:0034100	1-aminocyclopropane-1-c	0.18	1	0.37453184	26	0.075085916
10911	GO:0018871	1-aminocyclopropane-1-c	0.18	1	0.37453184	26	0.075085916
18579	GO:0042218	1-aminocyclopropane-1-c	0.18	1	0.37453184	26	0.075085916
30689	GO:0072330	monocarboxylic acid biosy	0.18	1	0.37453184	26	0.075085916
24968	GO:0050662	coenzyme binding	0.18	3	1.1235955	191	0.5515927
9541	GO:0016413	O-acetyltransferase activi	0.19	1	0.37453184	27	0.077973835
22340	GO:0046834	lipid phosphorylation	0.19	1	0.37453184	27	0.077973835
1029	GO:0001727	lipid kinase activity	0.19	1	0.37453184	27	0.077973835
9238	GO:0015980	energy derivation by oxida	0.19	1	0.37453184	28	0.080861755
19337	GO:0043021	ribonucleoprotein binding	0.20	1	0.37453184	29	0.083749674
2870	GO:0004064	arylesterase activity	0.21	1	0.37453184	30	0.086637594
3009	GO:0004322	ferroxidase activity	0.21	1	0.37453184	30	0.086637594
9785	GO:0016724	oxidoreductase activity, o	0.21	1	0.37453184	30	0.086637594
15414	GO:0033013	tetrapyrrole metabolic pro	0.21	1	0.37453184	30	0.086637594
15415	GO:0033014	tetrapyrrole biosynthetic p	0.21	1	0.37453184	30	0.086637594
3626	GO:0005083	small GTPase regulator a	0.21	2	0.7490637	111	0.3205591
14422	GO:0032012	regulation of ARF protein	0.21	1	0.37453184	31	0.08952551
14720	GO:0032312	regulation of ARF GTPase	0.21	1	0.37453184	31	0.08952551
29918	GO:0071554	cell wall organization or bi	0.21	2	0.7490637	112	0.32344702
3265	GO:0004630	phospholipase D activity	0.22	1	0.37453184	32	0.09241343
4757	GO:0006633|GO:0000037	fatty acid biosynthetic pro	0.22	1	0.37453184	32	0.09241343
6942	GO:0009674	potassium:sodium sympo	0.22	1	0.37453184	32	0.09241343
12522	GO:0022820	potassium ion symporter a	0.22	1	0.37453184	32	0.09241343
21041	GO:0045431	flavonol synthase activity	0.22	1	0.37453184	32	0.09241343
25836	GO:0051552	flavone metabolic process	0.22	1	0.37453184	32	0.09241343
25837	GO:0051553	flavone biosynthetic proce	0.22	1	0.37453184	32	0.09241343
25838	GO:0051554	flavonol metabolic proces	0.22	1	0.37453184	32	0.09241343
25839	GO:0051555	flavonol biosynthetic proc	0.22	1	0.37453184	32	0.09241343
7229	GO:0009987|GO:0008151	cellular process	0.22	88	32.9588	10601	30.614838
18956	GO:0042626	ATPase activity, coupled	0.22	3	1.1235955	211	0.6093511
9861	GO:0016820	hydrolase activity, acting	0.22	3	1.1235955	212	0.612239
24966	GO:0050660	flavin adenine dinucleotid	0.23	2	0.7490637	118	0.34077454
11206	GO:0019203	carbohydrate phosphatase	0.24	1	0.37453184	35	0.10107719
12817	GO:0030258	lipid modification	0.24	1	0.37453184	35	0.10107719
22012	GO:0046488|GO:0030384	phosphatidylinositol meta	0.24	1	0.37453184	35	0.10107719
4232	GO:0005992	trehalose biosynthetic pro	0.24	1	0.37453184	36	0.10396511
9393	GO:0016165	lipoxygenase activity	0.24	1	0.37453184	36	0.10396511
3260	GO:0004620	phospholipase activity	0.25	2	0.7490637	123	0.35521415
25485	GO:0051188	cofactor biosynthetic proc	0.25	2	0.7490637	123	0.35521415
20488	GO:0044238	primary metabolic process	0.25	76	28.46442	9167	26.47356
4231	GO:0005991	trehalose metabolic proce	0.25	1	0.37453184	38	0.10974095
7072	GO:0009813	flavonoid biosynthetic pro	0.27	1	0.37453184	40	0.1155168
4851	GO:0006733	oxidoreduction coenzyme	0.27	1	0.37453184	41	0.118404716
19758	GO:0043492	ATPase activity, coupled	0.27	3	1.1235955	235	0.67866117
3877	GO:0005516	calmodulin binding	0.27	3	1.1235955	236	0.6815491
2653	GO:0003723	RNA binding	0.27	8	2.9962547	797	2.3016722
4756	GO:0006631	fatty acid metabolic proce	0.28	1	0.37453184	42	0.121292636
6666	GO:0009312	oligosaccharide biosynthe	0.28	1	0.37453184	42	0.121292636
9370	GO:0016138	glycoside biosynthetic pro	0.28	1	0.37453184	42	0.121292636
21880	GO:0046351	disaccharide biosynthetic	0.28	1	0.37453184	42	0.121292636
14676	GO:0032268	regulation of cellular prote	0.28	1	0.37453184	43	0.124180555
25538	GO:0051246	regulation of protein meta	0.28	1	0.37453184	43	0.124180555
7071	GO:0009812	flavonoid metabolic proce	0.29	1	0.37453184	44	0.12706847
9753	GO:0016682	oxidoreductase activity, a	0.29	1	0.37453184	44	0.12706847
4942	GO:0006855	drug transmembrane trans	0.29	2	0.7490637	139	0.40142086
8642	GO:0015238|GO:0015239	drug transmembrane trans	0.29	2	0.7490637	139	0.40142086
9154	GO:0015893	drug transport	0.29	2	0.7490637	139	0.40142086
18832	GO:0042493|GO:0017035	response to drug	0.29	2	0.7490637	139	0.40142086
31431	GO:0080054	low affinity nitrate transme	0.29	1	0.37453184	45	0.1299564
13164	GO:0030695	GTPase regulator activity	0.30	2	0.7490637	142	0.4100846
20512	GO:0044262|GO:0006092	cellular carbohydrate meta	0.30	3	1.1235955	248	0.7162041
9396	GO:0016168	chlorophyll binding	0.30	1	0.37453184	46	0.13284431
4773	GO:0006650|GO:0006652	glycerophospholipid meta	0.31	1	0.37453184	47	0.13573223
8497	GO:0015079|GO:0022817	potassium ion transmemb	0.31	1	0.37453184	47	0.13573223
22010	GO:0046486	glycerolipid metabolic pro	0.31	1	0.37453184	47	0.13573223
3307	GO:0004675	transmembrane receptor	0.31	4	1.4981273	367	1.0598665
31421	GO:0080044	quercetin 7-O-glucosyltrar	0.32	1	0.37453184	49	0.14150807
9829	GO:0016779	nucleotidyltransferase act	0.32	2	0.7490637	148	0.42741212
8749	GO:0015399	primary active transmemb	0.32	3	1.1235955	260	0.75085914
8753	GO:0015405	P-P-bond-hydrolysis-drive	0.32	3	1.1235955	260	0.75085914
4897	GO:0006793	phosphorus metabolic pro	0.32	25	9.363296	2925	8.4471655
4900	GO:0006796	phosphate metabolic proc	0.32	25	9.363296	2925	8.4471655
3099	GO:0004428	inositol or phosphatidylino	0.33	1	0.37453184	51	0.14728391
3293	GO:0004659	prenyltransferase activity	0.33	1	0.37453184	51	0.14728391
8908	GO:0015629	actin cytoskeleton	0.33	1	0.37453184	51	0.14728391
18541	GO:0042180	cellular ketone metabolic	0.33	4	1.4981273	375	1.0829699
2809	GO:0003993	acid phosphatase activity	0.33	1	0.37453184	52	0.15017183
6965	GO:0009699	phenylpropanoid biosynth	0.33	1	0.37453184	52	0.15017183
6424	GO:0009055|GO:0009053	electron carrier activity	0.33	2	0.7490637	154	0.44473964
131	GO:0000166	nucleotide binding	0.33	11	4.11985	1213	3.5030468
9750	GO:0016679	oxidoreductase activity, a	0.34	1	0.37453184	53	0.15305975
9699	GO:0016616	oxidoreductase activity, a	0.34	3	1.1235955	266	0.7681867
3464	GO:0004857	enzyme inhibitor activity	0.34	2	0.7490637	157	0.4534034
17918	GO:0035556|GO:0007242	intracellular signal transdu	0.34	1	0.37453184	54	0.15594767
4224	GO:0005984	disaccharide metabolic pr	0.35	1	0.37453184	56	0.16172351
9369	GO:0016137	glycoside metabolic proce	0.35	1	0.37453184	56	0.16172351
9883	GO:0016846	carbon-sulfur lyase activit	0.35	1	0.37453184	56	0.16172351
6441	GO:0009072	aromatic amino acid famil	0.36	1	0.37453184	57	0.16461143
25483	GO:0051186	cofactor metabolic proces	0.36	2	0.7490637	164	0.47361887
6665	GO:0009311	oligosaccharide metabolic	0.36	1	0.37453184	58	0.16749935
8558	GO:0015145	monosaccharide transmen	0.36	1	0.37453184	58	0.16749935
9018	GO:0015749	monosaccharide transpor	0.36	1	0.37453184	58	0.16749935
18981	GO:0042651	thylakoid membrane	0.37	1	0.37453184	59	0.17038727
2980	GO:0004185	serine-type carboxypeptid	0.37	1	0.37453184	60	0.17327519
5652	GO:0008081|GO:0004434	phosphoric diester hydrola	0.37	1	0.37453184	60	0.17327519
6964	GO:0009698	phenylpropanoid metaboli	0.37	1	0.37453184	60	0.17327519
28375	GO:0070008	serine-type exopeptidase	0.37	1	0.37453184	60	0.17327519
4768	GO:0006644	phospholipid metabolic pr	0.38	1	0.37453184	61	0.1761631

8526	GO:0015112	nitrate transmembrane tra	0.38	1	0.37453184	61	0.1761631
8975	GO:0015706|GO:0006872	nitrate transport	0.38	1	0.37453184	61	0.1761631
3884	GO:0005524	ATP binding	0.38	5	1.8726592	524	1.51327
2978	GO:0004180	carboxypeptidase activity	0.38	1	0.37453184	62	0.17905103
17457	GO:0035091	phosphatidylinositol bindir	0.38	1	0.37453184	62	0.17905103
8675	GO:0015293	symporter activity	0.39	2	0.7490637	173	0.49961013
5704	GO:0008150|GO:0000004	biological_process	0.39	174	65.16854	22215	64.155136
22416	GO:0046915	transition metal ion transn	0.39	1	0.37453184	64	0.18482687
9697	GO:0016614	oxidoreductase activity, a	0.39	3	1.1235955	293	0.84616053
11616	GO:0019637	organophosphate metabo	0.40	1	0.37453184	65	0.18771479
25314	GO:0051015	actin filament binding	0.40	1	0.37453184	65	0.18771479
18953	GO:0042623	ATPase activity, coupled	0.40	5	1.8726592	537	1.550813
3180	GO:0004521	endoribonuclease activity	0.40	1	0.37453184	66	0.1906027
9765	GO:0016702	oxidoreductase activity, a	0.40	1	0.37453184	66	0.1906027
9837	GO:0016788	hydrolase activity, acting	0.40	13	4.8689137	1534	4.430069
4572	GO:0006412|GO:0006416	translation	0.40	2	0.7490637	180	0.5198256
32	GO:0000041	transition metal ion transp	0.41	1	0.37453184	68	0.19637854
5610	GO:0008026	ATP-dependent helicase	0.41	2	0.7490637	183	0.52848935
28401	GO:0070035	purine NTP-dependent he	0.41	2	0.7490637	183	0.52848935
20505	GO:0044255	cellular lipid metabolic pro	0.42	2	0.7490637	184	0.53137726
4844	GO:0006725	cellular aromatic compour	0.42	2	0.7490637	185	0.53426516
9486	GO:0016298	lipase activity	0.42	2	0.7490637	185	0.53426516
5726	GO:0008187	poly-pyrimidine tract bindi	0.42	1	0.37453184	70	0.20215438
5788	GO:0008266	poly(U) RNA binding	0.42	1	0.37453184	70	0.20215438
23522	GO:0048046	apoplast	0.42	1	0.37453184	70	0.20215438
13041	GO:0030554	adenyl nucleotide binding	0.42	5	1.8726592	550	1.5883559
14965	GO:0032559	adenyl ribonucleotide bind	0.42	5	1.8726592	550	1.5883559
6475	GO:0009108	coenzyme biosynthetic pr	0.43	1	0.37453184	72	0.20793022
9764	GO:0016701	oxidoreductase activity, a	0.43	1	0.37453184	72	0.20793022
3068	GO:0004386	helicase activity	0.43	2	0.7490637	189	0.54581684
3306	GO:0004674|GO:0004695	protein serine/threonine k	0.43	9	3.3707864	1062	3.0669708
3311	GO:0004683|GO:0004684	calmodulin-dependent pro	0.44	1	0.37453184	74	0.21370606
25510	GO:0051213	dioxygenase activity	0.44	1	0.37453184	75	0.21659398
28365	GO:0065008	regulation of biological qu	0.44	3	1.1235955	316	0.9125827
2897	GO:0004091|GO:0004302	carboxylesterase activity	0.44	2	0.7490637	194	0.5602564
2819	GO:0004004	ATP-dependent RNA heli	0.45	1	0.37453184	78	0.22525774
5725	GO:0008186|GO:0004010	RNA-dependent ATPase	0.45	1	0.37453184	78	0.22525774
30865	GO:0072509	divalent inorganic cation t	0.45	1	0.37453184	78	0.22525774
2654	GO:0003724	RNA helicase activity	0.46	1	0.37453184	80	0.2310336
23515	GO:0048037	cofactor binding	0.46	3	1.1235955	327	0.94434977
29202	GO:0070838	divalent metal ion transpo	0.47	1	0.37453184	82	0.23680943
16738	GO:0034357	photosynthetic membrane	0.47	1	0.37453184	83	0.23969735
4954	GO:0006869	lipid transport	0.48	2	0.7490637	208	0.6006873
8085	GO:0010876	lipid localization	0.48	2	0.7490637	208	0.6006873
4912	GO:0006813|GO:0015458	potassium ion transport	0.48	1	0.37453184	84	0.24258527
30168	GO:0071804	cellular potassium ion trar	0.48	1	0.37453184	84	0.24258527
30169	GO:0071805	potassium ion transmemb	0.48	1	0.37453184	84	0.24258527
30867	GO:0072511	divalent inorganic cation t	0.48	1	0.37453184	84	0.24258527
9295	GO:0016053	organic acid biosynthetic	0.49	2	0.7490637	211	0.6093511
21058	GO:0045454|GO:0030503	cell redox homeostasis	0.49	2	0.7490637	211	0.6093511
21922	GO:0046394	carboxylic acid biosynthet	0.49	2	0.7490637	211	0.6093511
5768	GO:0008238	exopeptidase activity	0.49	1	0.37453184	86	0.24836111
22376	GO:0046873	metal ion transmembrane	0.49	2	0.7490637	213	0.6151269
20624	GO:0044430	cytoskeletal part	0.49	1	0.37453184	88	0.25413695
20630	GO:0044436	thylakoid part	0.50	1	0.37453184	90	0.2599128
17617	GO:0035251	UDP-glucosyltransferase	0.50	2	0.7490637	218	0.62956655
3644	GO:0005102	receptor binding	0.51	1	0.37453184	91	0.2628007
3808	GO:0005351|GO:0005403	sugar:hydrogen symporte	0.52	1	0.37453184	94	0.27146447
3835	GO:0005402	cation:sugar symporter ac	0.52	1	0.37453184	94	0.27146447
6863	GO:0009579	thylakoid	0.52	1	0.37453184	94	0.27146447
10058	GO:0017111	nucleoside-triphosphatase	0.52	7	2.621723	878	2.5355935
3472	GO:0004866	endopeptidase inhibitor ac	0.52	1	0.37453184	95	0.27435237
8159	GO:0010951	negative regulation of enc	0.52	1	0.37453184	95	0.27435237
11712	GO:0019748	secondary metabolic proc	0.52	1	0.37453184	95	0.27435237
26712	GO:0052548	regulation of endopeptida	0.52	1	0.37453184	95	0.27435237
28101	GO:0061135	endopeptidase regulator a	0.52	1	0.37453184	95	0.27435237
6796	GO:0009507	chloroplast	0.53	2	0.7490637	229	0.6613336
13531	GO:0031072	heat shock protein binding	0.53	2	0.7490637	230	0.6642216
8677	GO:0015295	solute:hydrogen symporte	0.53	1	0.37453184	98	0.28301615
25379	GO:0051082	unfolded protein binding	0.53	1	0.37453184	98	0.28301615
4612	GO:0006468	protein phosphorylation	0.54	17	6.367041	2205	6.367863
11695	GO:0019725	cellular homeostasis	0.54	2	0.7490637	234	0.67577326
4320	GO:0006082	organic acid metabolic pro	0.54	3	1.1235955	369	1.0656425
11716	GO:0019752	carboxylic acid metabolic	0.54	3	1.1235955	369	1.0656425
19707	GO:0043436	oxoacid metabolic proces	0.54	3	1.1235955	369	1.0656425
11202	GO:0019199	transmembrane receptor	0.55	4	1.4981273	504	1.4555116
5874	GO:0008374	O-acyltransferase activity	0.55	1	0.37453184	102	0.29456782
18925	GO:0042592	homeostatic process	0.55	2	0.7490637	238	0.68732494
4405	GO:0006195	purine nucleotide catabol	0.55	6	2.247191	773	2.232362
6508	GO:0009143	nucleoside triphosphate c	0.55	6	2.247191	773	2.232362
6509	GO:0009144	purine nucleoside triphosp	0.55	6	2.247191	773	2.232362
6511	GO:0009146	purine nucleoside triphosp	0.55	6	2.247191	773	2.232362
6519	GO:0009154	purine ribonucleotide cata	0.55	6	2.247191	773	2.232362
6530	GO:0009166	nucleotide catabolic proce	0.55	6	2.247191	773	2.232362
6567	GO:0009203	ribonucleoside triphospha	0.55	6	2.247191	773	2.232362
6569	GO:0009205	purine ribonucleoside trip	0.55	6	2.247191	773	2.232362
6571	GO:0009207	purine ribonucleoside trip	0.55	6	2.247191	773	2.232362
6624	GO:0009261	ribonucleotide catabolic p	0.55	6	2.247191	773	2.232362
17034	GO:0034655	nucleobase, nucleoside, r	0.55	6	2.247191	773	2.232362
17035	GO:0034656	nucleobase, nucleoside a	0.55	6	2.247191	773	2.232362
22209	GO:0046700	heterocycle catabolic proc	0.55	6	2.247191	773	2.232362
30879	GO:0072523	purine-containing compou	0.55	6	2.247191	773	2.232362
4408	GO:0006200	ATP catabolic process	0.55	5	1.8726592	641	1.8511566
9918	GO:0016887|GO:0004002	ATPase activity	0.55	5	1.8726592	641	1.8511566
21600	GO:0046034	ATP metabolic process	0.55	5	1.8726592	641	1.8511566
19525	GO:0043226	organelle	0.55	23	8.614232	3013	8.701303
19528	GO:0043229	intracellular organelle	0.55	23	8.614232	3013	8.701303
9709	GO:0016627	oxidoreductase activity, a	0.55	1	0.37453184	104	0.30034366
20520	GO:0044270	cellular nitrogen compour	0.56	6	2.247191	777	2.2439137
2668	GO:0003779	actin binding	0.56	1	0.37453184	105	0.3032316
3195	GO:0004540	ribonuclease activity	0.56	1	0.37453184	106	0.3061195
9493	GO:0016310	phosphorylation	0.56	19	7.116105	2504	7.2313514
6824	GO:0009536	plastid	0.57	2	0.7490637	247	0.7133162
6563	GO:0009199	ribonucleoside triphospha	0.57	6	2.247191	790	2.2814567
6515	GO:0009150	purine ribonucleotide met	0.57	6	2.247191	791	2.2843447
7690	GO:0010466	negative regulation of per	0.57	1	0.37453184	110	0.31767118
12946	GO:0030414	peptidase inhibitor activity	0.57	1	0.37453184	110	0.31767118
25636	GO:0051346	negative regulation of hyd	0.57	1	0.37453184	110	0.31767118
6506	GO:0009141	nucleoside triphosphate m	0.58	6	2.247191	796	2.2987843
3304	GO:0004672|GO:0050222	protein kinase activity	0.58	10	3.7453184	1333	3.8495972
26711	GO:0052547	regulation of peptidase ac	0.58	1	0.37453184	112	0.32344702
28100	GO:0061134	peptidase regulator activit	0.58	1	0.37453184	112	0.32344702
4850	GO:0006732|GO:0006752	coenzyme metabolic proc	0.58	1	0.37453184	113	0.32633495

9535	GO:0016407	acetyltransferase activity	0.59	1	0.37453184	115	0.3321108
4373	GO:0006163	purine nucleotide metabol	0.60	6	2.247191	810	2.339215
26845	GO:0052689	carboxylic ester hydrolase	0.60	4	1.4981273	538	1.5537009
6622	GO:0009259|GO:0009121	ribonucleotide metabolic p	0.60	6	2.247191	812	2.344991
9285	GO:0016043	cellular component organ	0.60	2	0.7490637	261	0.7537471
25416	GO:0051119	sugar transmembrane tra	0.60	1	0.37453184	118	0.34077454
9824	GO:0016773	phosphotransferase activ	0.60	11	4.11985	1493	4.311664
9579	GO:0016462	pyrophosphatase activity	0.61	7	2.621723	954	2.7550755
9859	GO:0016818	hydrolase activity, acting	0.61	7	2.621723	956	2.7608514
19126	GO:0042803	protein homodimerization	0.61	2	0.7490637	265	0.7652988
22007	GO:0046483	heterocycle metabolic pro	0.61	8	2.9962547	1094	3.1593843
9858	GO:0016817	hydrolase activity, acting	0.61	7	2.621723	959	2.769515
6884	GO:0009607	response to biotic stimulu	0.61	1	0.37453184	122	0.3523262
30877	GO:0072521	purine-containing compou	0.62	6	2.247191	827	2.3883097
22049	GO:0046527	glucosyltransferase activi	0.62	2	0.7490637	270	0.77973837
4121	GO:0005856	cytoskeleton	0.62	1	0.37453184	126	0.3638779
19125	GO:0042802	identical protein binding	0.63	5	1.8726592	697	2.01288
4649	GO:0006520|GO:0006519	cellular amino acid metab	0.63	2	0.7490637	277	0.7999538
9823	GO:0016772	transferase activity, trans	0.63	14	5.243446	1932	5.579461
3874	GO:0005509	calcium ion binding	0.64	2	0.7490637	279	0.8057296
2758	GO:0003924	GTPase activity	0.64	1	0.37453184	132	0.3812054
4394	GO:0006184	GTP catabolic process	0.64	1	0.37453184	132	0.3812054
21605	GO:0046039	GTP metabolic process	0.64	1	0.37453184	132	0.3812054
4215	GO:0005975	carbohydrate metabolic p	0.64	7	2.621723	990	2.8590407
3490	GO:0004888|GO:0004926	transmembrane receptor a	0.64	4	1.4981273	571	1.6490022
20532	GO:0044282	small molecule catabolic	0.65	6	2.247191	854	2.4662836
30205	GO:0071841	cellular component organ	0.65	2	0.7490637	285	0.8230572
9811	GO:0016757|GO:0016932	transferase activity, trans	0.66	5	1.8726592	729	2.1052935
6046	GO:0008610	lipid biosynthetic process	0.67	1	0.37453184	141	0.4071967
8676	GO:0015294	solute:cation symporter a	0.67	1	0.37453184	141	0.4071967
8557	GO:0015144	carbohydrate transmembr	0.67	1	0.37453184	143	0.41297254
16602	GO:0034219	carbohydrate transmembr	0.67	1	0.37453184	143	0.41297254
3873	GO:0005507	copper ion binding	0.68	1	0.37453184	146	0.42163628
20356	GO:0044106	cellular amine metabolic p	0.68	2	0.7490637	303	0.8750397
6071	GO:0008643|GO:0006861	carbohydrate transport	0.68	1	0.37453184	147	0.42452422
20517	GO:0044267	cellular protein metabolic	0.68	23	8.614232	3225	9.313541
9801	GO:0016747	transferase activity, trans	0.69	3	1.1235955	456	1.3168914
11426	GO:0019438	aromatic compound biosy	0.69	1	0.37453184	149	0.43030006
20498	GO:0044248	cellular catabolic process	0.69	6	2.247191	894	2.5818002
9488	GO:0016301	kinase activity	0.69	12	4.494382	1740	5.0249805
3200	GO:0004553|GO:0016800	hydrolase activity, hydroly	0.70	2	0.7490637	312	0.90103096
5791	GO:0008270	zinc ion binding	0.70	13	4.8689137	1880	5.4292893
8517	GO:0015103	inorganic anion transmem	0.70	1	0.37453184	154	0.44473964
11224	GO:0019222	regulation of metabolic pr	0.70	22	8.2397	3115	8.995871
6662	GO:0009308	amine metabolic process	0.70	2	0.7490637	314	0.9068068
13656	GO:0031224	intrinsic to membrane	0.70	8	2.9962547	1193	3.4452884
2975	GO:0004175|GO:0016809	endopeptidase activity	0.71	2	0.7490637	320	0.9241344
4867	GO:0006753	nucleoside phosphate me	0.71	6	2.247191	914	2.6395588
6484	GO:0009117	nucleotide metabolic proc	0.71	6	2.247191	914	2.6395588
5732	GO:0008194	UDP-glycosyltransferase	0.71	2	0.7490637	322	0.9299102
18582	GO:0042221	response to chemical stim	0.72	2	0.7490637	328	0.94723773
12572	GO:0022890|GO:0015082	inorganic cation transmen	0.73	2	0.7490637	332	0.9587894
12516	GO:0022804	active transmembrane tra	0.73	5	1.8726592	787	2.2727928
20618	GO:0044424	intracellular part	0.73	27	10.11236	3853	11.127155
6077	GO:0008652	cellular amino acid biosyn	0.73	1	0.37453184	169	0.48805845
26932	GO:0055086	nucleobase, nucleoside a	0.74	6	2.247191	944	2.7261963
9800	GO:0016746	transferase activity, trans	0.74	3	1.1235955	498	1.438184
4067	GO:0005783	endoplasmic reticulum	0.74	1	0.37453184	174	0.50249803
20619	GO:0044425	membrane part	0.74	10	3.7453184	1523	4.398302
8967	GO:0015698	inorganic anion transport	0.74	1	0.37453184	176	0.5082739
20533	GO:0044283	small molecule biosynthet	0.75	2	0.7490637	347	1.0021082
6166	GO:0008757	S-adenosylmethionine-de	0.75	1	0.37453184	180	0.5198256
5195	GO:0007165|GO:0023033	signal transduction	0.75	6	2.247191	961	2.775291
12602	GO:0023052|GO:0023046	signaling	0.75	6	2.247191	961	2.775291
6663	GO:0009309	amine biosynthetic proces	0.75	1	0.37453184	181	0.5227135
9818	GO:0016765|GO:0016766	transferase activity, trans	0.76	1	0.37453184	185	0.53426516
22479	GO:0046983	protein dimerization activi	0.76	3	1.1235955	517	1.4930545
3476	GO:0004872|GO:0019041	receptor activity	0.76	4	1.4981273	672	1.9406822
2657	GO:0003727|GO:0003728	single-stranded RNA bind	0.77	1	0.37453184	188	0.54292893
30204	GO:0071840	cellular component organ	0.77	2	0.7490637	362	1.045427
30066	GO:0071702	organic substance transp	0.78	3	1.1235955	529	1.5277096
20521	GO:0044271	cellular nitrogen compoun	0.78	2	0.7490637	367	1.0598665
31465	GO:0080090	regulation of primary meta	0.78	18	6.741573	2710	7.826263
28378	GO:0070011	peptidase activity, acting	0.79	3	1.1235955	537	1.550813
12608	GO:0030001	metal ion transport	0.79	2	0.7490637	374	1.080082
5840	GO:0008324	cation transmembrane tra	0.79	3	1.1235955	538	1.5537009
10244	GO:0018130	heterocycle biosynthetic p	0.79	1	0.37453184	199	0.57469606
9836	GO:0016787	hydrolase activity	0.80	25	9.363296	3723	10.751725
8943	GO:0015672	monovalent inorganic cati	0.80	2	0.7490637	383	1.1060733
9266	GO:0016020	membrane	0.80	25	9.363296	3741	10.803708
13753	GO:0031323	regulation of cellular meta	0.81	18	6.741573	2770	7.999538
5766	GO:0008236	serine-type peptidase acti	0.81	1	0.37453184	214	0.6180149
8495	GO:0015077	monovalent inorganic cati	0.81	1	0.37453184	214	0.6180149
25094	GO:0050789|GO:0050791	regulation of biological pr	0.81	29	10.8614235	4315	12.461374
4609	GO:0006464	protein modification proce	0.81	18	6.741573	2781	8.031305
15436	GO:0033036	macromolecule localizatio	0.81	2	0.7490637	397	1.1465042
5763	GO:0008233	peptidase activity	0.81	3	1.1235955	565	1.6316746
9845	GO:0016798	hydrolase activity, acting	0.82	2	0.7490637	401	1.1580559
4558	GO:0006396|GO:0006394	RNA processing	0.82	1	0.37453184	219	0.63245445
20531	GO:0044281	small molecule metabolic	0.82	11	4.11985	1788	5.1636004
10103	GO:0017171	serine hydrolase activity	0.82	1	0.37453184	220	0.63534236
6425	GO:0009056	catabolic process	0.82	6	2.247191	1051	3.0352037
8673	GO:0015291|GO:0015290	secondary active transme	0.82	2	0.7490637	407	1.1753833
11221	GO:0019219	regulation of nucleobase,	0.83	17	6.367041	2675	7.7251854
25468	GO:0051171	regulation of nitrogen con	0.83	17	6.367041	2675	7.7251854
4990	GO:0006915|GO:0008632	apoptosis	0.83	1	0.37453184	227	0.6555578
28364	GO:0065007	biological regulation	0.83	29	10.8614235	4376	12.637537
4755	GO:0006629	lipid metabolic process	0.84	3	1.1235955	590	1.7038727
9906	GO:0016874	ligase activity	0.84	3	1.1235955	597	1.7240882
11828	GO:0019899	enzyme binding	0.84	1	0.37453184	237	0.684437
5756	GO:0008219	cell death	0.84	1	0.37453184	239	0.69021285
8209	GO:0012501|GO:0016244	programmed cell death	0.84	1	0.37453184	239	0.69021285
9462	GO:0016265	death	0.84	1	0.37453184	239	0.69021285
25098	GO:0050794|GO:0051244	regulation of cellular proc	0.84	26	9.737827	3987	11.514136
11522	GO:0019538|GO:0006411	protein metabolic process	0.85	27	10.11236	4134	11.938661
5712	GO:0008168|GO:0004480	methyltransferase activity	0.85	1	0.37453184	244	0.7046524
5981	GO:0008509	anion transmembrane tran	0.85	1	0.37453184	245	0.70754033
4917	GO:0006820|GO:0006822	anion transport	0.85	1	0.37453184	247	0.7133162
5661	GO:0008092	cytoskeletal protein bindir	0.86	1	0.37453184	249	0.719092
12805	GO:0030246	carbohydrate binding	0.86	1	0.37453184	251	0.7248679
3475	GO:0004871|GO:0005062	signal transducer activity	0.86	4	1.4981273	787	2.2727928
27063	GO:0060089	molecular transducer activ	0.86	4	1.4981273	787	2.2727928
19684	GO:0043412	macromolecule modificati	0.86	18	6.741573	2900	8.374968

9795	GO:0016740	transferase activity	0.86	24	8.988764	3764	10.870131
9796	GO:0016741	transferase activity, transf	0.87	1	0.37453184	261	0.7537471
25998	GO:0051716	cellular response to stimu	0.88	6	2.247191	1138	3.2864528
8494	GO:0015075	ion transmembrane transp	0.88	4	1.4981273	827	2.3883097
16603	GO:0034220	ion transmembrane transp	0.89	4	1.4981273	833	2.4056373
4911	GO:0006812|GO:000681	cation transport	0.89	3	1.1235955	662	1.9118029
14667	GO:0032259	methylation	0.89	1	0.37453184	285	0.8230572
4916	GO:0006818	hydrogen transport	0.90	1	0.37453184	292	0.84327257
9246	GO:0015992	proton transport	0.90	1	0.37453184	292	0.84327257
9812	GO:0016758	transferase activity, transf	0.90	2	0.7490637	501	1.4468478
22415	GO:0046914	transition metal ion bindin	0.91	15	5.6179776	2599	7.5057034
7141	GO:0009889	regulation of biosynthetic	0.91	15	5.6179776	2605	7.523031
7772	GO:0010556	regulation of macromolec	0.91	15	5.6179776	2605	7.523031
13756	GO:0031326	regulation of cellular biosy	0.91	15	5.6179776	2605	7.523031
32213	GO:2000112	regulation of cellular macr	0.91	15	5.6179776	2605	7.523031
20640	GO:0044446	intracellular organelle part	0.91	3	1.1235955	710	2.0504231
20616	GO:0044422	organelle part	0.91	3	1.1235955	712	2.0561988
7692	GO:0010468	regulation of gene expres	0.92	15	5.6179776	2635	7.6096687
4849	GO:0006730|GO:001975	one-carbon metabolic pro	0.92	1	0.37453184	325	0.93857396
3449	GO:0004842|GO:000484	ubiquitin-protein ligase ac	0.92	1	0.37453184	326	0.94146186
27228	GO:0060255	regulation of macromolec	0.92	15	5.6179776	2660	7.6818666
5909	GO:0008415	acyltransferase activity	0.92	1	0.37453184	330	0.95301354
2643	GO:0003700|GO:000013	sequence-specific DNA b	0.92	7	2.621723	1417	4.0921826
716	GO:0001071	nucleic acid binding trans	0.92	7	2.621723	1417	4.0921826
9654	GO:0016567	protein ubiquitination	0.92	1	0.37453184	332	0.9587894
14854	GO:0032446	protein modification by sm	0.92	1	0.37453184	332	0.9587894
29011	GO:0070647	protein modification by sm	0.92	1	0.37453184	332	0.9587894
3903	GO:0005575|GO:000837	cellular_component	0.93	56	20.973783	8515	24.590637
22375	GO:0046872	metal ion binding	0.93	17	6.367041	2985	8.6204405
11747	GO:0019787|GO:000863	small conjugating protein	0.93	1	0.37453184	339	0.9790048
9267	GO:0016021	integral to membrane	0.93	5	1.8726592	1103	3.1853757
19470	GO:0043167	ion binding	0.93	17	6.367041	2997	8.655096
19472	GO:0043169	cation binding	0.93	17	6.367041	2997	8.655096
2674	GO:0003824	catalytic activity	0.94	72	26.966291	10750	31.045137
3178	GO:0004519	endonuclease activity	0.94	1	0.37453184	356	1.0280994
4910	GO:0006811	ion transport	0.94	4	1.4981273	959	2.769515
4530	GO:0006355|GO:003258	regulation of transcription	0.94	14	5.243446	2591	7.4826
25544	GO:0051252	regulation of RNA metabo	0.94	14	5.243446	2593	7.488376
4641	GO:0006508	proteolysis	0.94	4	1.4981273	965	2.7868426
12547	GO:0022857|GO:000538	transmembrane transporte	0.94	7	2.621723	1483	4.282785
22409	GO:0046906	tetrapyrrole binding	0.95	2	0.7490637	596	1.7212002
3723	GO:0005215|GO:000547	transporter activity	0.95	9	3.3707864	1844	5.325324
9913	GO:0016881	acid-amino acid ligase ac	0.95	1	0.37453184	391	1.1291766
3945	GO:0005622	intracellular	0.95	28	10.486892	4751	13.720507
26931	GO:0055085	transmembrane transport	0.96	7	2.621723	1548	4.4705
25195	GO:0050896|GO:005186	response to stimulus	0.96	9	3.3707864	1881	5.432177
3946	GO:0005623	cell	0.96	52	19.475655	8227	23.758917
20657	GO:0044464	cell part	0.96	52	19.475655	8227	23.758917
19533	GO:0043234	protein complex	0.96	3	1.1235955	848	2.448956
3177	GO:0004518	nuclease activity	0.96	1	0.37453184	427	1.2331418
12574	GO:0022892	substrate-specific transpo	0.97	6	2.247191	1463	4.2250266
9911	GO:0016879	ligase activity, forming ca	0.97	1	0.37453184	456	1.3168914
4909	GO:0006810|GO:001545	transport	0.97	11	4.11985	2315	6.6855345
25529	GO:0051234	establishment of localizat	0.97	11	4.11985	2315	6.6855345
25476	GO:0051179	localization	0.97	11	4.11985	2319	6.697086
3863	GO:0005488	binding	0.98	92	34.45693	13903	40.15075
9869	GO:0016829	lyase activity	0.98	1	0.37453184	488	1.4093049
11931	GO:0020037	heme binding	0.99	1	0.37453184	550	1.5883559
3955	GO:0005634	nucleus	0.99	5	1.8726592	1433	4.138389
12573	GO:0022891	substrate-specific transme	0.99	4	1.4981273	1241	3.5839086
3872	GO:0005506	iron ion binding	0.99	1	0.37453184	563	1.6258988
19526	GO:0043227	membrane-bounded orga	0.99	8	2.9962547	2142	6.185924
19530	GO:0043231	intracellular membrane-bo	0.99	8	2.9962547	2142	6.185924
3876	GO:0005515|GO:004530	protein binding	1.00	34	12.734082	6933	20.021948
2626	GO:0003674|GO:000555	molecular_function	1.00	157	58.8015	24394	70.447914
